# No Evidence of the Effect of Extreme Weather Events on Annual Occurrence of Four Groups of Ectothermic Species

**DOI:** 10.1371/journal.pone.0110219

**Published:** 2014-10-17

**Authors:** Agnieszka H. Malinowska, Arco J. van Strien, Jana Verboom, Michiel F. WallisdeVries, Paul Opdam

**Affiliations:** 1 Spatial Planning Group, Wageningen University, Wageningen, the Netherlands; 2 Statistics Netherlands, The Hague, the Netherlands; 3 Policy and Biodiversity Group, ALTERRA Wageningen UR, Wageningen, the Netherlands; 4 De Vlinderstichting/Dutch Butterfly Conservation, Wageningen, the Netherlands; 5 Laboratory of Entomology, Wageningen University, Wageningen, the Netherlands; 6 Nature and Society Group, ALTERRA Wageningen UR, Wageningen, the Netherlands; University of Waikato (National Institute of Water and Atmospheric Research), New Zealand

## Abstract

Weather extremes may have strong effects on biodiversity, as known from theoretical and modelling studies. Predicted negative effects of increased weather variation are found only for a few species, mostly plants and birds in empirical studies. Therefore, we investigated correlations between weather variability and patterns in occupancy, local colonisations and local extinctions (metapopulation metrics) across four groups of ectotherms: Odonata, Orthoptera, Lepidoptera, and Reptilia. We analysed data of 134 species on a 1×1 km-grid base, collected in the last 20 years from the Netherlands, combining standardised data and opportunistic data. We applied dynamic site-occupancy models and used the results as input for analyses of (i) trends in distribution patterns, (ii) the effect of temperature on colonisation and persistence probability, and (iii) the effect of years with extreme weather on all the three metapopulation metrics. All groups, except butterflies, showed more positive than negative trends in metapopulation metrics. We did not find evidence that the probability of colonisation or persistence increases with temperature nor that extreme weather events are reflected in higher extinction risks. We could not prove that weather extremes have visible and consistent negative effects on ectothermic species in temperate northern hemisphere. These findings do not confirm the general prediction that increased weather variability imperils biodiversity. We conclude that weather extremes might not be ecologically relevant for the majority of species. Populations might be buffered against weather variation (e.g. by habitat heterogeneity), or other factors might be masking the effects (e.g. availability and quality of habitat). Consequently, we postulate that weather extremes have less, or different, impact in real world metapopulations than theory and models suggest.

## Introduction

Given the failure of post-Kyoto negotiations, an effective halting of the global climate change seems unrealistic within the next decades. The climate is expected to change and one of the most apparent strategy of biodiversity conservation will be by adapting the landscape (e.g. creating new habitat patches, increasing heterogeneity or abiotic quality of existing patches, increasing connectivity between patches). For adequate conservation measures, knowledge is required on the impact of climatic changes on the populations’ dynamics in time and space [1], [2].

Many studies have been published on the latitudinal and elevational range shifts of species [3]–[5], phenological advancements [5]–[8] and changes in community structure, all in relation to climate change [9], [10]. However, to understand how we can adapt landscapes to facilitate species persistence, we need to investigate how climate change impacts interfere with population dynamics at the regional scale [11]. These effects are more difficult to grasp [12] and can potentially interact with habitat fragmentation [13], [14].

Regional population dynamics, i.e. within areas of several hundreds to a few thousand km^2^, are important for conservation, especially in temperate Europe, where natural habitats are highly fragmented and immersed in inhospitable landscape matrix [15]. The dynamics of animals in fragmented landscapes can be described by a metapopulation theory where, for some species, local populations exist in a dynamic equilibrium of local extinctions and colonisations (classical metapopulations) [16], [17]. Even more species live in spatially-structured populations with source-sink dynamics [18] or rescue effects [19], where nonetheless extinctions and colonisations play an important role for species survival. In this paper, we extend the definition of metapopulation to accommodate both classical metapopulations and spatially-structured populations. Local extinctions and colonisations can be affected by many factors, such as demographical factors, multispecies interactions, habitat configuration, environmental stochasticity and habitat quality [20]. In this paper, we focus on a relatively unknown aspect of environmental stochasticity: extreme weather events related to climate change. At a global scale climate models project more intense hot extremes, less intense cold extremes, more intense precipitation and longer dry spells [21], although at a local scale there are many uncertainties in the projections of climate models. In northwest Europe this will mean more heat waves, more heavy precipitation and more winter storms [22].

Literature offers some good examples of the influence of weather events on colonisation and extinction patterns, but in most cases these impacts have not been related to climate change. Colonisation of available habitats has been found to follow the occurrence of favourable environmental conditions e.g. warm and sunny weather for Lepidoptera [23]–[25]. In terrestrial ectothermic animals the probability to colonise new habitat patches can increase along with mean temperature increase, because these animals are known to be more active at higher temperatures [26], leading to better dispersal [27]. Rising temperatures may as well increase habitat availability, enhancing colonisations [28]. Similarly, extinction frequency has been related to the occurrence of extreme weather events. Drought, for example is known to affect the survival of butterflies negatively, due to desiccation of the nectar and host plants [29]–[31]. Extinctions of two local populations of *Euphydryas editha bayensis* butterfly were linked to increased variability in precipitation [32]. Most of the research on the effects of weather extremes has concentrated on plants [33], [34] or birds [35]–[38].

Although strategies for landscape adaptation to mitigate climate effects on metapopulations in landscapes with fragmented habitat have been proposed [2], [11], [39], they are mostly based on evidence of animals responding to changes in temperature averages (e.g. by shifting ranges polewards). Studies on the effects of weather extremes are still limited to specific effects and single species [30], [31], [40]. However, for successful conservation of biodiversity there is a need for a broader view on how climate change affects various groups of species. In this paper, we concentrate on ectothermic species, which are expected to be most rapidly affected by increased weather variability resulting from anthropogenic climate change [41].

The objective of this paper is, to investigate correlations between weather variability and patterns in local colonisations and extinctions of low-altitude temperate populations of four ectothermic groups: Odonata (dragonflies and damselflies), Orthoptera (grasshoppers and crickets), Lepidoptera (butterflies), and Reptilia (reptiles). We used extensive data sets from The Netherlands and tested three specific predictions: 1) given that the average summer temperature in the Netherlands in the last 20 years showed a positive trend, general trends in ectotherms should be positive; 2) colonisation and persistence probabilities should be higher during periods of high temperature; and 3) extreme weather events are expected to be reflected in higher extinction risks and lower occupancy and colonisation probabilities.

We analysed three metapopulation metrics: occupancy, local persistence and local colonisation in relation to weather variability. The combination of standardised data from monitoring schemes and opportunistic data from citizen science databases, gave us an unprecedented number of observations to analyse.

## Materials and Methods

### Study design

We investigated how weather variability affects the probabilities of occupancy, colonisation and persistence (the complement of extinction), hereafter called metapopulation metrics. These three metapopulation metrics are widely used in metapopulation theory [16], [17], [42]. We used 1×1 km grid-cell occupancy as a proxy for patch occupancy. Data analysis was done in two steps. First, in order to obtain estimates of metapopulation metrics per species, per year, per grid-cell, we ran dynamic site-occupancy models which take imperfect detection into account [43]. These models are described here only shortly. Second, we used the results of these occupancy models as an input for latter analyses on the effects of weather variability. These latter analyses are the core of this paper. Starting from a coarse prediction that global climate change will be beneficial for low-altitude temperate ectotherms, we examined general changes in distribution patterns in the last two decades. Next, we focused on the effect of temperature on colonisation and persistence probability, because of the prediction of increased dispersal and better survival in warm years. Finally, we explored the effect of years with extreme weather on all the three metapopulation metrics, in case that these effects are not gradual, but of threshold nature.

### Materials

We analysed four groups of organisms: Odonata (58 dragonfly and damselfly species), Orthoptera (32 grasshopper and cricket species), Lepidoptera (37 butterfly species), and Reptilia (7 reptile species). The main sources of data were opportunistic observations, i.e. not collected using a standardized field method. These data were mainly recorded by volunteers at the online data entry facilities (www.waarneming.nl and telmee.nl) and retrieved from the National Database Flora and Fauna. In addition, standardized monitoring data were available for butterflies, dragonflies and reptiles and these were added to the data (for the details on these monitoring schemes see [44]–[46]). All records were validated by species experts. For the above-mentioned groups, we were able to cover their whole range in the Netherlands on a 1×1 km-grid base, with the oldest records from year 1990, on average we have covered 3954 grid cells for dragonflies and damselflies, 2173 grid cells for grasshoppers and crickets, 8796 grid cells for butterflies and 928 grid cells for reptiles, what gave us more than three millions individual records from opportunistic observations and an average of one thousand standardised transects per year. Because of the intensity of recording in the Netherlands, we assume it unlikely that grid cells with no observations during the entire study period contain the species studied. Weather data were retrieved from Royal Netherlands Meteorological Institute (www.knmi.nl) for the meteorological station of De Bilt, which is used as a national reference of the general weather pattern. Indices of weather extremes were retrieved from European Climate Assessment and Dataset for the meteorological station of De Bilt (ECA&D, www.ecad.eu, [47]). Occupancy models were run with JAGS software [48]. All post-processing analyses were performed with software R v. 2.14 [49], unless stated otherwise.

### Site-occupancy models

**Figure 1:**
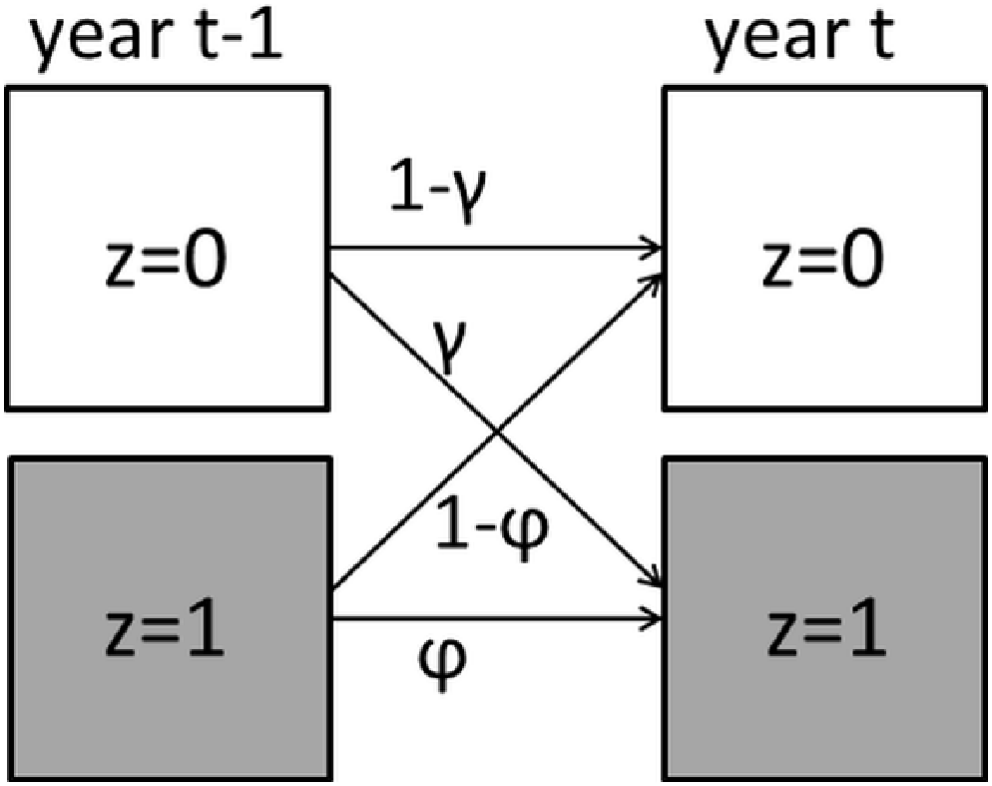
Dynamic site-occupancy model. *z* is true occupancy (0 or 1) of a site, *φ* is local persistence probability, *1-φ* is local extinction probability, *γ* is local colonisation probability and *1-γ* is the probability that the site stays unoccupied.

To estimate the occupancy probability per species, per year, per site (grid-cell), we ran dynamic site-occupancy models for each species separately, accounting for imperfect detection (Fig. 1). These models estimate metapopulation parameters reliably, even when data is not standardised [50]. Because many opportunistic observations were presence-only (or rather detections-only), data was augmented by generating zeros for non-observations (see e.g. [51]). The model (Fig. 1,[43]) describes each site in each year as either occupied (*z* = 1) or not (*z* = 0), occupied sites can persist being occupied with a certain persistence probability (*φ*) regardless of colonisation probability in that year and unoccupied sites can be colonised with a certain colonisation probability (*γ*) regardless of persistence probability in that year. The true occupancy is formulated as follows:
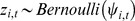
(1.1)

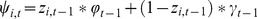
(1.2)where *z_i,t_* is true occupancy (0 or 1) of site *i* in year *t*, *ψ_i,t_* is occupancy probability of site *i* in a year *t*, *φ_t-1_* is the probability of persistence from year *t-1* to year *t*, *γ_t-1_* is the probability of local colonisation from year *t-1* to year *t*.

The hierarchical nature of the model allows separating the ecological process that results in true occupancy (Eq. 1.1 and 1.2) from the observation process that results in a detection of a species (Eq. 1.3 and 1.4). In our approach, each species has its own detection probability (*p*) that may differ per year, per day in the season and per data quality category; e.g. species detection is more likely at its peak abundance in the season or from a standardised monitoring than from opportunistic observations. If occupied grid-cells are surveyed using multiple visits and methods, the probability to detect the species at least once will also be higher. When a grid was not surveyed the dataset contained a missing value; these were taken into account during the analysis. The observation model is formulated as follows:
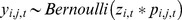
(1.3)

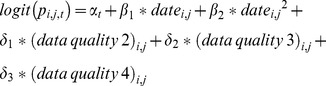
(1.4)where *y_i,j,t_* is the detection/non-detection data (0 or 1) of site *i* in year *t* on visit *j*, *p_i,j,t_* is the detection probability at a site *i* in year *t* on visit *j*, *α_t_* is a yearly intercept of detection probability, *β’s* are the effects of date of the visit and *δ’s* are the effects of data quality categories. Data quality depends on the length of the day lists and the degree of field method standardisation; categories are: 1 = single observations, 2 = short day lists, 3 = long day lists, 4 = standardised monitoring data. The models were fitted using a Bayesian mode of inference. We used uninformative priors for all parameters, among which priors with mean 0.5 for annual colonisation and persistence. The model and the computational procedures are described in detail in [52].

The occurrence probabilities of all years, except the first one, are defined recursively as a combination of colonisation and persistence probabilities, and therefore this model is very suitable to test predictions in metapopulation theory [43]. The output of these models are time series of occupancy, colonisation and persistence probabilities per year, as well as number of occupied and empty sites per year. We used the results of these models as input for our further analyses.

### Changes in distribution

Our first prediction was that global climate change should be beneficial for ectotherms, i.e. that the metapopulation metrics would increase. Using the estimated metapopulation metrics obtained from site-occupancy models across all sites we checked if there was a significant trend in occupancy, colonisation and persistence probability per species. To see if the frequency distribution of trends was universal, we performed Pearson’s chi-squared test for all taxonomic groups except reptiles because of their small sample size.

### Effect of temperature on colonisation probability

According to our second prediction, colonisation should be more frequent during periods of high temperature. To assess the effect of temperature on colonisation probabilities, we related colonisation probabilities per species per year to the mean daily average temperature of the period within the year that the species was active. From the occupancy models we obtained the number of colonised and extinct sites per species per year, which we then analysed with generalised linear models (Eq. 1.5, 1.6 and 1.7) using logit link and quasibinomial error distribution to account for overdispersion [53]. As explanatory variables we included: the mean daily average temperature during the active period of an adult of a species, square term of the aforementioned temperature to account for possible quadratic effects and the number of occupied sites, thus sites not available for colonisation. The reason of the latter was to account for density dependence: when density of occupied sites is high, then there are many dispersers available that could potentially colonise unoccupied sites. Two models were tested for each species: for year t (Eq. 1.6) and for year t-1 (Eq. 1.7), because the colonisations in year t could result either from individuals dispersing in year t-1 or from increased population growth and subsequent dispersal in year t. The best fitting model was chosen by the removal of non-significant terms from the model until all the terms were significant. The models had the following structure:
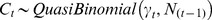
(1.5)

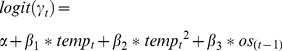
(1.6)

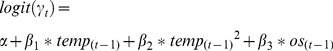
(1.7)where *γ_t_* is colonisation probability from year t-1 to year t, *C_t_* is the number of sites successfully colonised in year t, *N_(t-1)_* is the number of empty sites at year t-1 (thus all sites available for colonisation), *temp_t_* is the mean daily average temperature during the active period of an adult of a species at year t, *temp_t_^2^* is square of this mean daily average temperature for year t, *os_(t-1)_* is the standardised number of occupied sites, thus sites not available for colonisation. The second model is analogical for temperature in year t-1. We performed an analogical analysis for persistence, testing the prediction that higher temperatures improve survival.

### Effect of extreme years on metapopulation metrics

The effect of weather on metapopulation metrics may be non-linear, but of a threshold nature, e.g. bad weather conditions may have a continuously non-measurable effect until the threshold is exceeded and the whole local population gets extinct. Therefore, we searched for coincidence between years with extreme weather and extreme values of metapopulation metrics and the opposite: can extreme values of metapopulation metrics be explained by extreme weather in those years?

Because we were interested in the annual variation in metapopulation metrics, we de-trended the time series for species that showed positive or negative trends, with TrendSpotter software [54], [55]. We discarded earlier years (1990–1996) because of high uncertainty in the metapopulation metrics estimates, due to low number of observations. For each species, we considered years extreme if metapopulation metrics were higher or lower than the mean value by the arbitrary value of 1.5 times standard deviation. These years were added up in a histogram to see if any year was particularly affecting metapopulation metrics.

Alternatively, we identified meteorologically extreme years based on information given at www.knmi.nl/klimatologie/lijsten to be years with: mild winters as winters with Hellmann (knmi.nl/klimatologie/lijsten/hellmann.html) cold index <5, cold winters as winters with Hellmann cold index >100, dry springs as in first 10 springs with the lowest precipitation sum since year 1901, wet springs as in first 10 springs with the highest precipitation sum since year 1901 and hot summers as in first 3 summers with the highest average temperature since year 1901.

Finally, we identified extreme years based on indices of extremes of European Climate Assessment & Dataset (ECA&D). To detect the most important climate related indices we used principal component analysis (PCA) applying CANOCO 5.02 [56], [57]. We used indices for the summer months (April-September) of the years 1906–2011. We reduced the initial number of 64 indices (Table S1) to 10 by excluding highly correlated ones and the ones explaining least variance. Because the indices were standardised, the years with average weather are grouped around the origin of PCA axes, and years with more extreme weather are situated at the peripheries of the graph. Based on the results of PCA (Fig. S1), we identified years with extreme summers to be: 1998 - rainy, 2003 - hot, but also with low temperatures, high daily temperature range, 2011 & 2007– years with heavy rainfalls, 2006 - hot. Although summers in the last decades in the Netherlands got warmer with more precipitation, years with extreme weather (see Fig. S1 and Table S1) did not get more frequent.

## Results

### Changes in distribution

**Figure 2:**
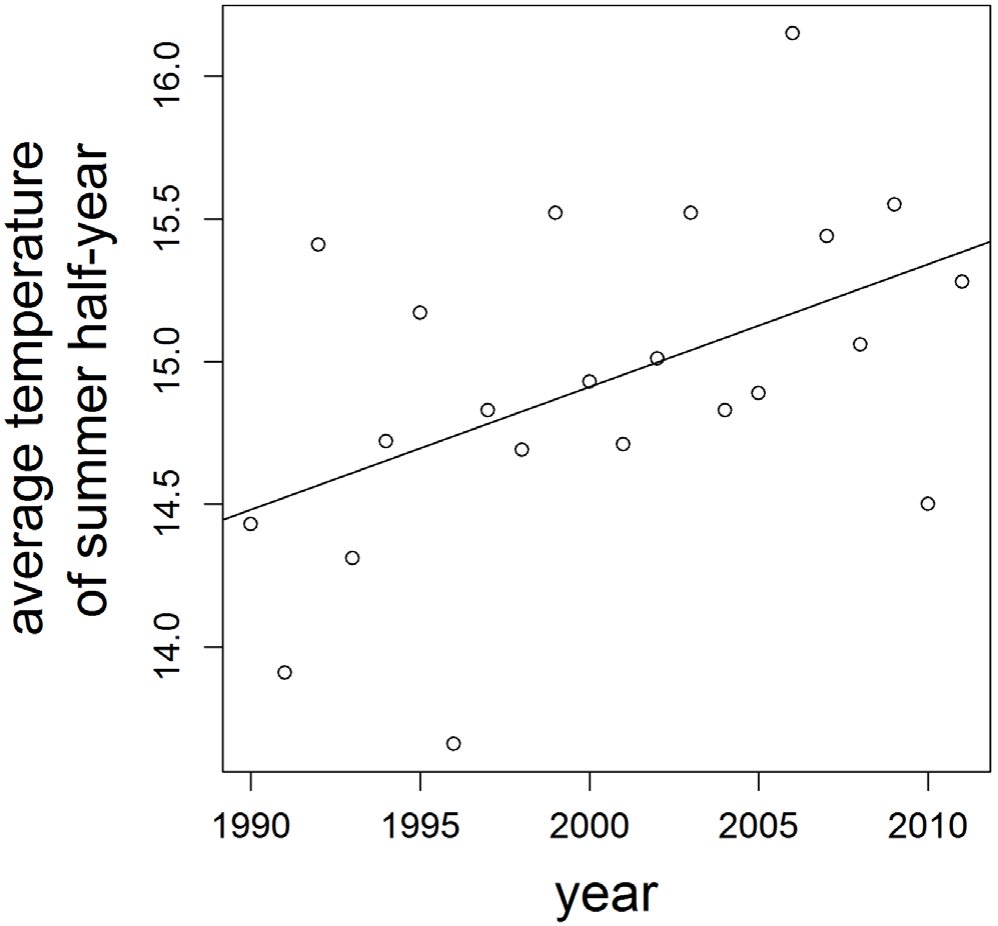
The average summer half-year temperature of the last 20 years in the Netherlands.

Roughly two-thirds of all species showed a trend in occupancy probability and about half of all species in colonisation and persistence (the complement of extinction) probabilities. All groups, except butterflies, showed more positive than negative trends in metapopulation metrics (Table 1). This was consistent with our prediction, that in a warming world ectotherm occupancy will increase, as summer half-year (months April–September) temperatures in the Netherlands rose in the last 15 years (Fig. 2, R^2^ = 0.238, p = 0.021). In contrast to the other species groups, butterflies showed more negative than positive trends in colonisation and occupancy probabilities. Trend patterns in persistence did not differ between taxonomic groups (Table 1, Χ^2^ = 8.0737, df = 4, p = 0.089, reptiles excluded because of small sample size), with very few species showing negative trends. The trends differed however for both occupancy (Χ^2^ = 26.329, df = 4, p<0.001), and colonisation (Χ^2^ = 19.1015, df = 4, p<0.001). Negative colonisation trends were seen for 29 species, especially among butterflies –16 species out of 37, and dragonflies –10 species out of 58. For butterflies, negative trends in colonisation probabilities were reflected in negative trends in occupancy probabilities (as occupancy is net of colonisation and persistence), but this was not the case for dragonflies.

**Table 1:** Number of species by group showing significantly increasing trend, significantly decreasing trend or no significant trend in metapopulation metrics.

		species groups				
metapopulationmetric	trend	Odonata(n = 58)	Orthoptera(n = 32)	Lepidoptera(n = 37)	Reptilia(n = 7)	total(n = 134)
occupancy	positive	39	12	10	4	63
	negative	4	4	15	1	24
	no trend	15	16	12	2	45
colonisation	Positive	22	7	7	2	36
	Negative	10	2	16	1	29
	no trend	26	23	14	4	67
persistence	Positive	30	14	11	0	55
	Negative	3	2	7	0	12
	no trend	25	16	19	7	67

### Effect of temperature on colonisation probability

To zoom in at the possible mechanisms of positive trends found in the metapopulation metrics, we examined the relation between colonisation probability and the mean daily average temperature during the active period of an adult species. Our prediction, that higher temperatures promote colonisation, was not supported (Table 2, Figure S2). A small fraction of species showed a positive relation between colonisation probability and temperature. For only one species we found a non-linear effect suggesting that there exists a temperature within summer temperatures range for which colonisation probability is the highest (thermal optimum). The majority of species did not show any relation with temperature, showed a linear negative relationship or a quadratic positive relationship (without thermal optimum). These species were denoted in table 2 as: other than expected. Colonisation probability was thus rarely correlated with summer temperature. The same applies to local persistence (Table 3, Figure S2). See Figure S2 for species-specific response curves.

**Table 2:** Number of species by group that show given relationships between colonisation probability and temperature in current or preceding year.

		relationship between colonisationprobability and temperature		
species group	year	positiverelation	thermaloptimum	other thanexpected
Odonata (n = 58)	t	9	1	48
	t-1	5	1	52
Orthoptera (n = 32)	t	0	0	32
	t-1	2	0	30
Lepidoptera (n = 37)	t	5	0	32
	t-1	1	0	36
Reptilia (n = 7)	t	1	0	6
	t-1	1	0	6
total (n = 134)	t	15	1	118
	t-1	9	1	124

**Table 3:** Number of species by group that show given relationships between persistence probability and temperature in current or preceding year.

		relationship between persistenceprobability and temperature		
species group	year	positiverelation	thermaloptimum	other thanexpected
Odonata (n = 58)	t	6	5	47
	t-1	2	10	46
Orthoptera (n = 32)	t	0	1	31
	t-1	1	10	21
Lepidoptera (n = 37)	t	1	3	33
	t-1	2	1	34
Reptilia (n = 7)	t	1	0	6
	t-1	0	0	7
total (n = 134)	t	8	9	117
	t-1	5	21	108

### Effect of extreme years on metapopulation metrics

**Figure 3:**
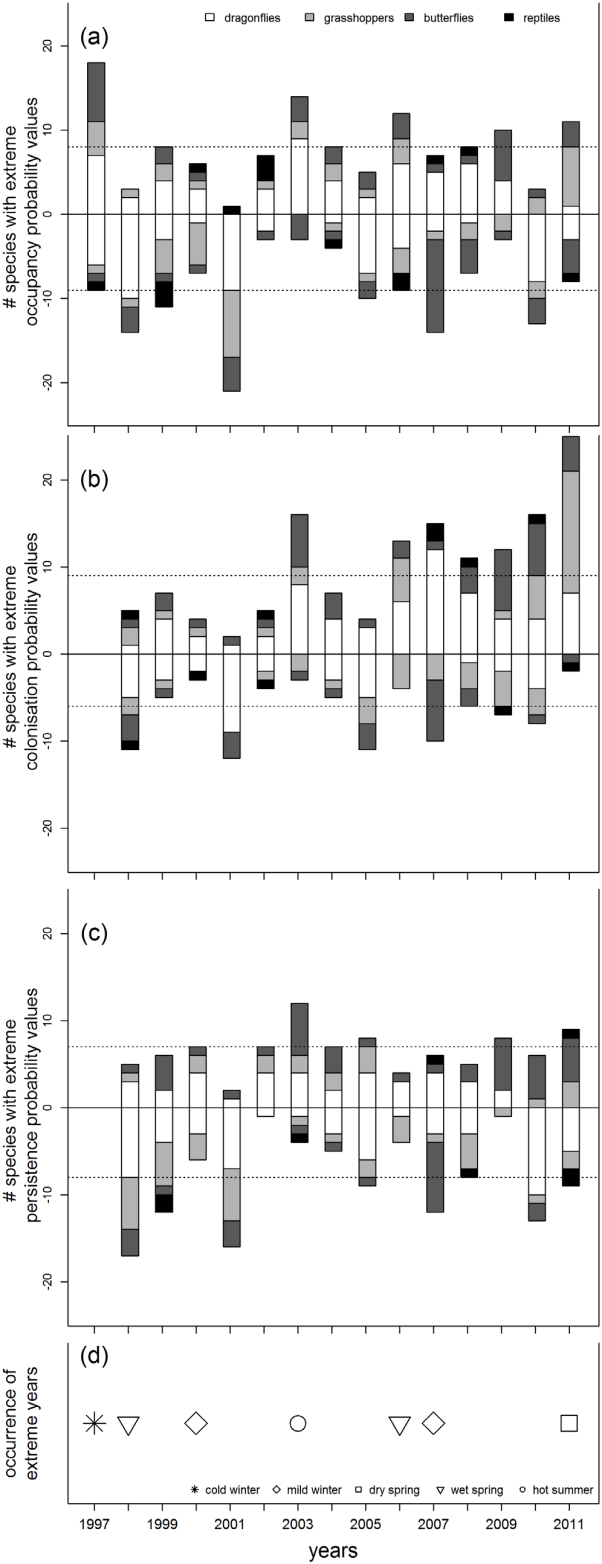
Number of species per year that show extreme values of metapopulation metrics (de-trended). Positive frequencies refer to years with values greater than mean value +1.5 times standard deviation, negative frequencies refer to years with values smaller than mean value - 1.5 times standard deviation. Dashed line marks the range if the frequencies were distributed uniformly. a) occupancy probability, b) colonisation probability, c) persistence probability, d) occurrence of extreme years. Years with extreme weather are marked as follows:○ hot summer, □ dry spring, ▿ wet spring, ◊ mild winter, 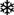 cold winter.

It is also possible that species do not react to changes in weather in a gradual mode, but rather that they are only sensitive to extreme weather events. We compared, therefore, years with extreme weather or with extreme changes in metapopulation metrics. Most extreme values for population metrics were in year 2011: 19% of species had very high occupancy and in 2001: 16% of species had very low persistence (Fig. 3a,b,c). We identified the years that could potentially trigger extreme ecological response (as defined by Chambert et al. [58]) to be: 2000 and 2007 - mild winters, 1997 - cold winter, 2011 - dry spring, 1998, 2006 - wet springs, and 2003 and 2006 - hot summers (Fig. 3d). Highest occupancy probabilities were seen for year 1997 and lowest in 2001, which were years with average weather. Colonisation probability was highest in 2011, which was characterised by a very dry spring (but: spring of 1996 was also very dry) and lowest in 2001, which was a year with average weather. Persistence probability was highest in 2003 when a very dry and hot summer occurred, and lowest in 1998 which was characterised by a very wet spring (but: spring of 2006 was also wet).

## Discussion

The objective of this paper was to find general patterns in metapopulation metrics (probabilities of occupancy, local colonisation and local persistence) across four groups of ectothermic organisms in relation to weather variability. Using the combination of standardised and opportunistic data for over 20 years for 134 species, we found the positive effect of incremental changes in temperature on local colonisation and persistence probability only for a few species. We did not find any evidence that extreme weather events are reflected in higher extinction risks. This finding does not corroborate the general prediction that increased weather variability imperils biodiversity.

It has been frequently suggested that weather extremes have a strong effect on biodiversity [59], [60]. The theoretical and modelling studies (e.g. [61], [62]) that predict negative effects of increased weather variation are, nevertheless, backed up with empirical data for a few species only [31], [32], [40]. Studies in ecosystems with low α biodiversity, few species interactions, or abiotic conditions driven by few focal variables (e.g. [58], [63]) are more successful in proving the effects of weather extremes than studies of complex ecosystems in temperate climates with many interacting factors. Moreover, correlative and theoretical models predicting extinctions from climate change either do not account for population dynamics at all (e.g. climate envelopes; [64], [65]) or simplify the systems studied by excluding possible effects of habitat heterogeneity, microclimate or multispecies interactions. Therefore, we propose that current weather extremes have less, or different, impact in real world metapopulations of low-altitude temperate ectotherms than theory and models suggest.

The lack of clear impact of weather variability might be due to inadequacy of our methods to capture the relation between weather extremes and metapopulation metrics, inadequate definition of weather extremes, potential buffering mechanisms mitigating impacts of weather variability in the temperate ecosystems we examined, or other factors overriding the effects of weather extremes on metapopulation dynamics. In the following paragraphs we discuss whether limitations in our approach could explain why we did not find the strong effects of extreme weather variability which we expected considering the widespread evidence in literature [34], [59], [60]. Subsequently, we address possible ecological mechanisms masking such effects in our data set.

### Limitations in the method

Opportunistic data, collected by amateurs, may seem to be less trustworthy than monitoring data collected by expert volunteers. However, opportunistic data have been shown to be as good at detecting distribution trends as data gathered with a standardised sampling protocol, provided appropriately analysed. Van Strien et al. [50] showed that opportunistic data for butterflies and dragonflies produced similar estimates of trends in occupancy as standardized data, when analysed by a site-occupancy model. That is because a site-occupancy model is able to adjust for variation in recorder effort by taking into account the detection probability of species in sites. Not only long-term trends but also annual colonisation and persistence estimates were strongly correlated between the datasets used by van Strien et al. [50] (R = 0,85 and 0,79 for colonisation and persistence for butterflies and R = 0,89 and 0,55 for dragonflies). Therefore, we believe that opportunistic data may also produce reliable estimates of colonisation and persistence. Hence, we can fully use the advantages of large dataset with opportunistic observations.

Our results were not biased by rare species. According to Mckann et al. [66], when the number of sites is limited, as often for rare species, the estimates of colonisation and persistence may be biased, approaching the mean of priors (which in our case is 0.5). We repeated therefore all the analyses excluding rare species that occurred in less than 120 sites (following Mckann et al. [66]) and these new results (Table S2, S3, S4 and Fig. S3, S5) were in line with our conclusions. Neither were our analyses biased by the lack of data in earlier years. Our conclusions were still valid, after we repeated the analyses with years 1997–2011 only, discarding years with high uncertainty of metapopulation estimates (Table S5, S6, S7 and Fig. S4).

Whereas in most studies on the effects of extreme weather on animals abundance data are used, we use occupancy data. Abundance data seem to be more sensitive to environmental variation than occupancy data [67], [68], because these data carry more information. Weather extremes can cause a drop in abundance in the short run (see [31]), but our results show that these effects disappear on a scale of 1×1 km occupancy. That is, even though populations may decrease in abundance, they do not necessarily become locally extinct following the extreme year. Still, substantial short-term effects of weather extremes should have resulted in a significant occurrence of extinctions that would have been detected in our analysis. Consequently, we see the merit to use occupancy data for conservation purposes.

Our results are bound to the temporal and spatial scale that we worked on. Our time series covers 15–20 years, which might not be sufficiently long to encompass the full range of climate change, including the extremes. We assume however, that climatic effects emerge from the immediate effects of weather variability on the species studied. The spatial scale of observation 1×1 km^2^ grid is not often used in research on the effects of weather or climate, most effects found are observed by analysing individual transects, patches or specific study areas [40], [69]. While these studies are valuable in capturing local ecological processes, we doubt if they can be translated into species long-term survival on a greater scale. There are also effects found on continental or global scale (e.g. effects of NAO or ENSO; [70], [71]), however, for policy-making purposes our scale is more appropriate, because most nature management policies lie in the responsibility of regional or national authorities.

Dynamic occupancy models require a “closure assumption”: we assume that during the seasons sites either stay occupied, or stay empty. This assumption may not always be appropriate, as seen for example in good dispersers, such as dragonflies. Negative trends in colonisation for dragonflies are not followed by negative trends in occupancy (Table 1). Good dispersal capacities of dragonflies might suggest a rescue effect here – extinct sites can be recolonized during the same season, leaving no trace in our data, hence no extra negative trends in occupancy probabilities. We also assume that sites with no observation of a certain species throughout the years analysed do not contain this species. Theoretically it would be possible that a species was non-detected in a site for 15 years, although we consider it very unlikely.

### Definition and relevancy of weather extremes

The definition of extreme weather events is not straightforward; extreme weather events, as perceived by humans, do not necessarily cause an extreme ecological response (as defined by Chambert et al. [58]). That is why we not only examined years defined meteorologically as extreme, but also years in which we saw the greatest variability in metapopulation metrics. Moreover, as suggested by Gutschick and BassiriRad [72], to trigger an extreme ecological response the whole sequence of events could be more important than a single extreme value in one of the conditions. This is illustrated in our case by the very poor persistence (especially for butterflies) from year 2006 to 2007. This season began with the hot summer of 2006 with a wet June, dry July and wet August, followed by the extremely mild winter of 2007 and a very warm spring of 2007 with extremely high precipitation deficit in April (>100 mm) and very wet May. It could be that the combination of these factors, especially variation in dry and wet periods, is what species have difficulty to cope with. Some species might only be susceptible to species-specific extremes (e.g. [37]), or the incremental changes in weather conditions might be more important than the extremes. Nonetheless, we used broadly accepted indicators of extreme climate [47] that should be sufficient to grasp the effects on biodiversity if they were large enough. Even the extreme weather of year 2003 [73] was not reflected in our data. We deliberately did not consider specific extremes for specific species, because we were interested in finding general patterns, as conservation policy is usually not based on single species. This leaves us to conclude that, contrary to our expectation, weather extremes do not yet have visible and consistent negative effects on trends in annual occurrence, colonisation and persistence probabilities of ectothermic species in fragmented landscapes of temperate Europe. Consequently, we propose that conservation efforts can be focused on other biodiversity threats than weather extremes, until new evidence arises.

### Many populations may be ecologically buffered against weather extremes

When so many authors raise concerns about weather extremes [34], [59], [60], do they overestimate the effect? We suggest that one reason that effects are smaller than expected could be that many populations are buffered against weather variation either by evolutionary adaptation or by flexible use of microhabitats in heterogeneous environment [74].

Species may be able to cope with extreme conditions, because their realised climatic niche may be narrower than the potential niche. The study by Martin and Huey [75] is an example. They showed that many lizards keep their body temperature below the temperature maximising fitness. This is due to the fact that thermal dependence of fitness in ectotherms is highly asymmetric, and deviation of the body temperature to higher temperatures has higher fitness costs than the same deviation to the lower temperatures. When confronted with exceptionally high temperatures, these species are actually in their thermal optimum.

Many species inhabit a mosaic of various habitat patches, which contribute to spreading the risk by containing diverse microhabitats. This can dampen local population fluctuations caused by, for instance, weather disturbances, thus decreasing extinction risk [76], [77]. In a heterogeneous landscape thermal variation can often exceed the predicted rise in temperature due to climate change and species can actively choose appropriate microclimates [74], [78]. Especially species living in heathlands and grasslands may therefore be adapted to very abrupt changes in temperature and moisture; with an air temperature of +11.8°C at a heathland, for example, soil temperature can vary from −2°C in the shade of a juniper bush up to +62°C on dead grass tussocks perpendicular to the sun rays [79]. Whereas the difference between the maximum monthly temperature for months April-September and the minimum monthly temperature for months April-September for the years 1990 till 2012 is 25.9°C (source: ECA&D, www.ecad.eu). Thus even in extremely hot years, there are many microhabitats to choose from.

### Other factors are masking the effects

Alternatively, there might be other factors, of greater importance, that mask the effects of weather variation. One such factor could be habitat quality: species may already occupy all the sparse fragments of habitat of a good quality, and colonisations of areas of poorer quality may be unsuccessful. In a recent study of sparrows, the dispersal was temperature-dependent only in areas with poor habitat quality (more exposed to temporal fluctuations in weather and food availability) as opposed to habitats of good quality, with enough food and shelter [80]. A British study of butterflies [81] revealed that populations at the leading distribution edge do not utilise a broader range of habitat types as the climate warms, but rather that their habitat width contracts. The authors suggest that the degradation of habitat quality poses far larger threats to population conservation than climate change. Habitat fragmentation can also be a hindrance to dispersal [82] and hence colonisation, but this mechanism is not apparent from our data, as our results are similar for good and poor dispersers. Spatial analysis of our data could help disentangle the effects of habitat quality and climate change. Although we did not find any immediate extreme ecological responses to weather extremes, we cannot exclude that extreme weather events have some negative long-term consequences by cumulative or recurring effects [58] or that time lags in species response and their corresponding extinction debt follow some non-linear patterns, perhaps even with tipping points, that we were not able to detect.

### Implications

In this study we have not been able to confirm the often-suggested significant impact of climate change-induced weather extremes on ectothermic species at a regional scale. Interesting questions are why the effects of weather extremes found on local scales by other authors (e.g. [30], [32], [83]) are not reflected at the regional or national level, impacting the persistence of species as a whole; and under which circumstances do abundance effects of weather extremes found by e.g. WallisDeVries et al. [31] translate to occupancy effects. While there was no extreme event that affected the whole group of the species studied, some specific species or groups of species might be vulnerable and future efforts should find out which conditions or traits are responsible for this high vulnerability. Confronting the documented effects of weather extremes with our results, we propose that further research should concentrate on the interference with habitat and spatial scale. Effects of habitat quality, heterogeneity and microclimate might interfere with the effects of extreme weather and these effects could be scale-dependent.

## Supporting Information

Figure S1 **Multivariate climate pattern of summers in the Netherlands.**
(DOCX)

Figure S2 **Response curves of colonisation and persistence probability in relation to temperature per species.**
(PDF)

Figure S3 **Response curves of colonisation and persistence probability in relation to temperature per species, excluding rare species.**
(PDF)

Figure S4 **Response curves of colonisation and persistence probability in relation to temperature per species, for years 1997–2011.**
(PDF)

Figure S5 **Number of species per year that show extreme values of metapopulation metrics, excluding rare species.**
(DOCX)

Table S1 **Indices of extremes used in the PCA and their trends.**
(DOCX)

Table S2 **Trends excluding rare species.**
(DOCX)

Table S3 **Relationship between colonisation and temperature, excluding rare species.**
(DOCX)

Table S4 **Relationship between persistence and temperature, excluding rare species.**
(DOCX)

Table S5 **Trends for years 1997–2011.**
(DOCX)

Table S6 **Relationship between colonisation and temperature for years 1997–2011.**
(DOCX)

Table S7 **Relationship between persistence and temperature for years 1997–2011.**
(DOCX)
