# Supplementary material for: No Evidence of the Effect of Extreme Weather Events on Annual Occurrence of Four Groups of Ectothermic Species
Source: PLoS One. 2014 Oct 17;9(10):e110219. doi: 10.1371/journal.pone.0110219 (PMC4201516; doi:10.1371/journal.pone.0110219)

Figure S1 Multivariate climate pattern of summers in the Netherlands. Made by unconstrained PCA (Canoco 5.02, [56,57] based on correlation matrix. Eigenvalue for the first axis is 0.45, for the second 0.28. Circles are years, empty circles are years between 1906 and 1989, filled circles are years between 1990 and 2011. Only the latter years are given in the figure and allowed for further investigation. Indices of extremes are: RR - precipitation sum, R95p – number of very wet days, TN – mean of daily minimum temperature, TG – mean of daily mean temperature, TX – mean of daily maximum temperature, SS – sunshine duration, DTR – mean of diurnal temperature range, PP – mean of daily surface air pressure, FD – number of frost days, HD17 – heating degree days, for complete indices definitions see: [www.ecad.eu](http://www.ecad.eu).


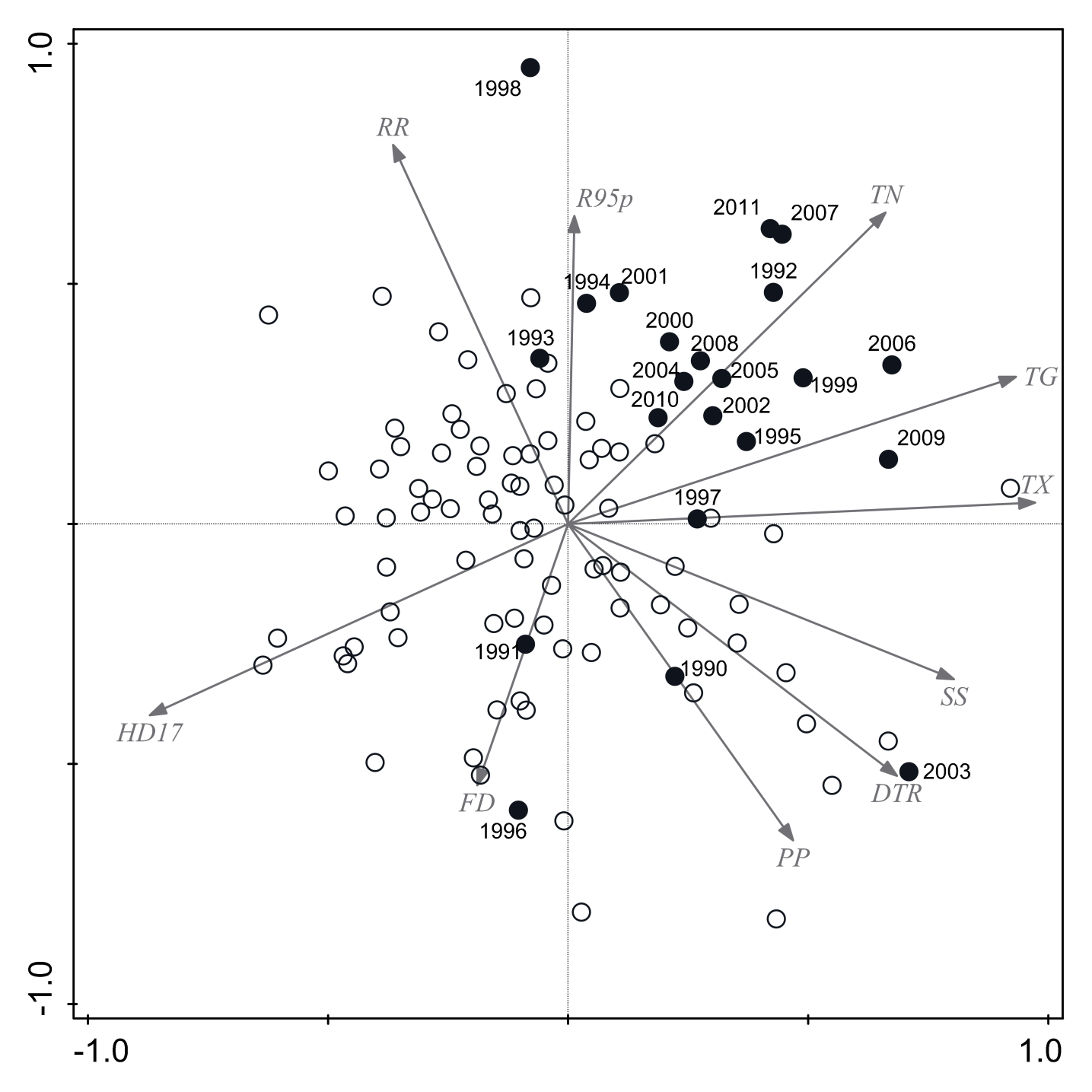

Supplement: Figure S1 — Multivariate climate pattern of summers in the Netherlands. (DOCX) [file pone.0110219.s001.docx]
