# Supplementary figures and images for: No Evidence of the Effect of Extreme Weather Events on Annual Occurrence of Four Groups of Ectothermic Species
Source: PLoS One. 2014 Oct 17;9(10):e110219. doi: 10.1371/journal.pone.0110219 (PMC4201516; doi:10.1371/journal.pone.0110219)

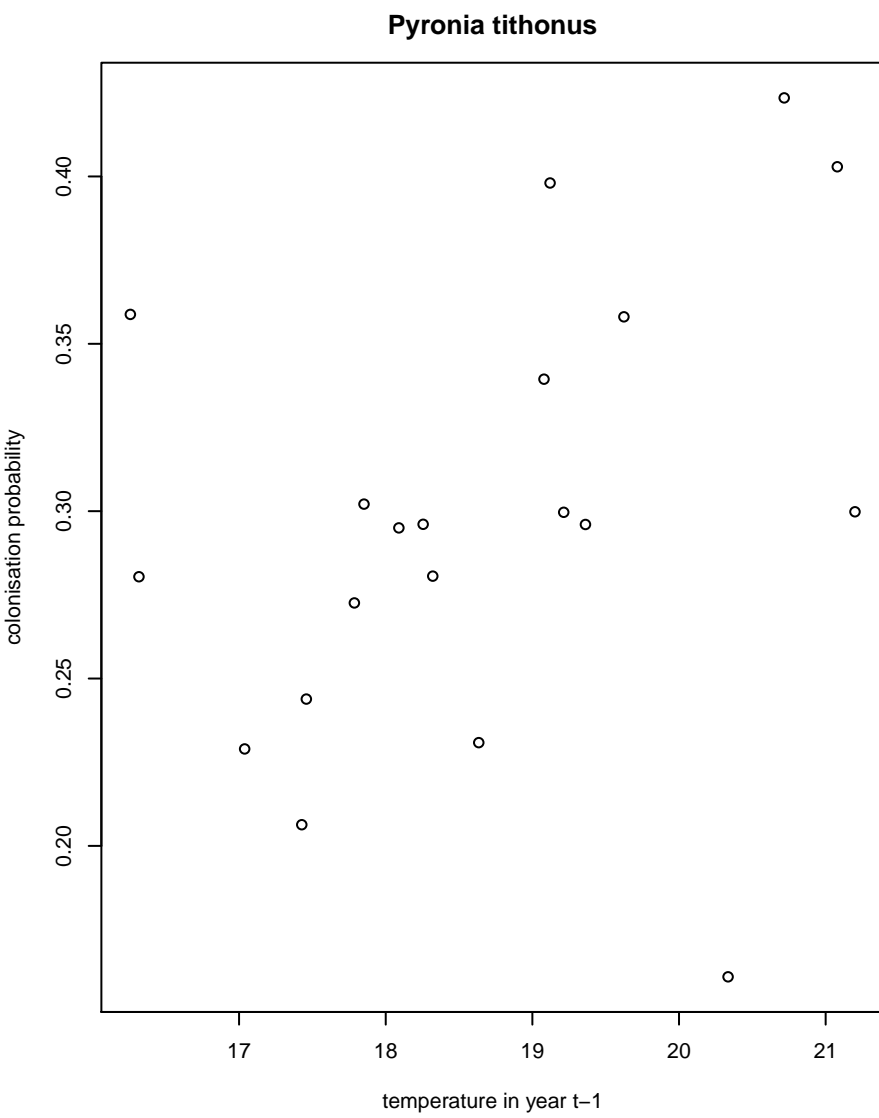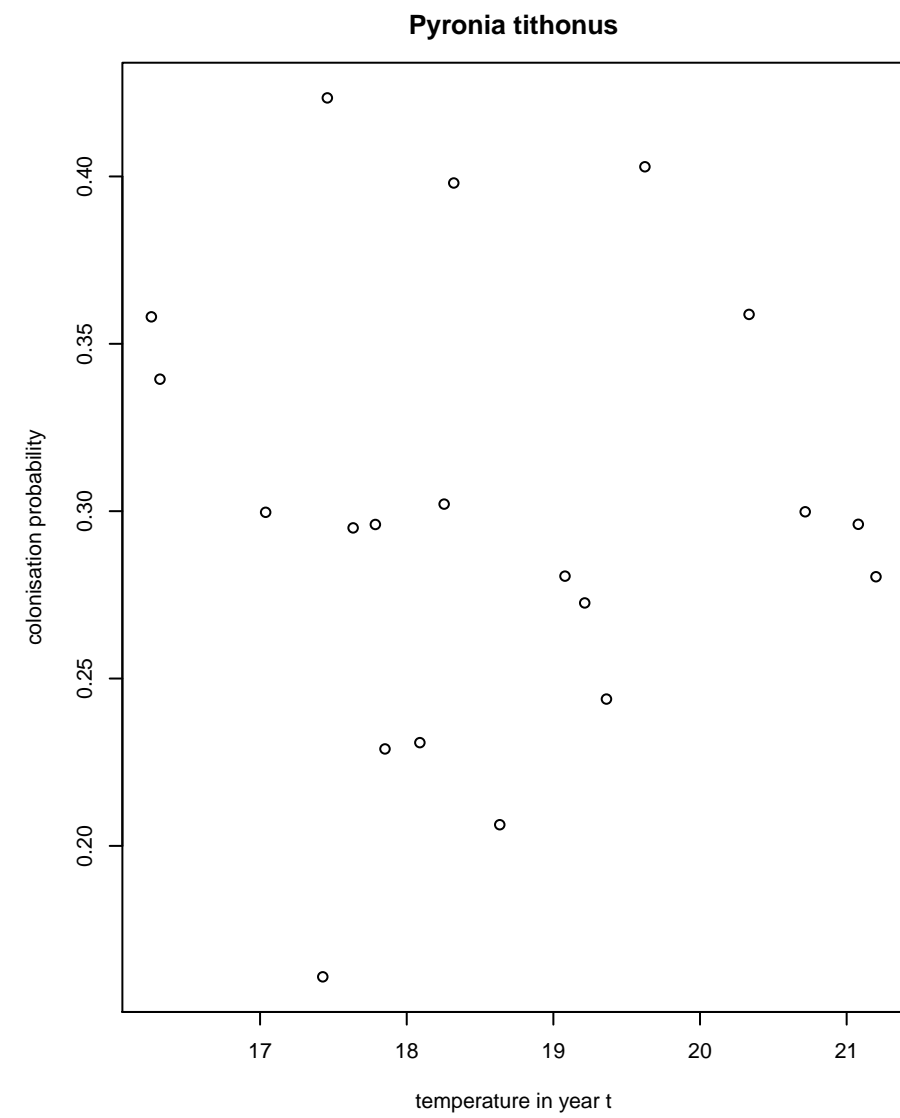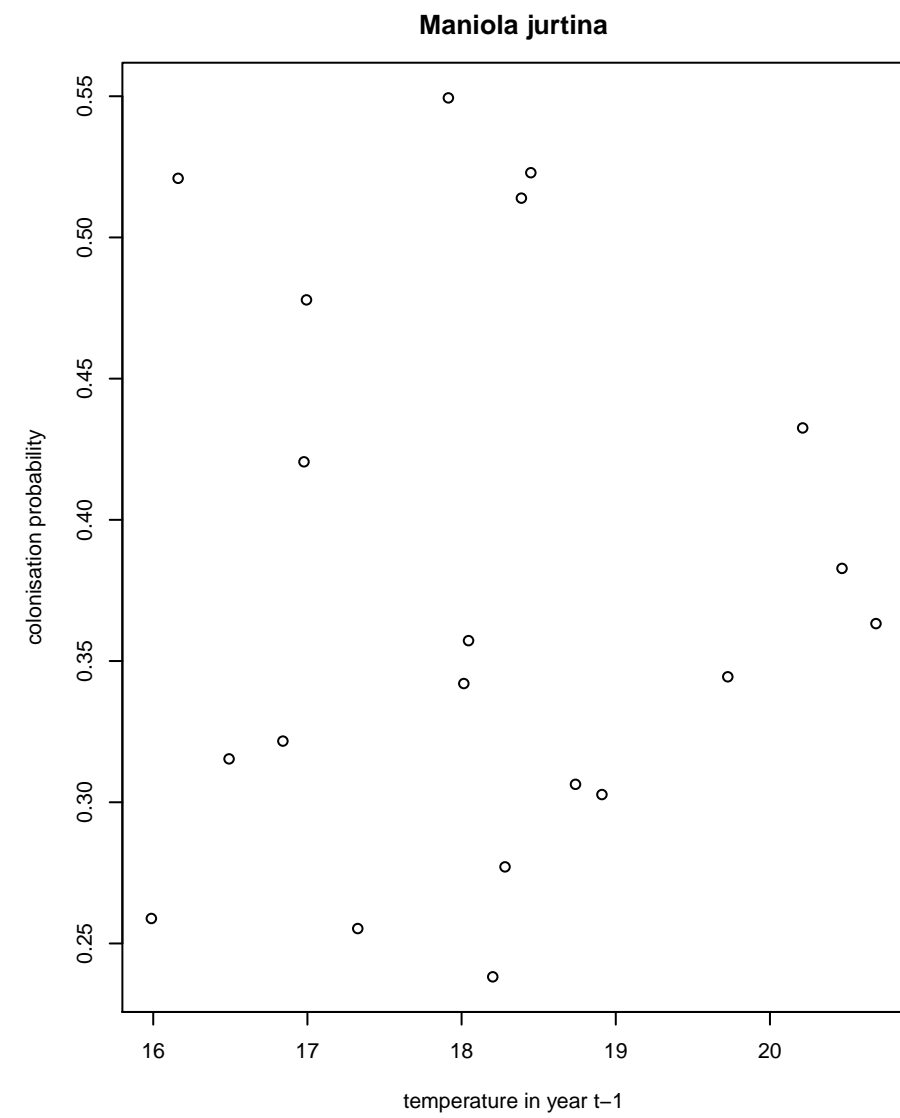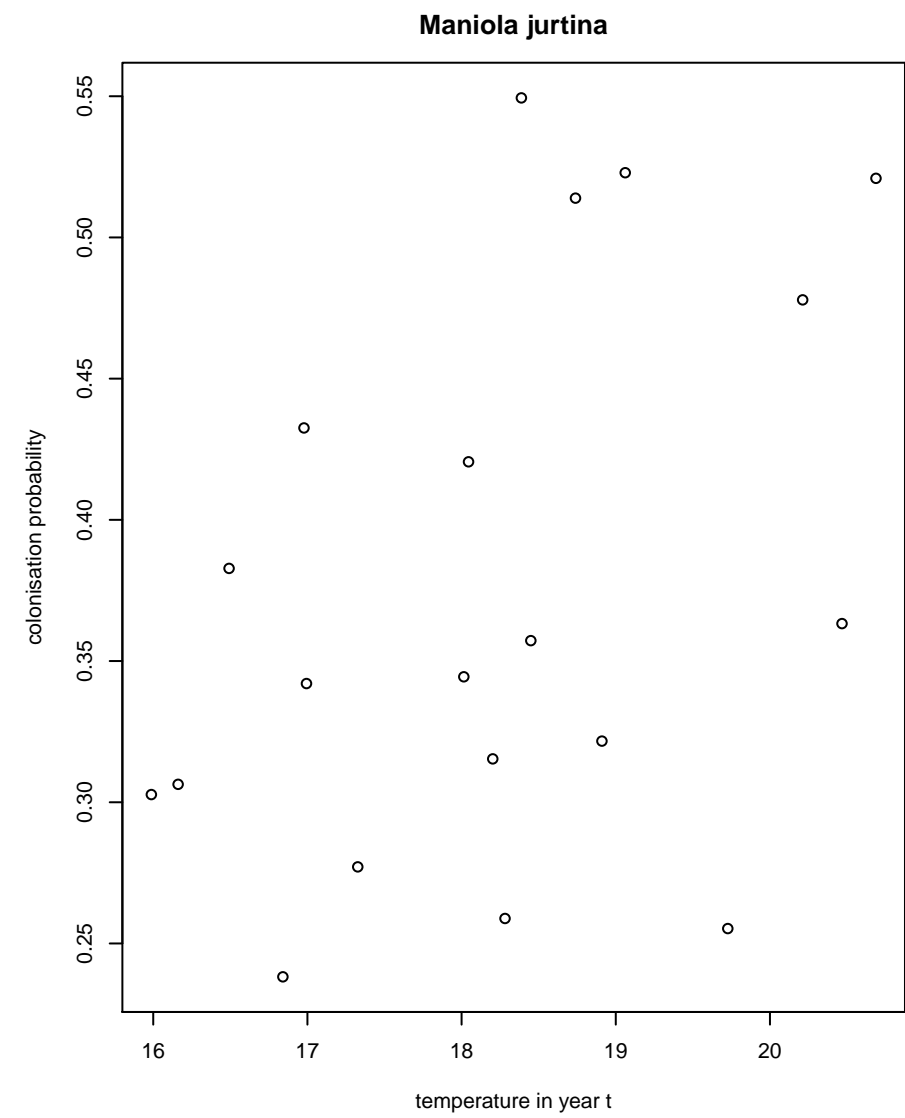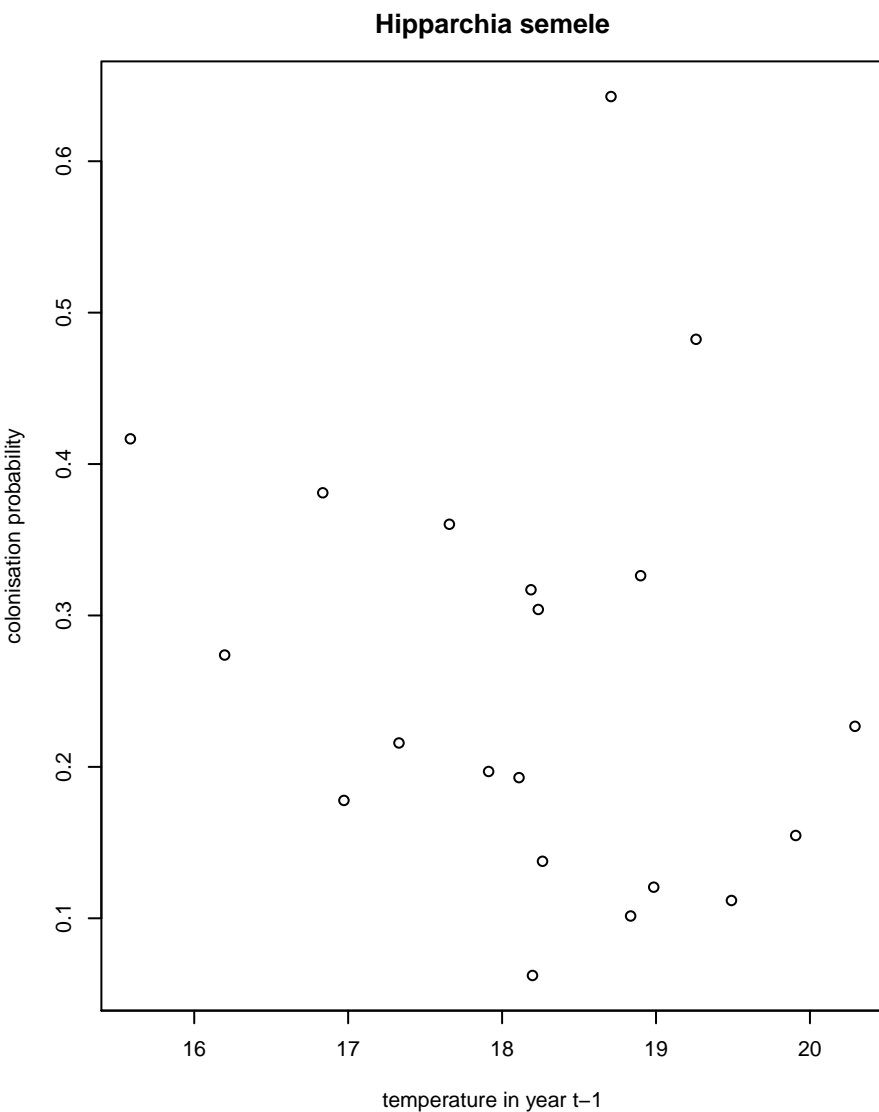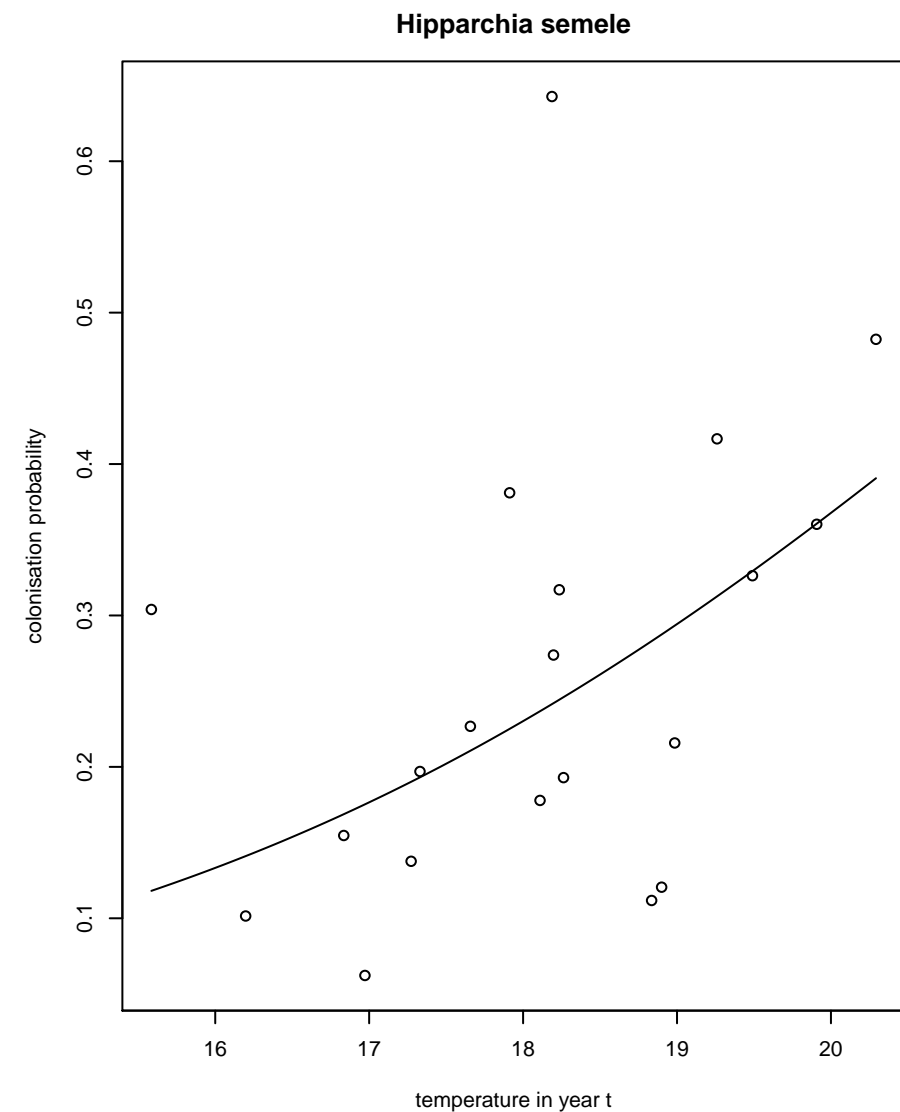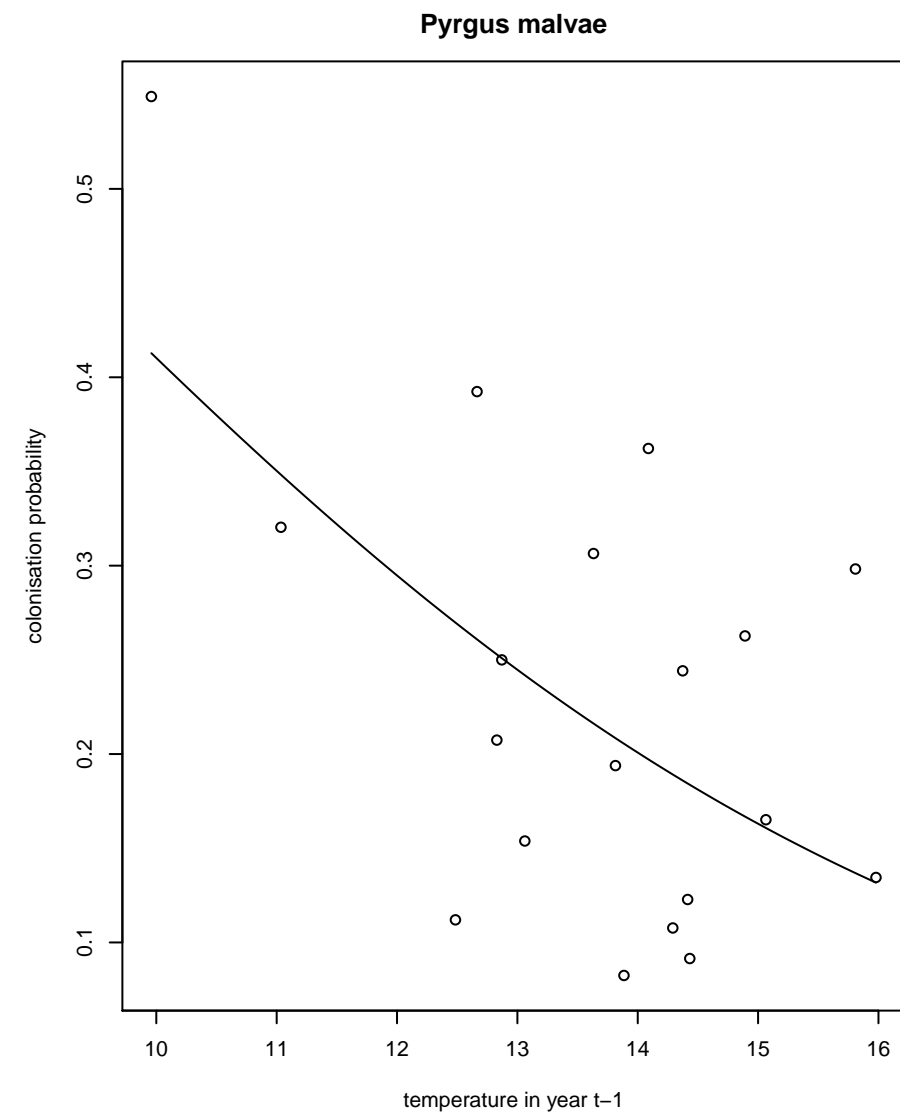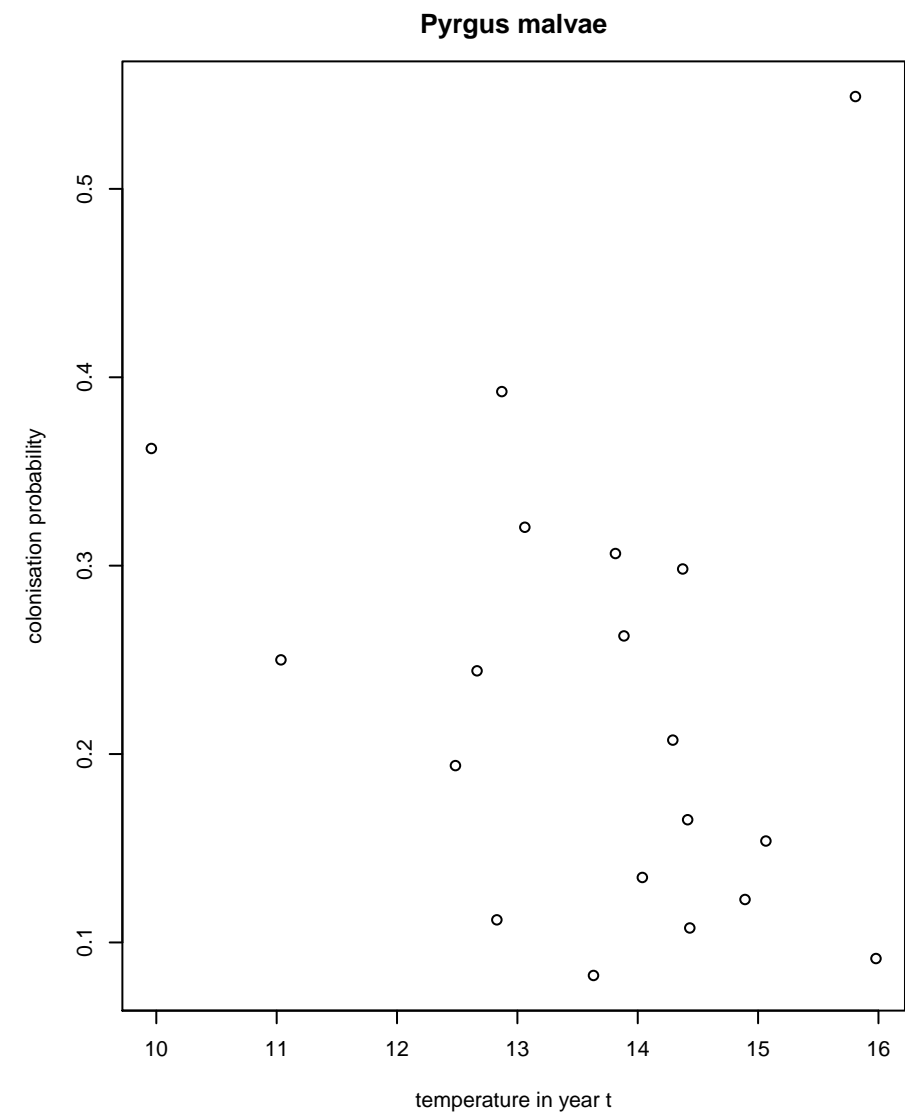

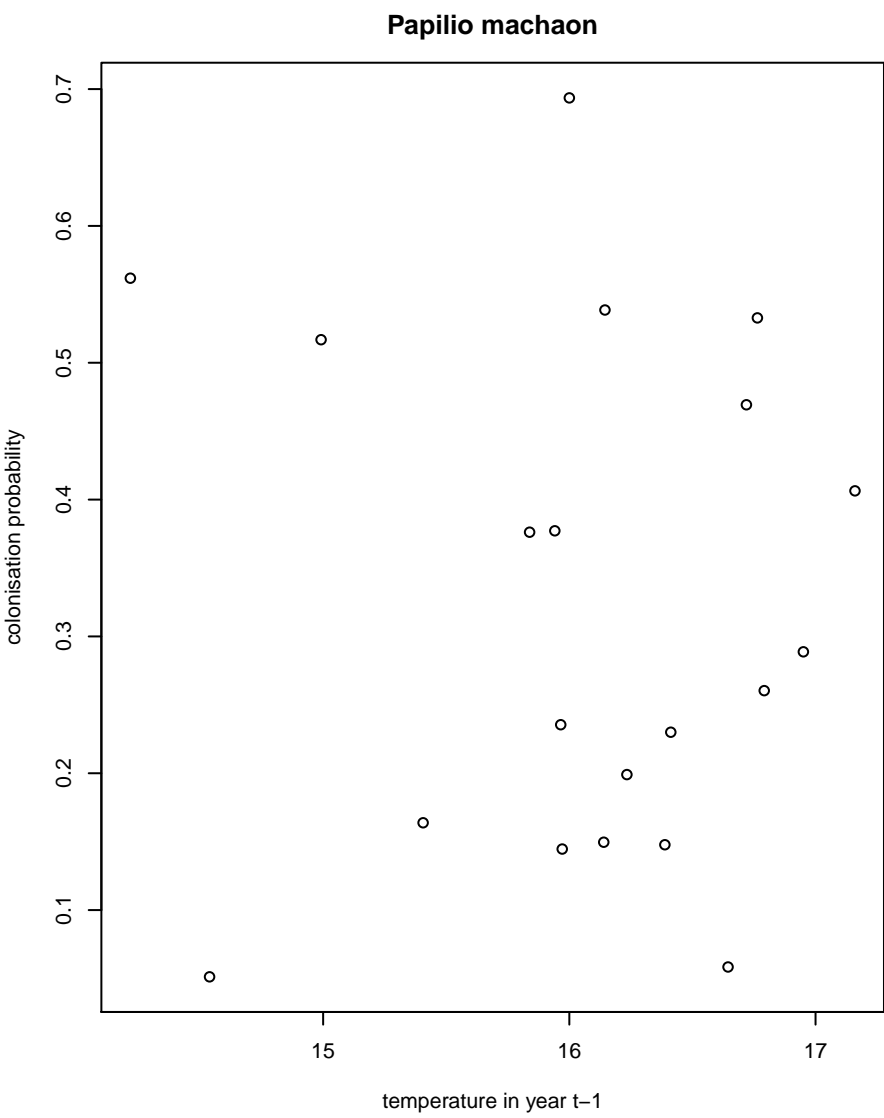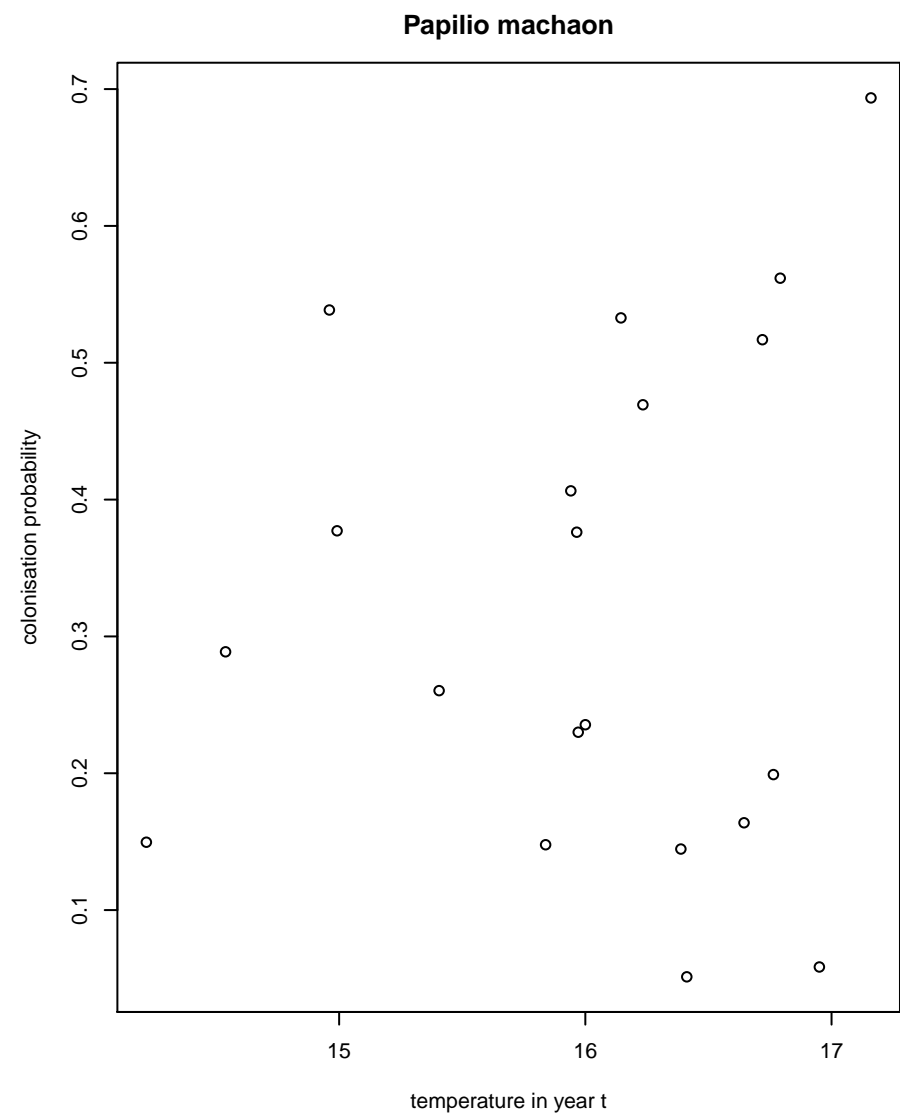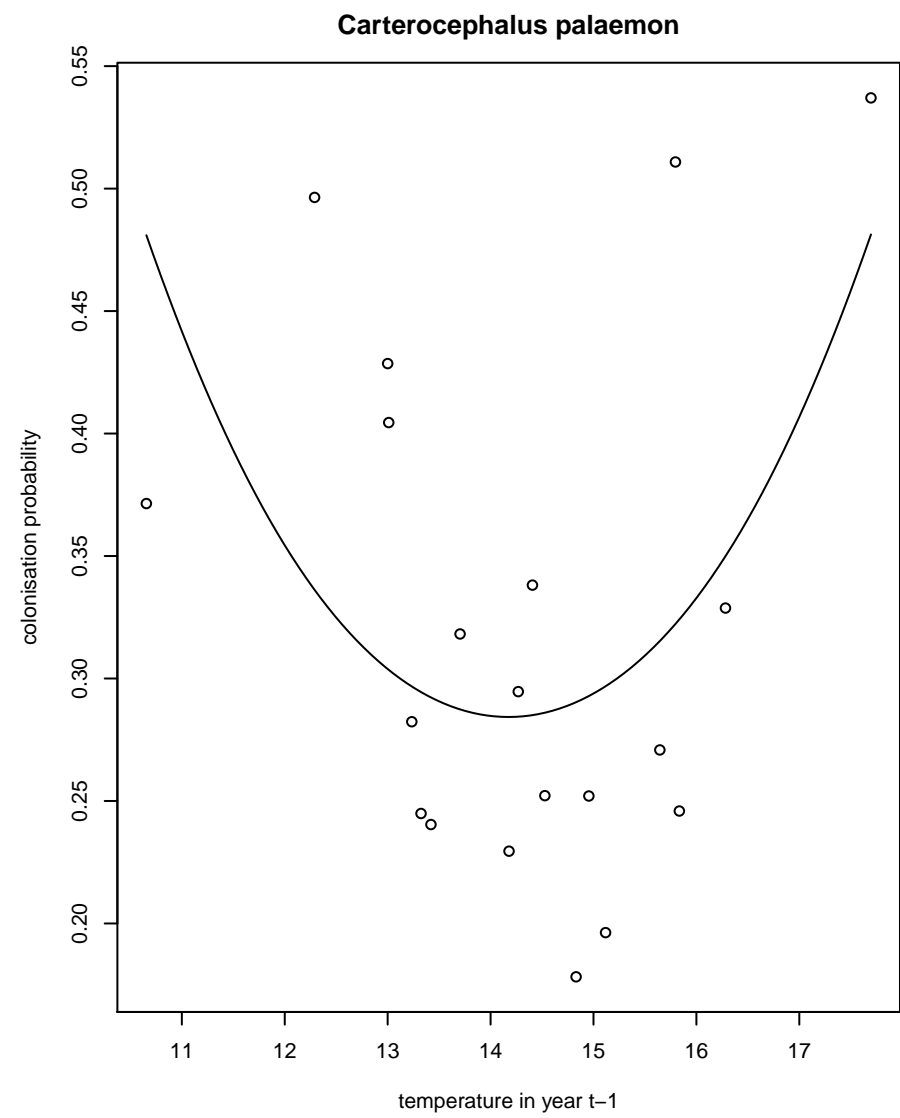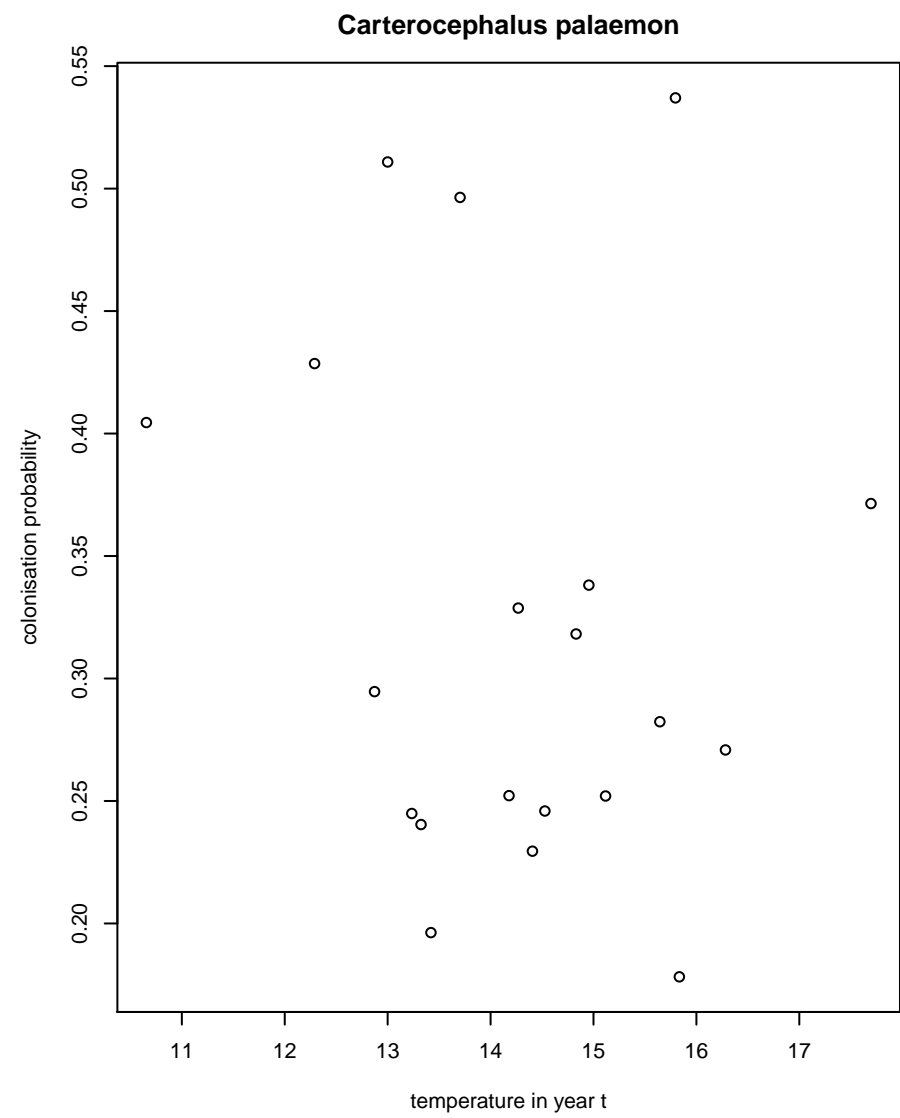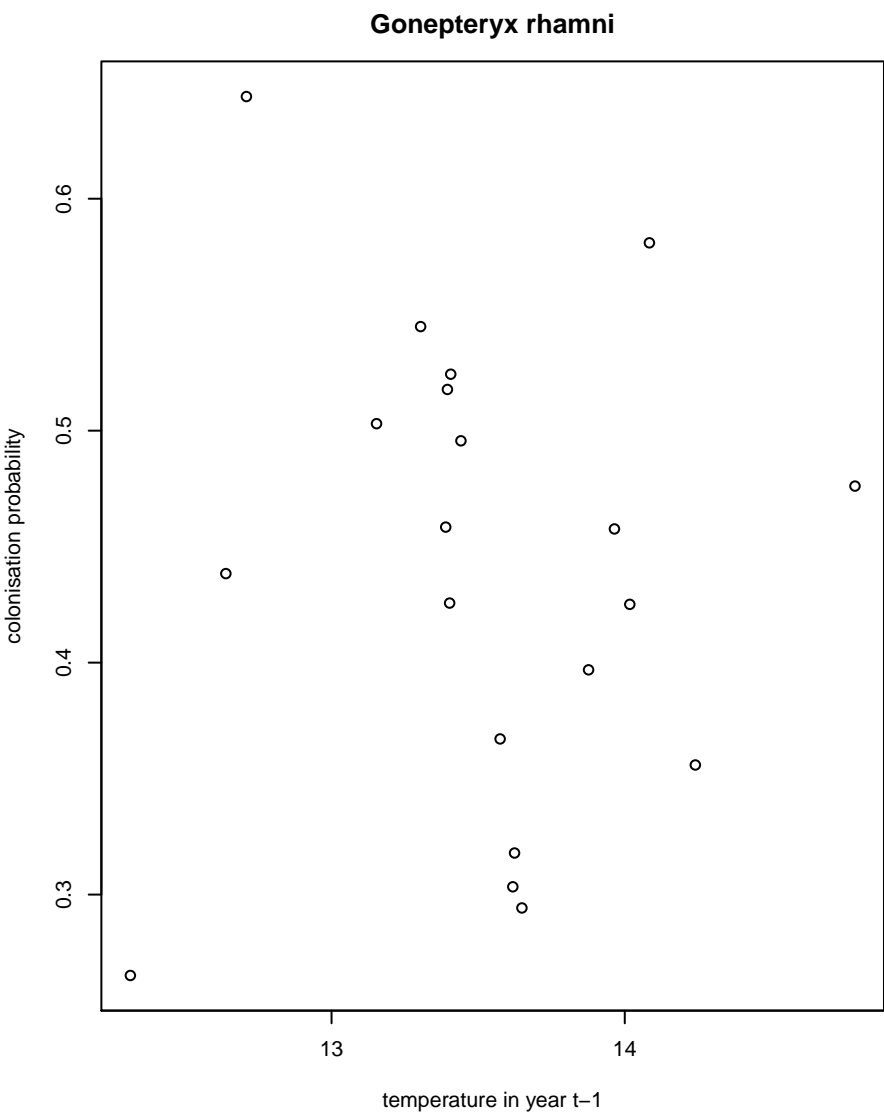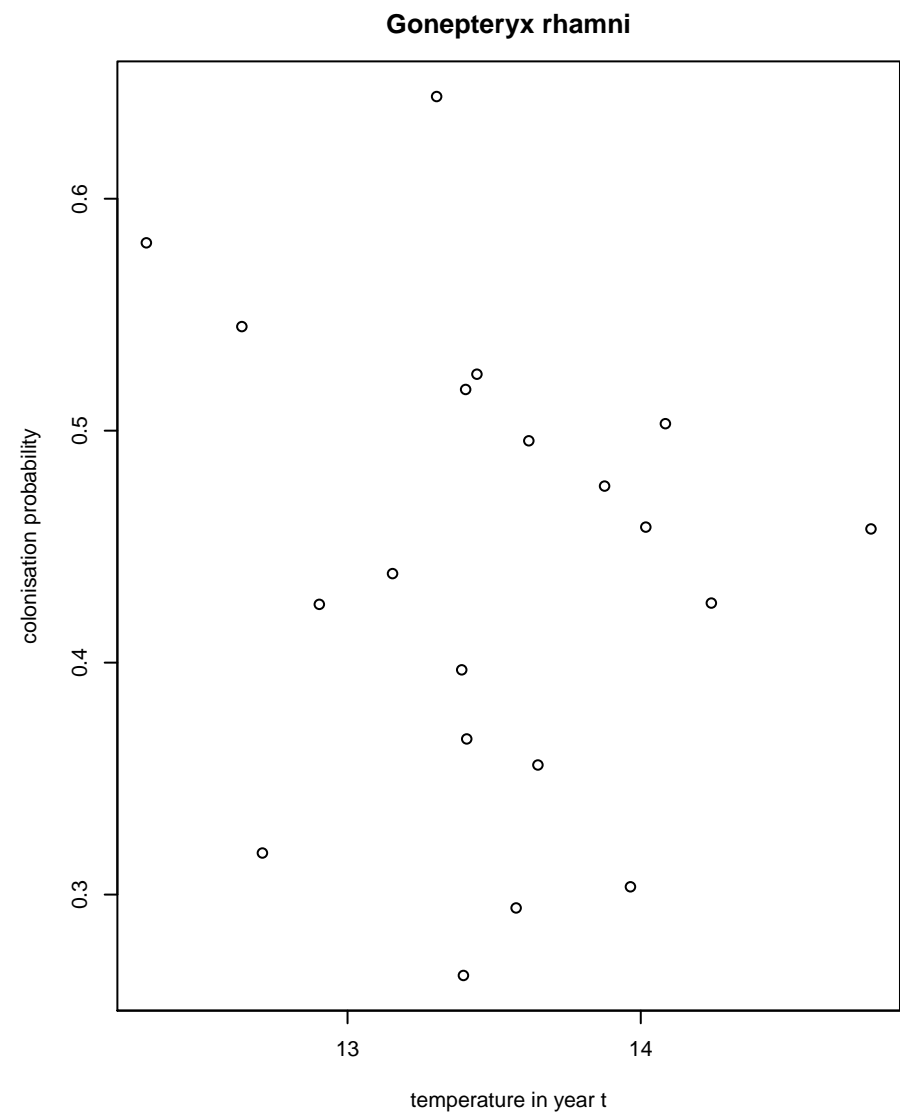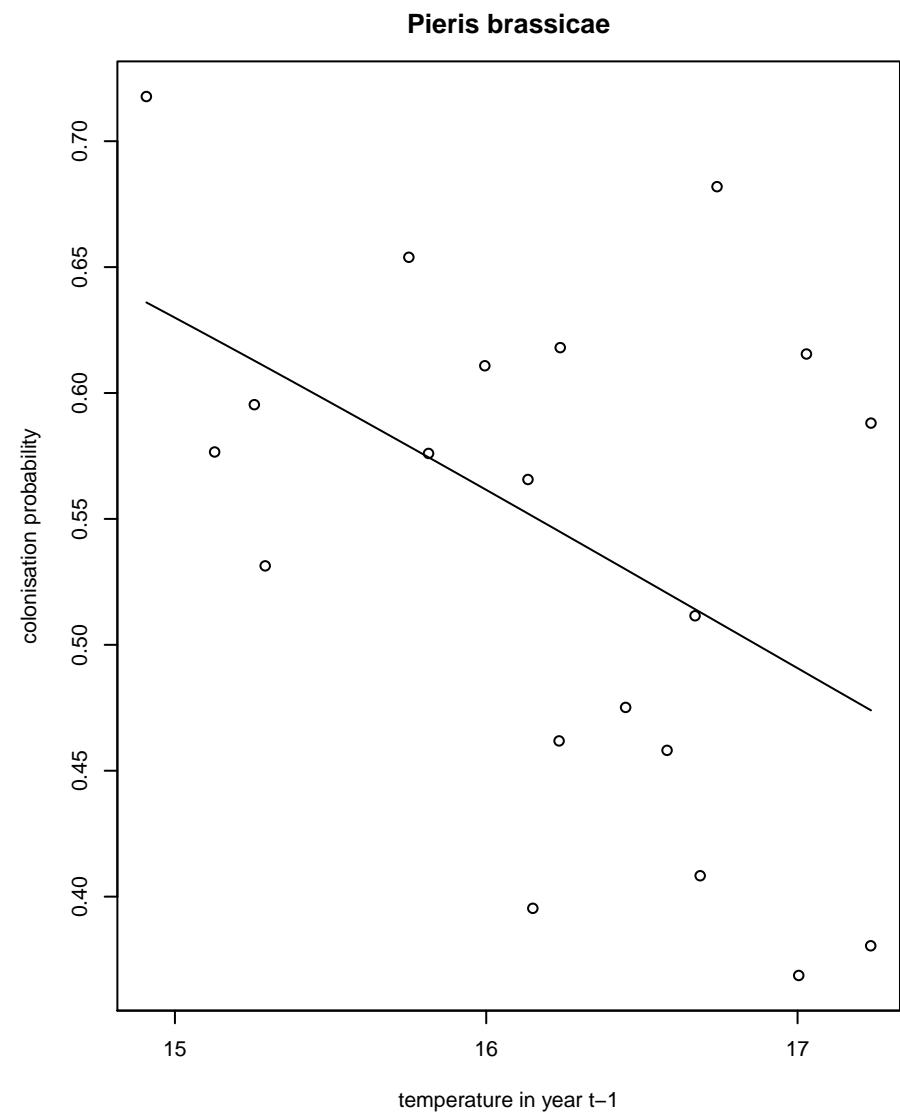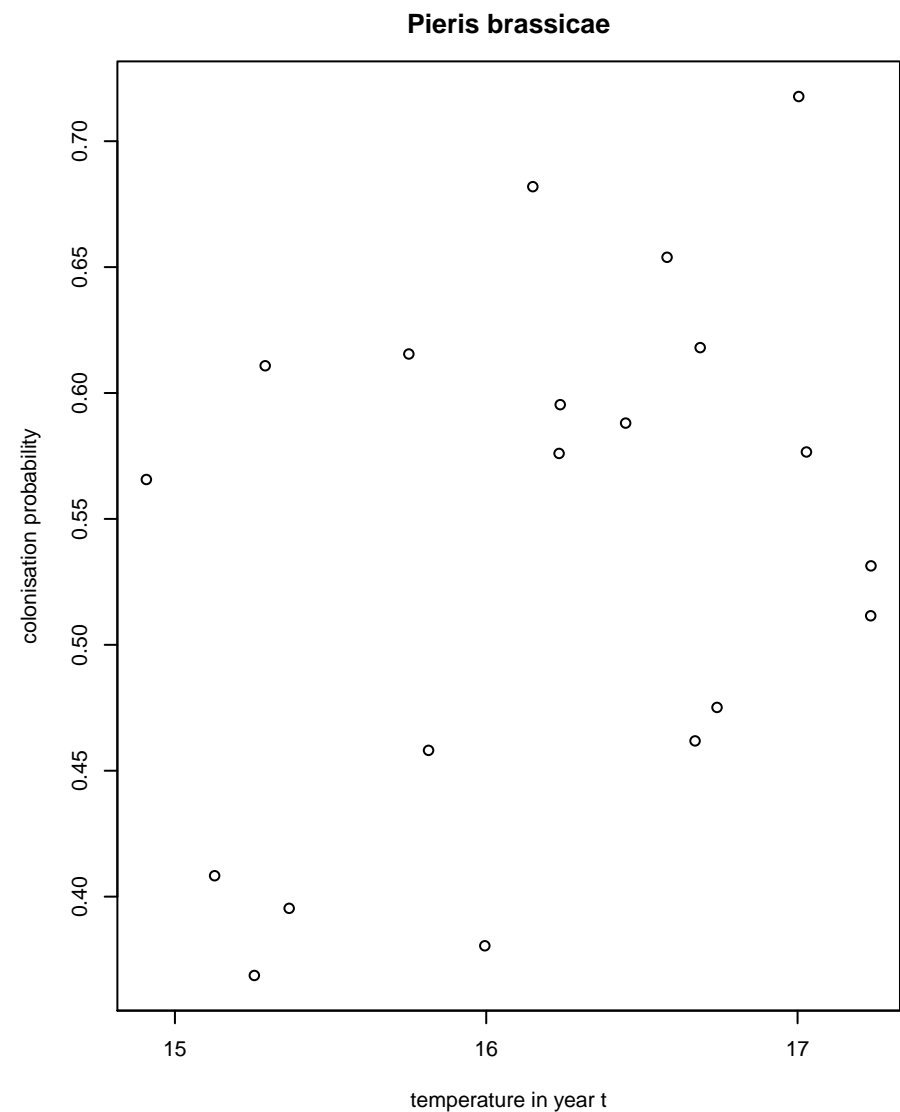

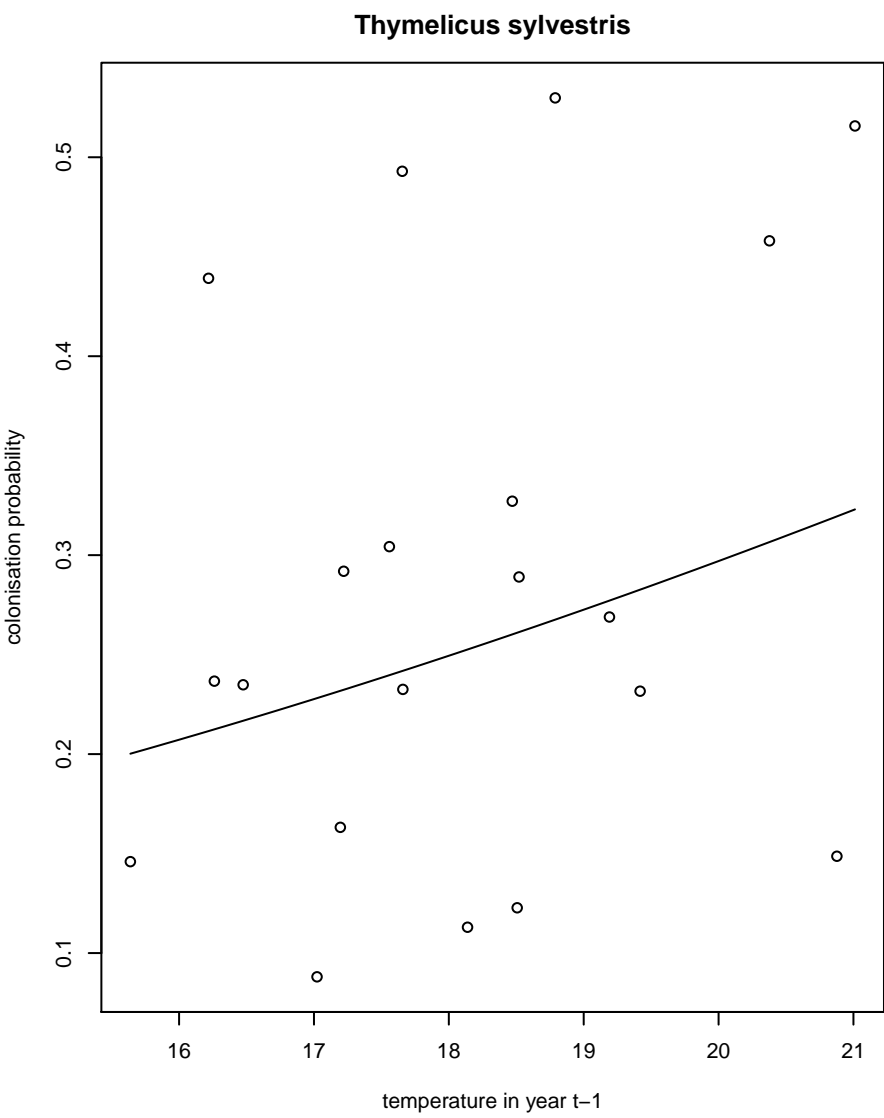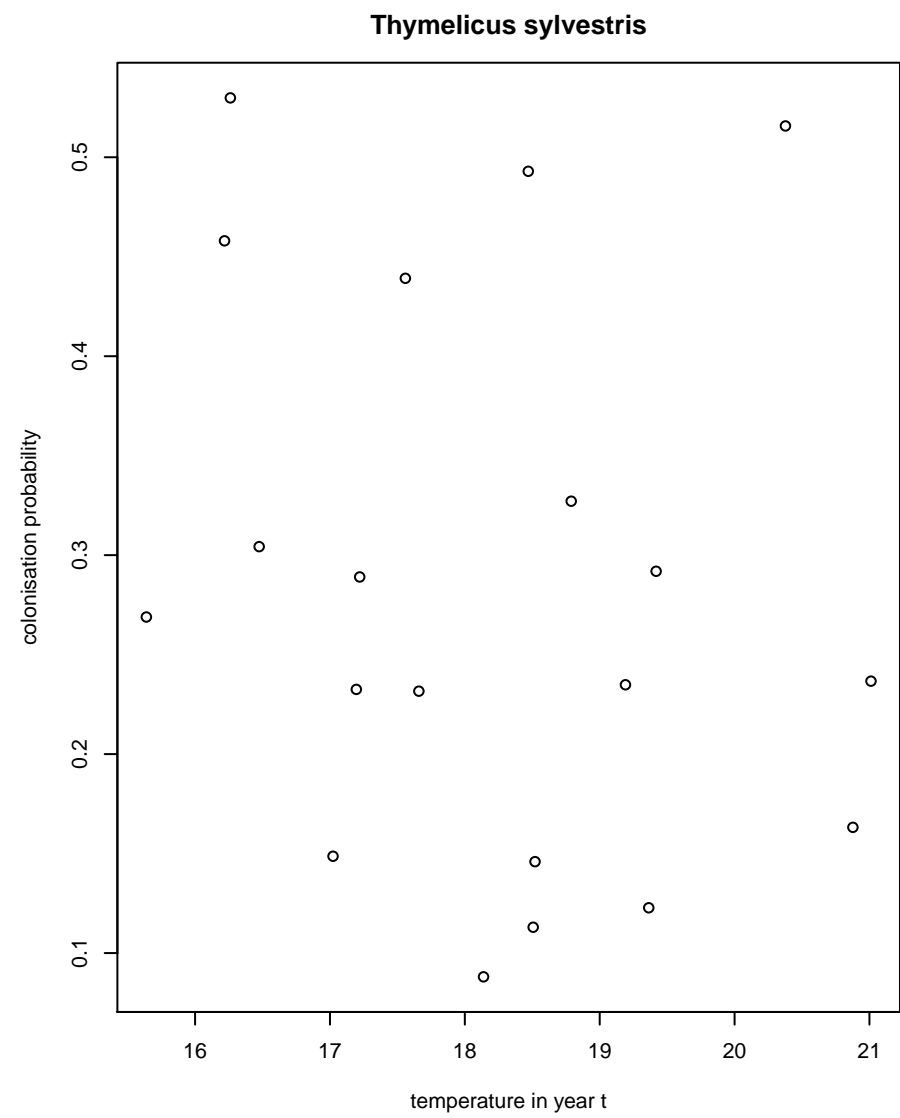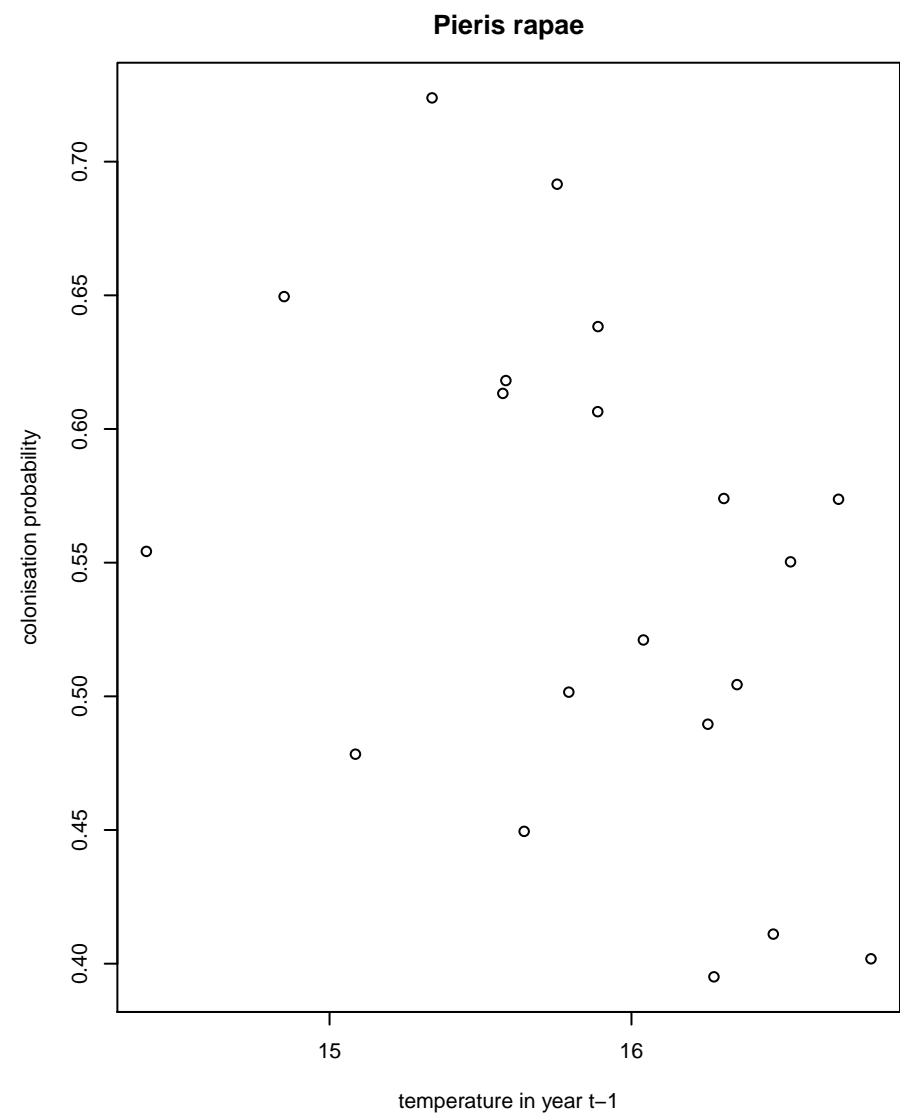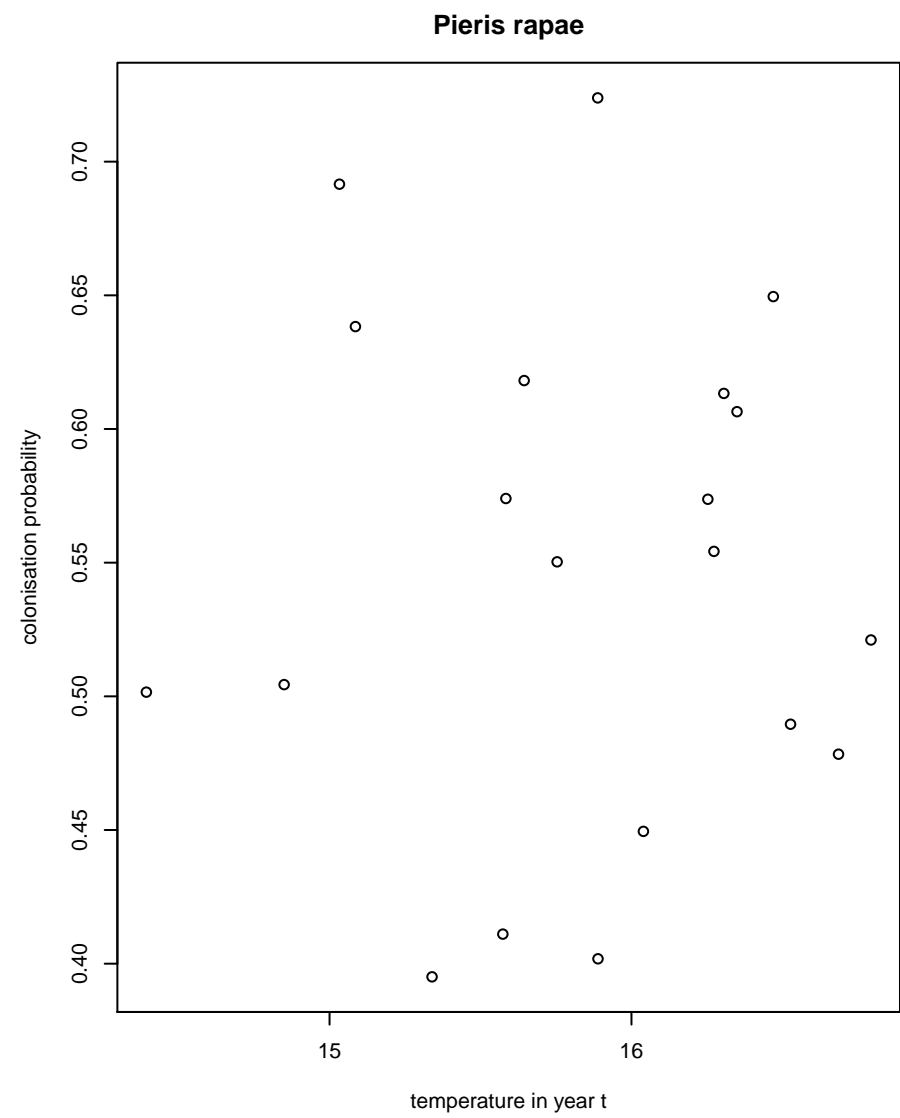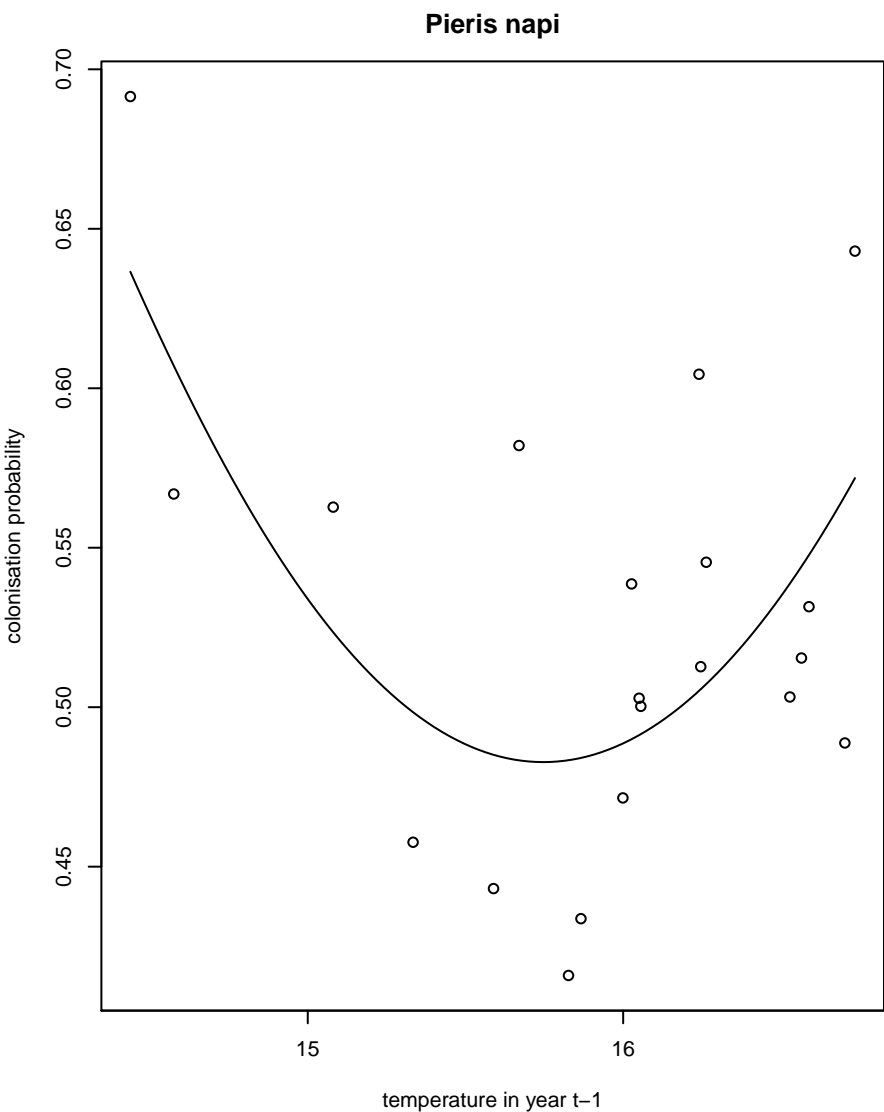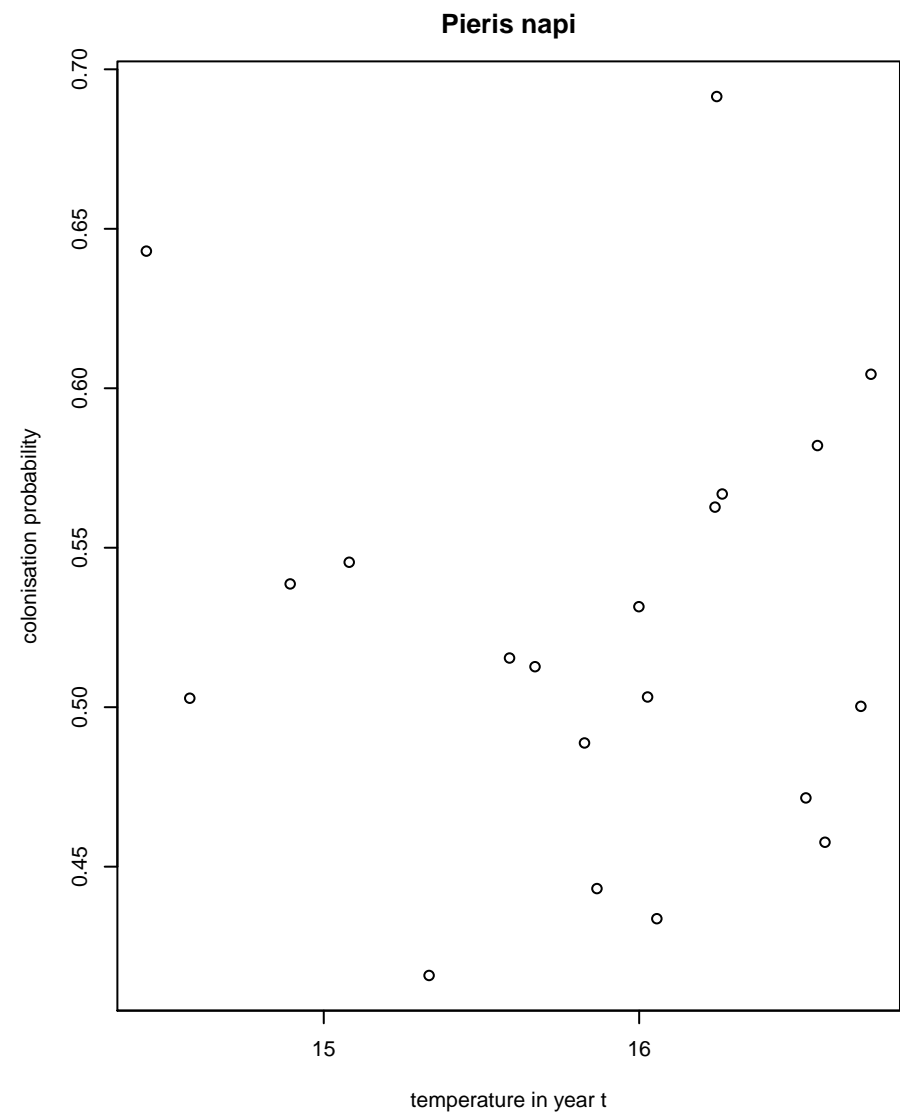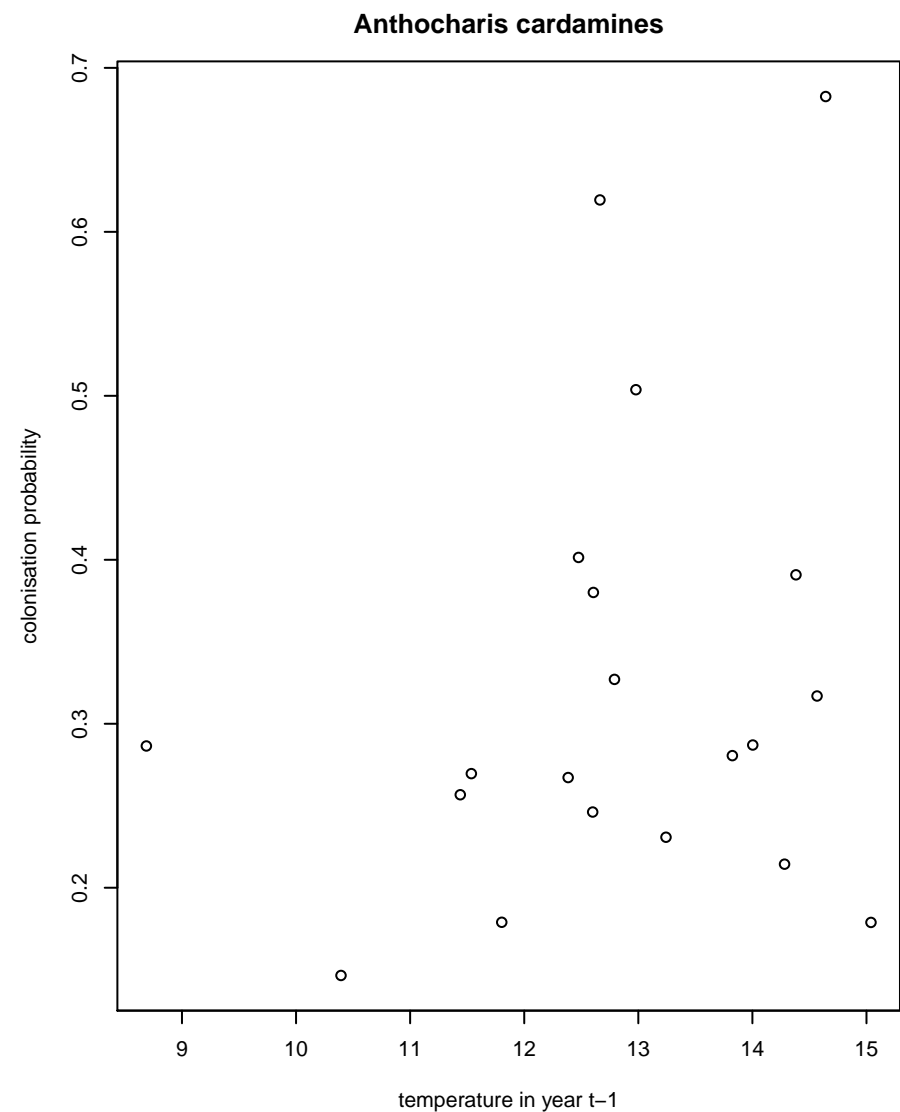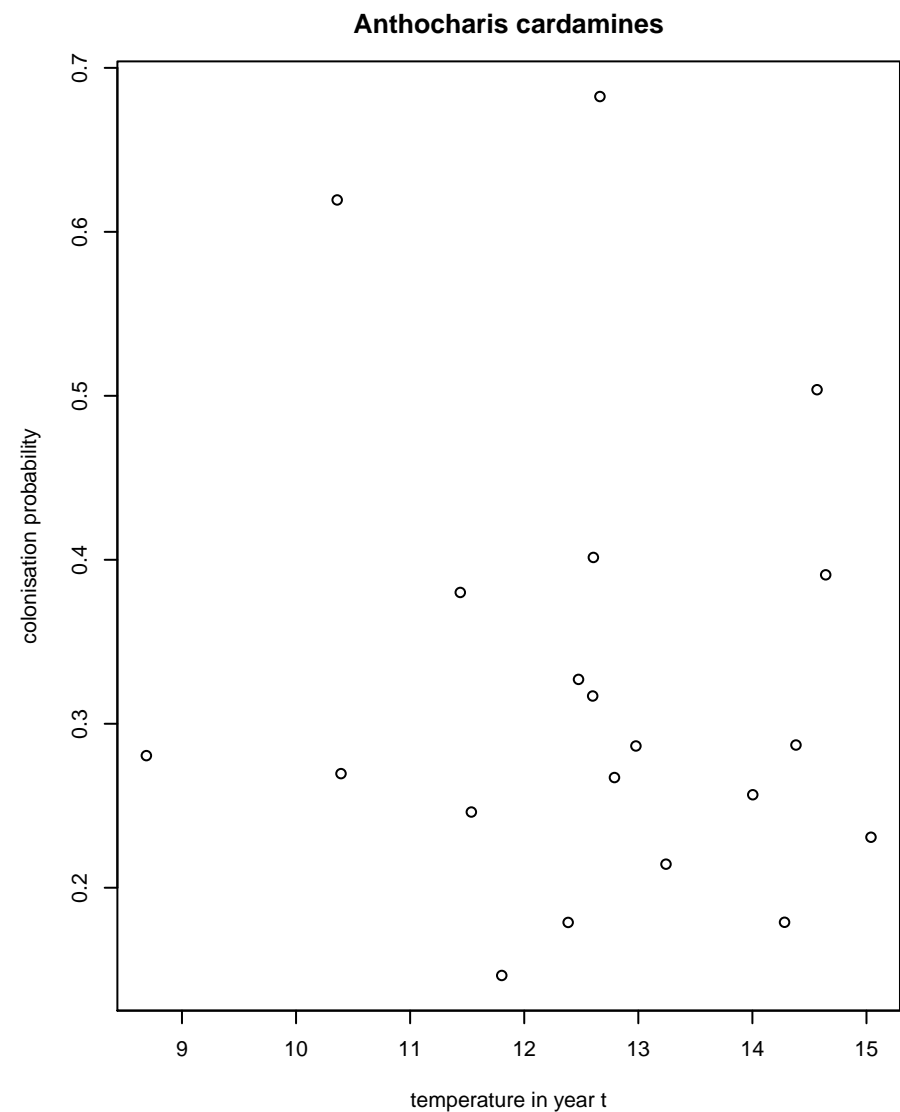

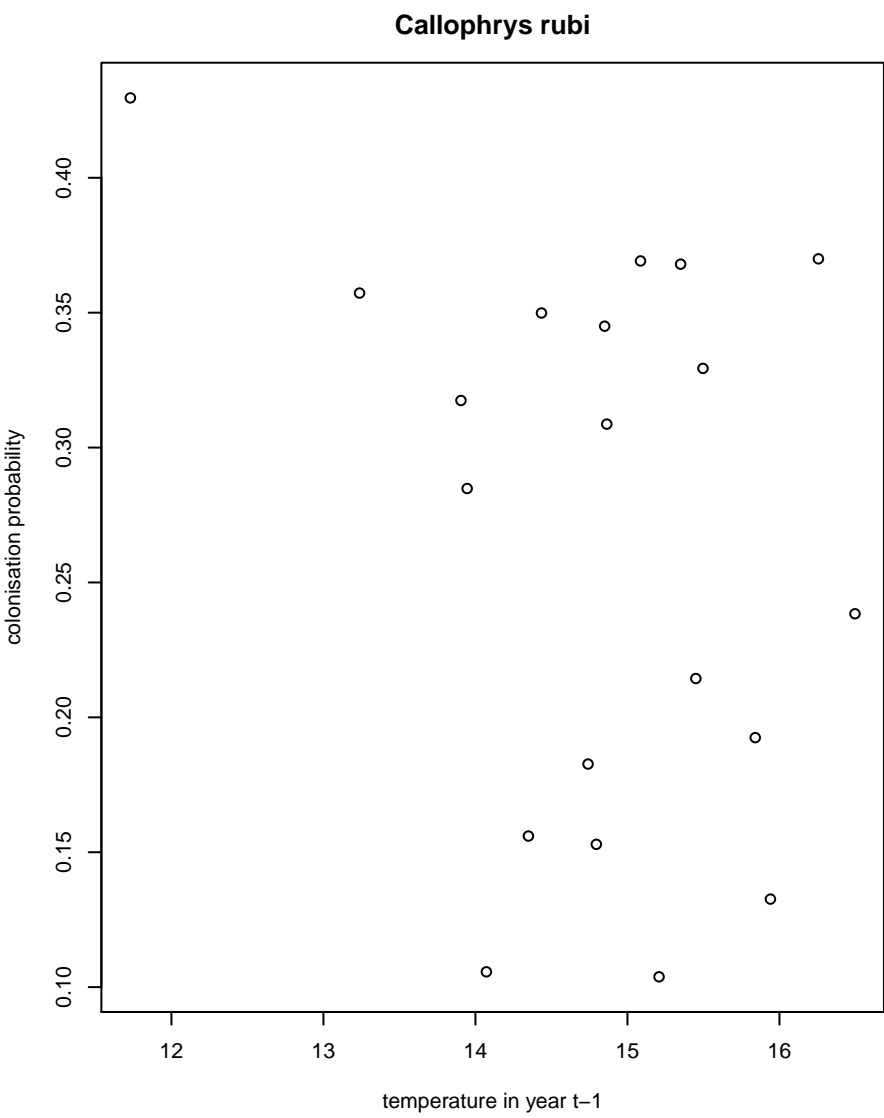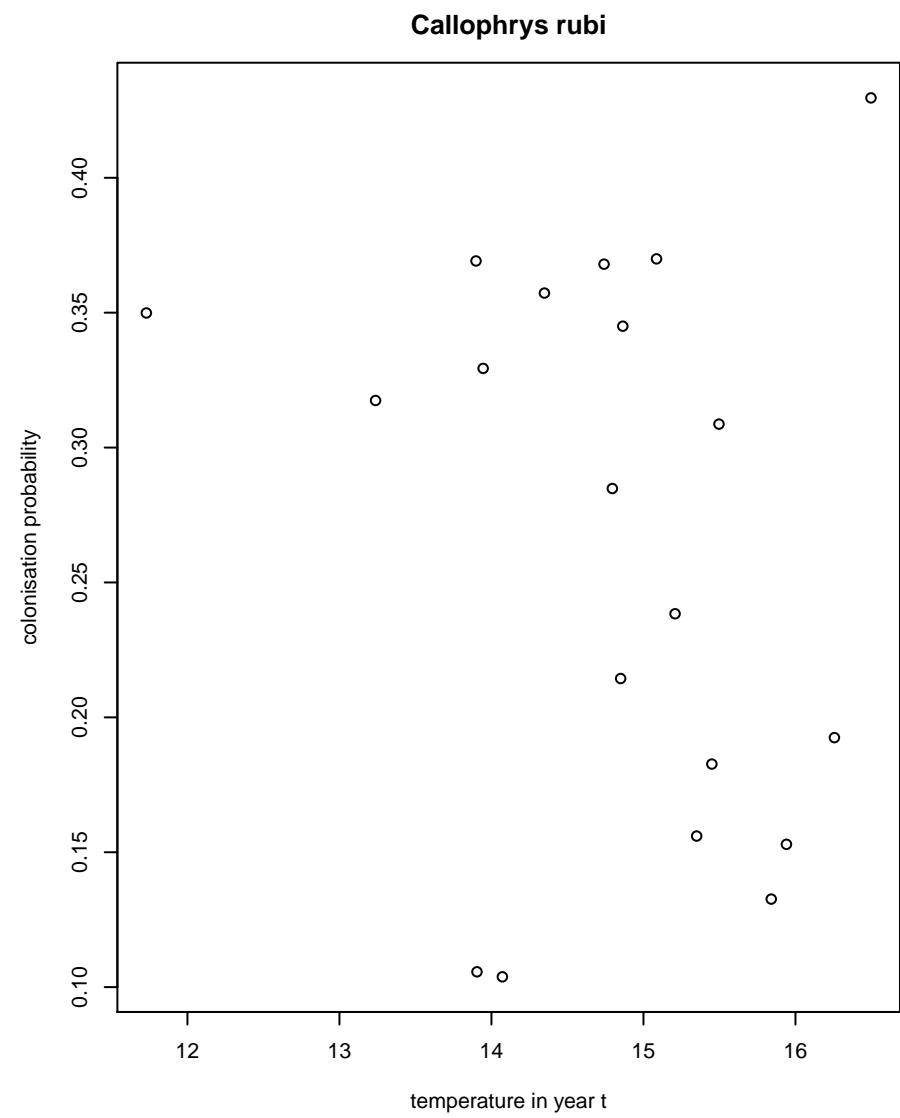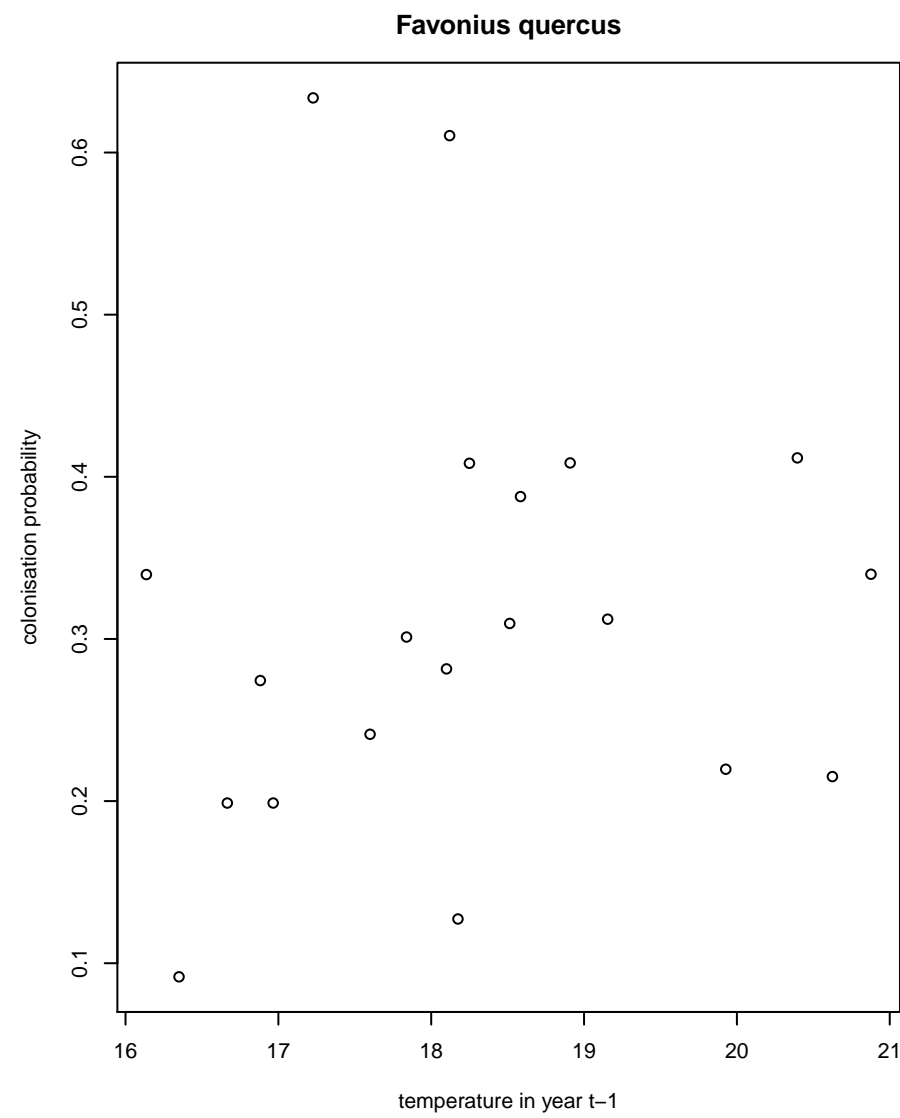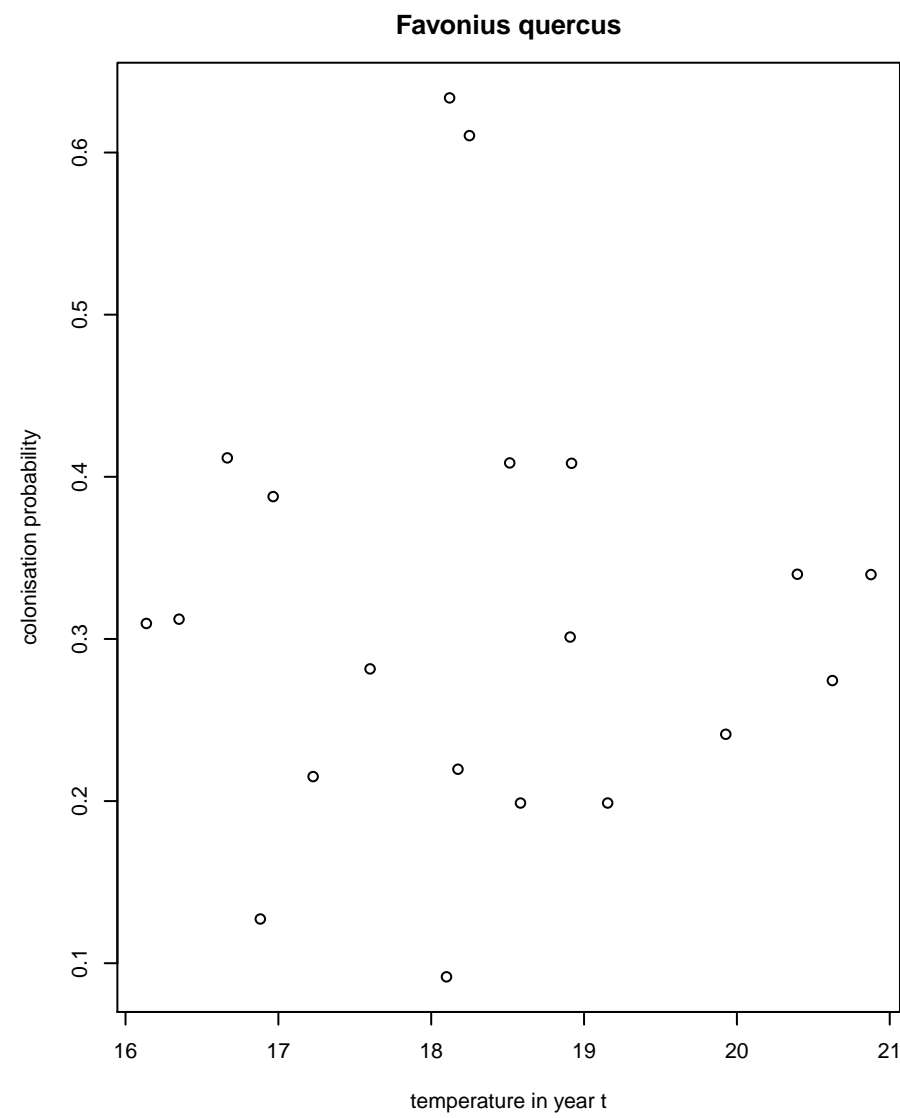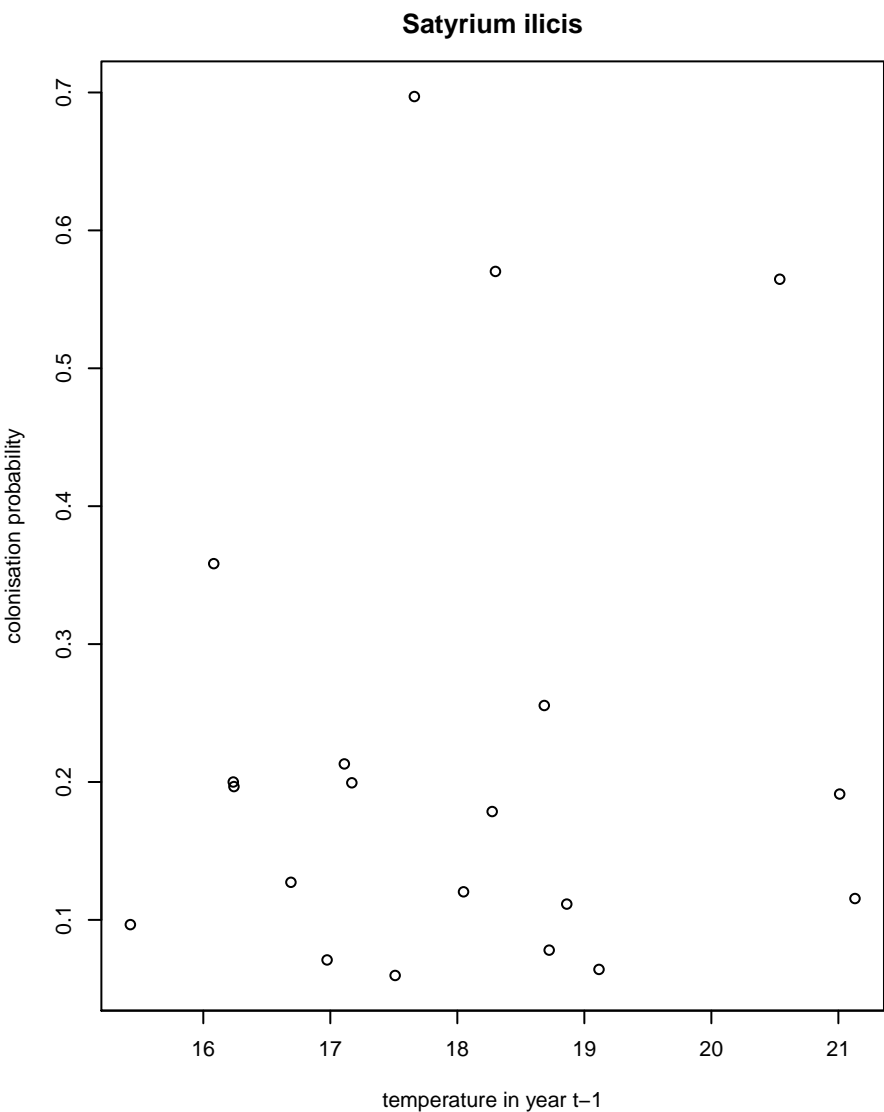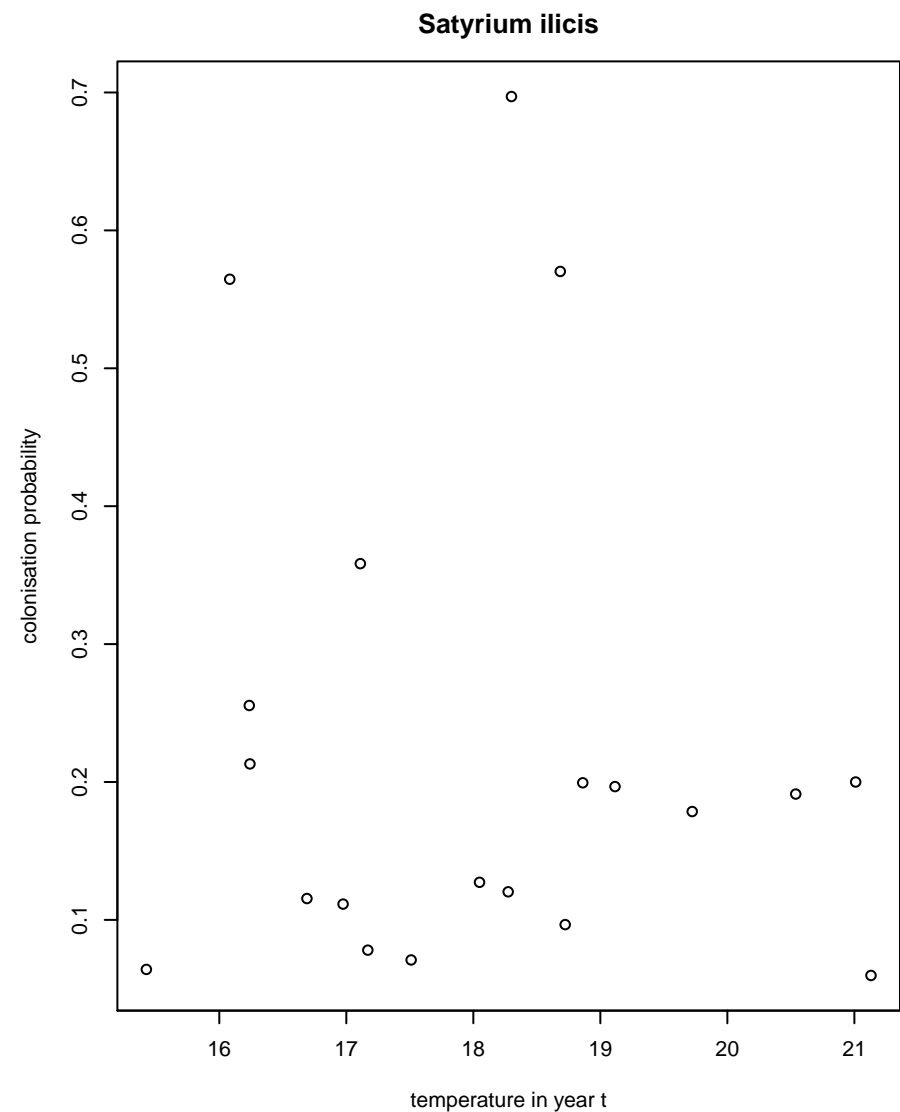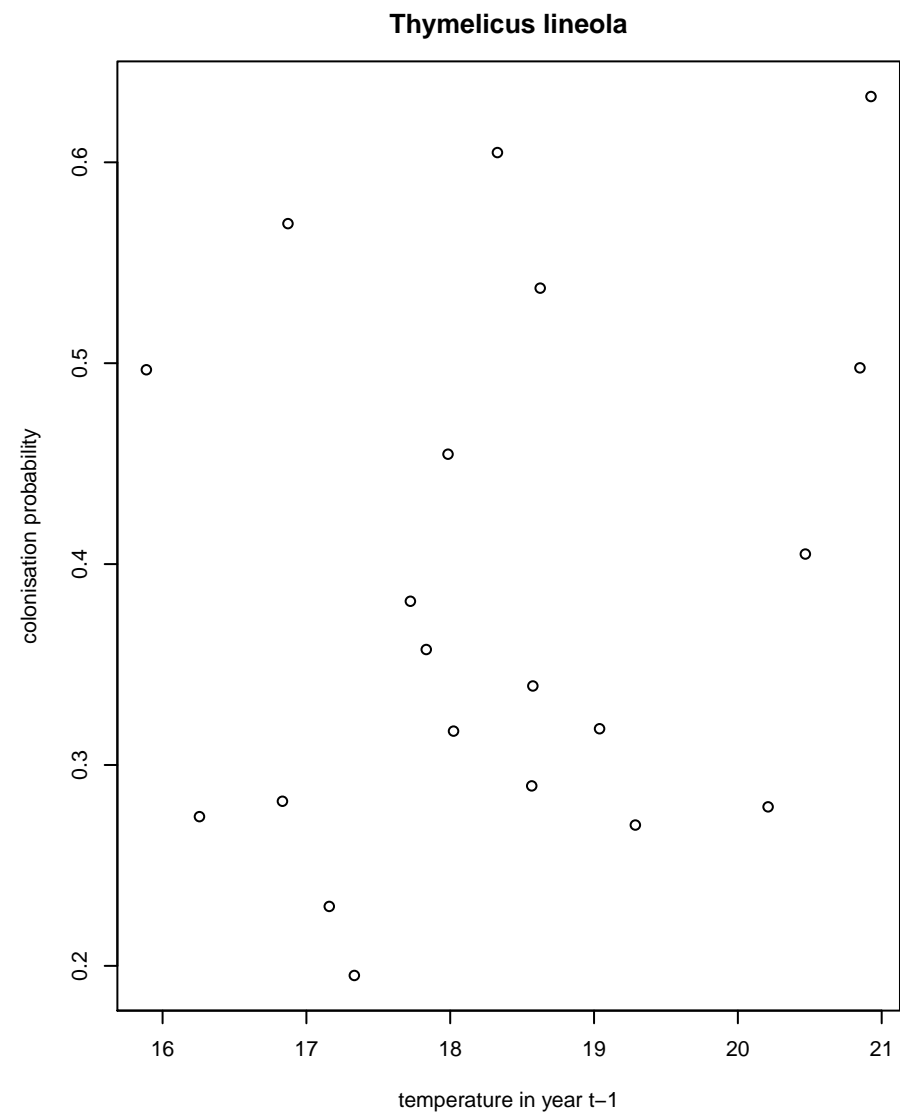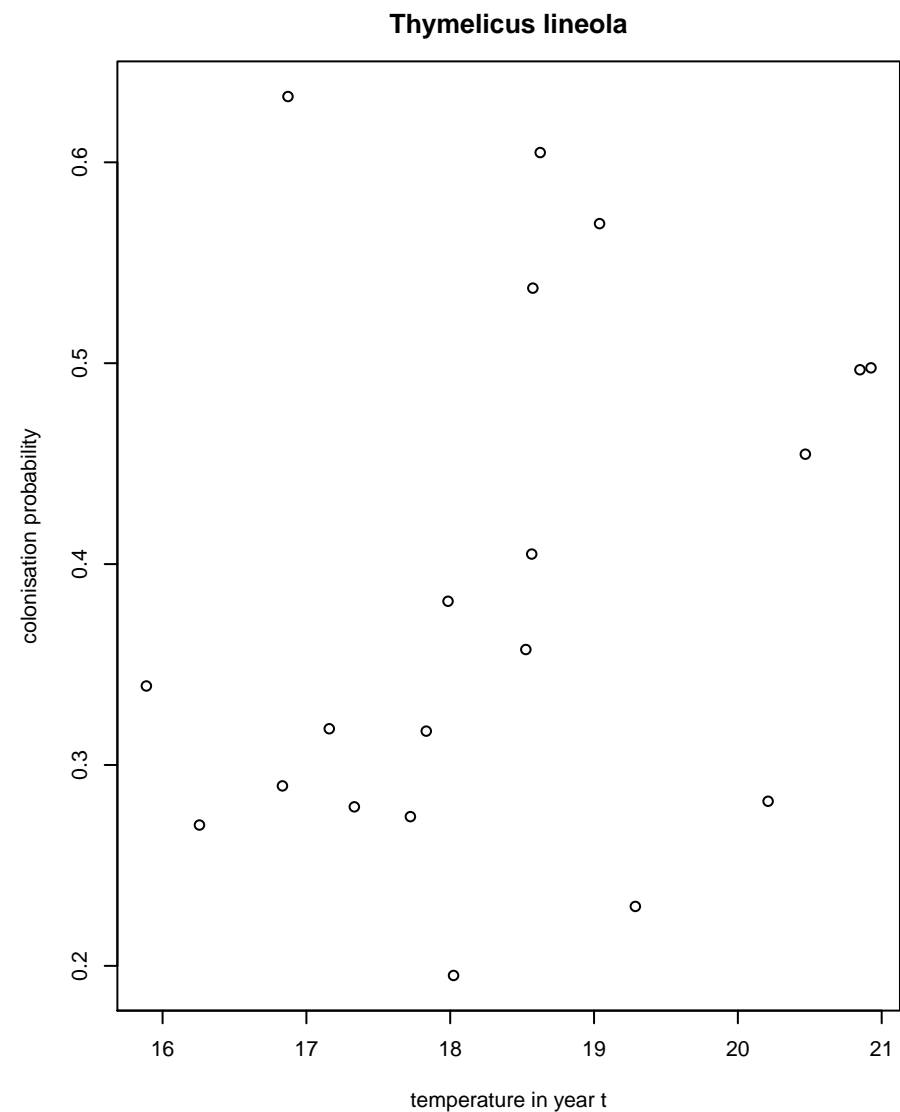

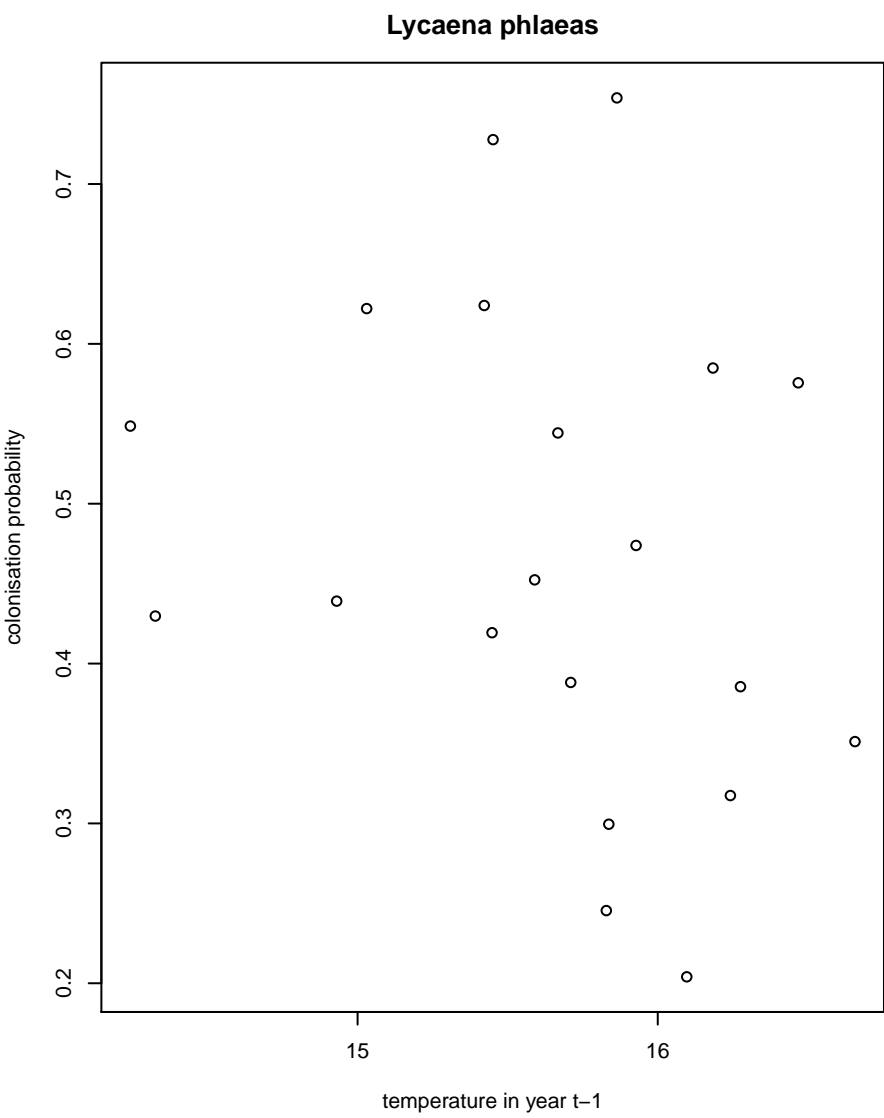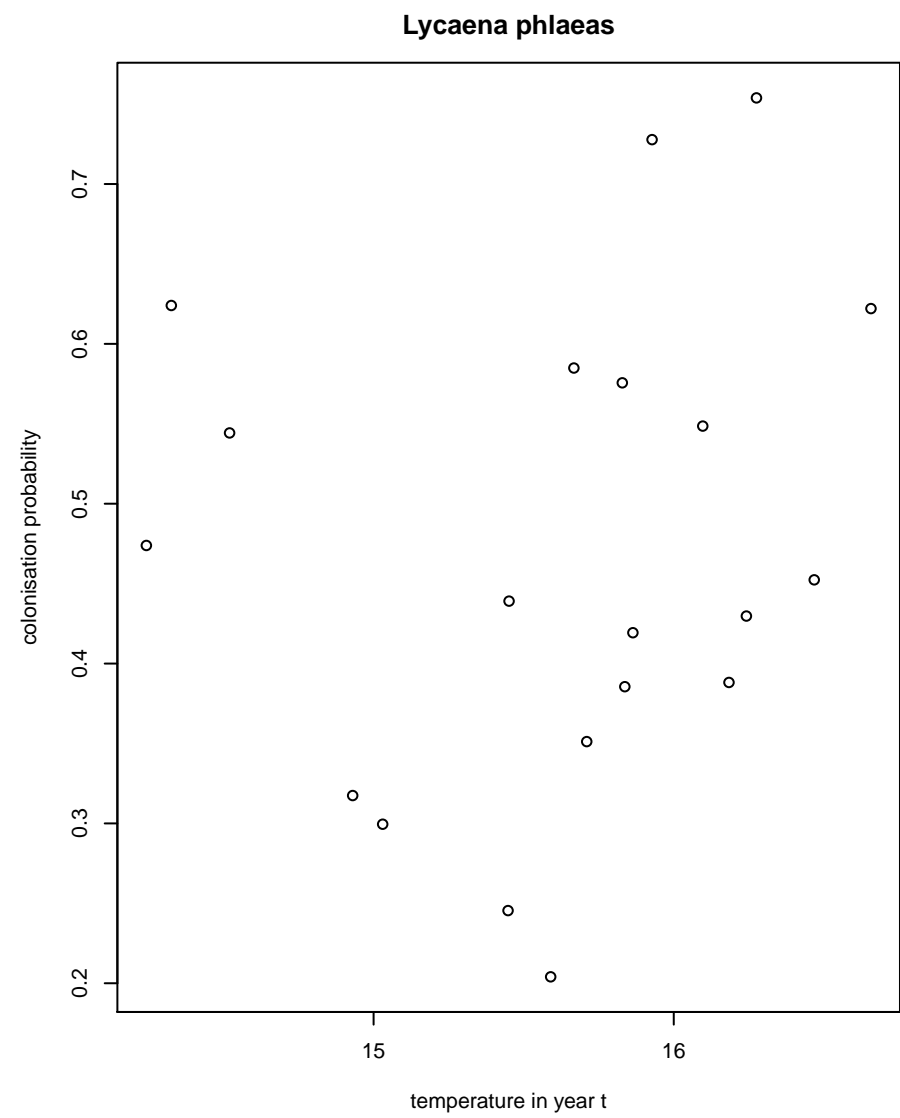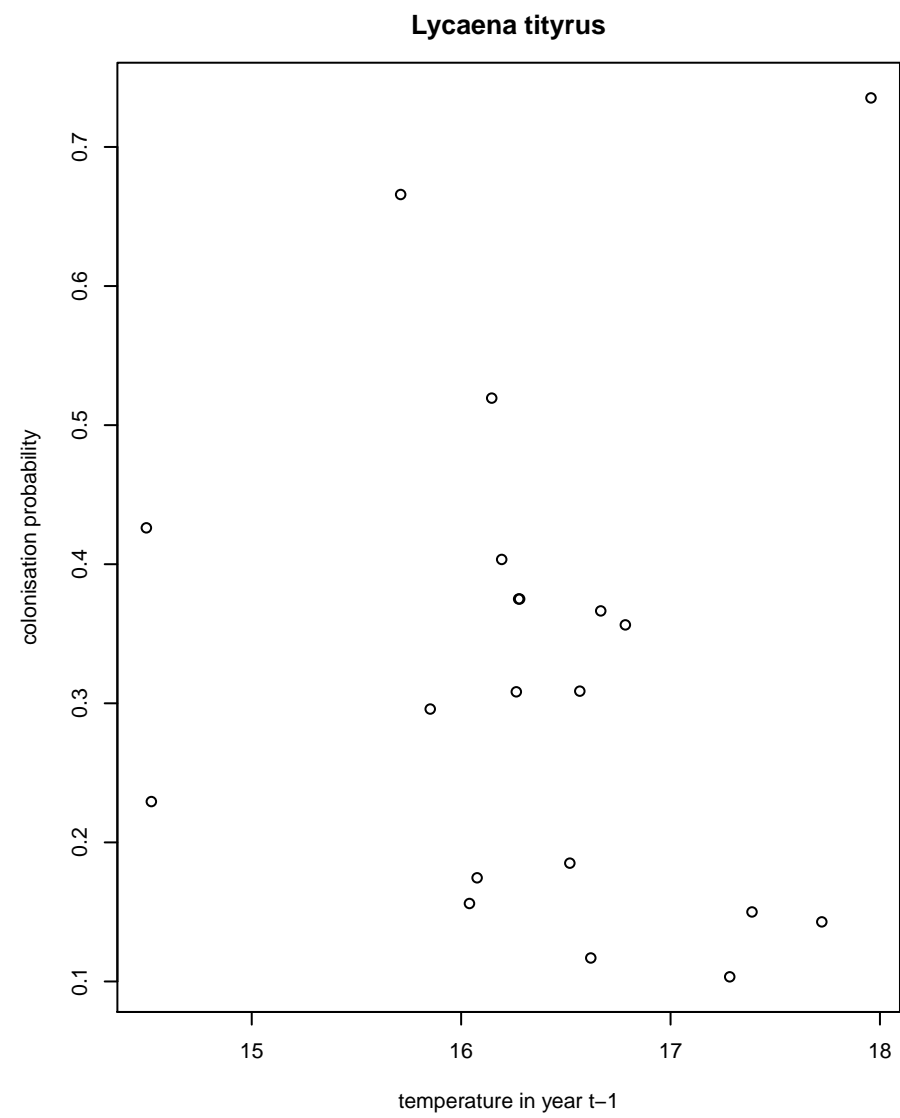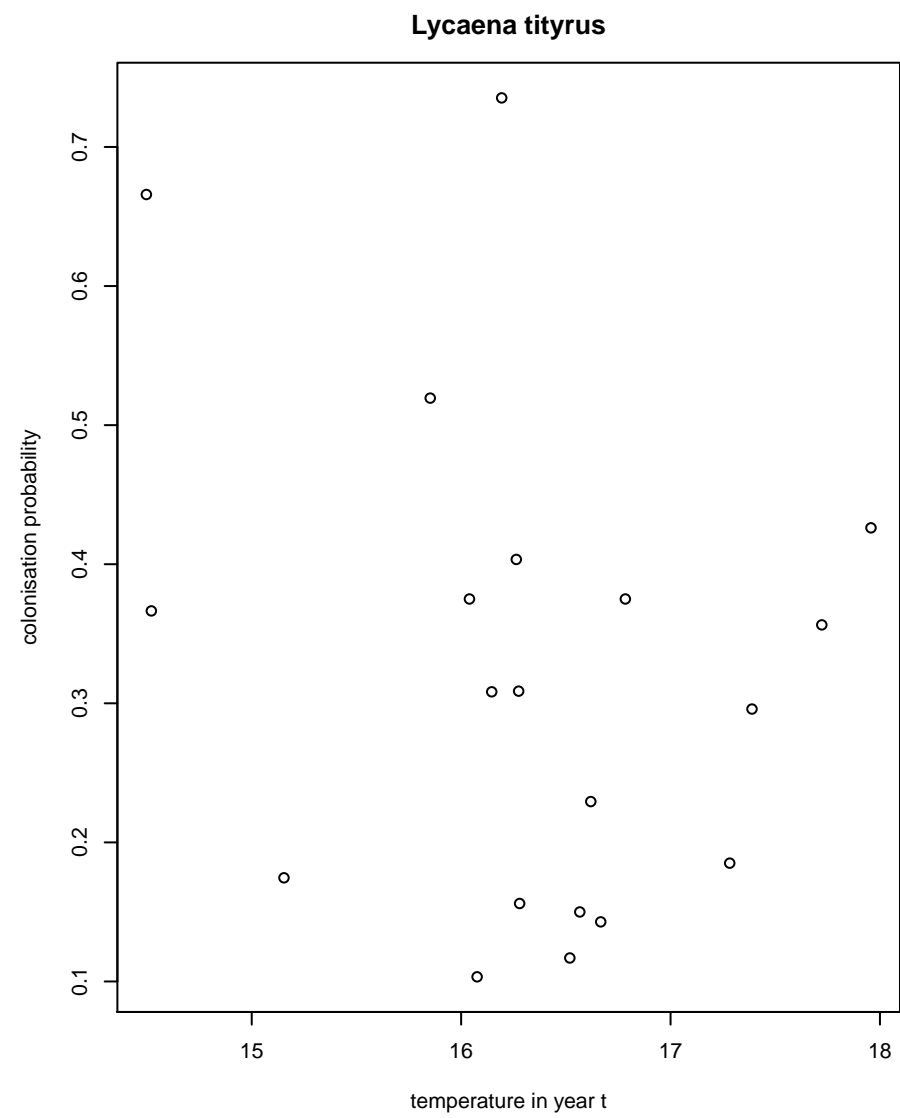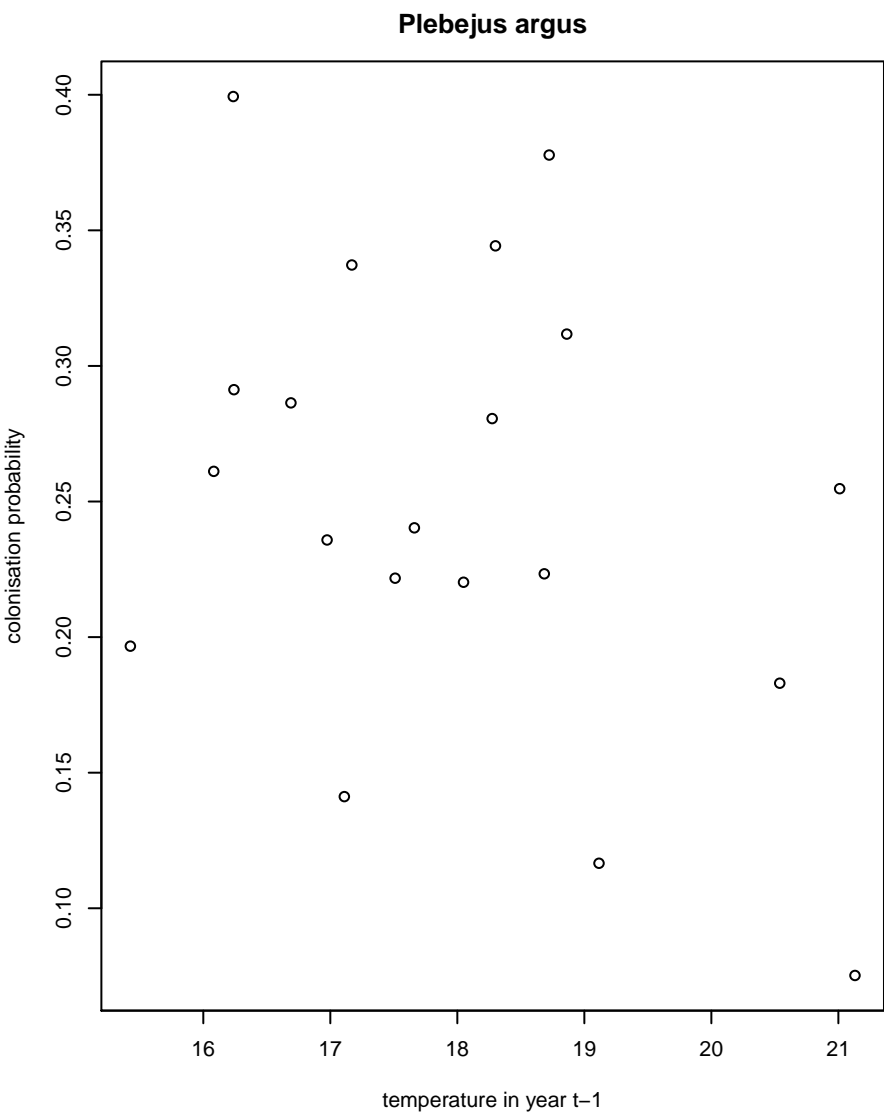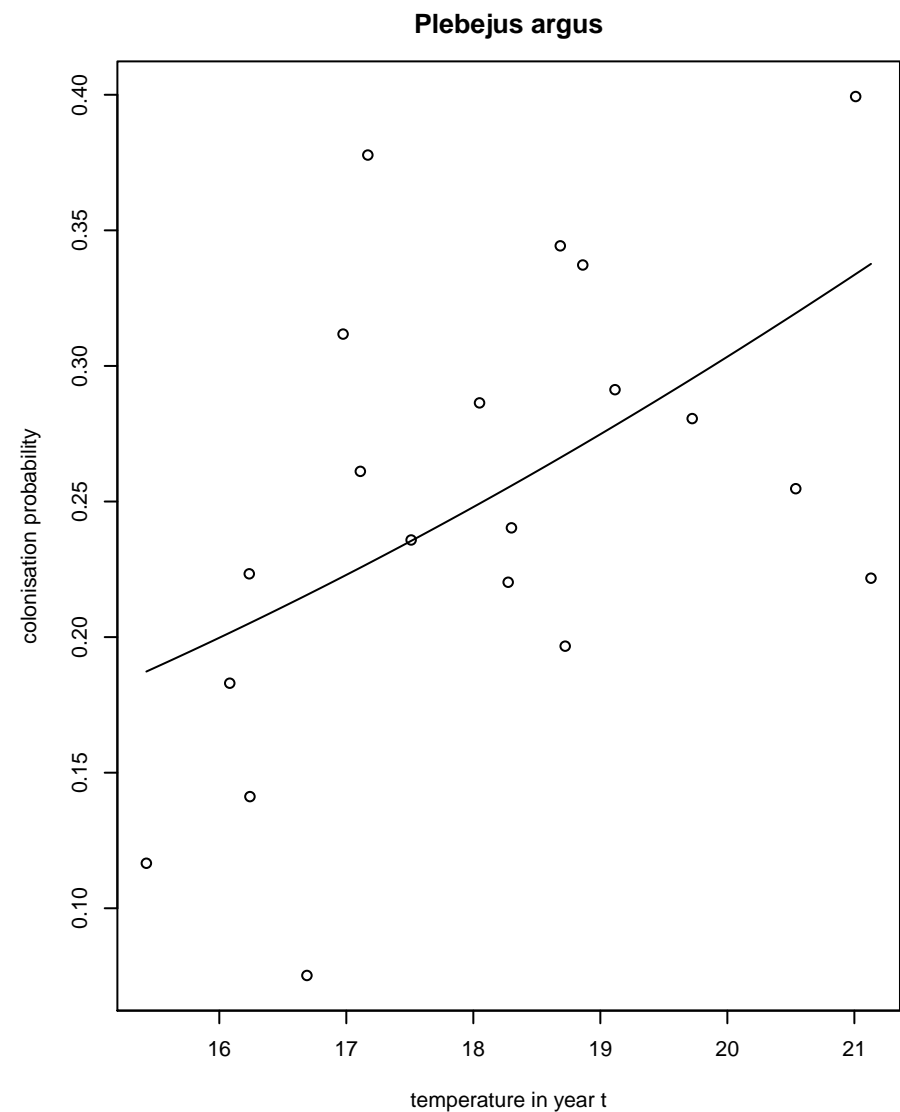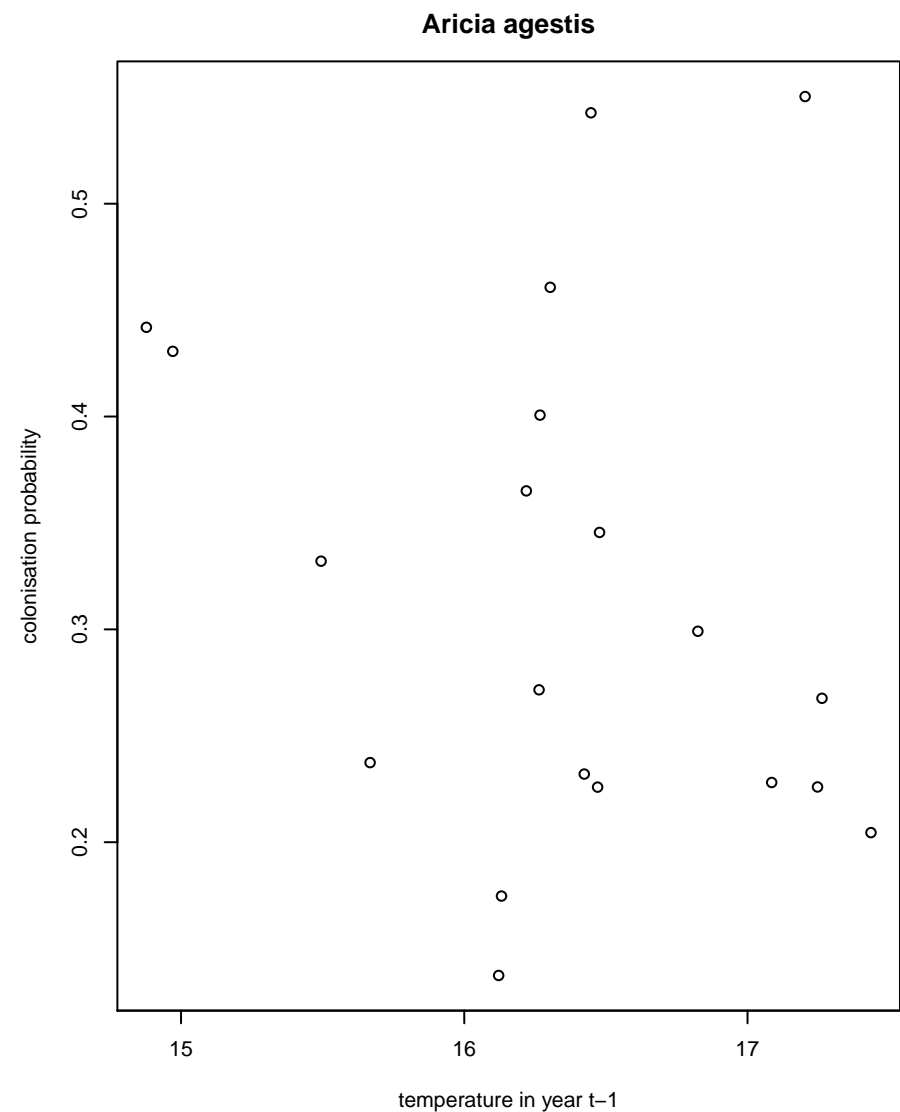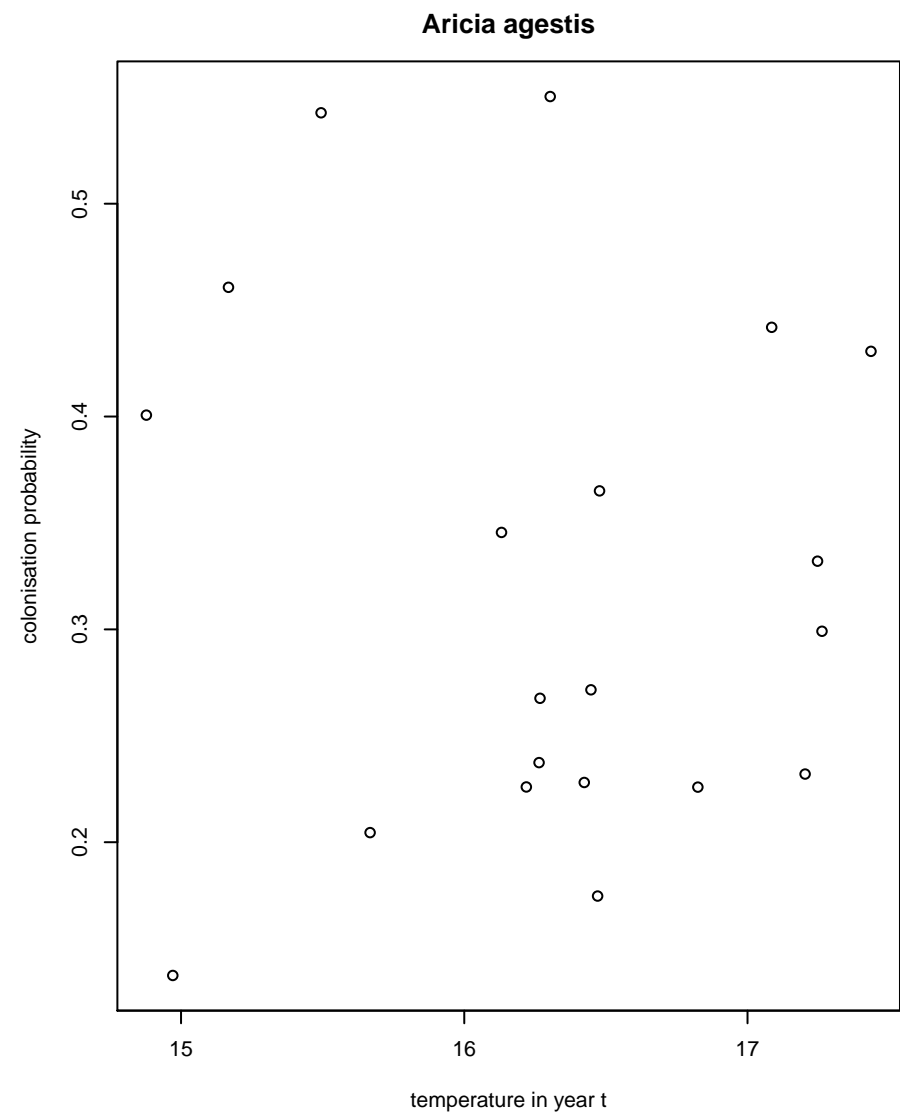

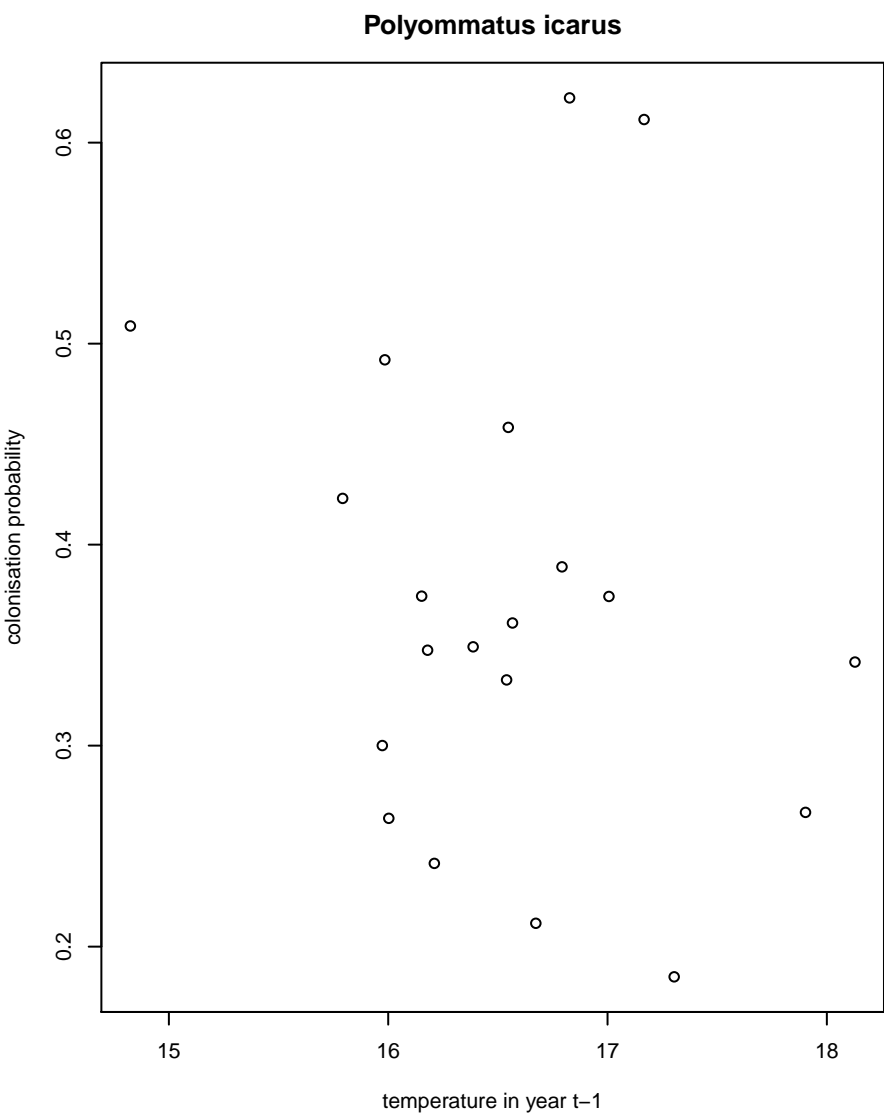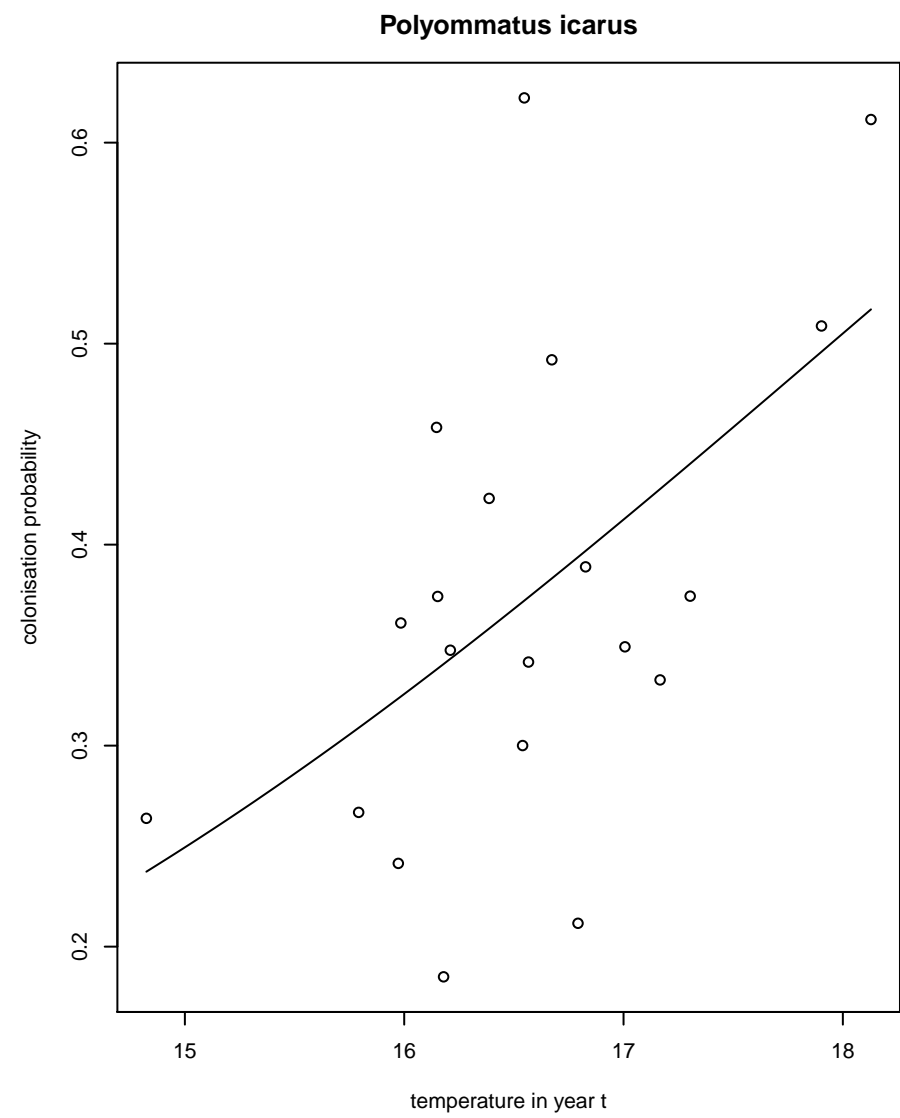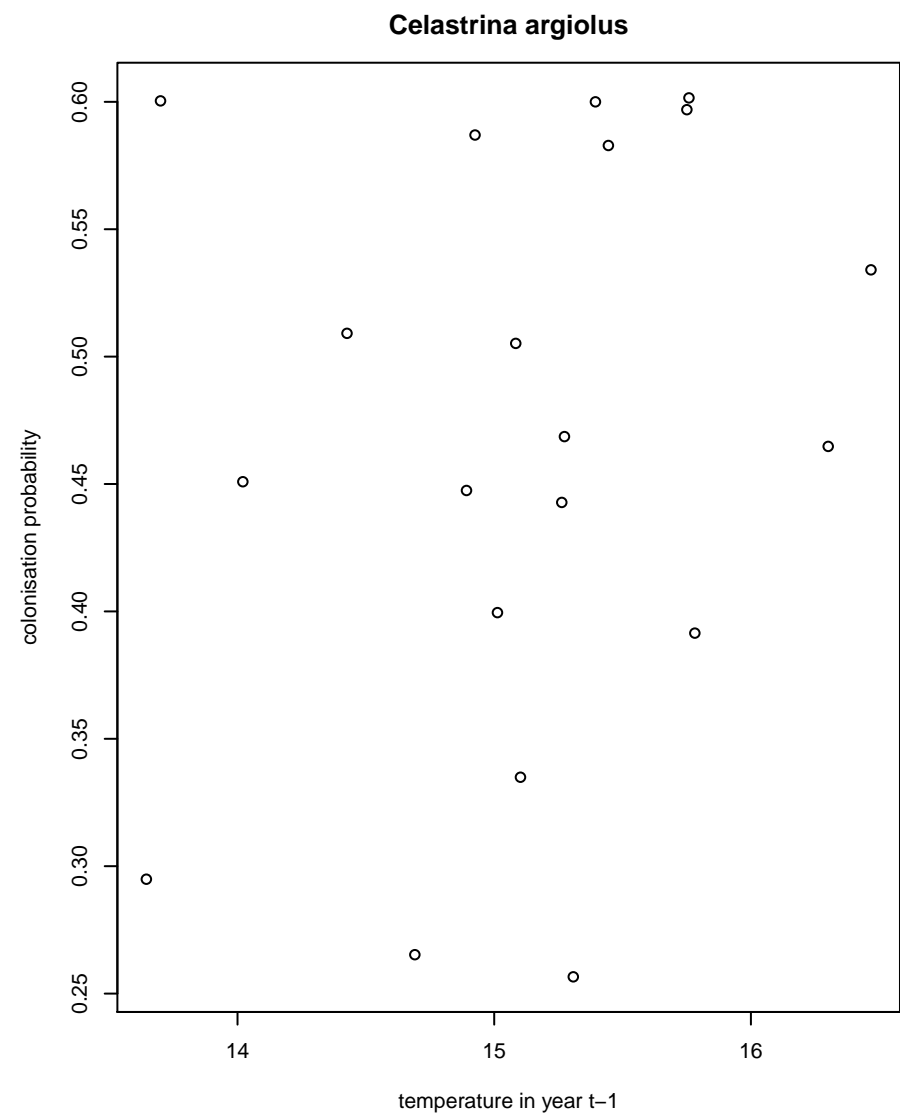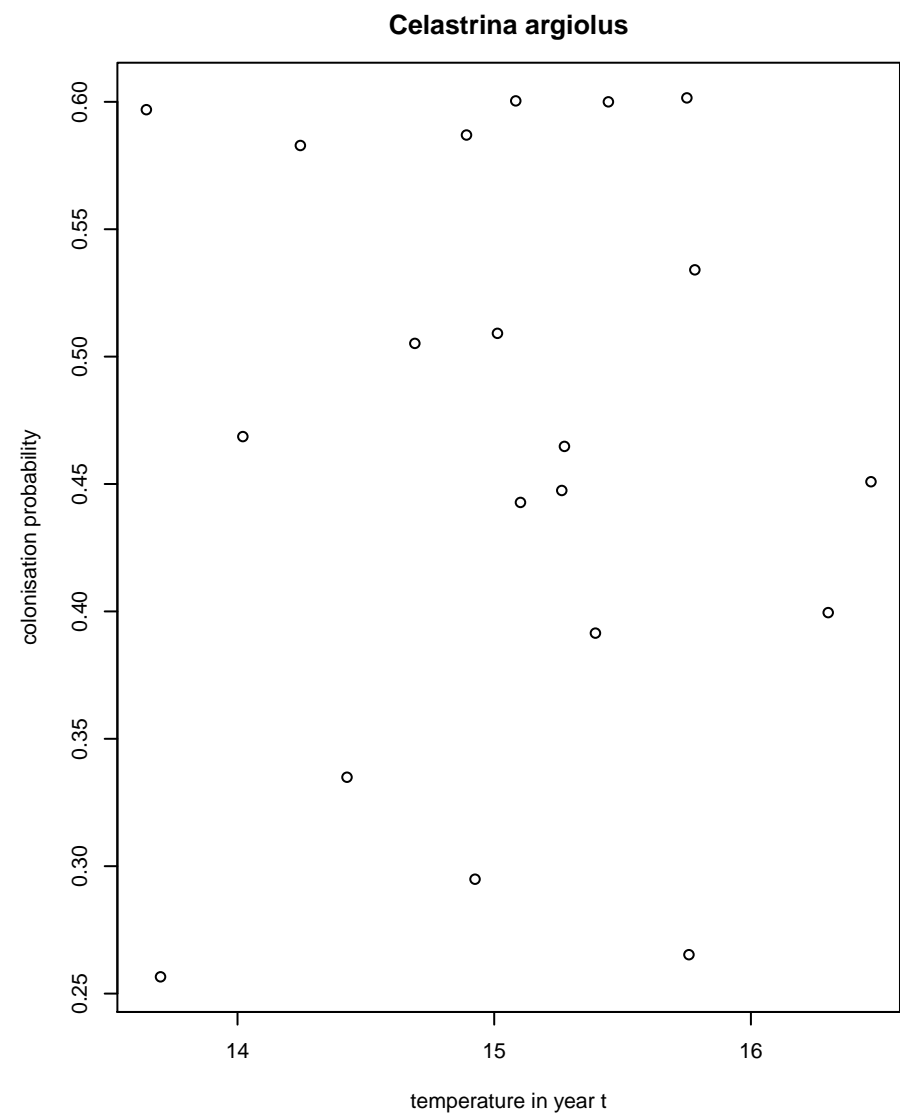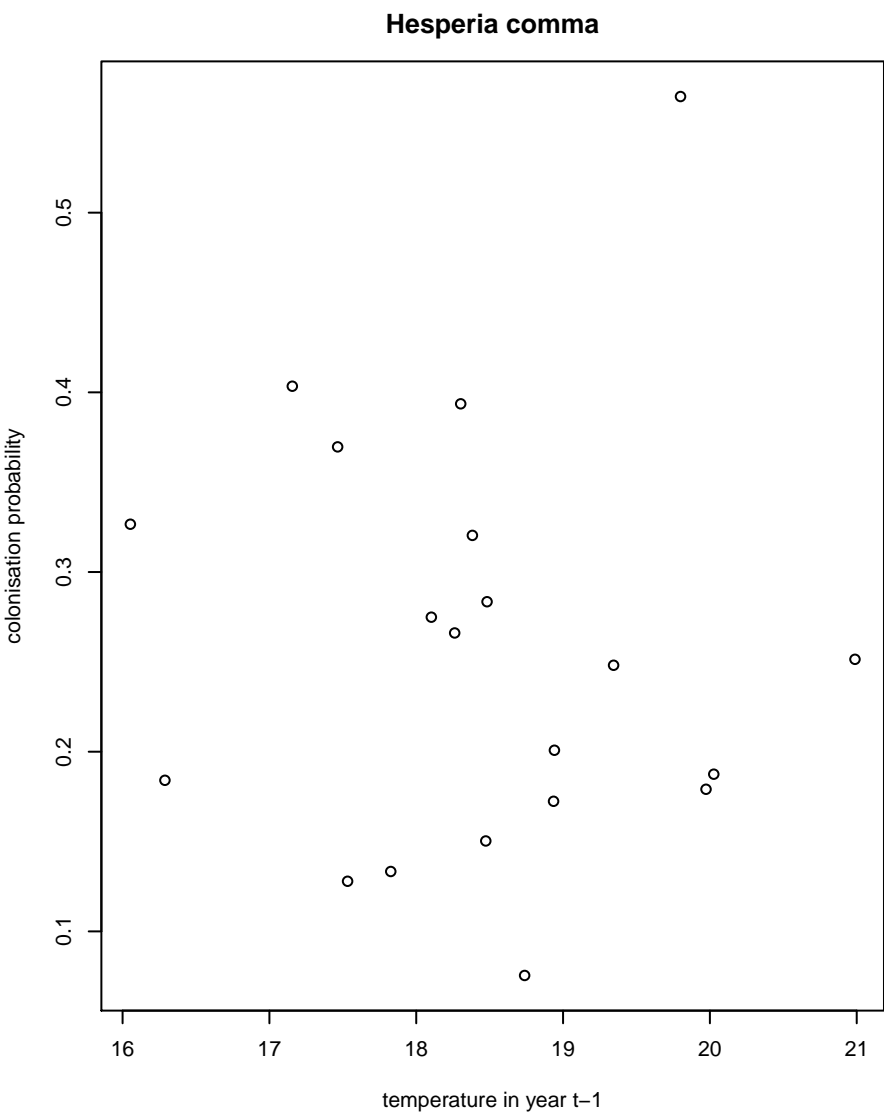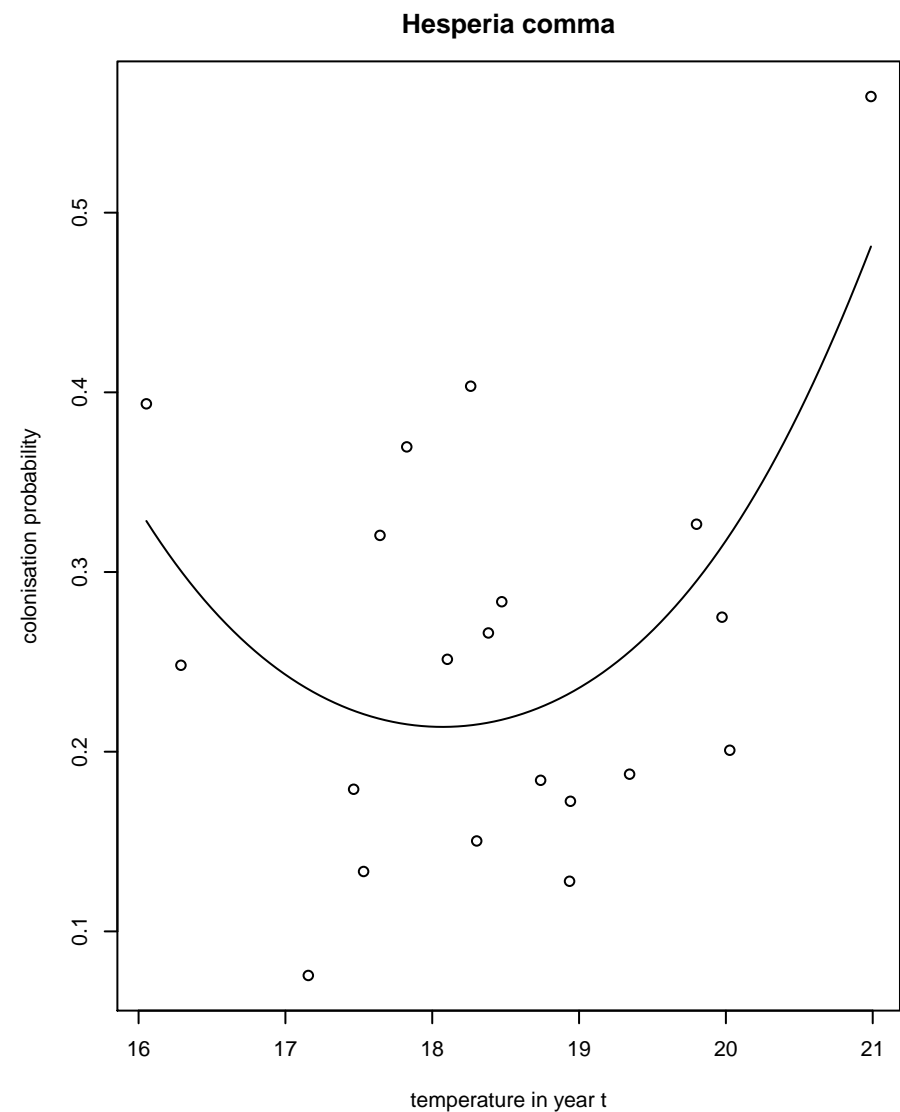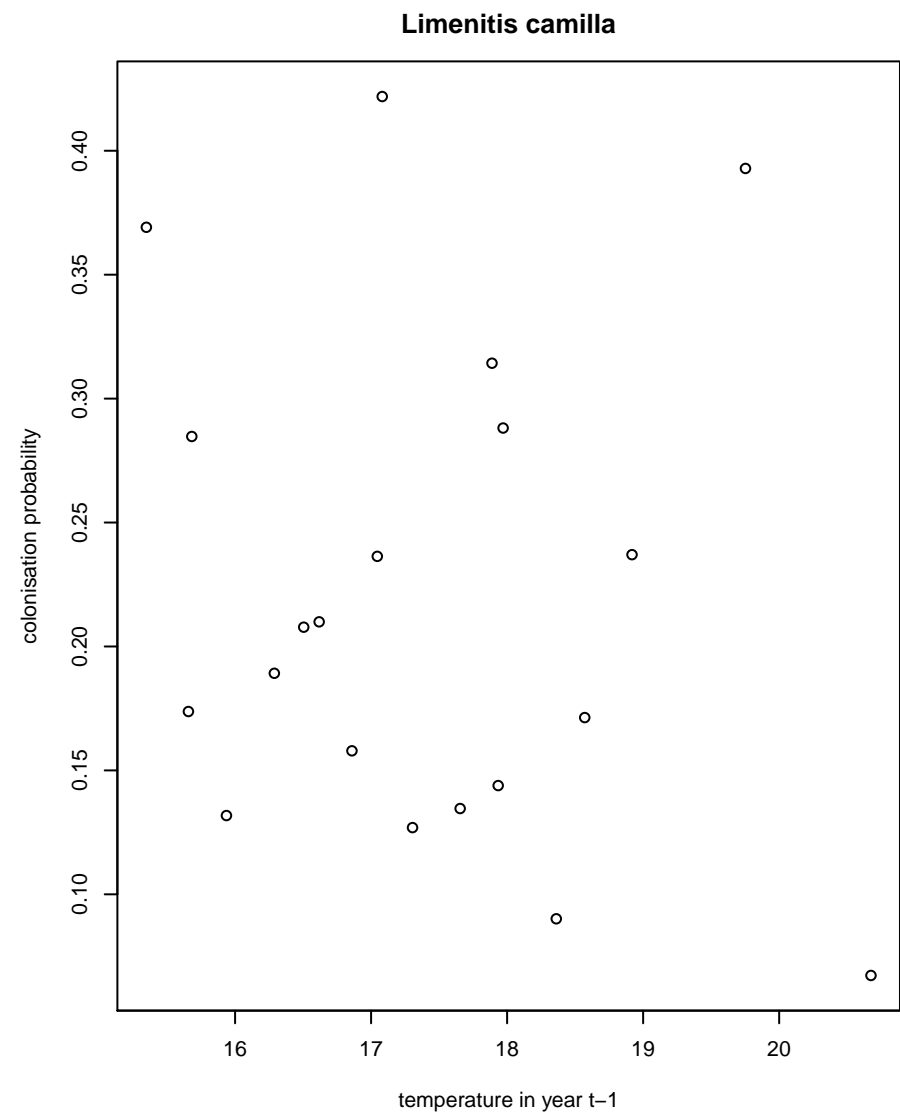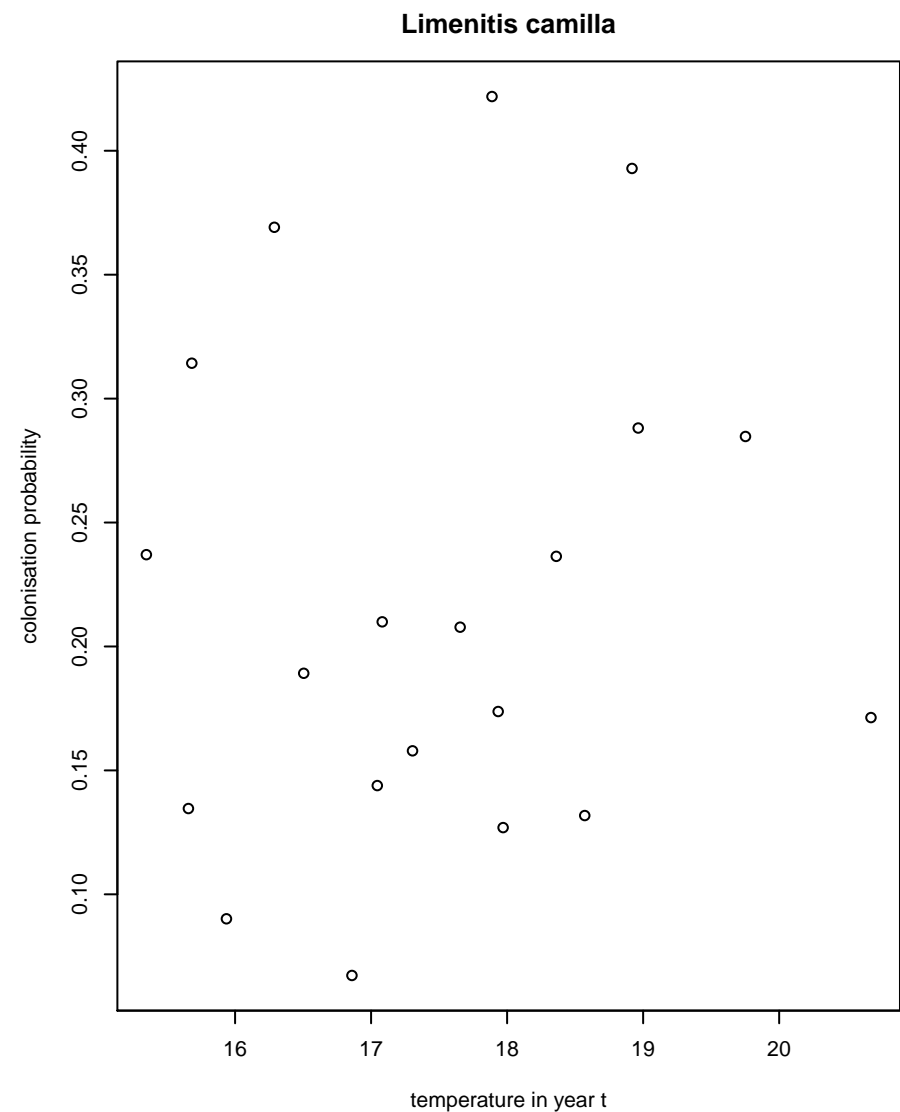

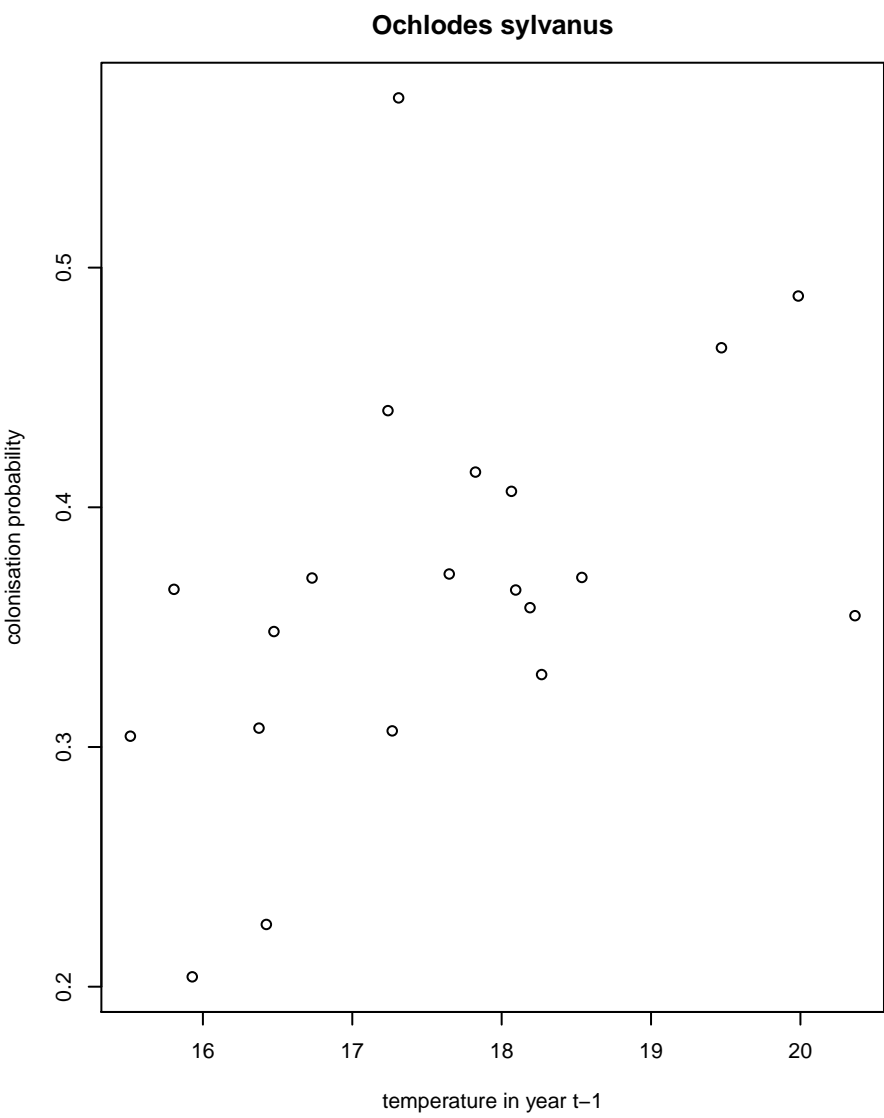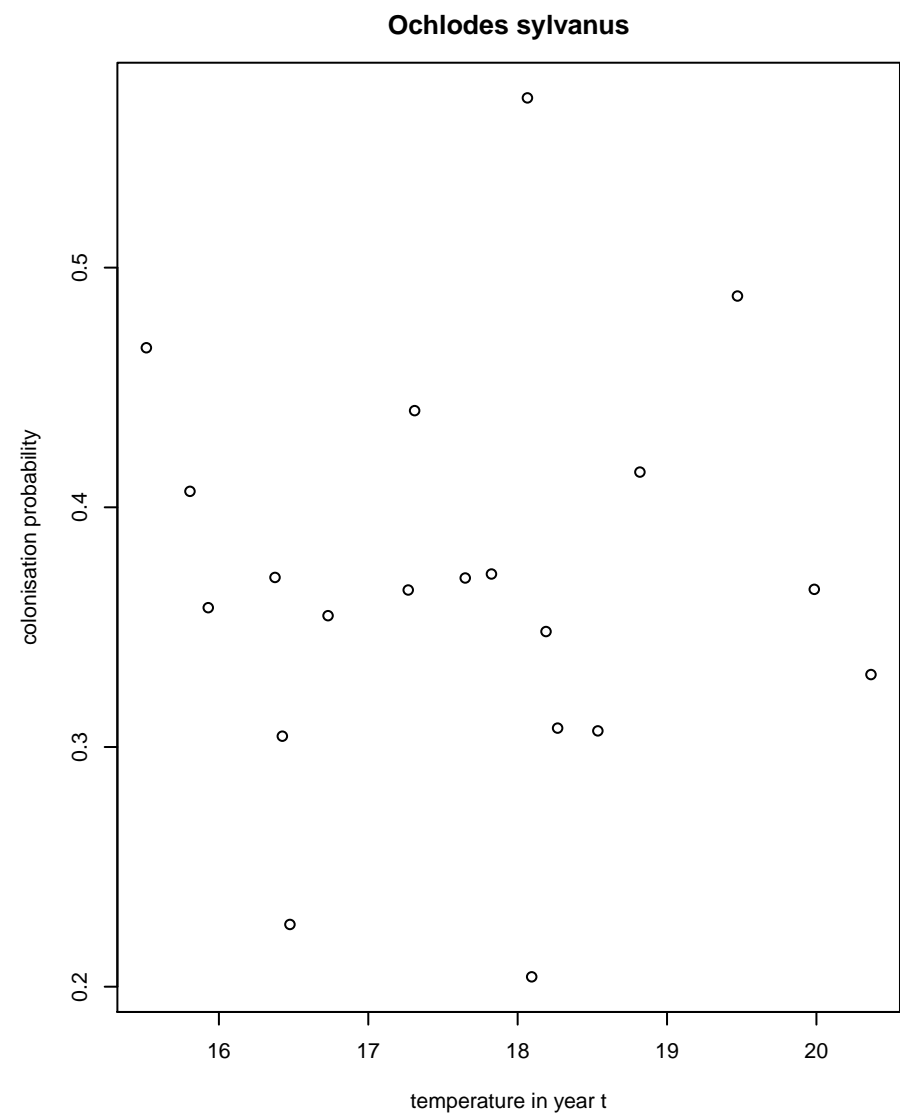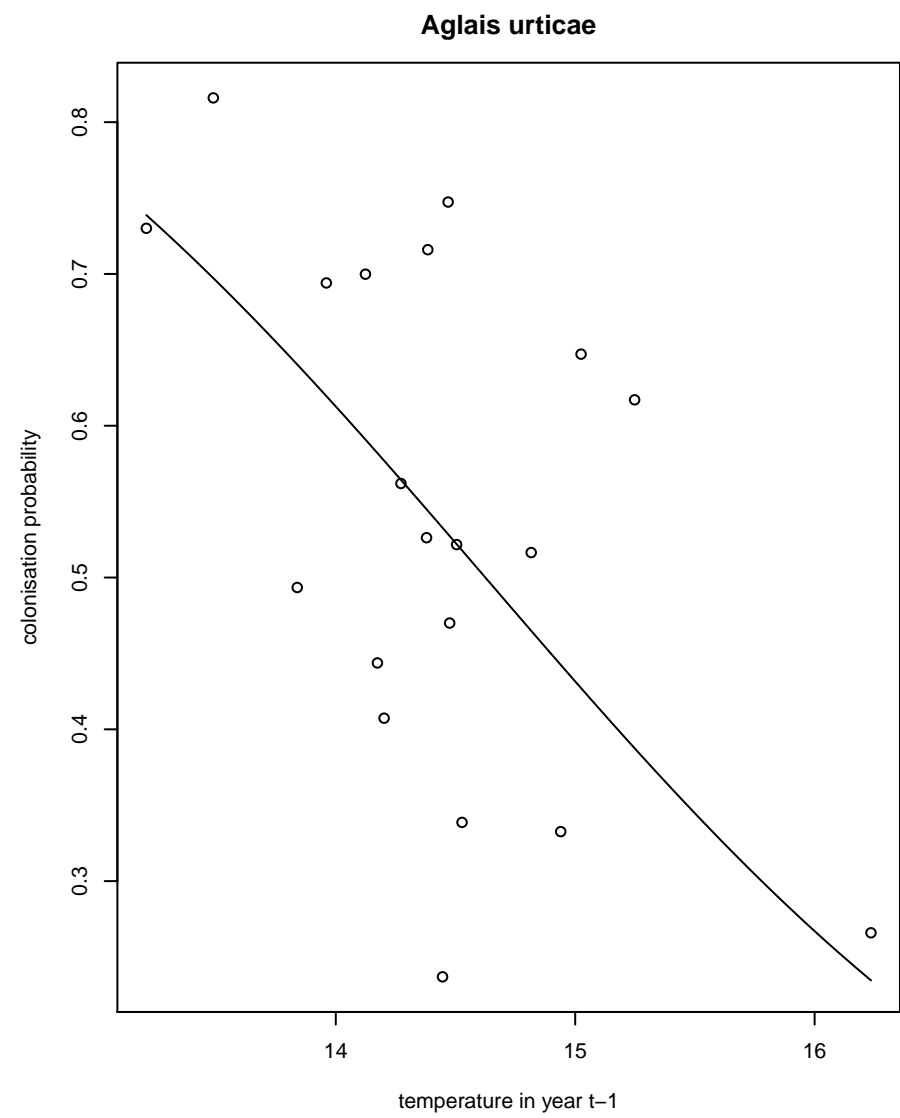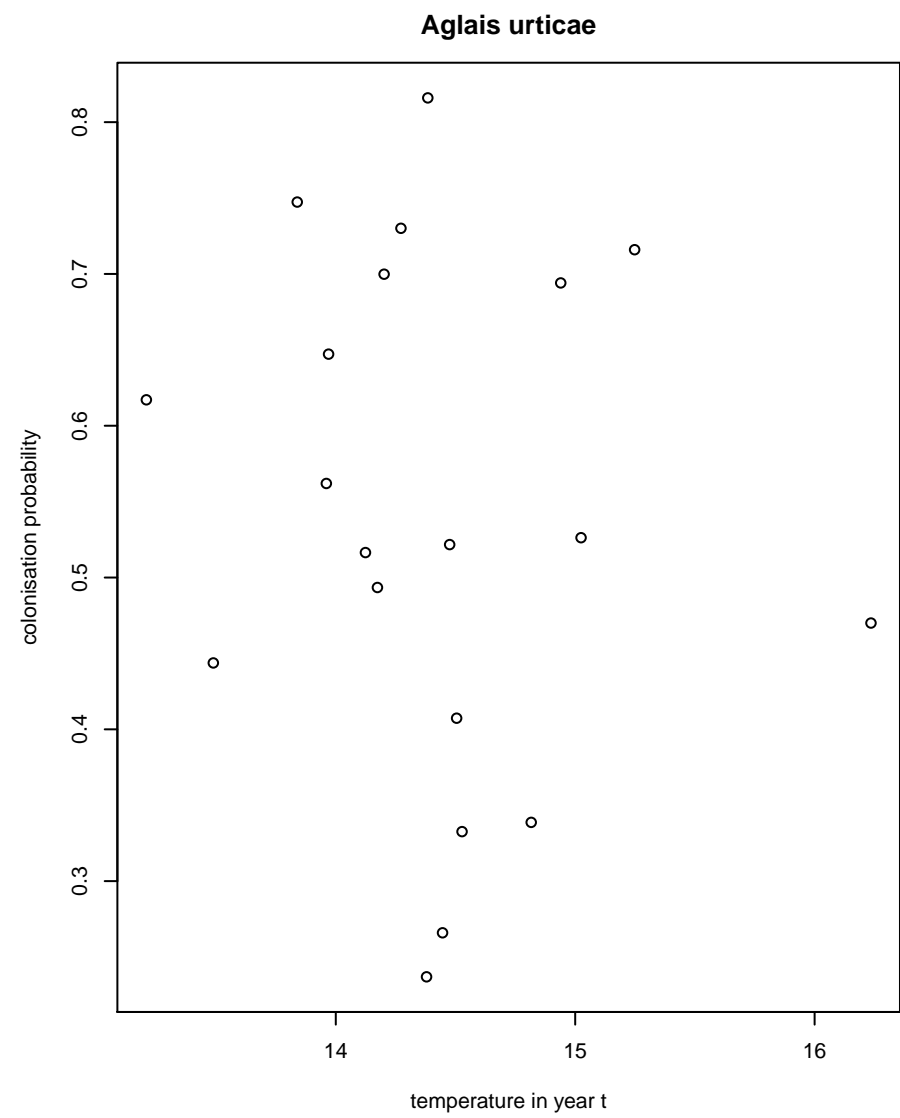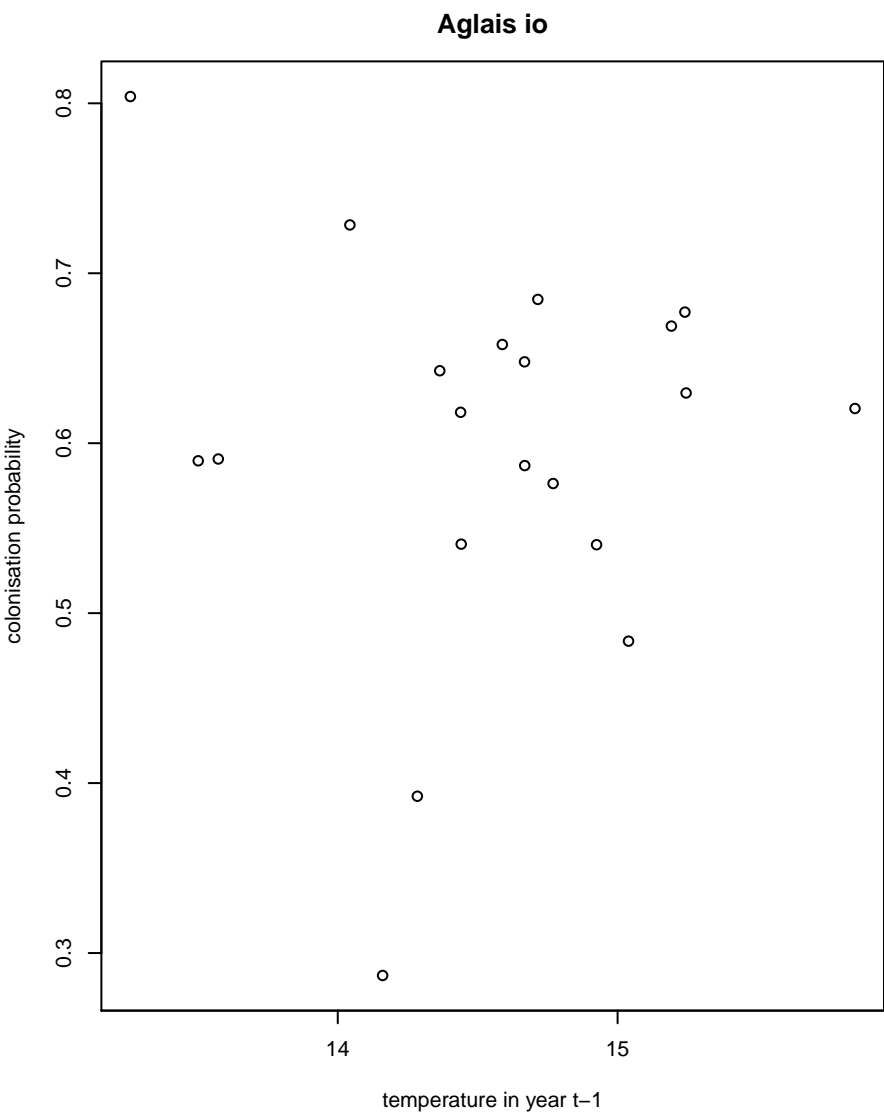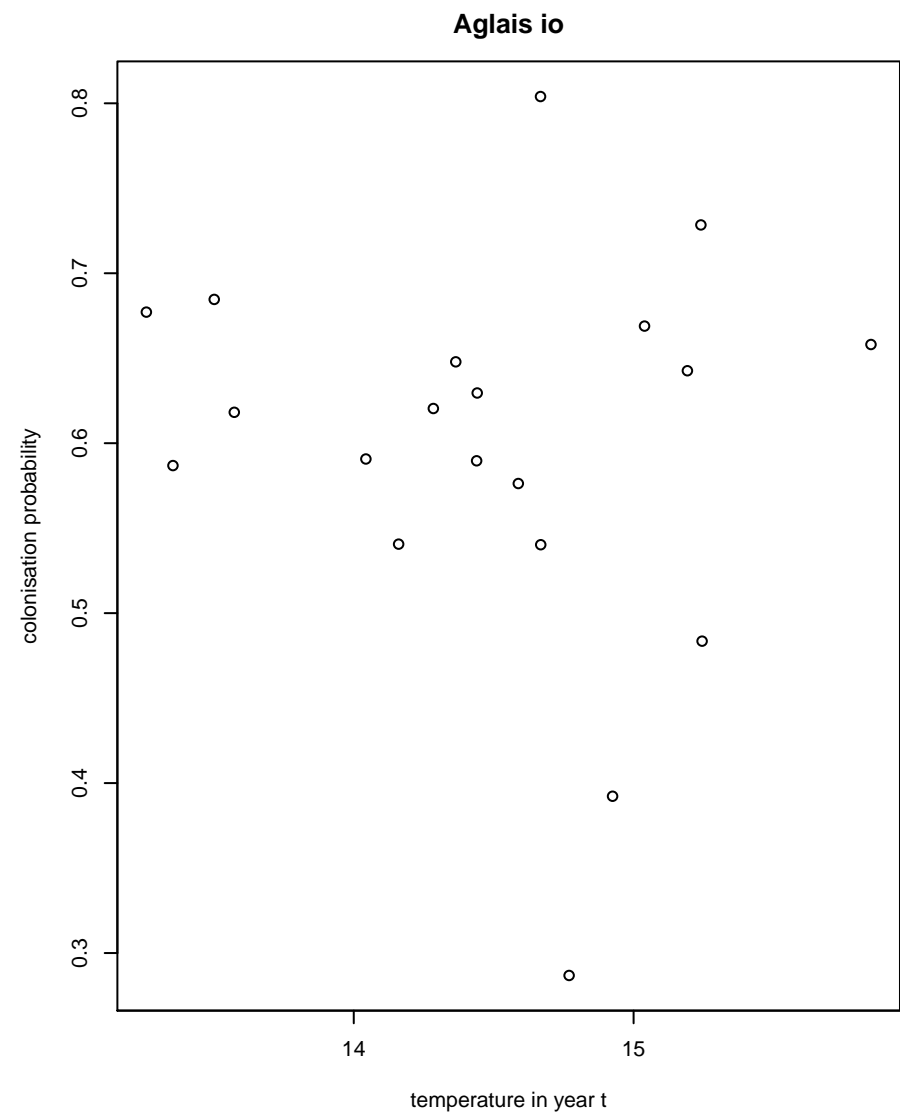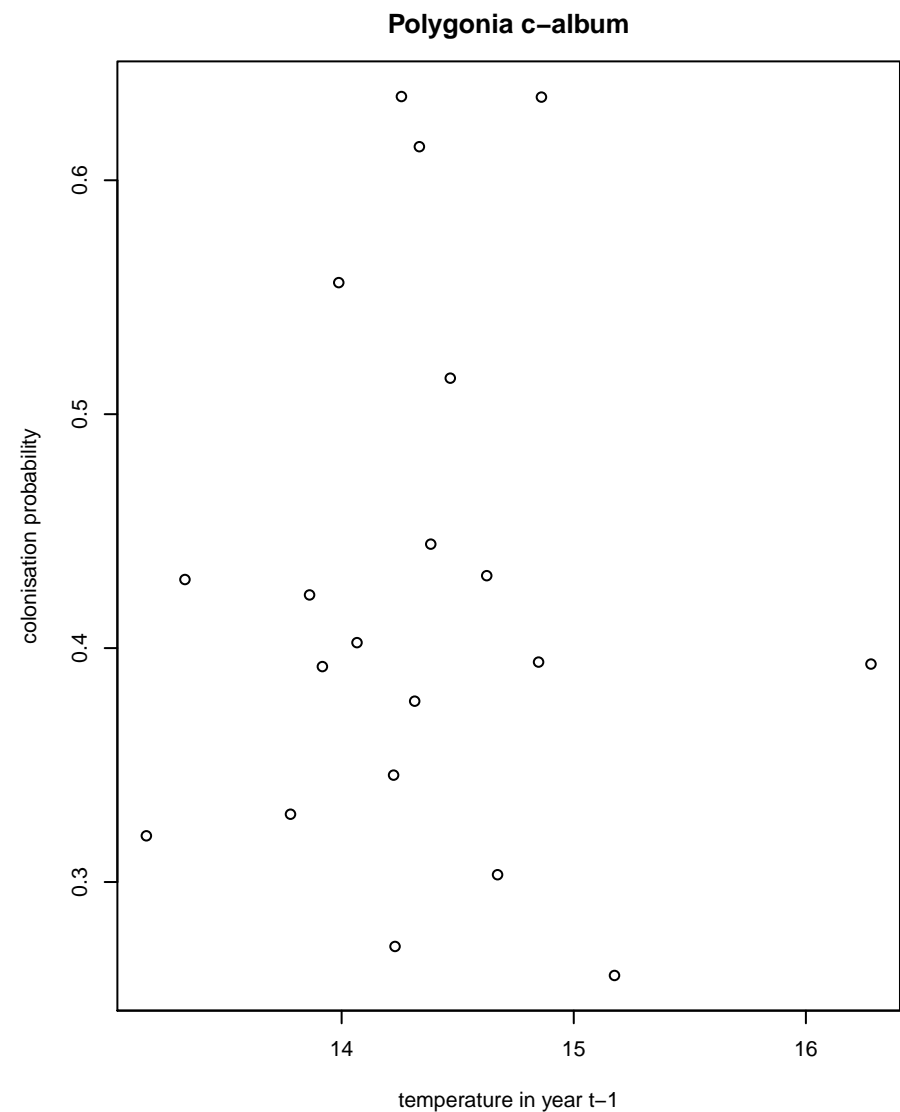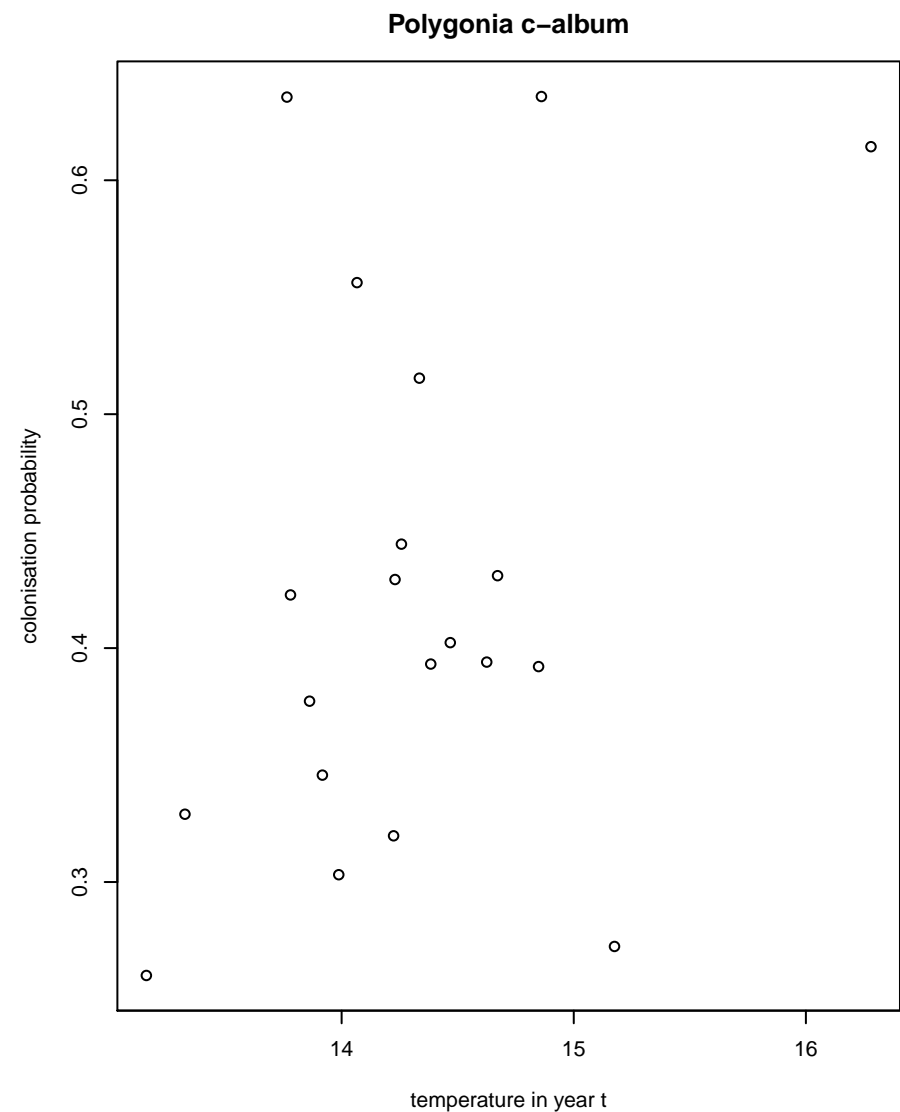

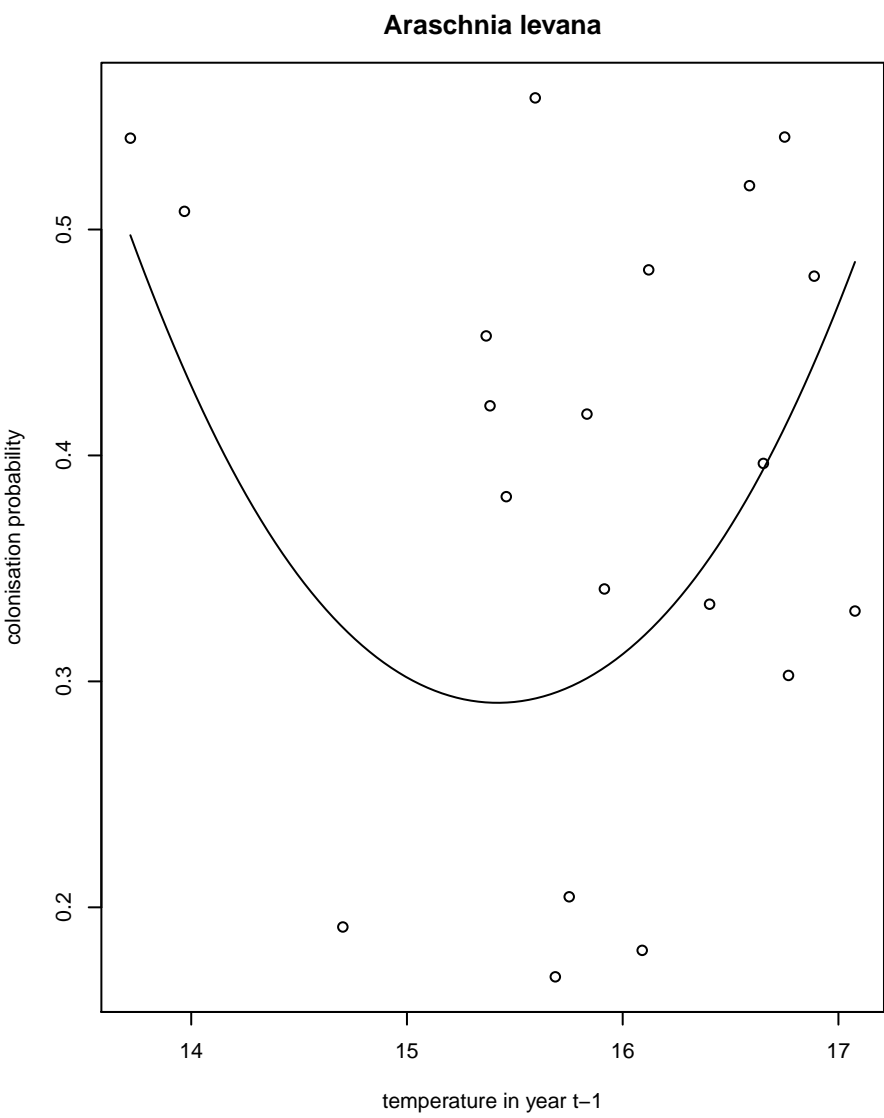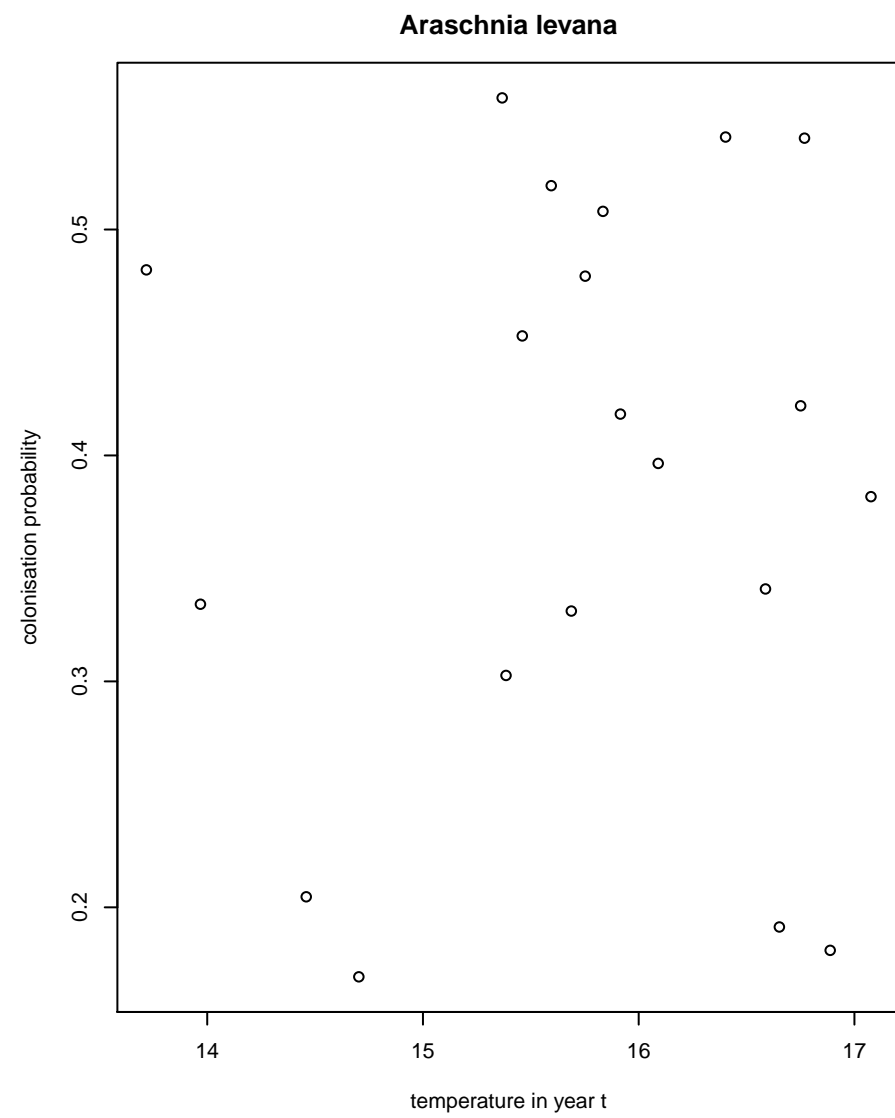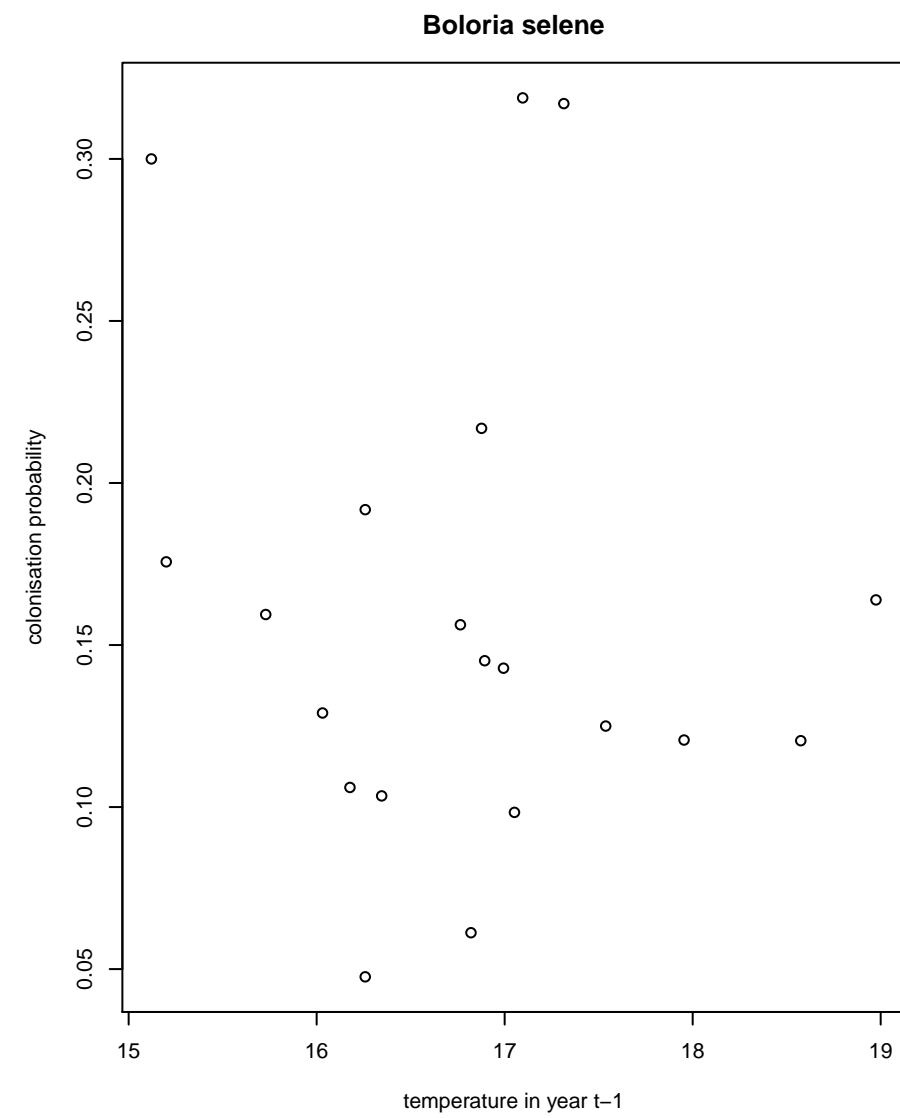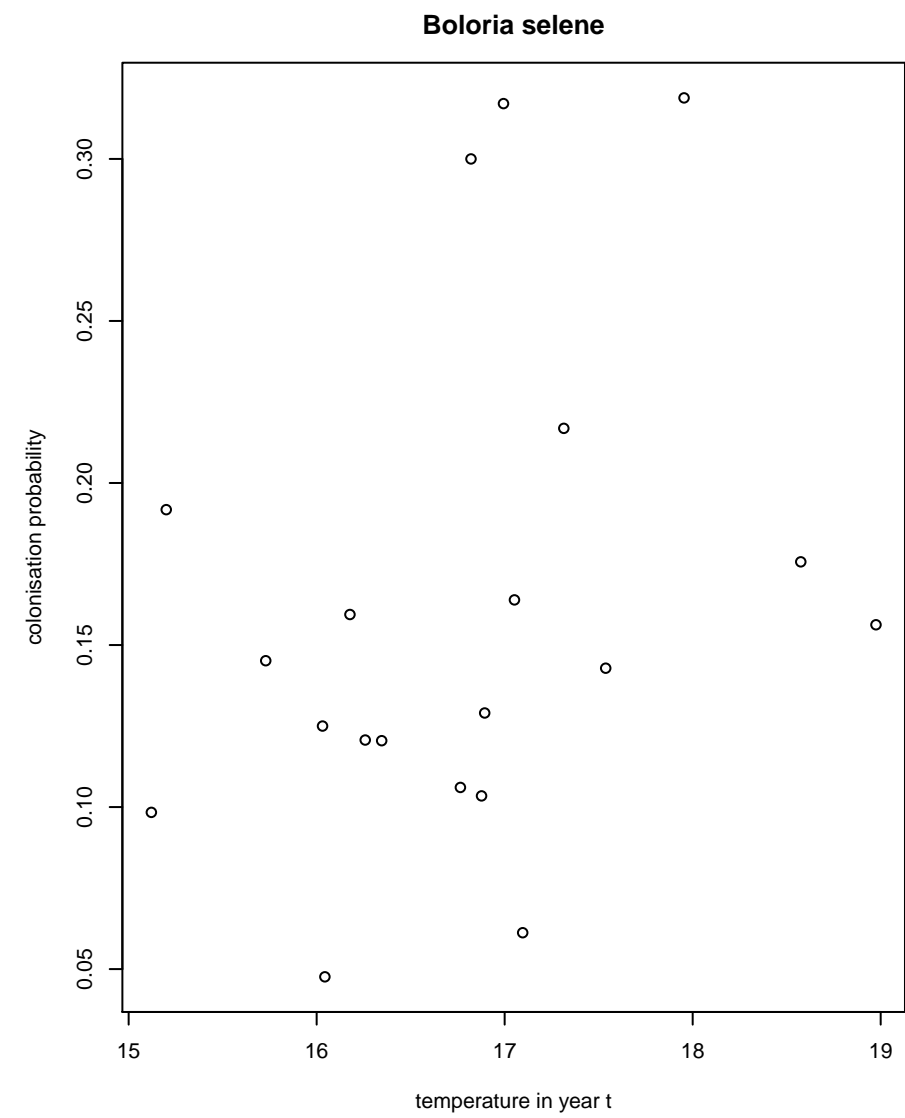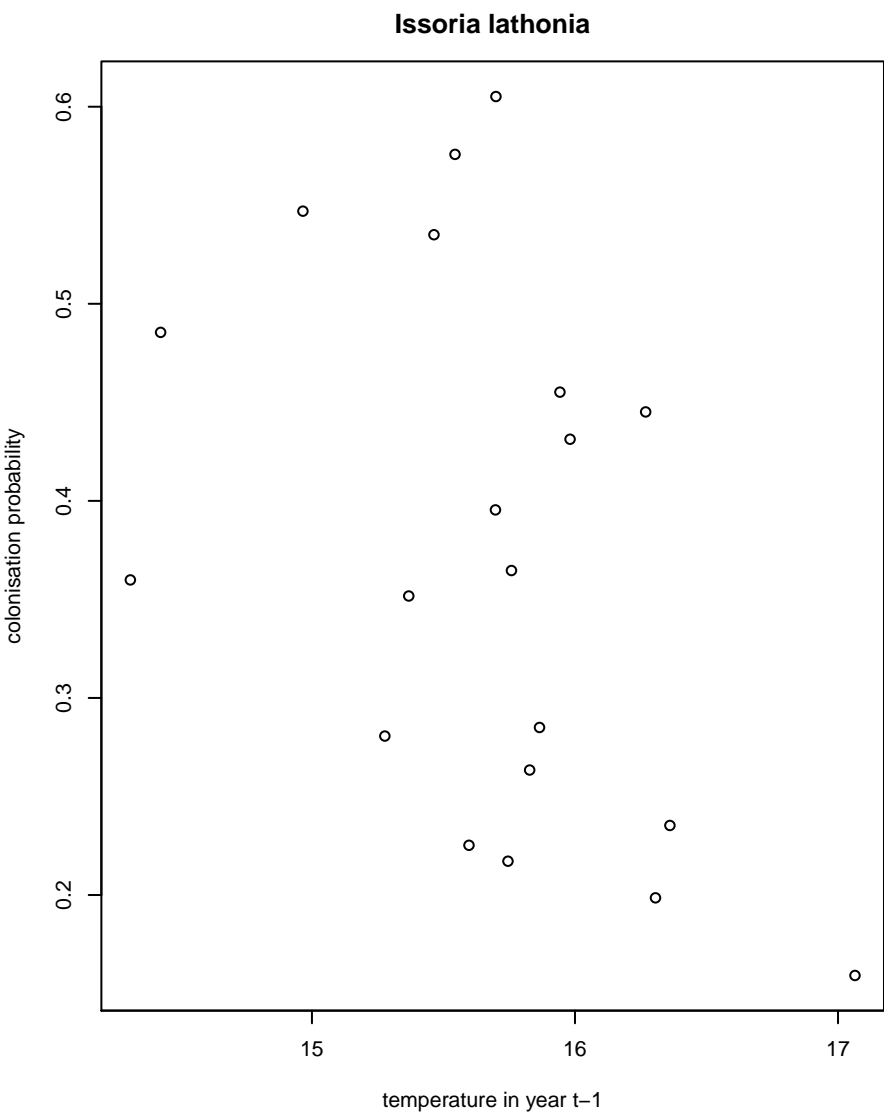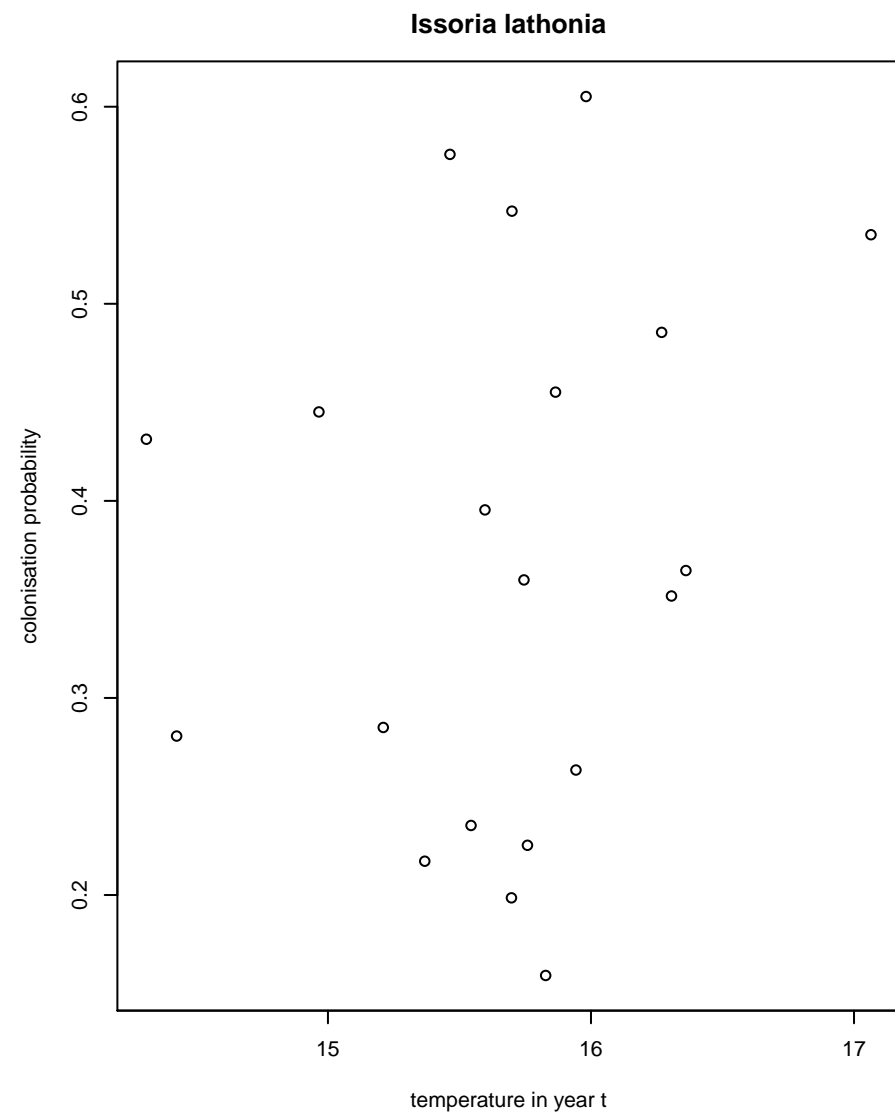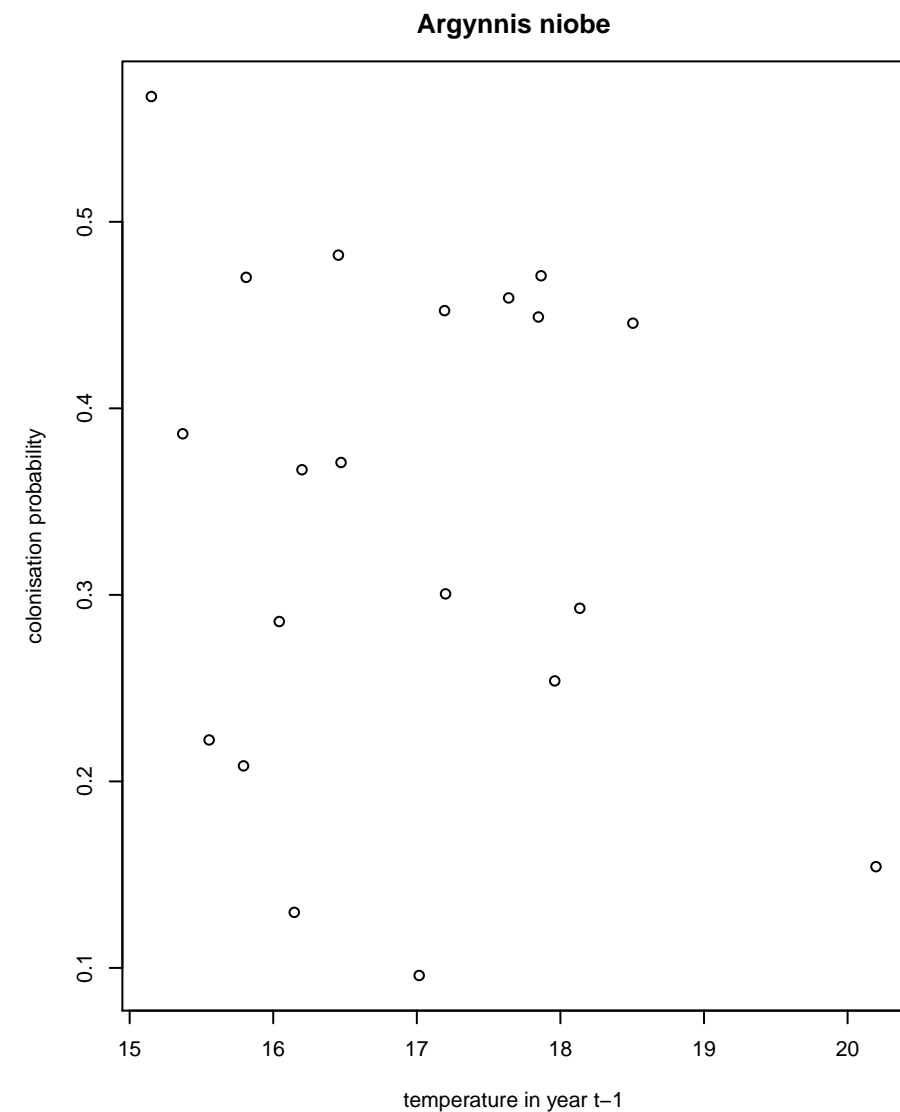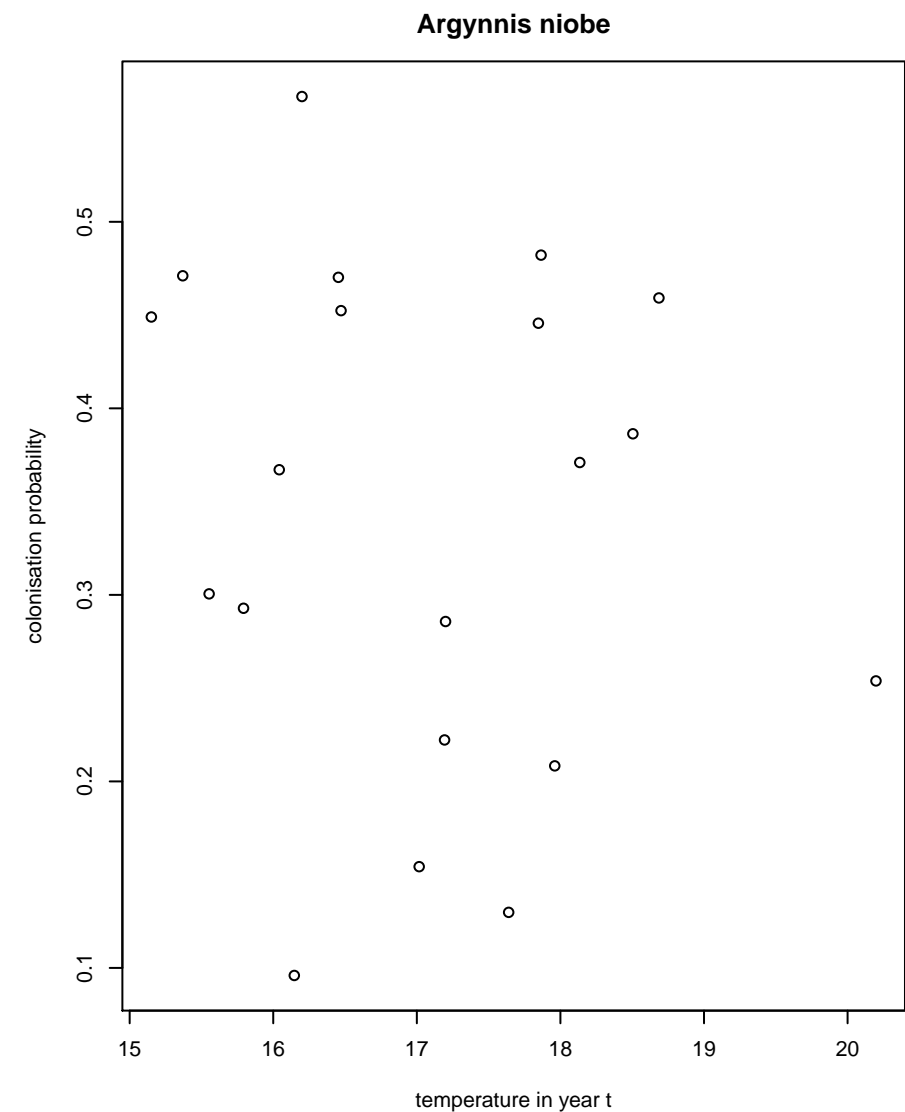

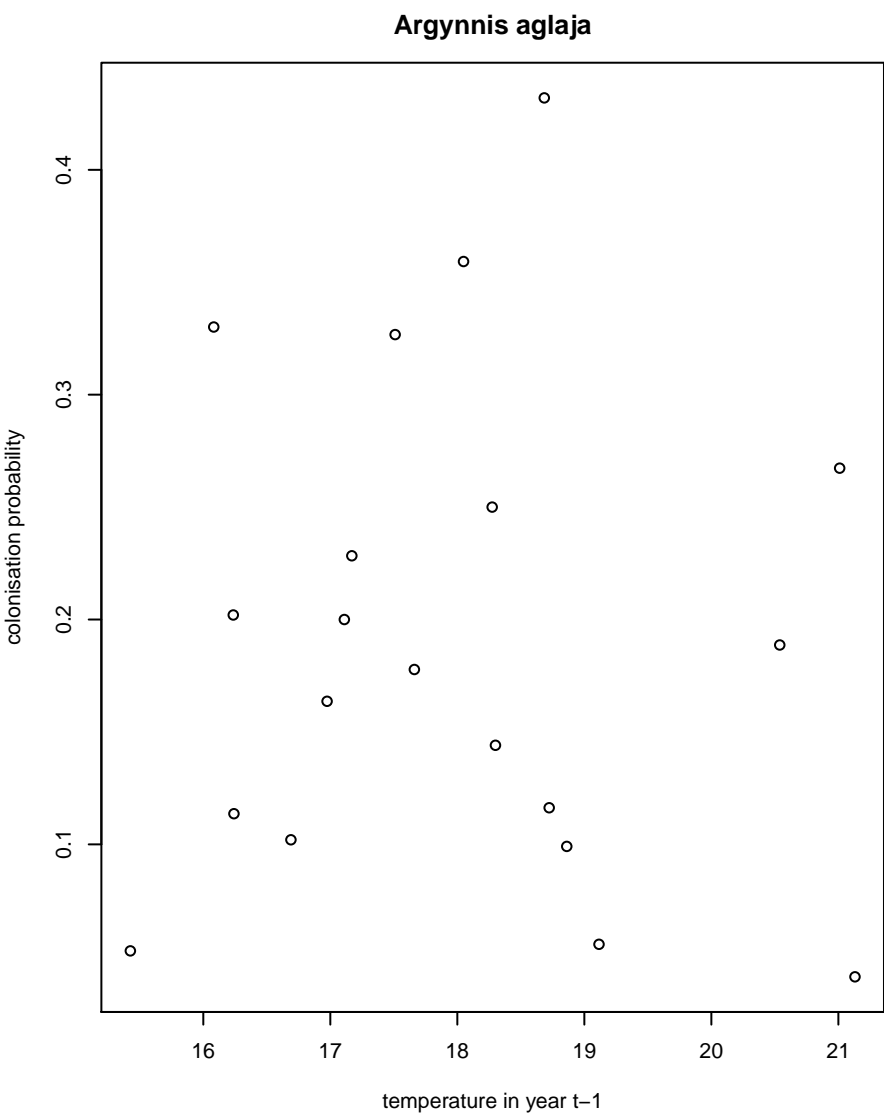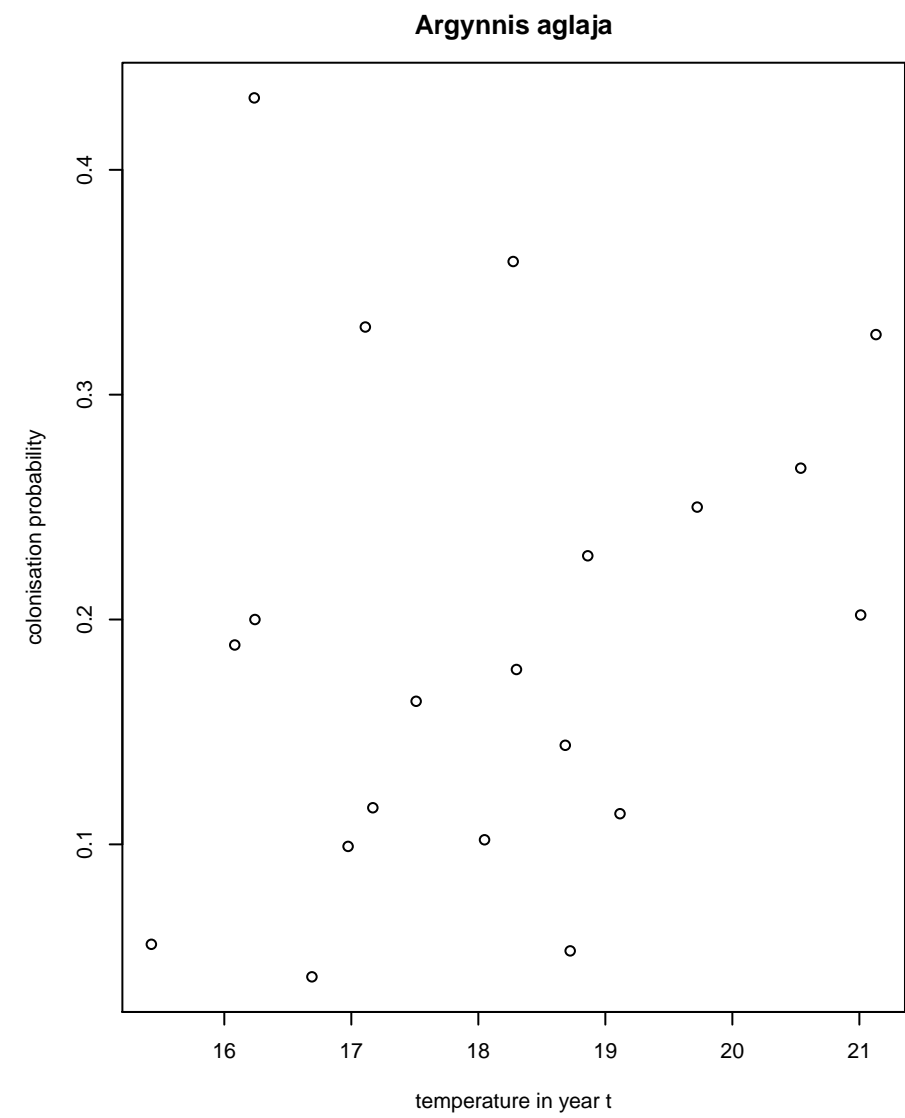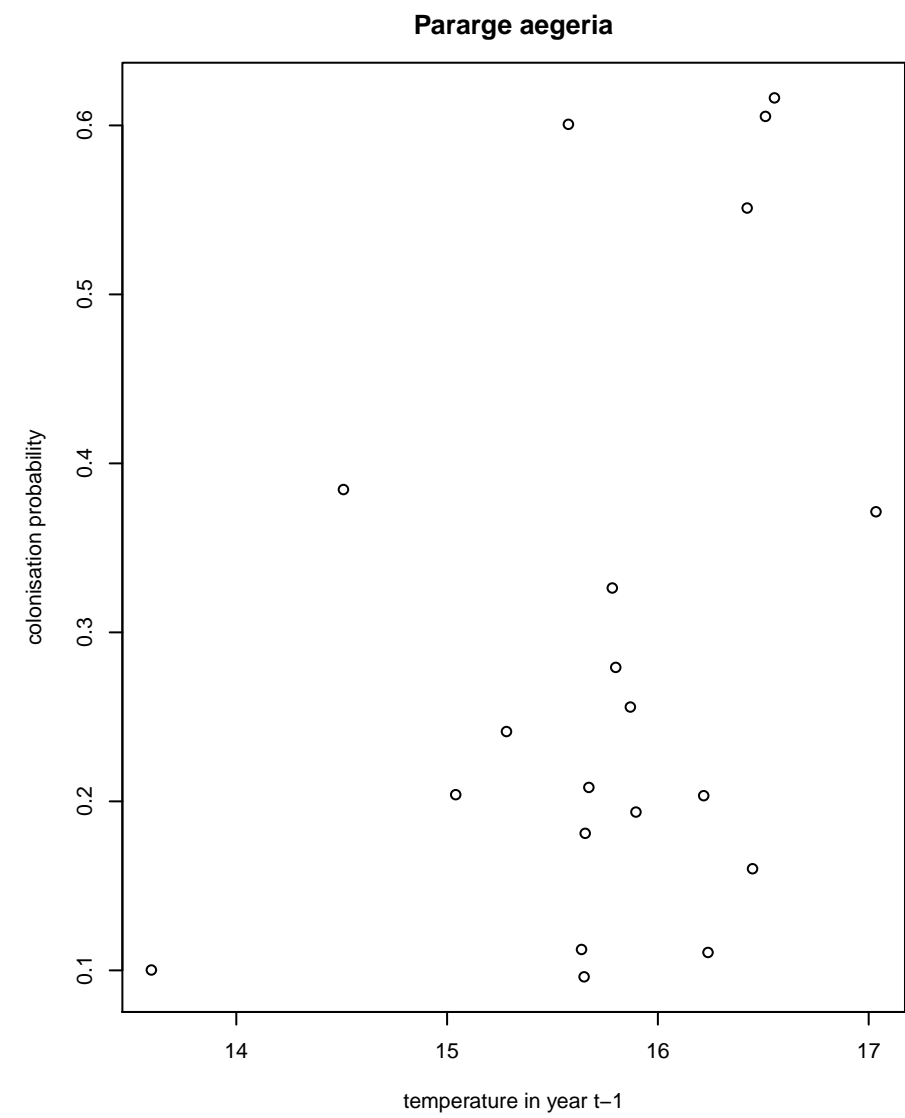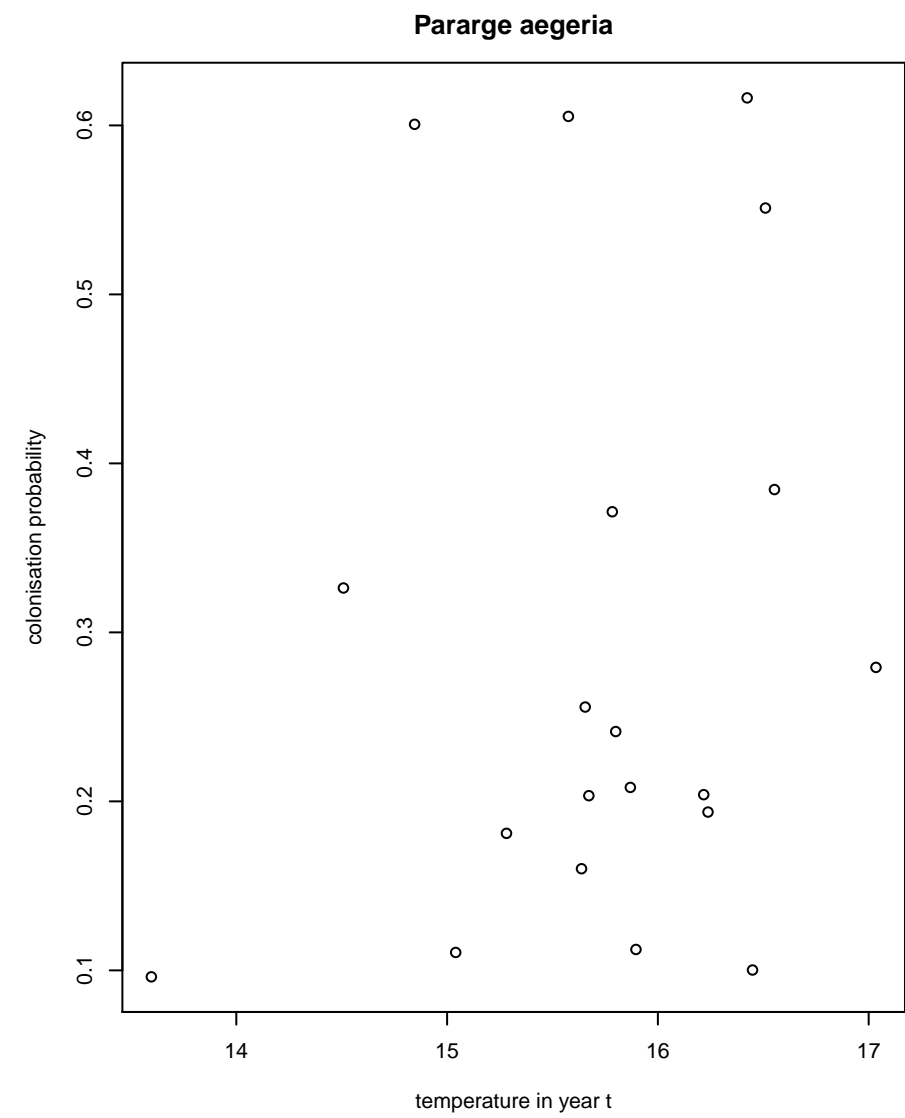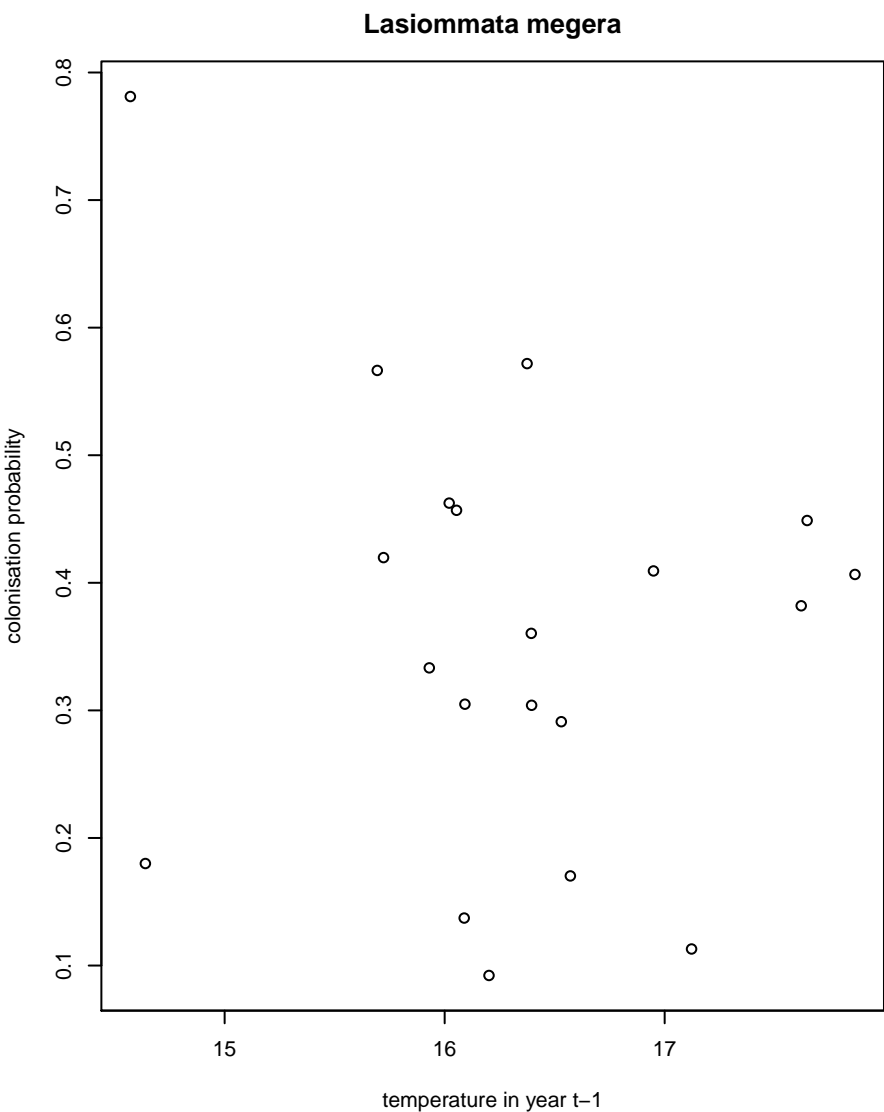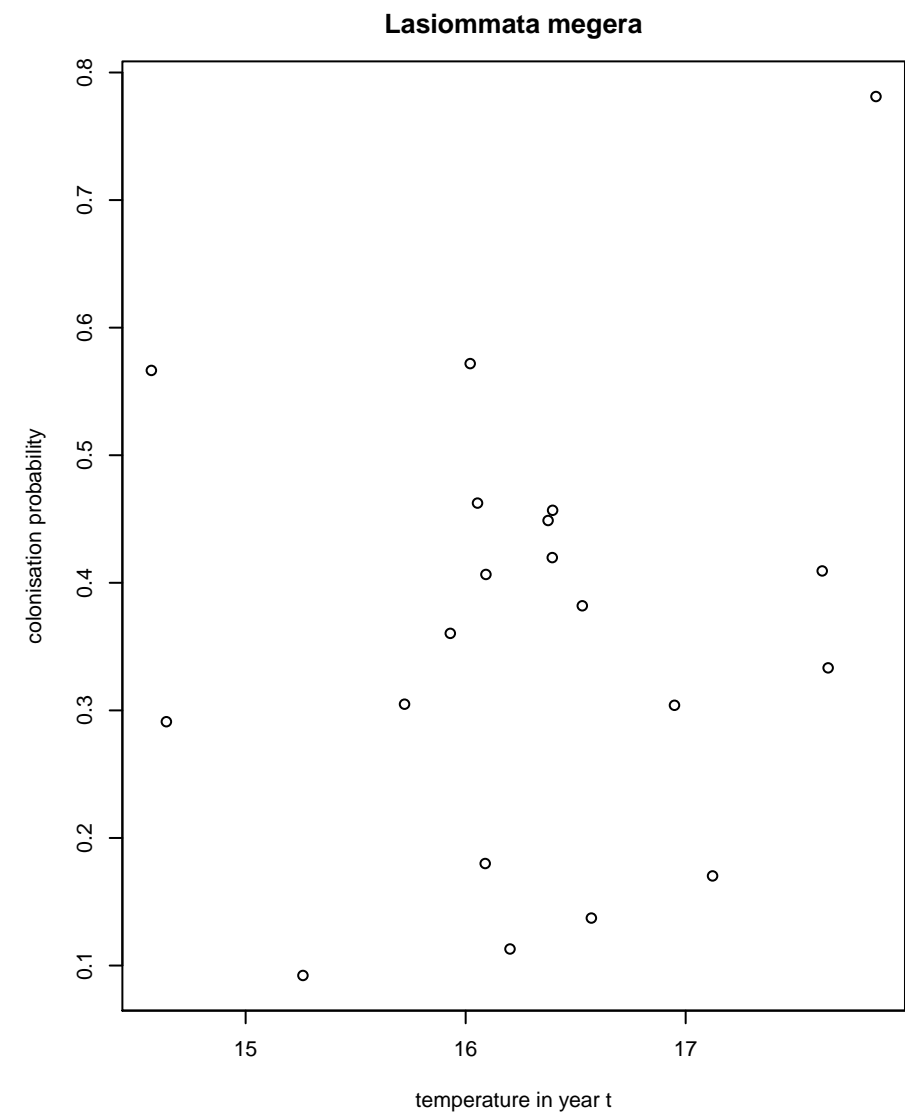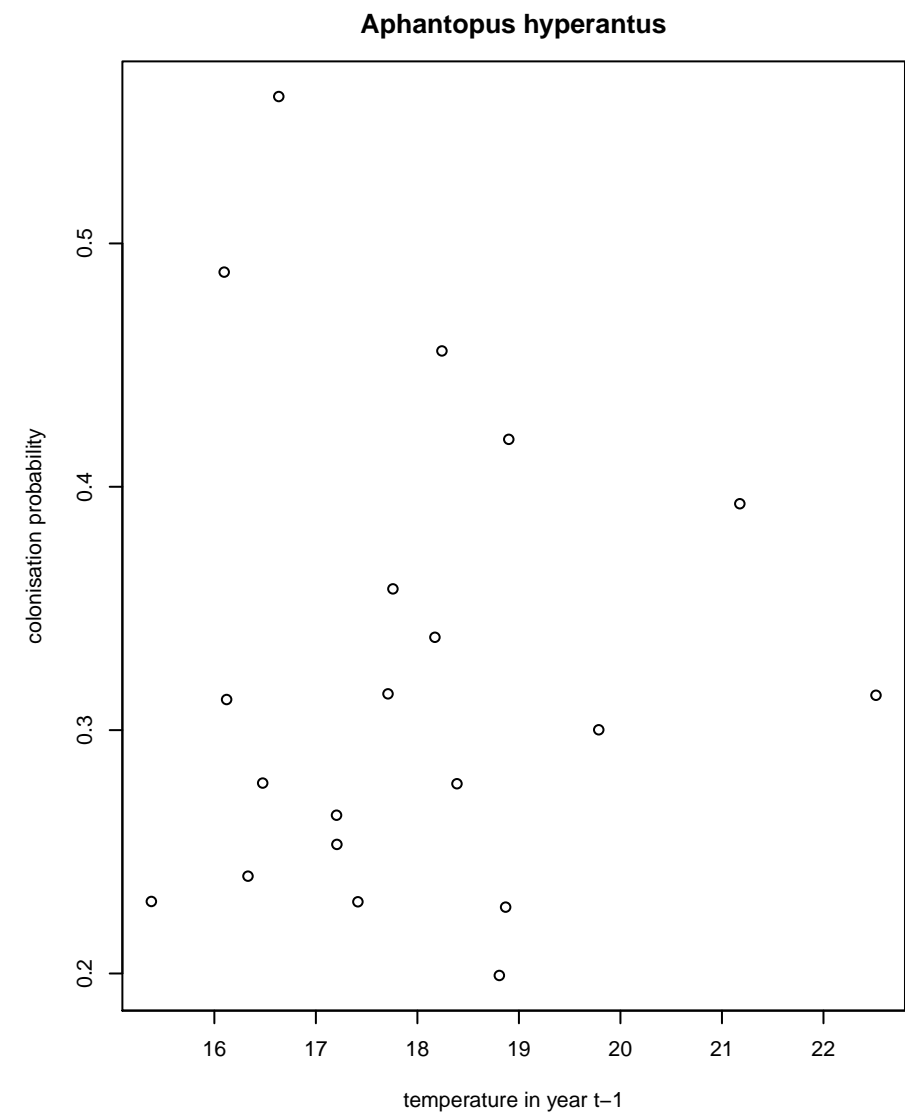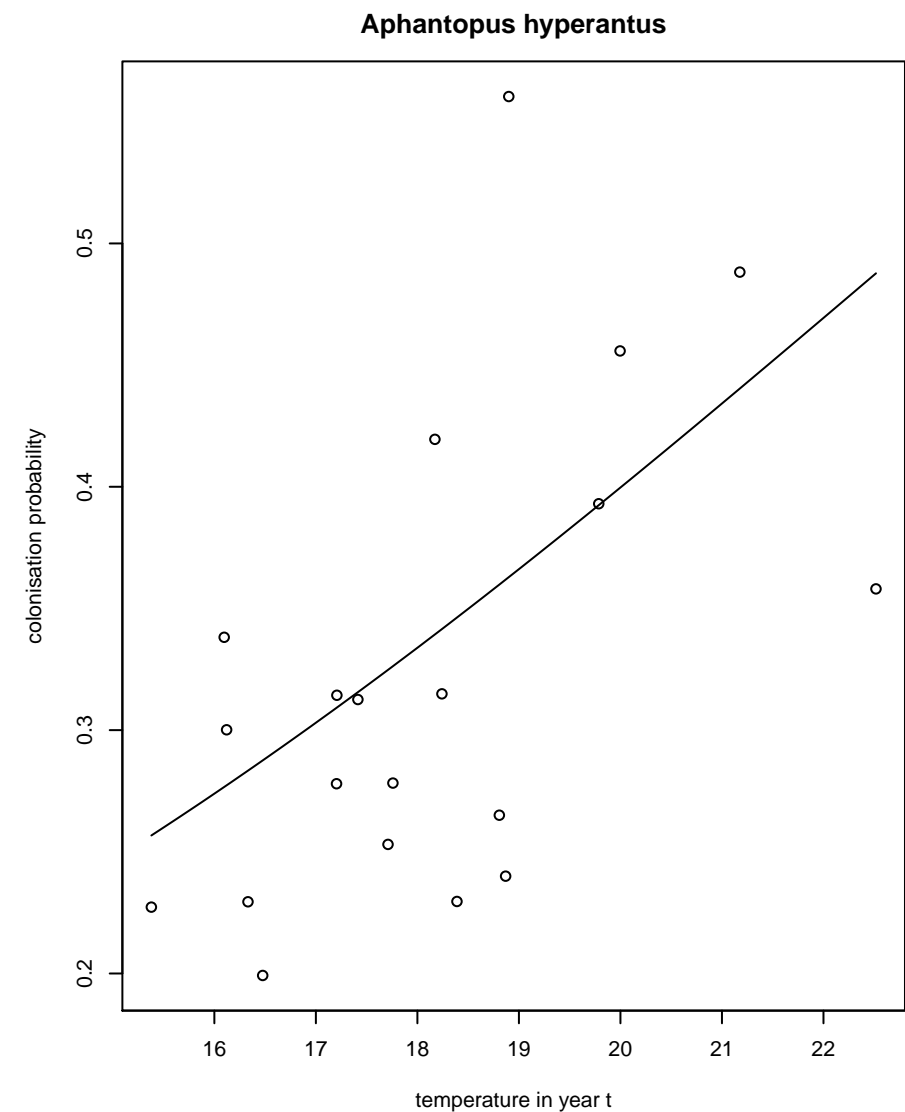

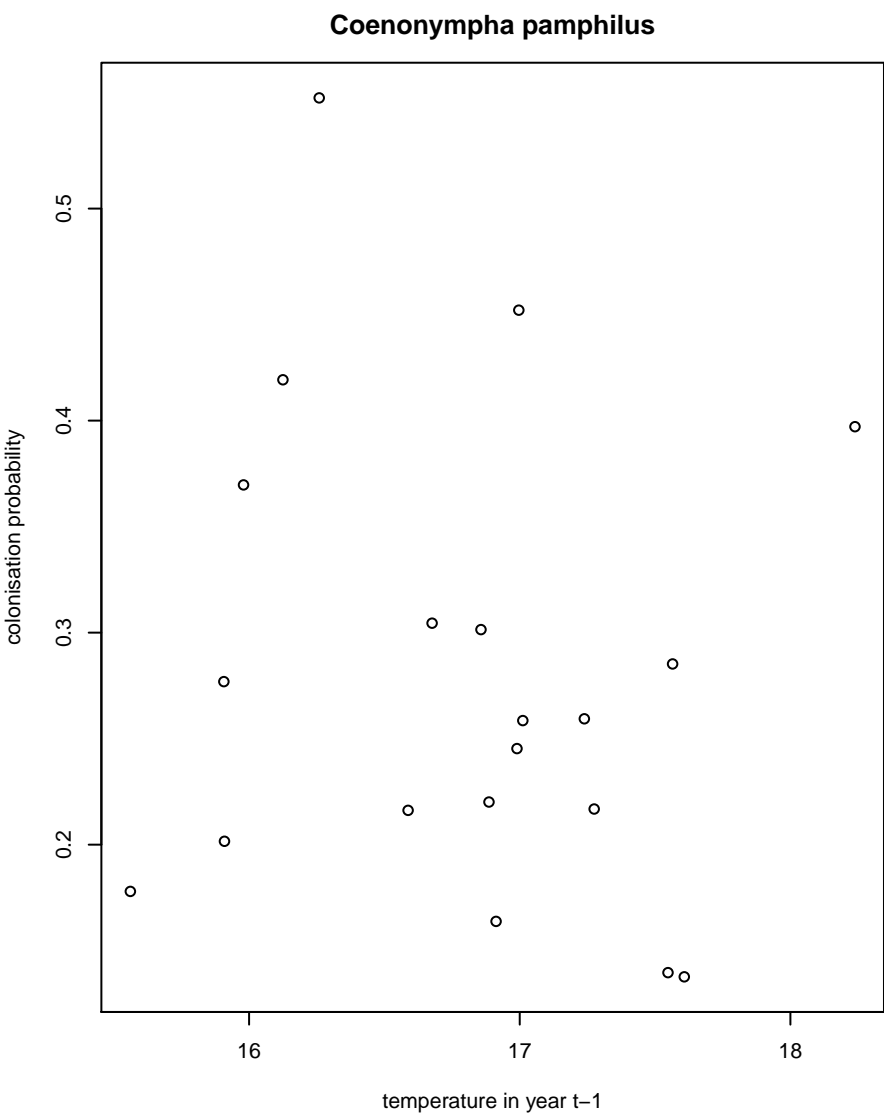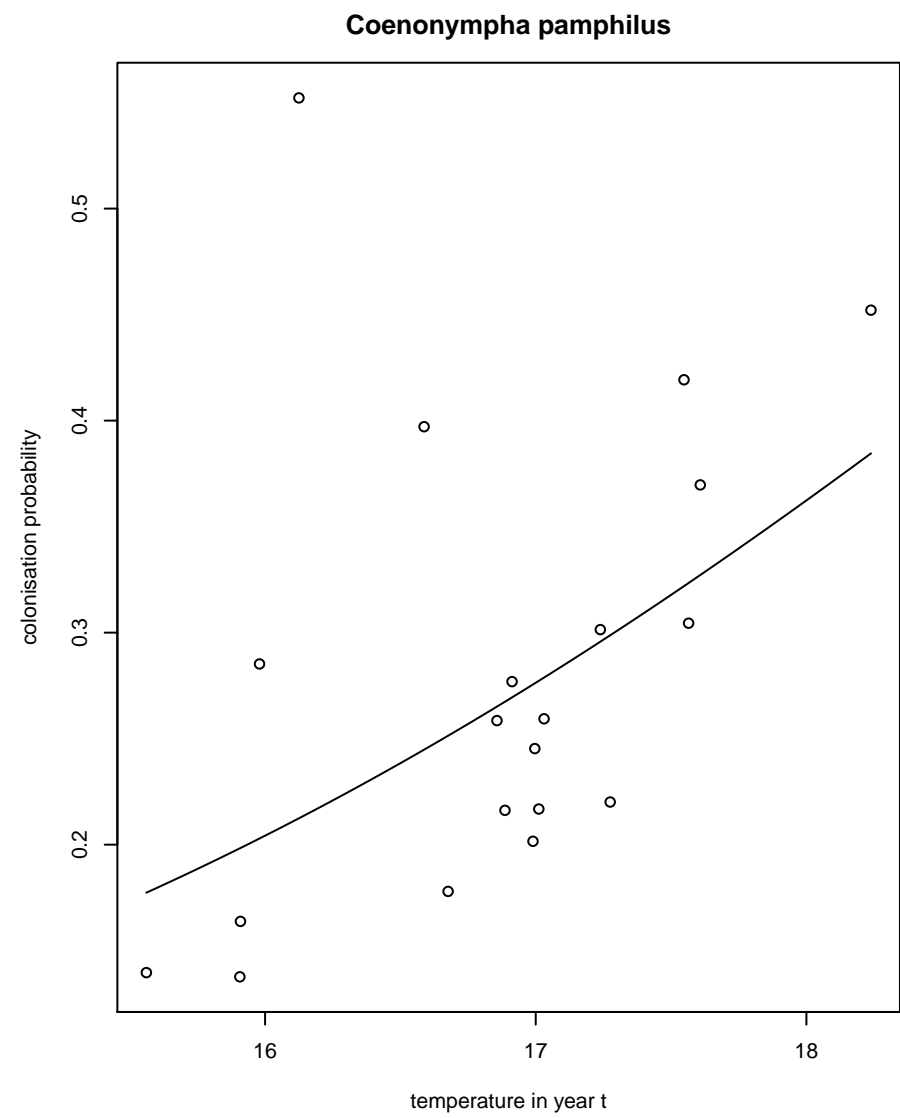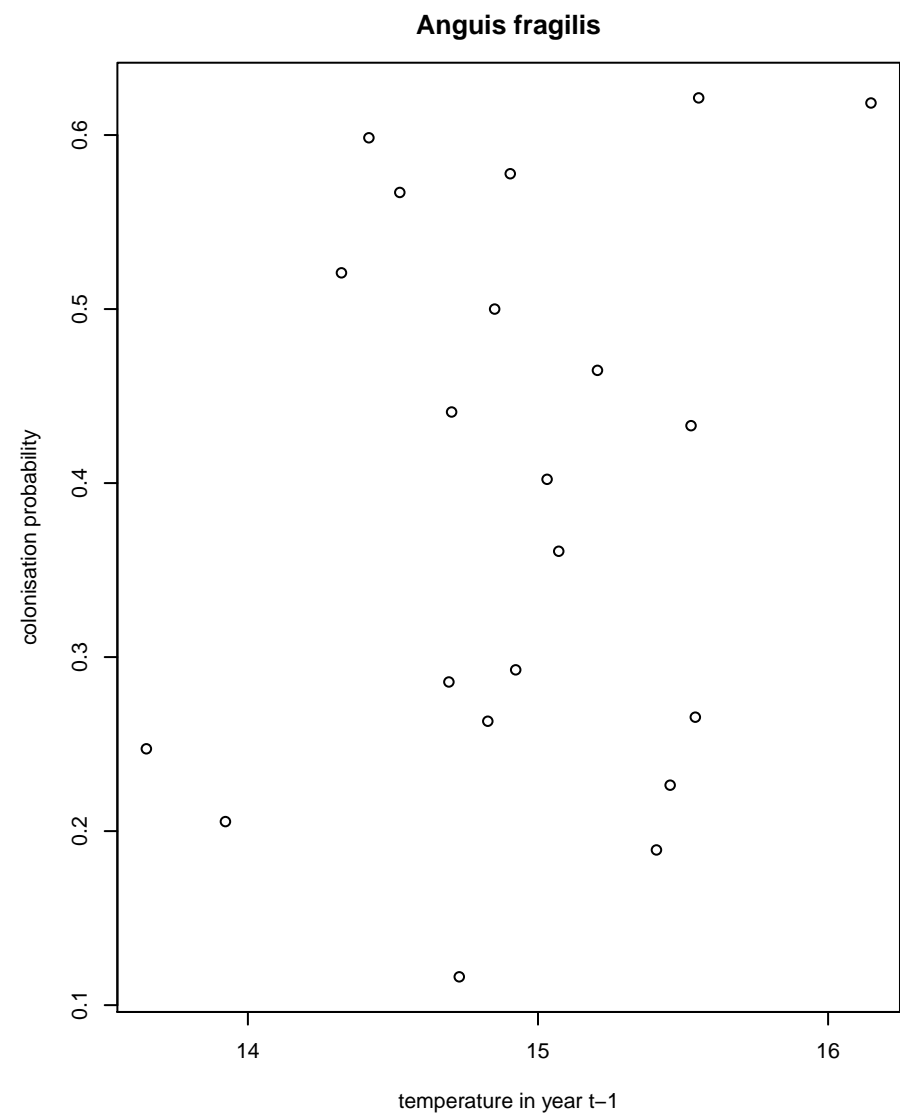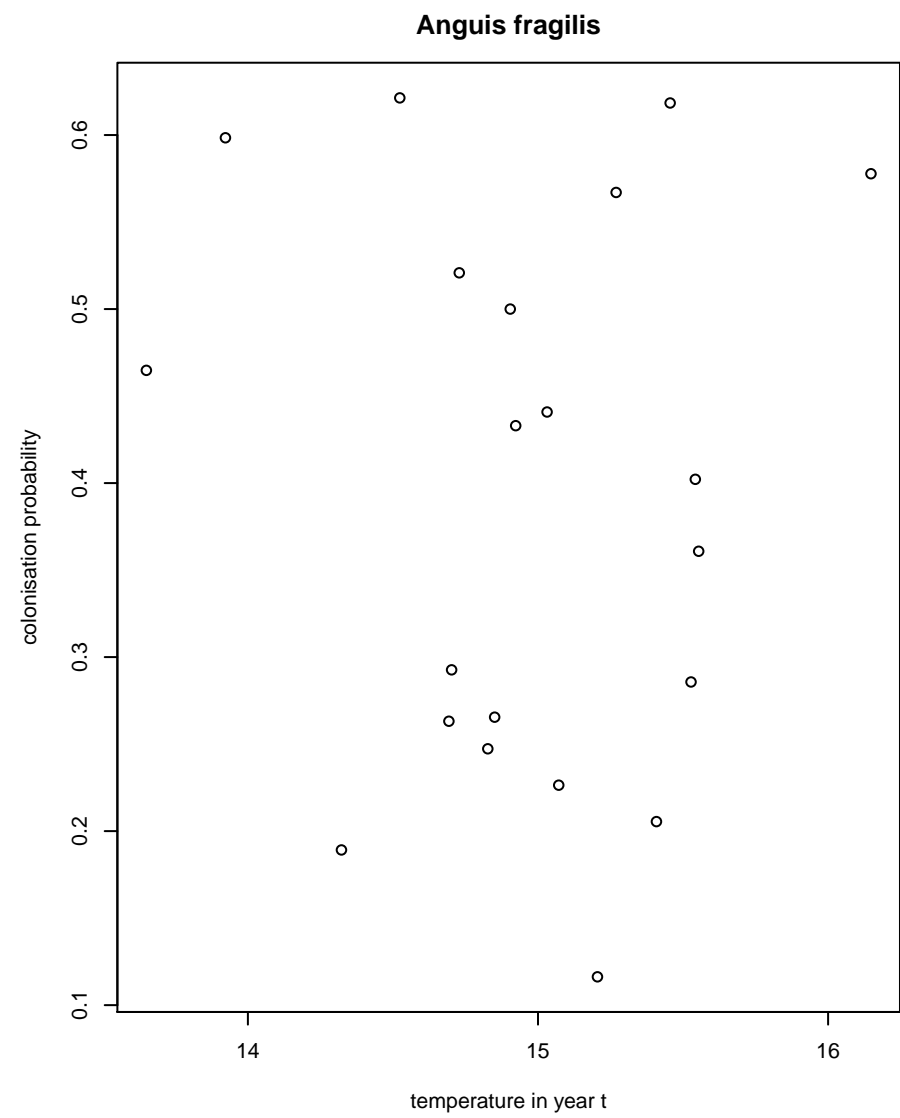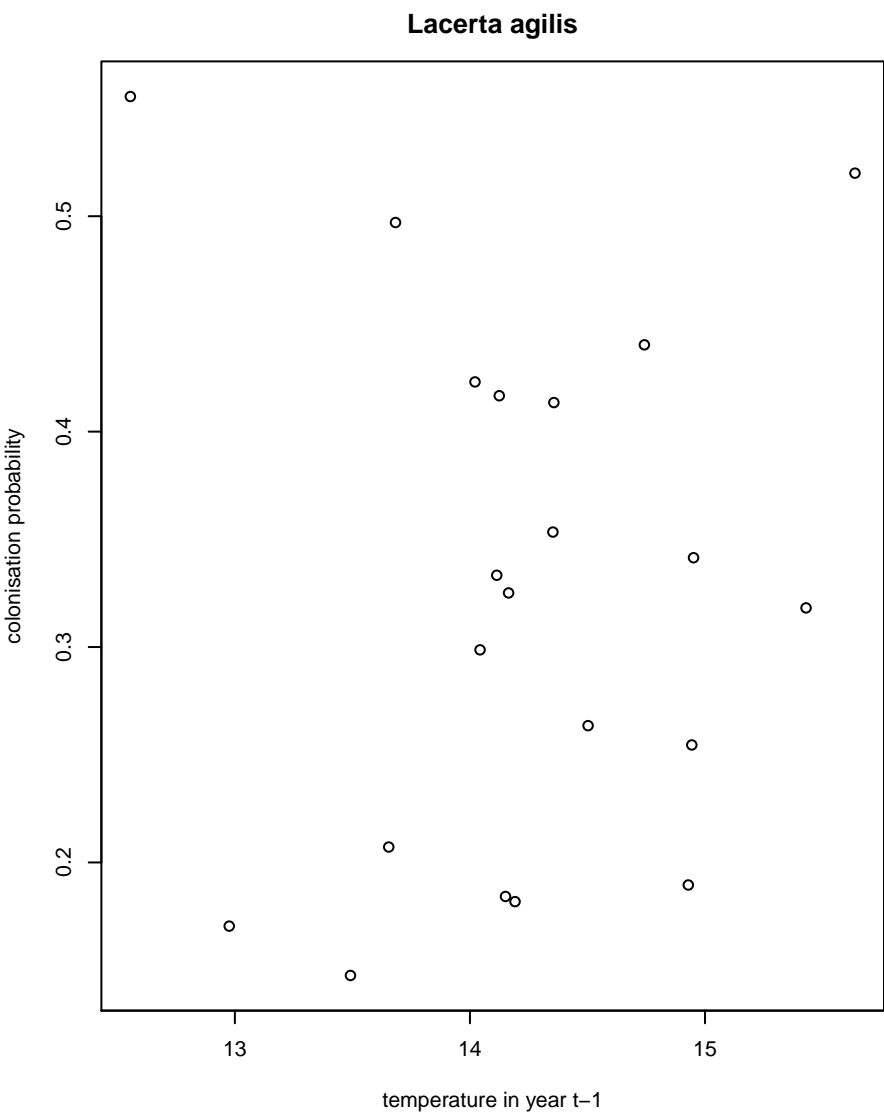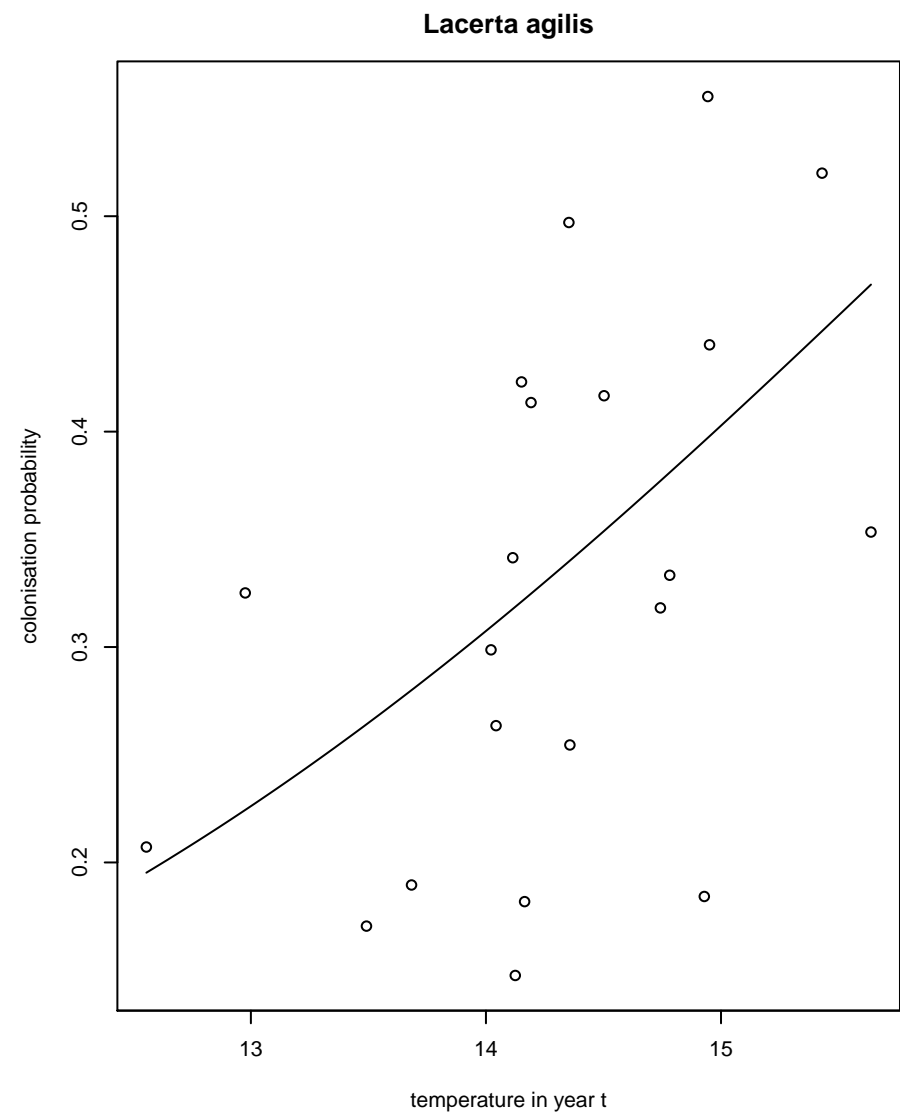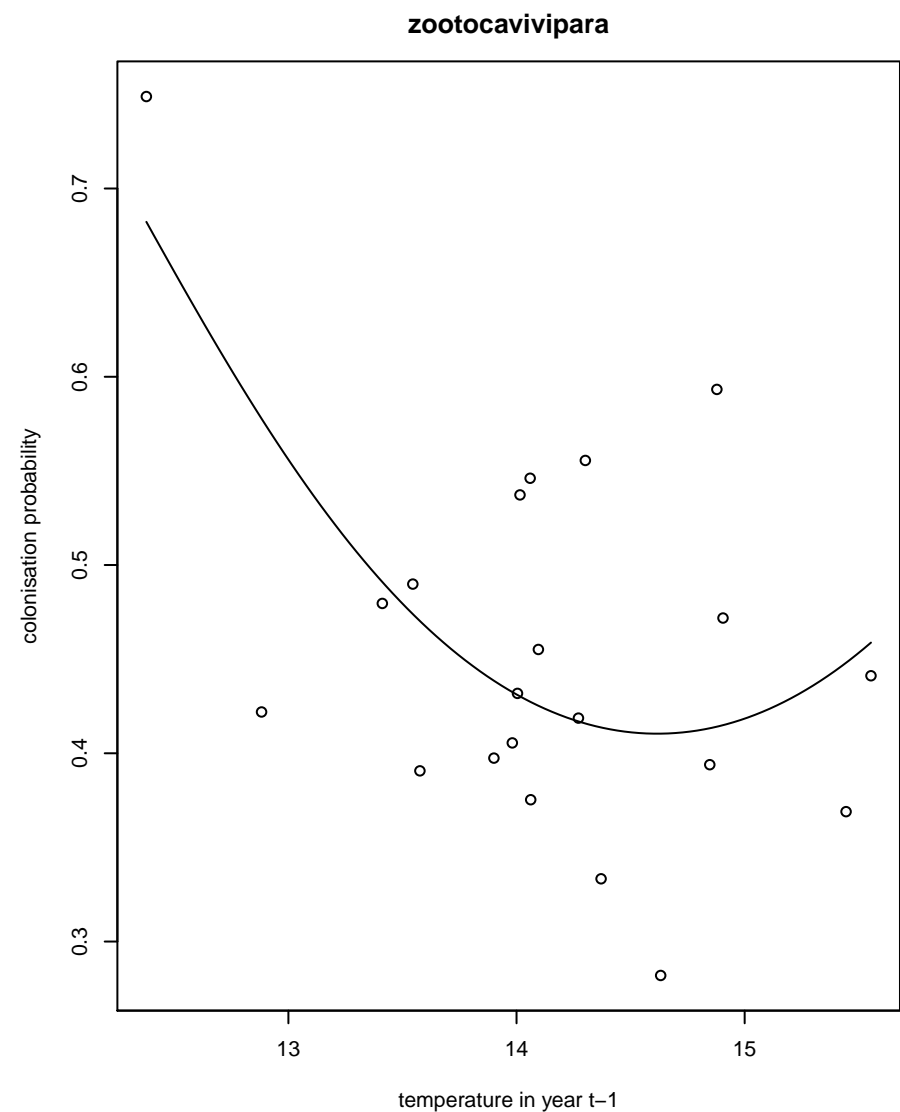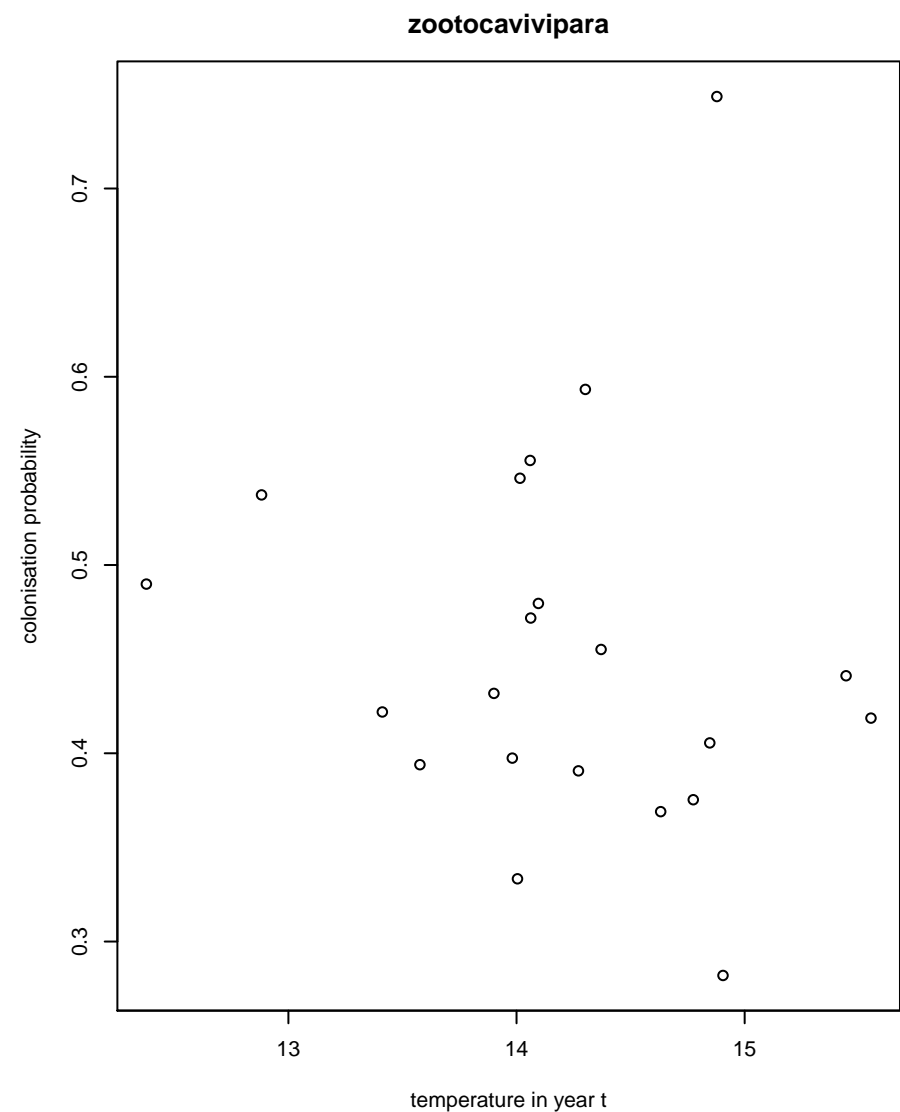

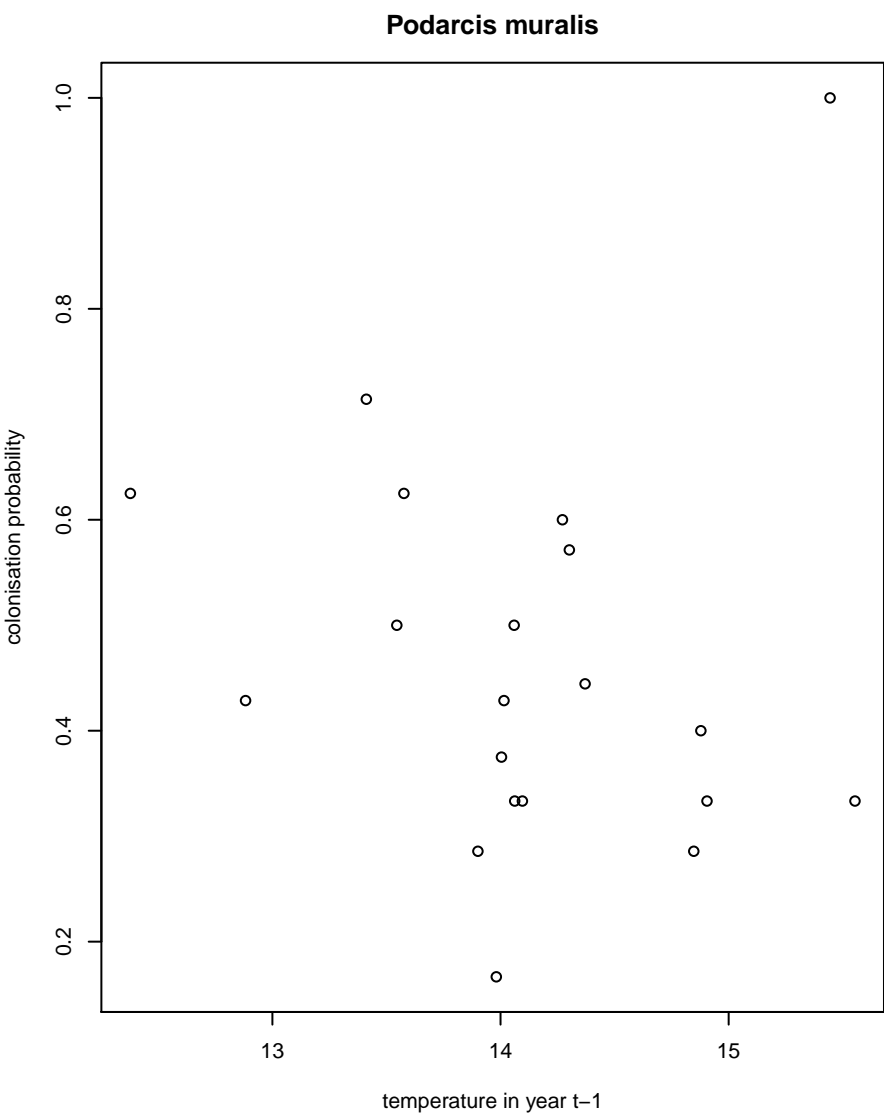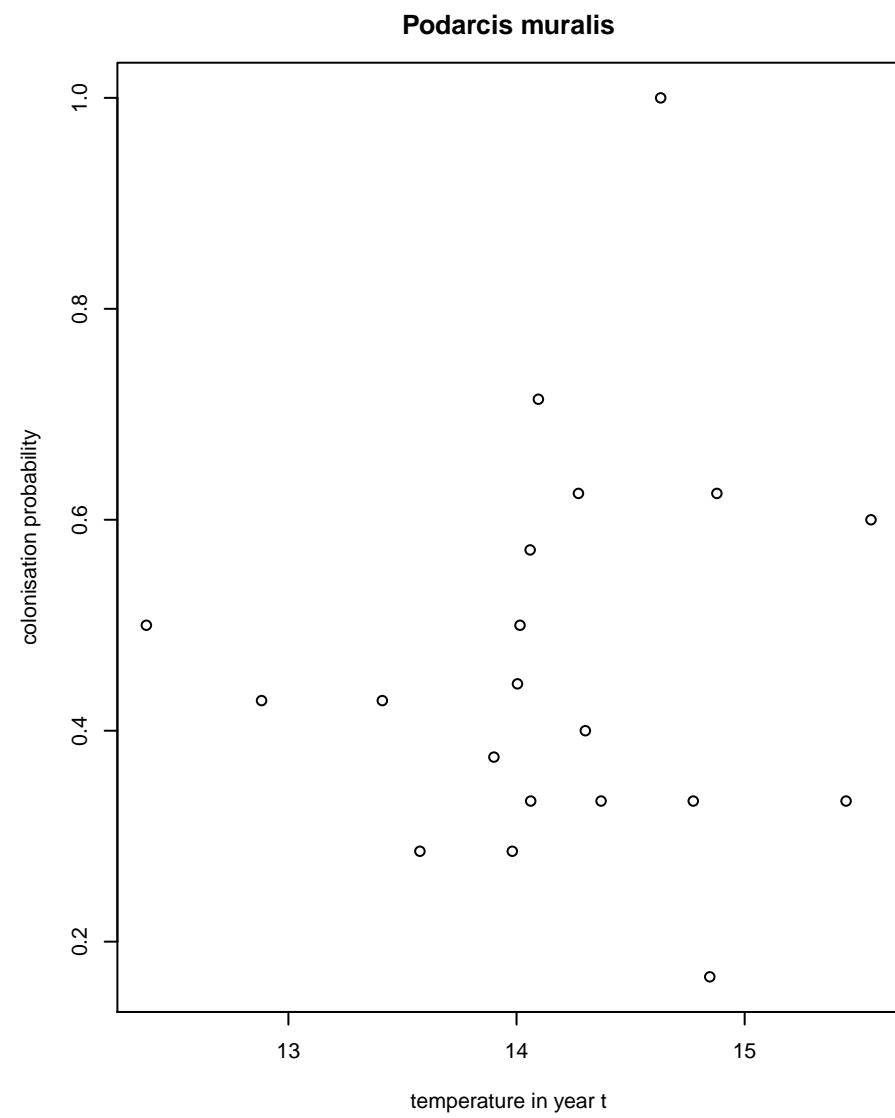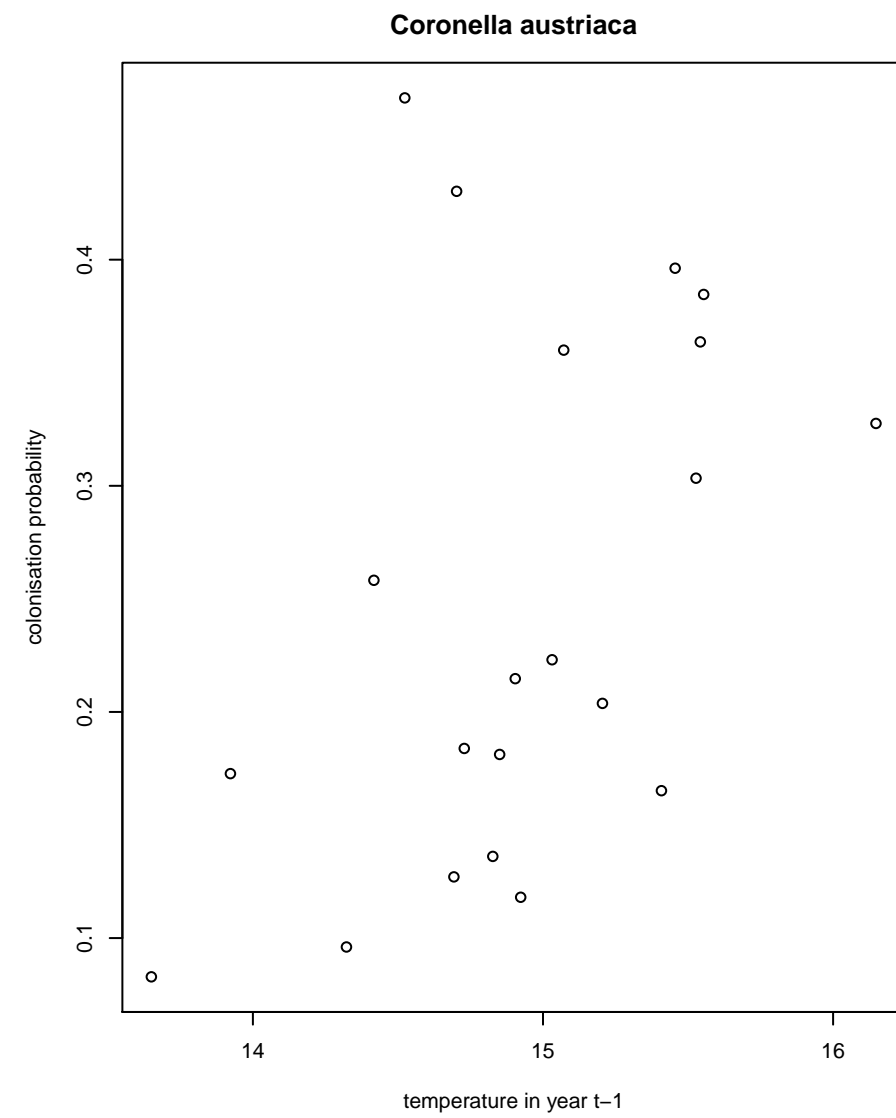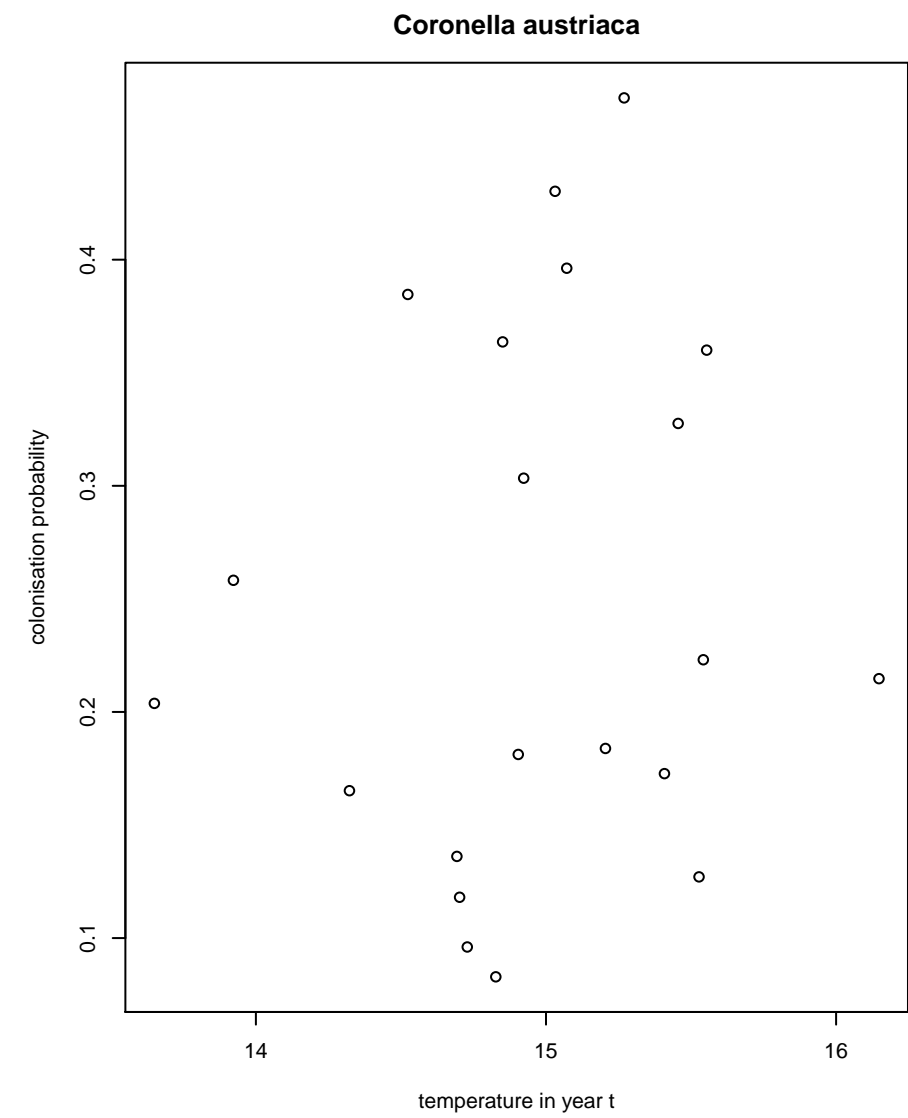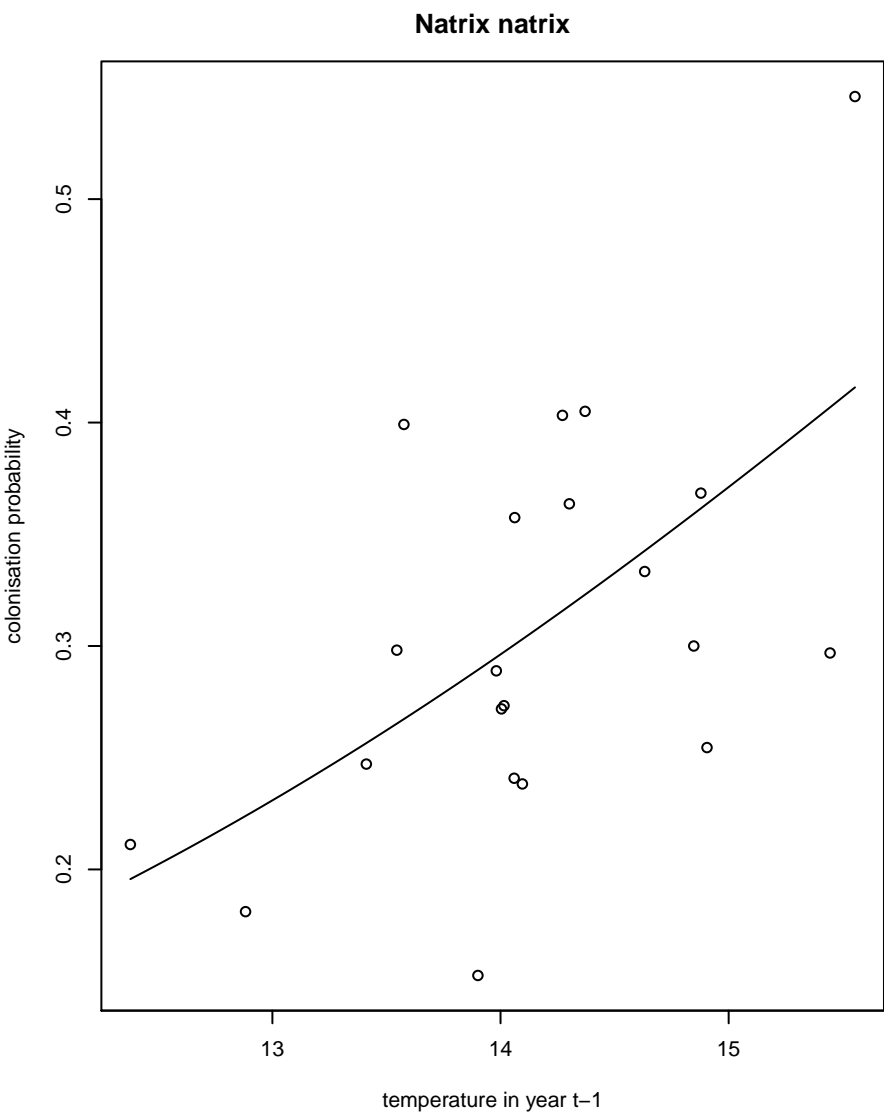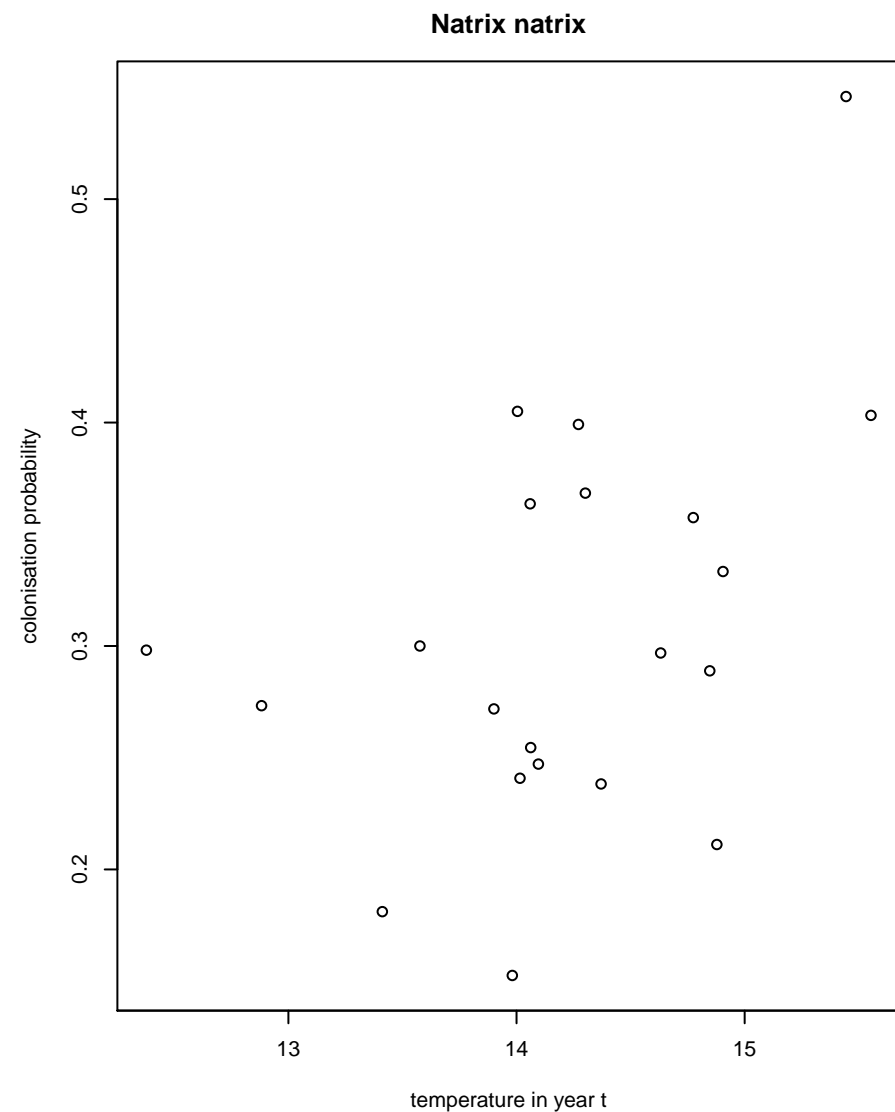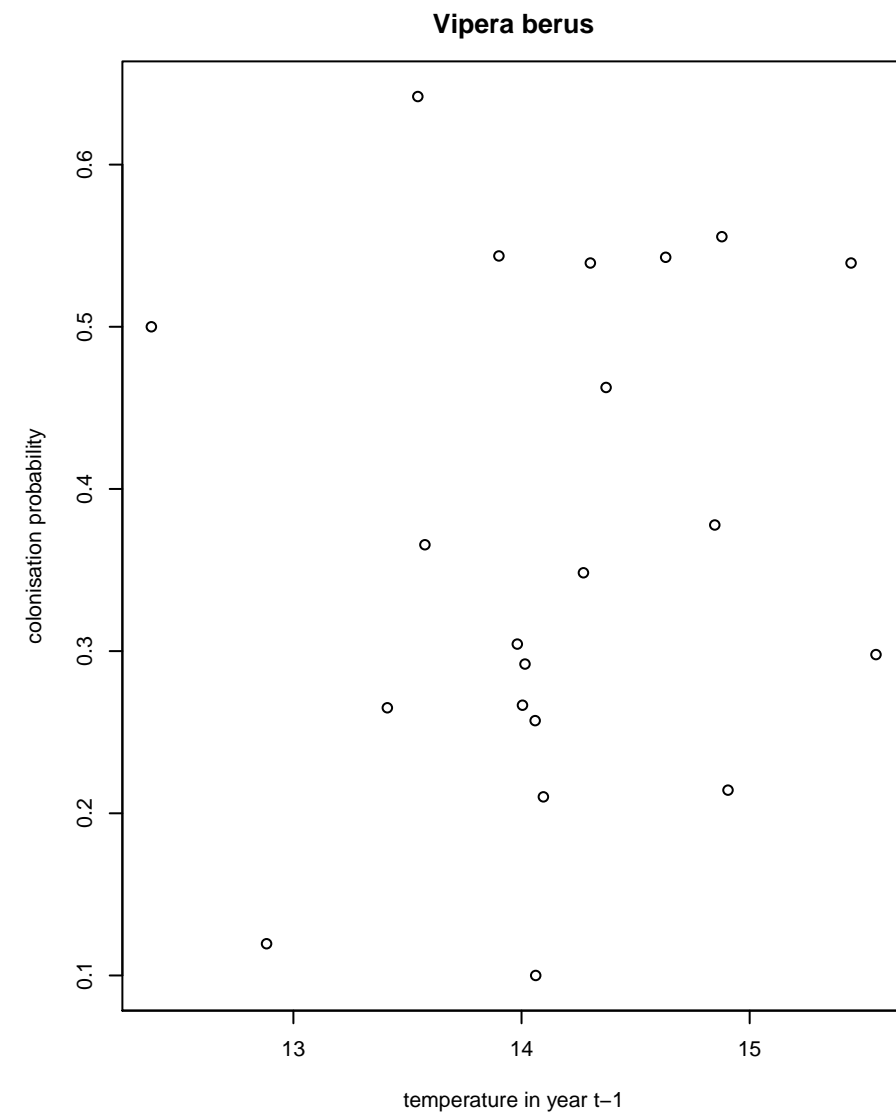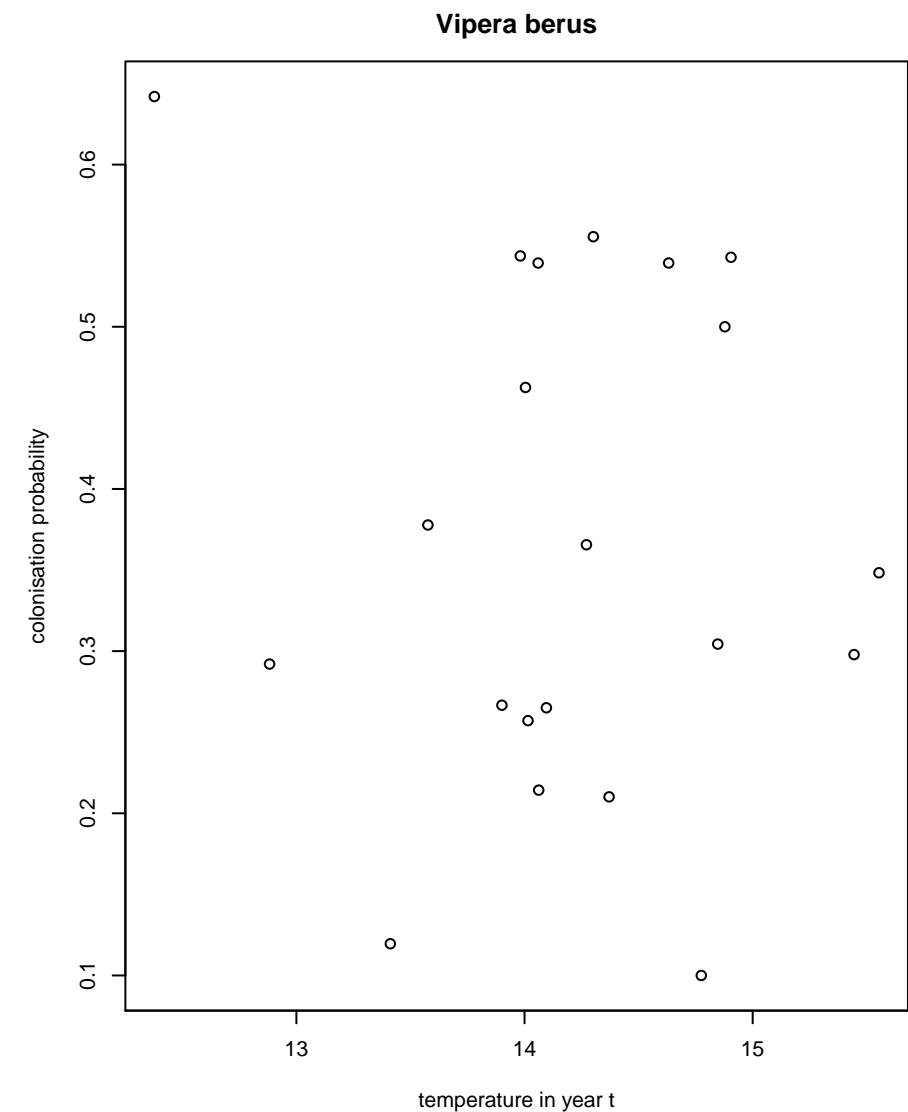

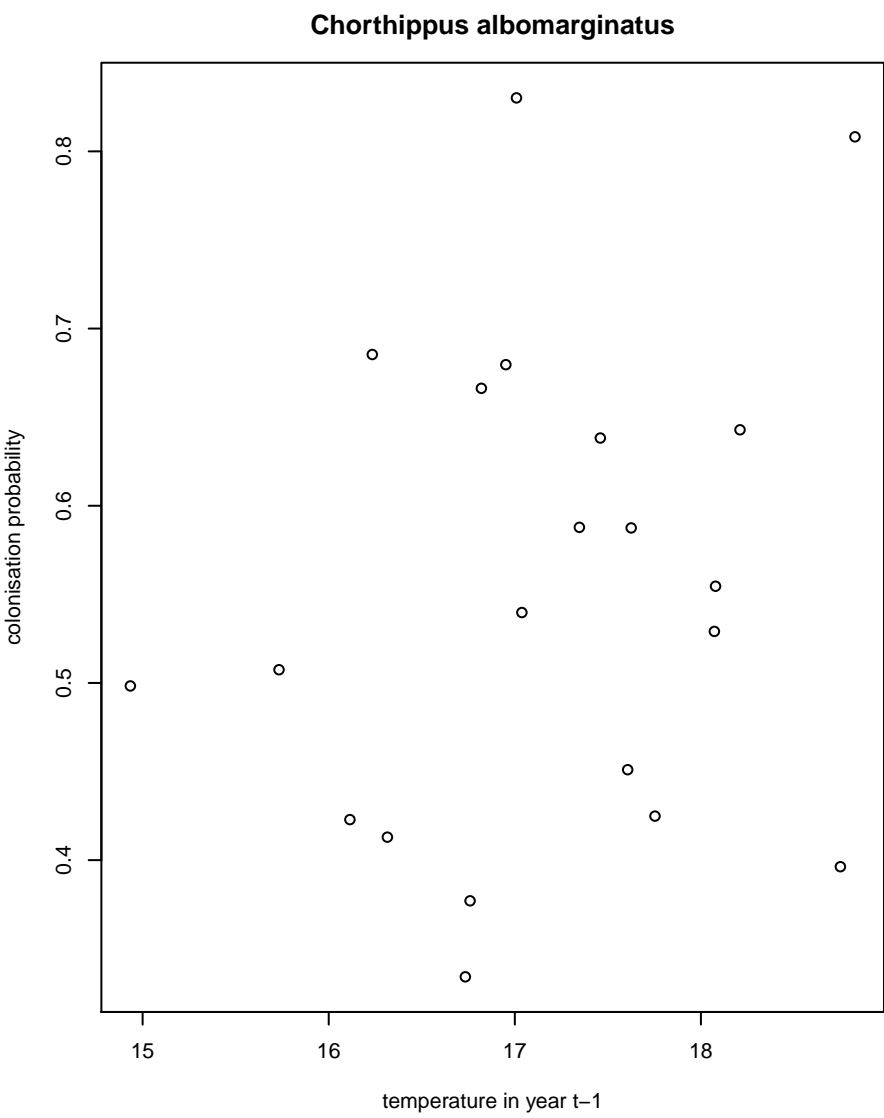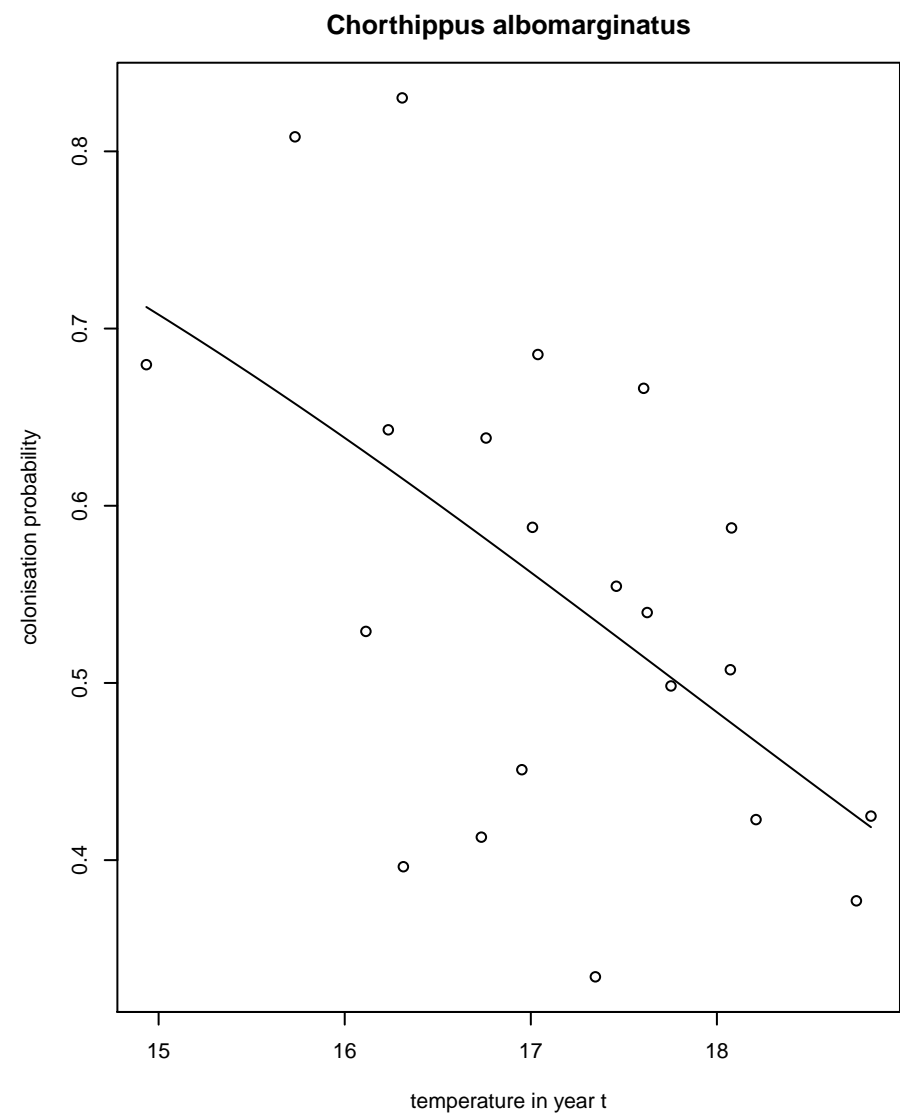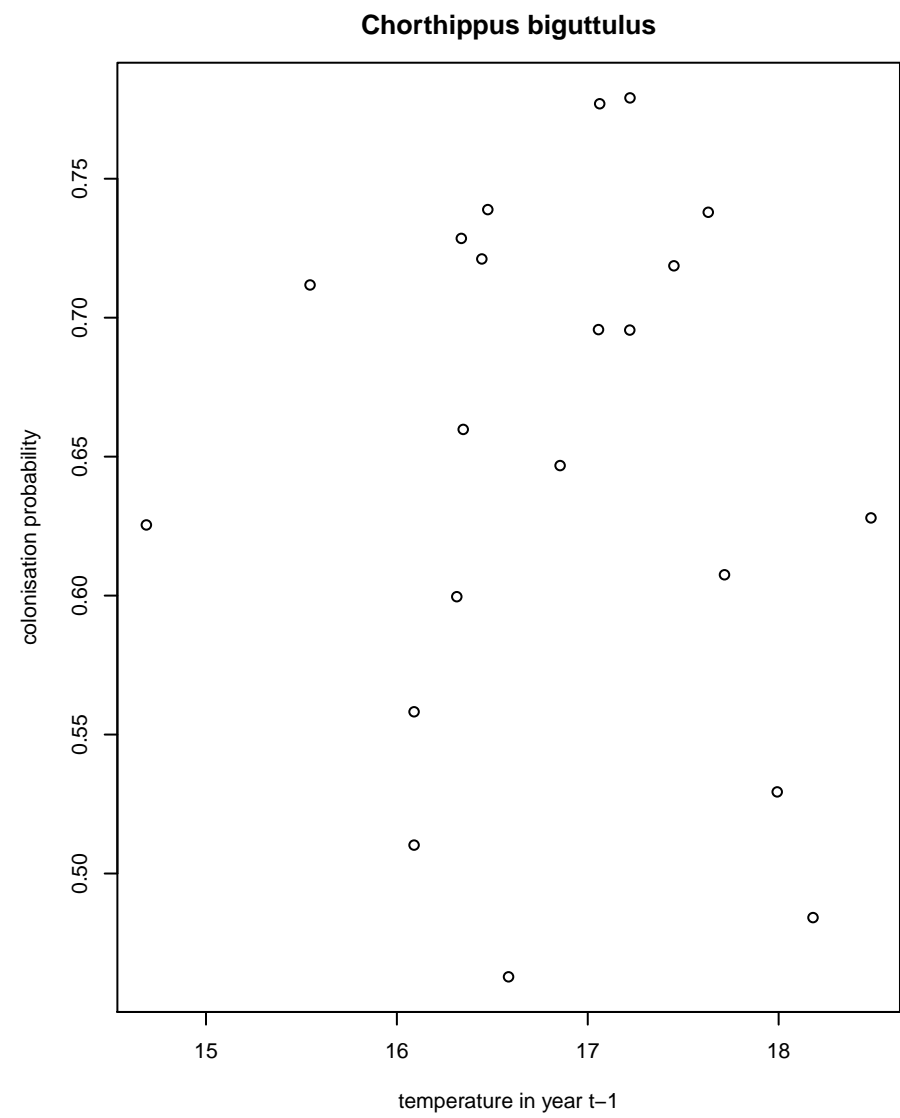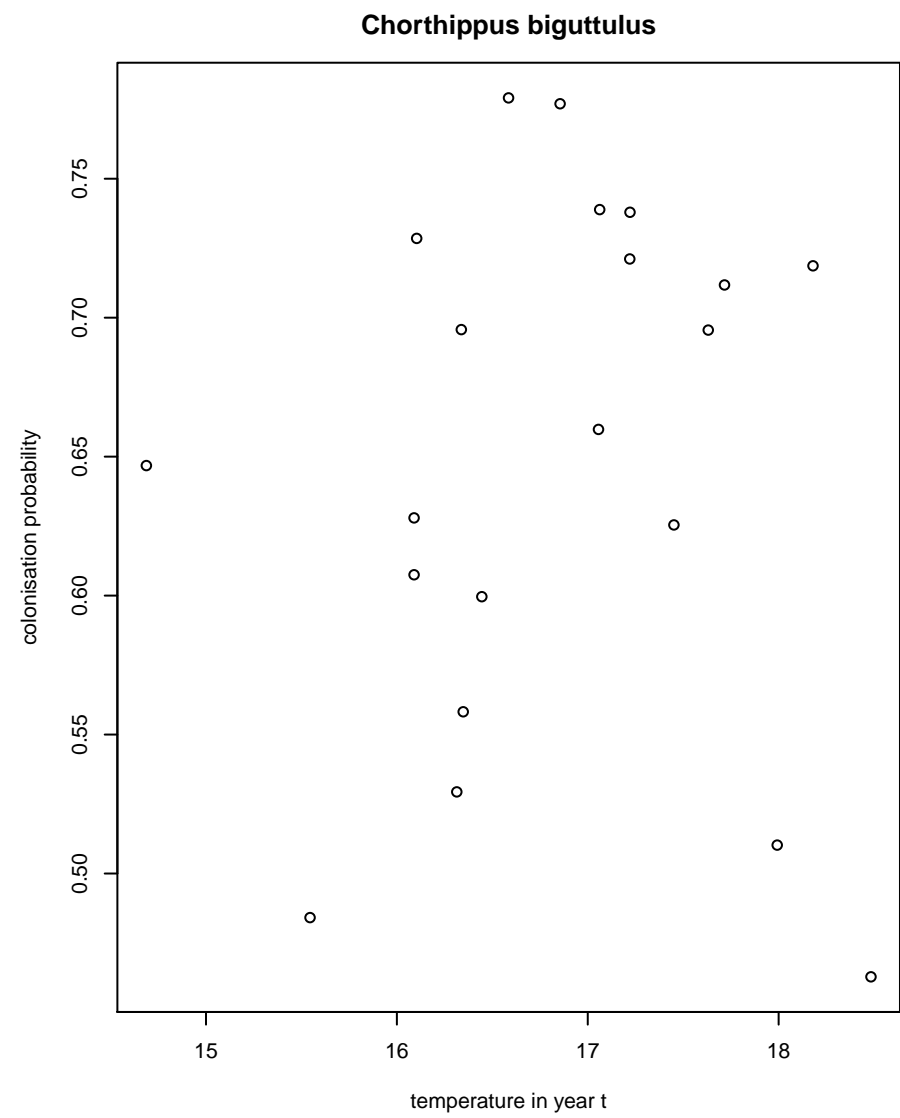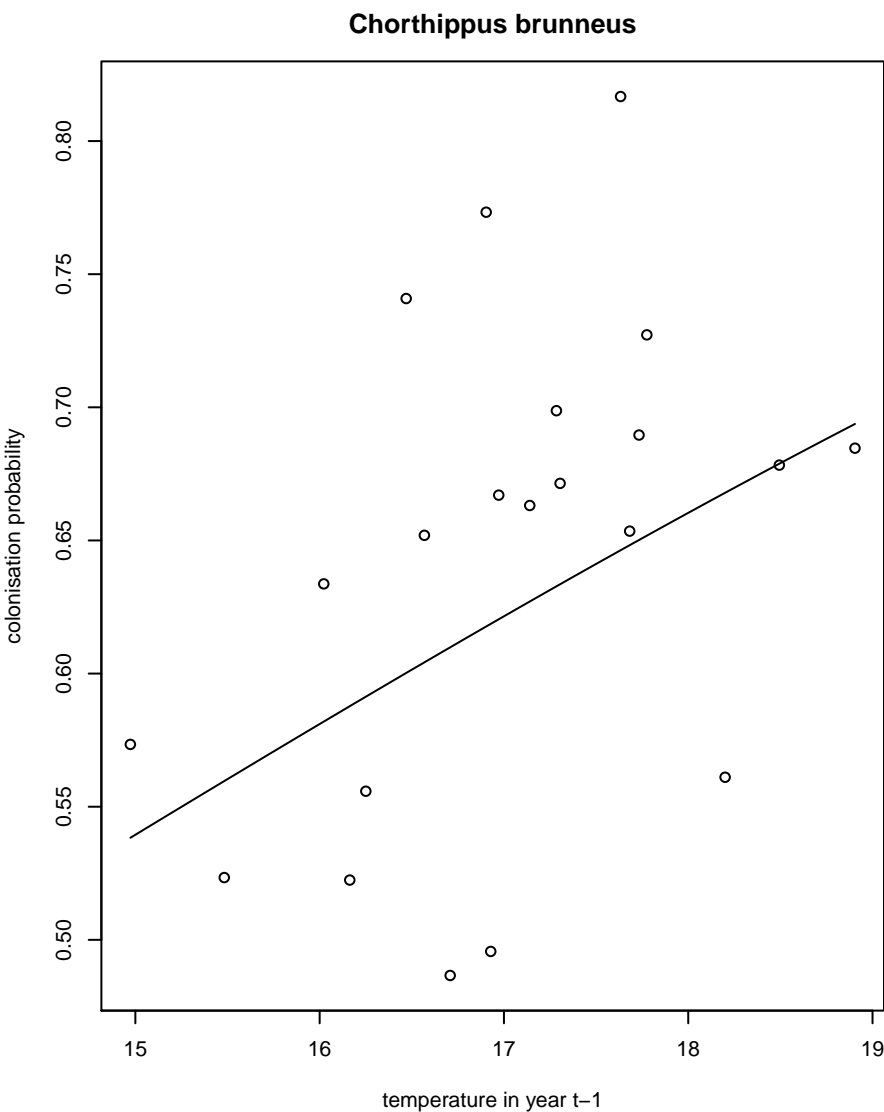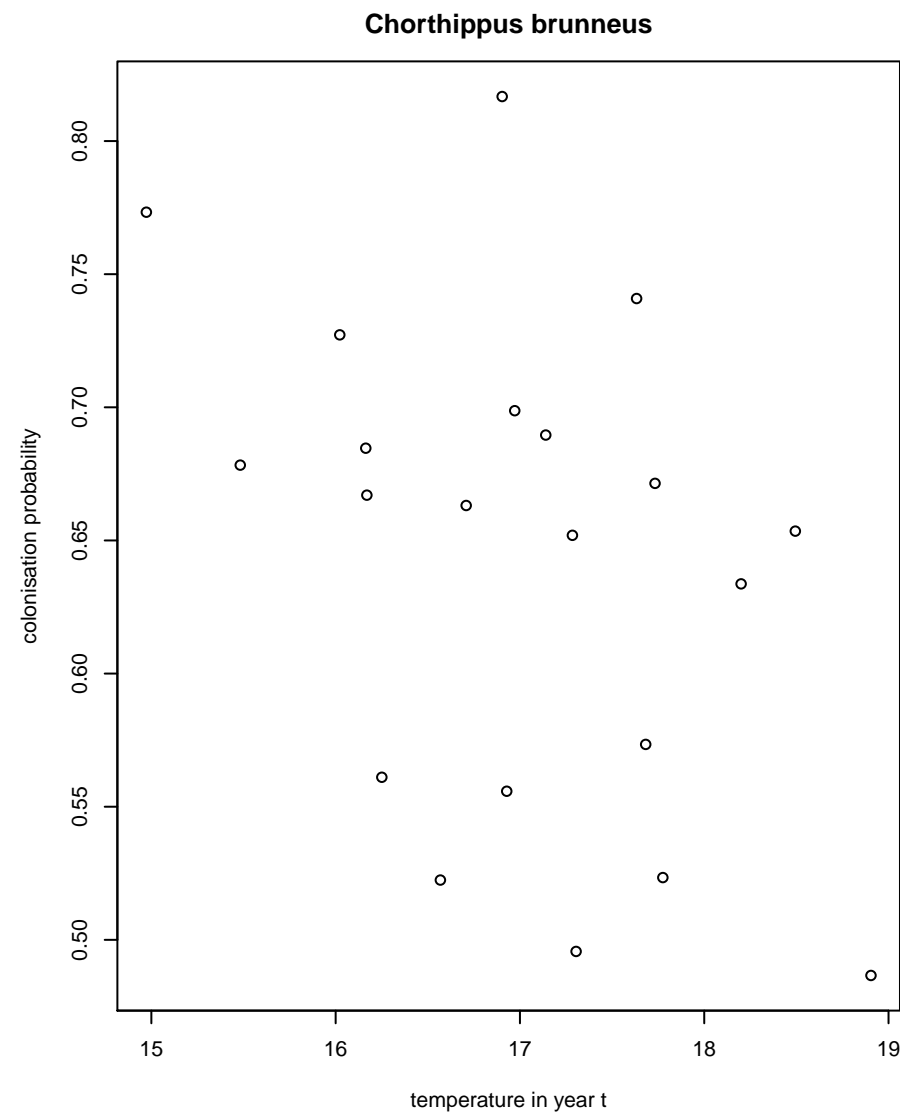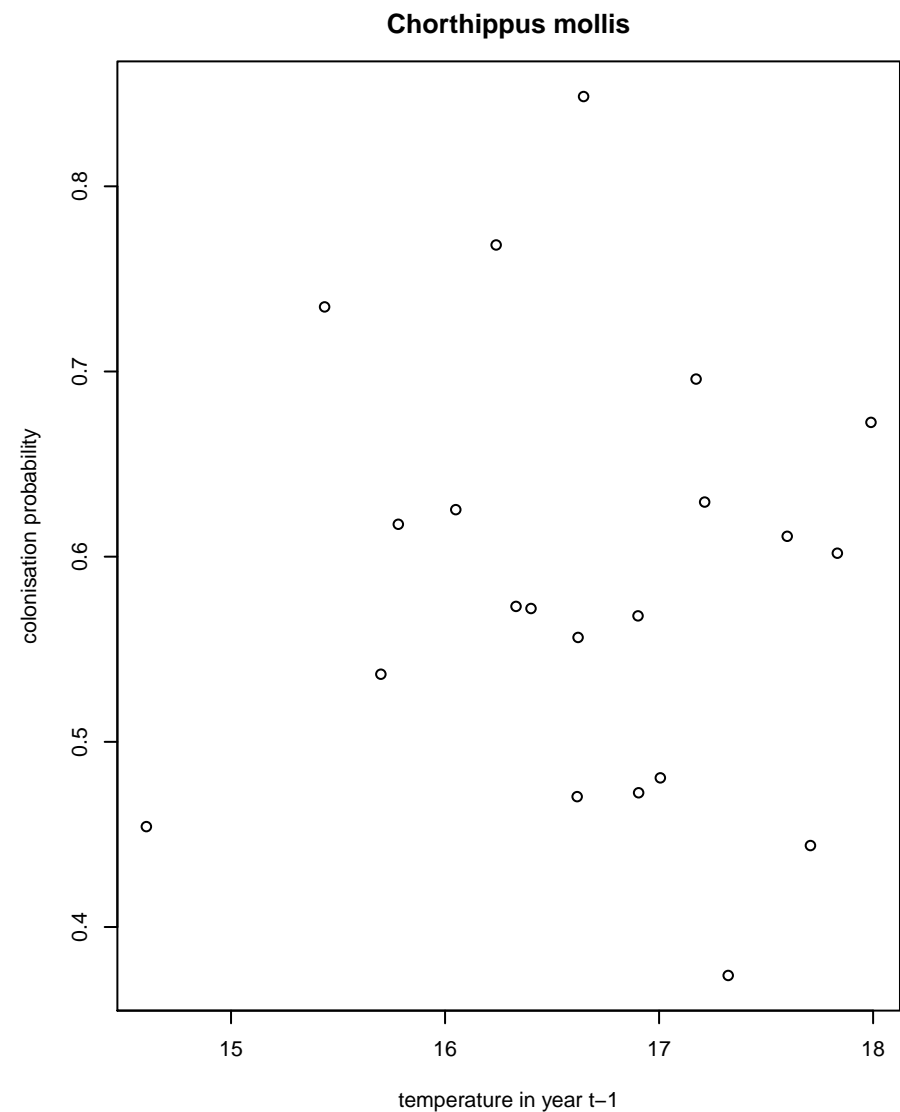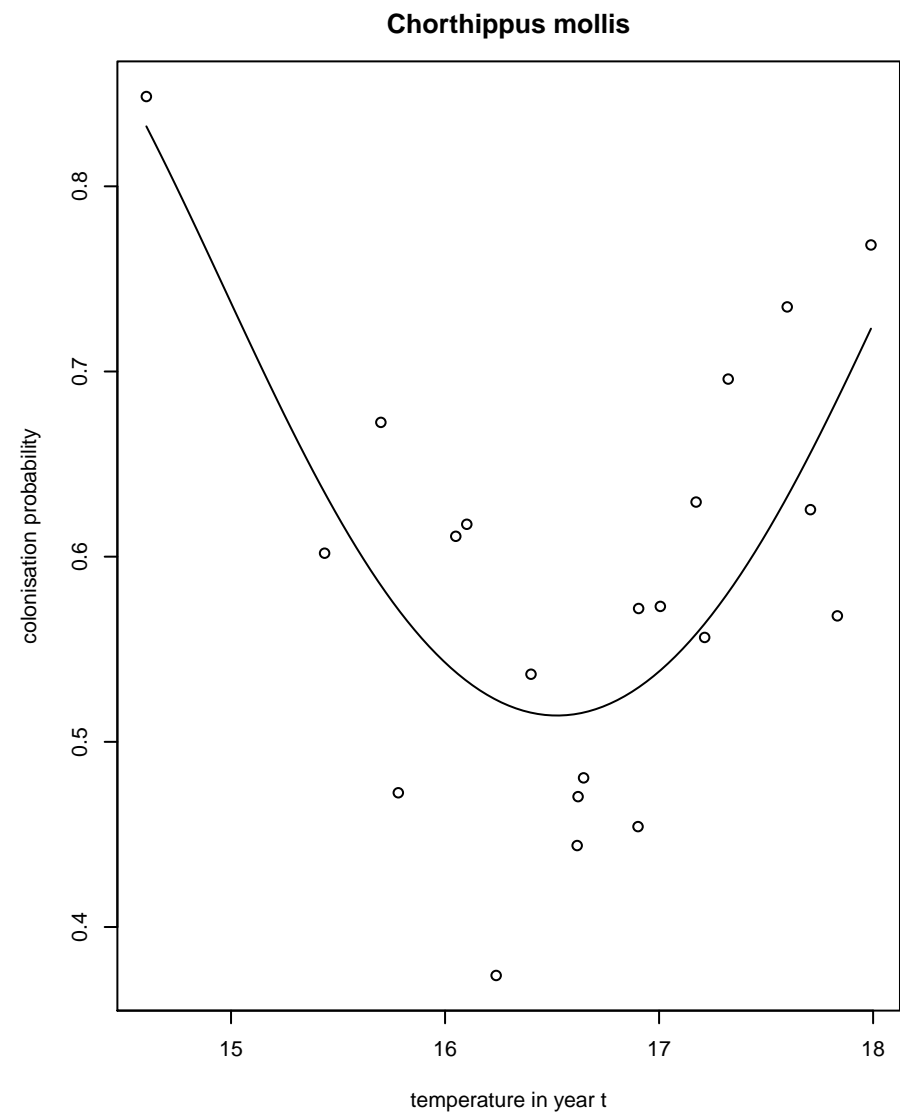

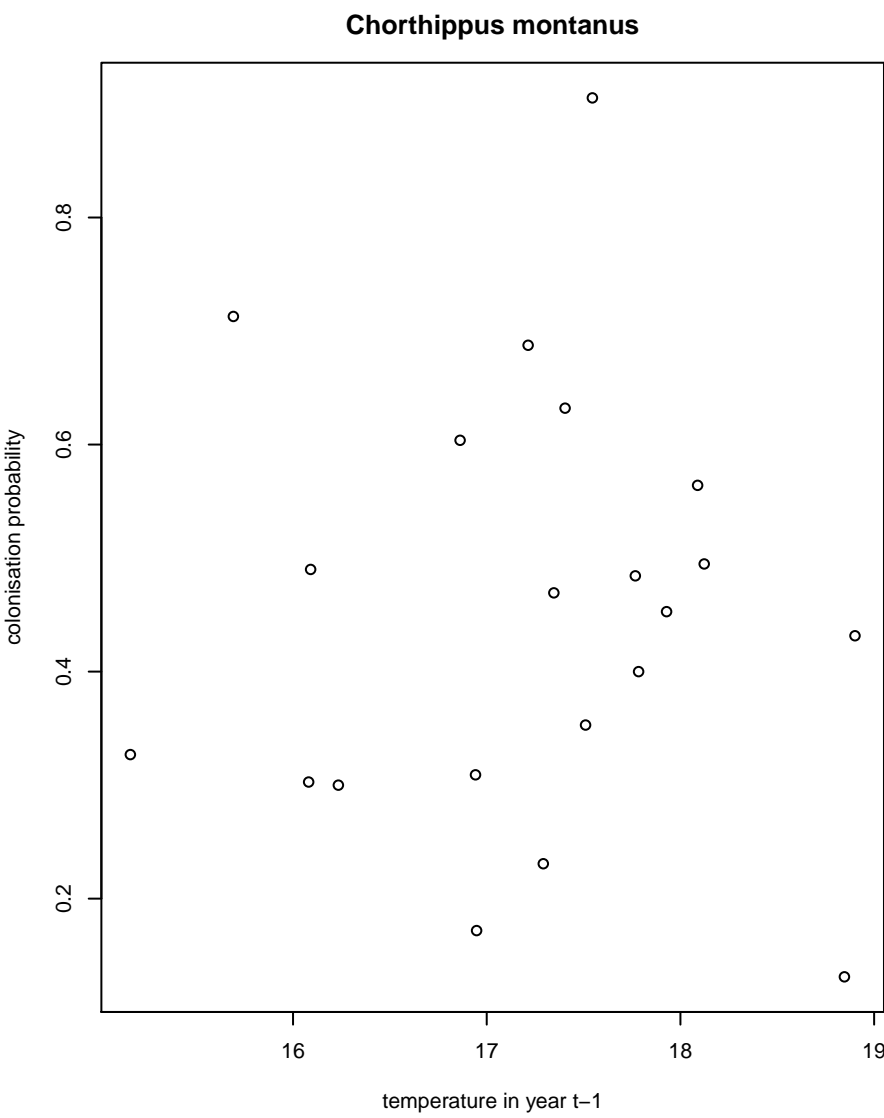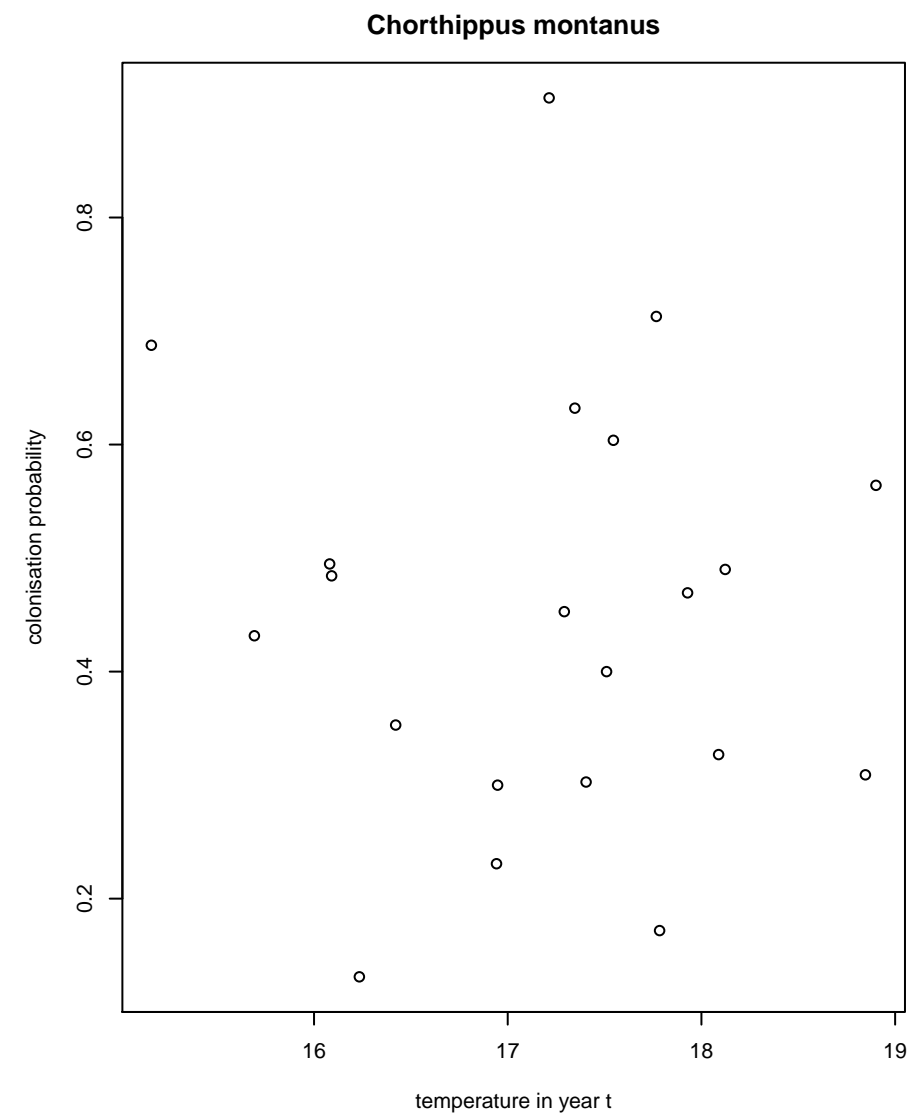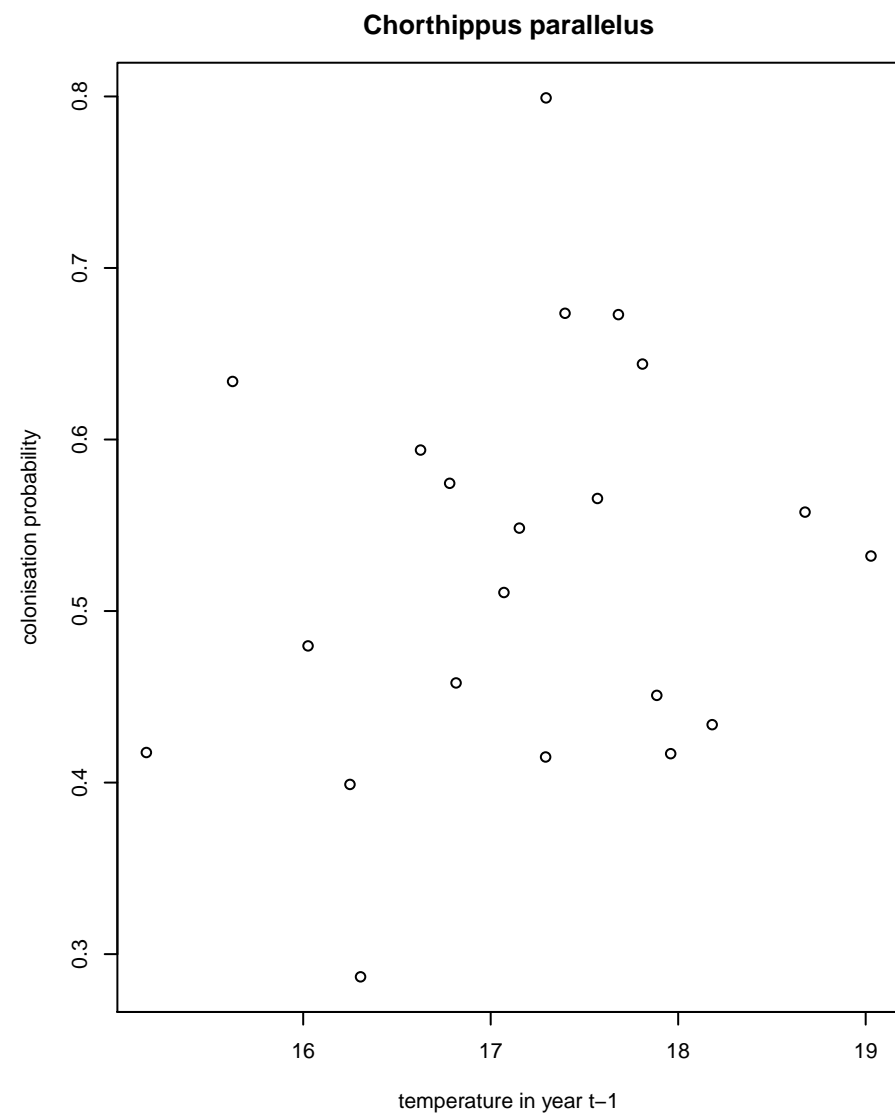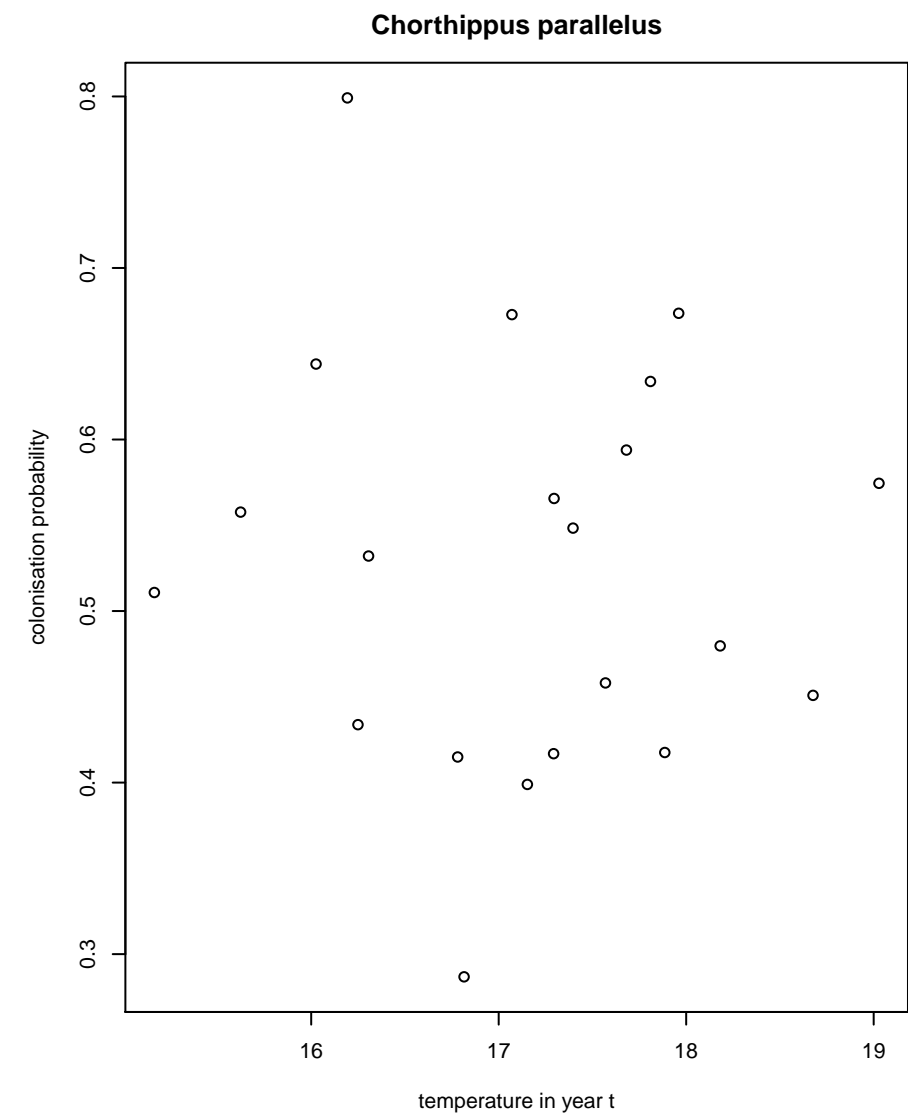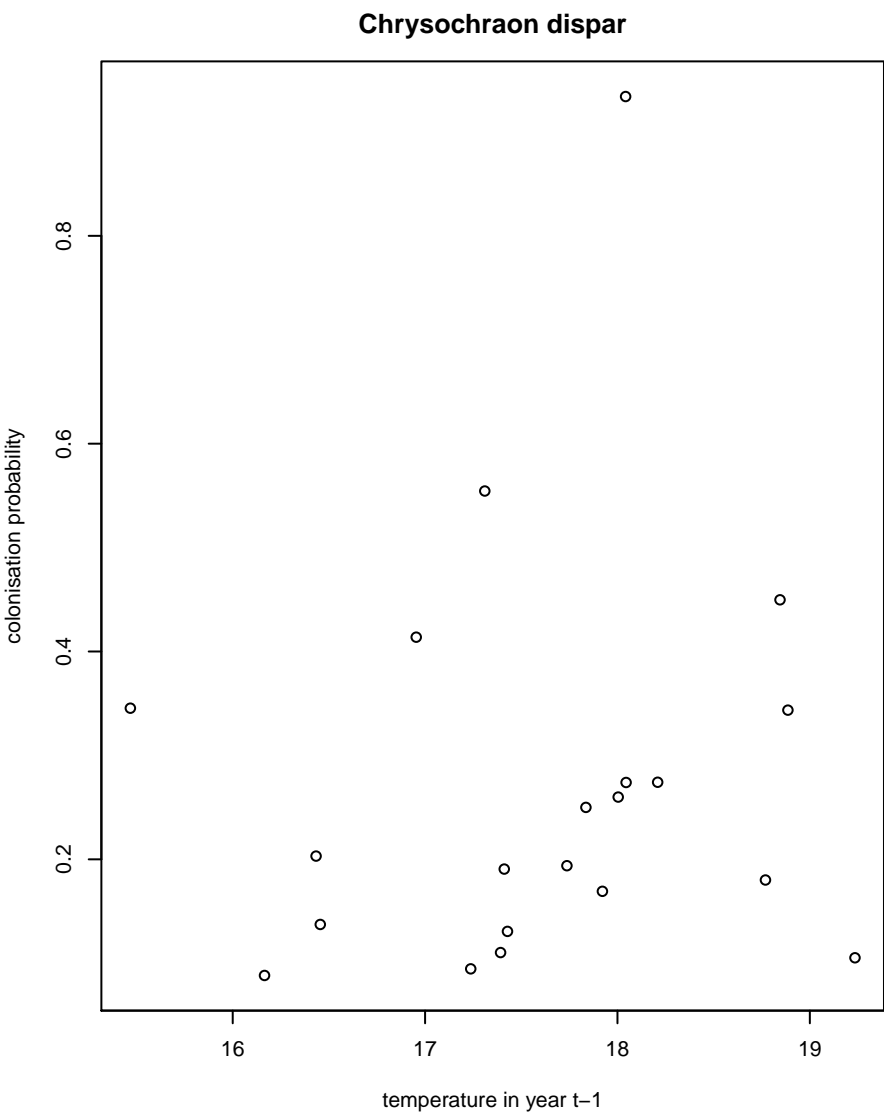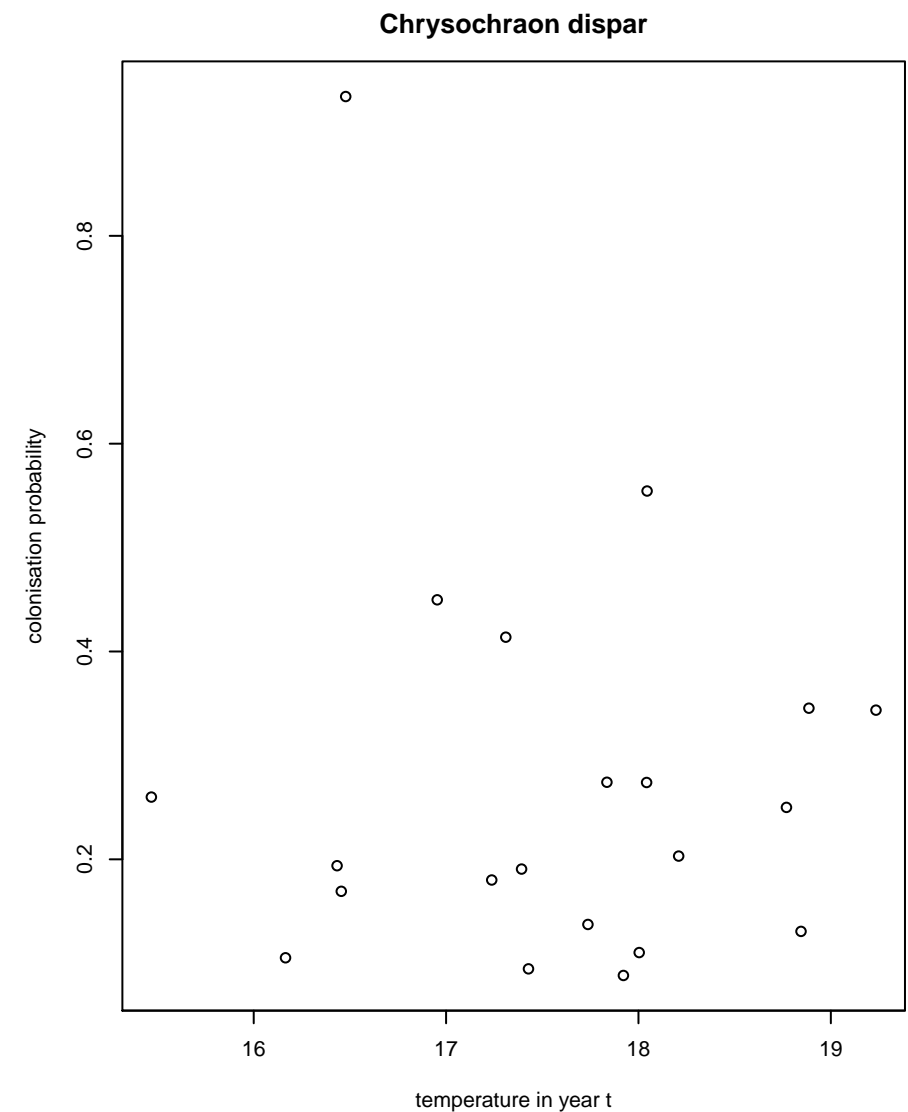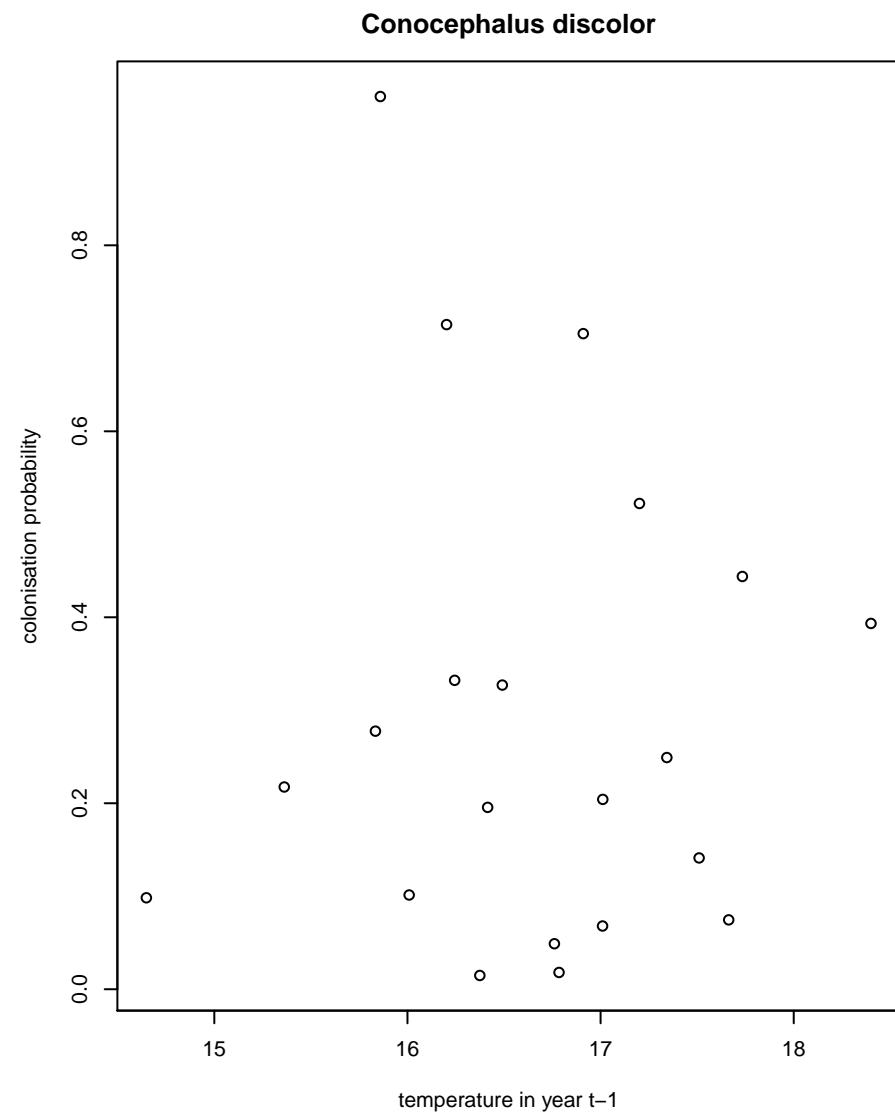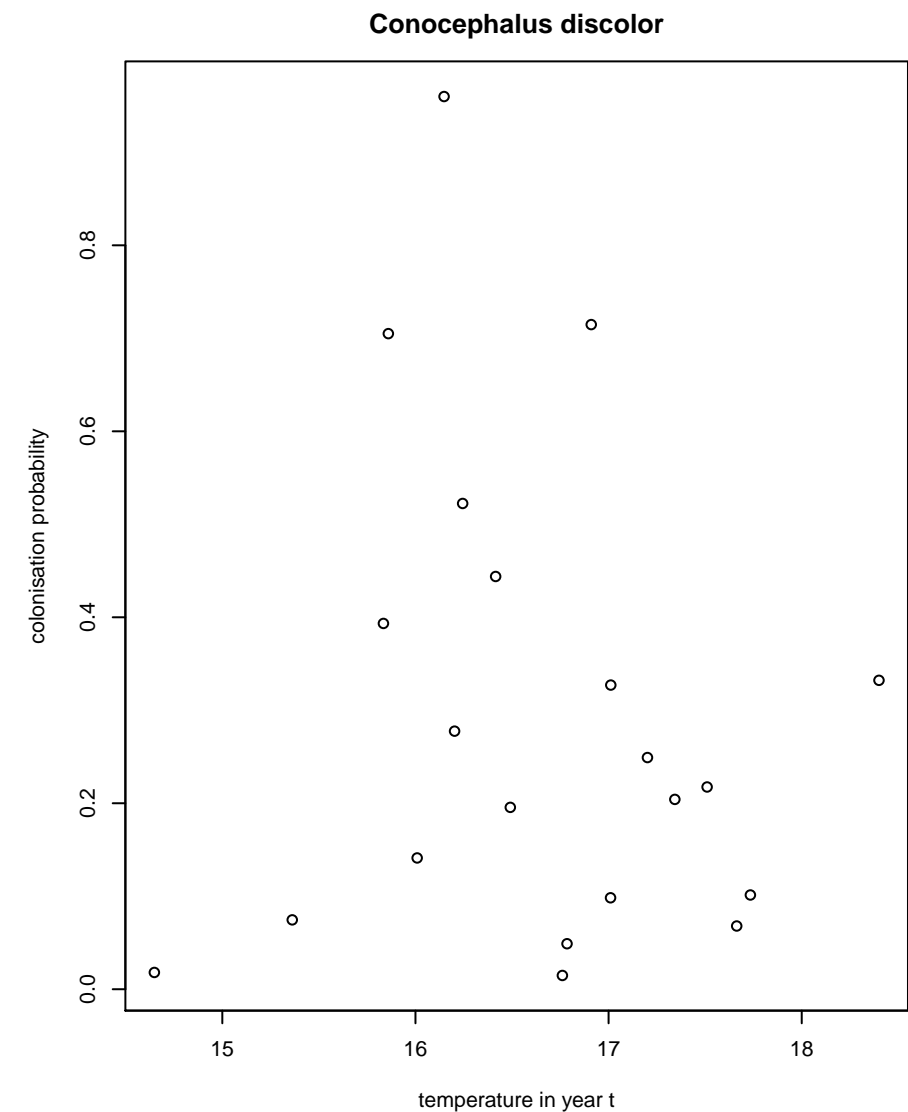

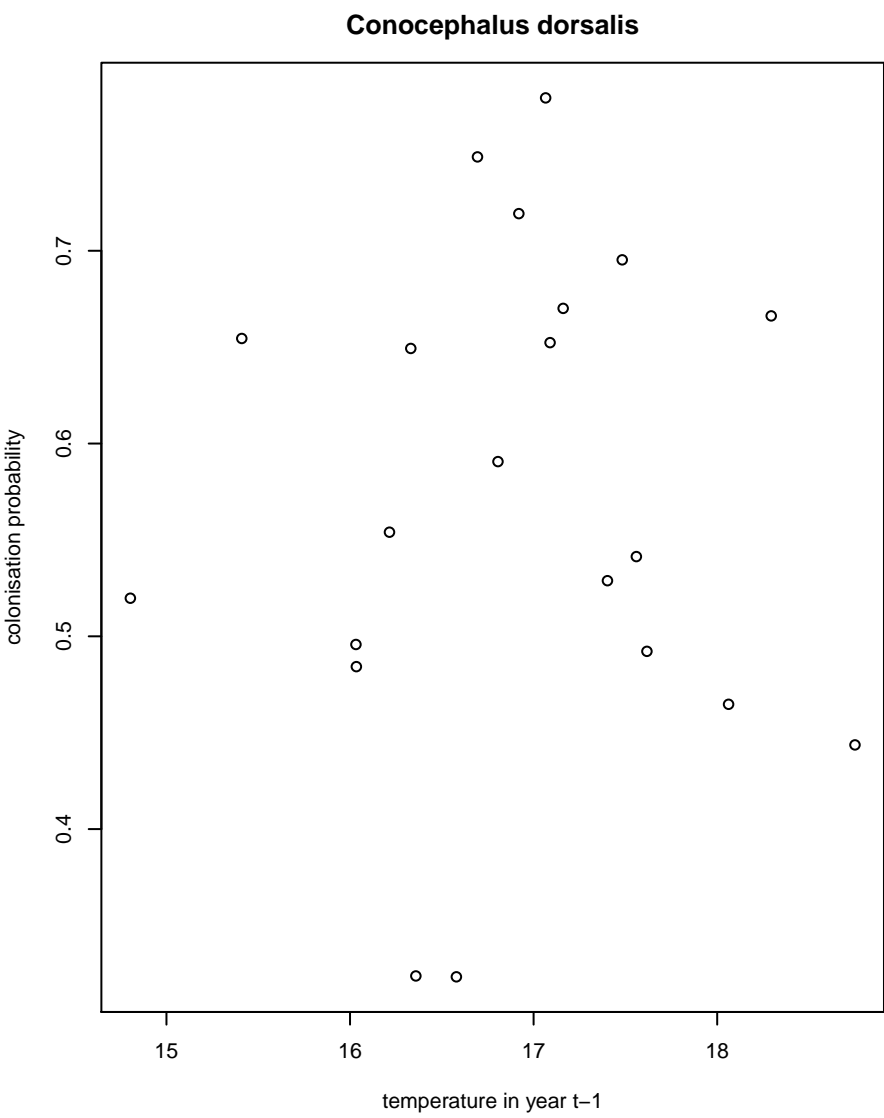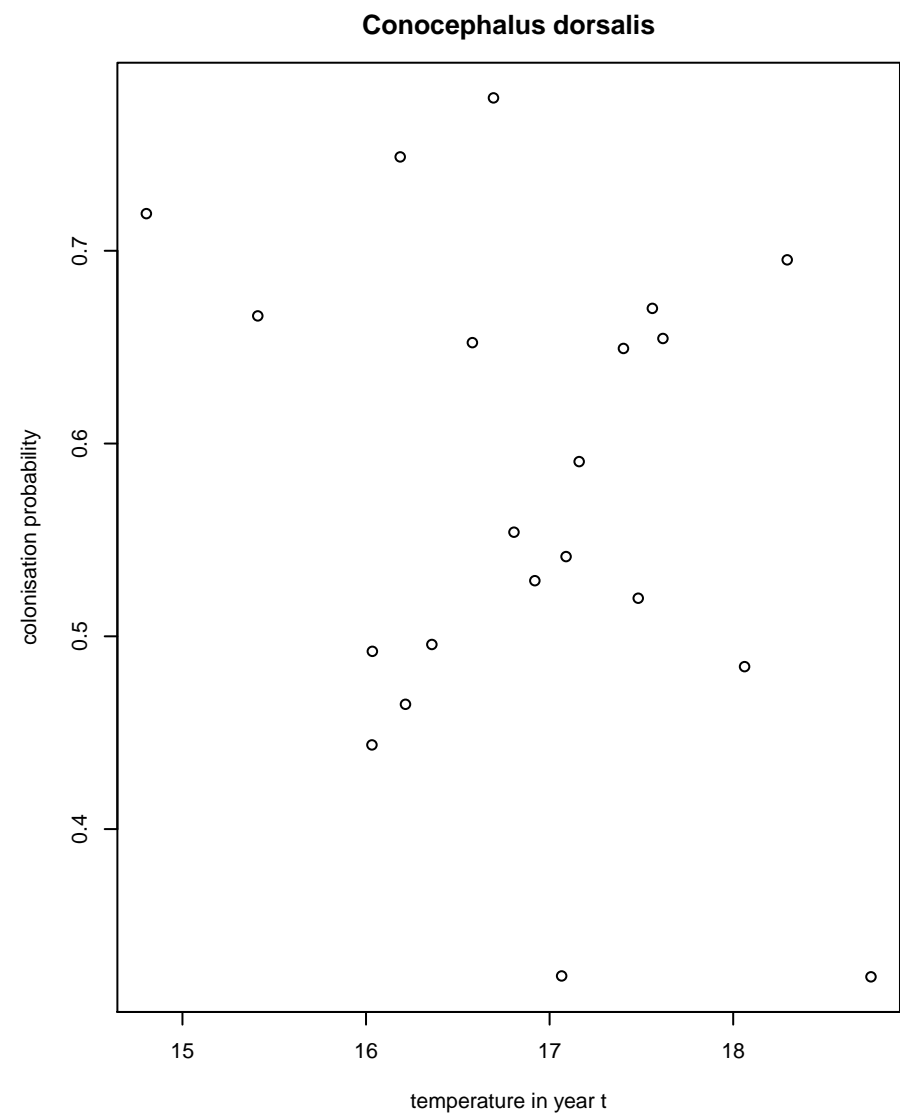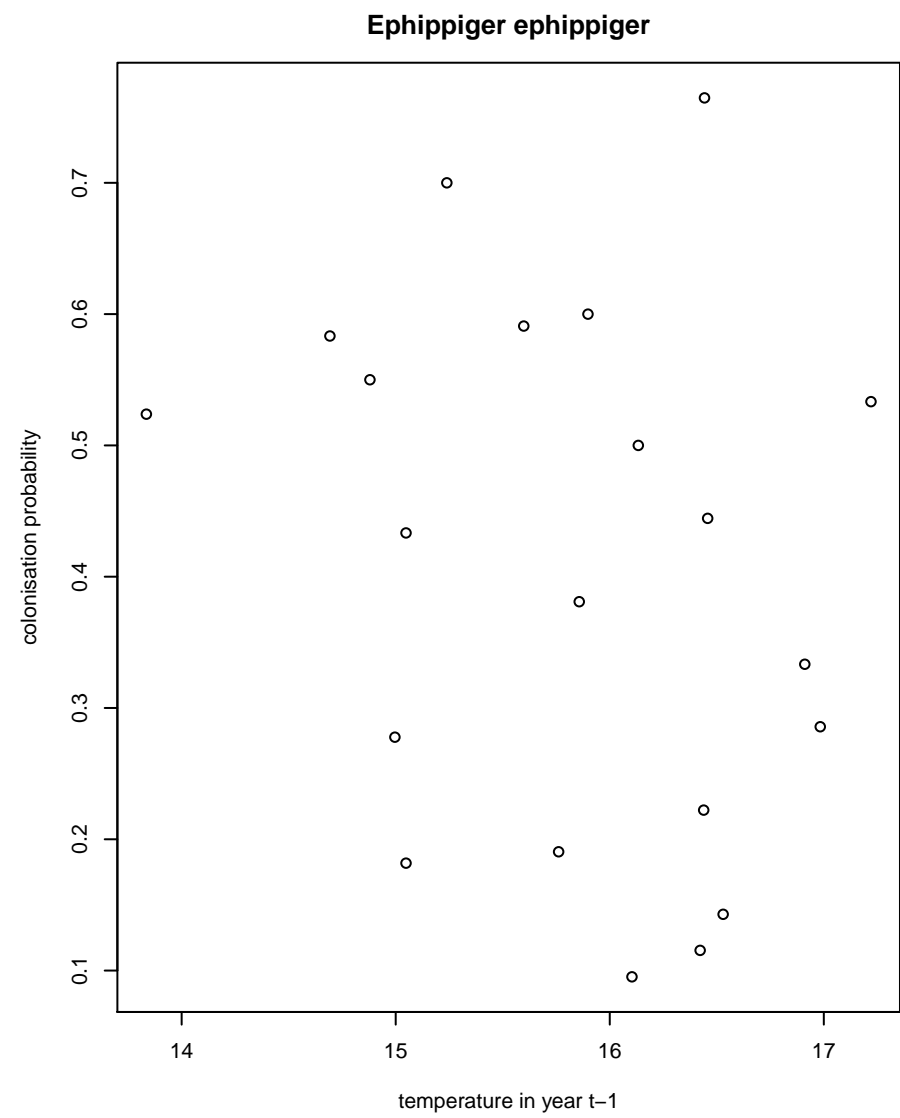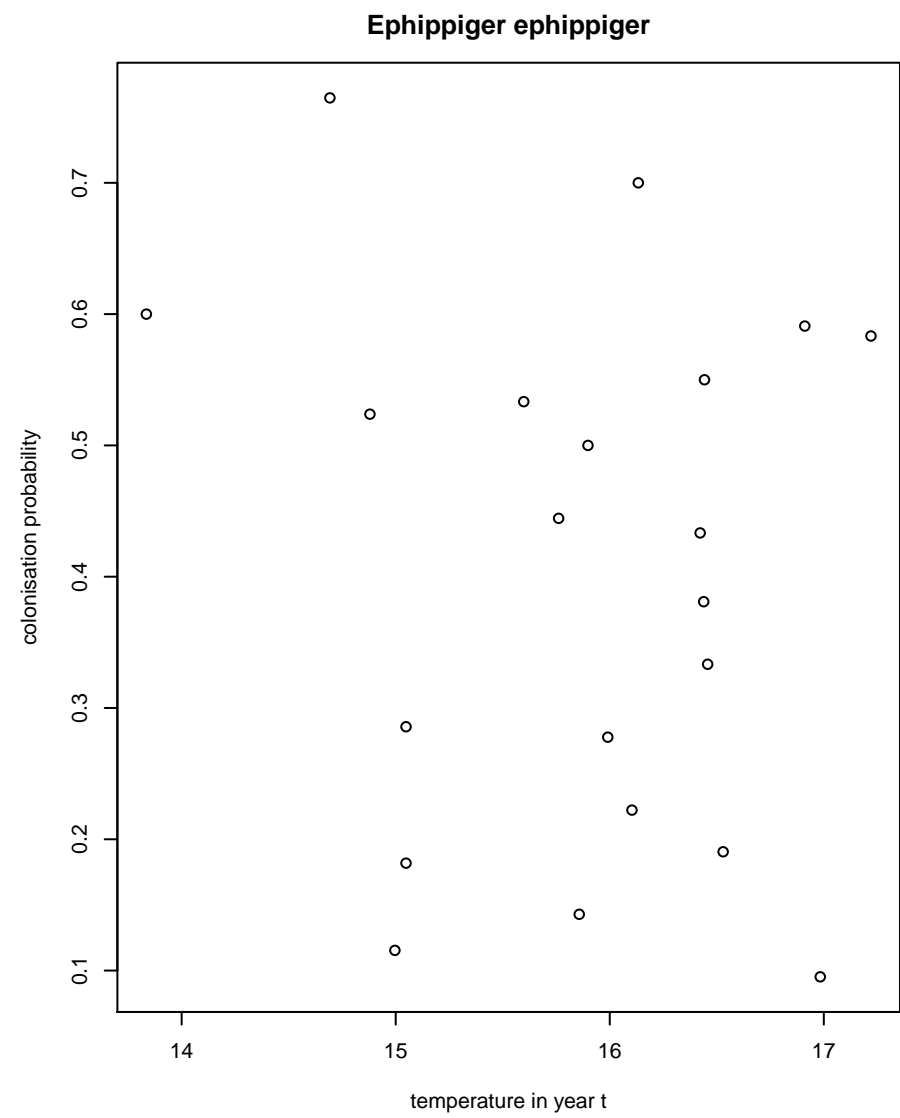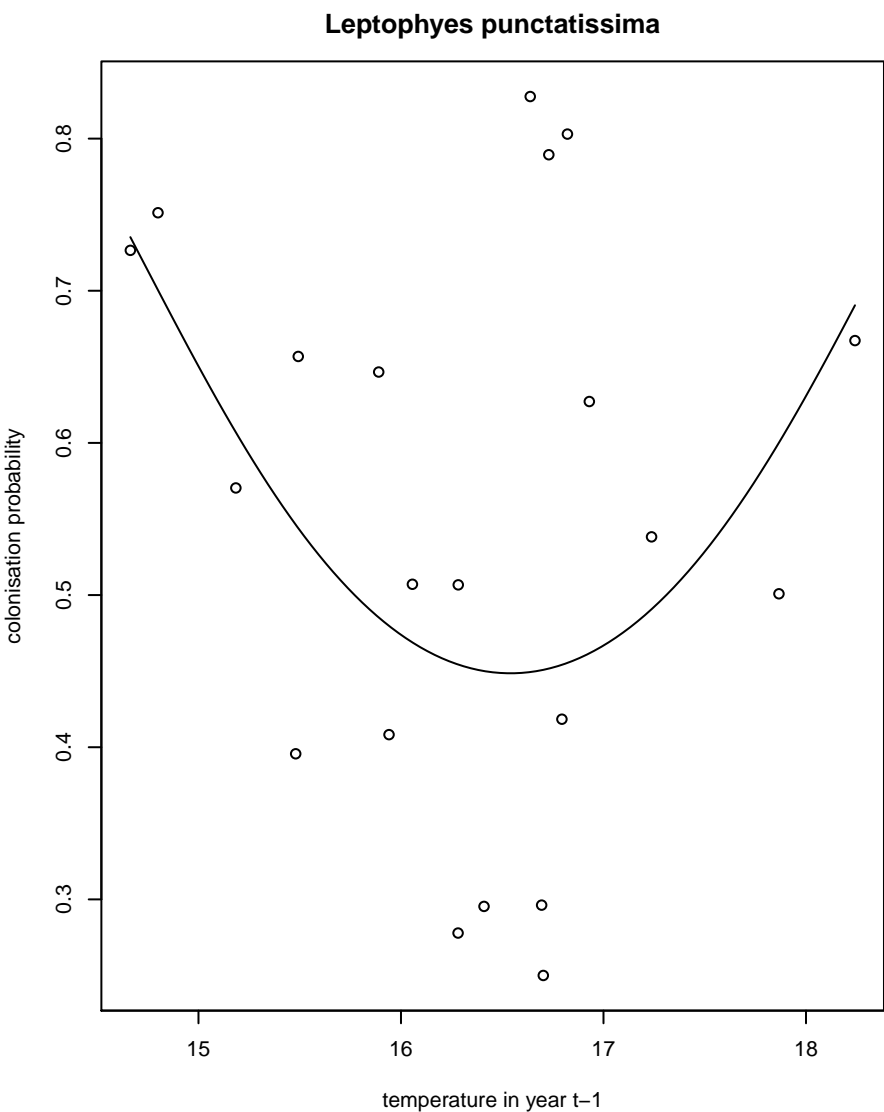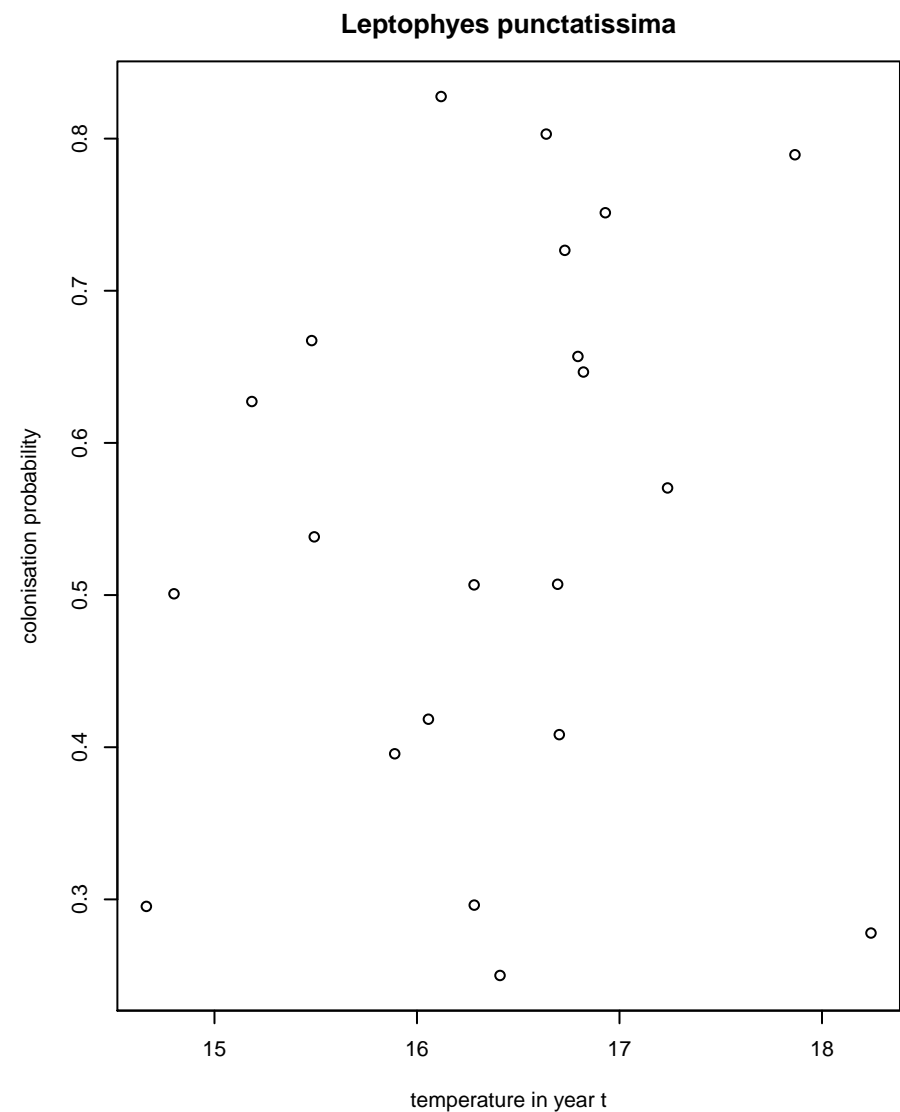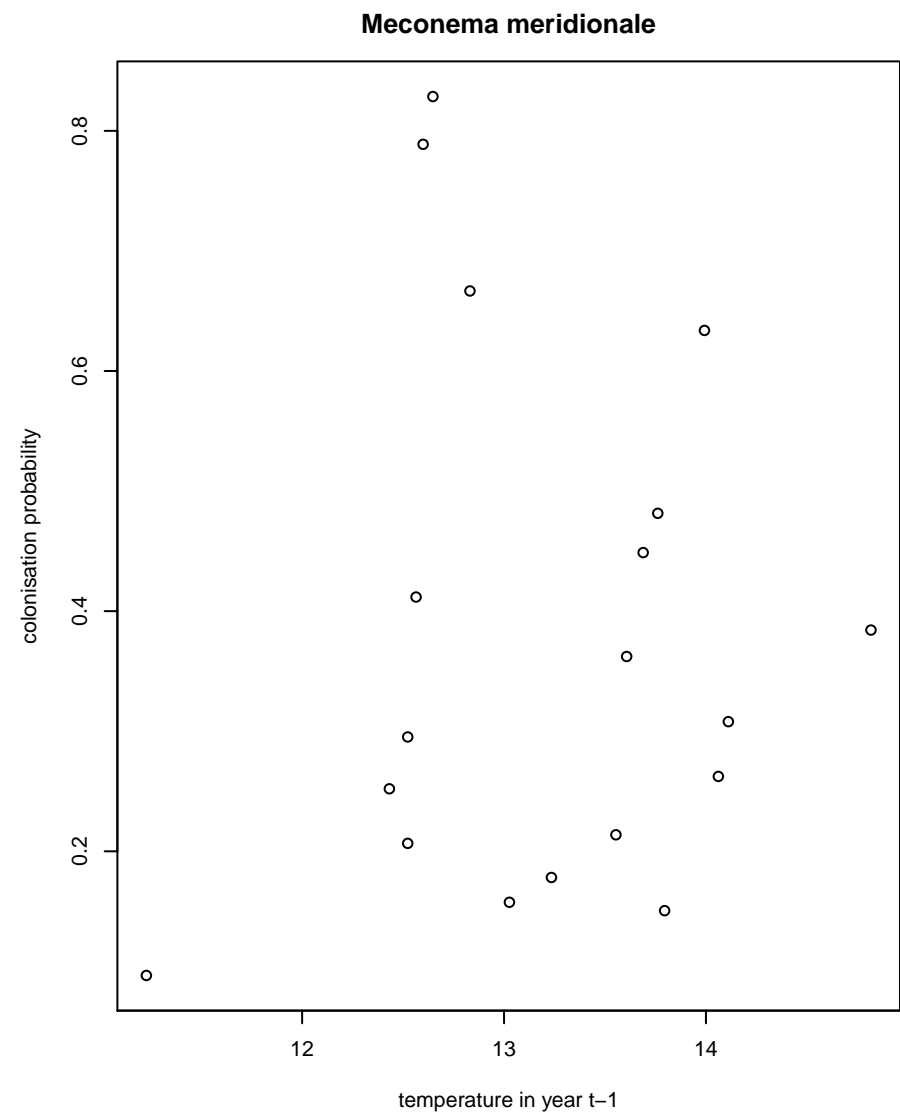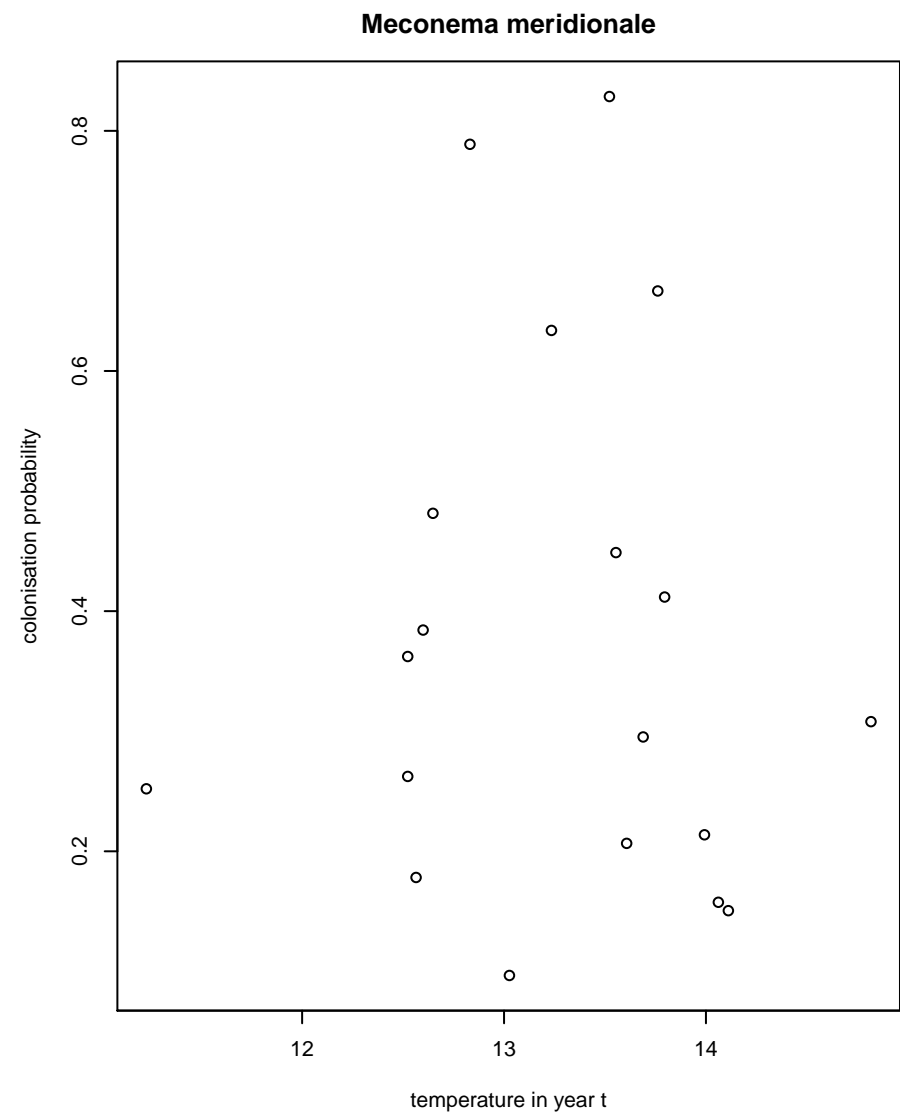

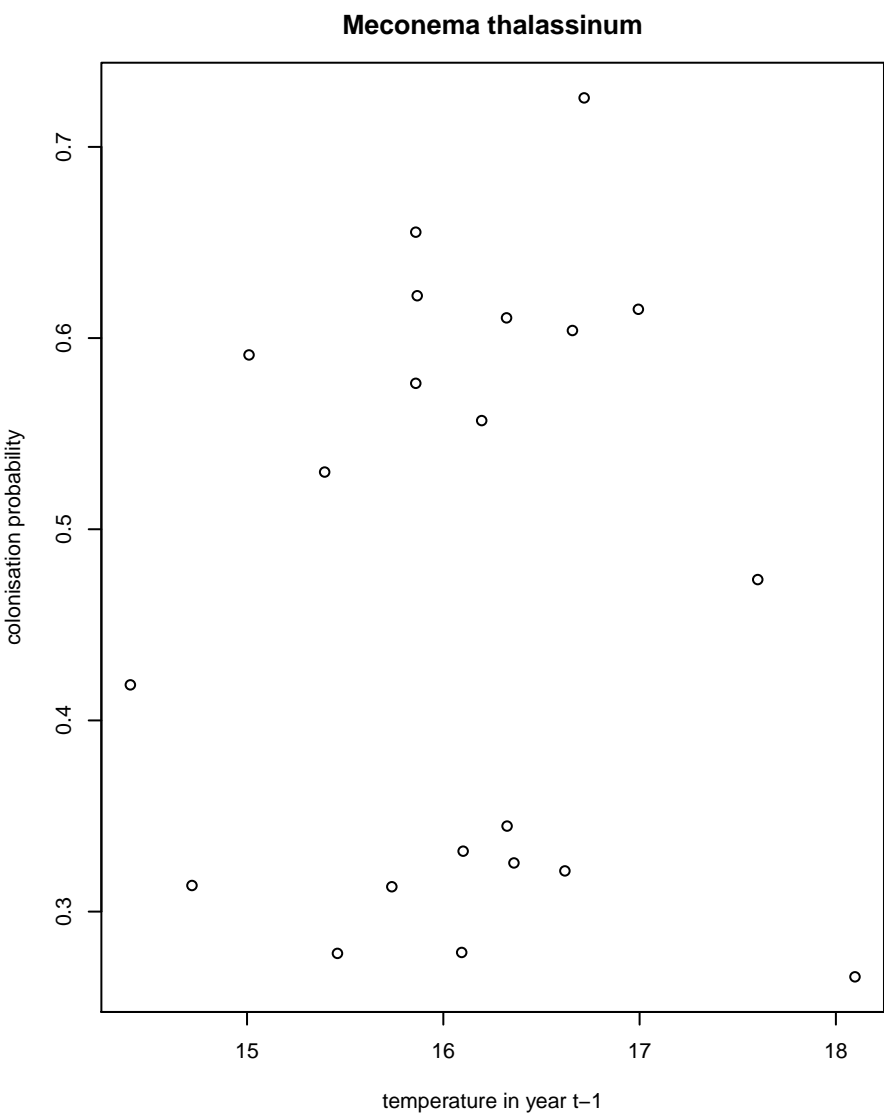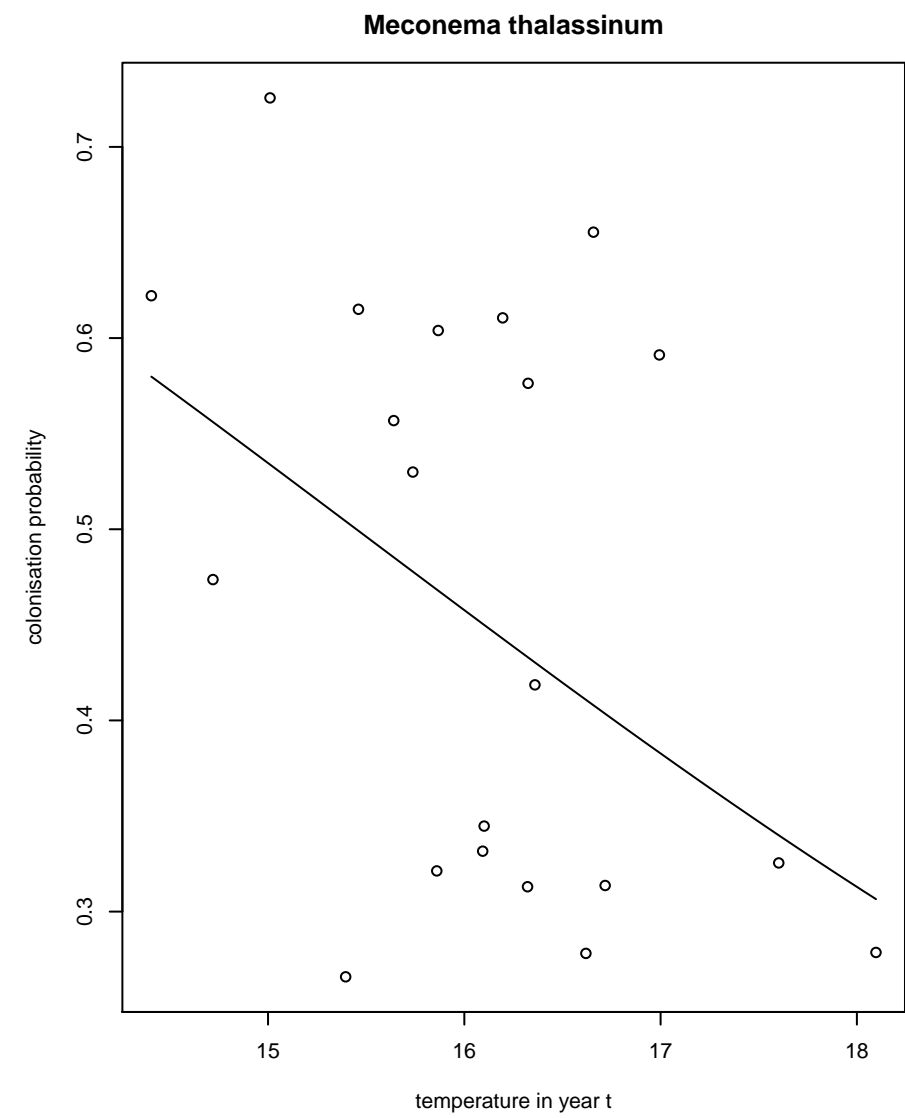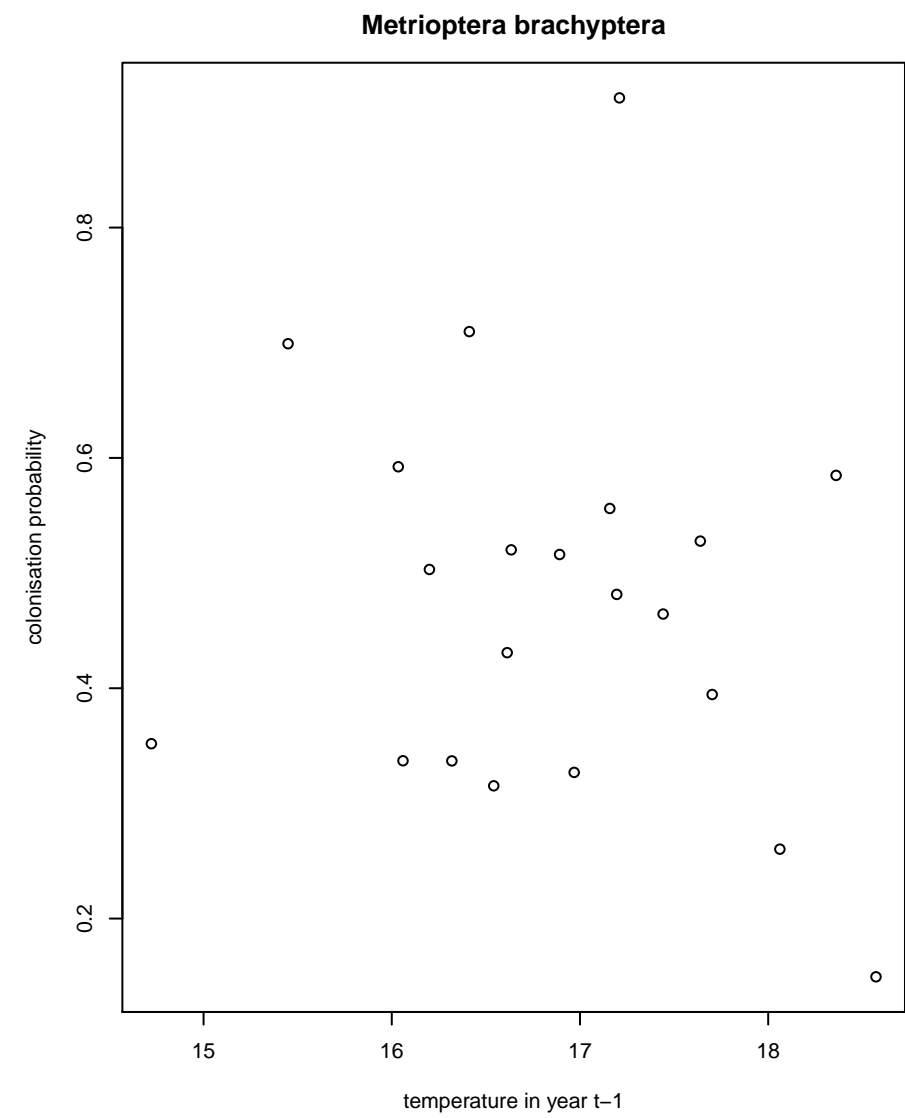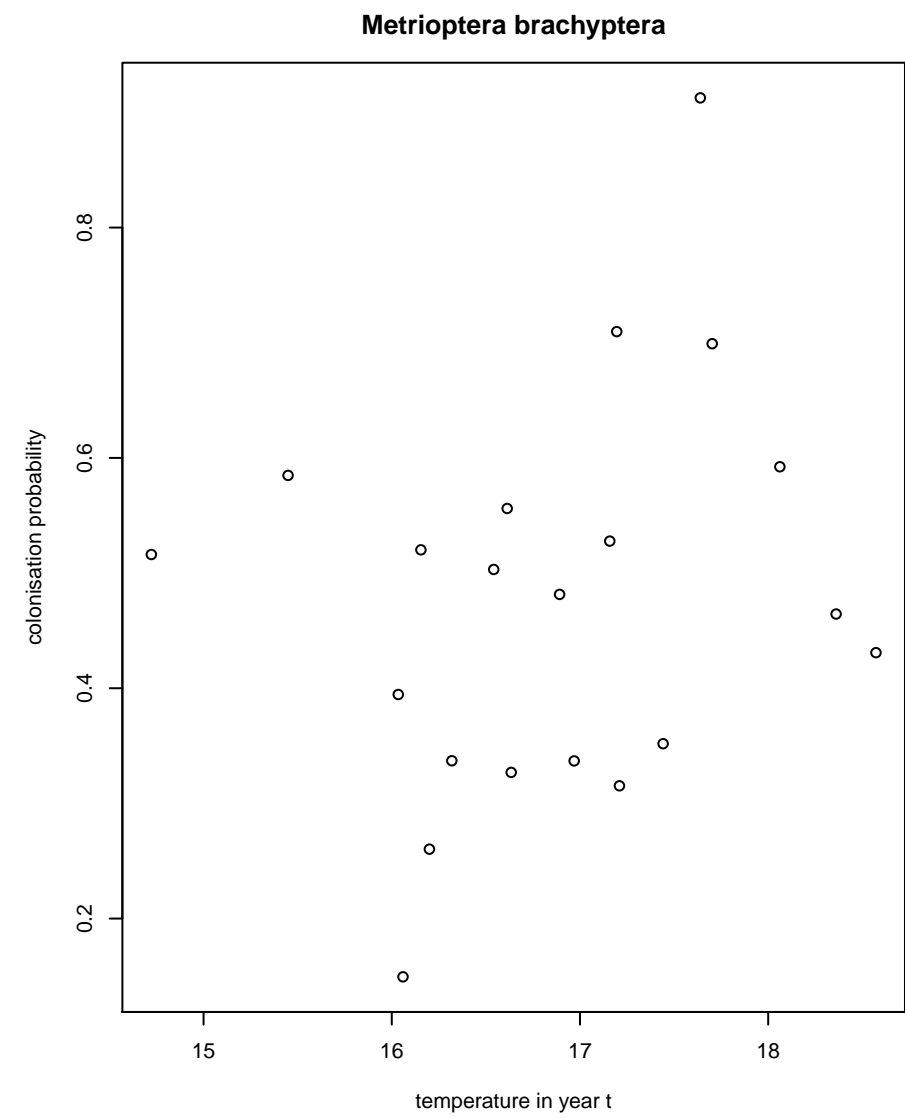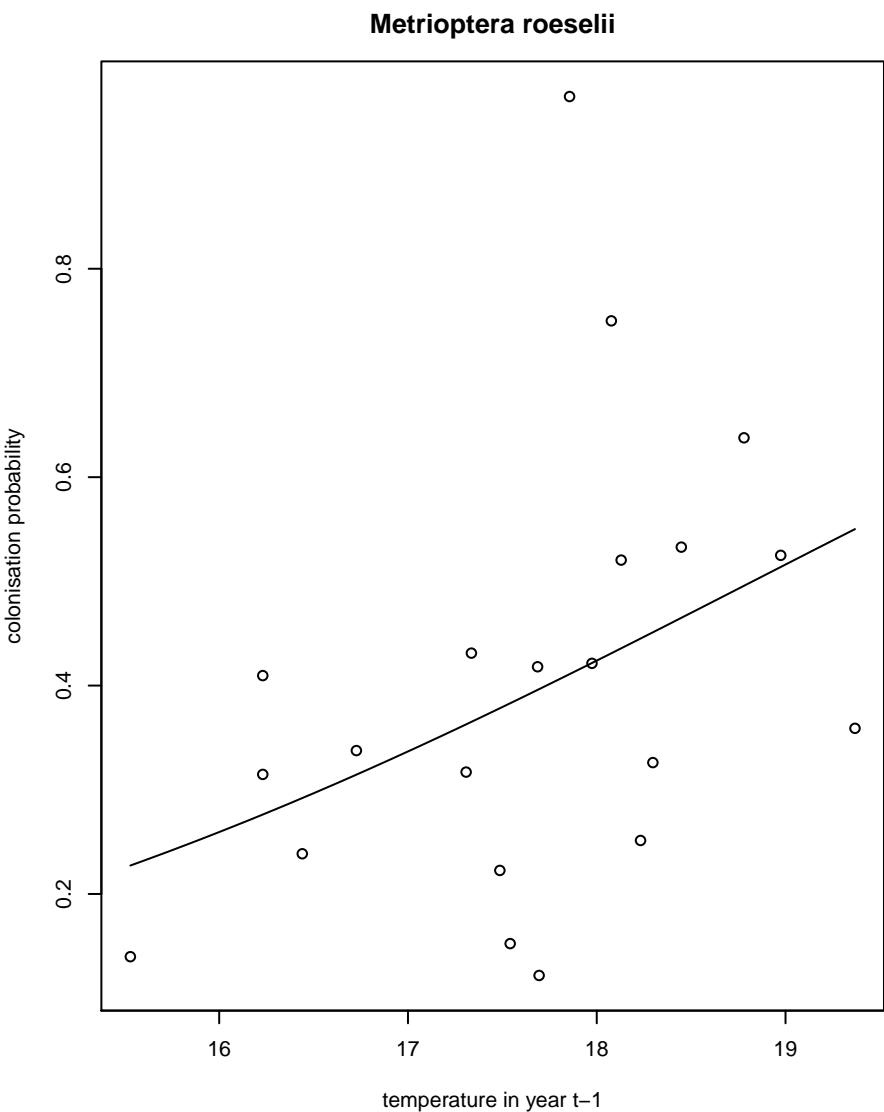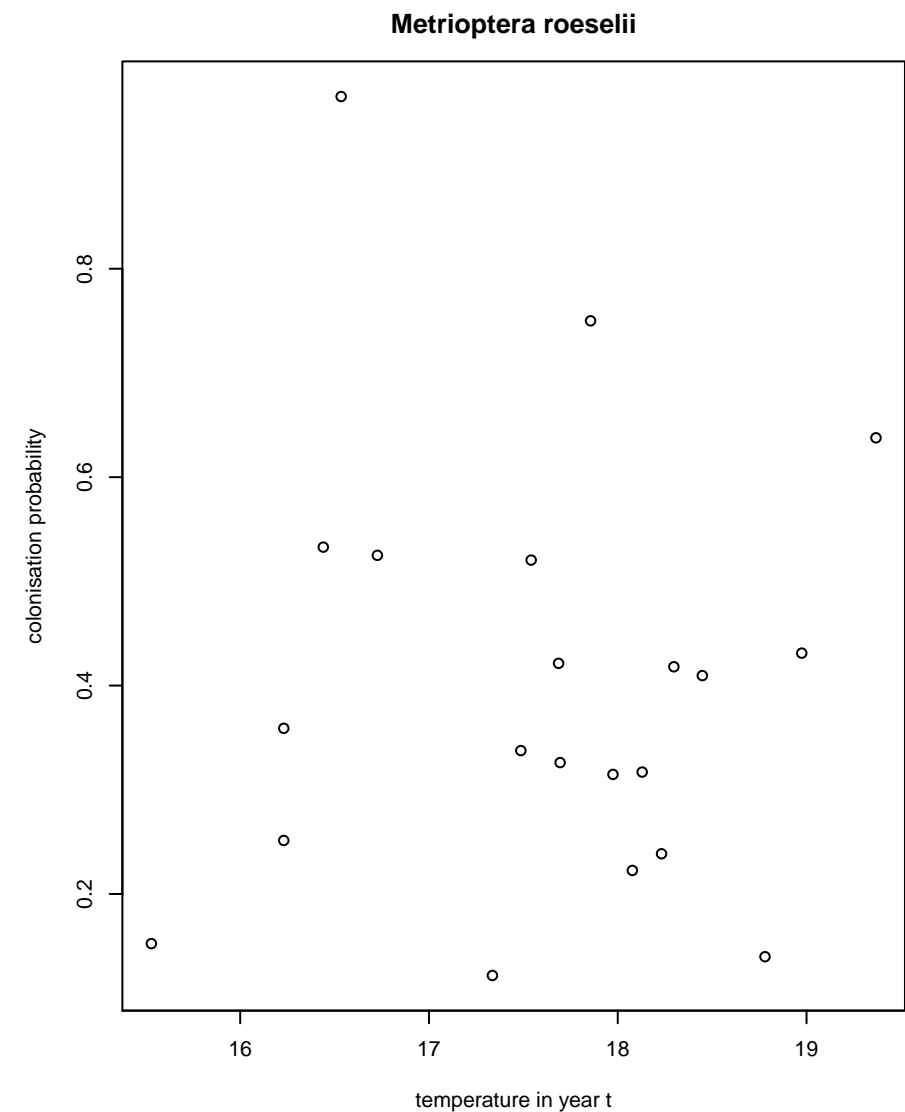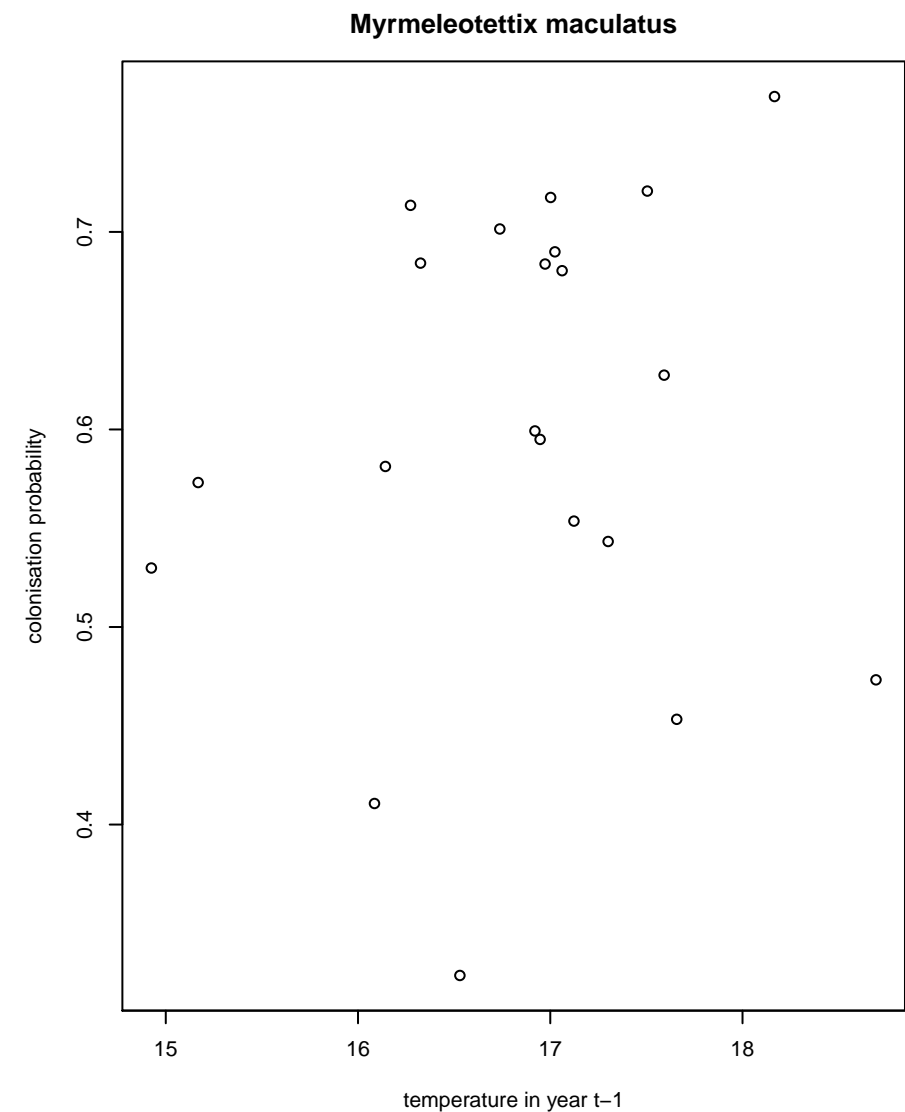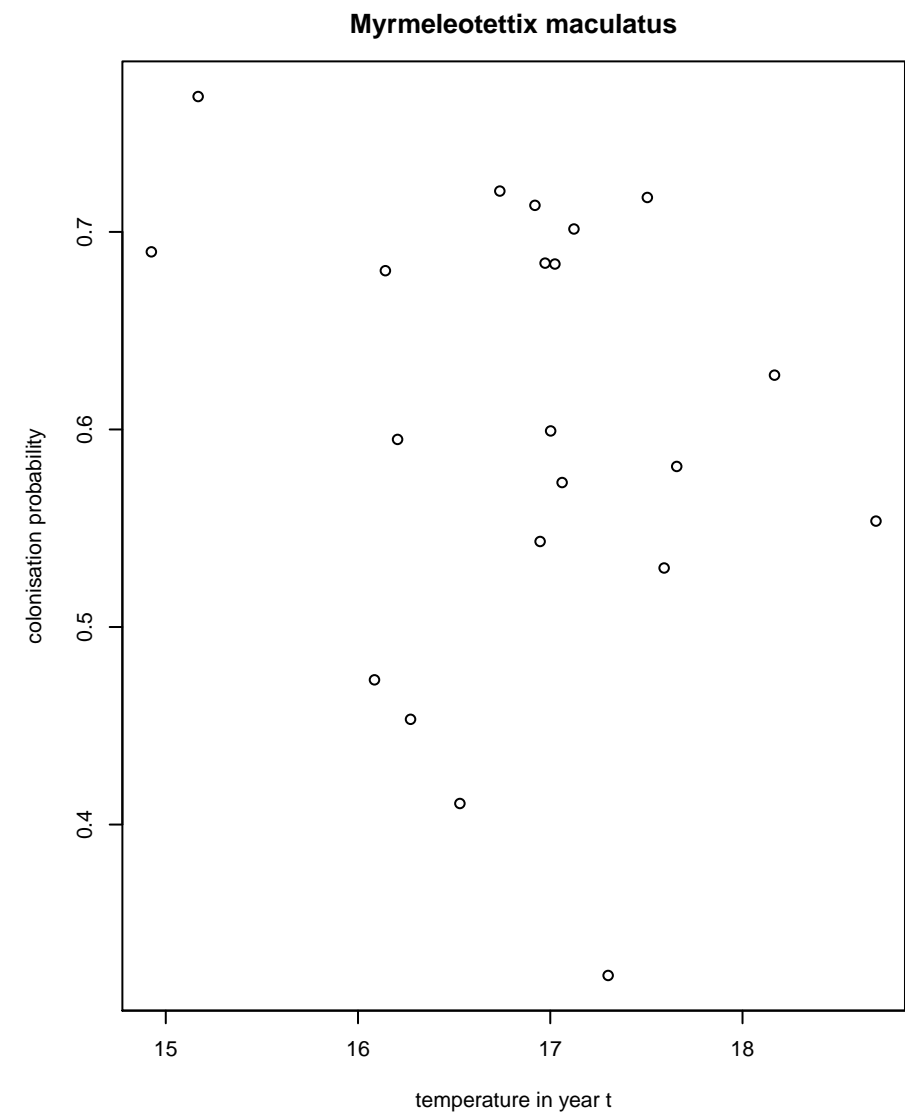

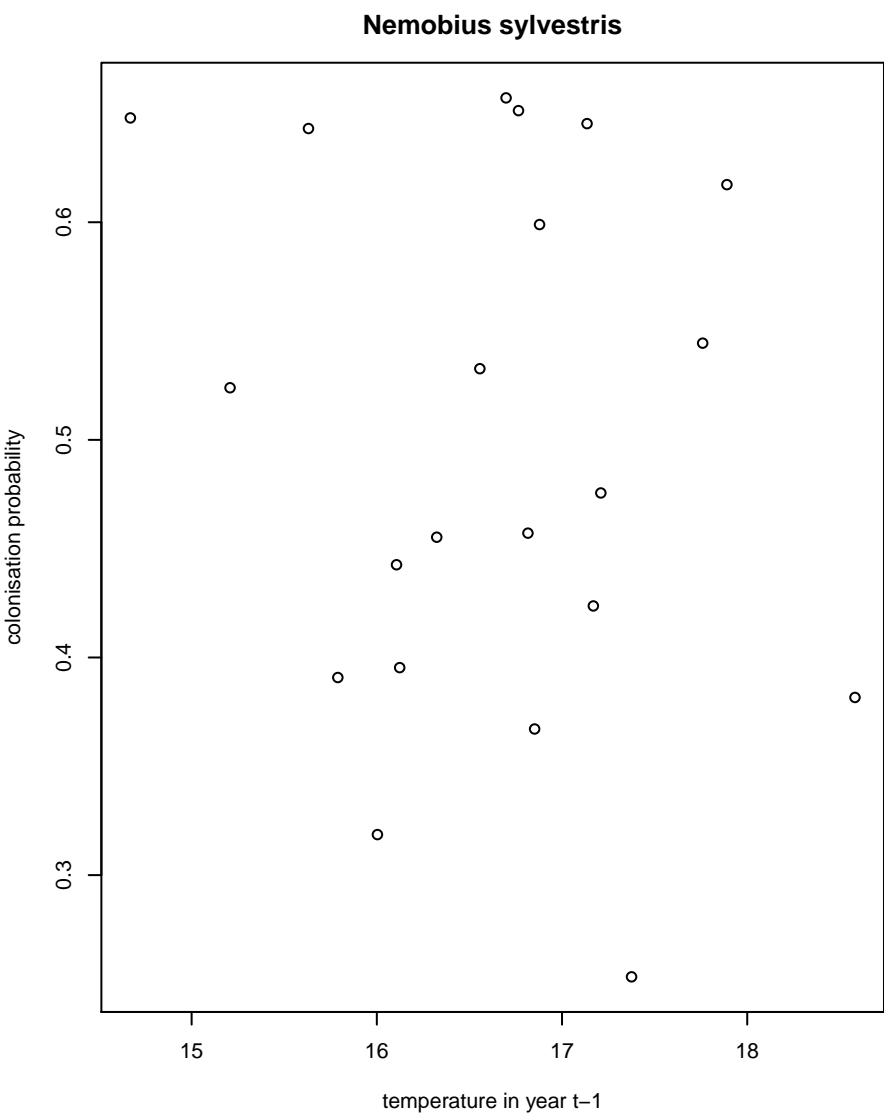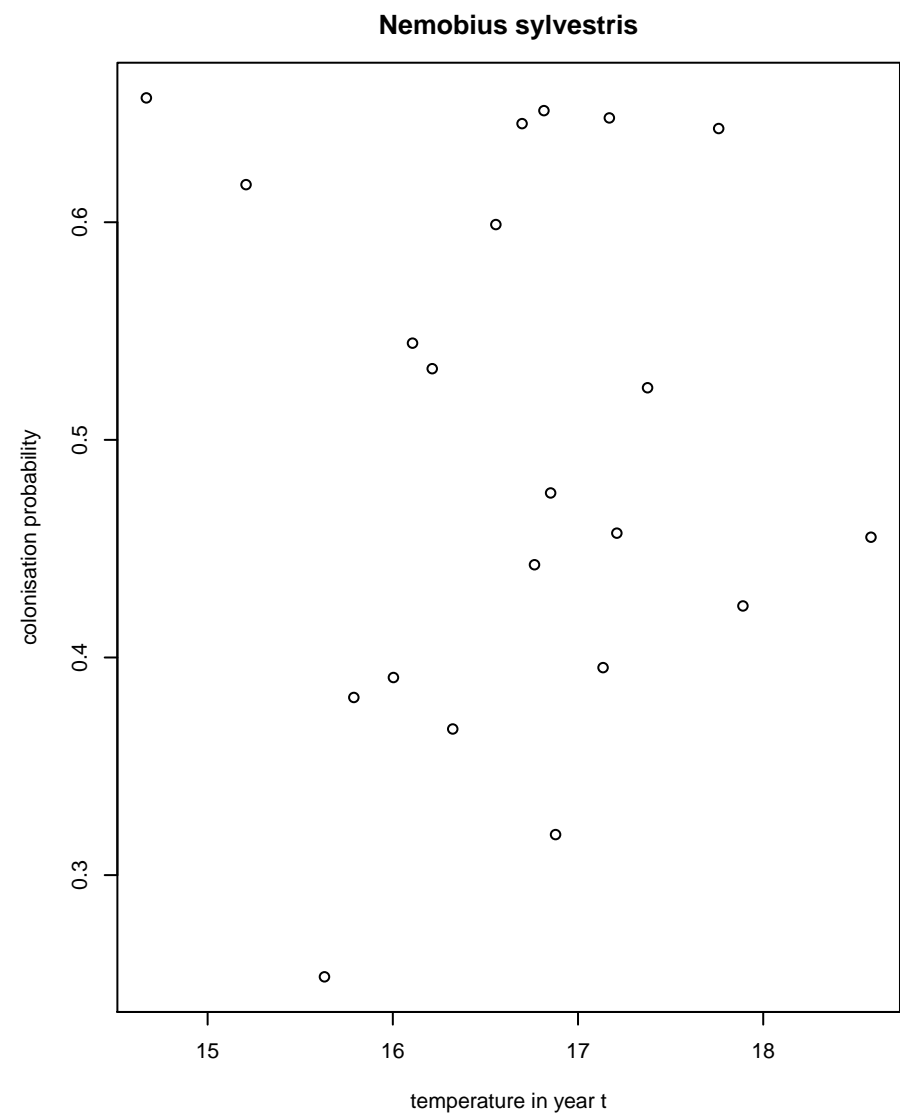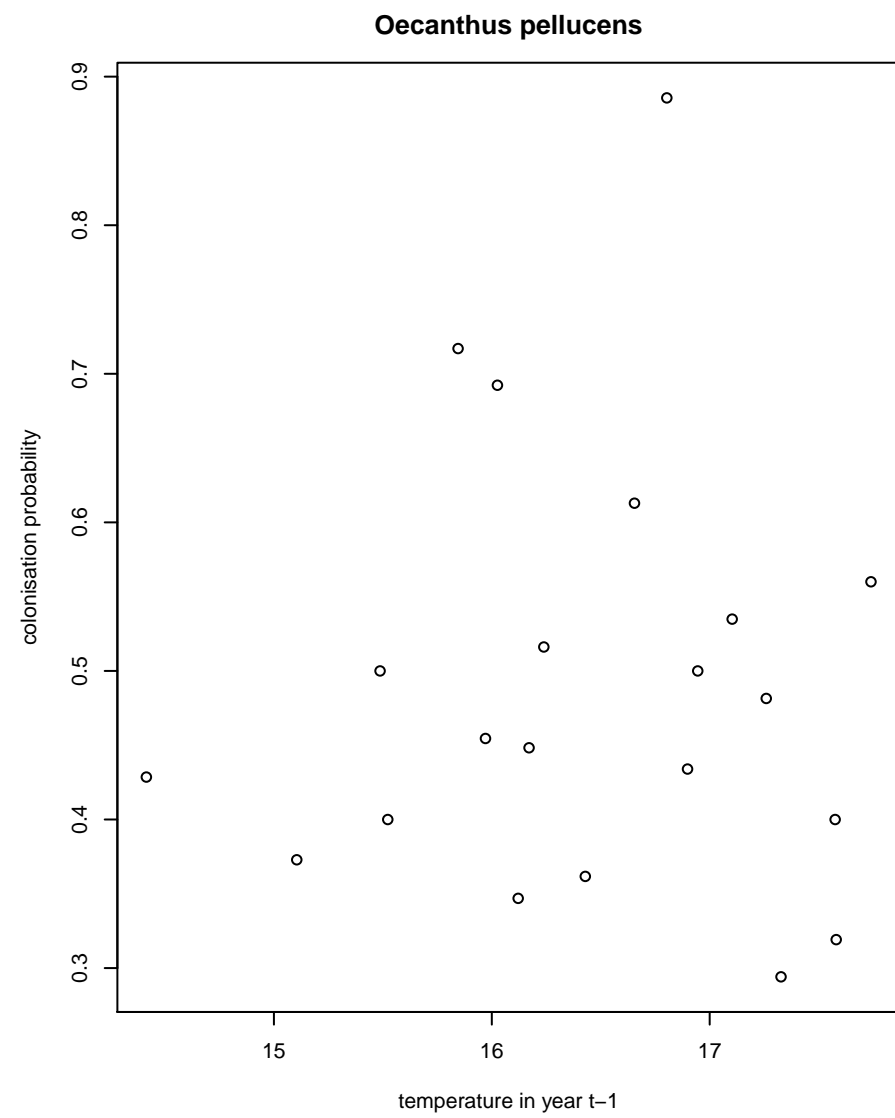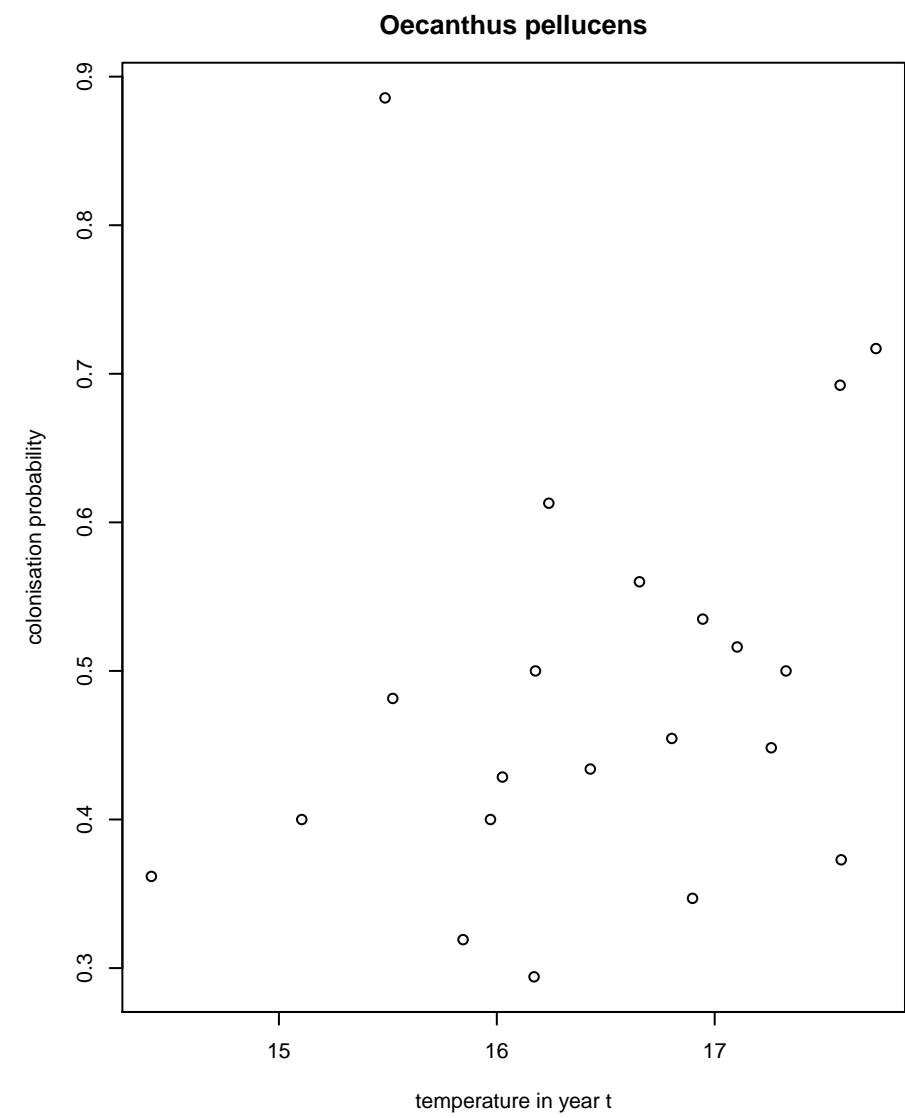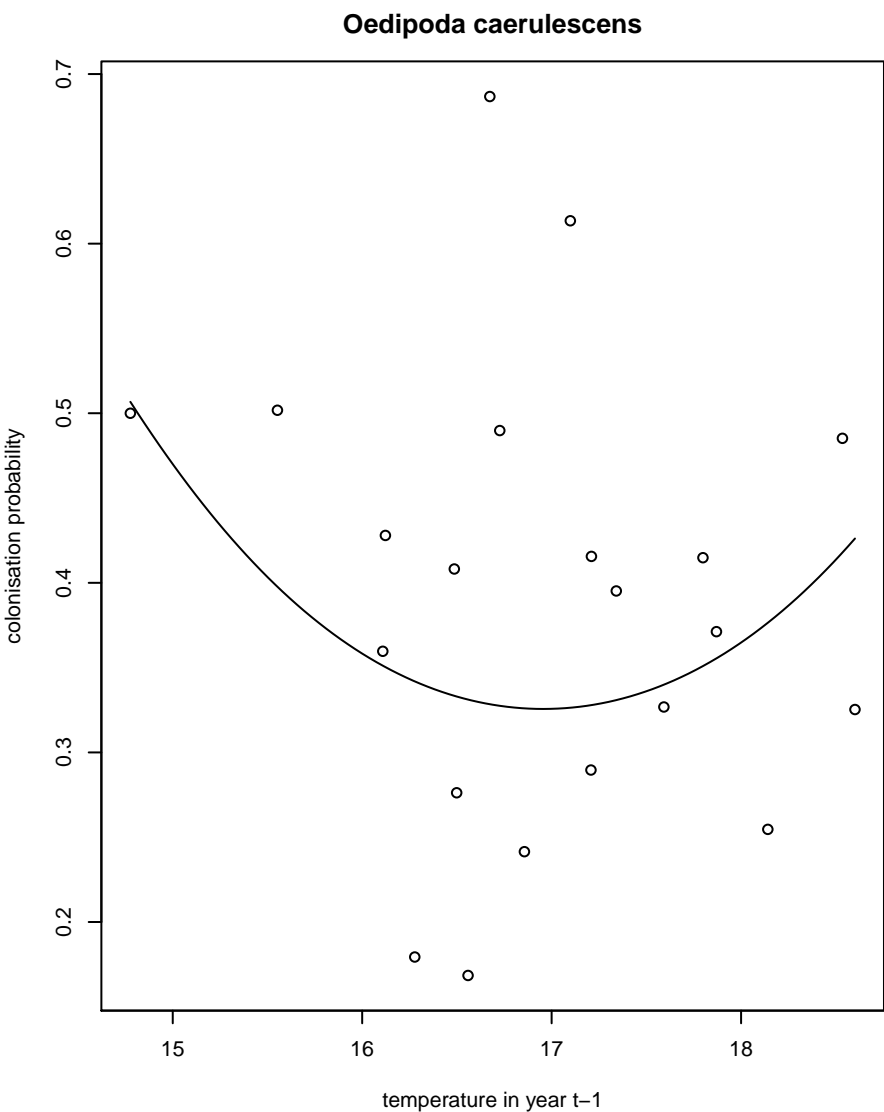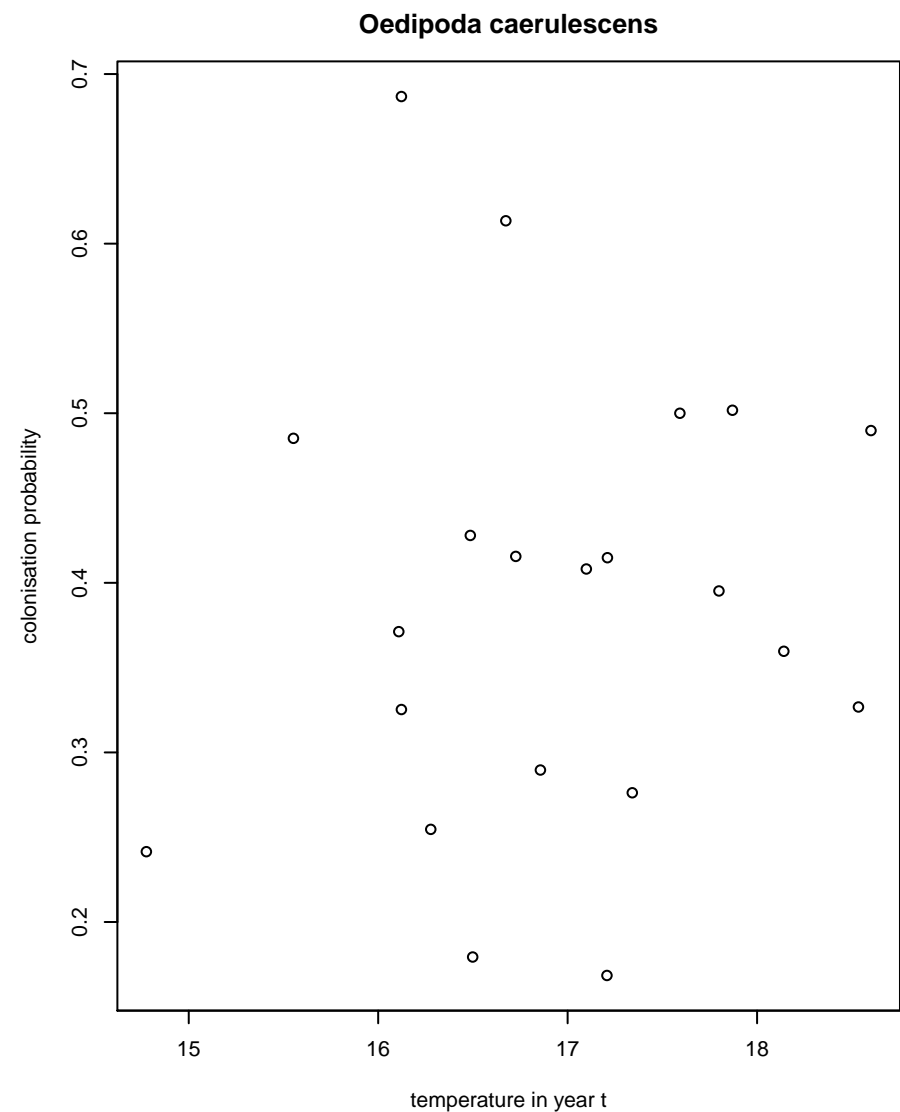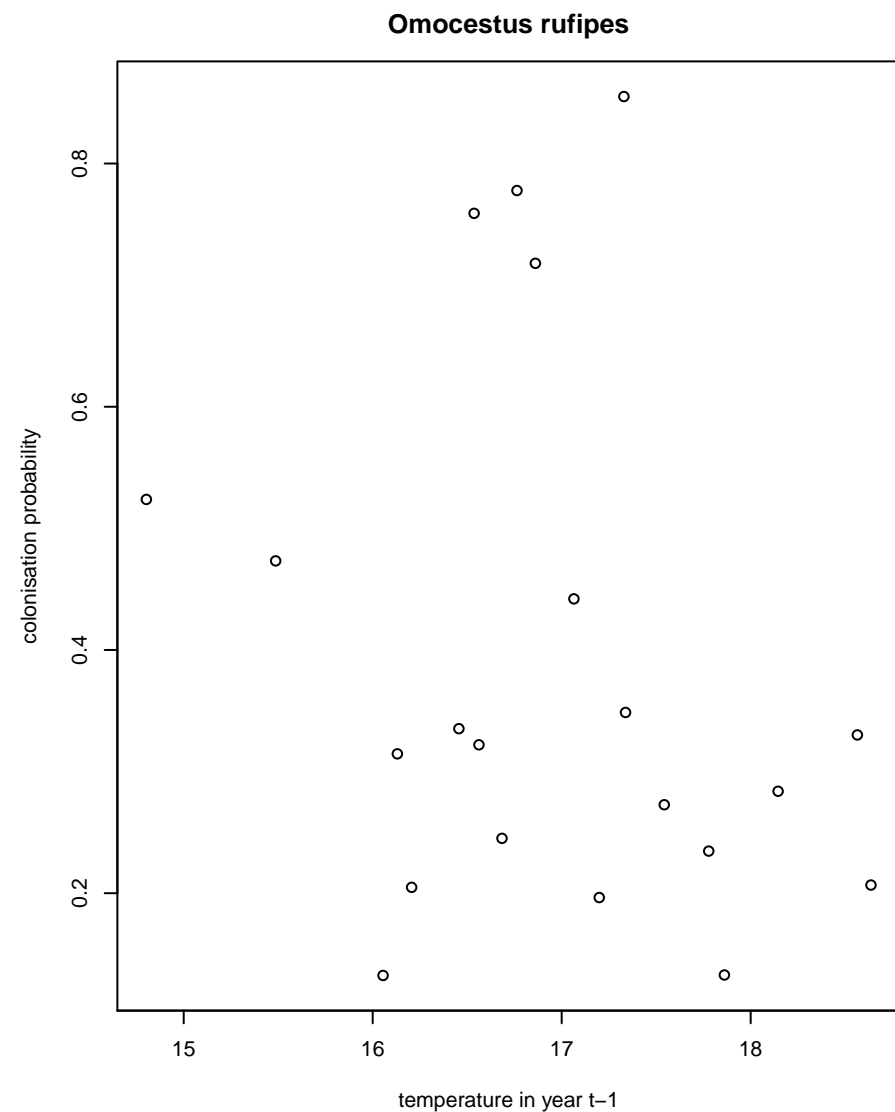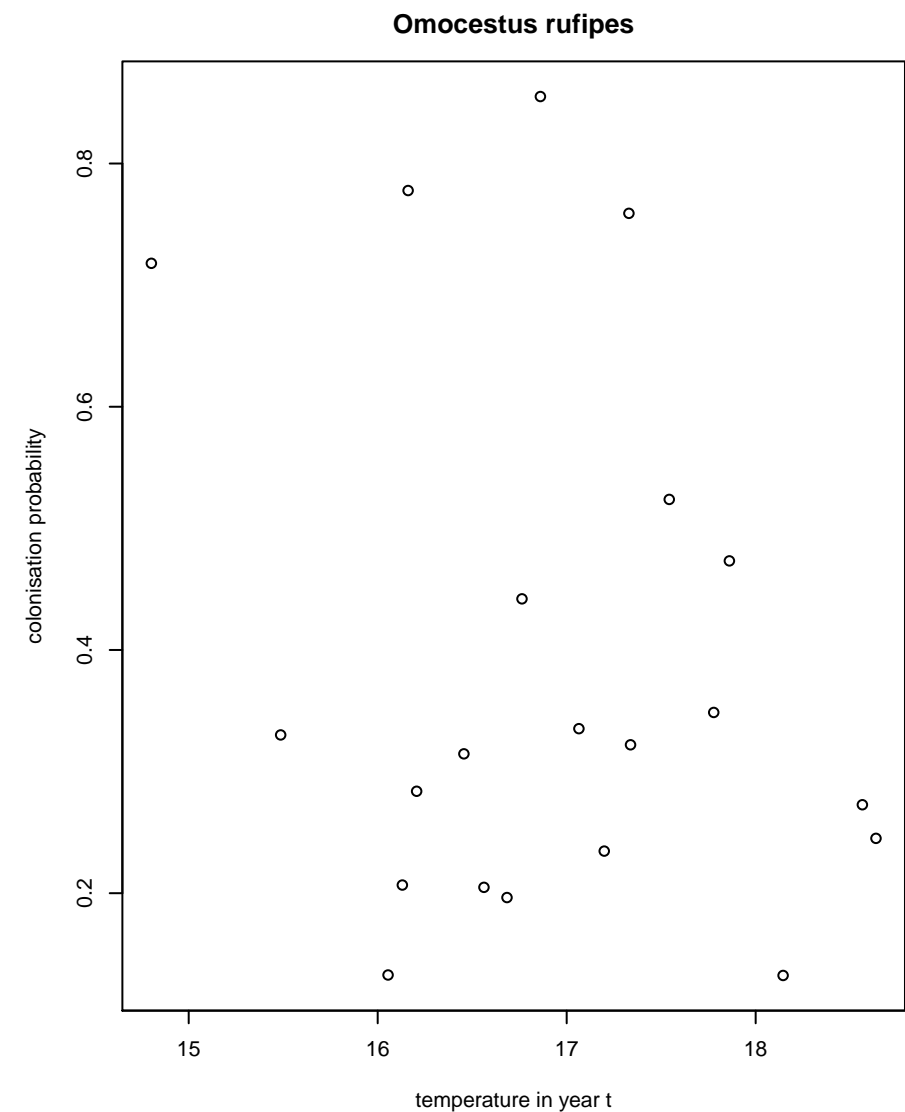

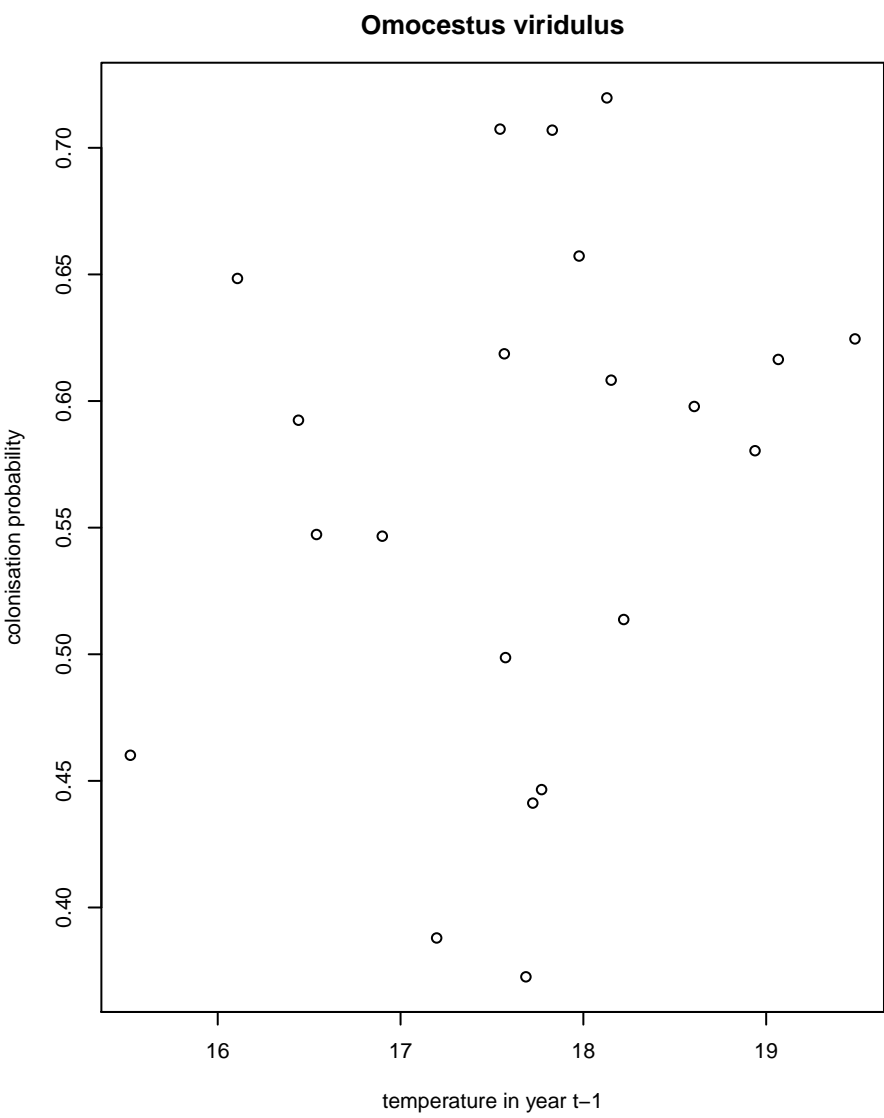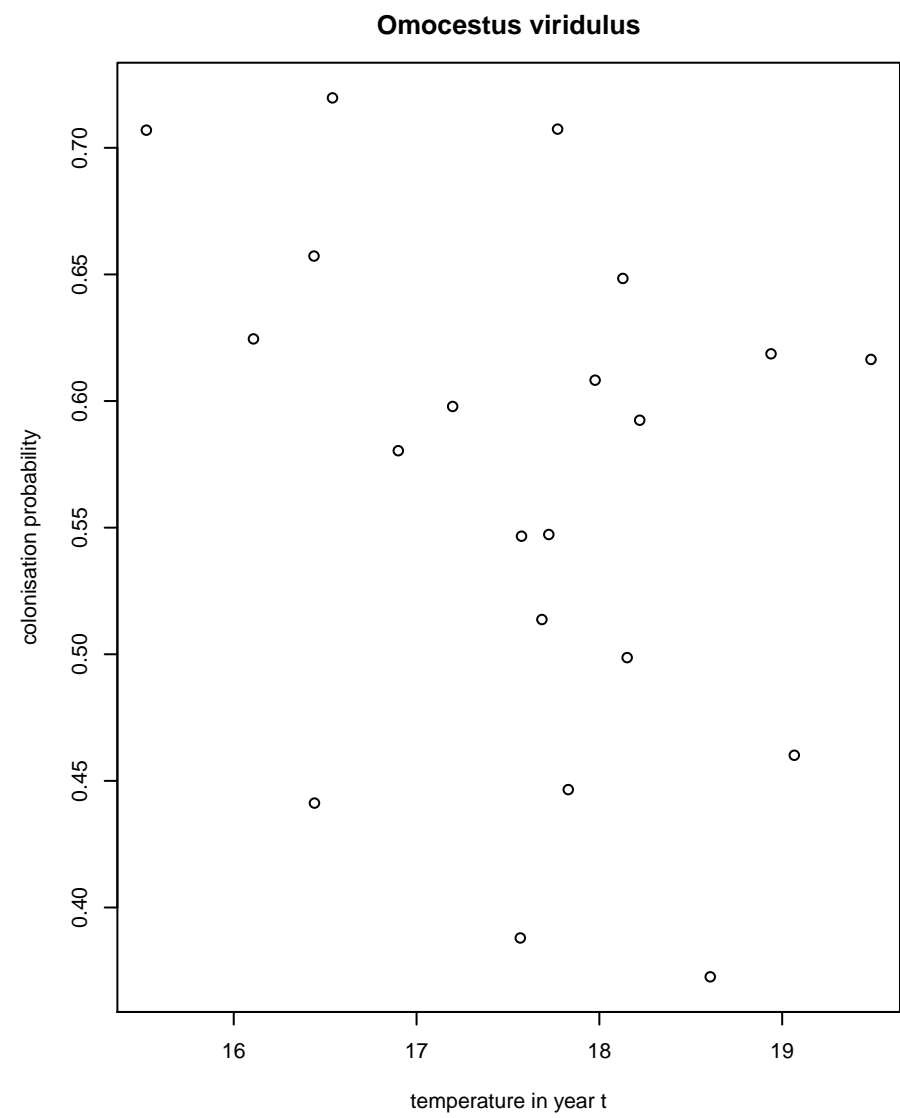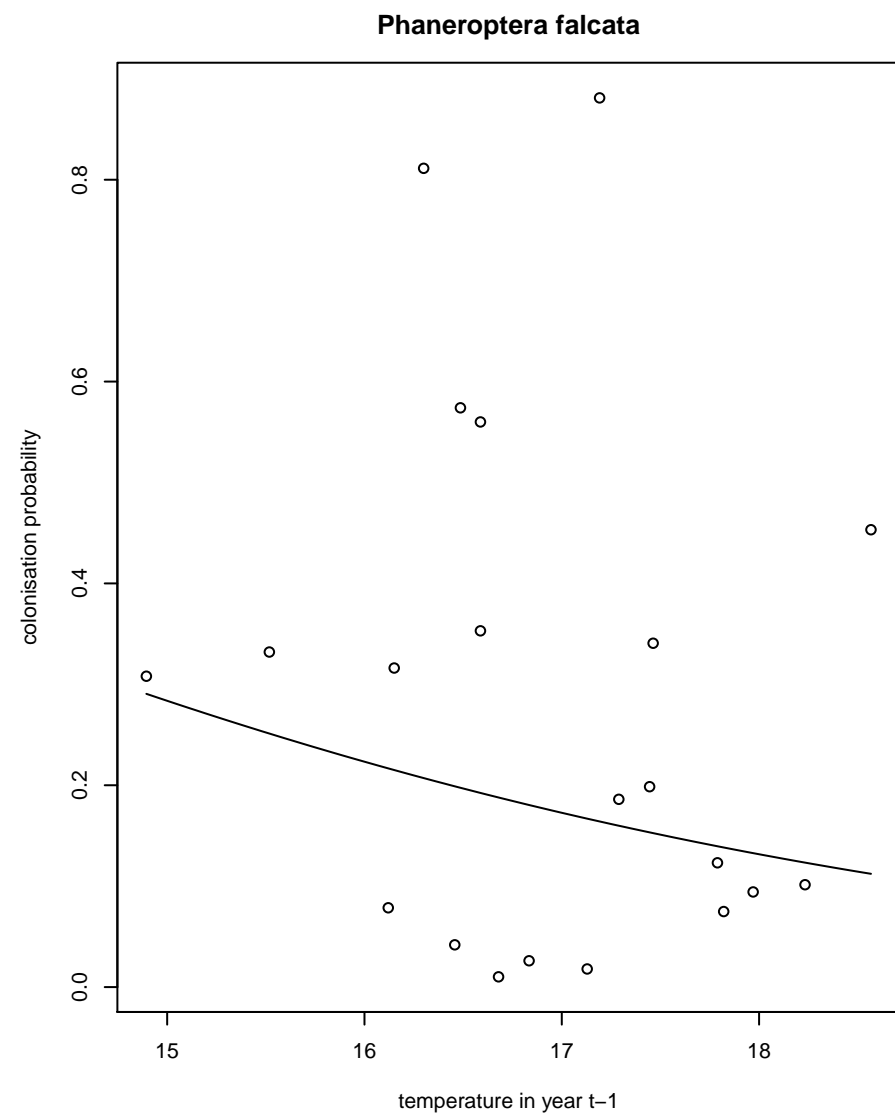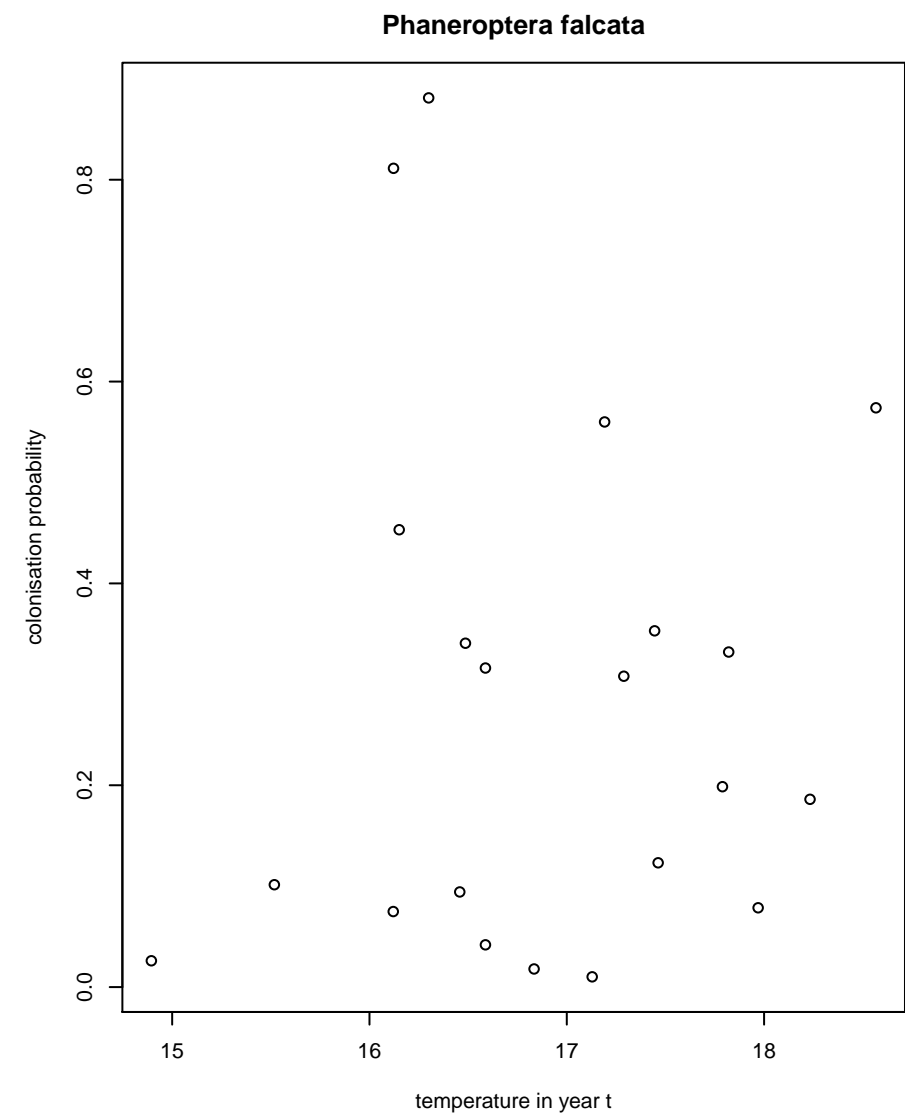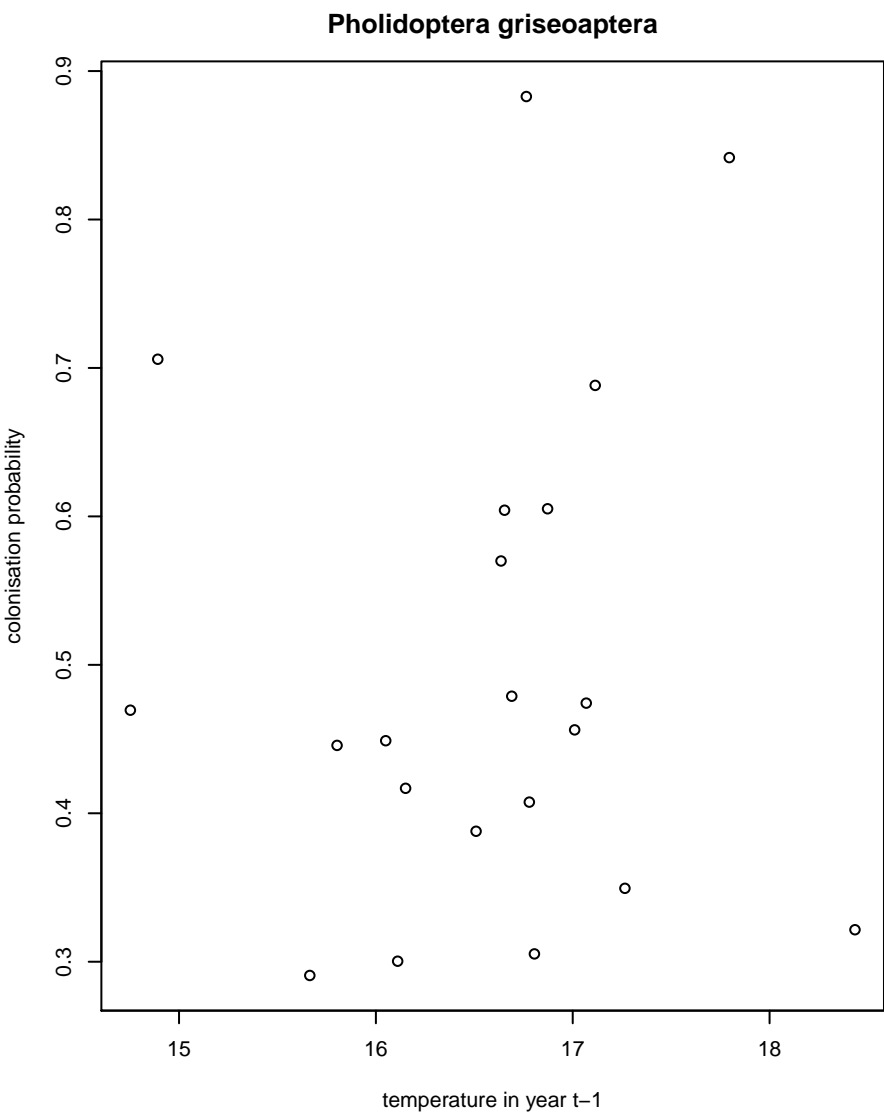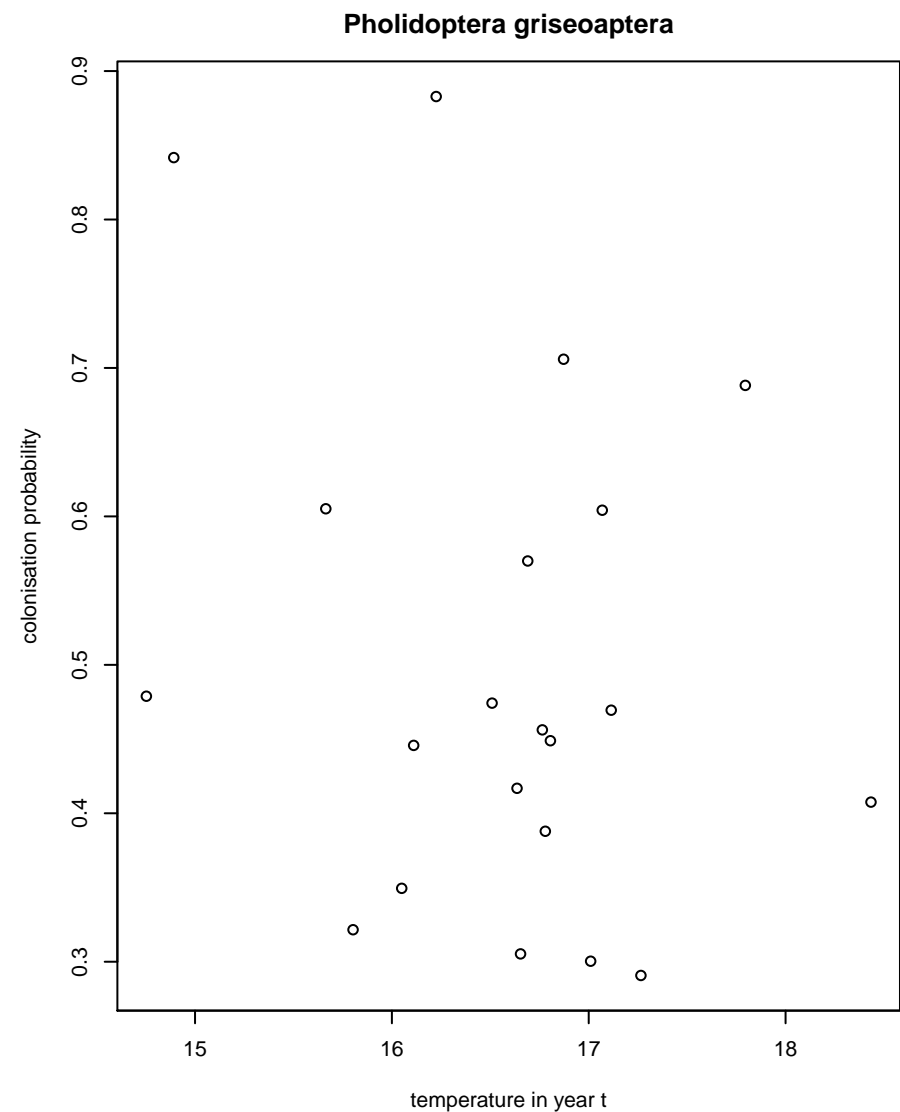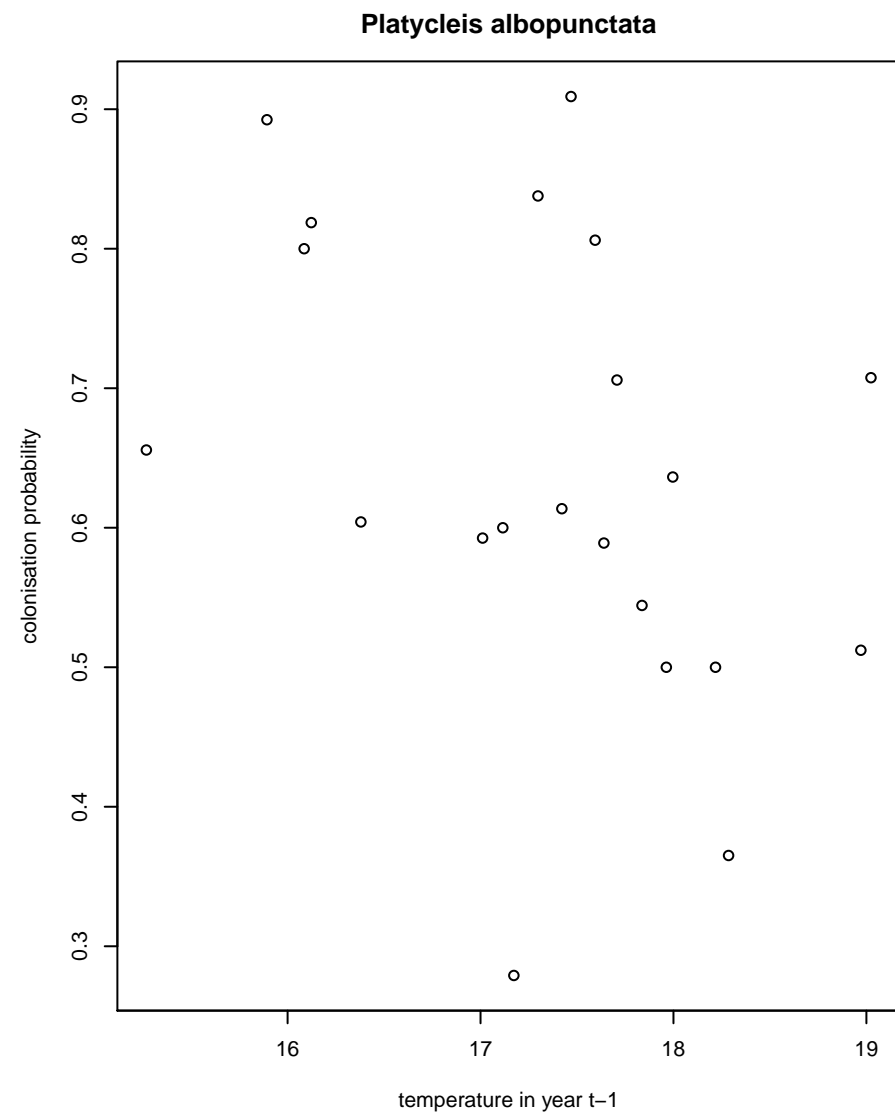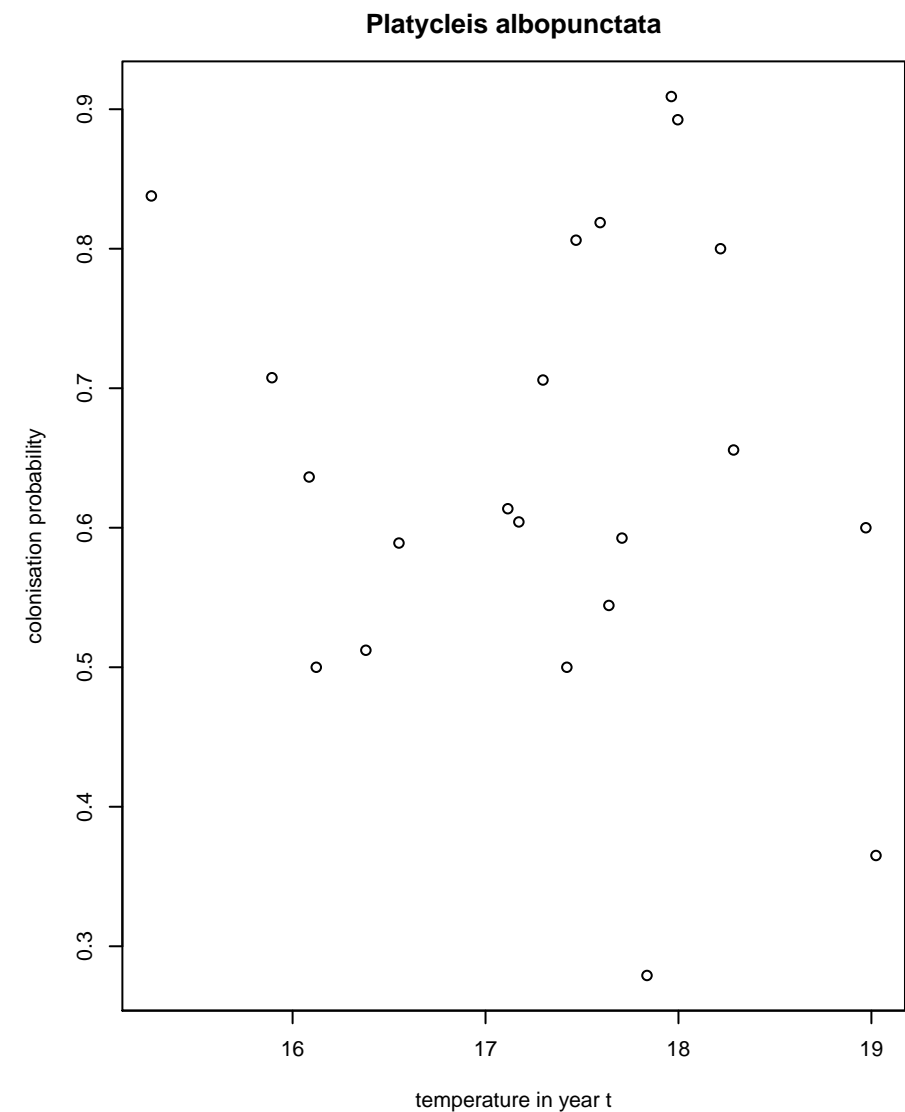

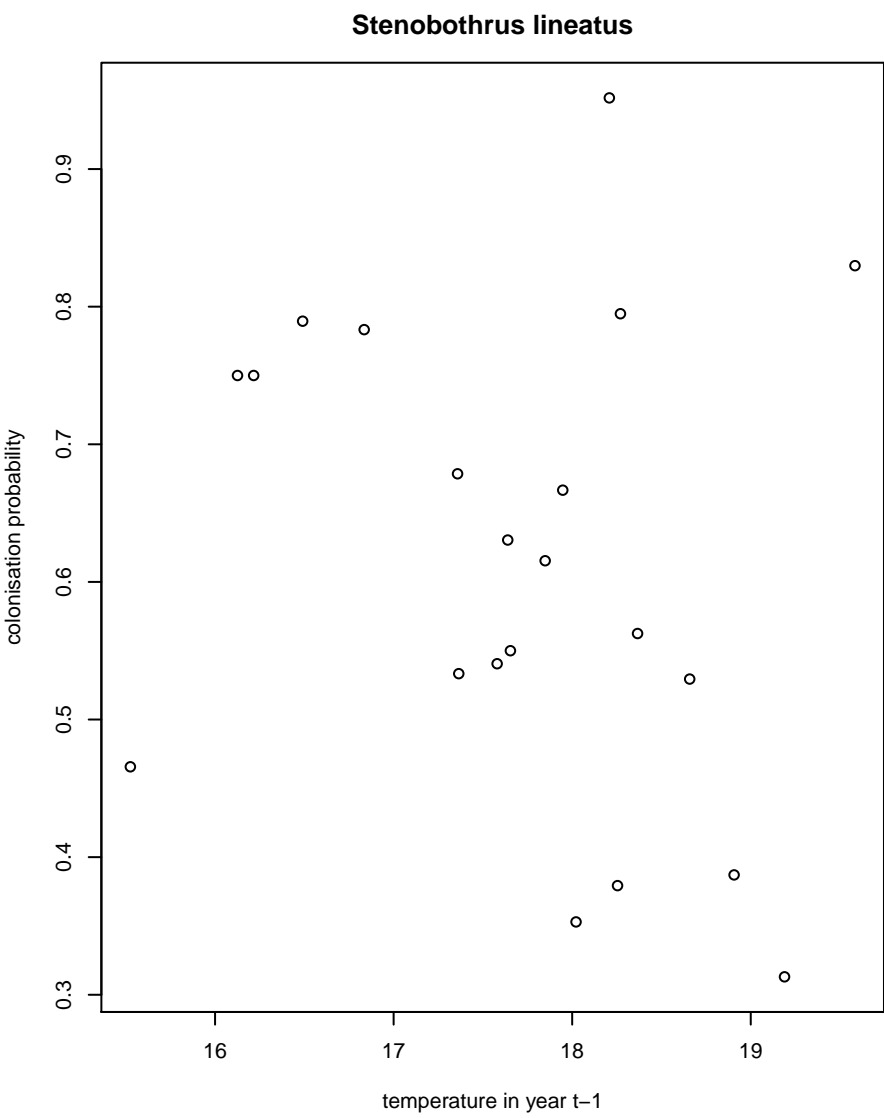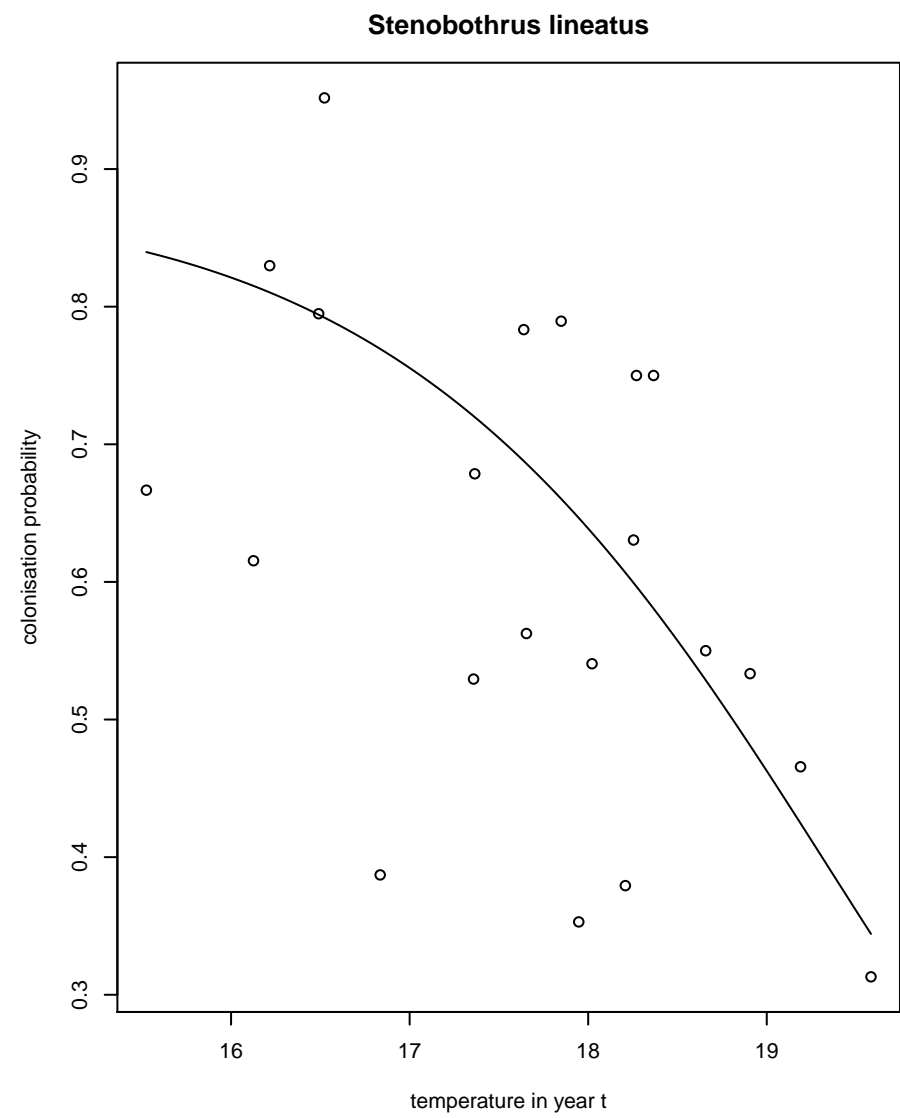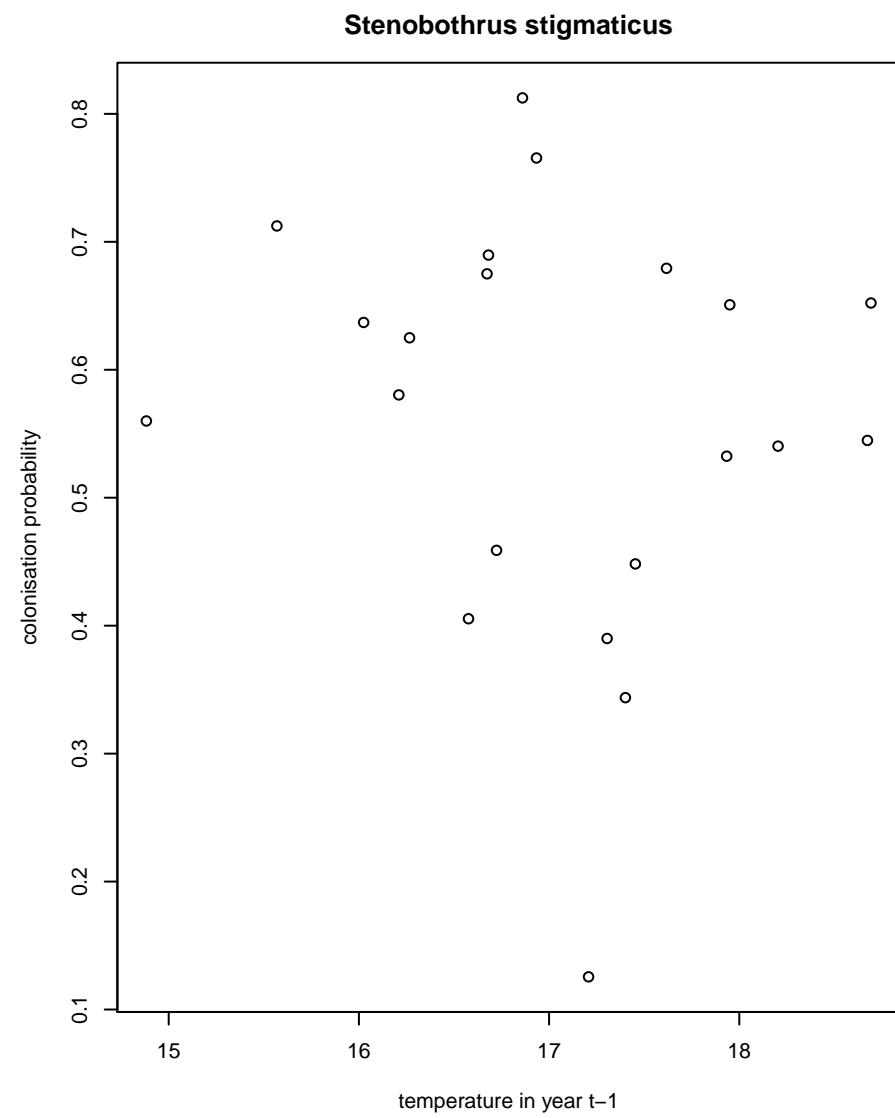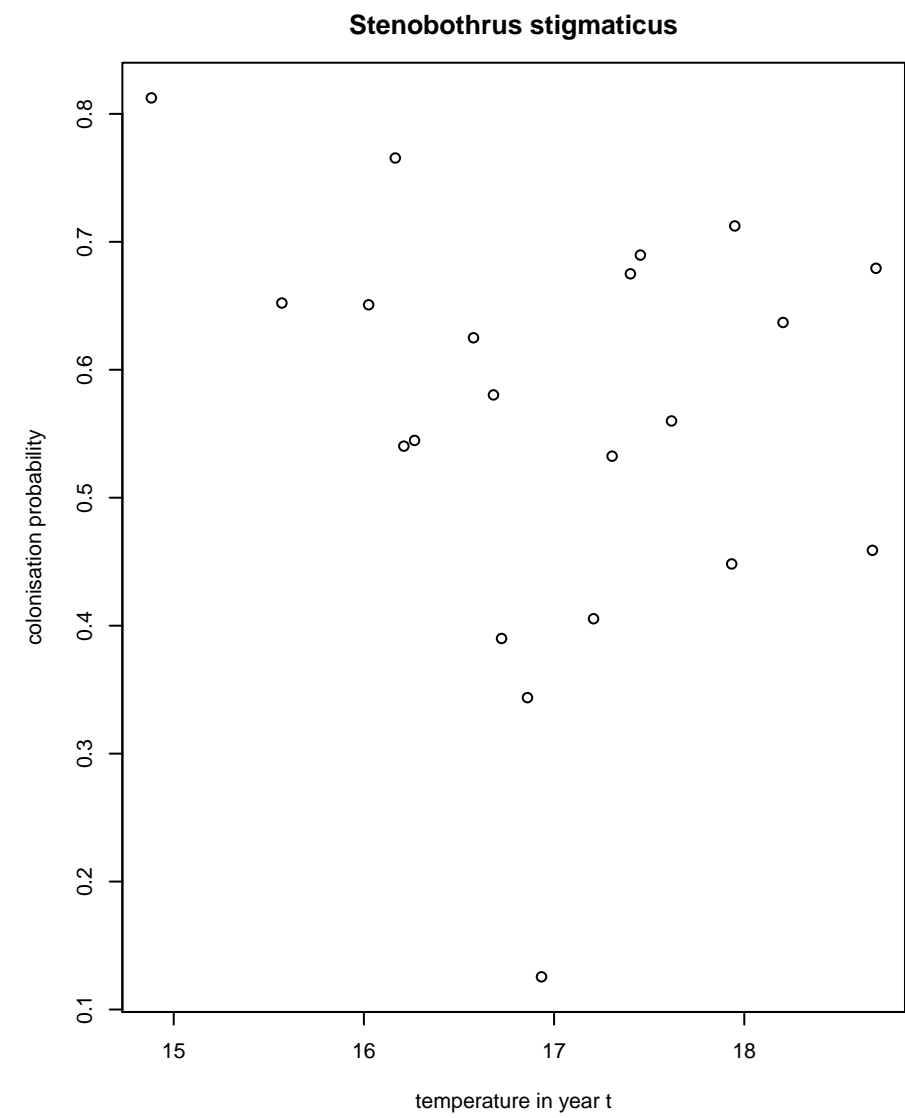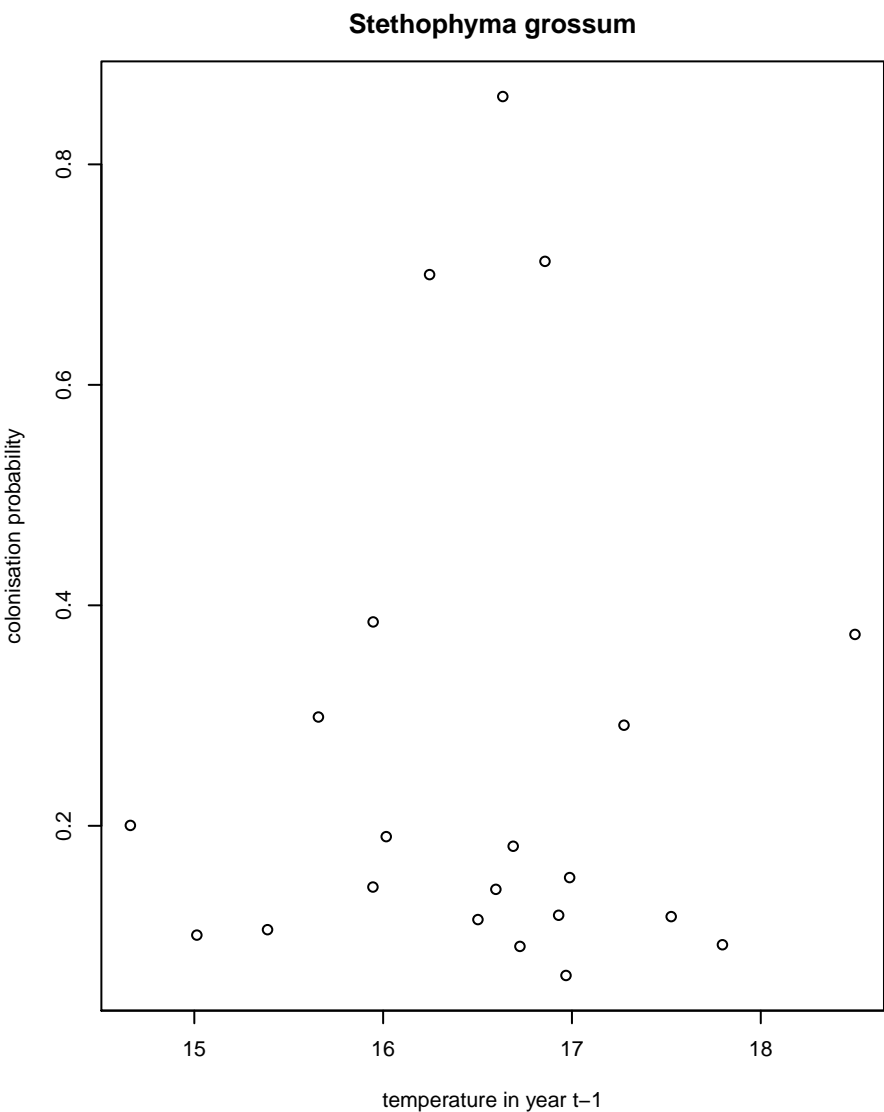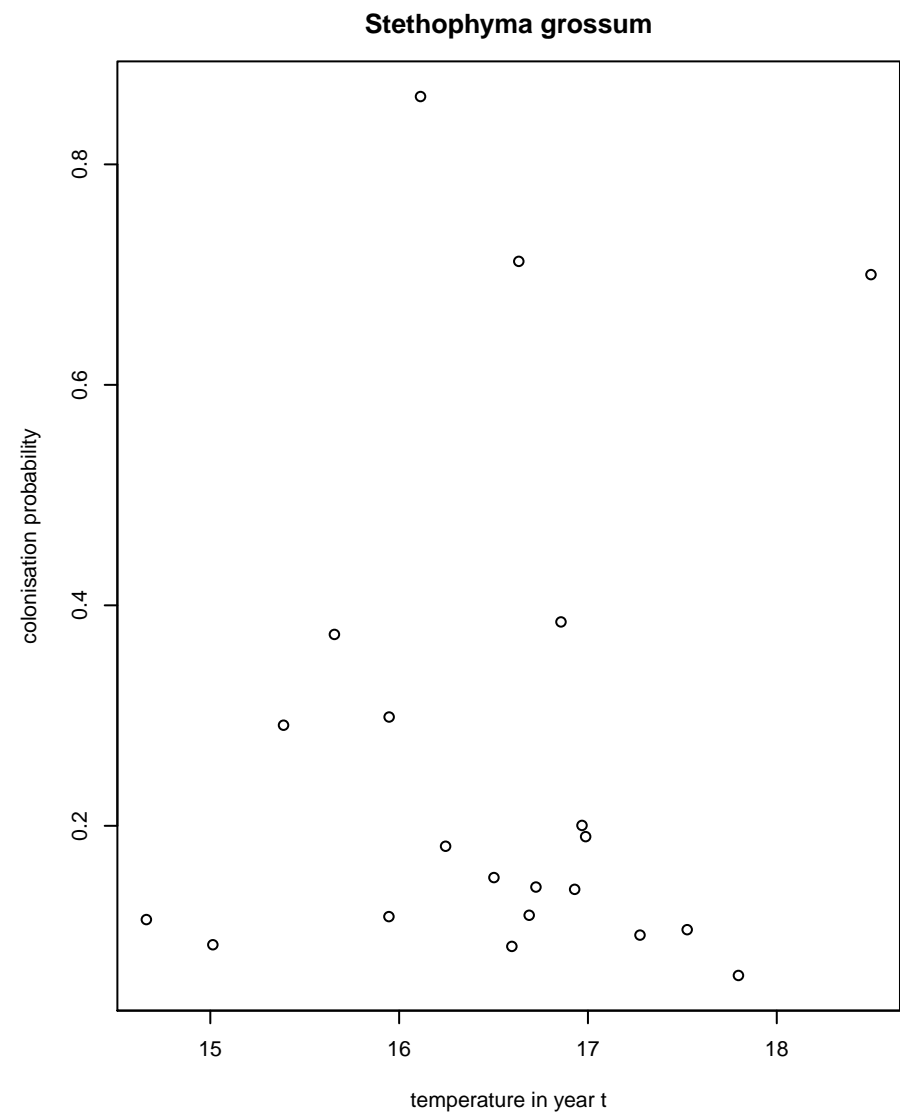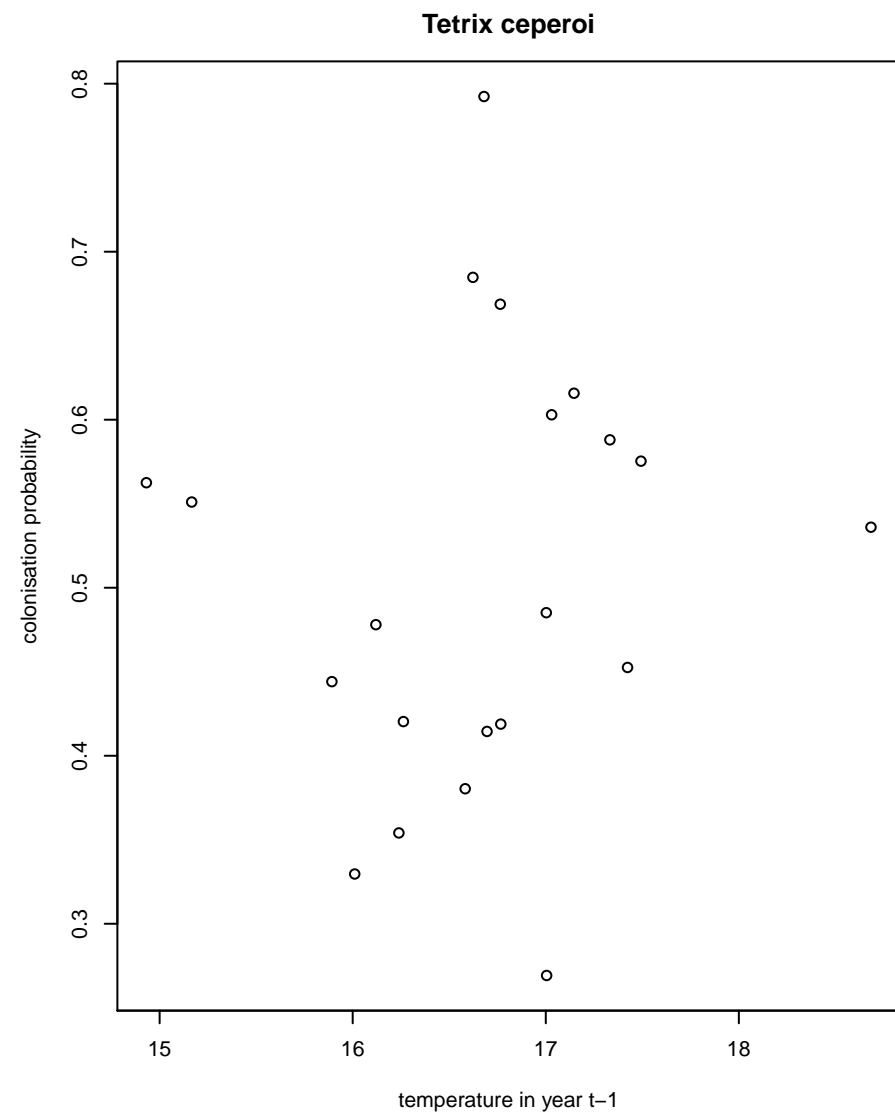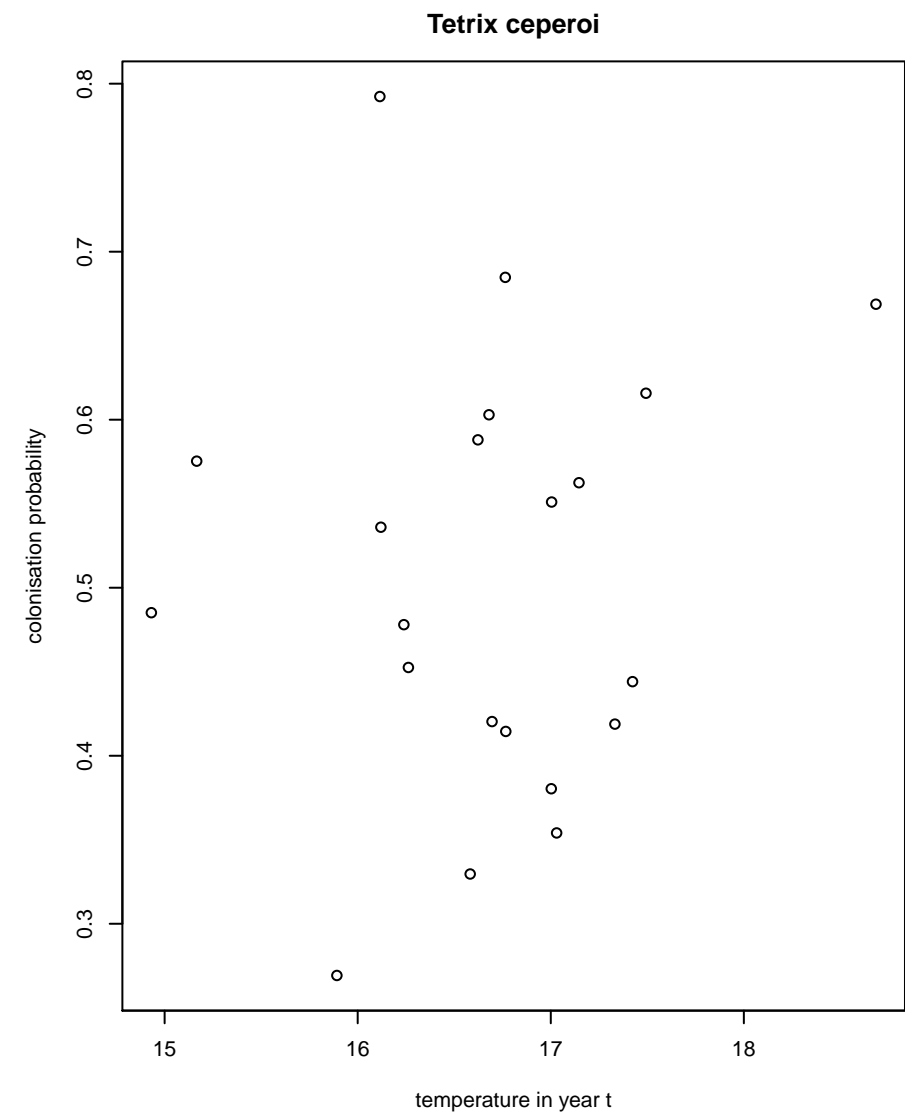

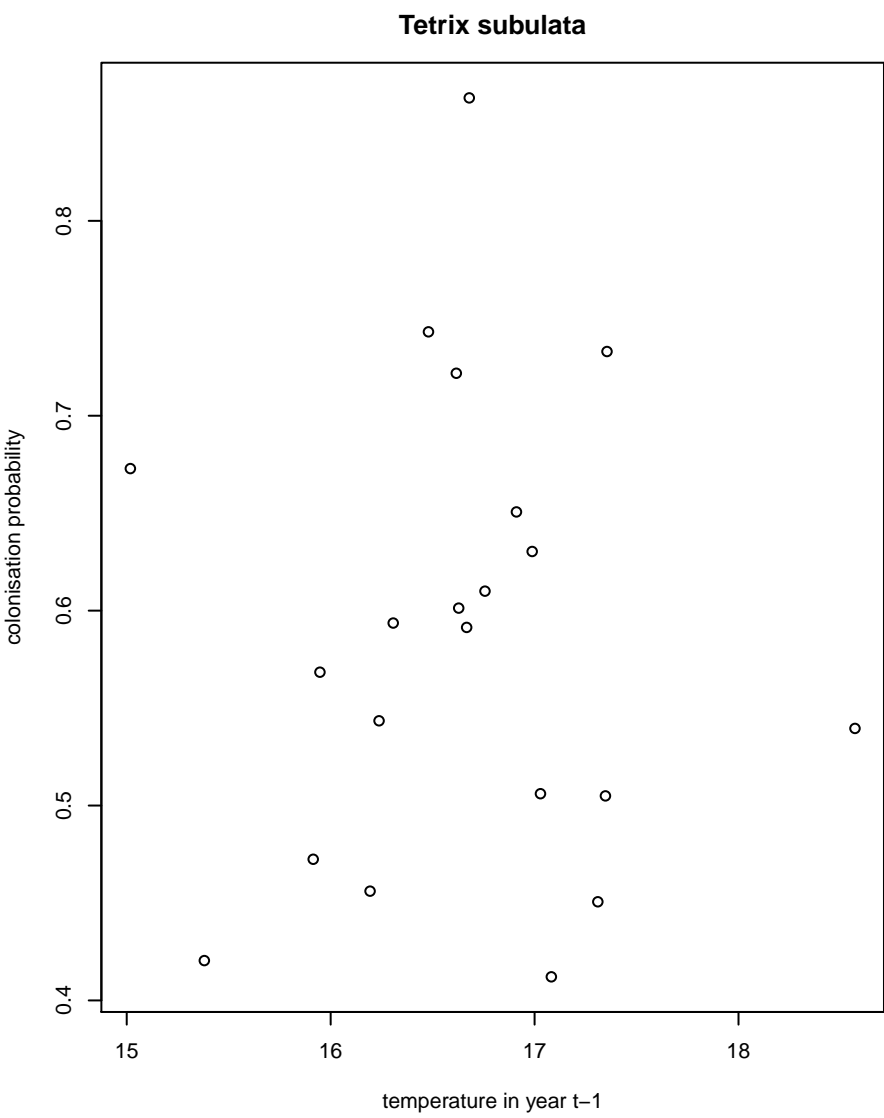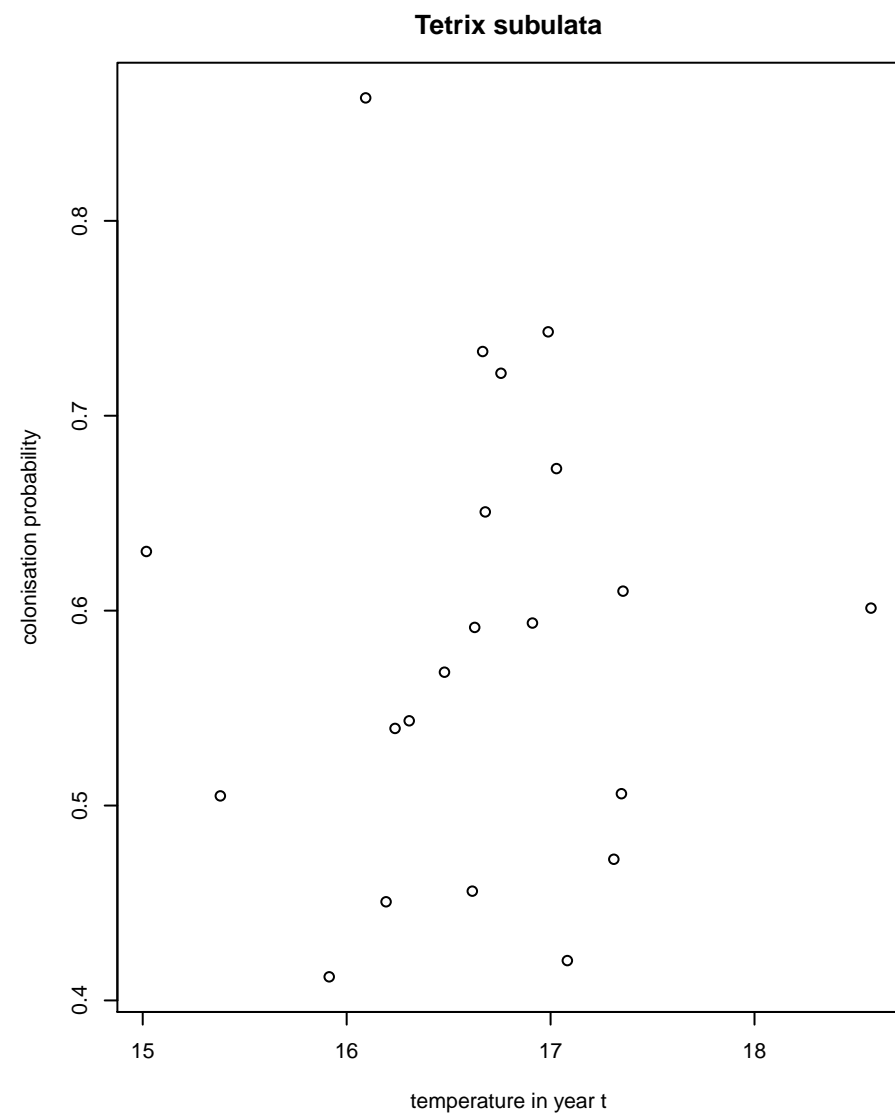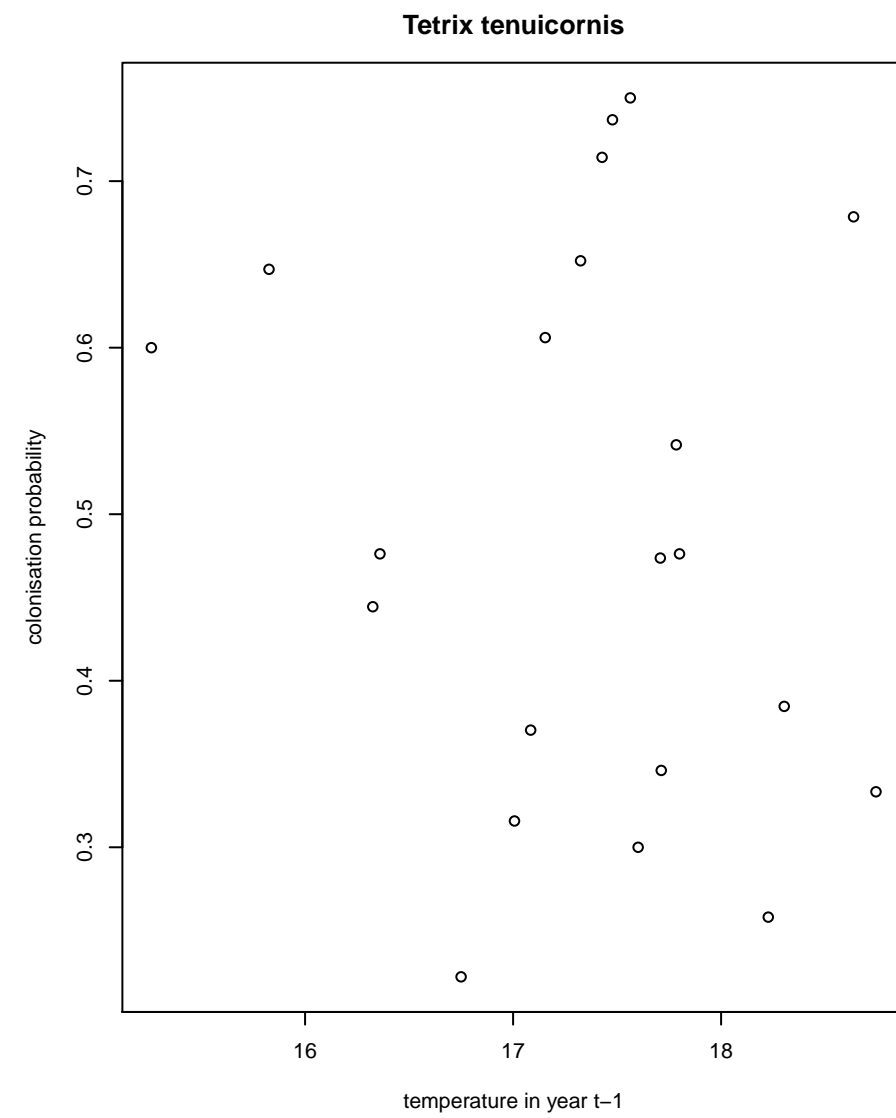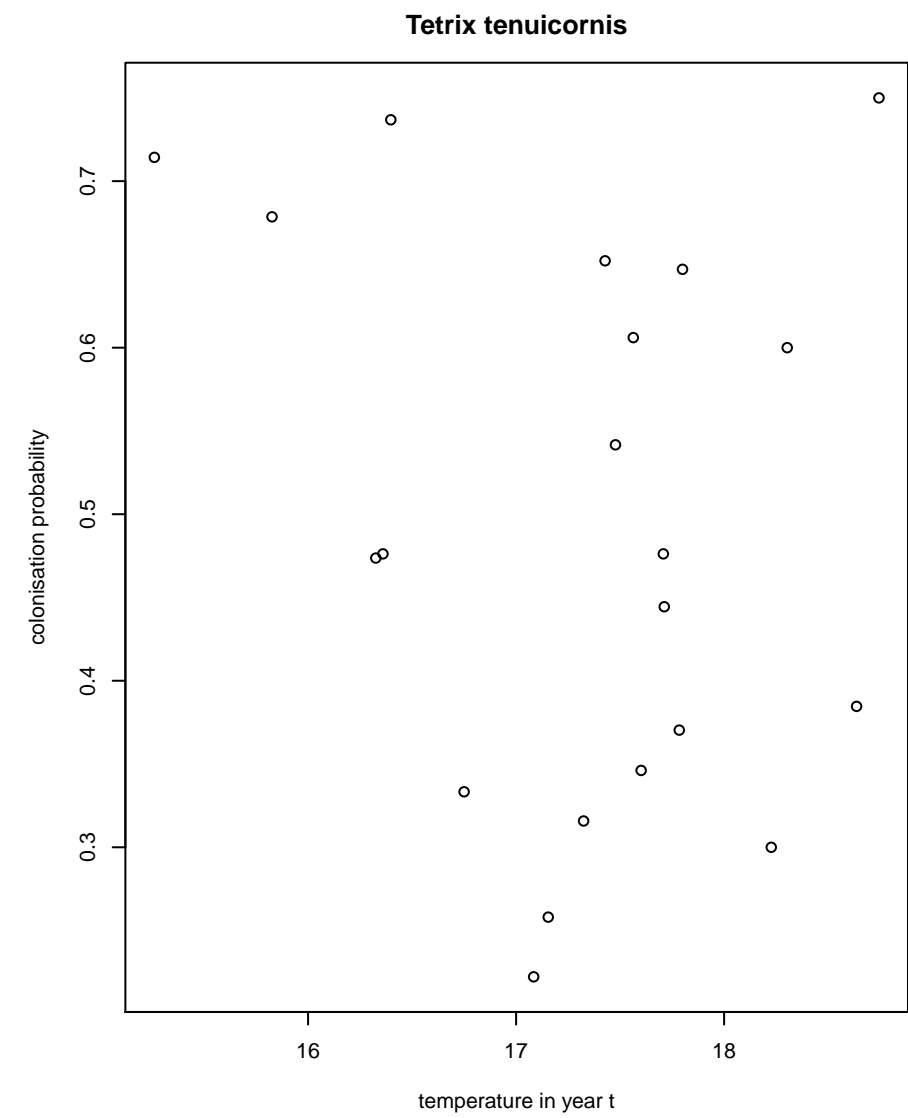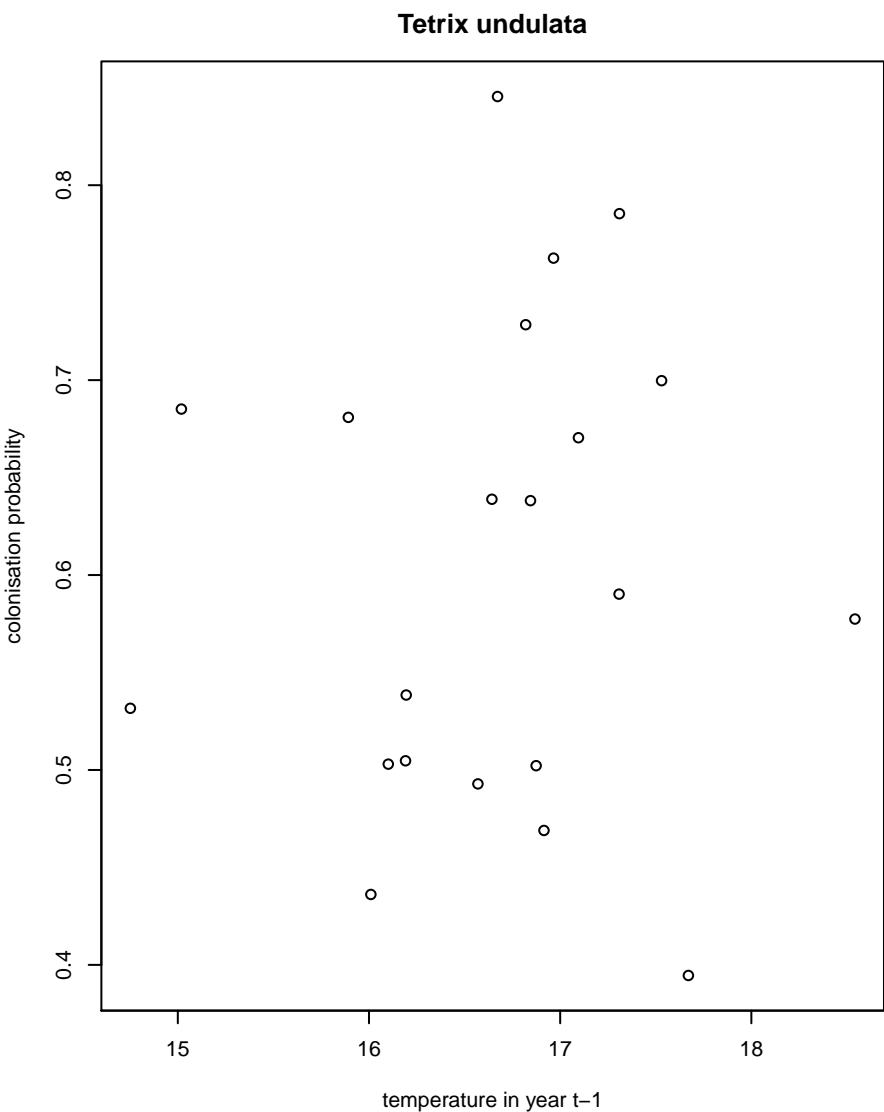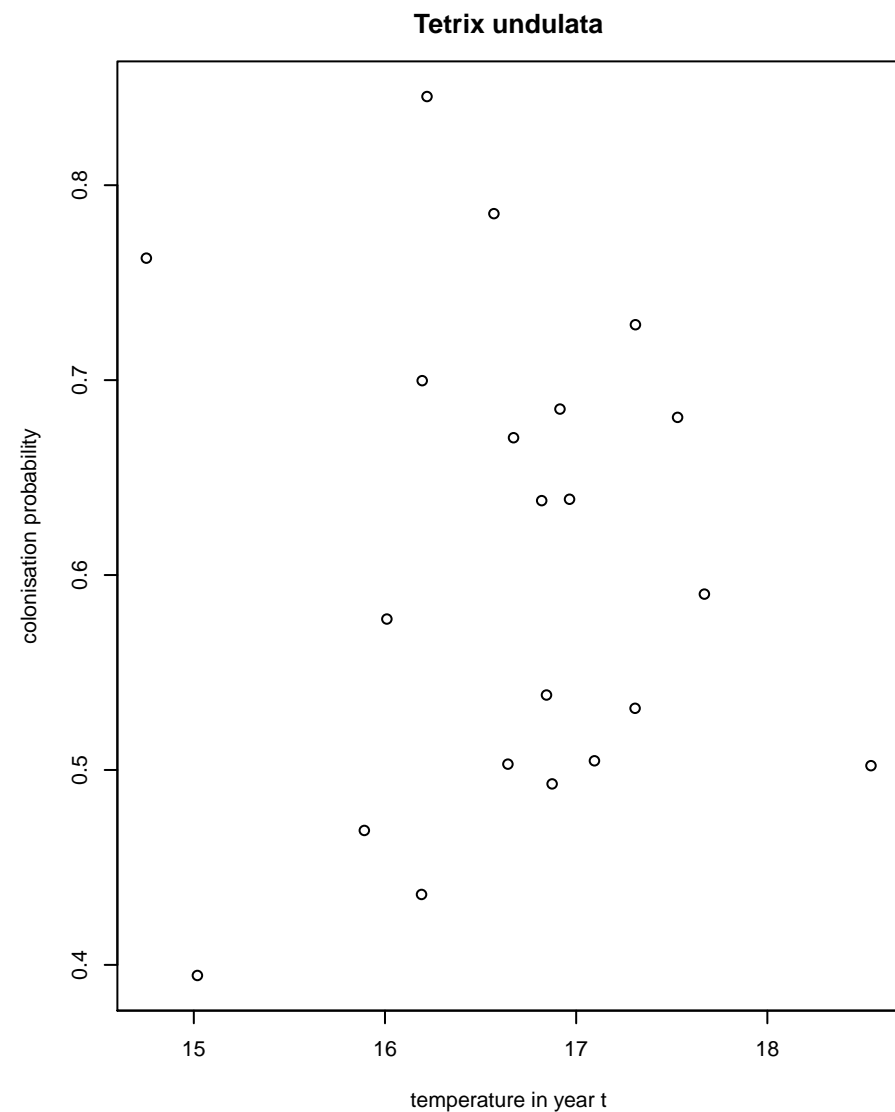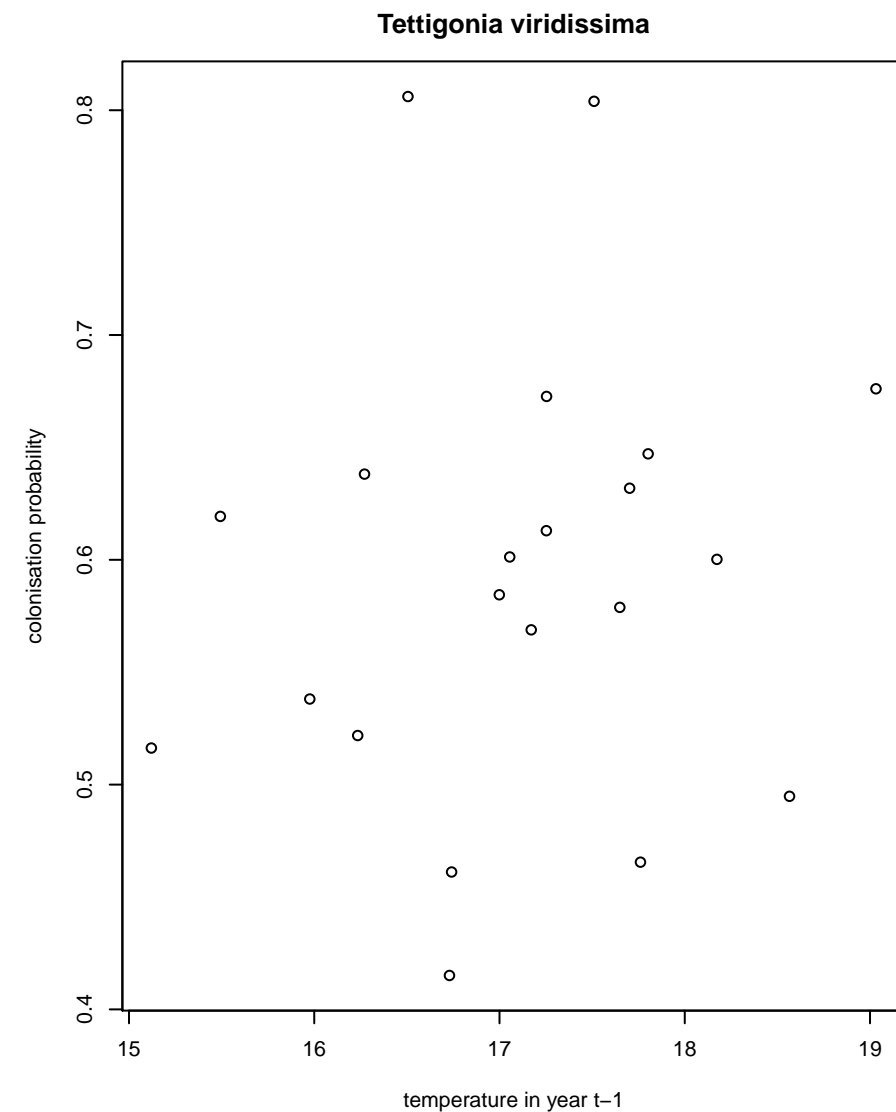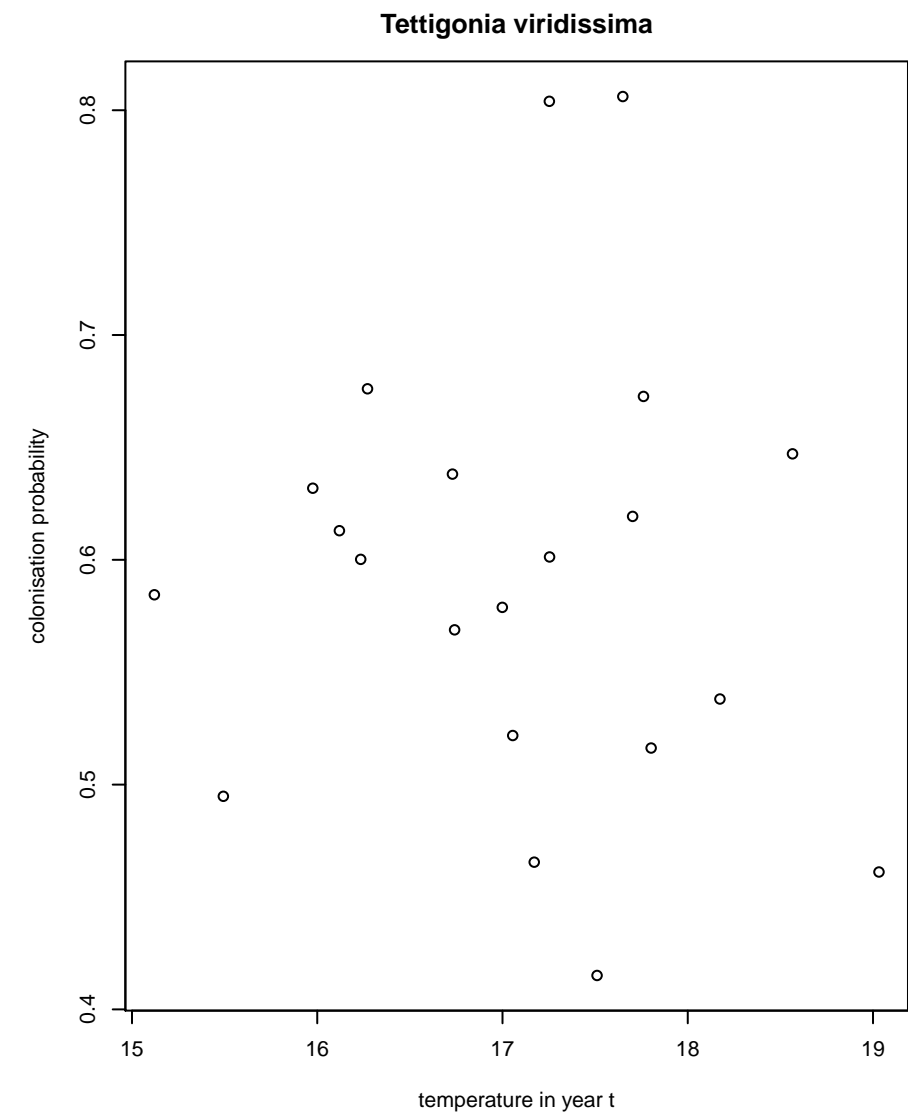

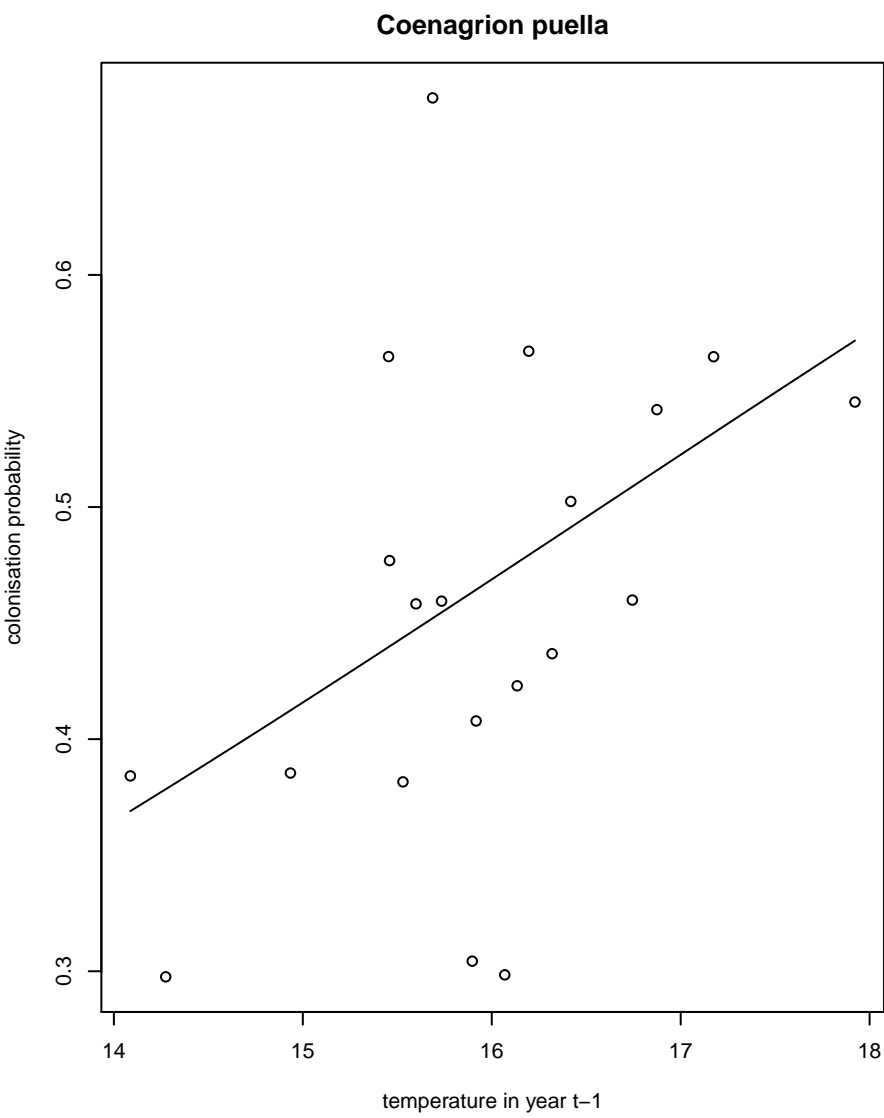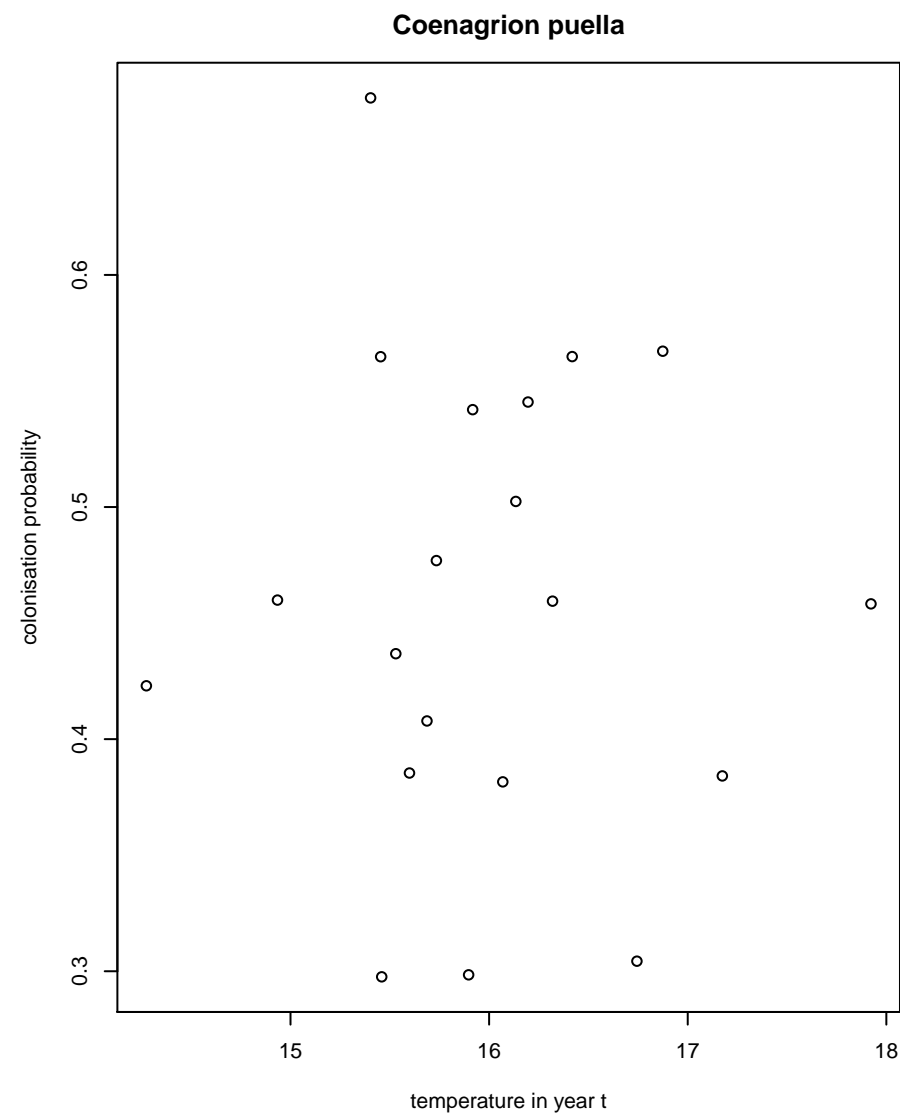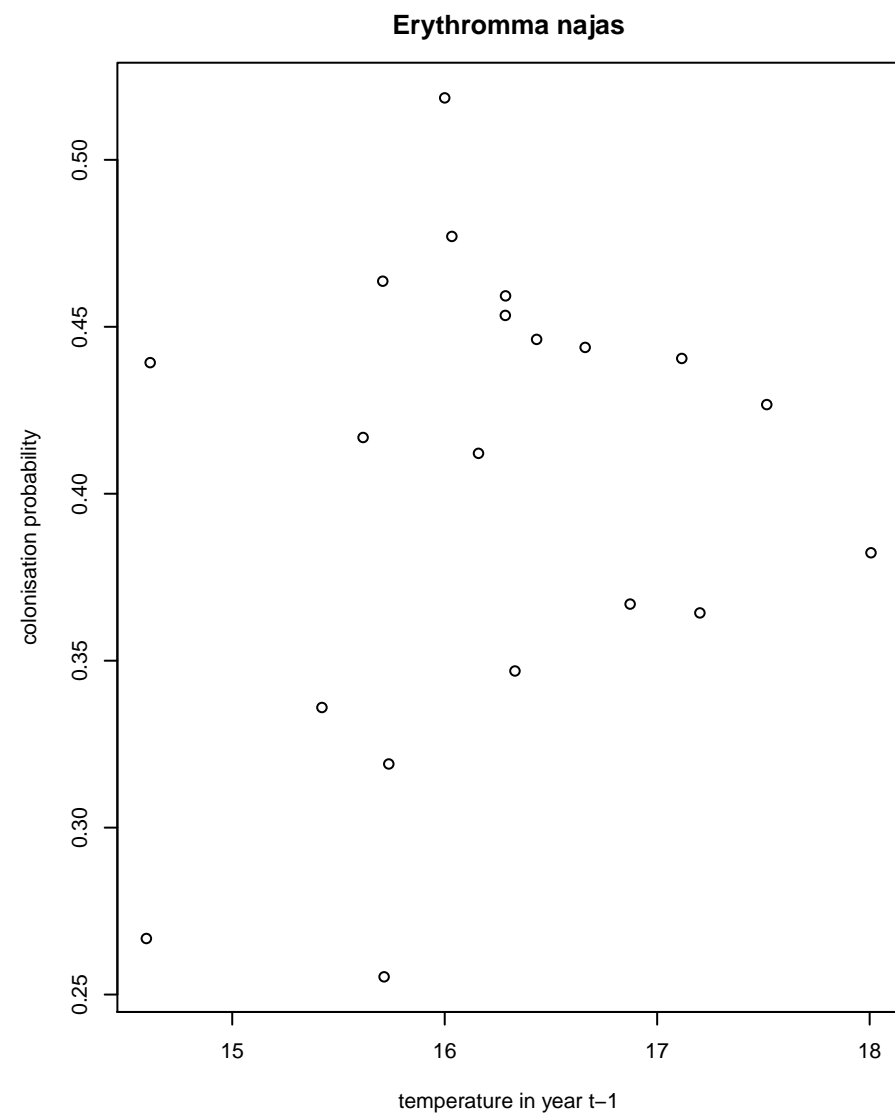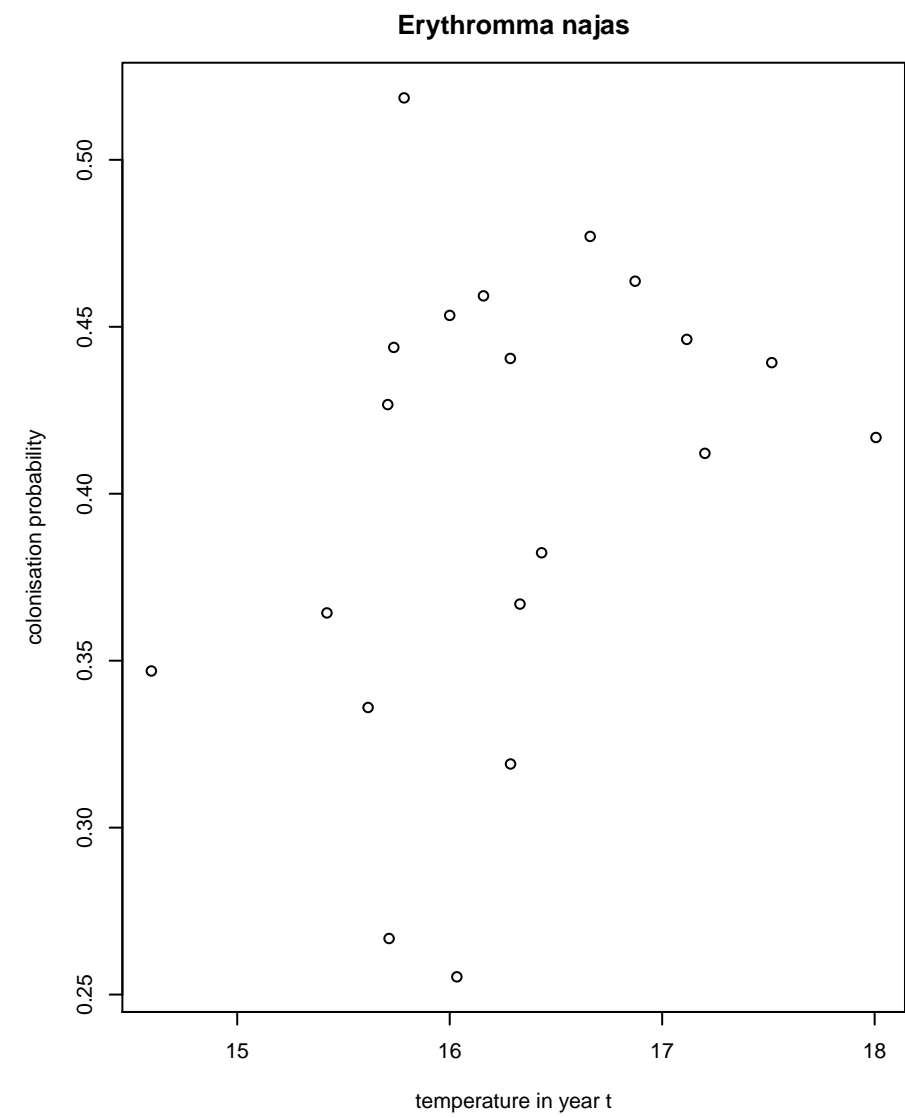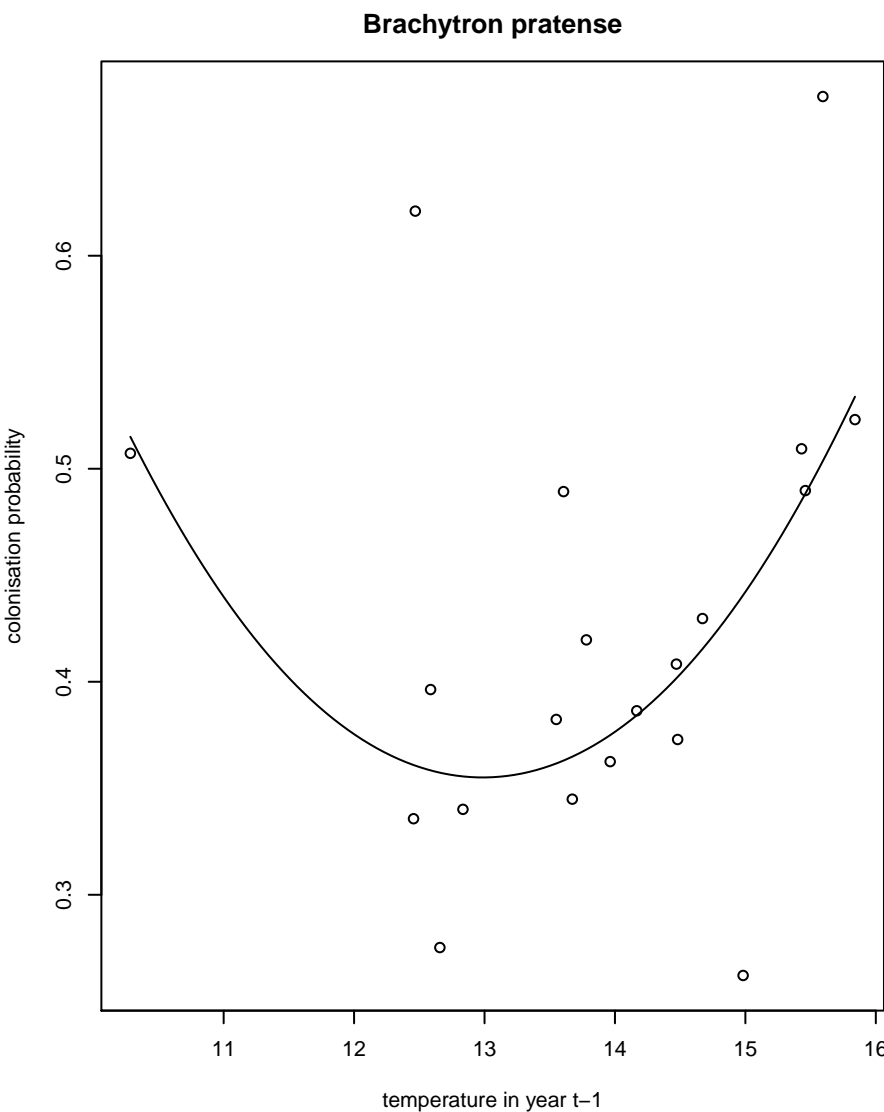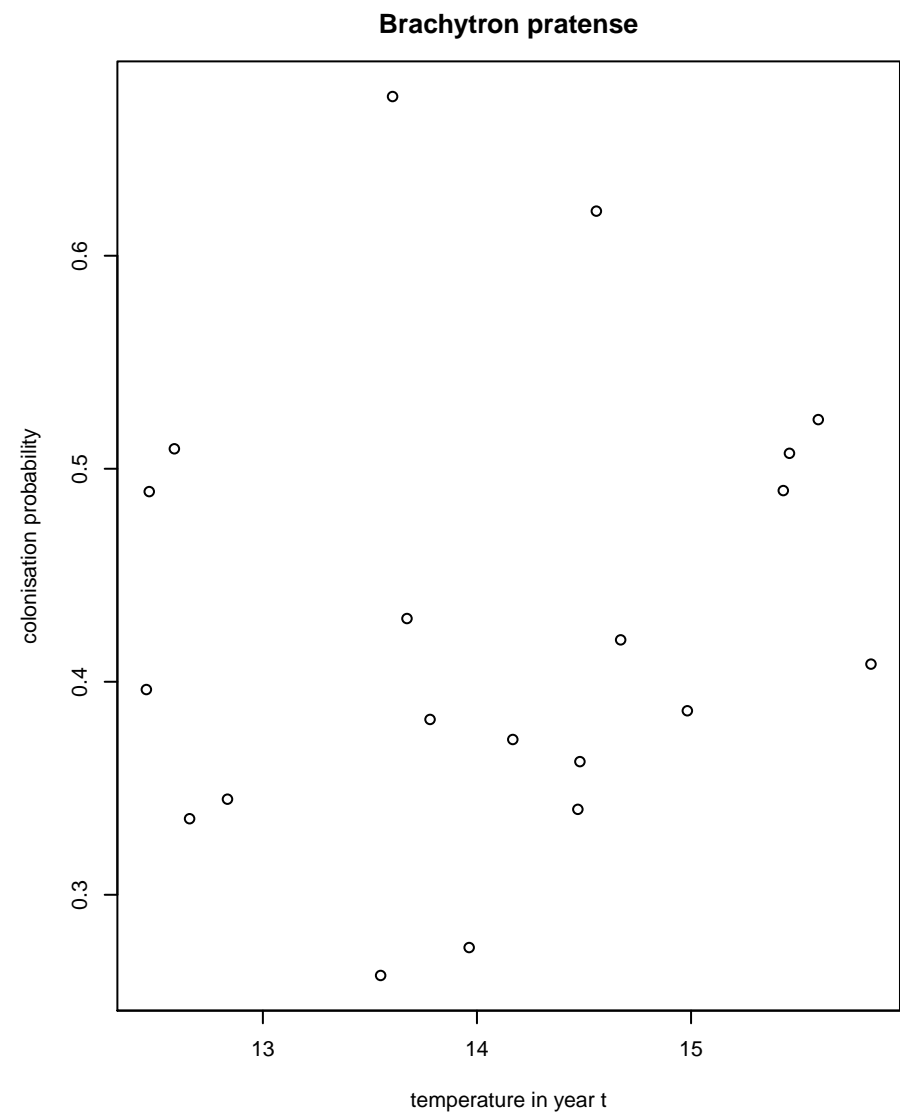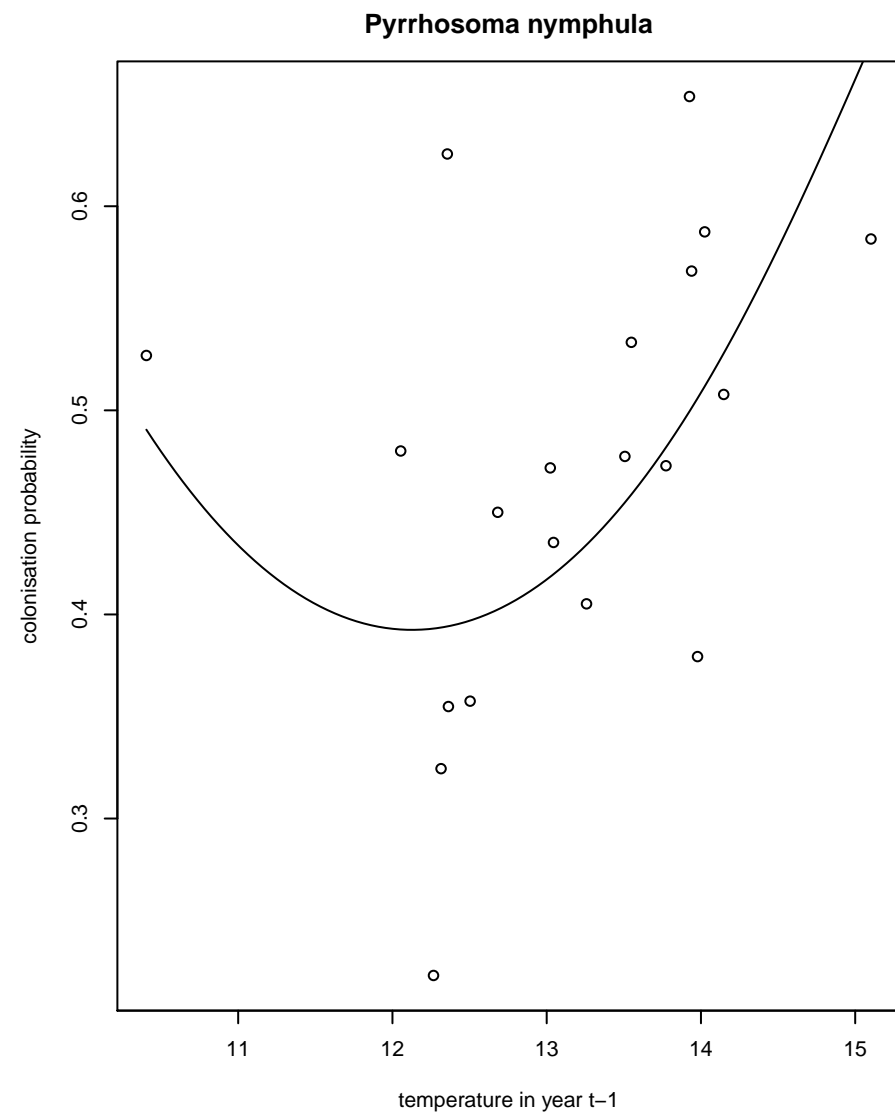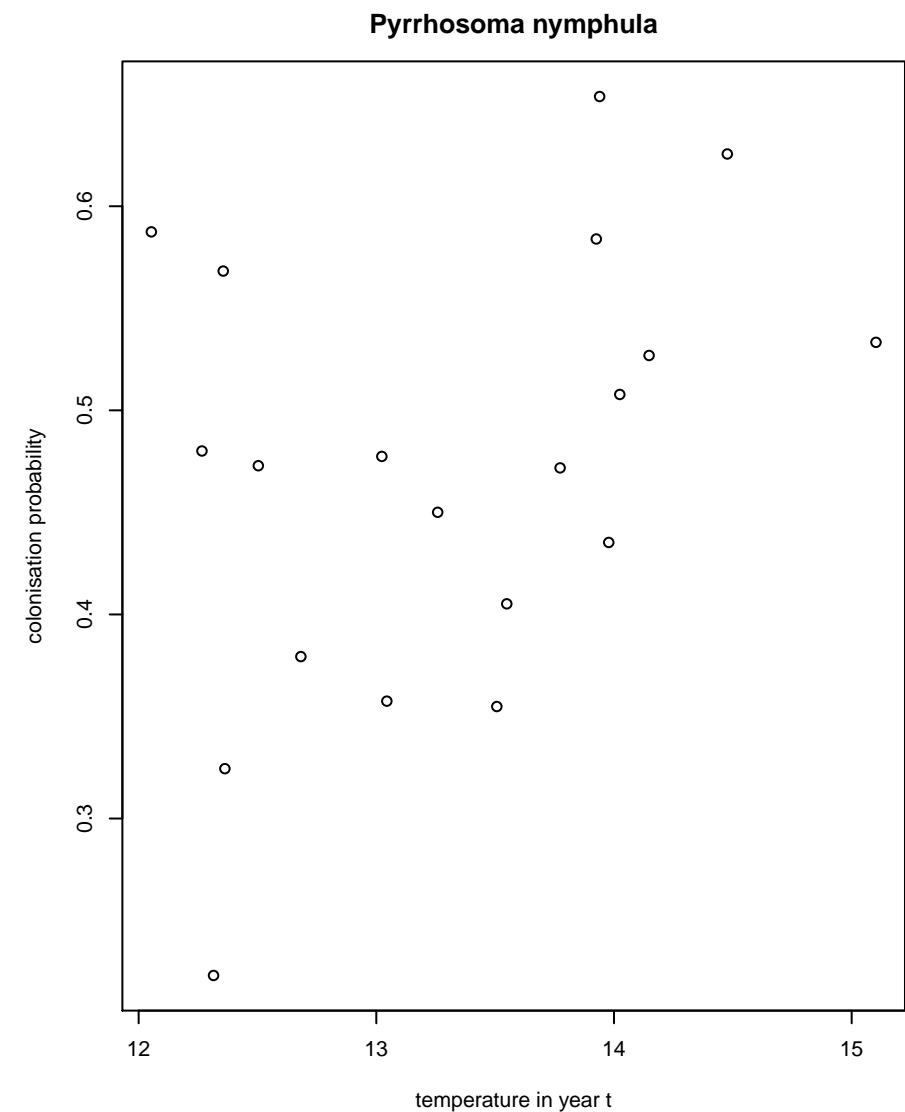

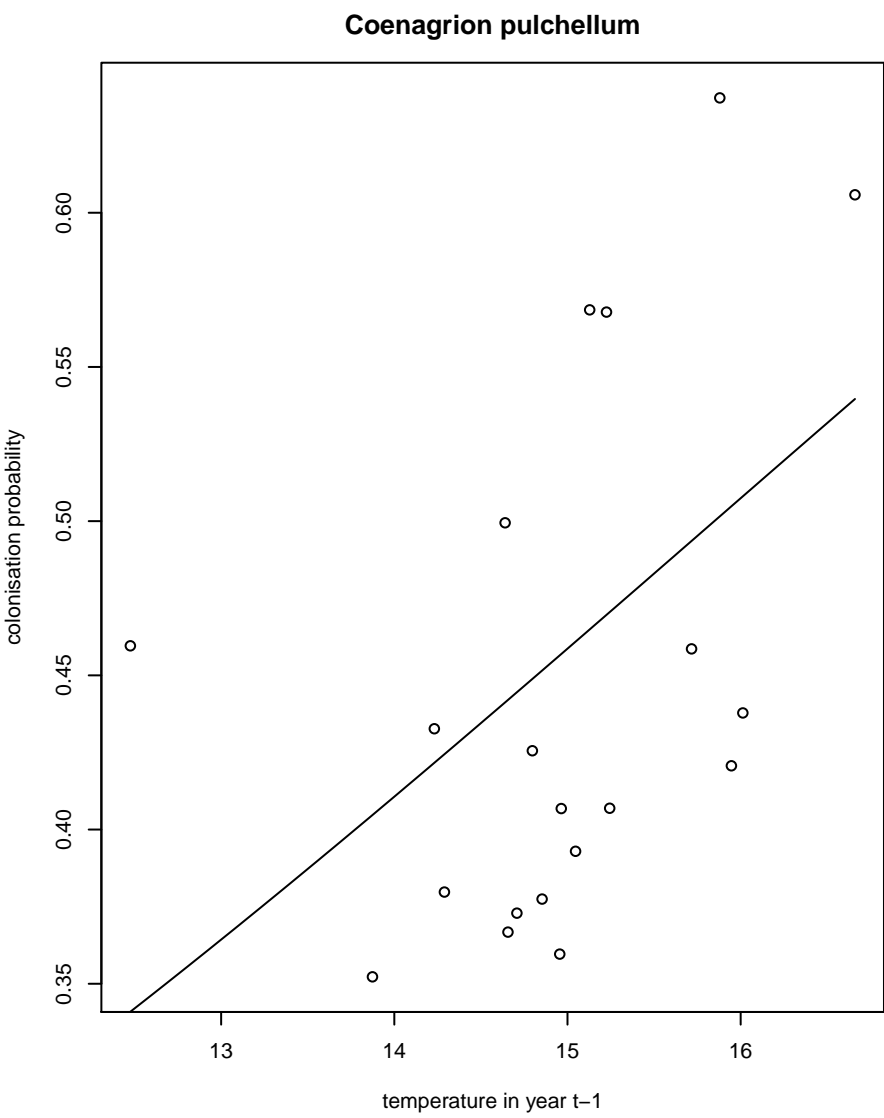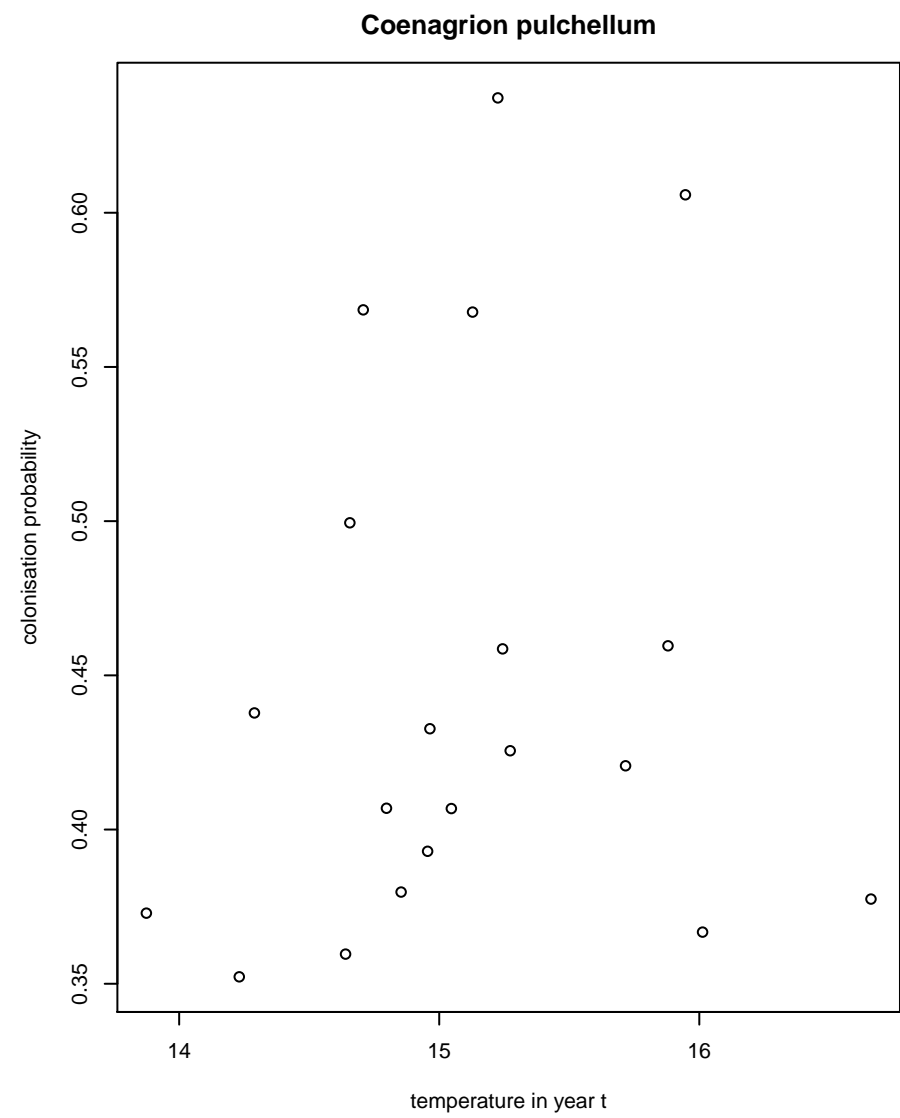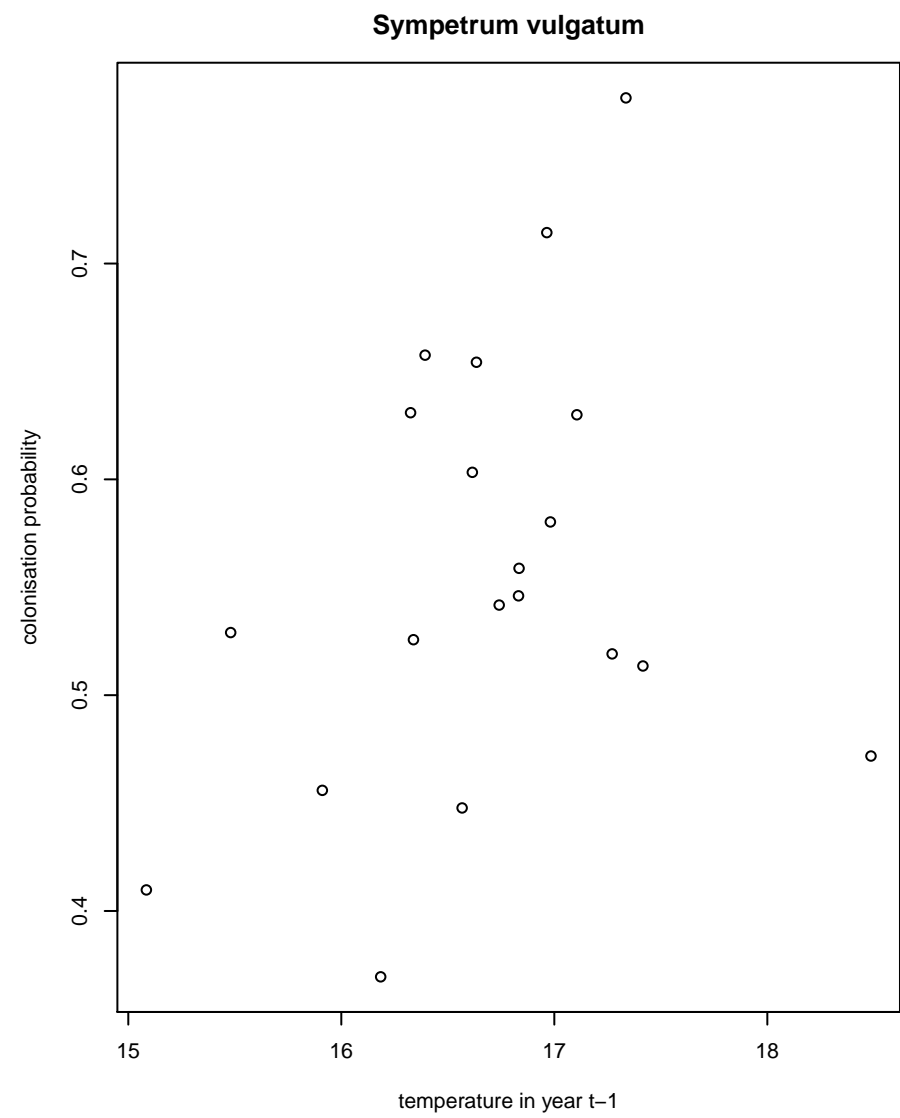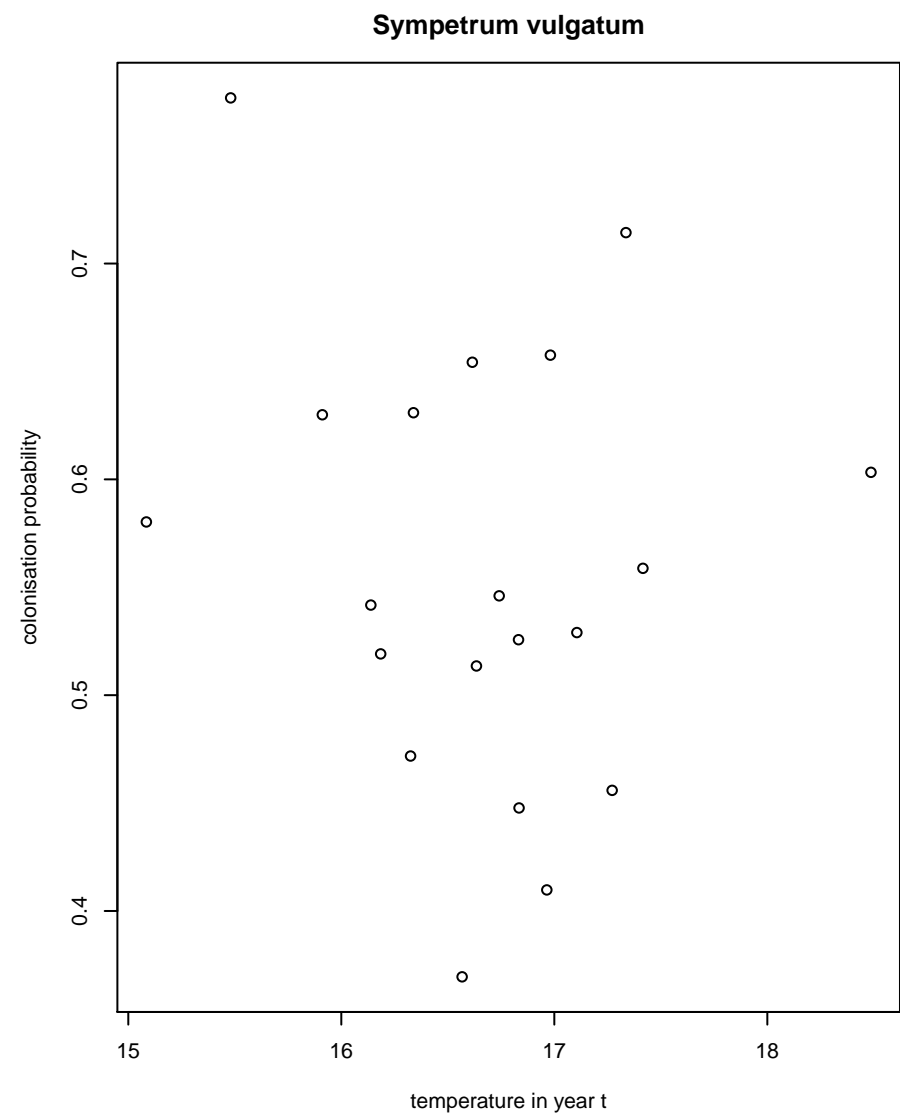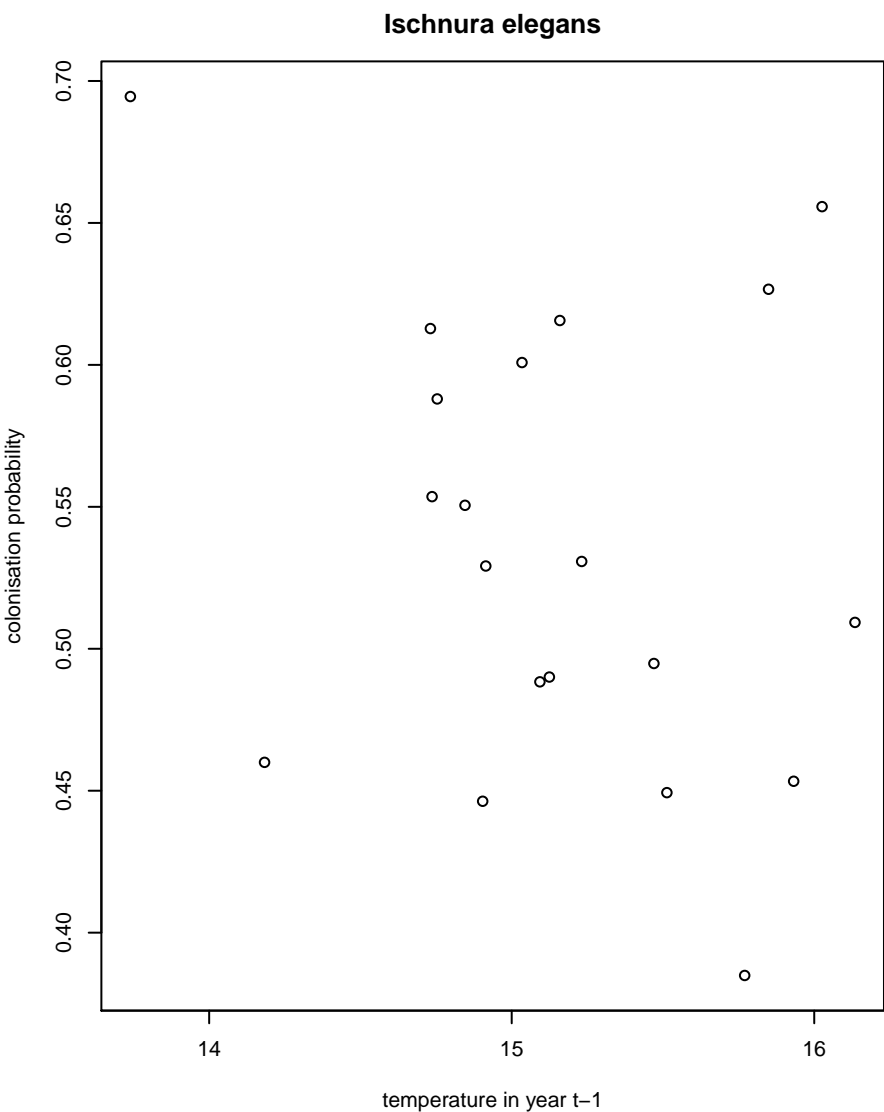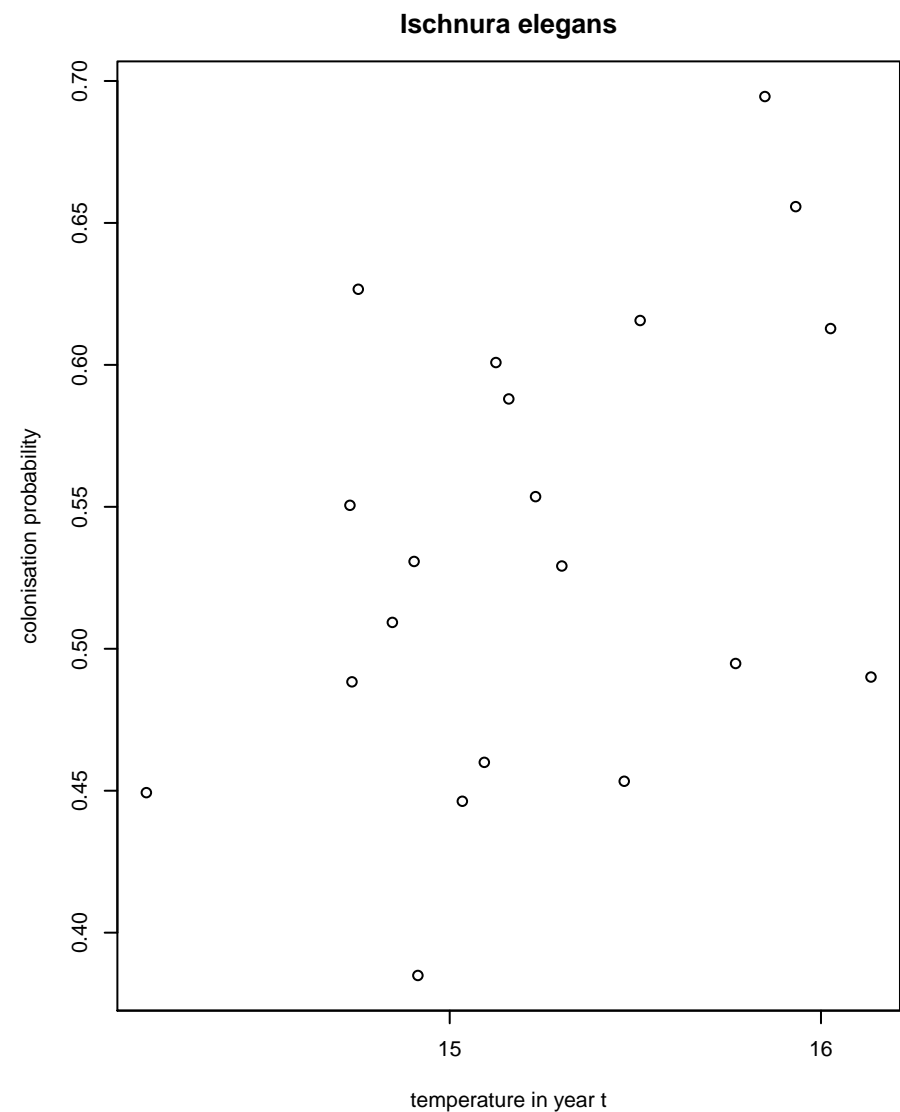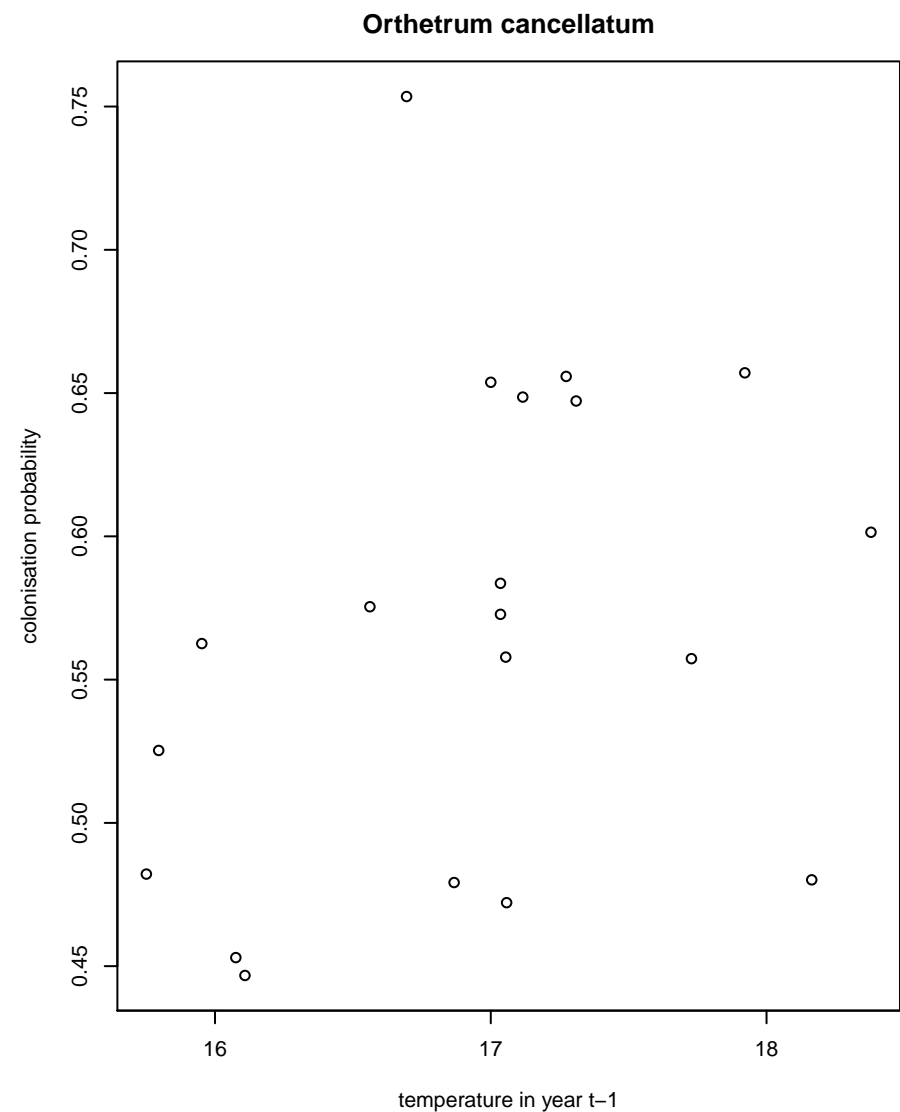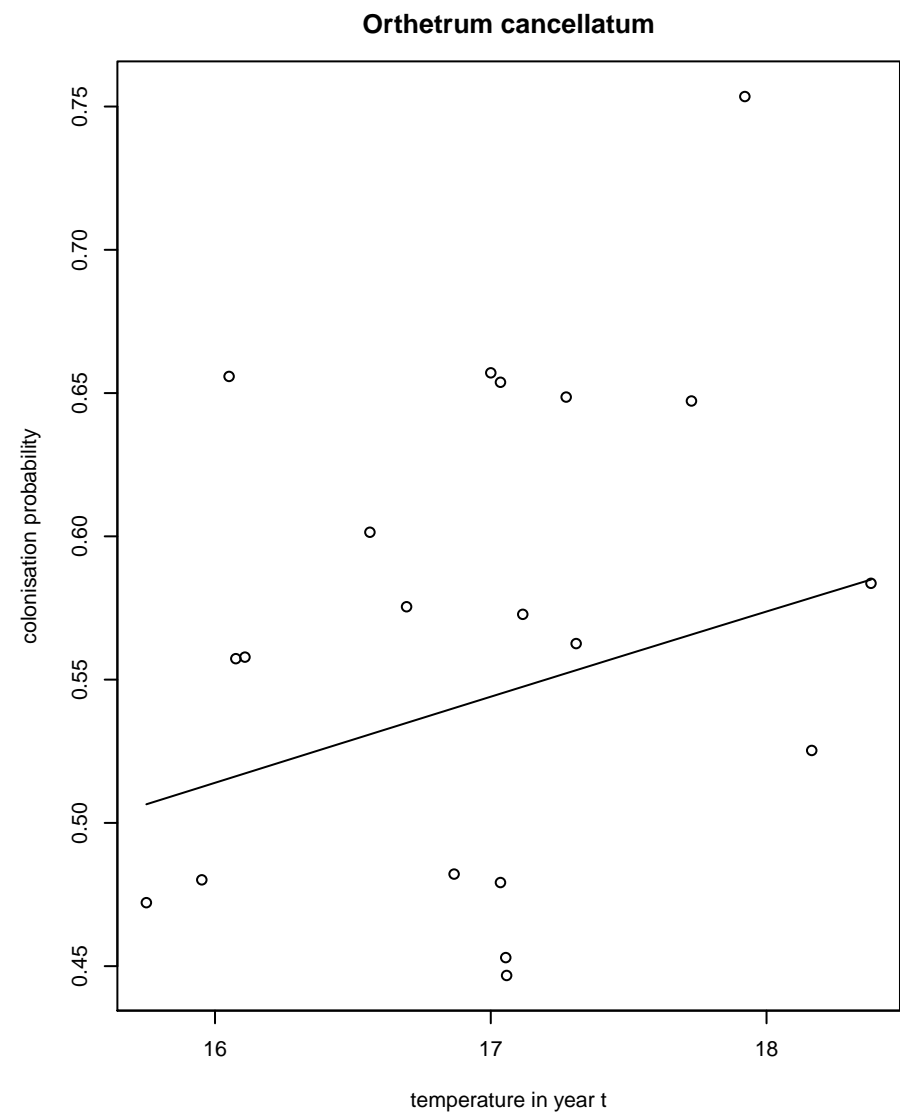

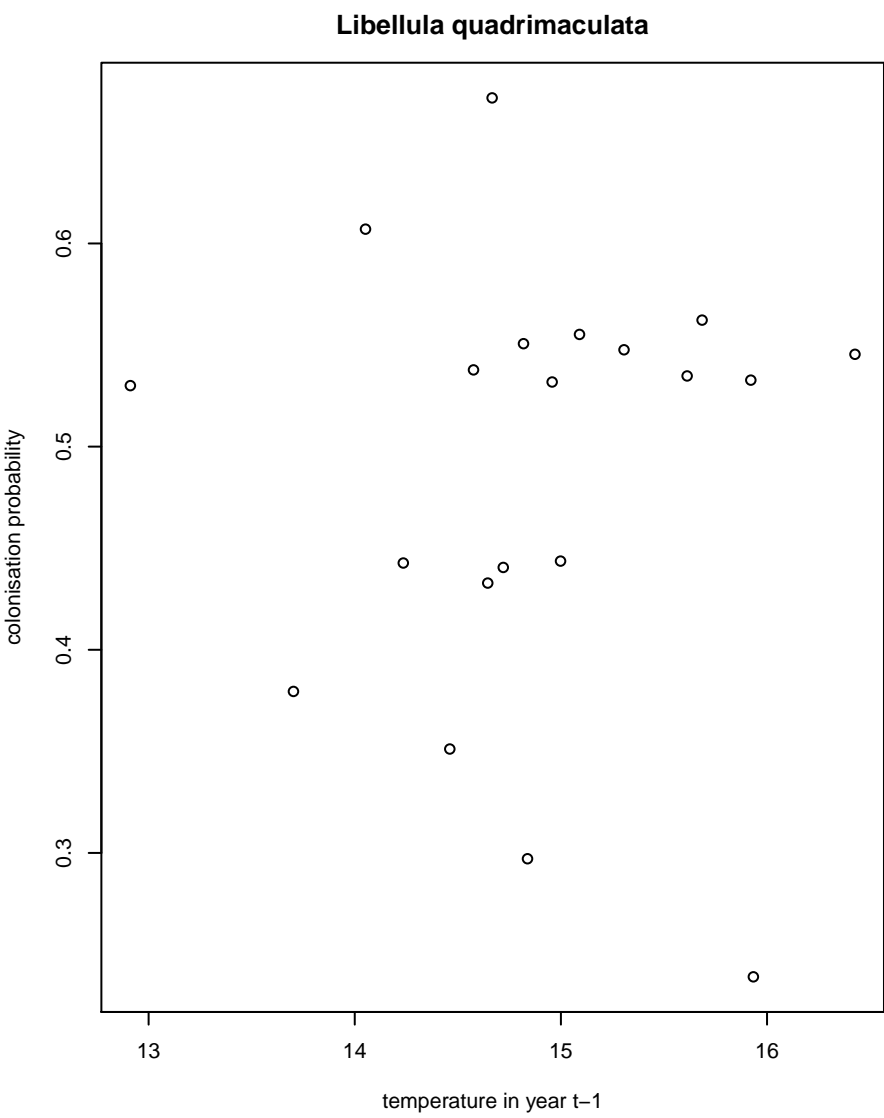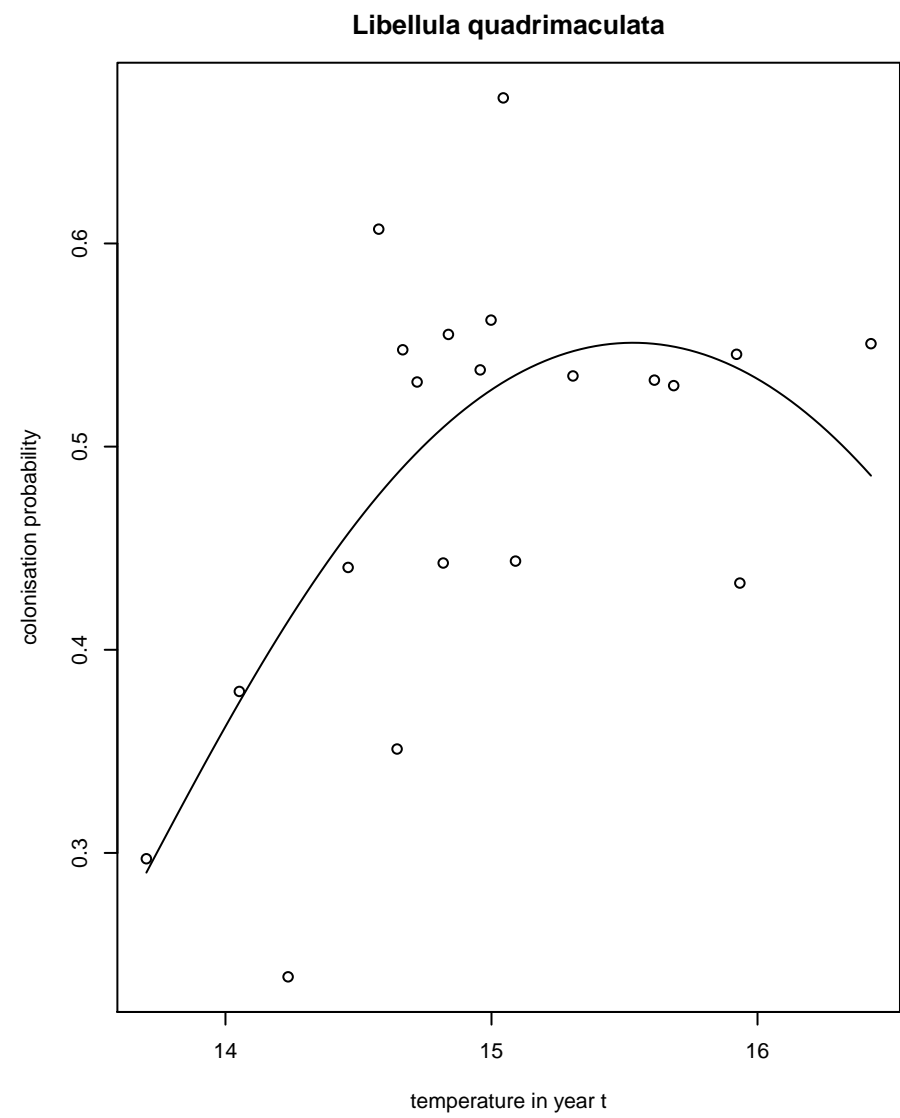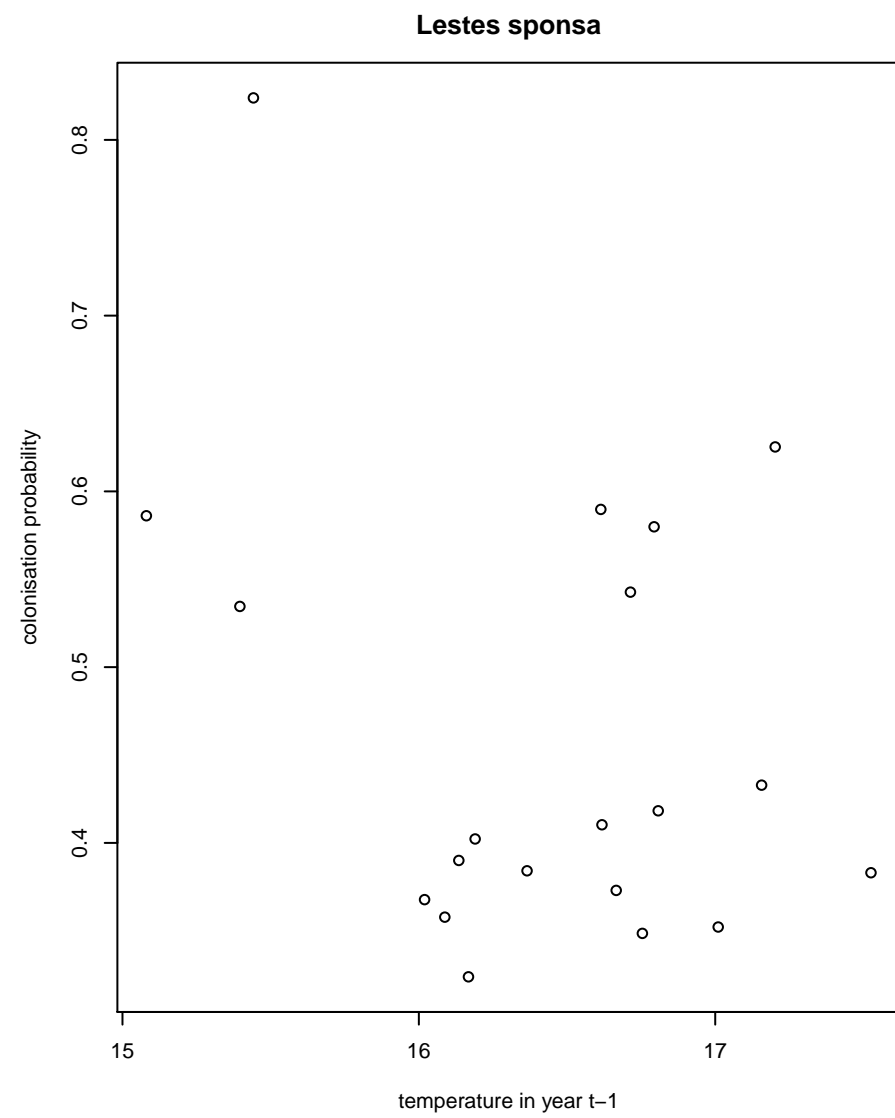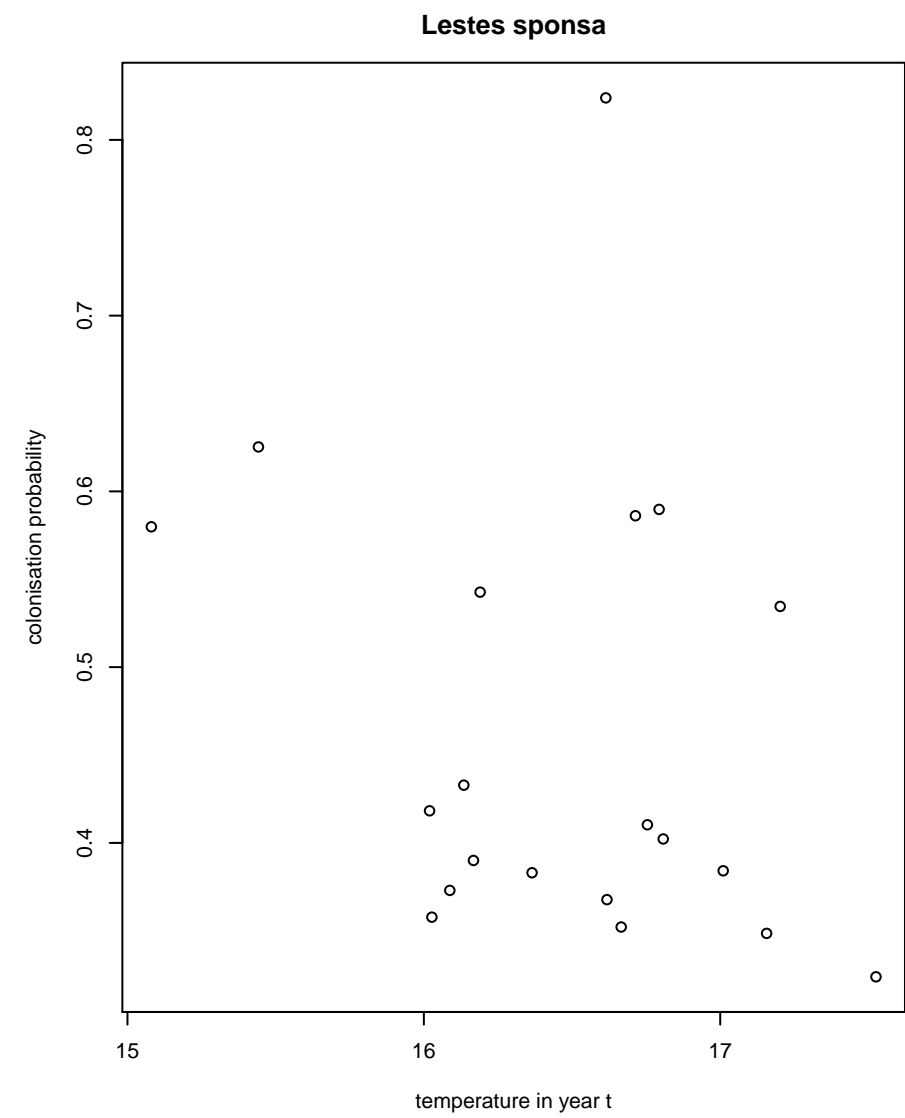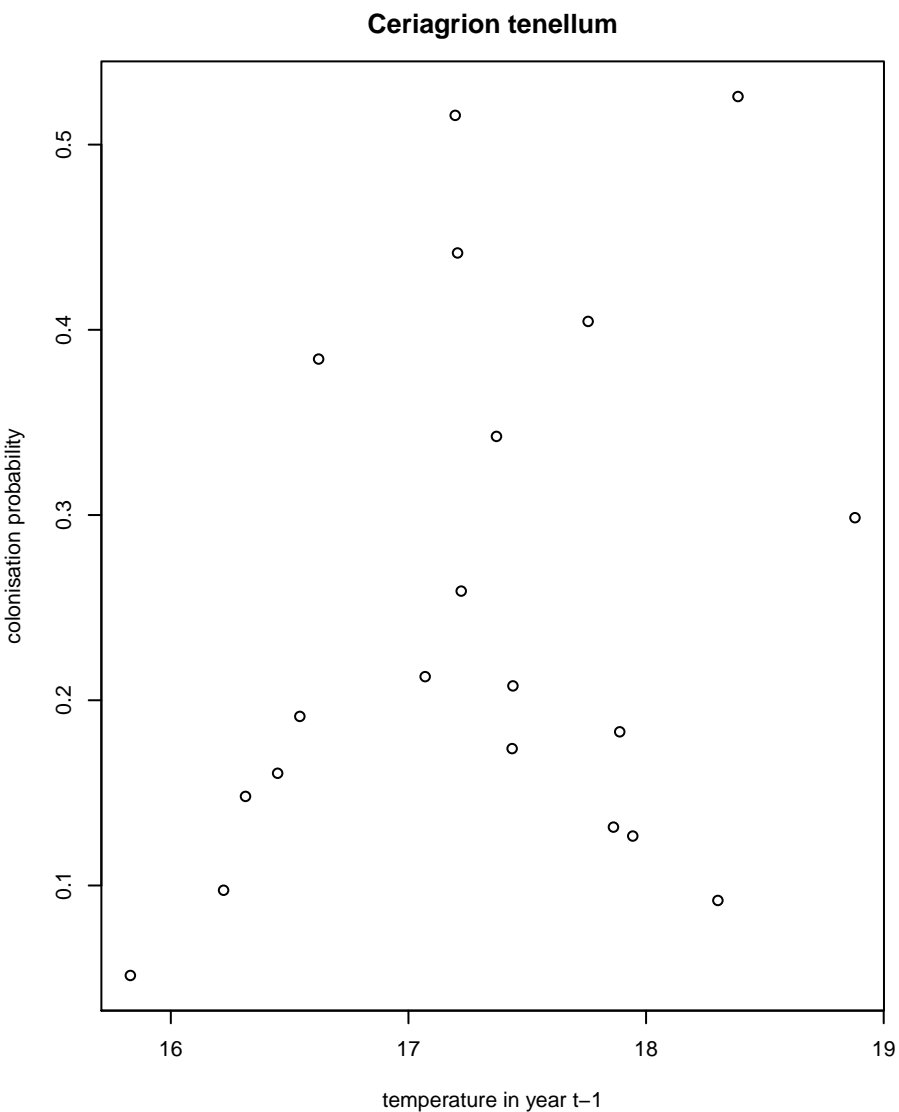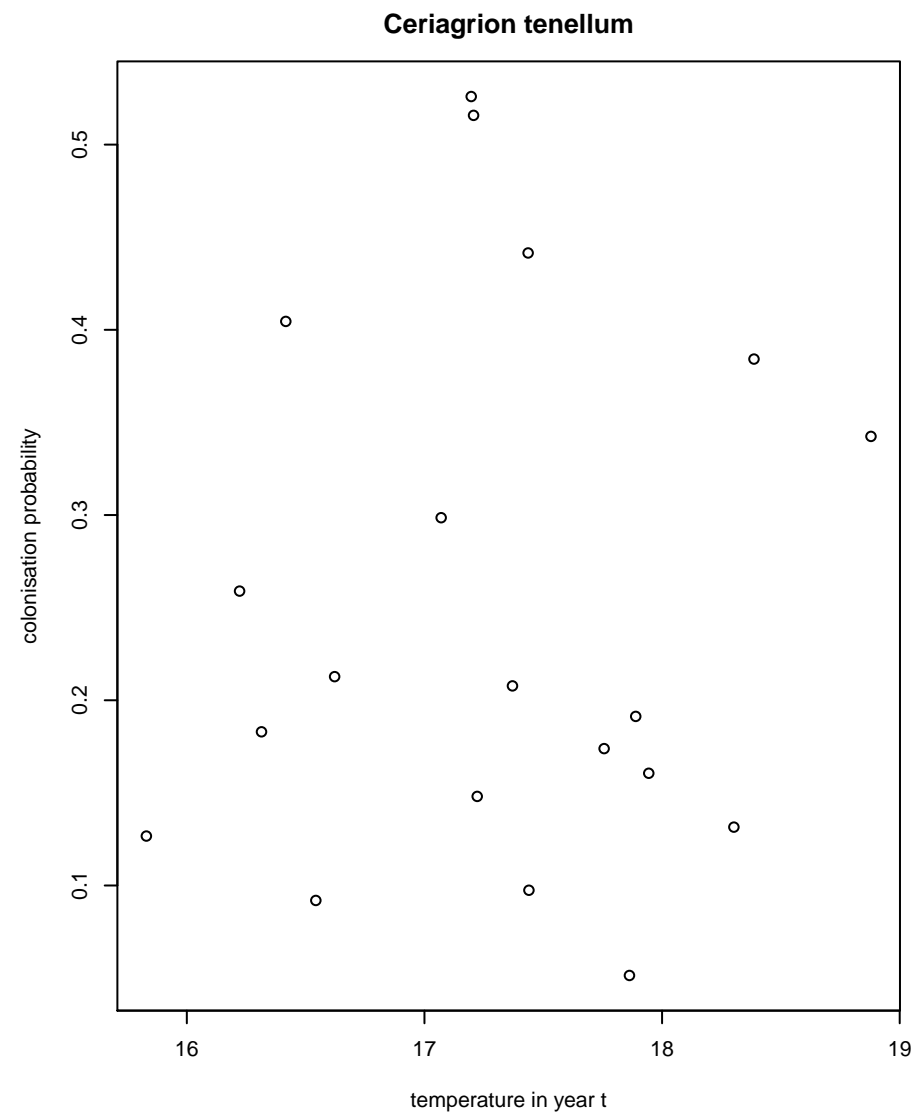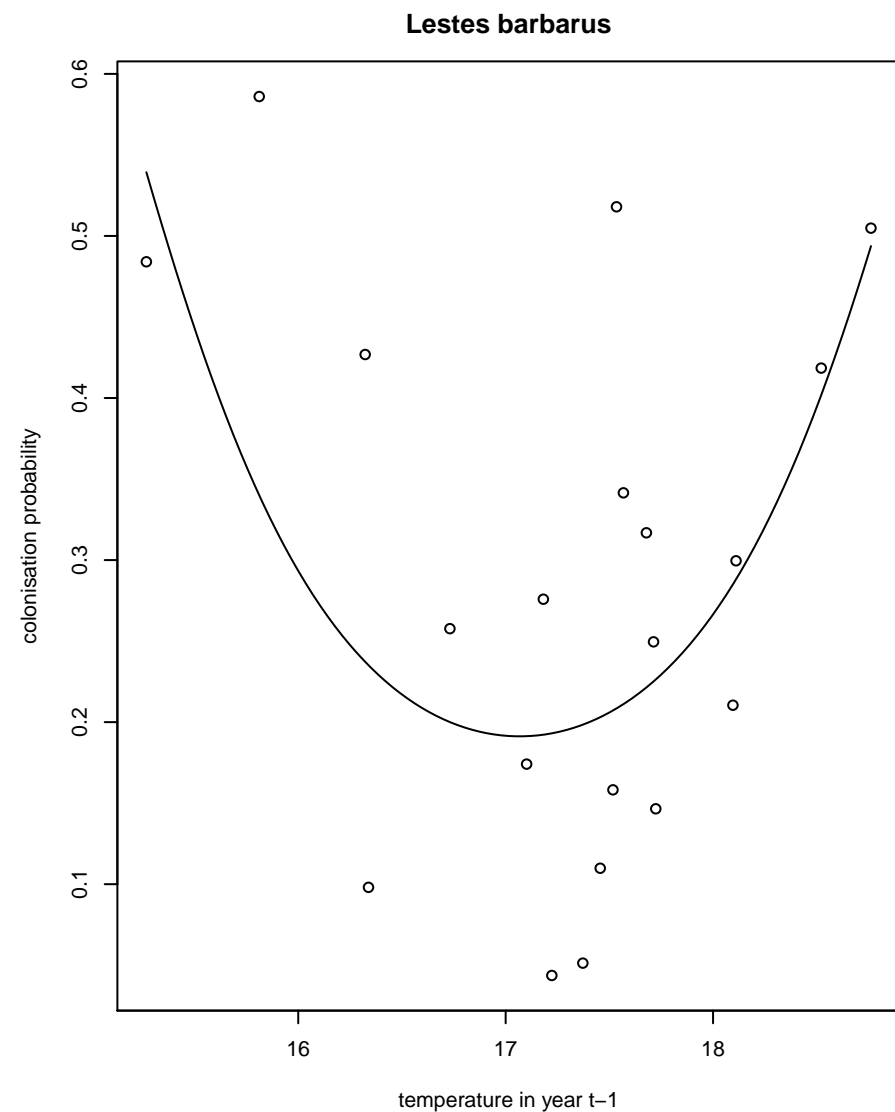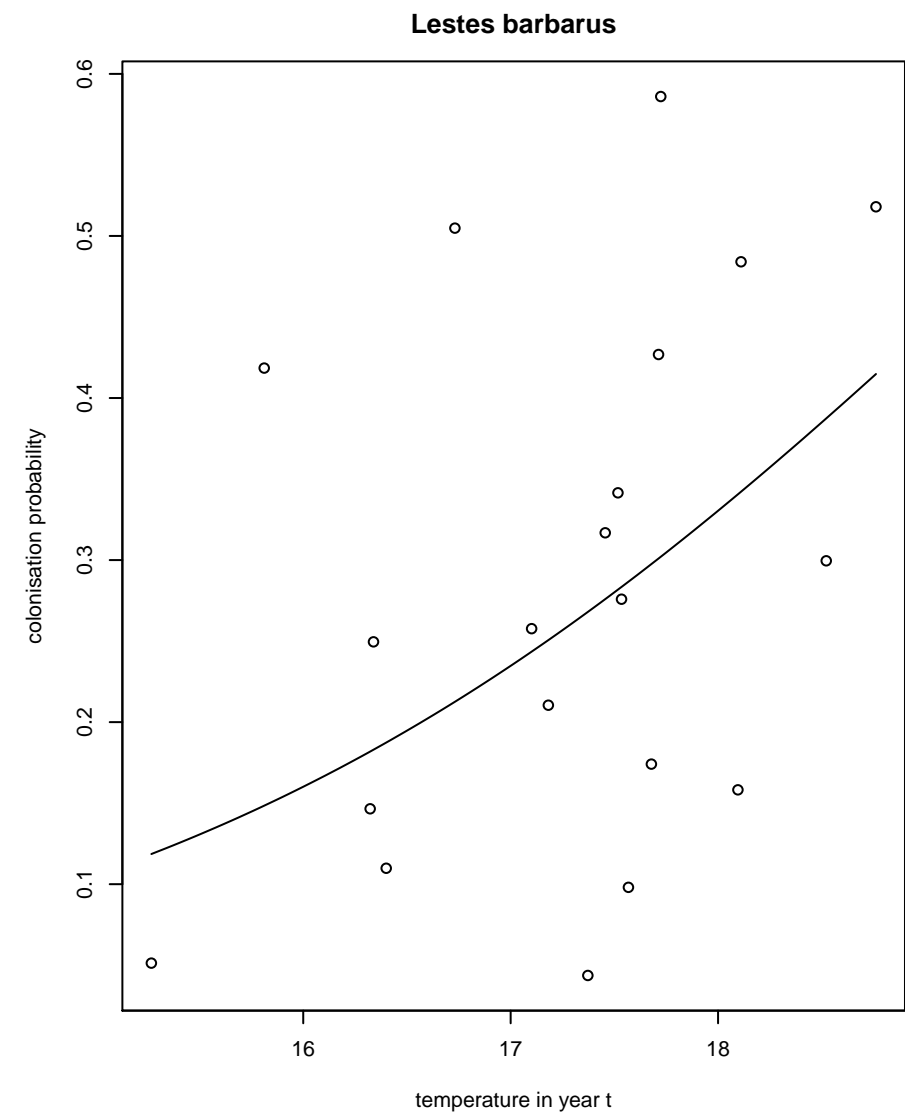

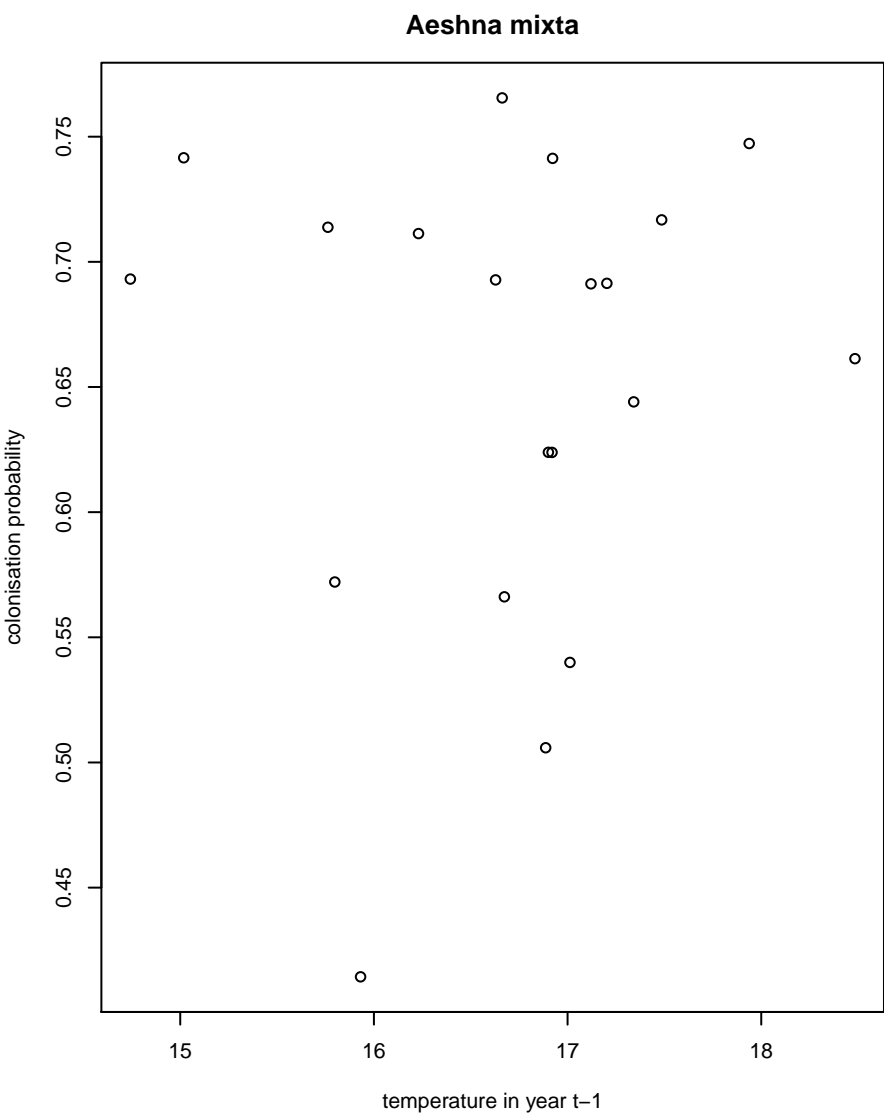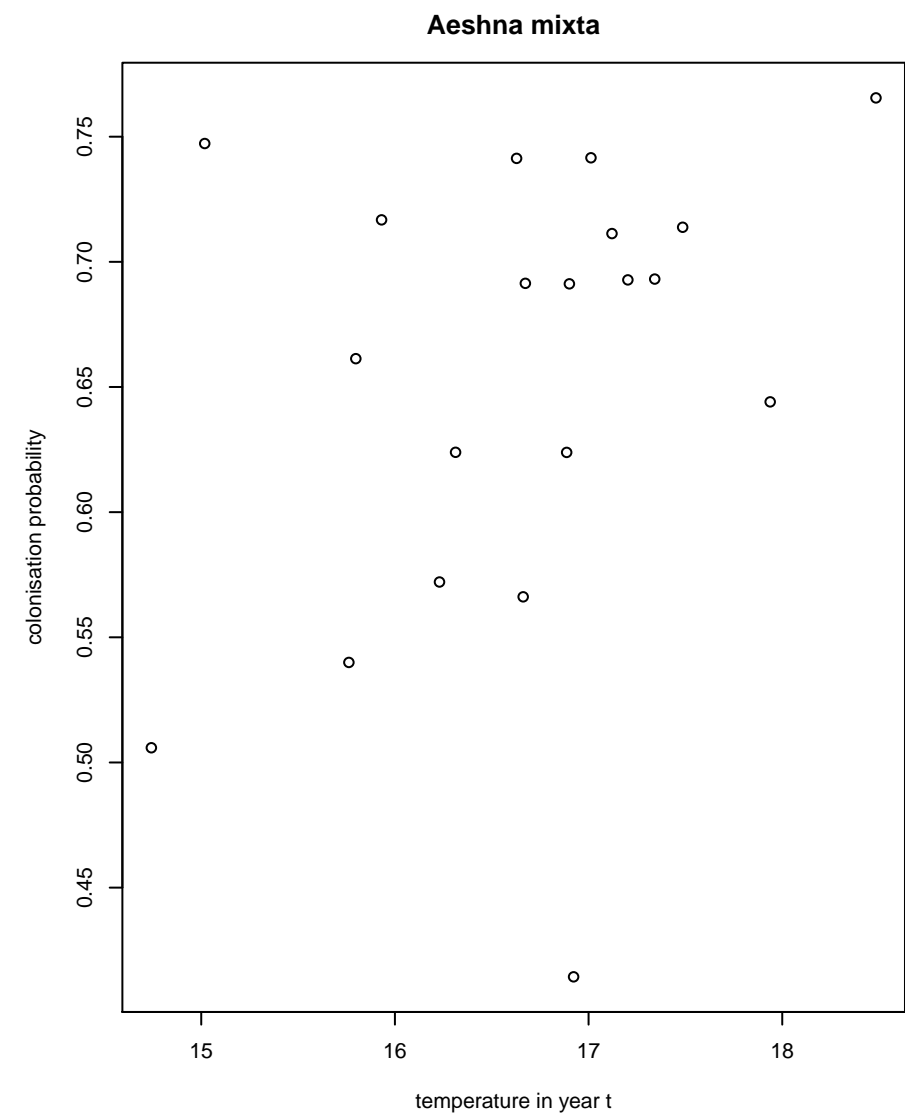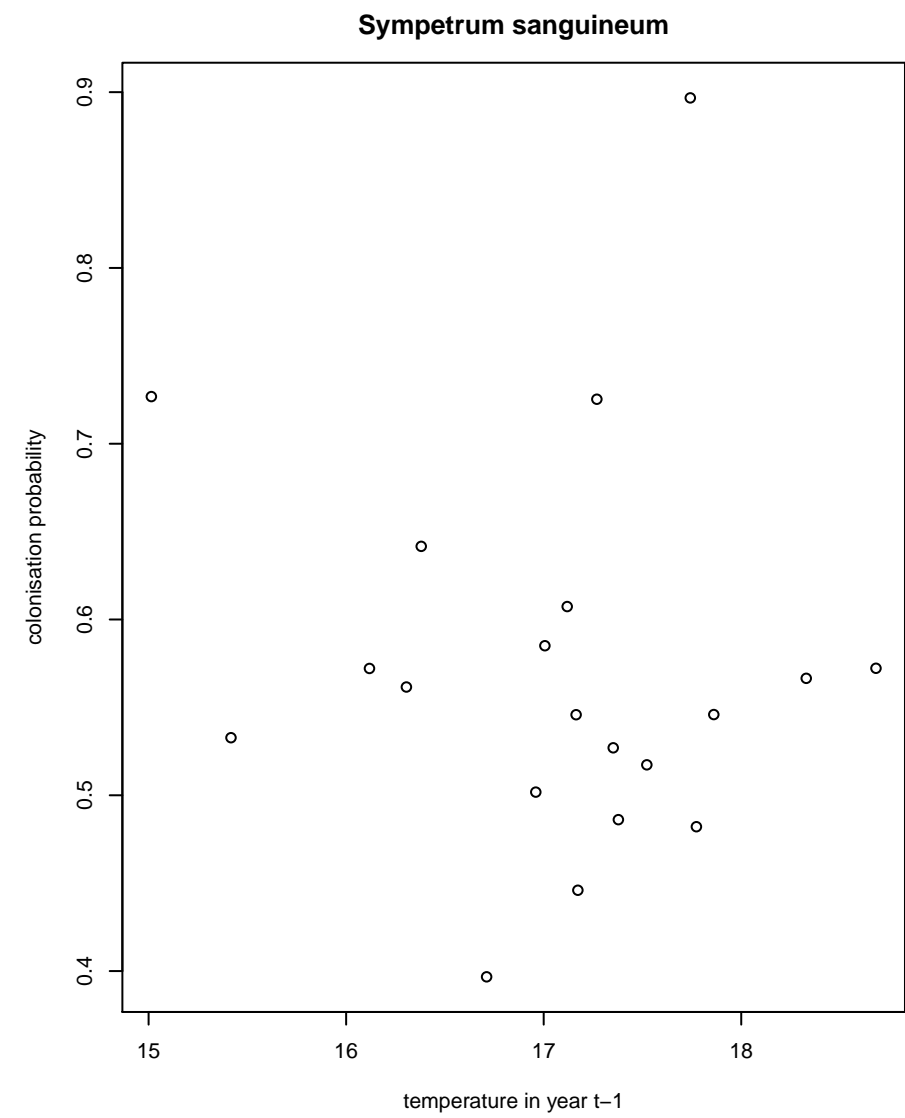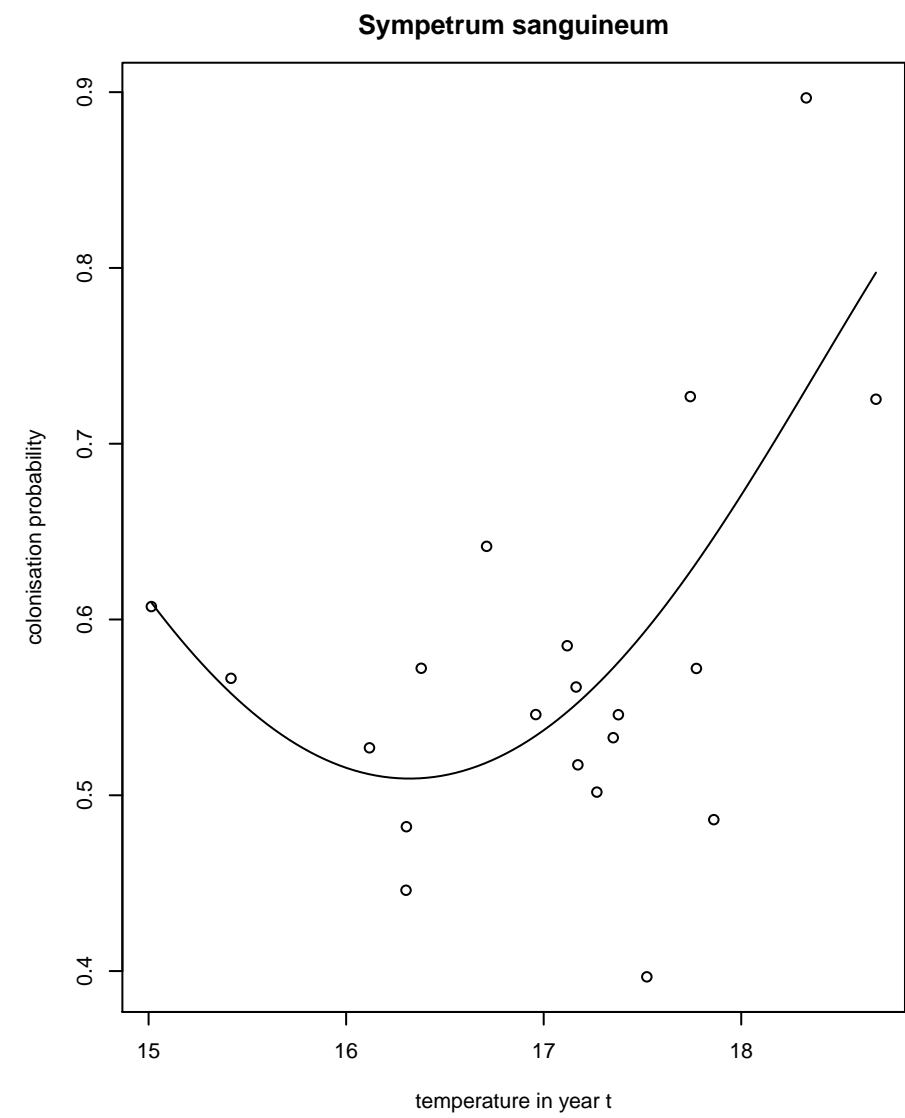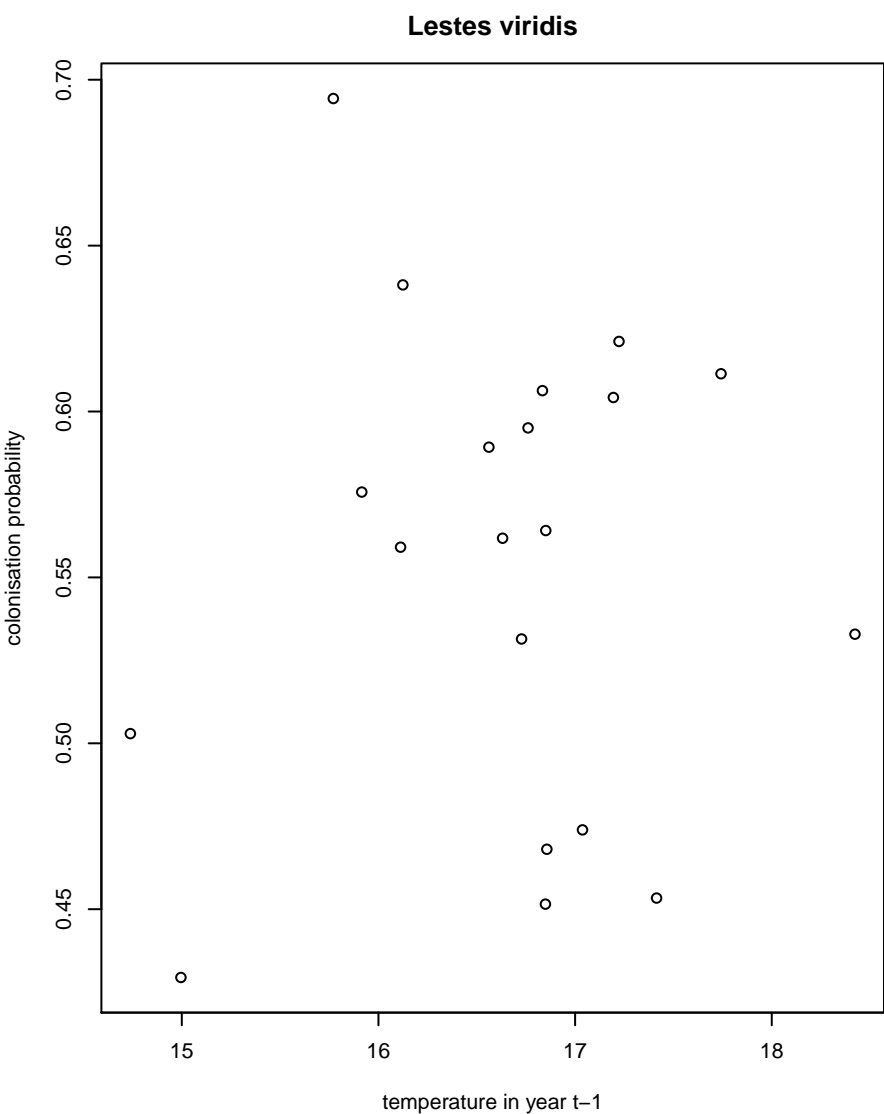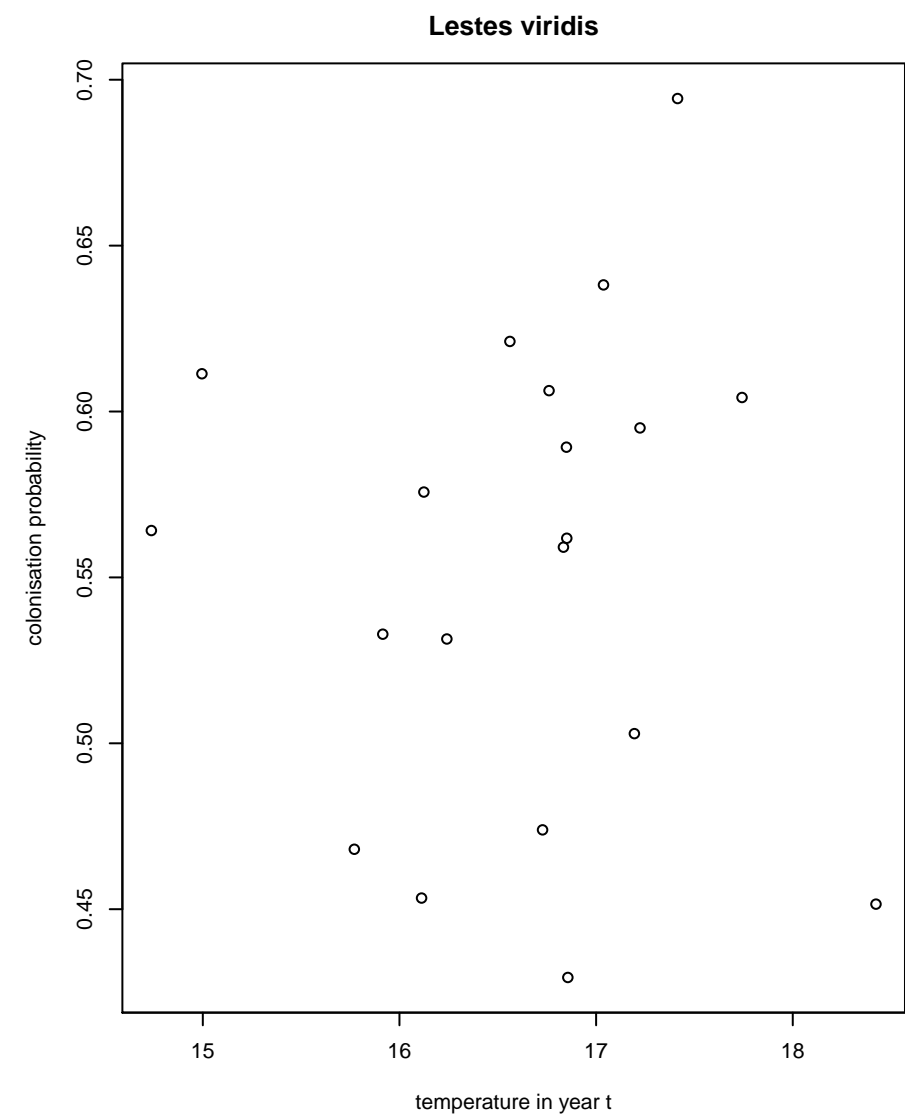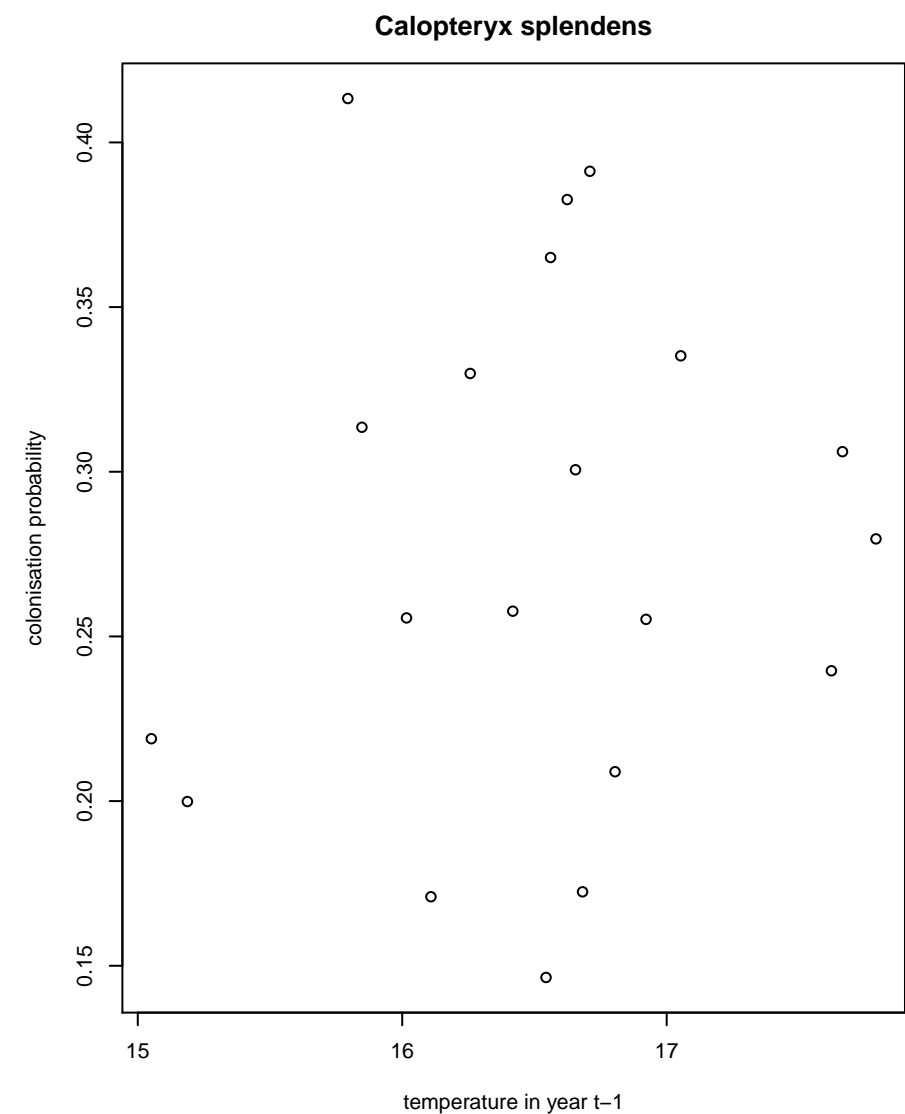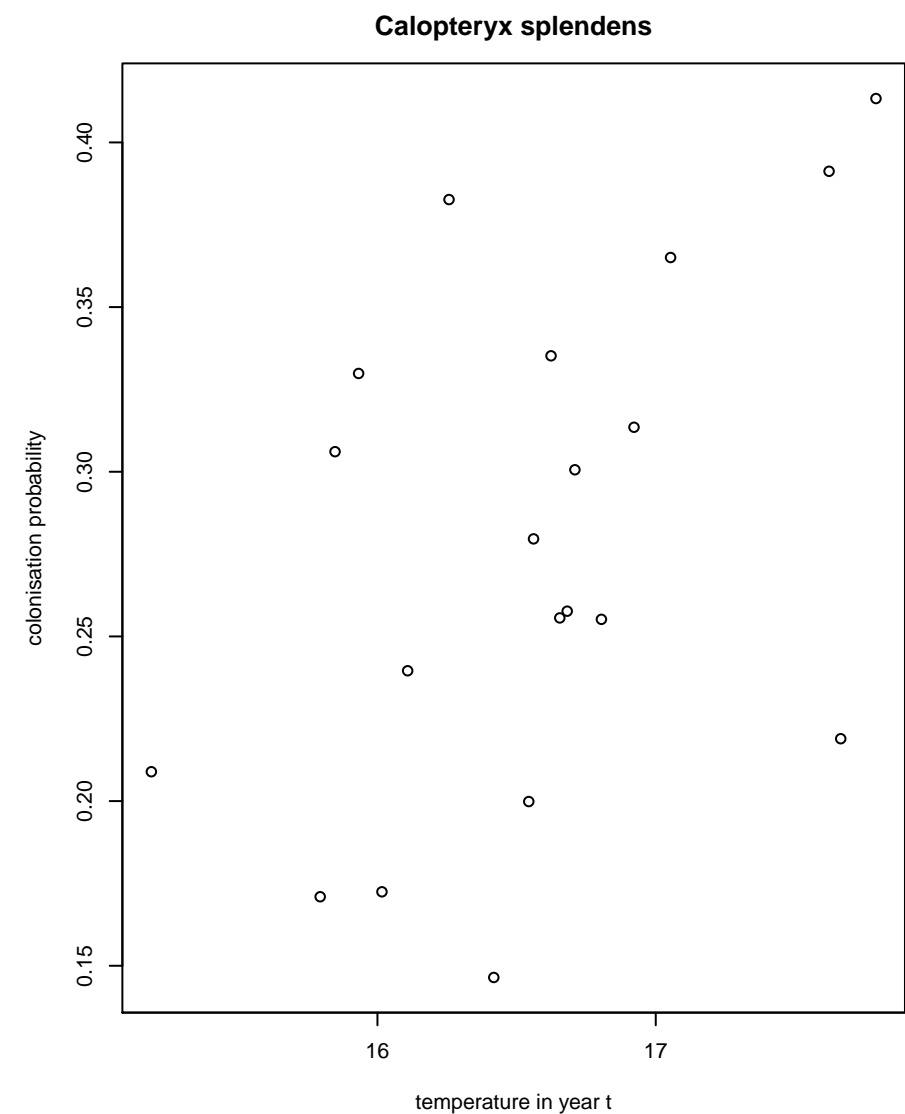

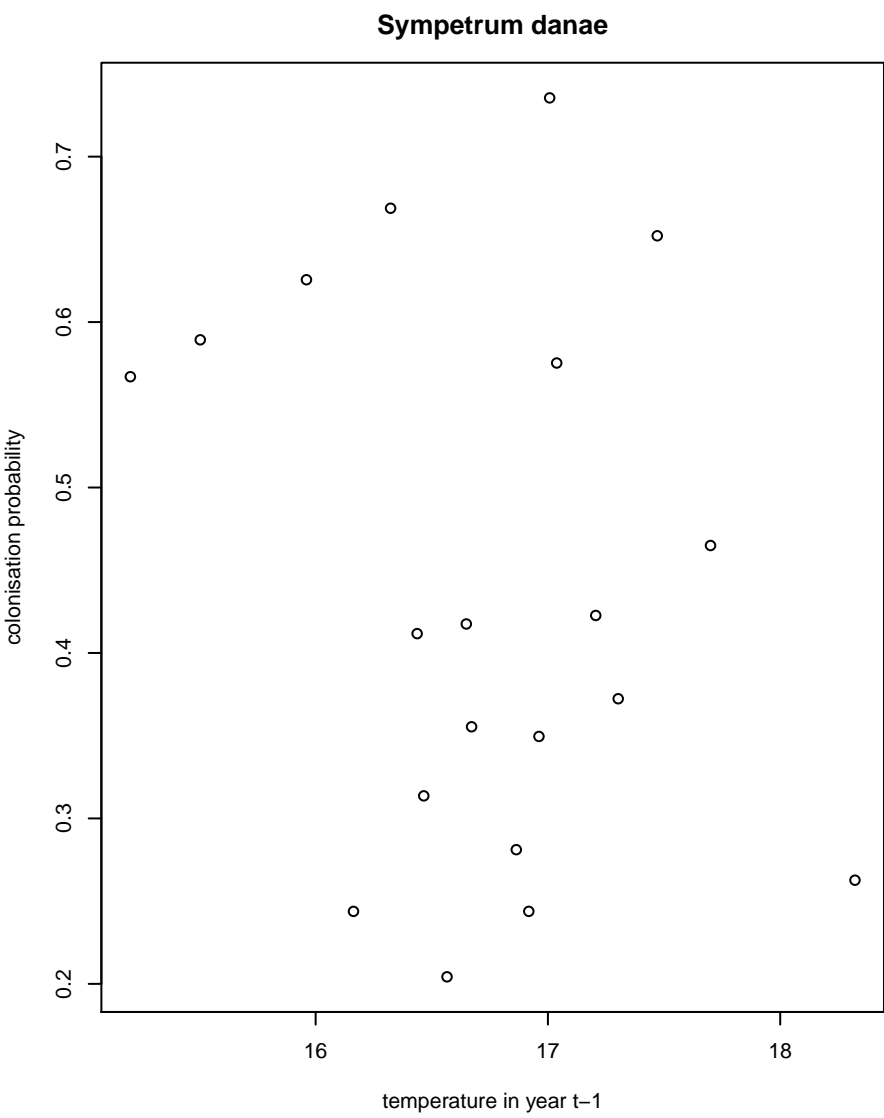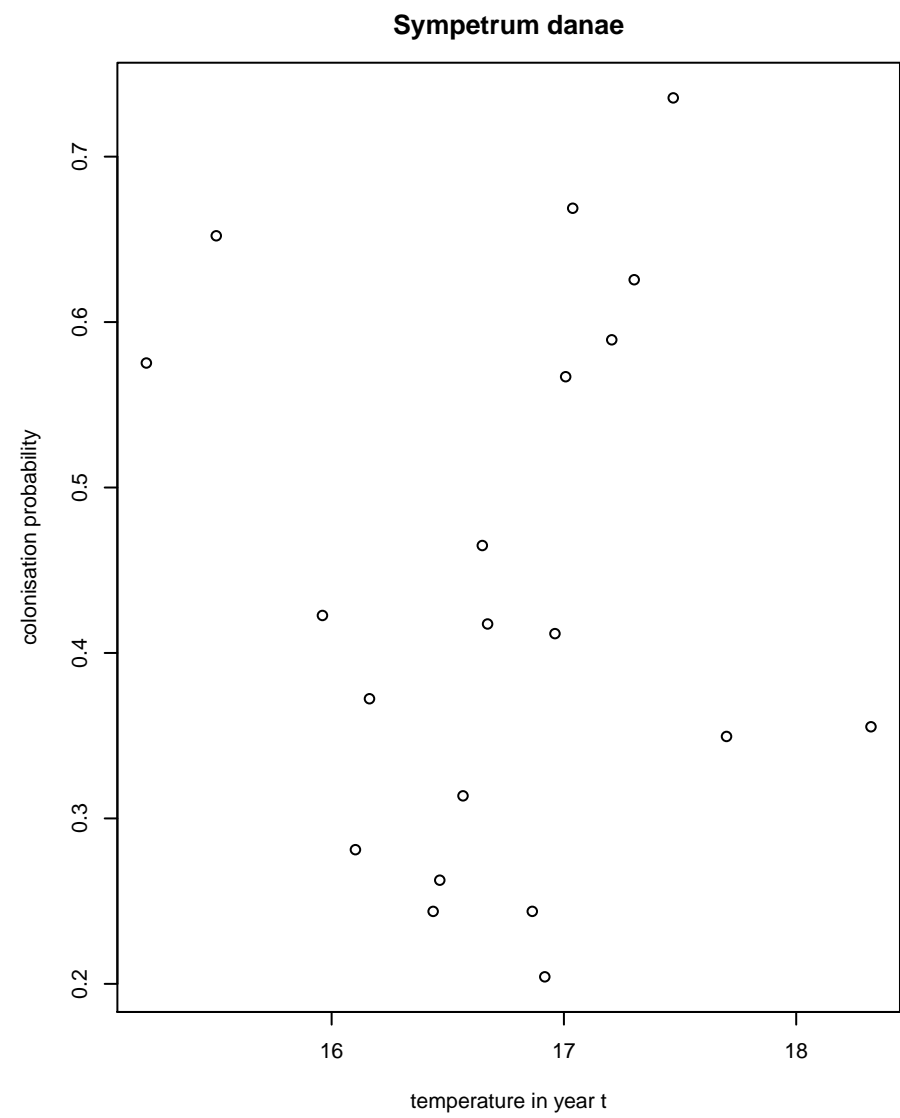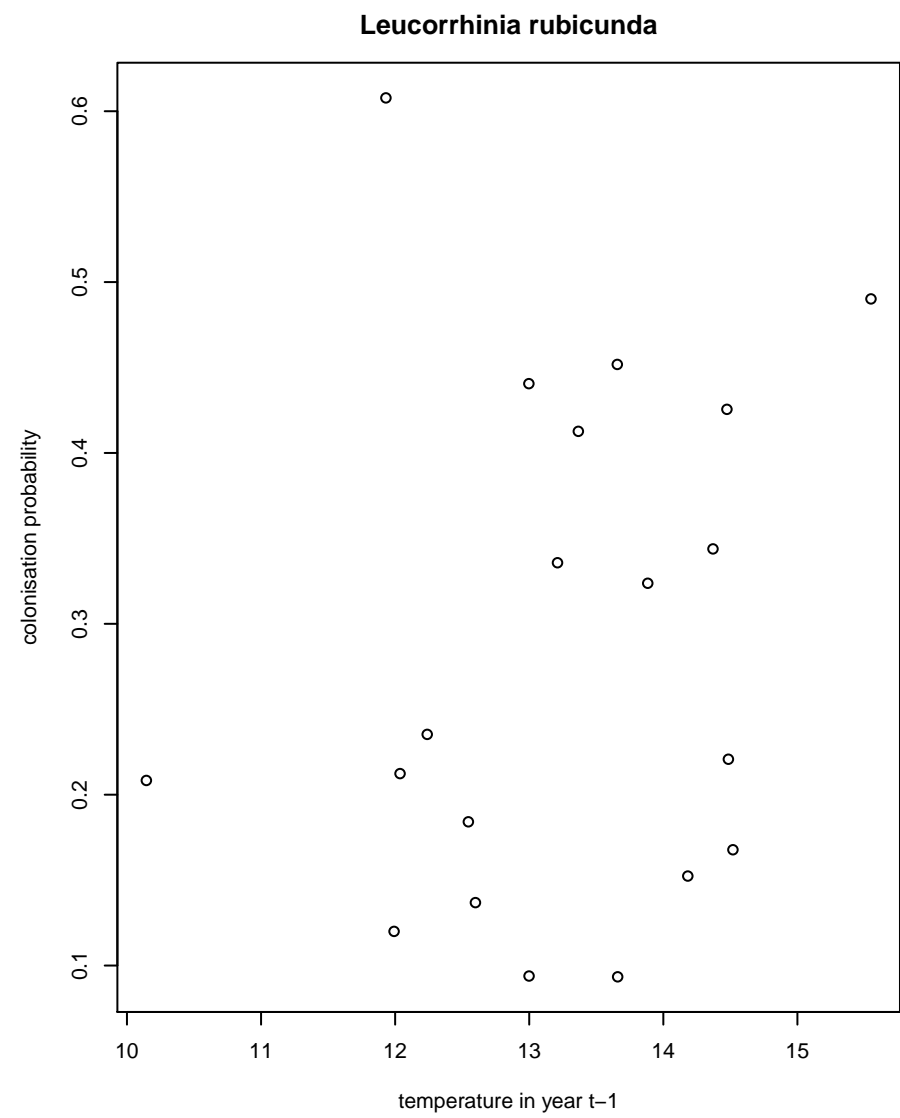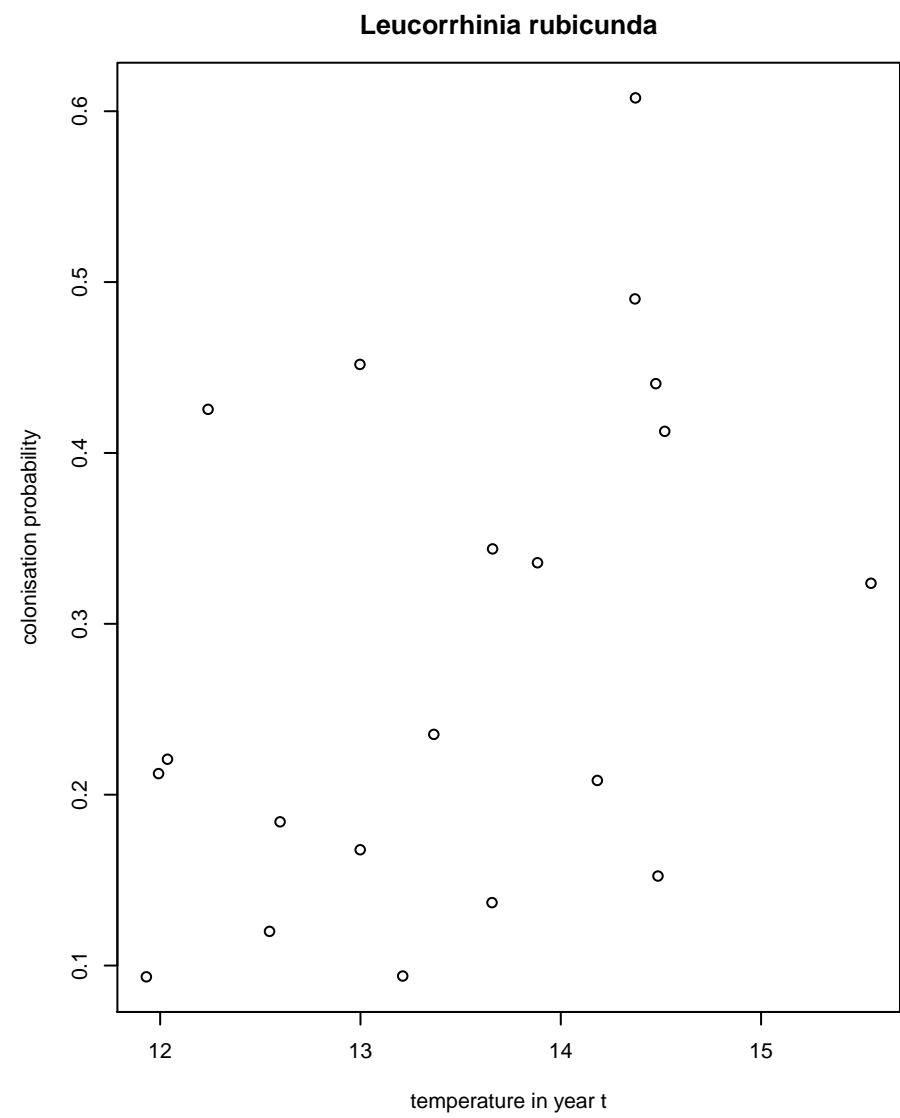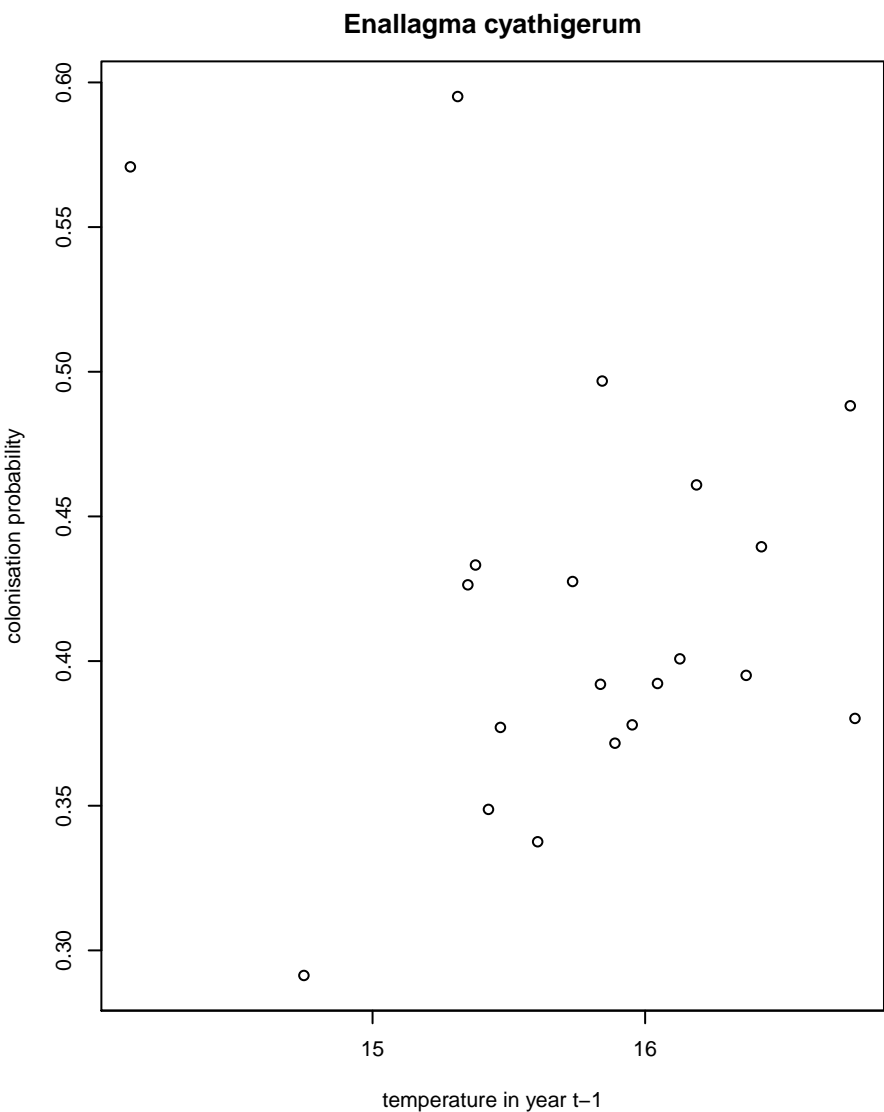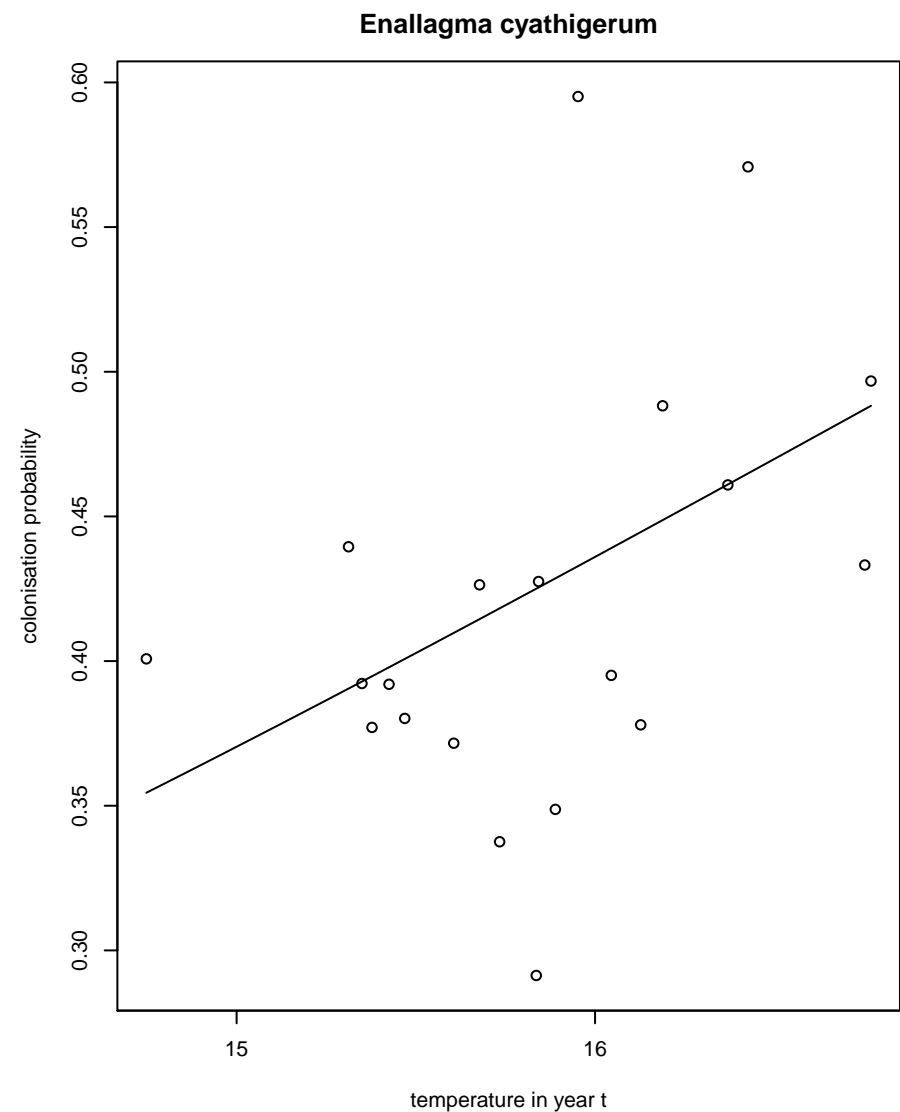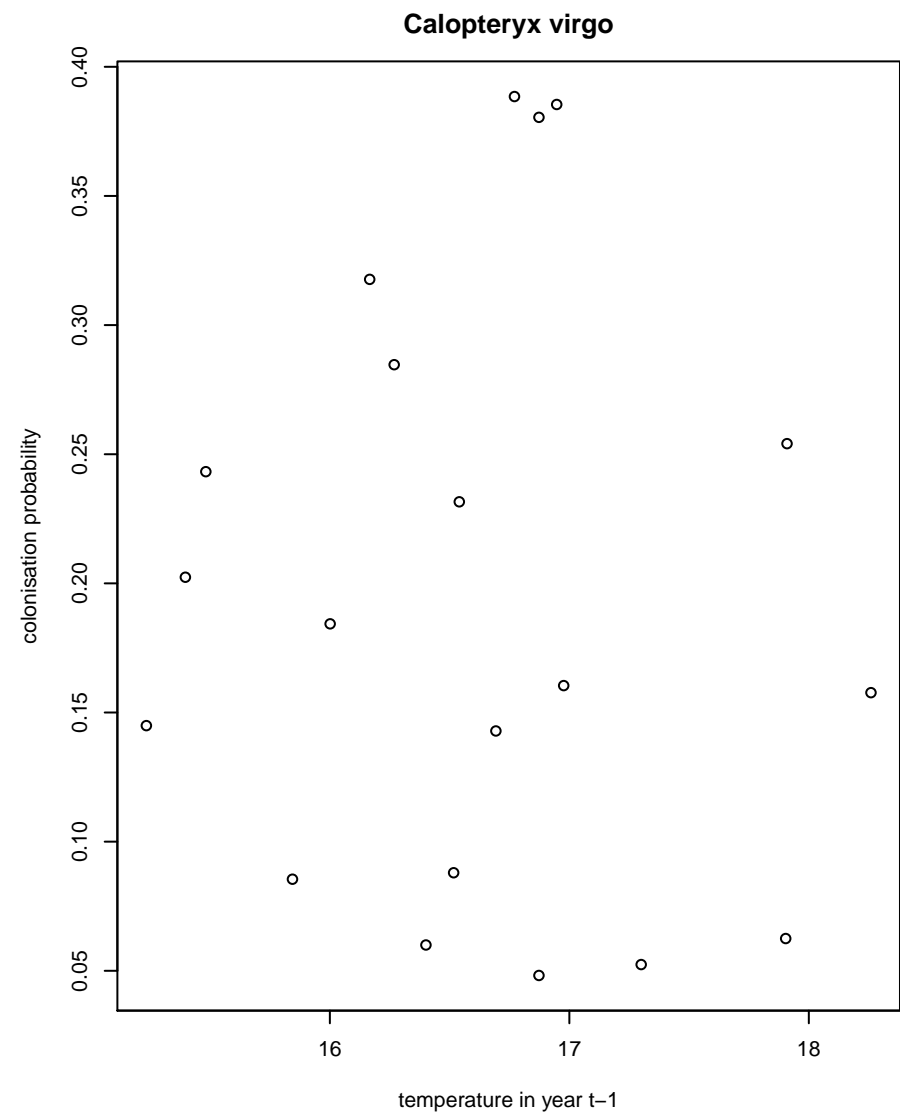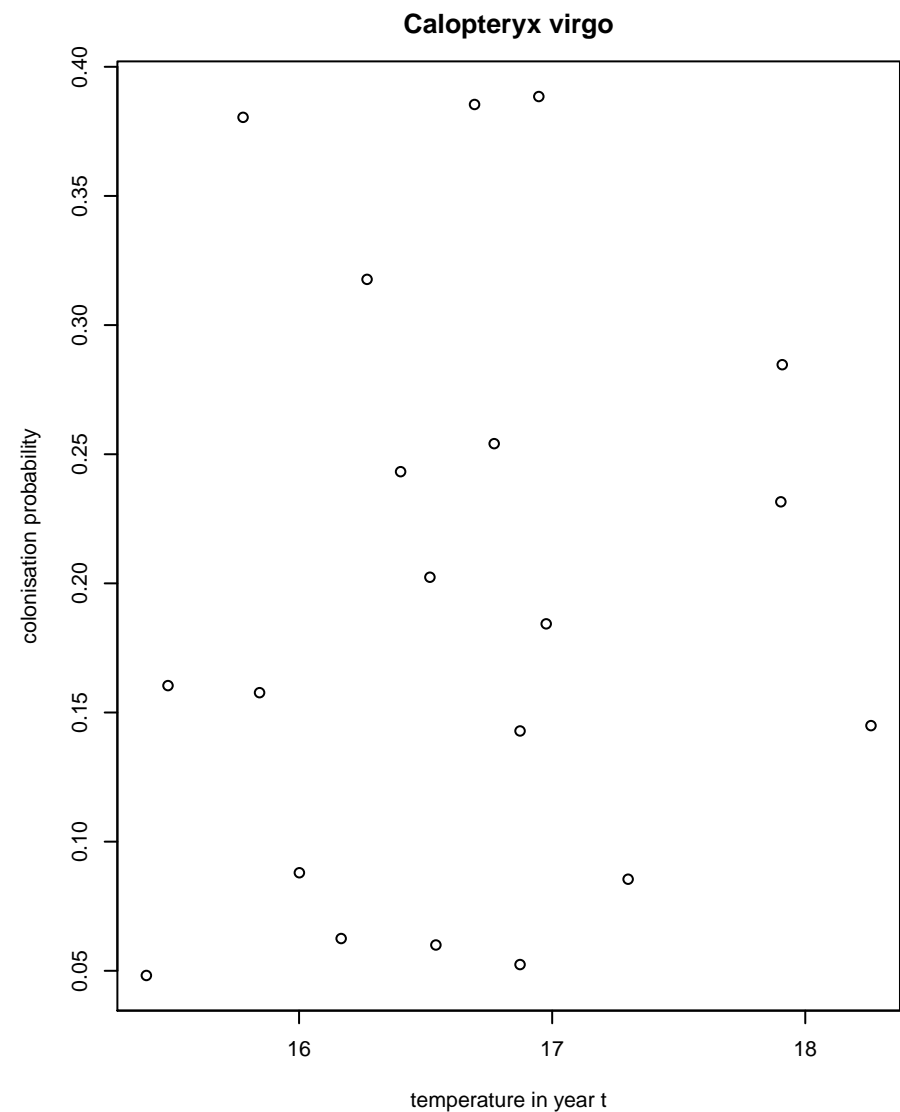

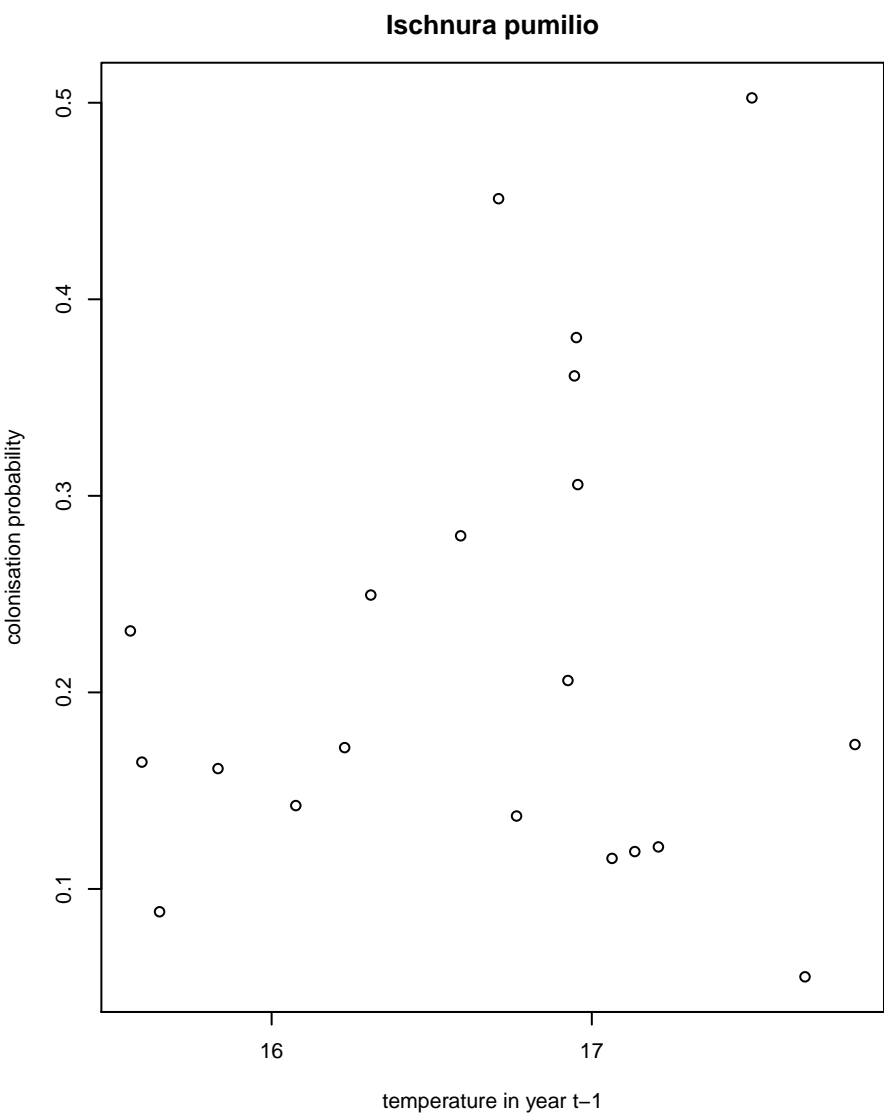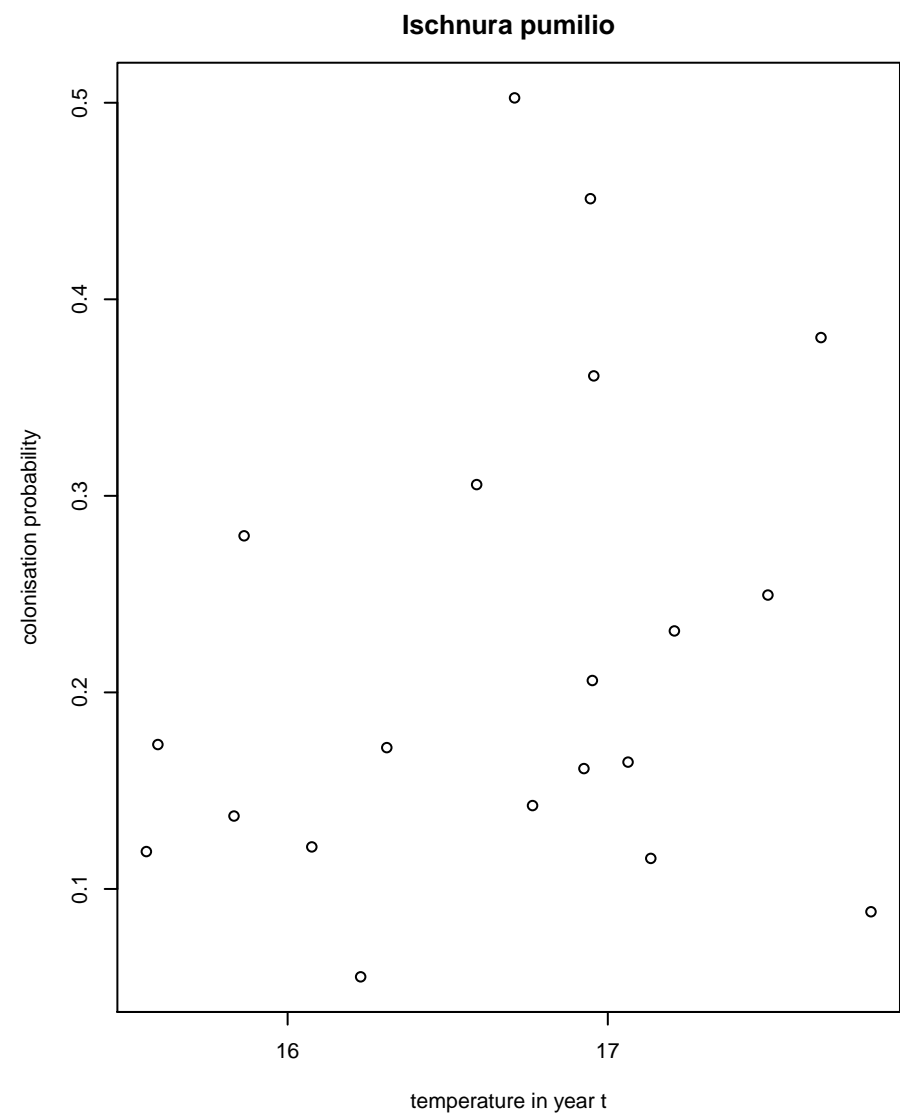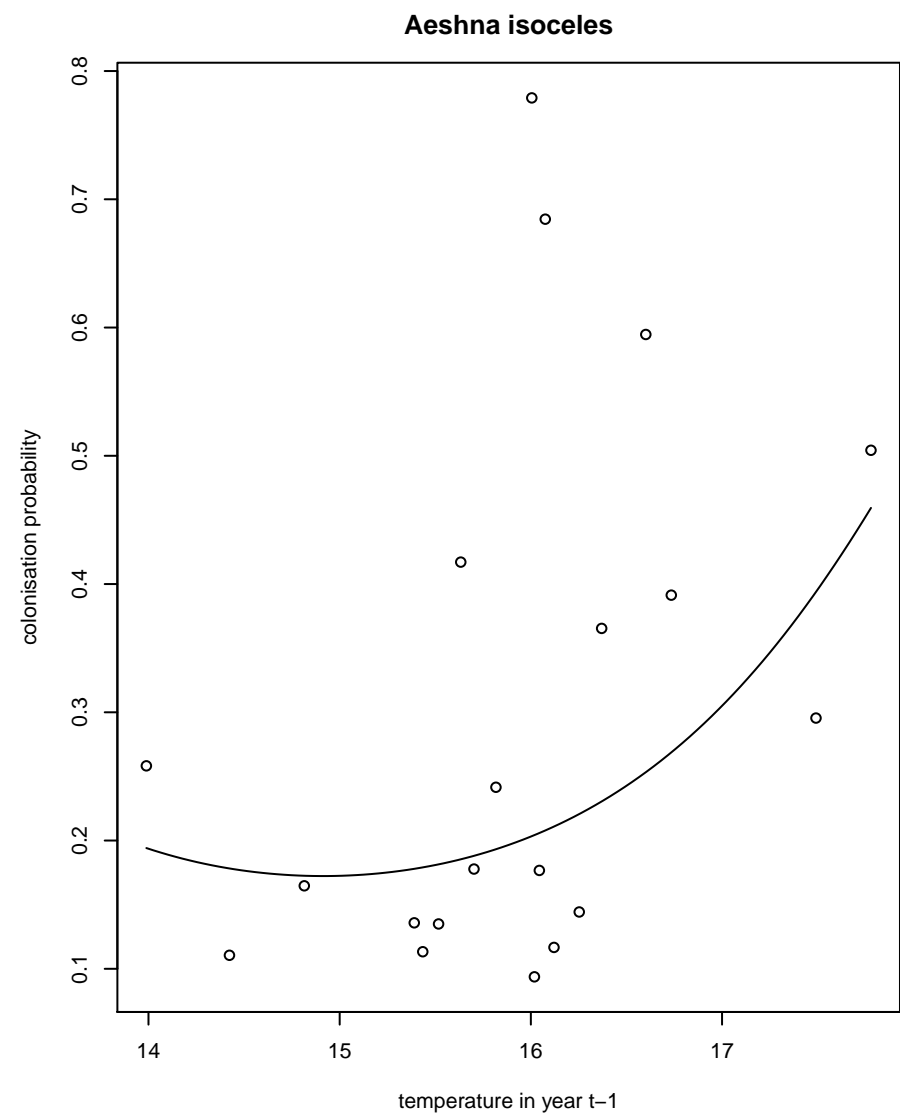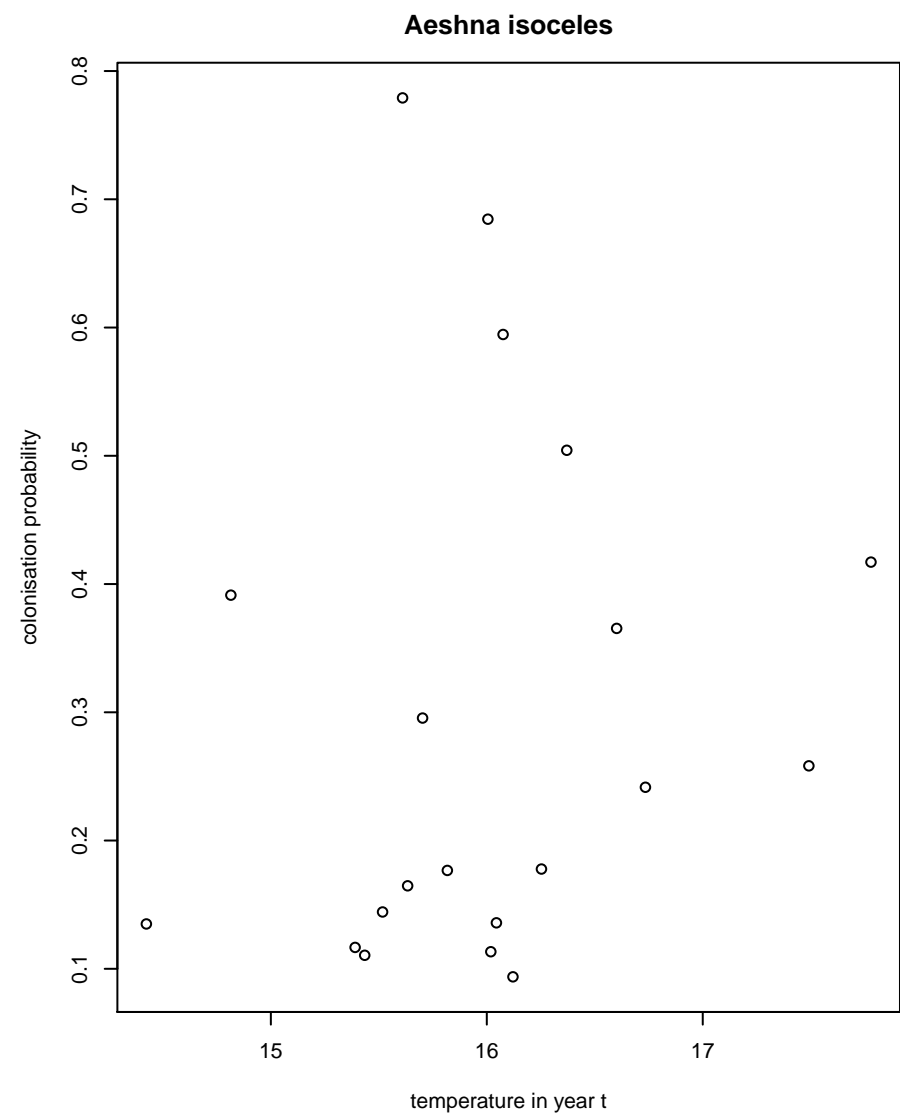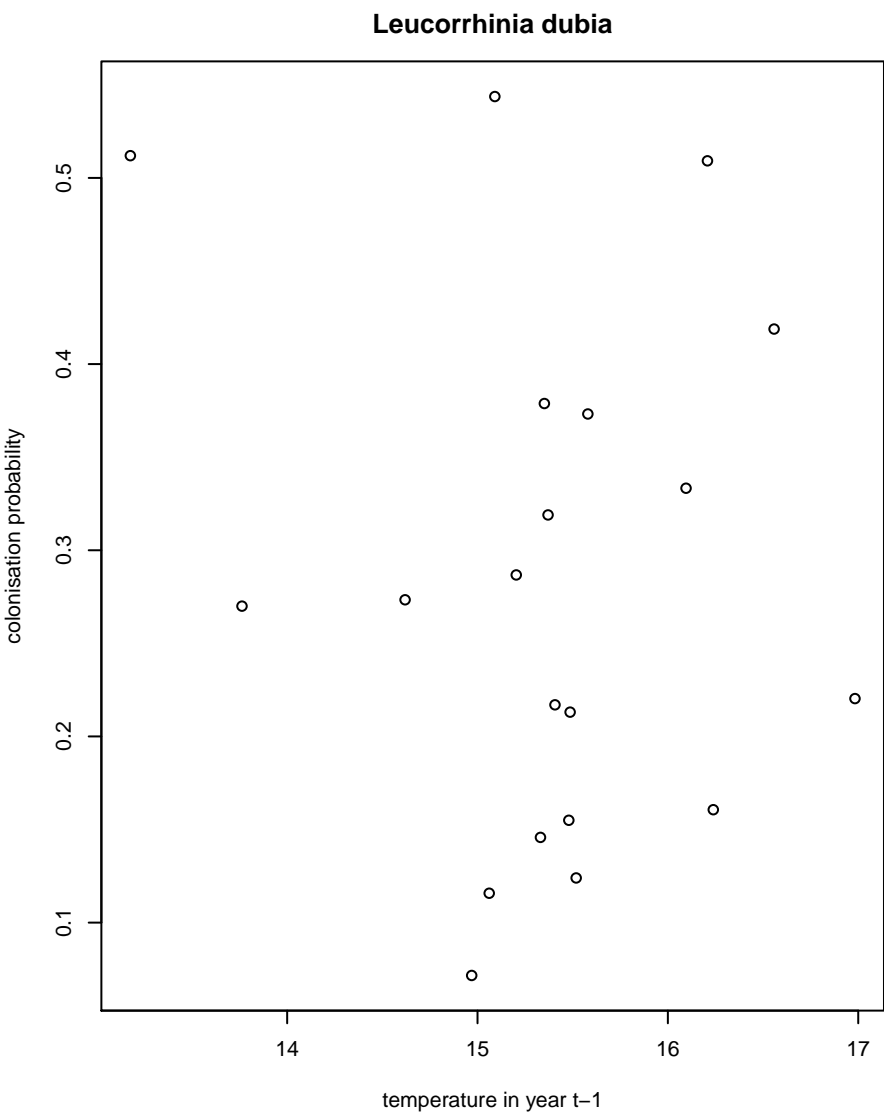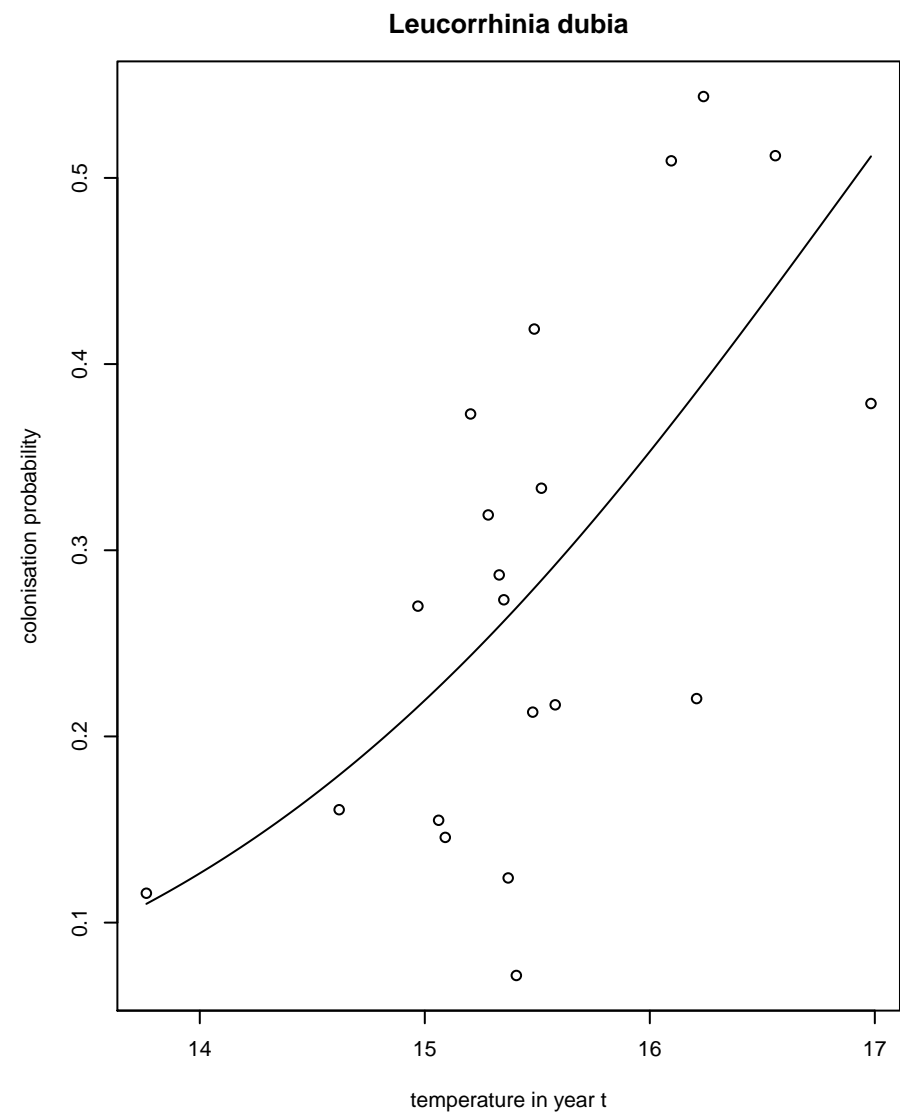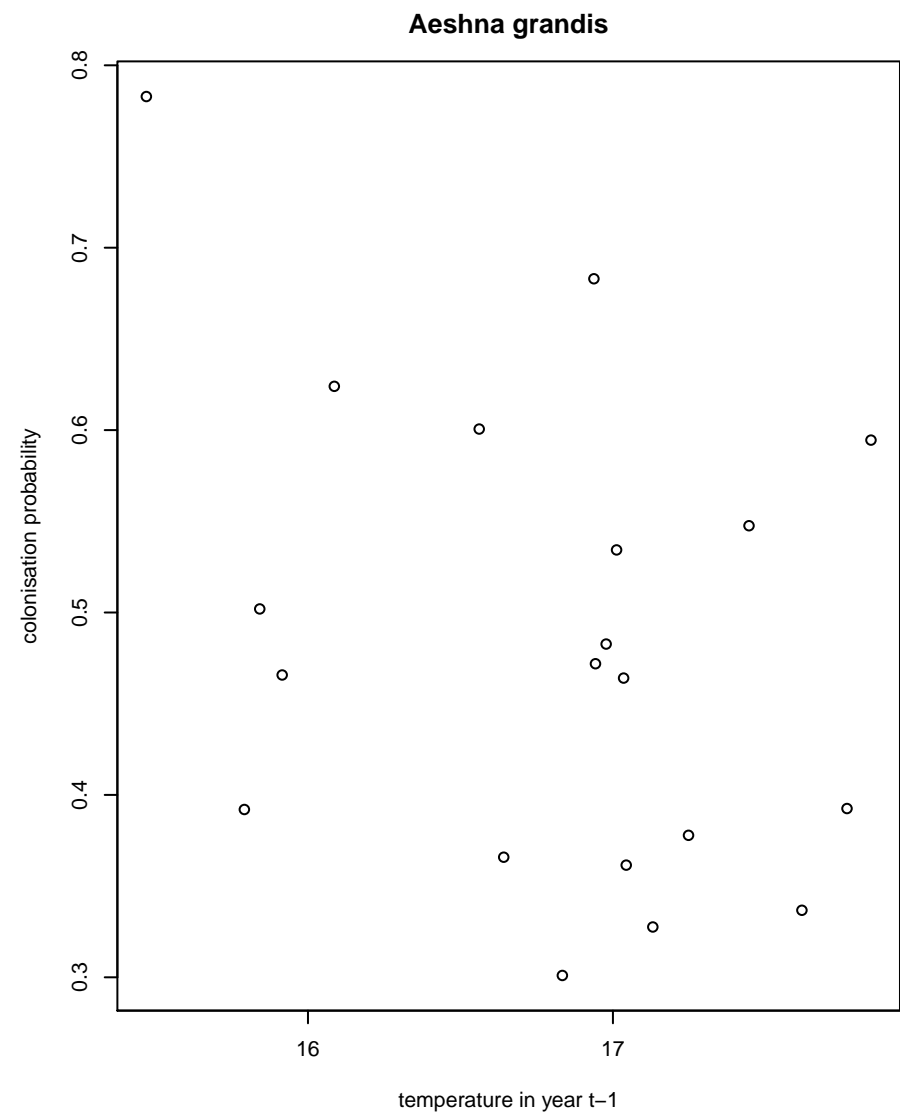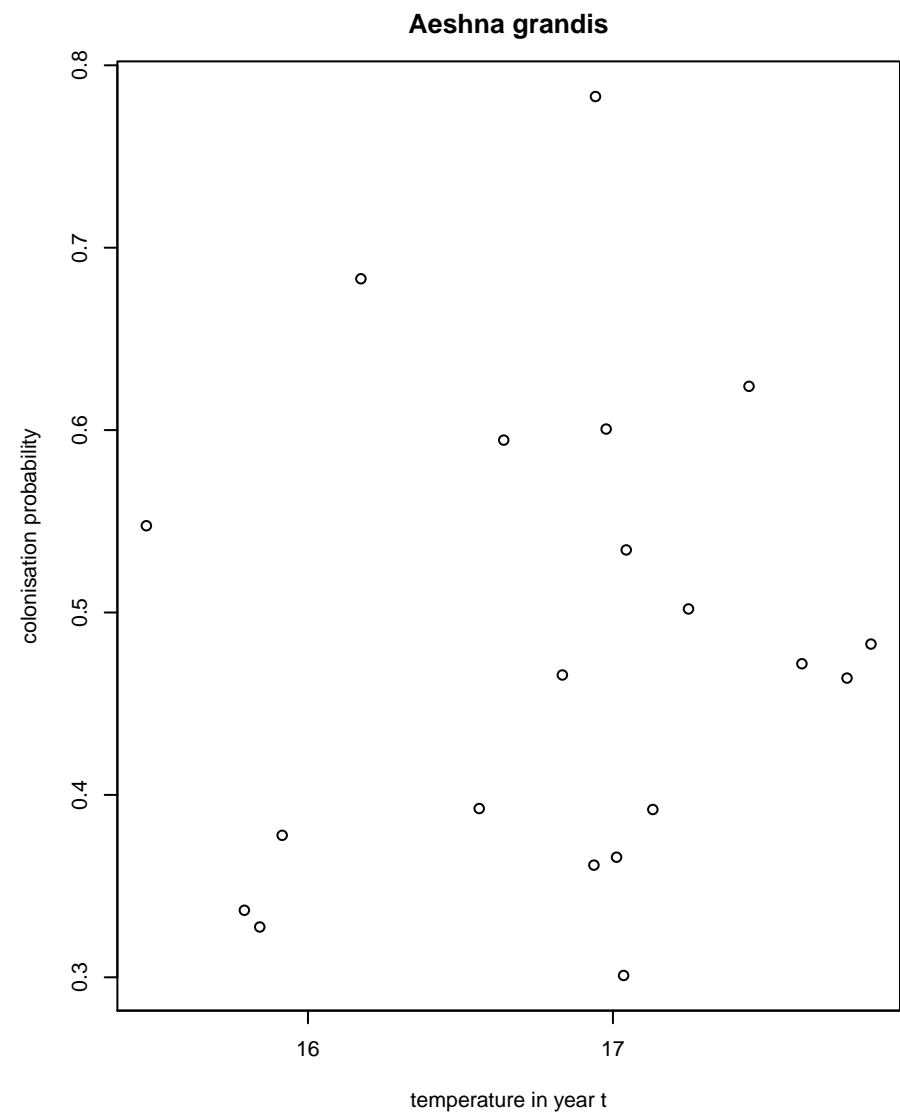

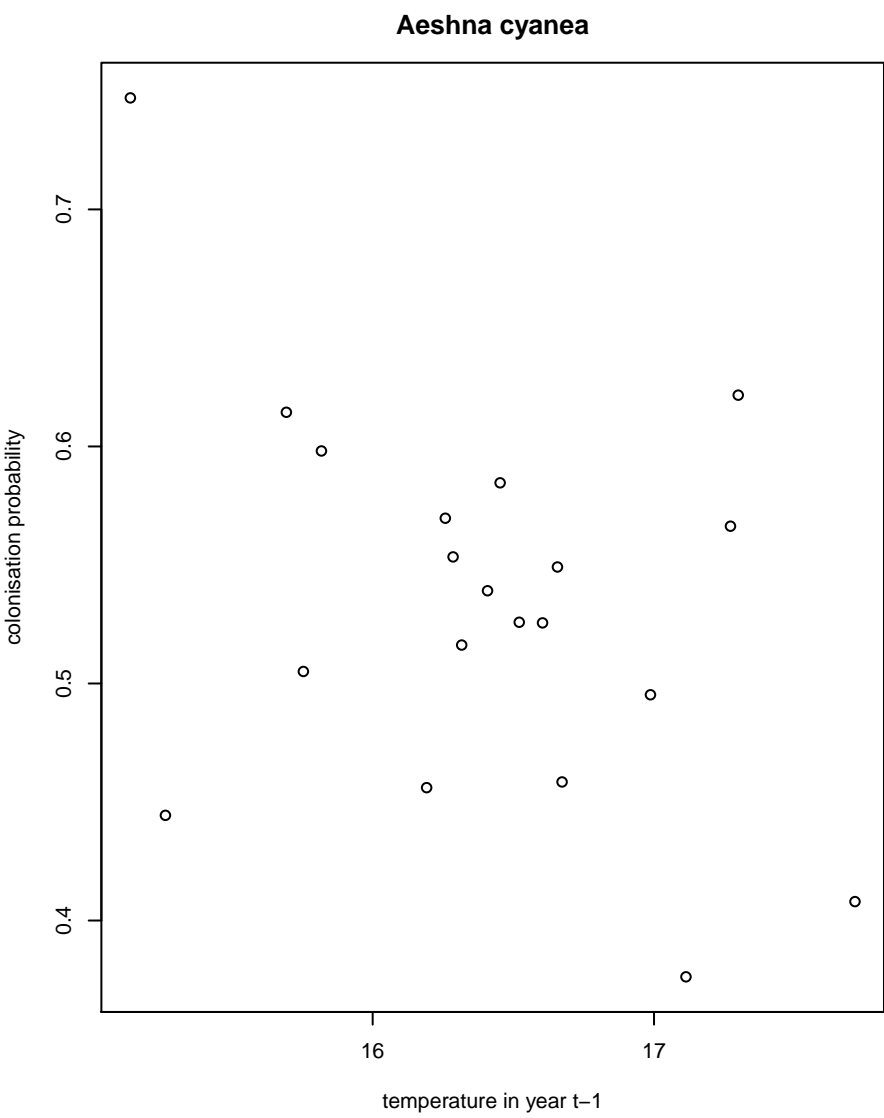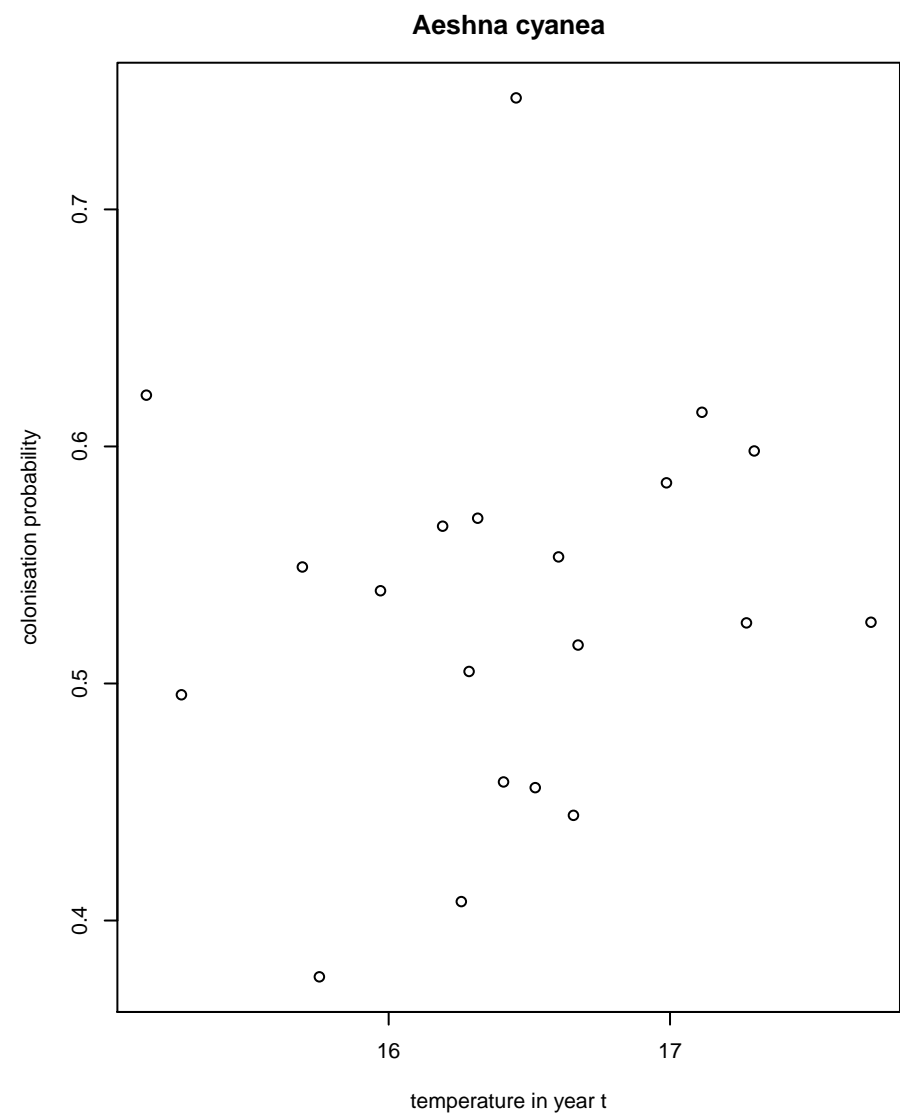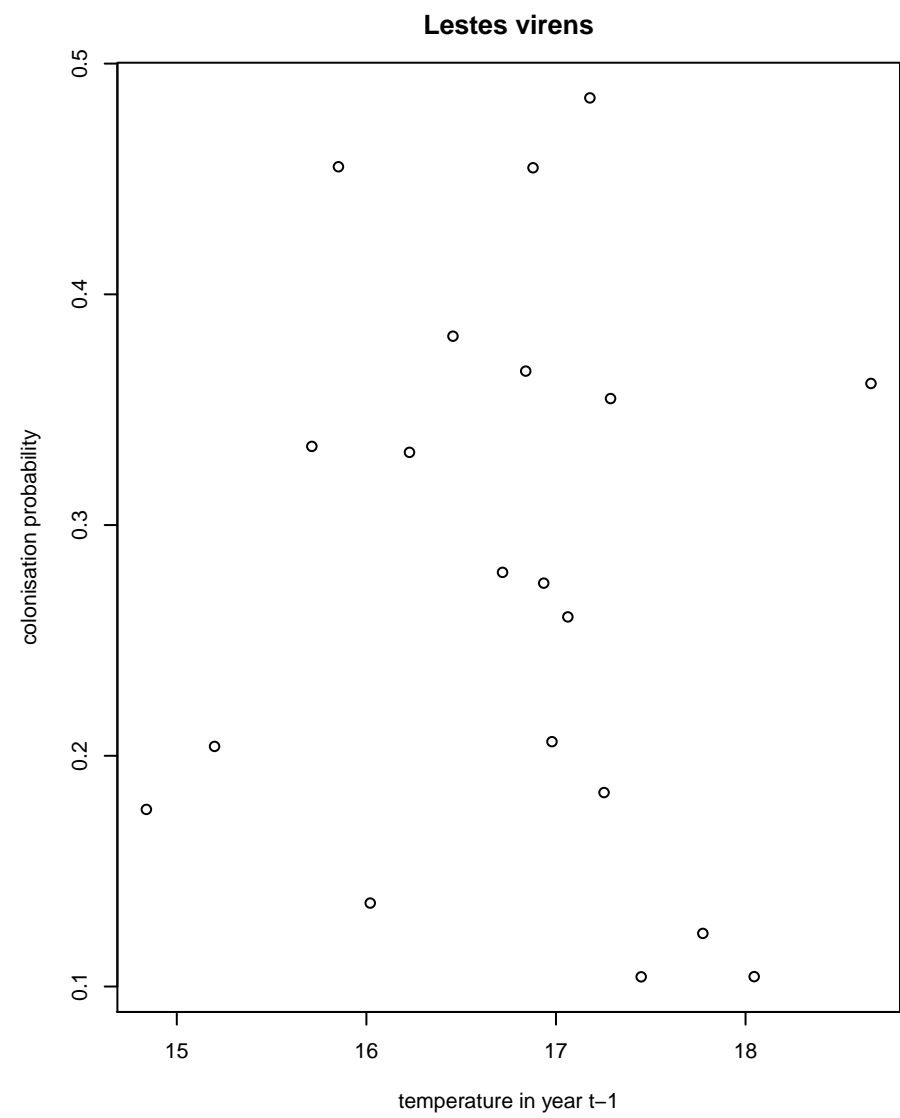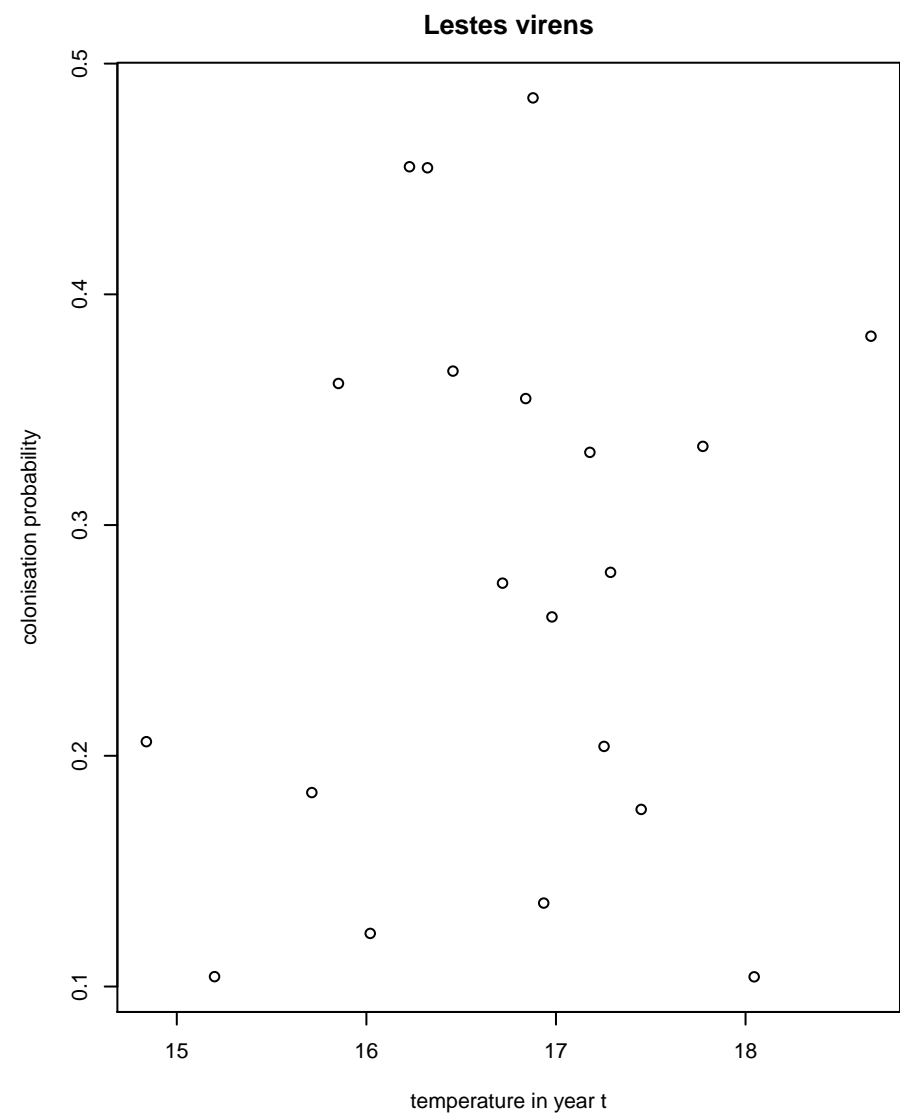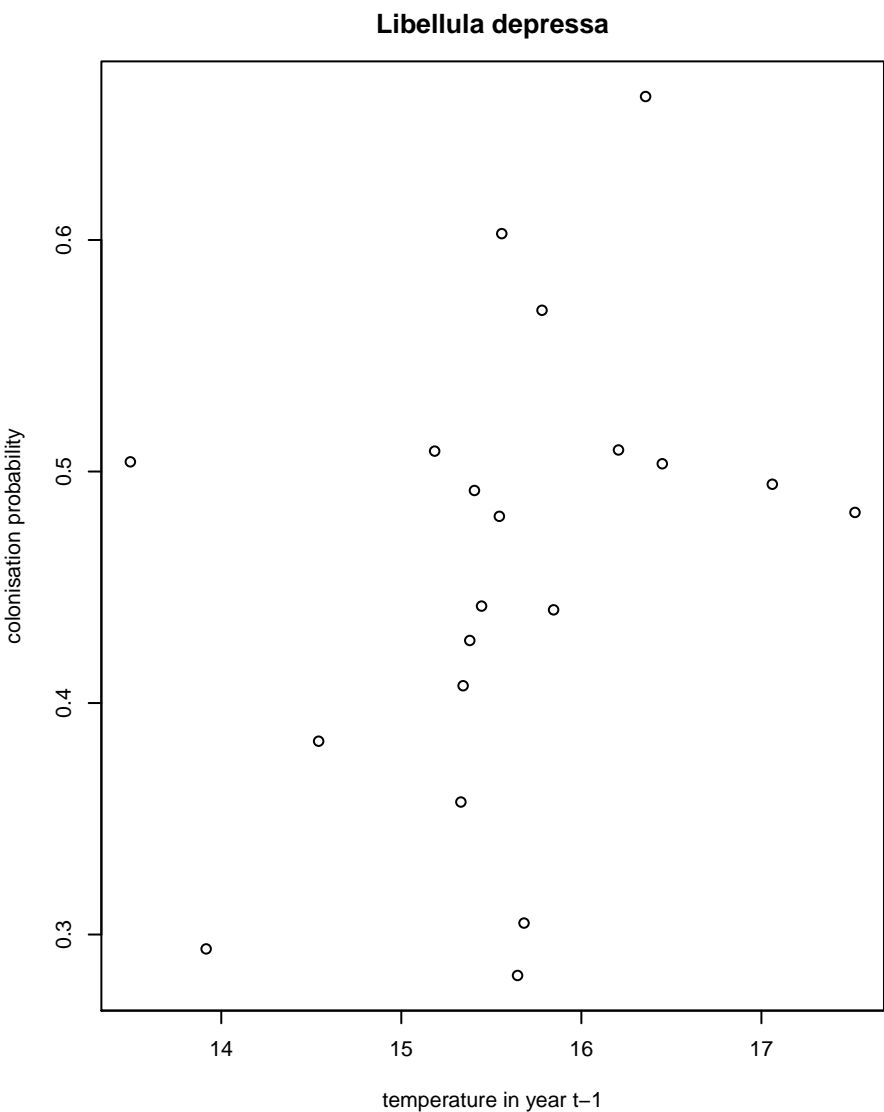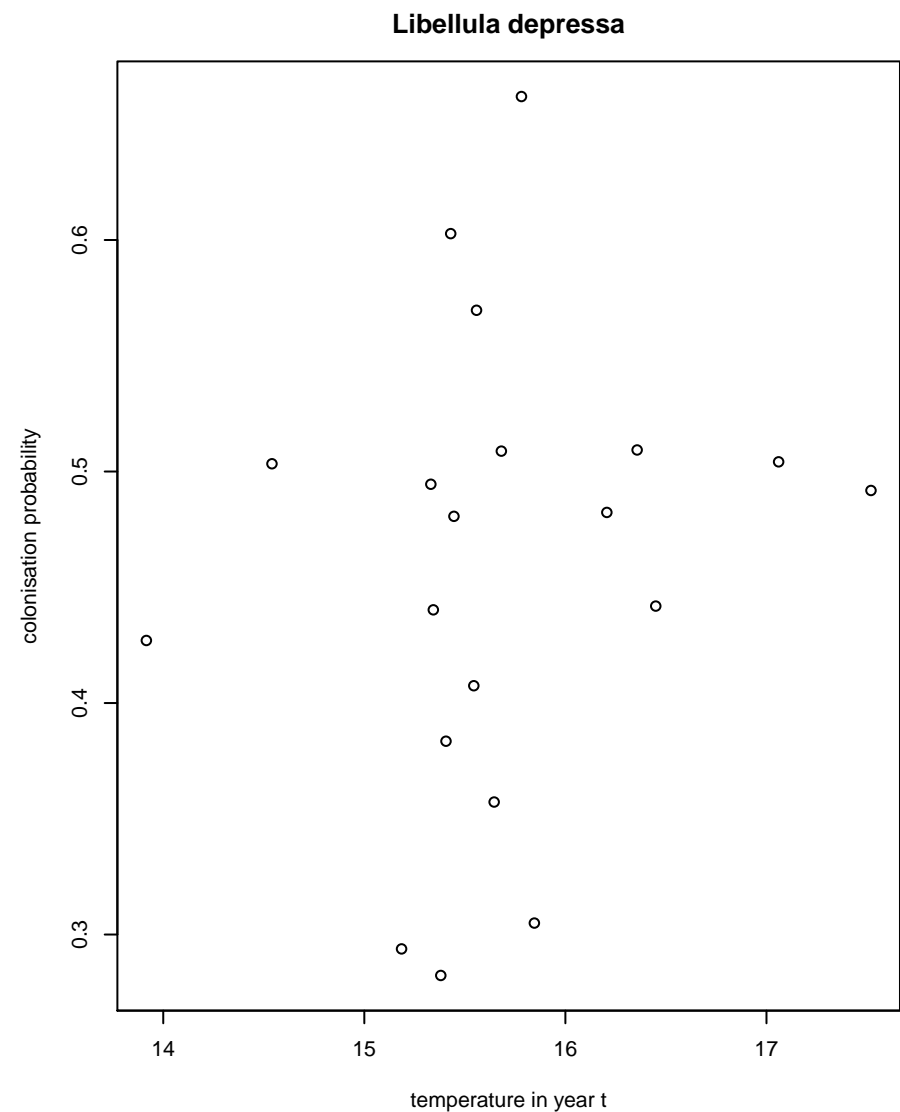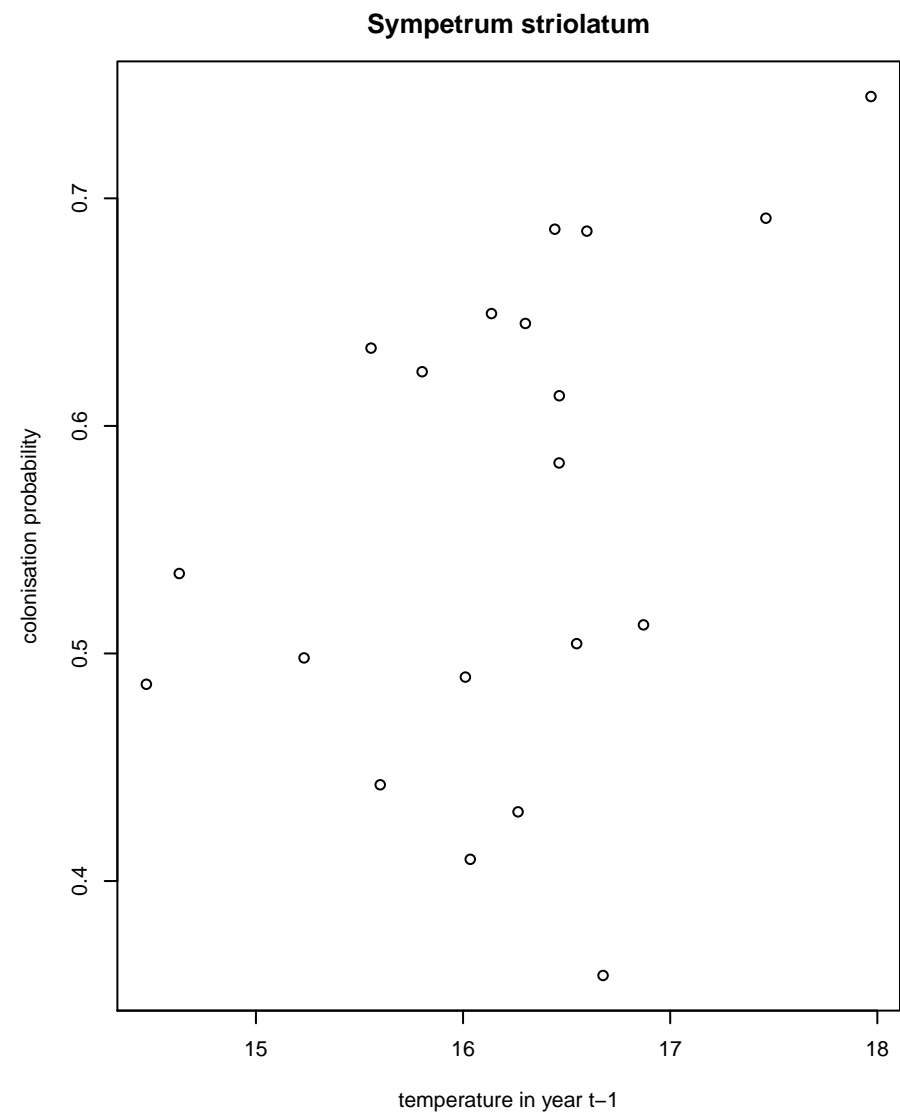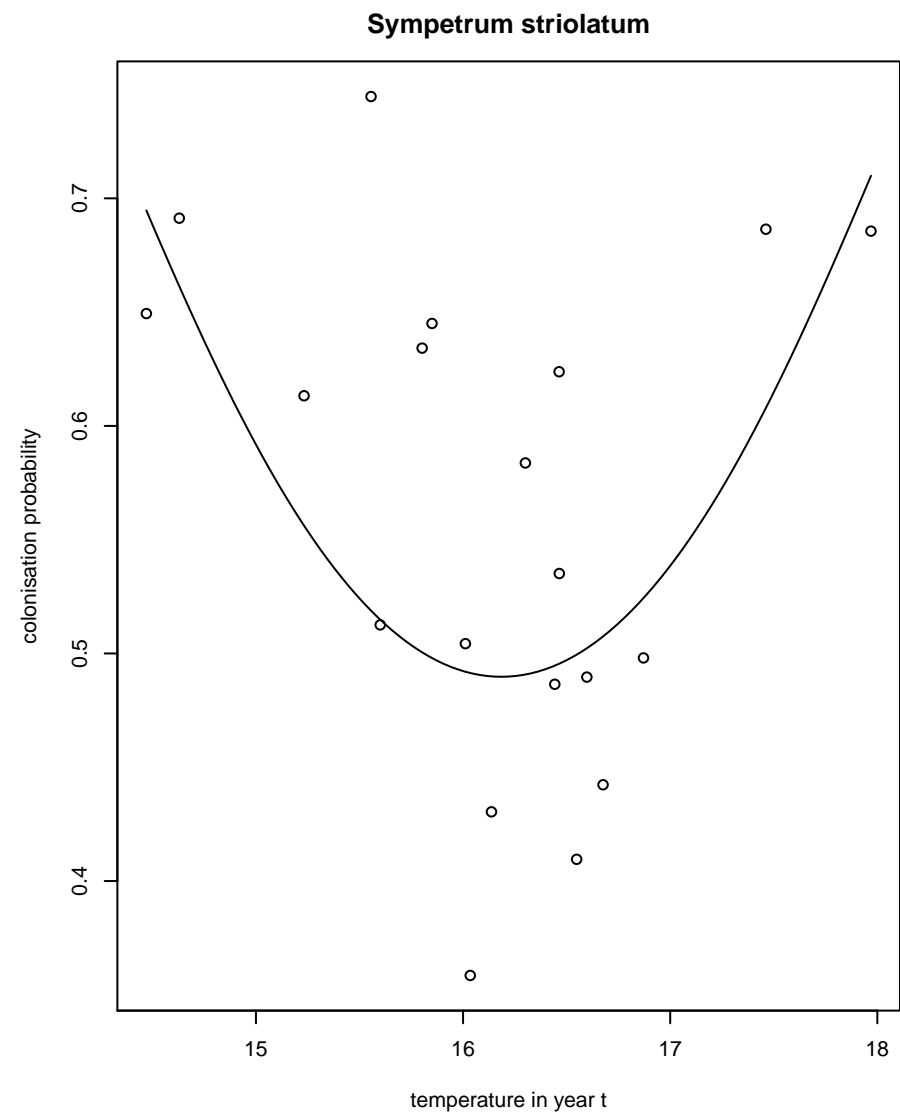

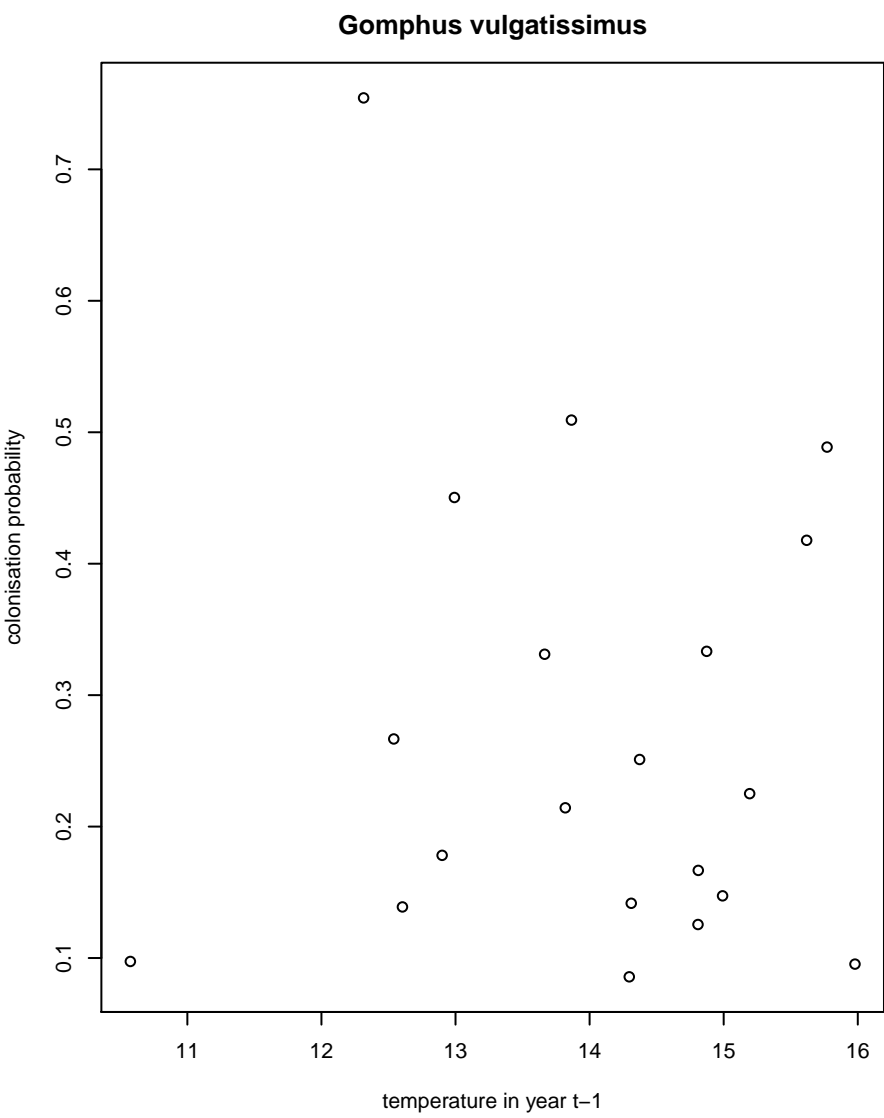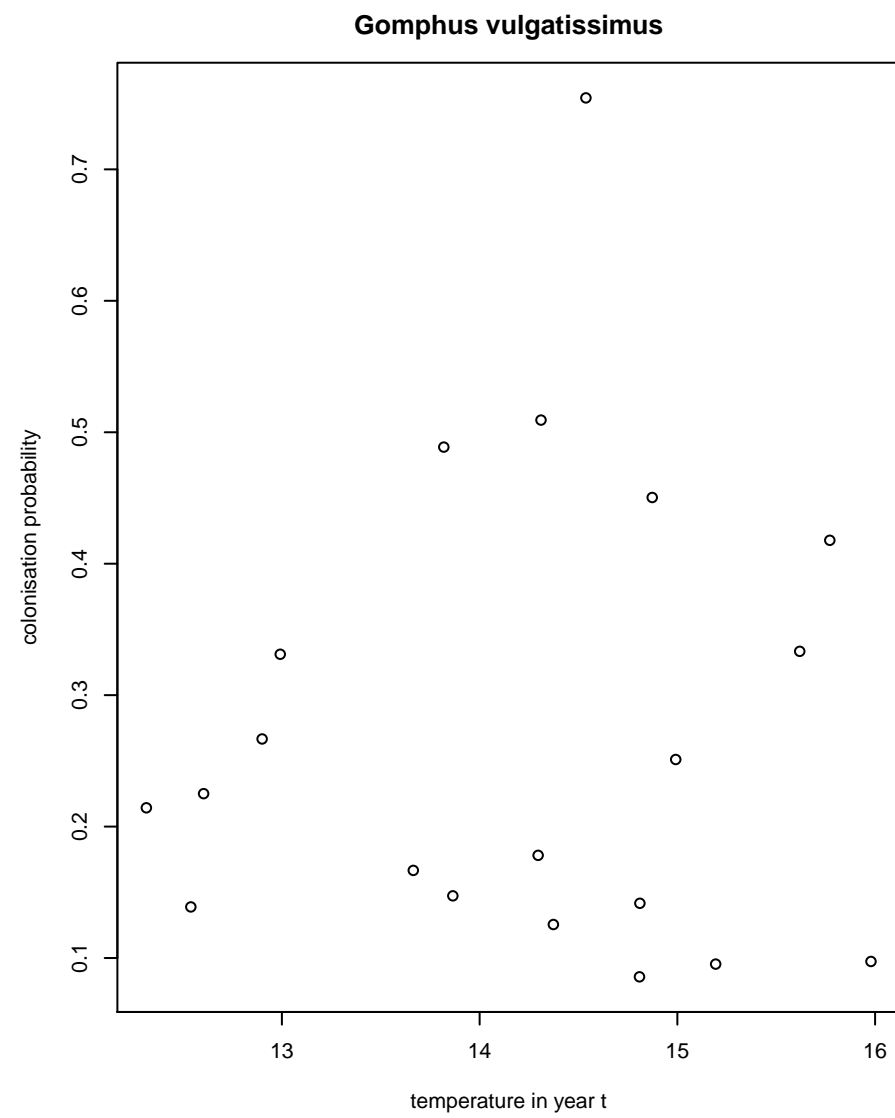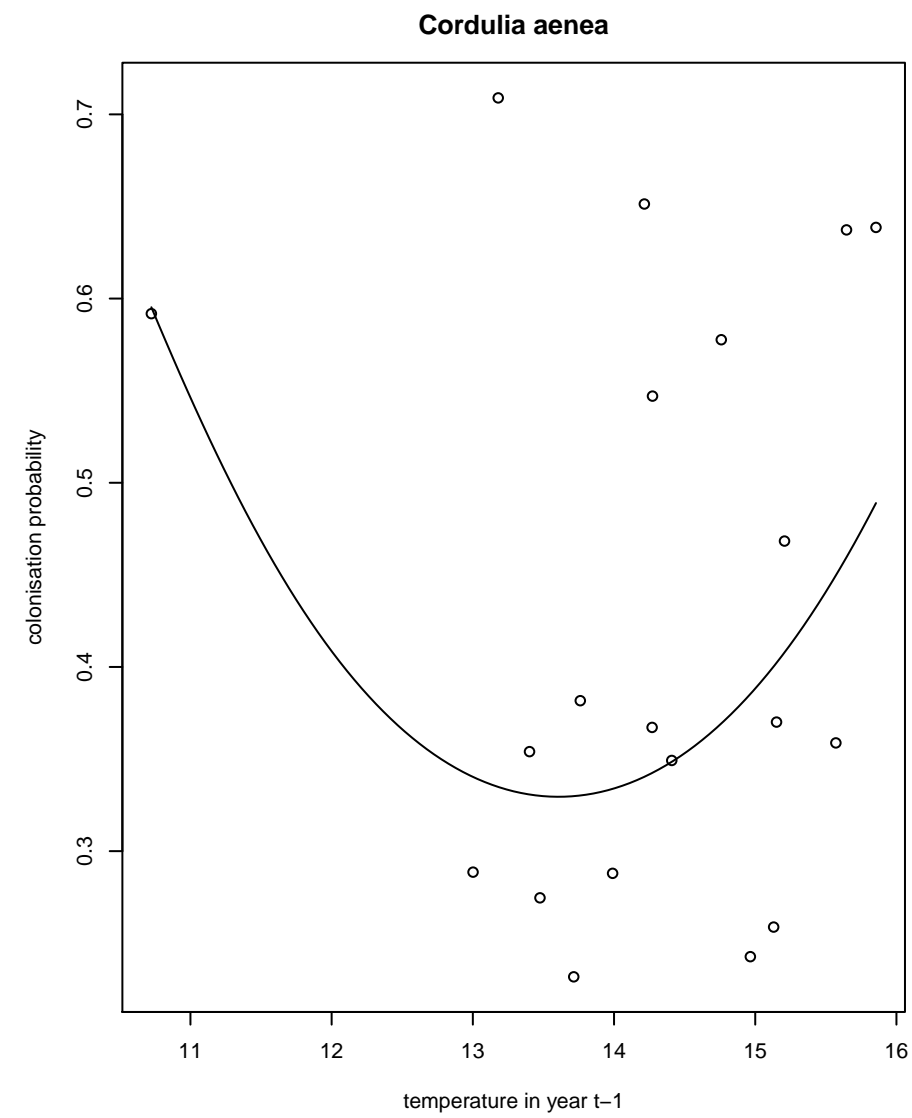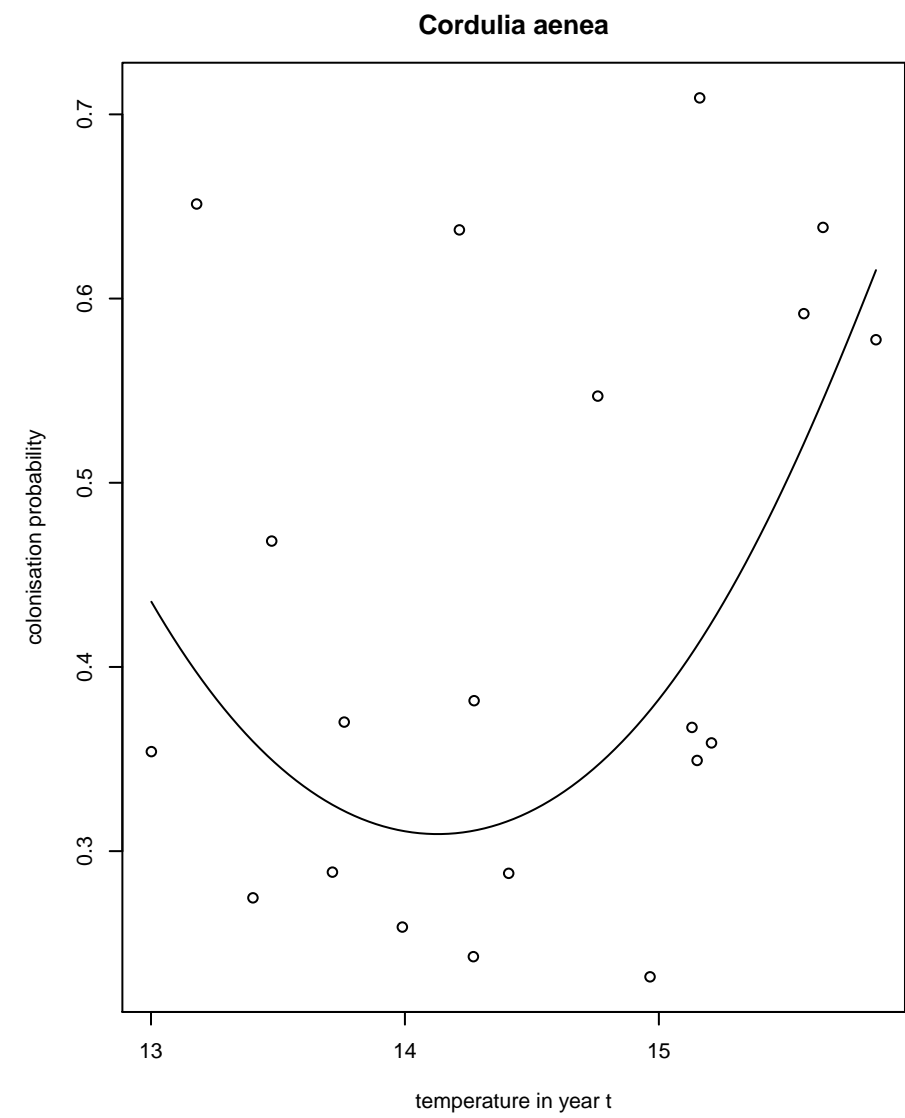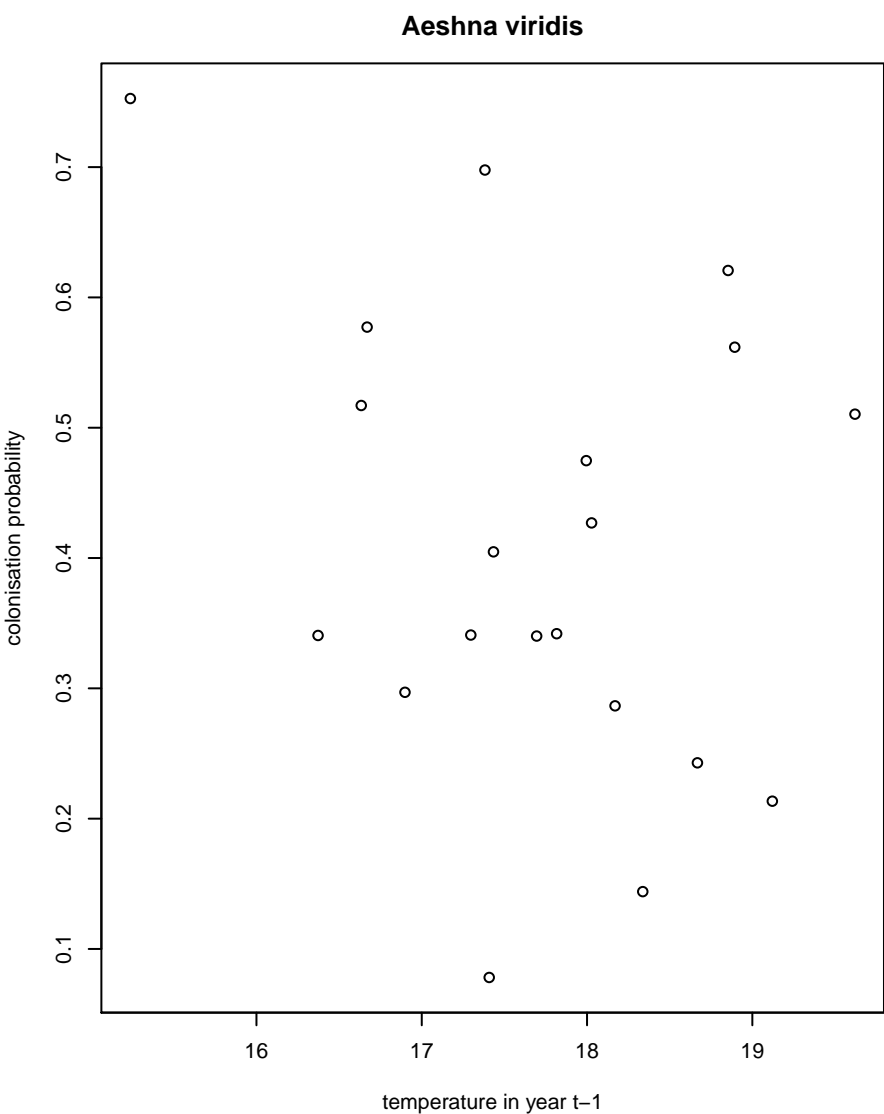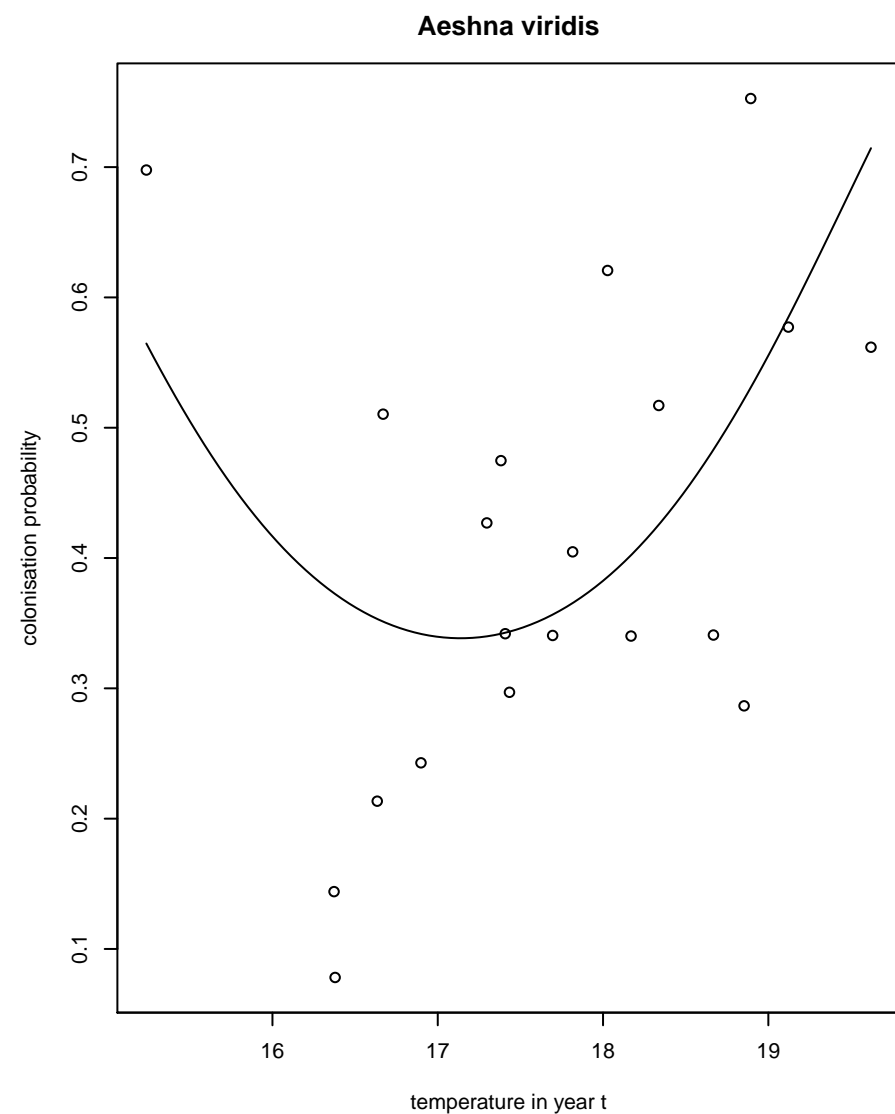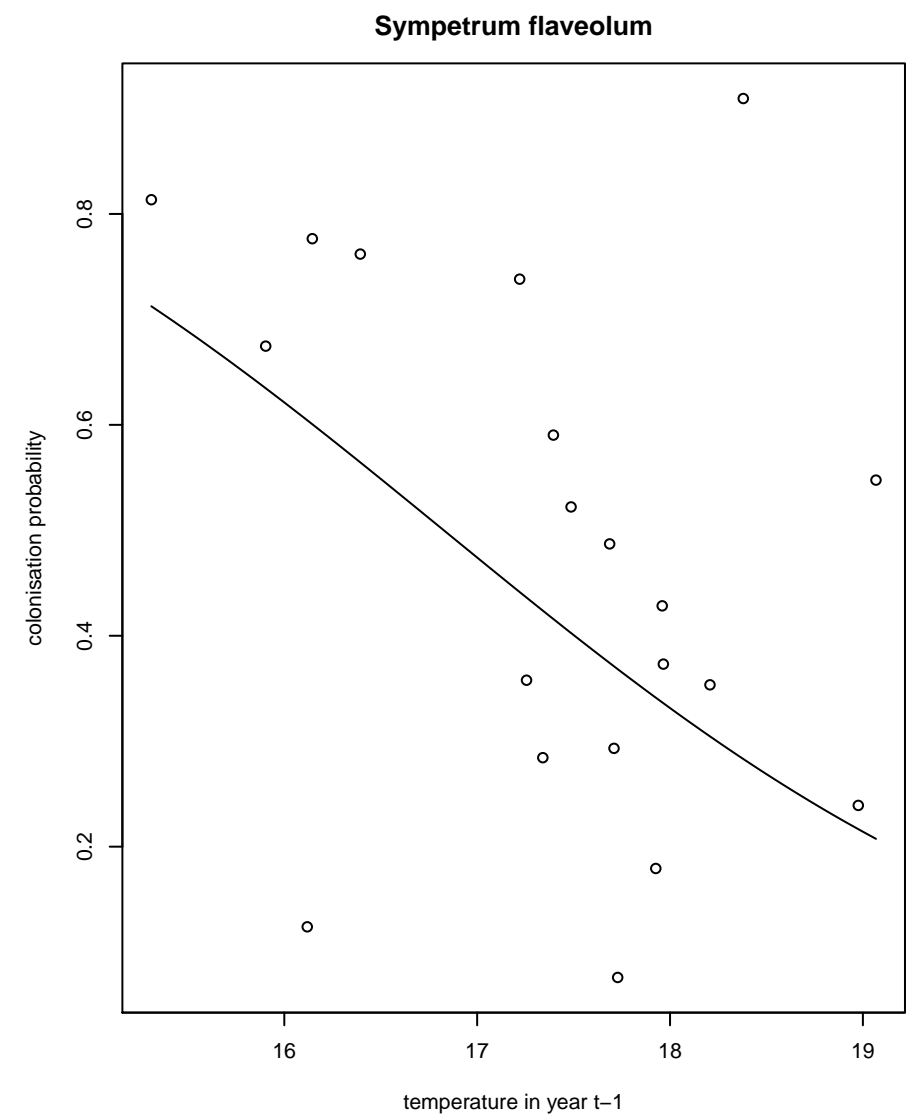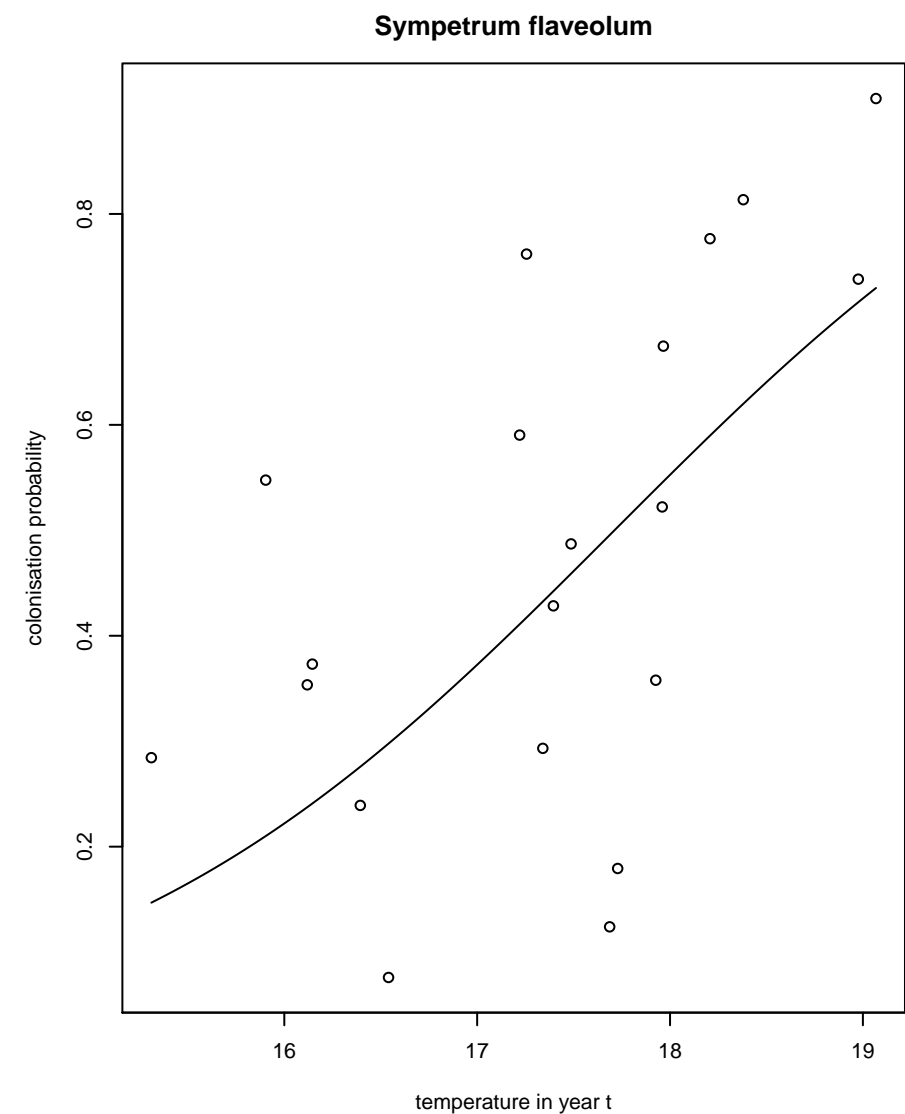

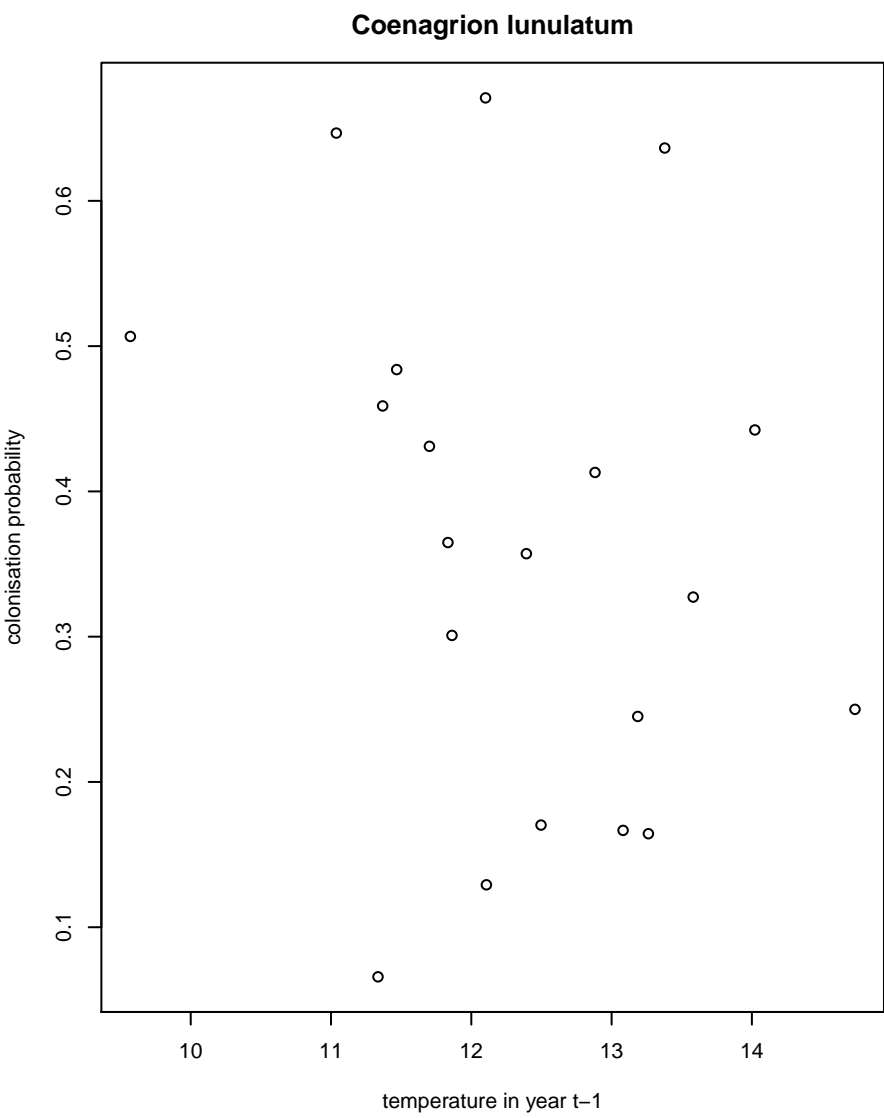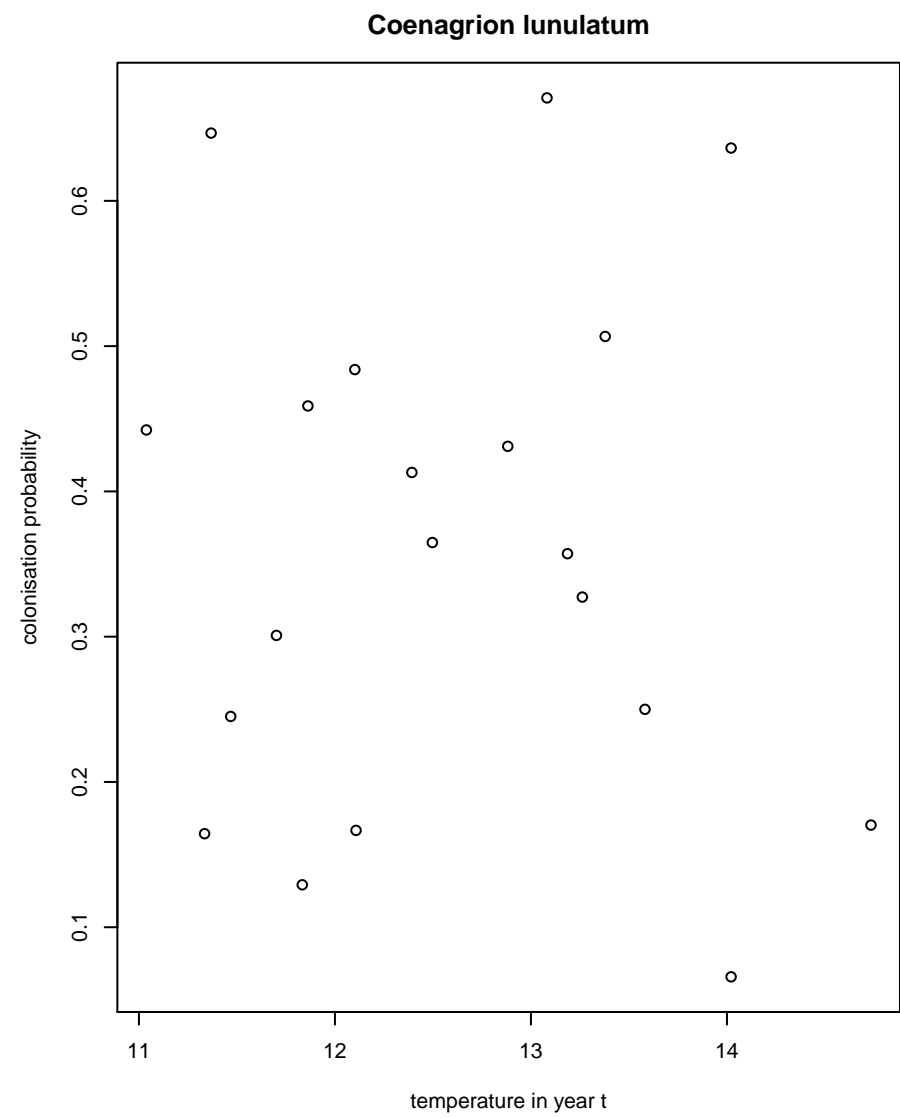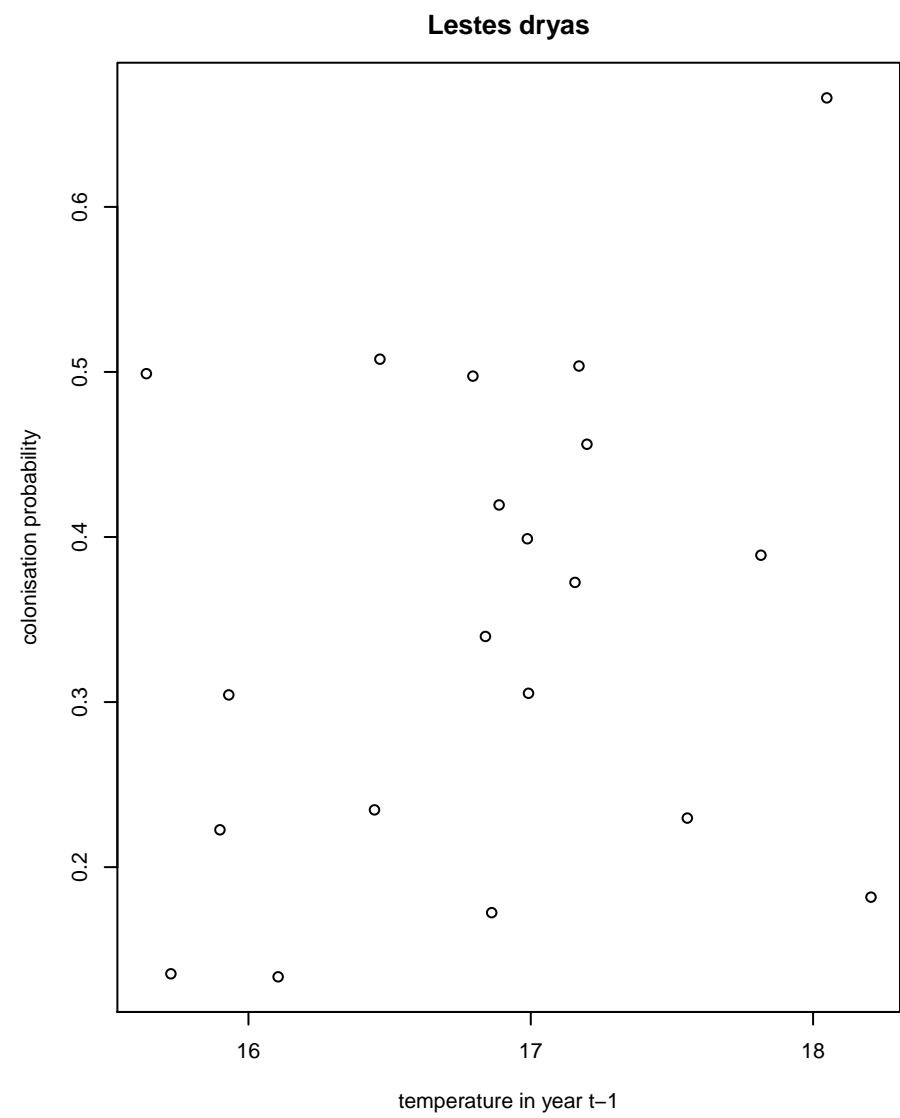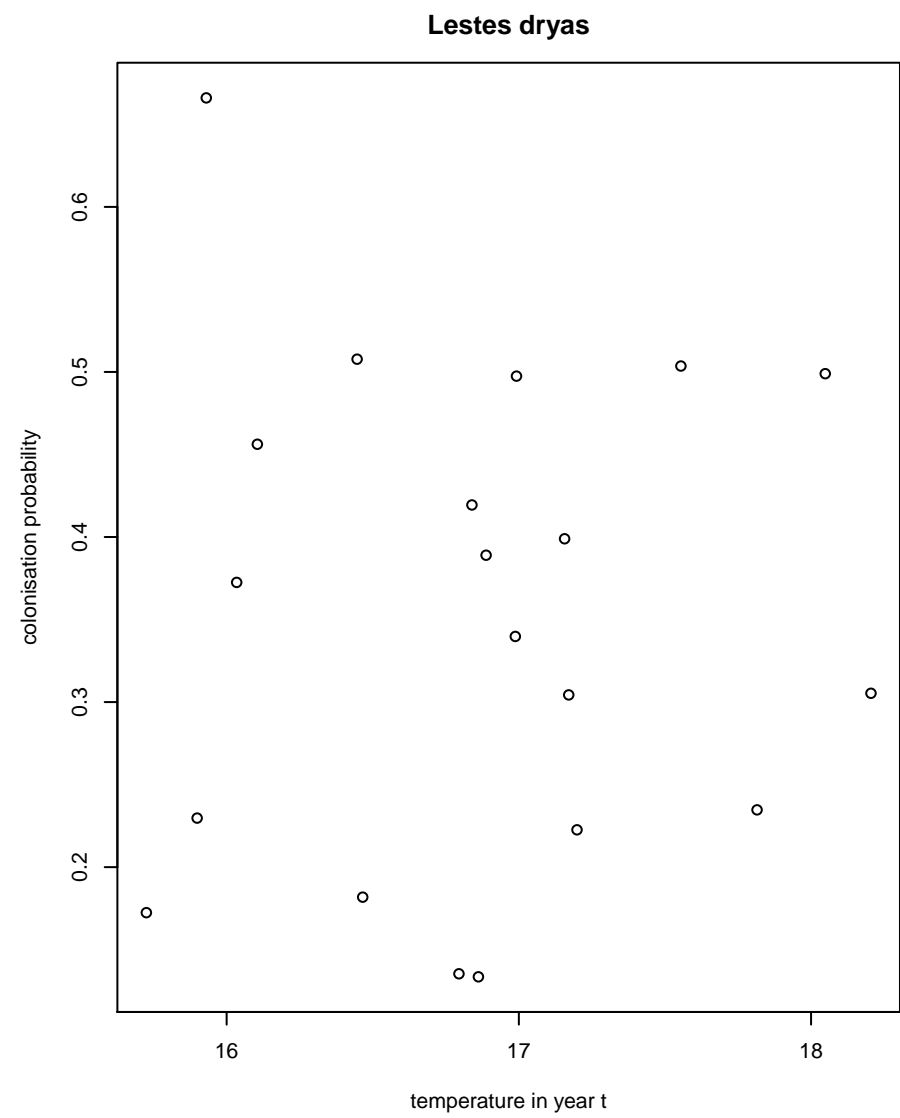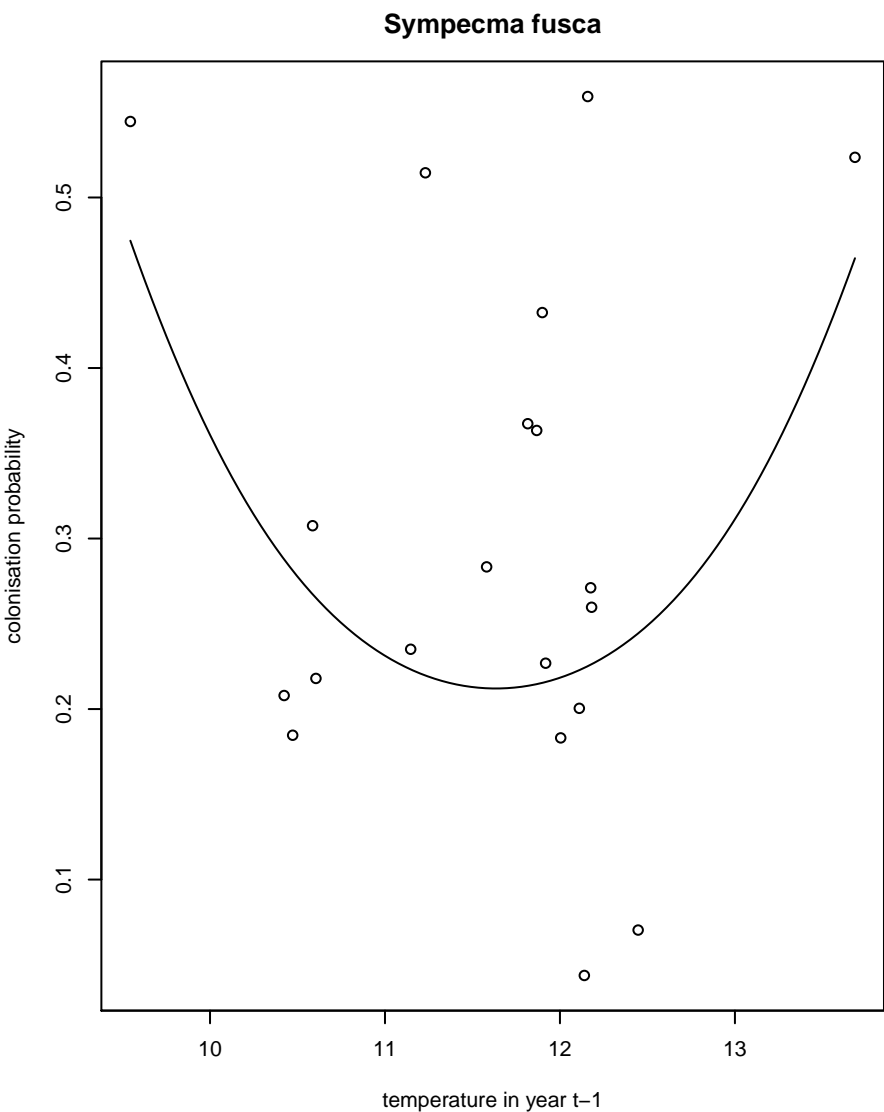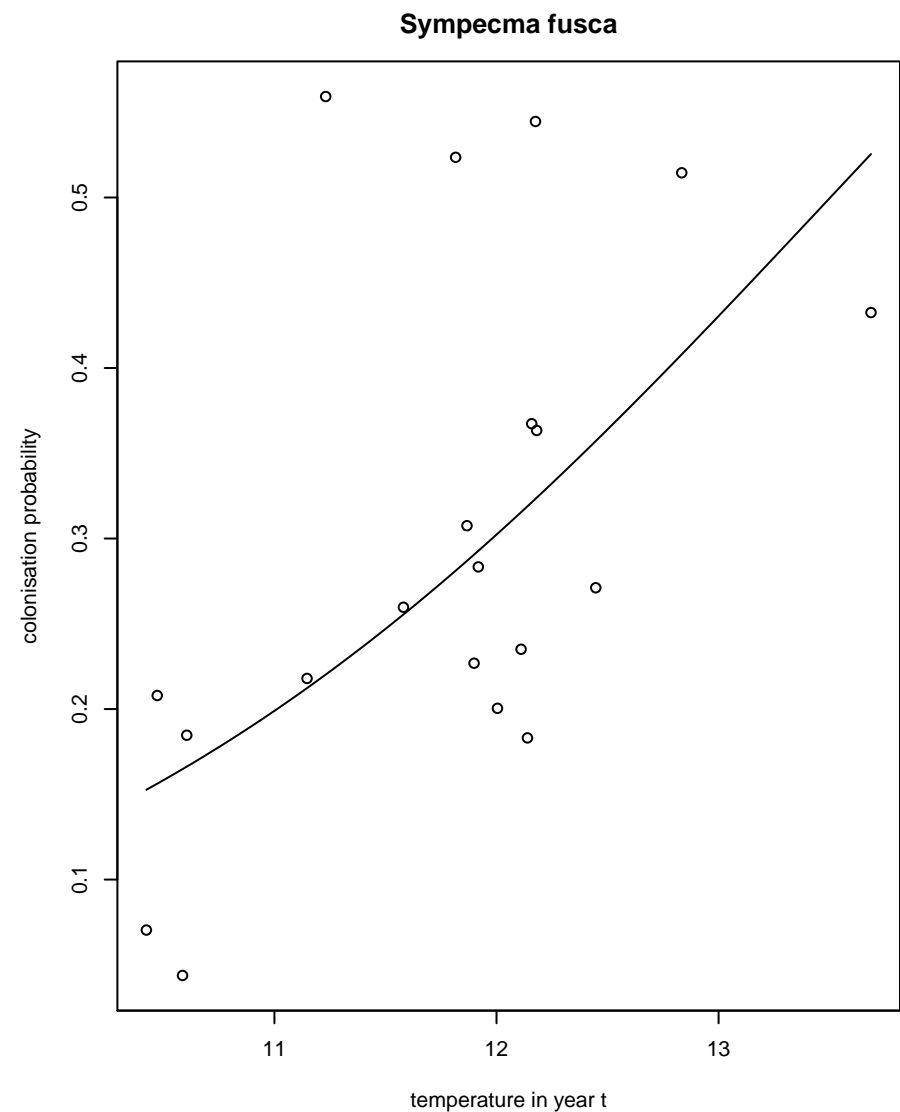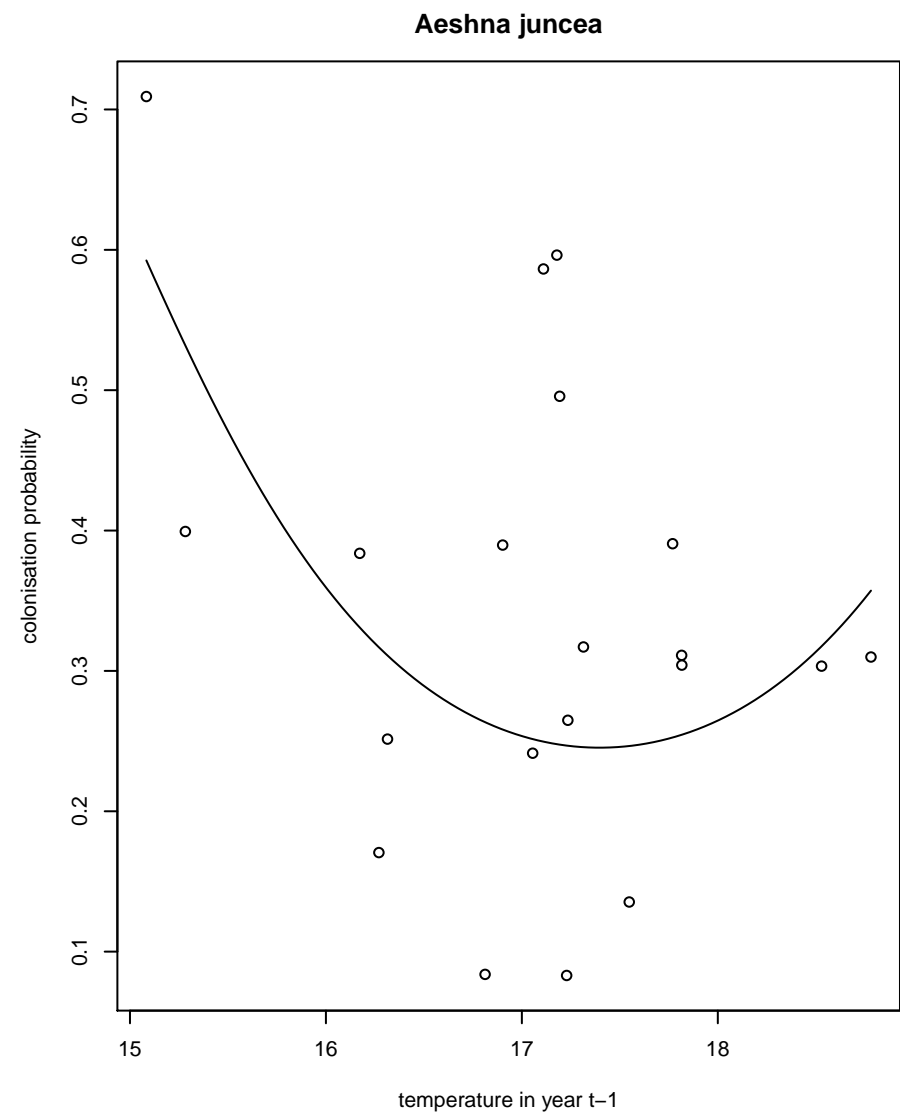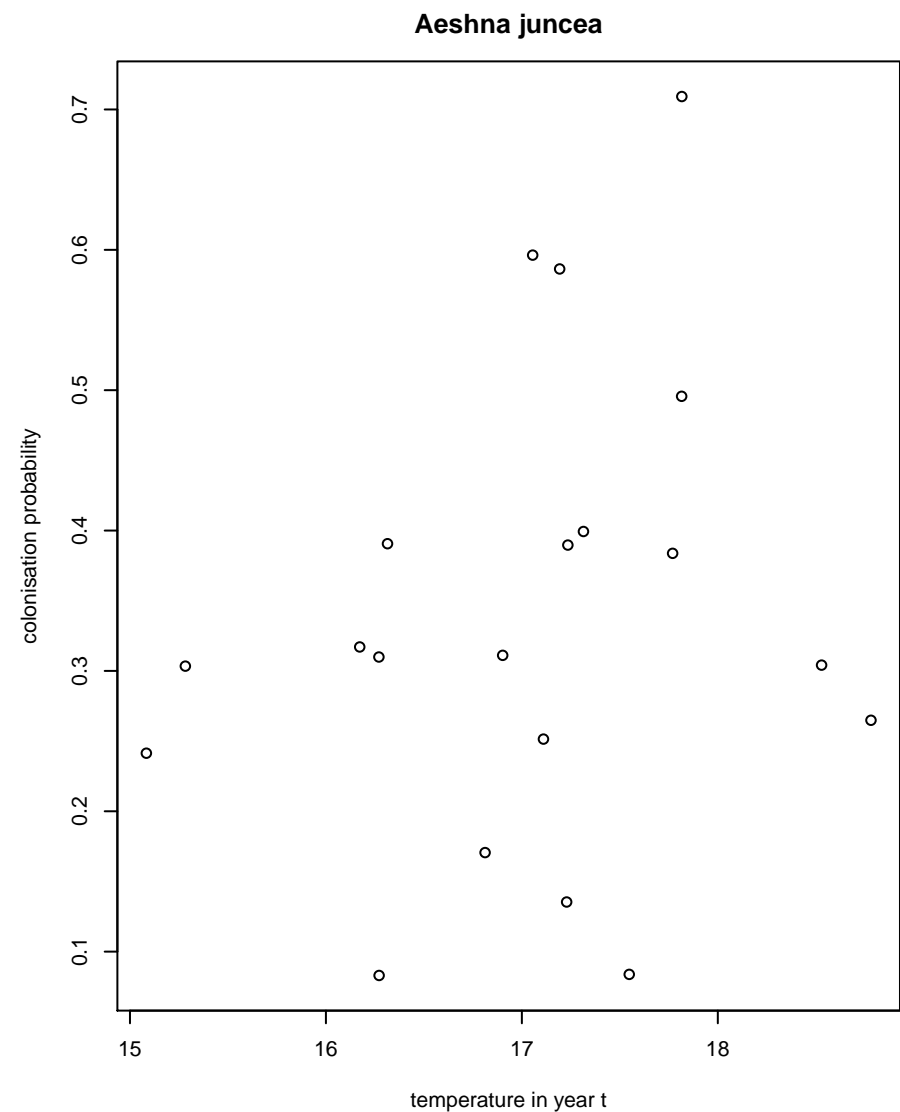

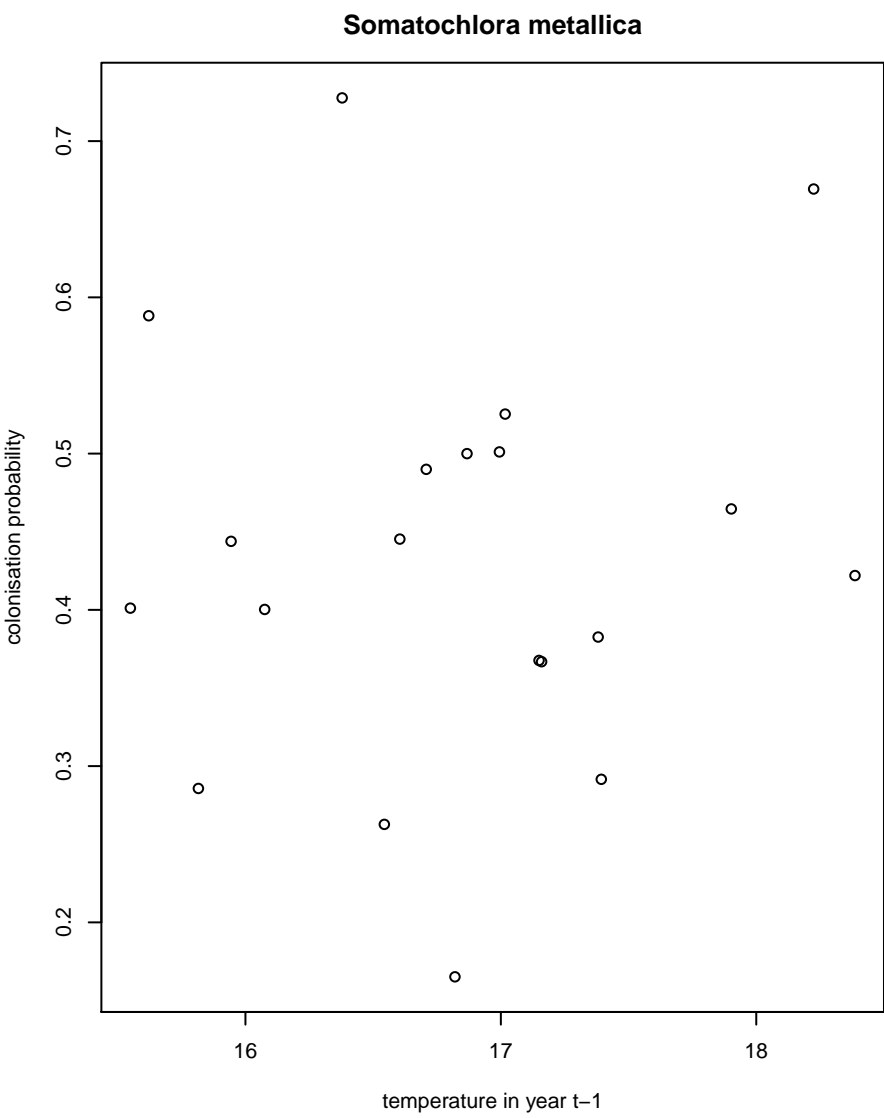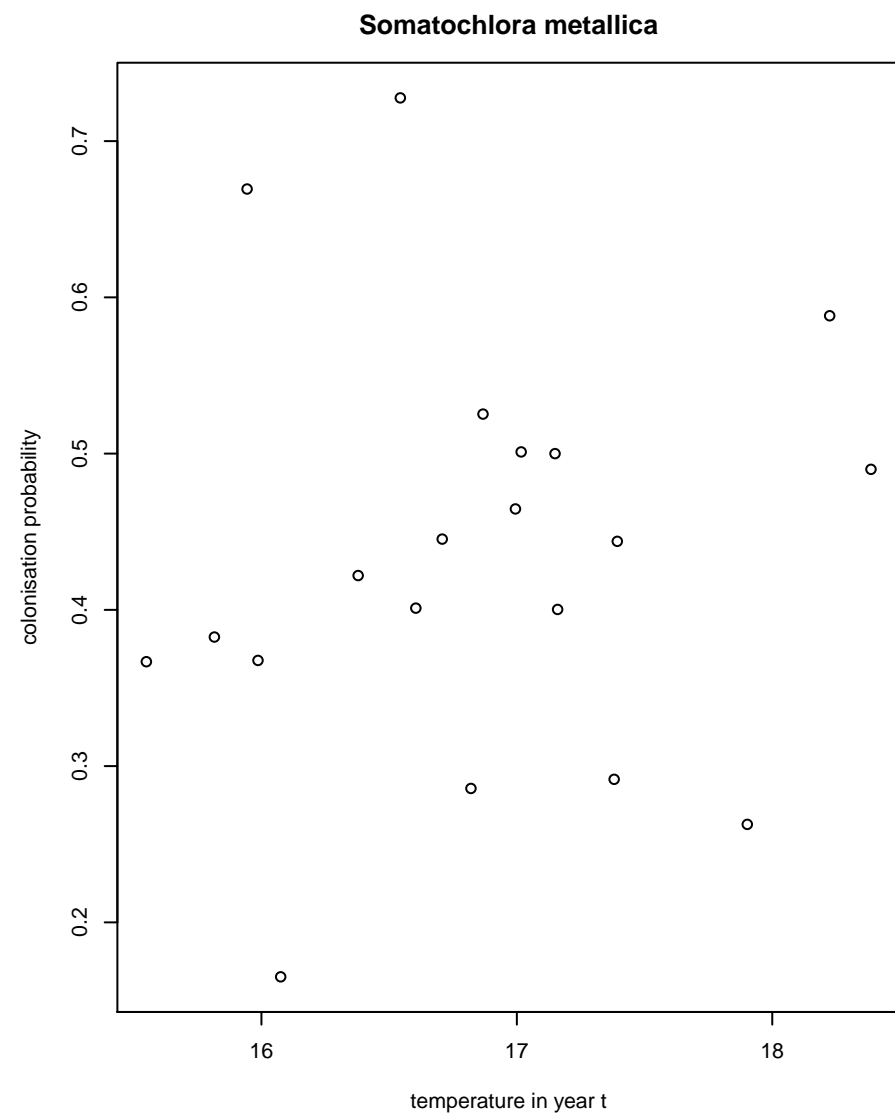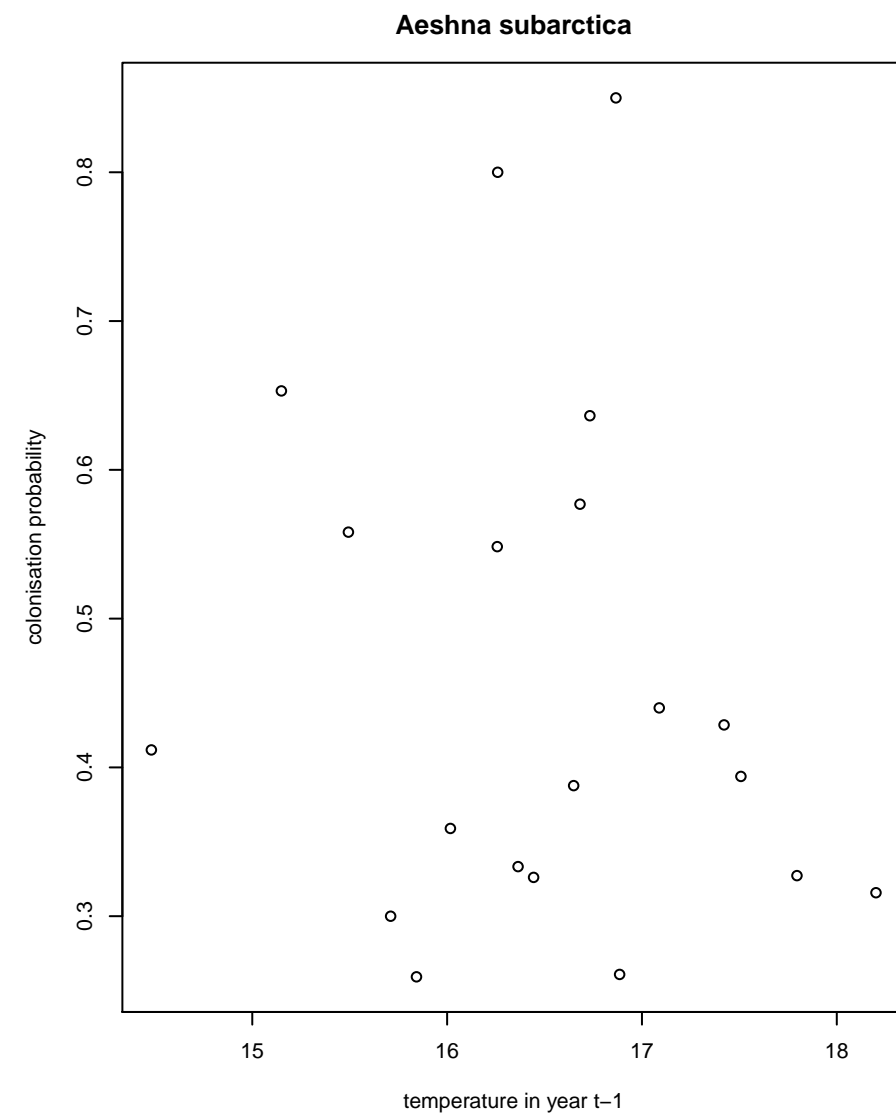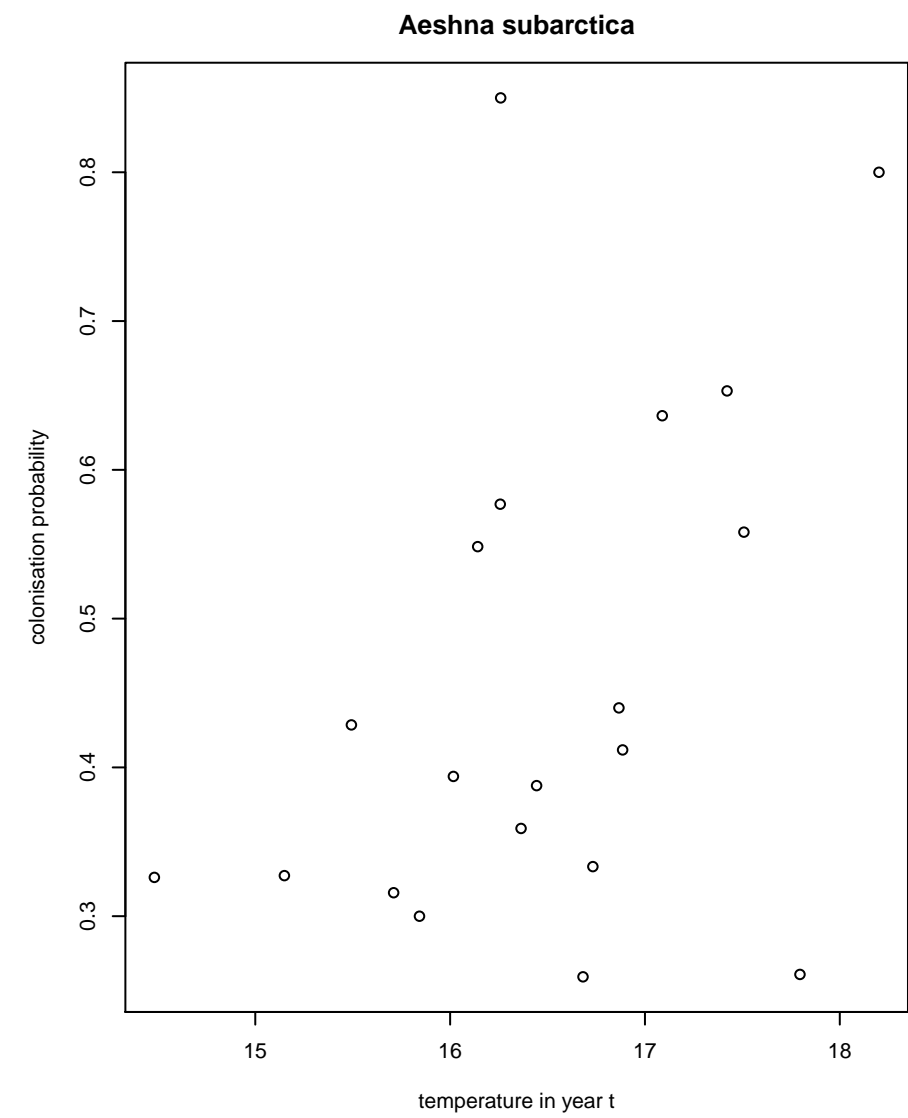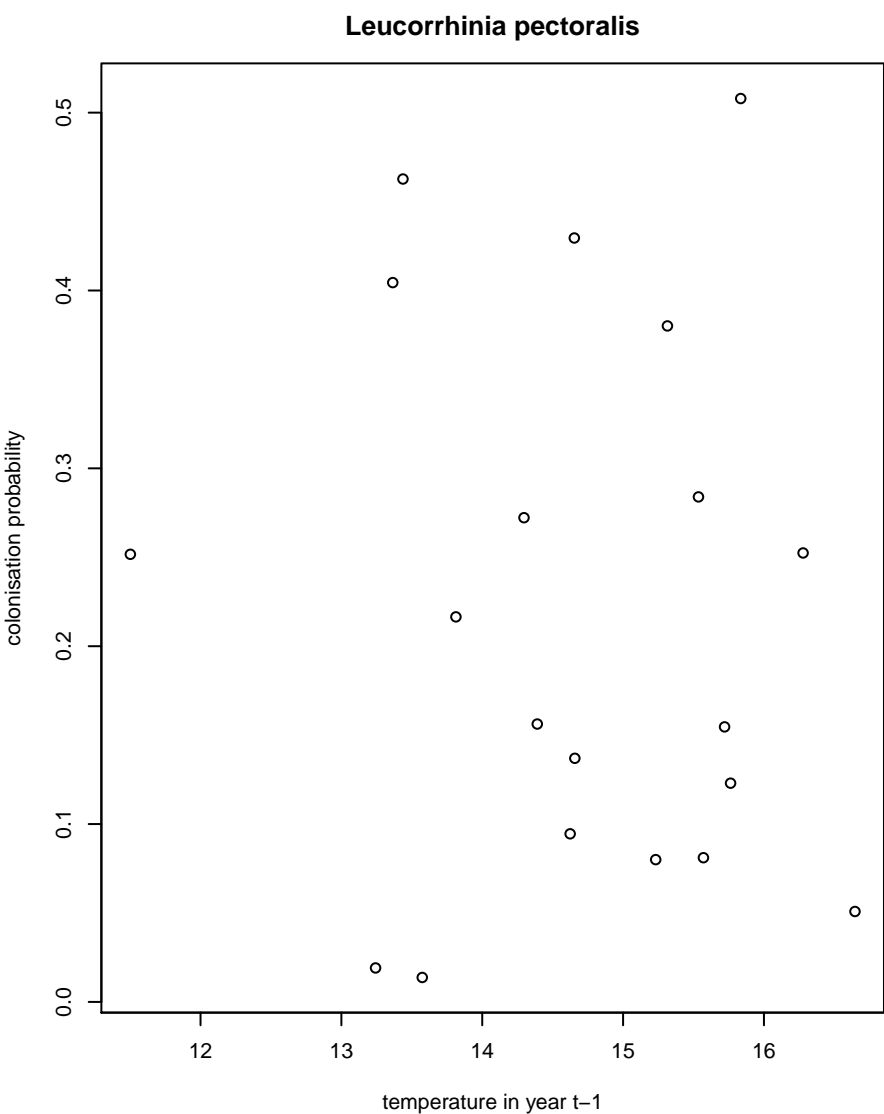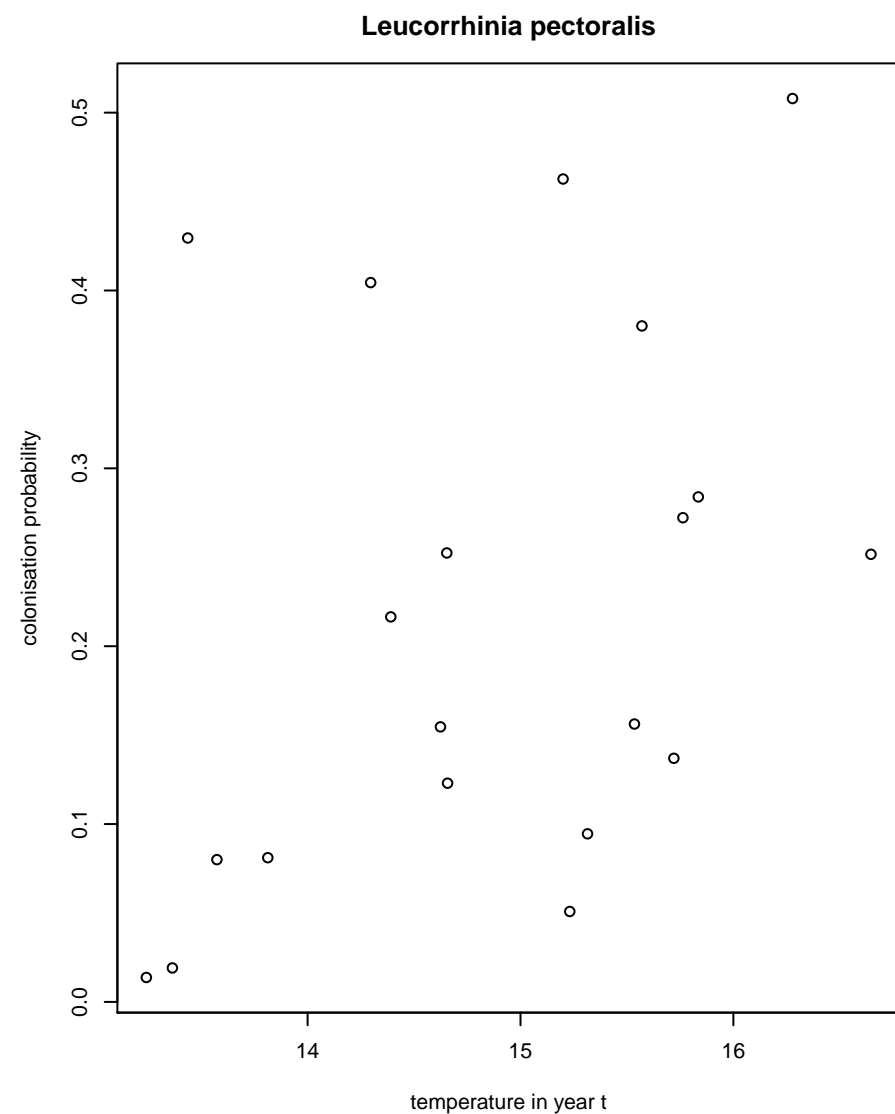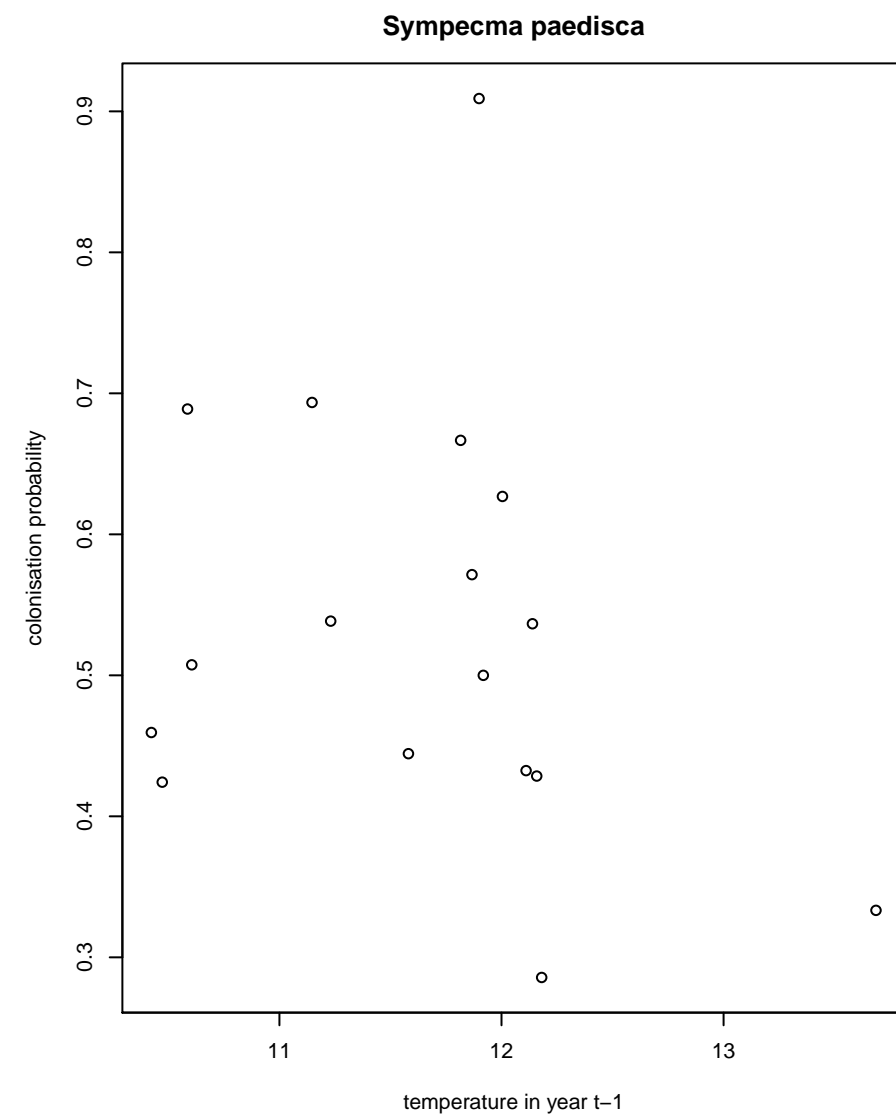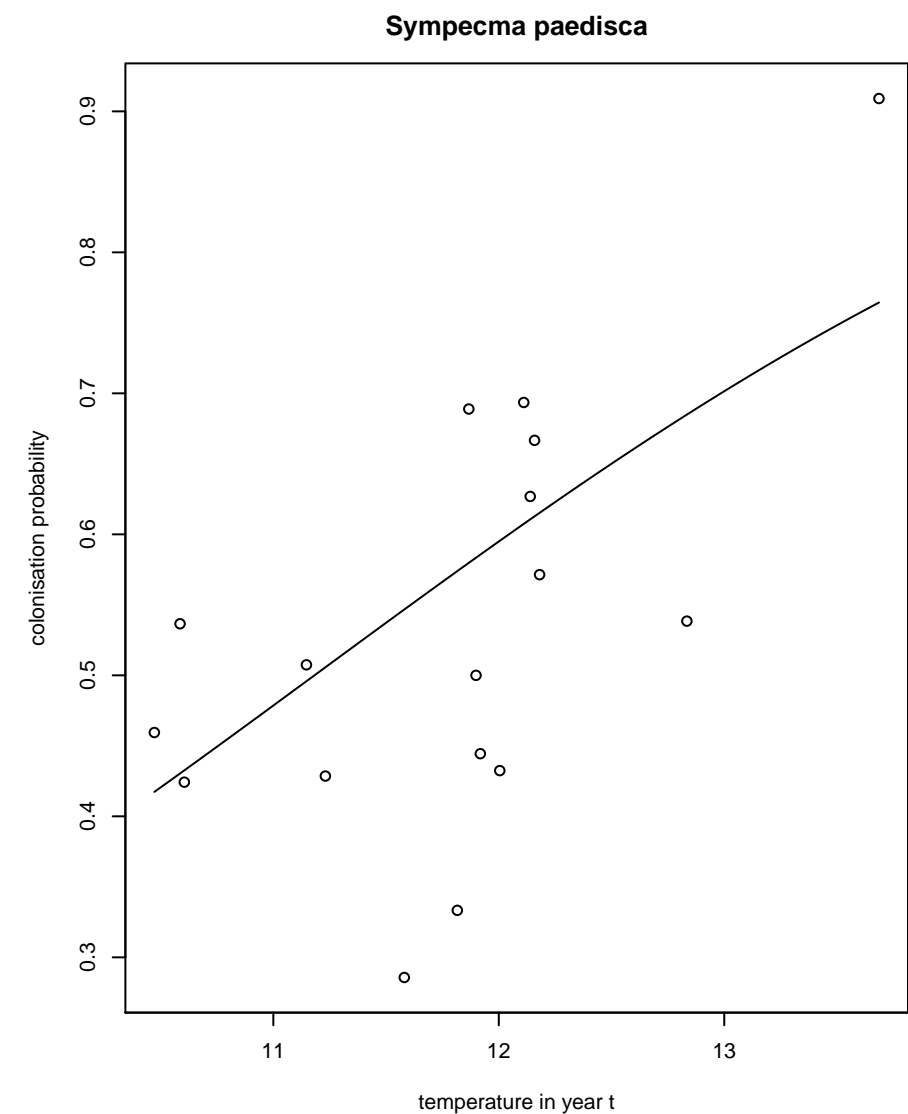

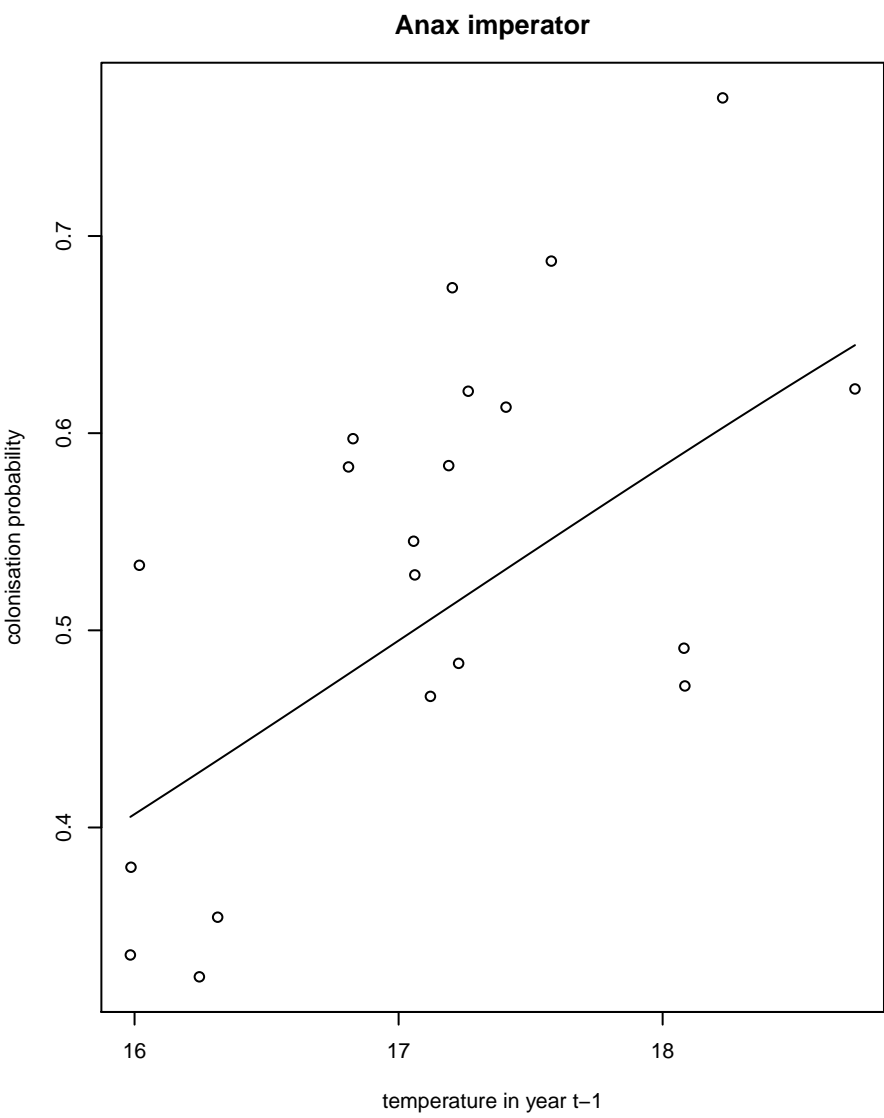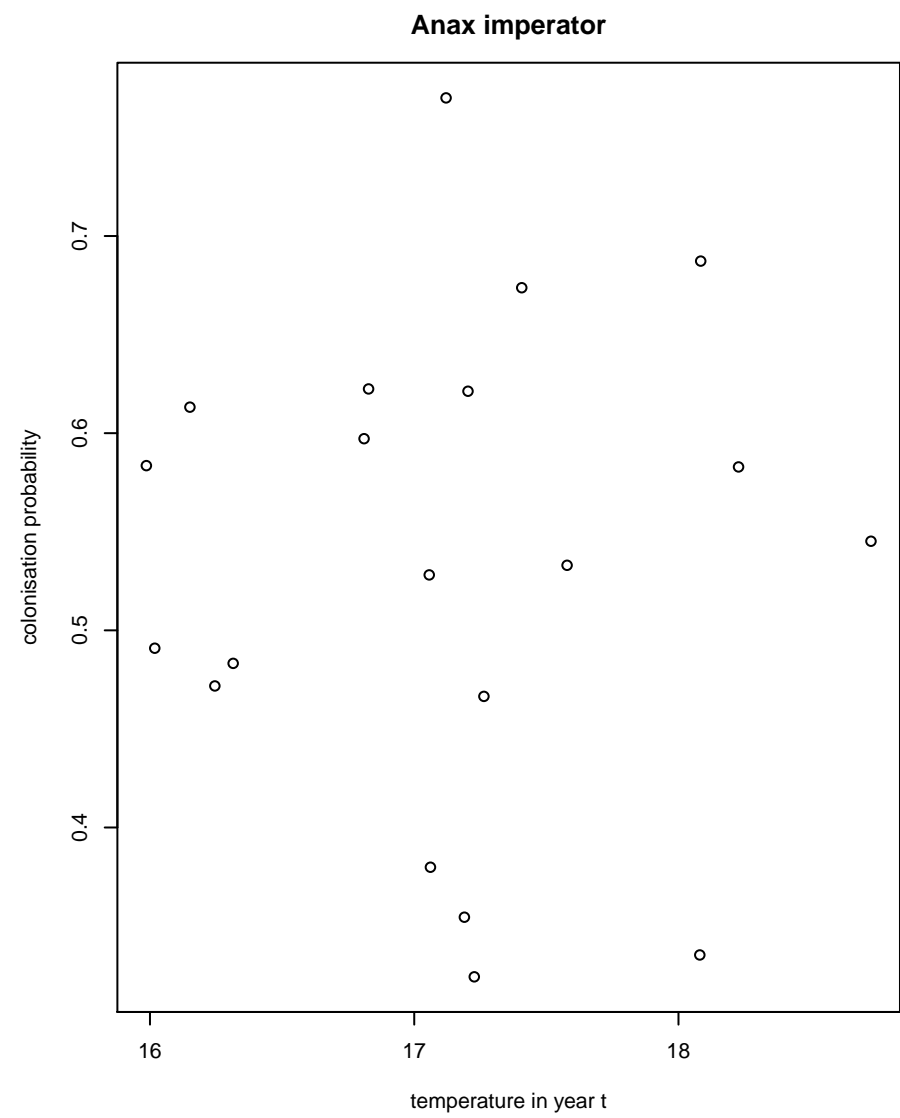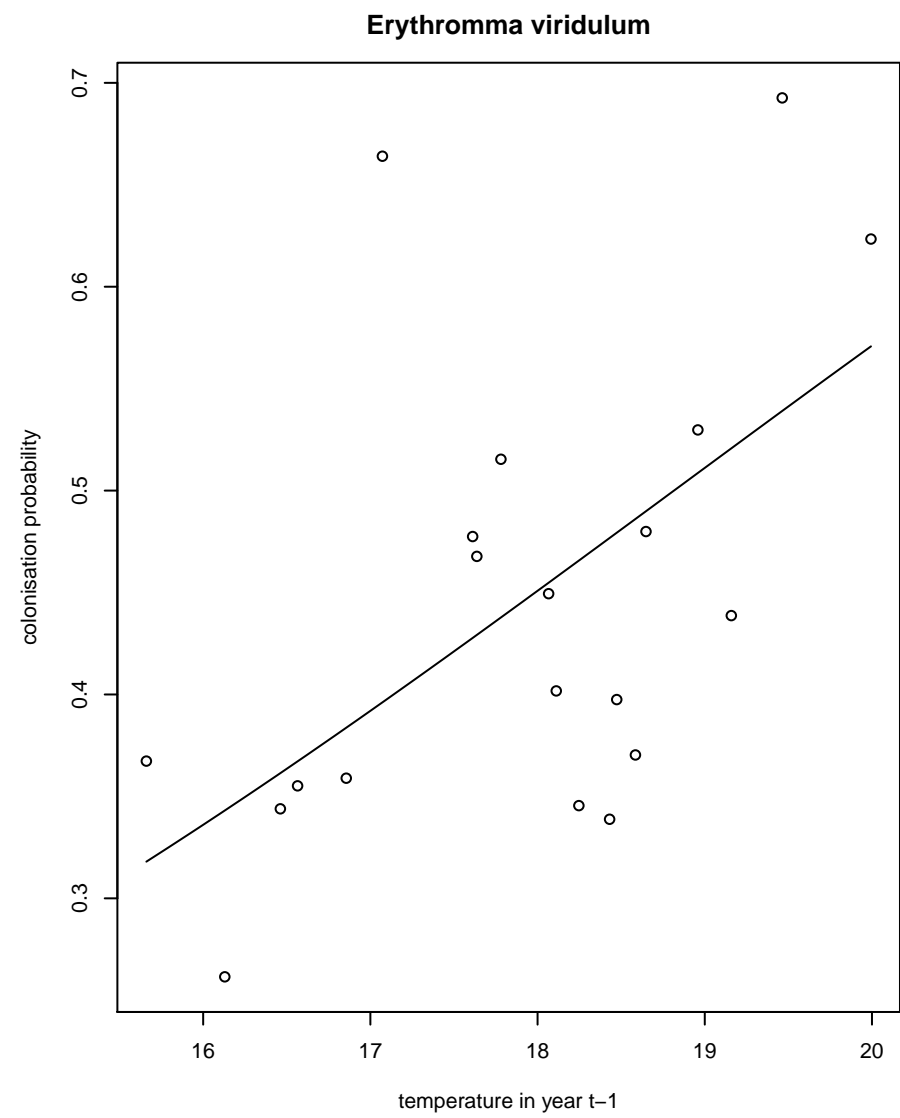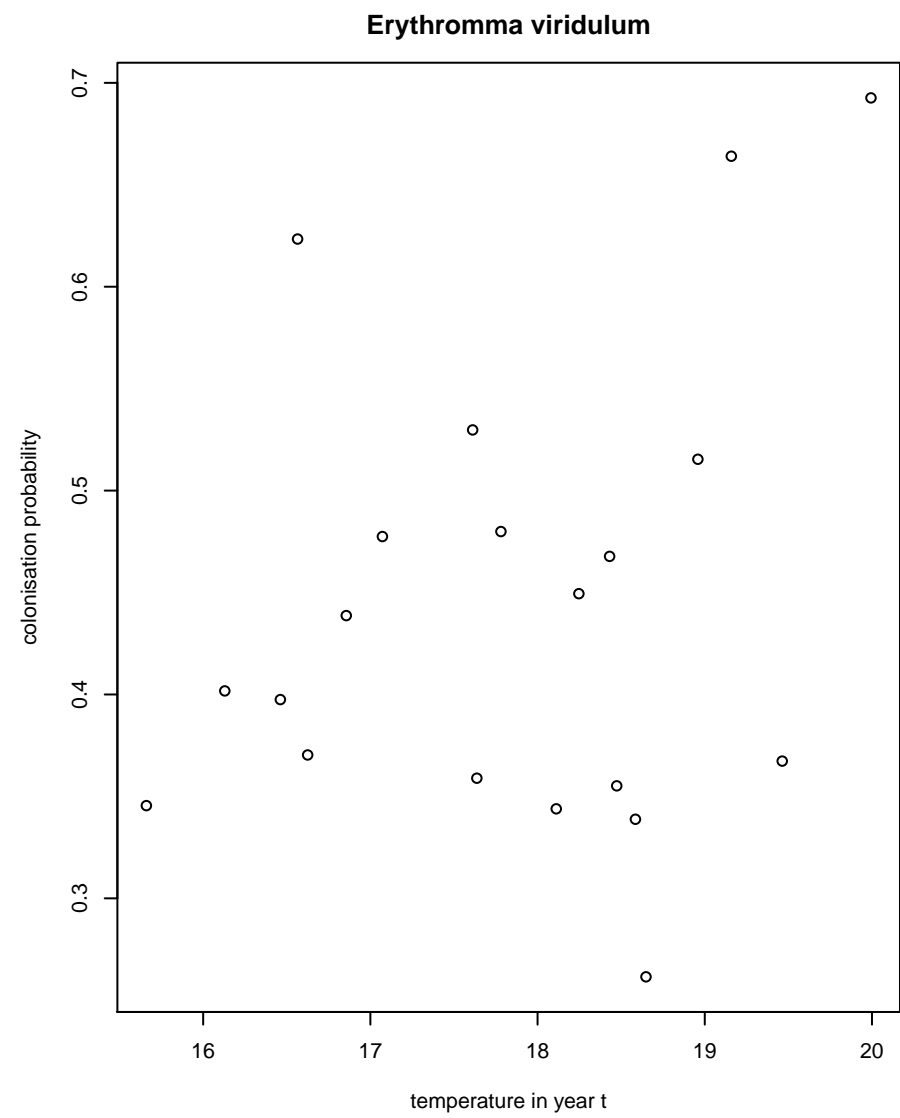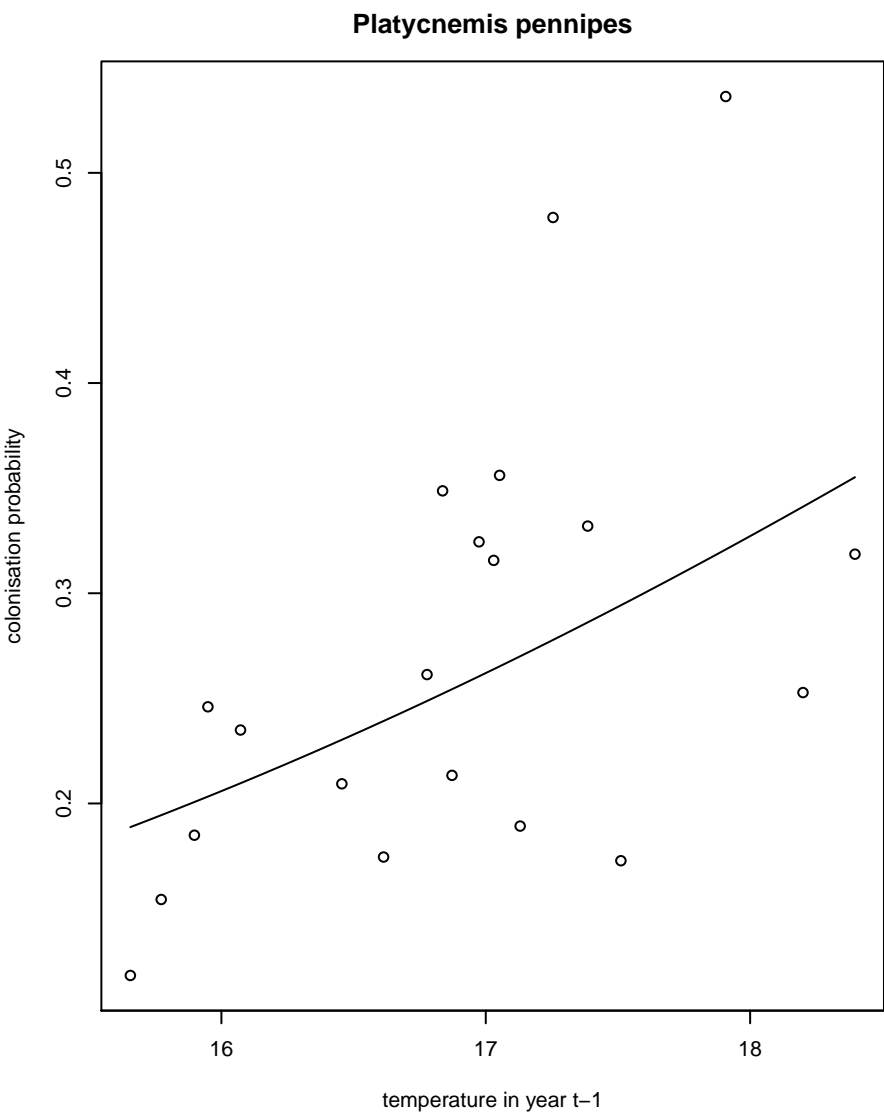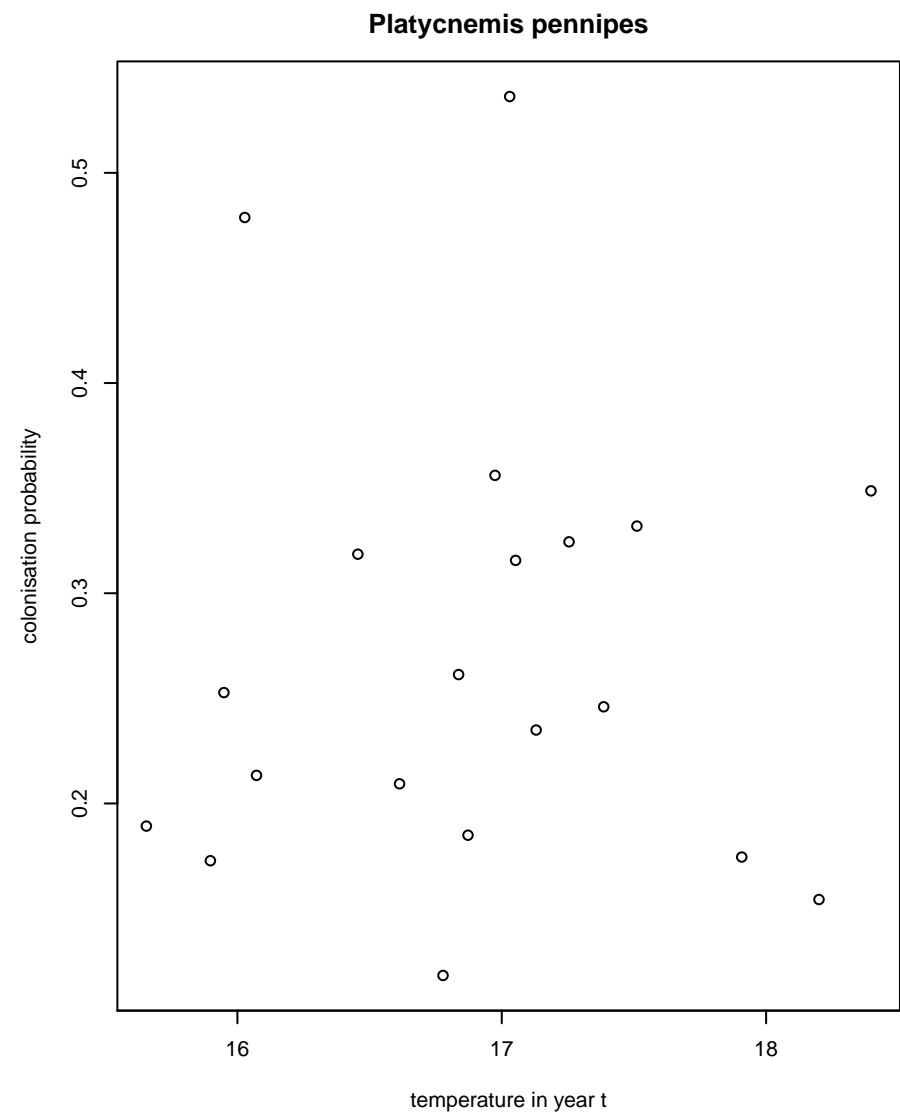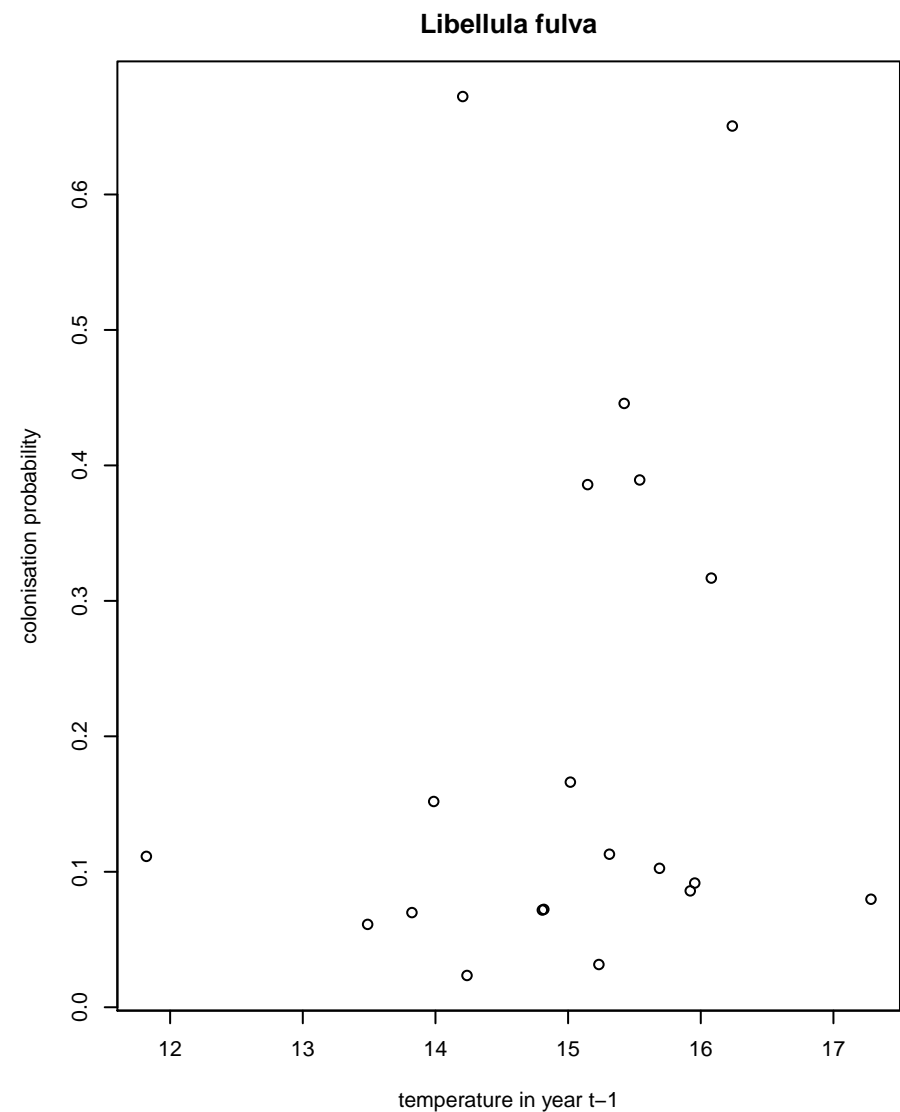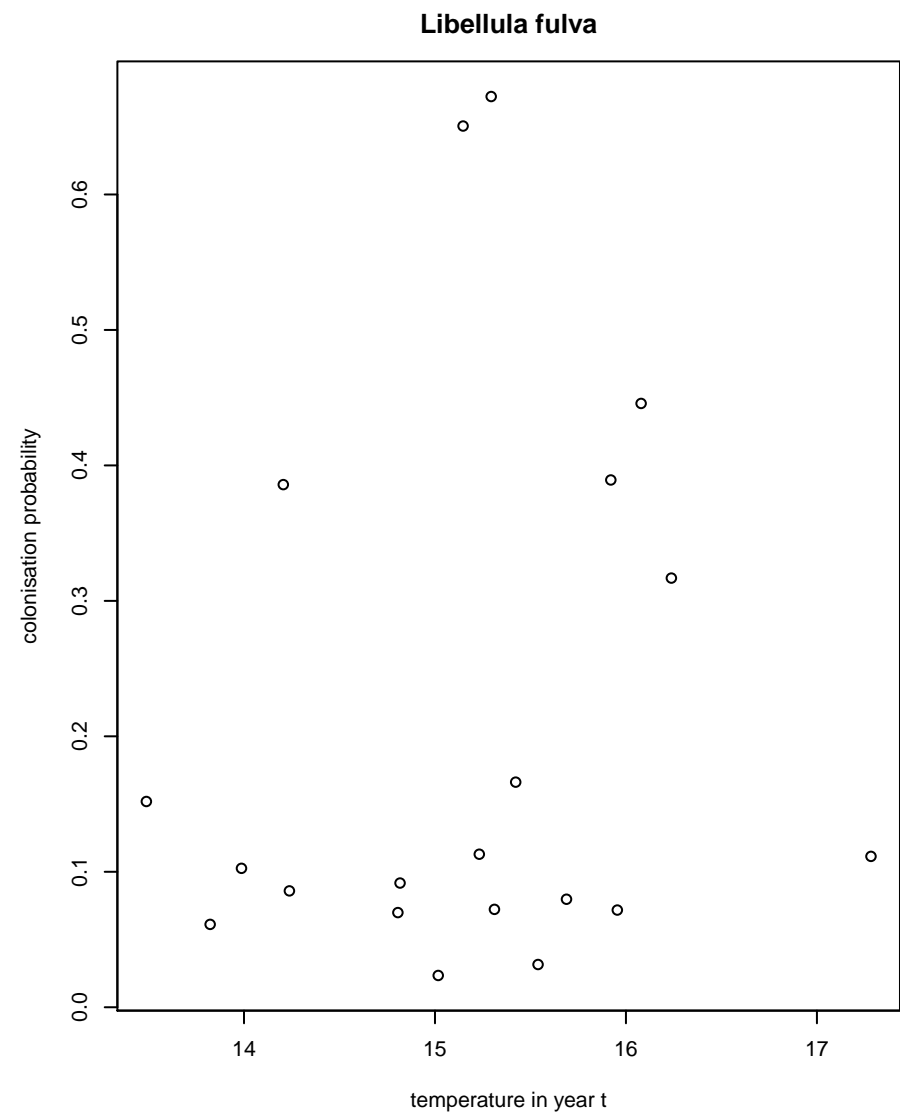

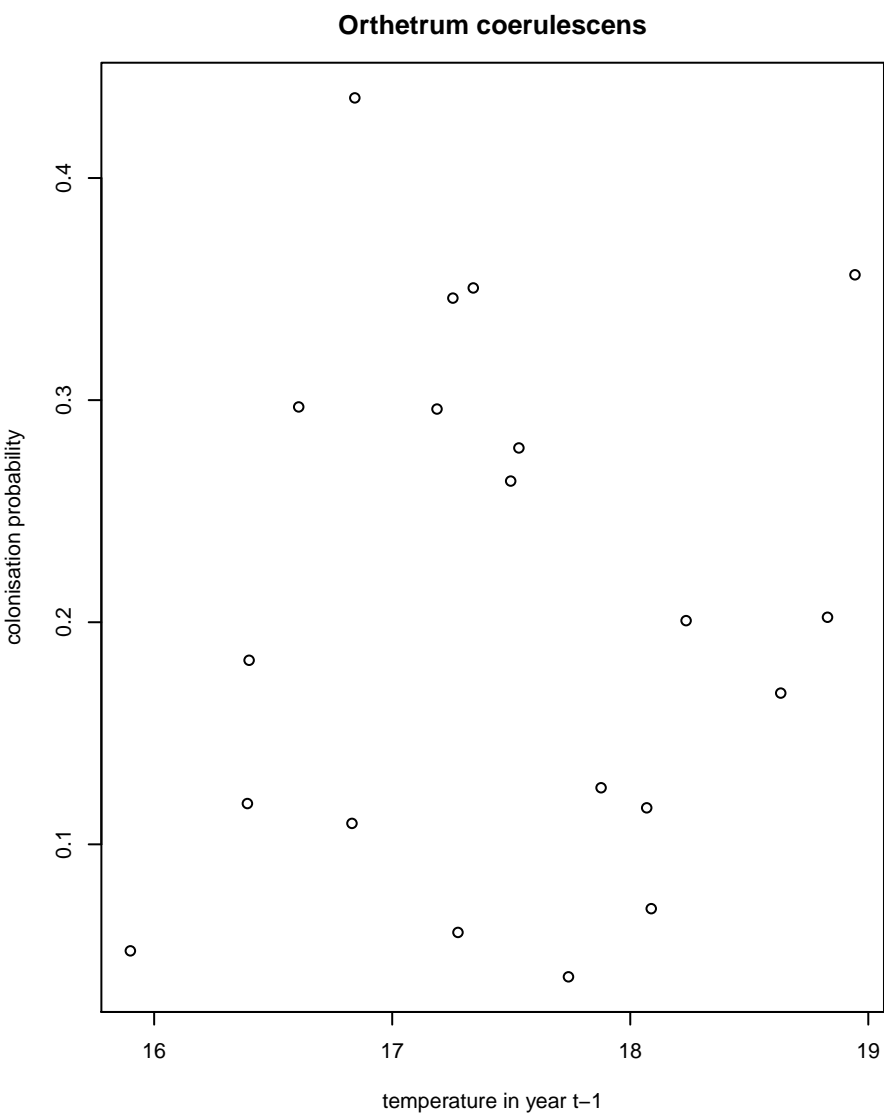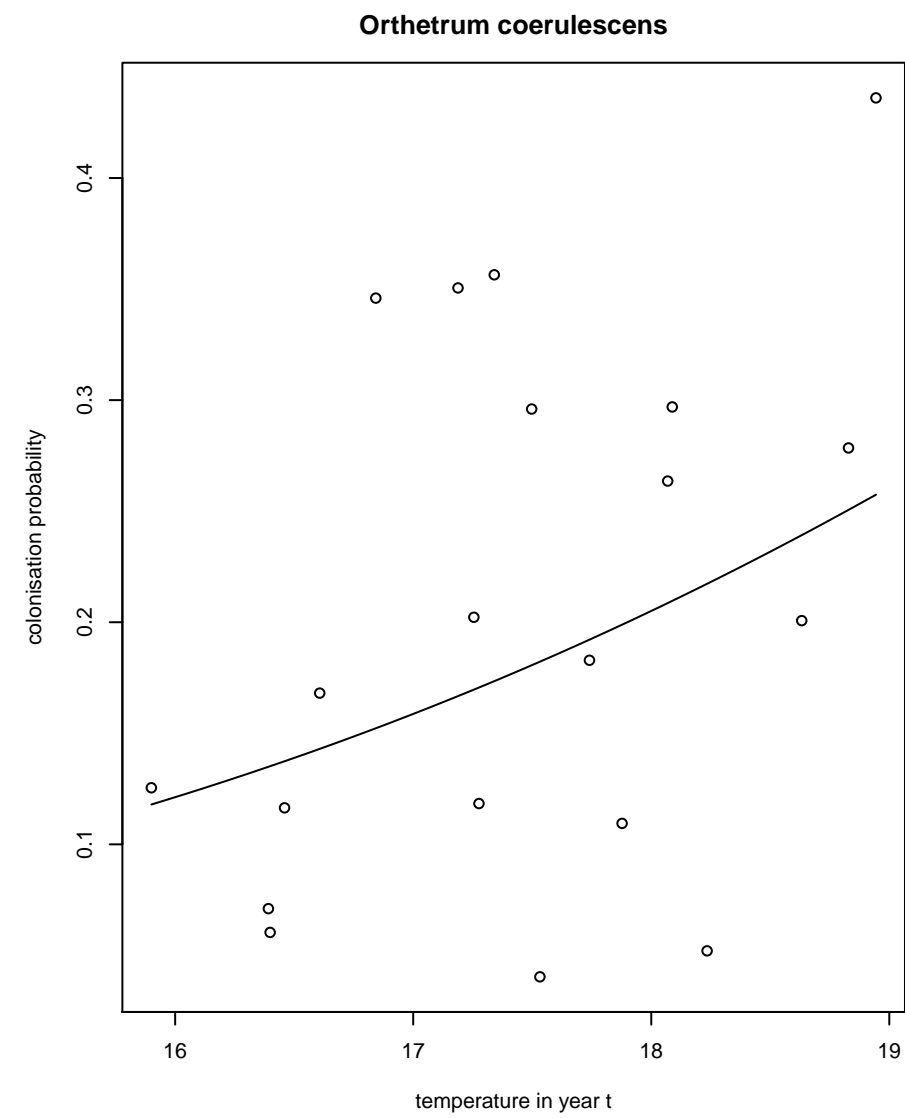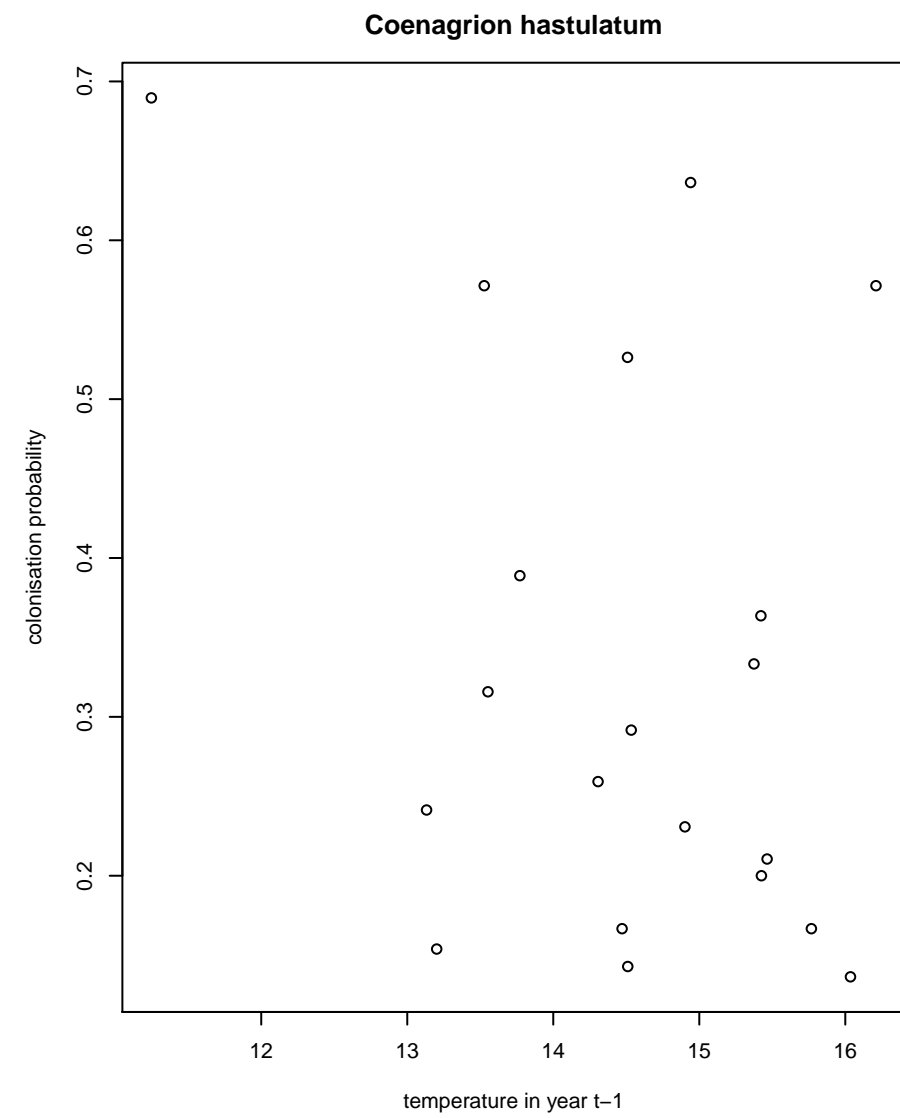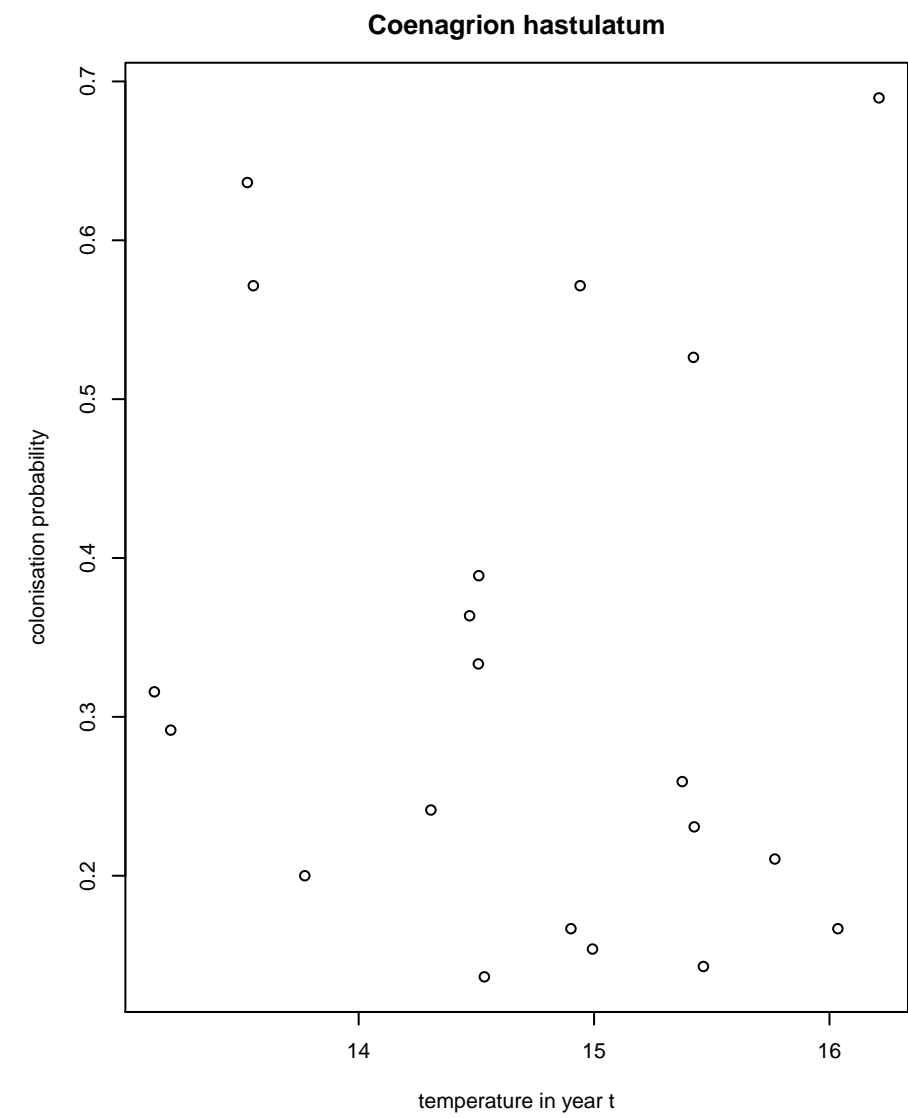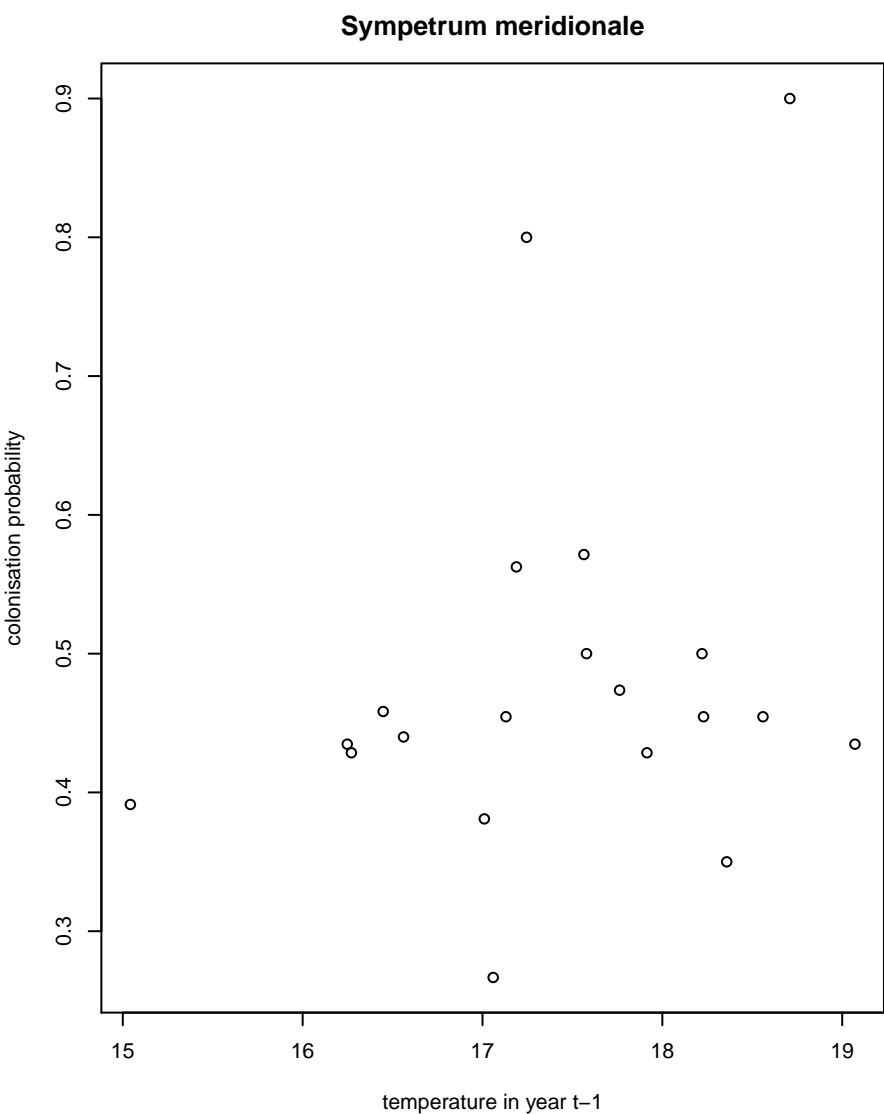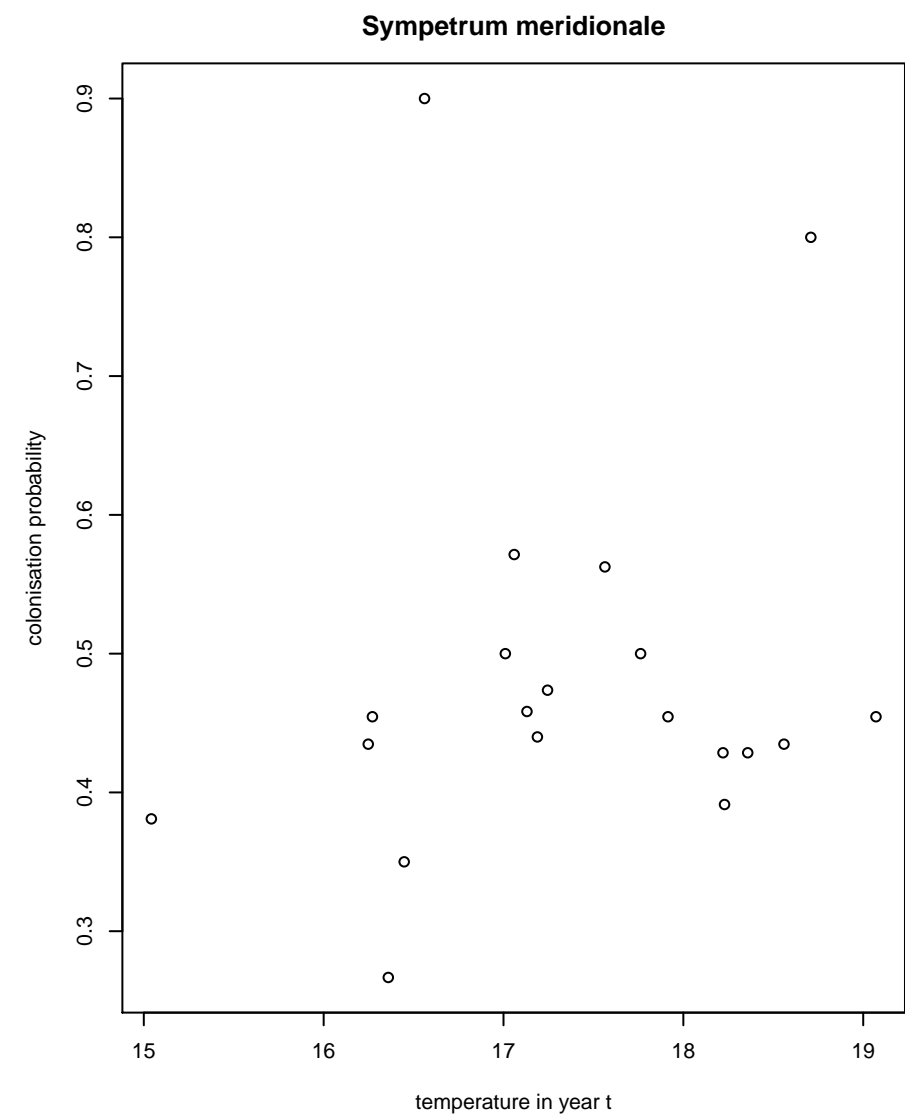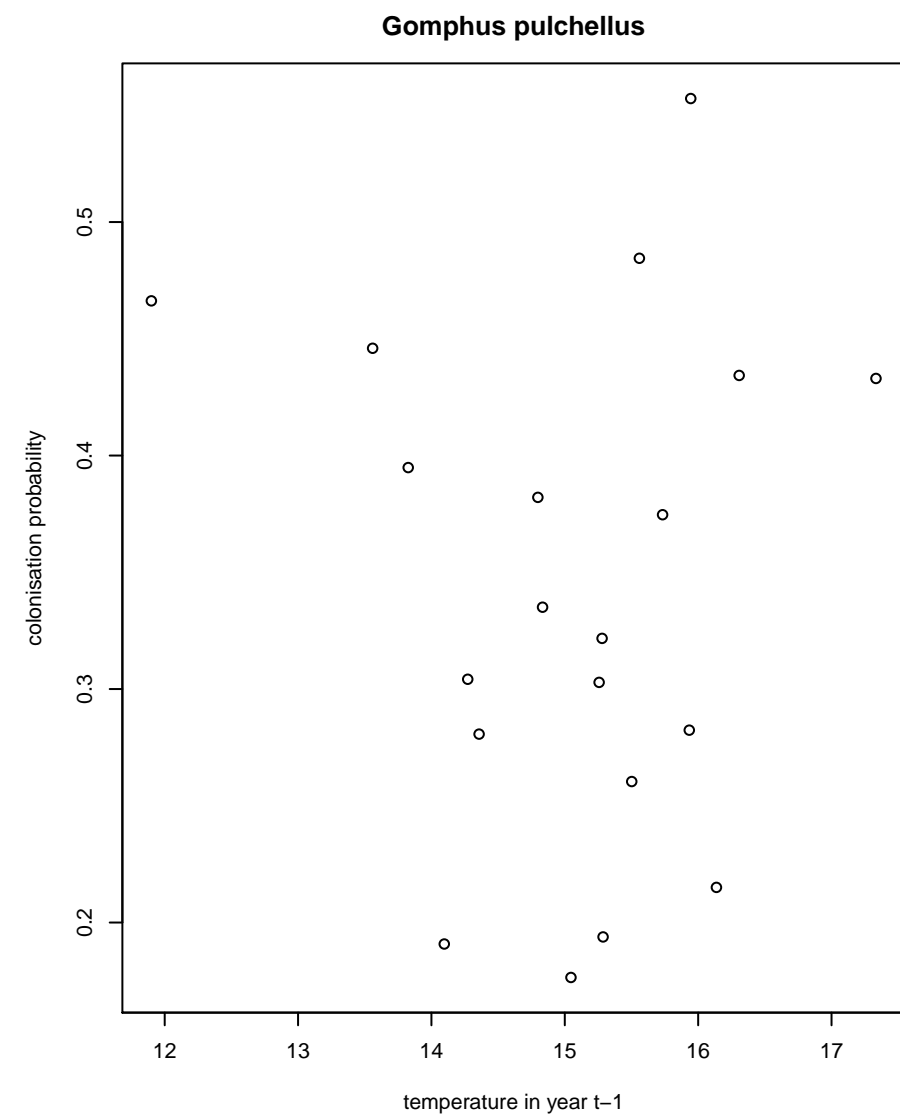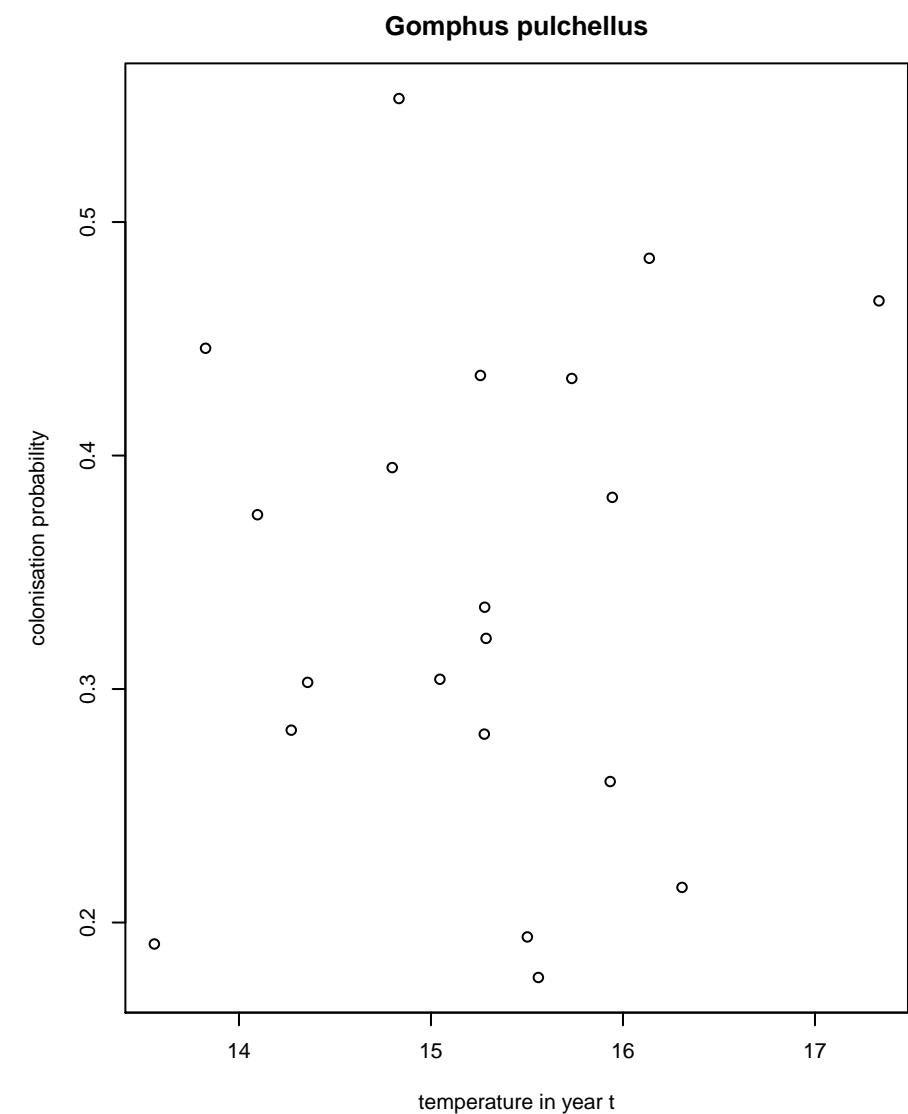

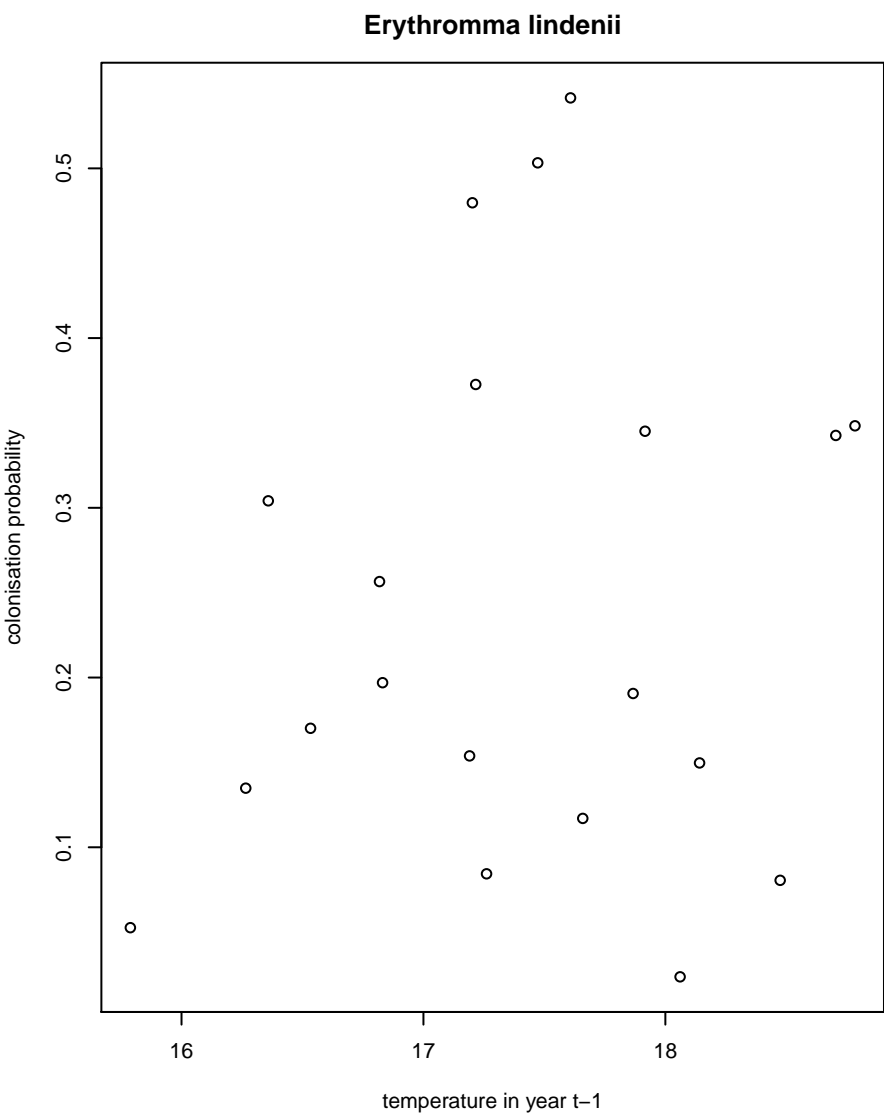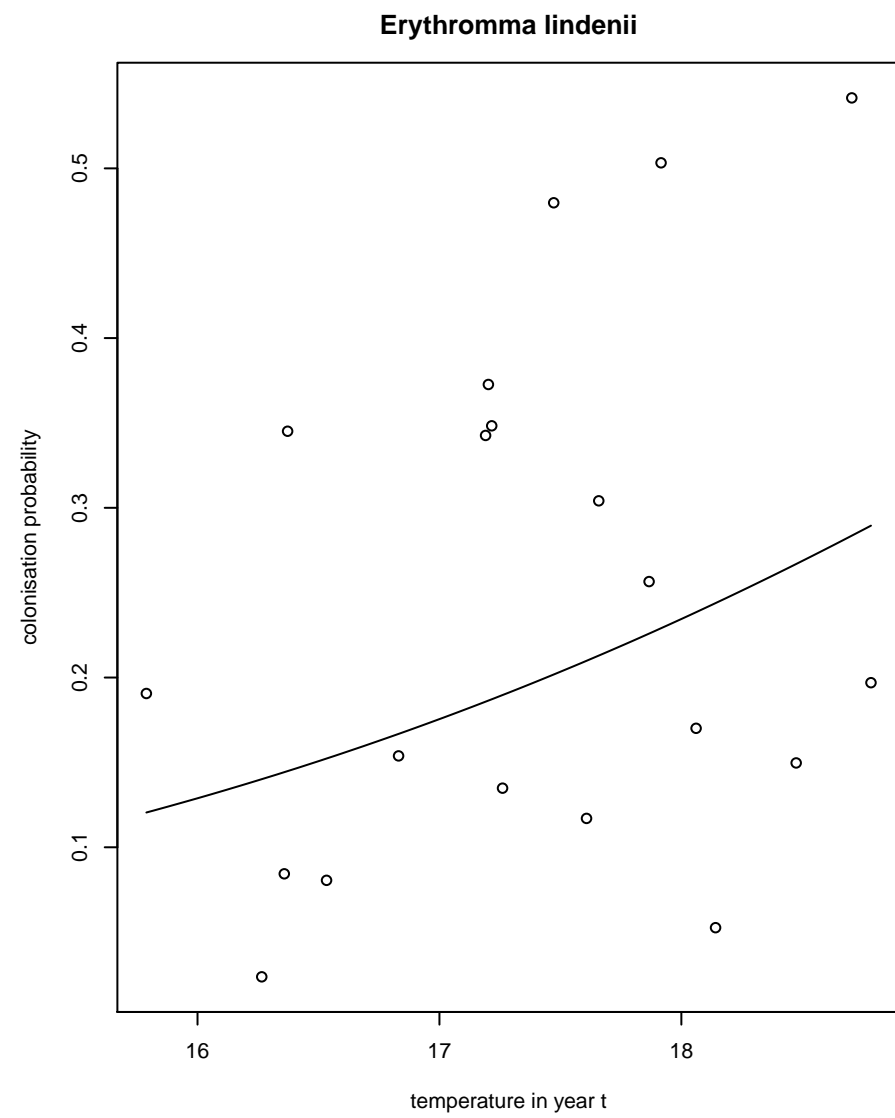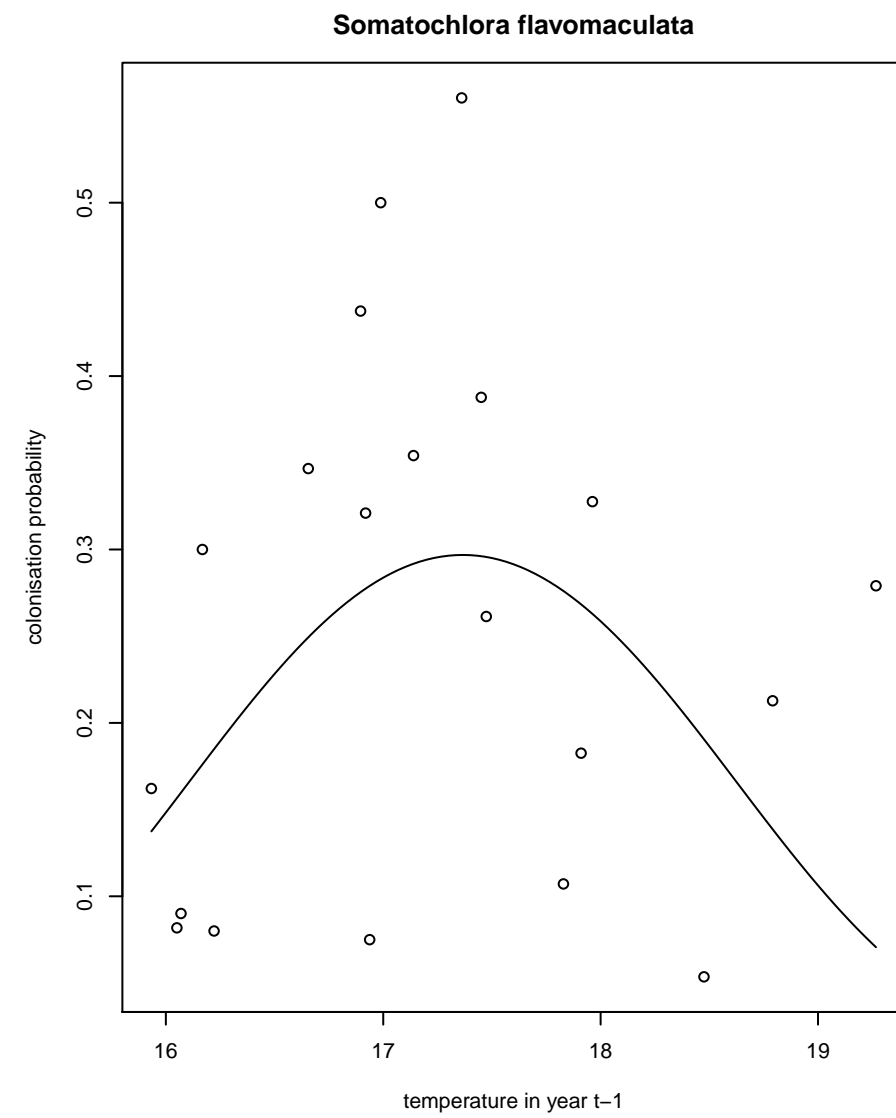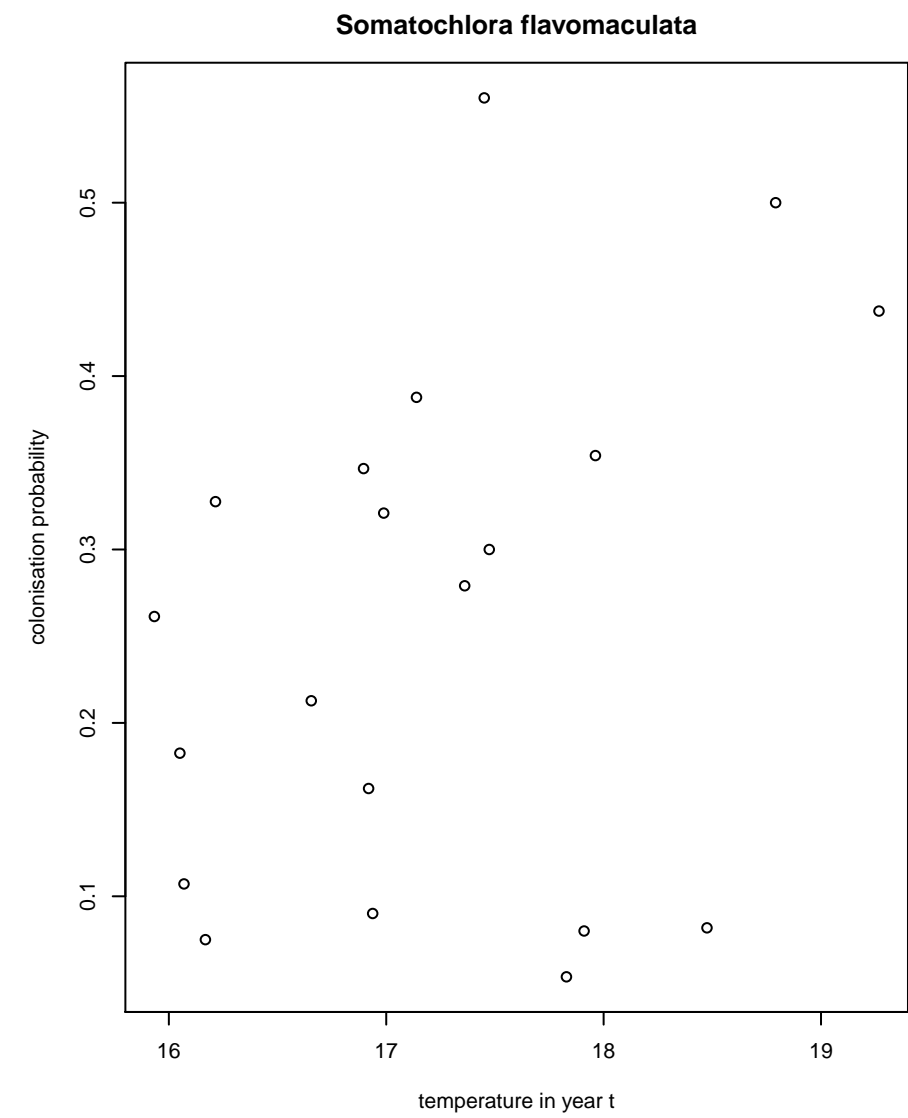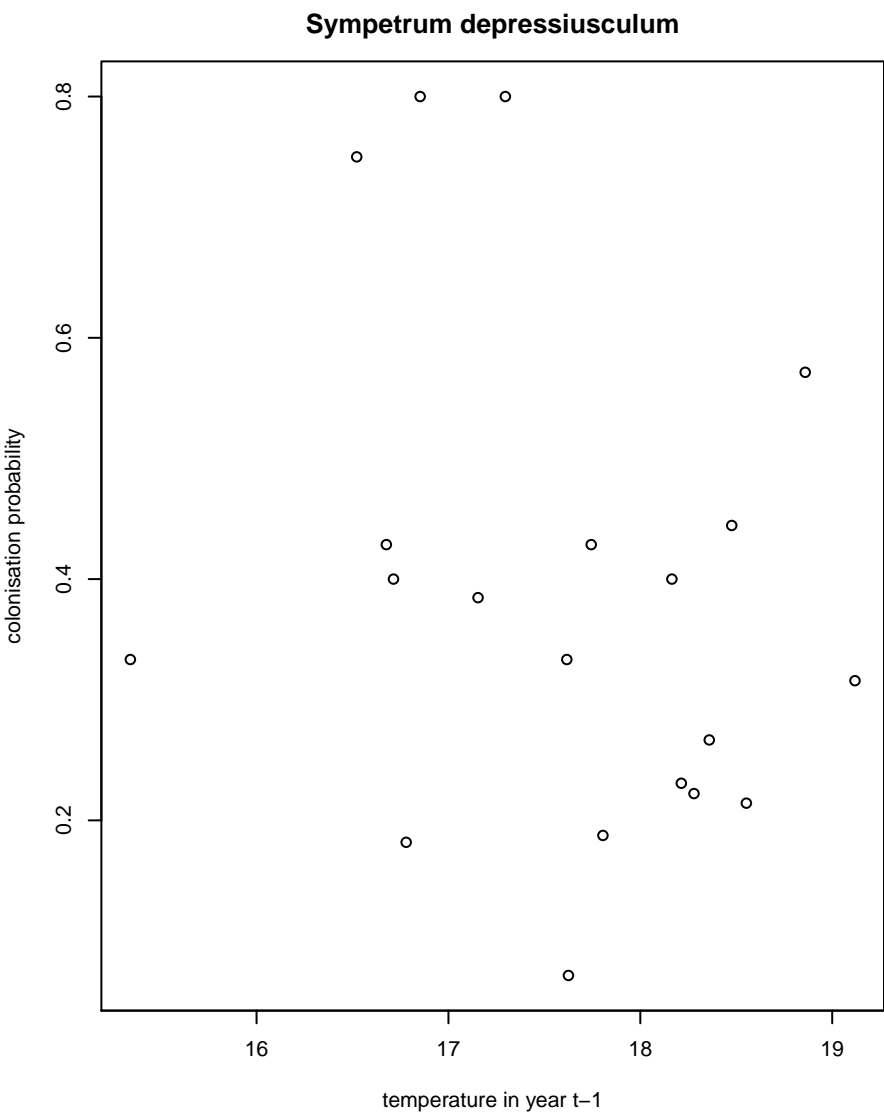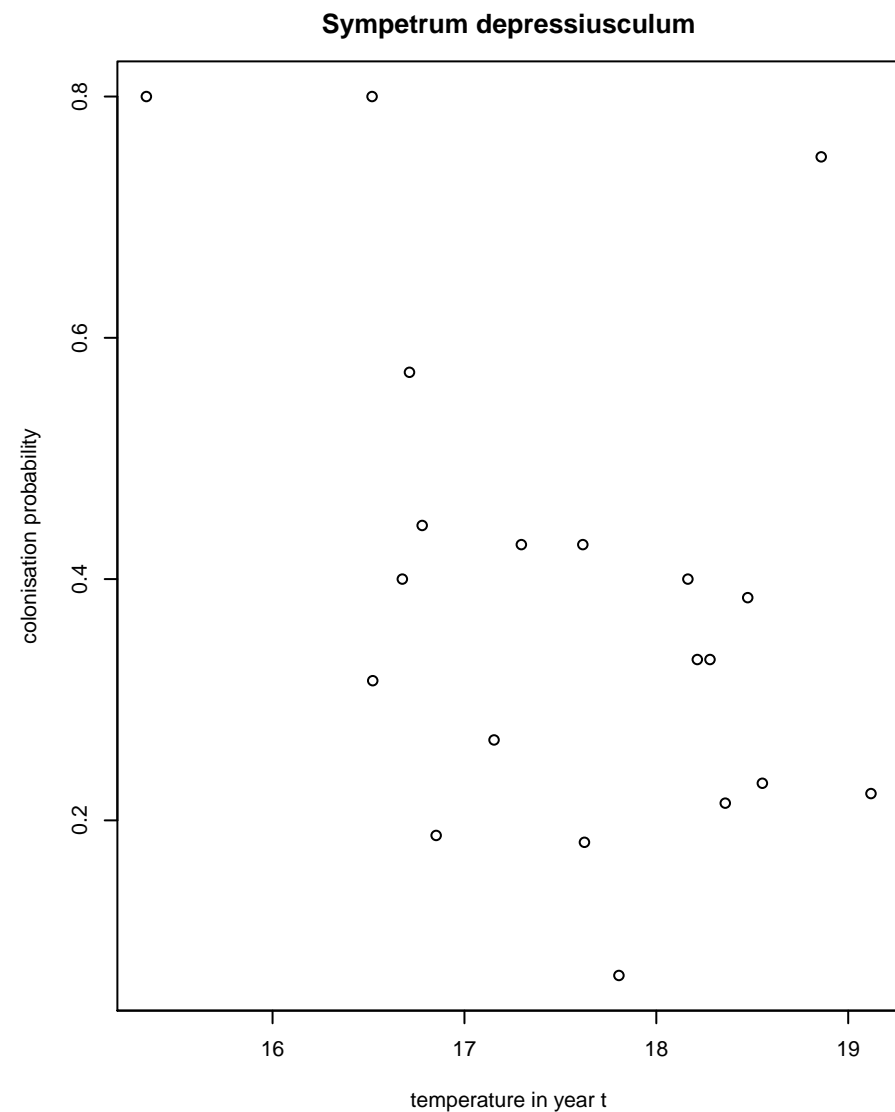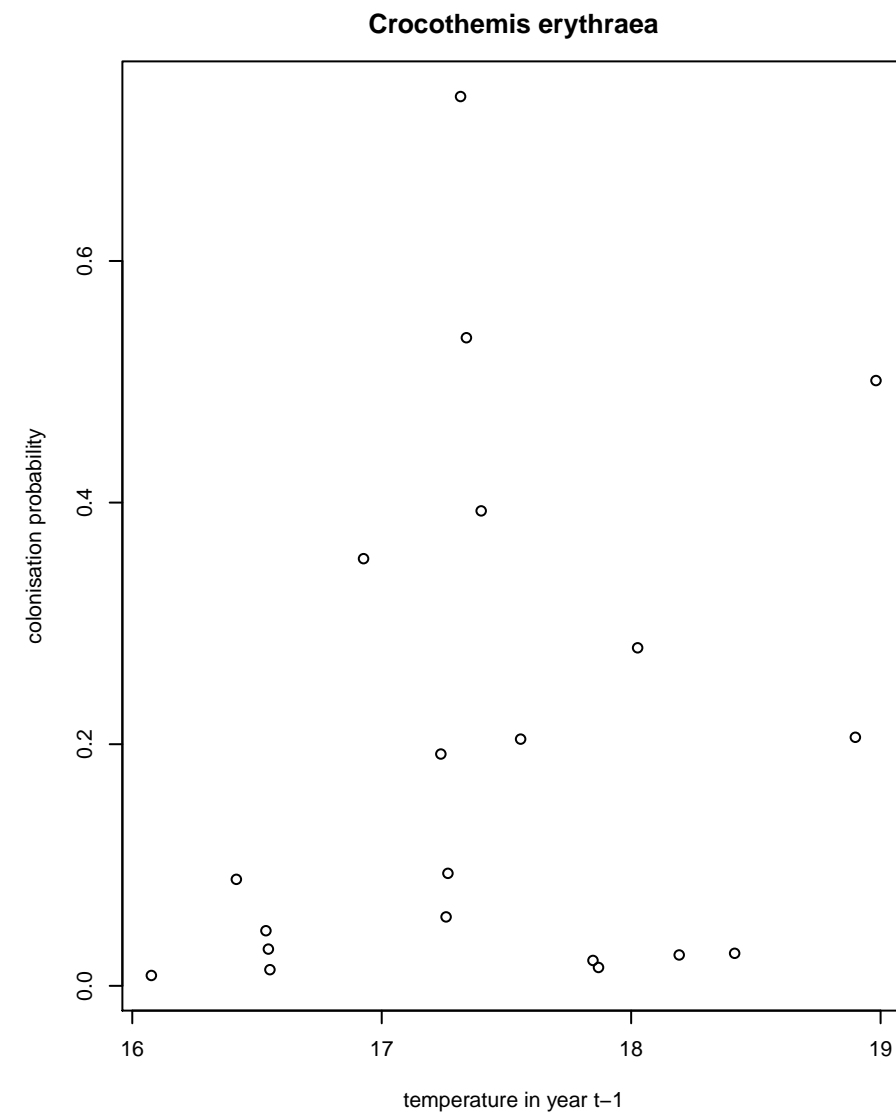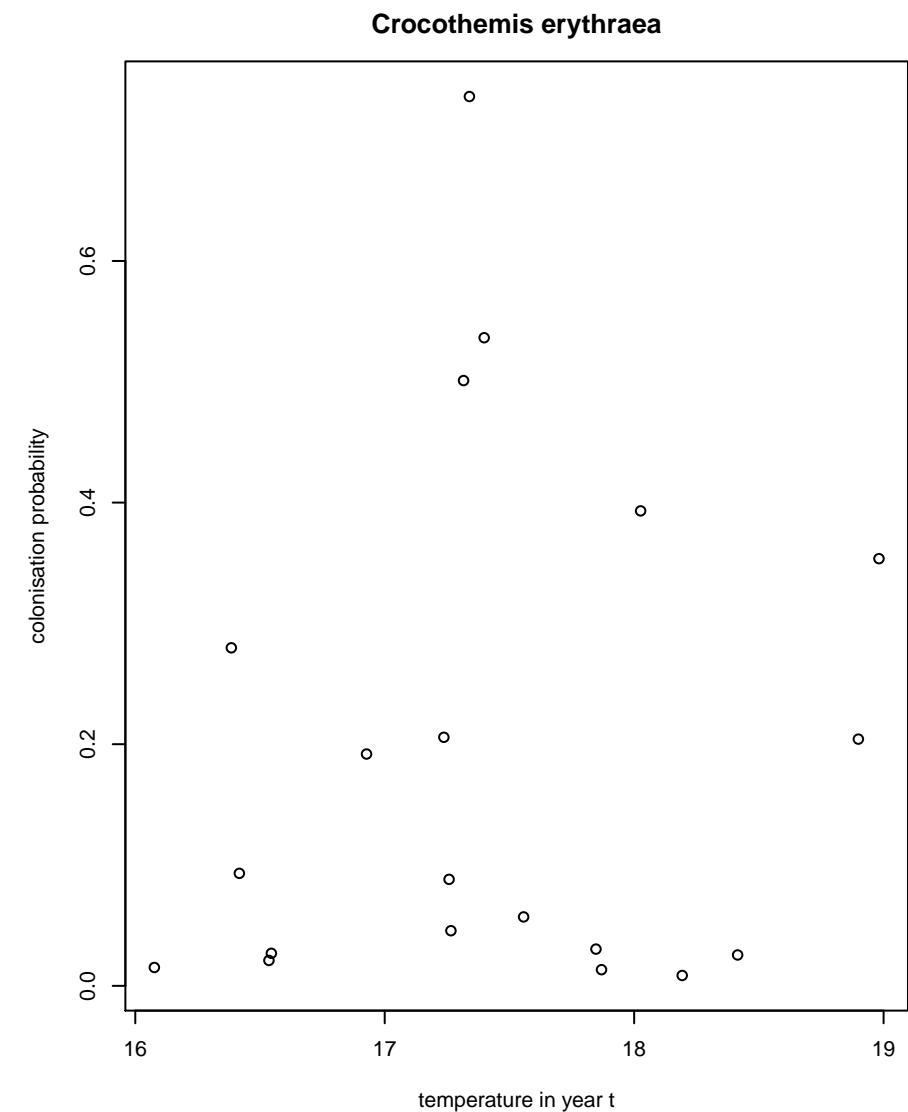

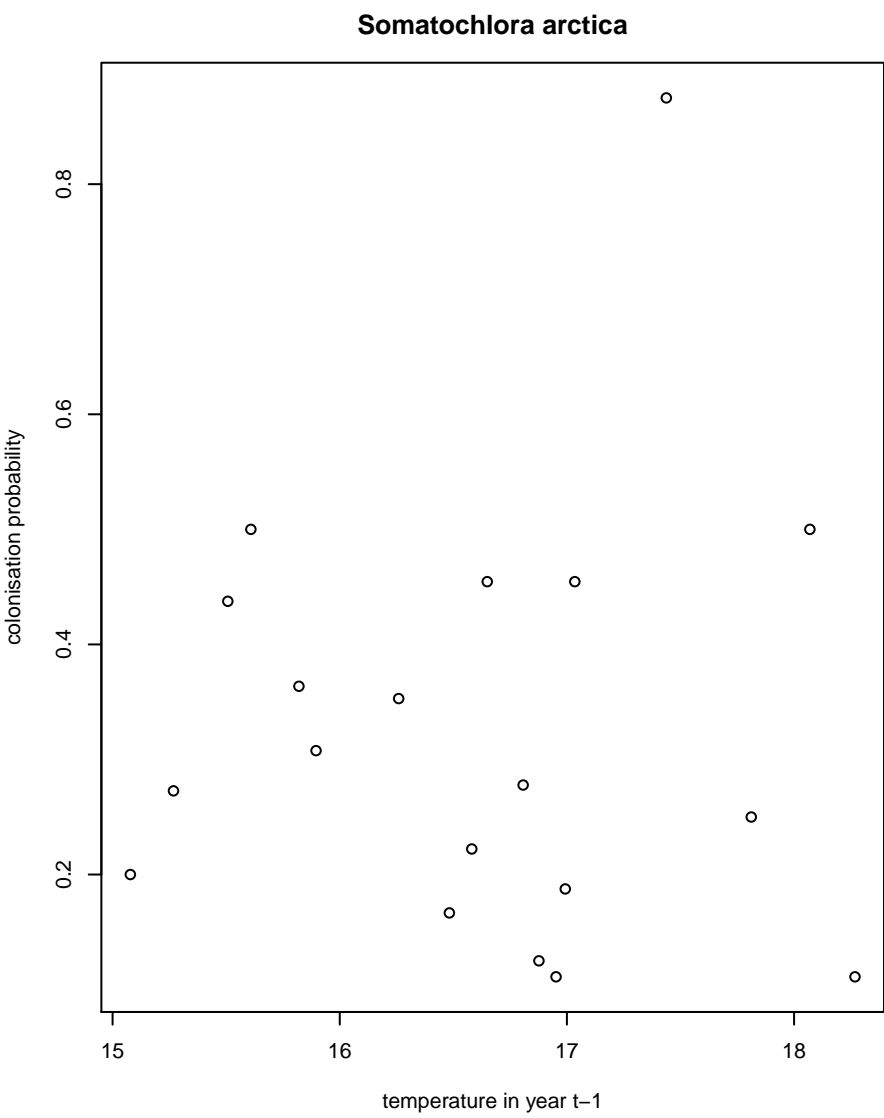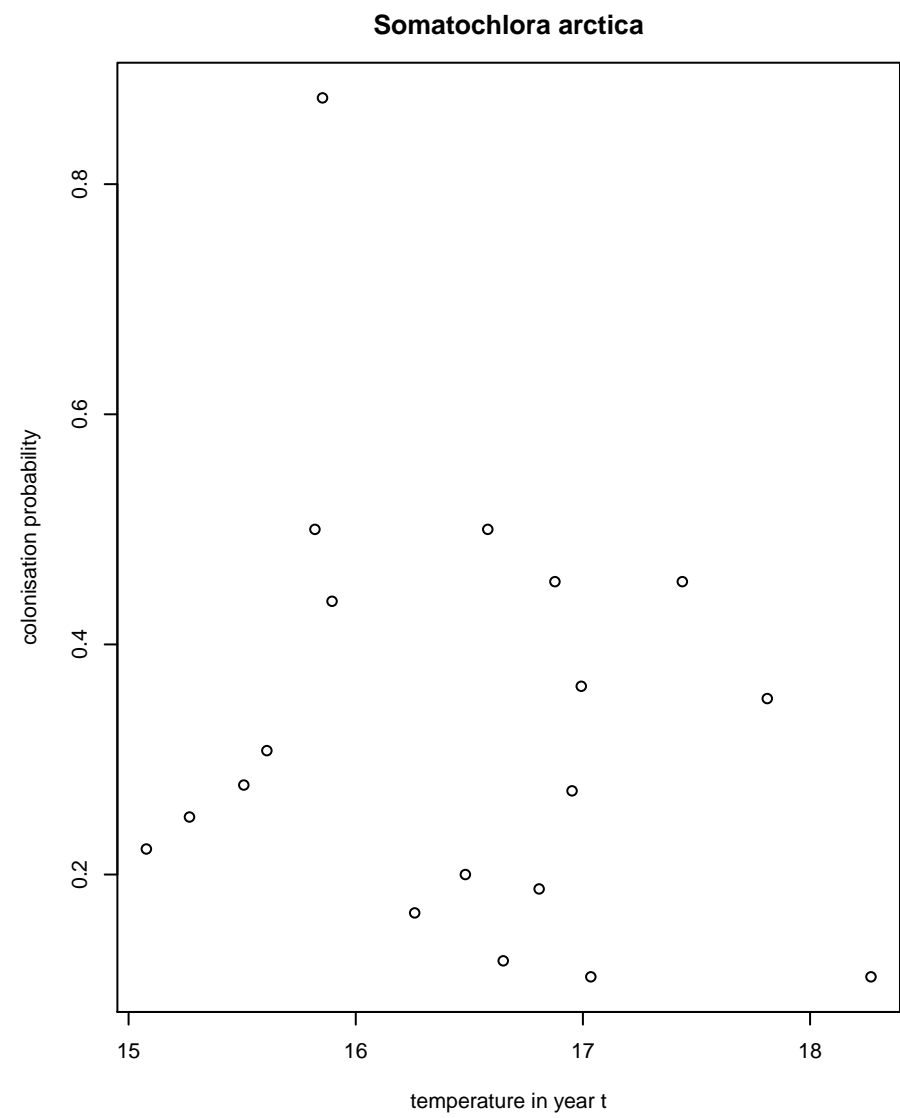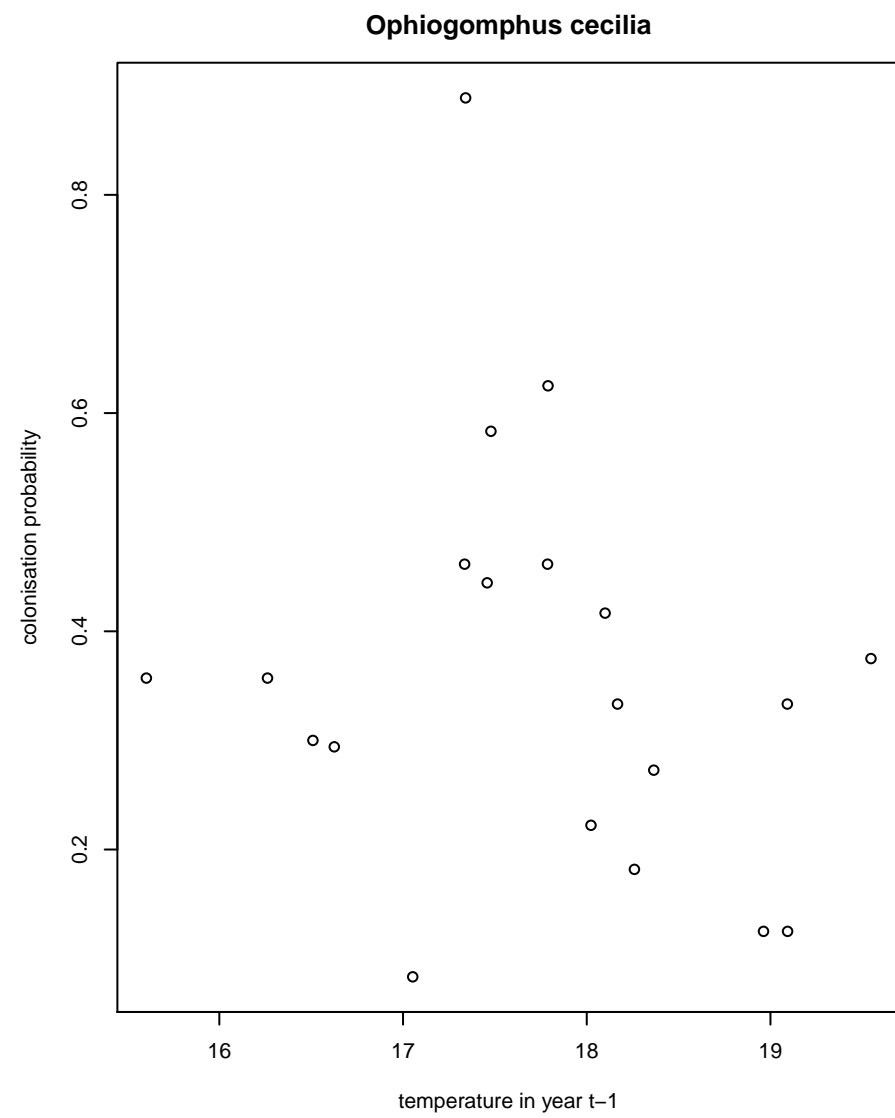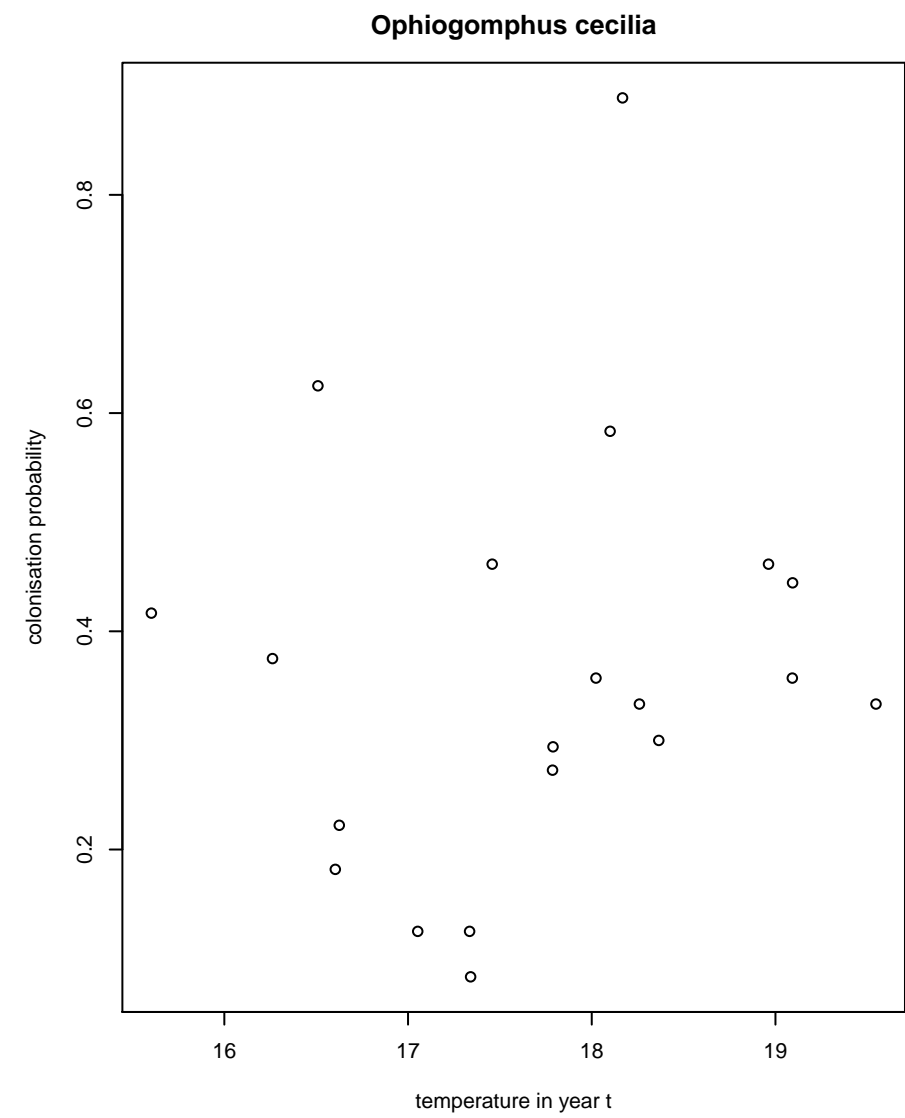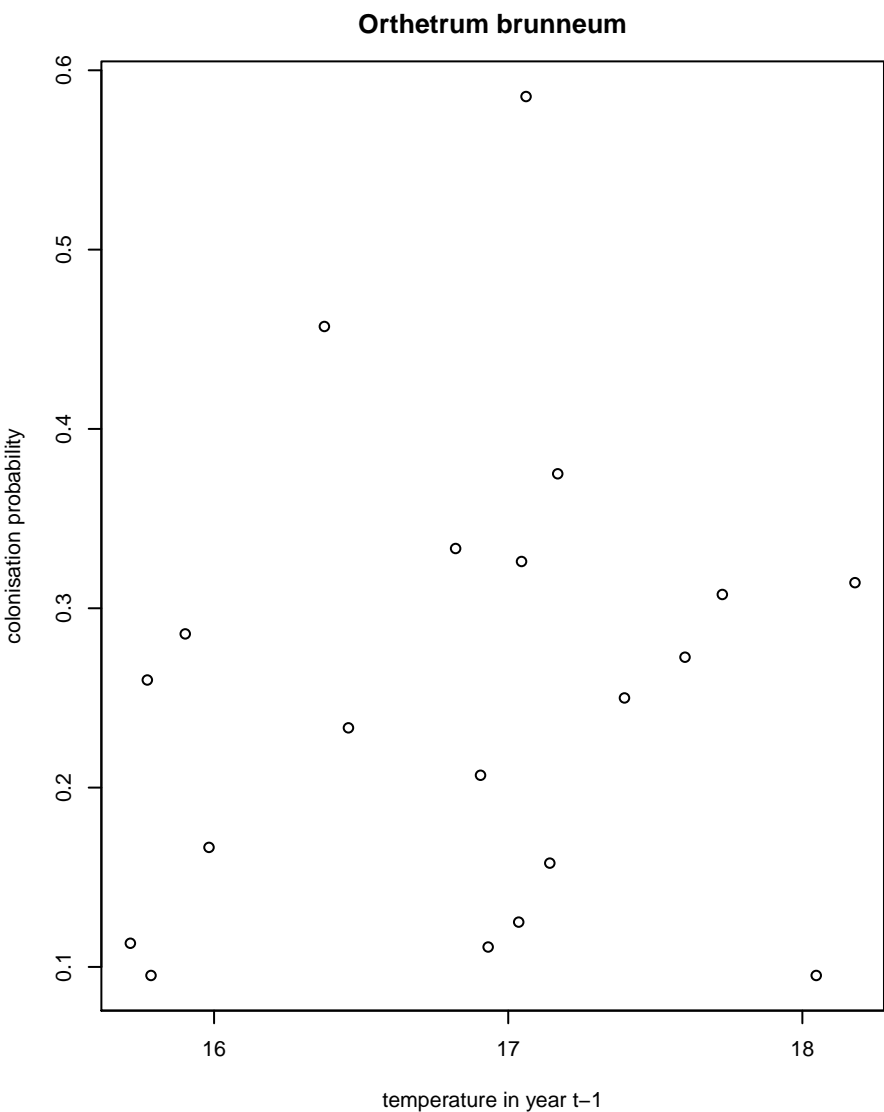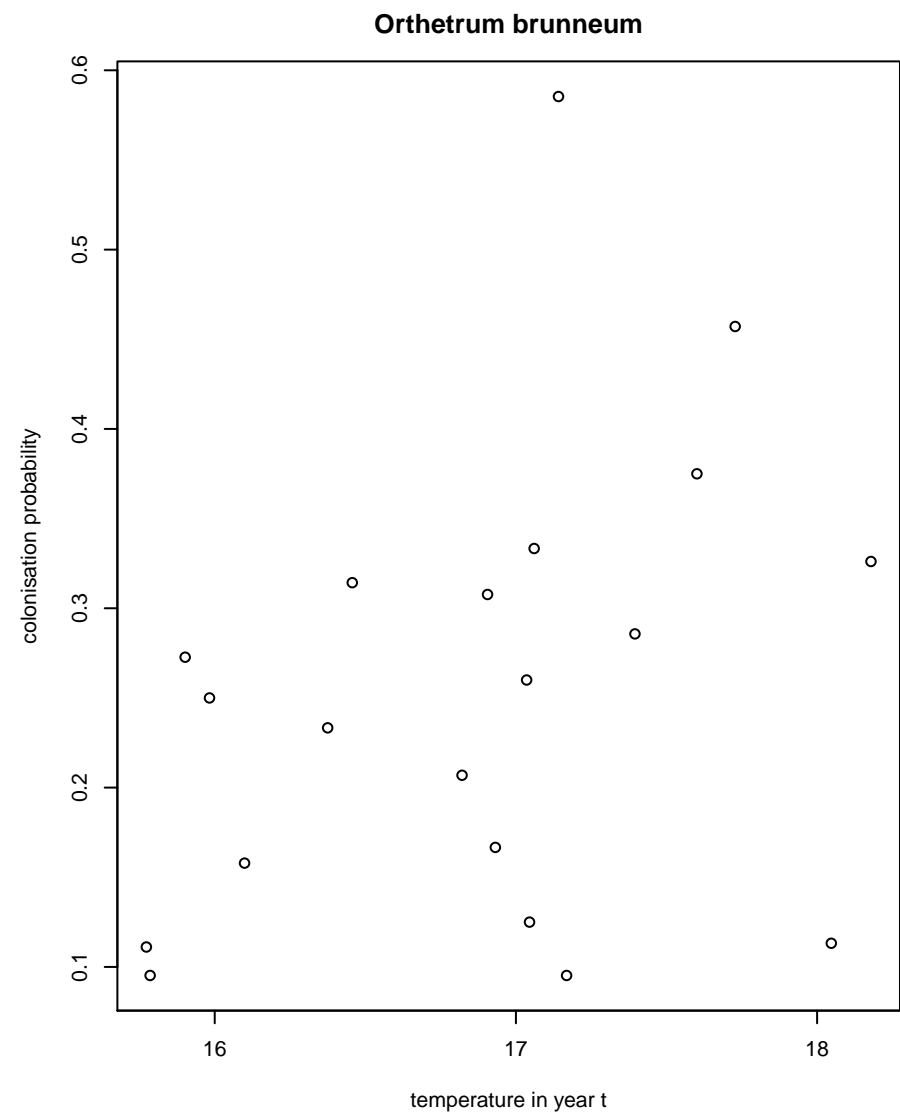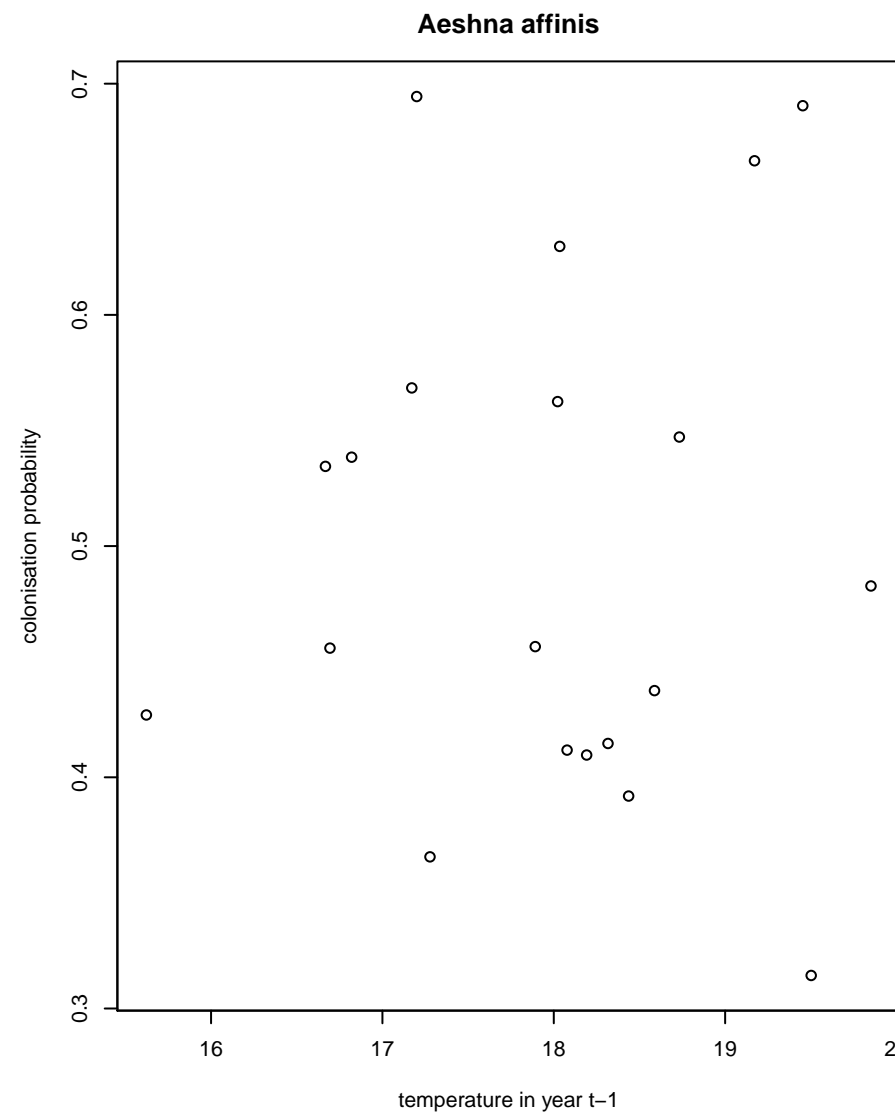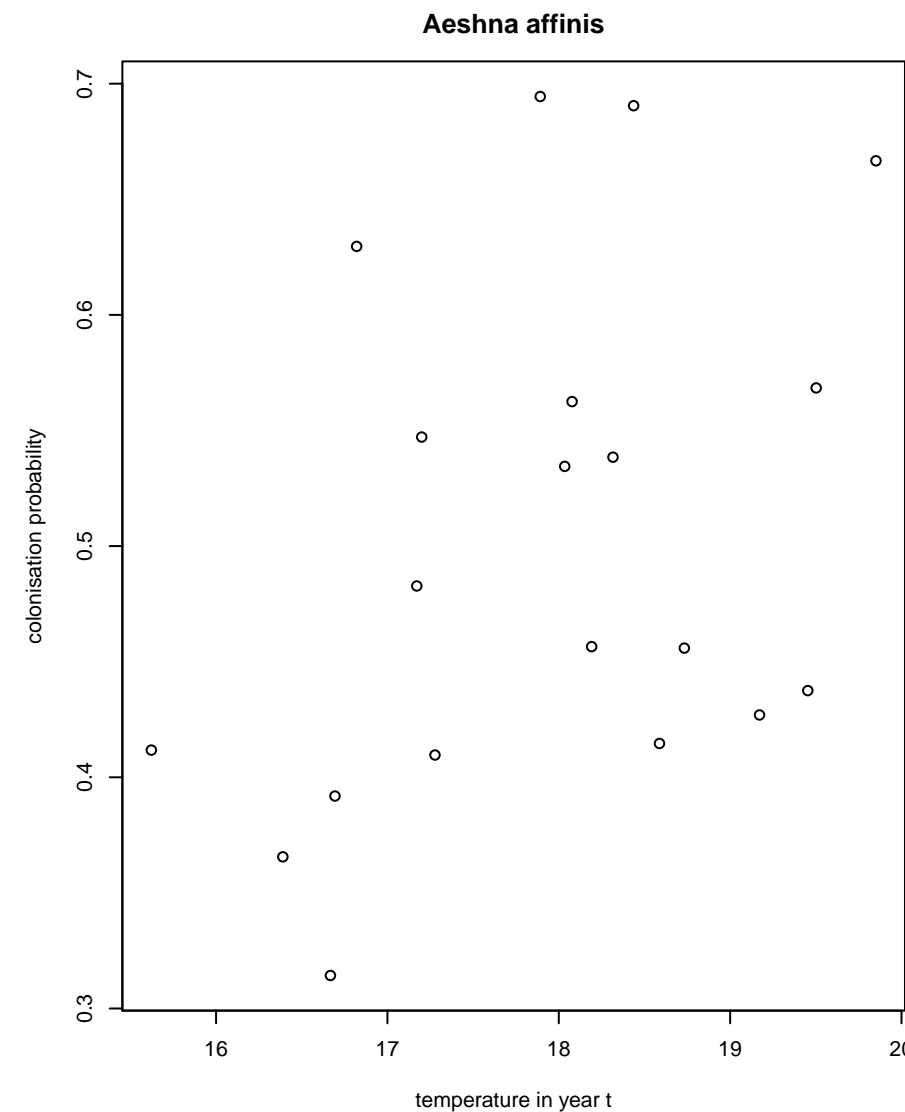

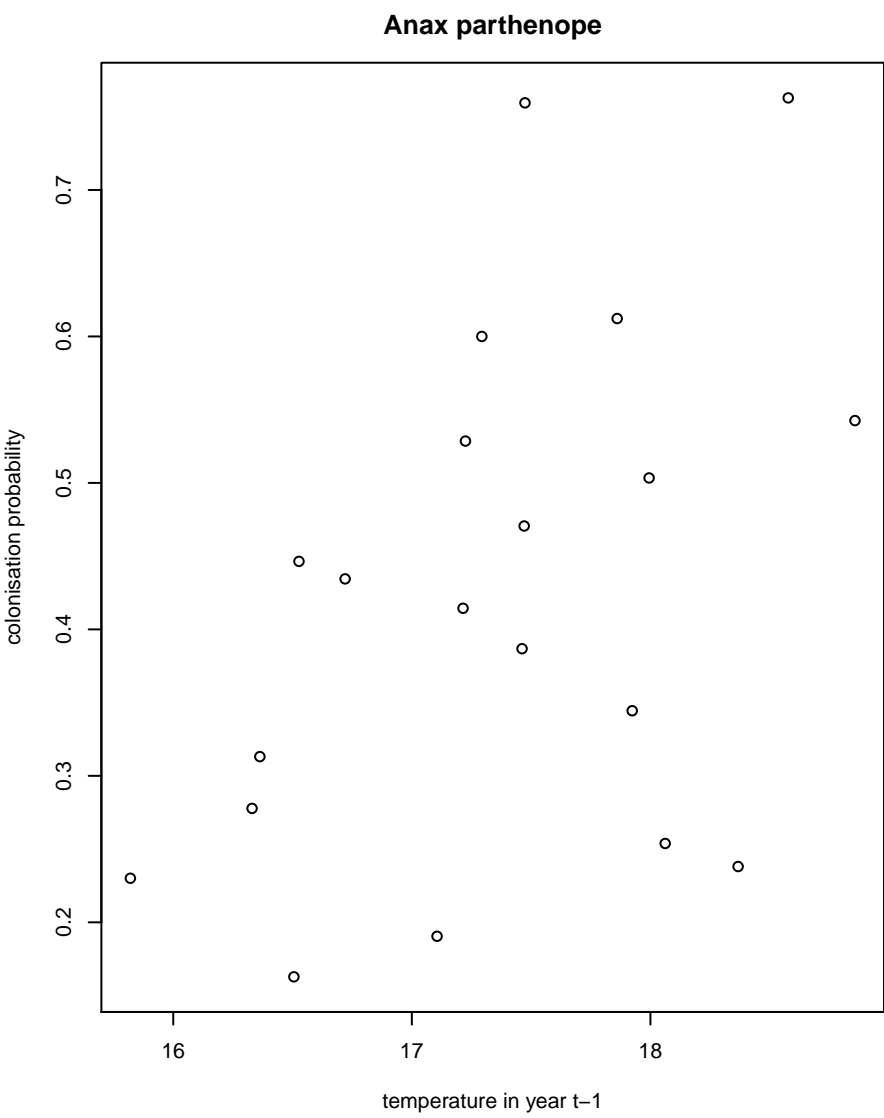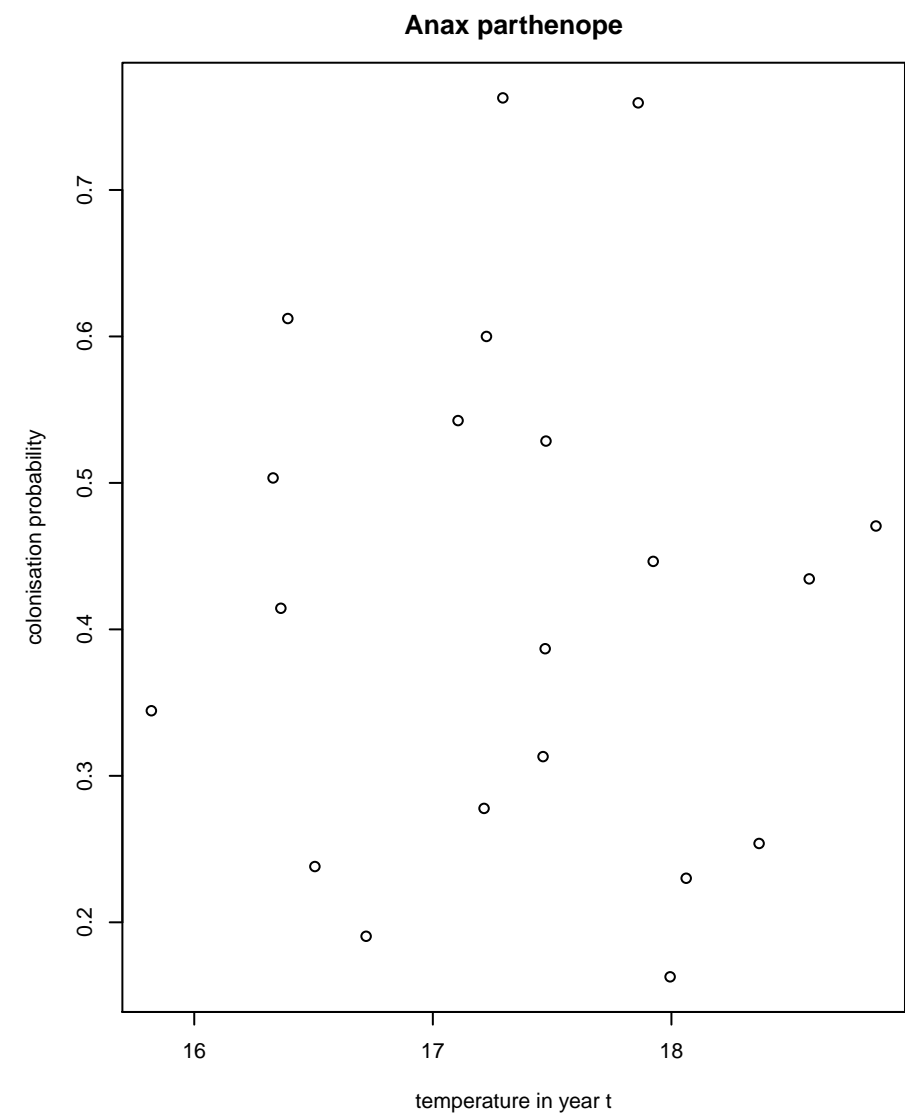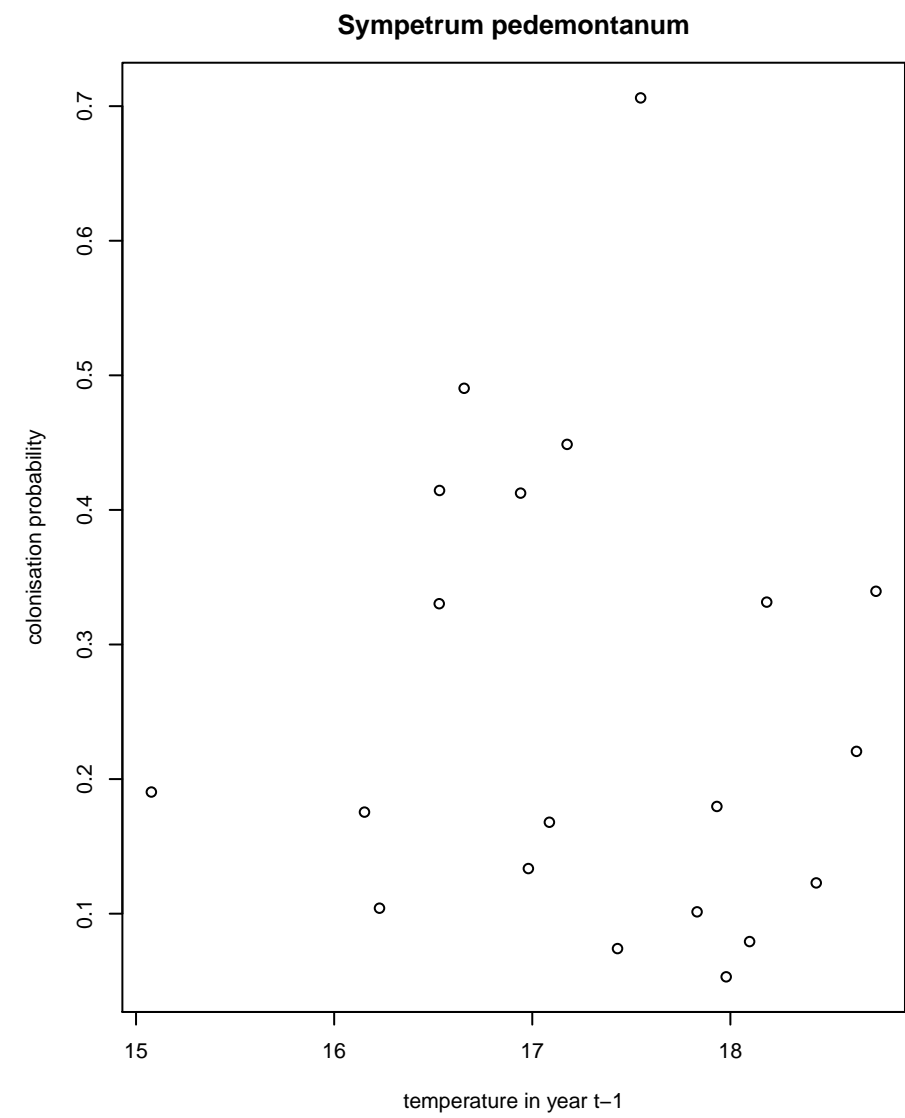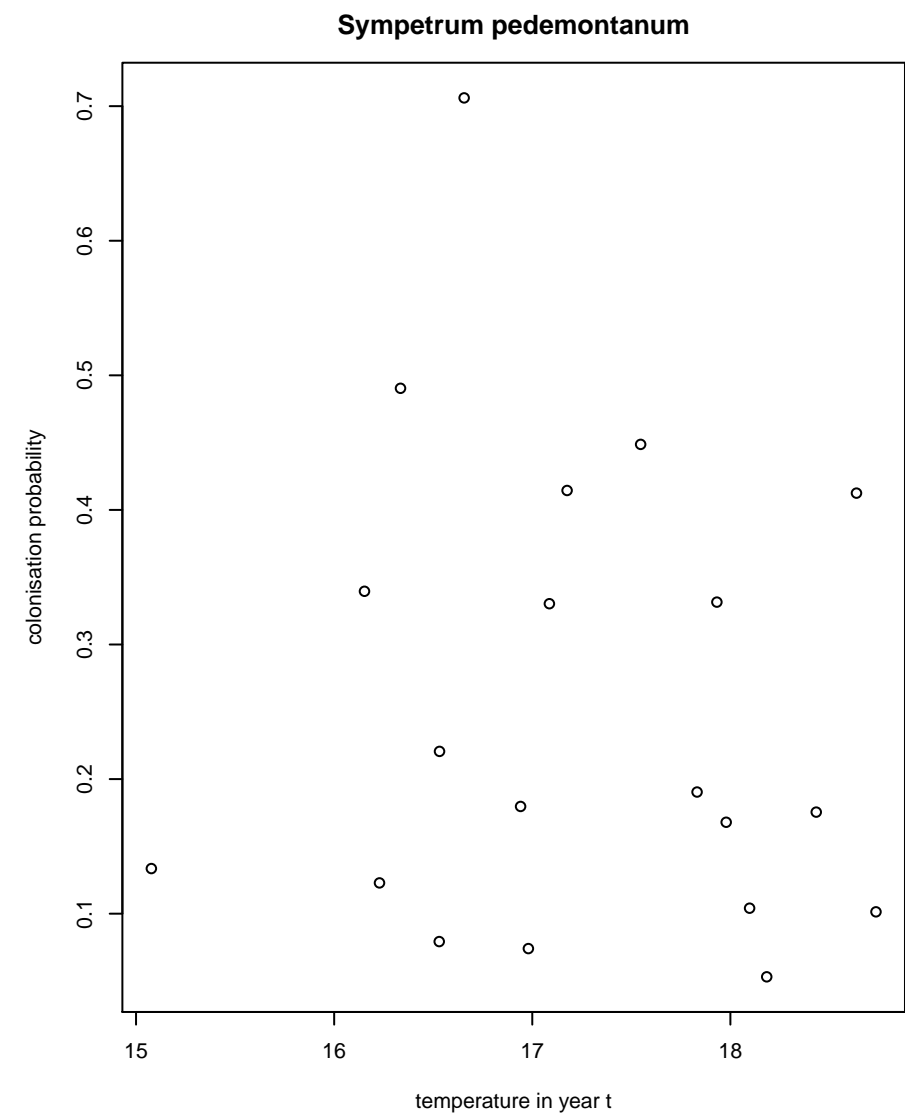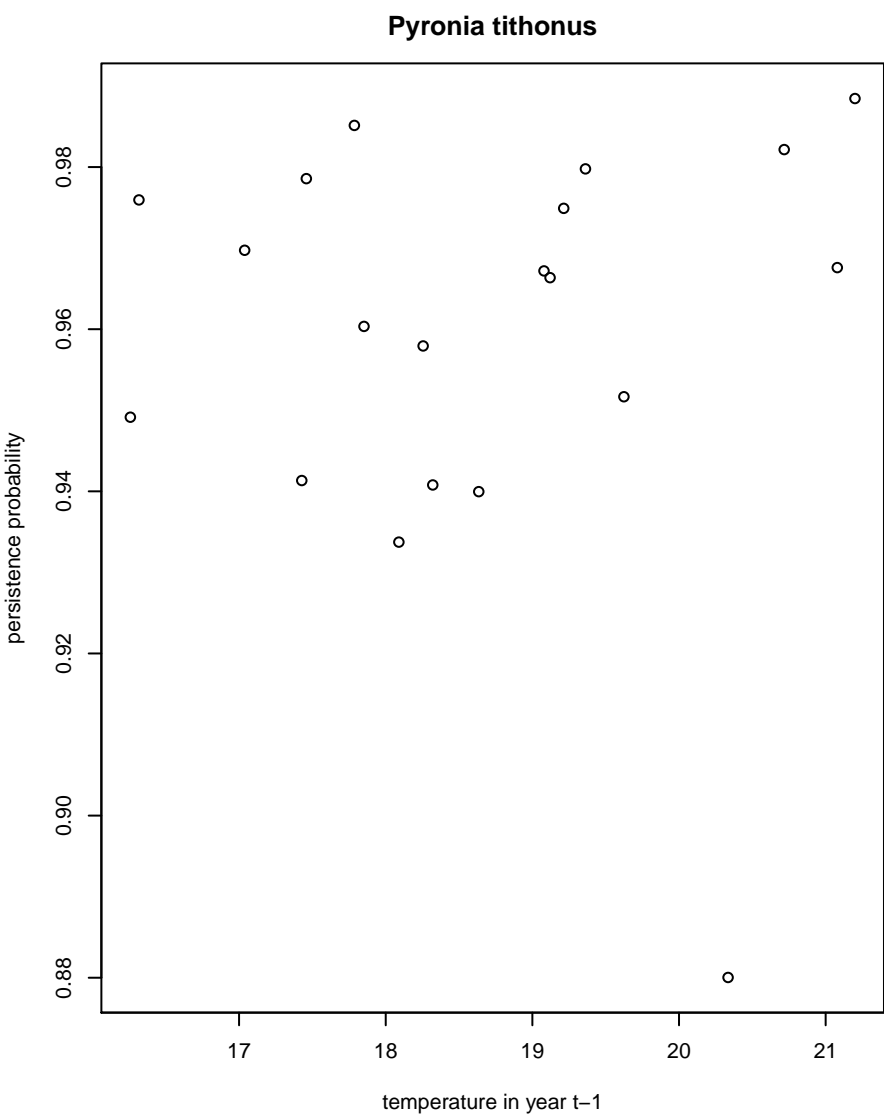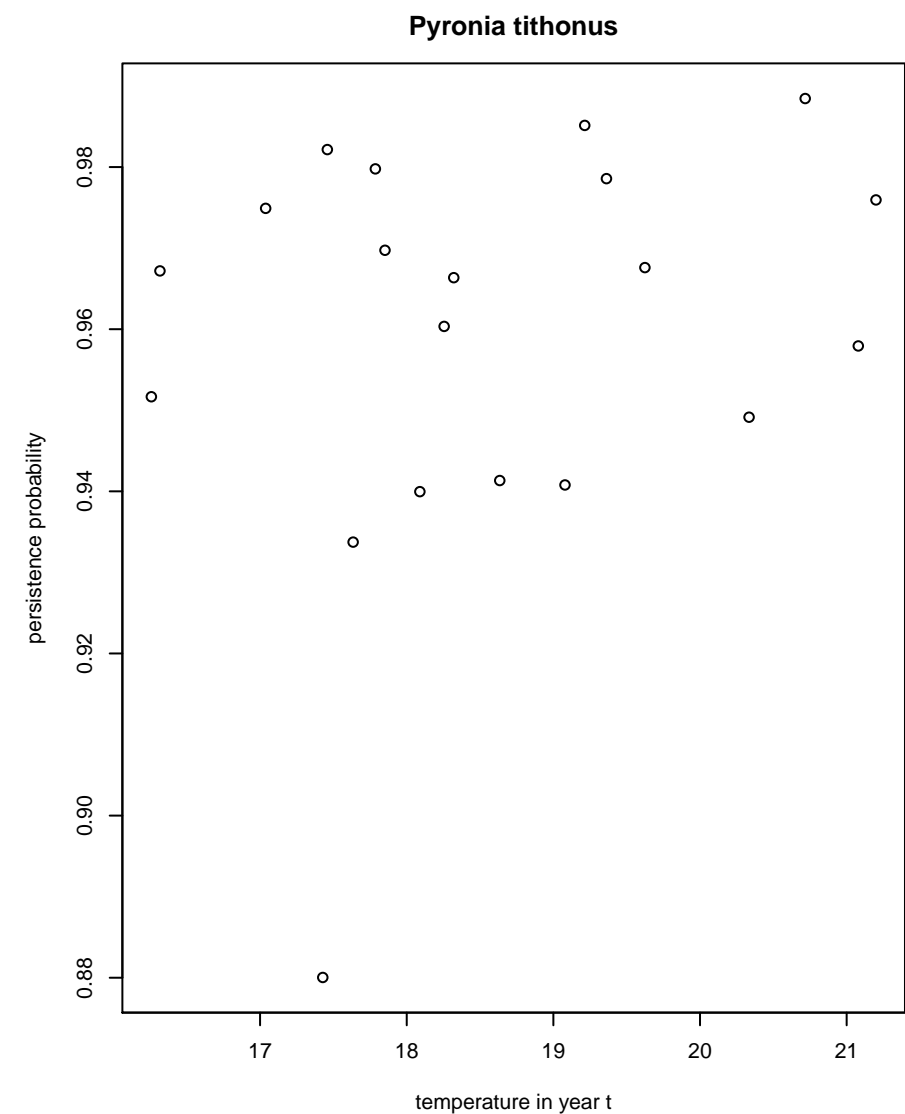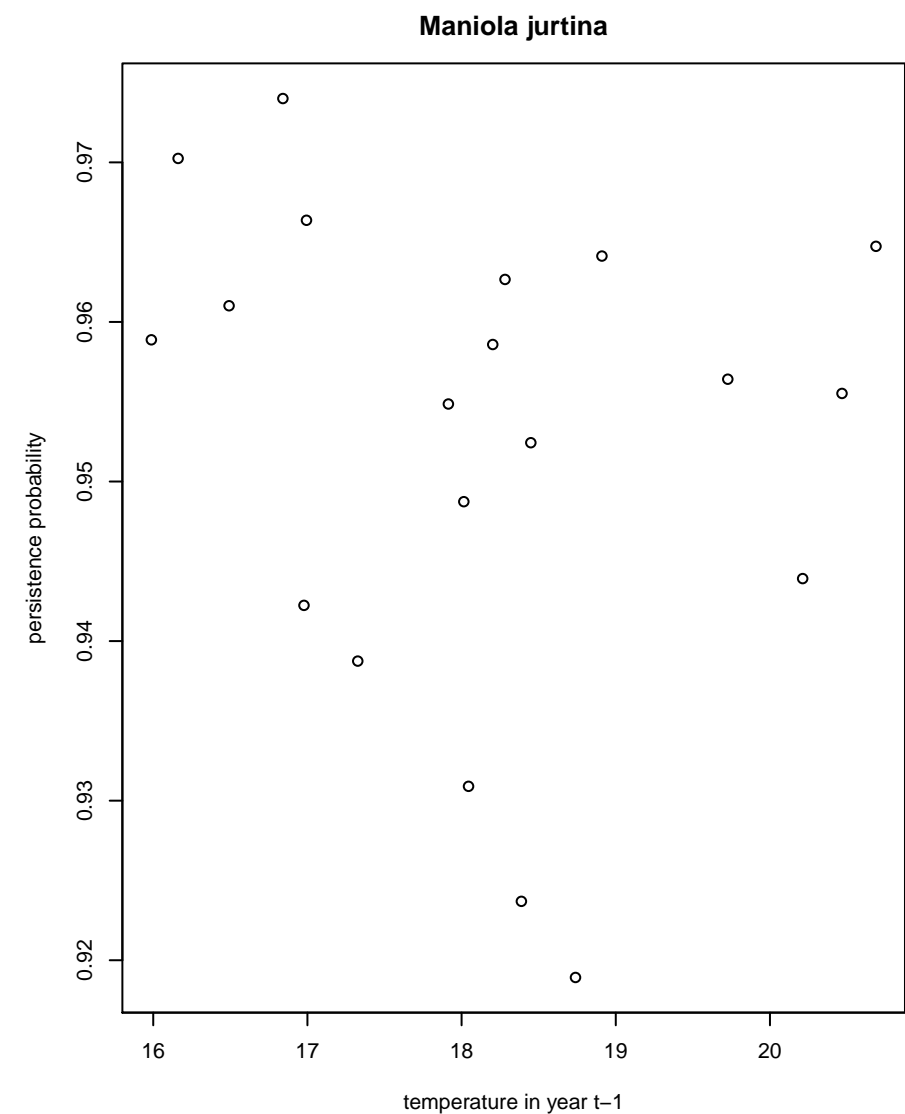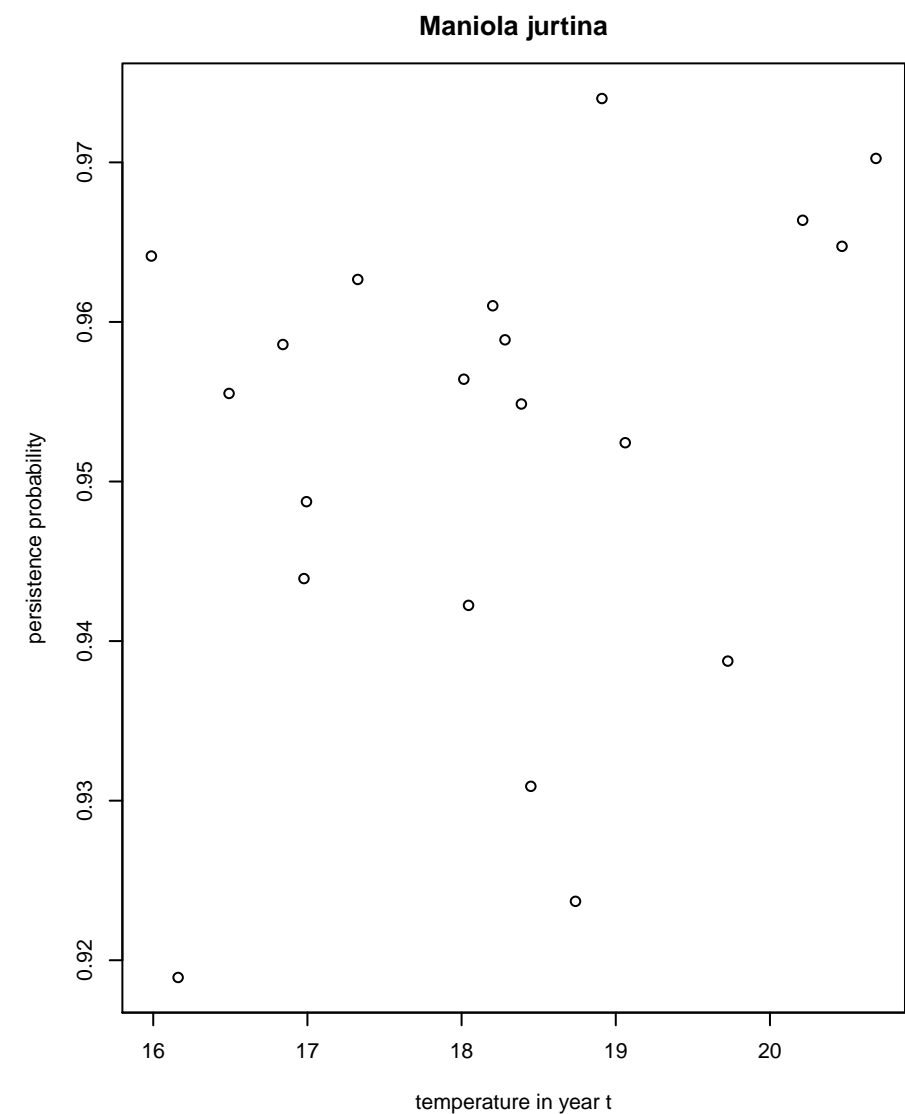

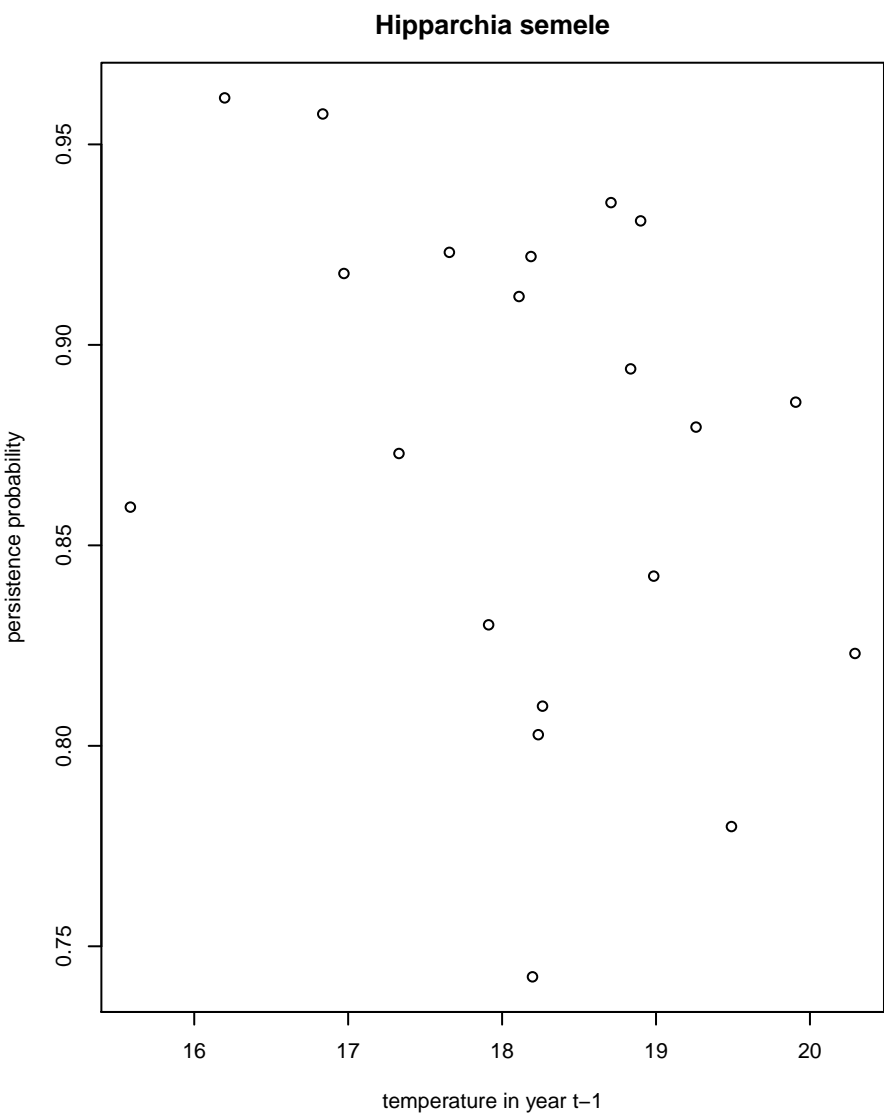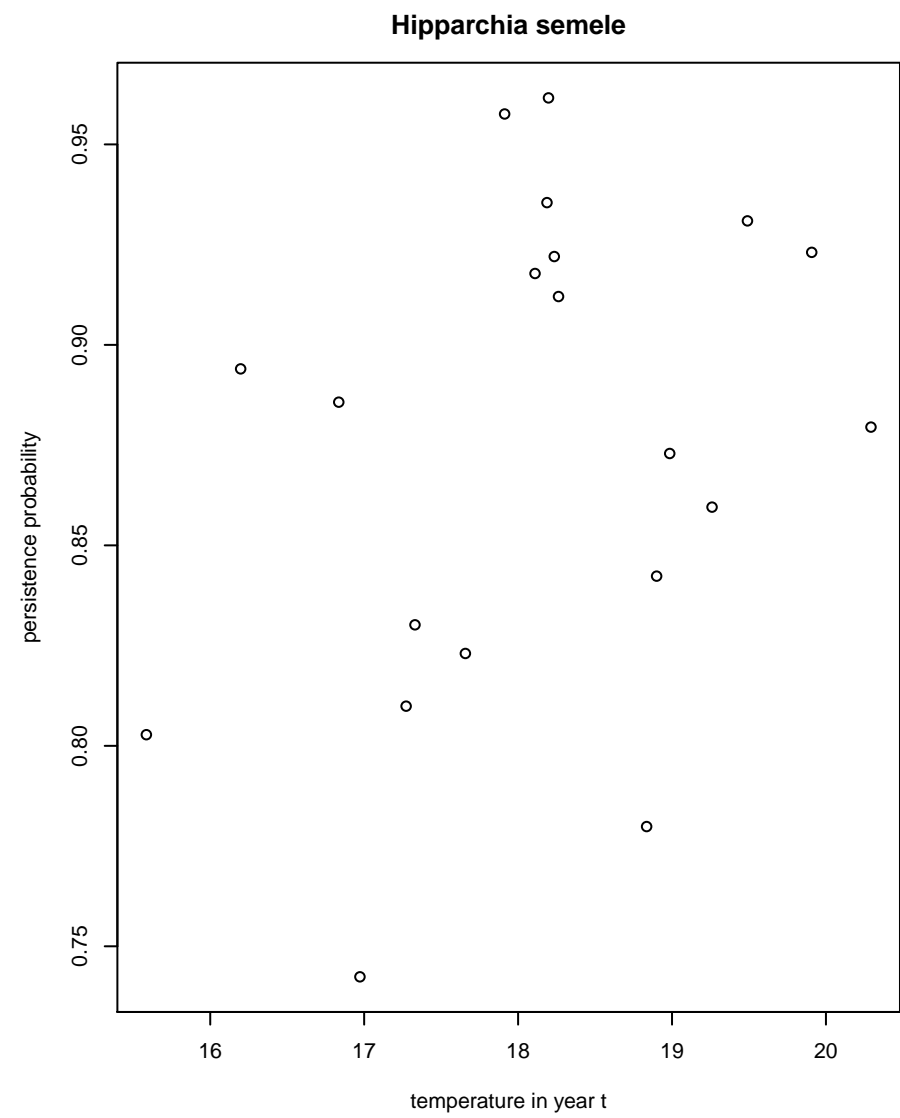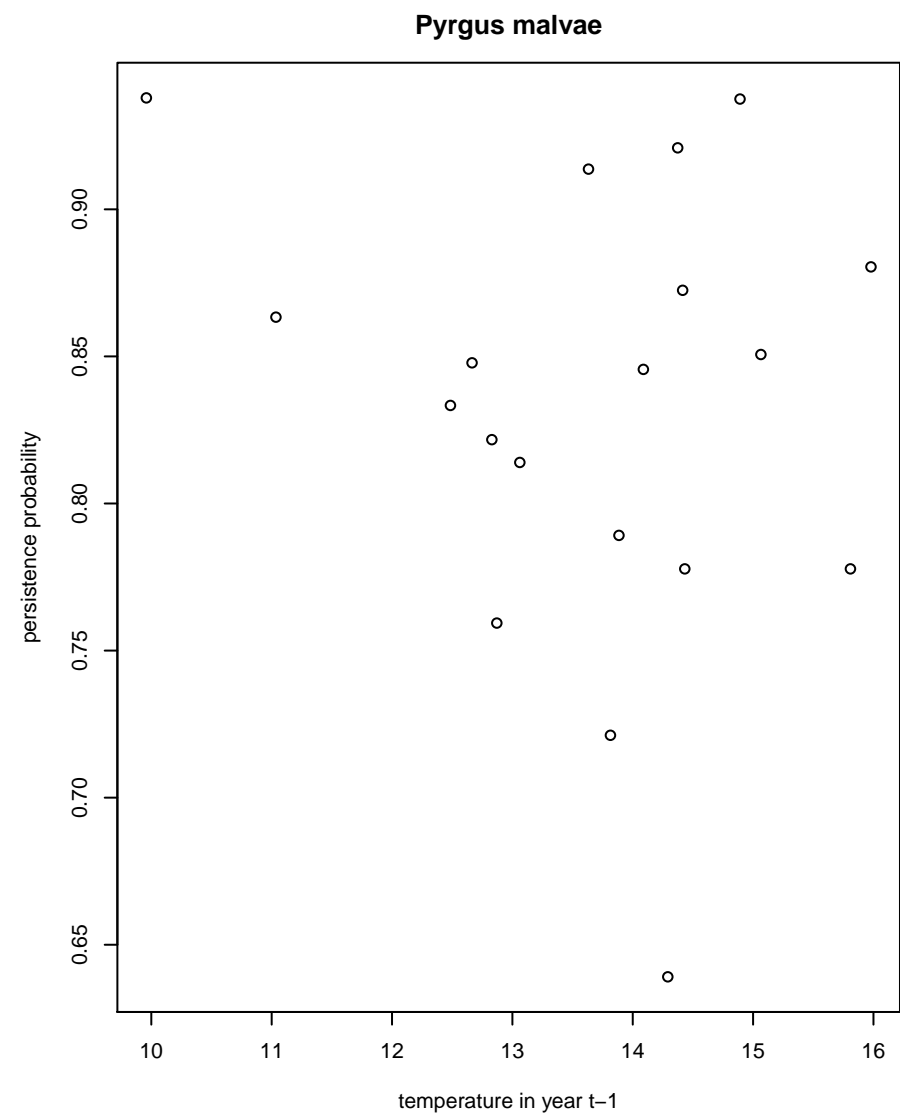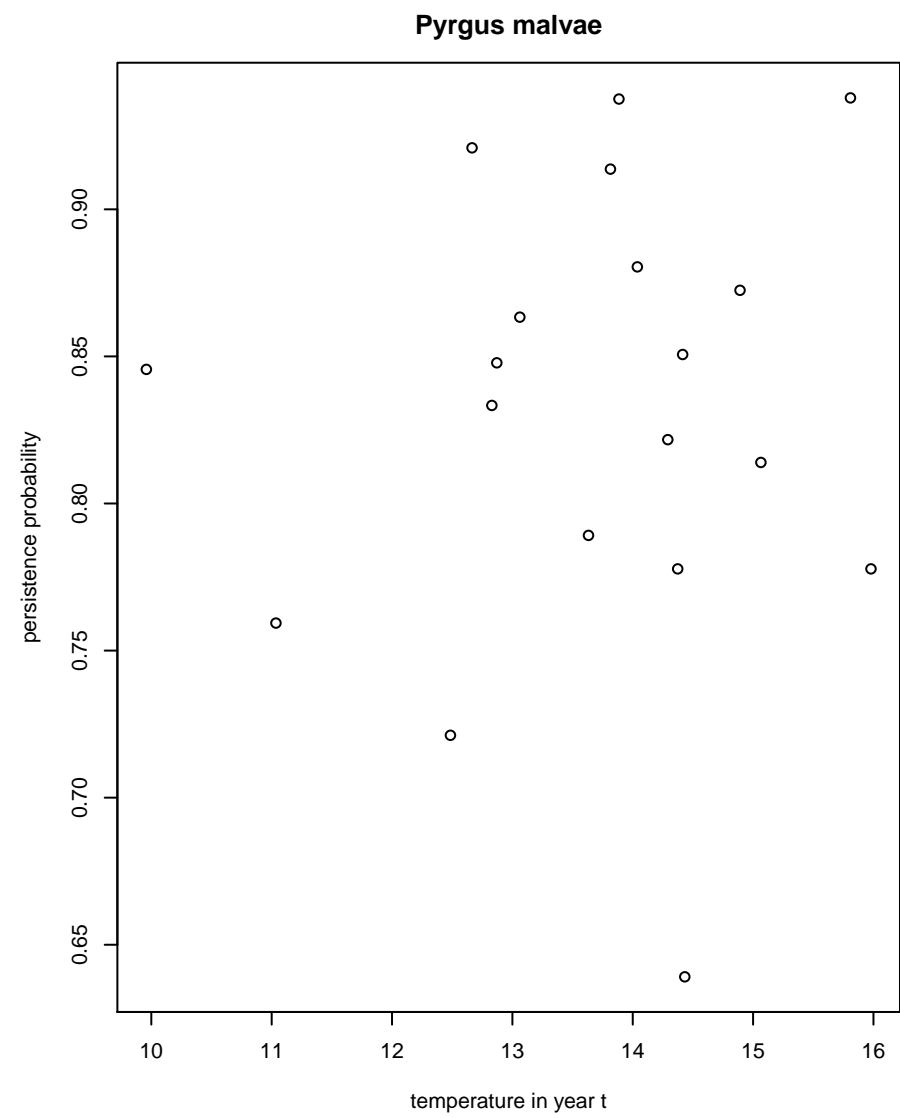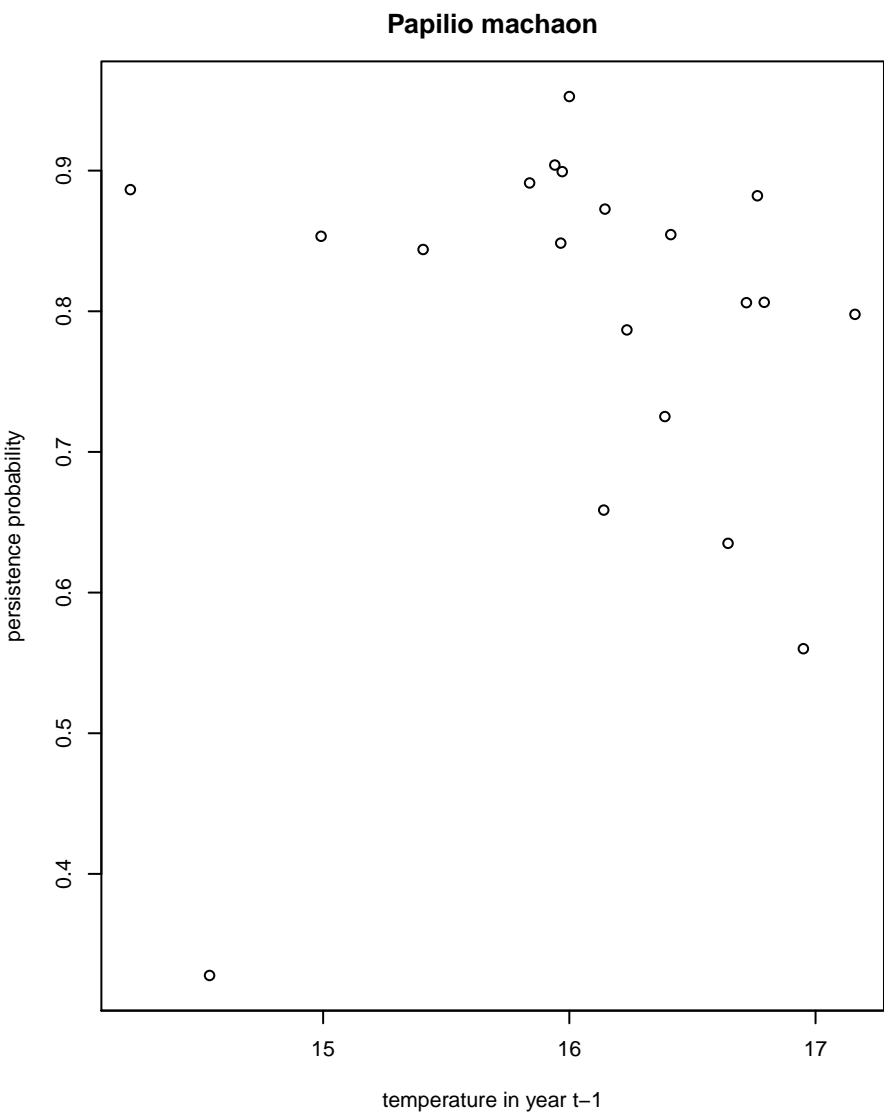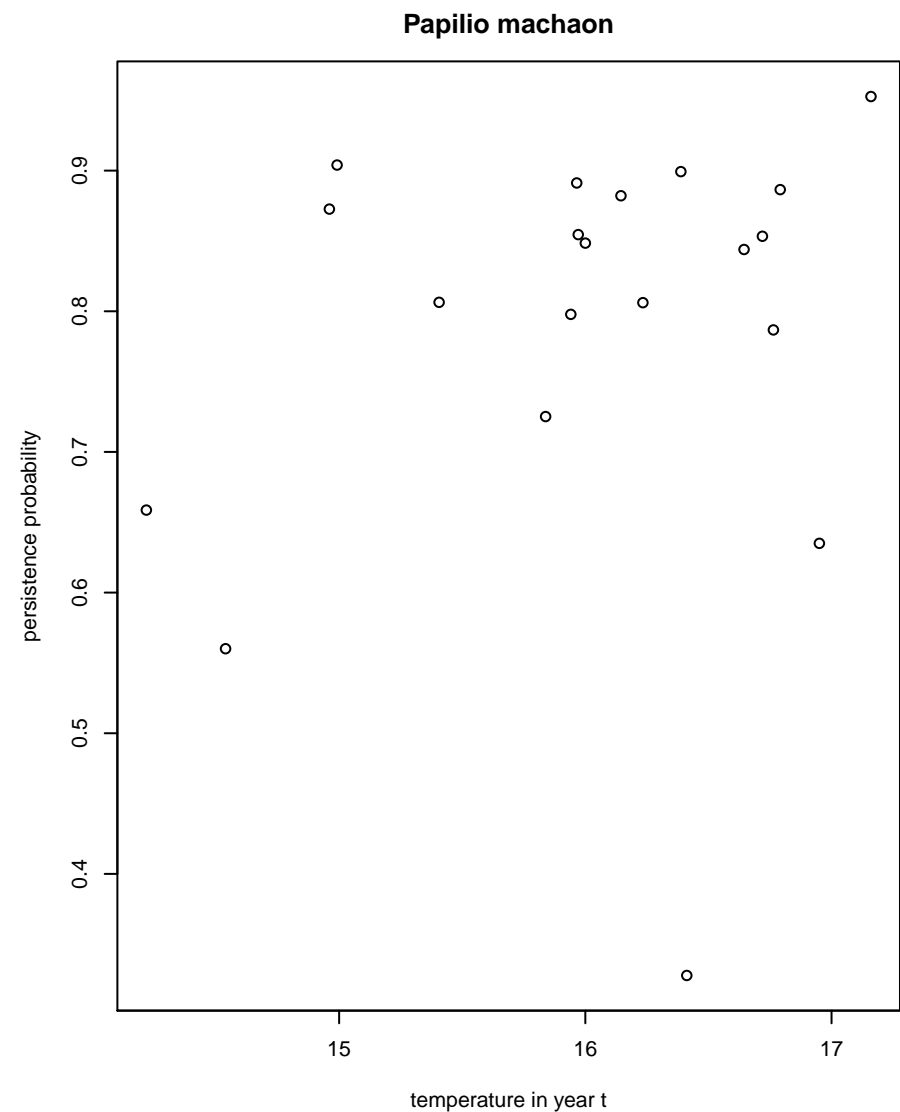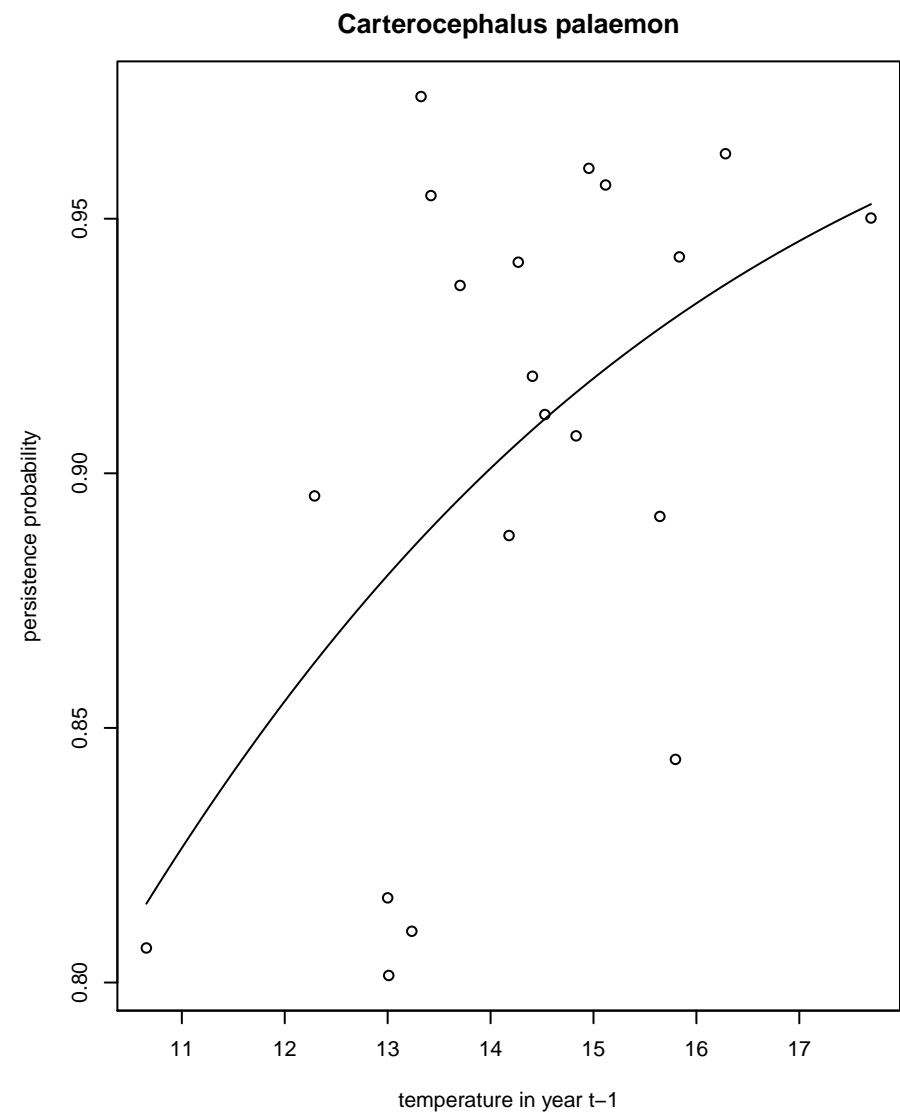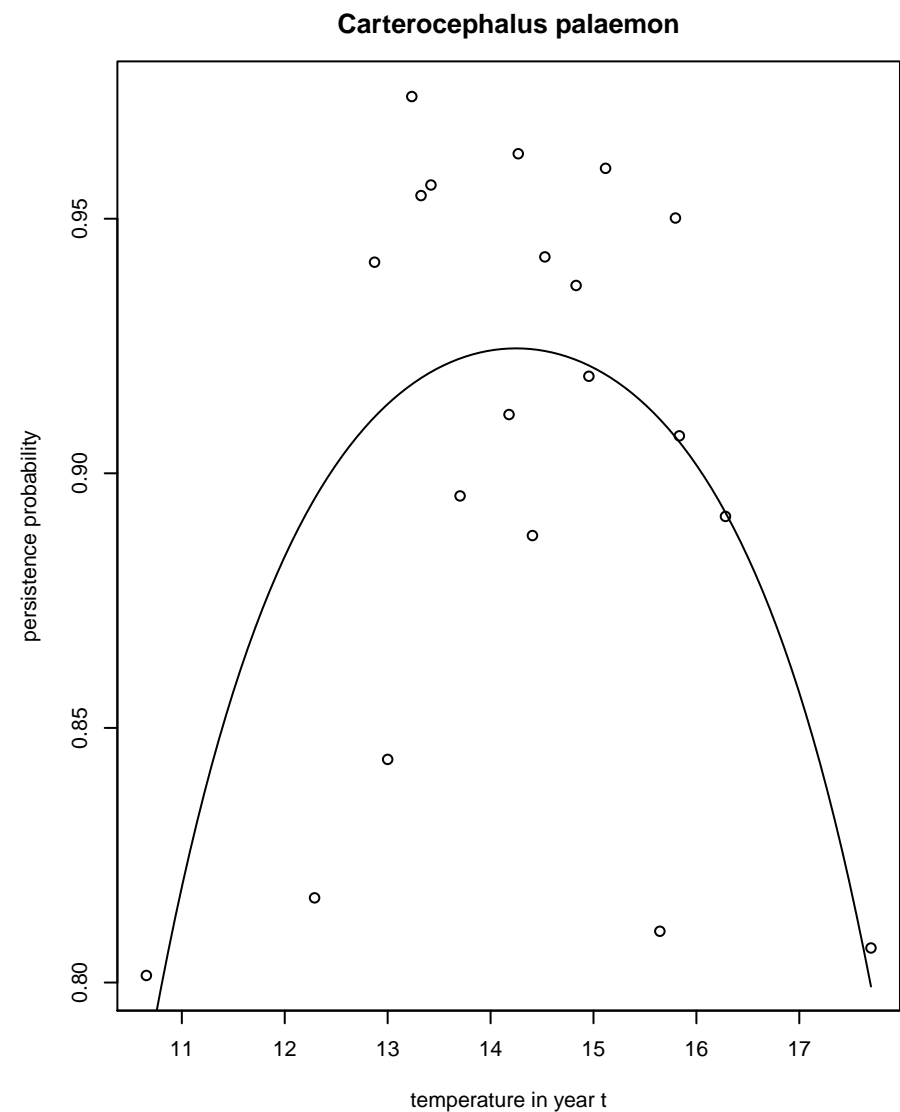

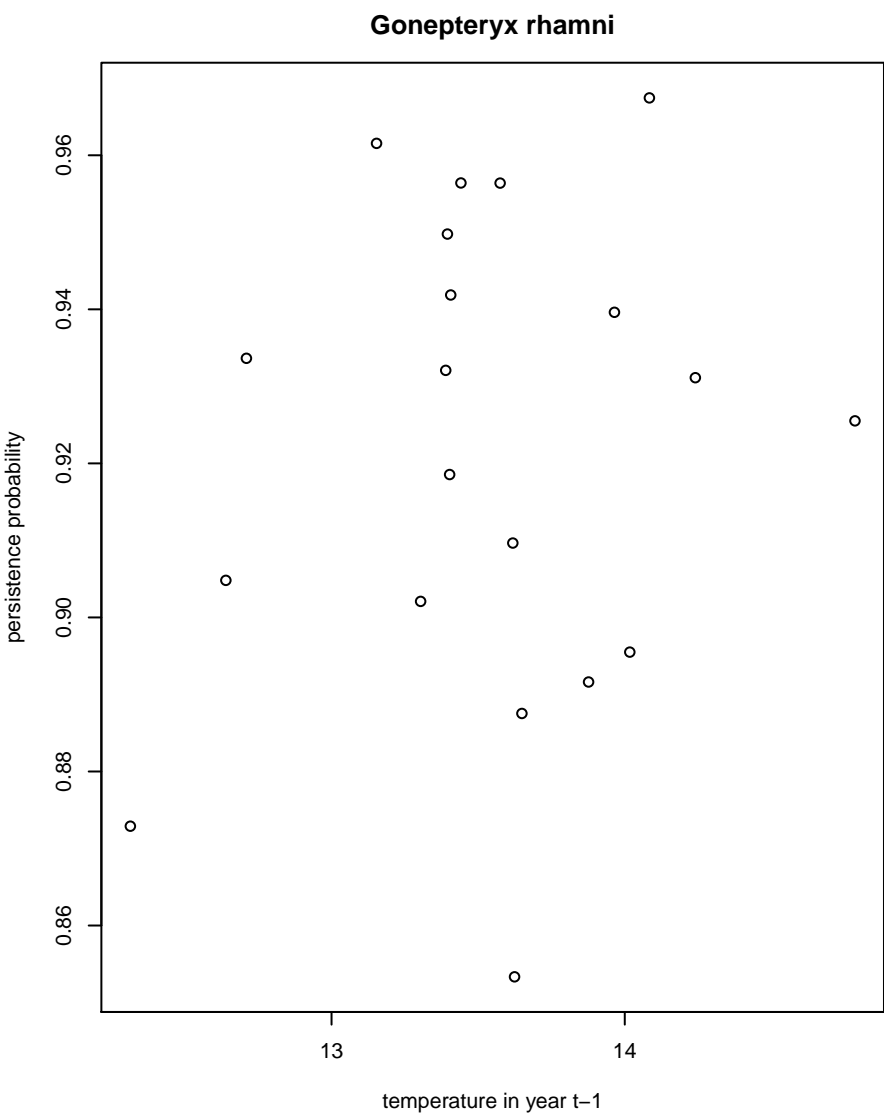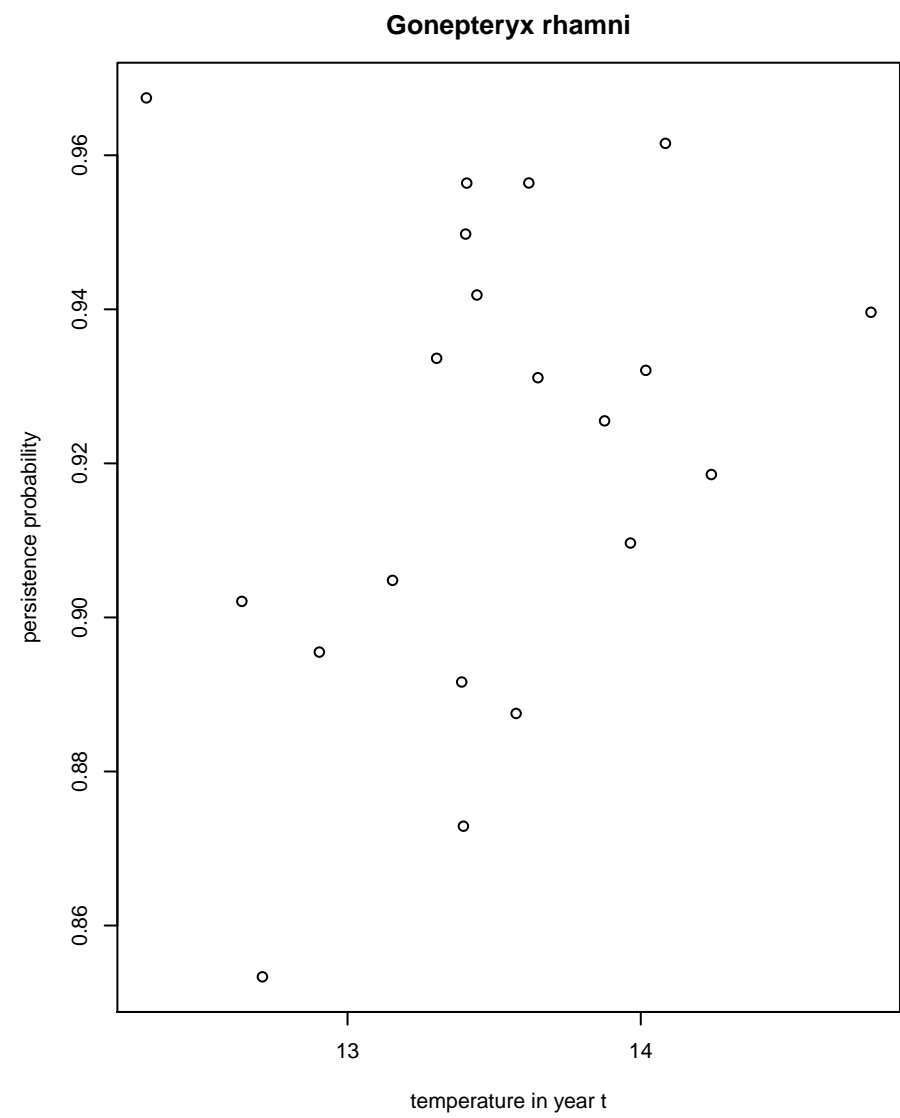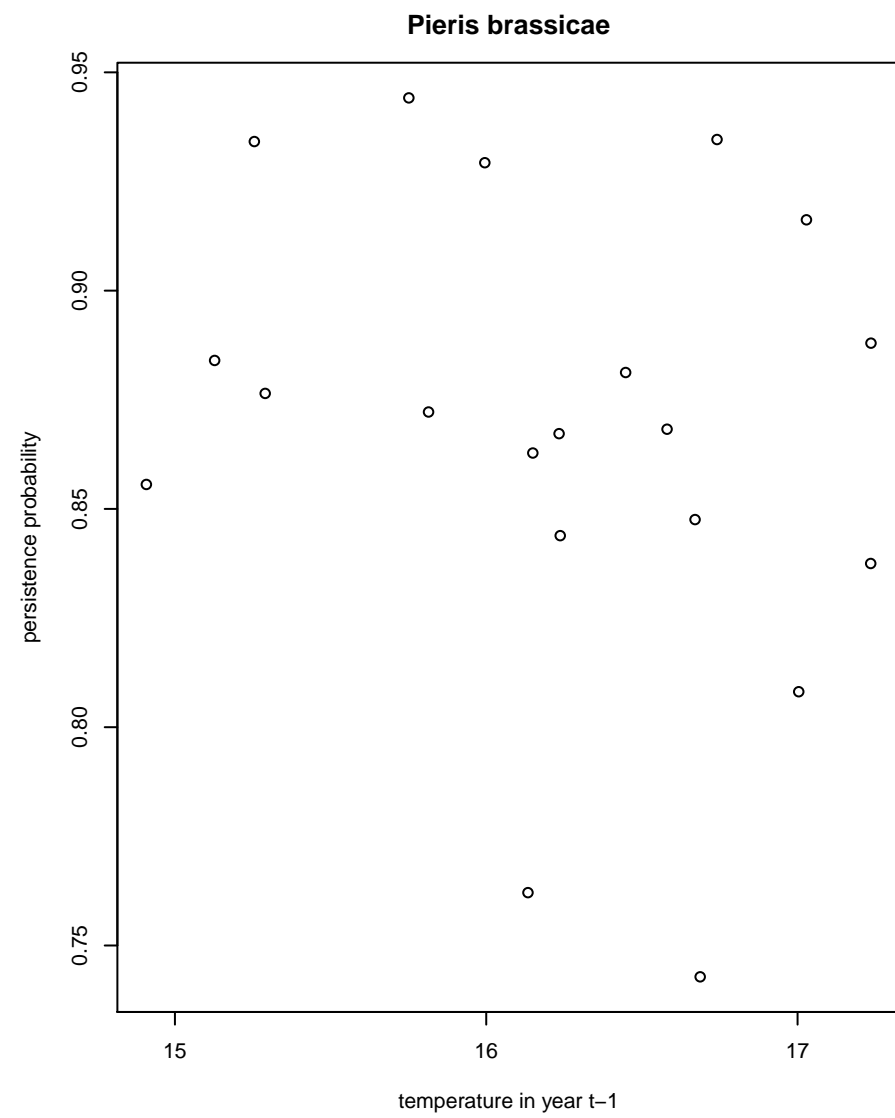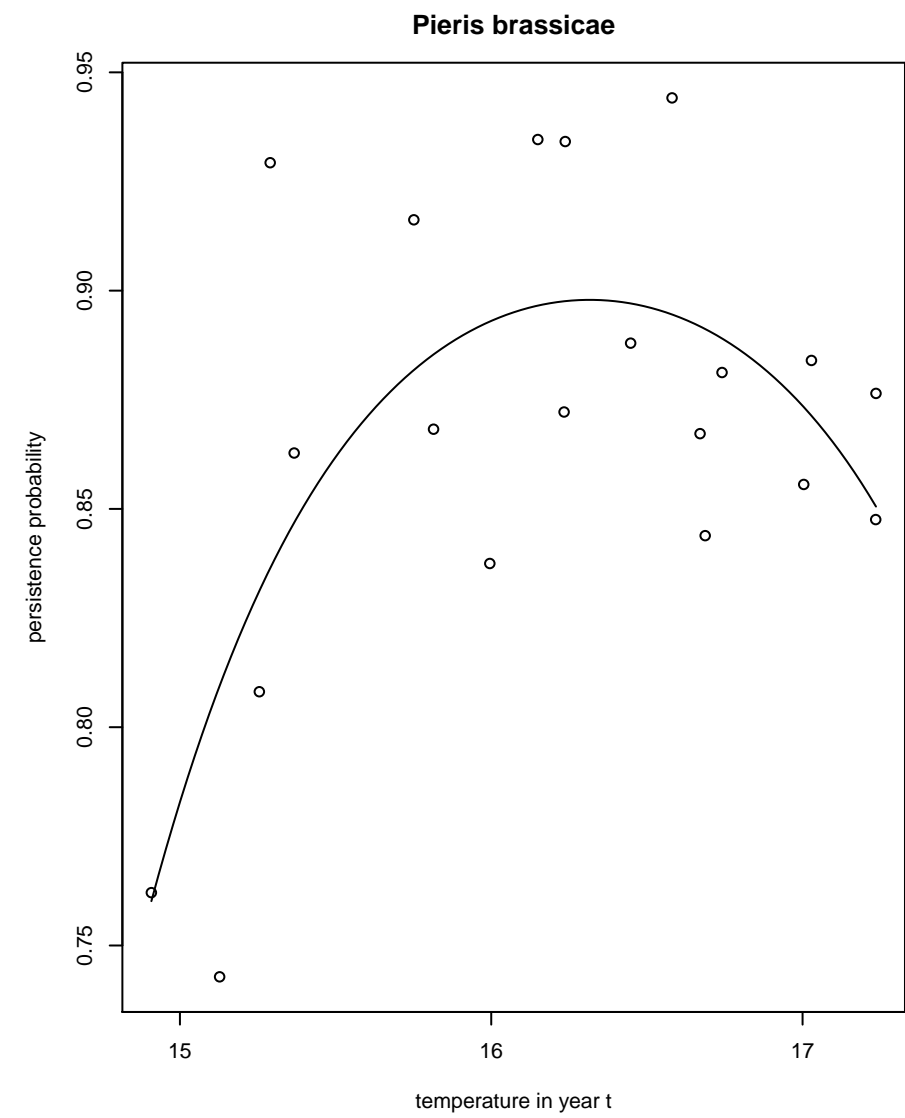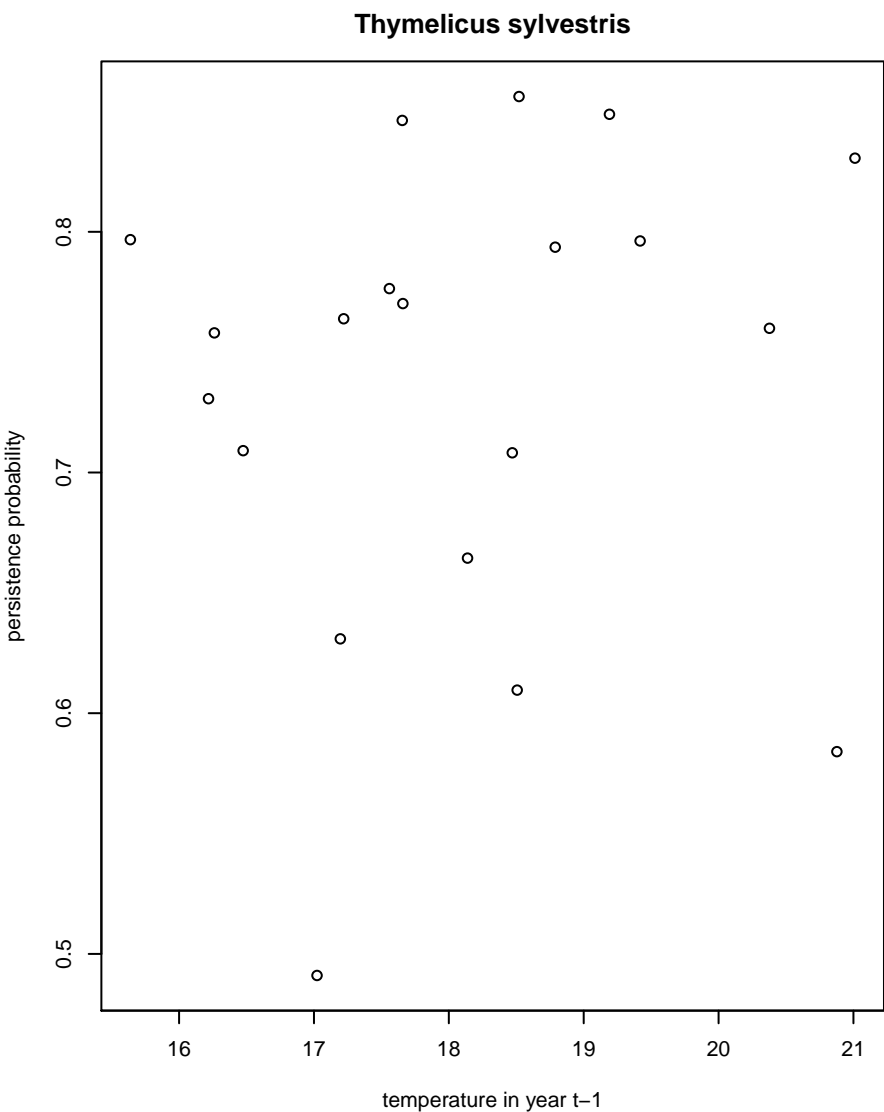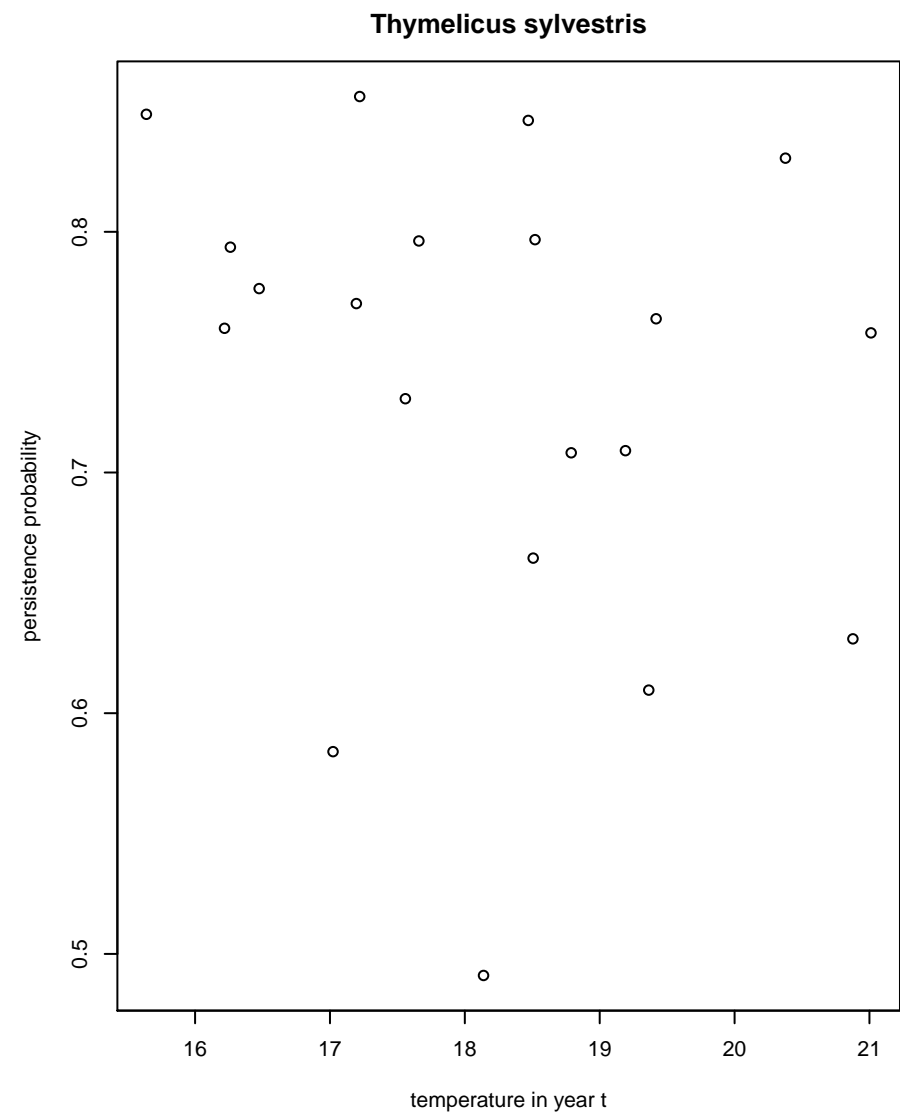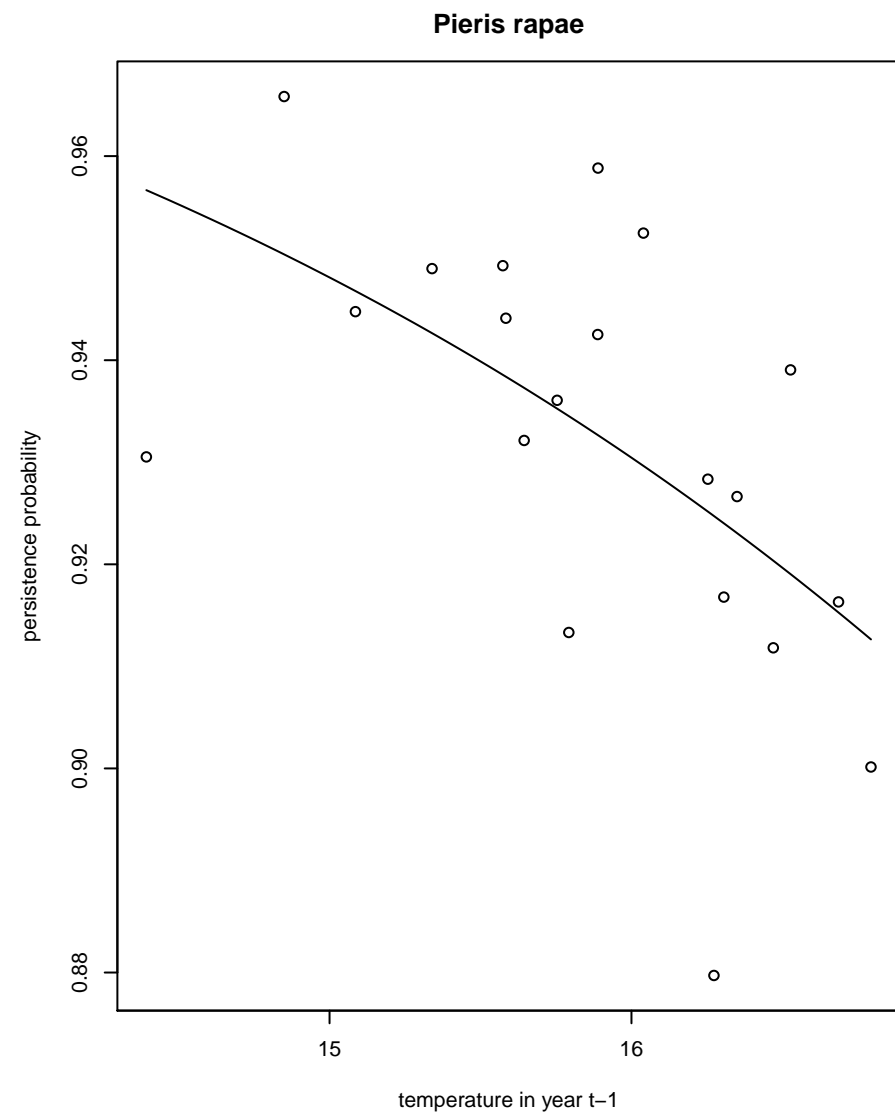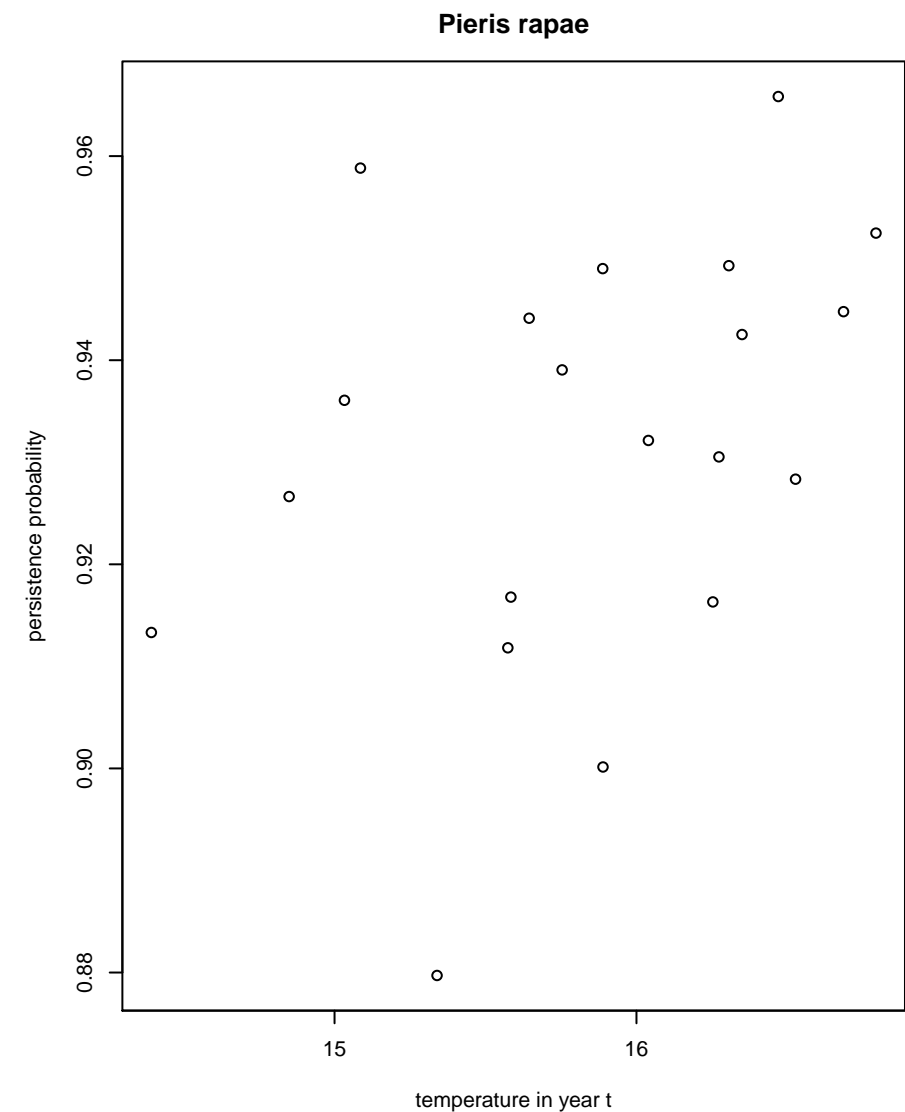

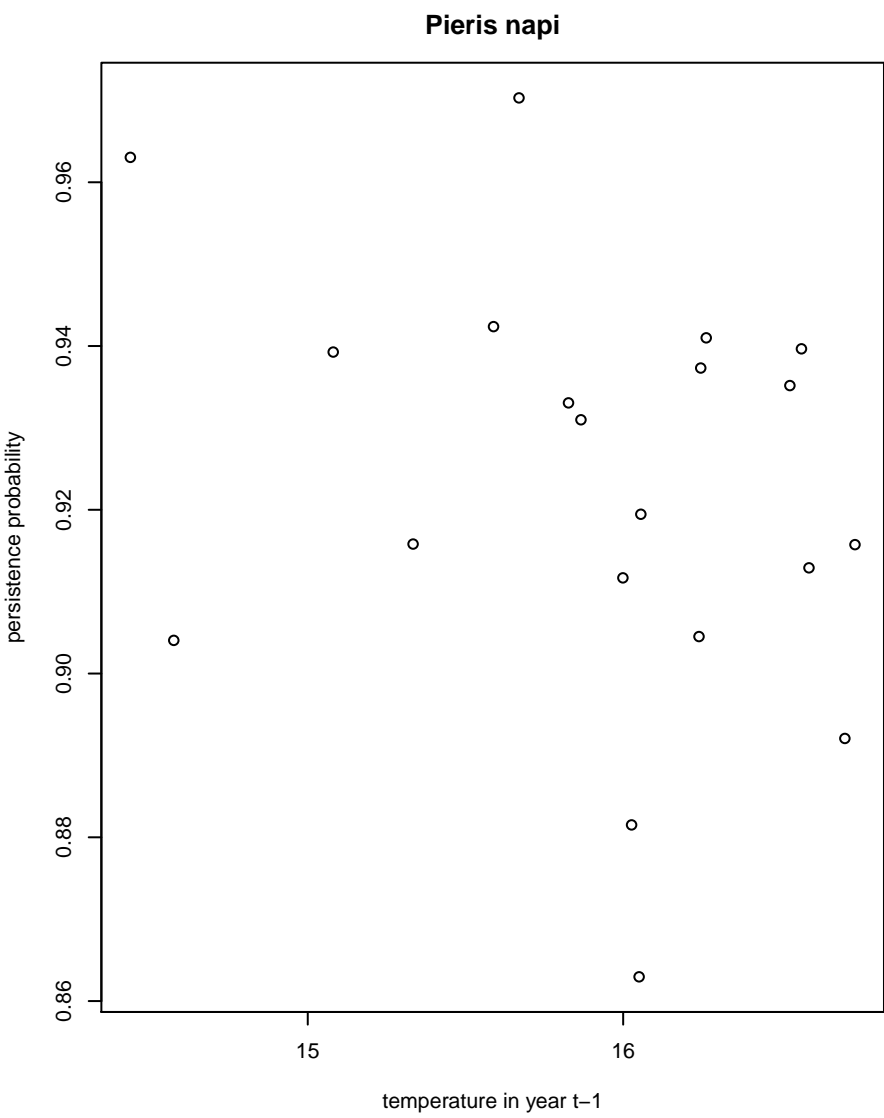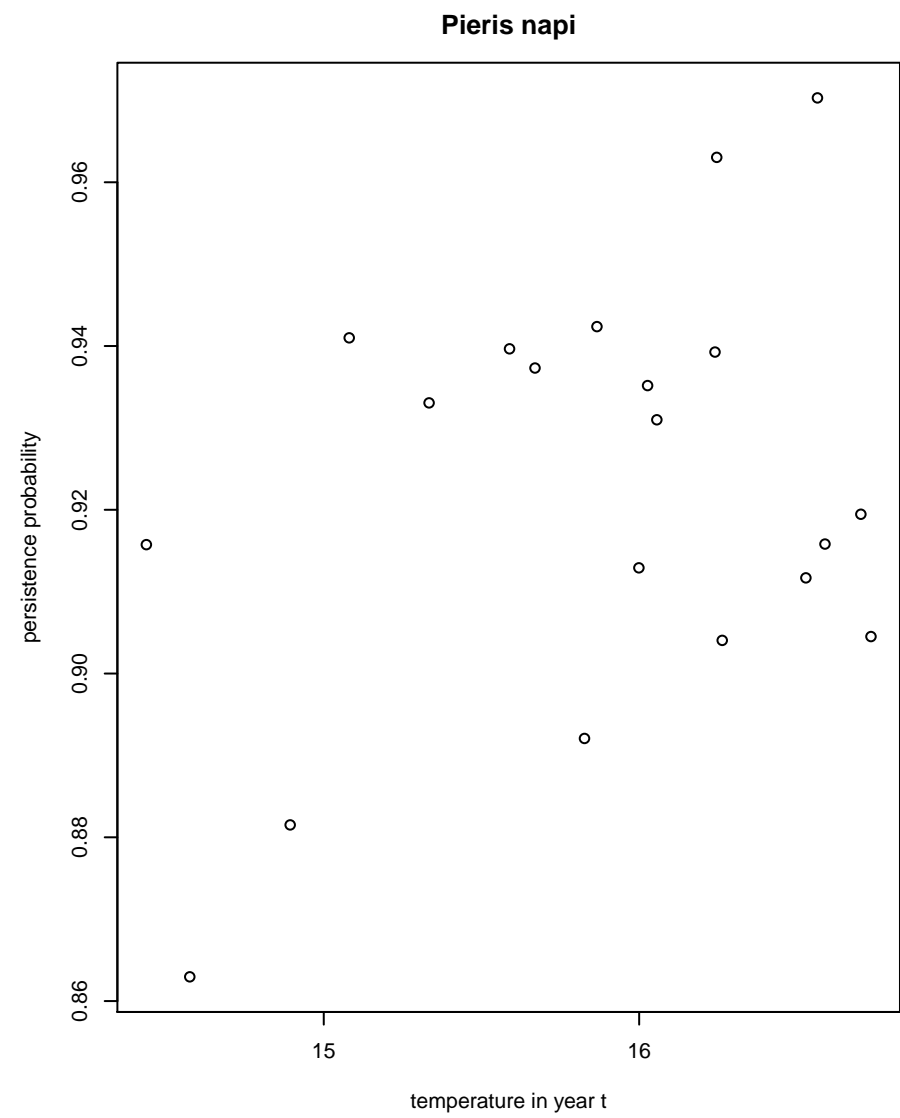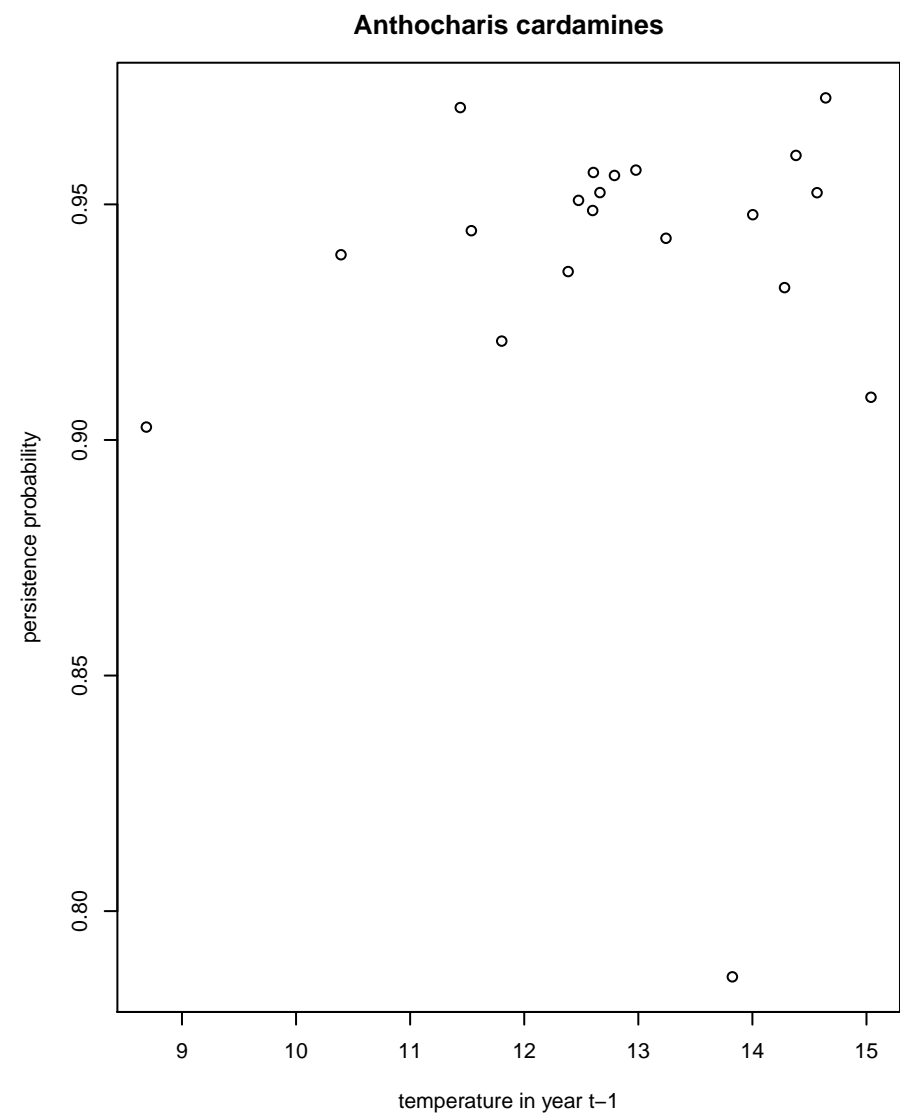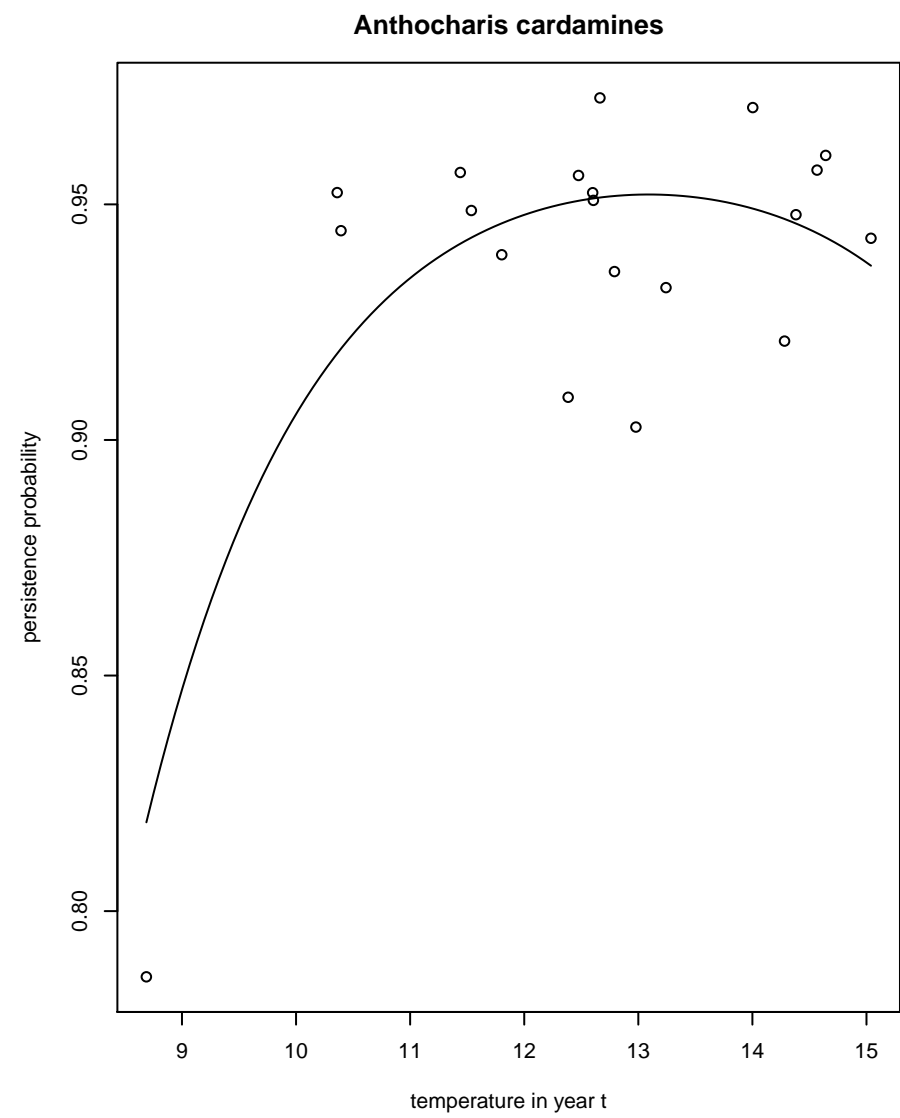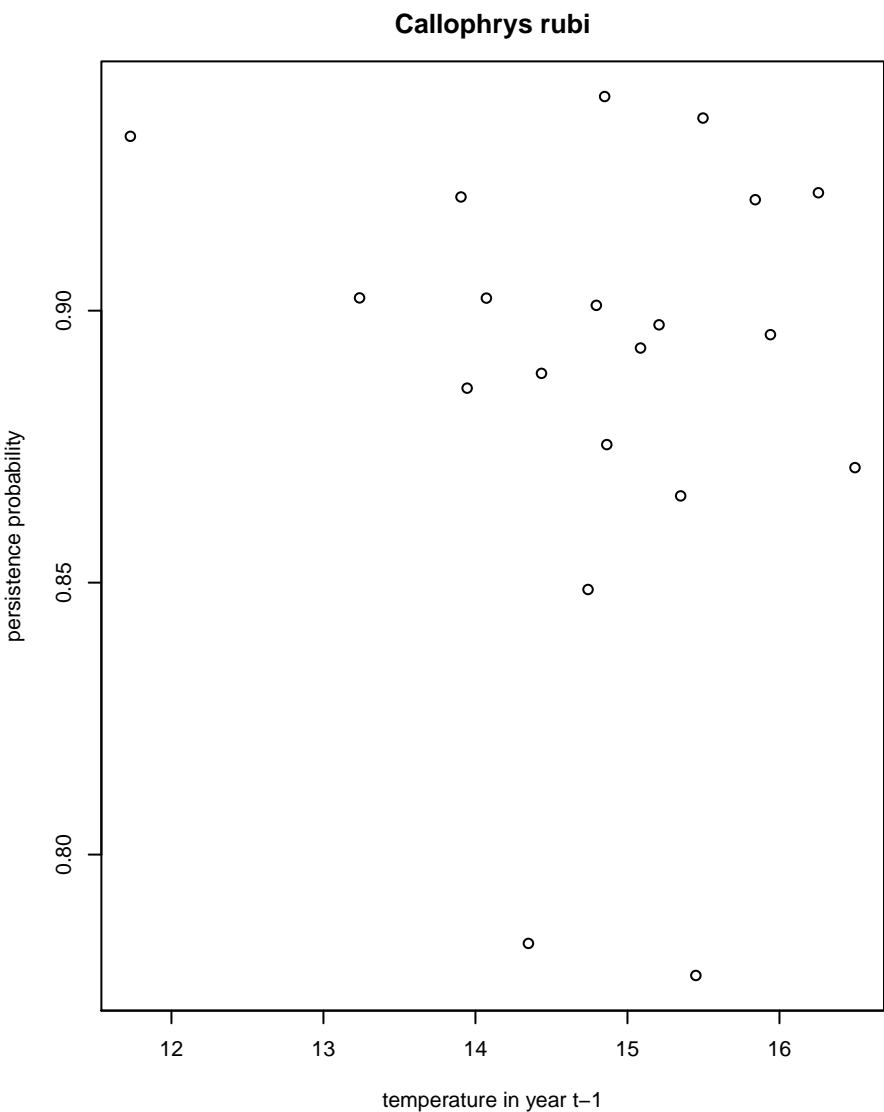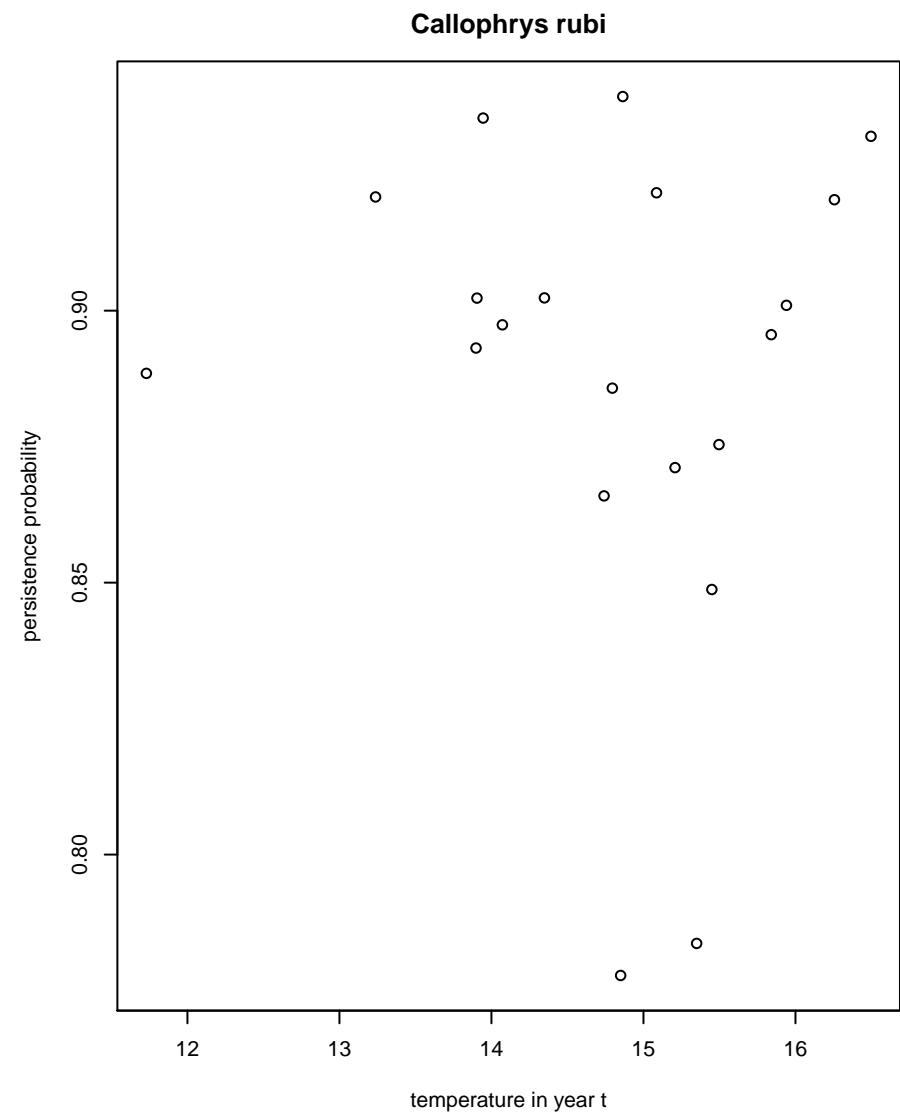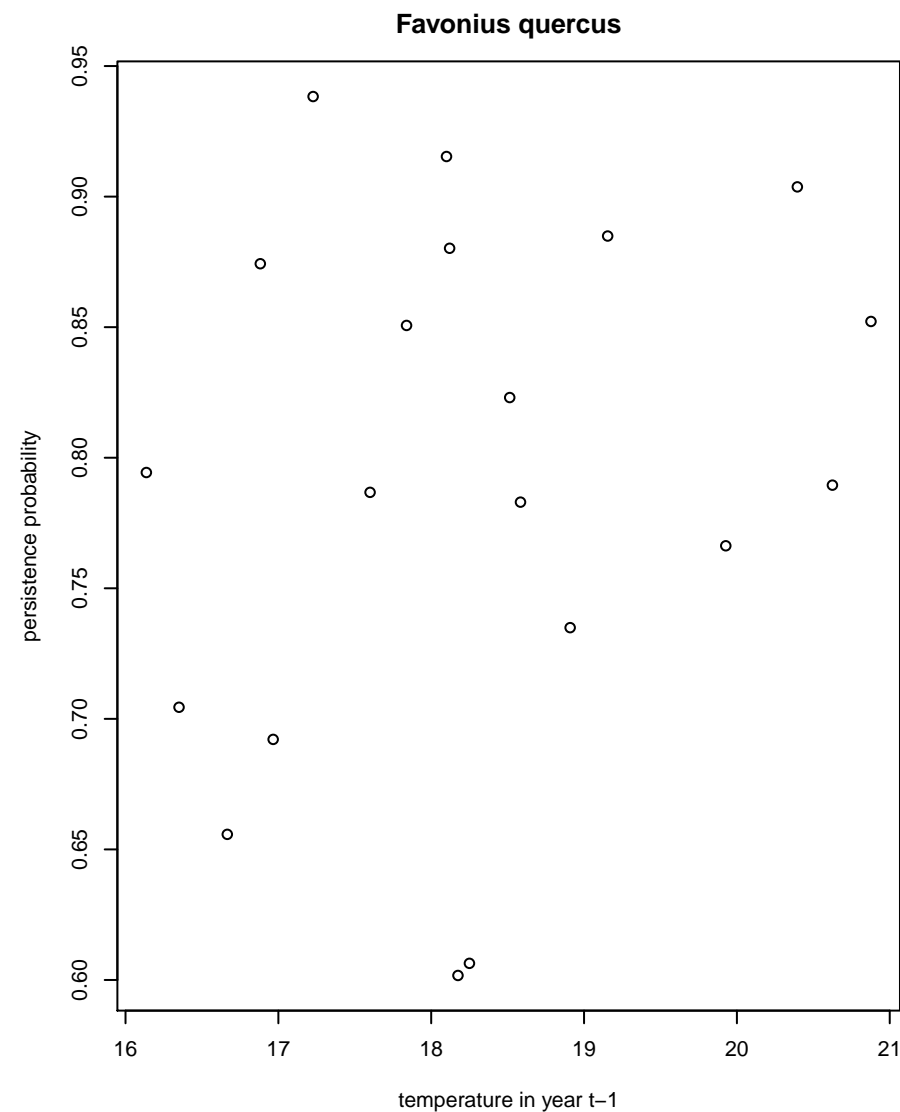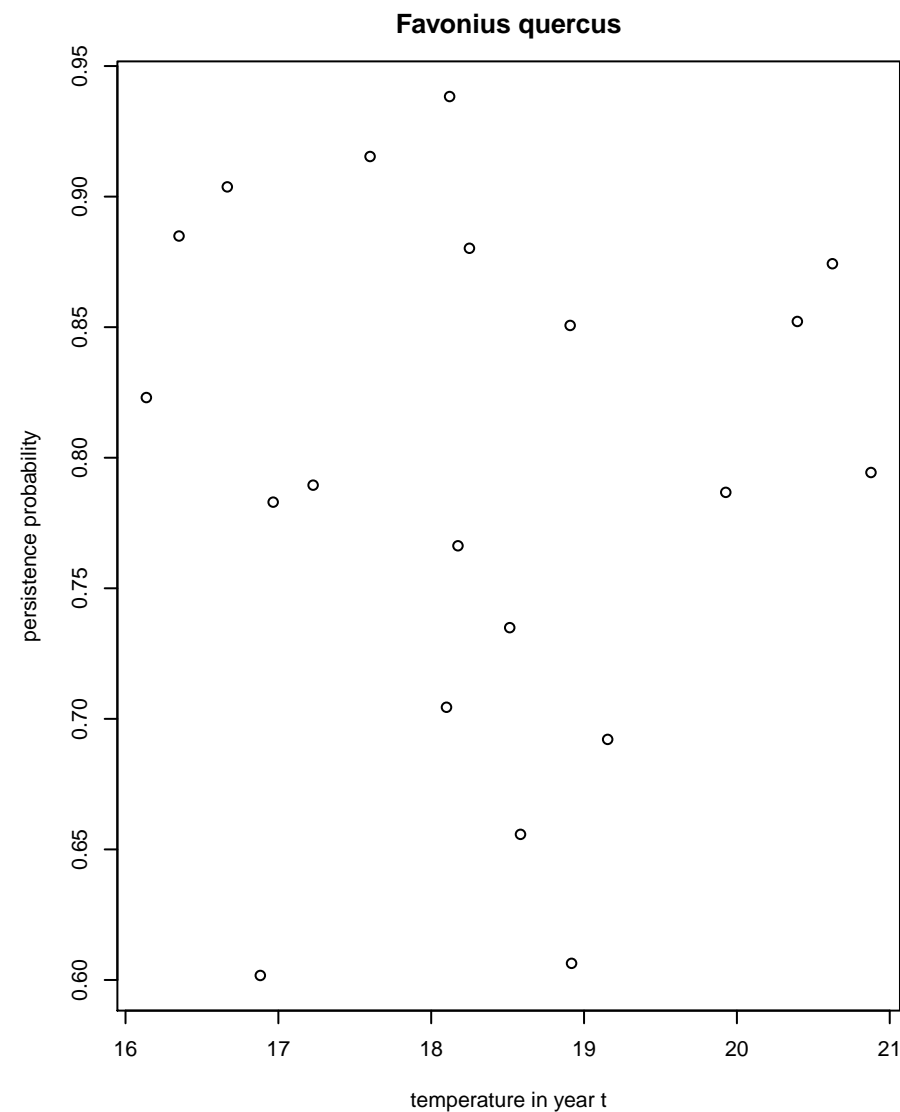

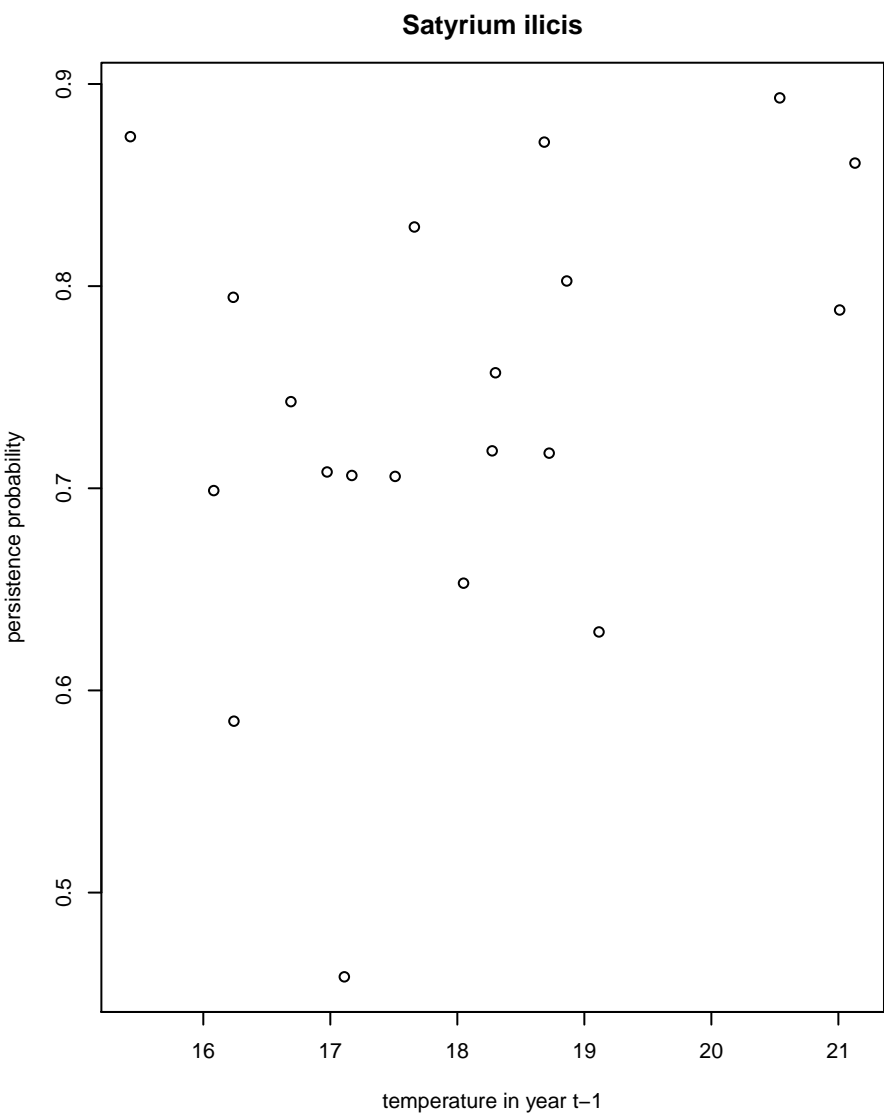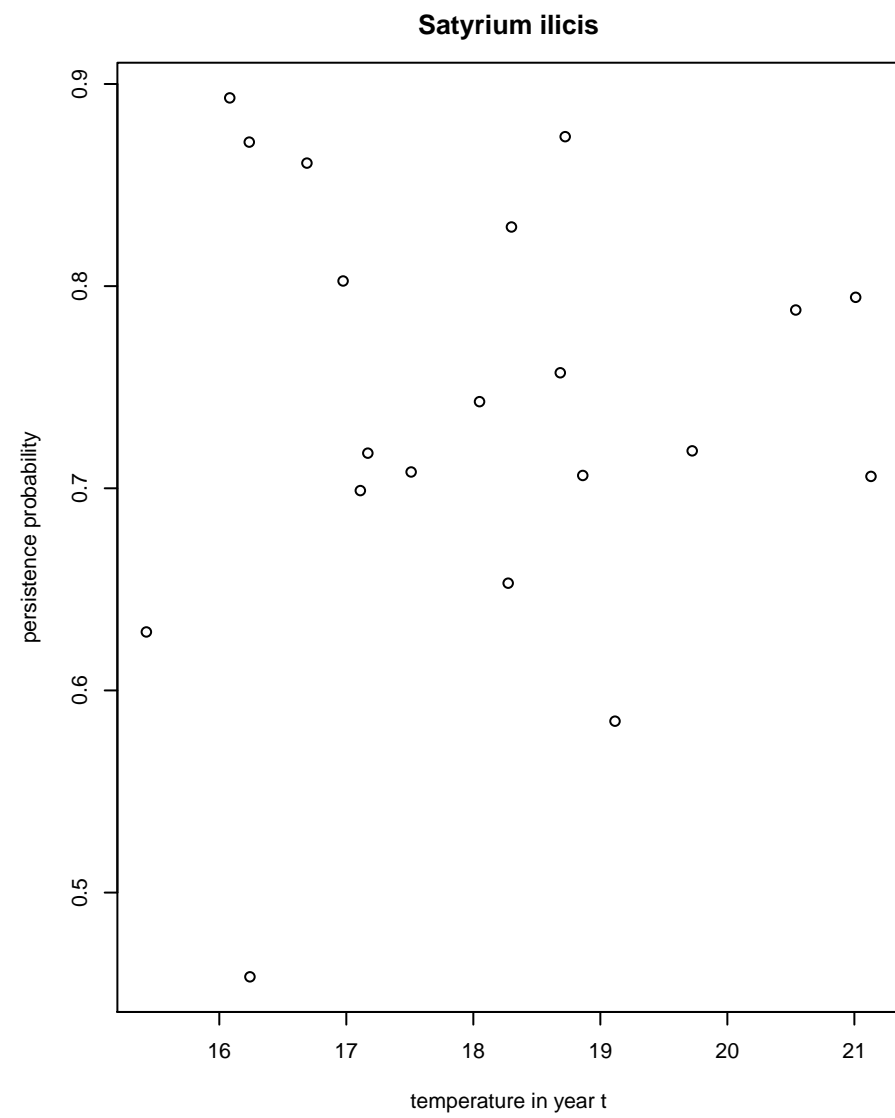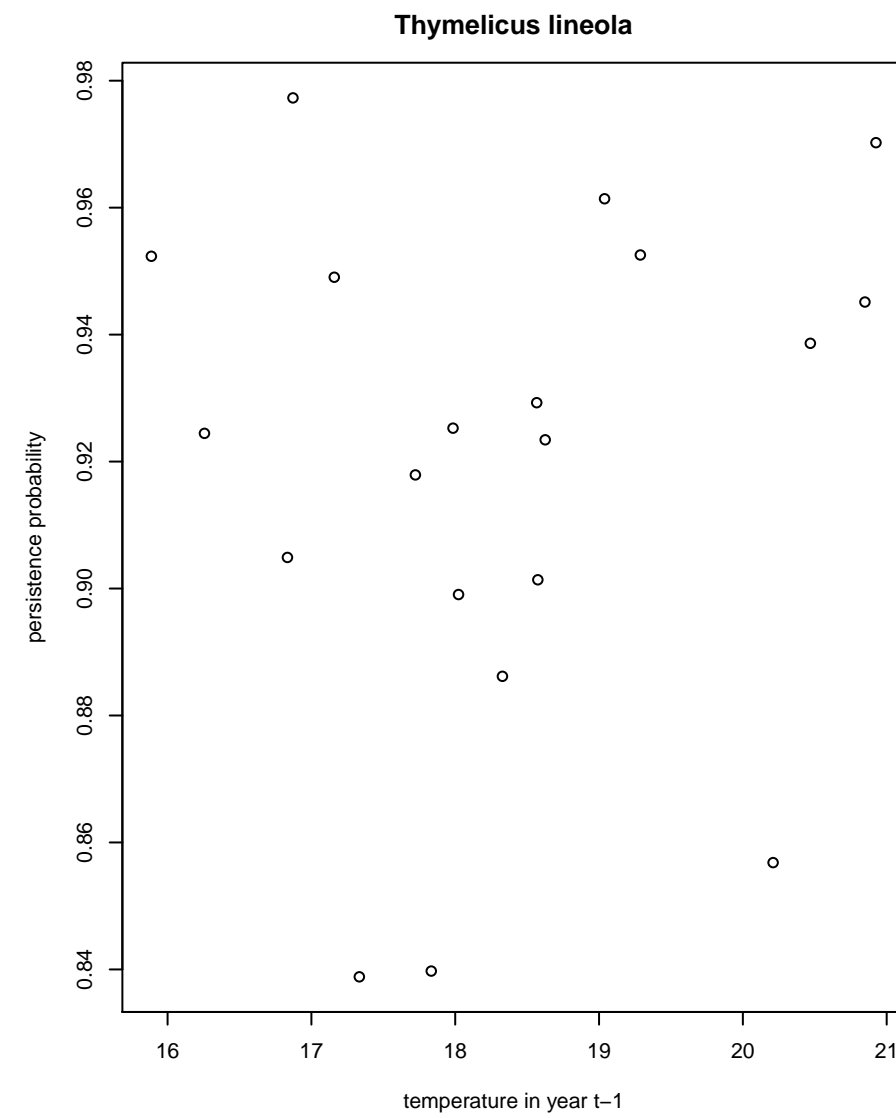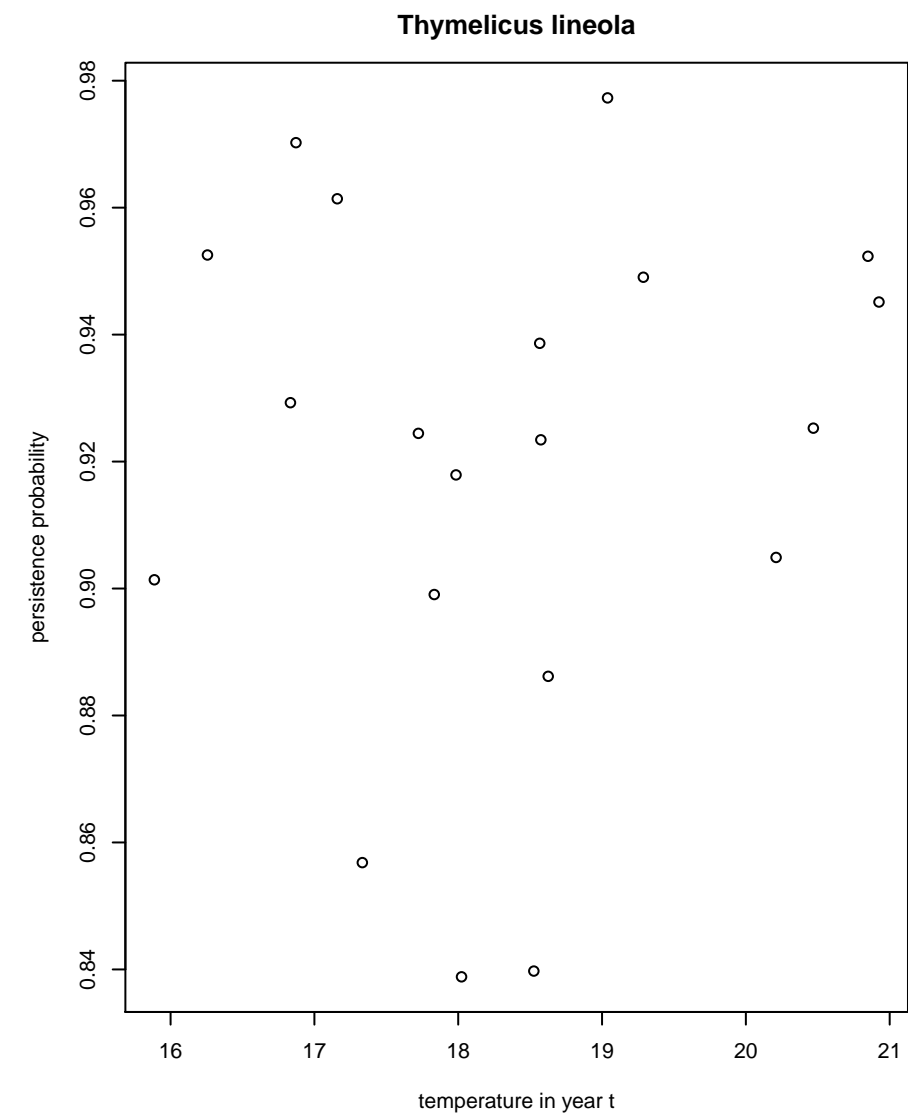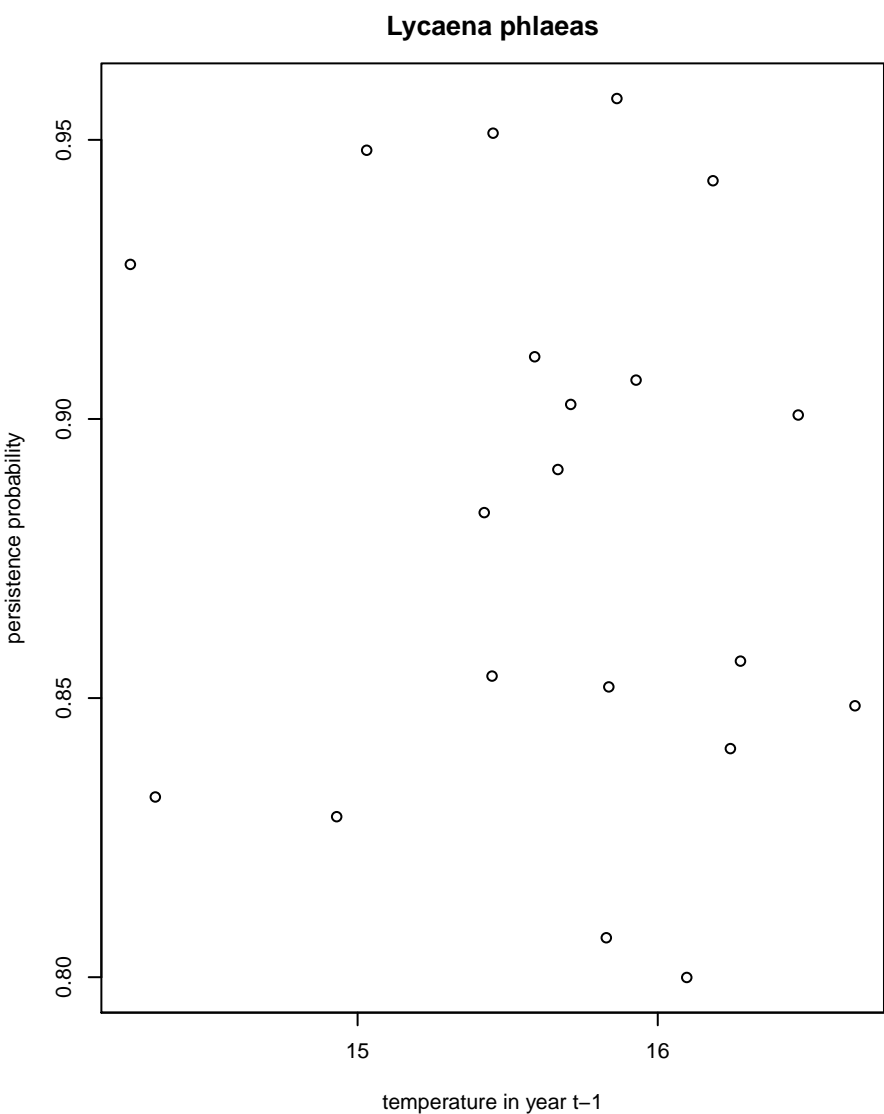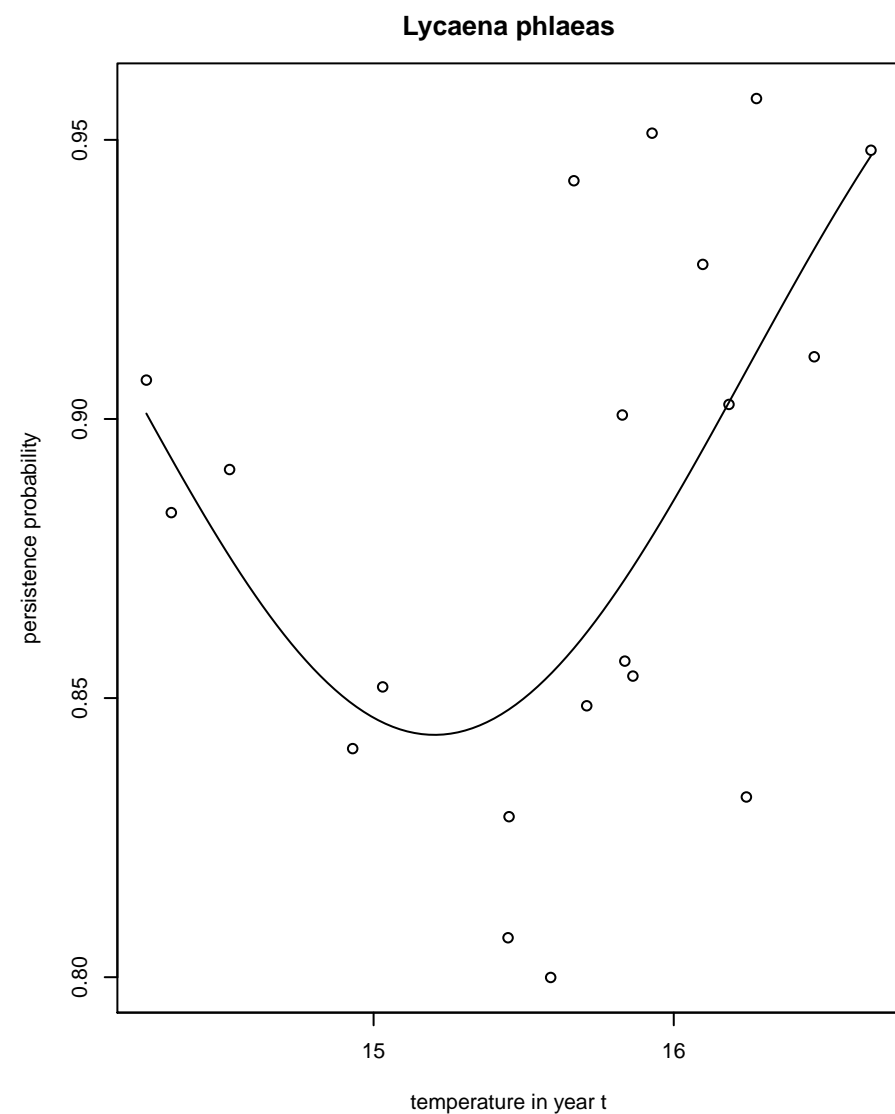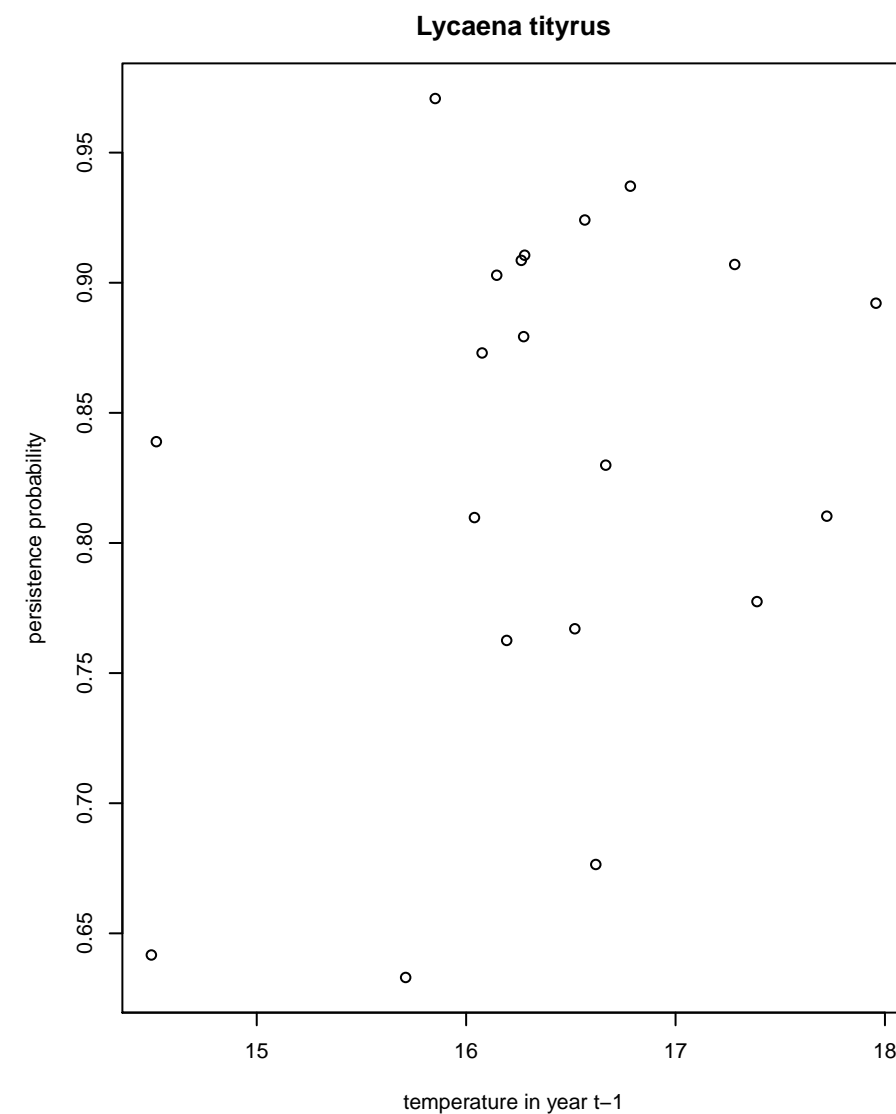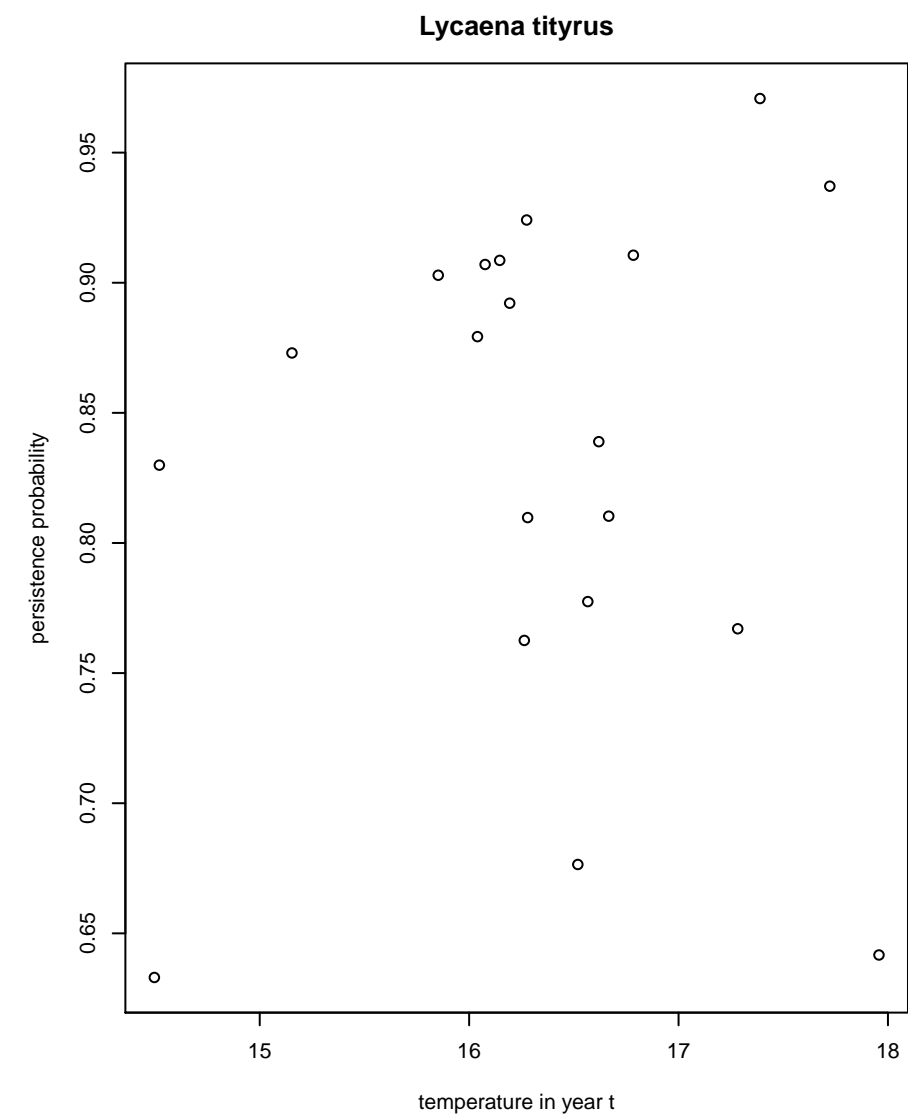

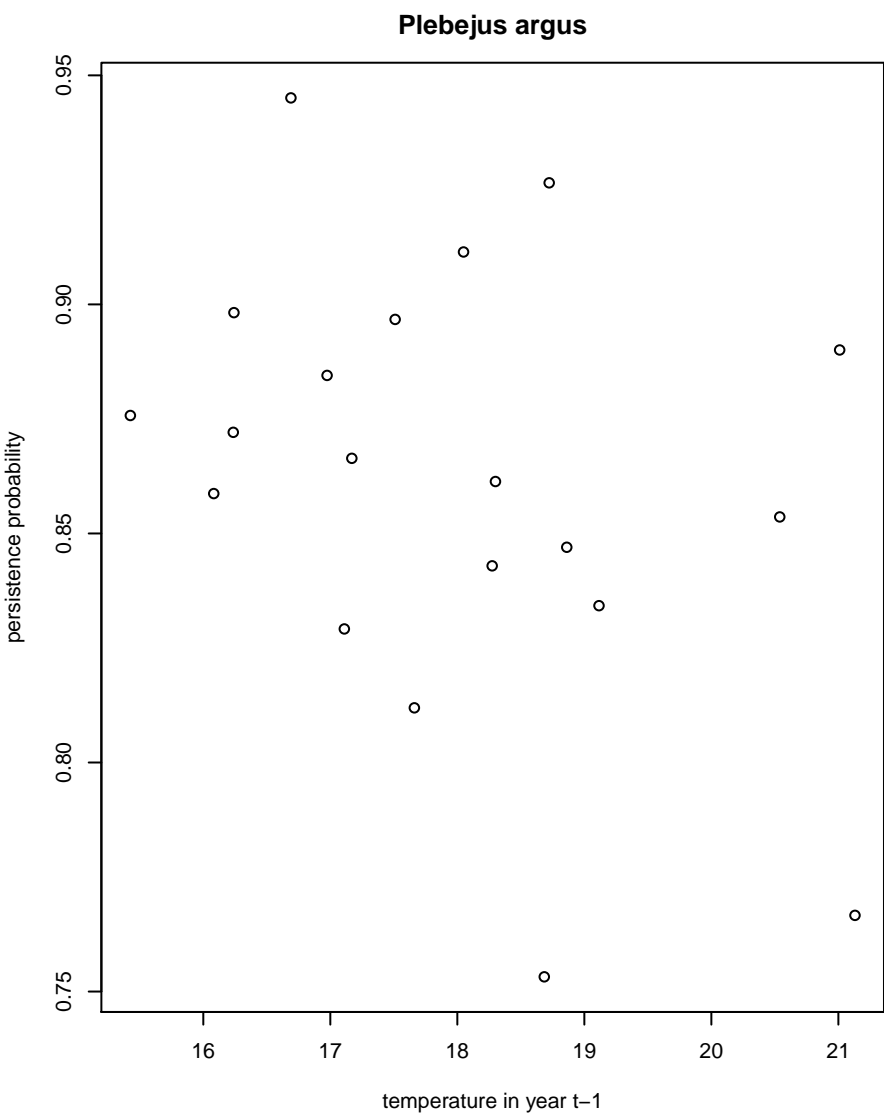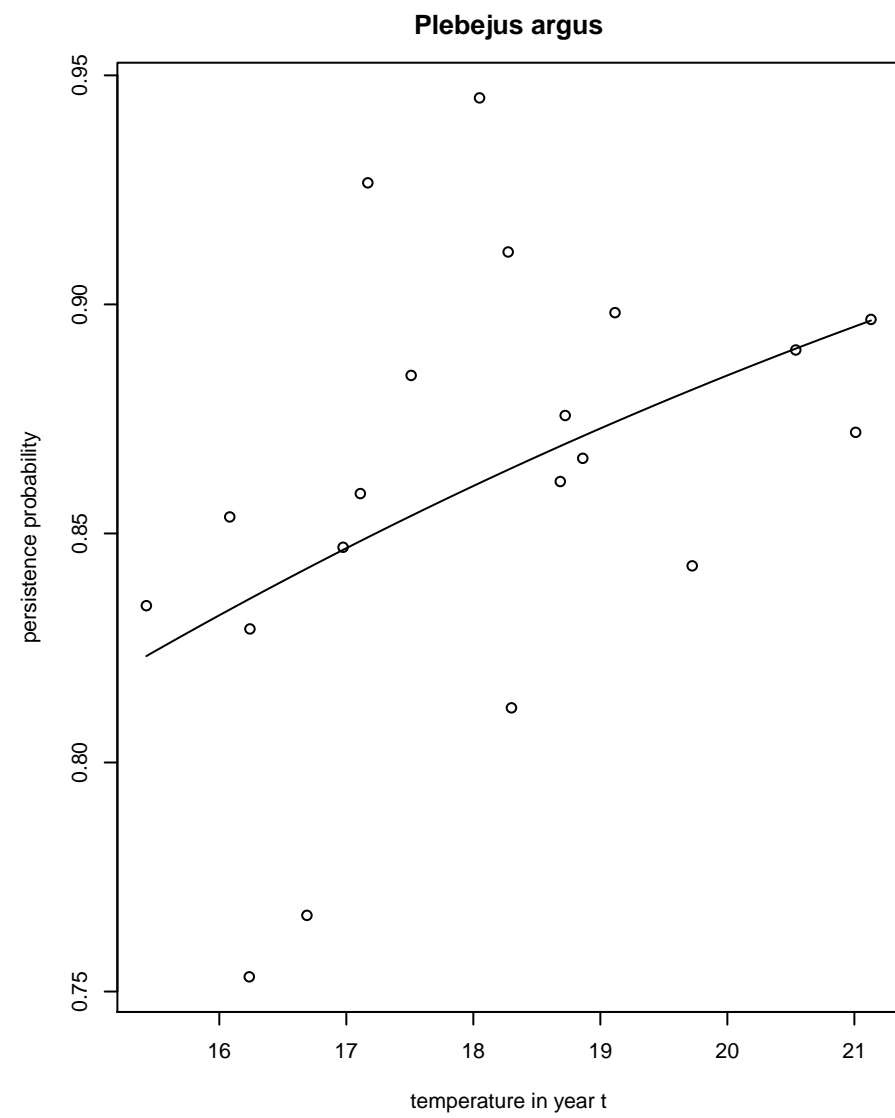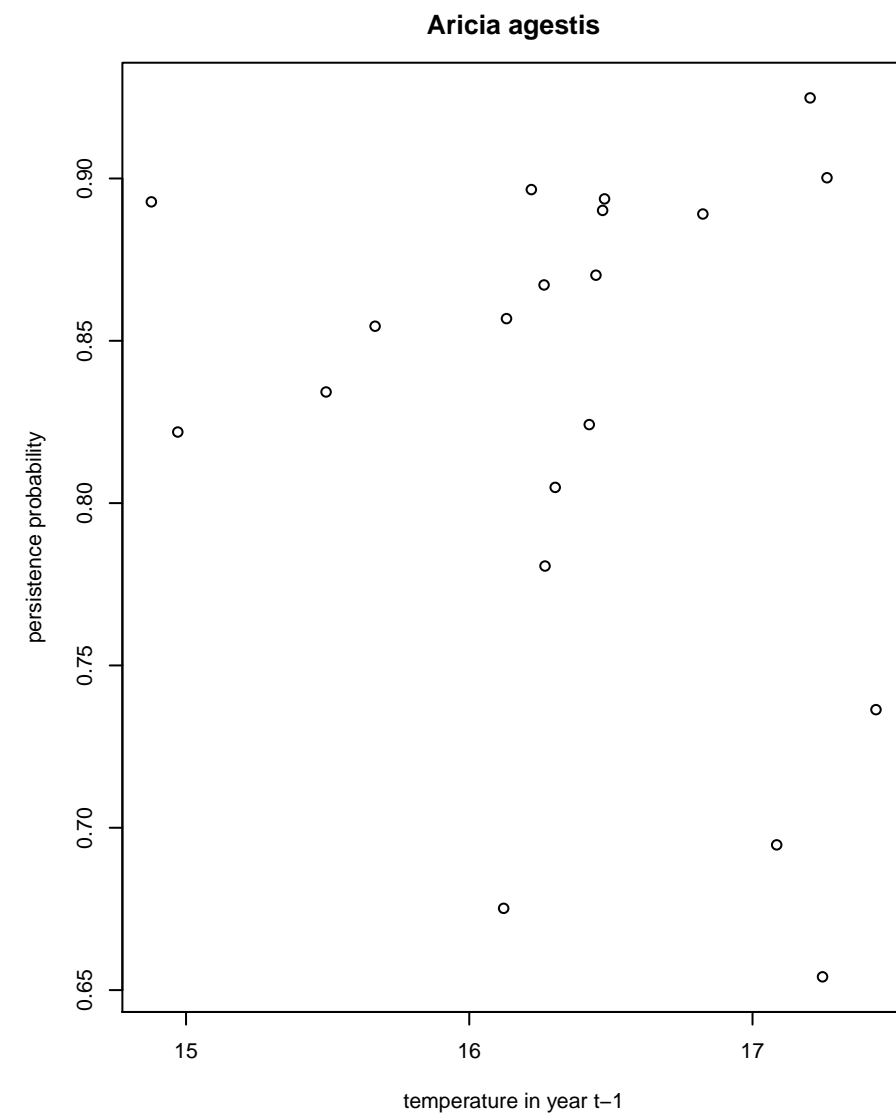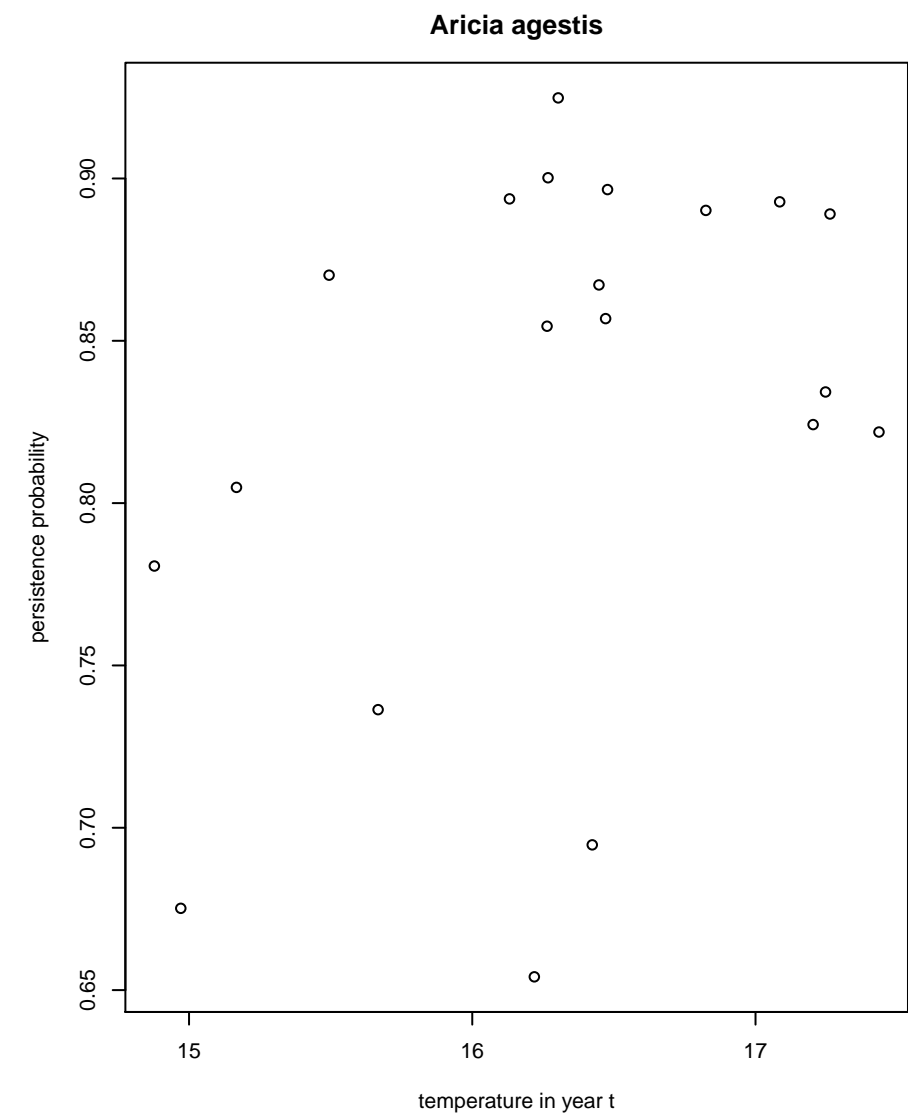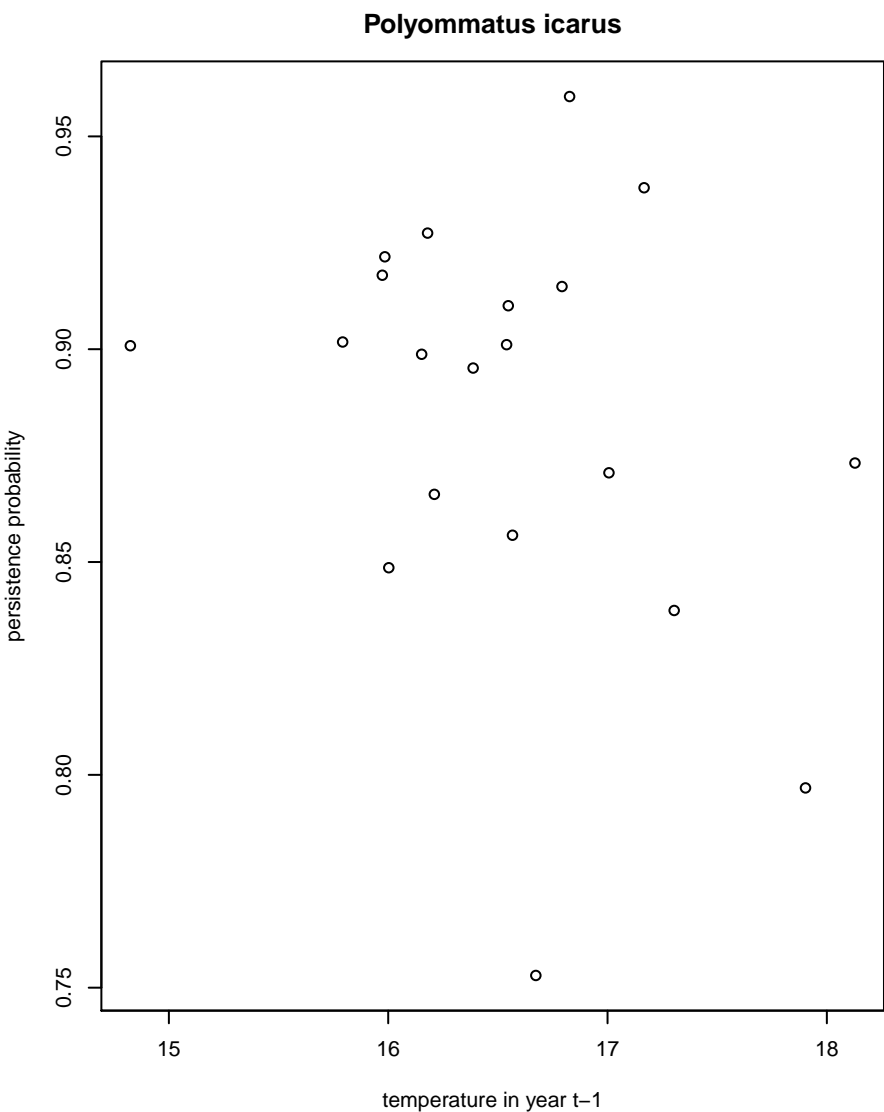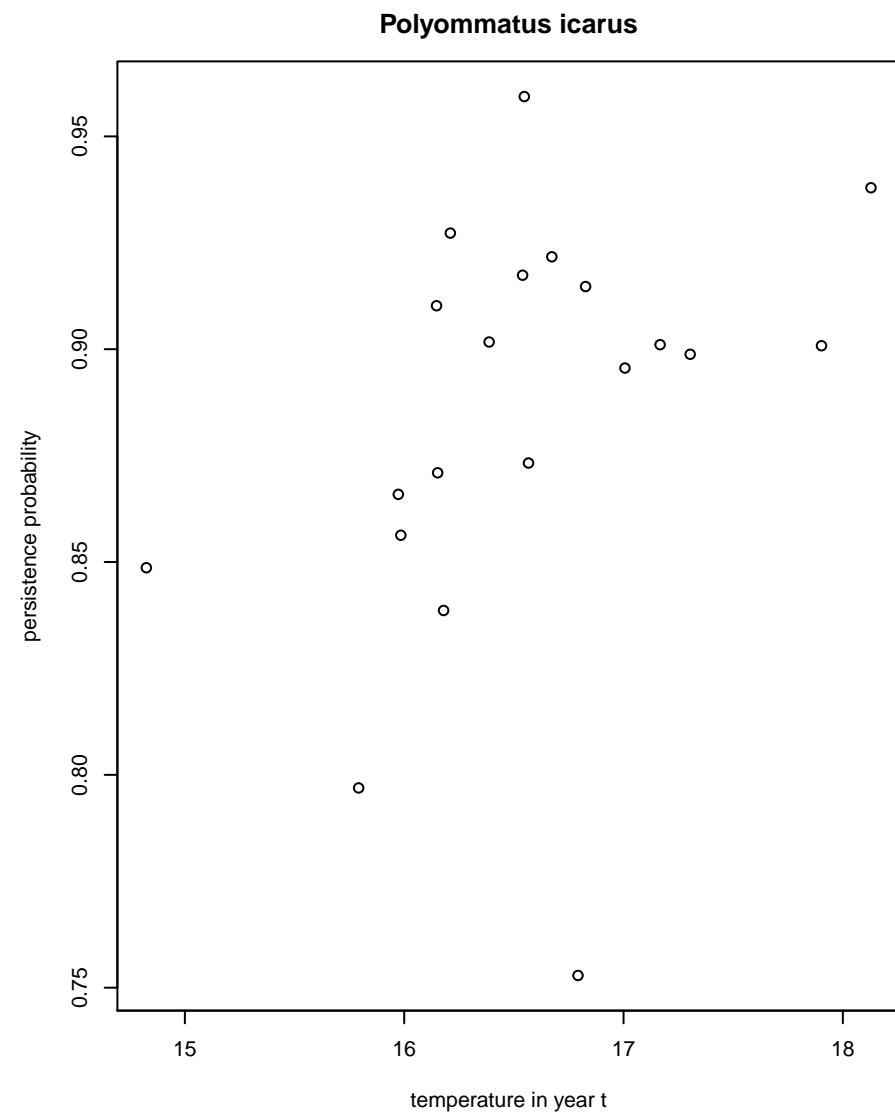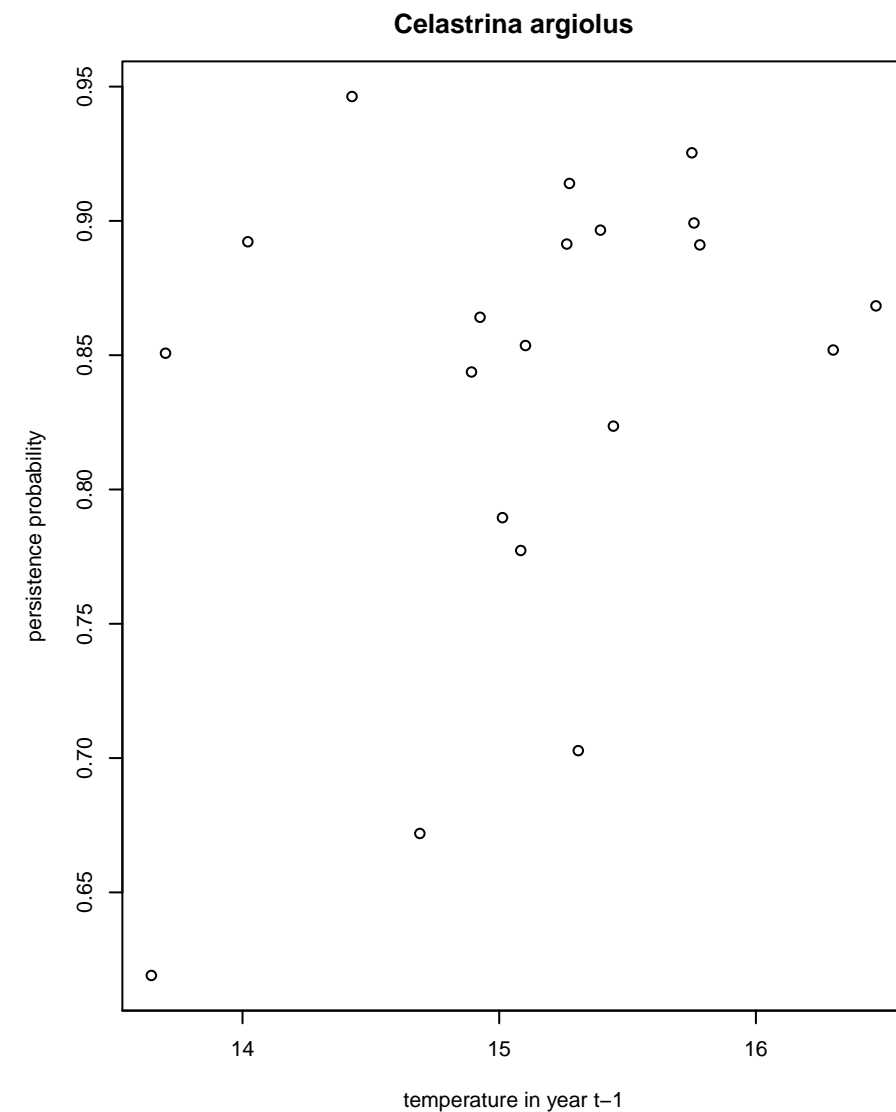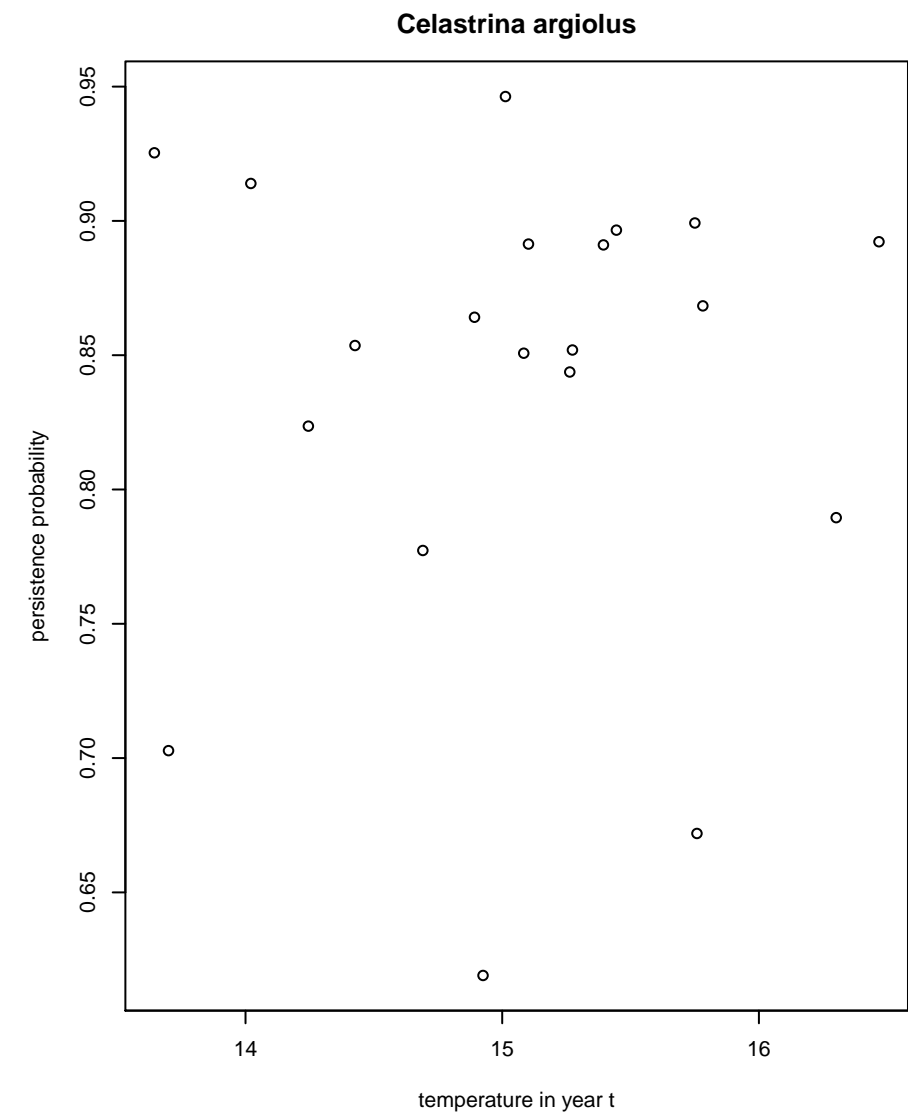

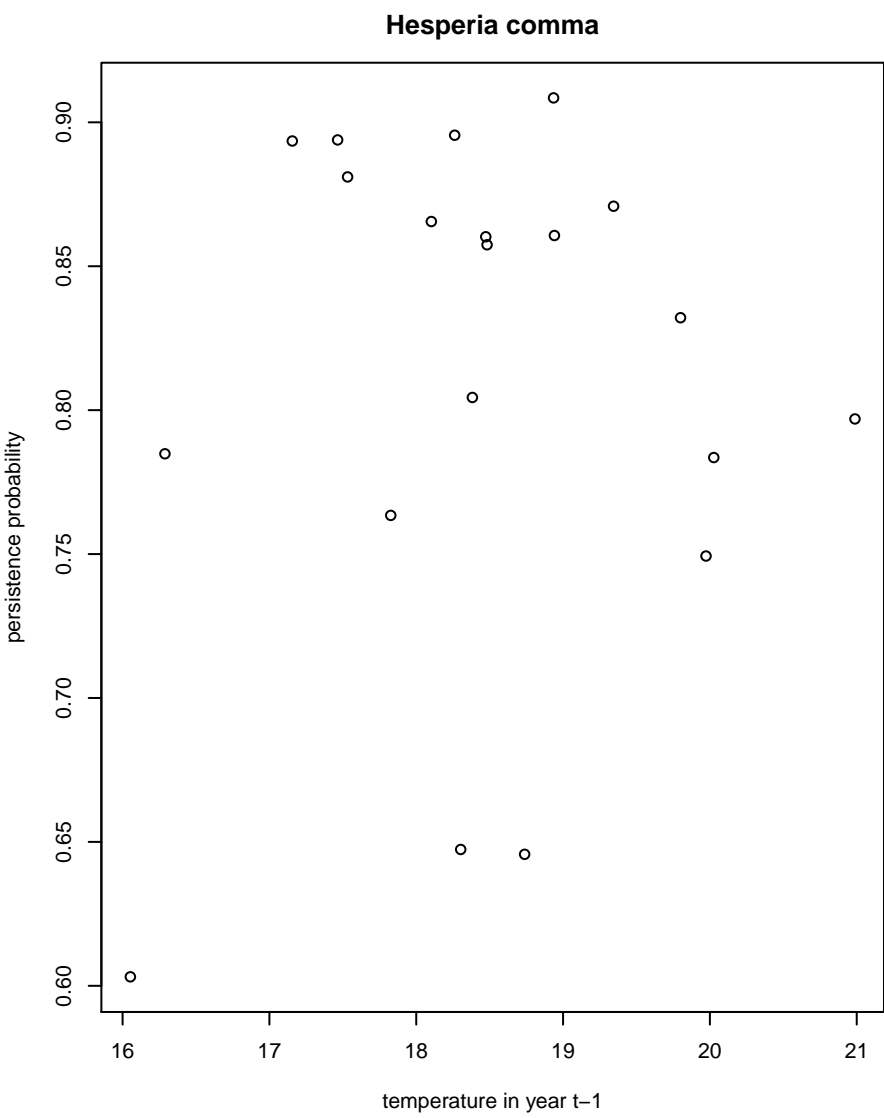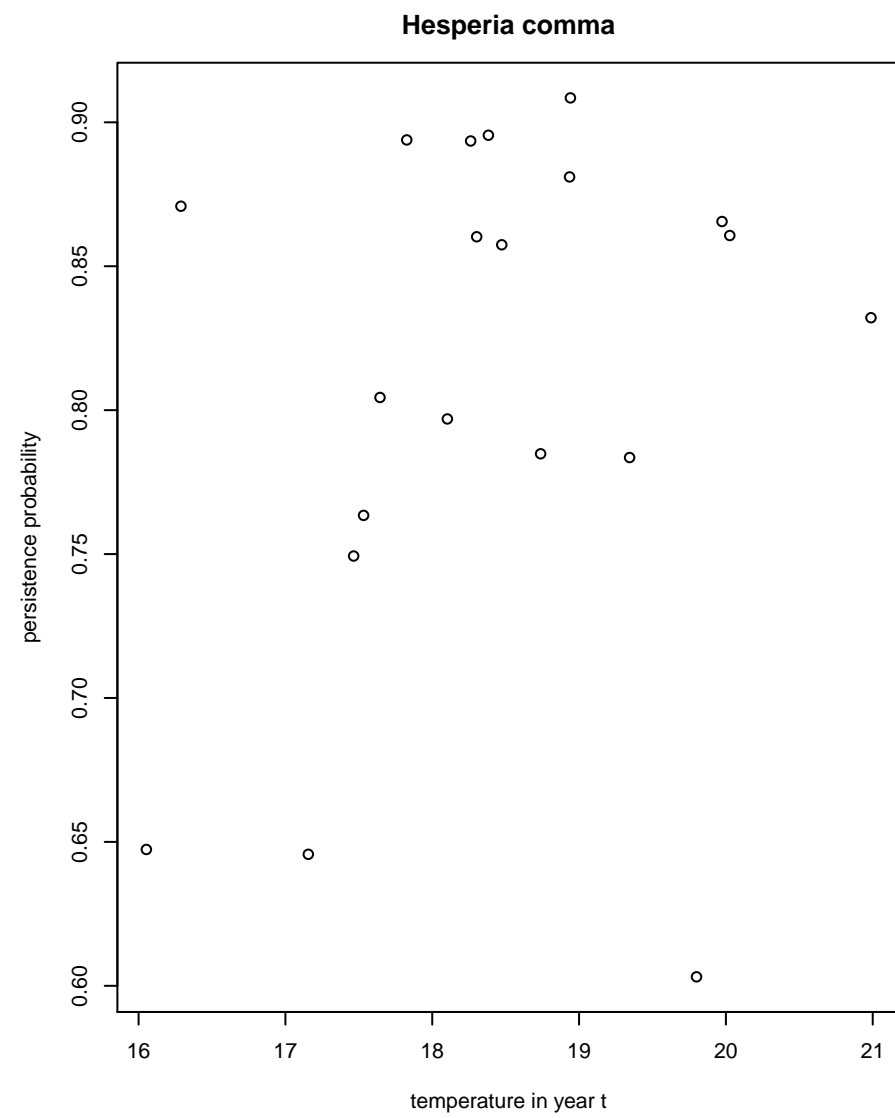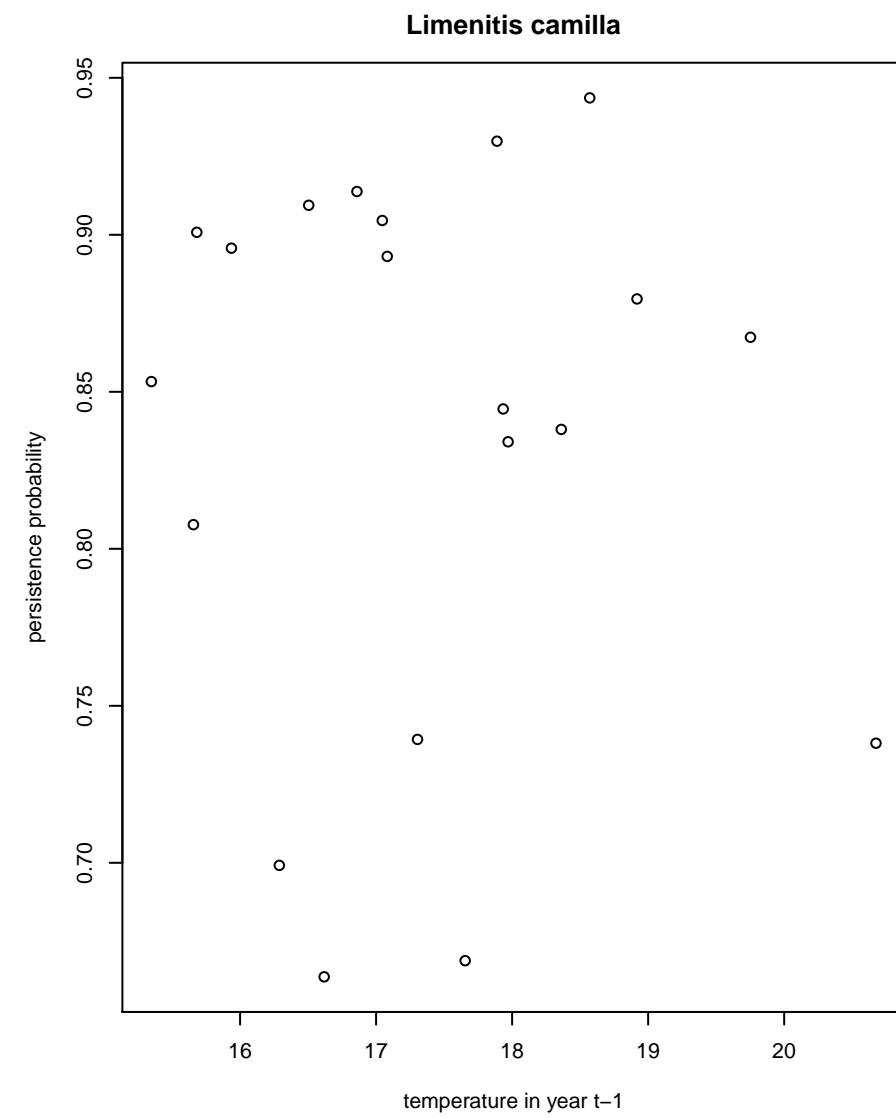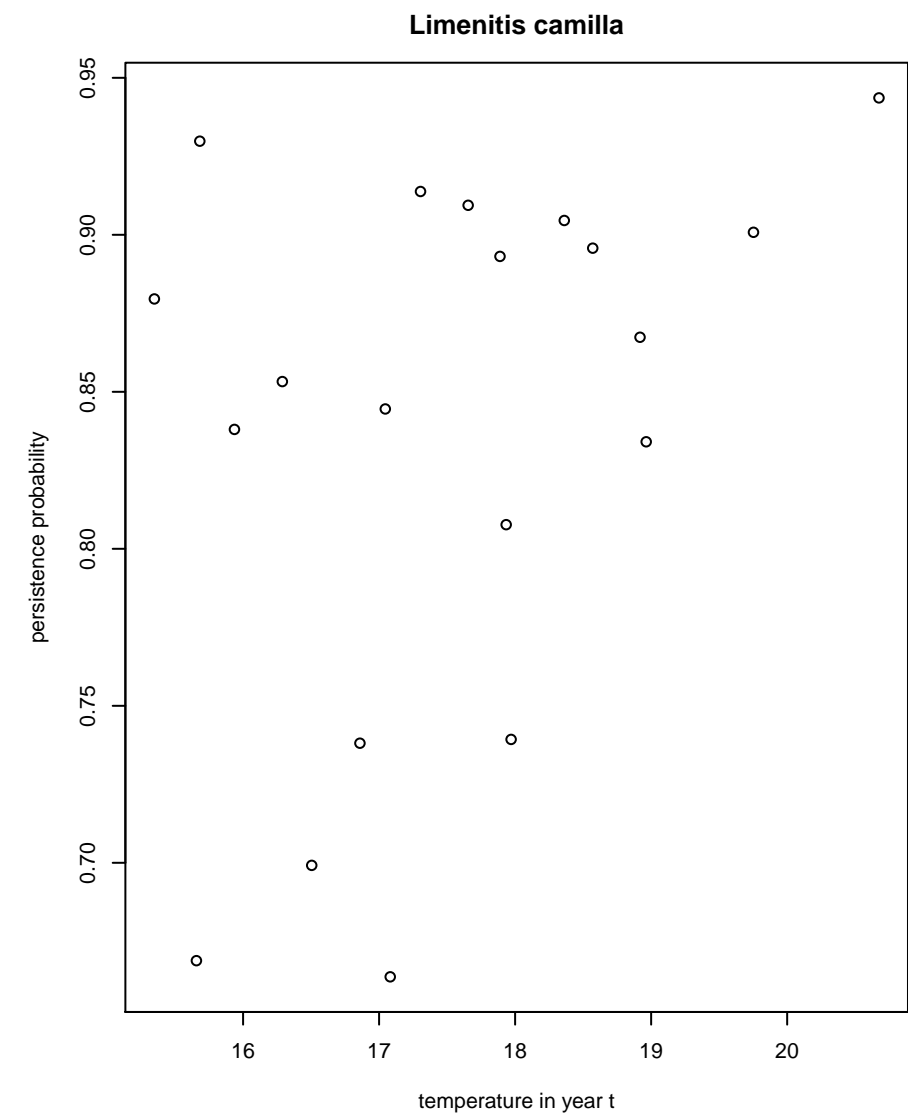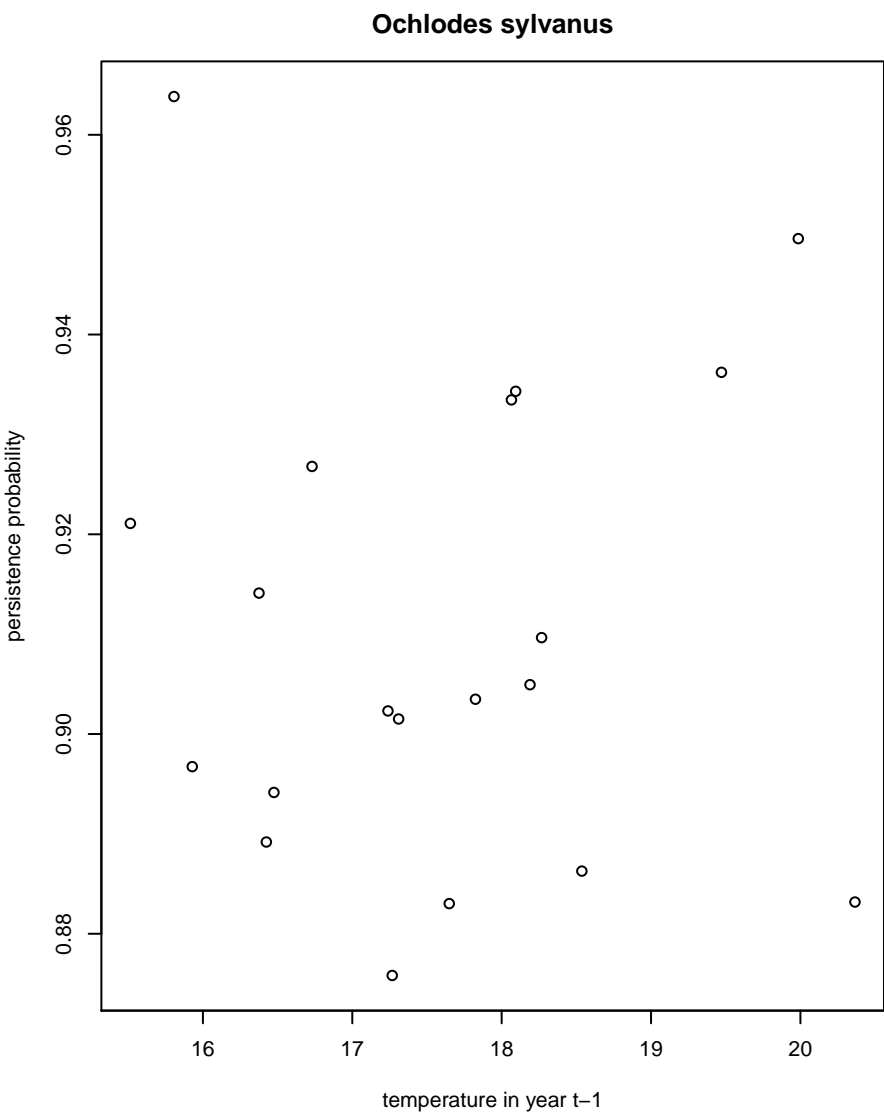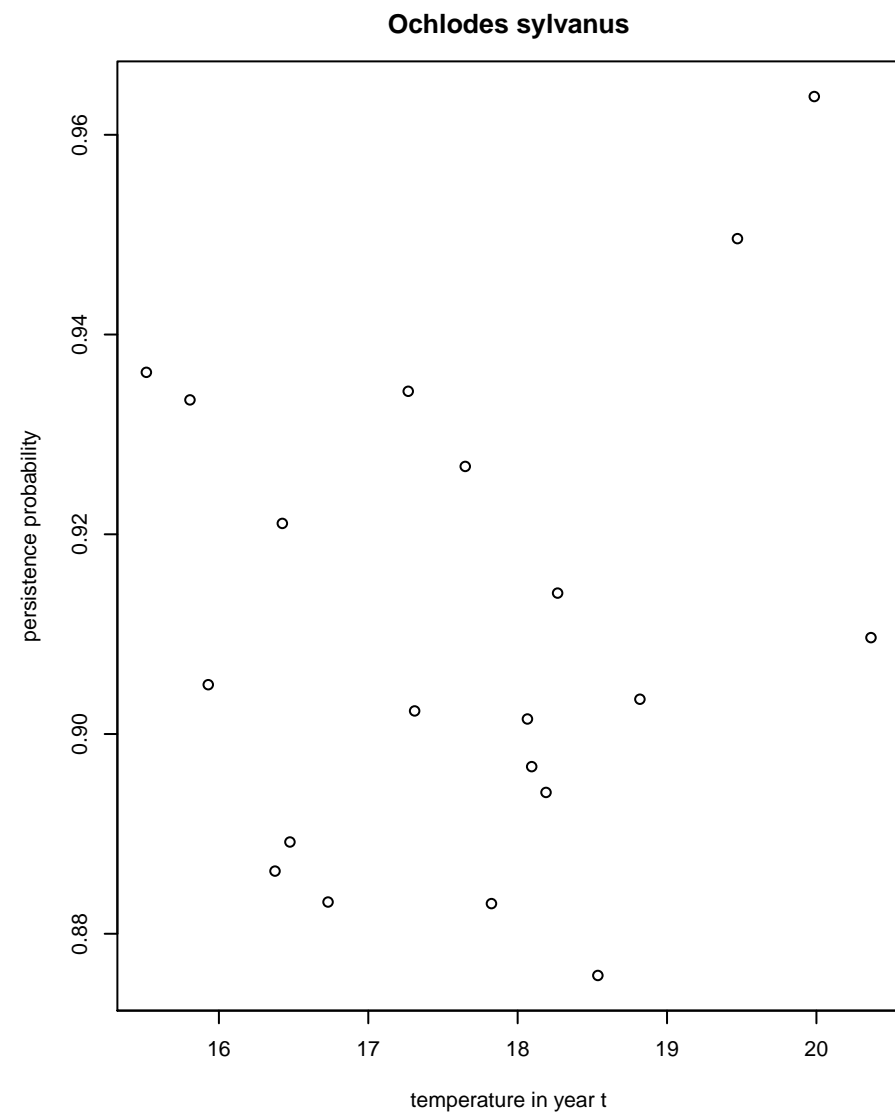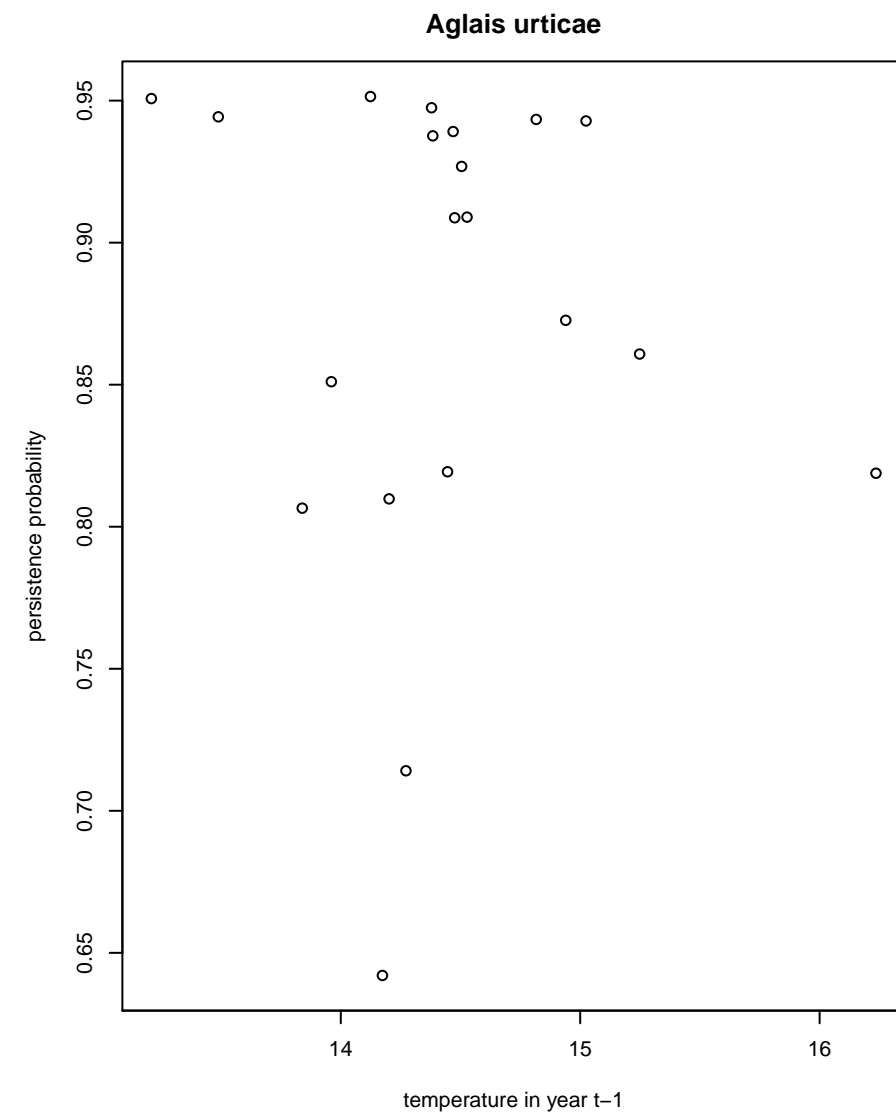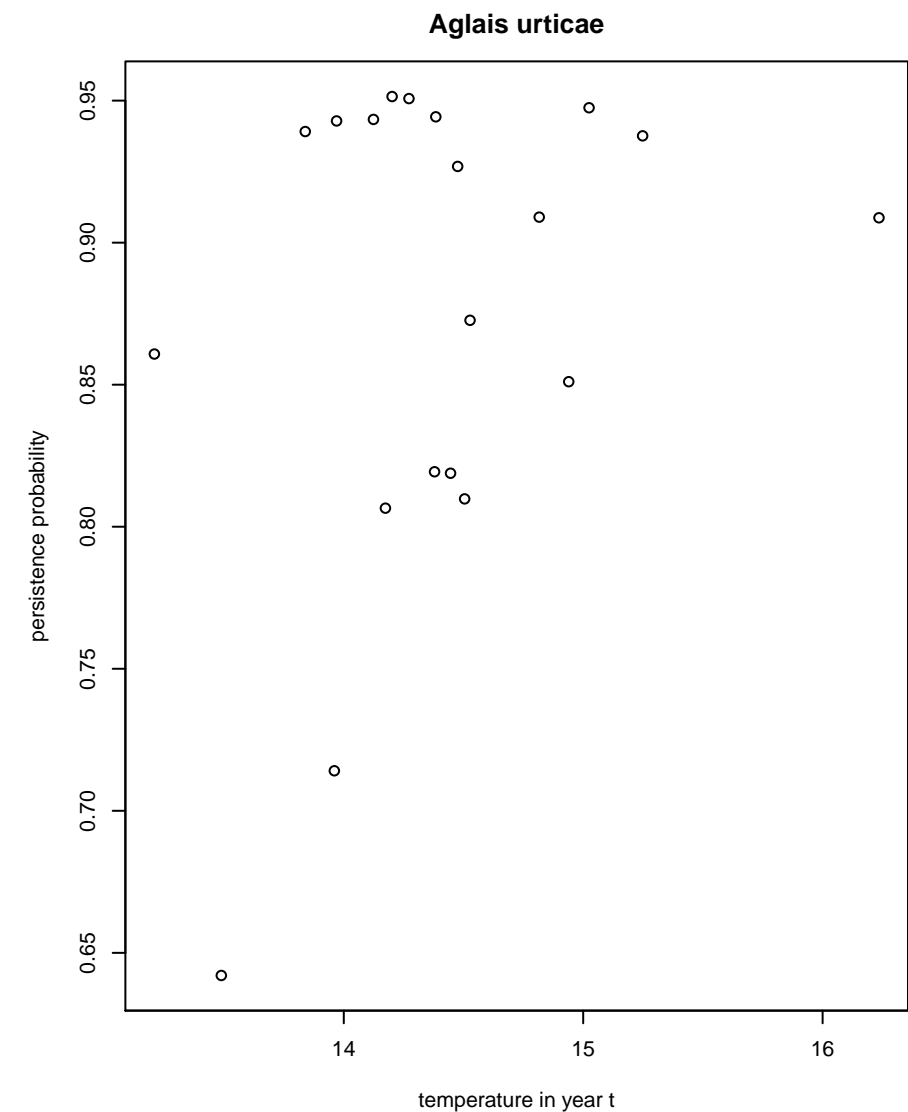

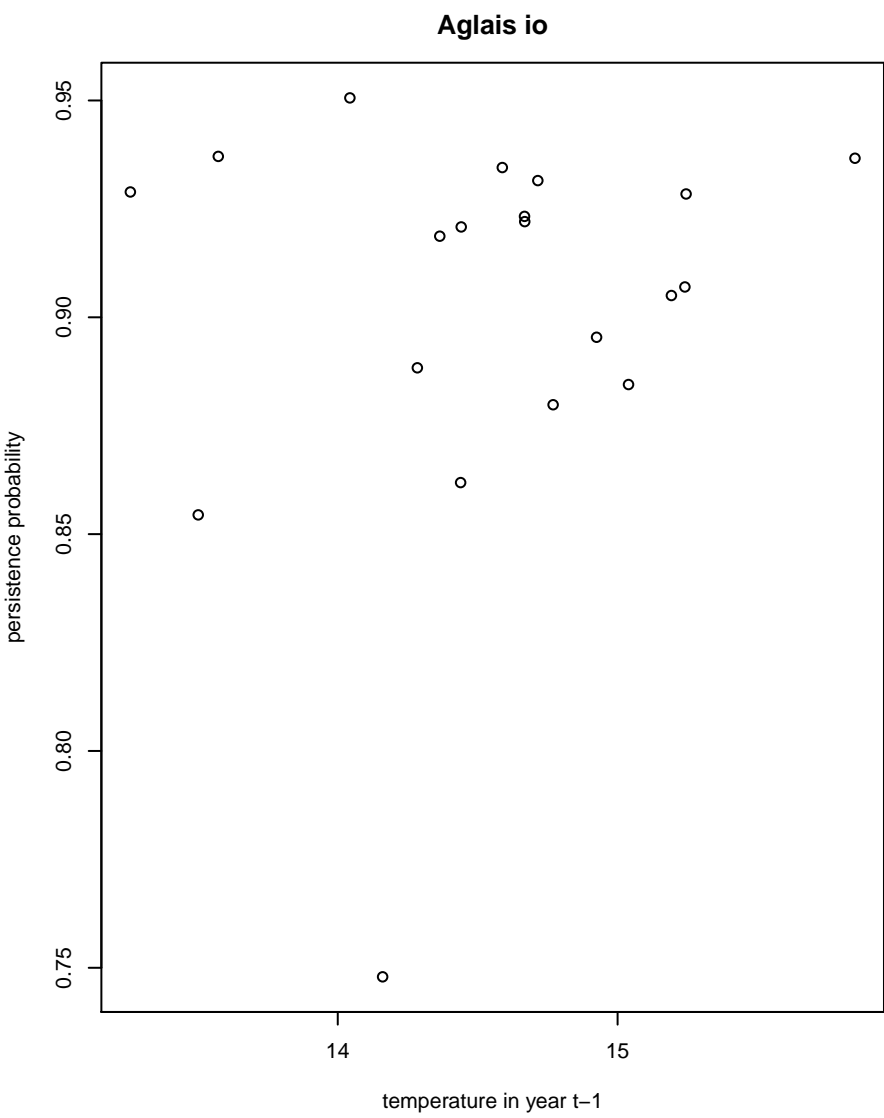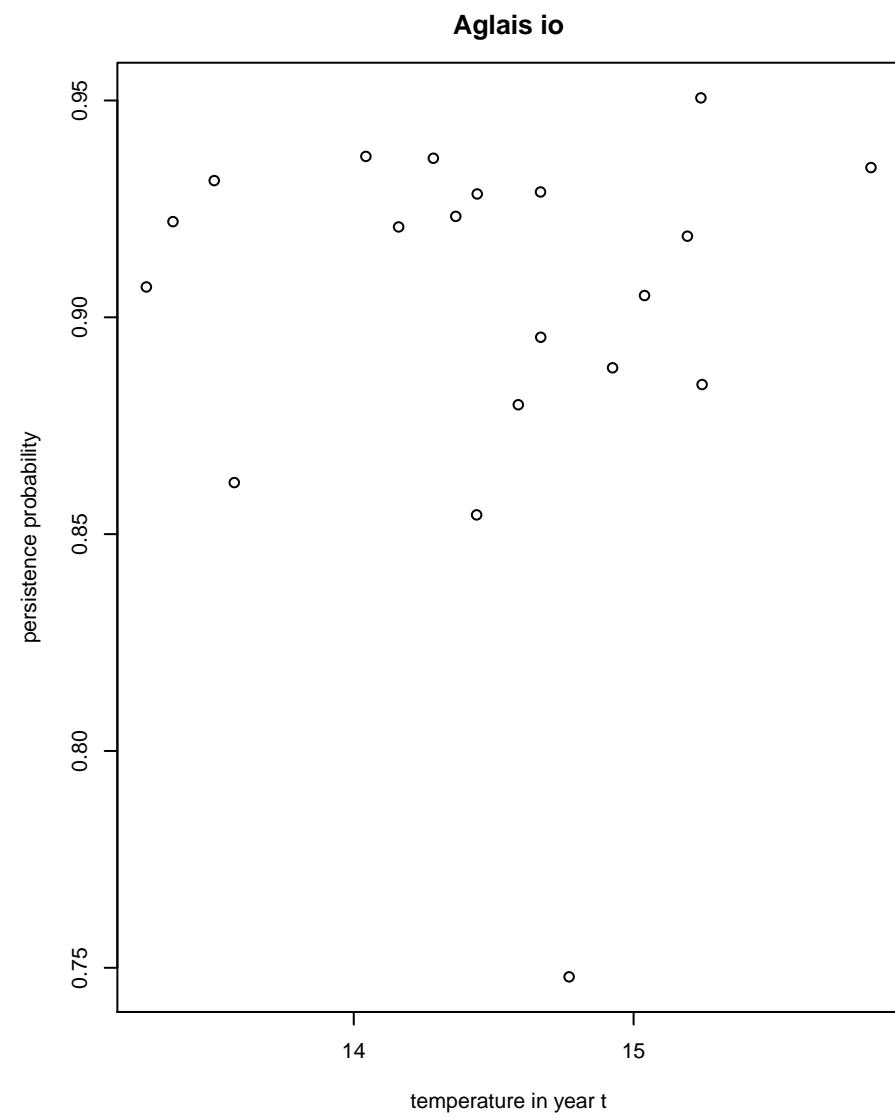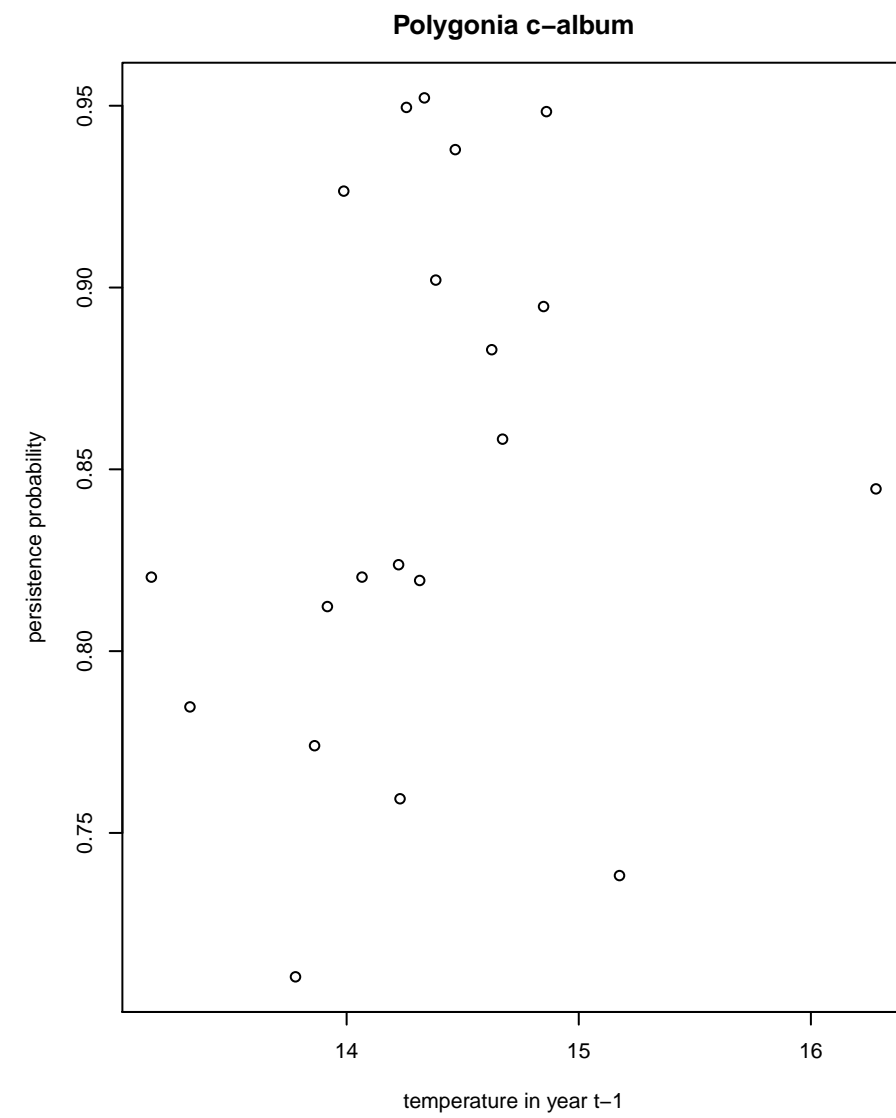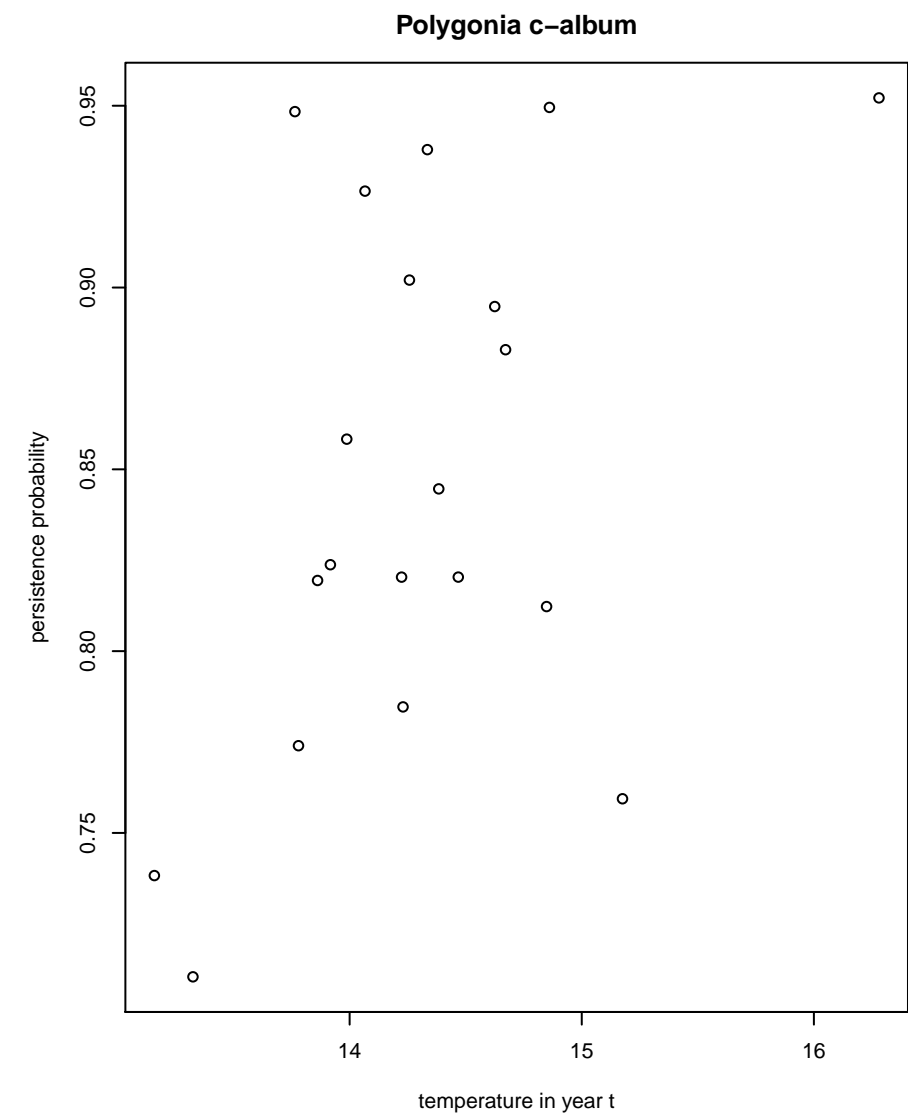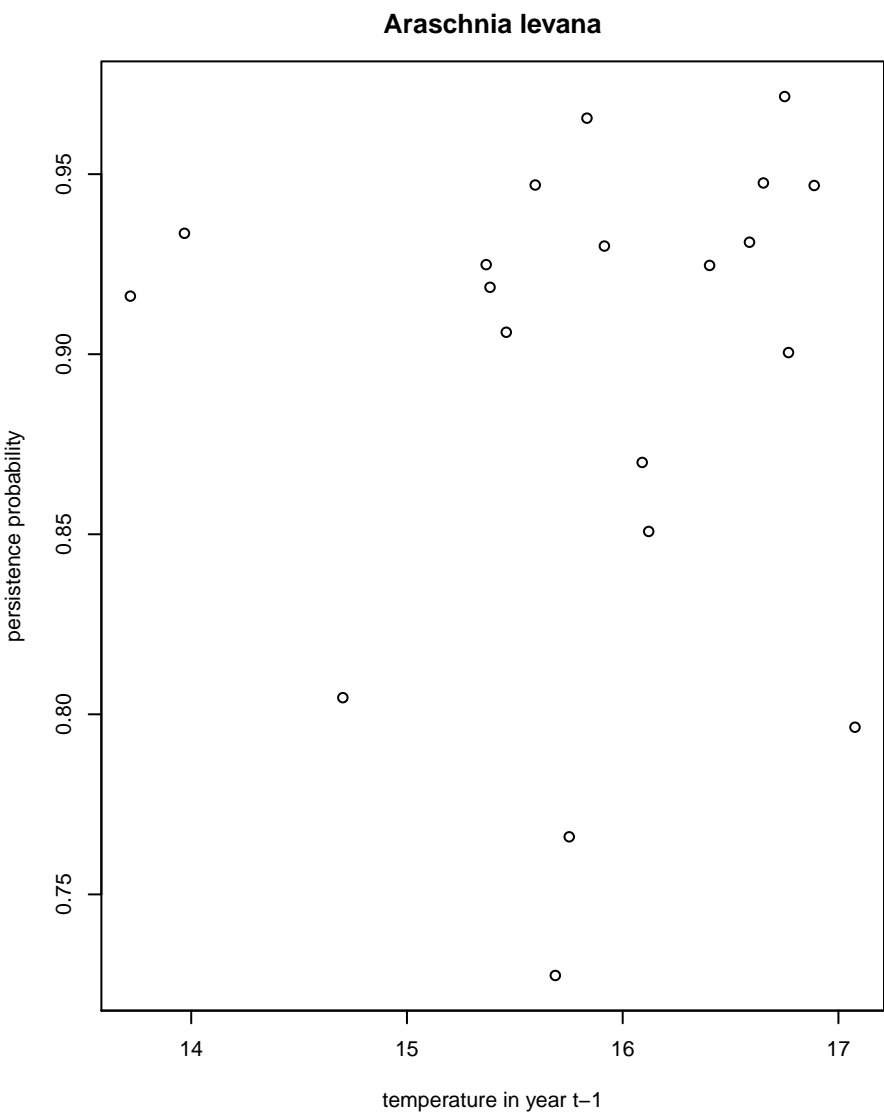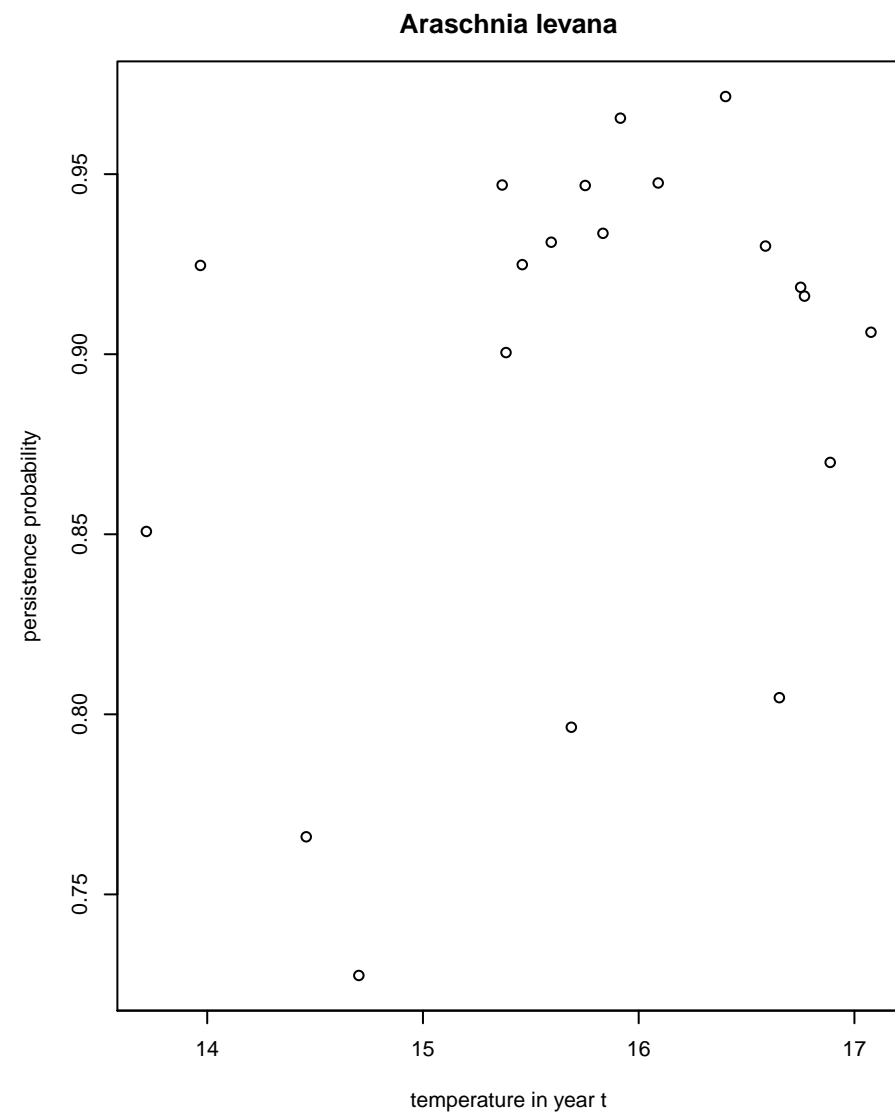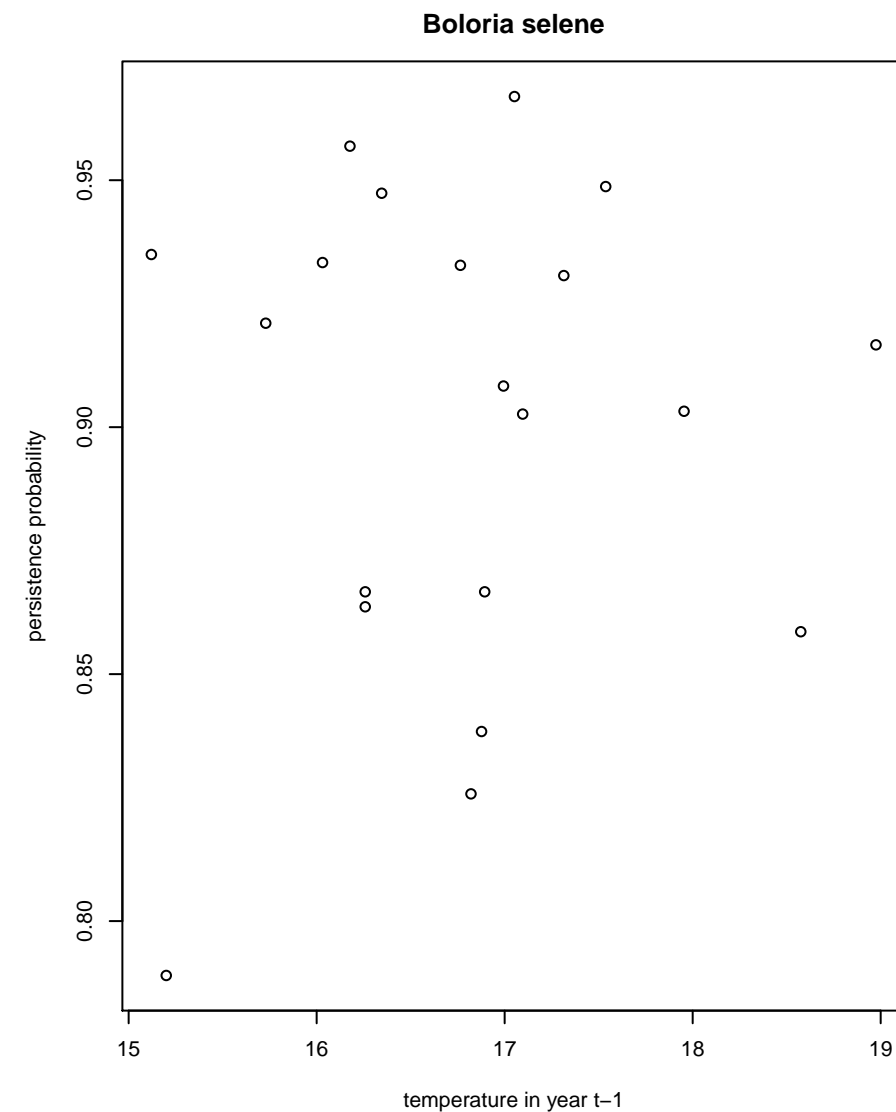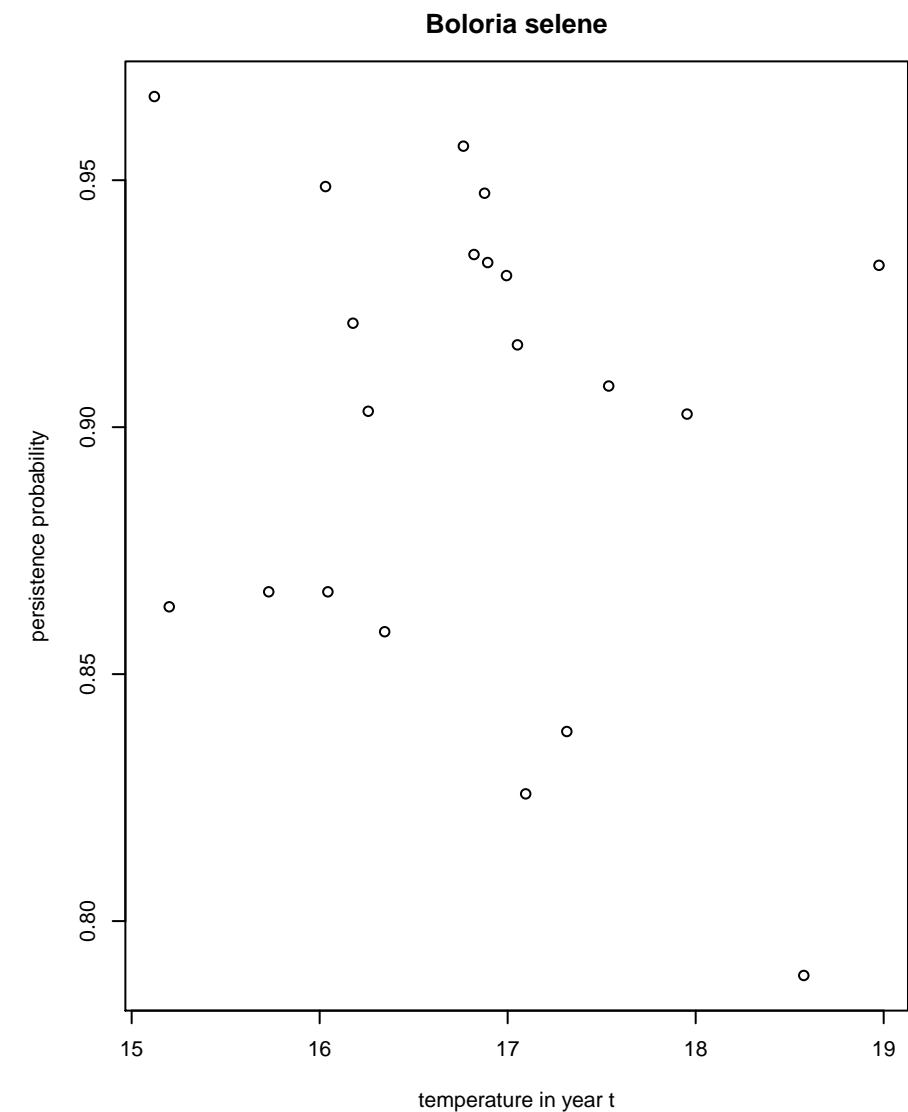

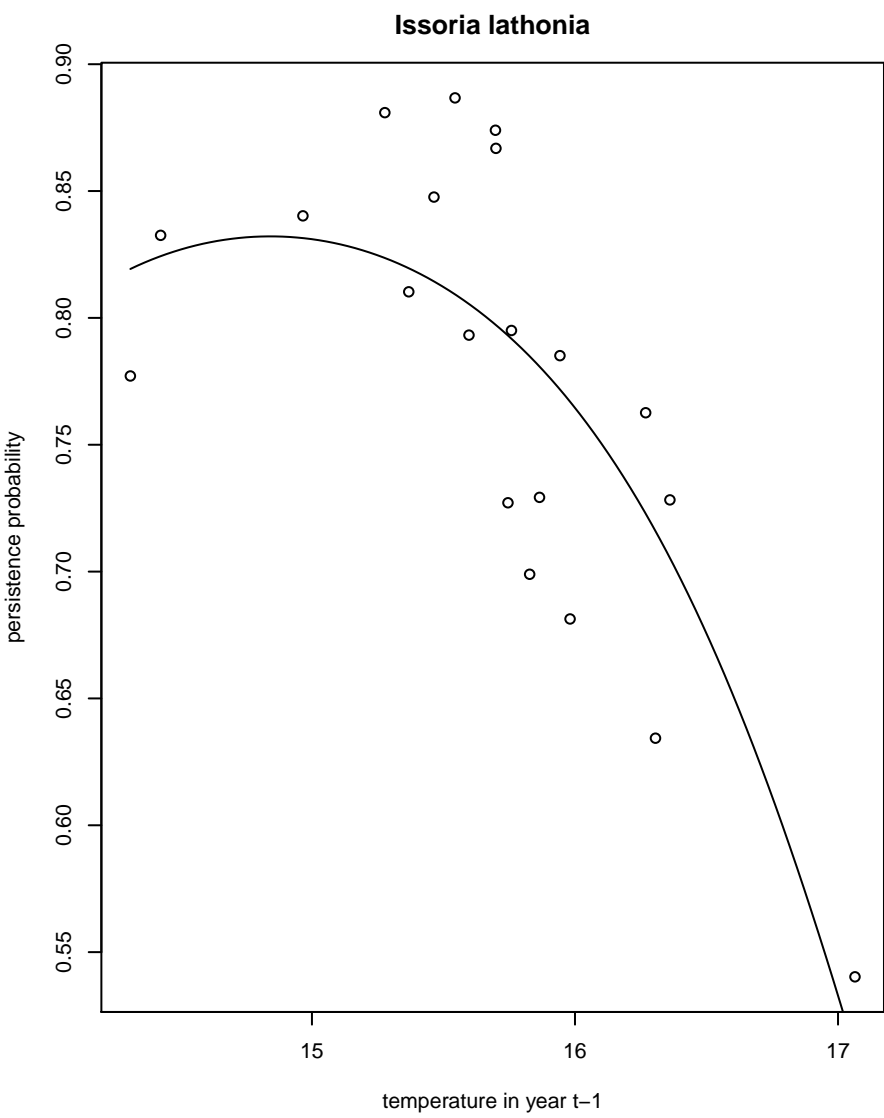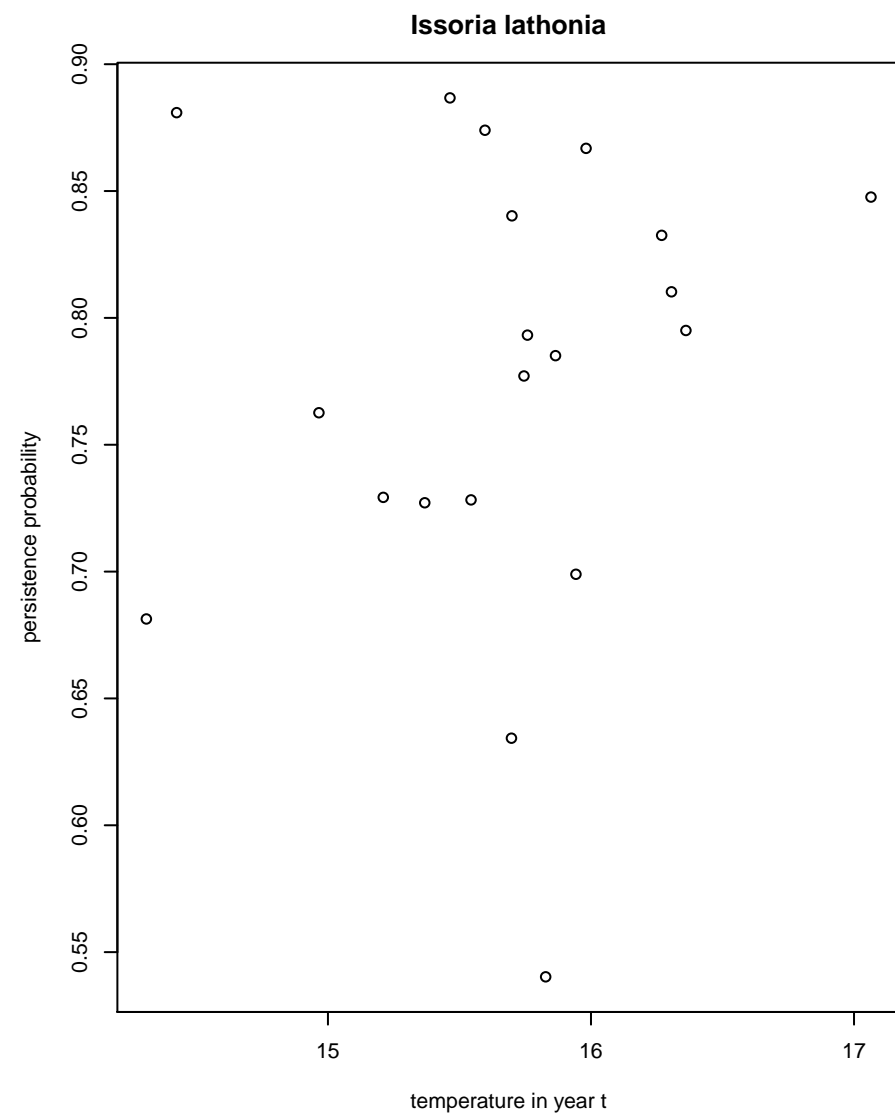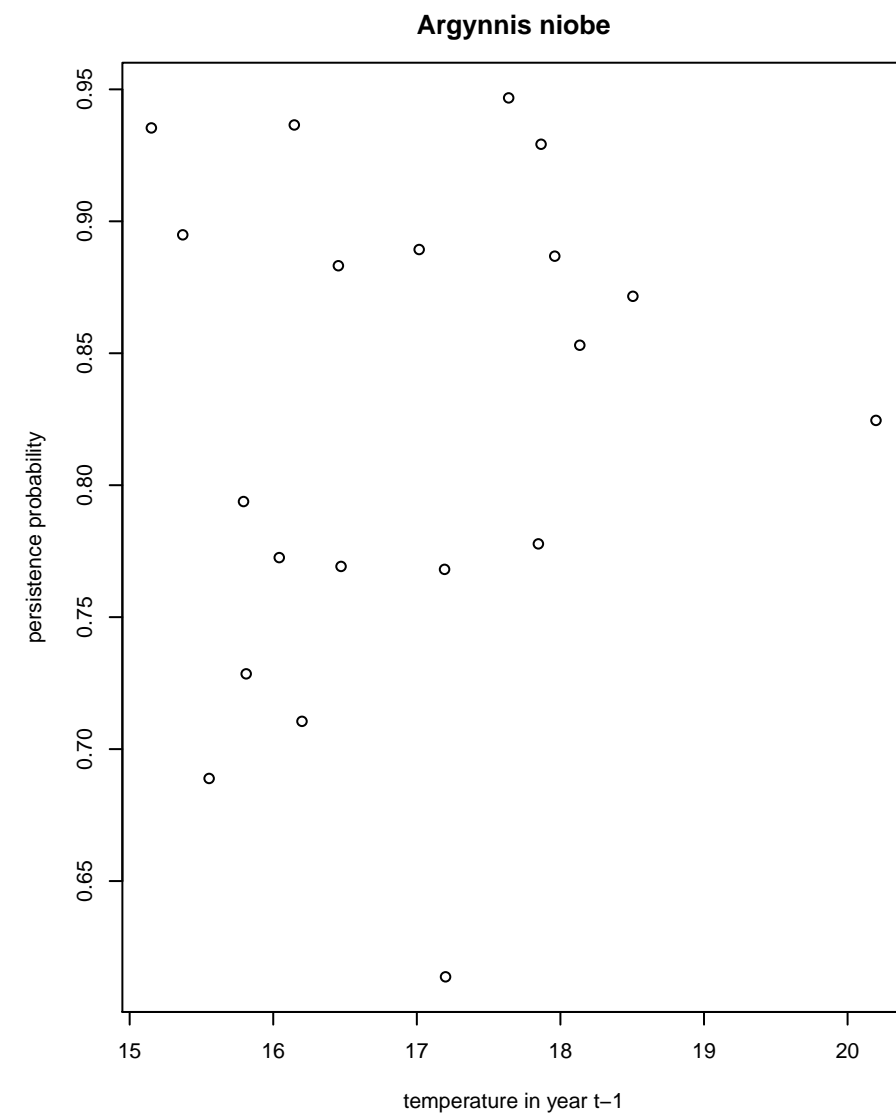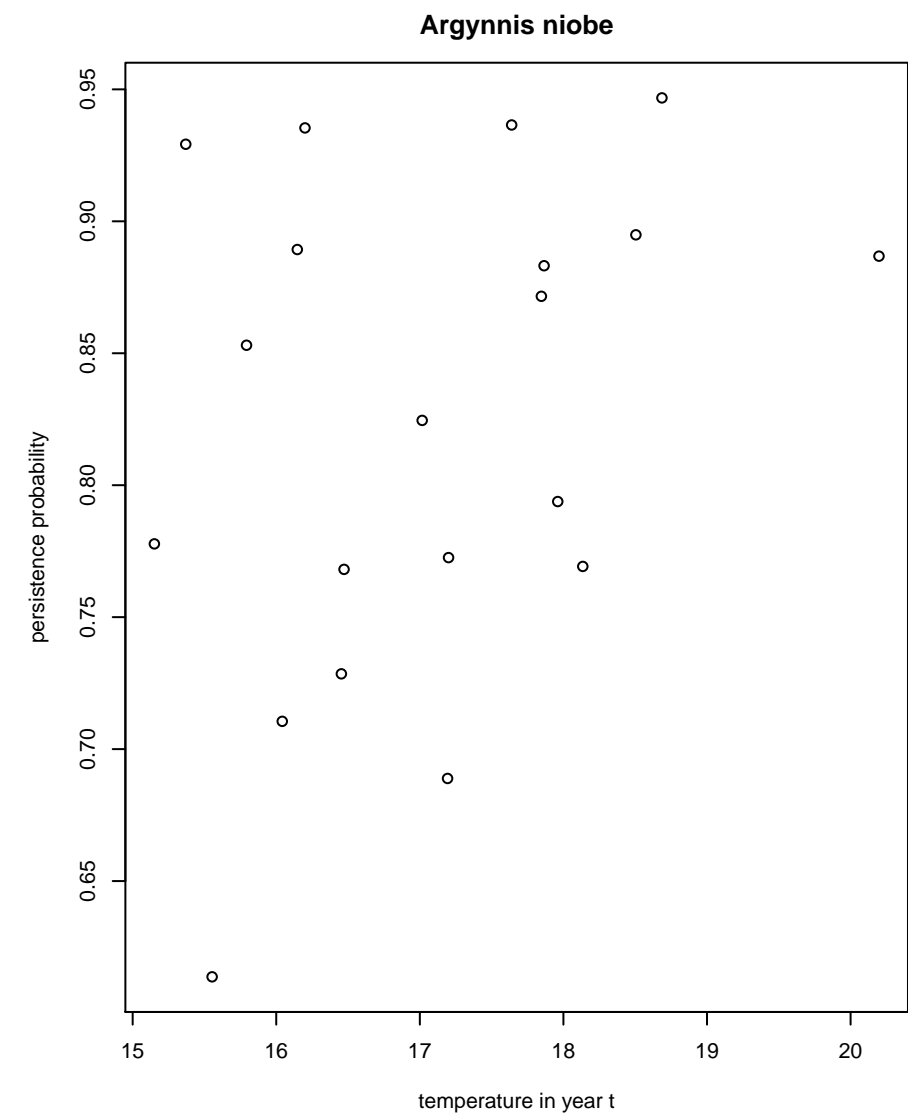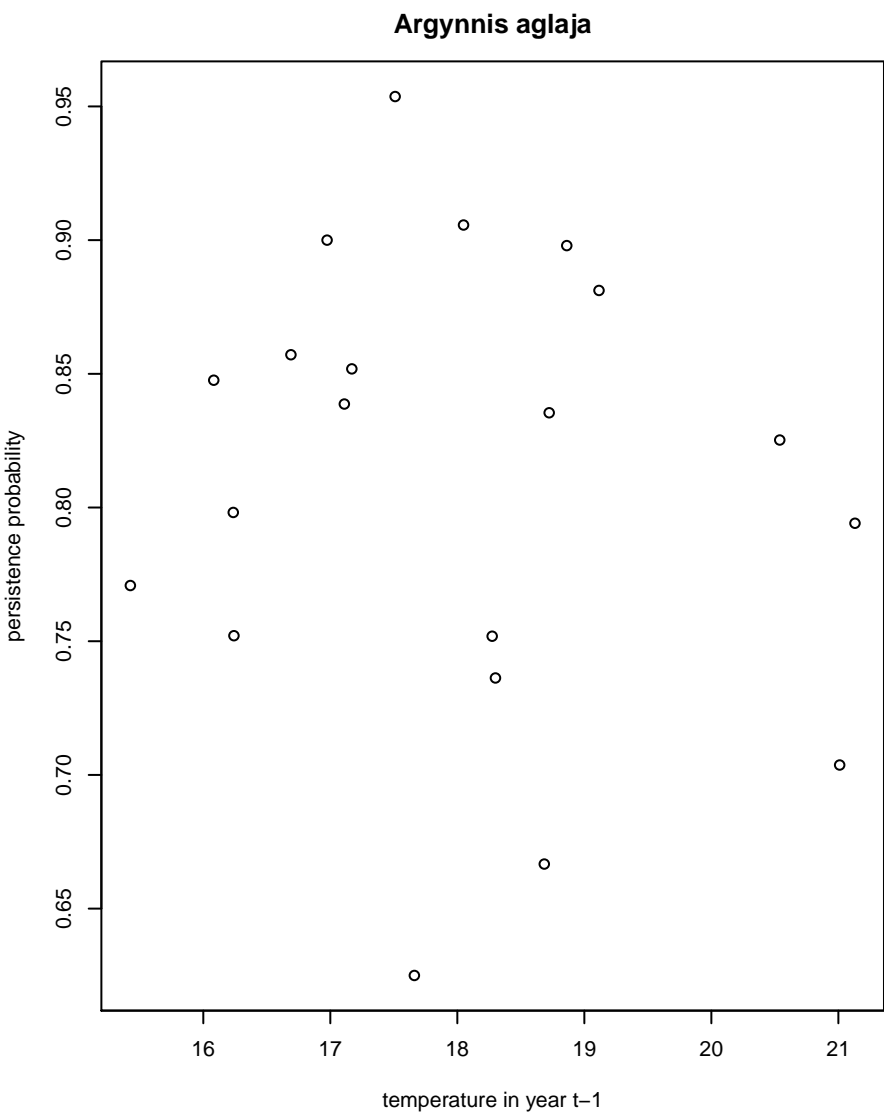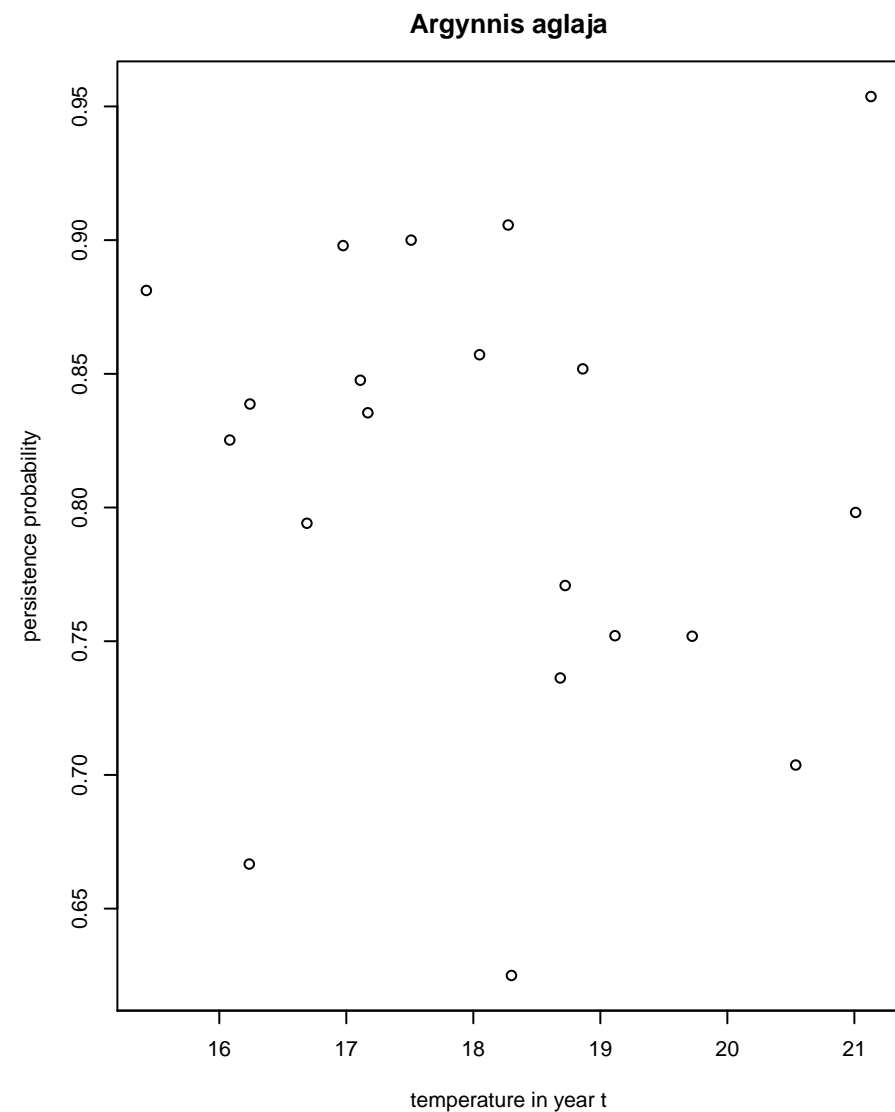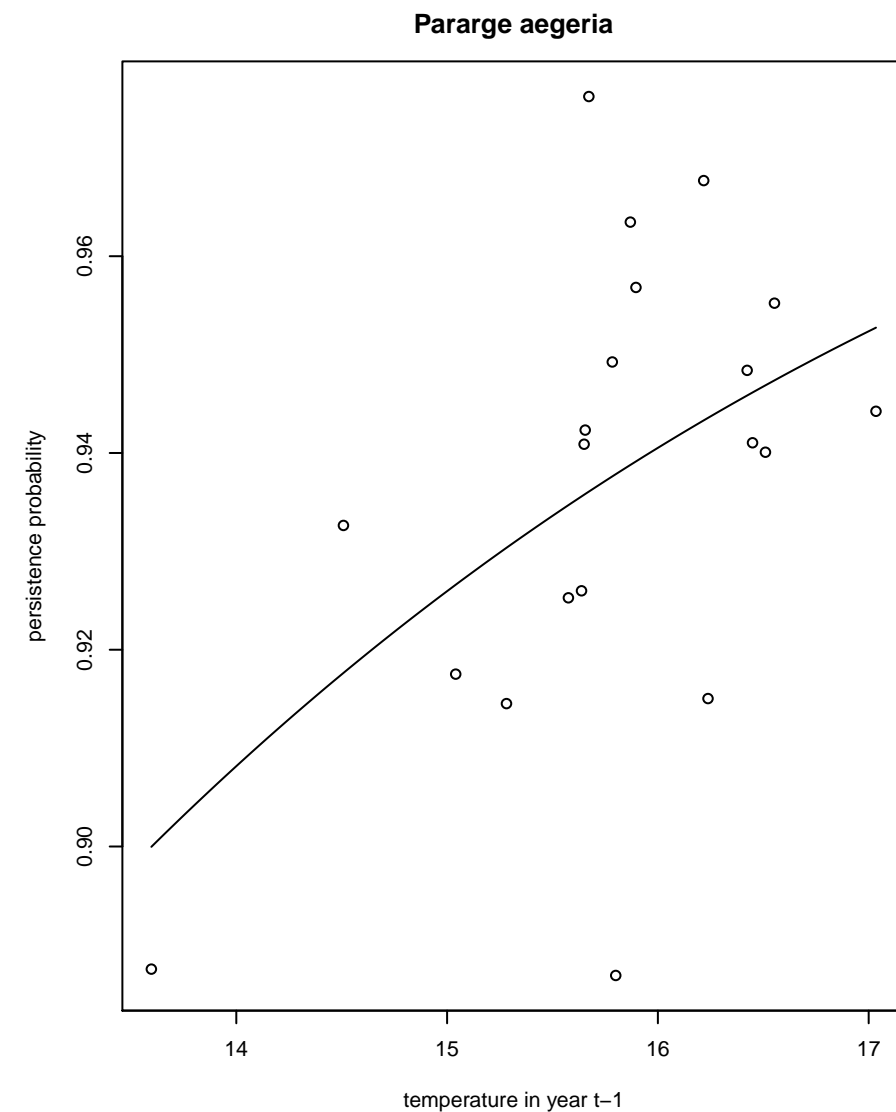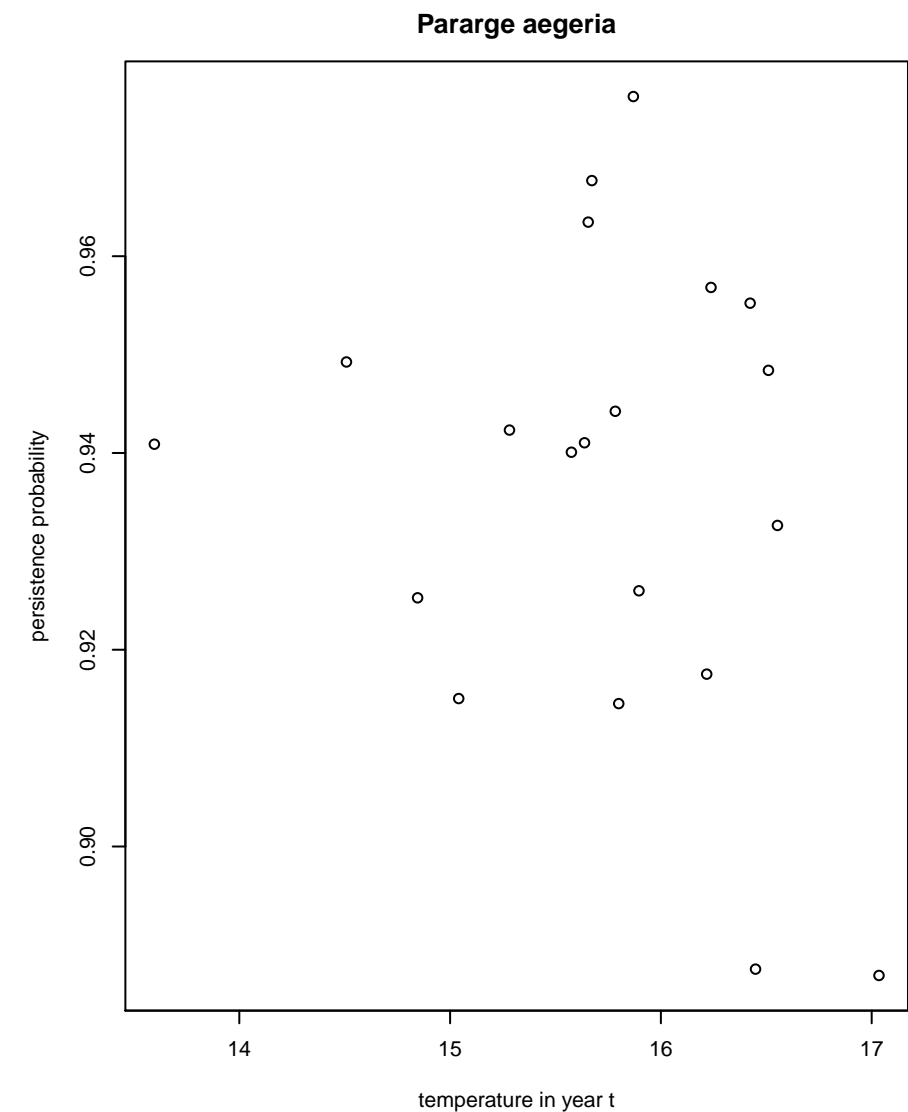

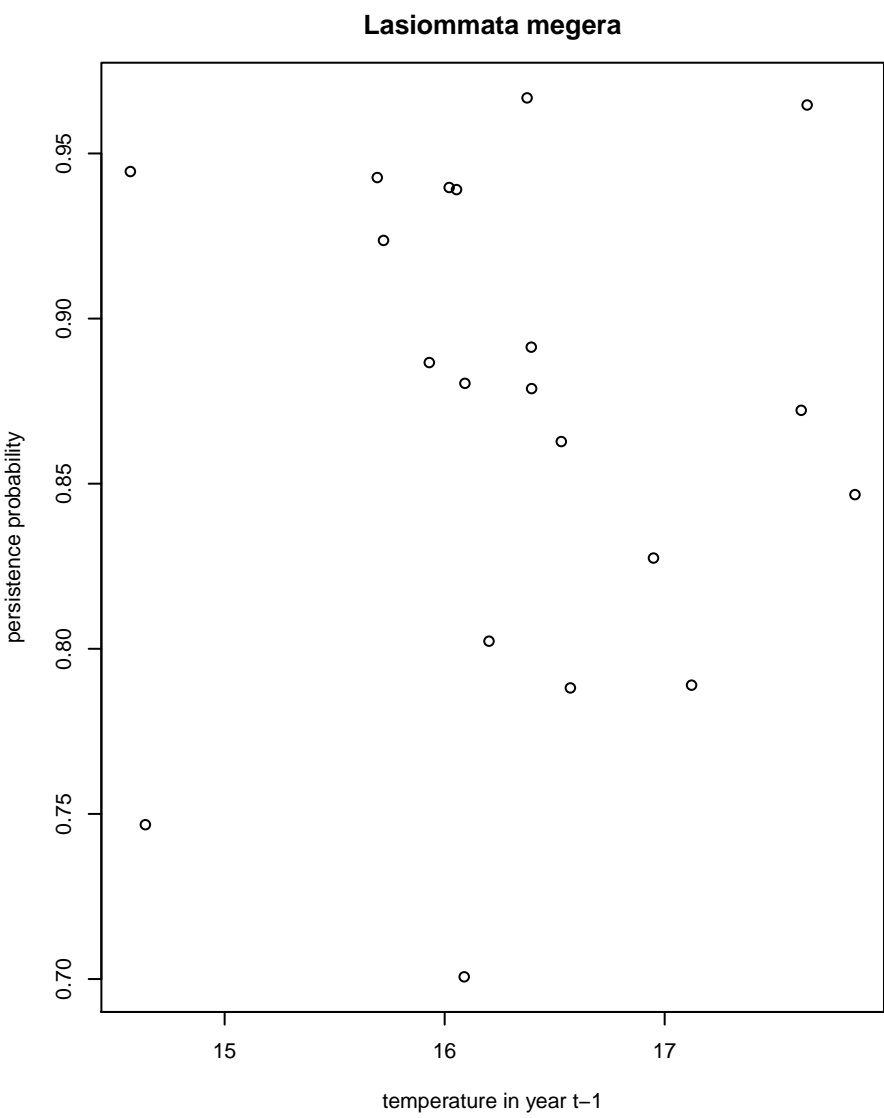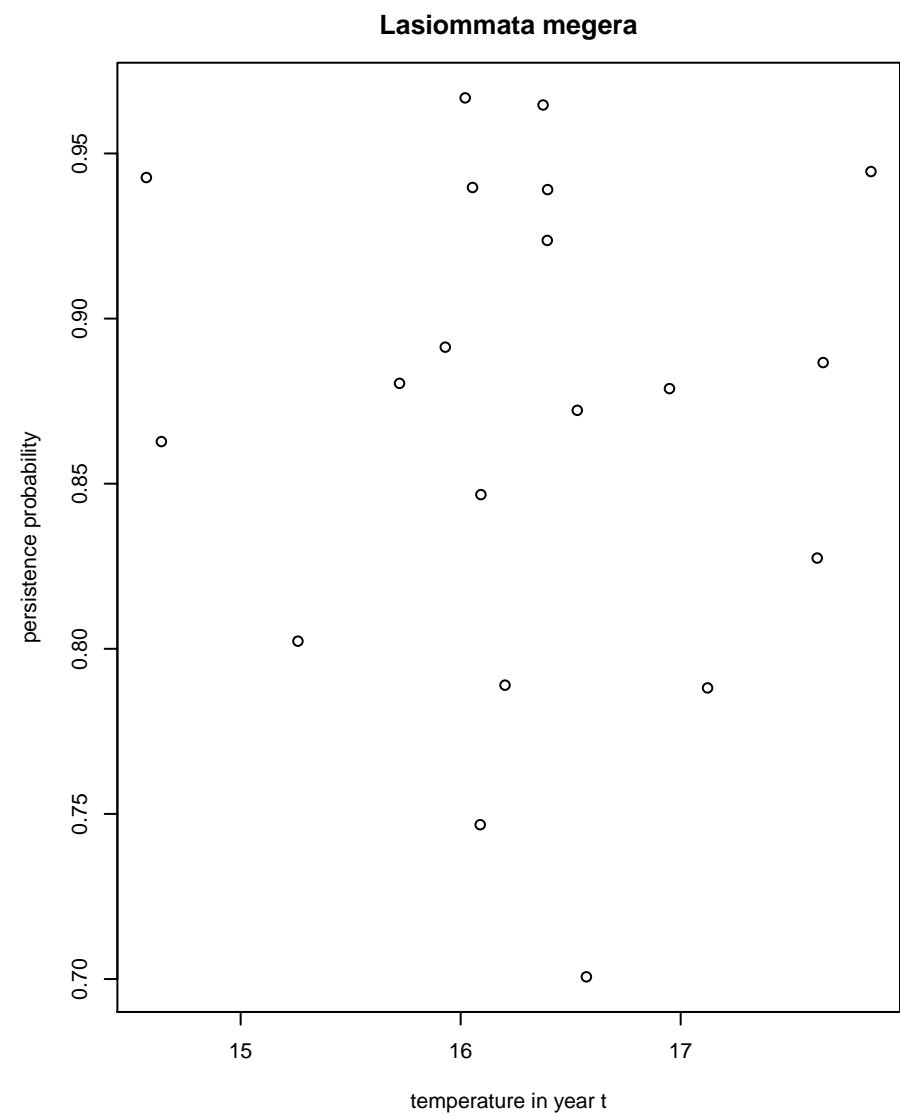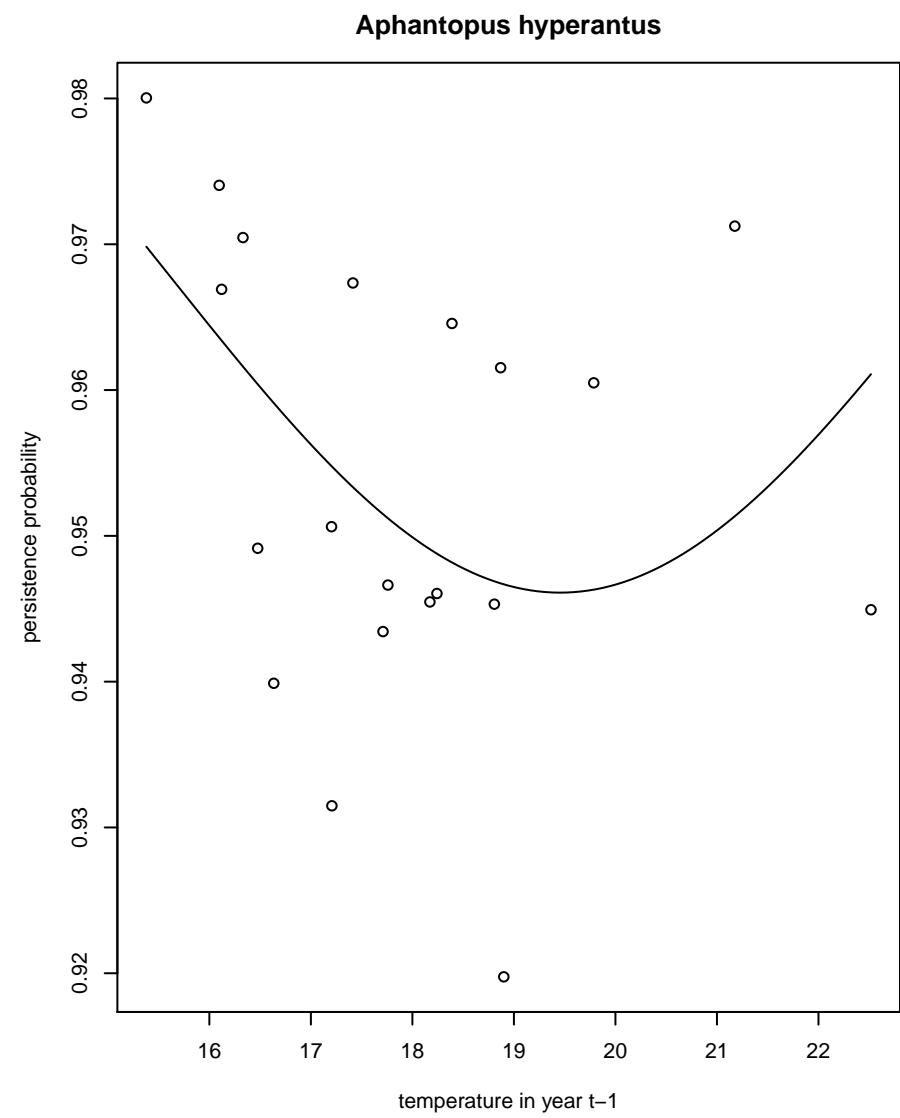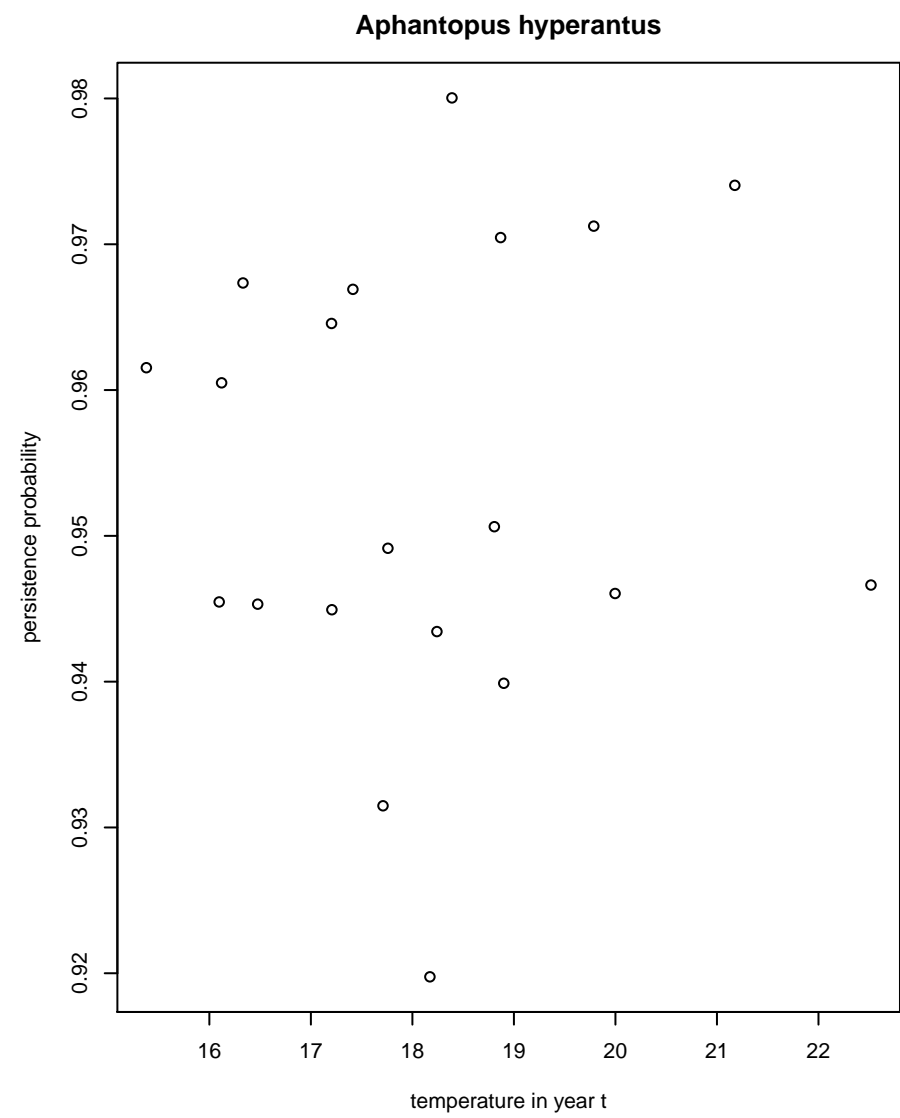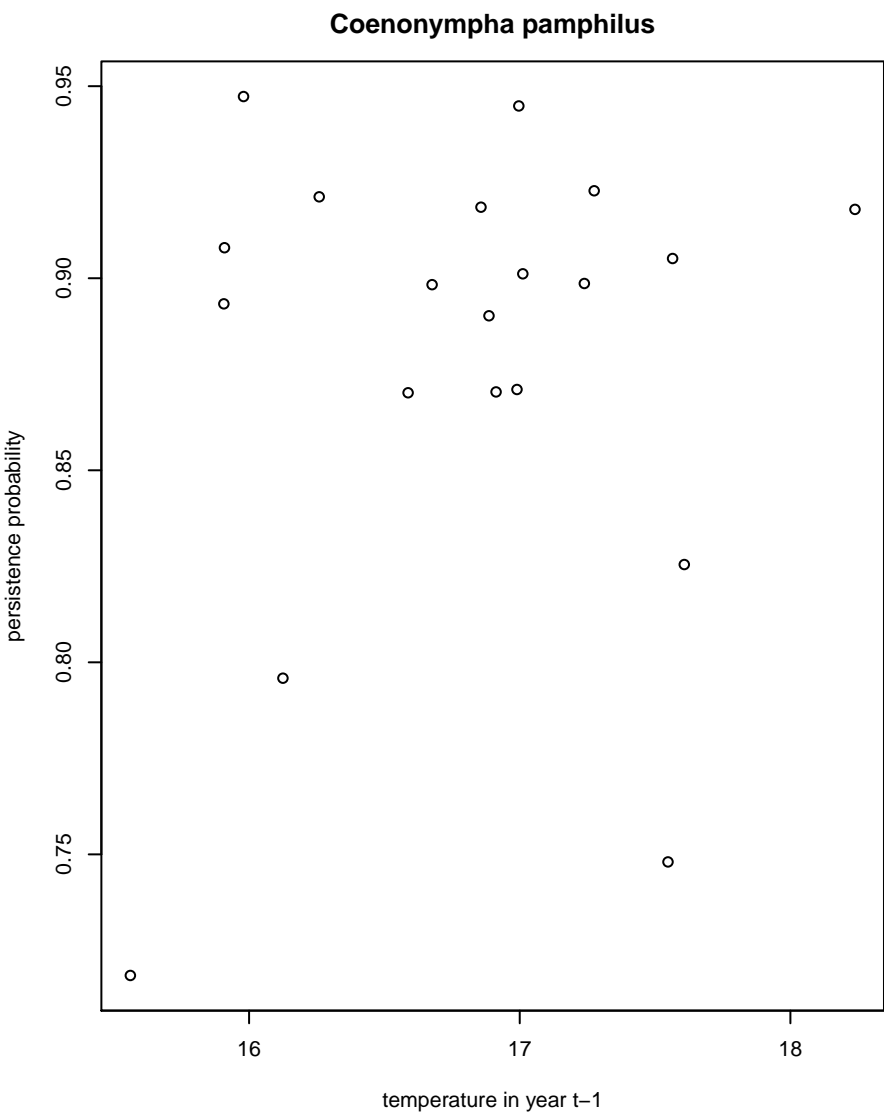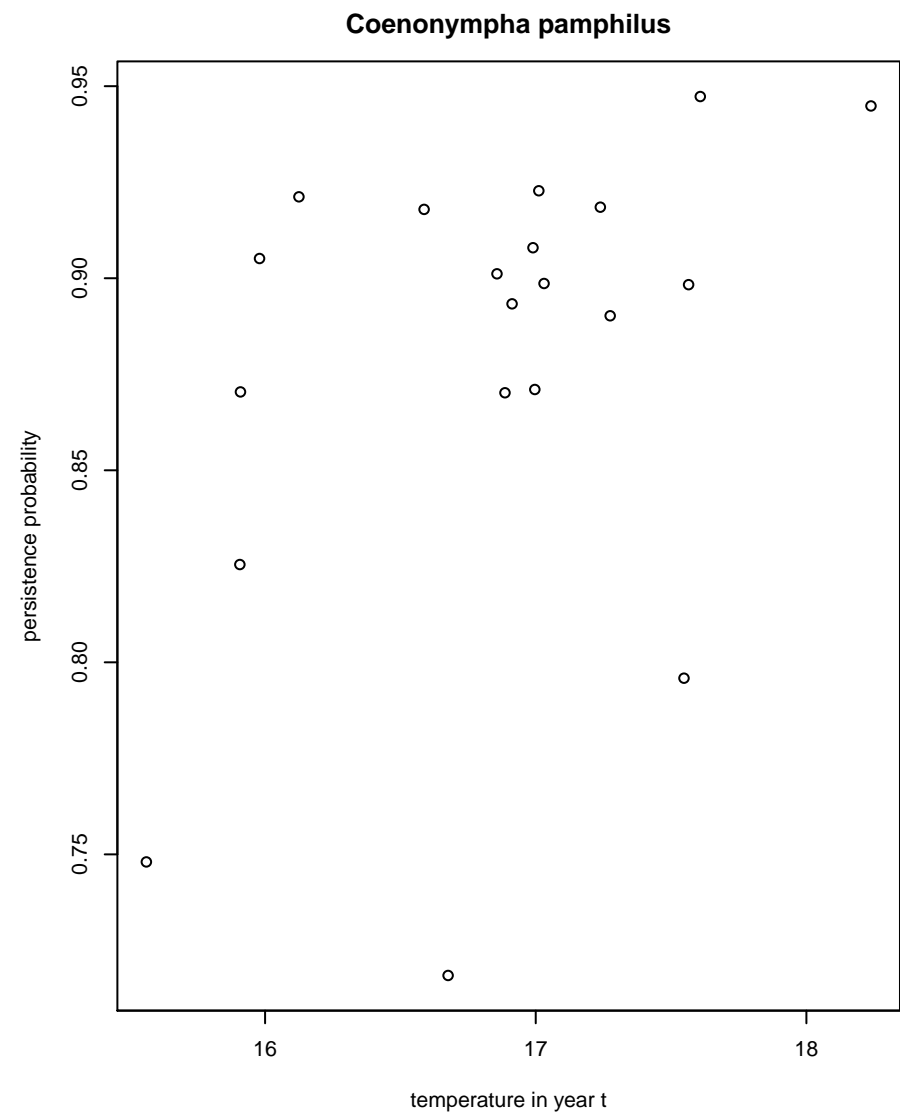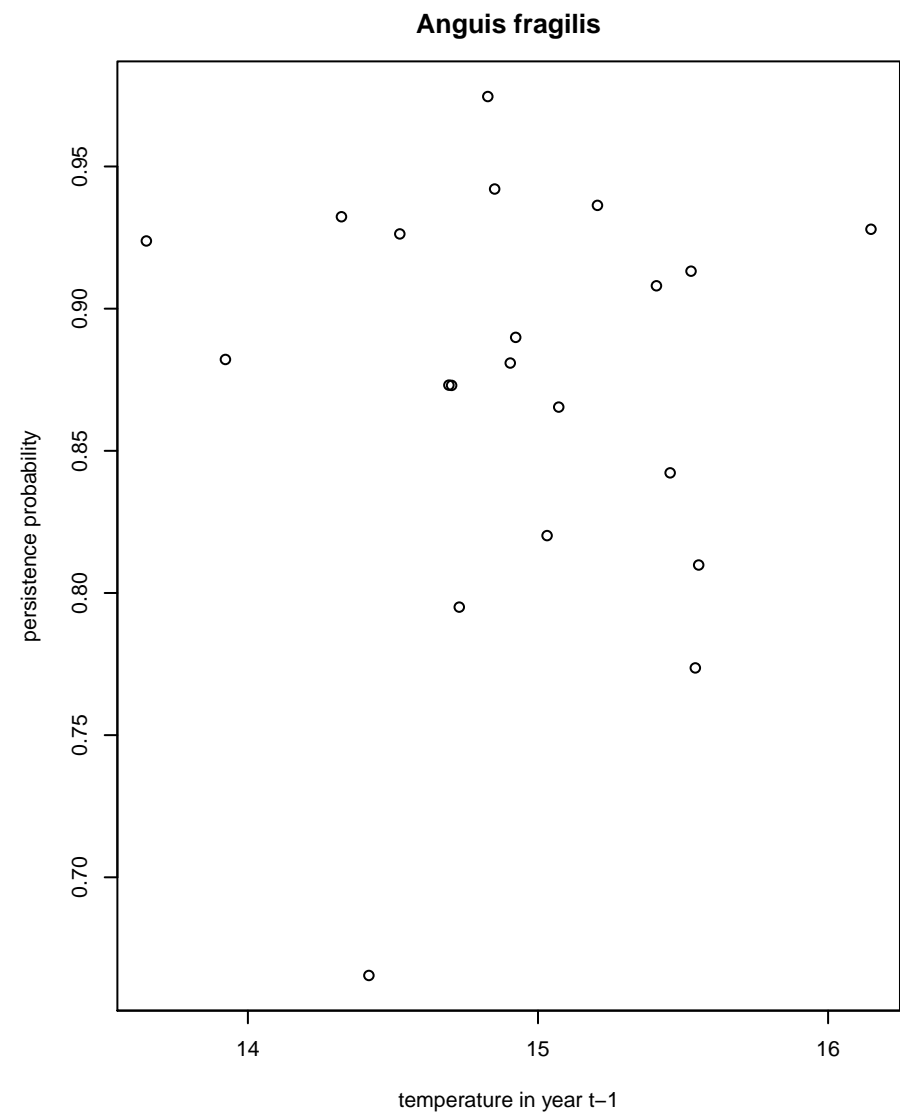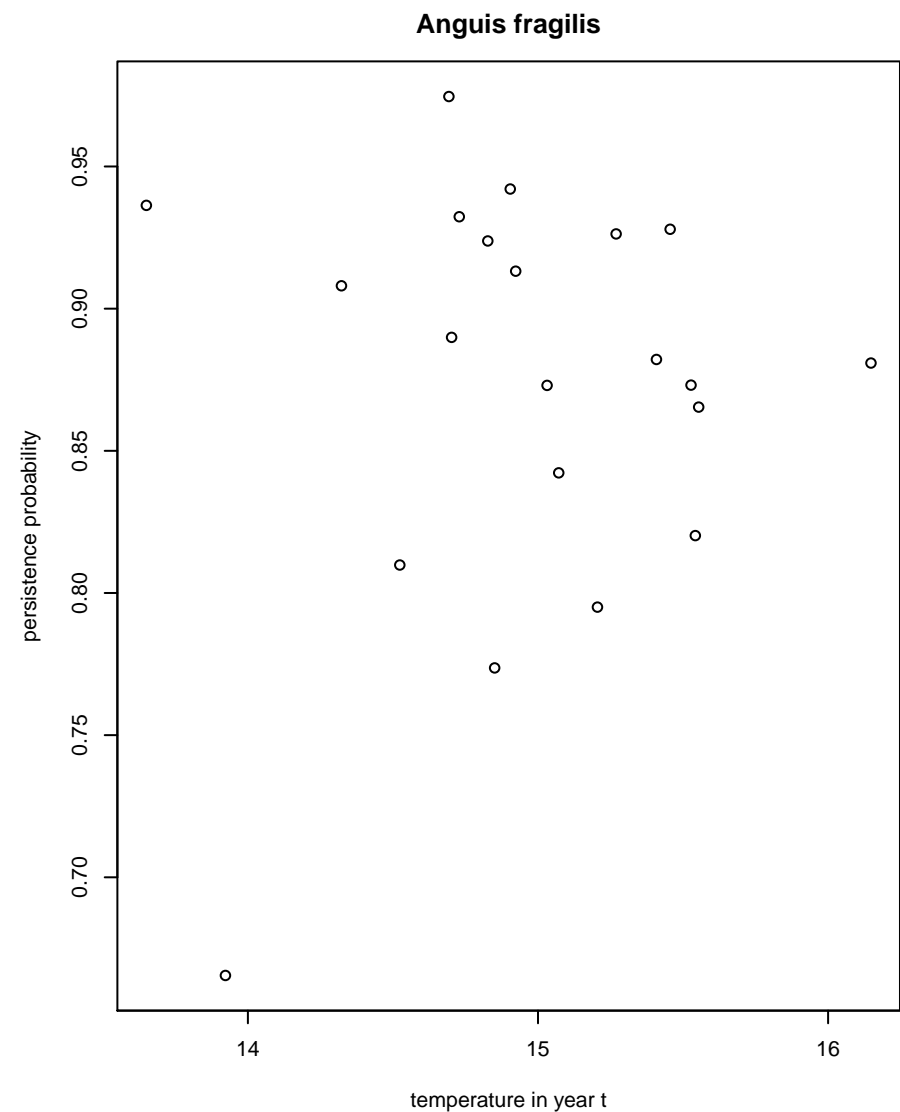

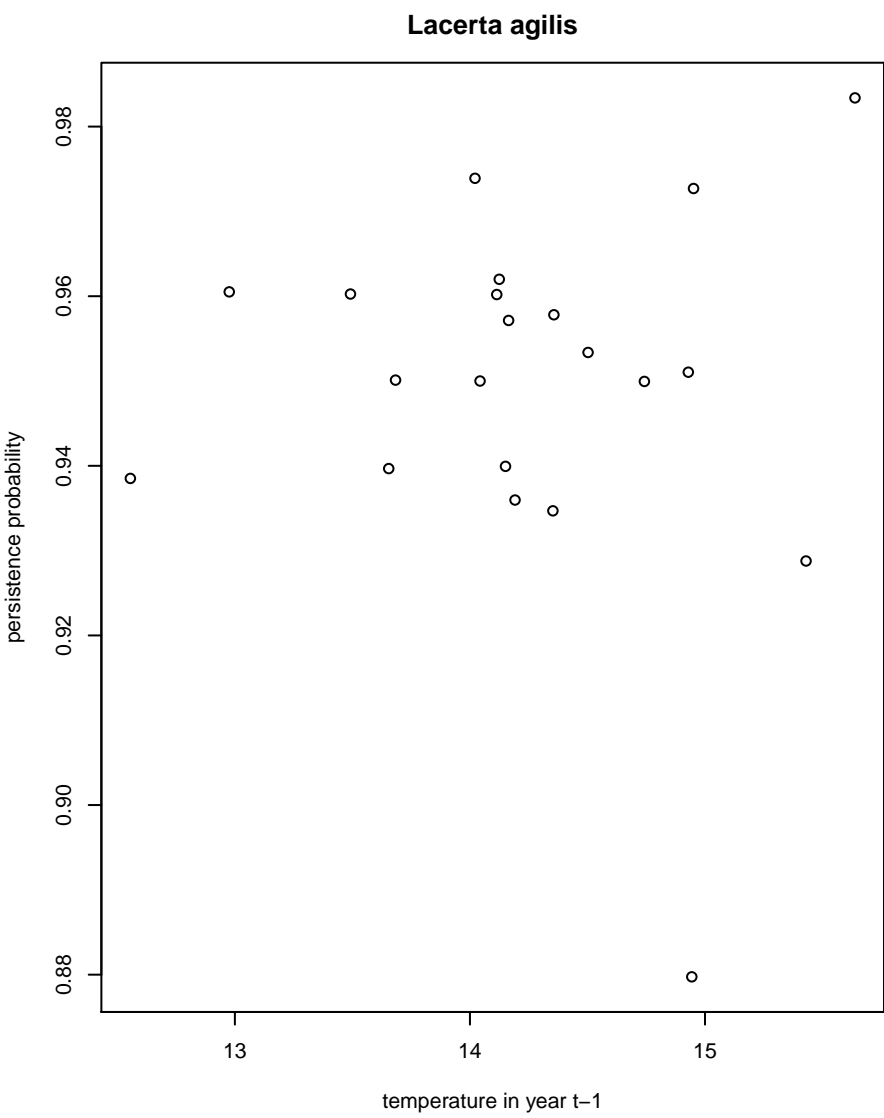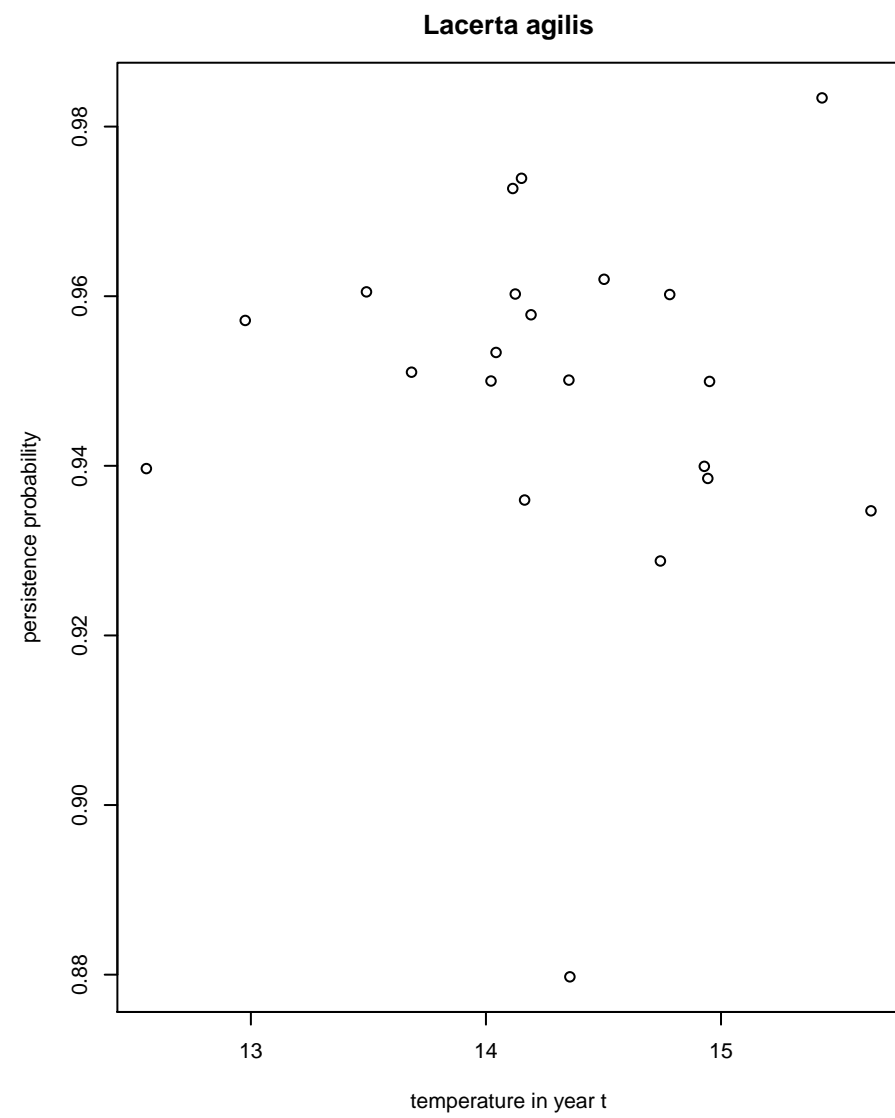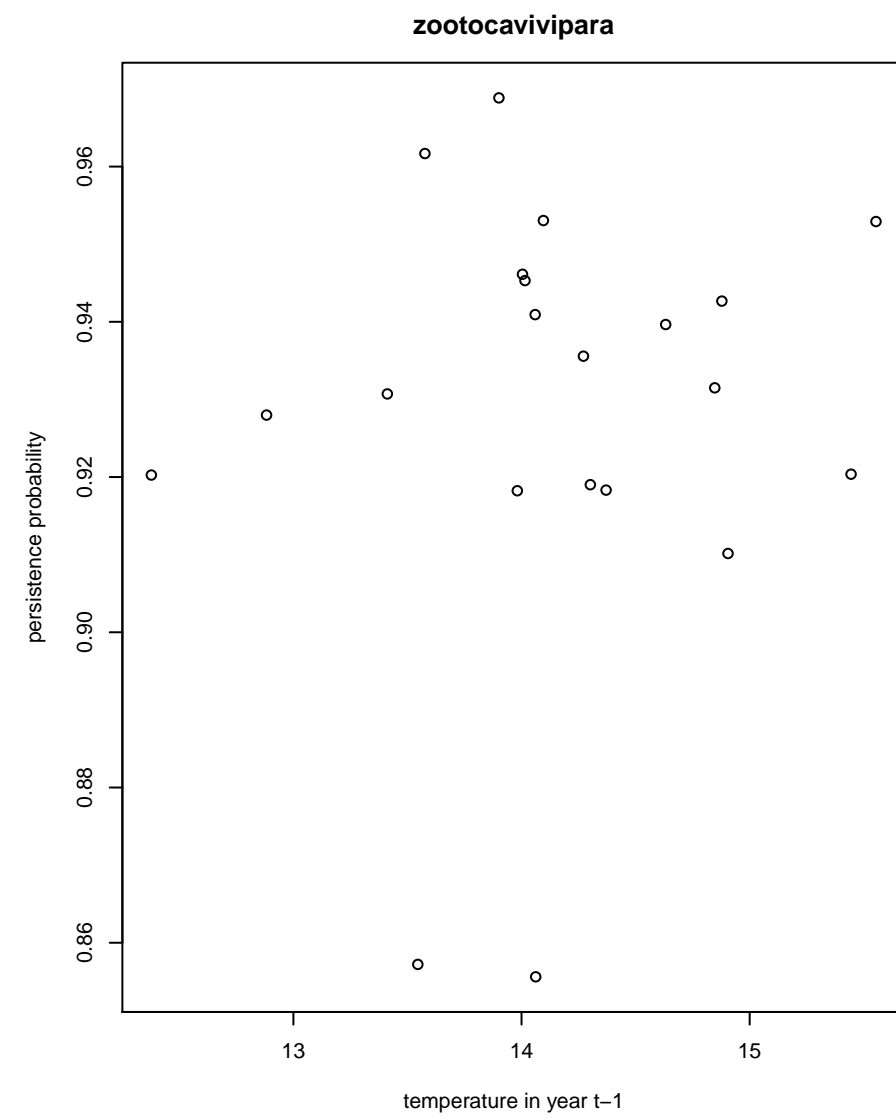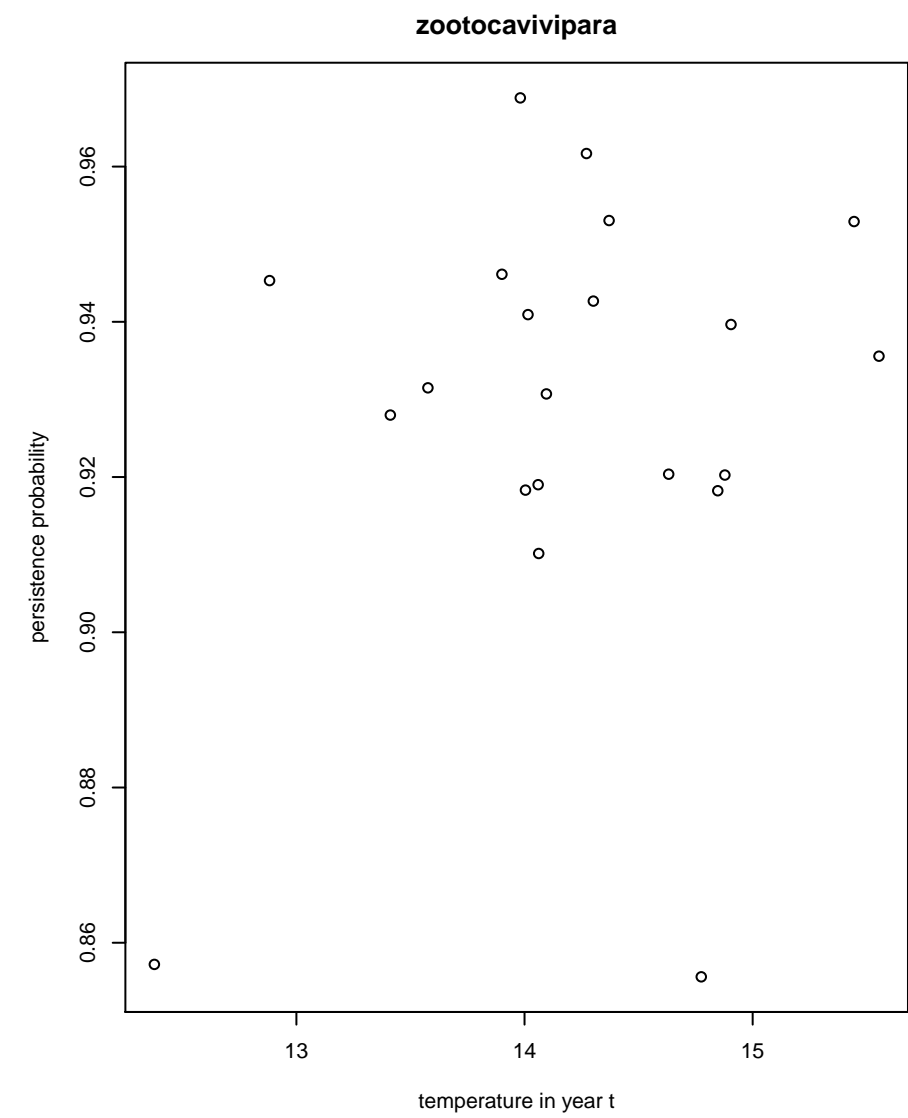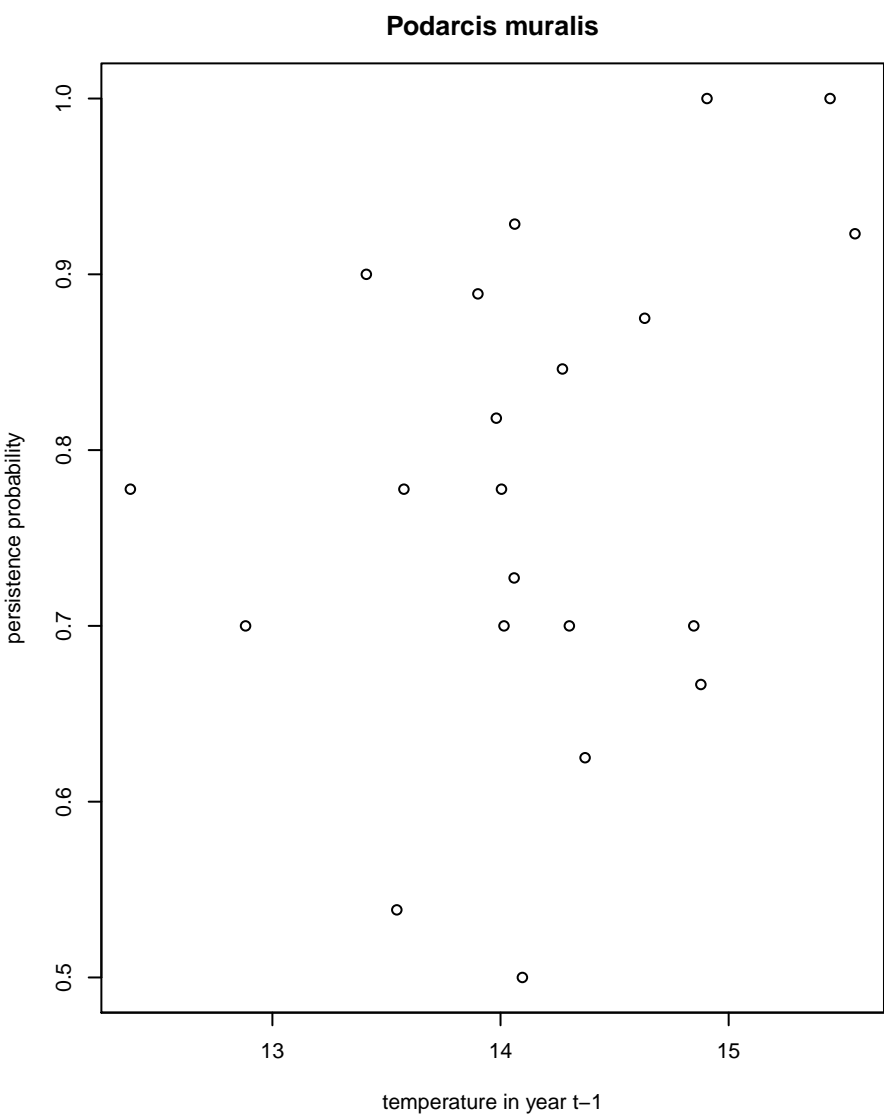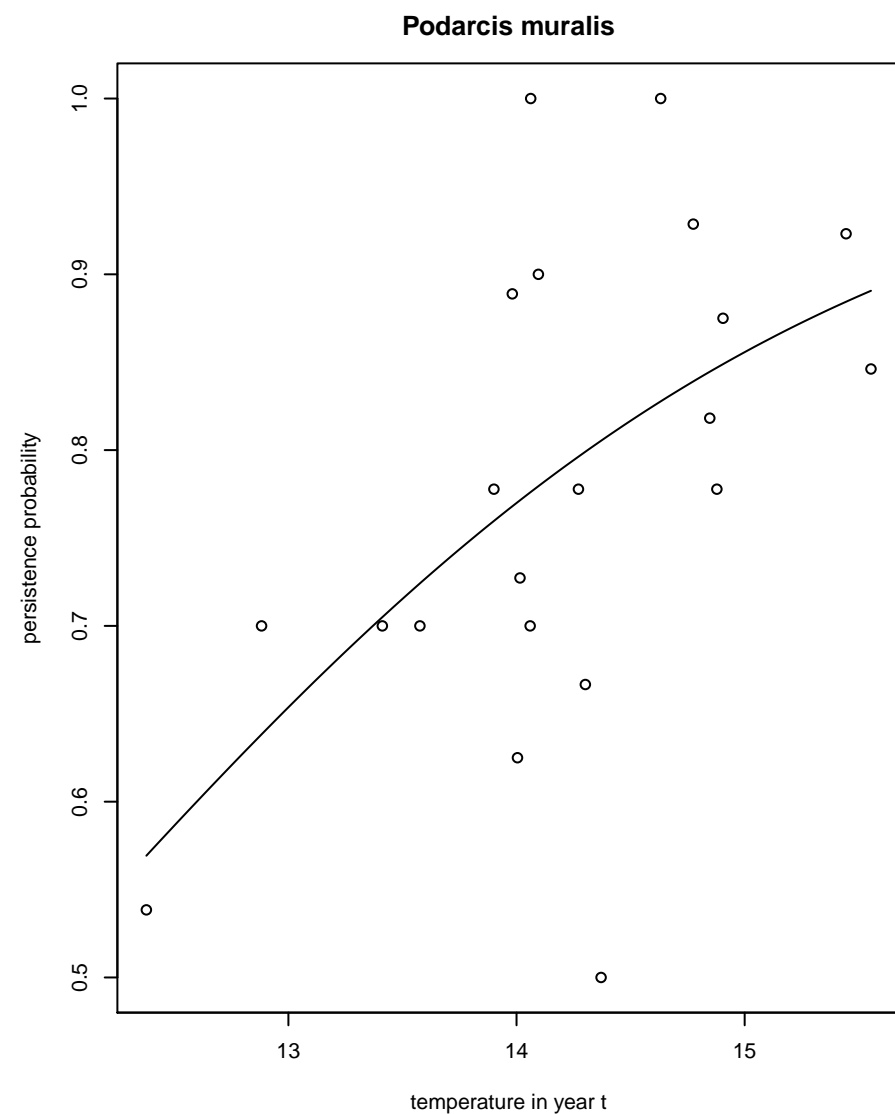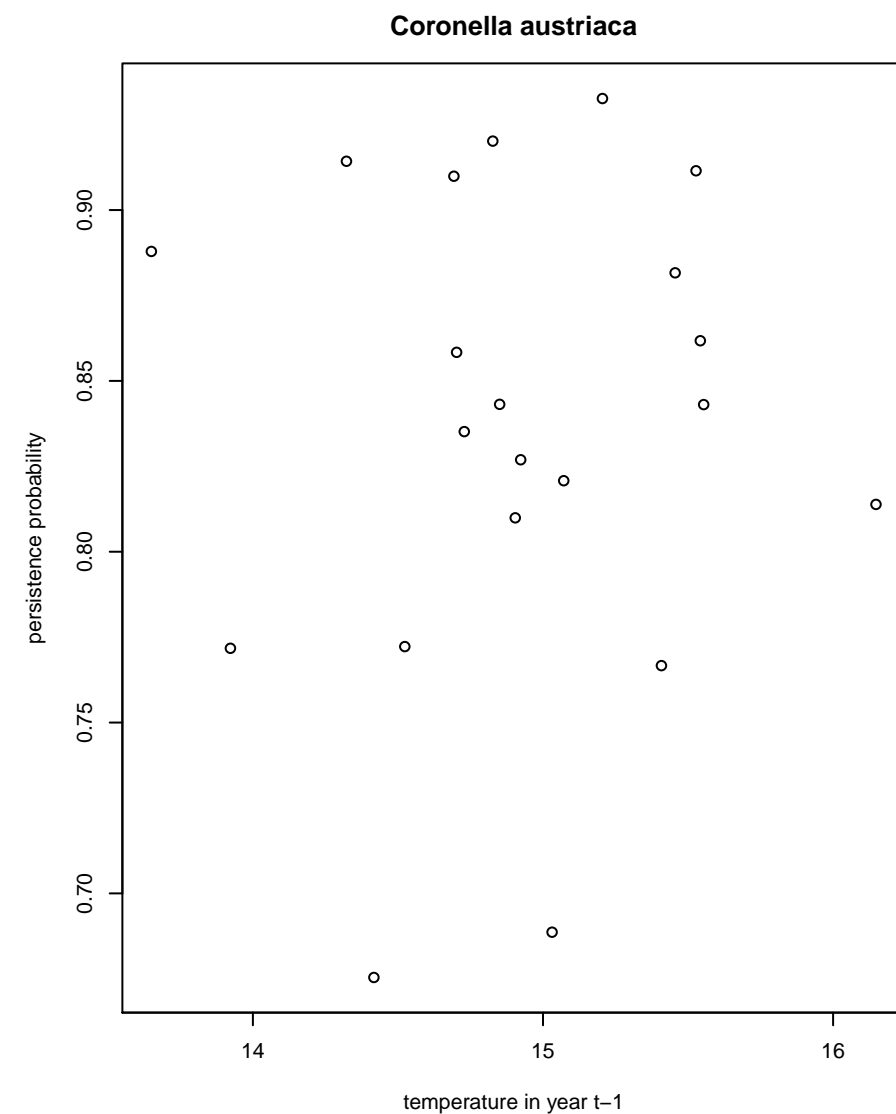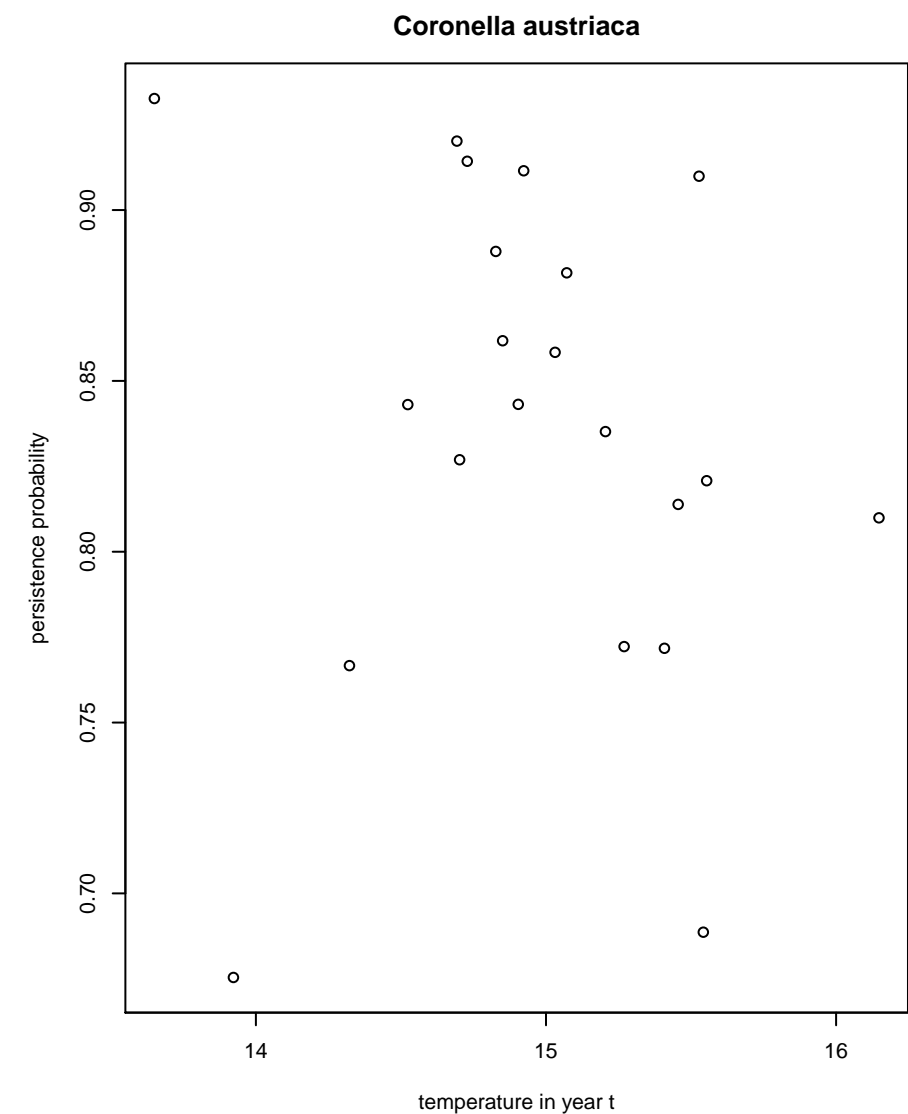

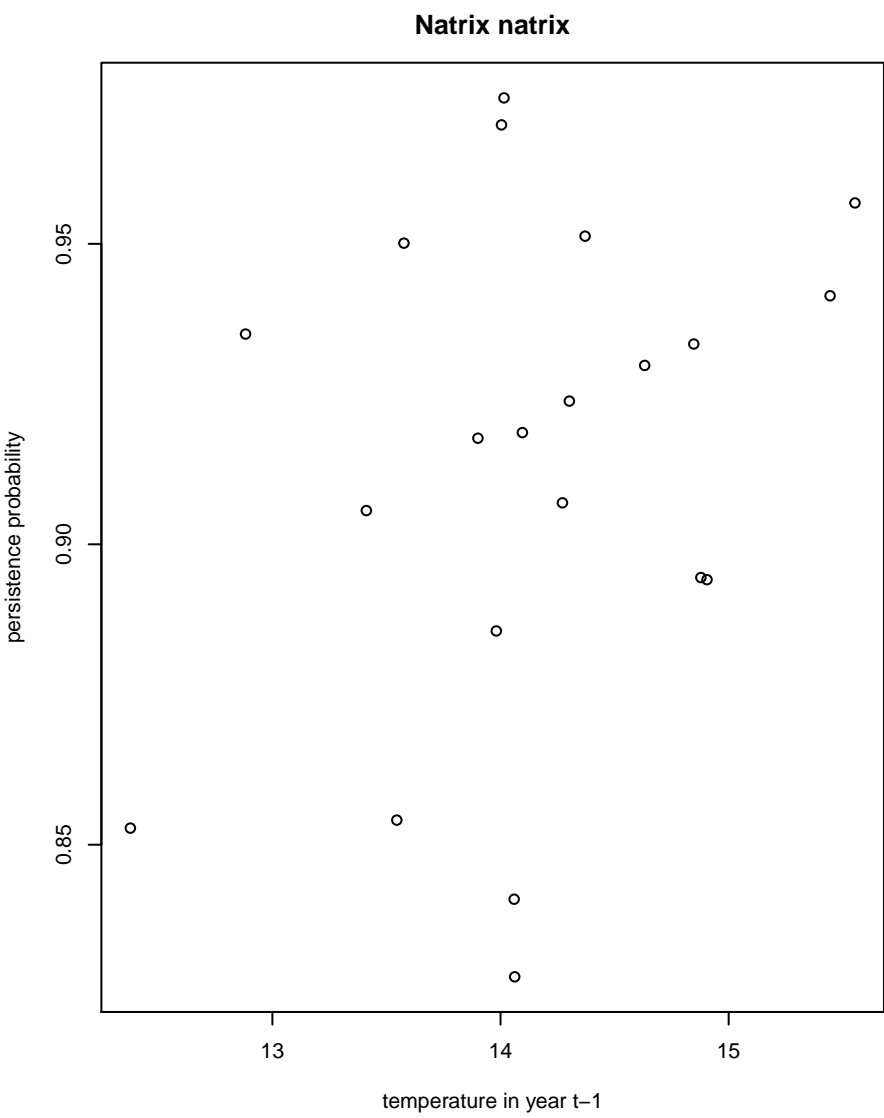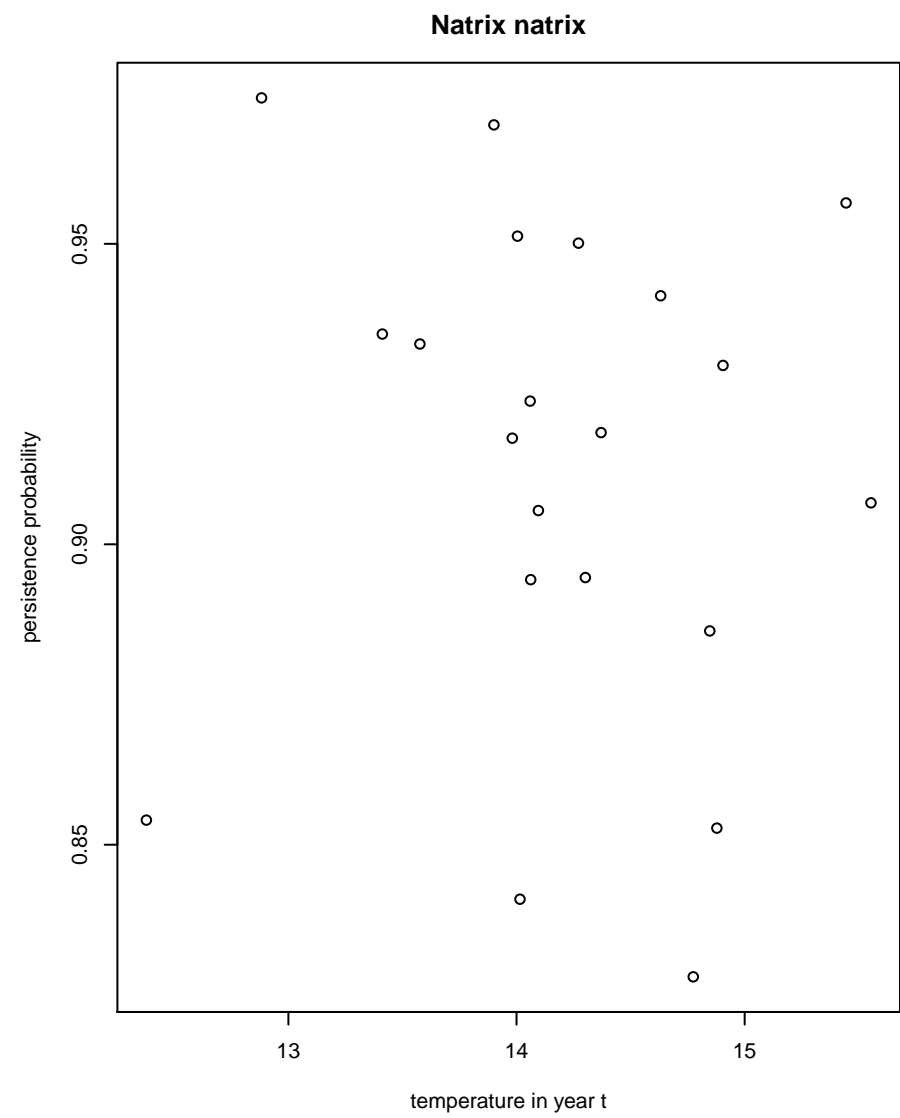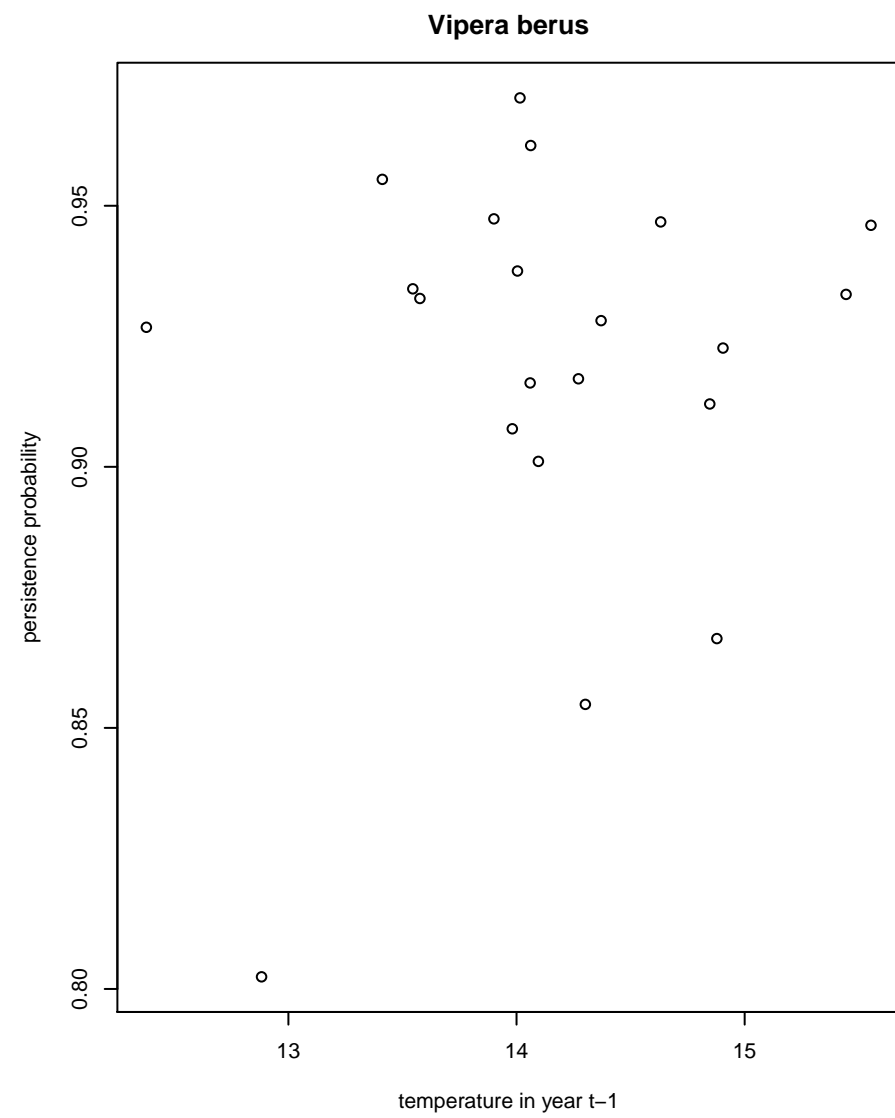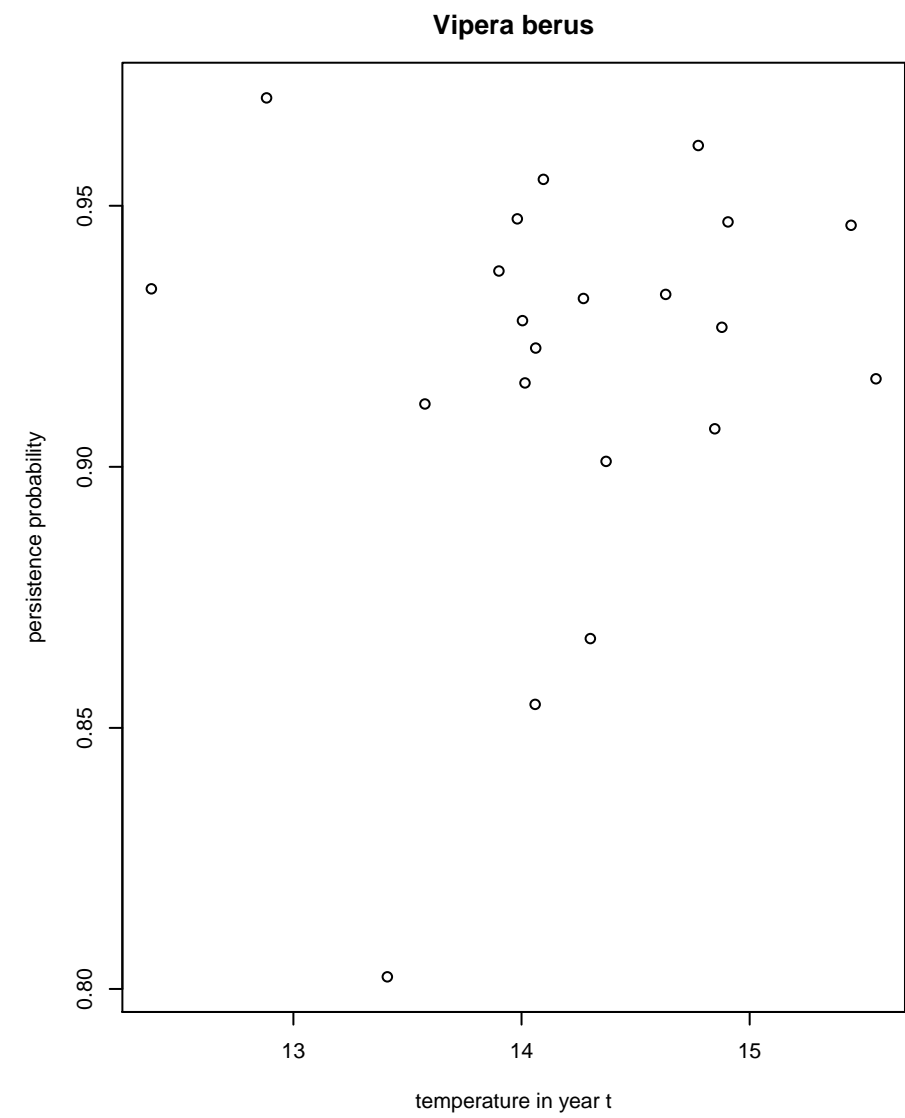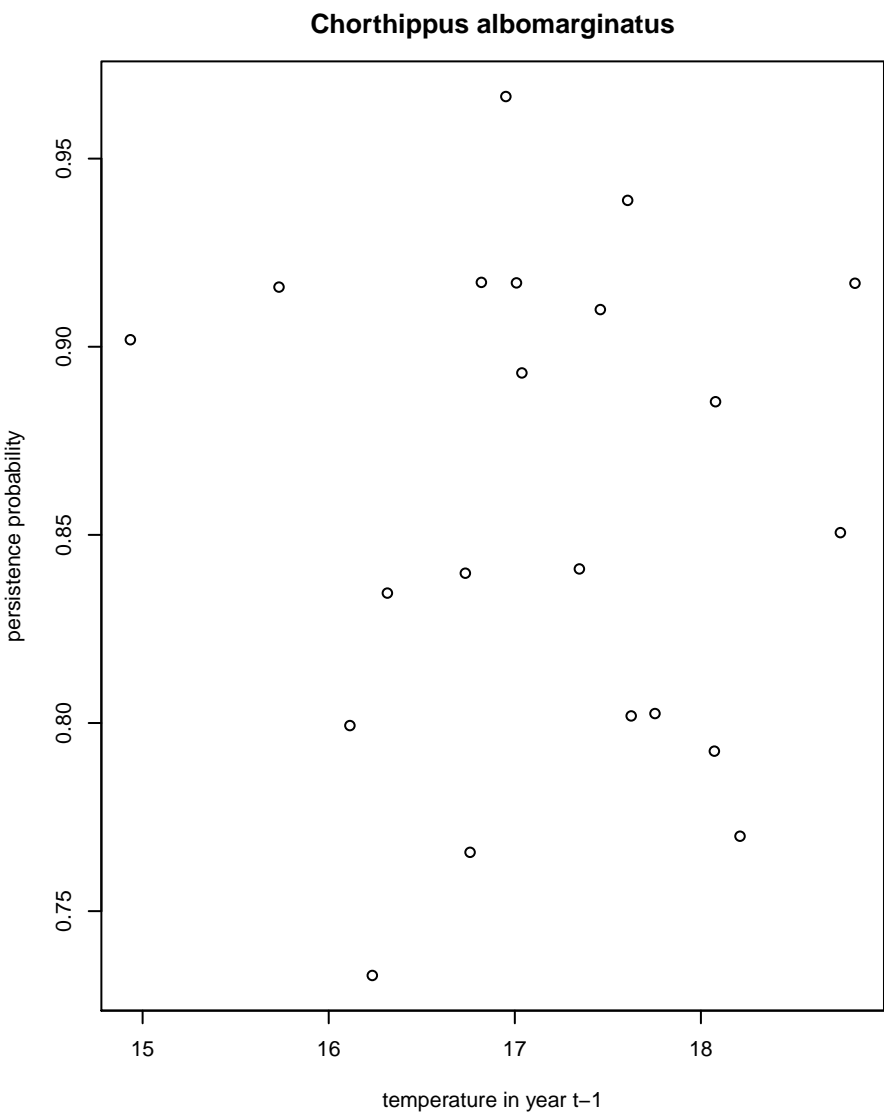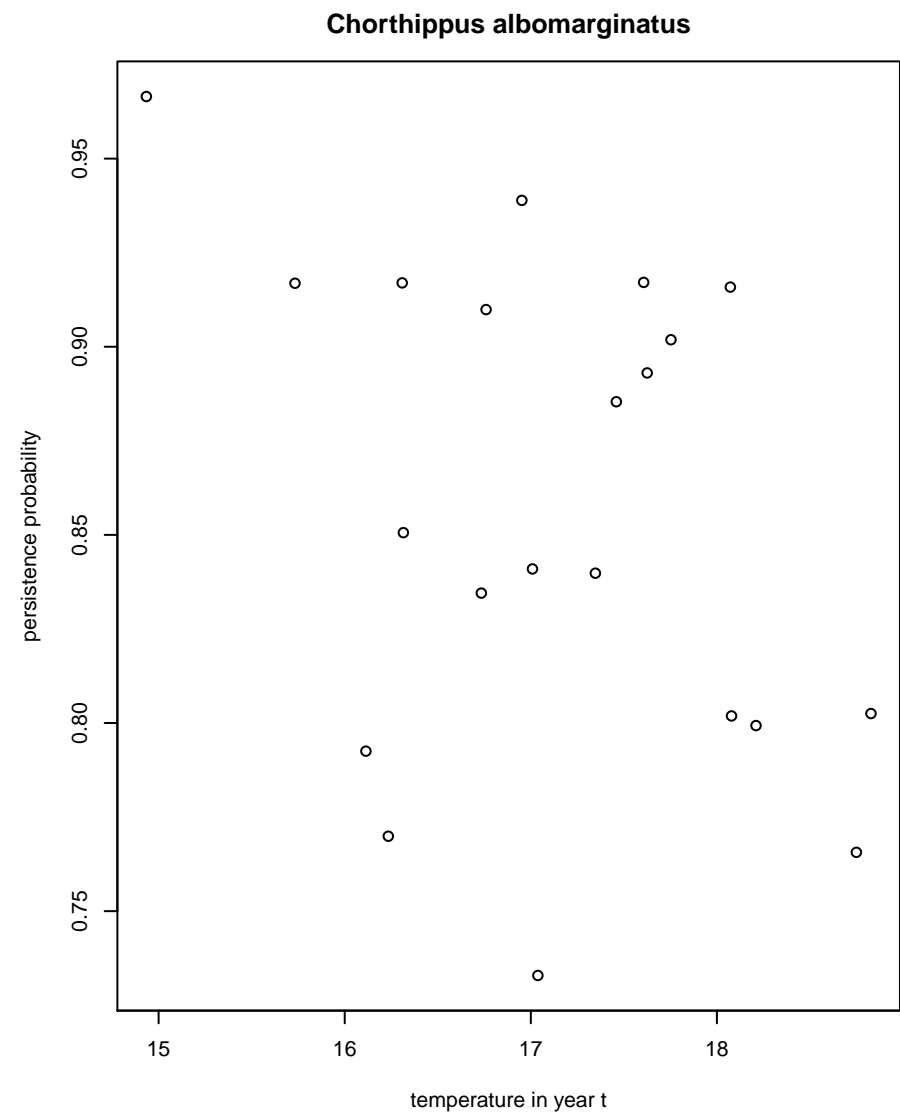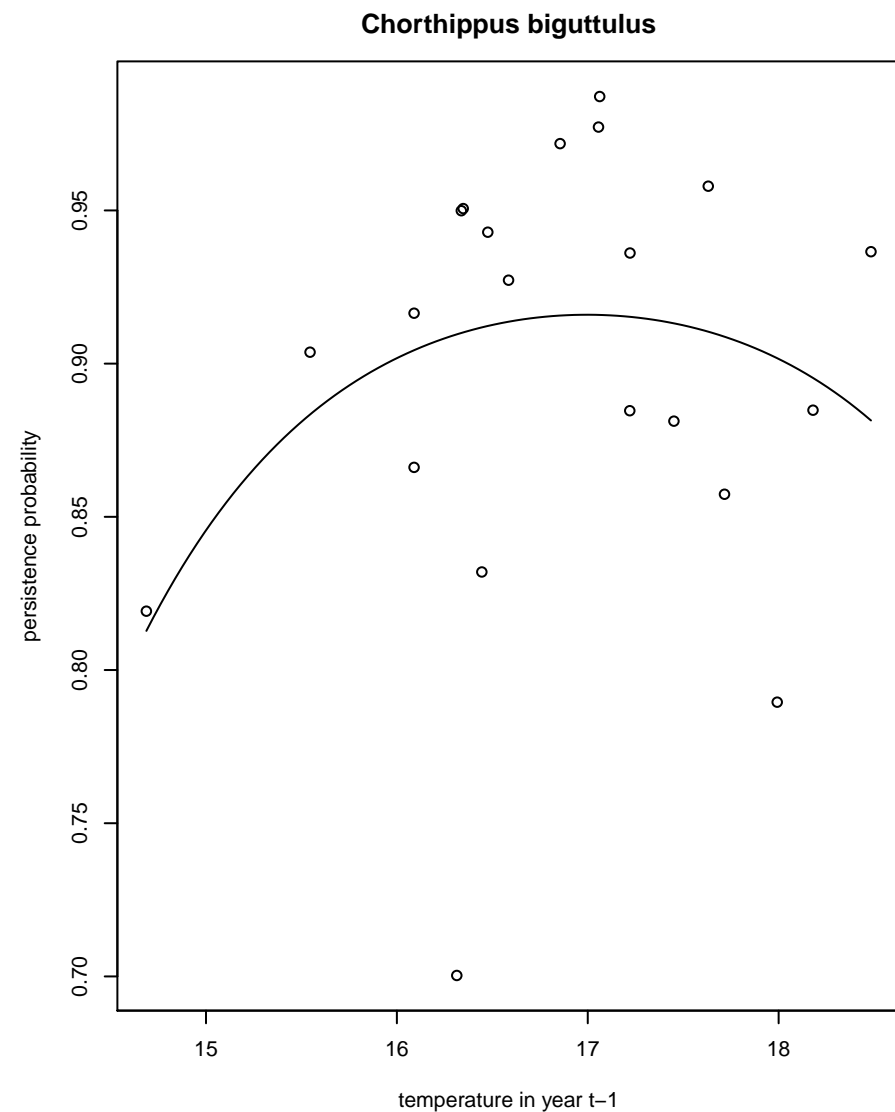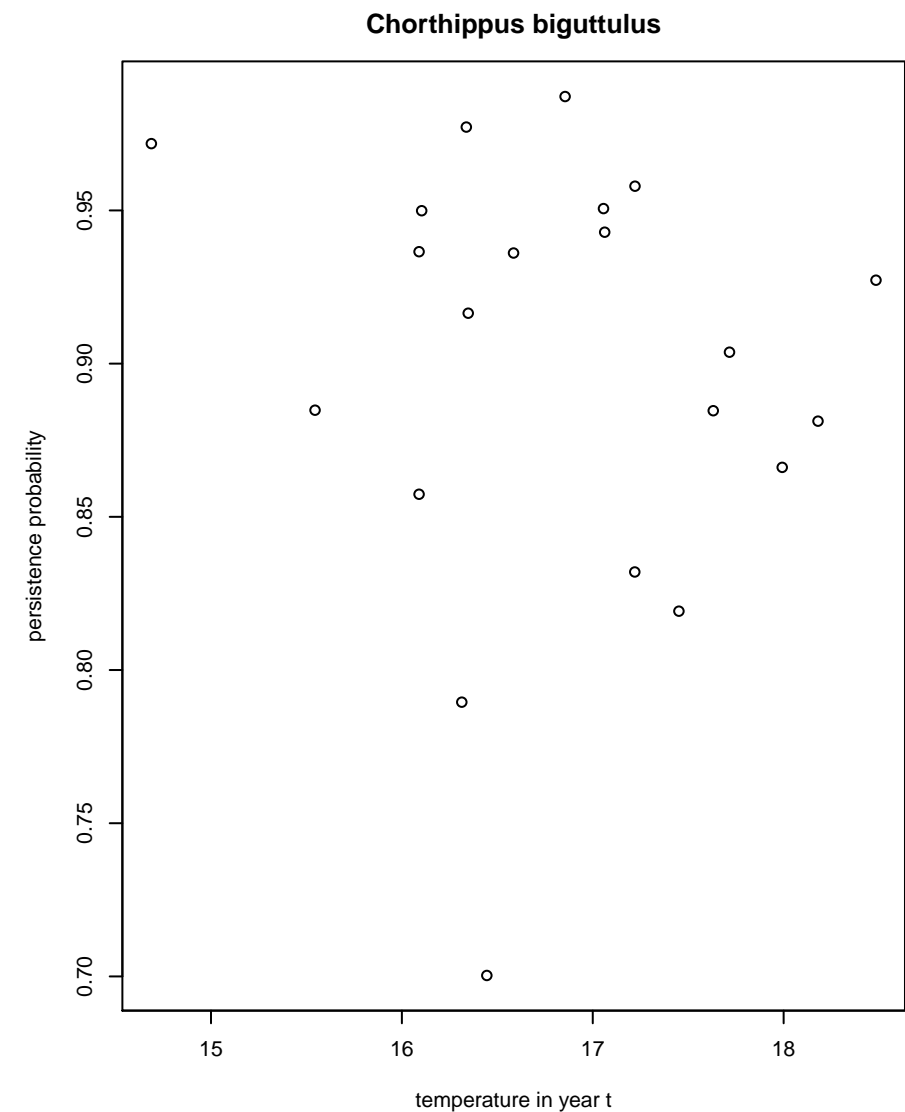

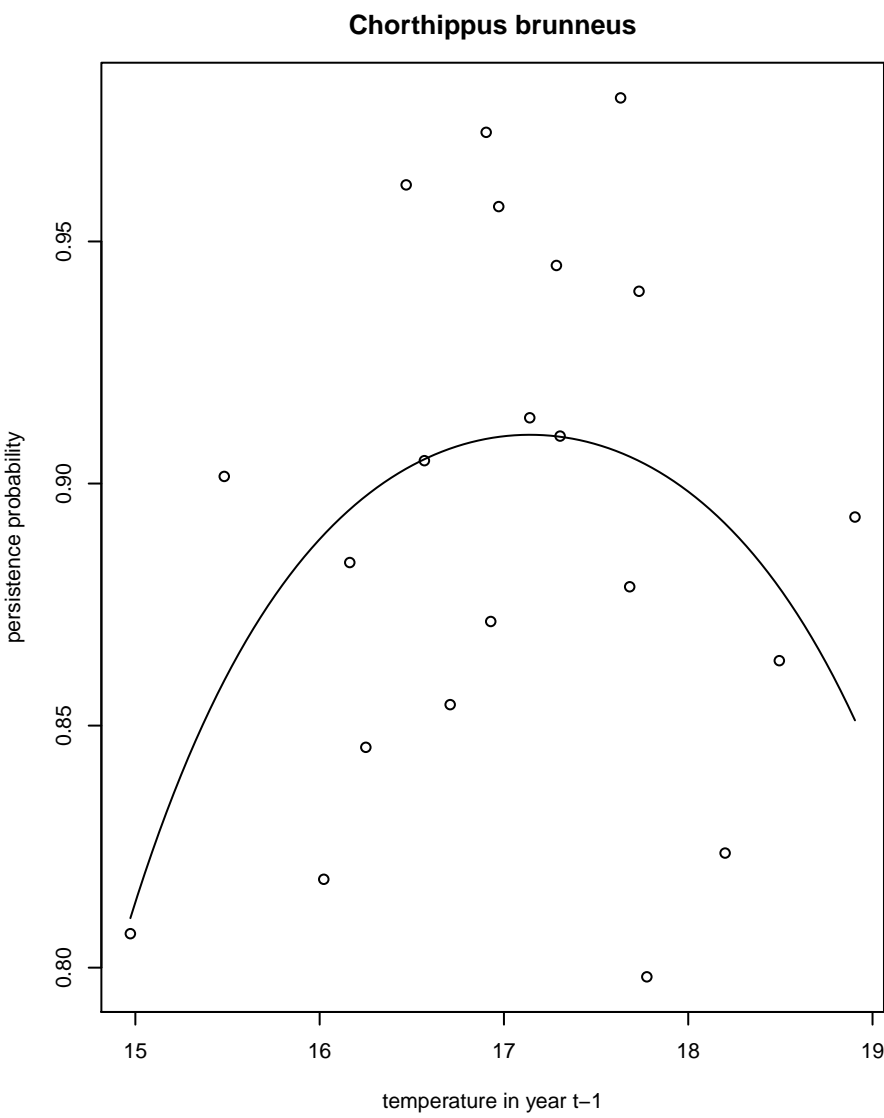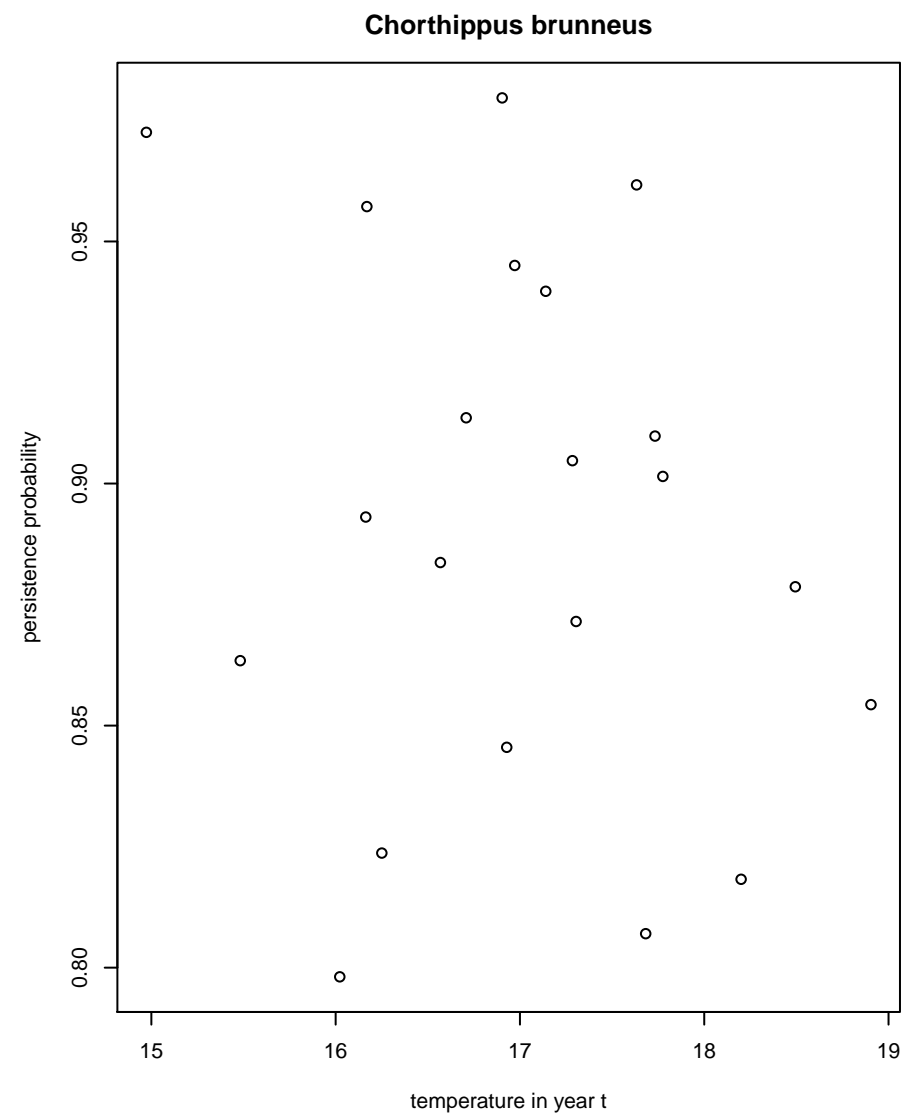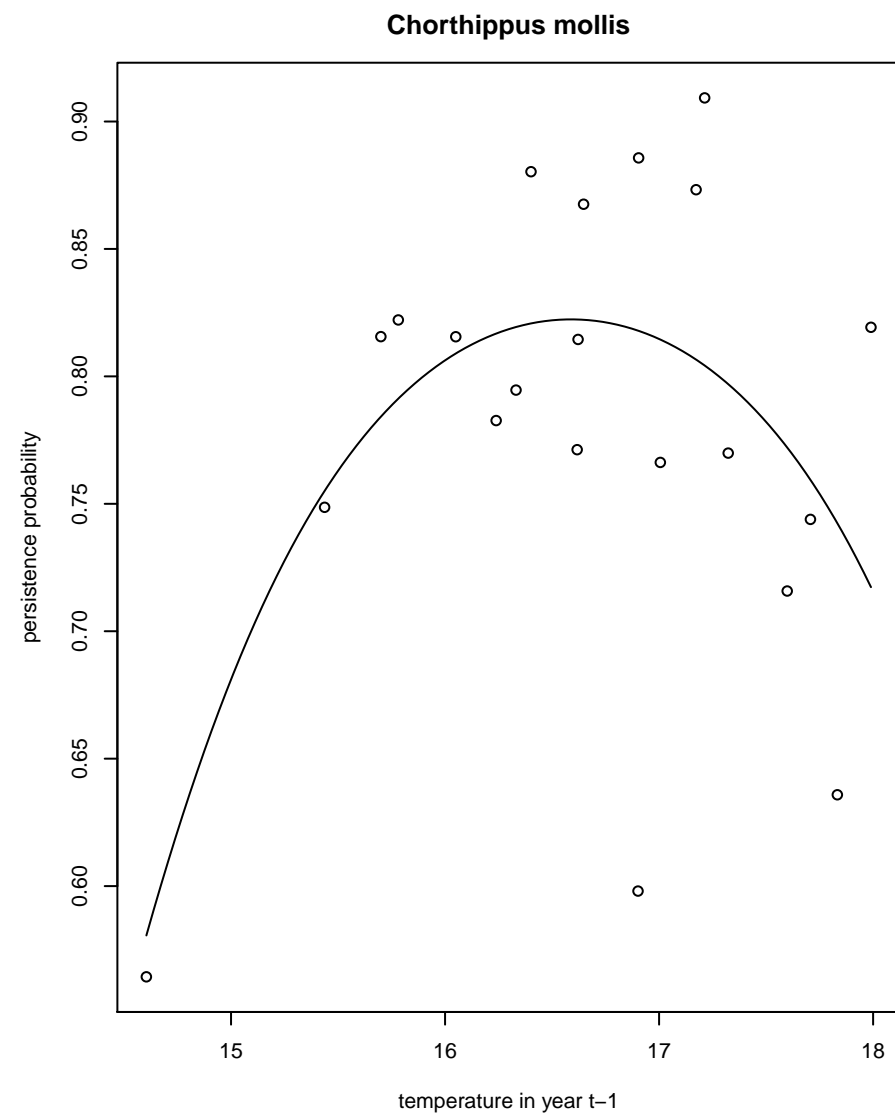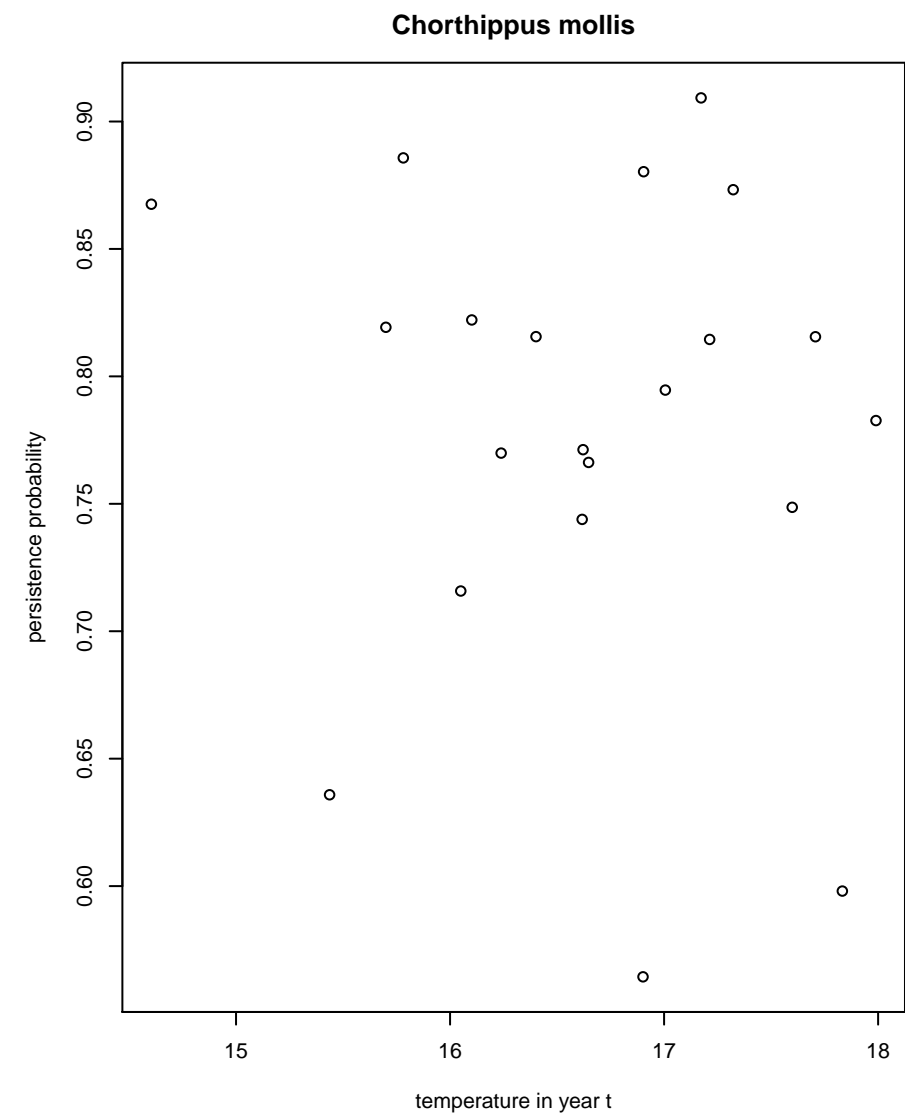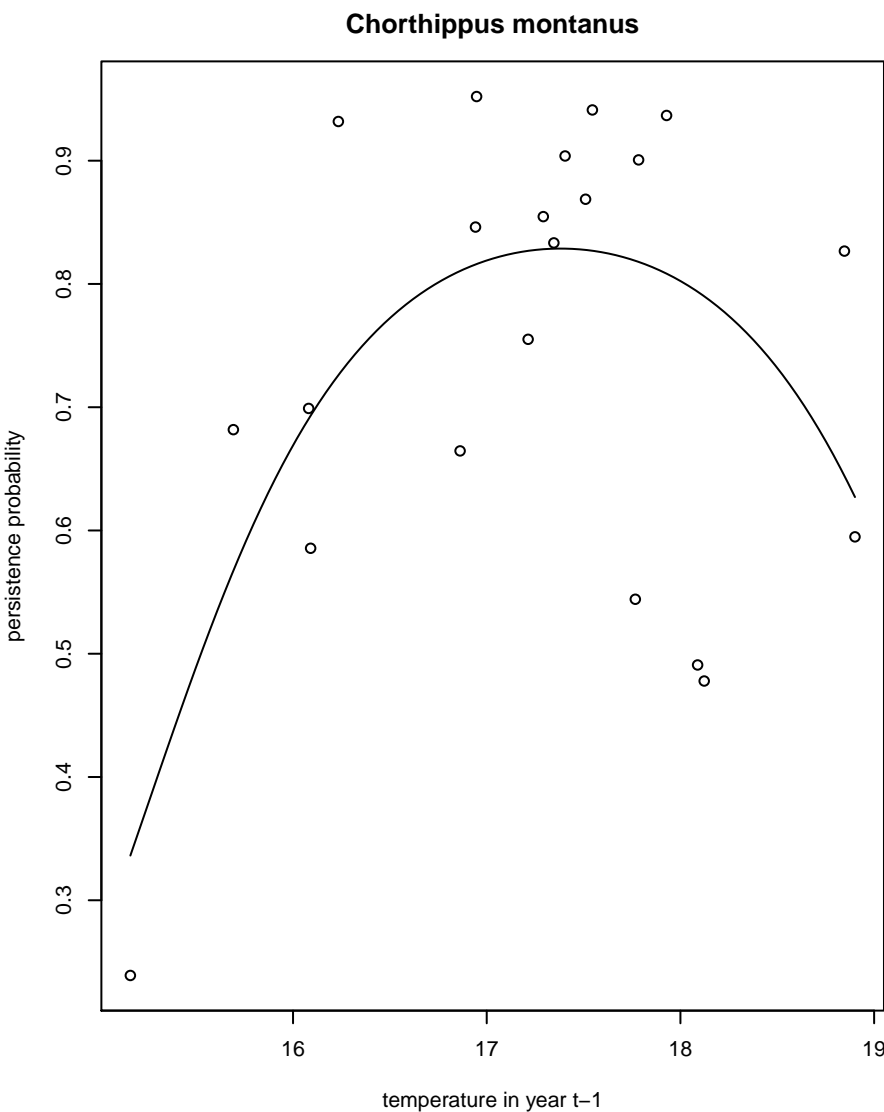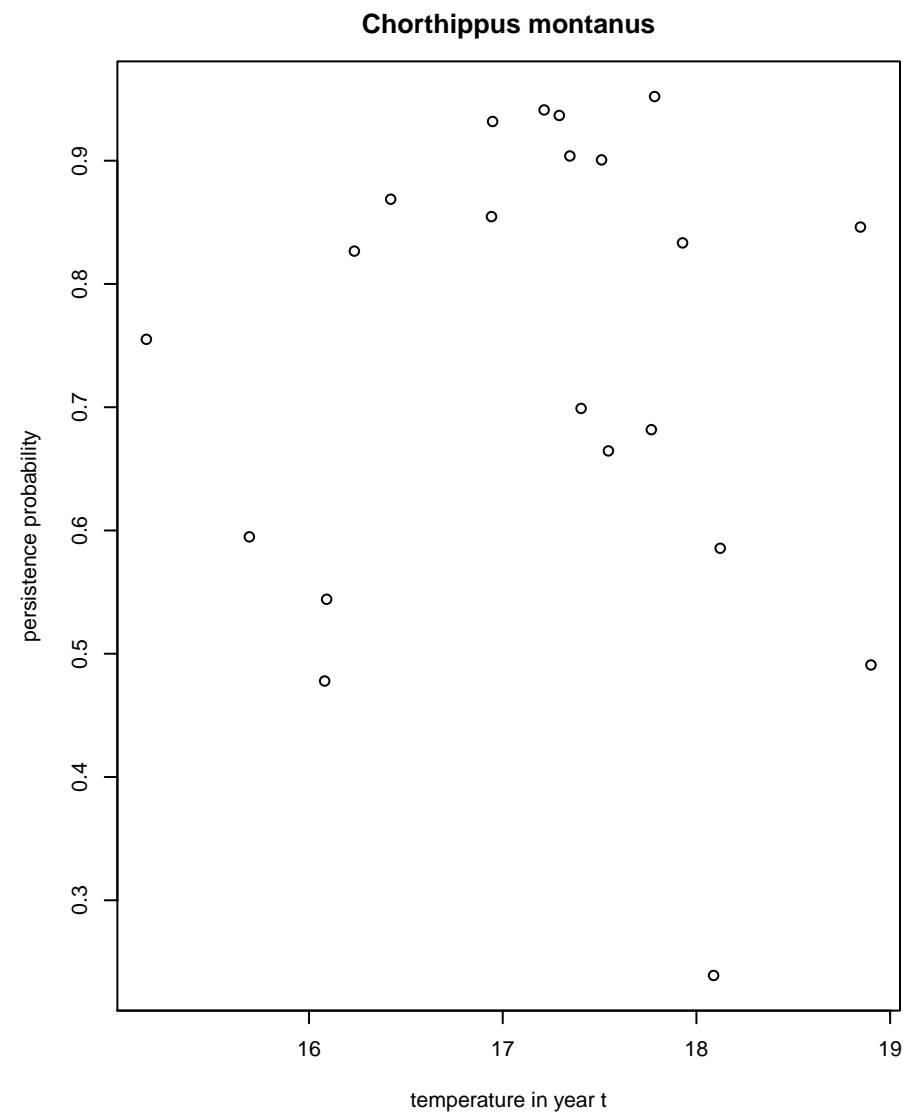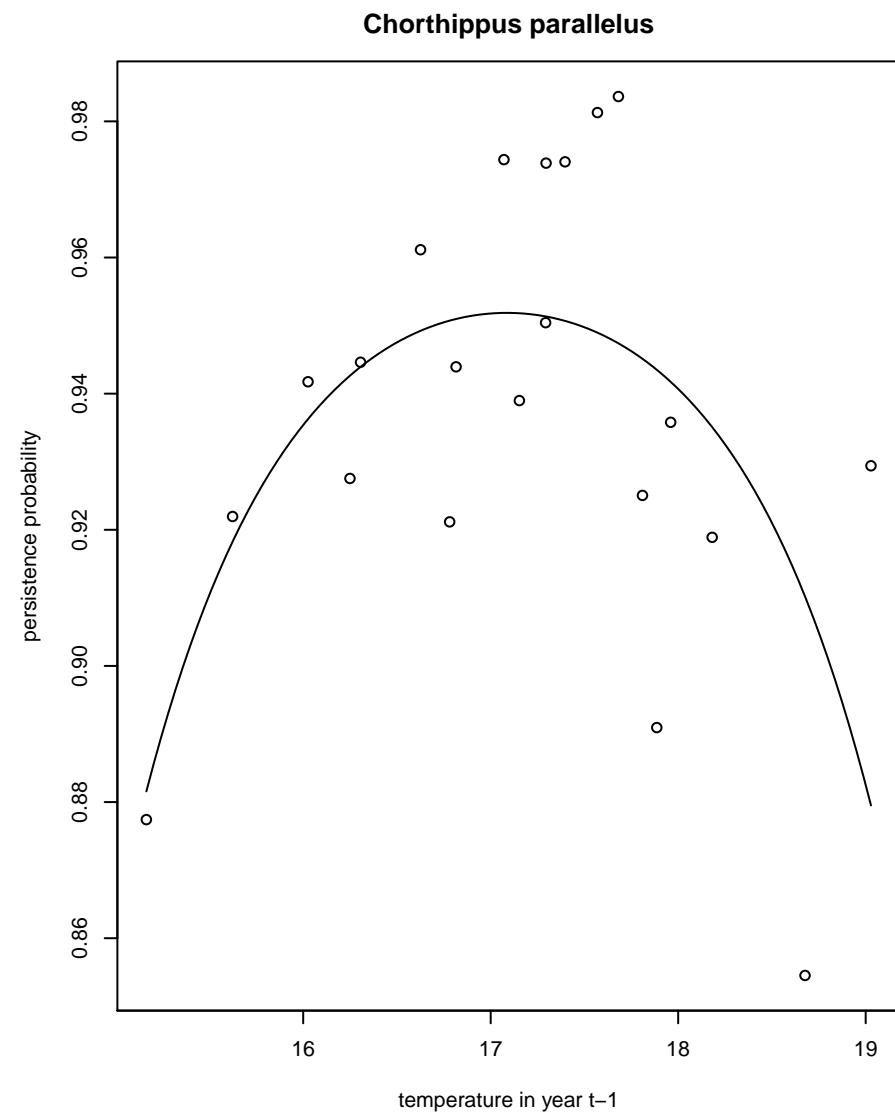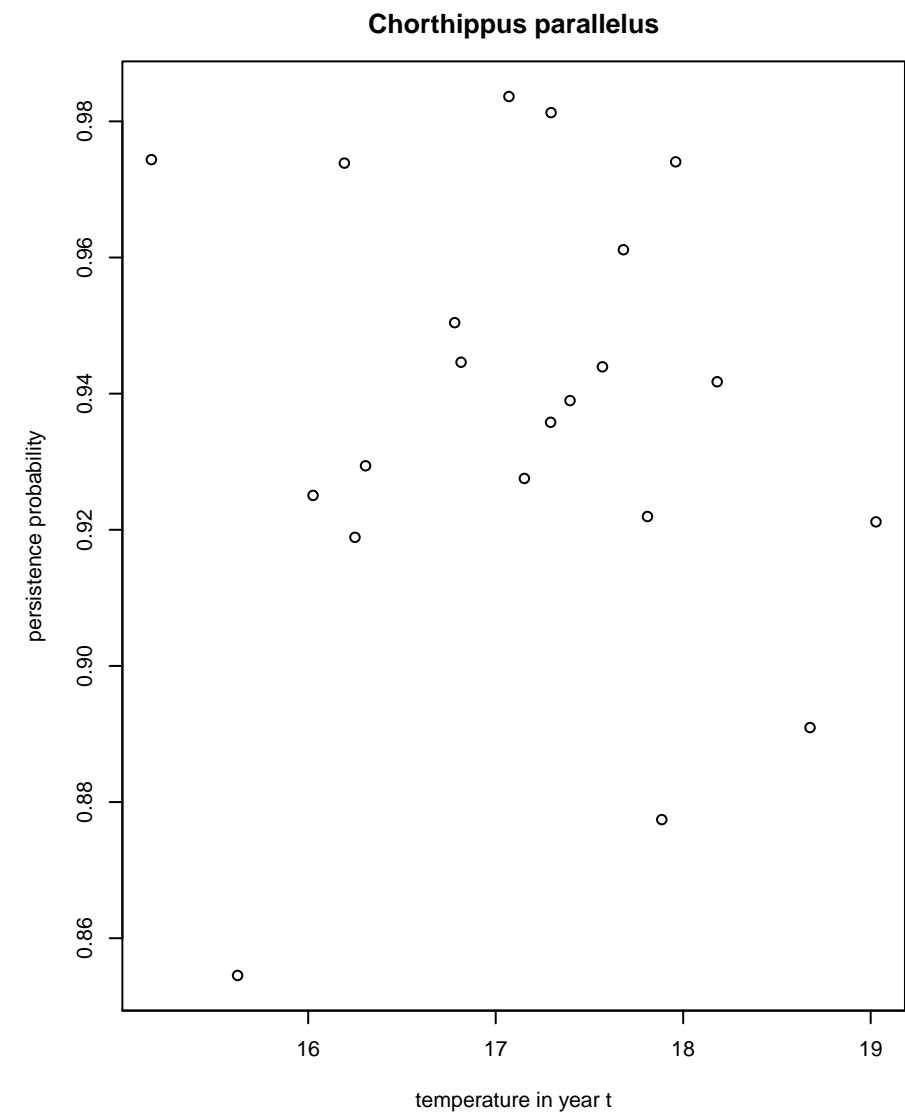

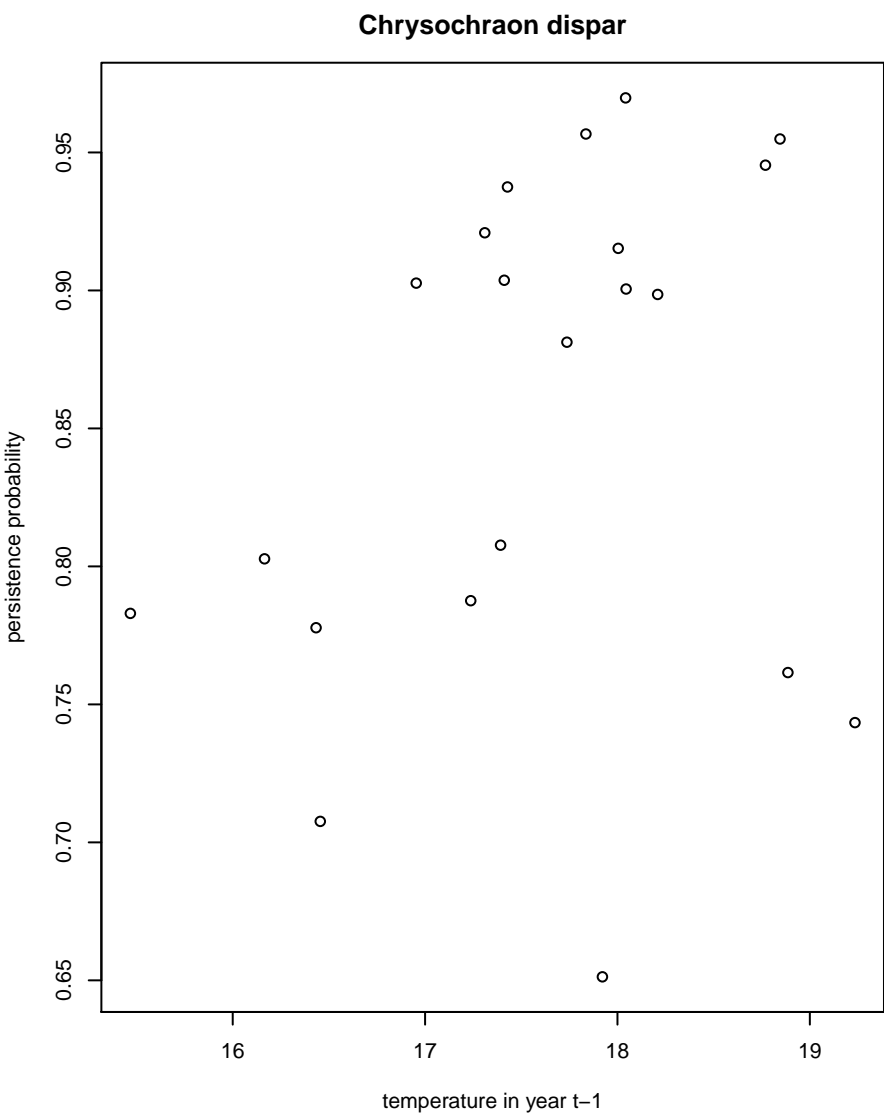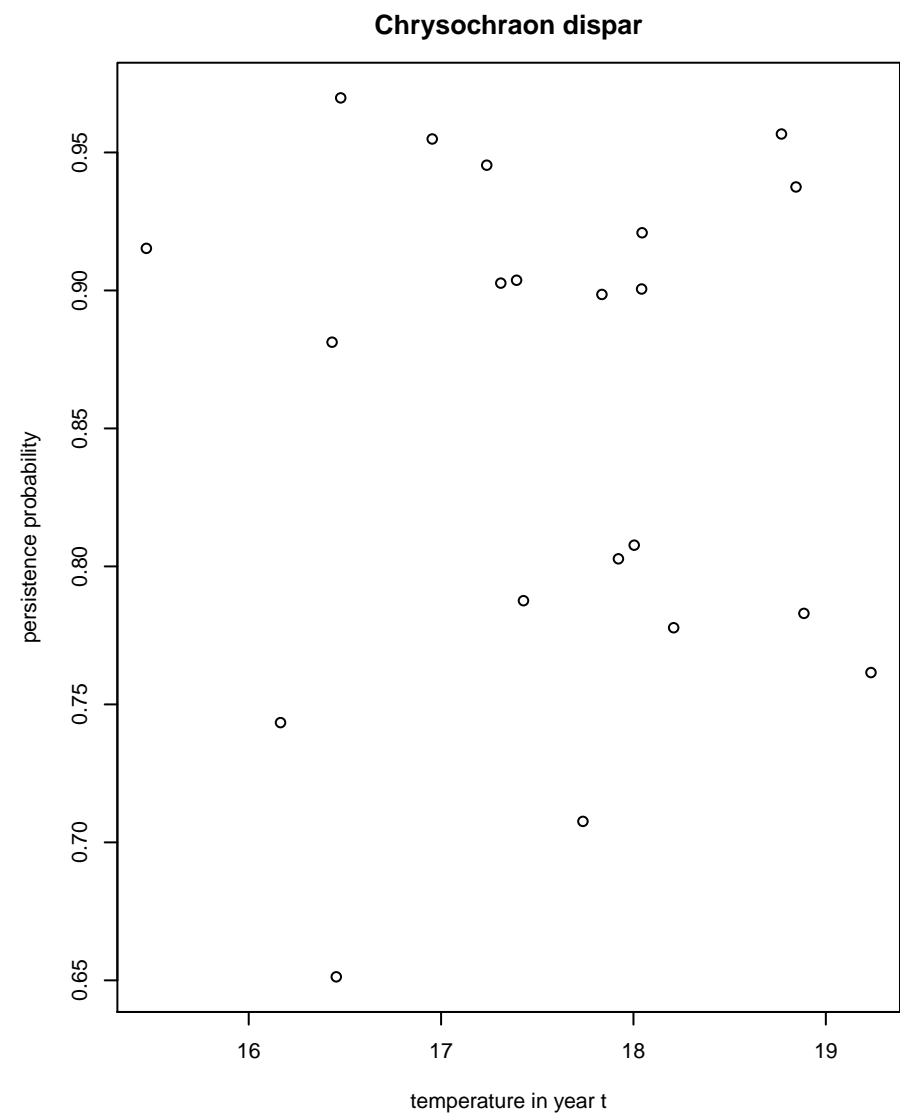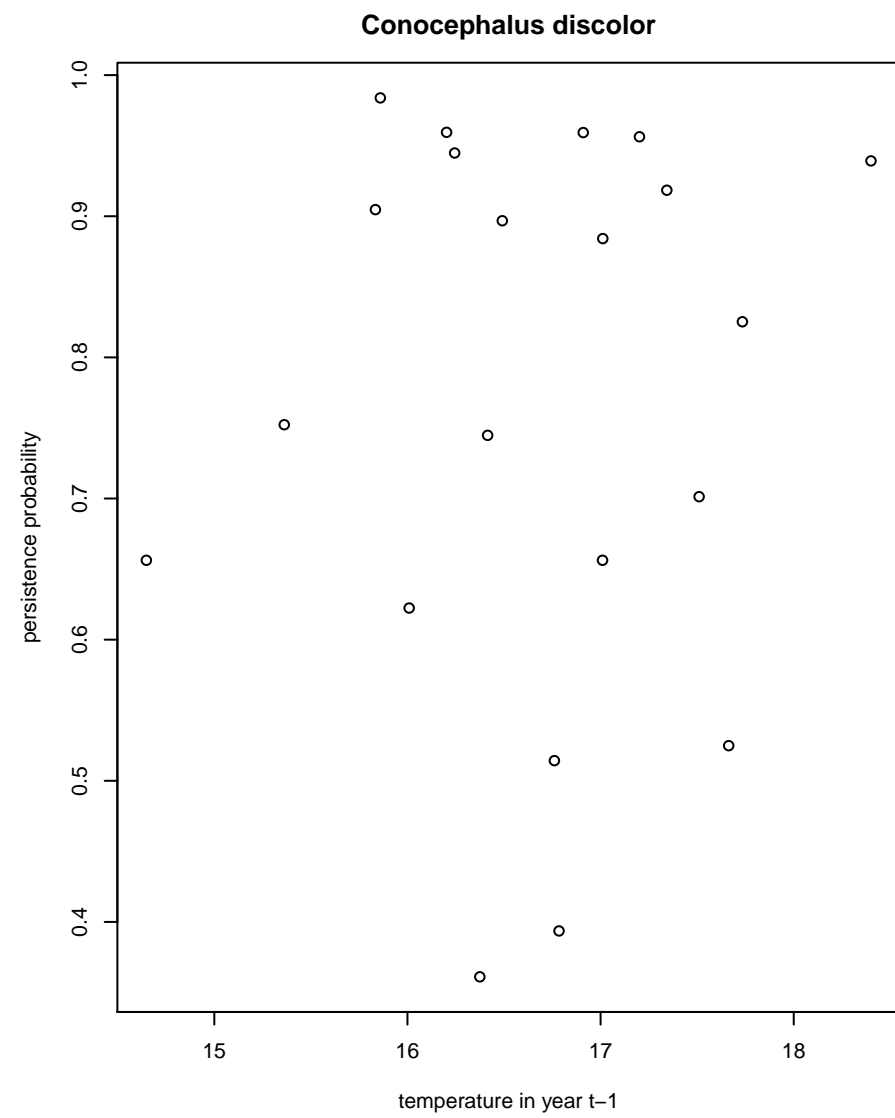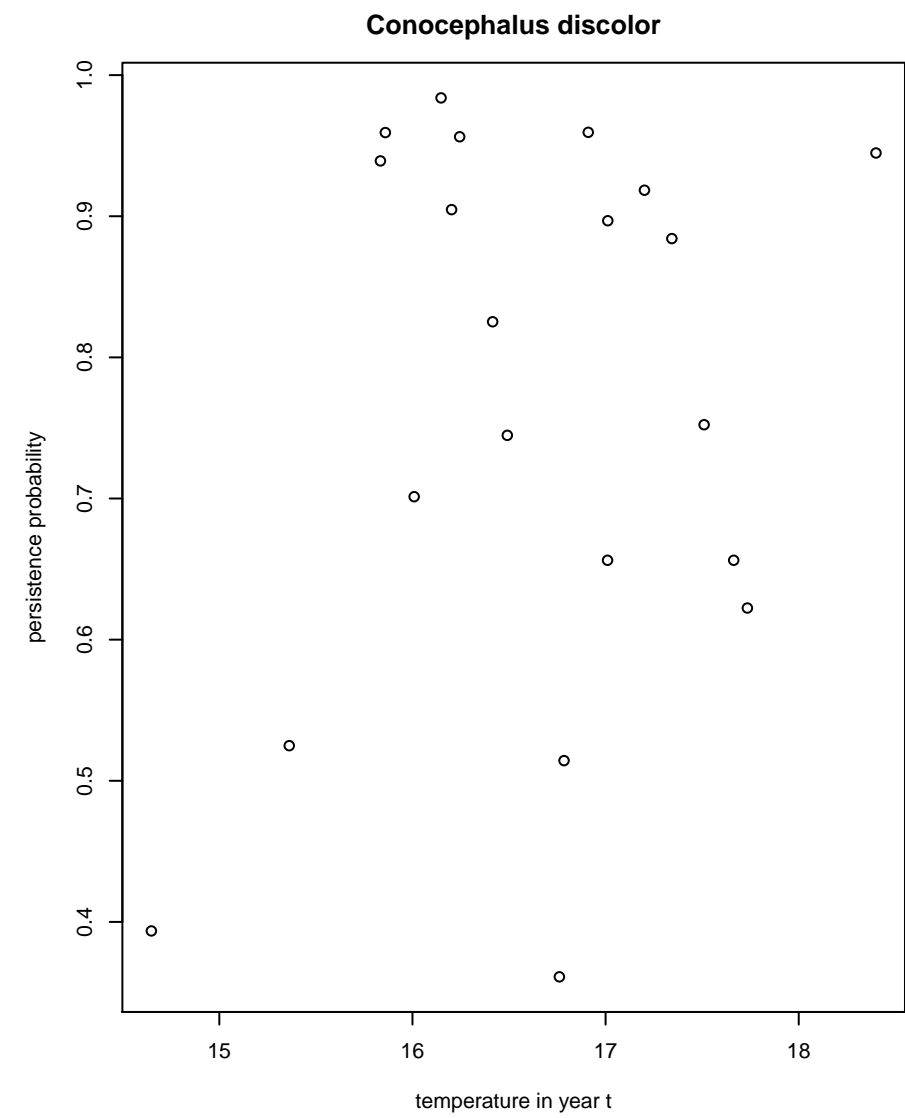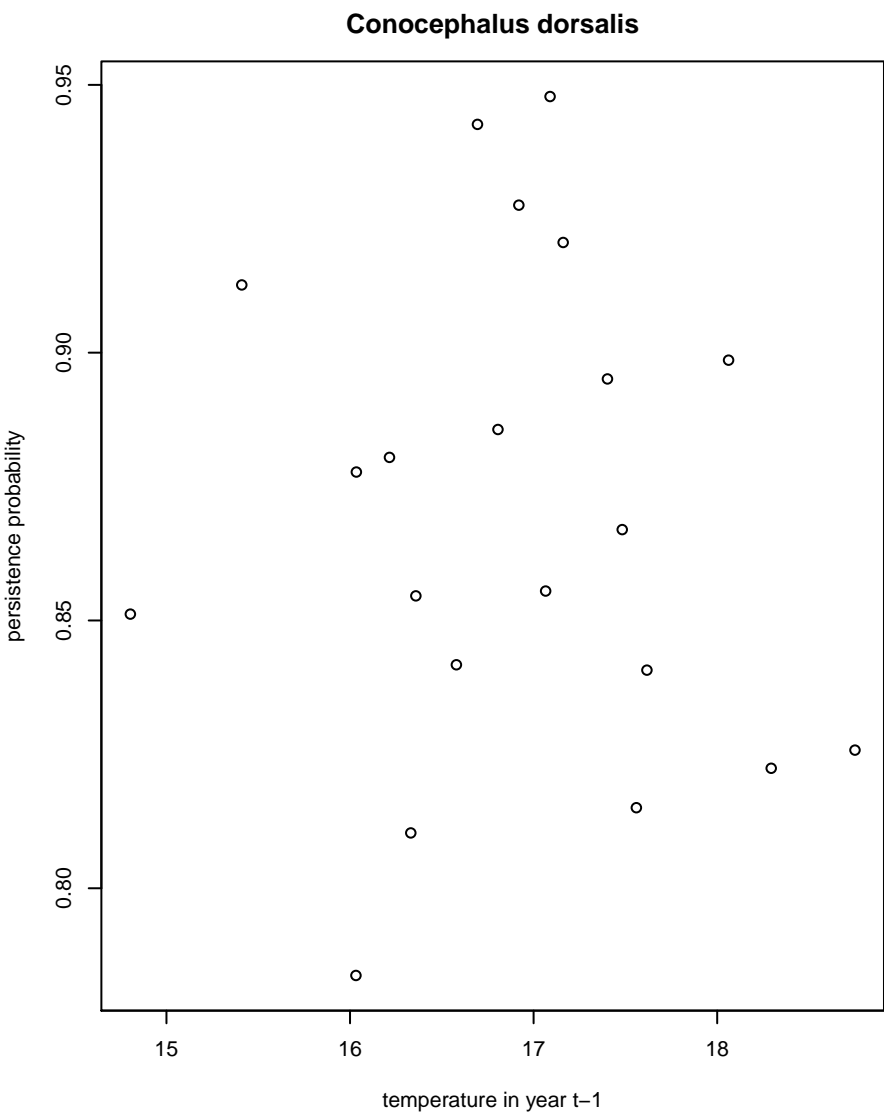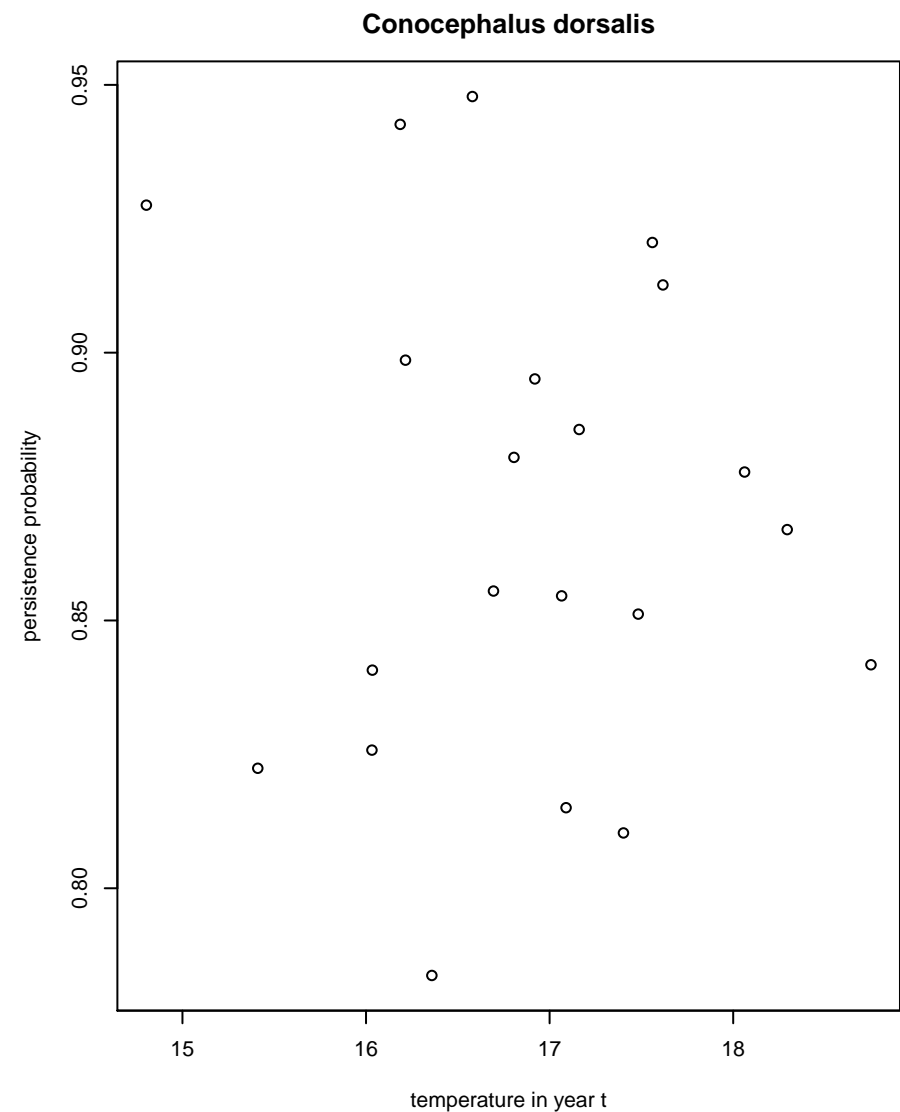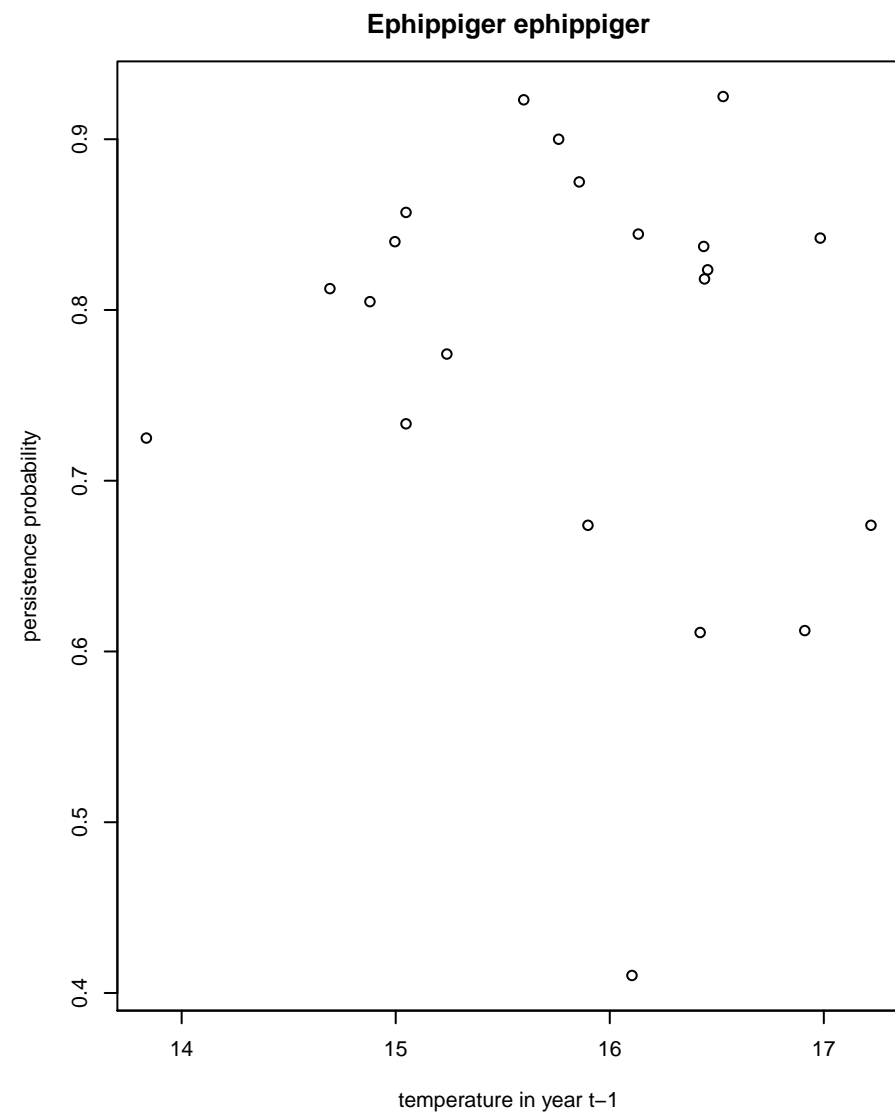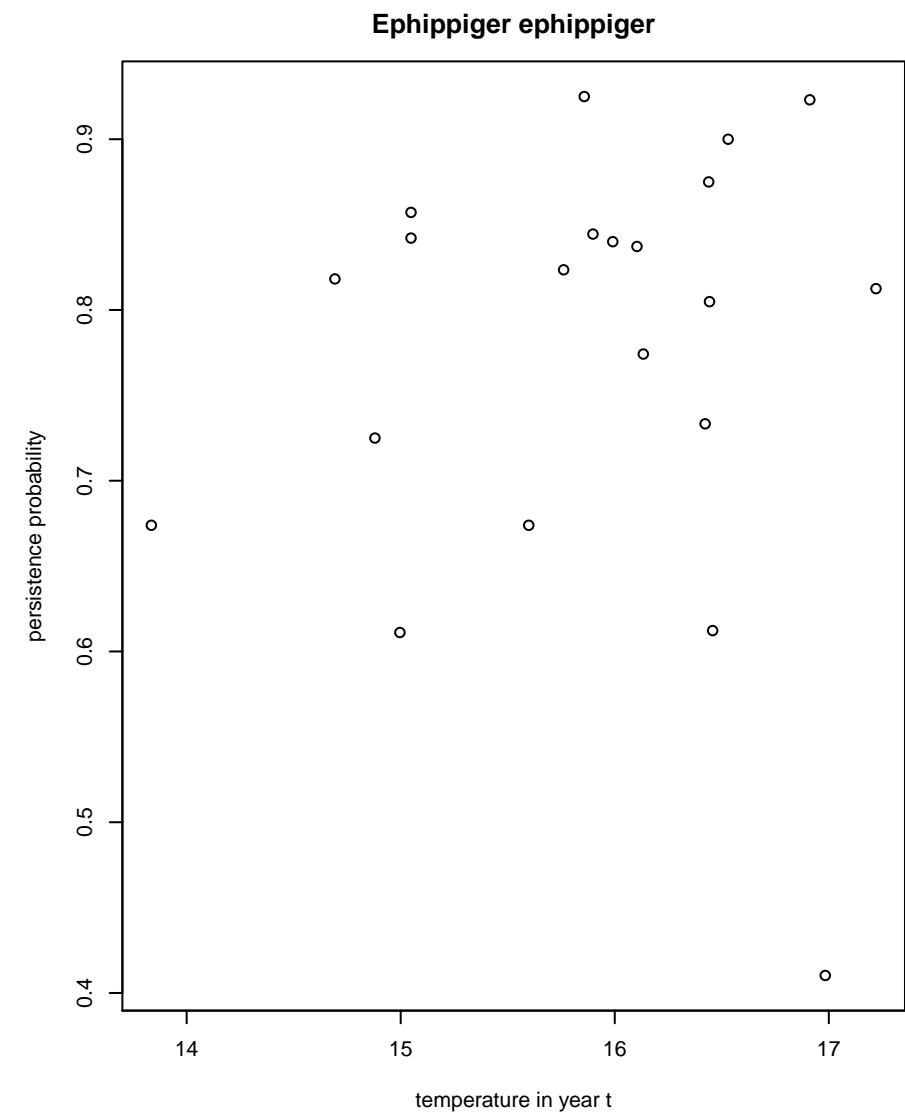

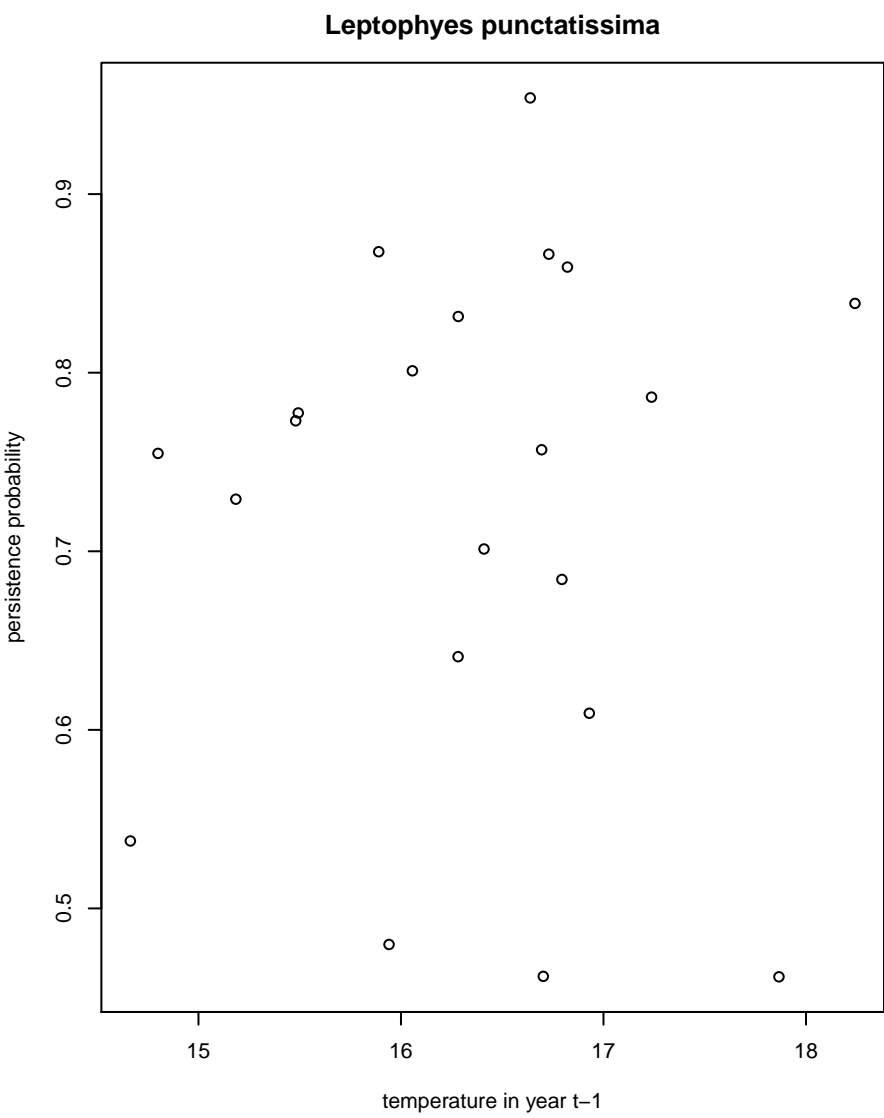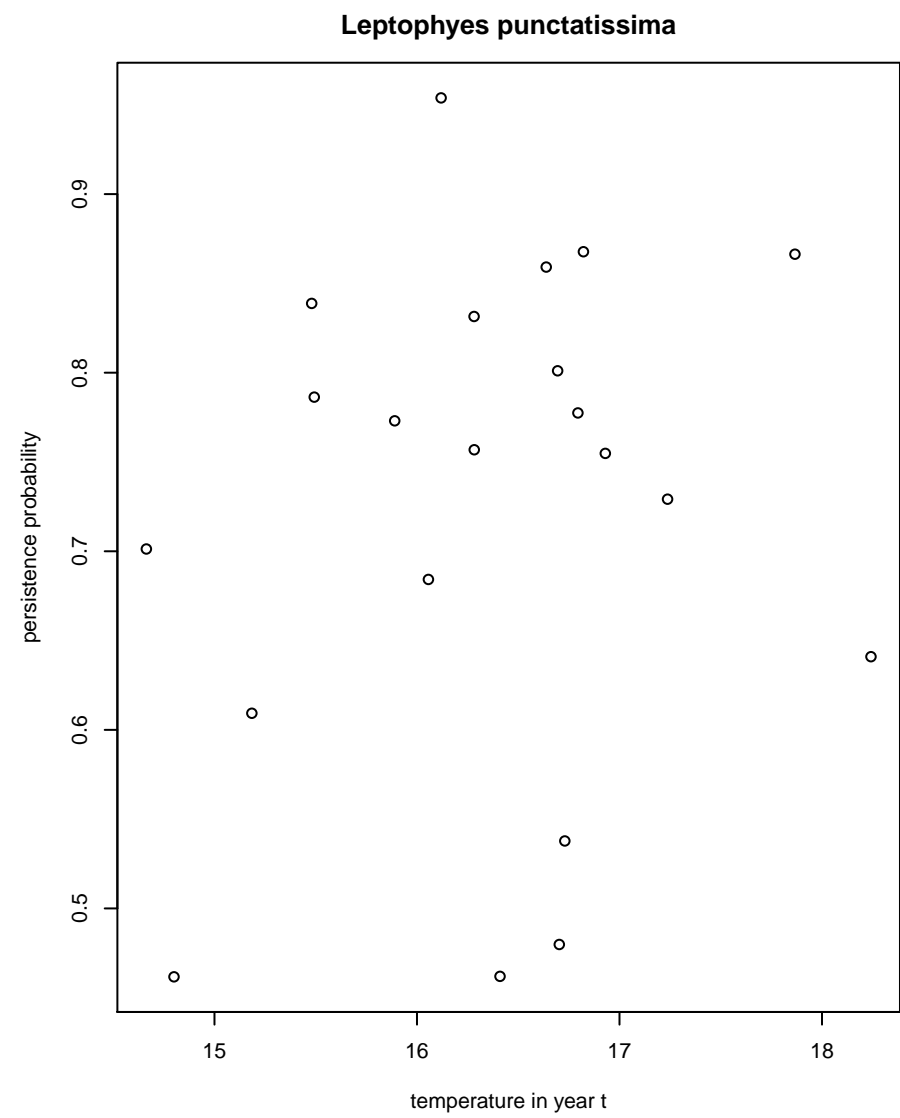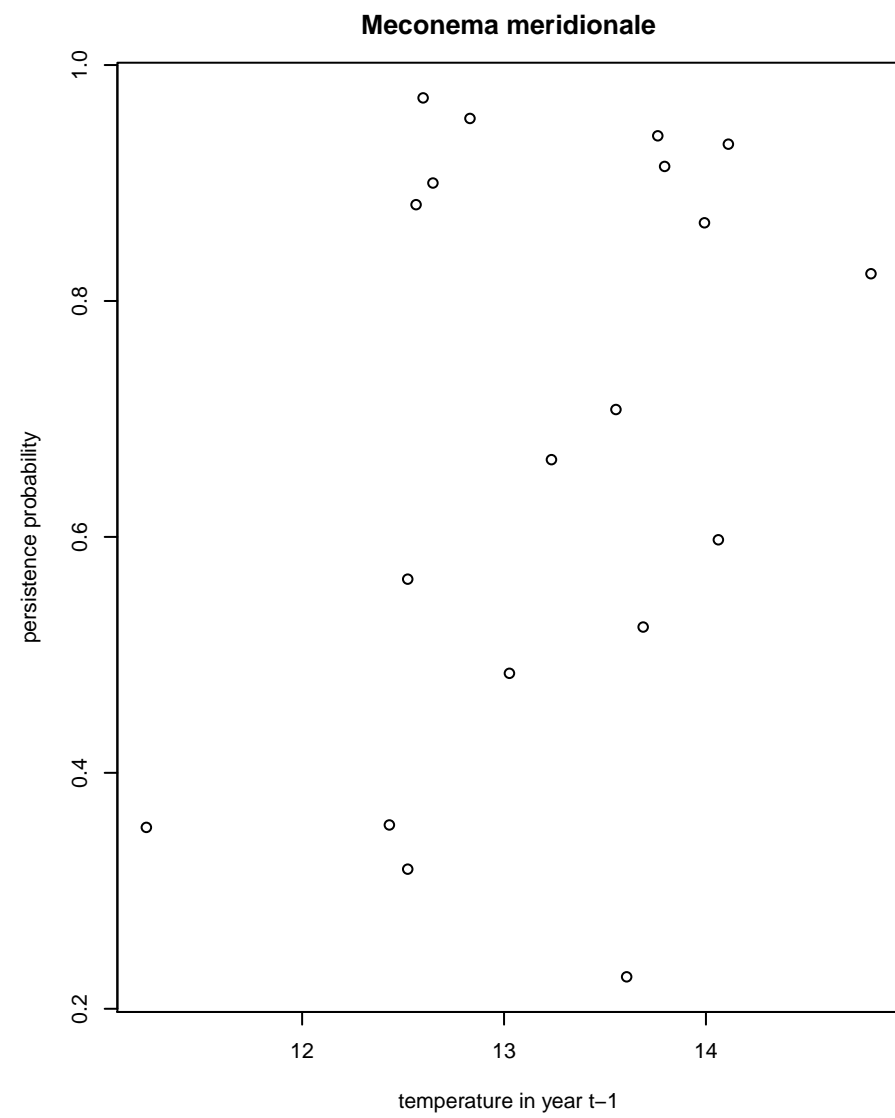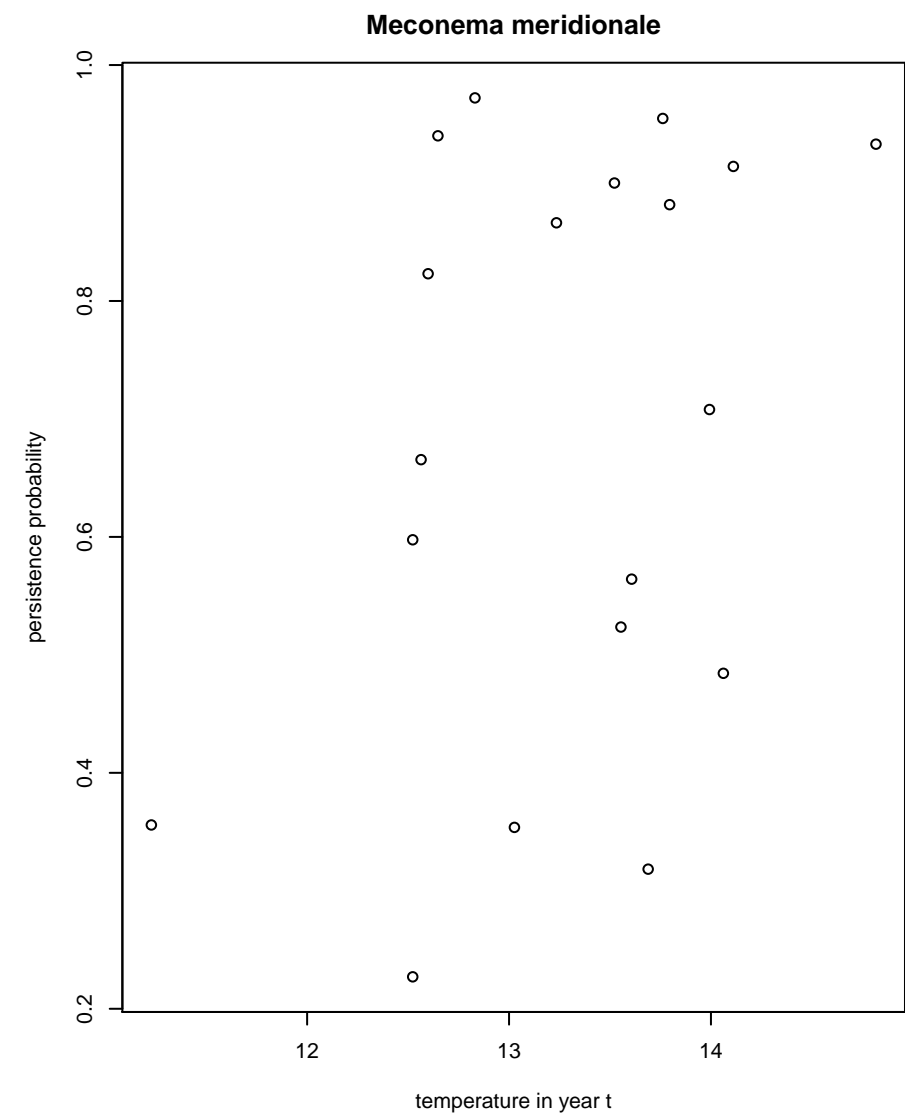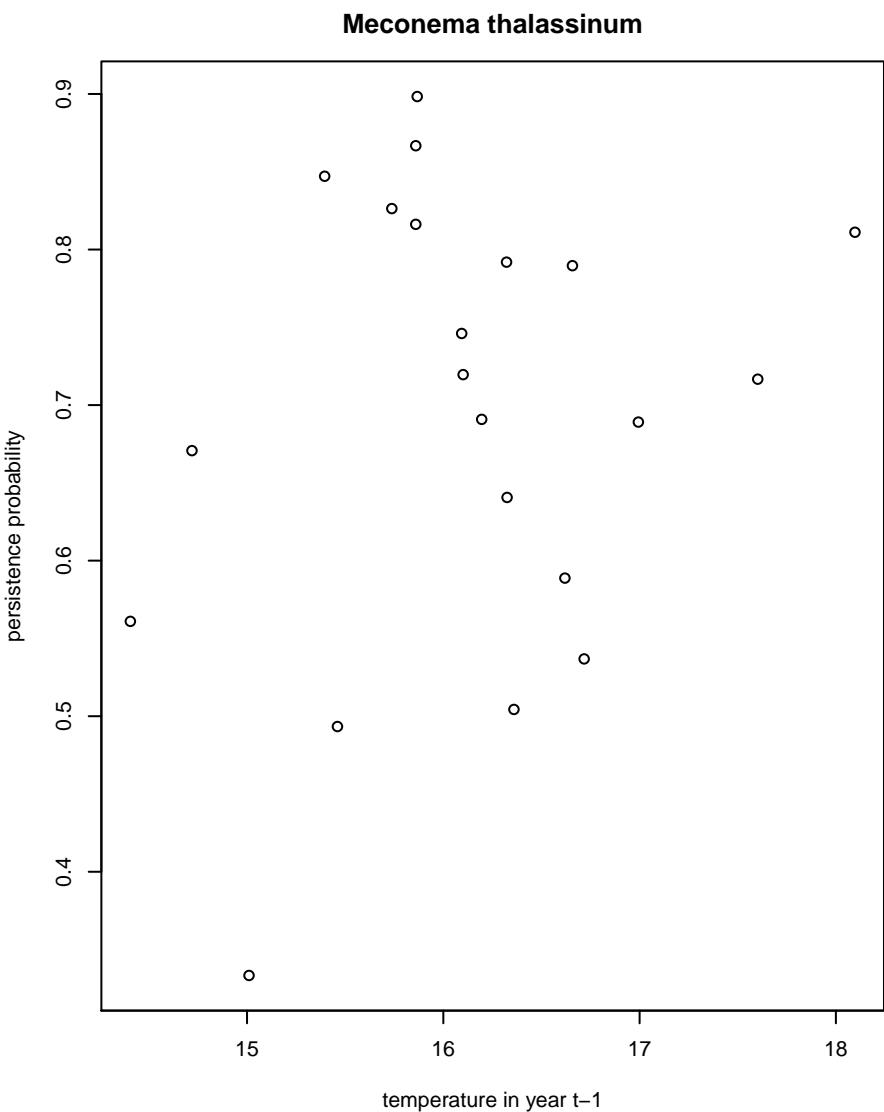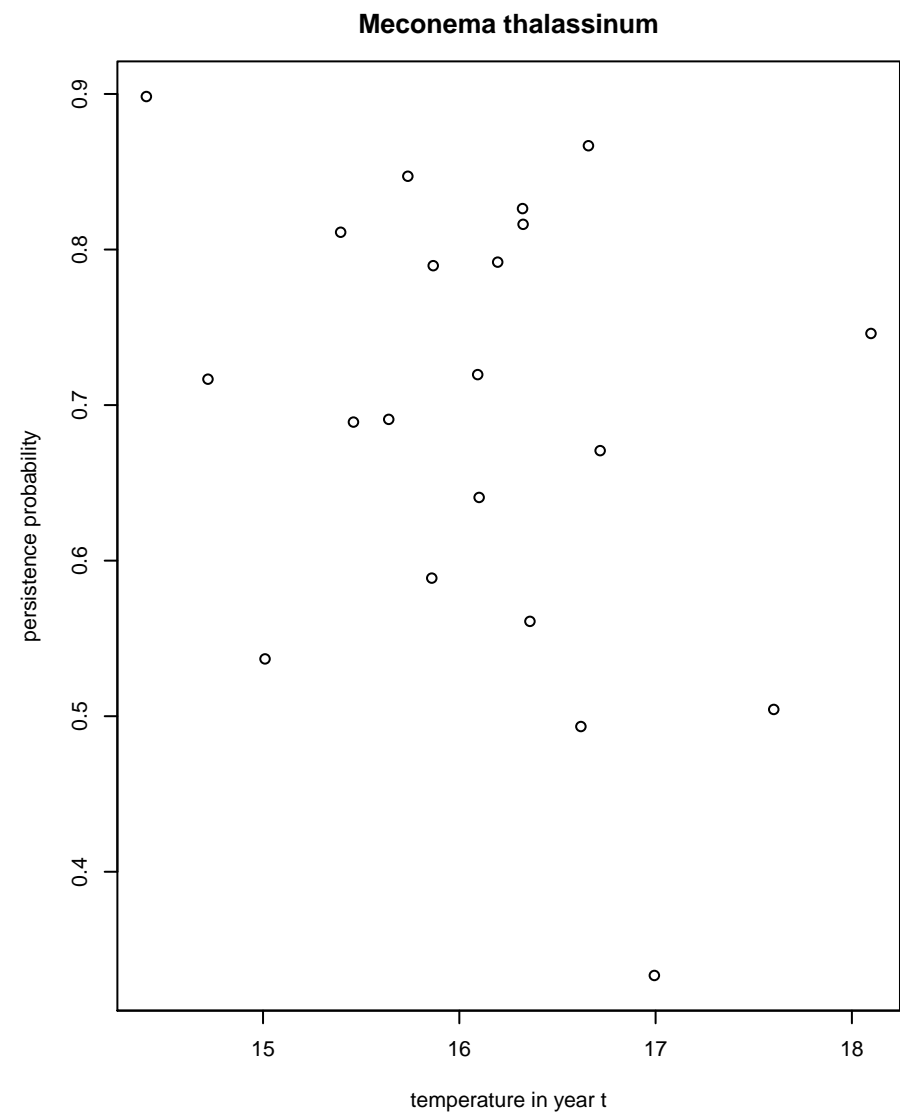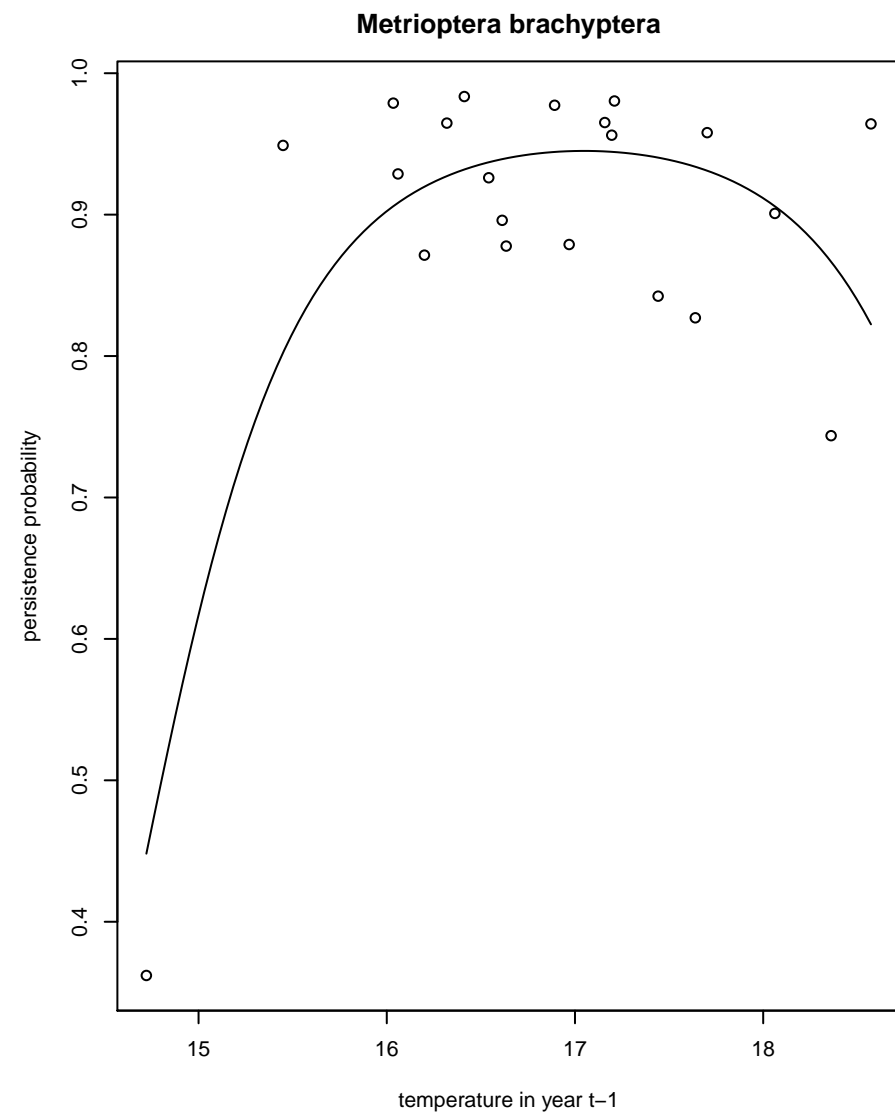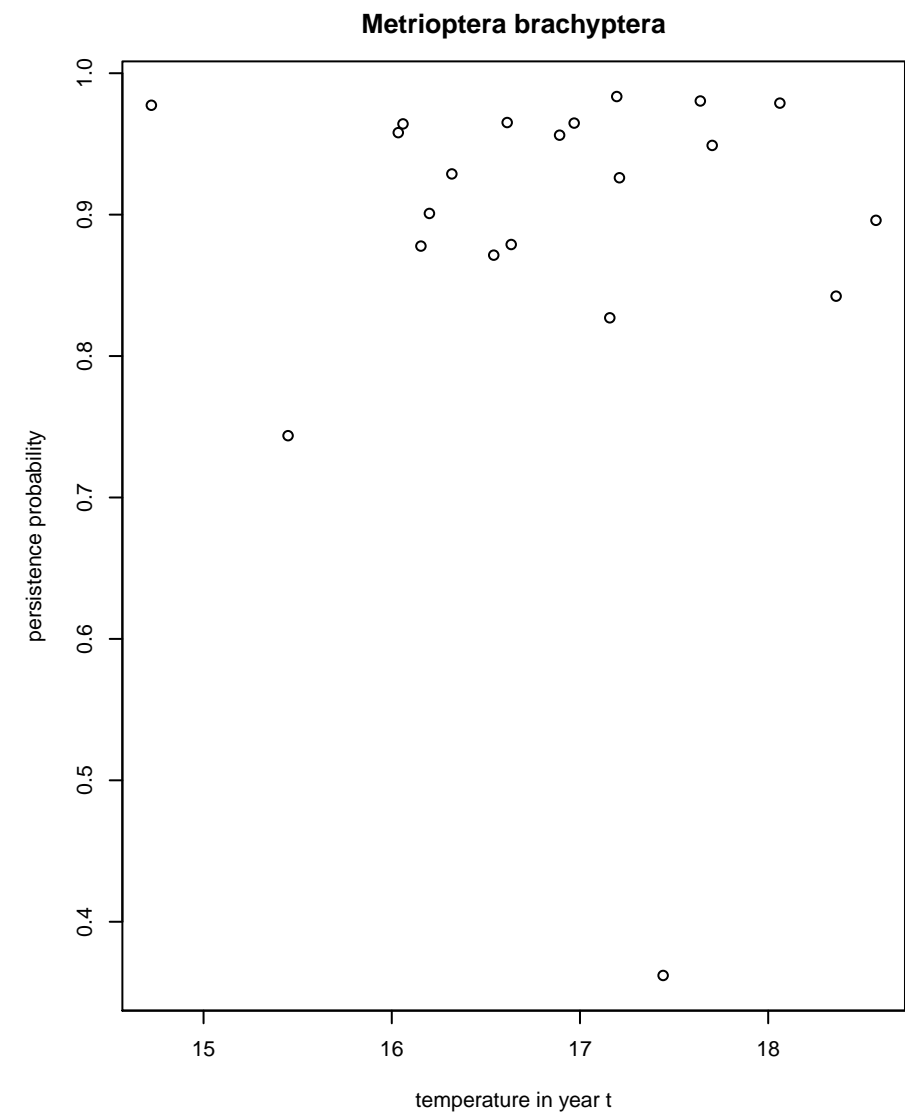

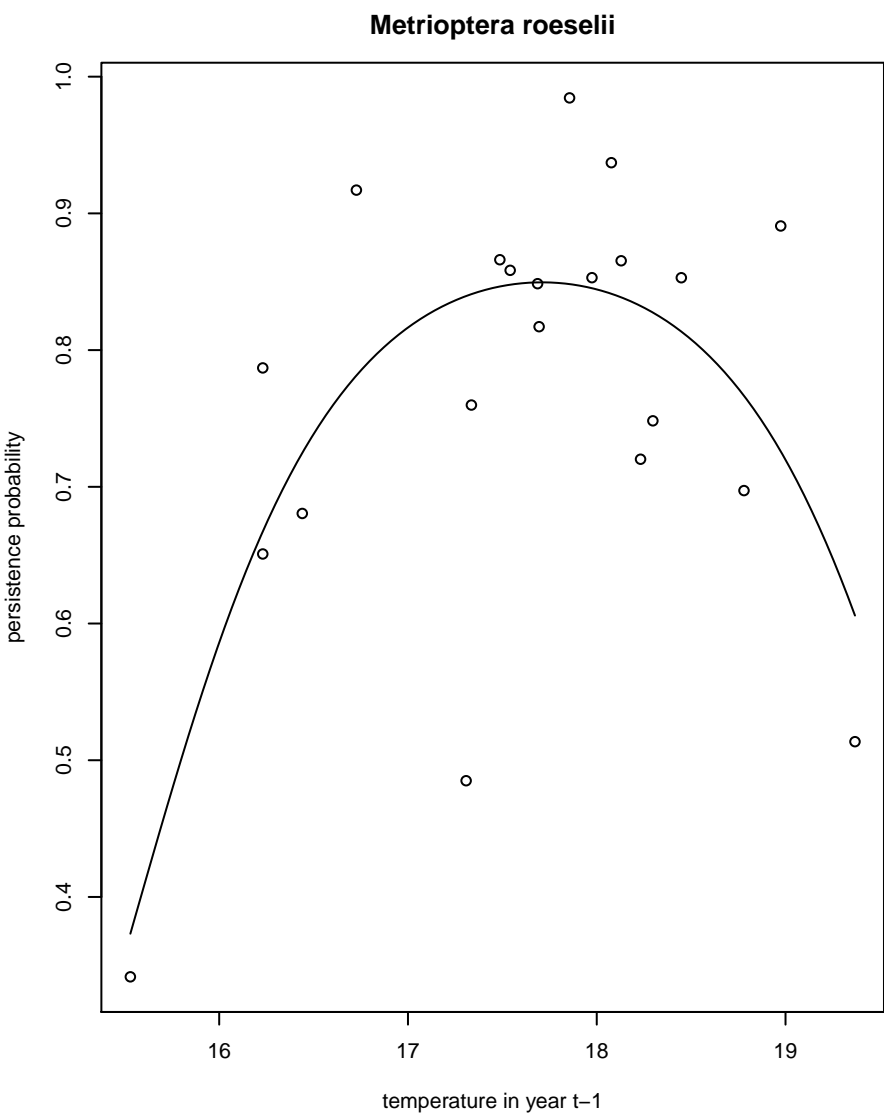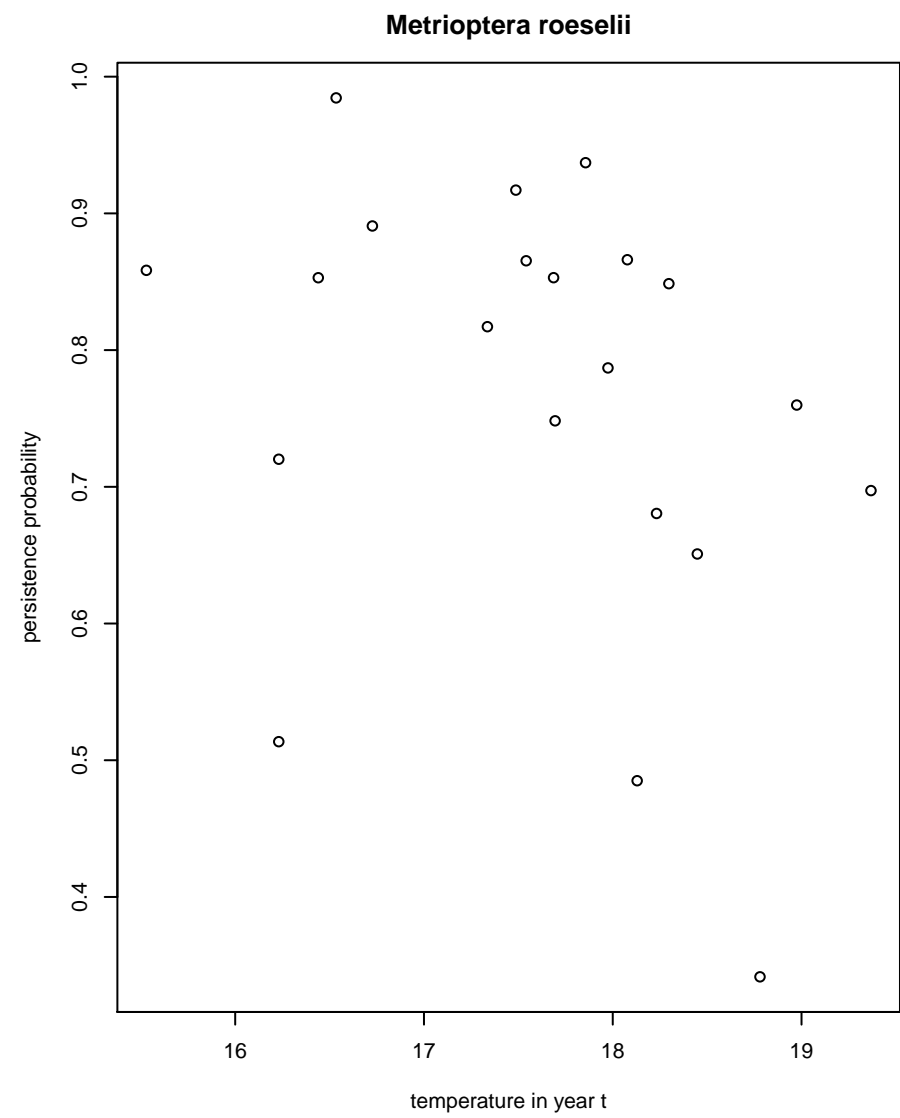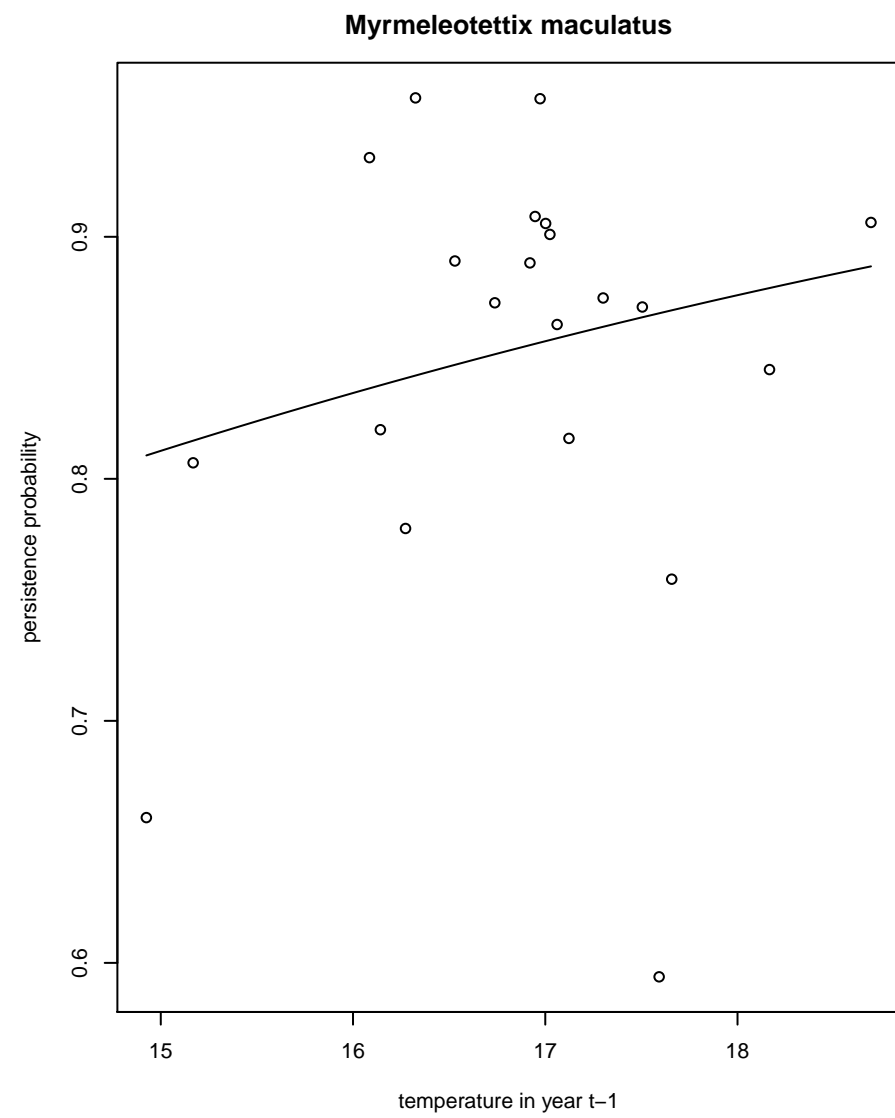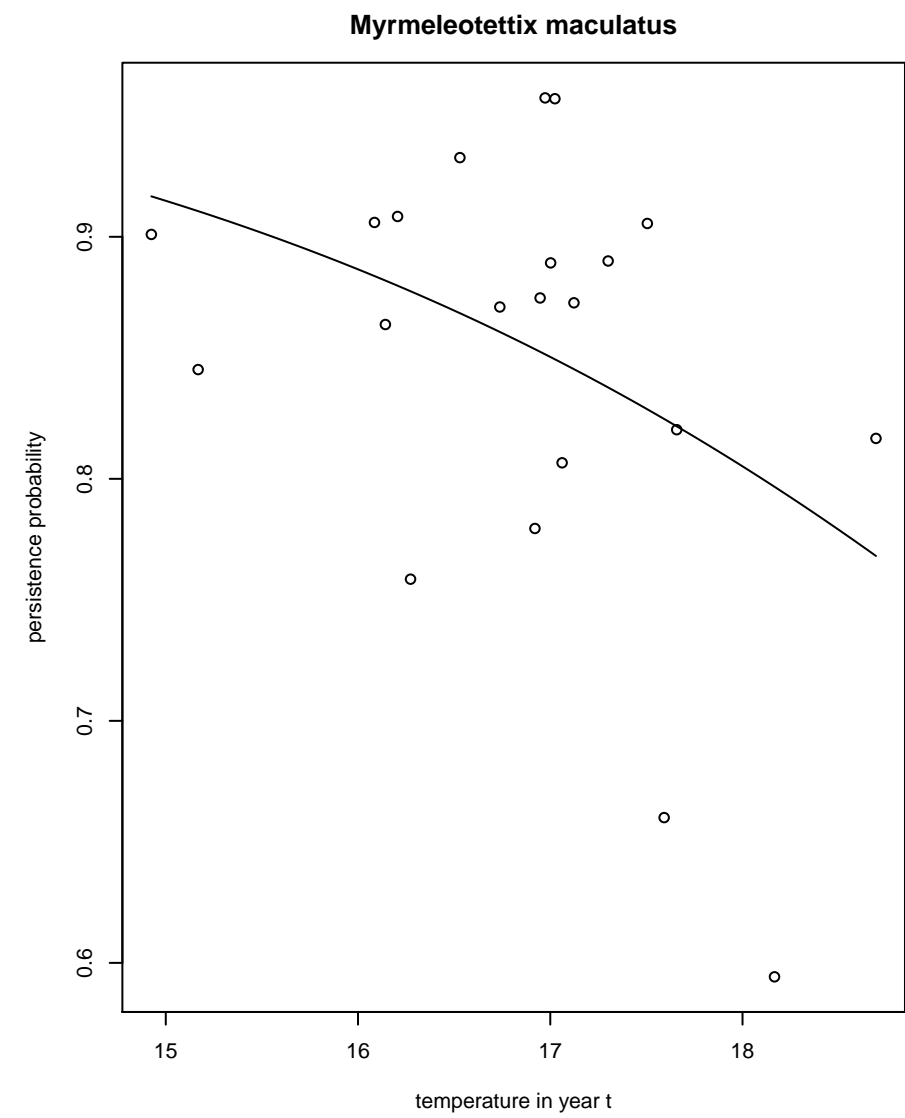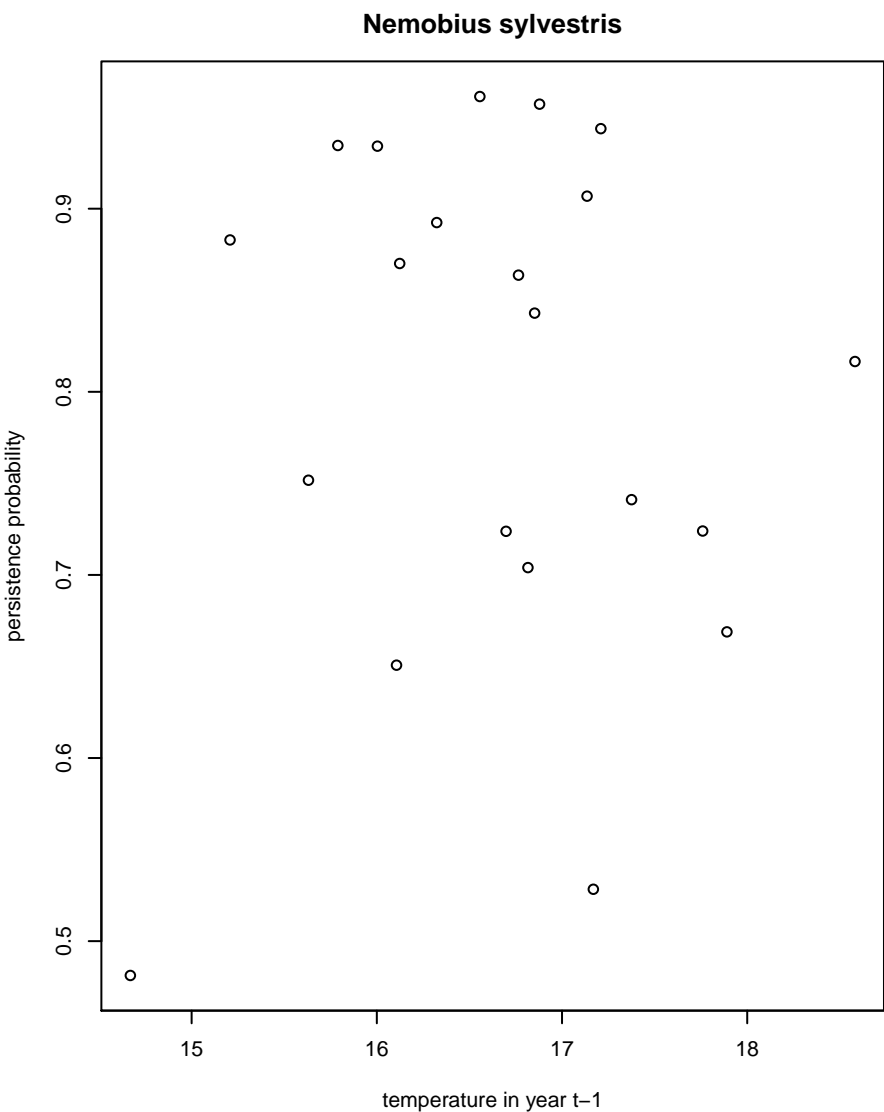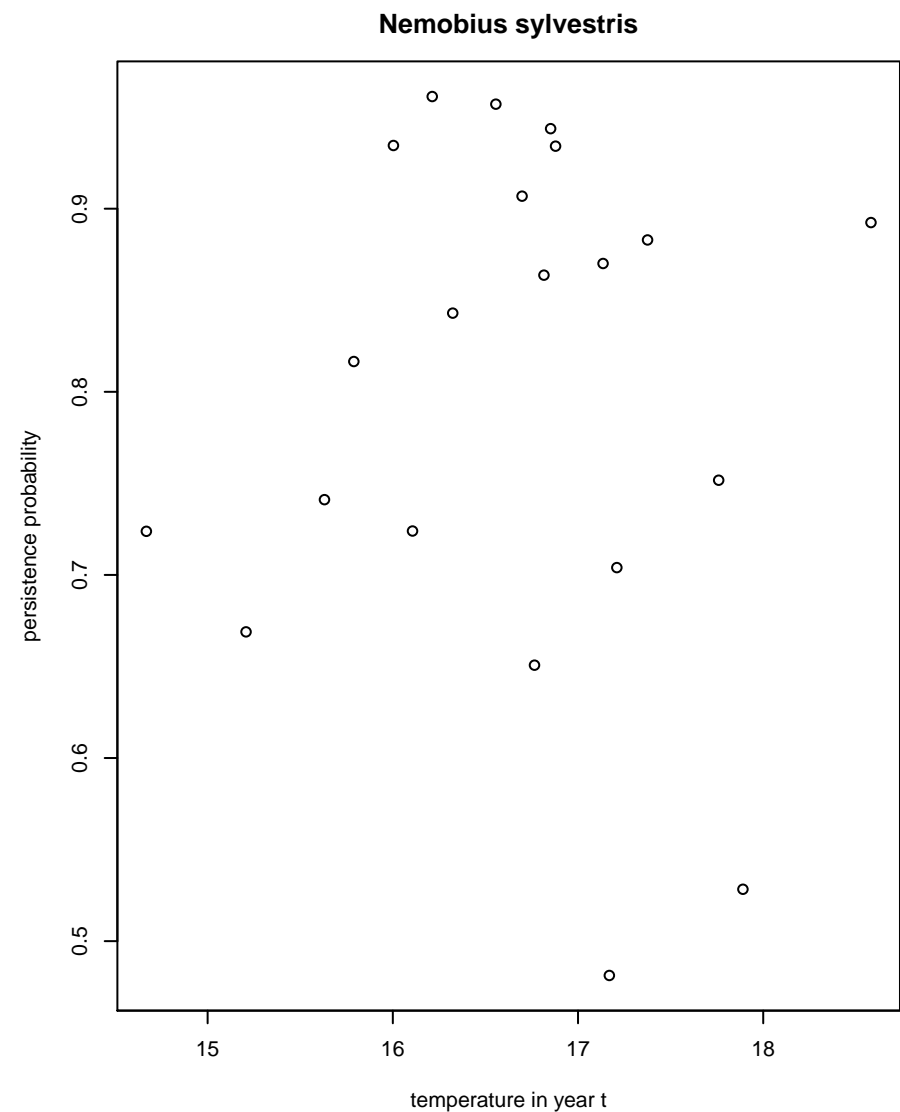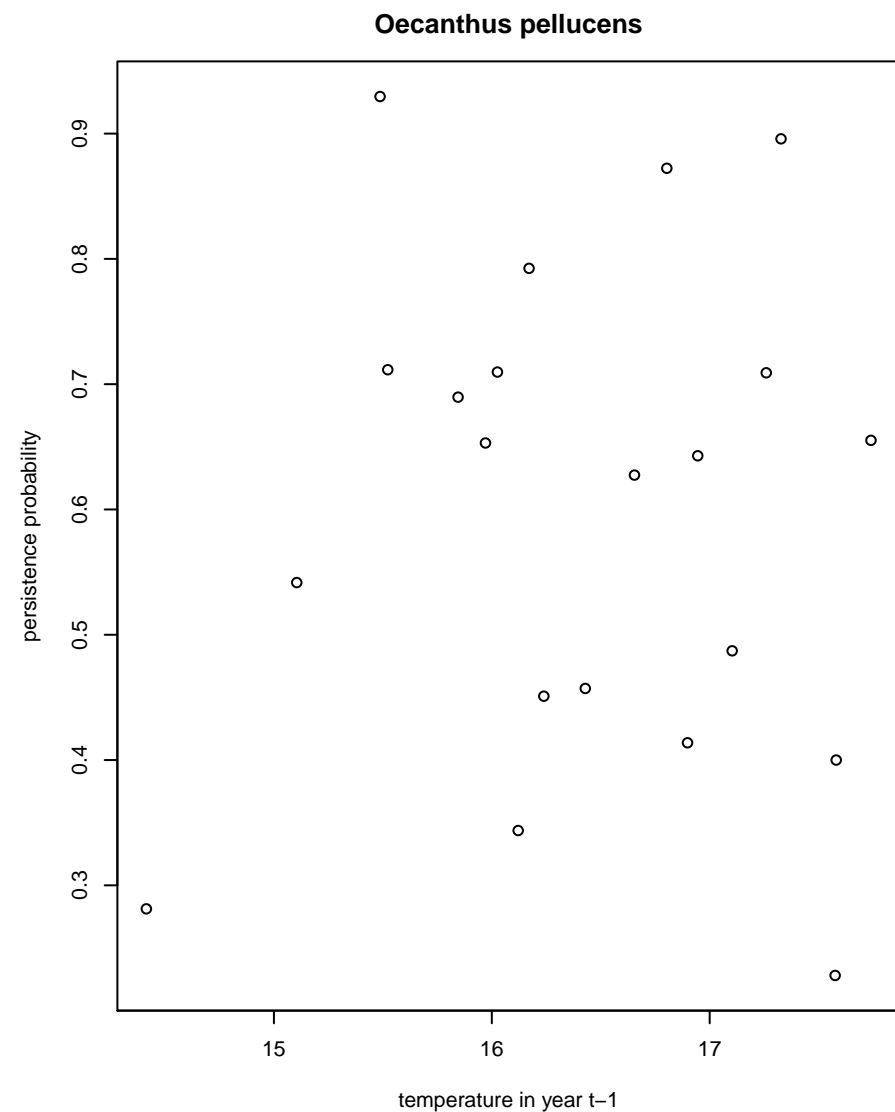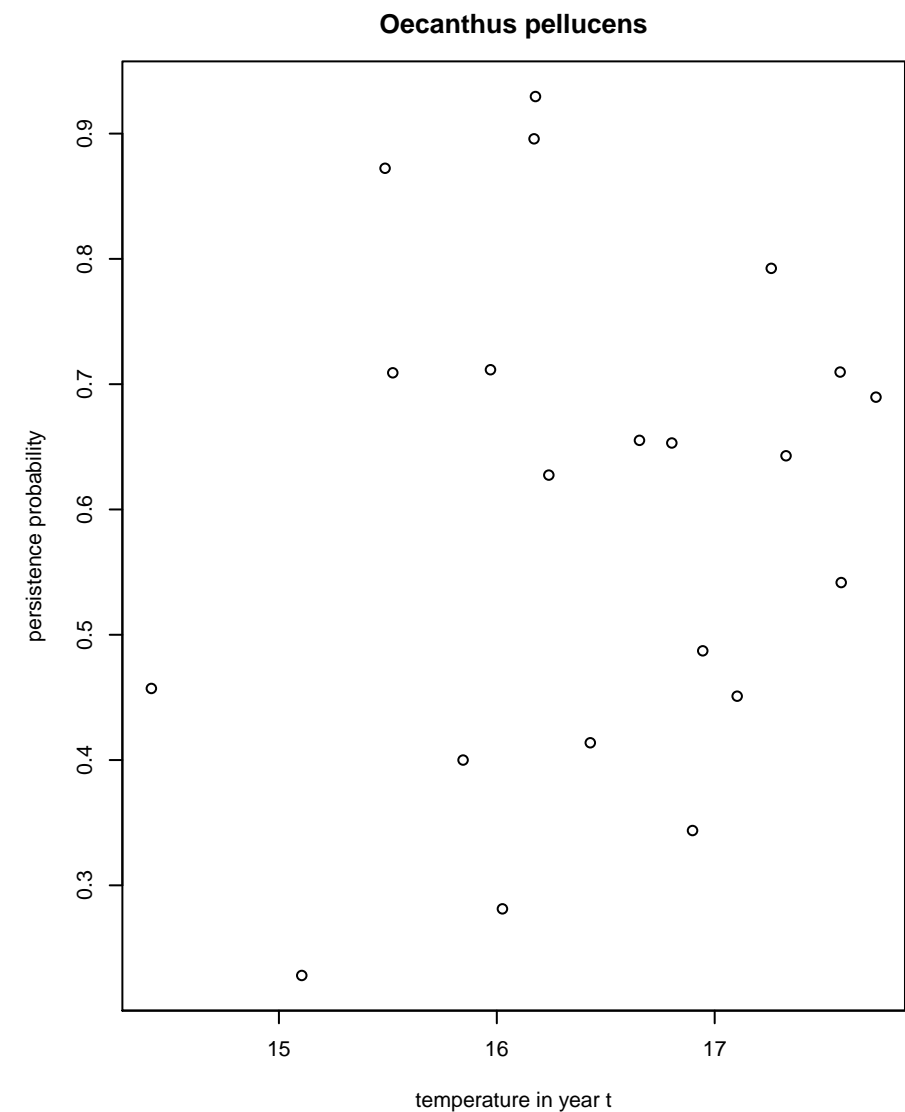

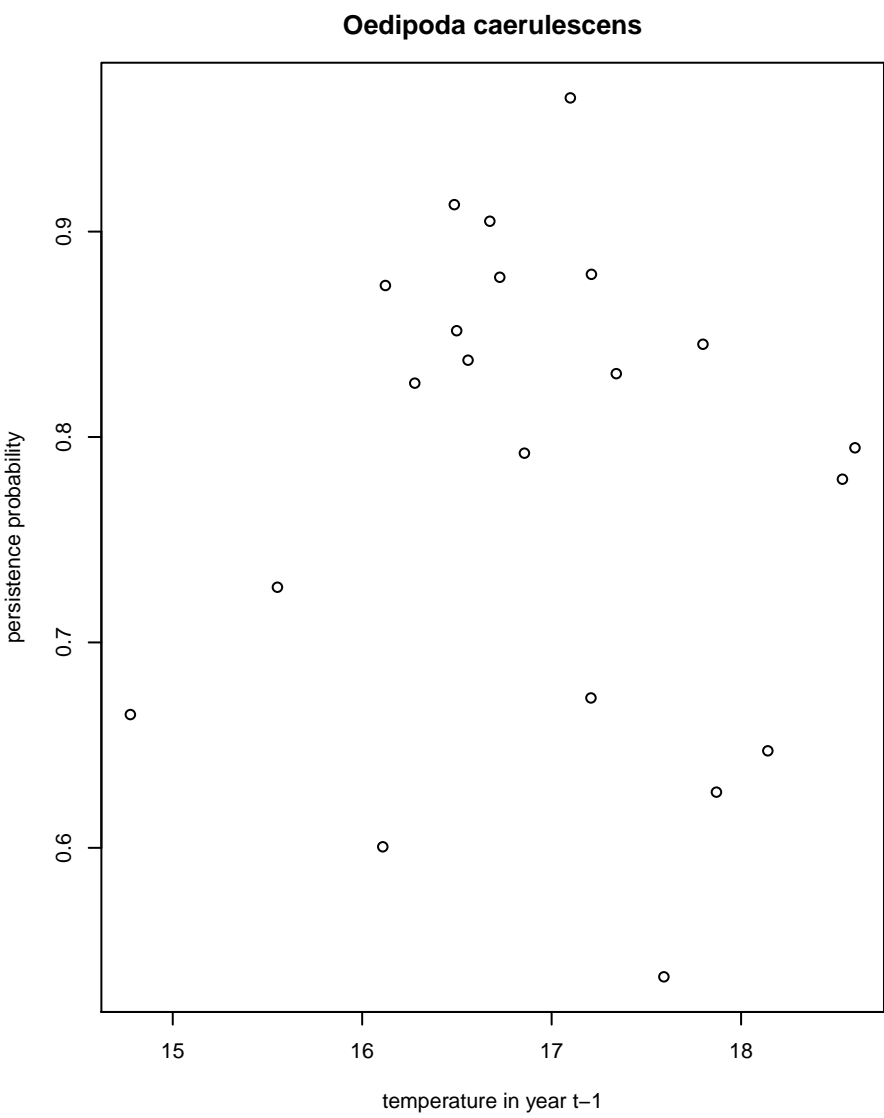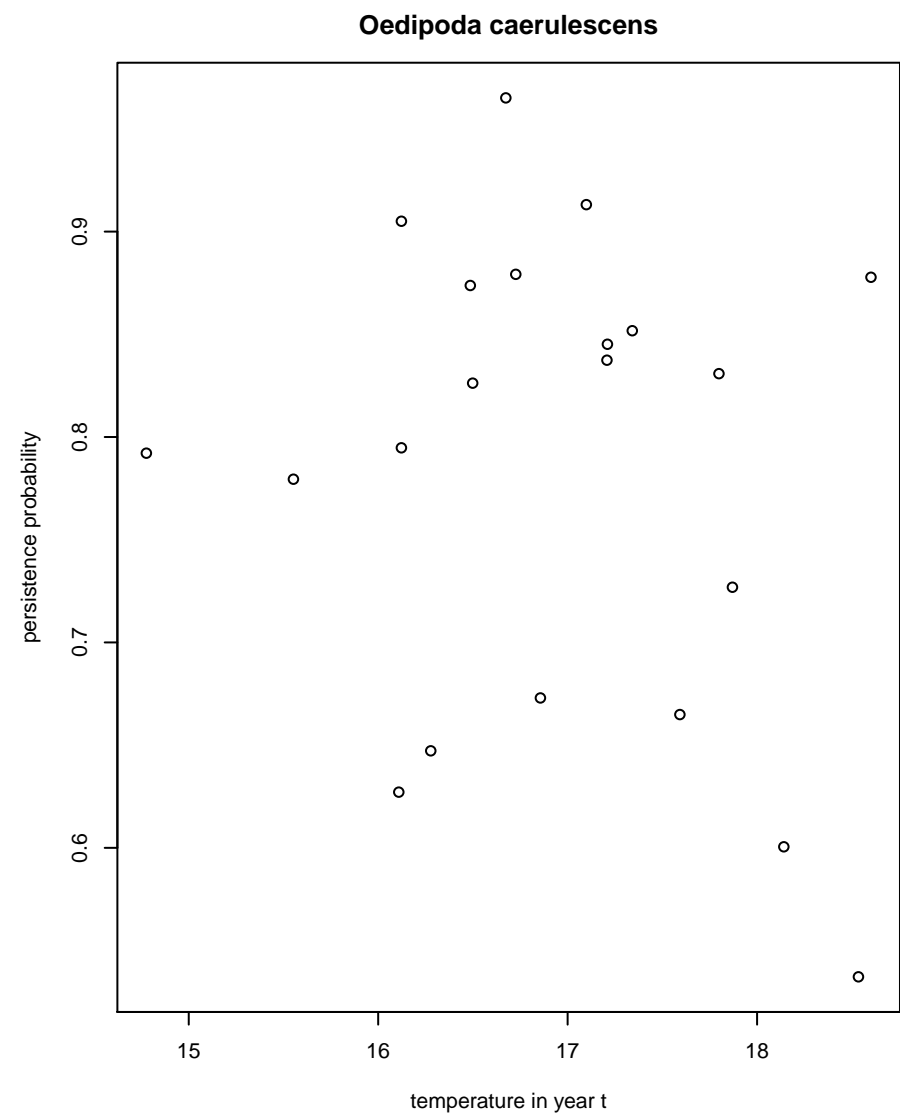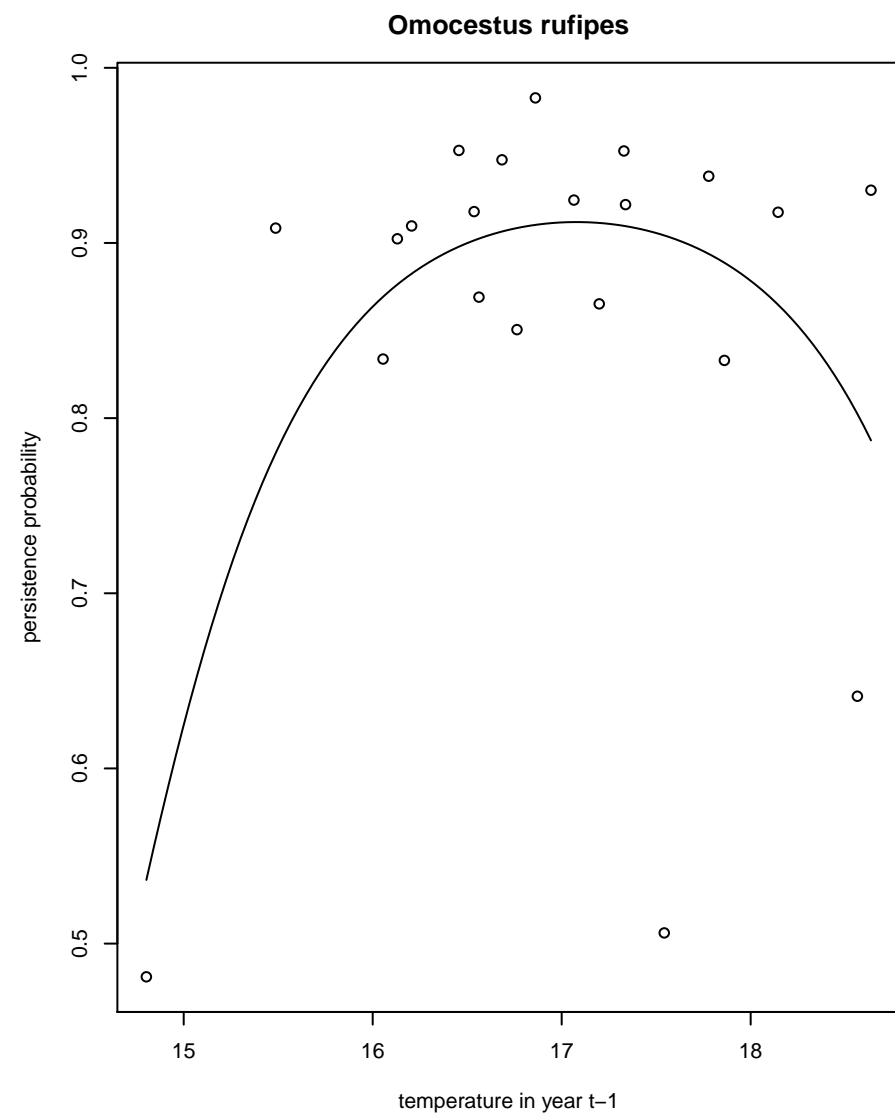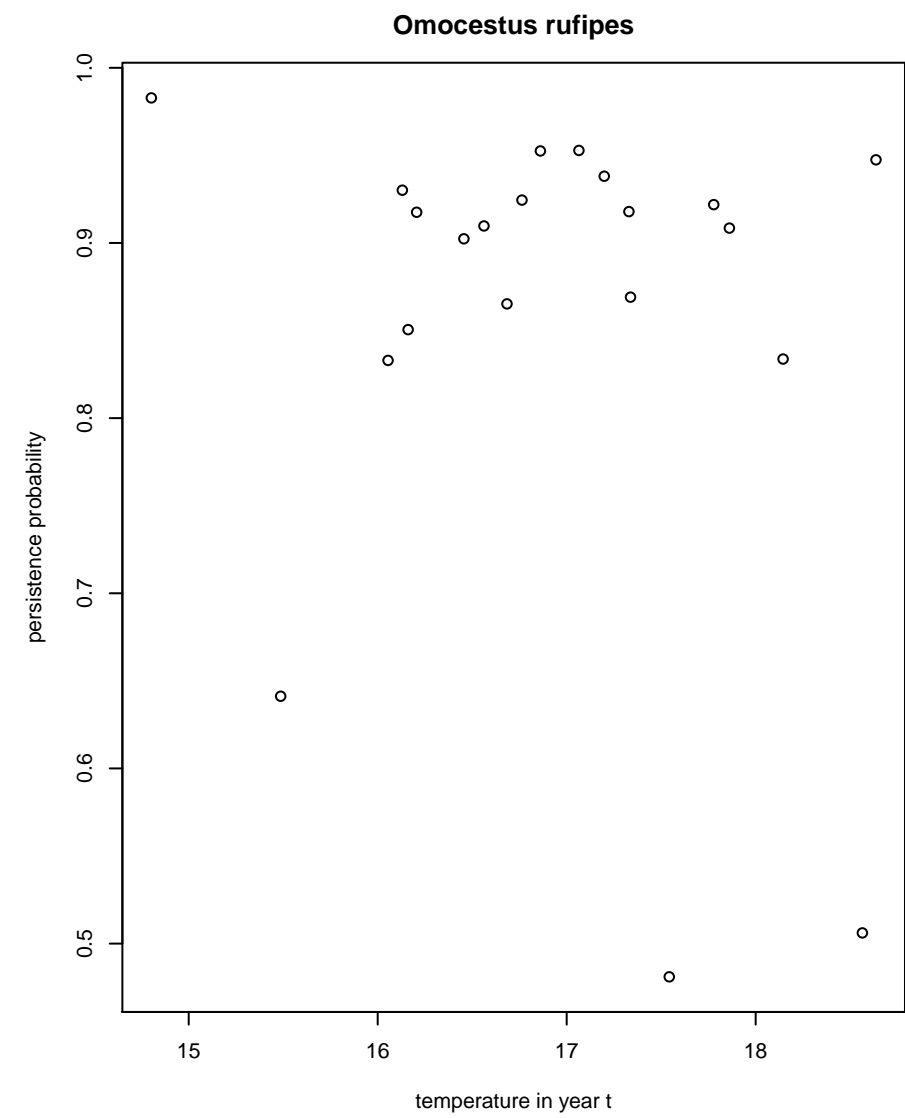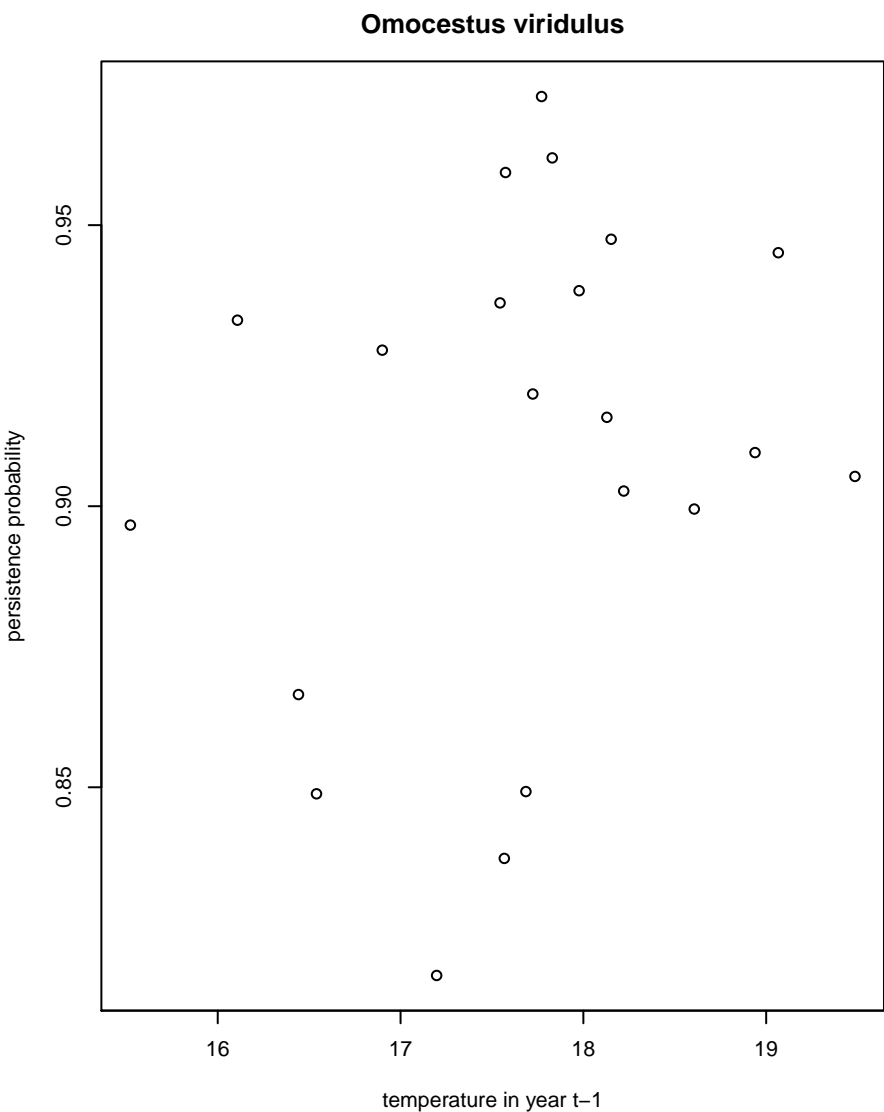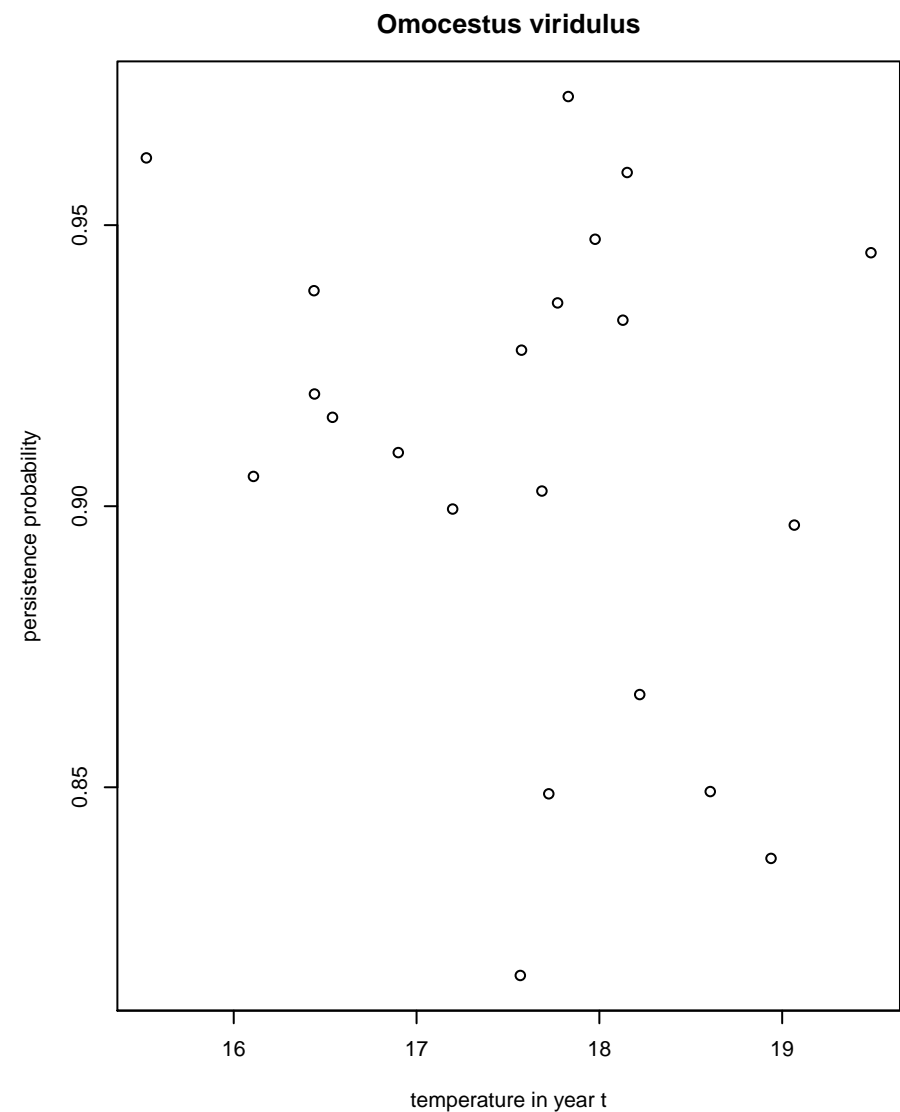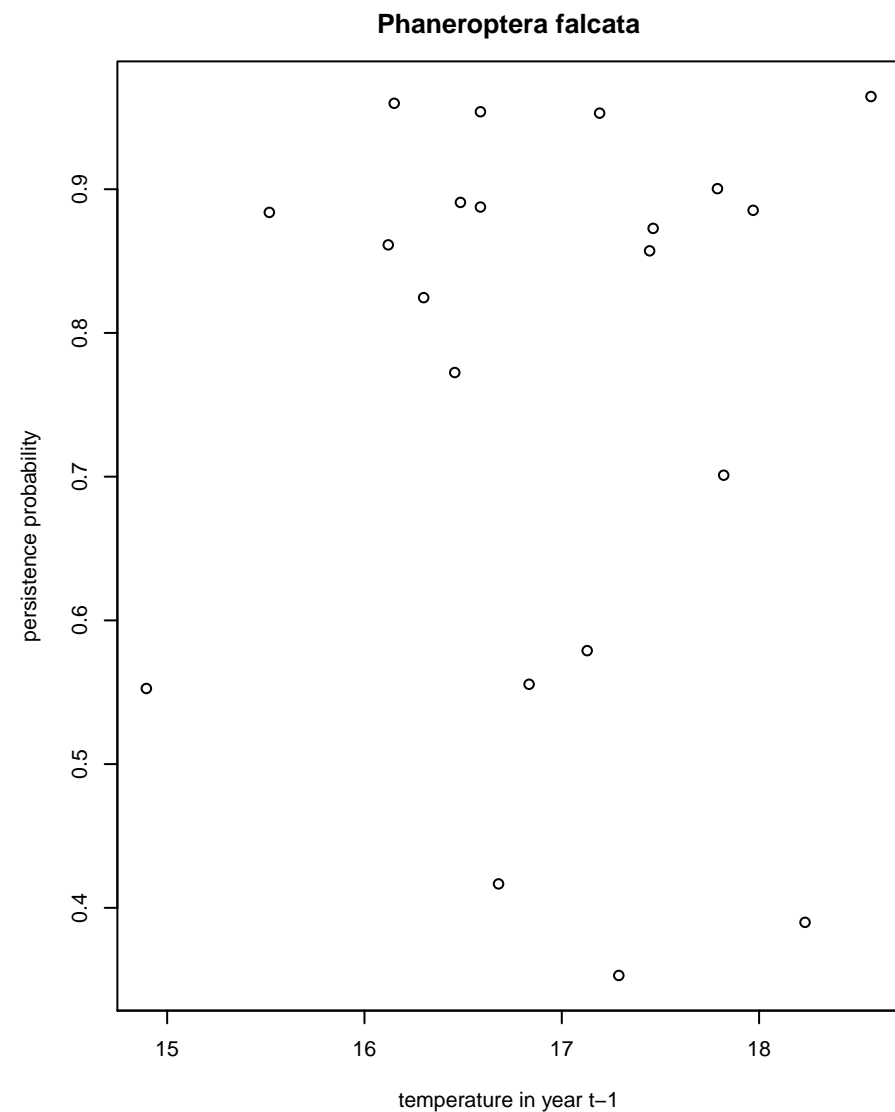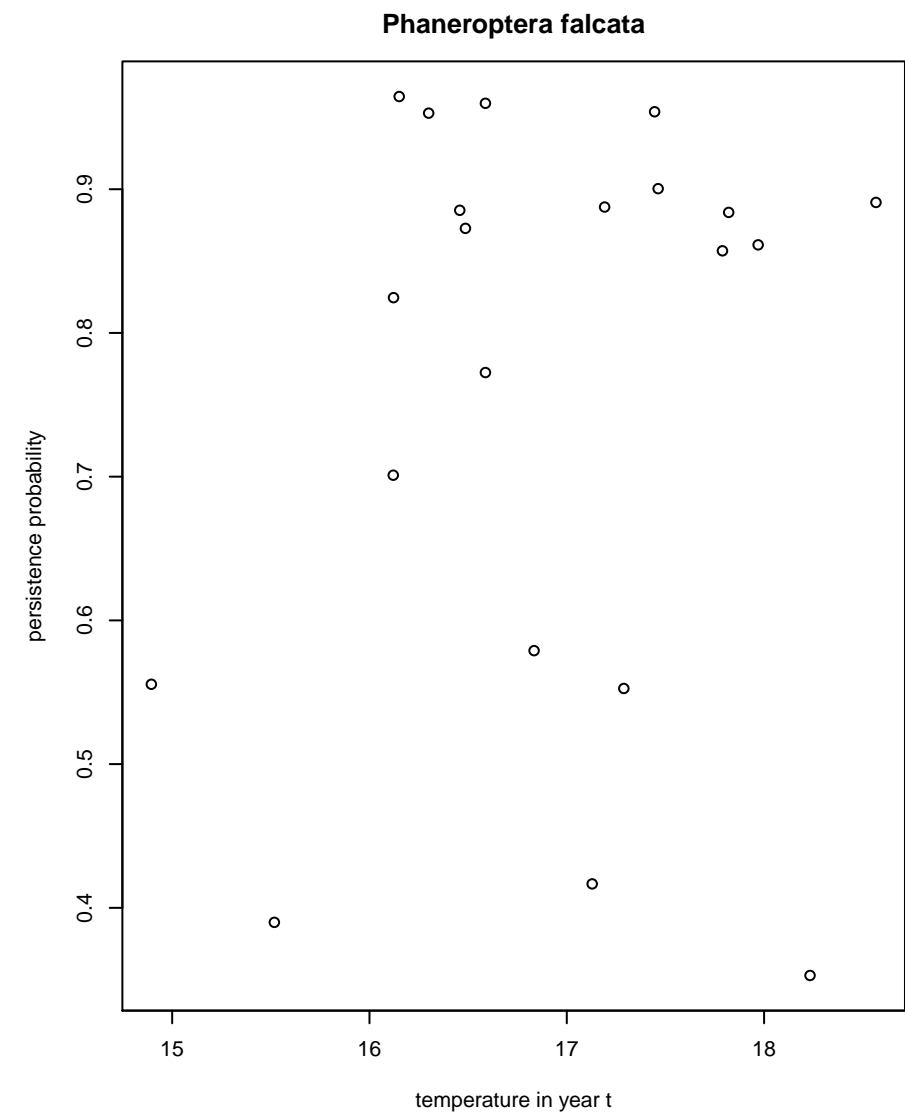

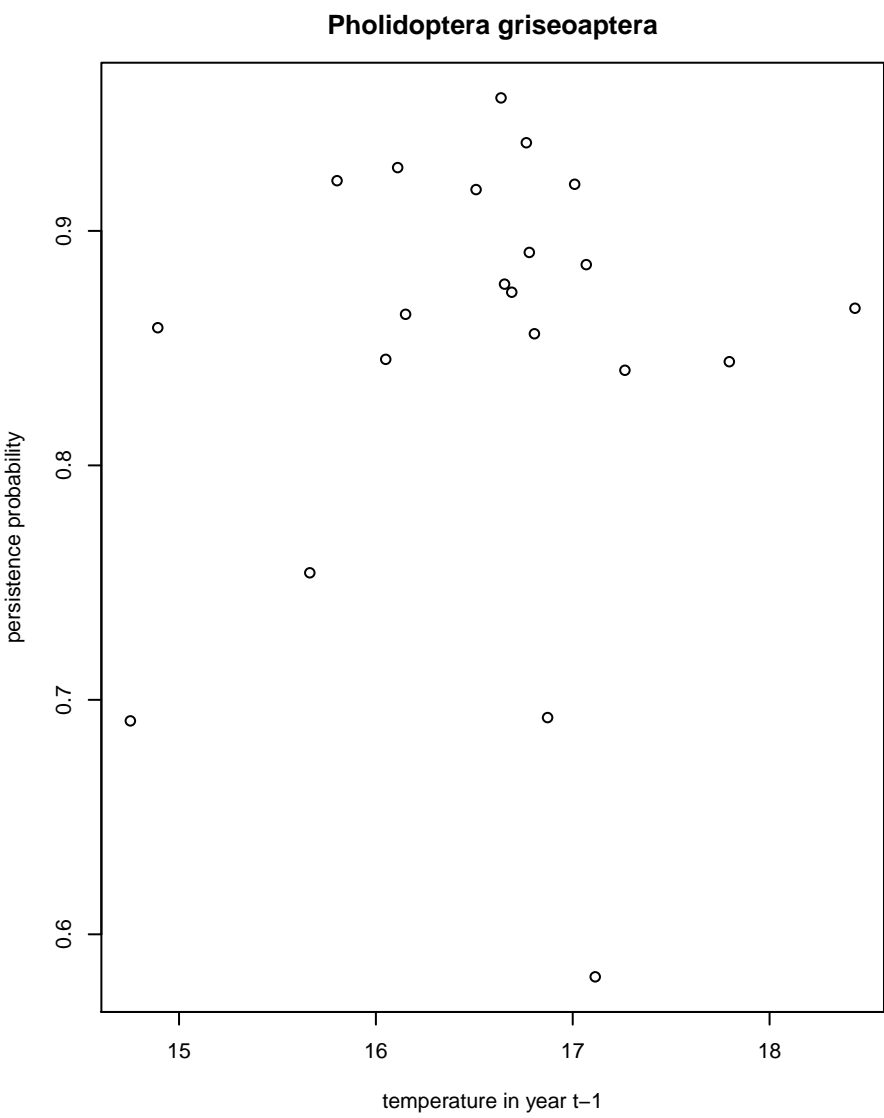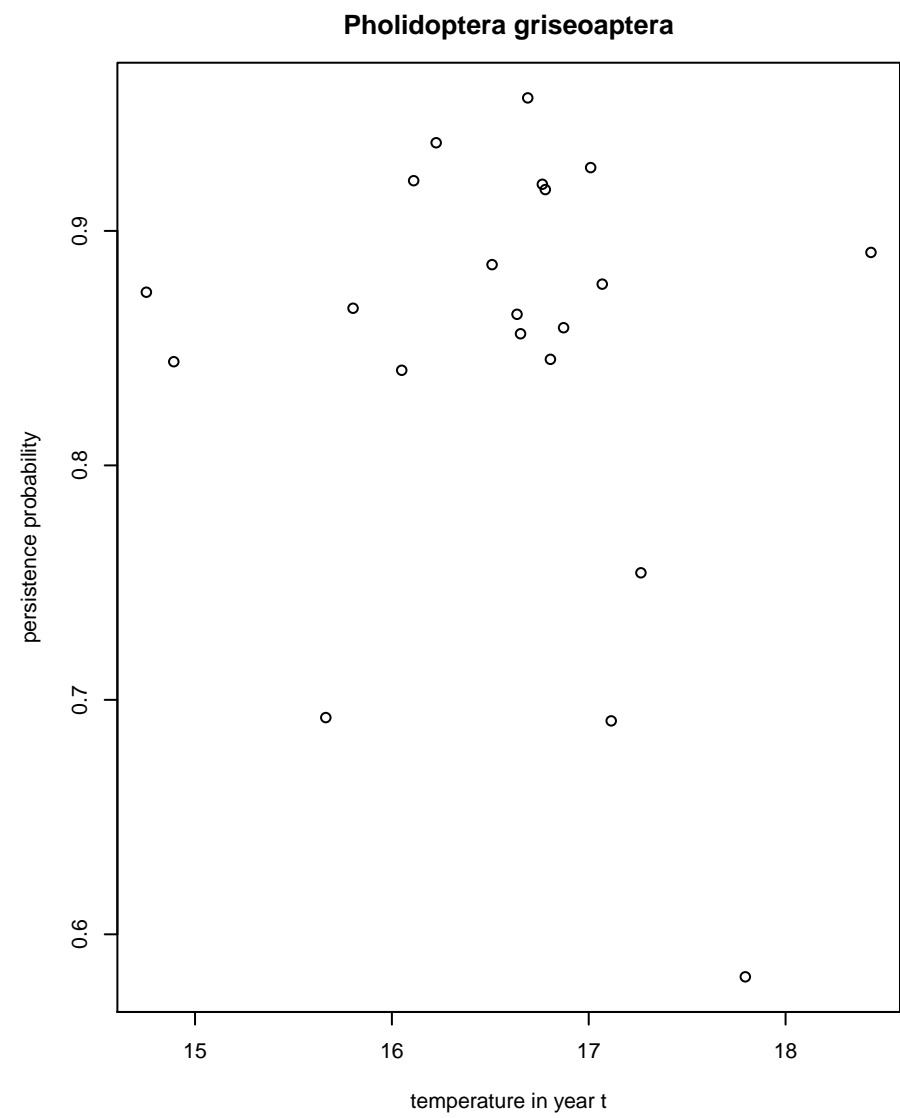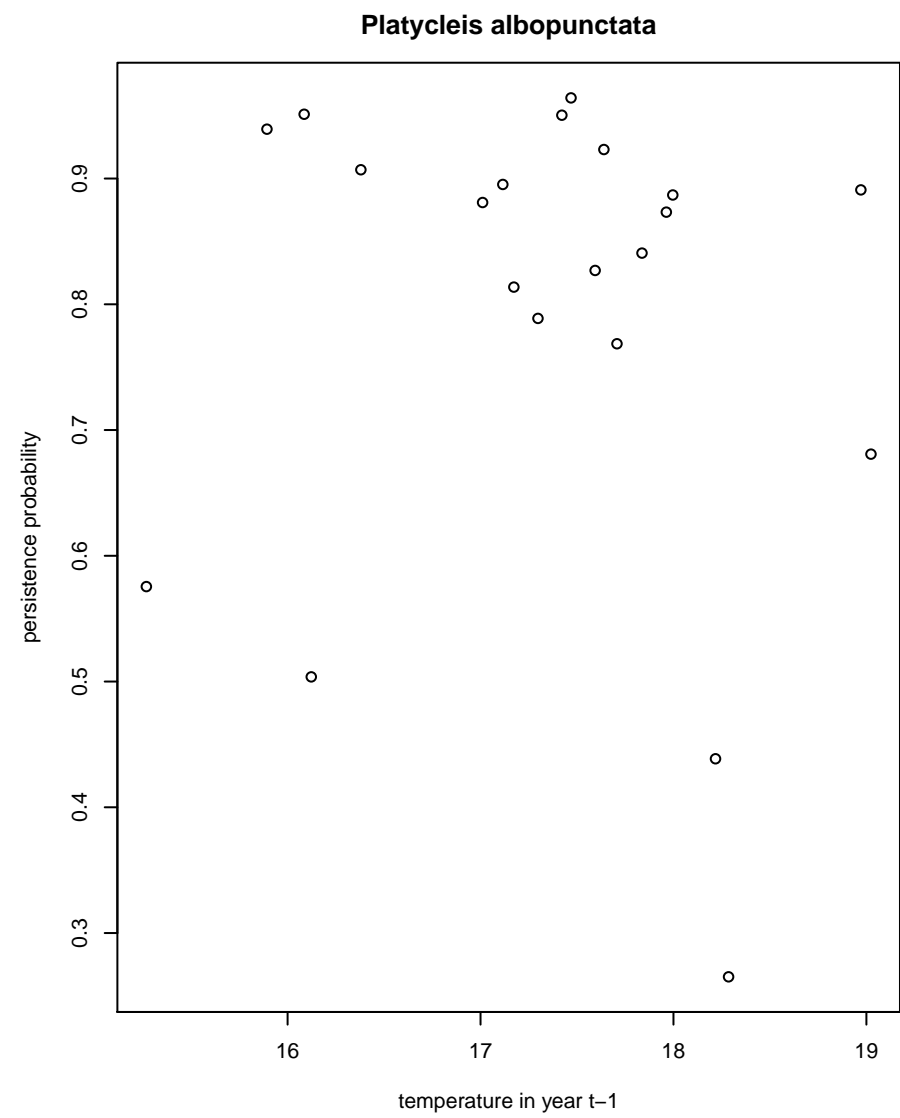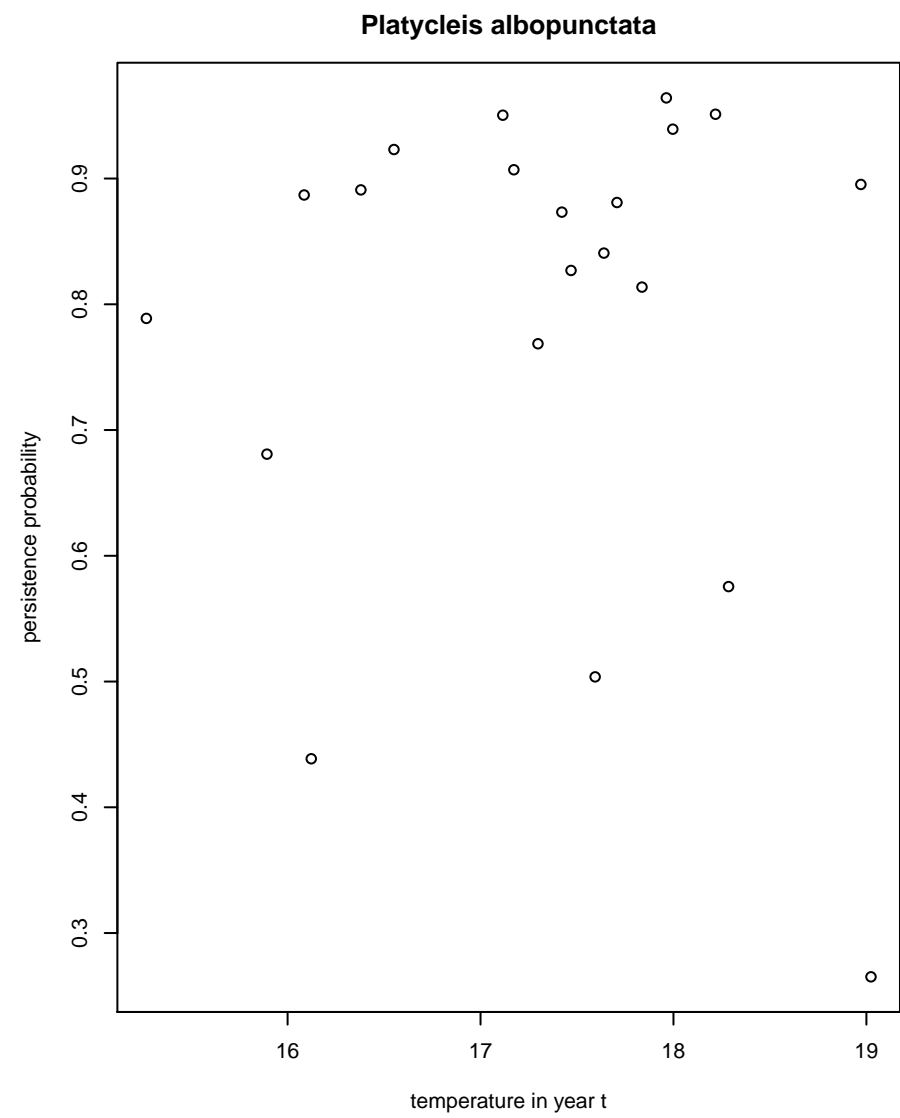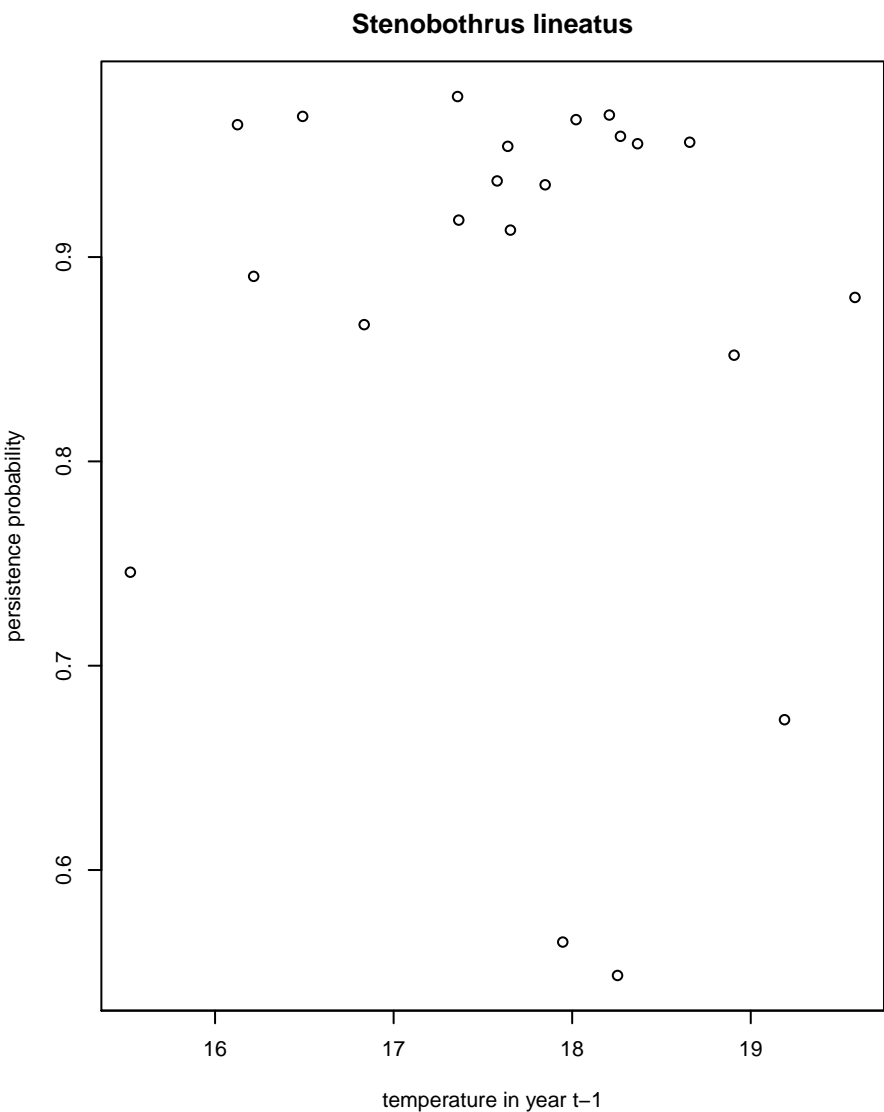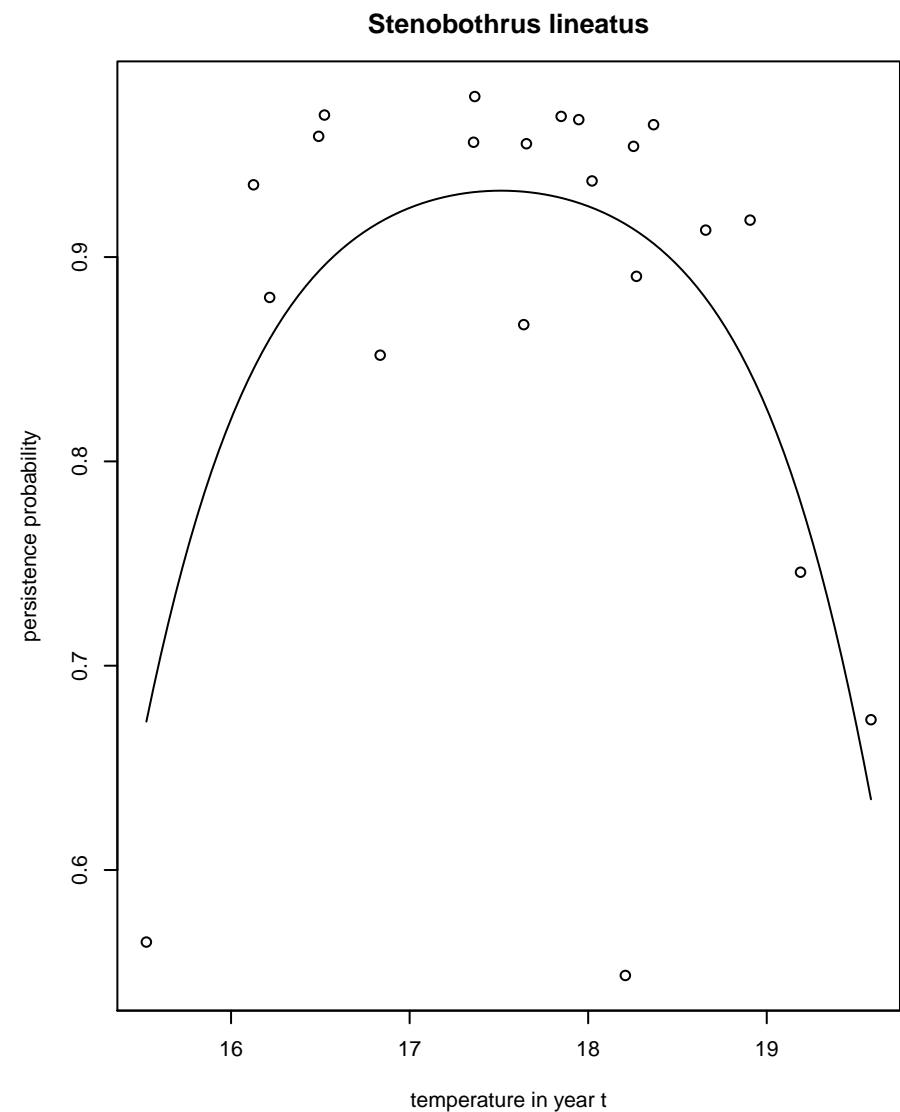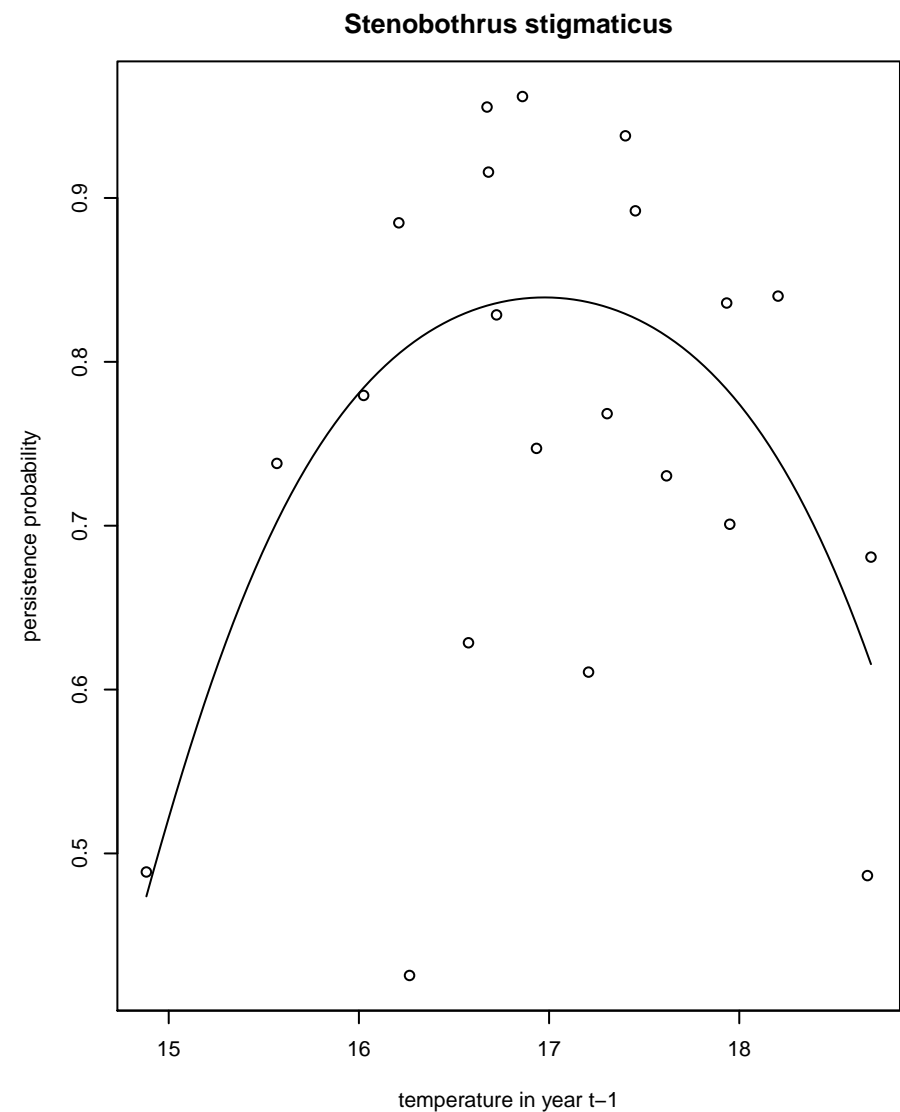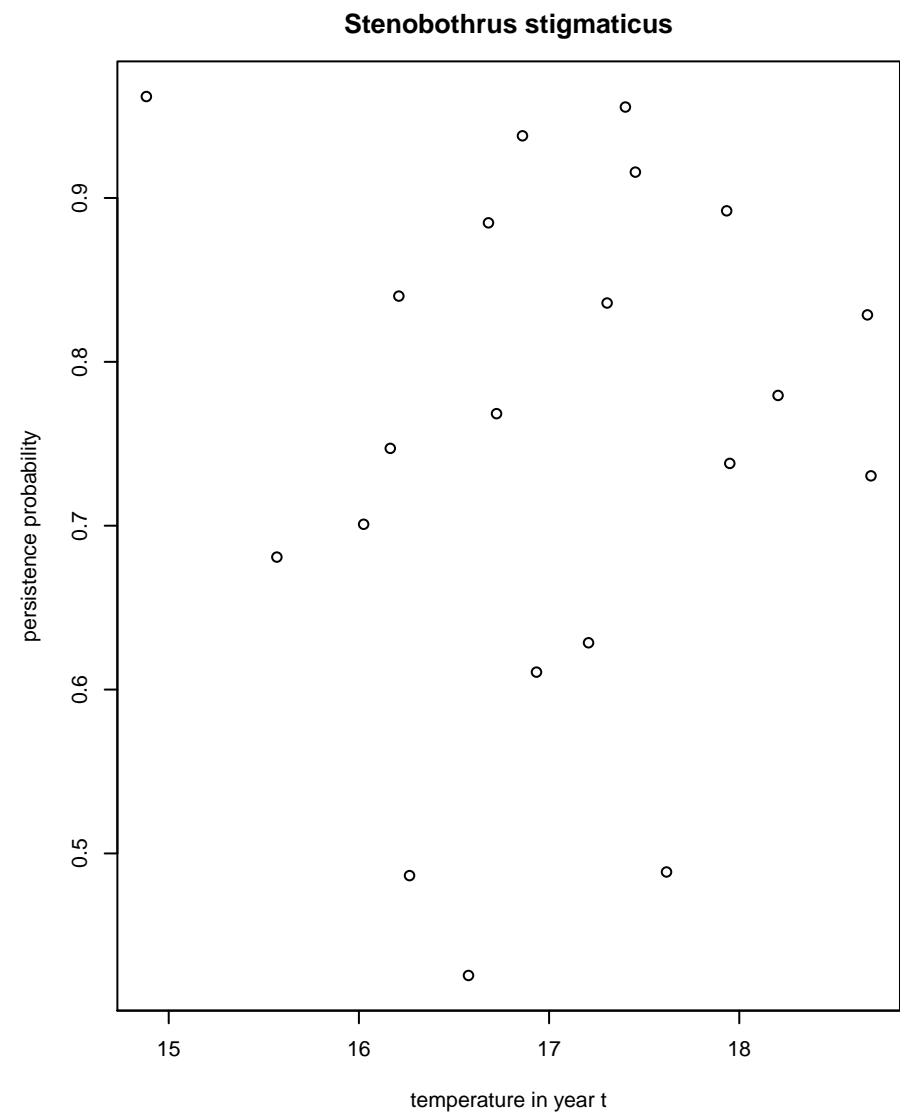

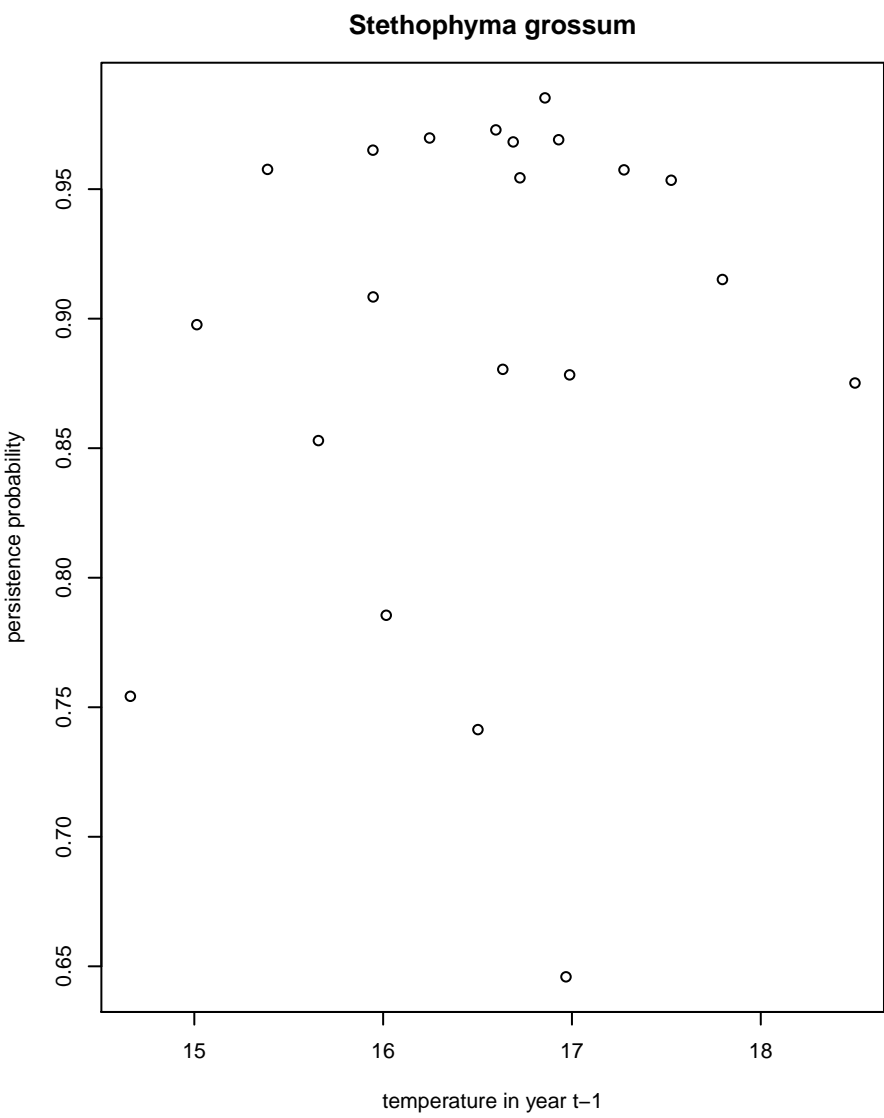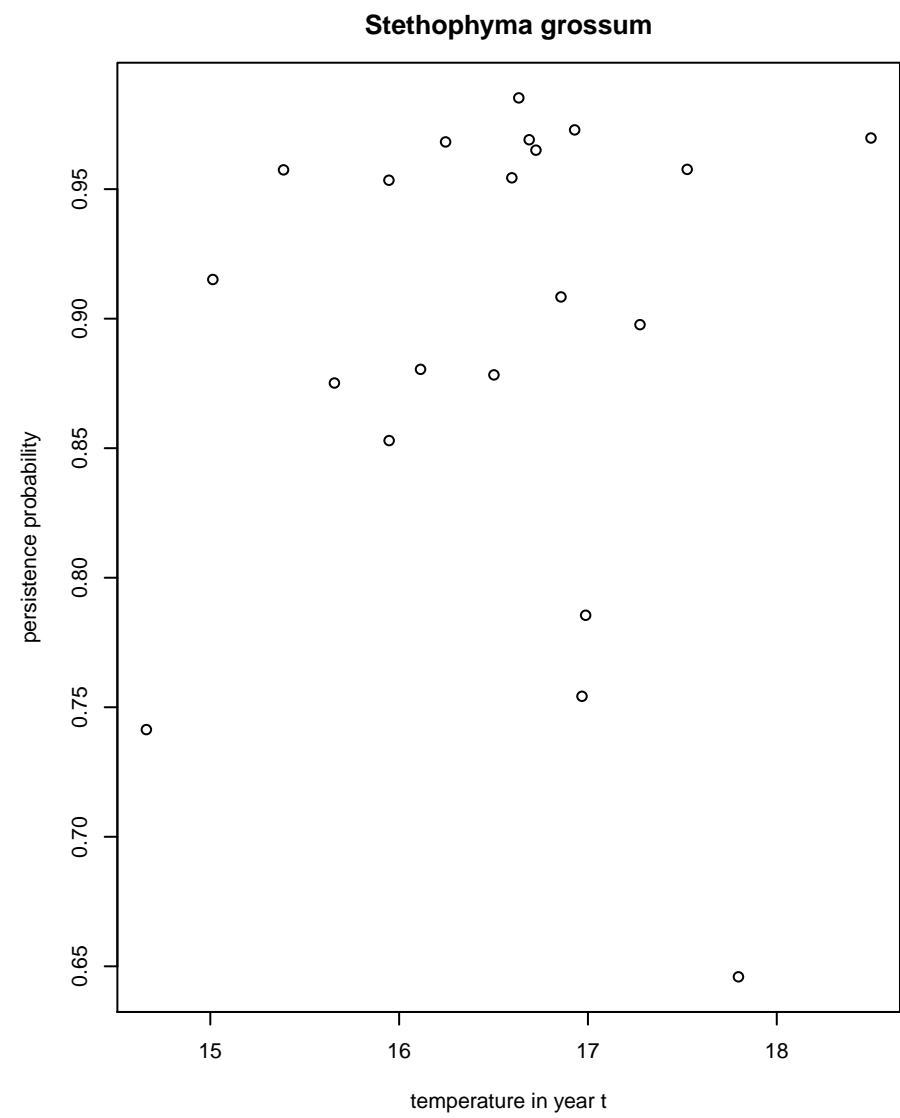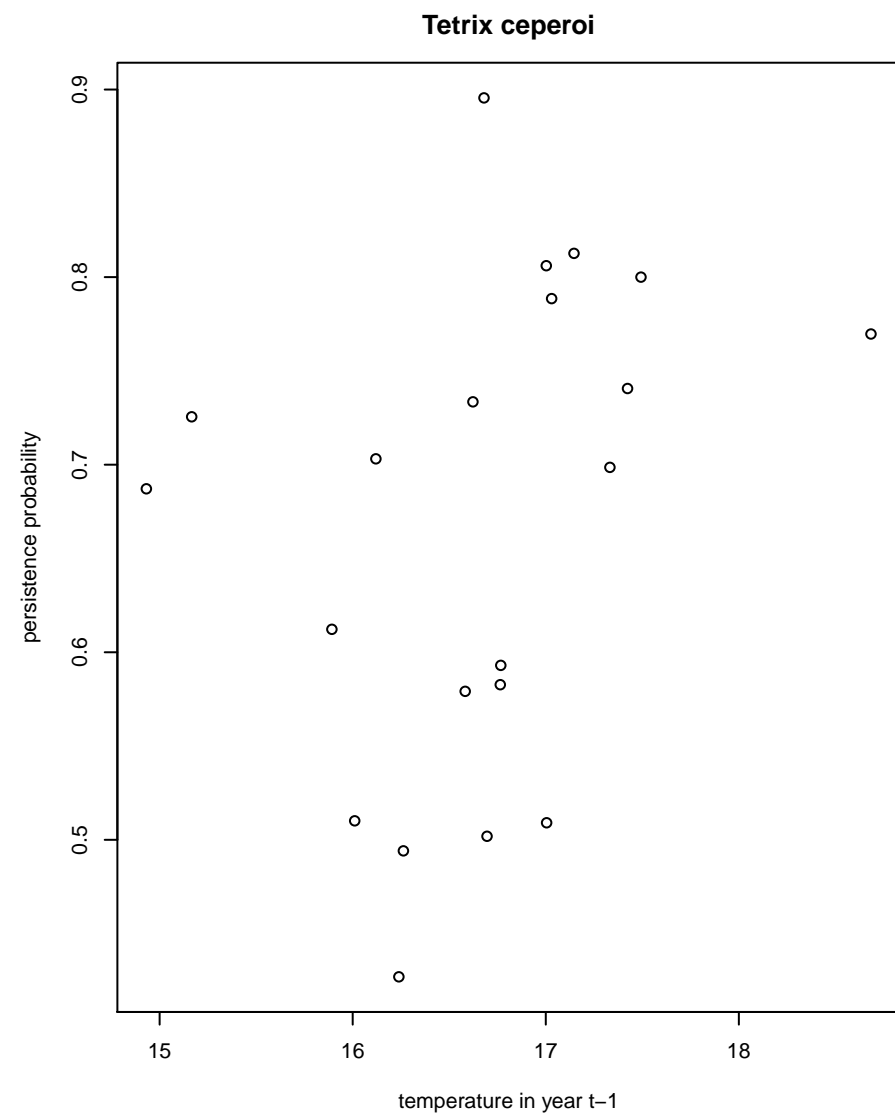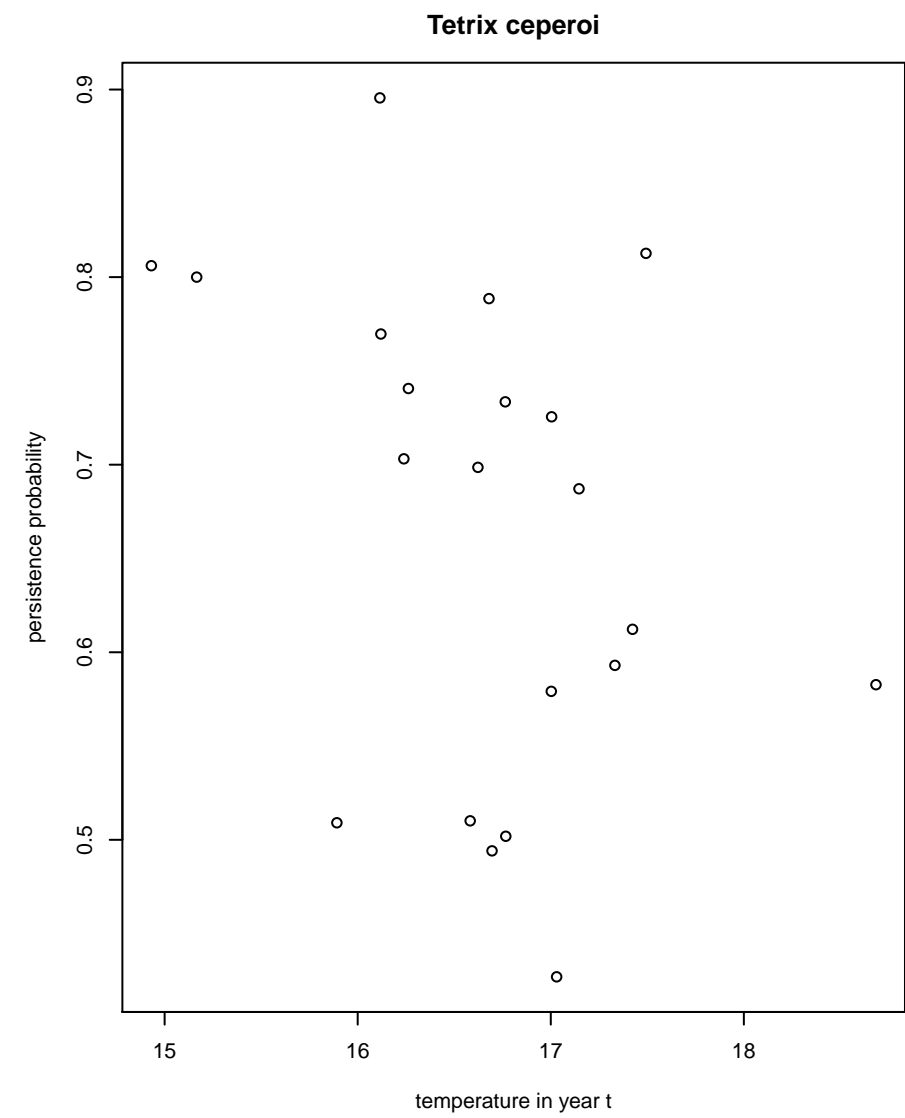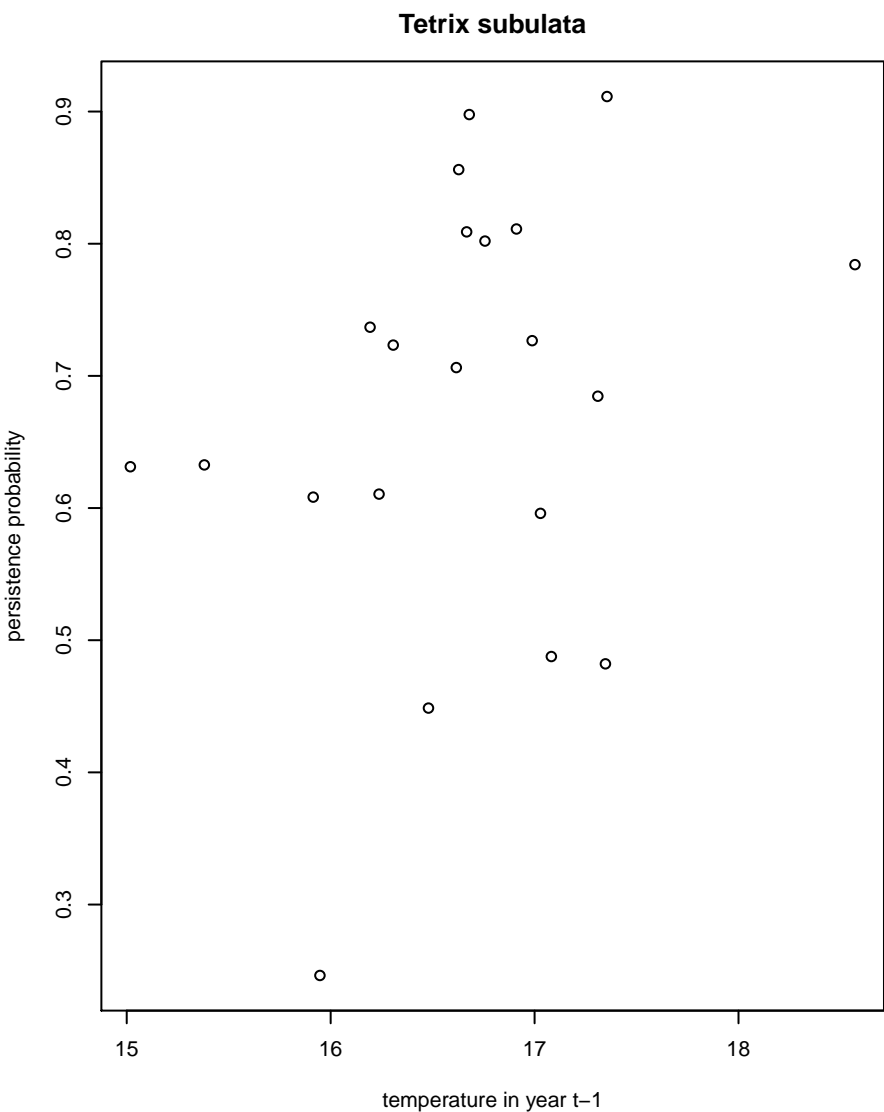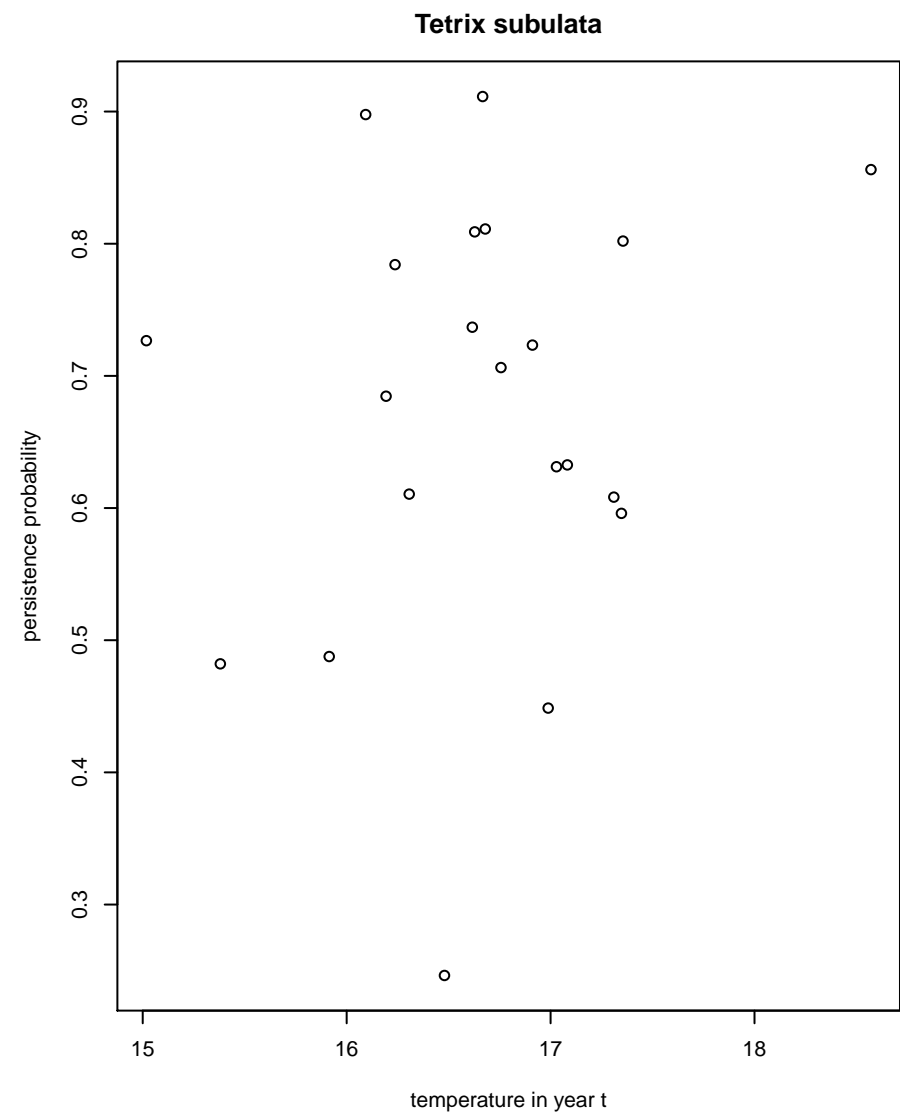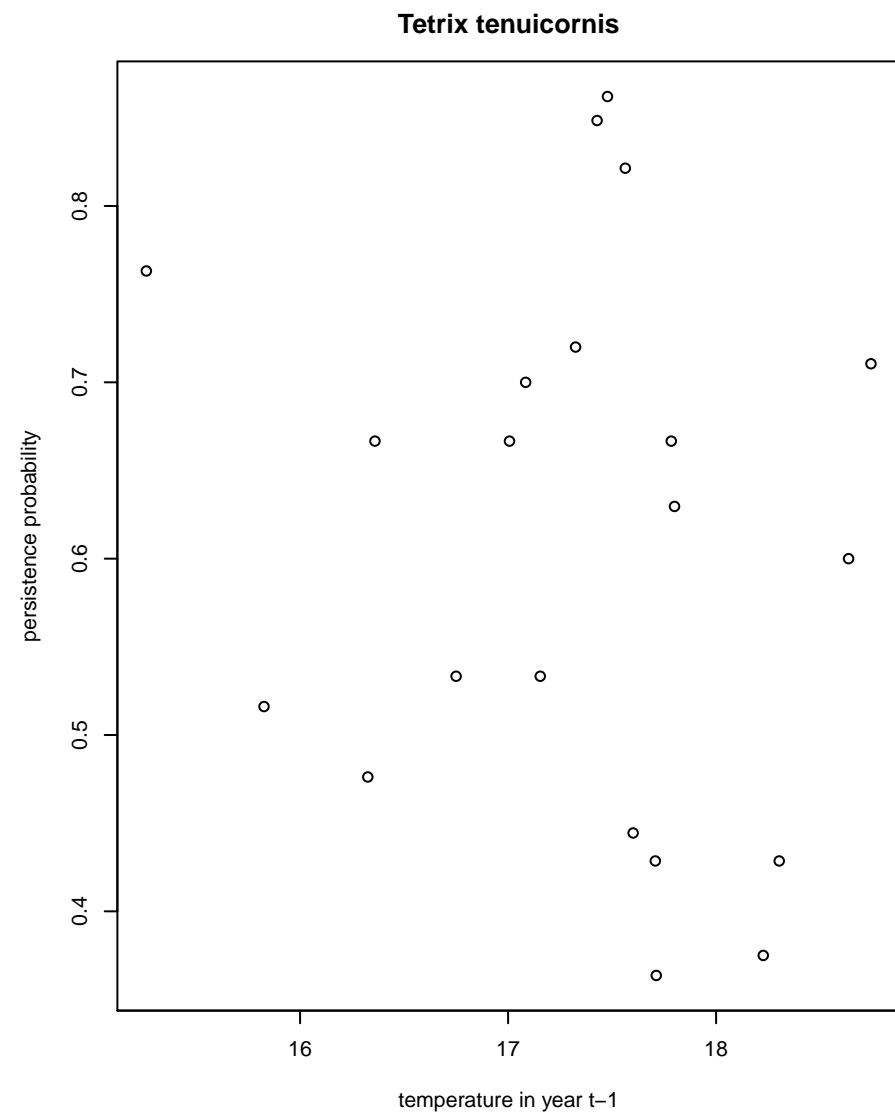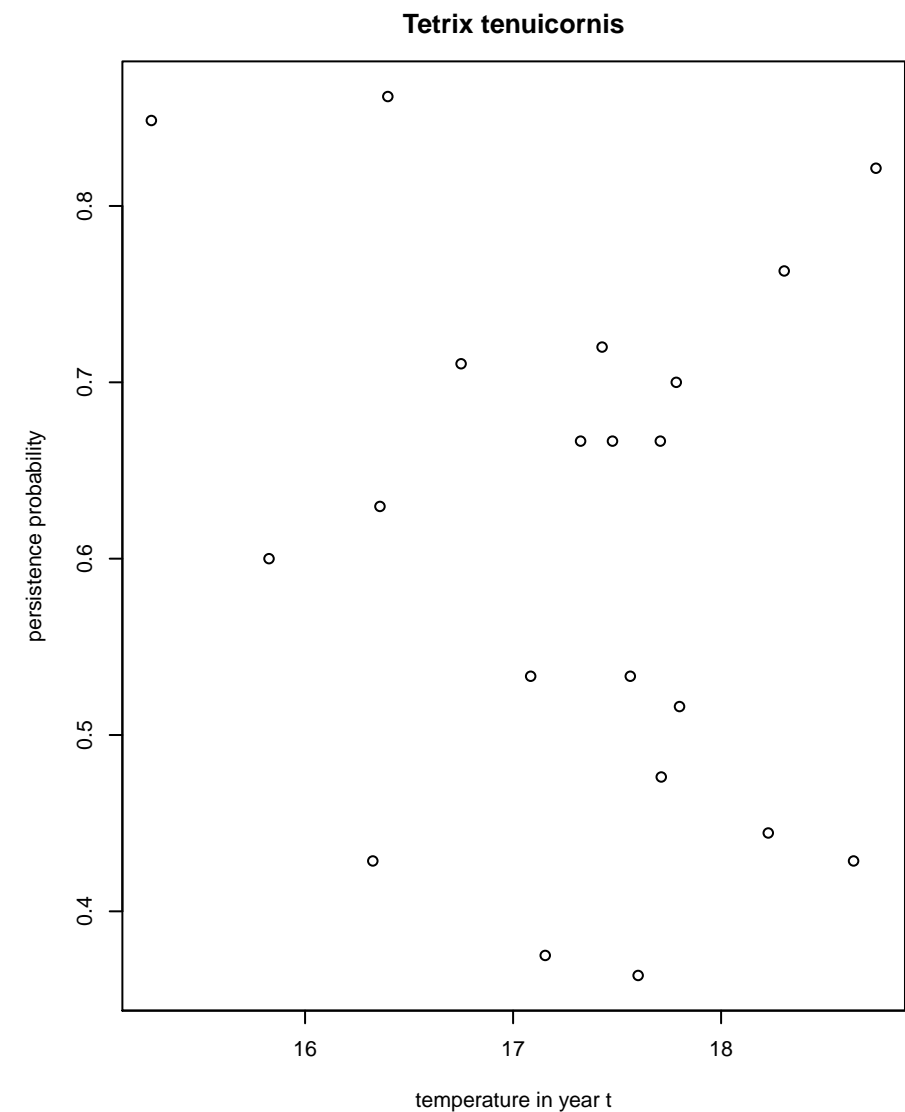

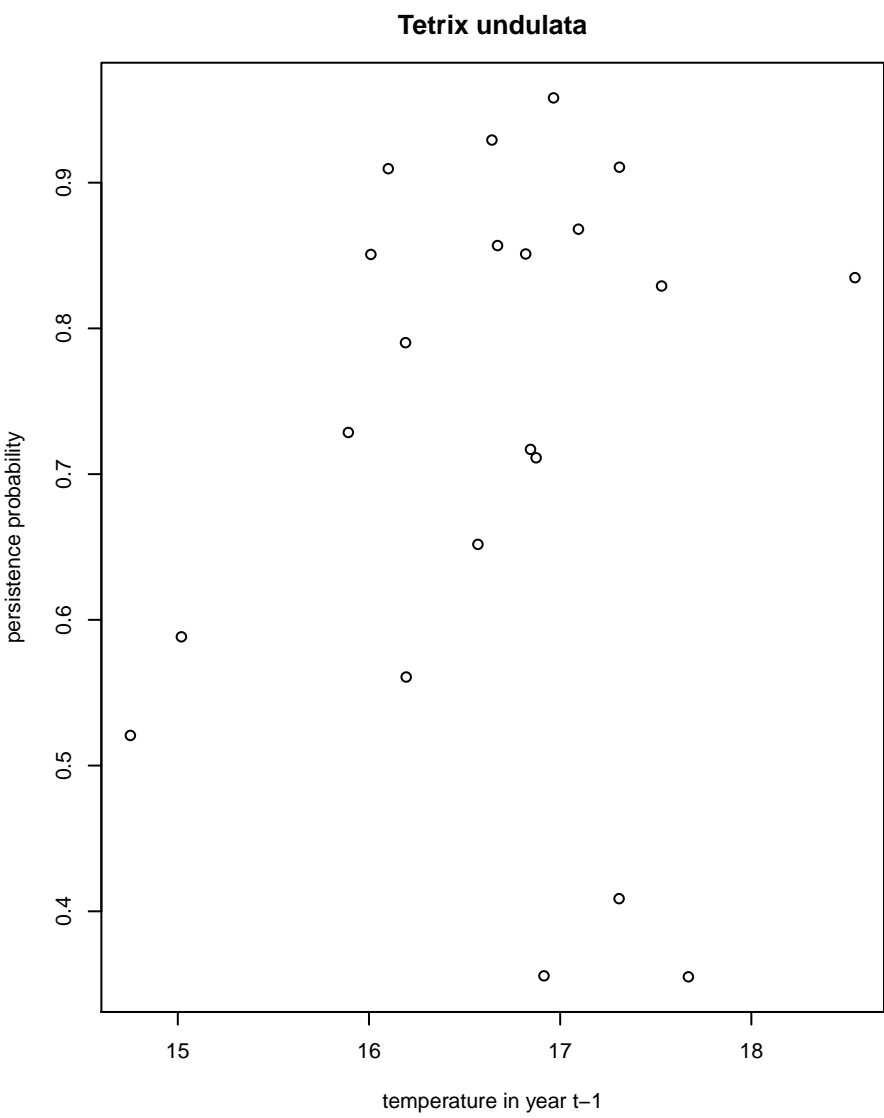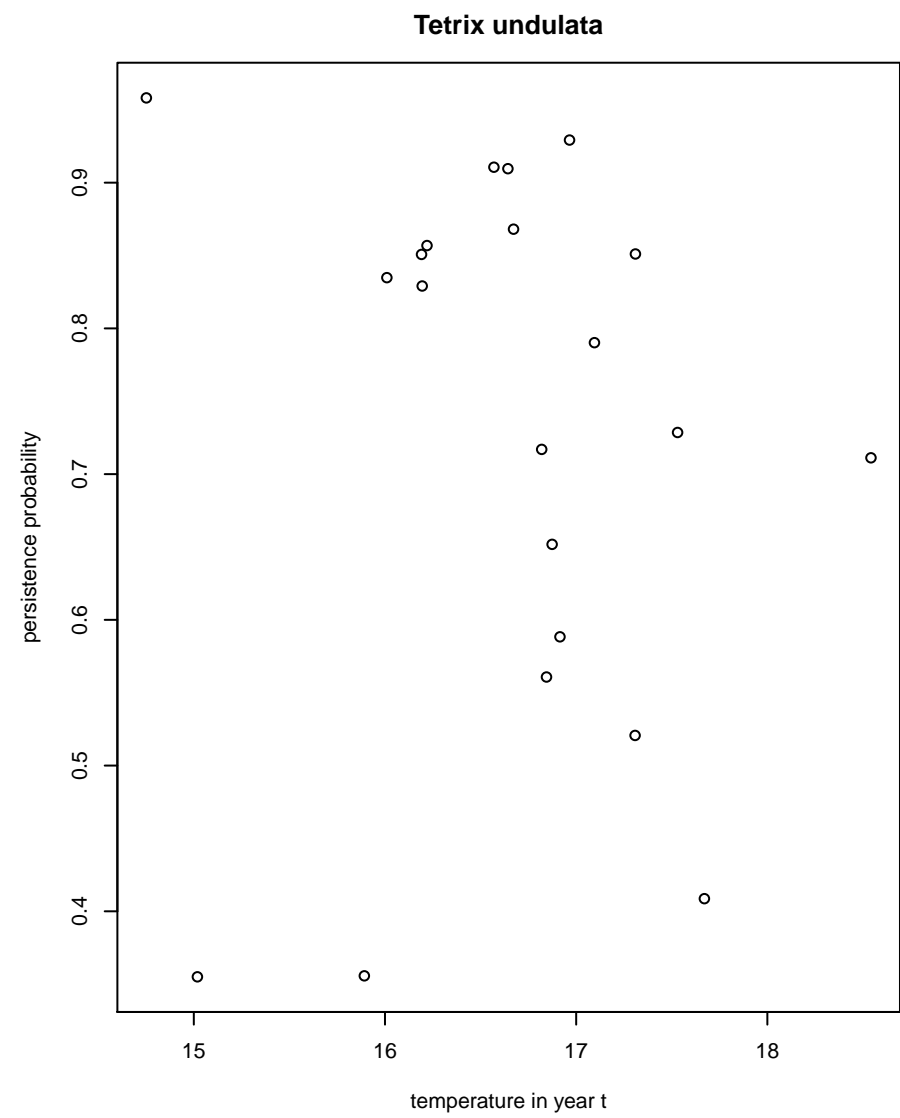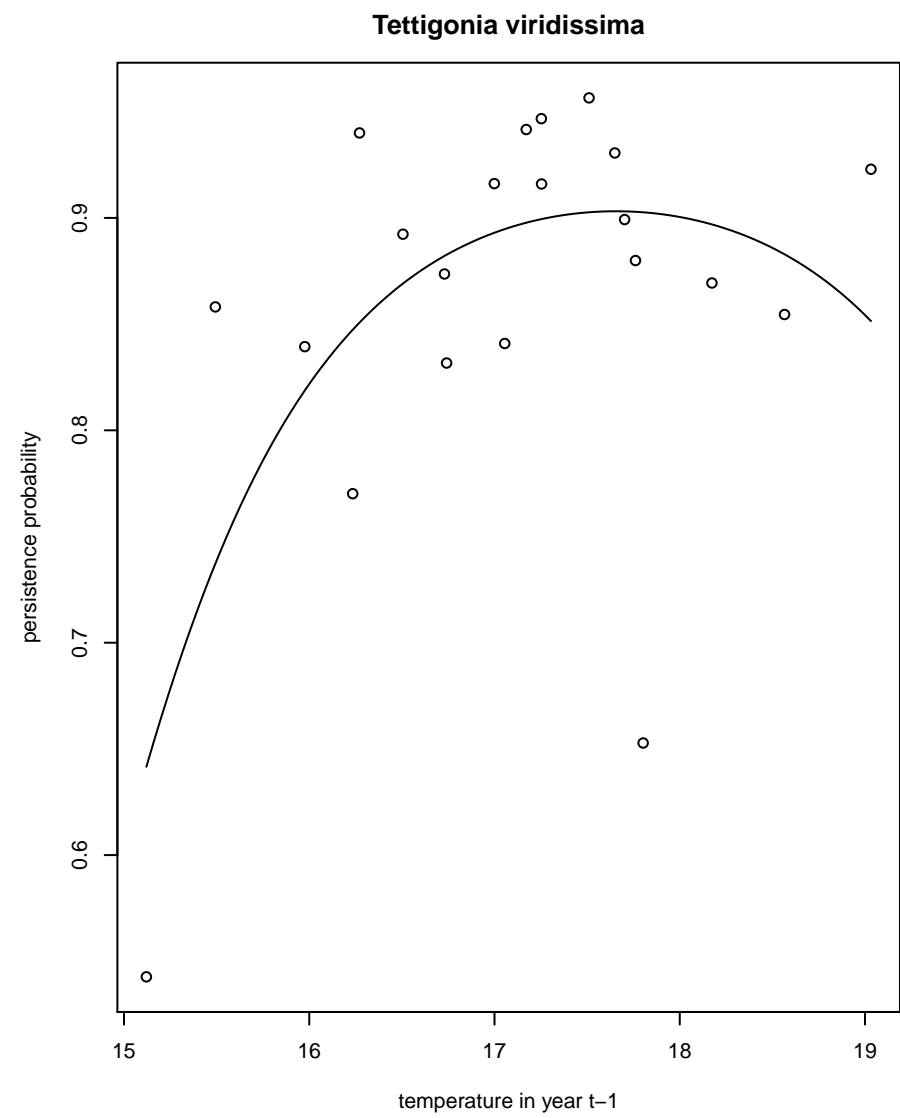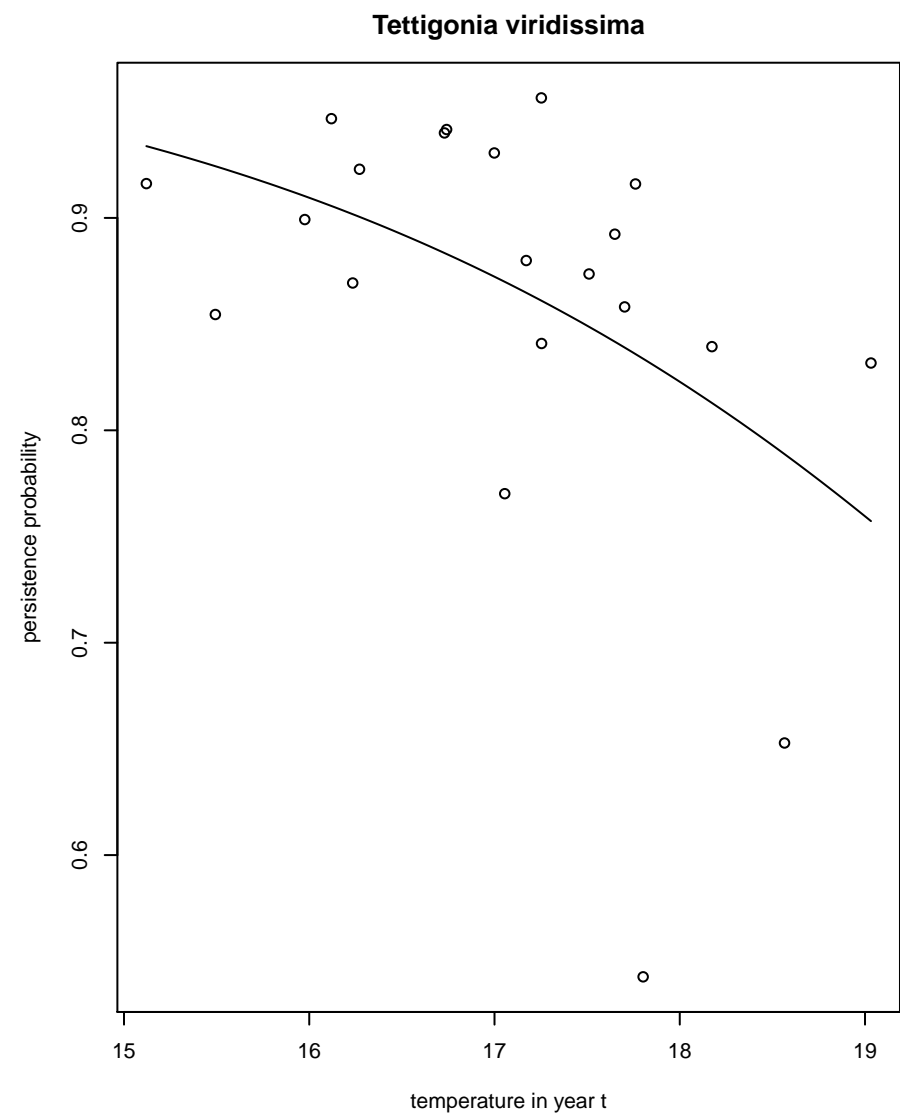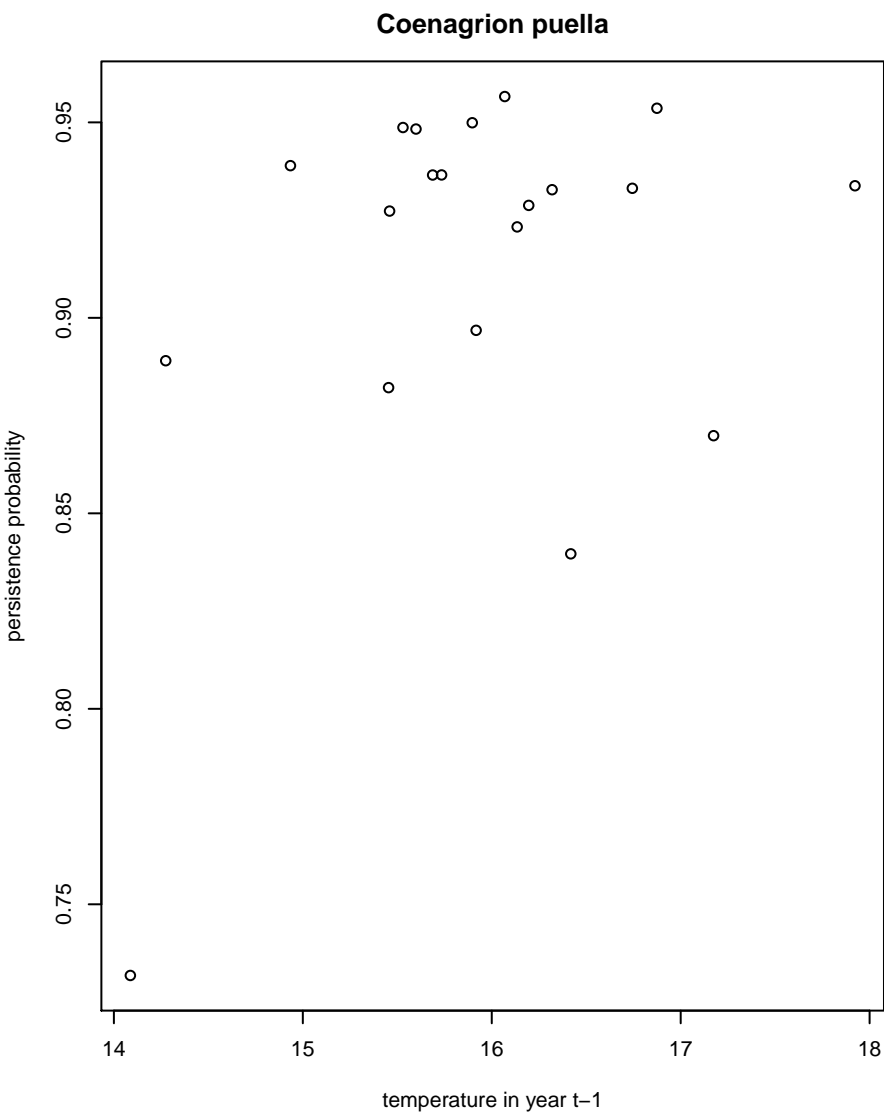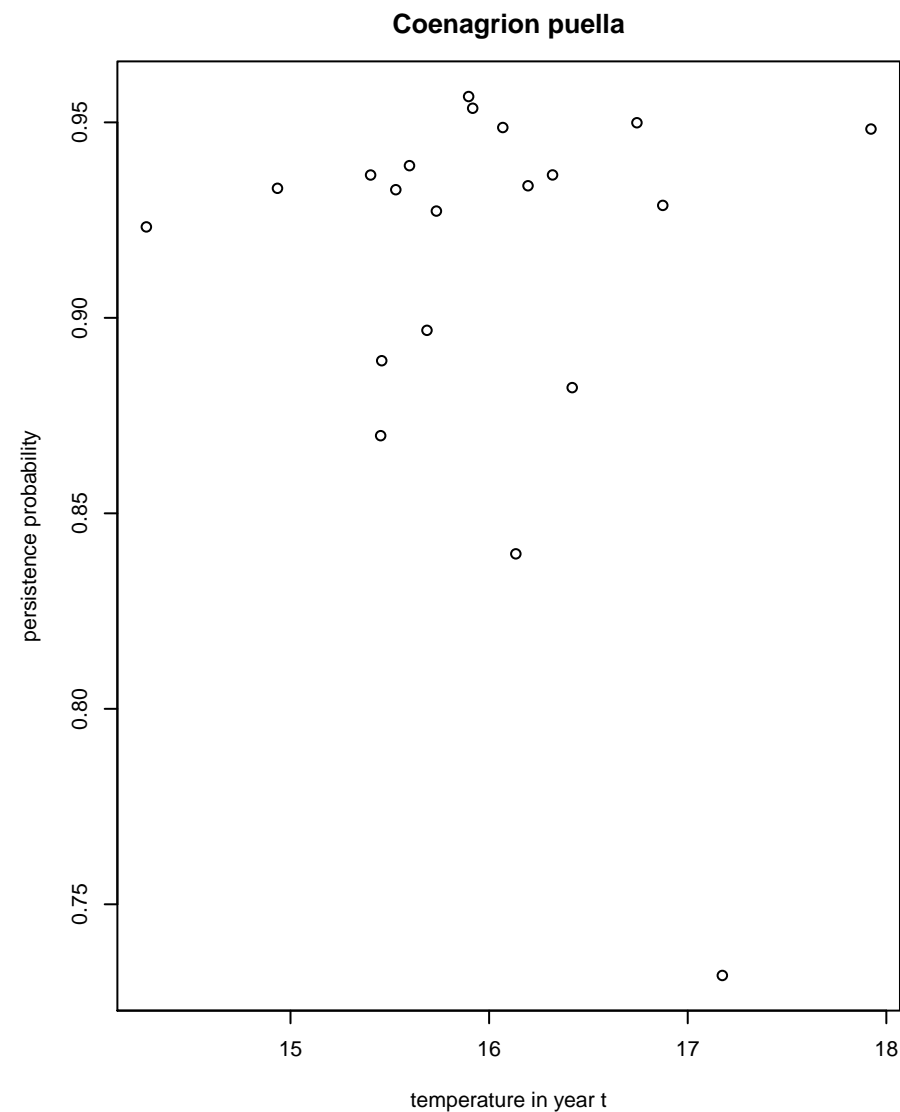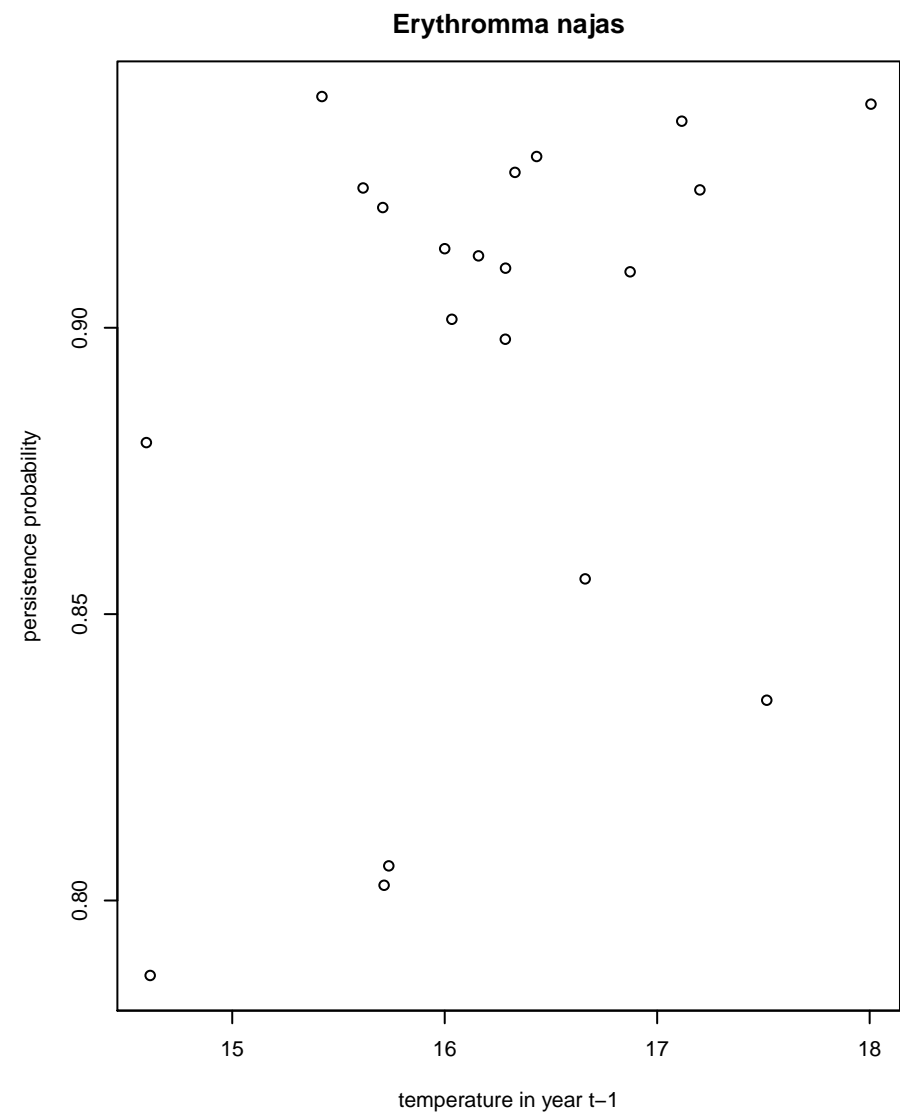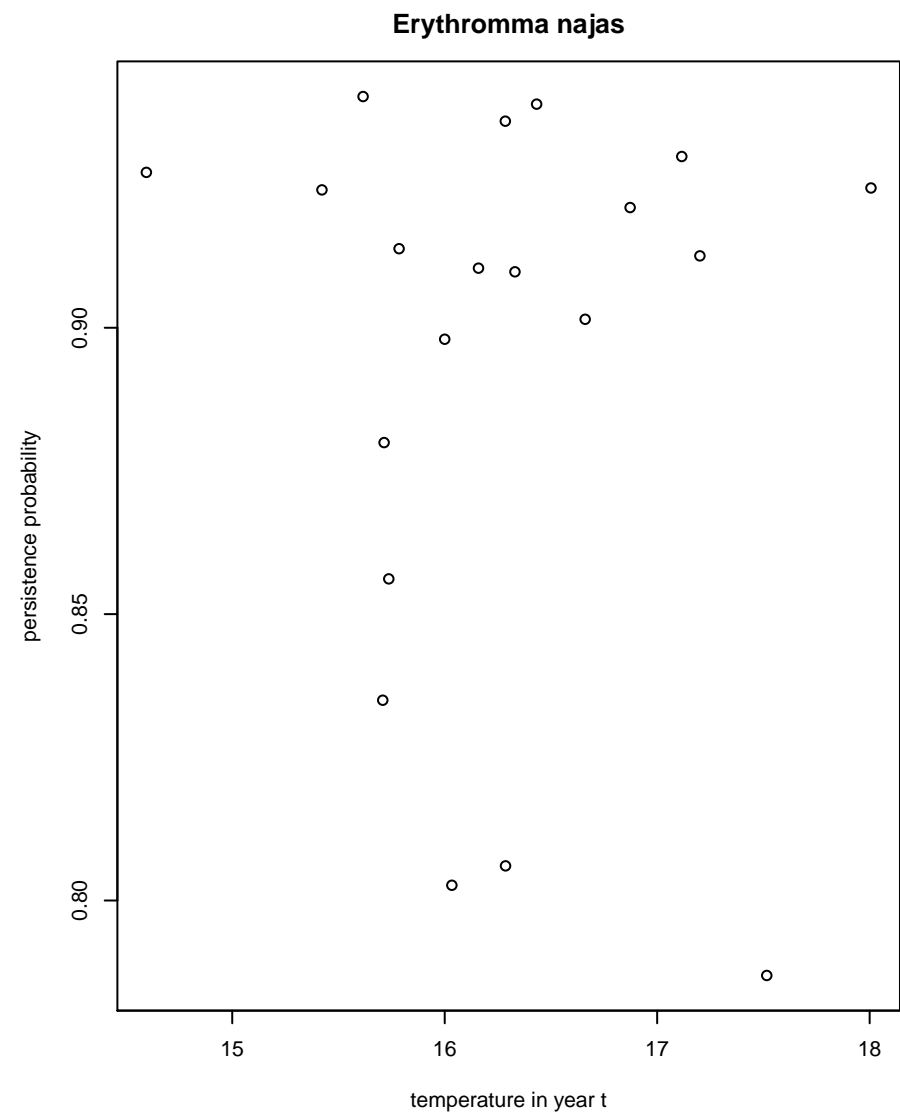

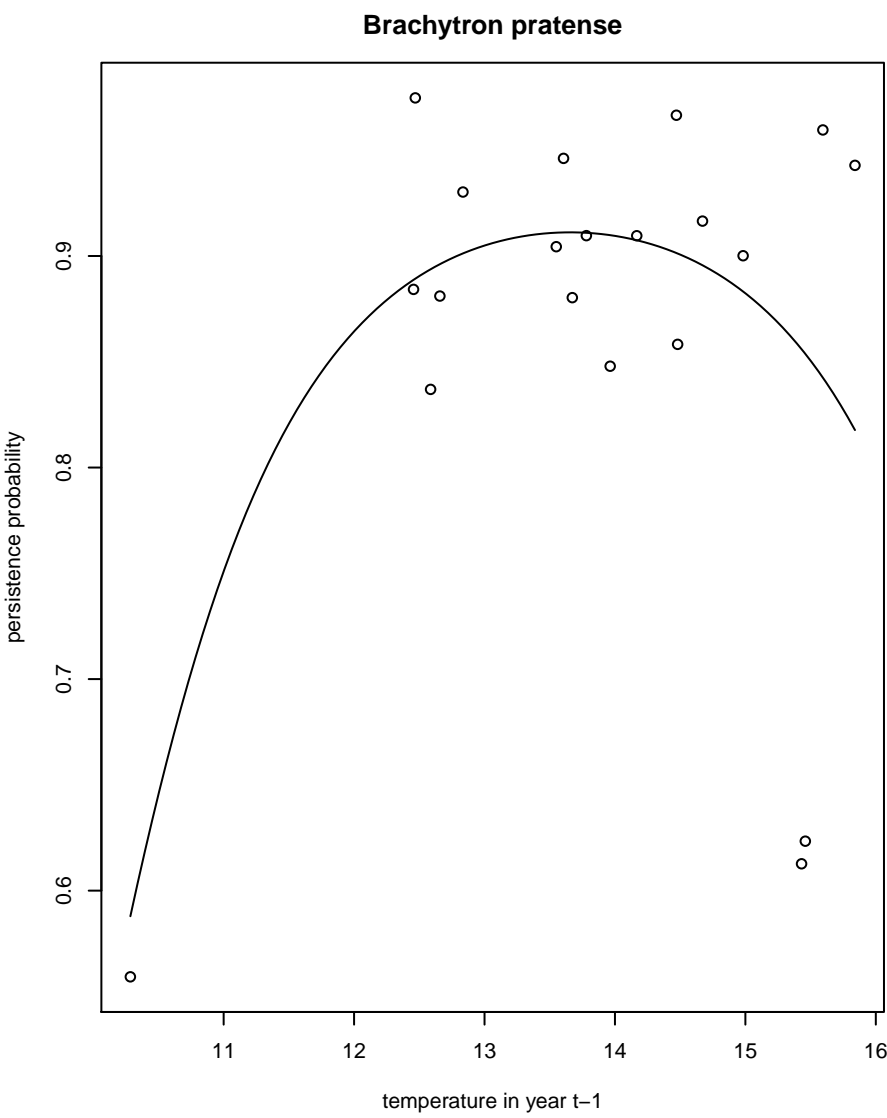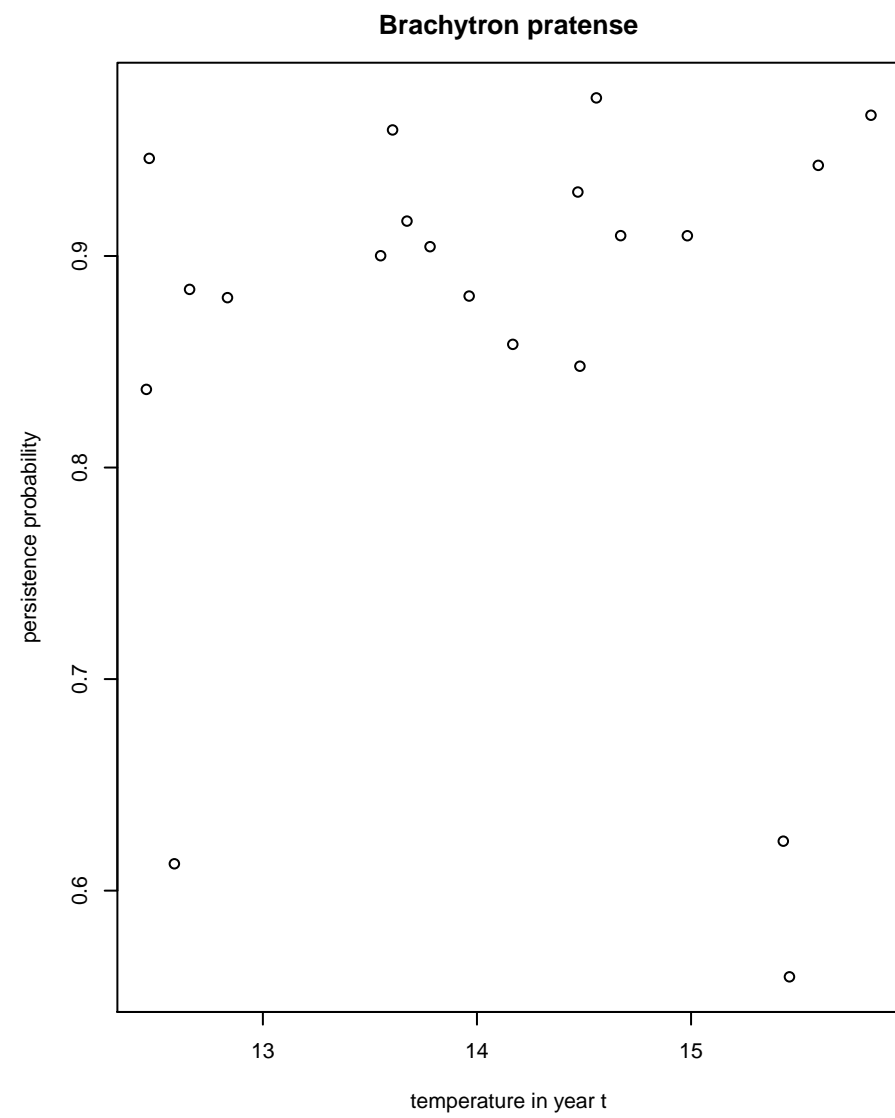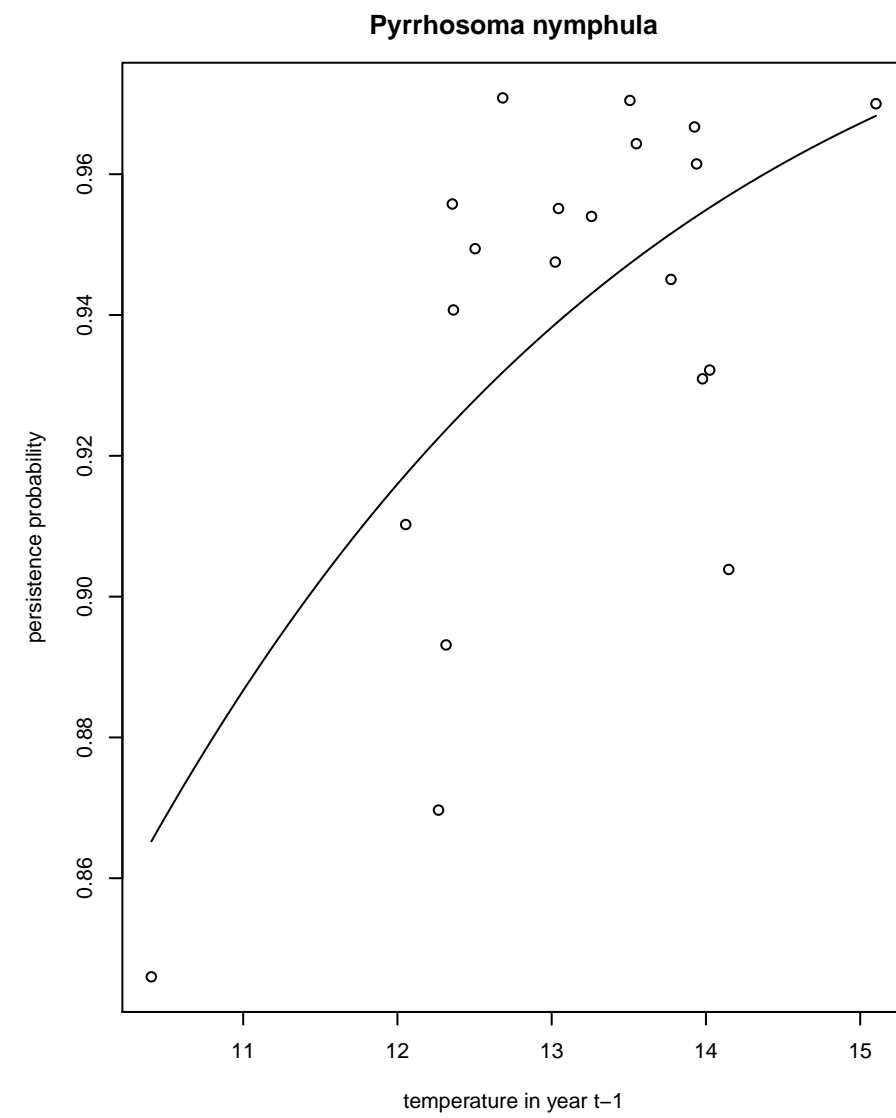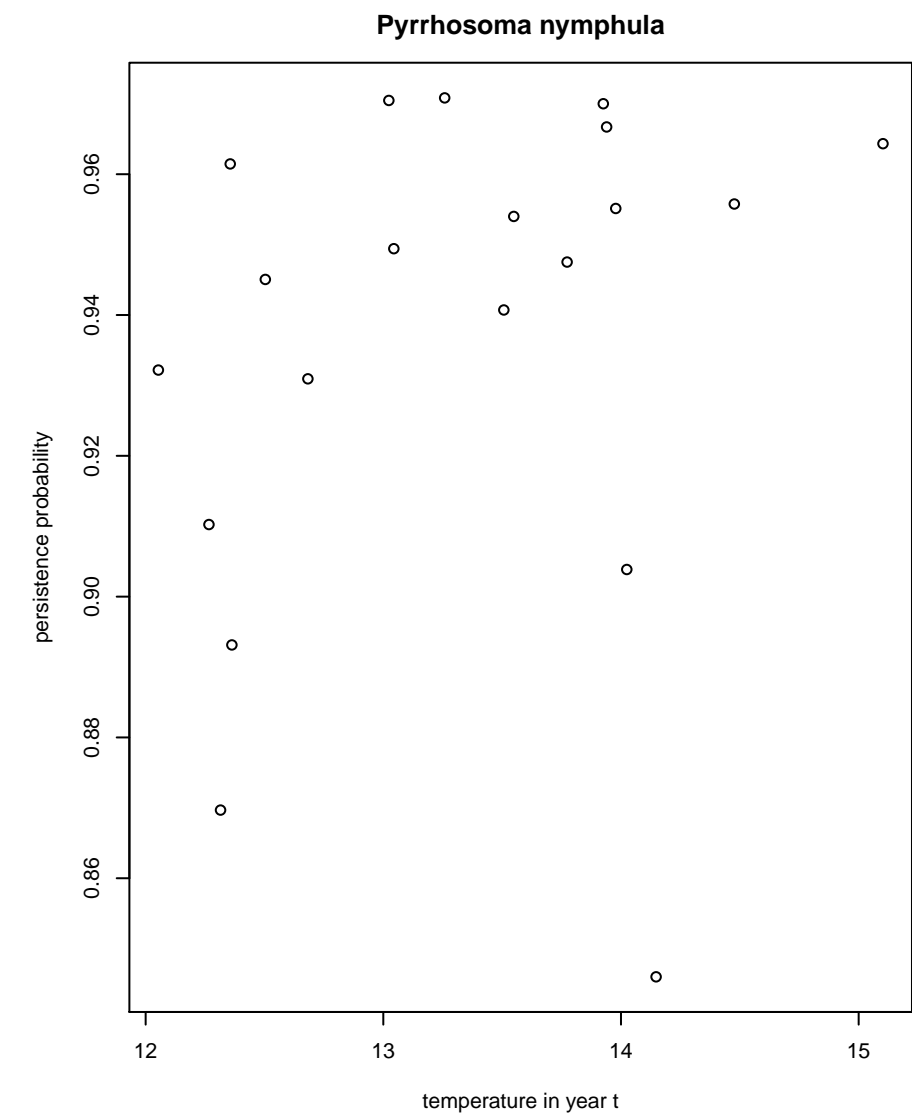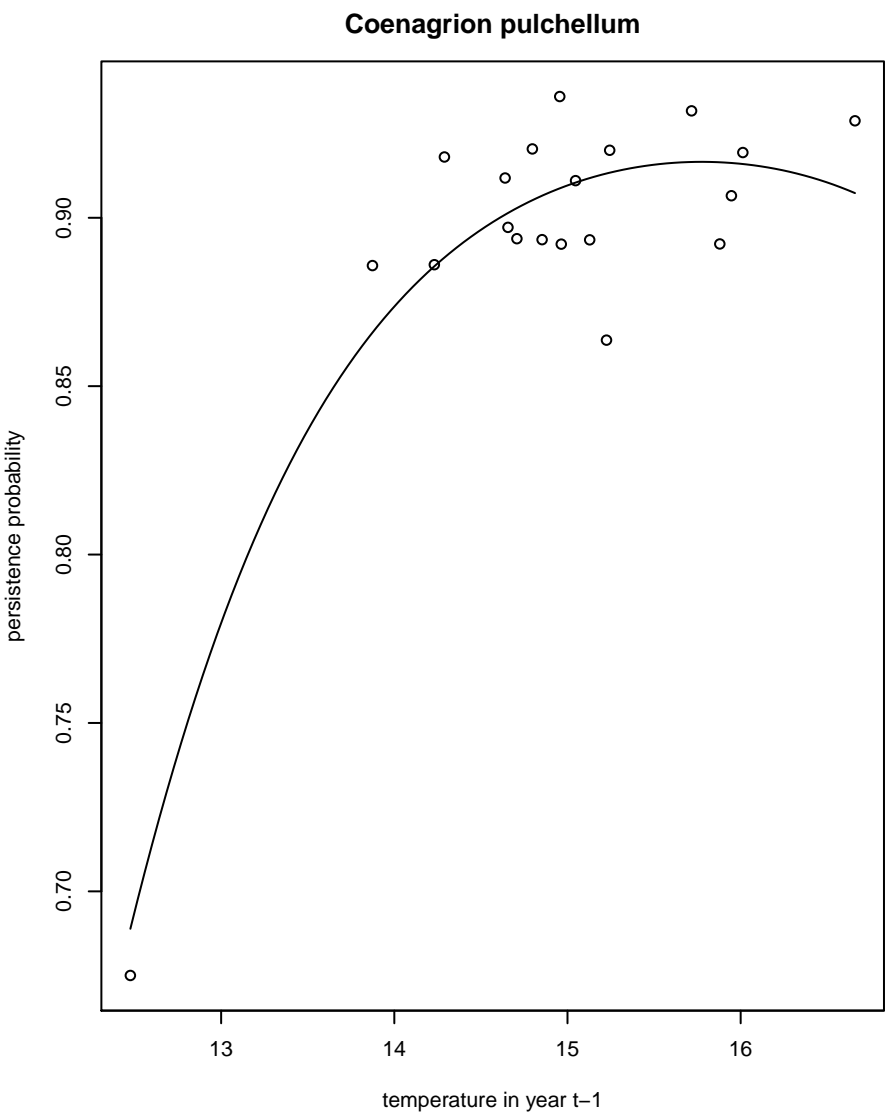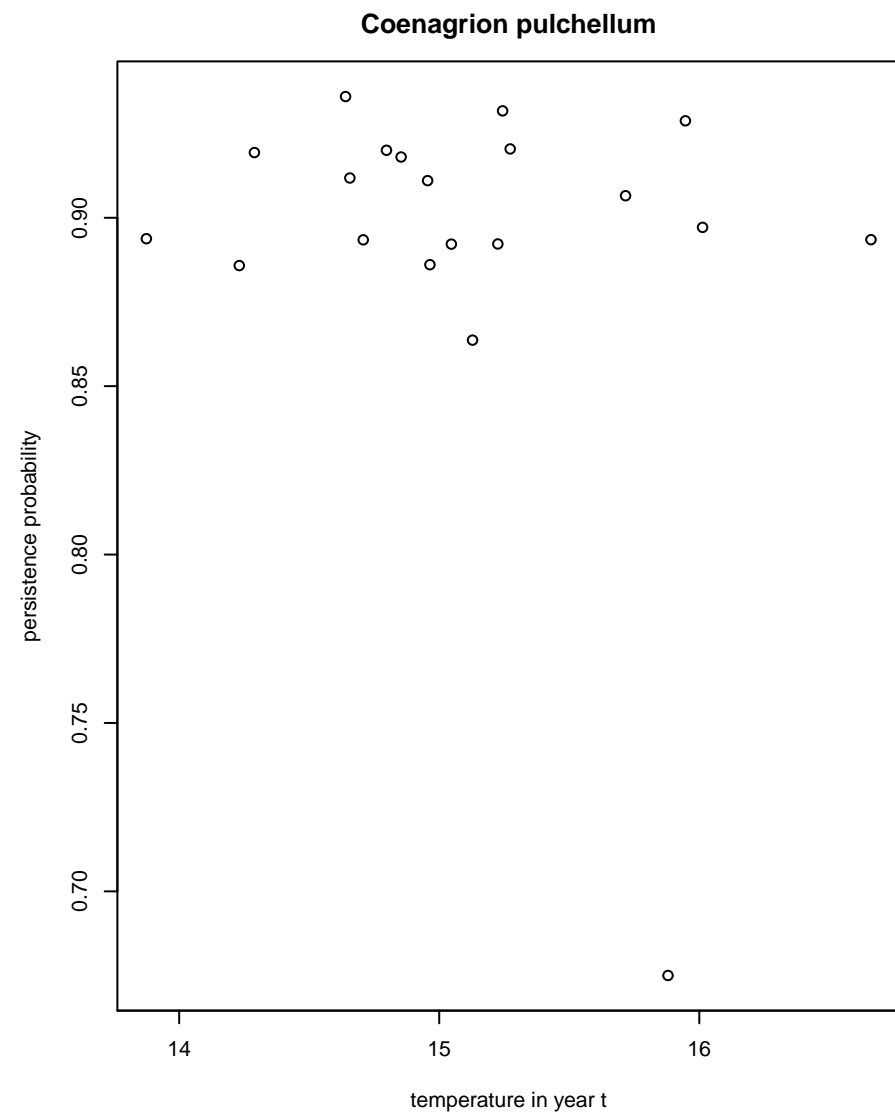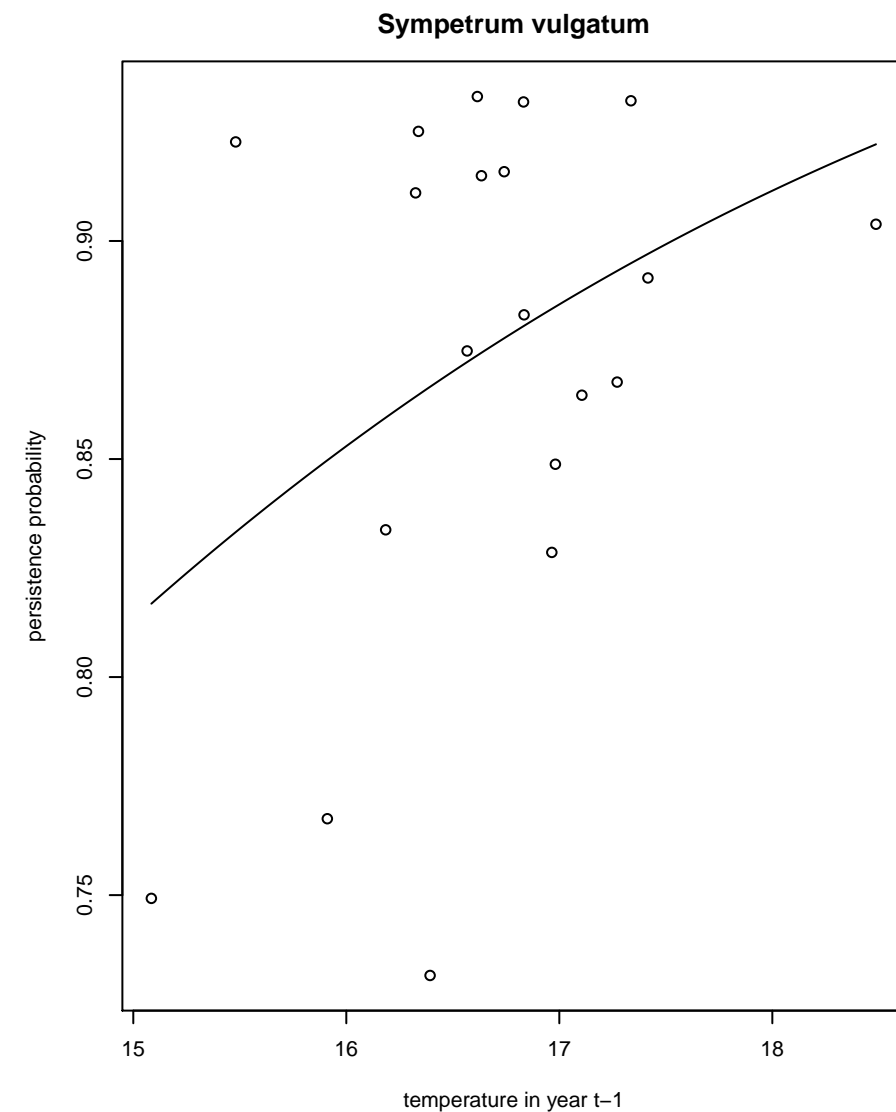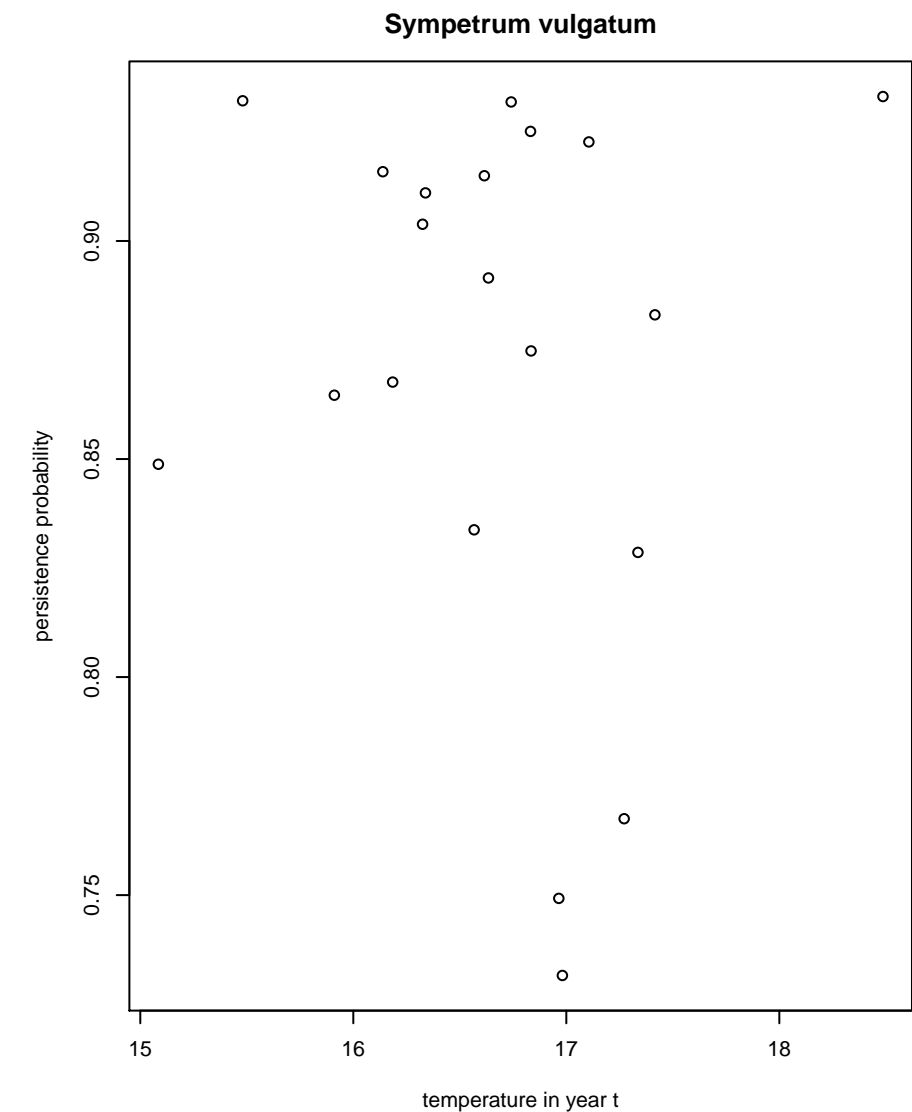



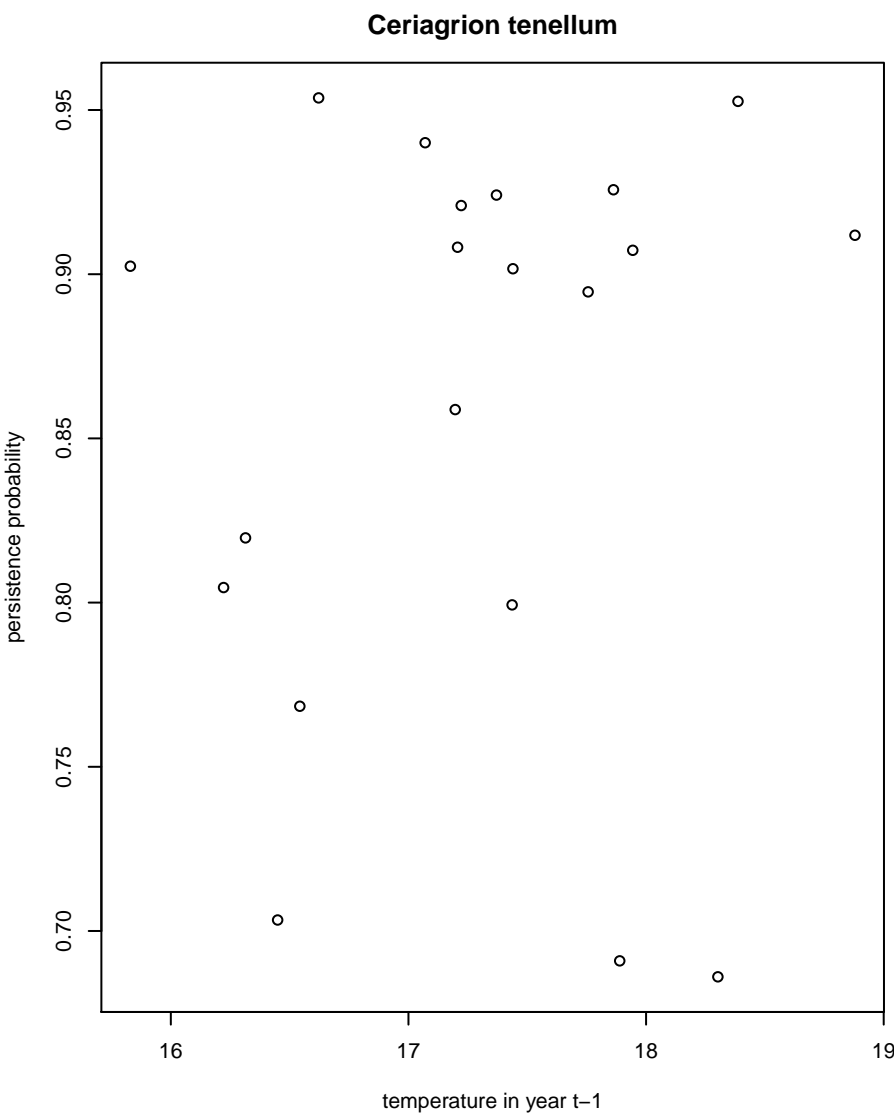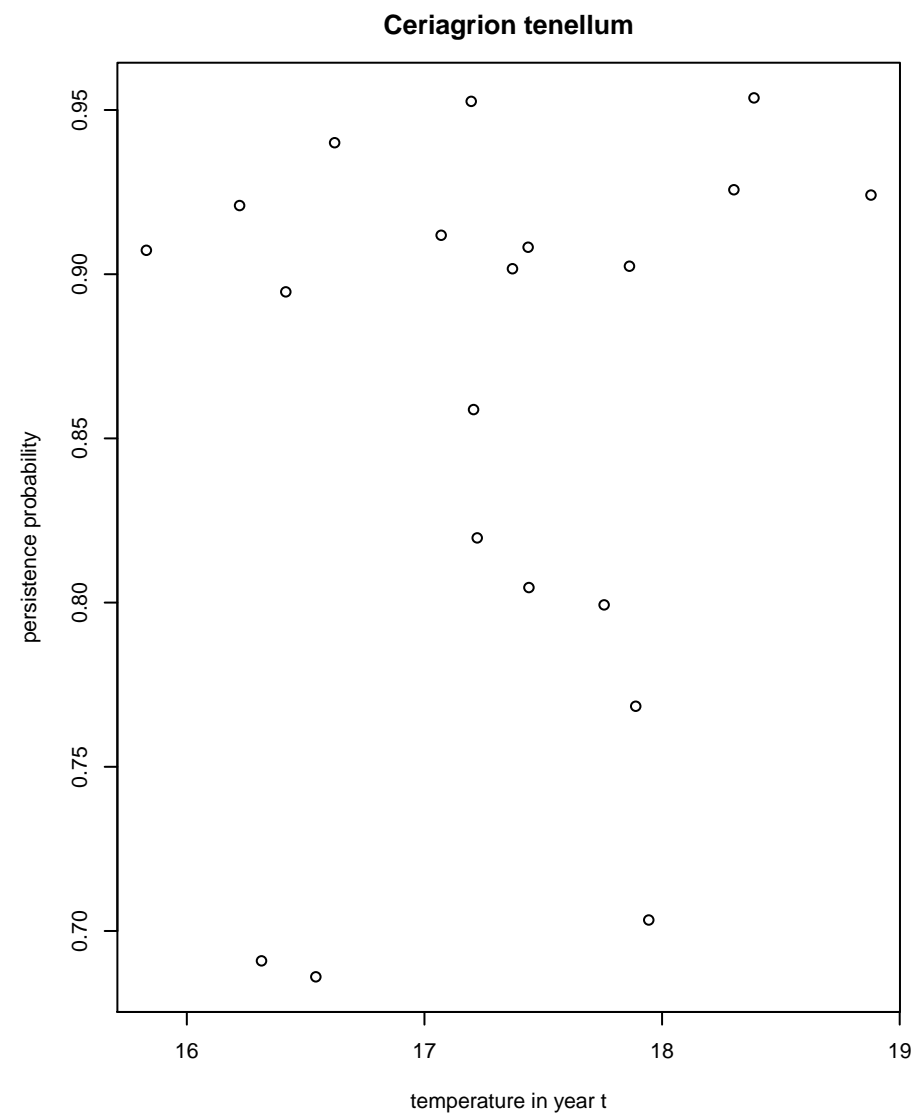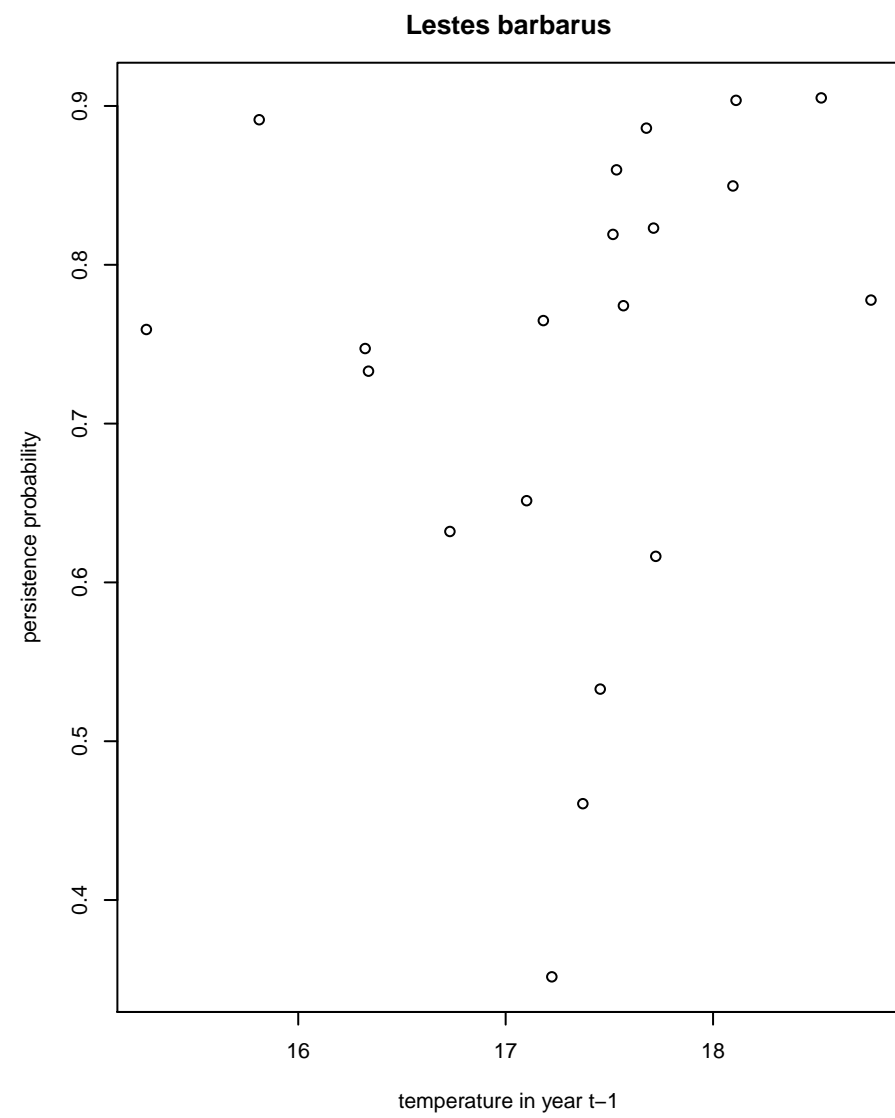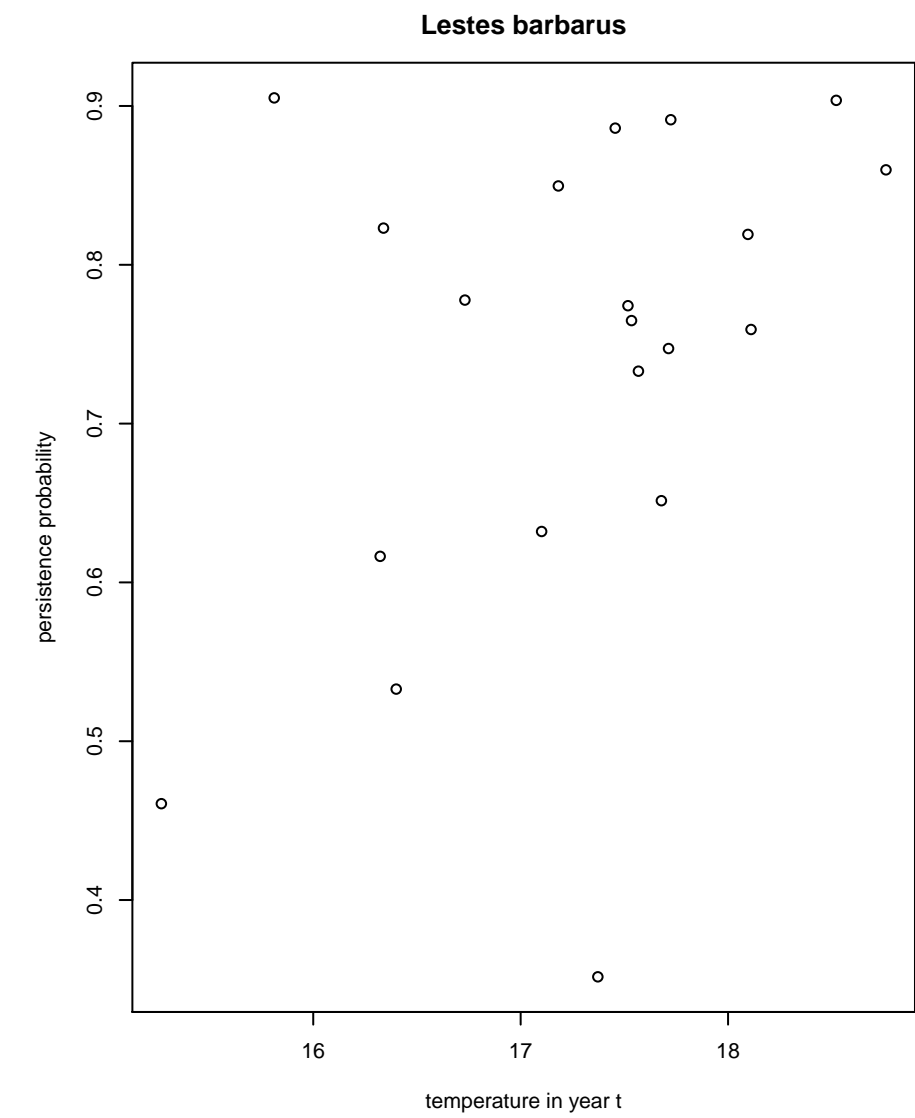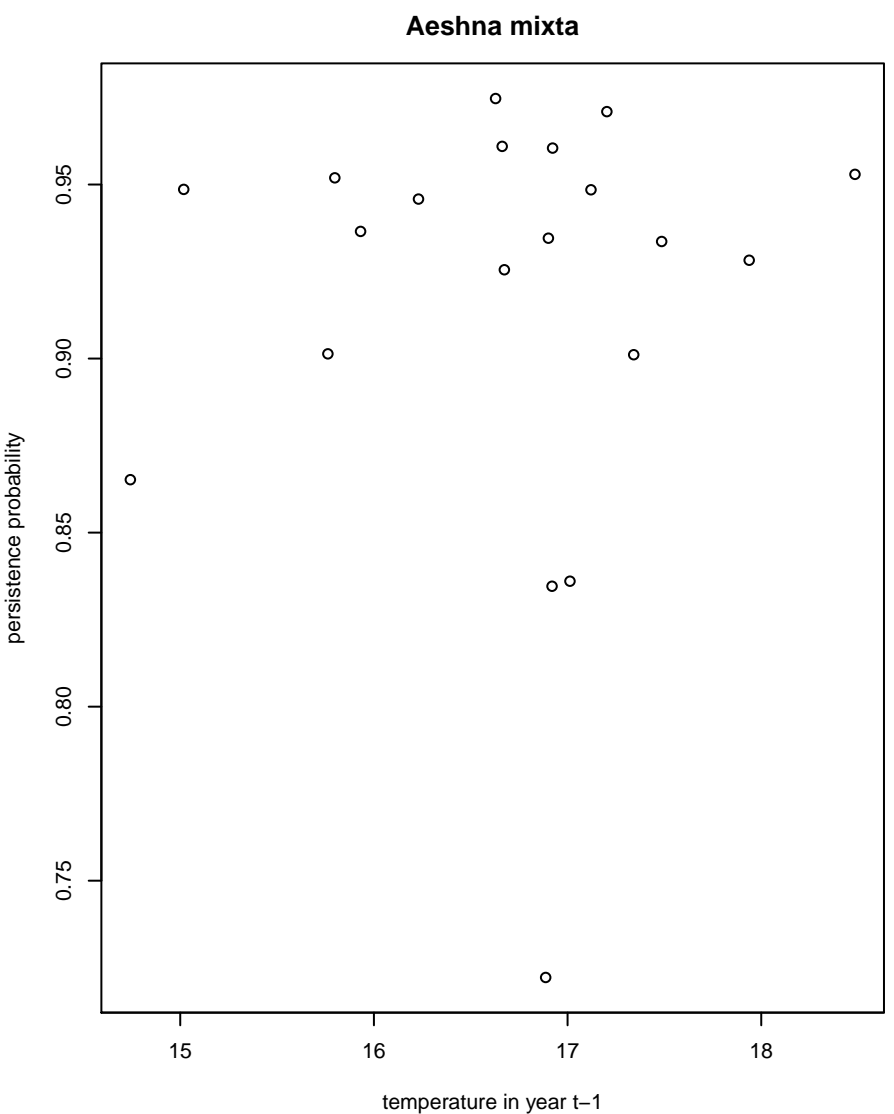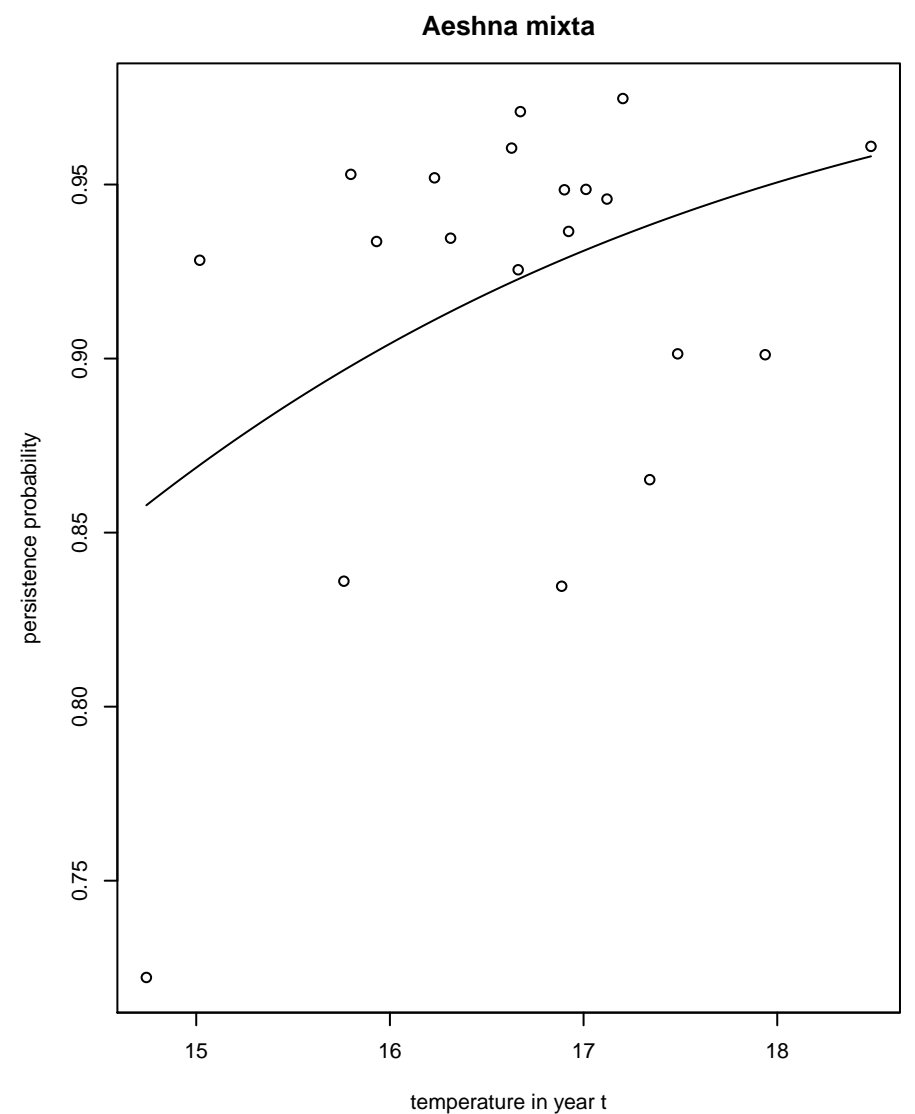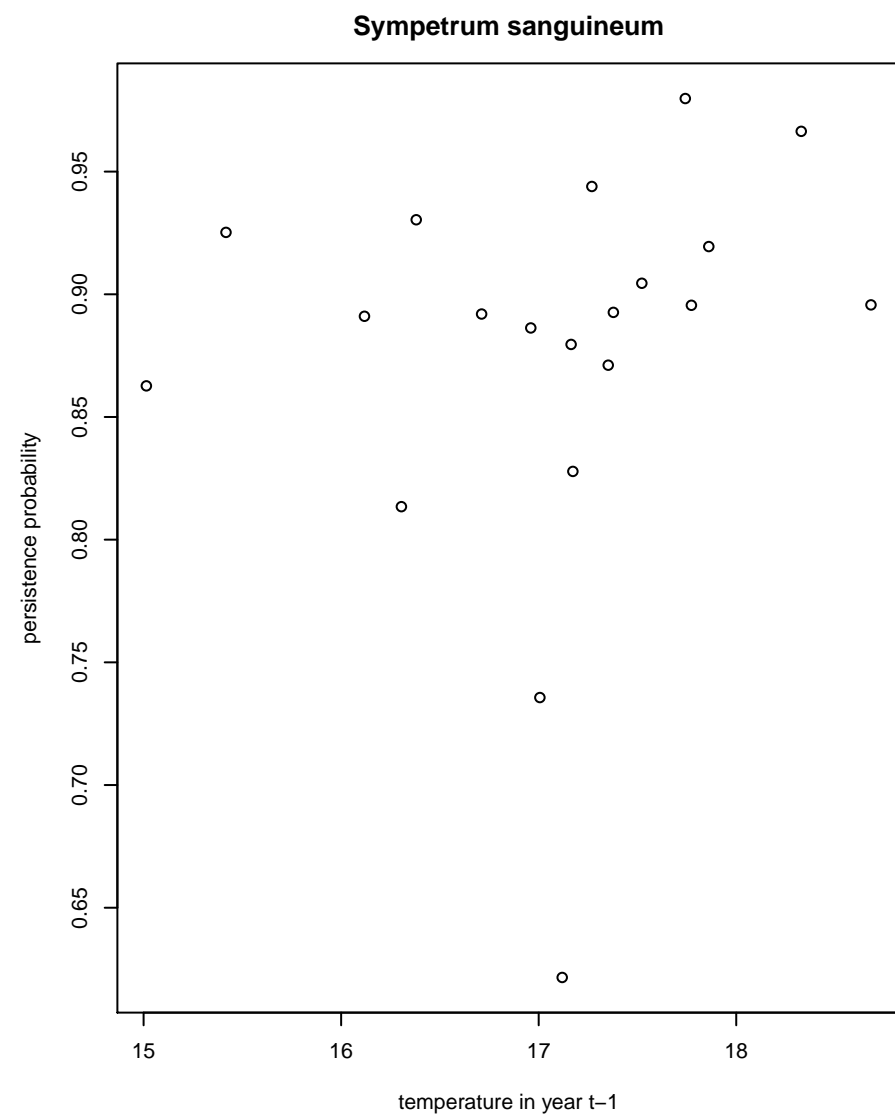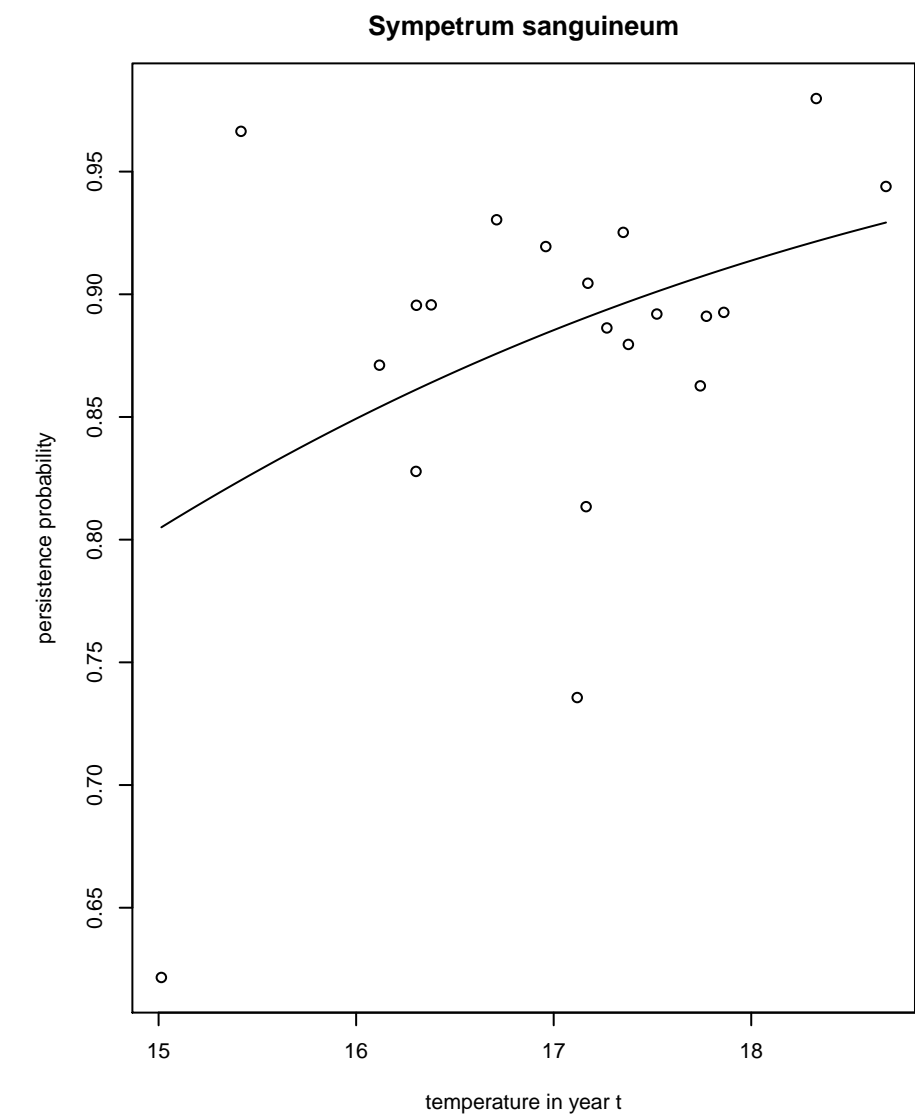

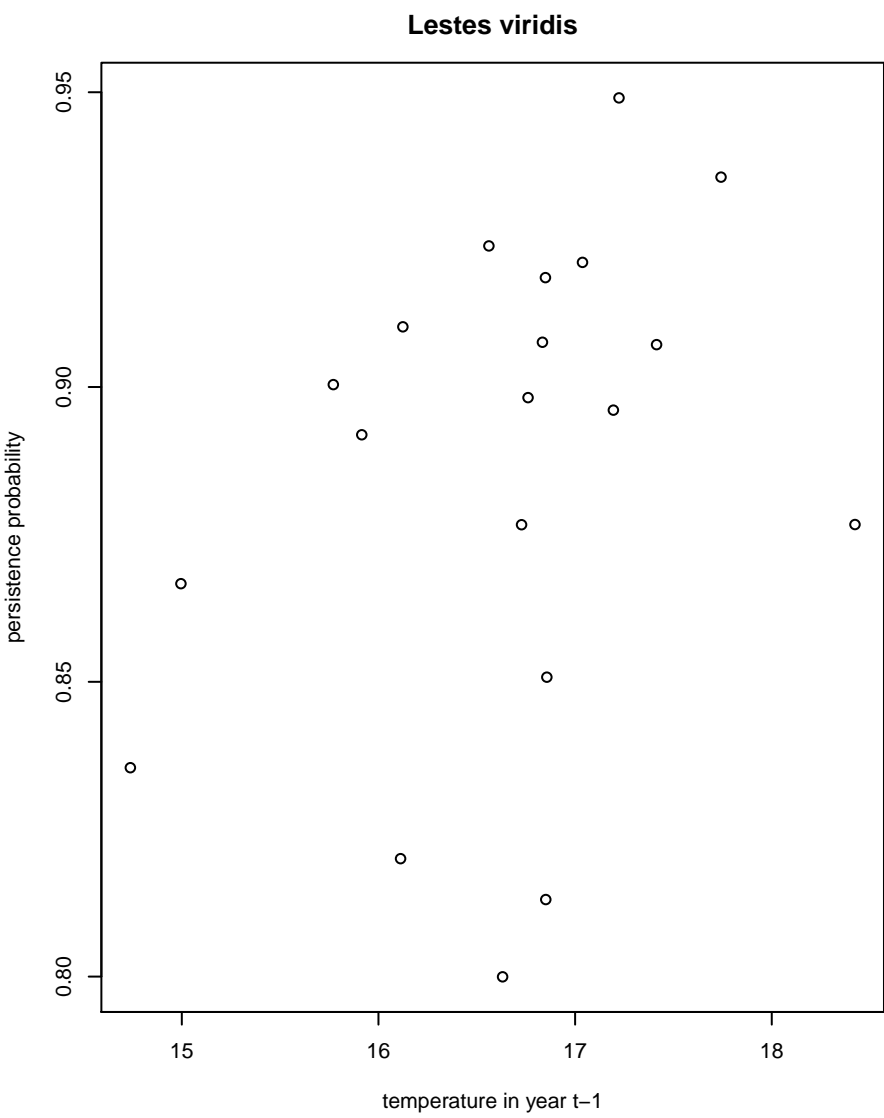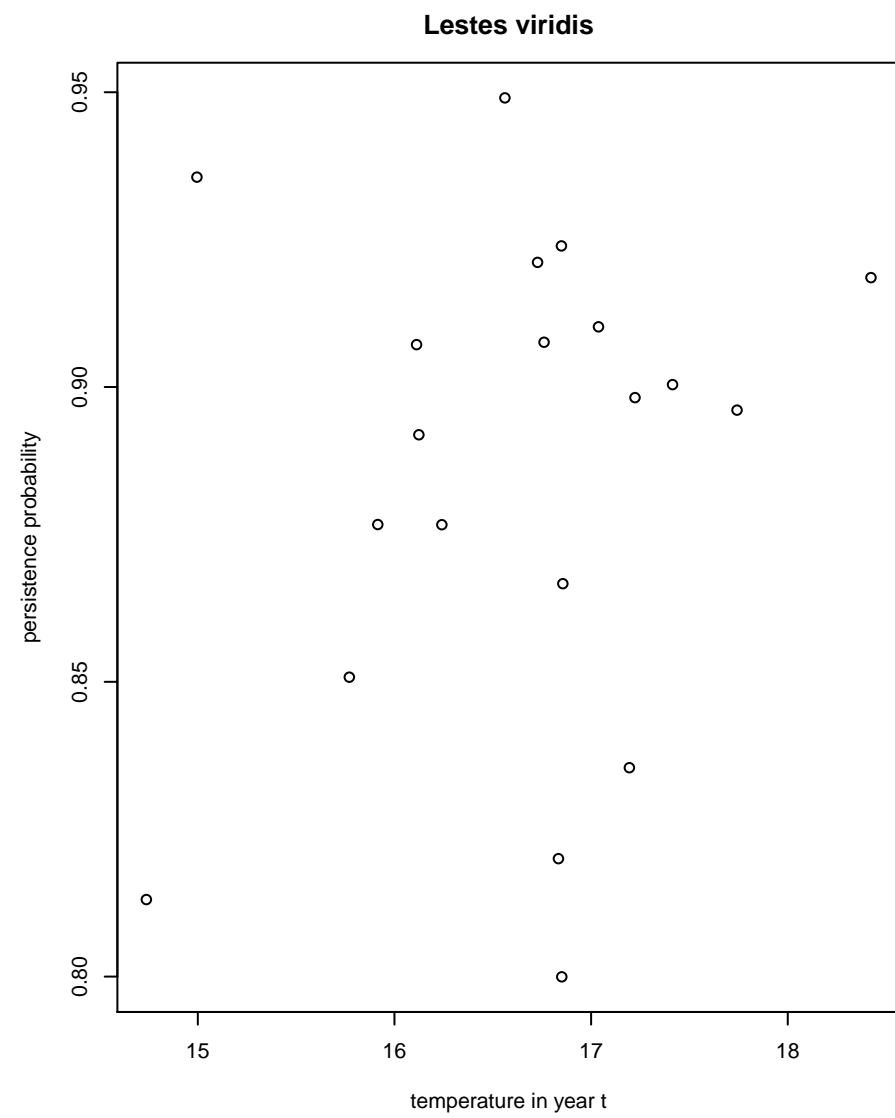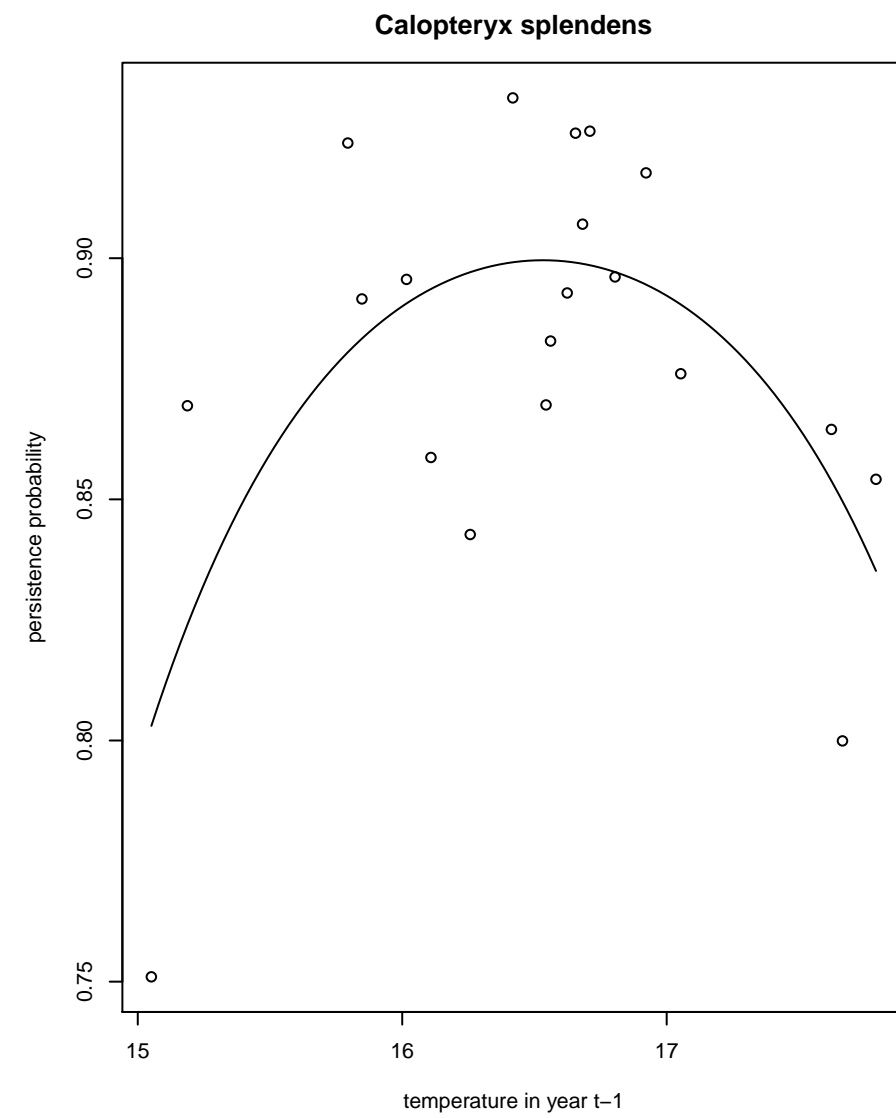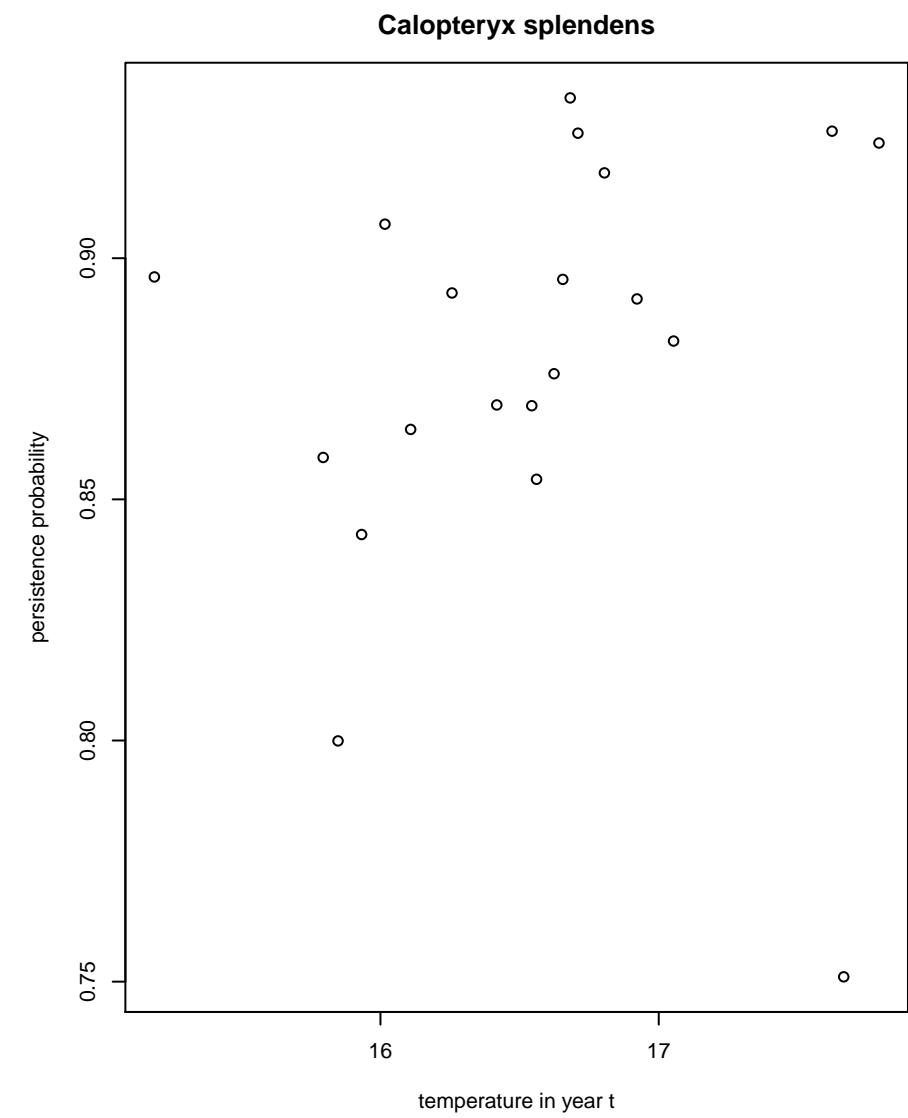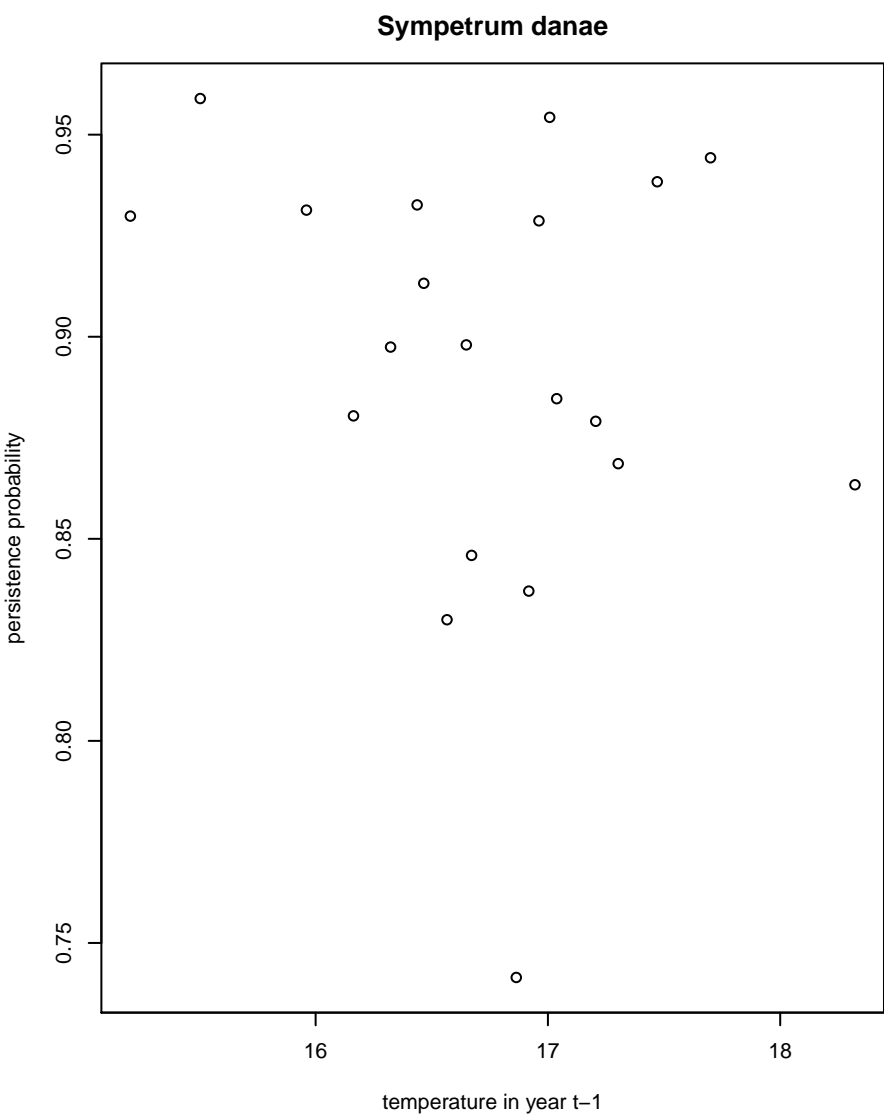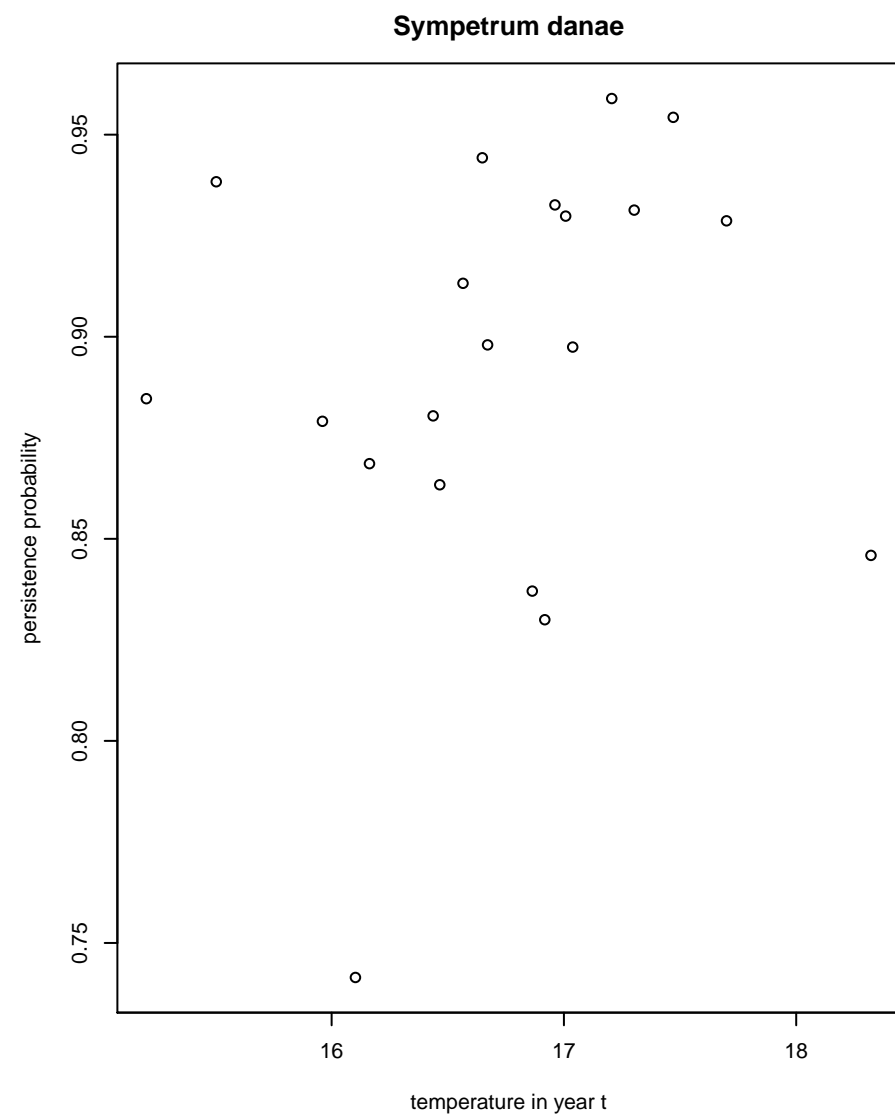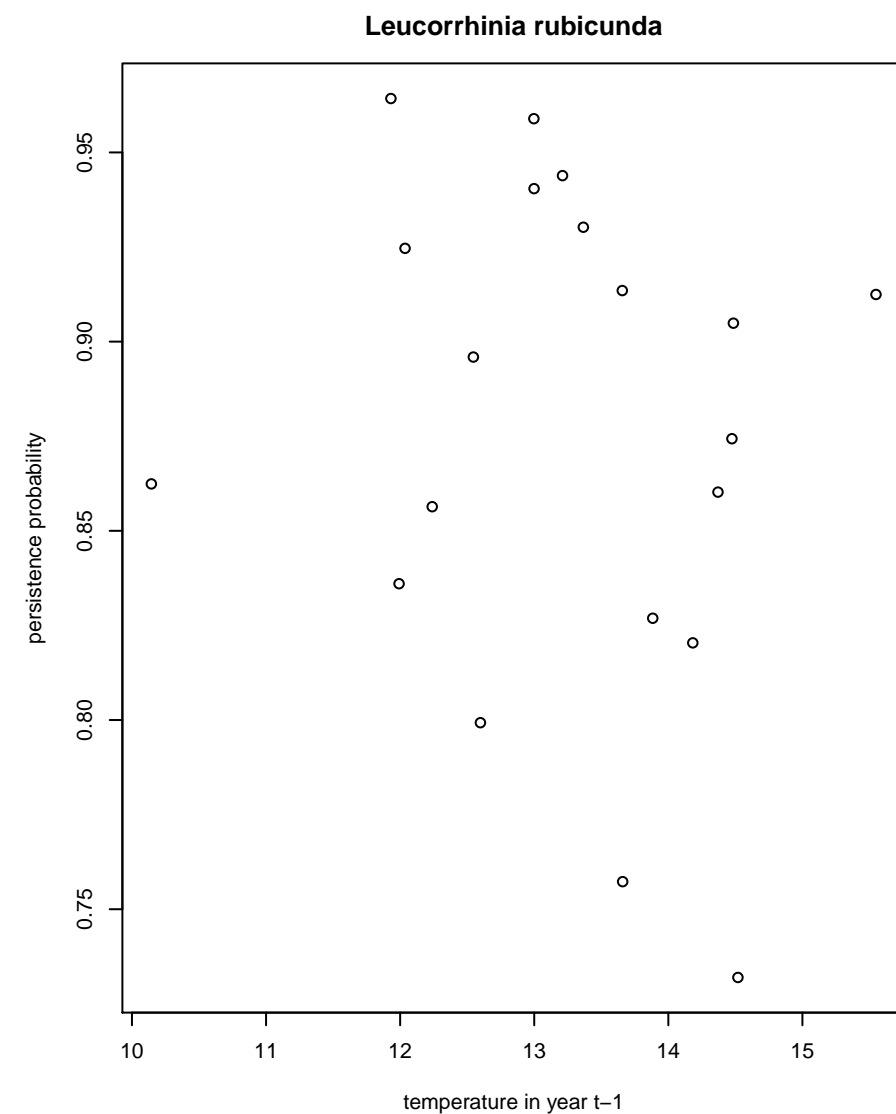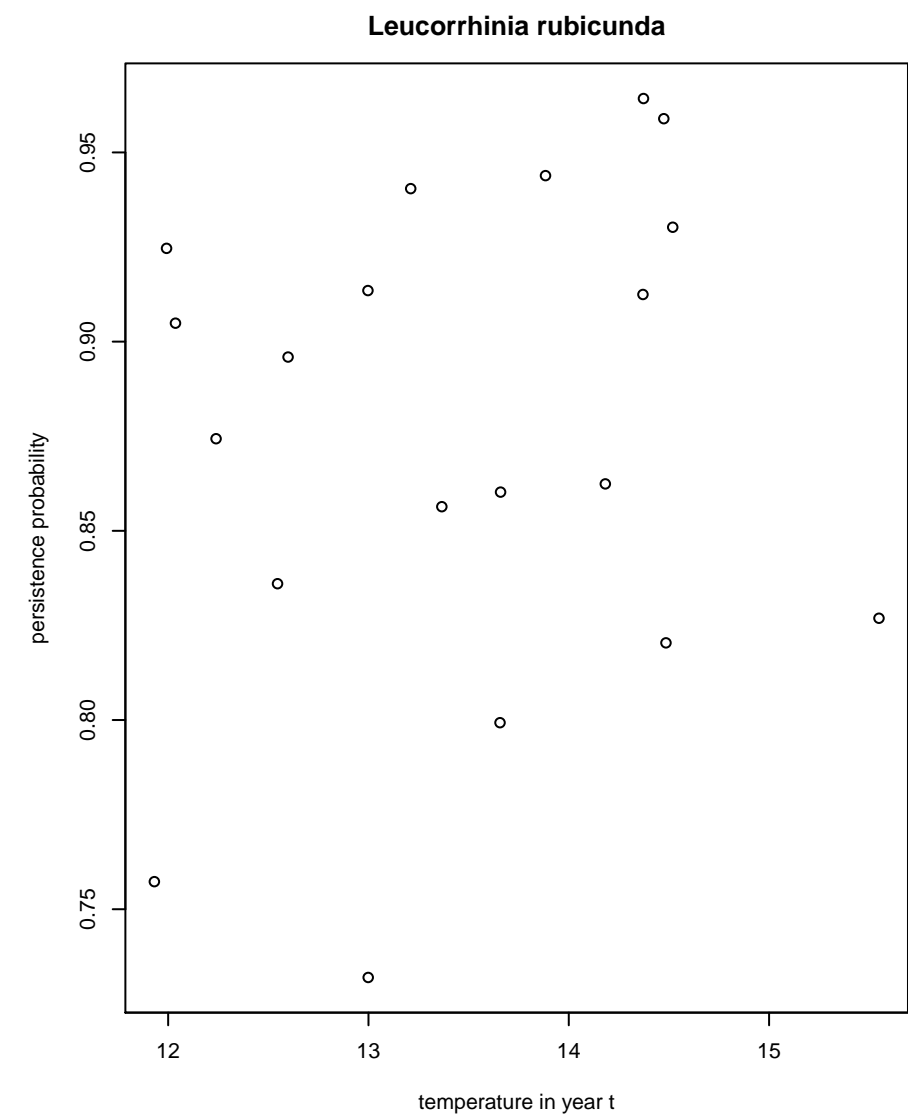

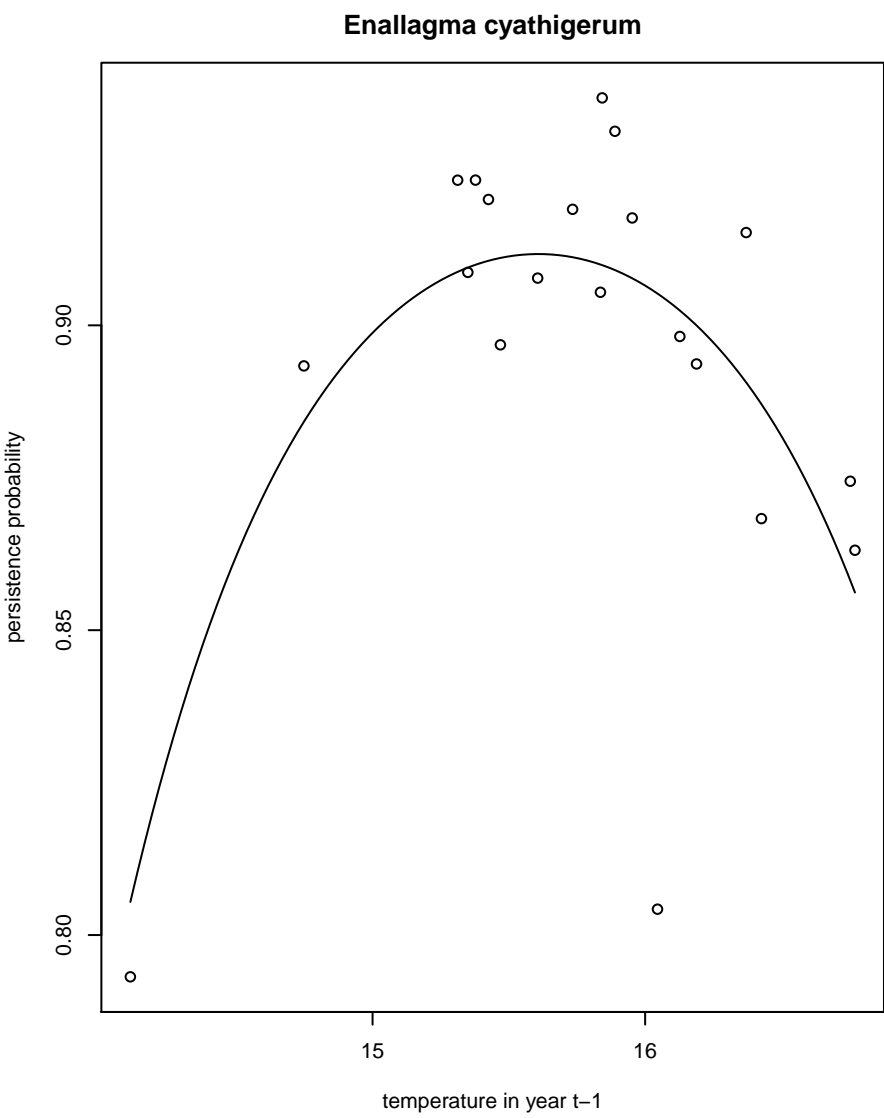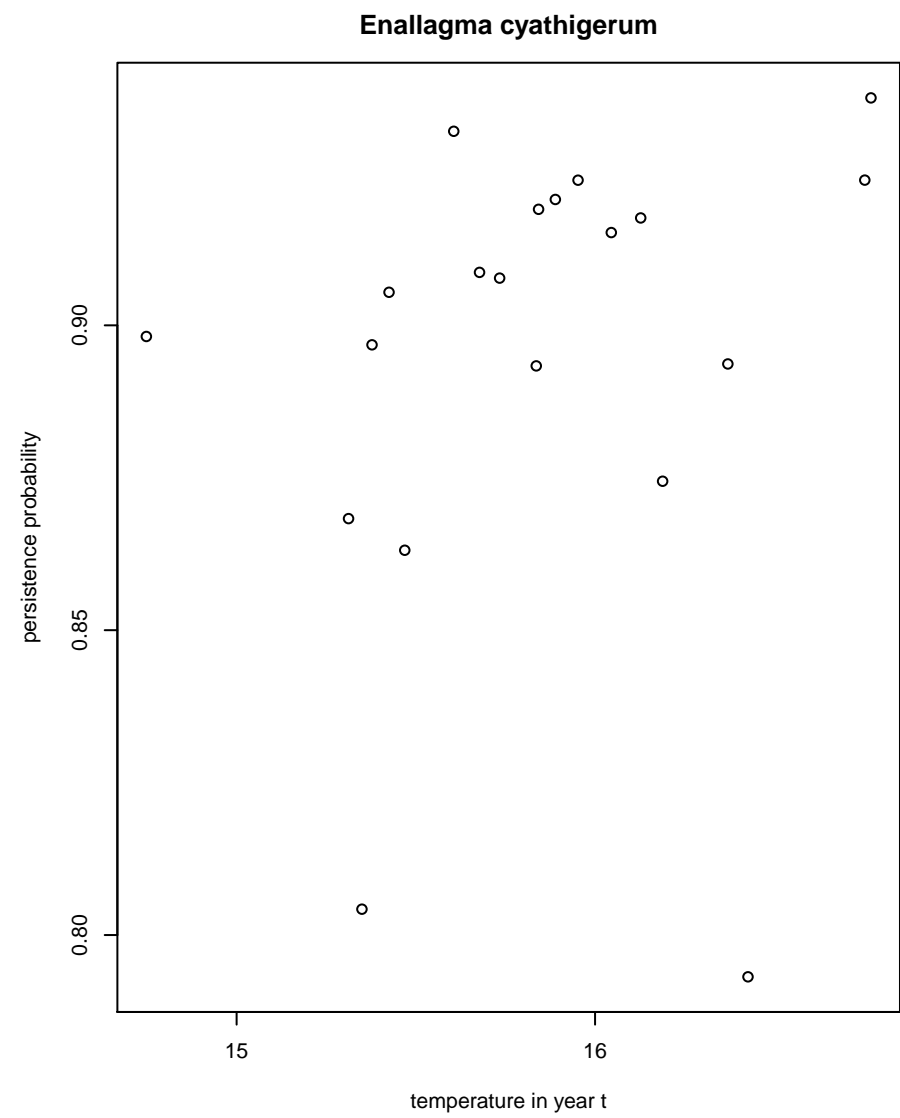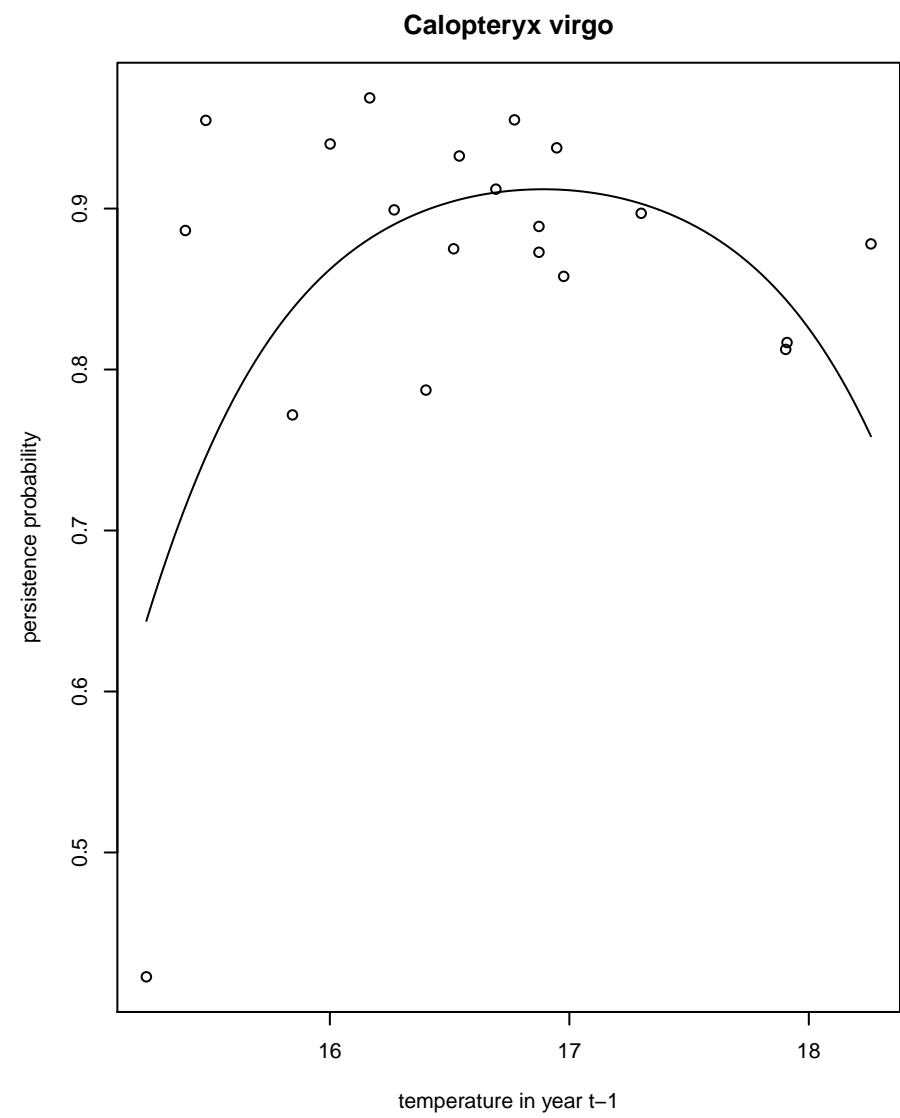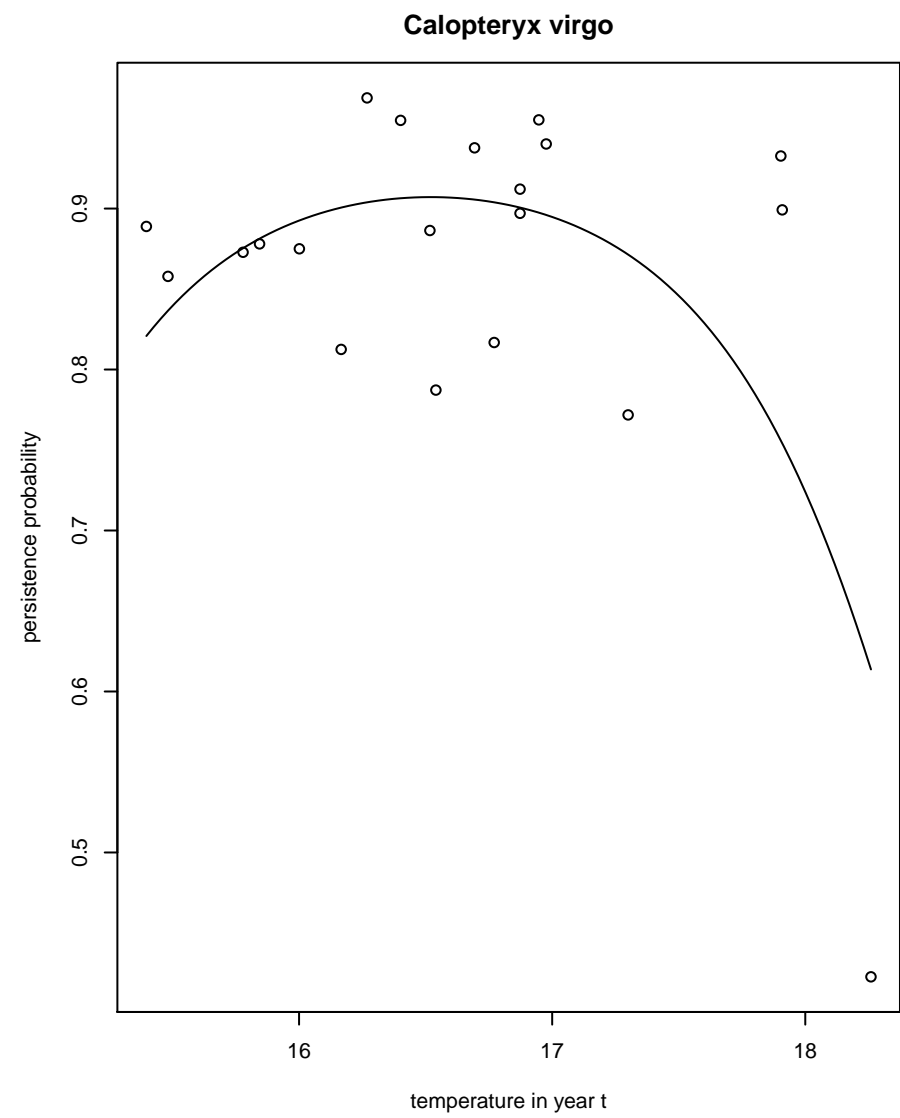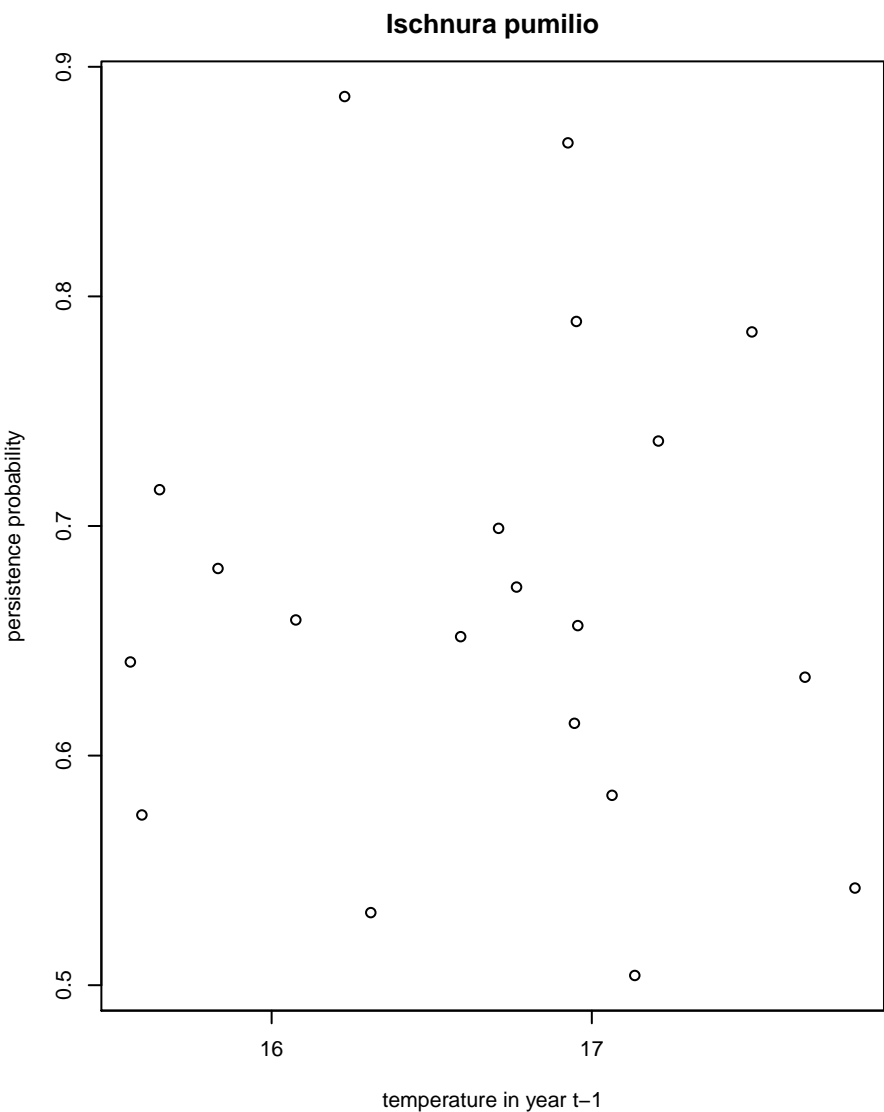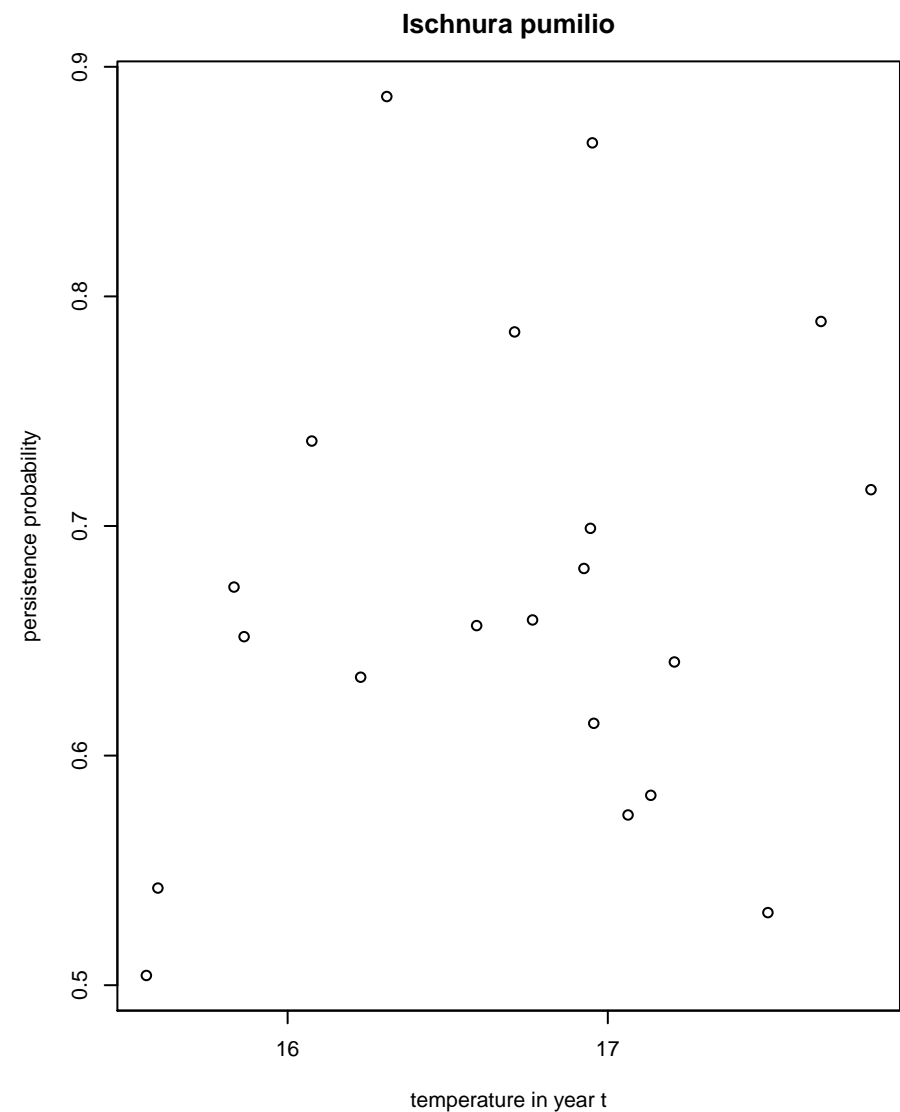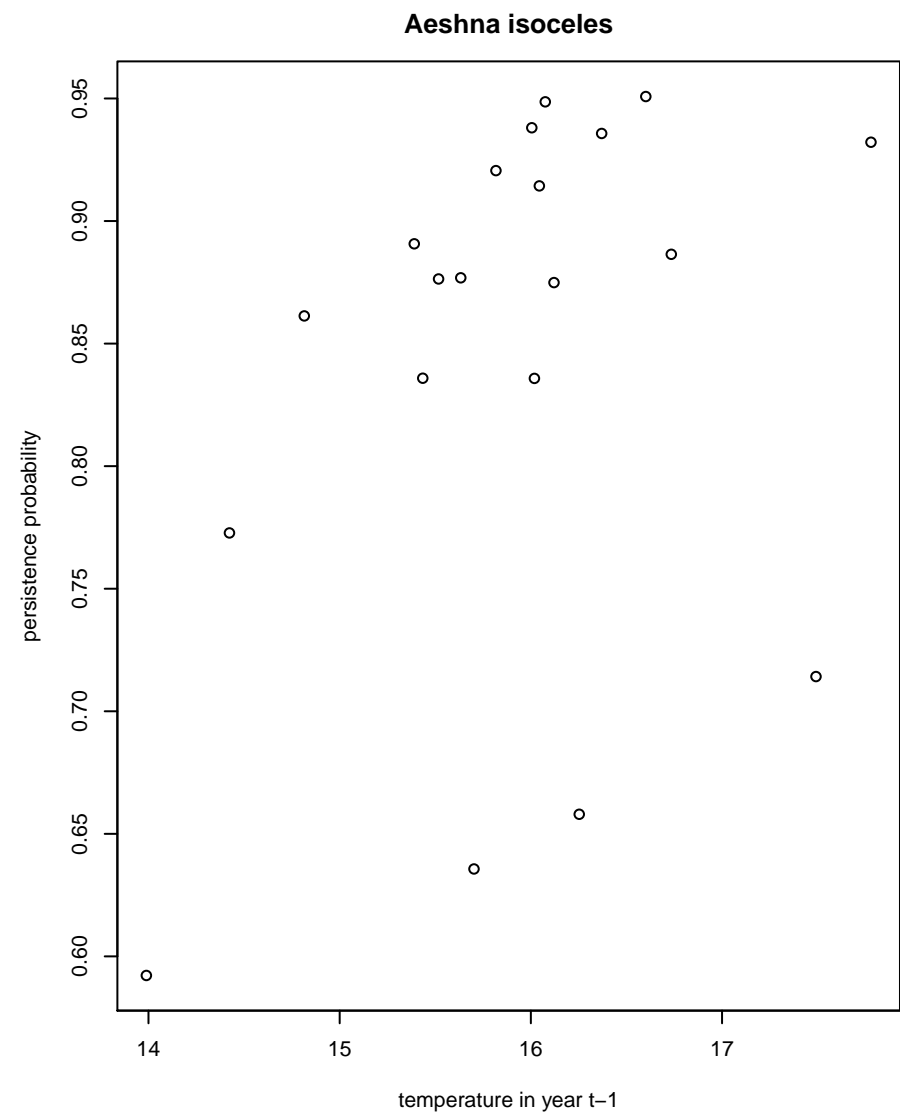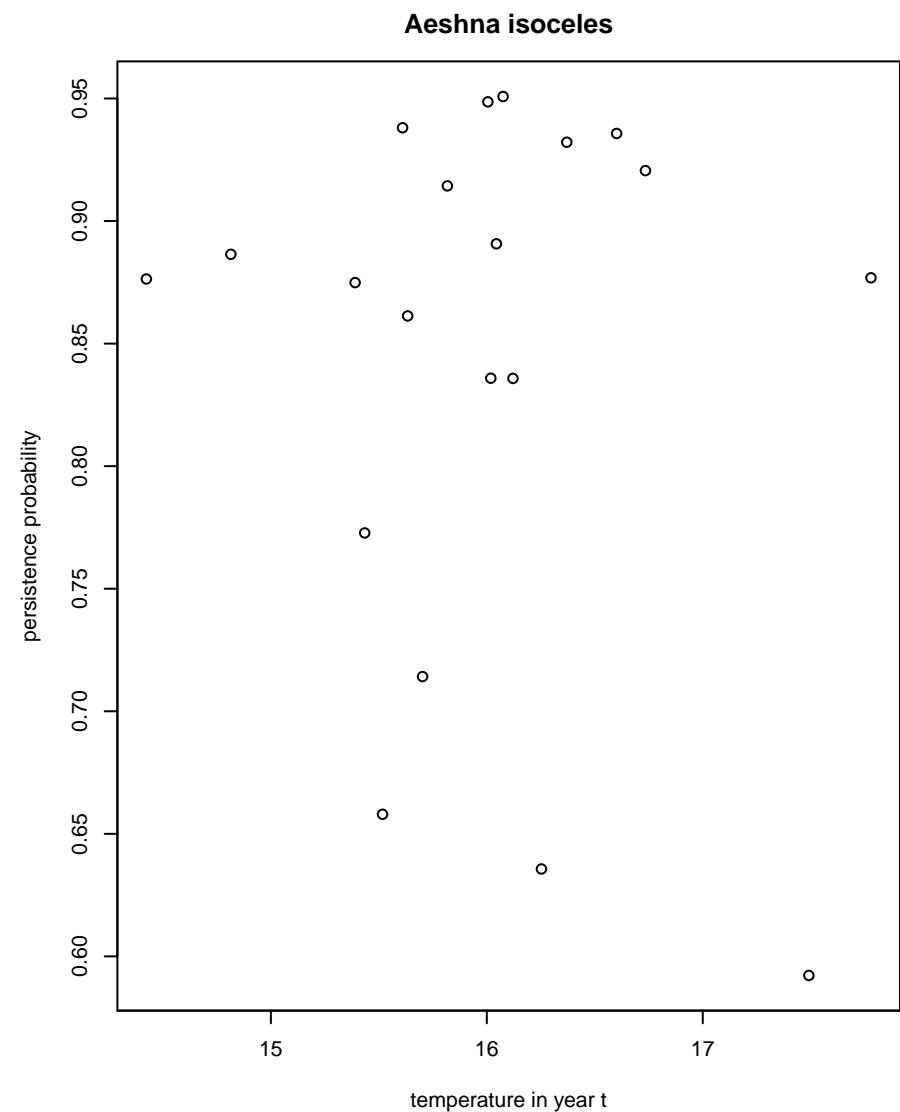

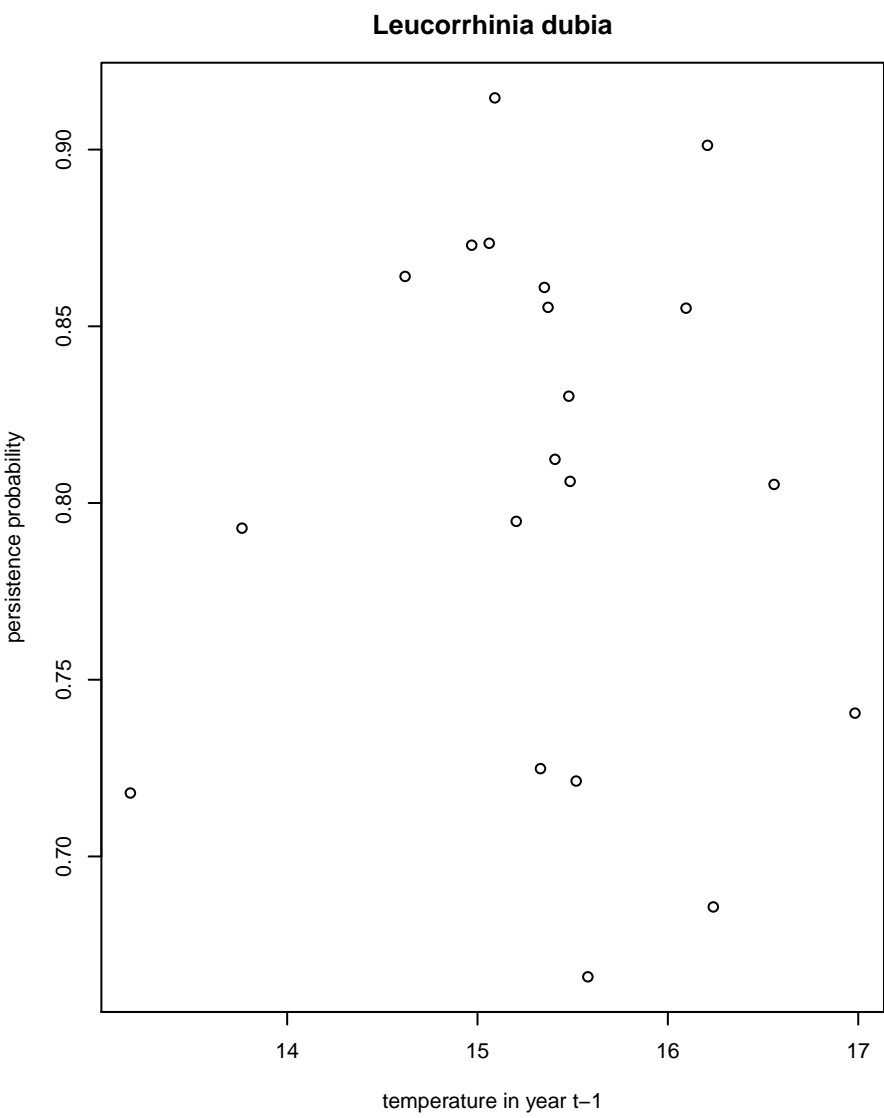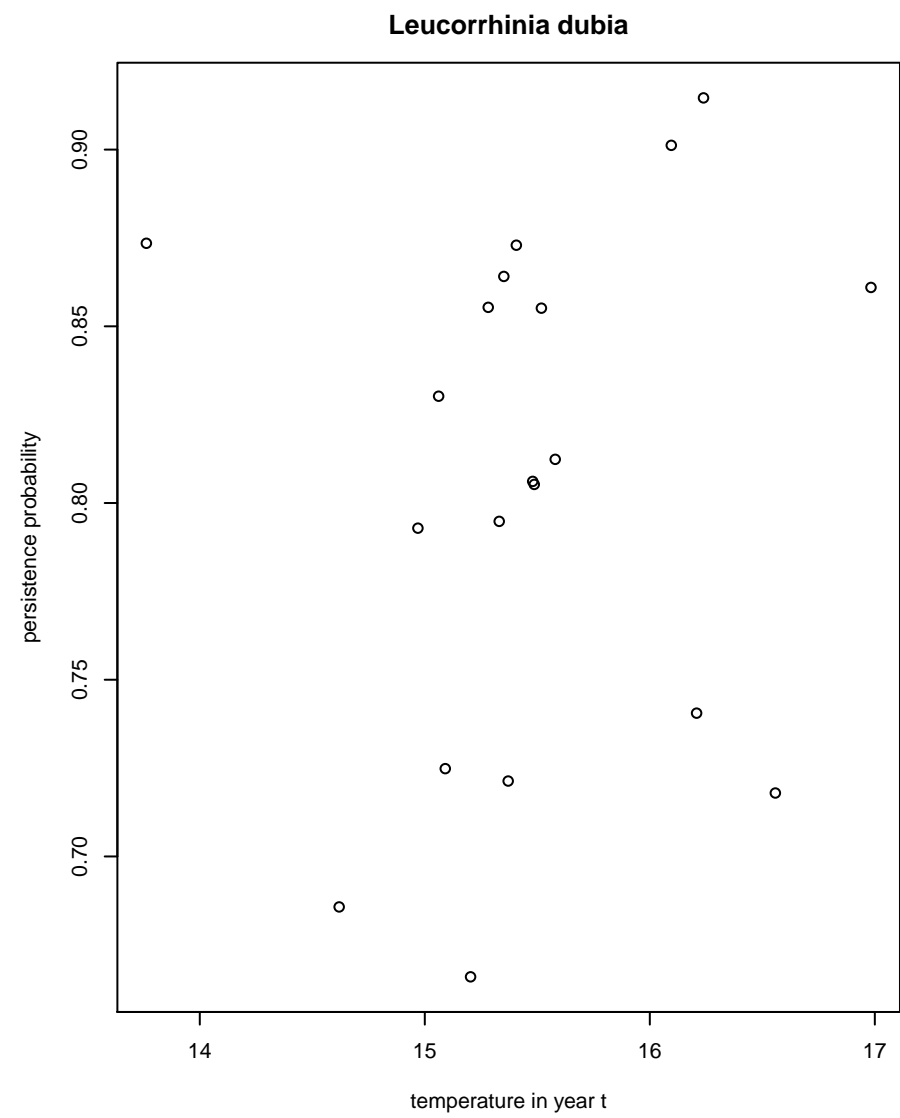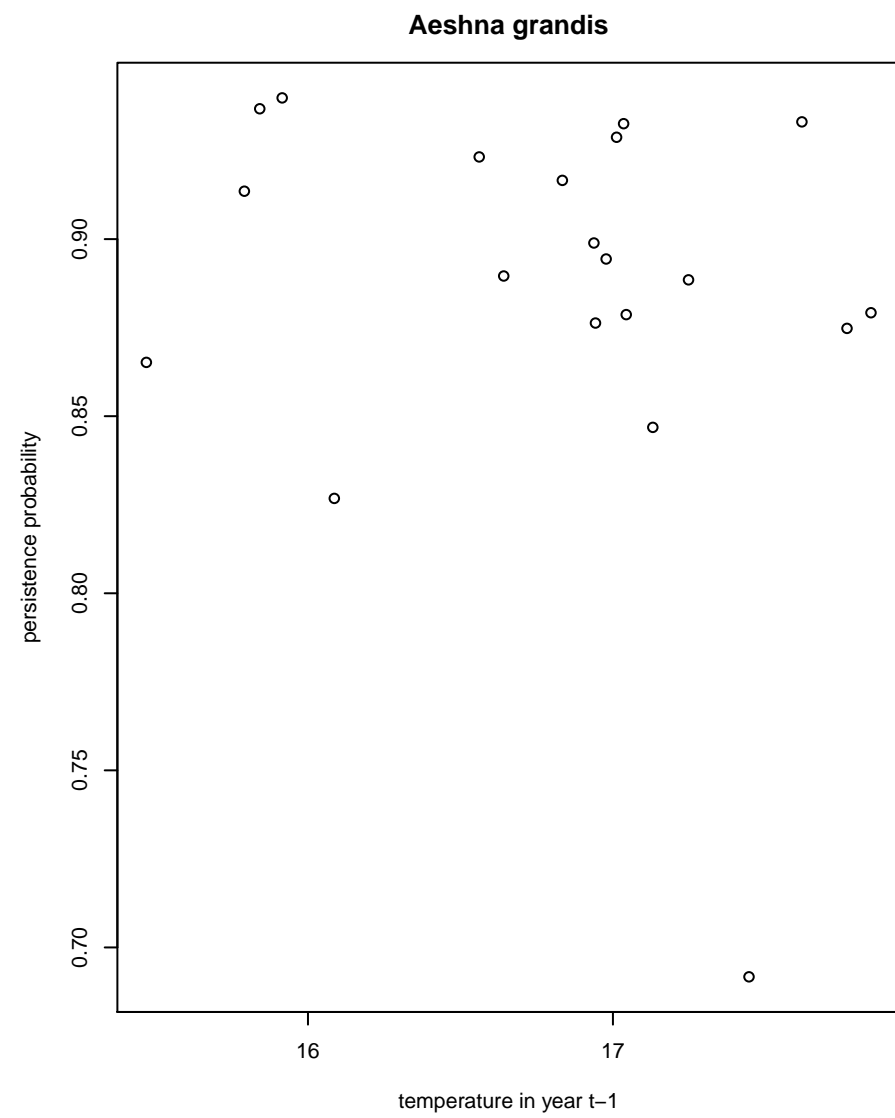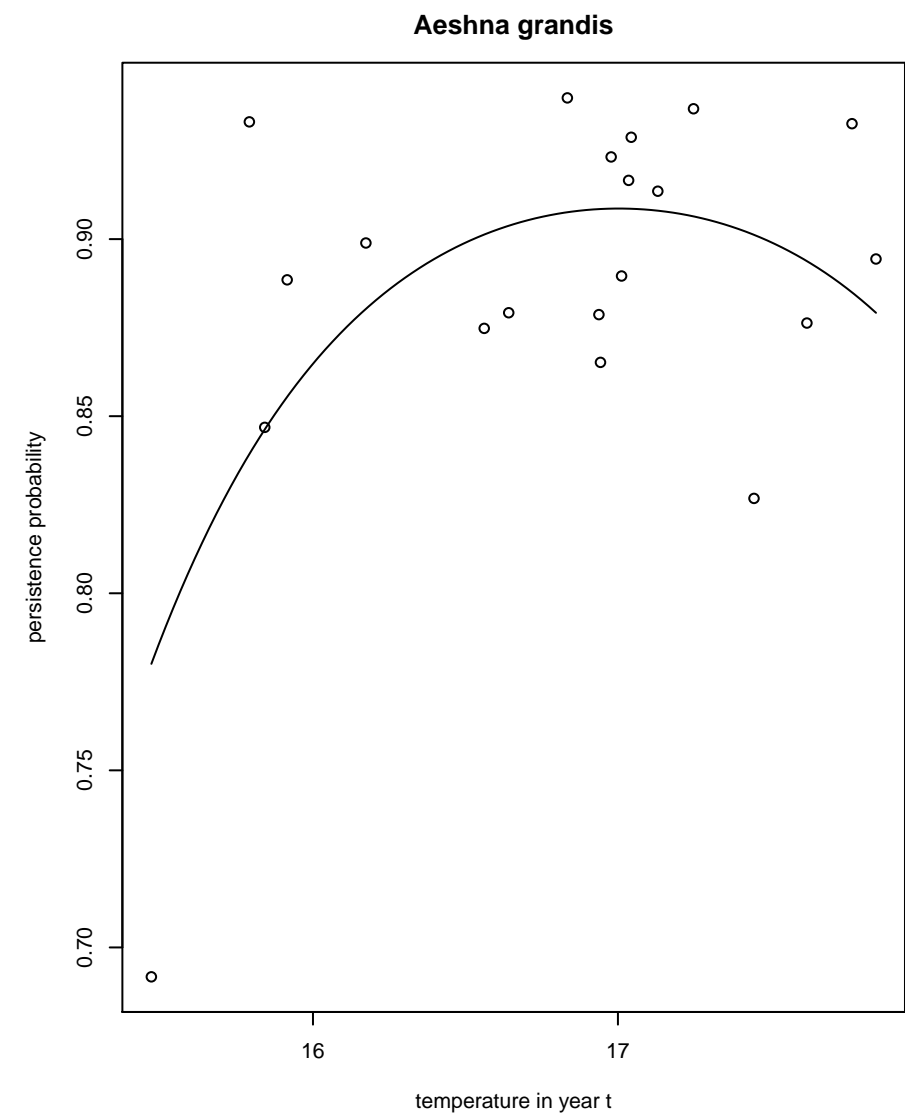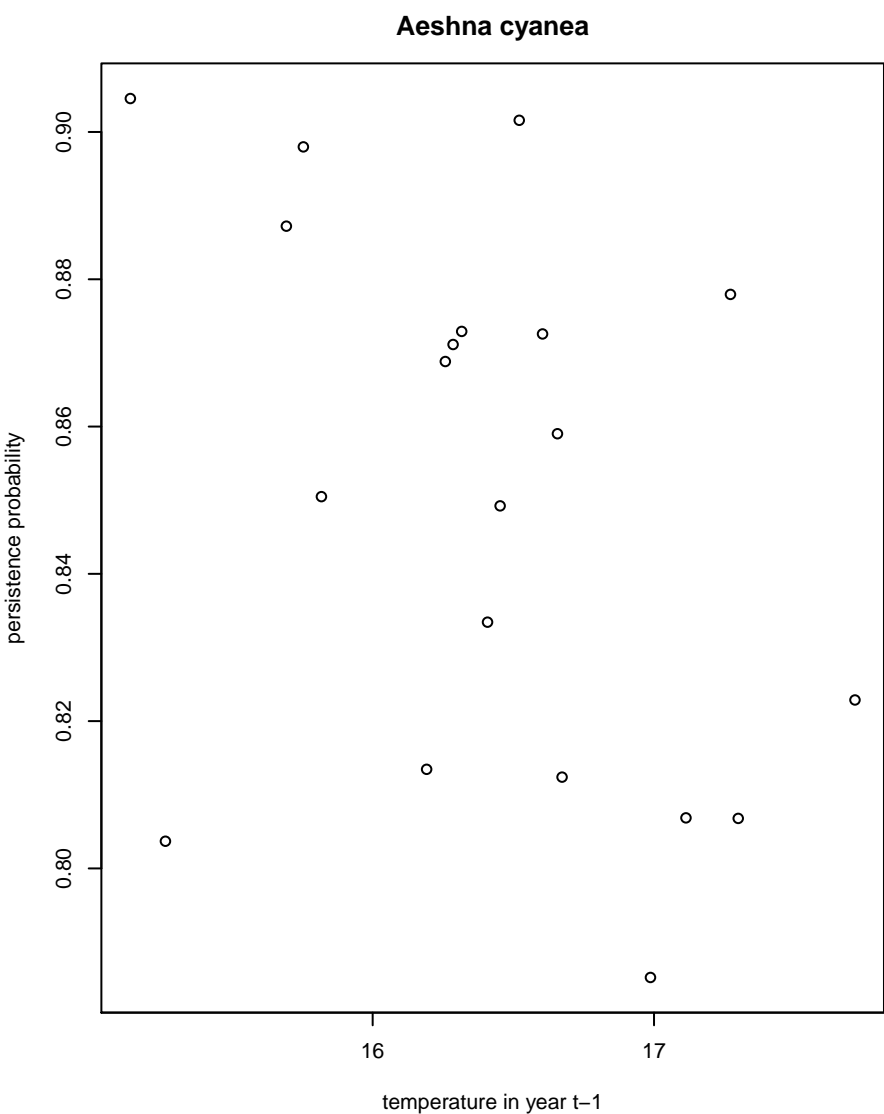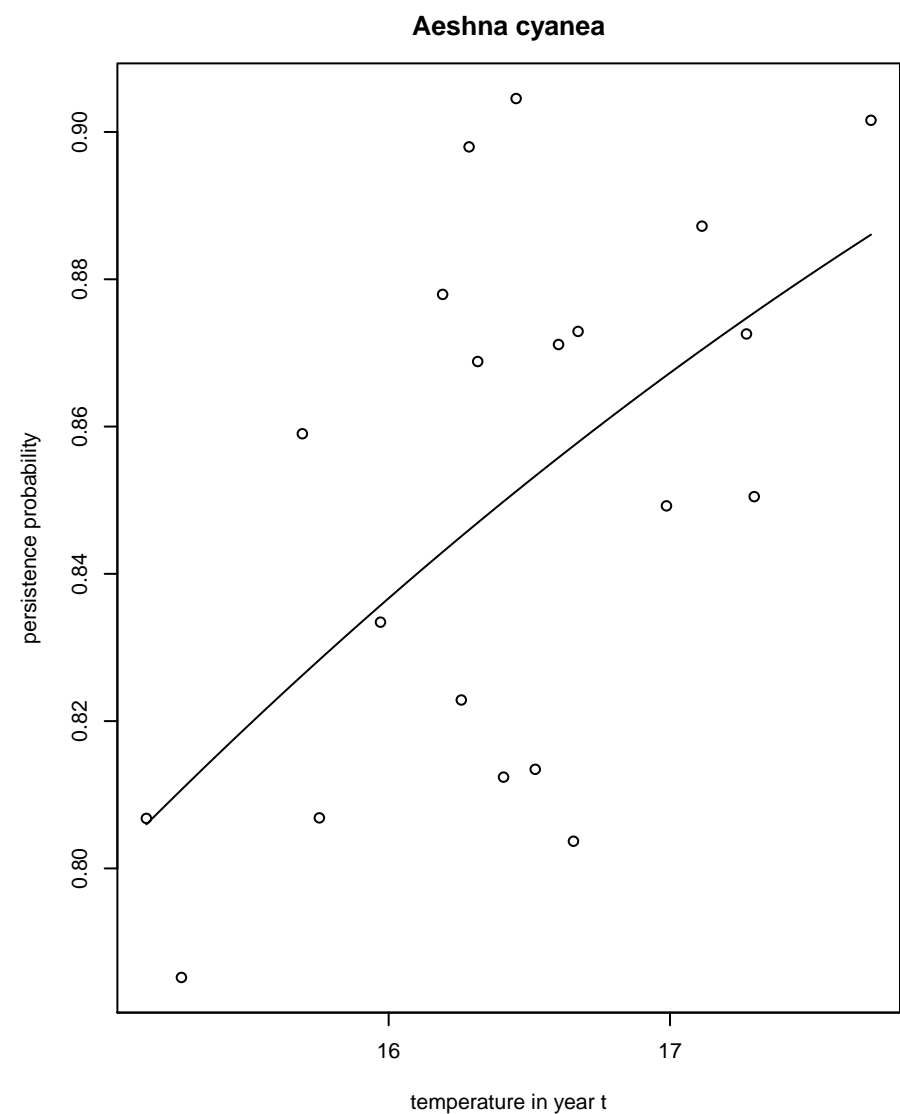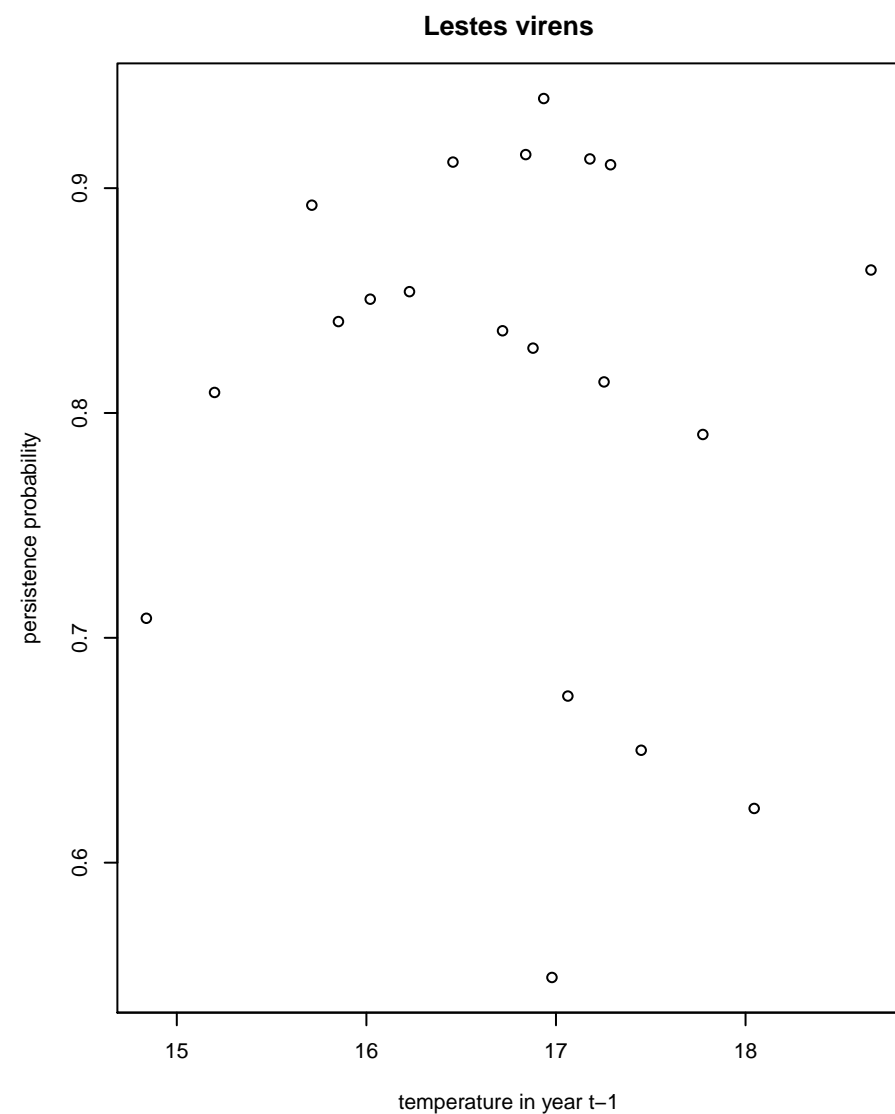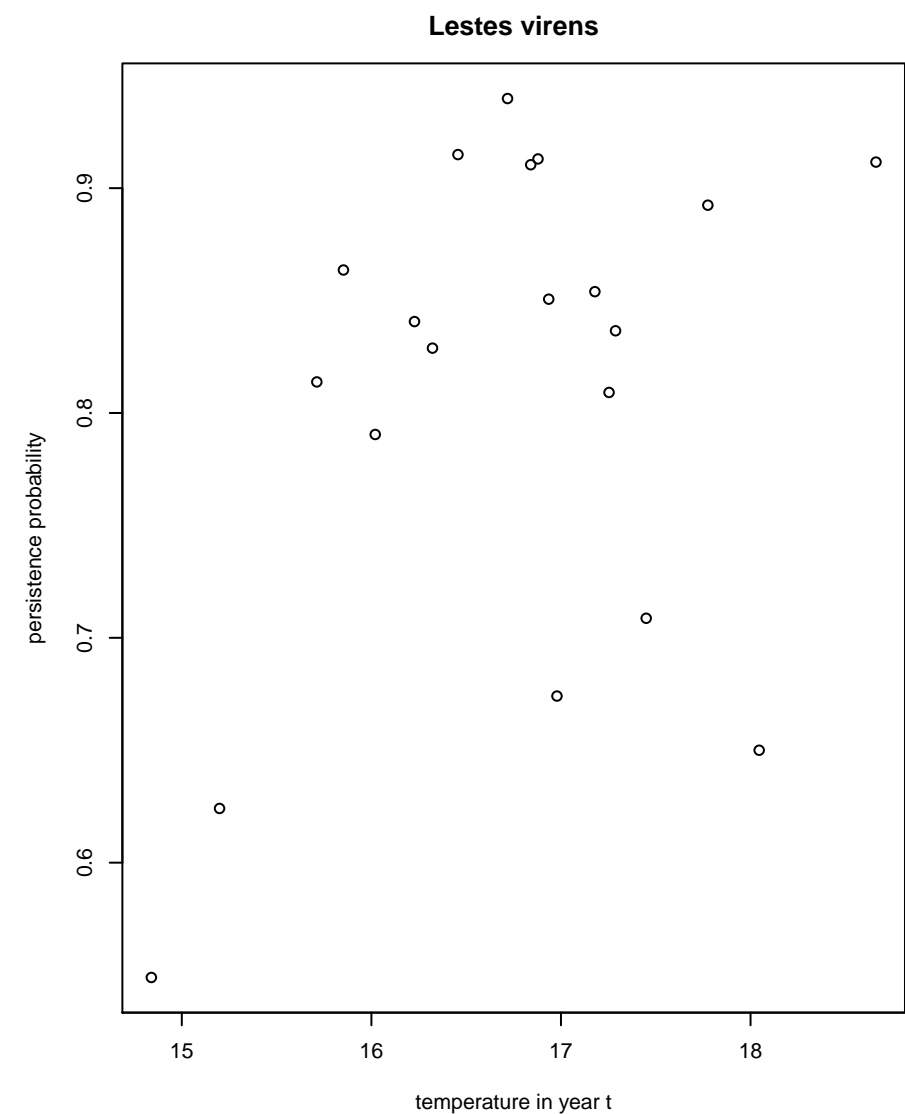

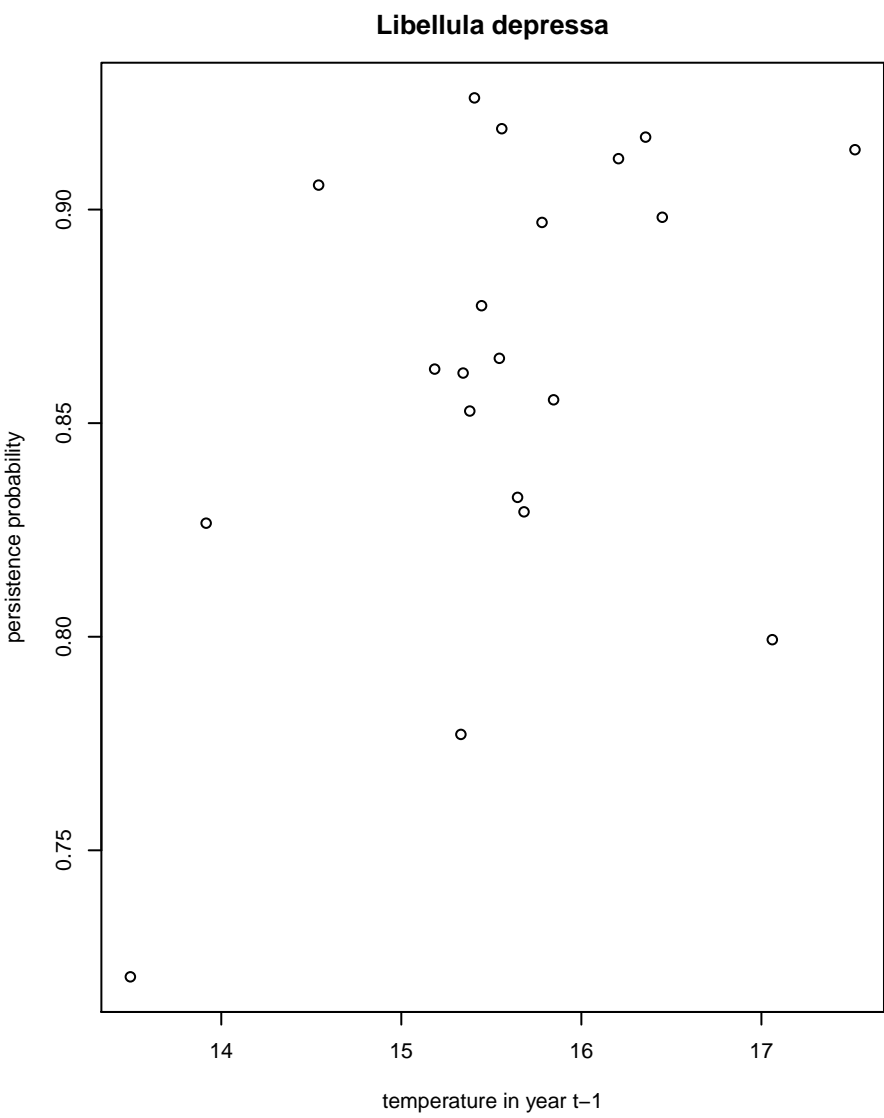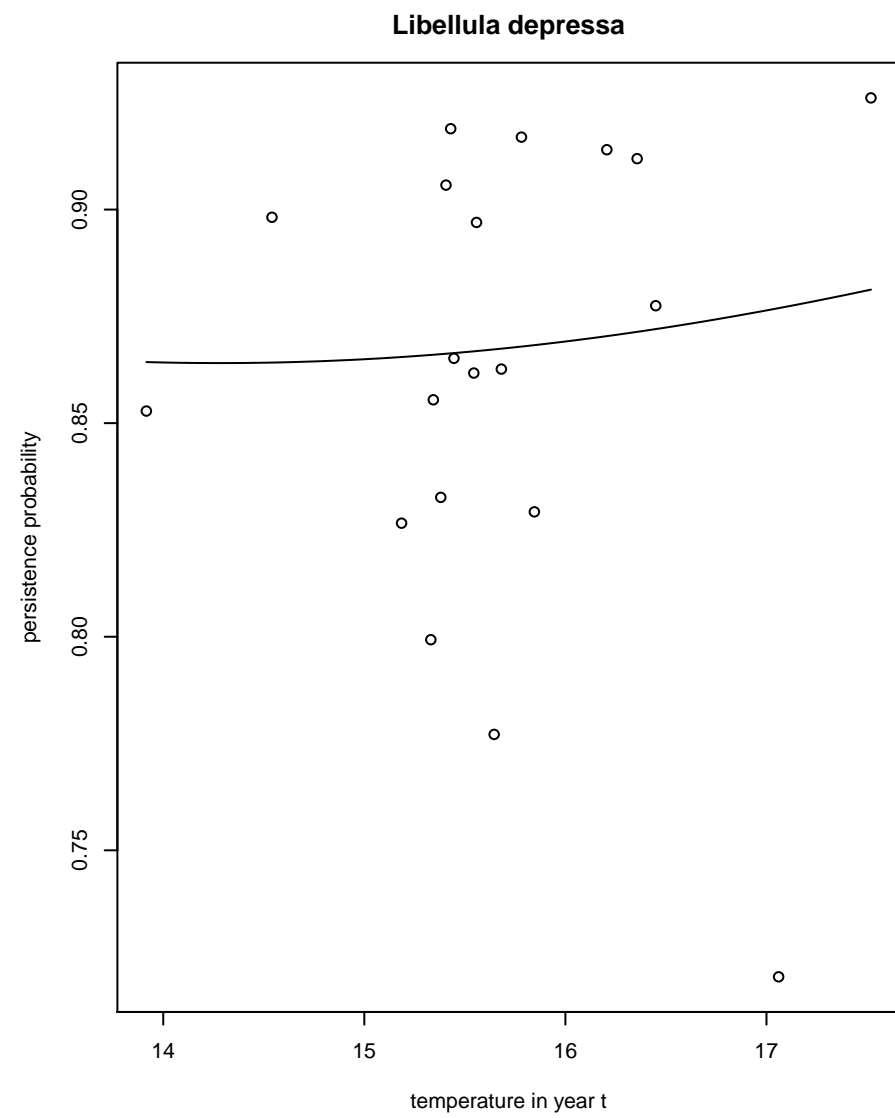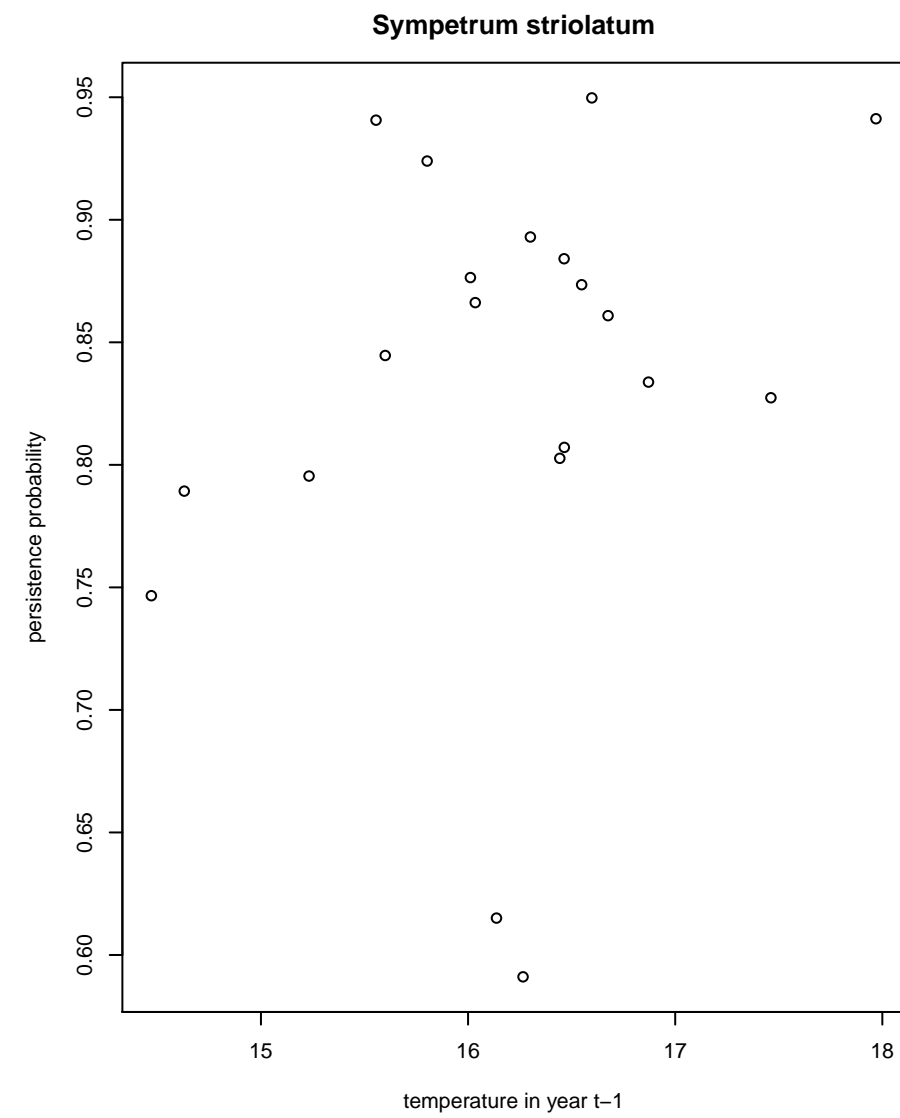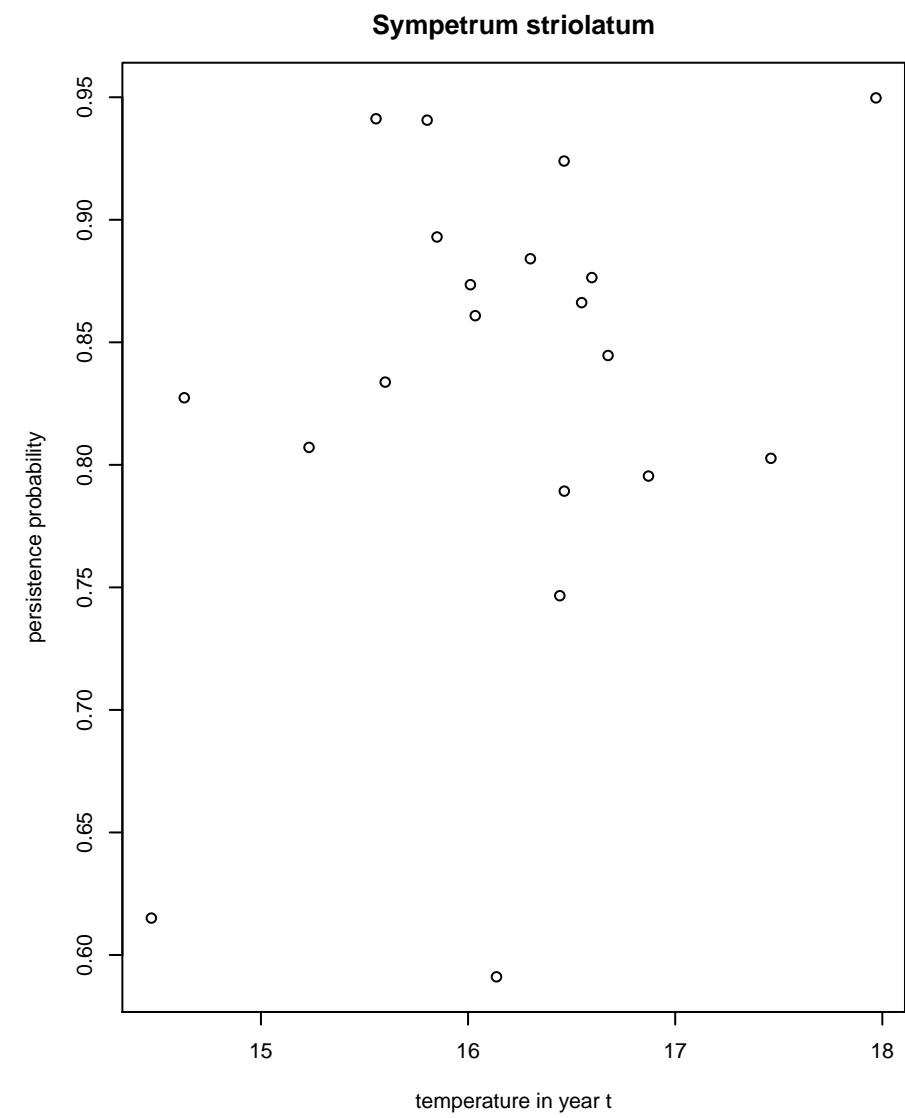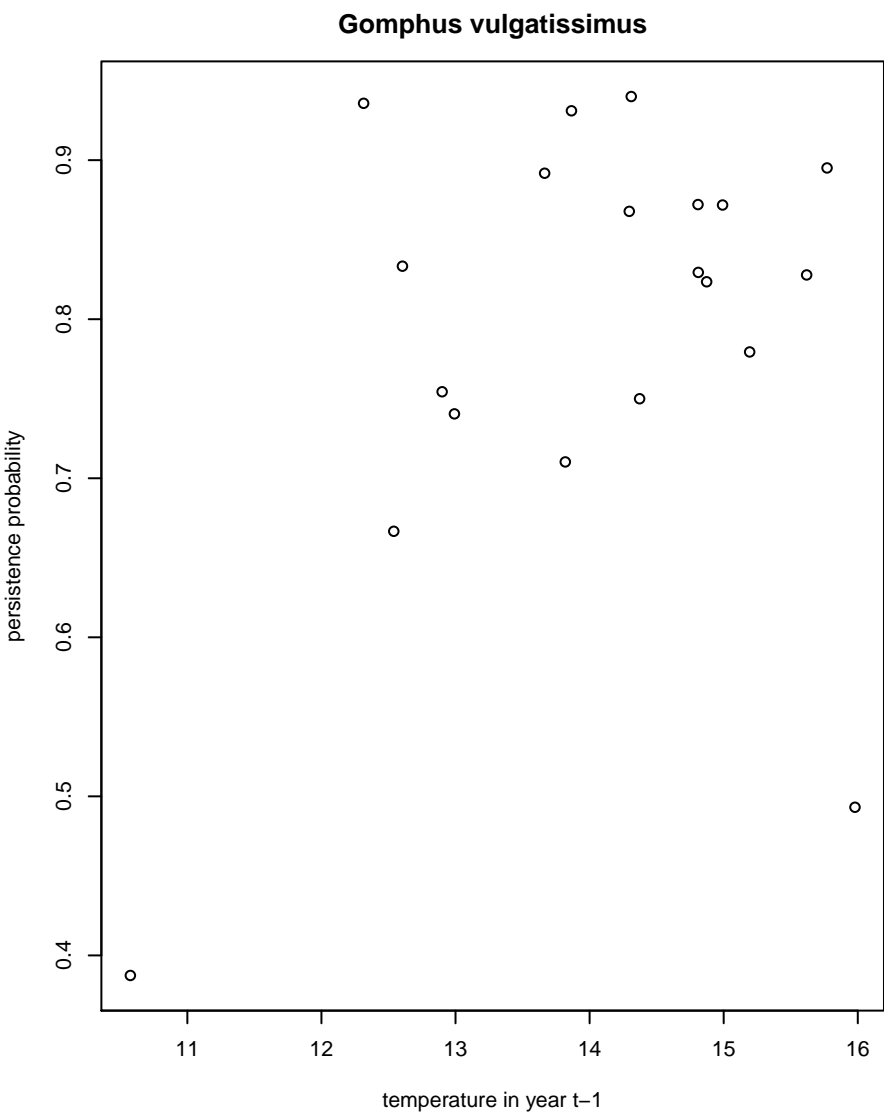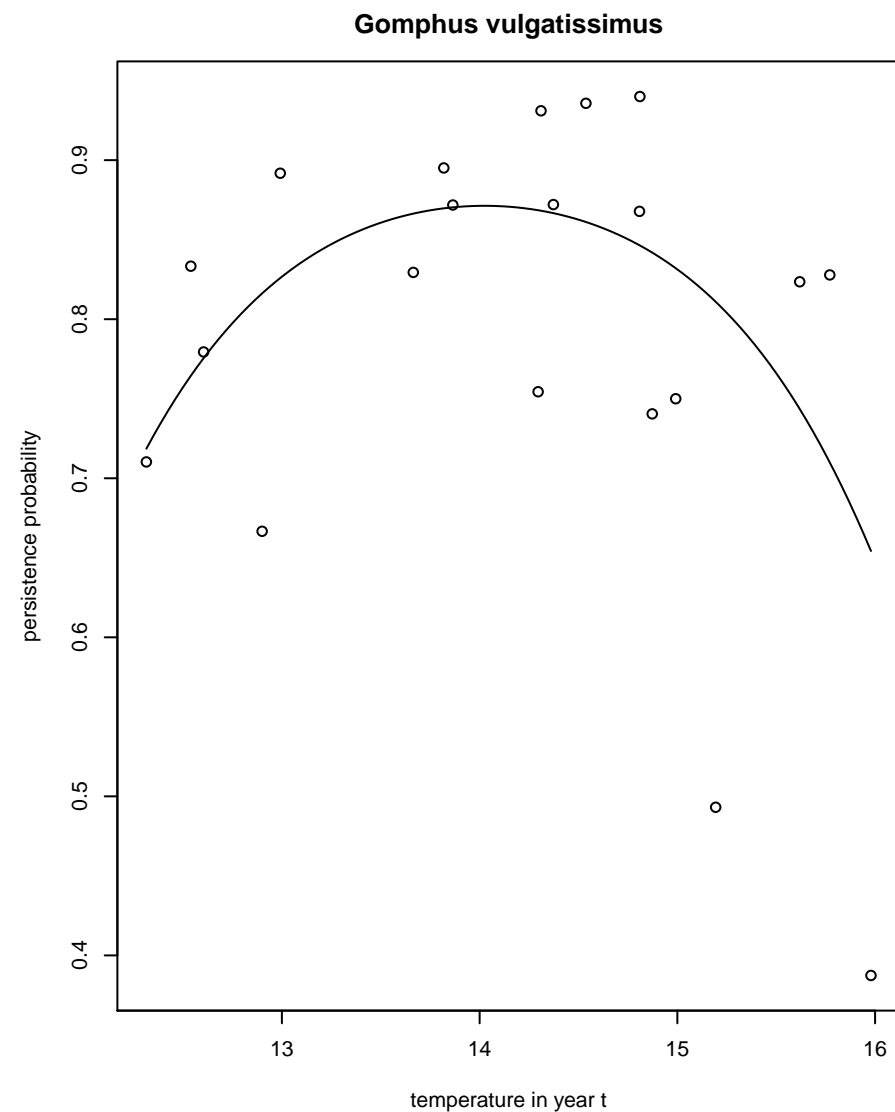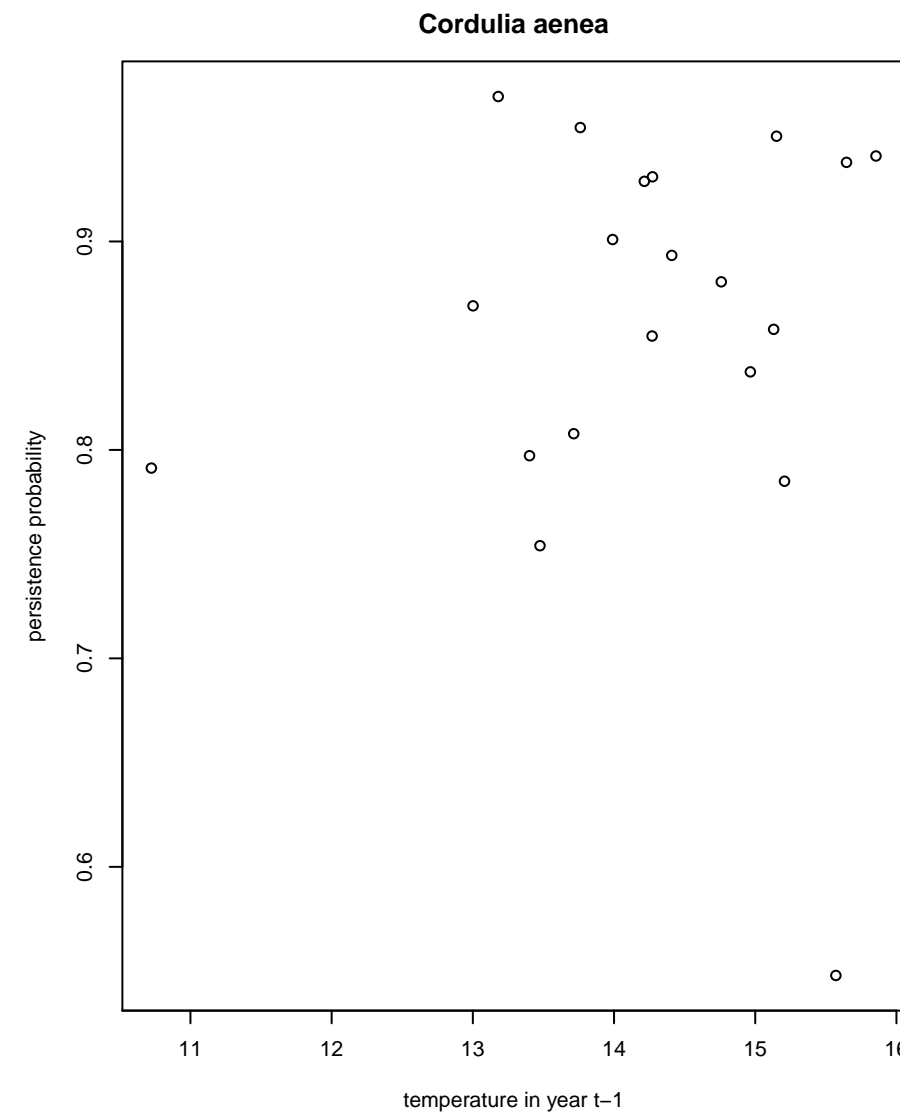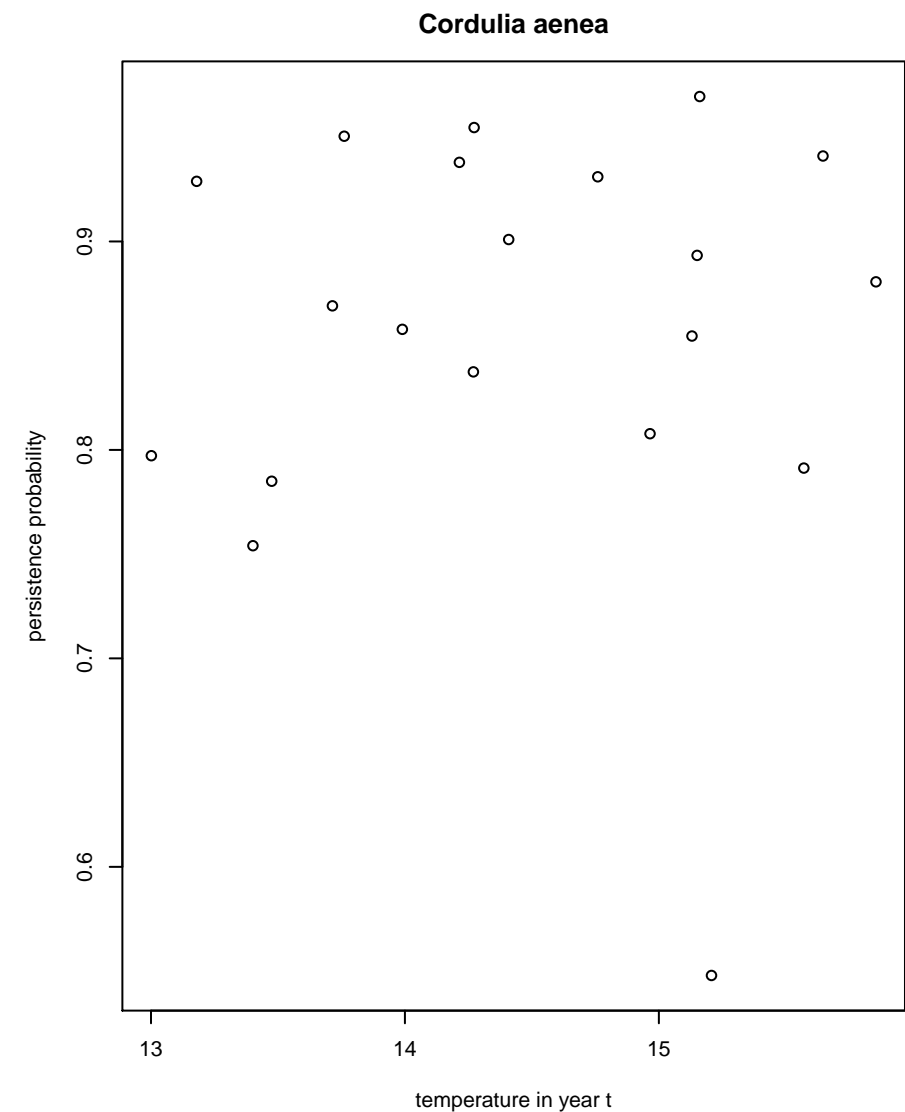

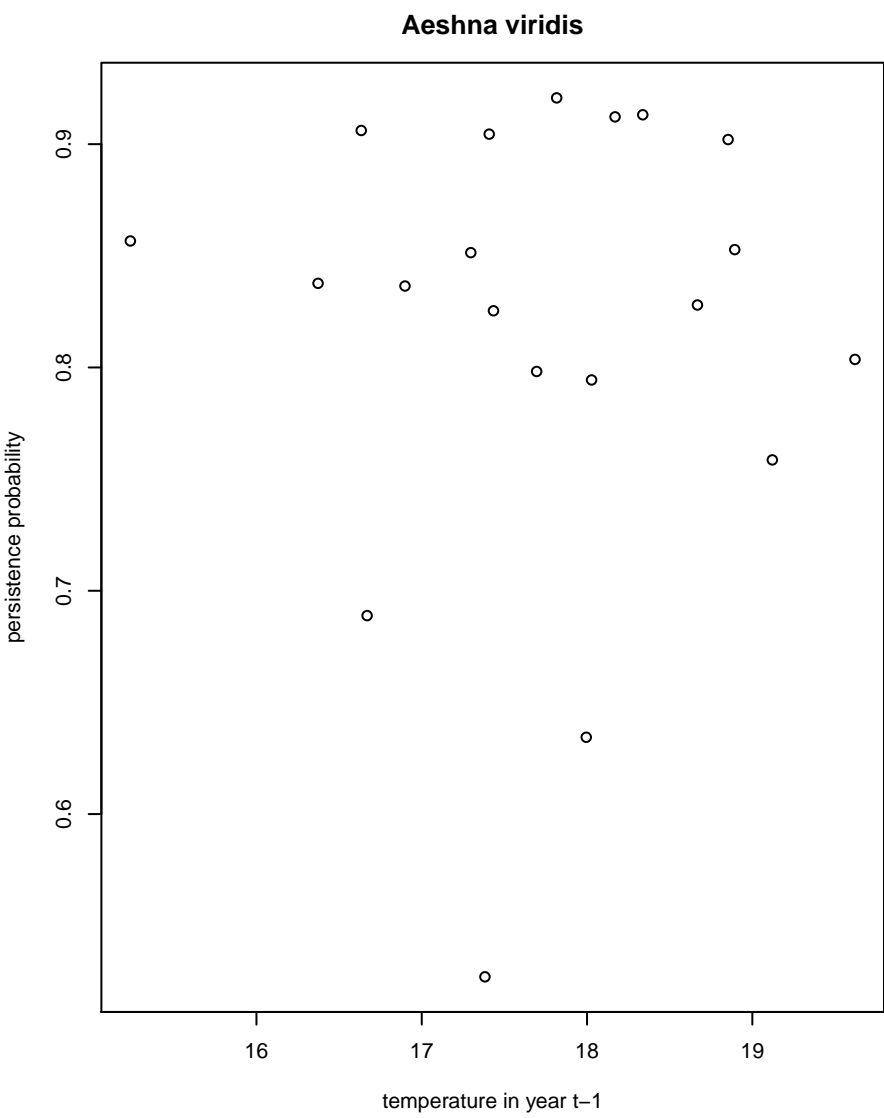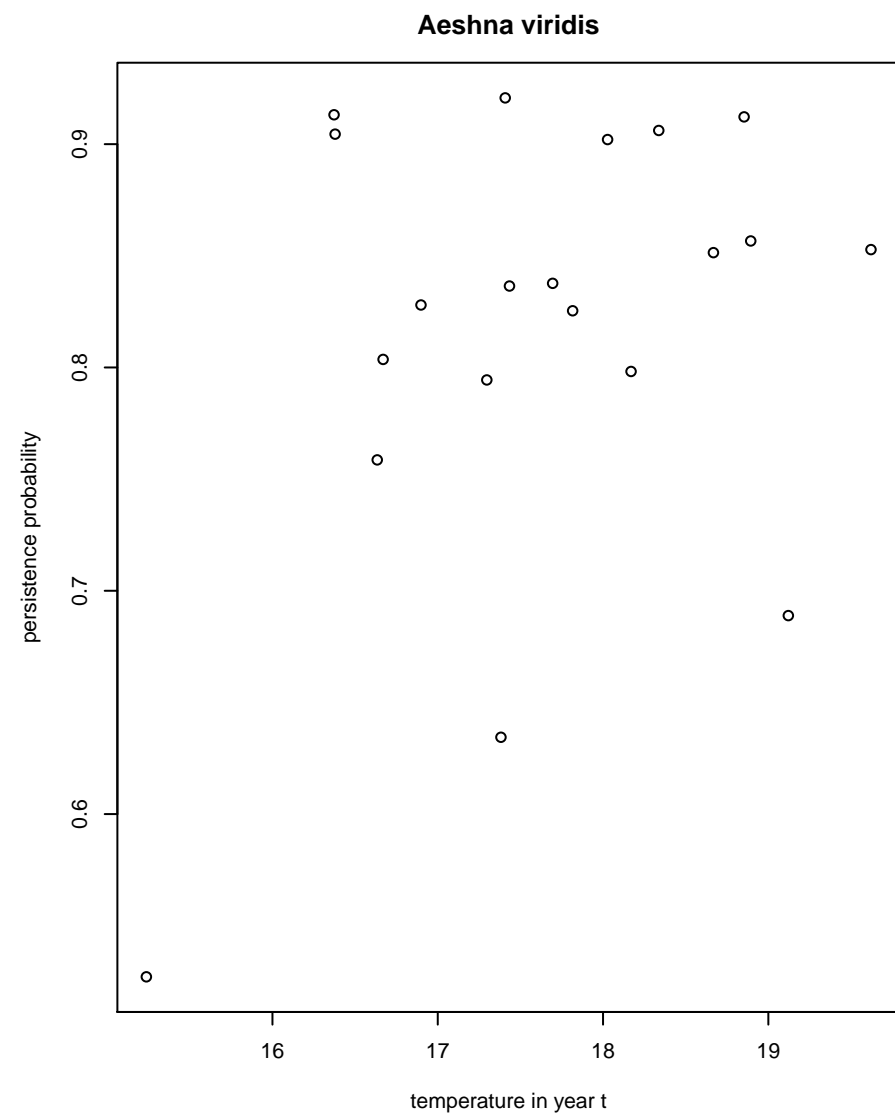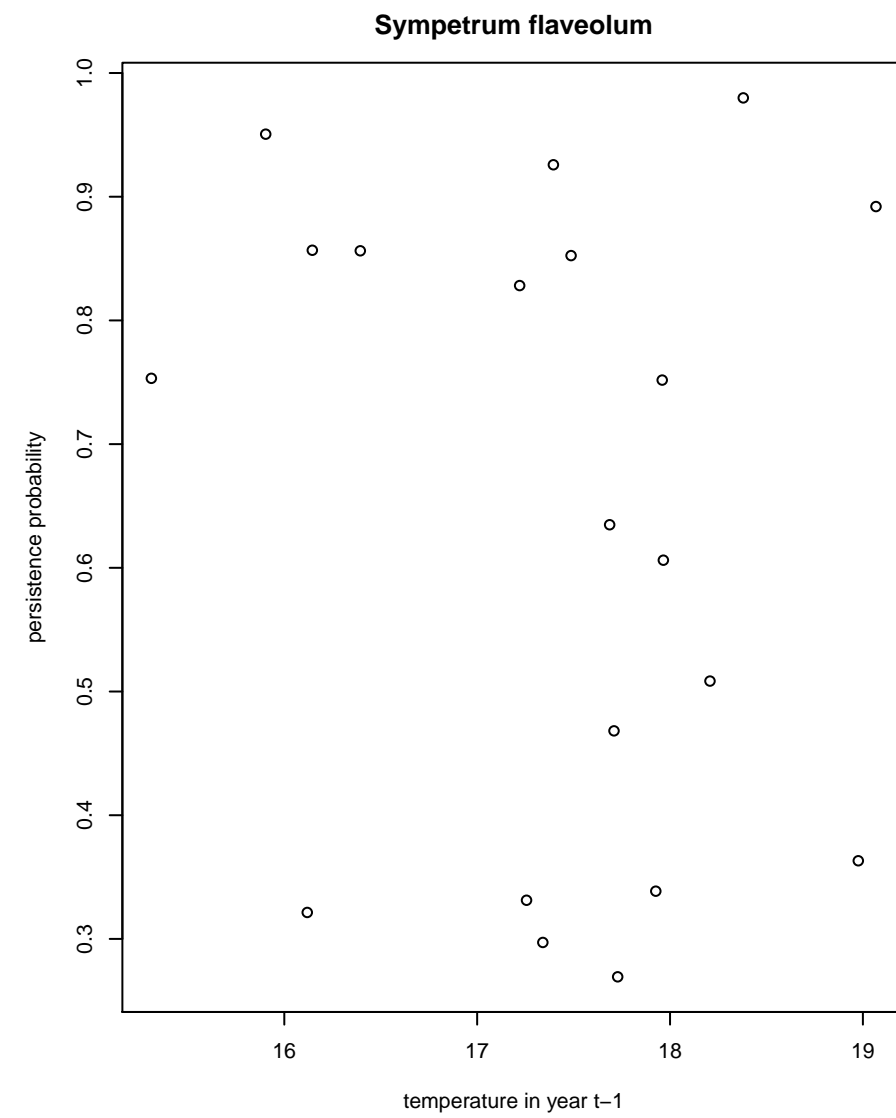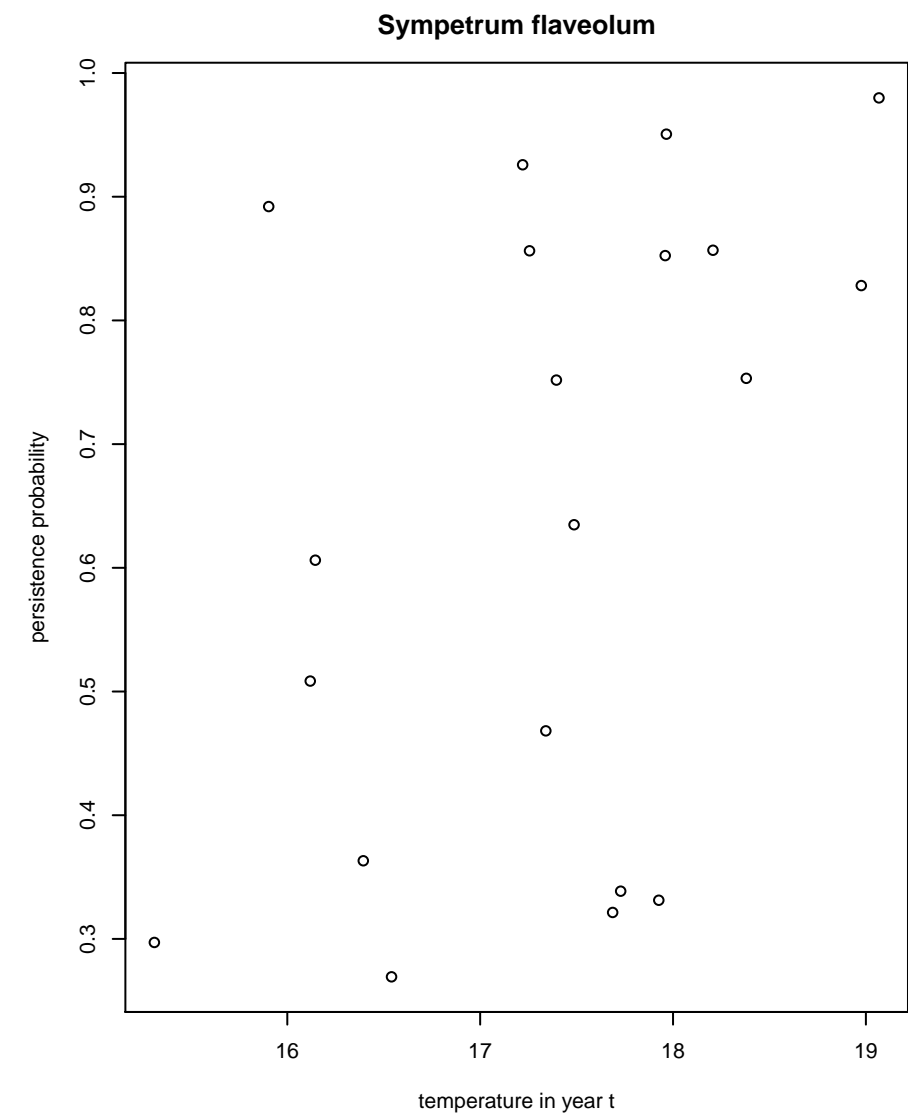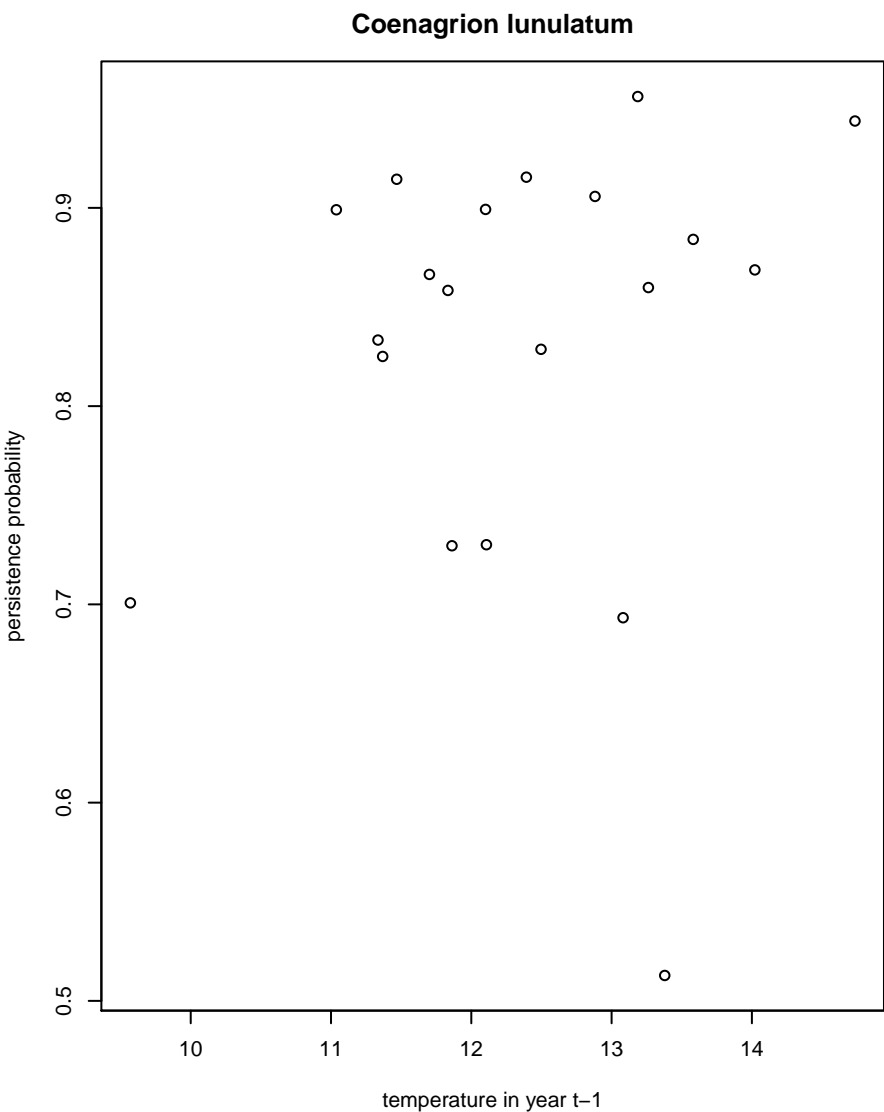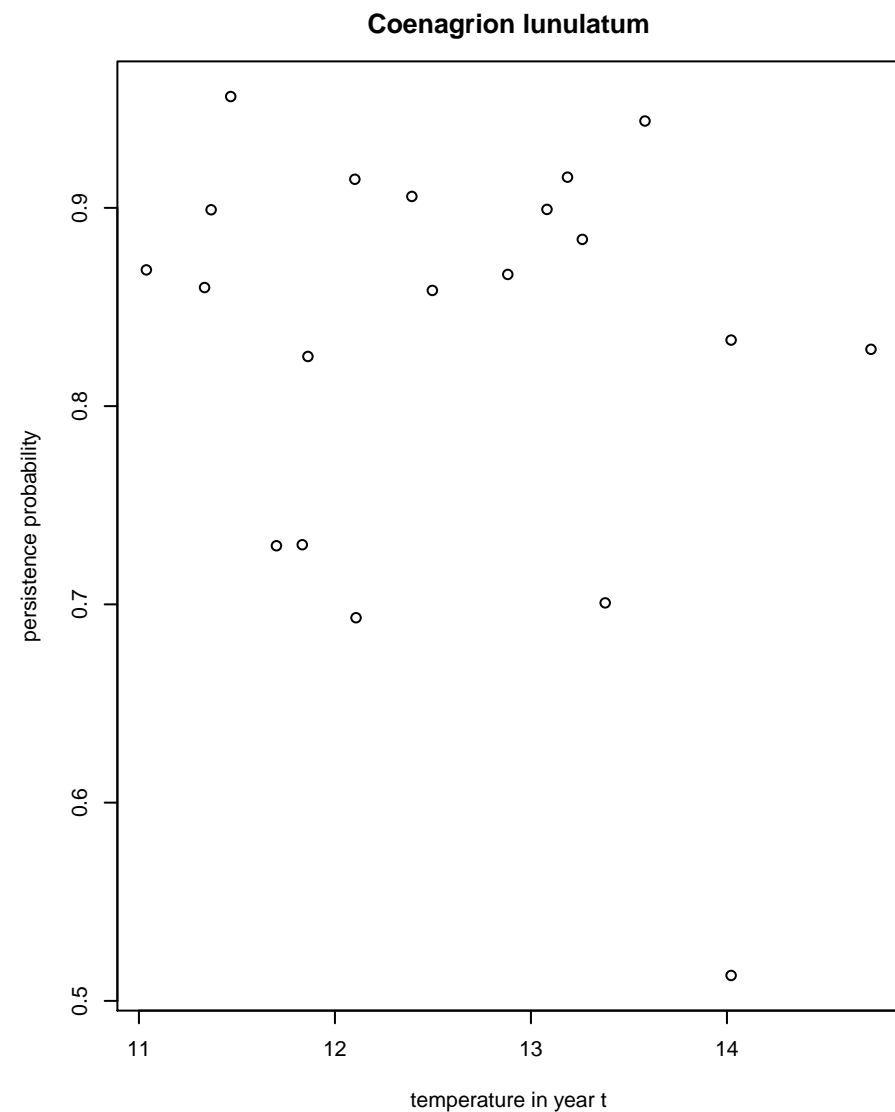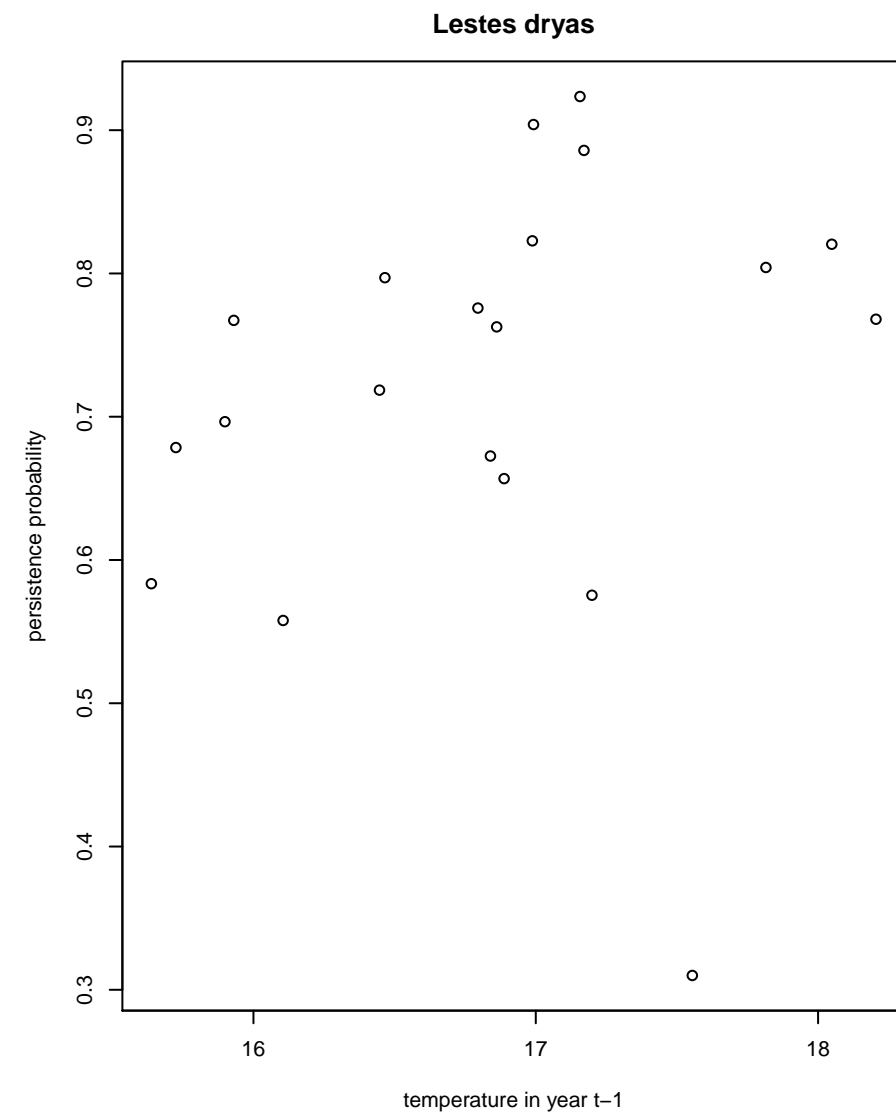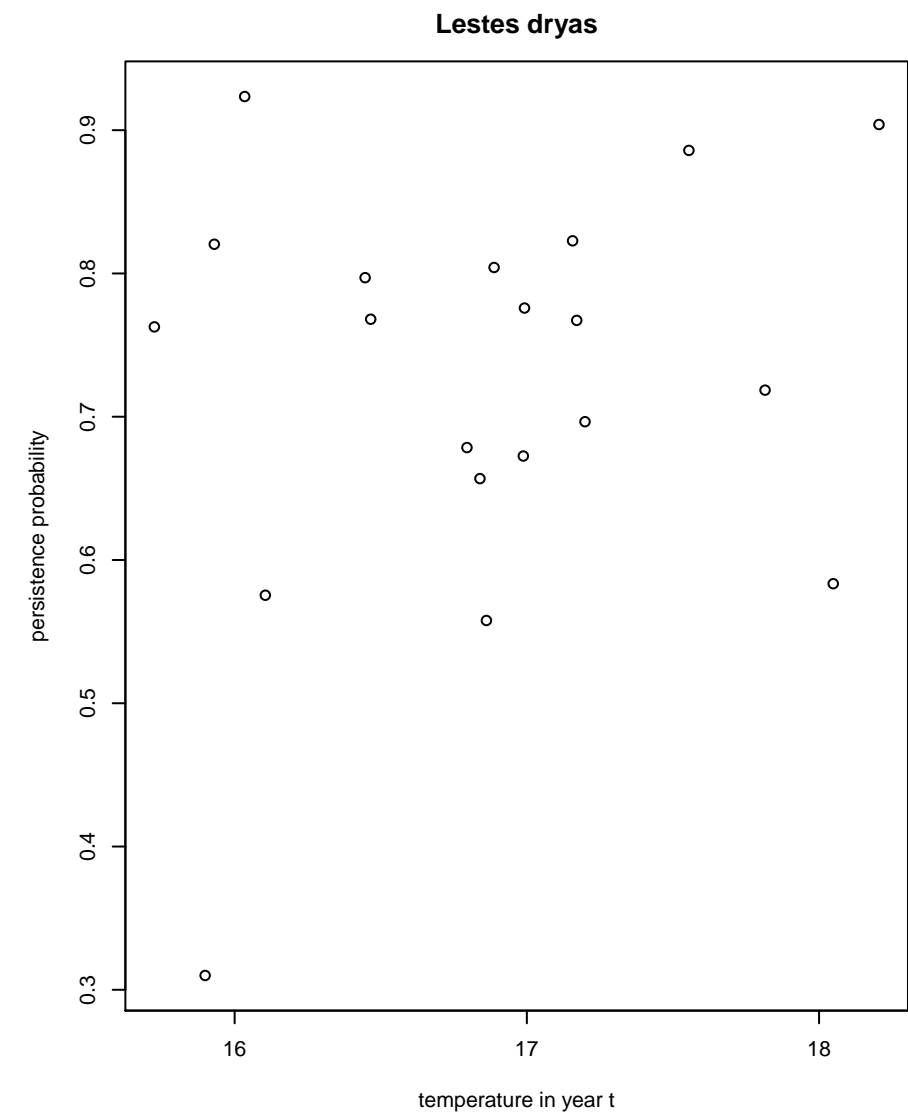

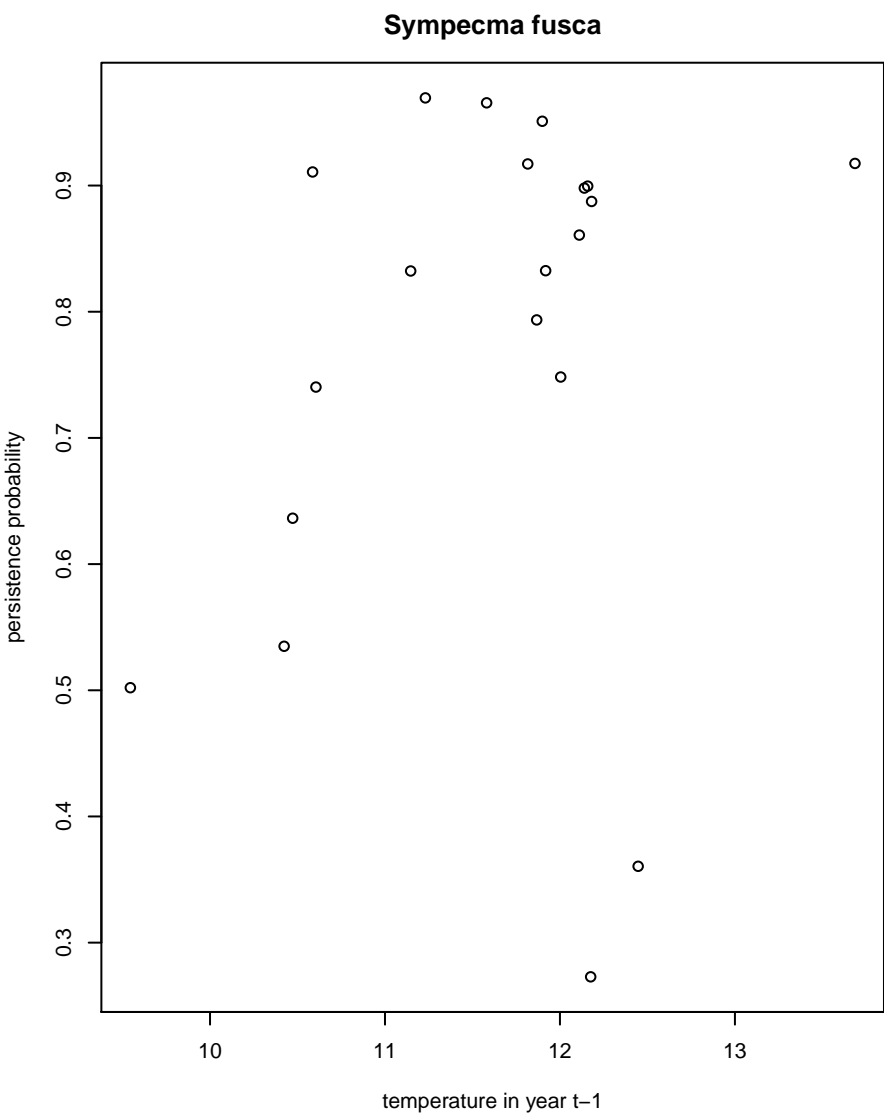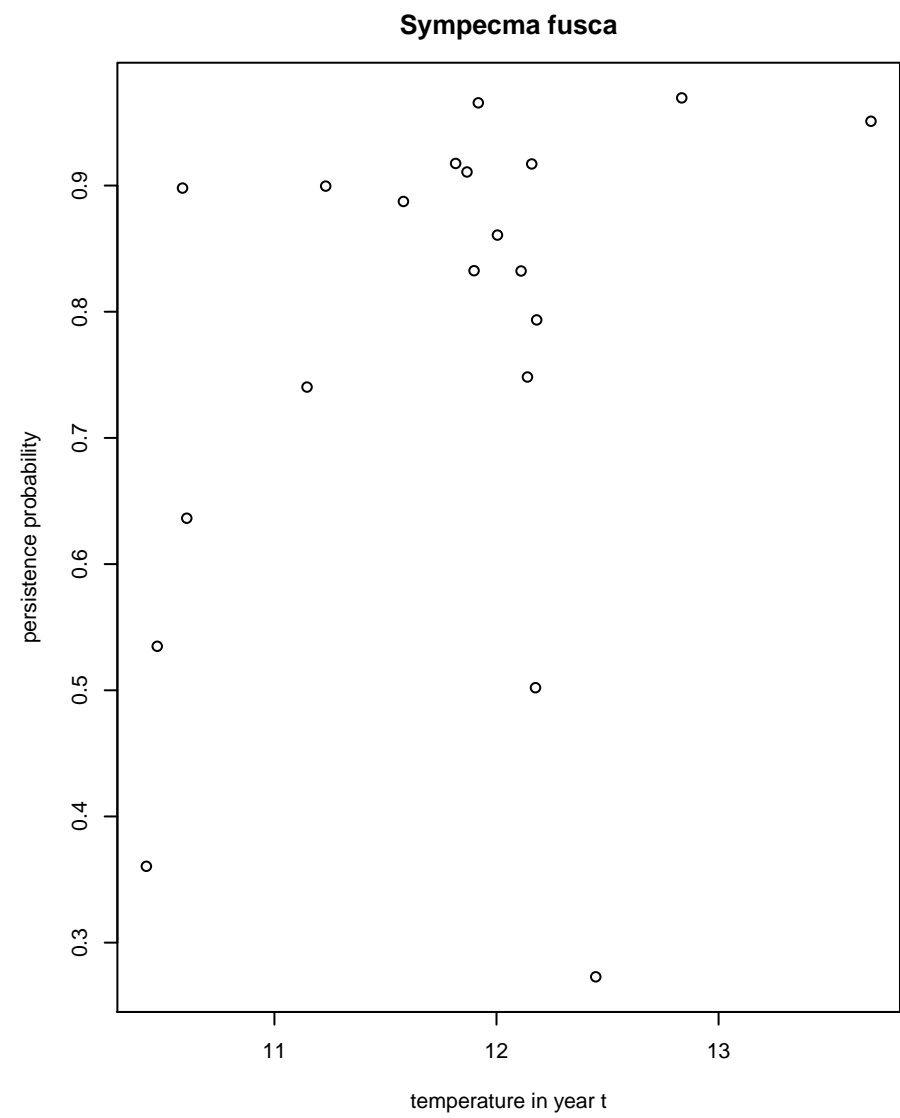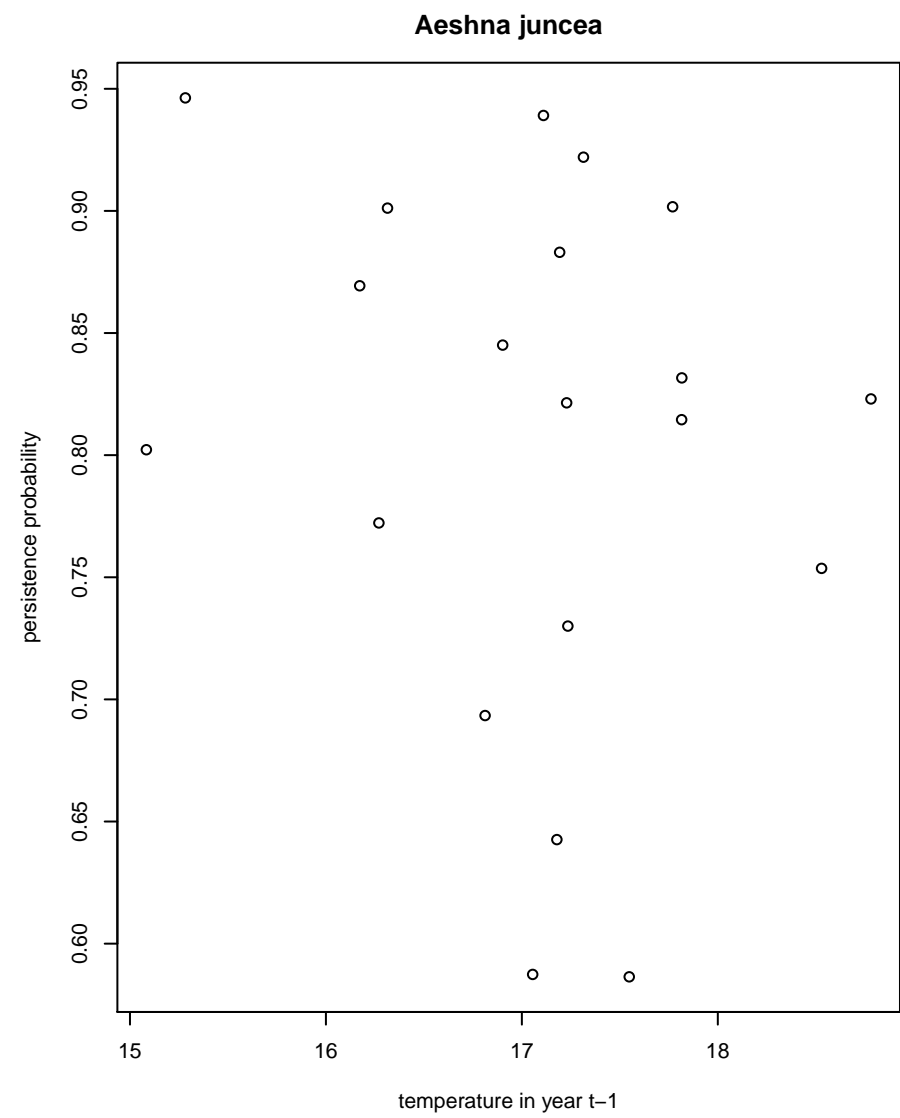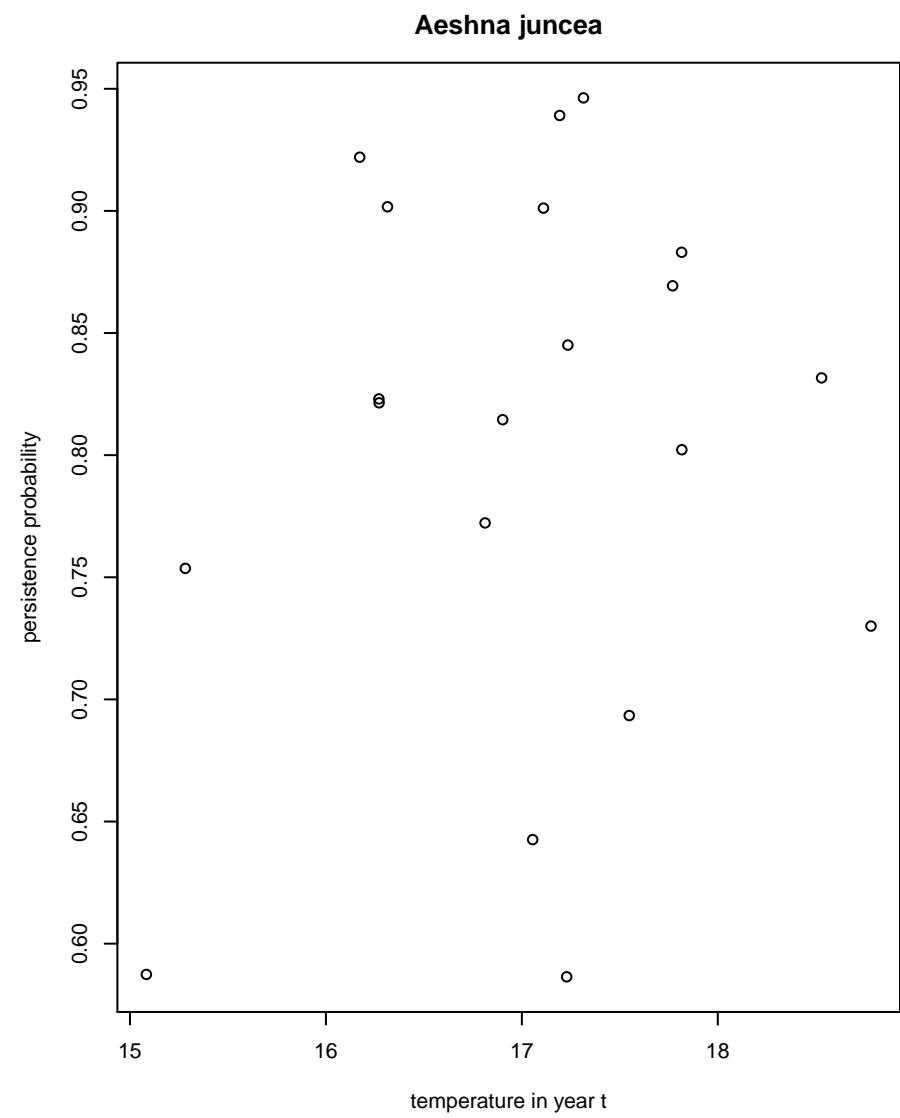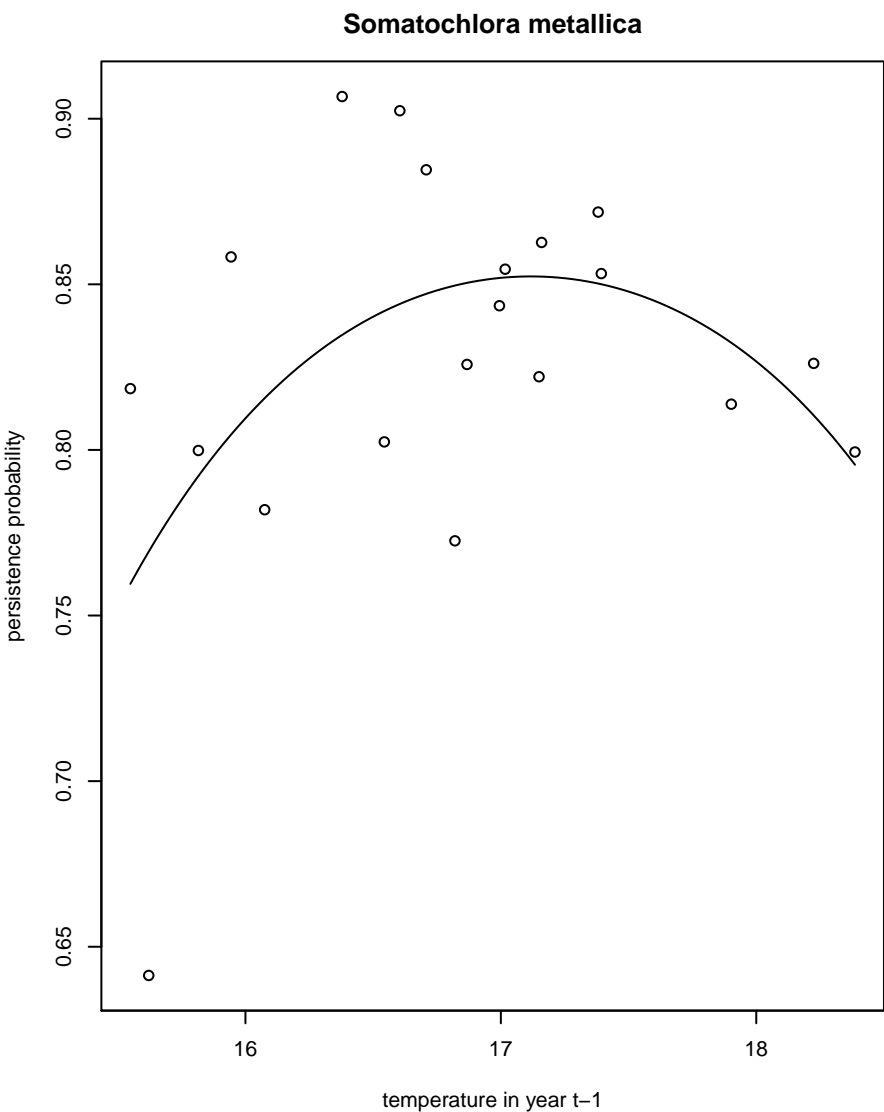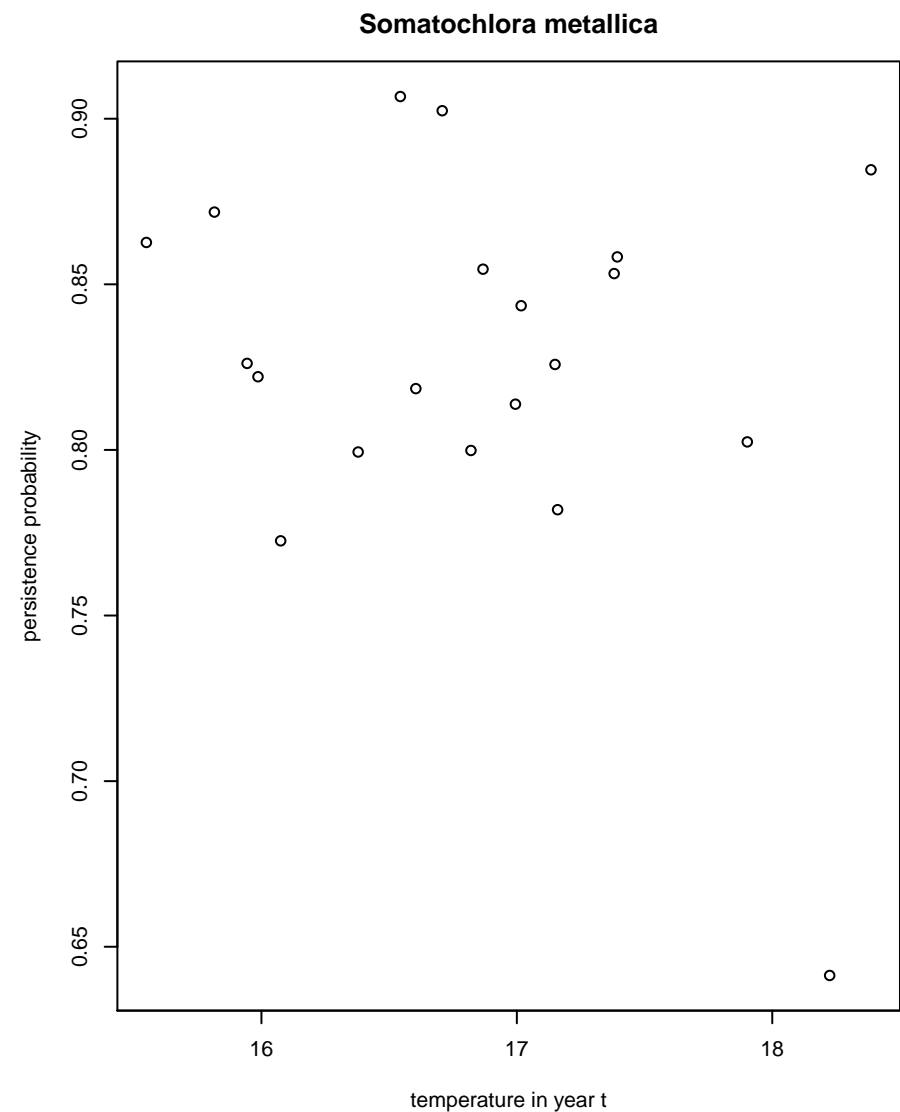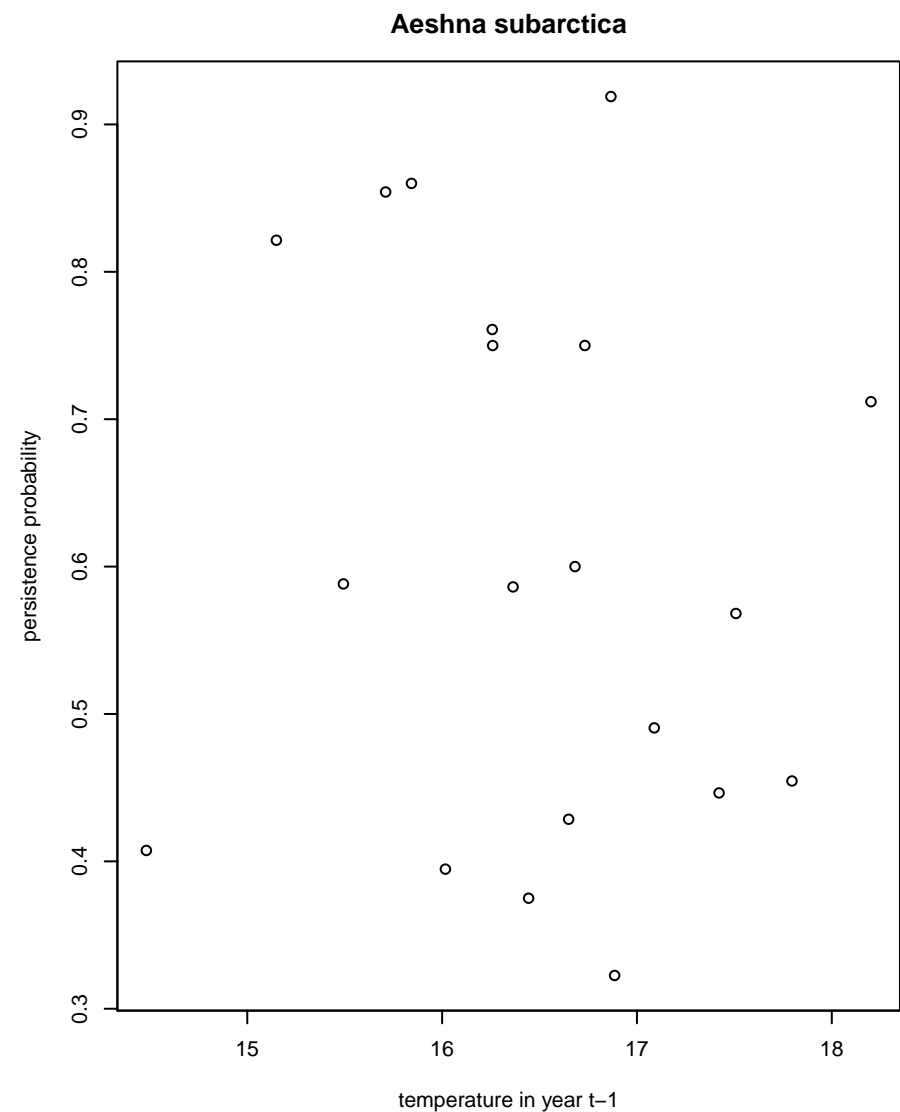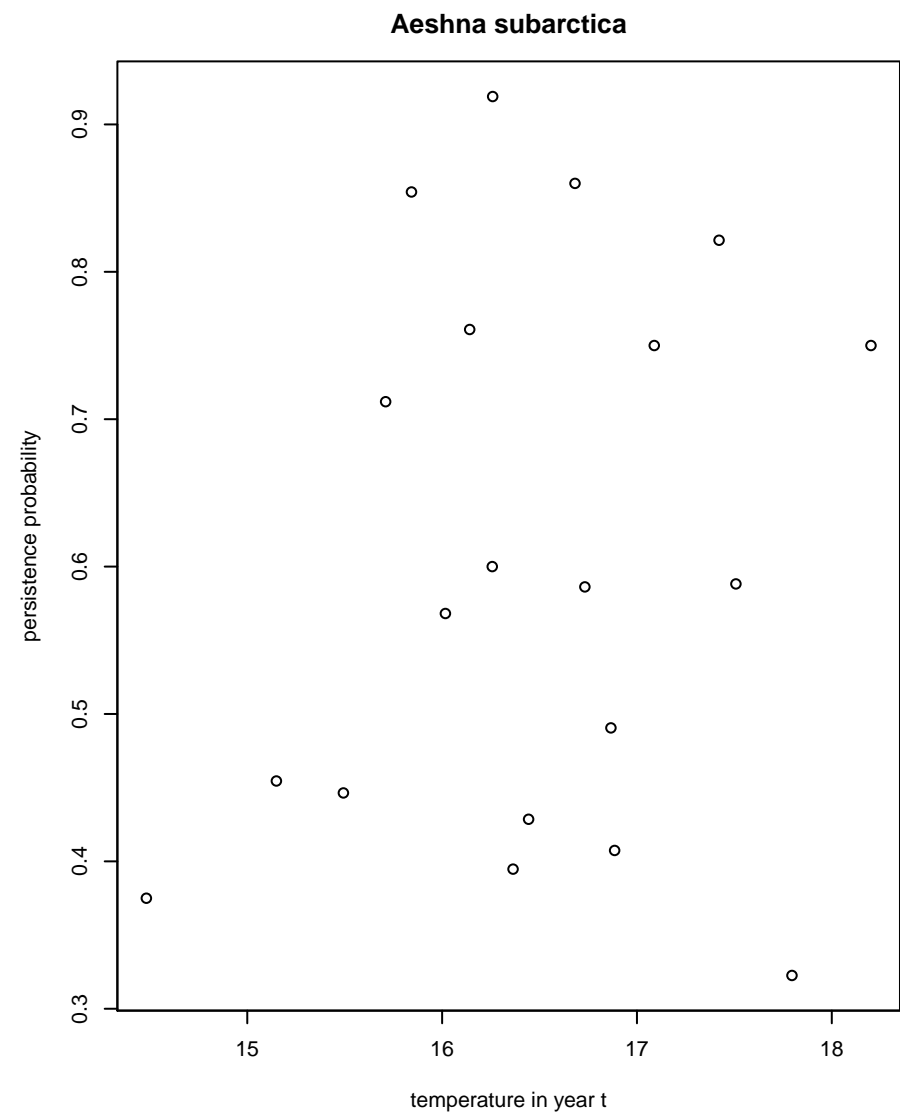

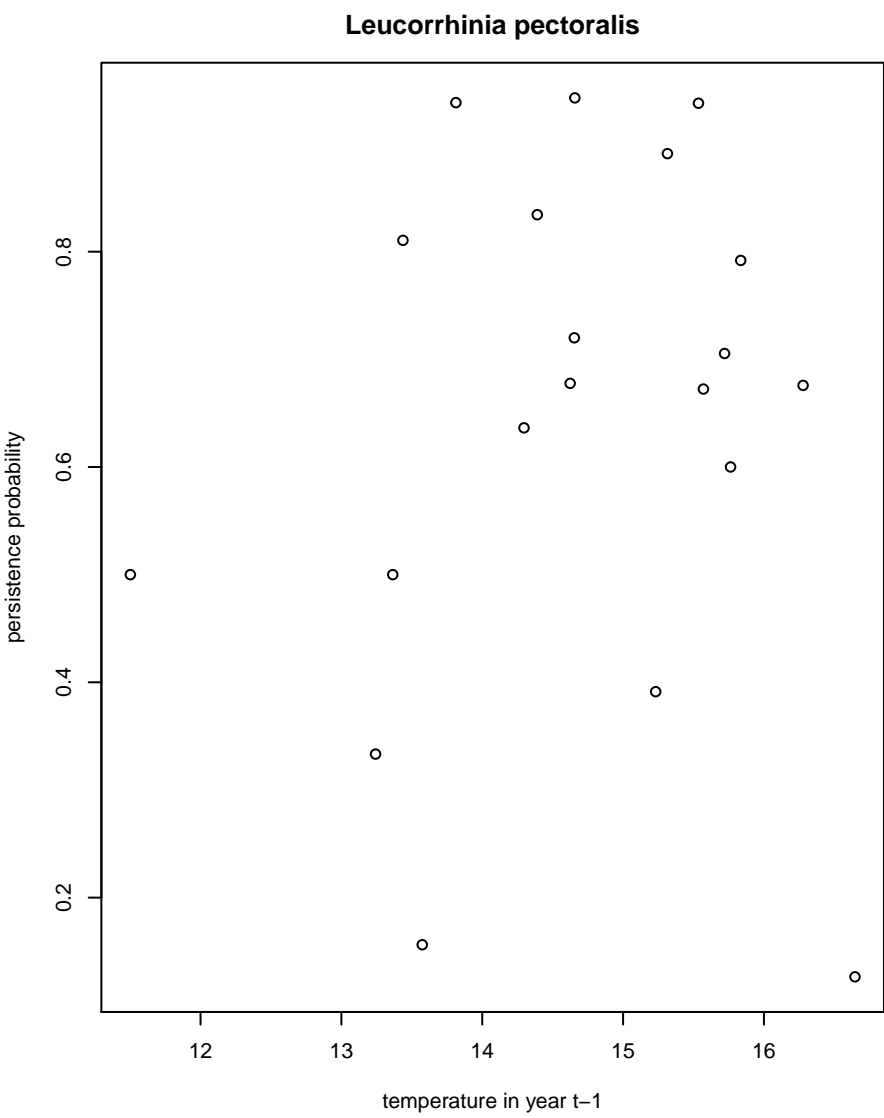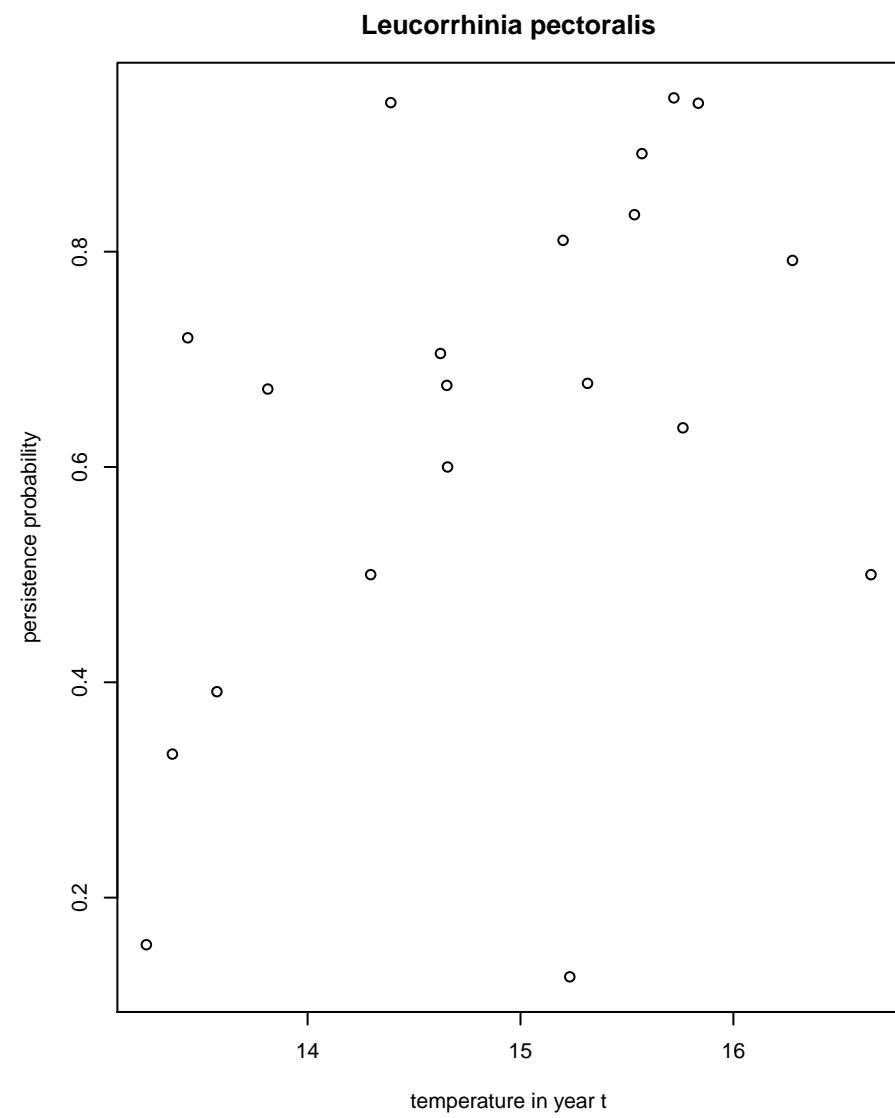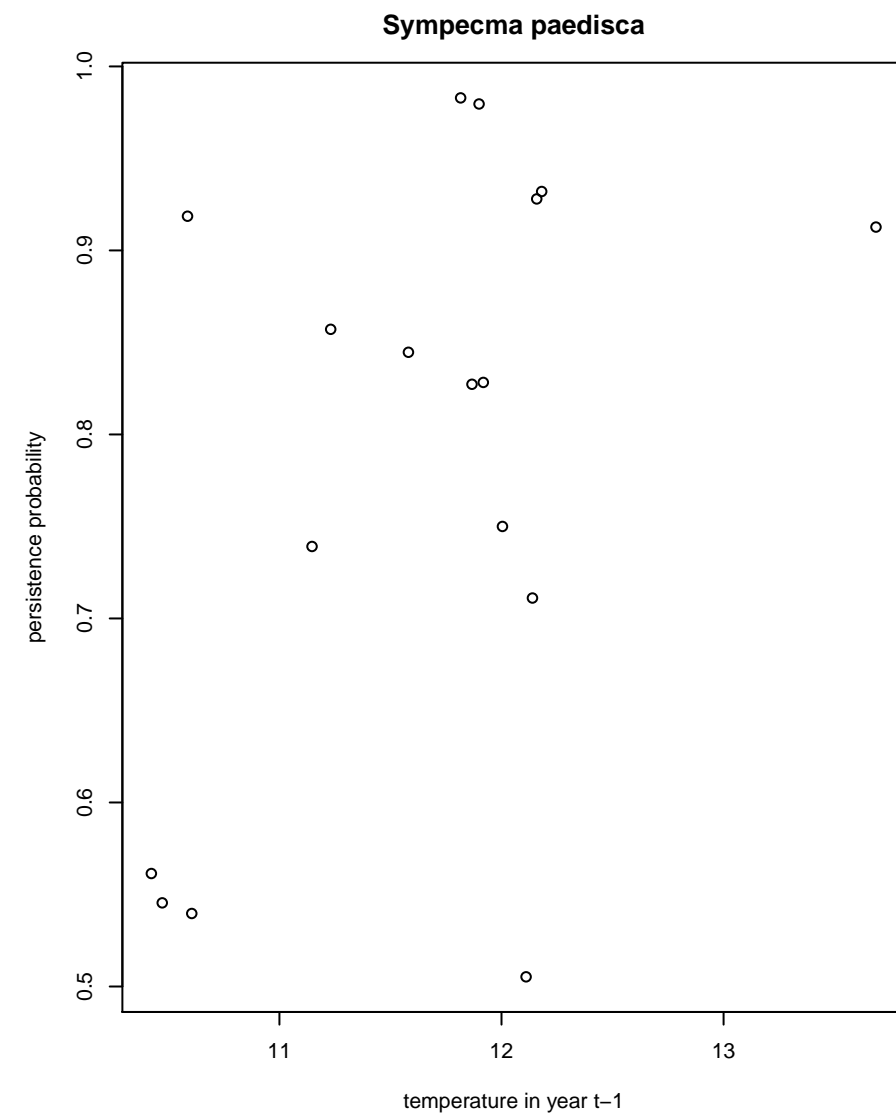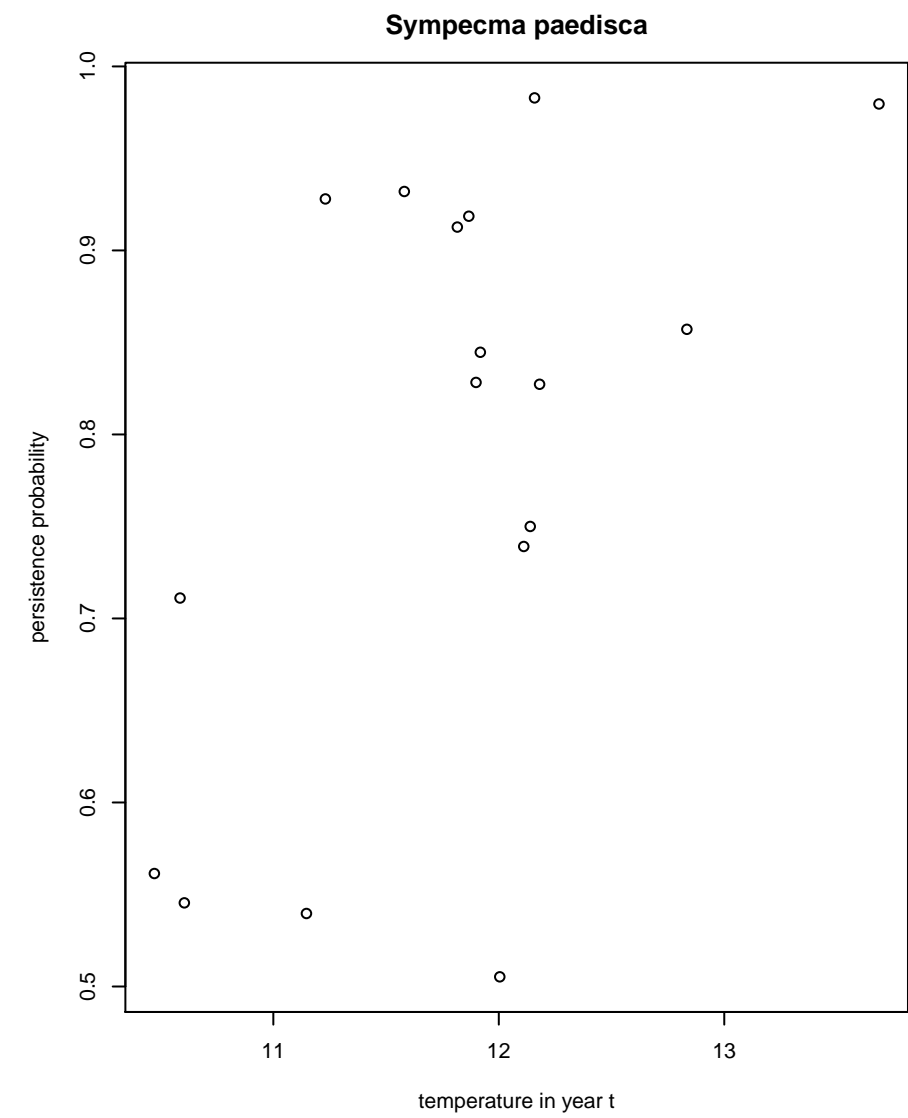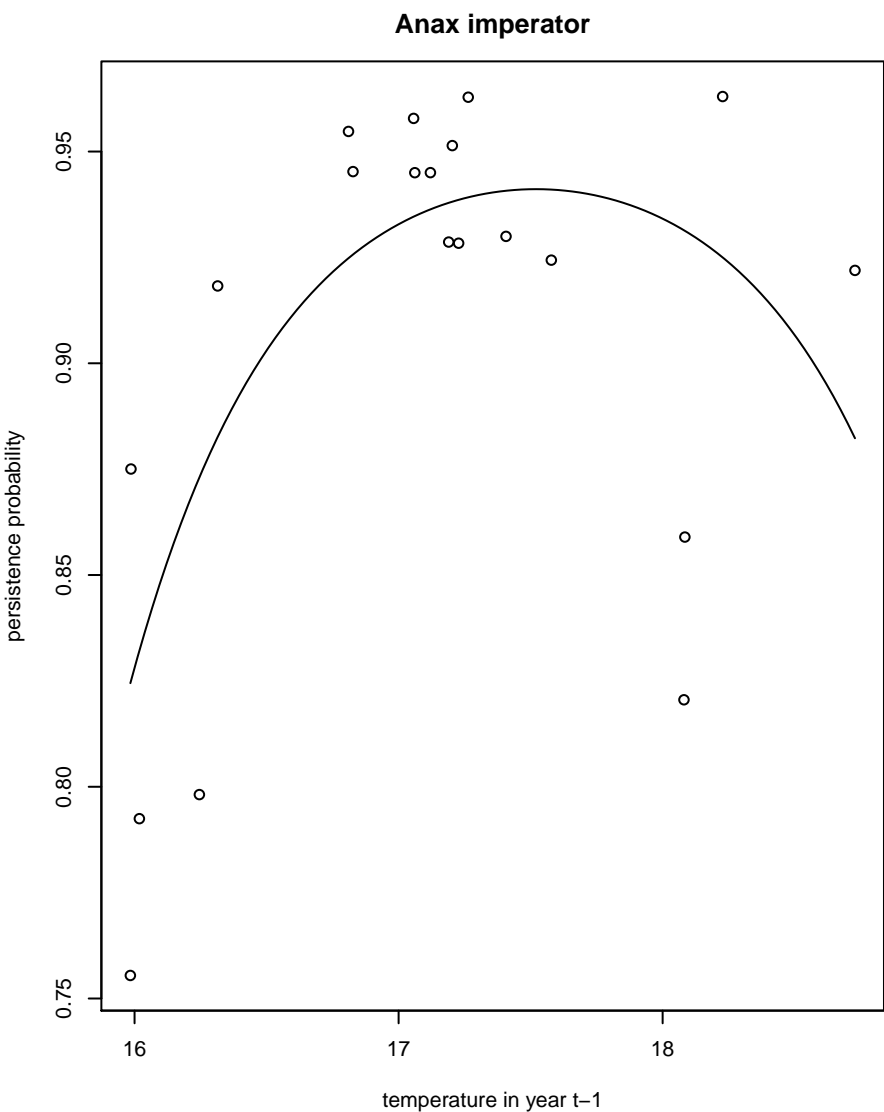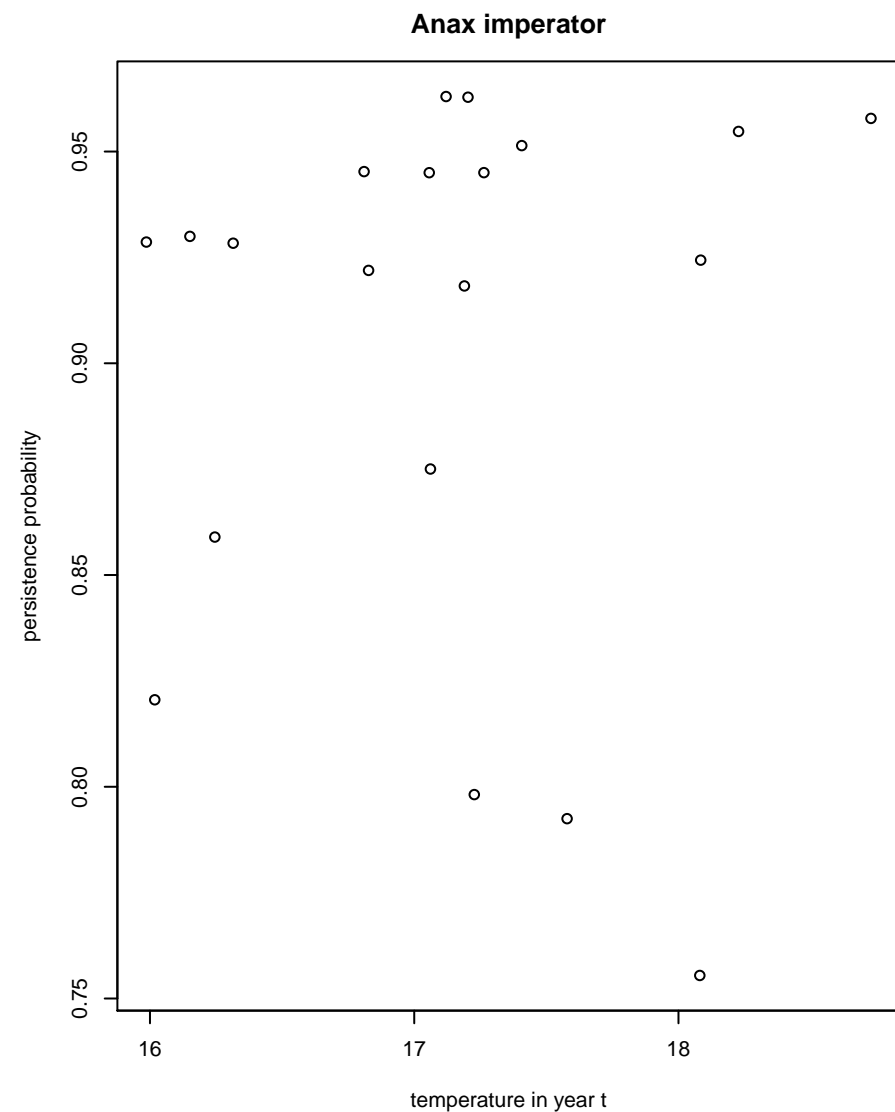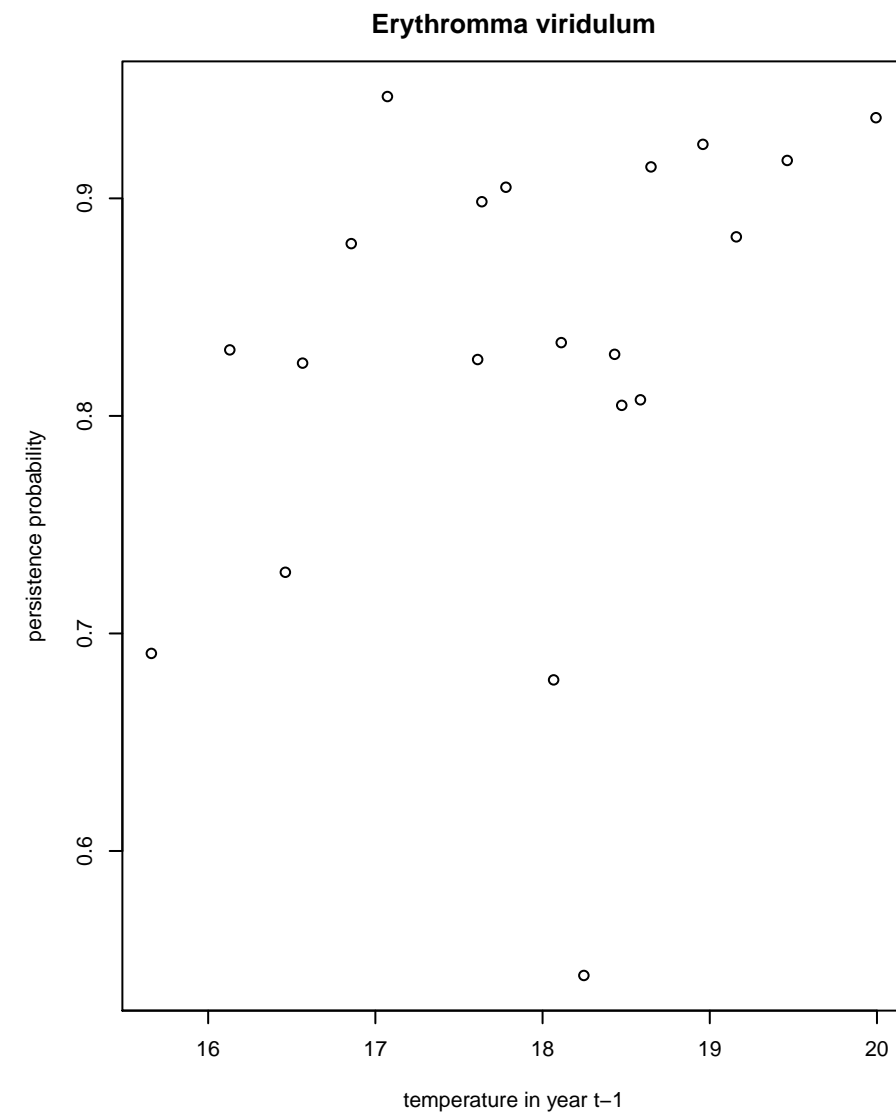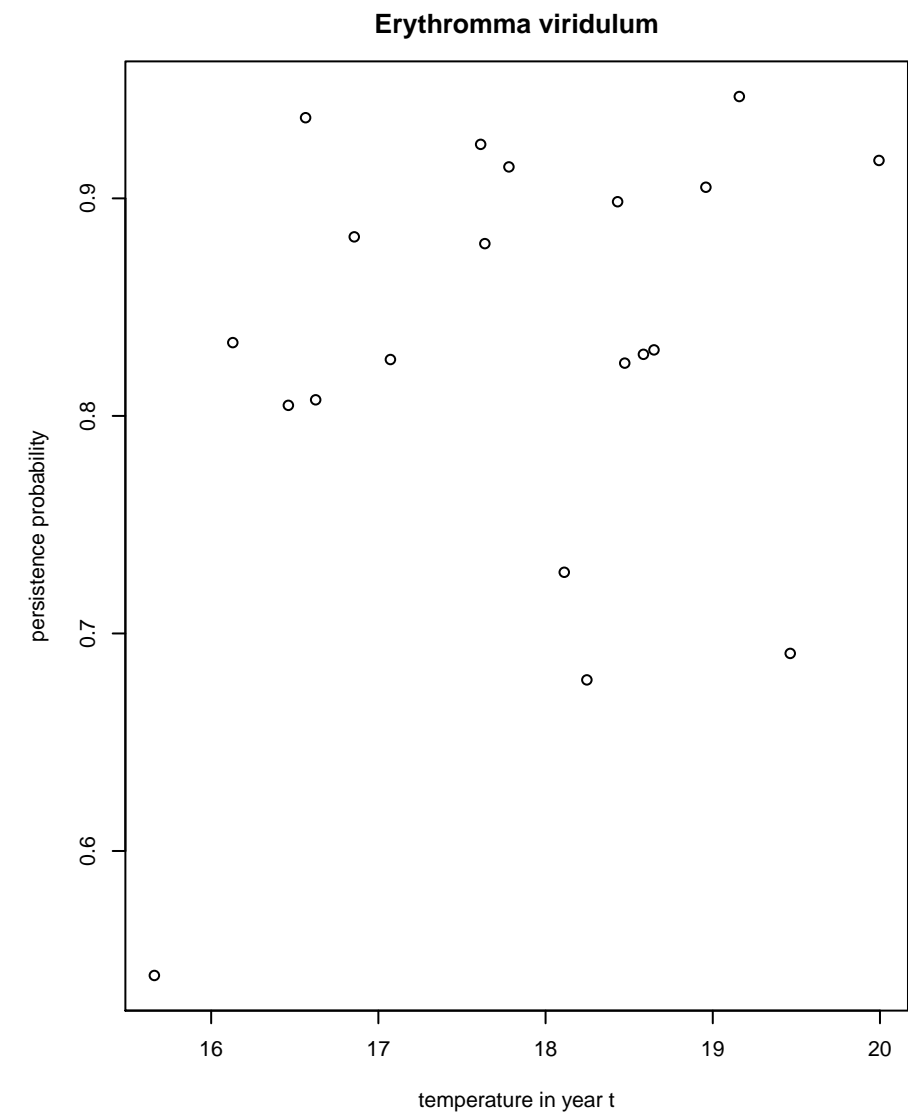

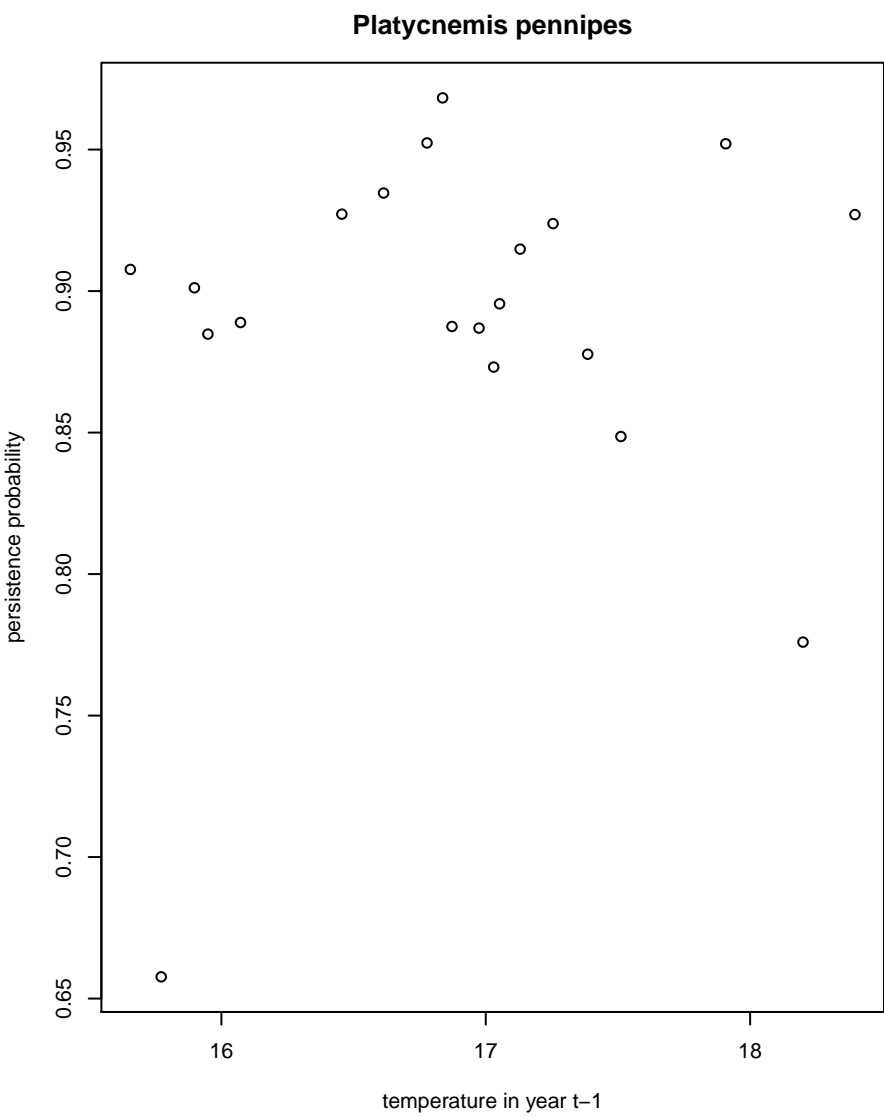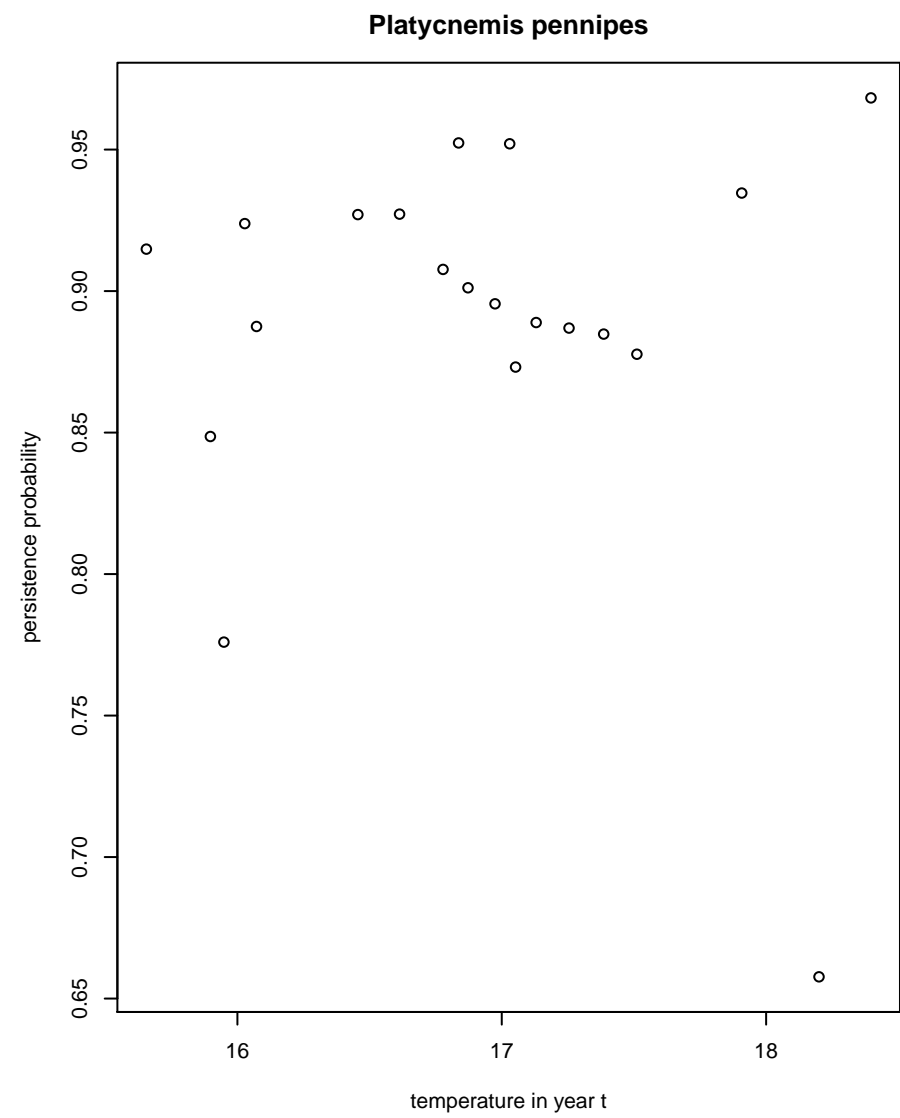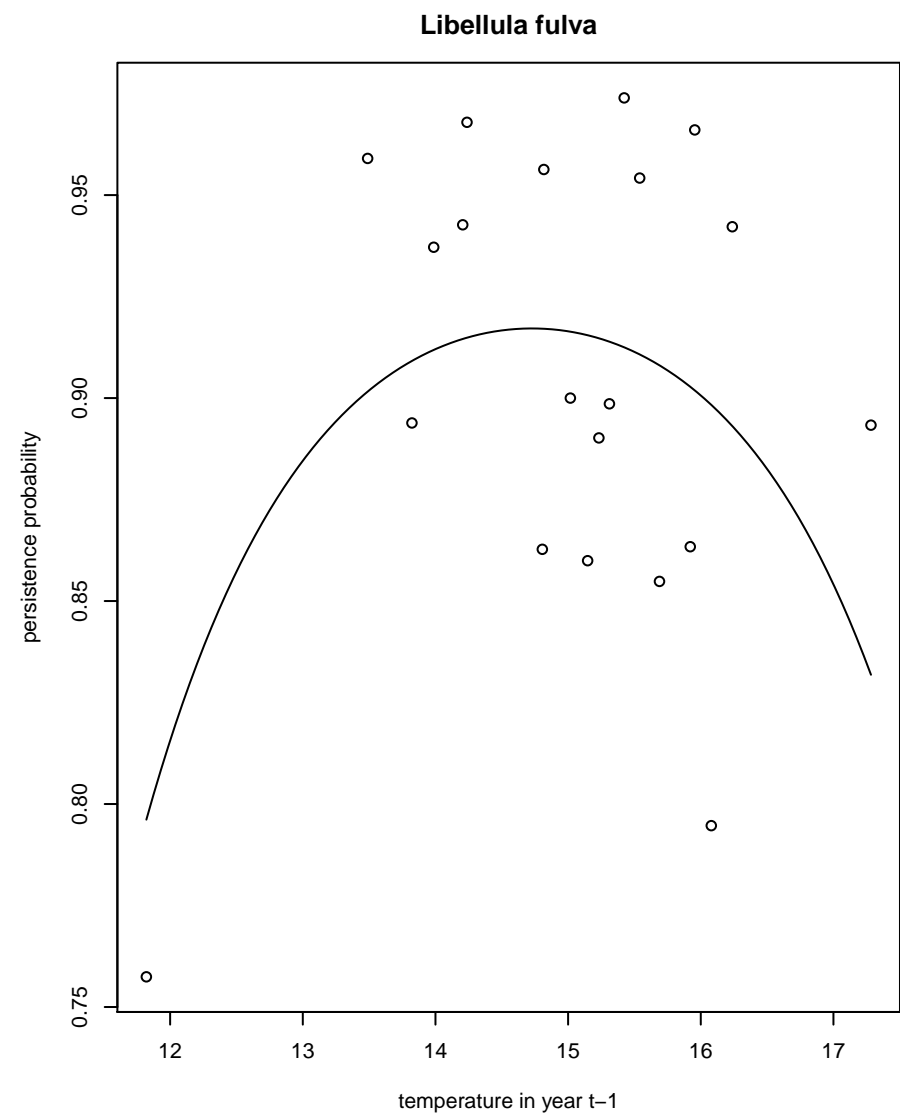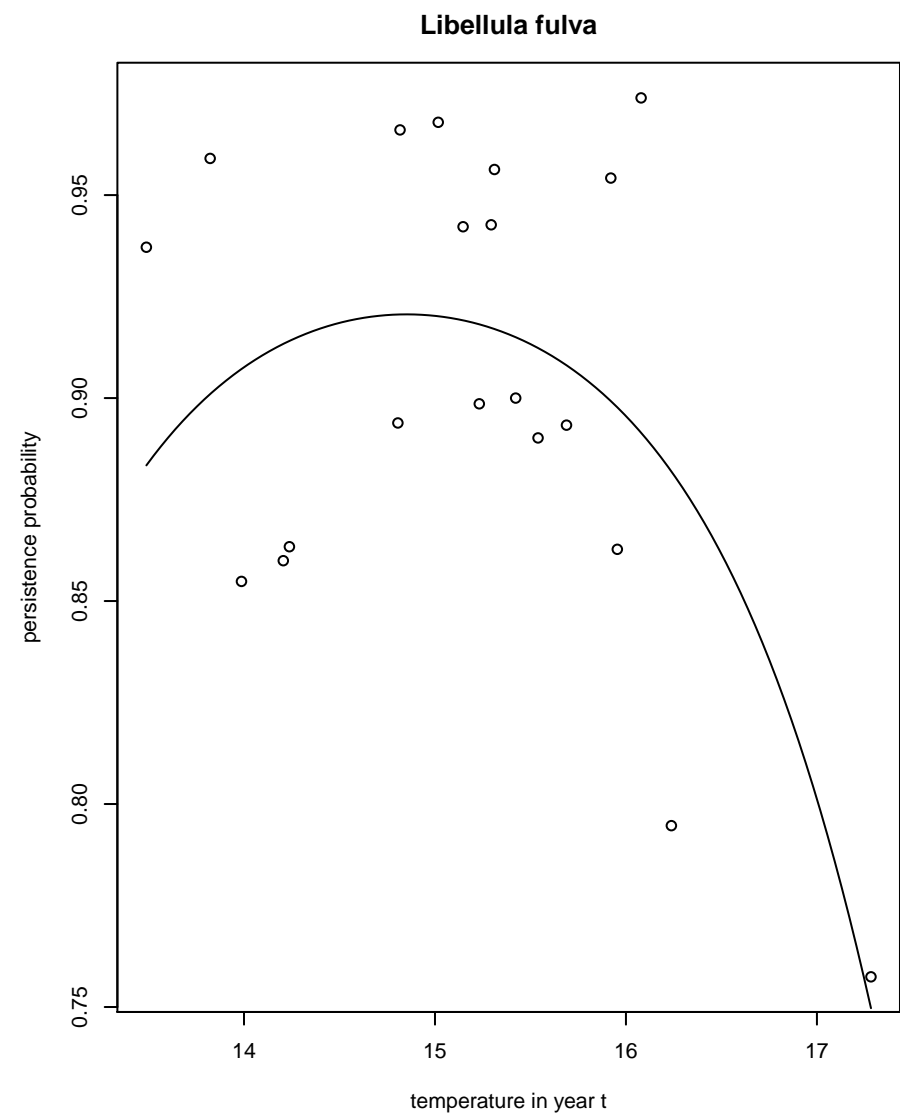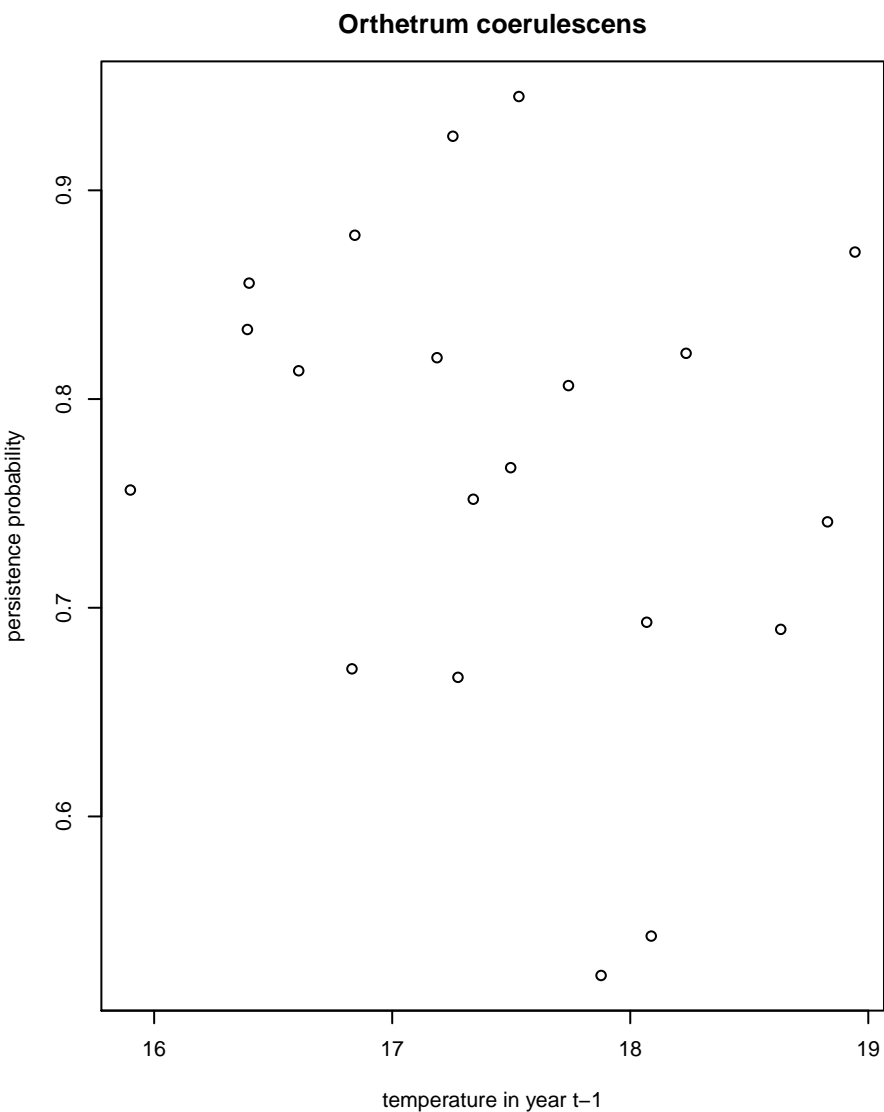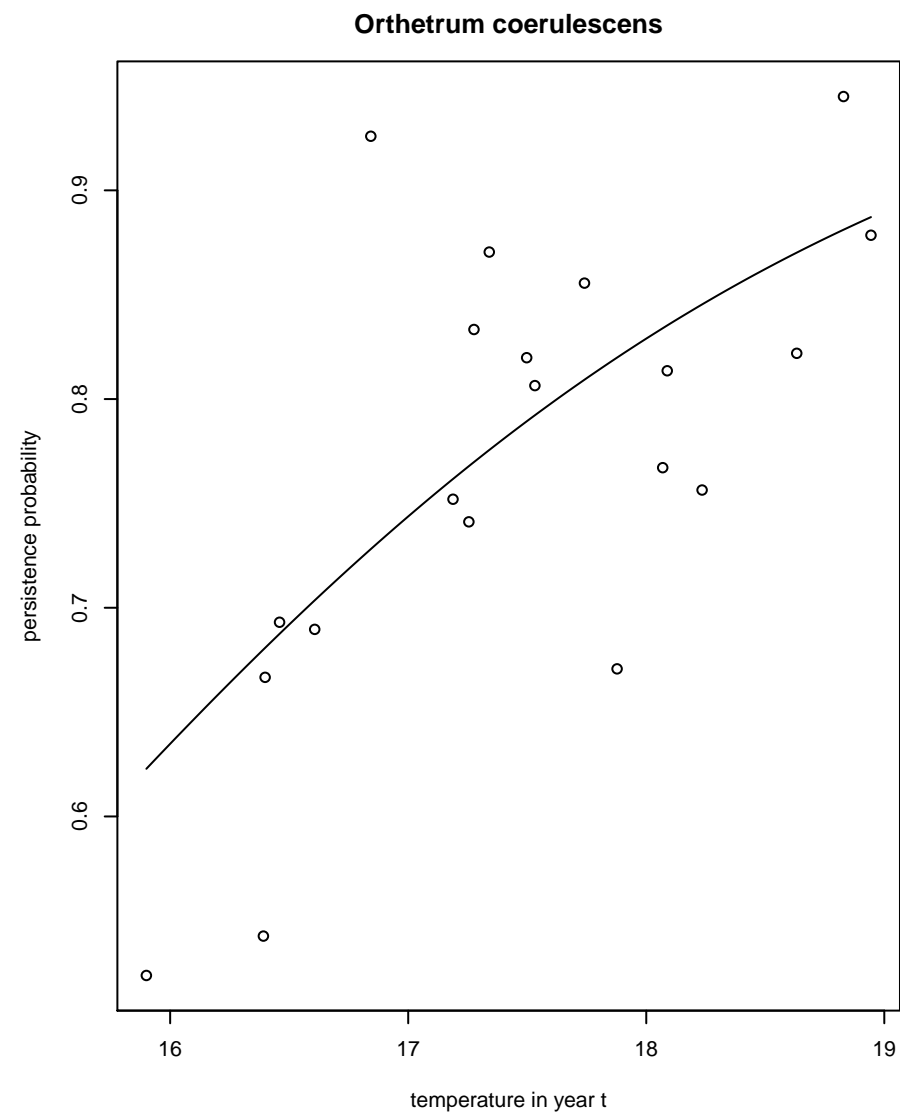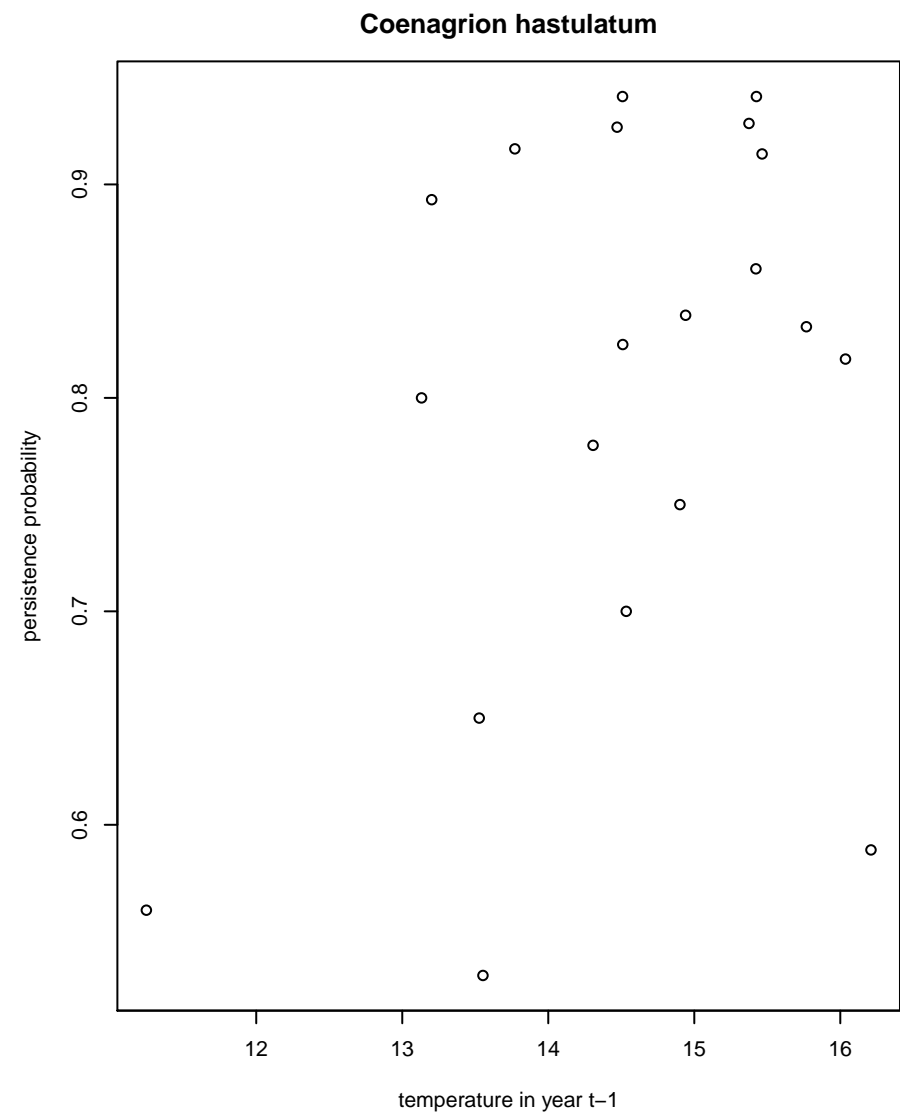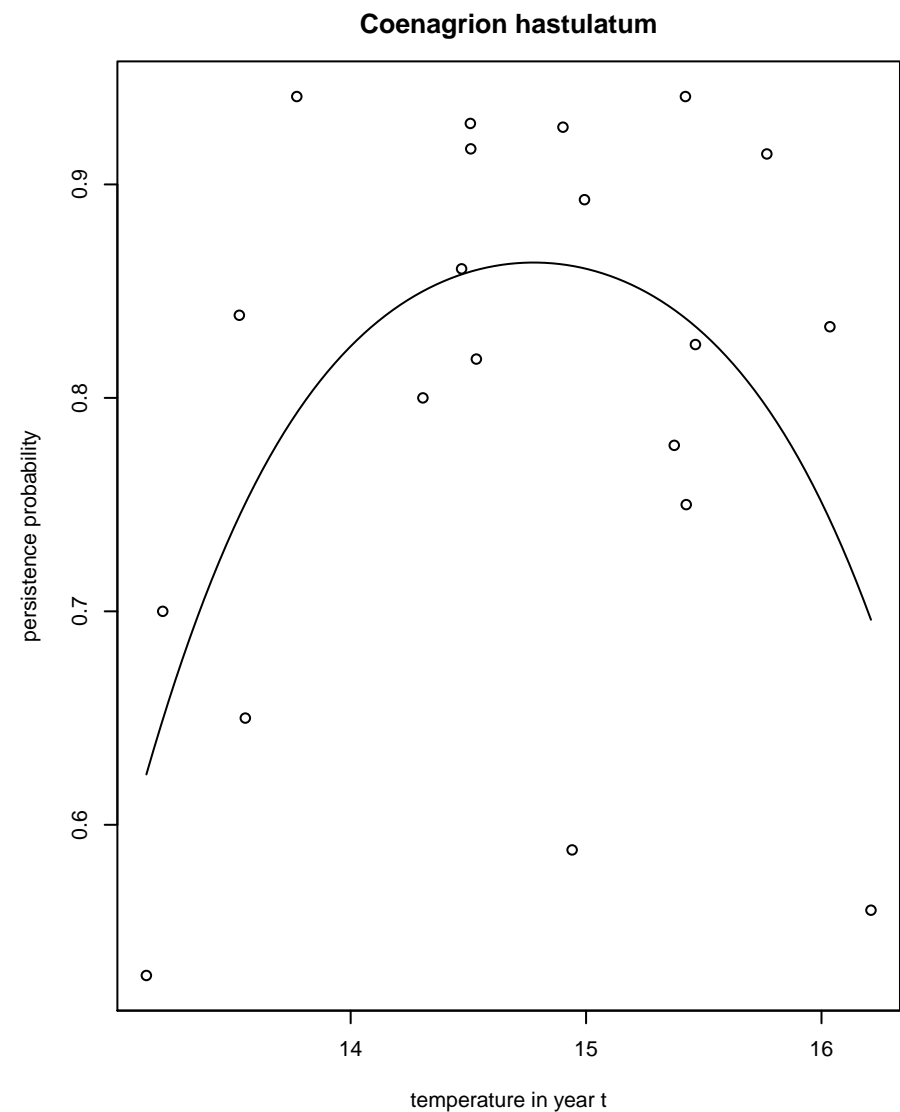

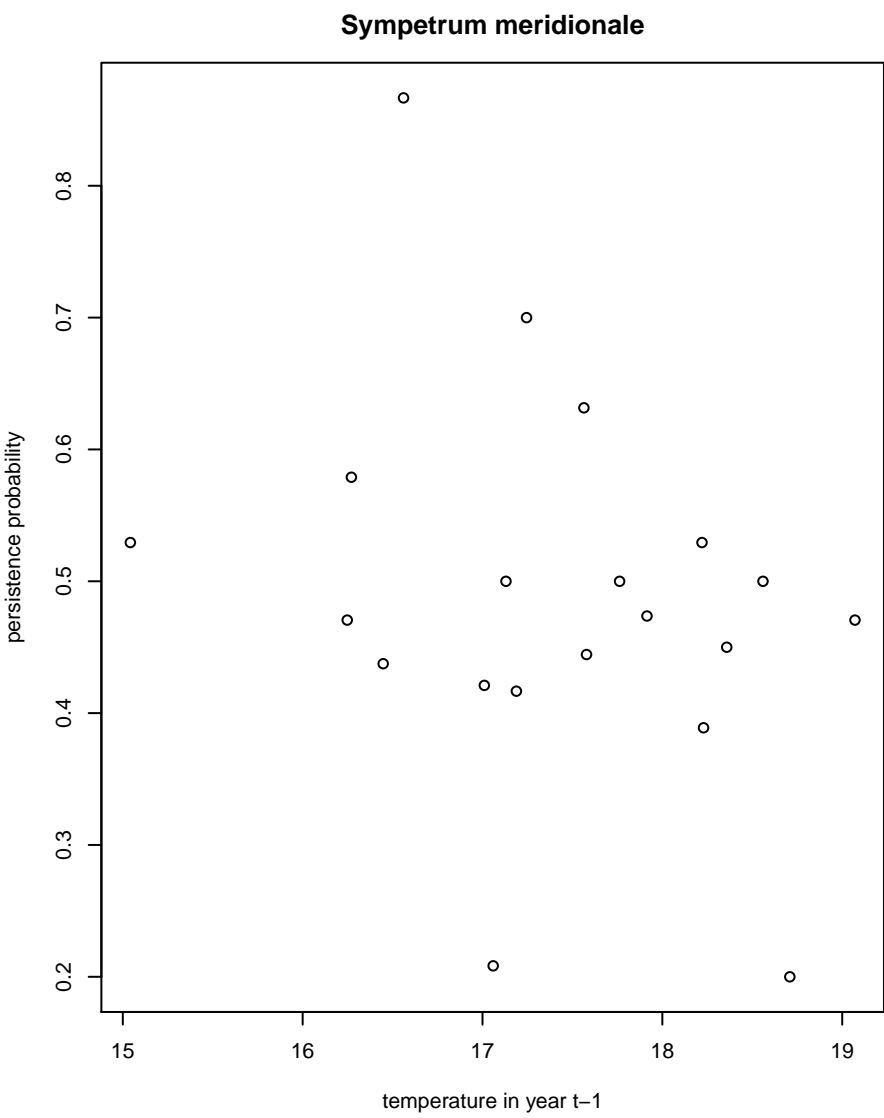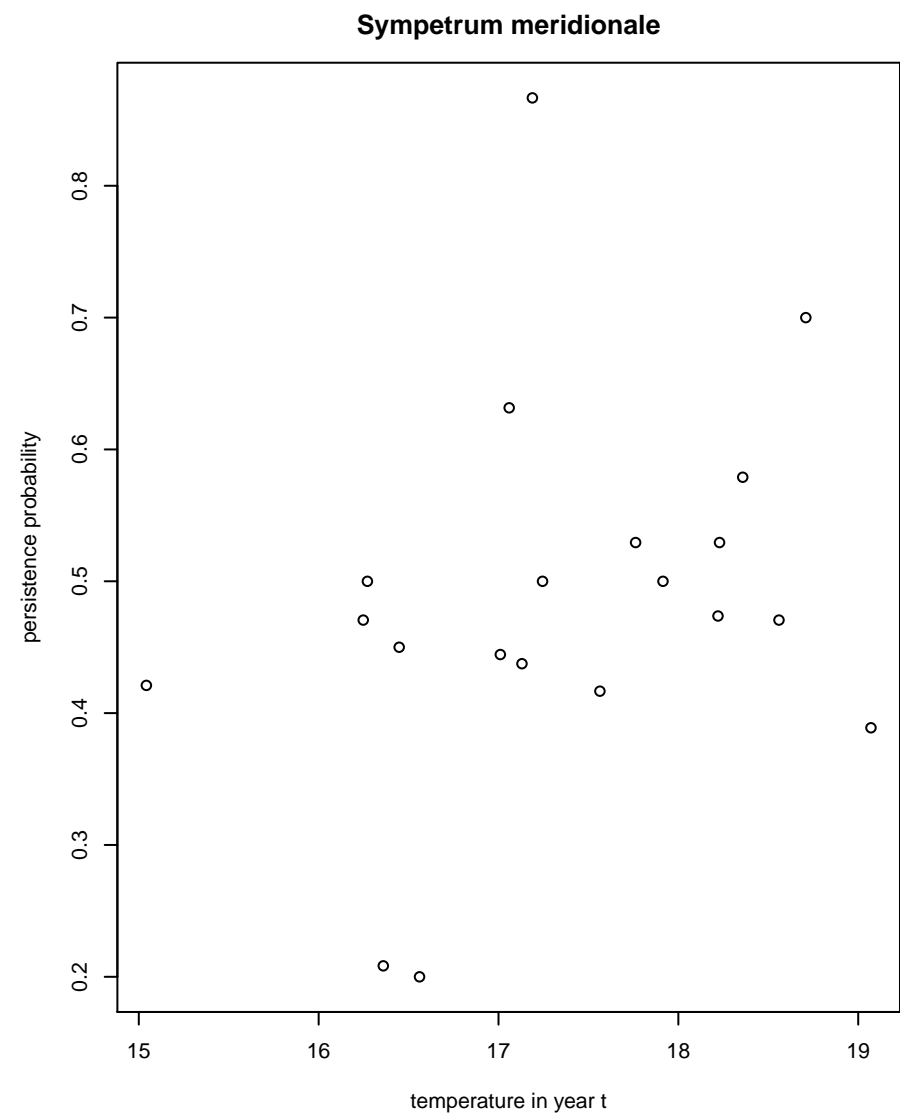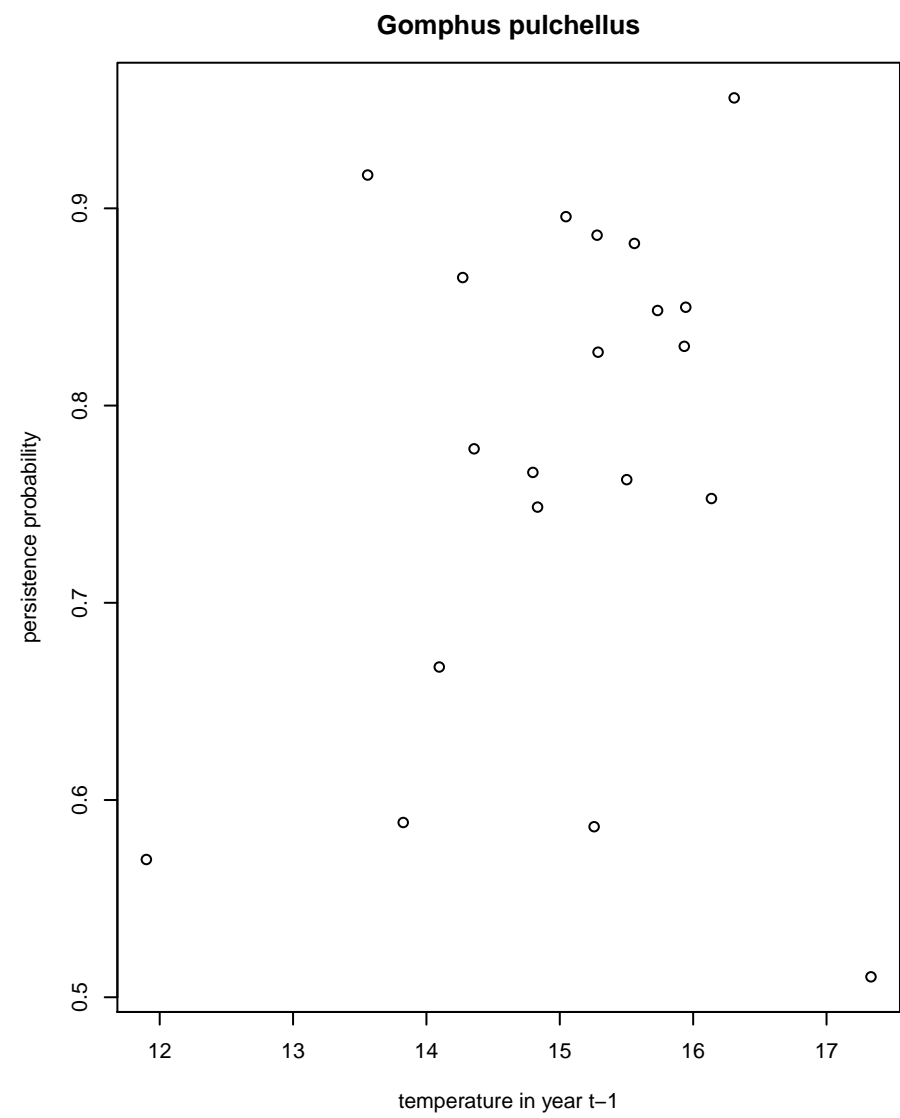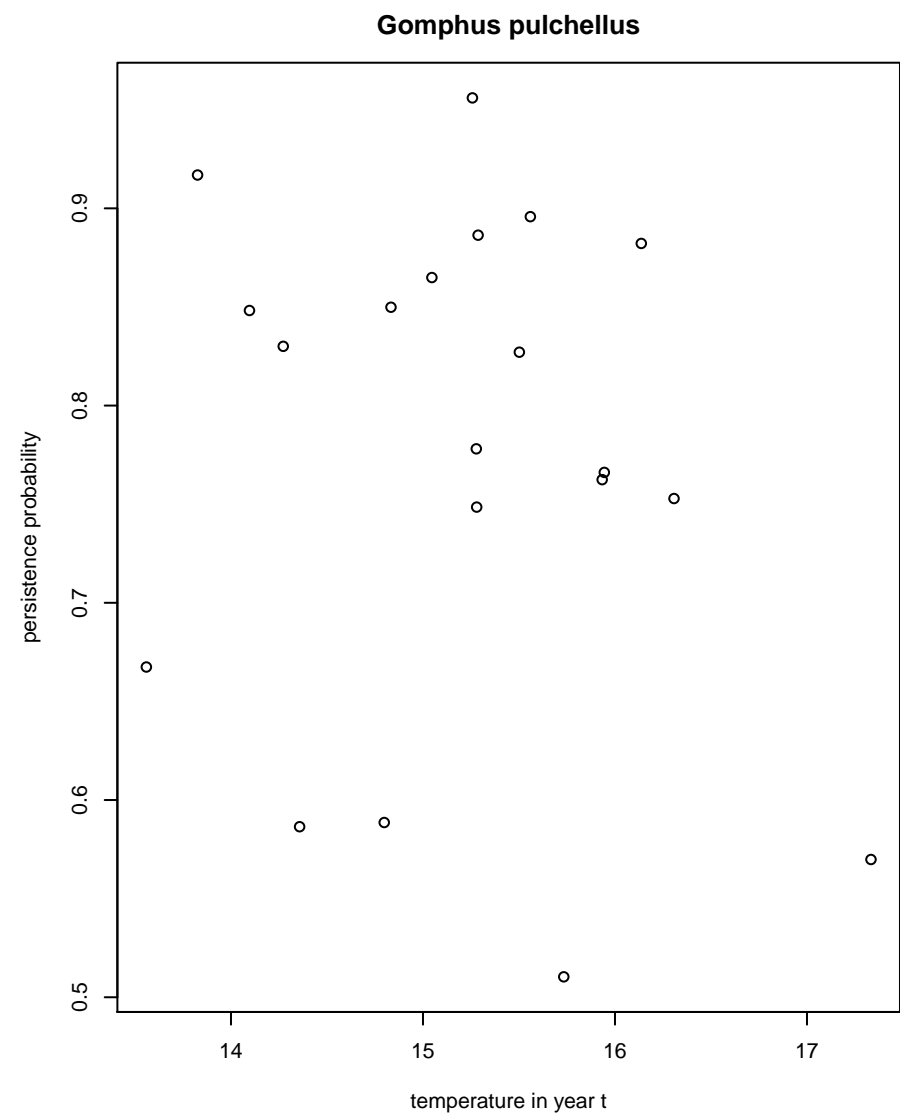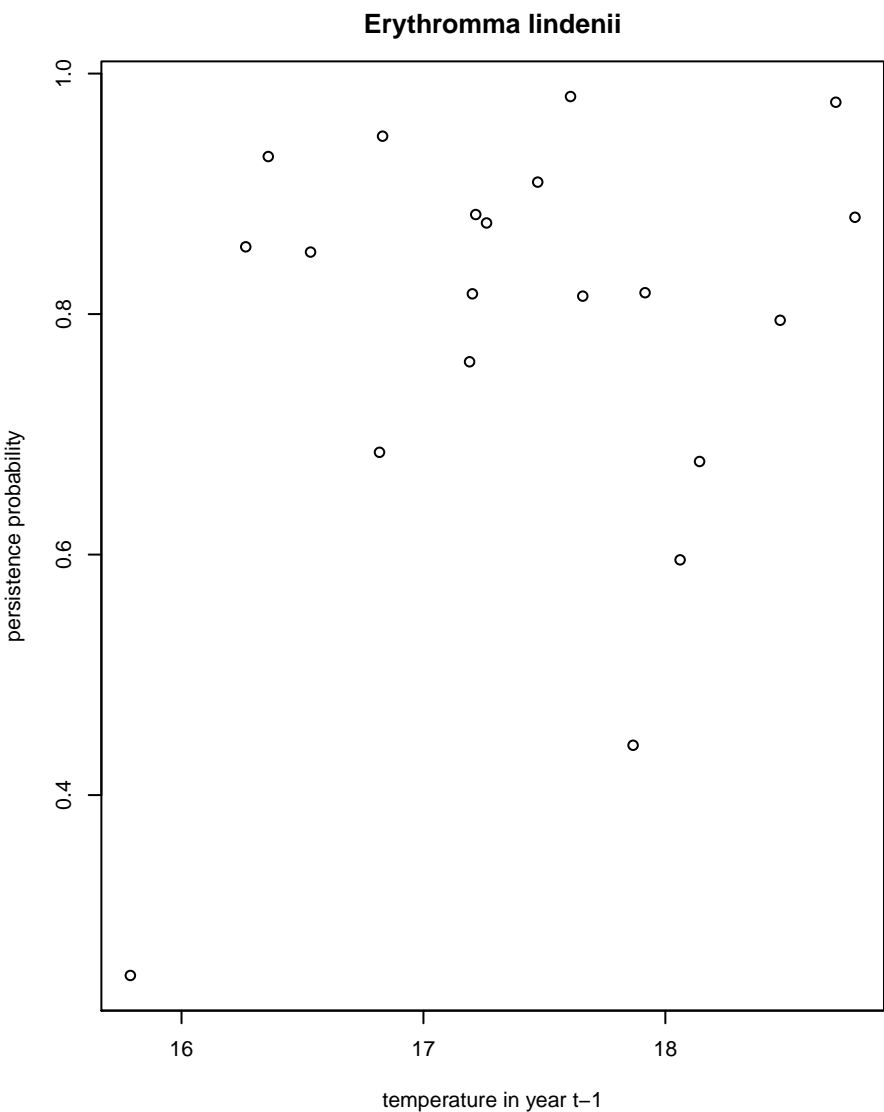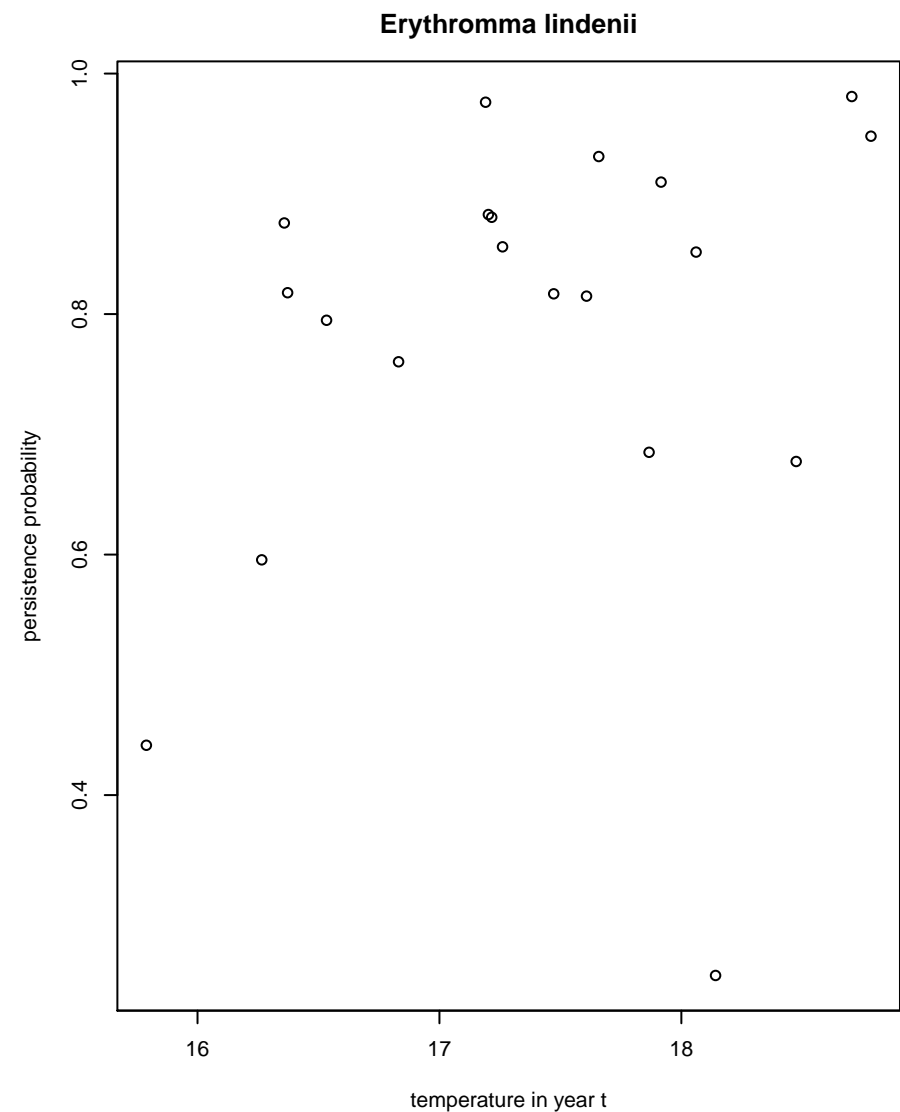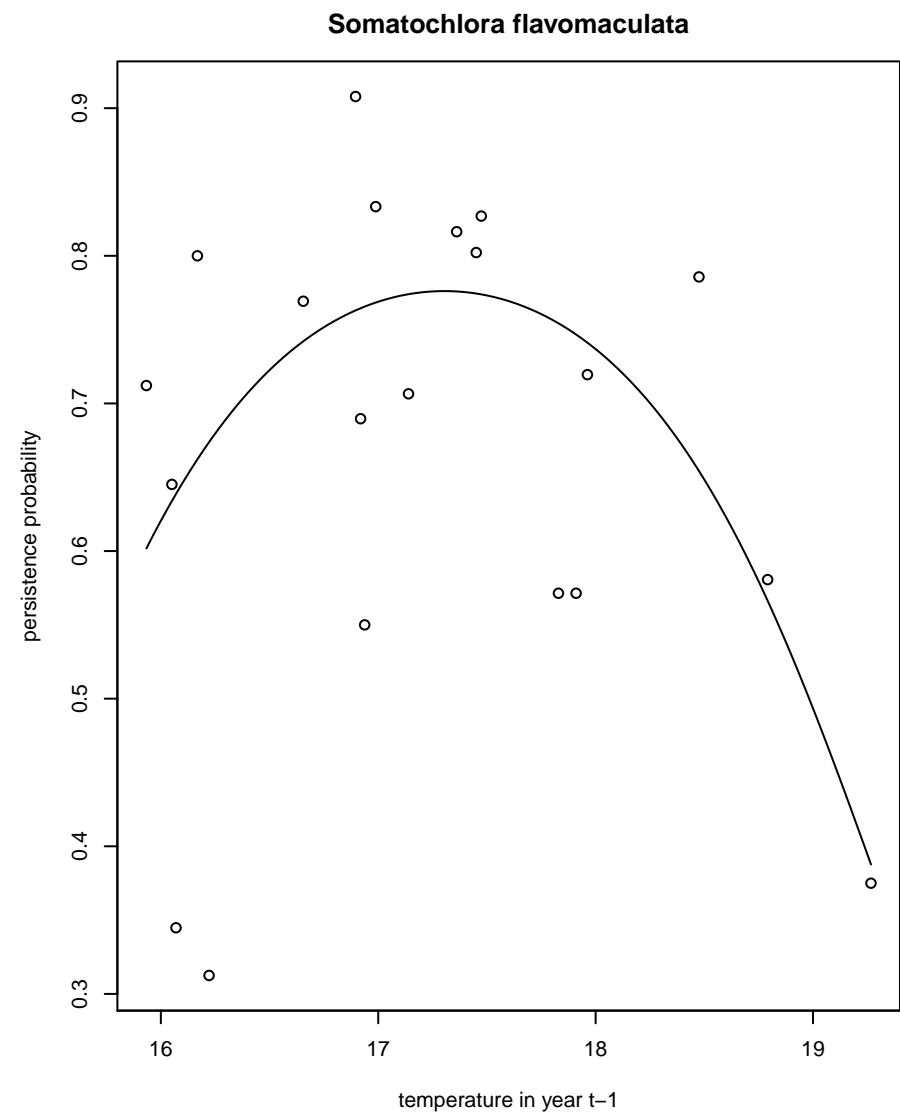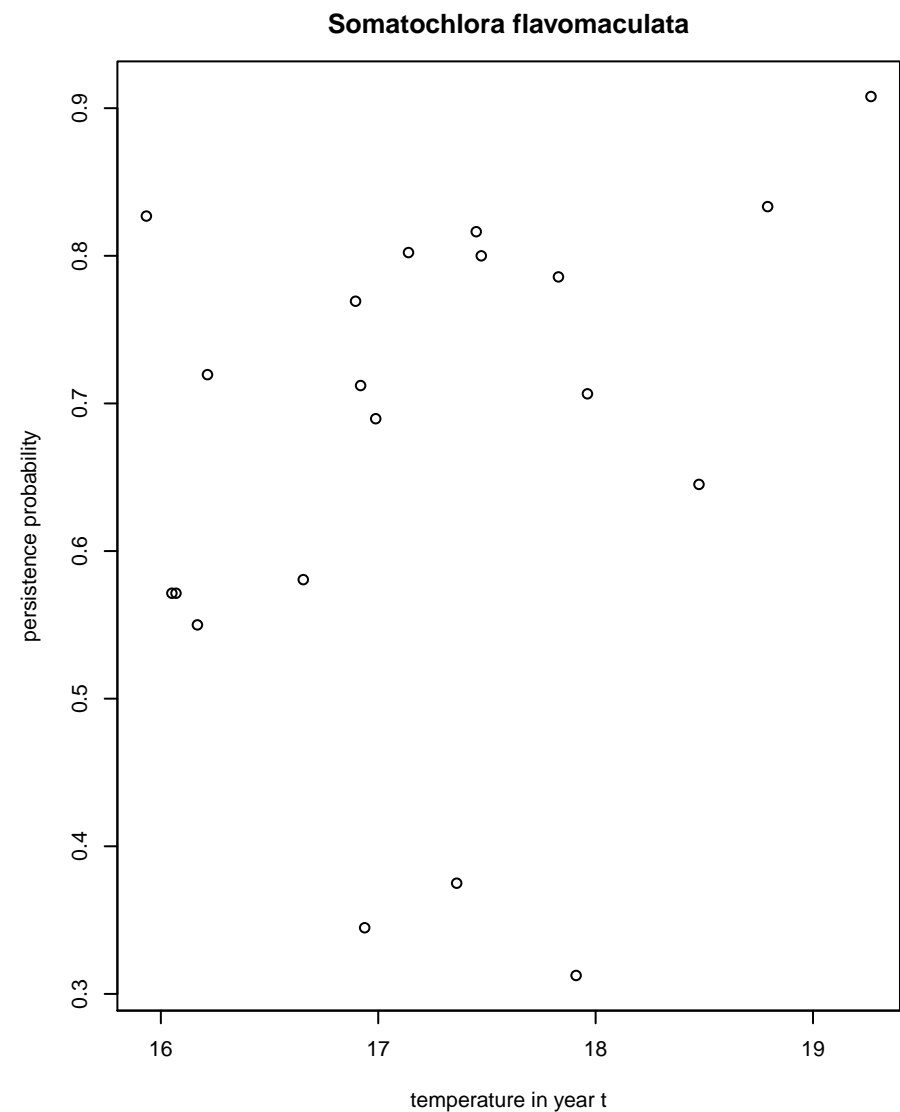

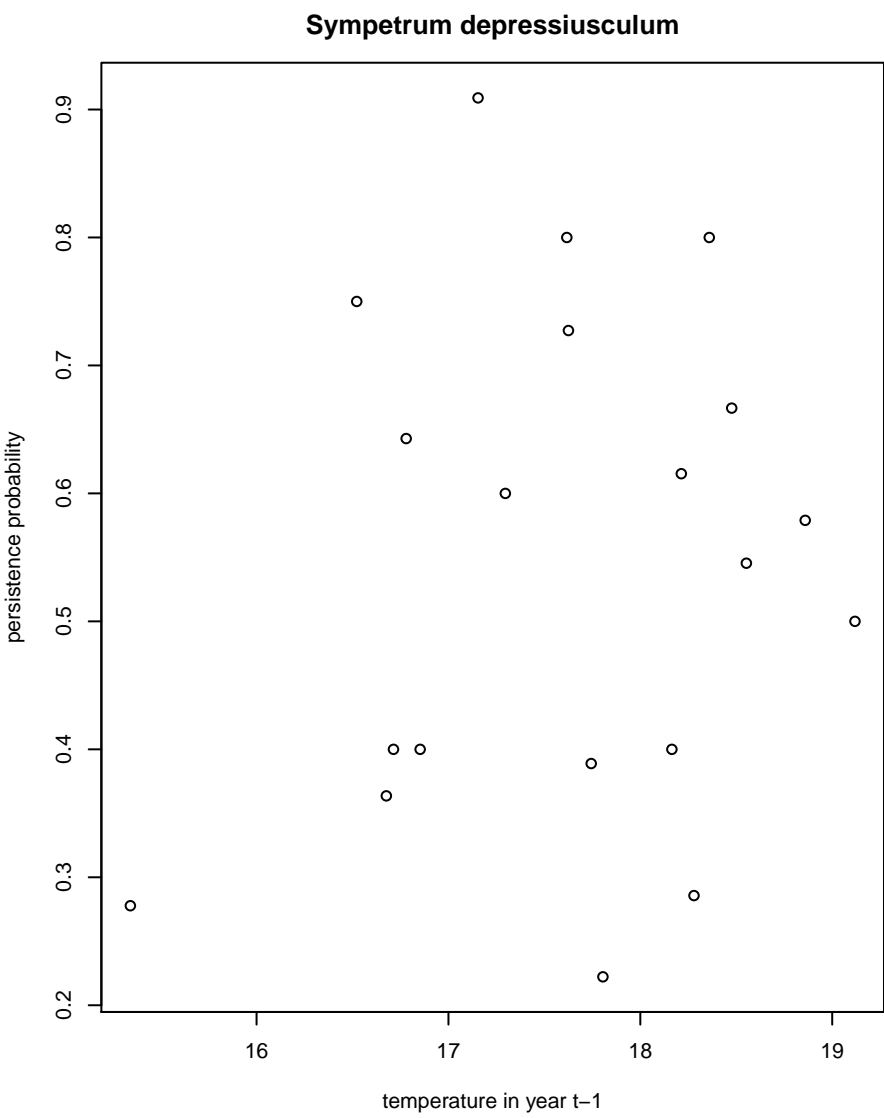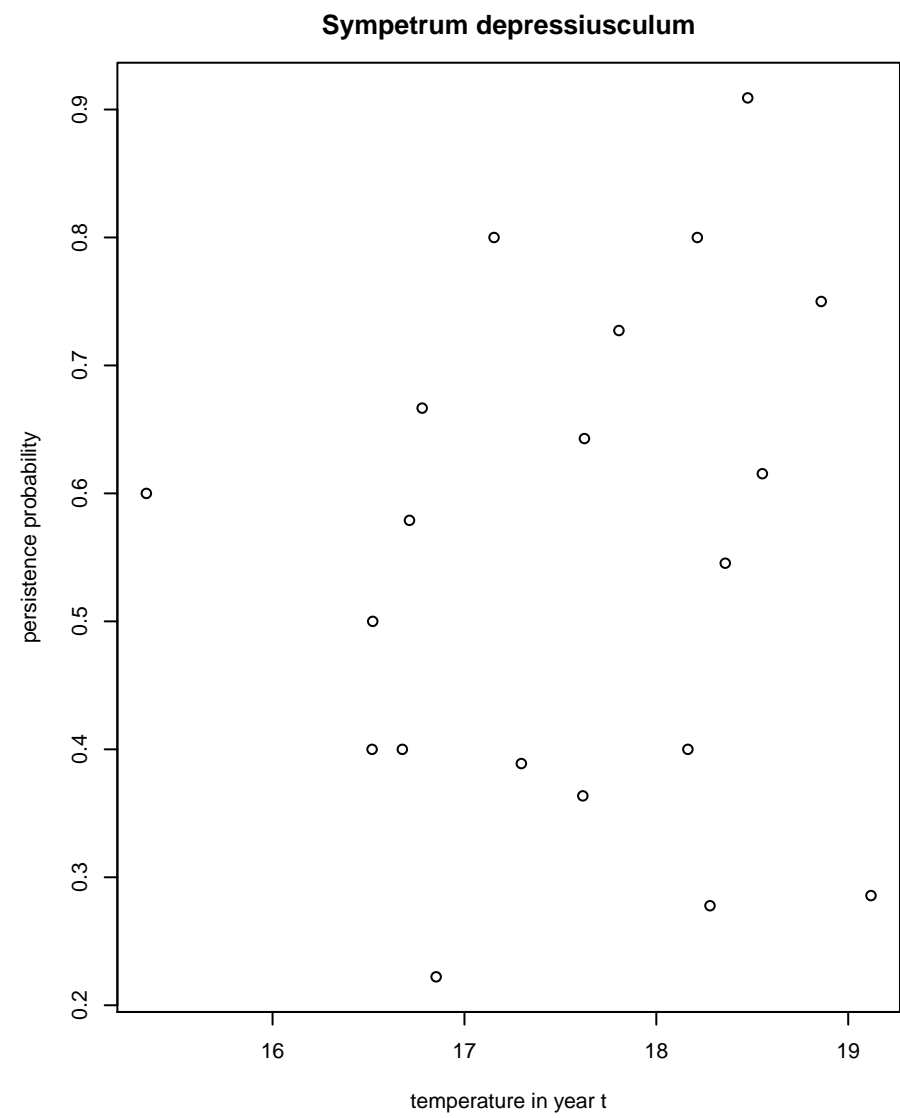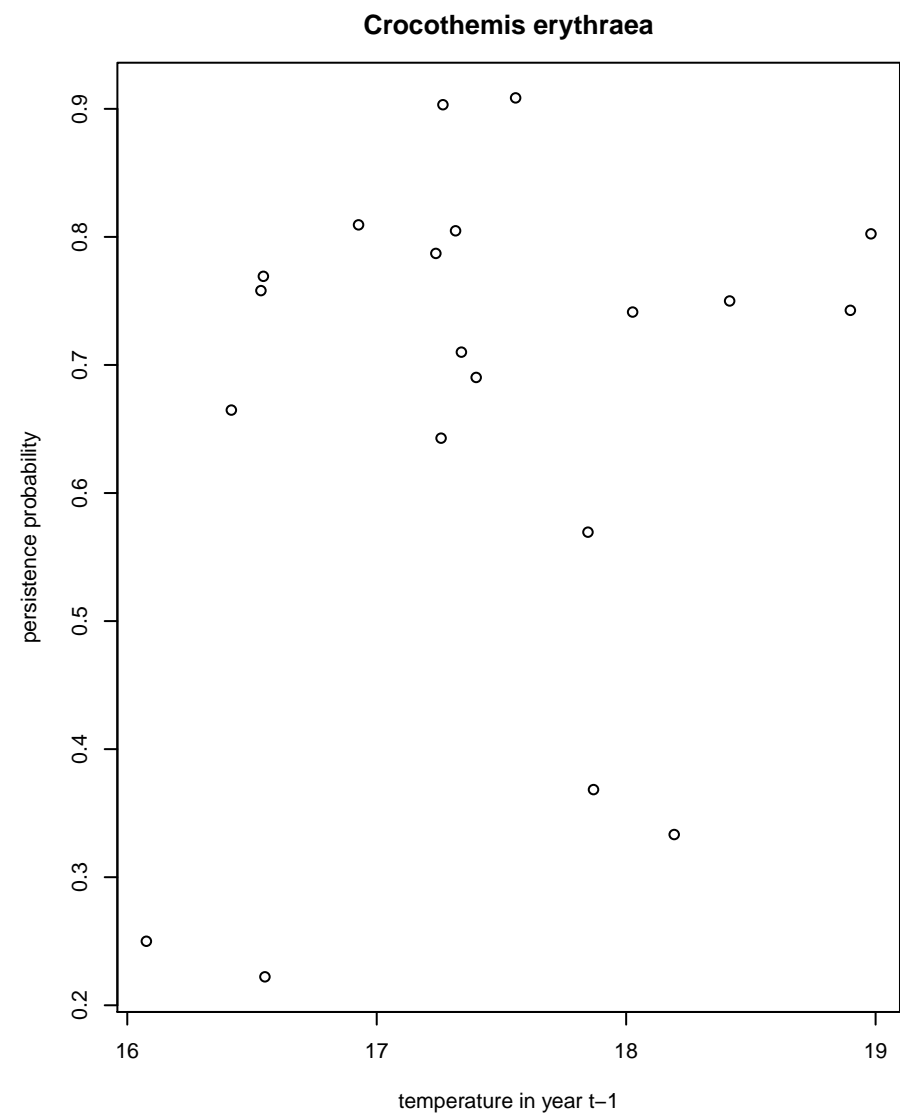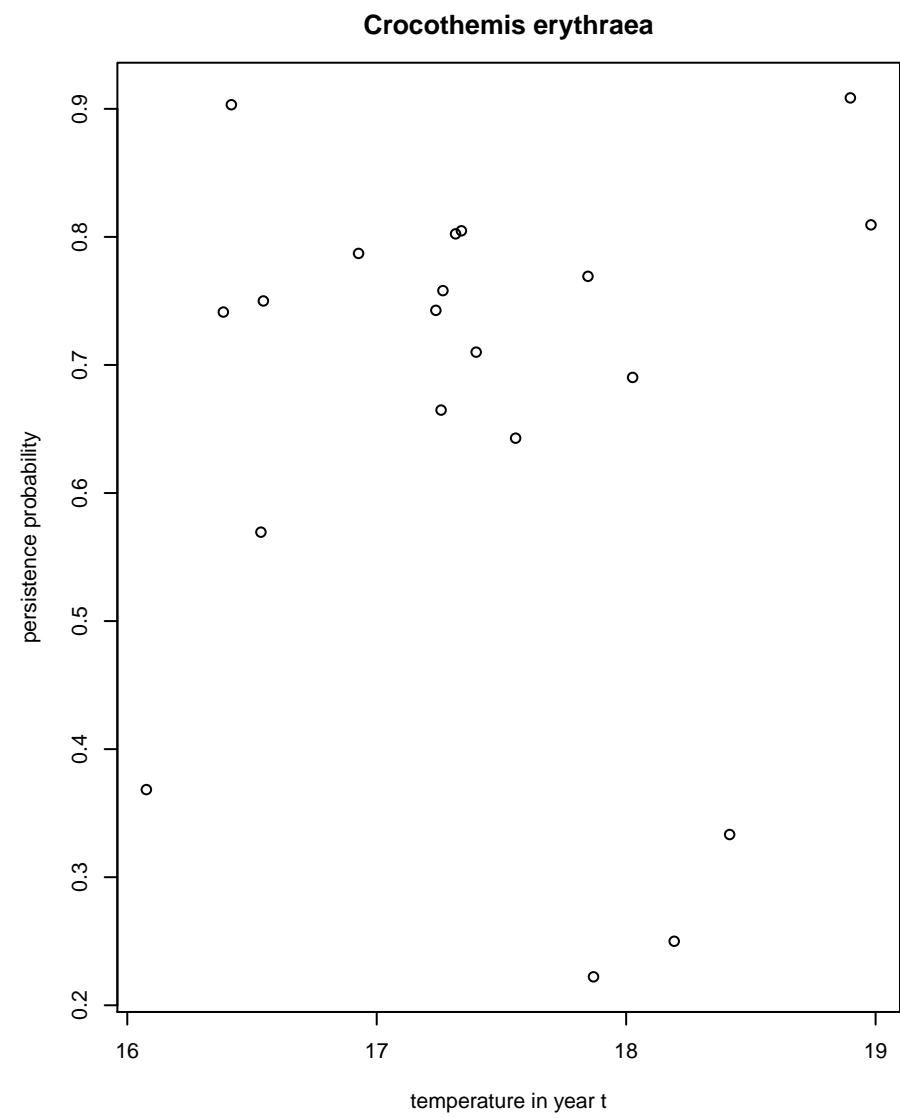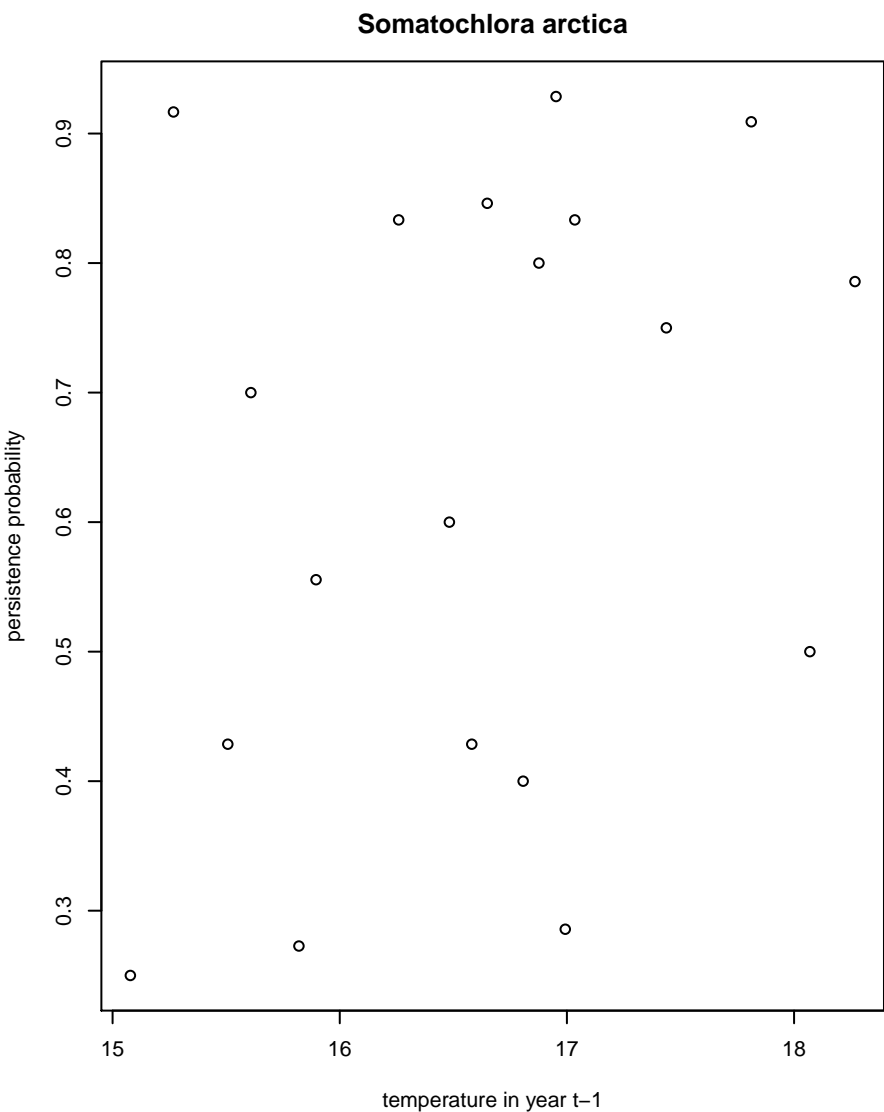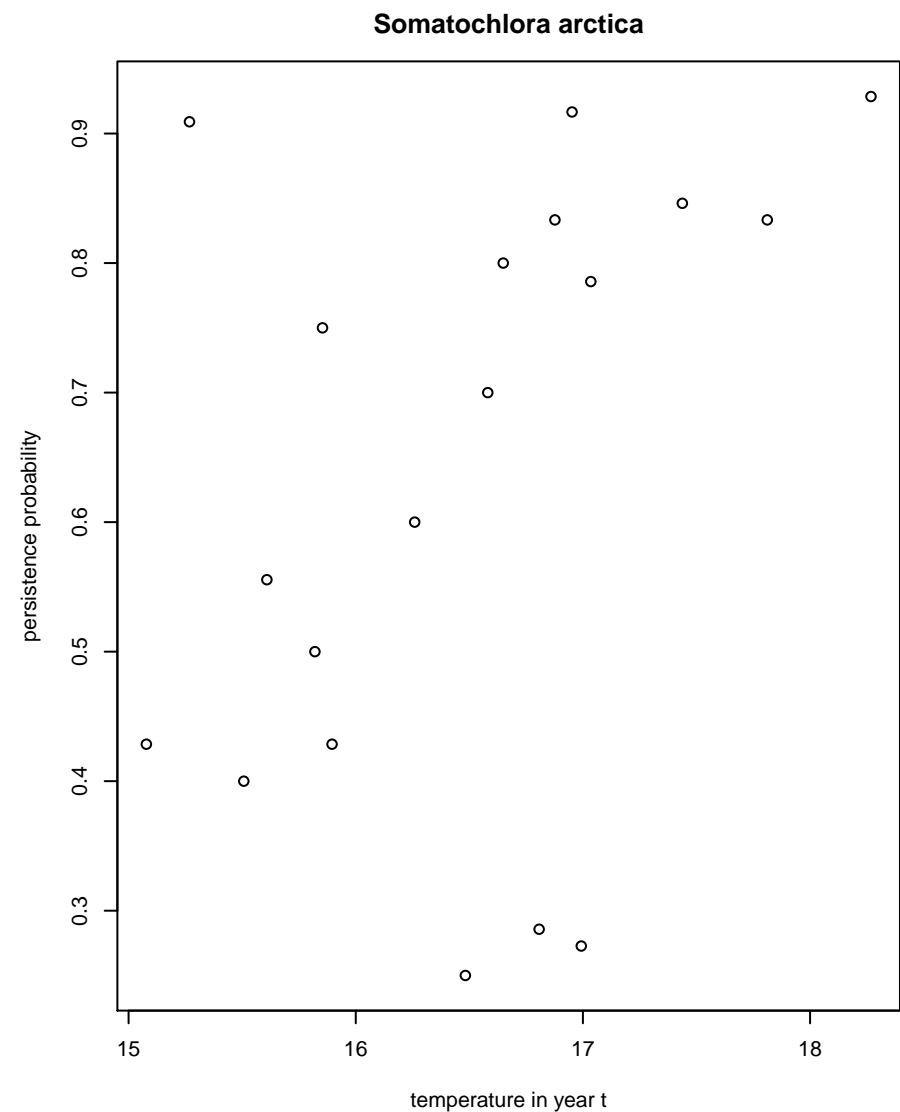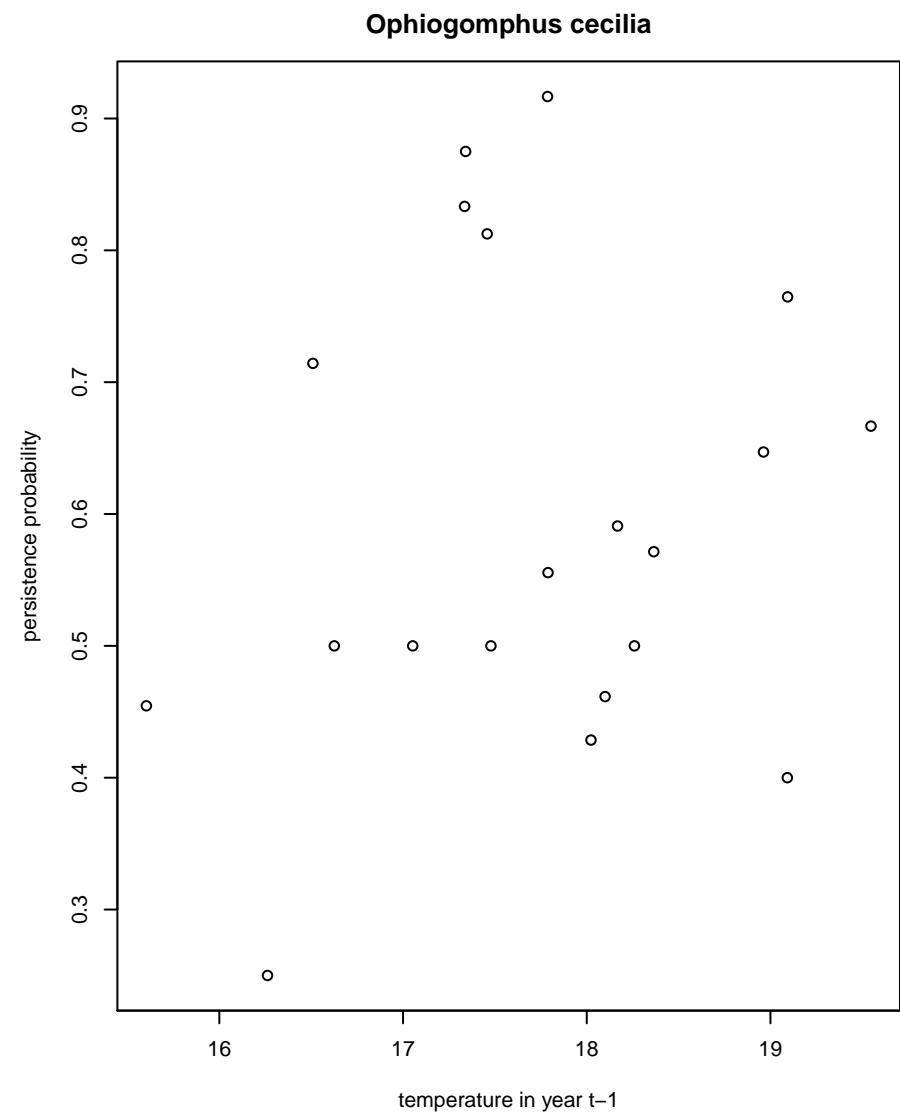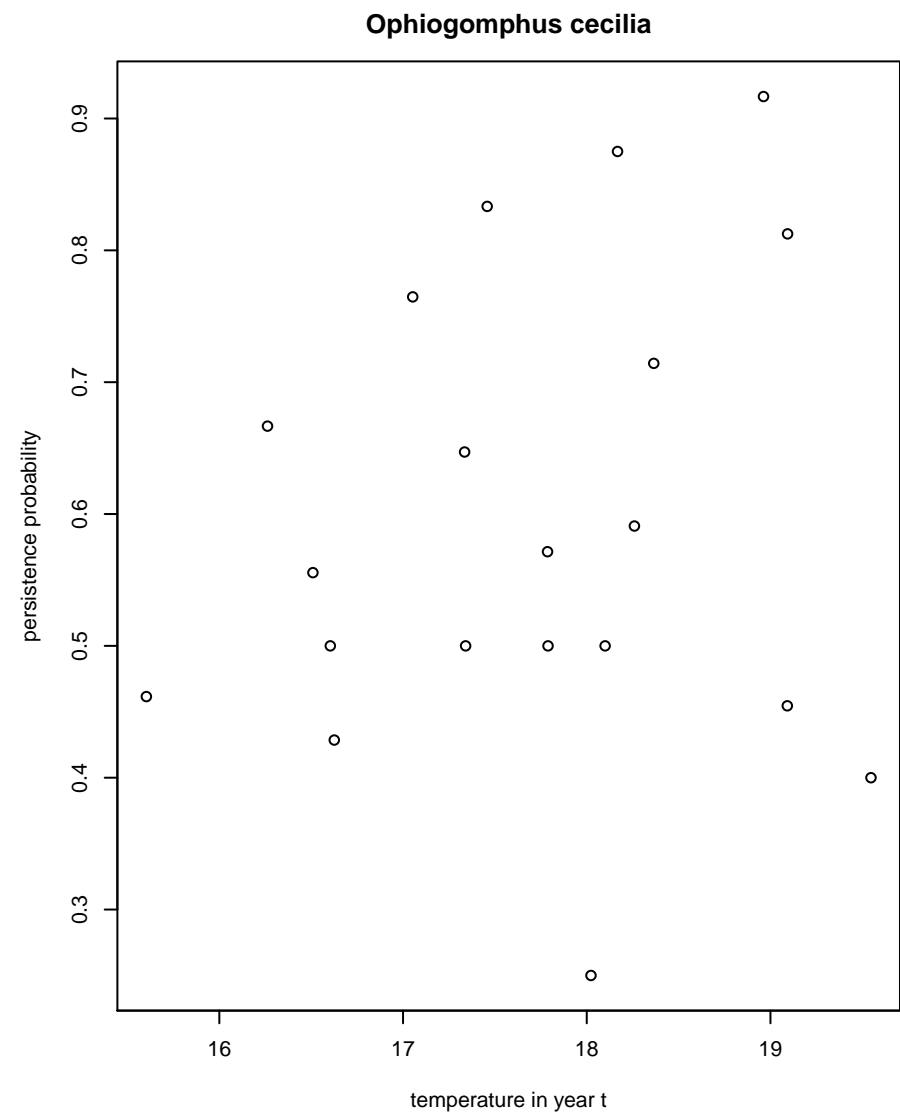

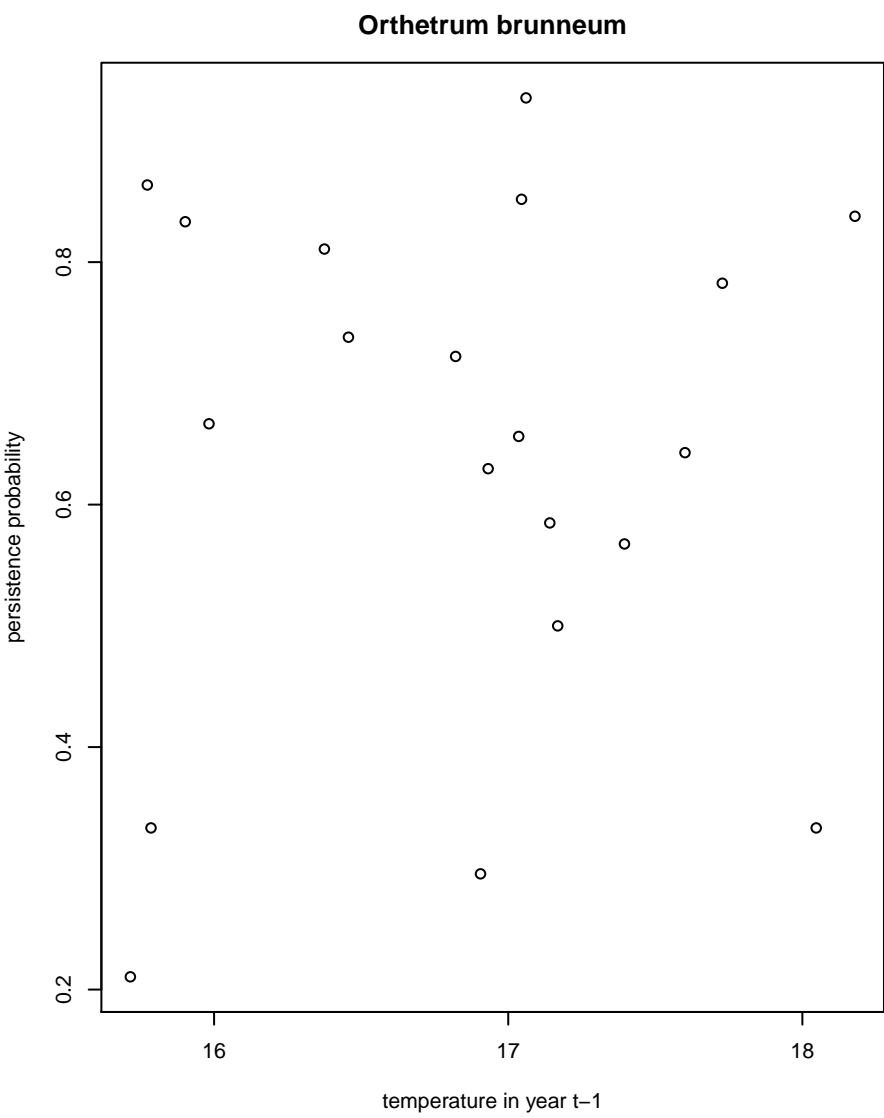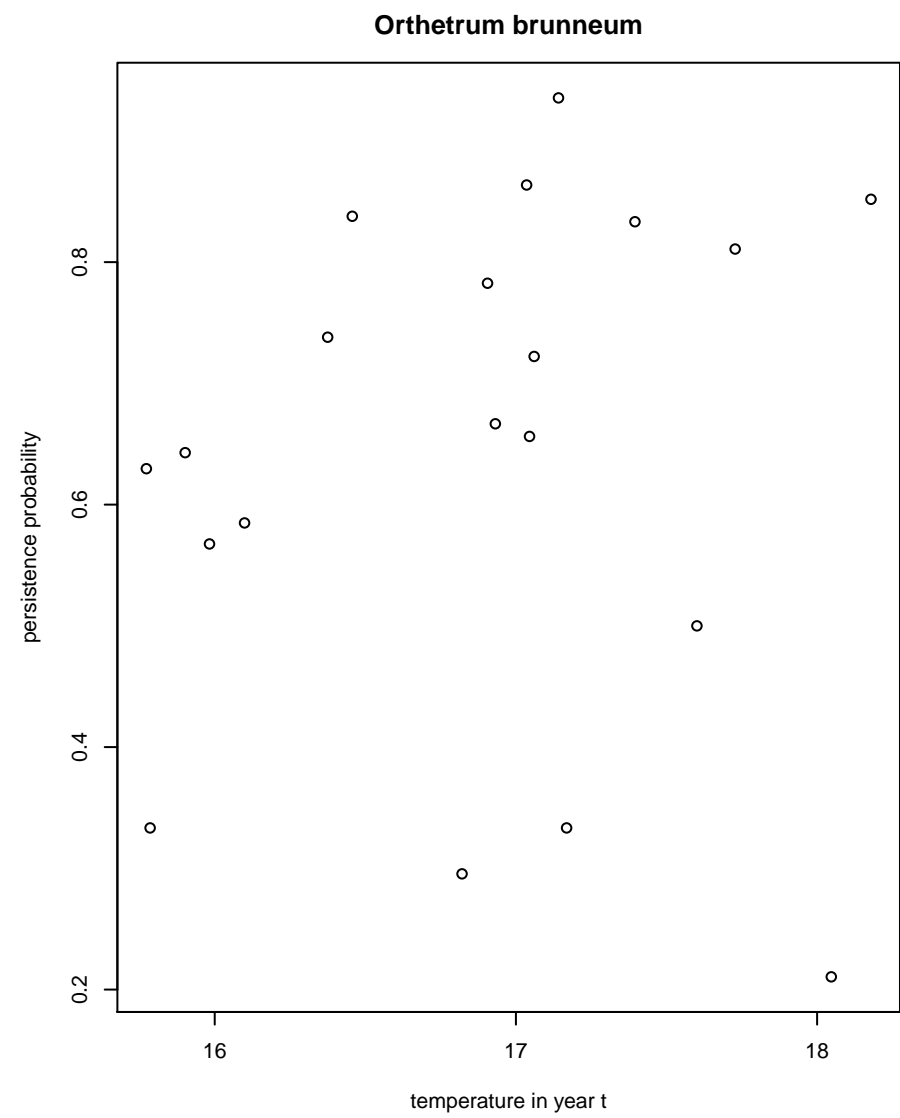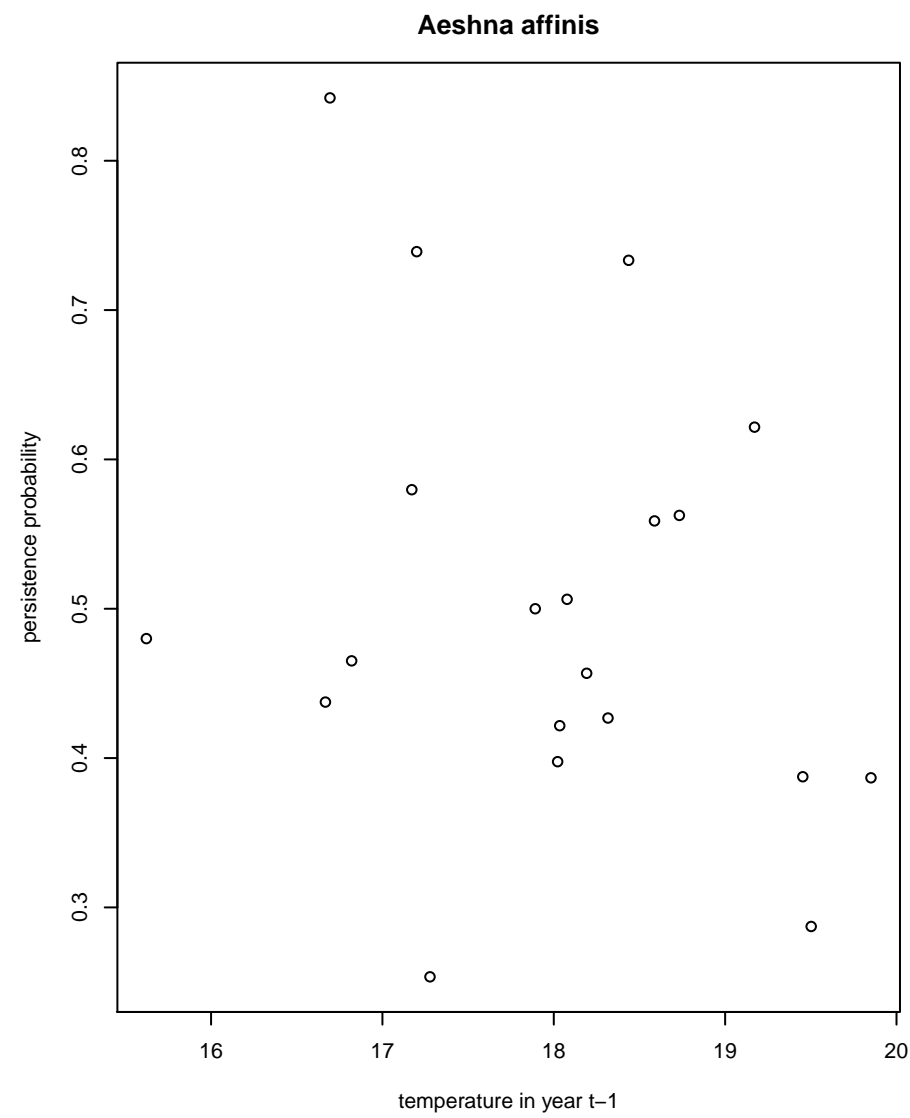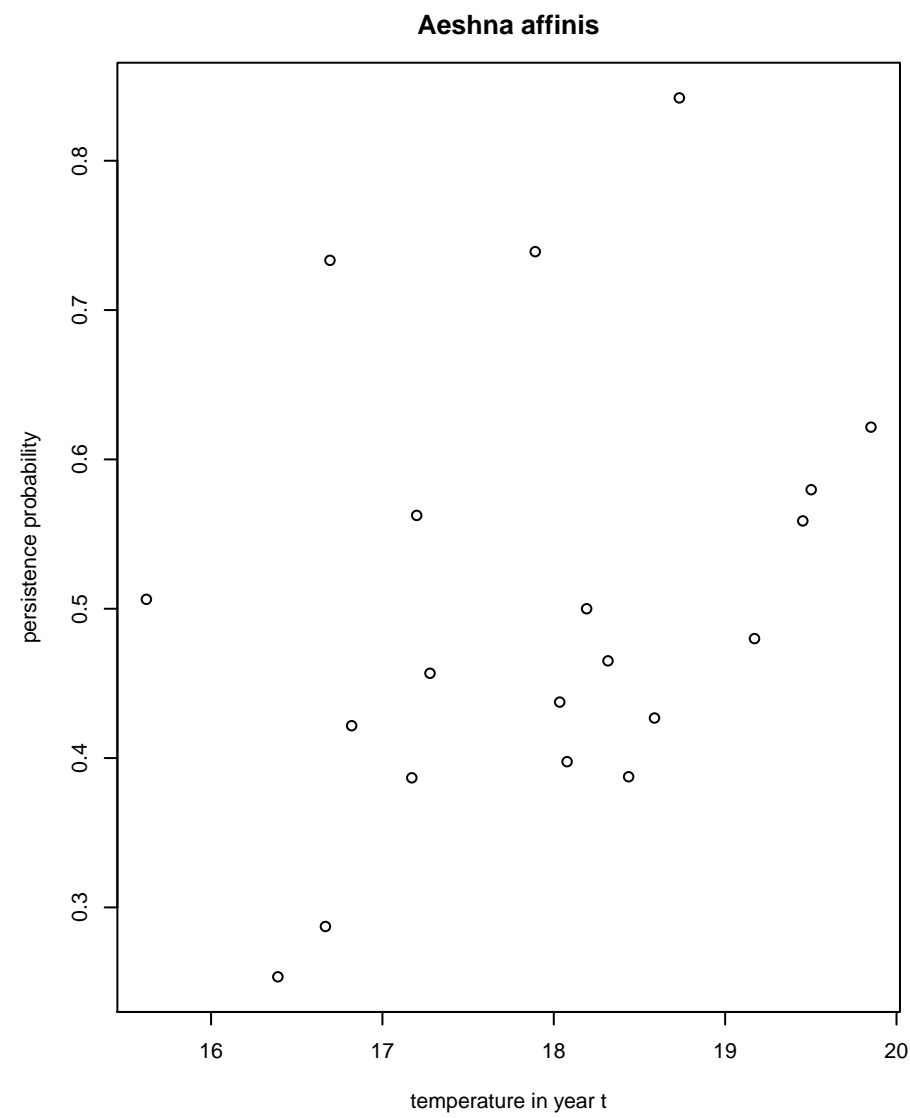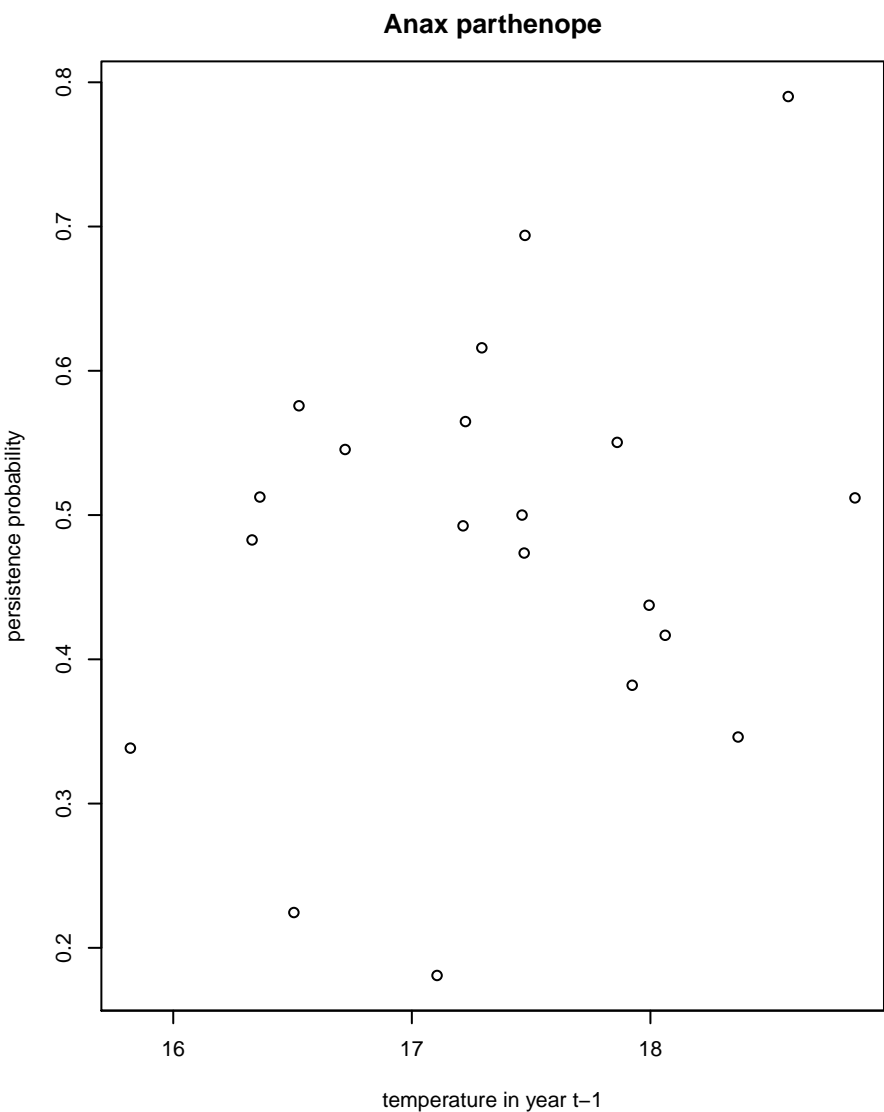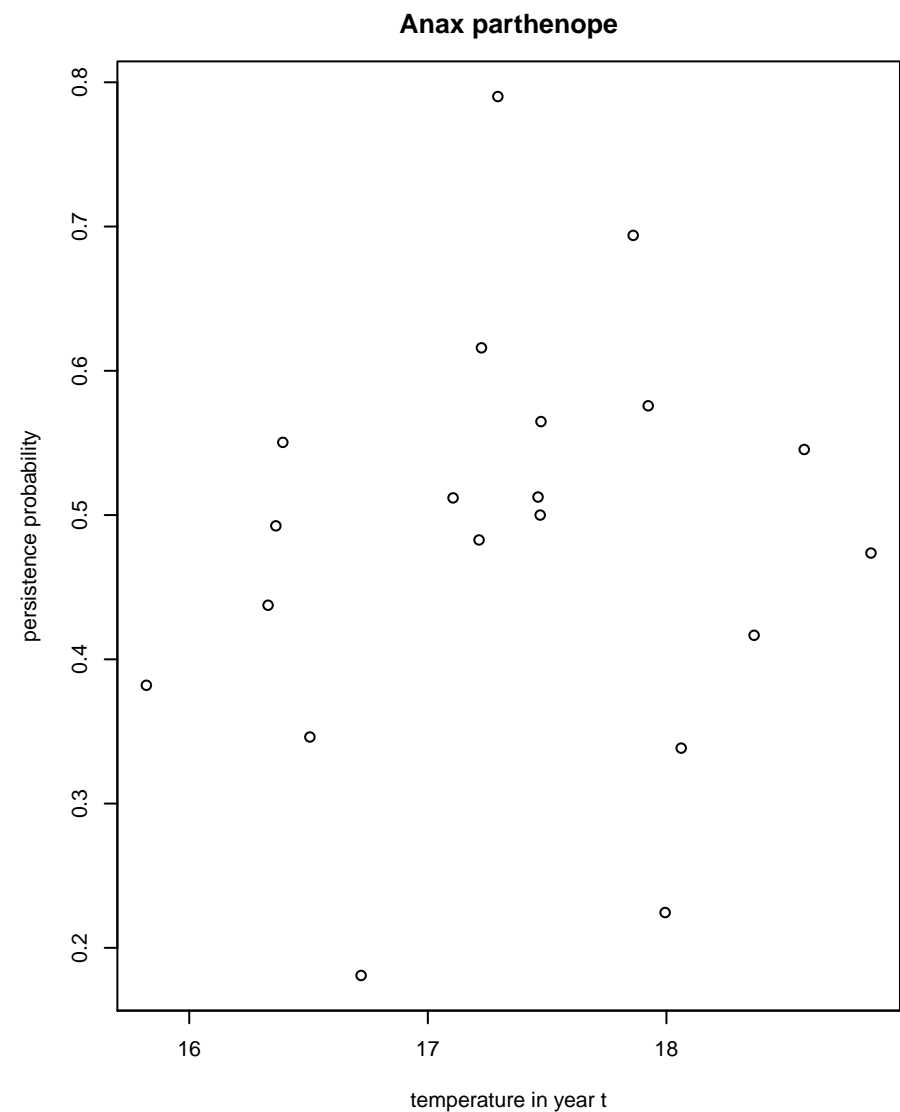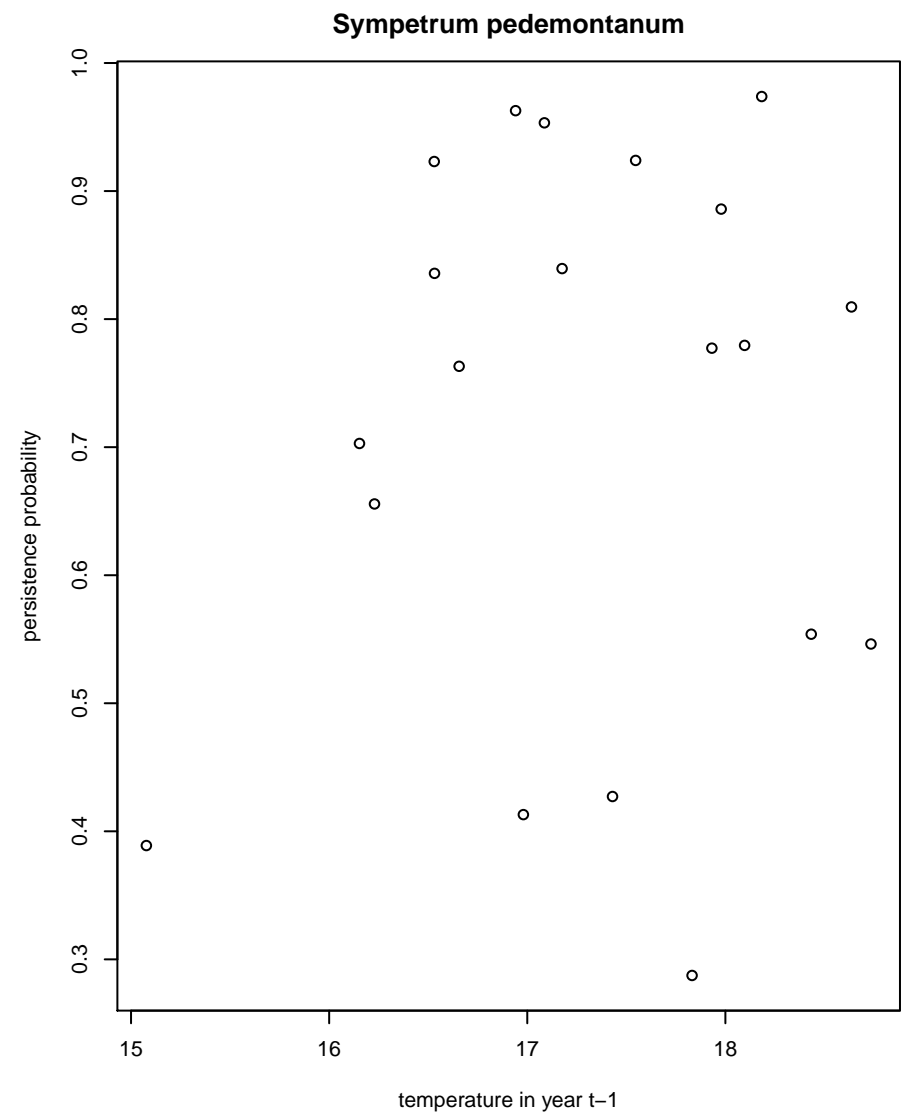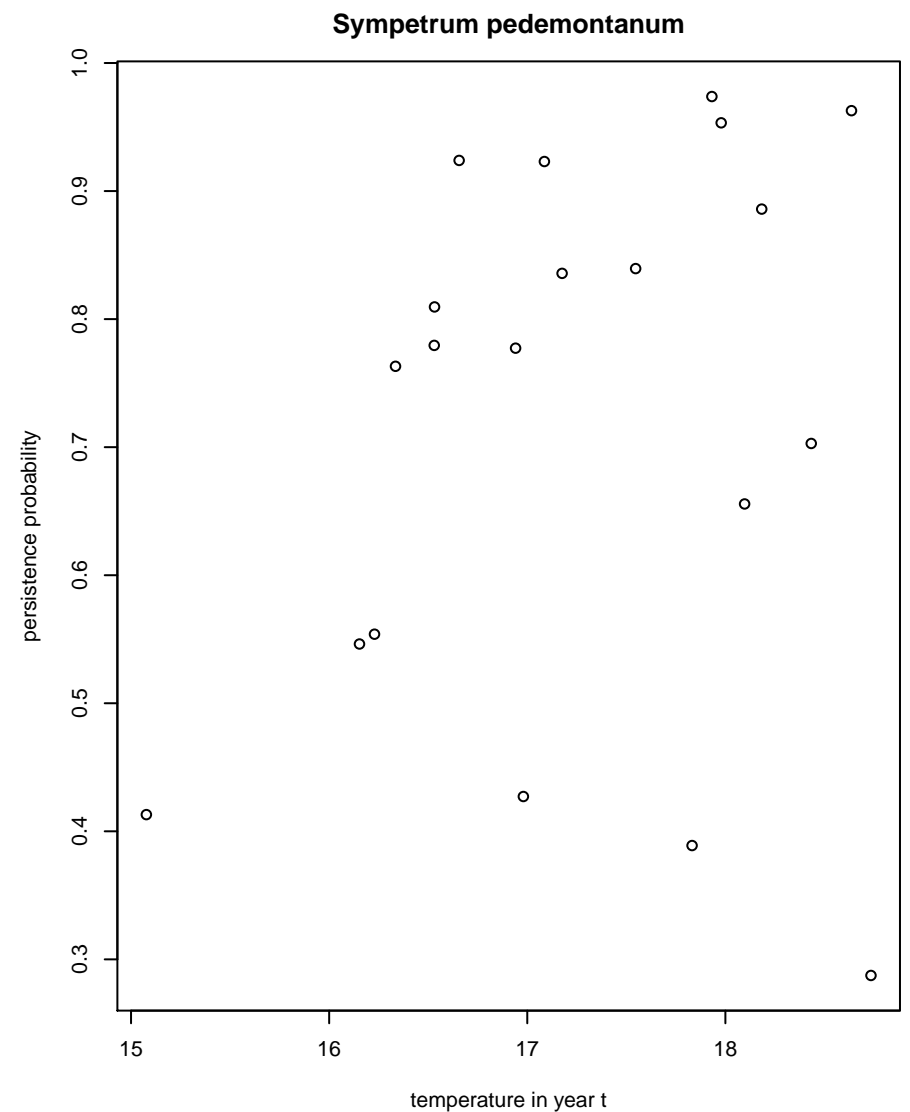

Supplement: Figure S2 — Response curves of colonisation and persistence probability in relation to temperature per species. (PDF) [file pone.0110219.s002.pdf]

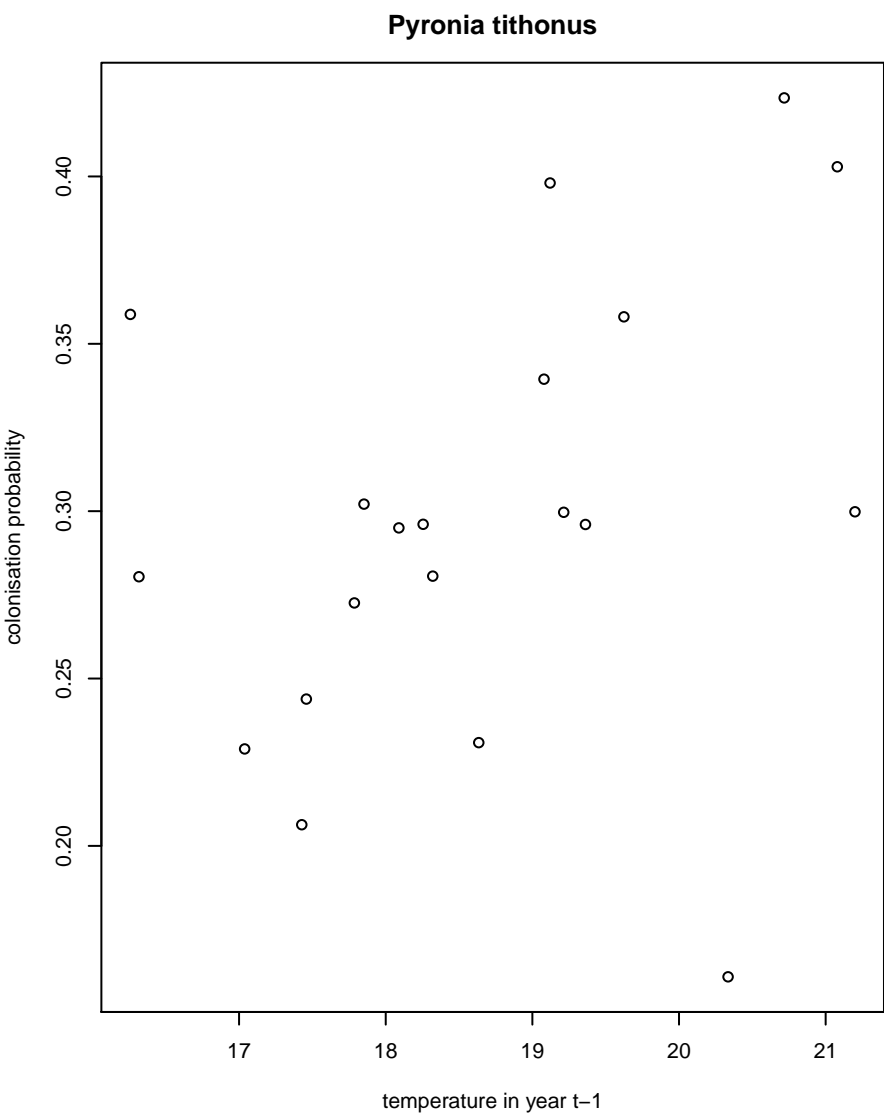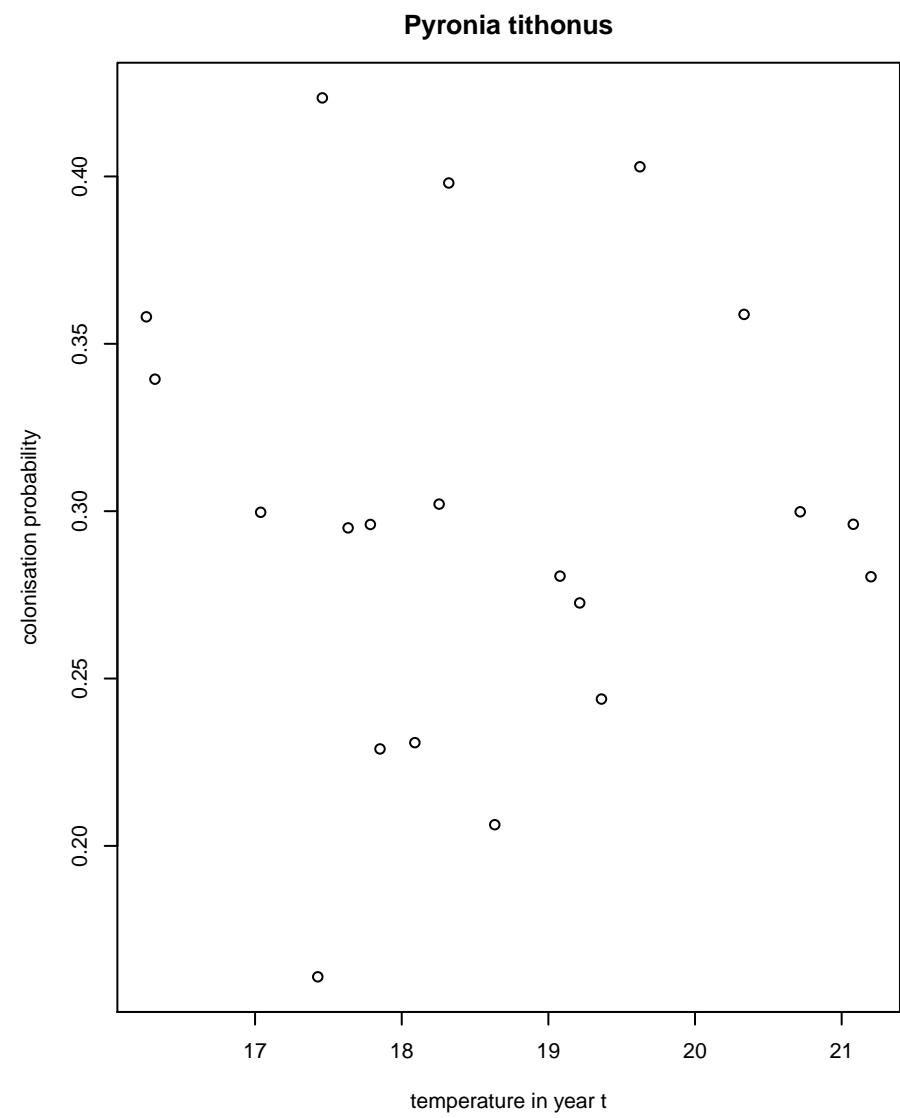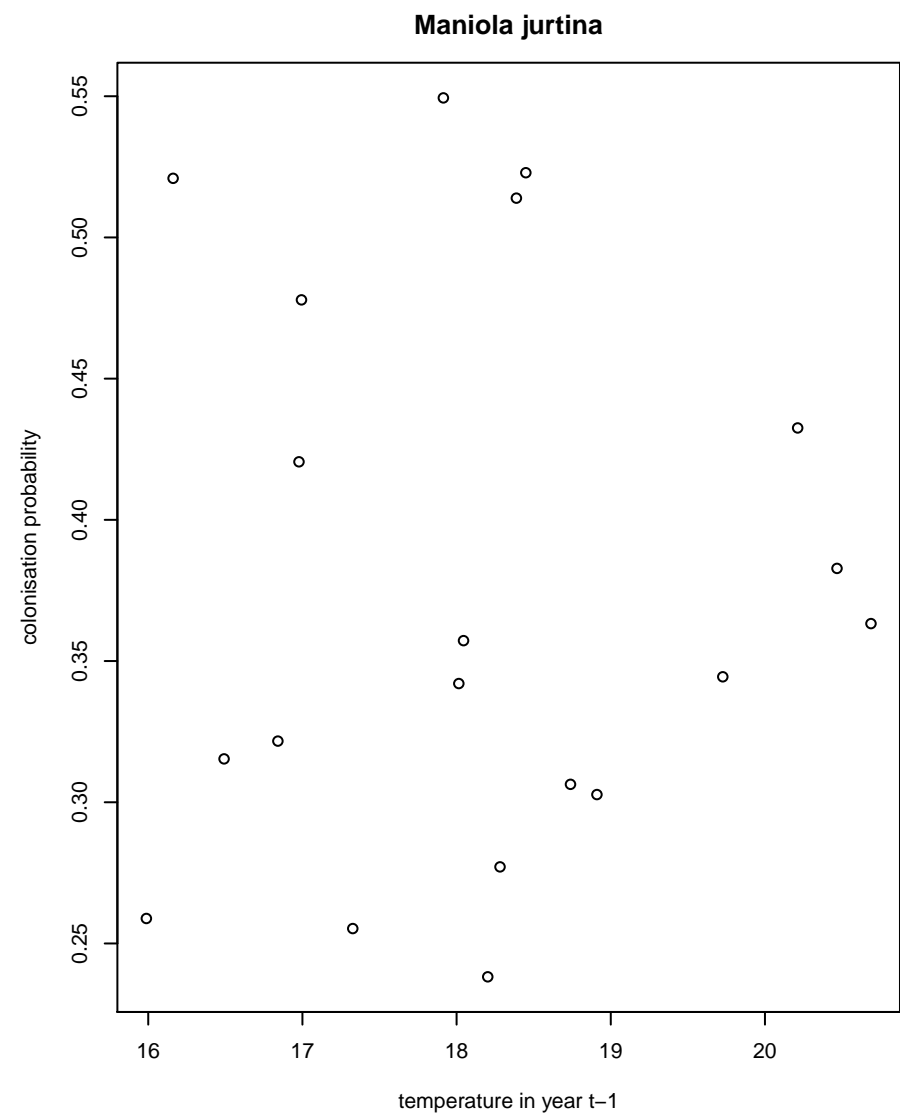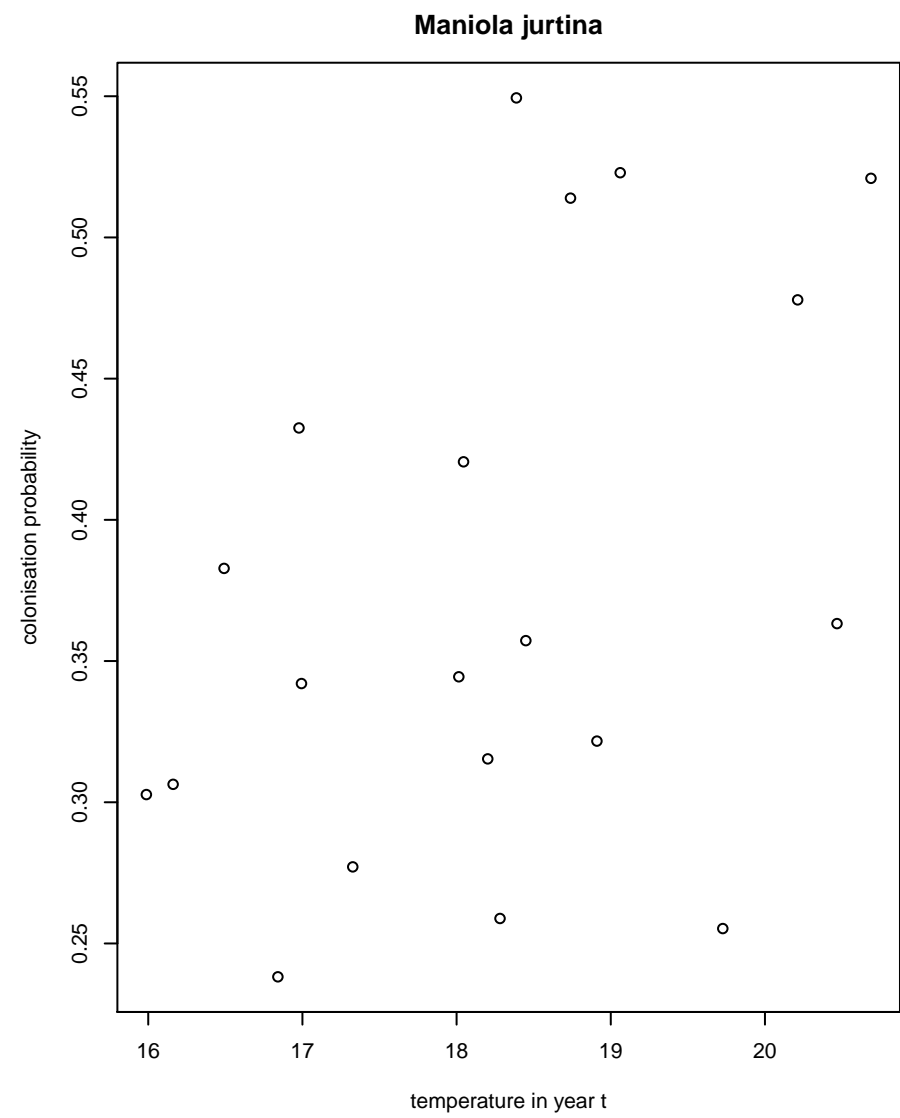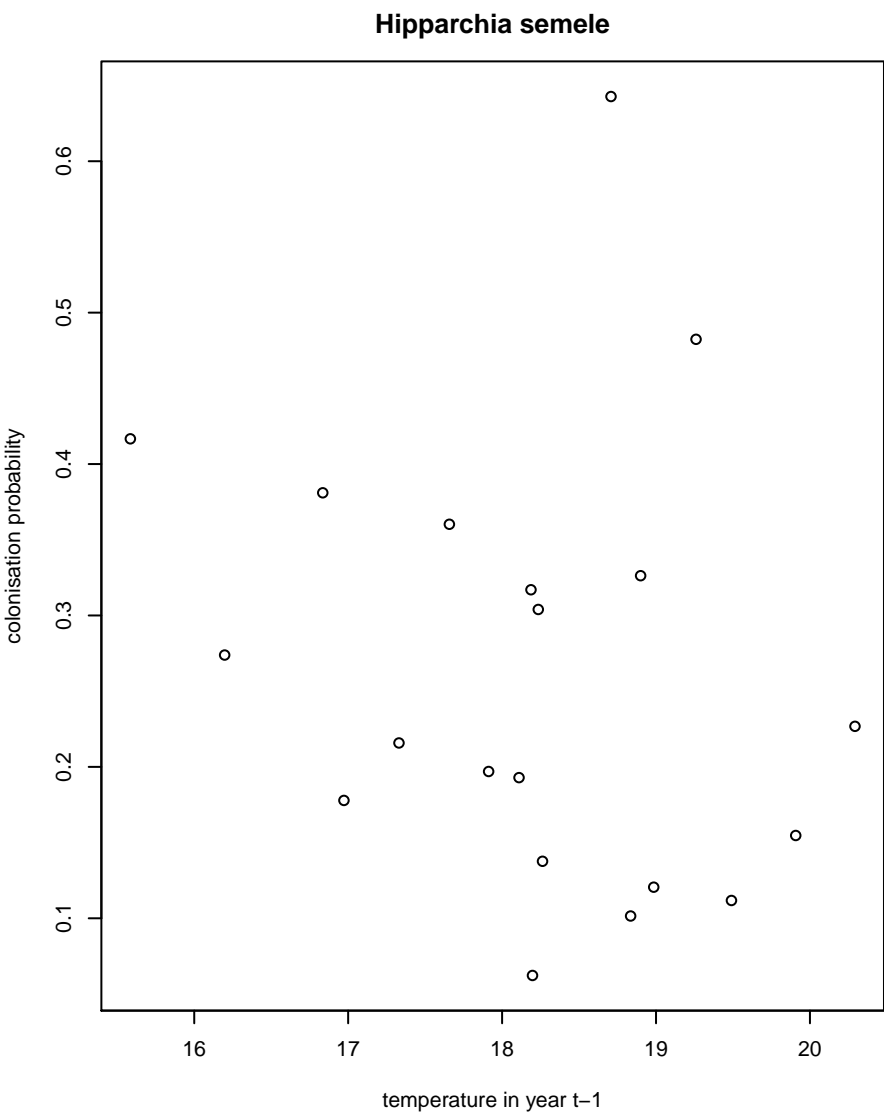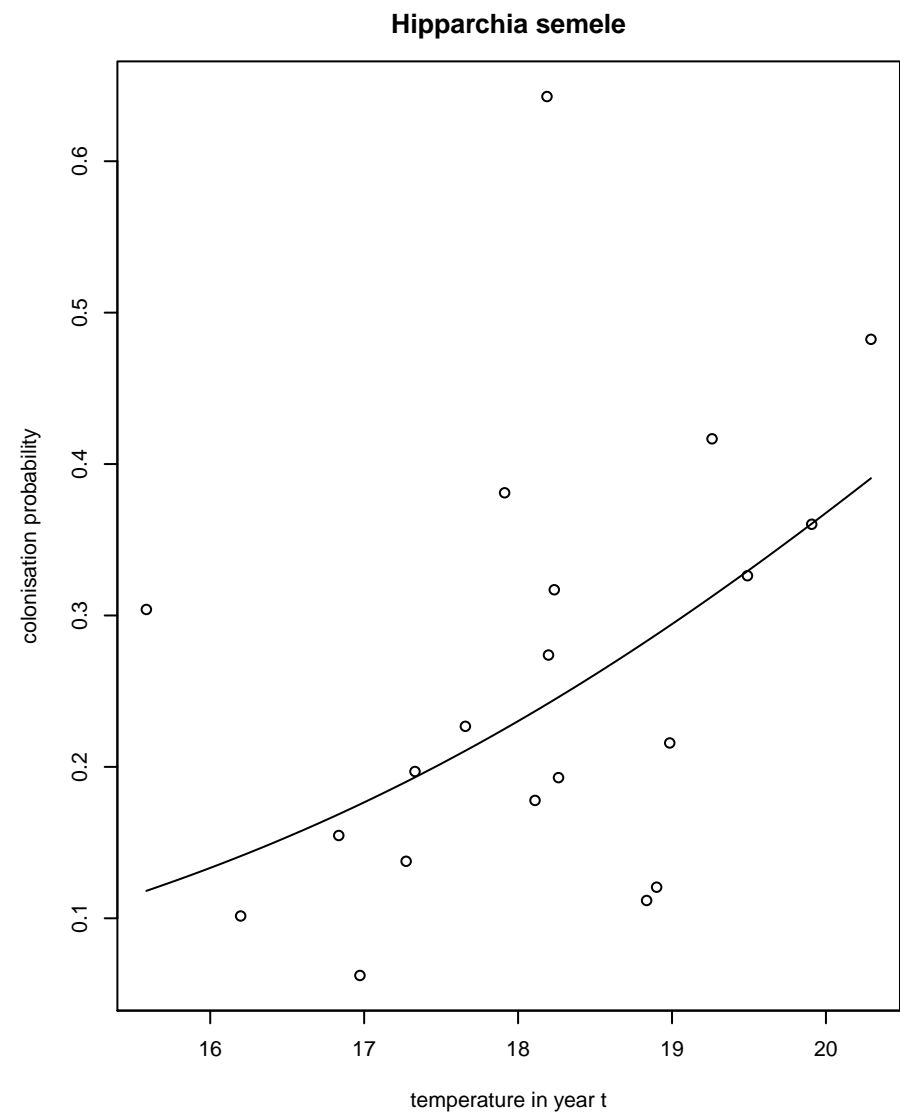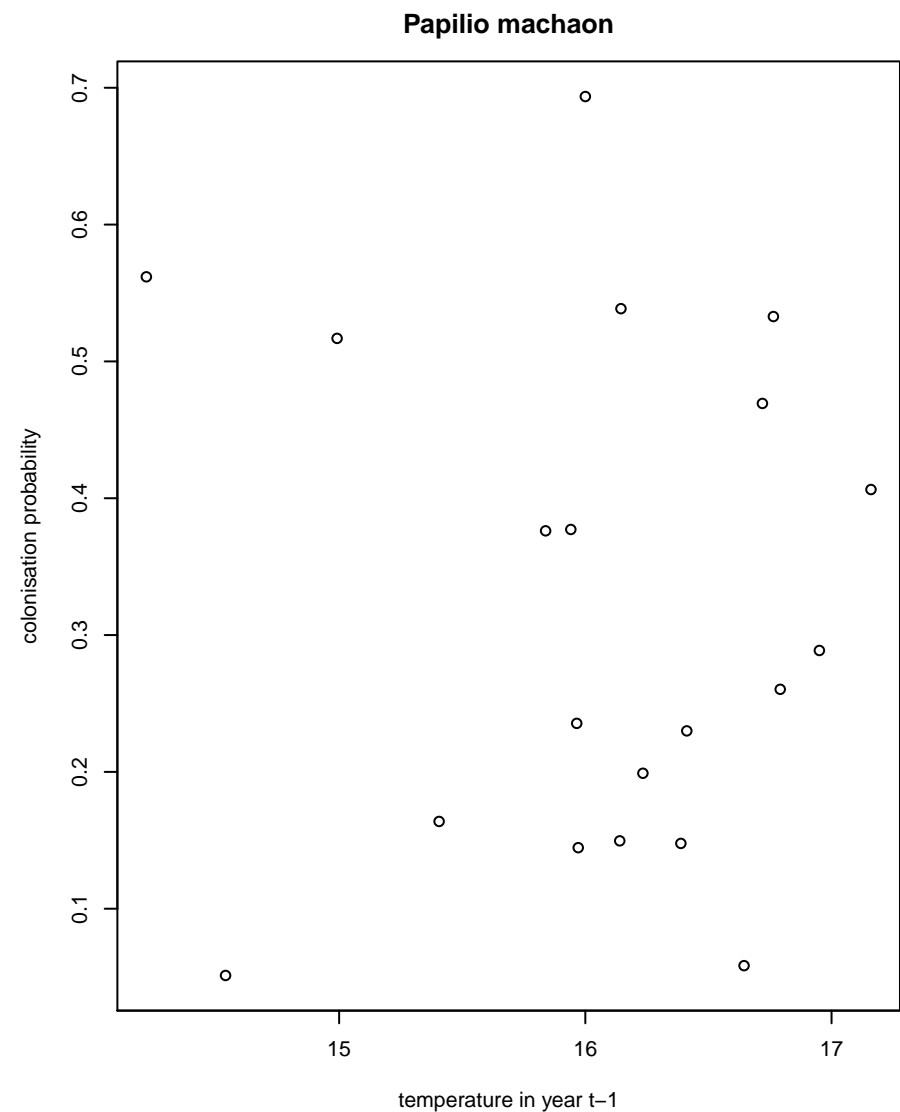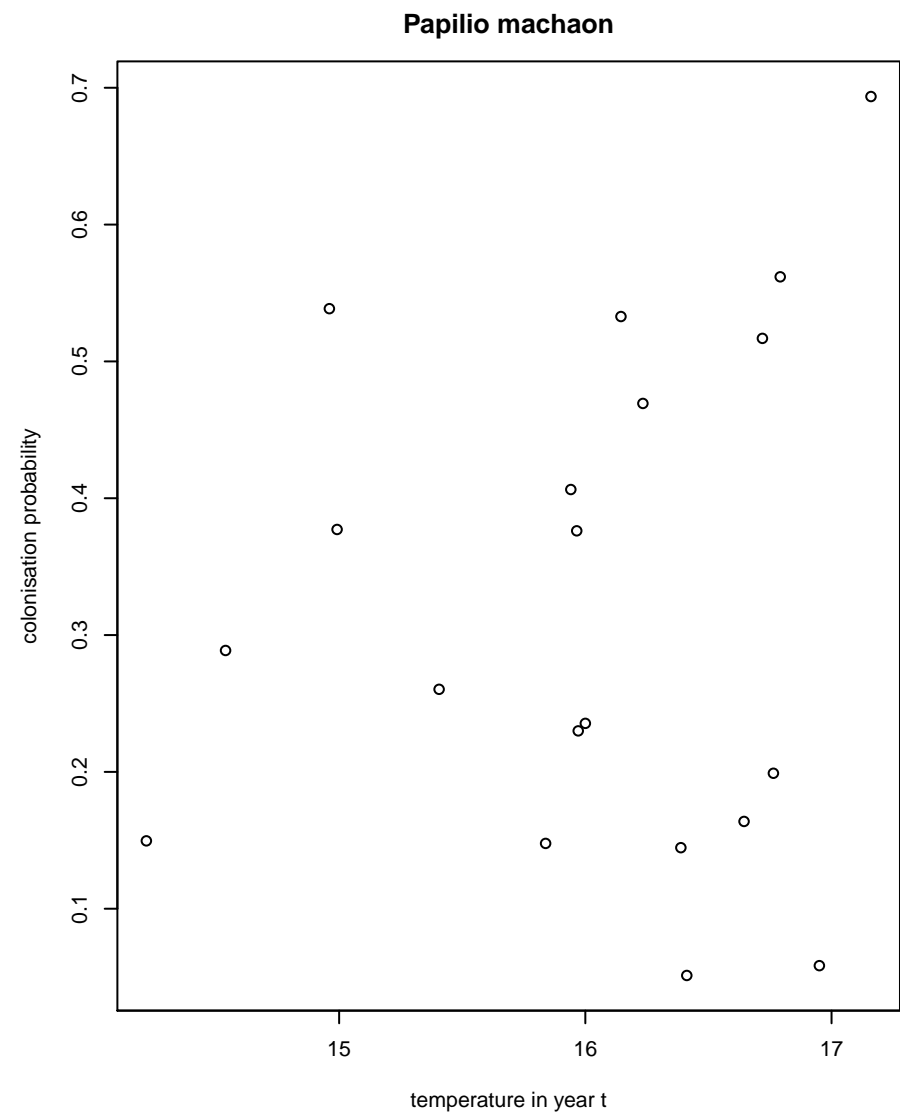

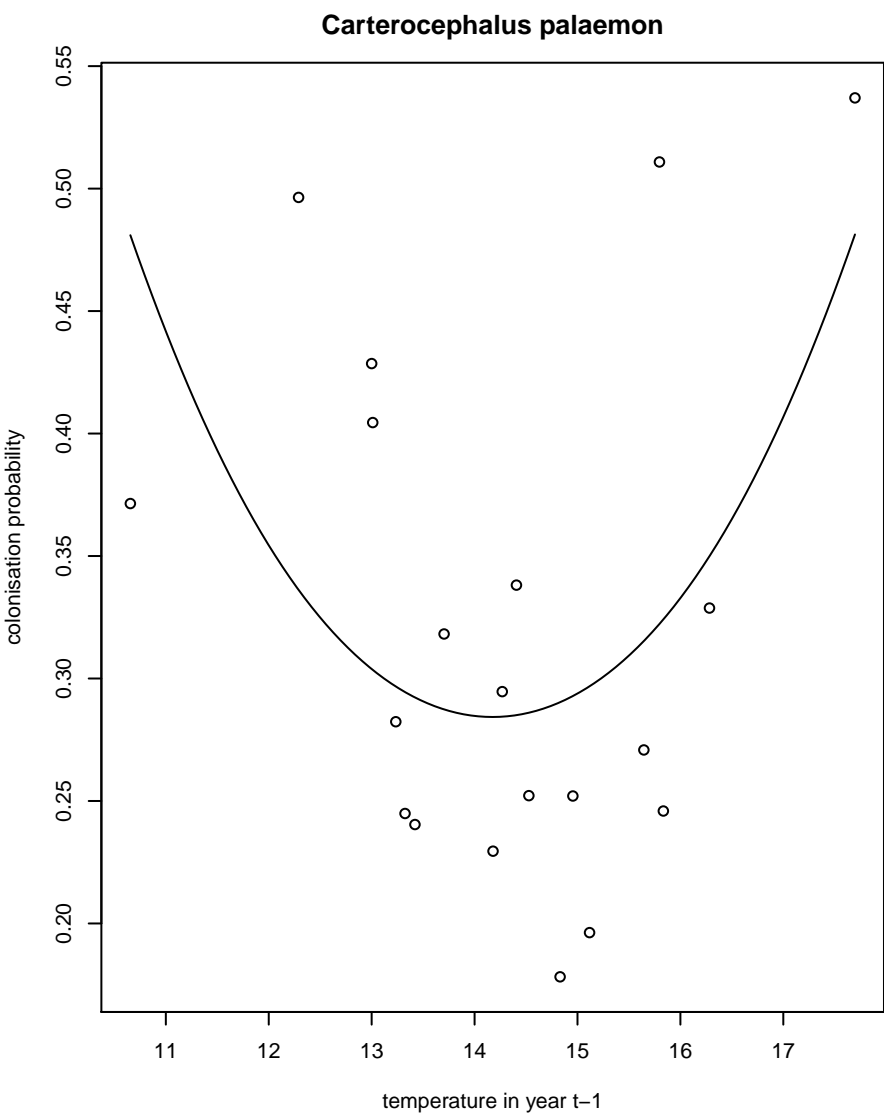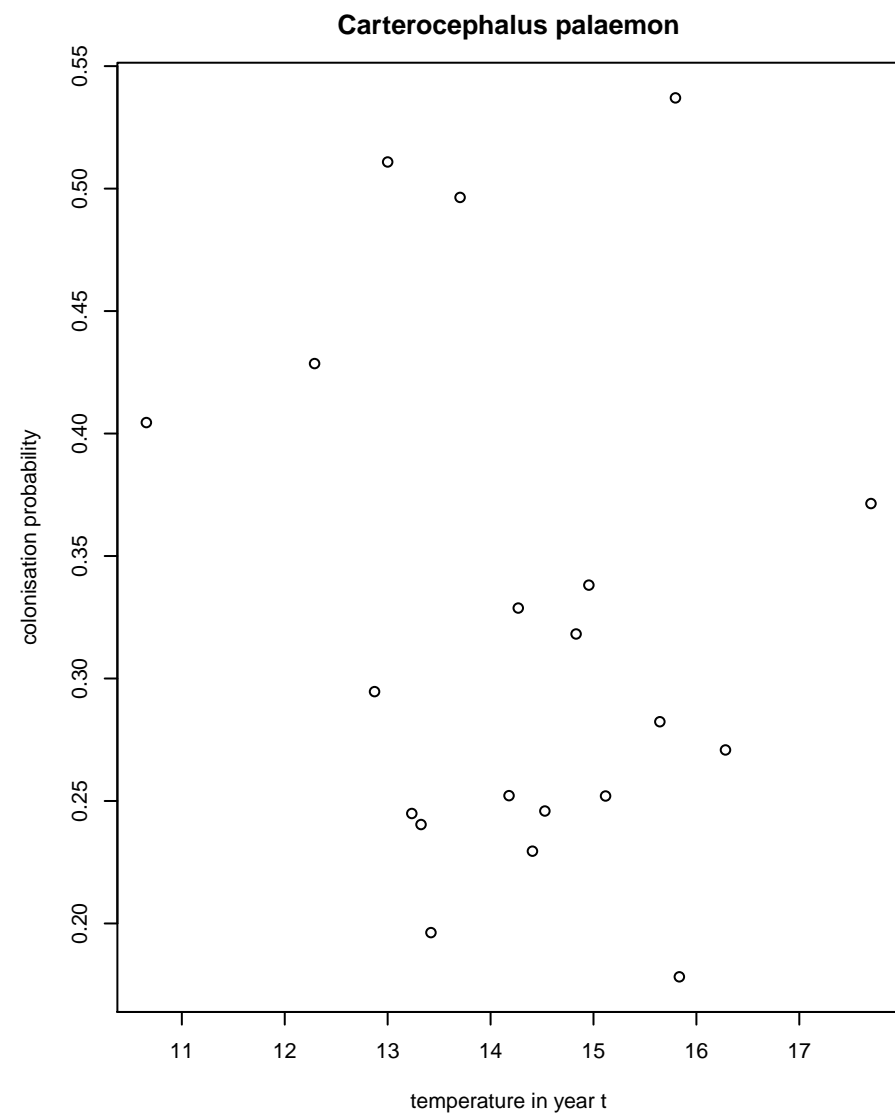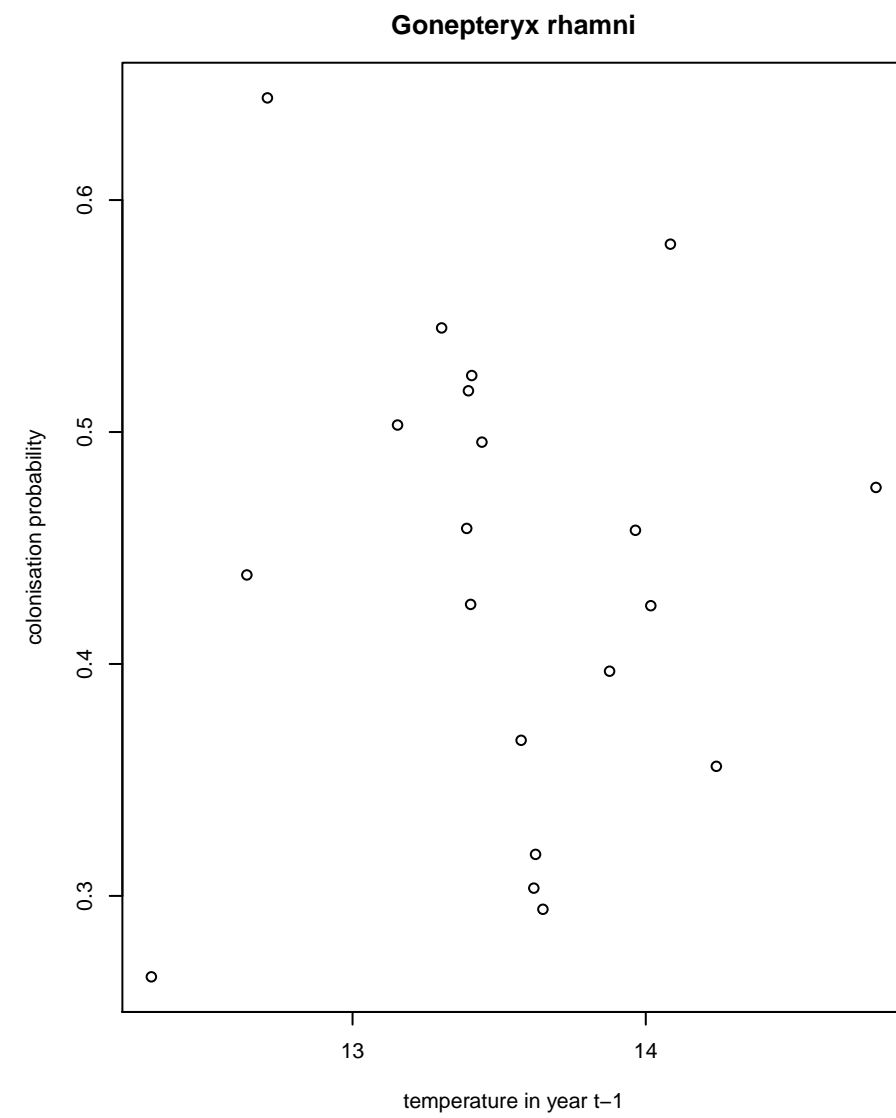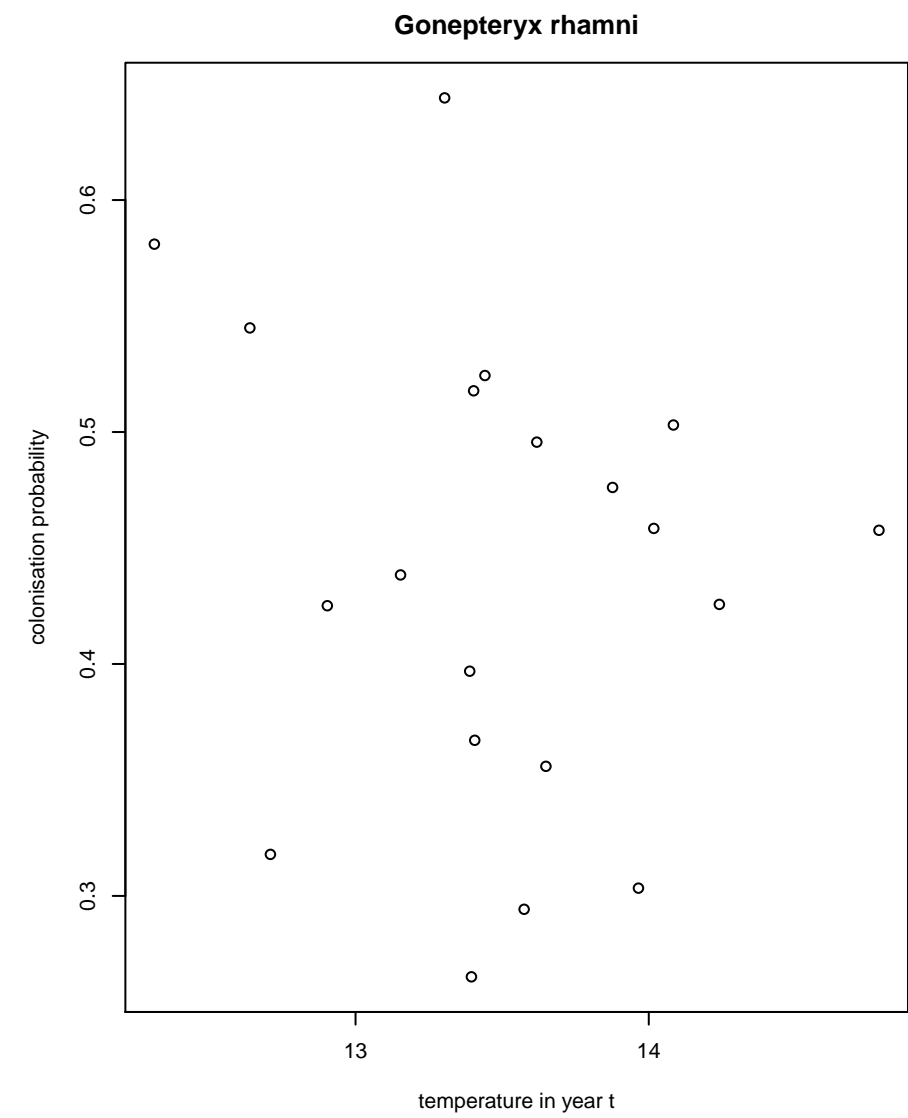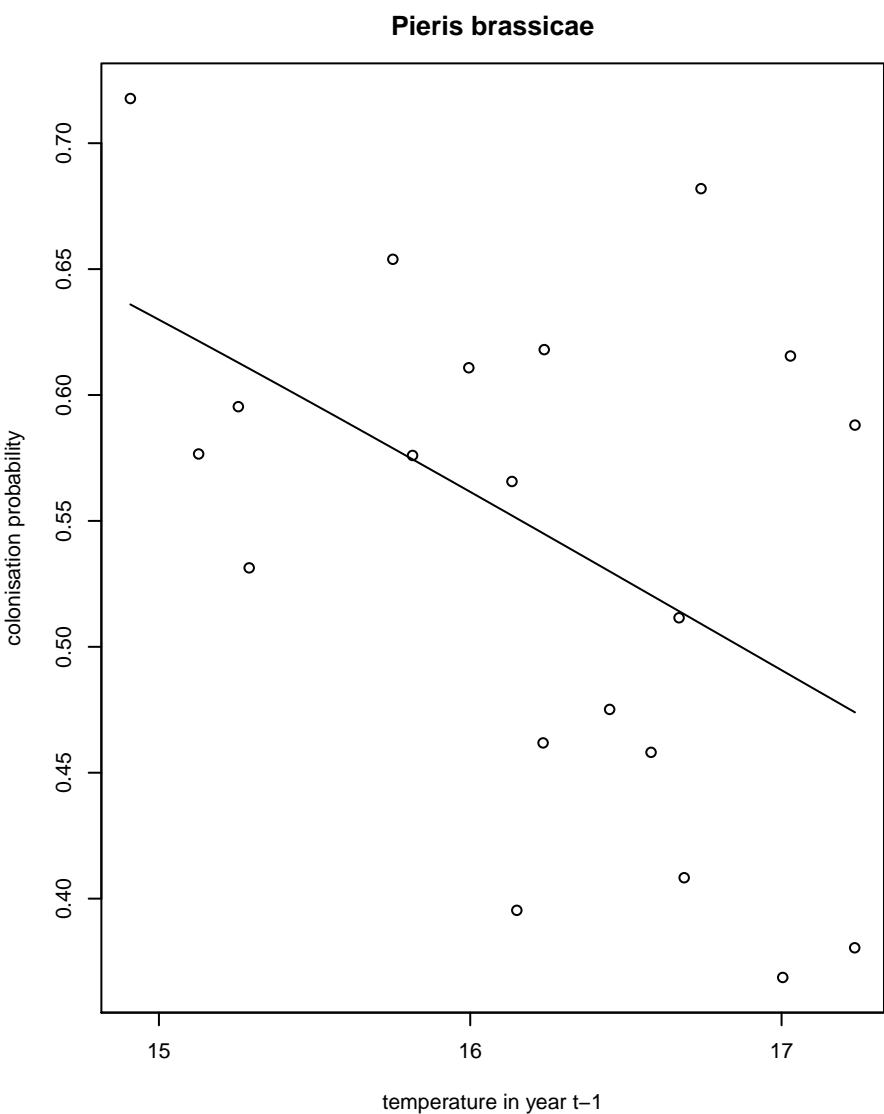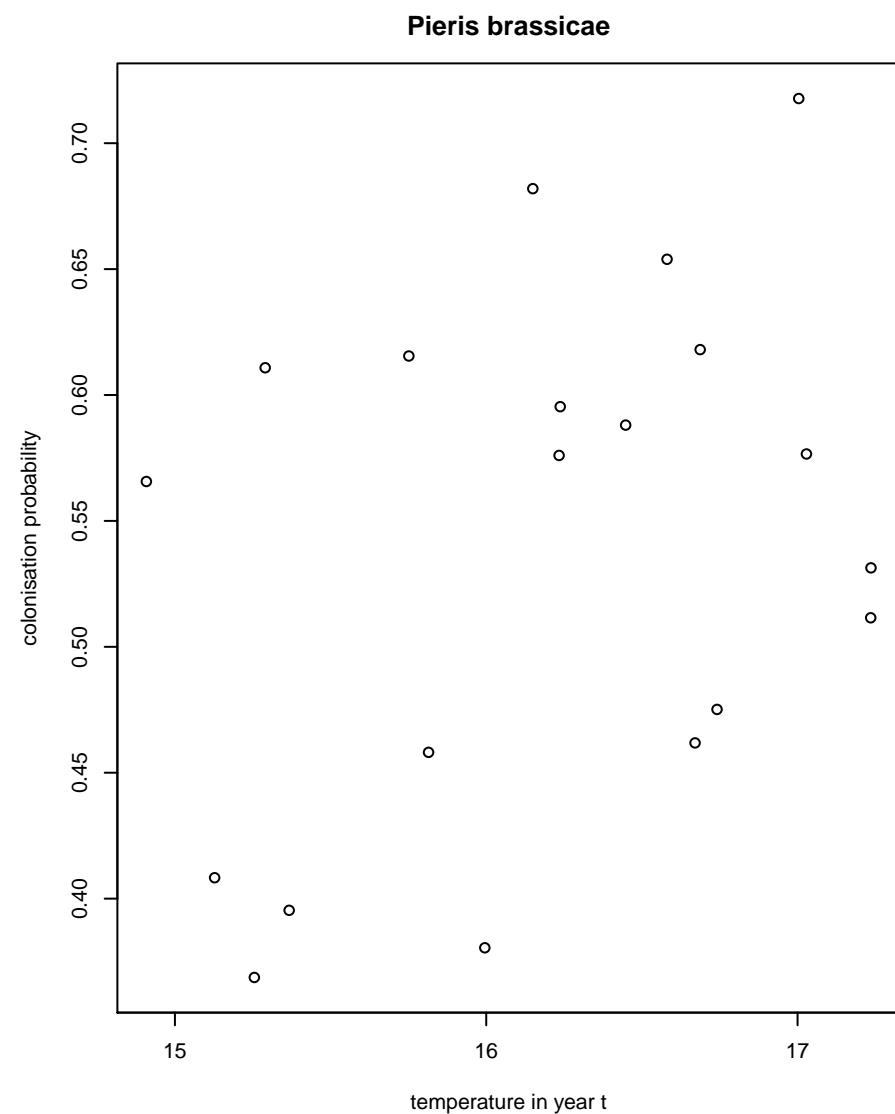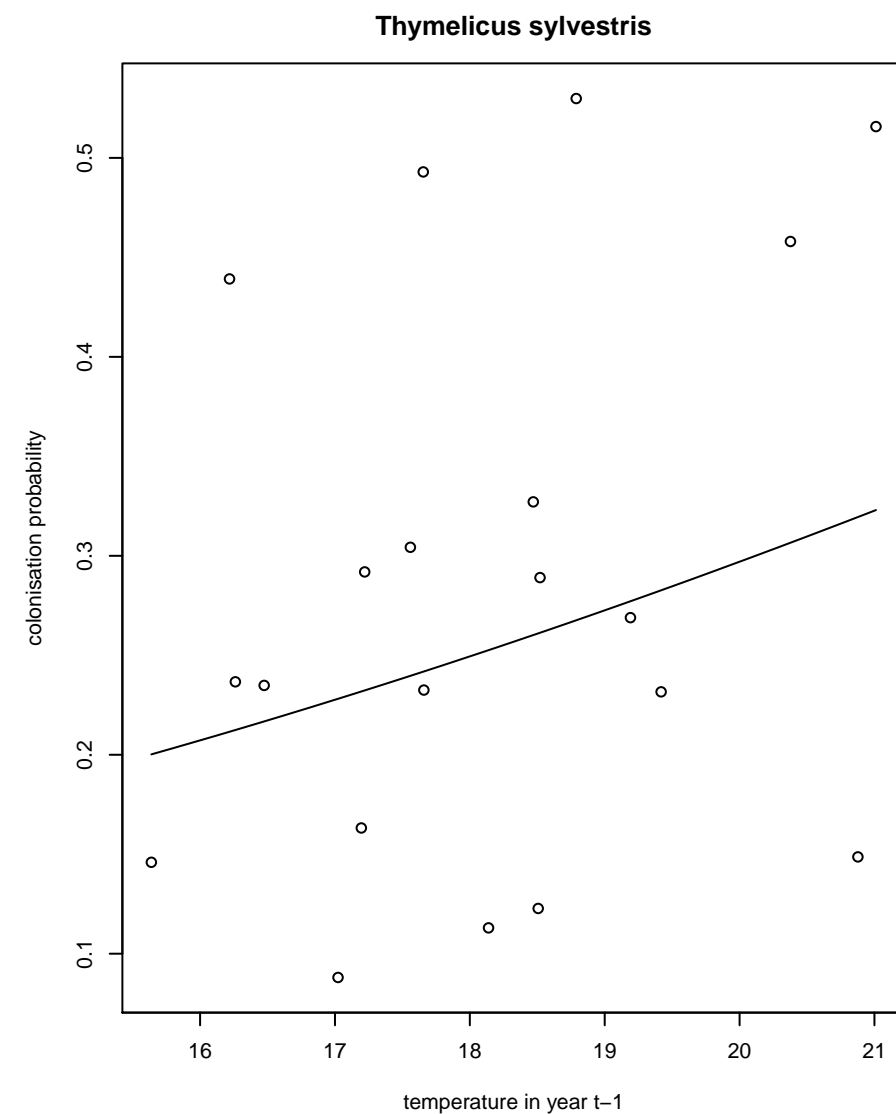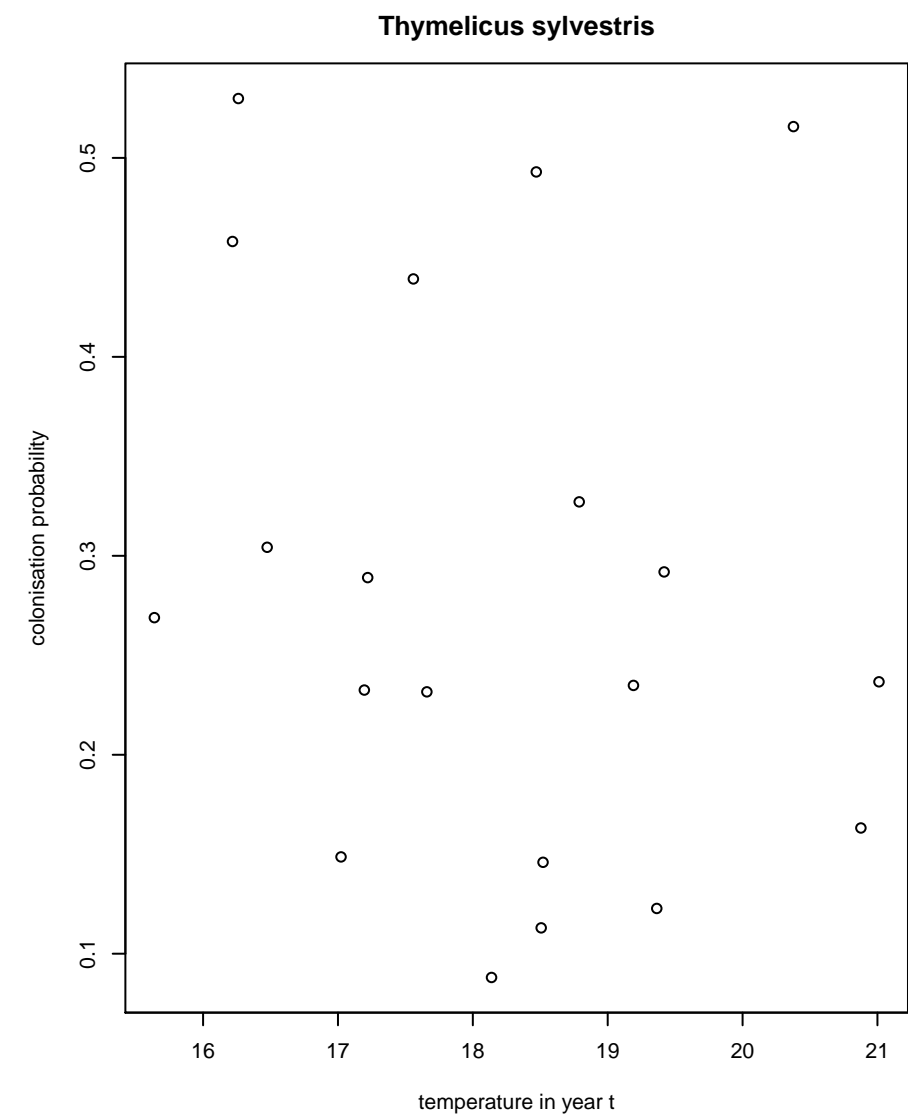

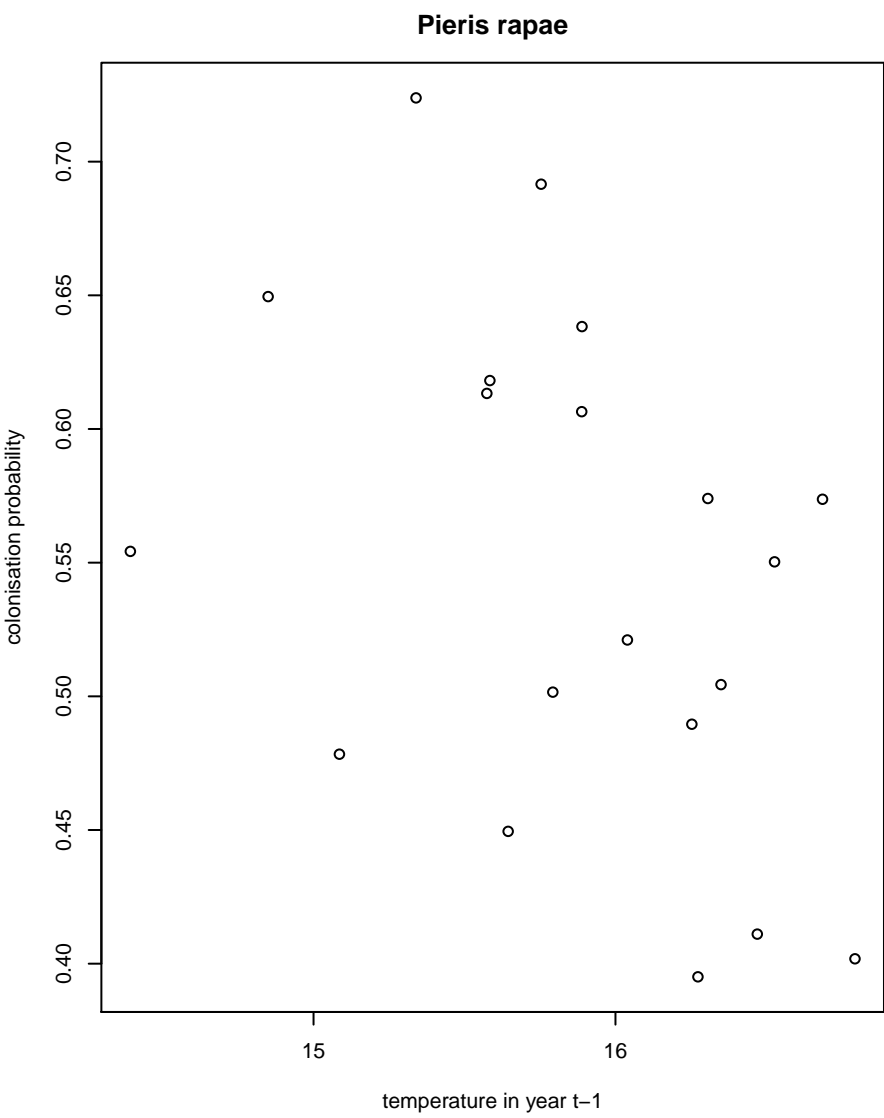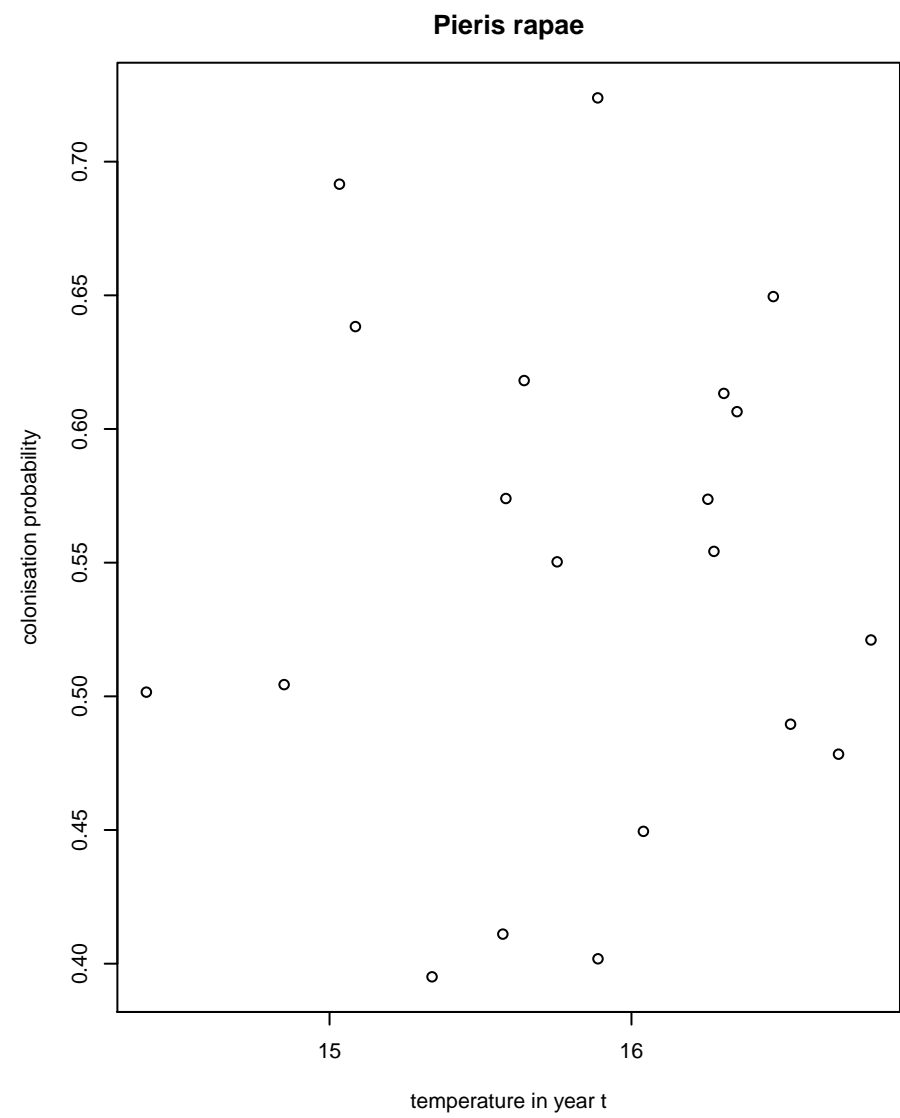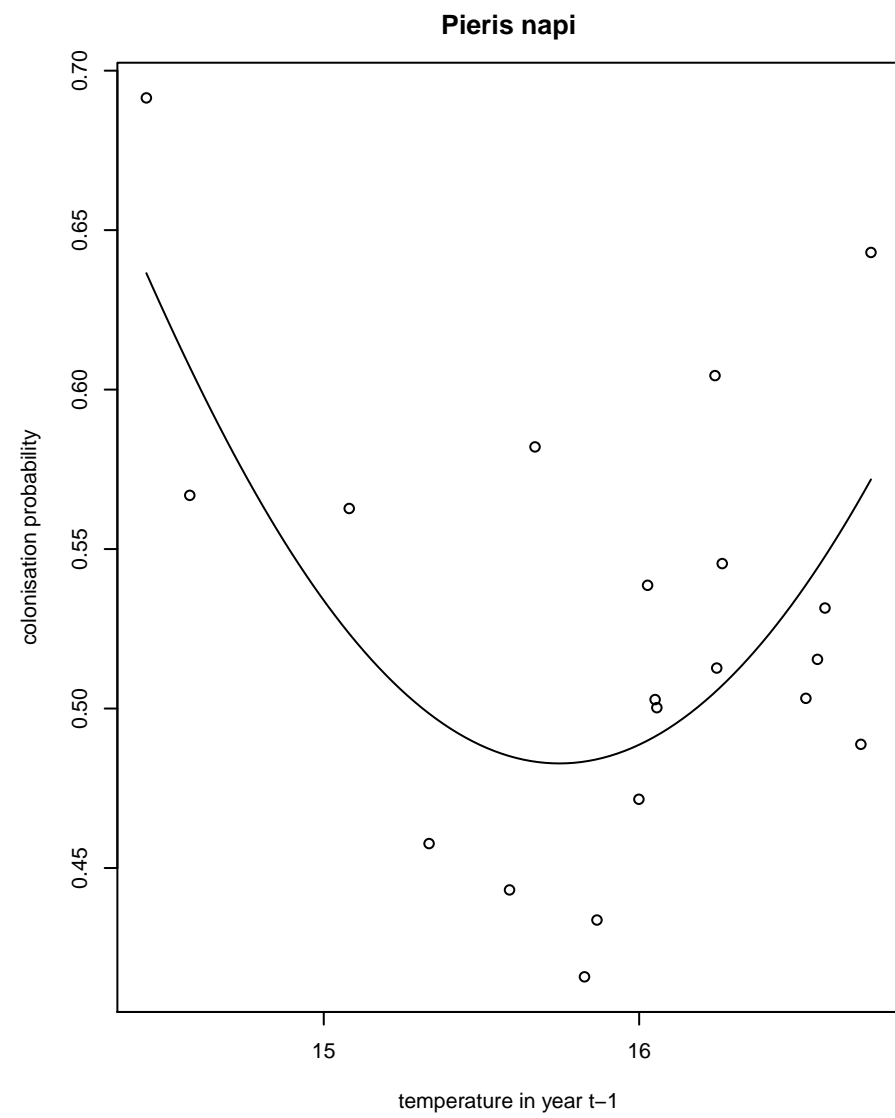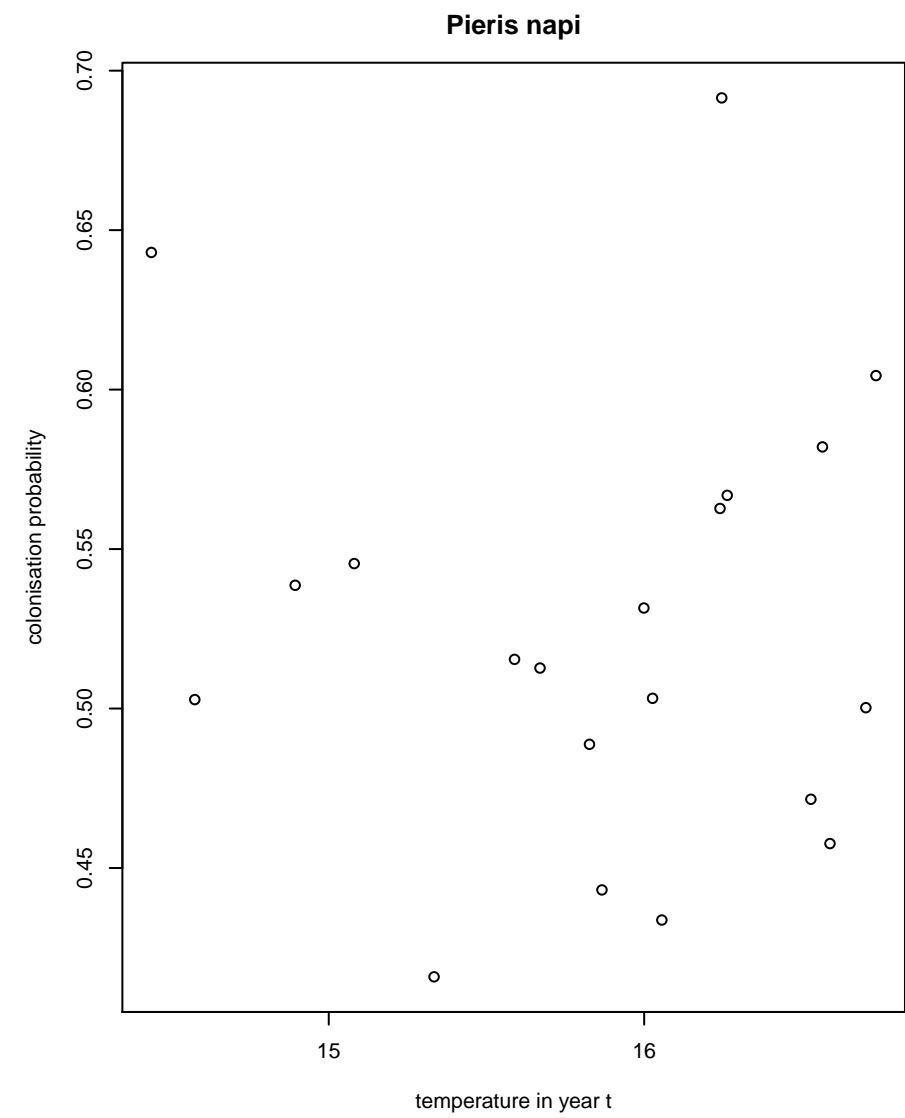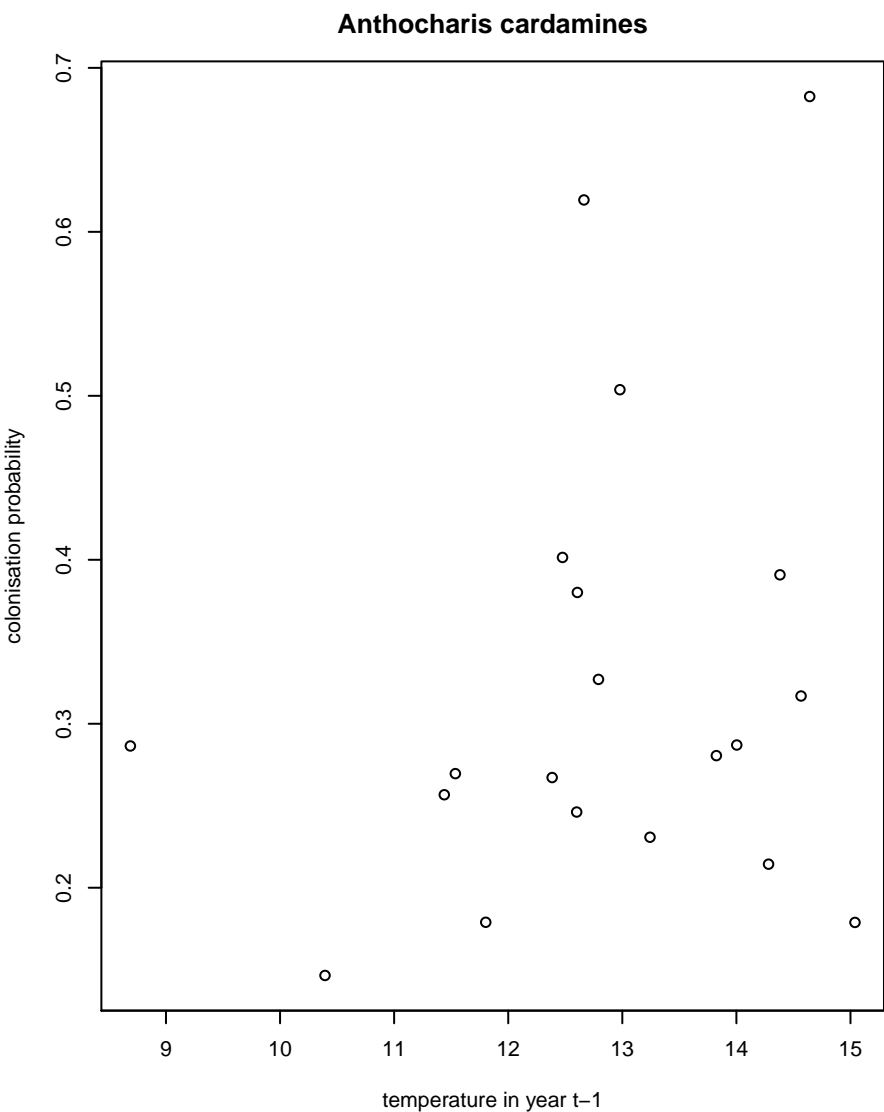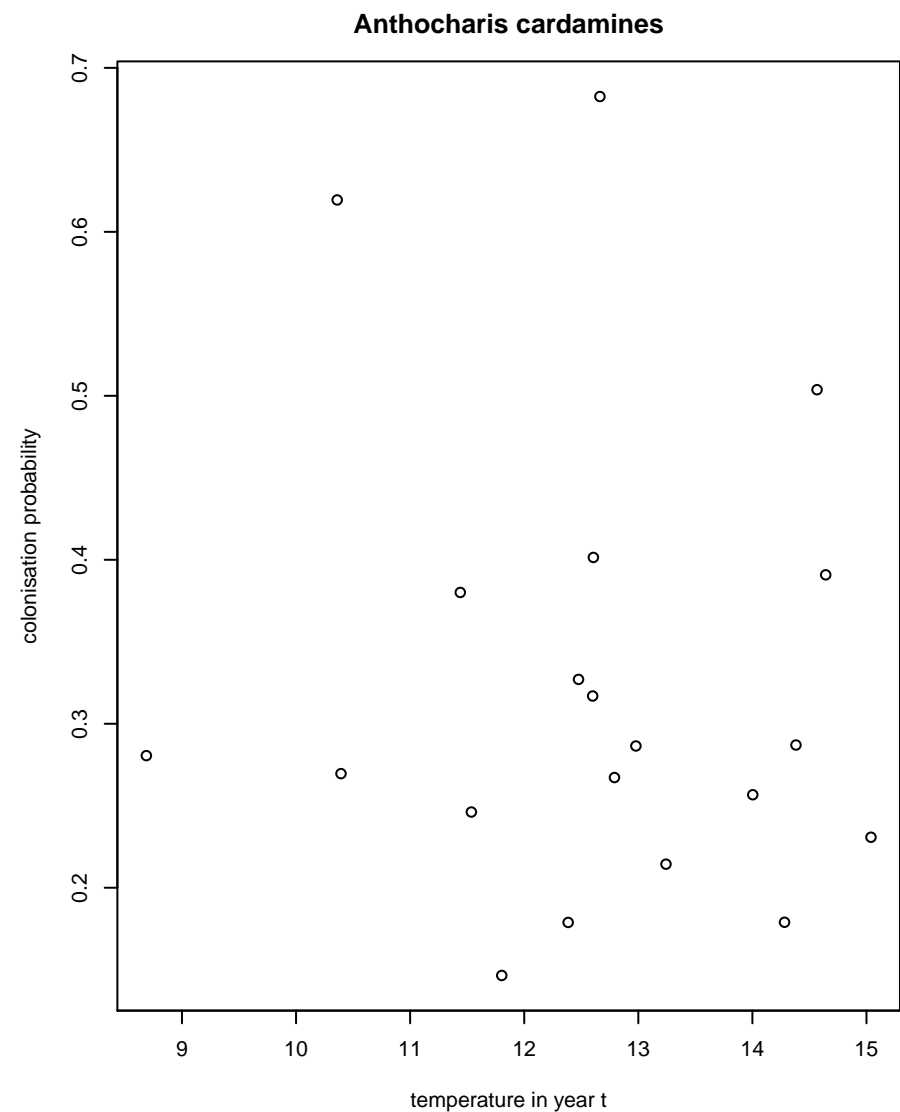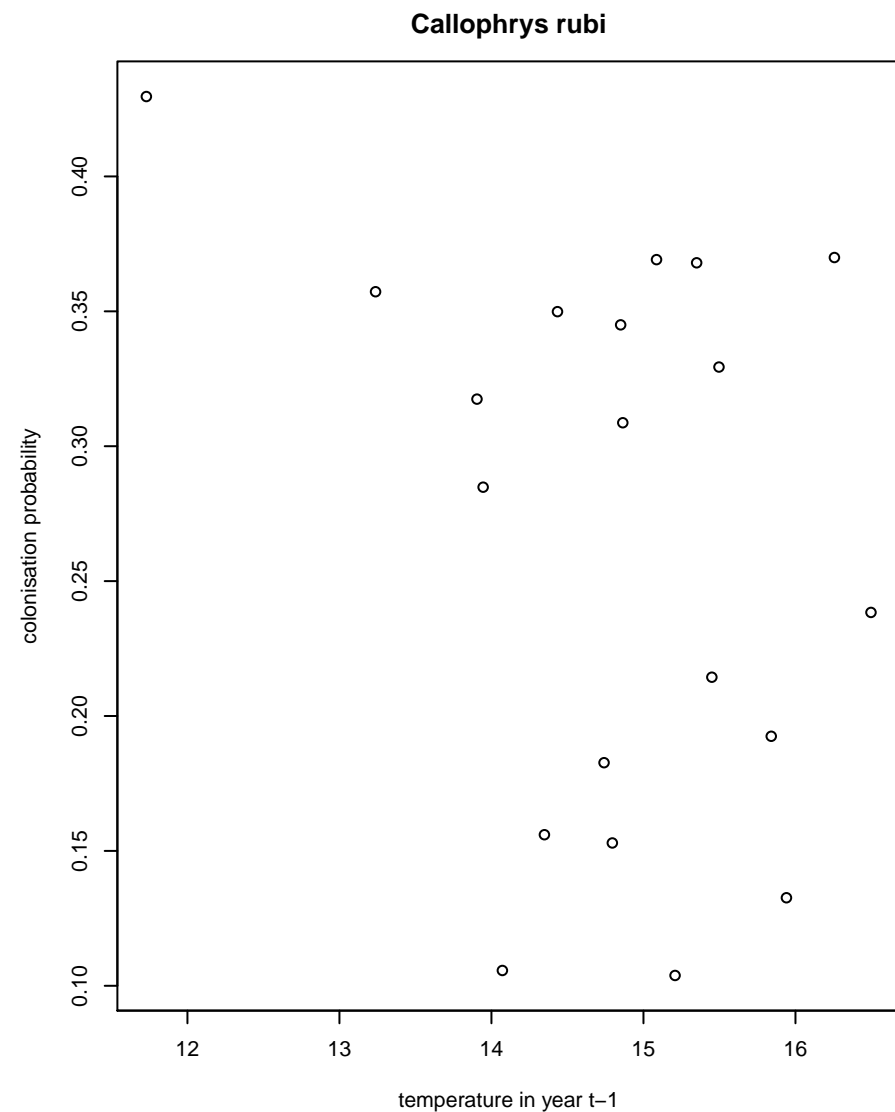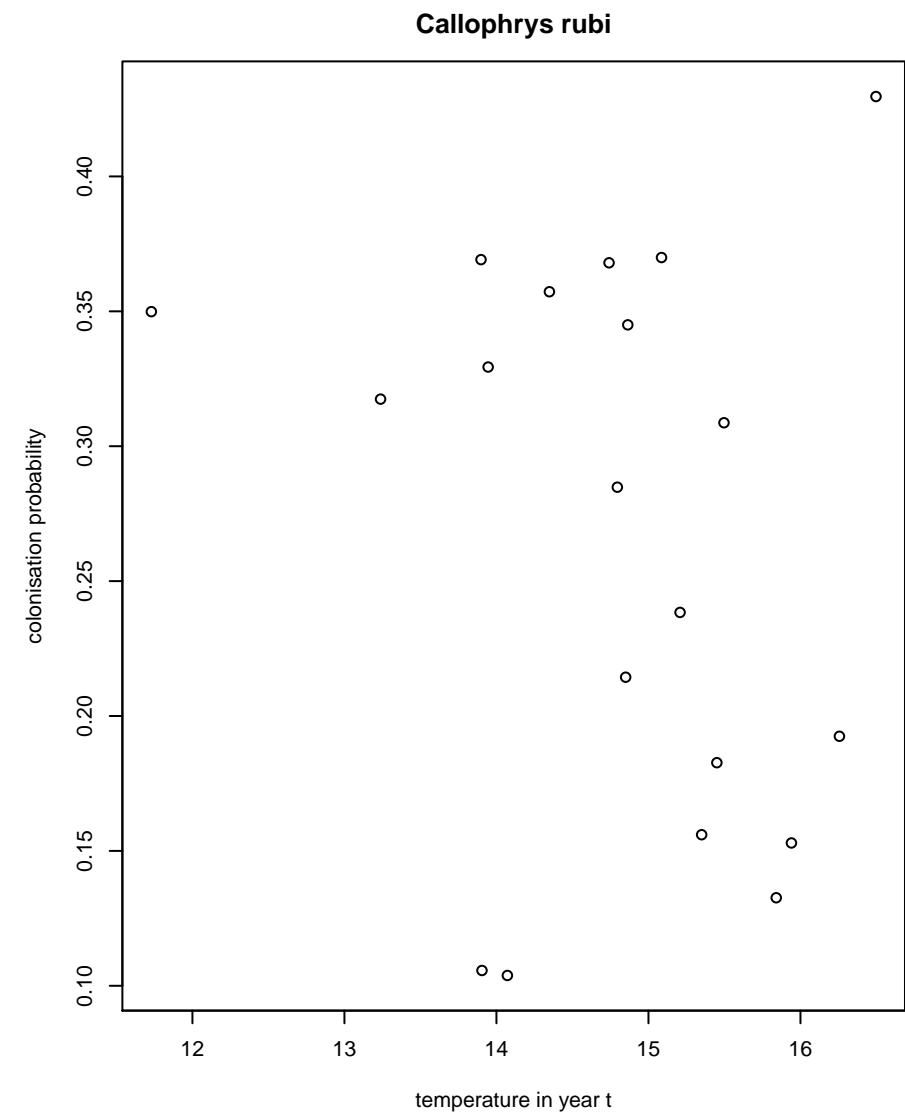

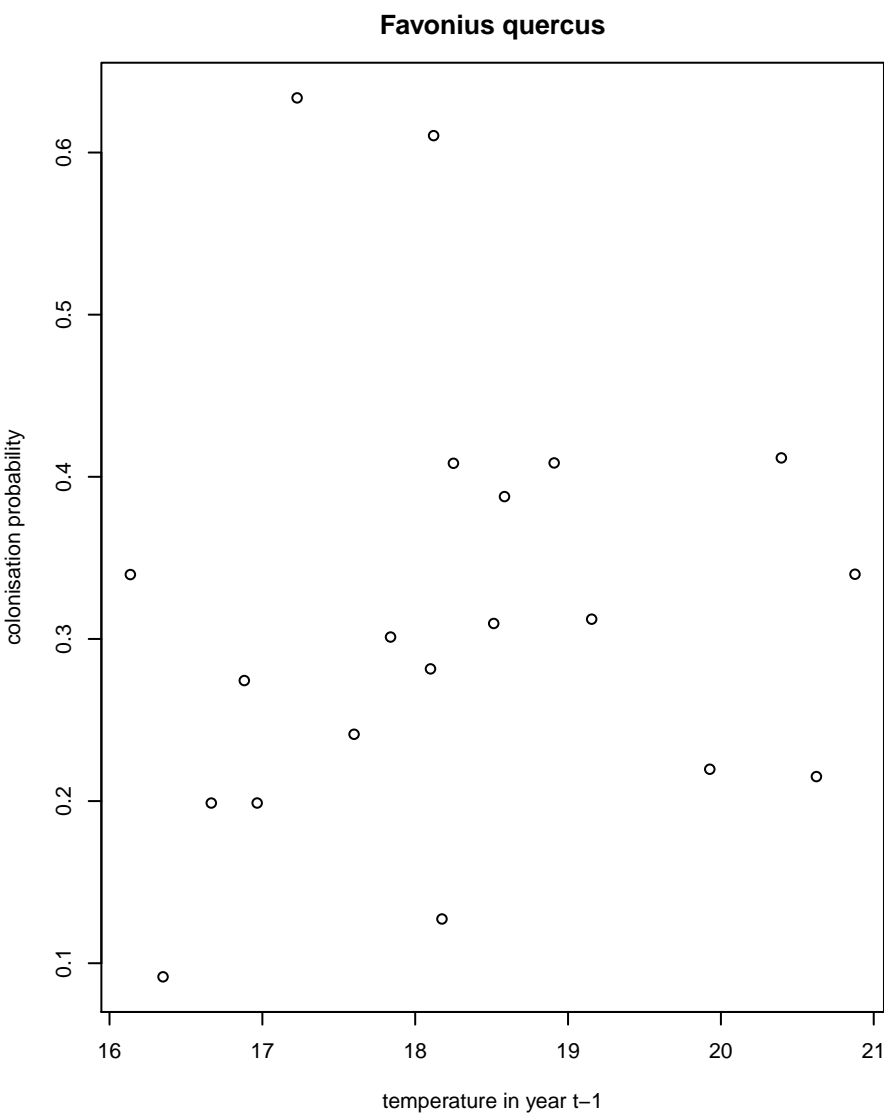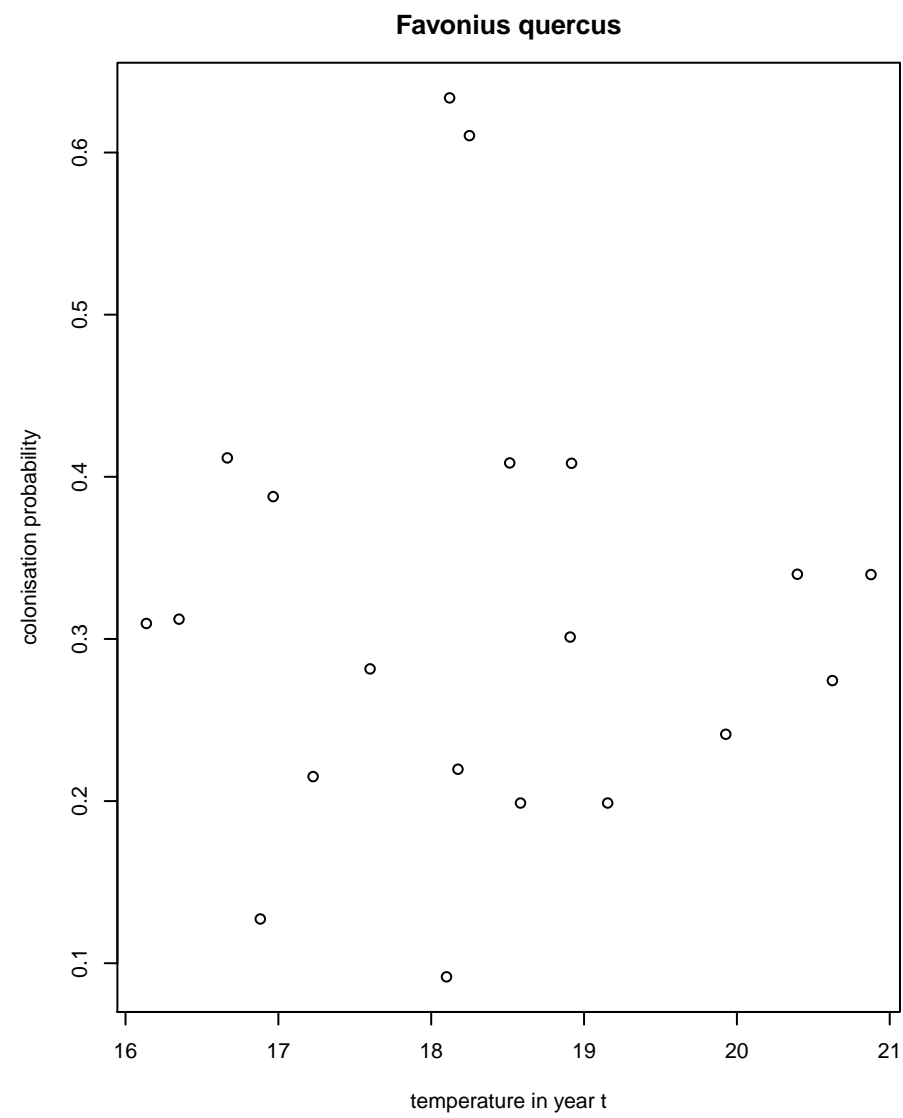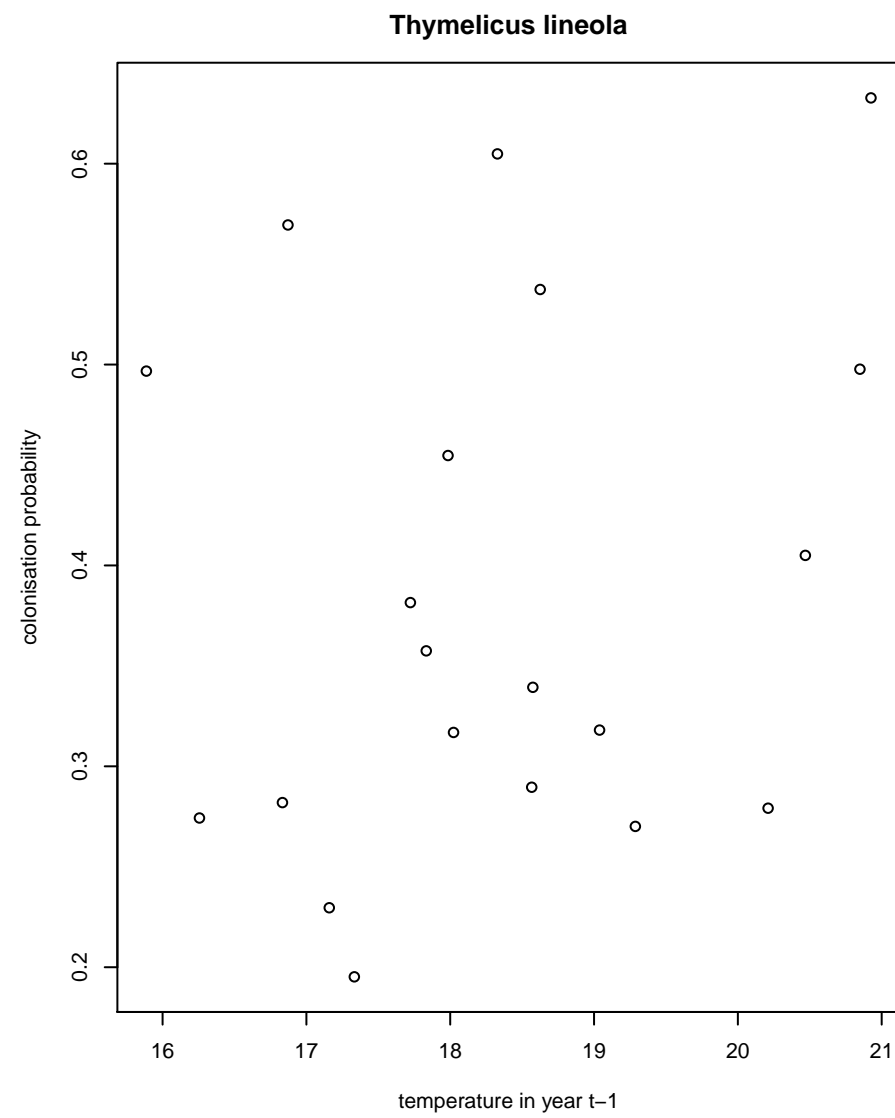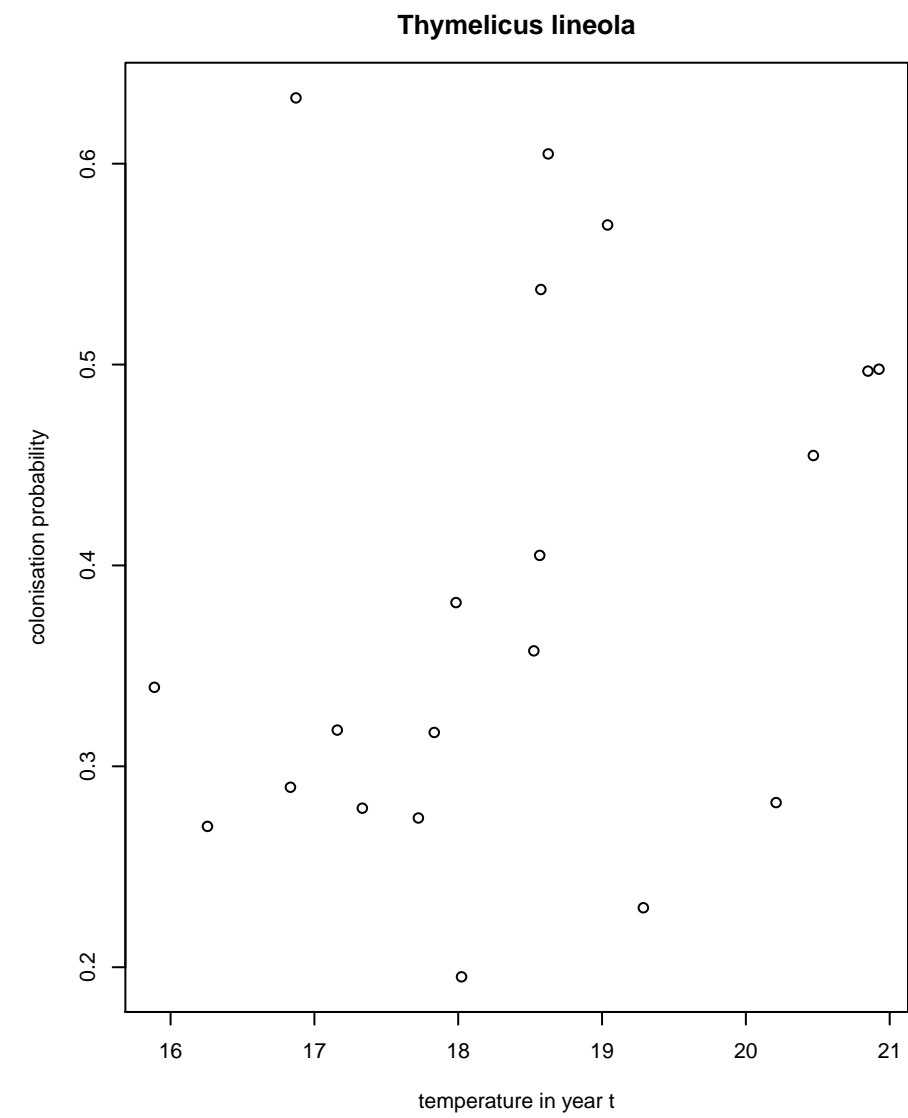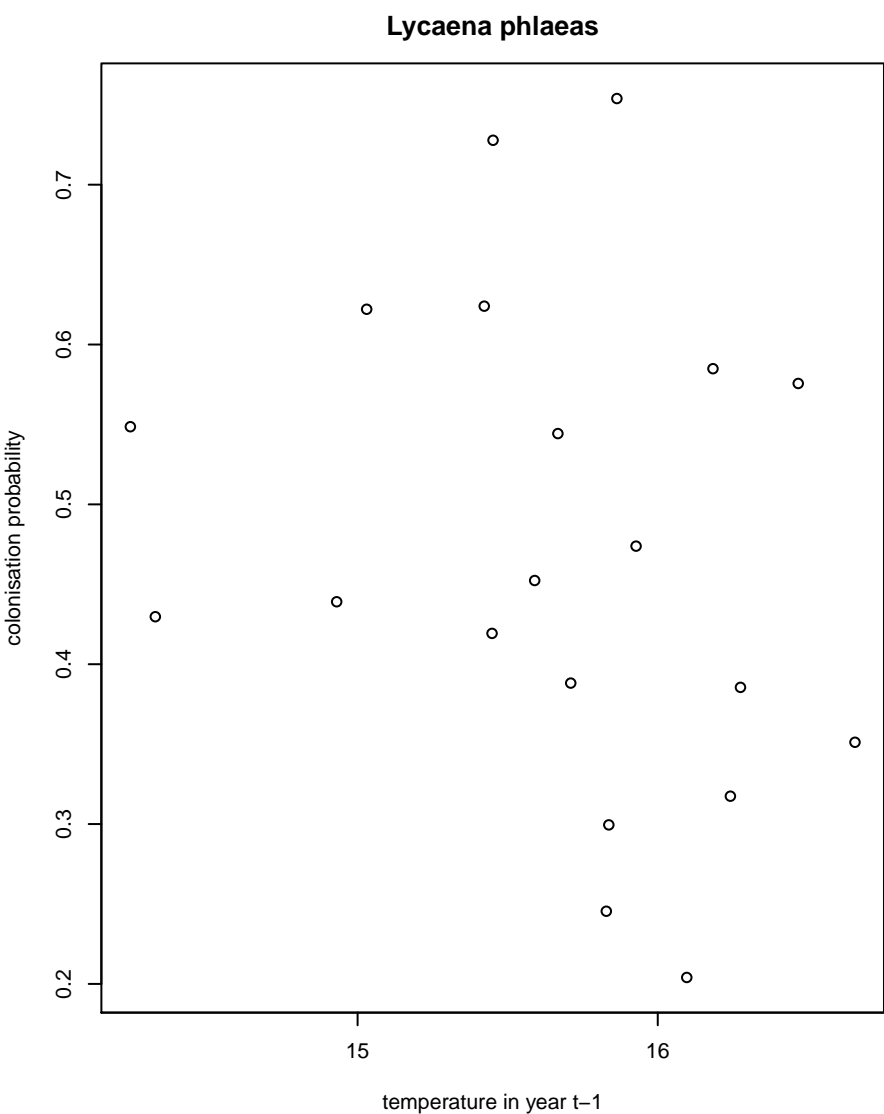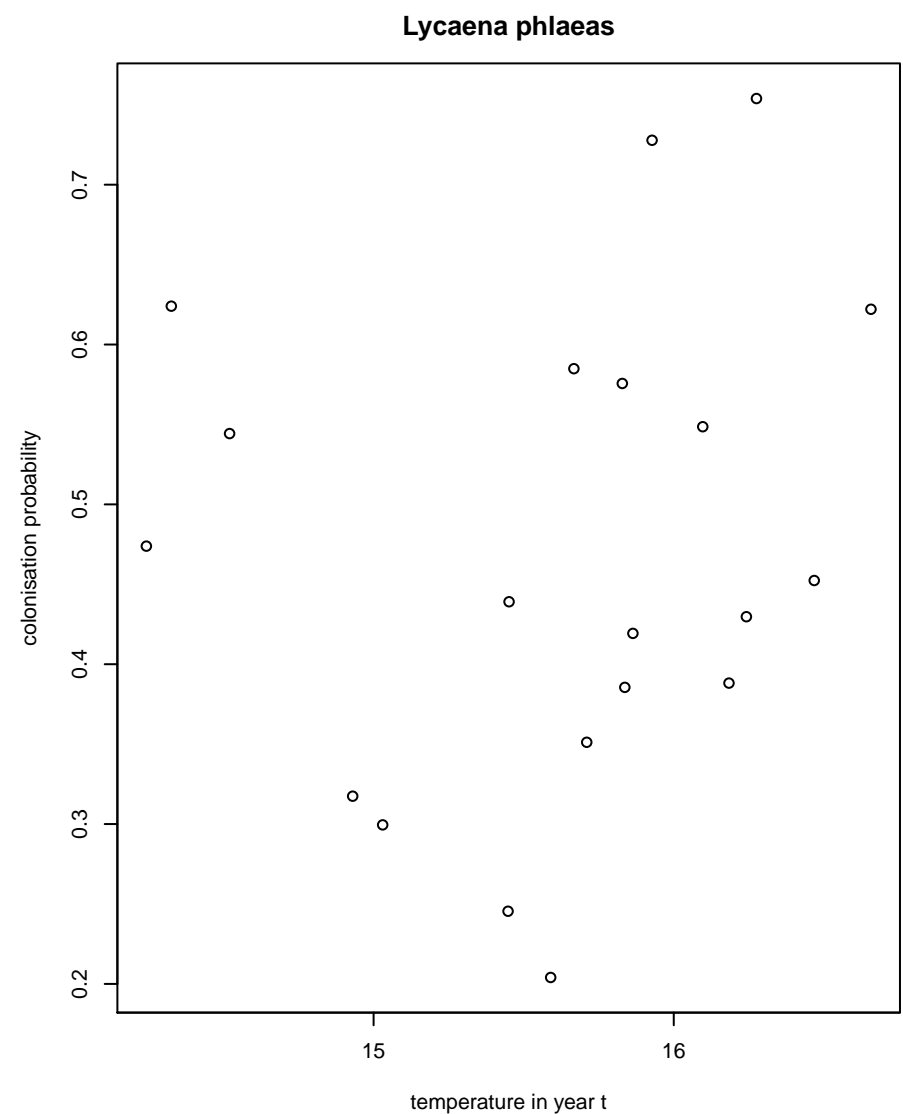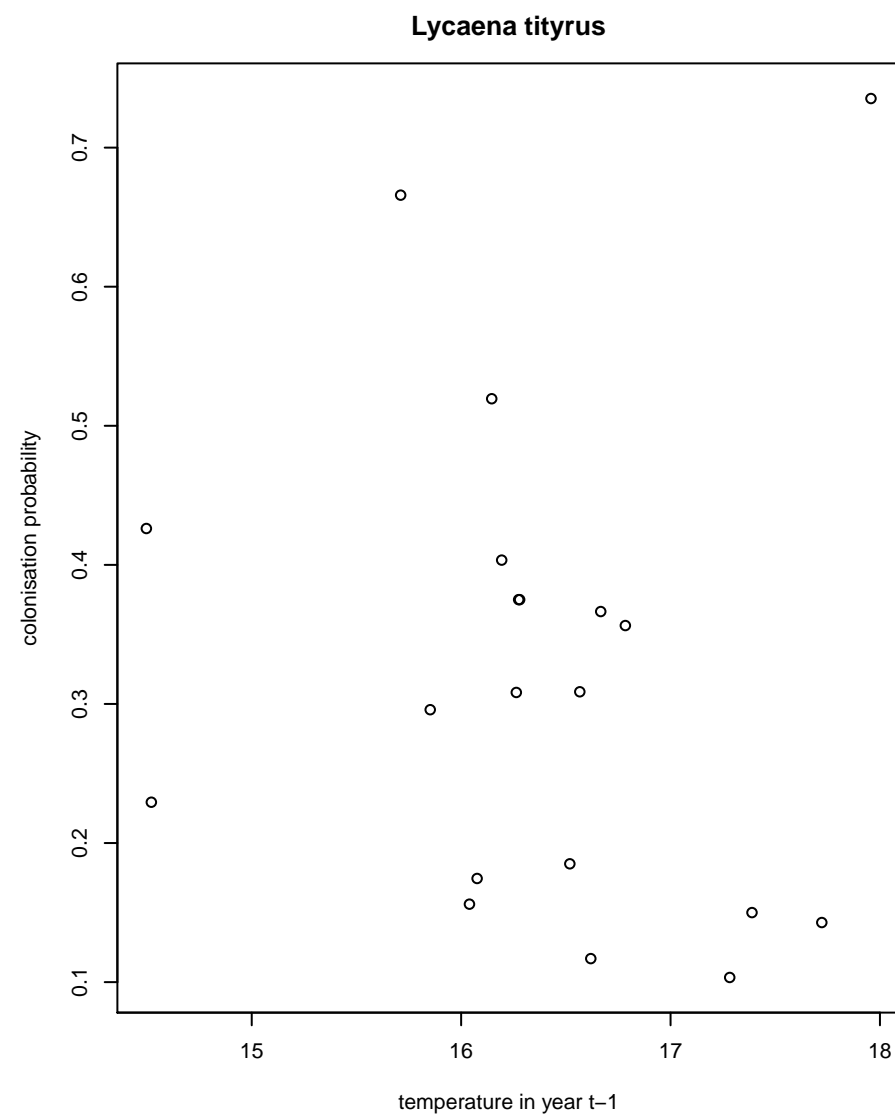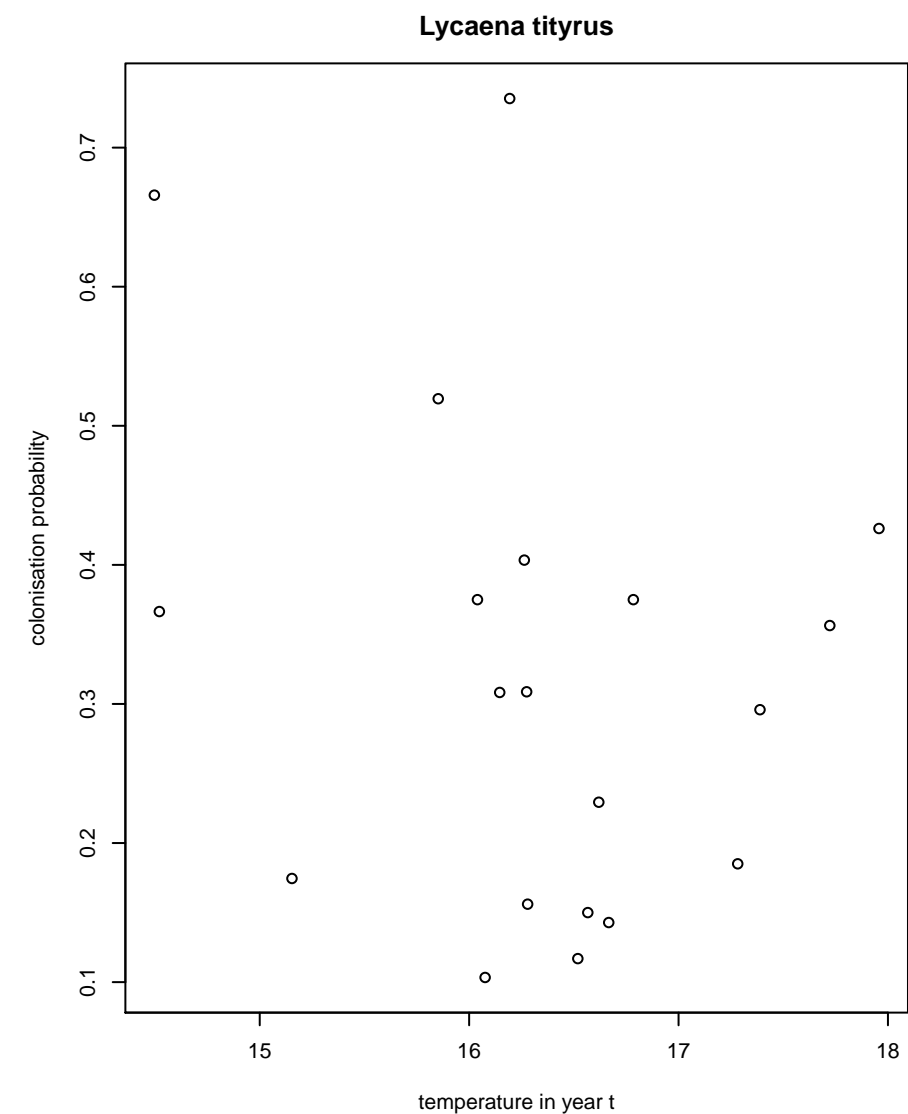

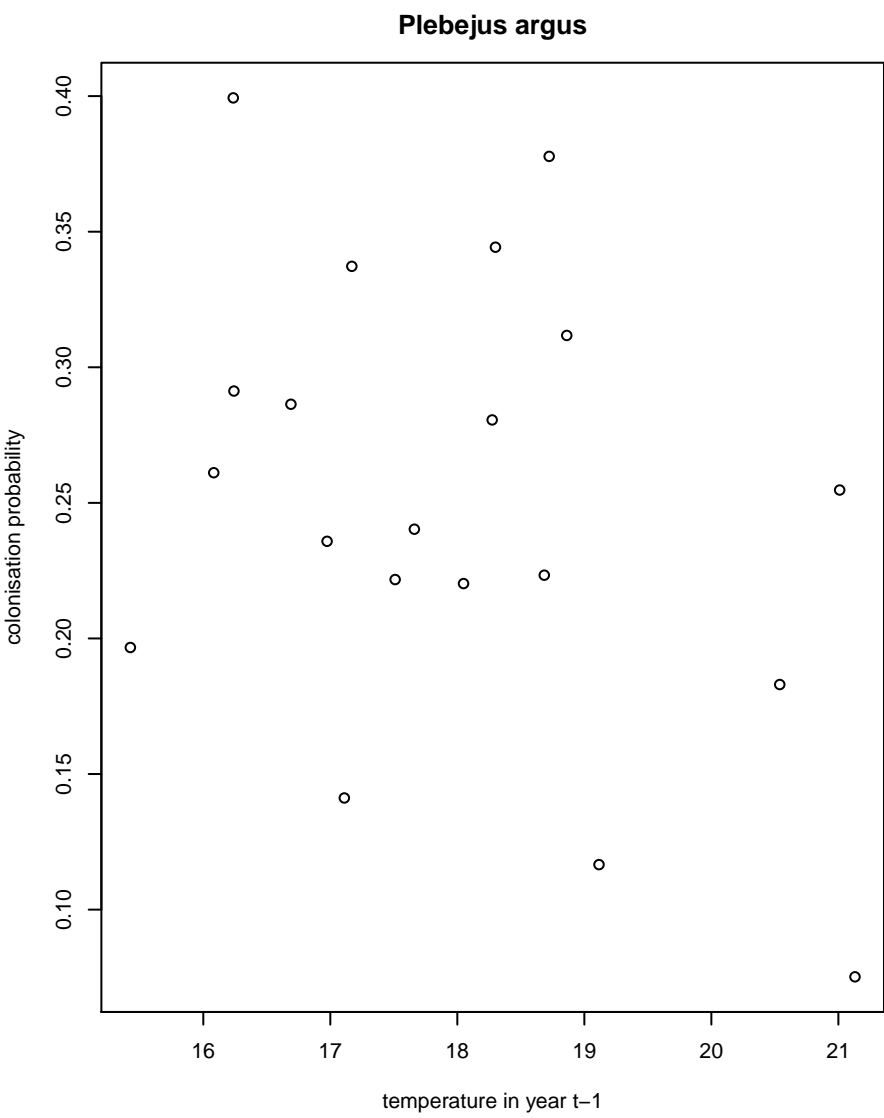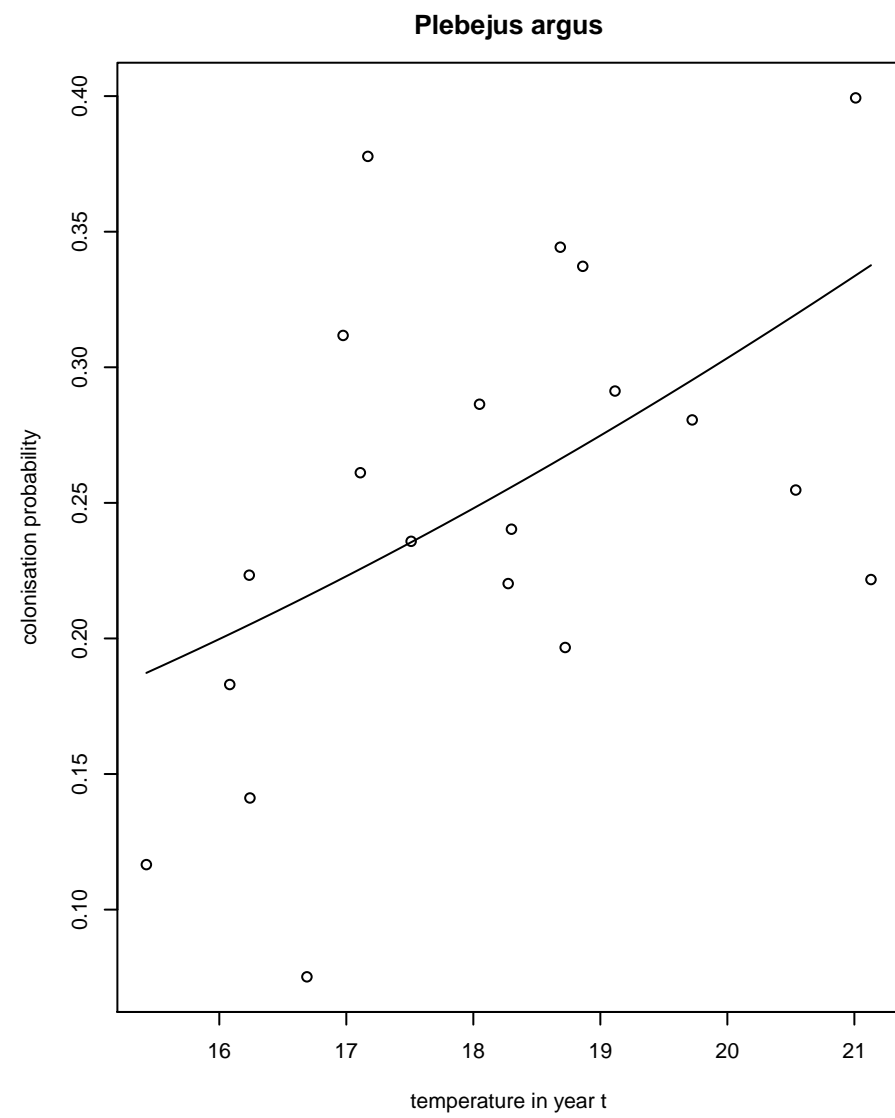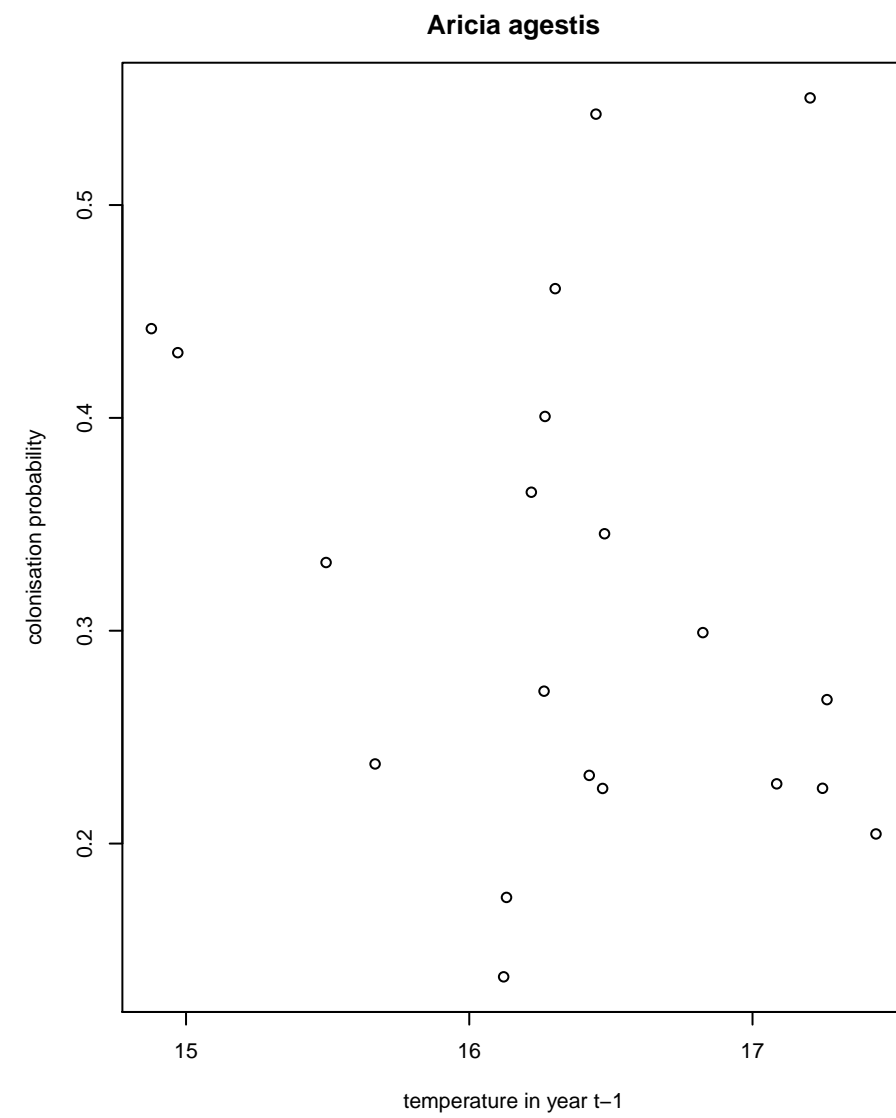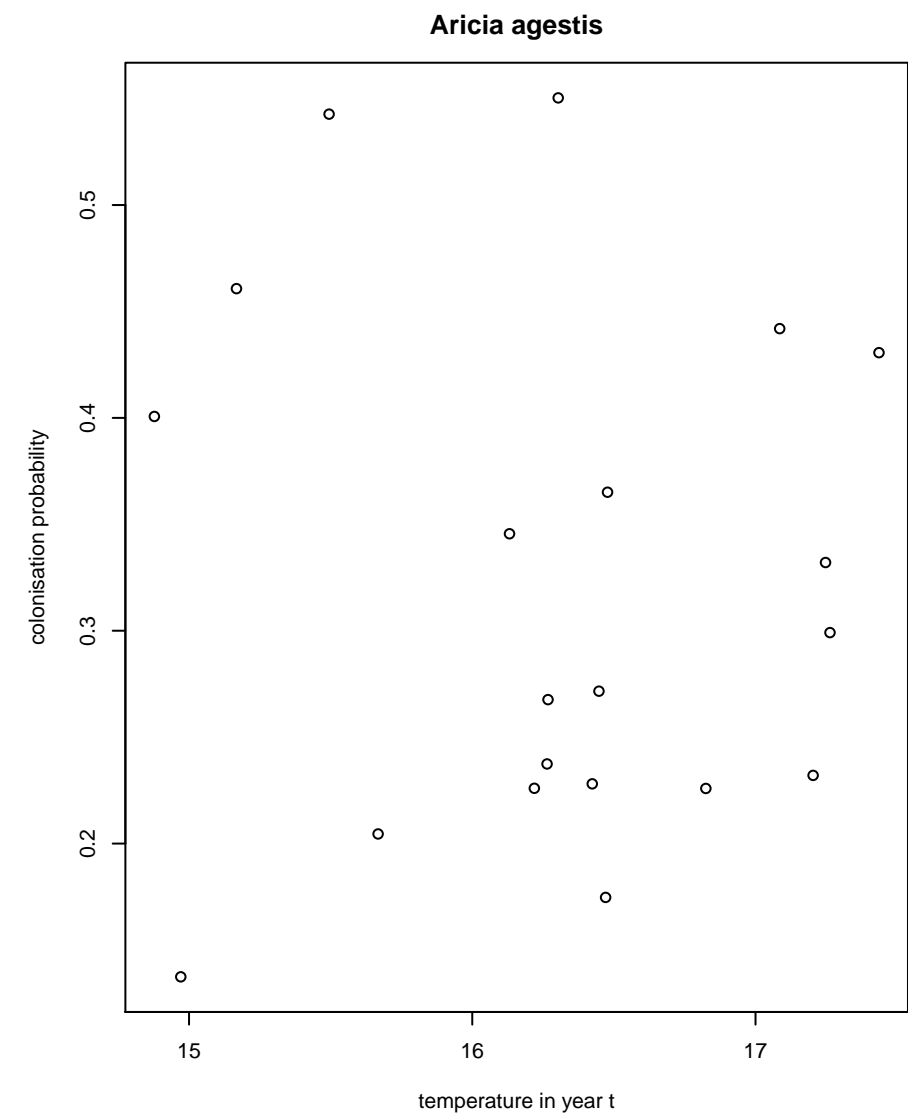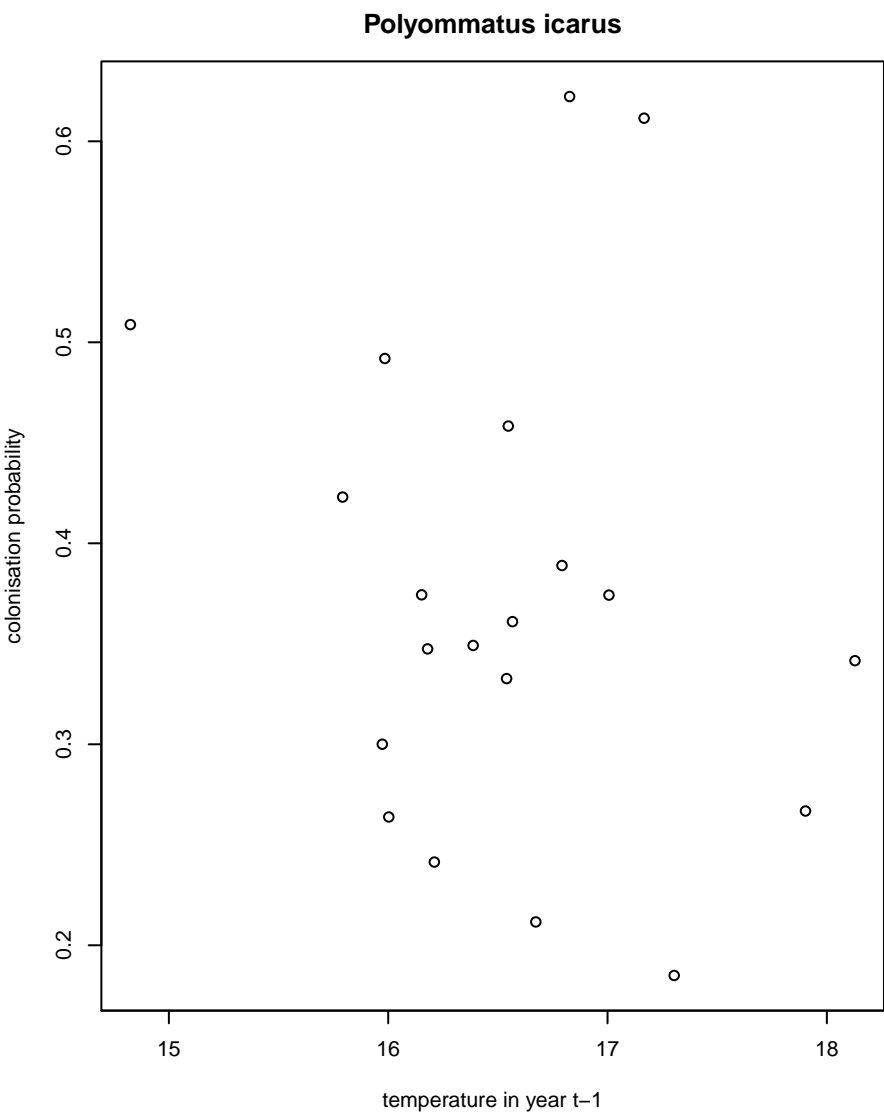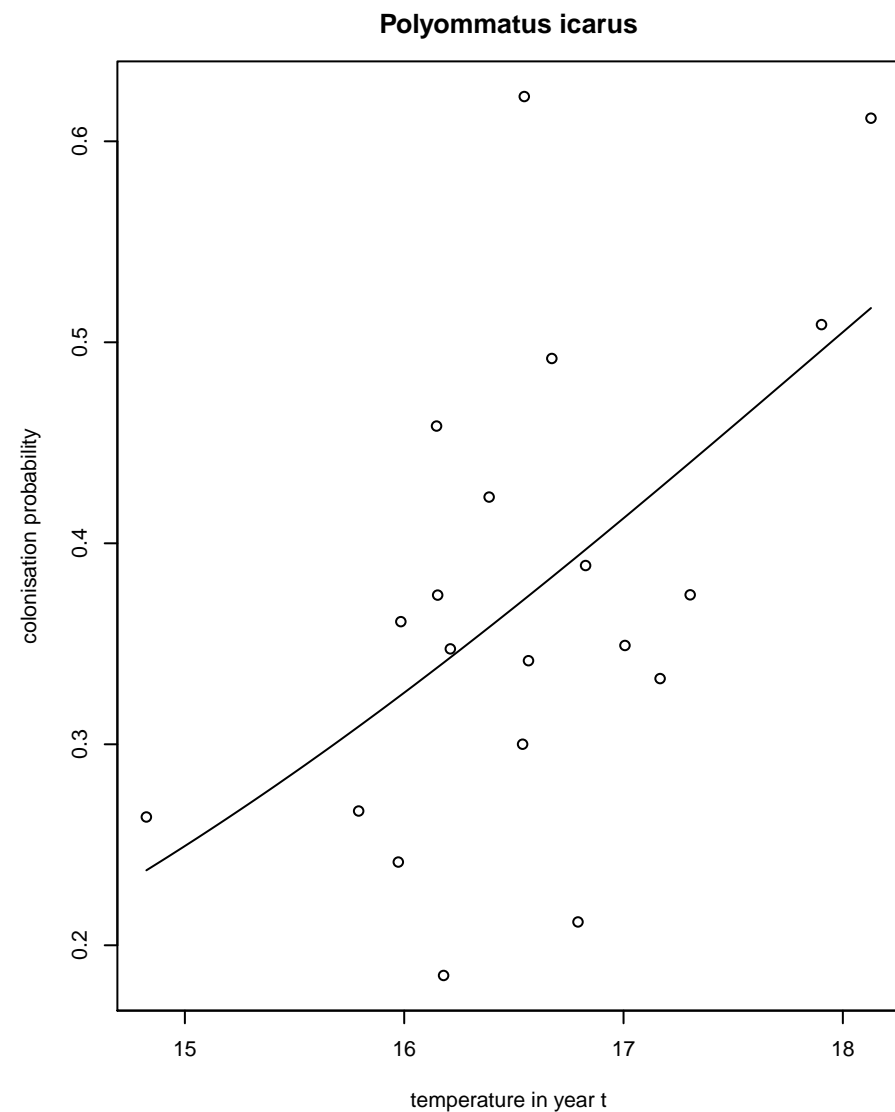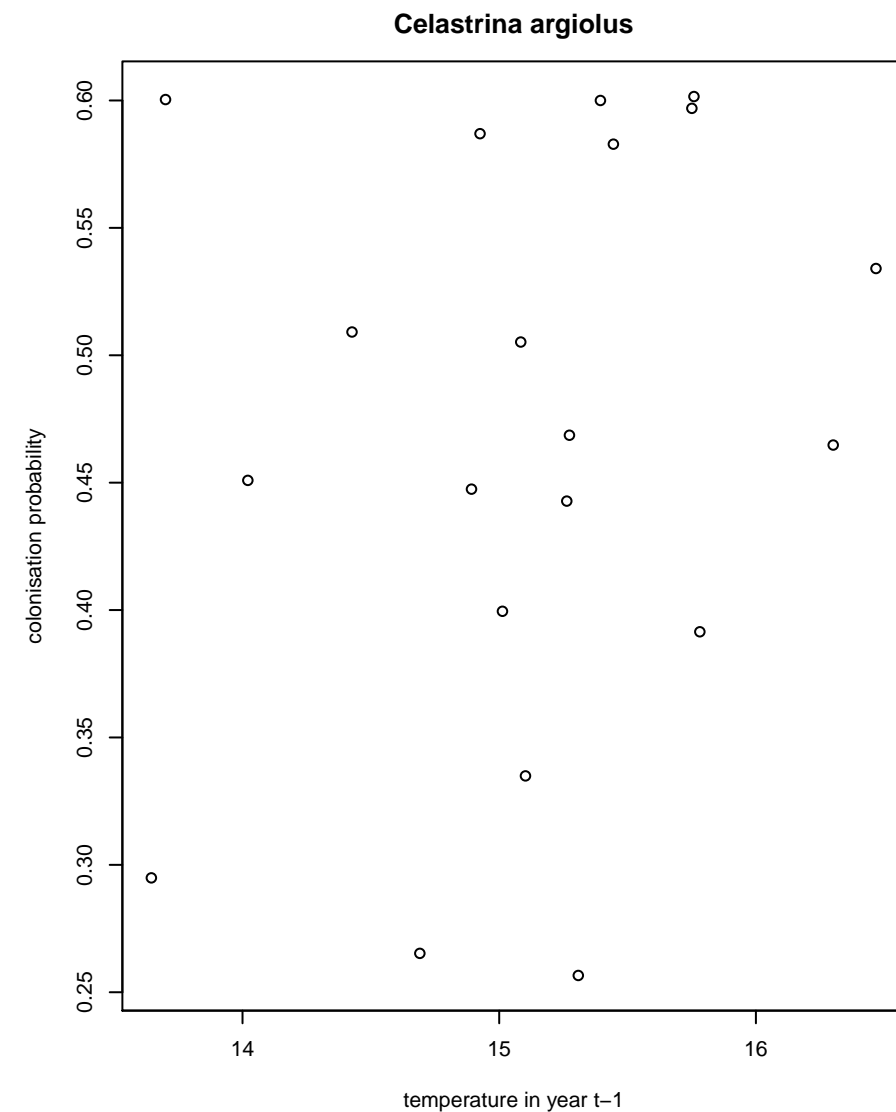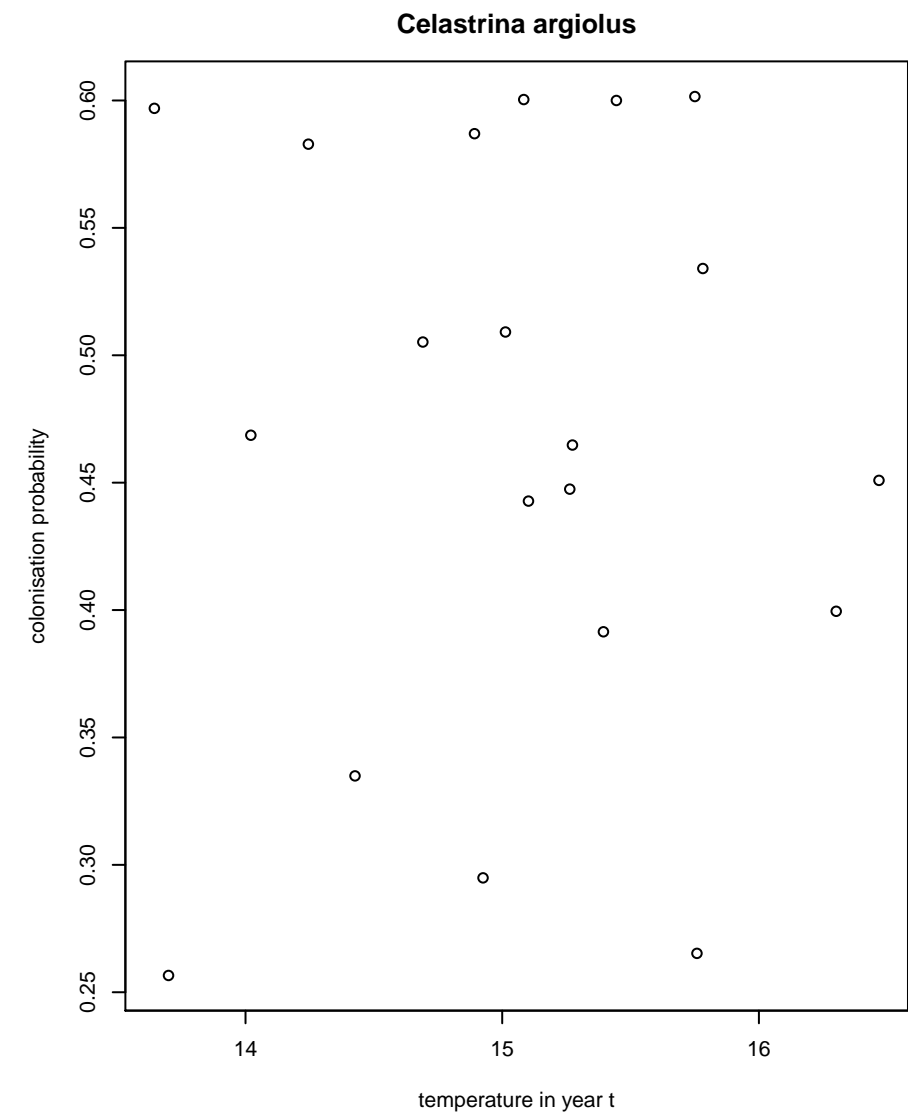

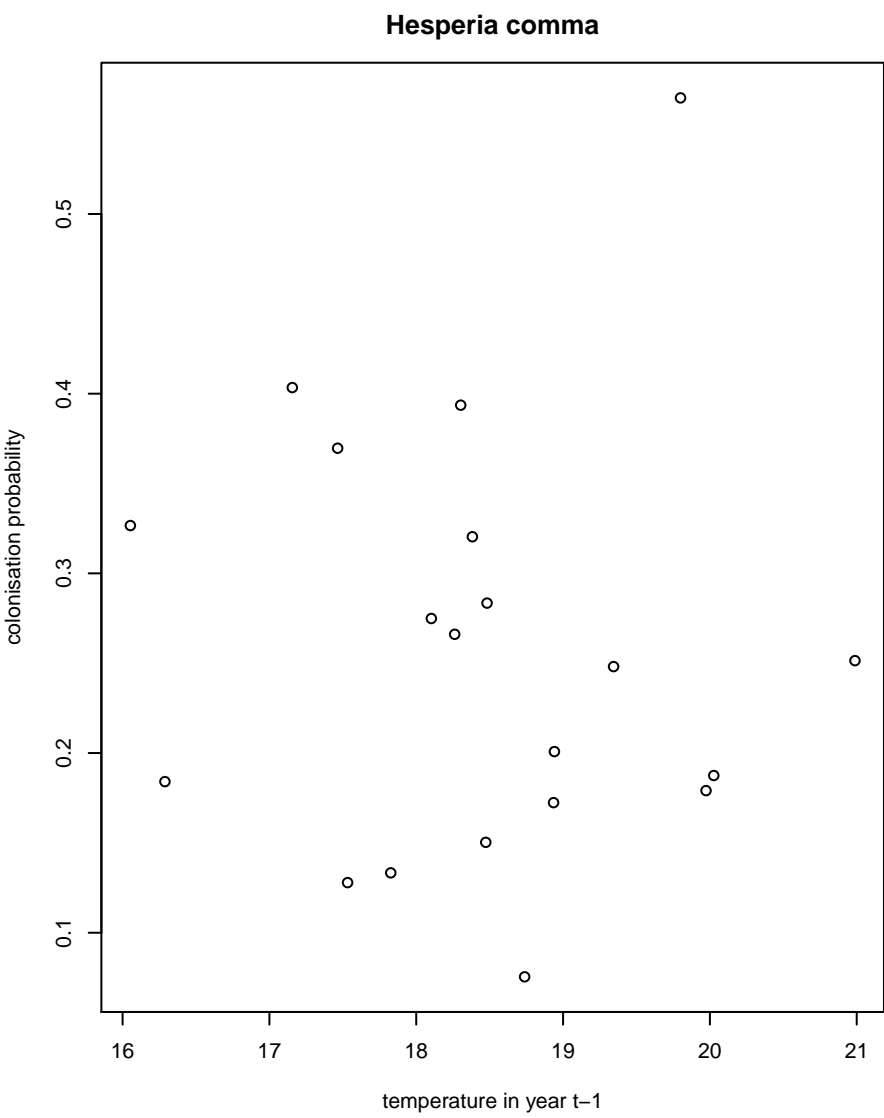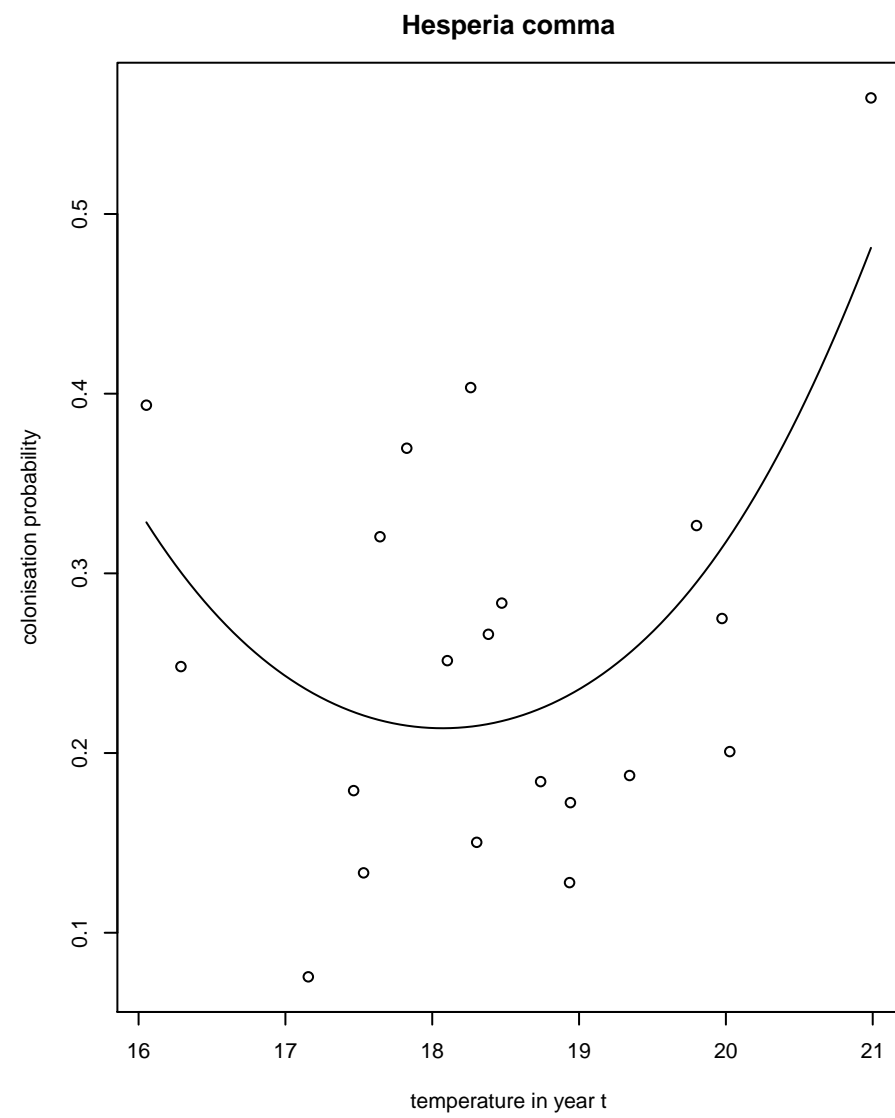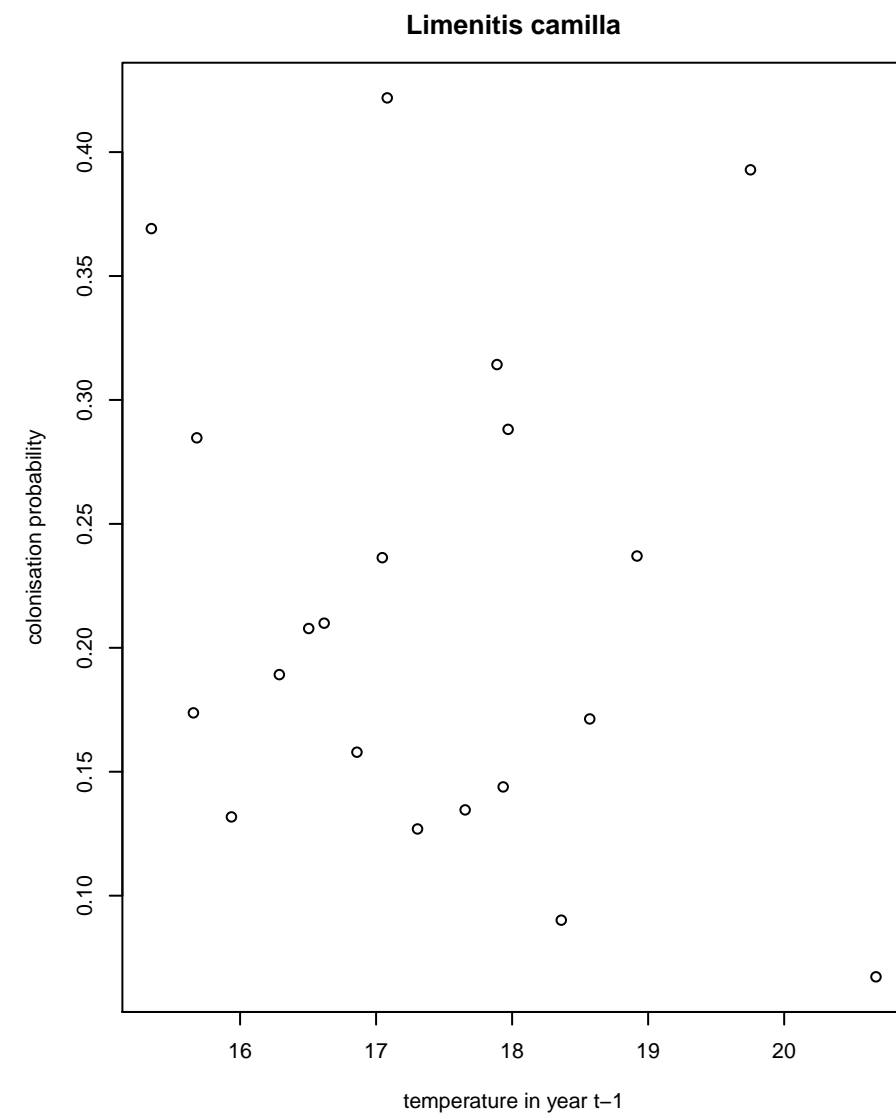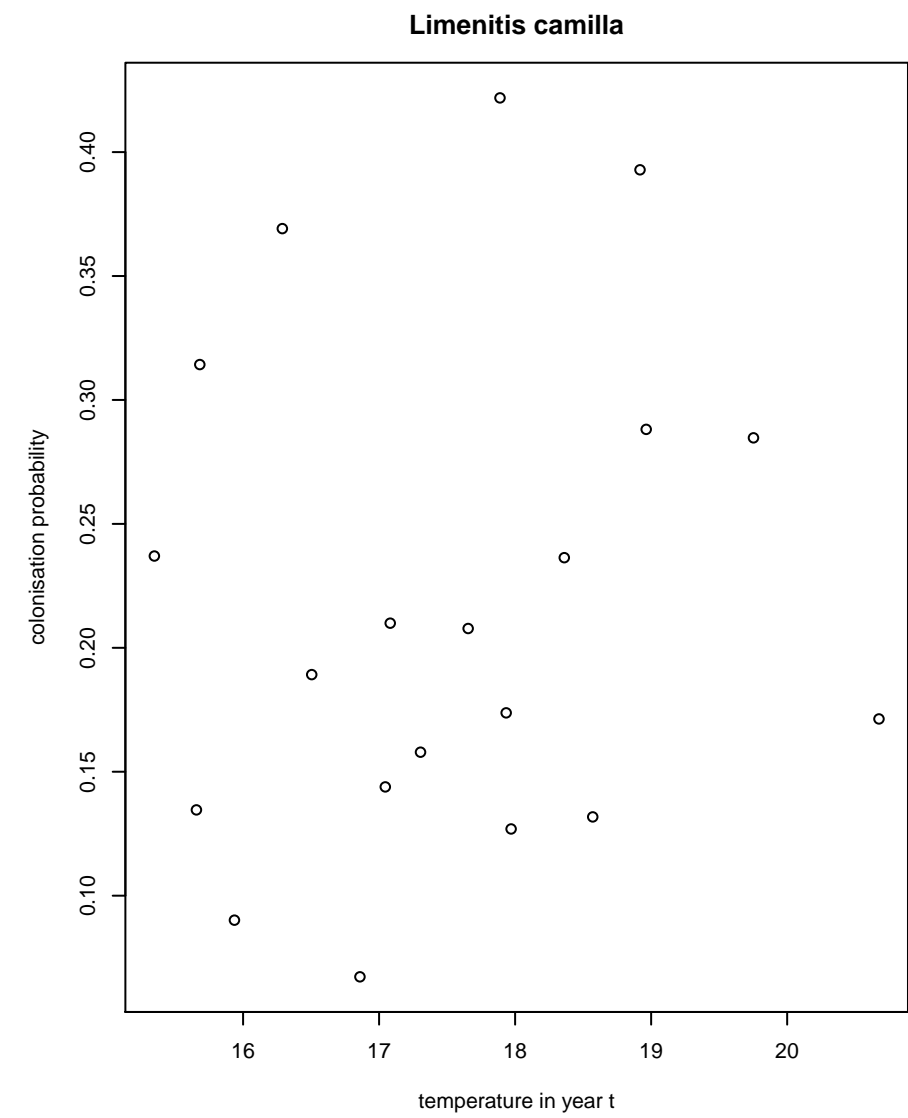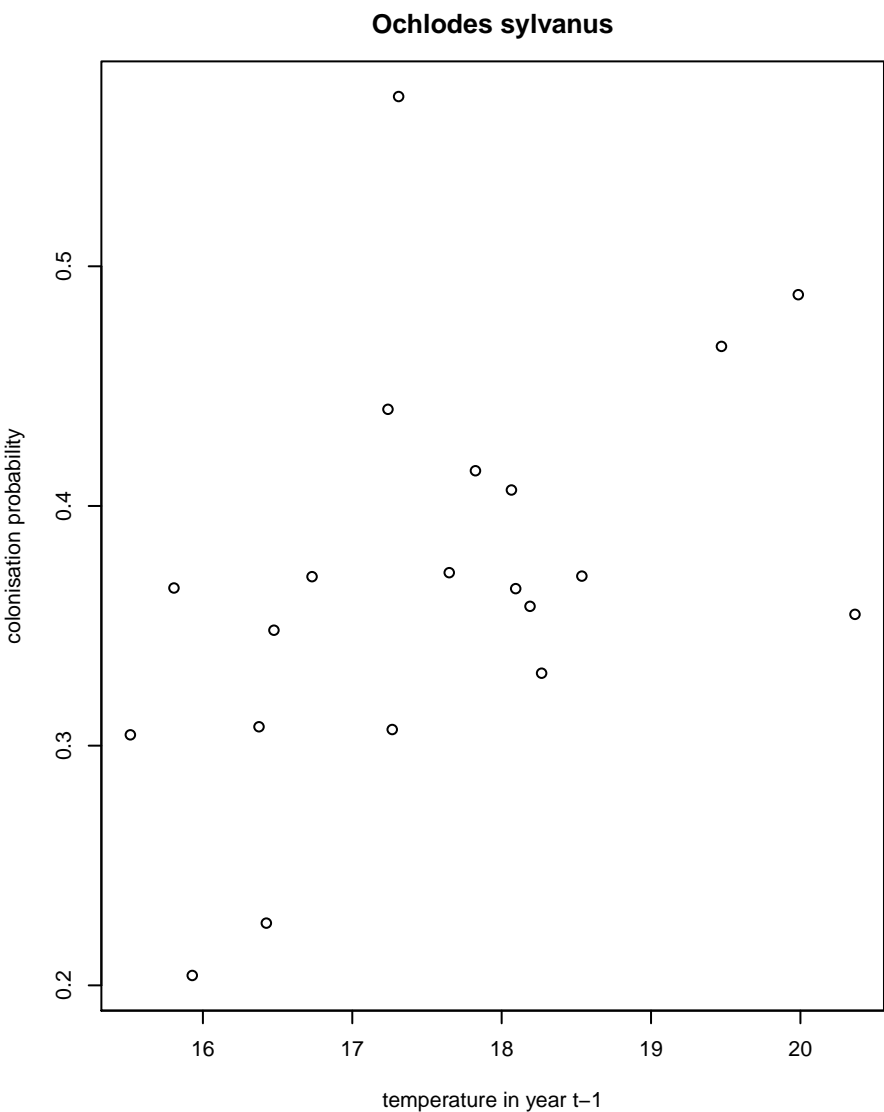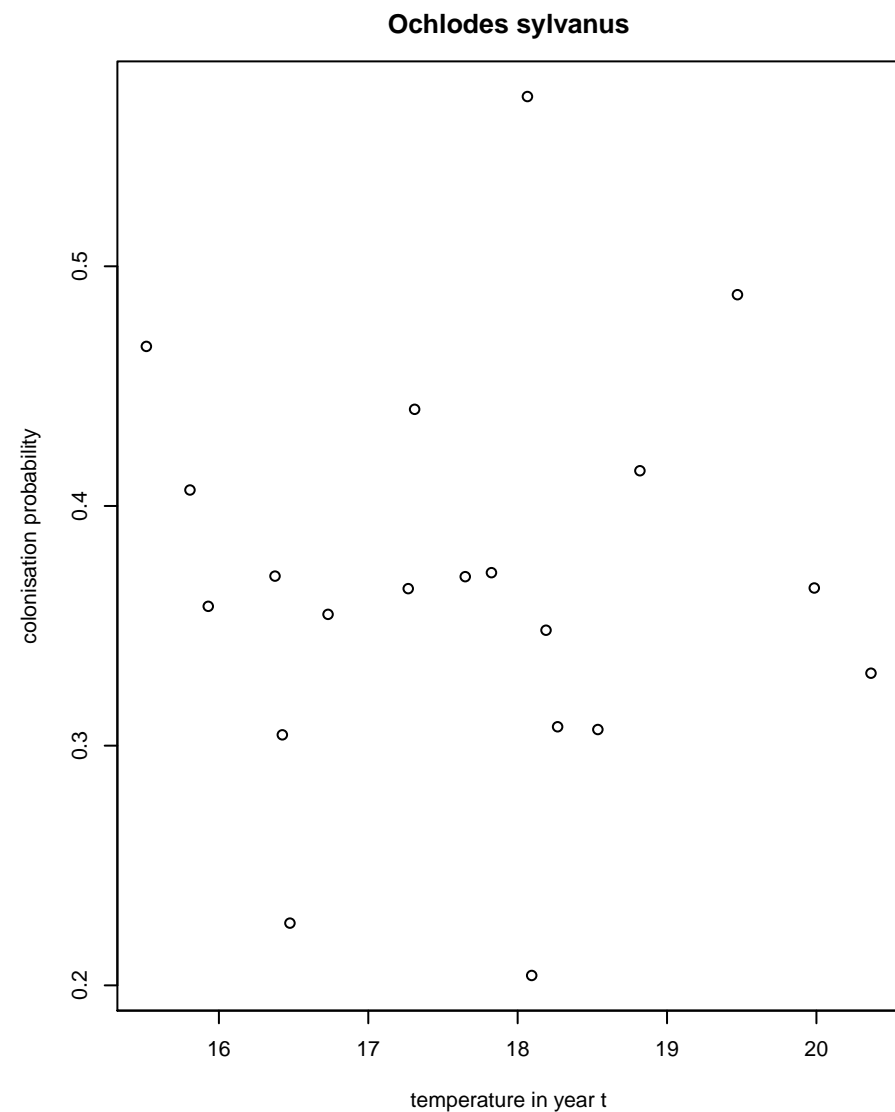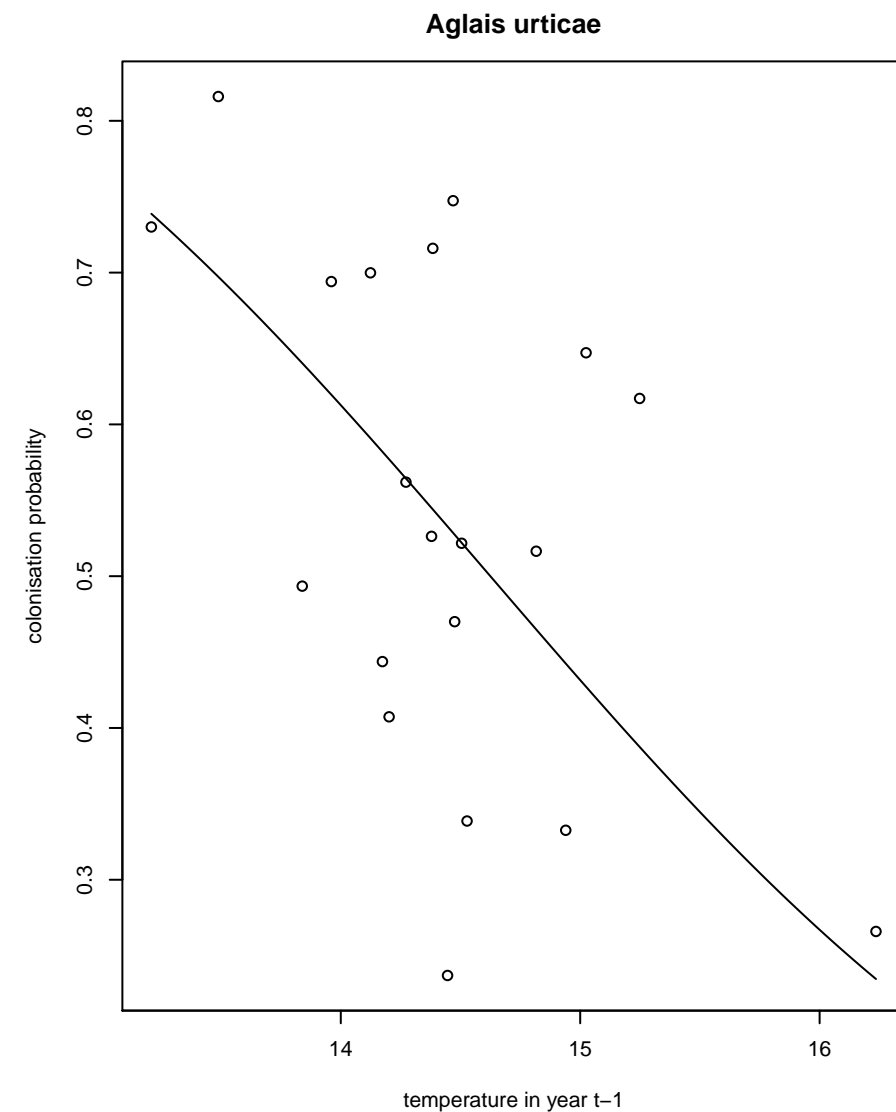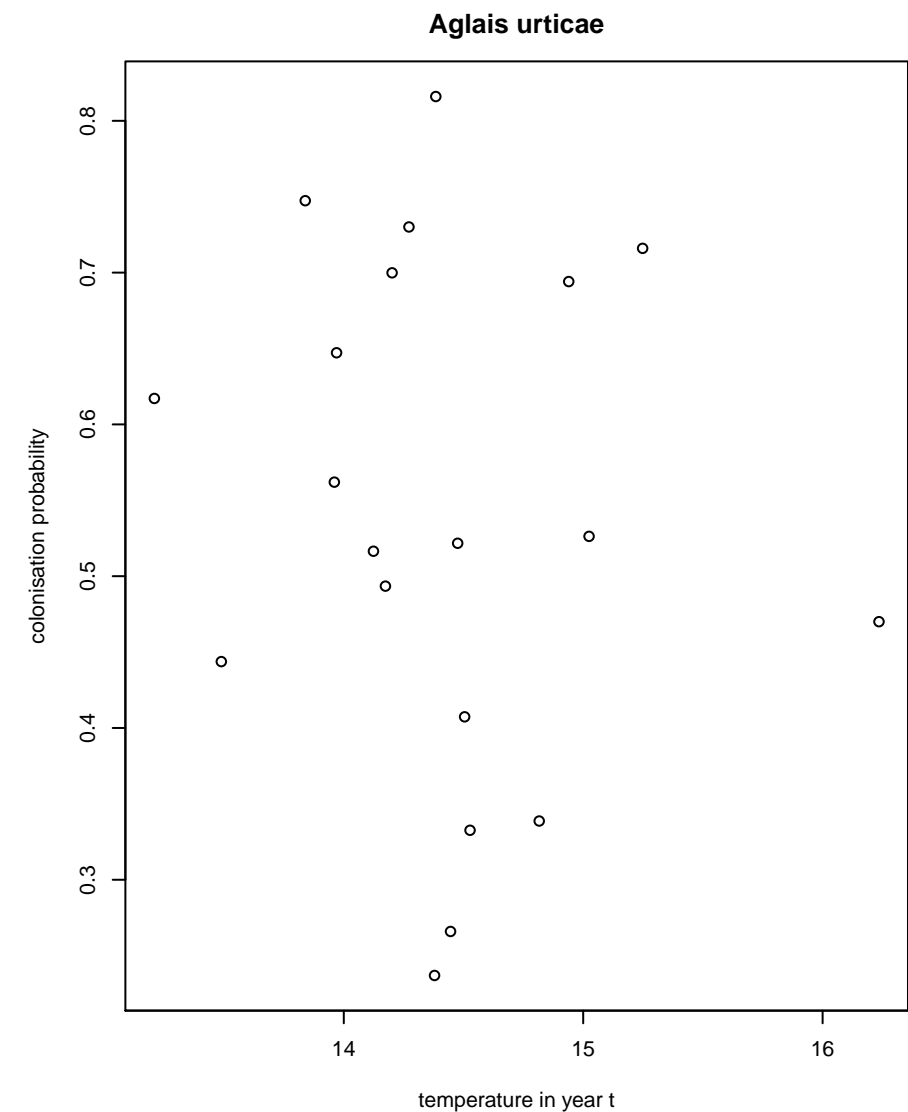

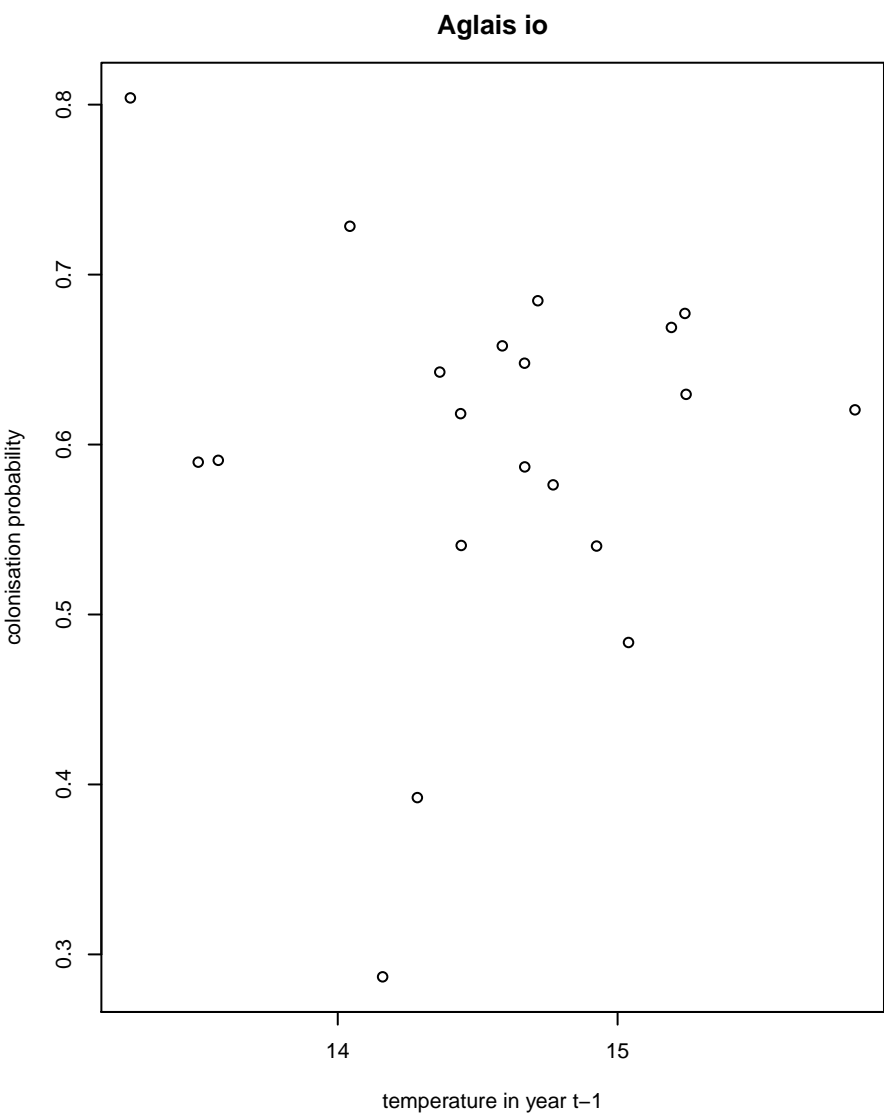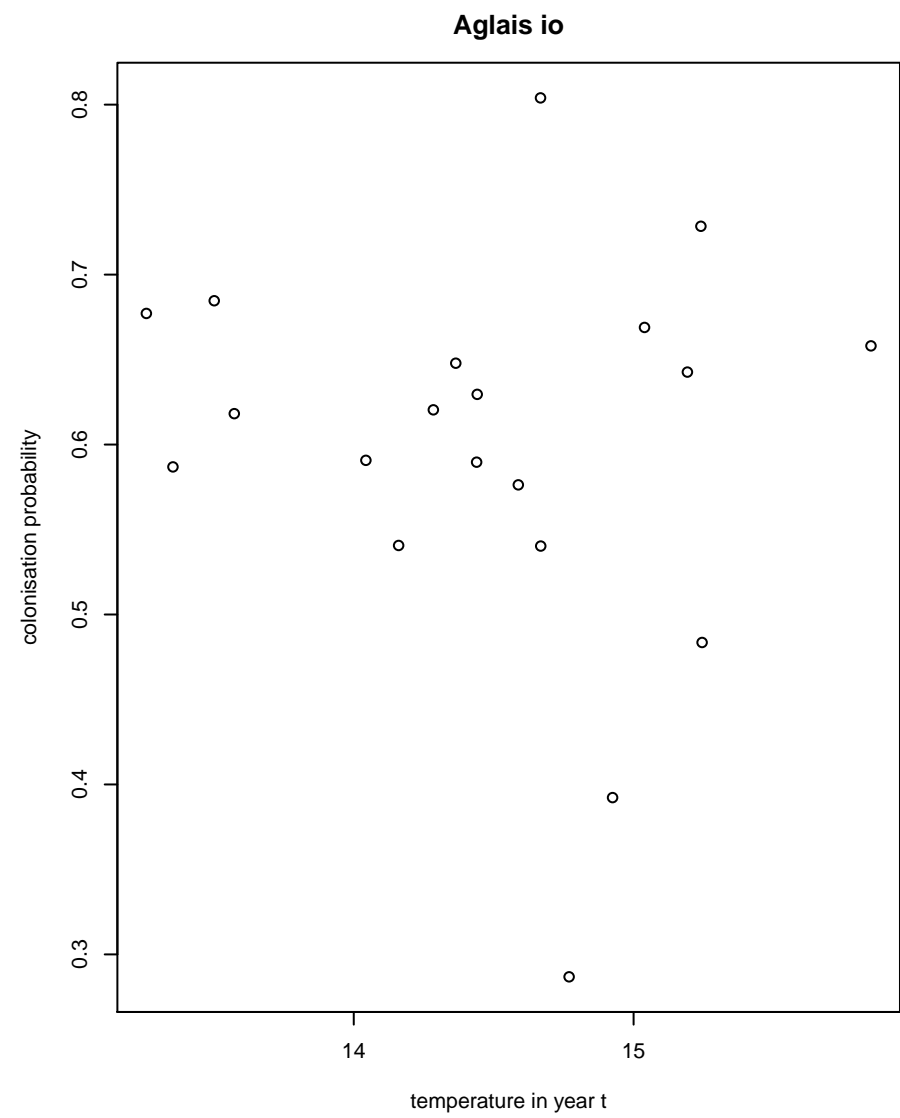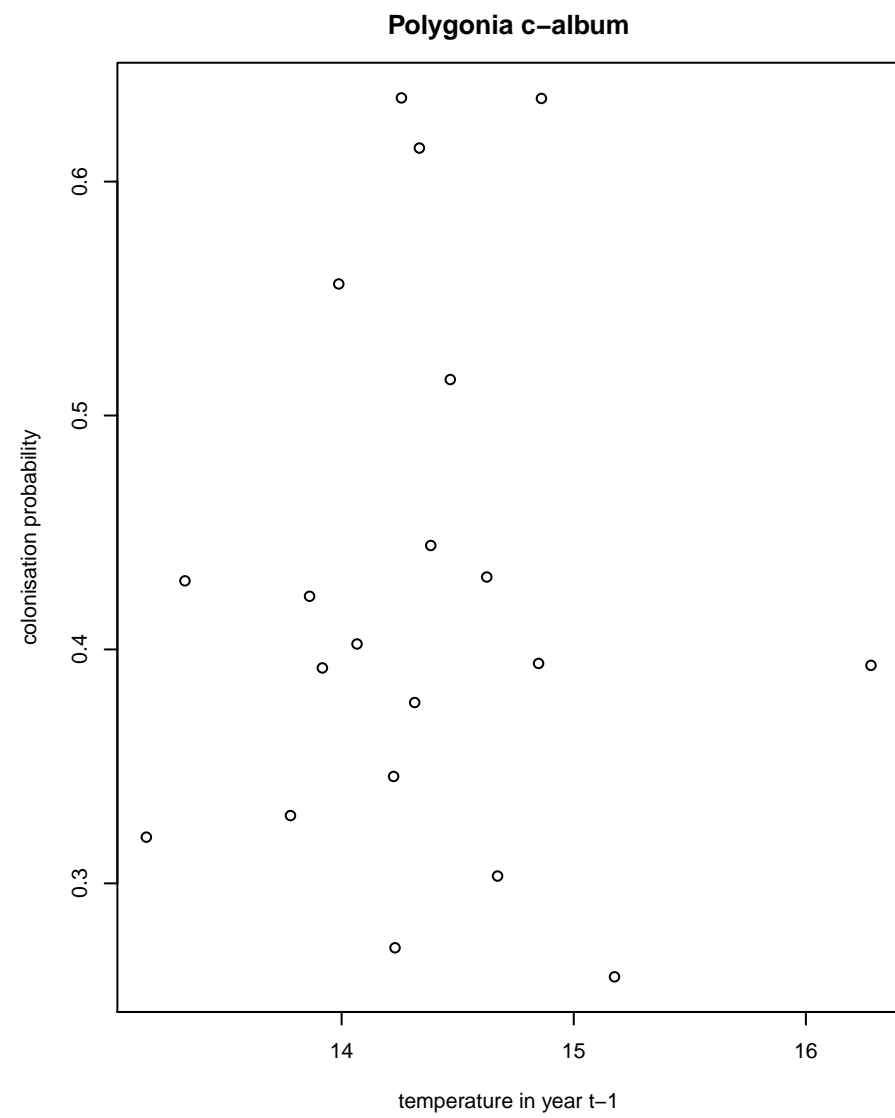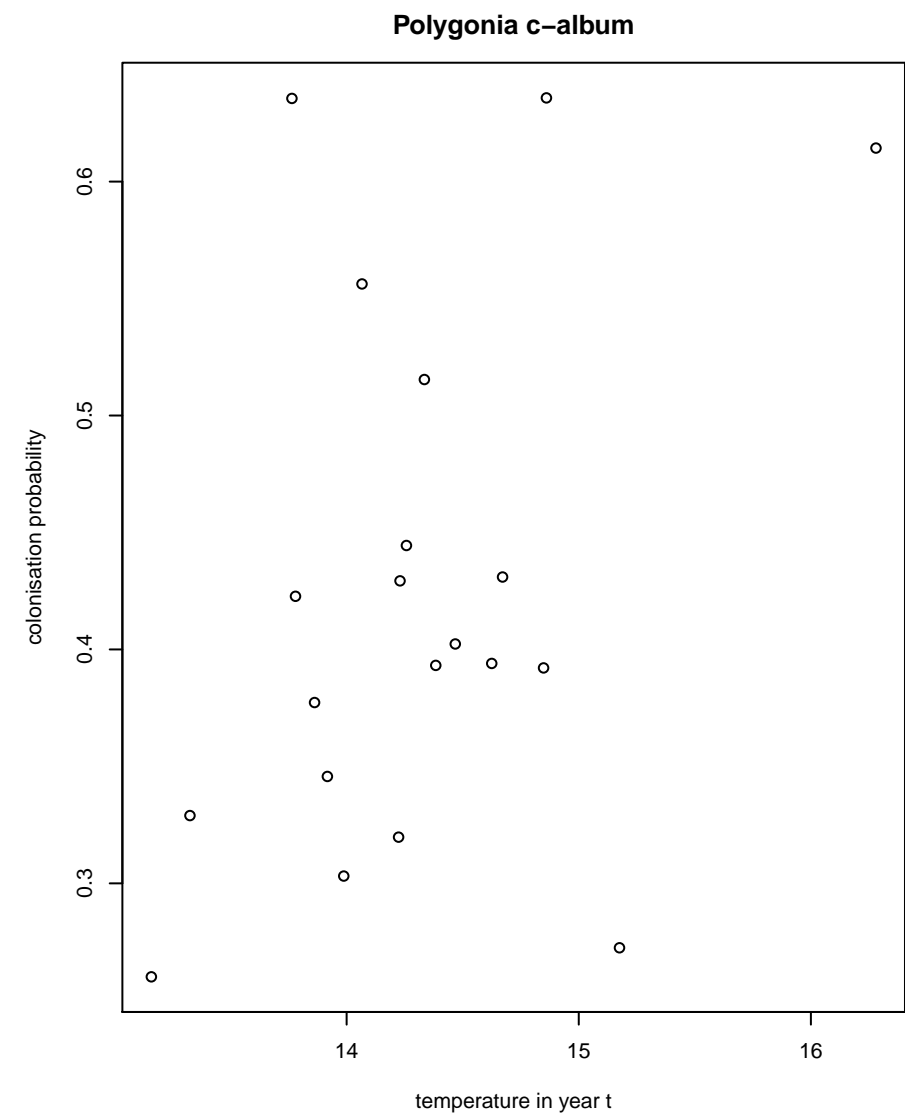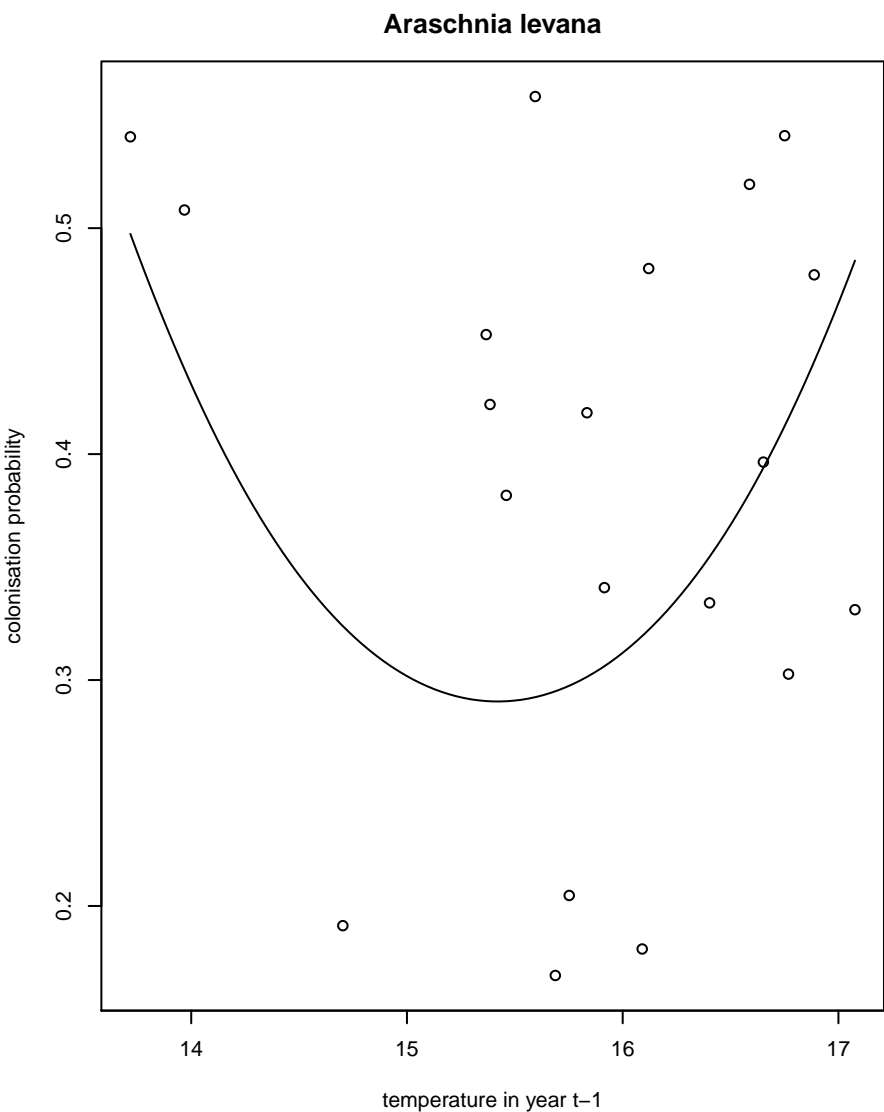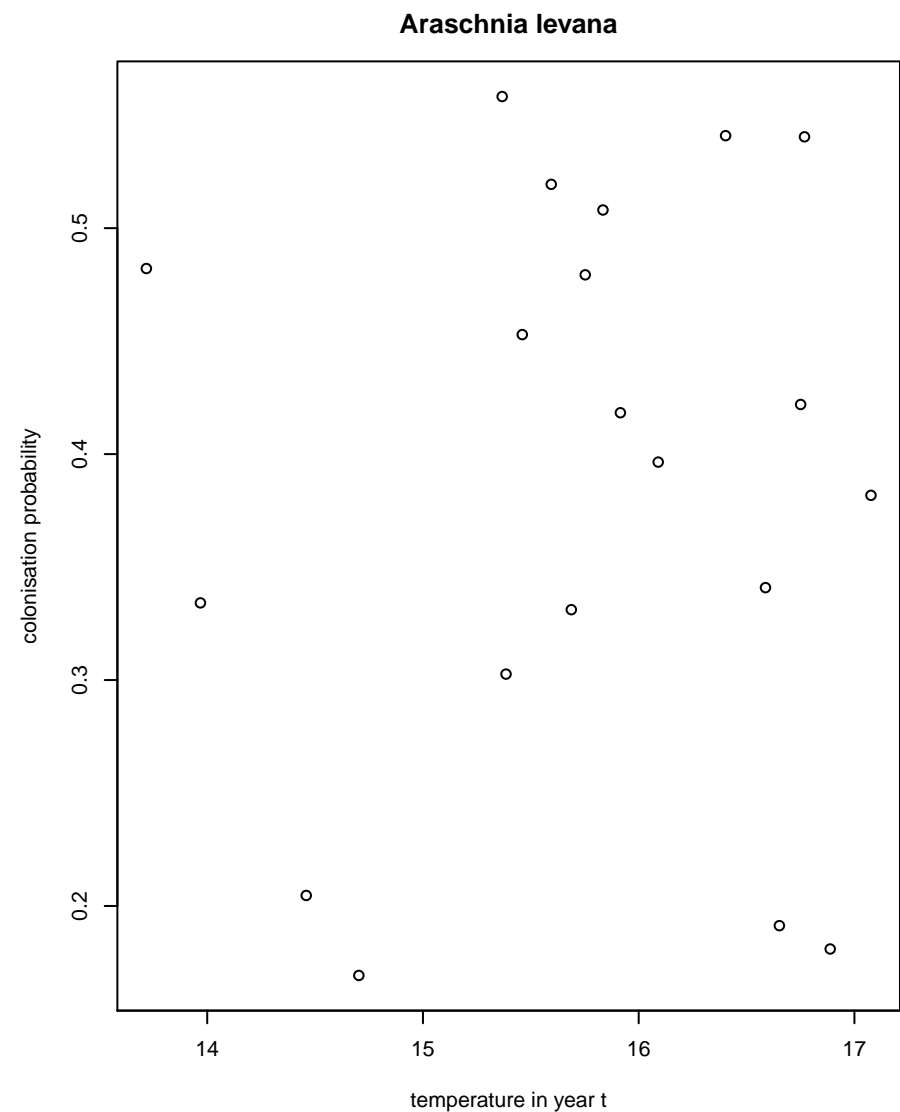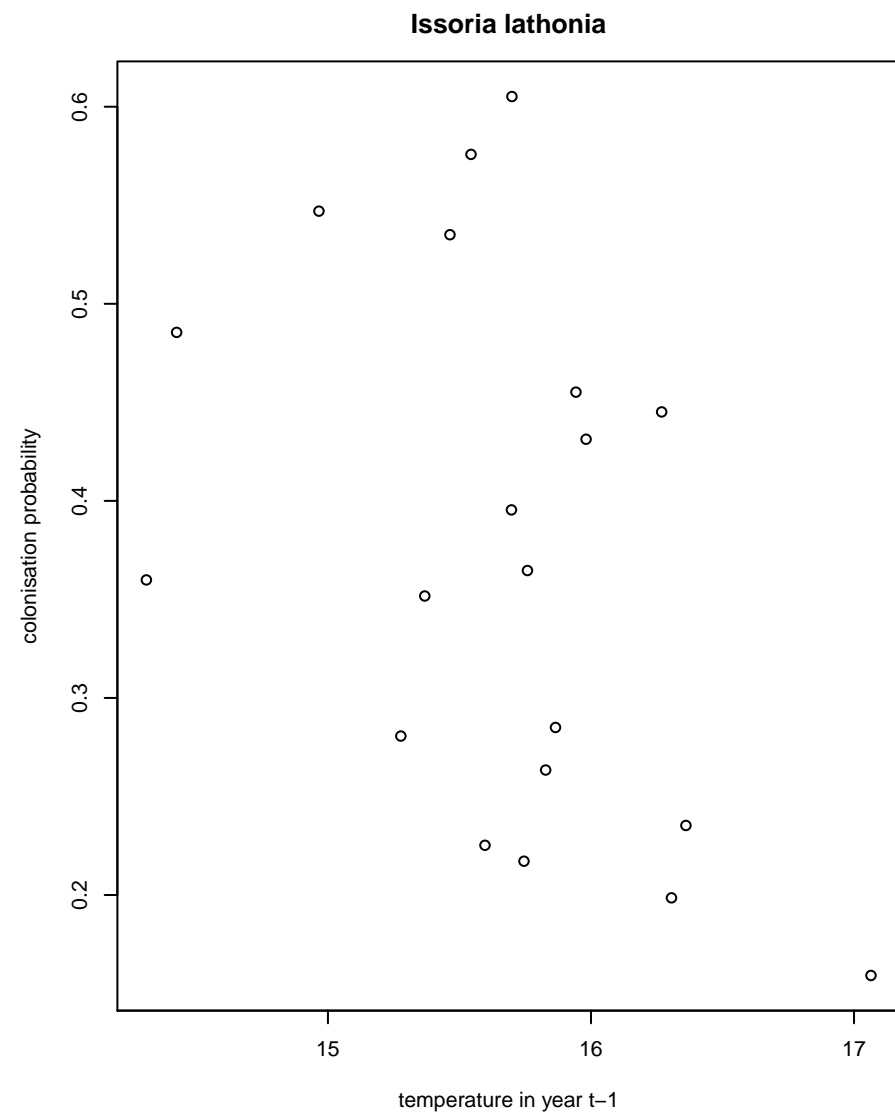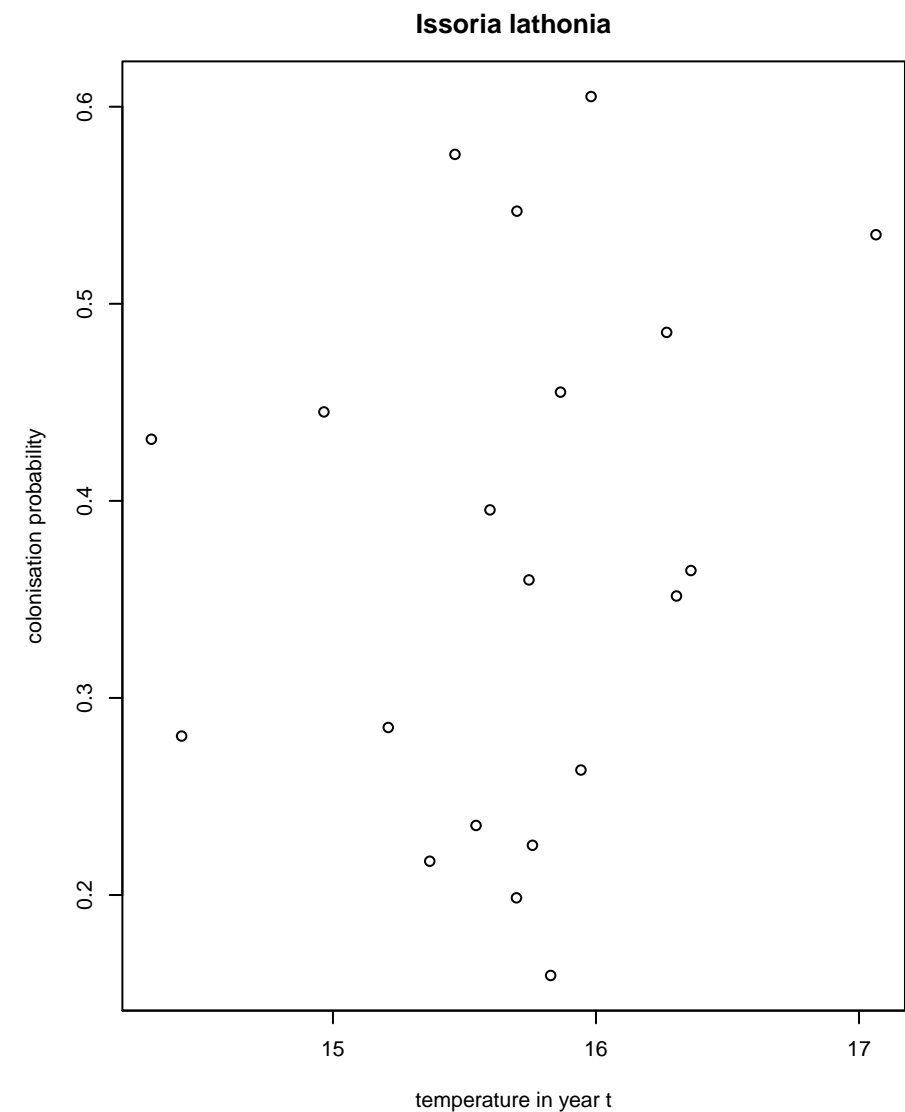

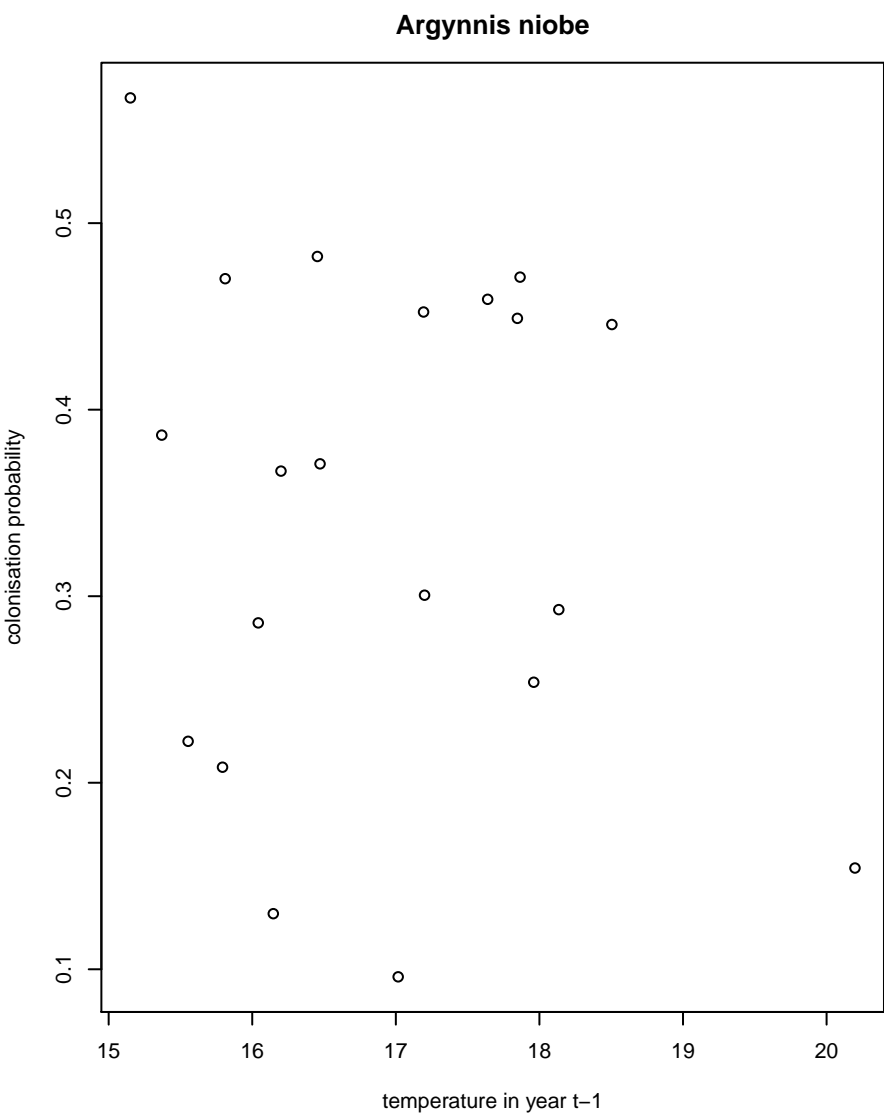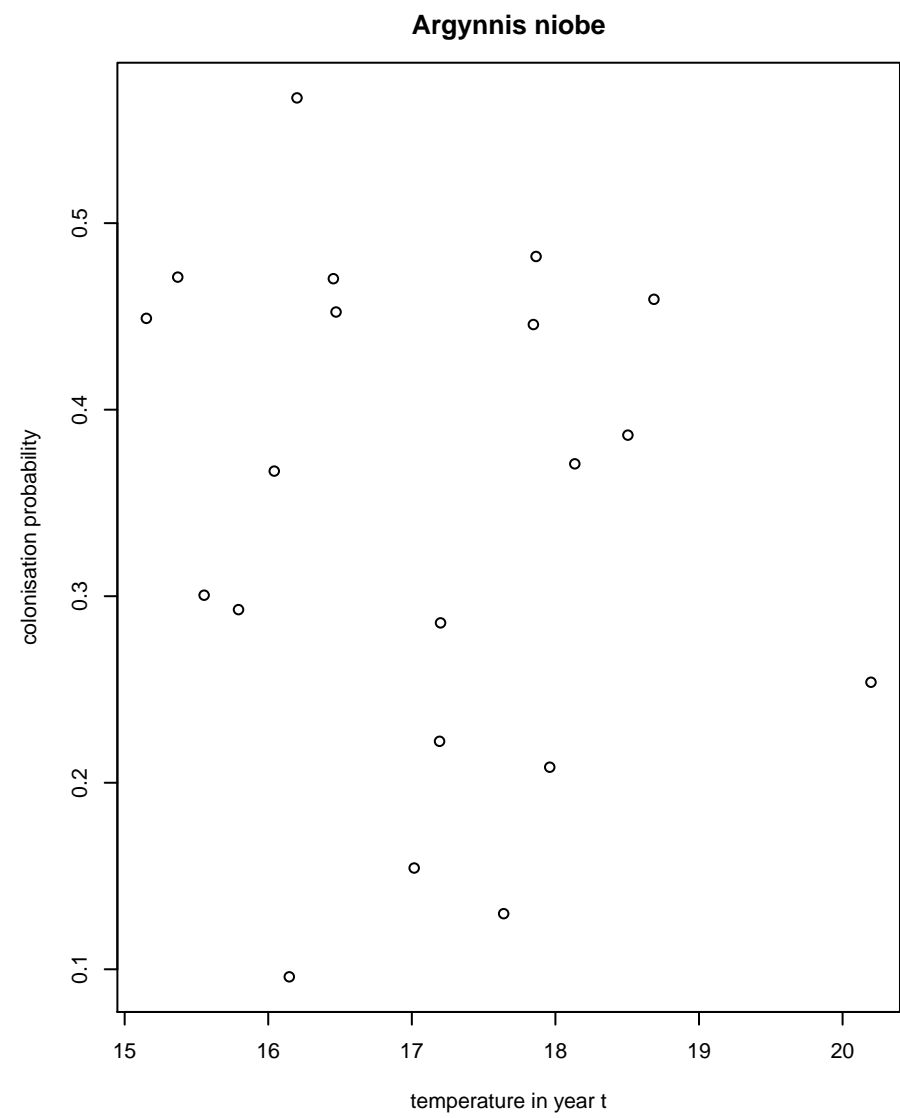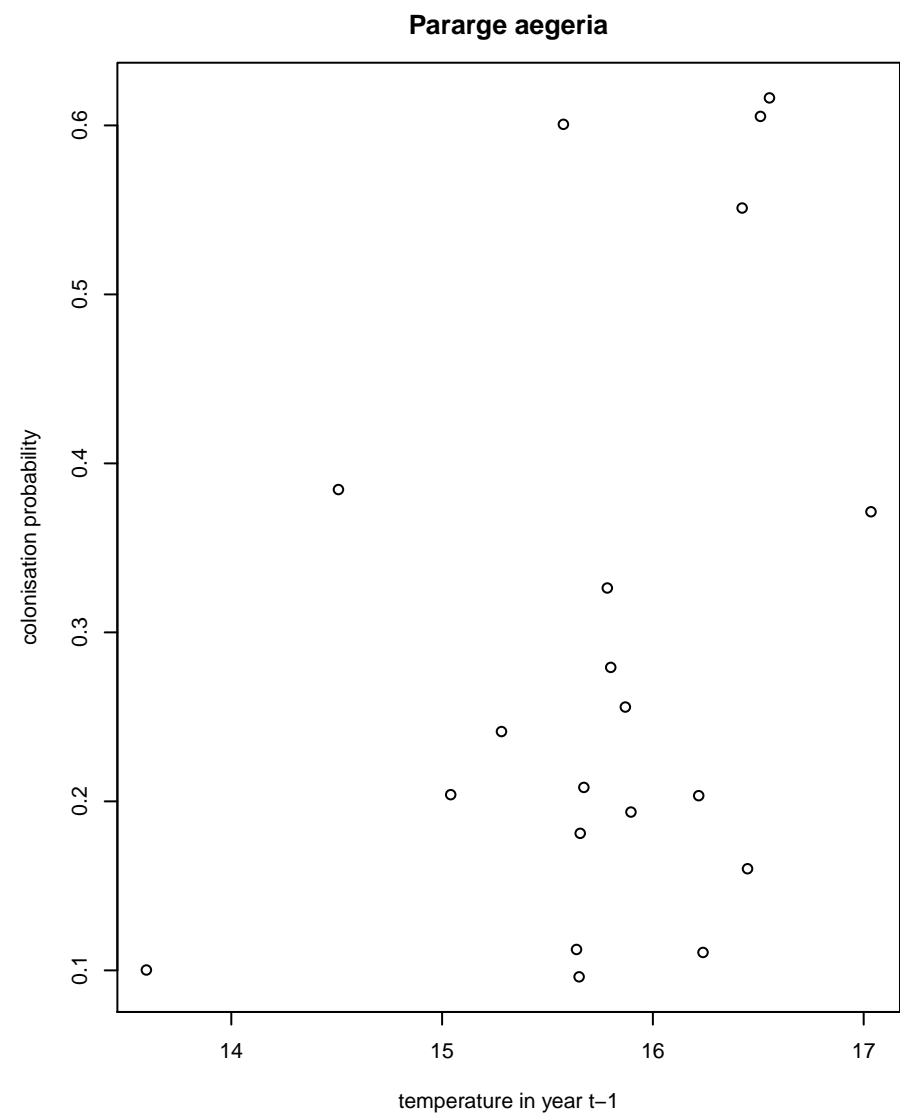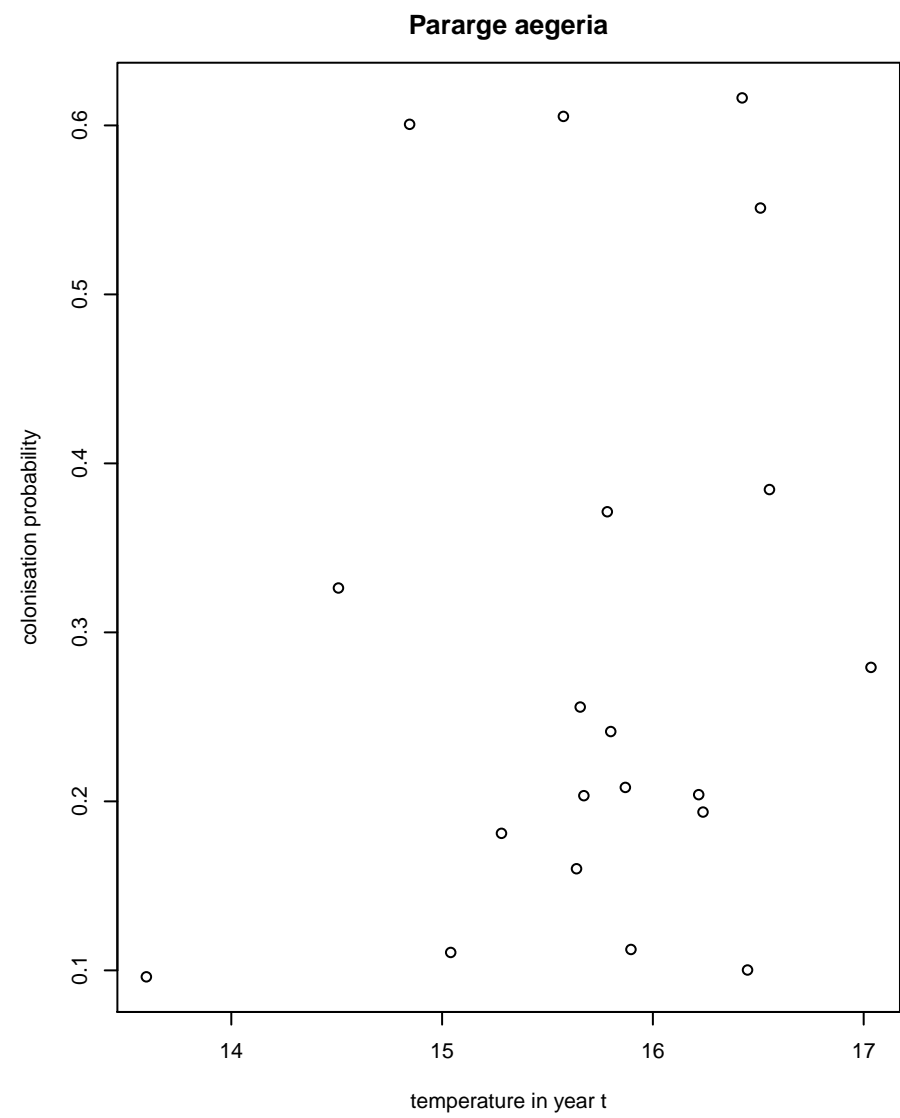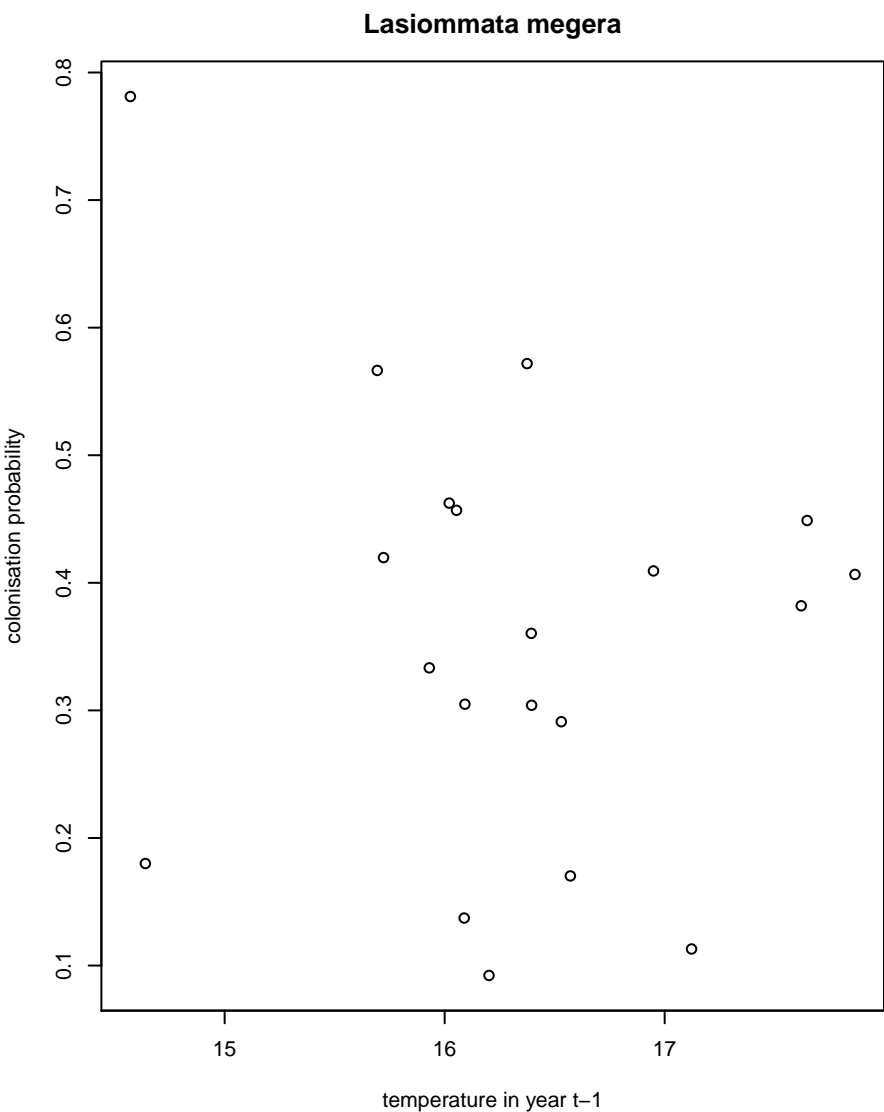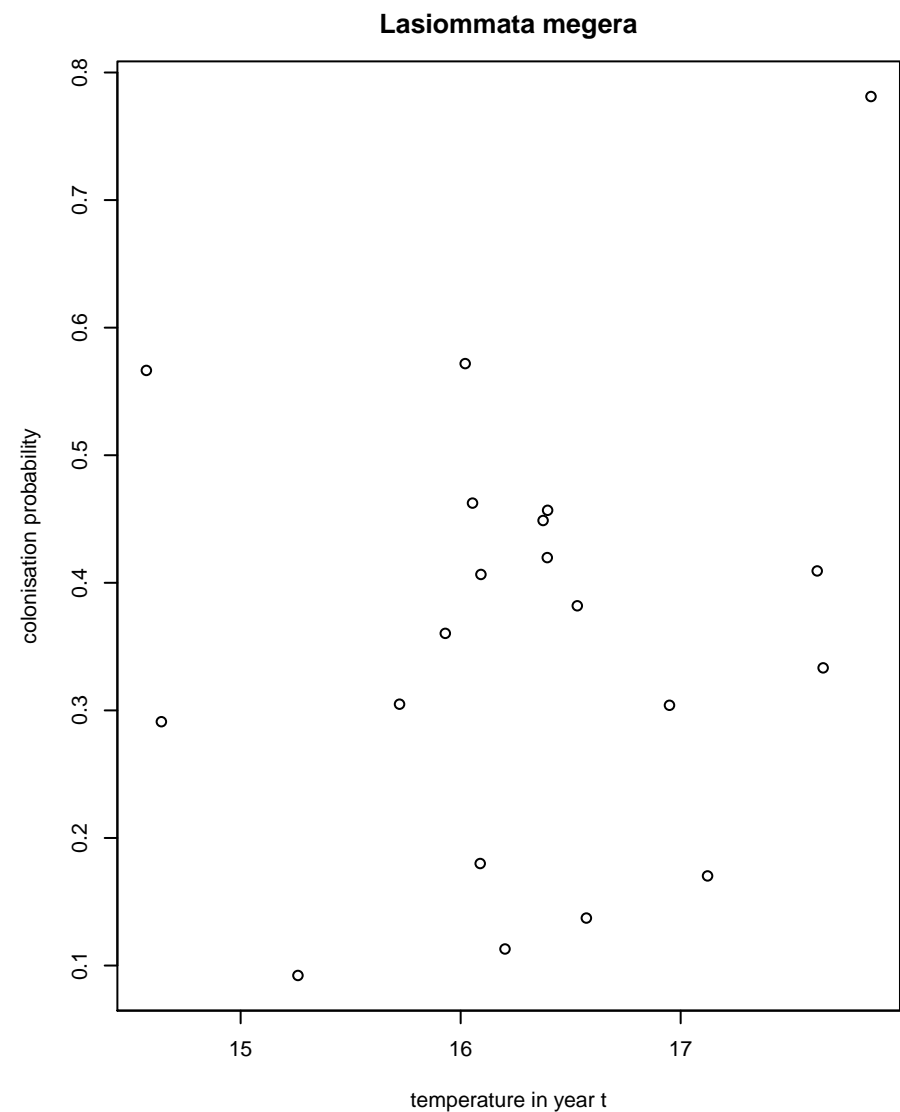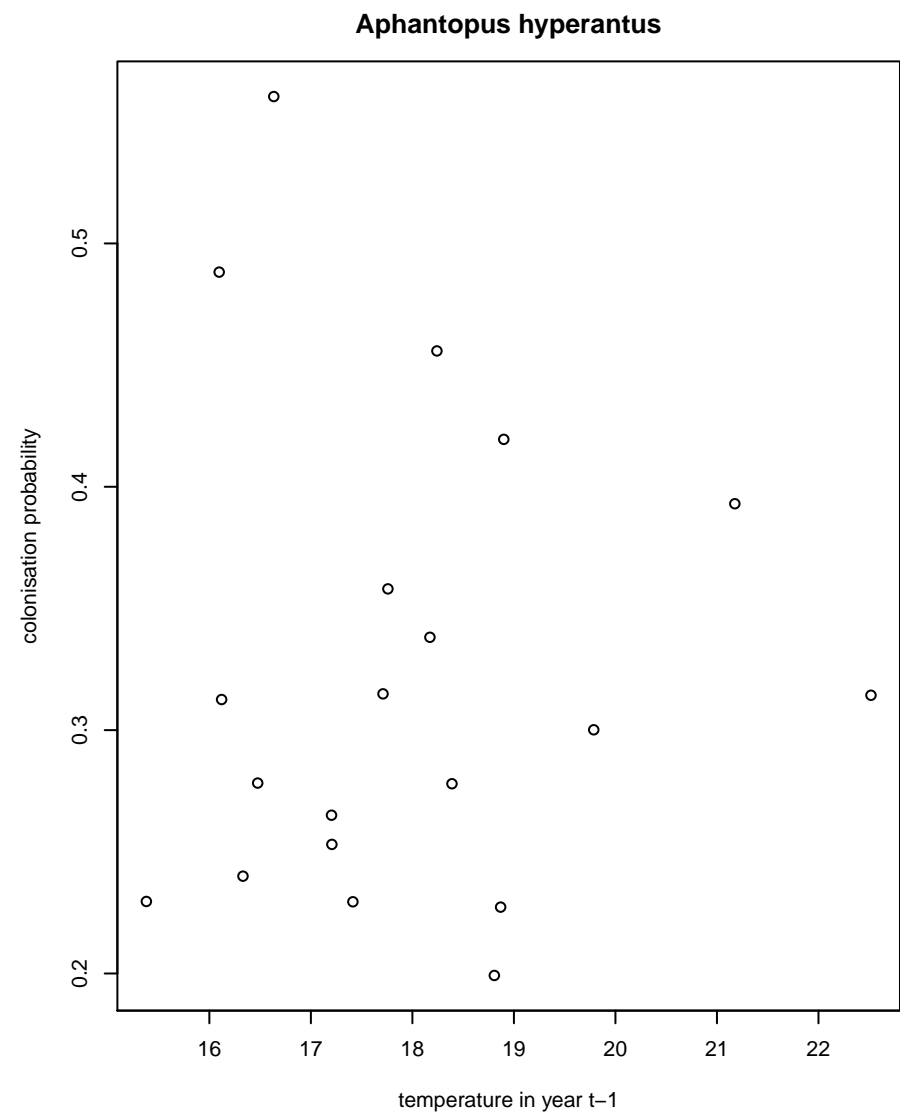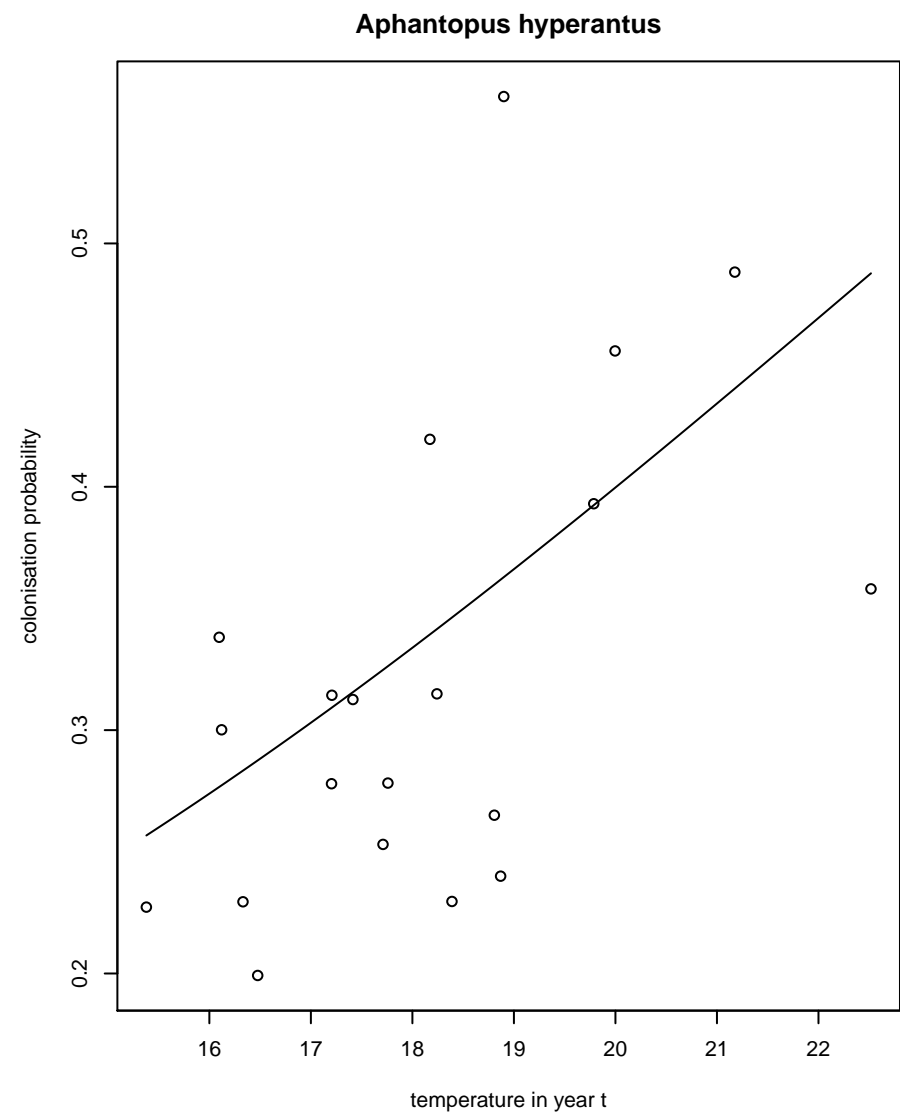

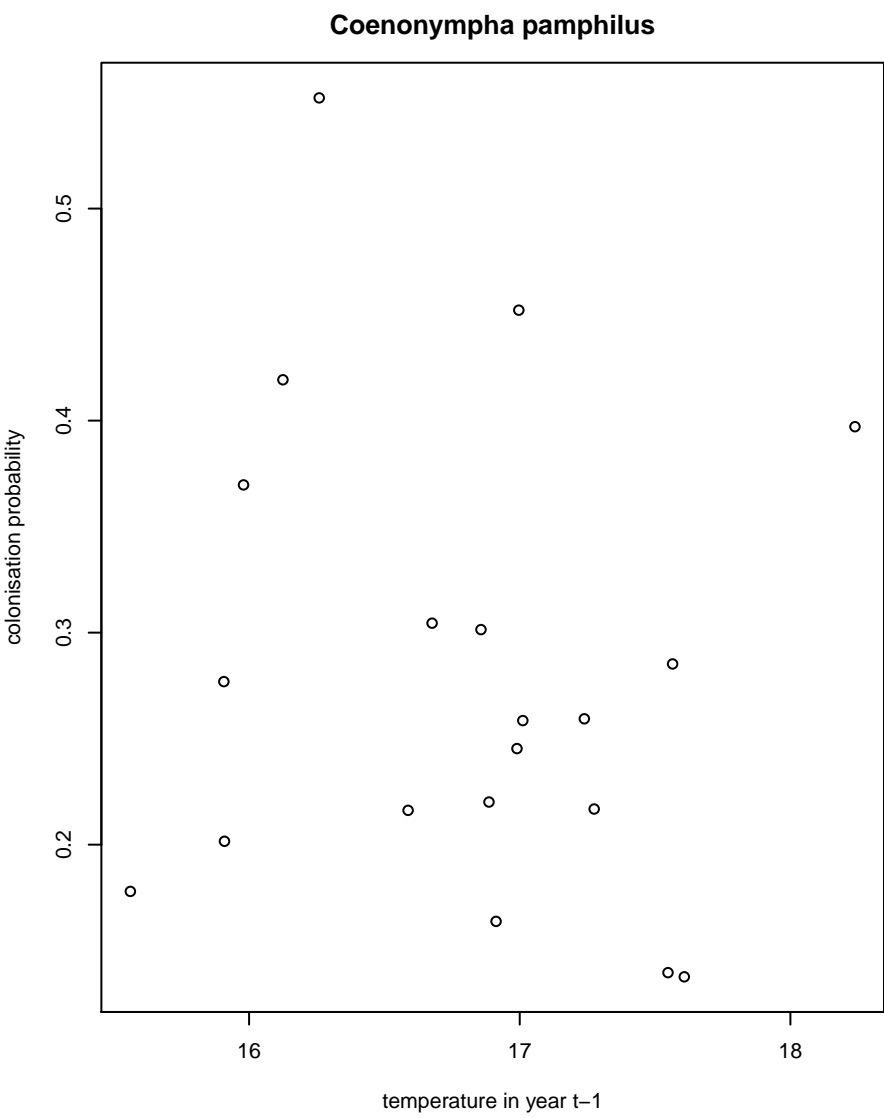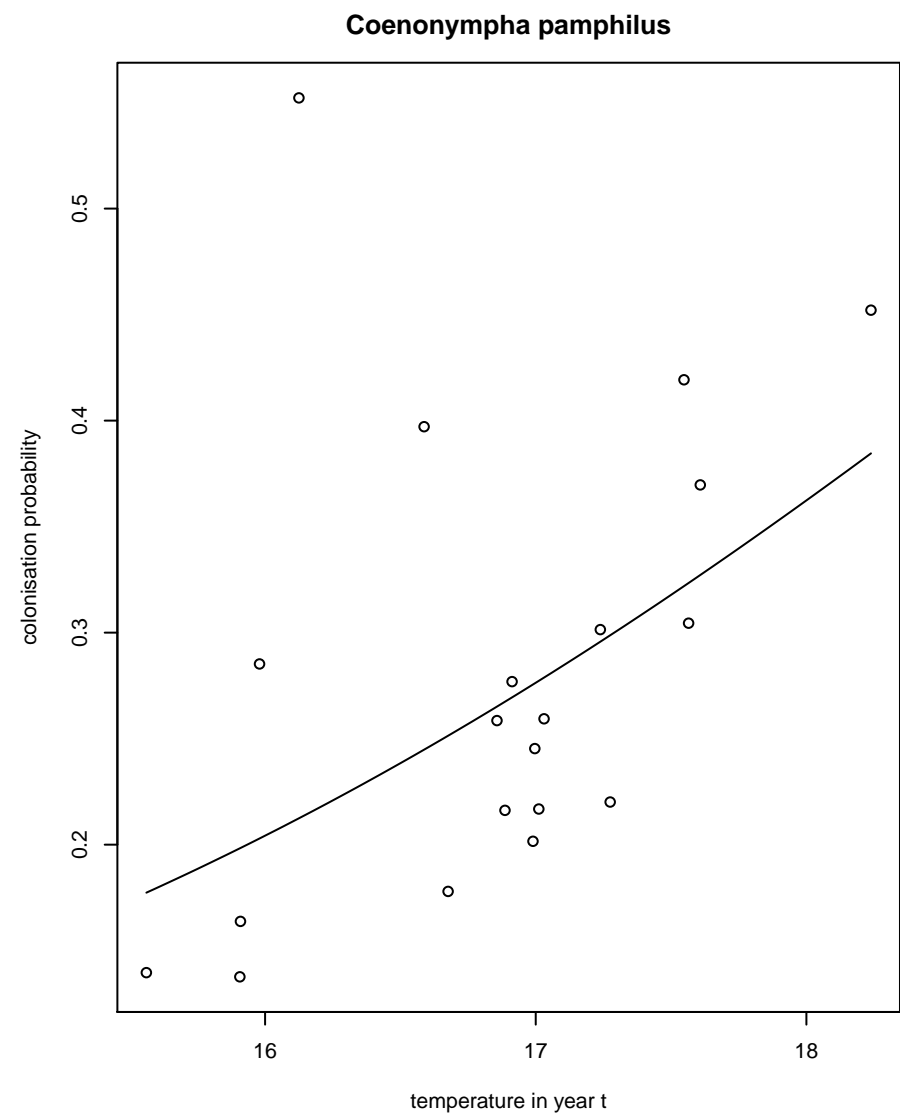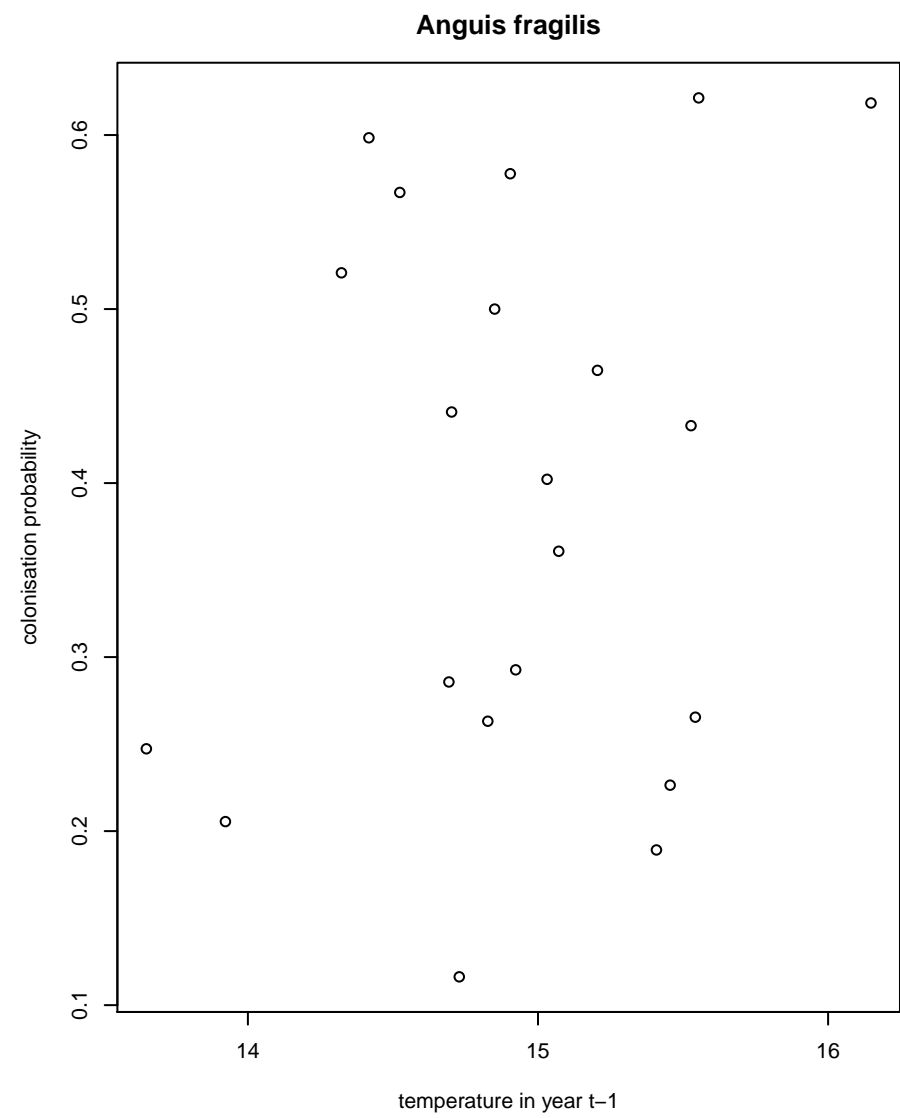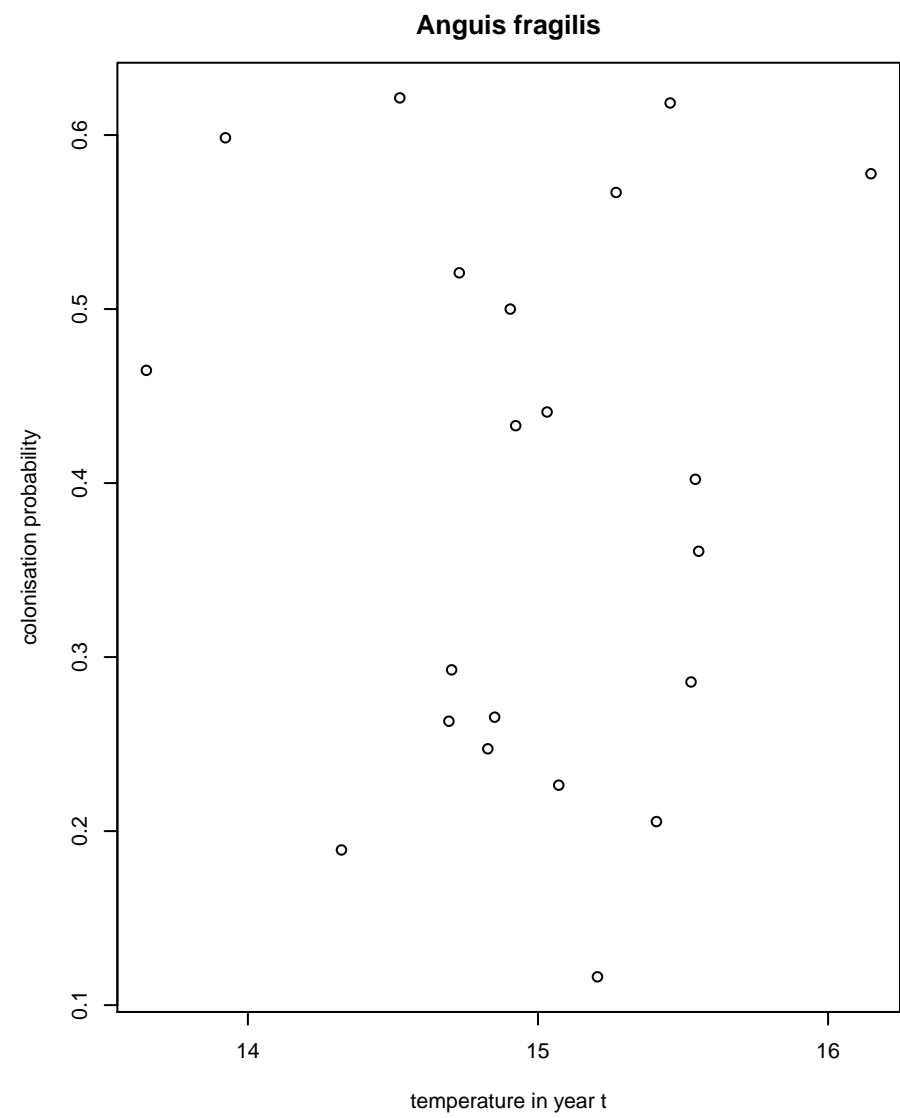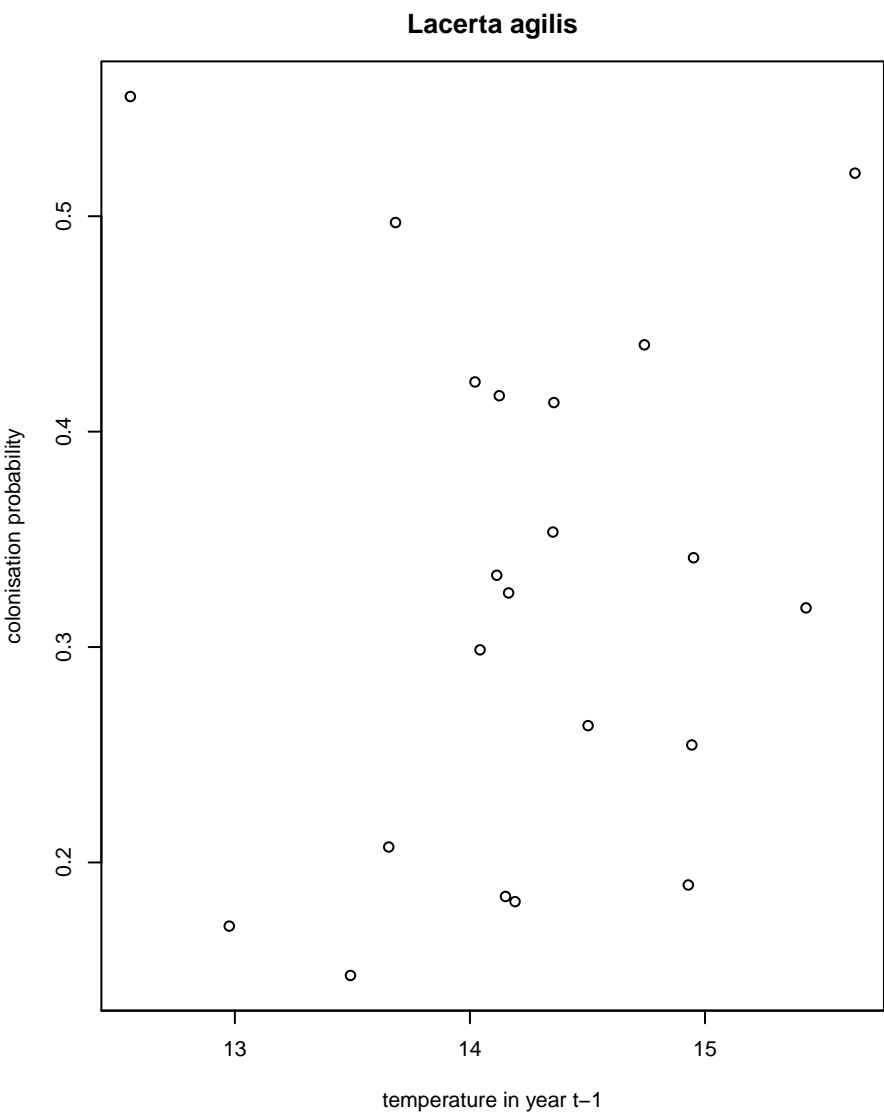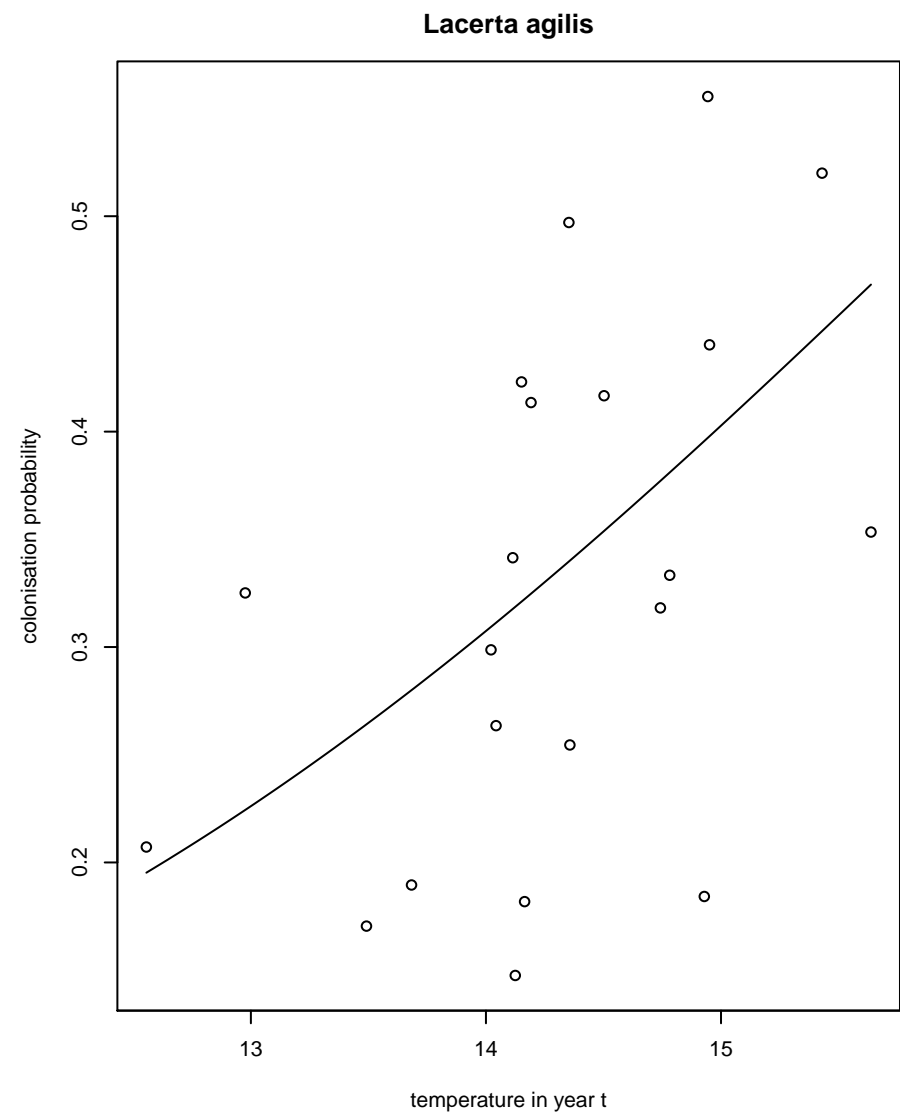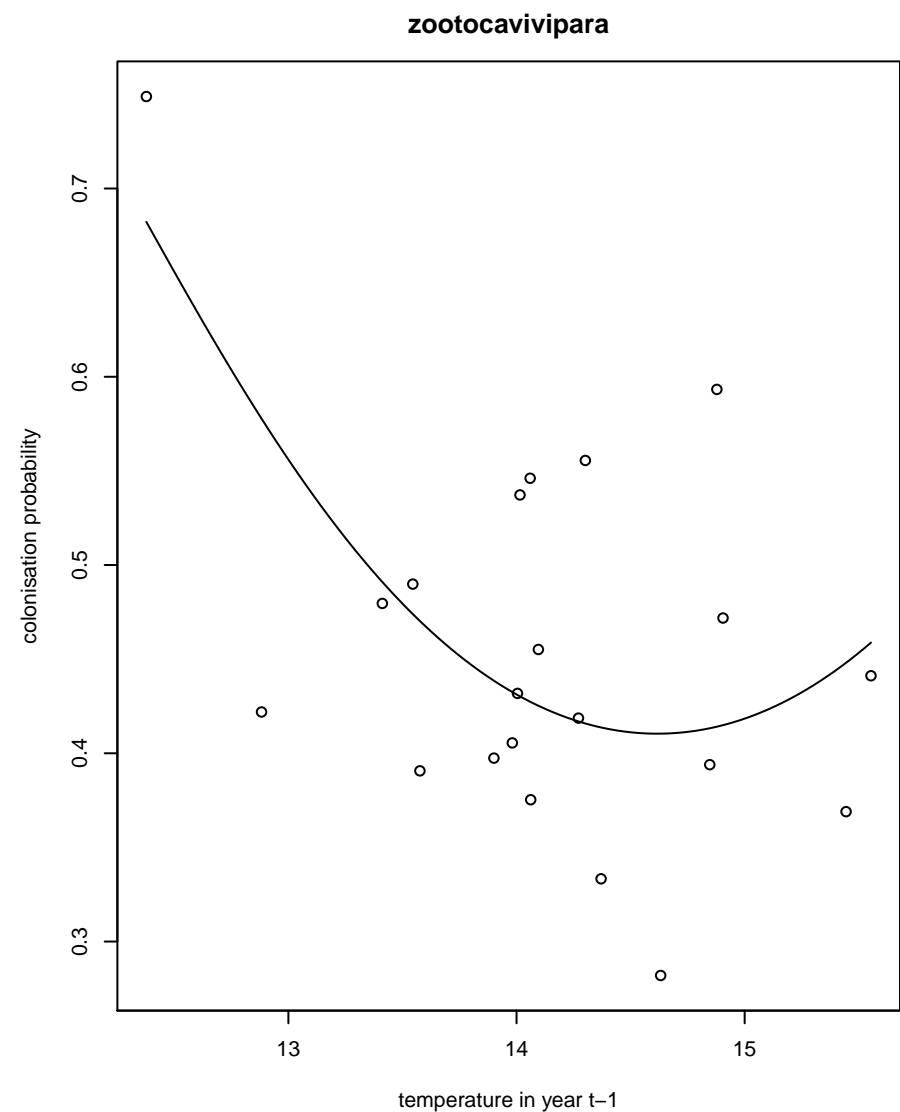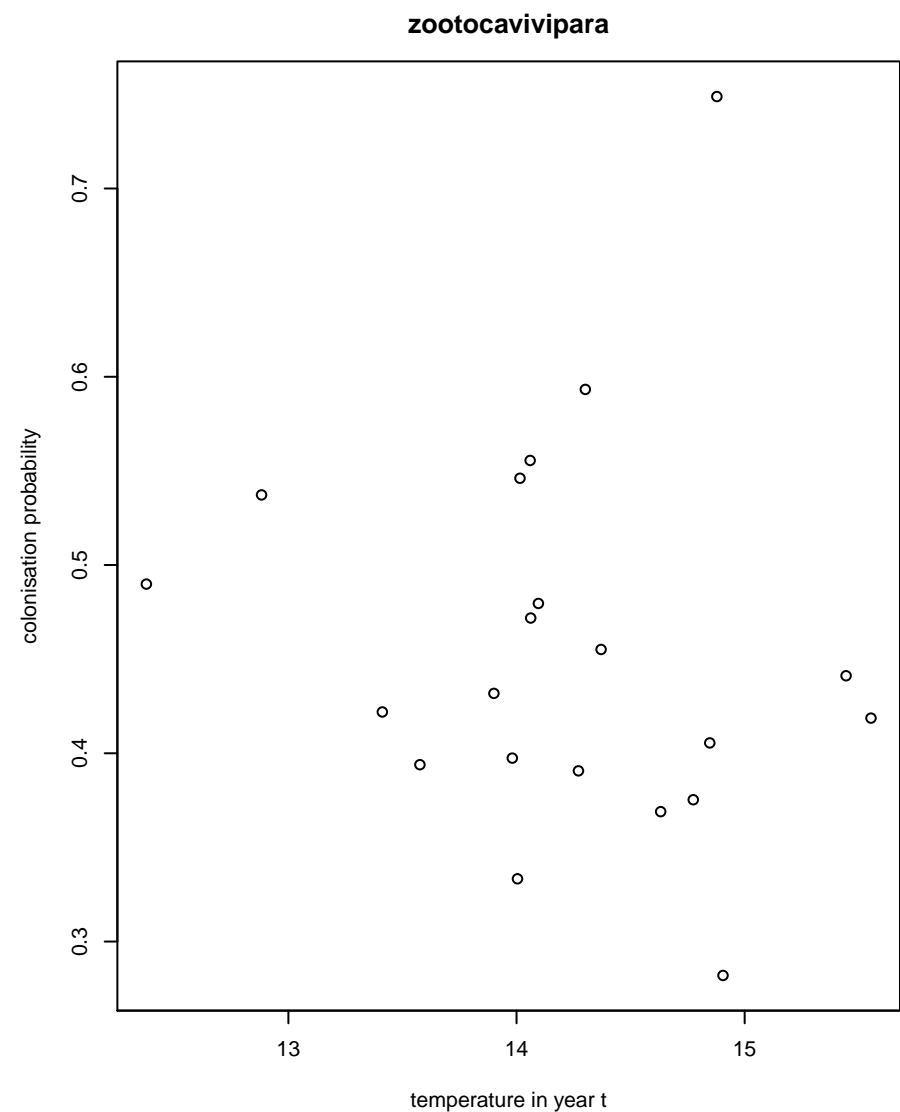

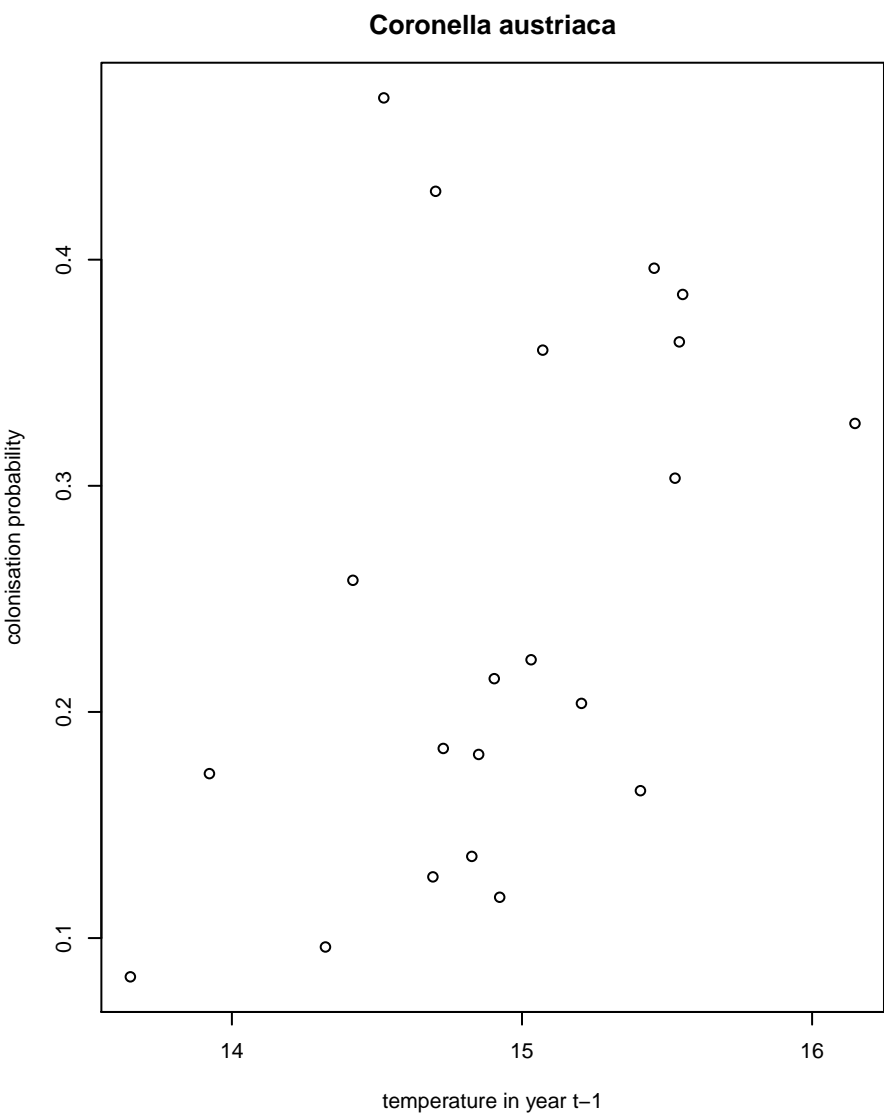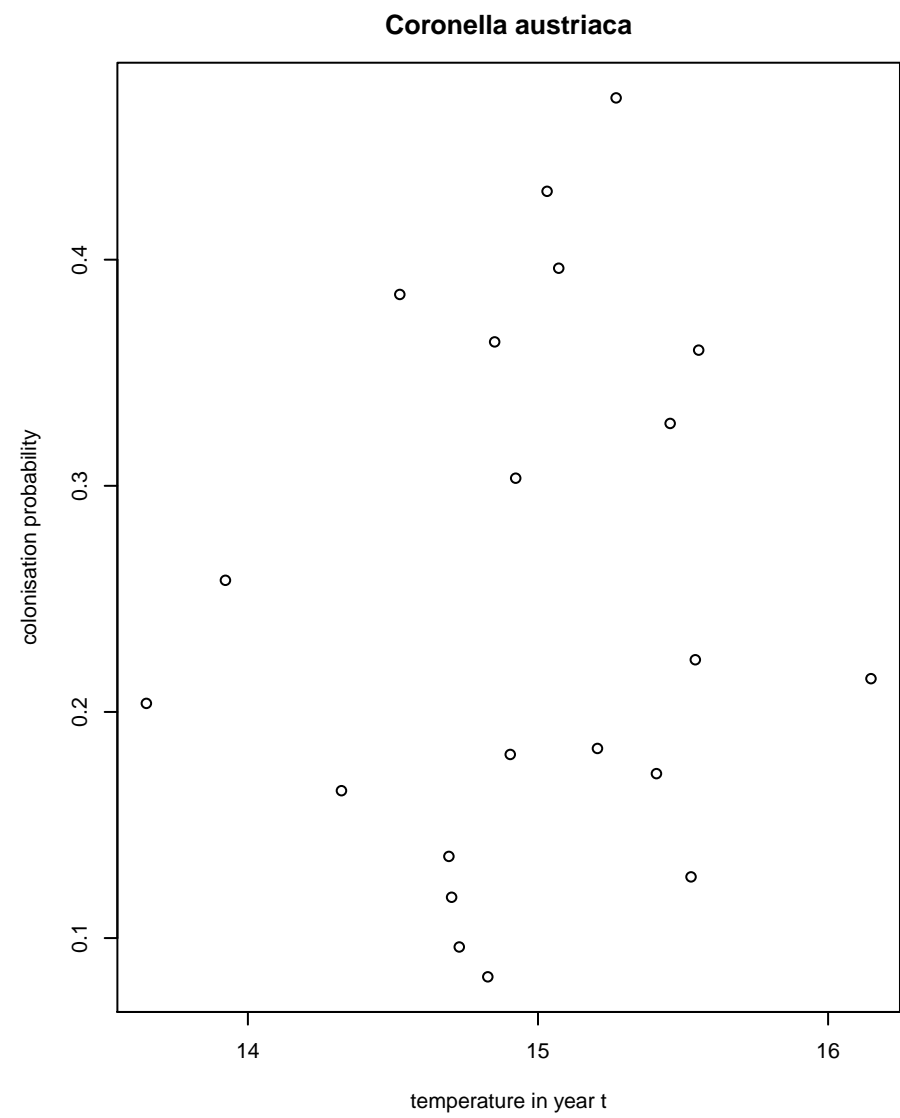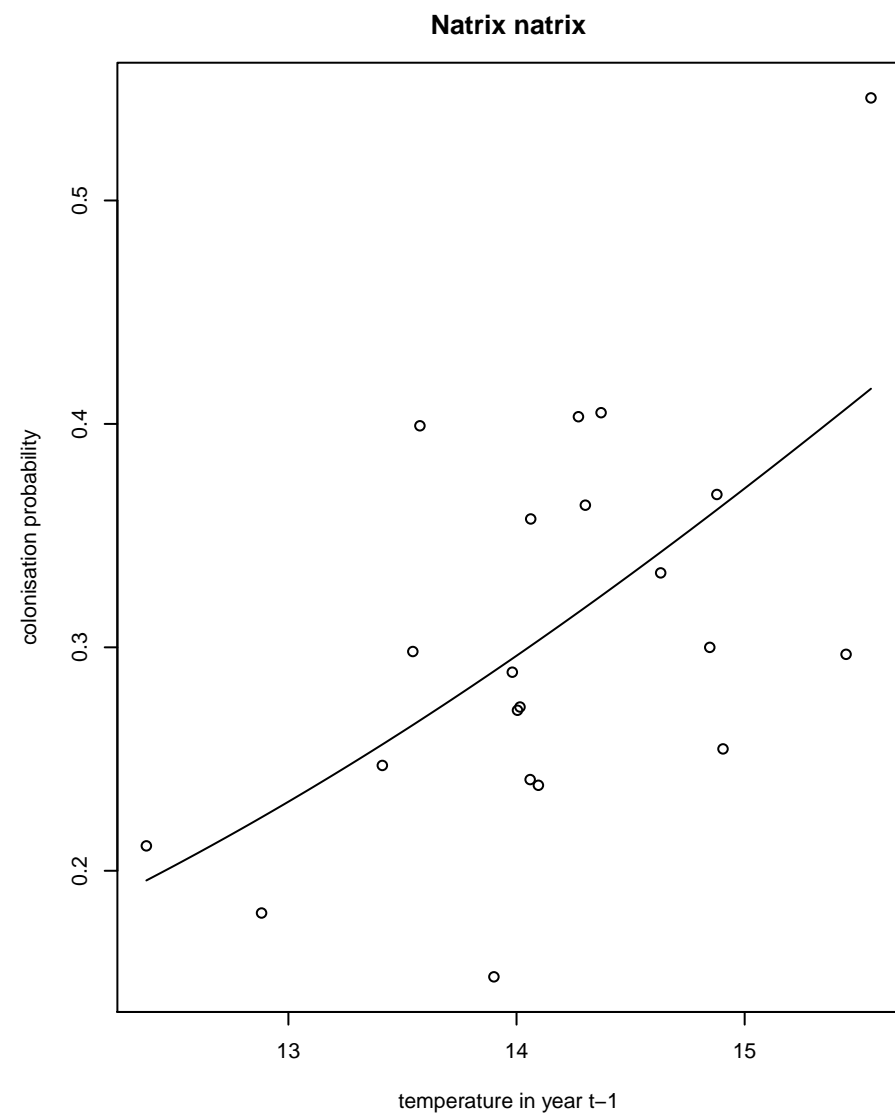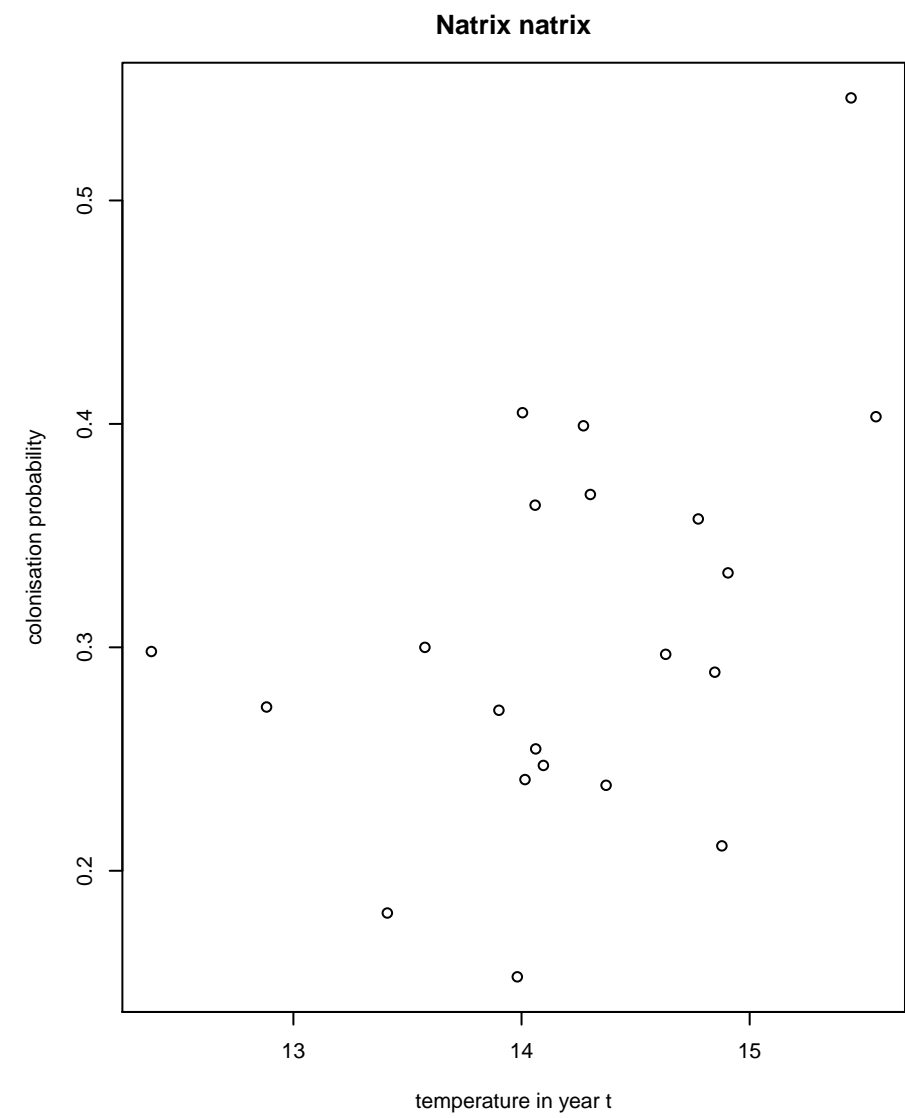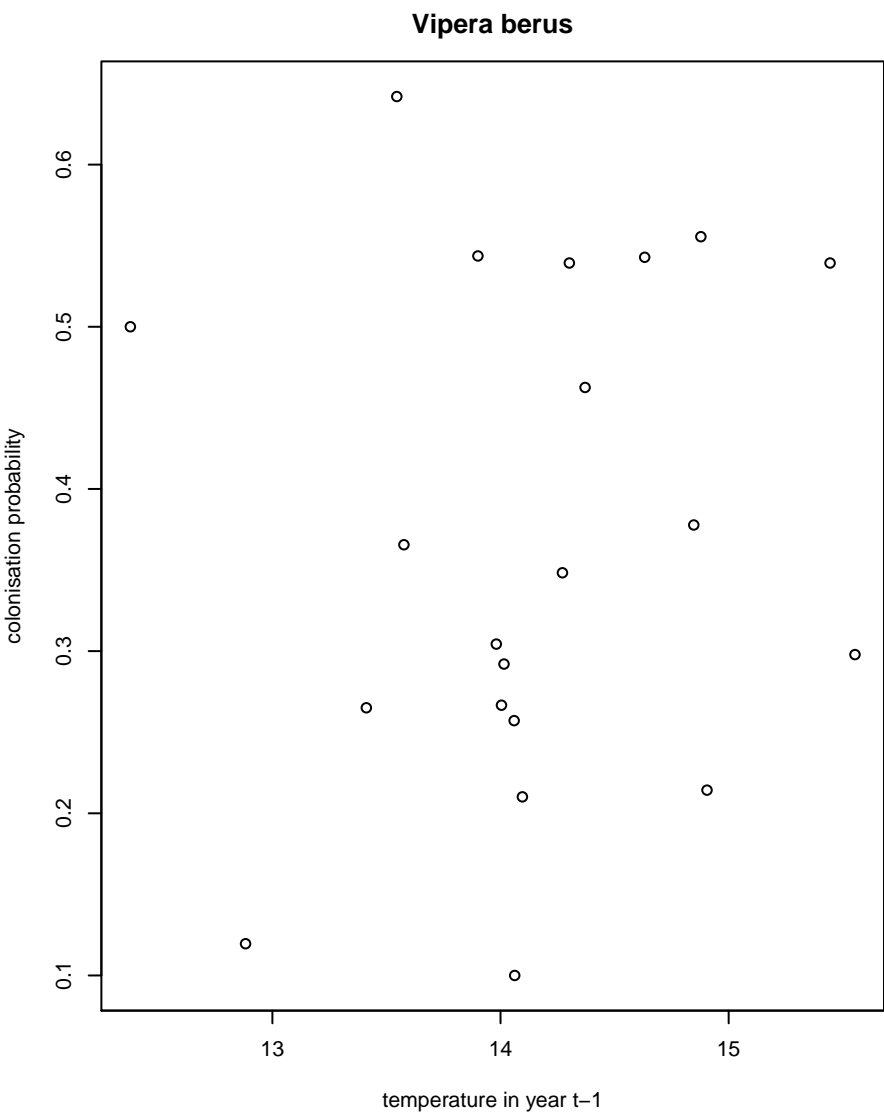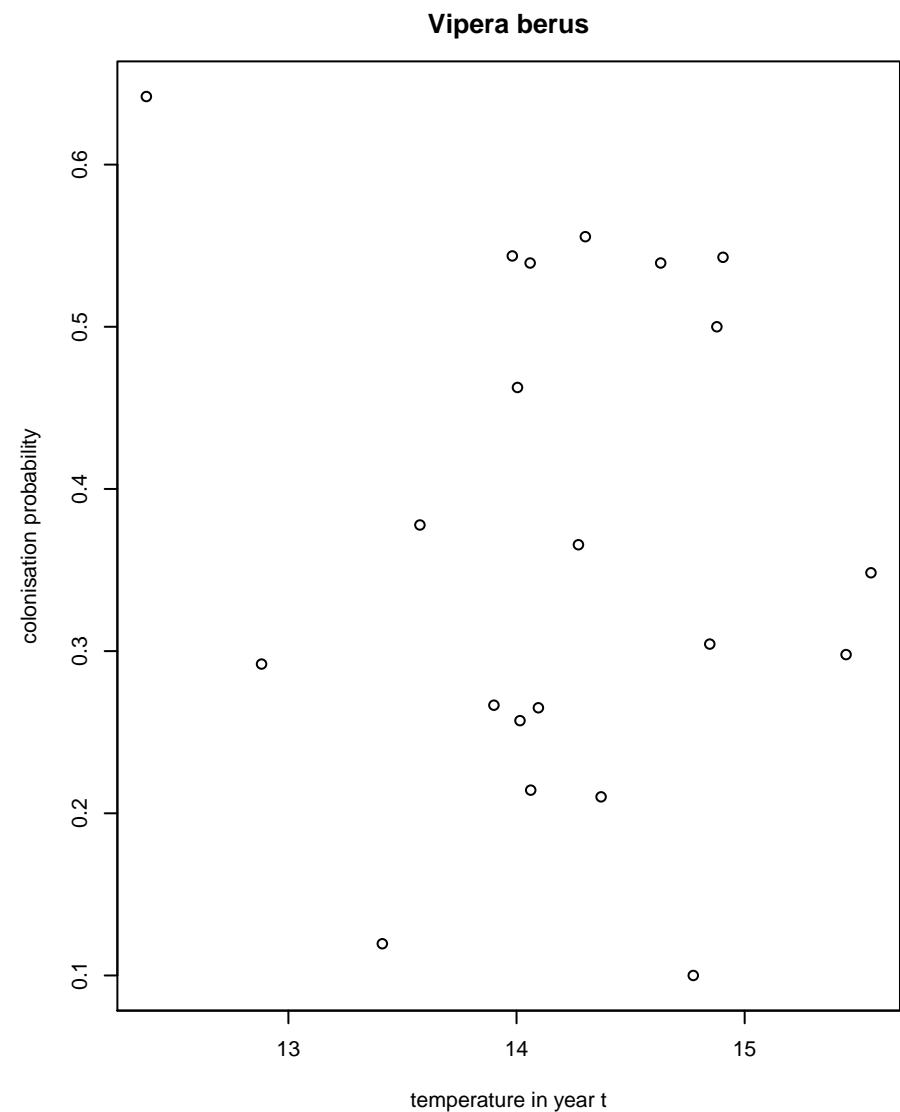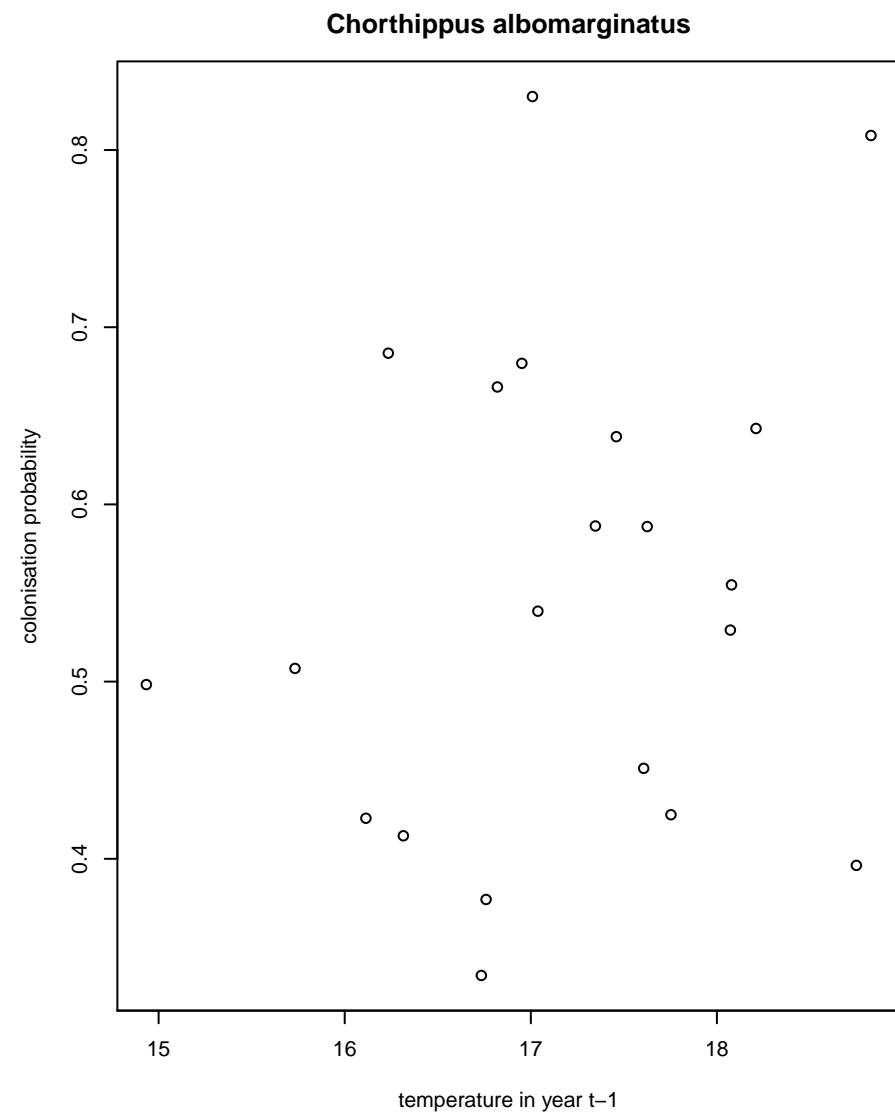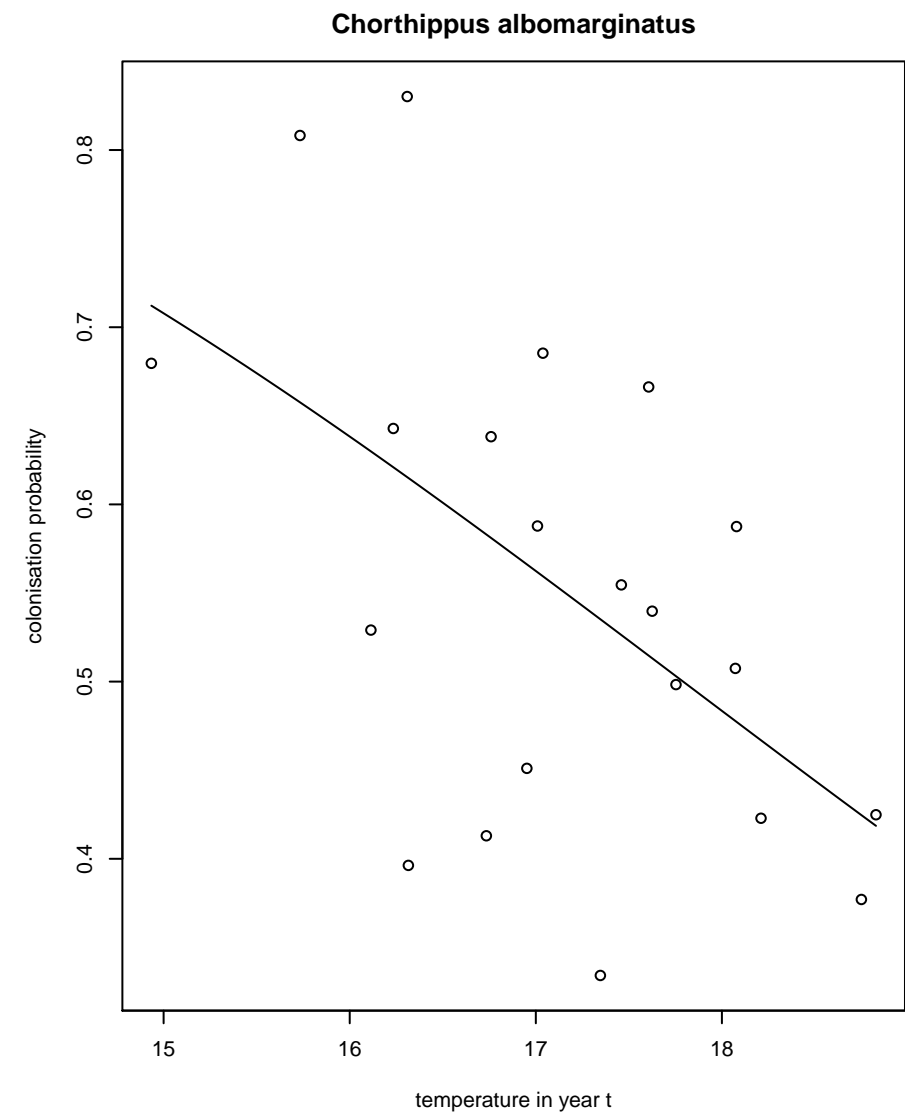

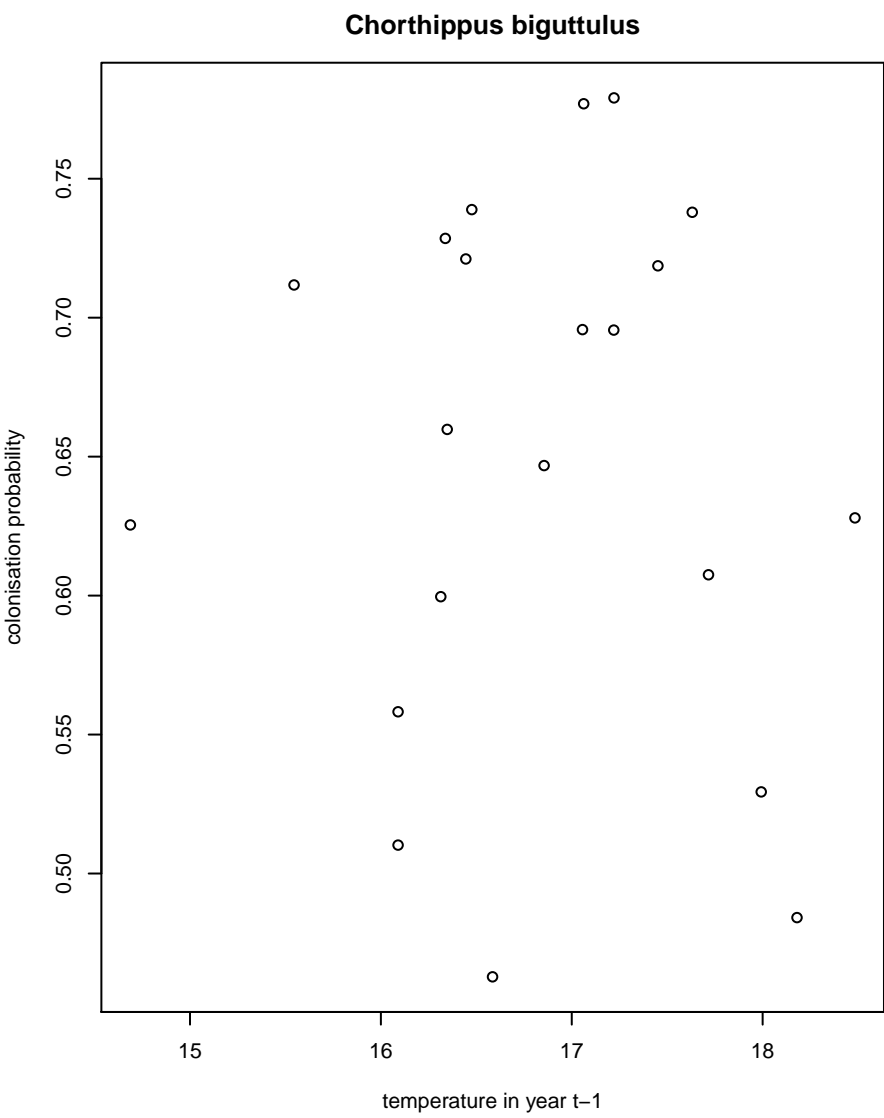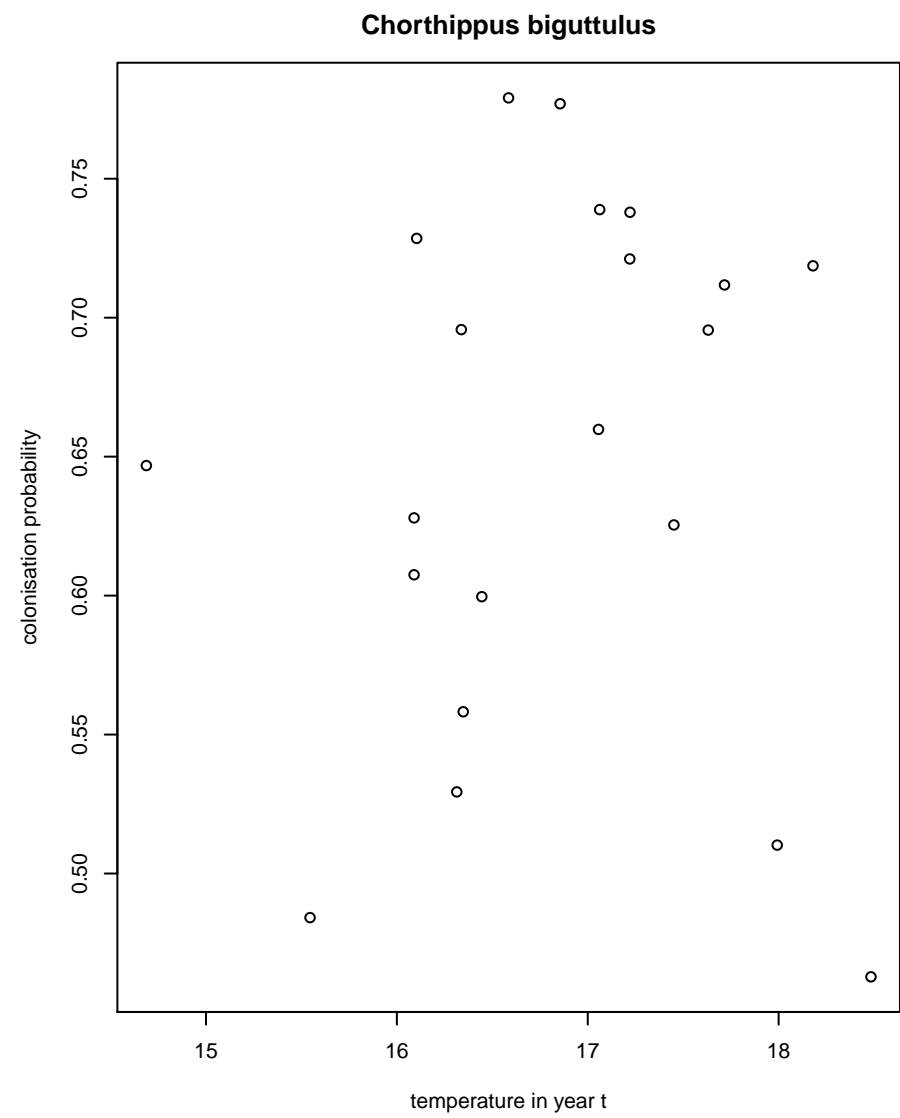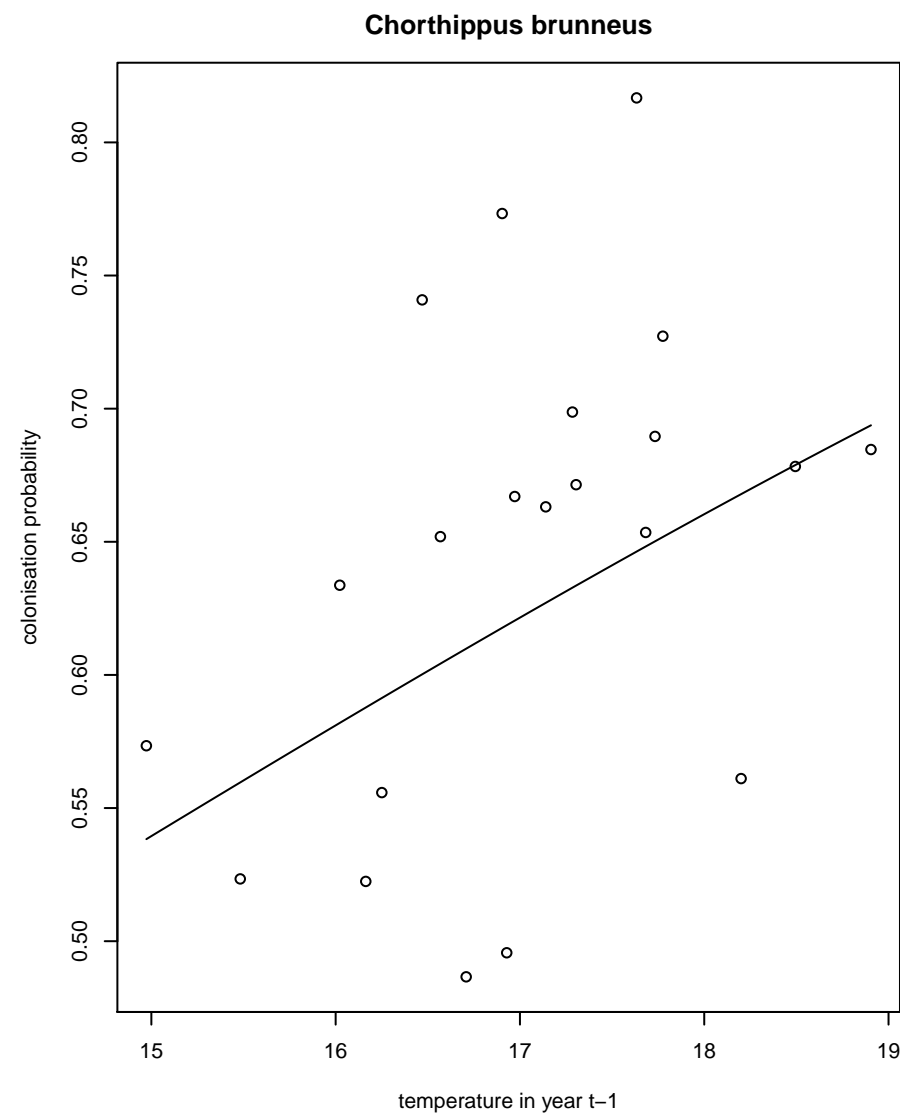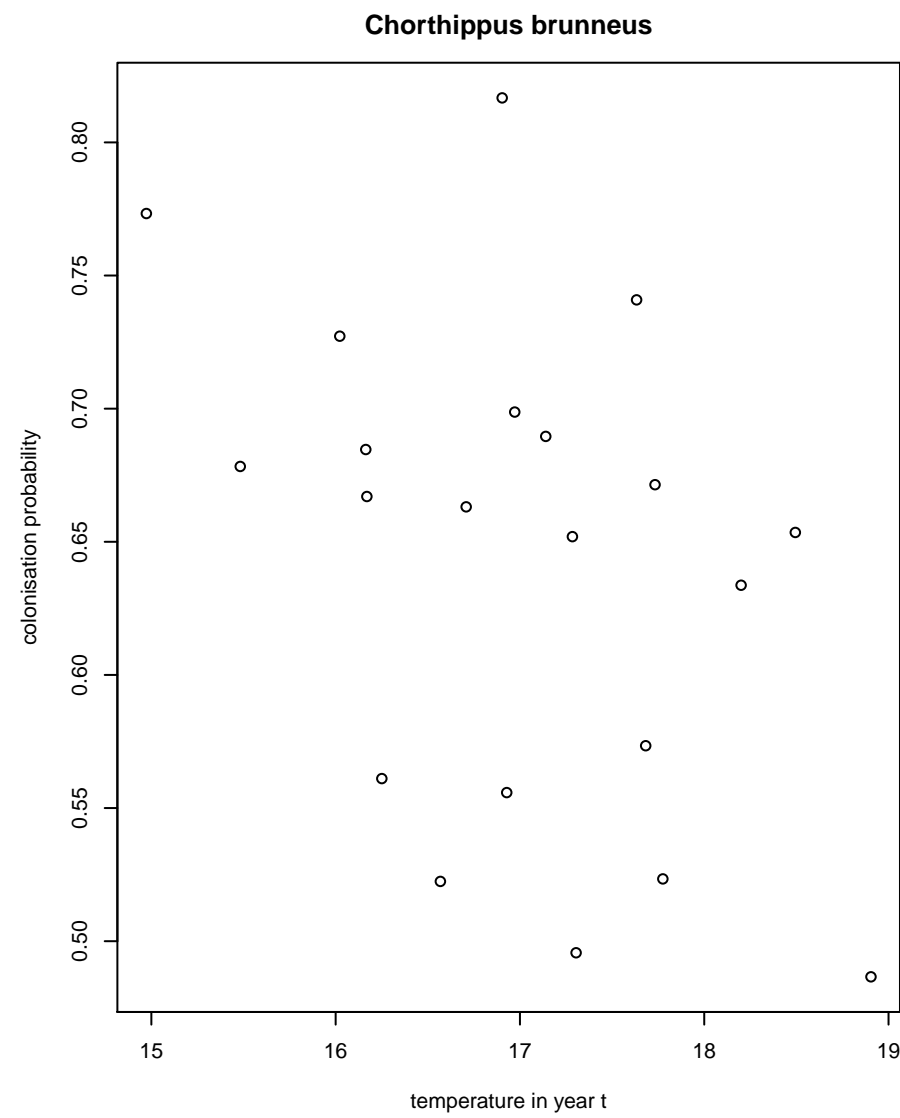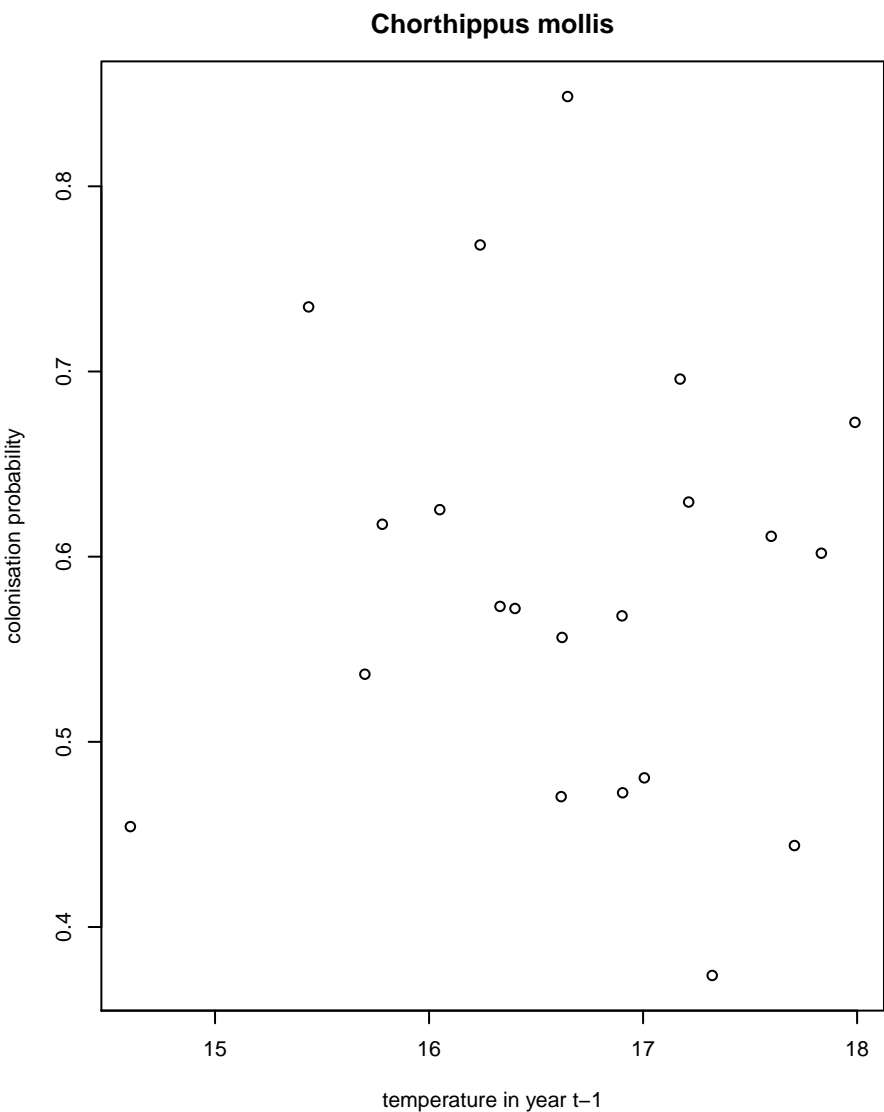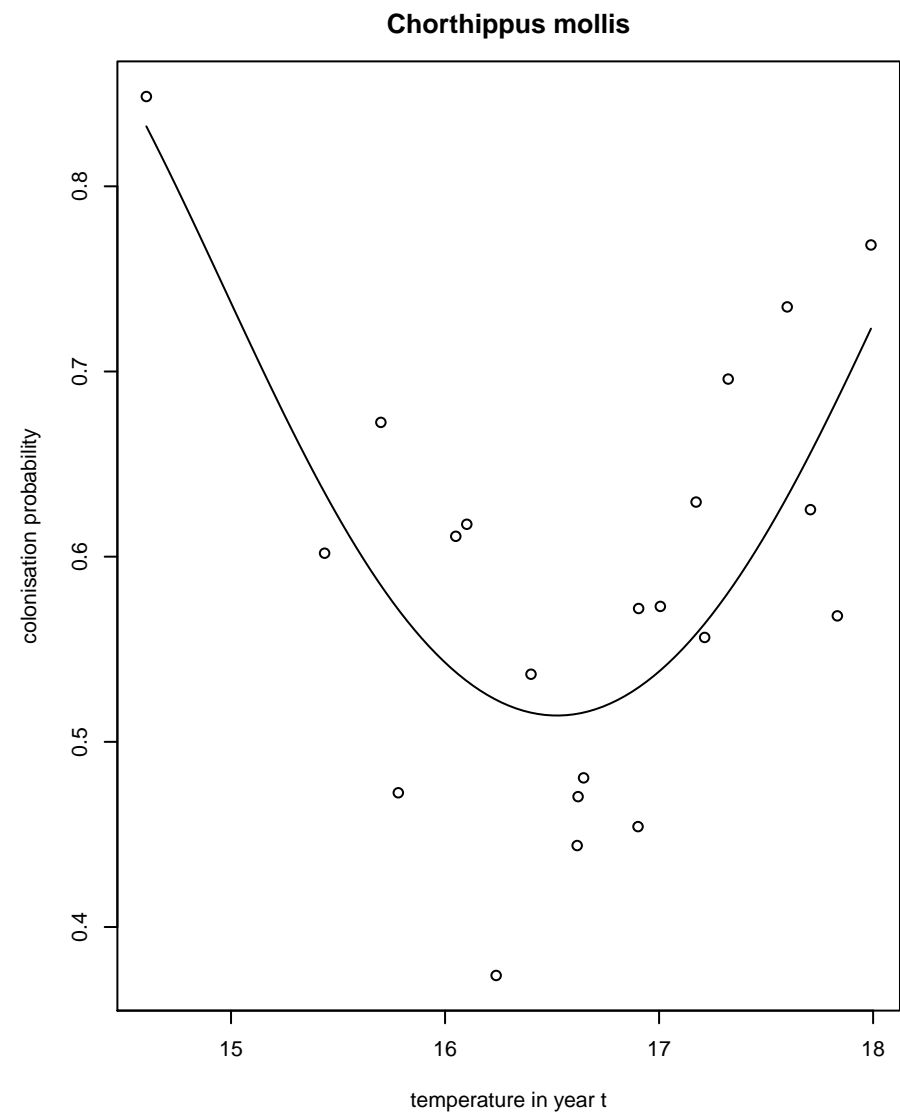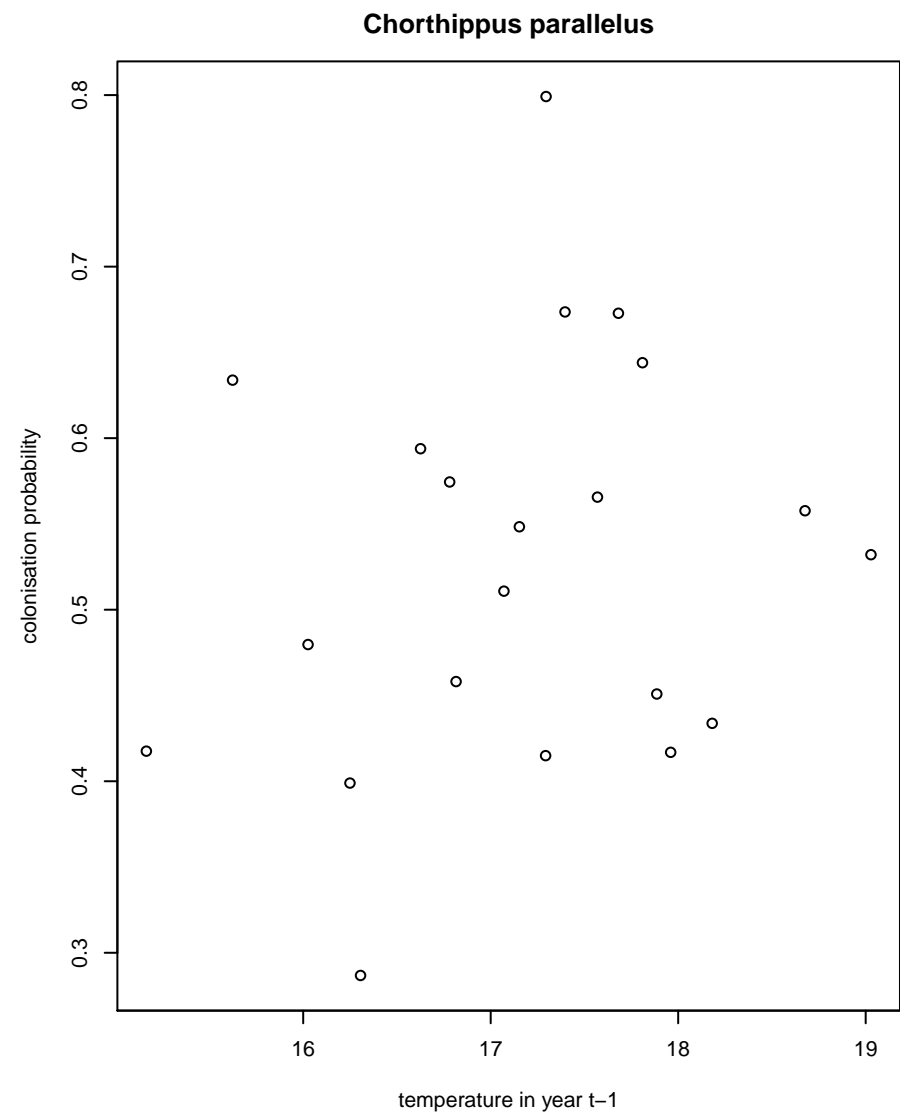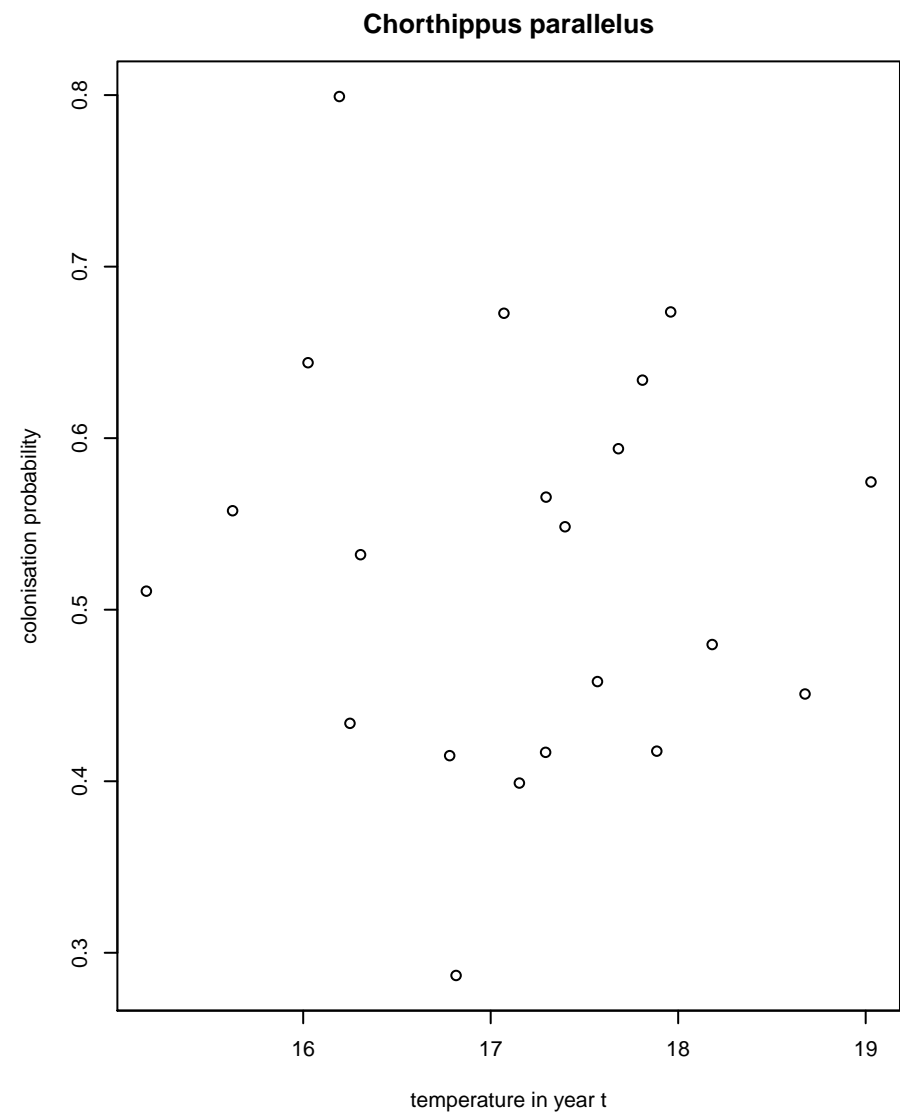

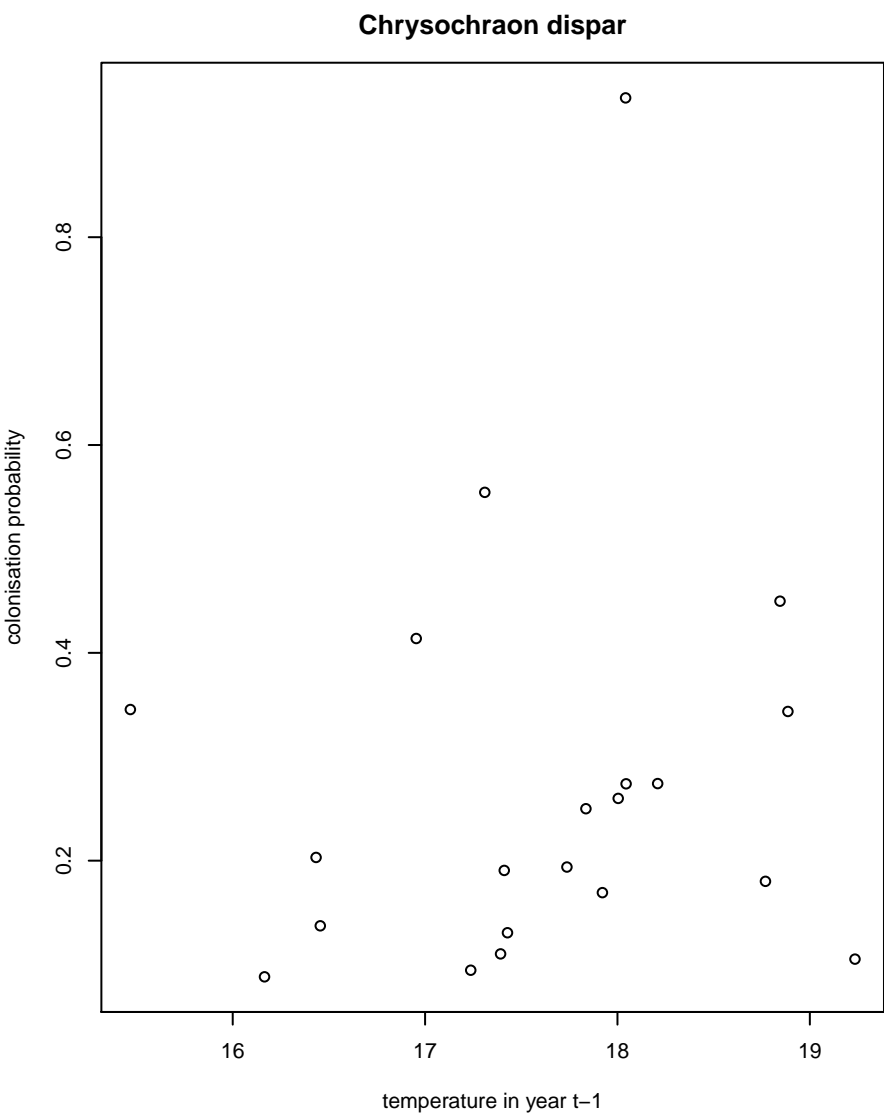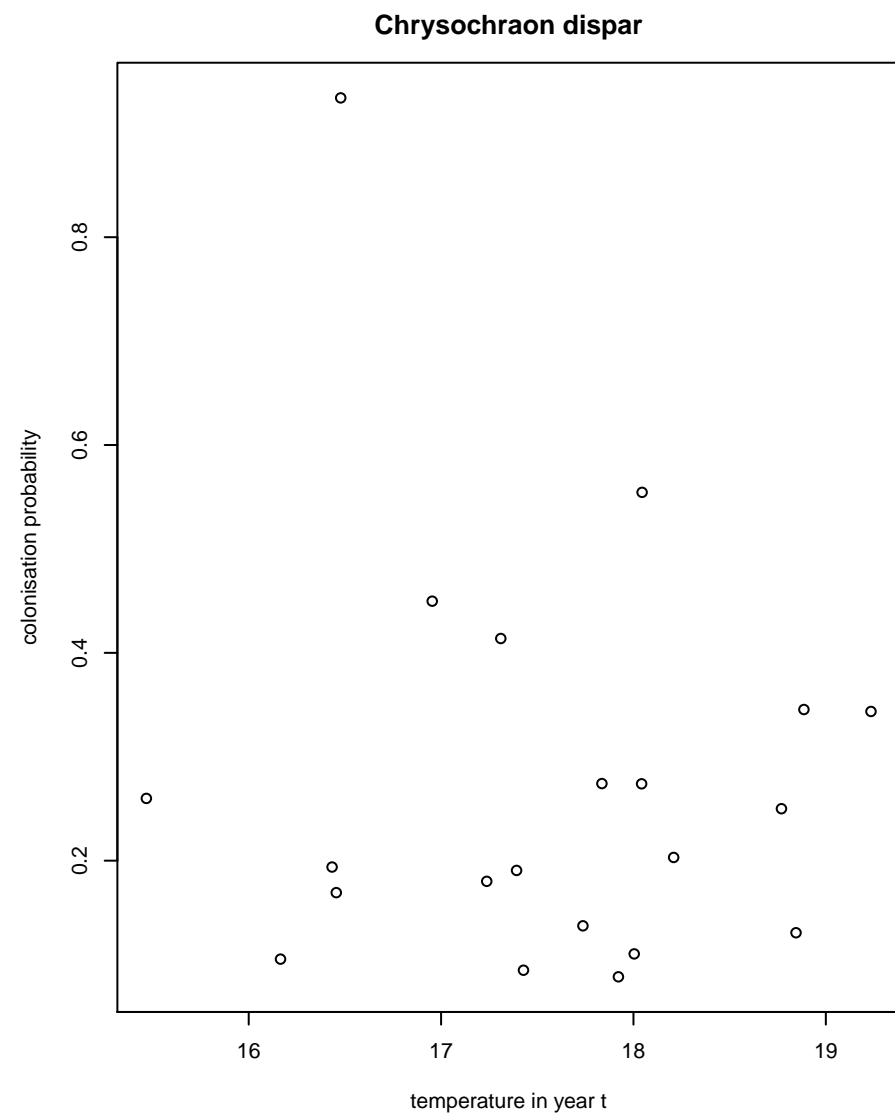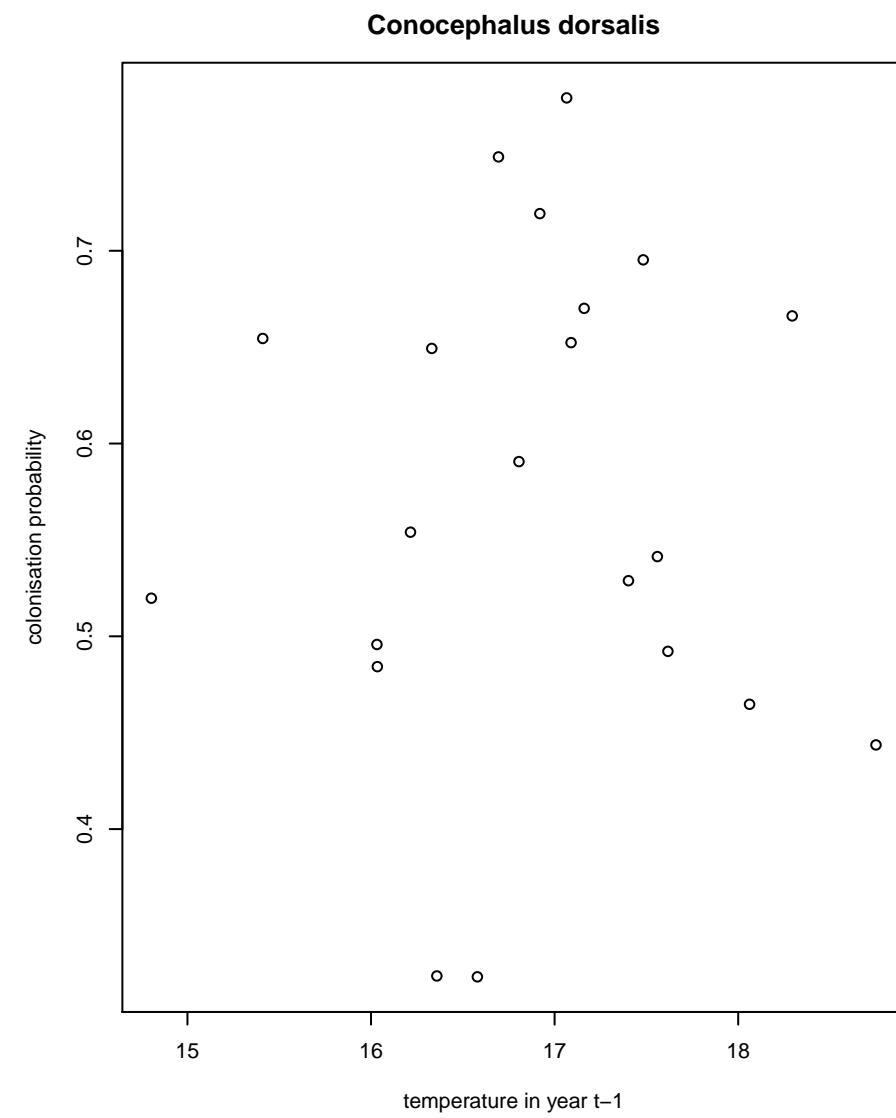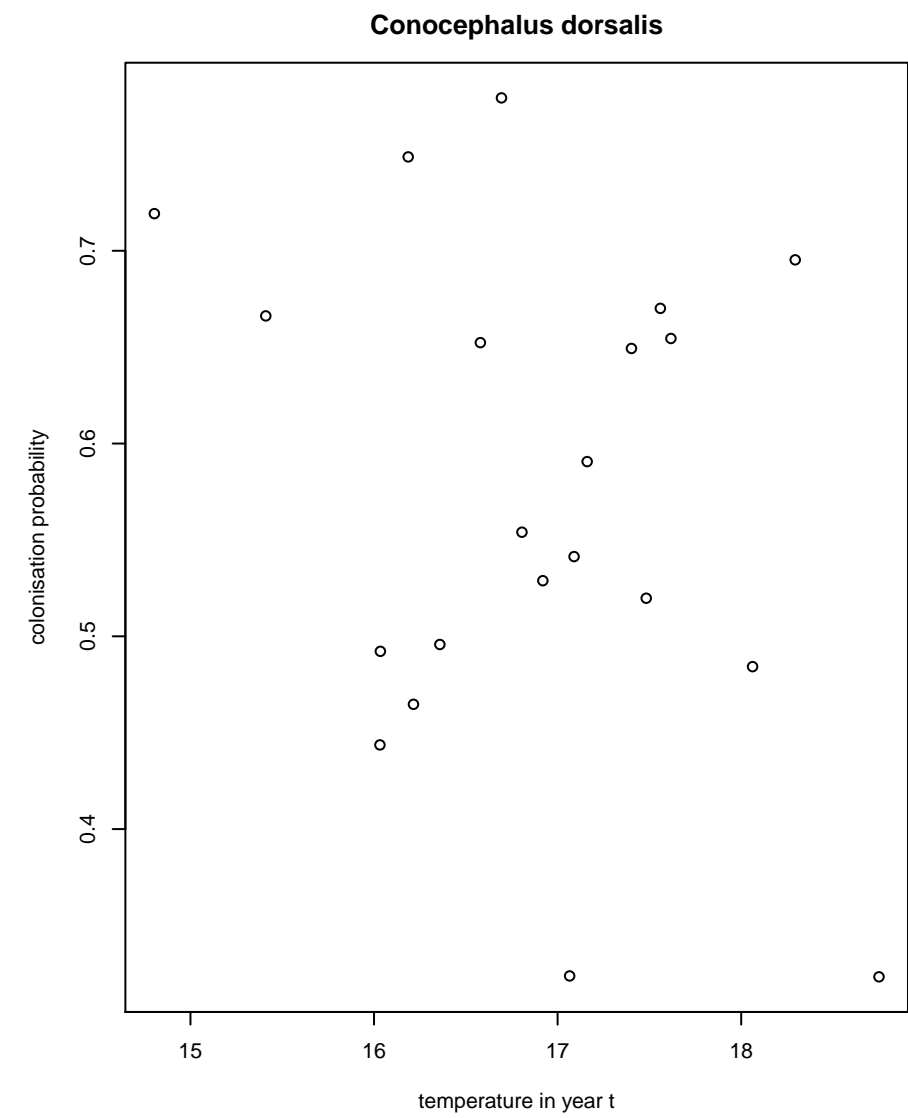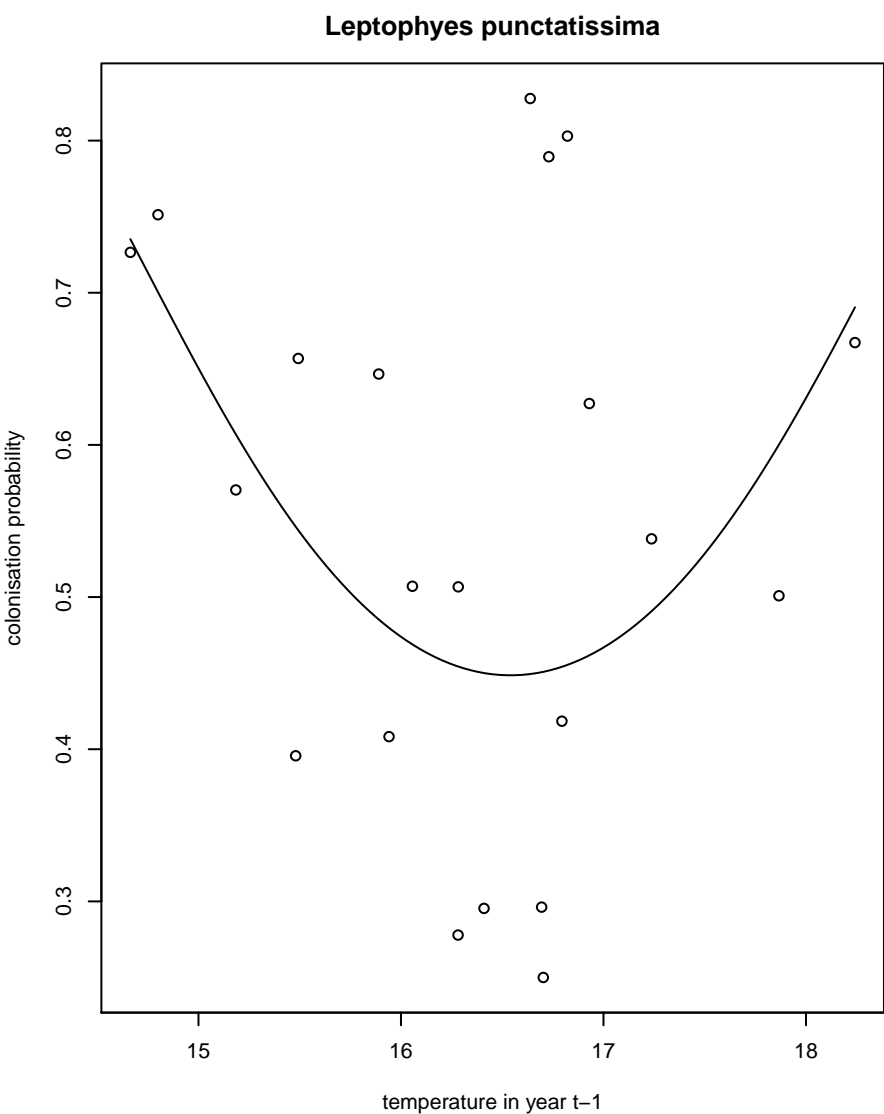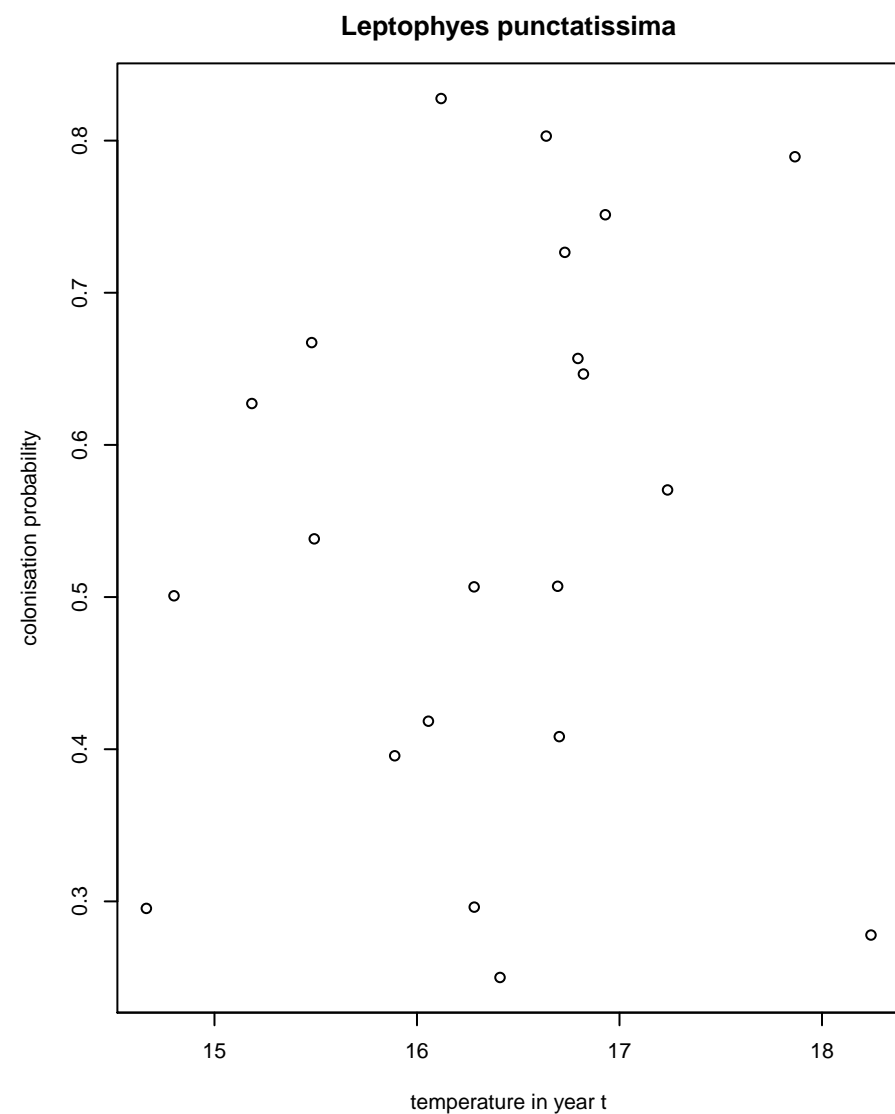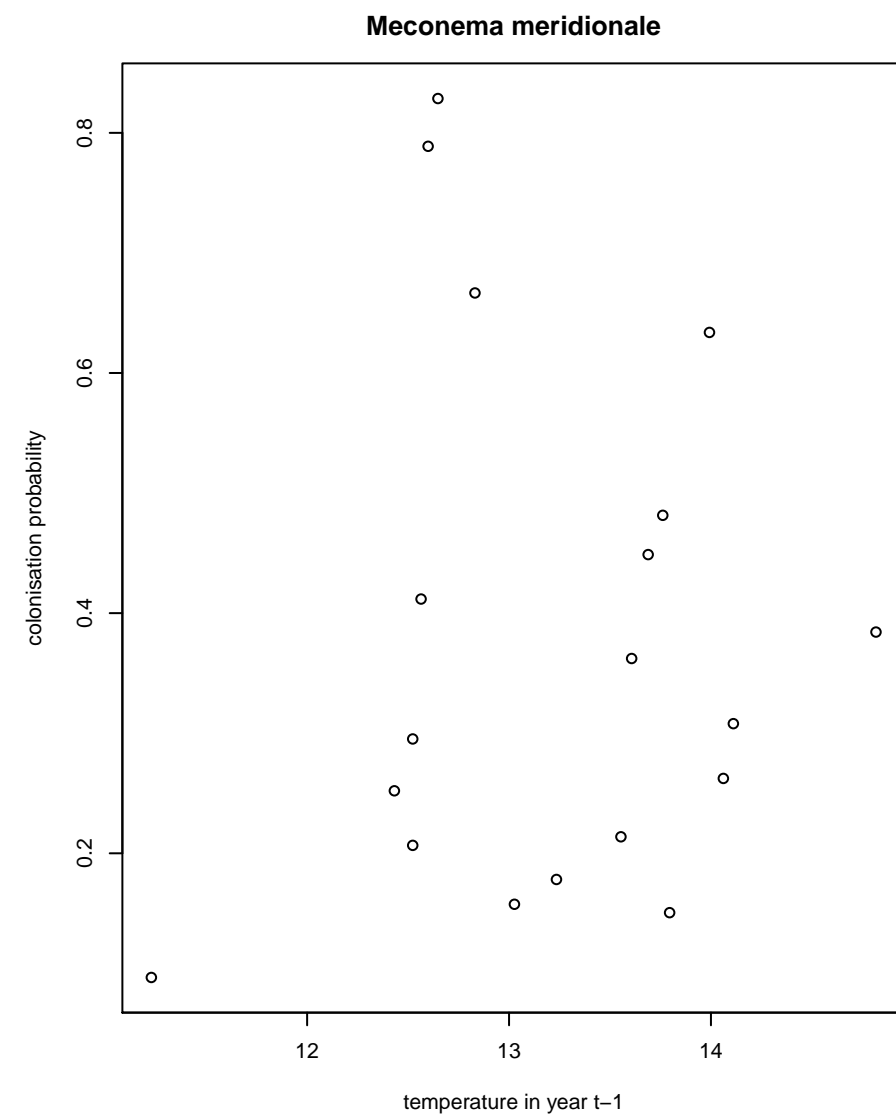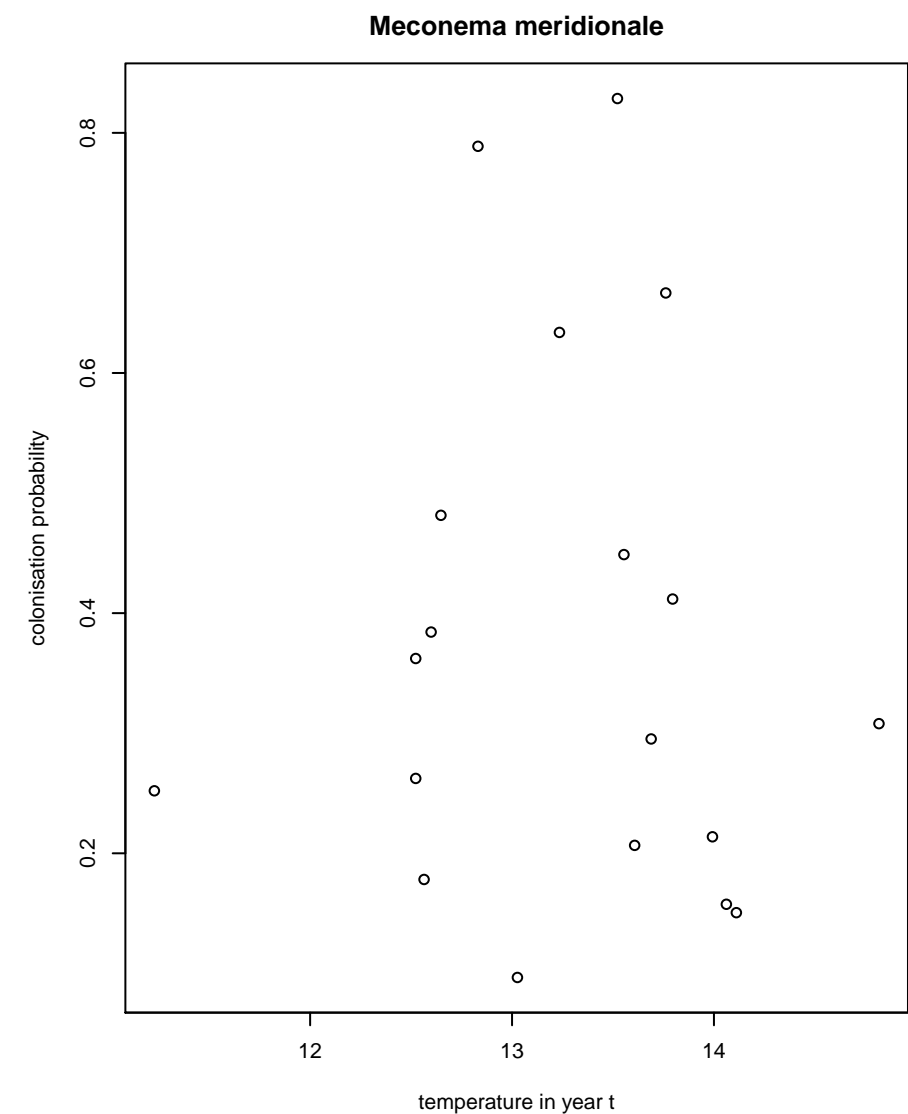

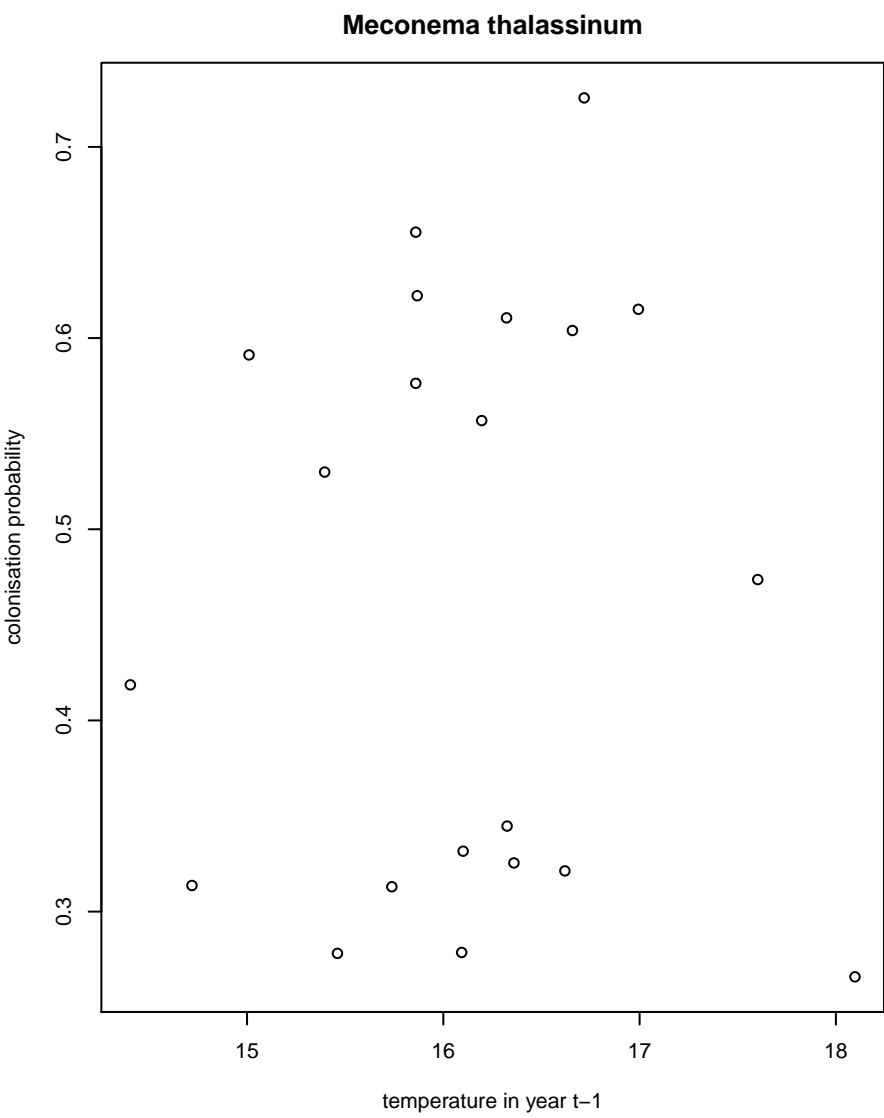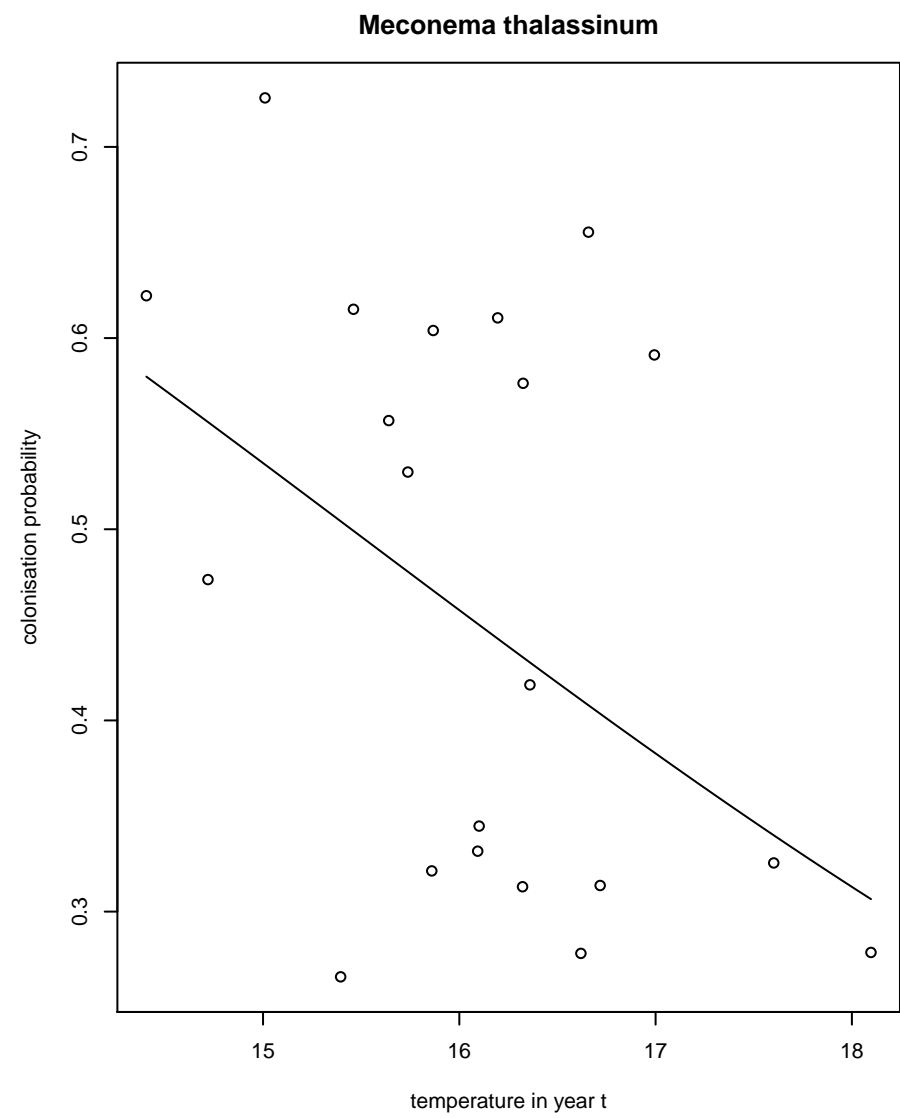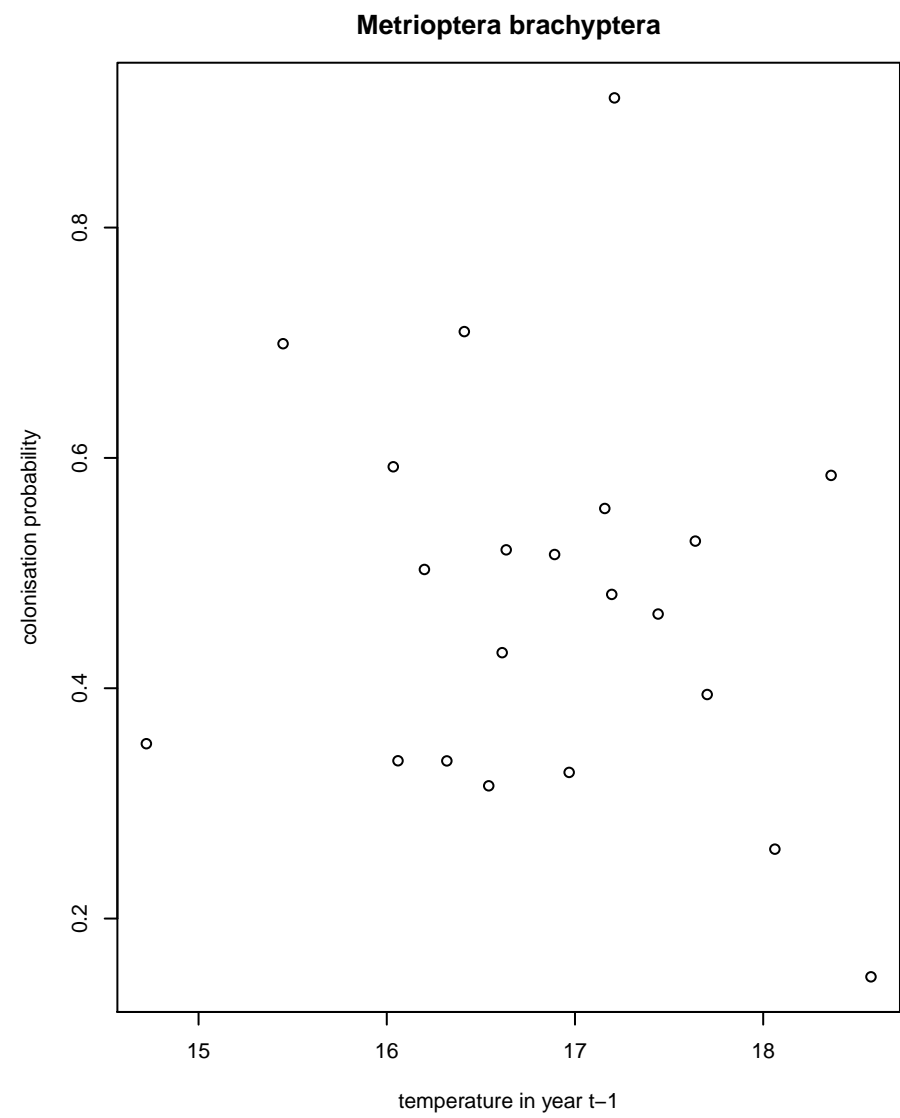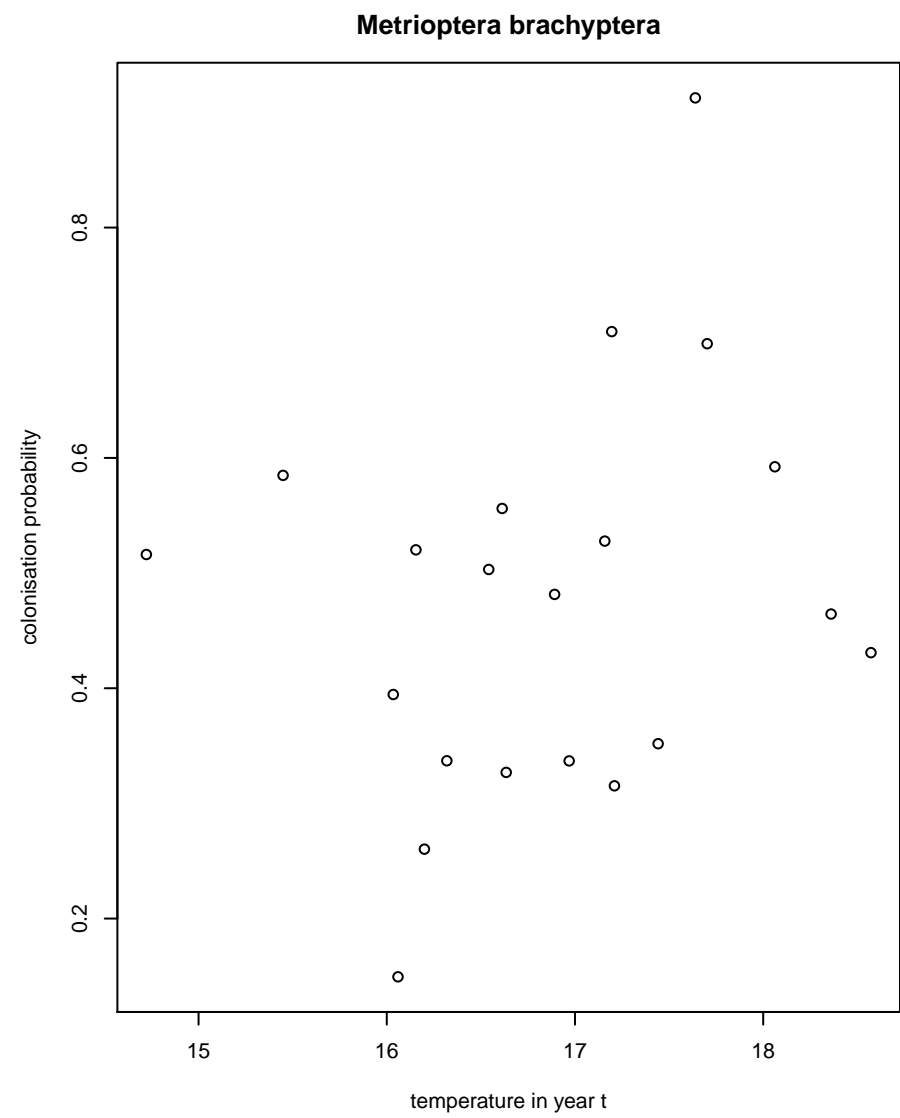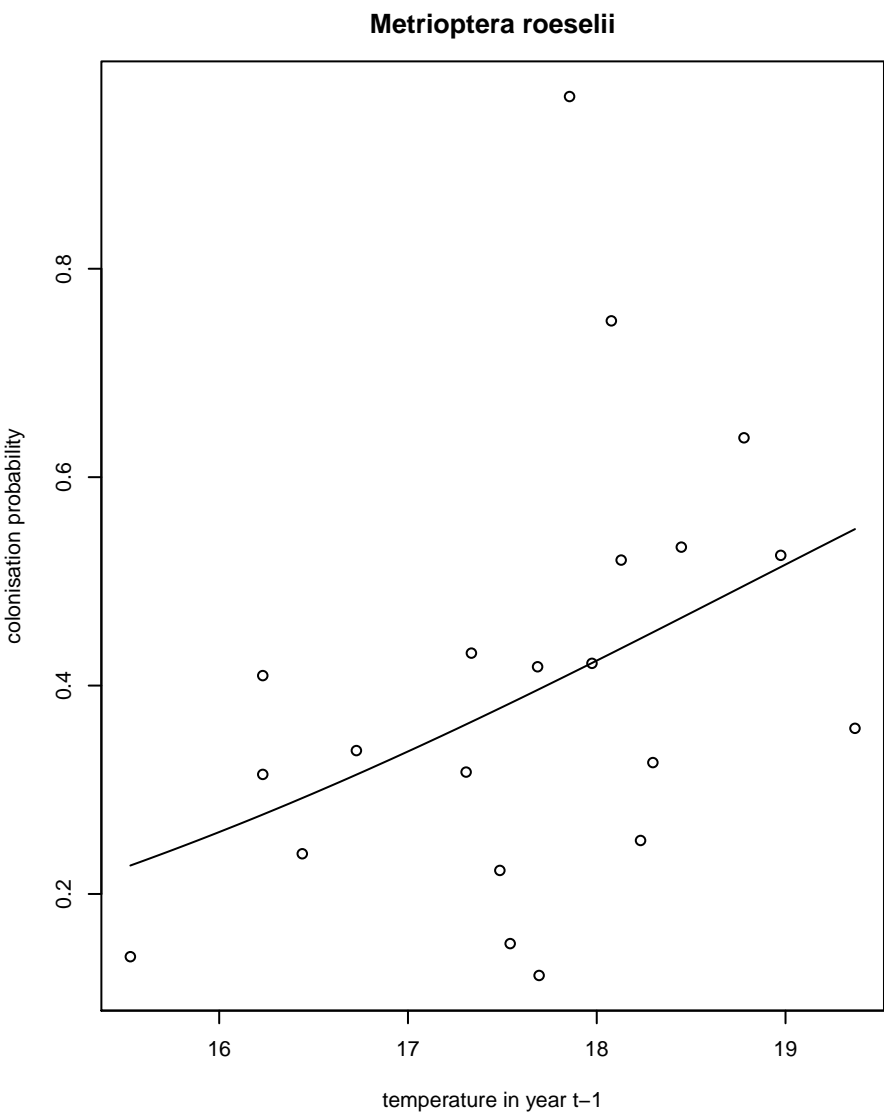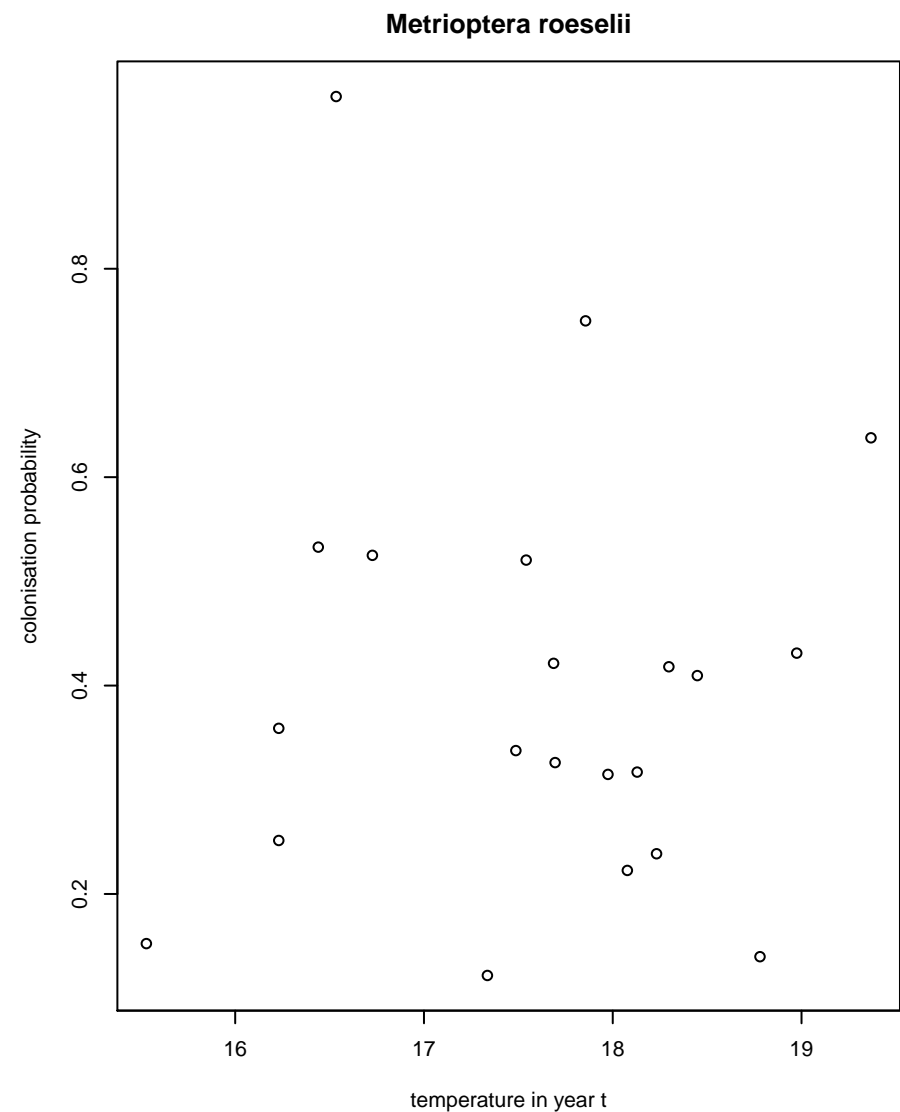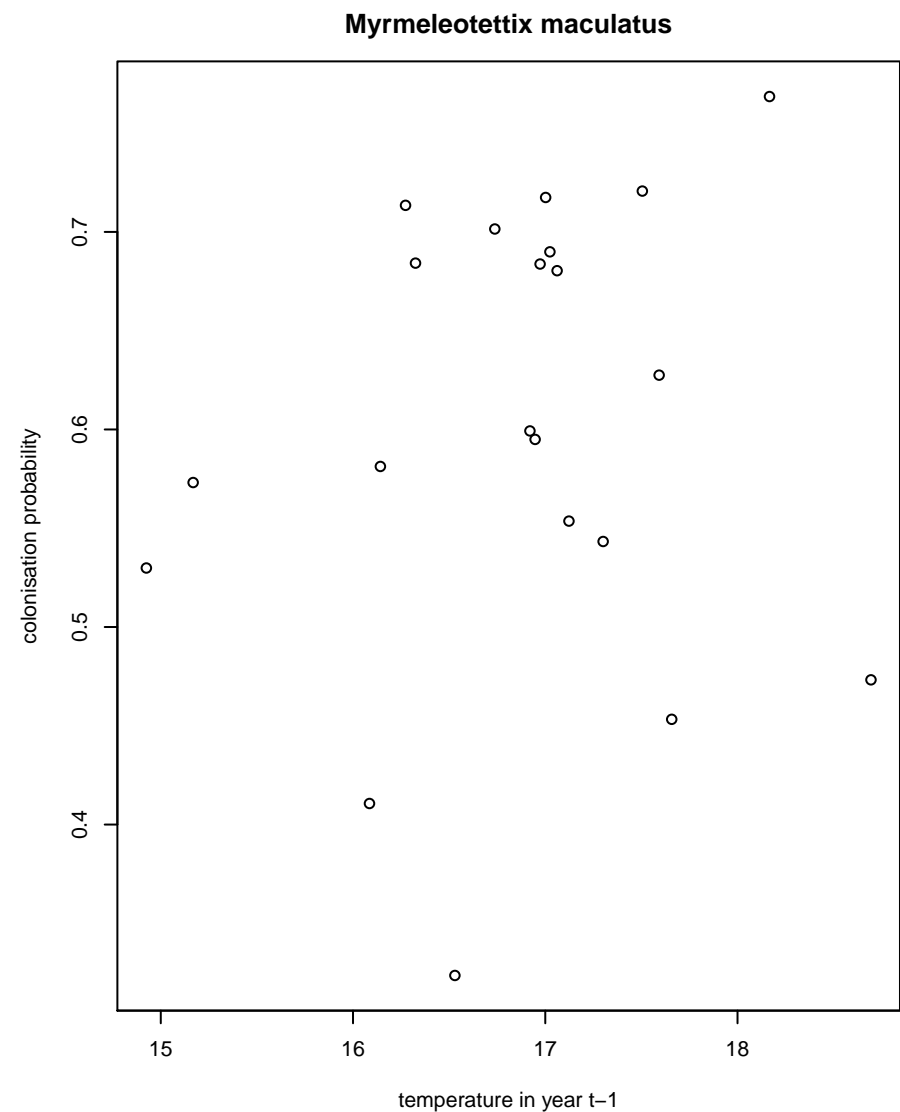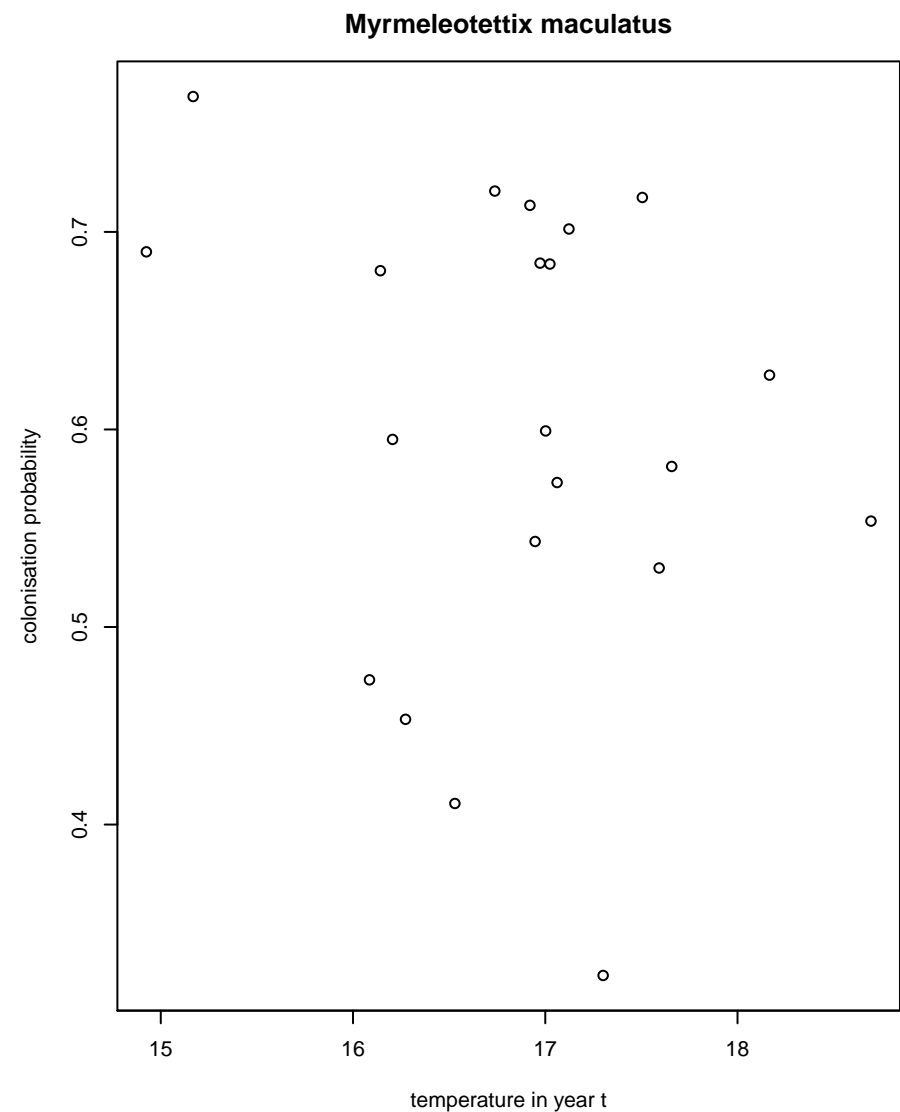

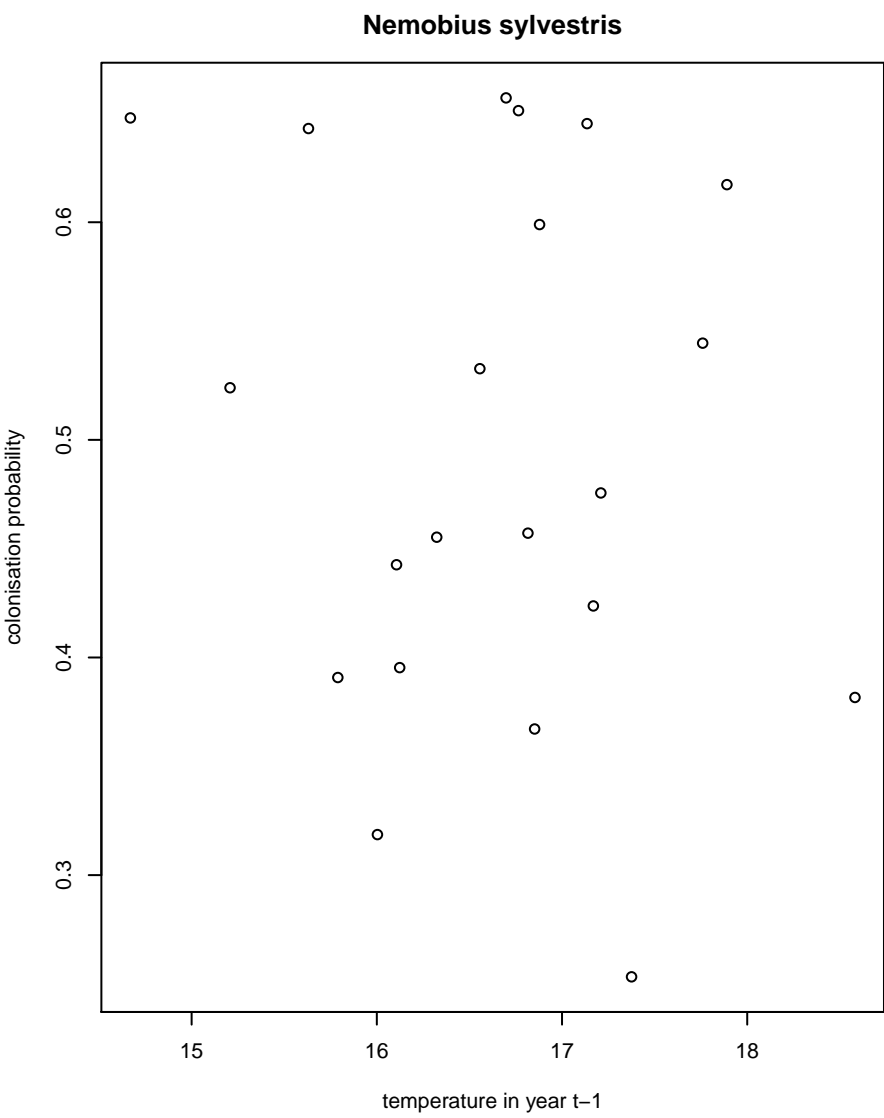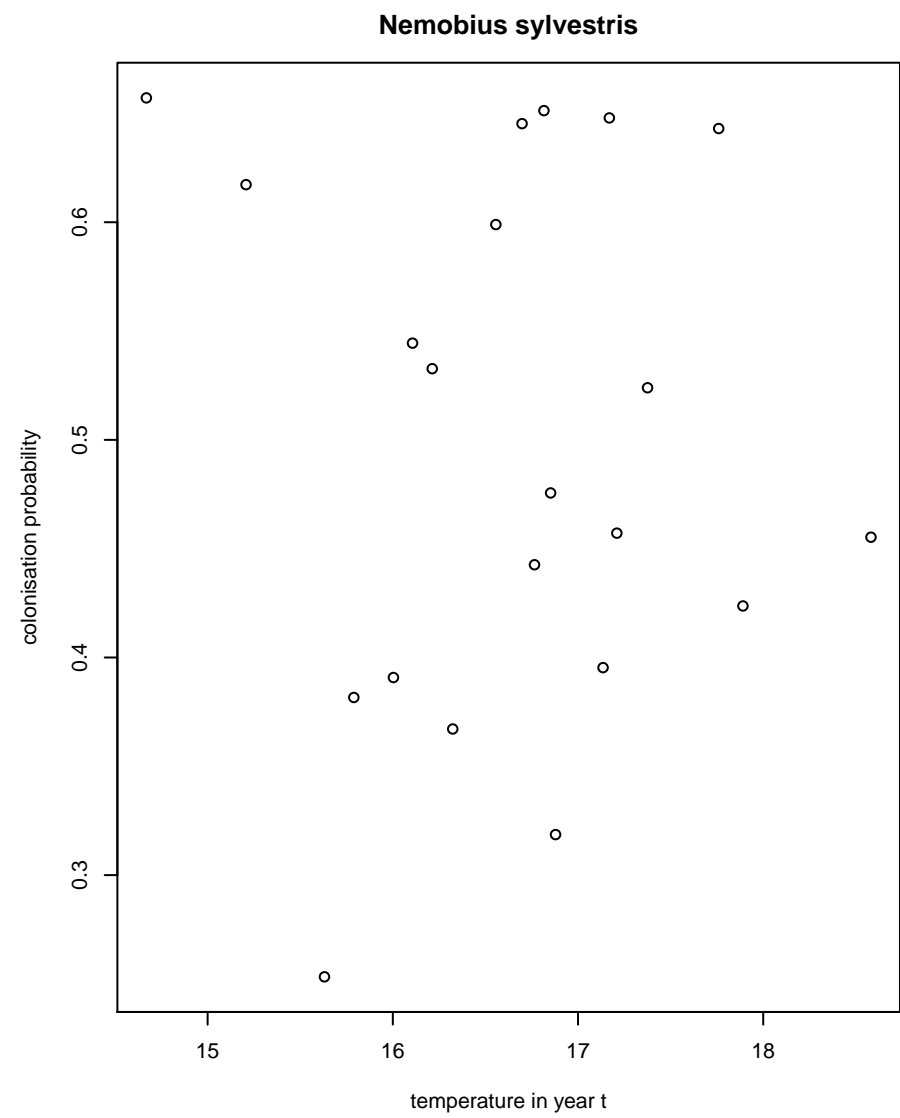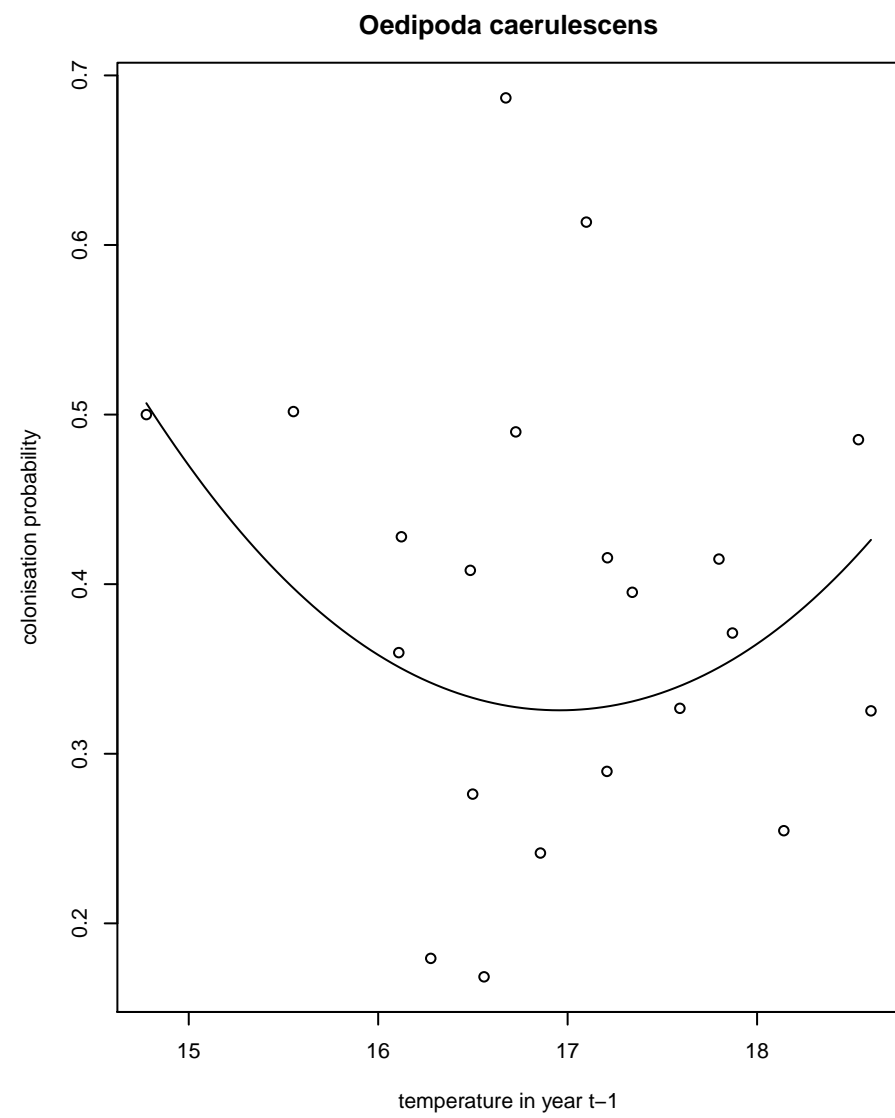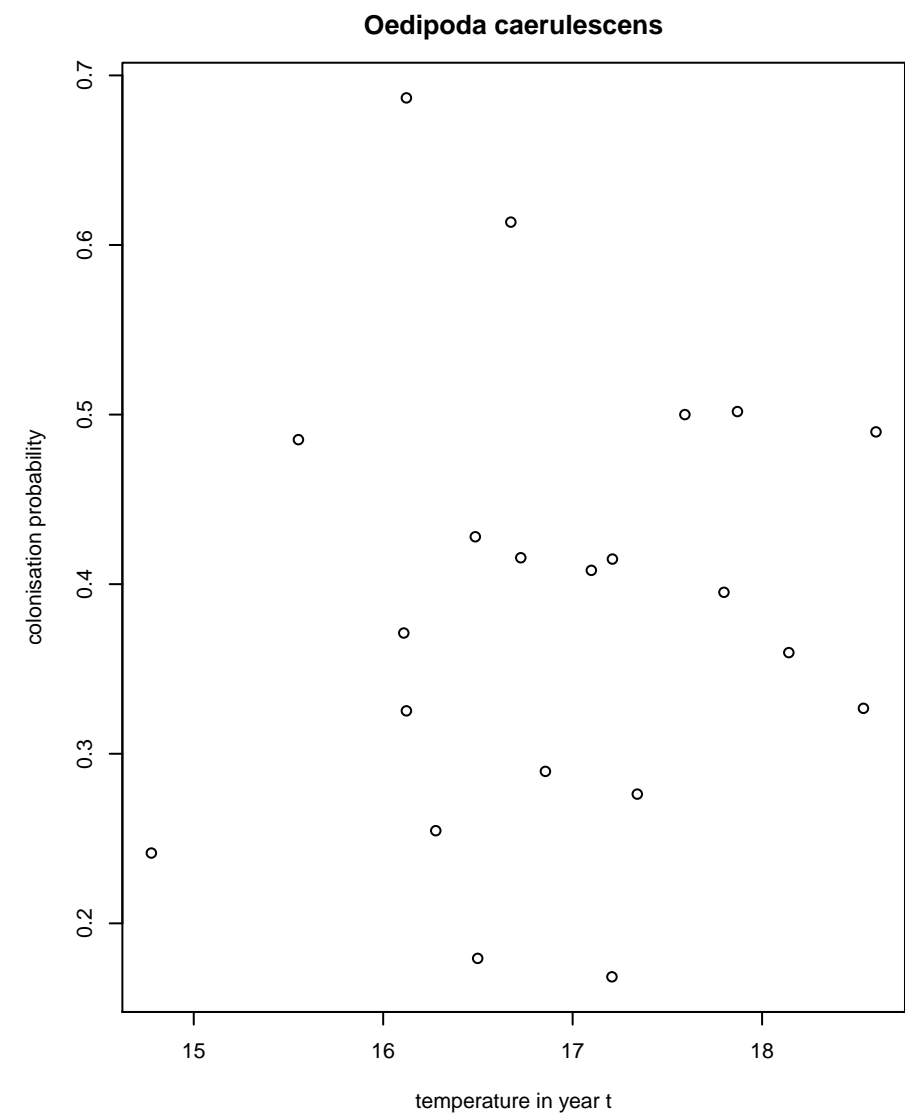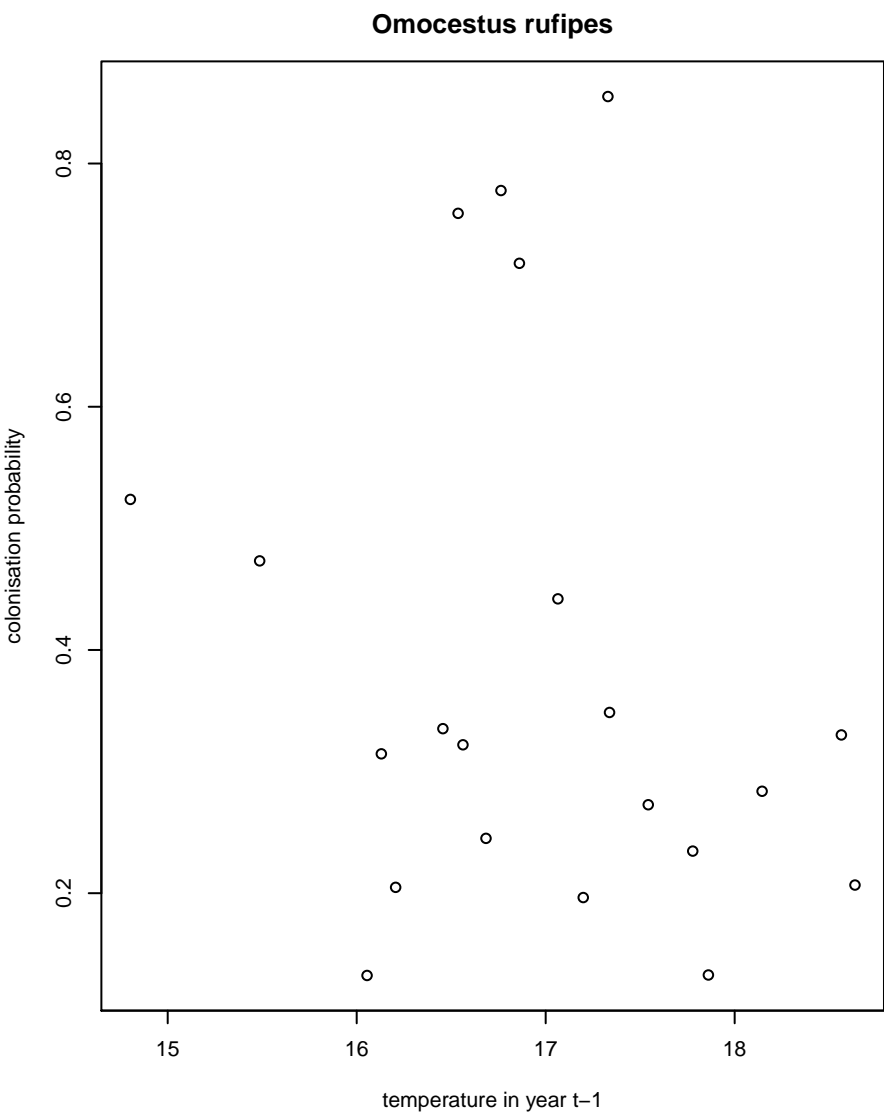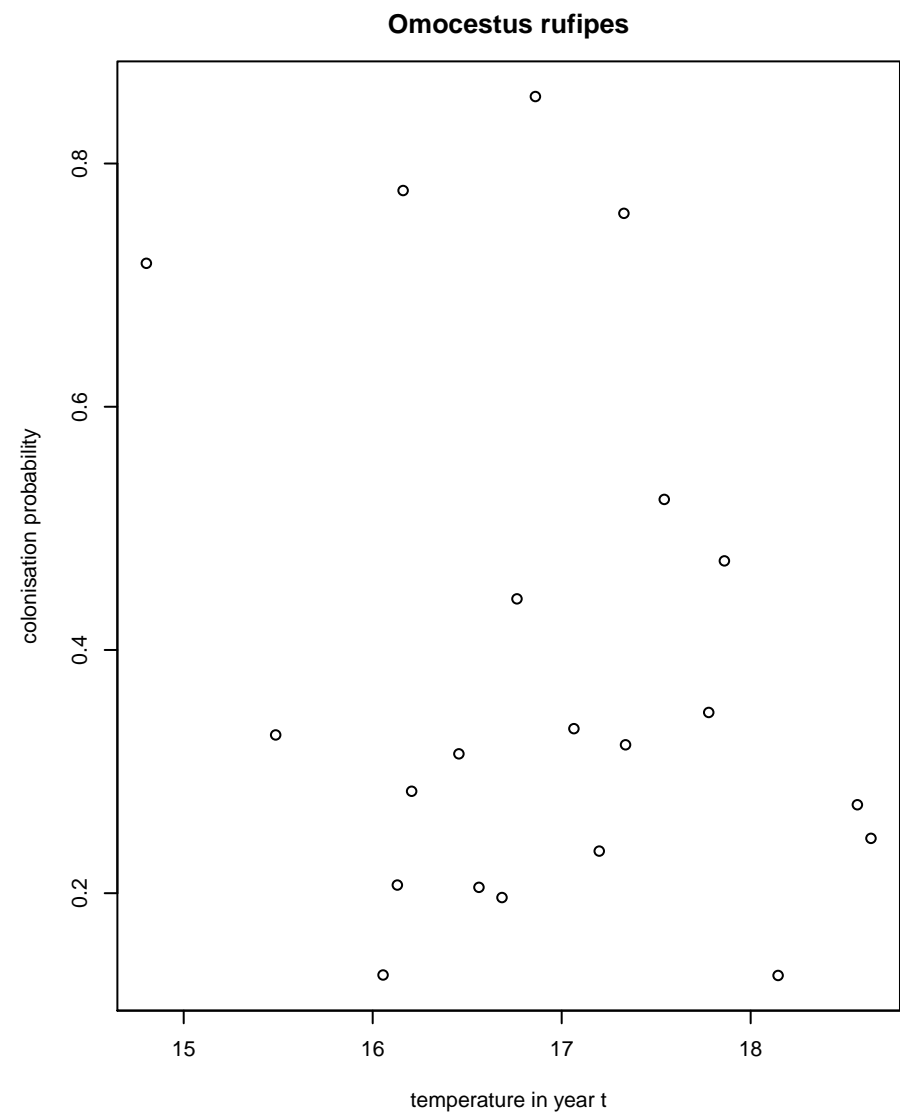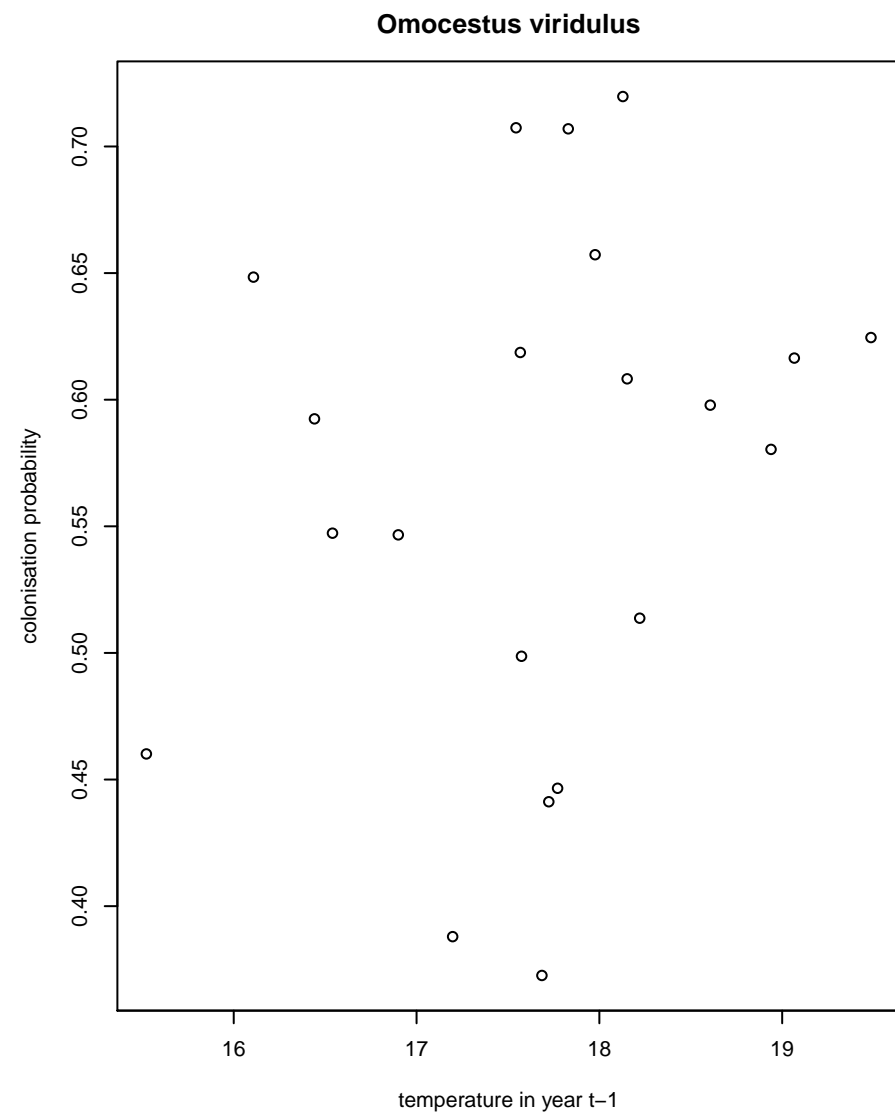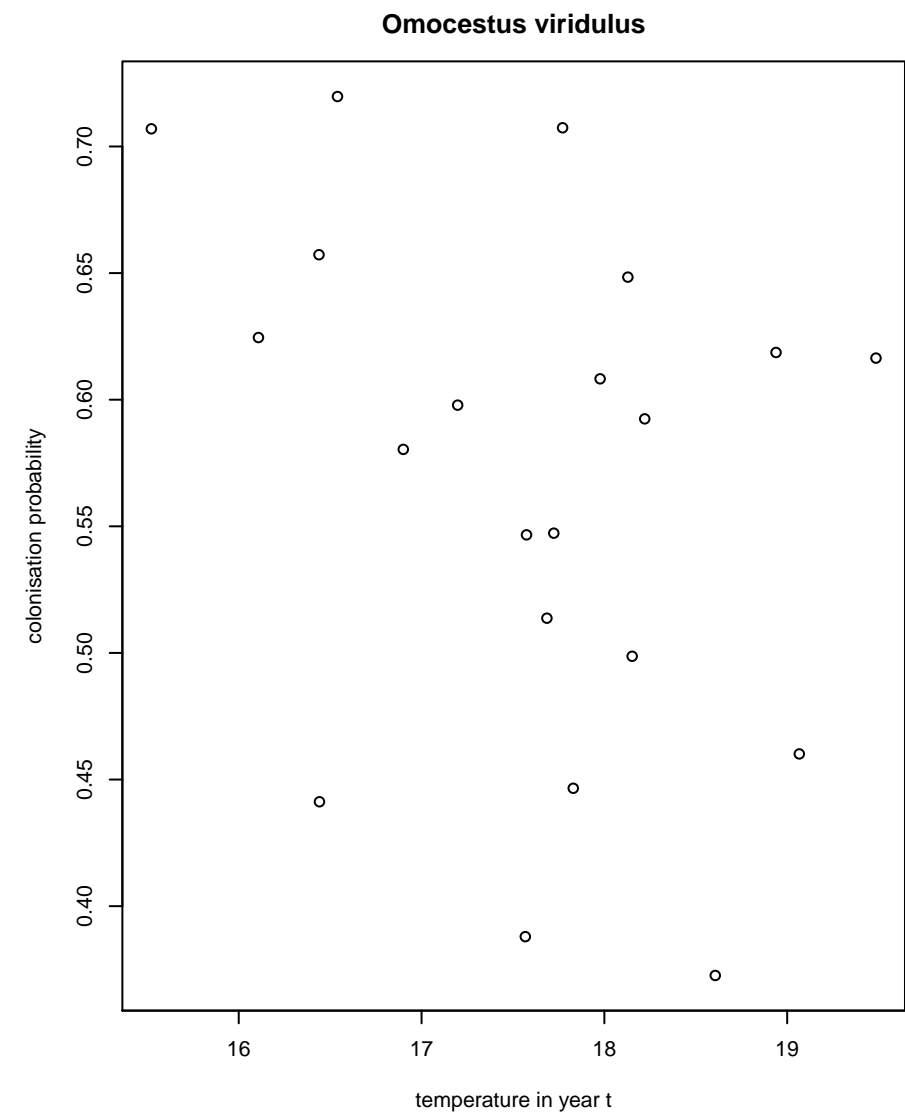

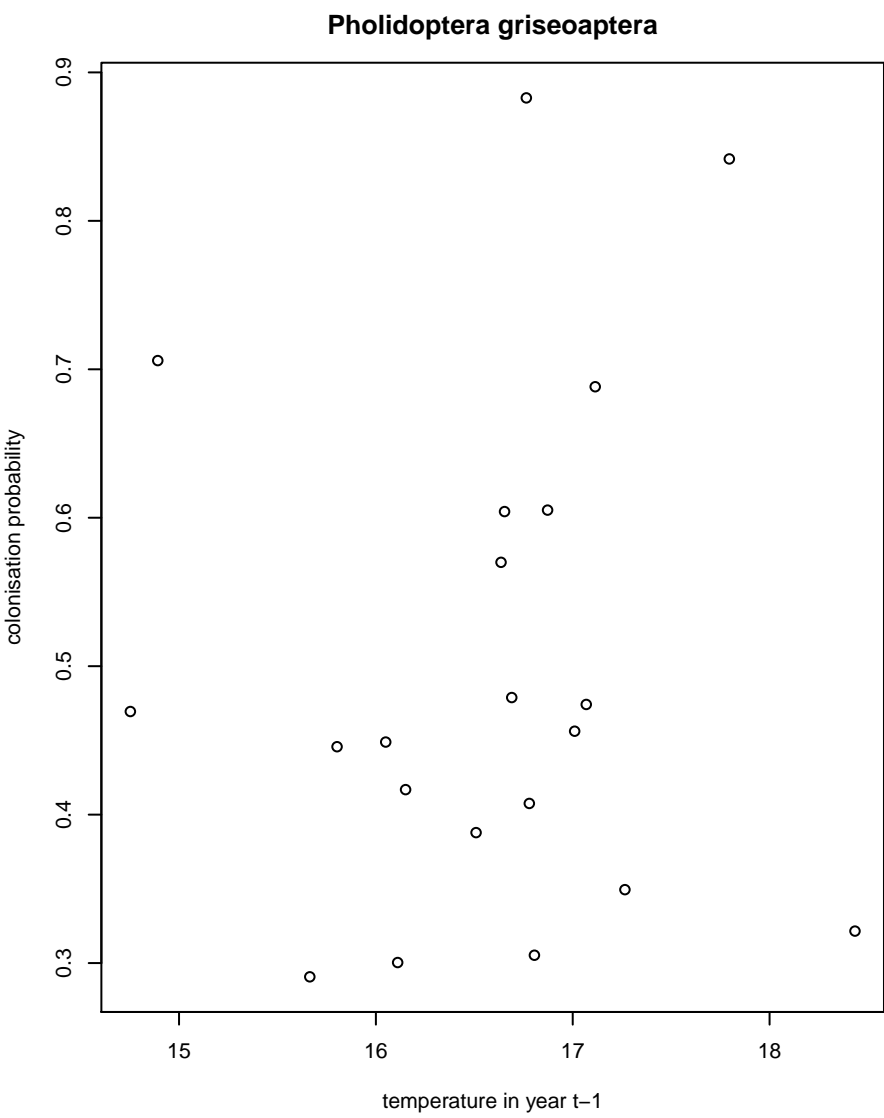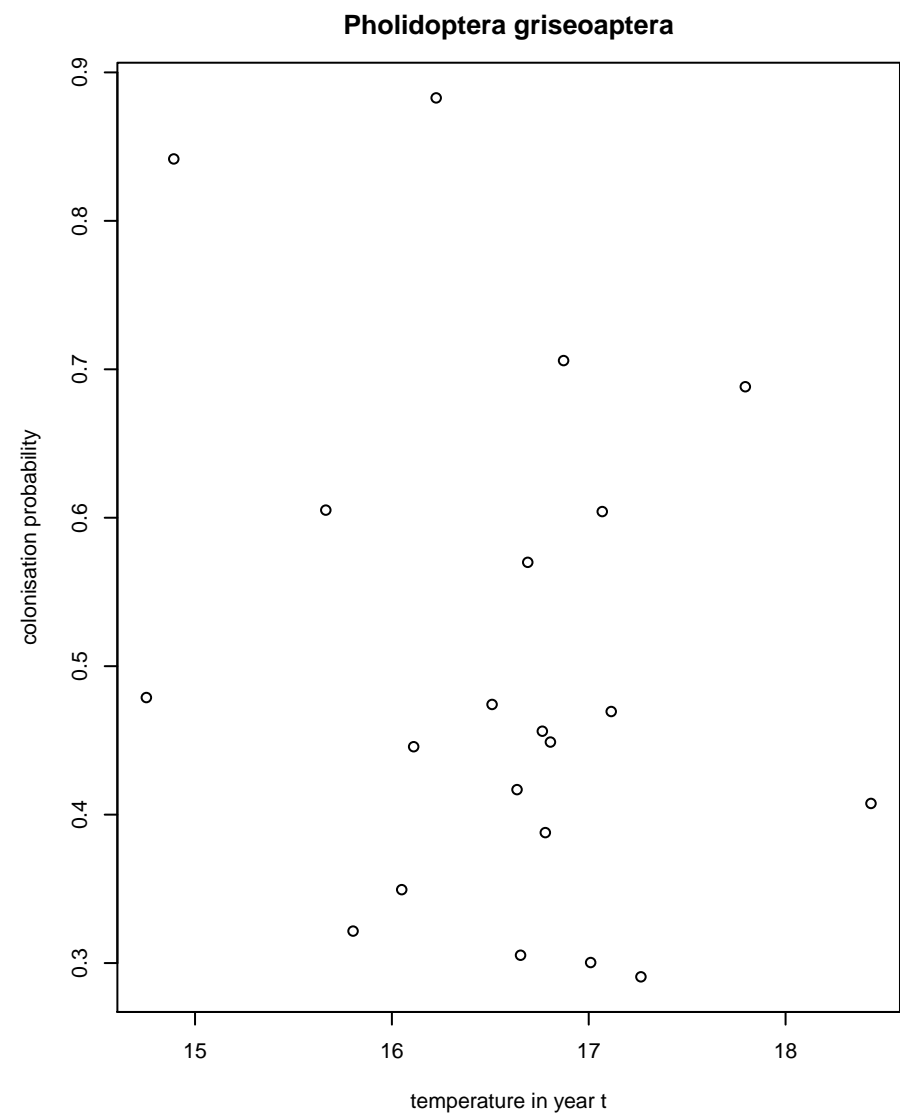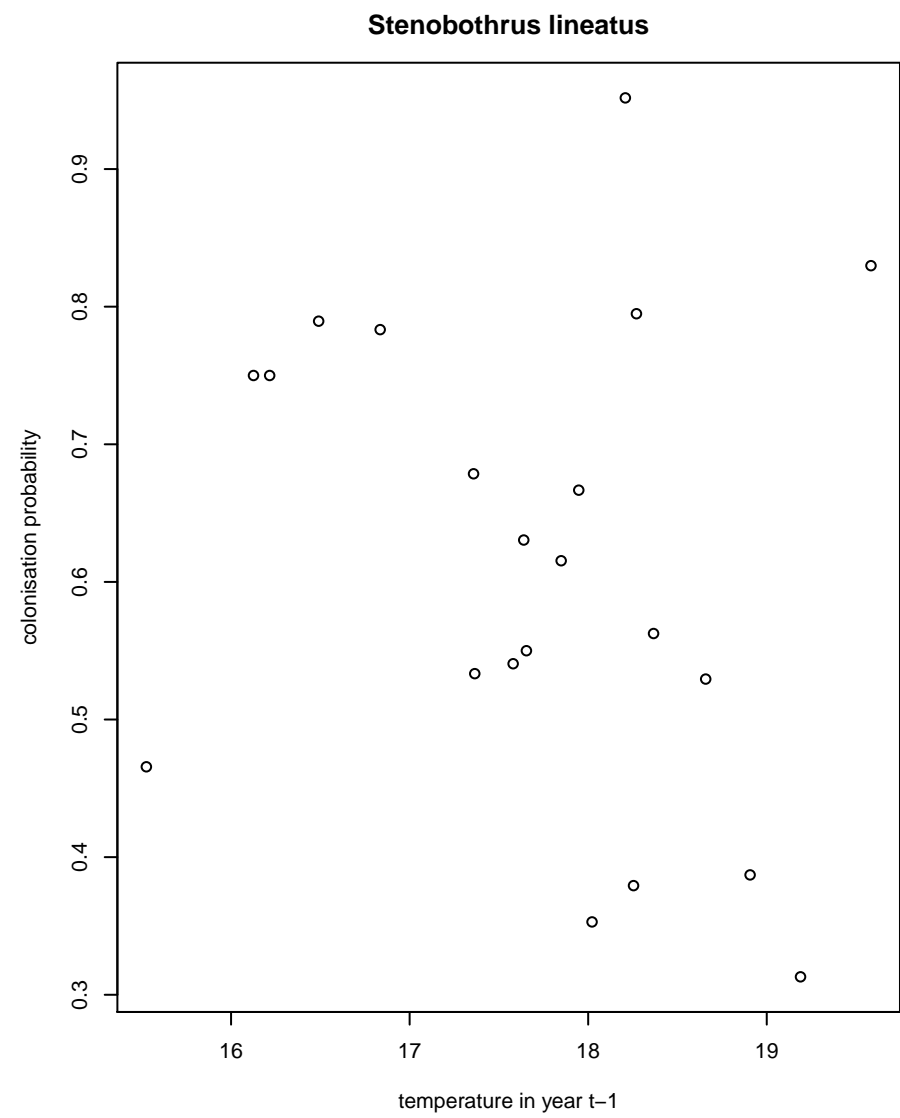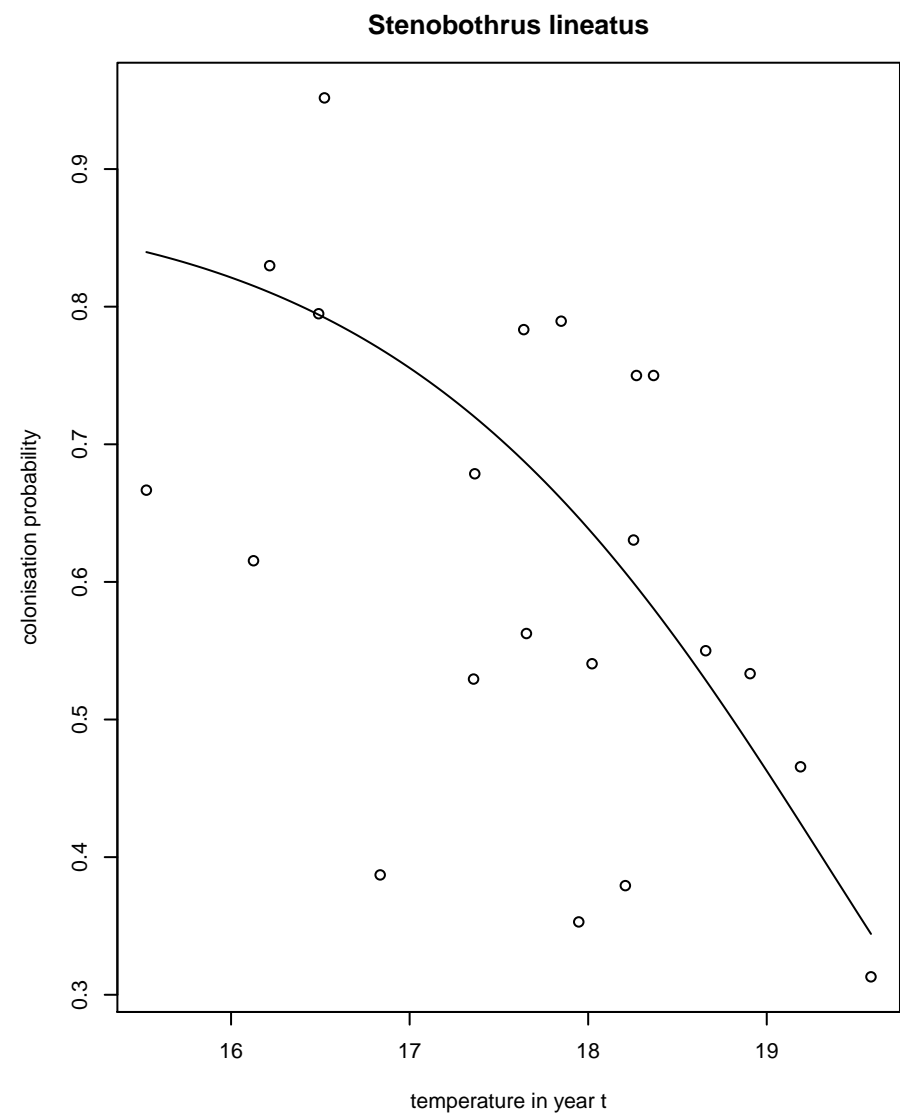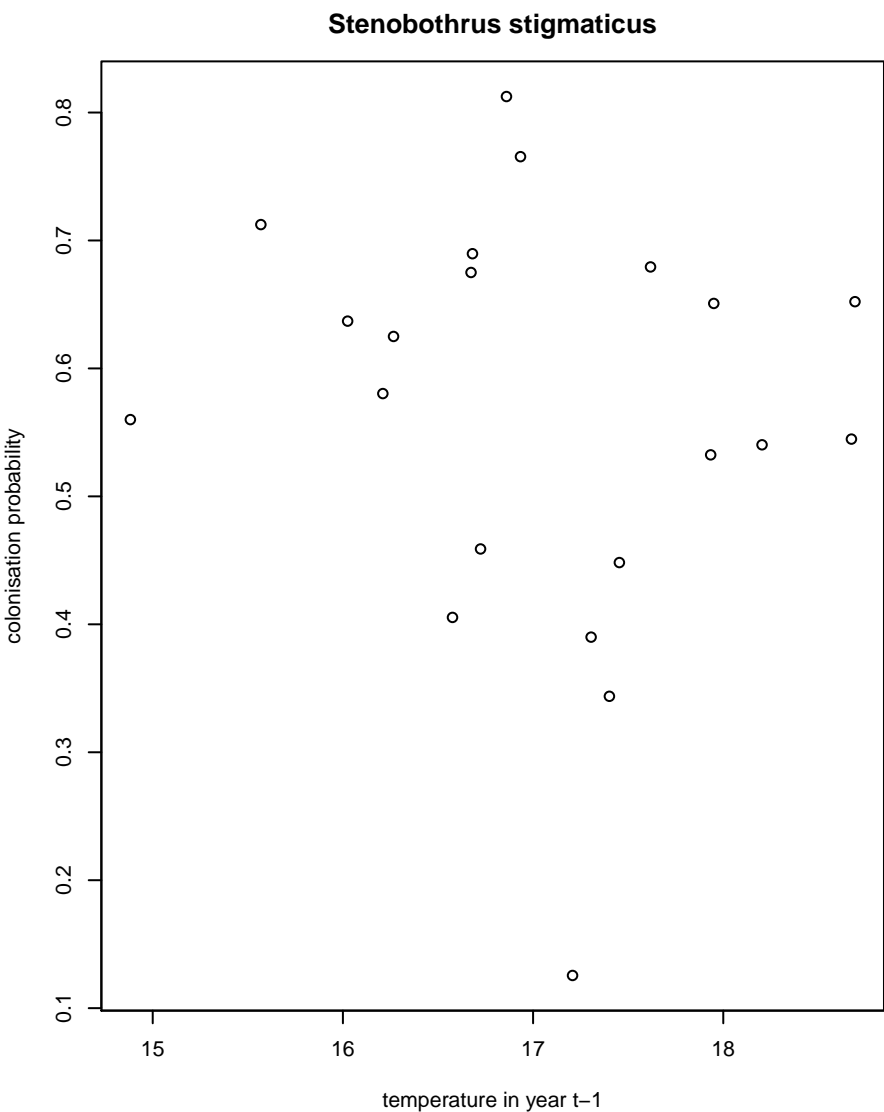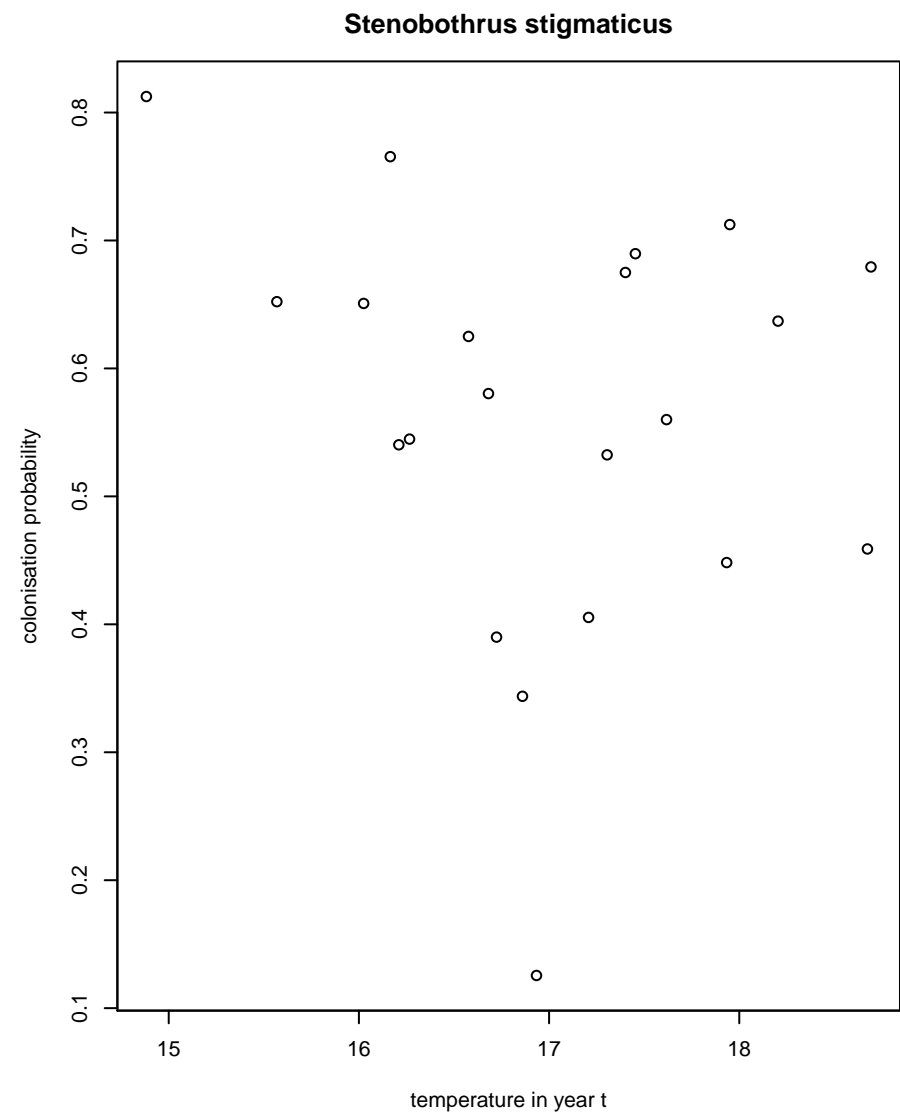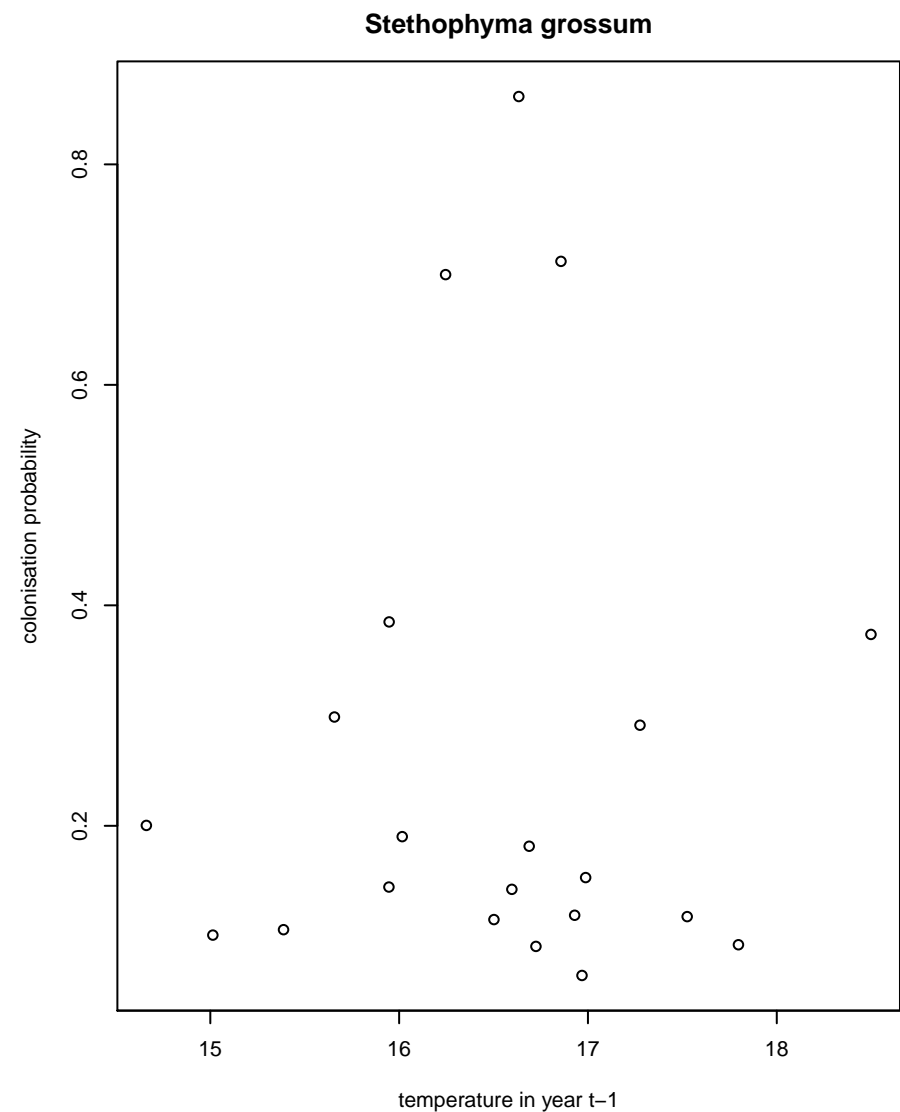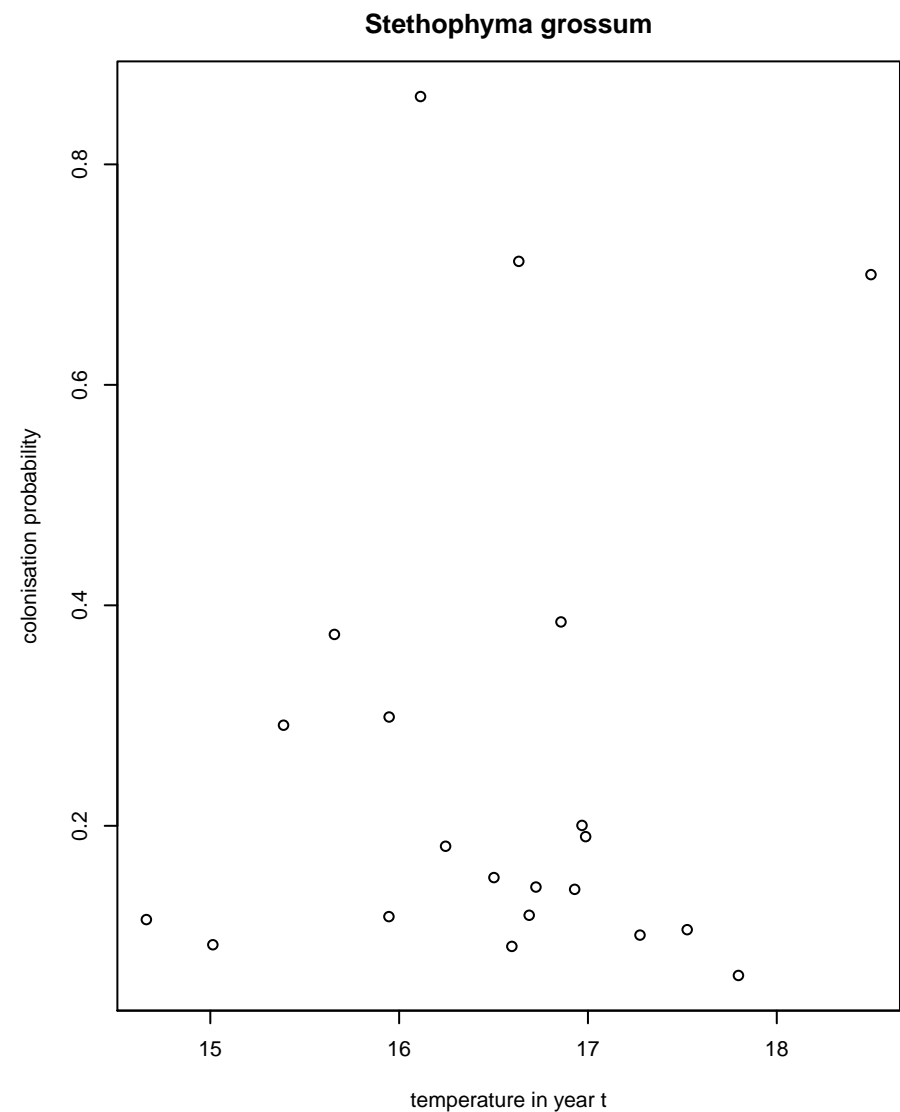

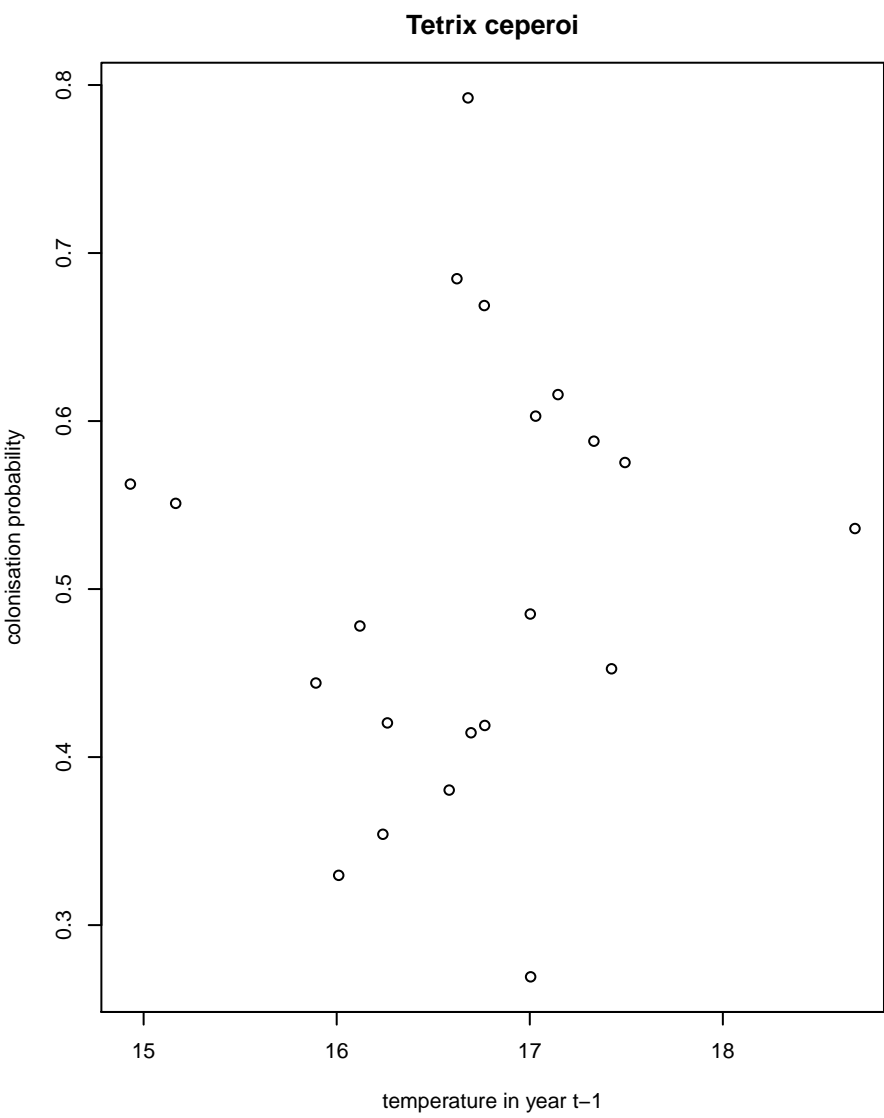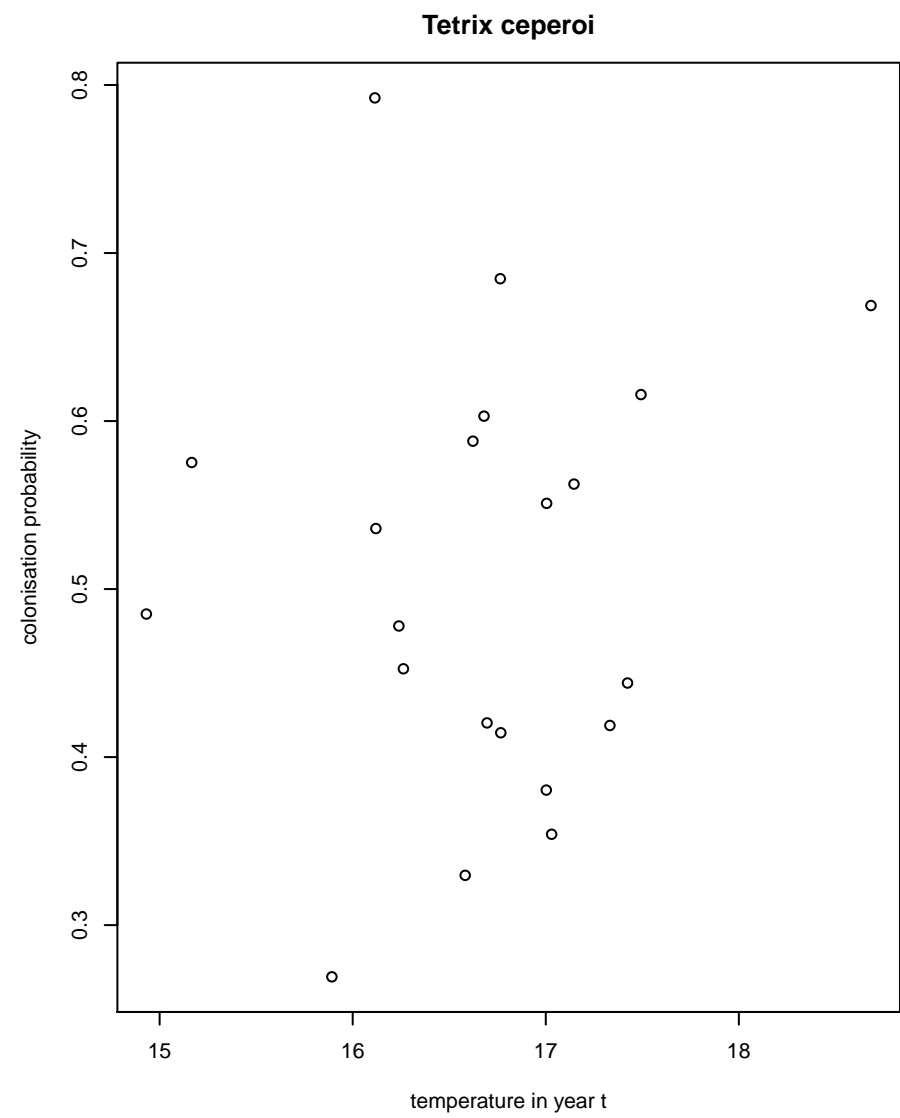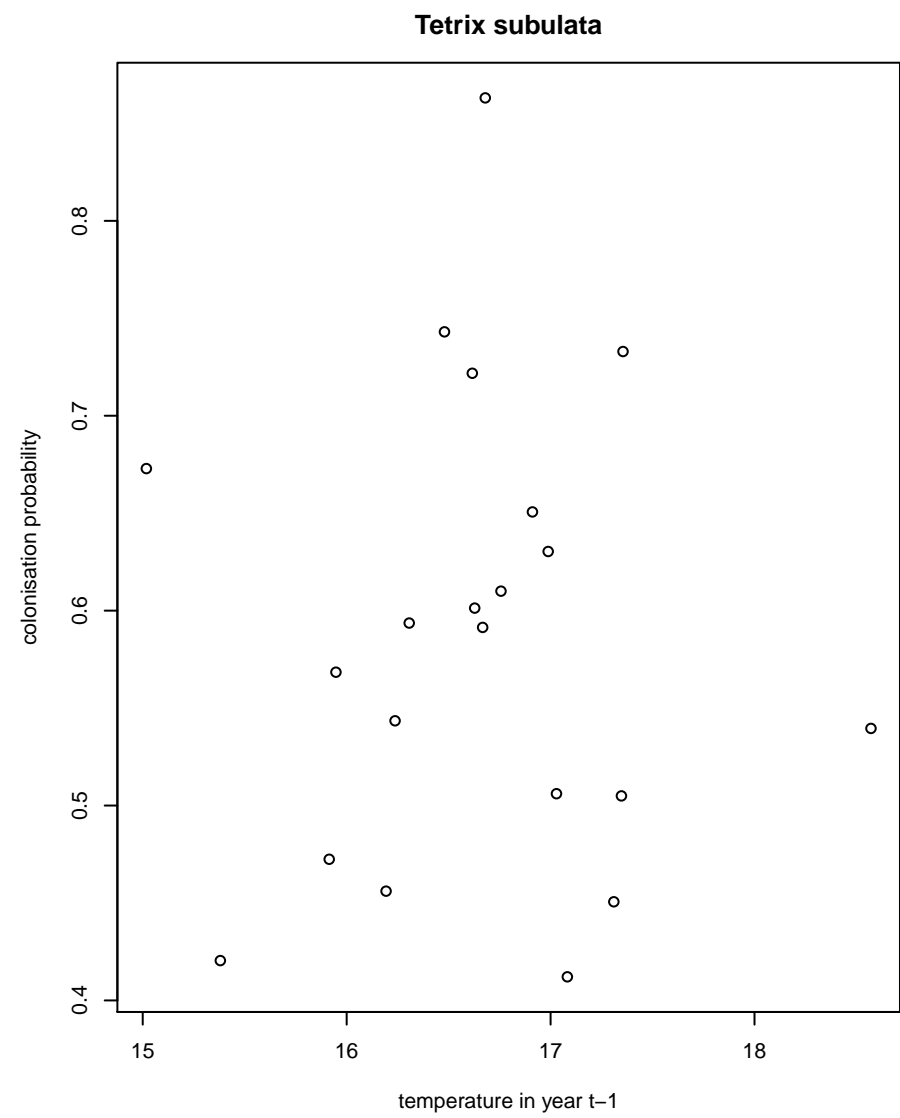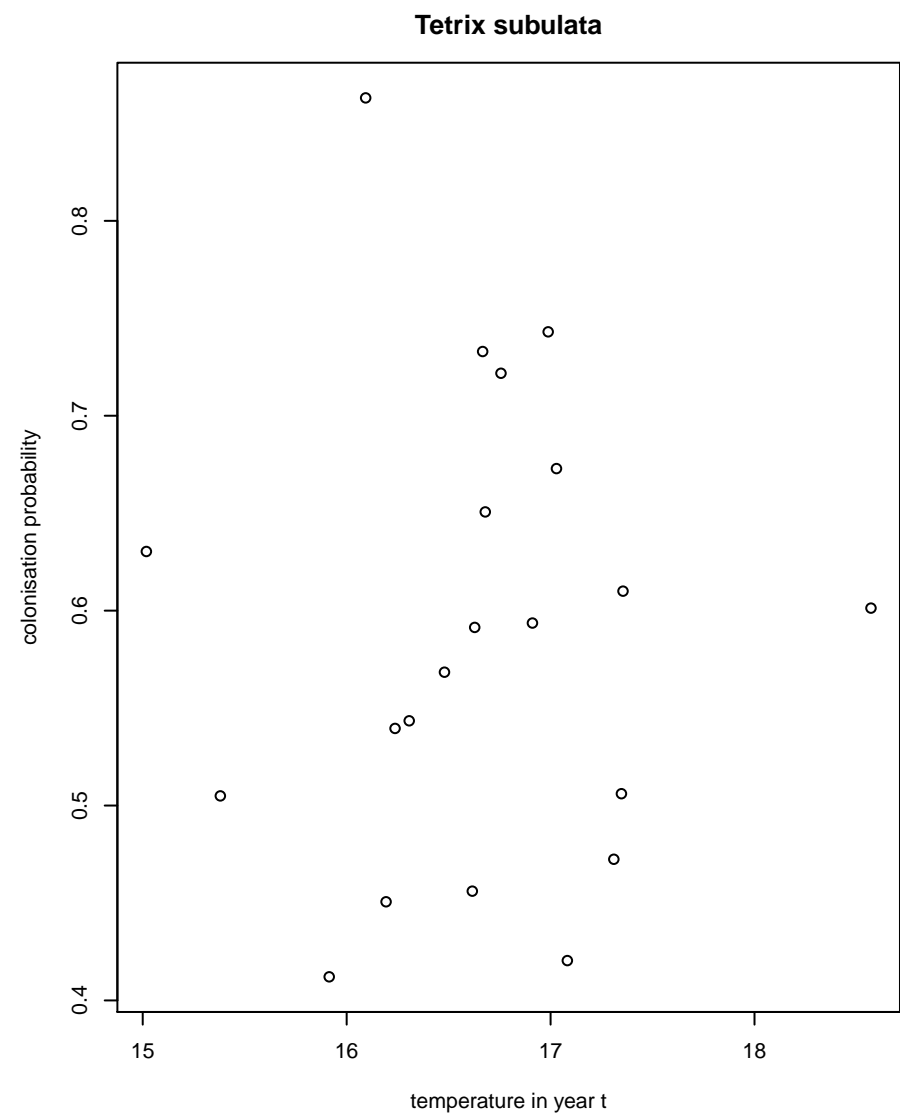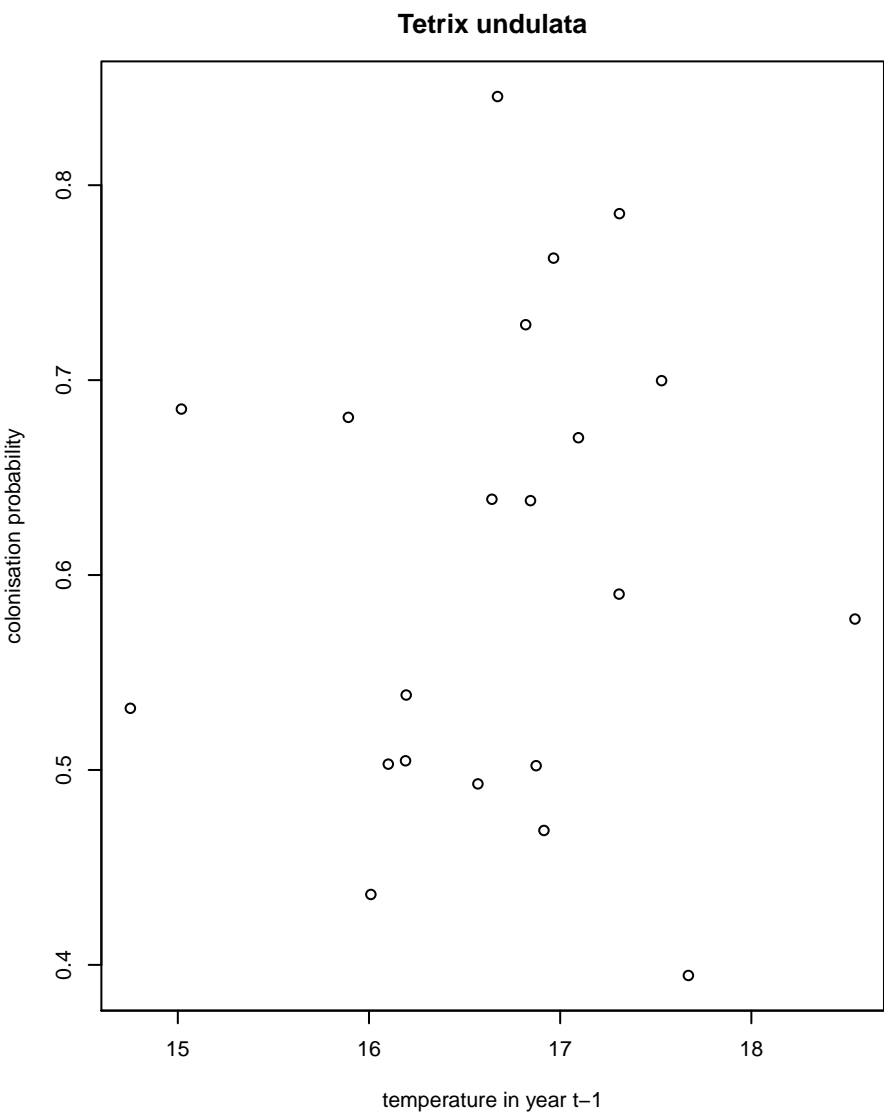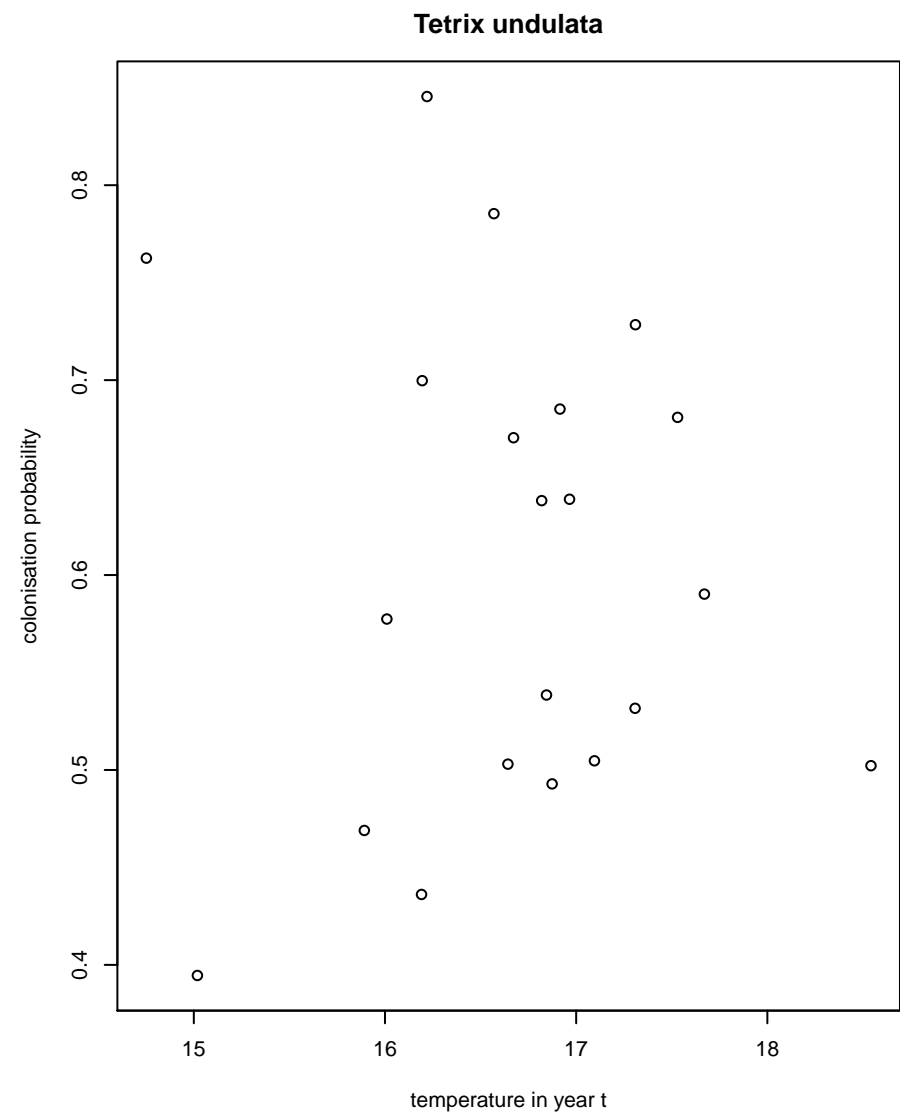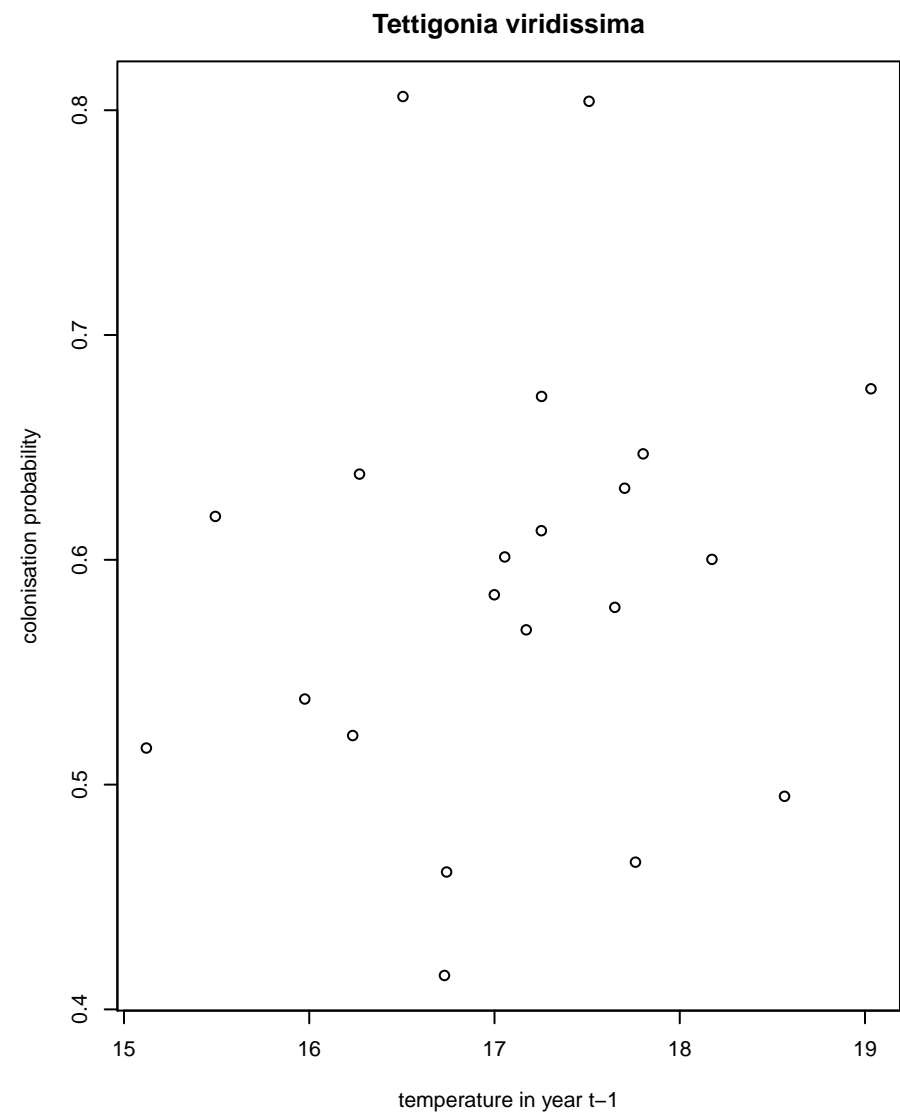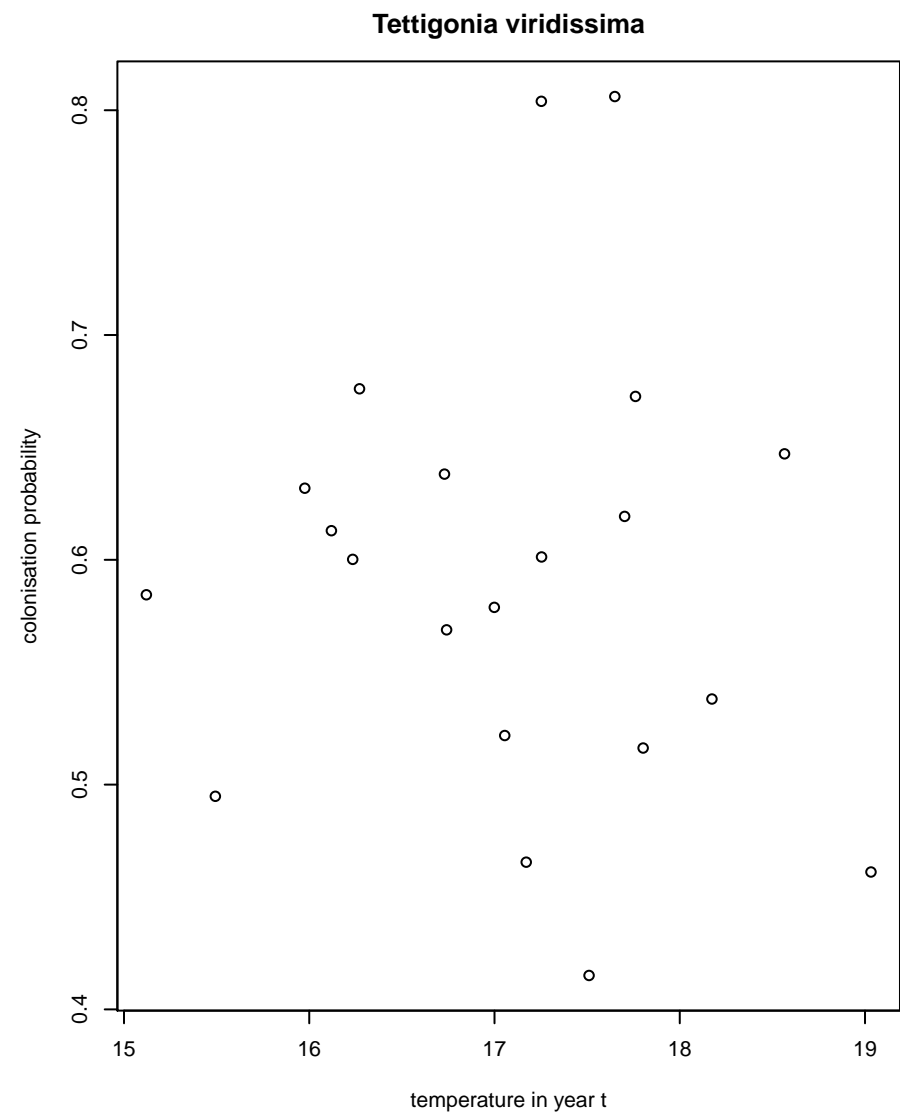

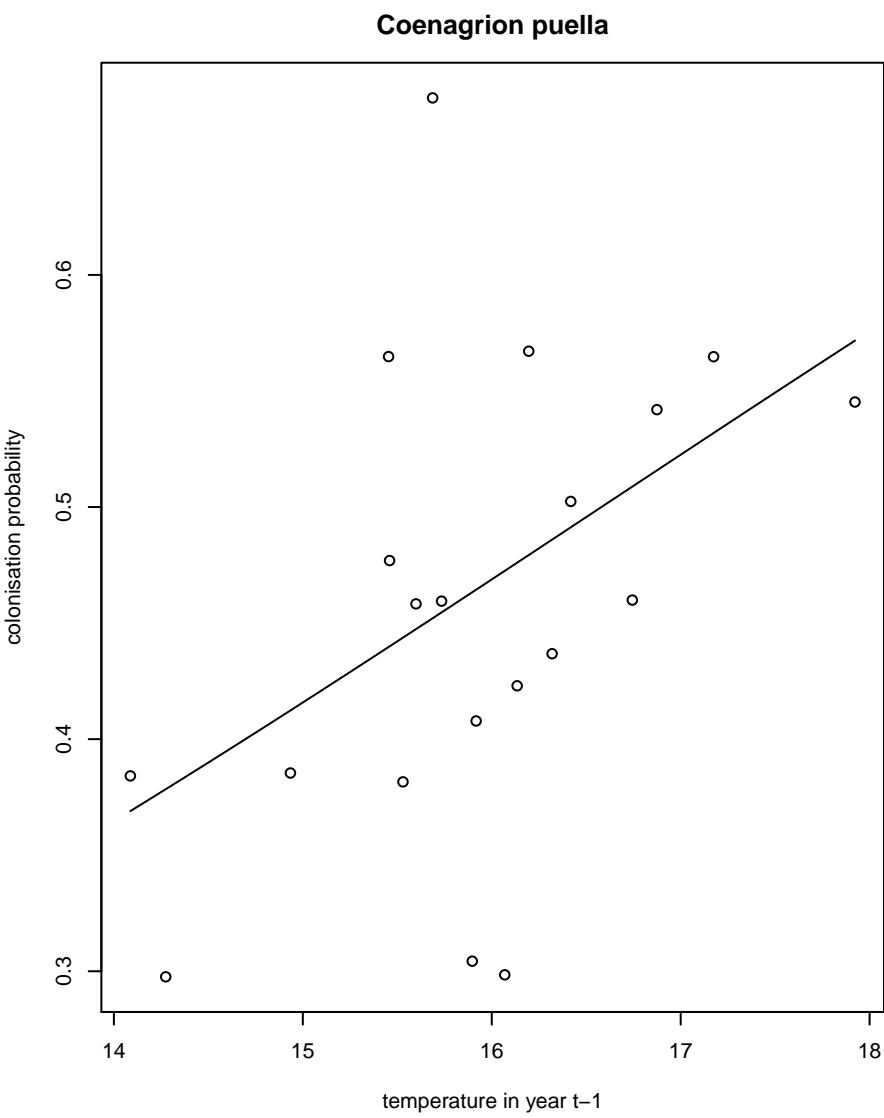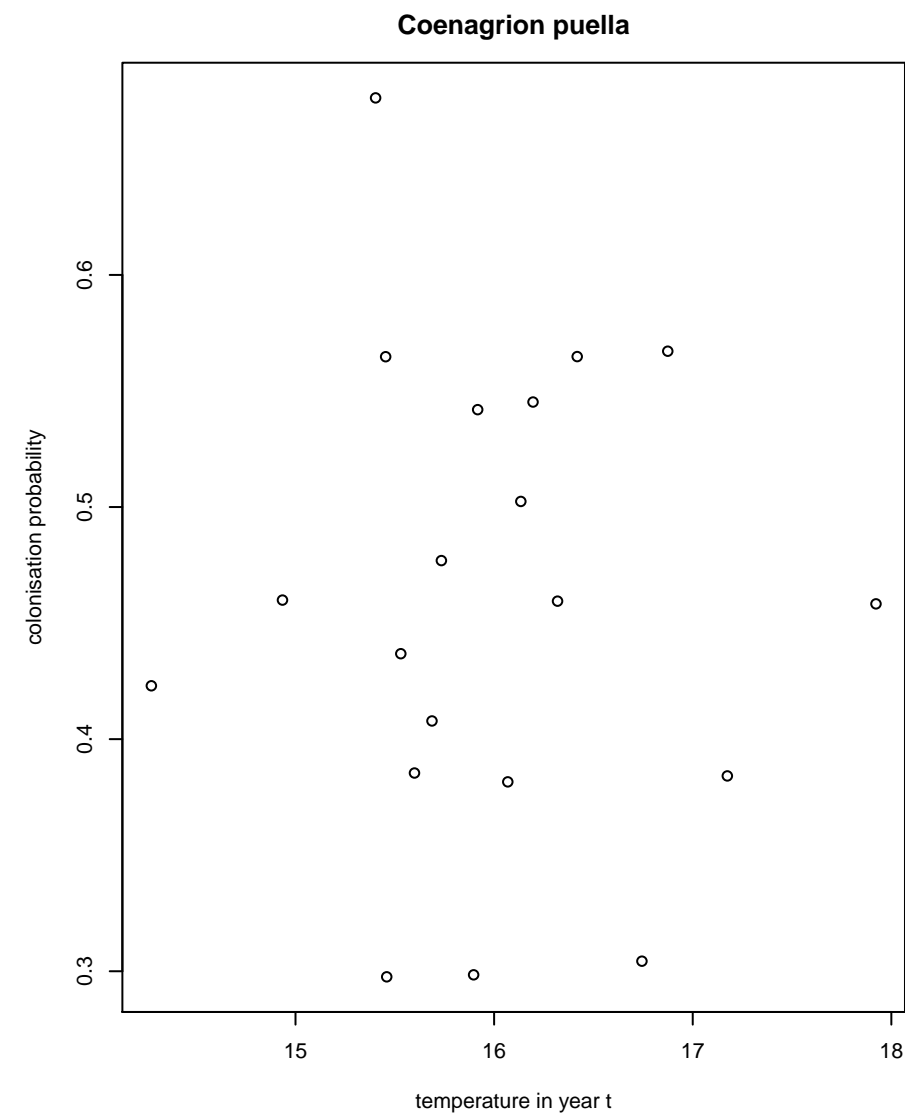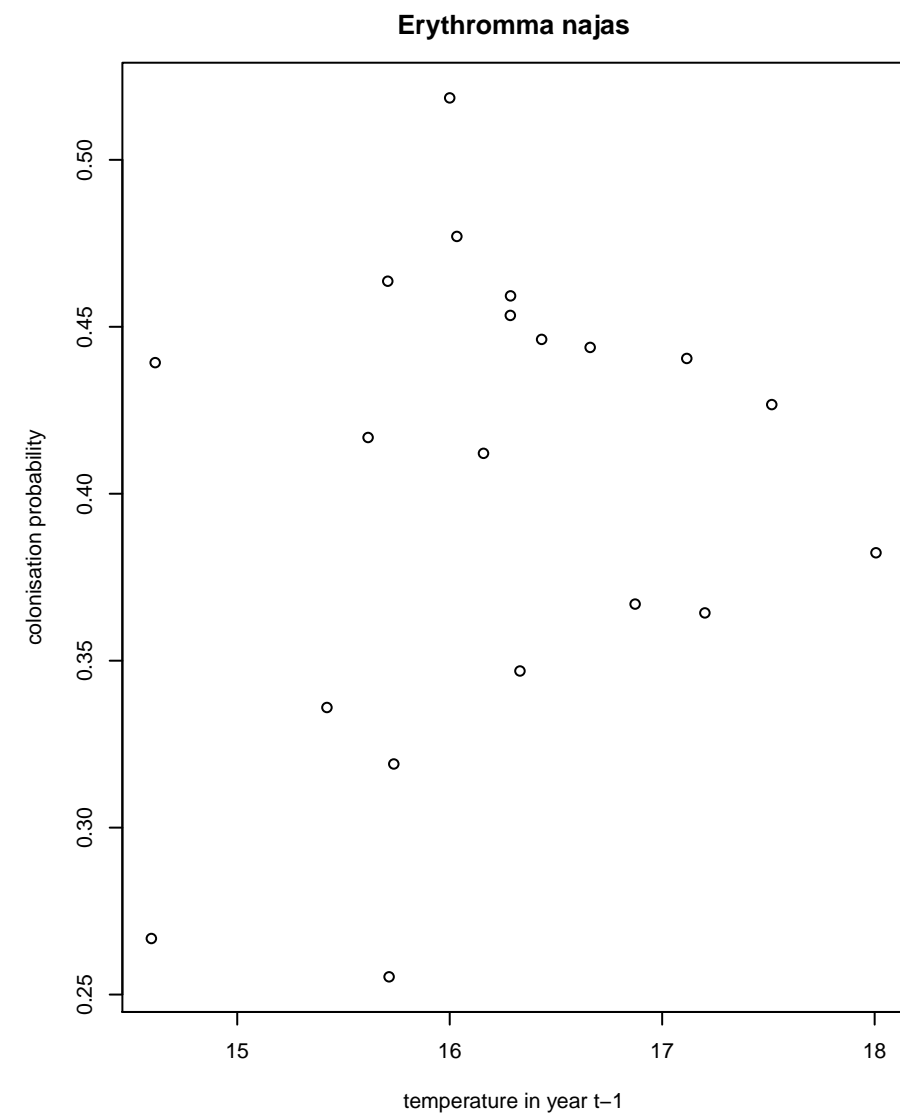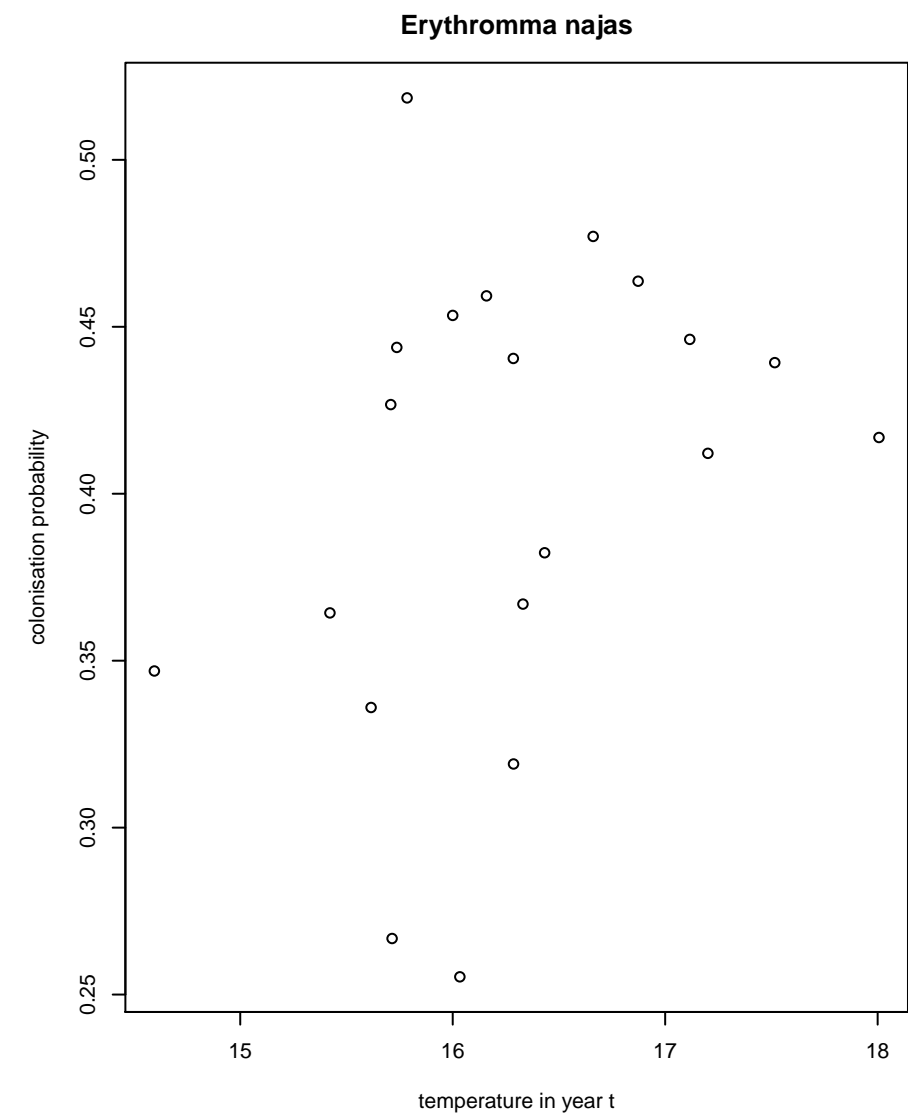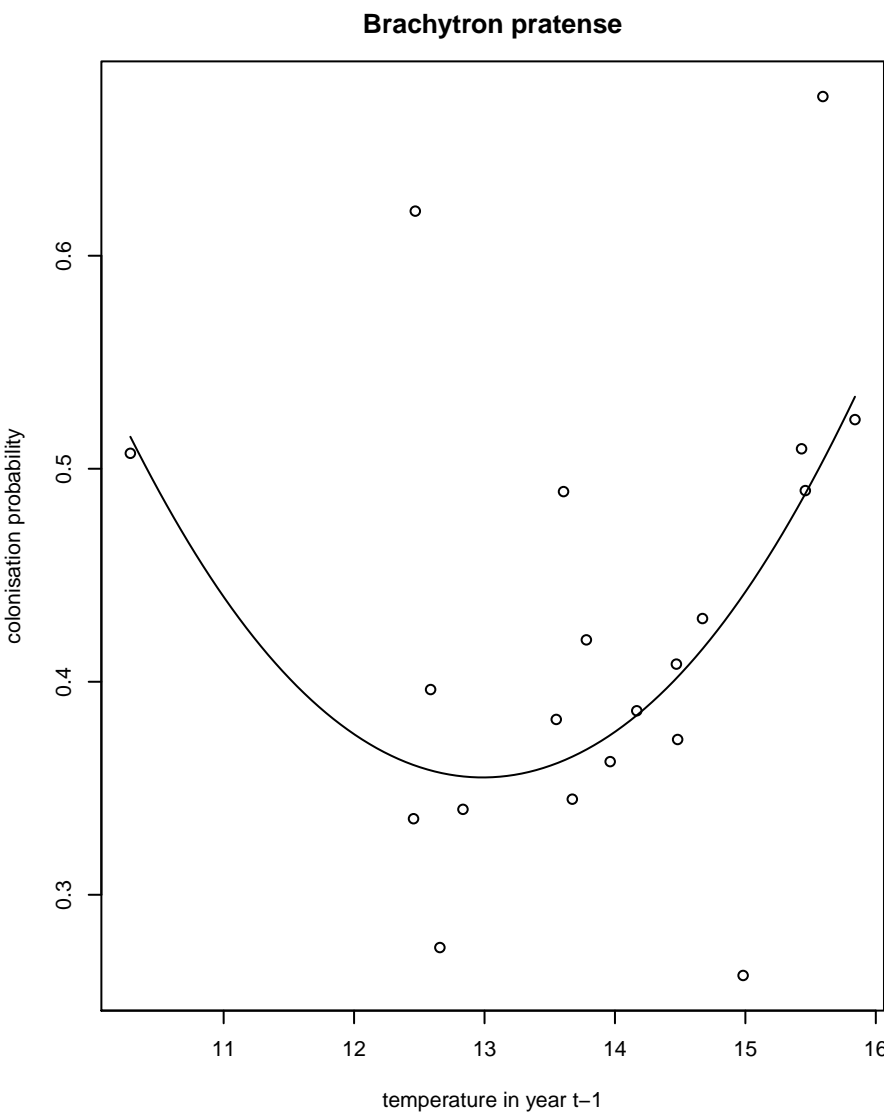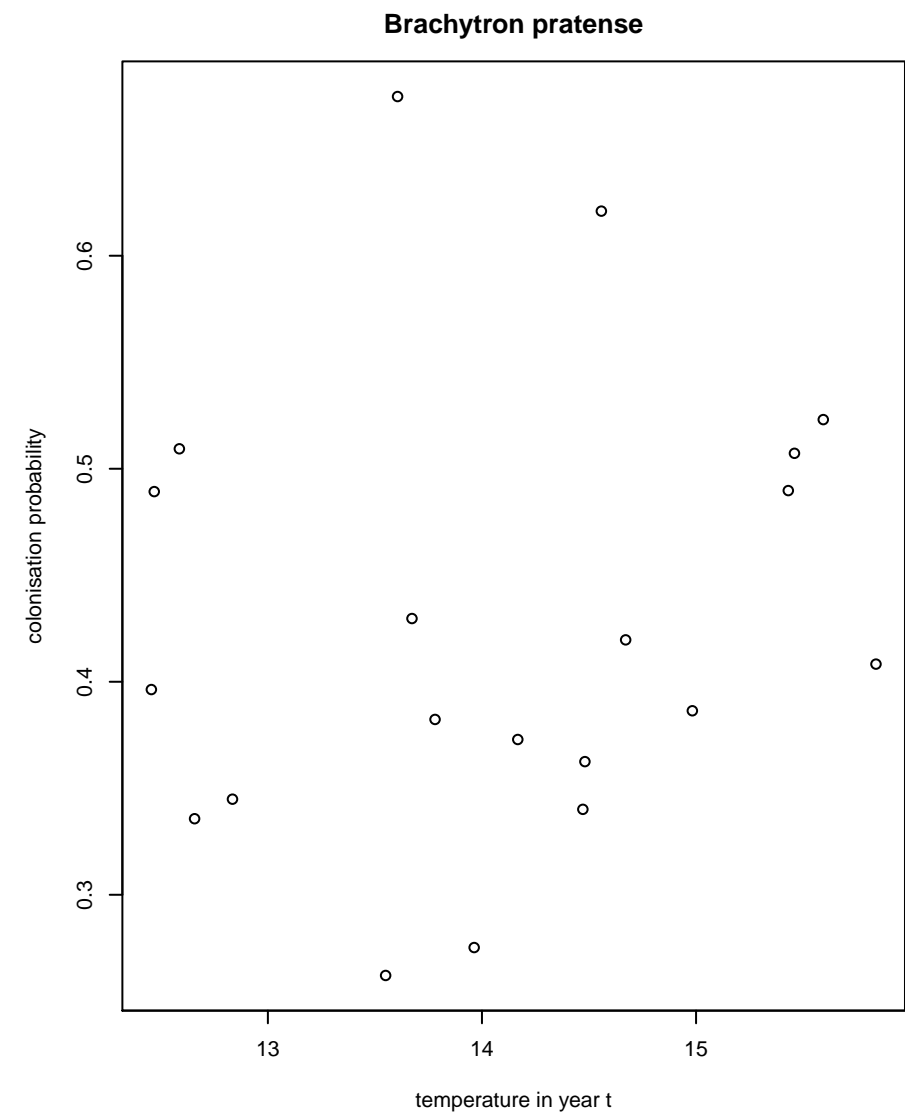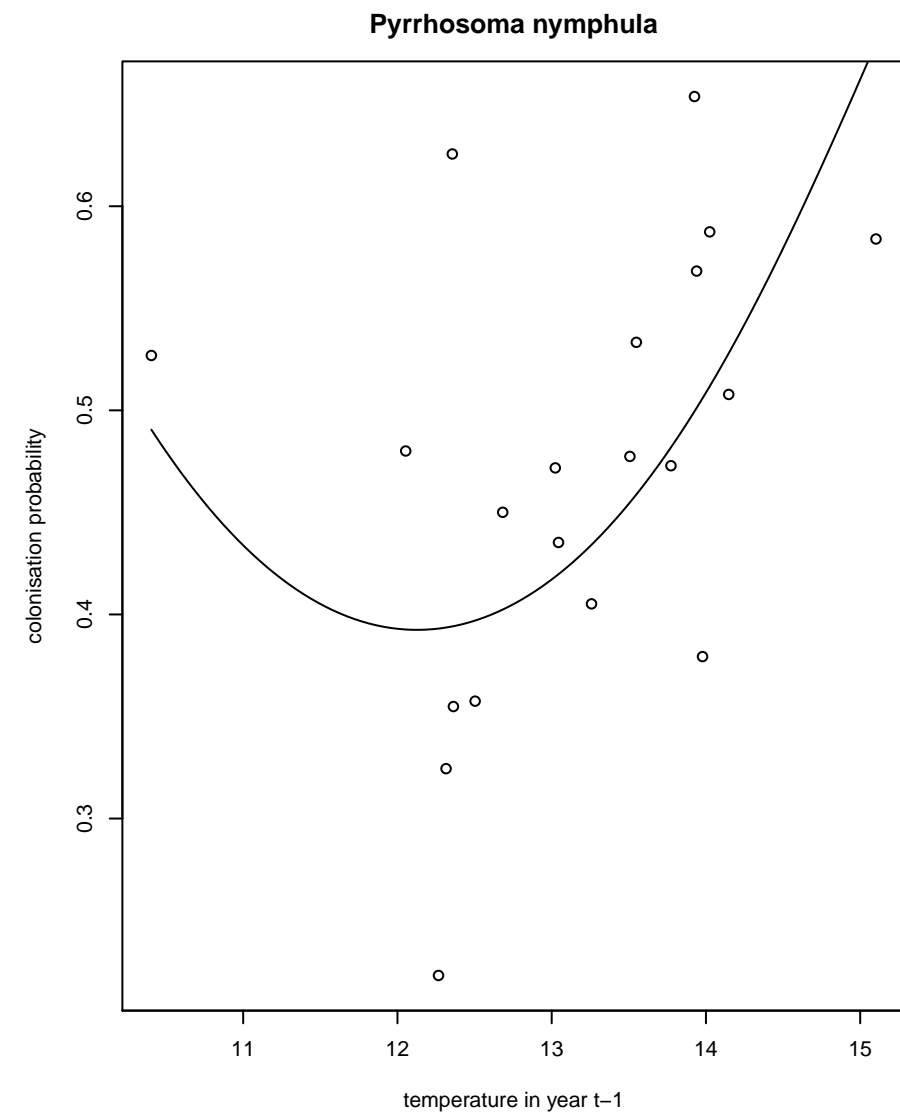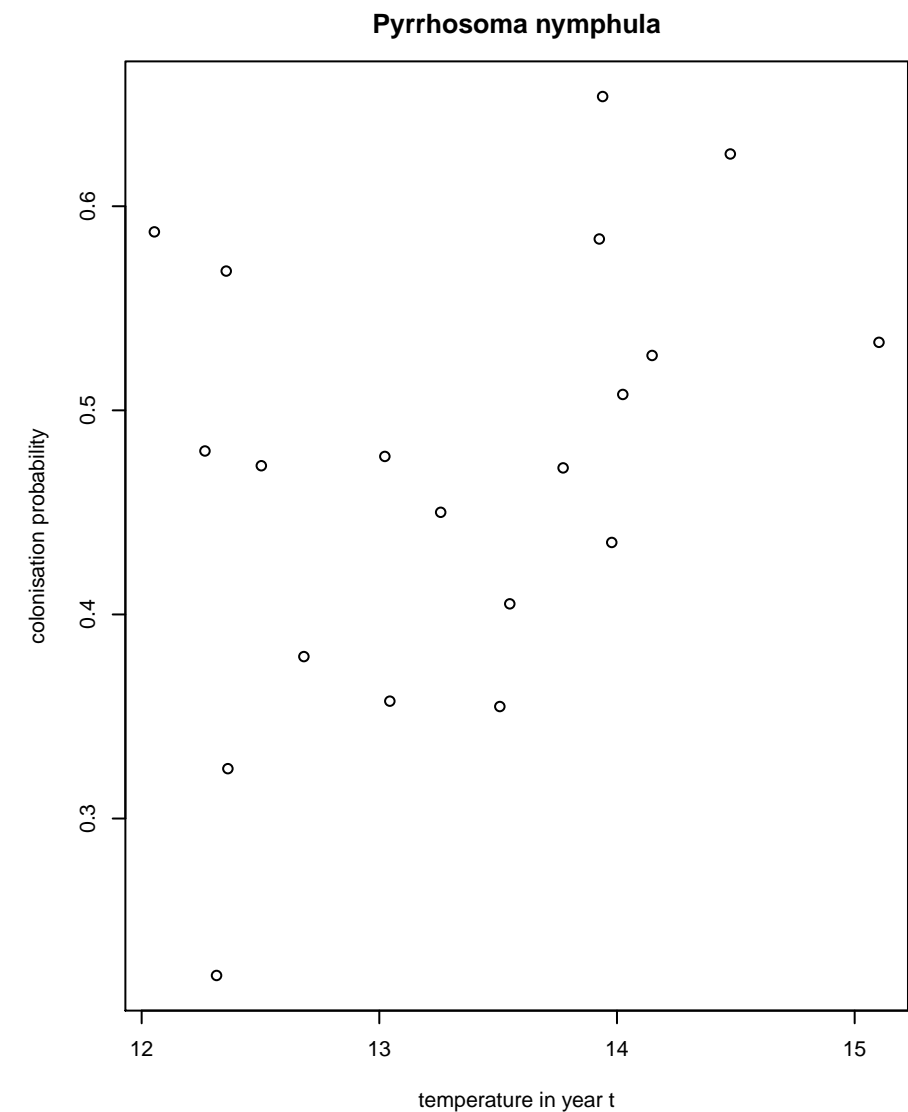

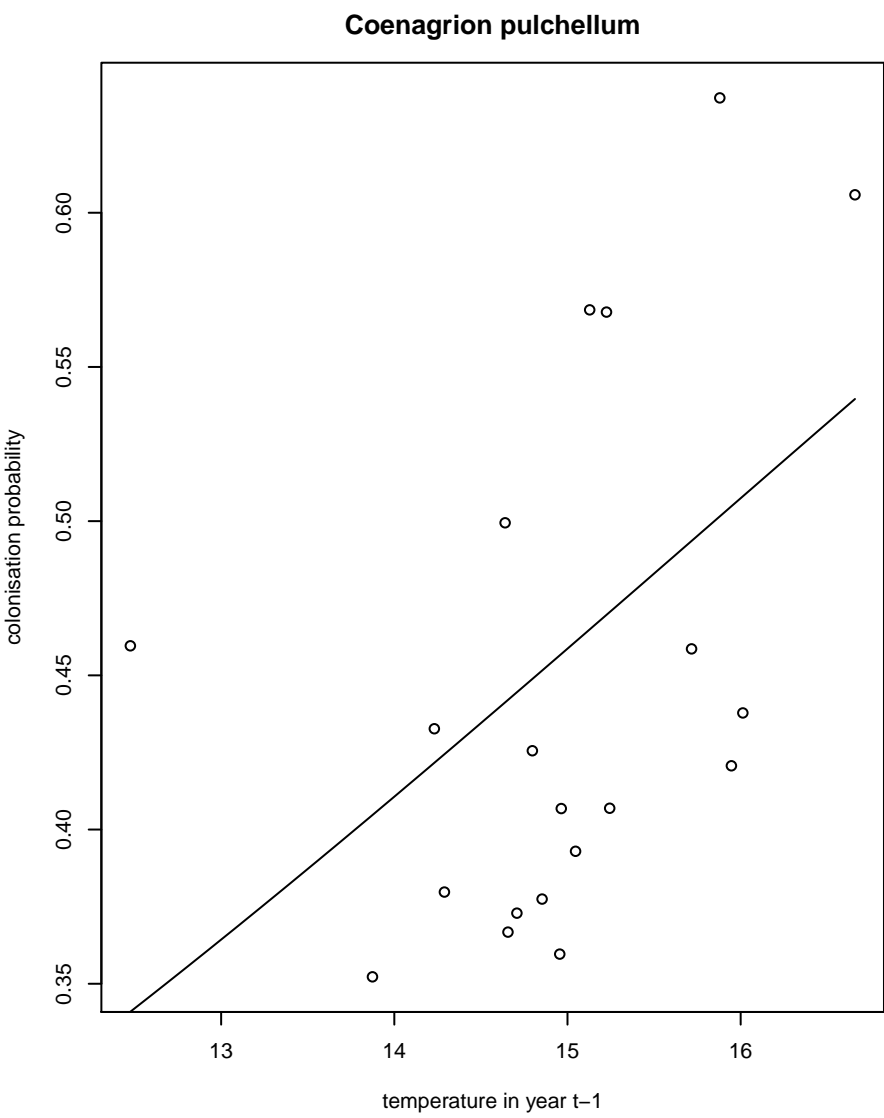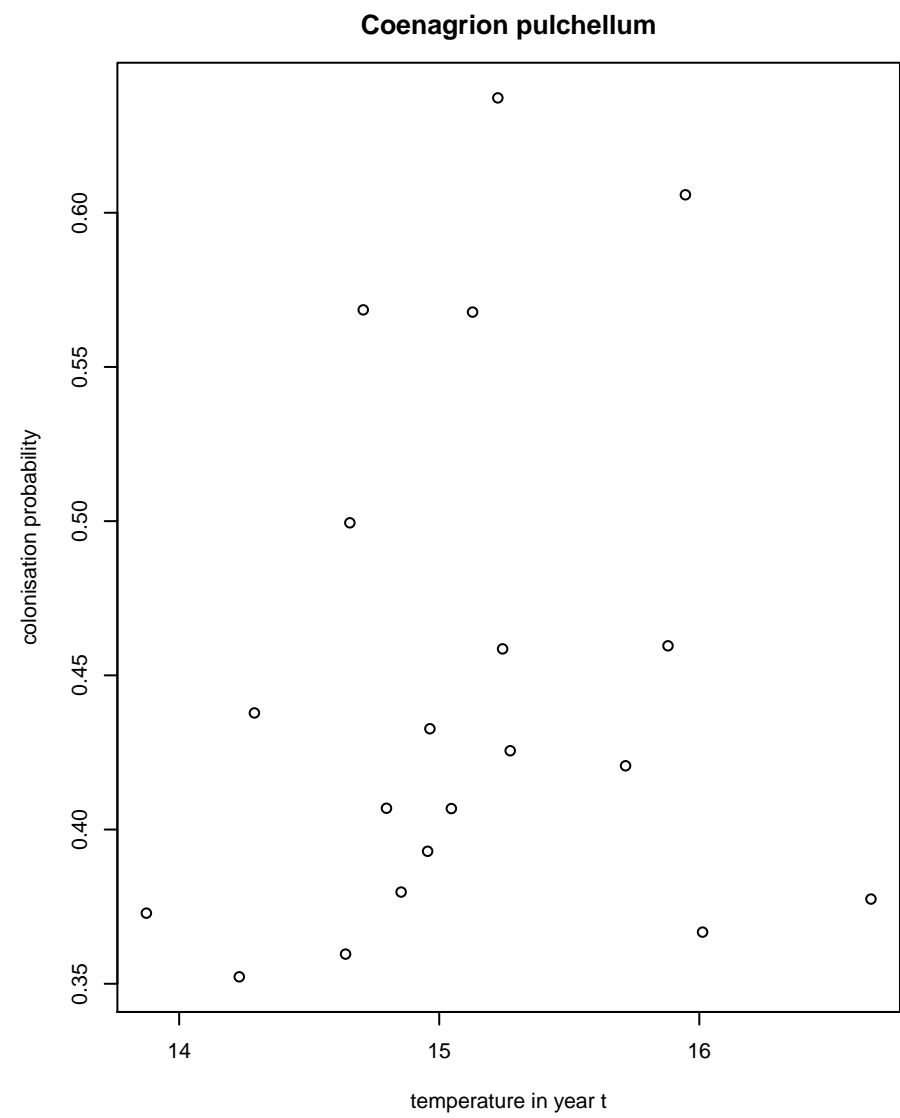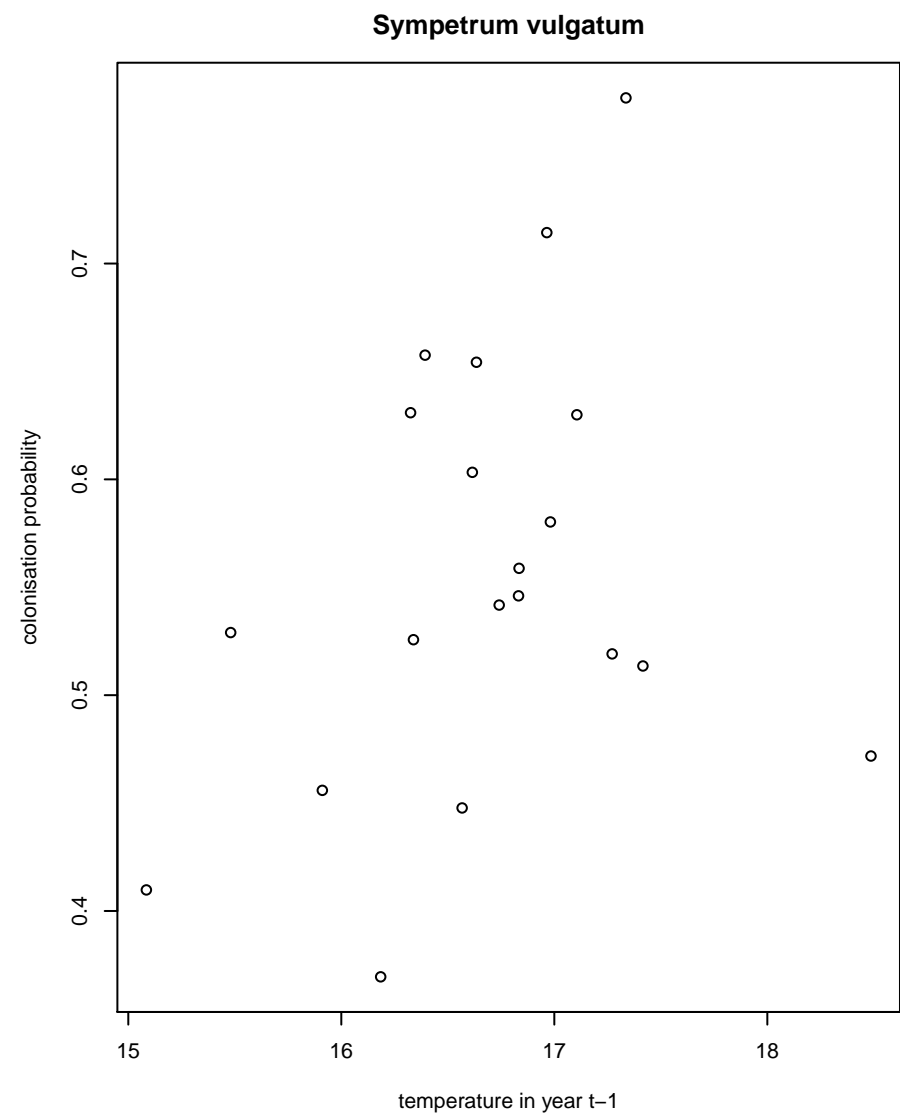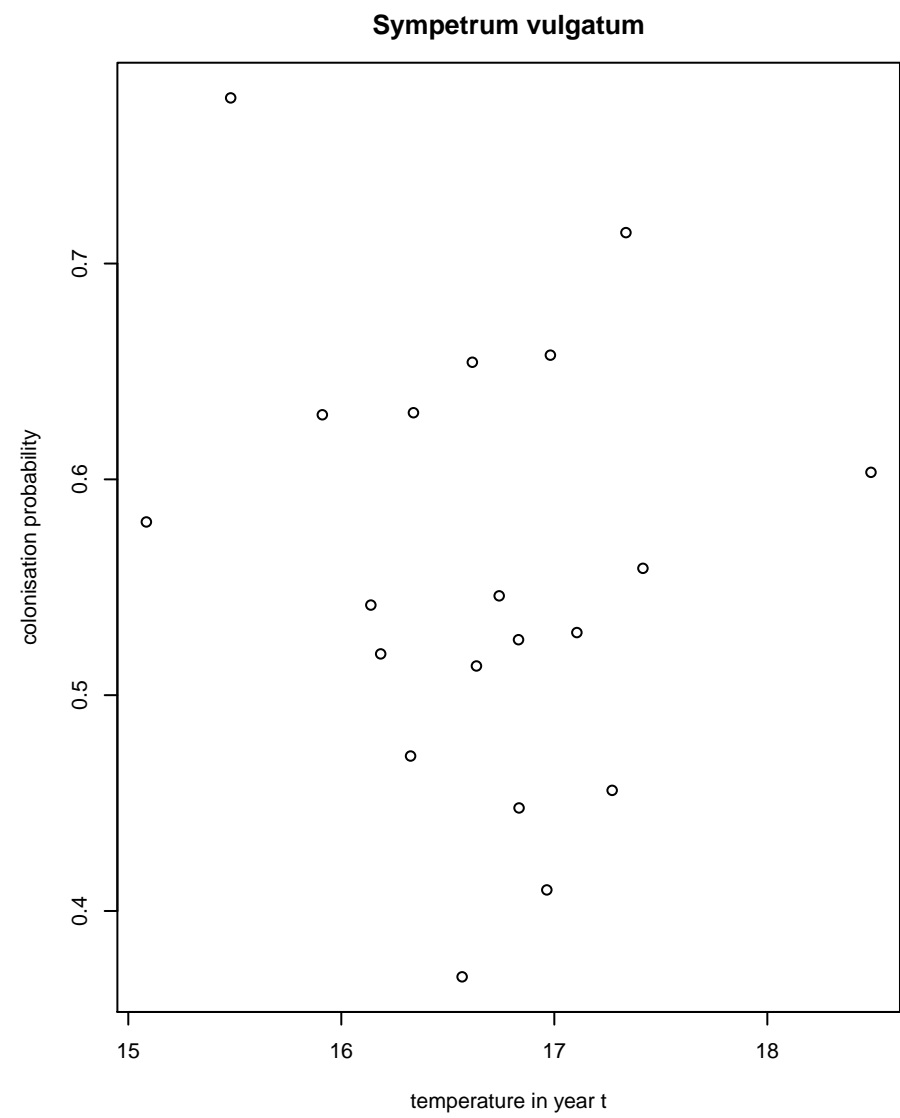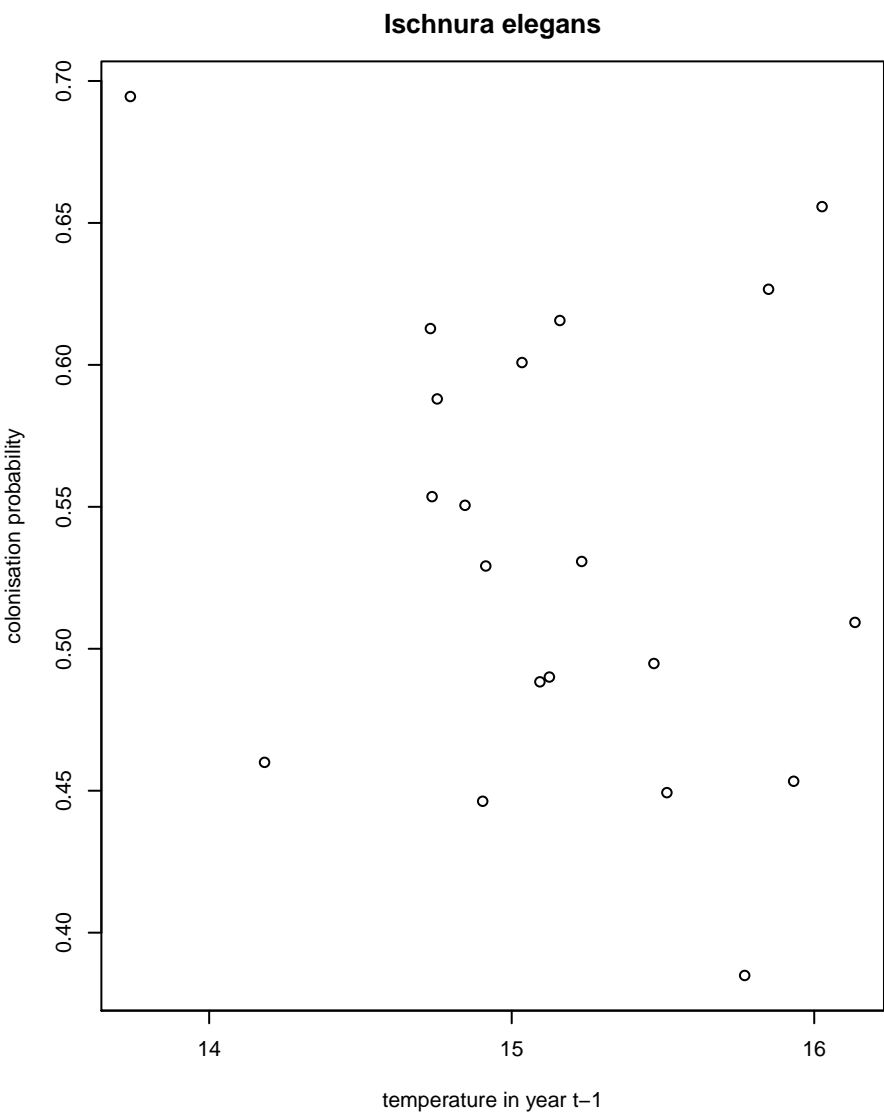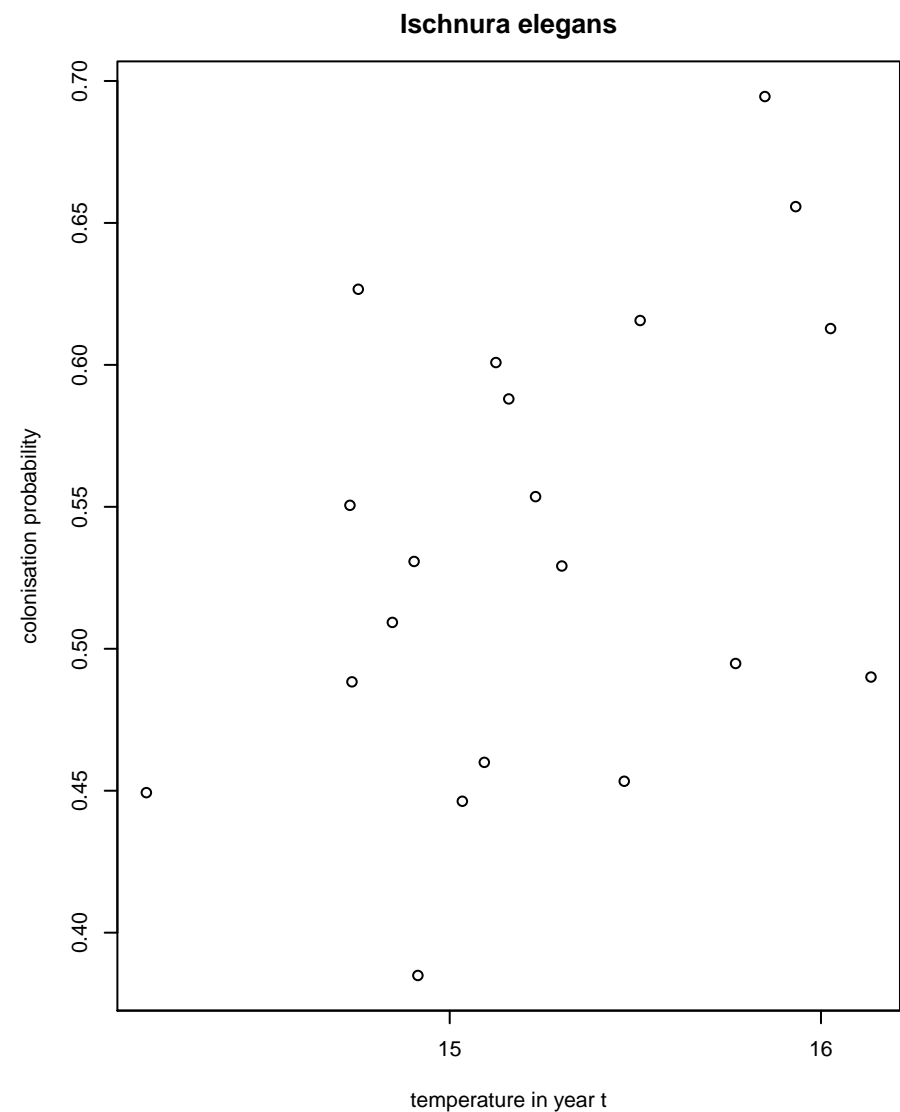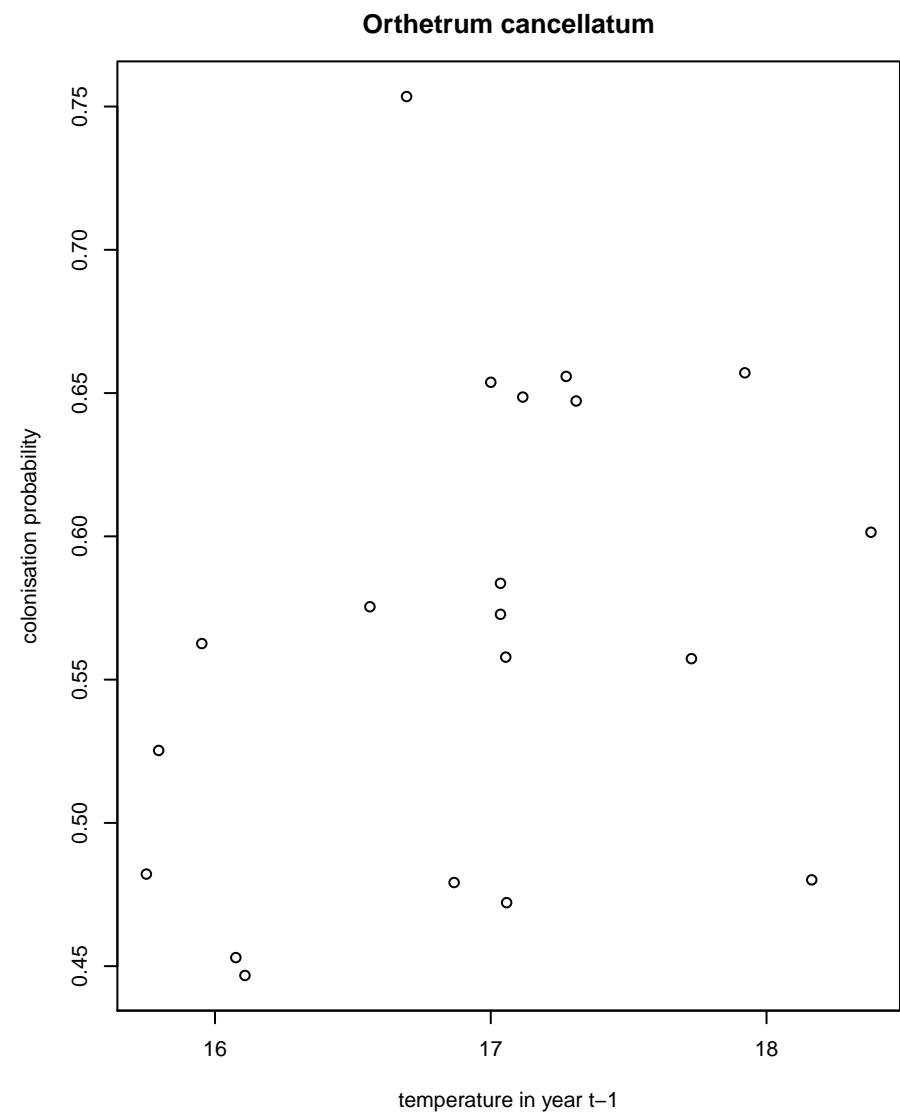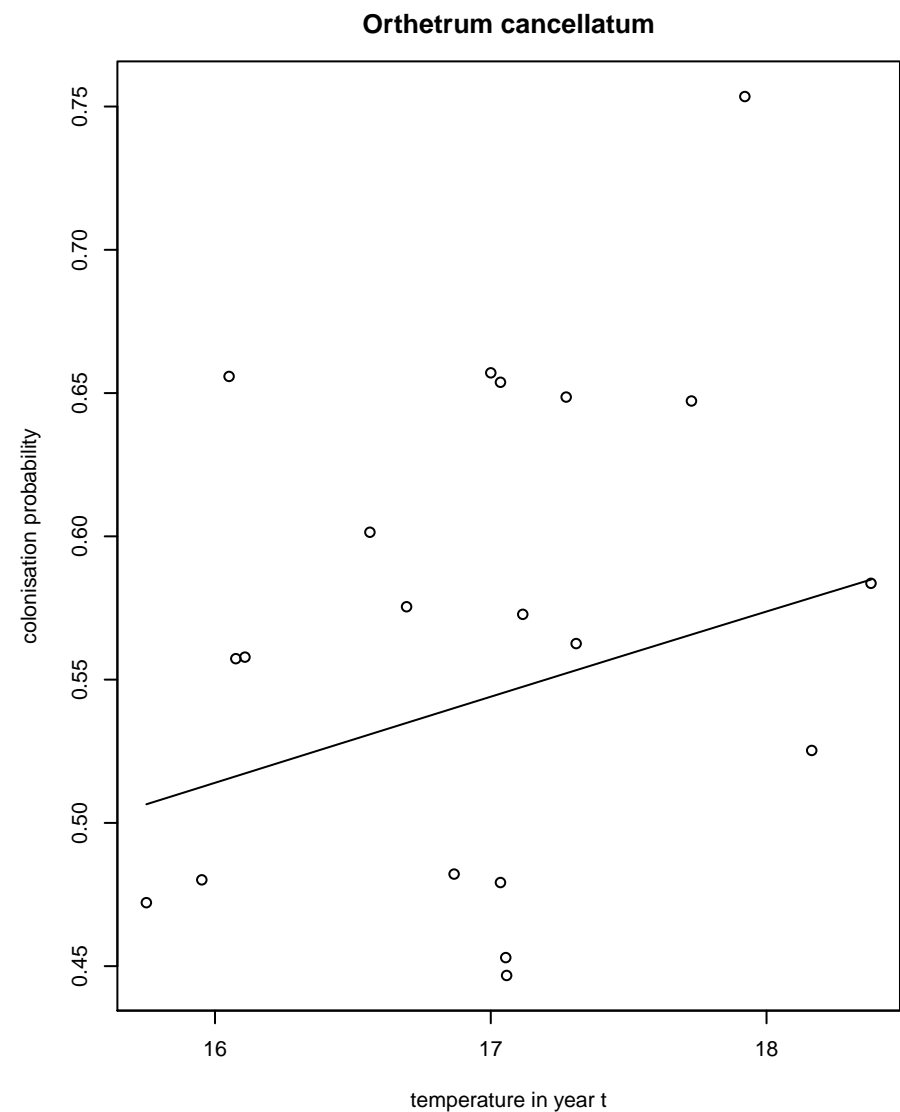

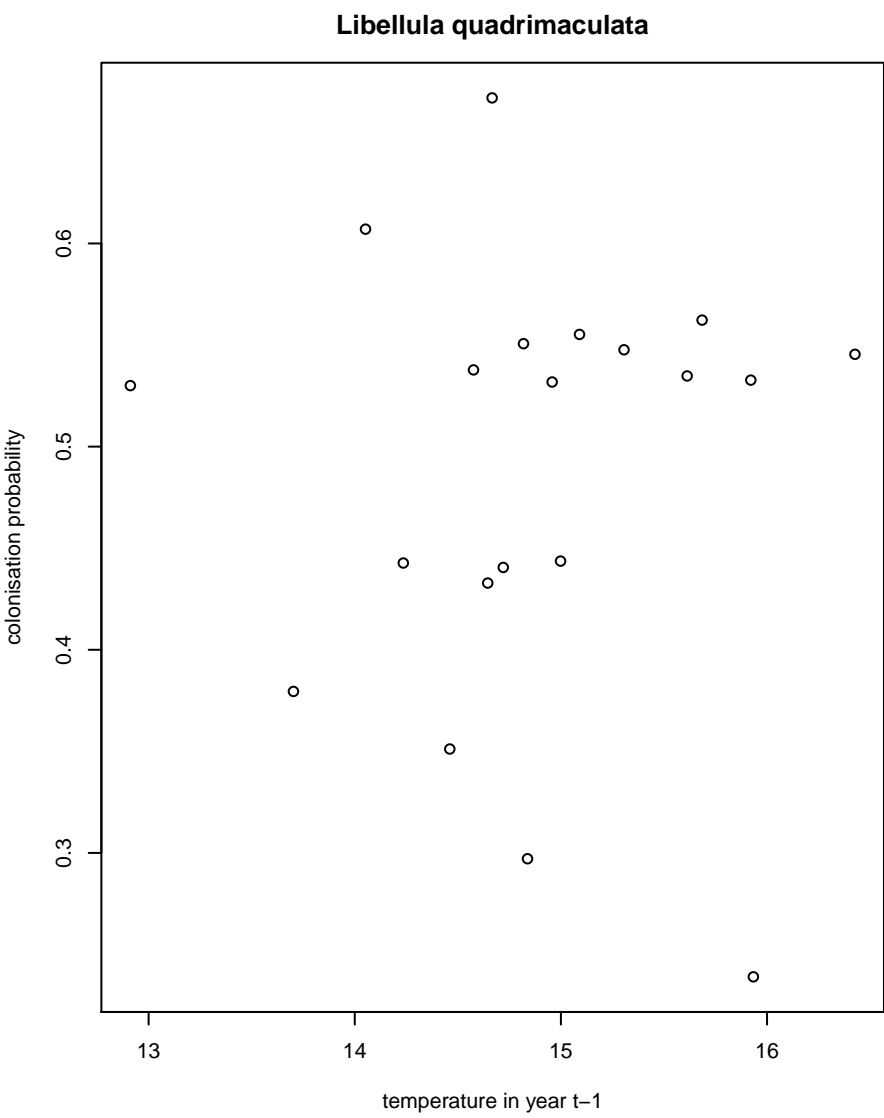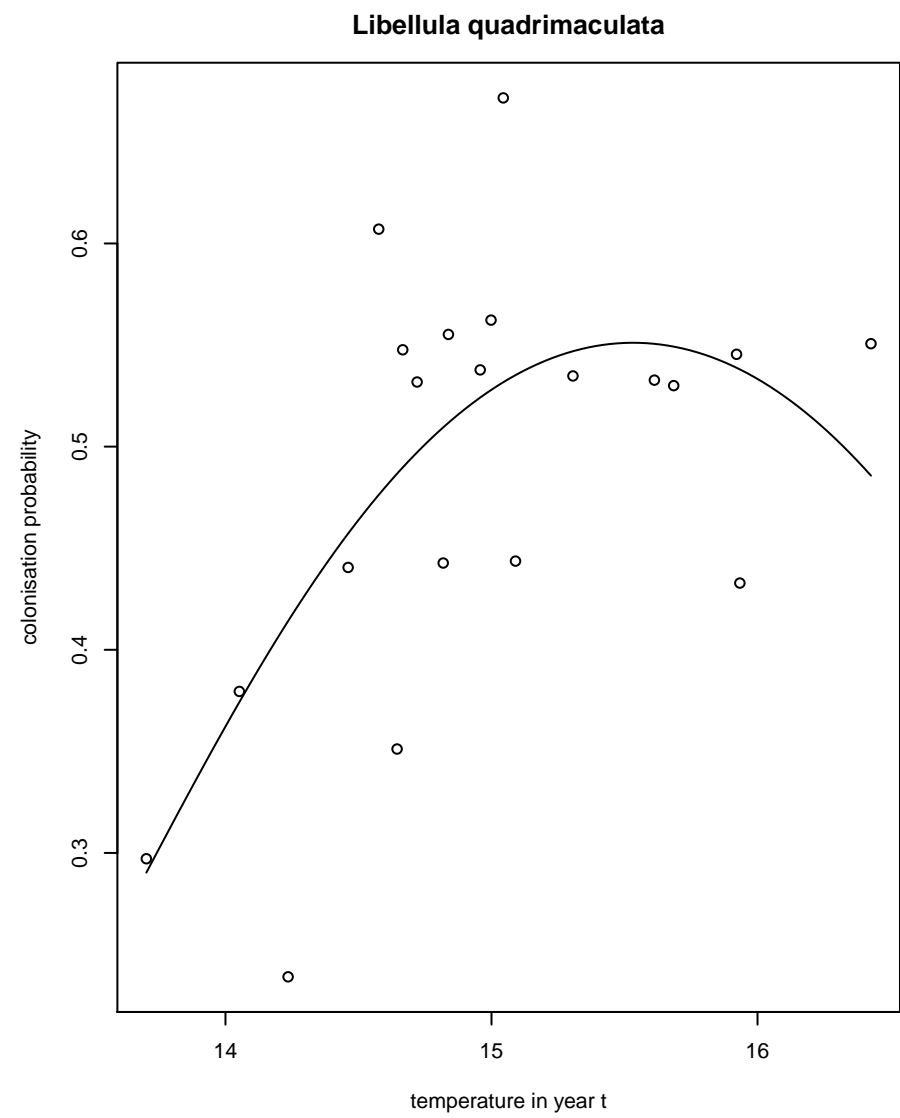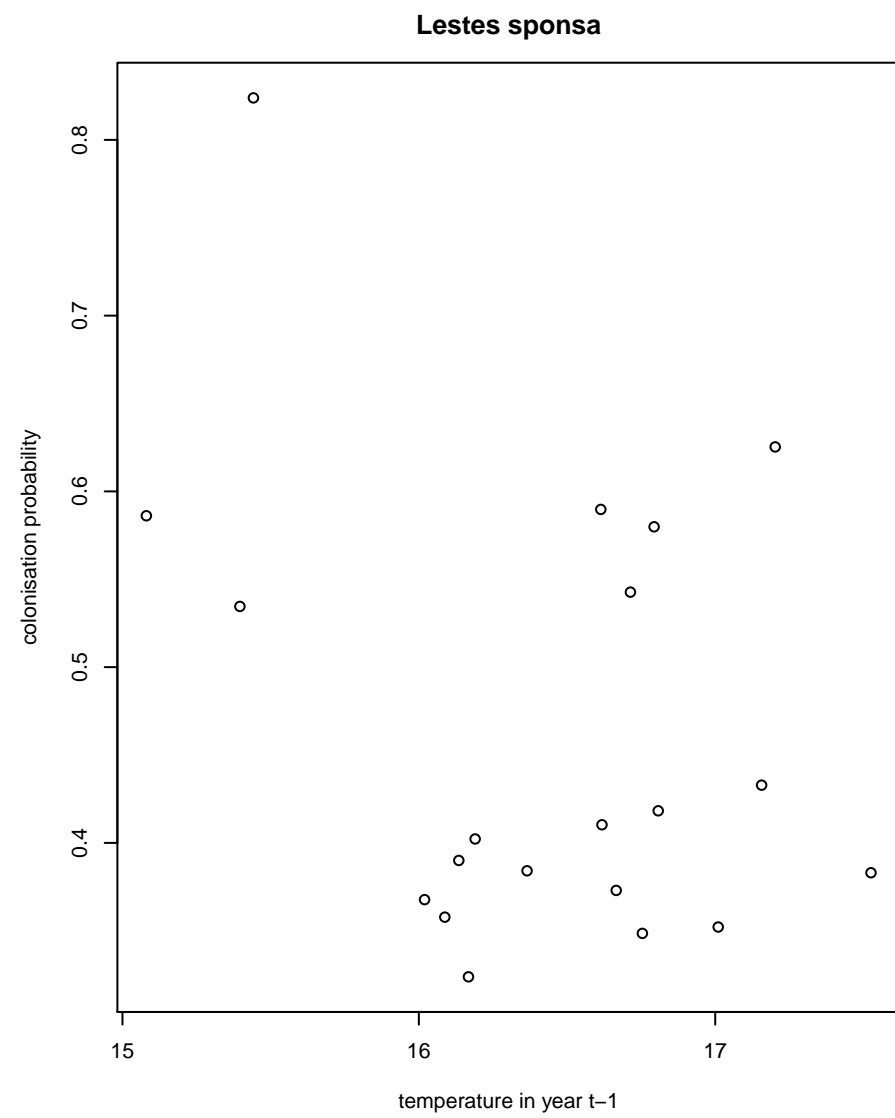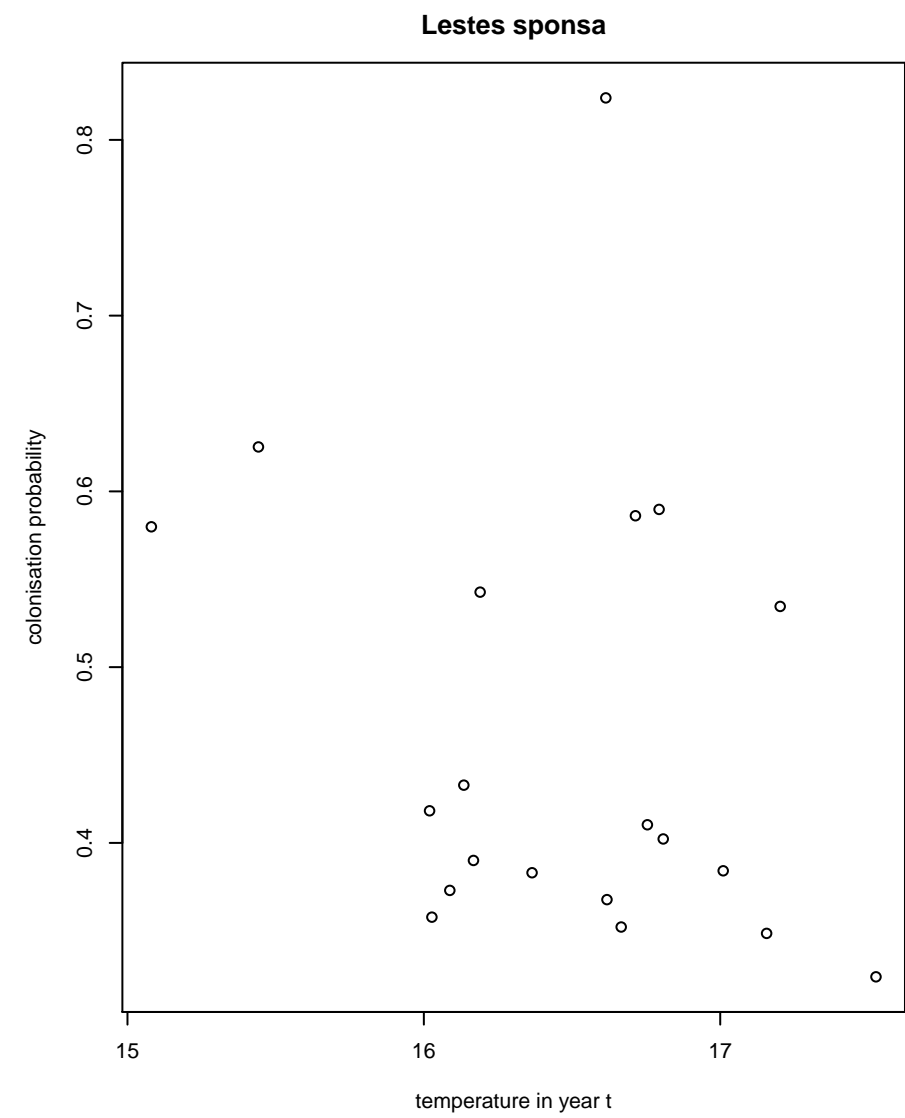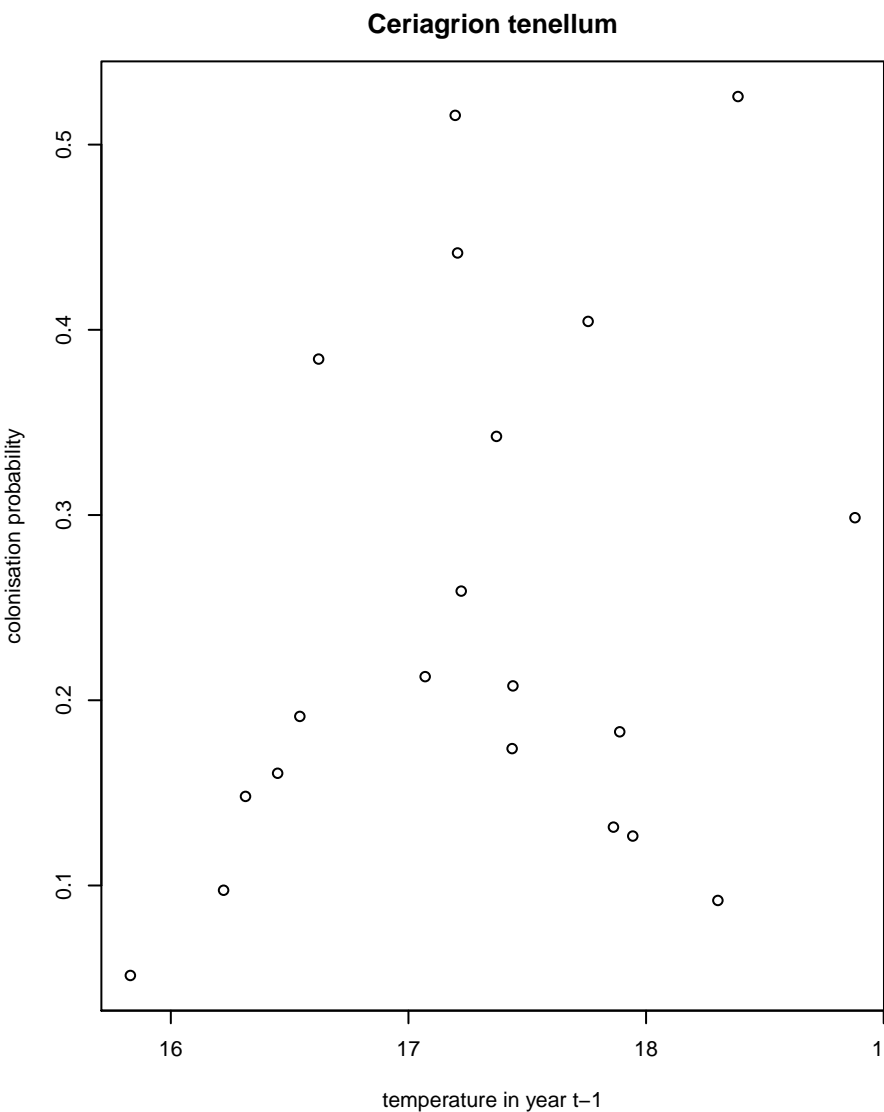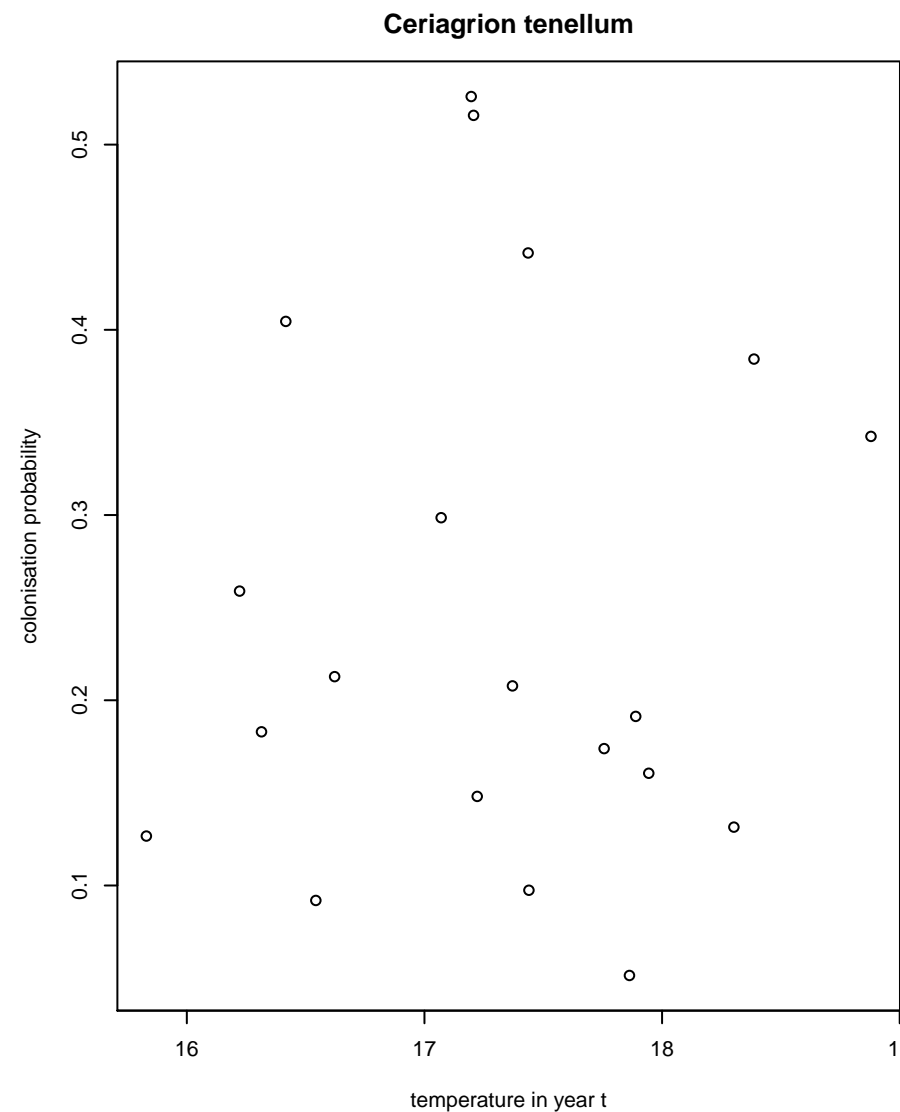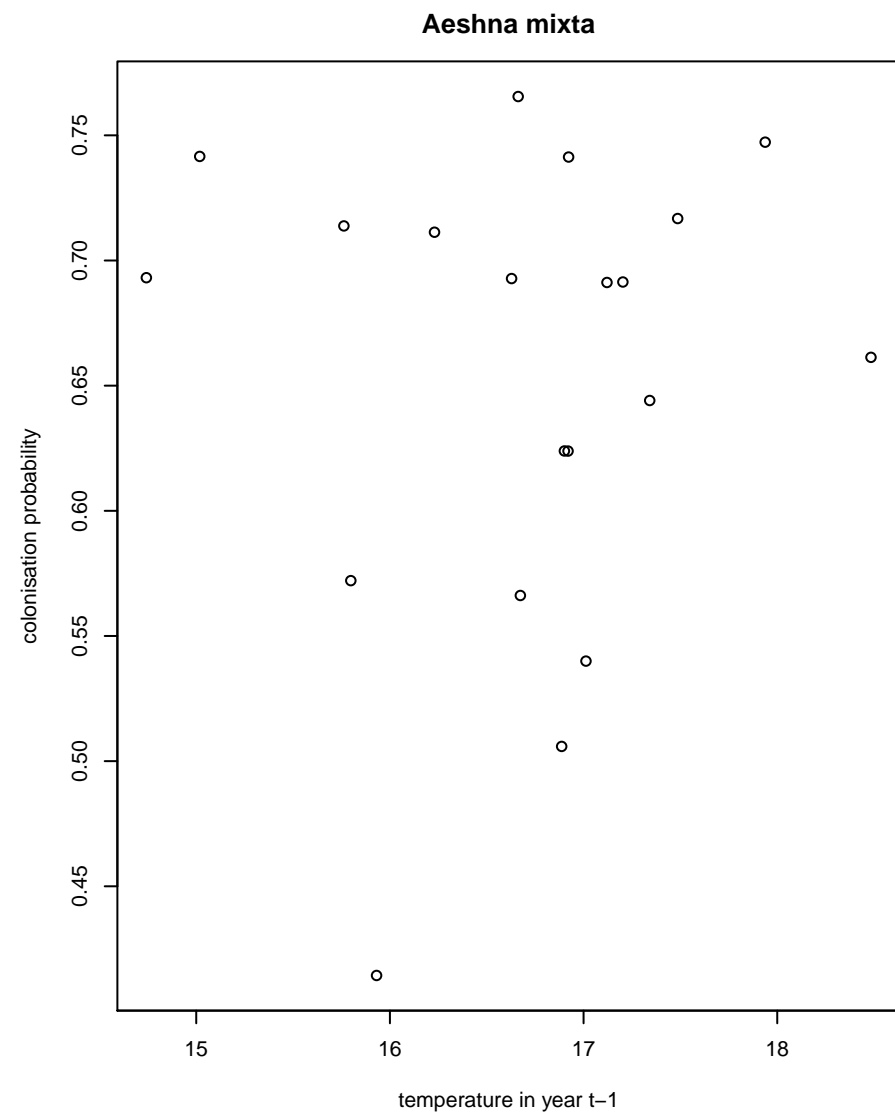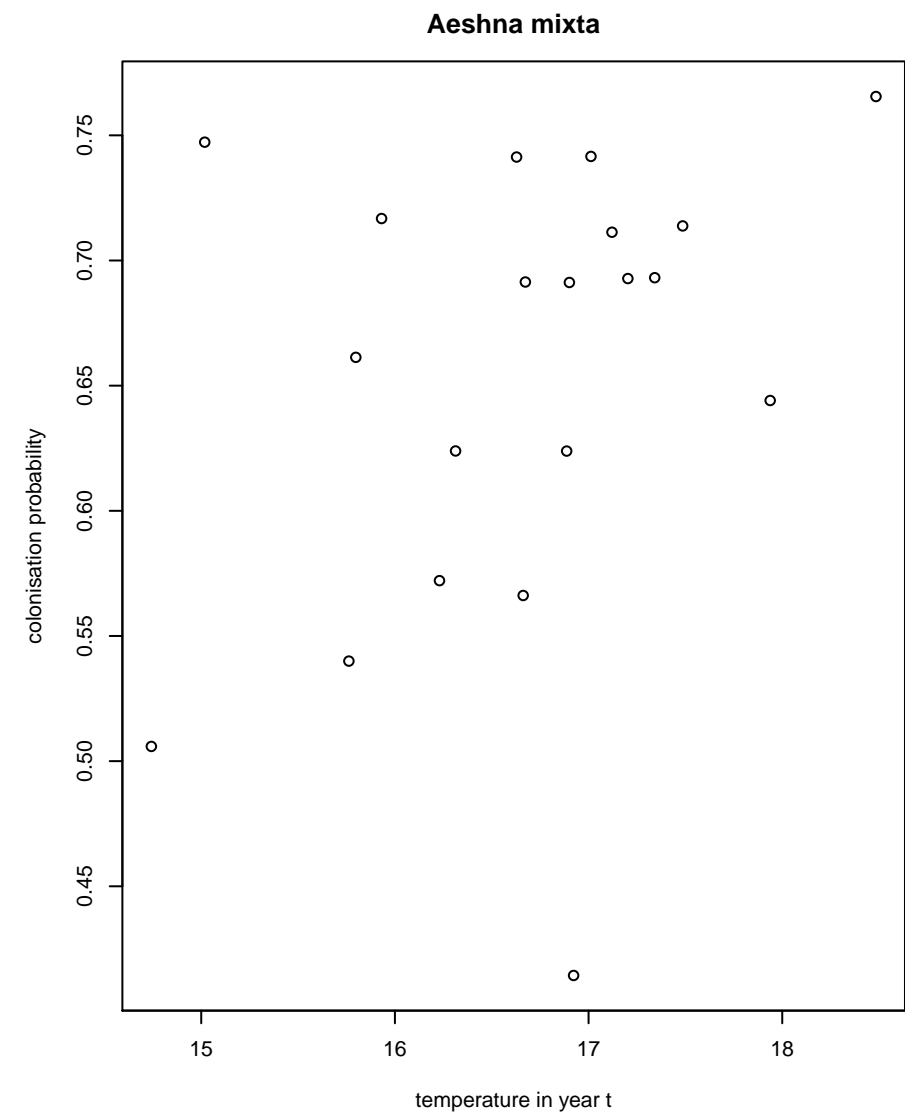

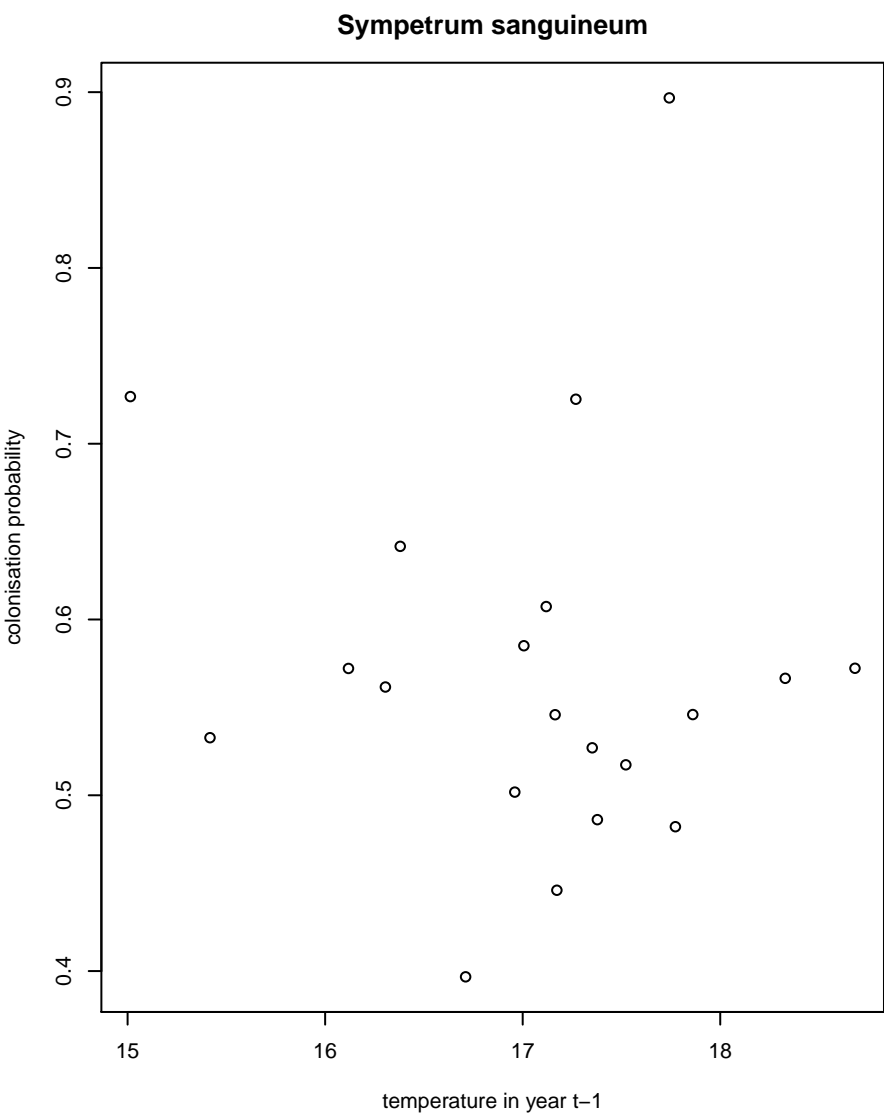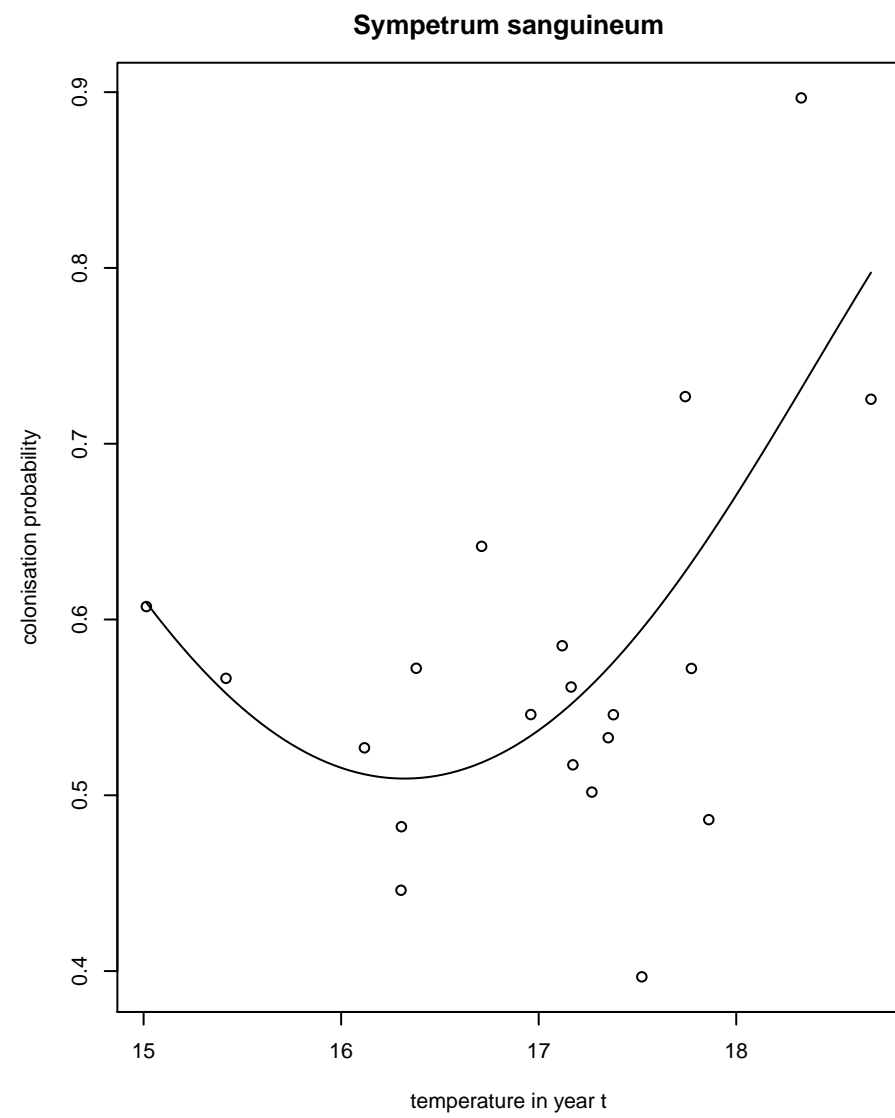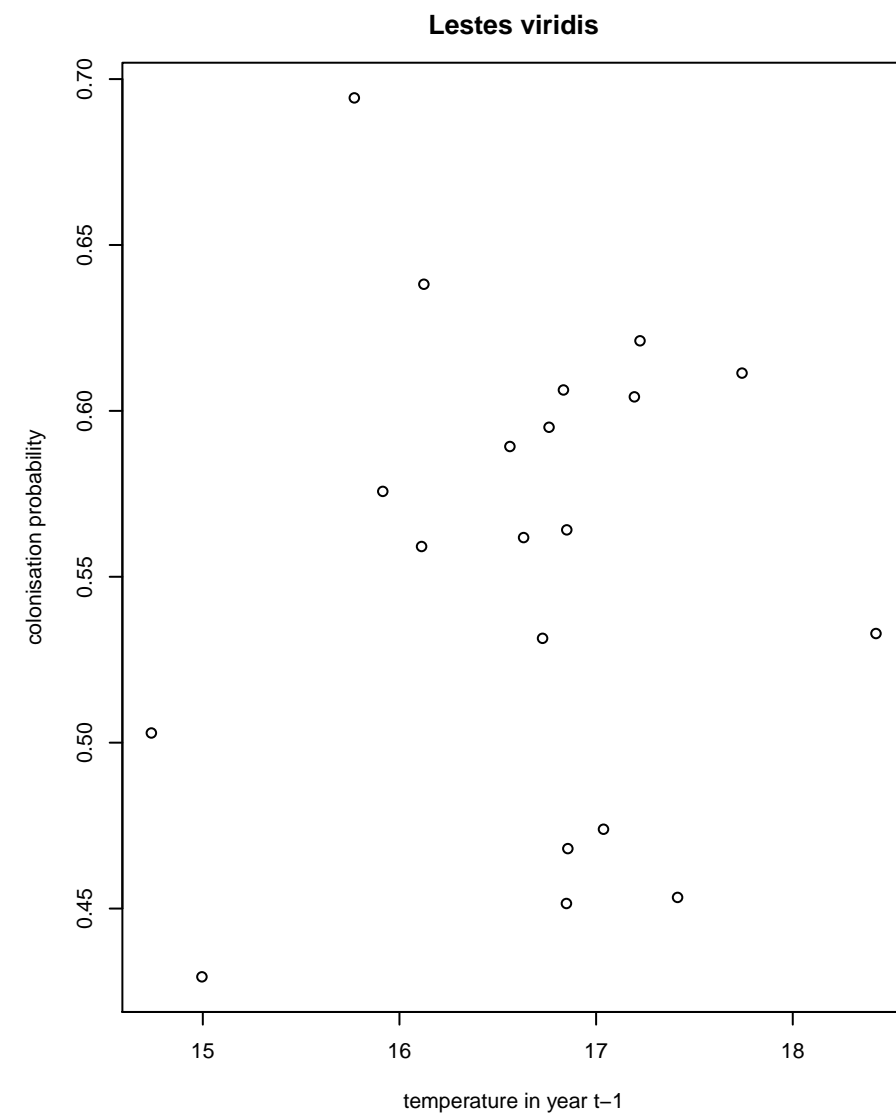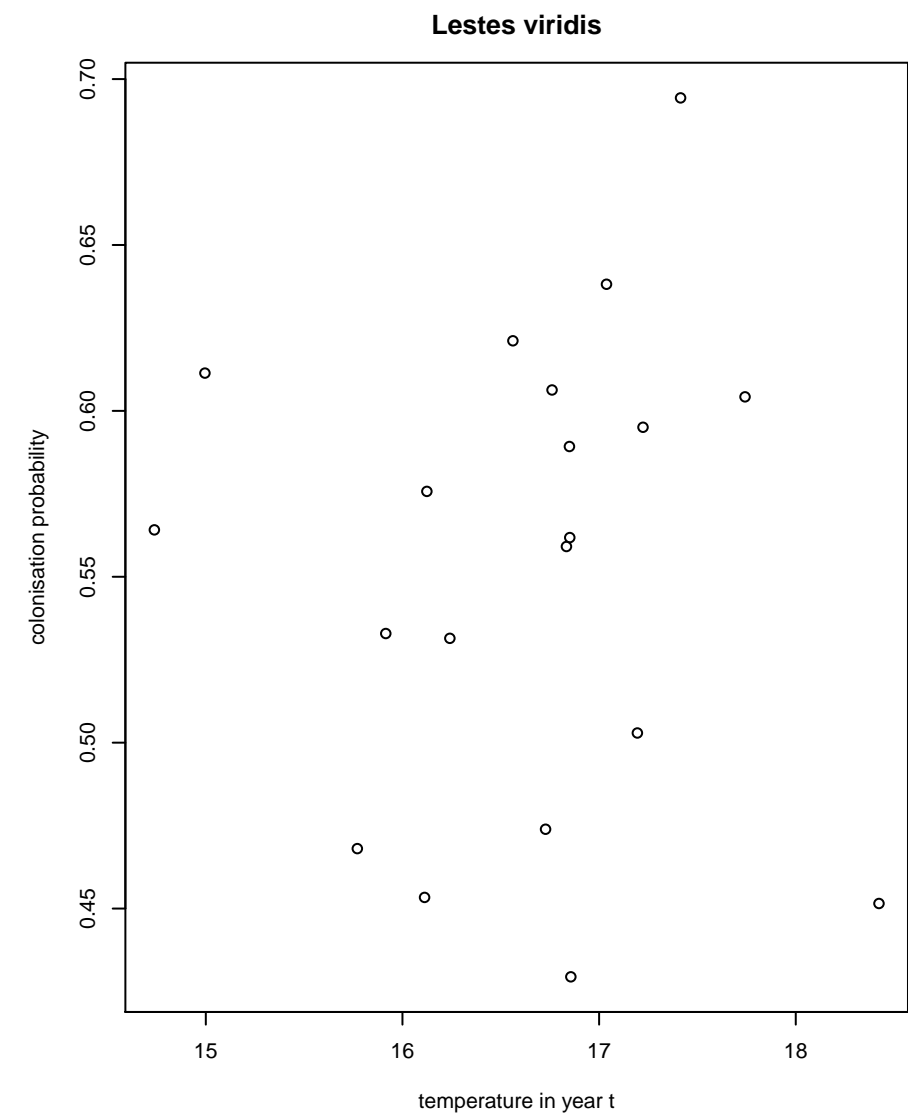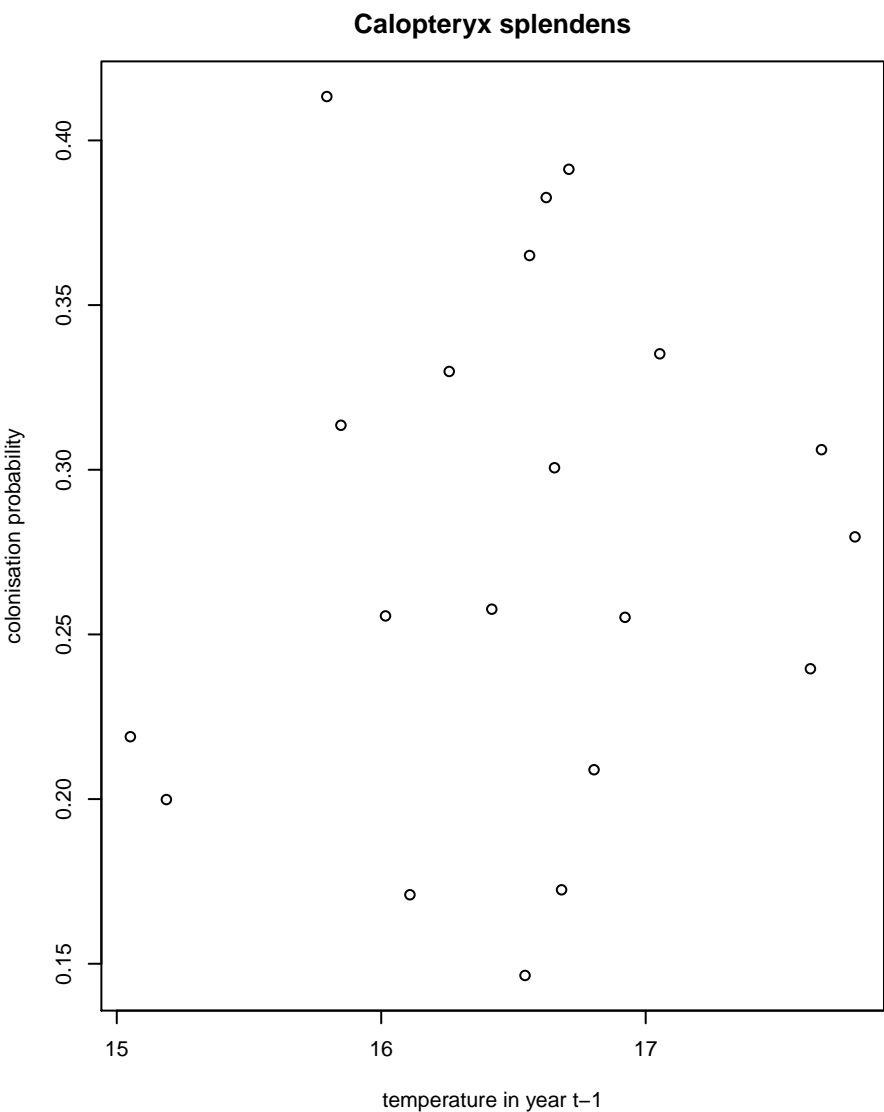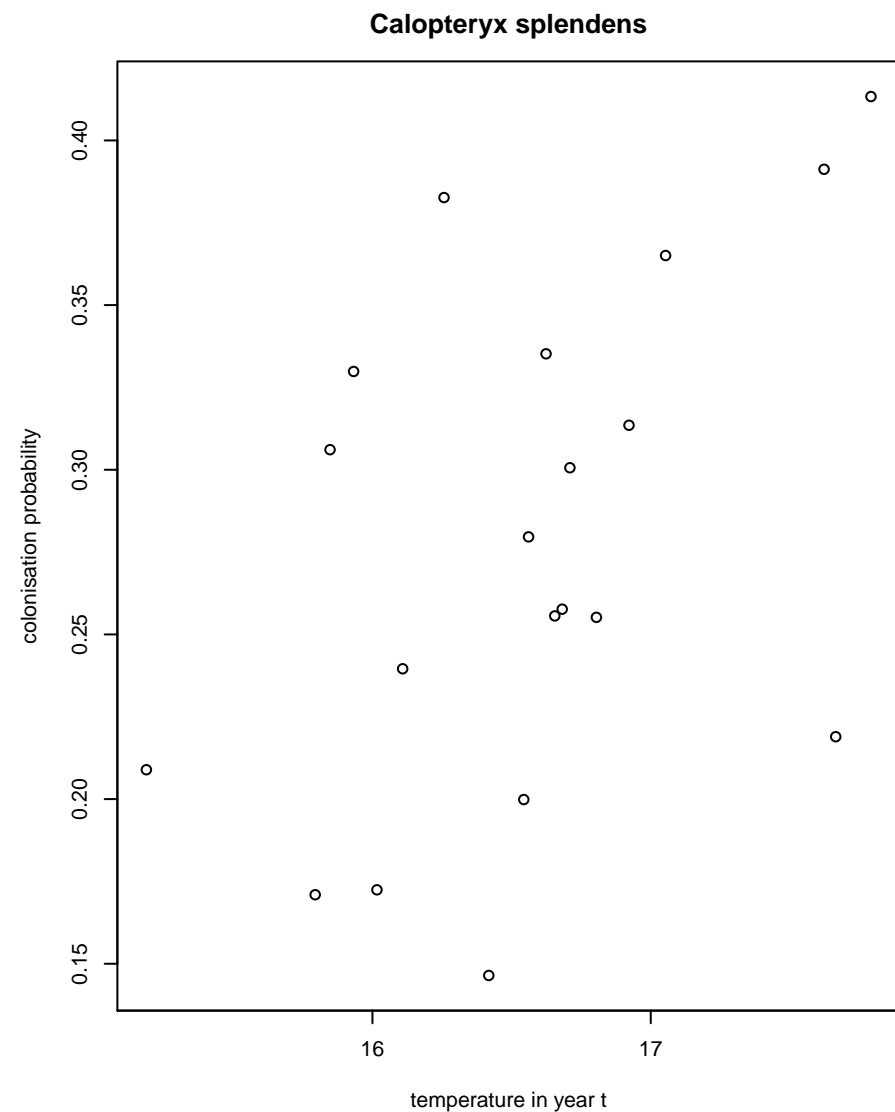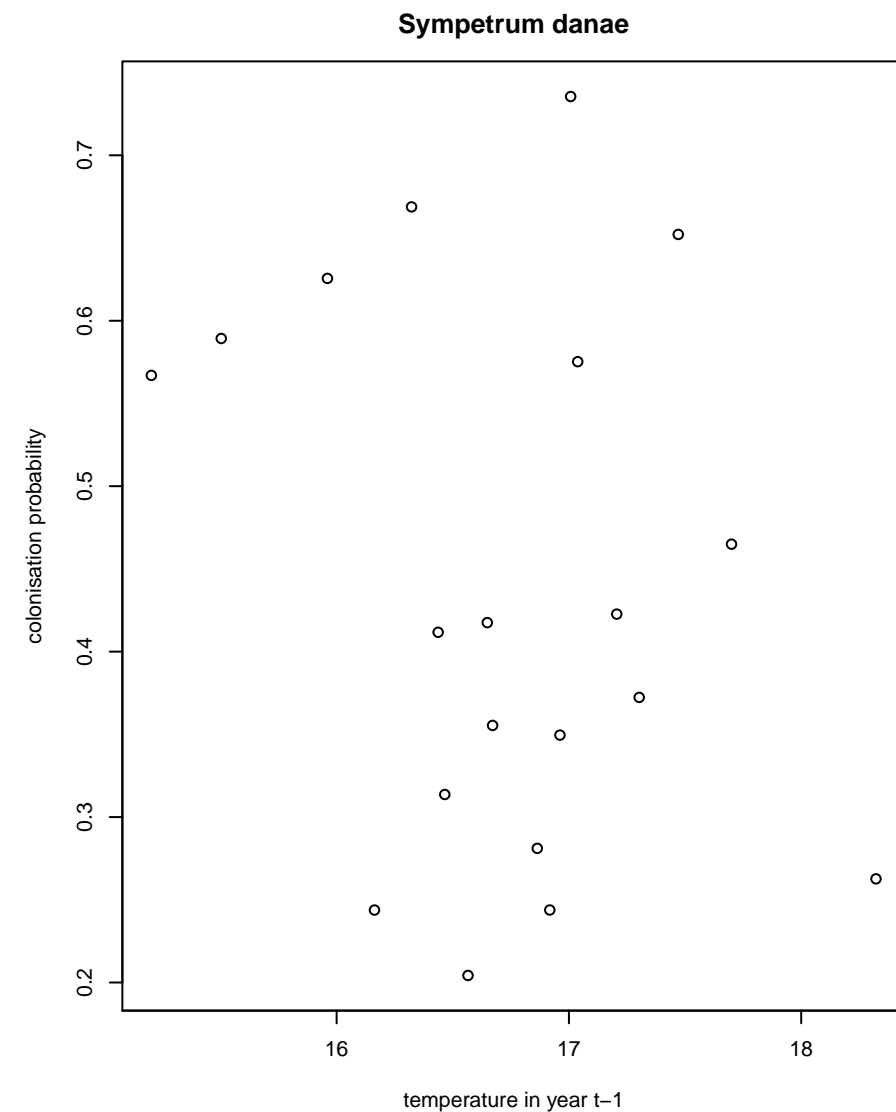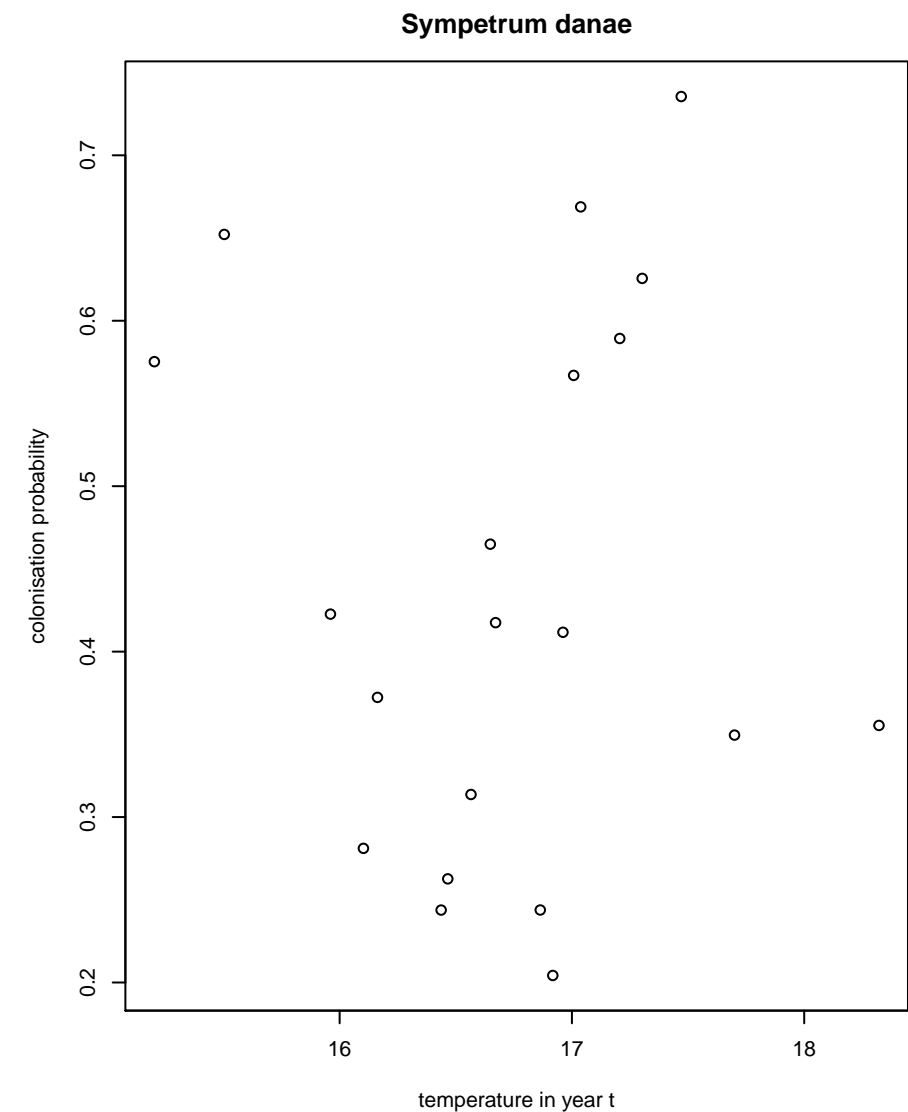

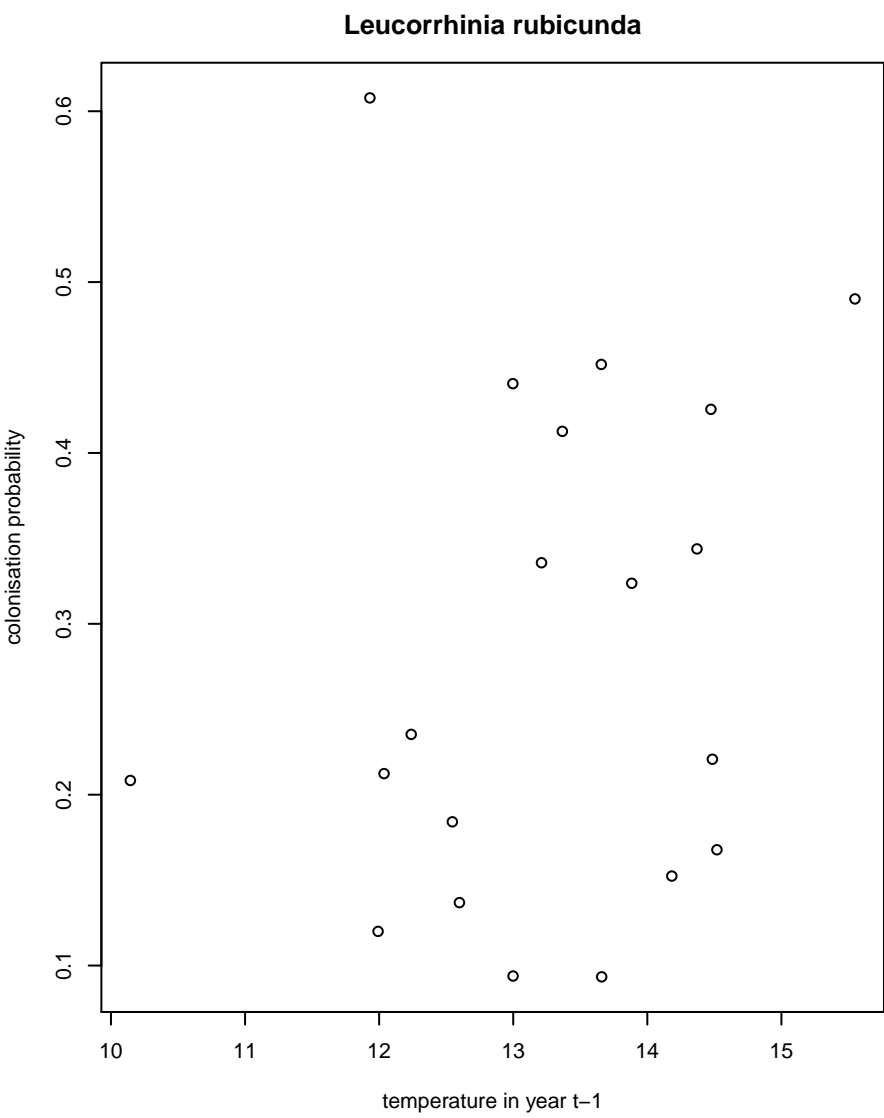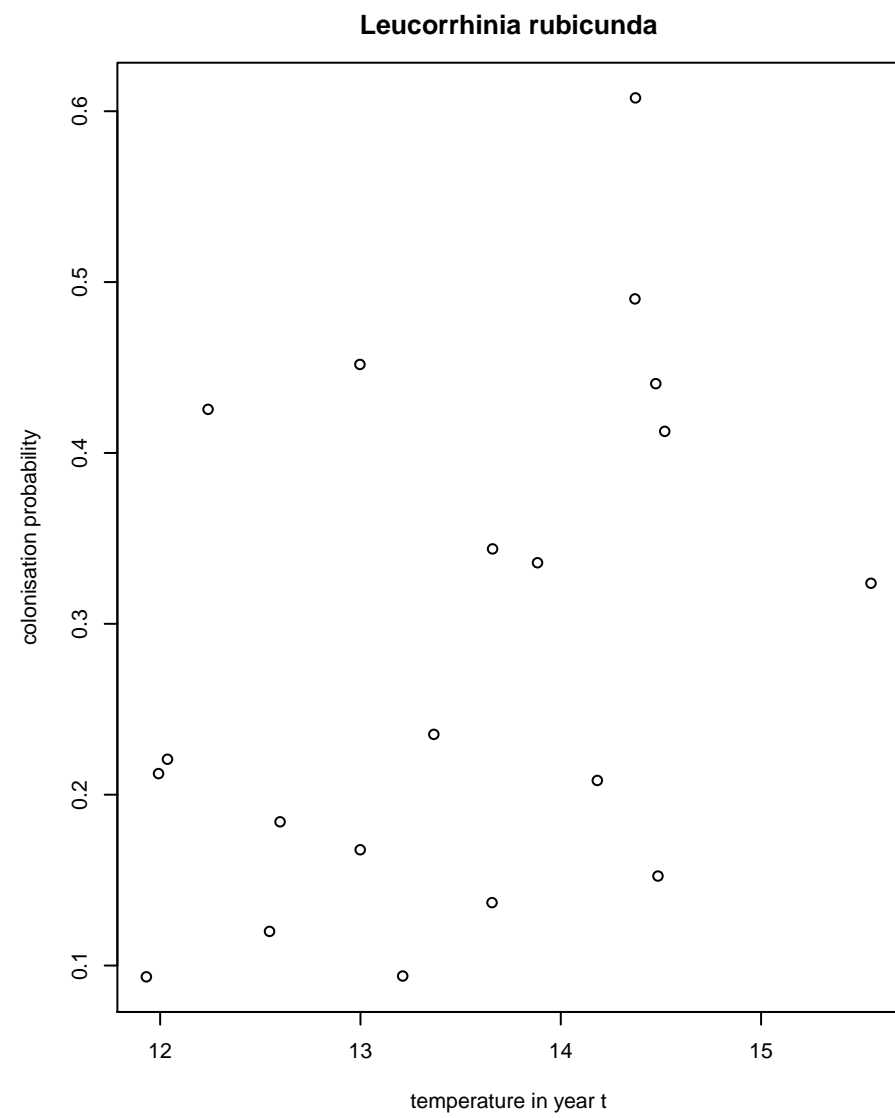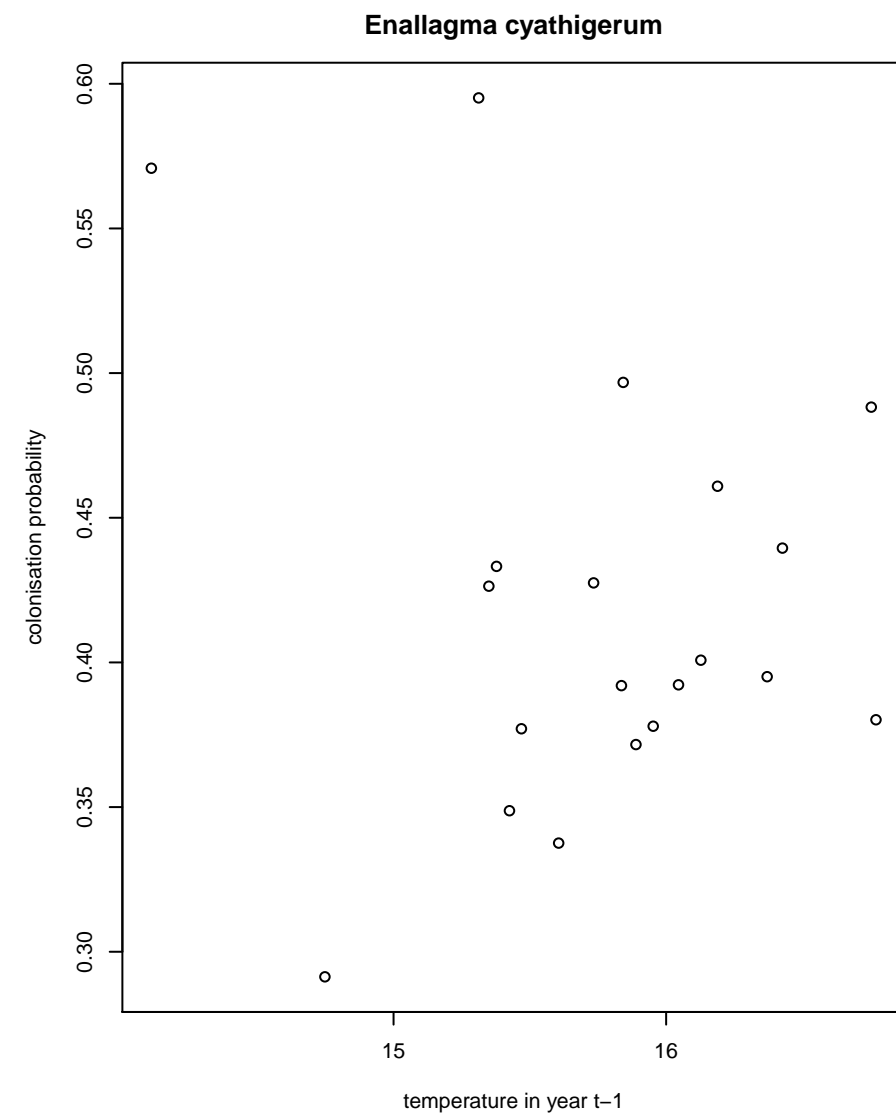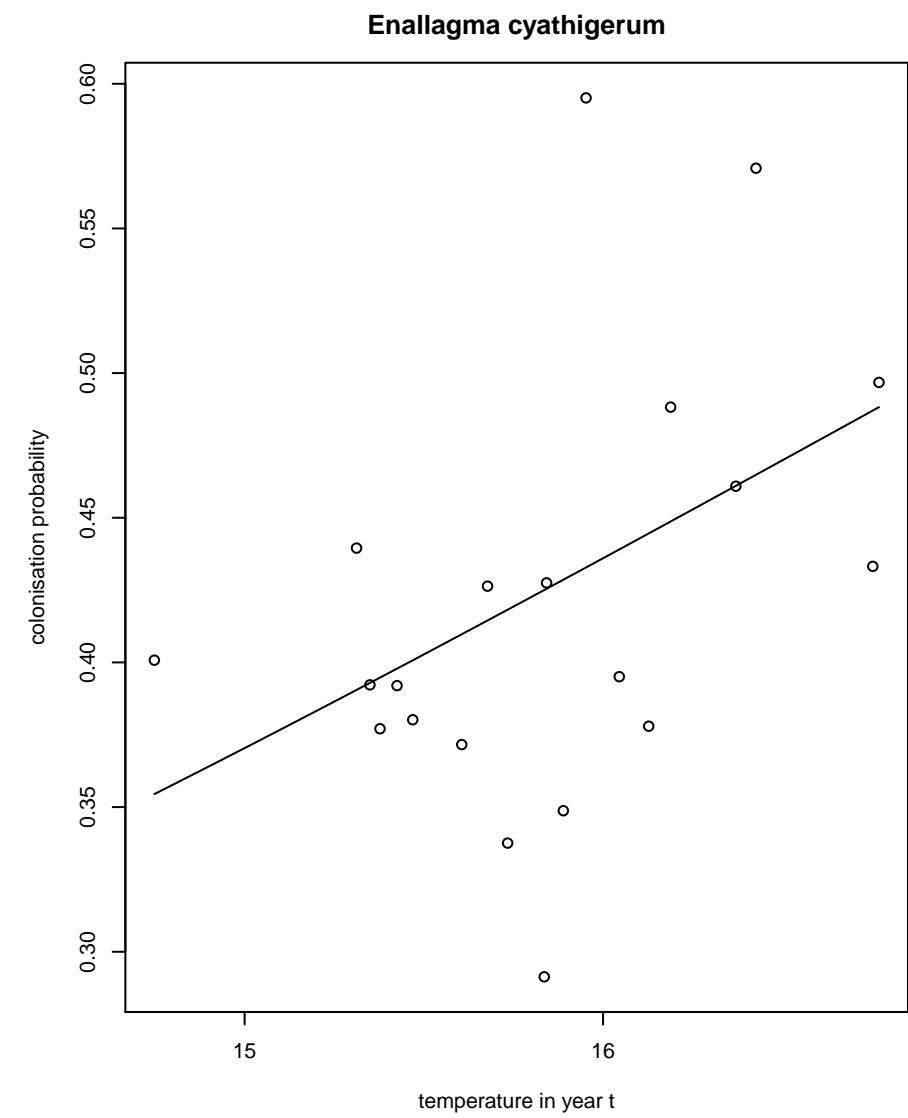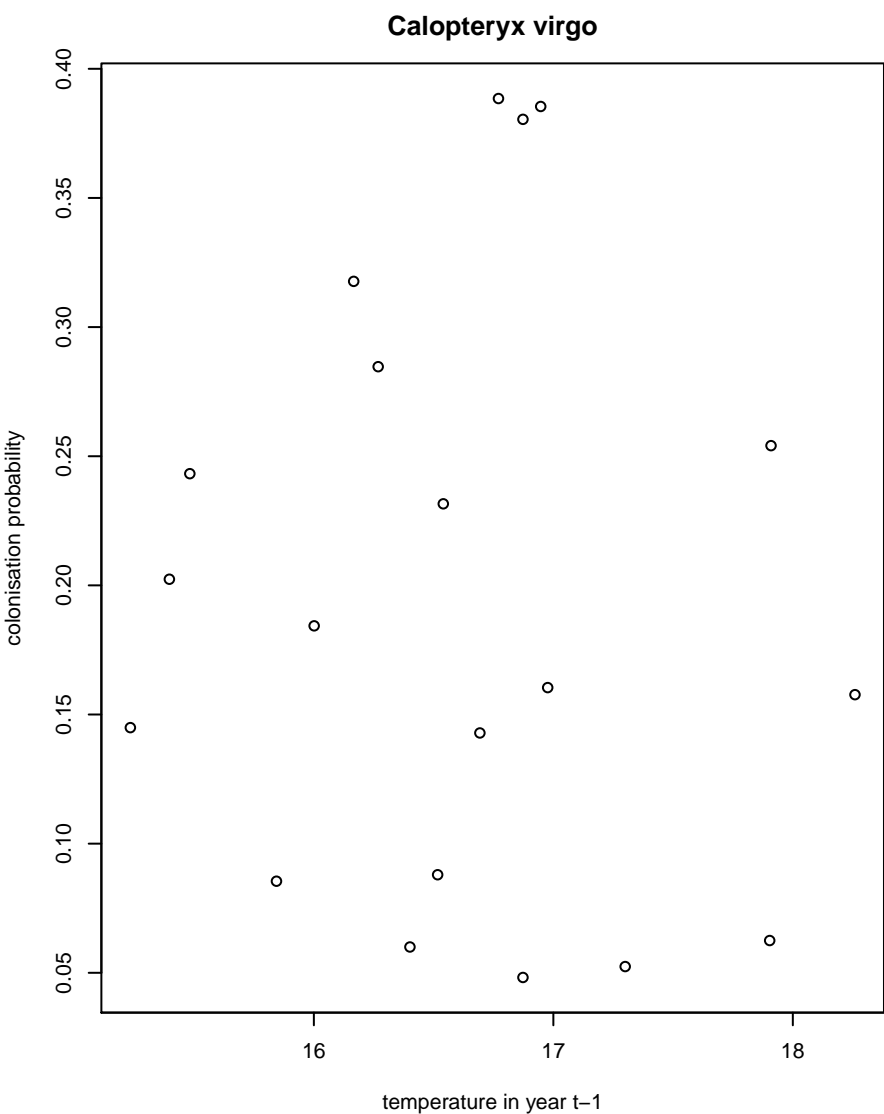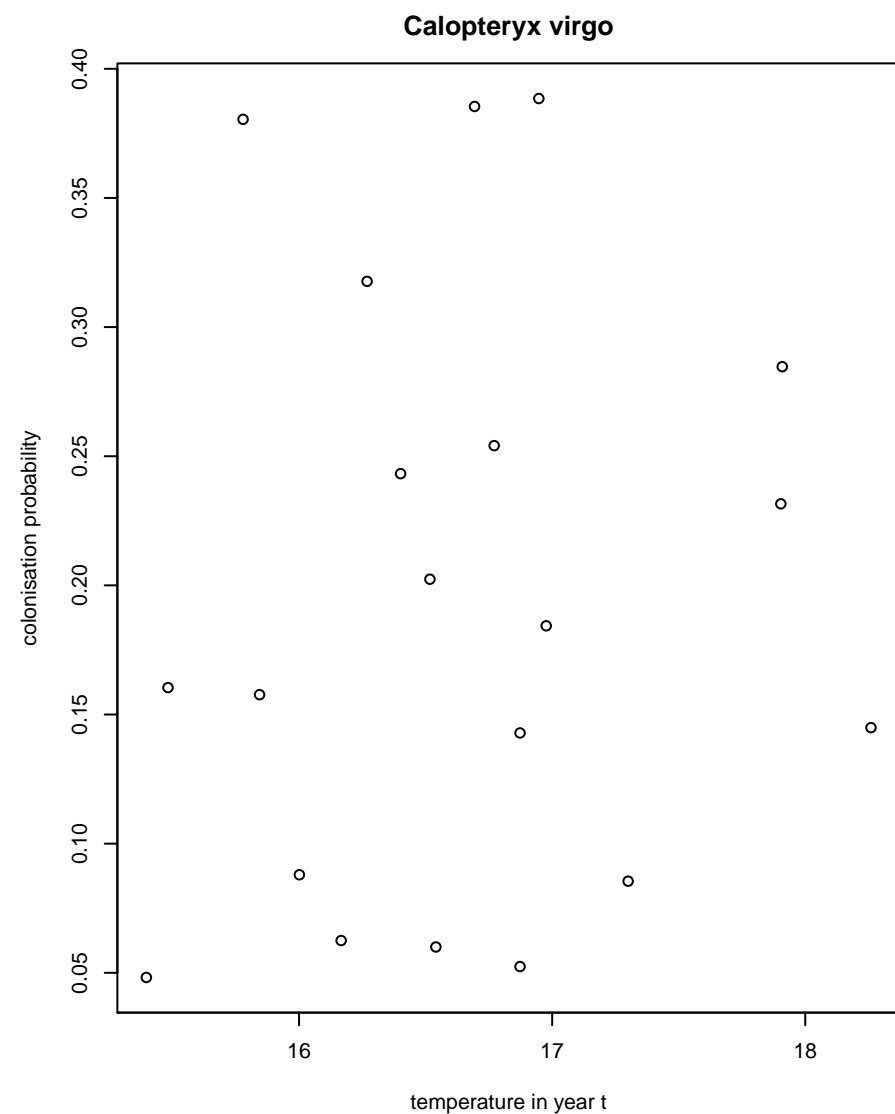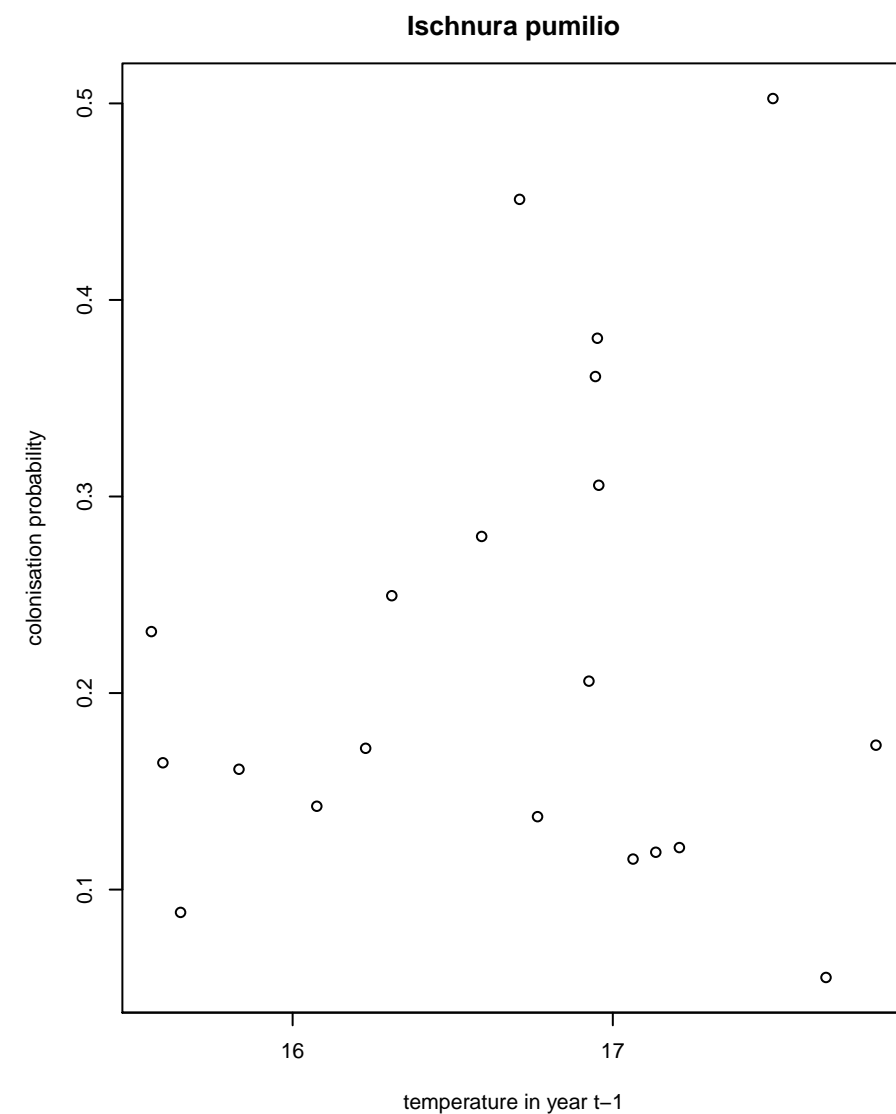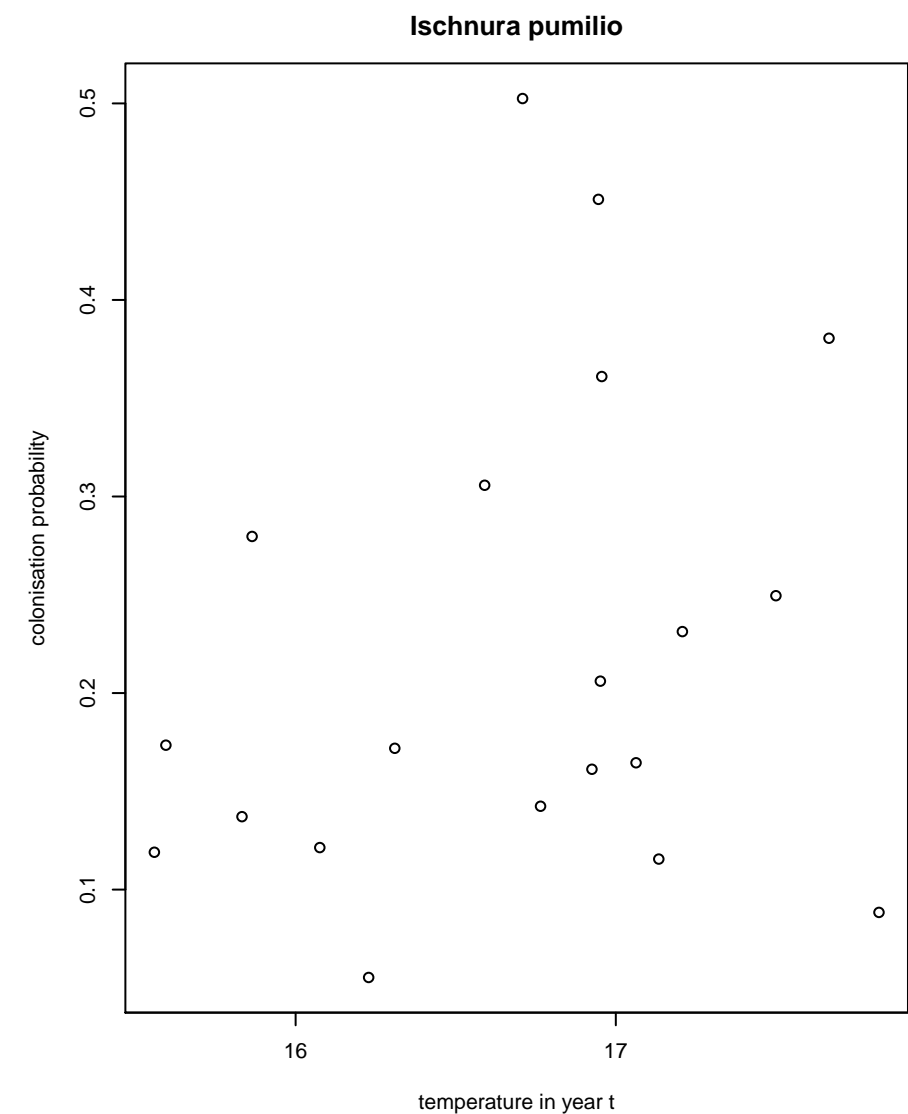

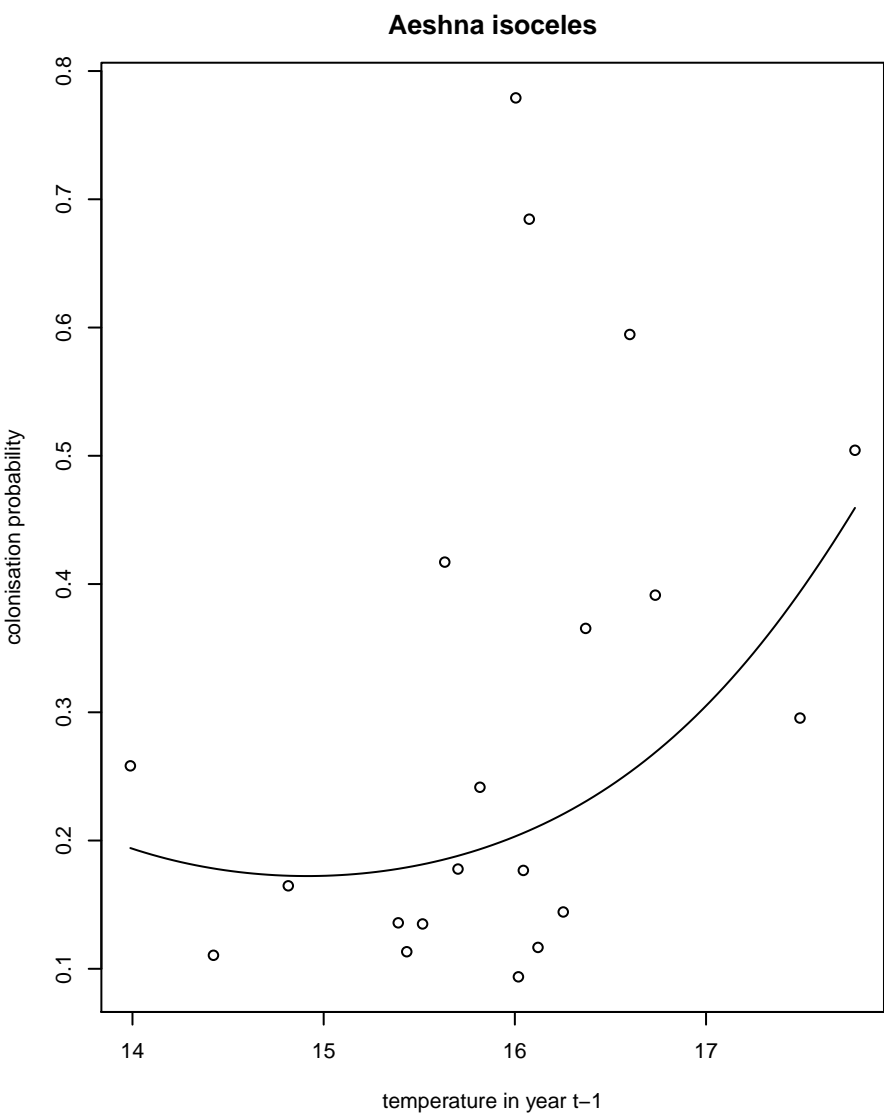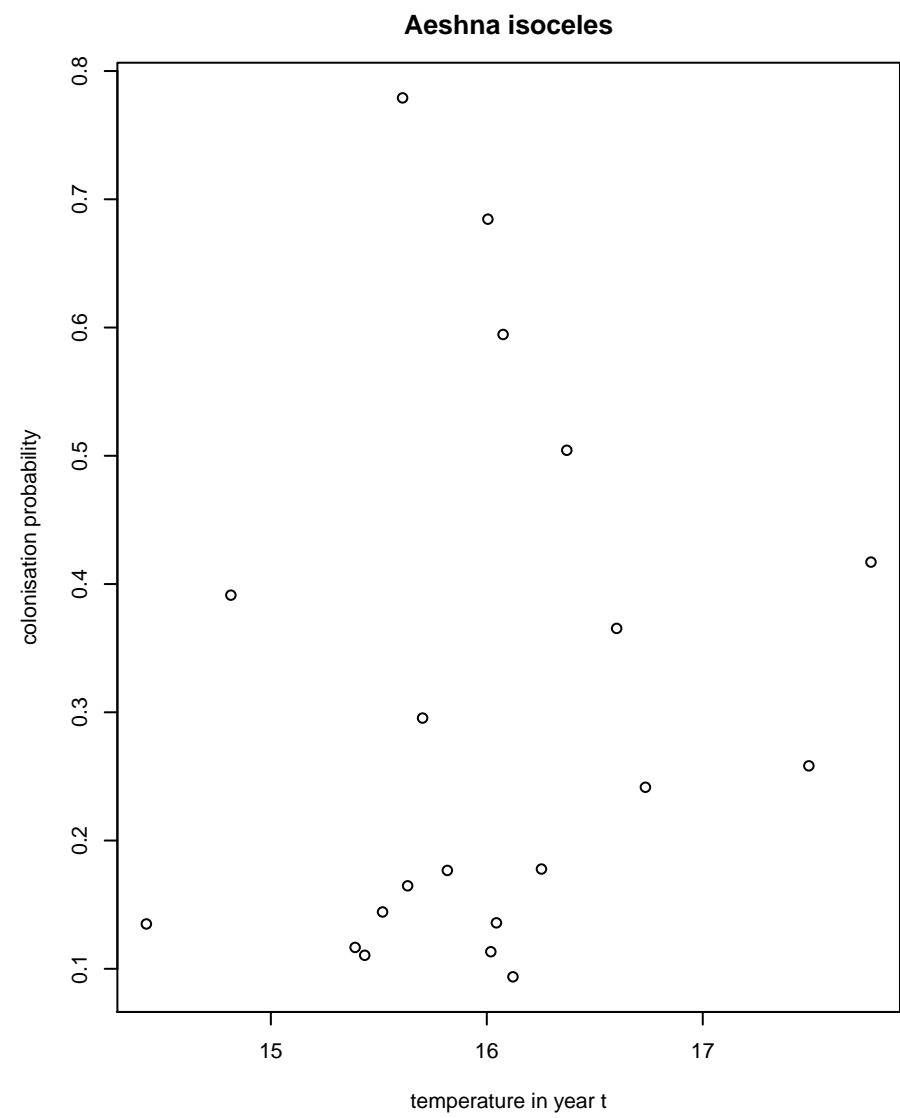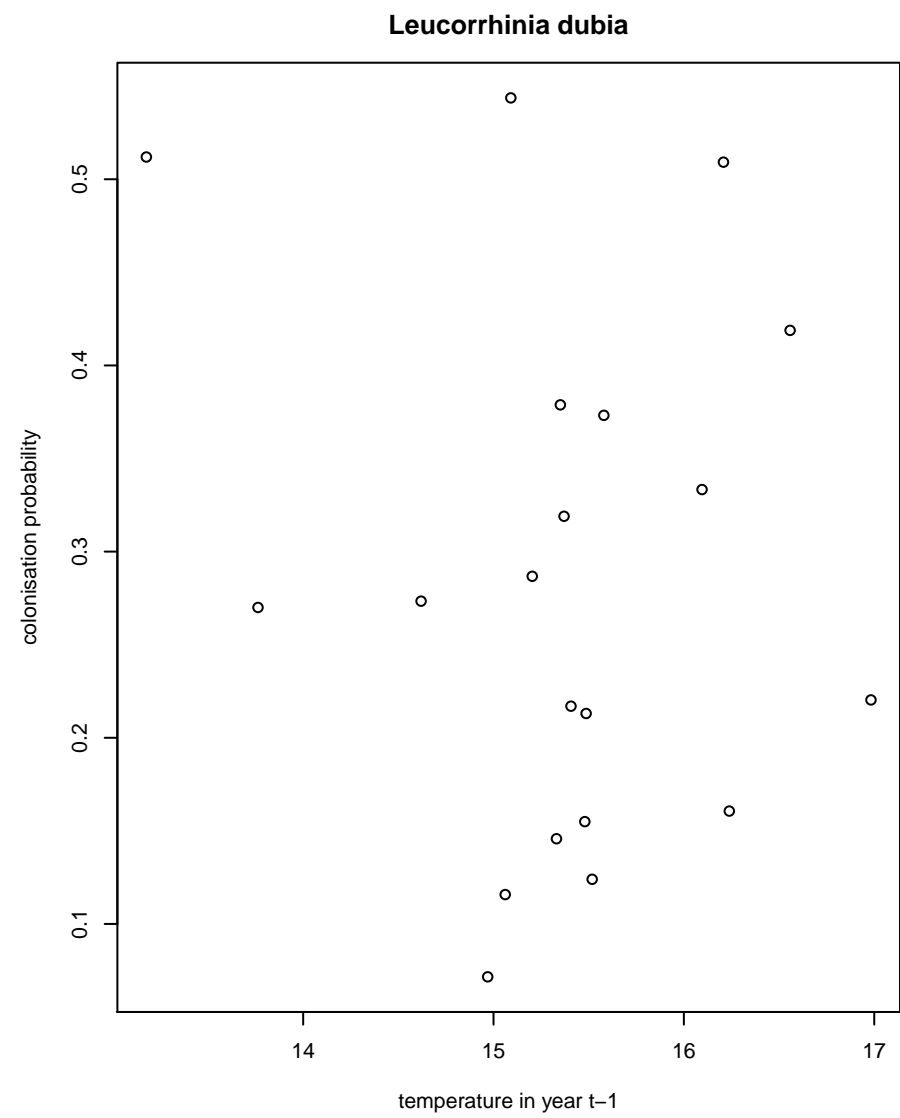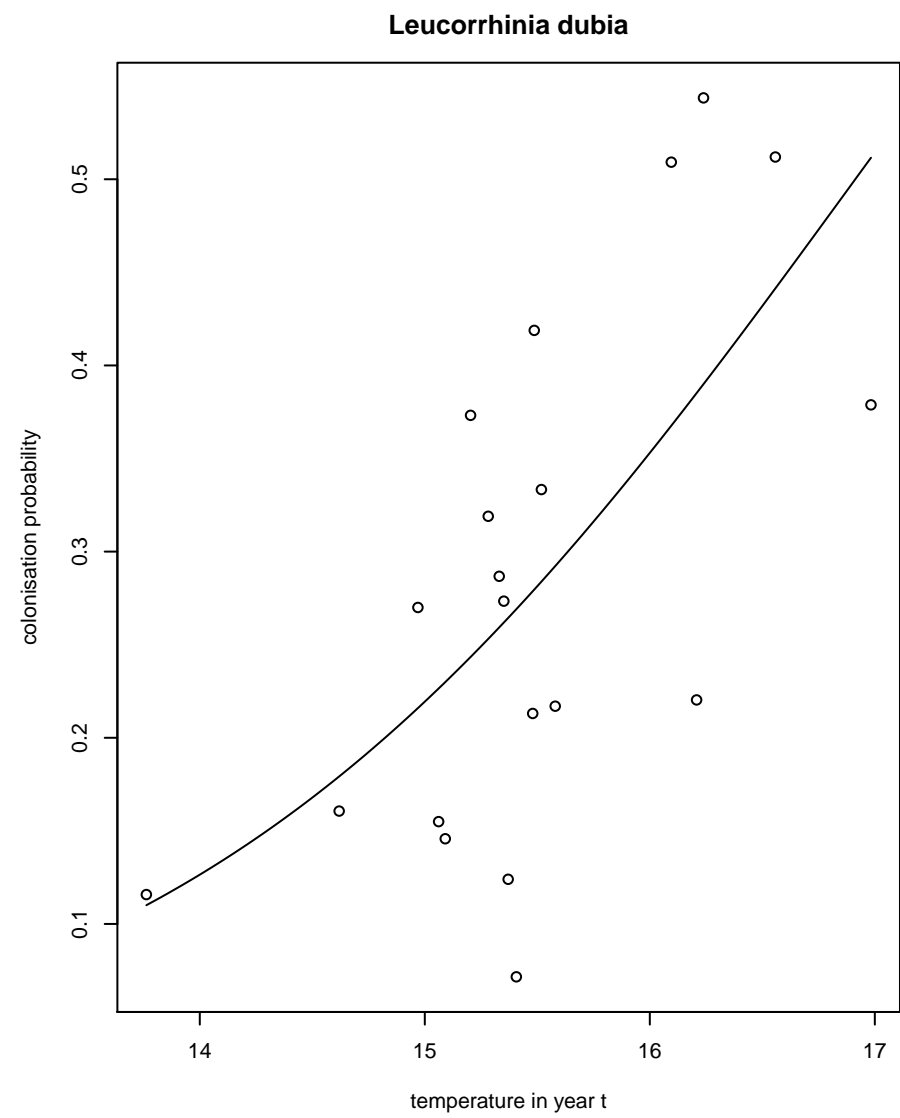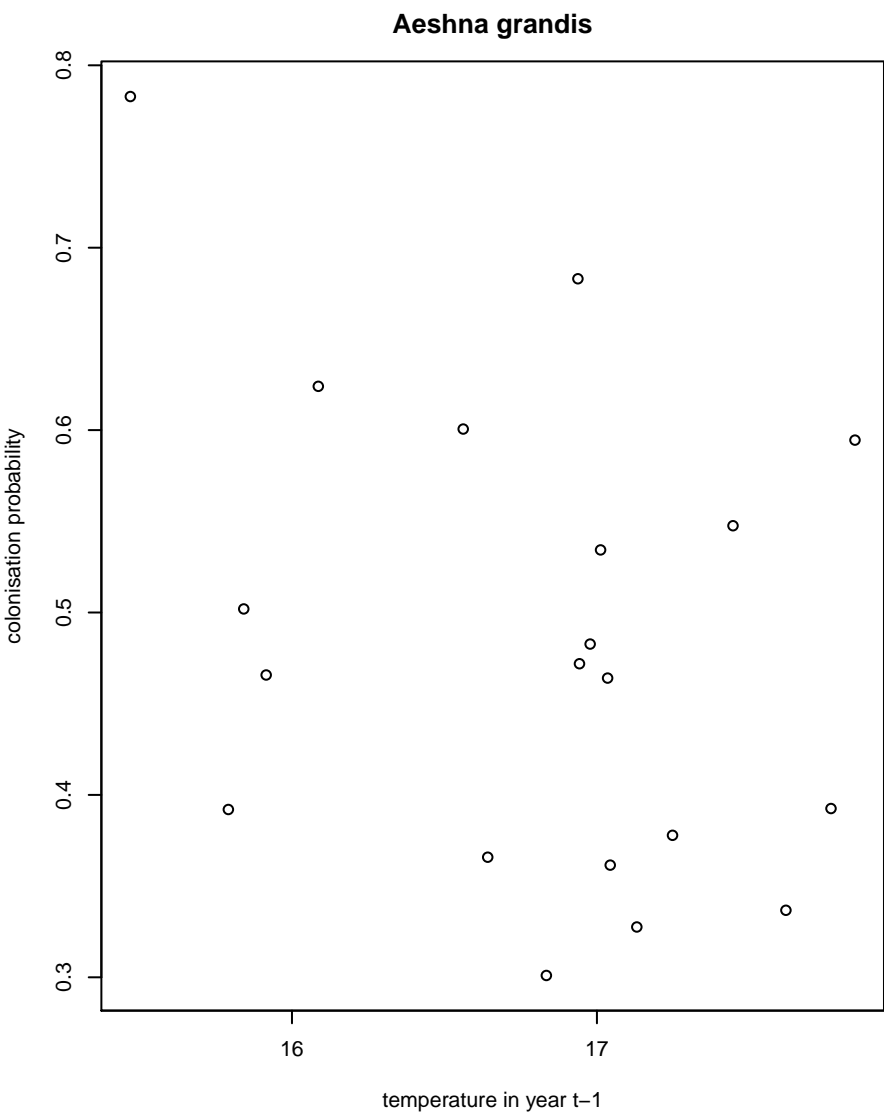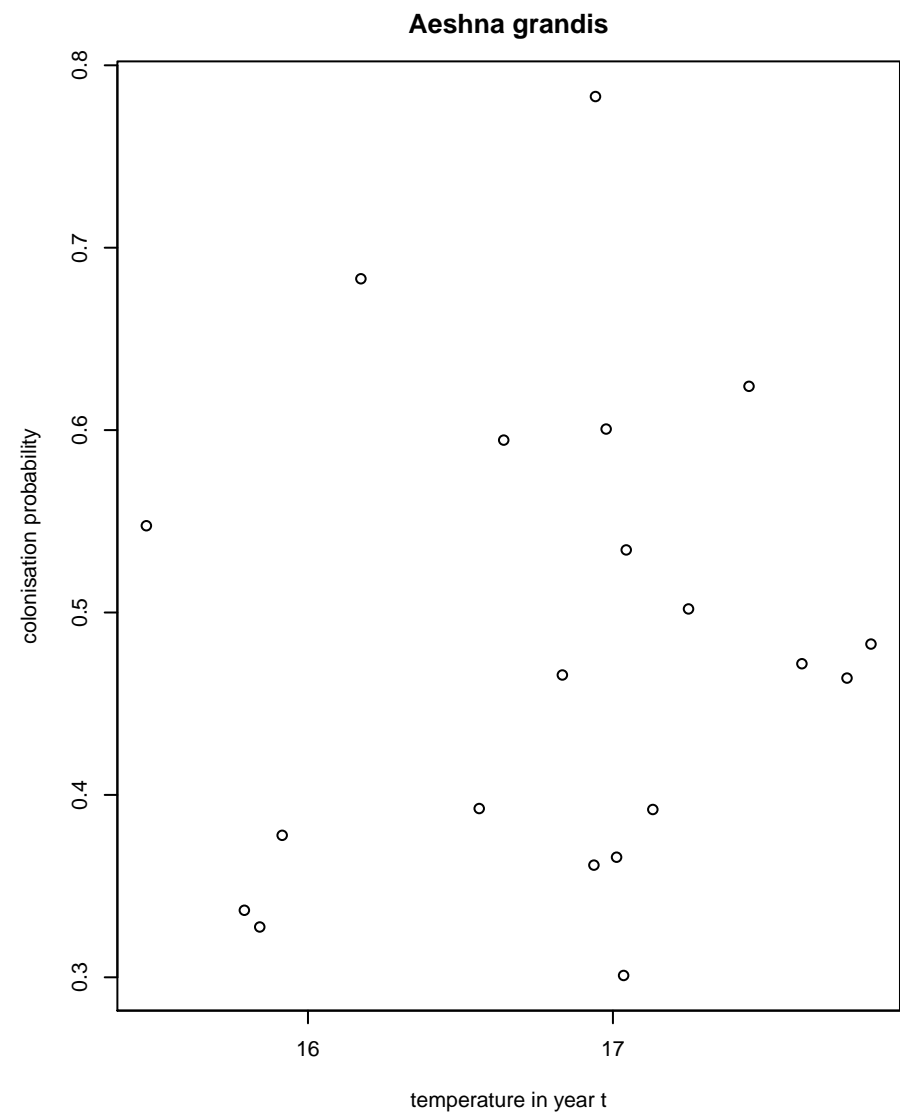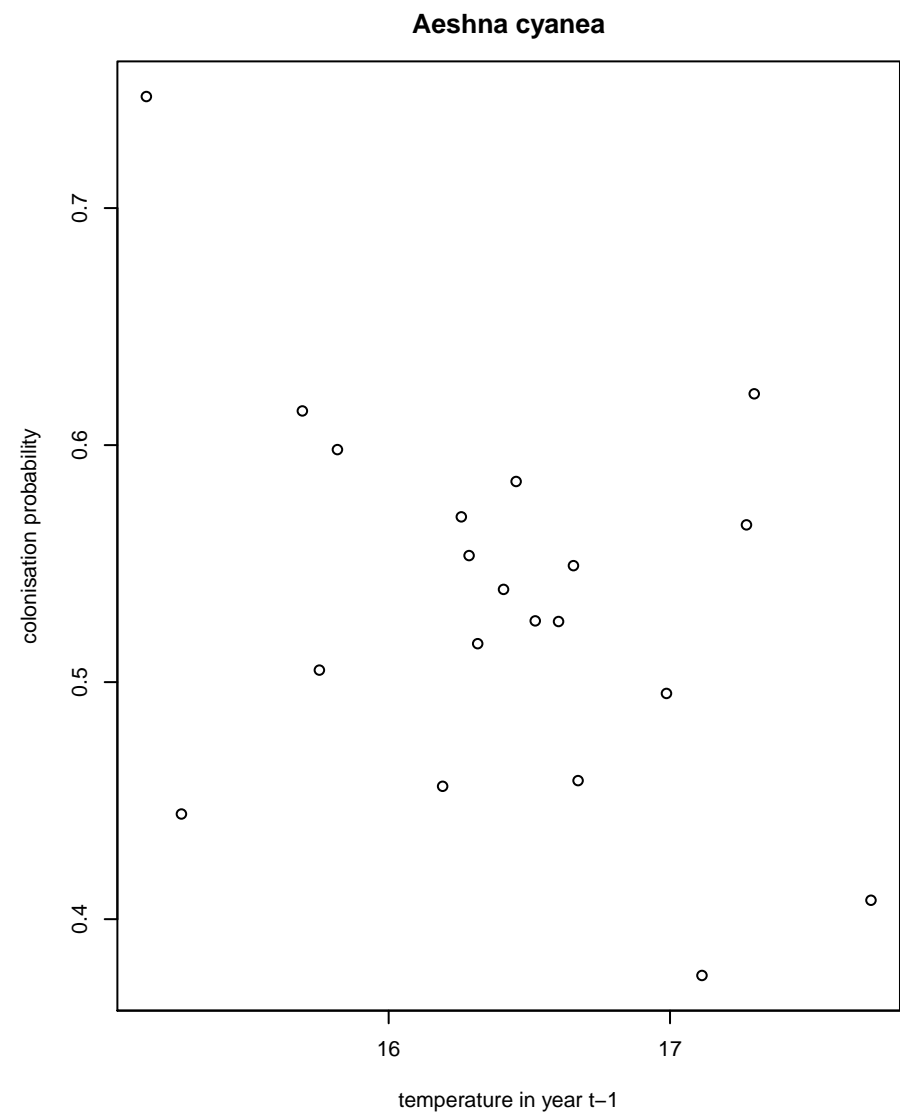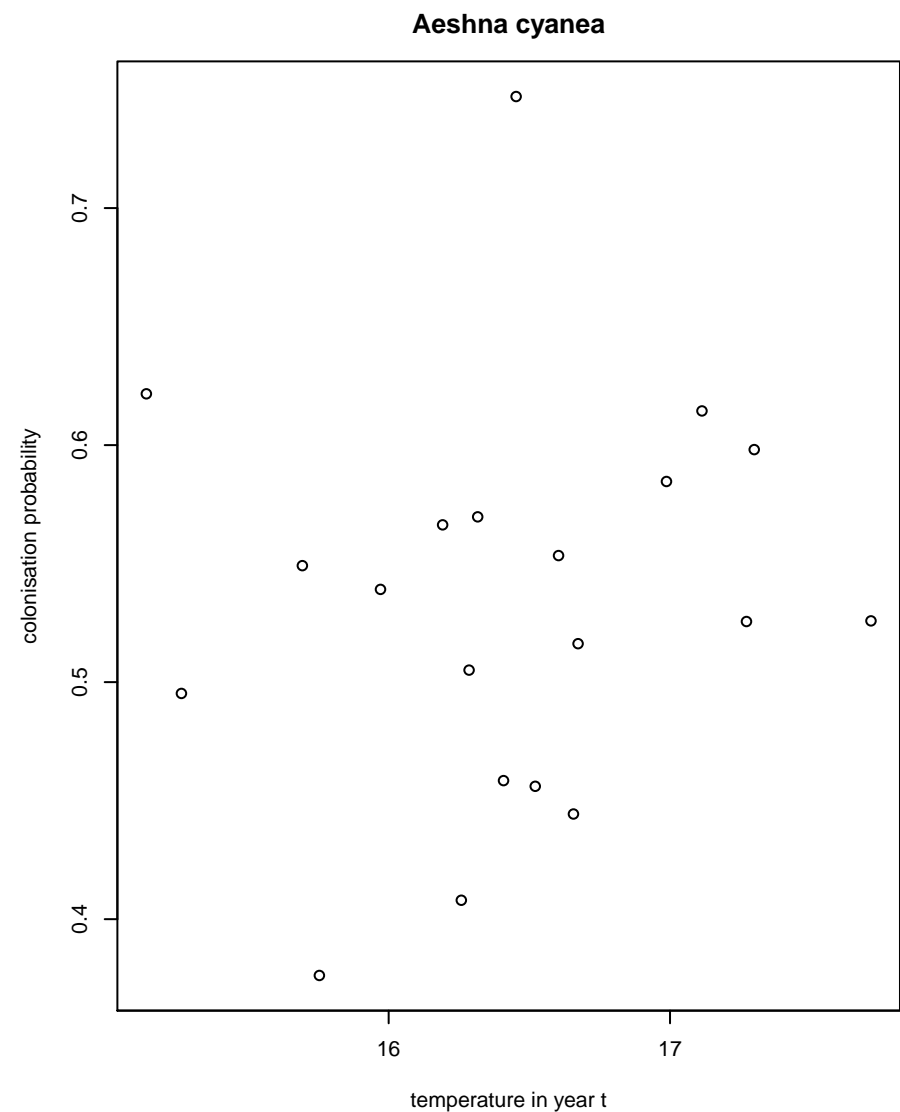

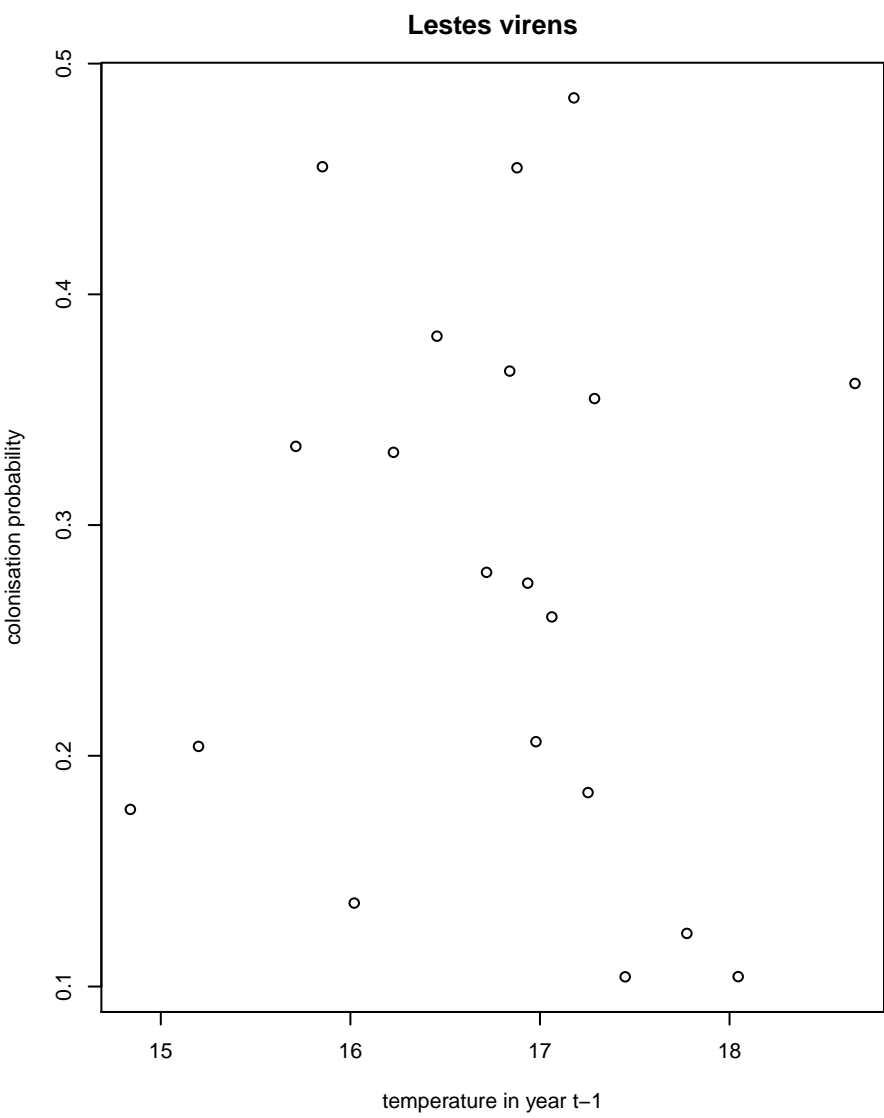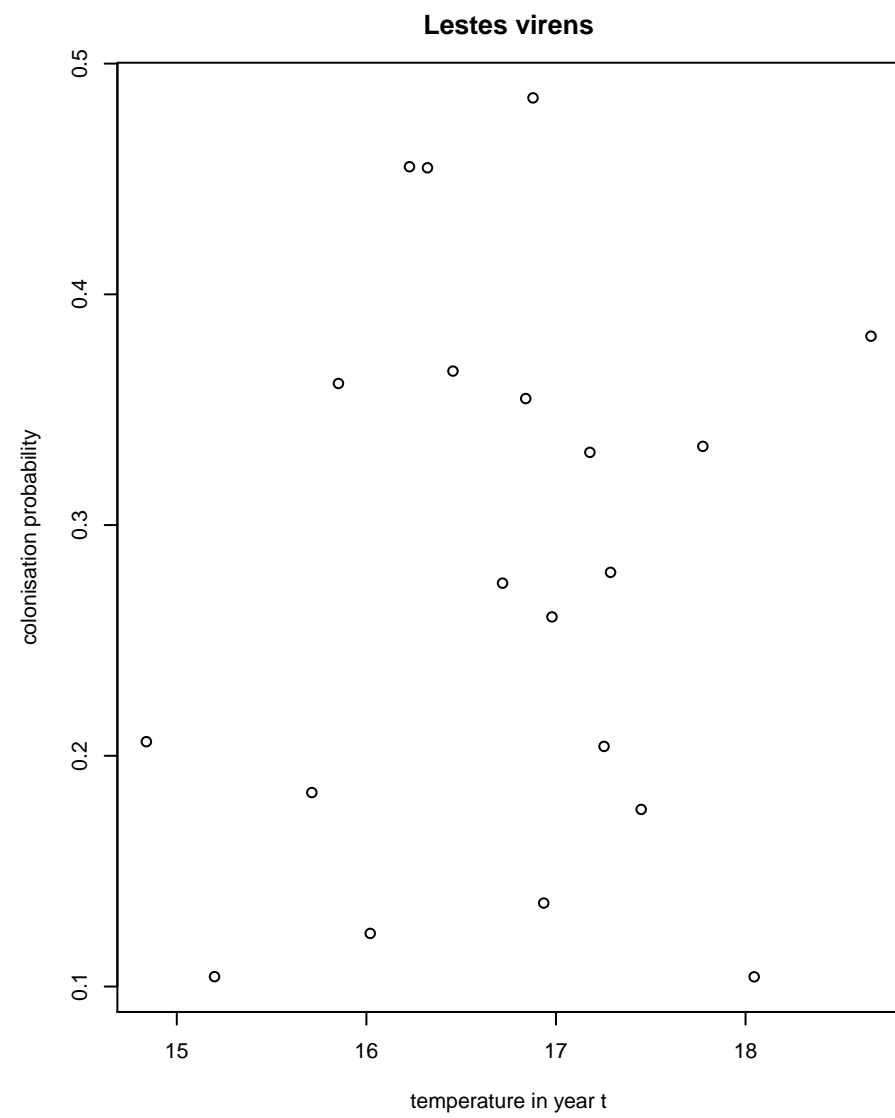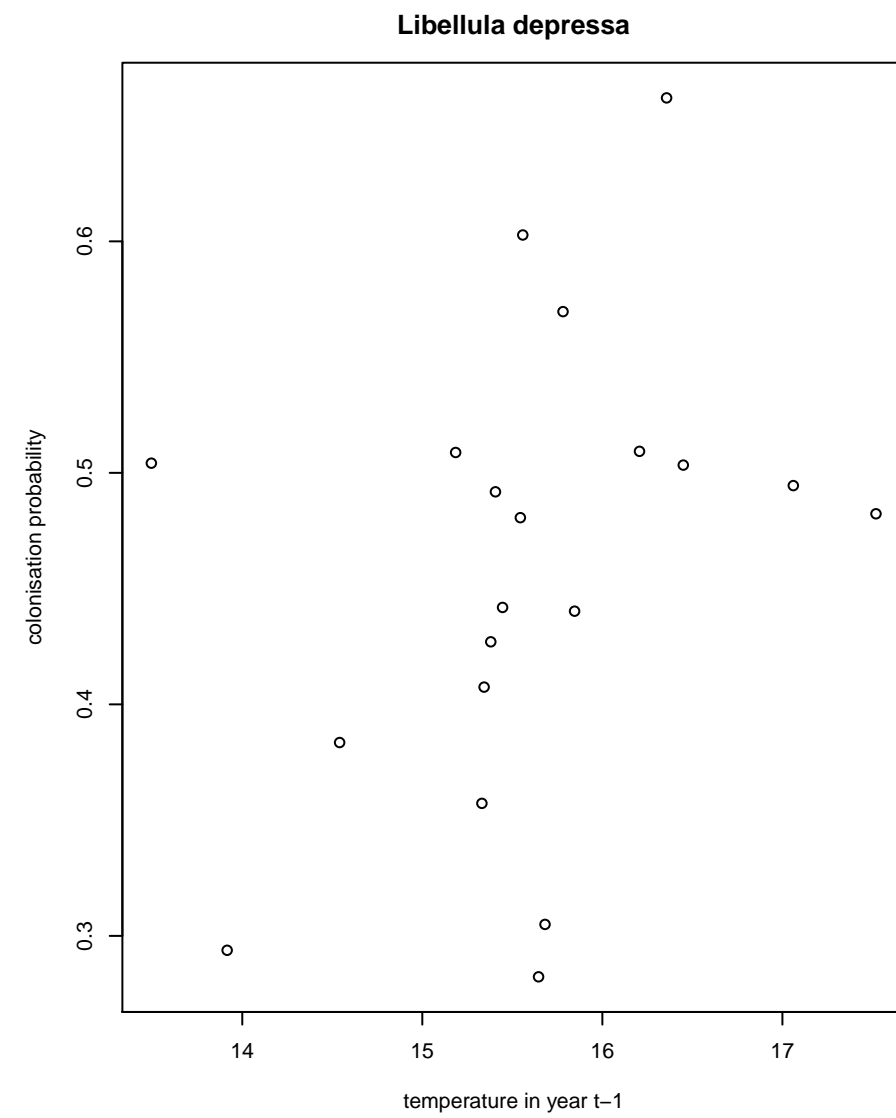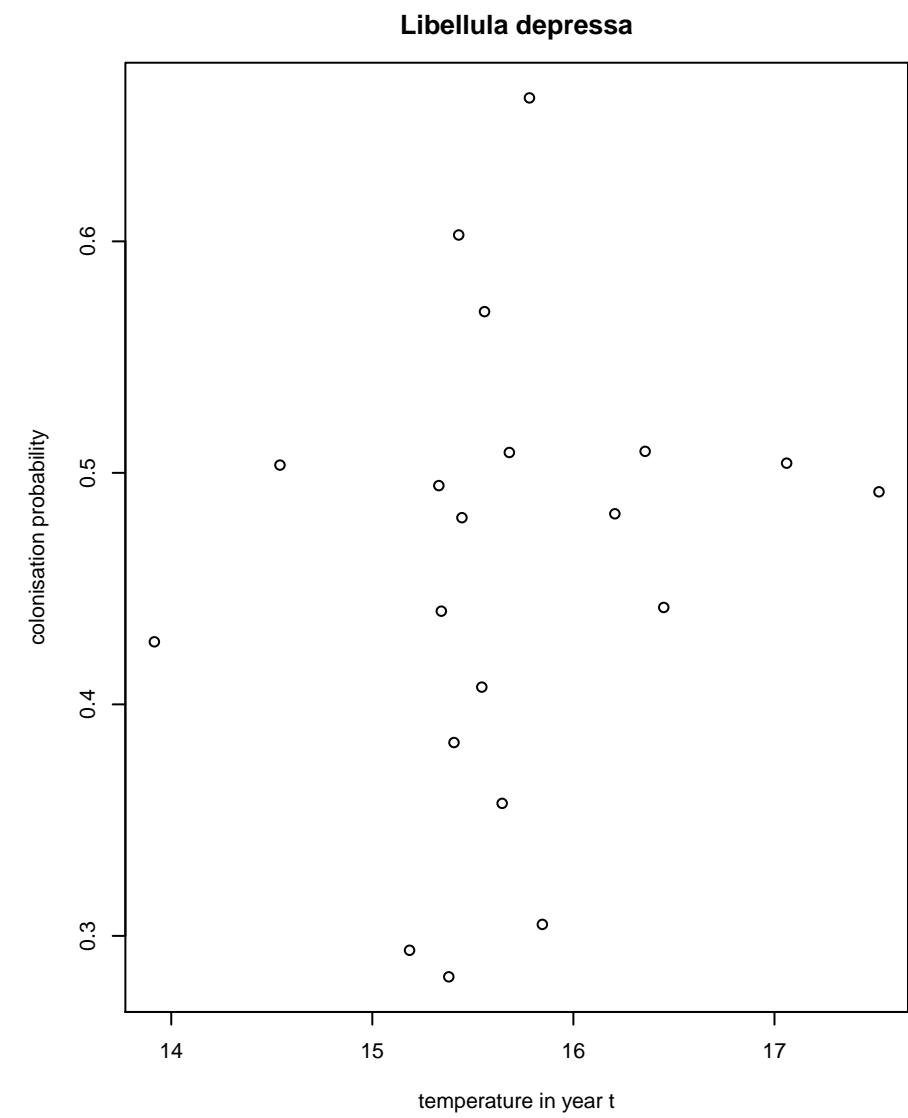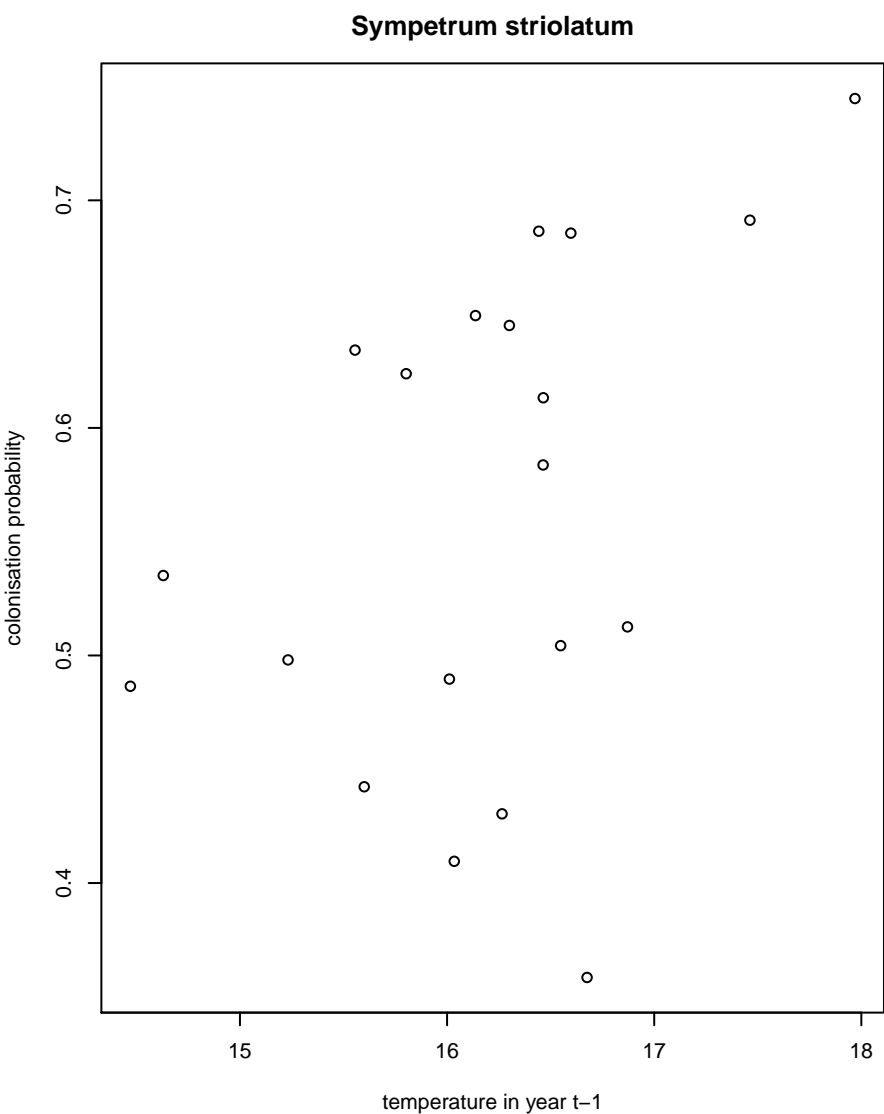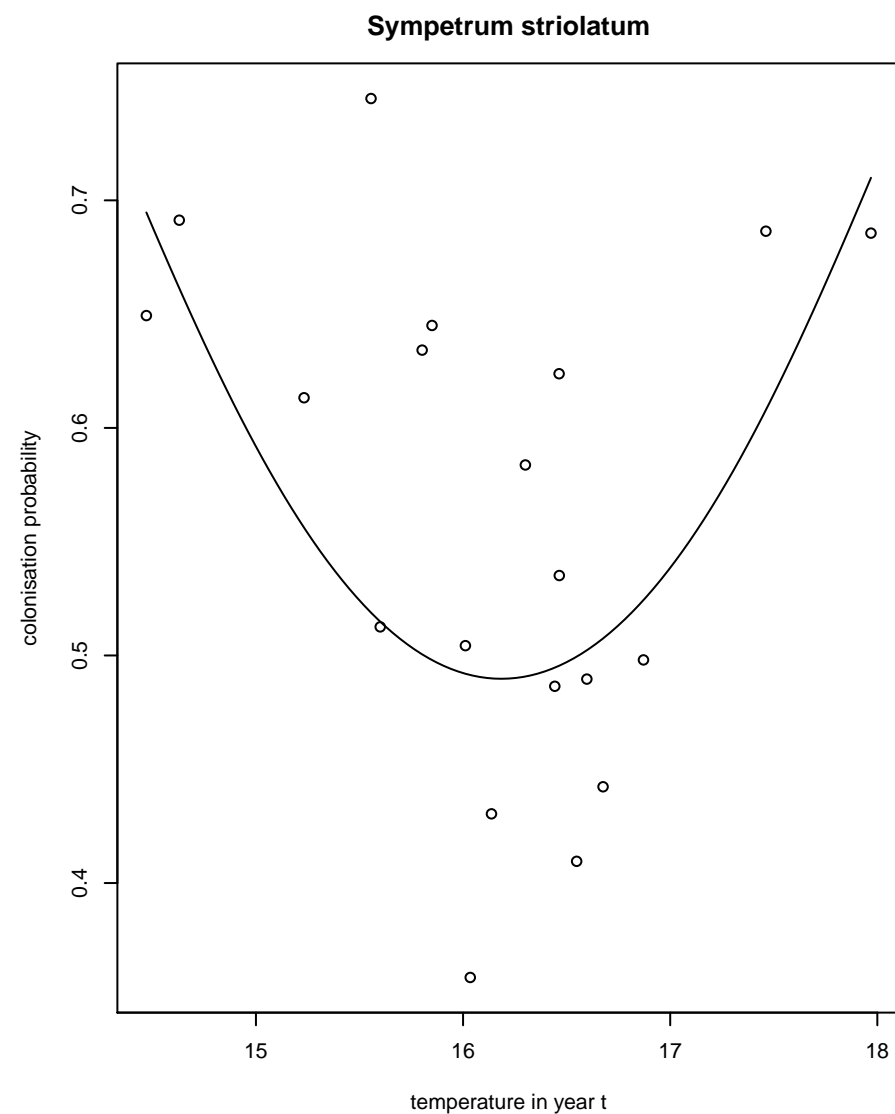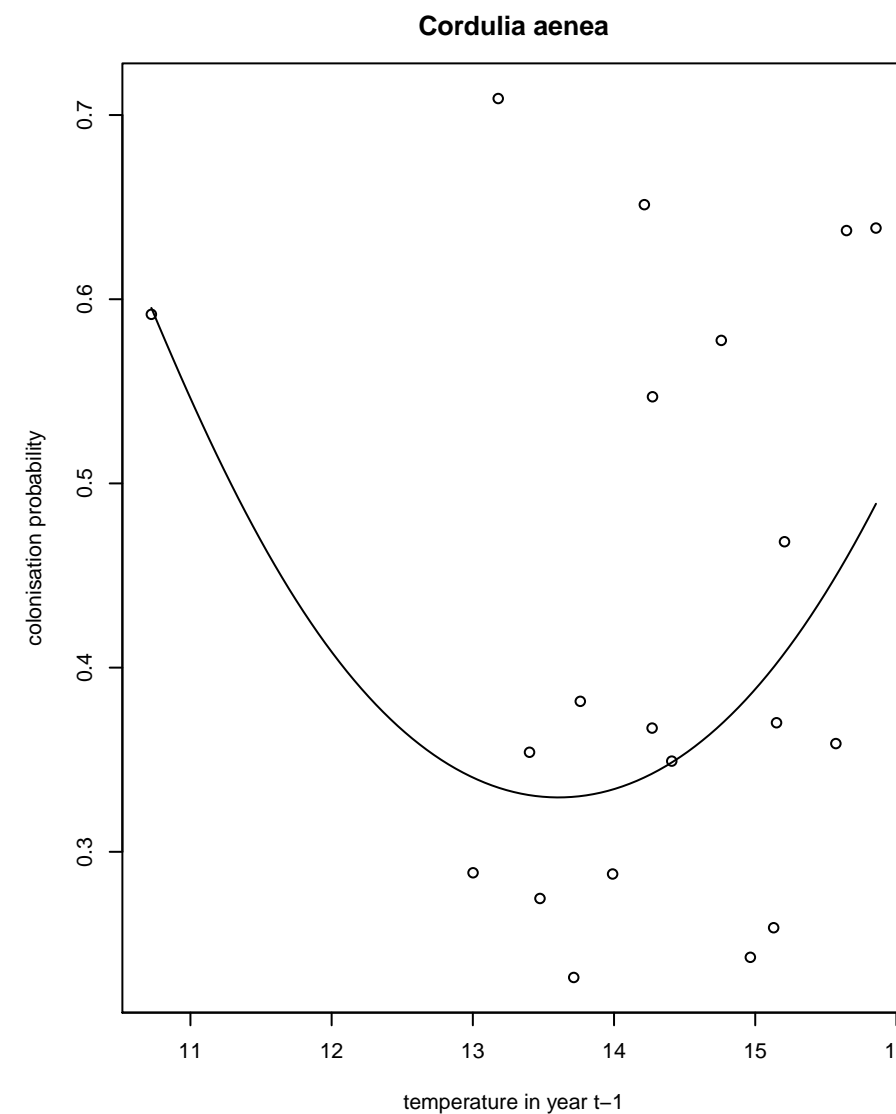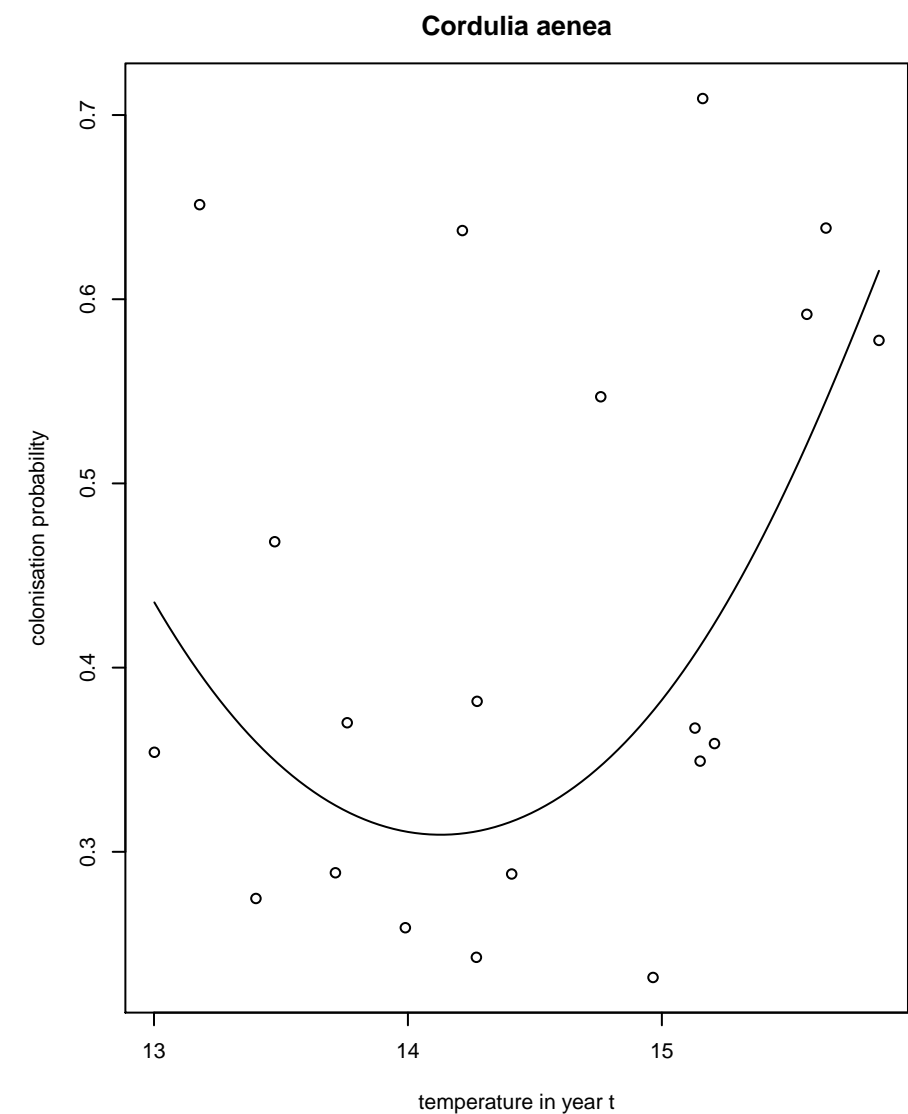

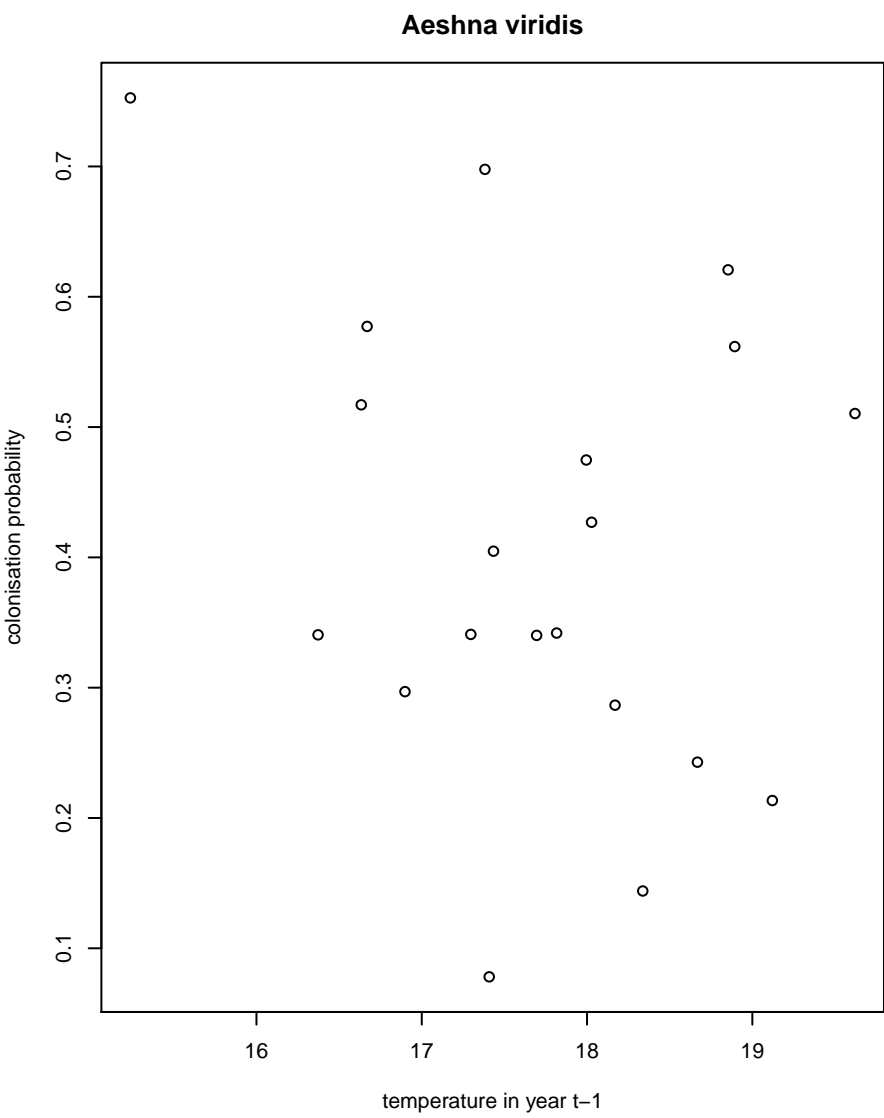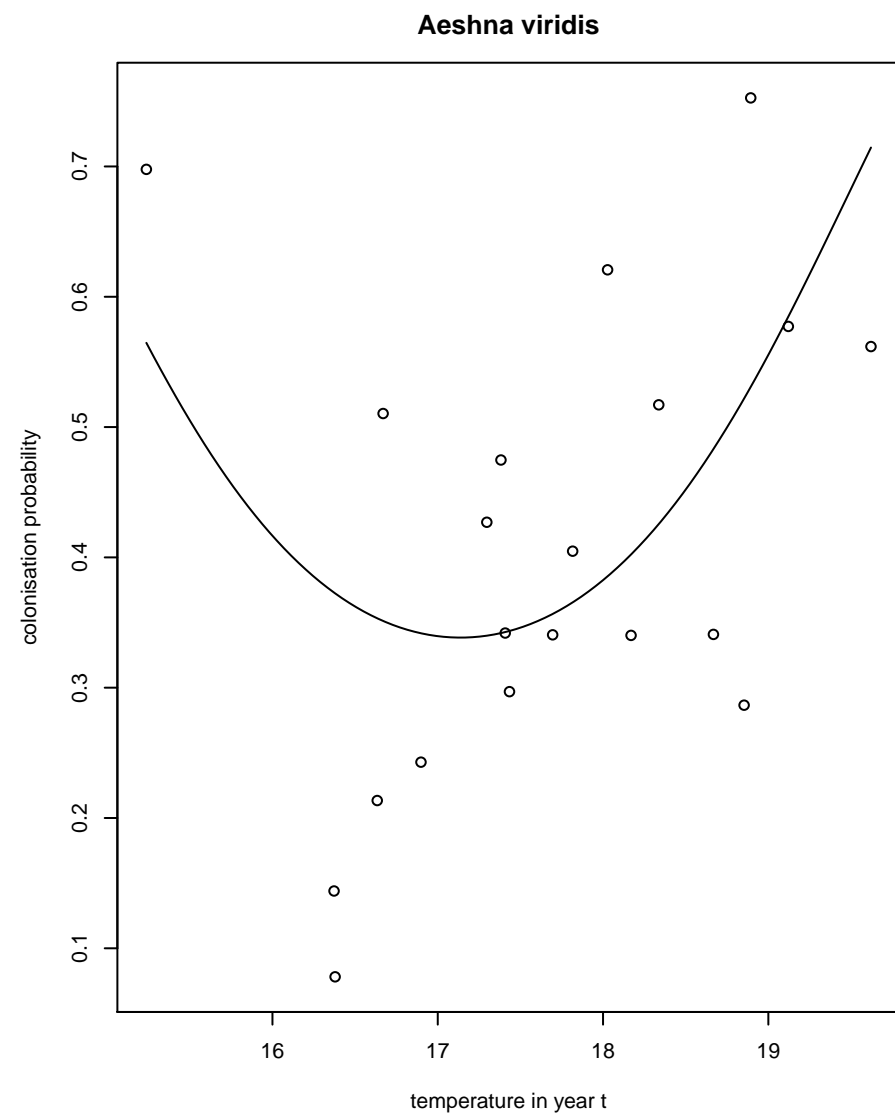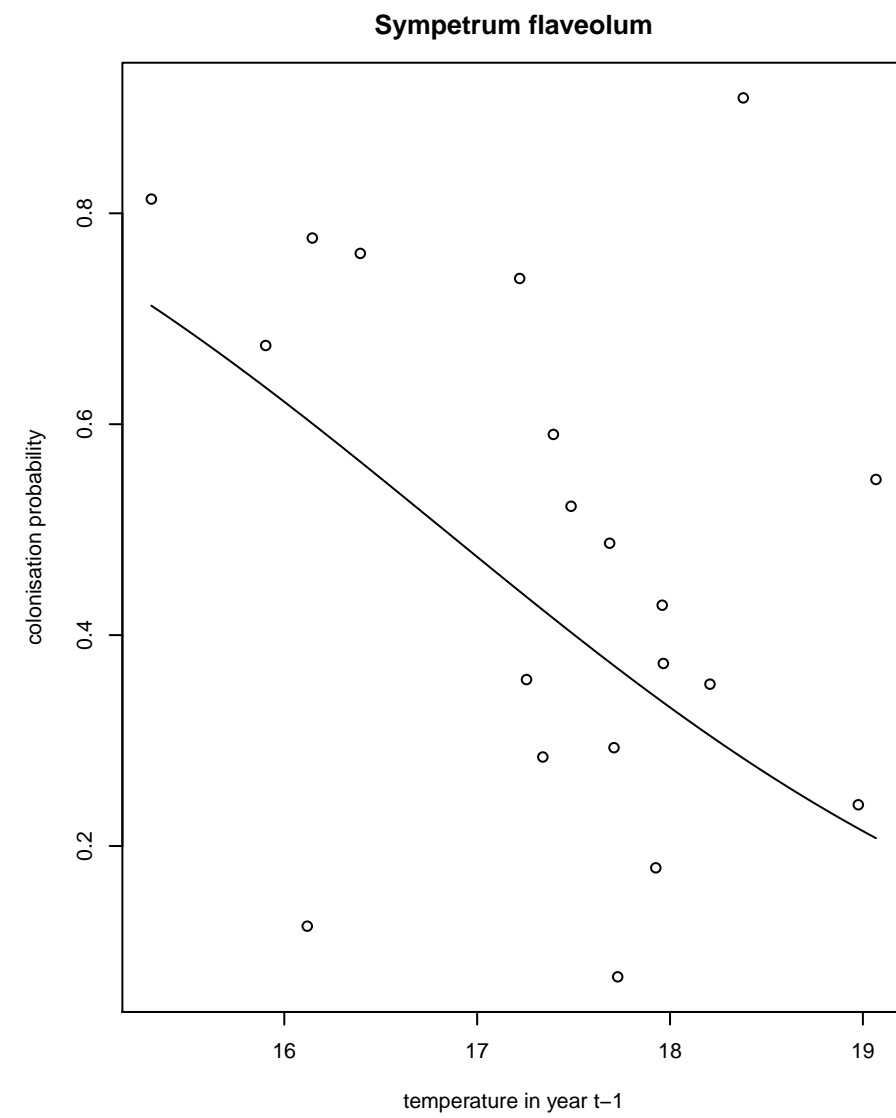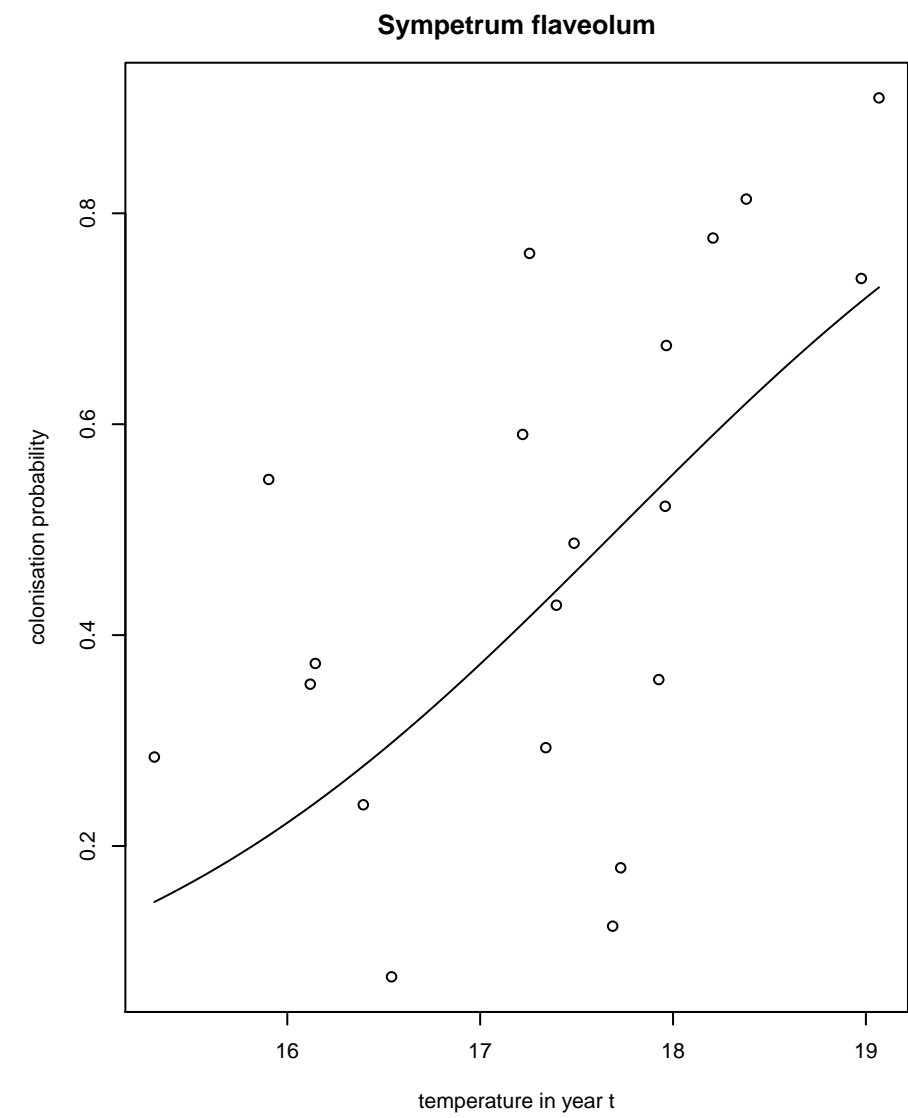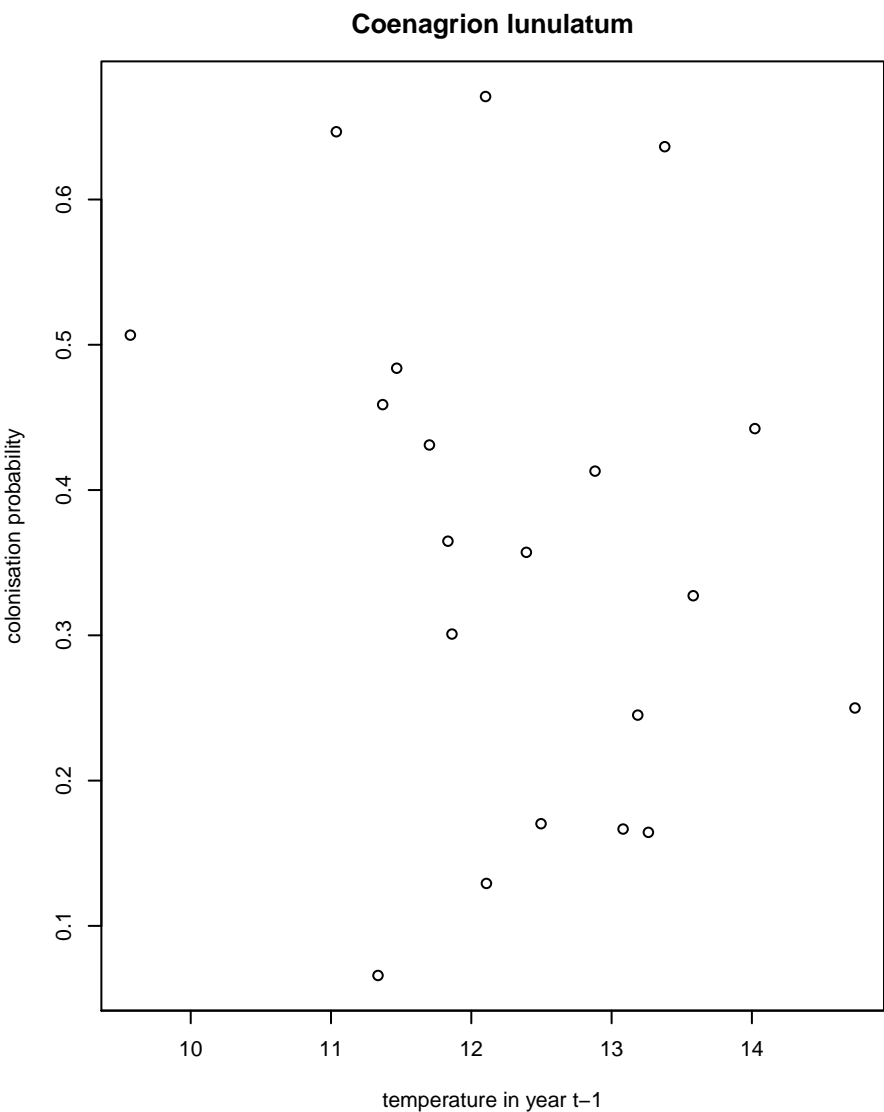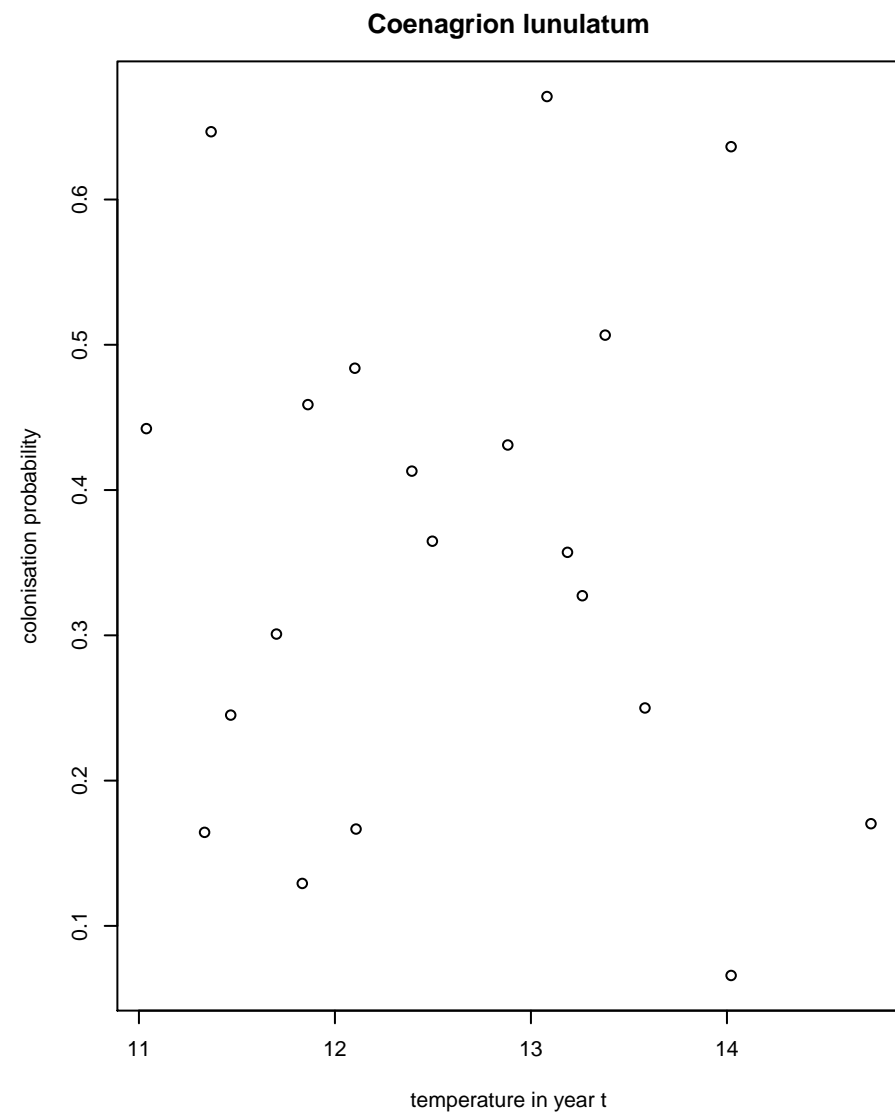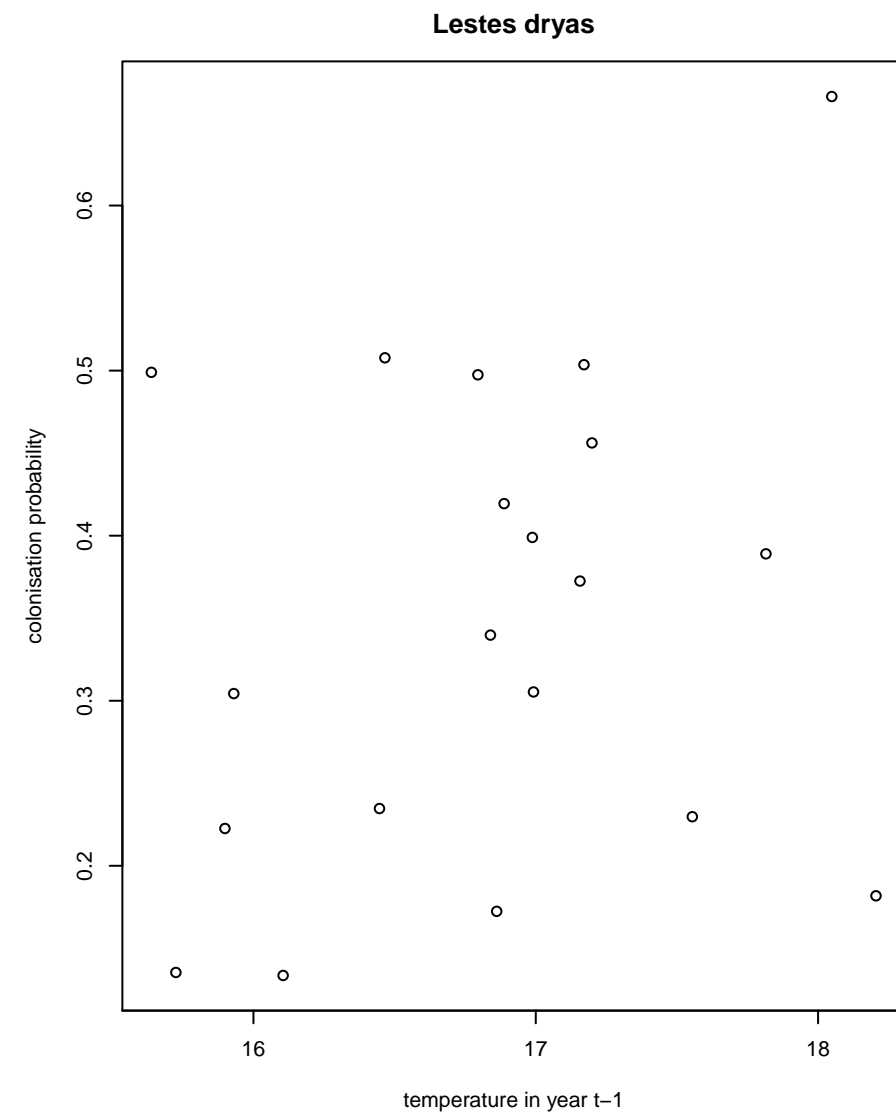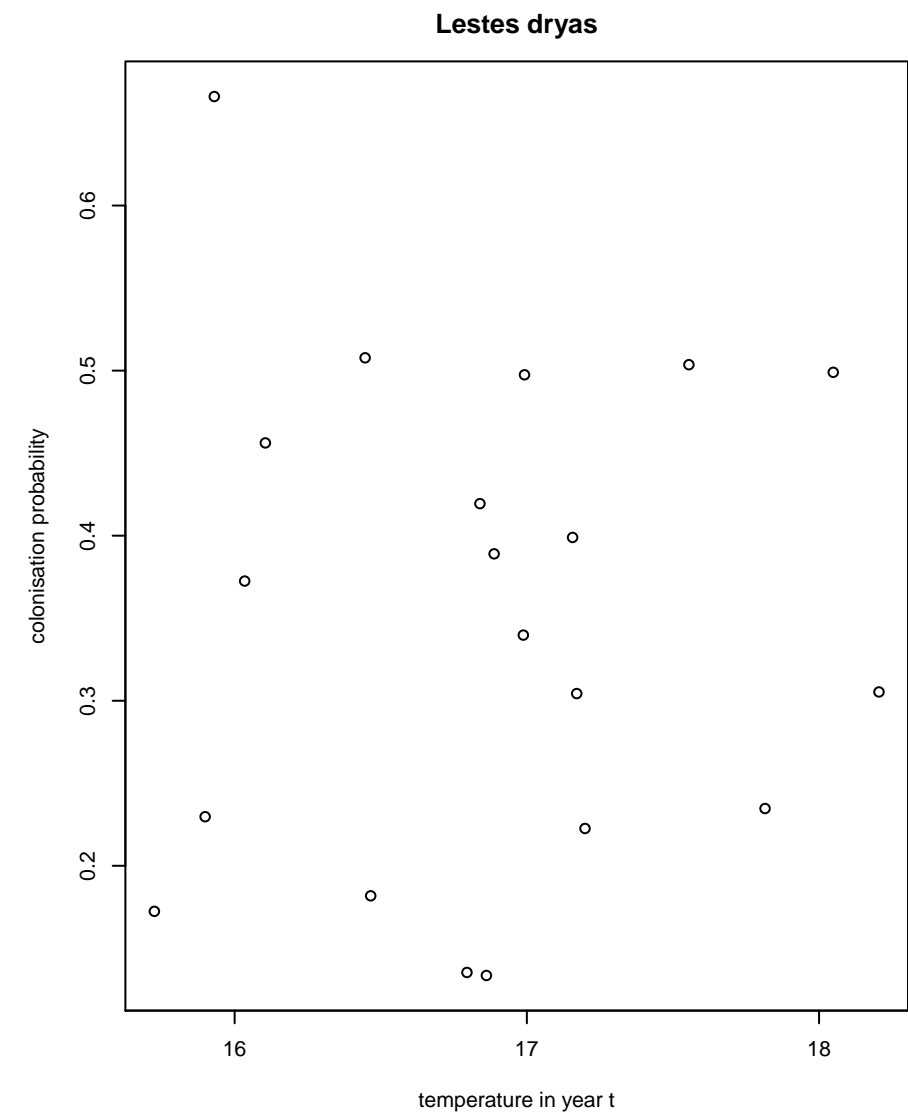

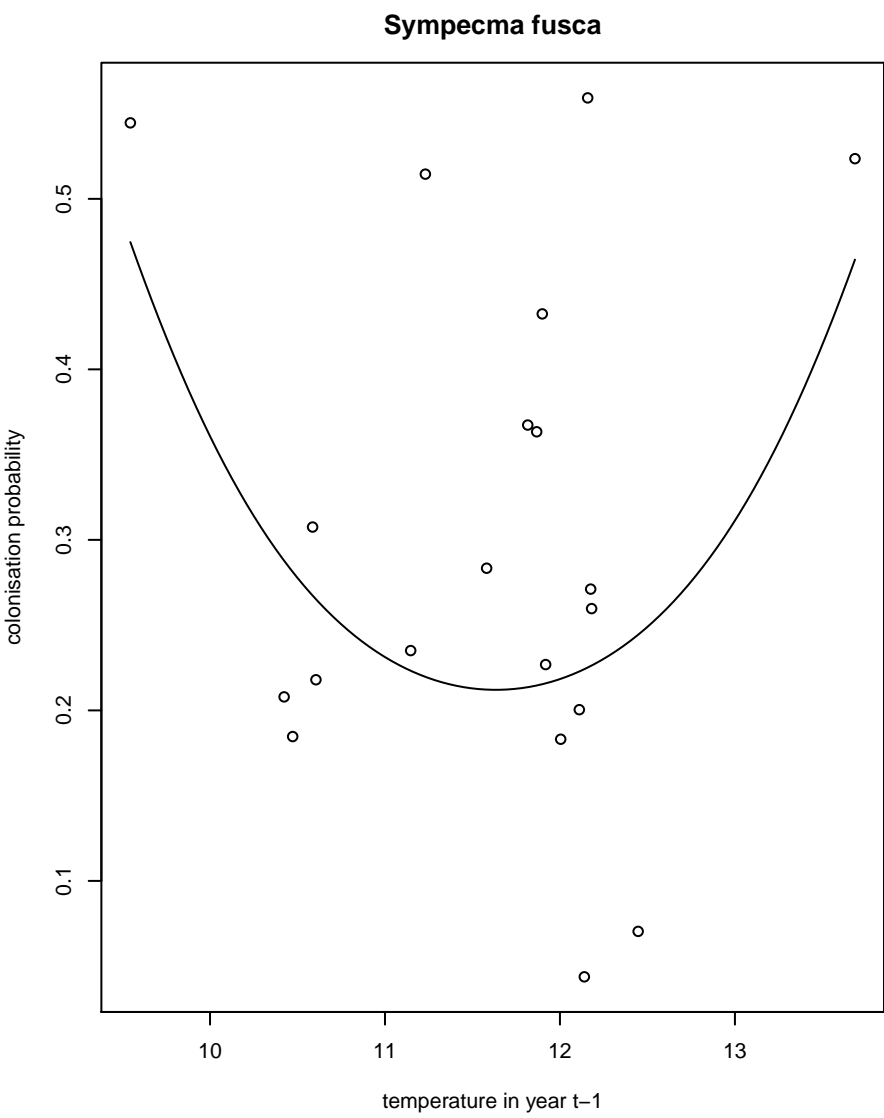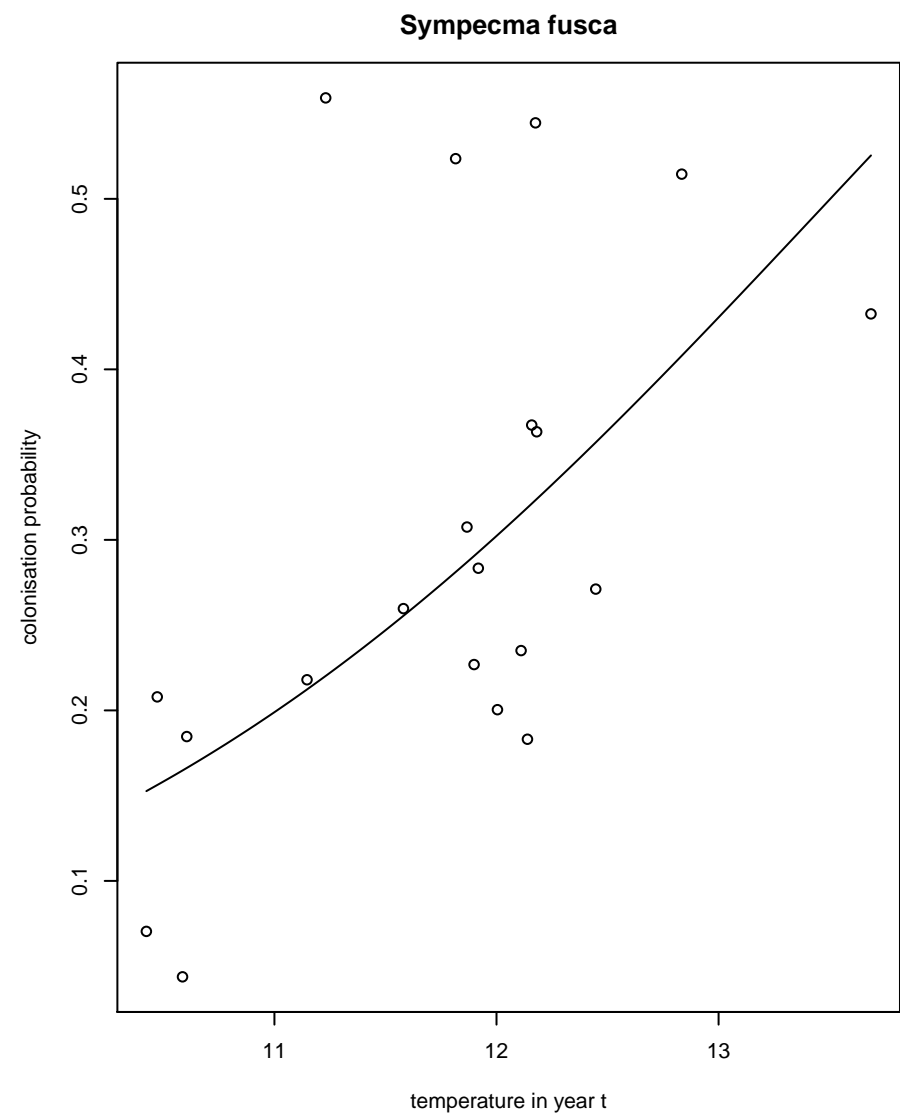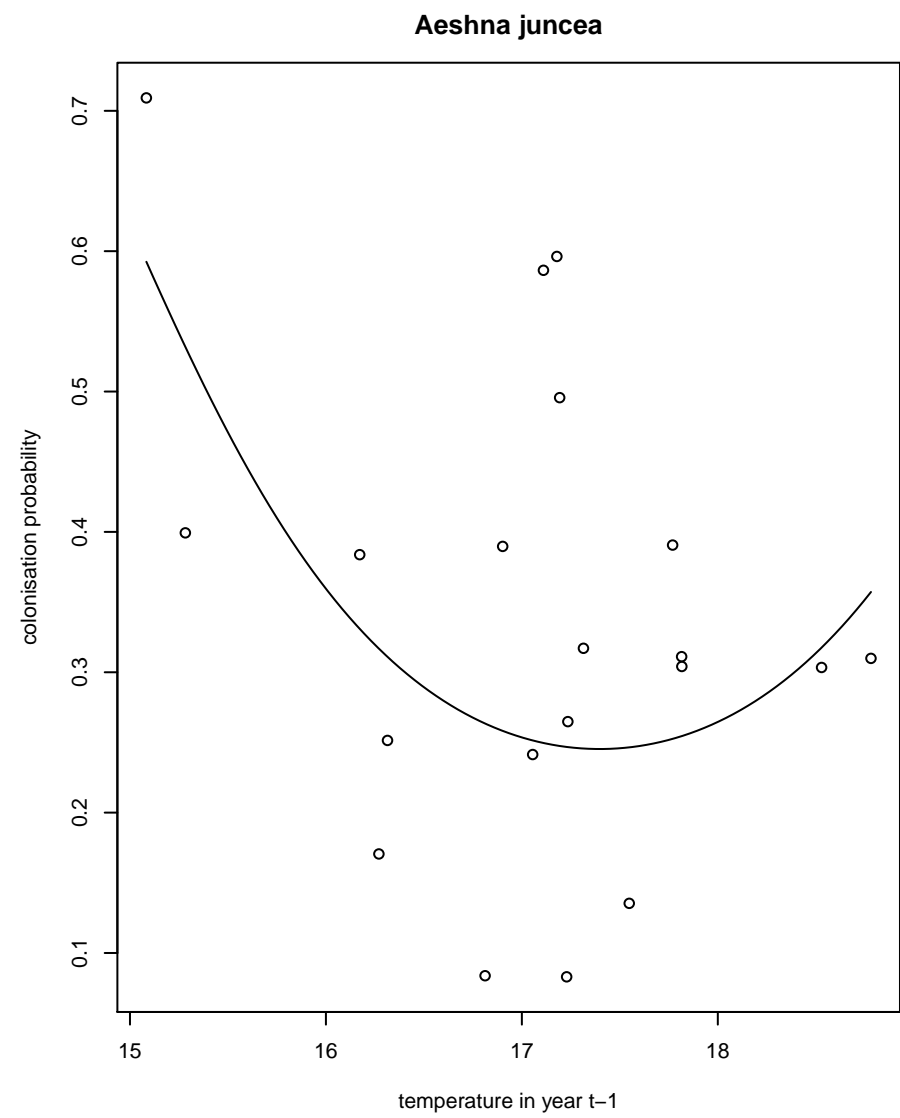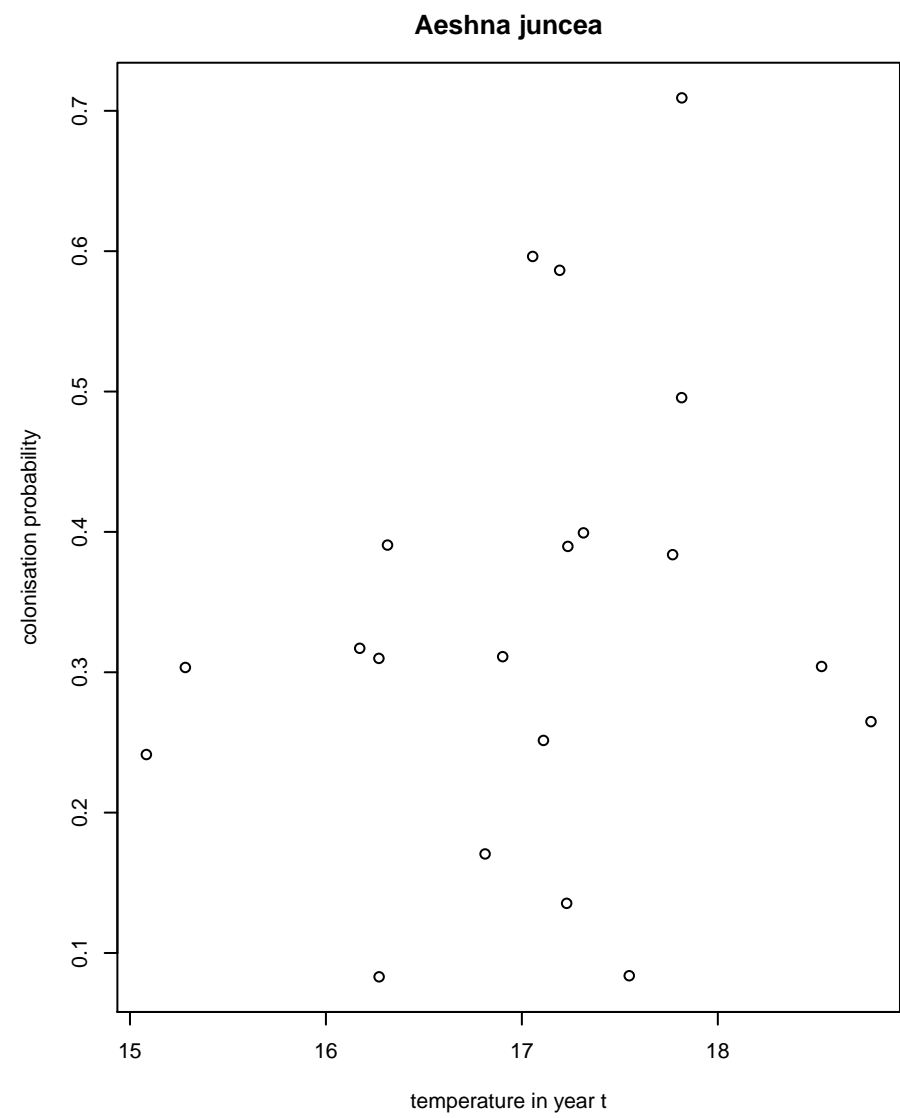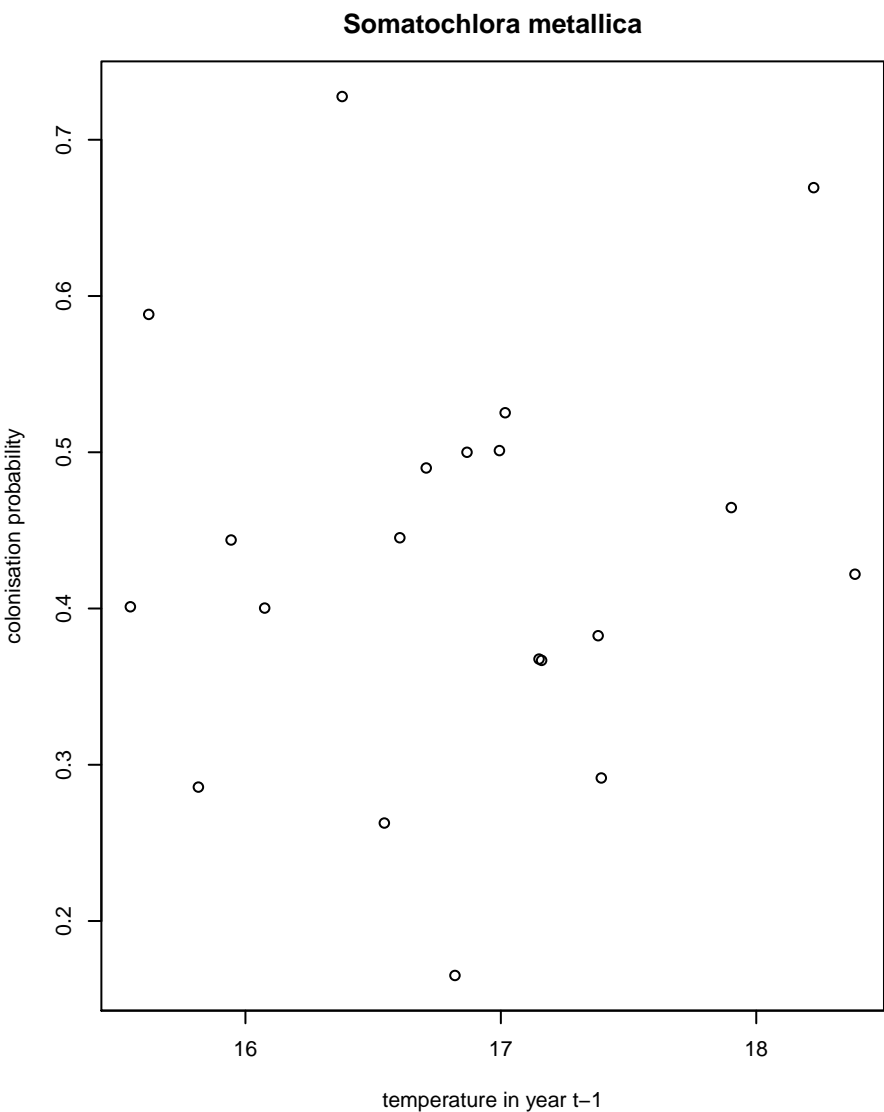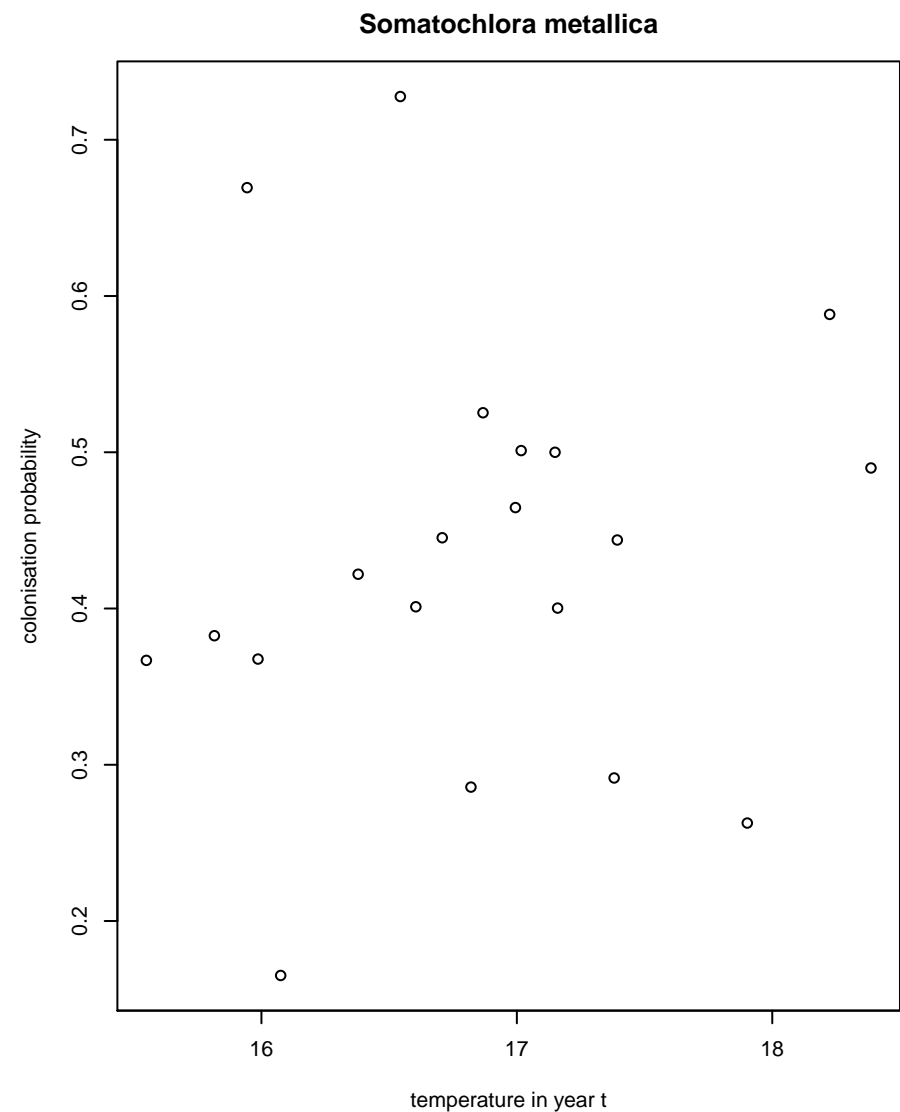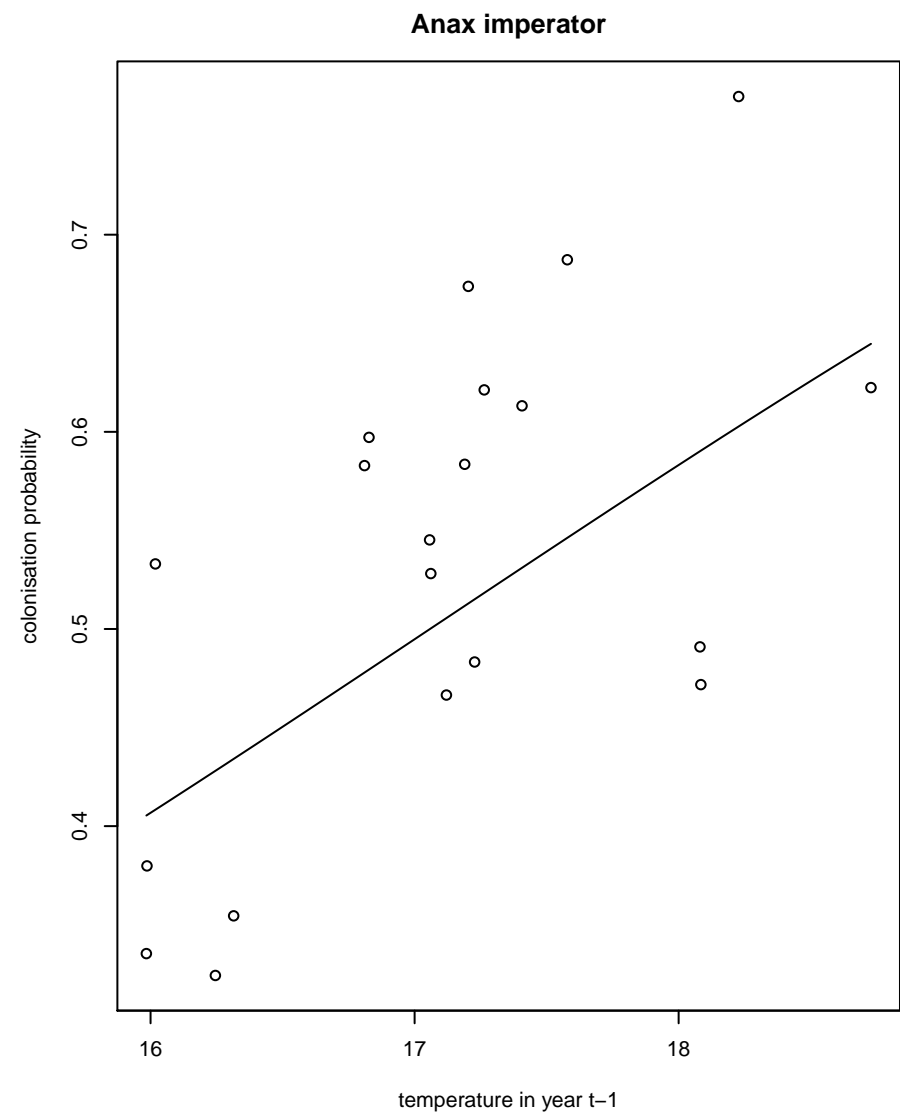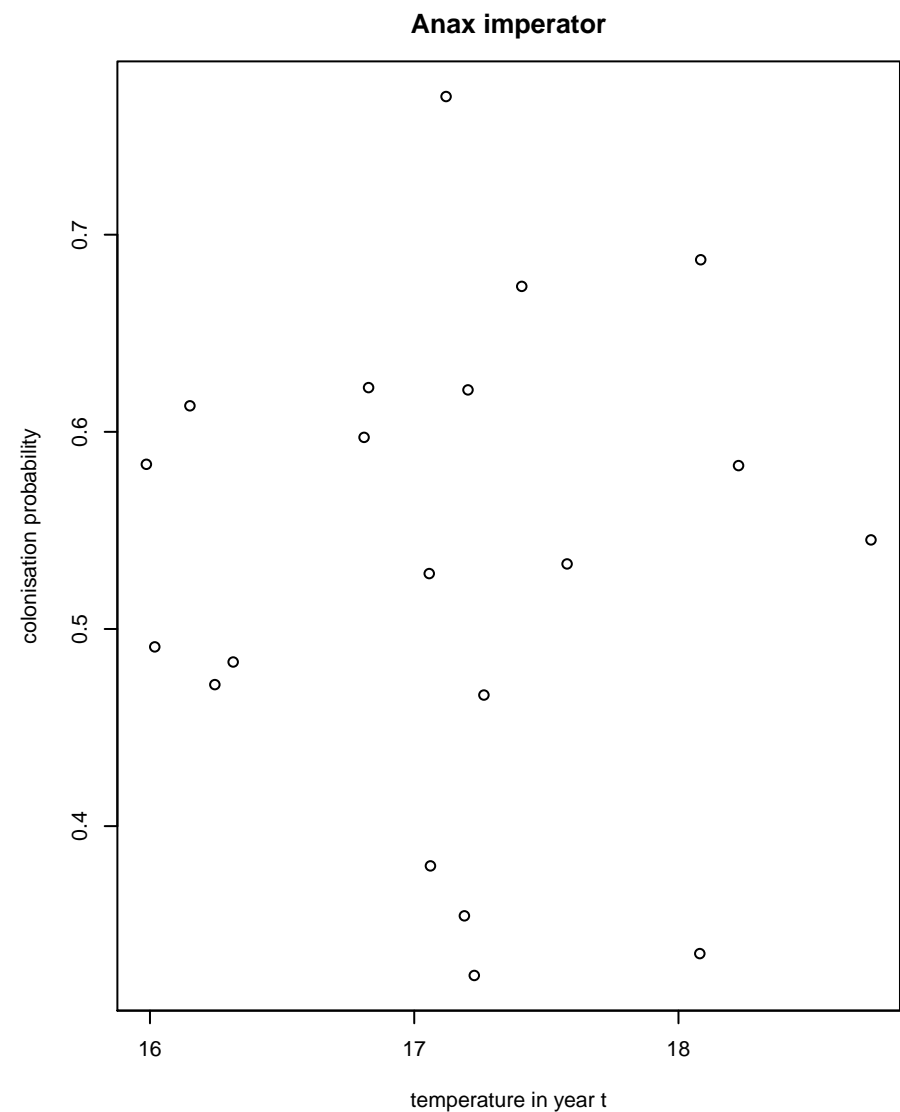

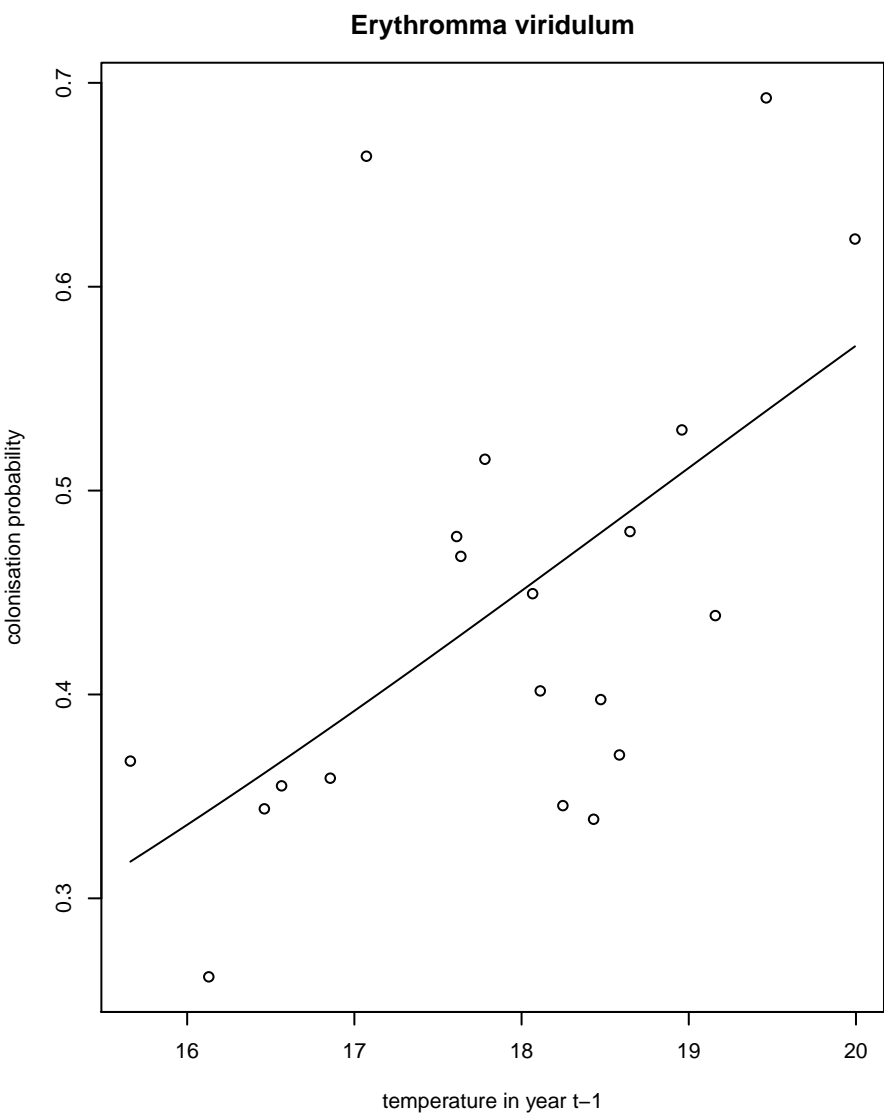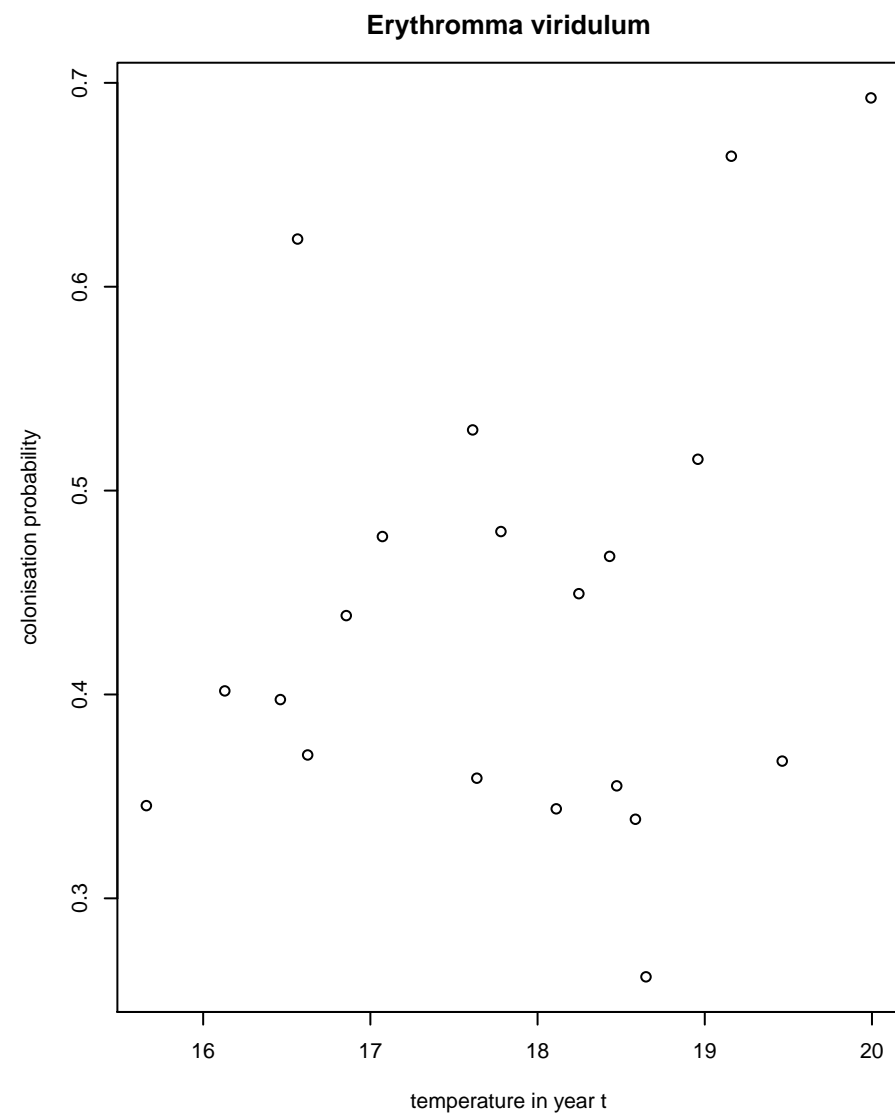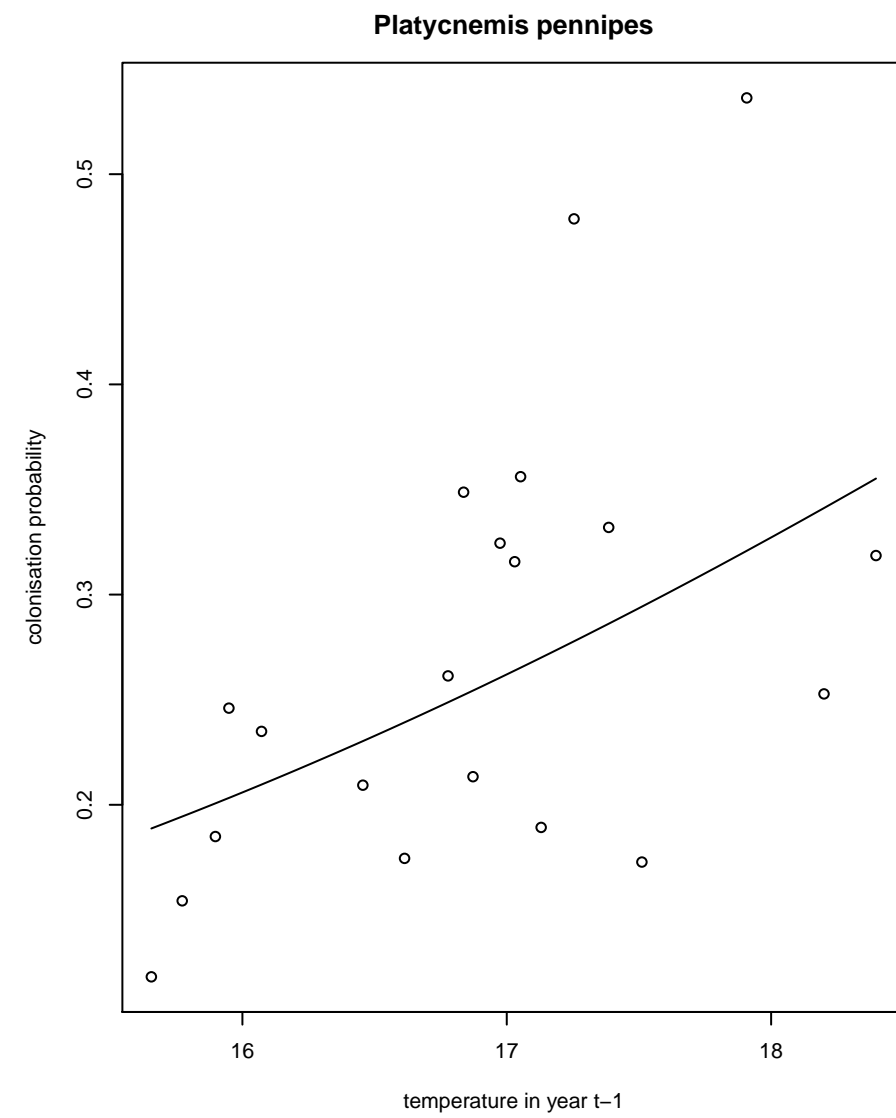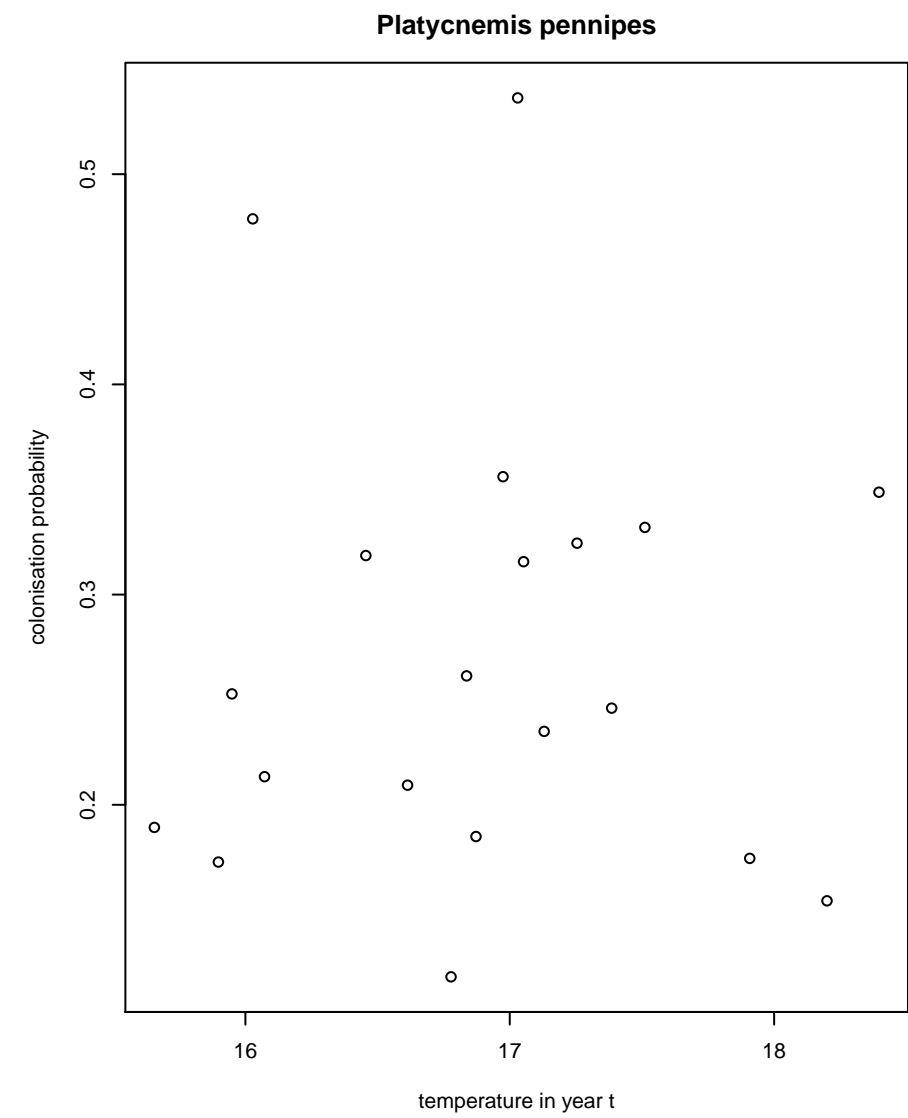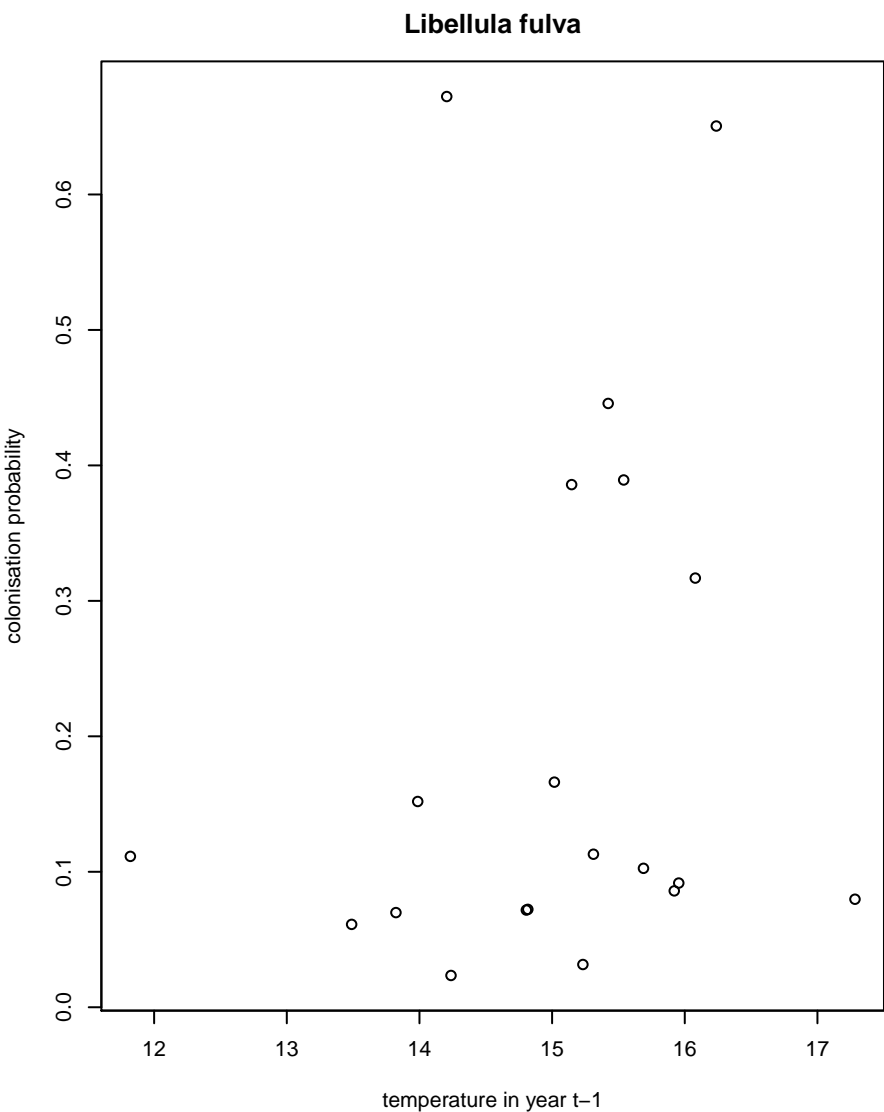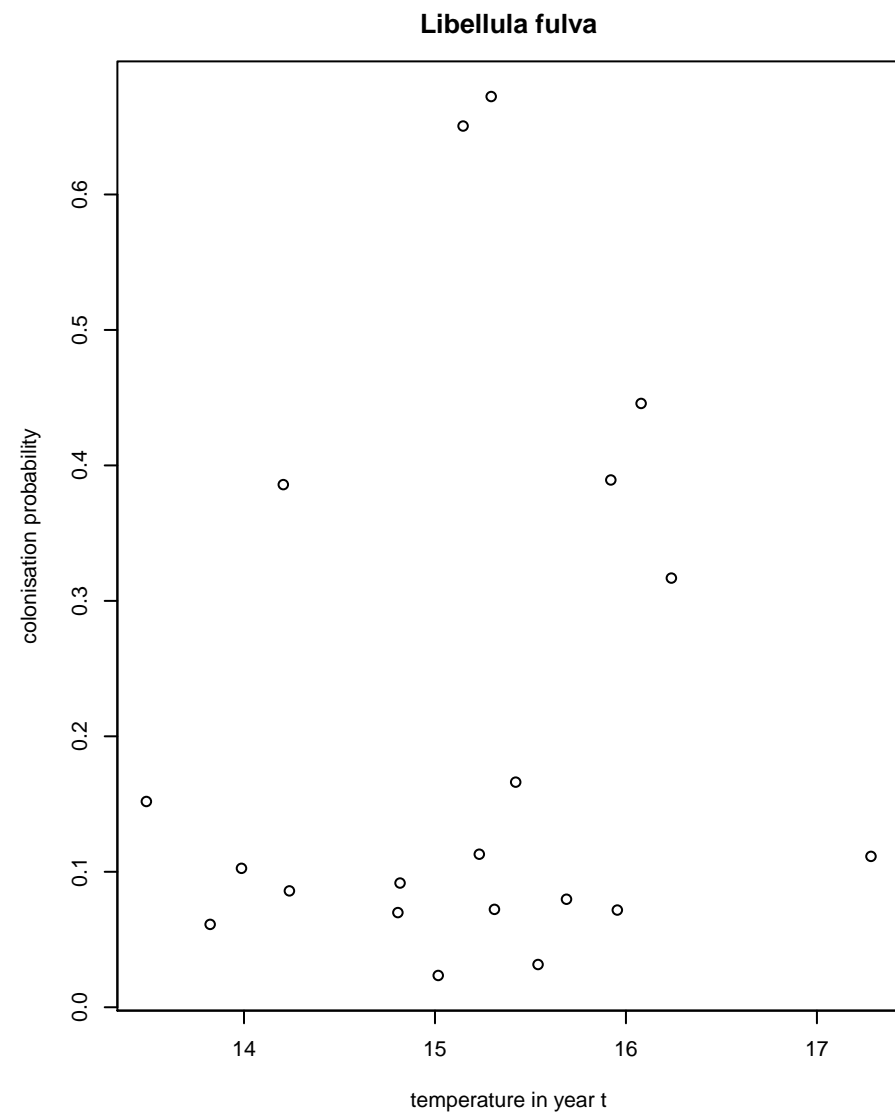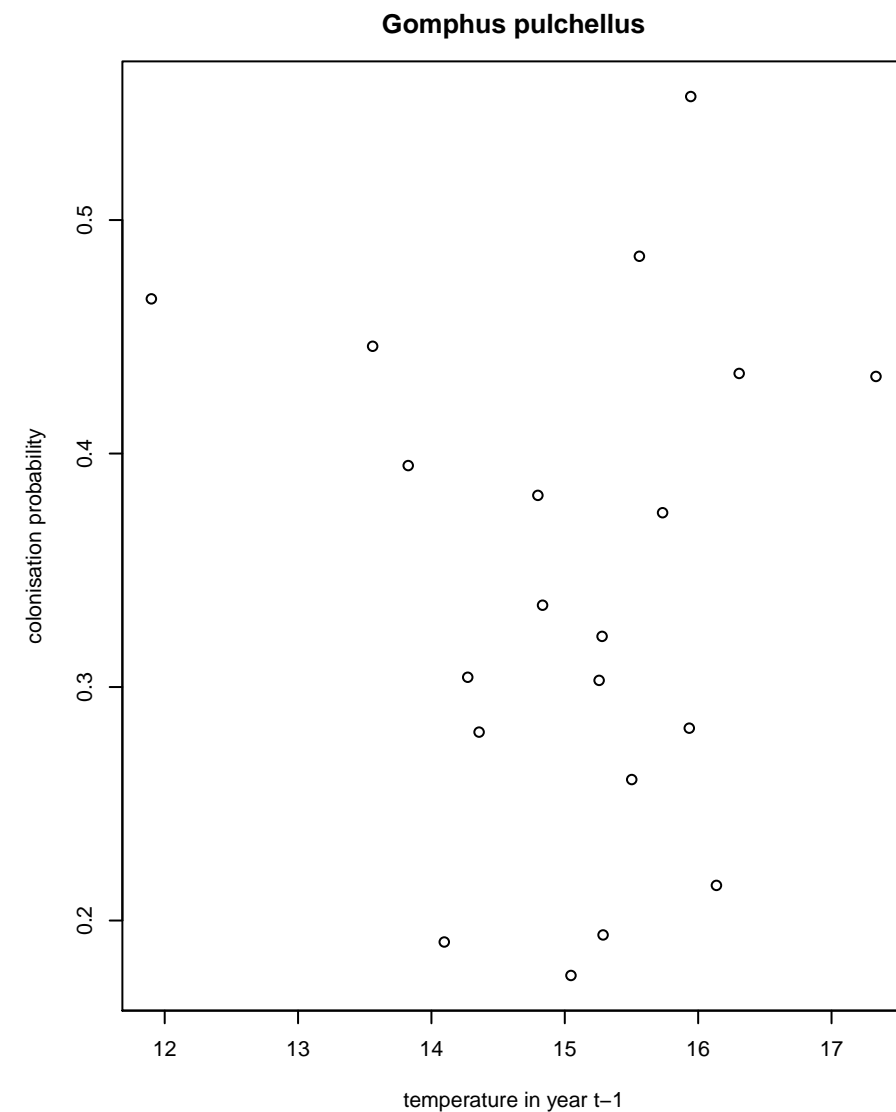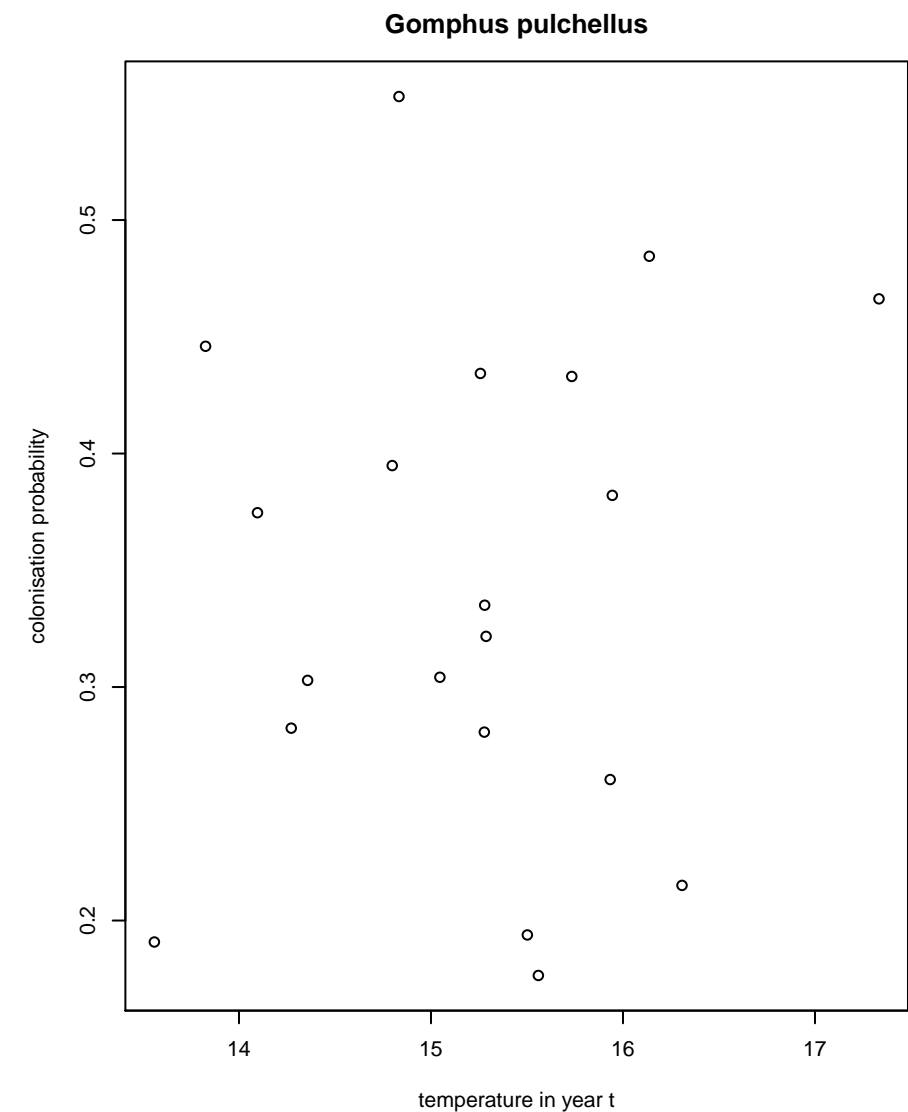

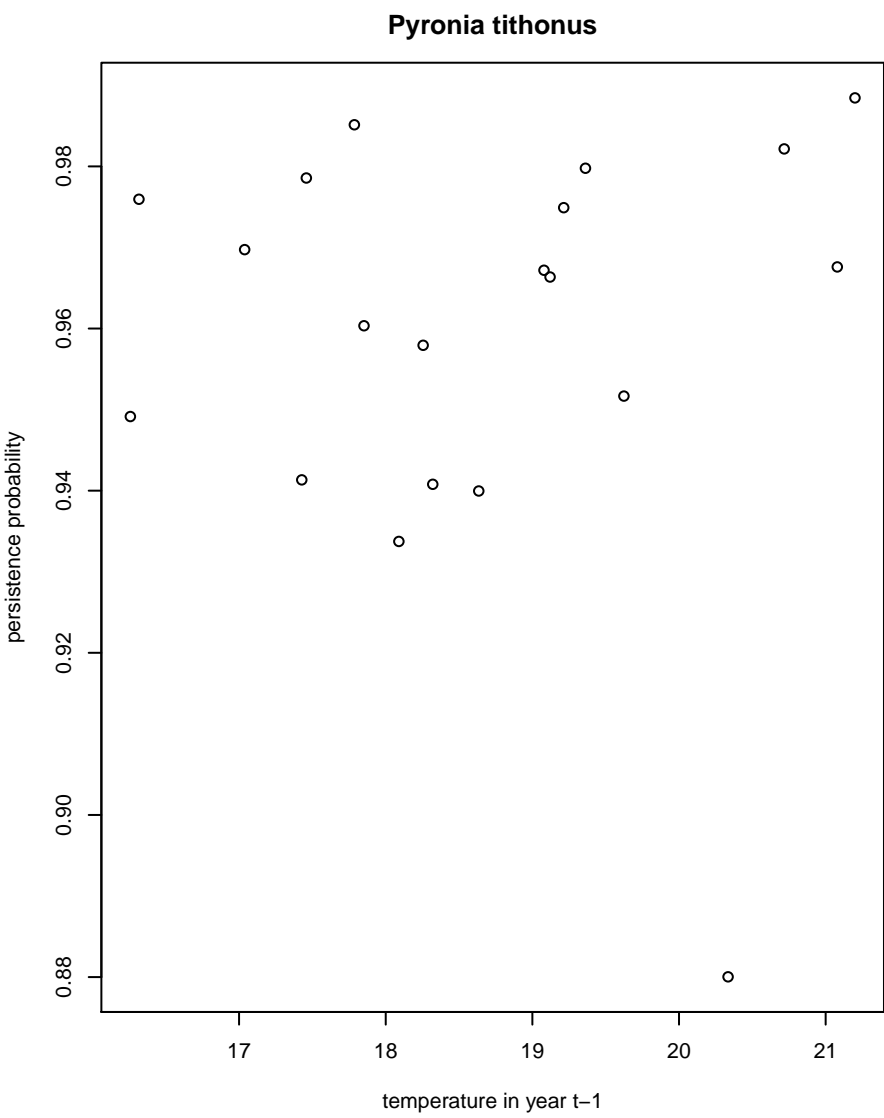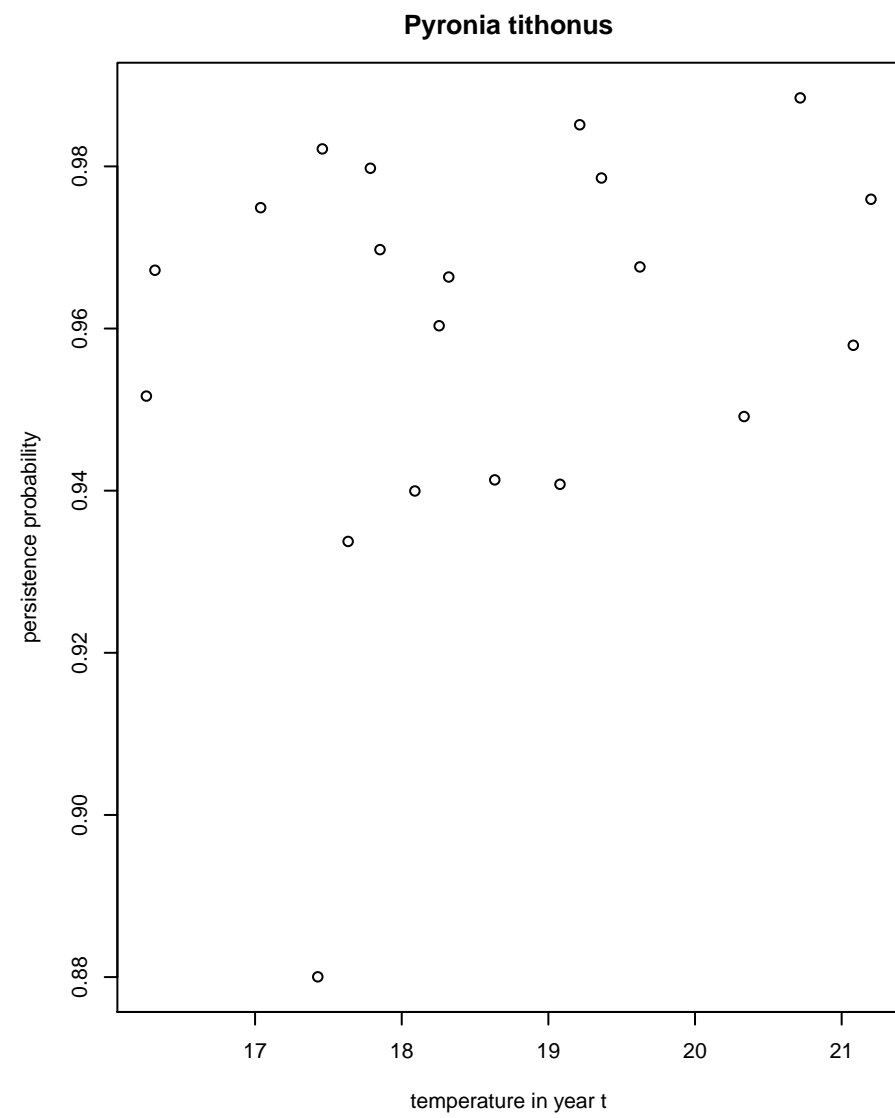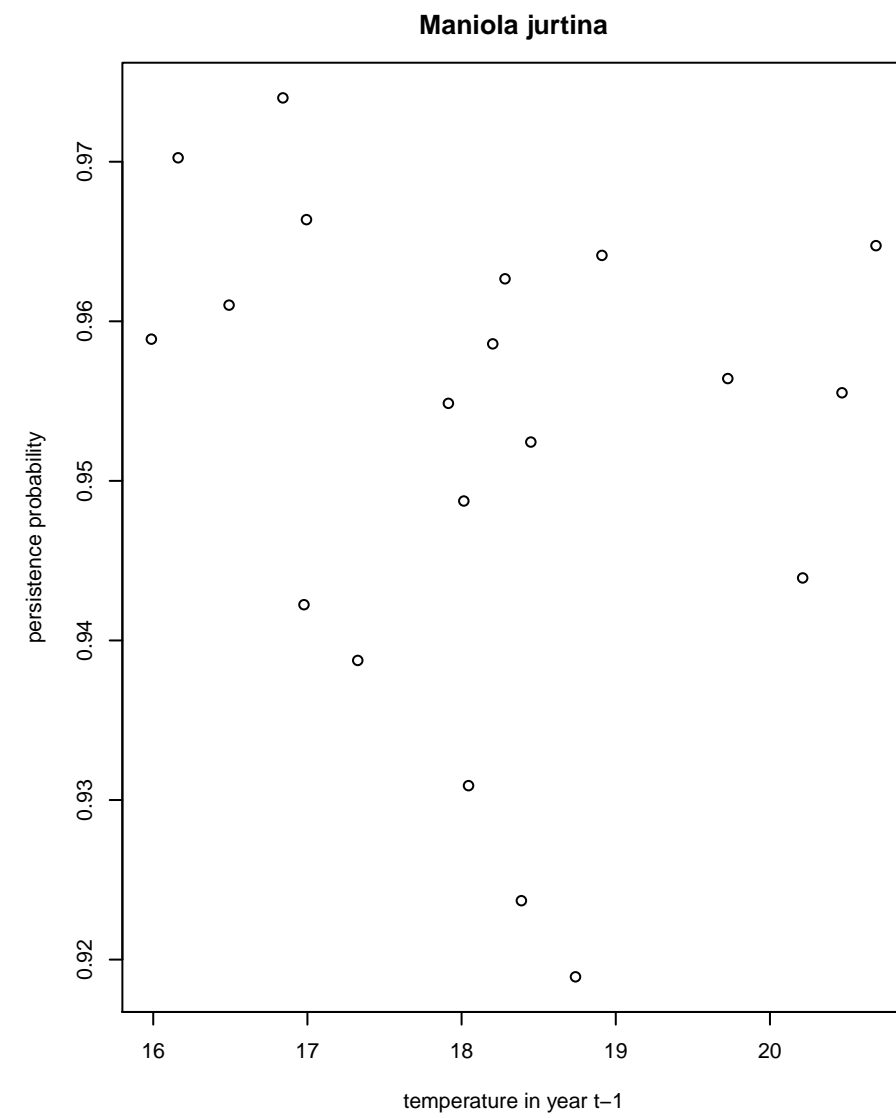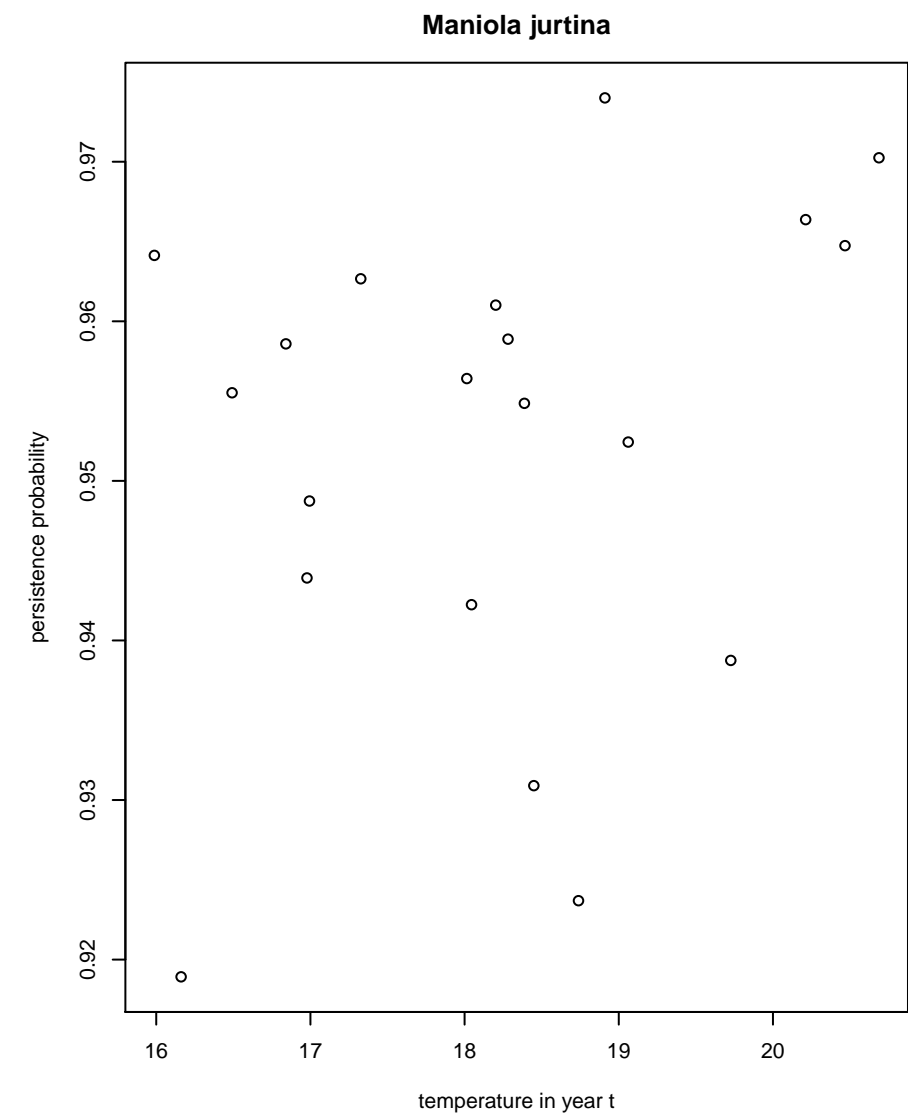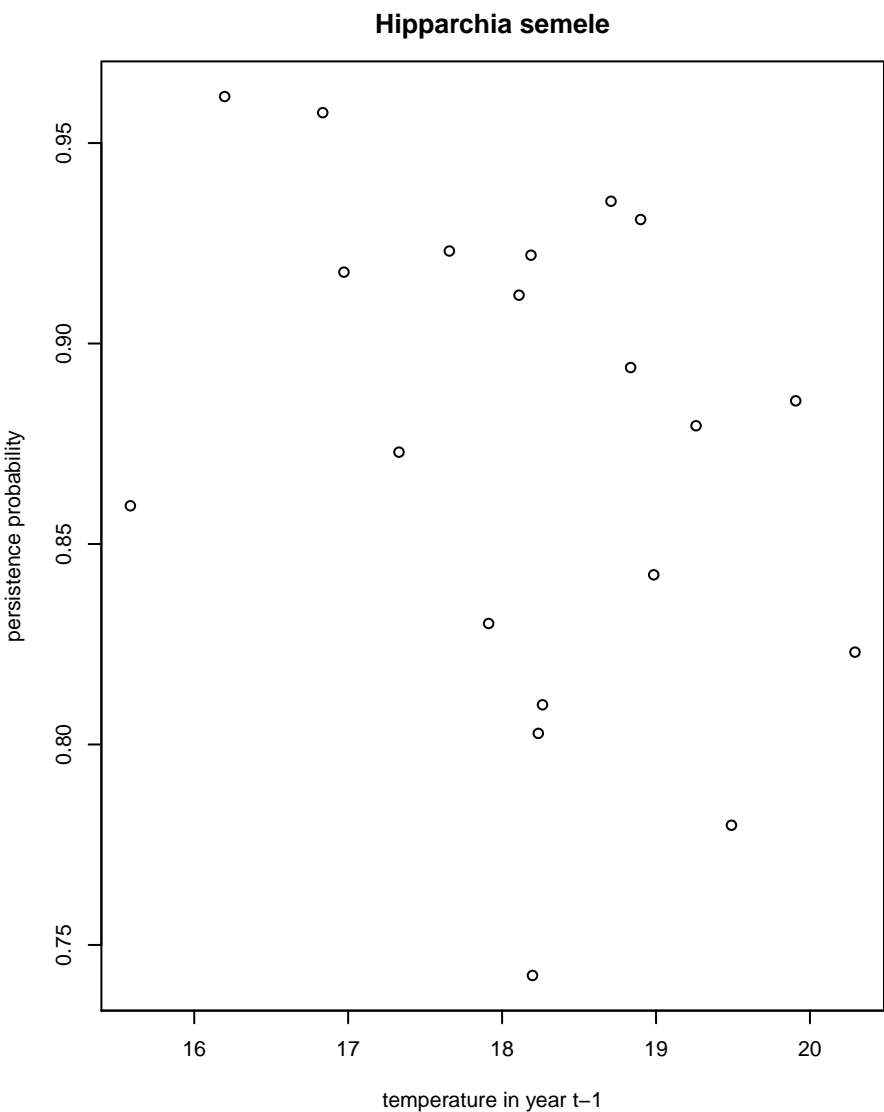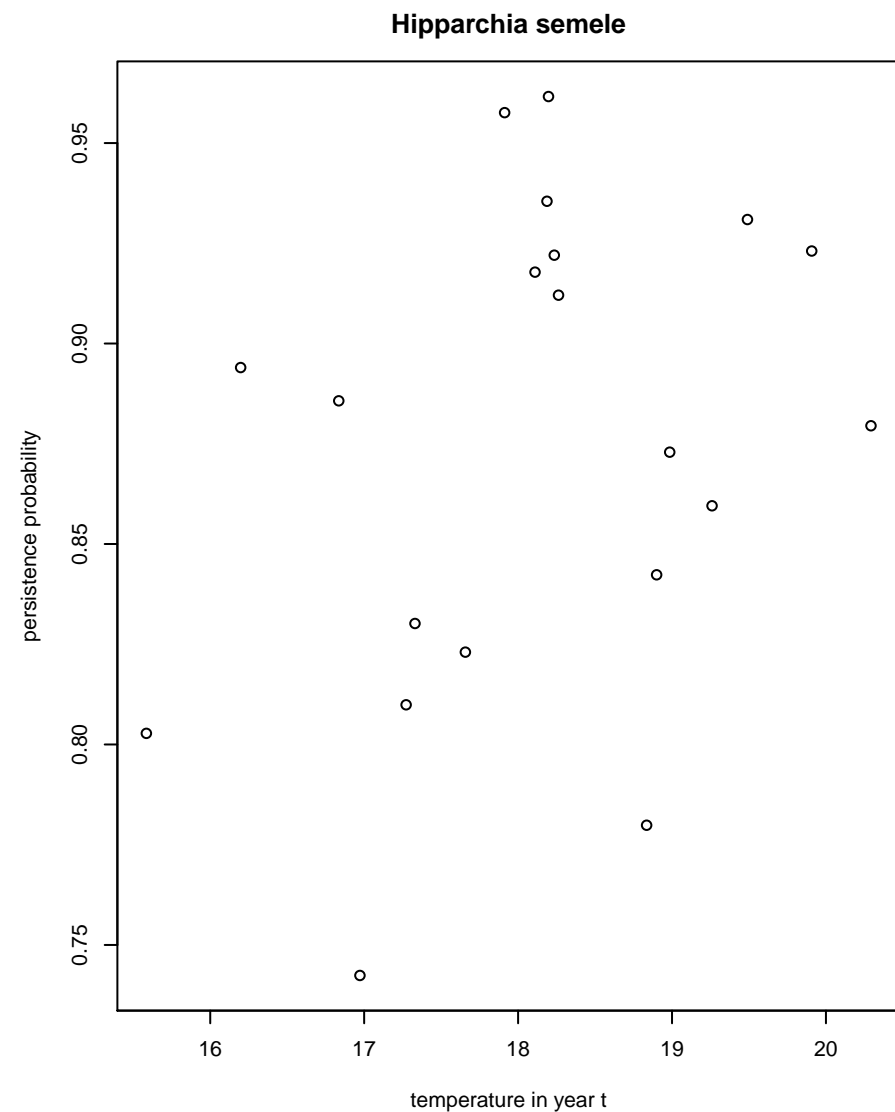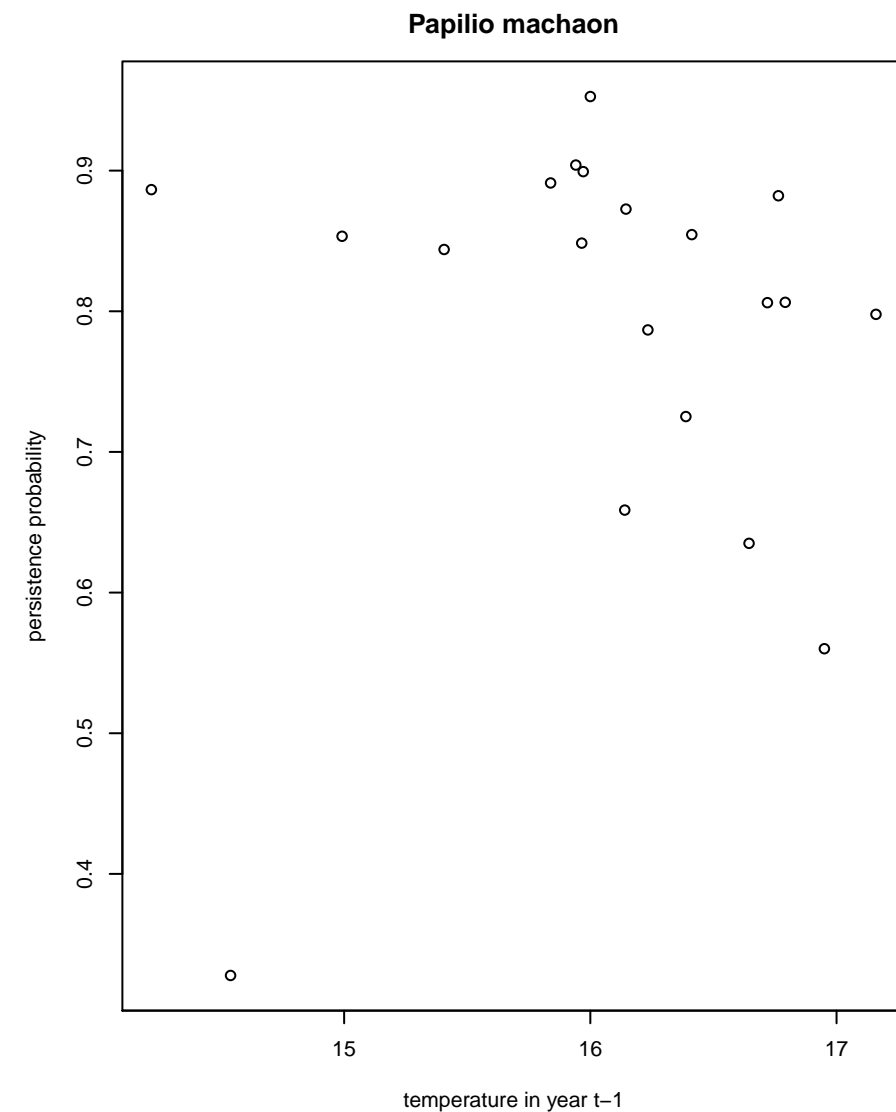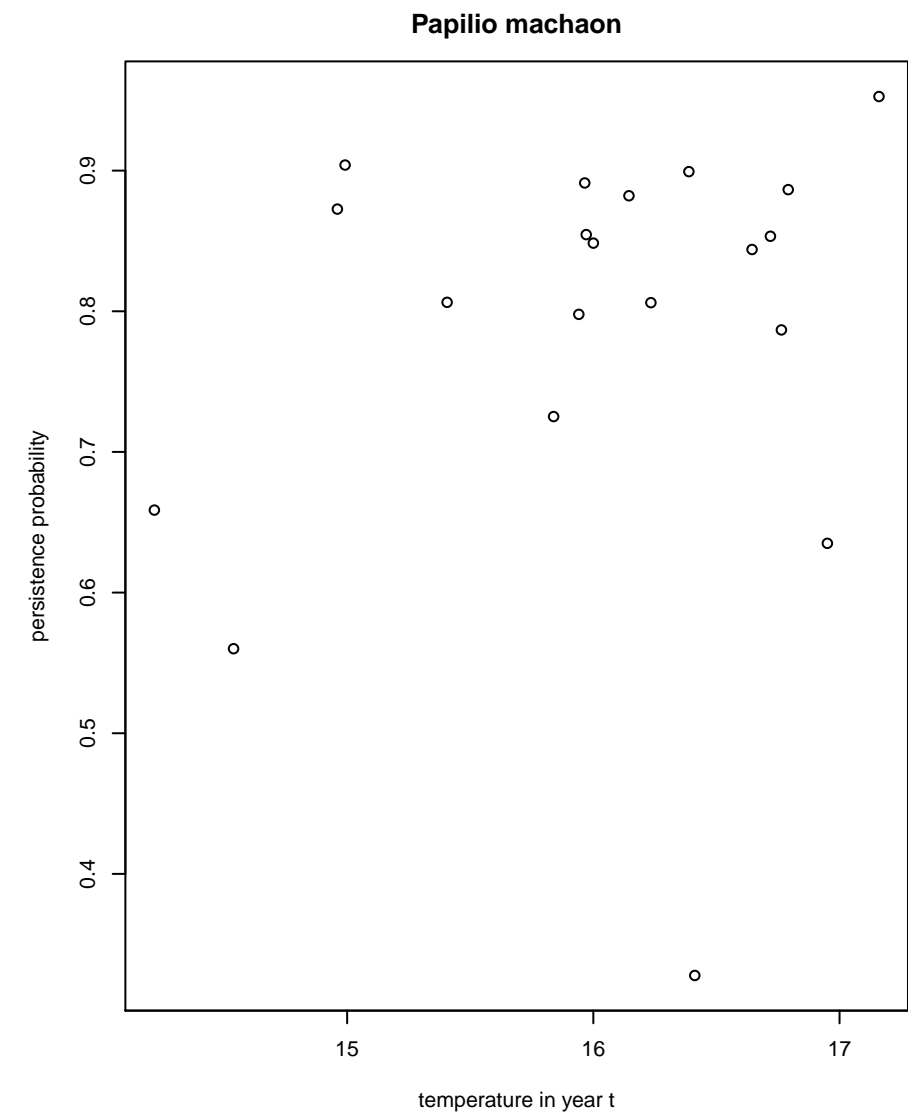

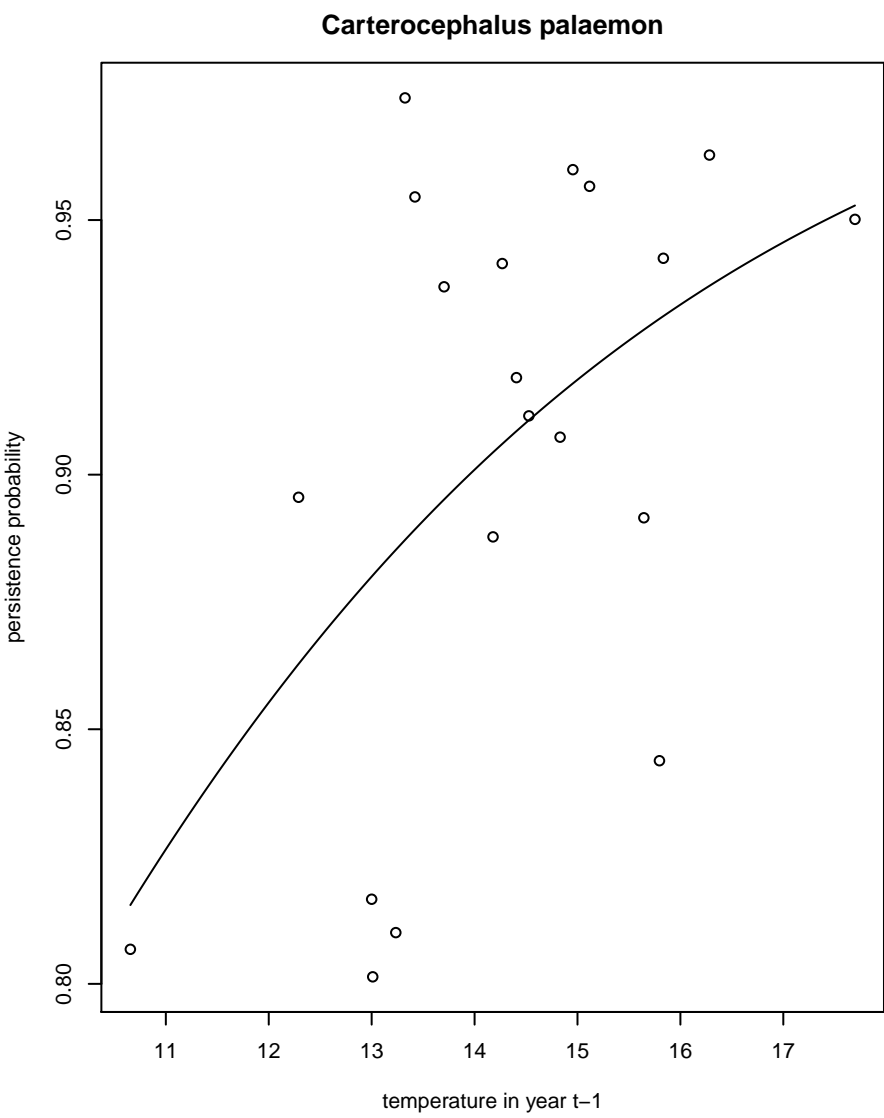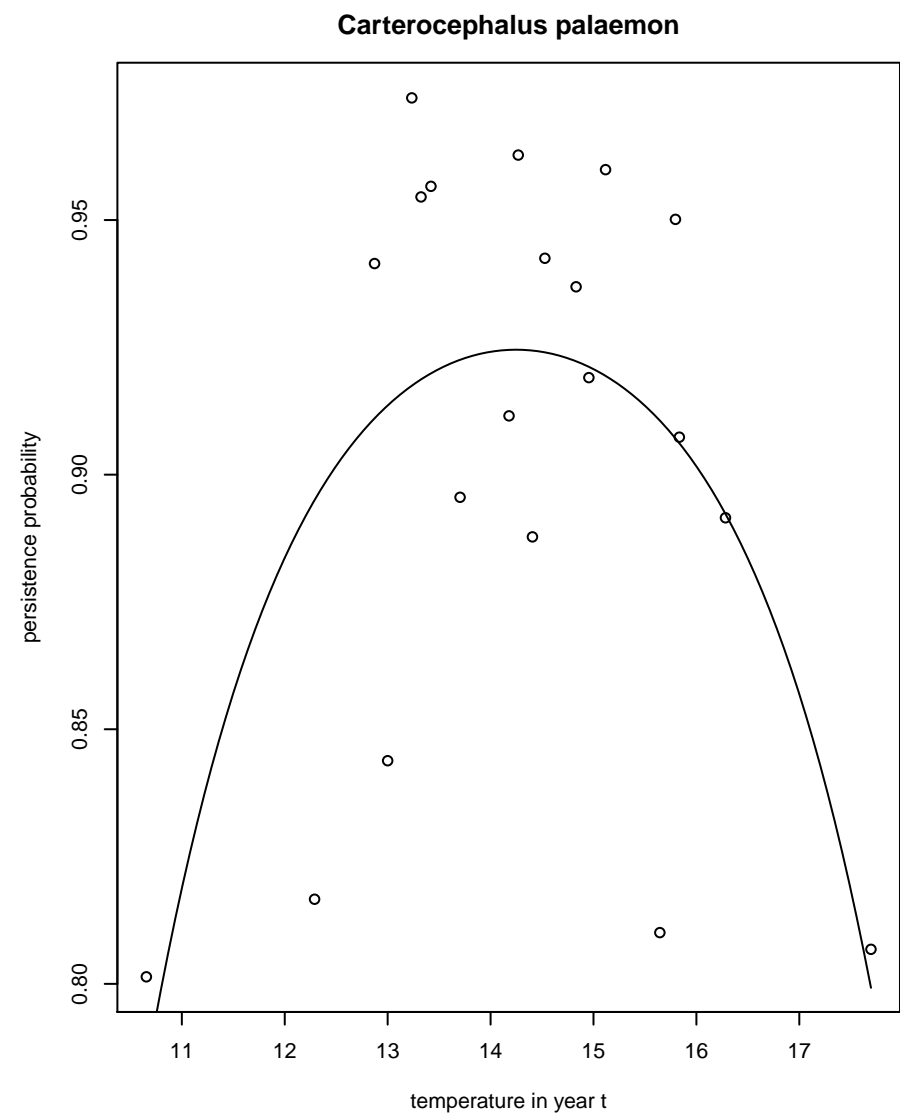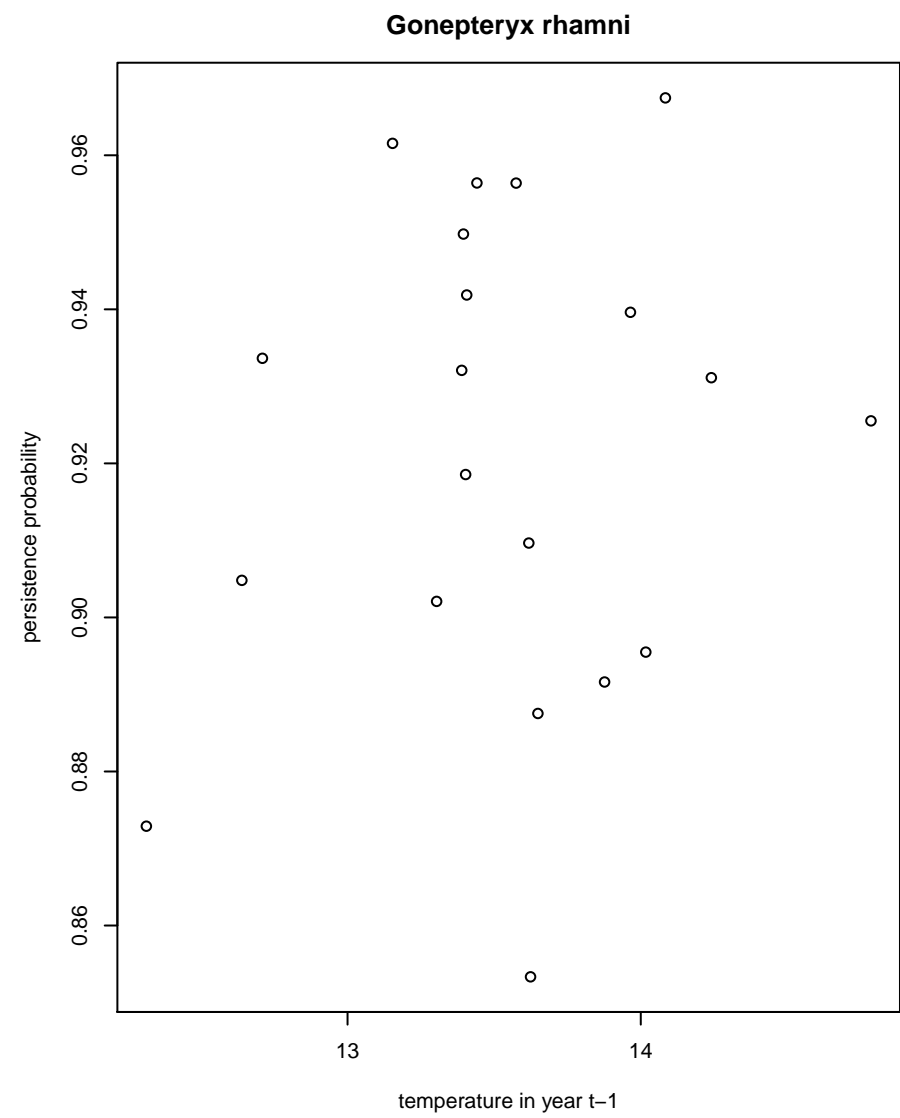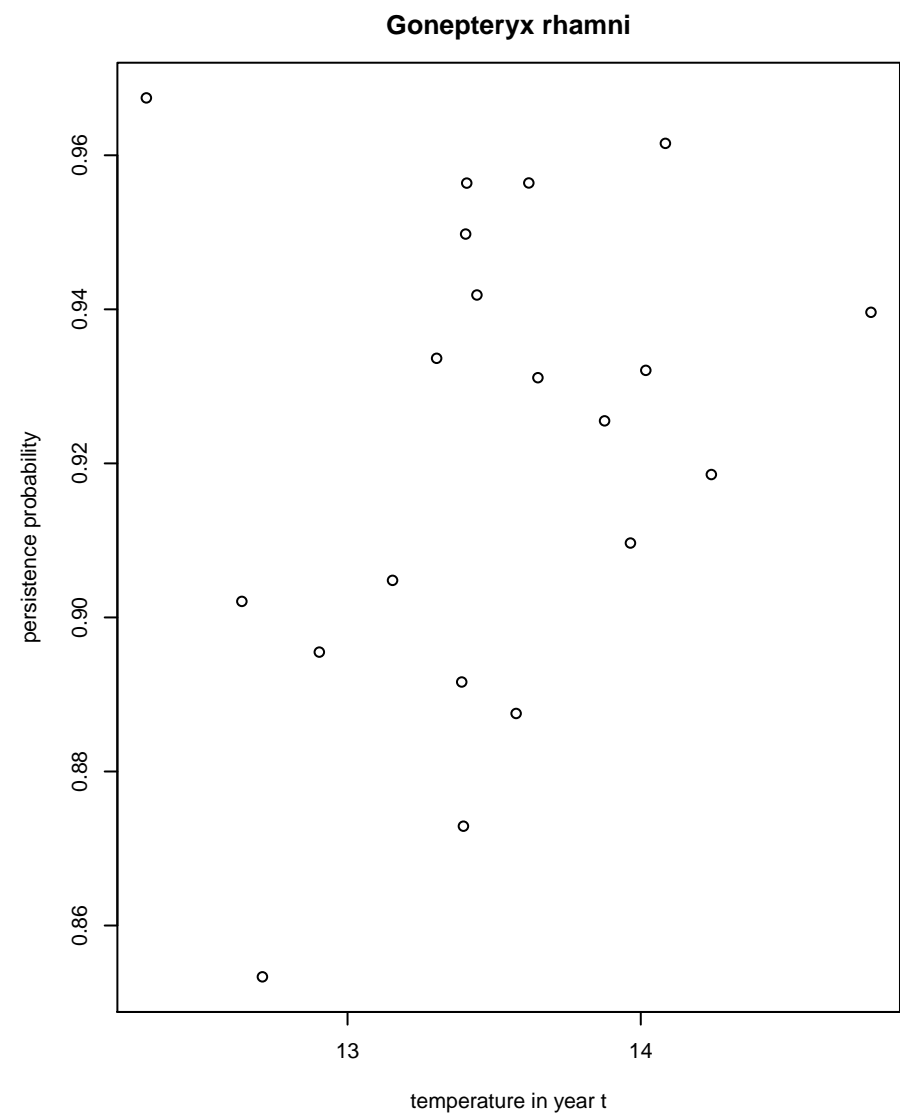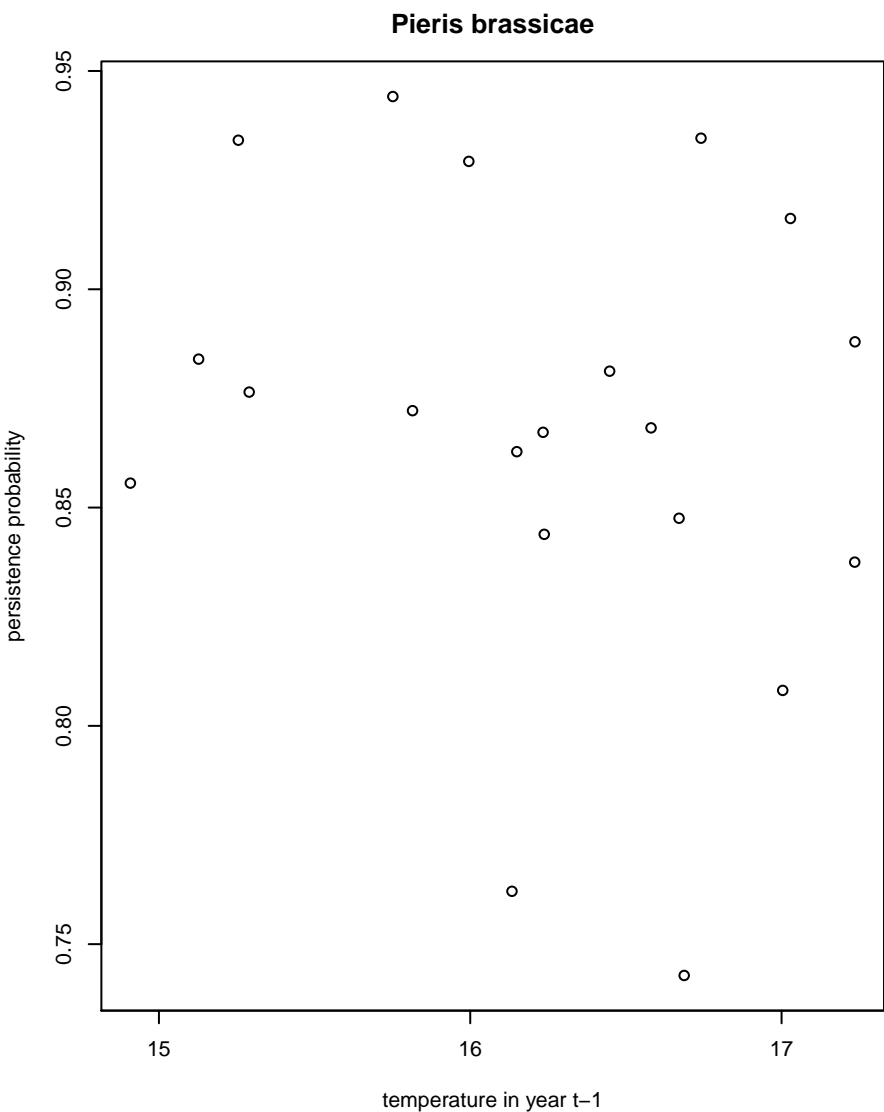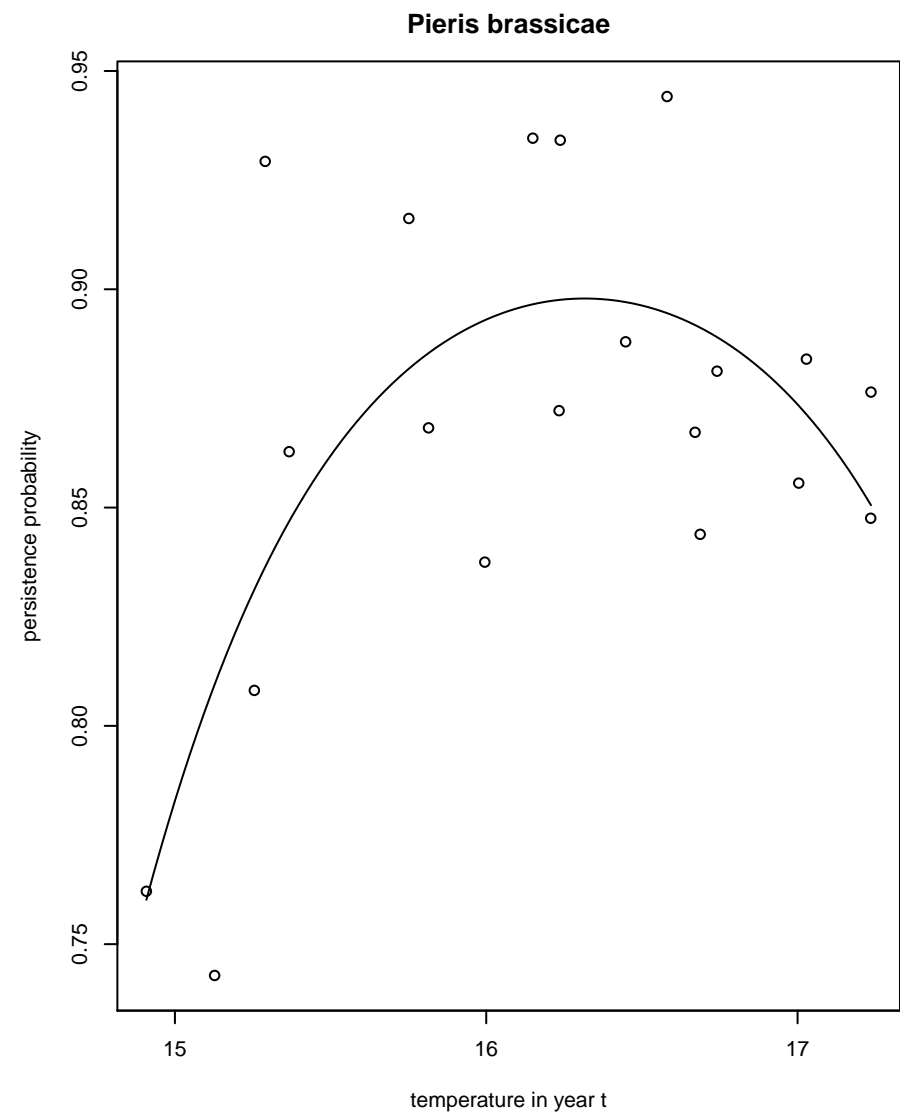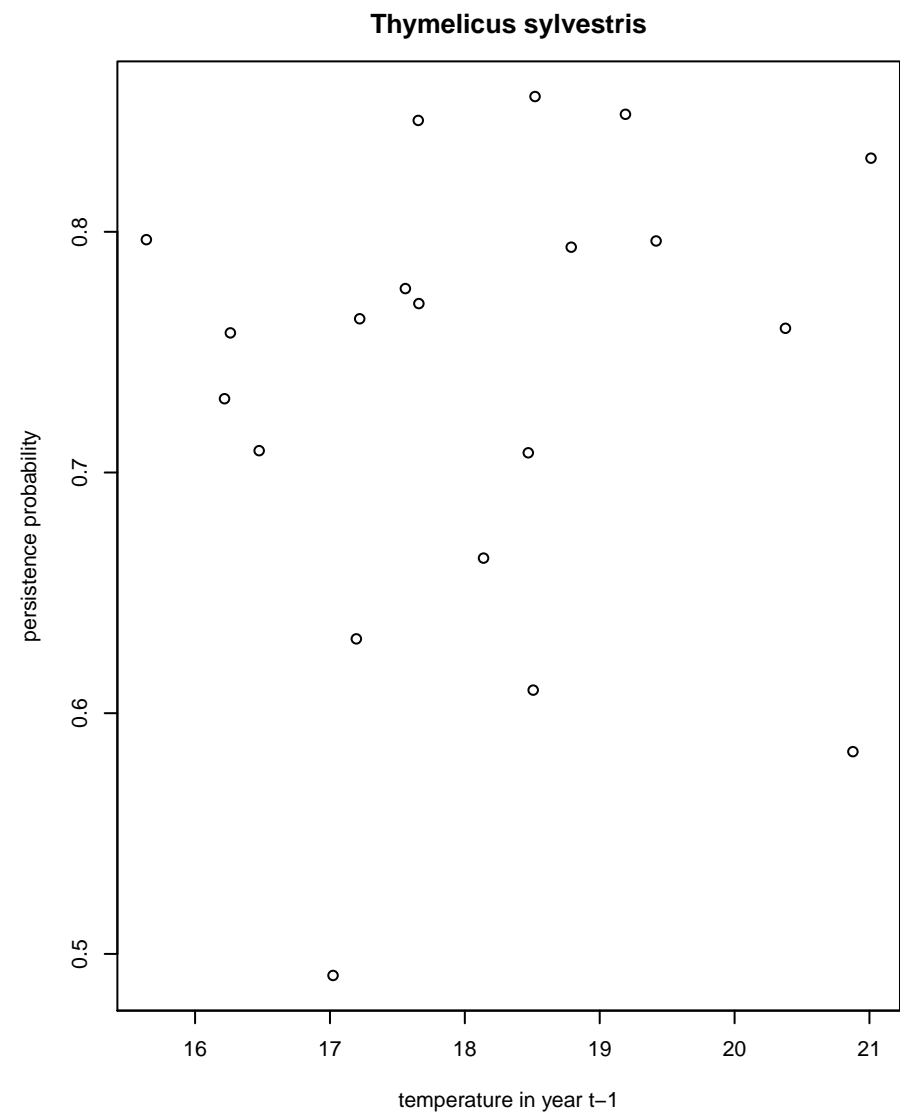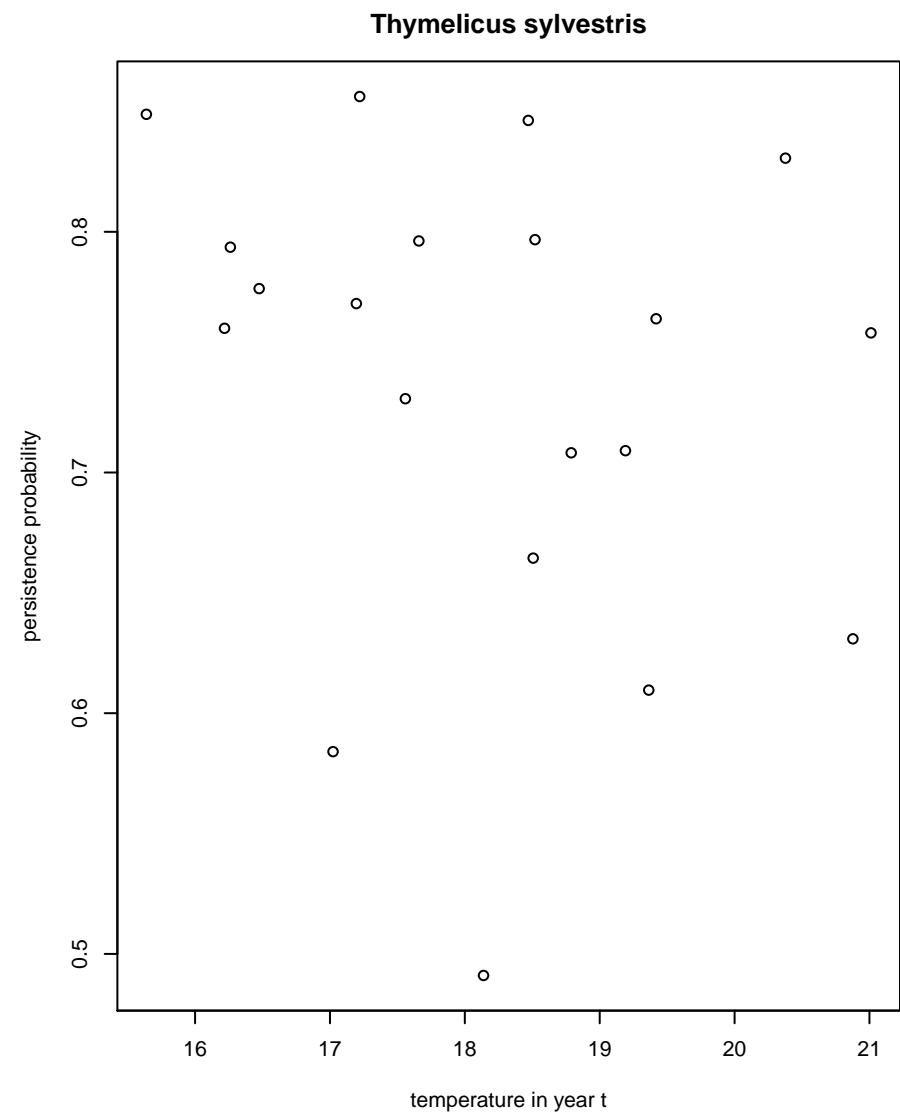

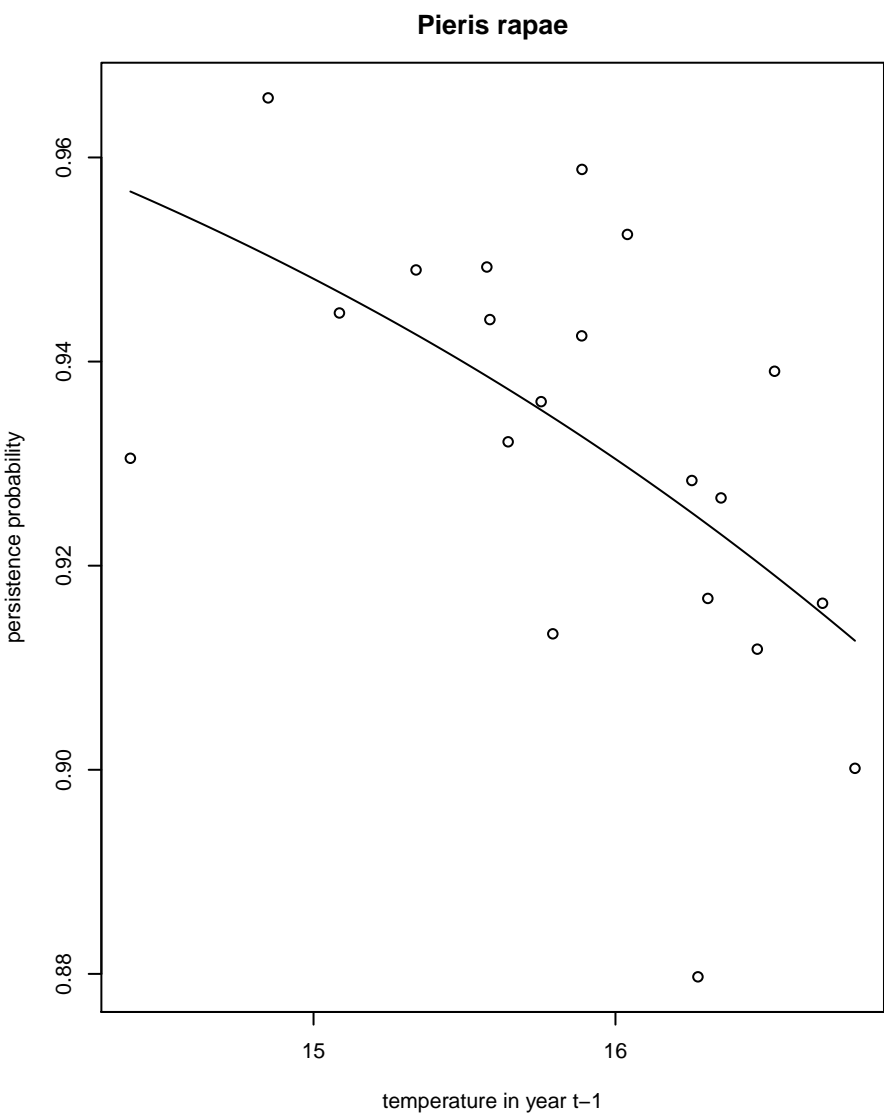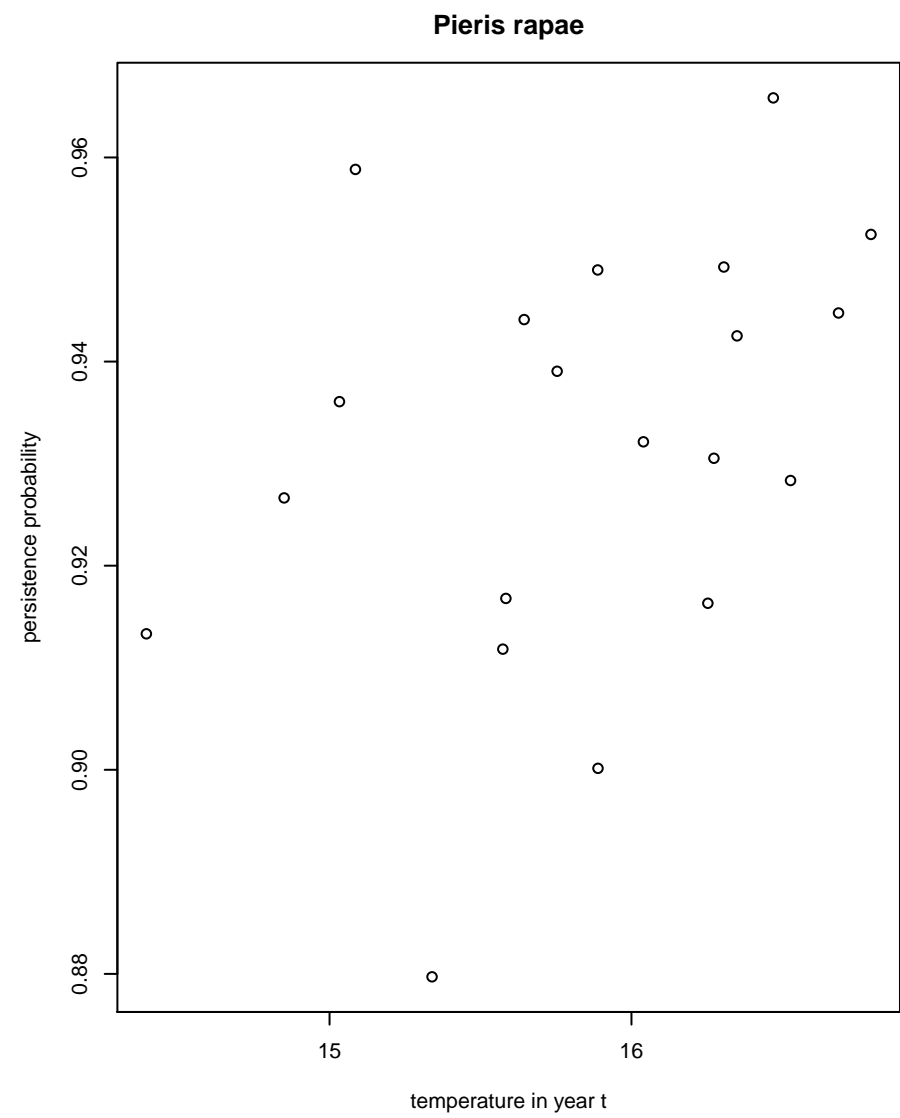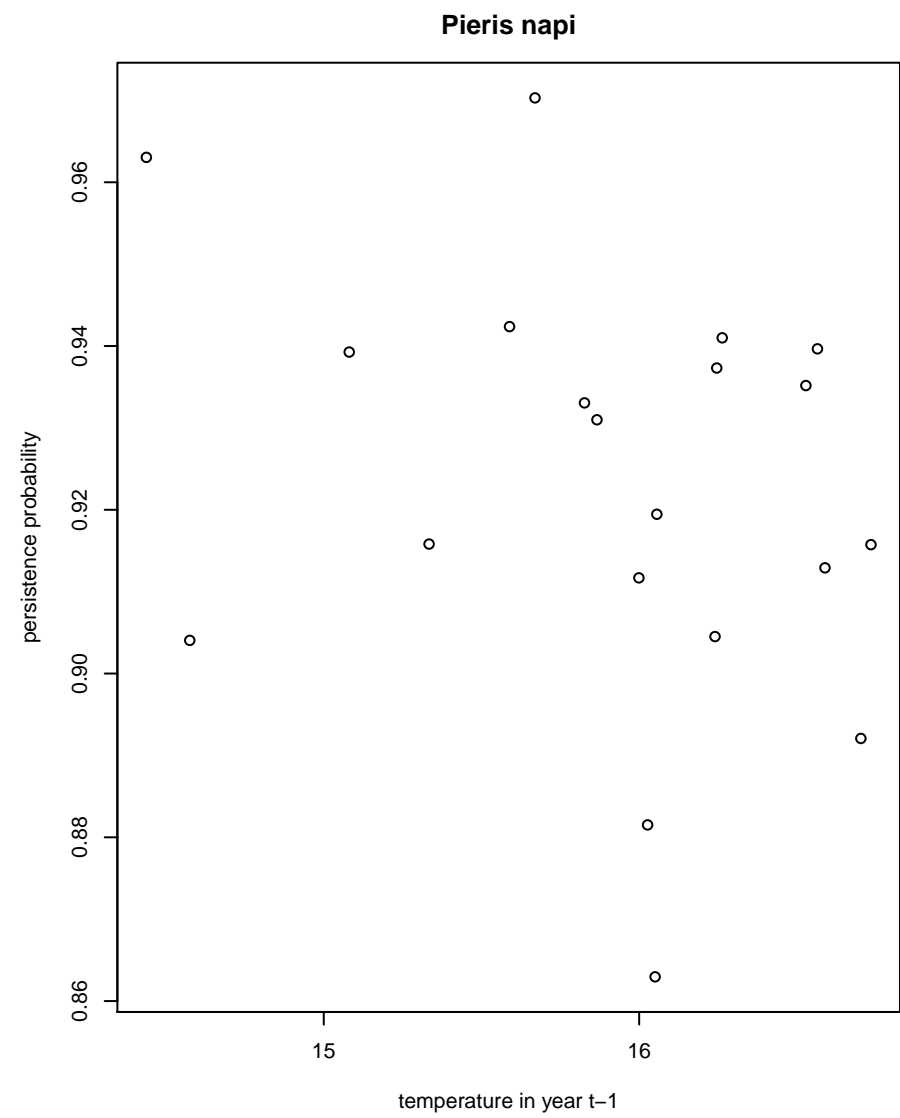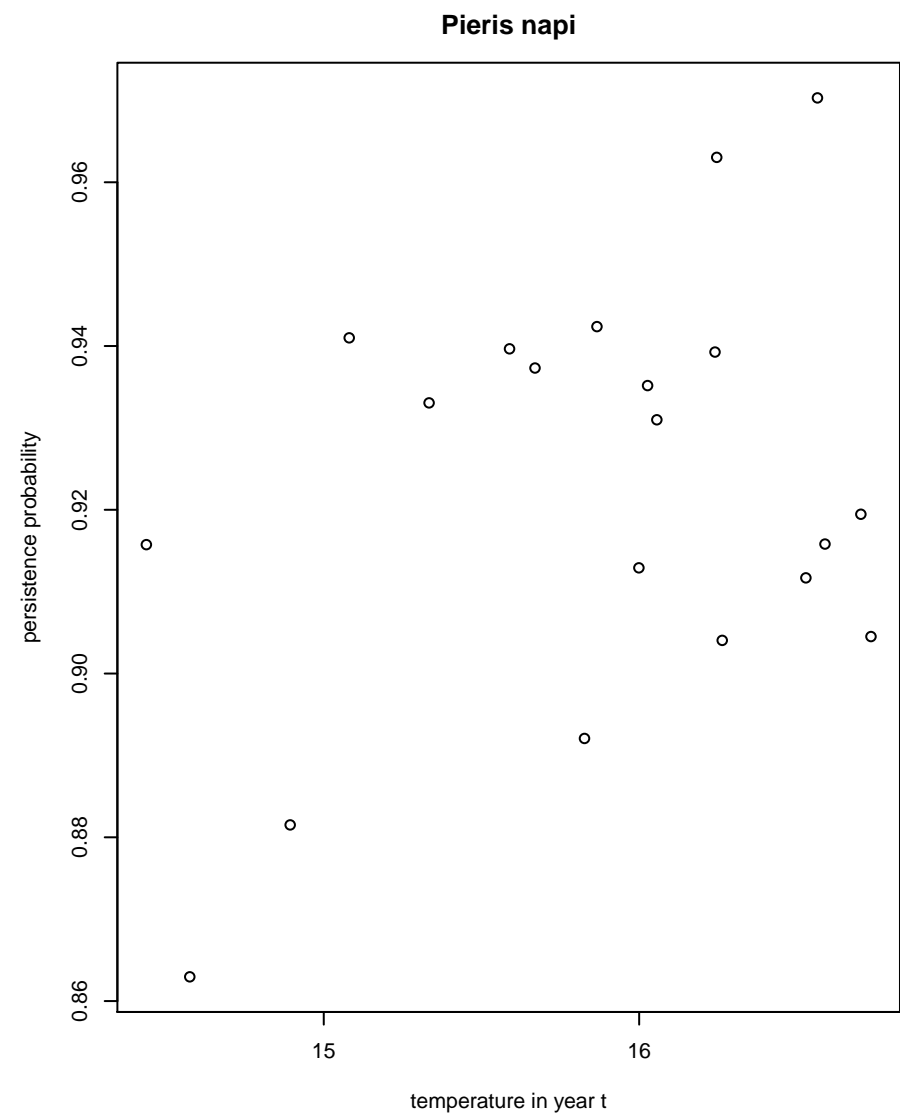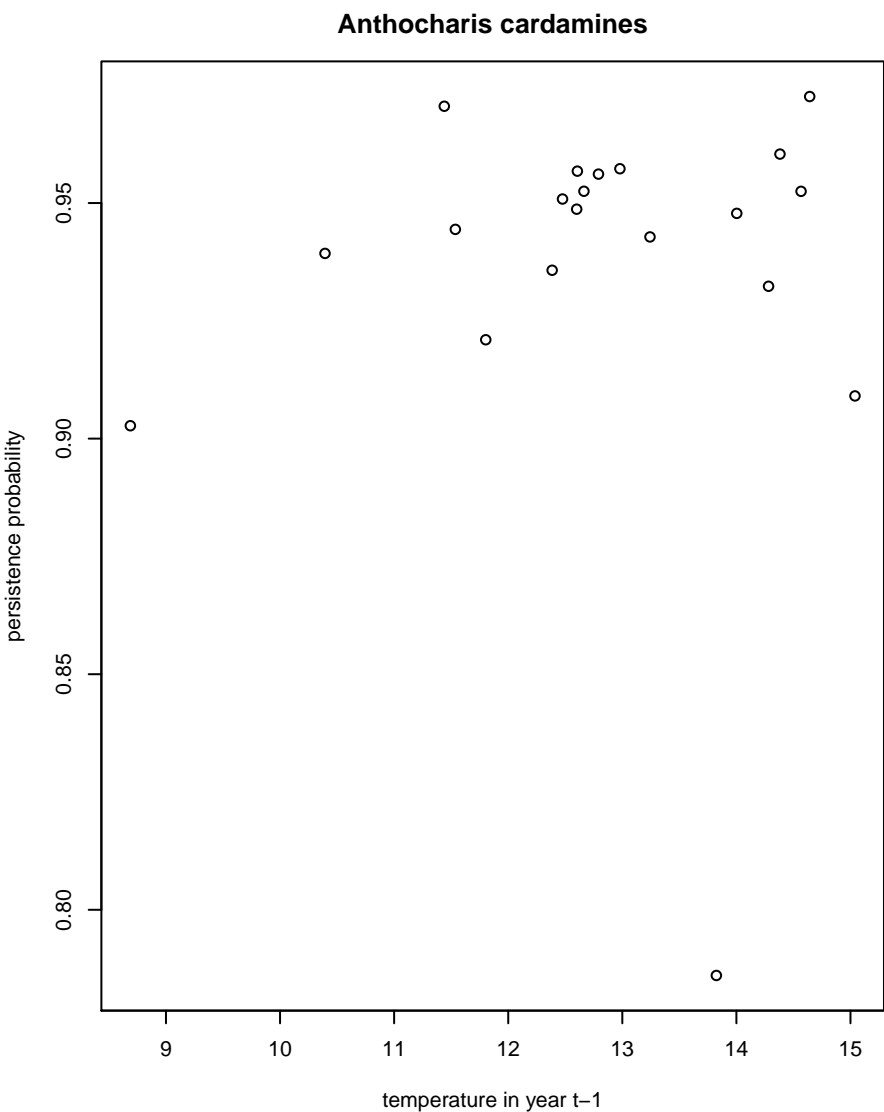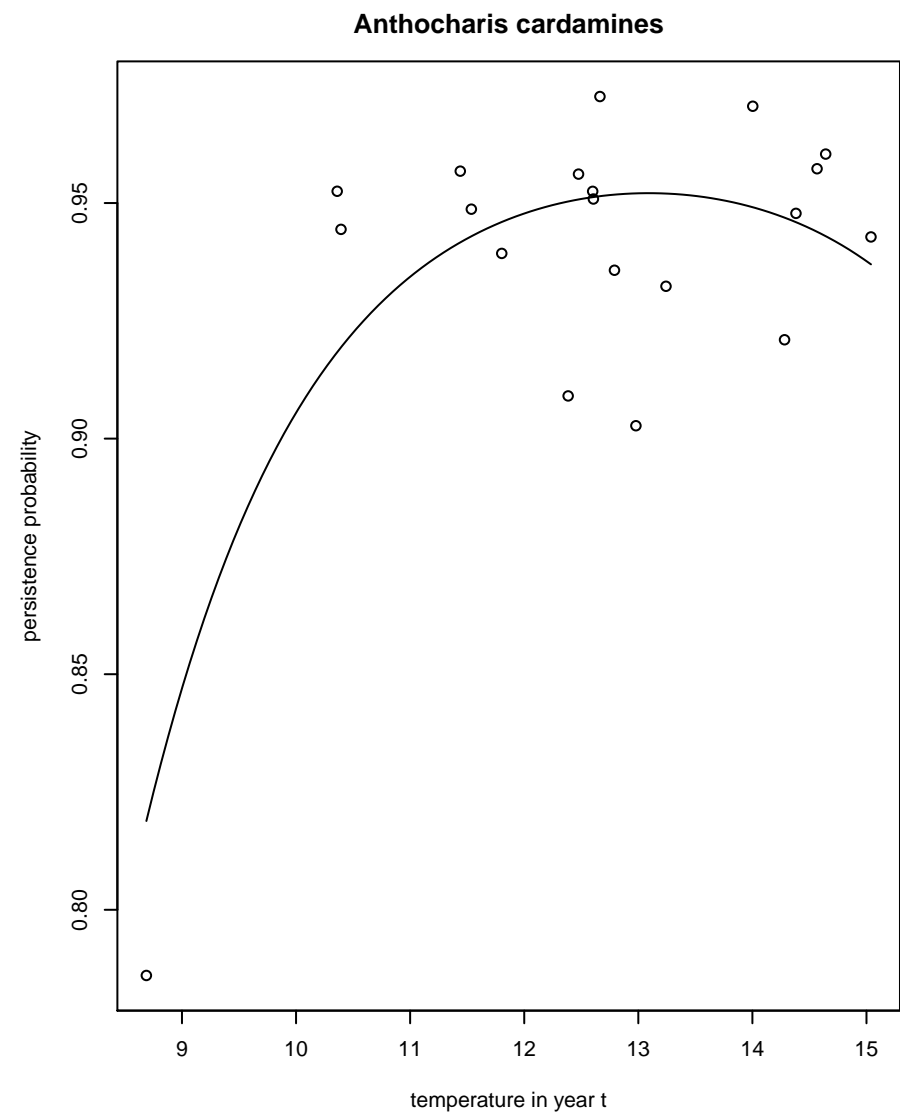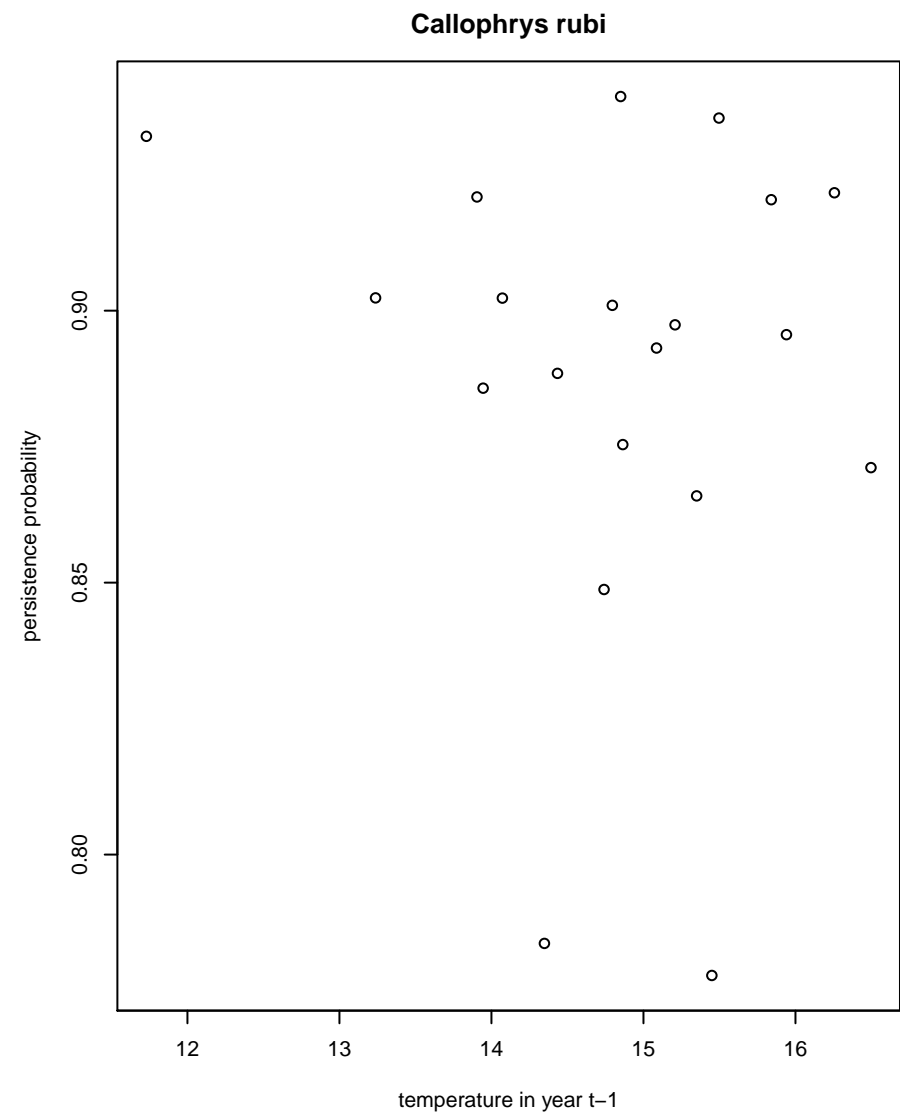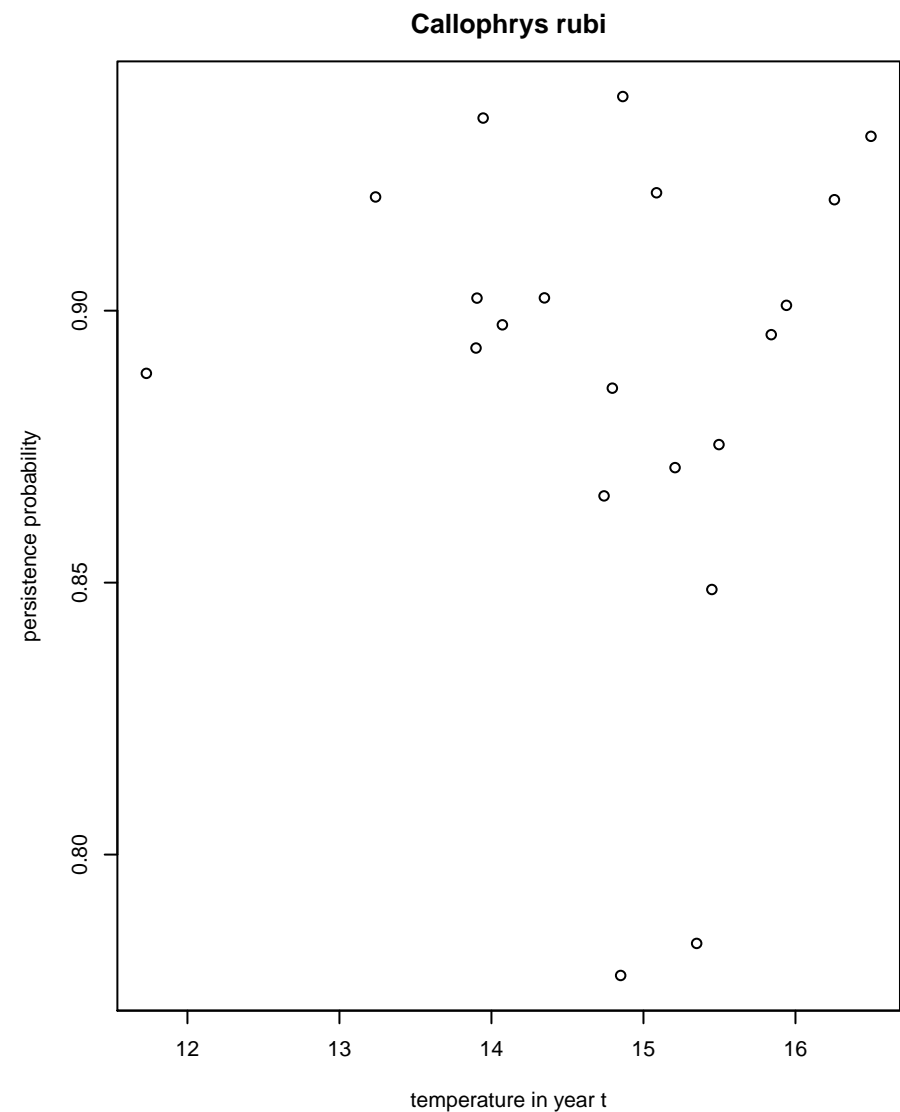

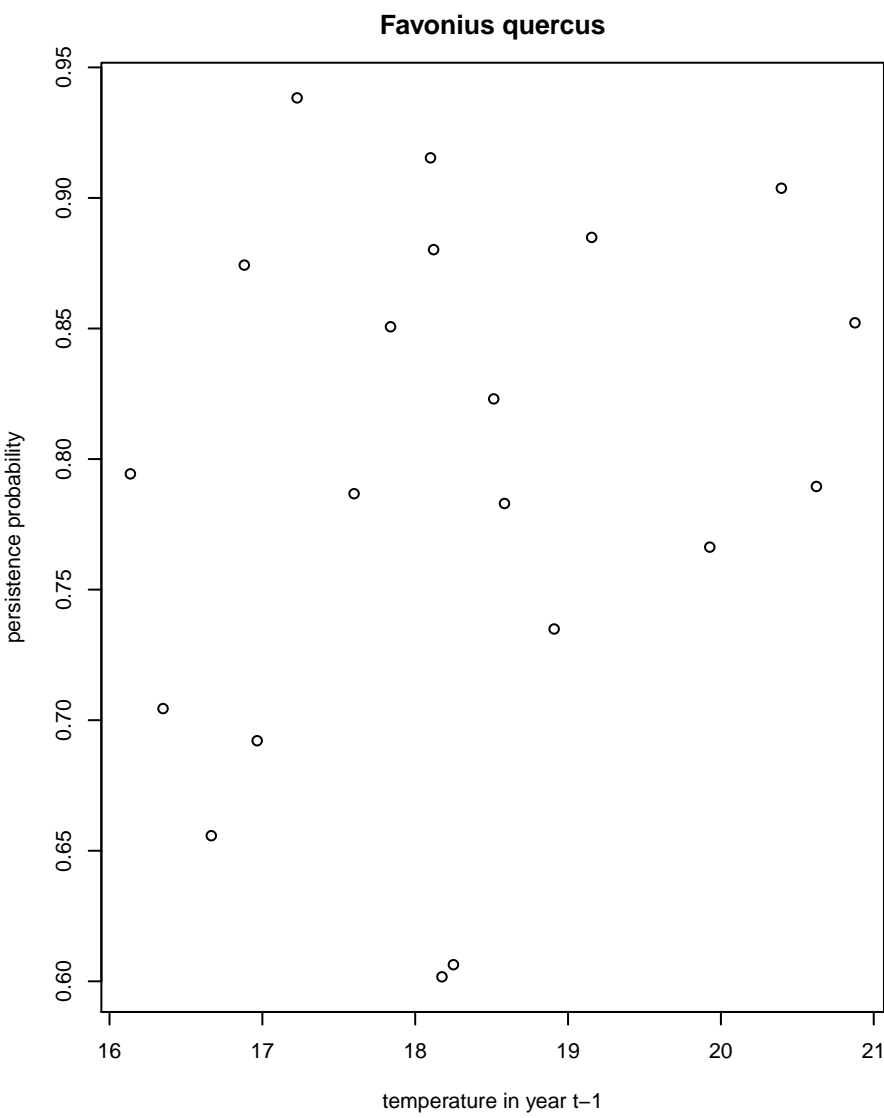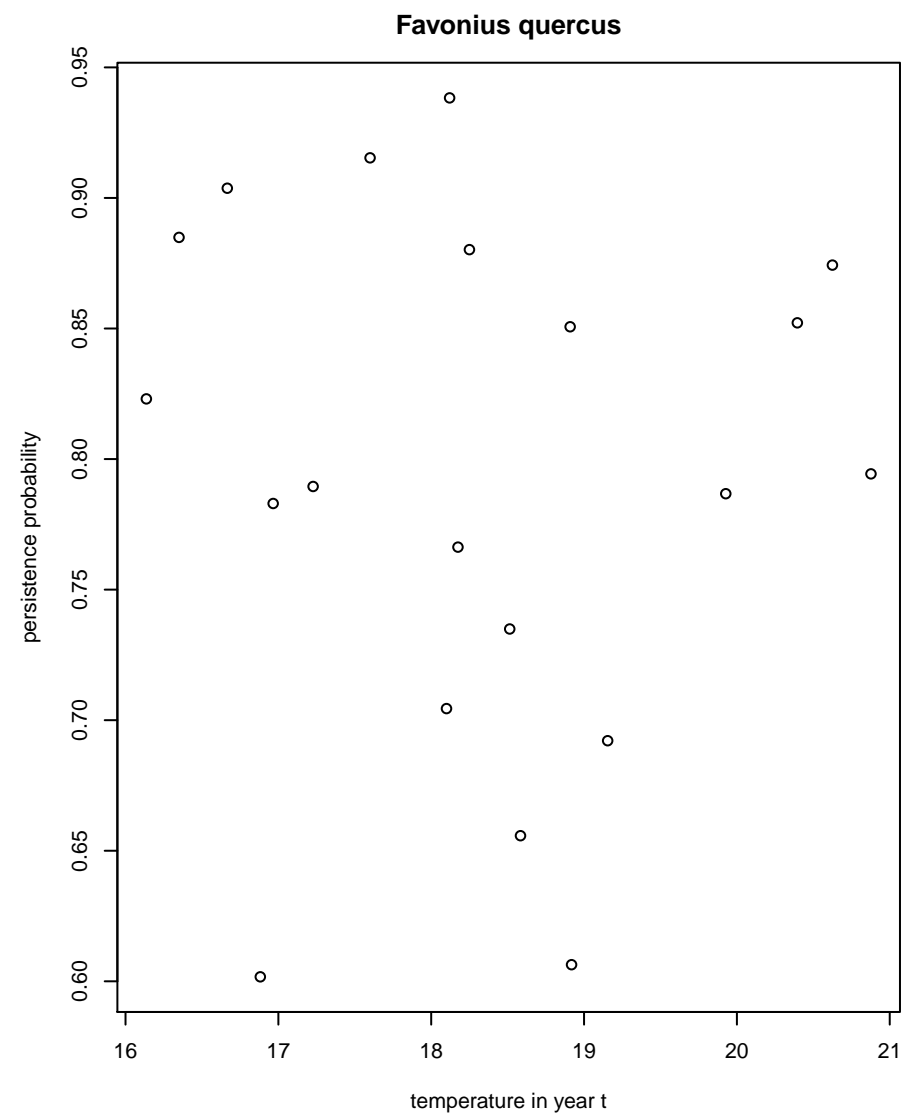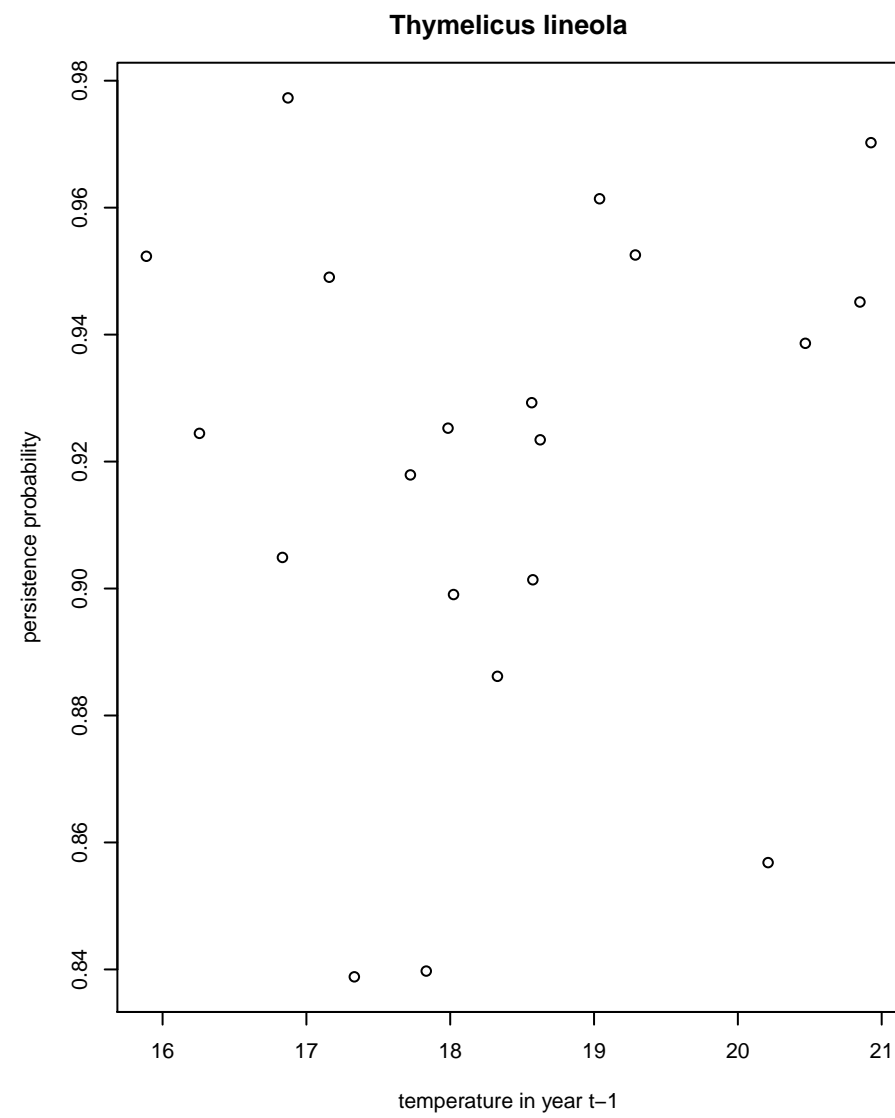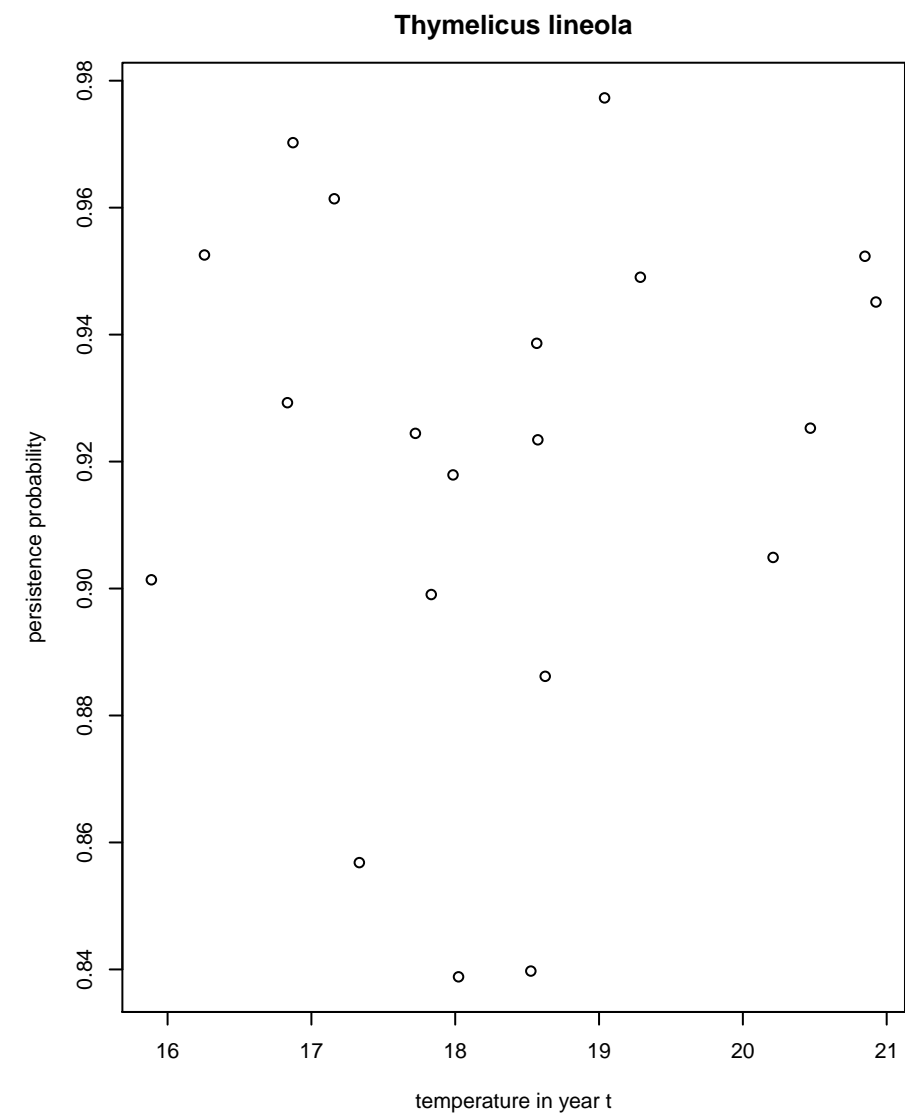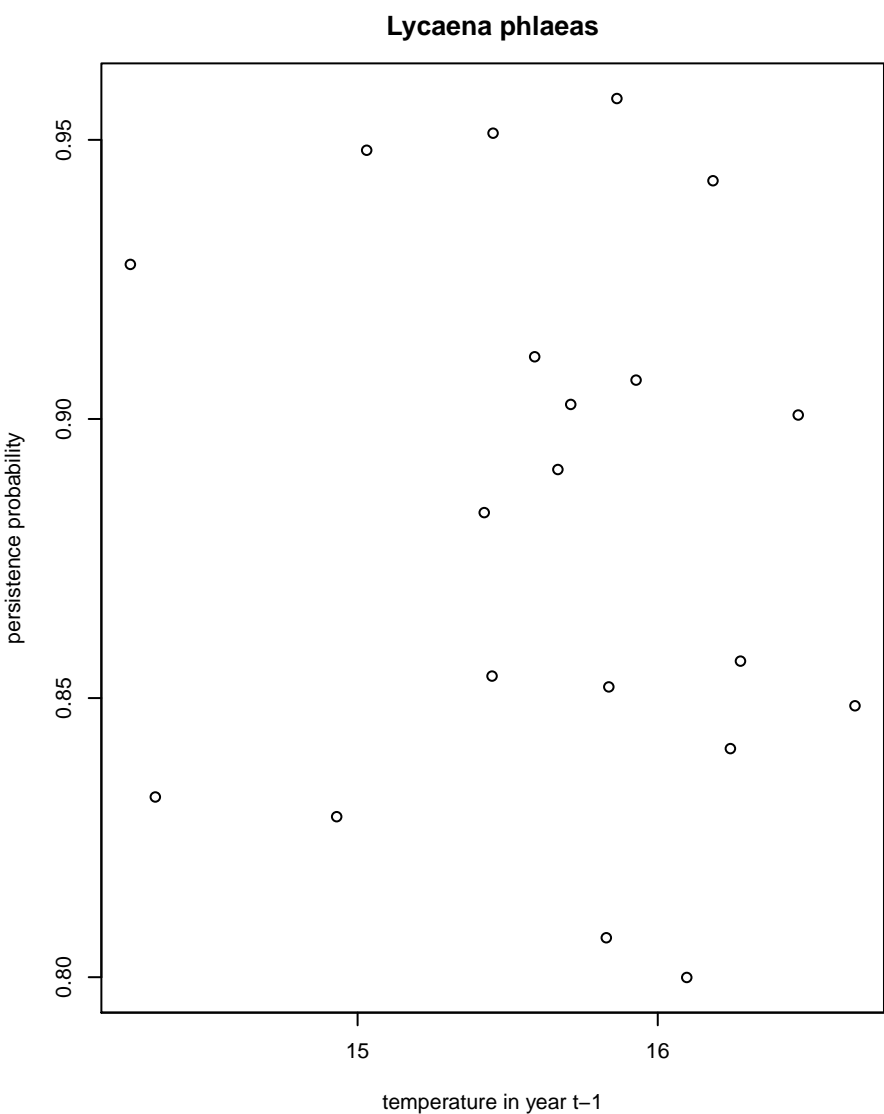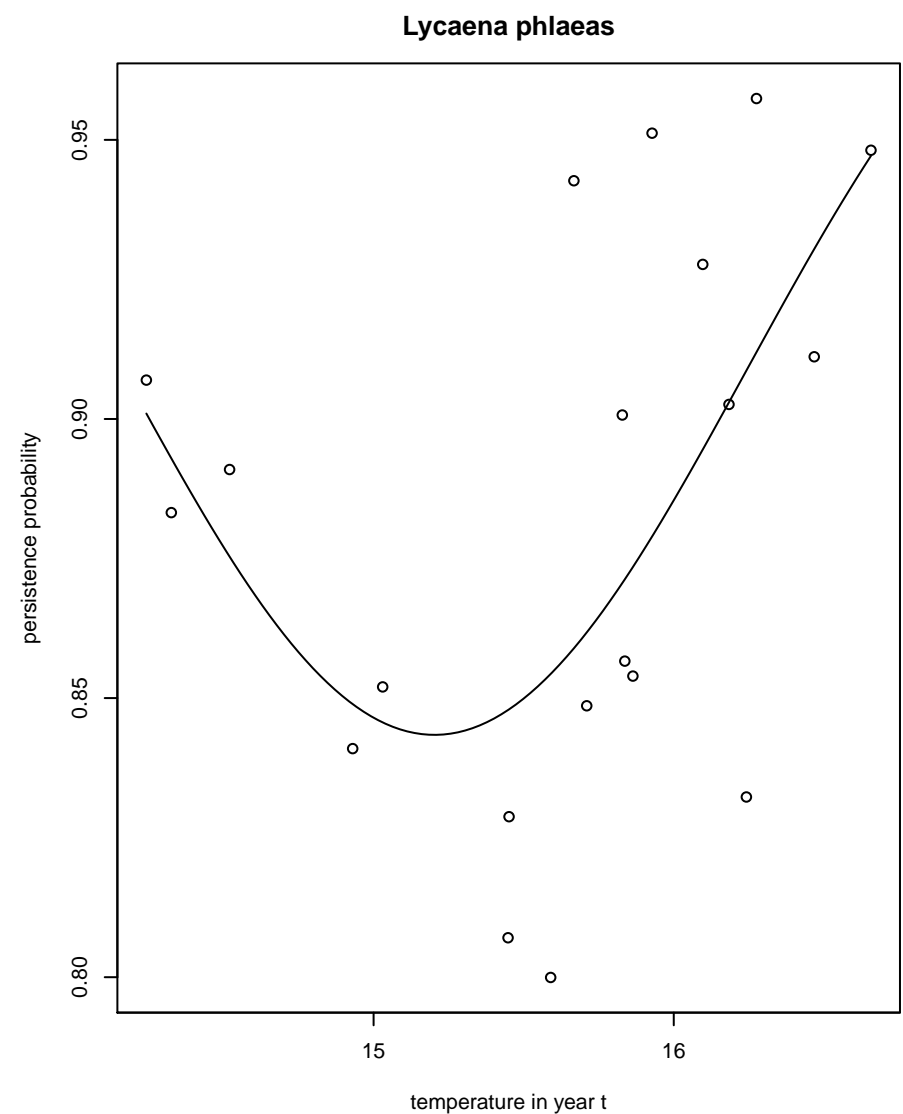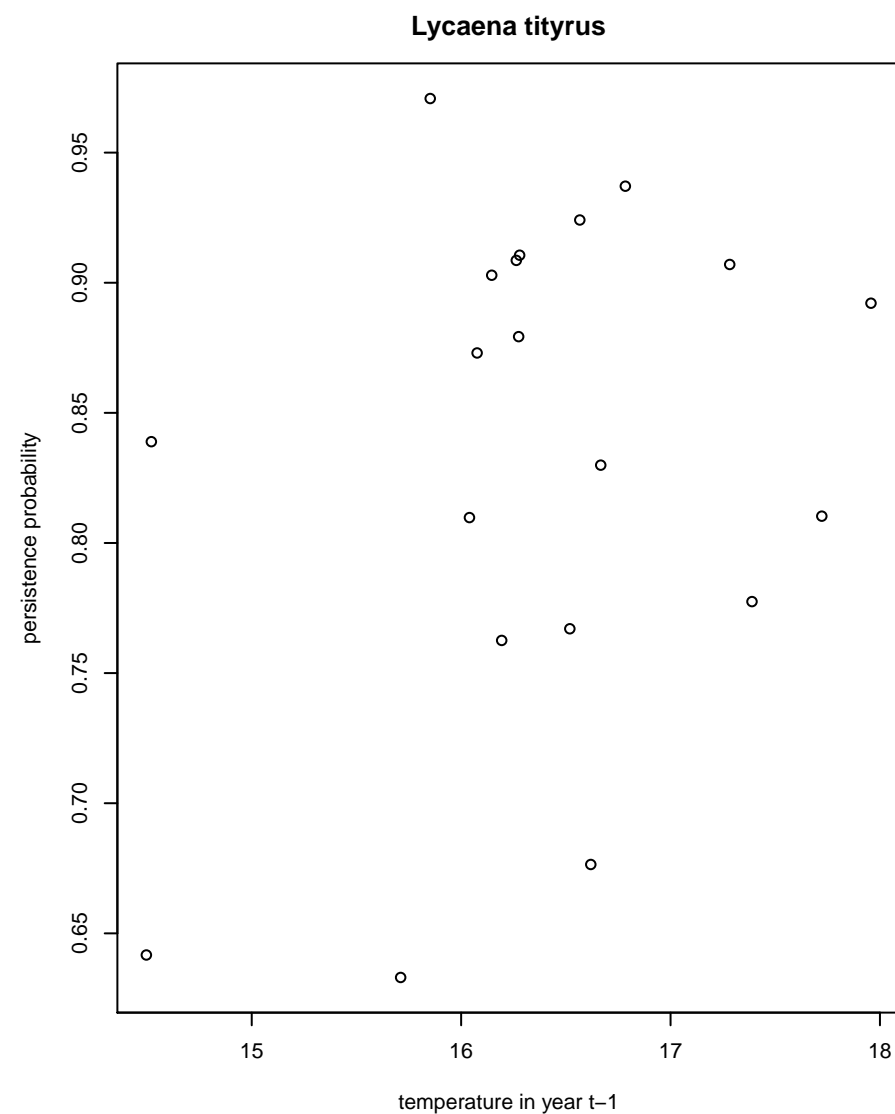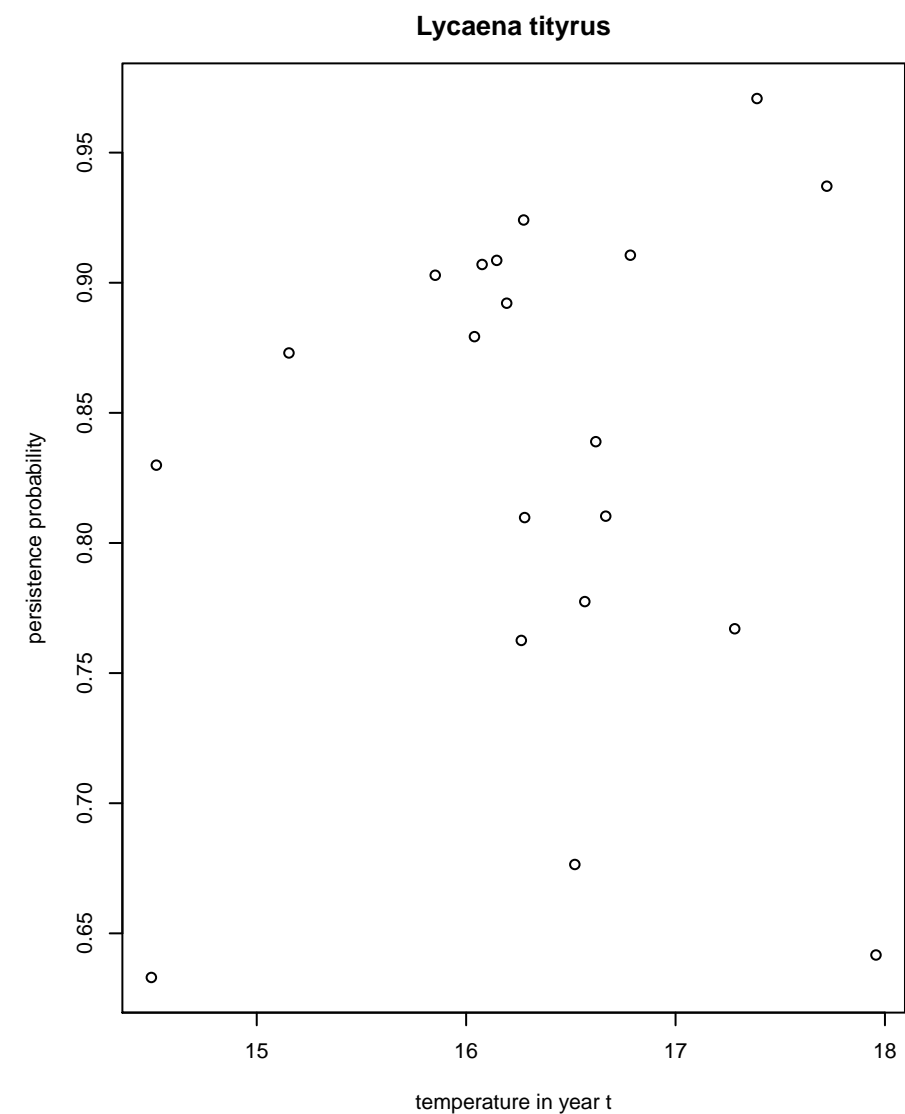

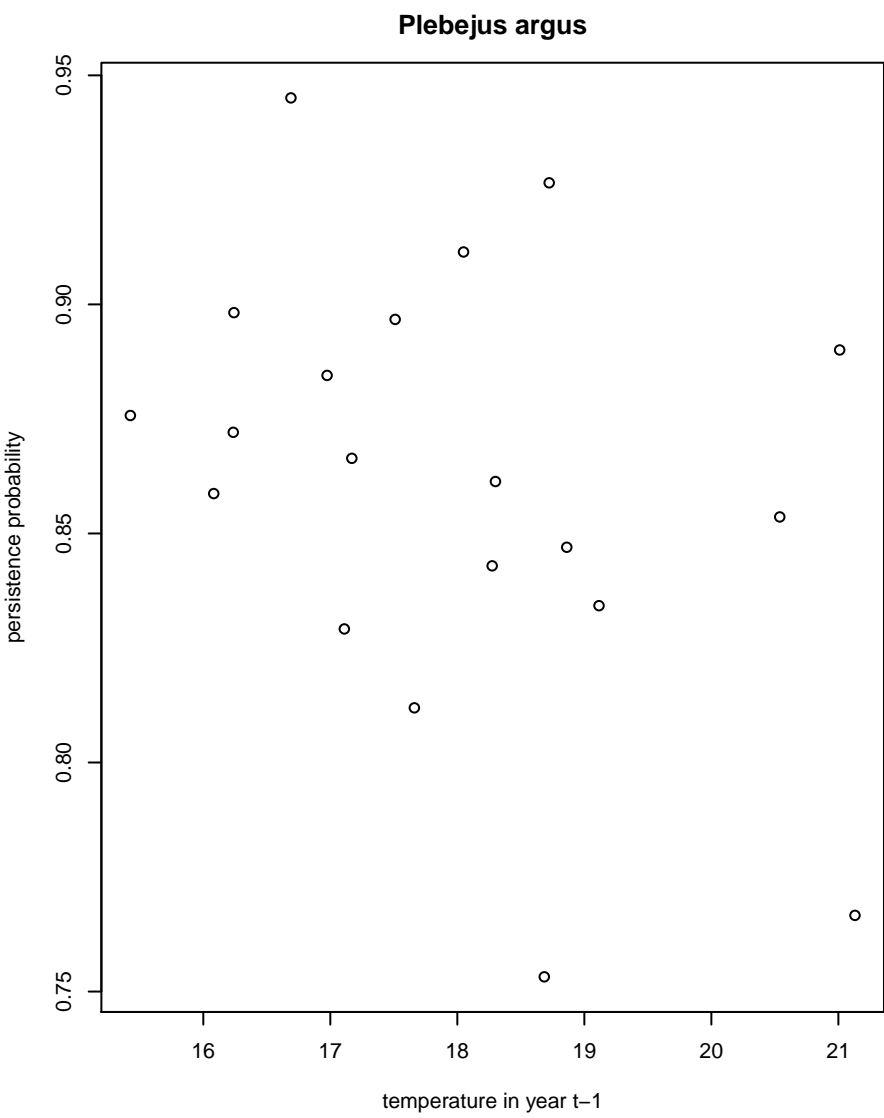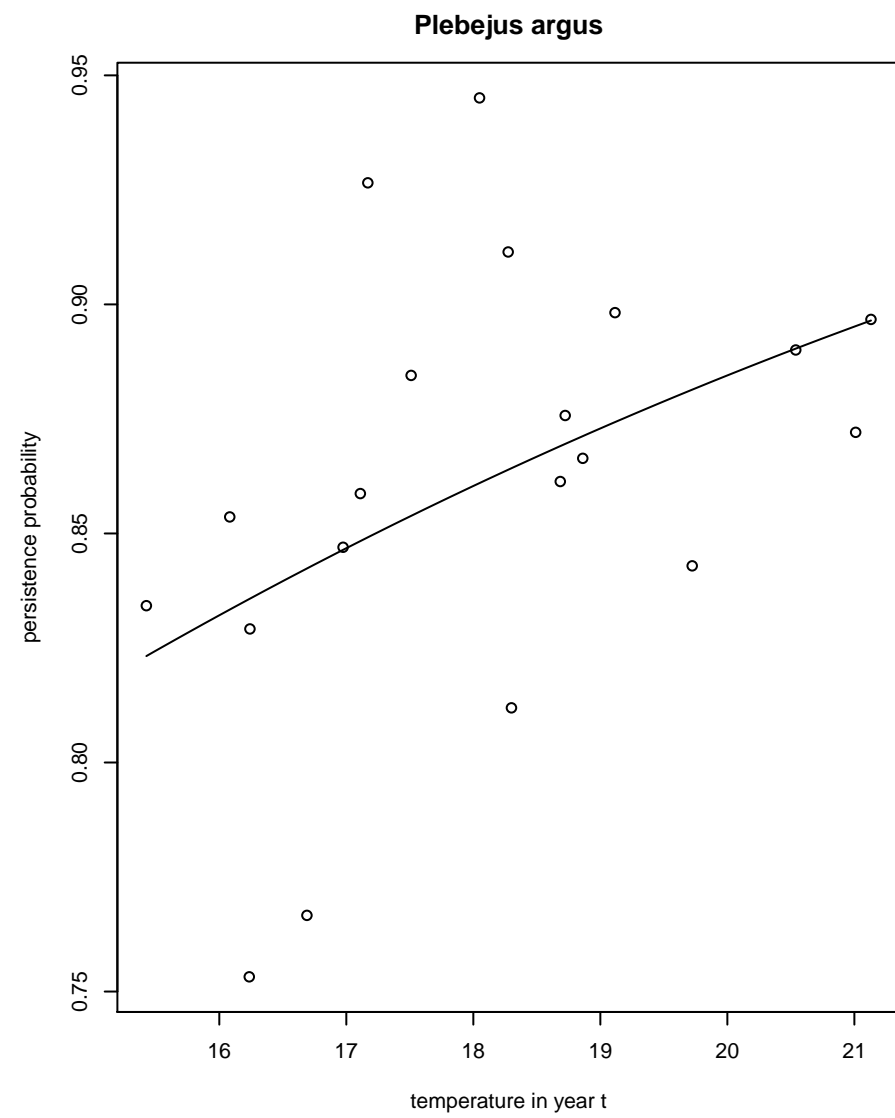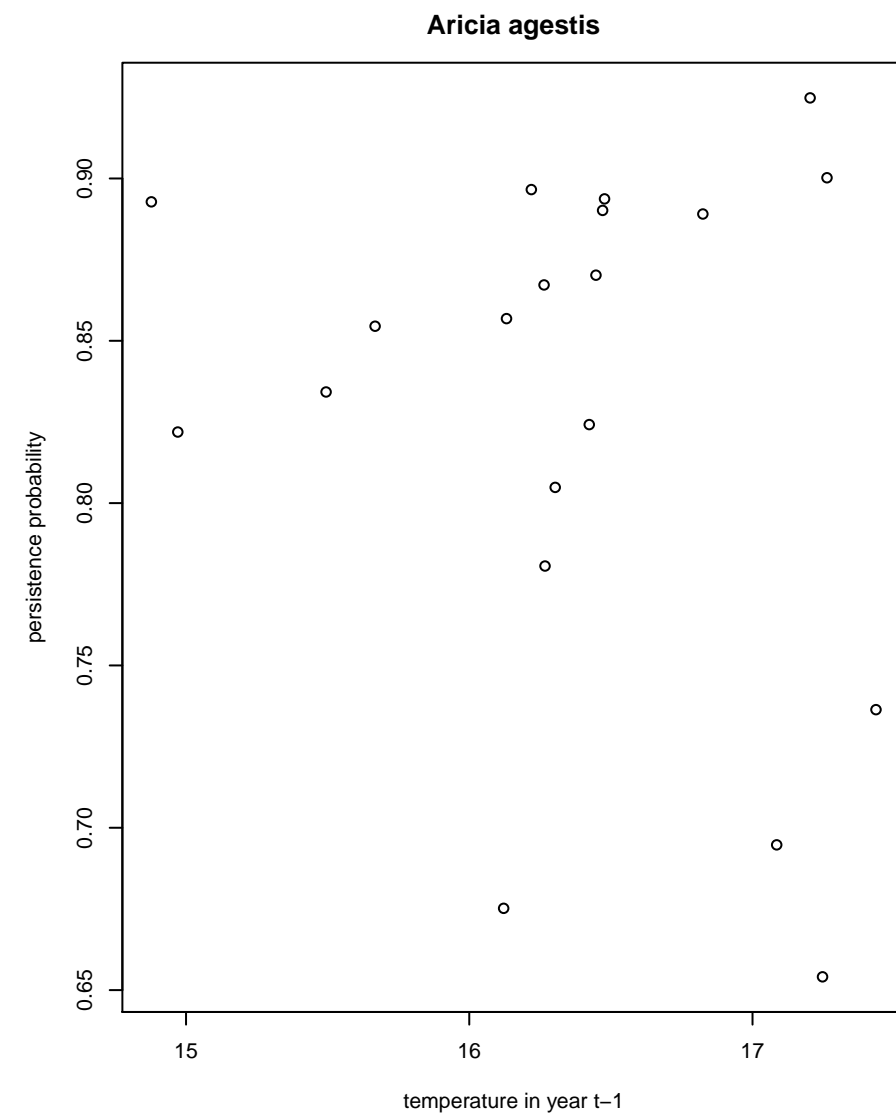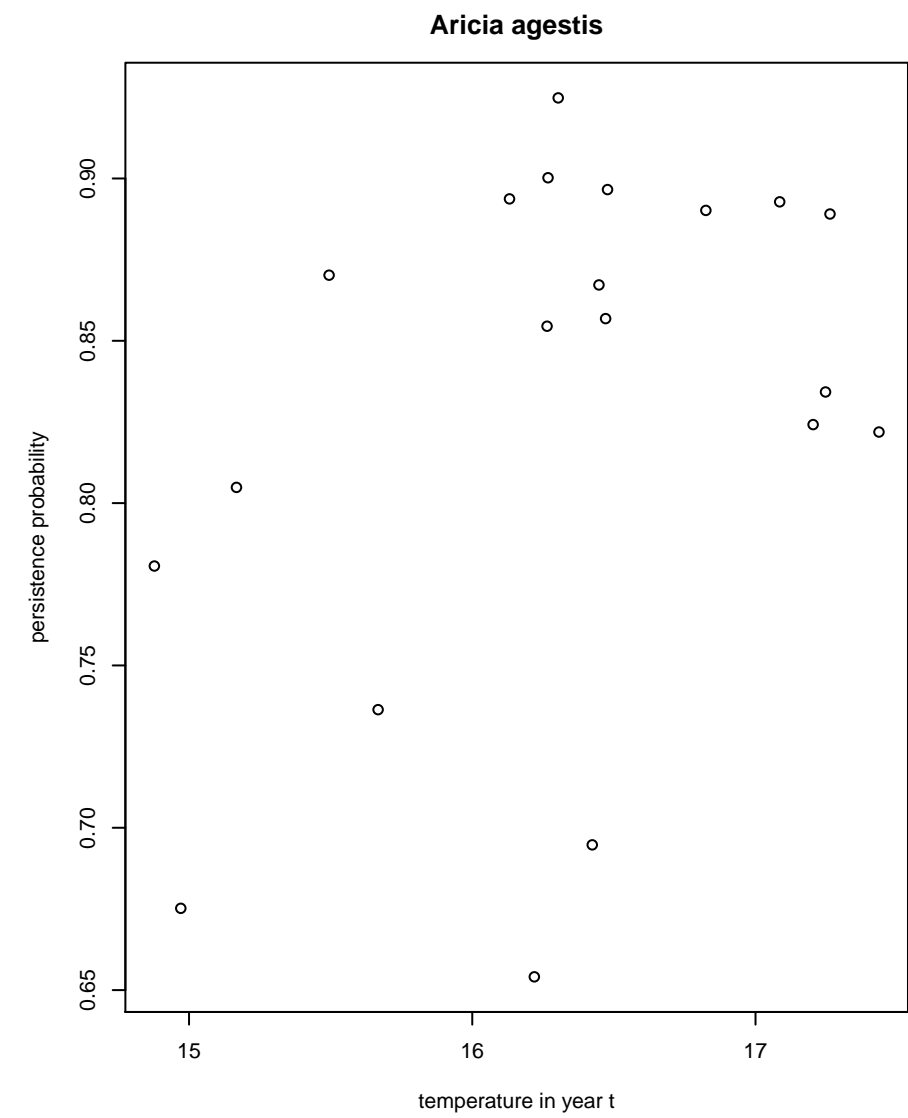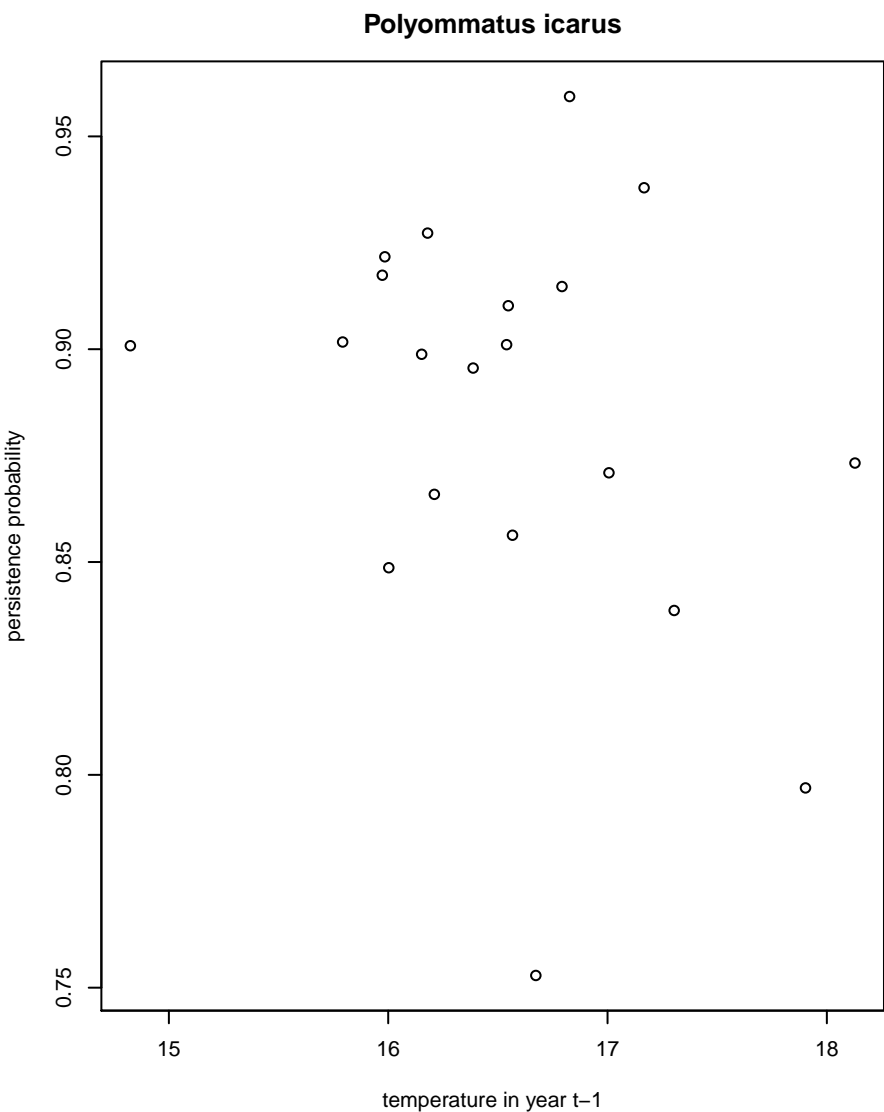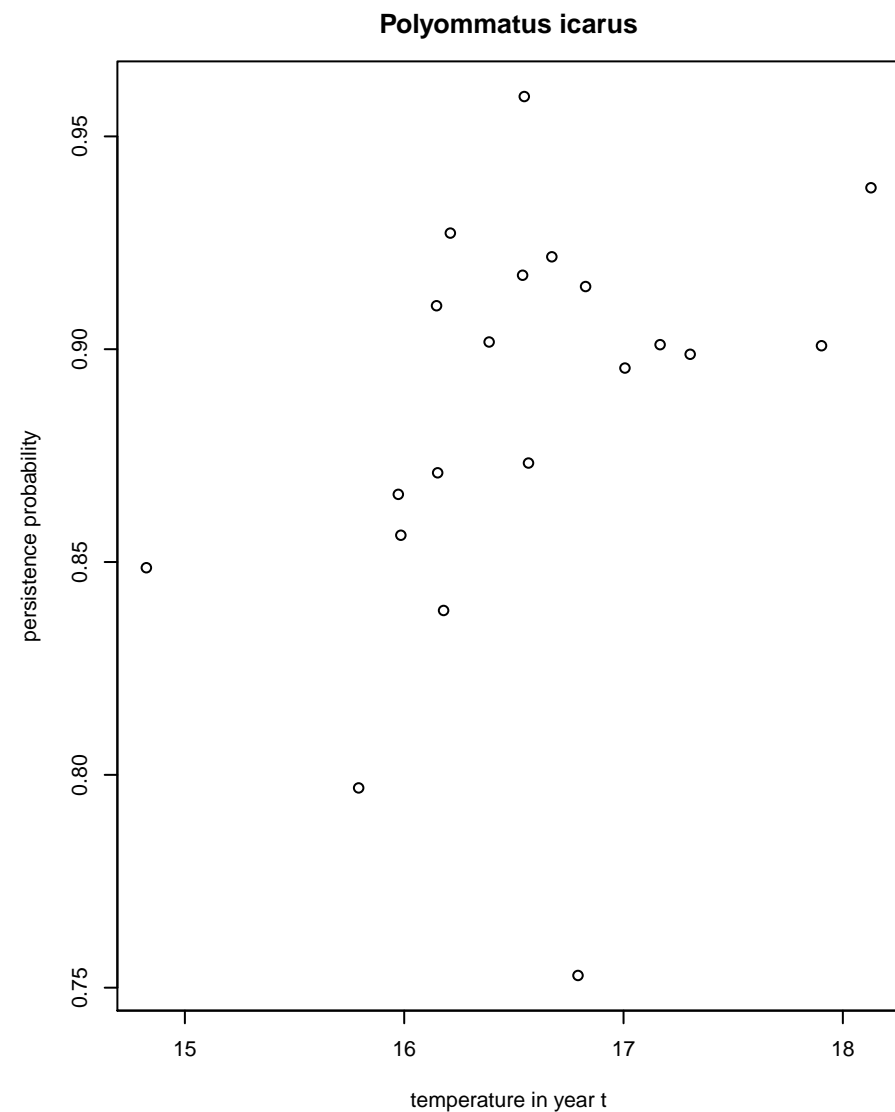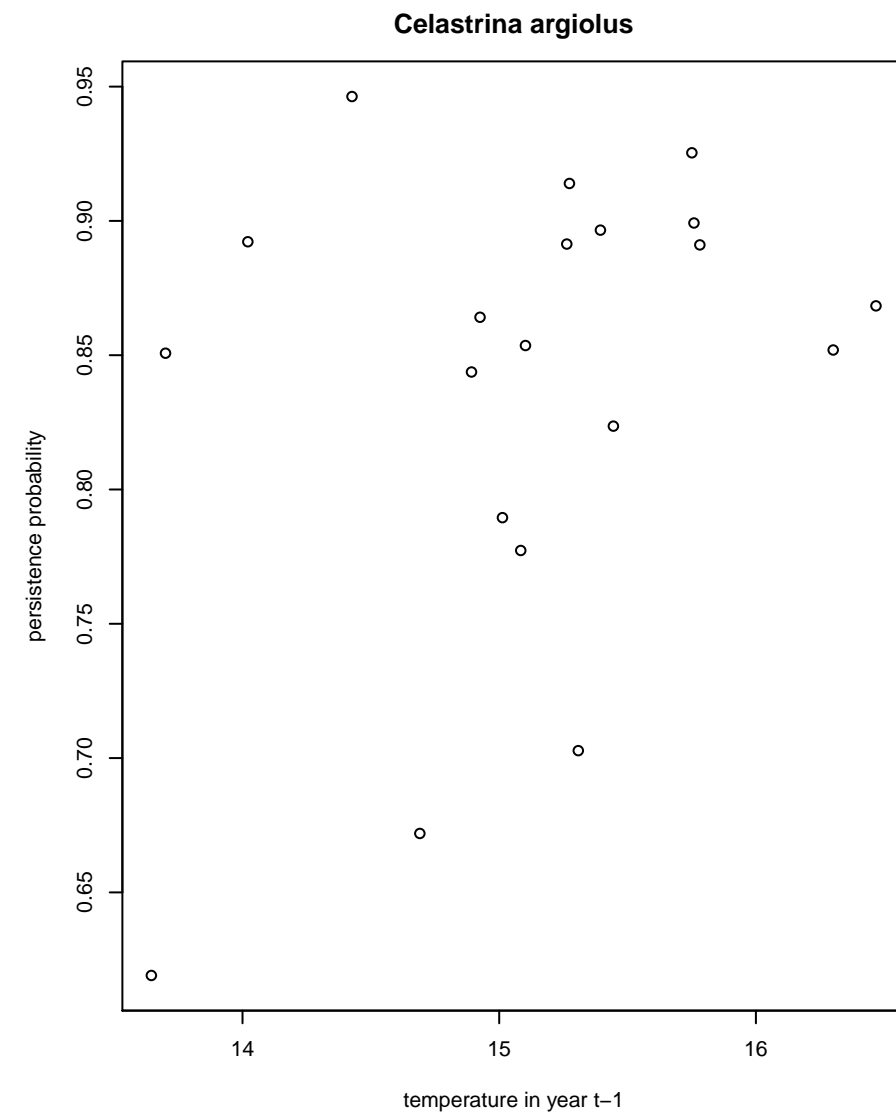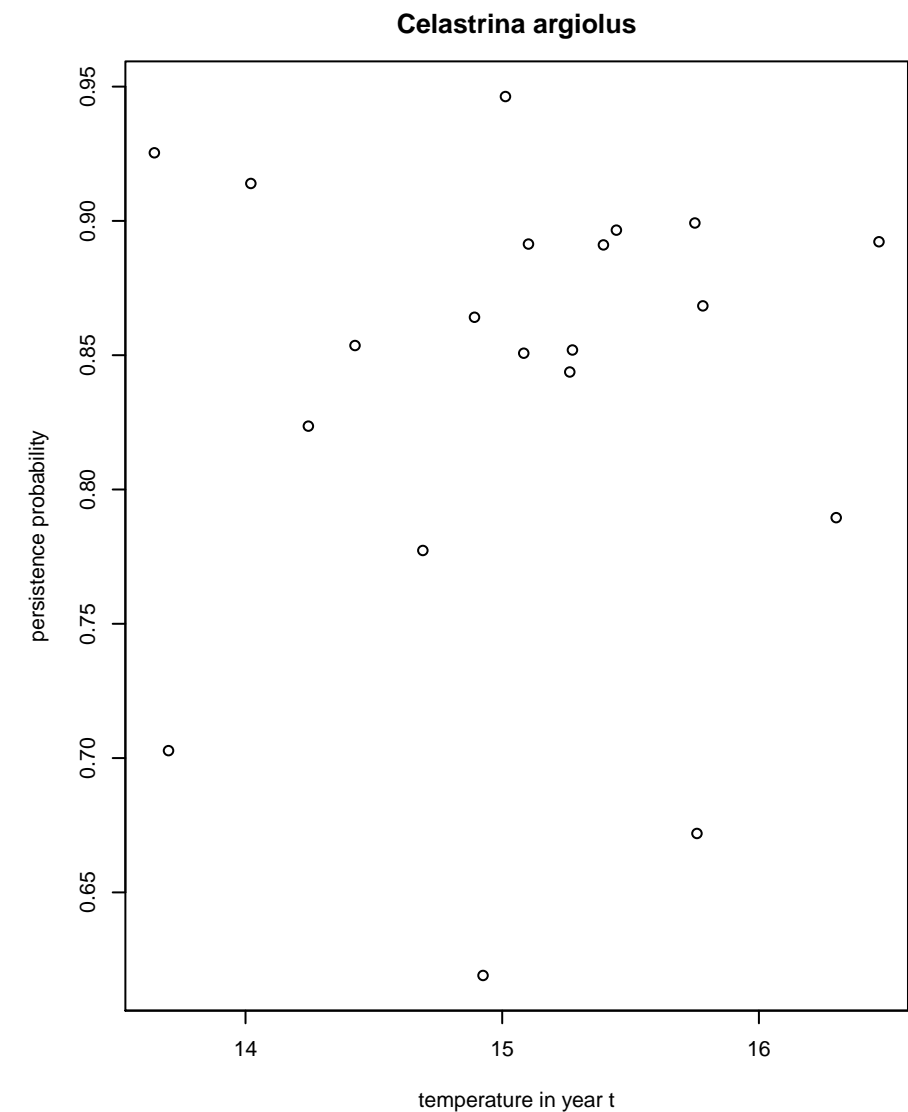

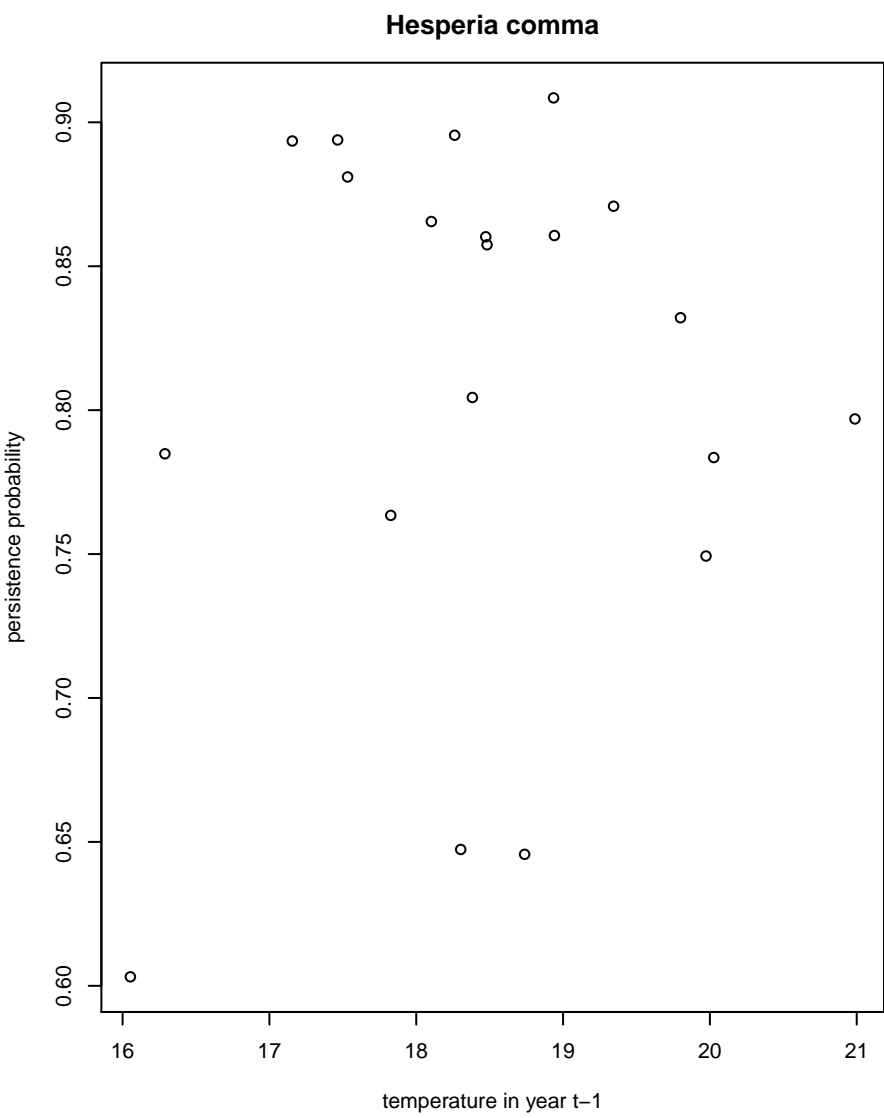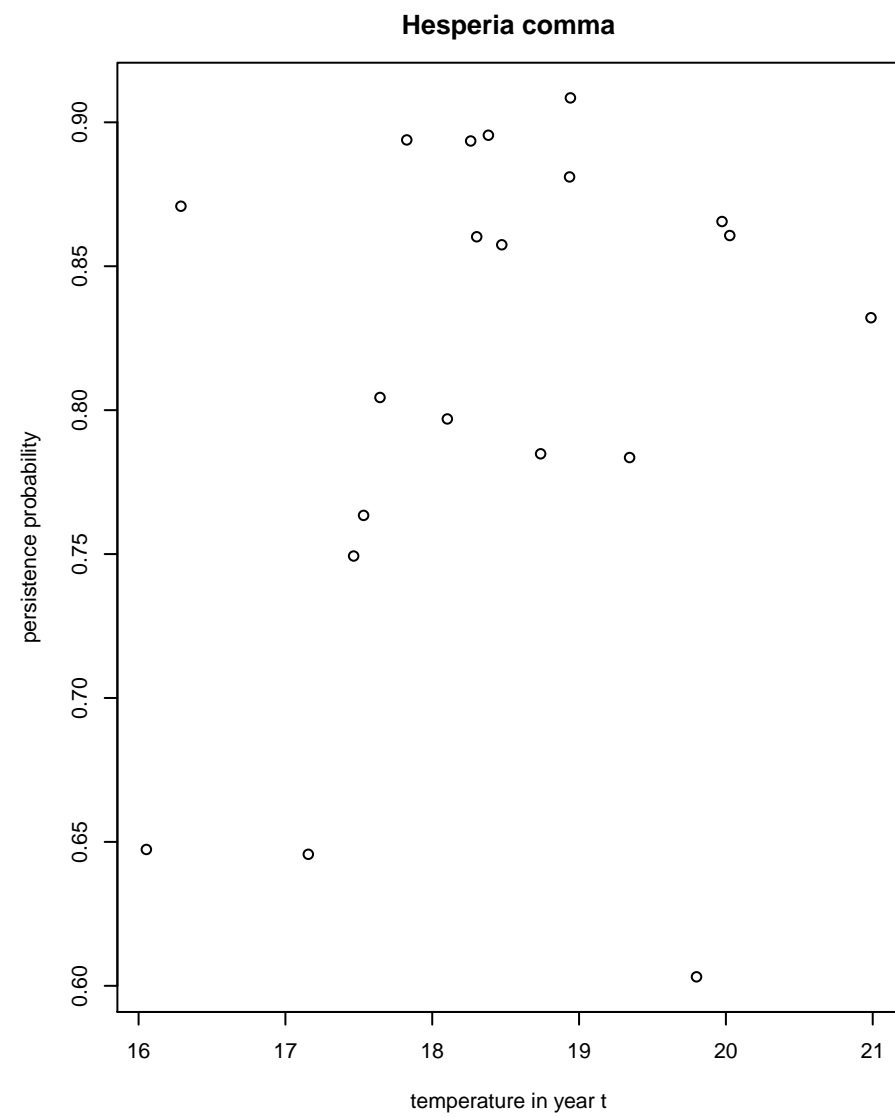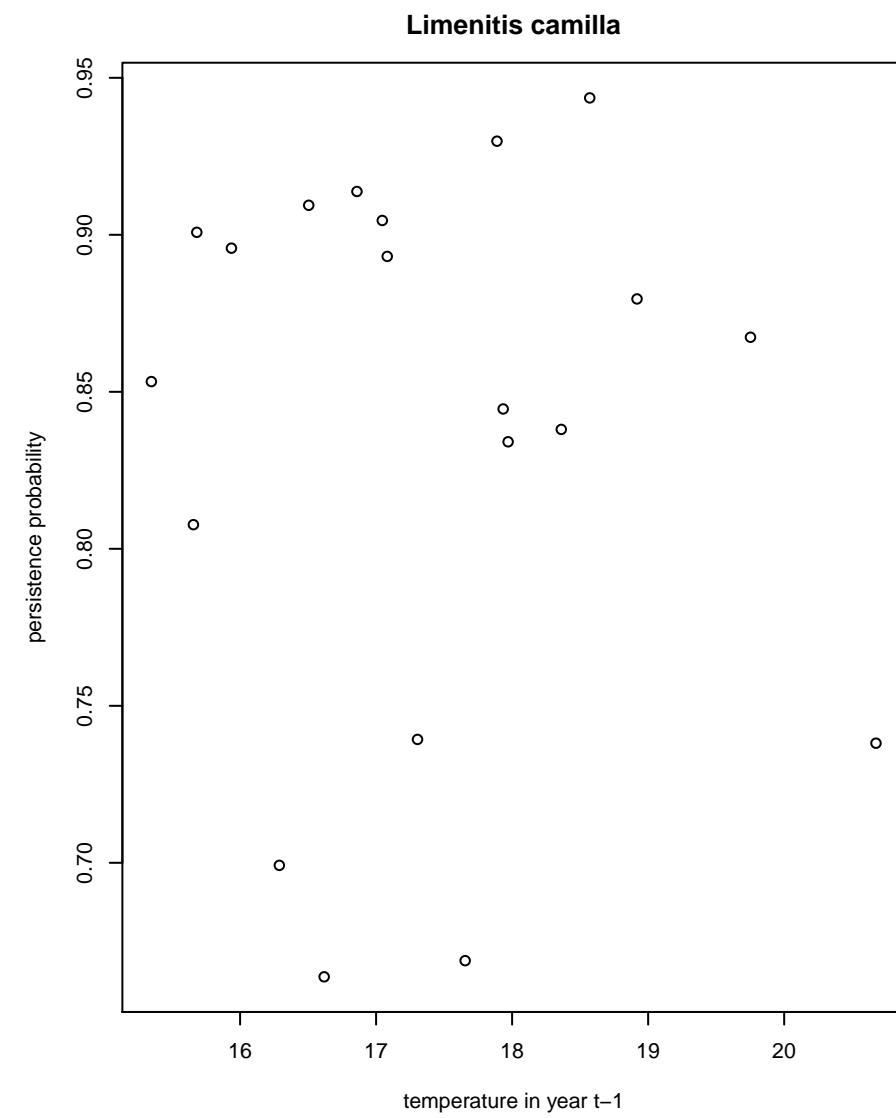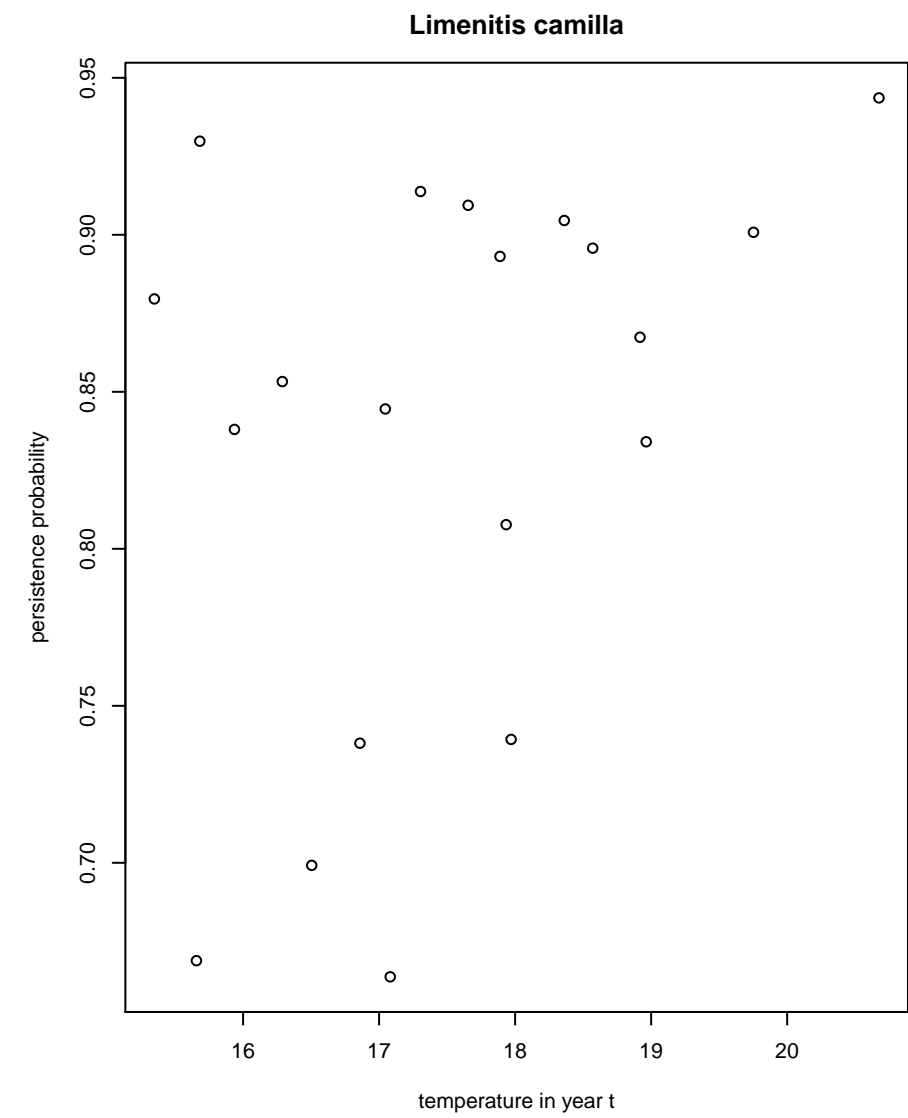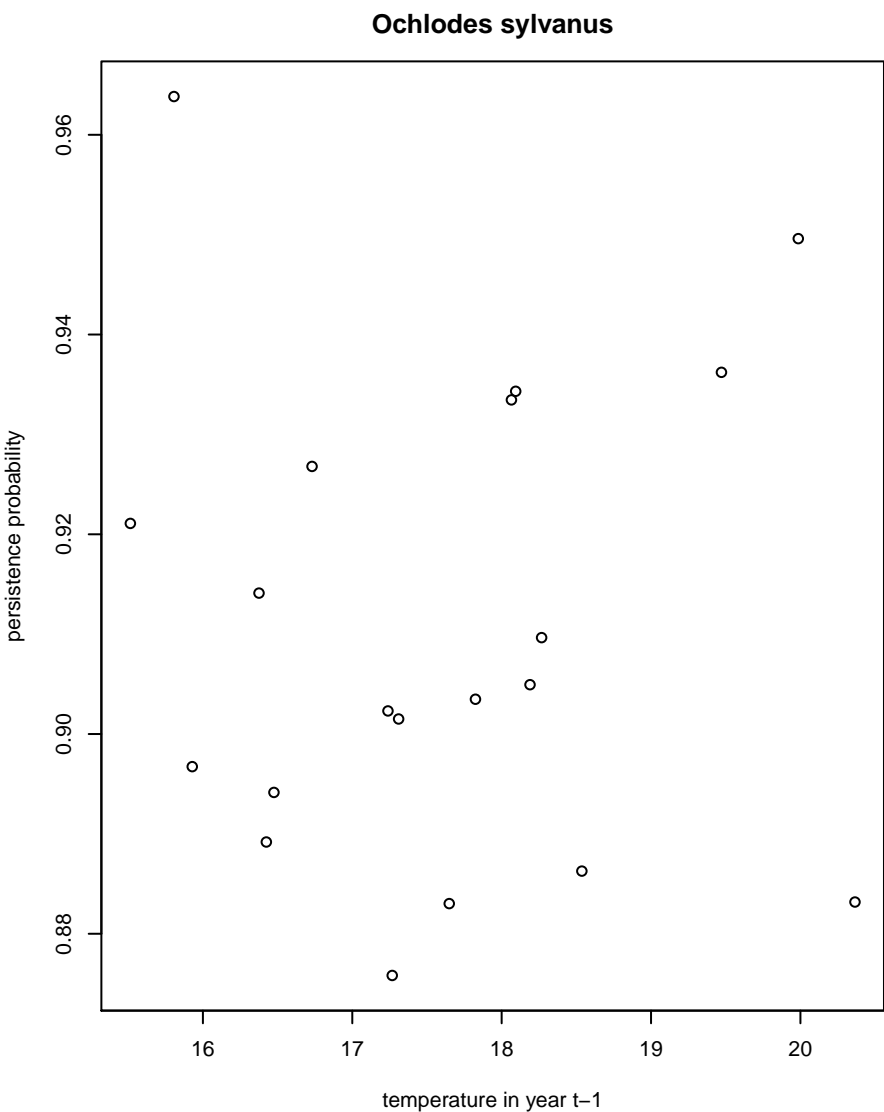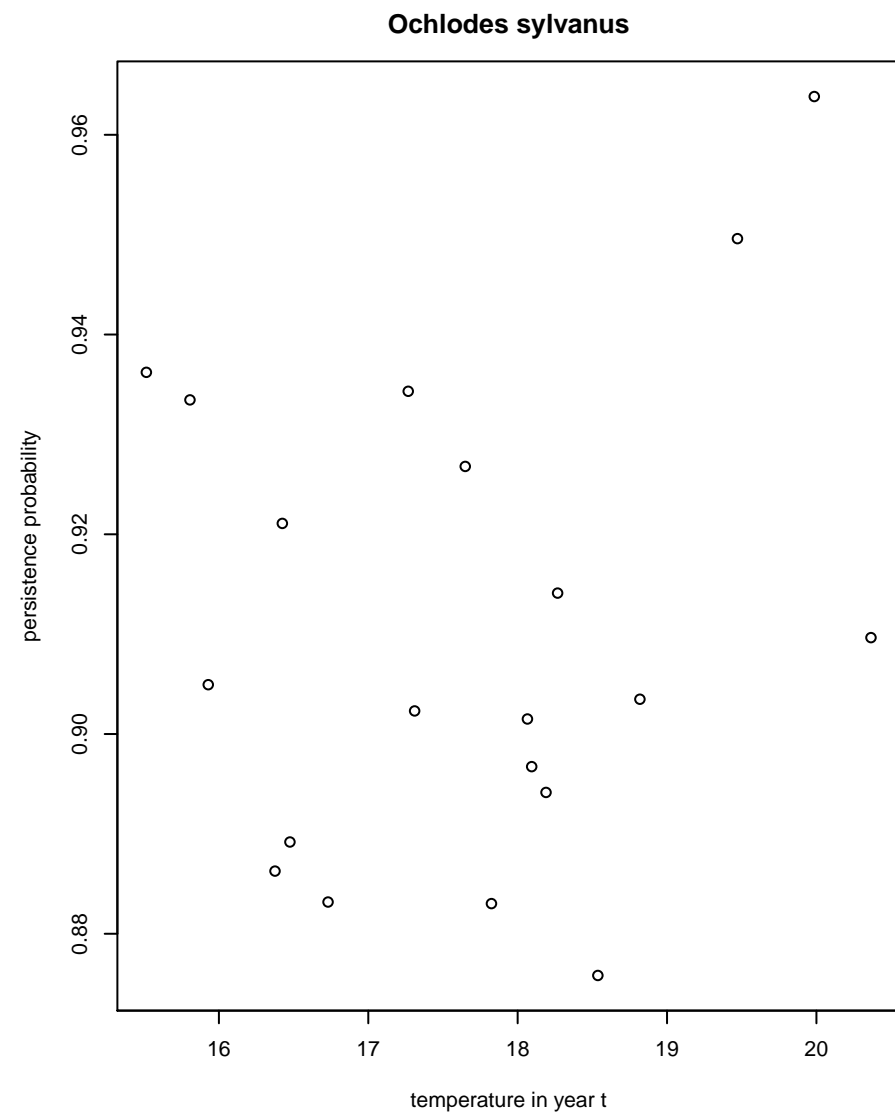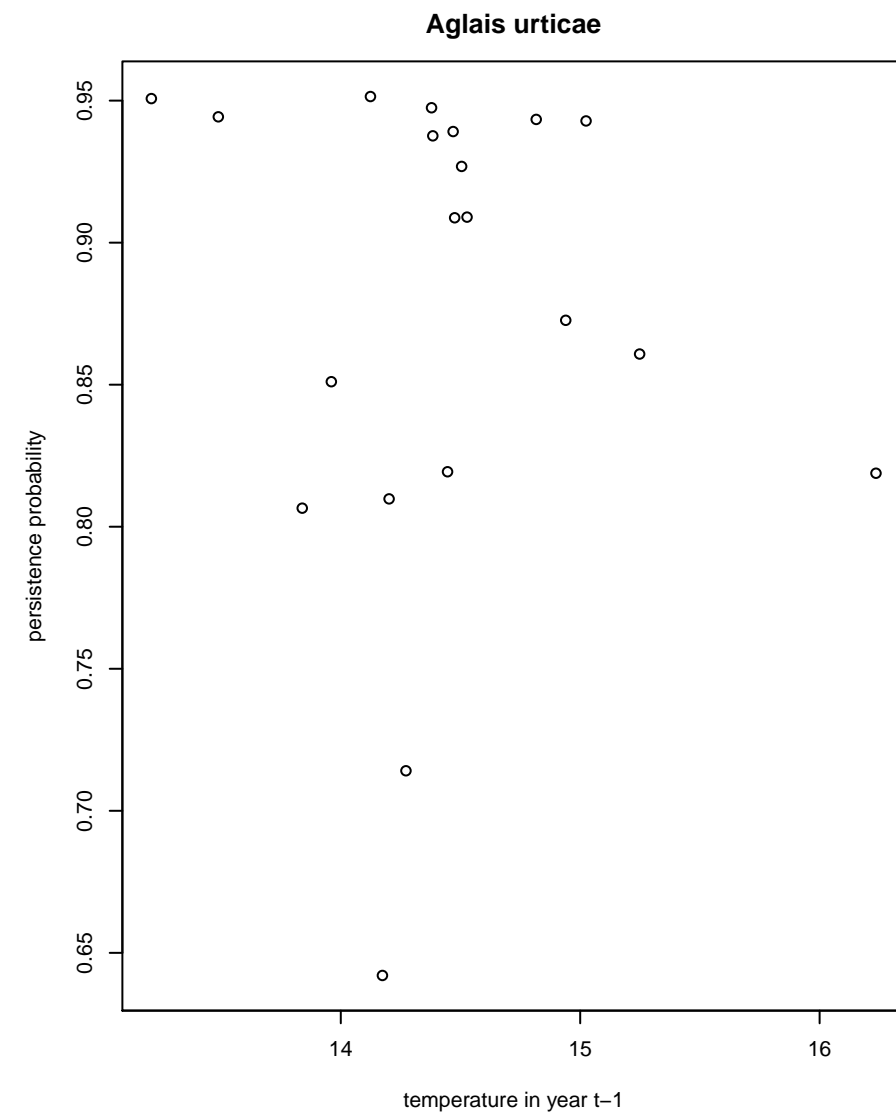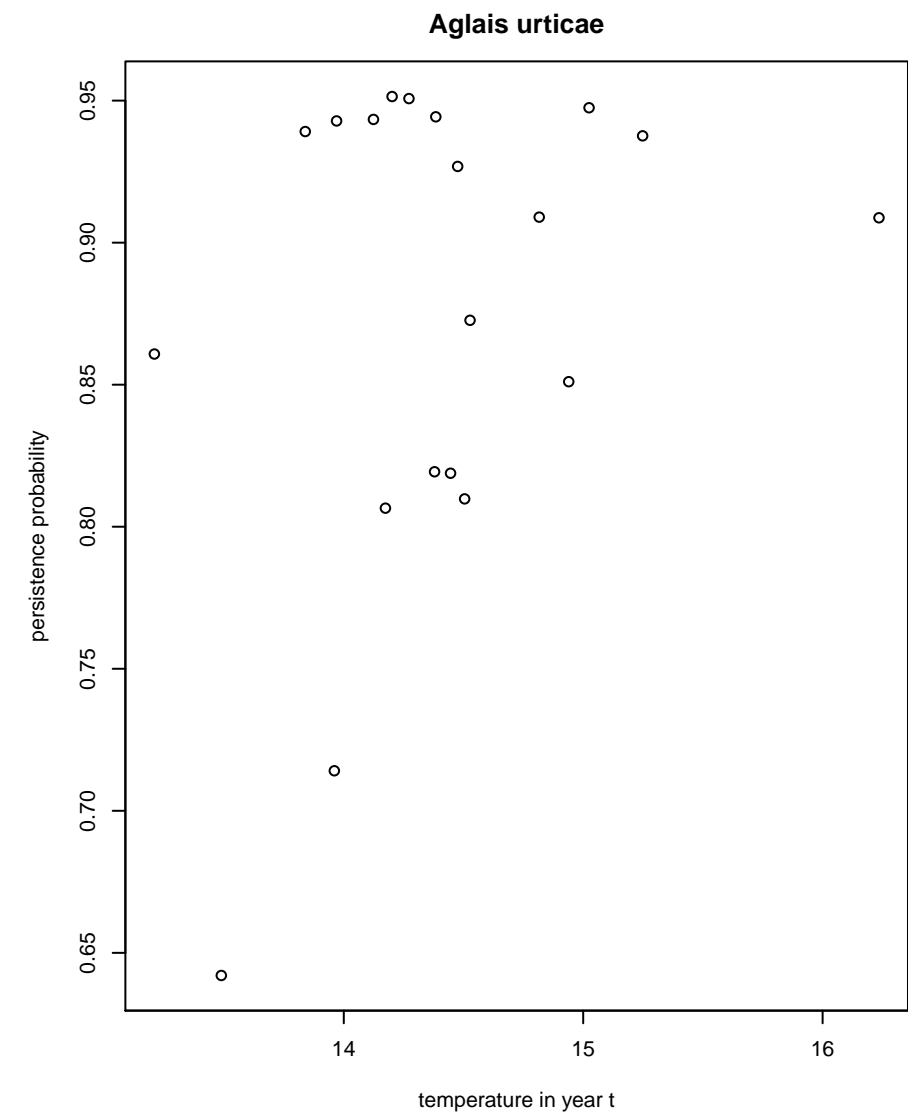

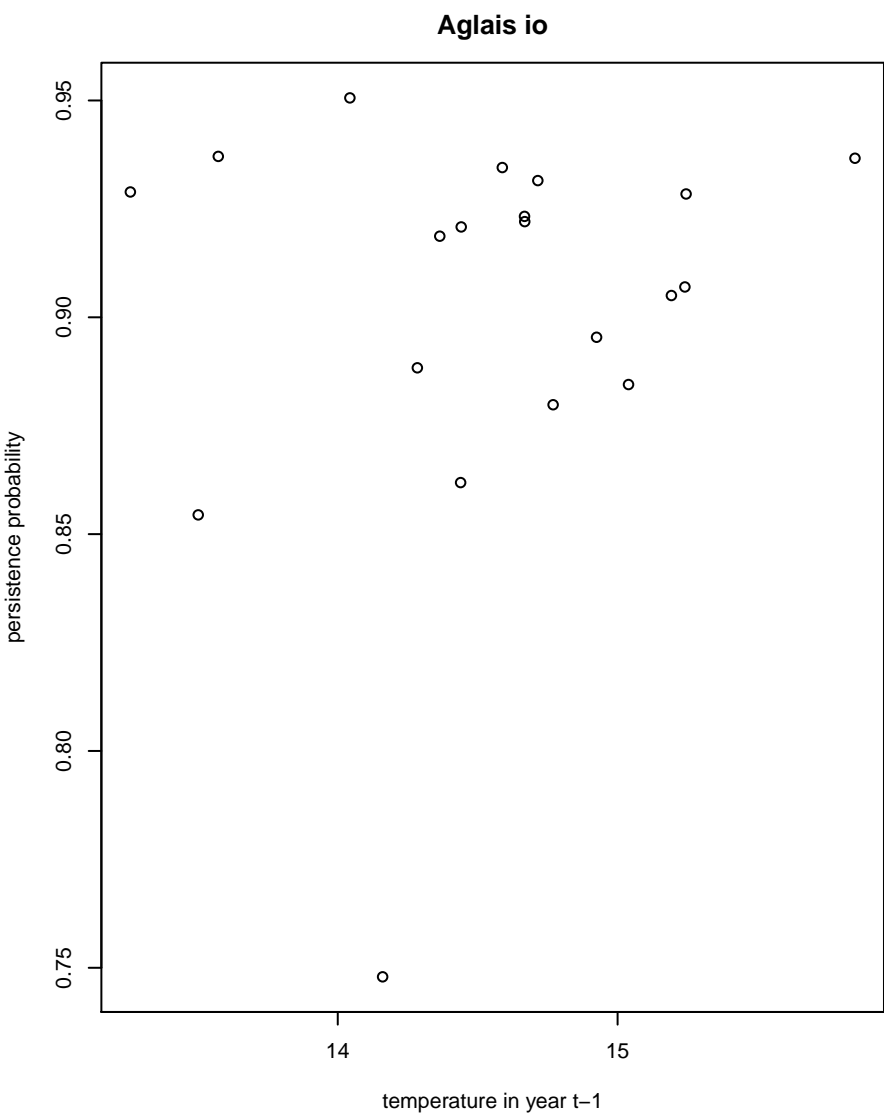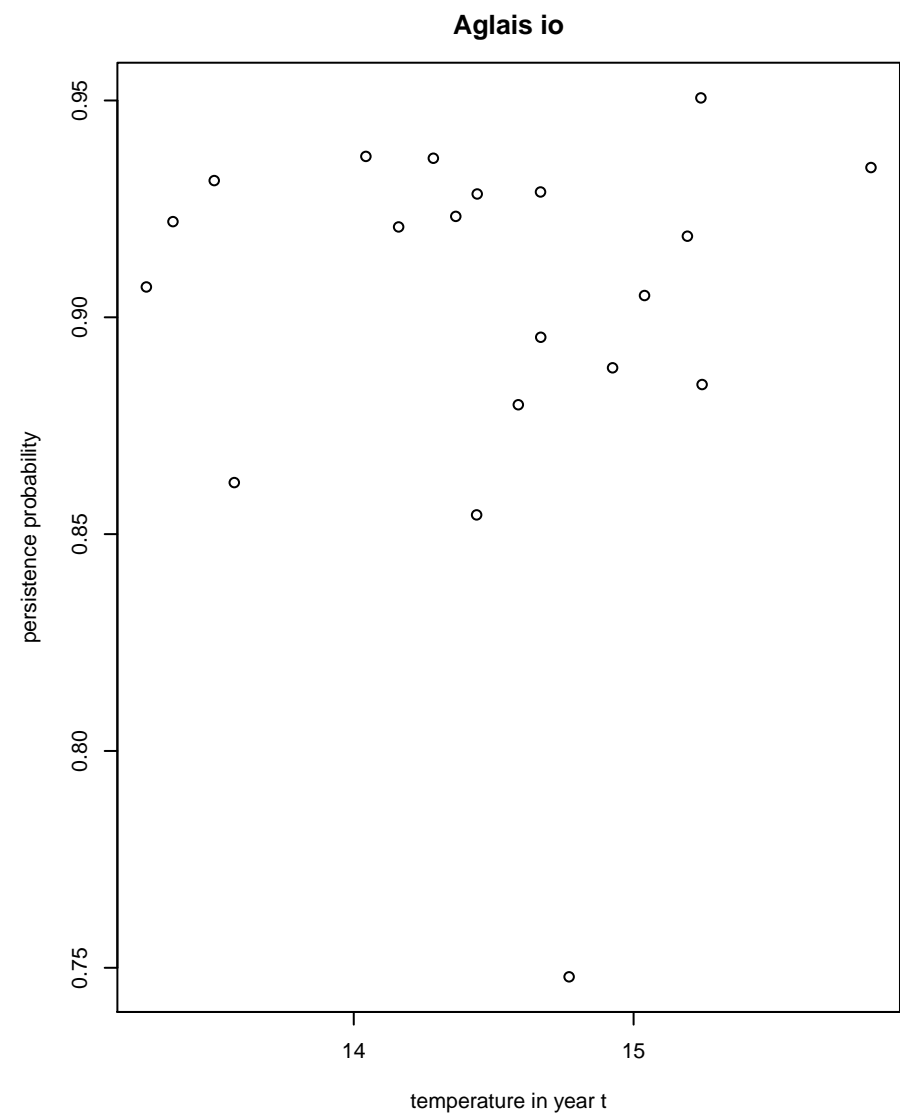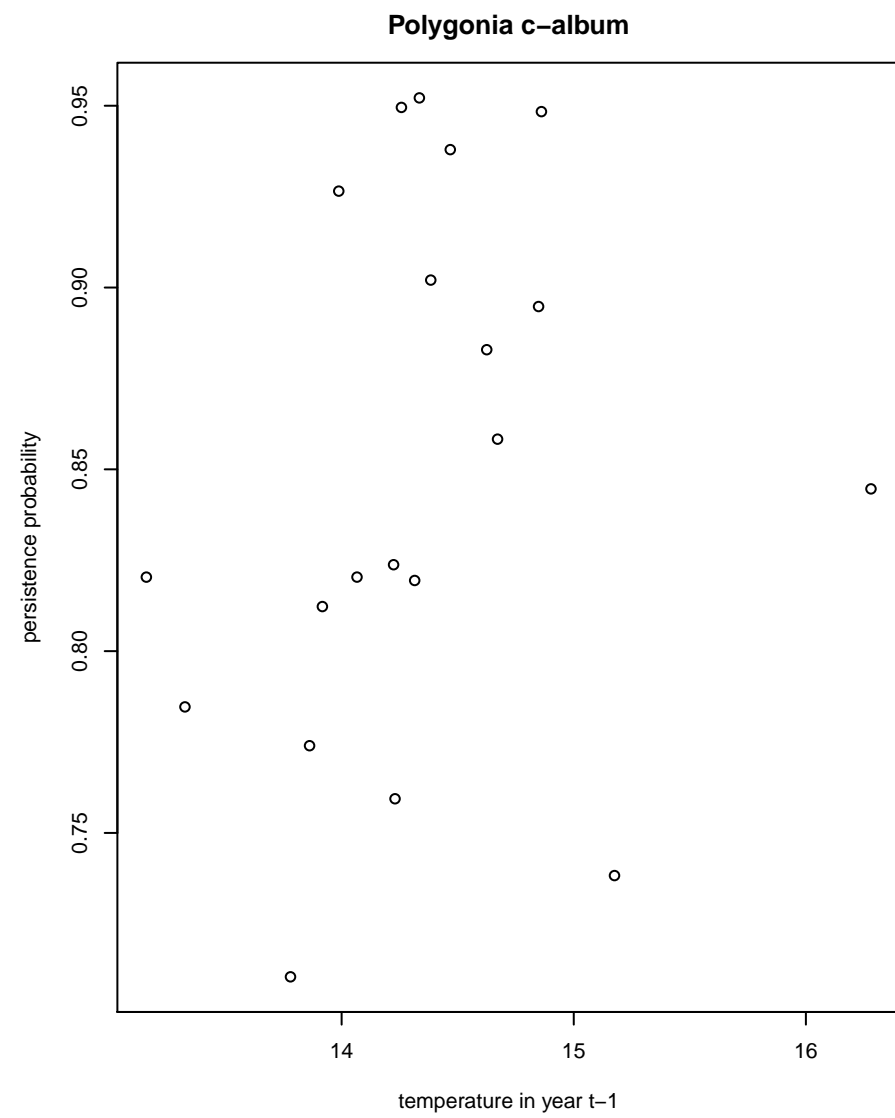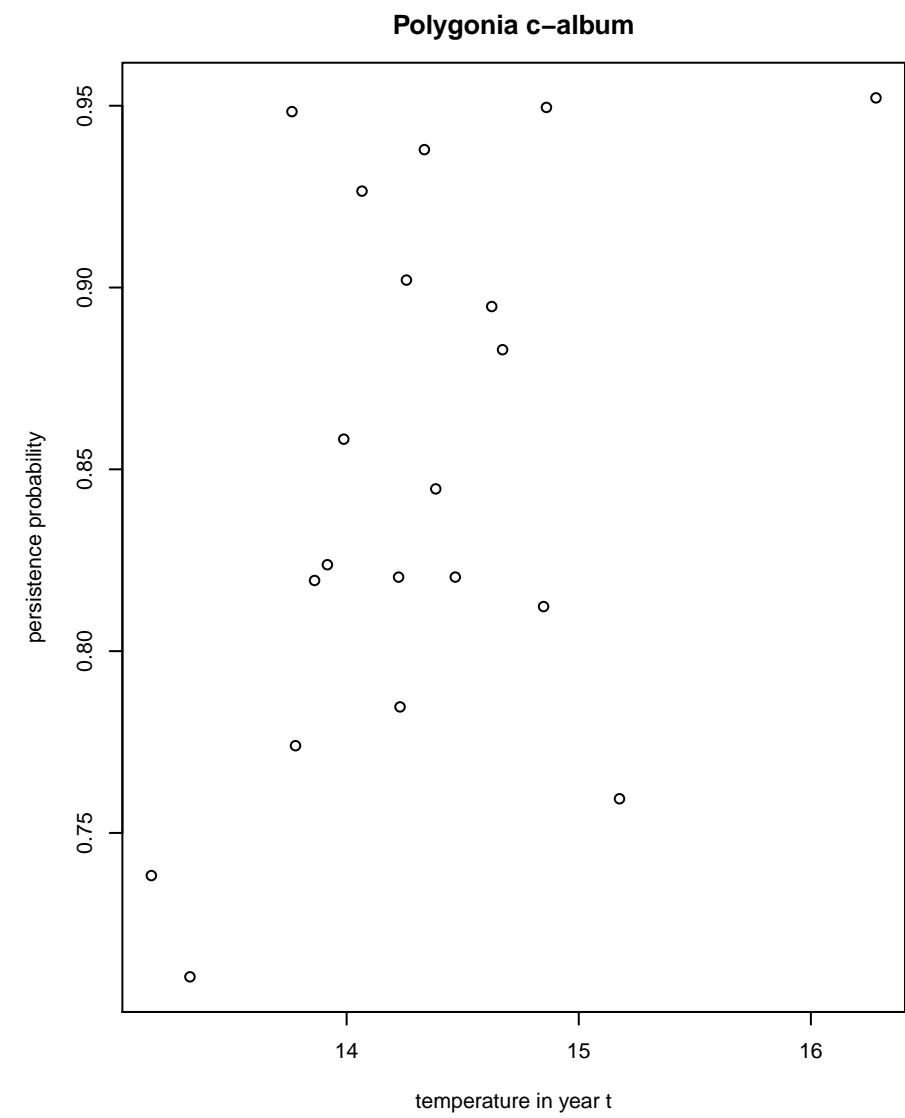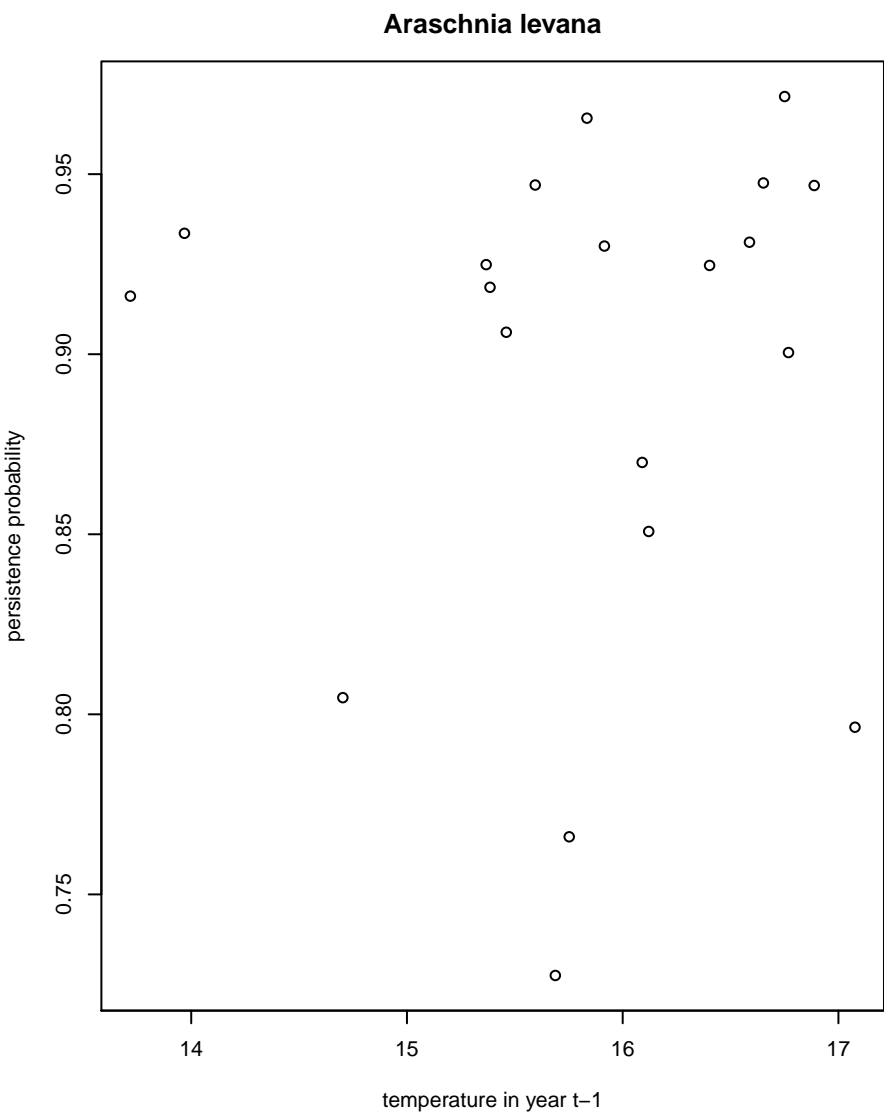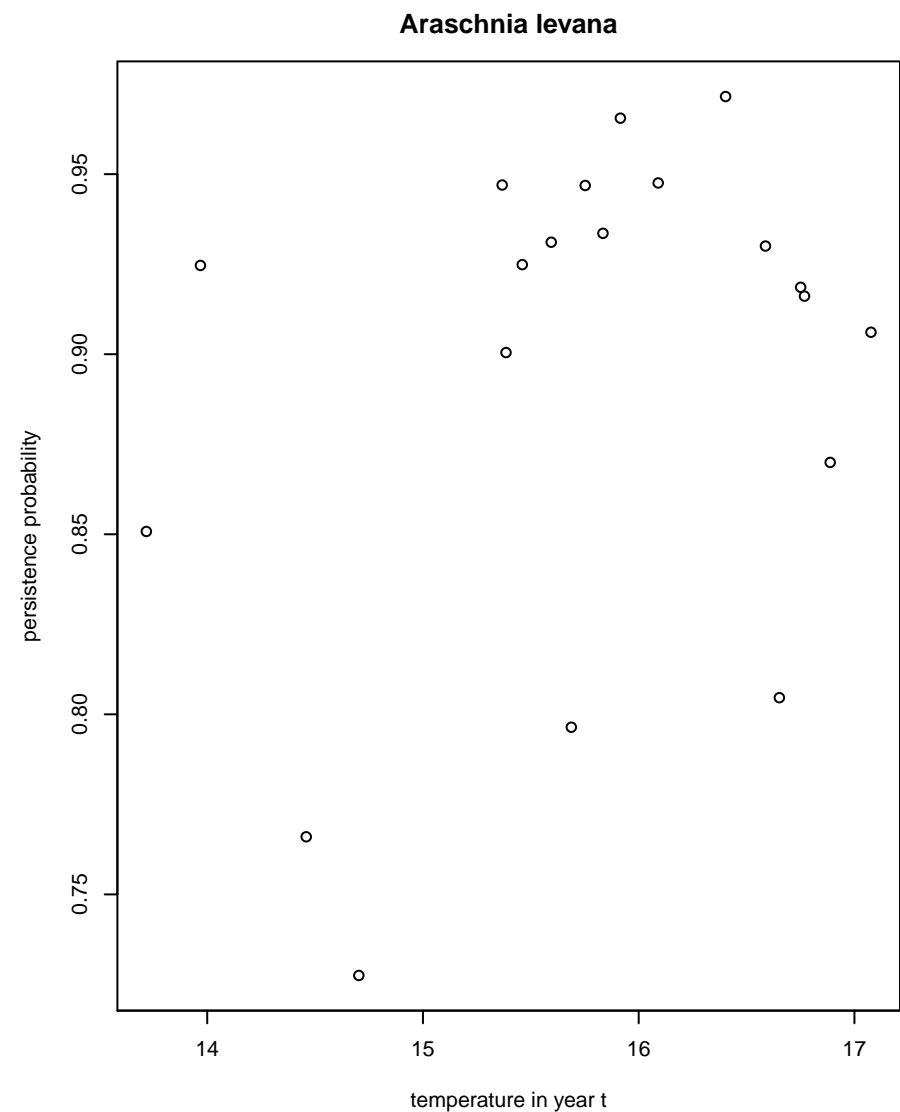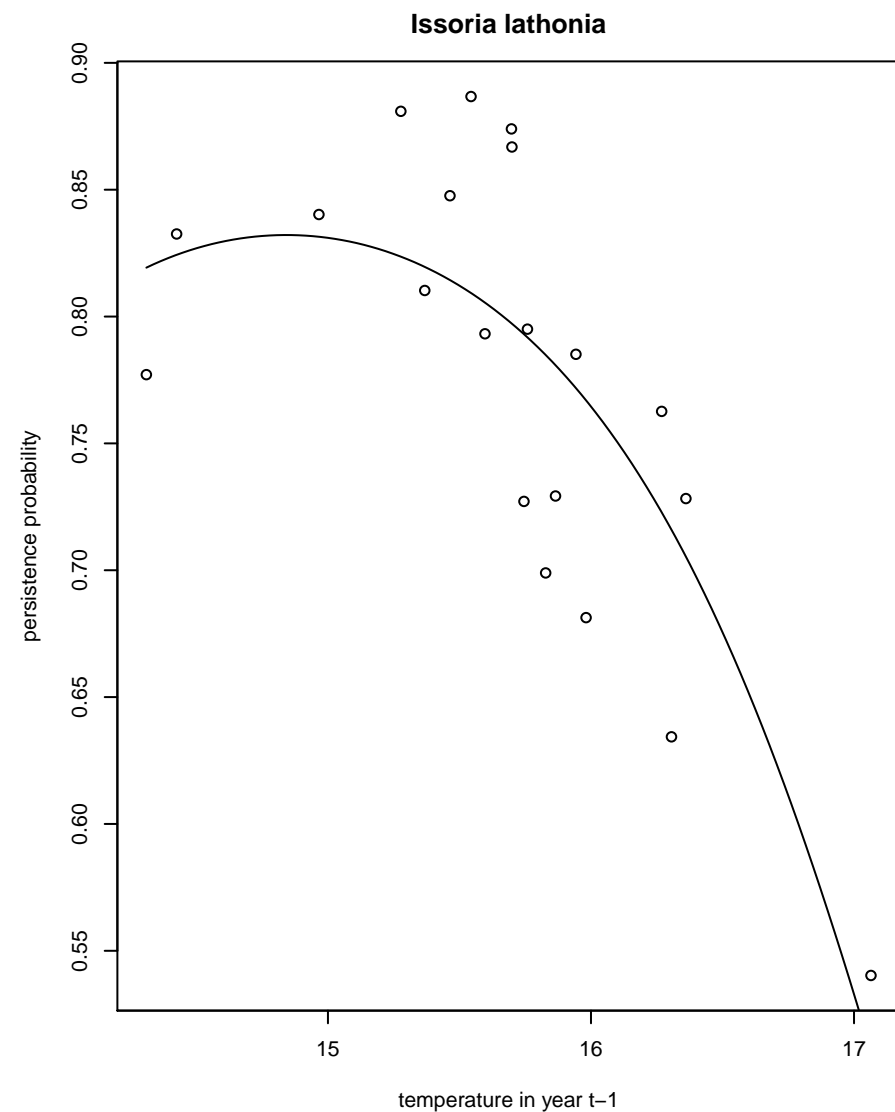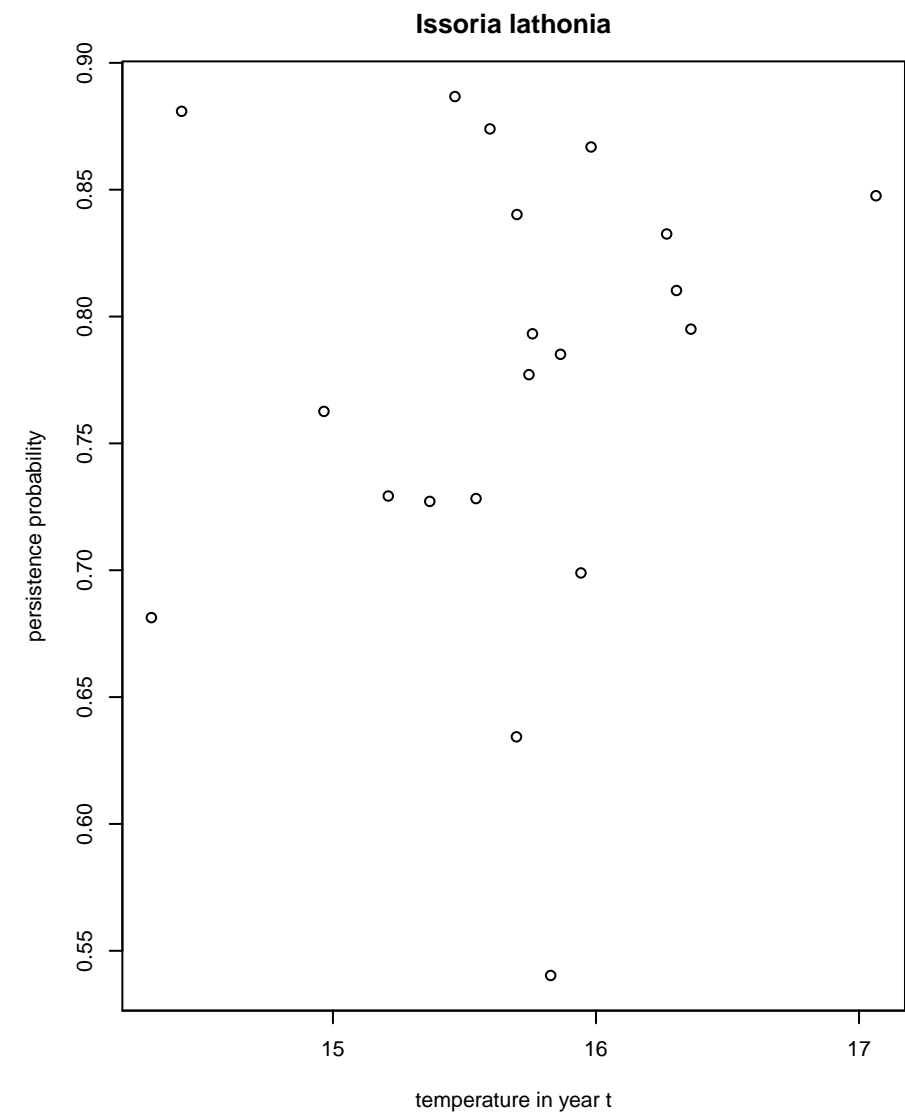

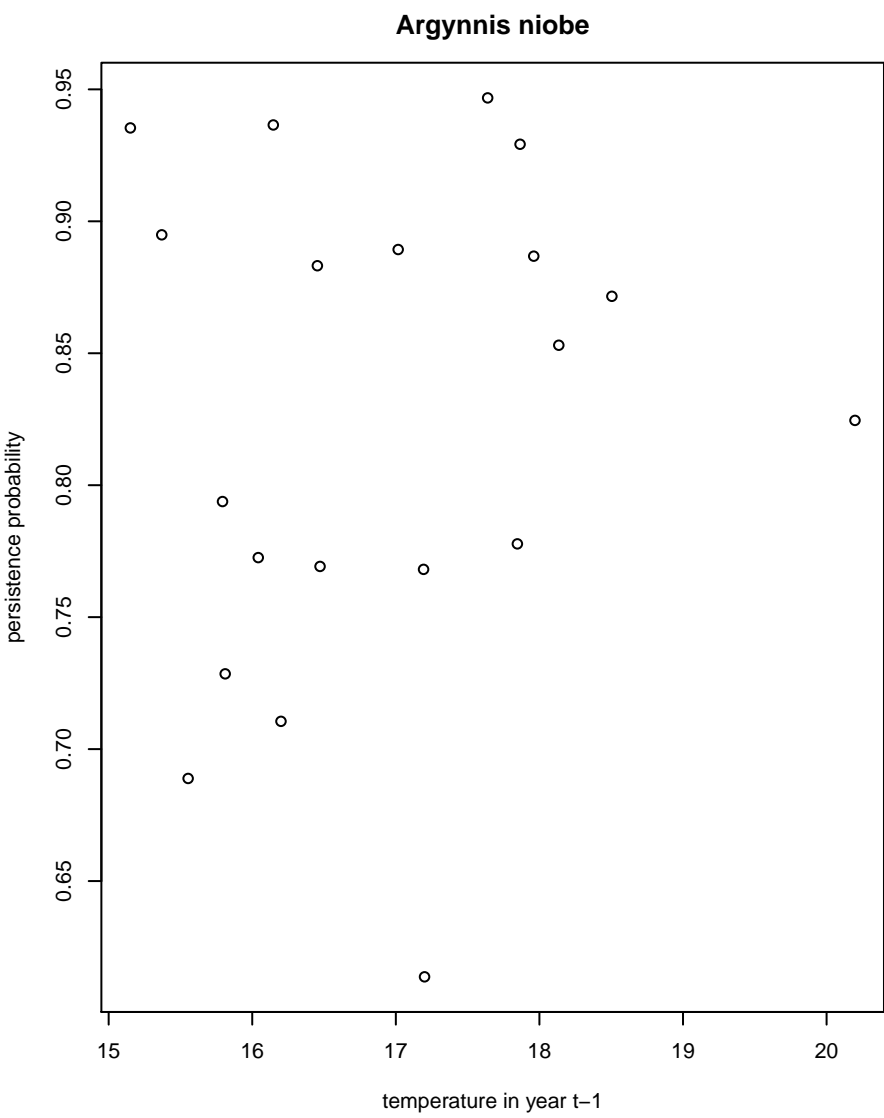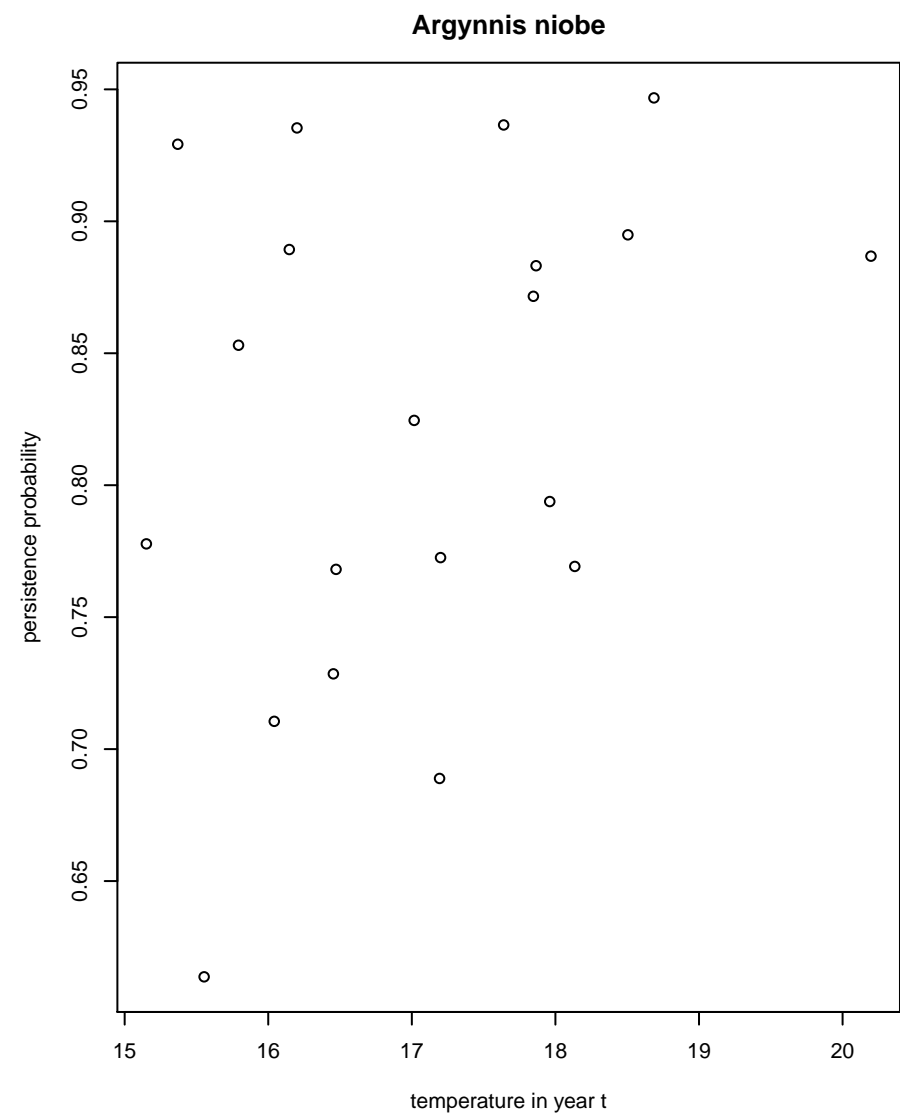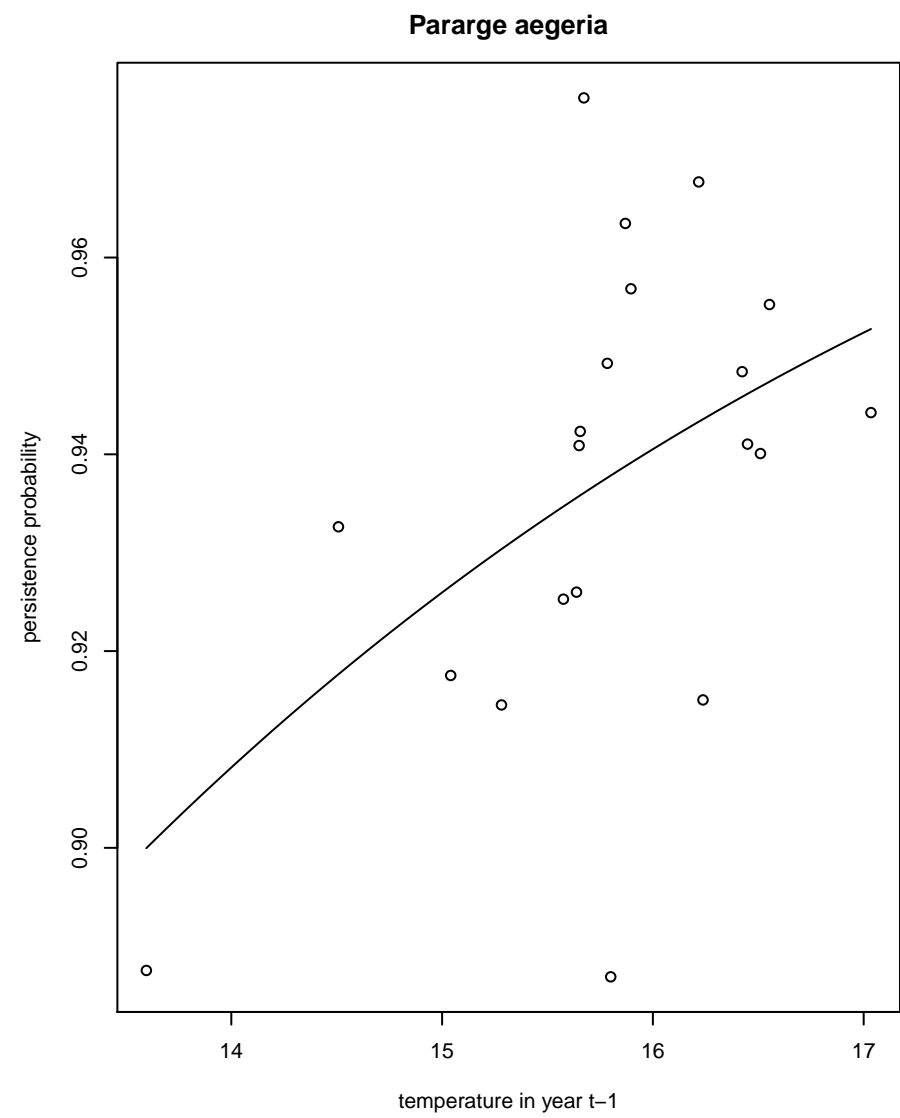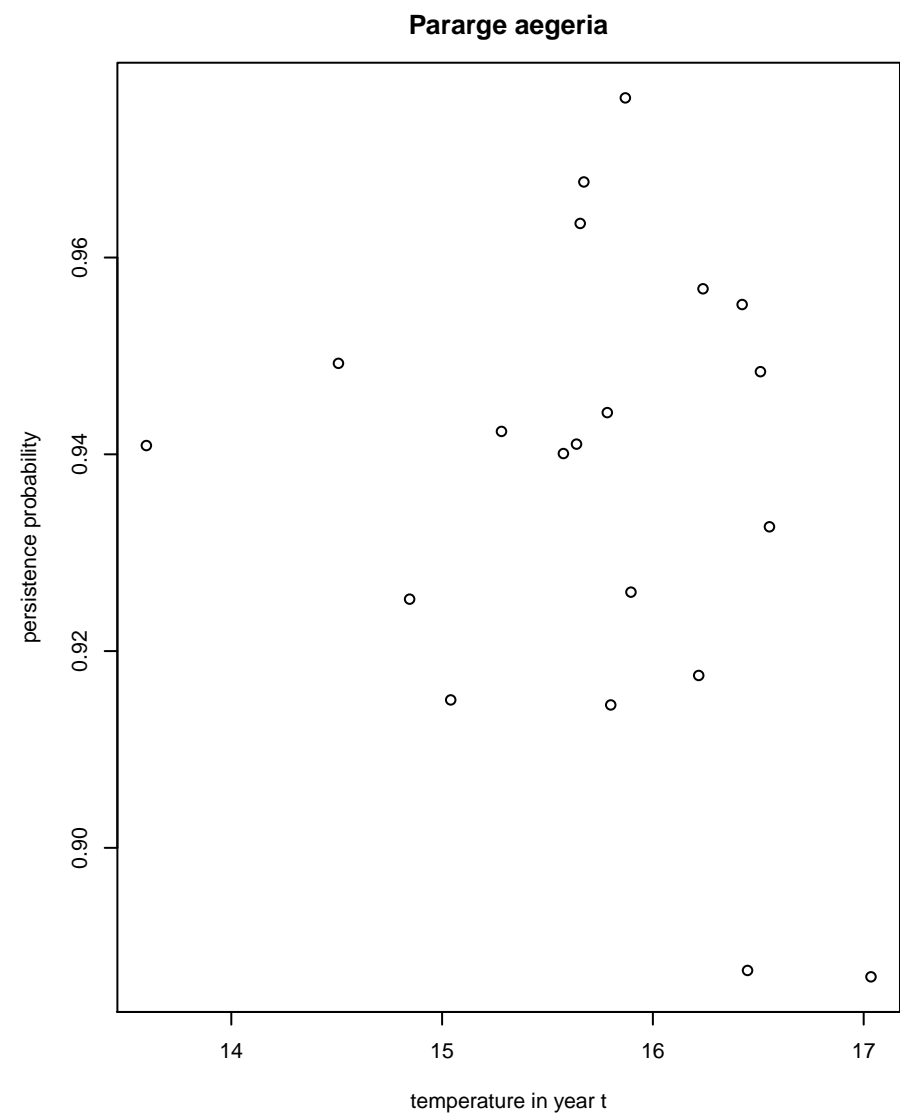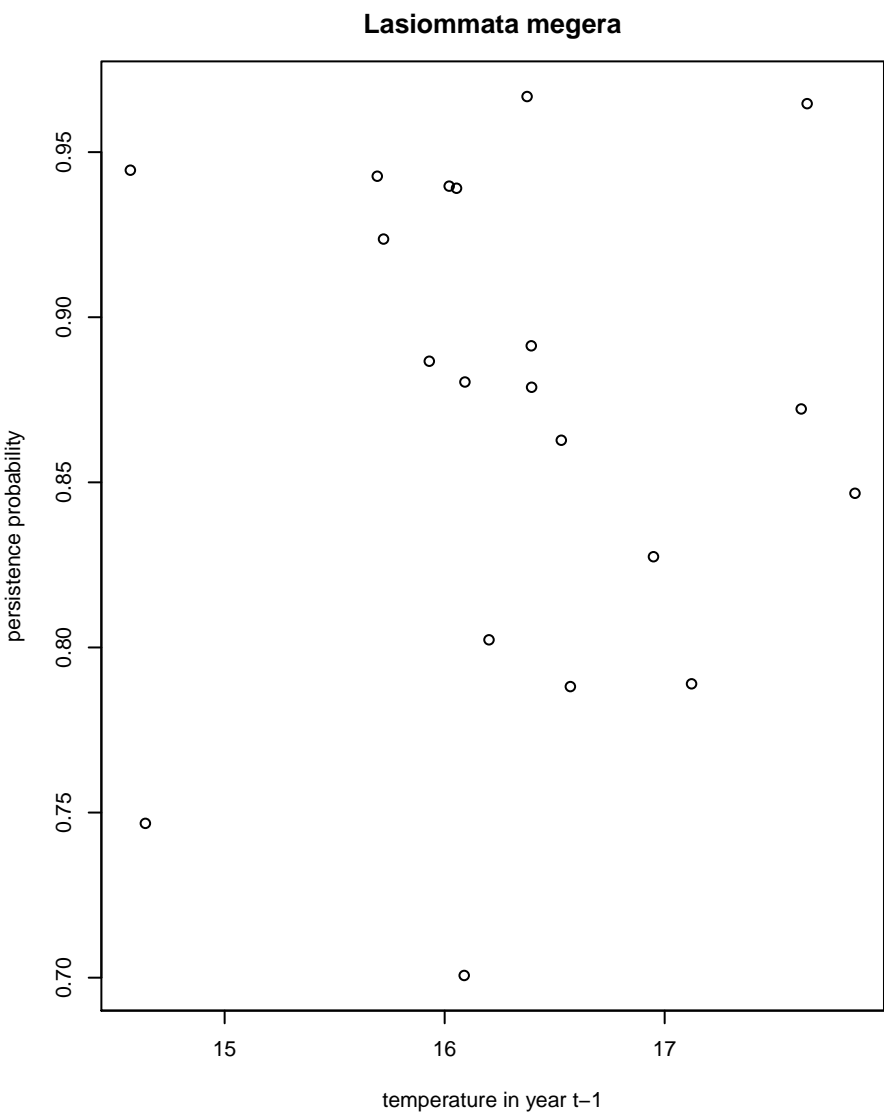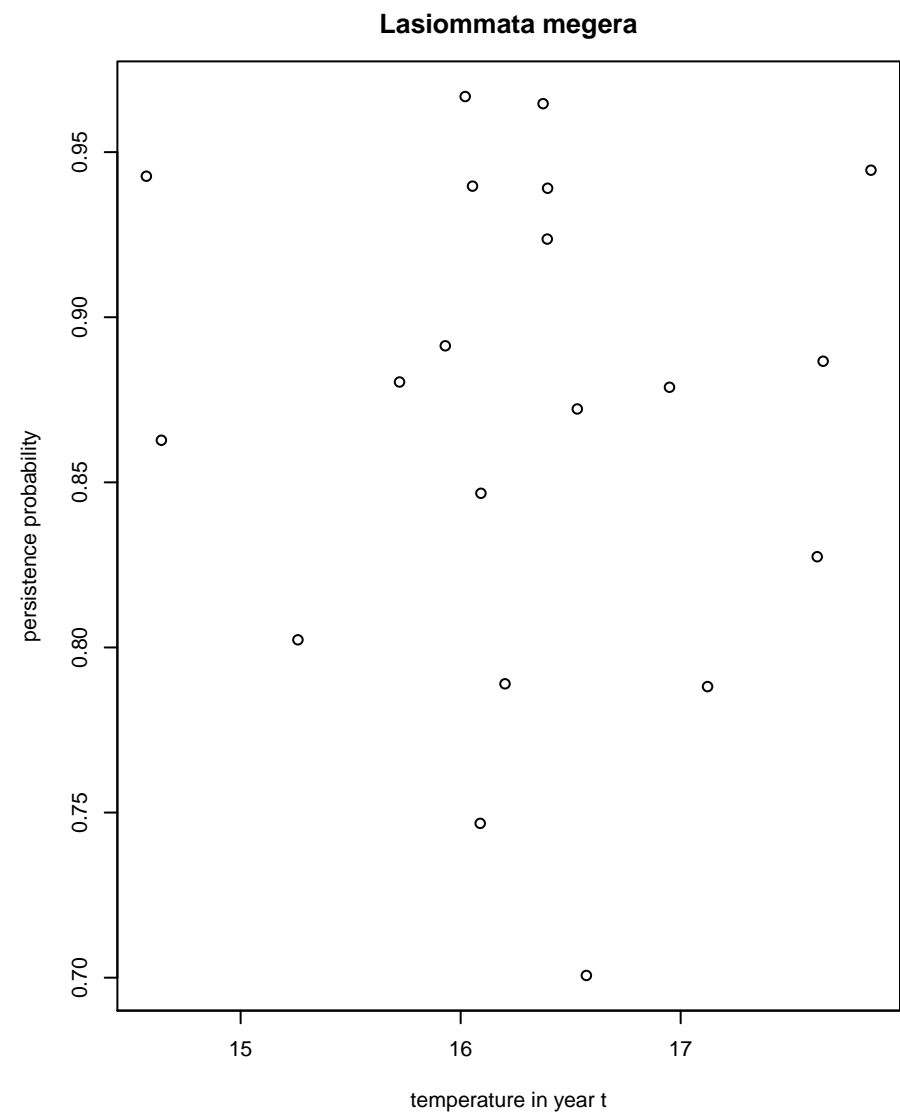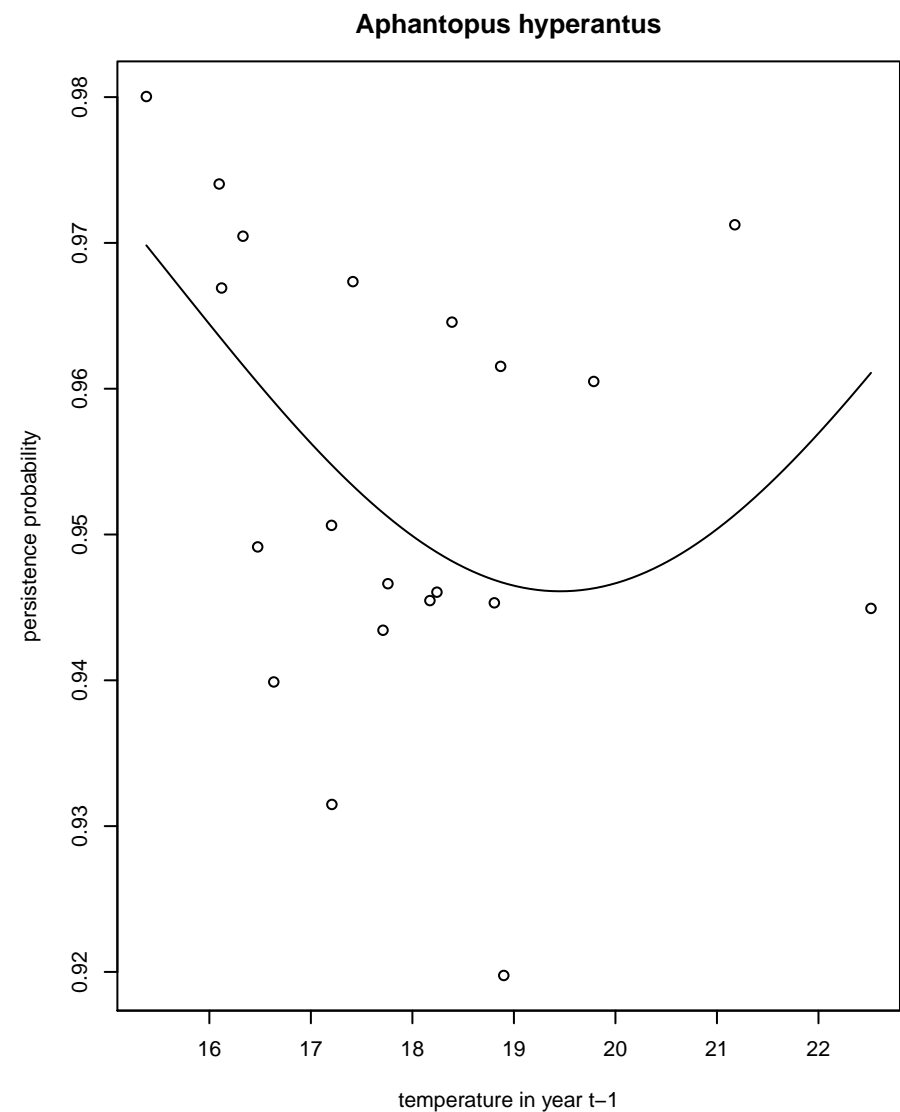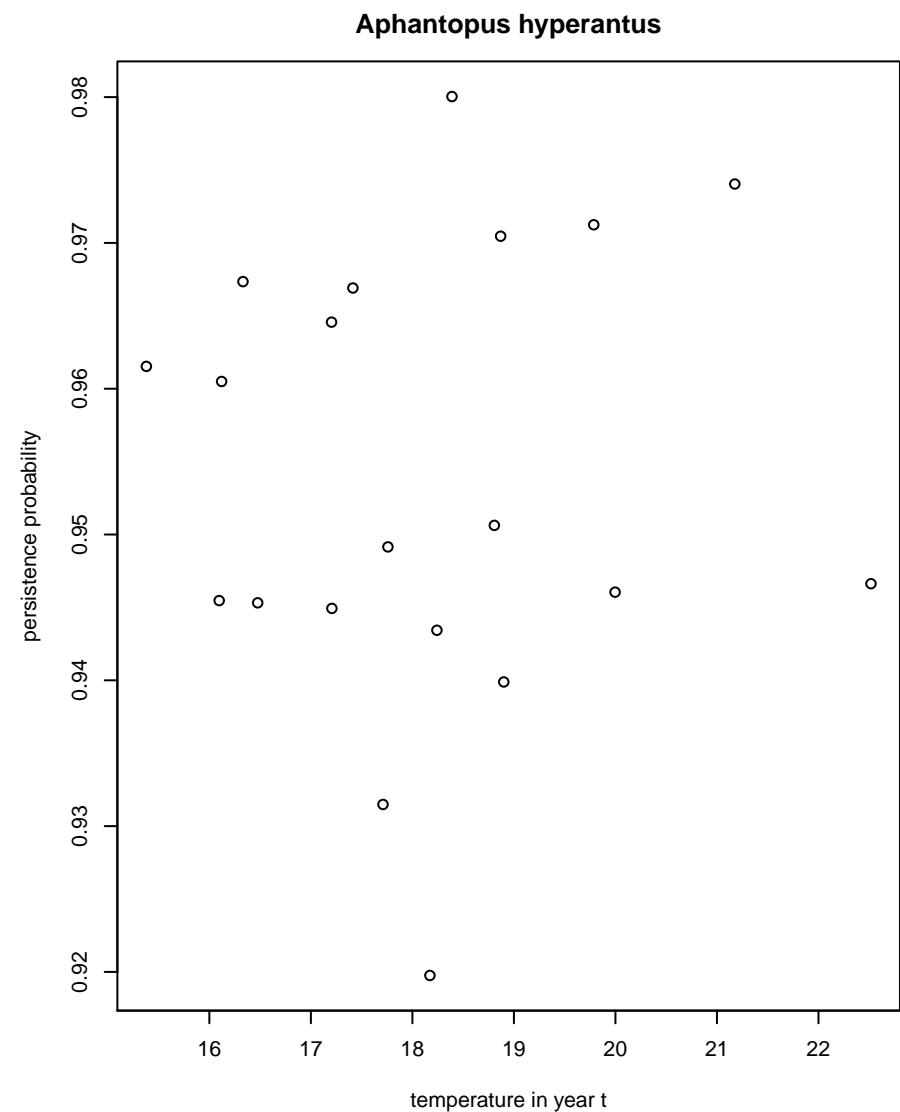

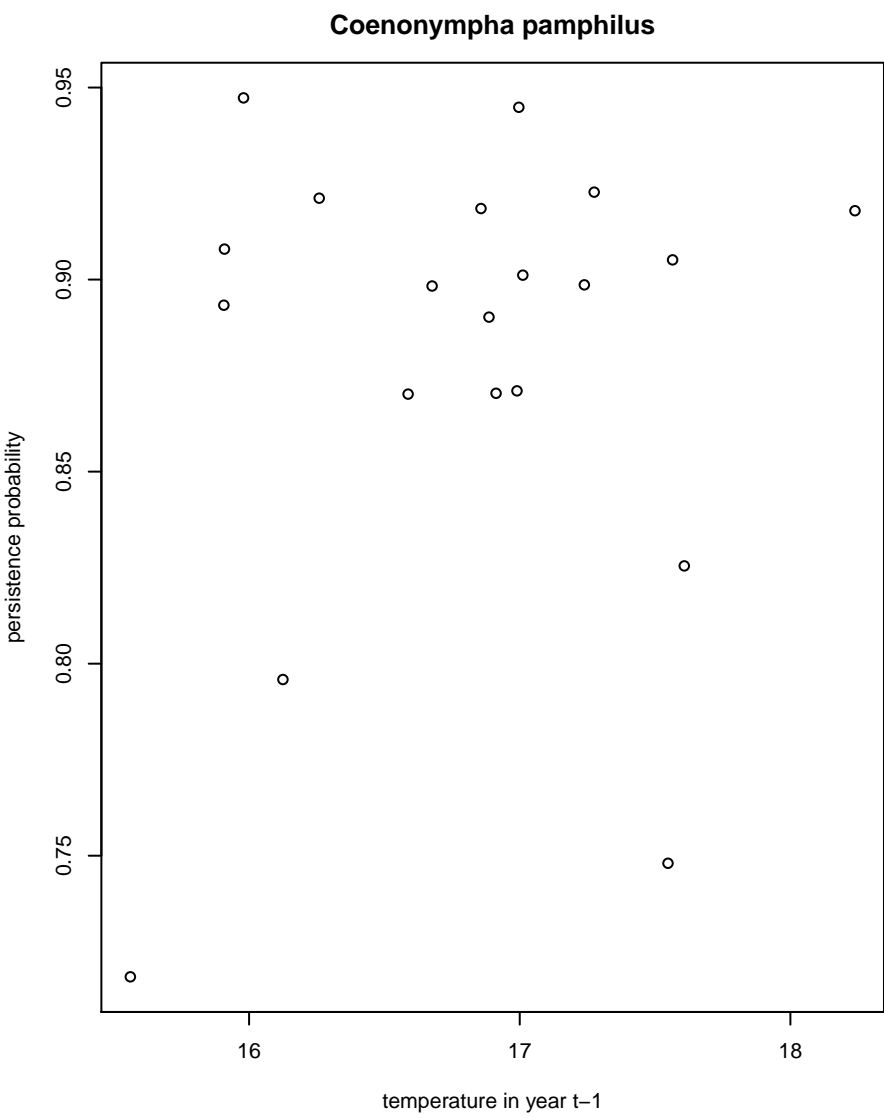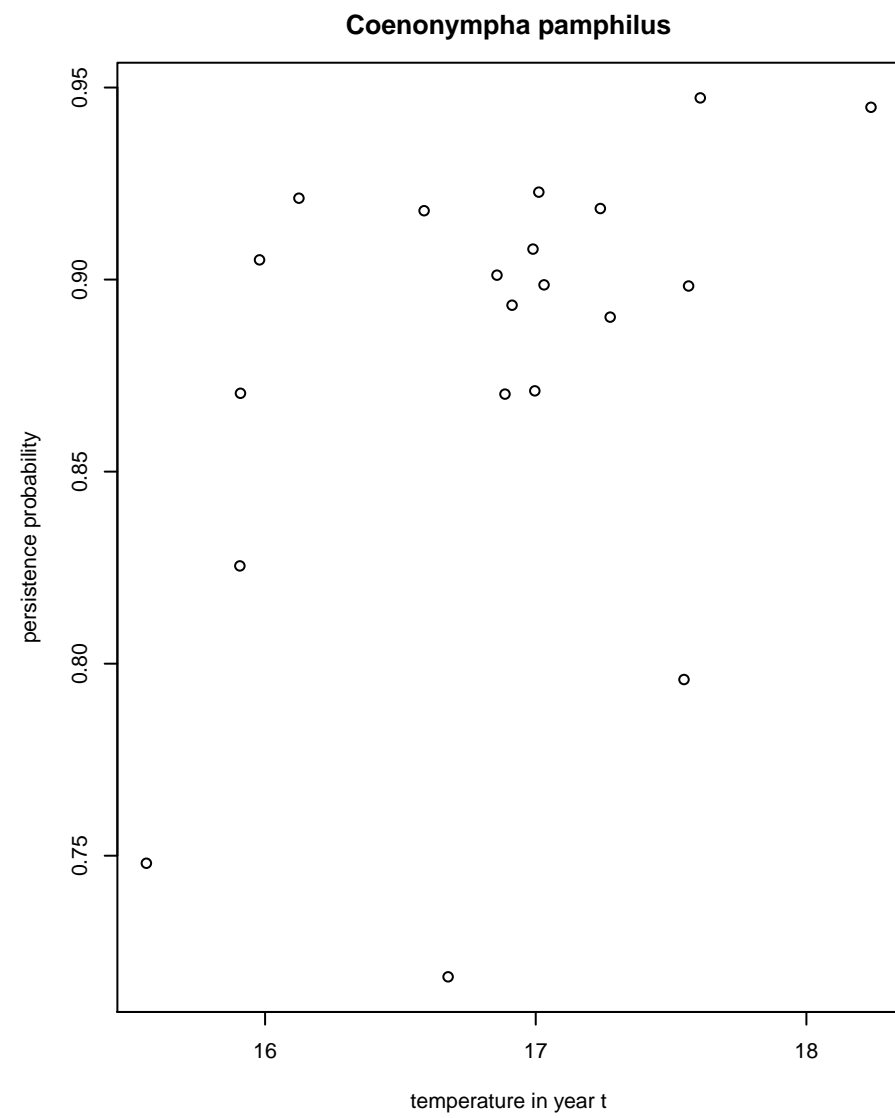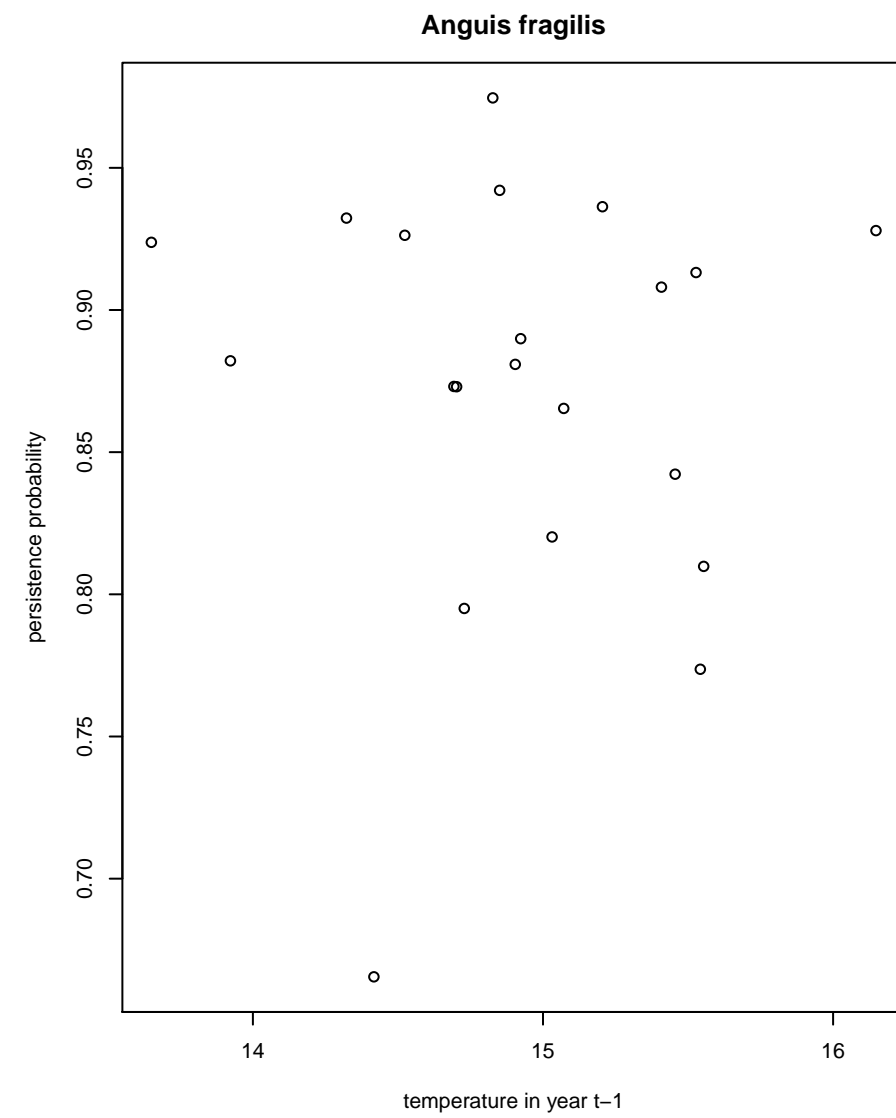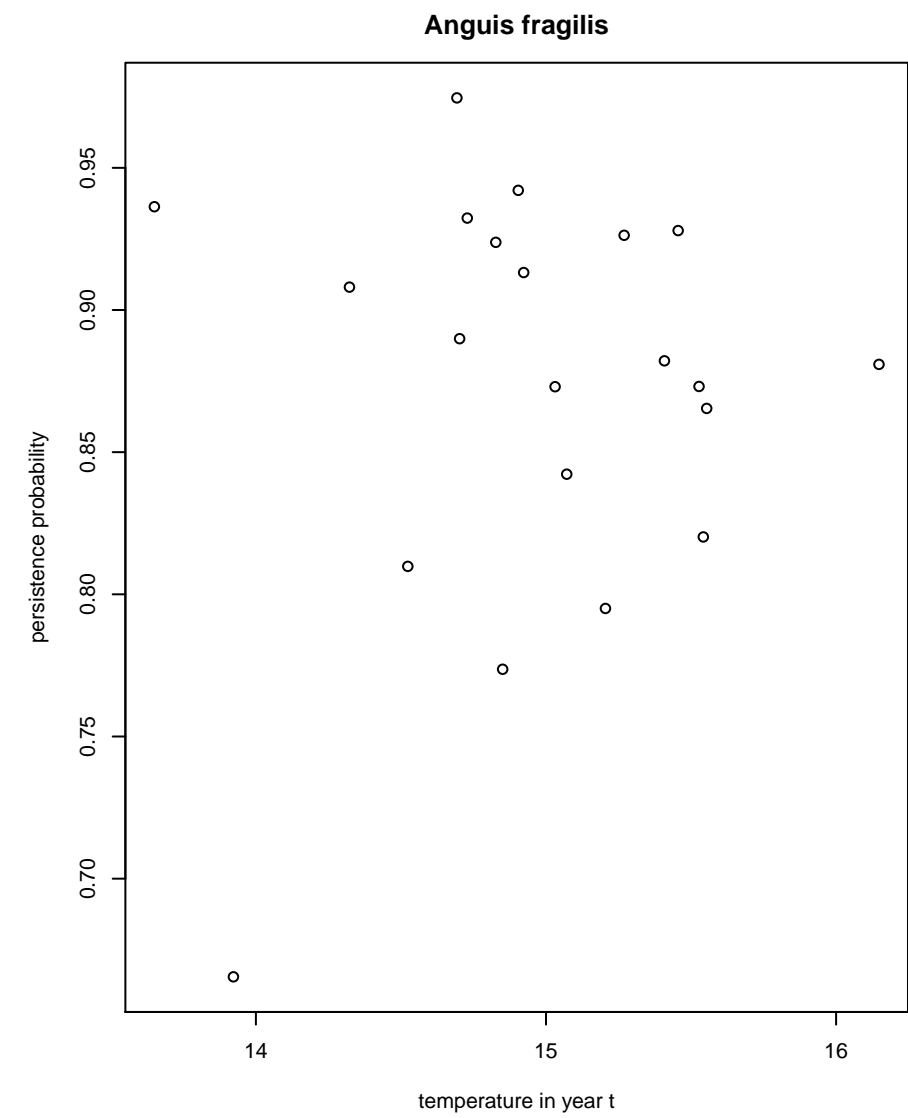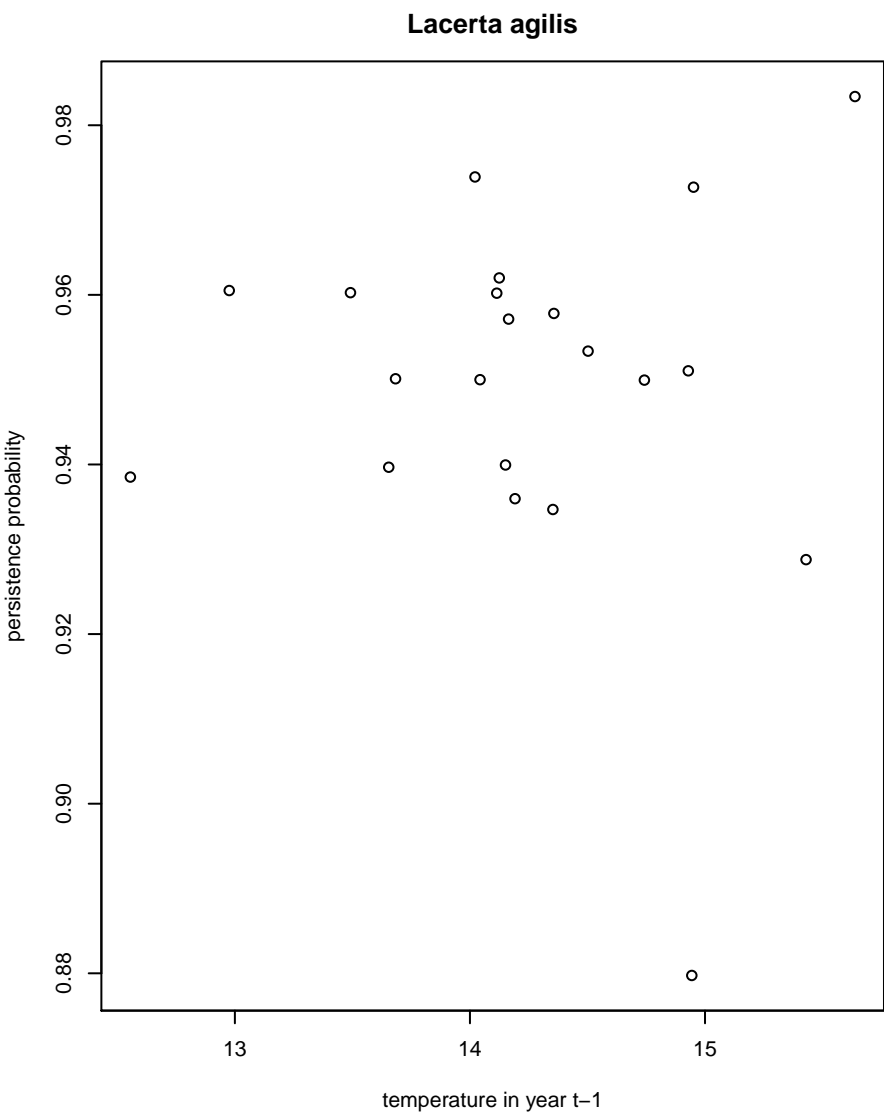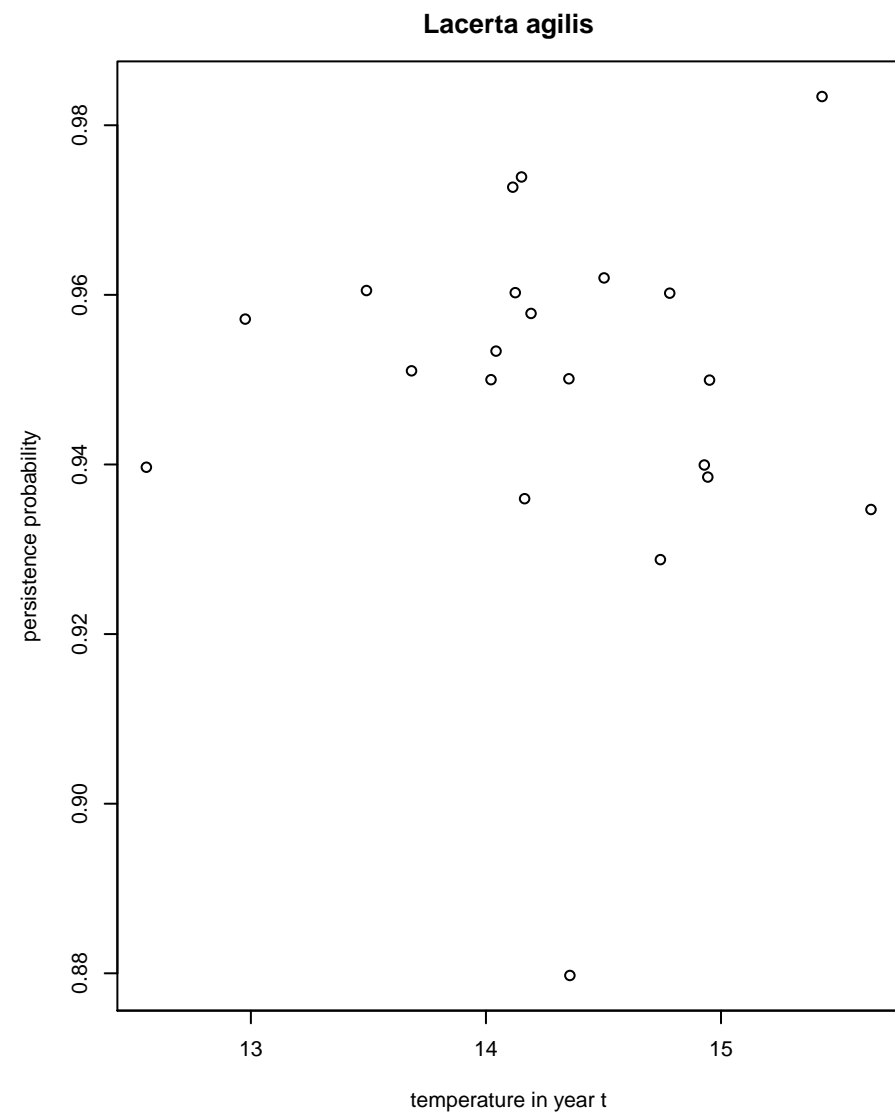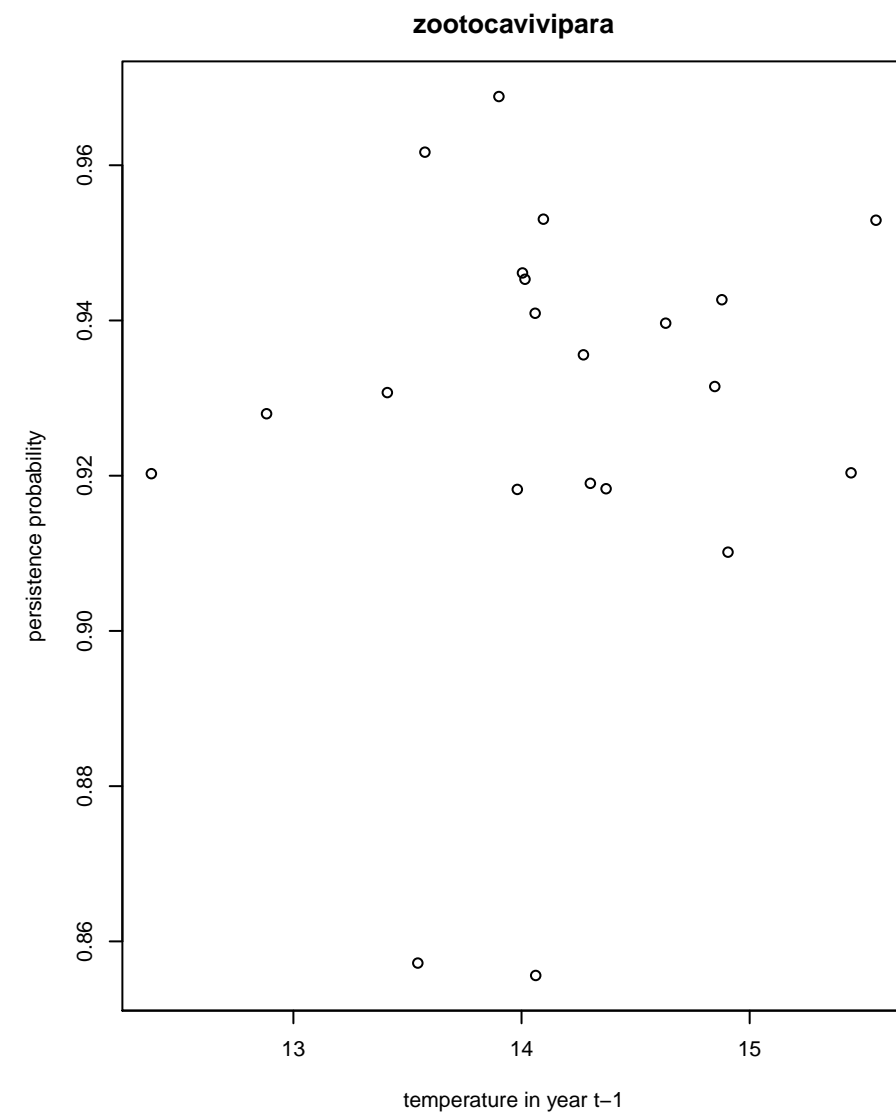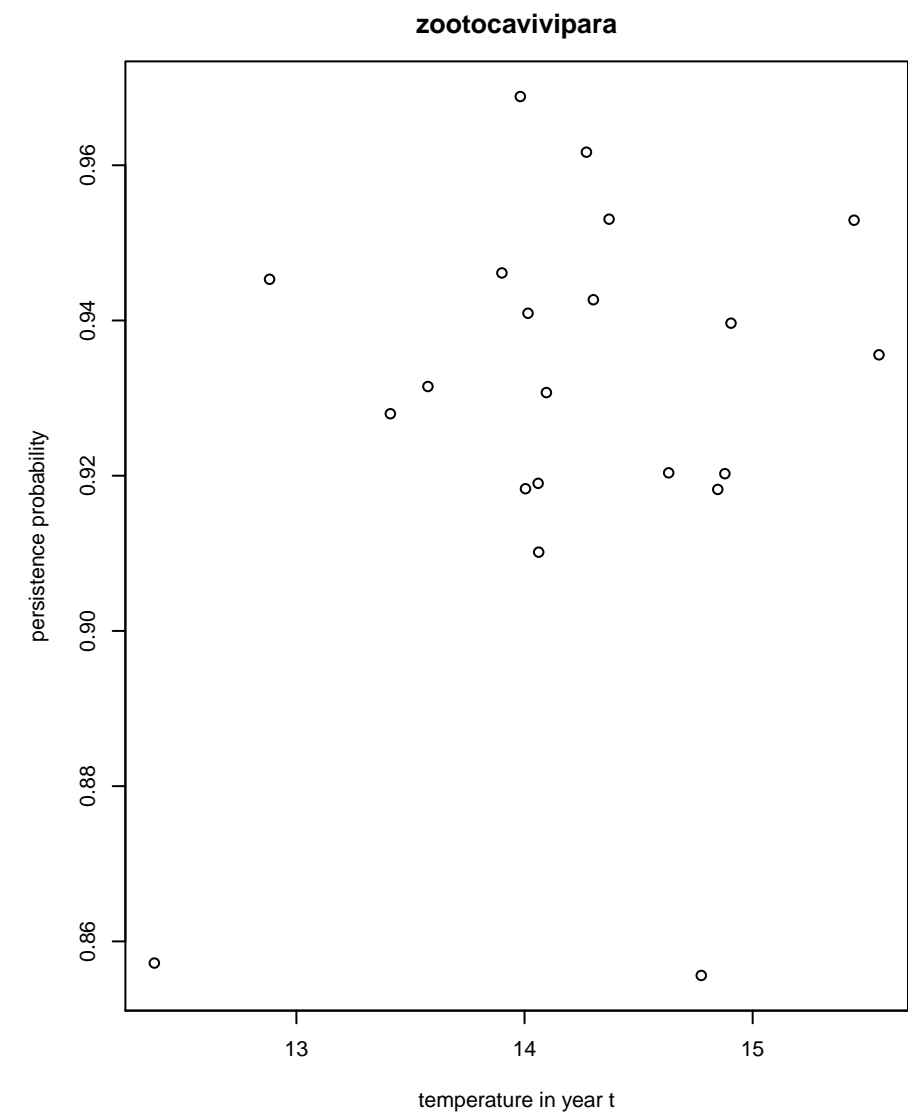

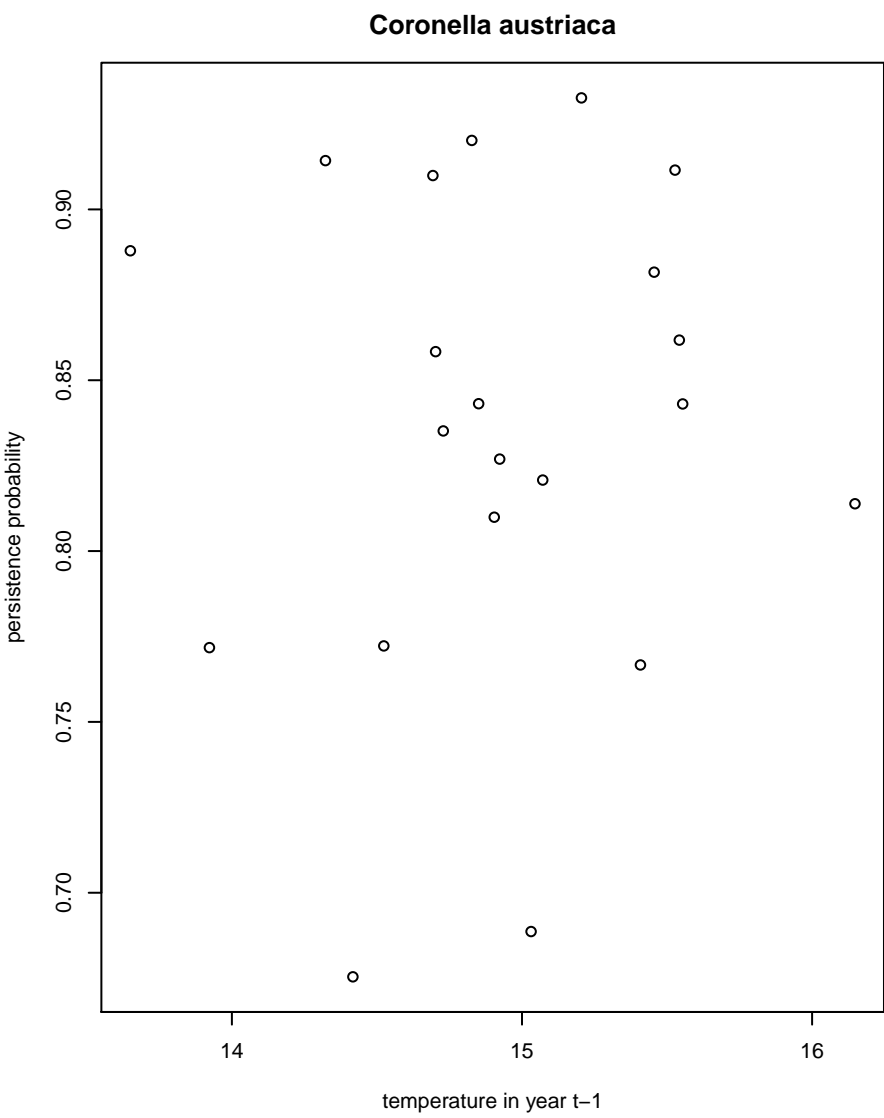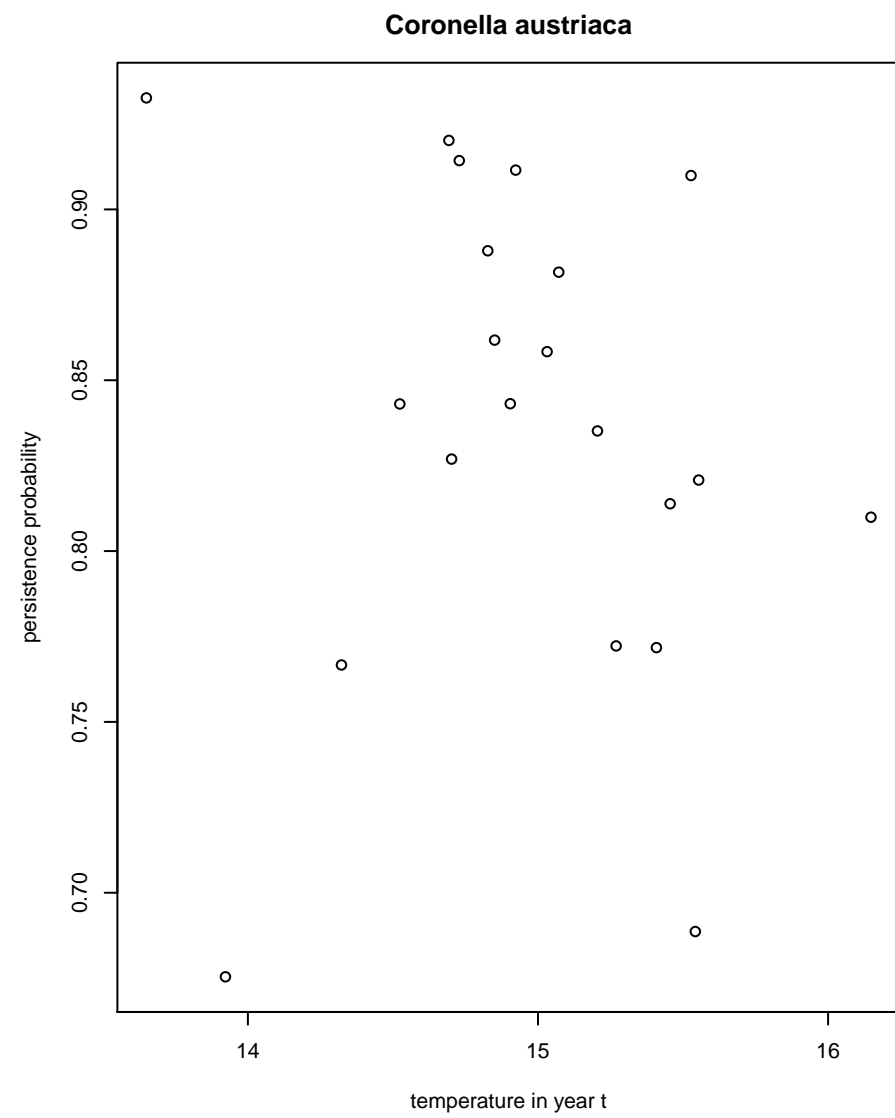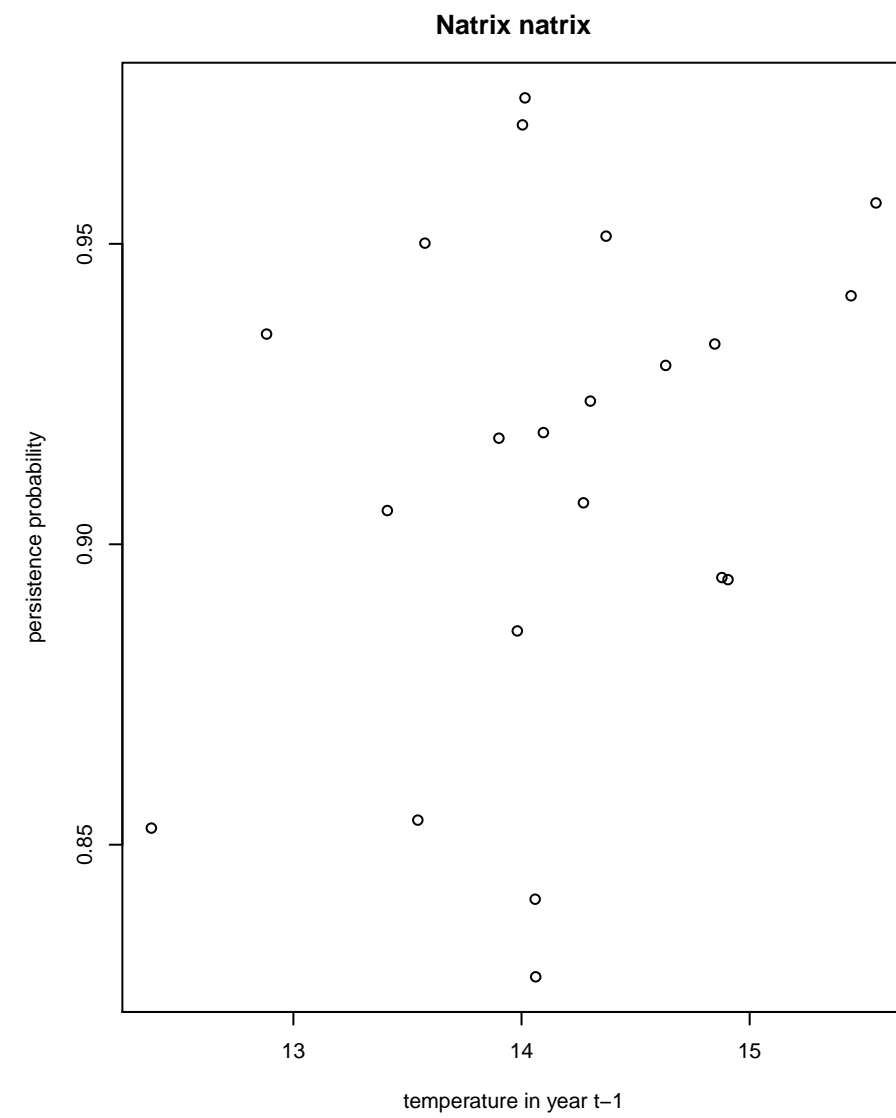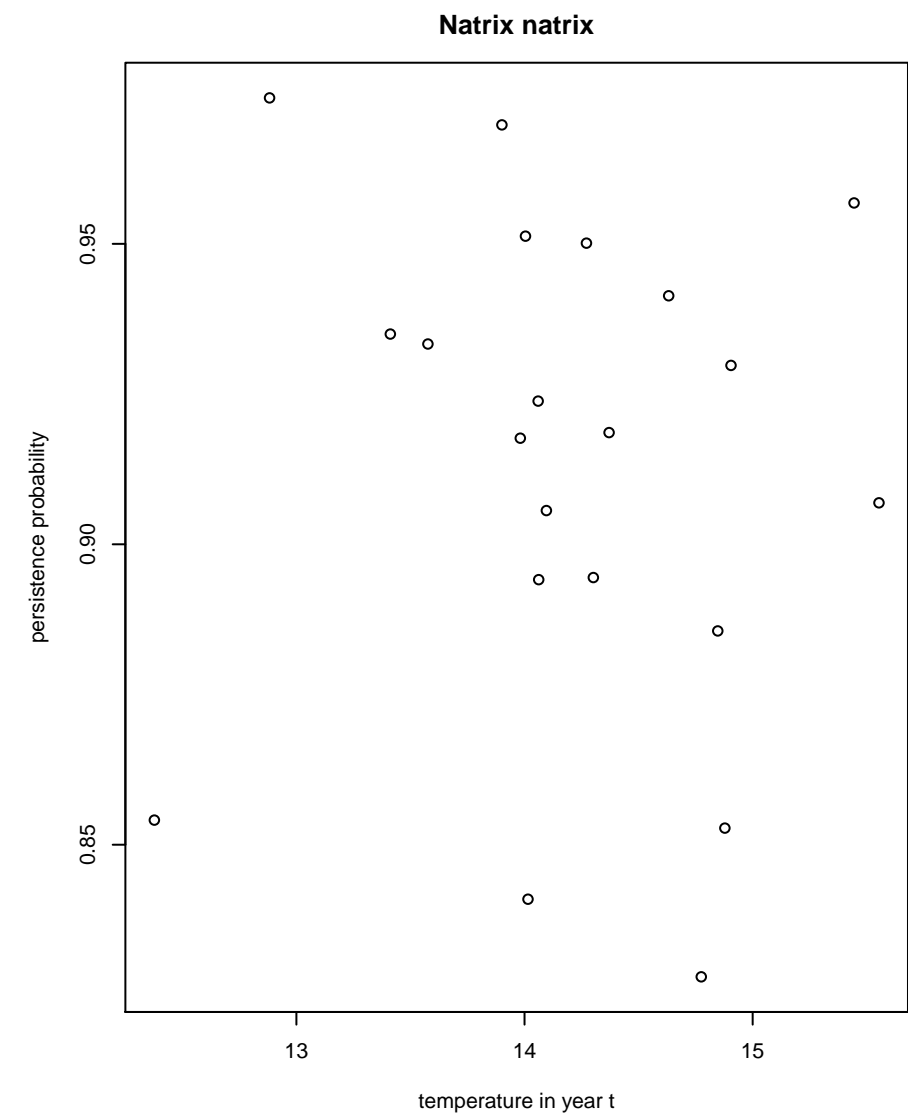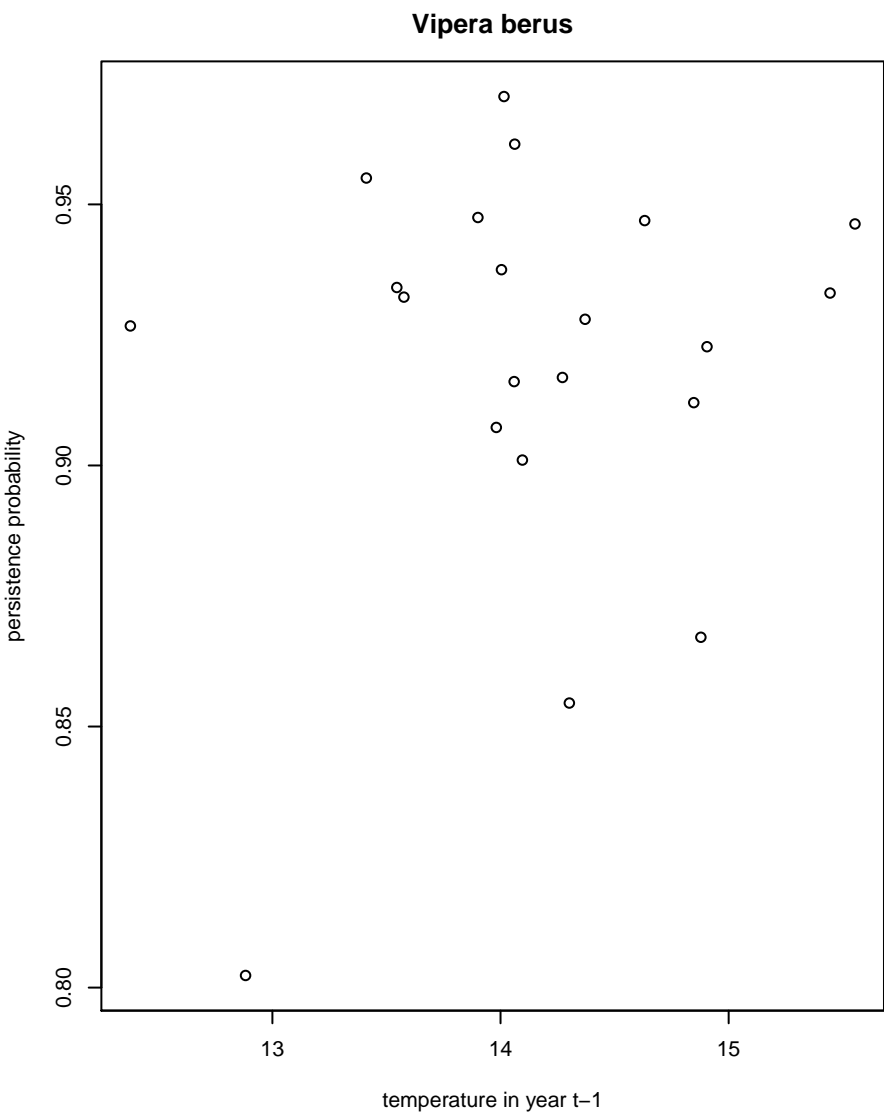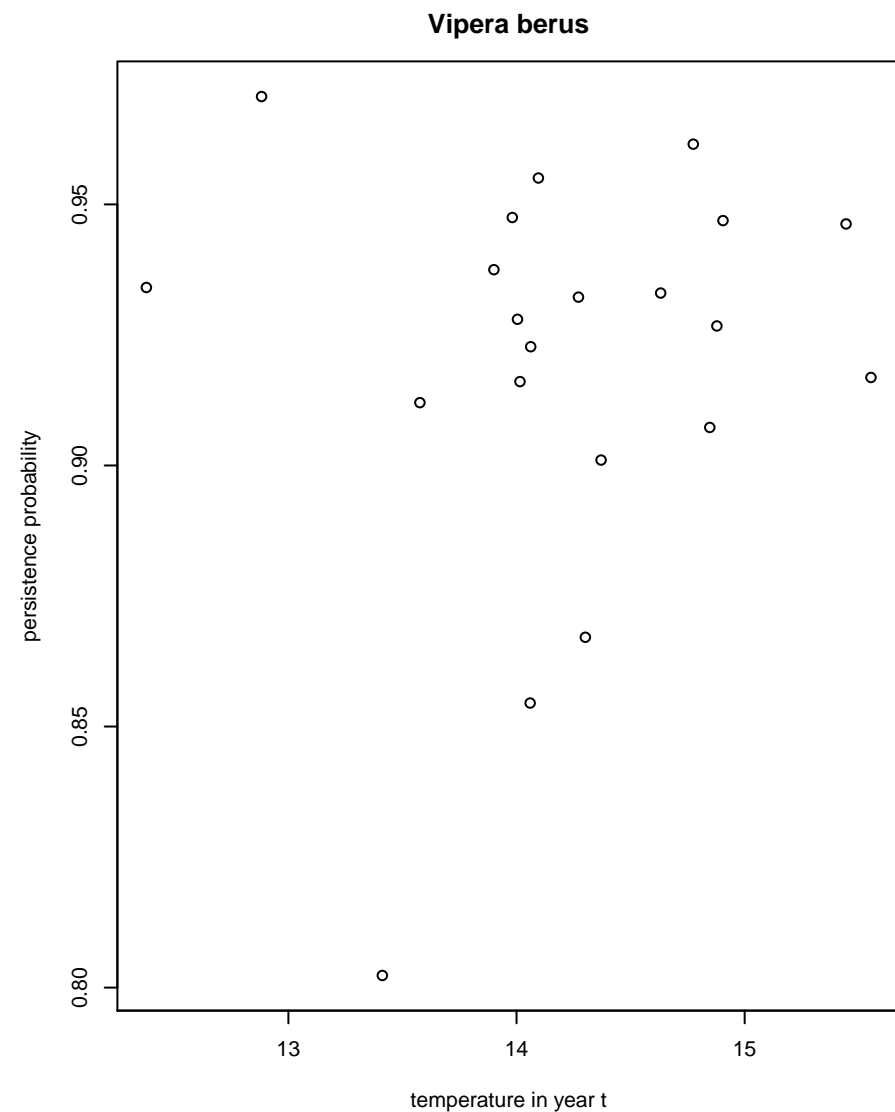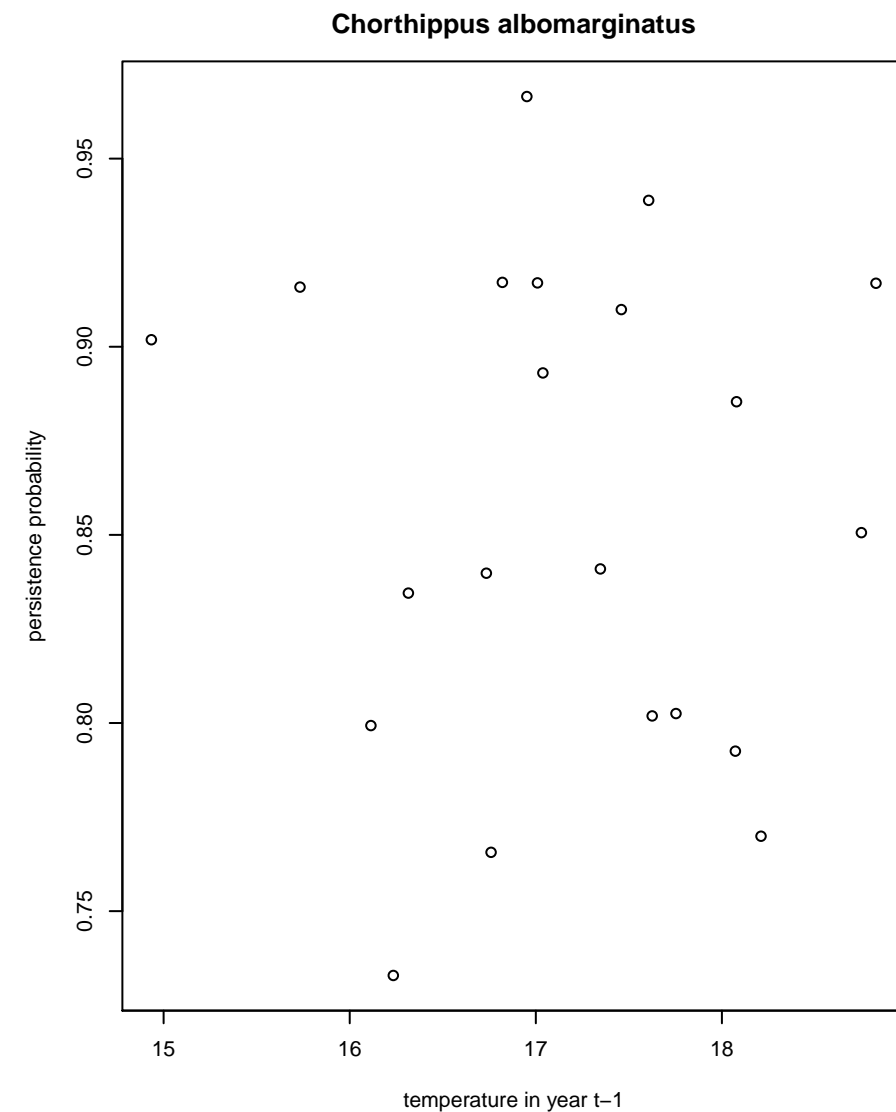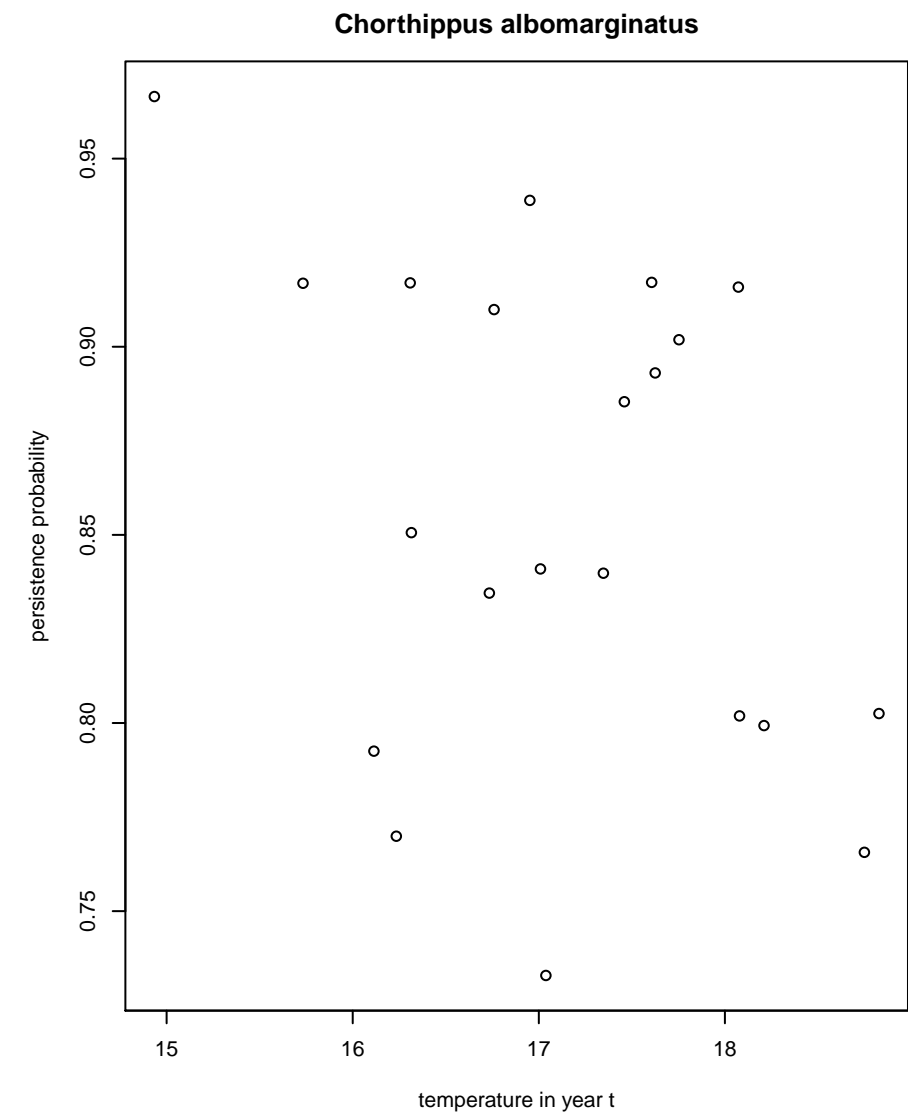

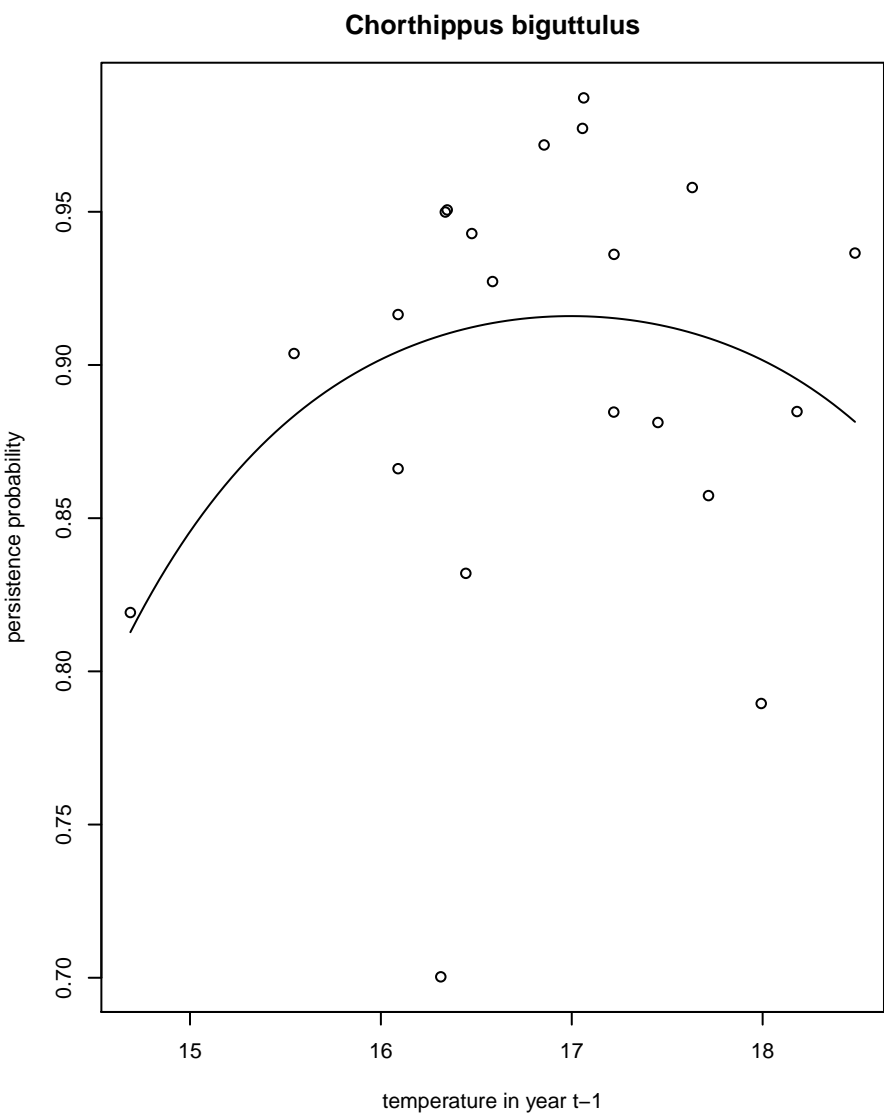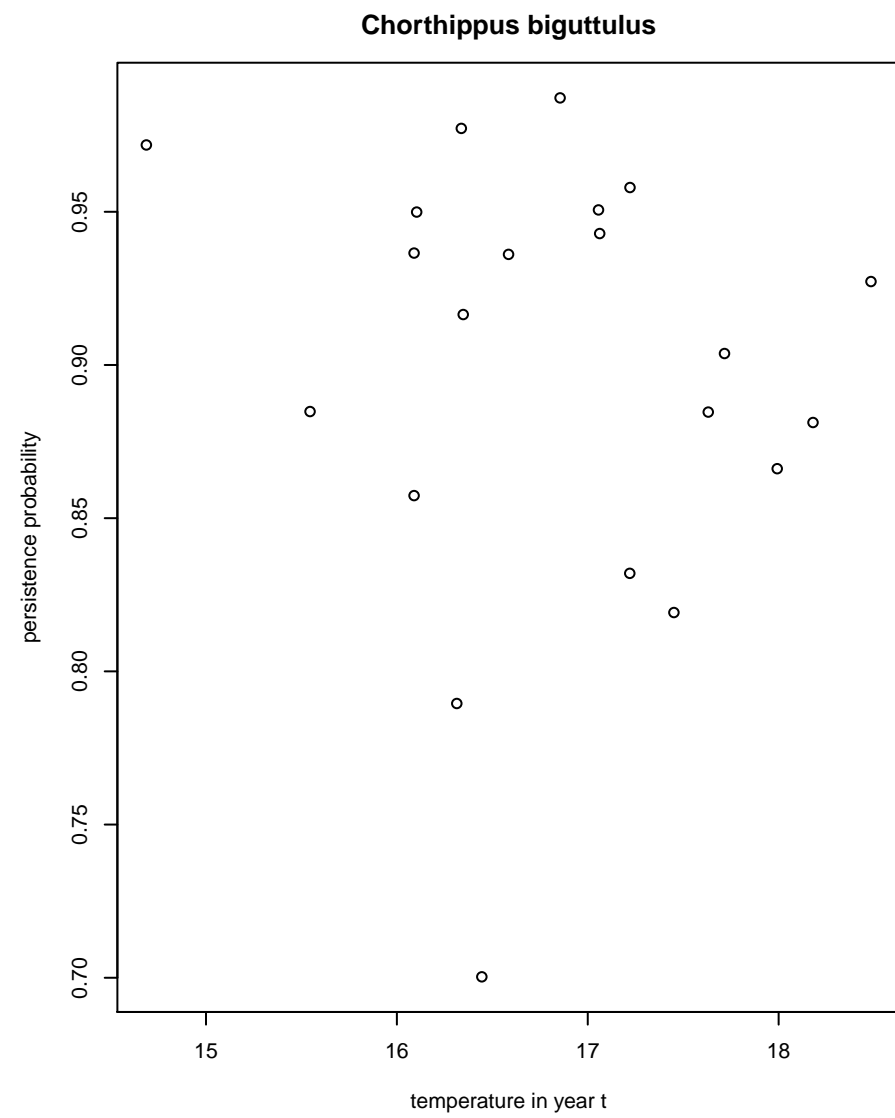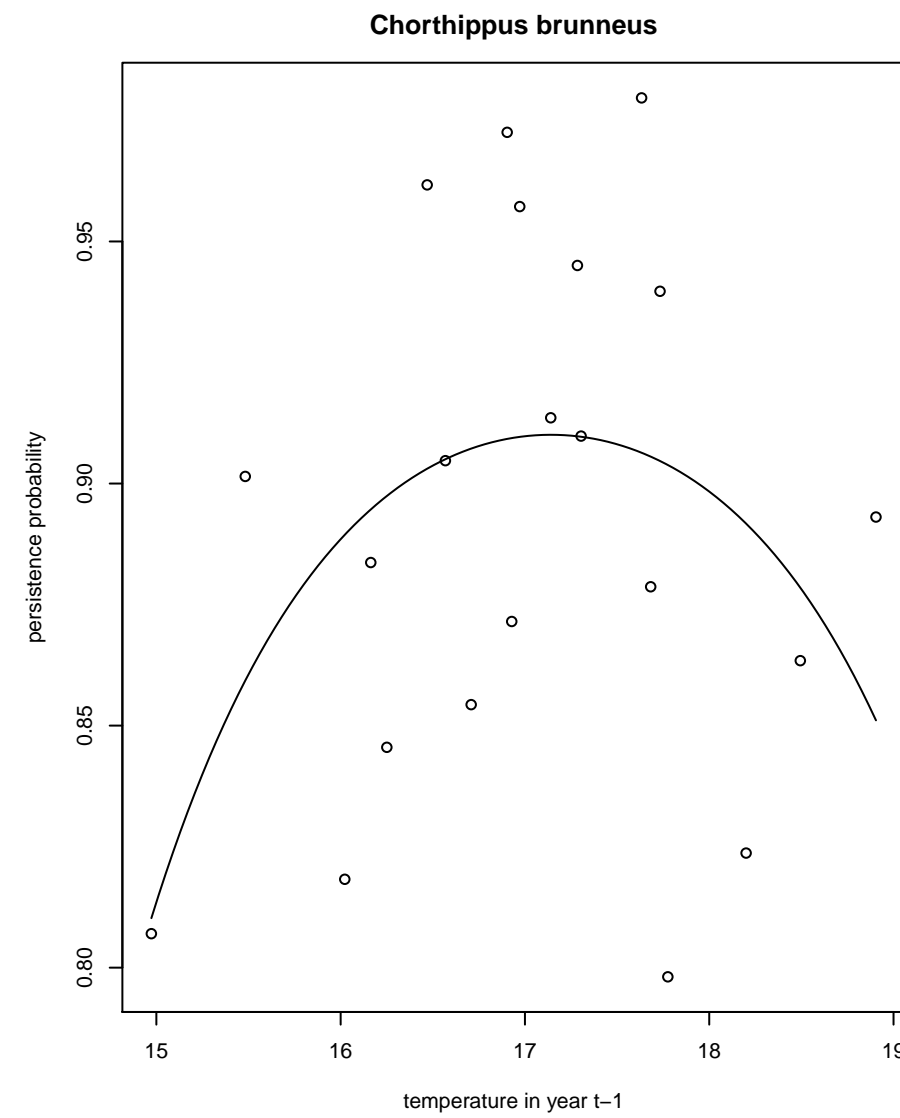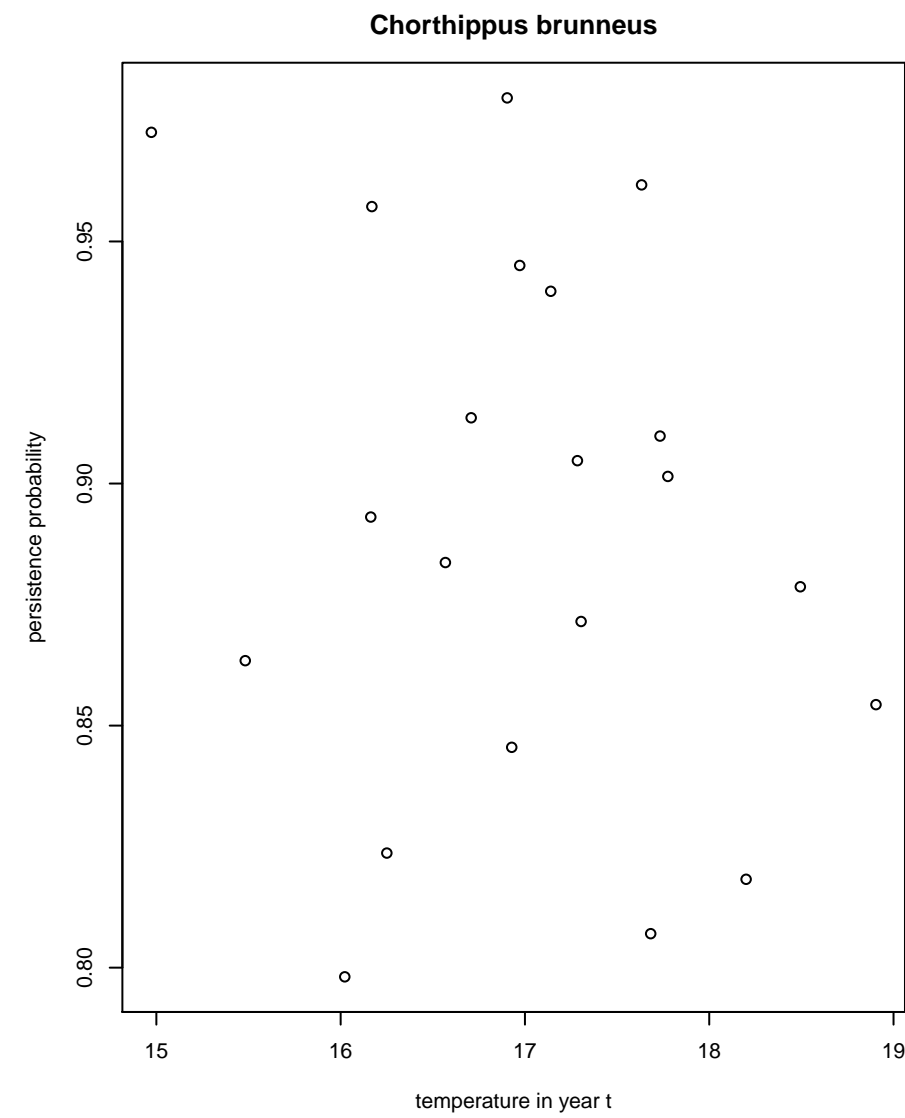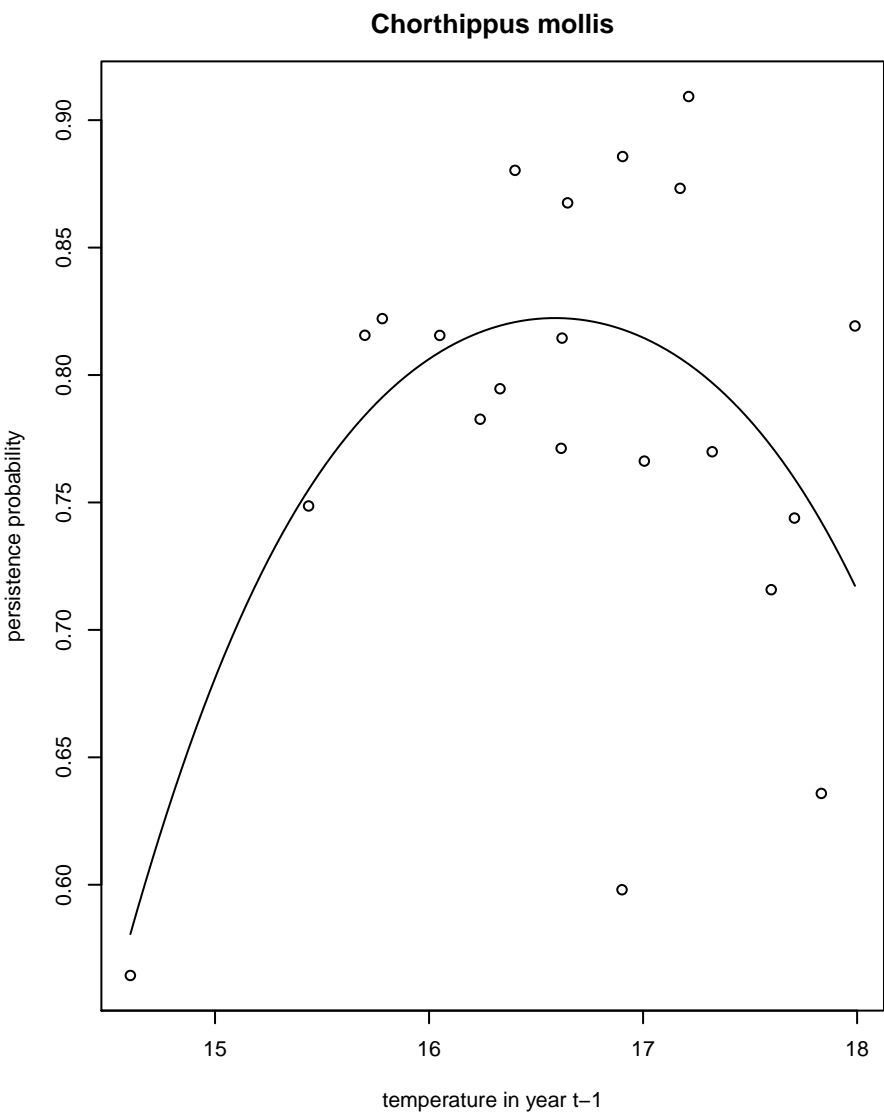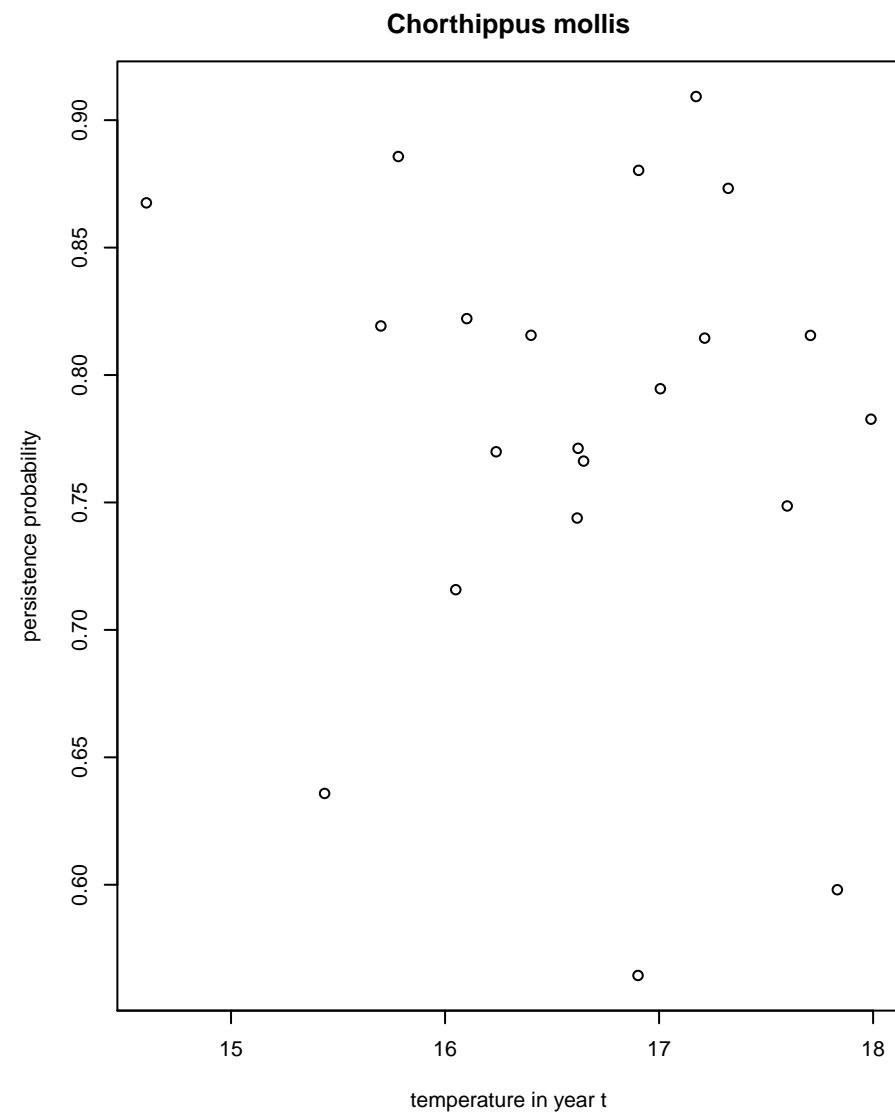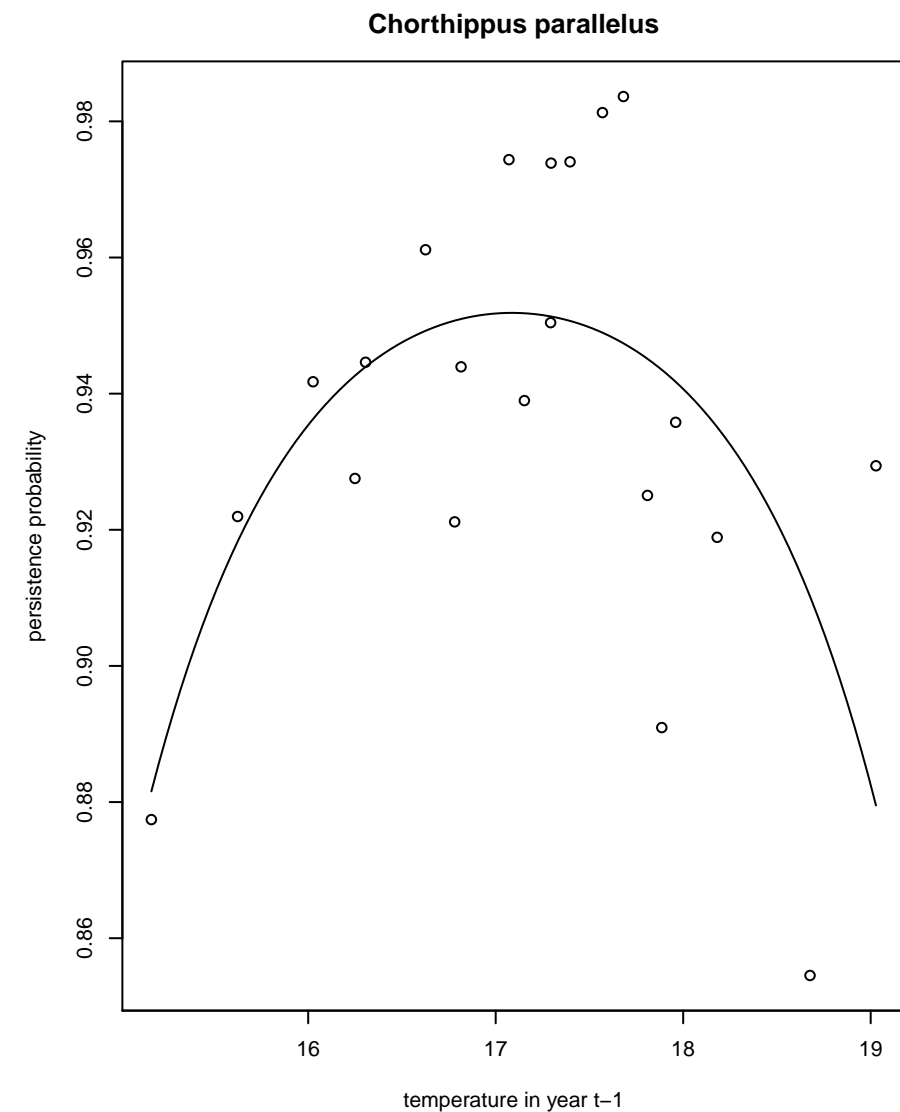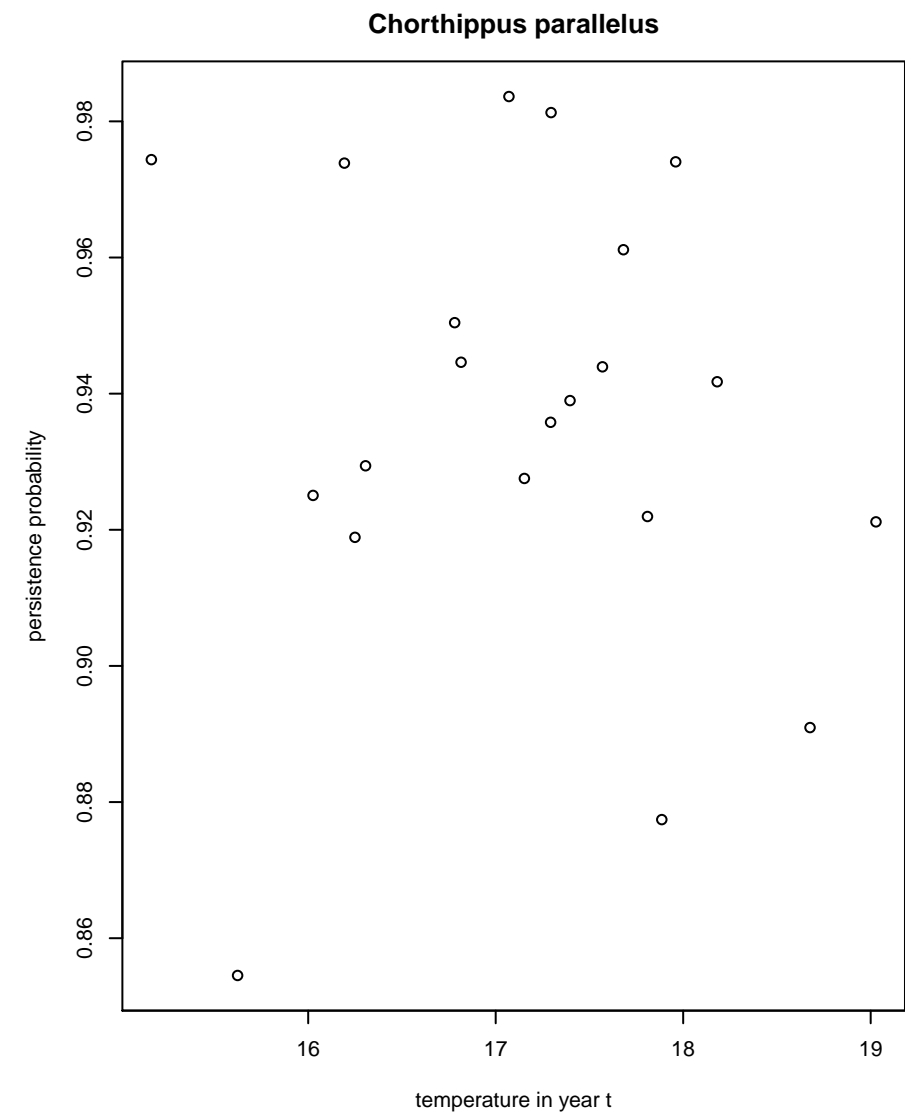

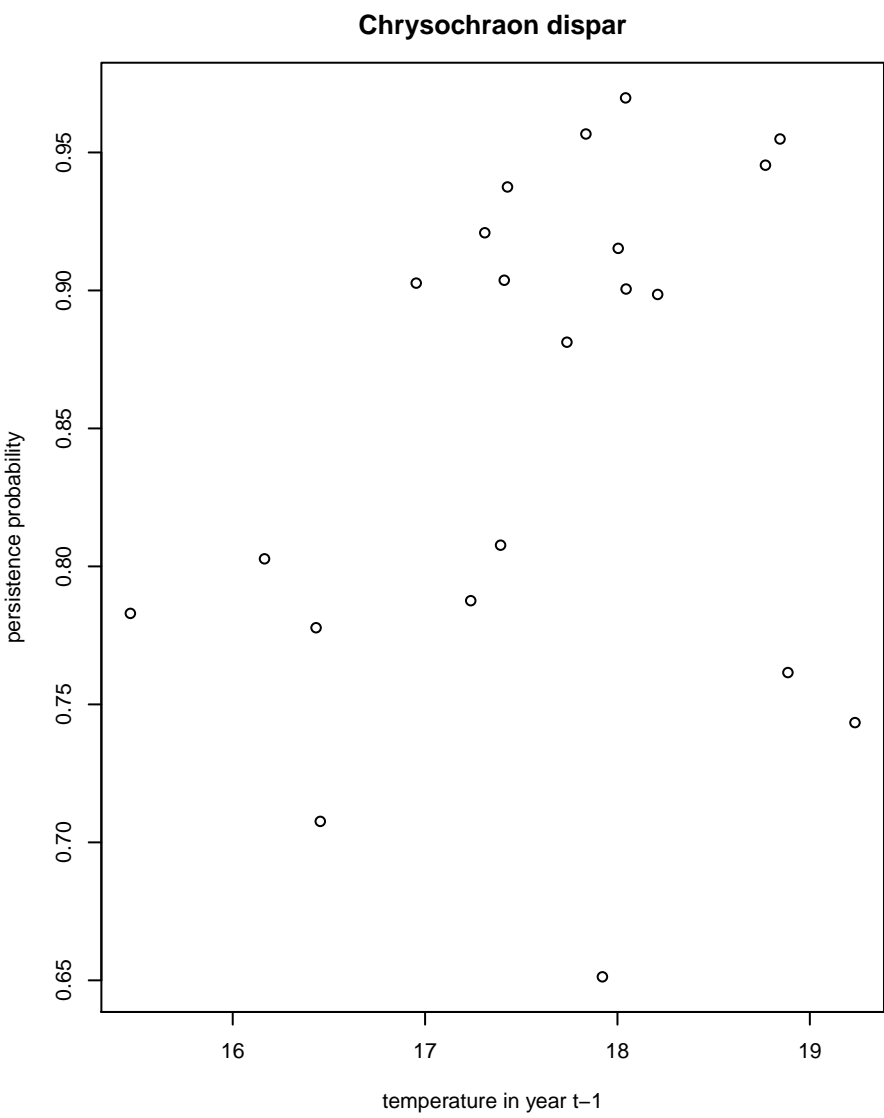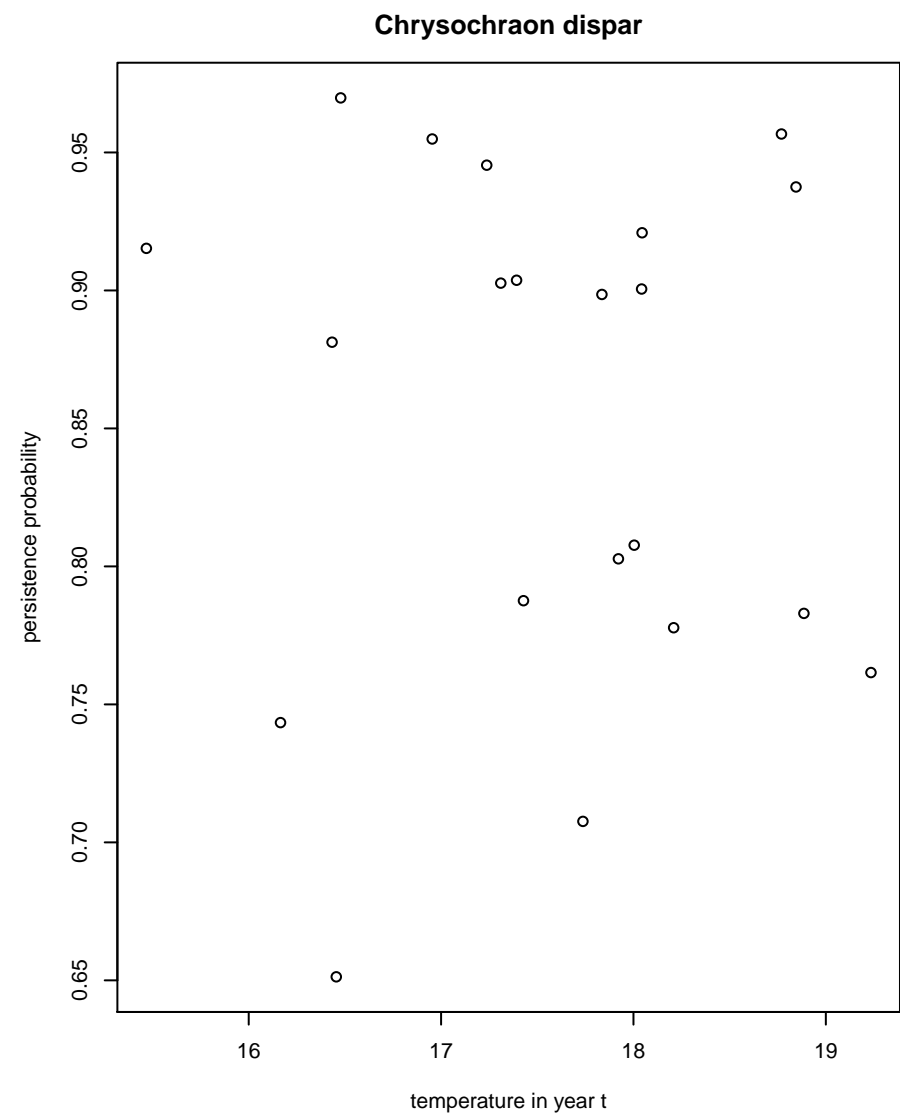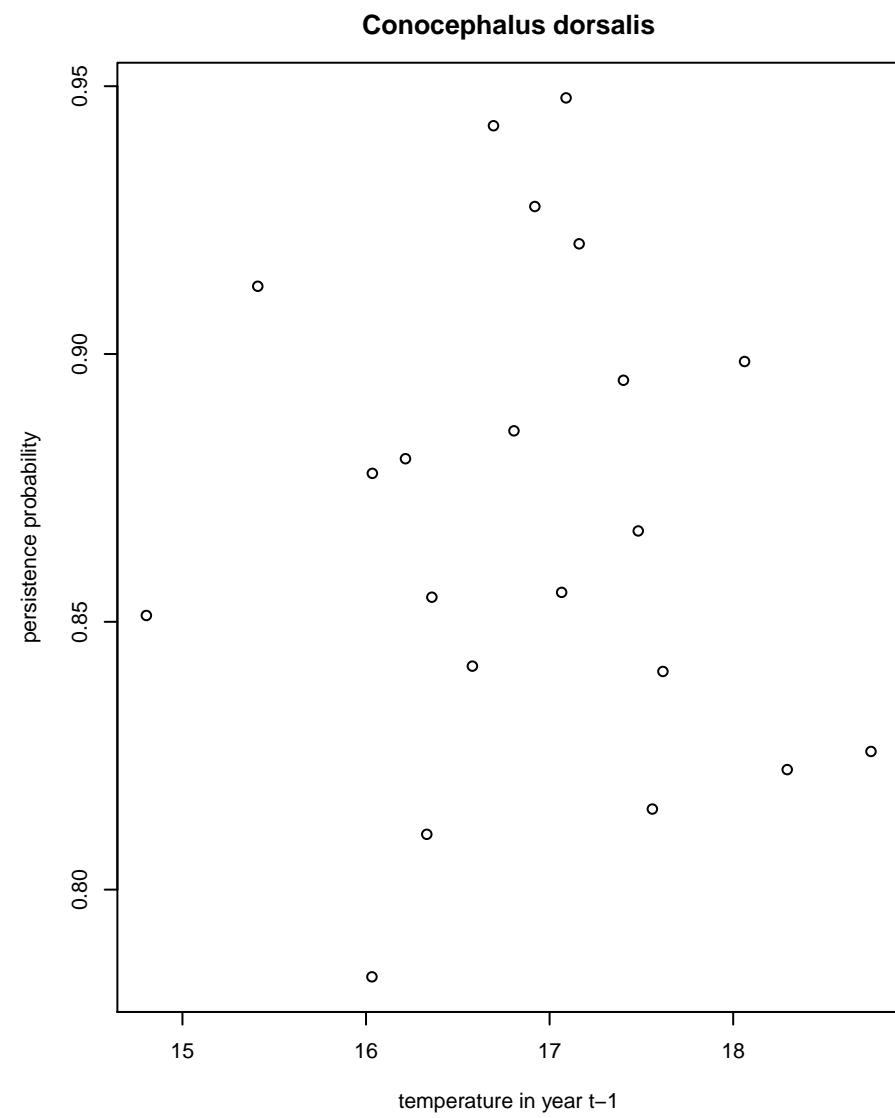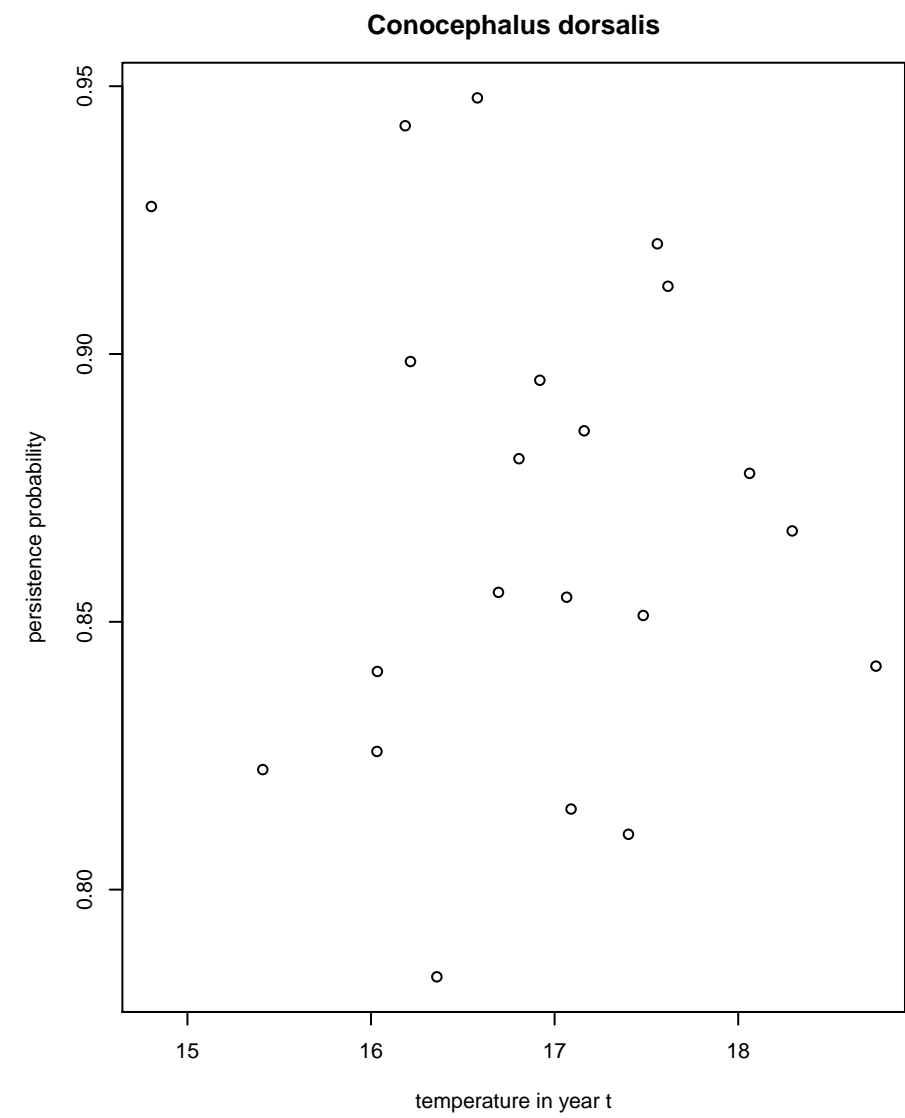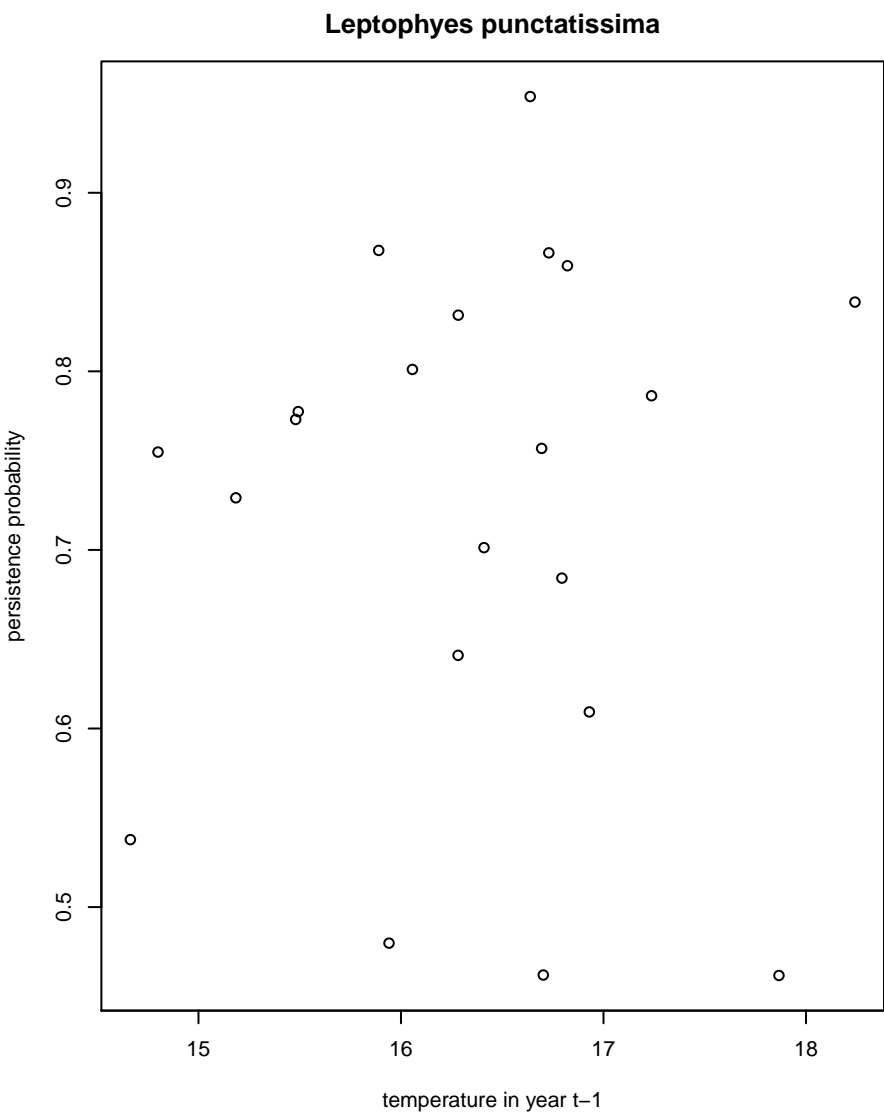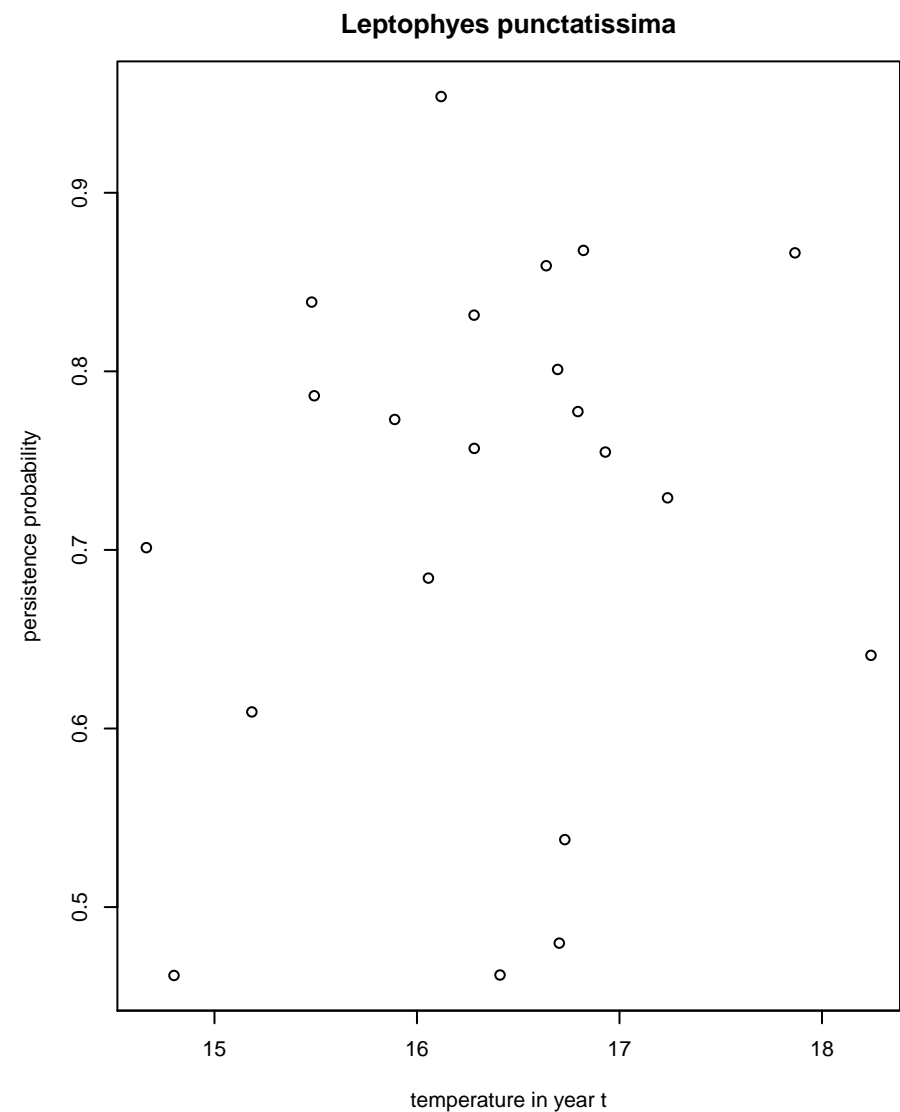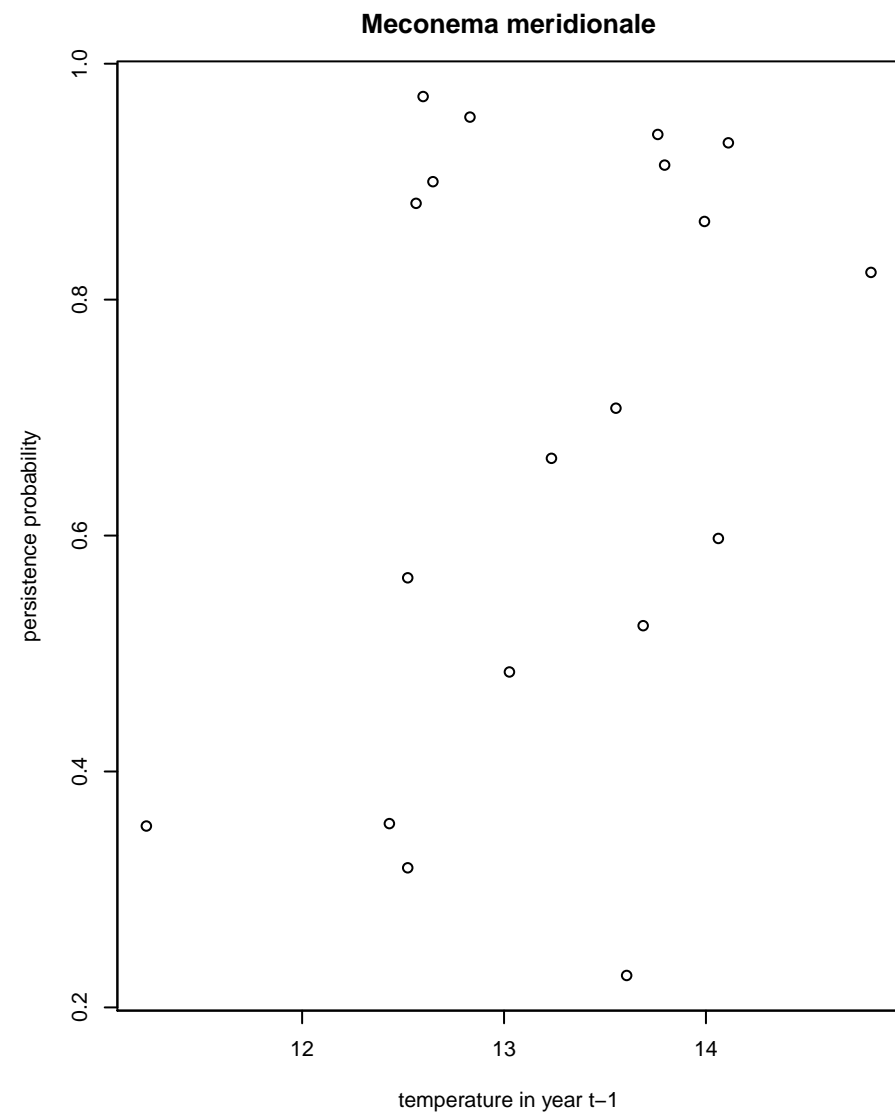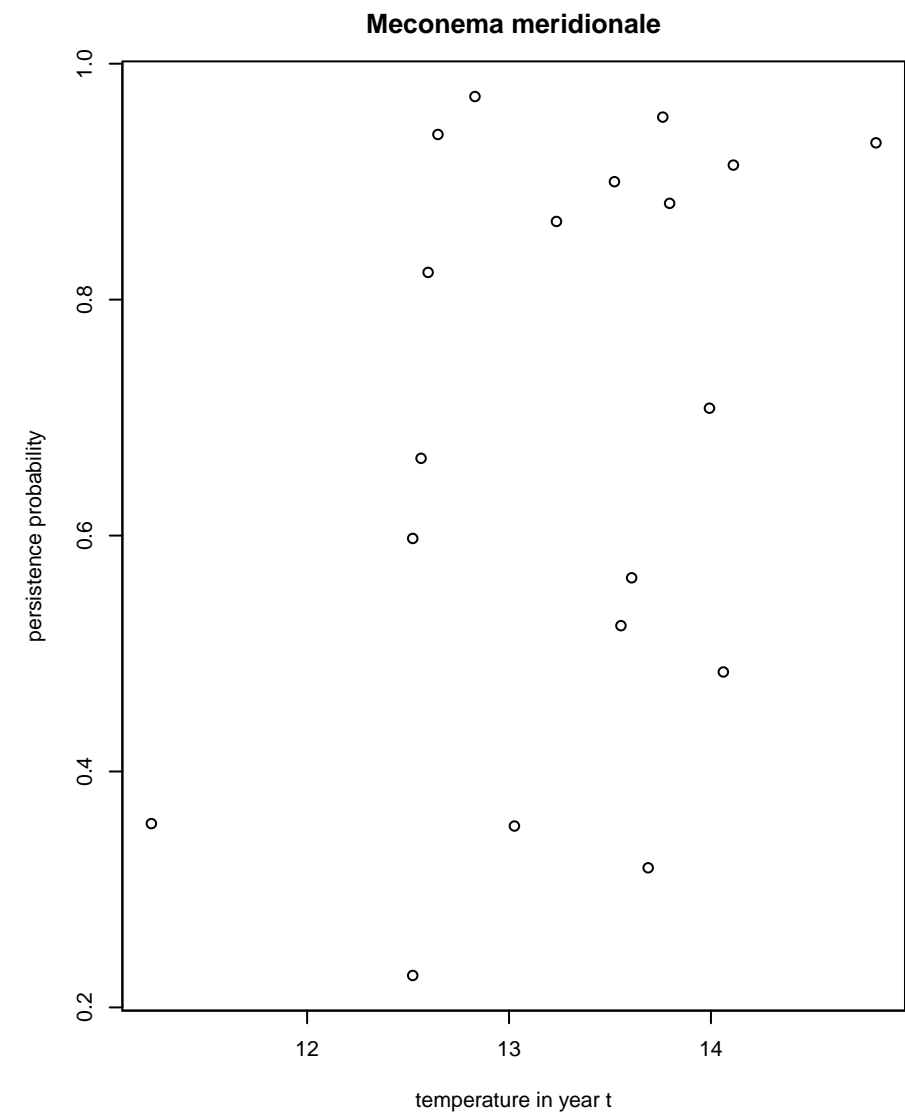

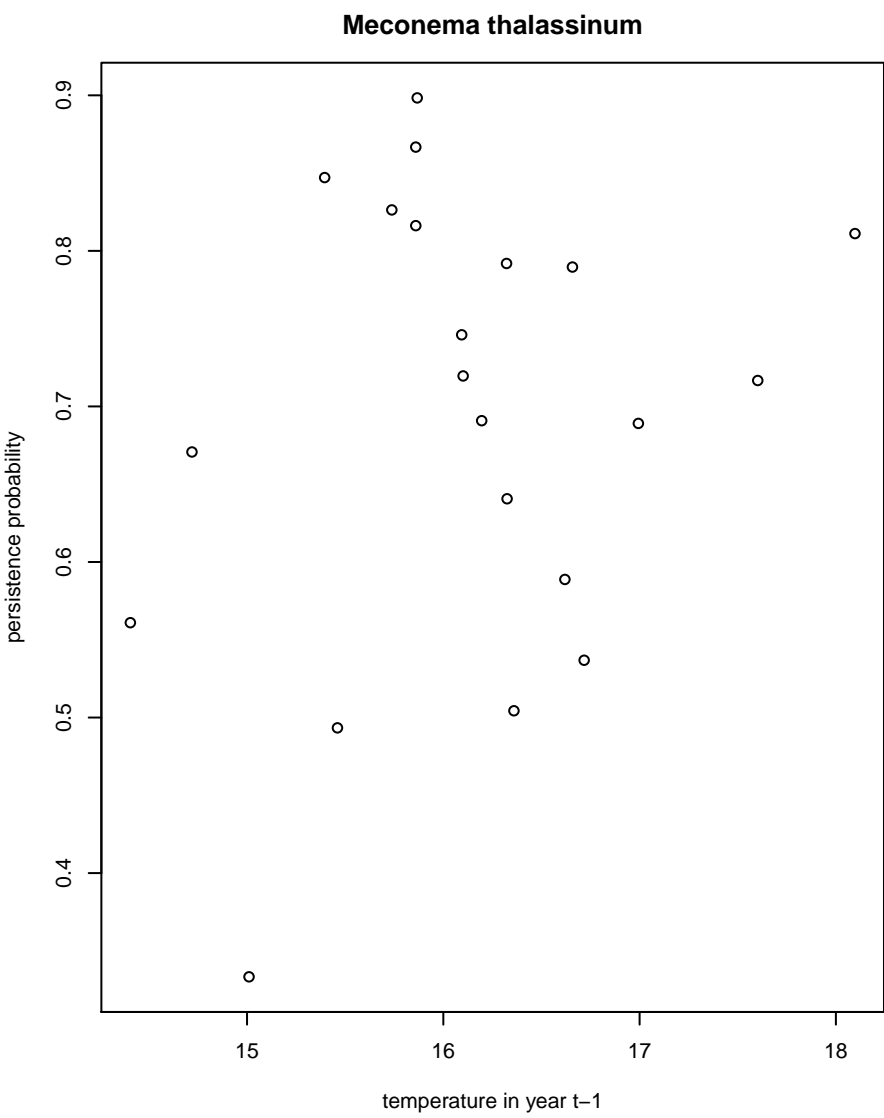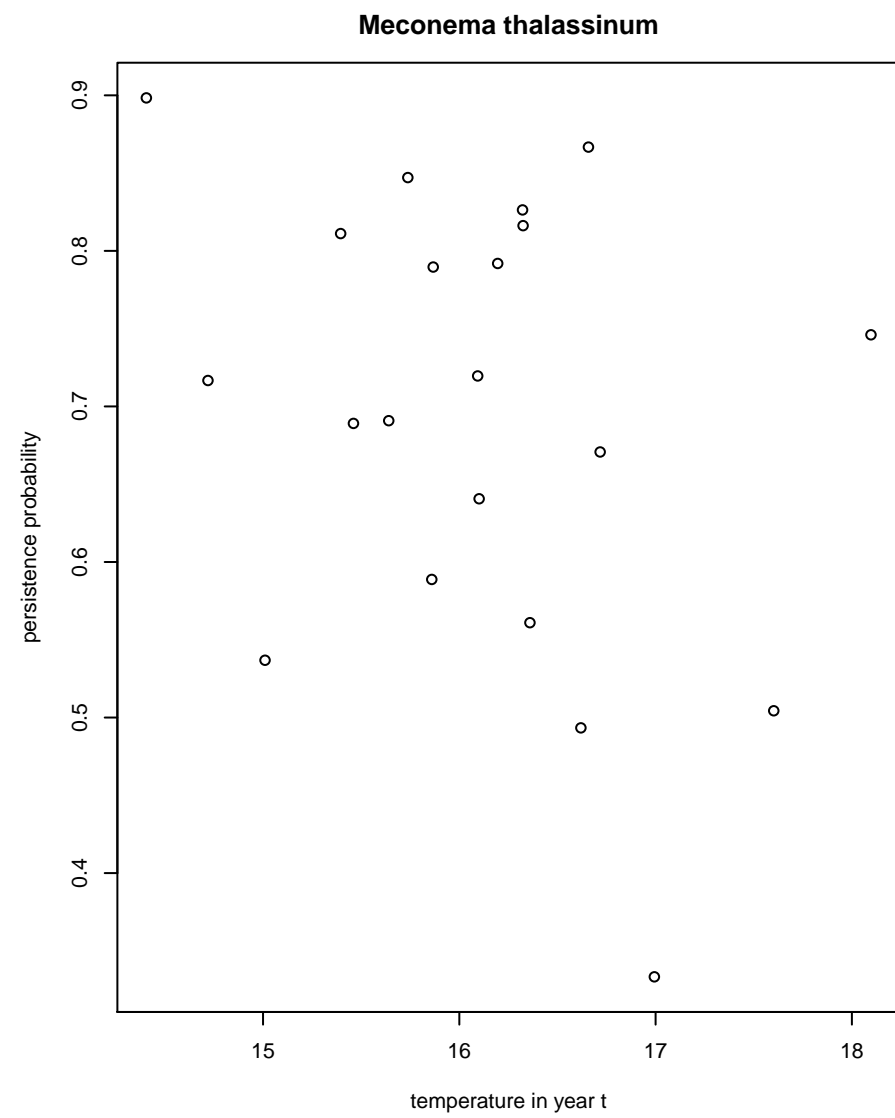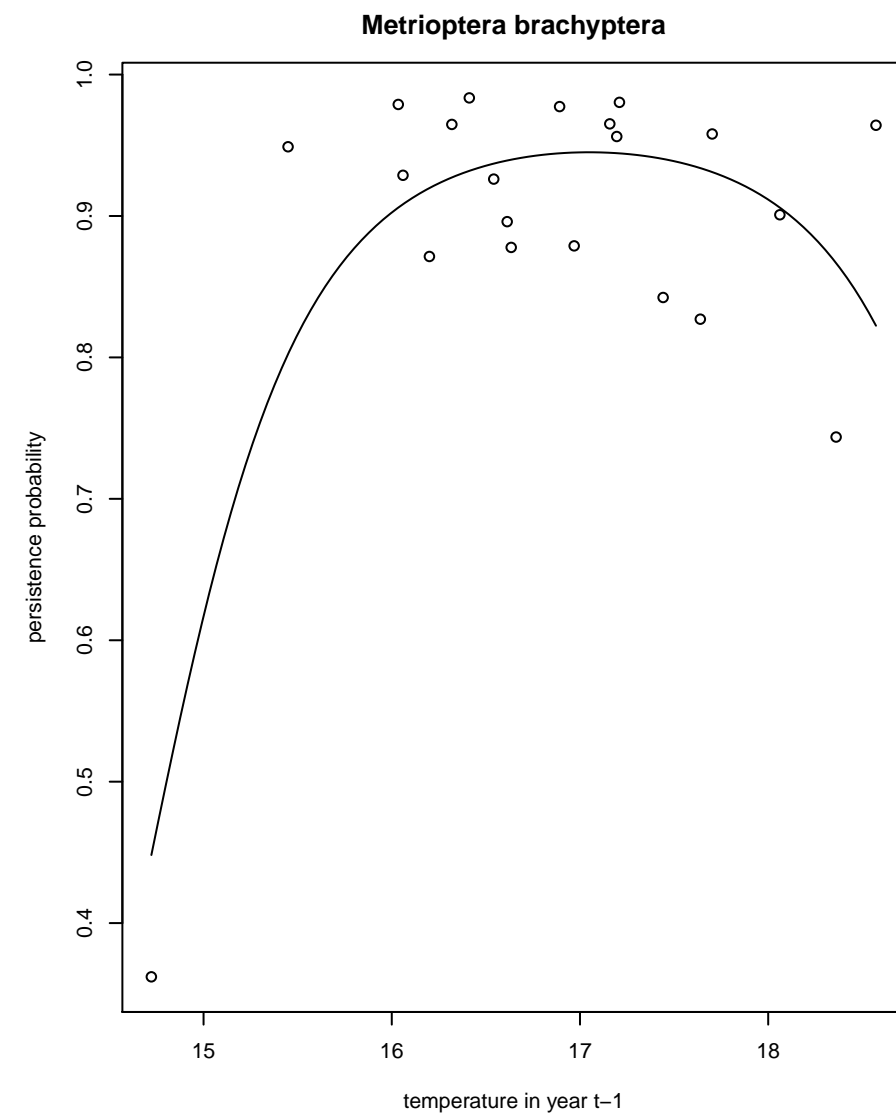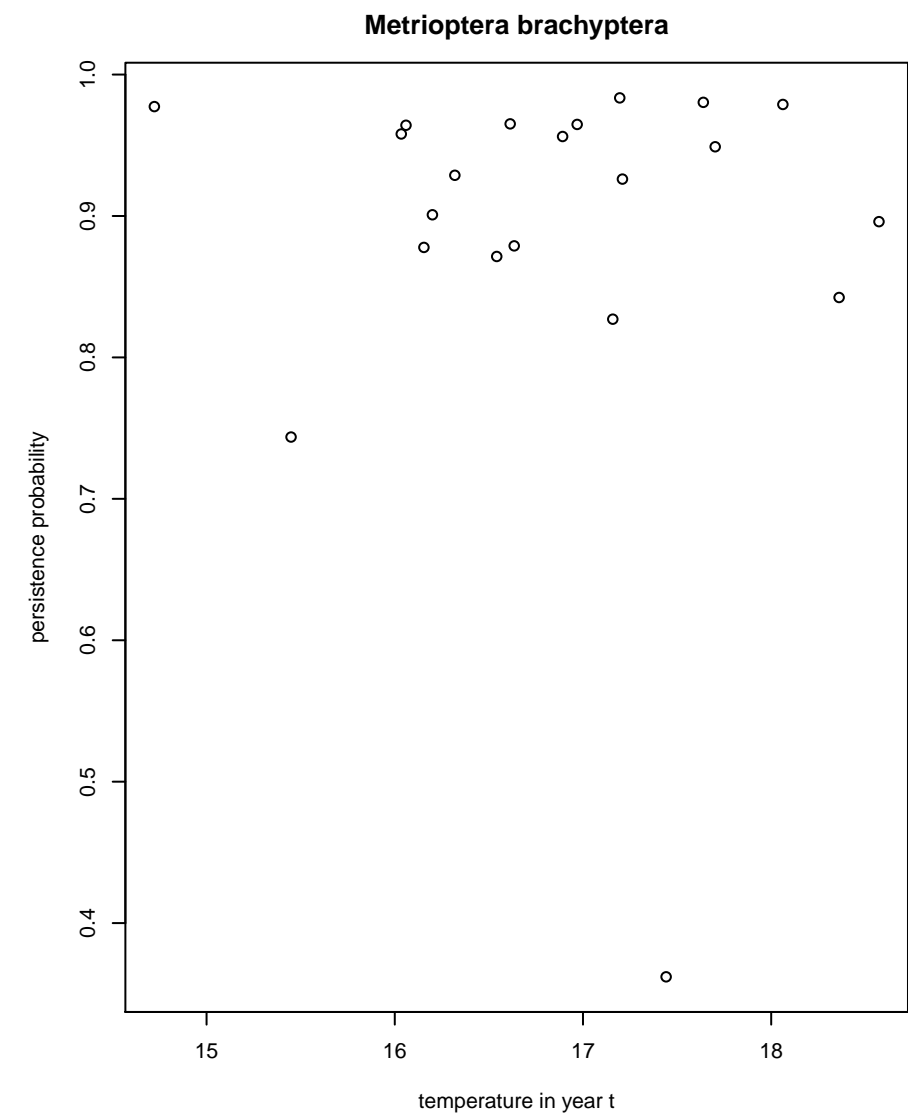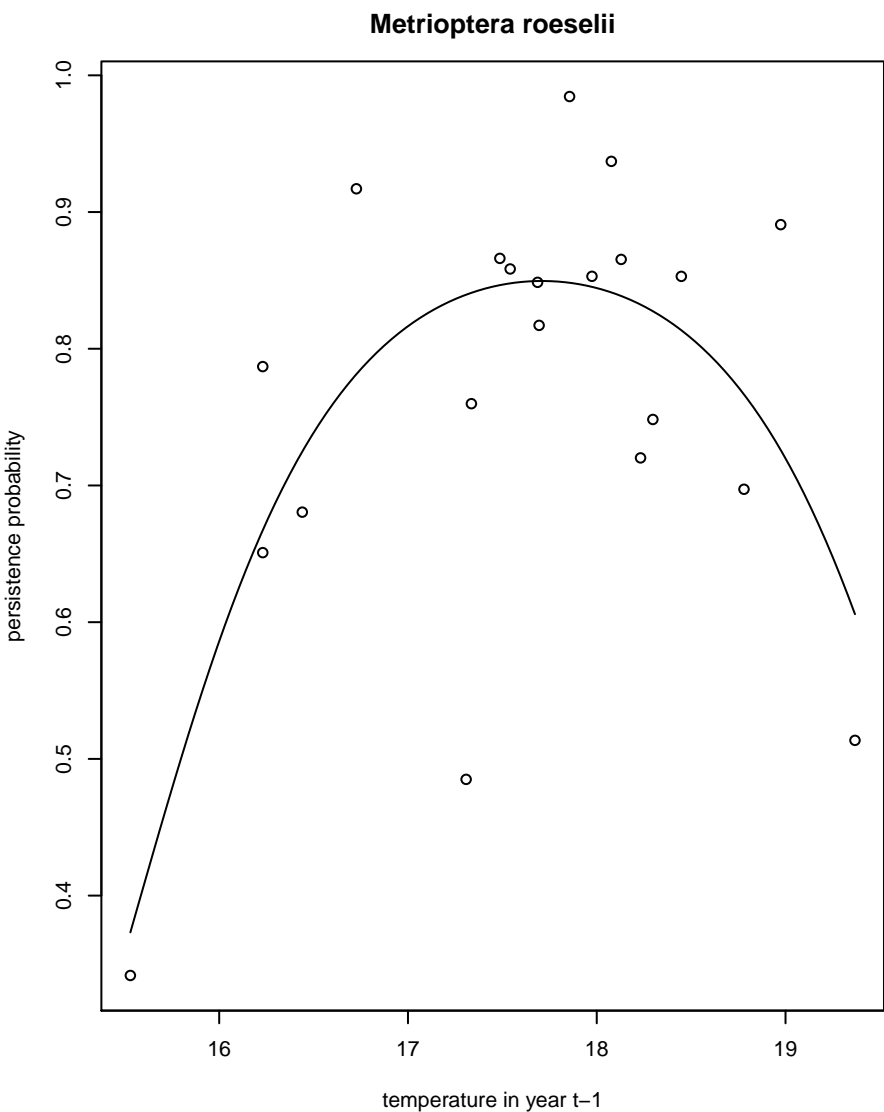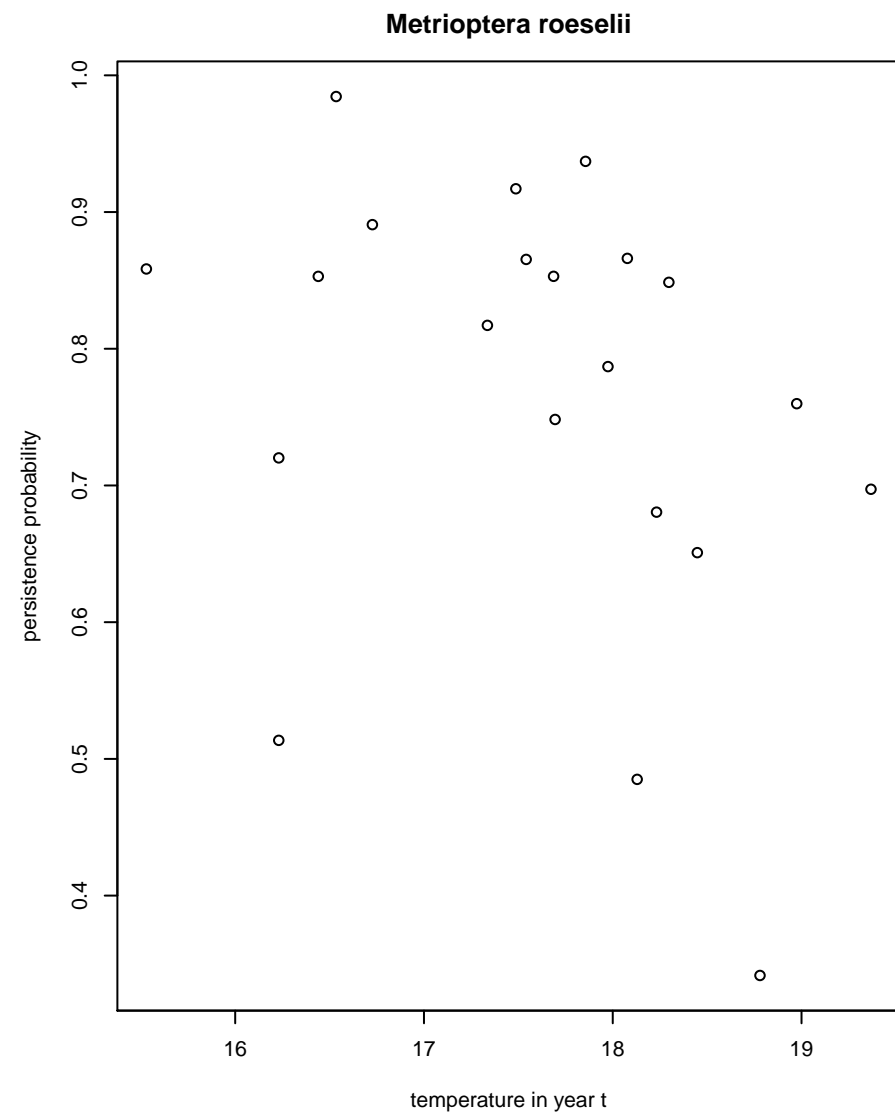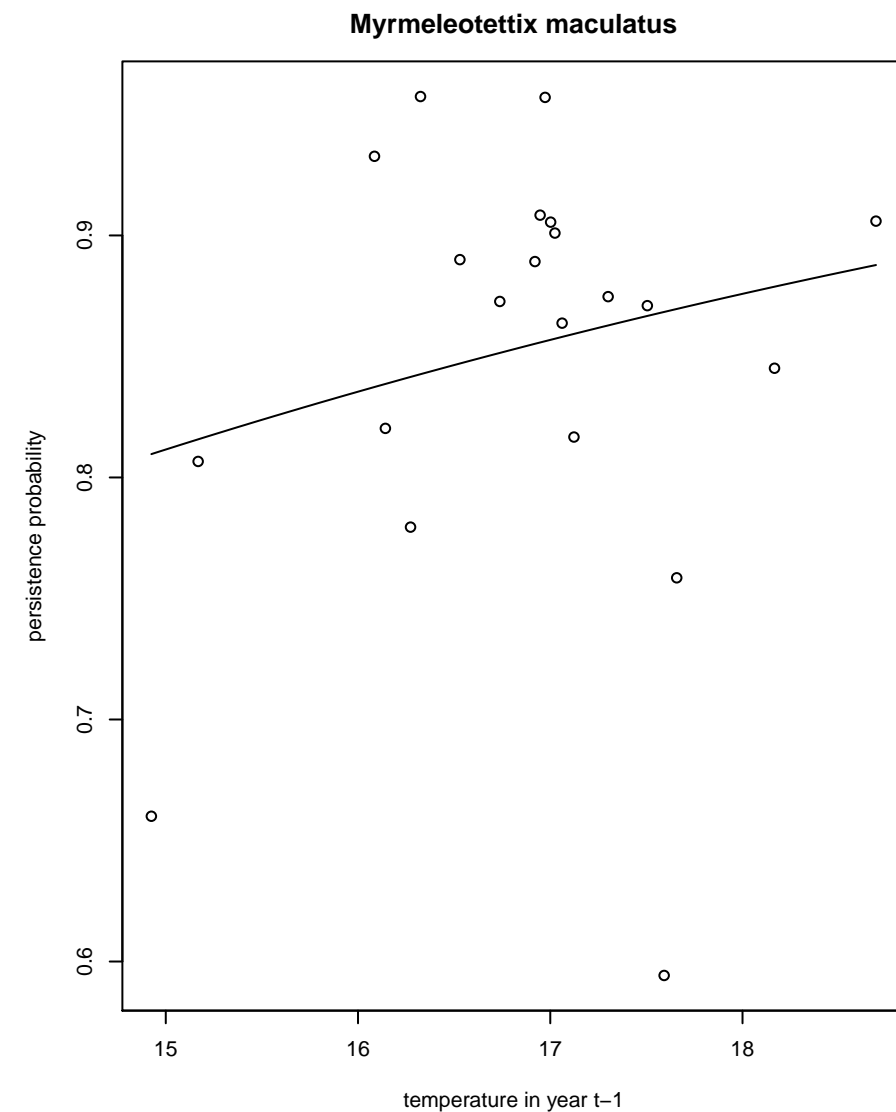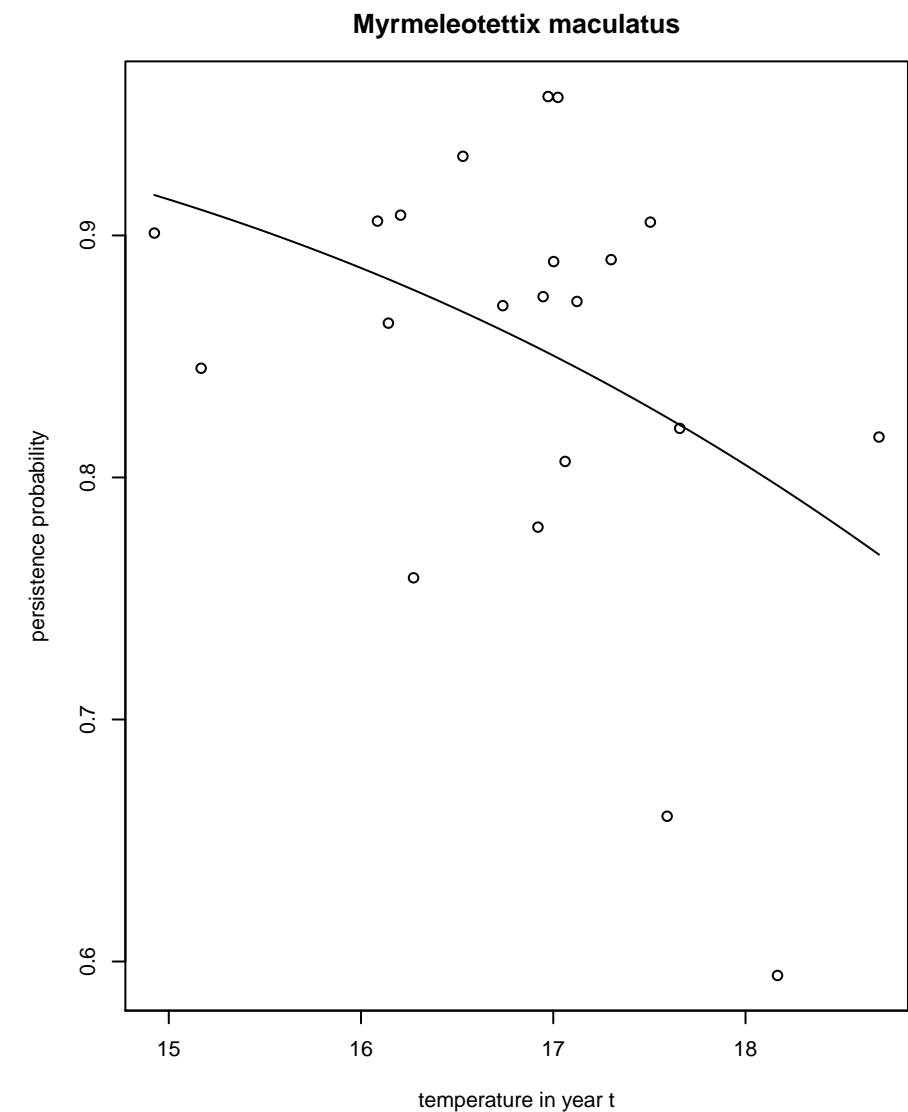

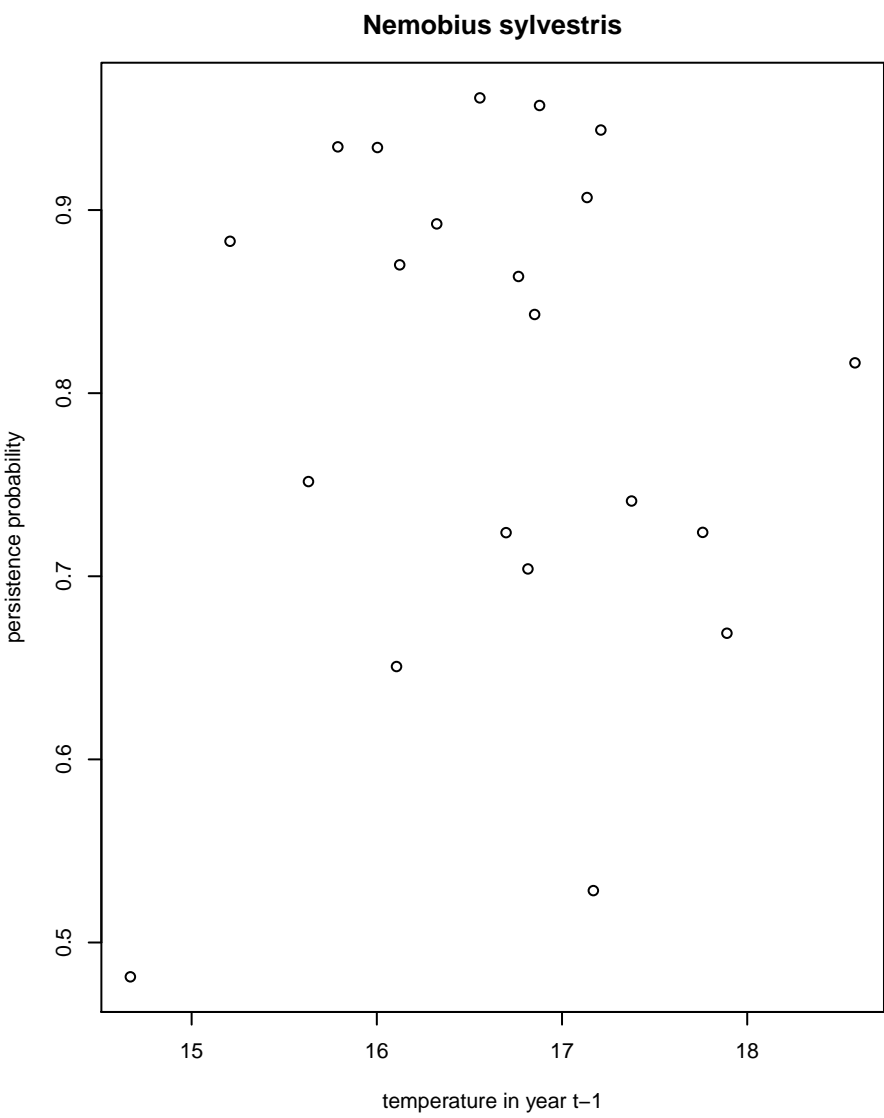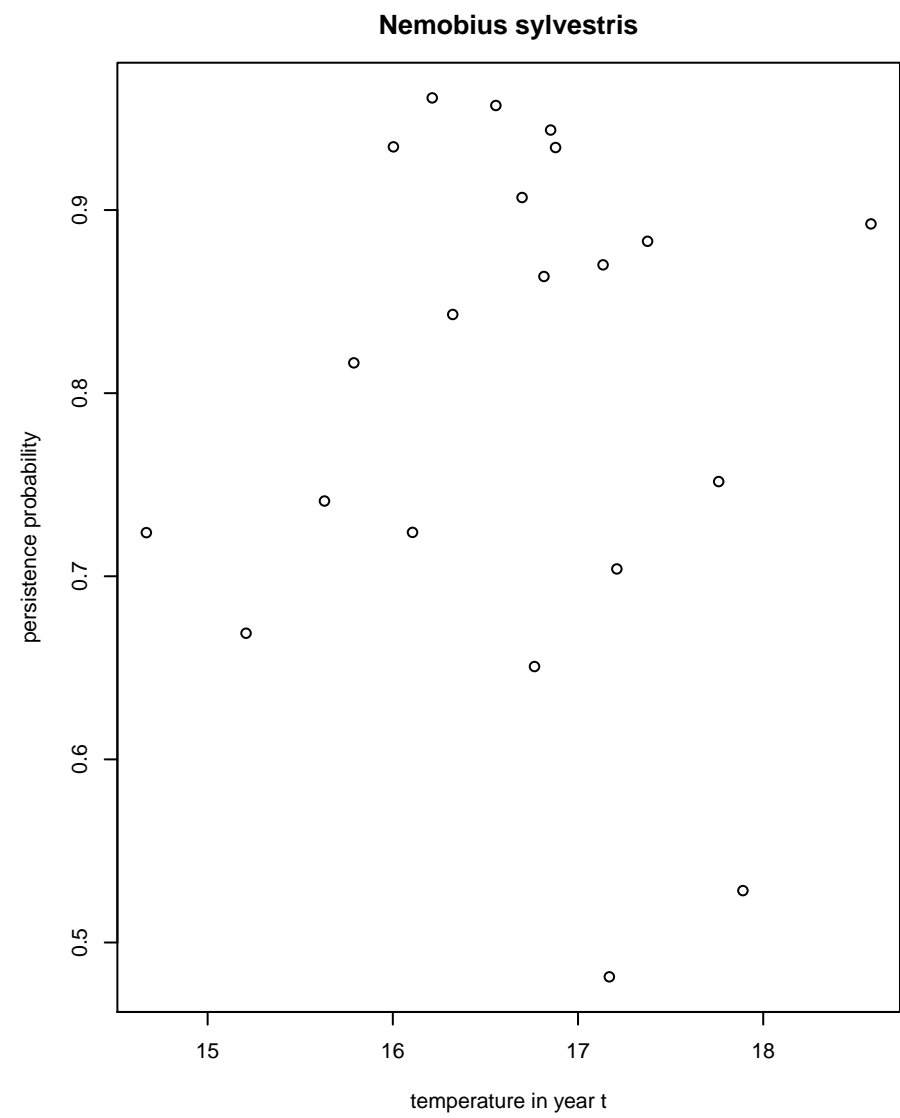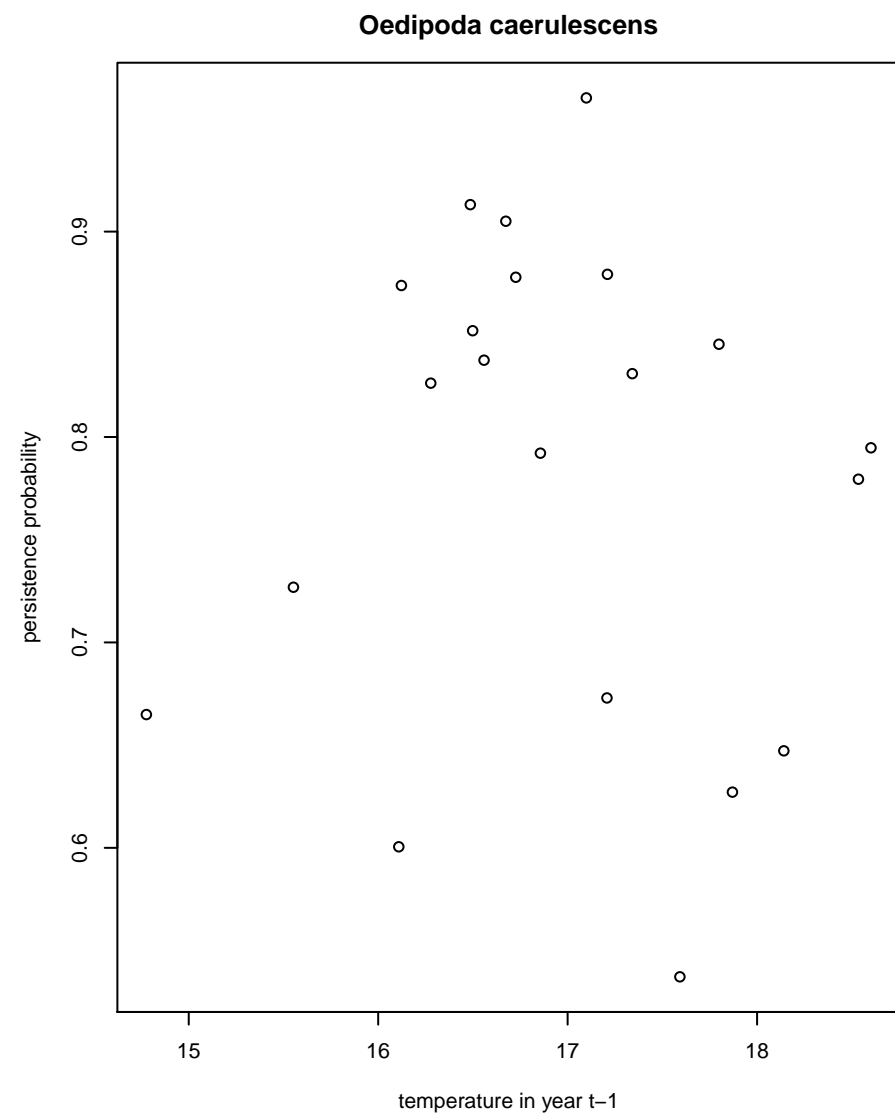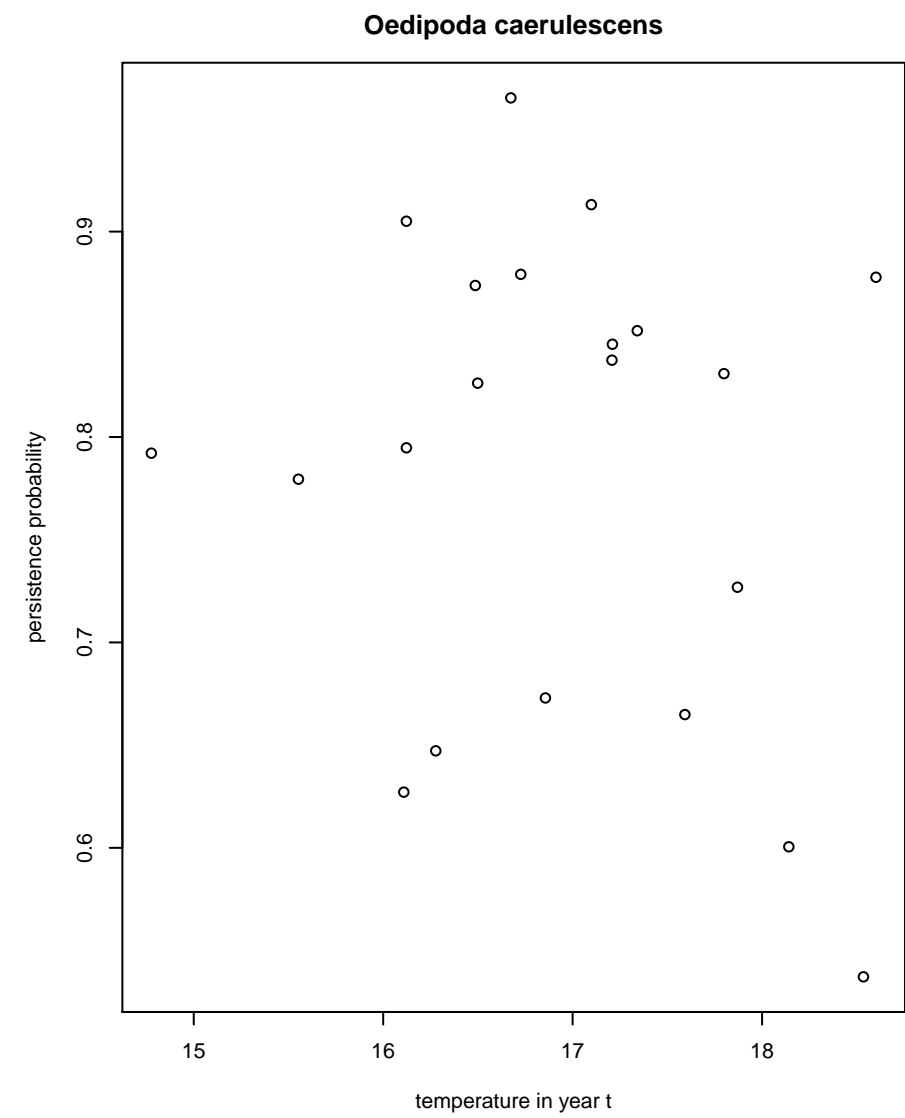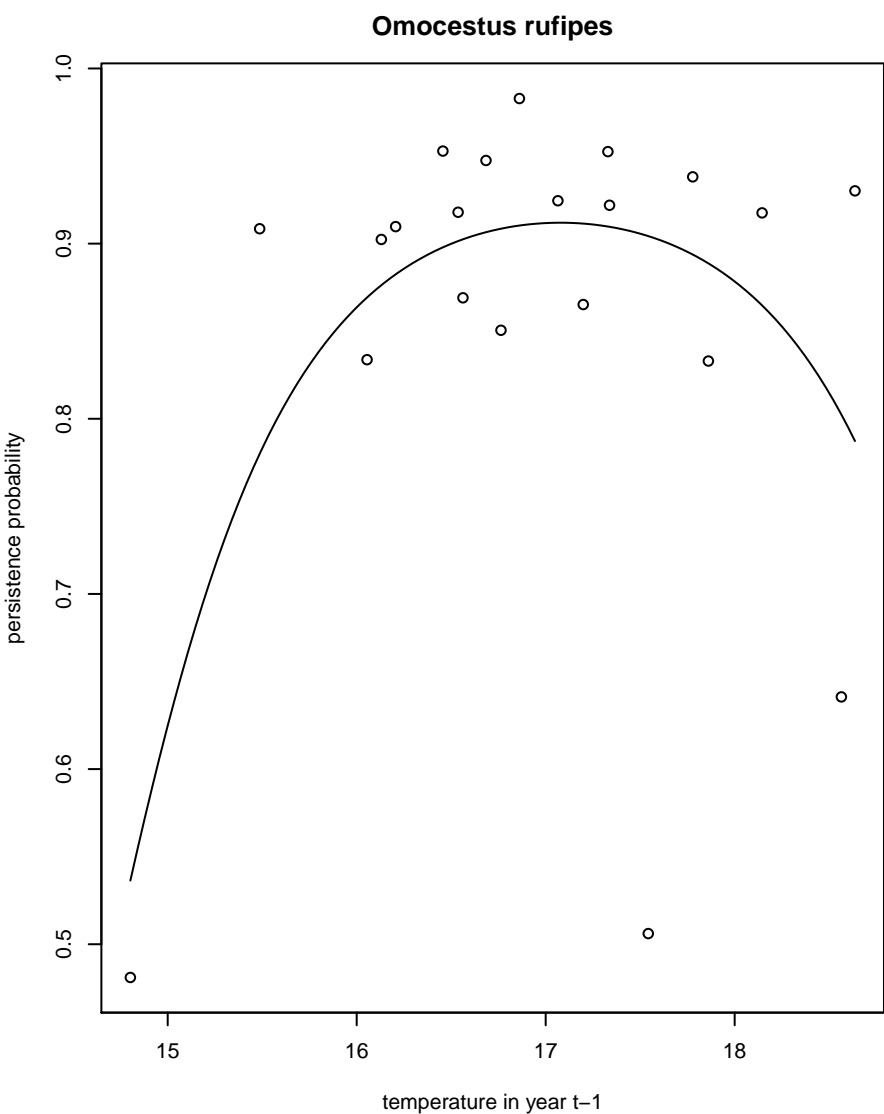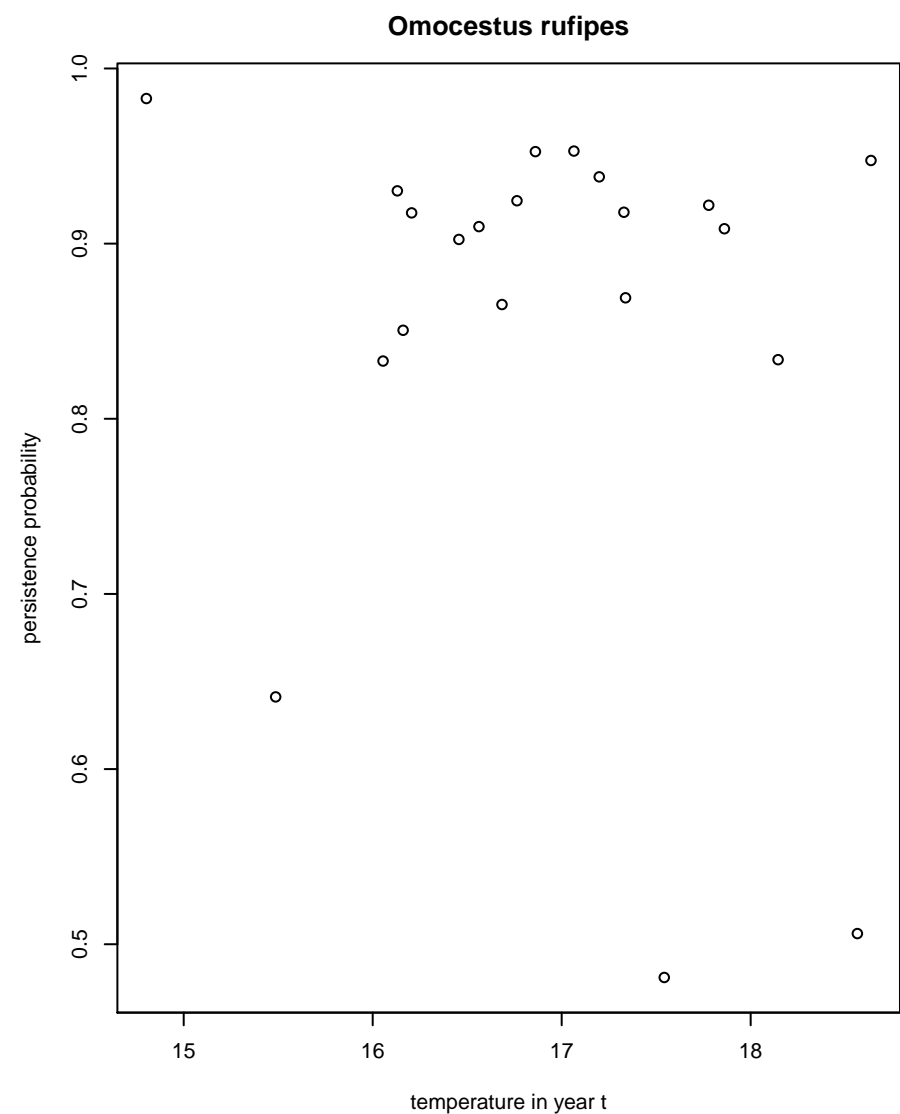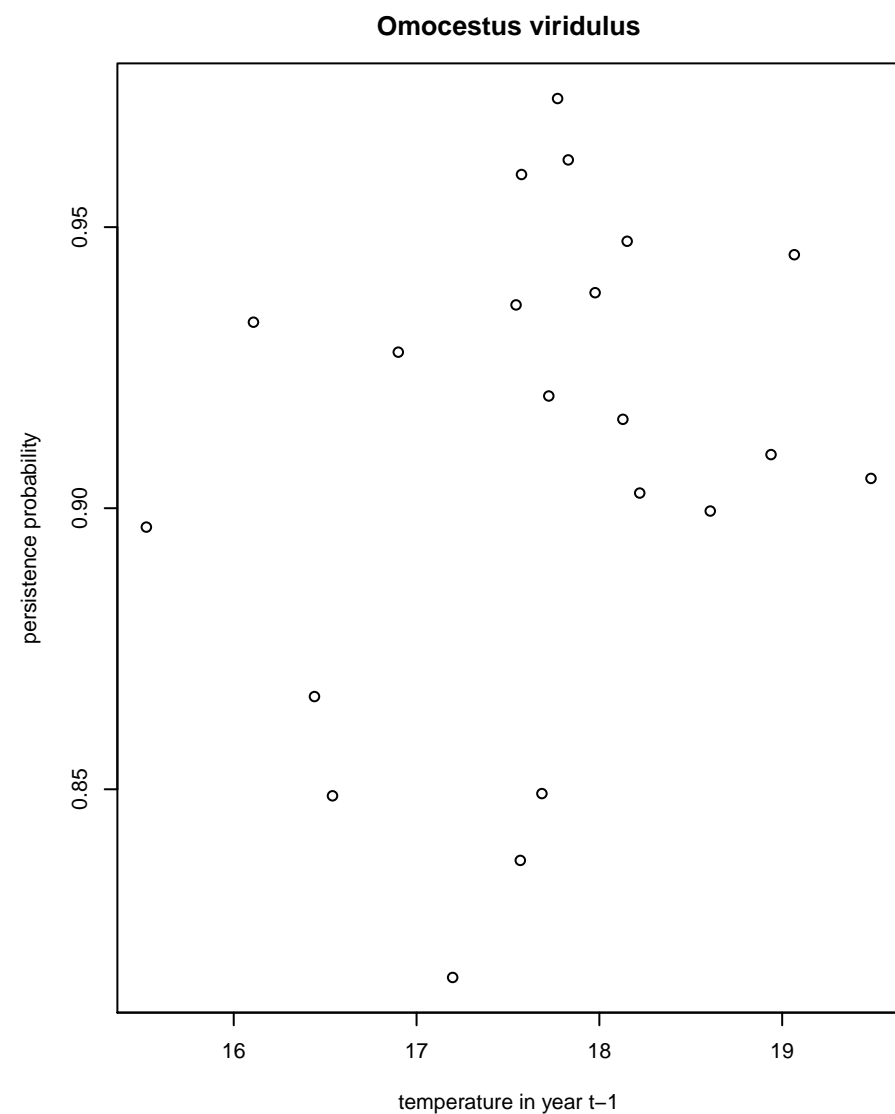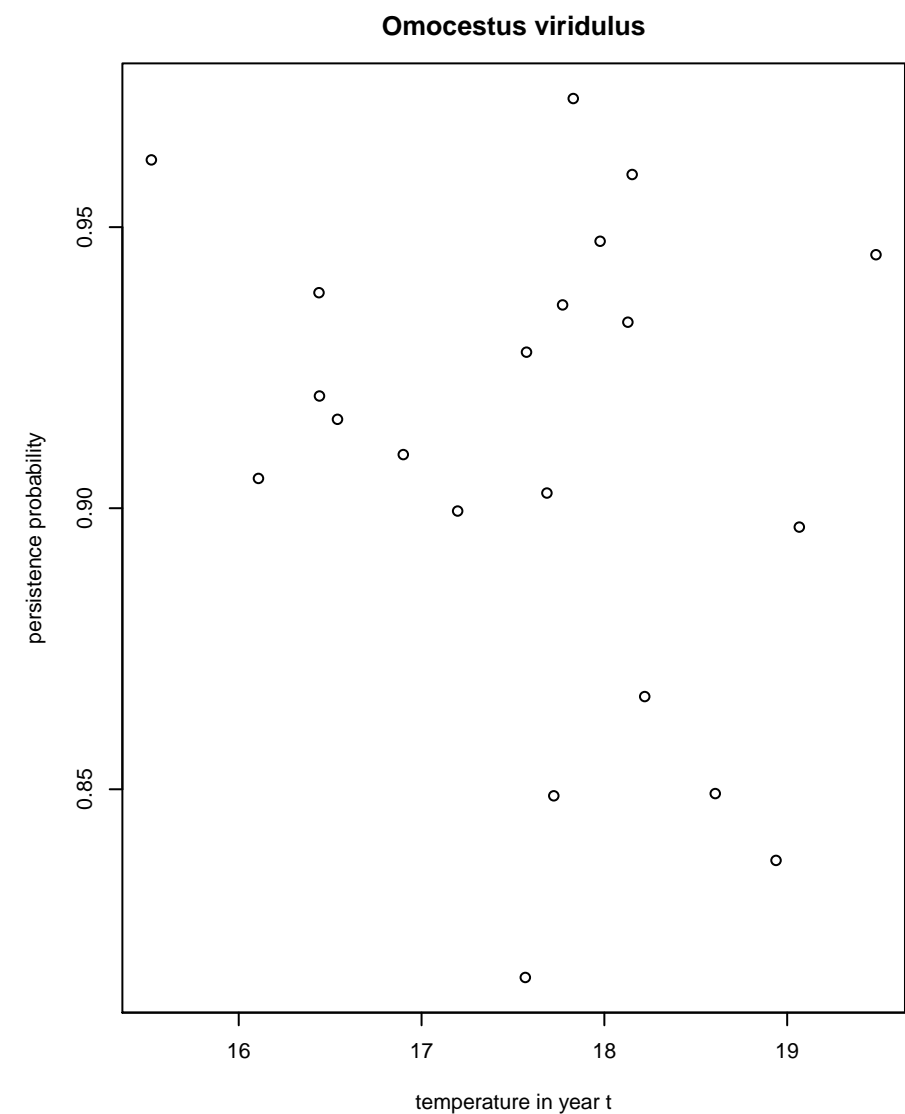

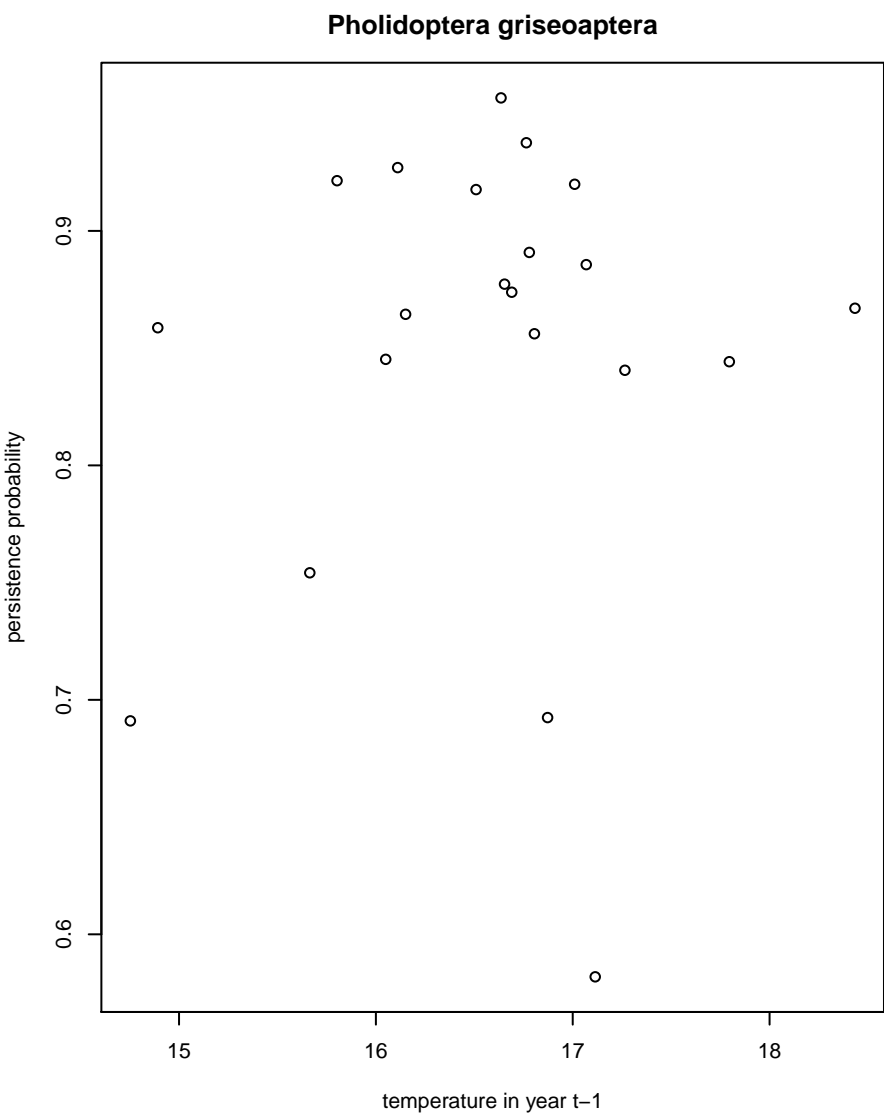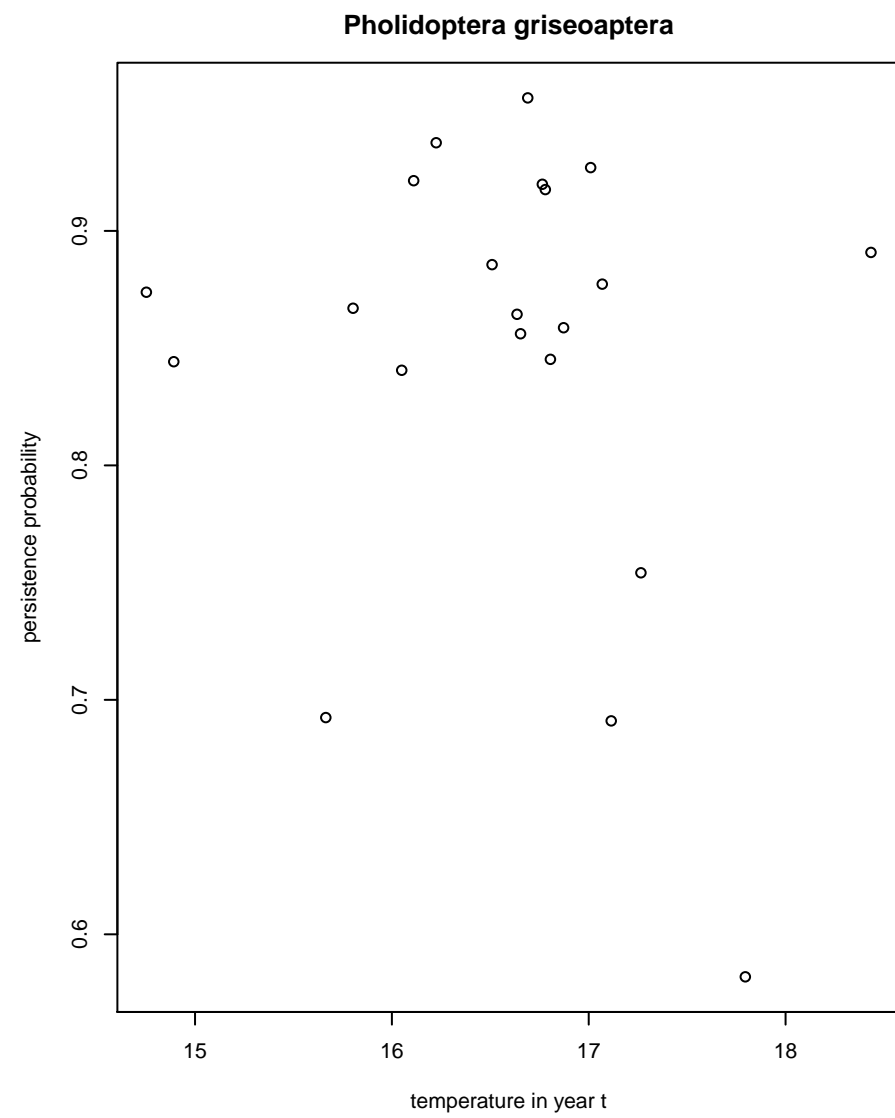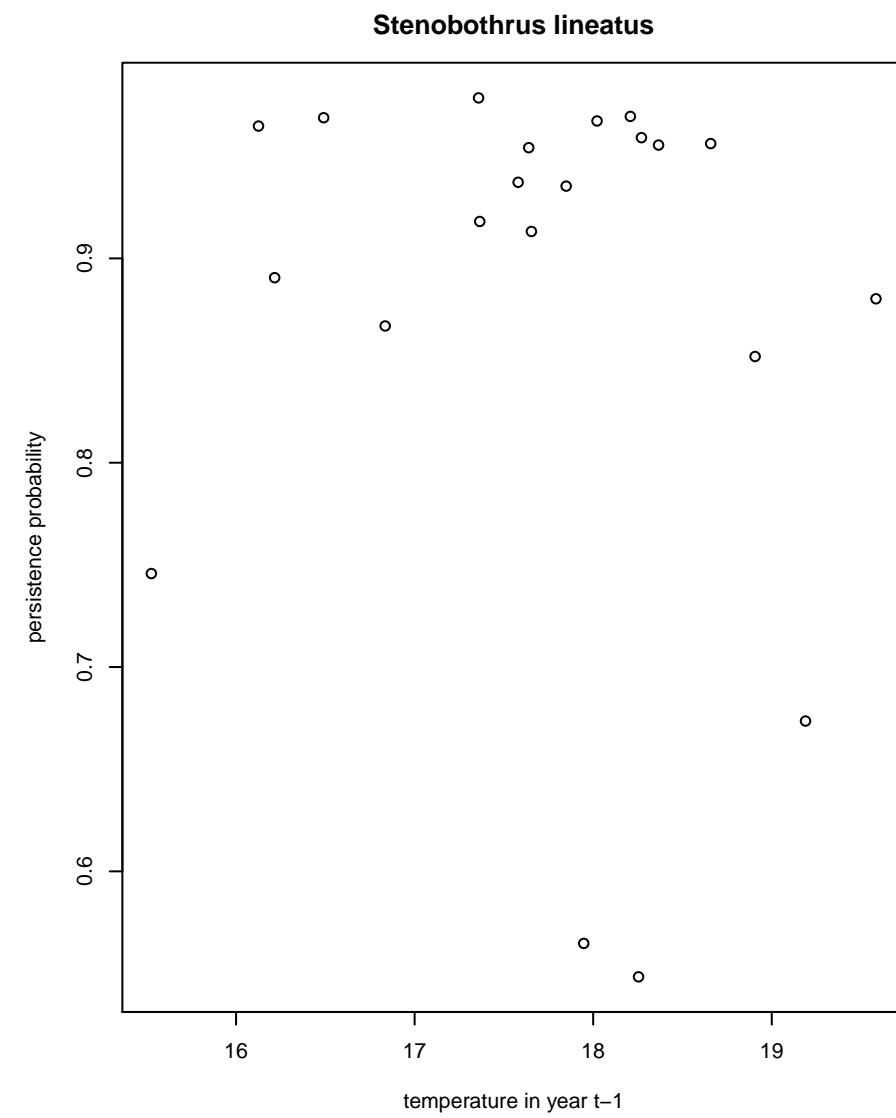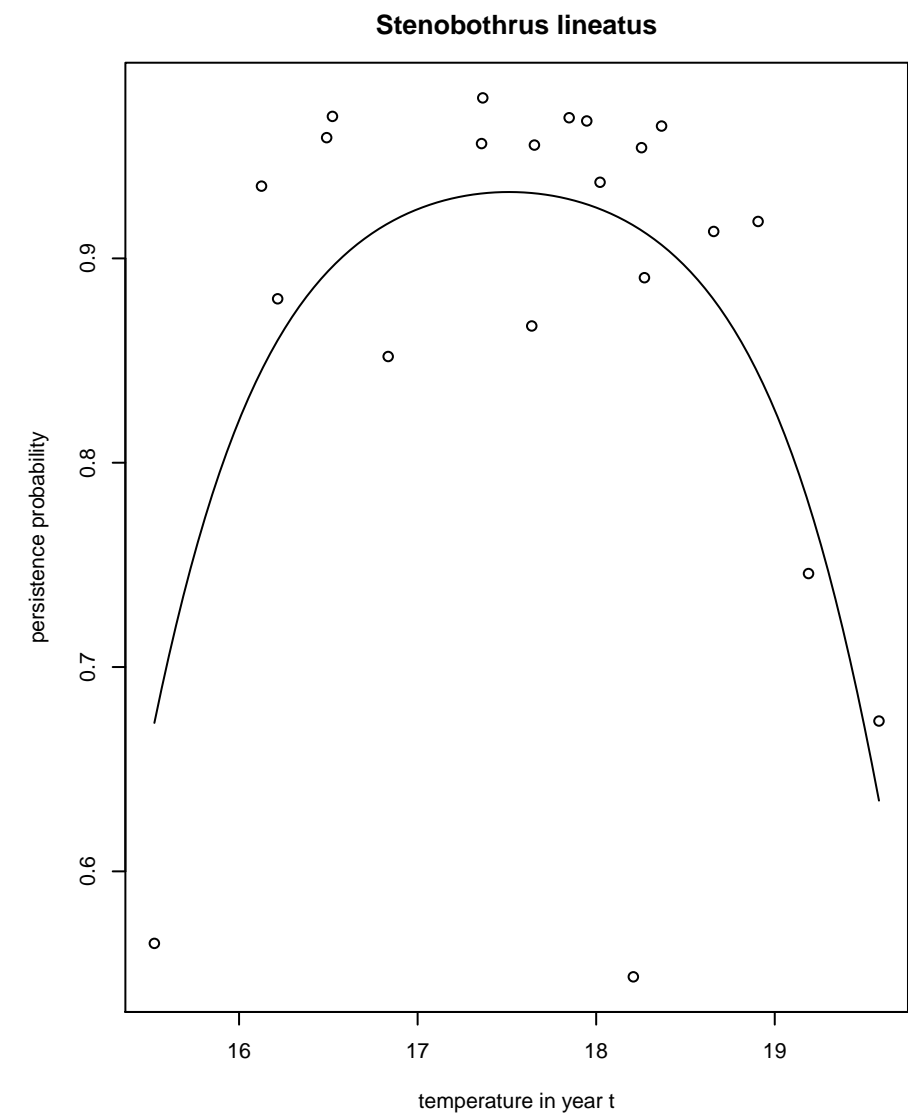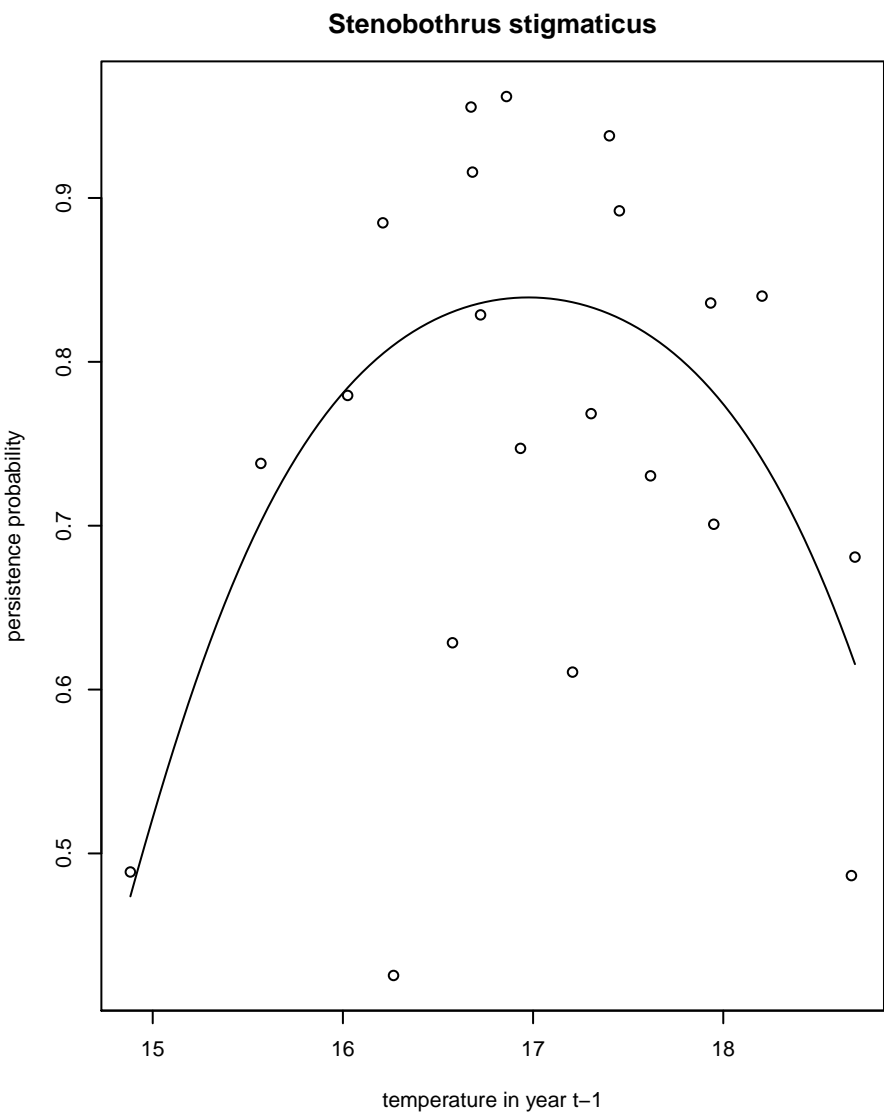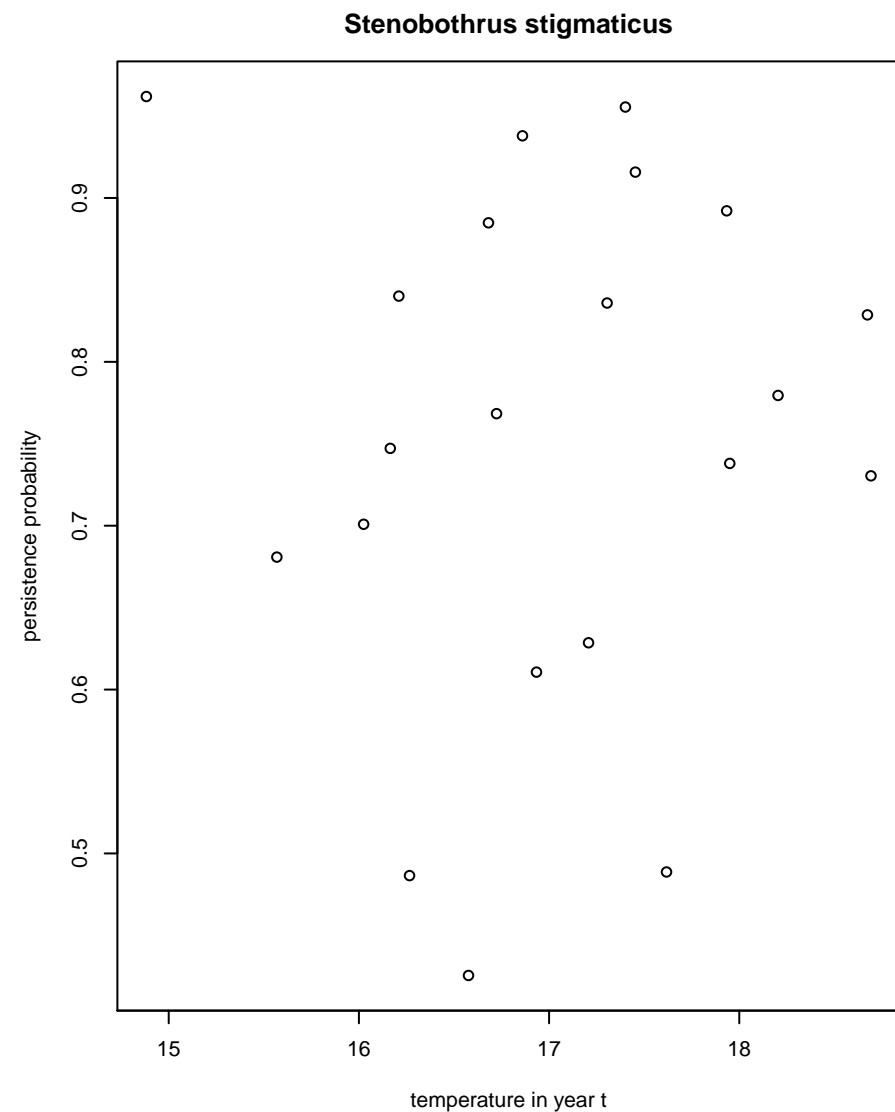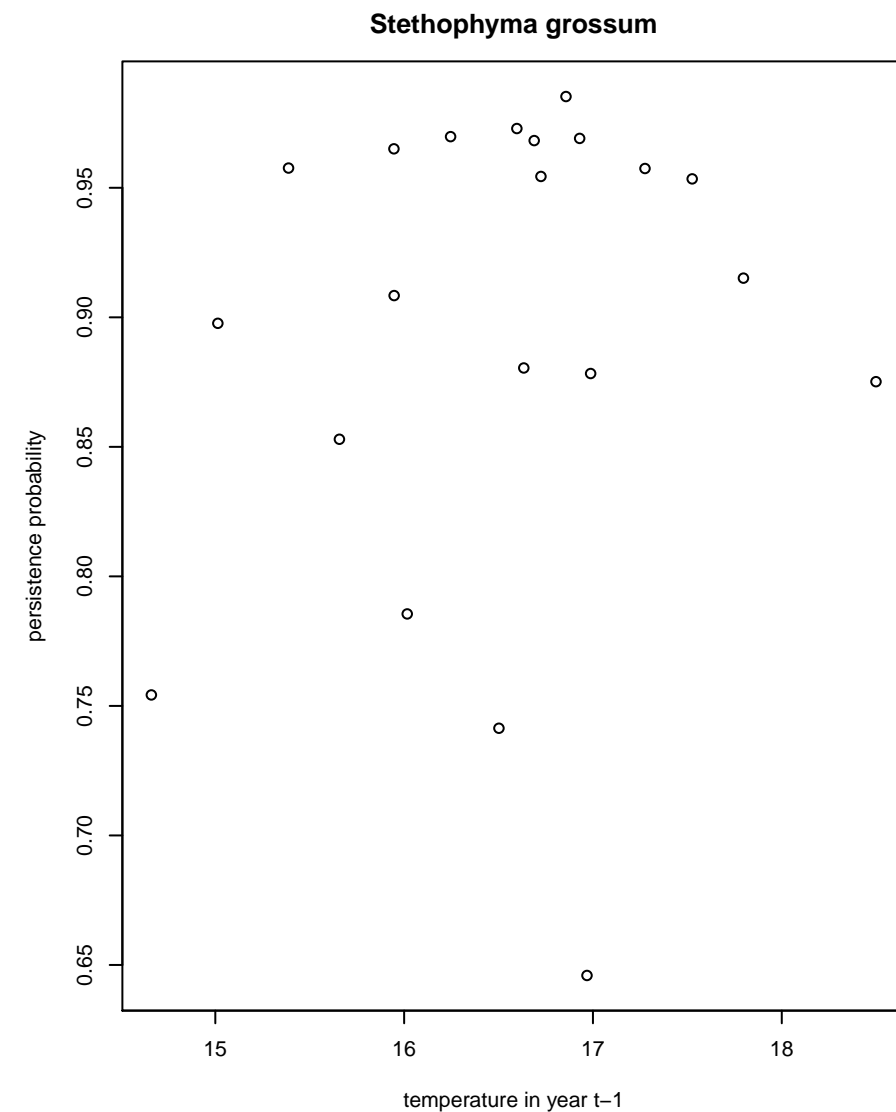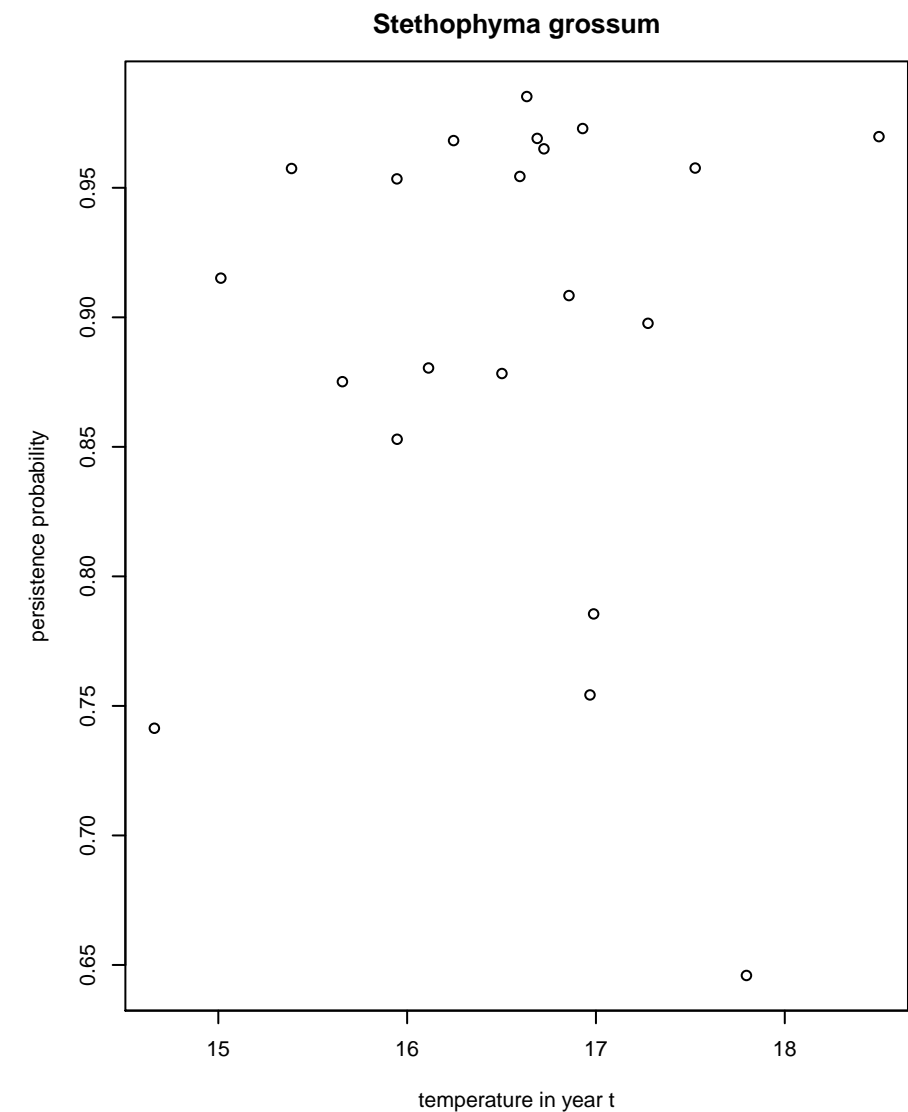

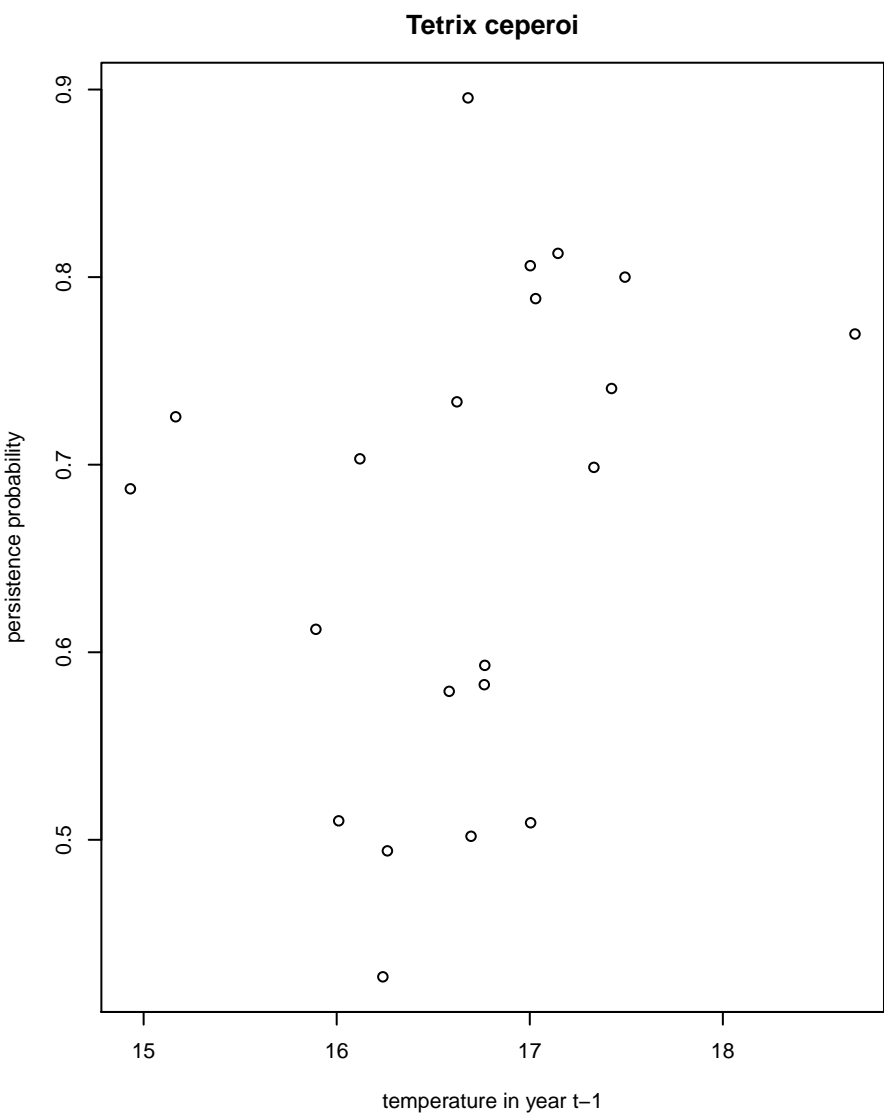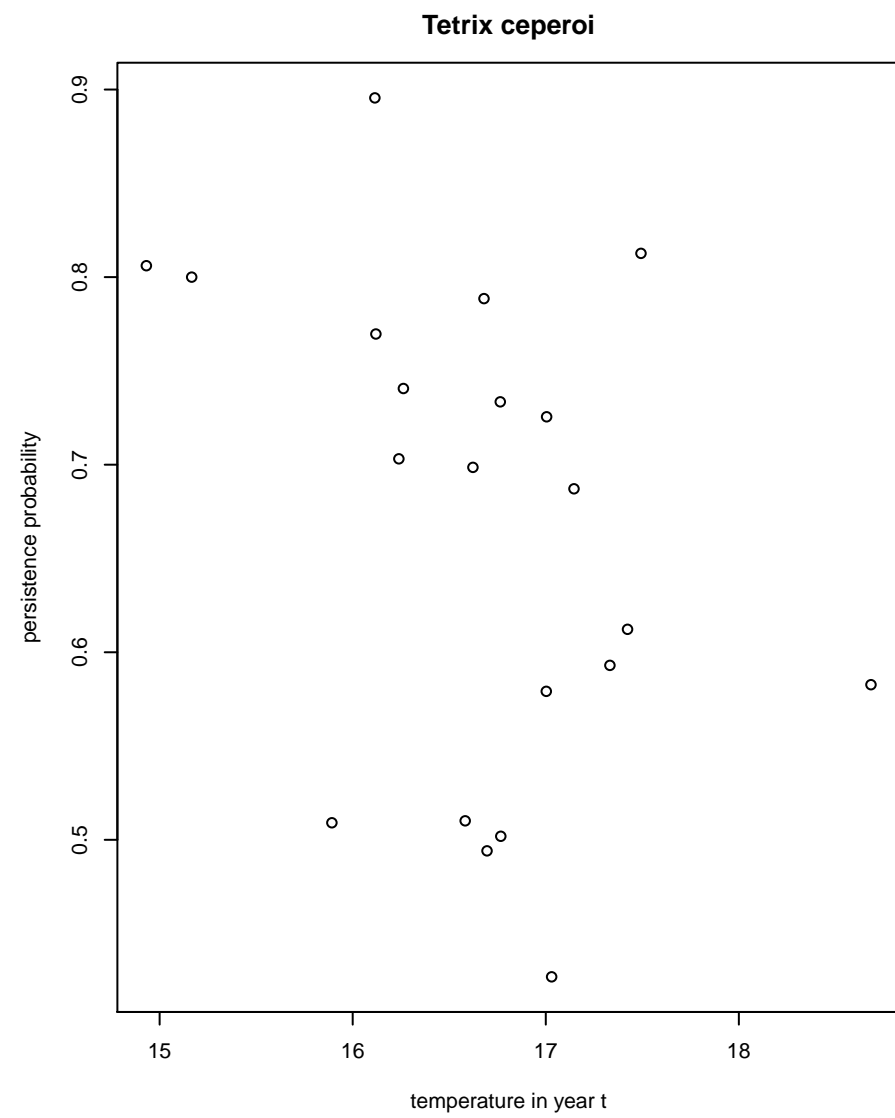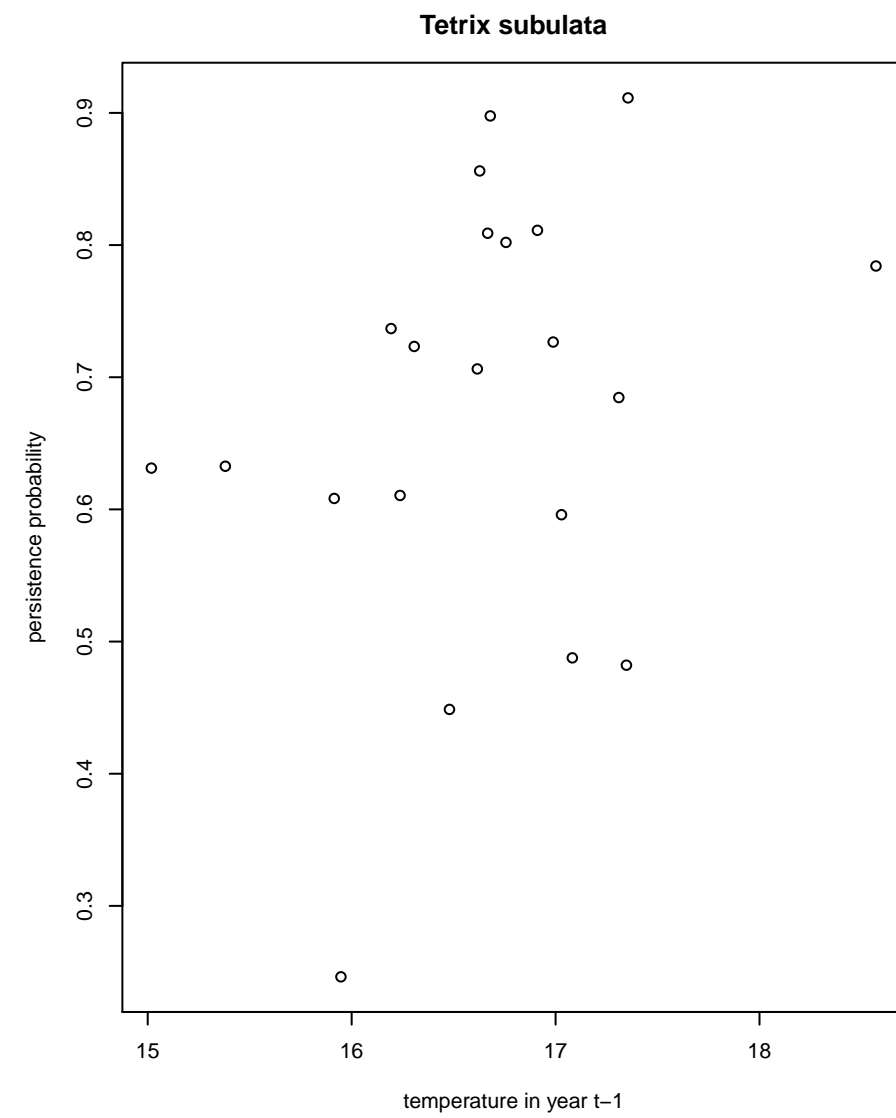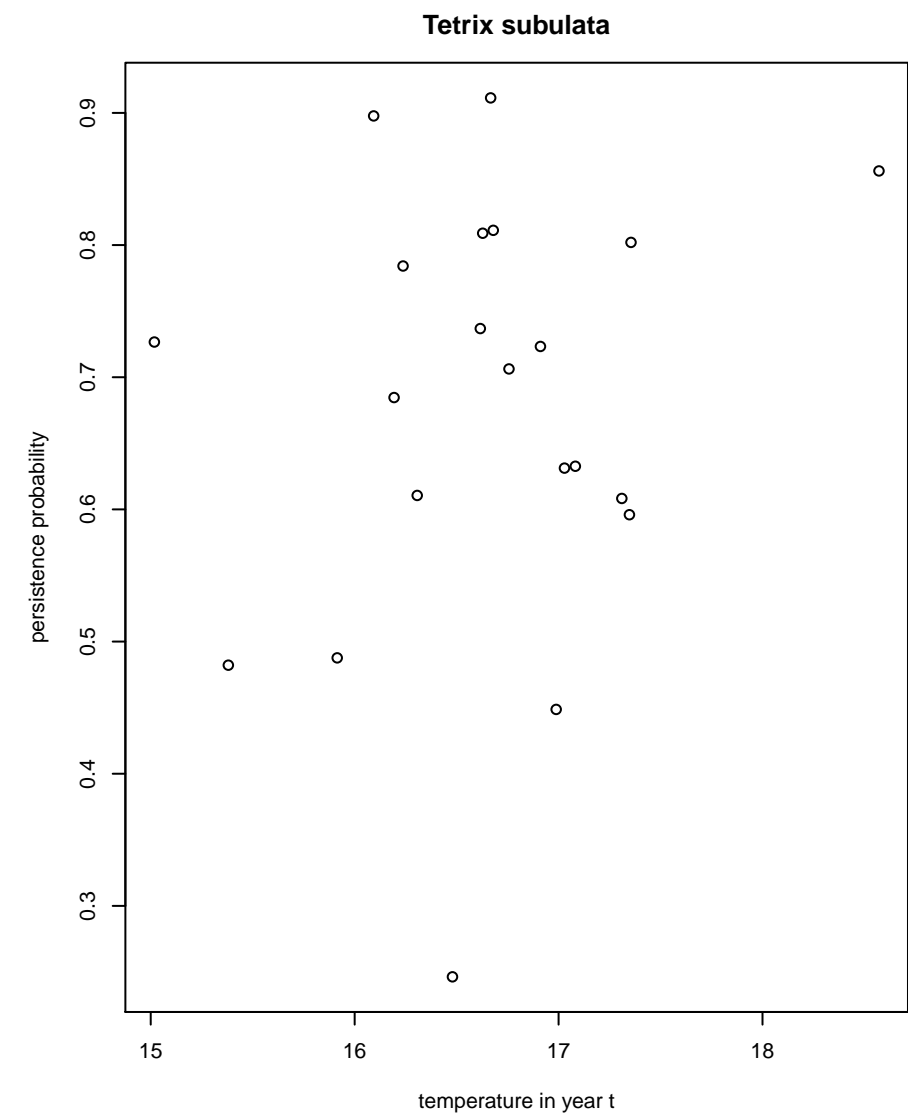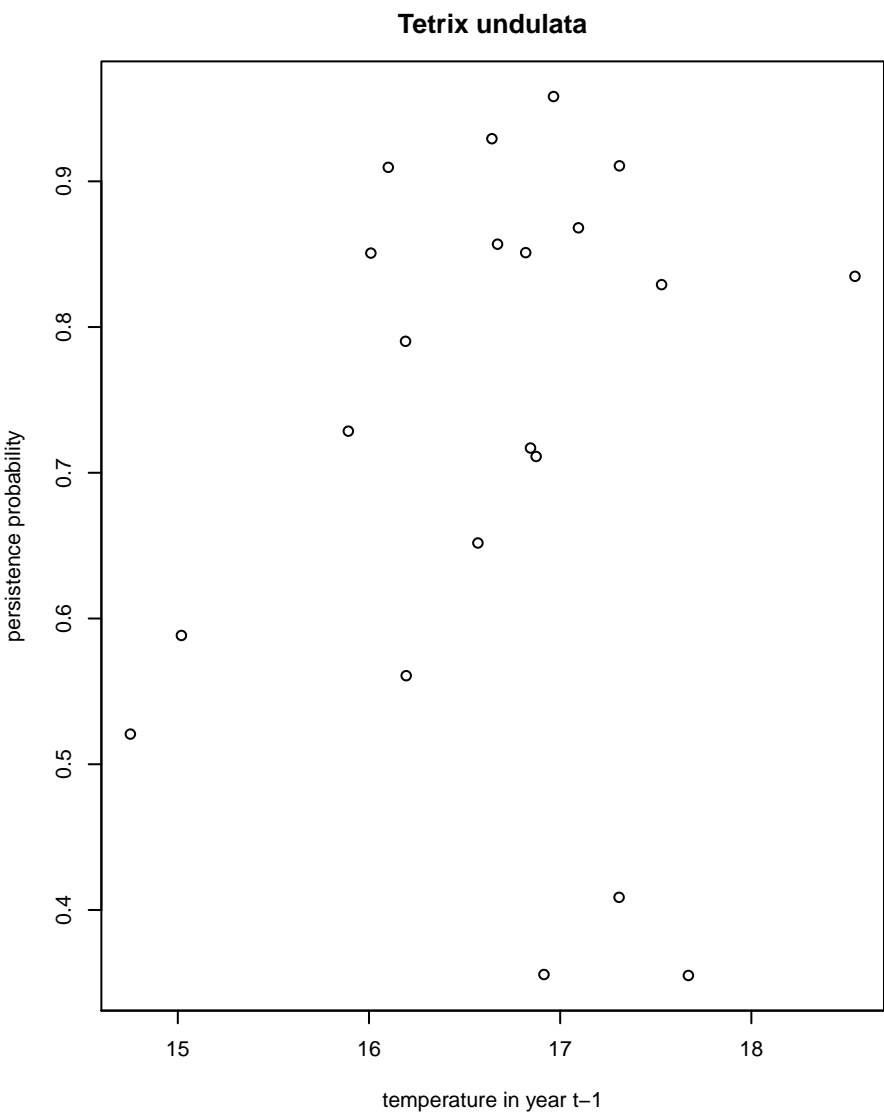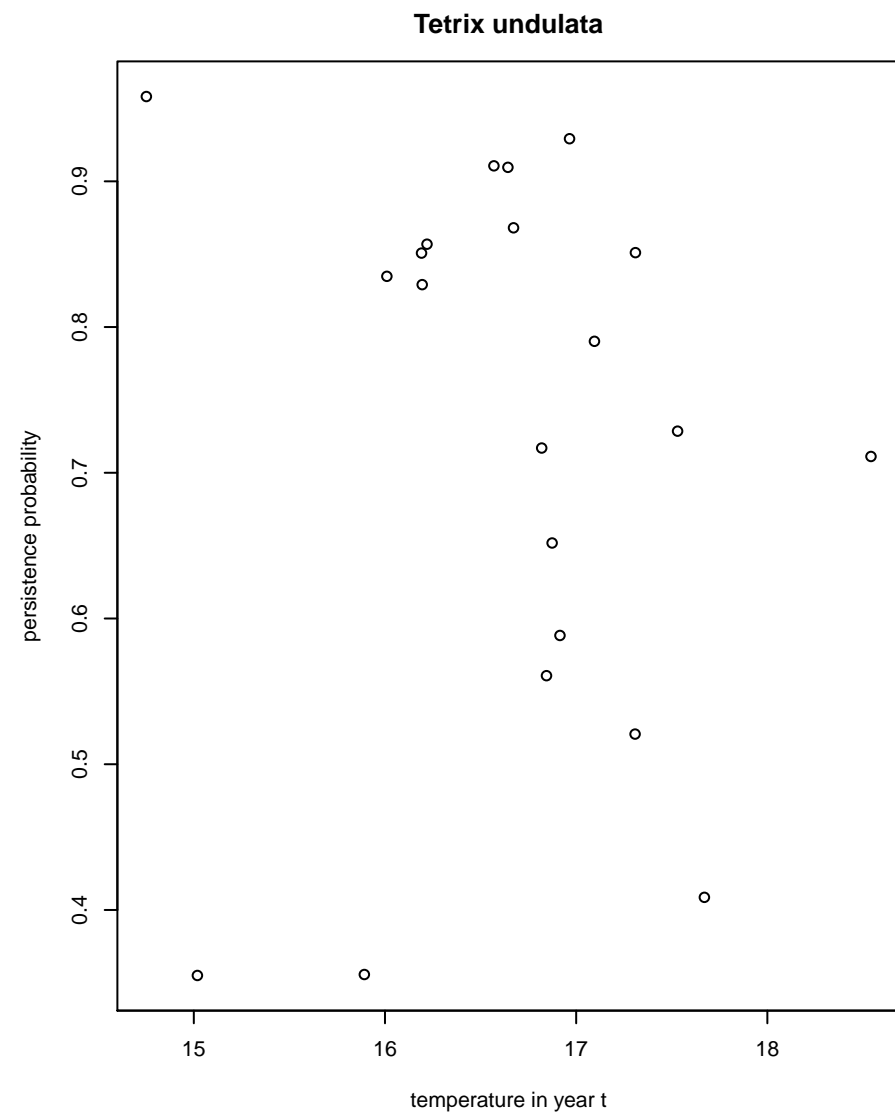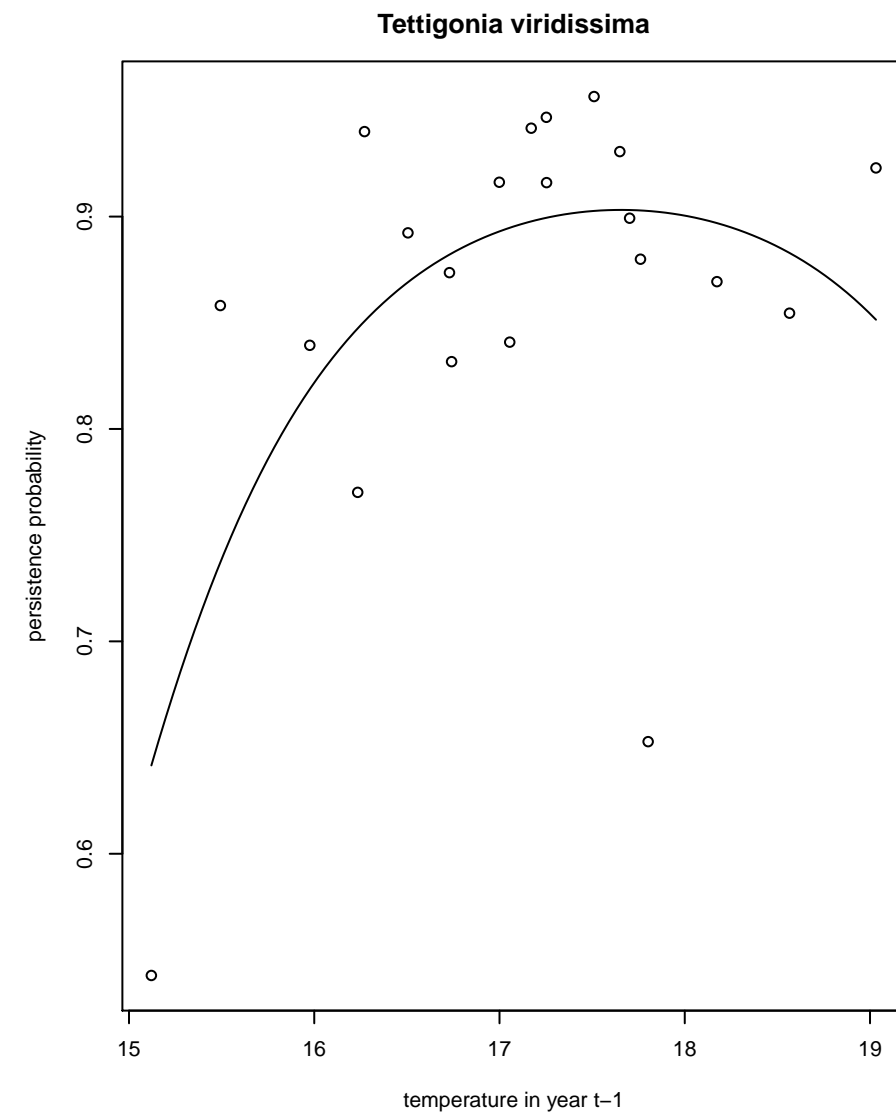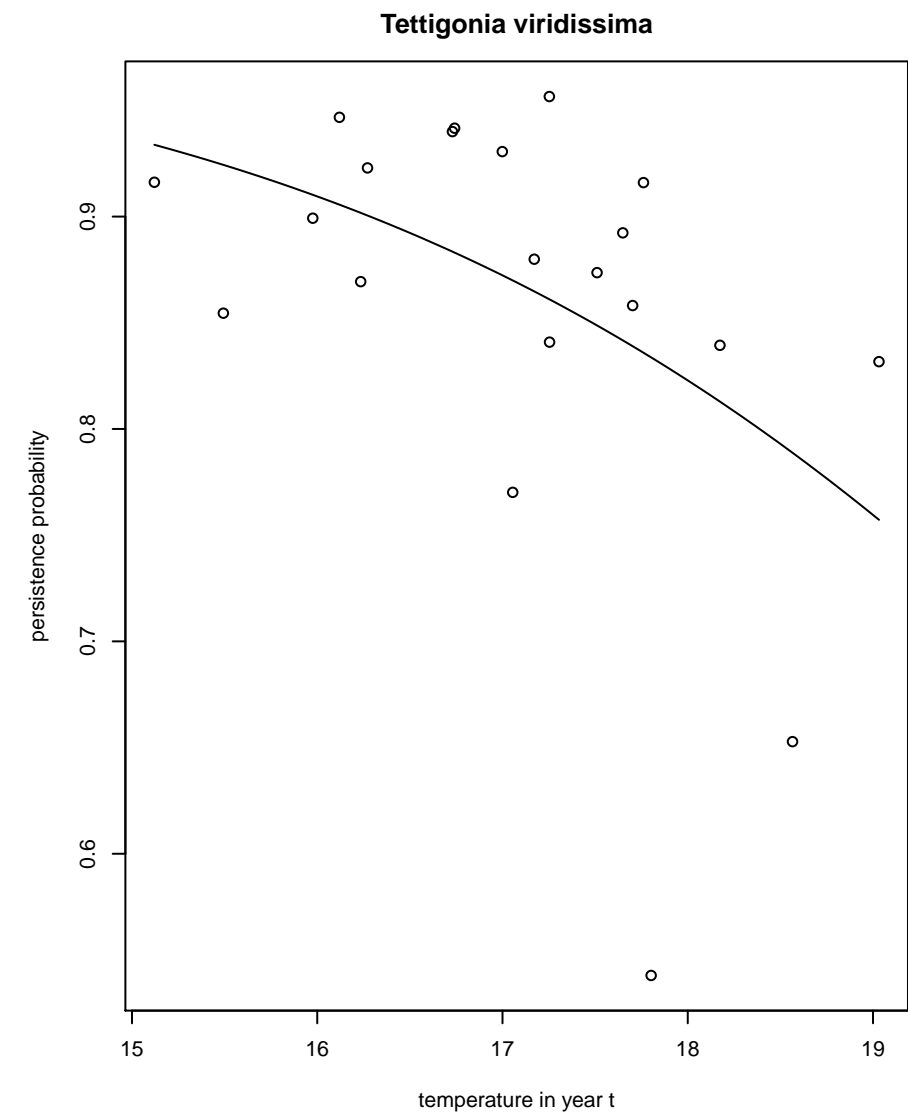

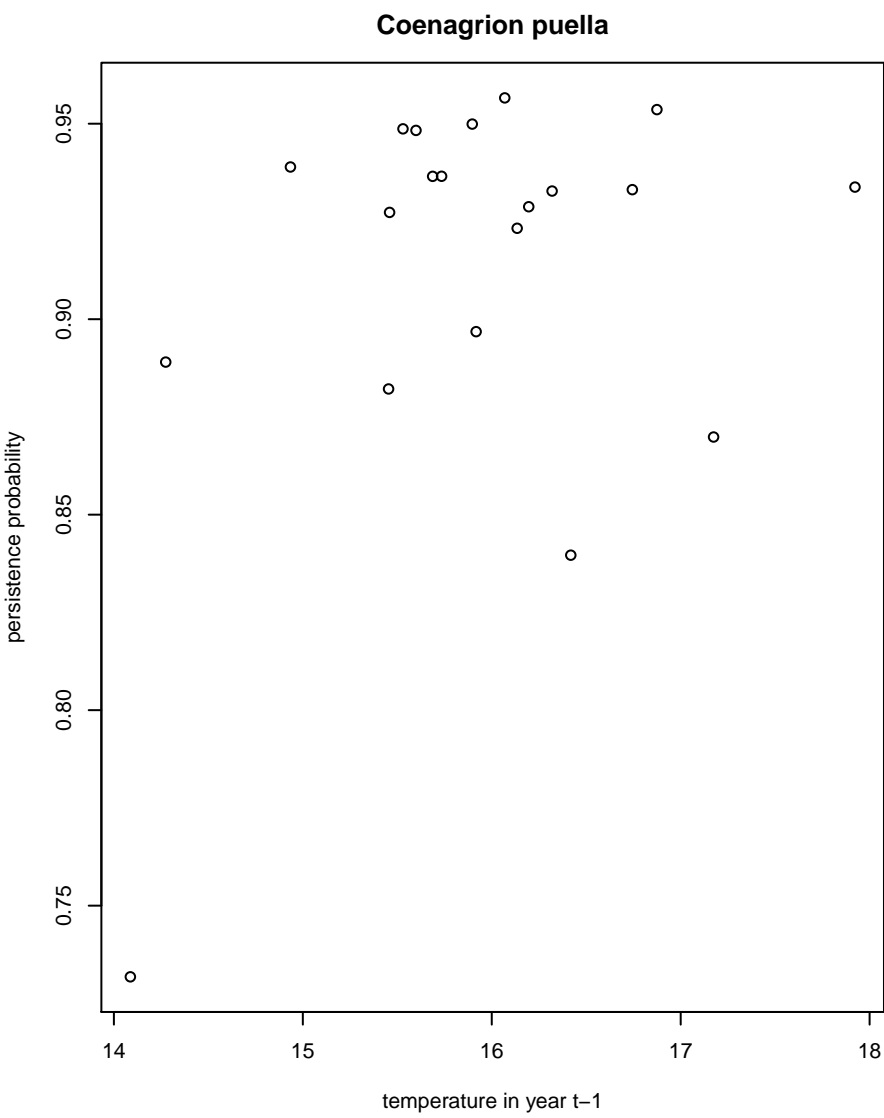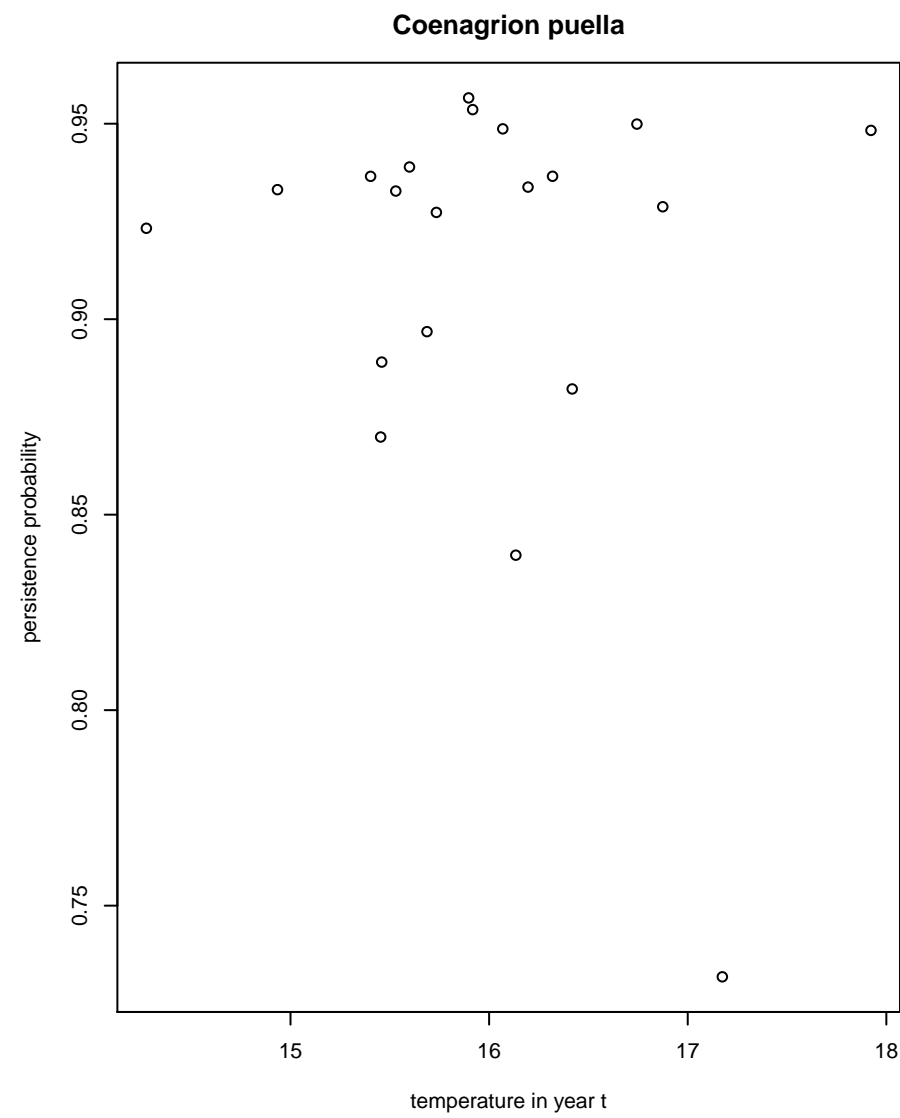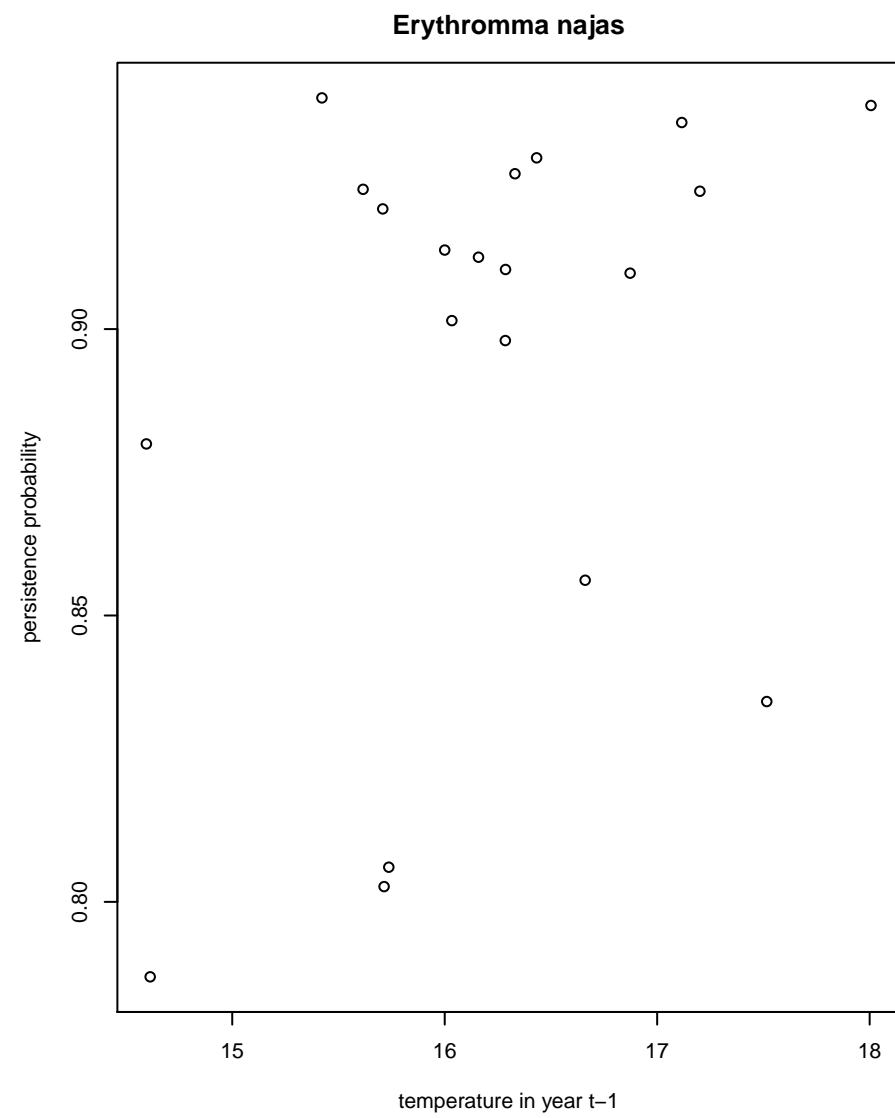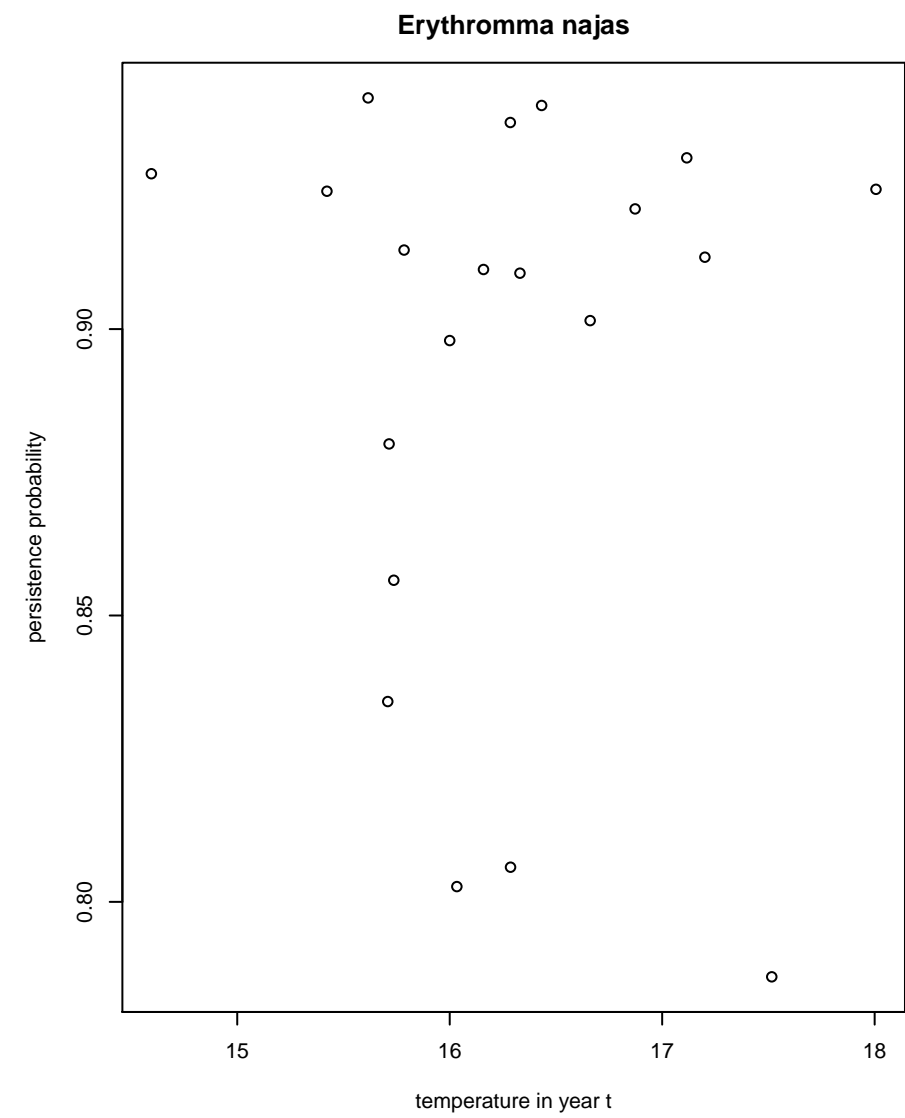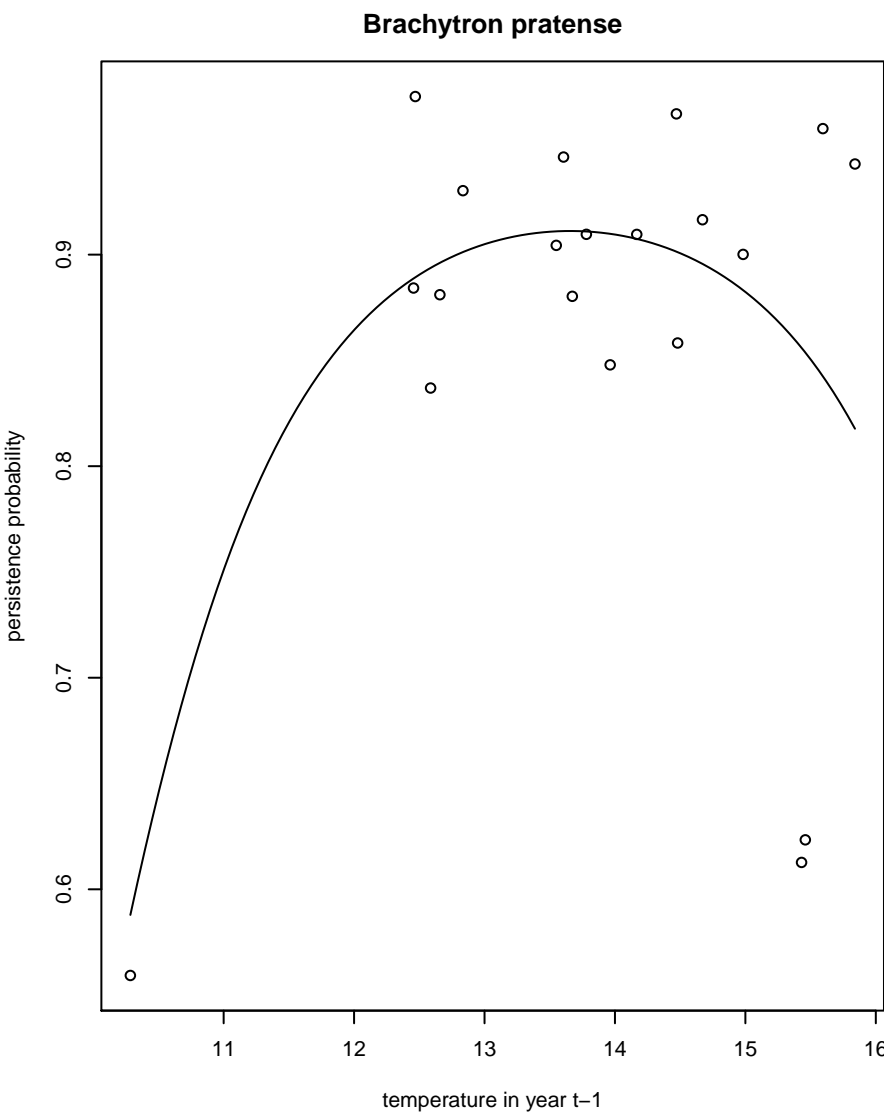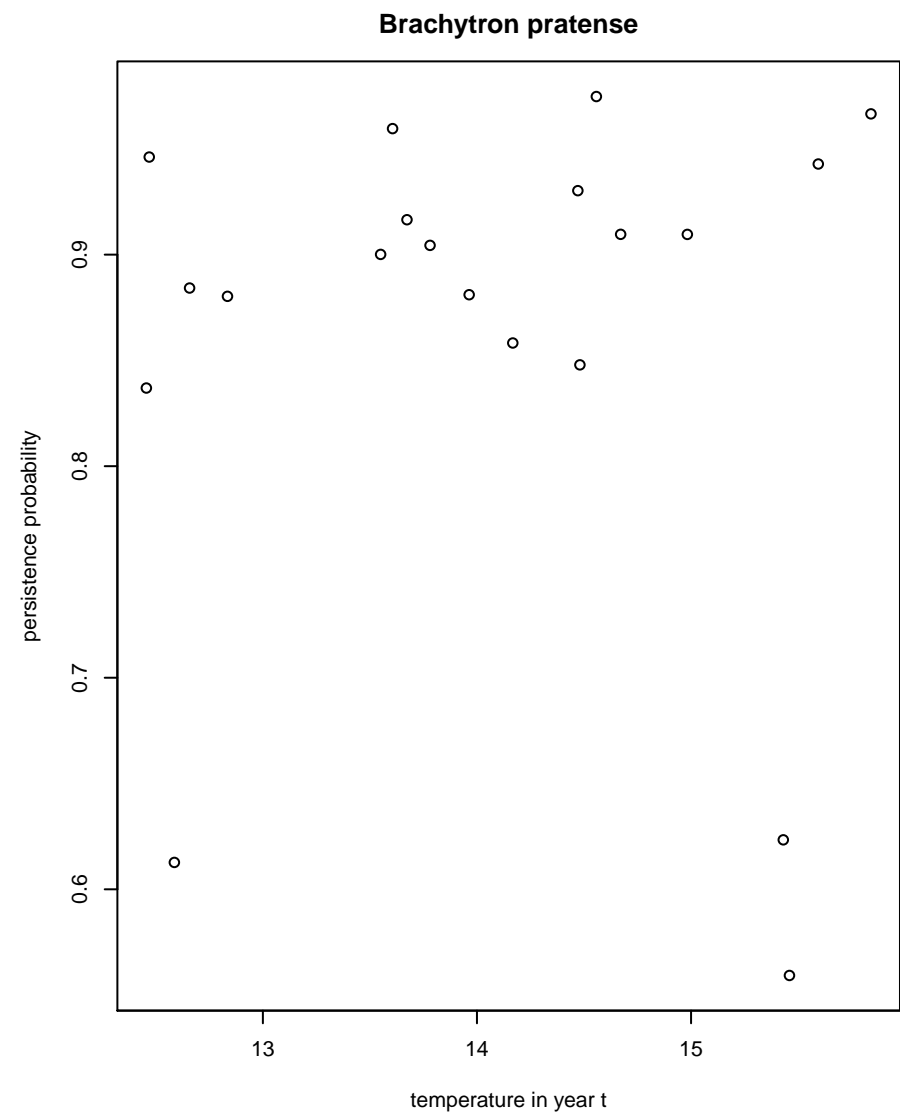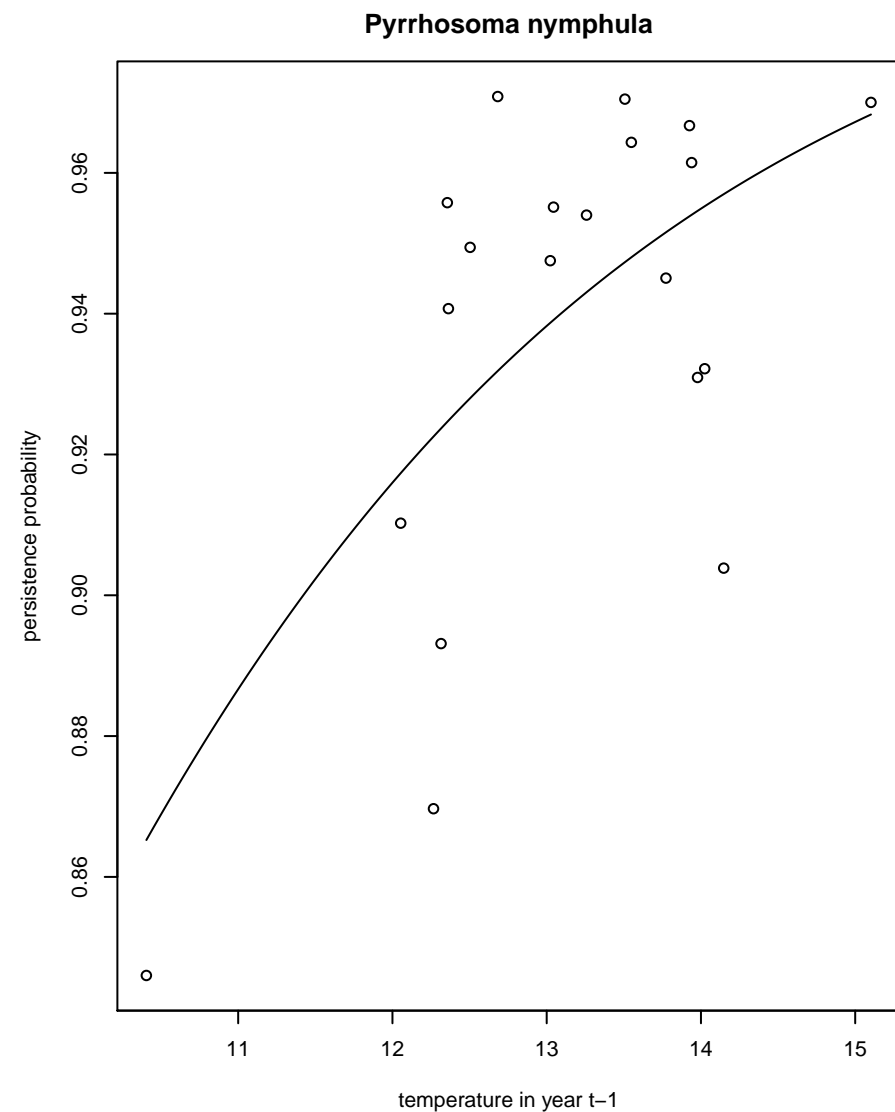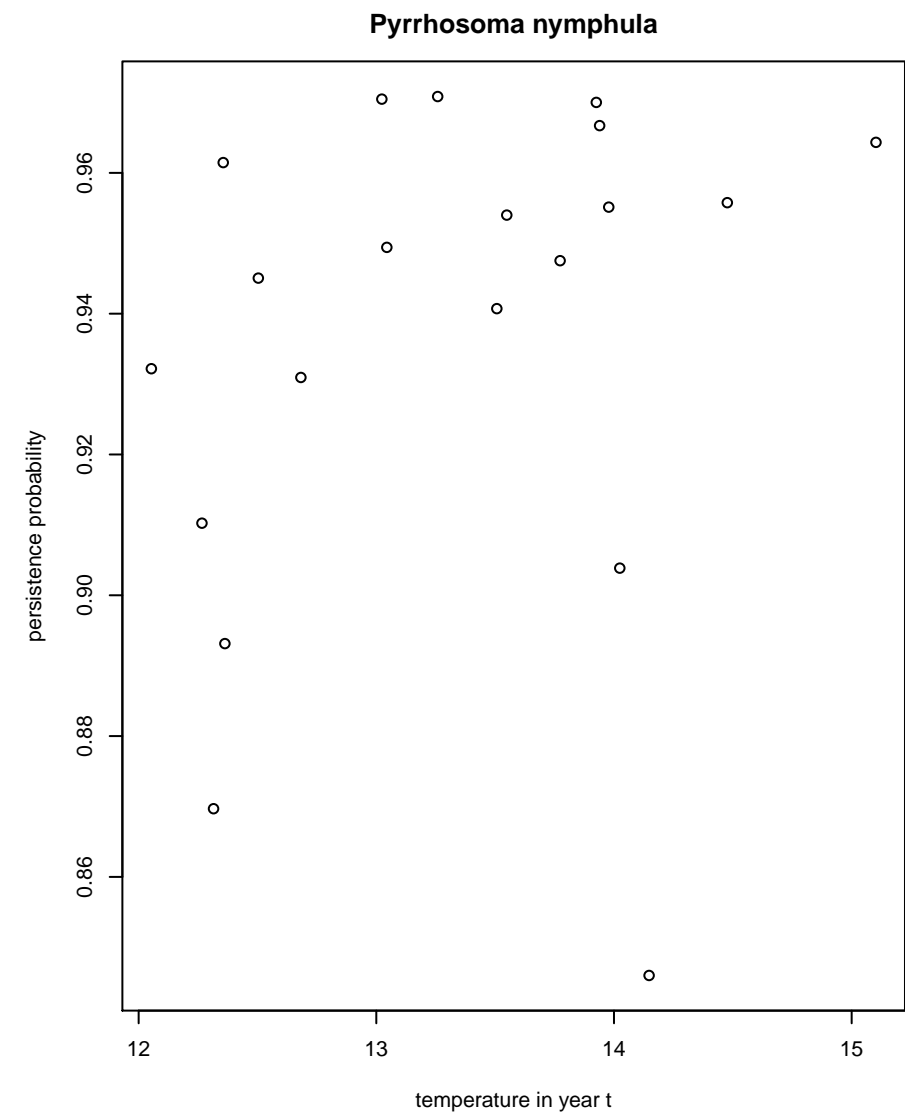

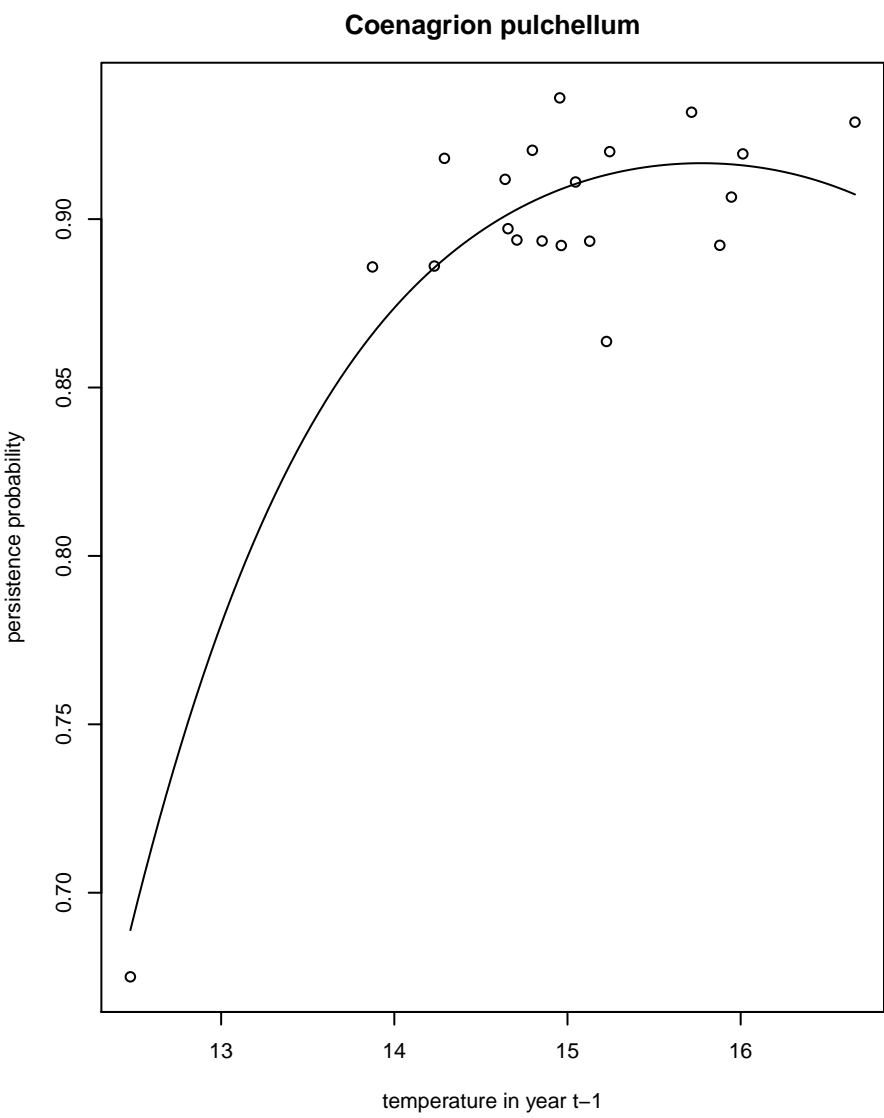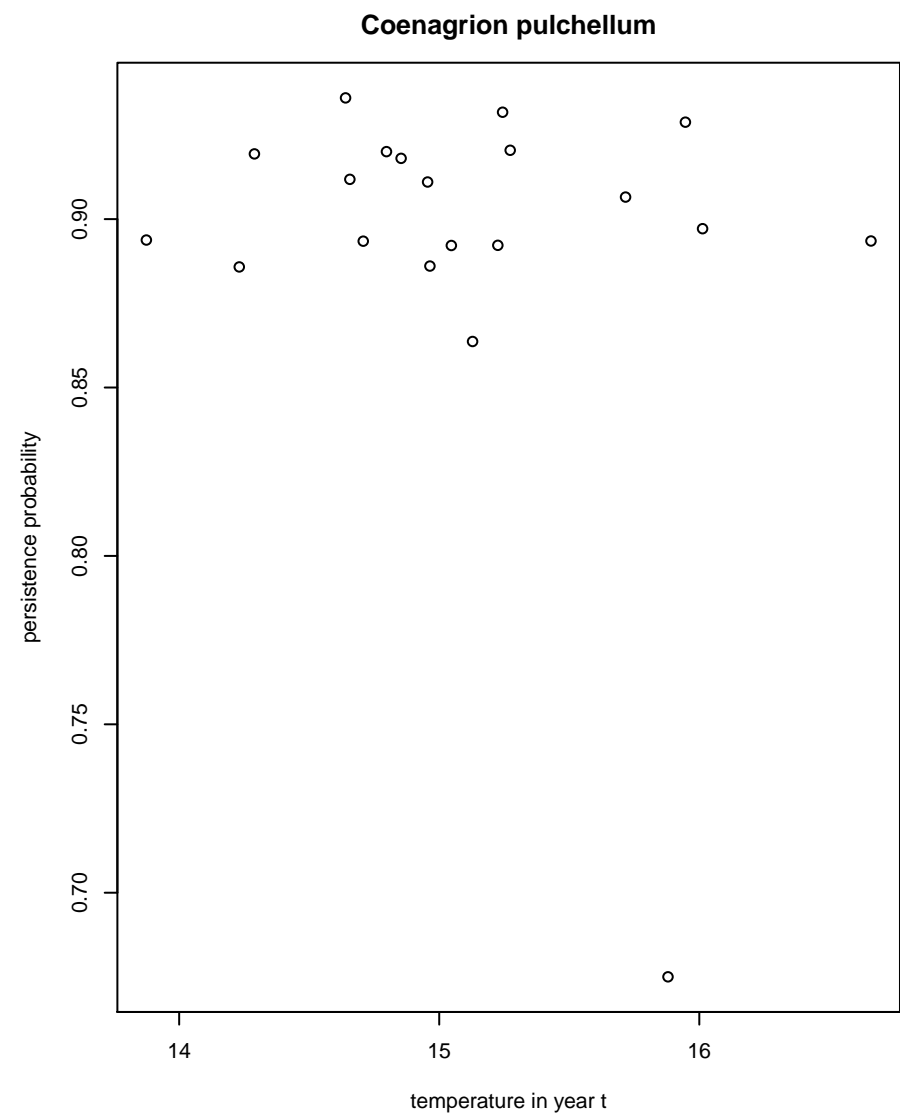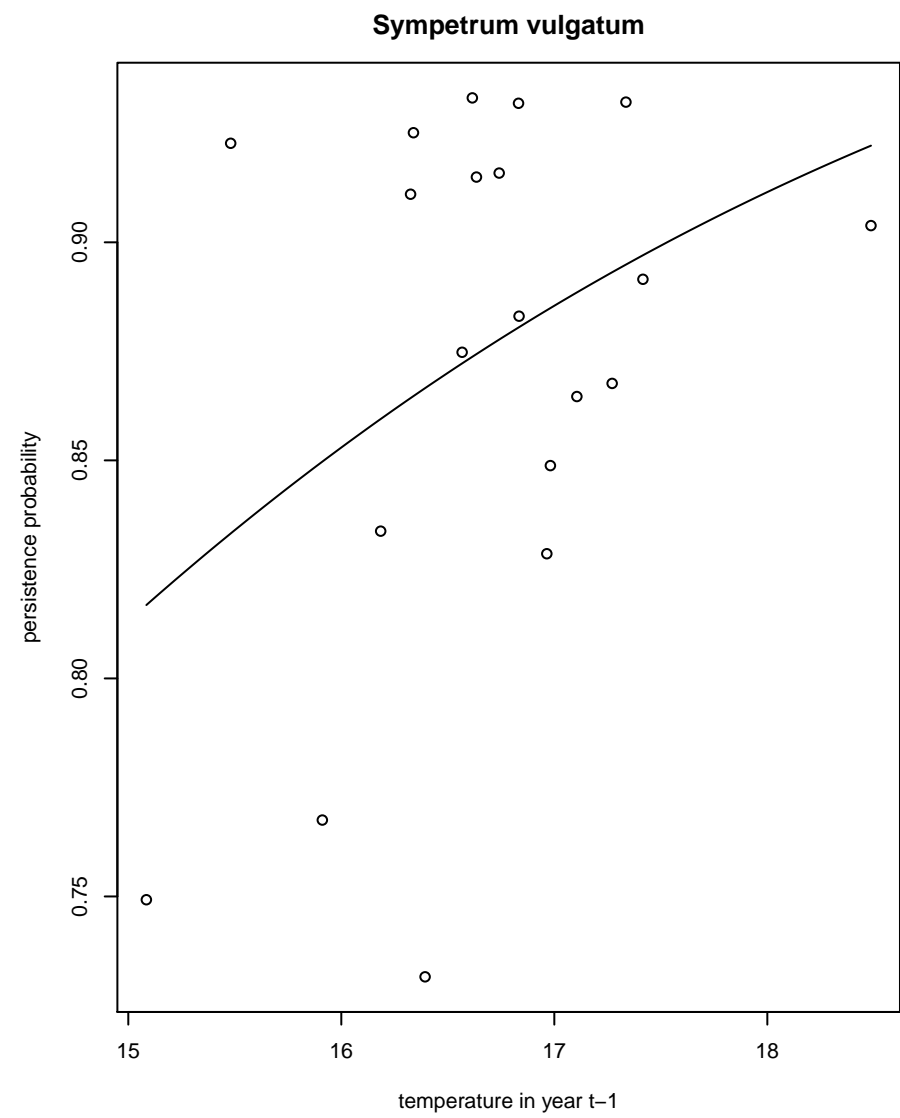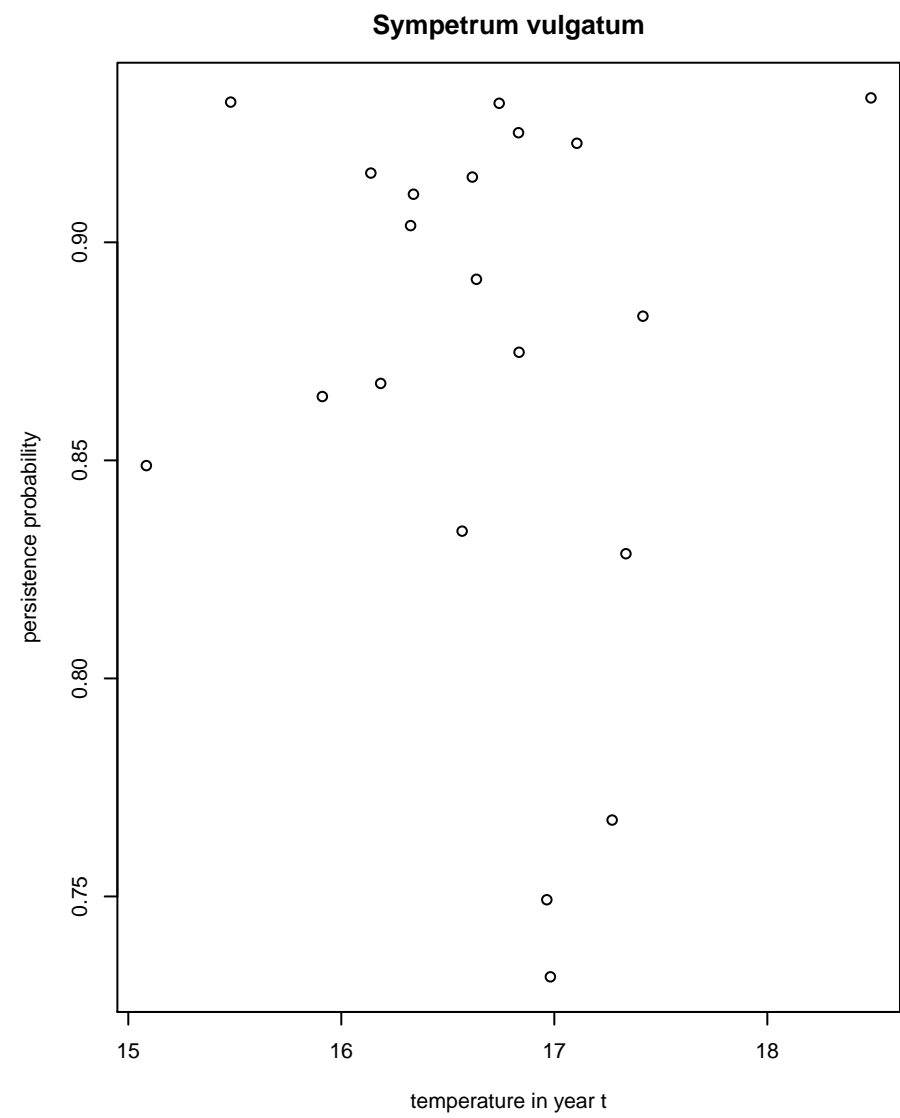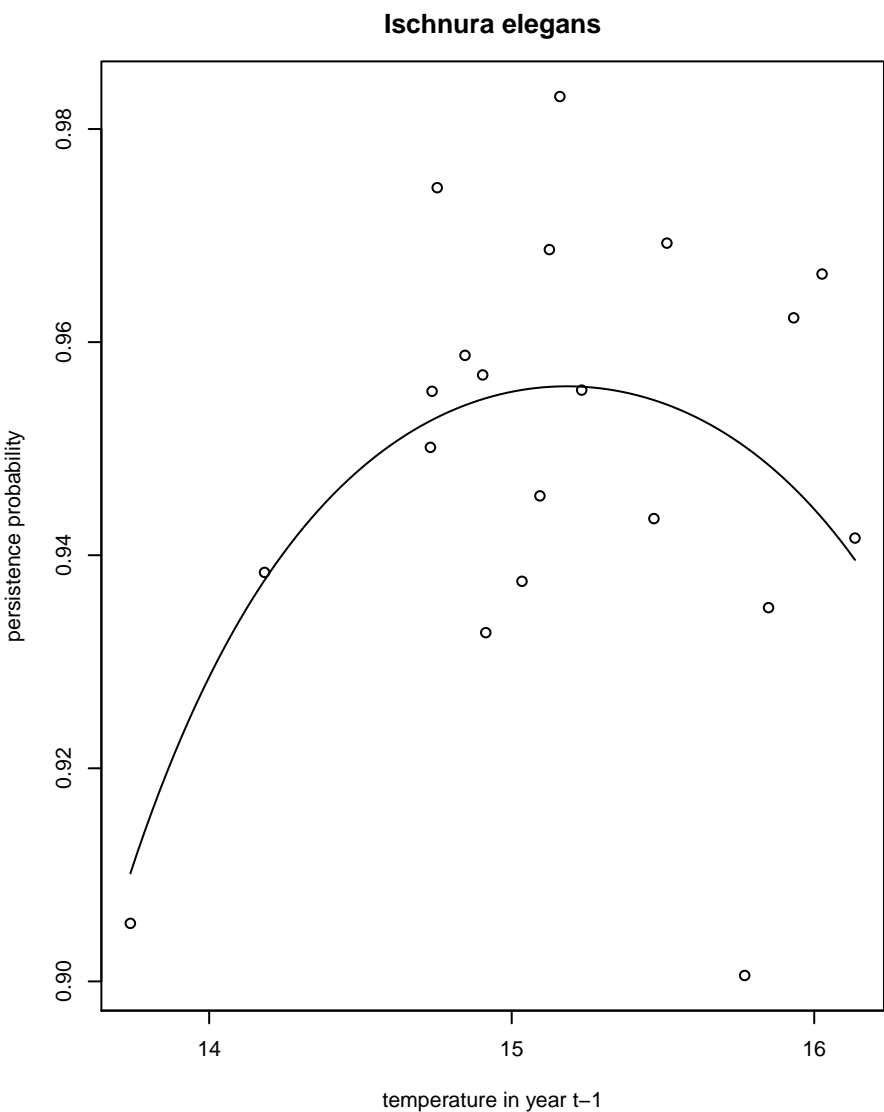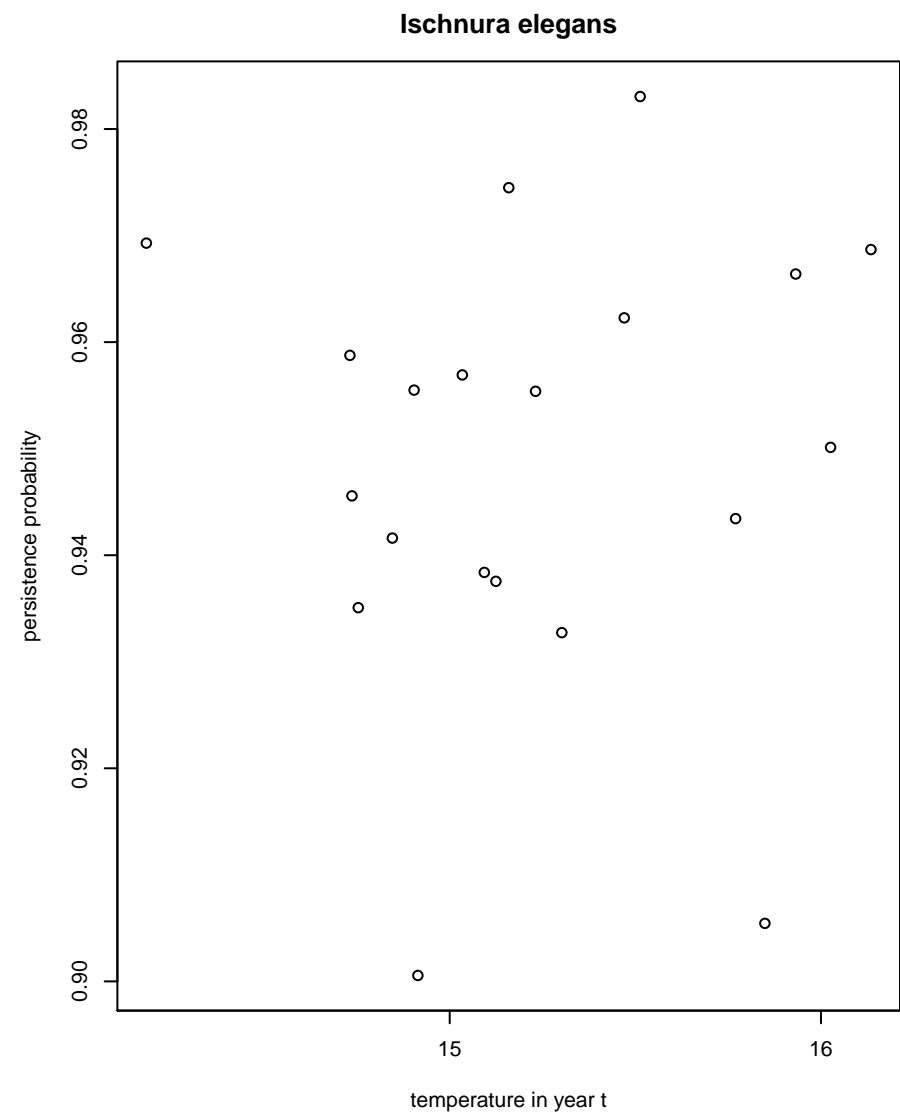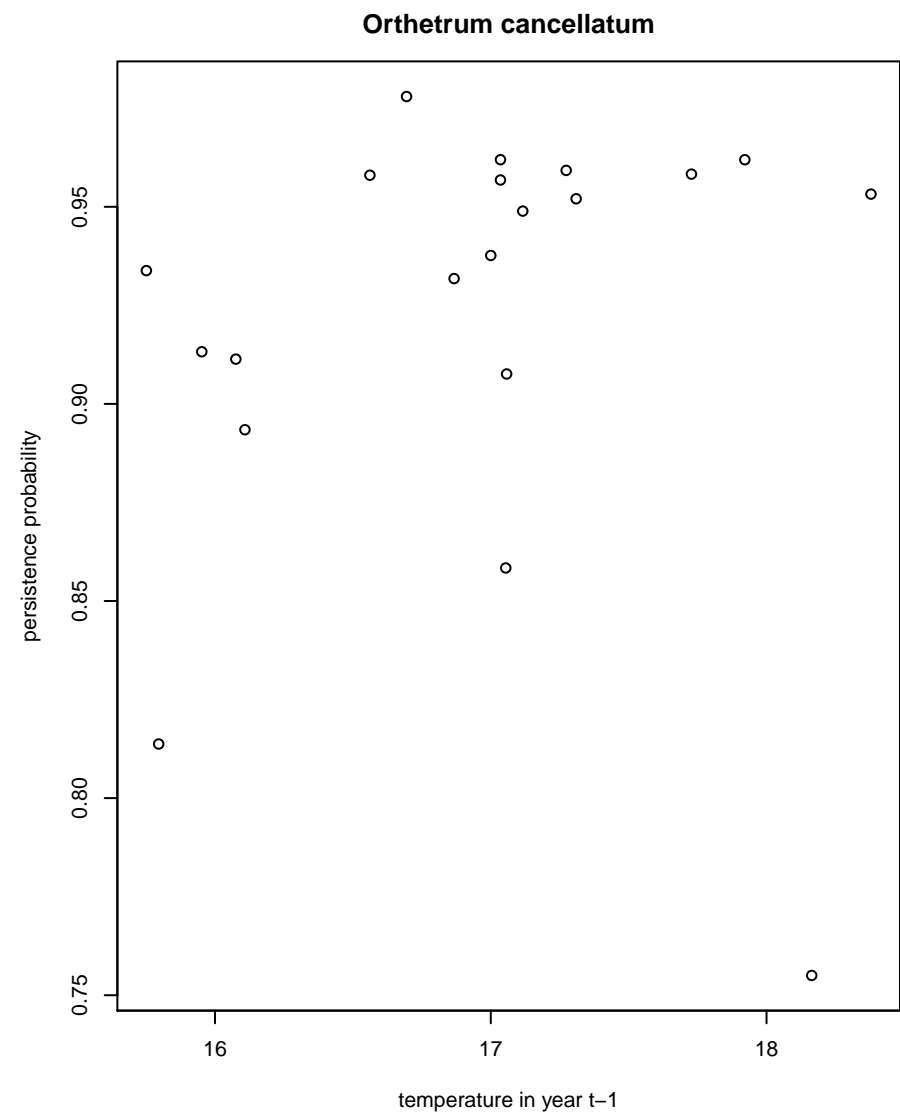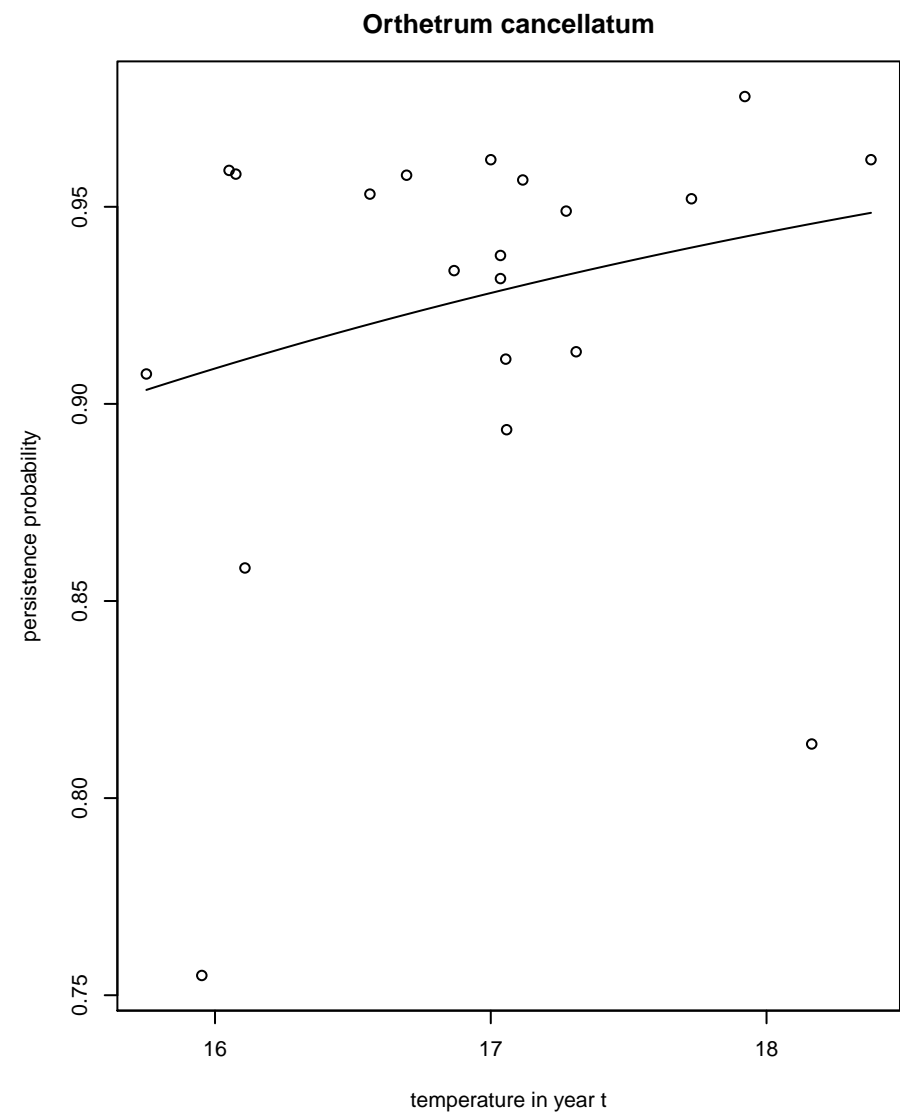

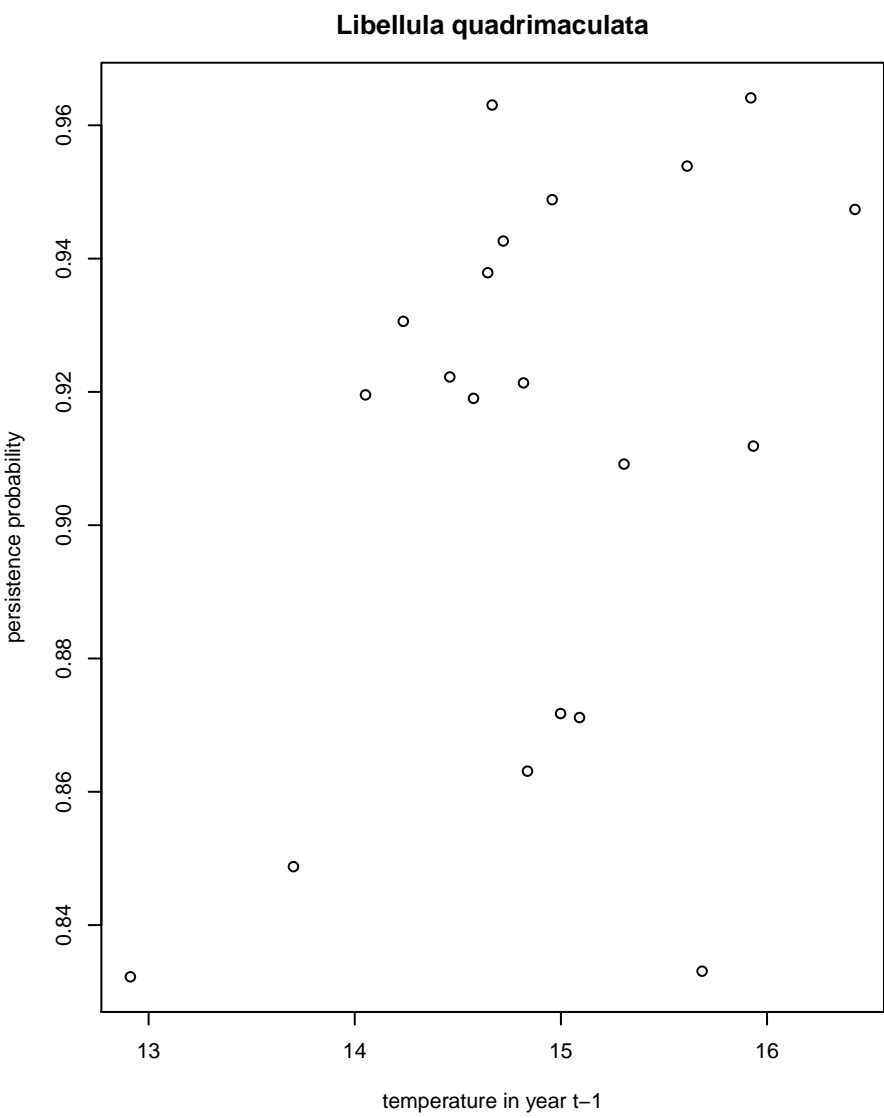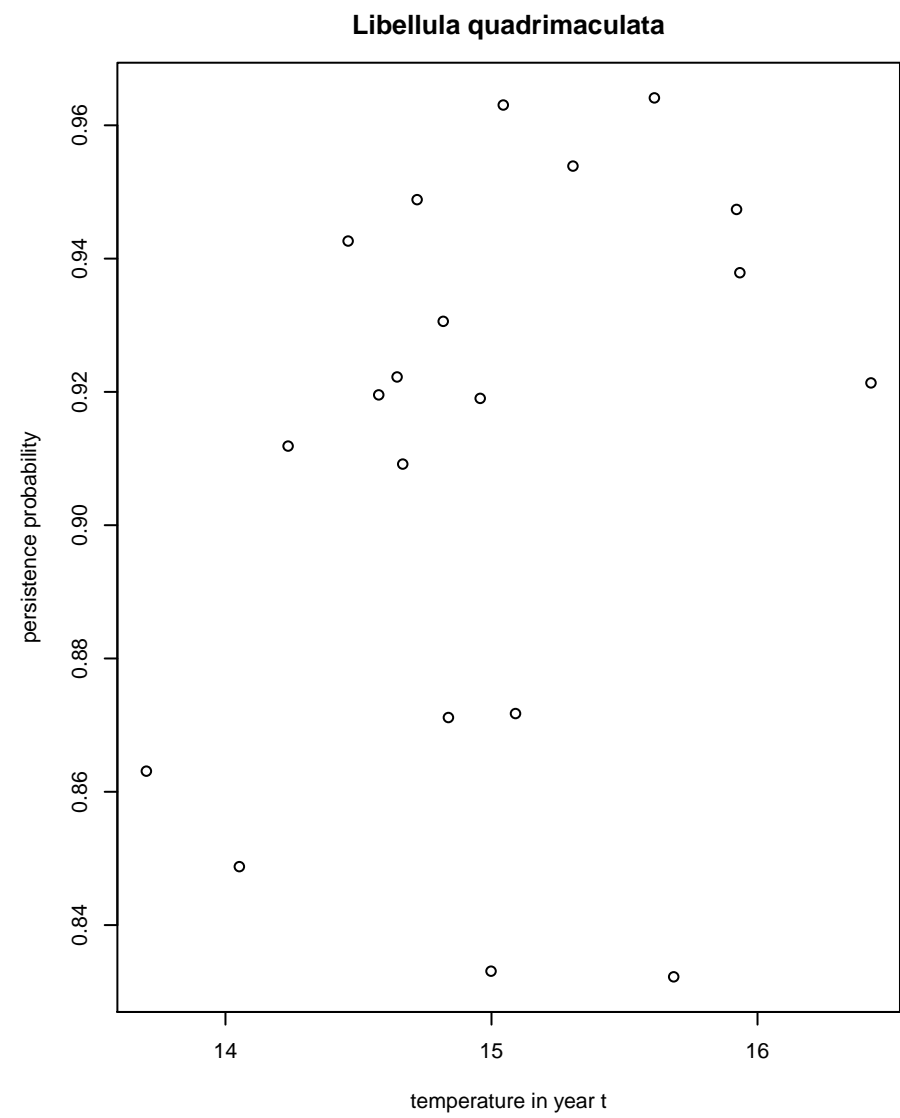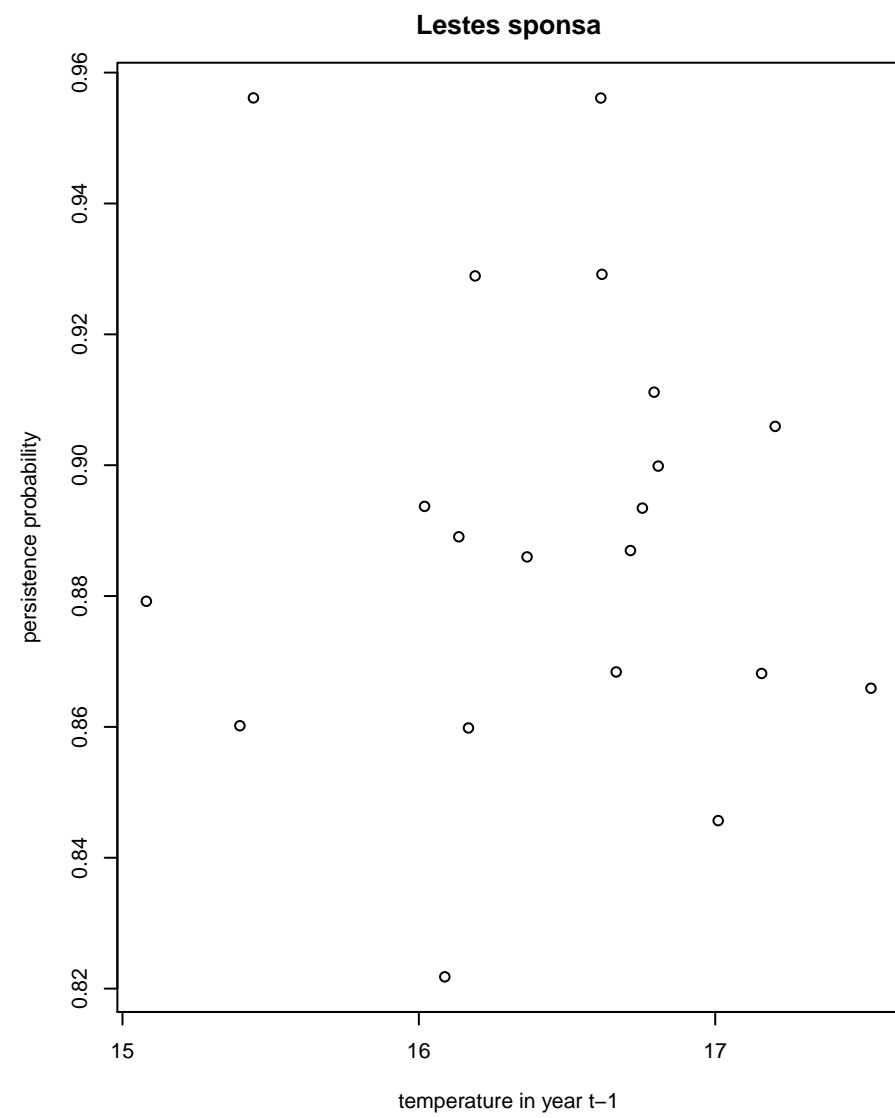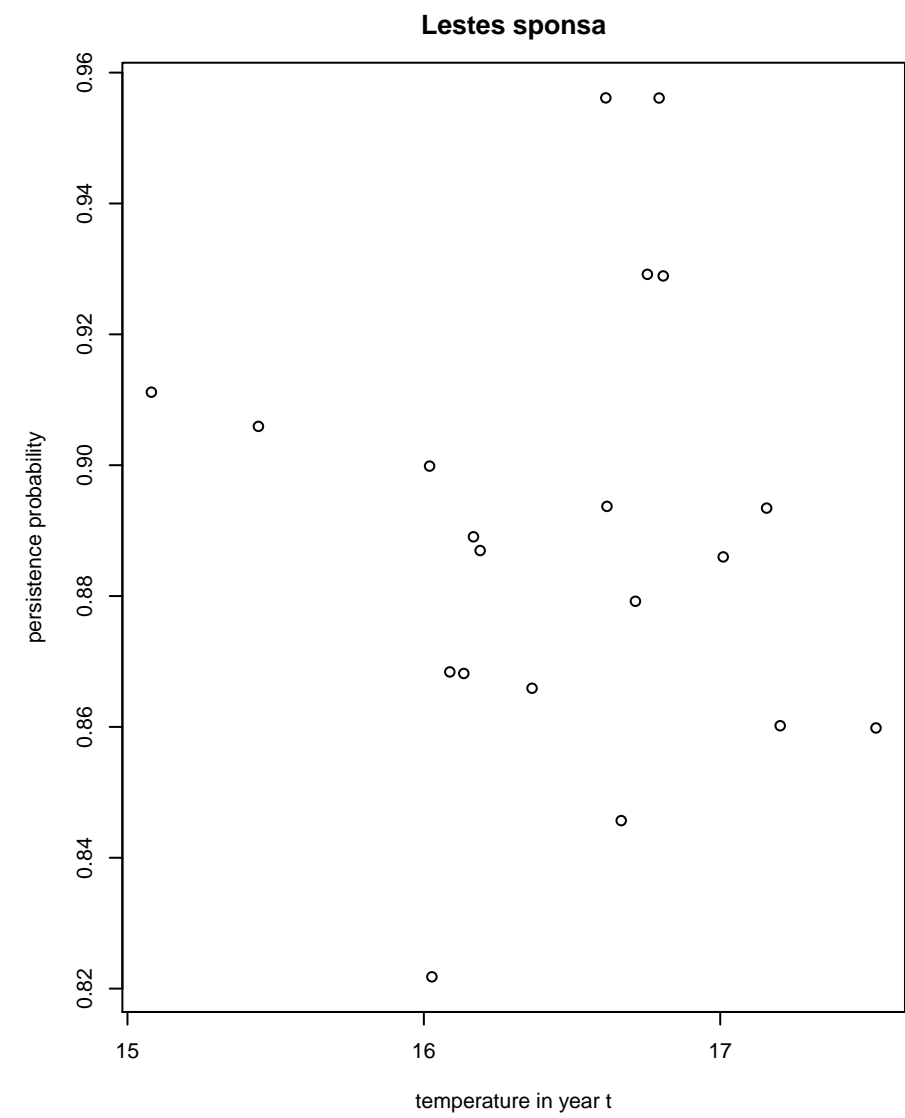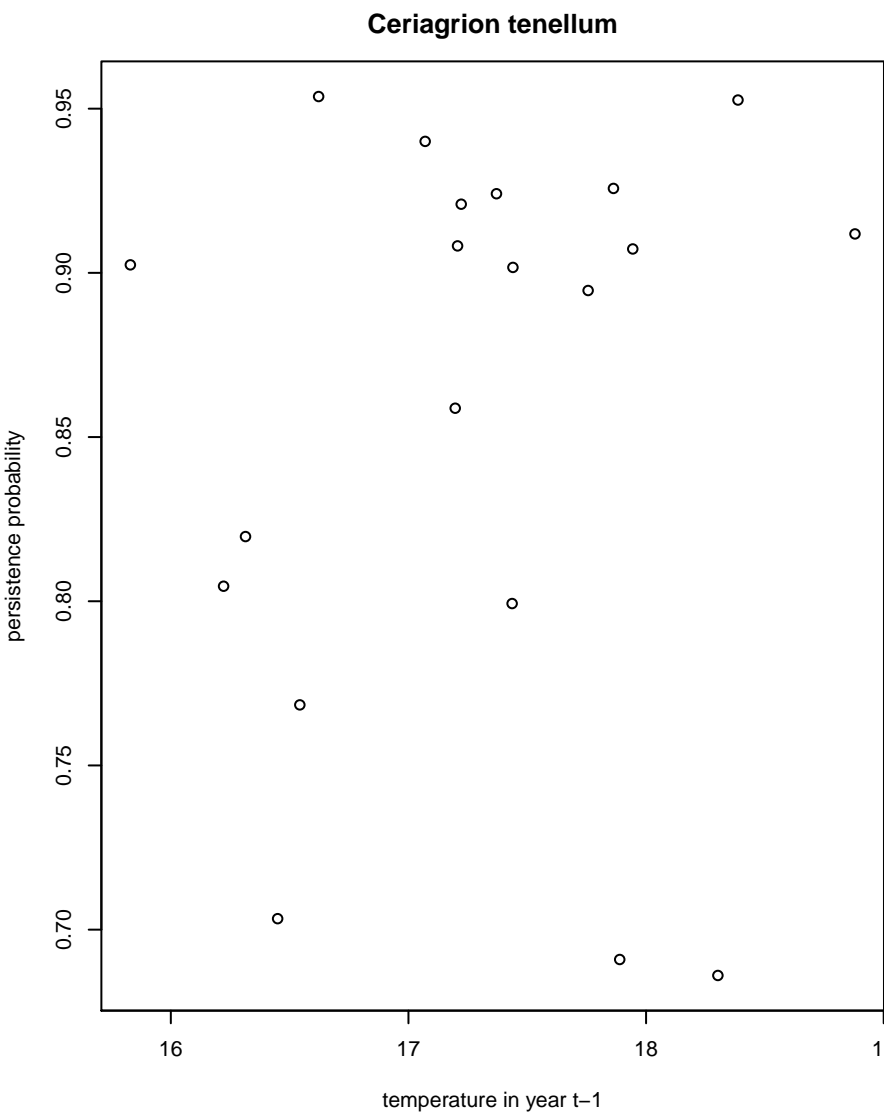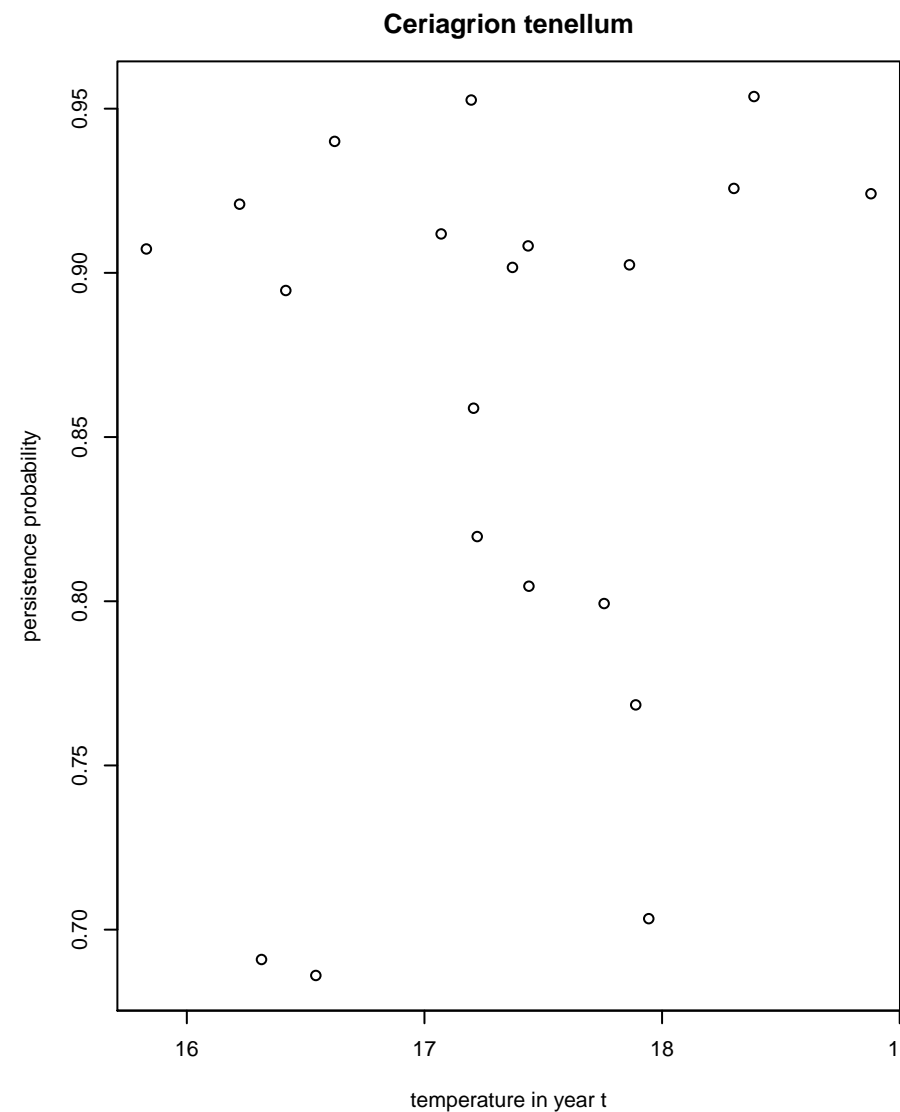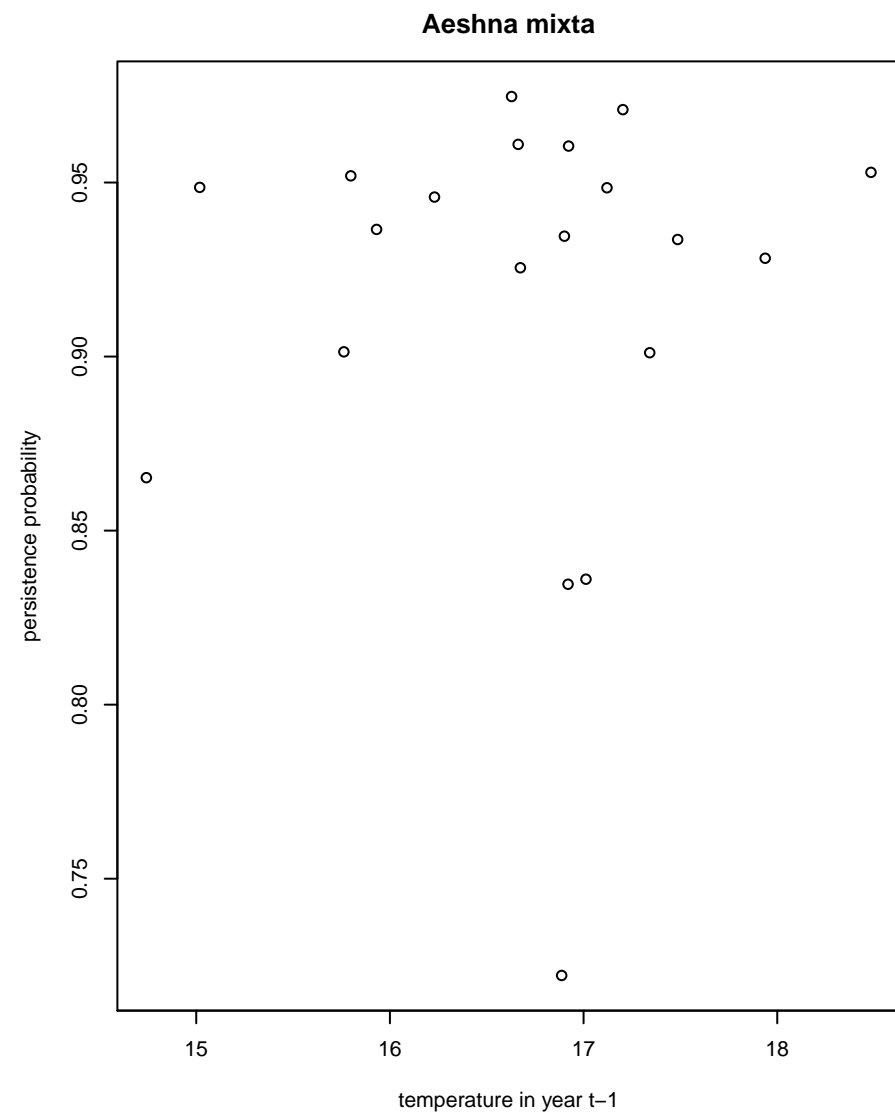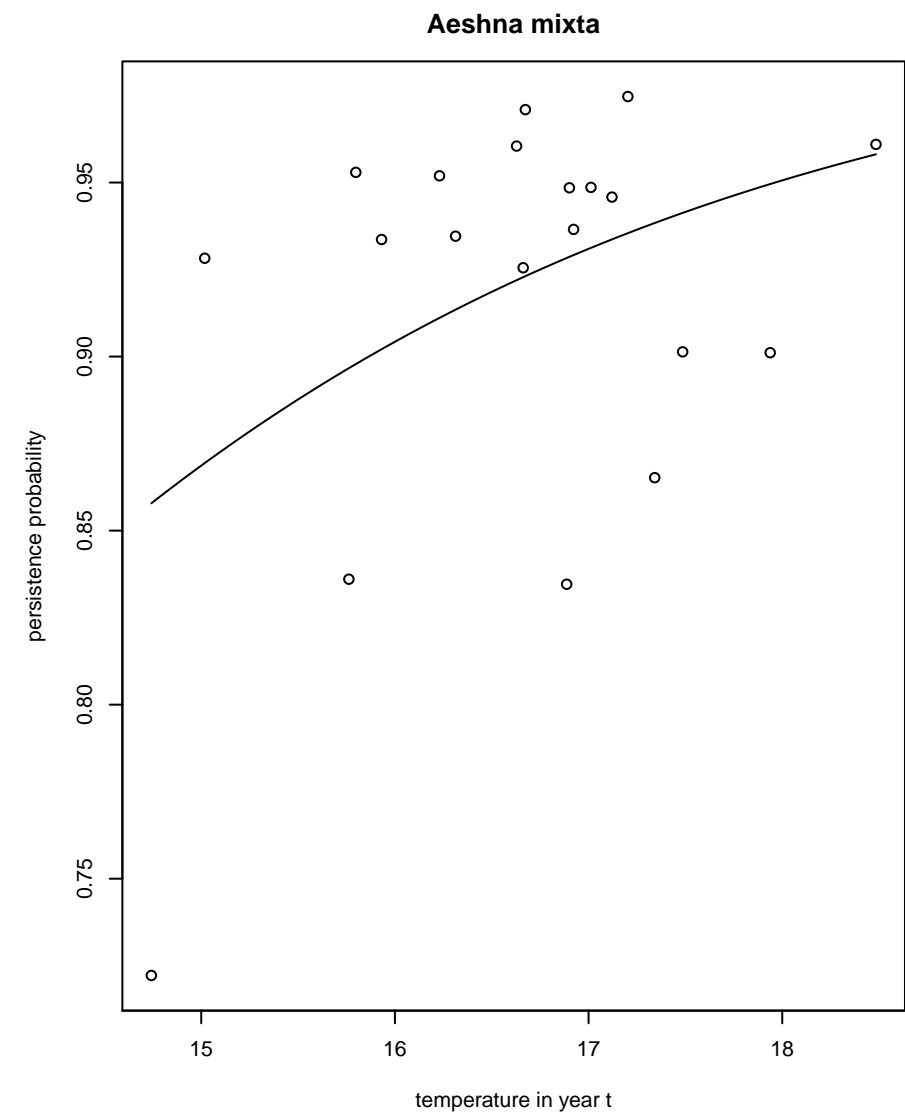

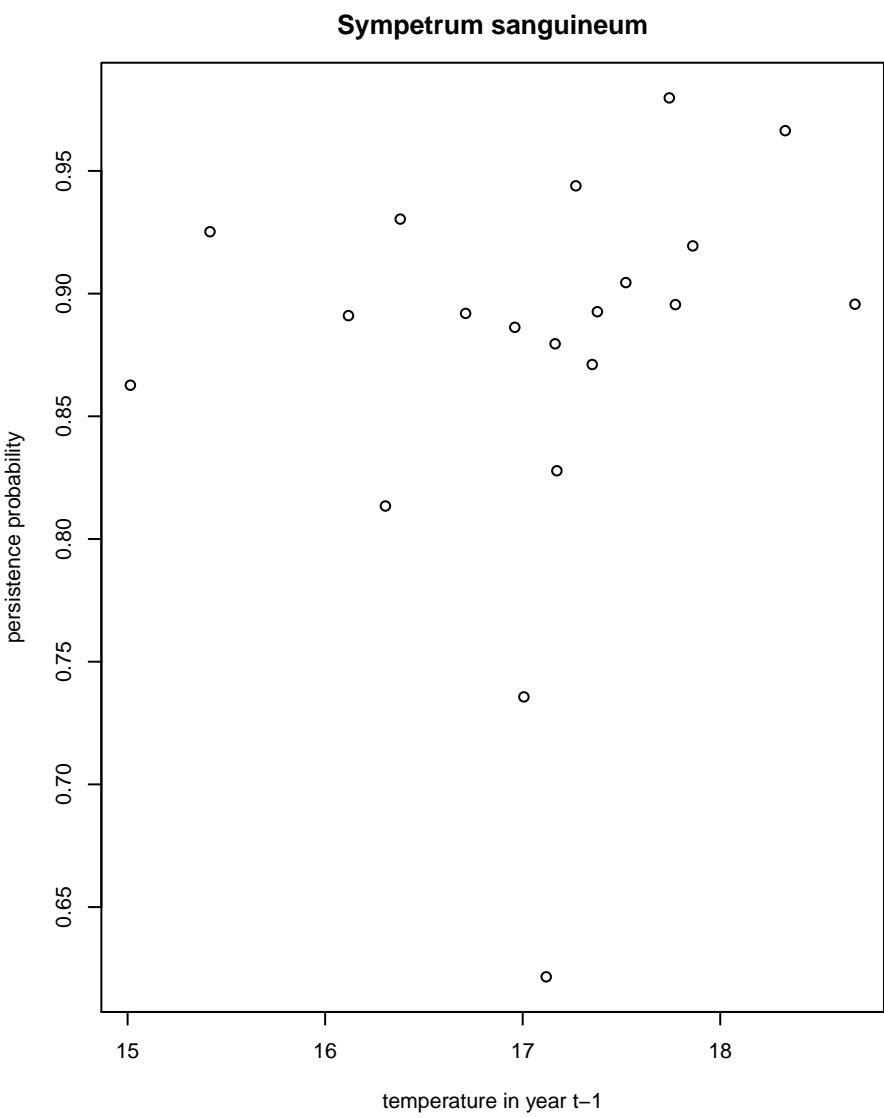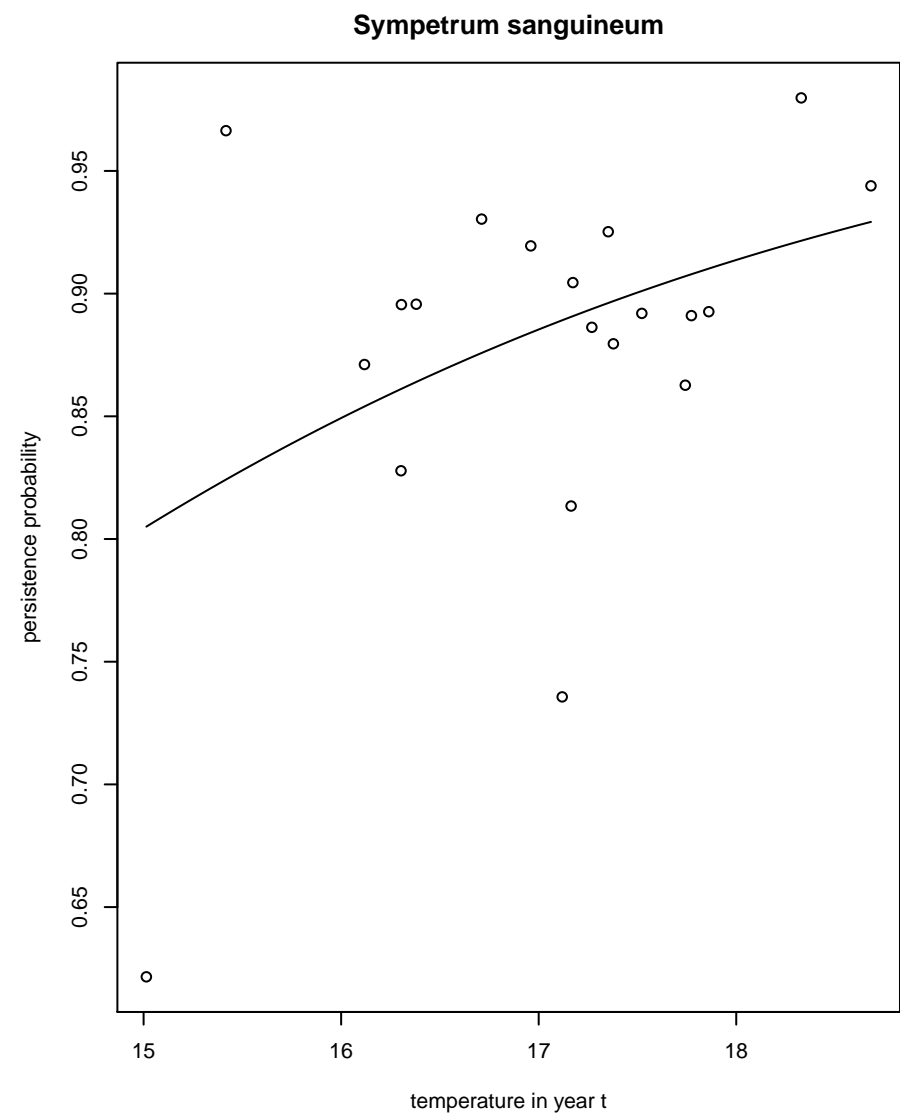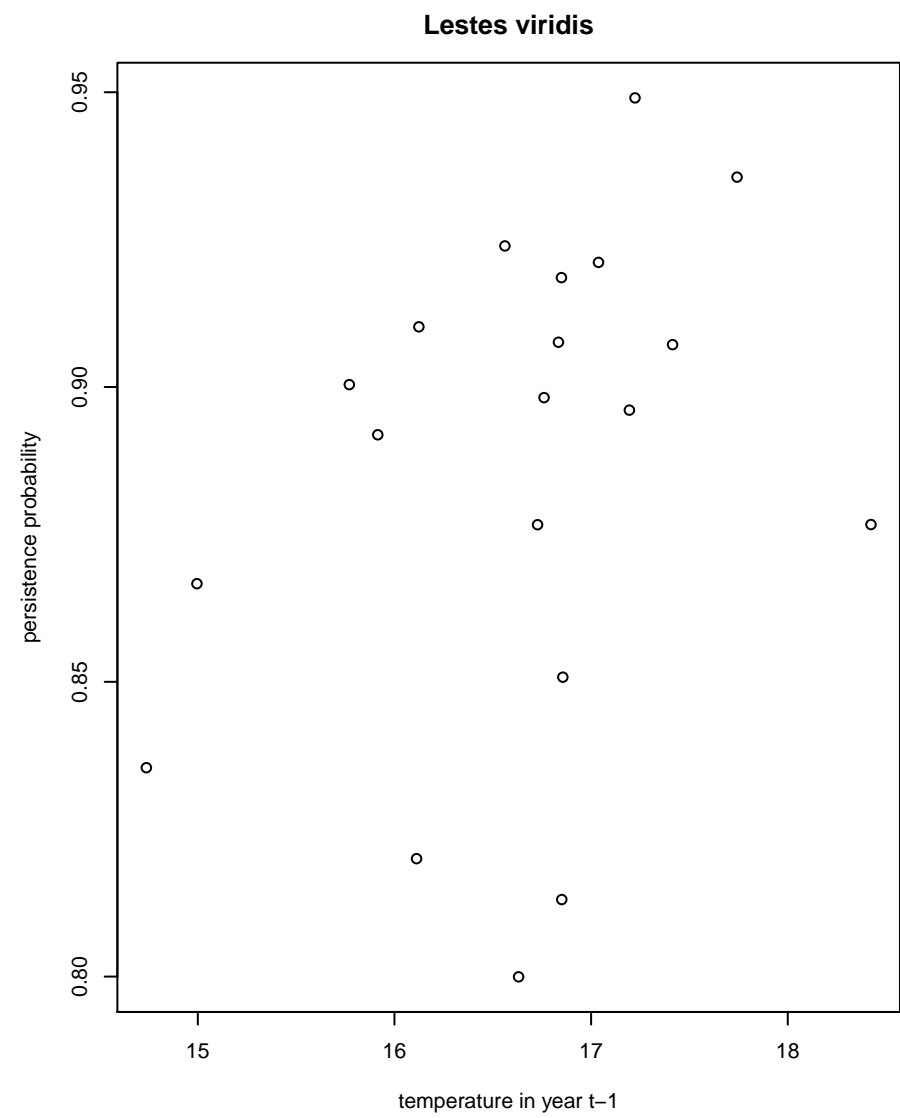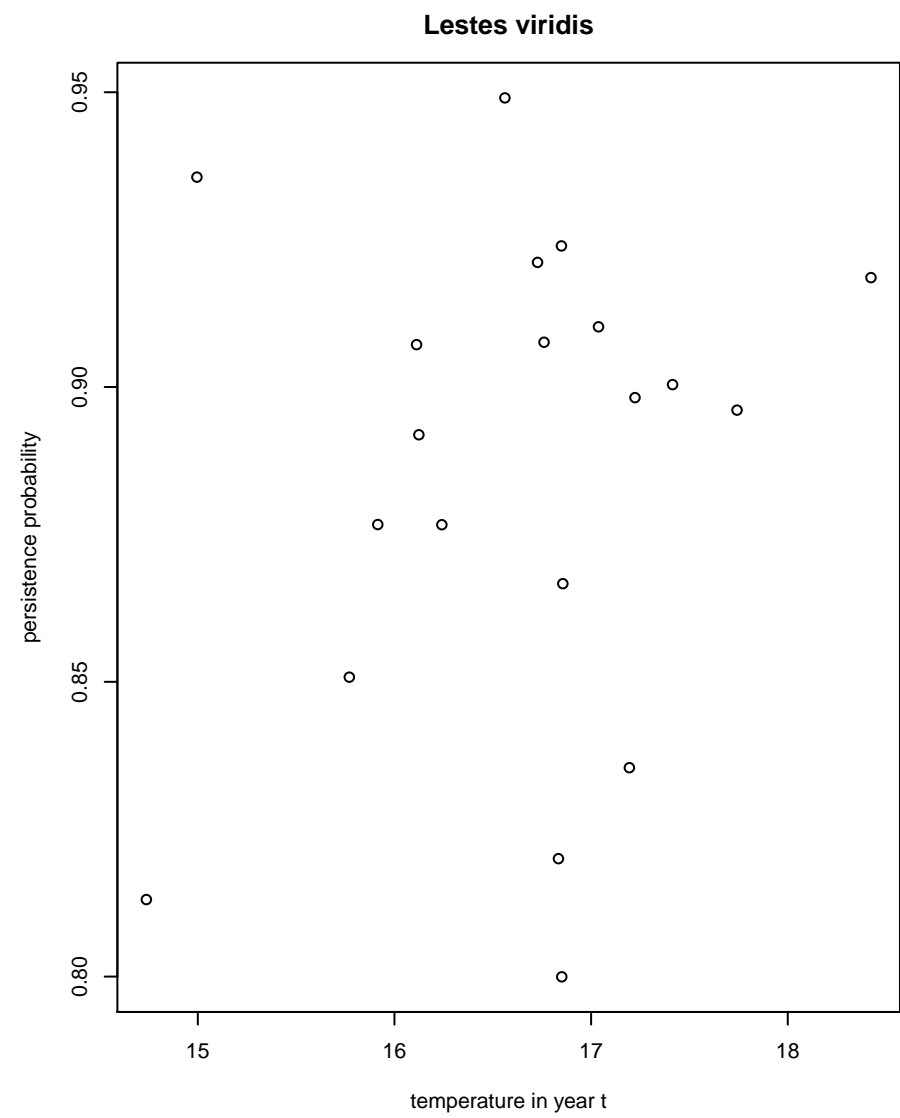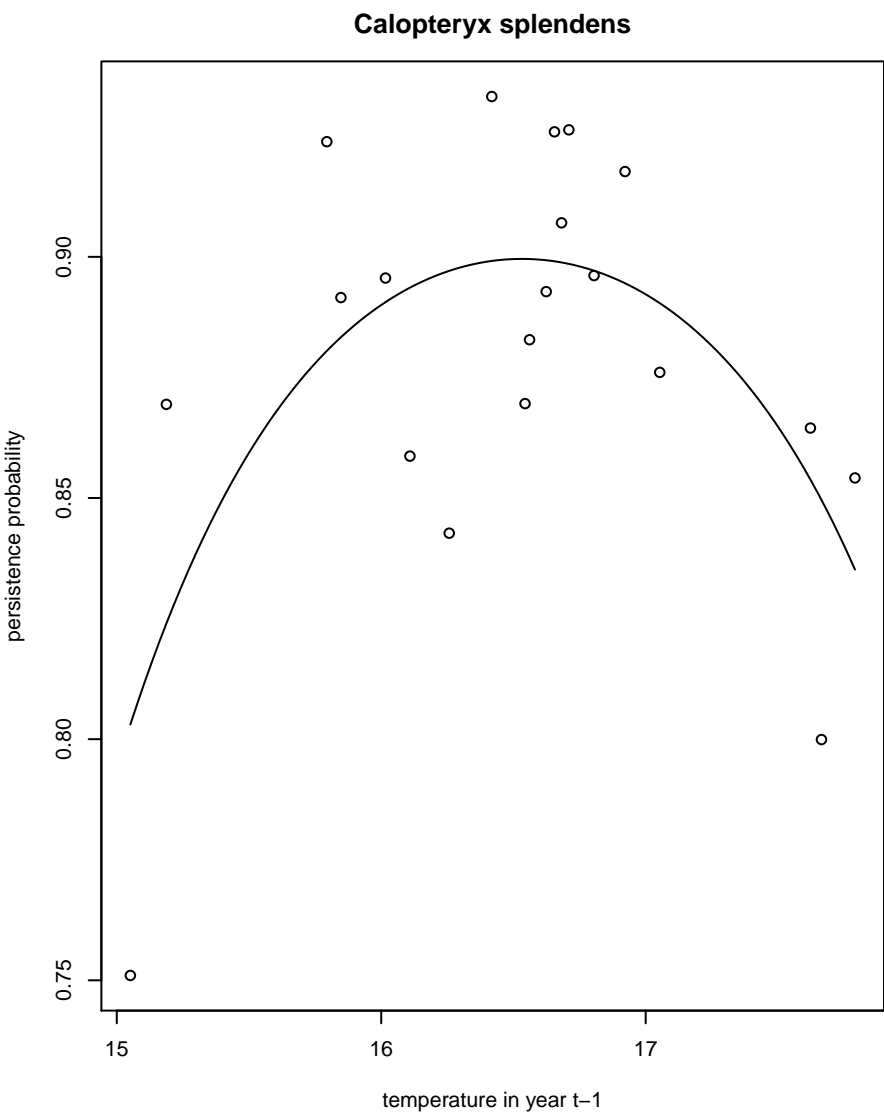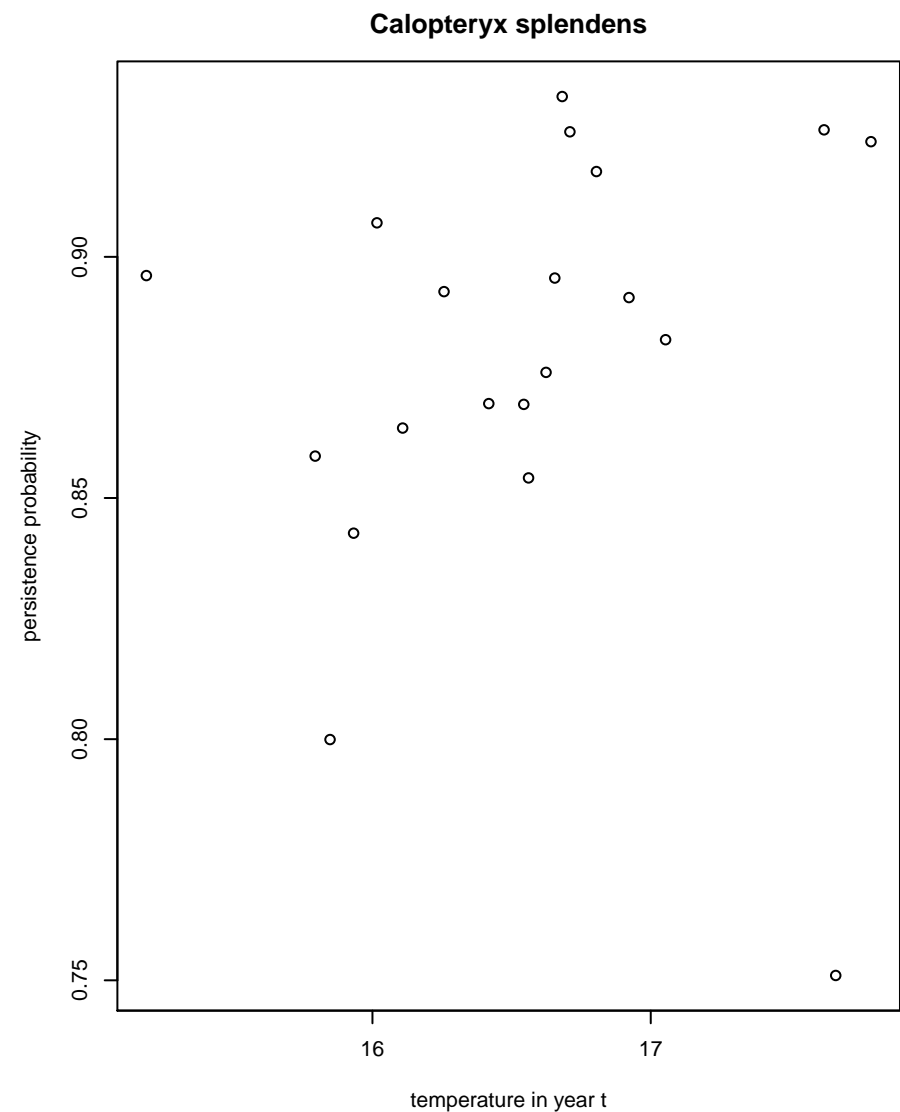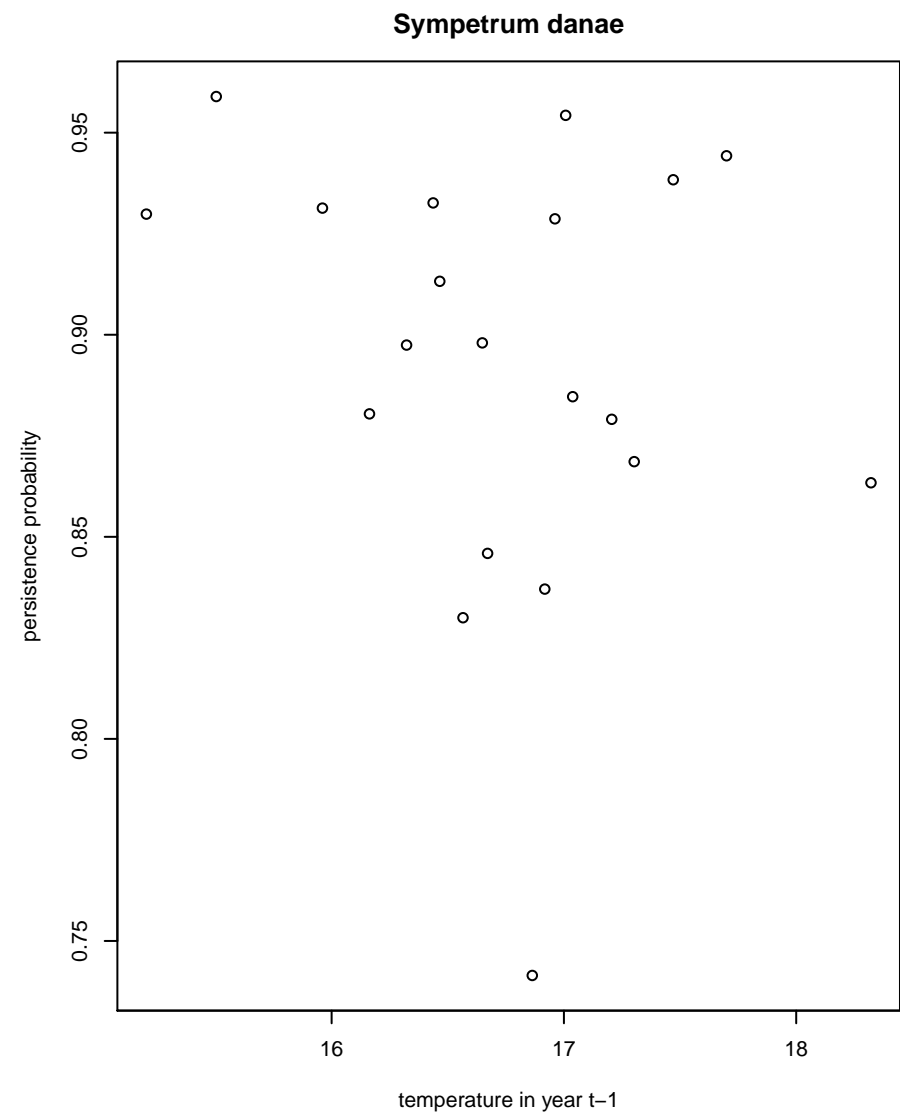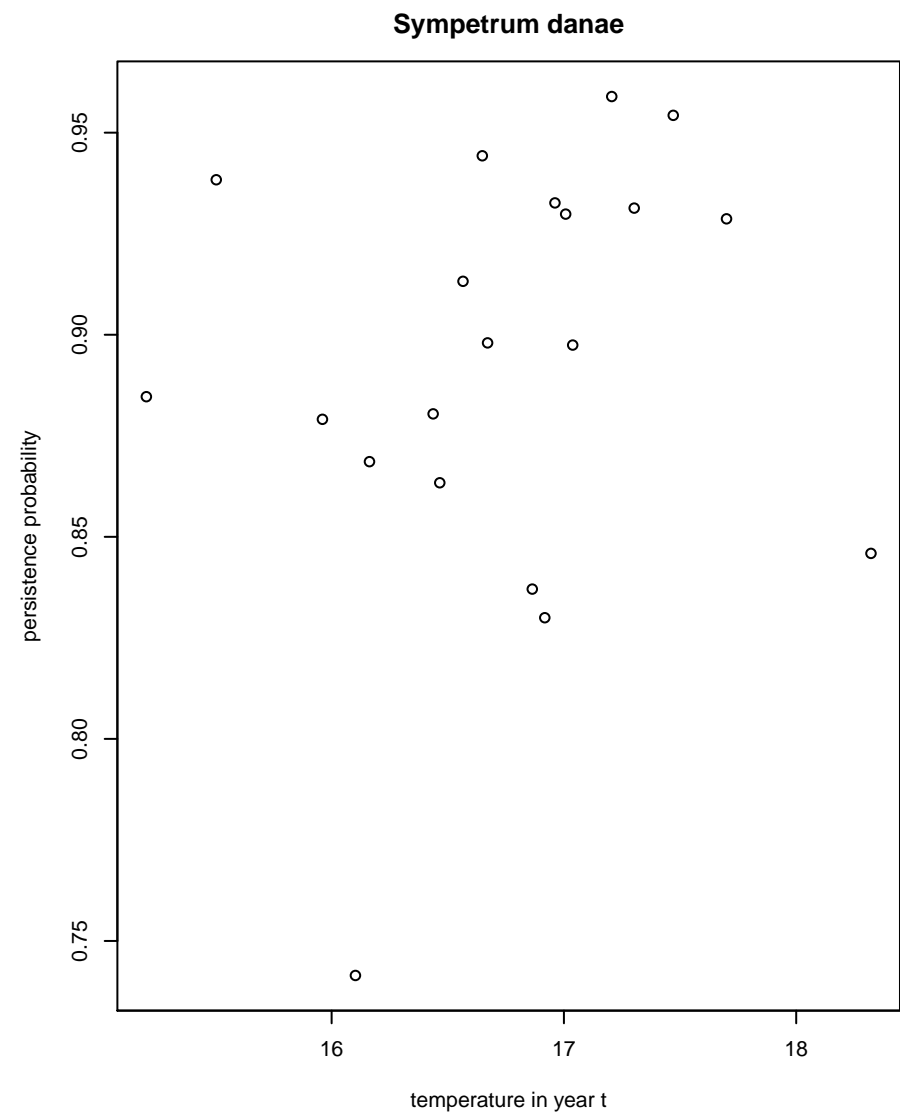

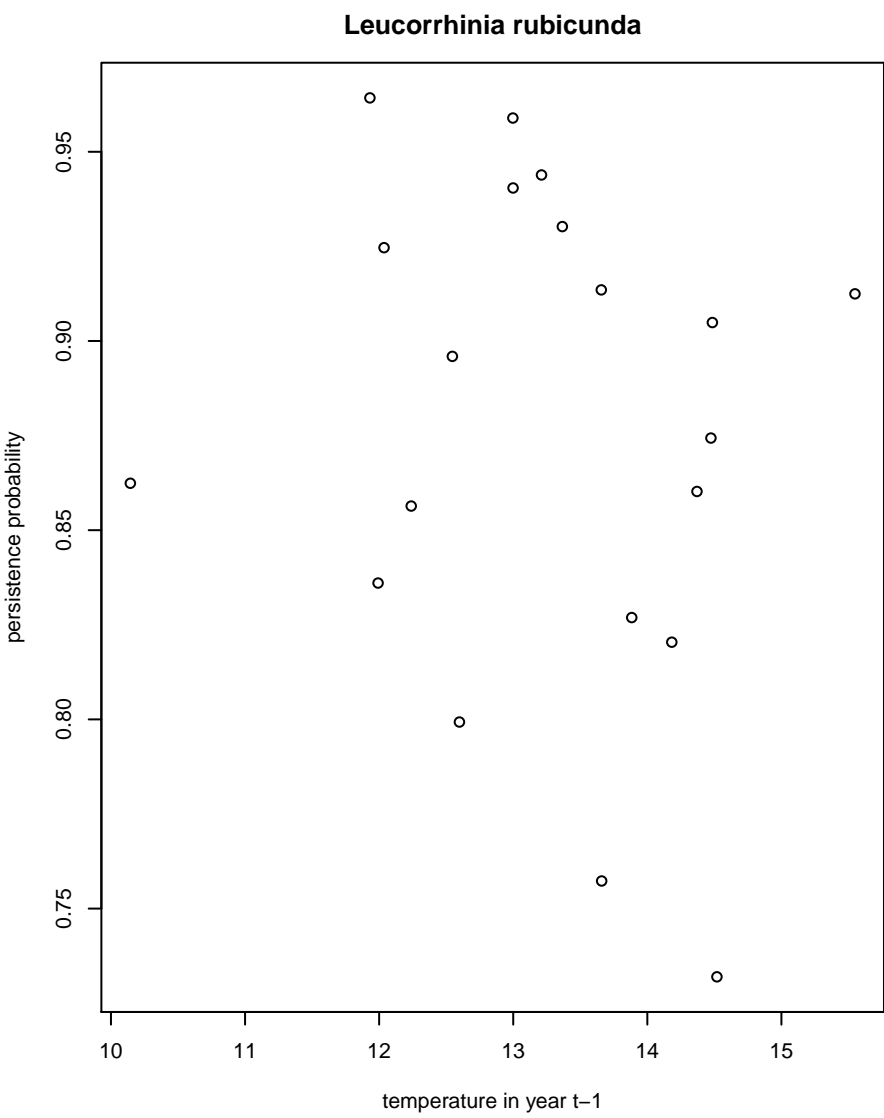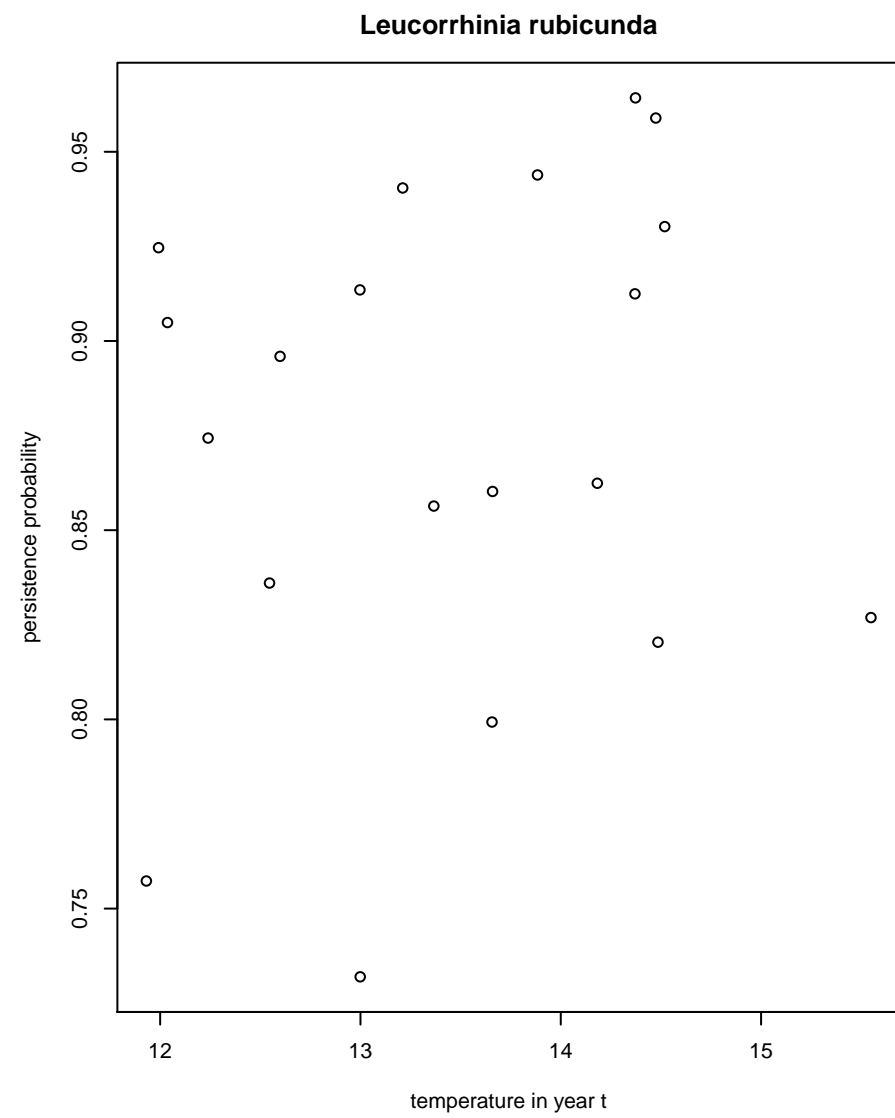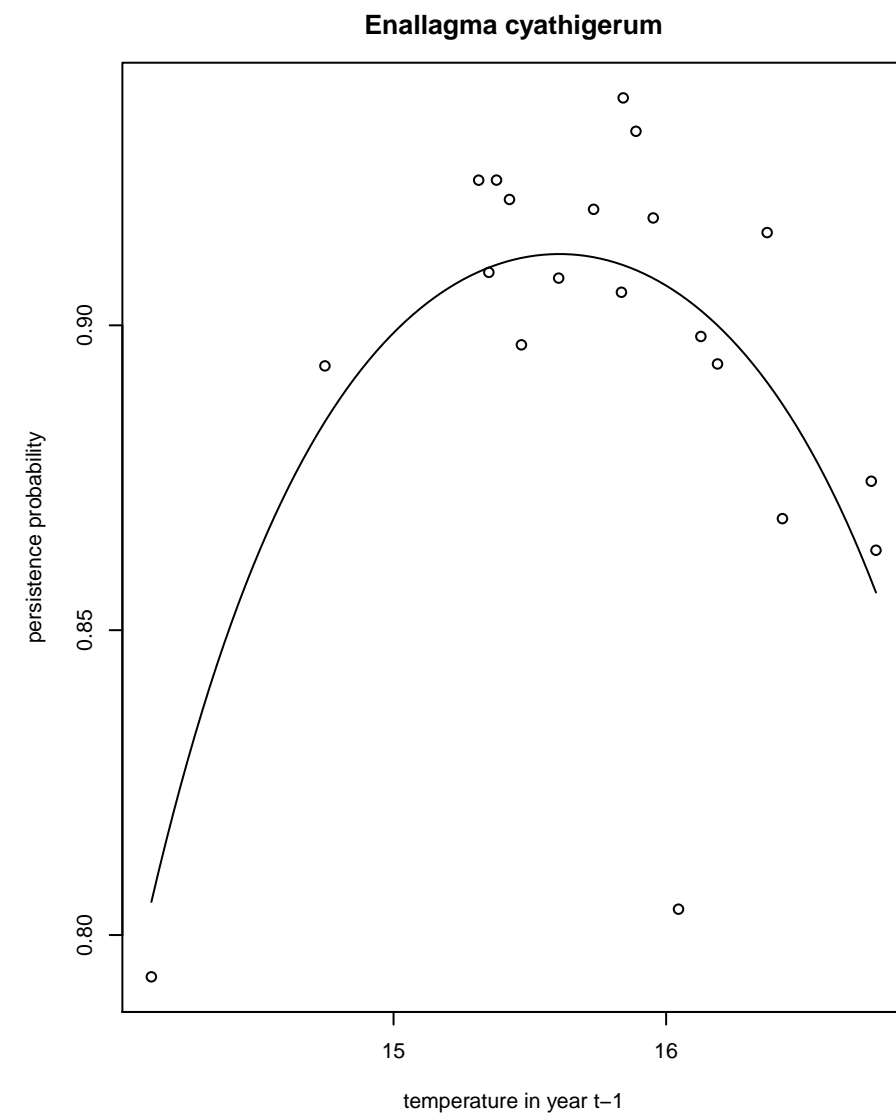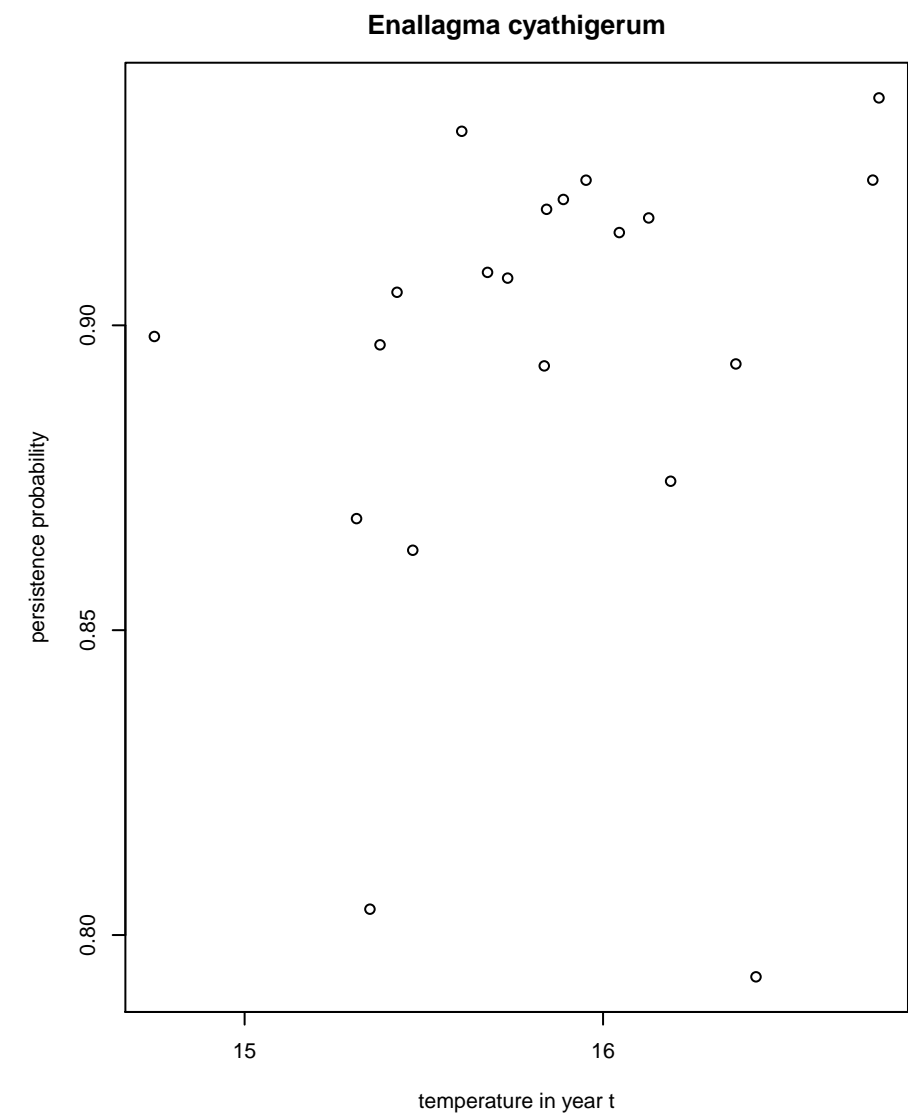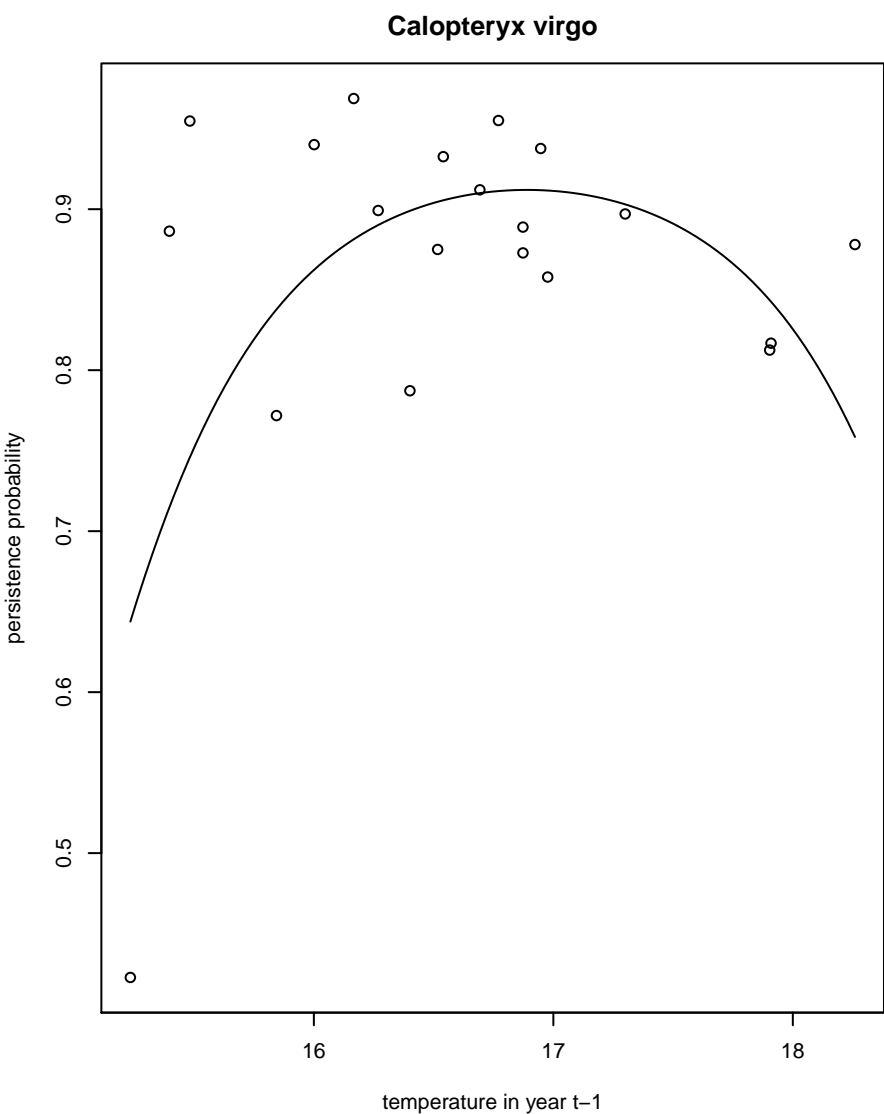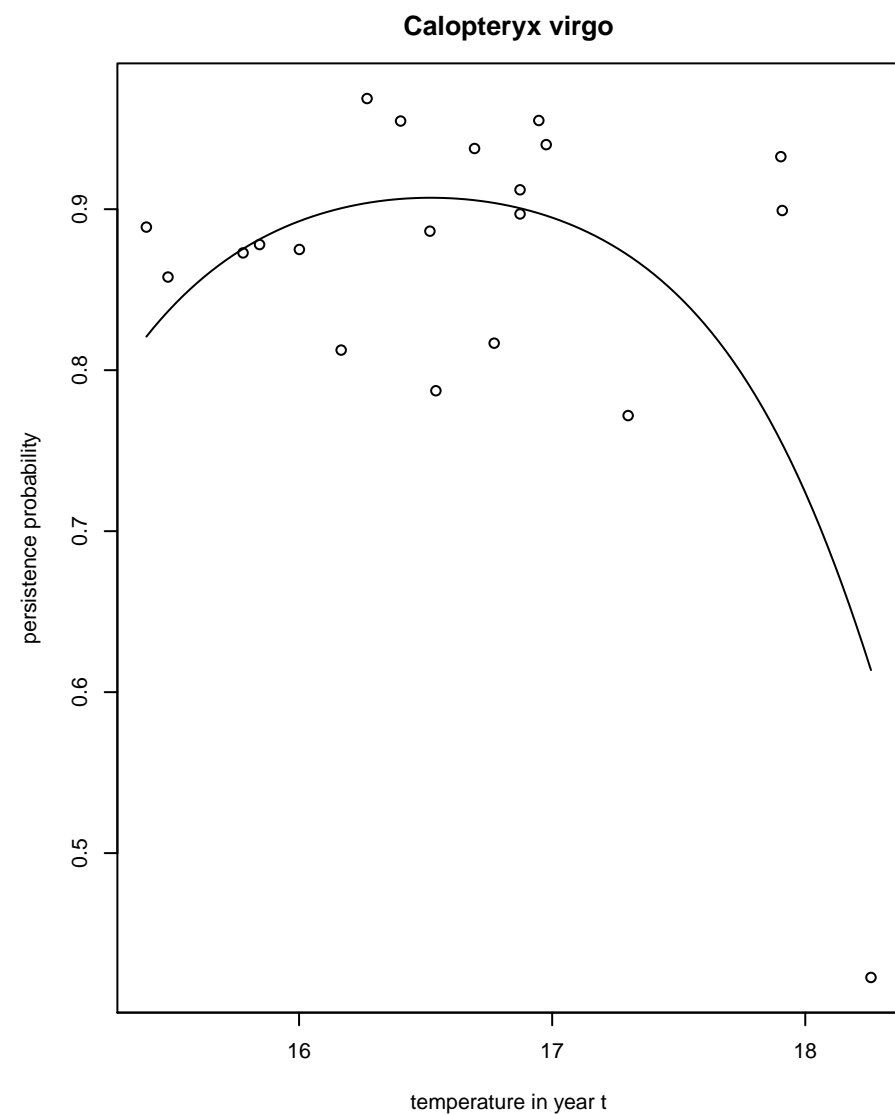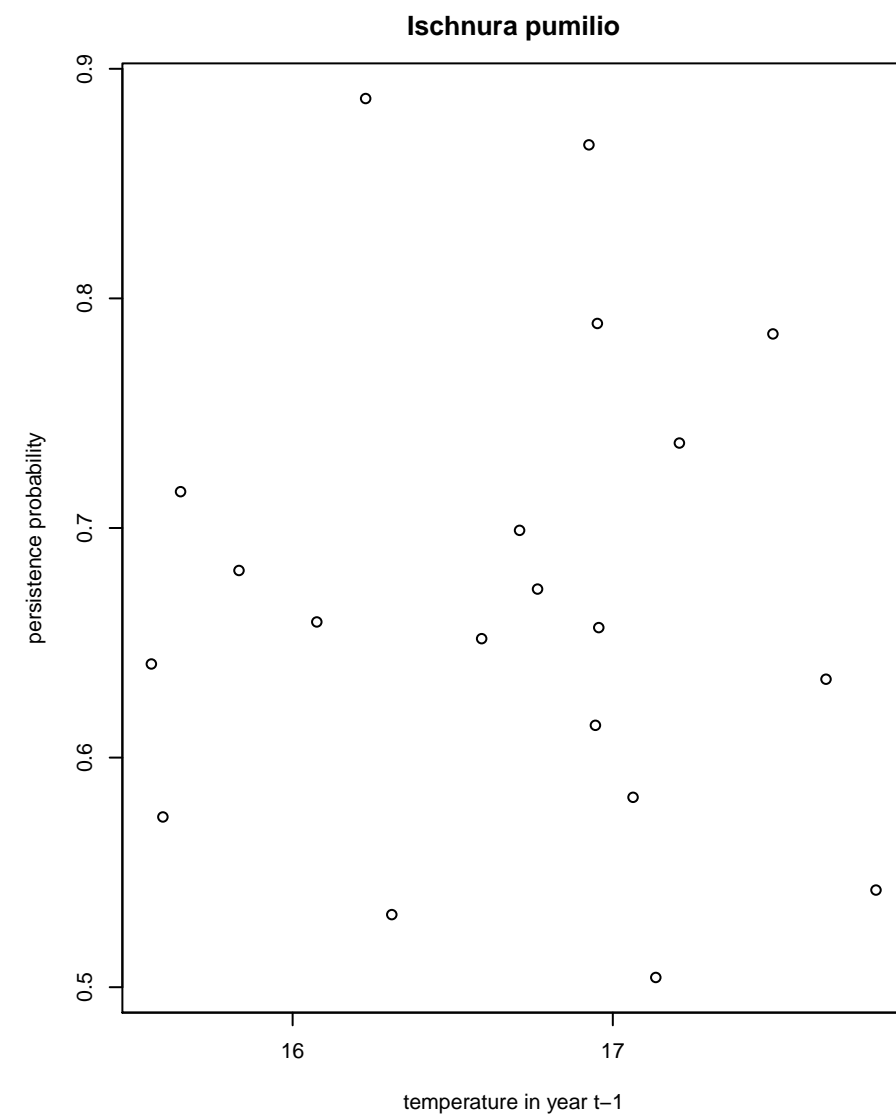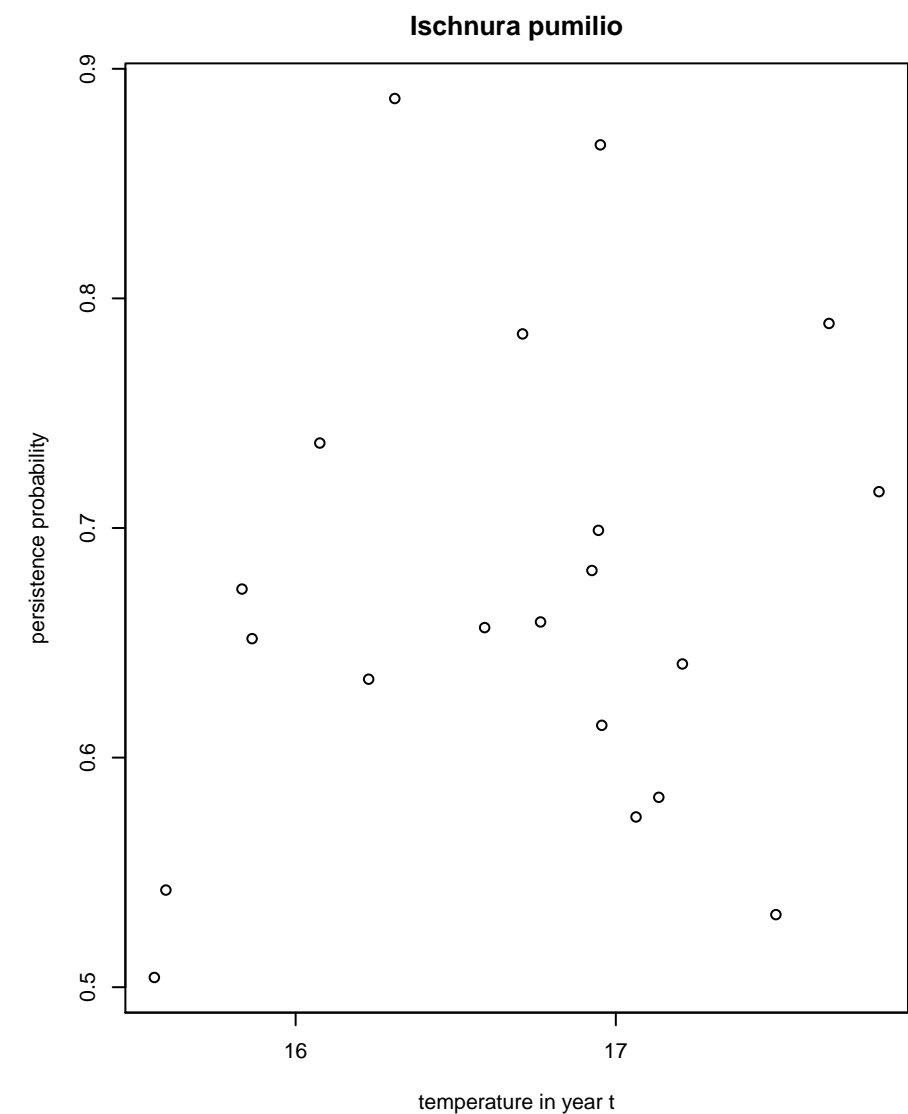

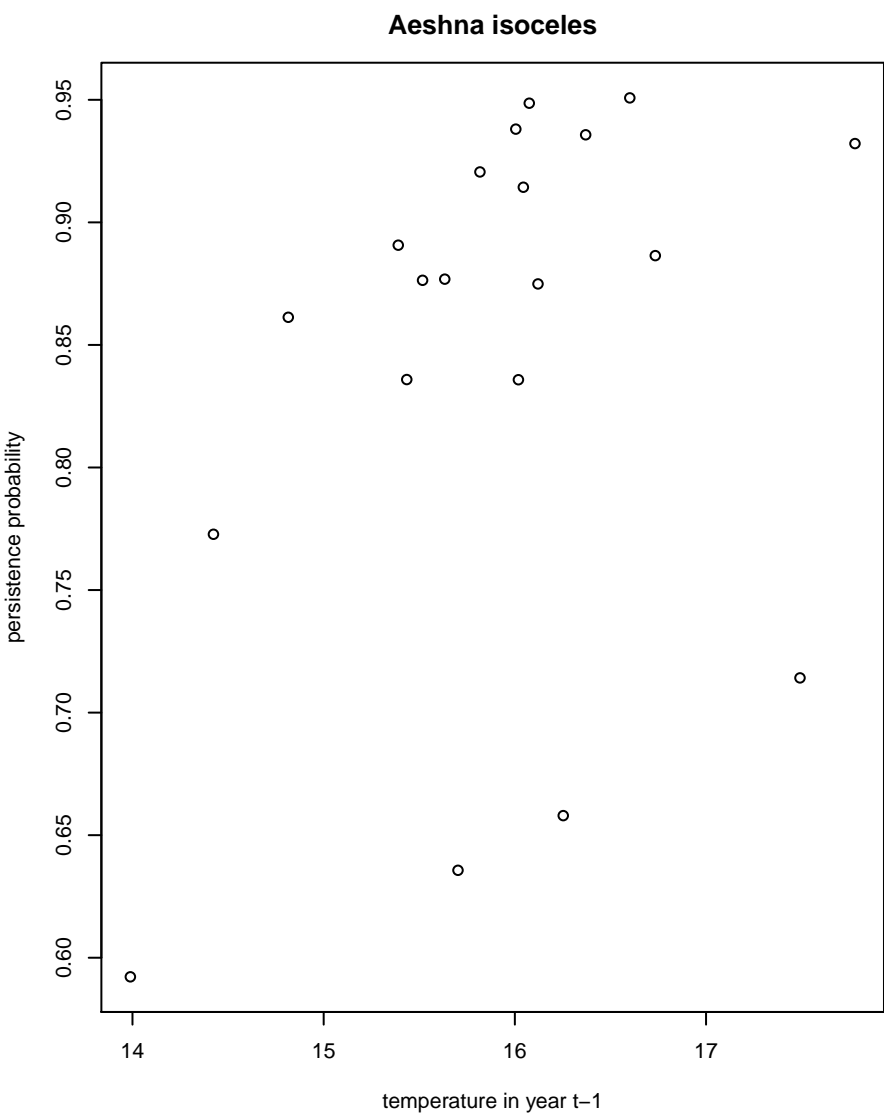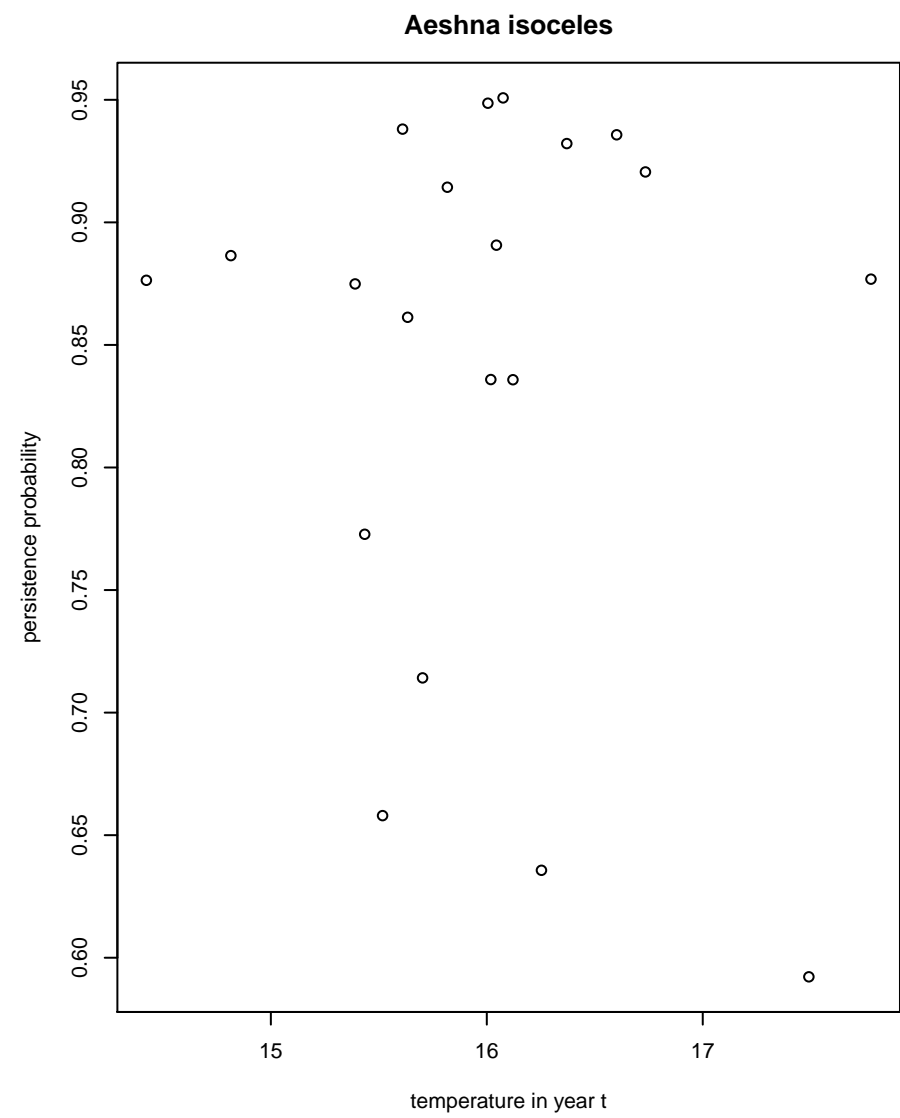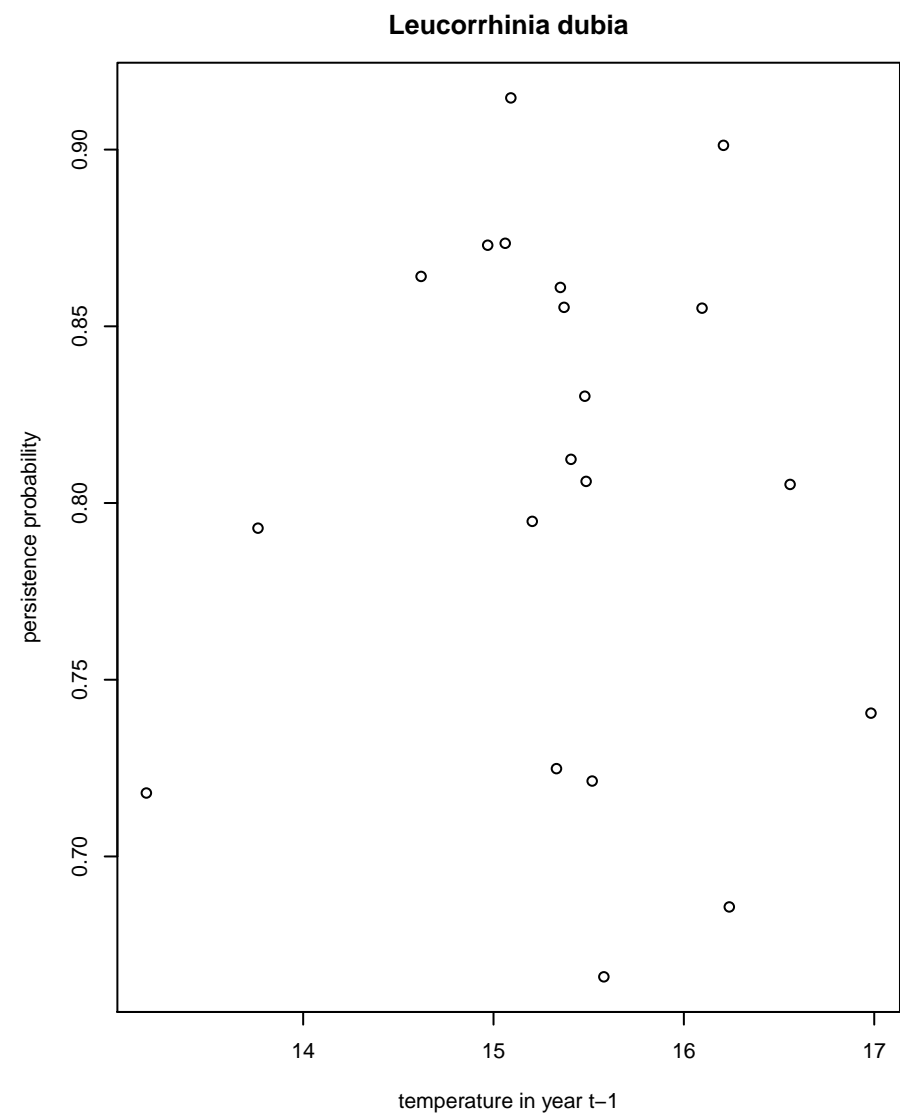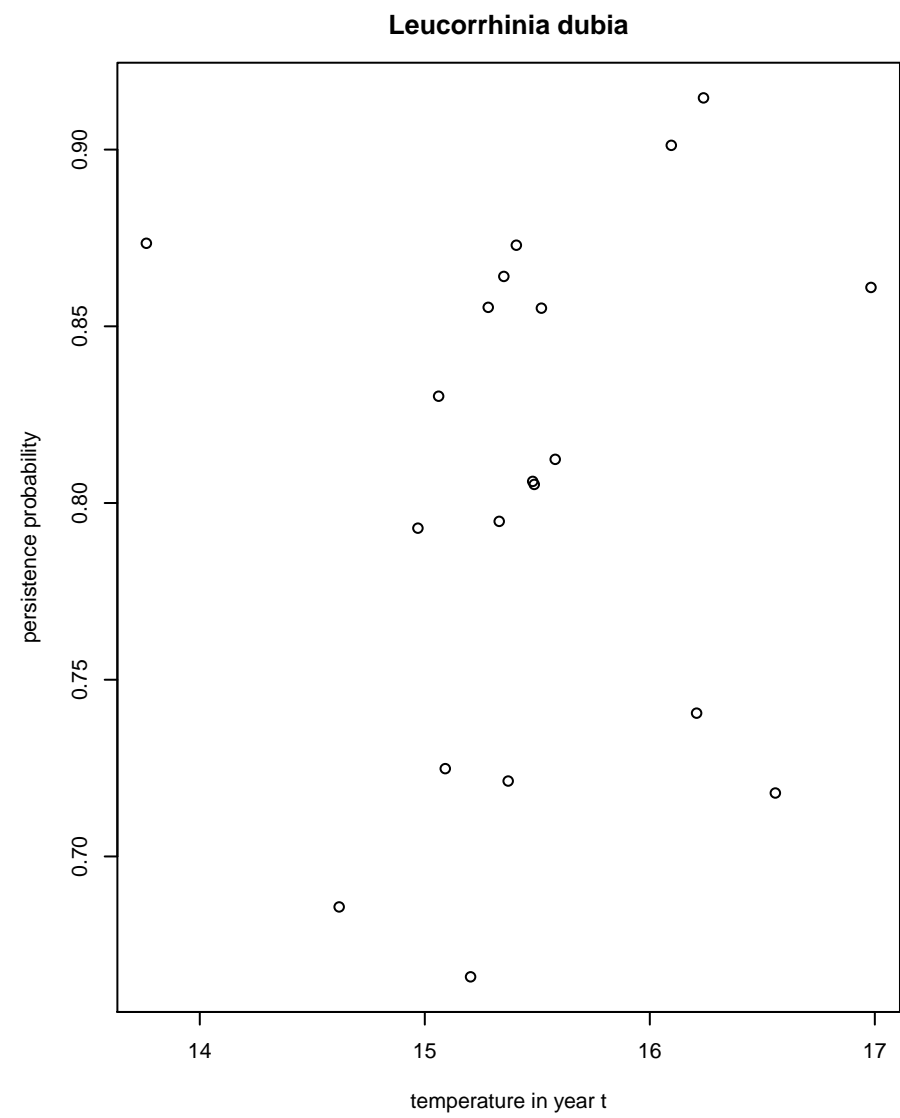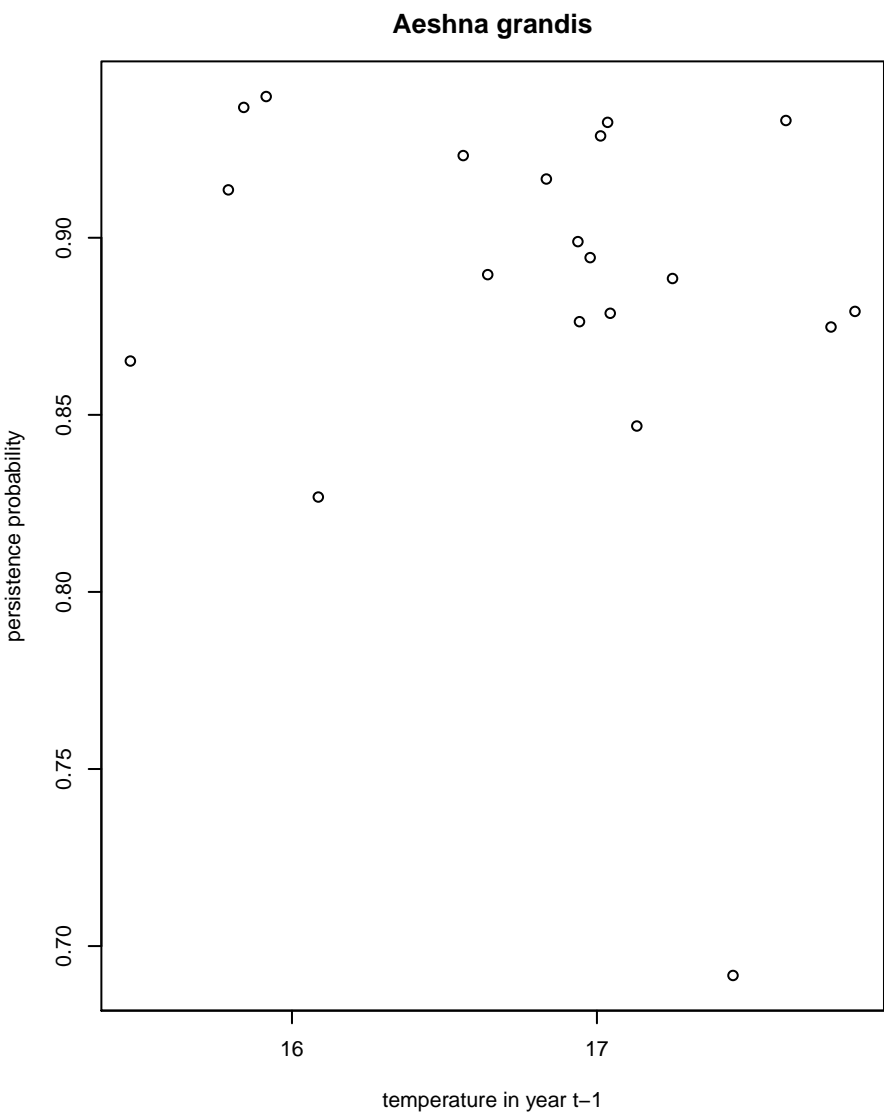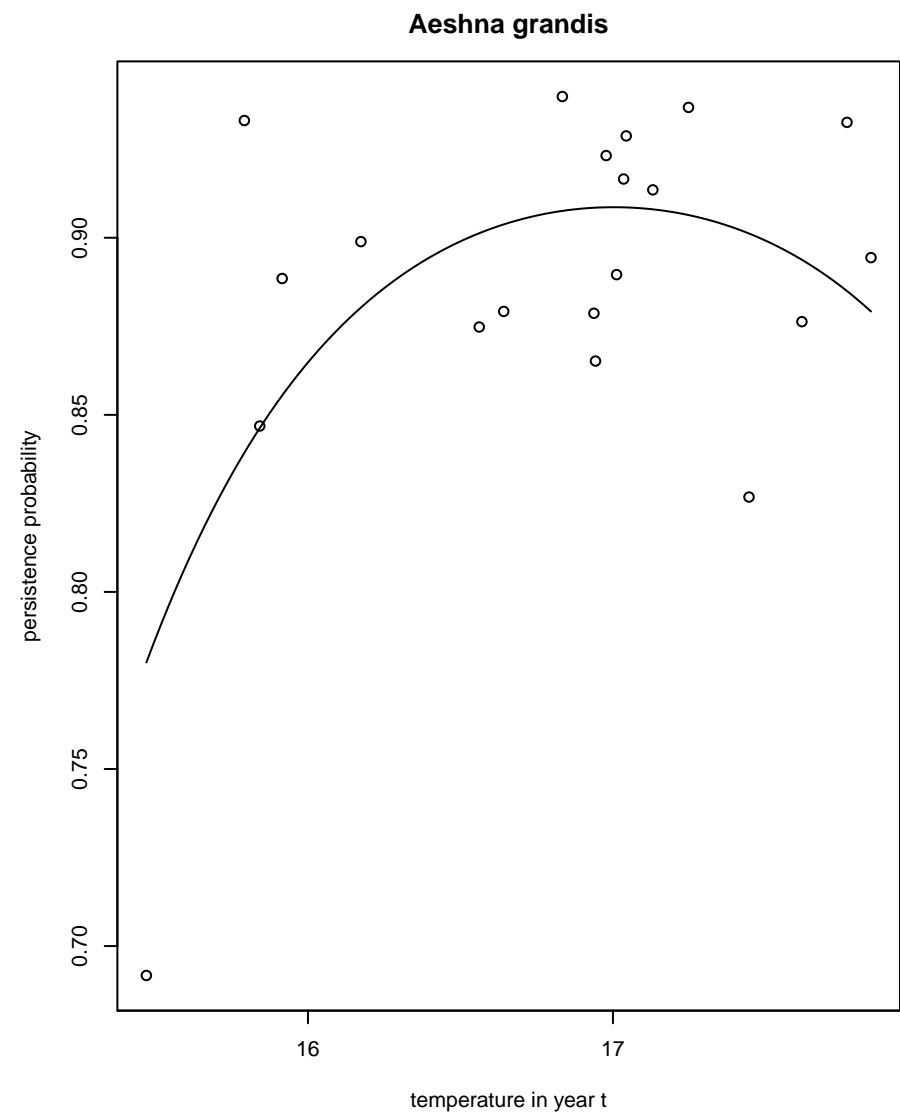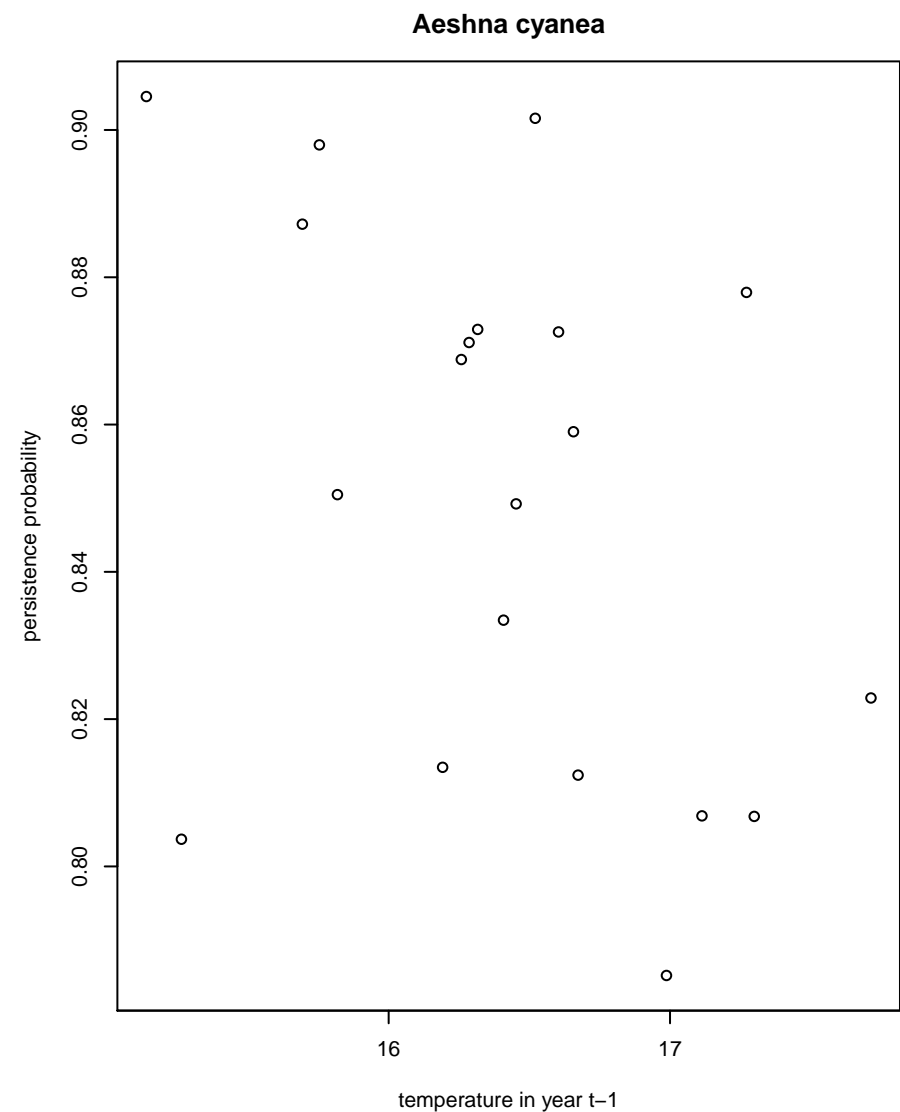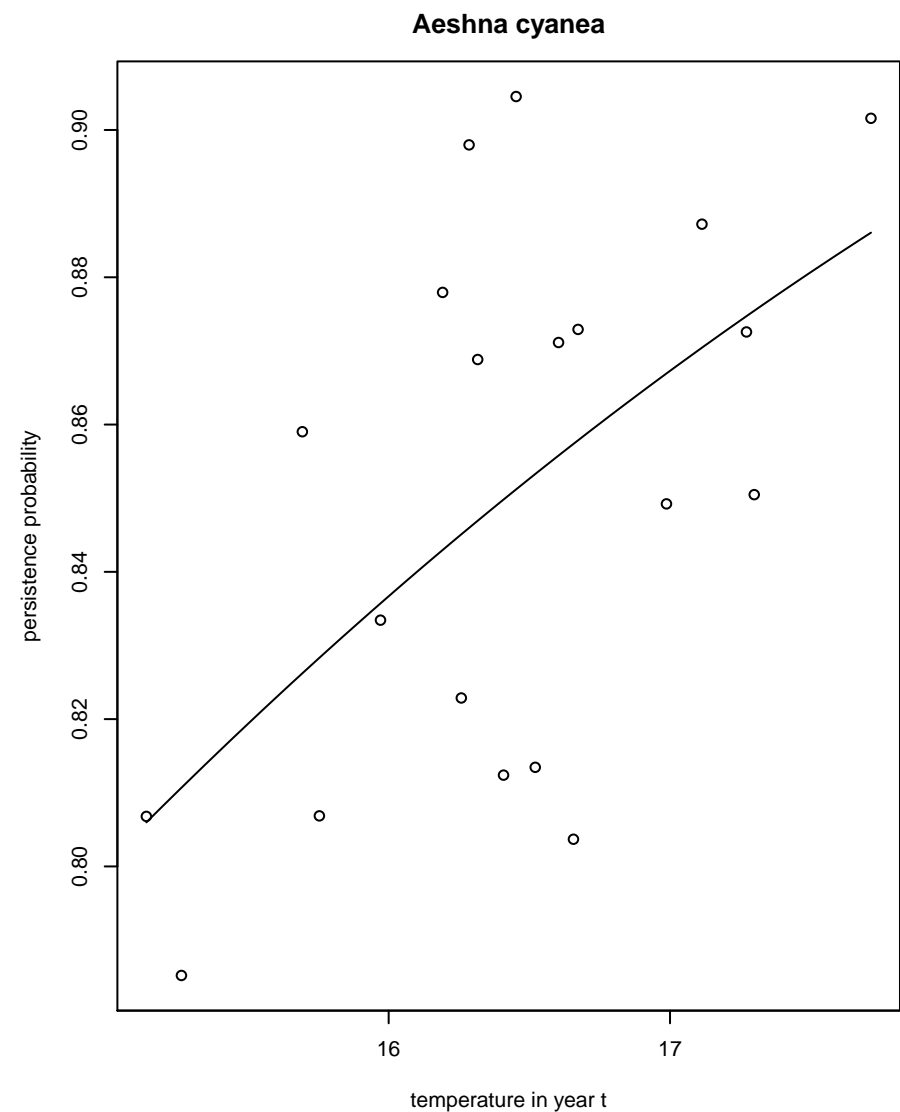

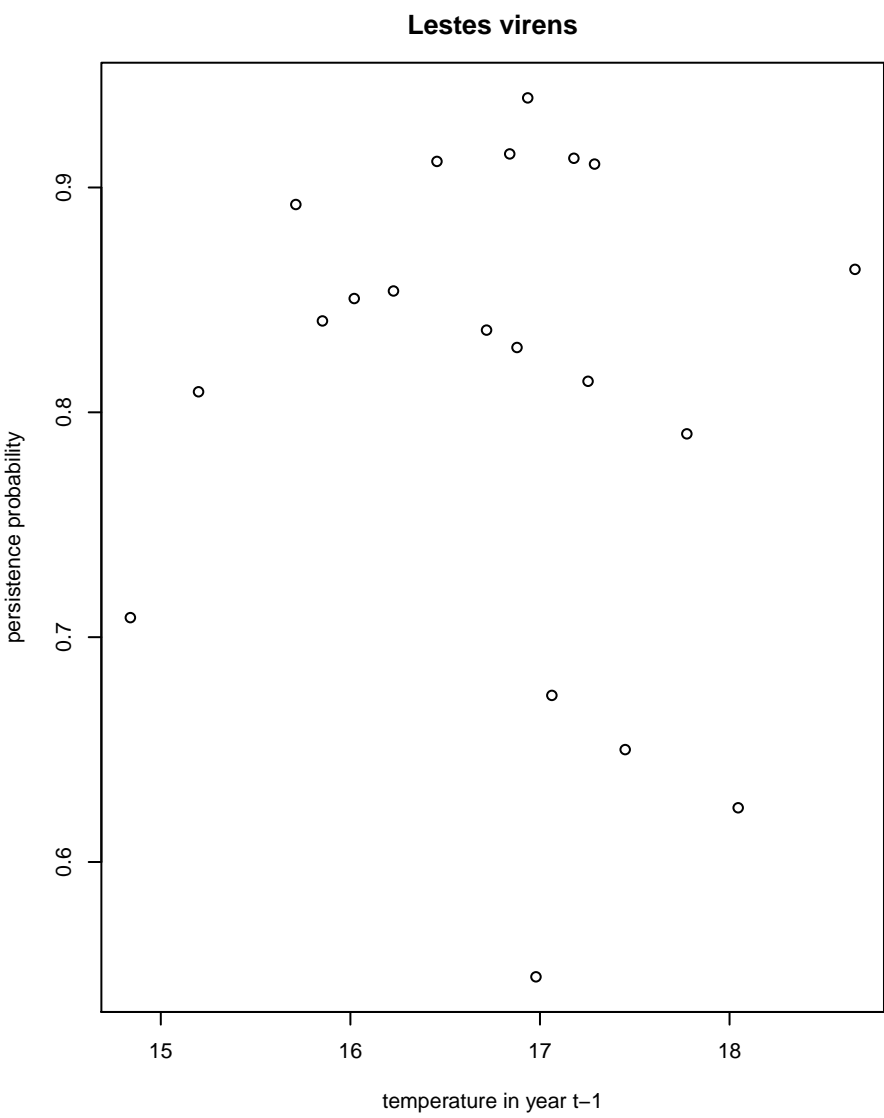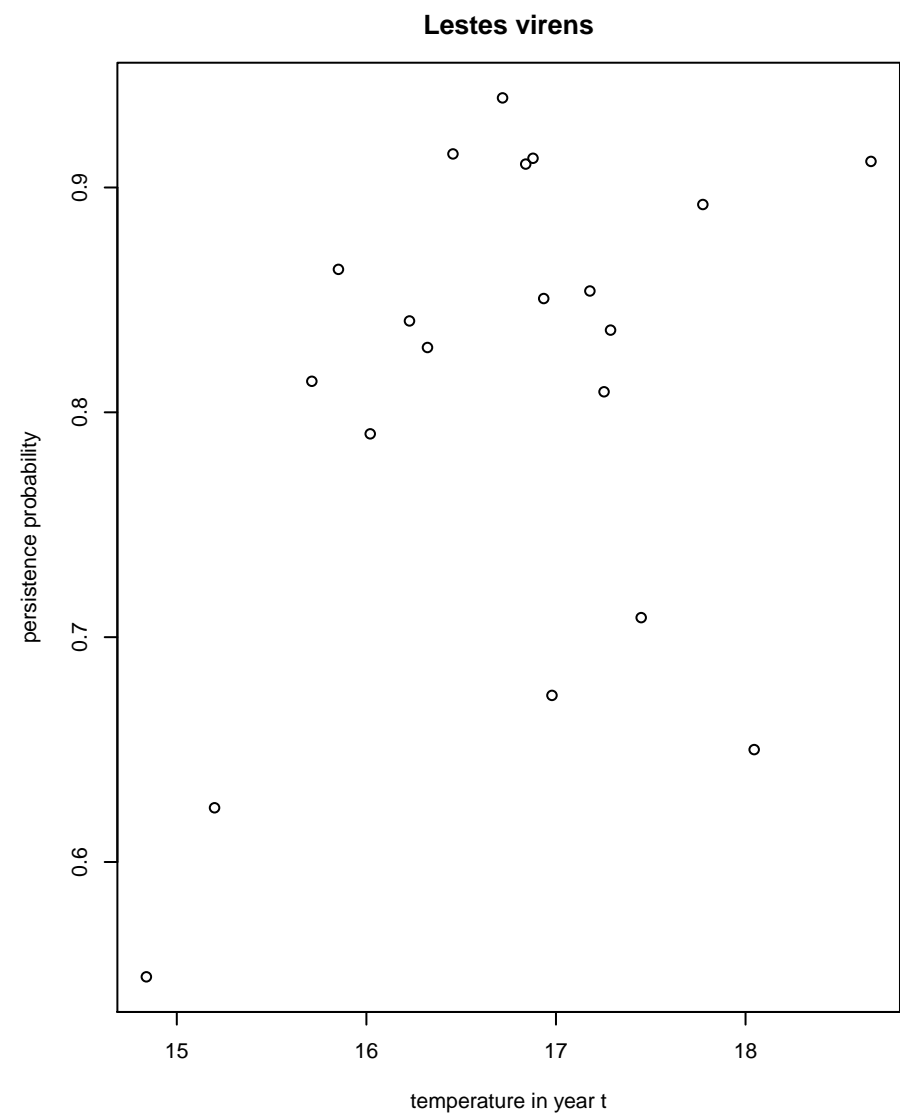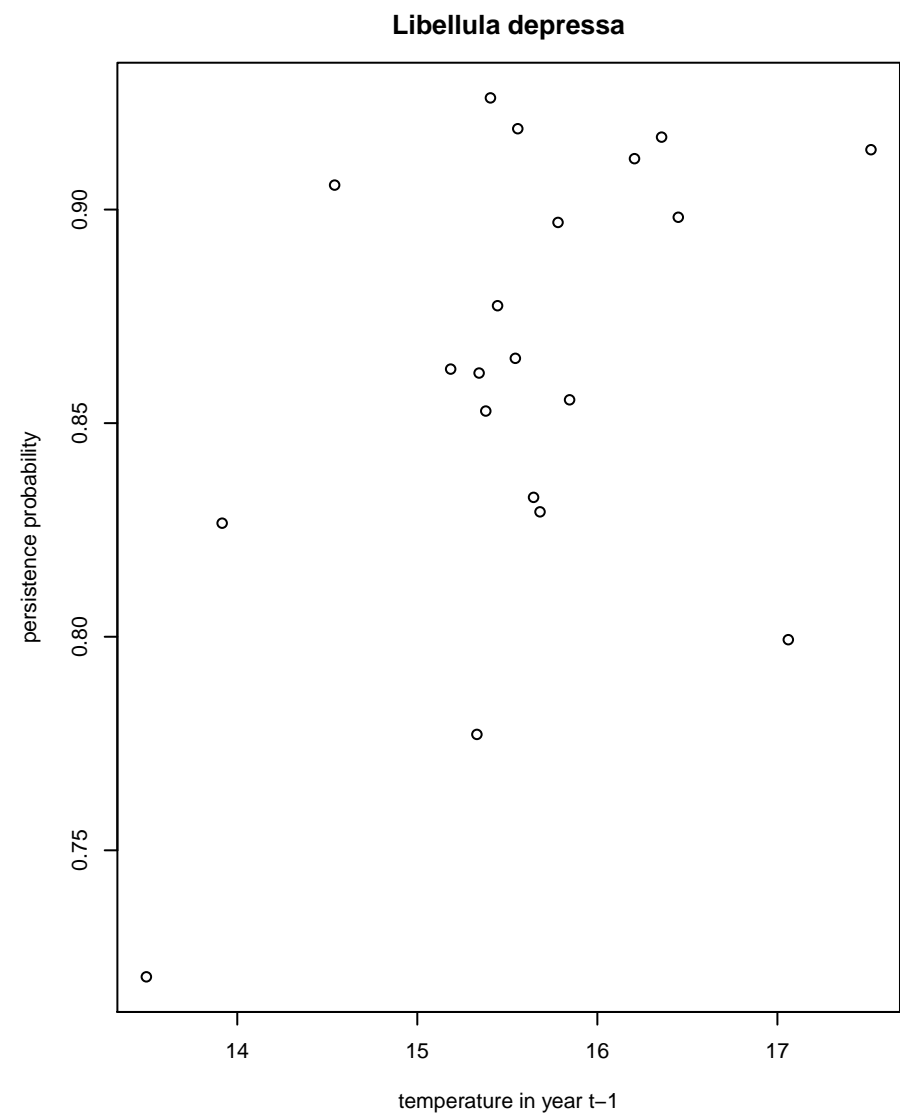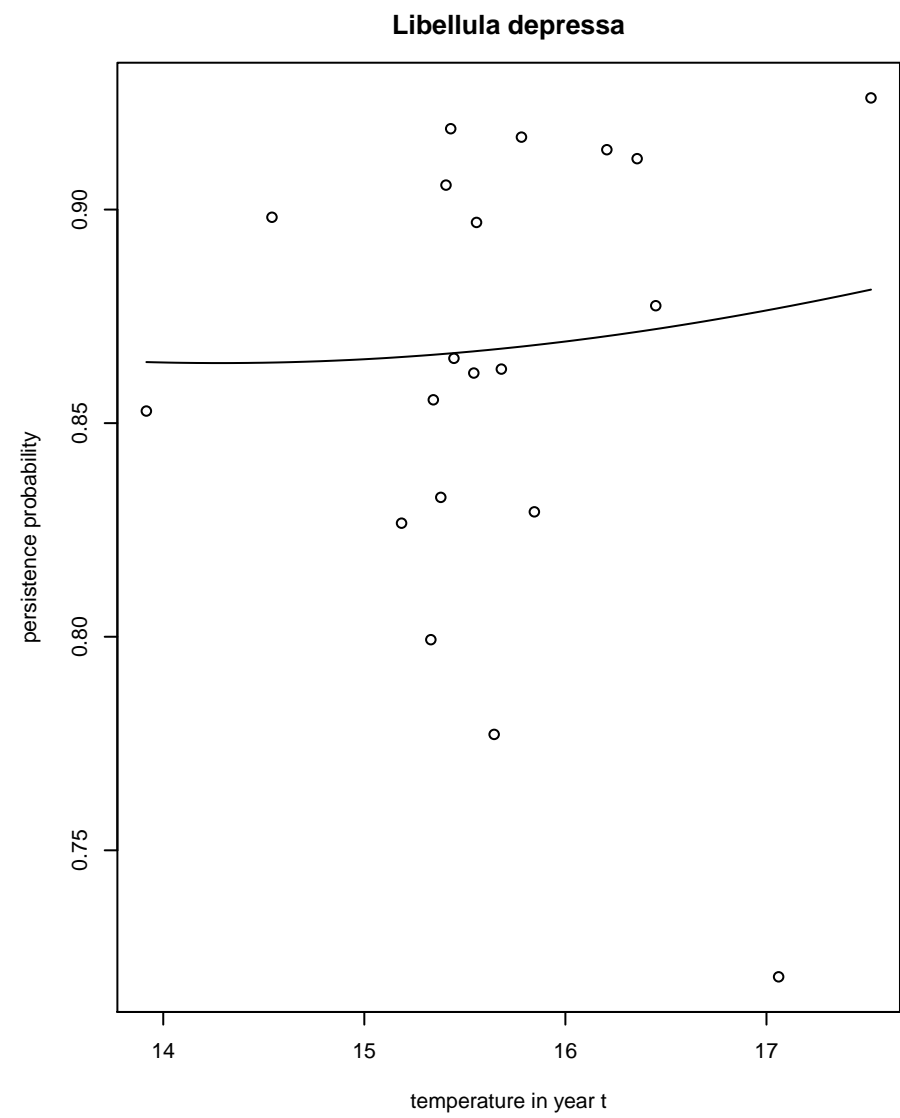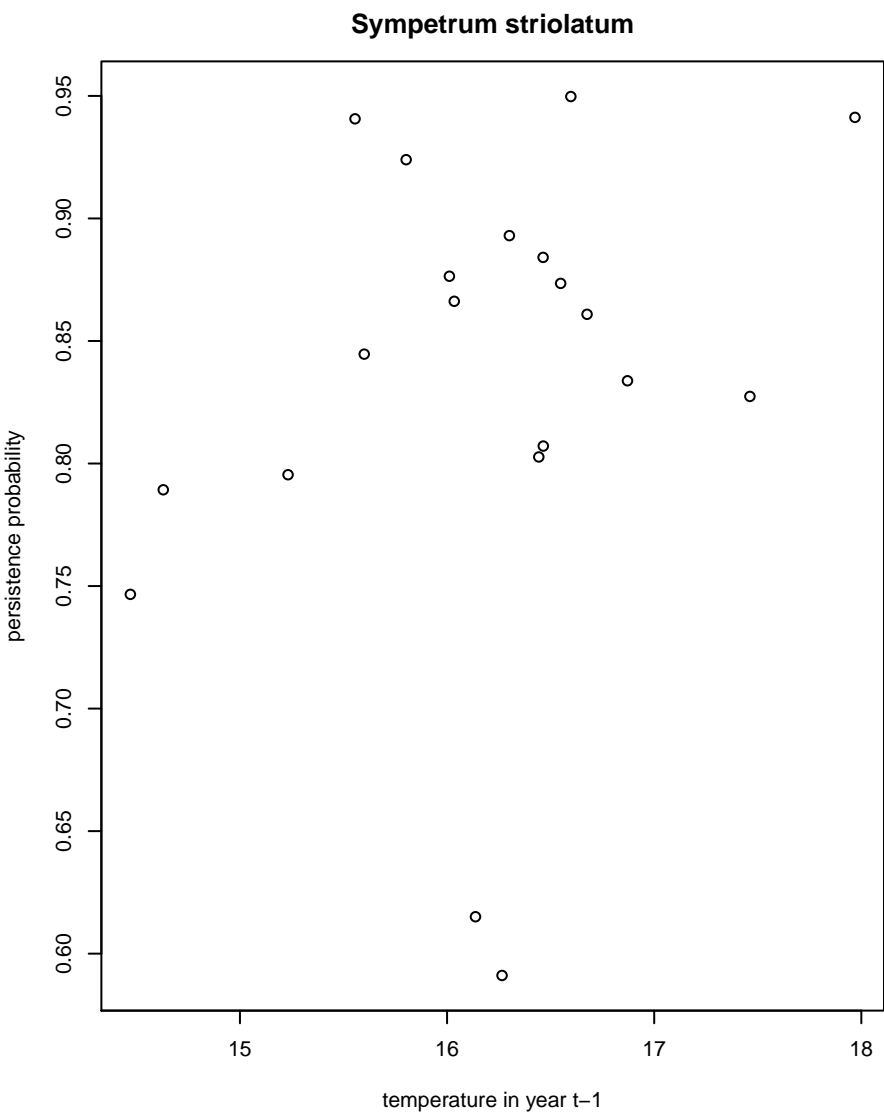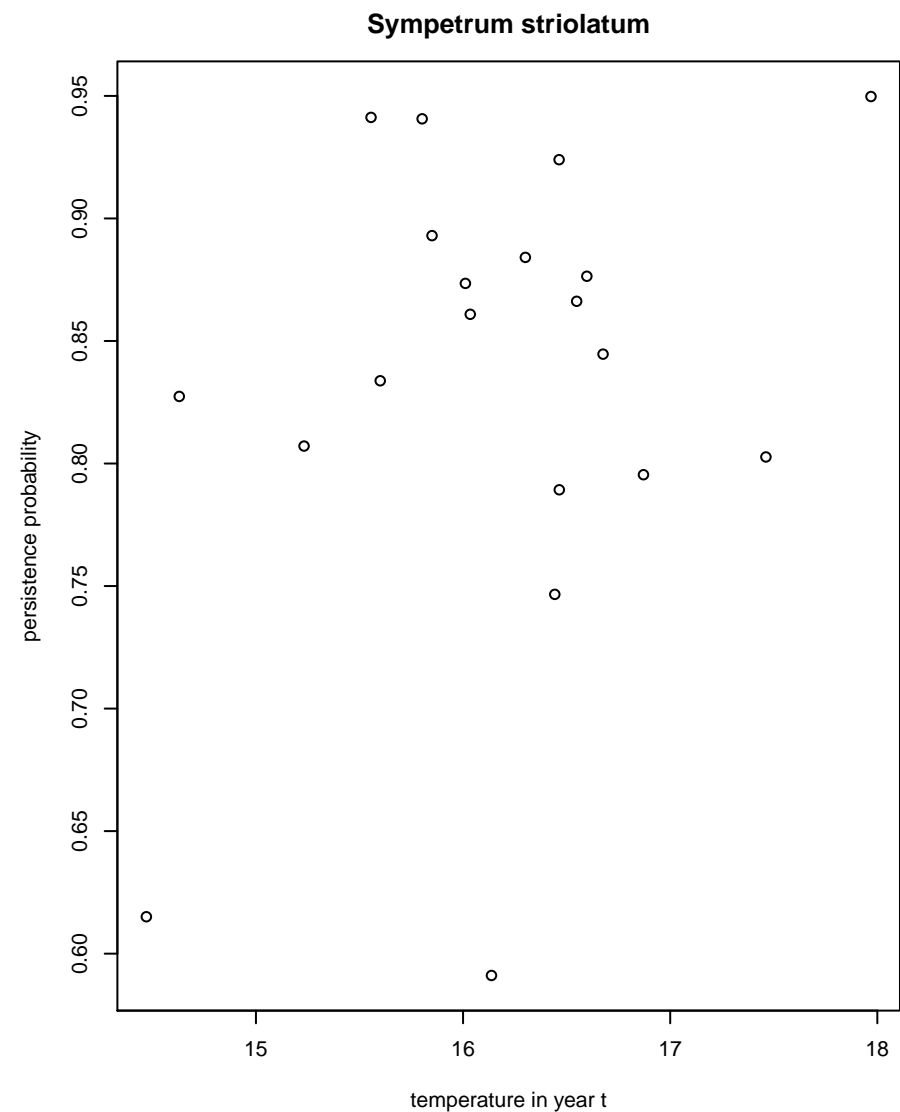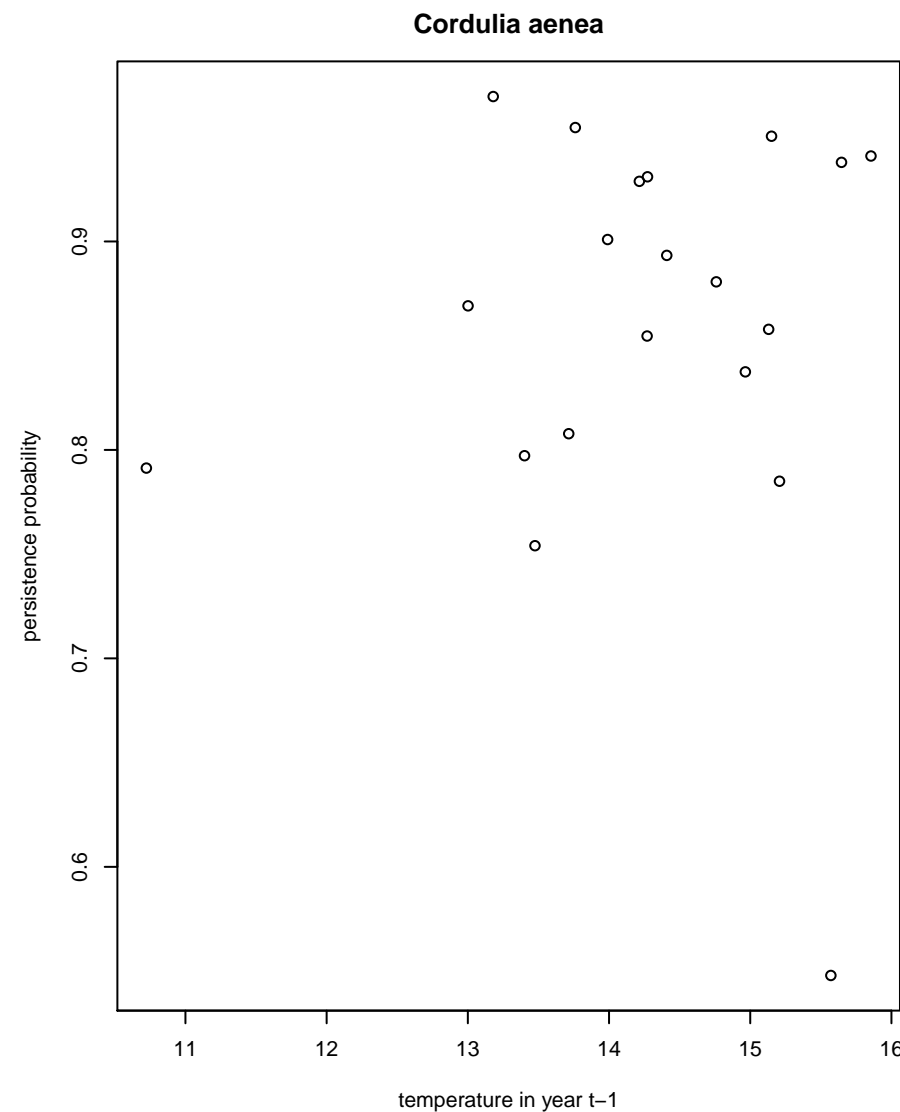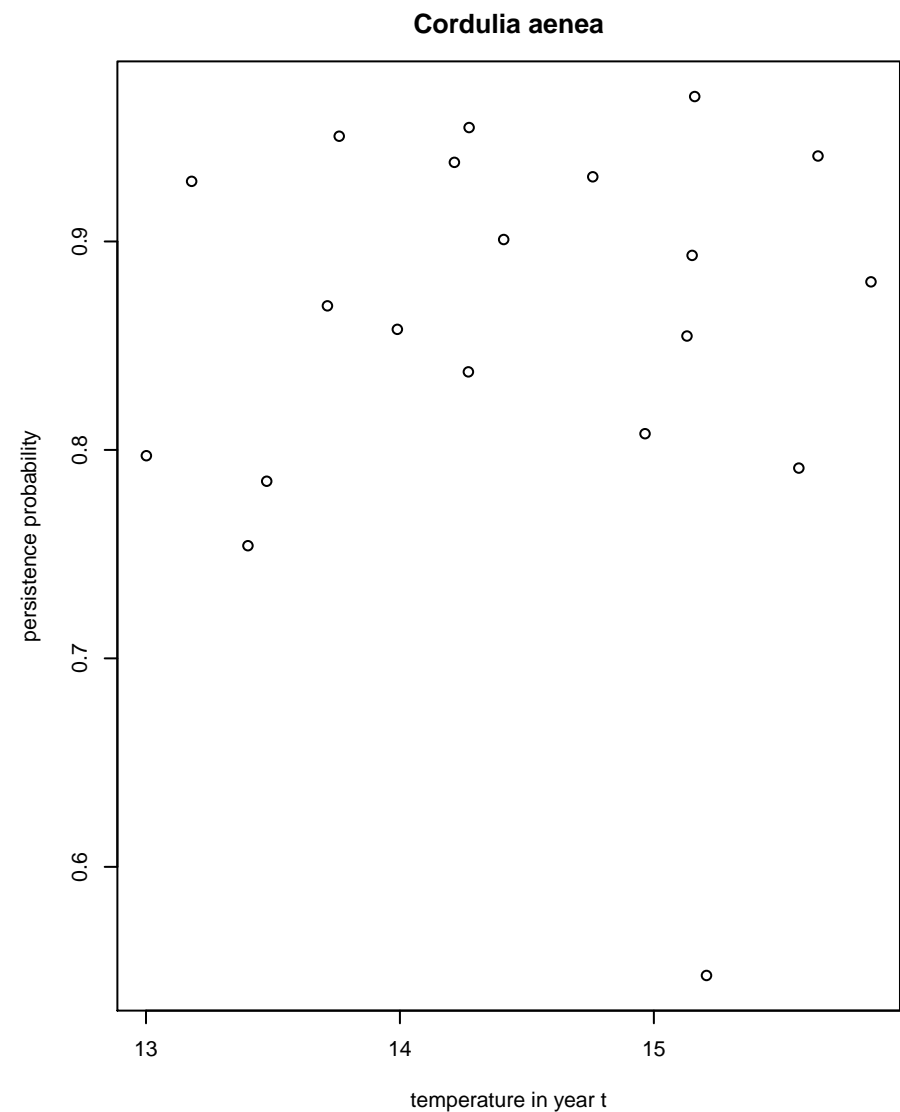

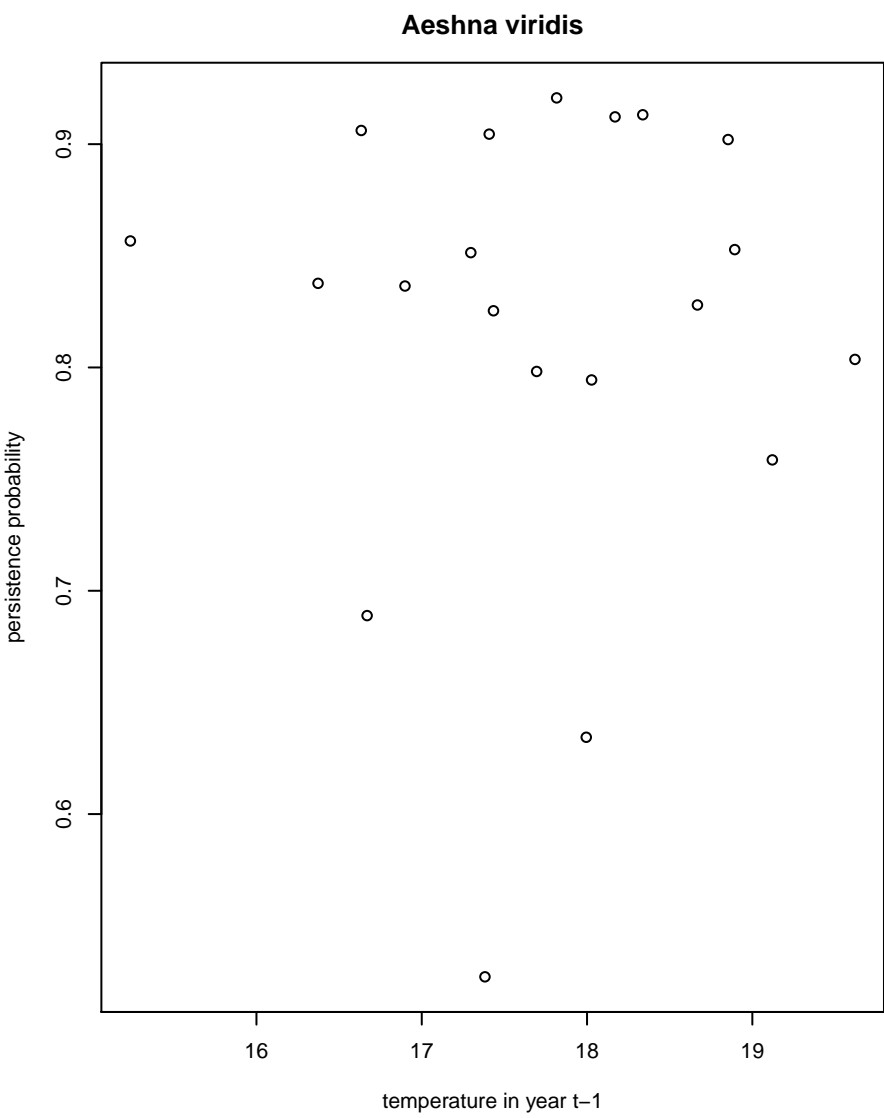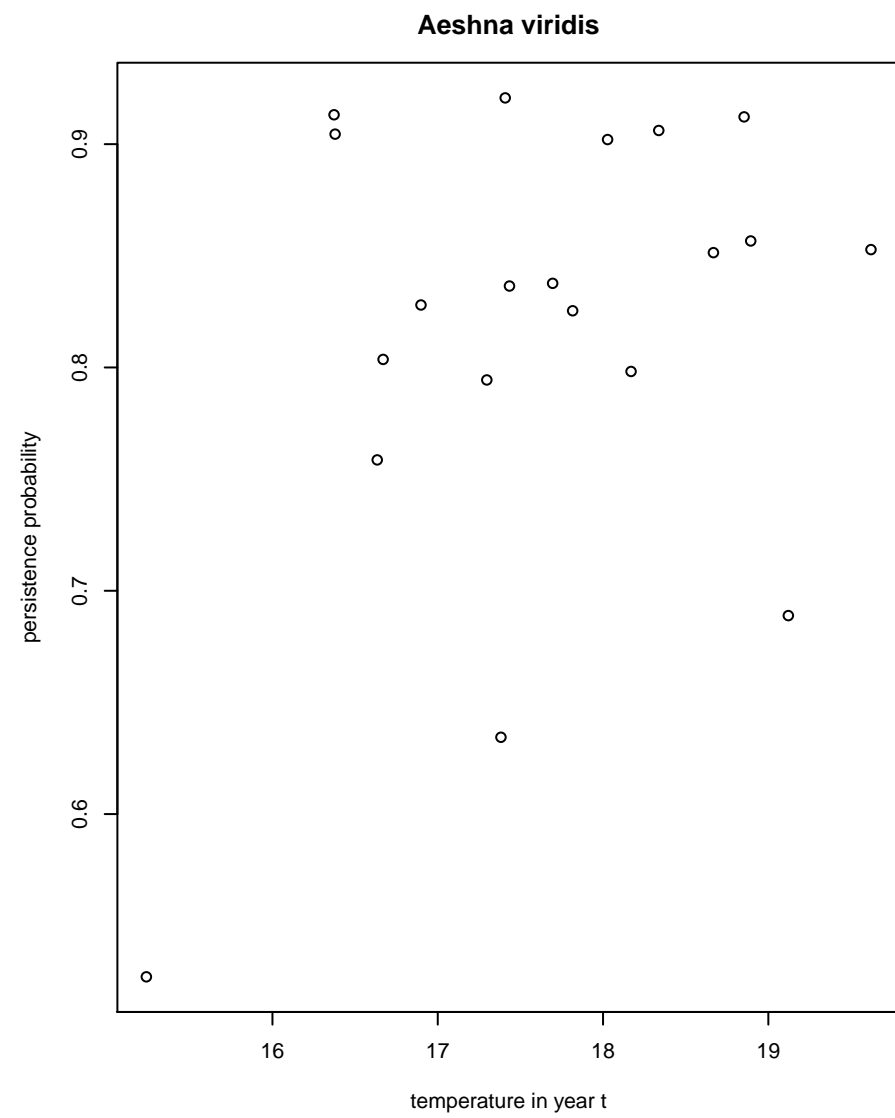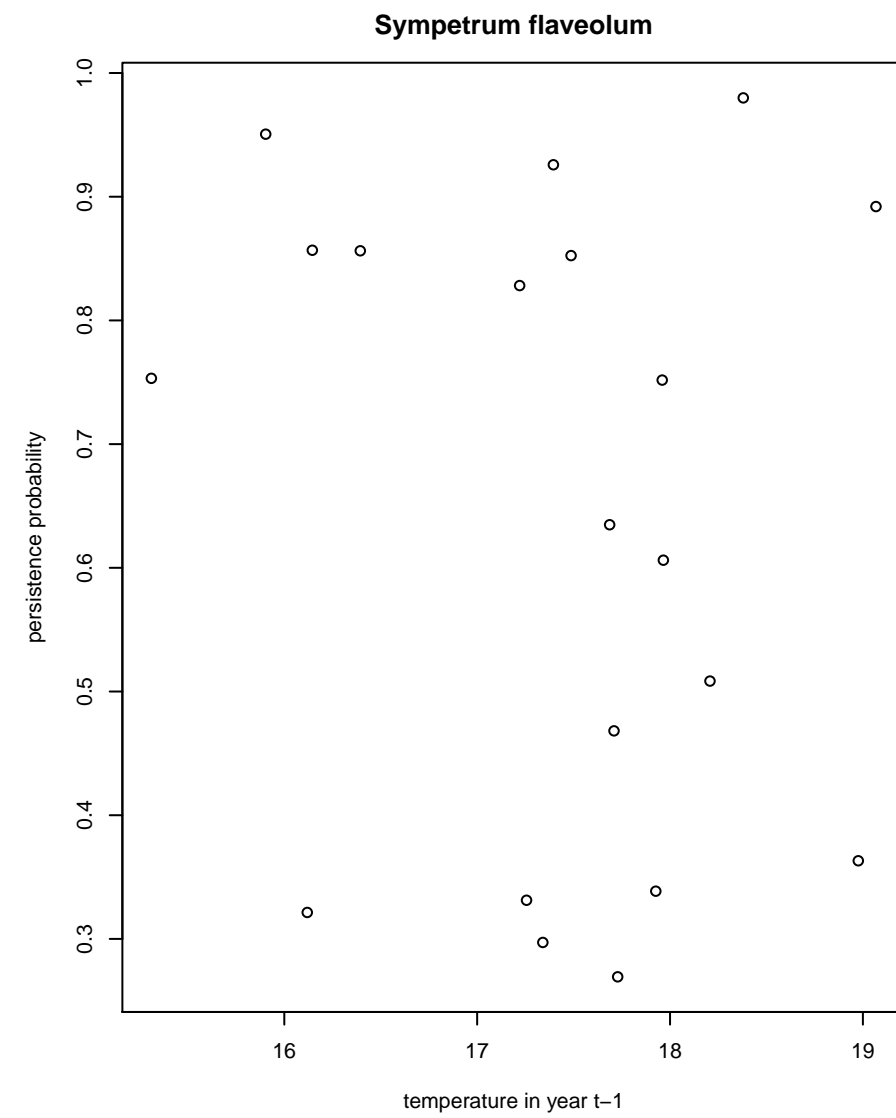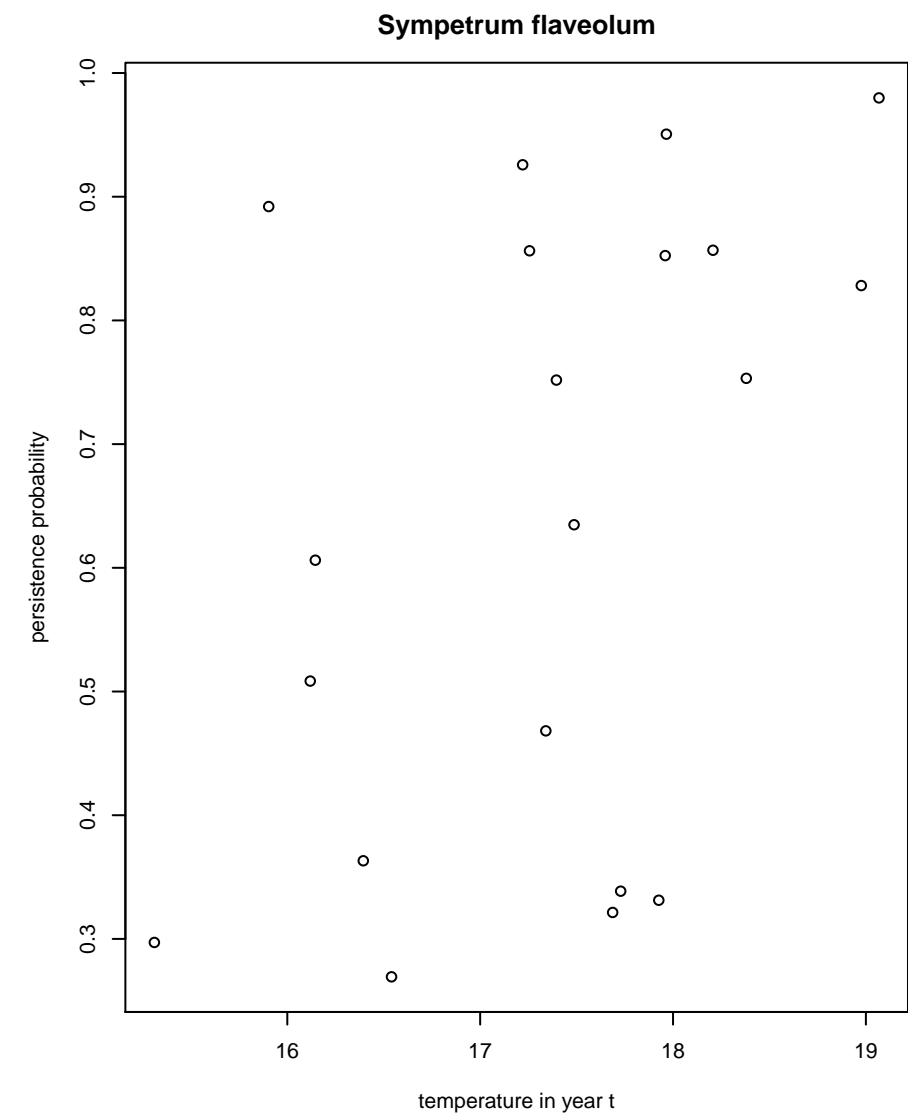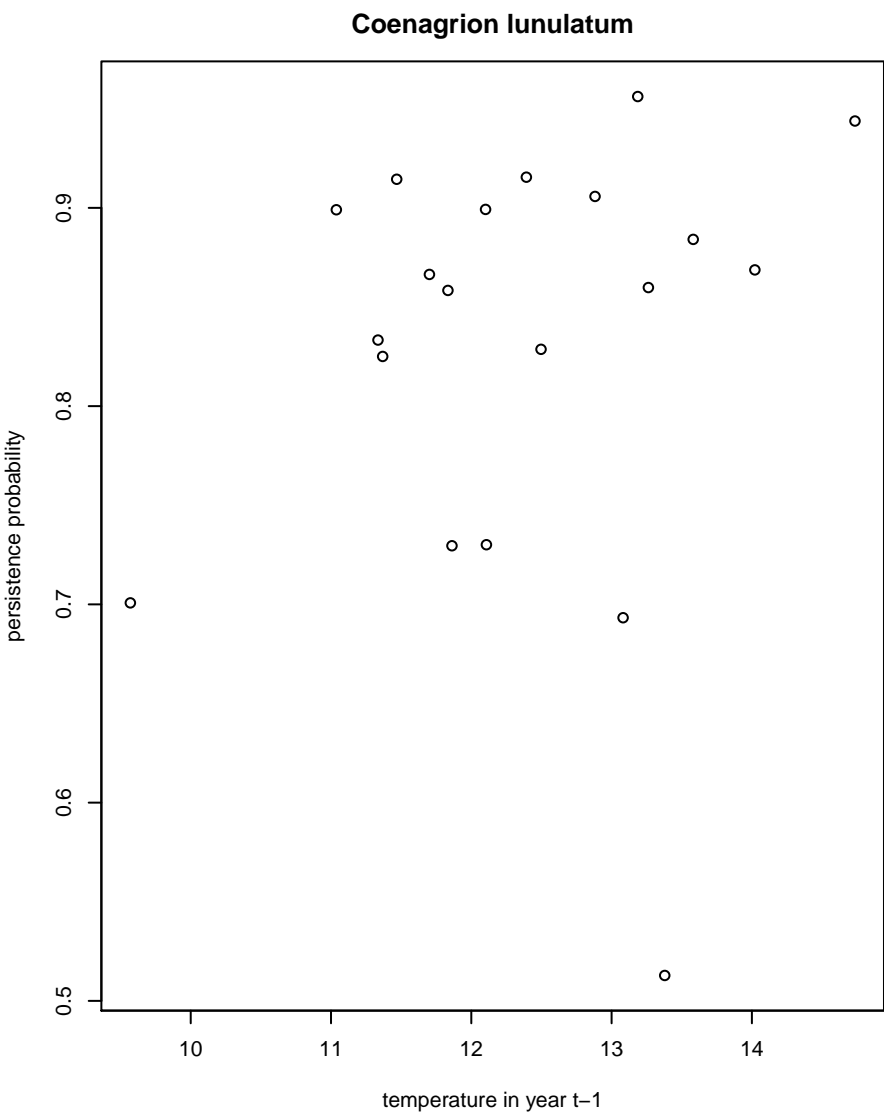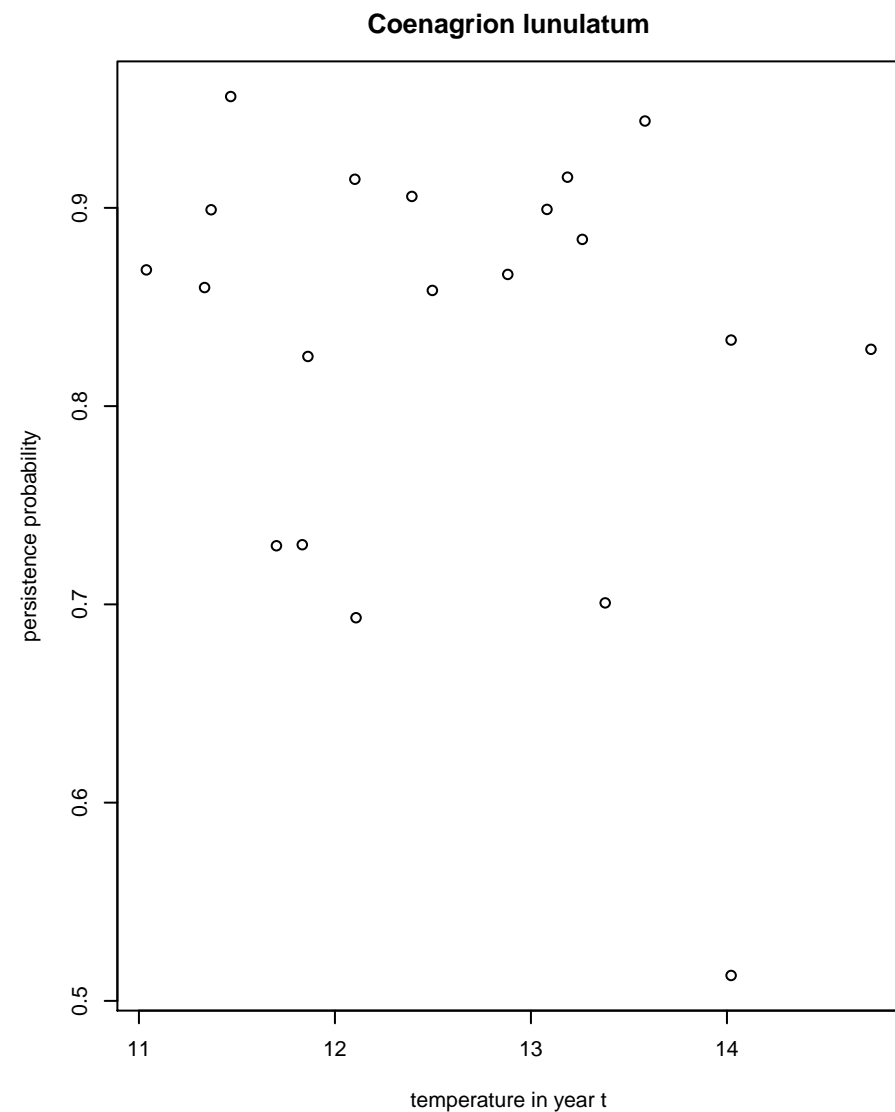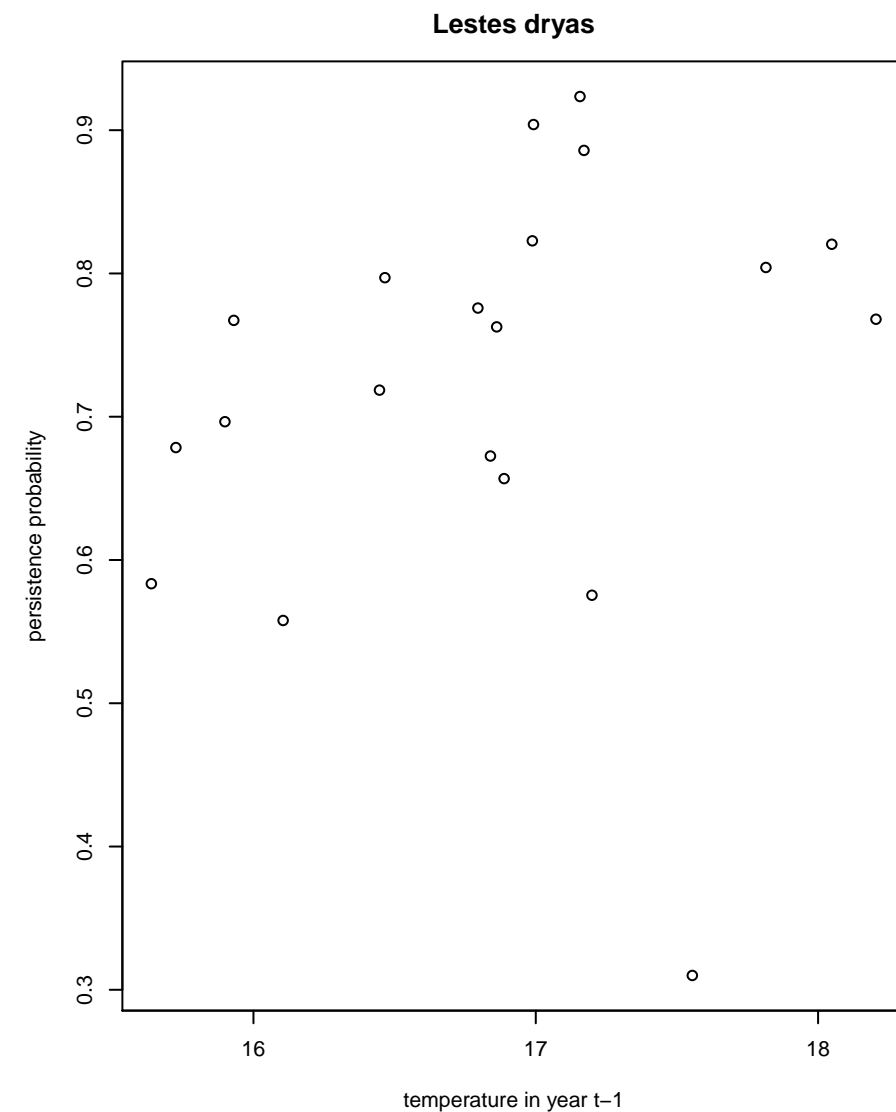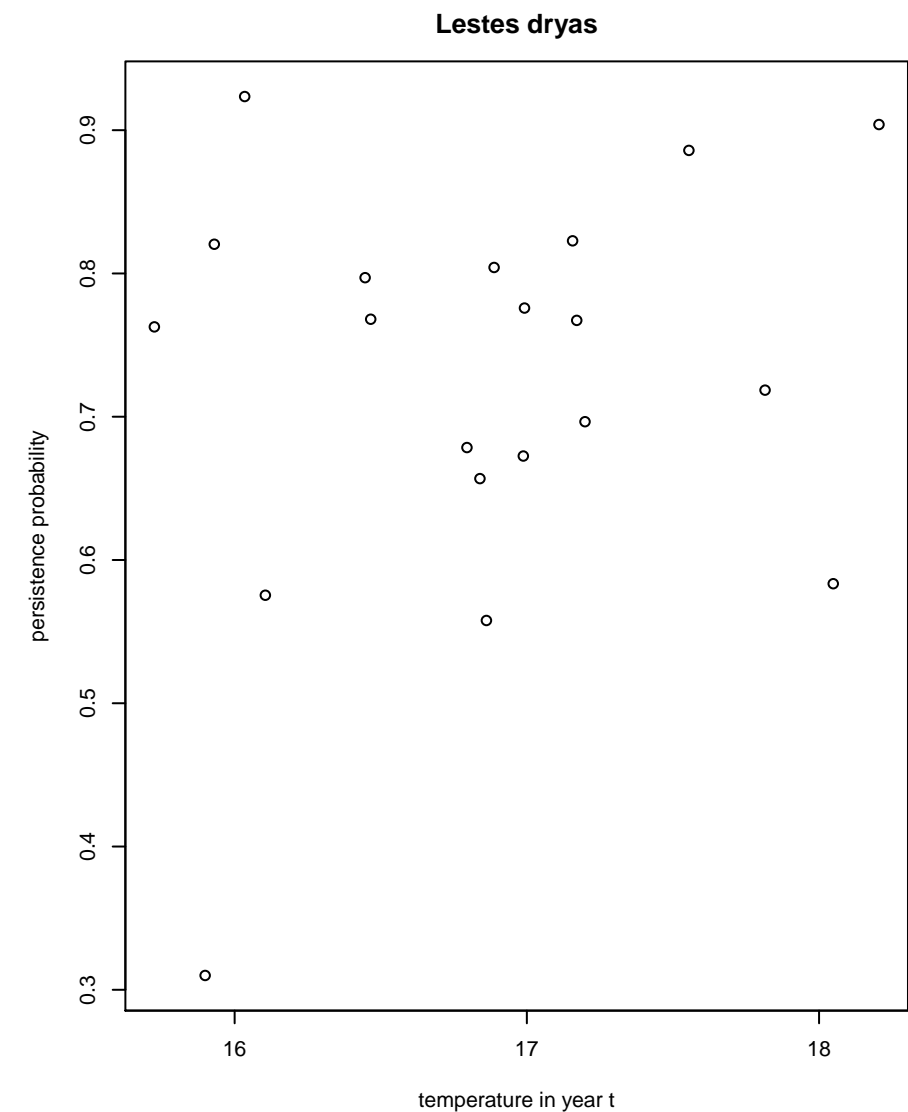

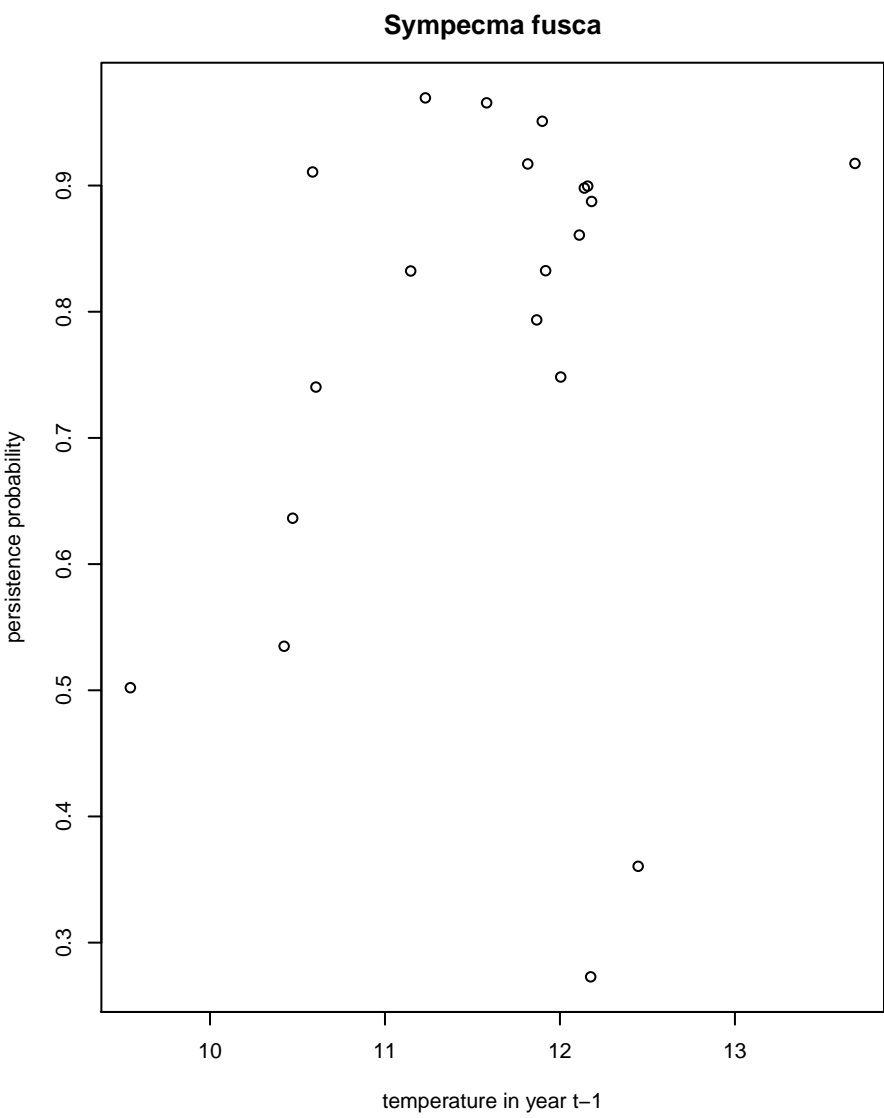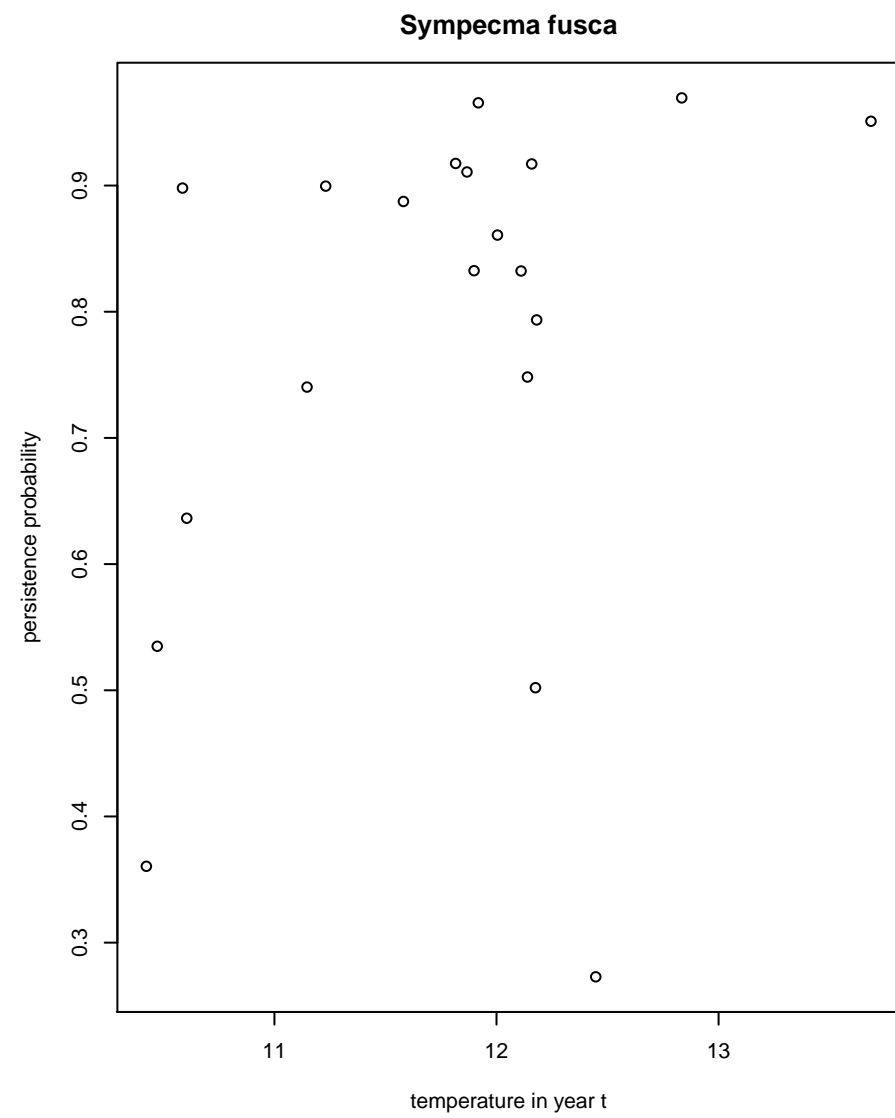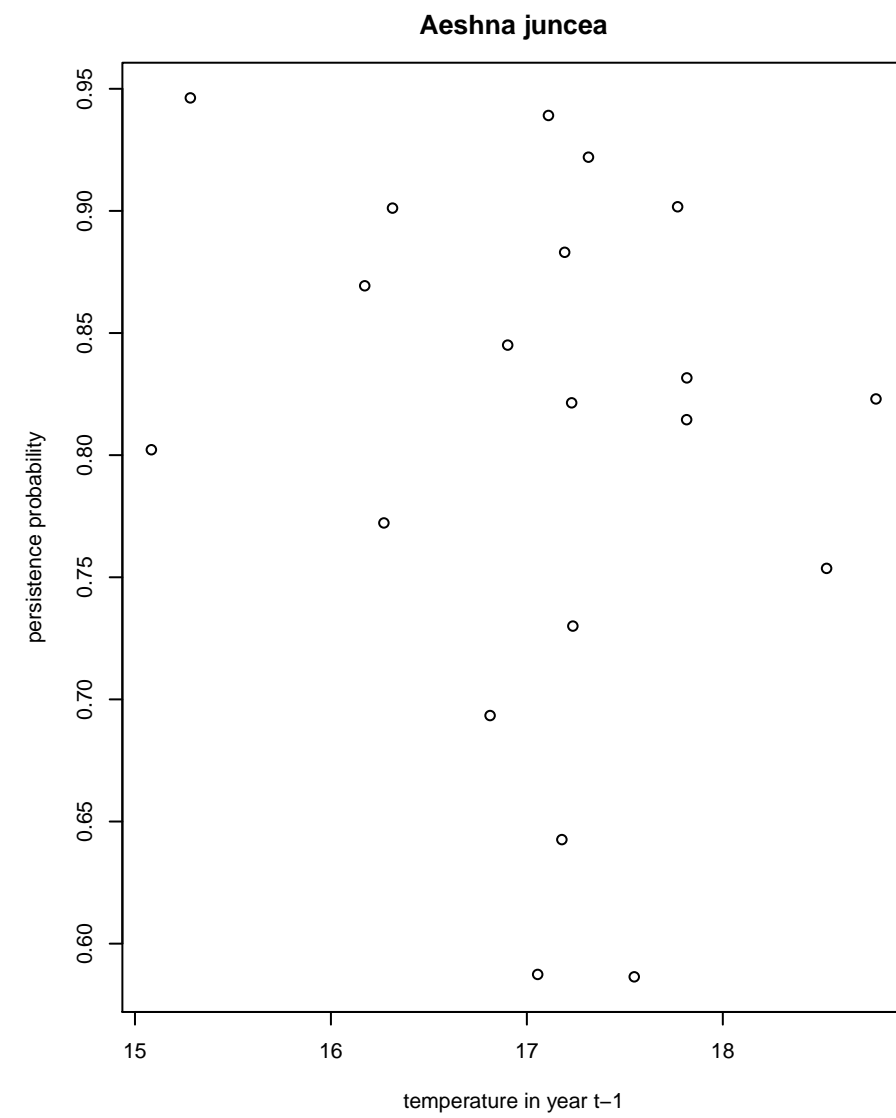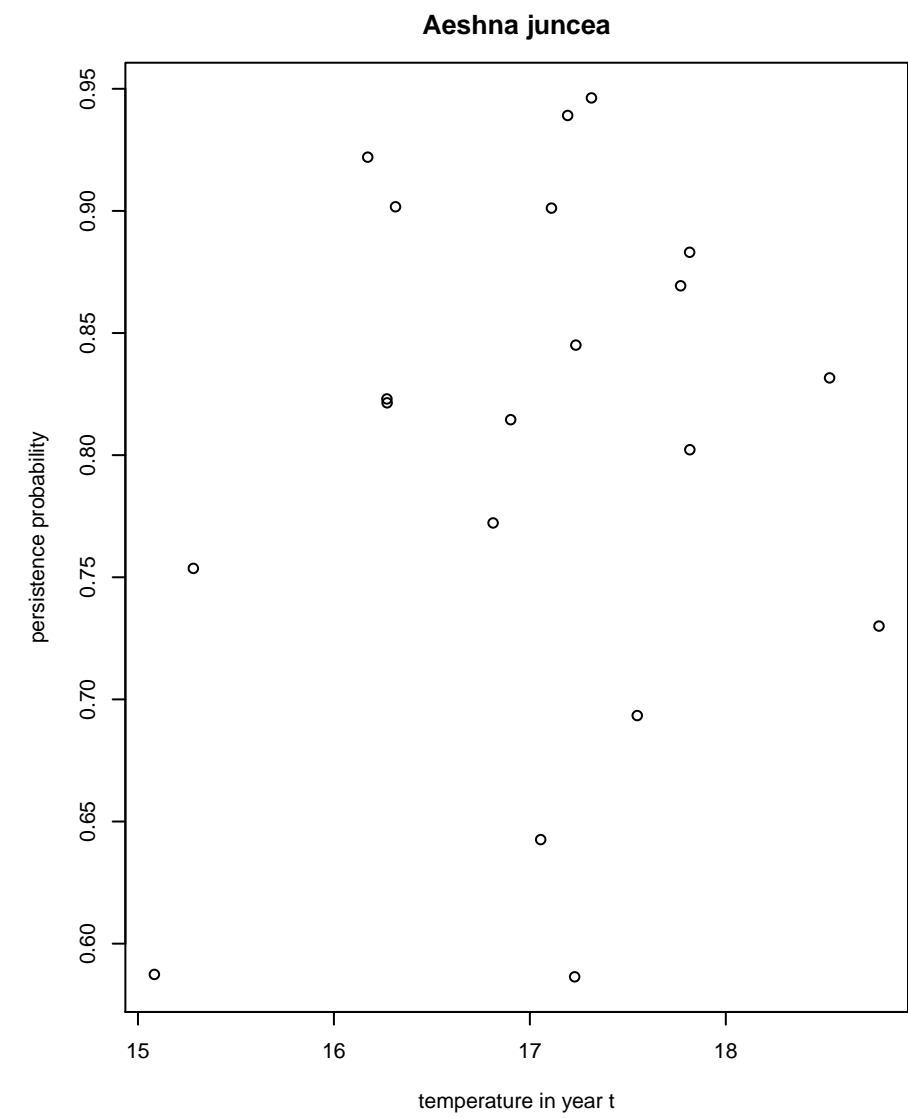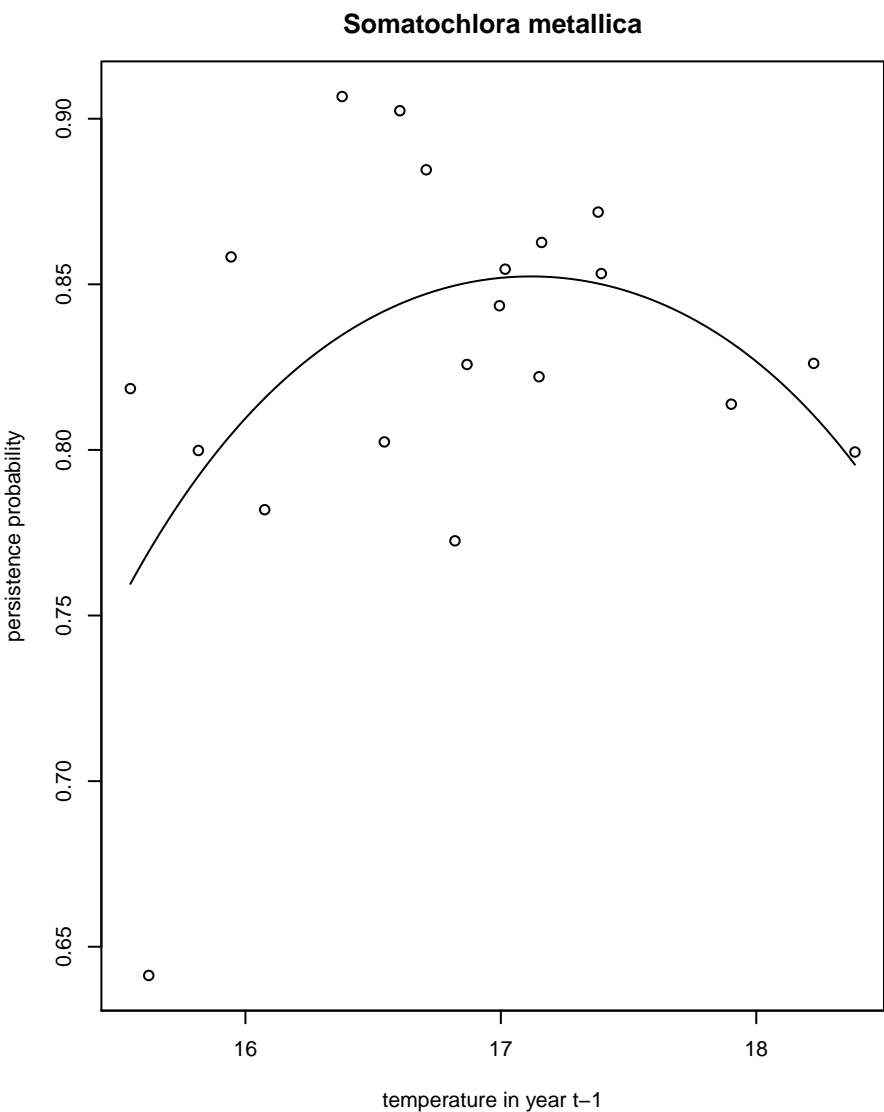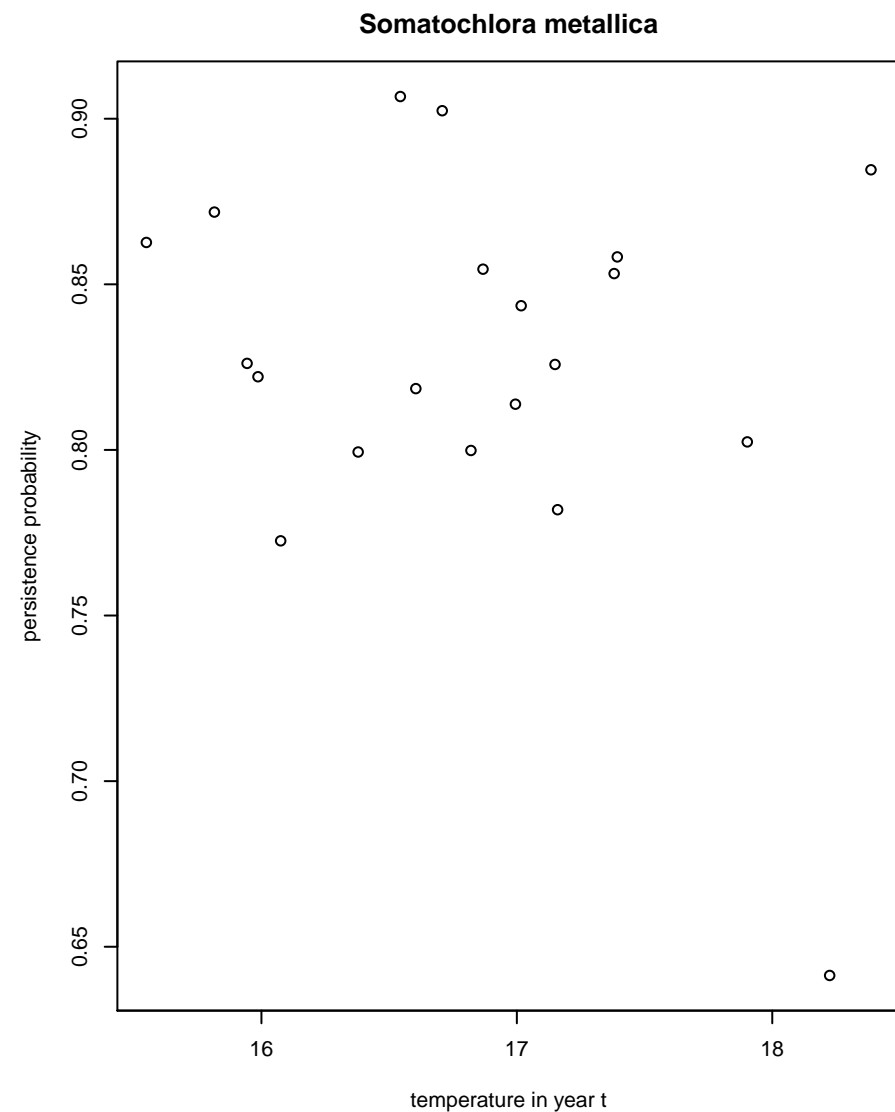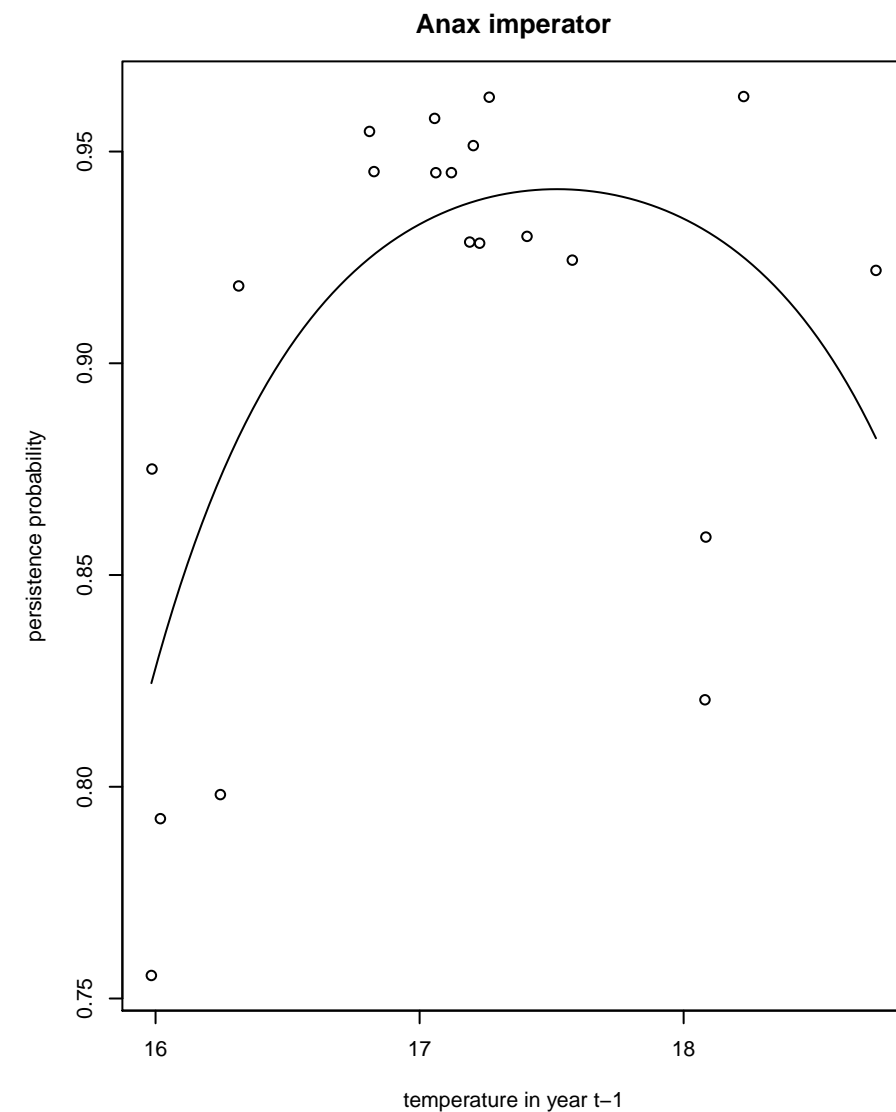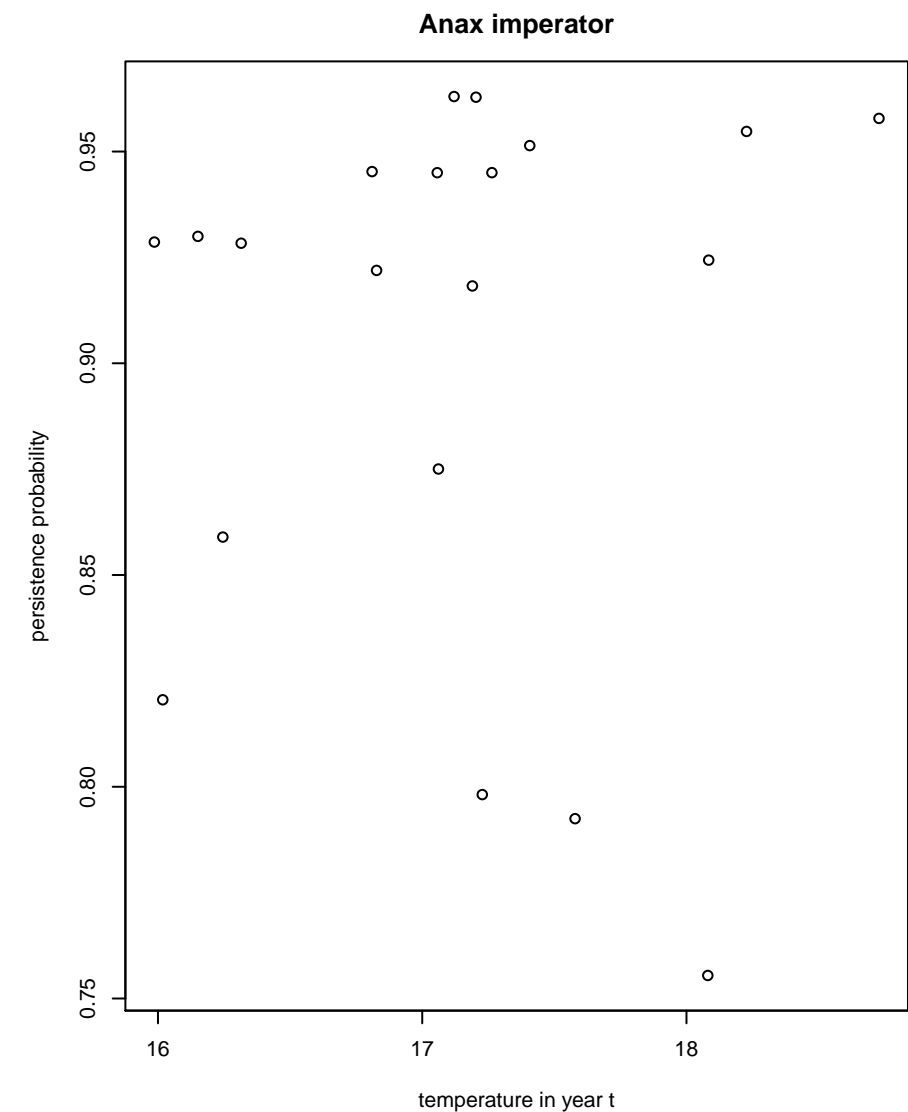

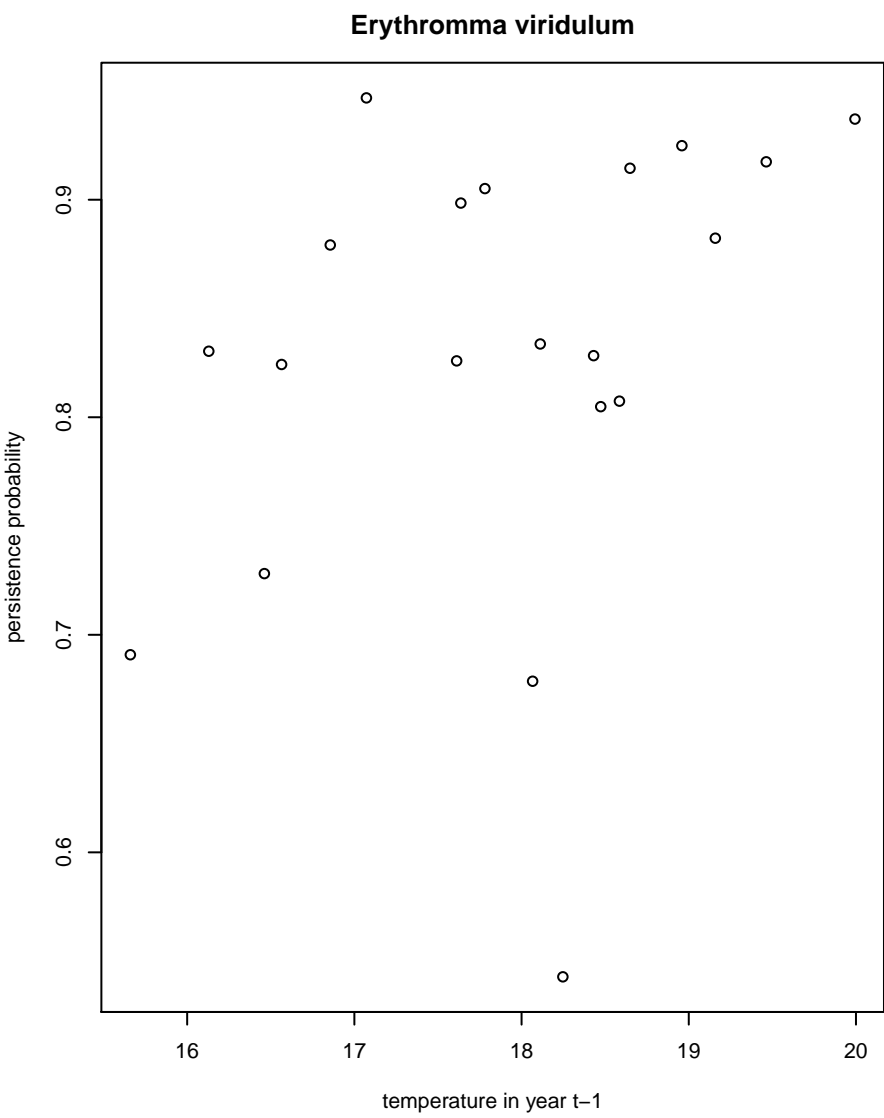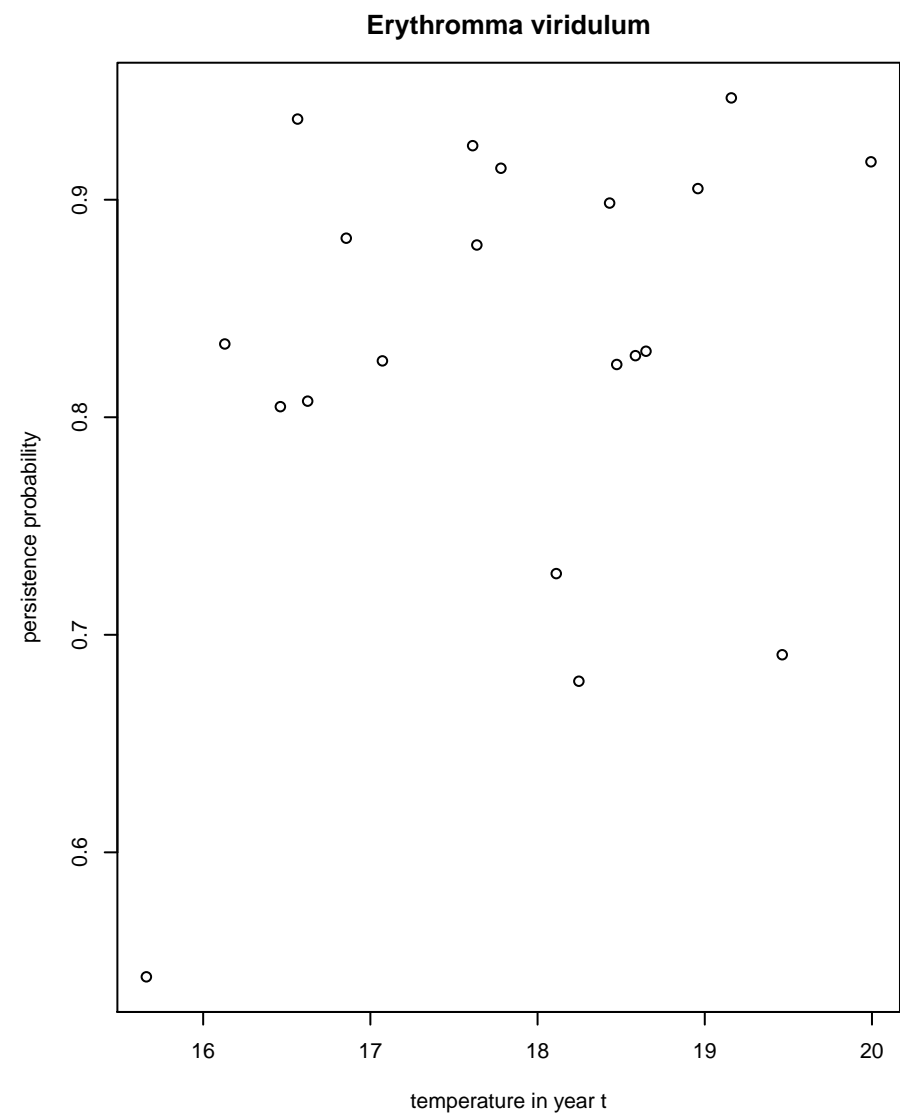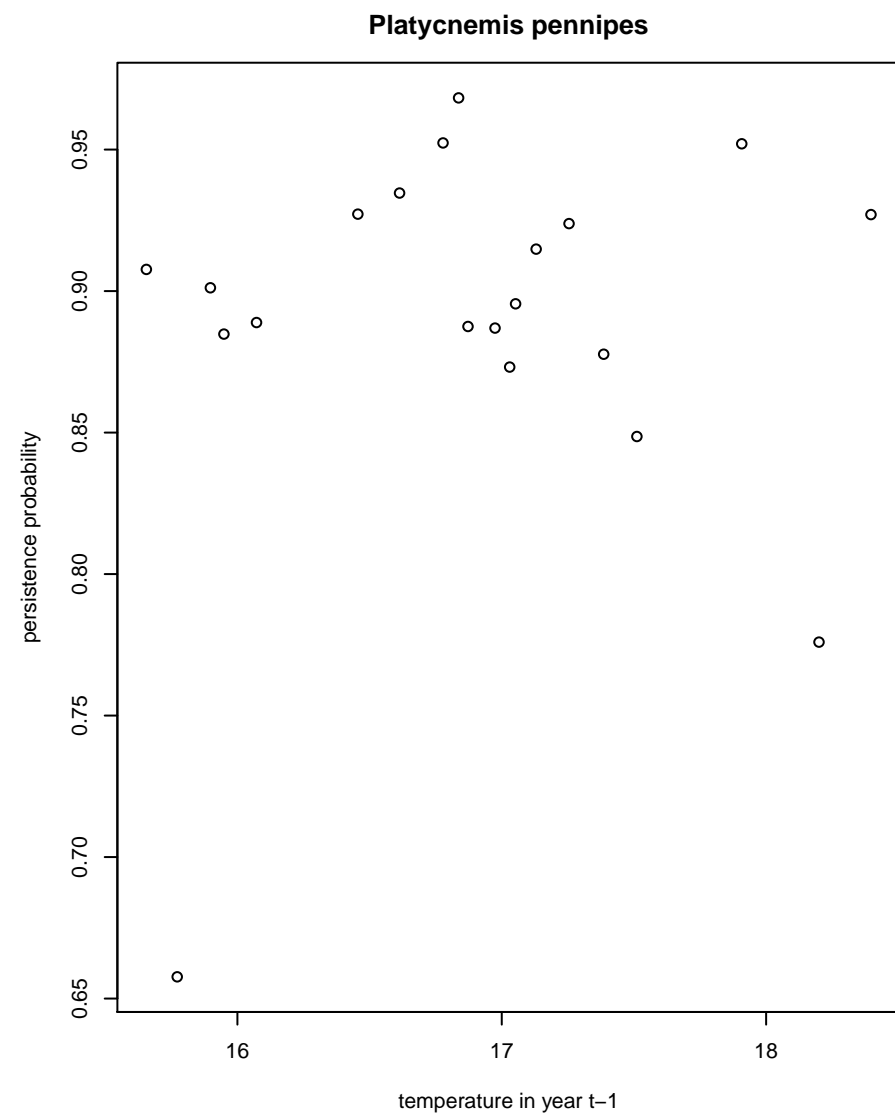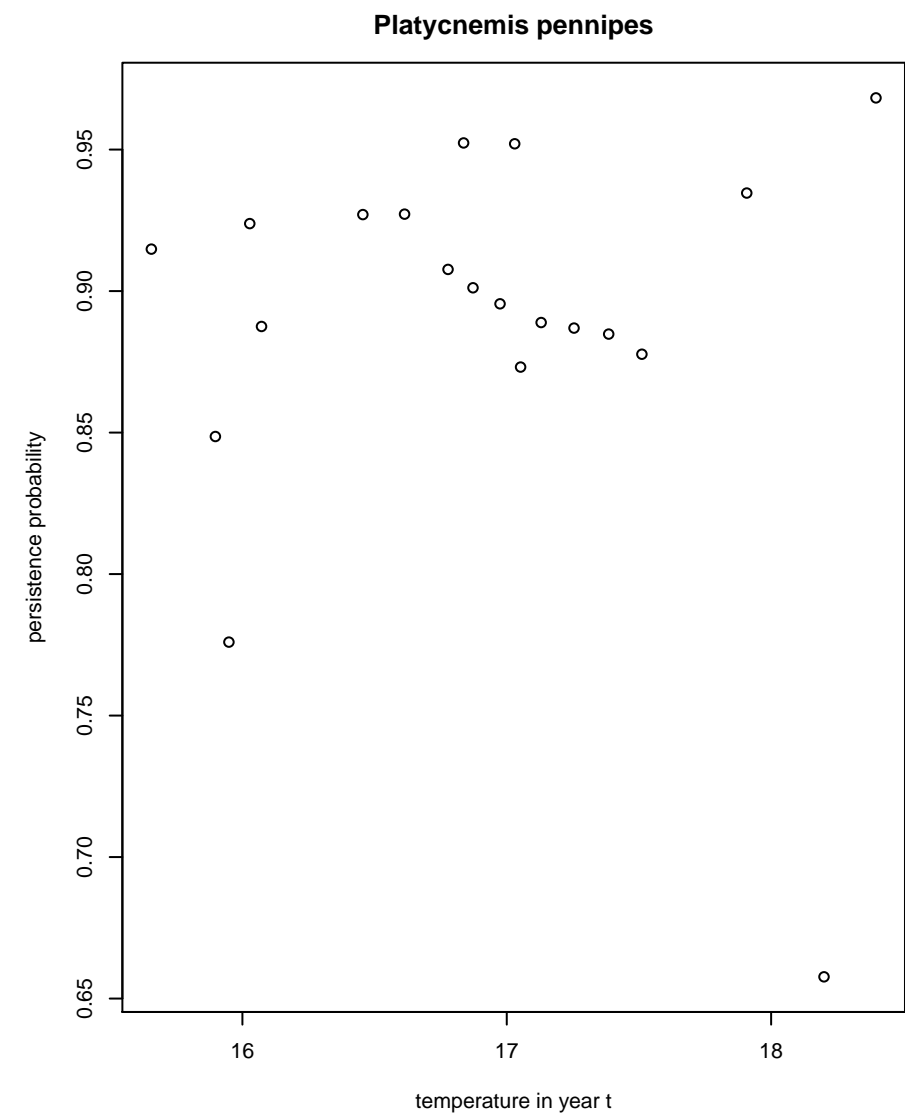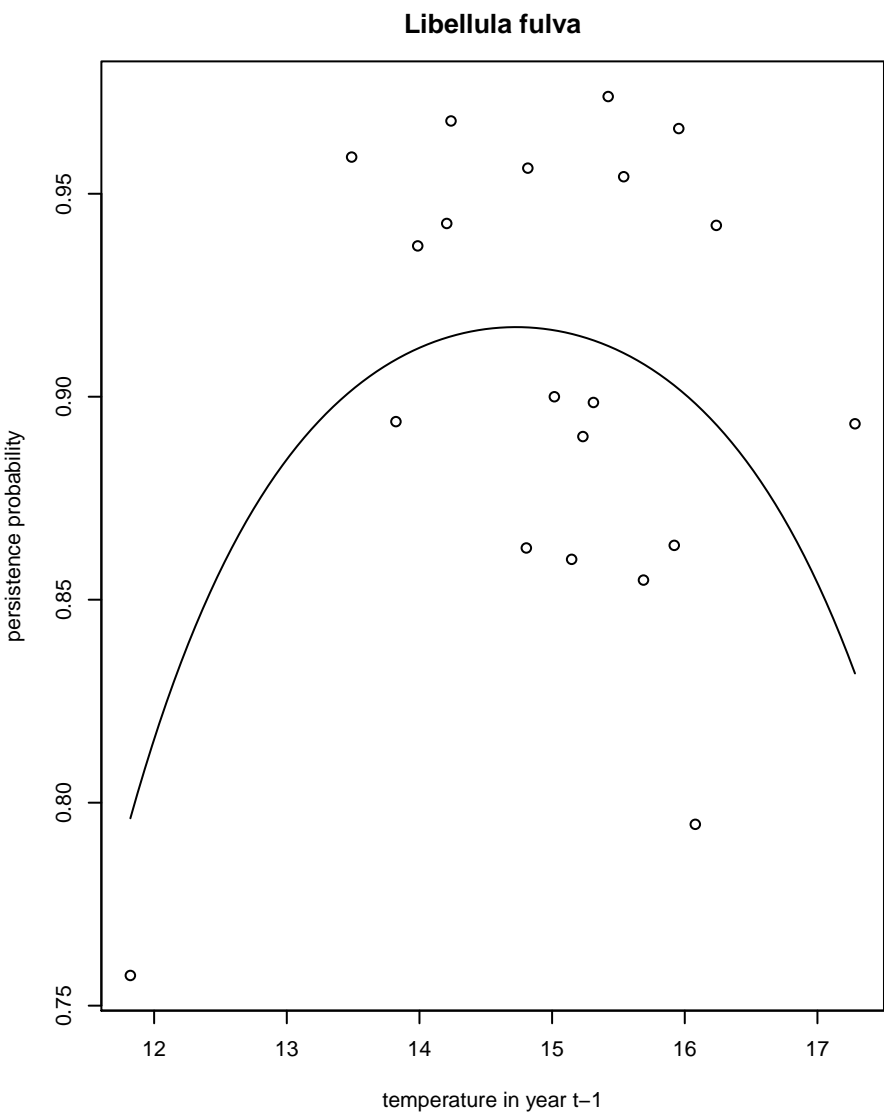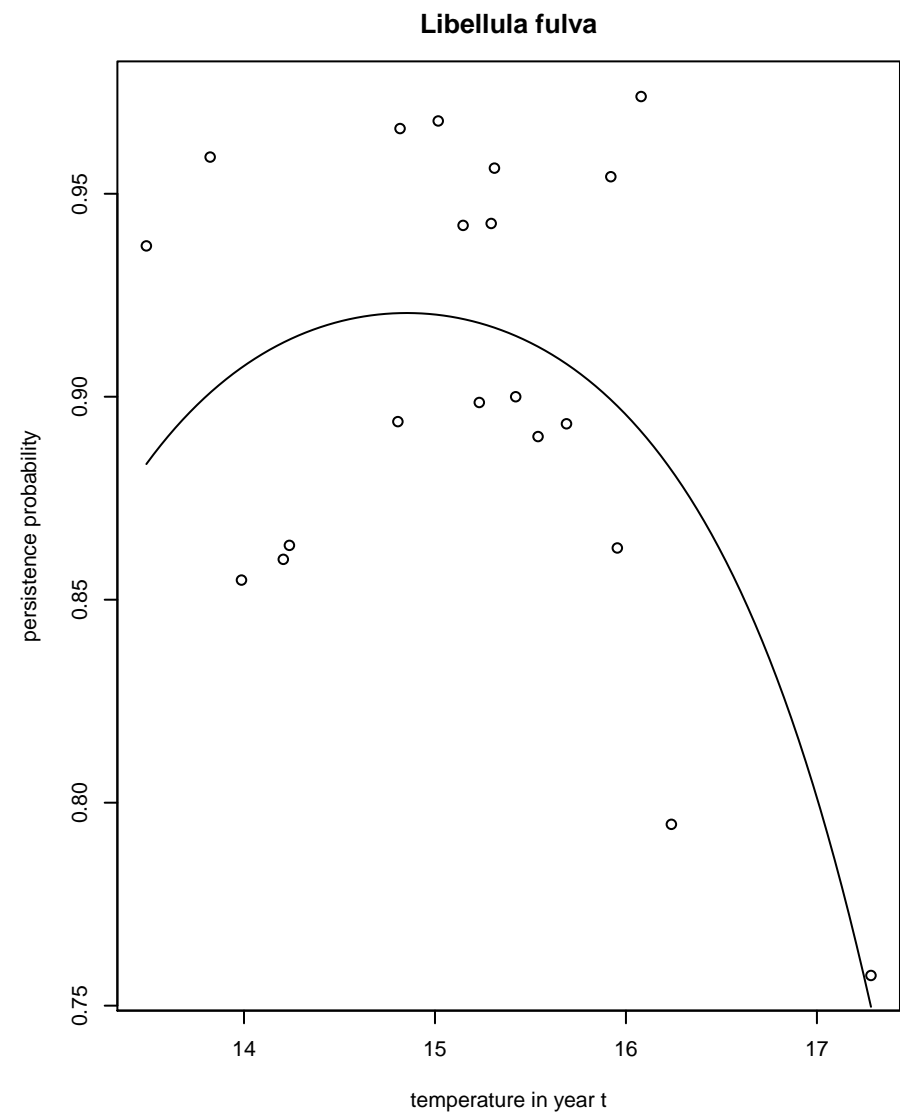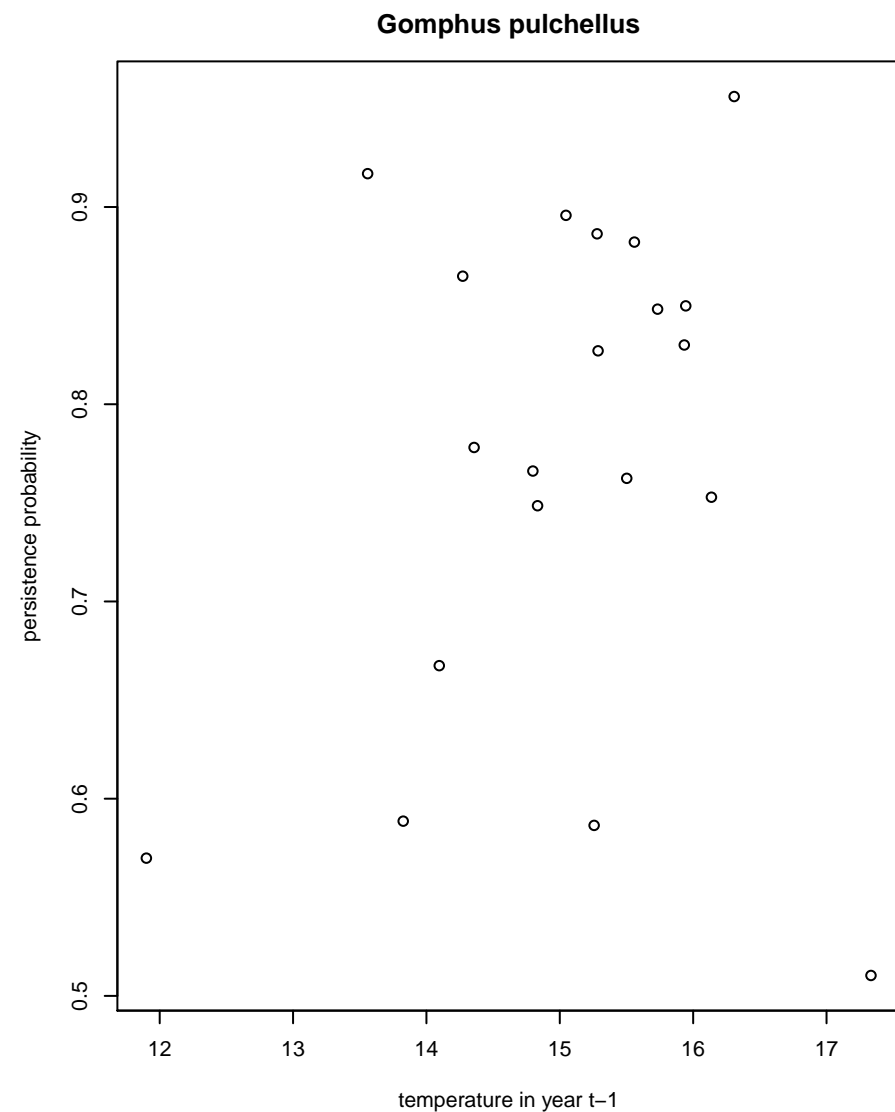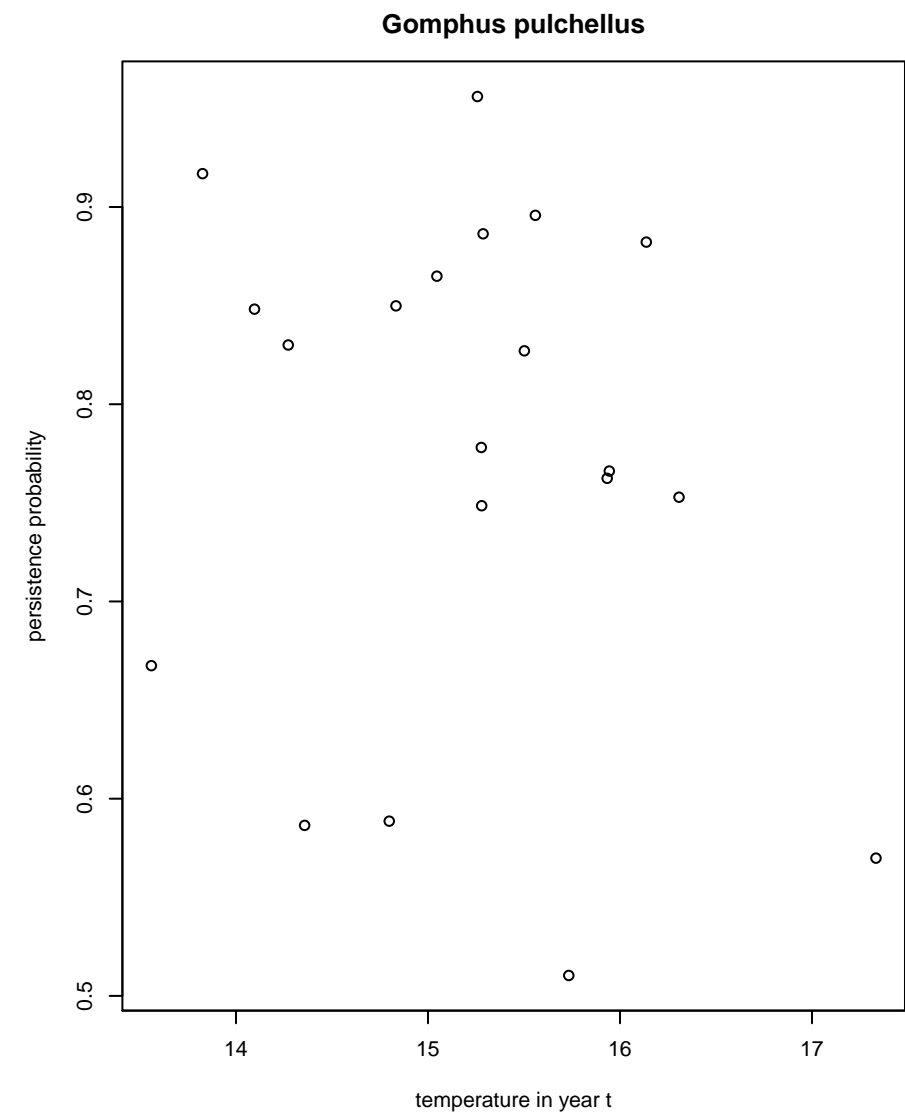

Supplement: Figure S3 — Response curves of colonisation and persistence probability in relation to temperature per species, excluding rare species. (PDF) [file pone.0110219.s003.pdf]

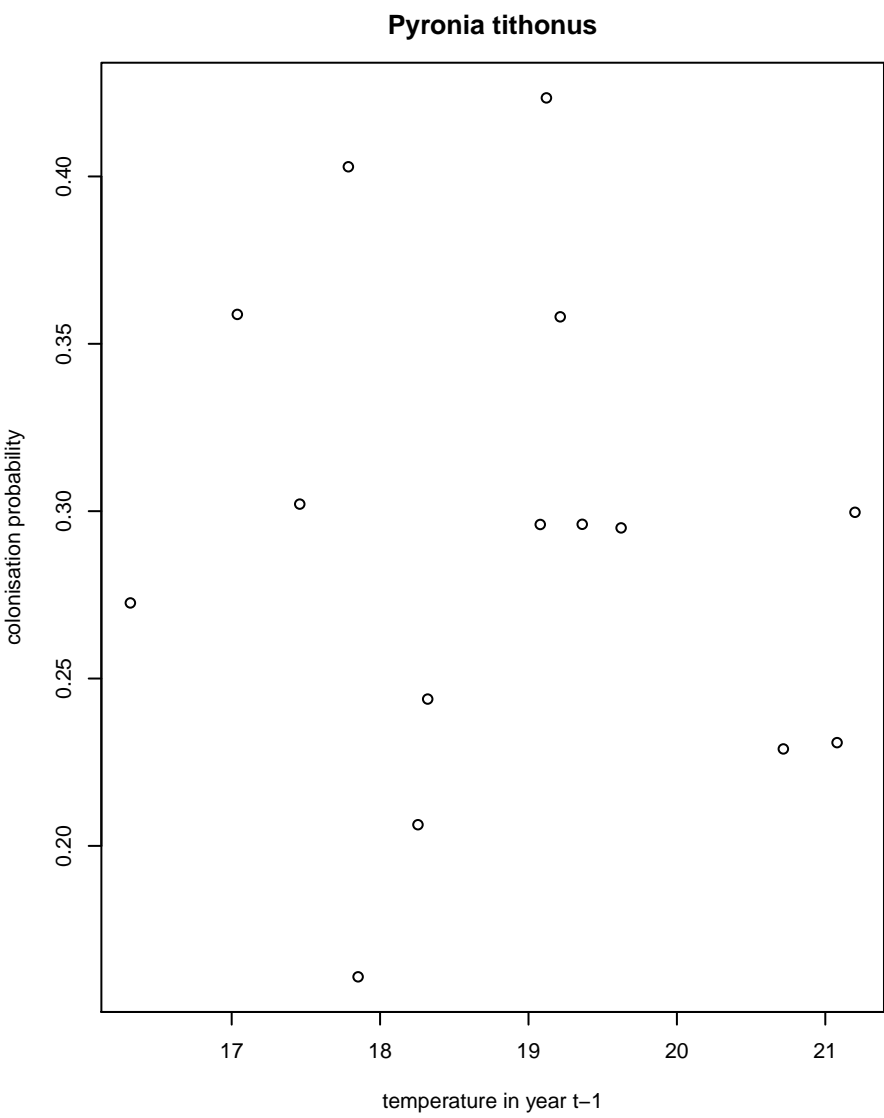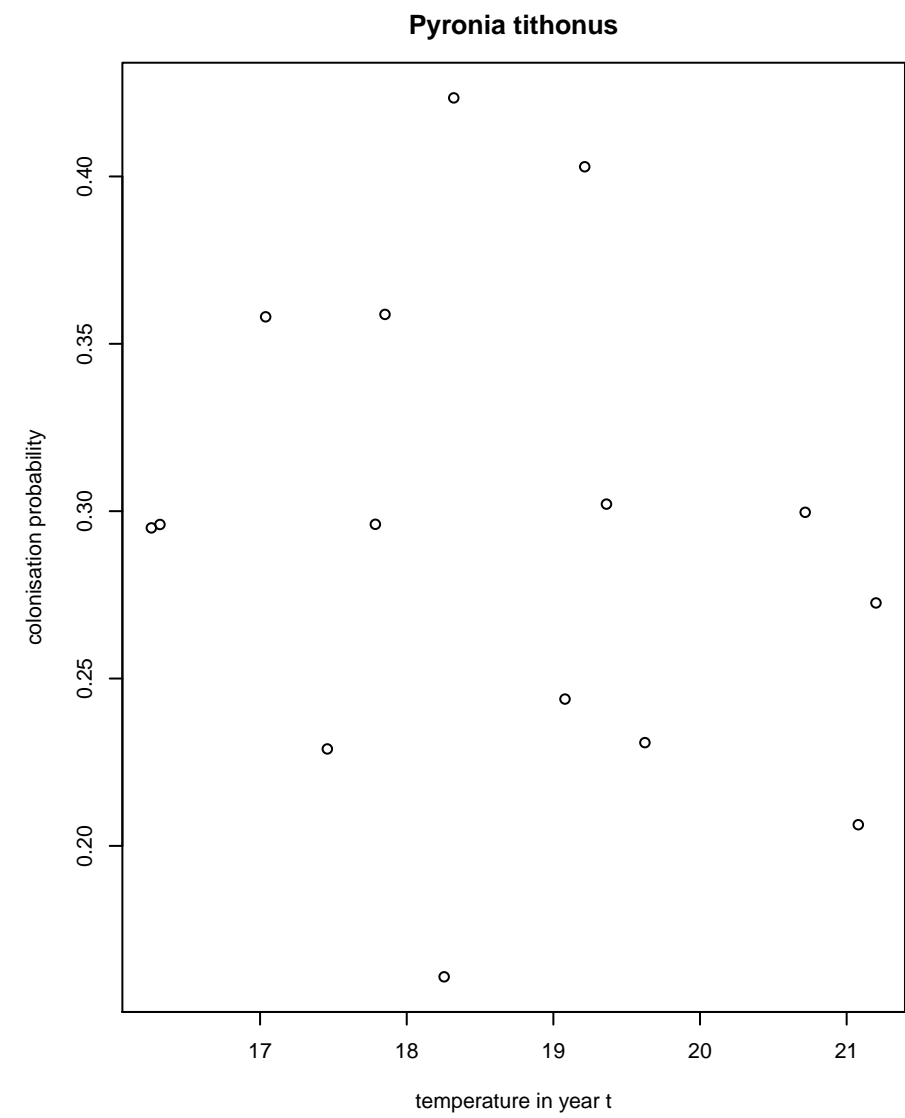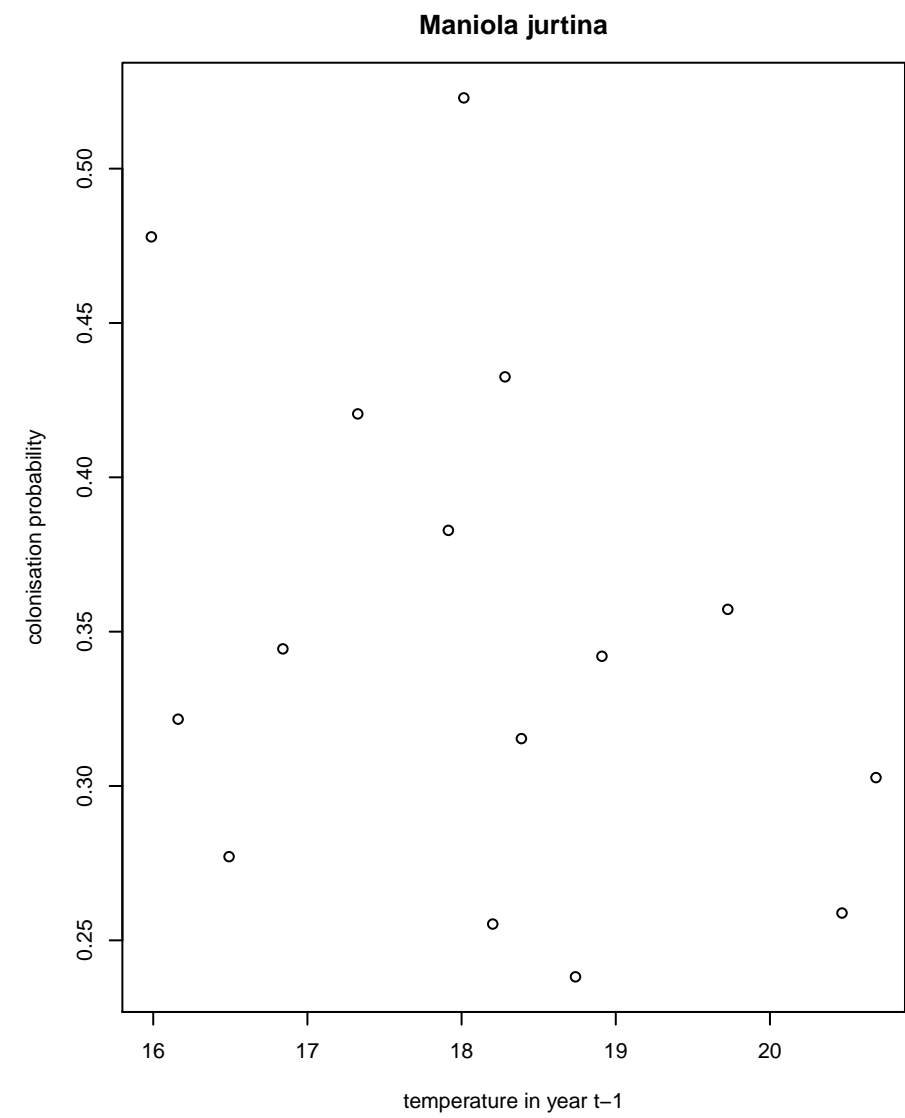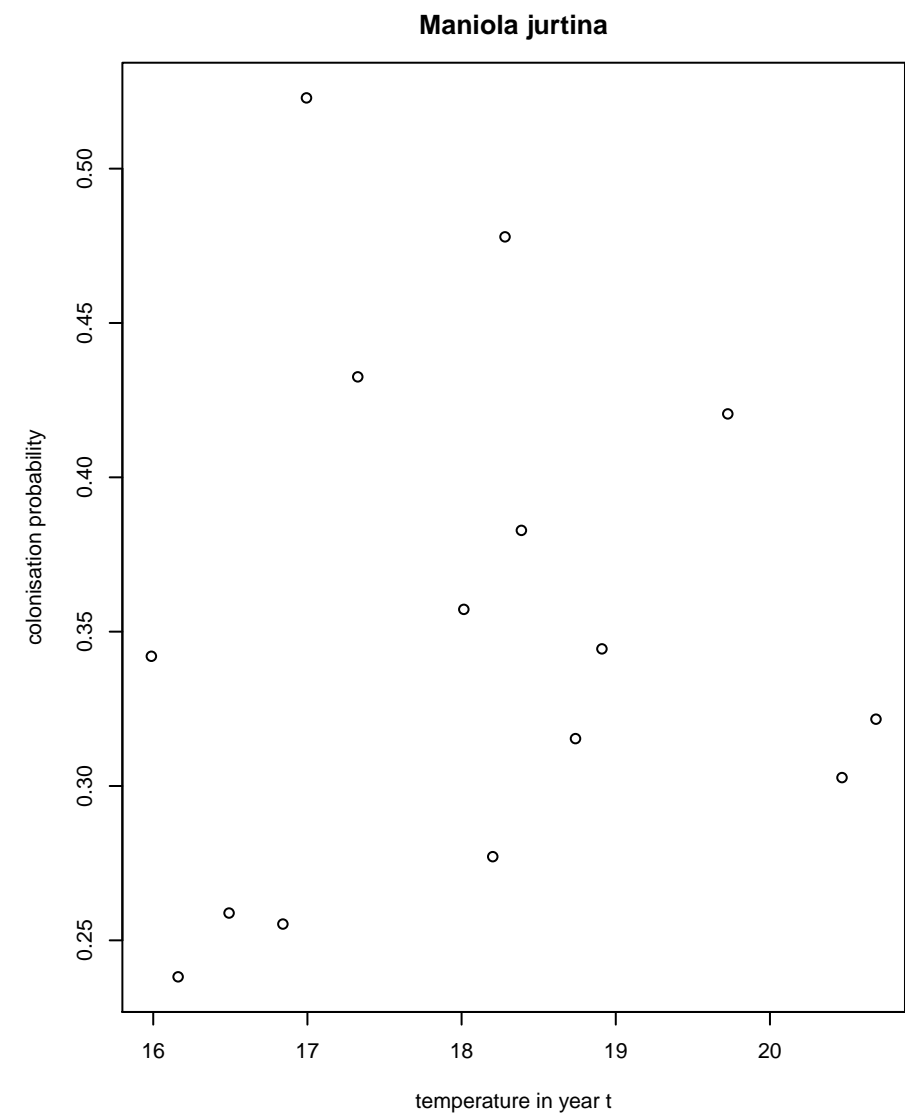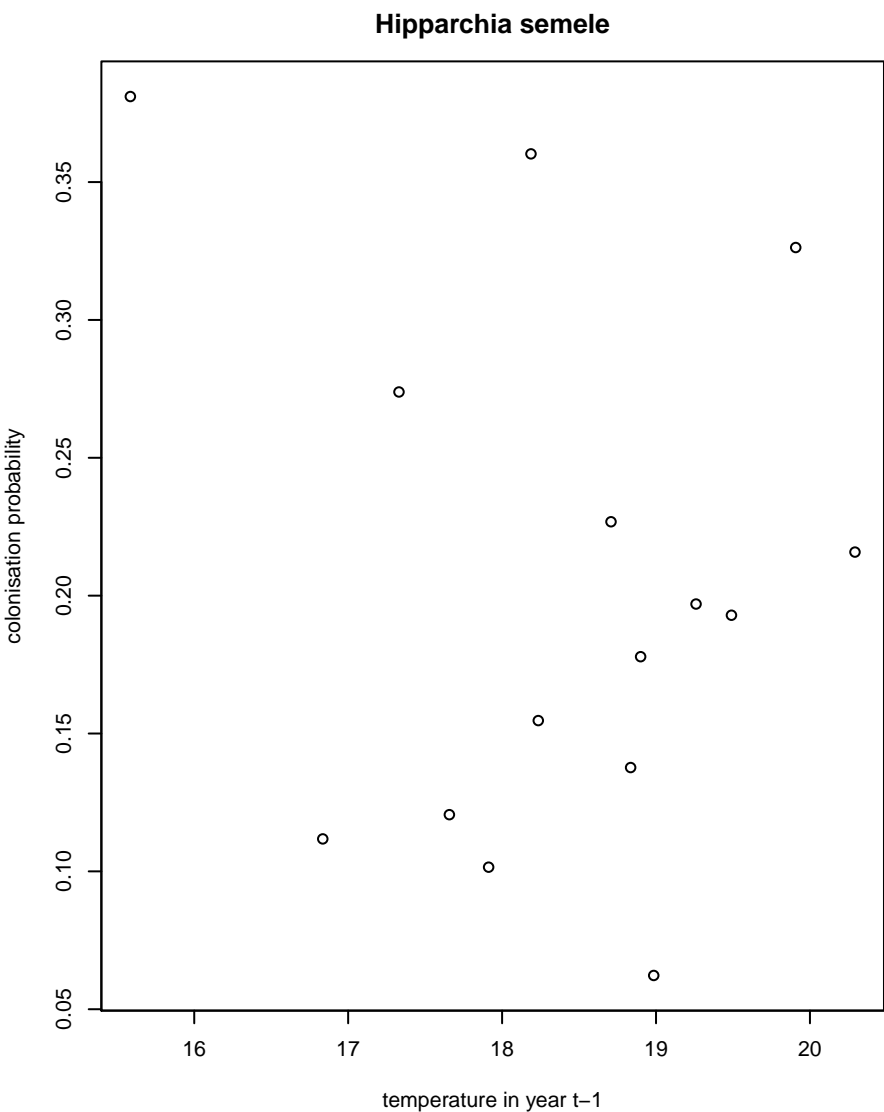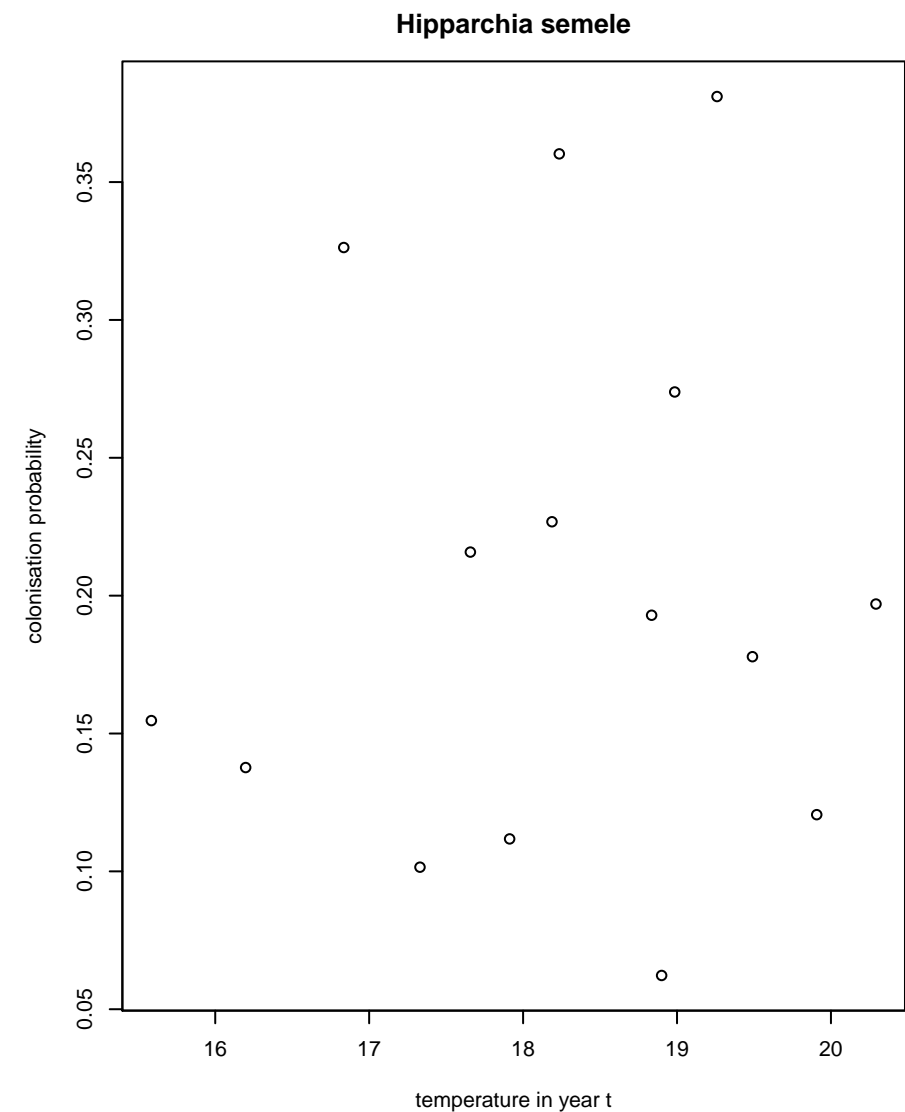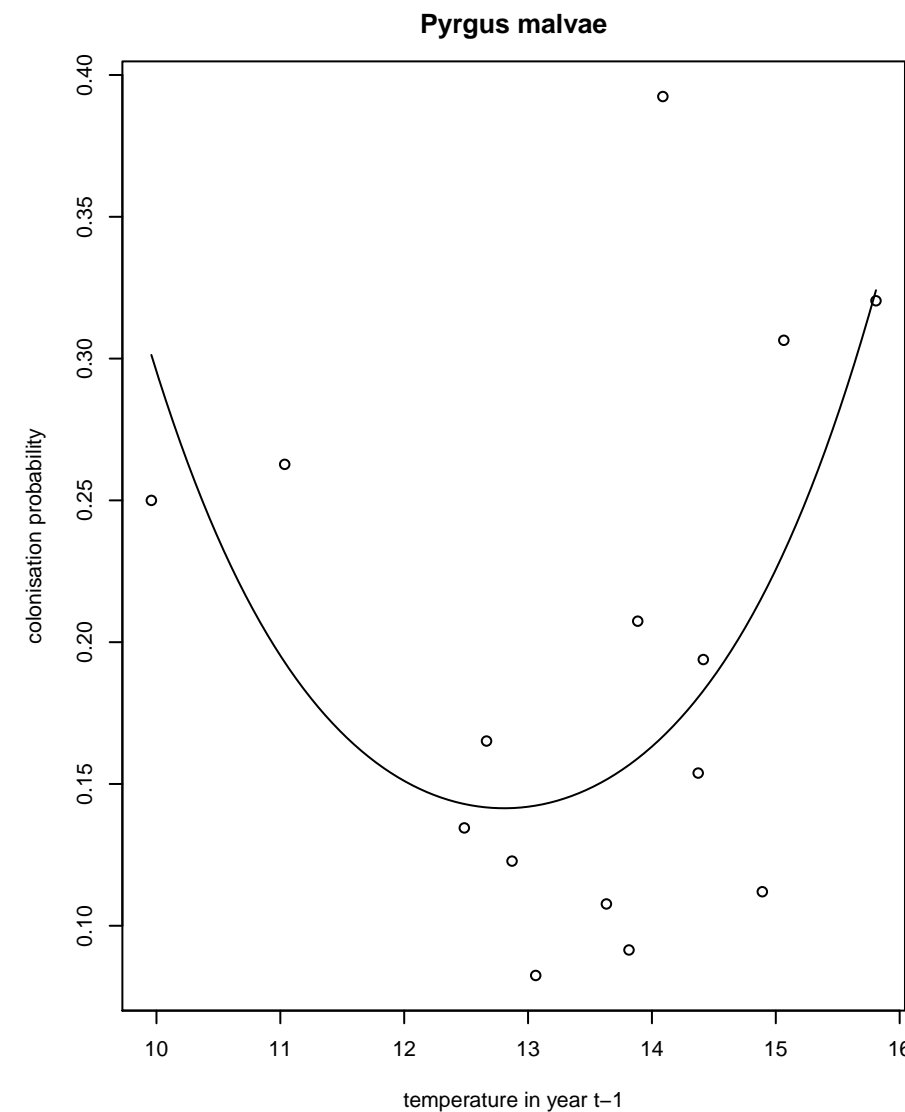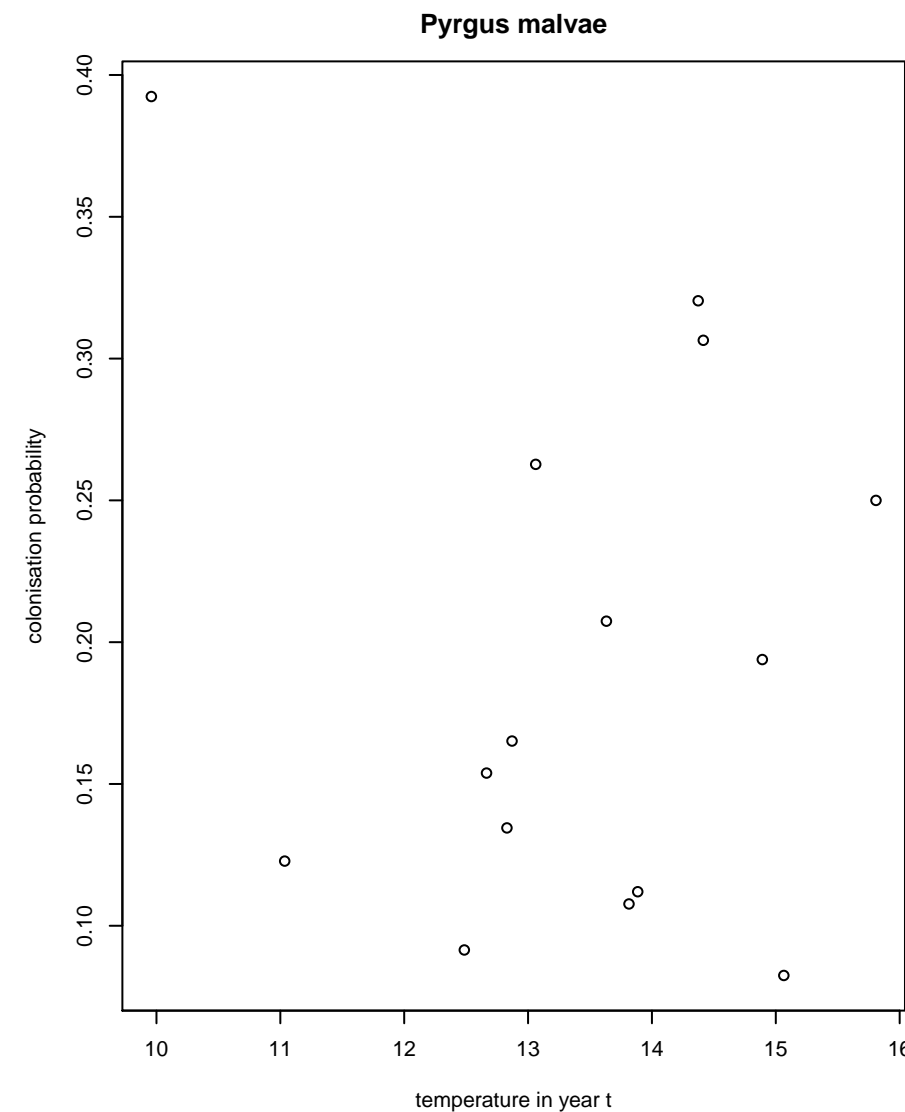

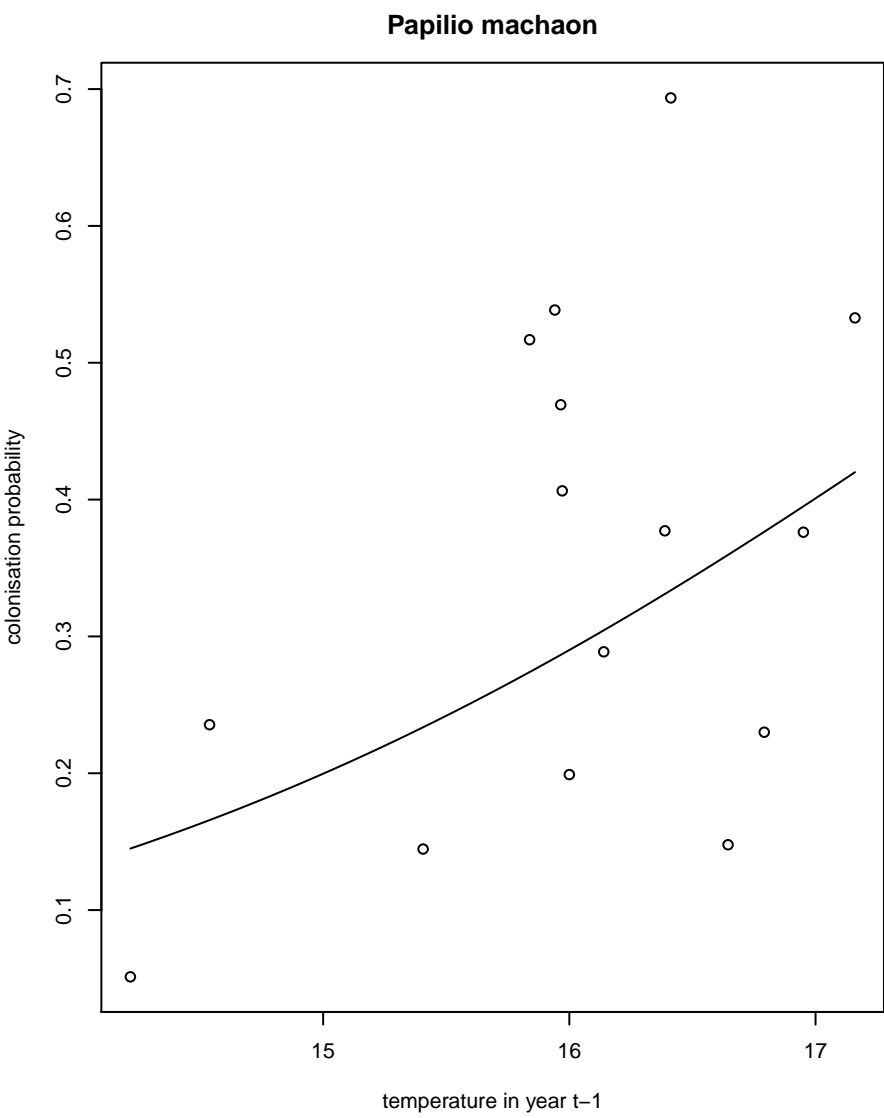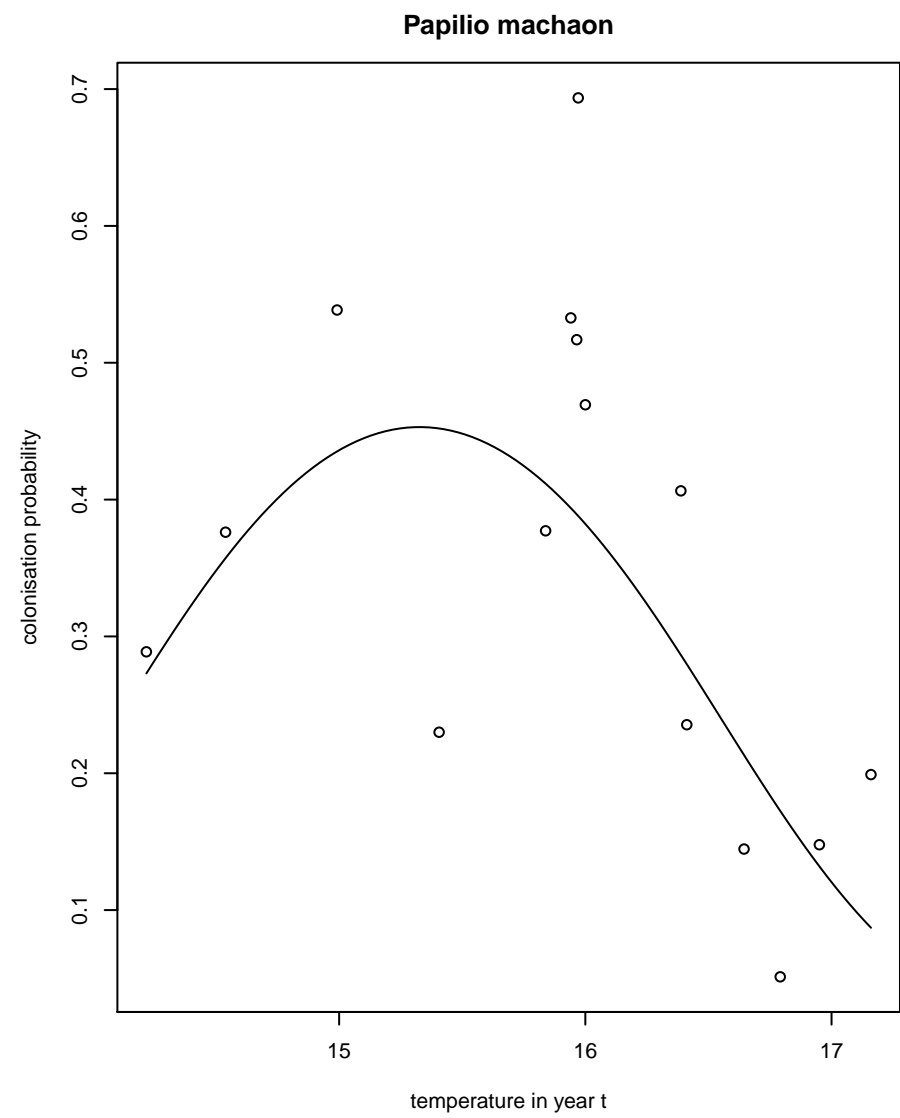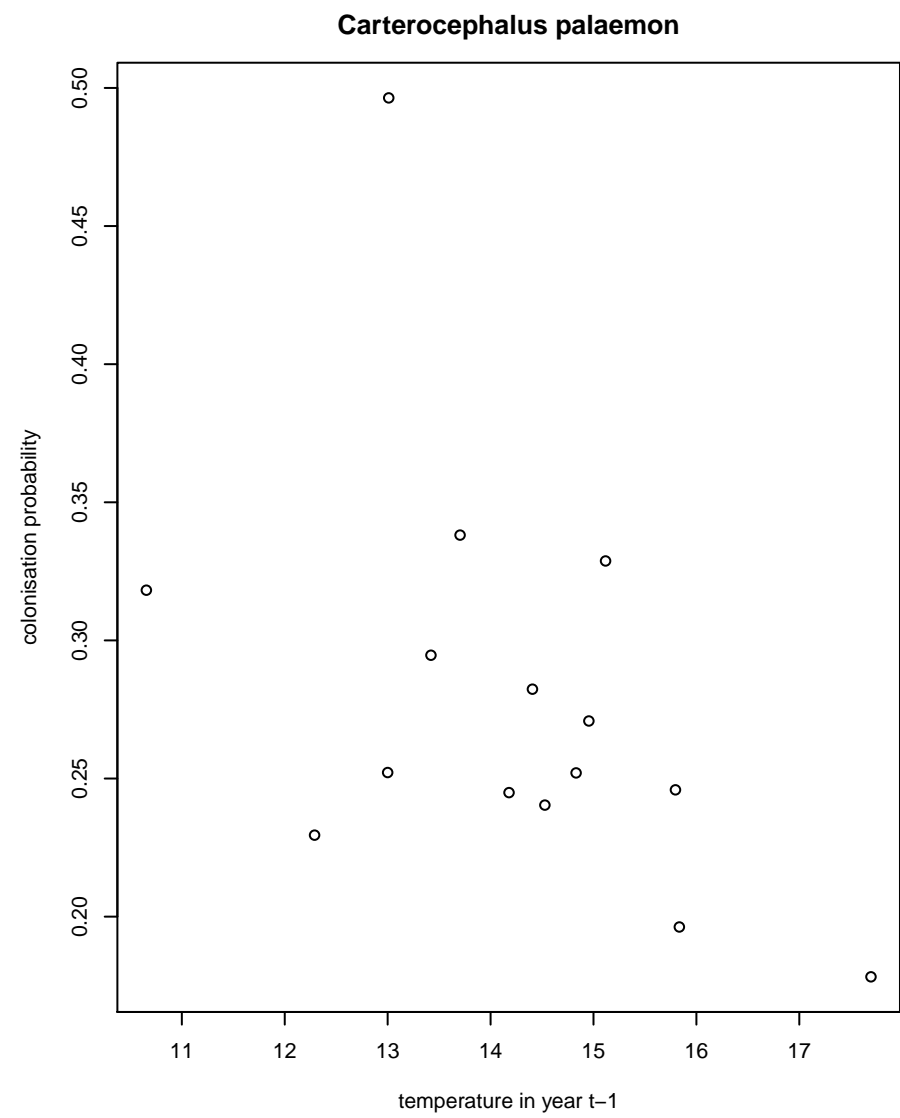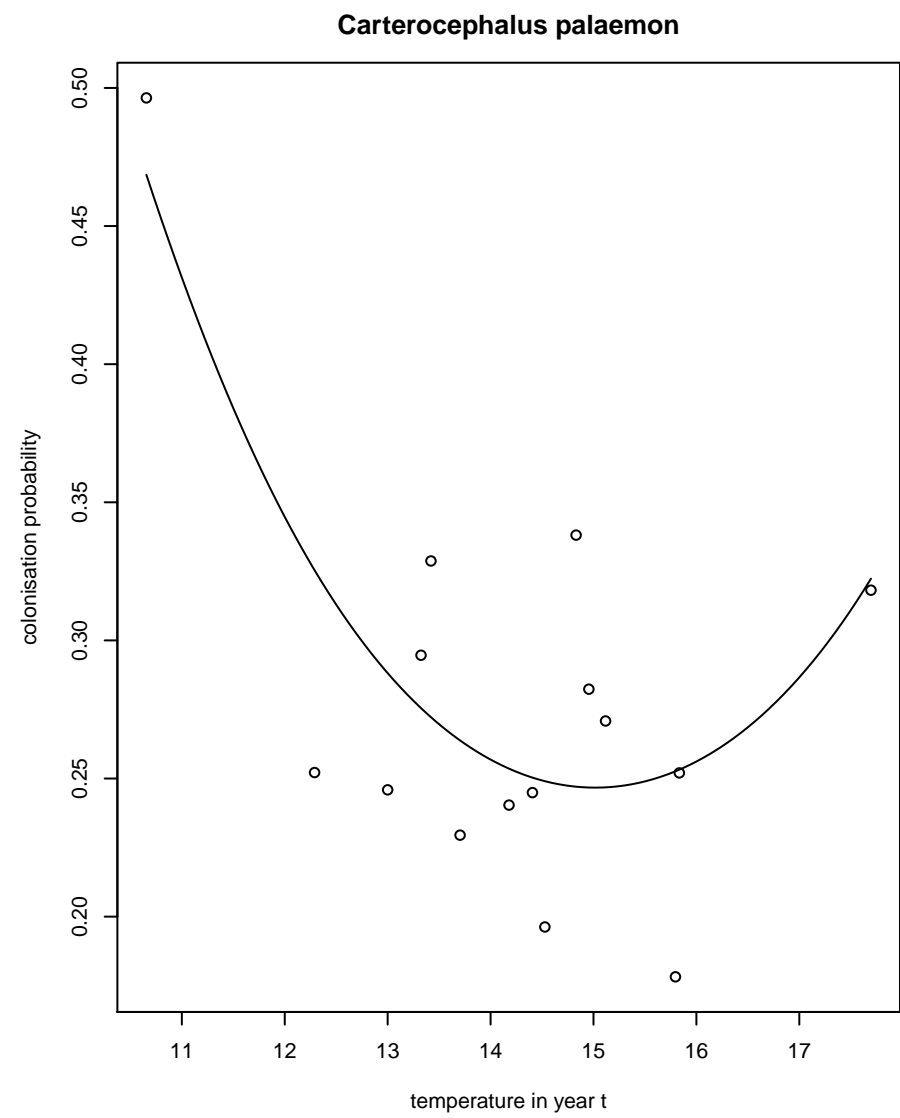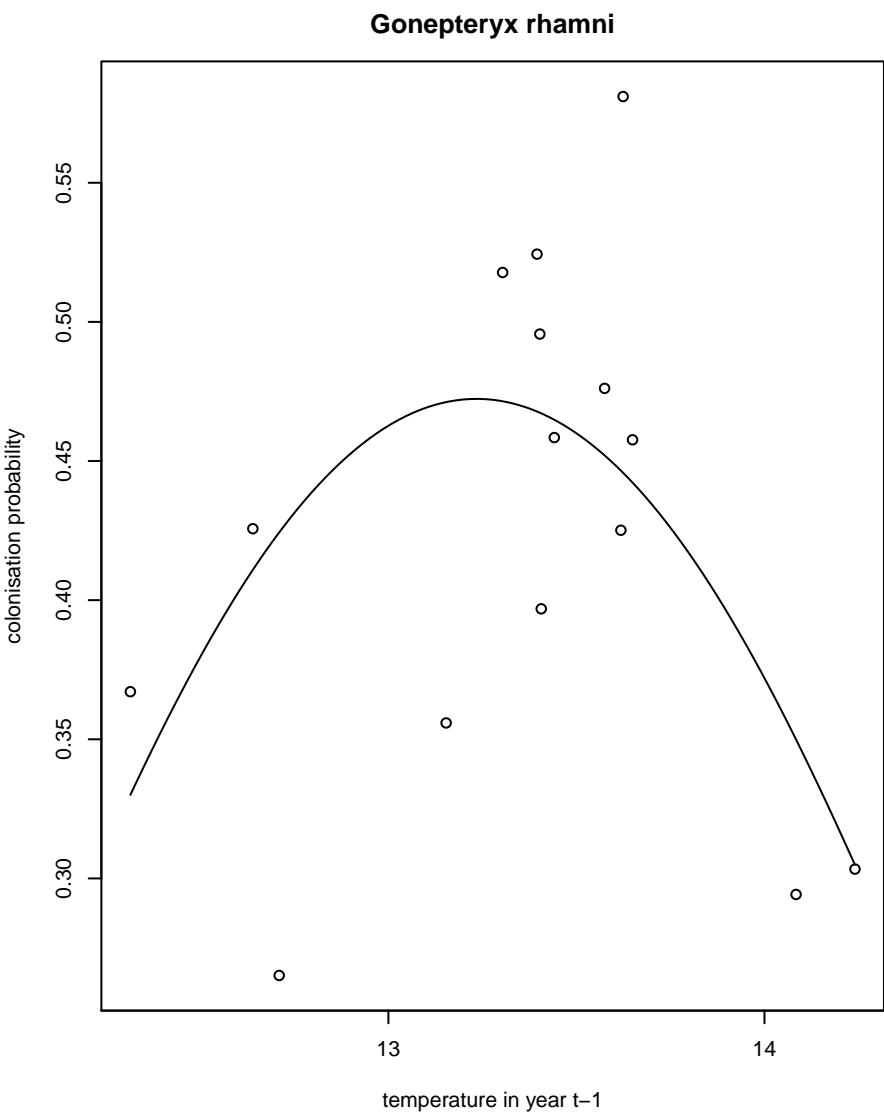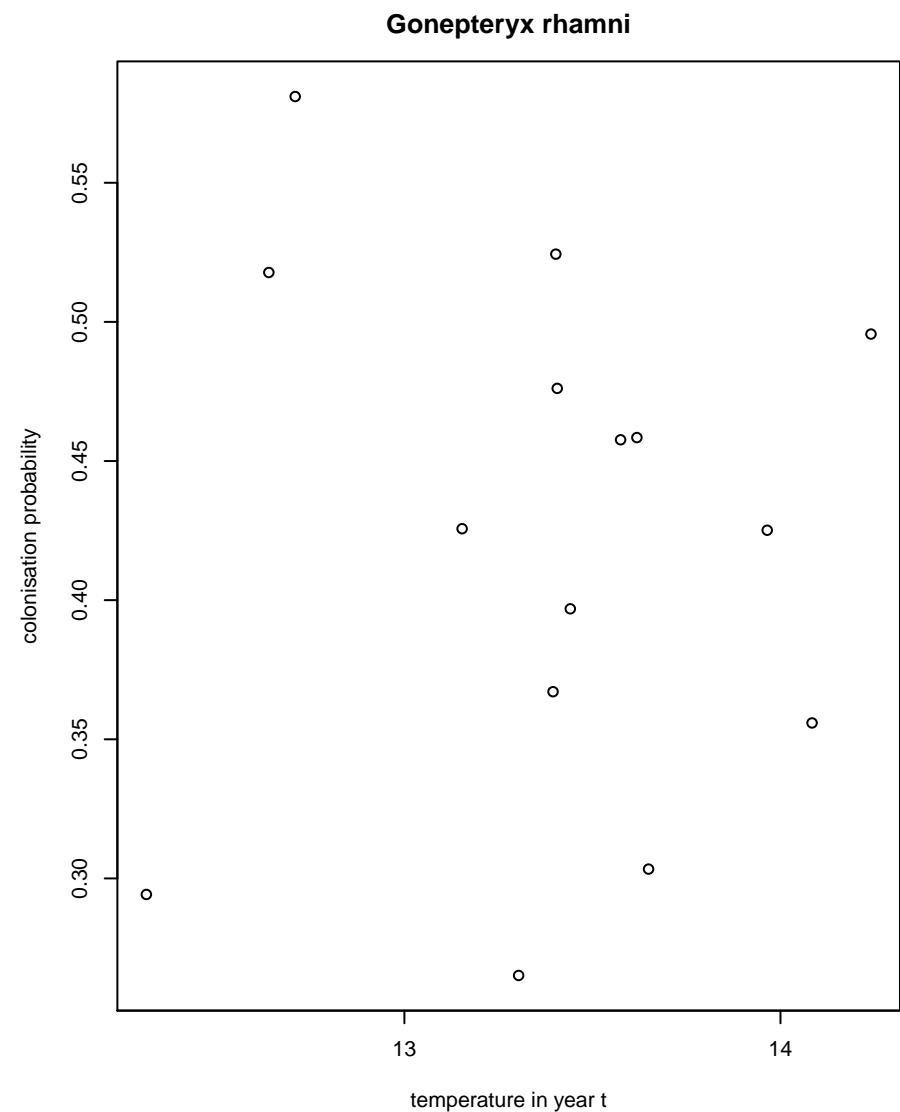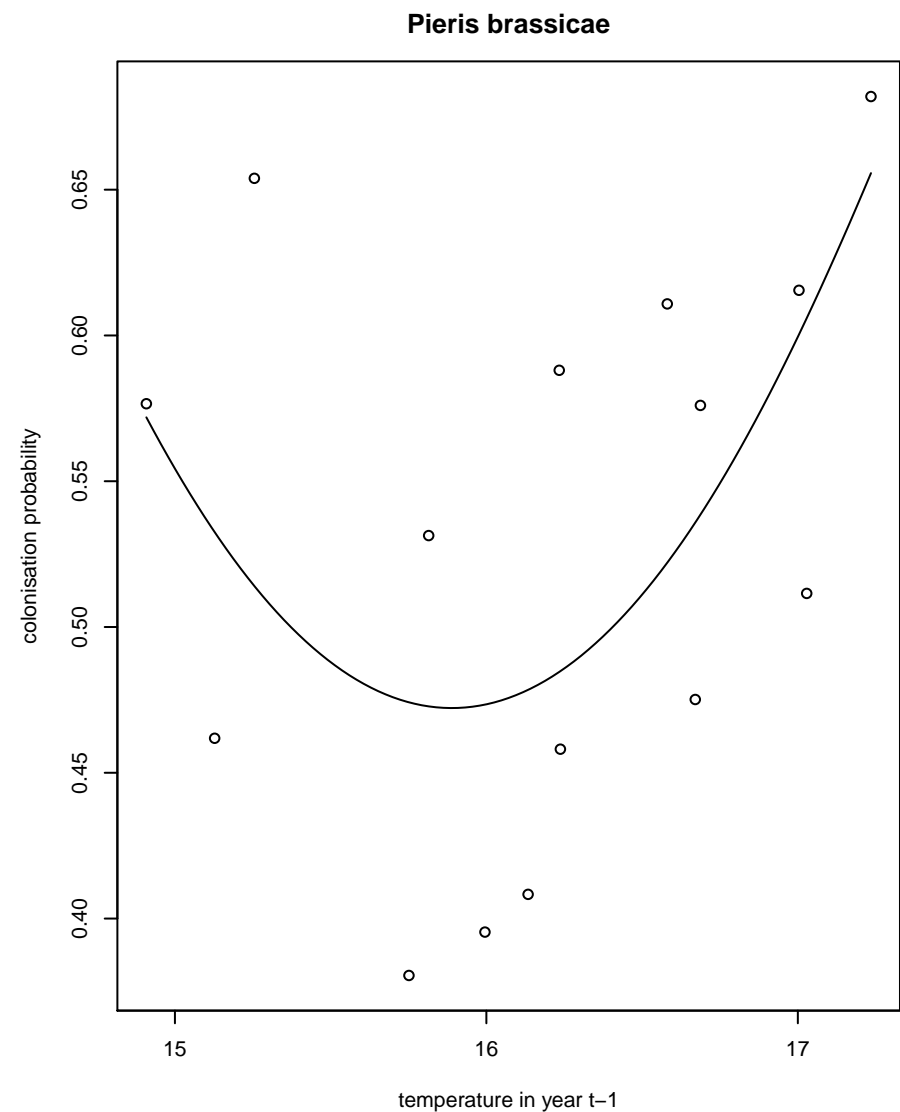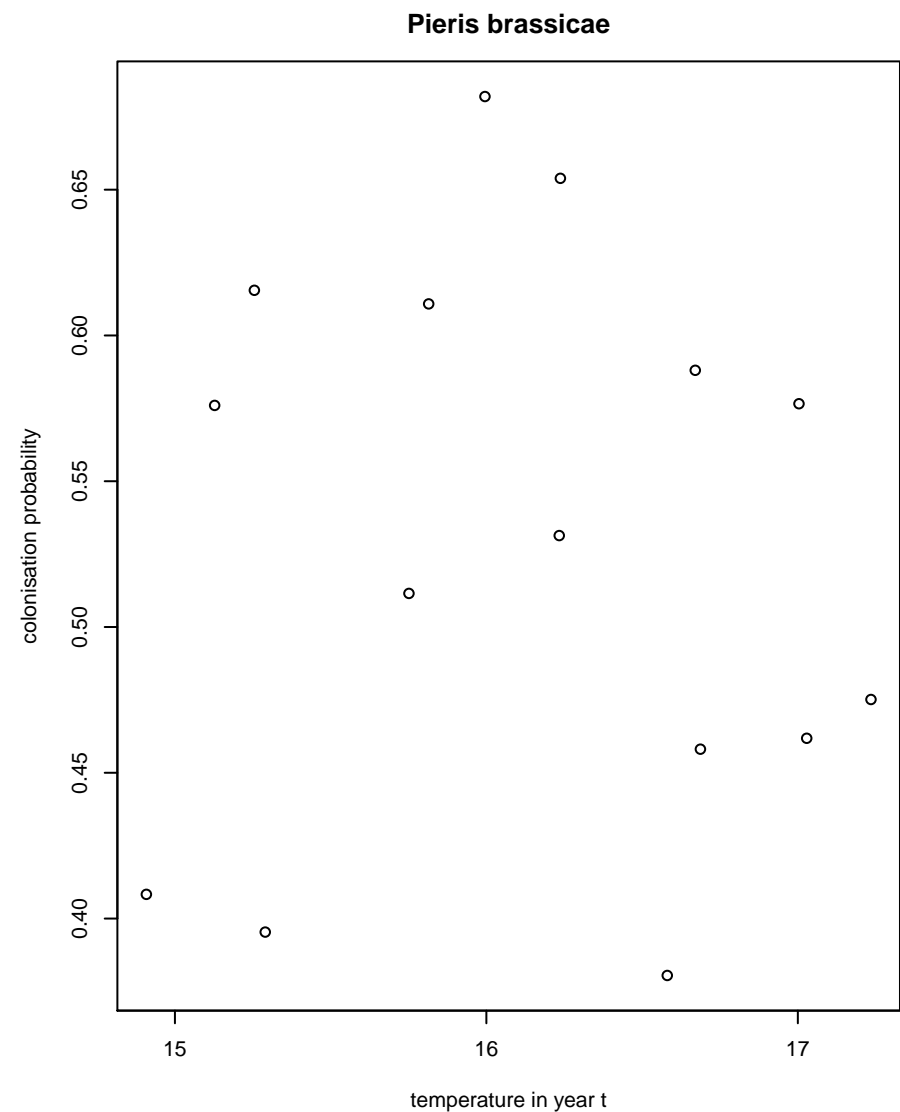

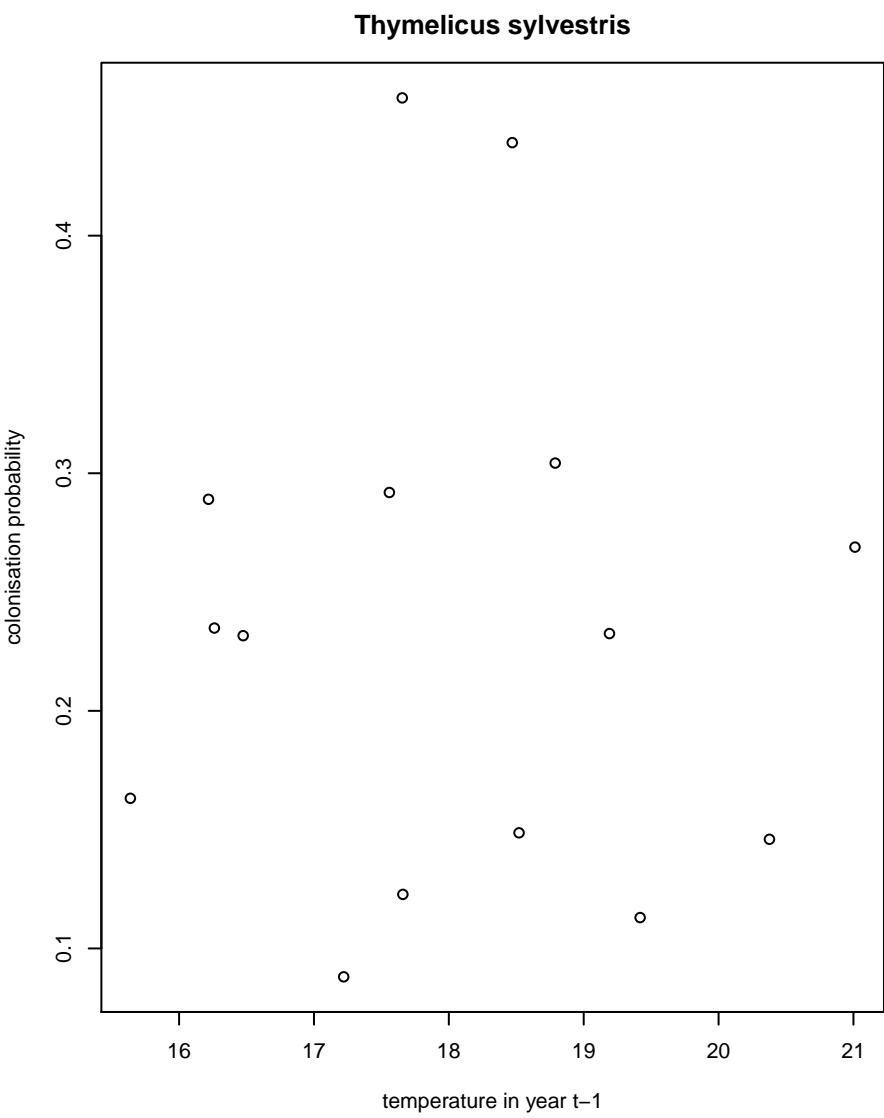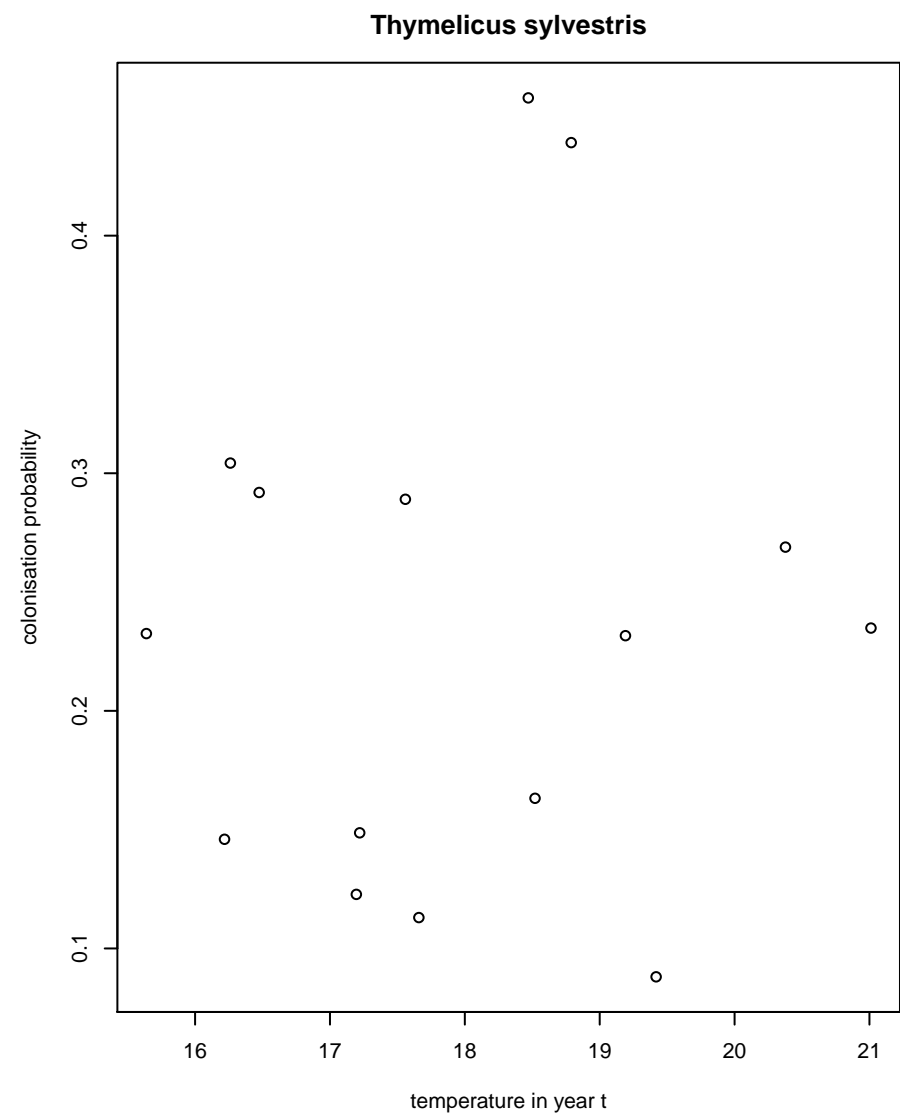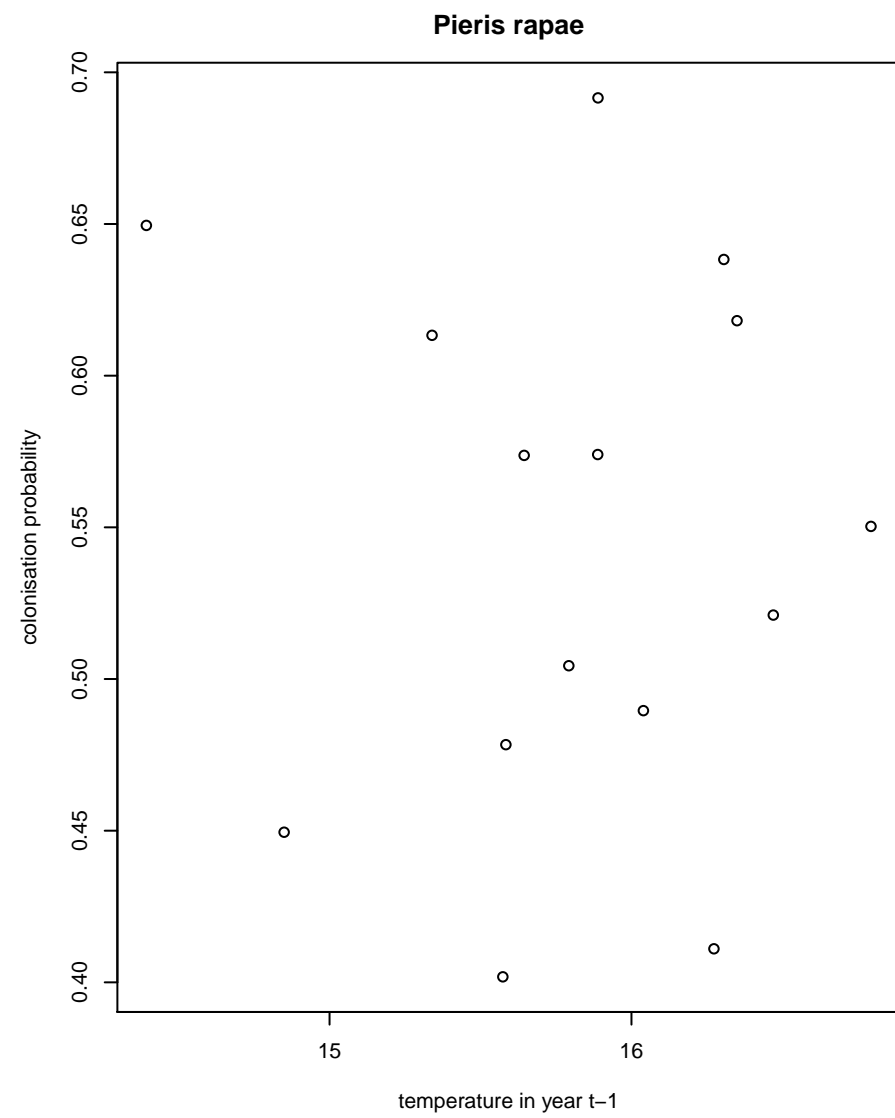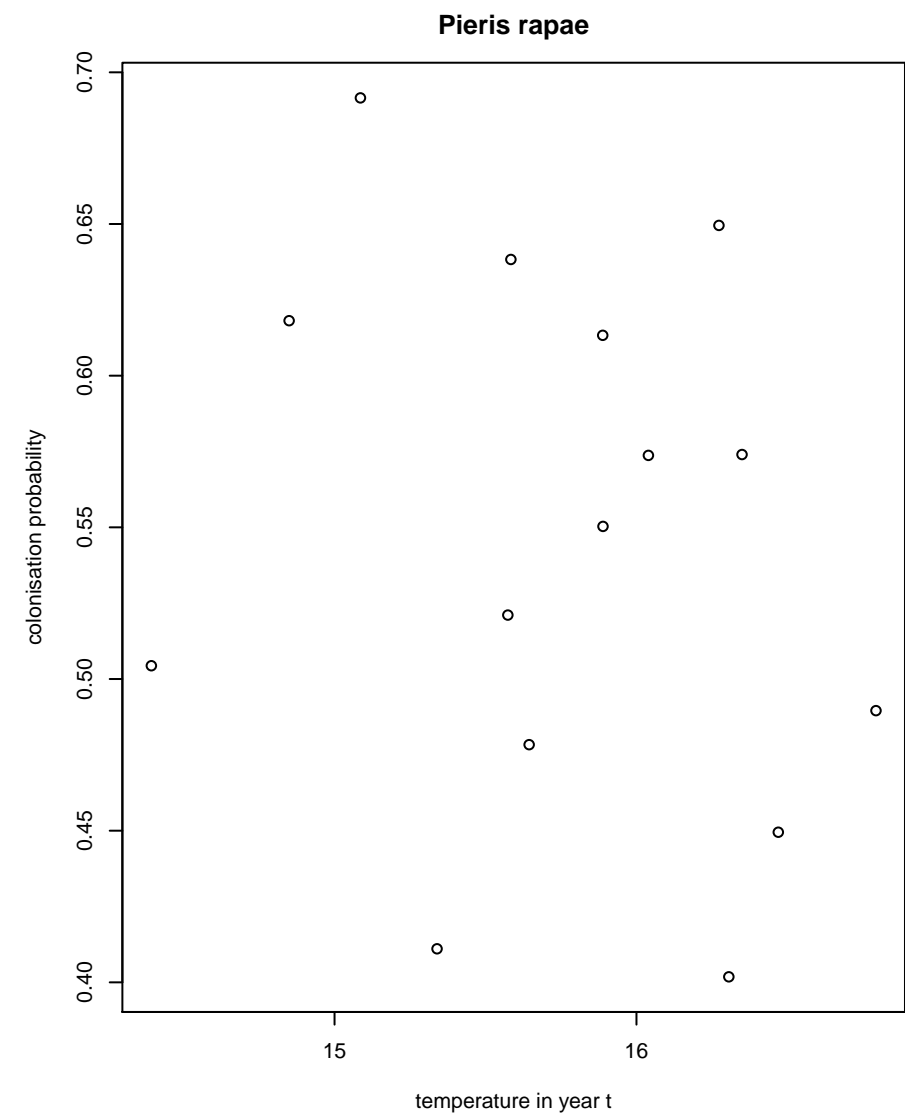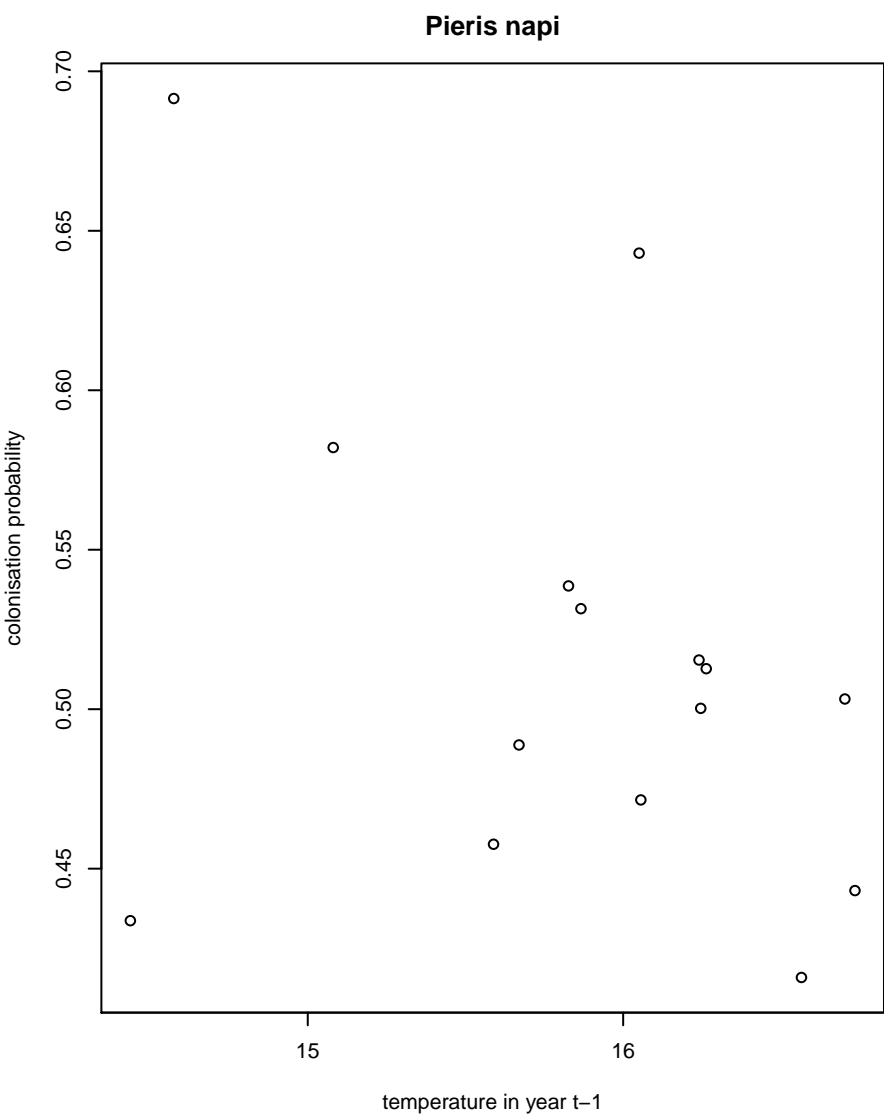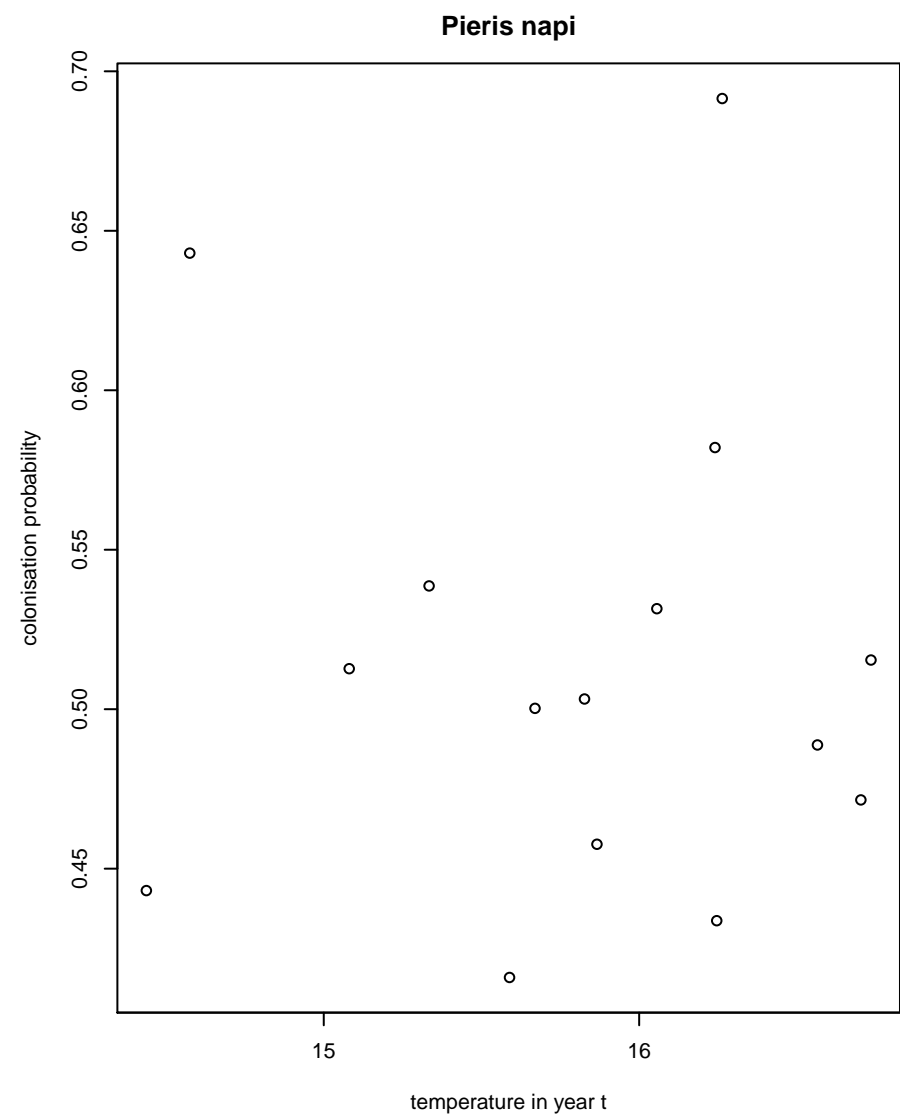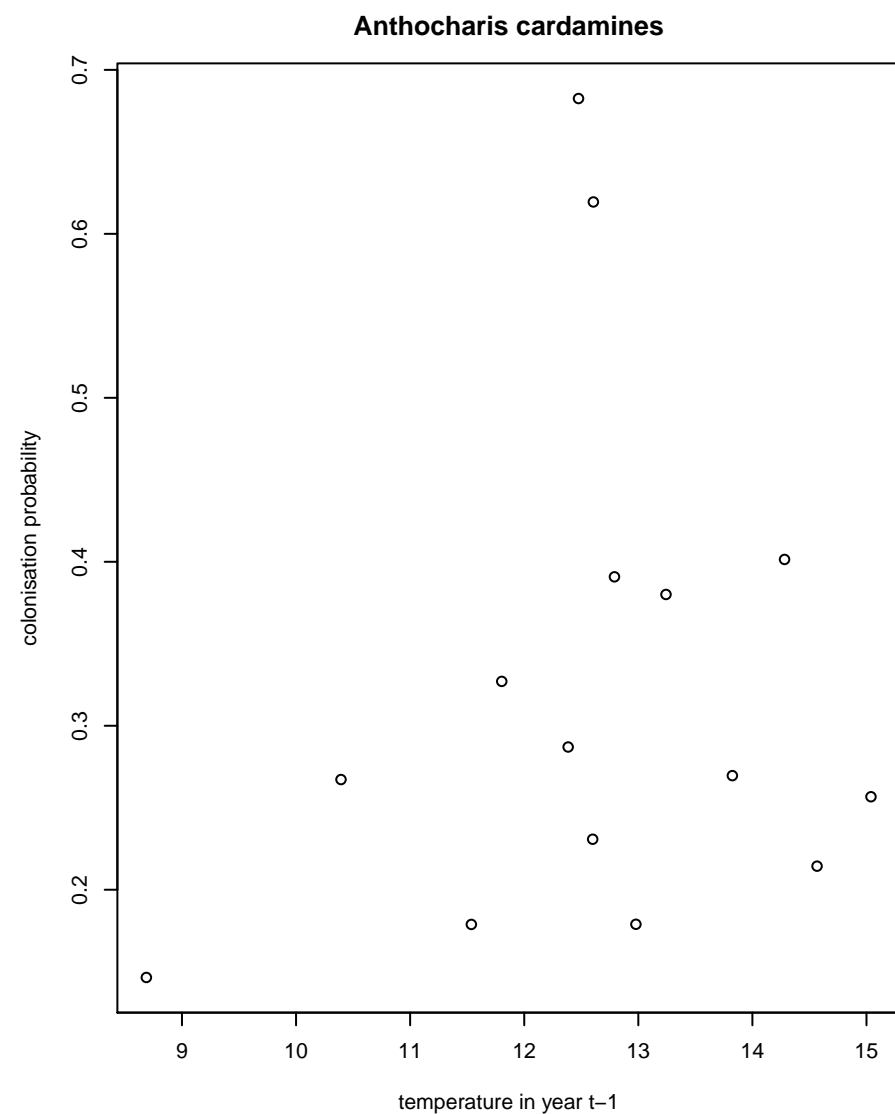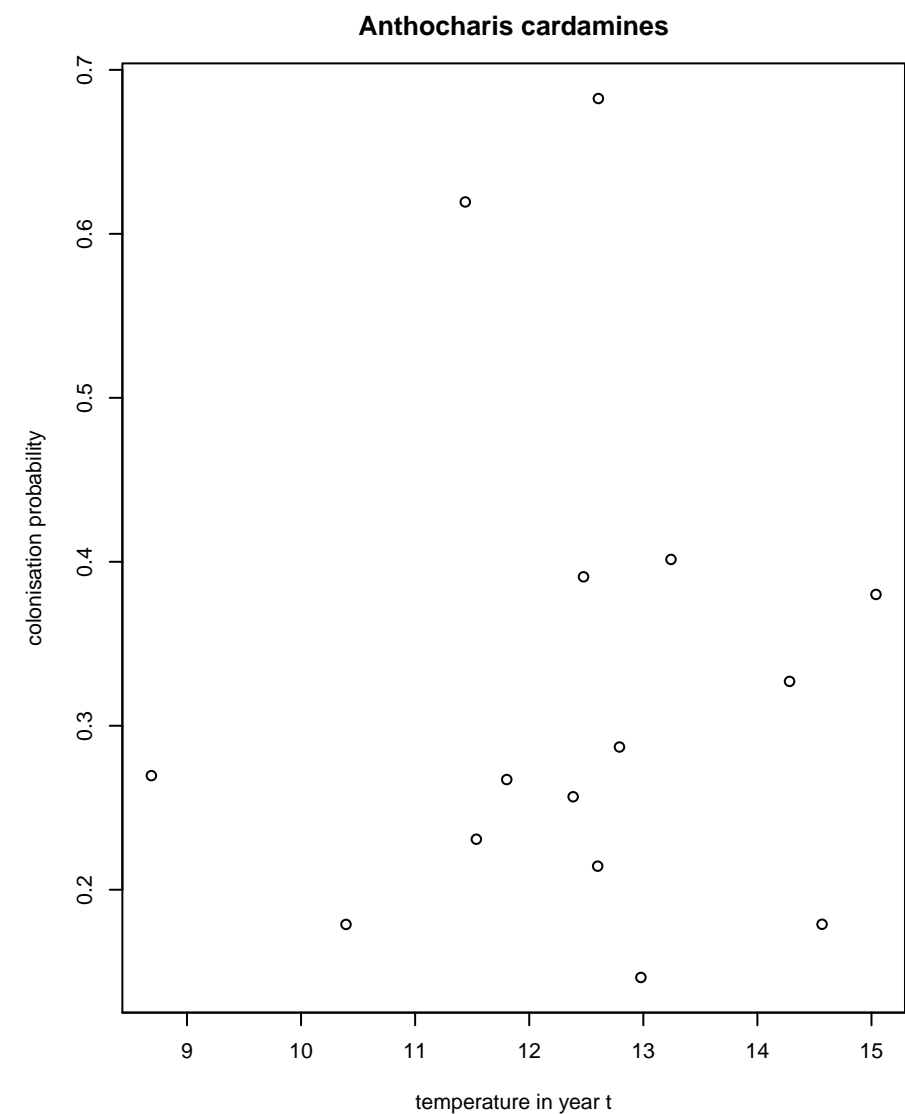

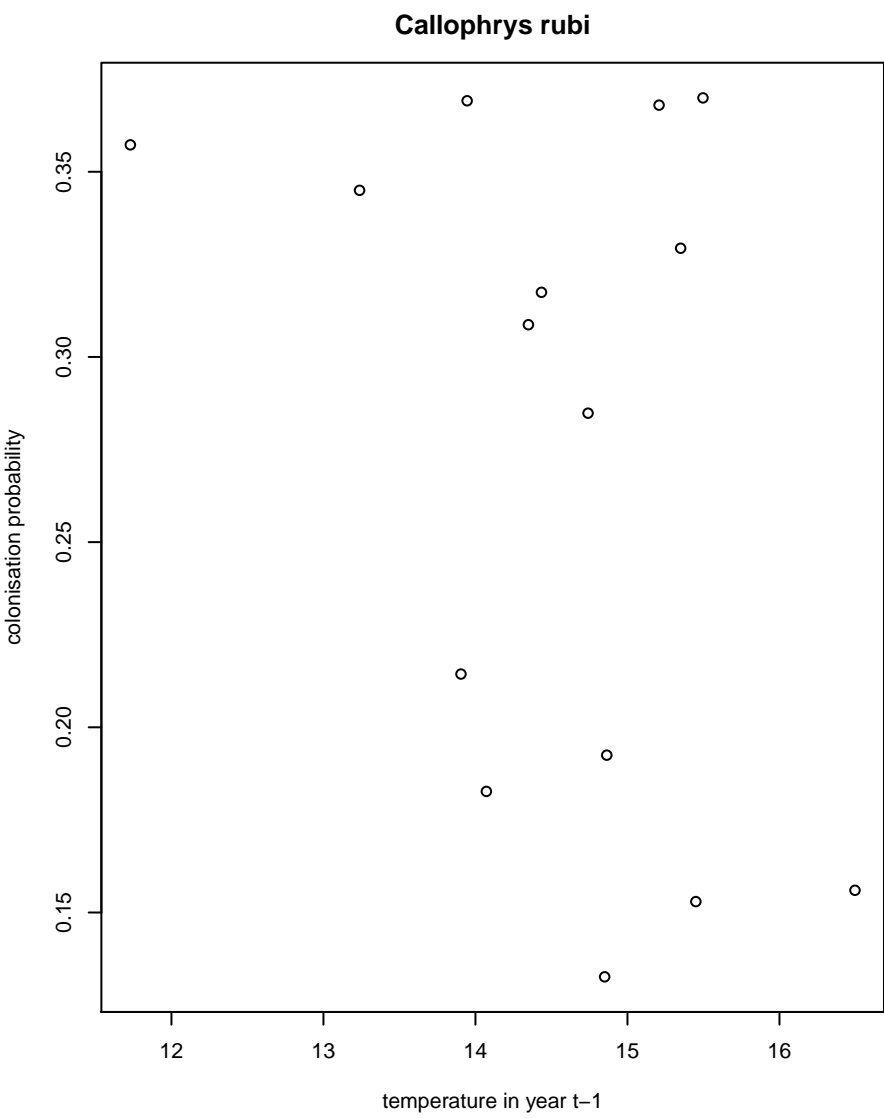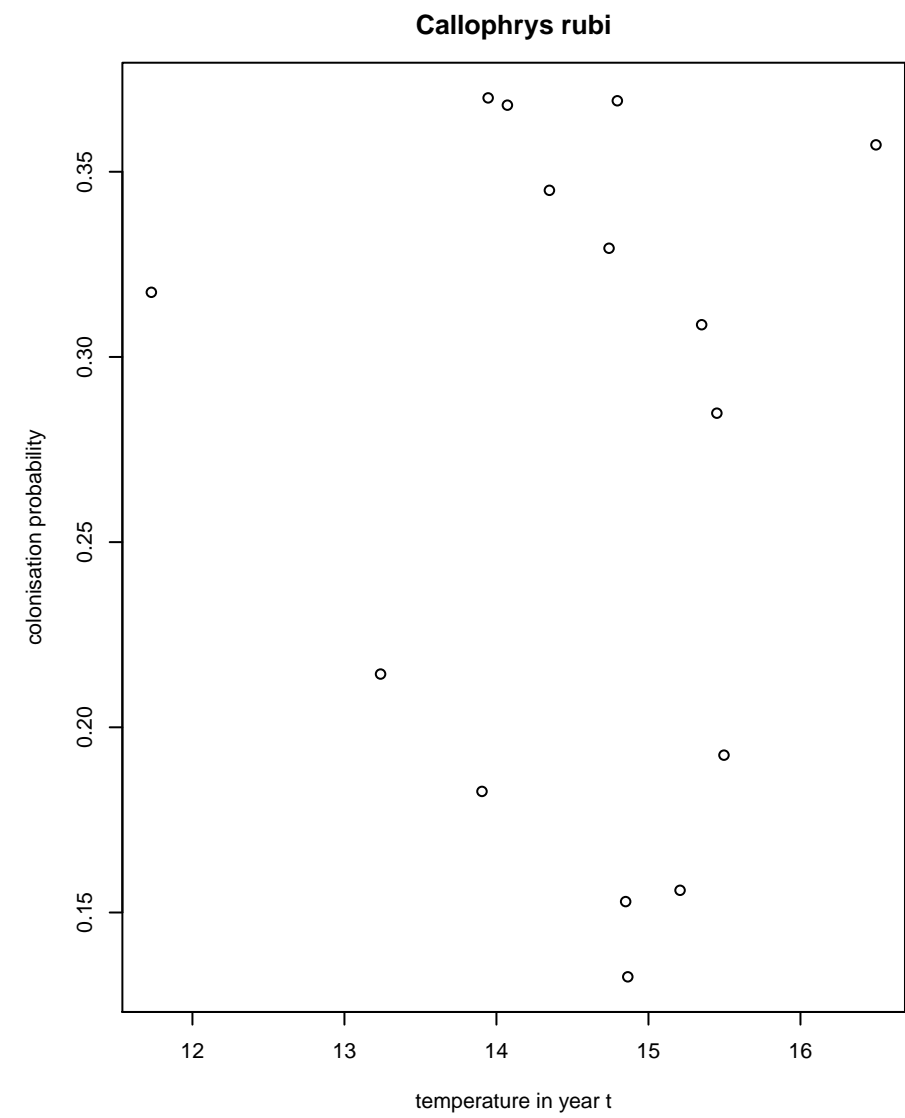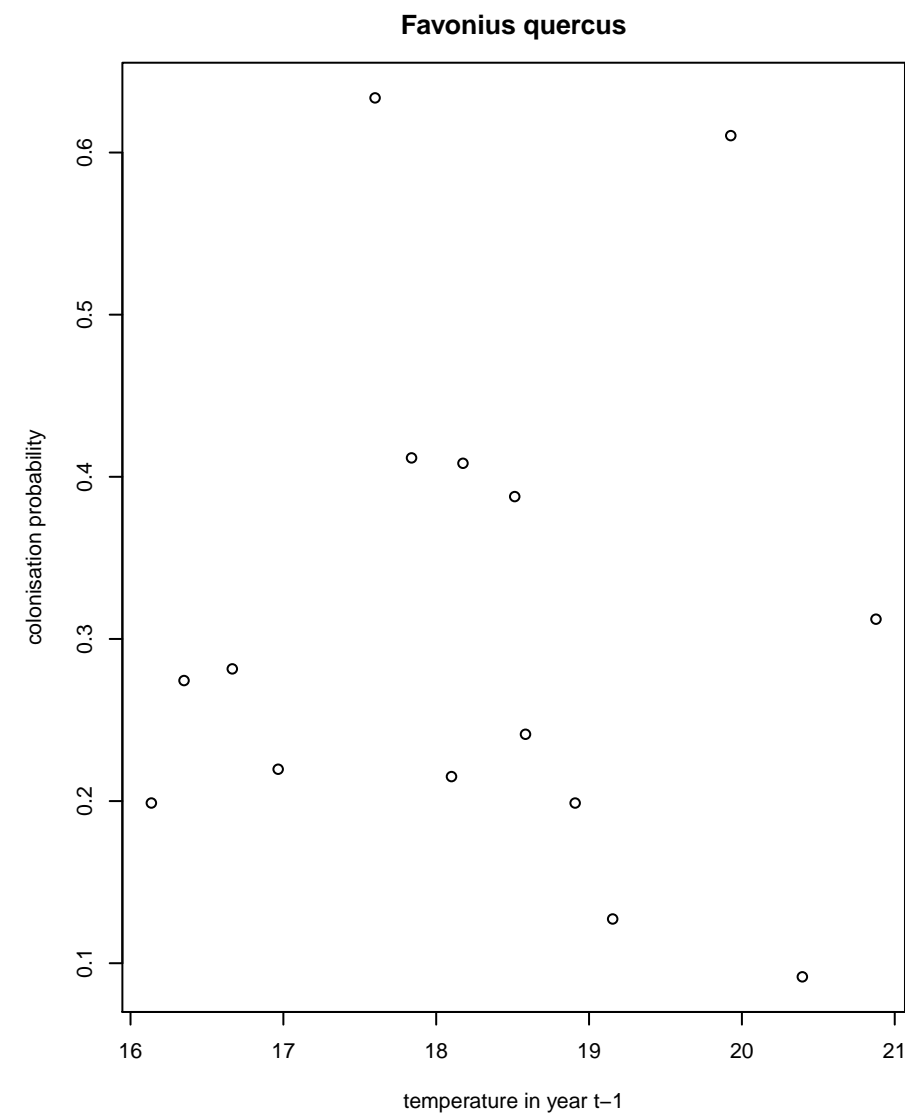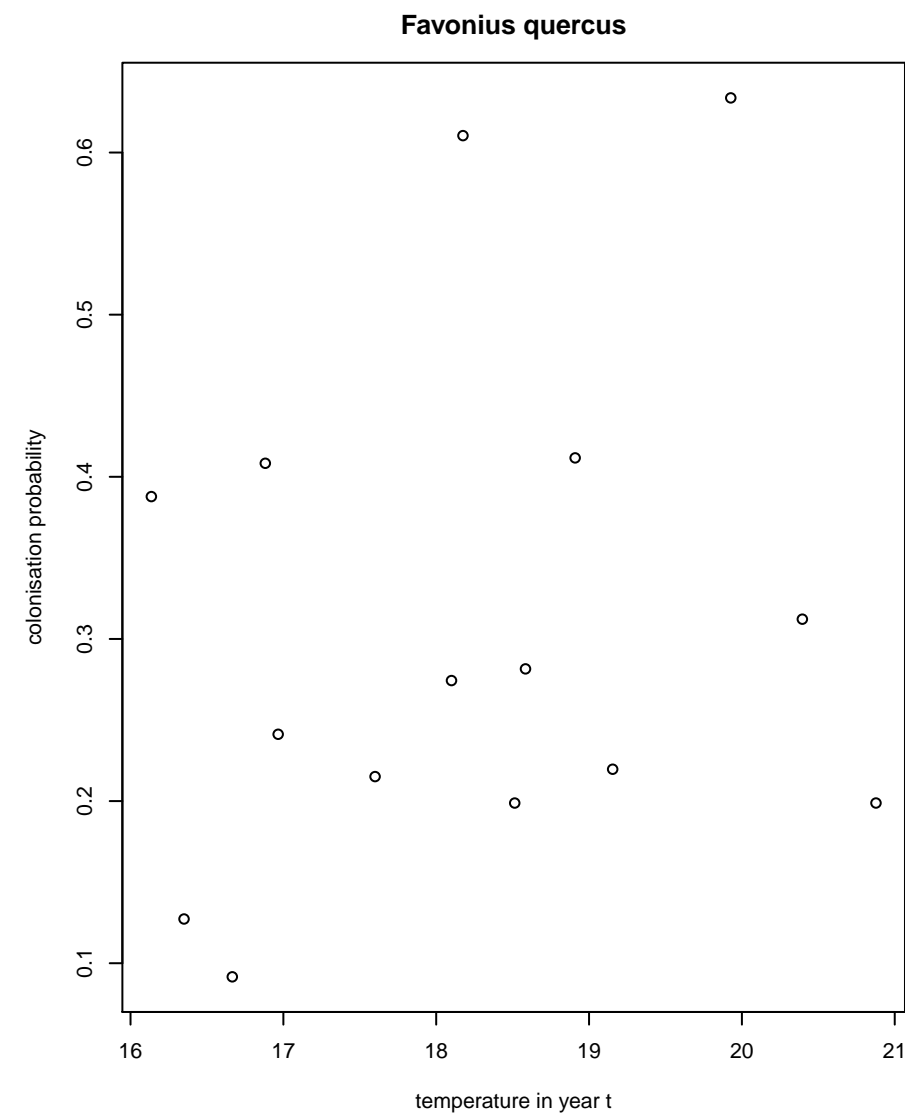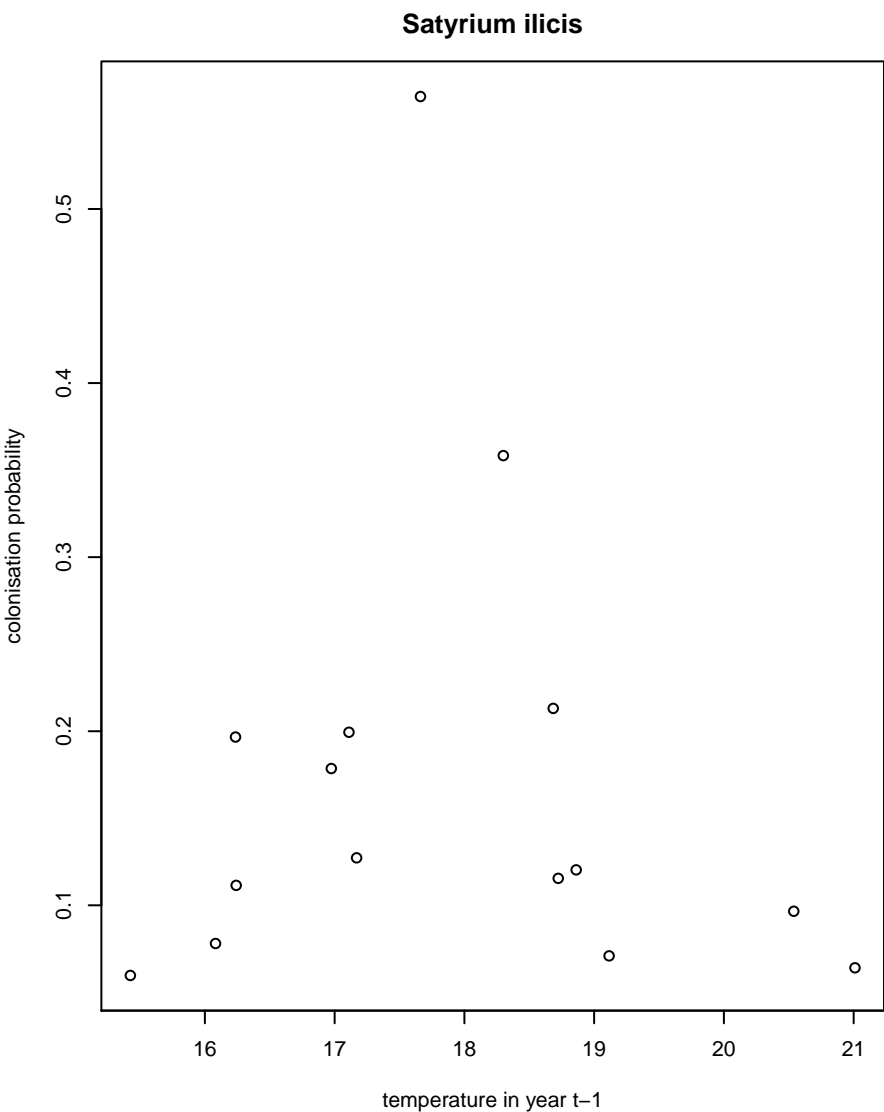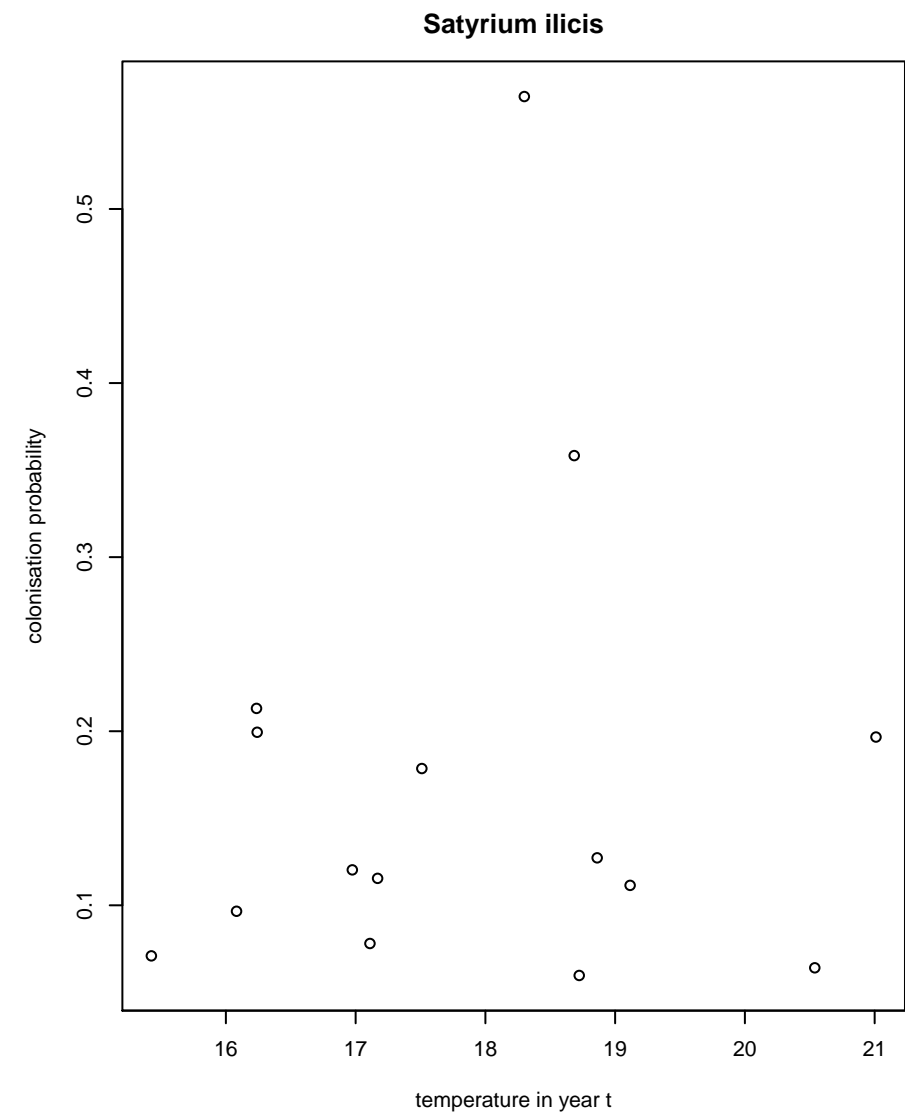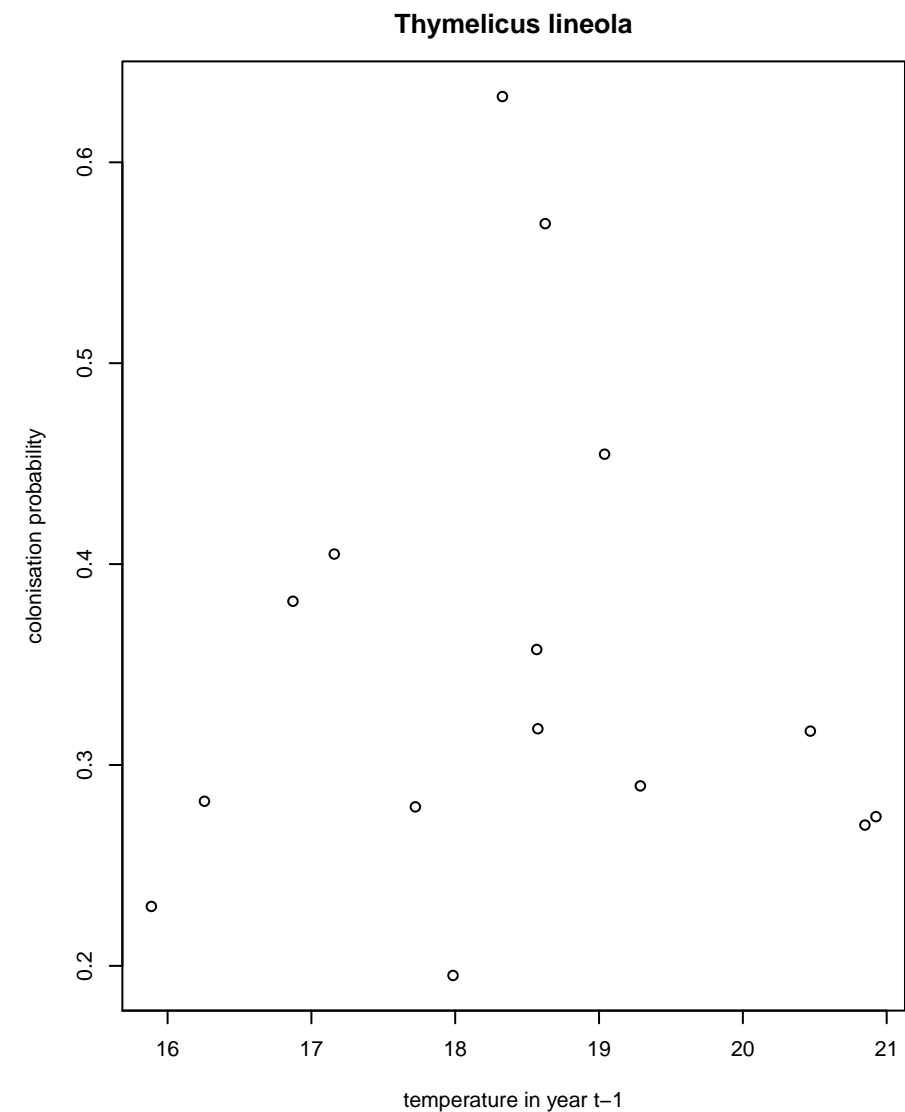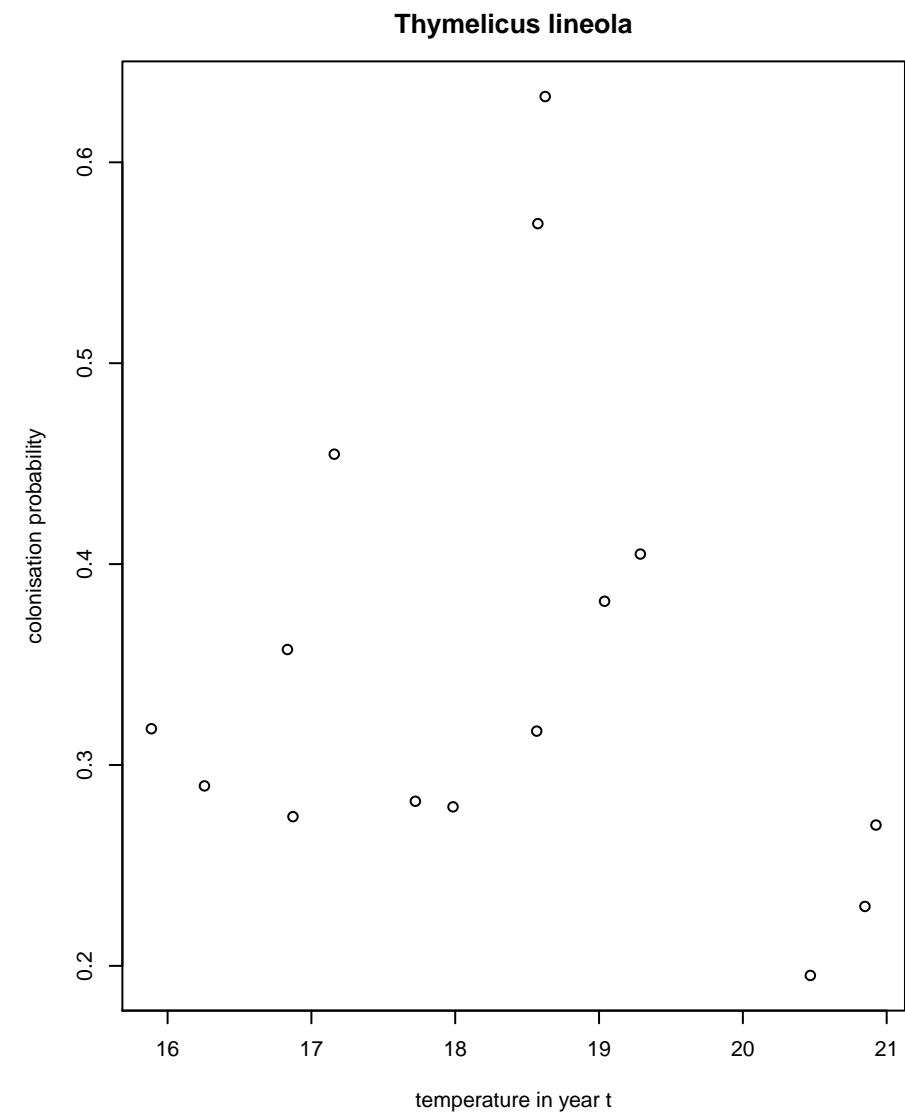

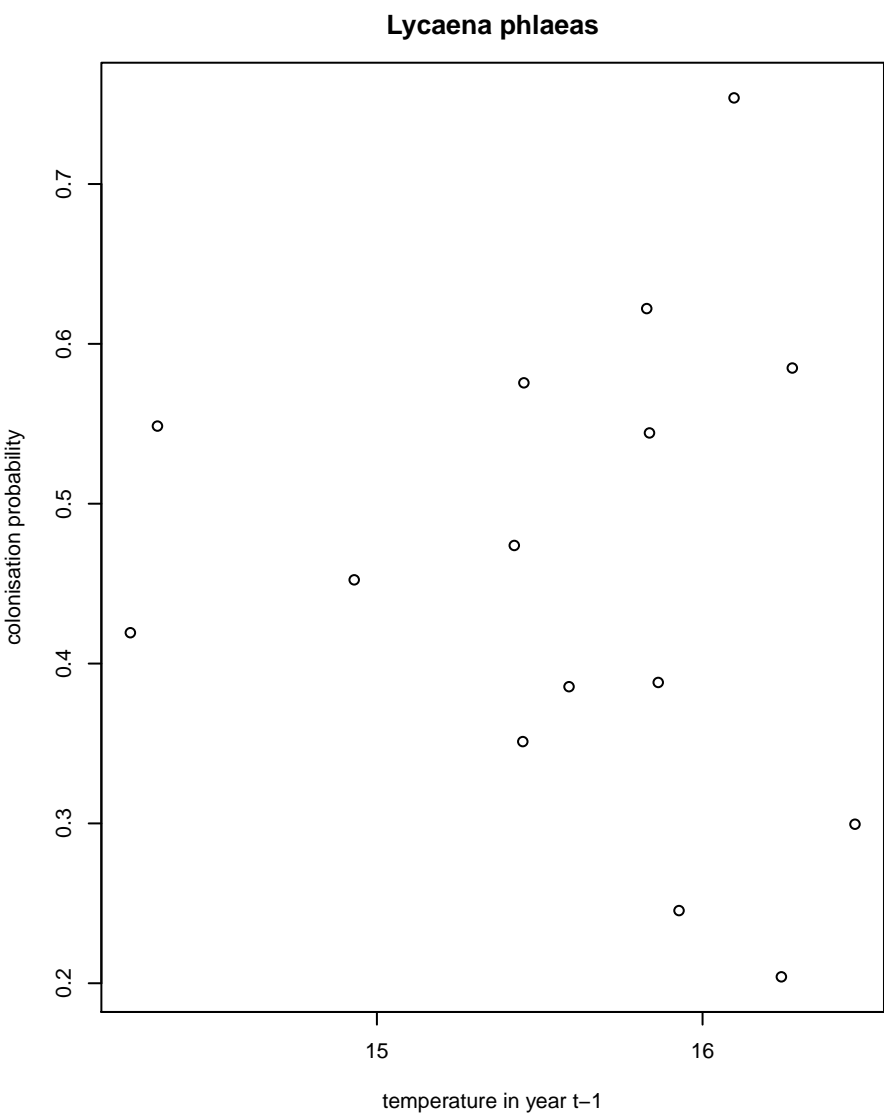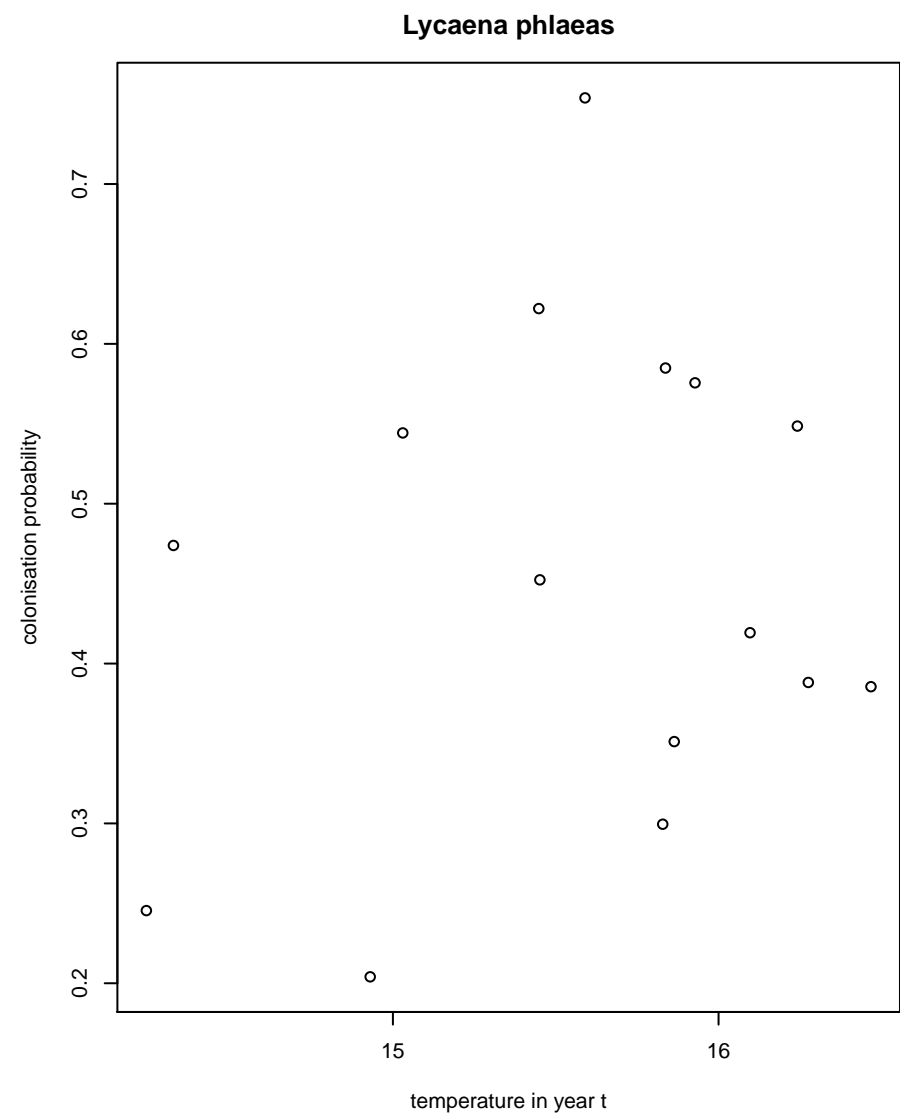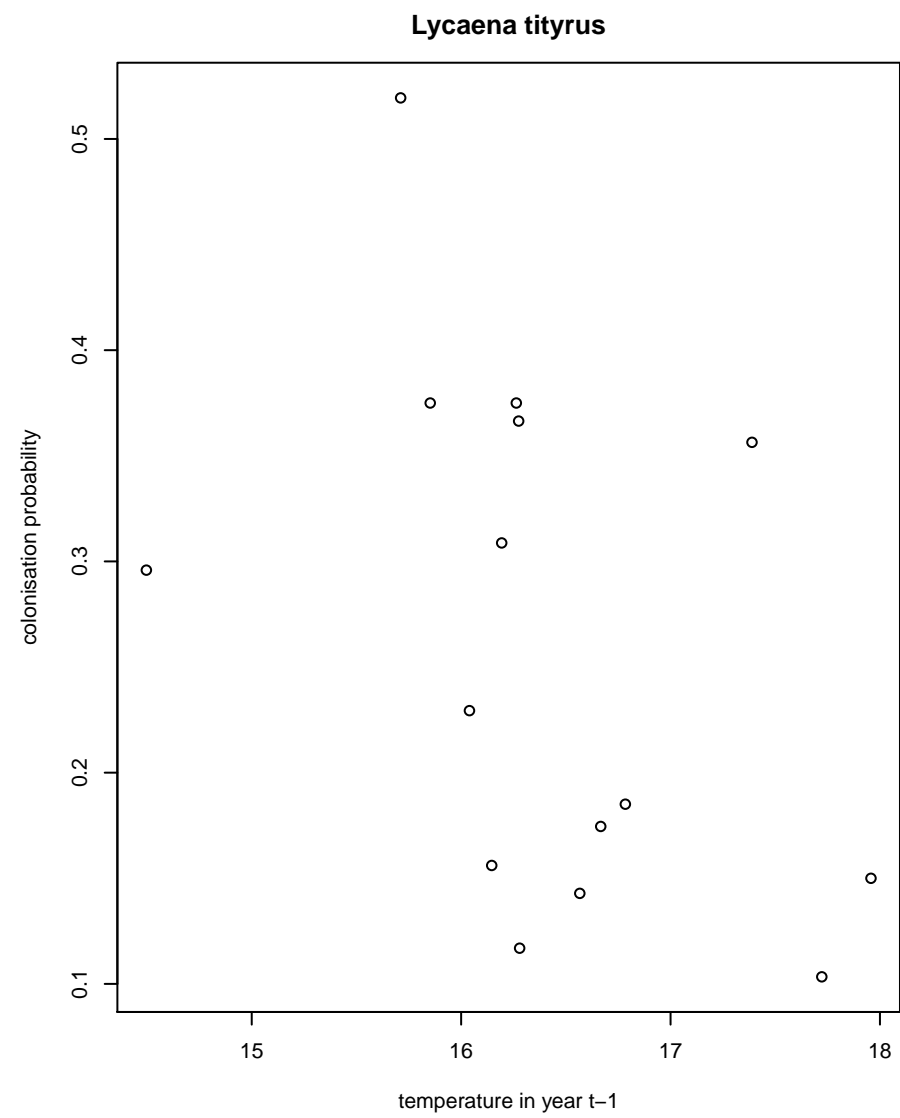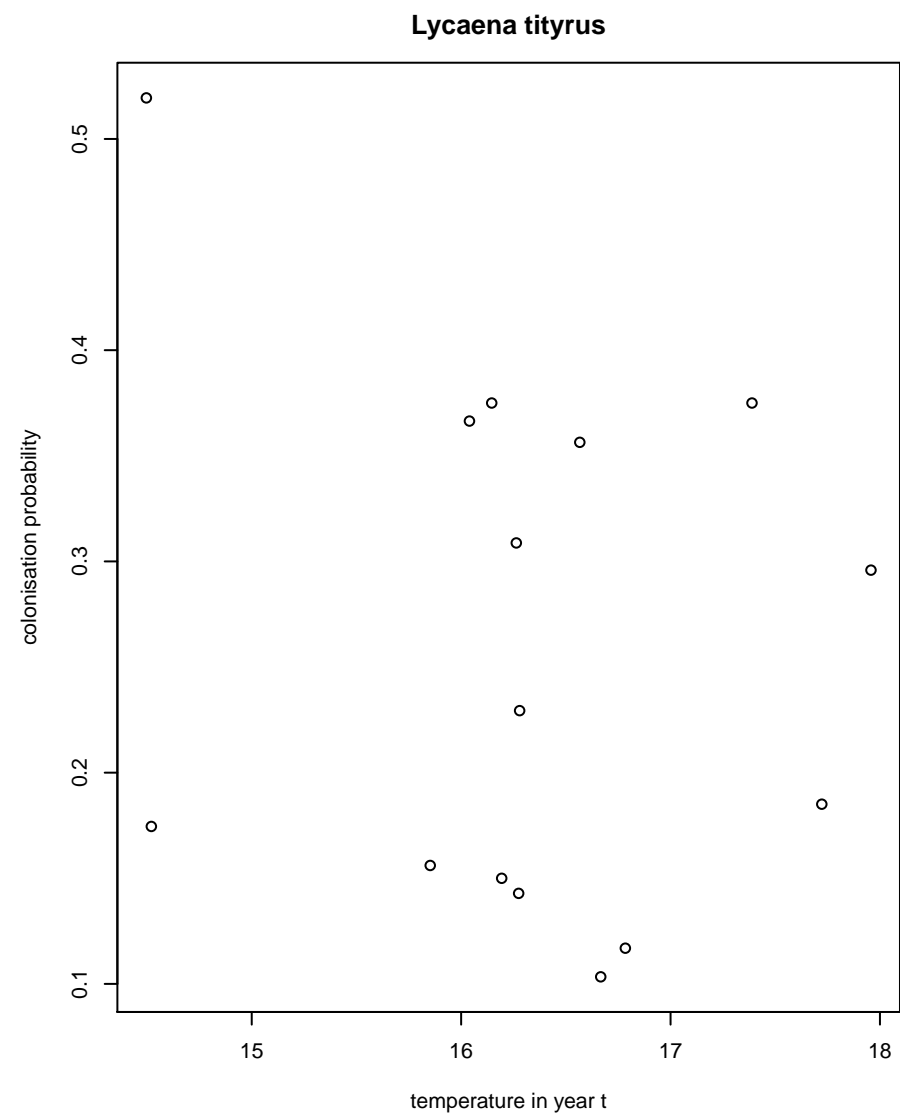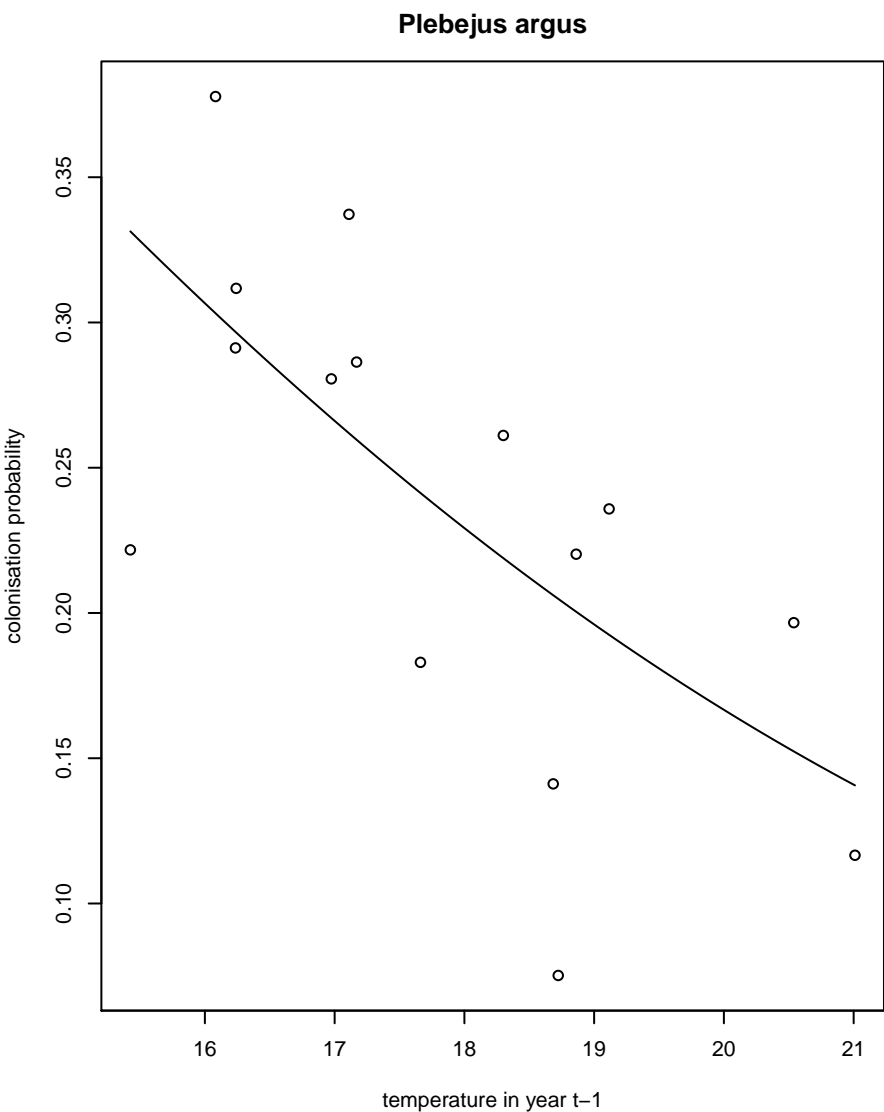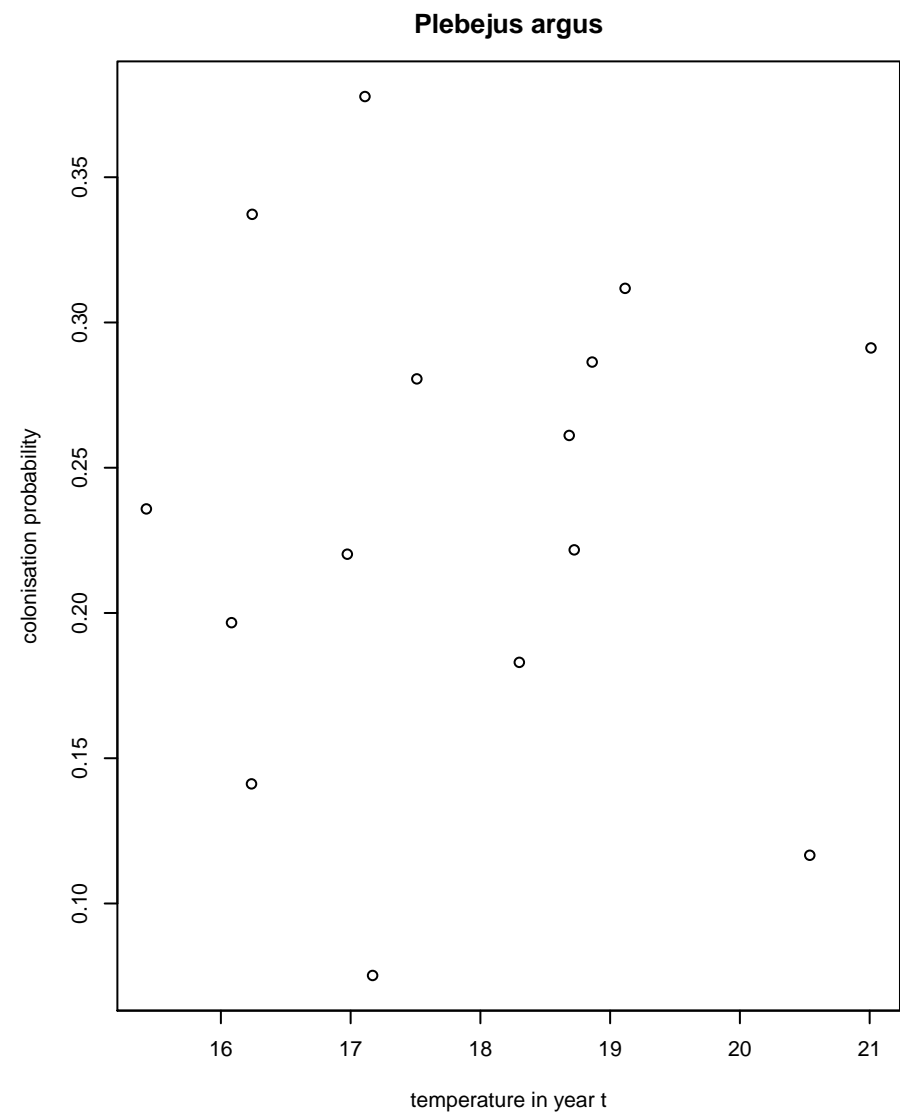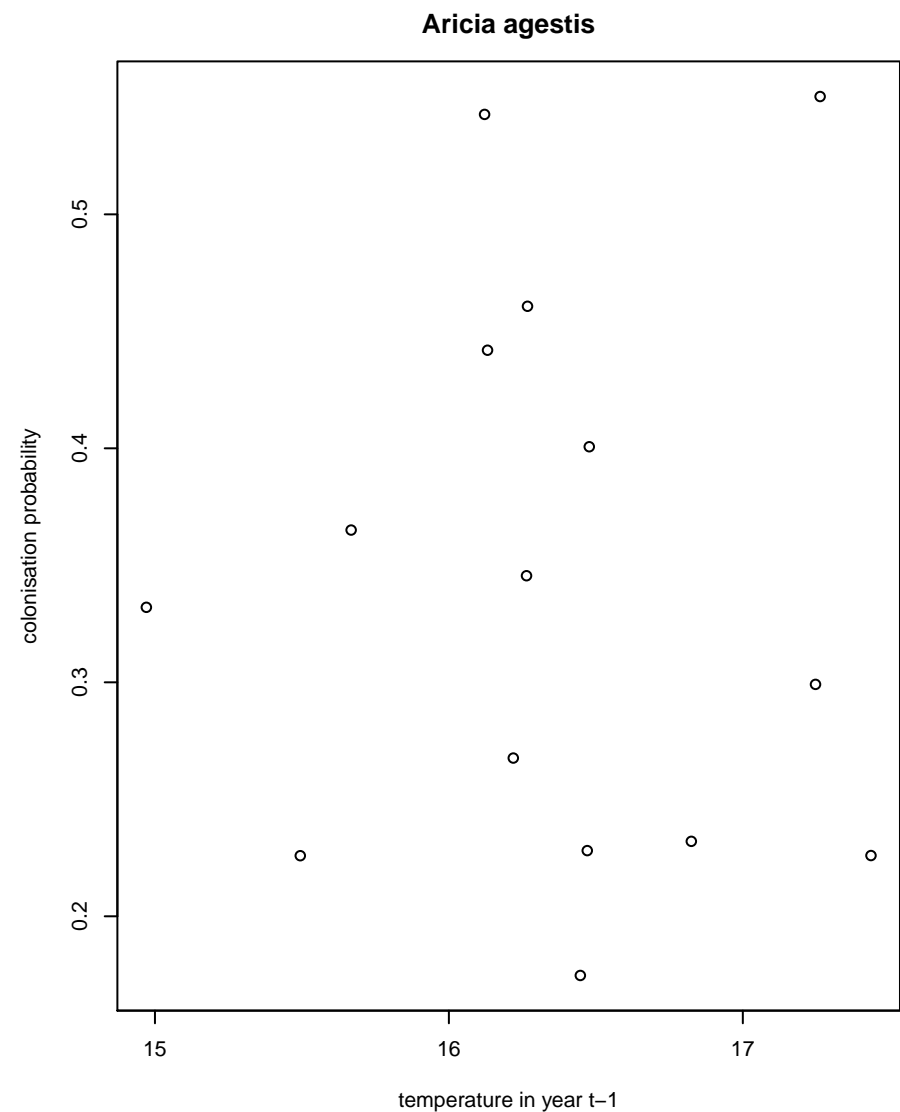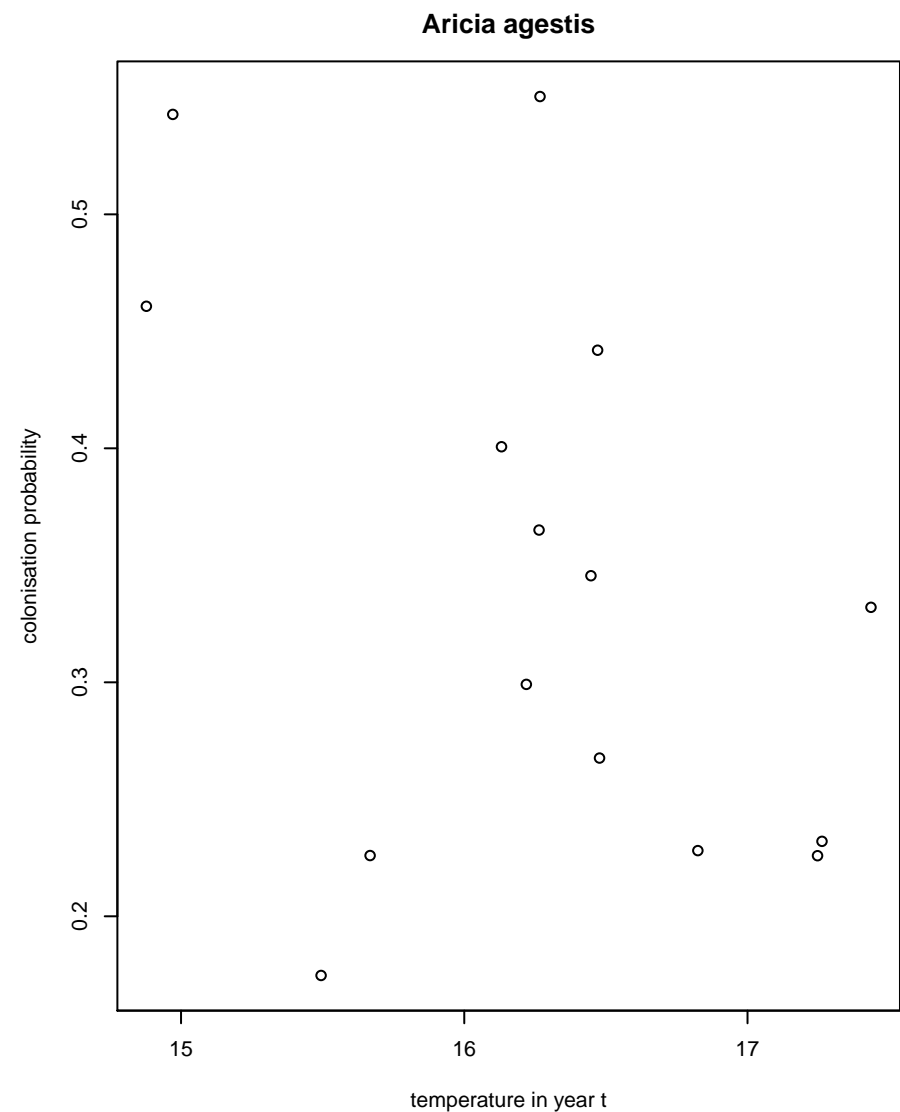

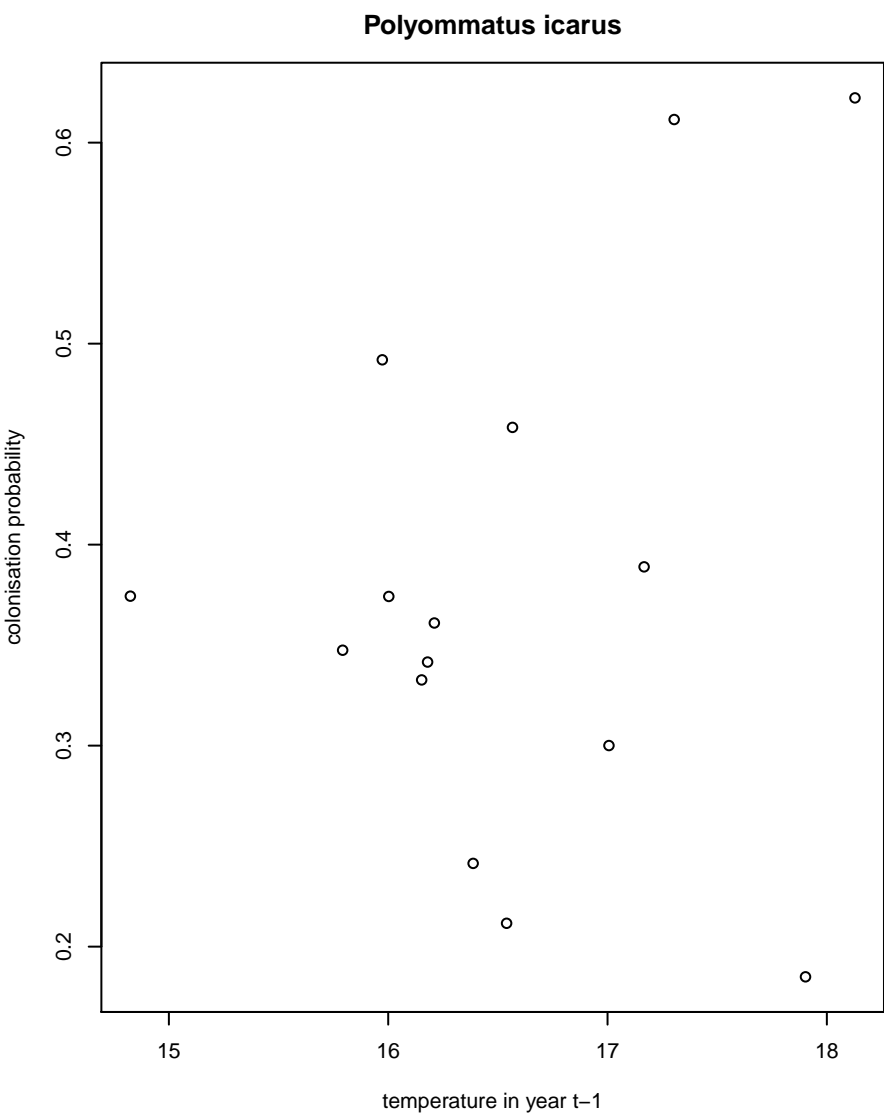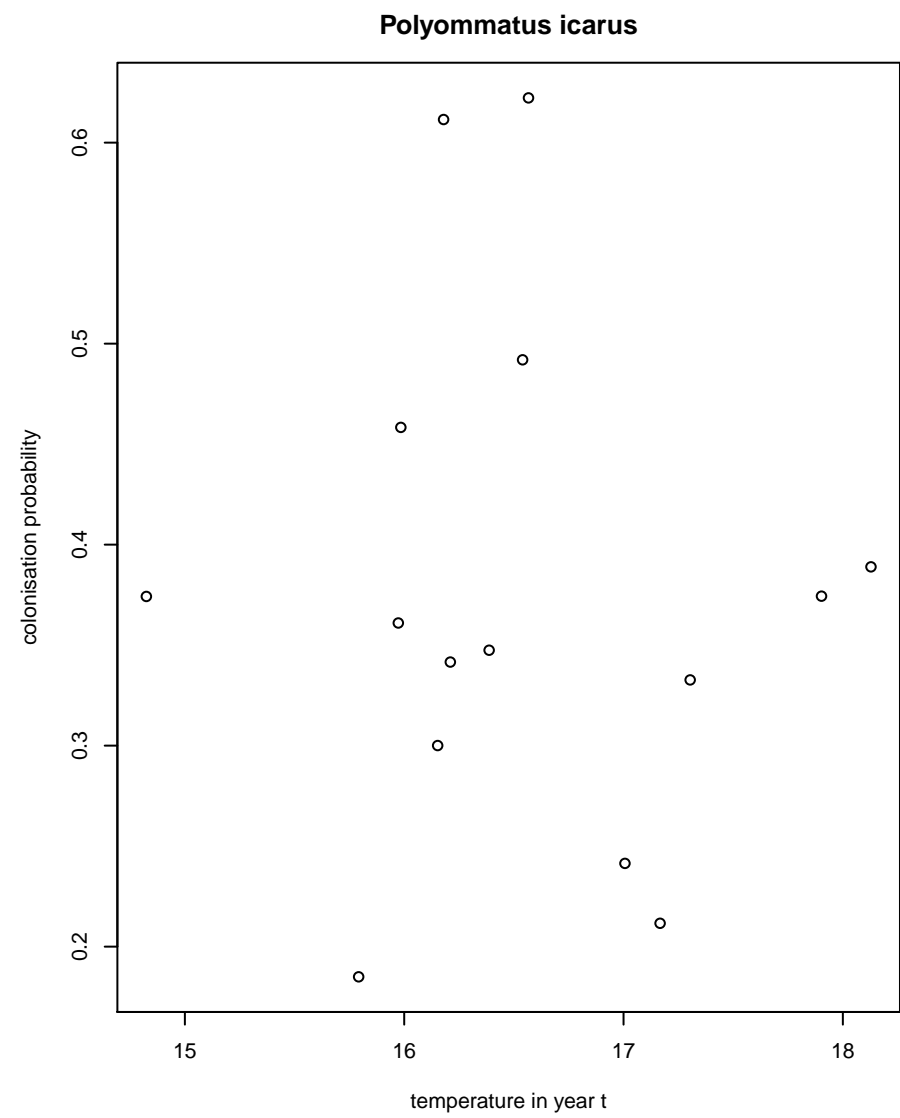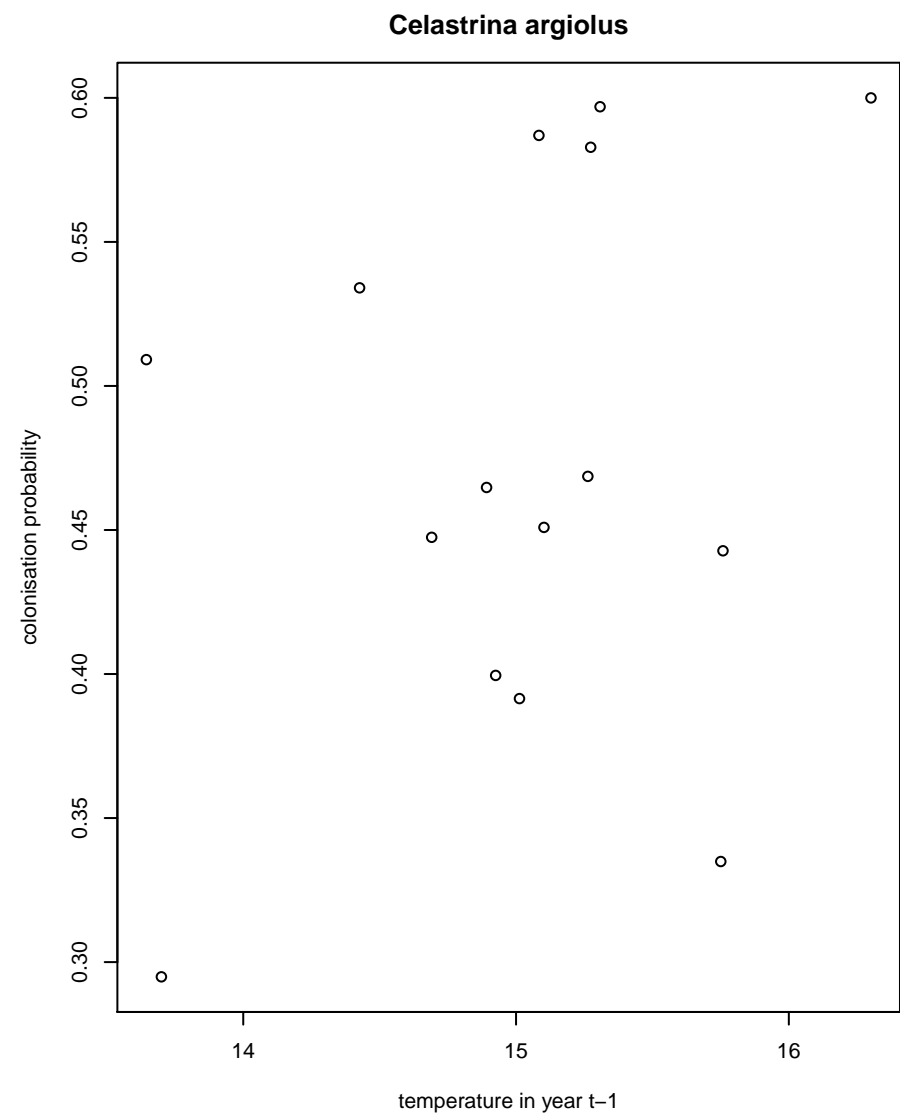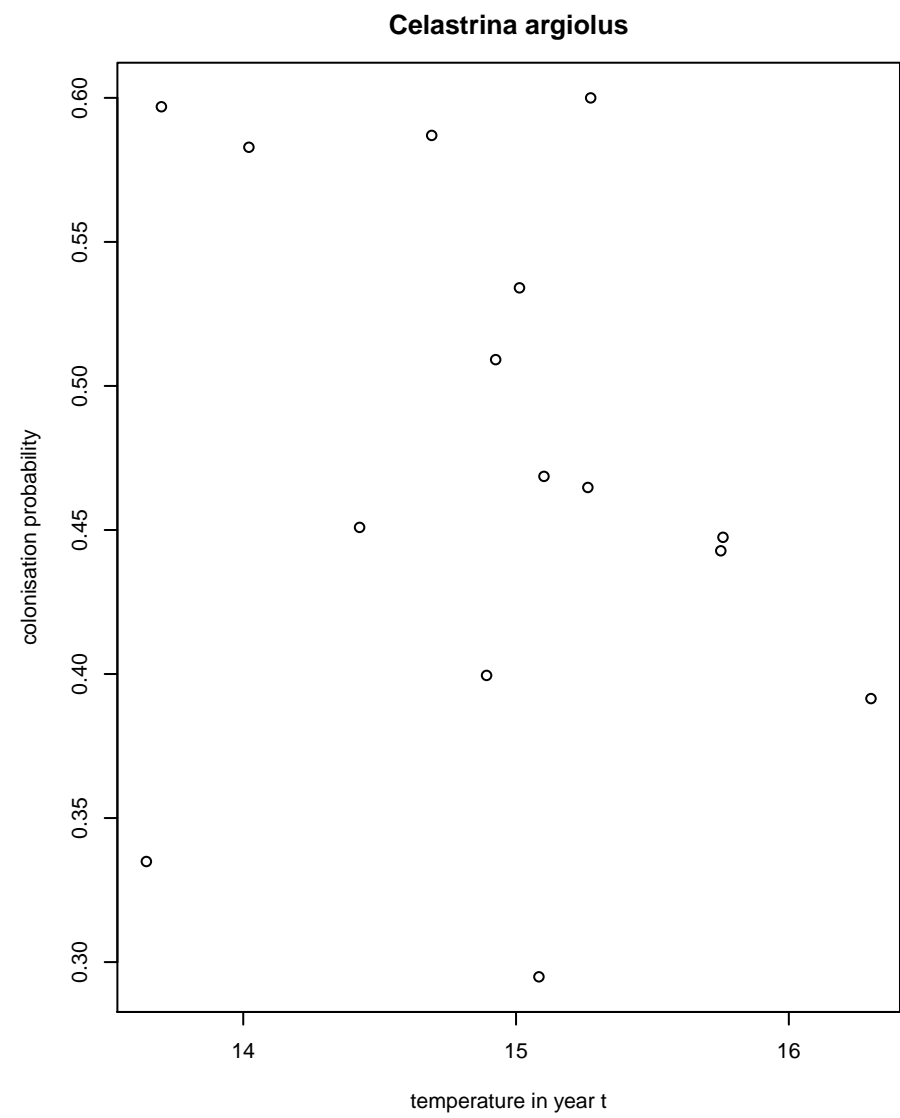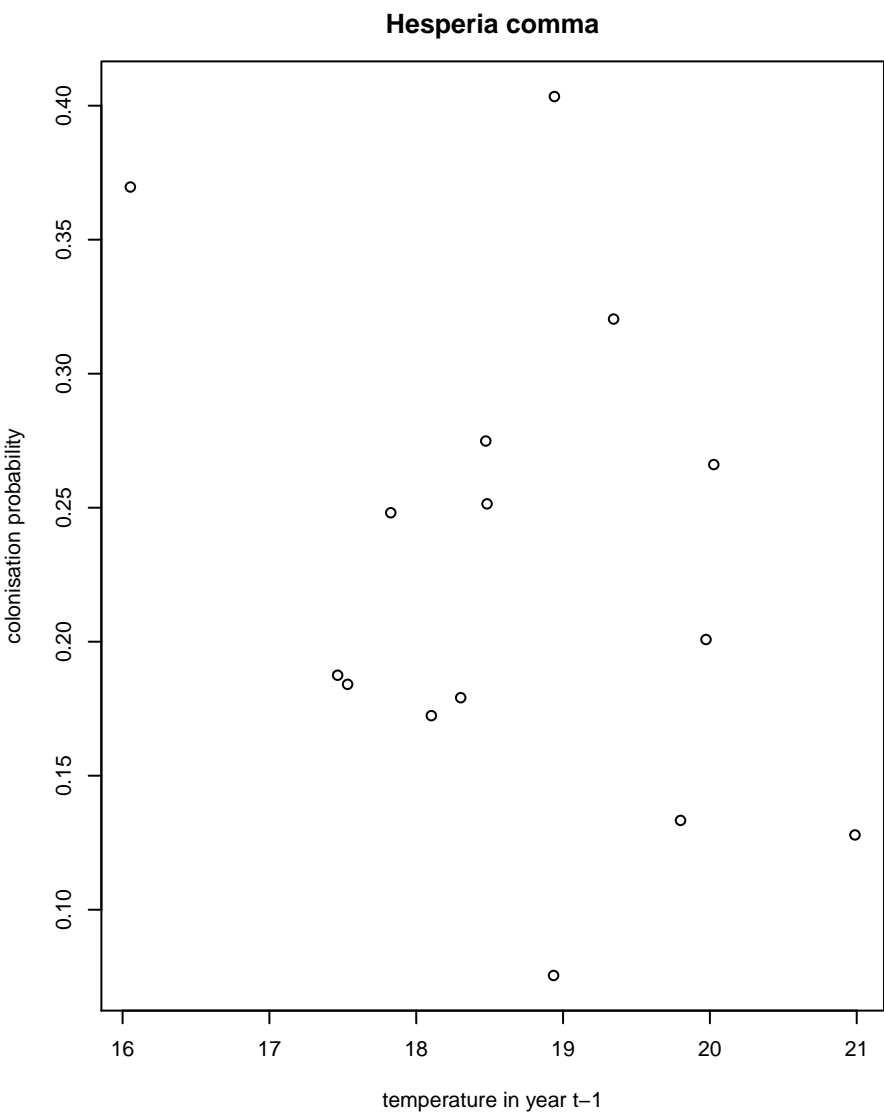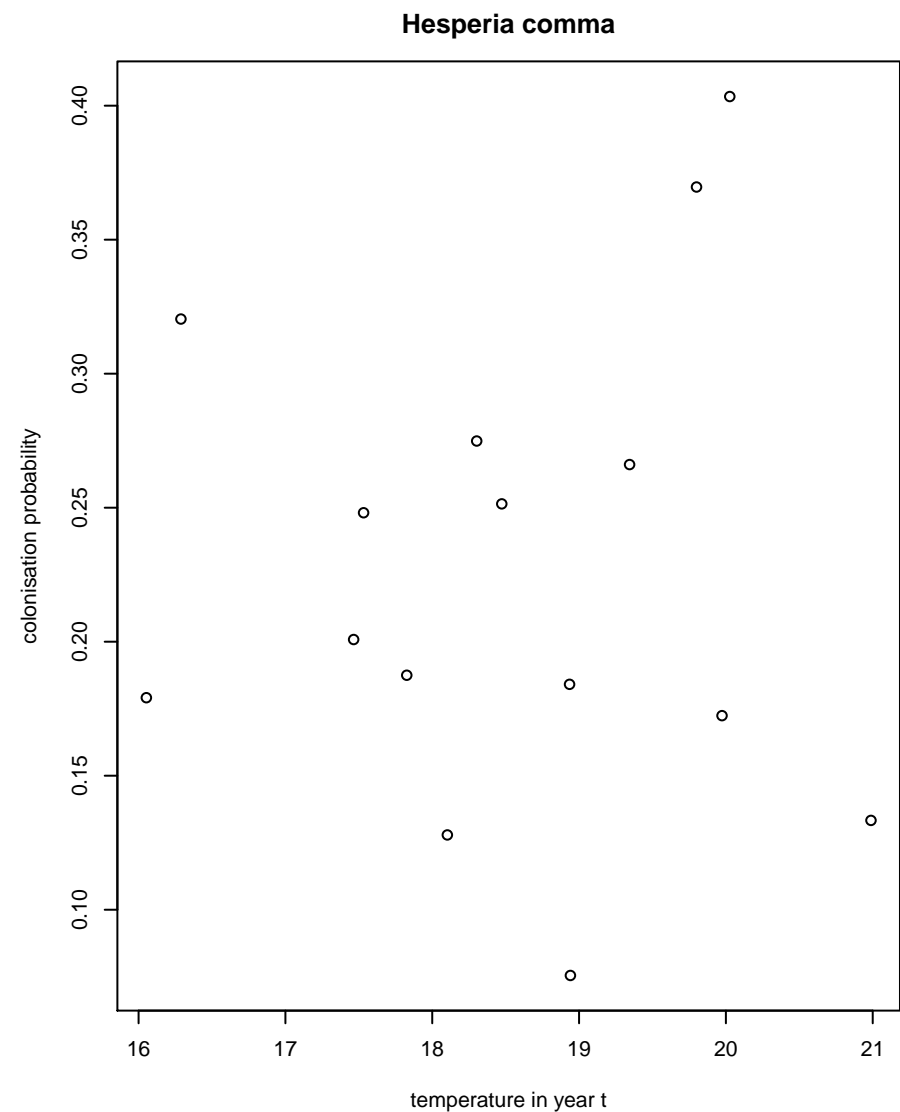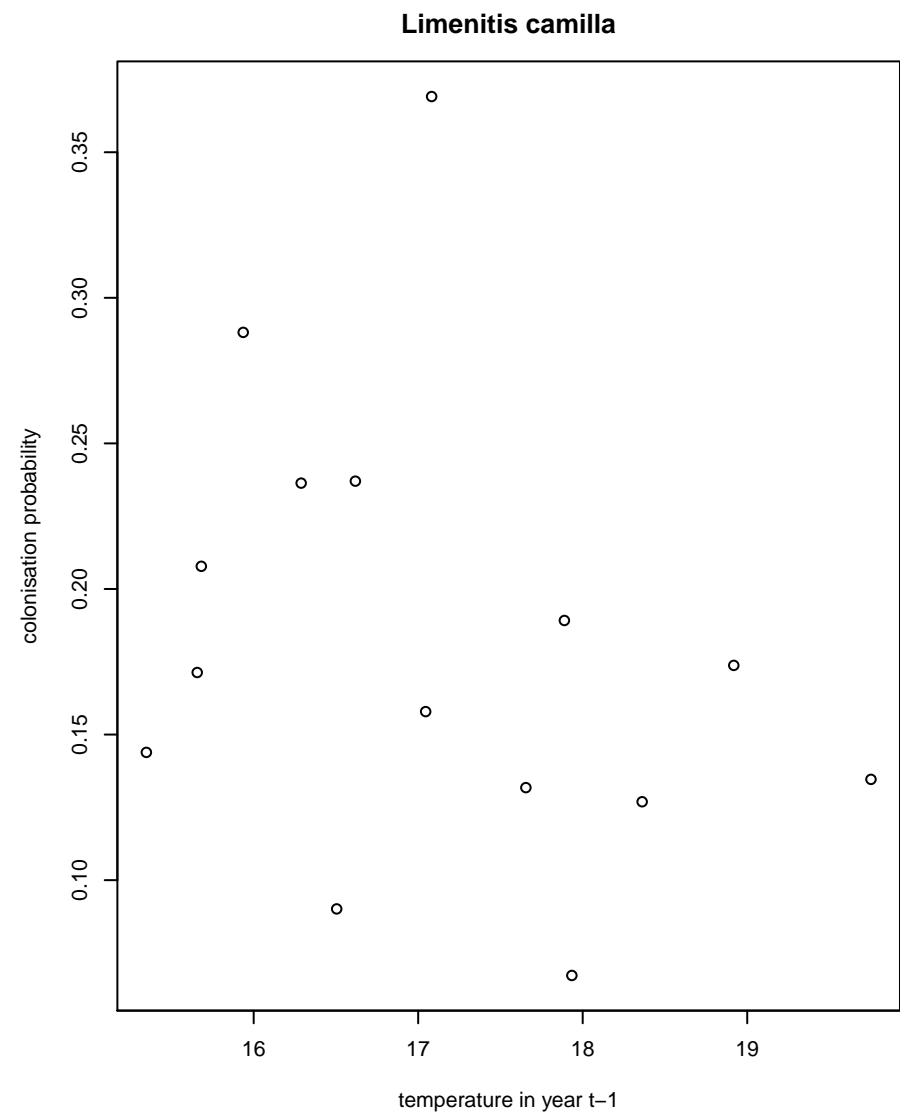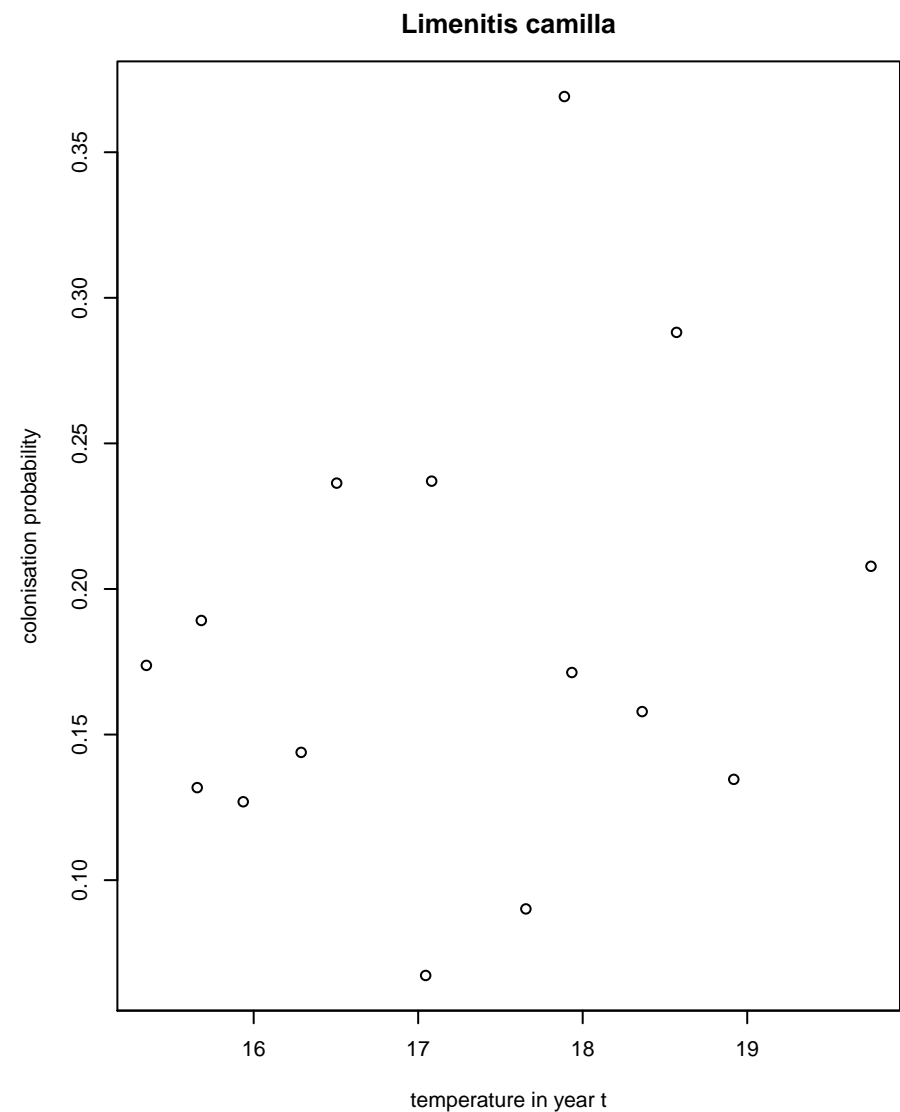

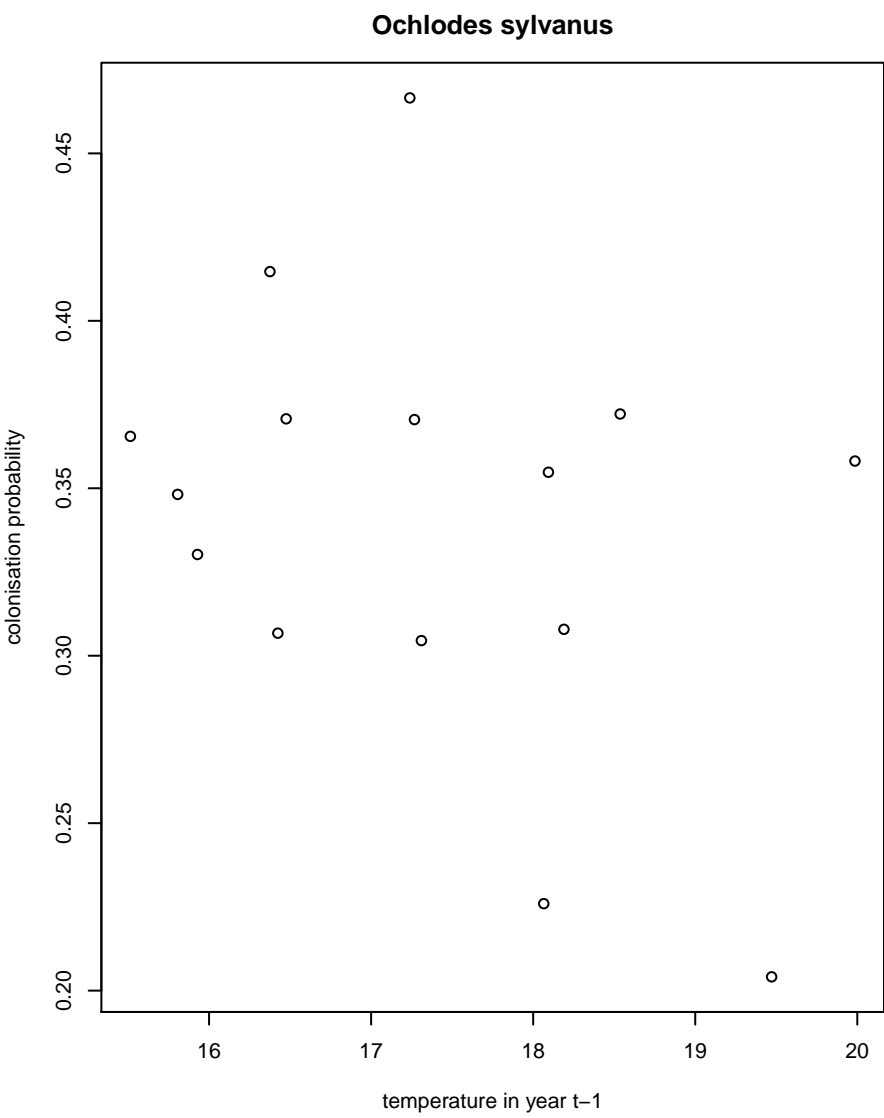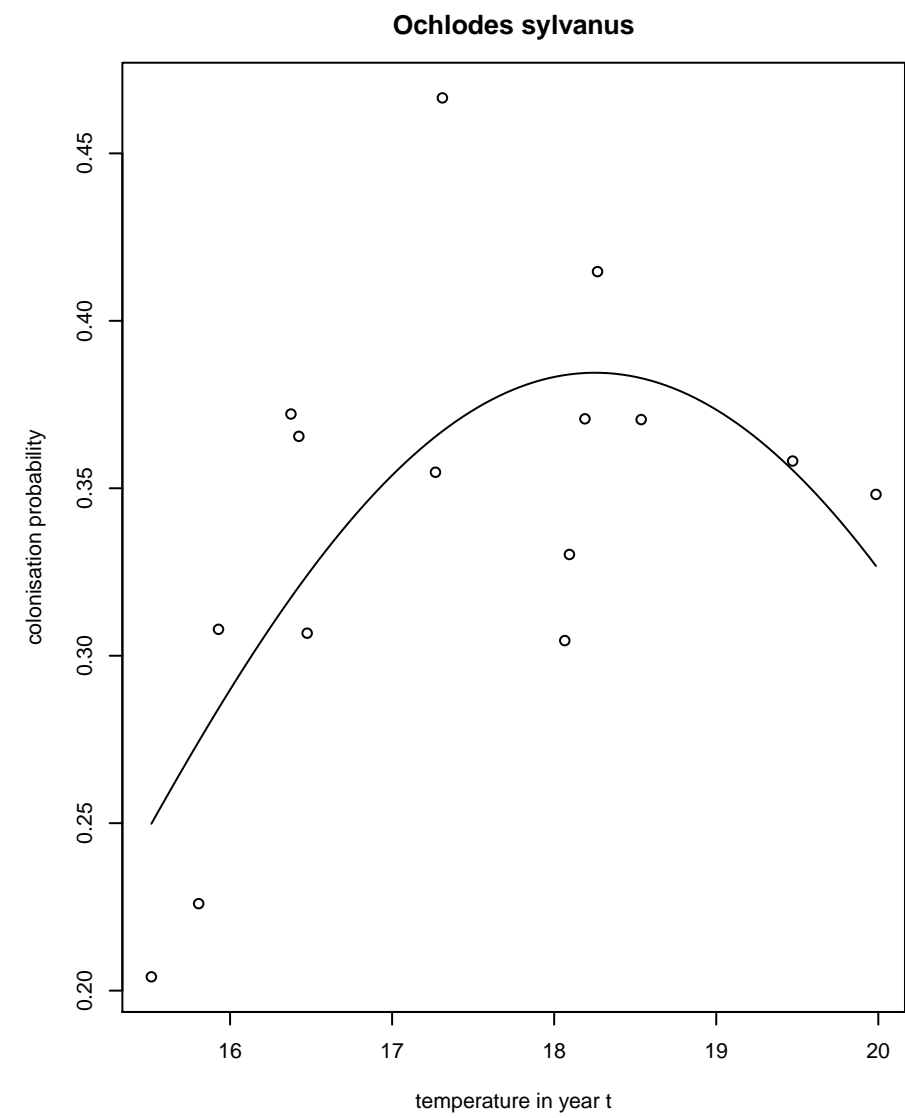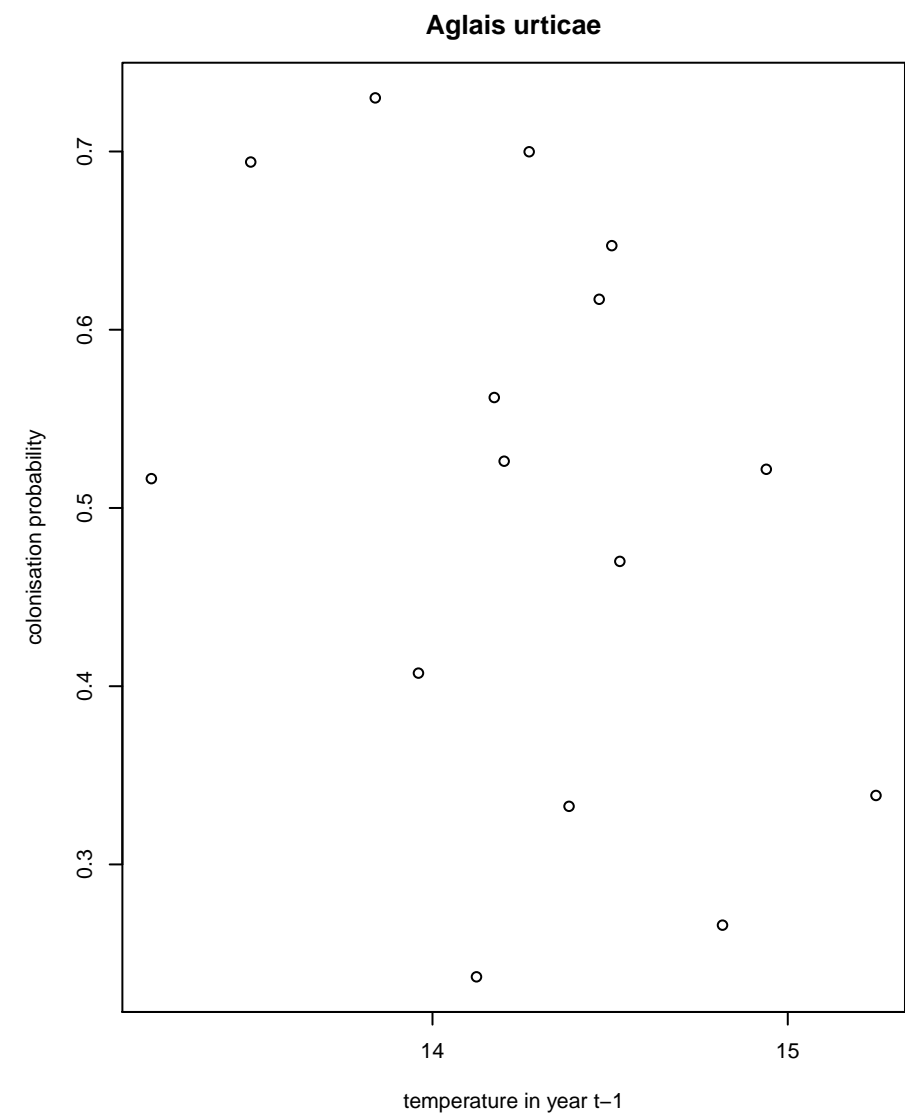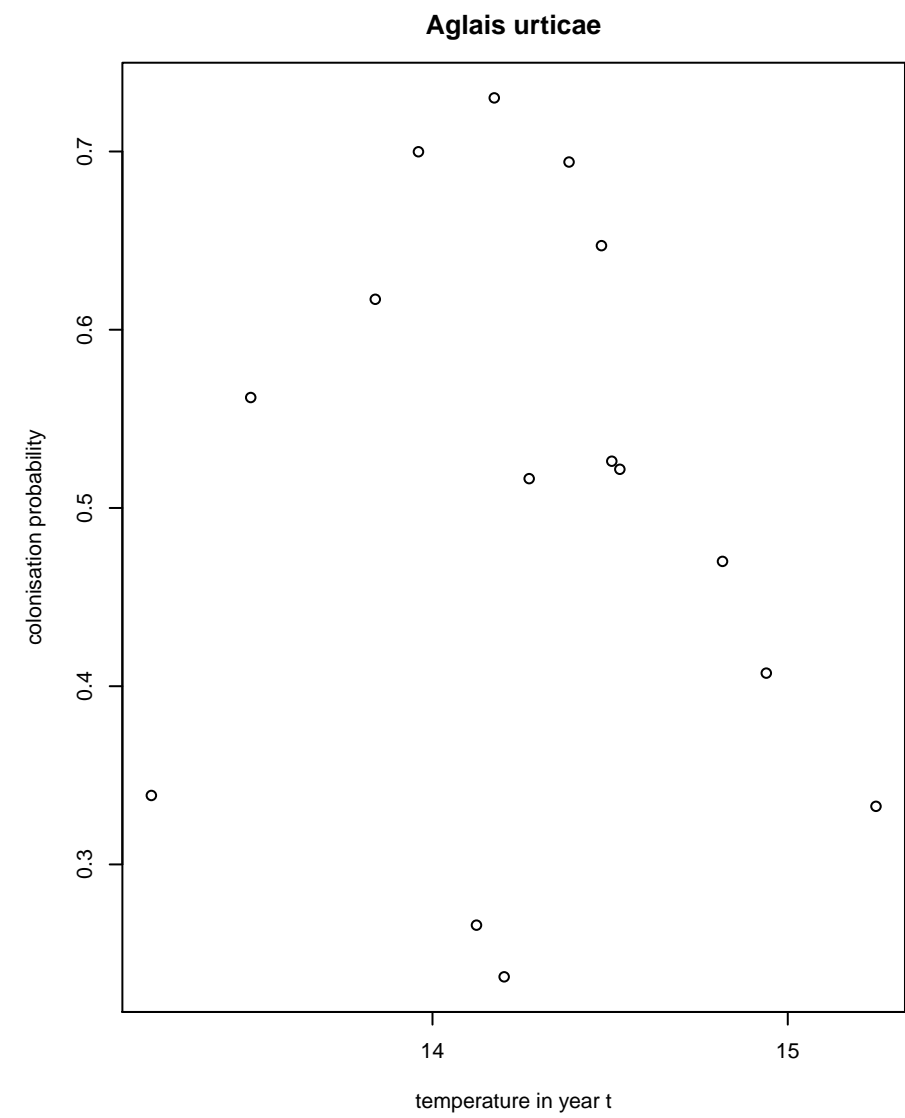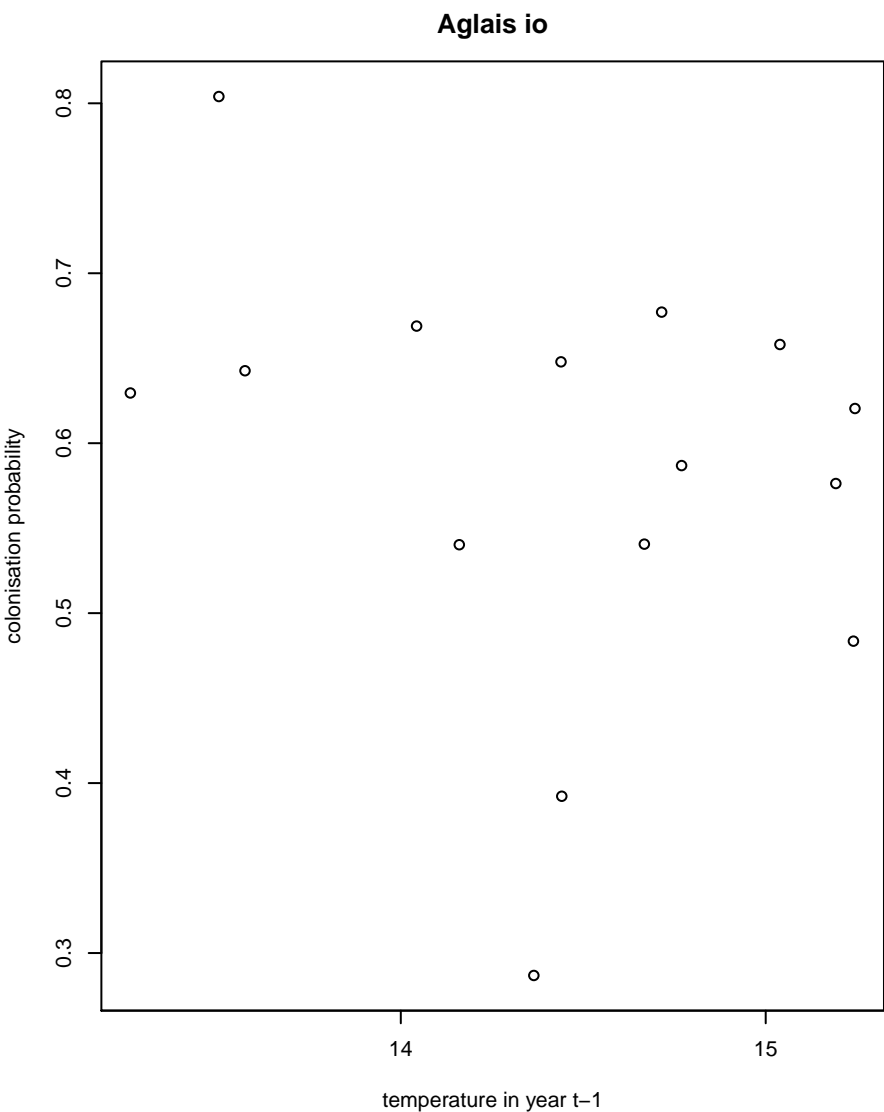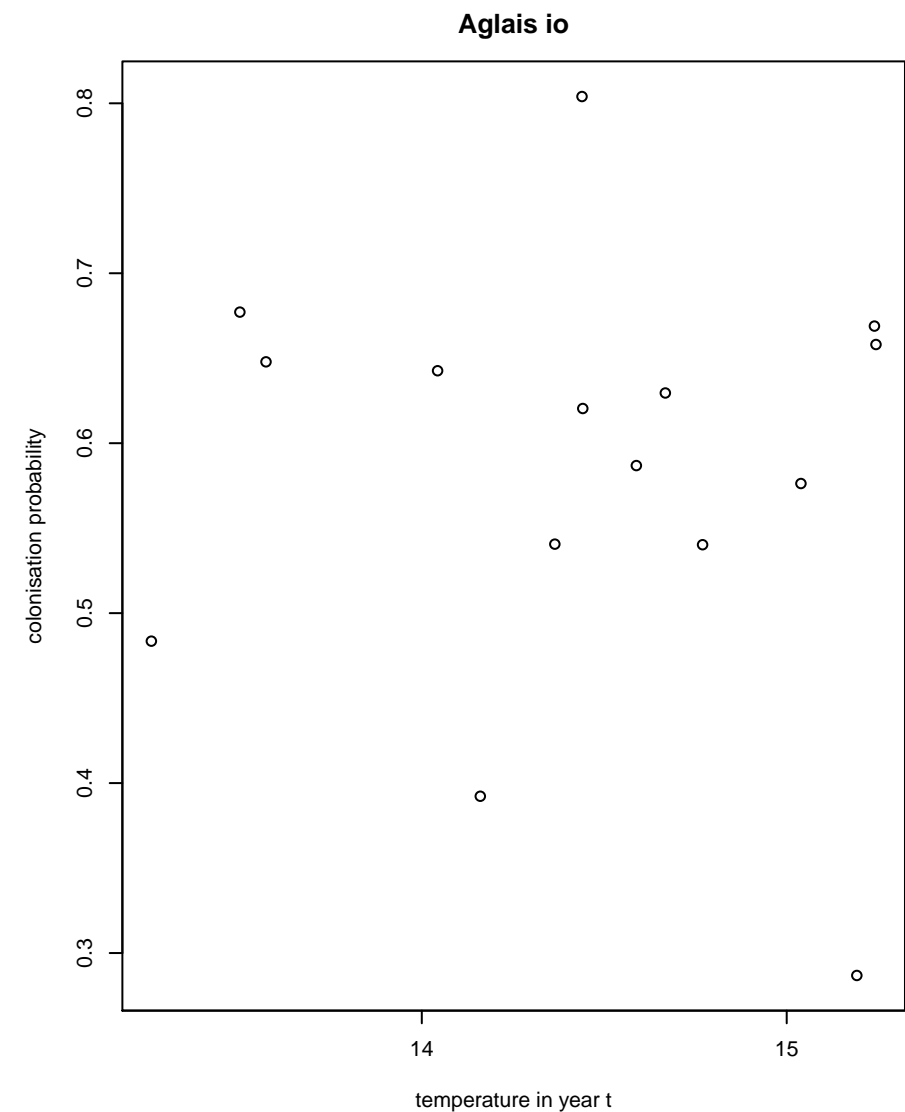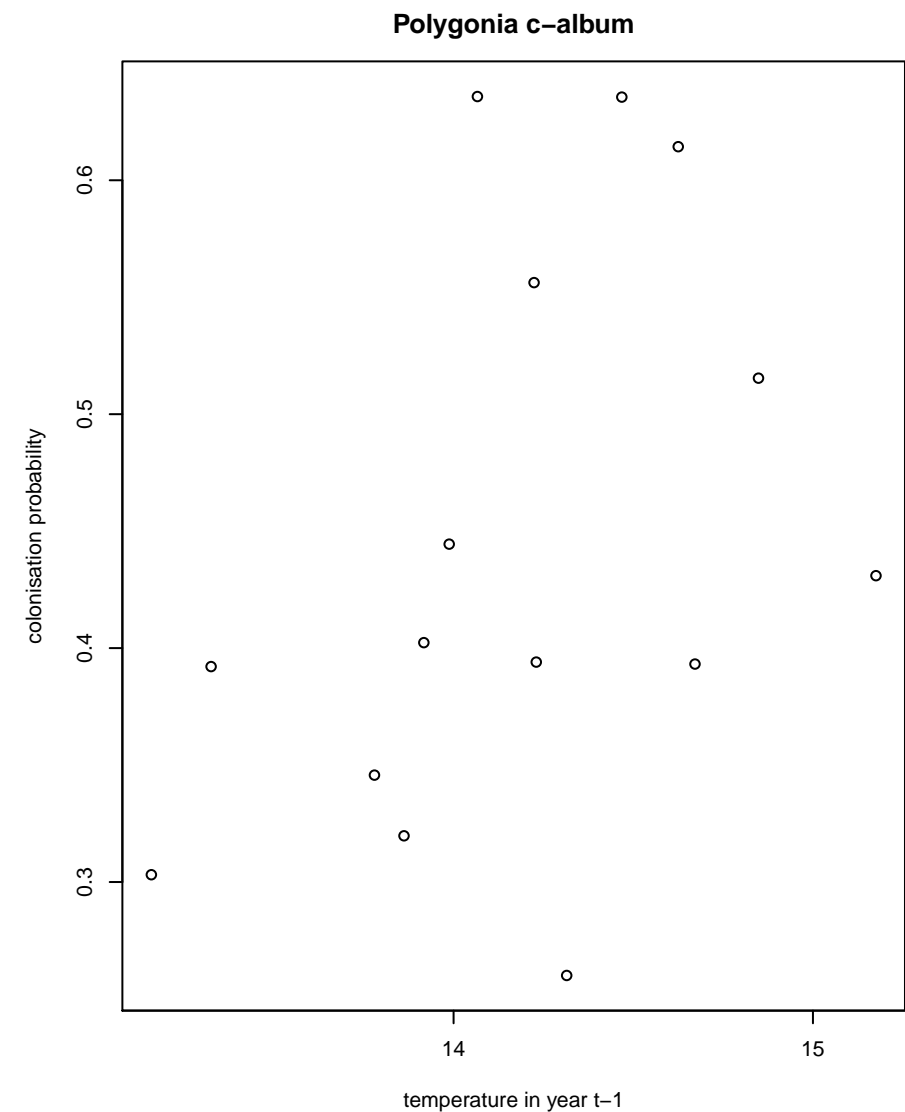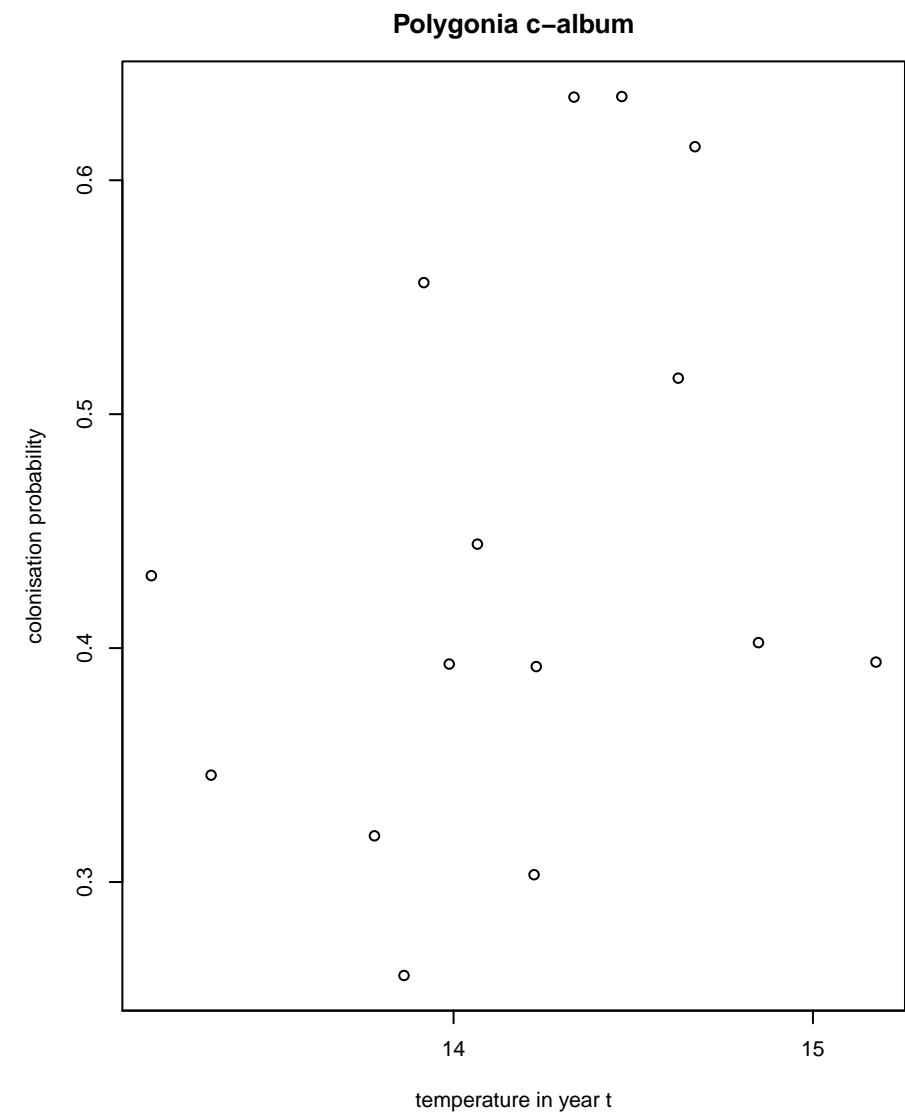

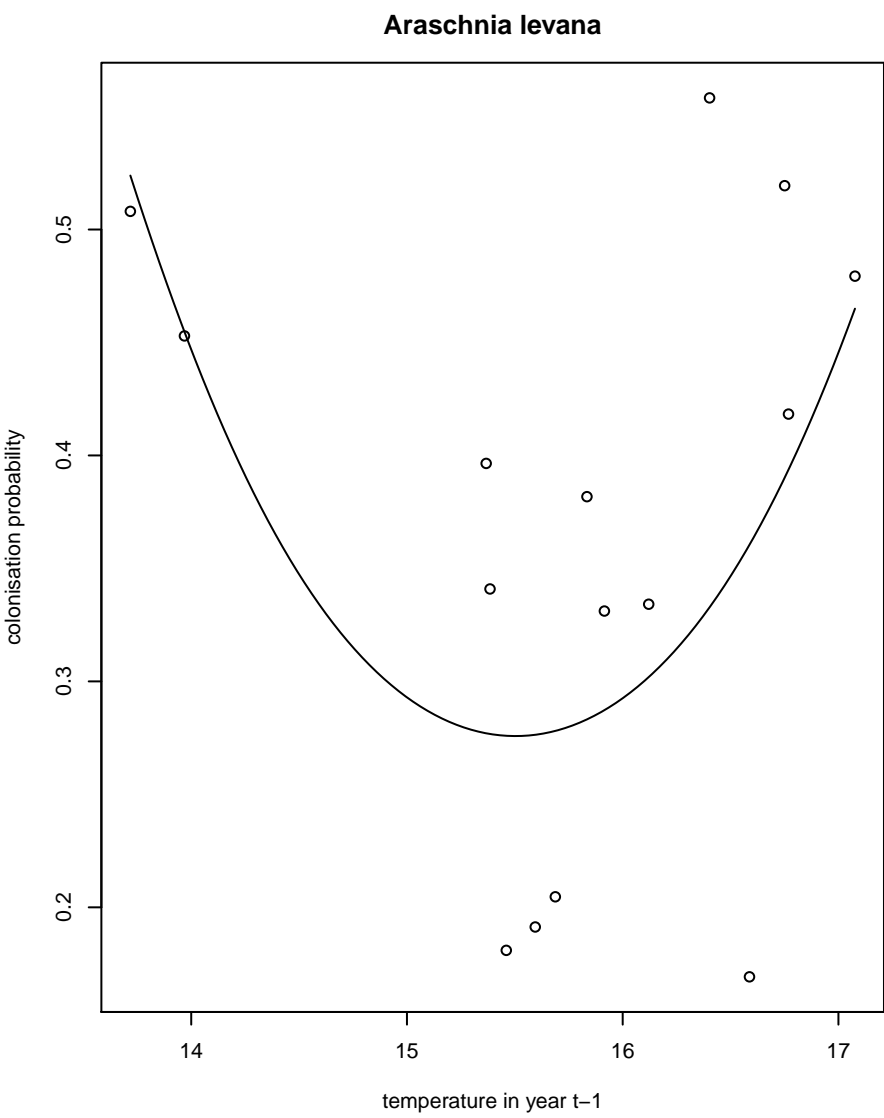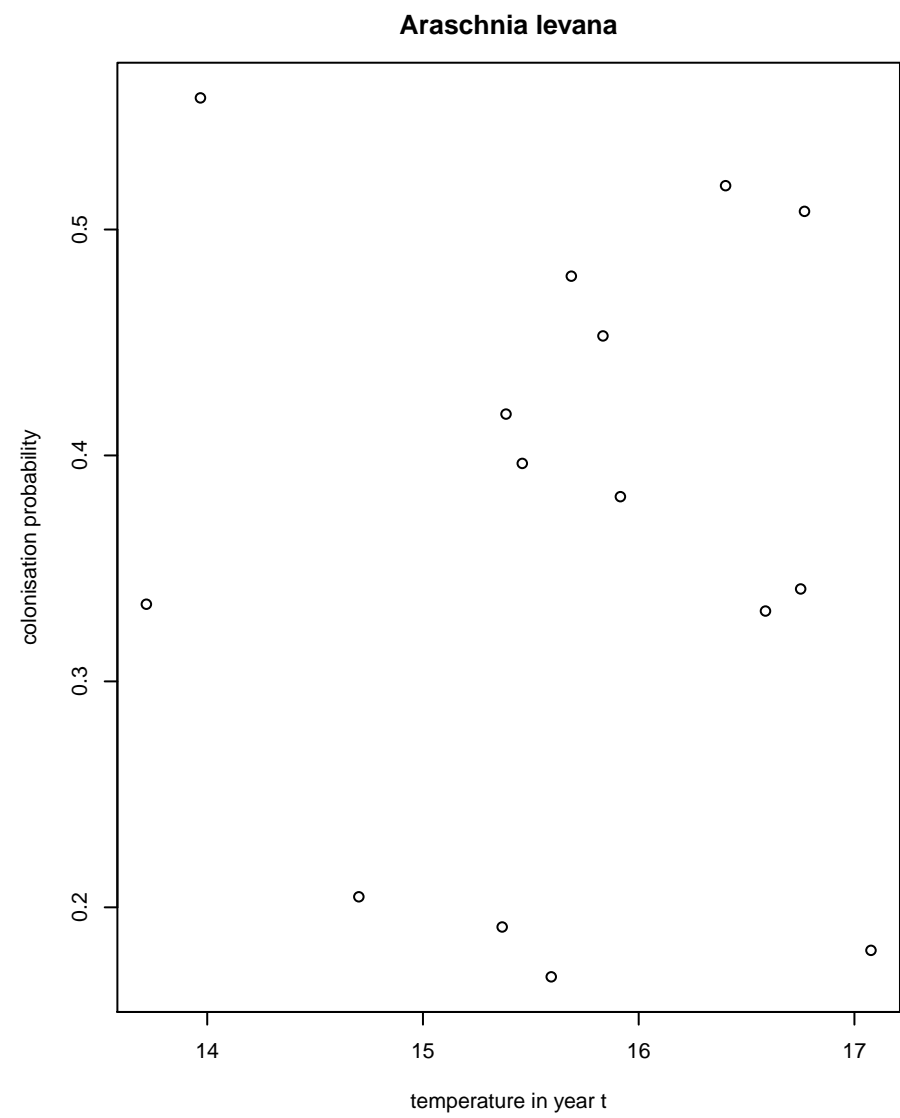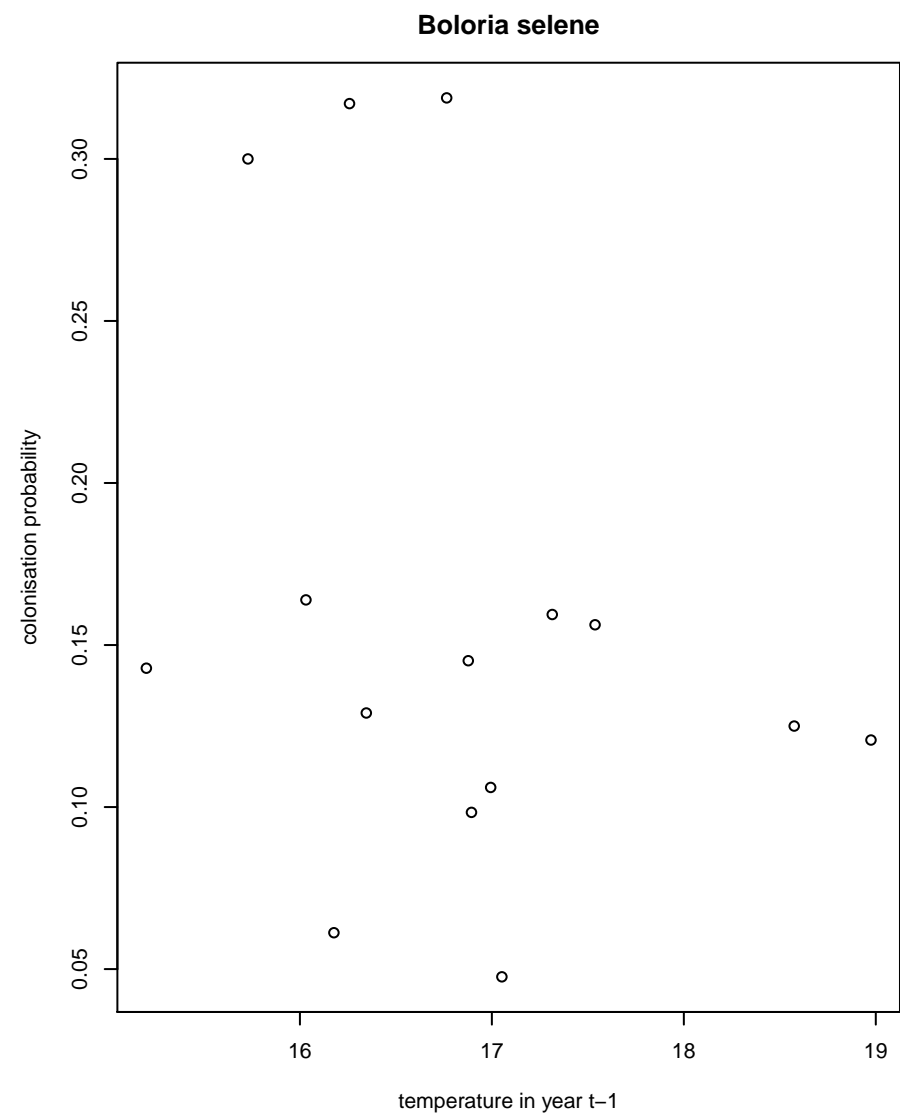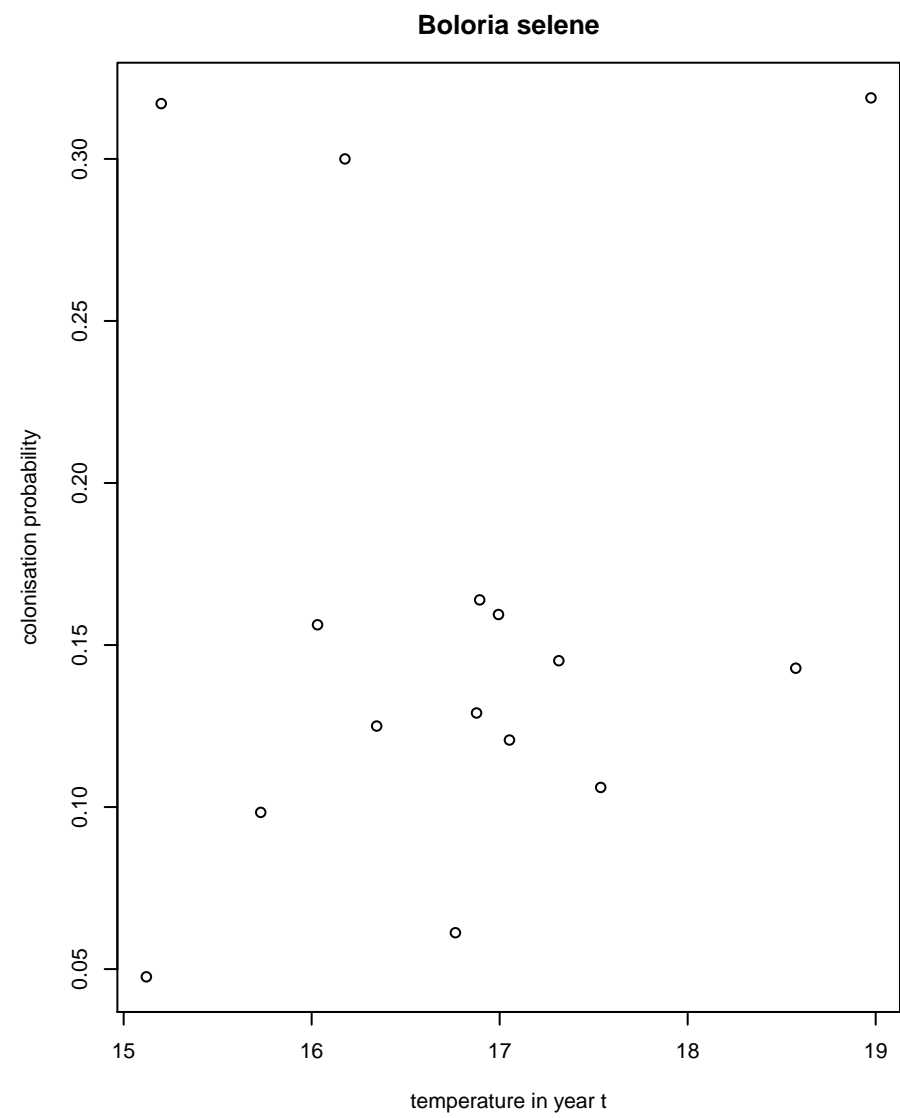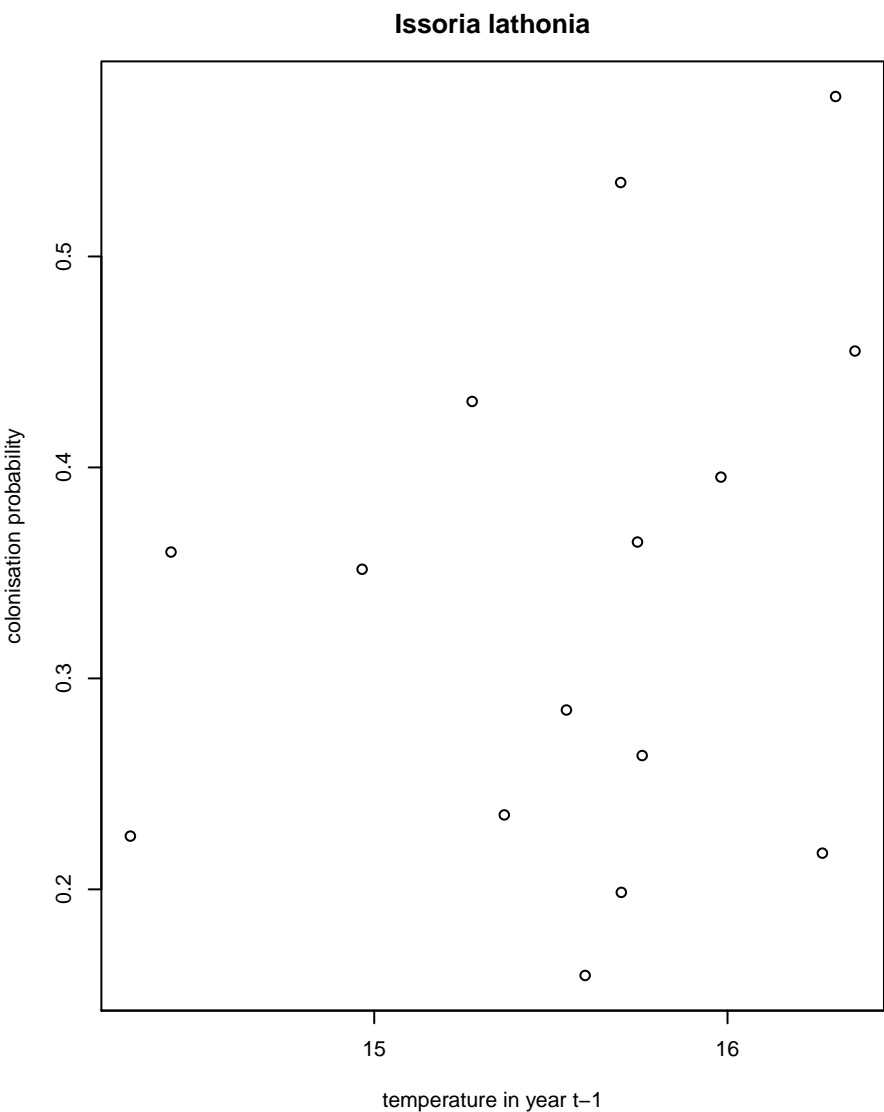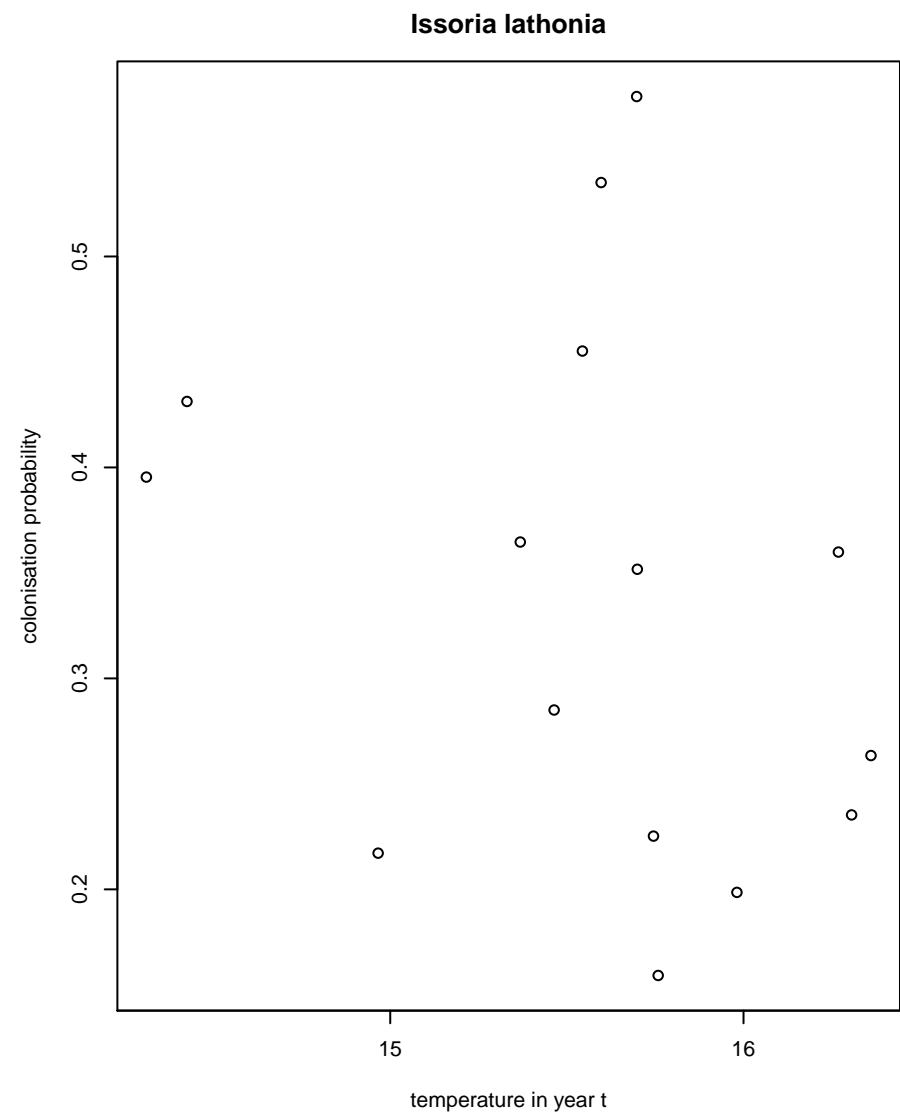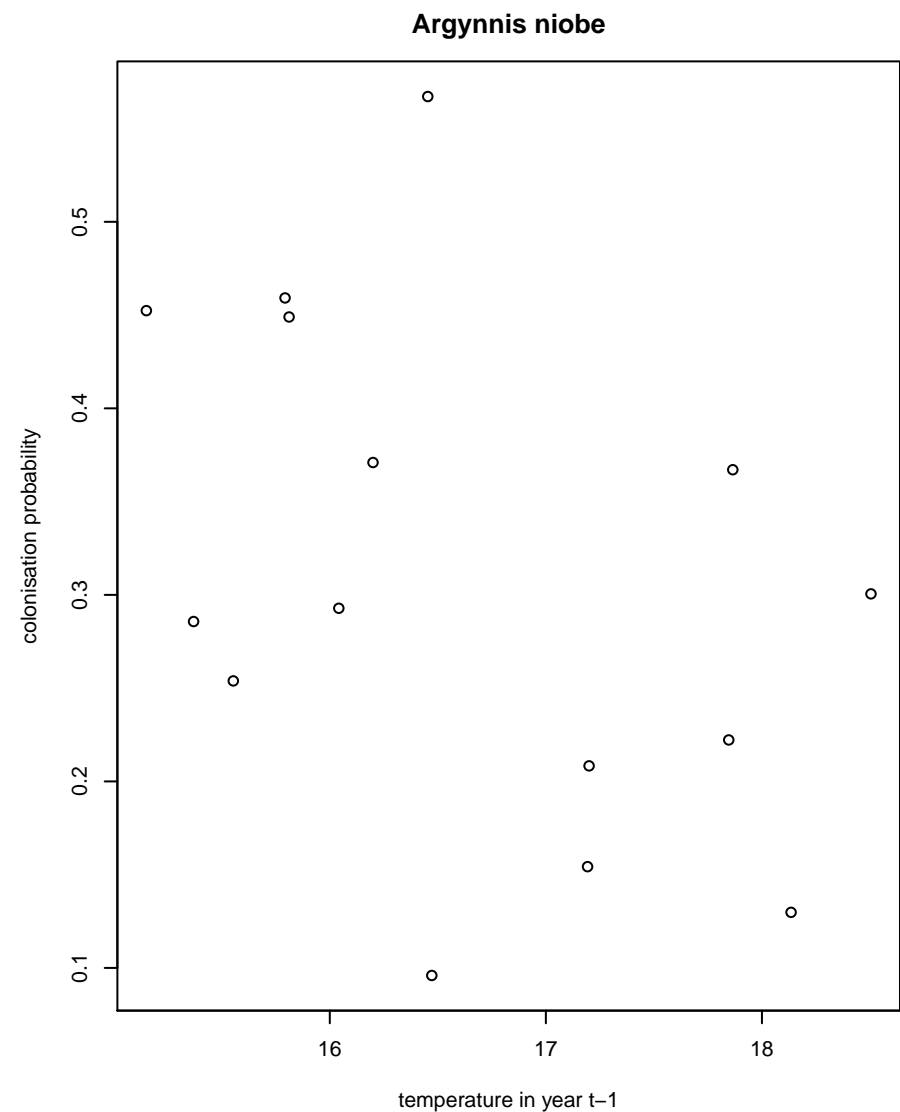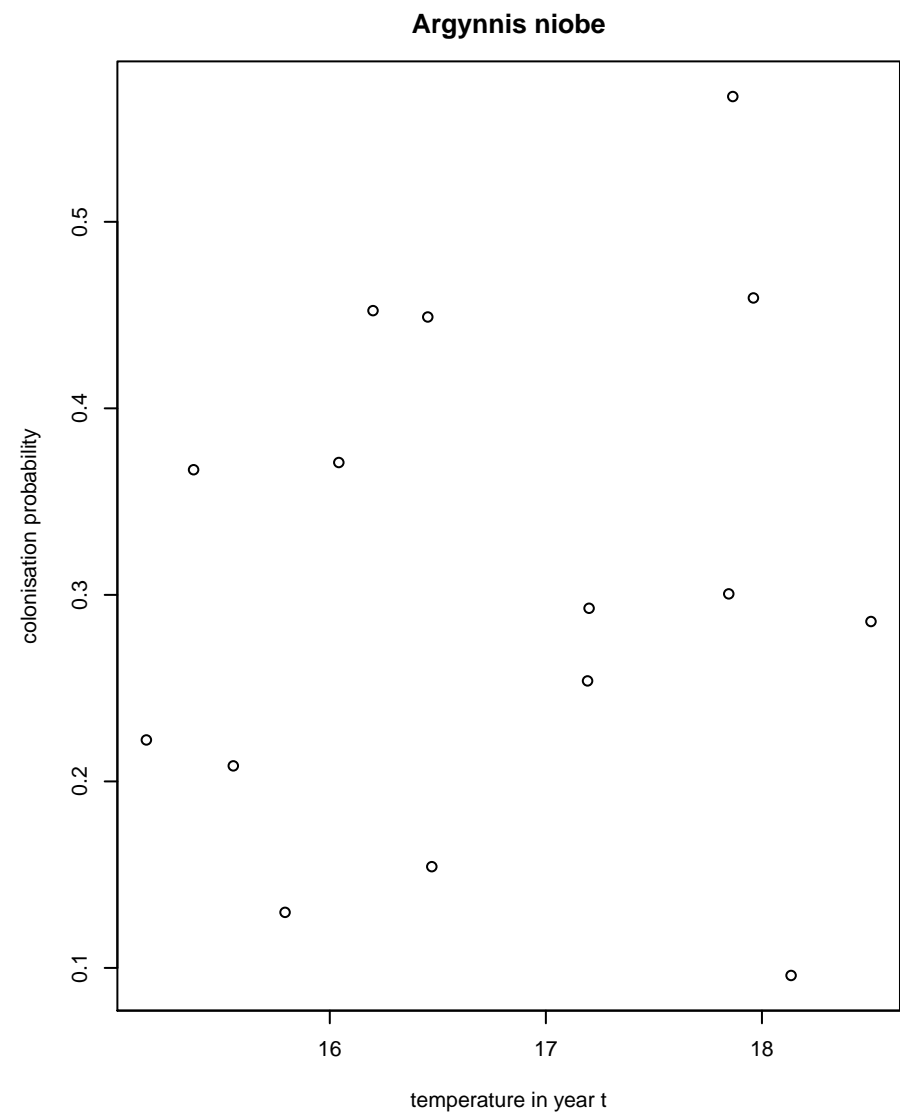

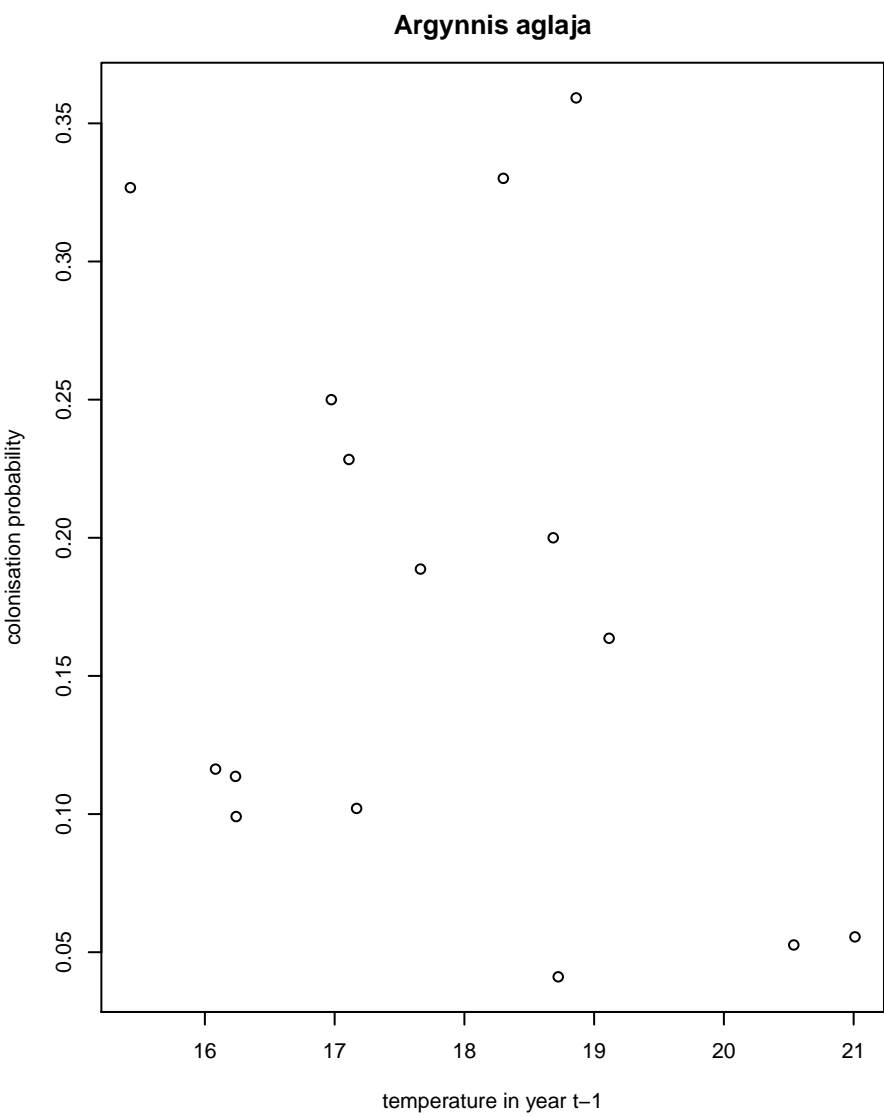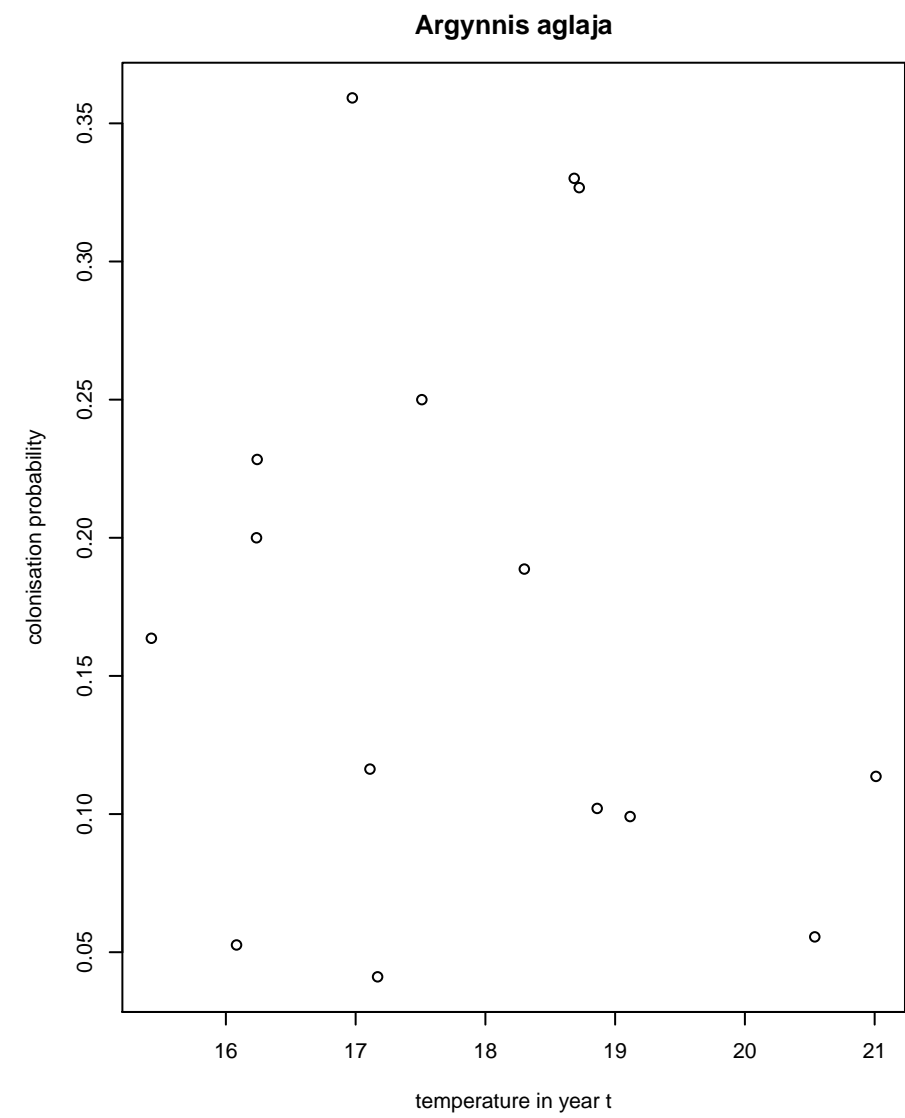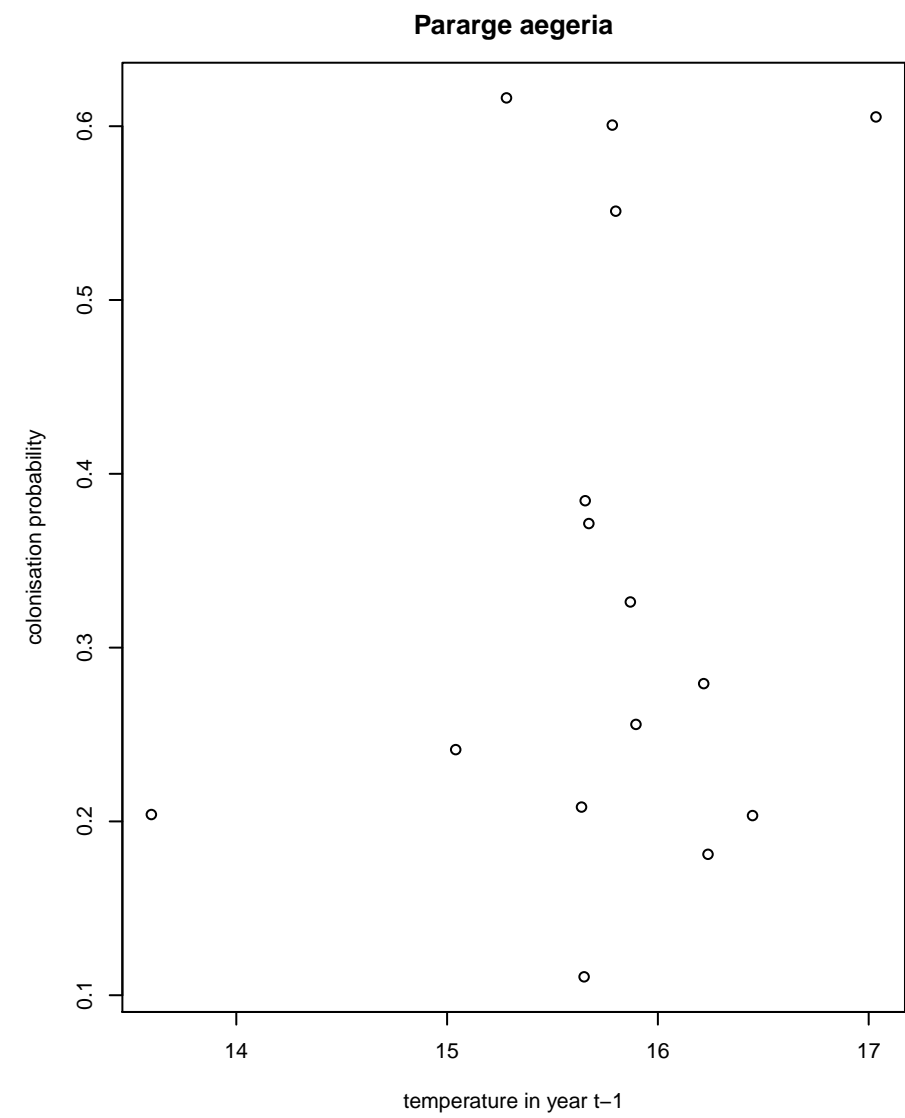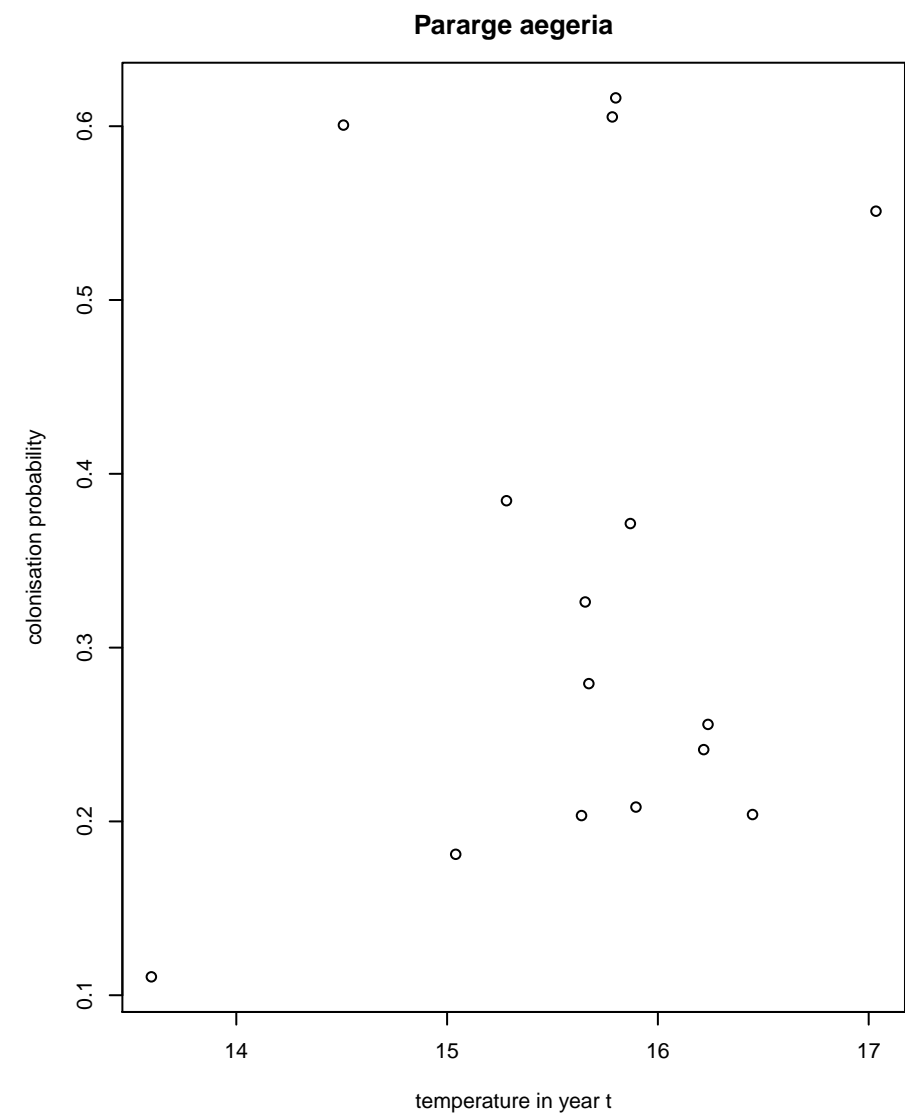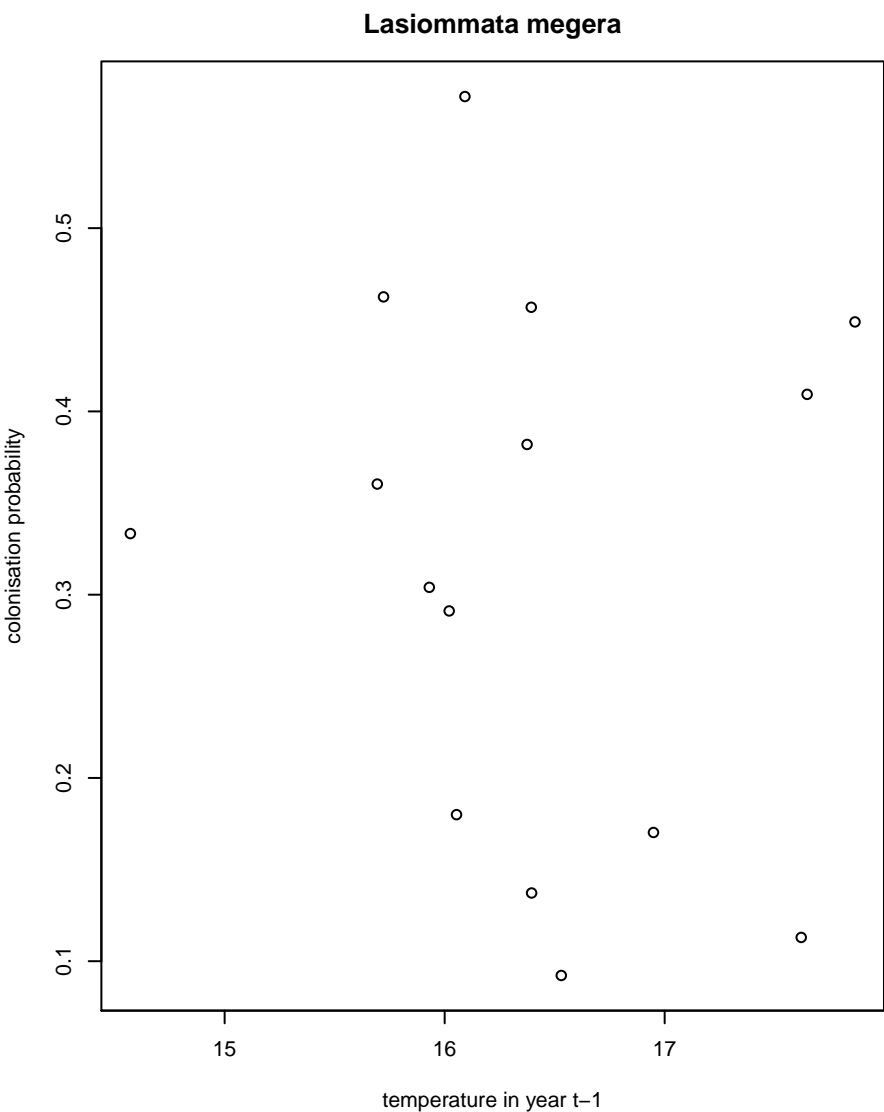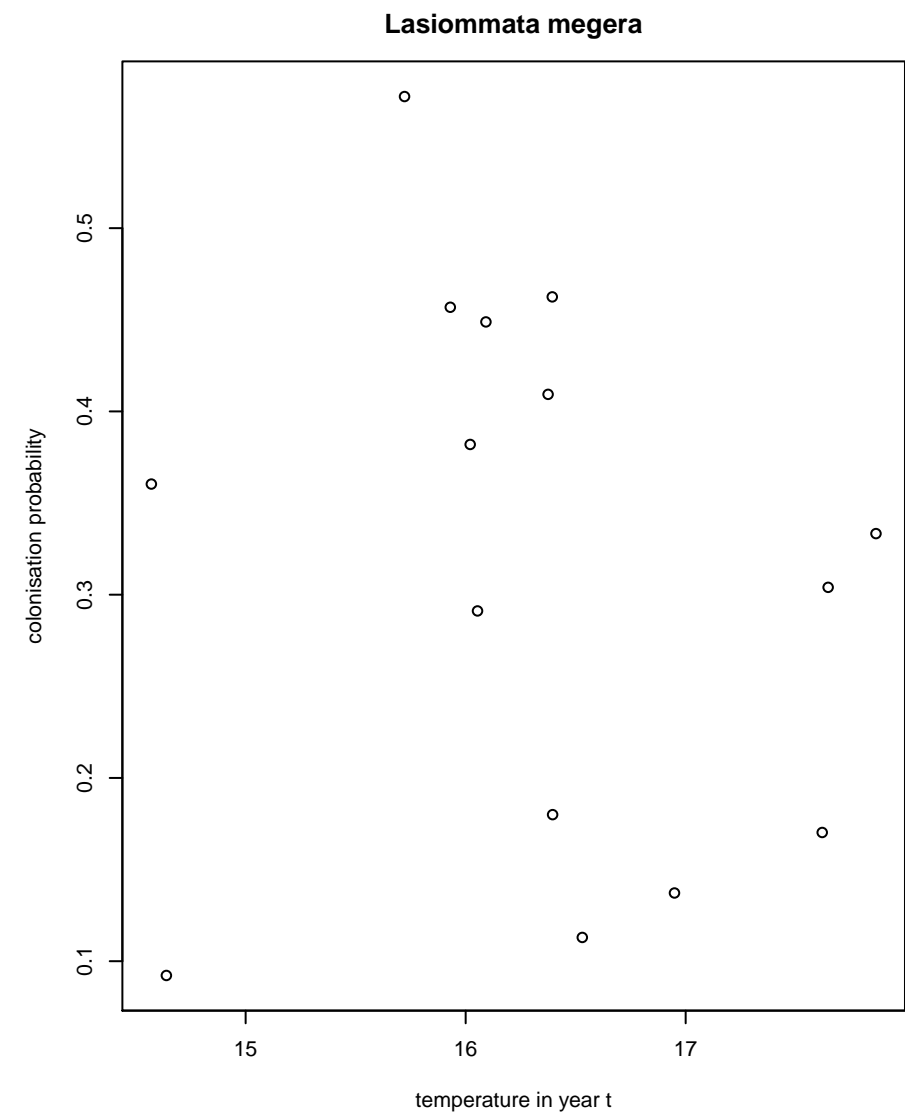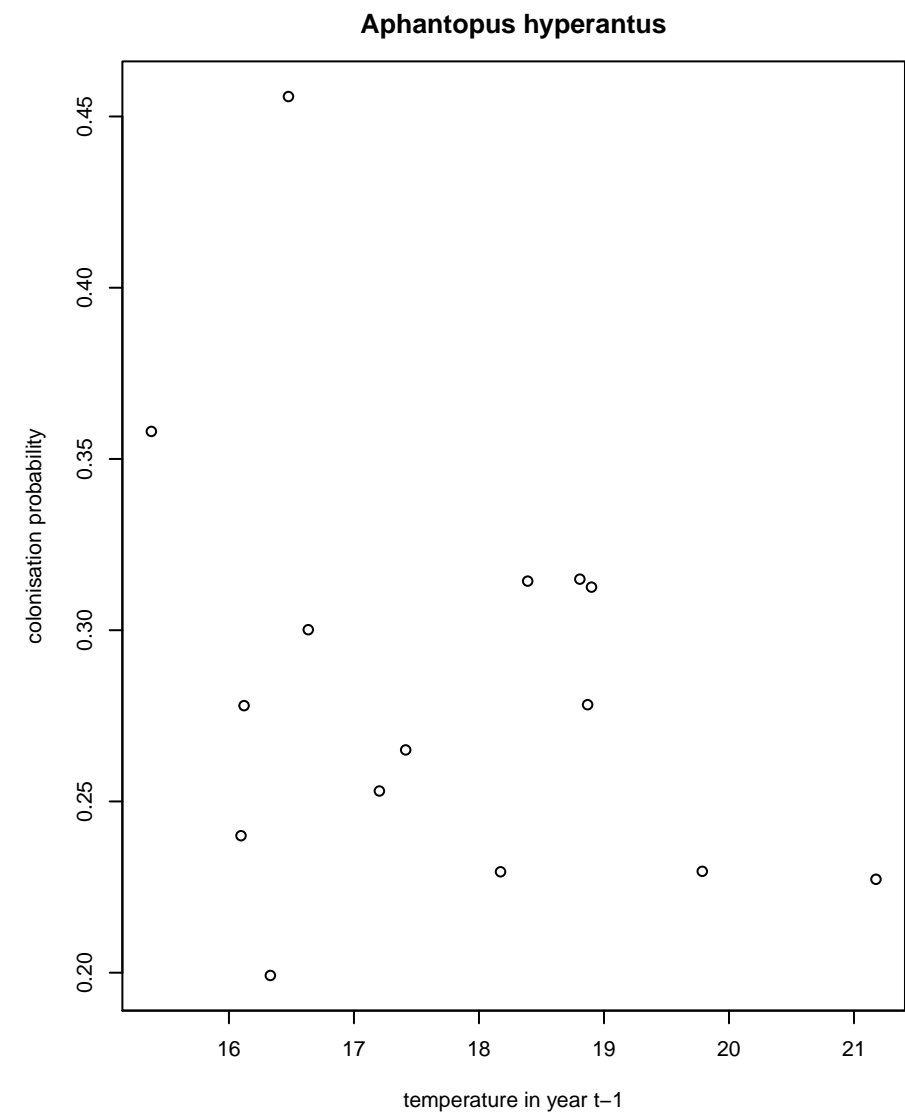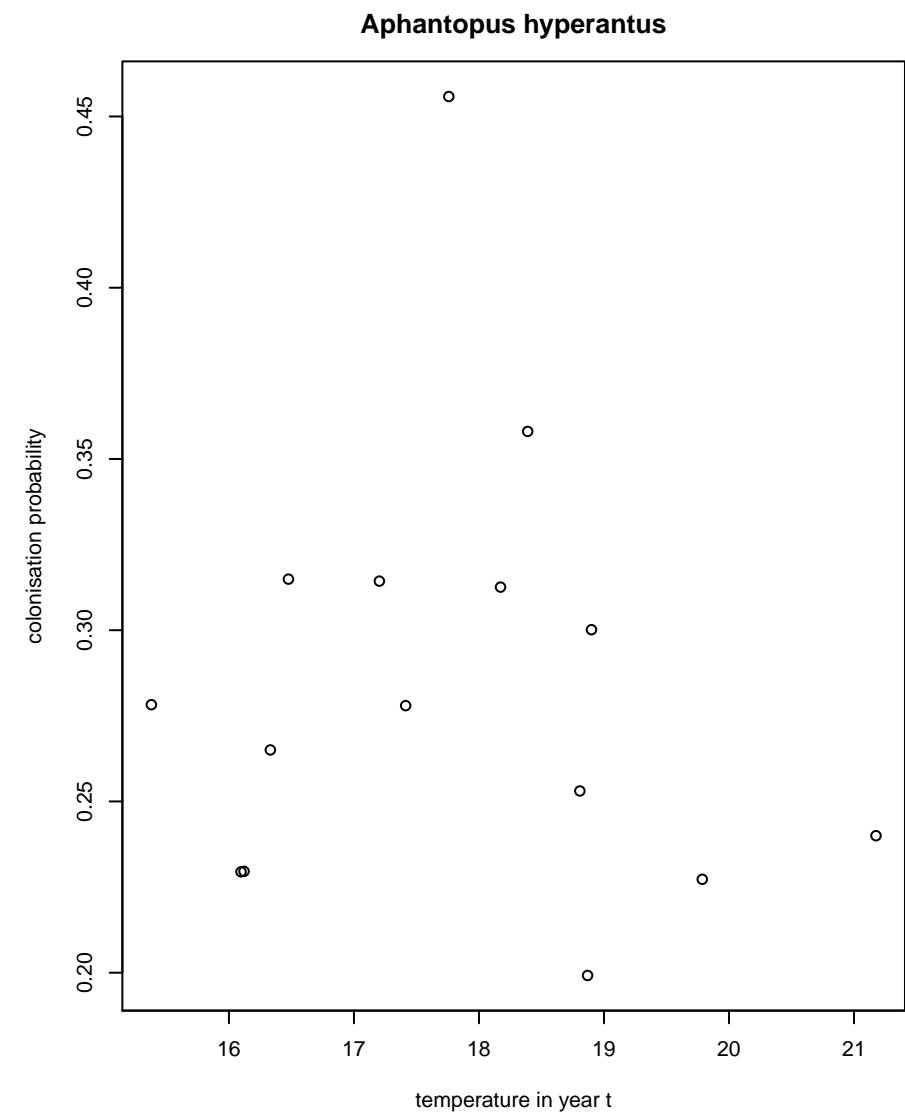

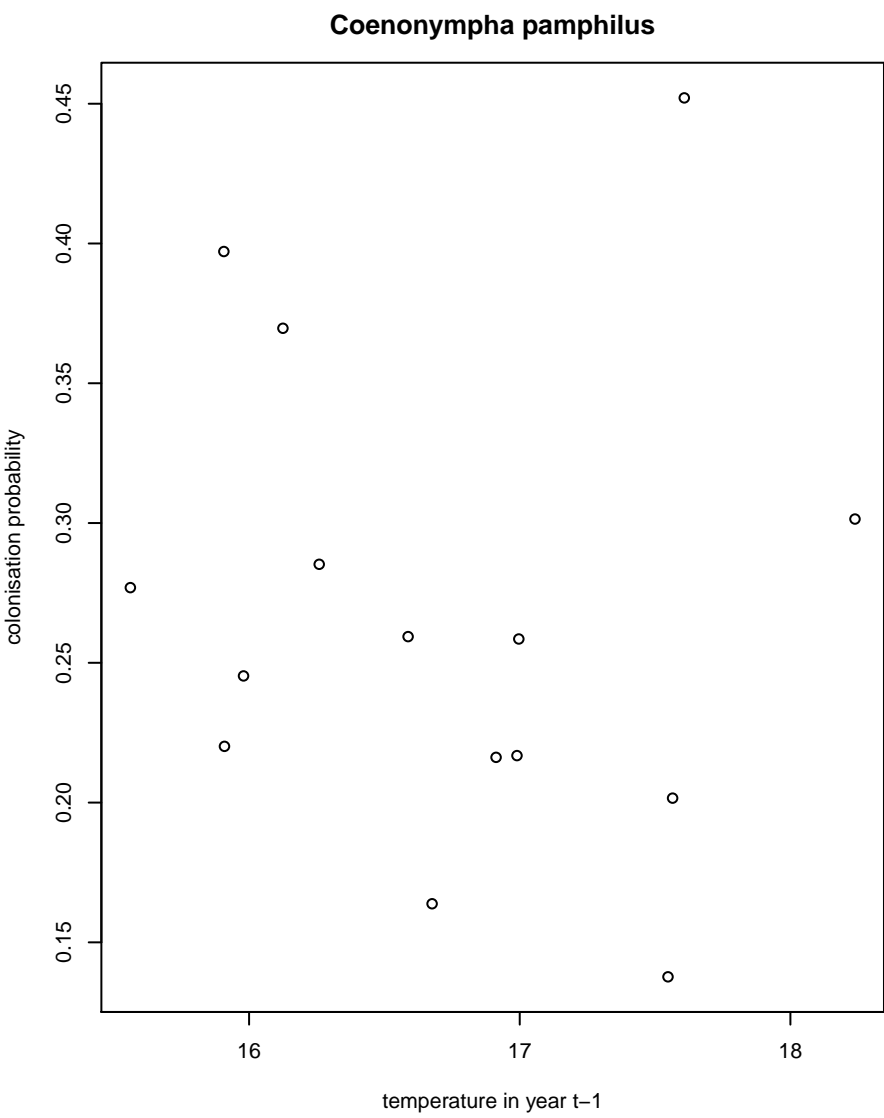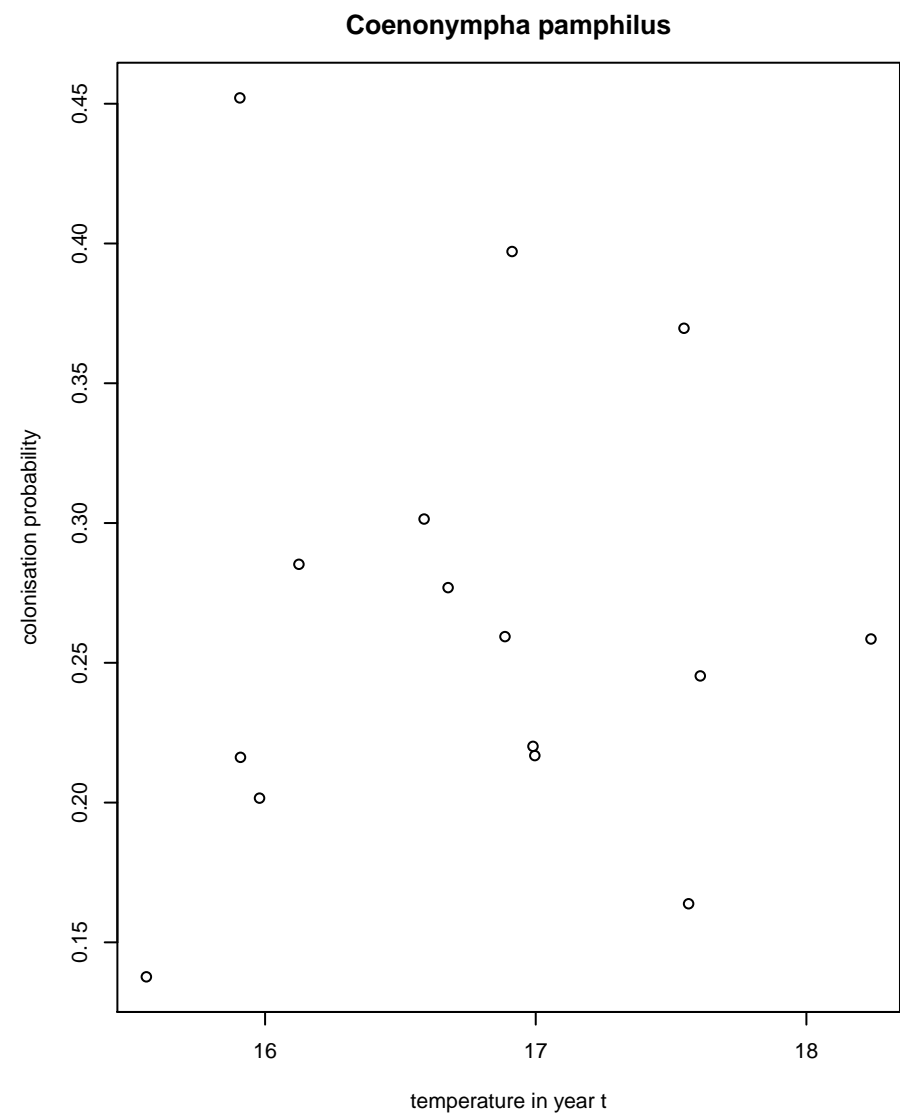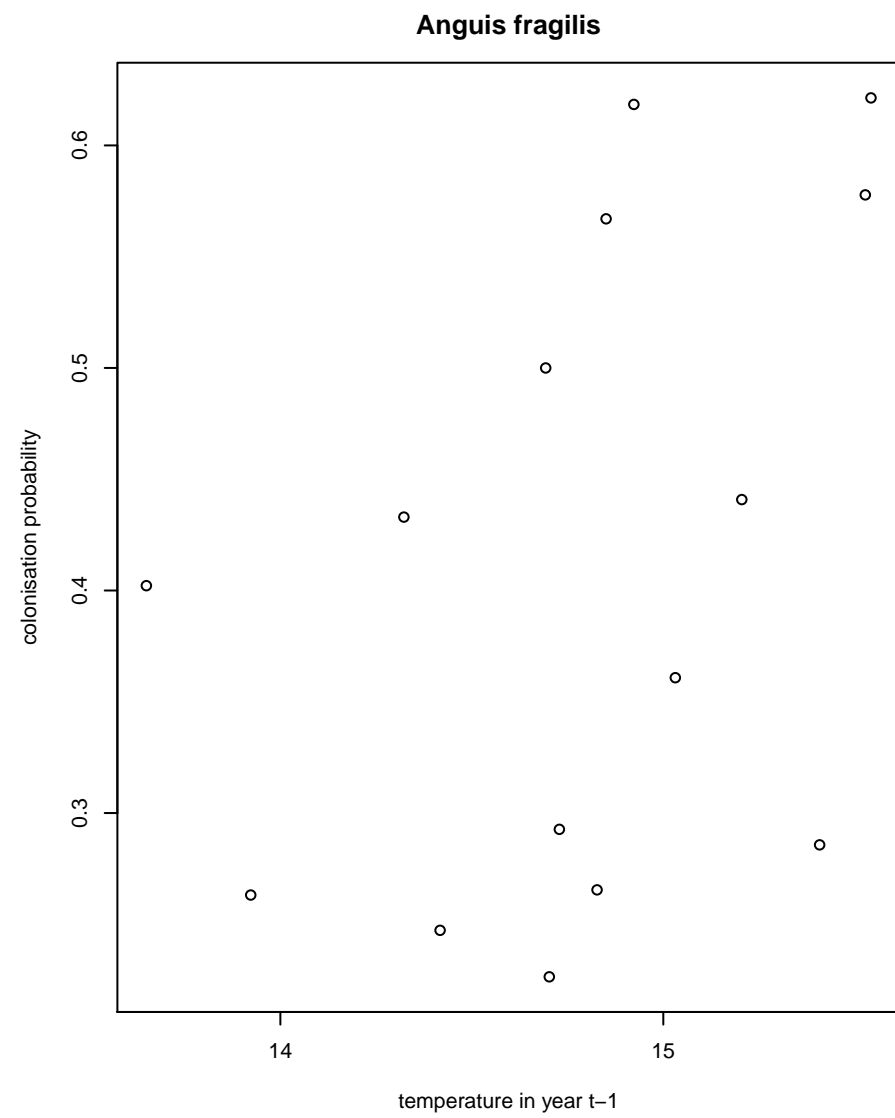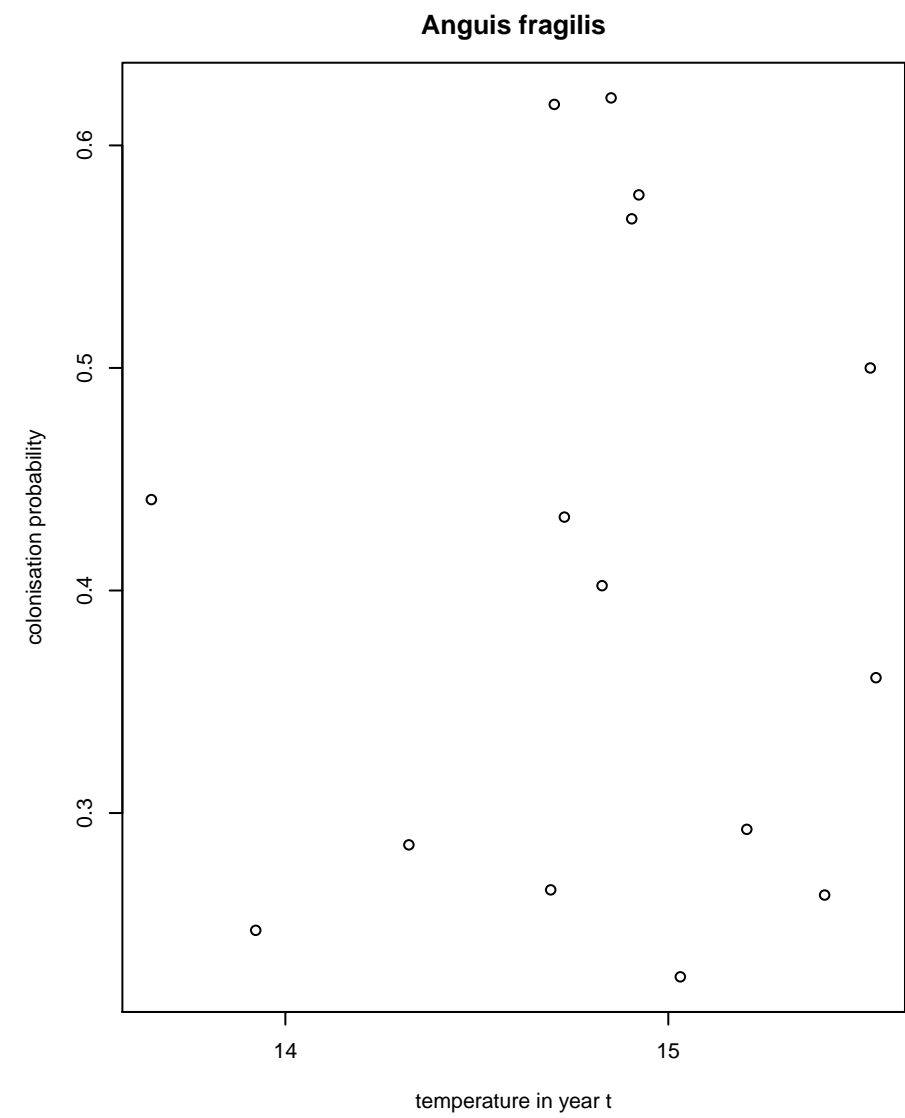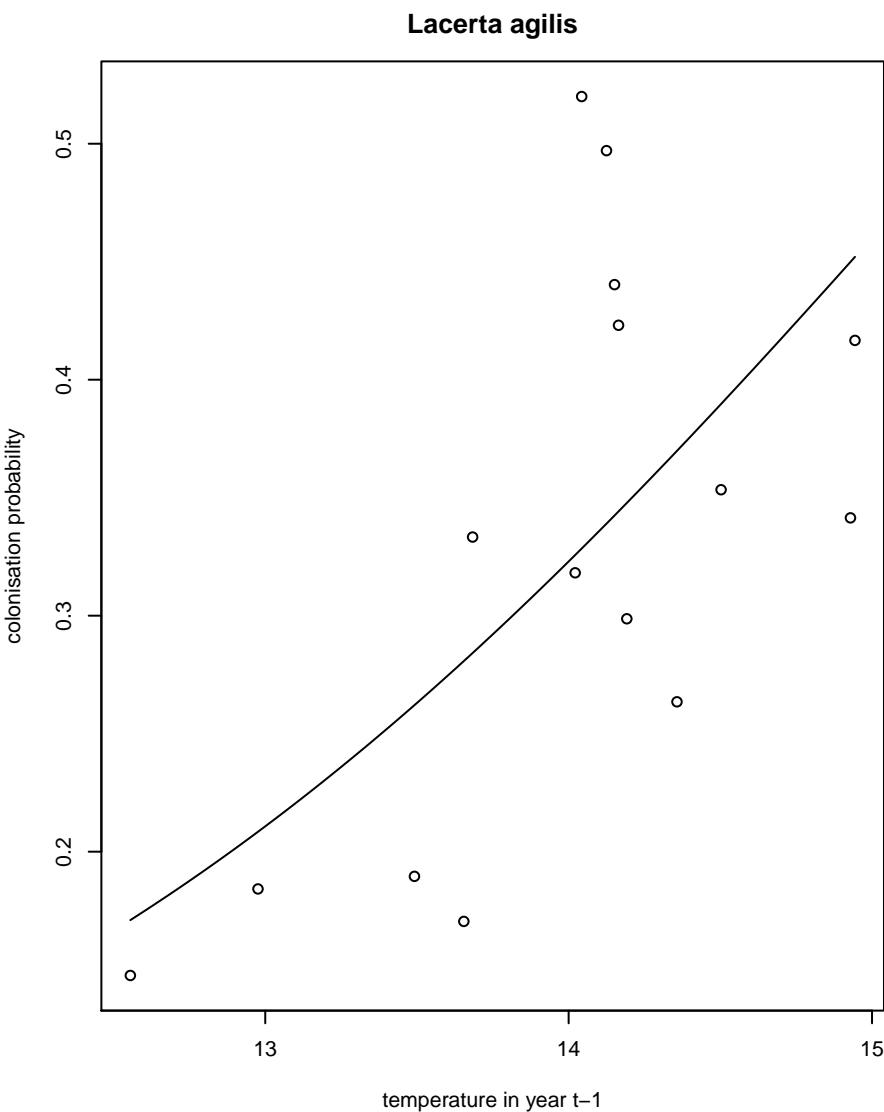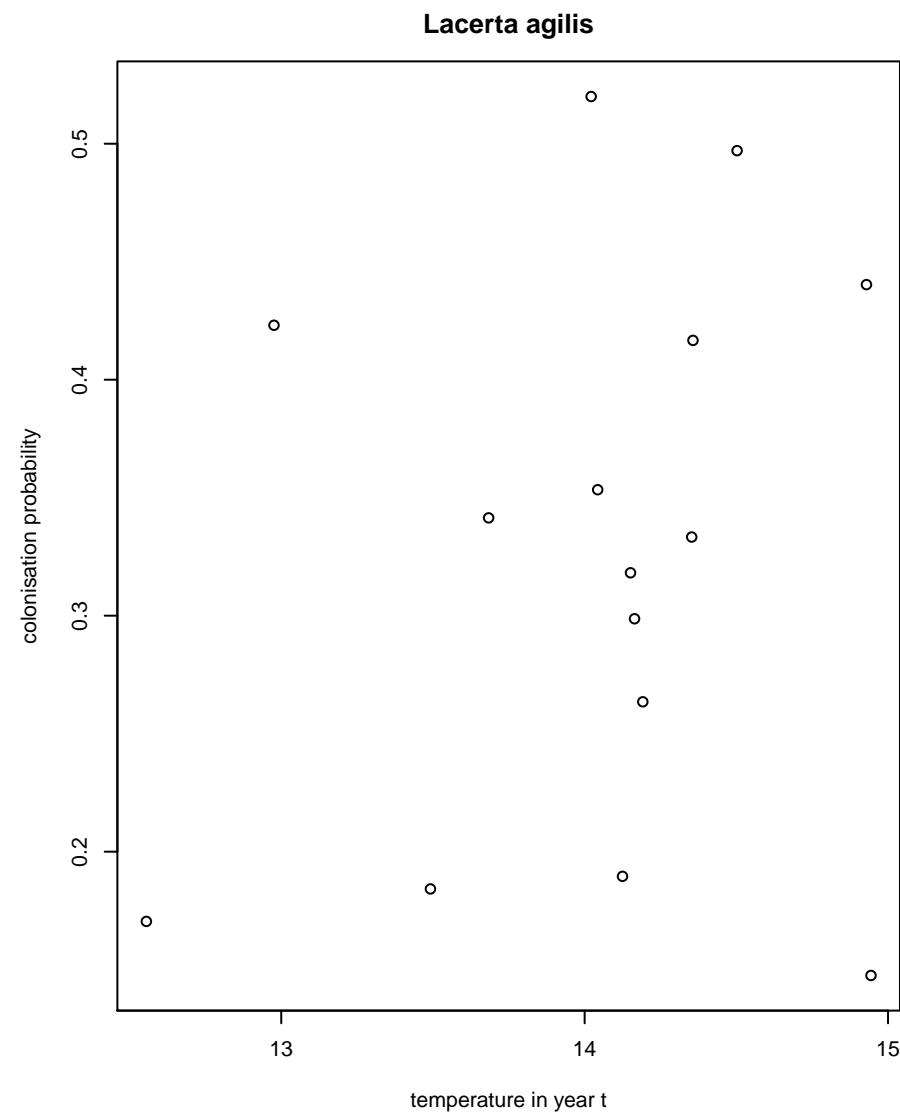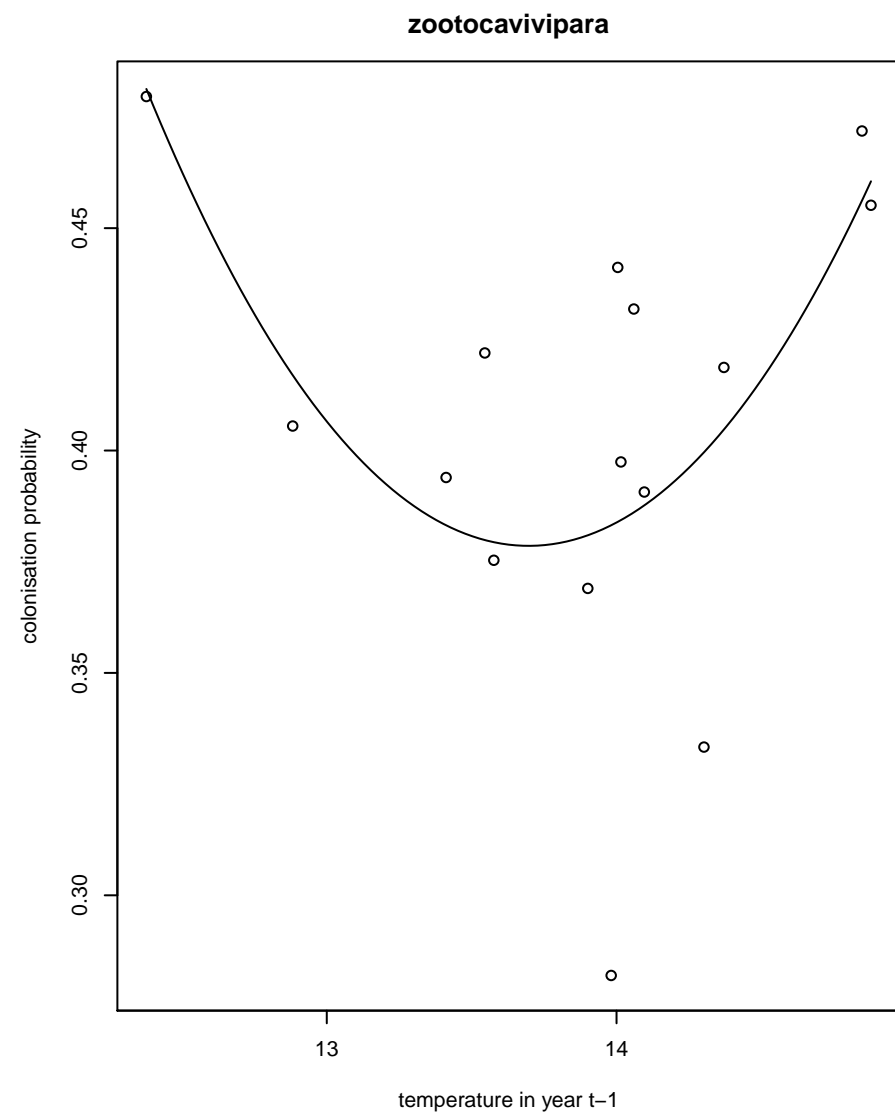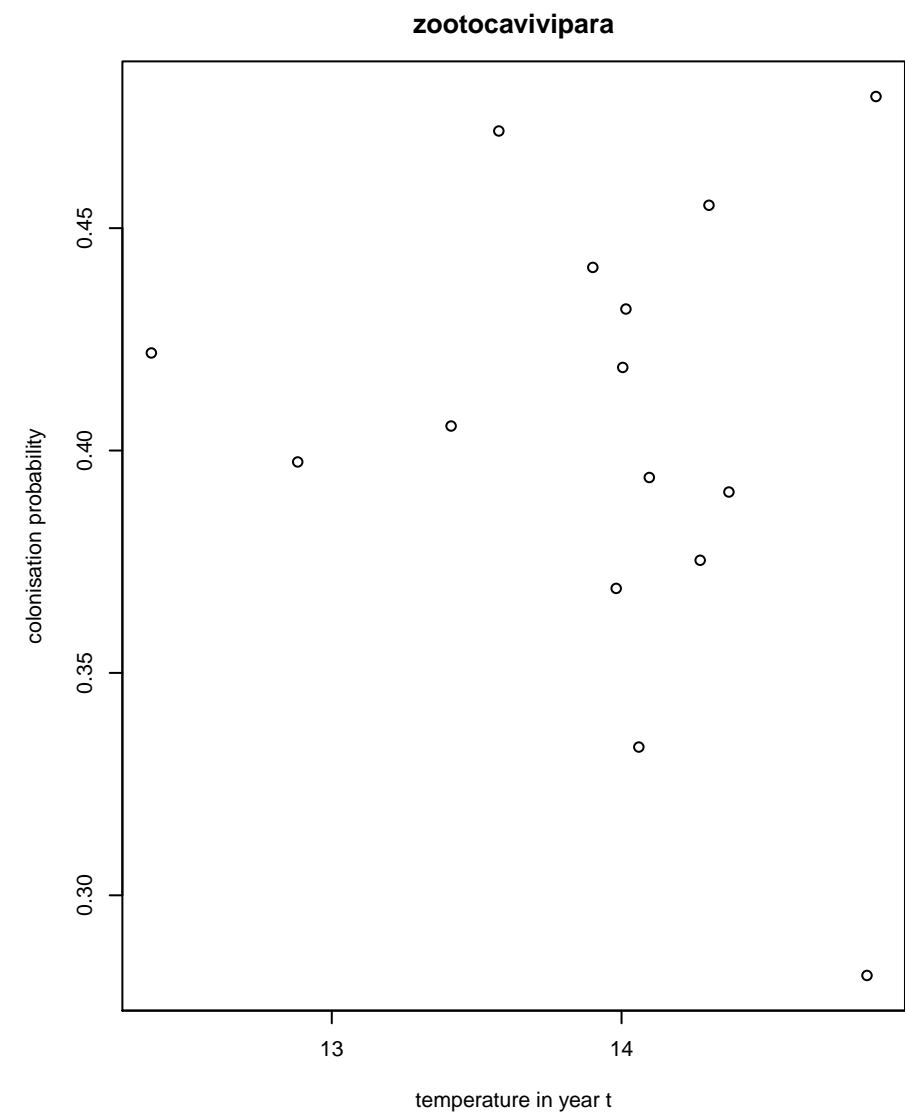

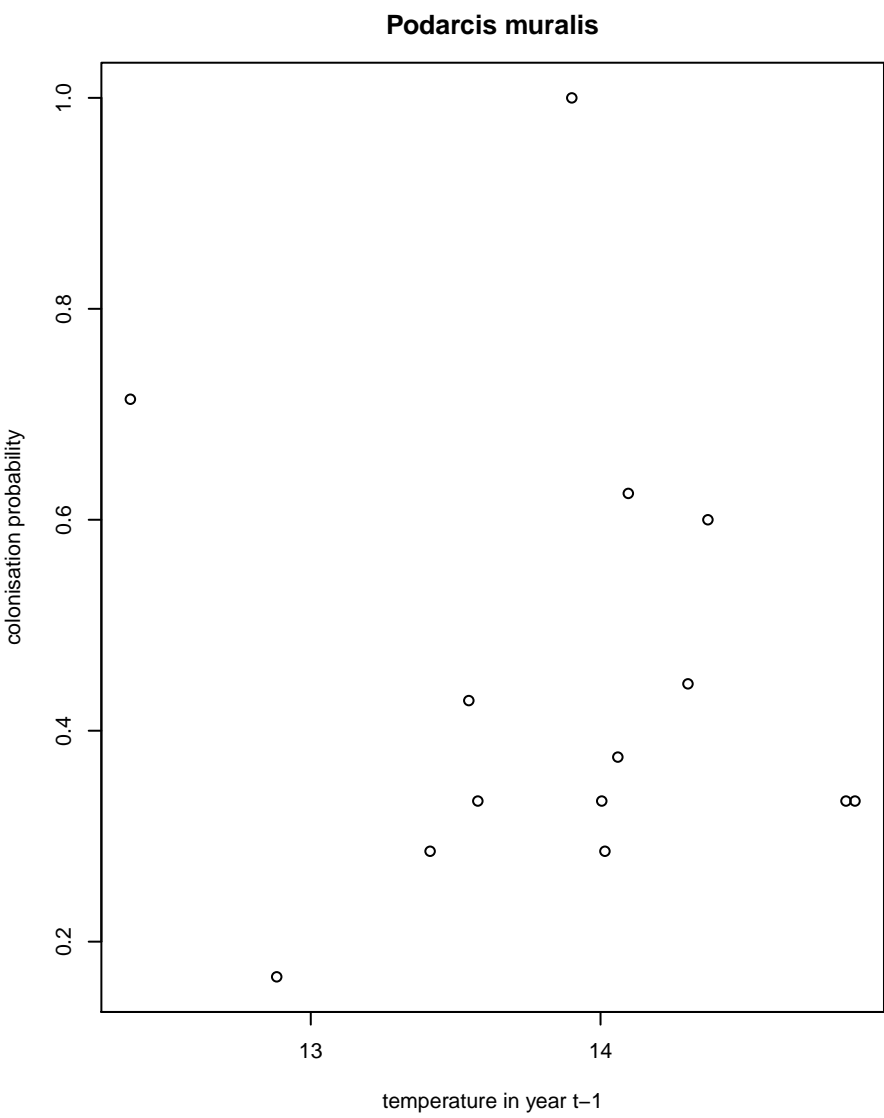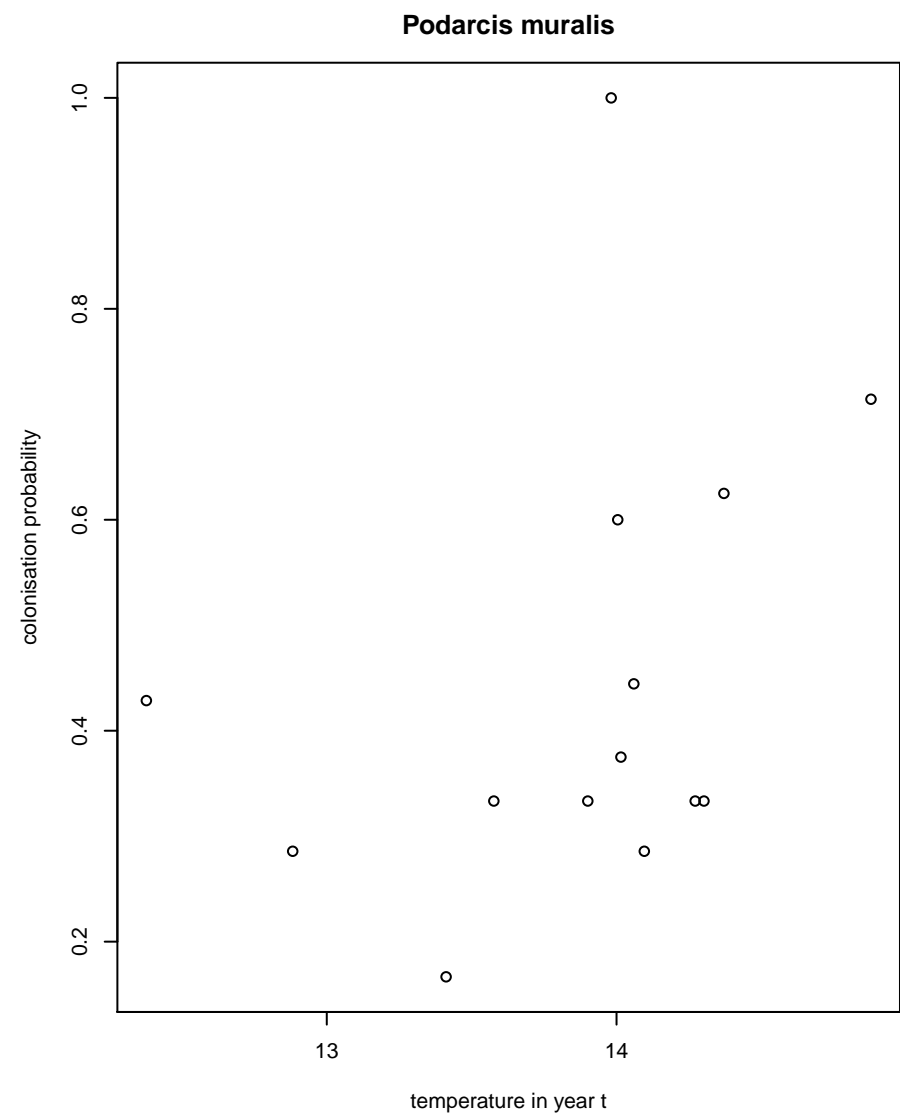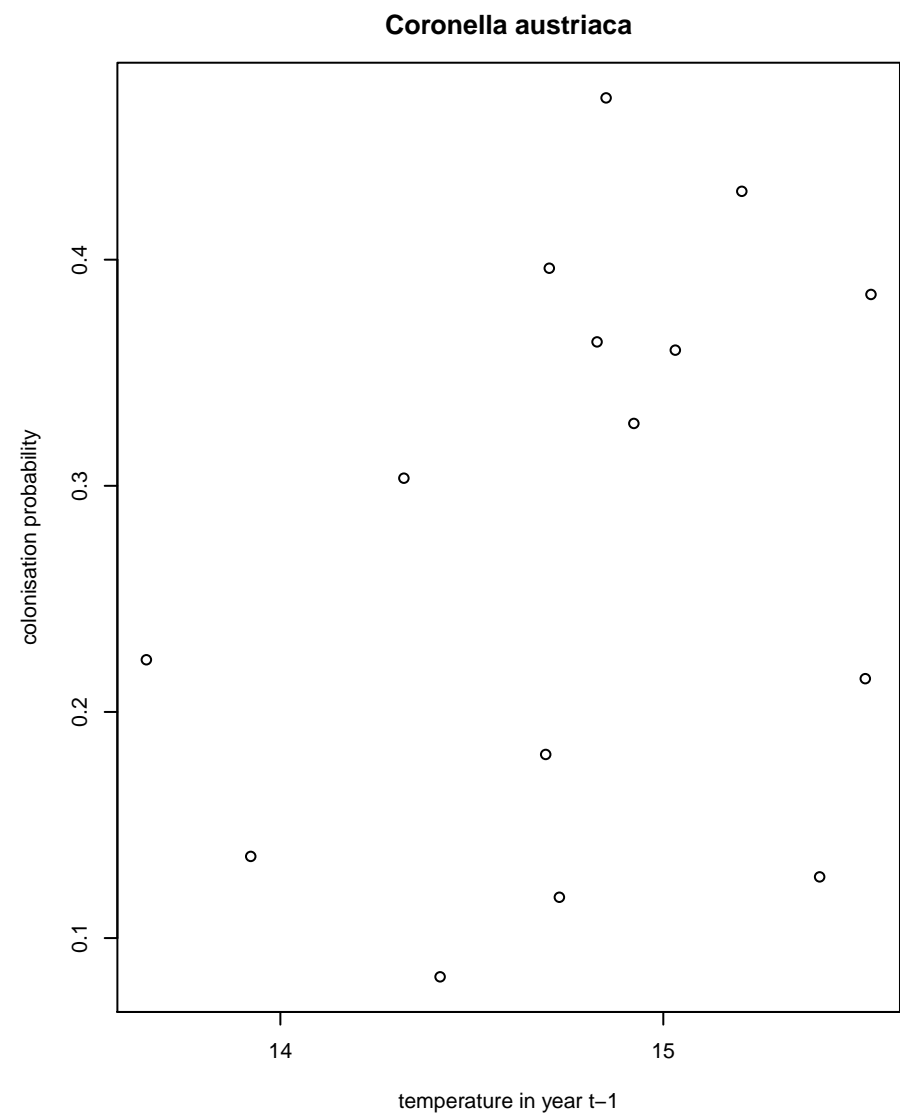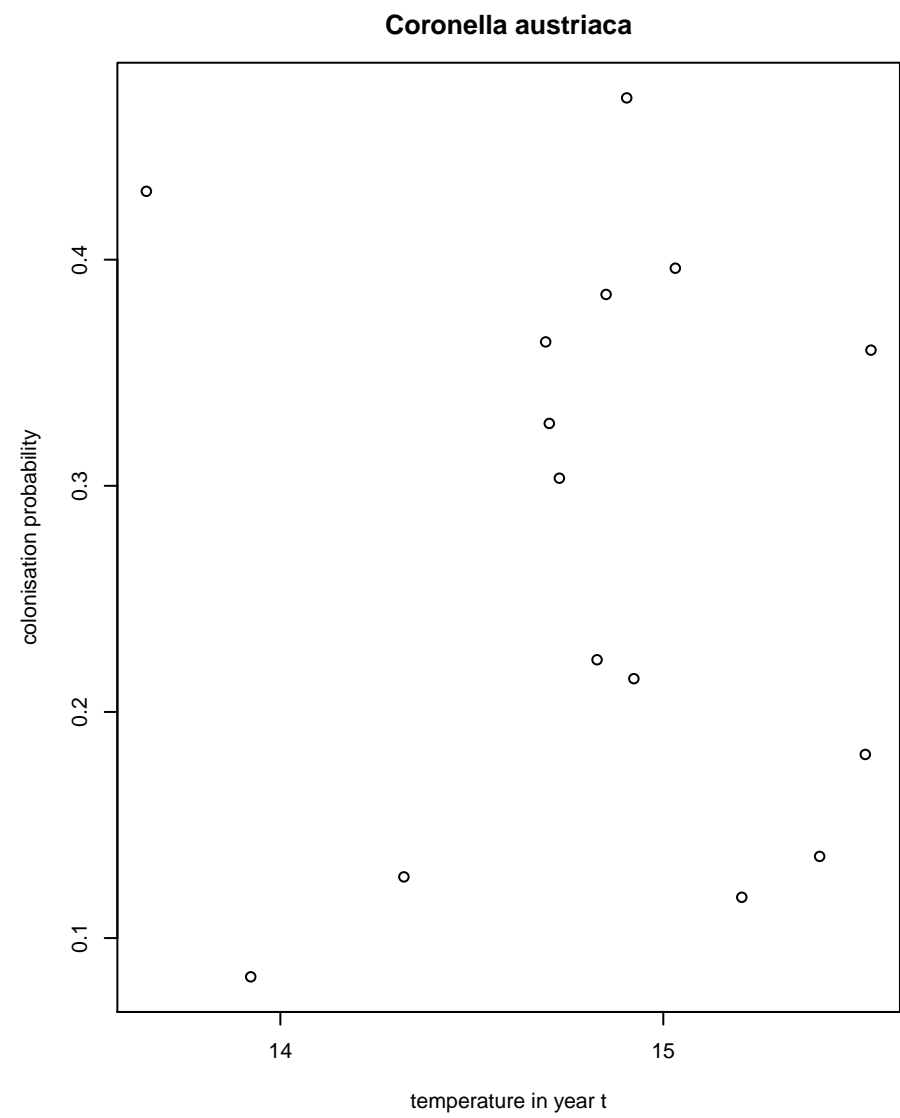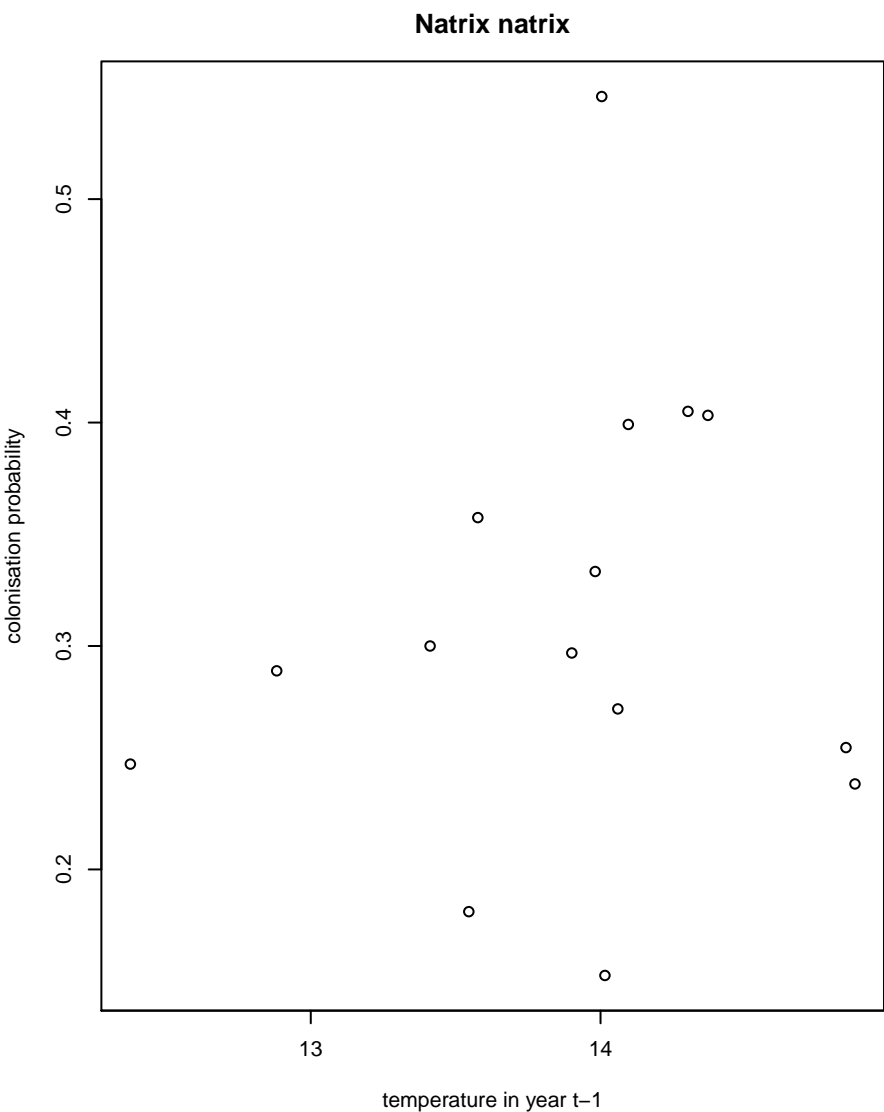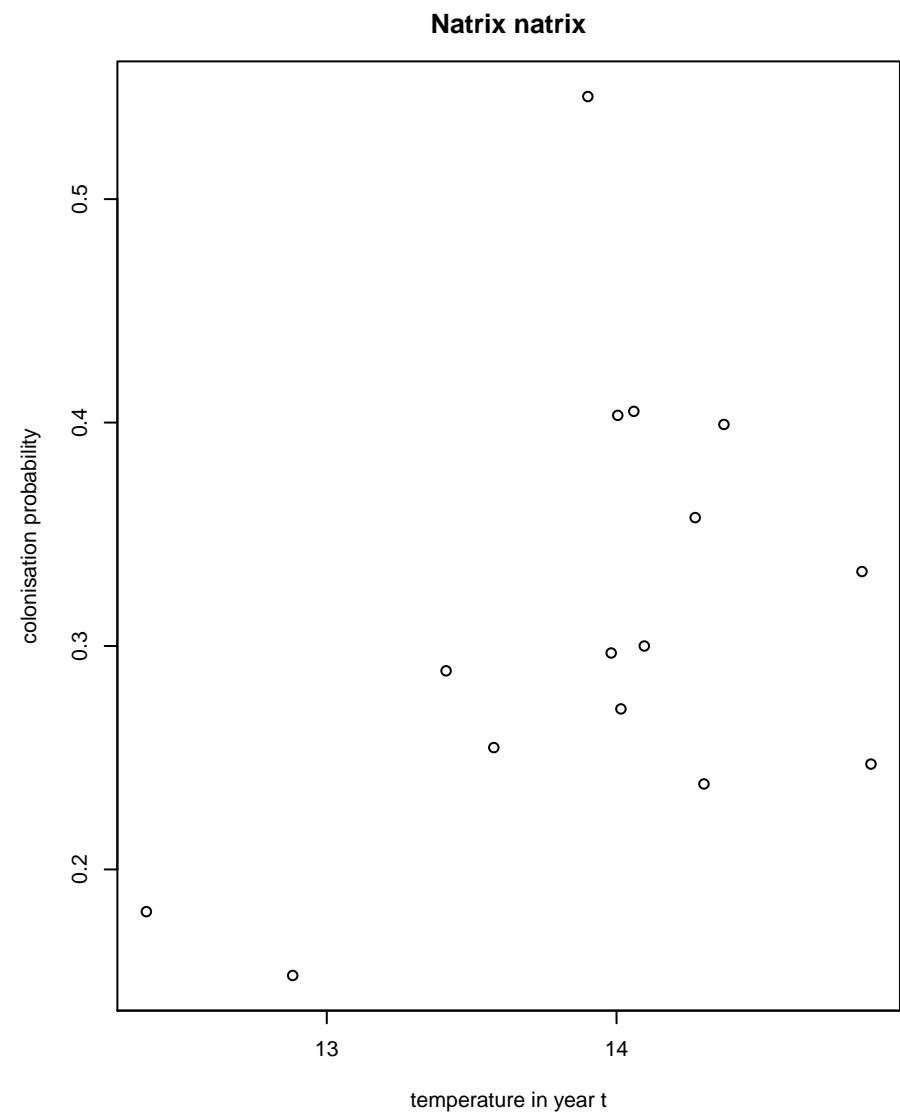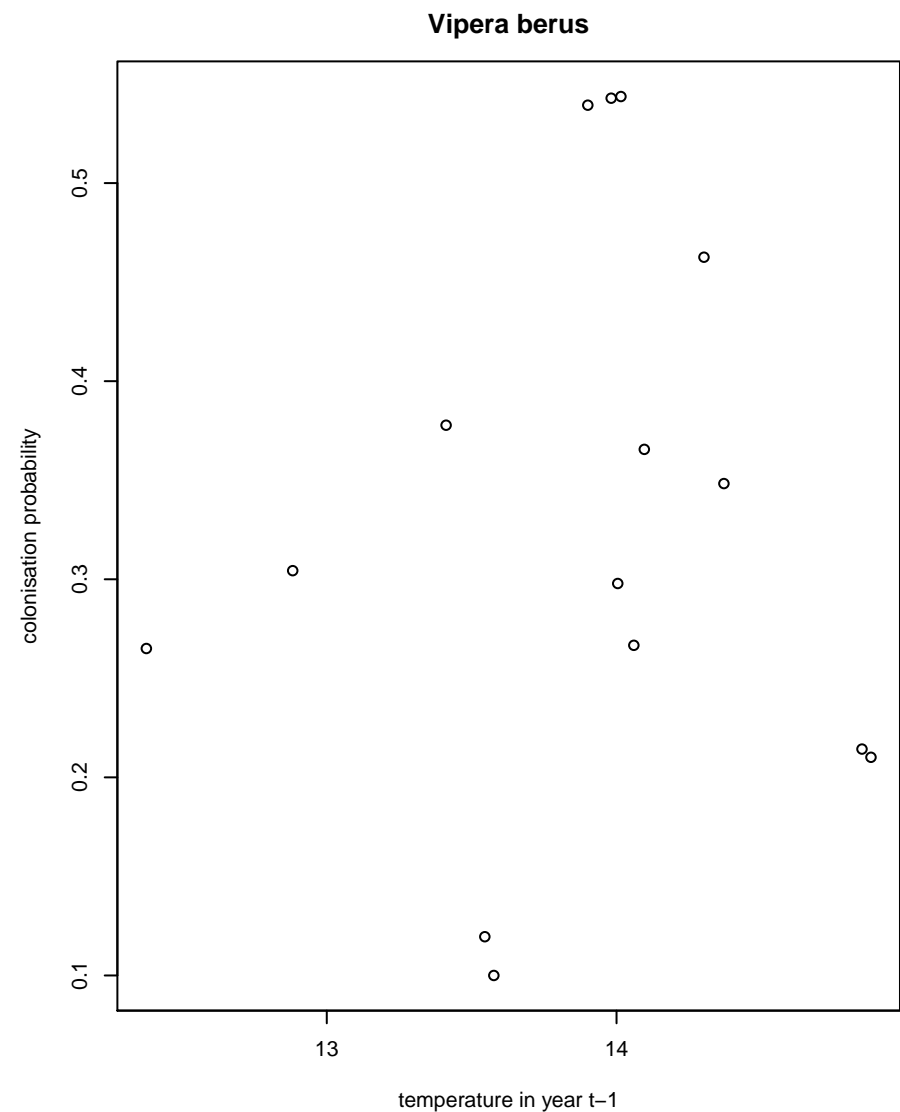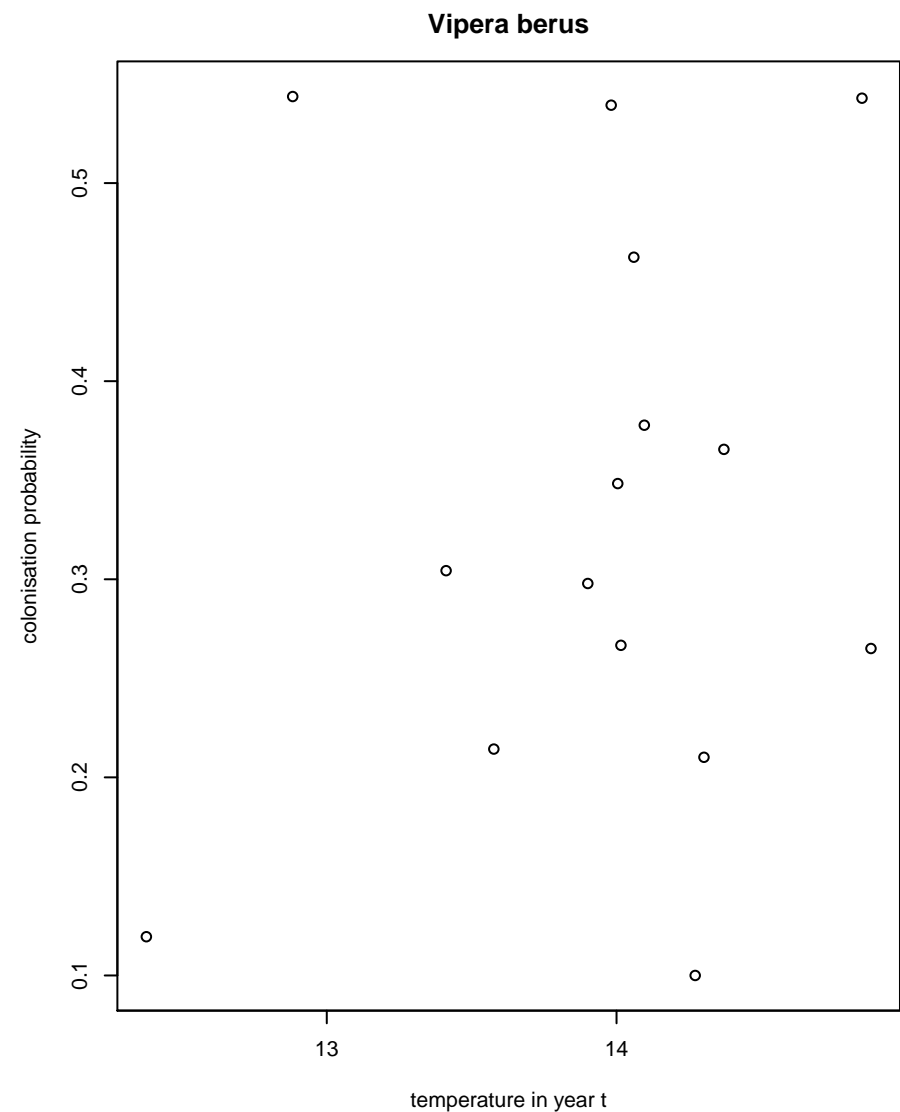

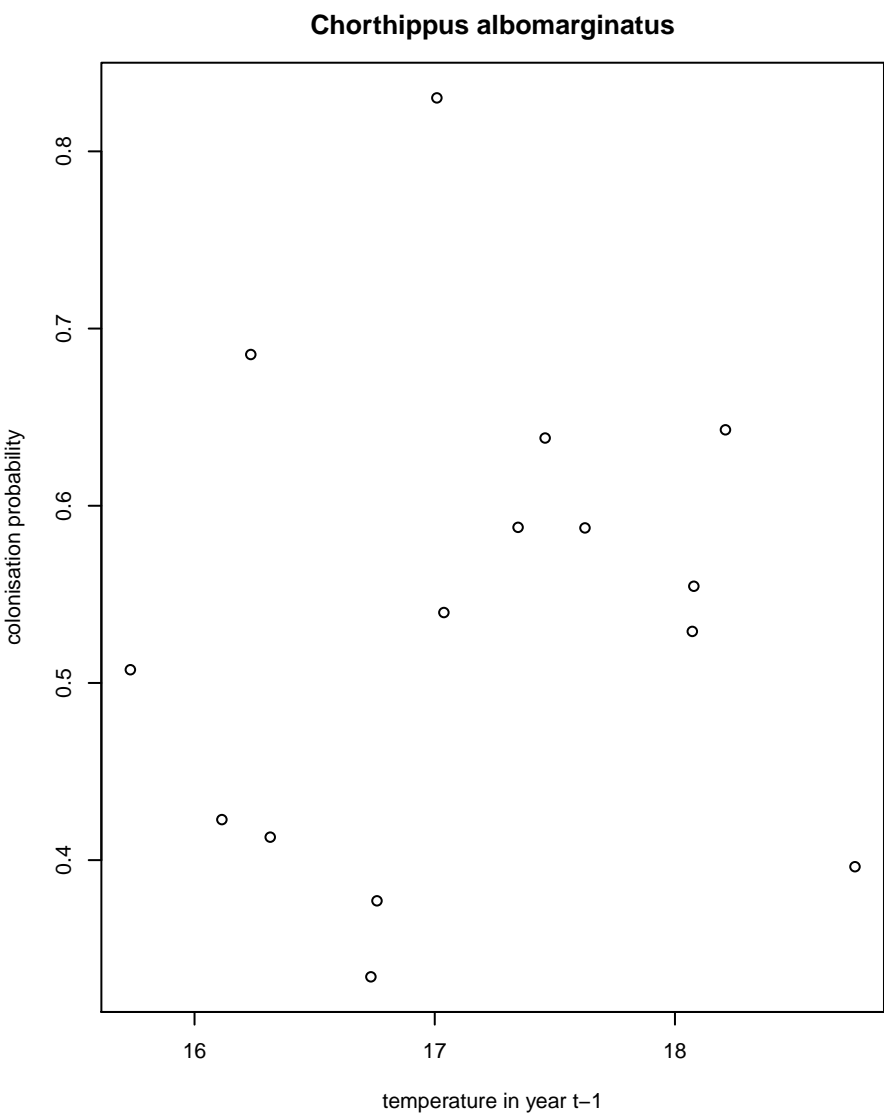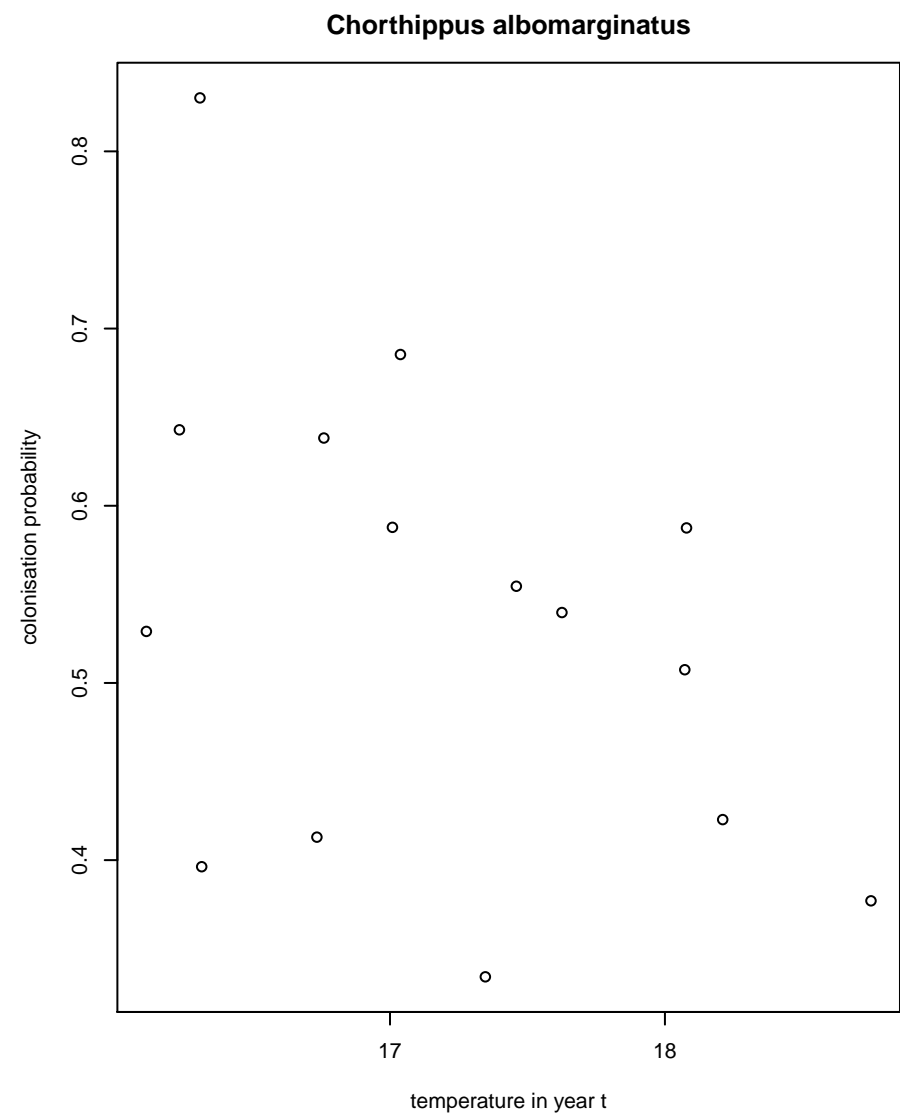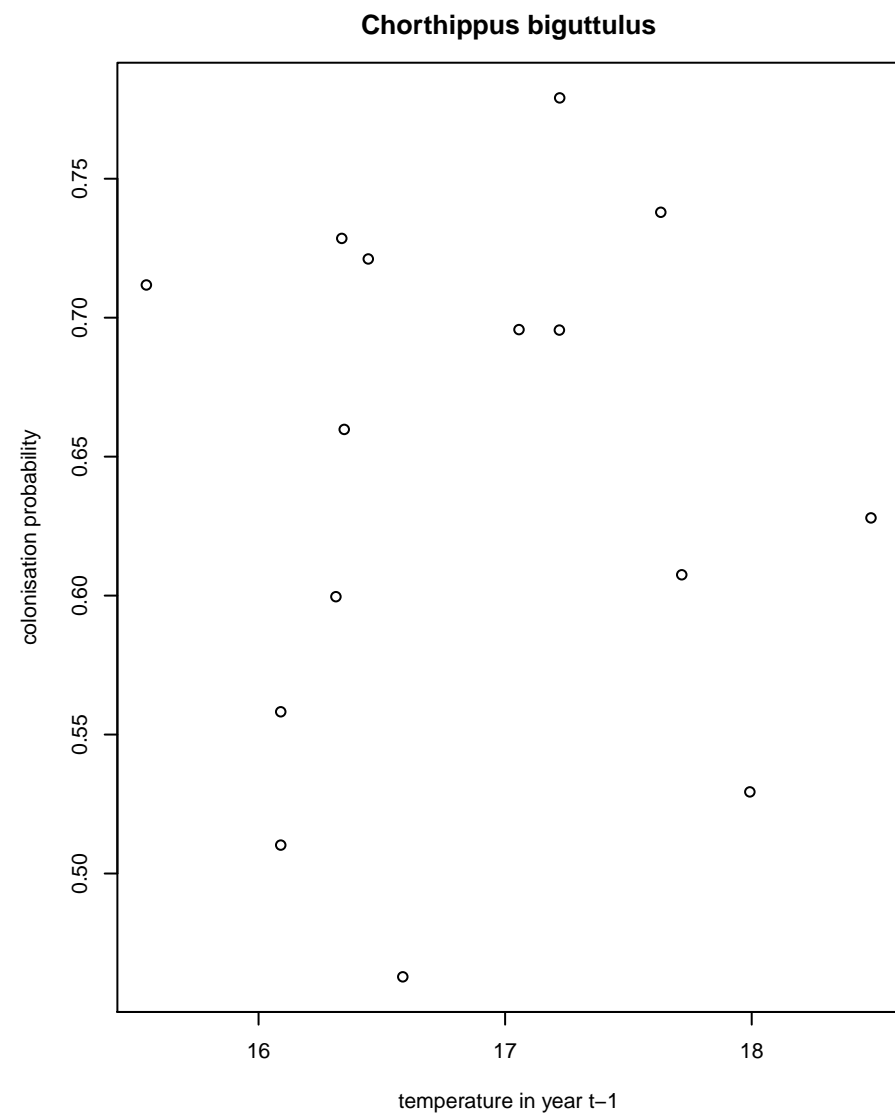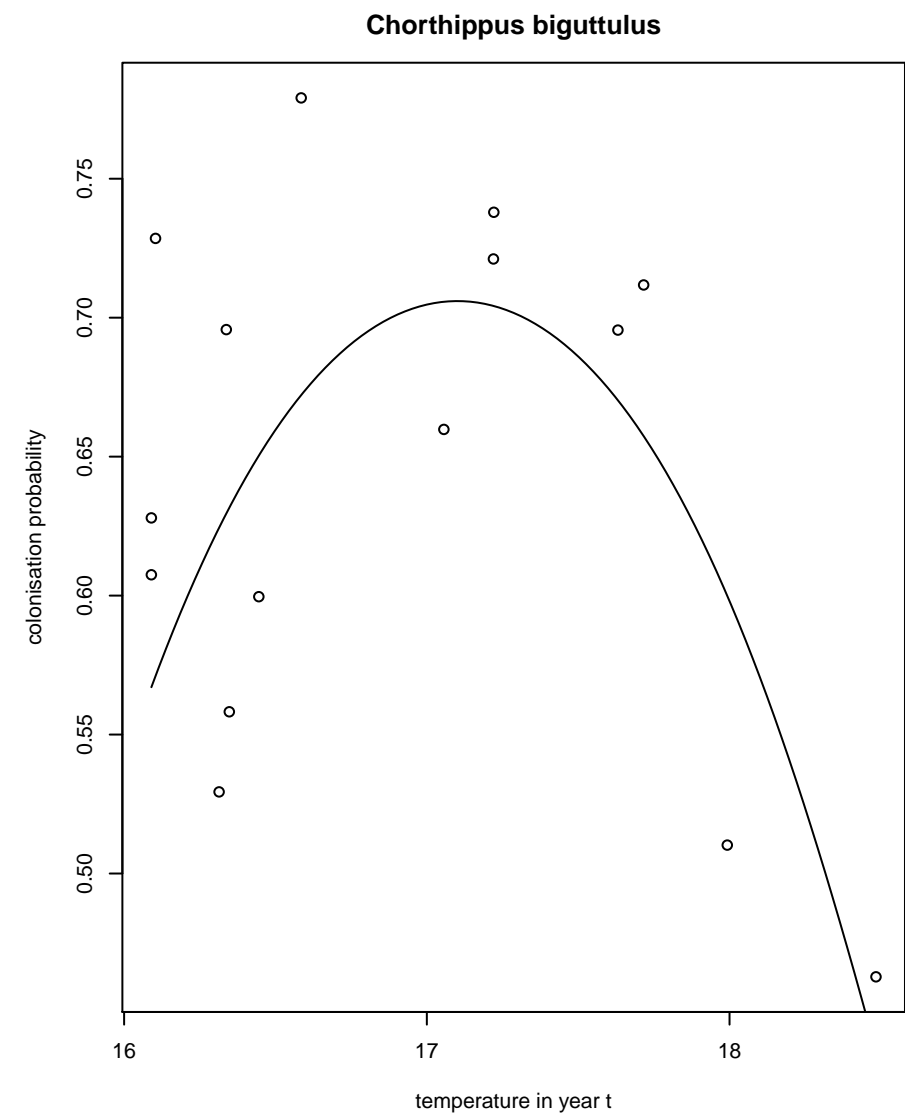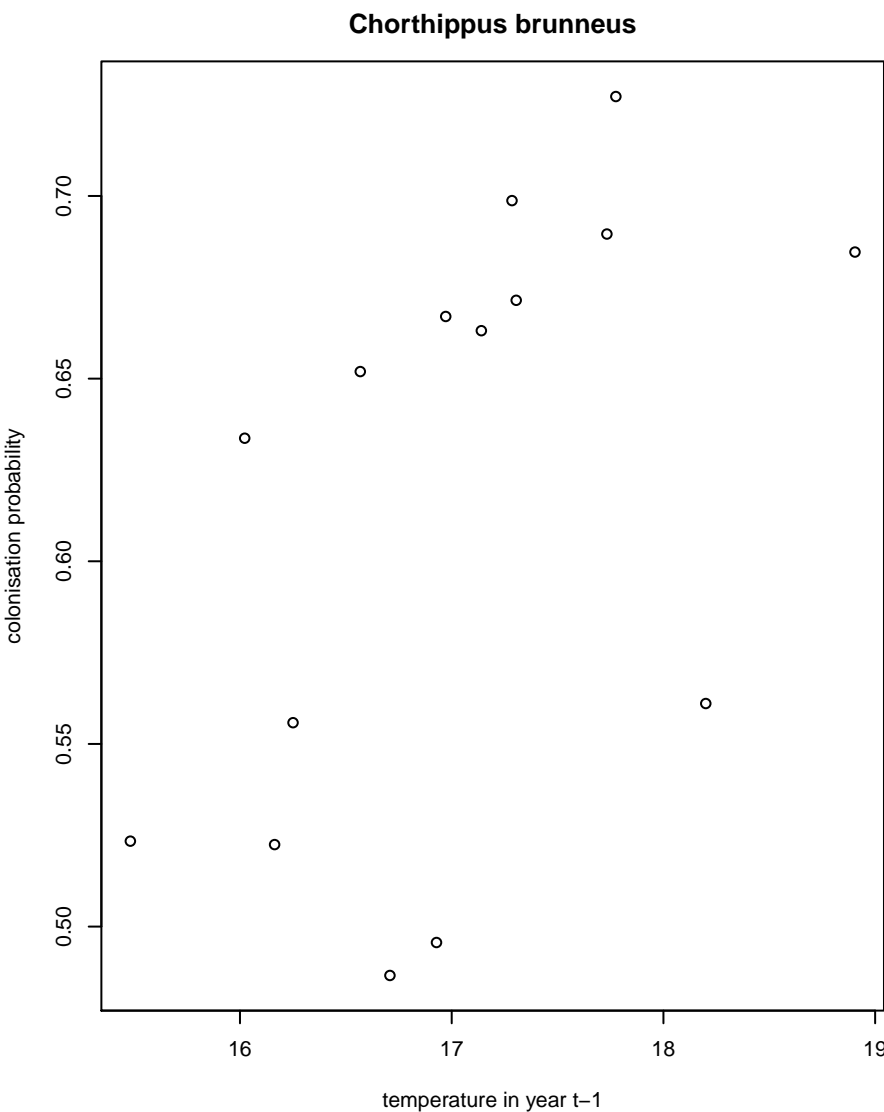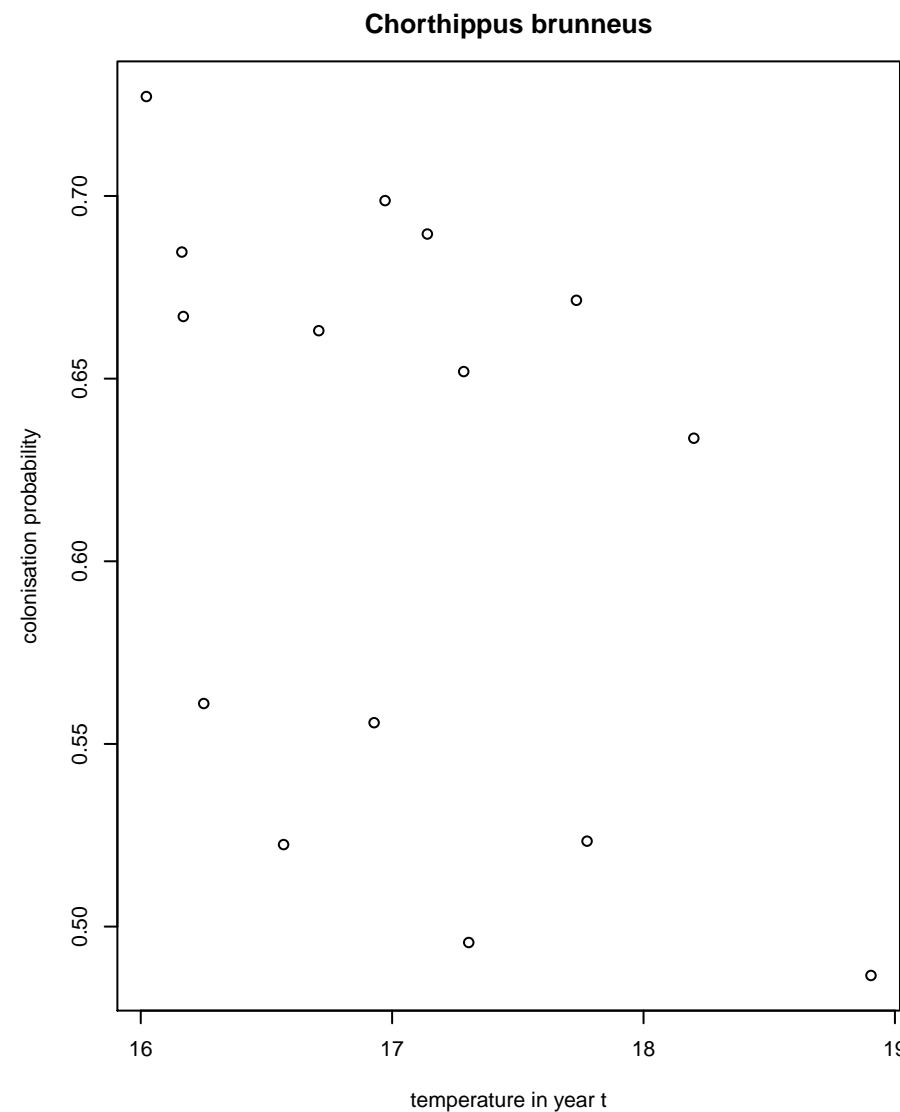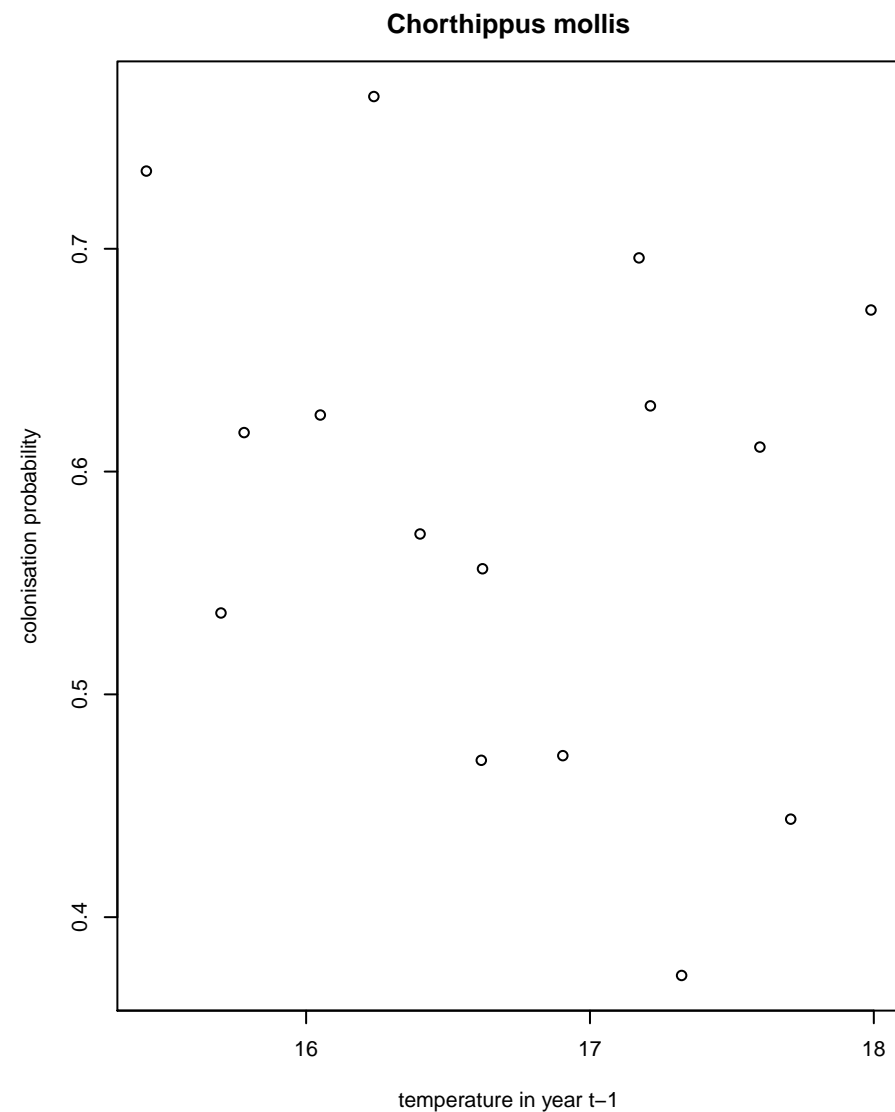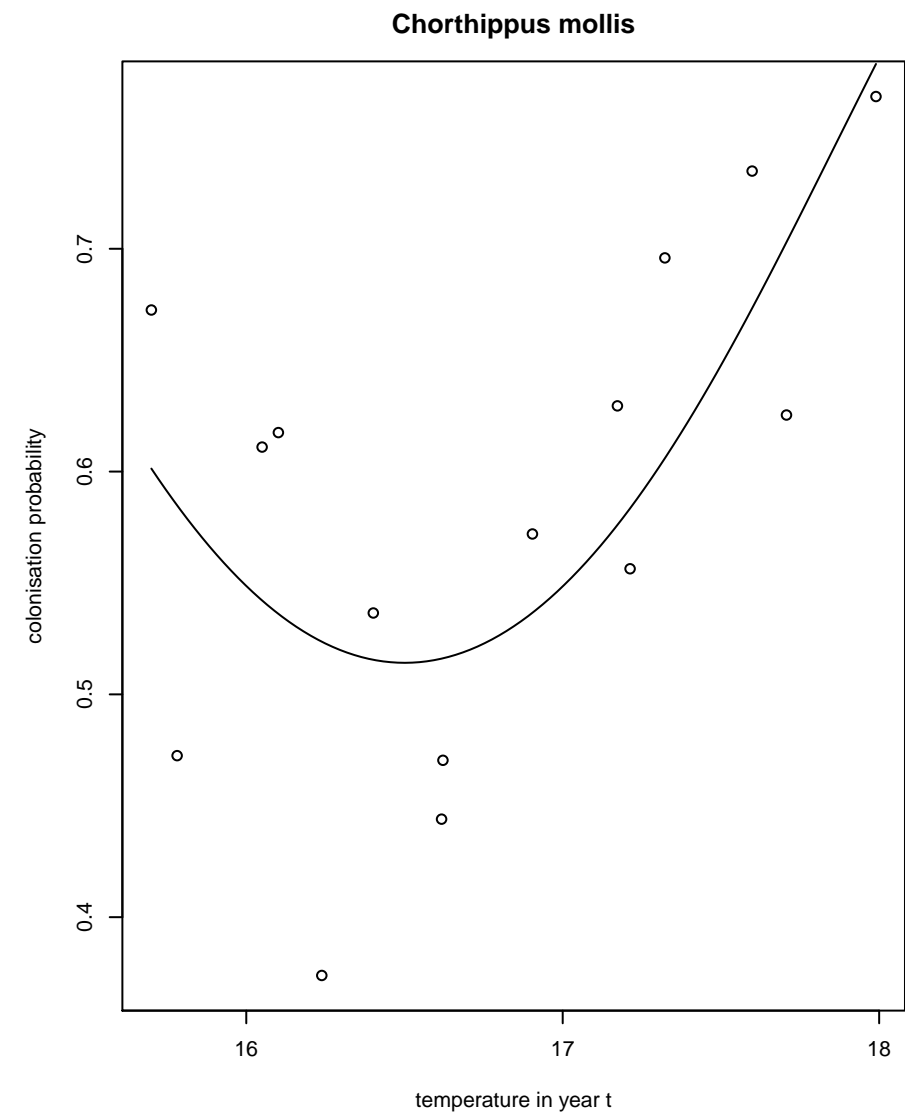

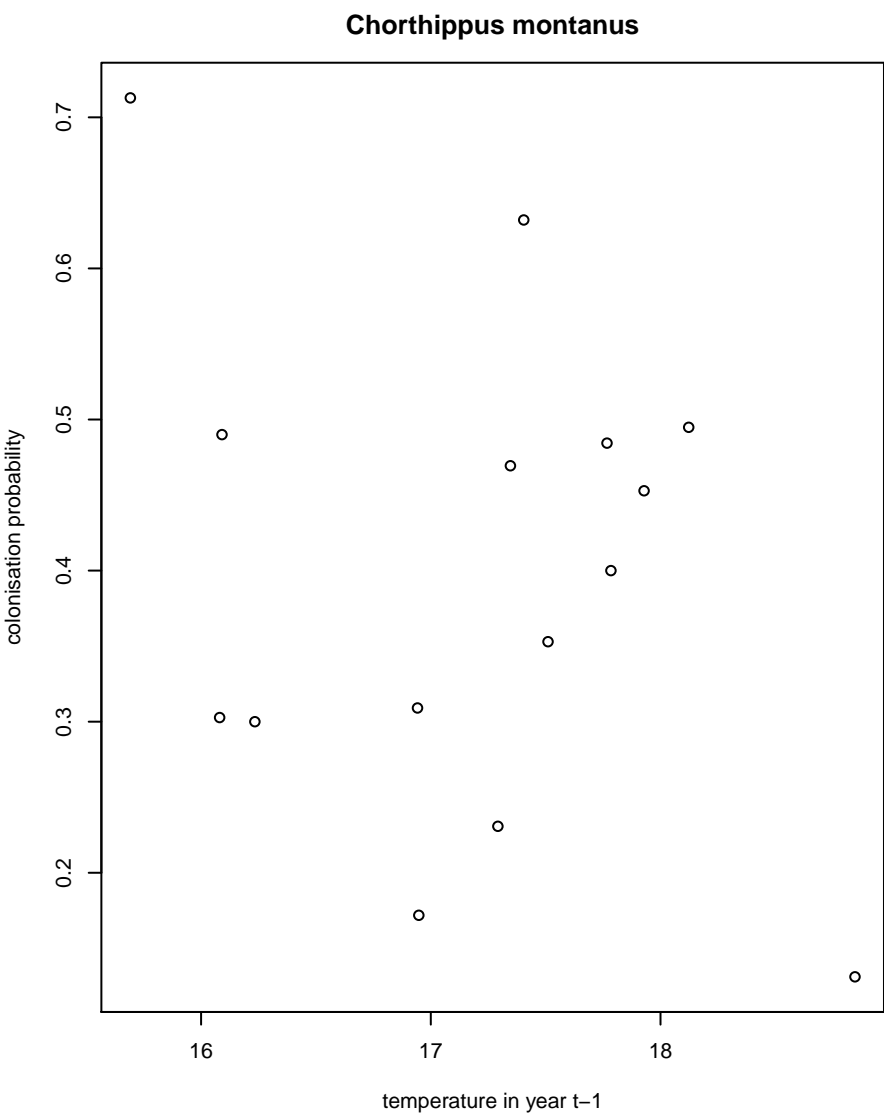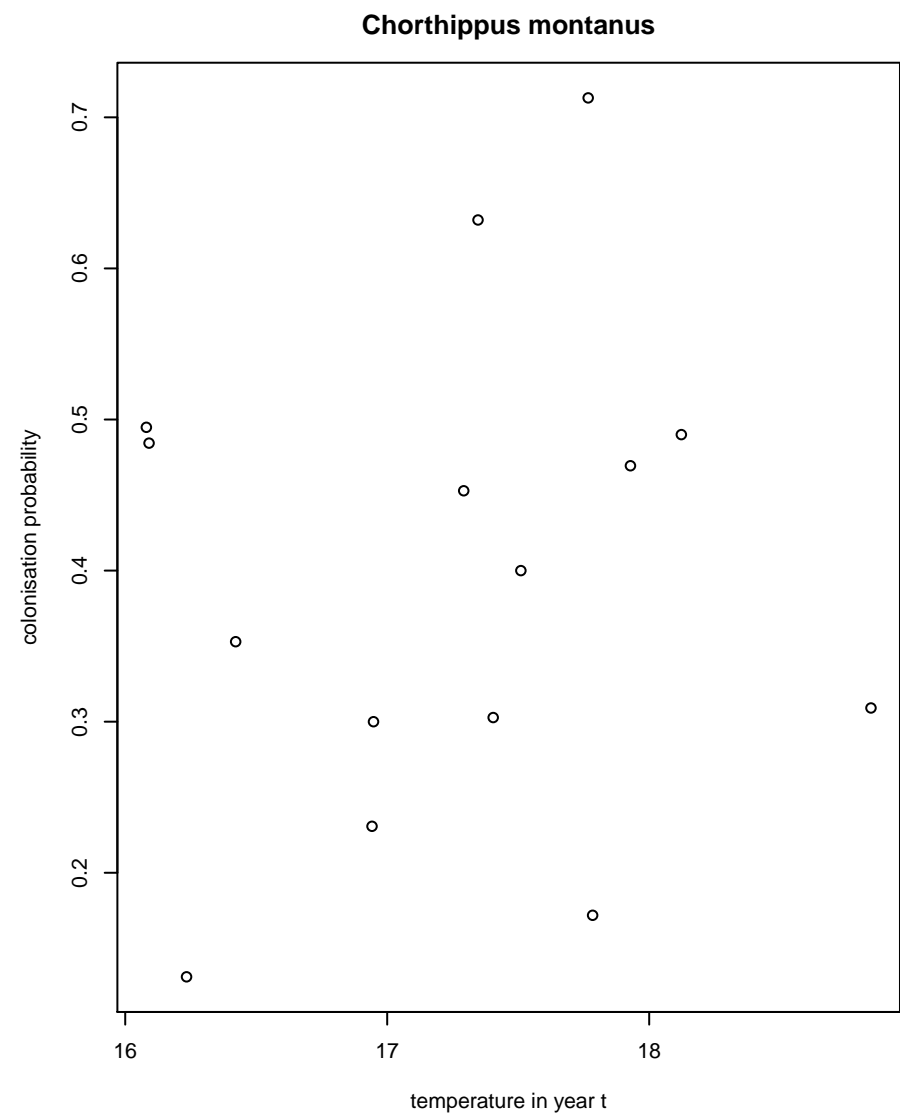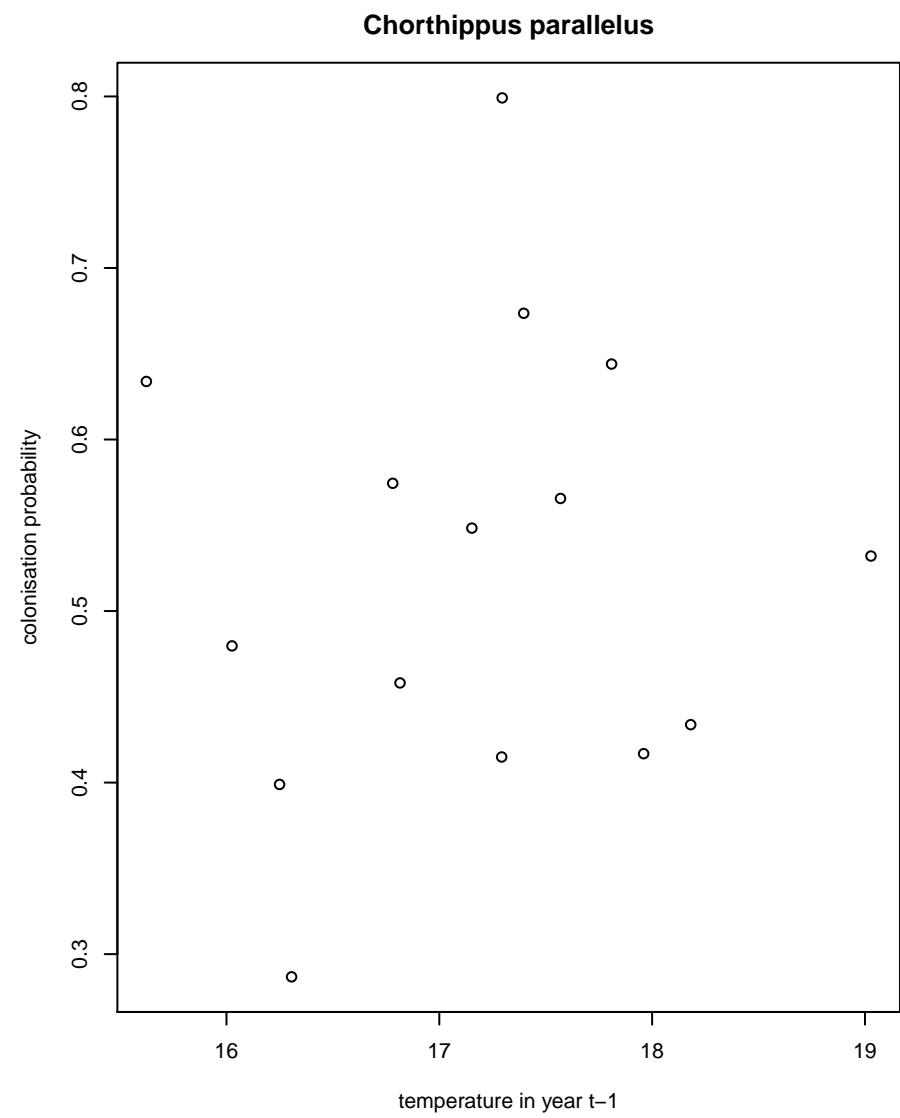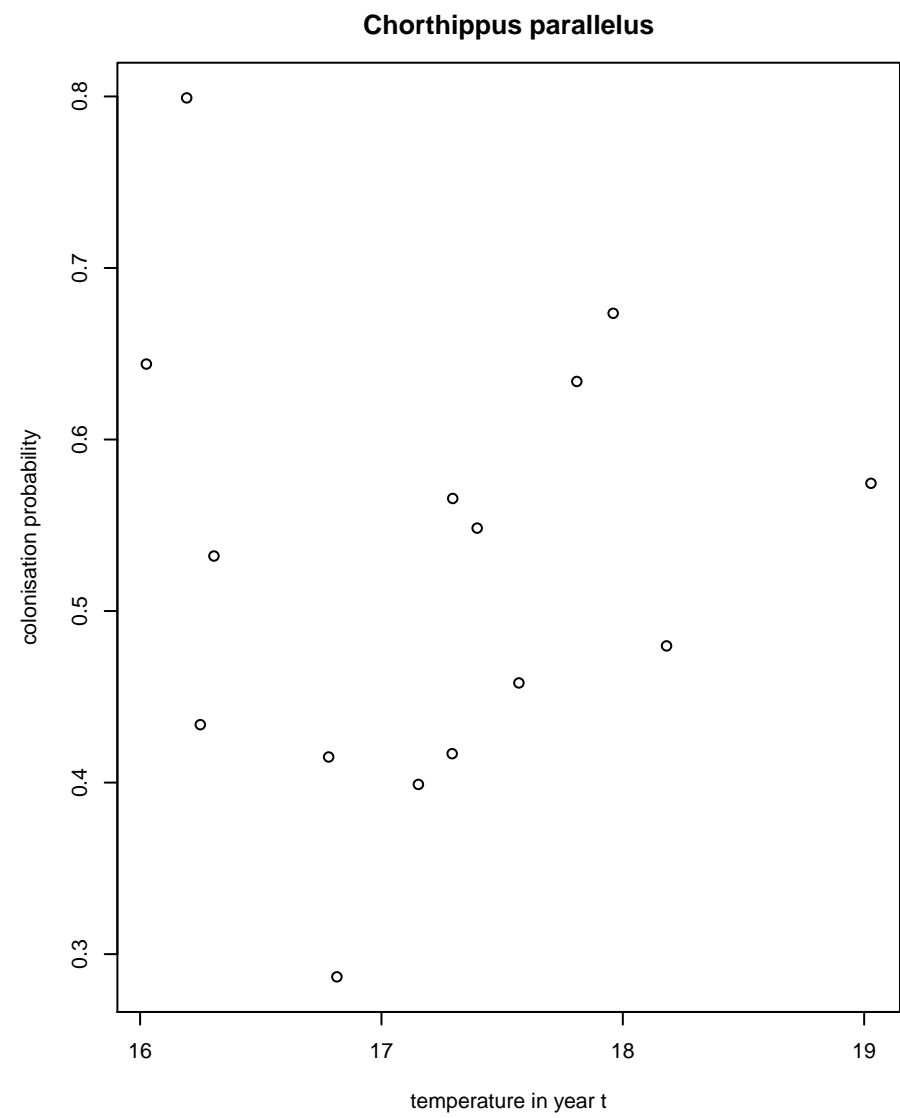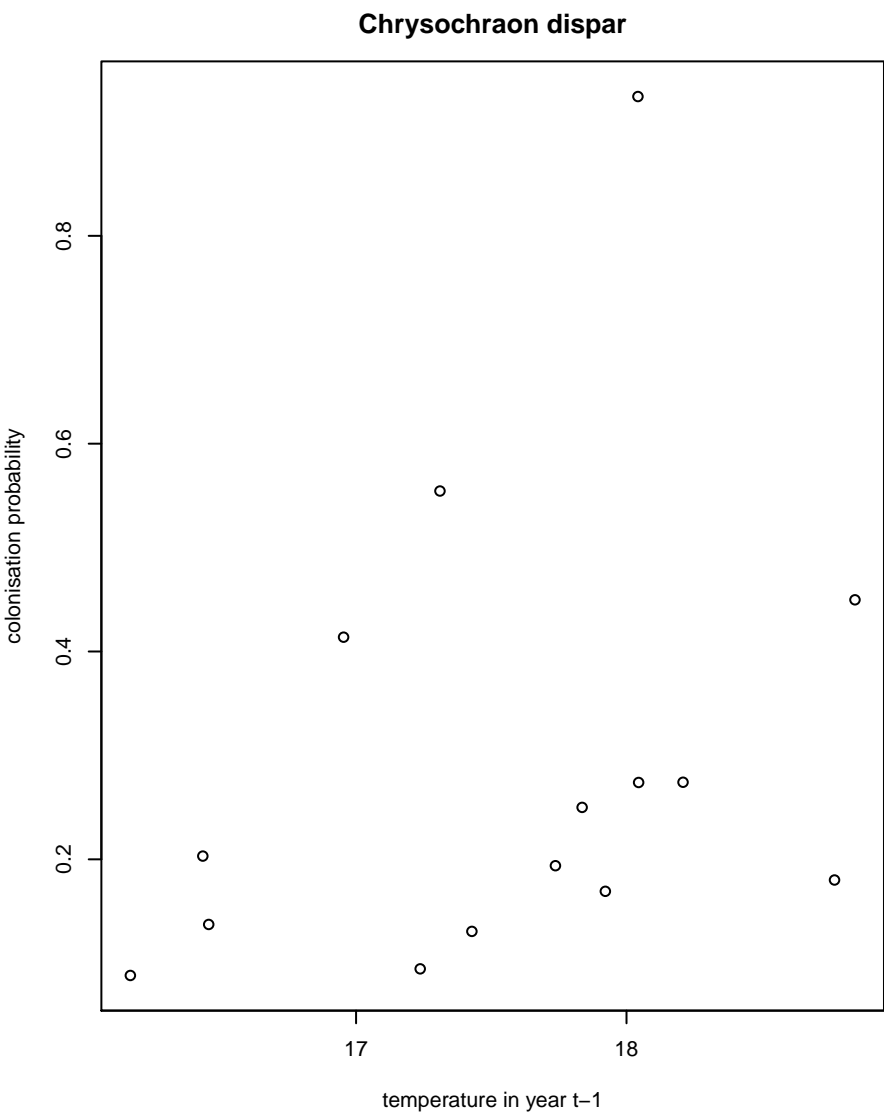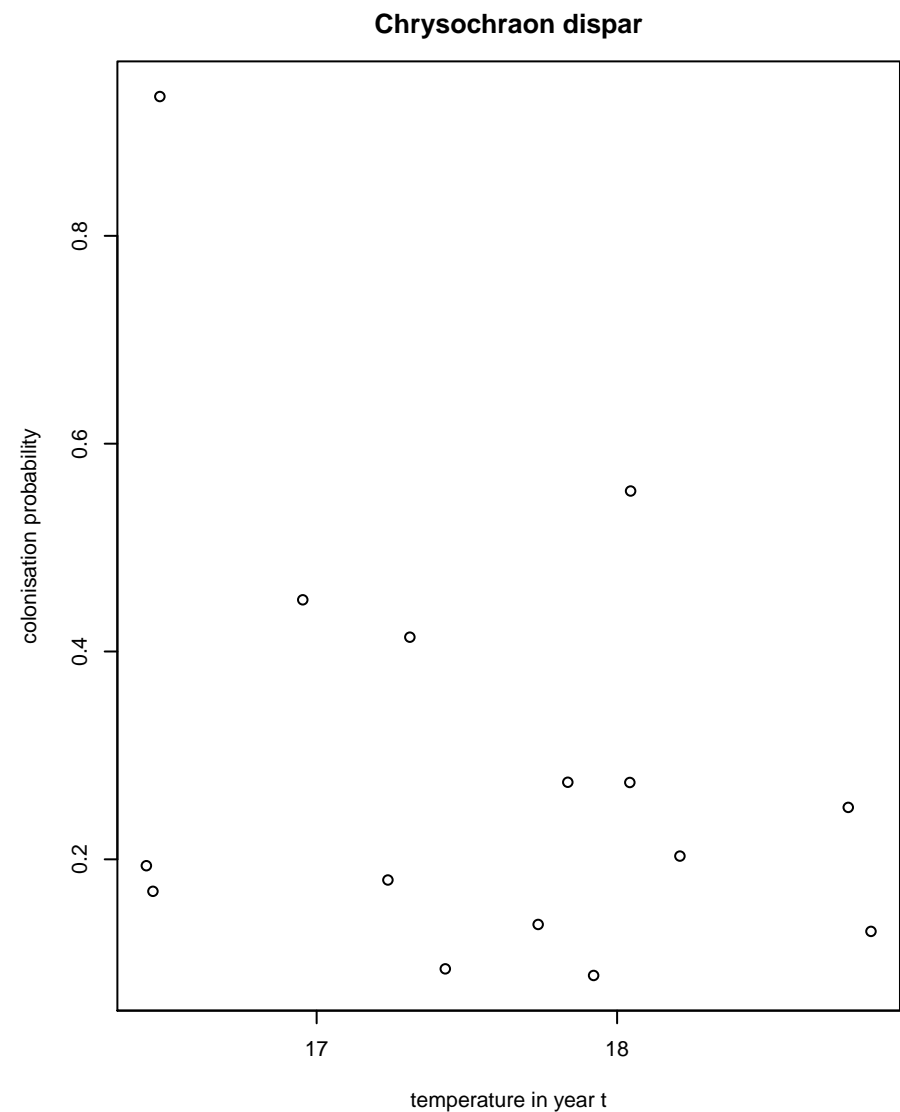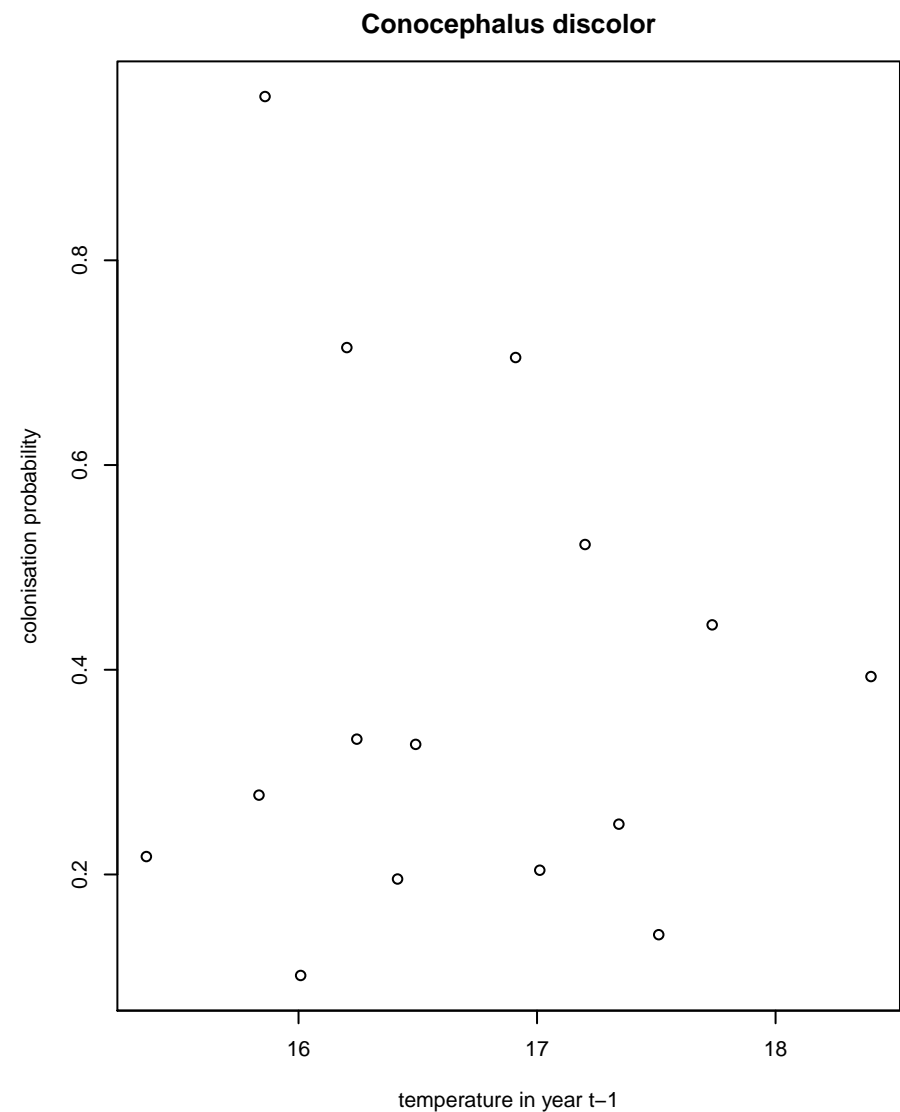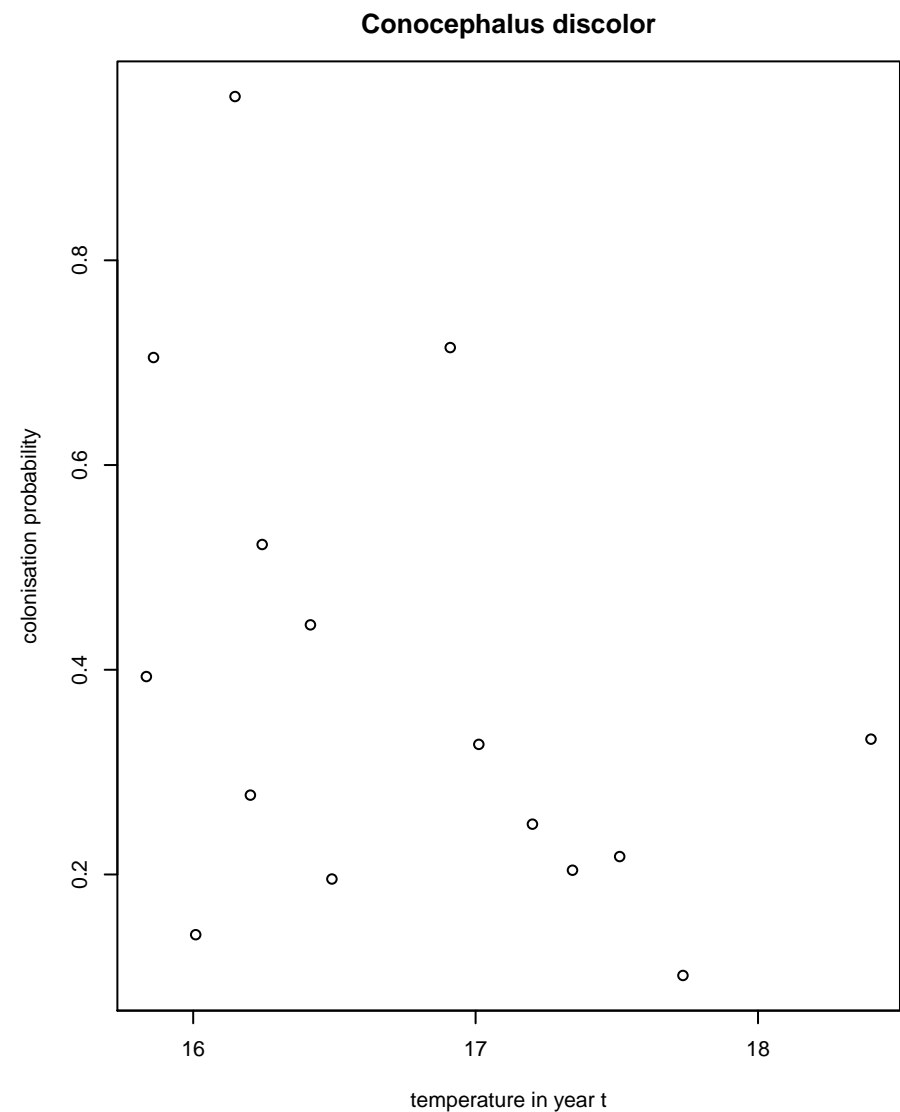

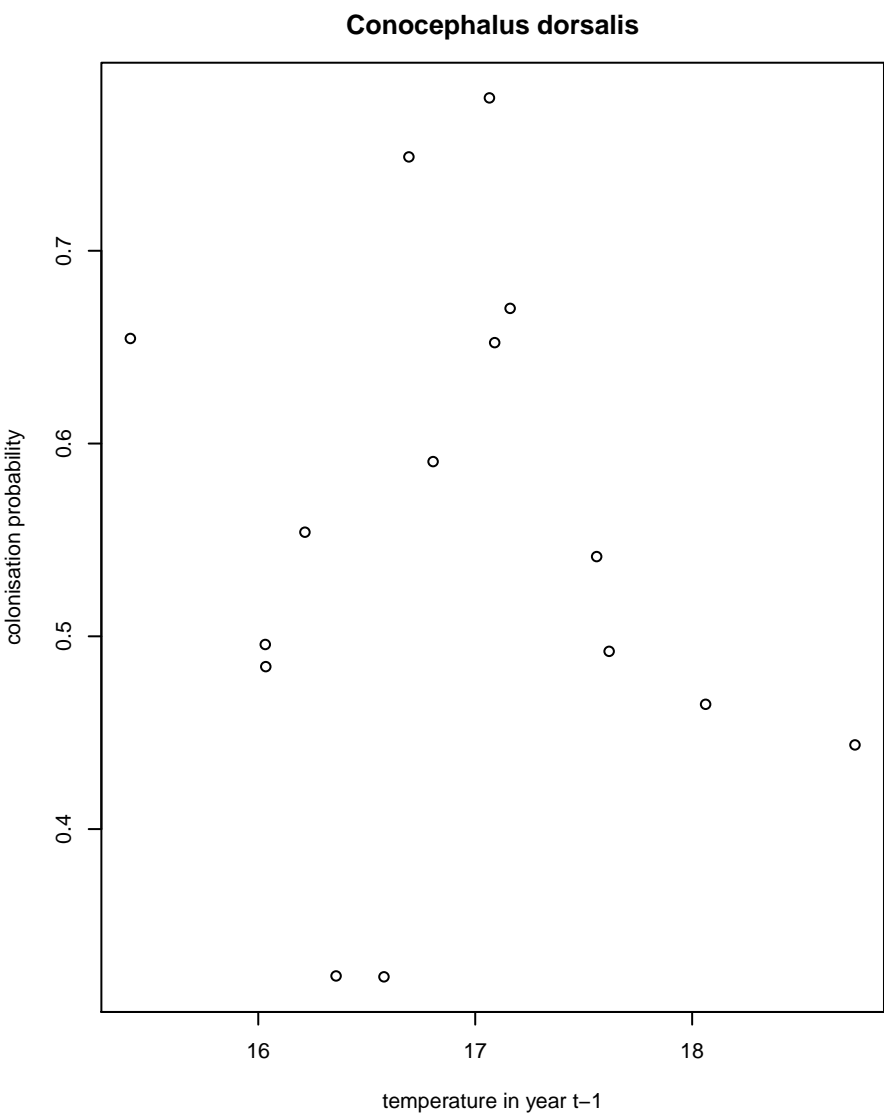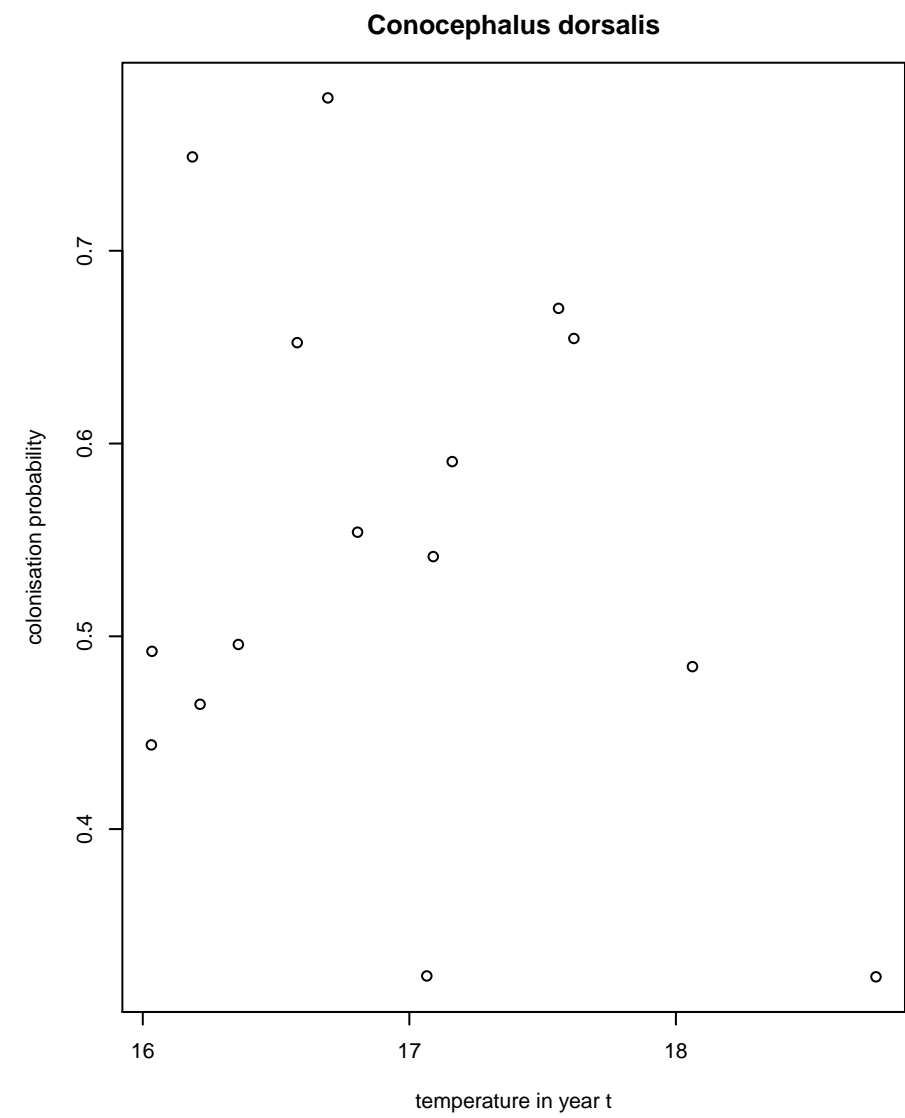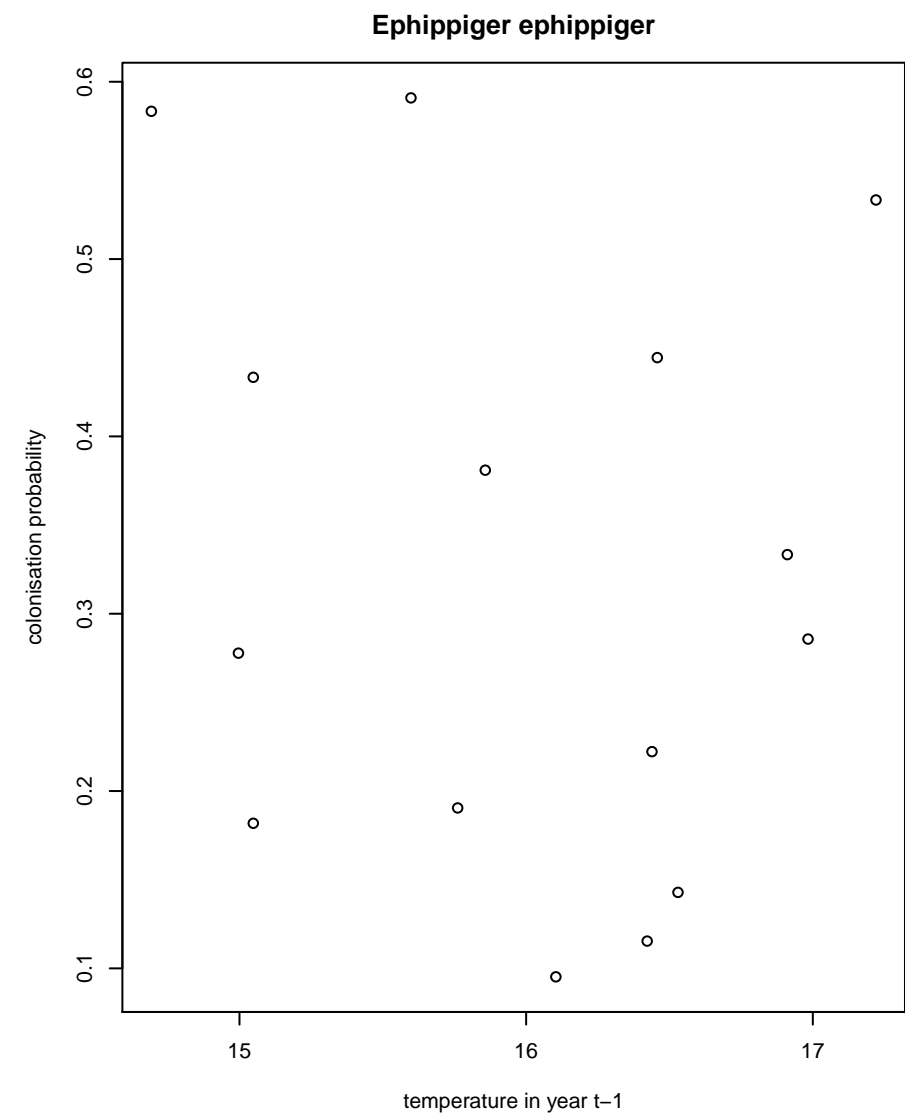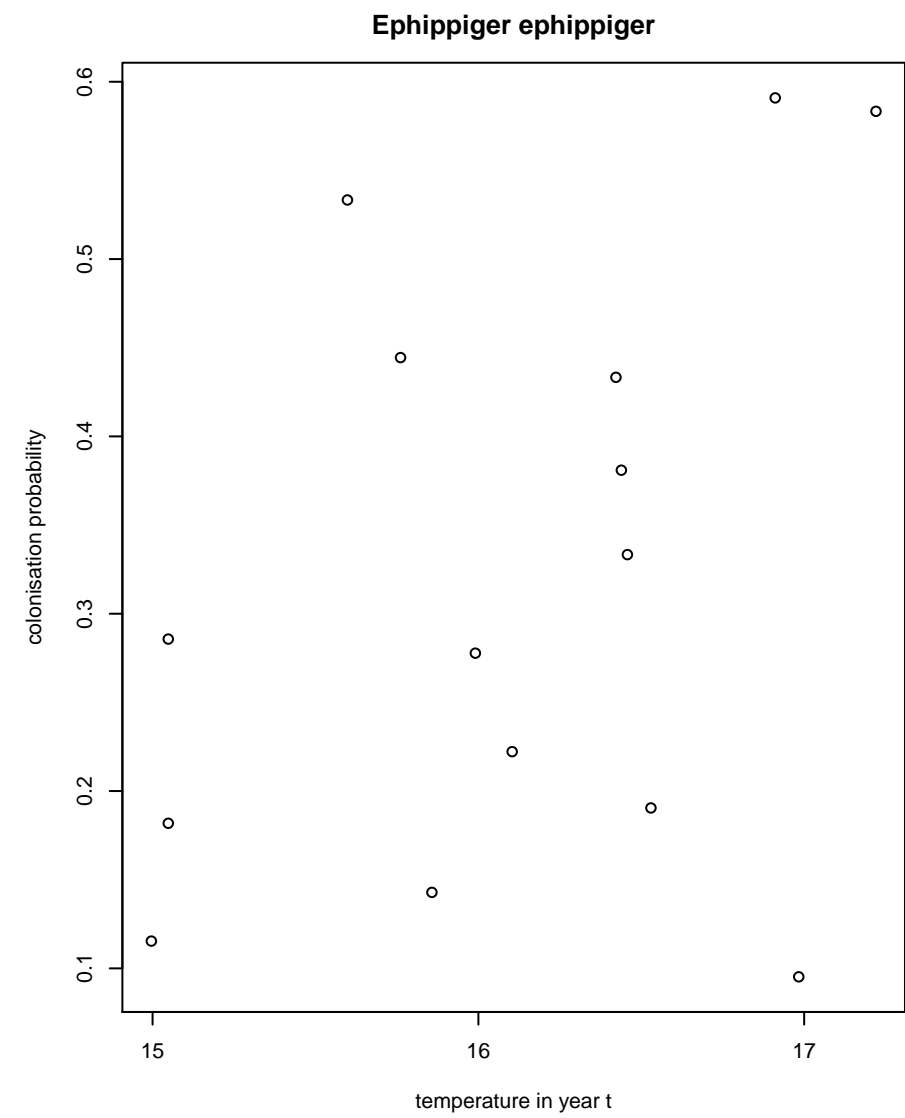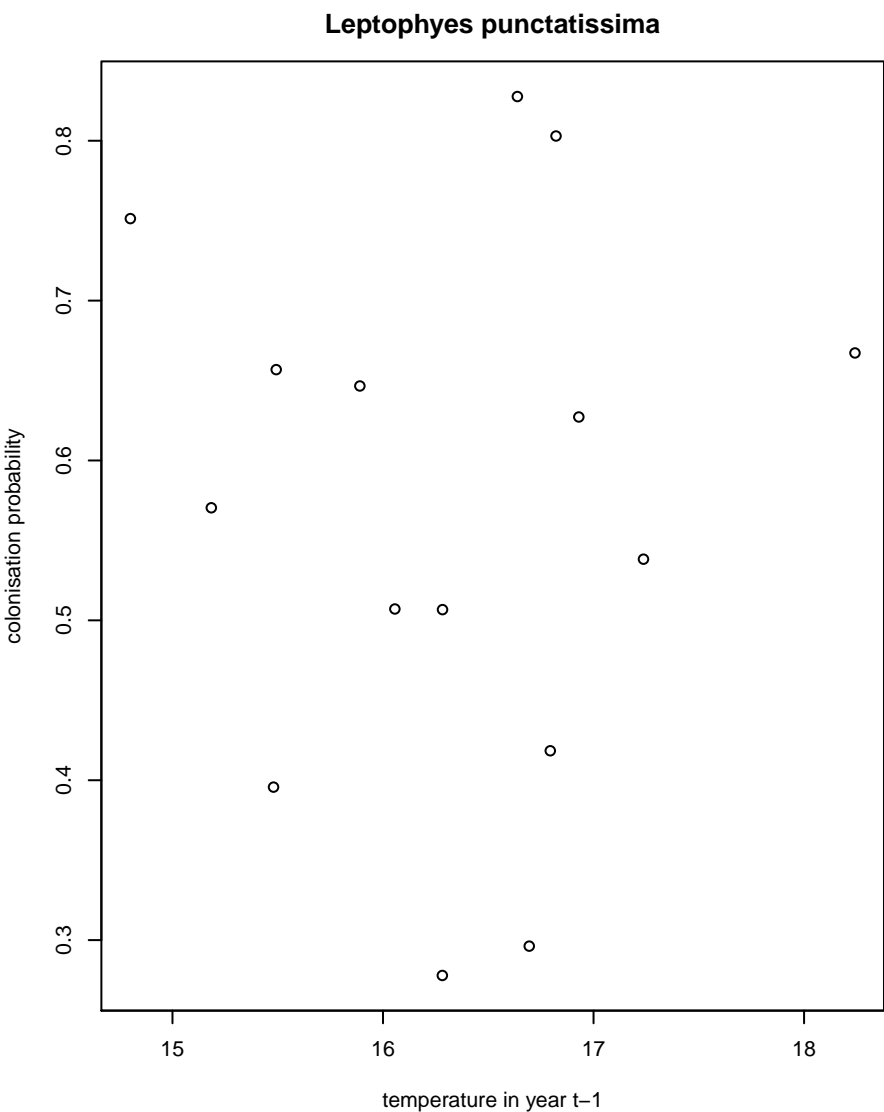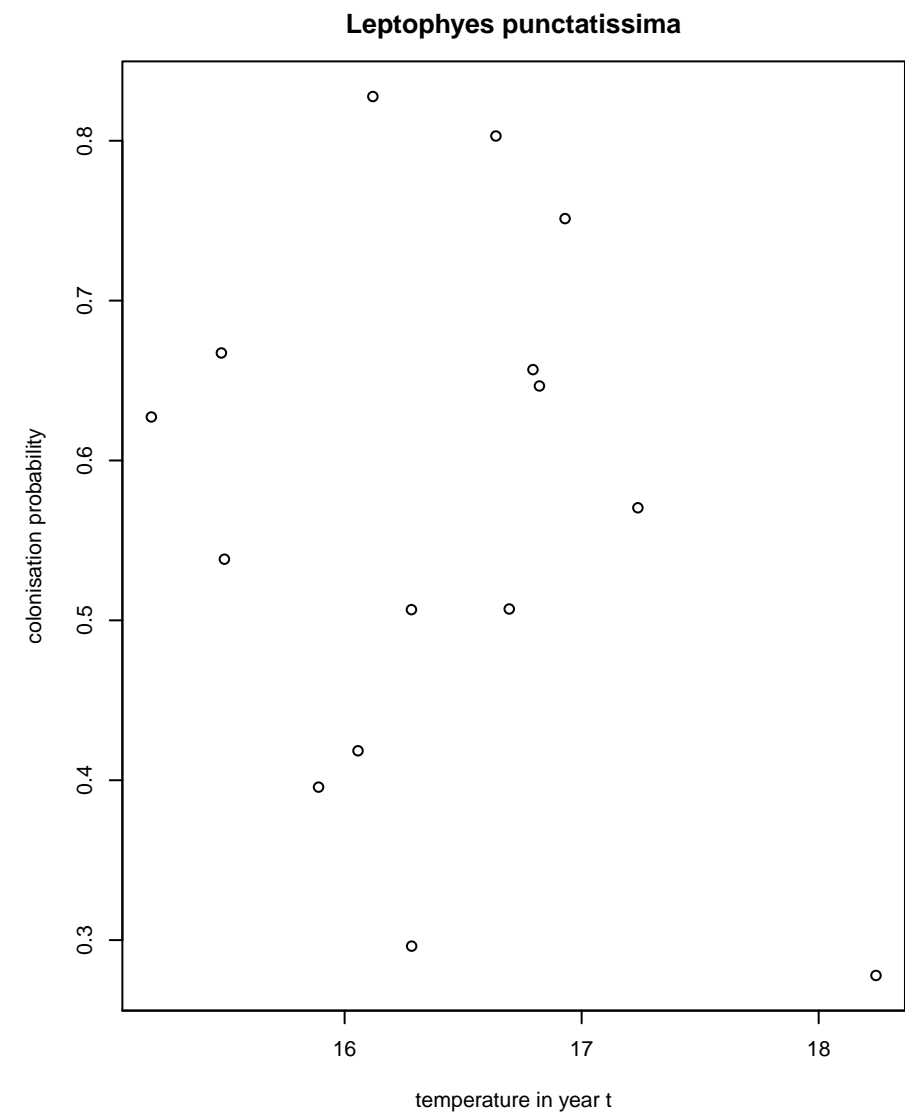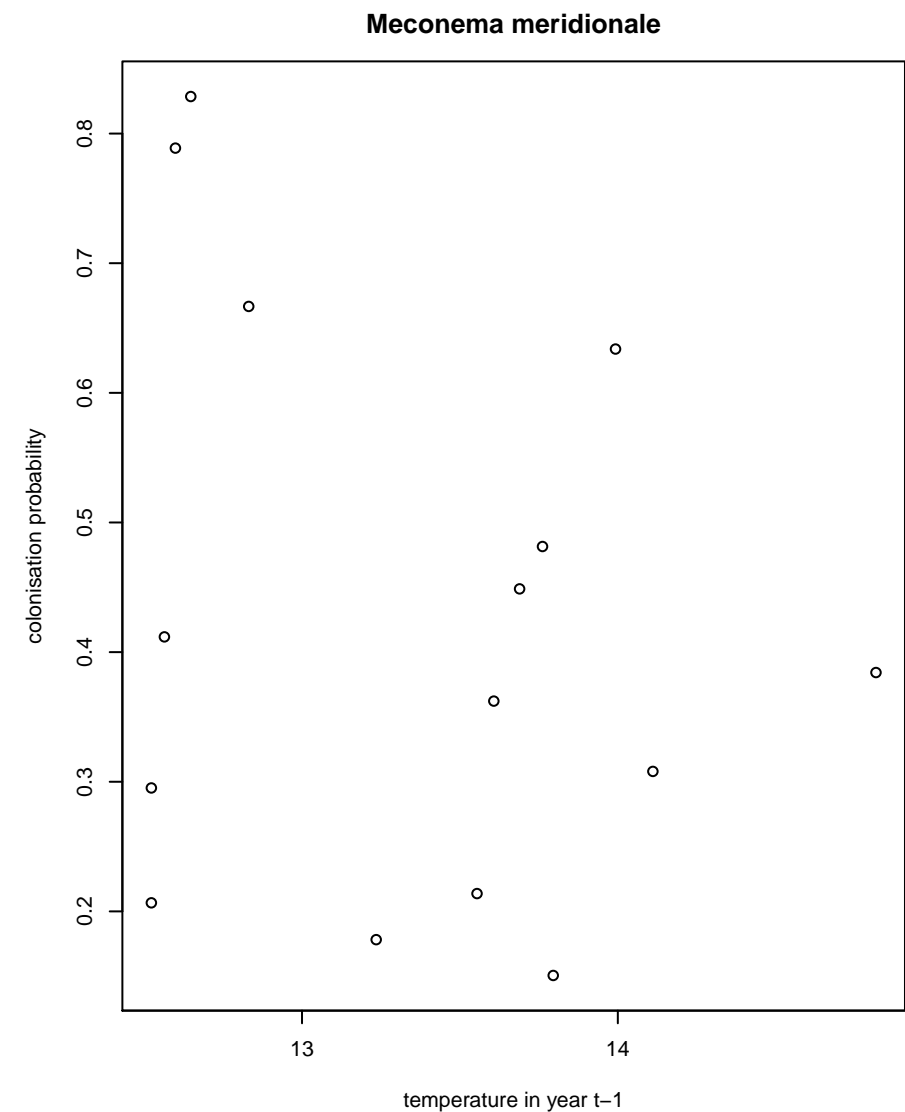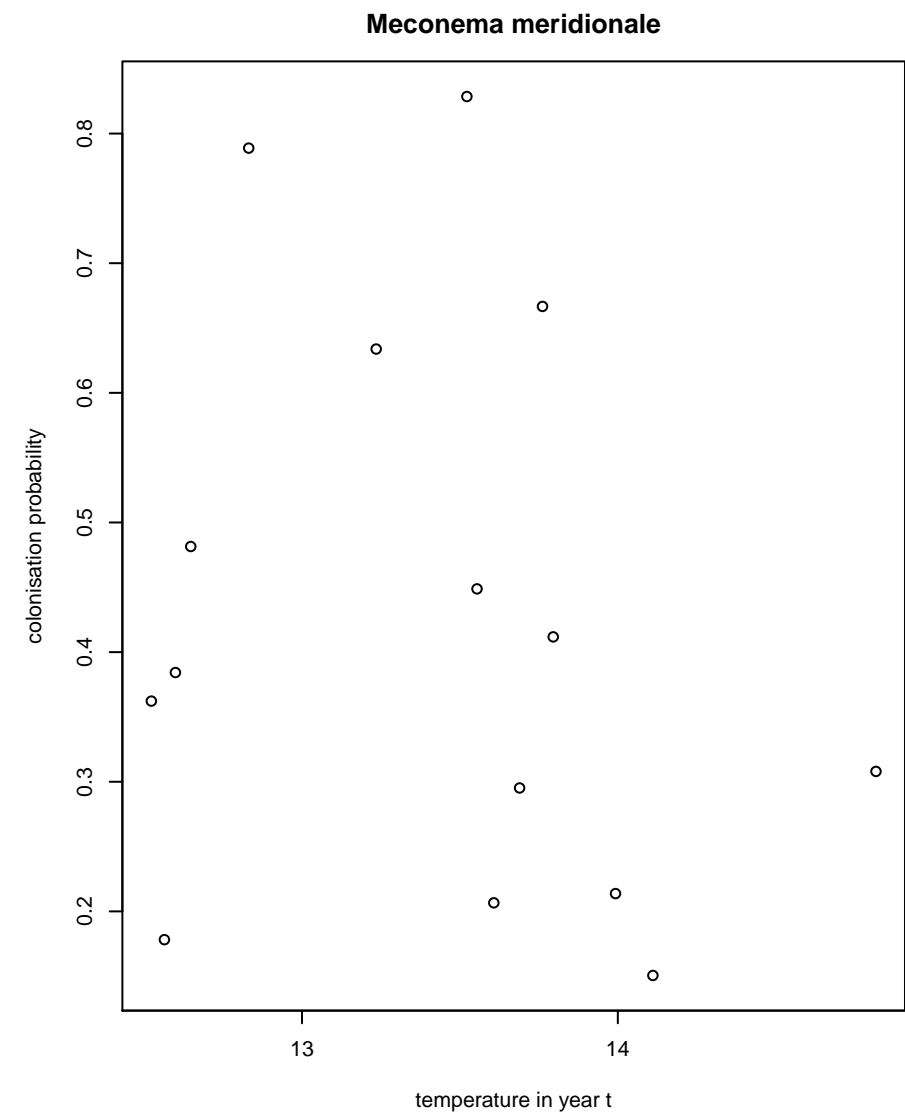

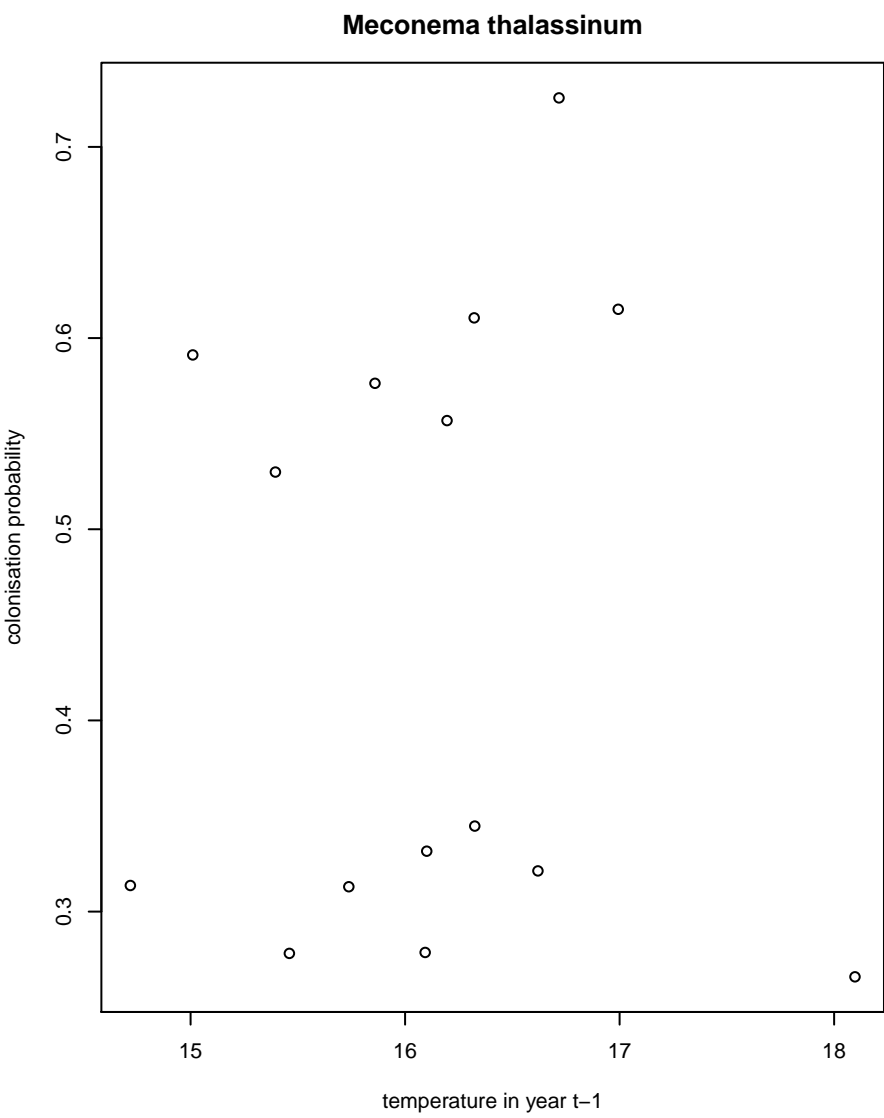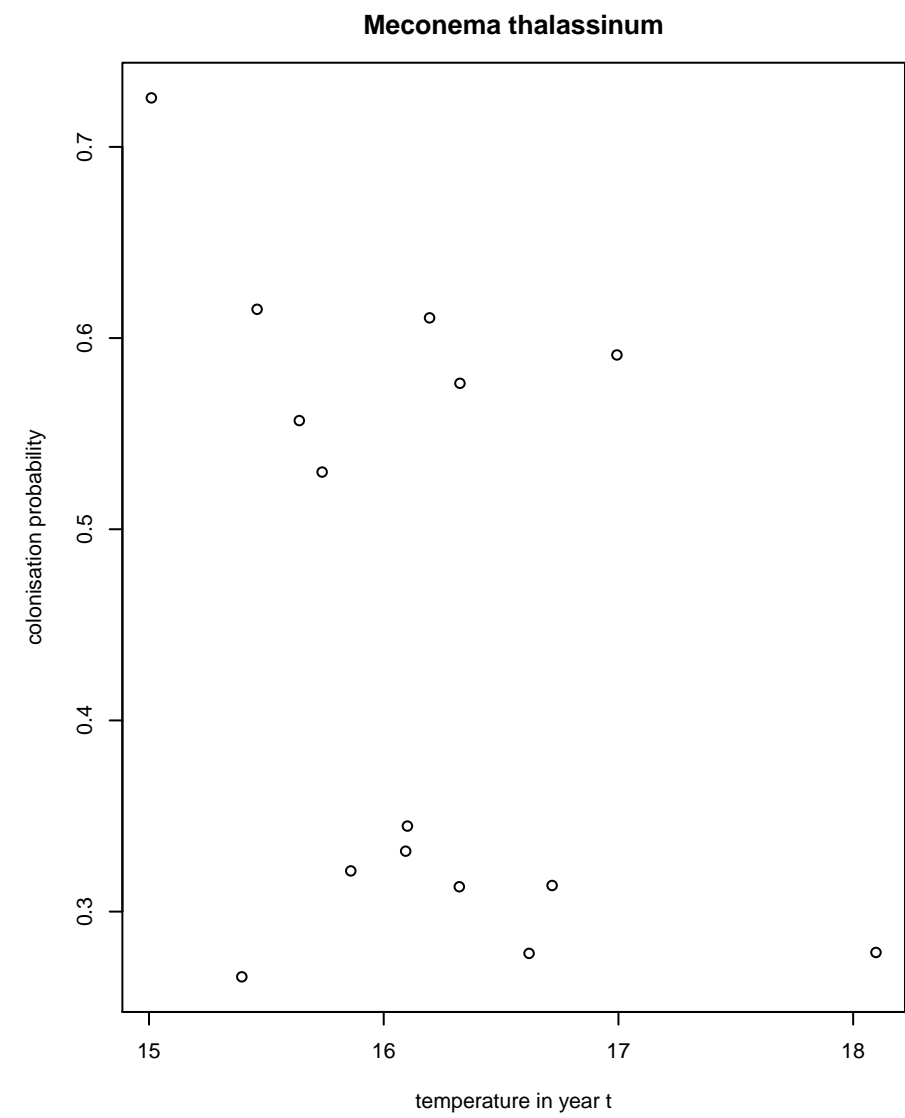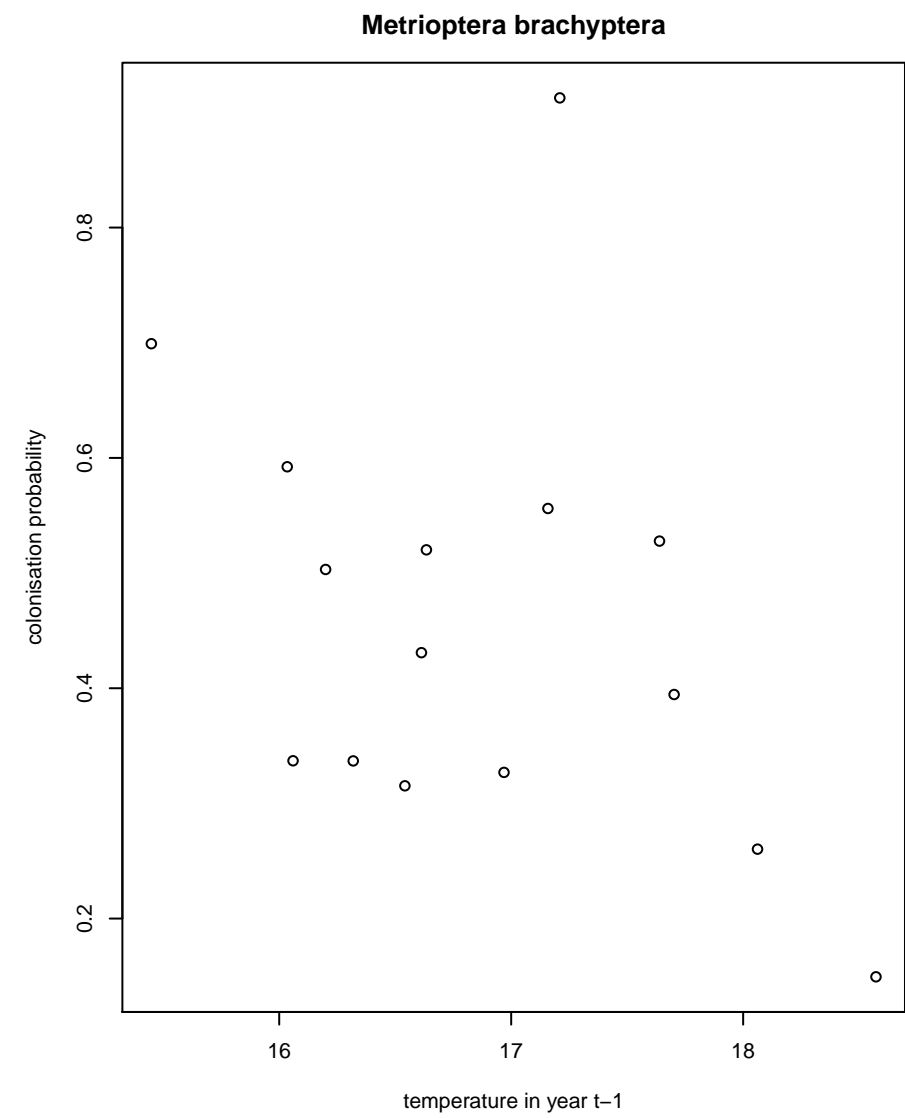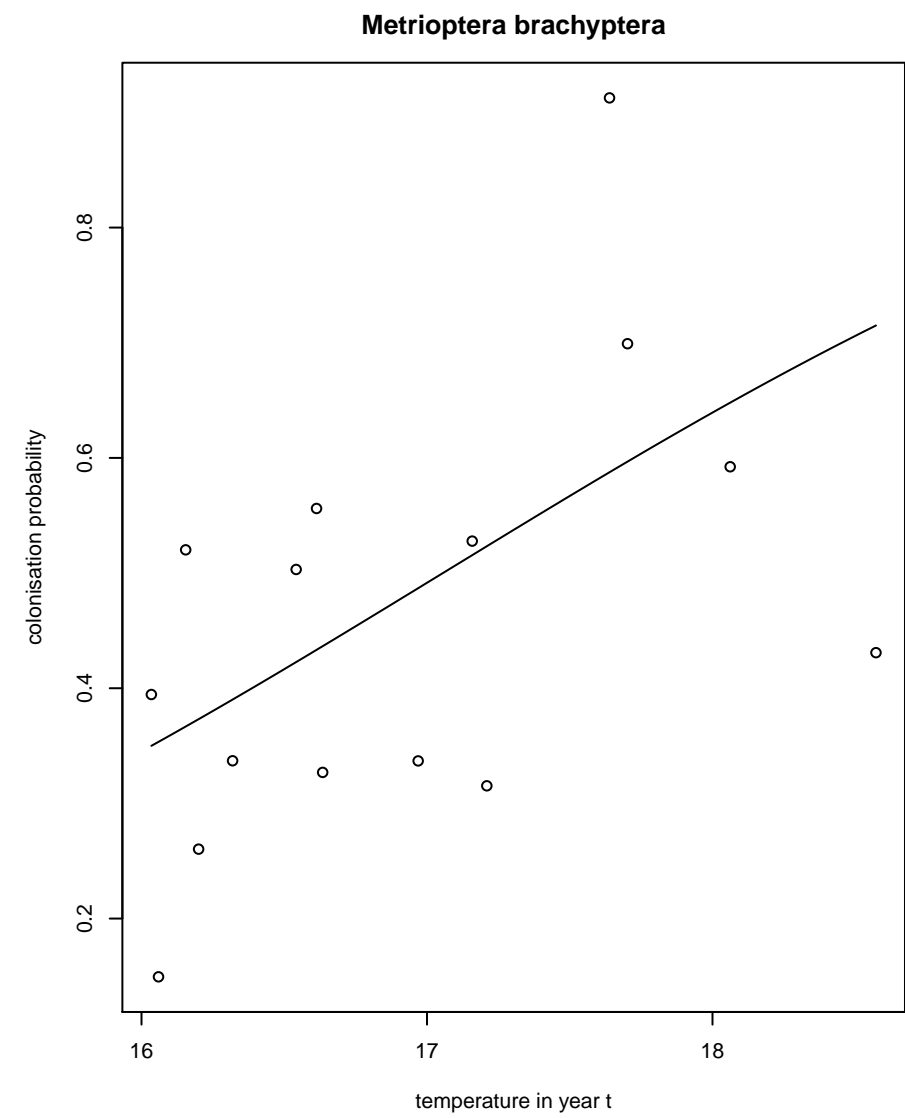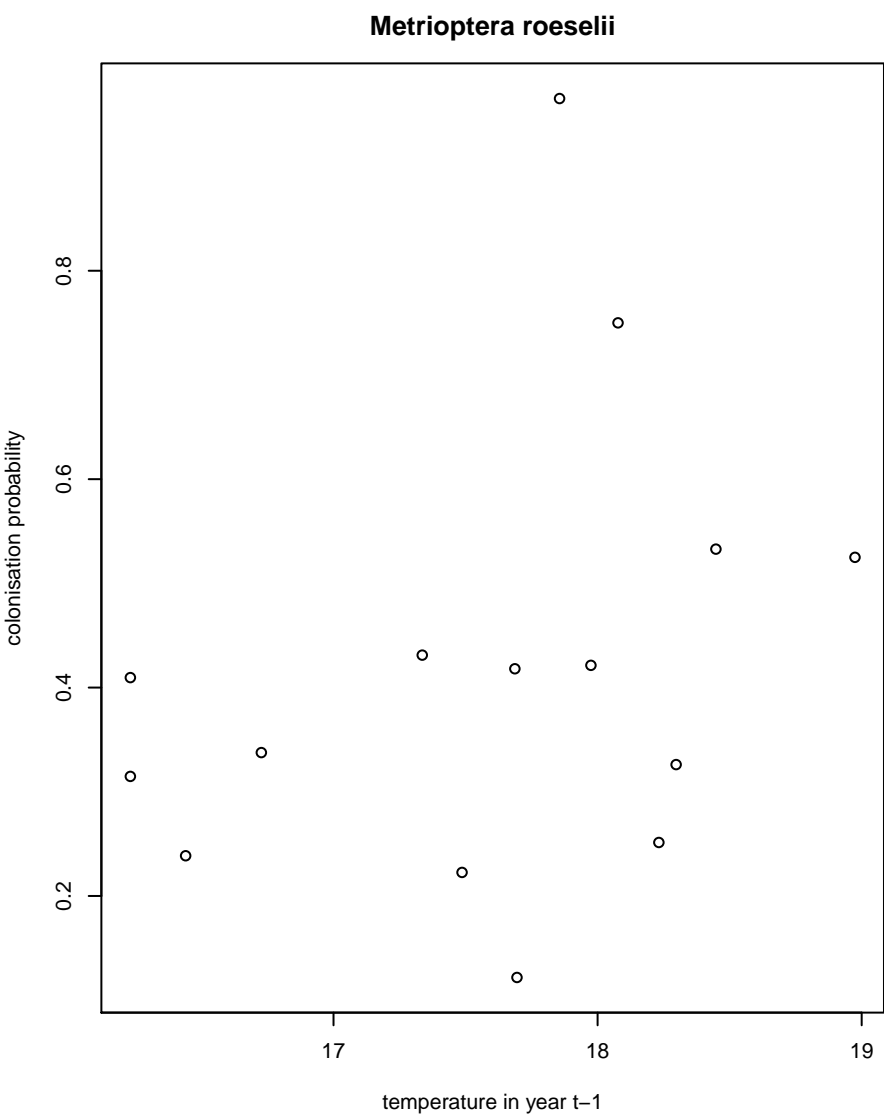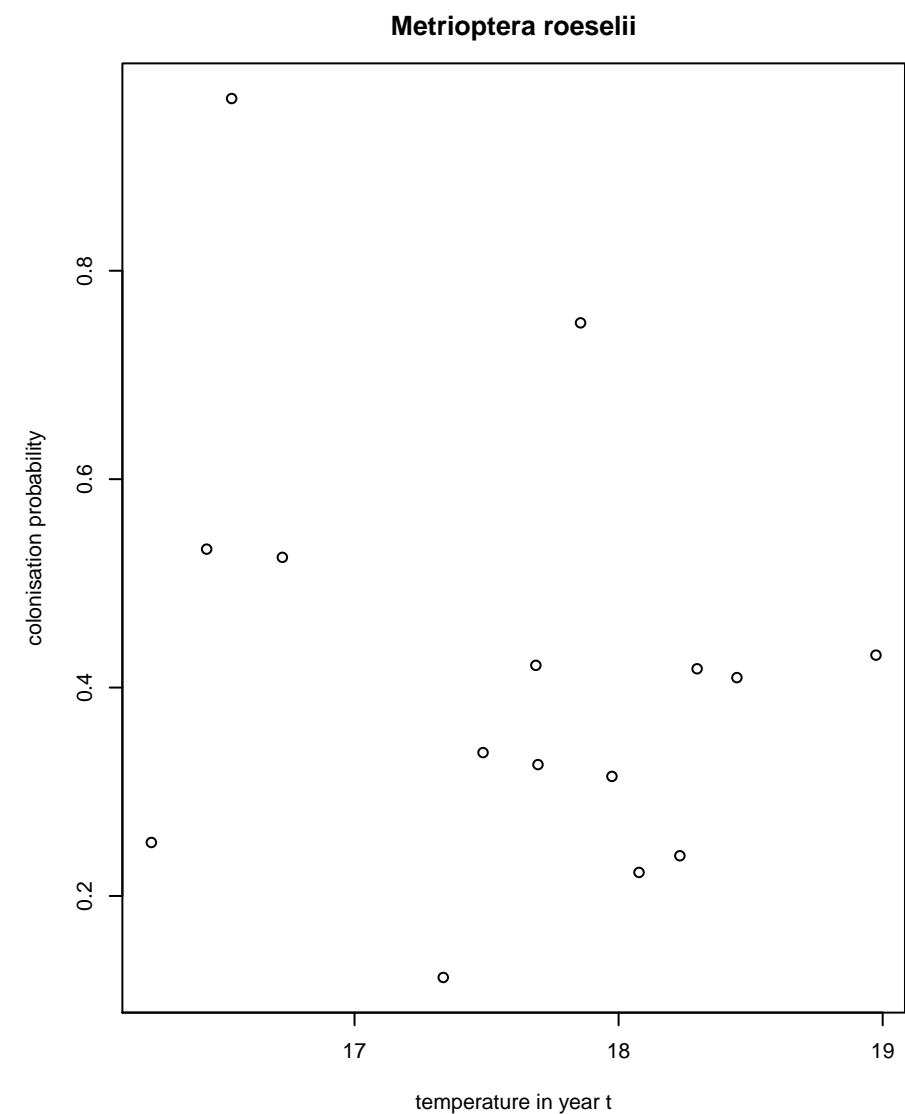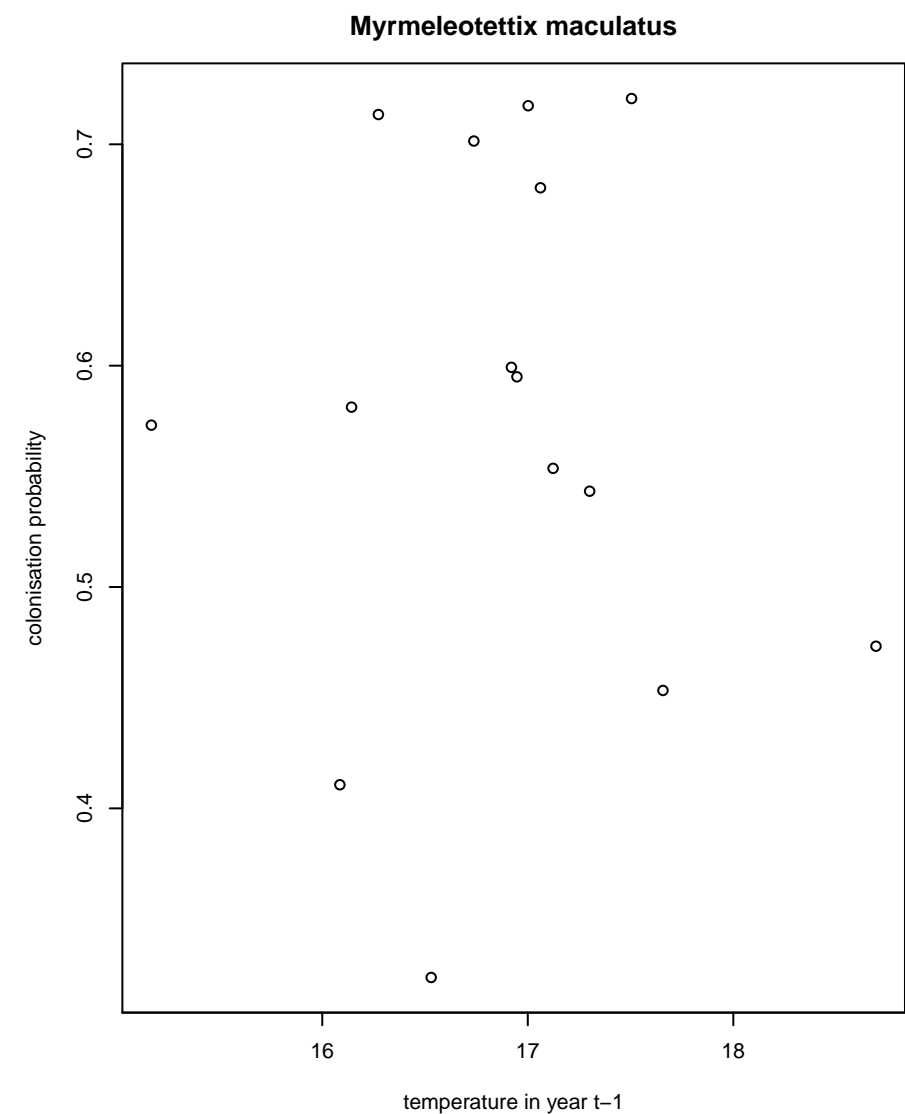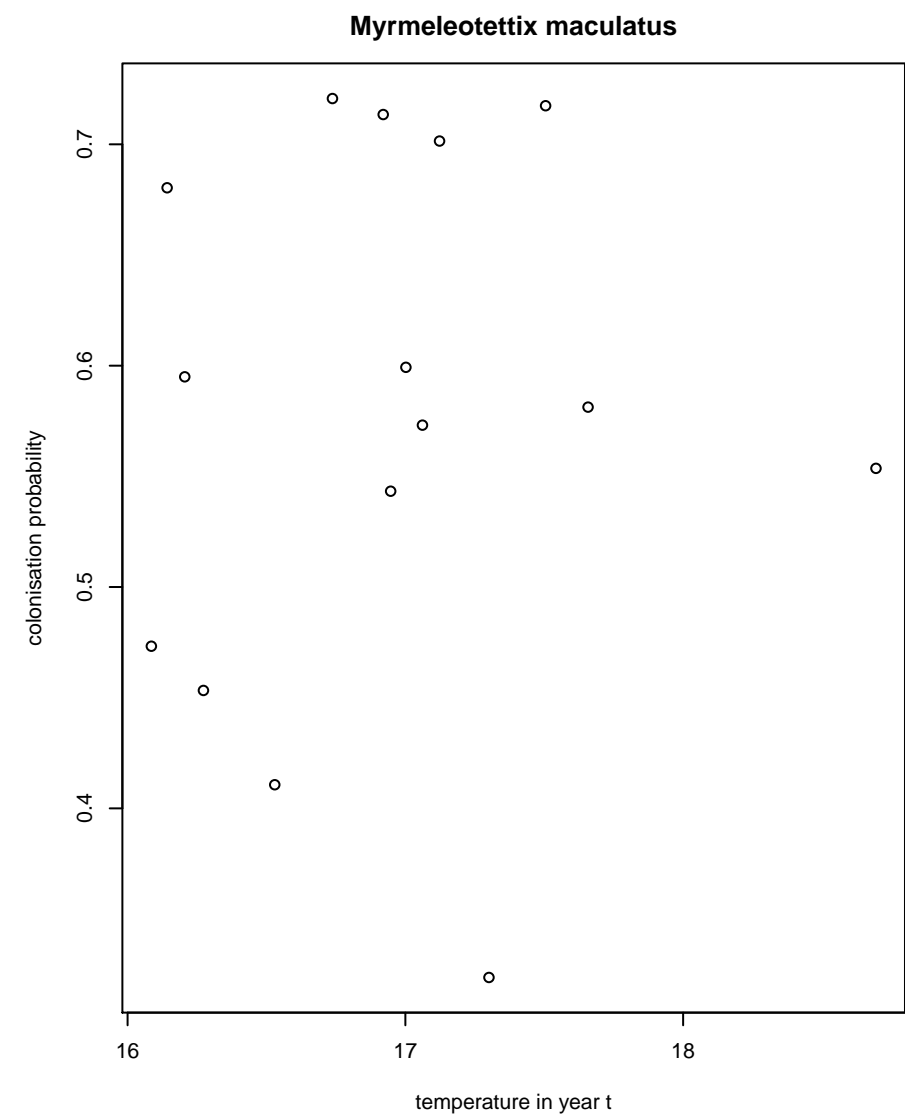

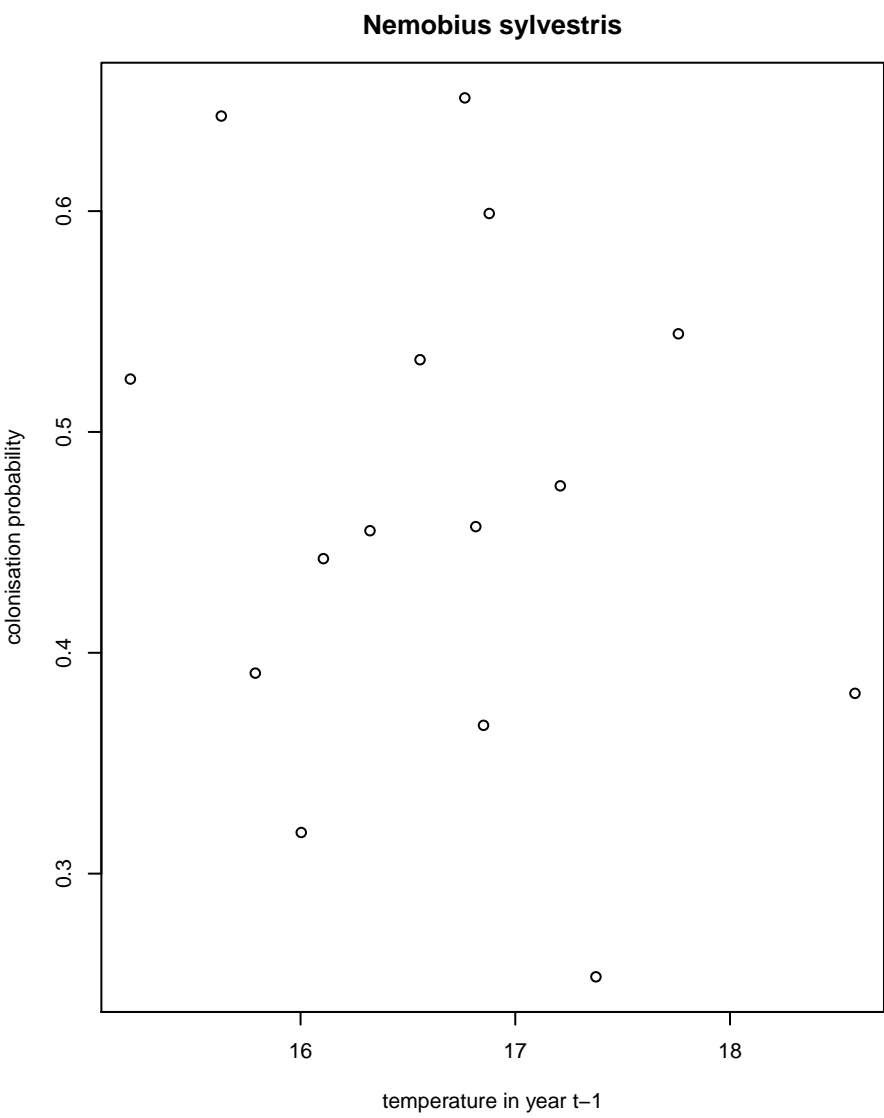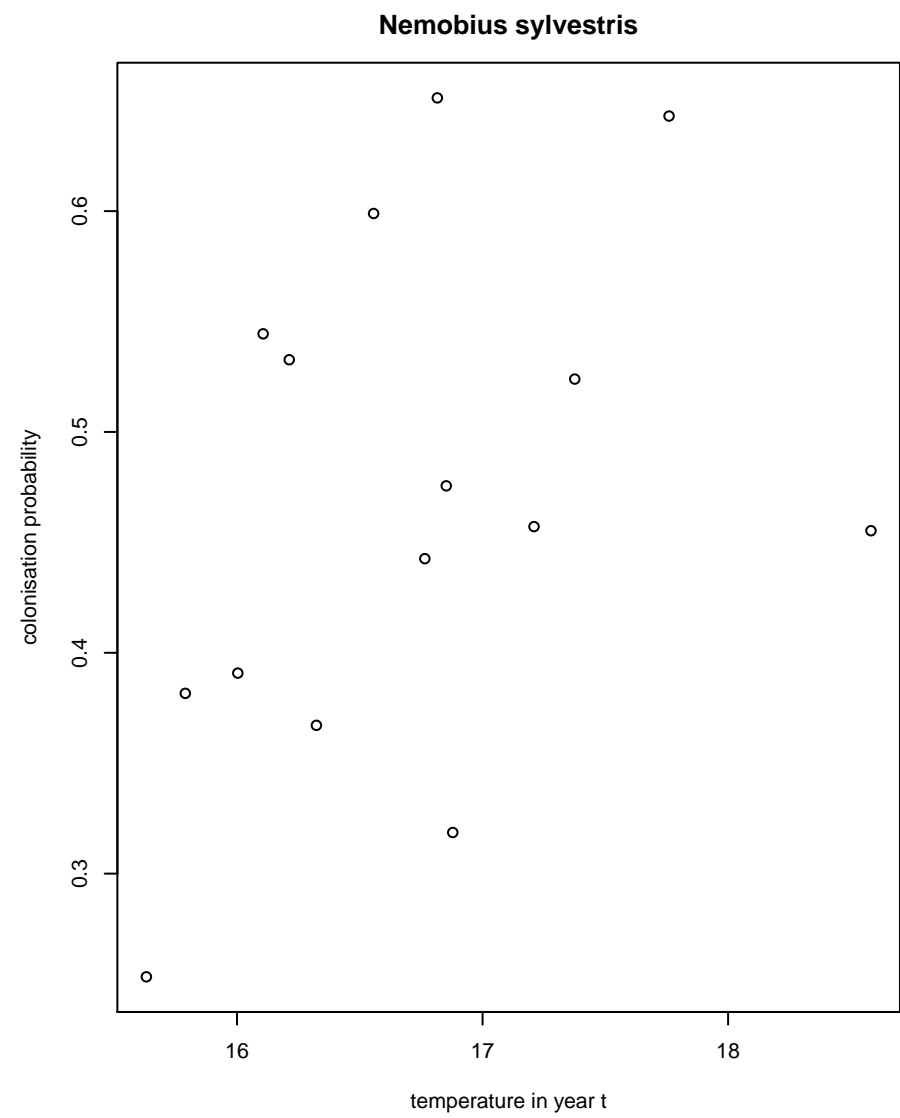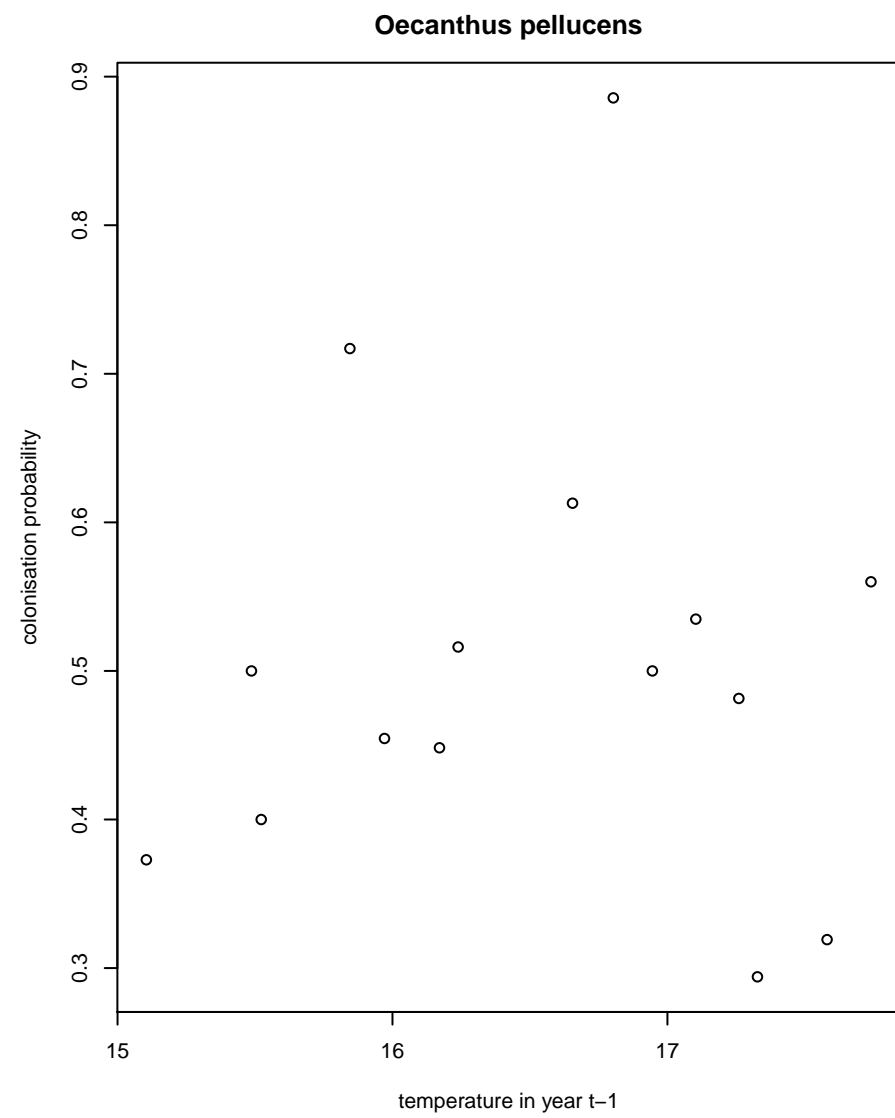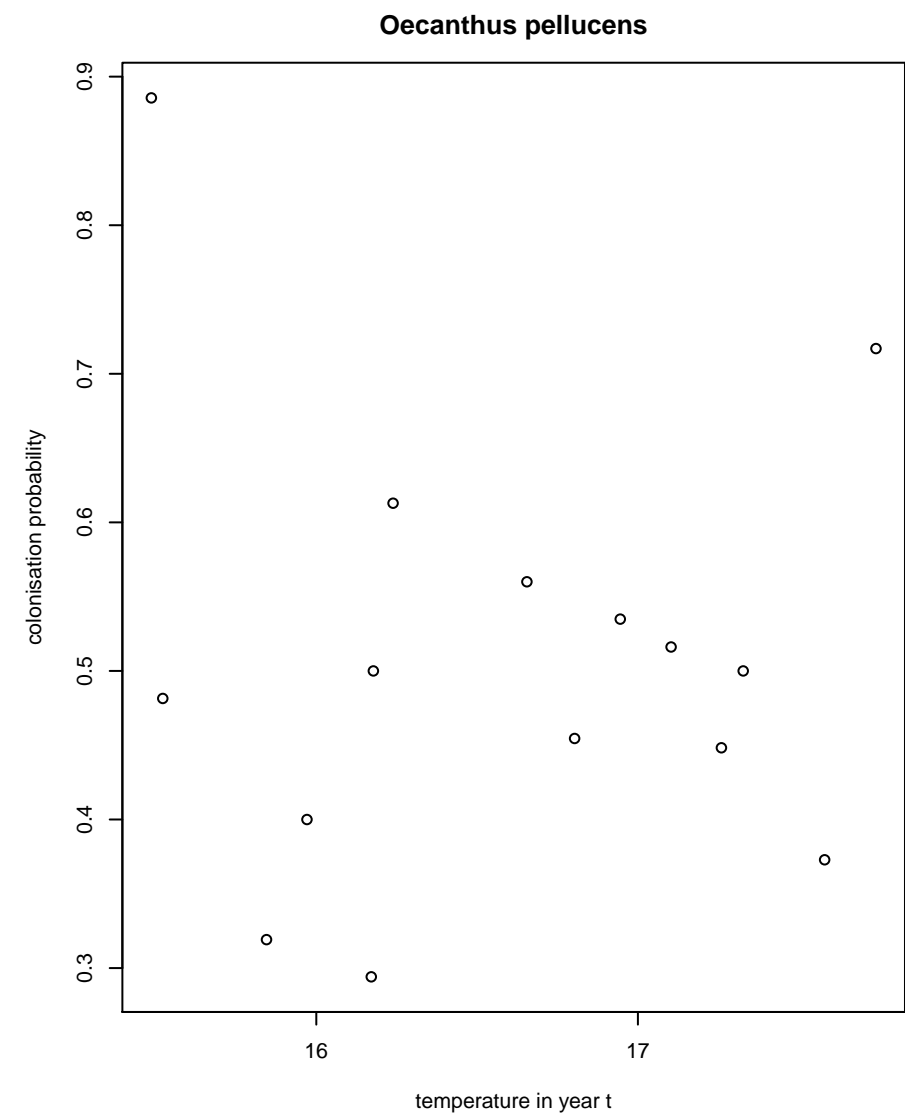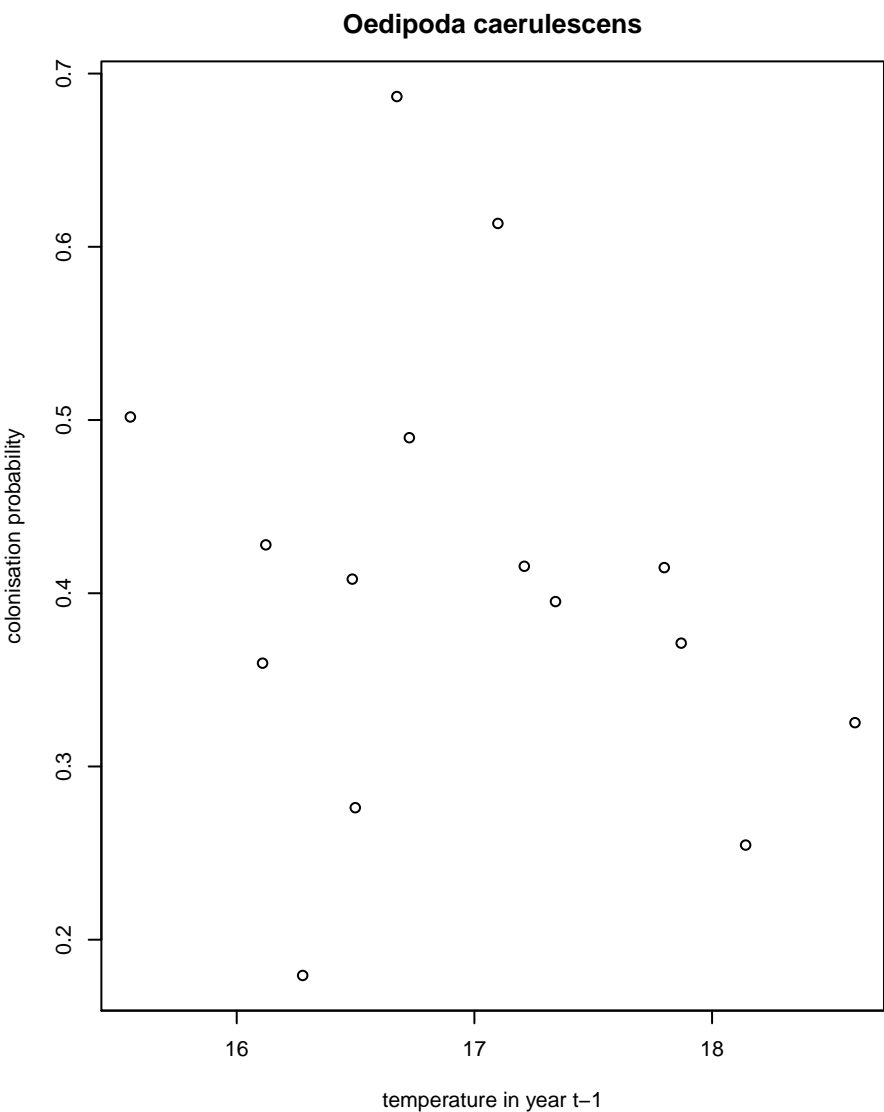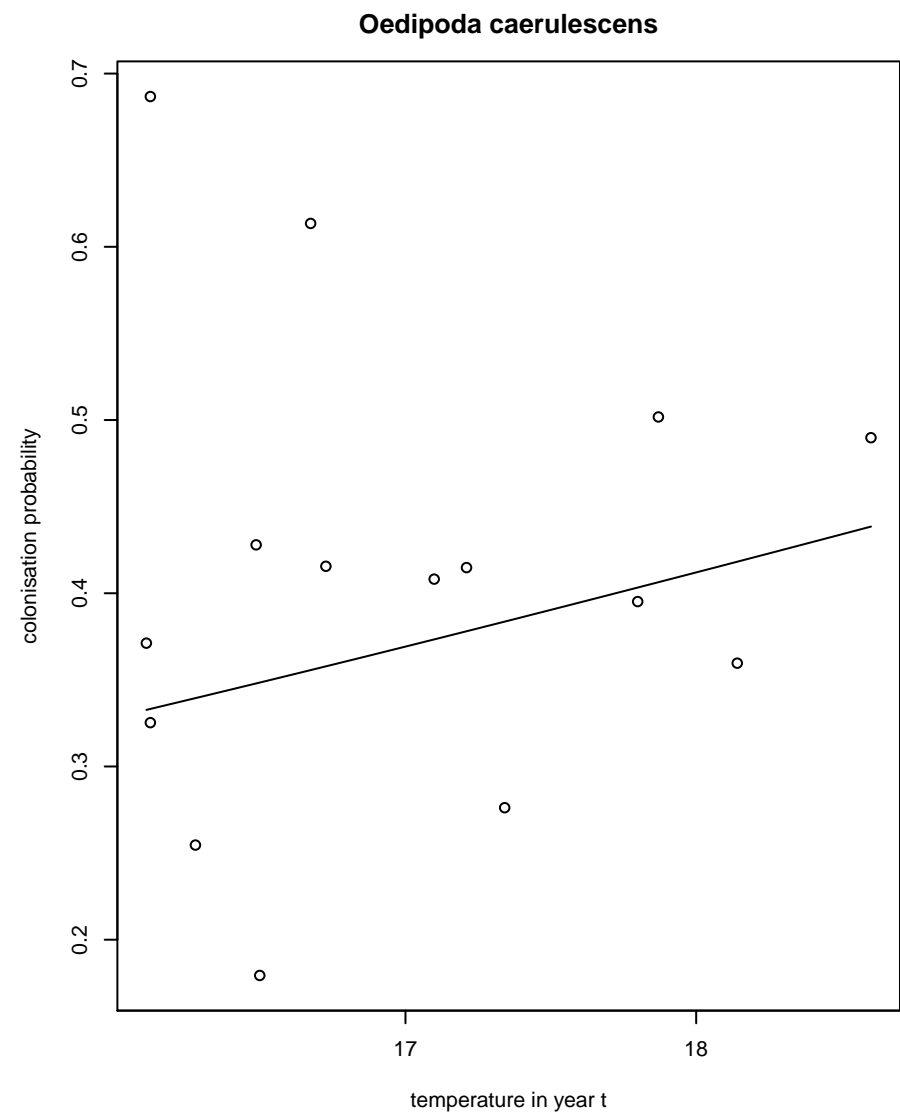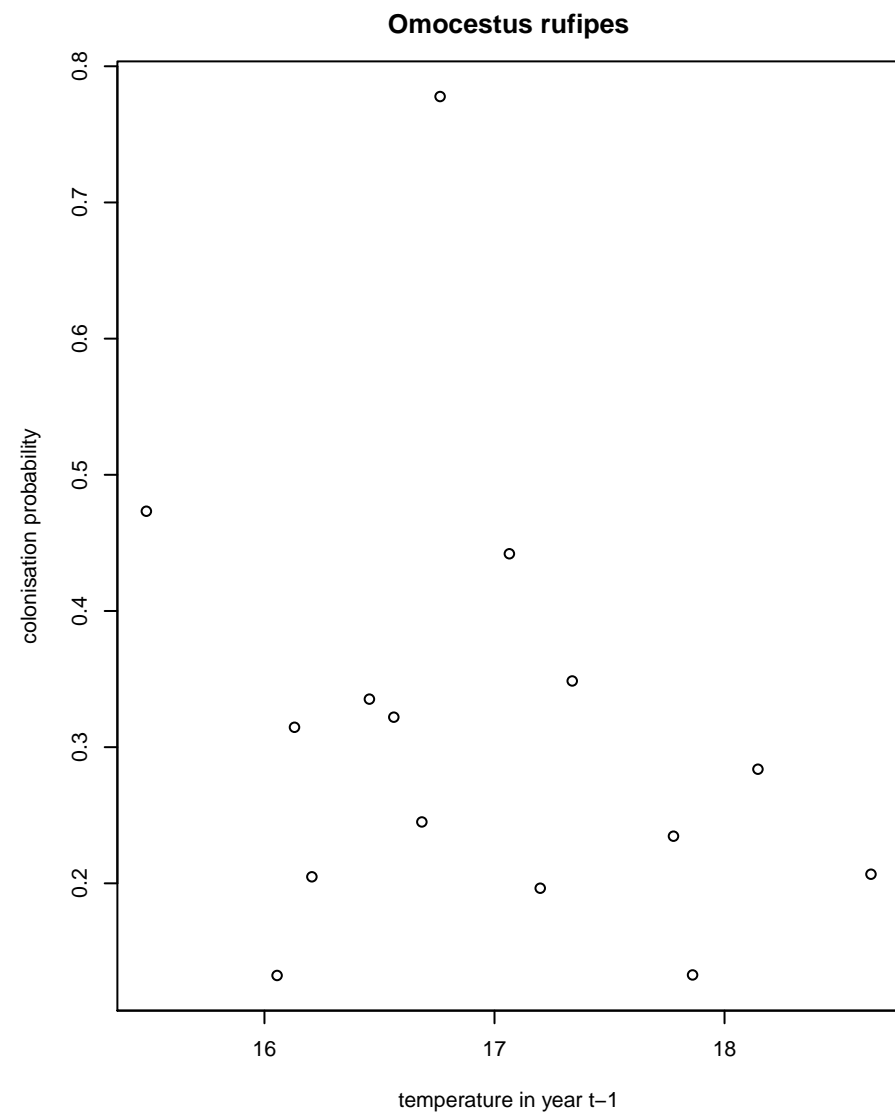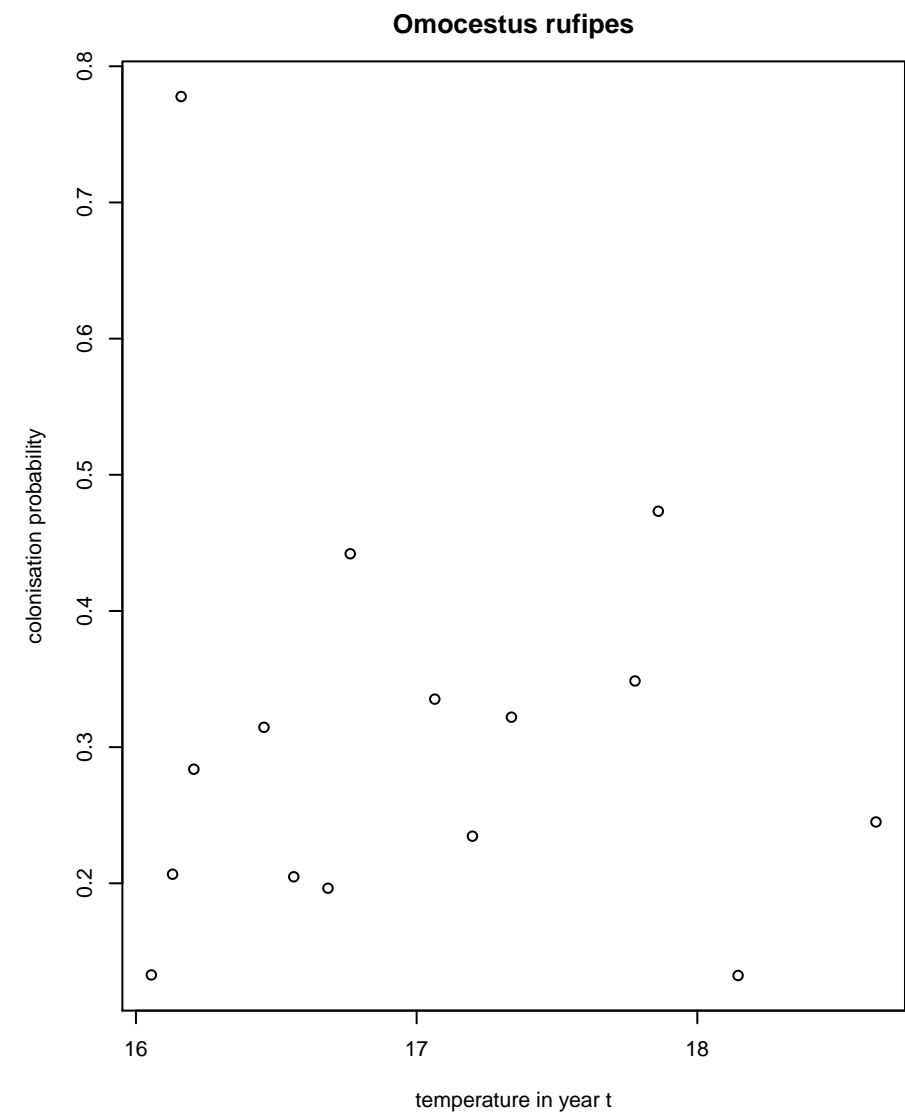

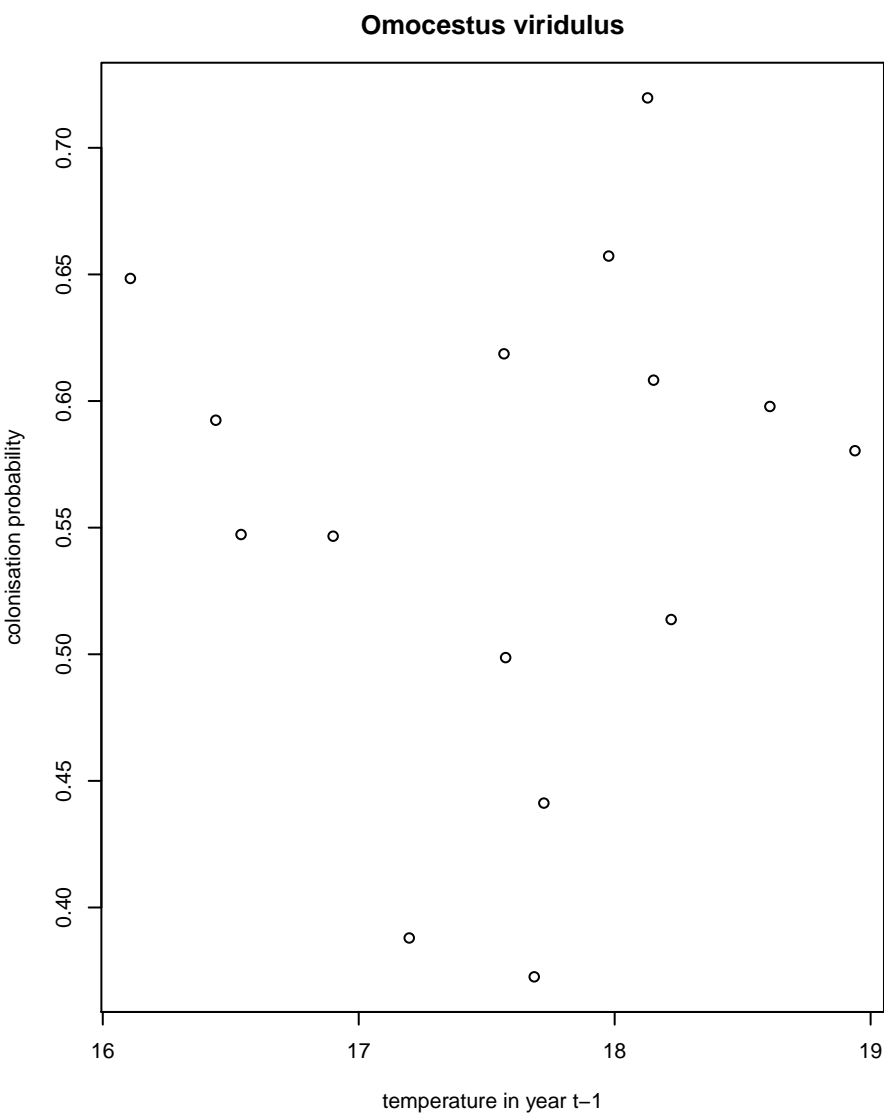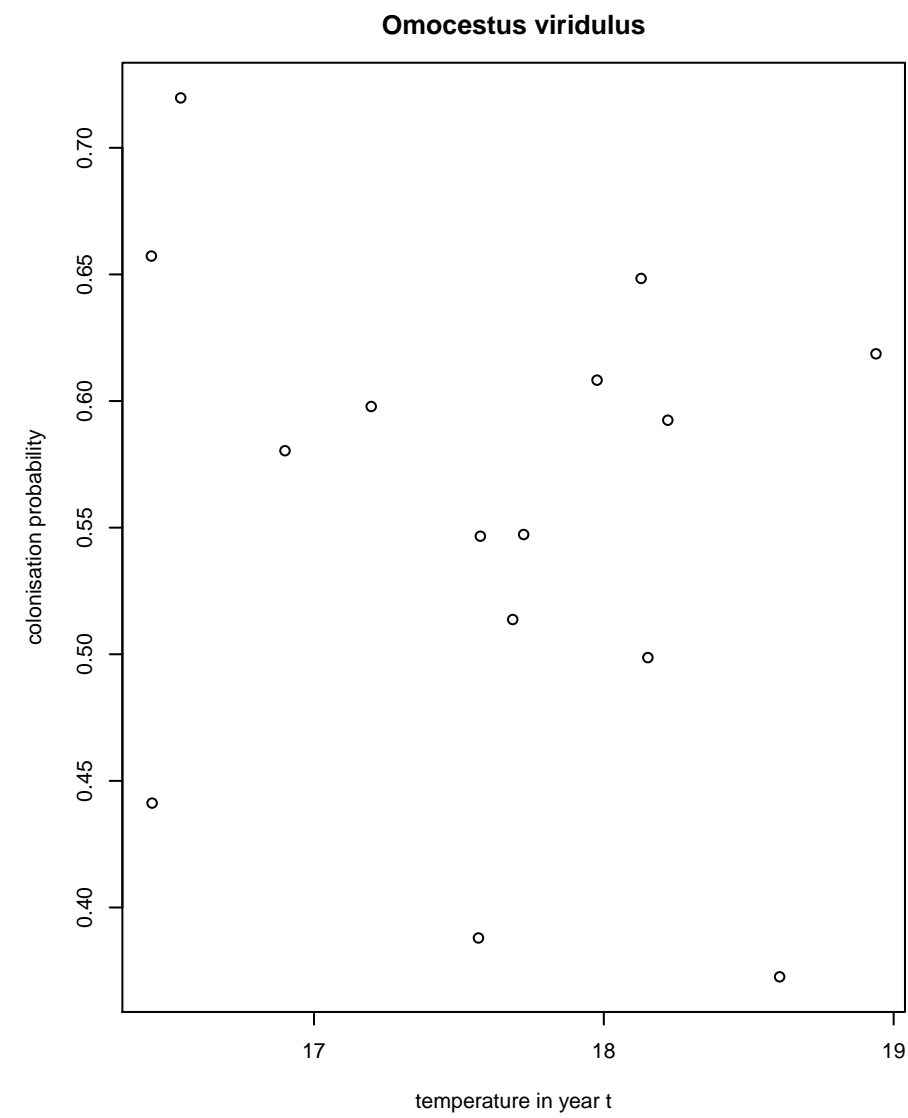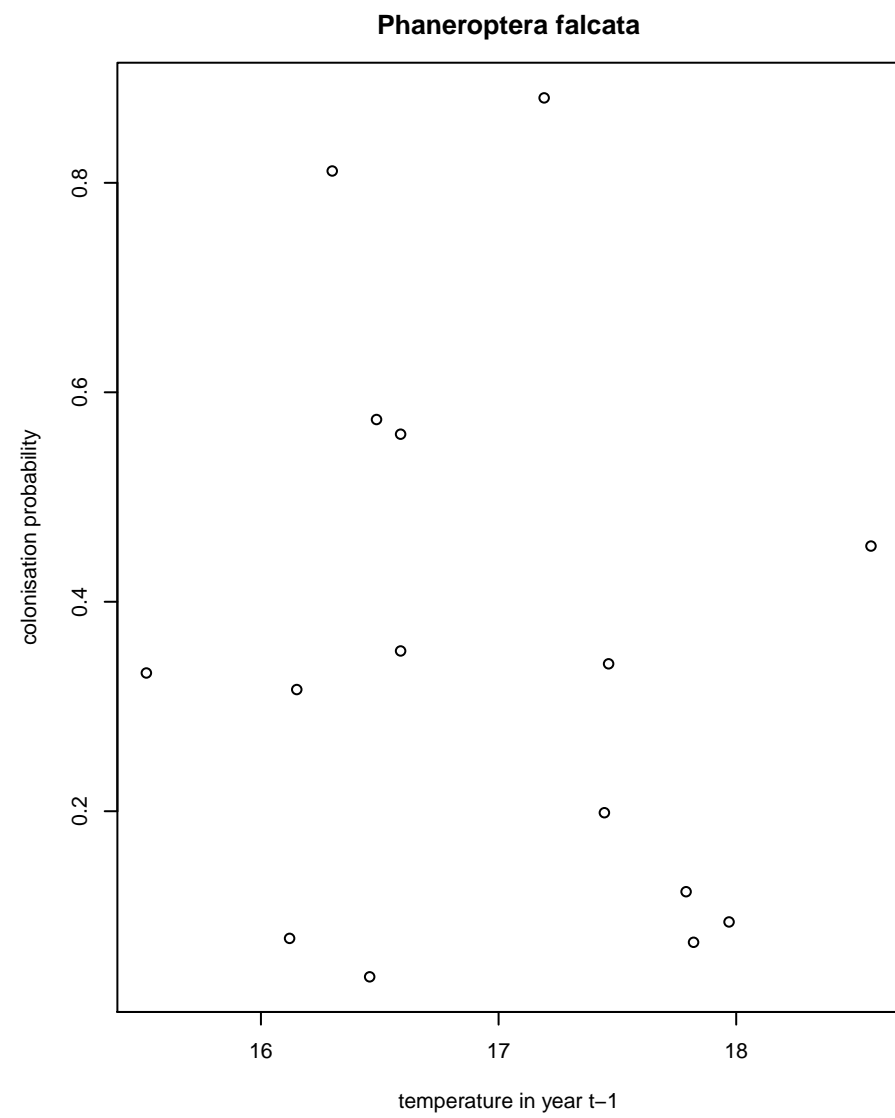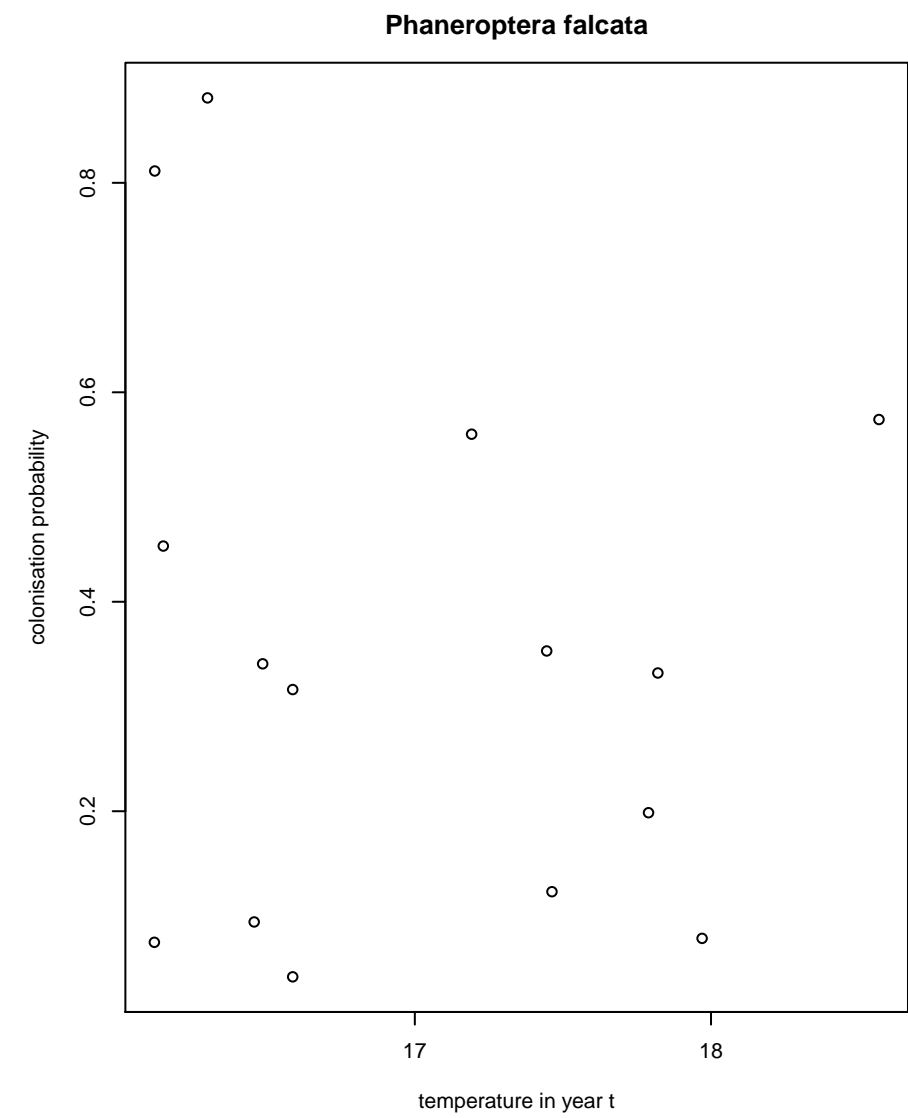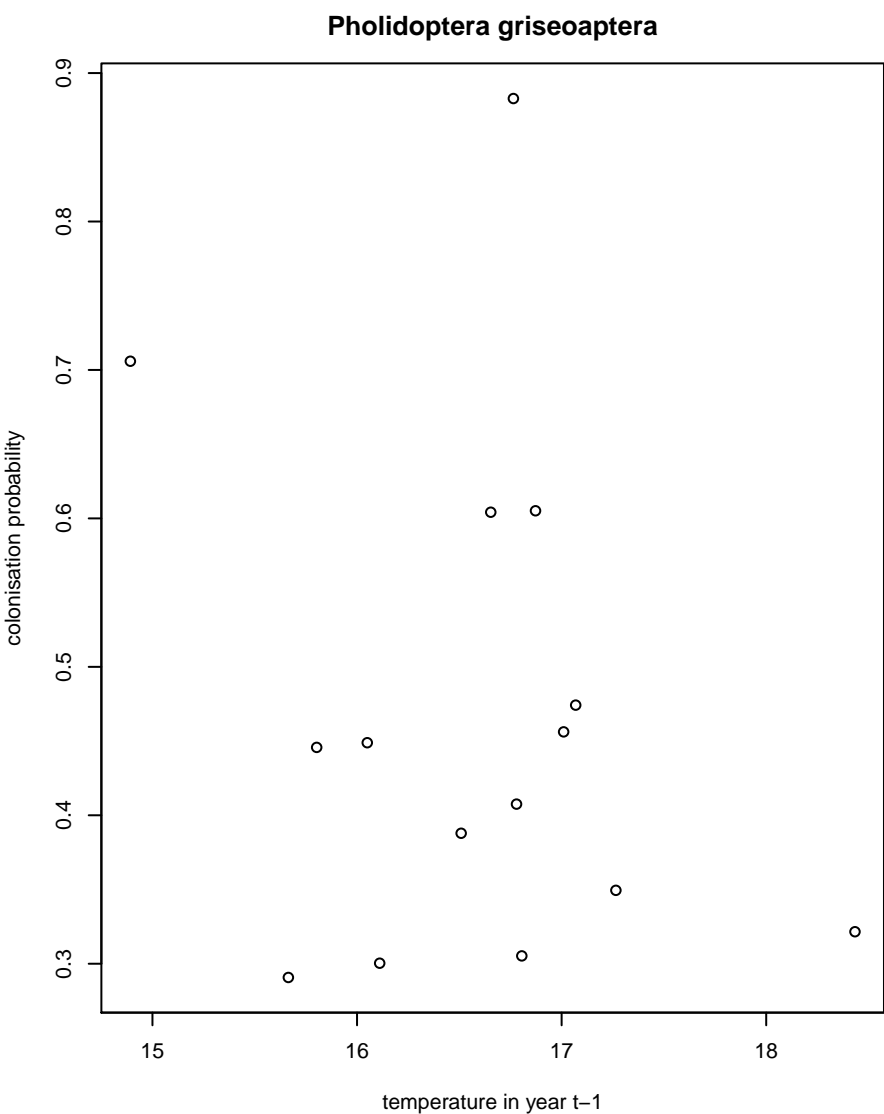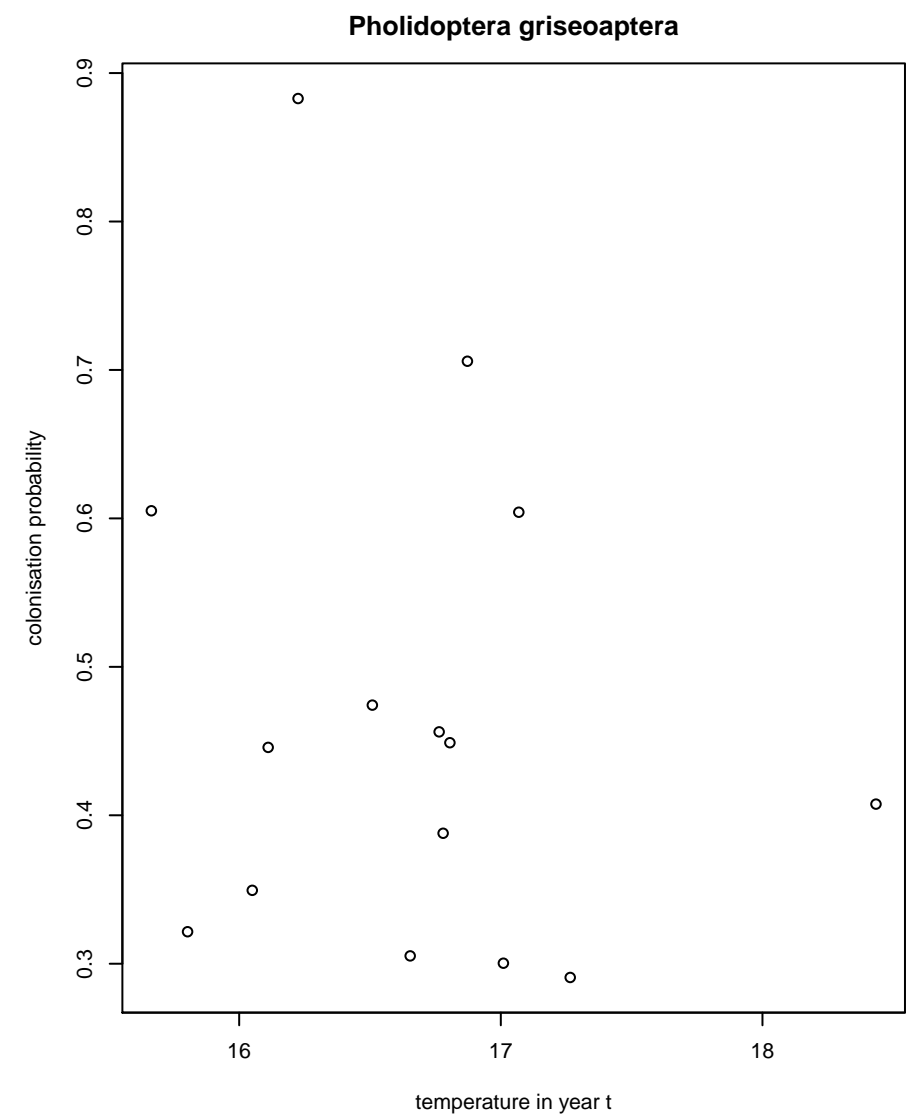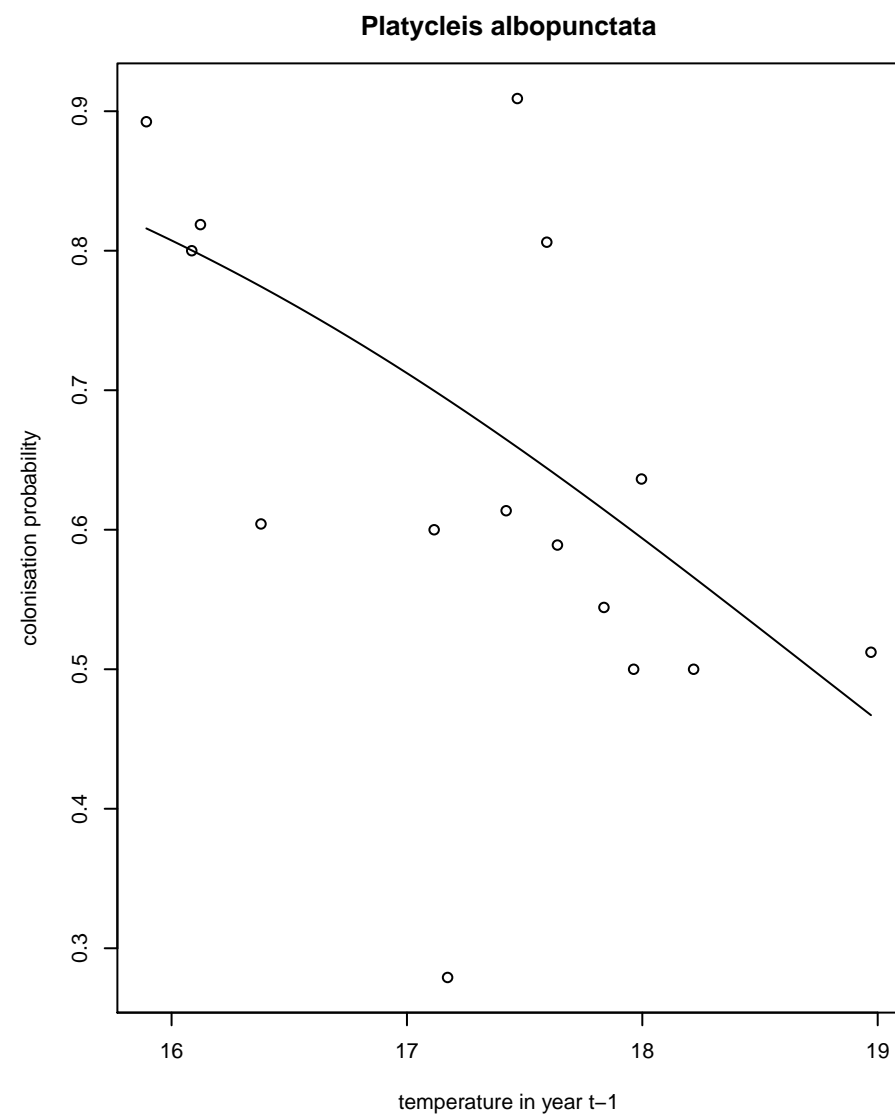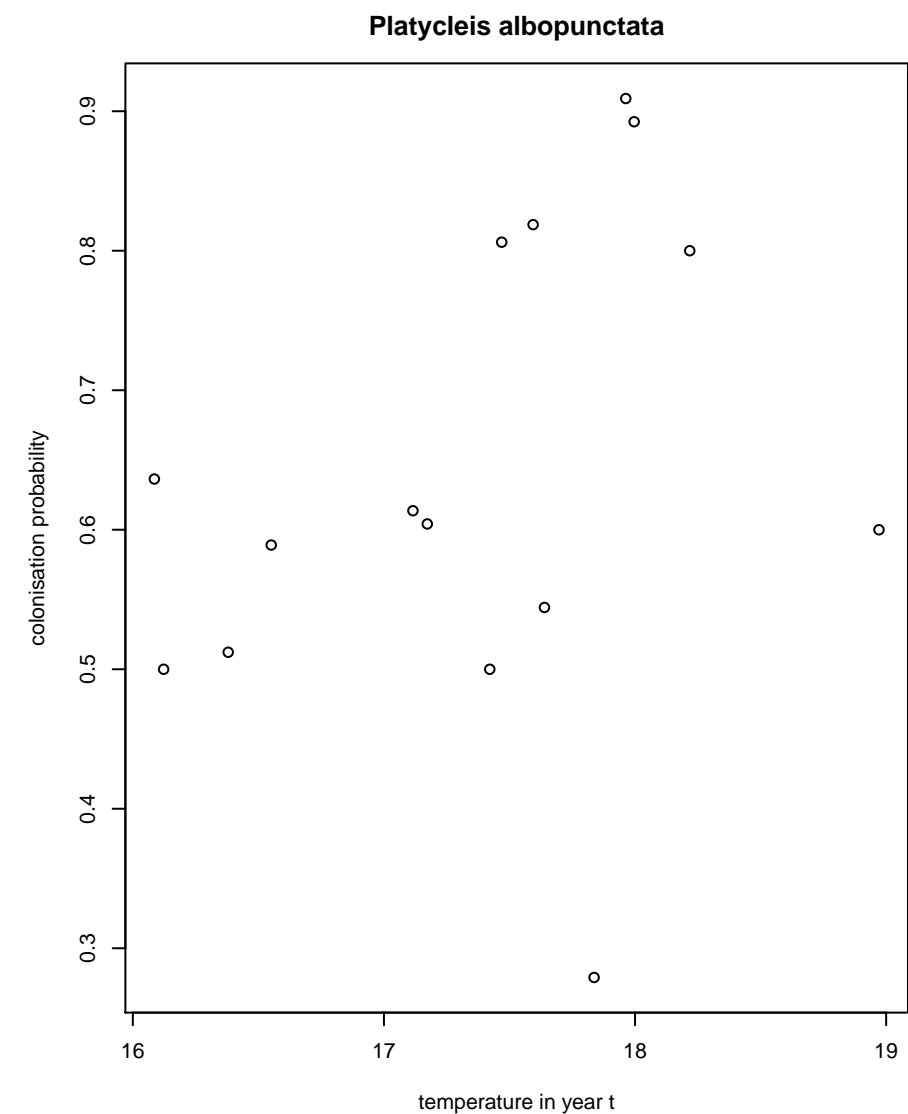

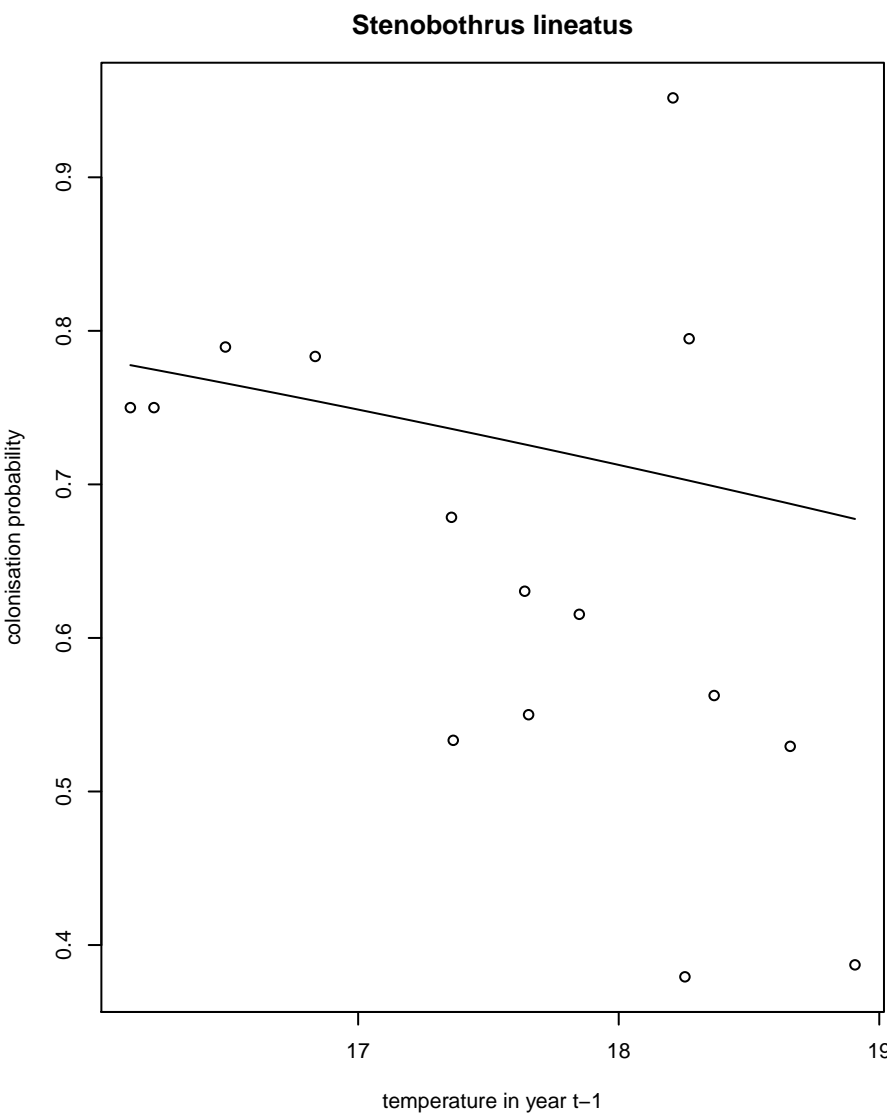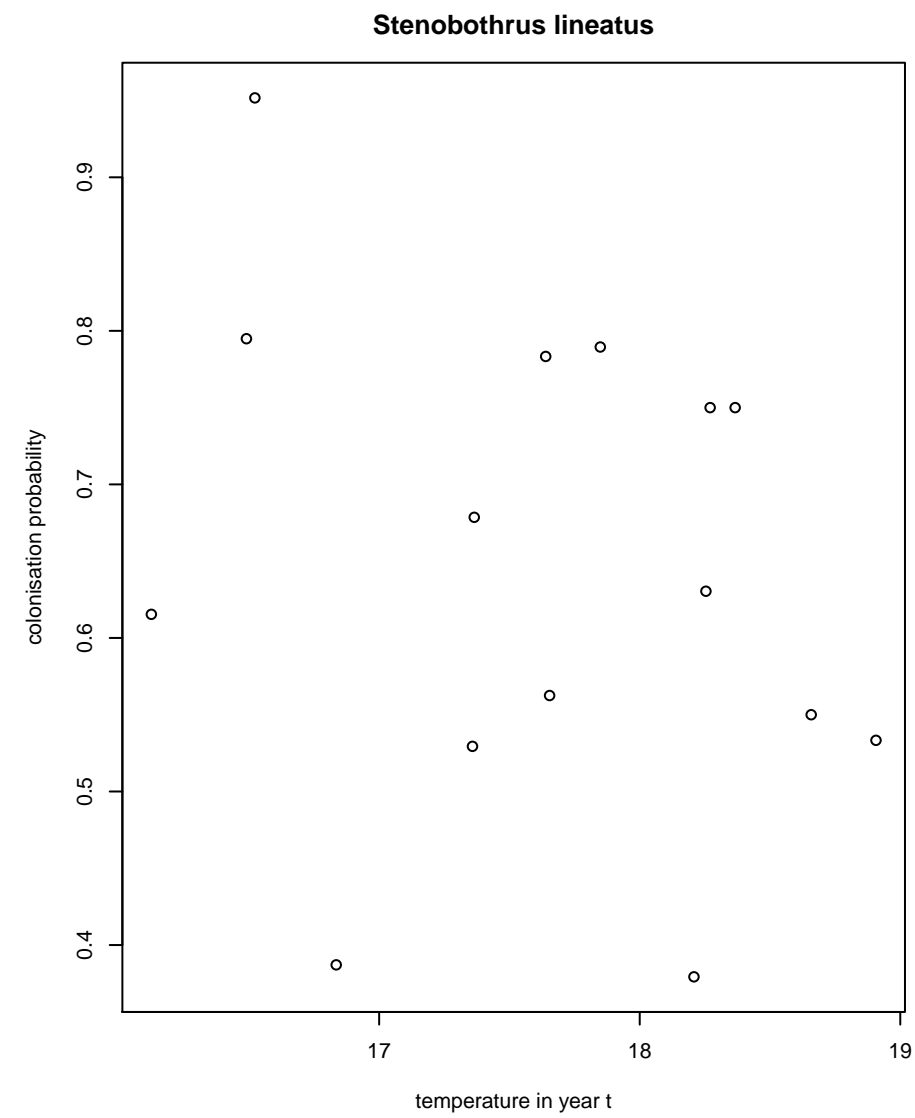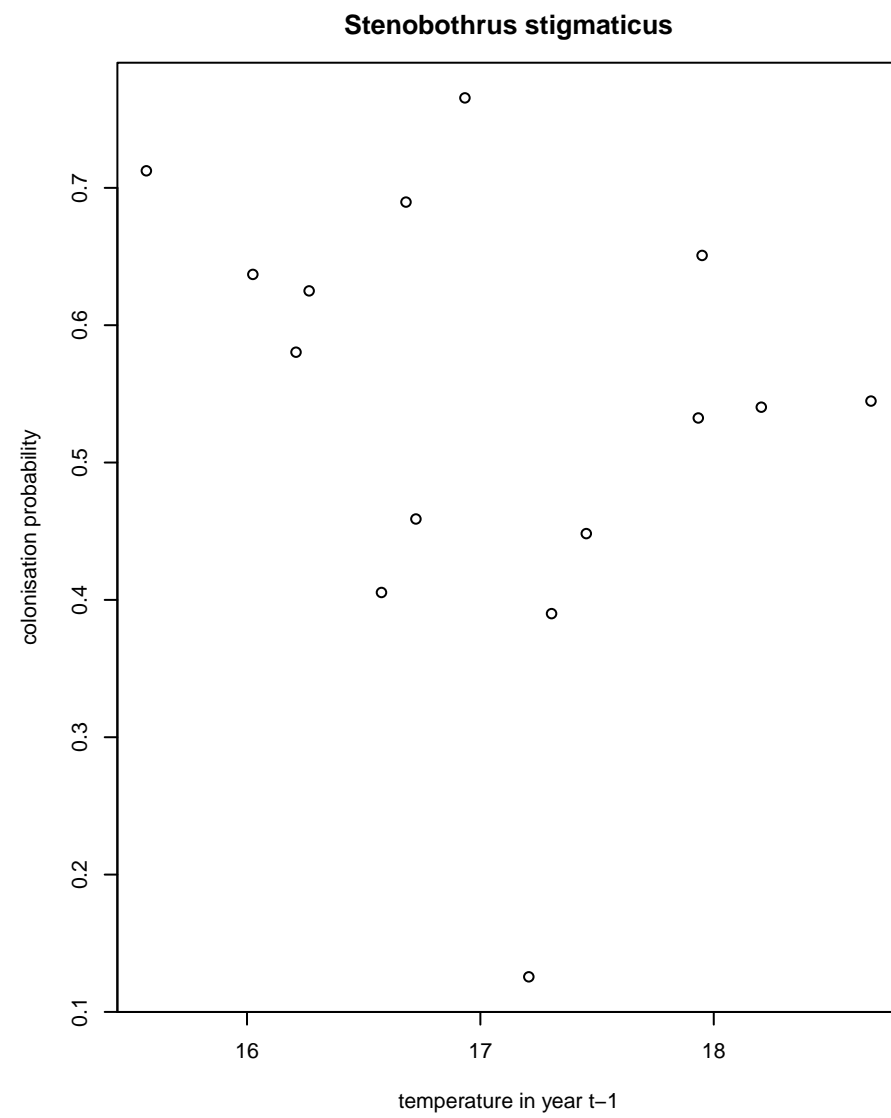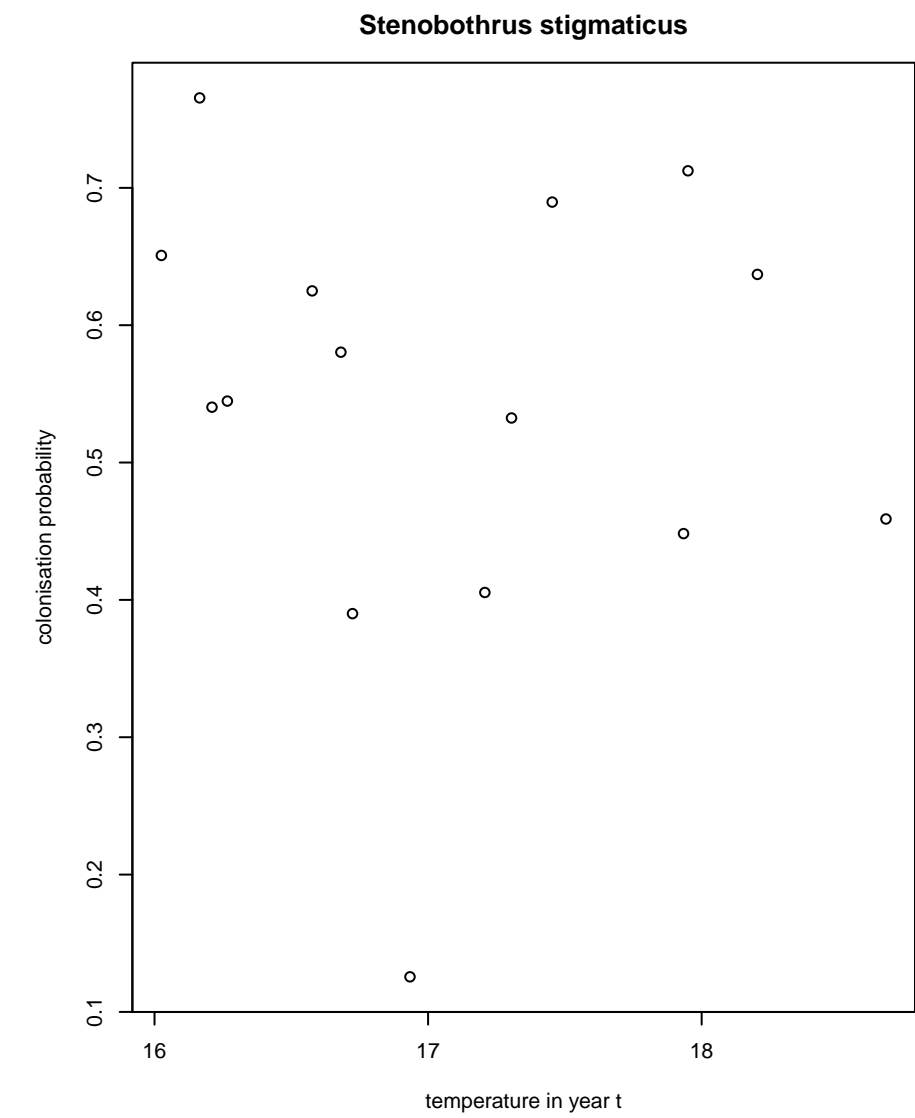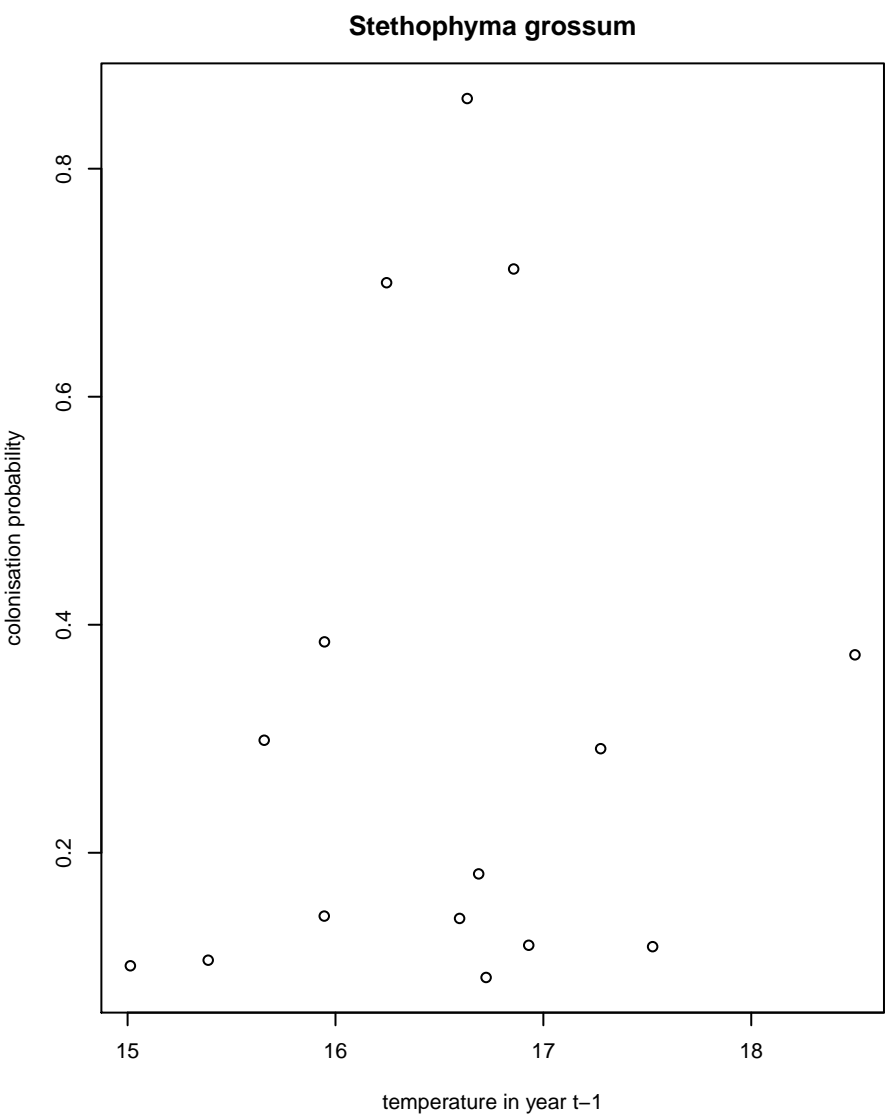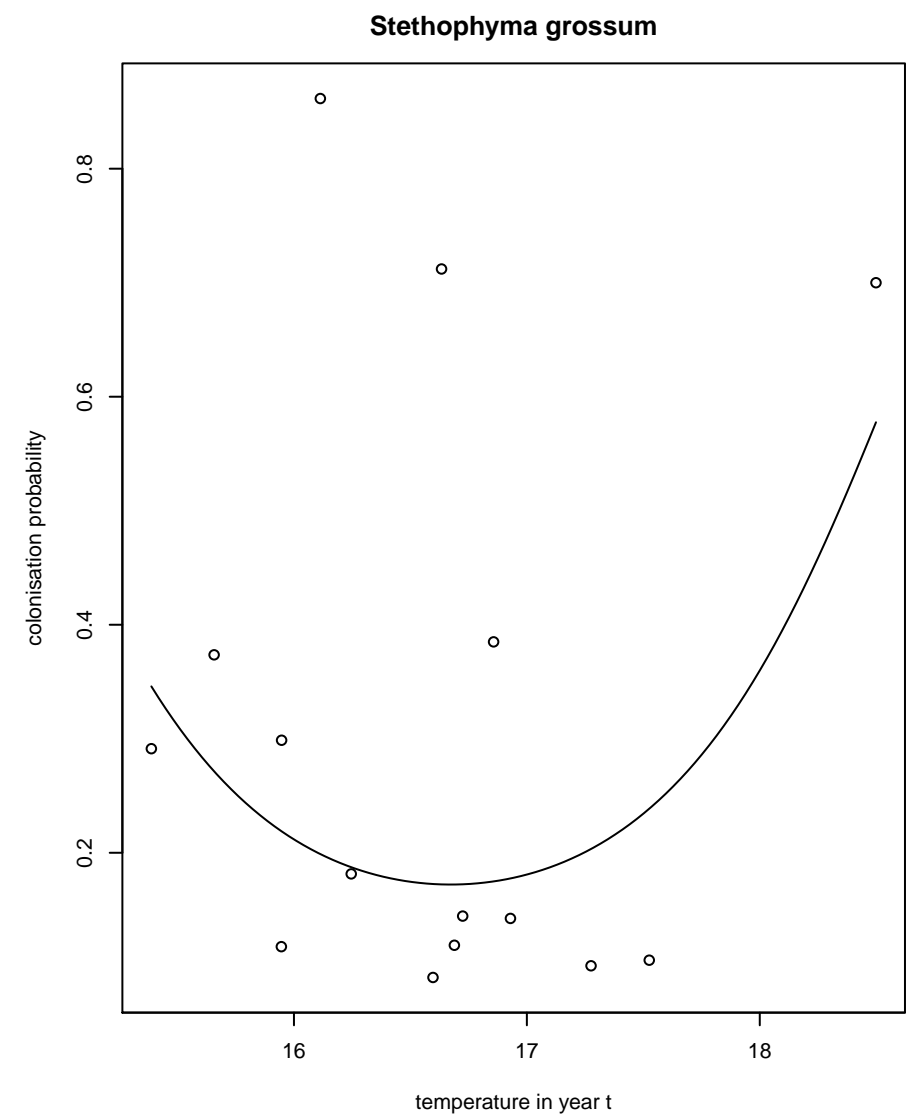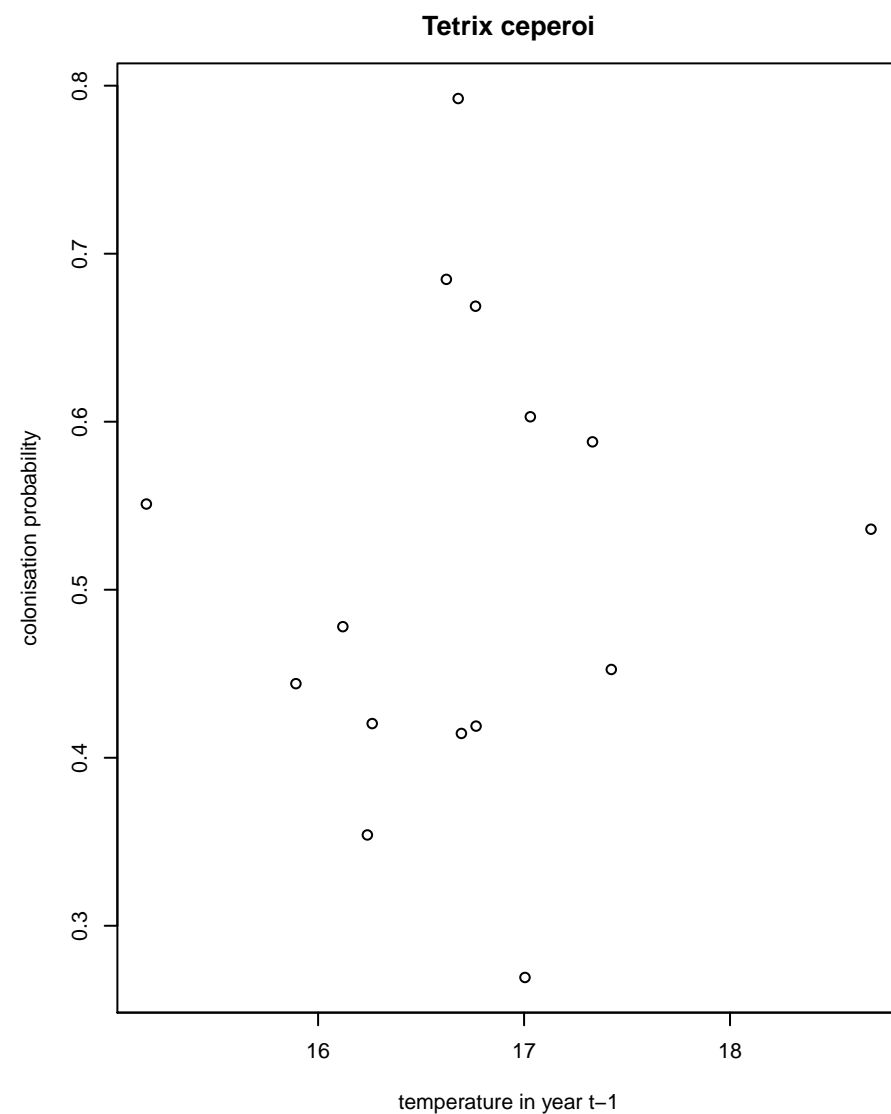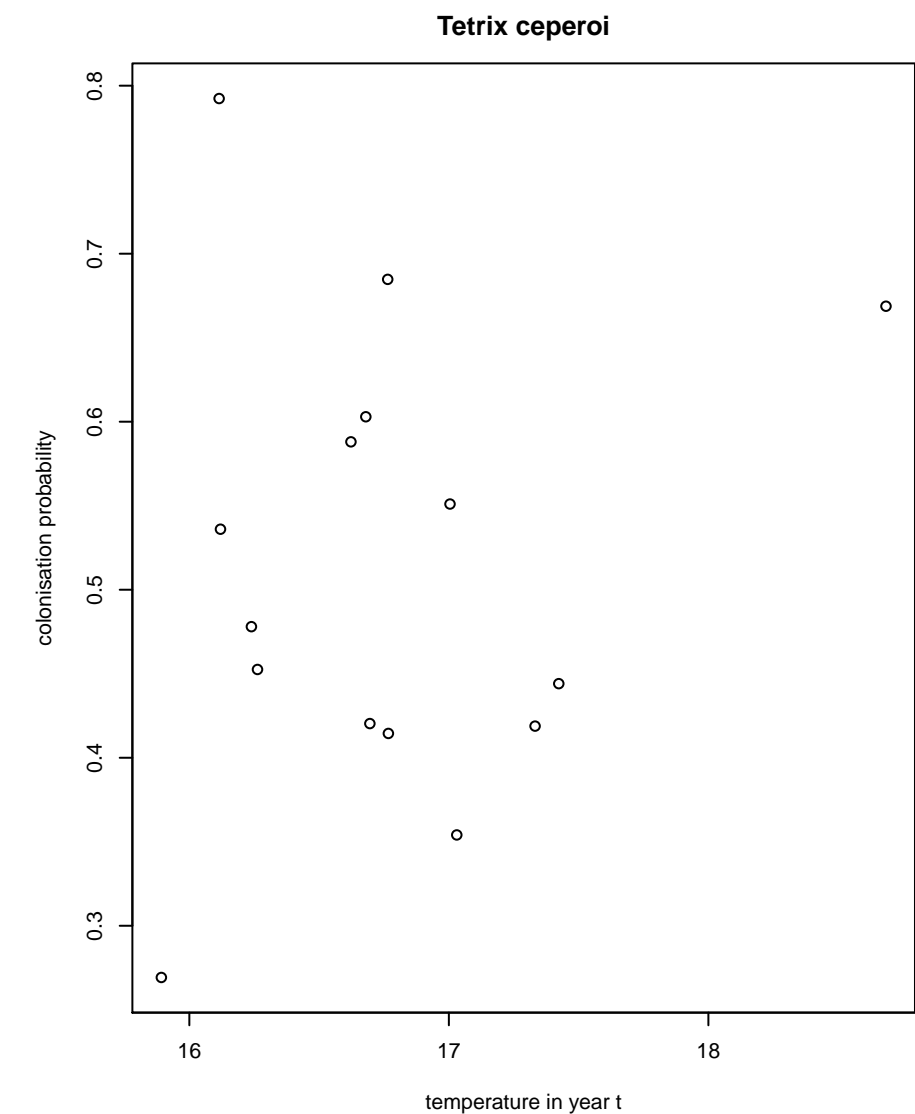

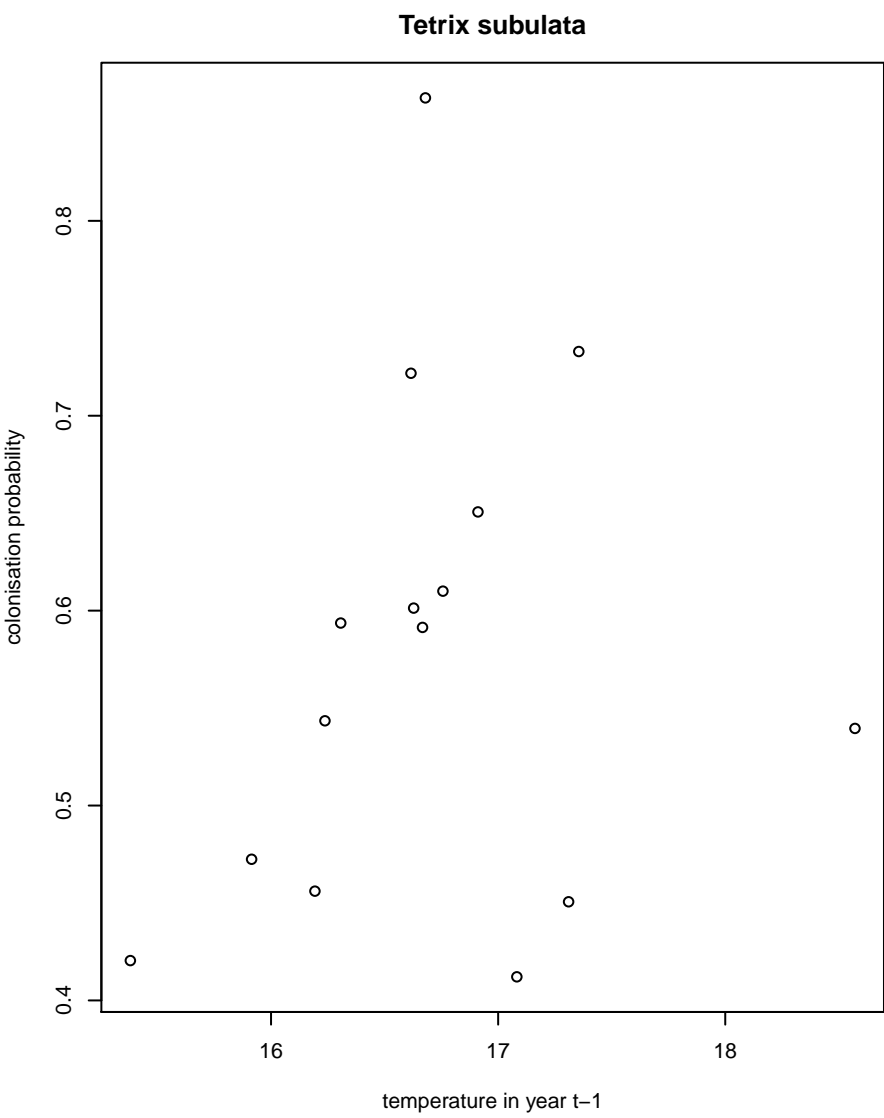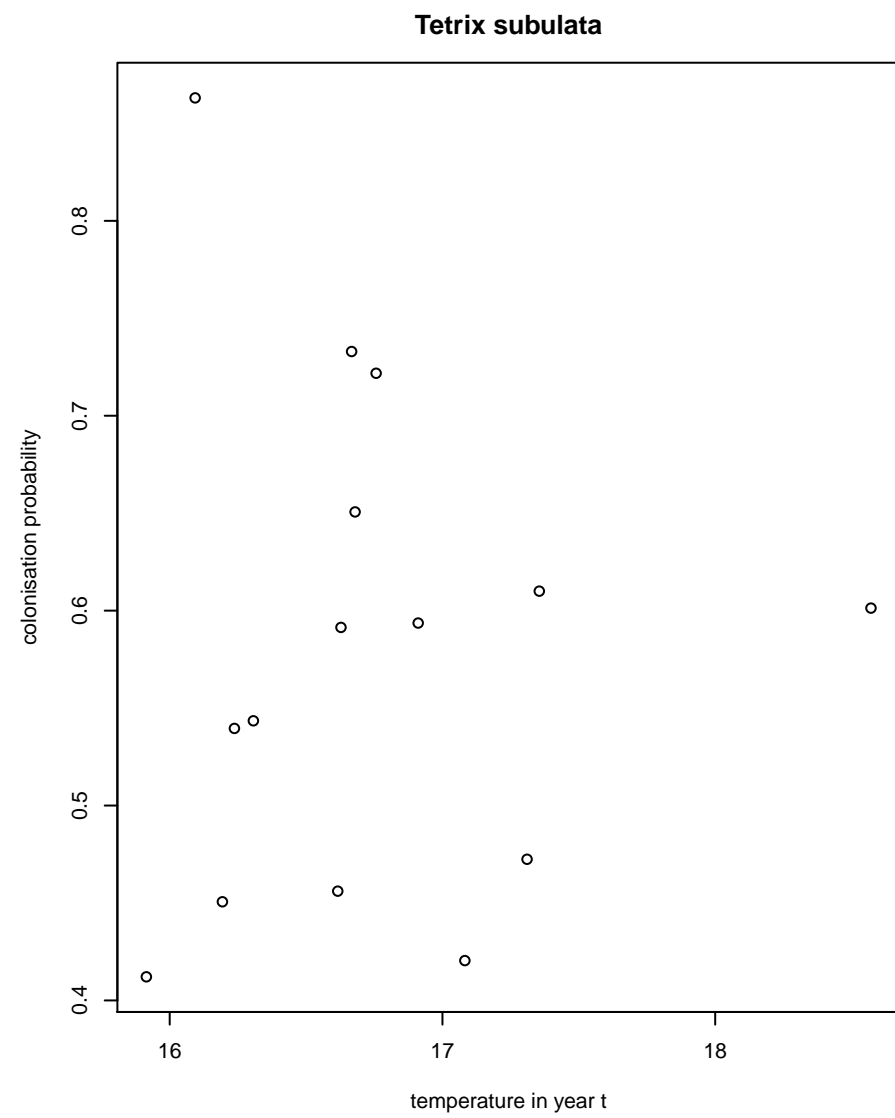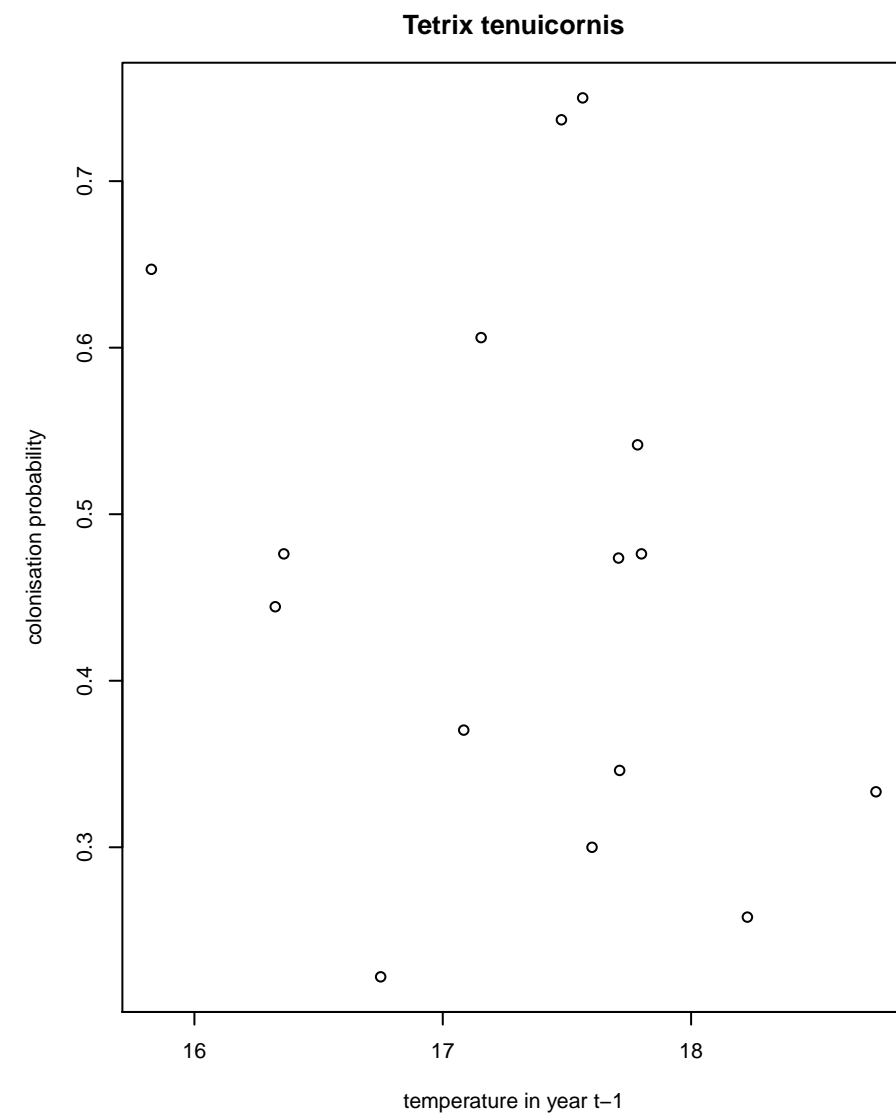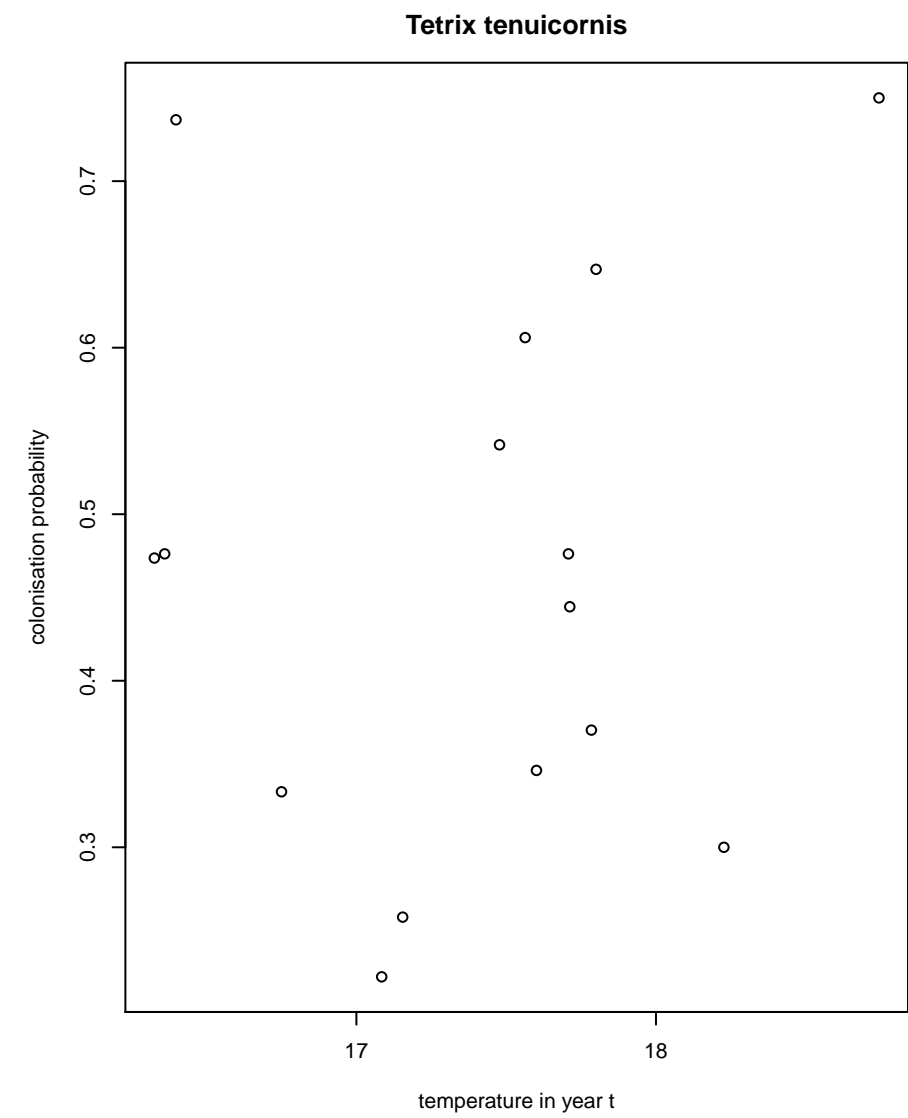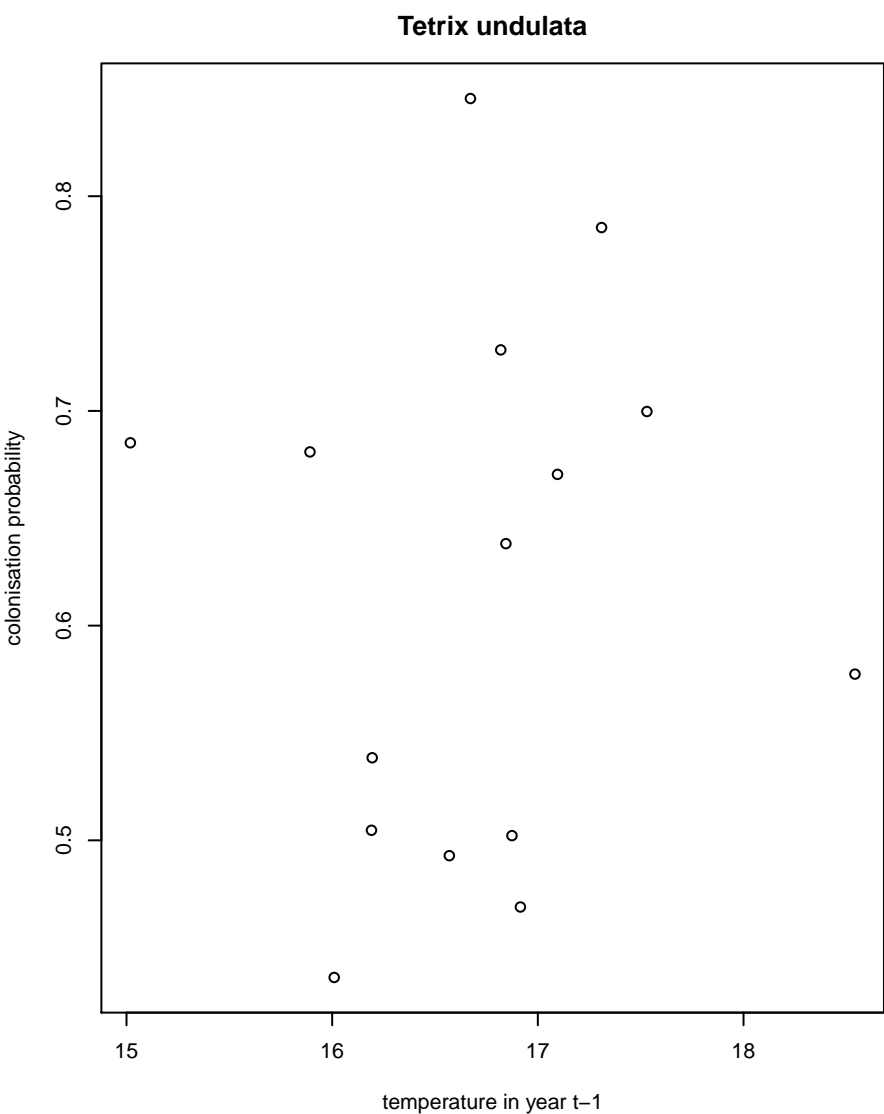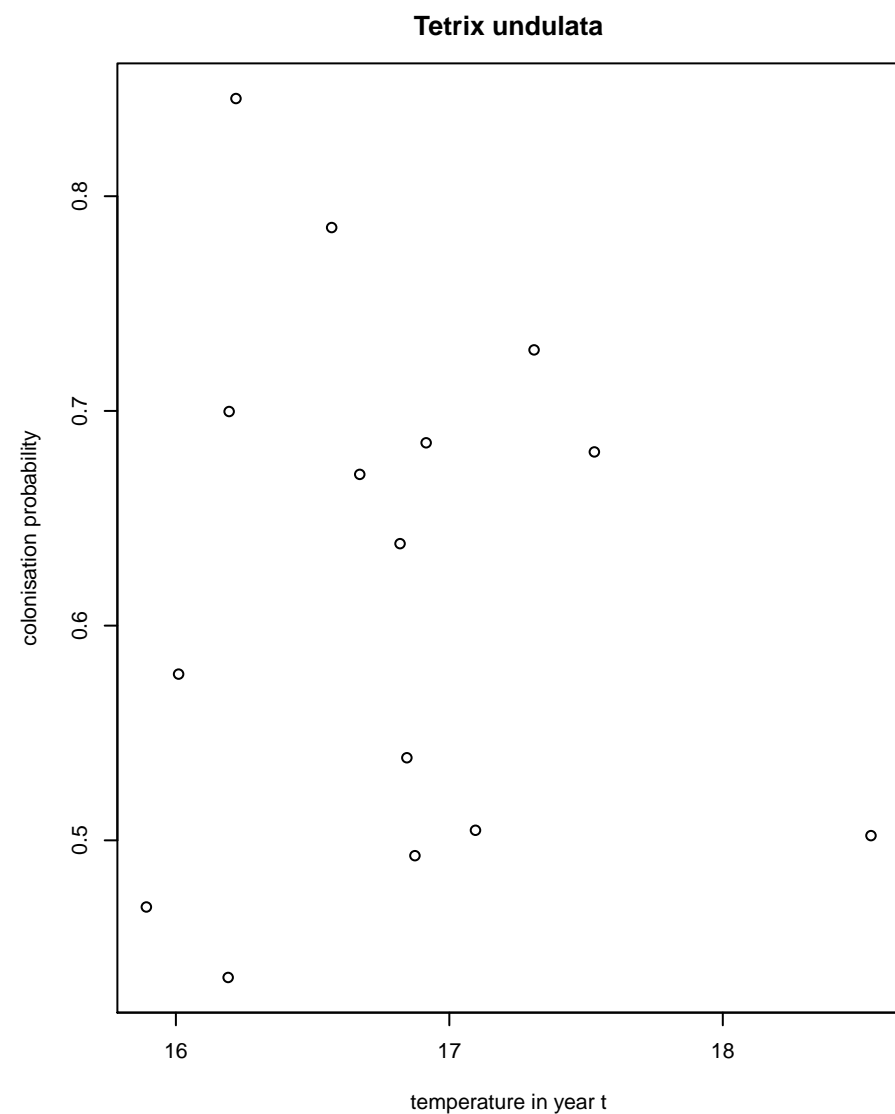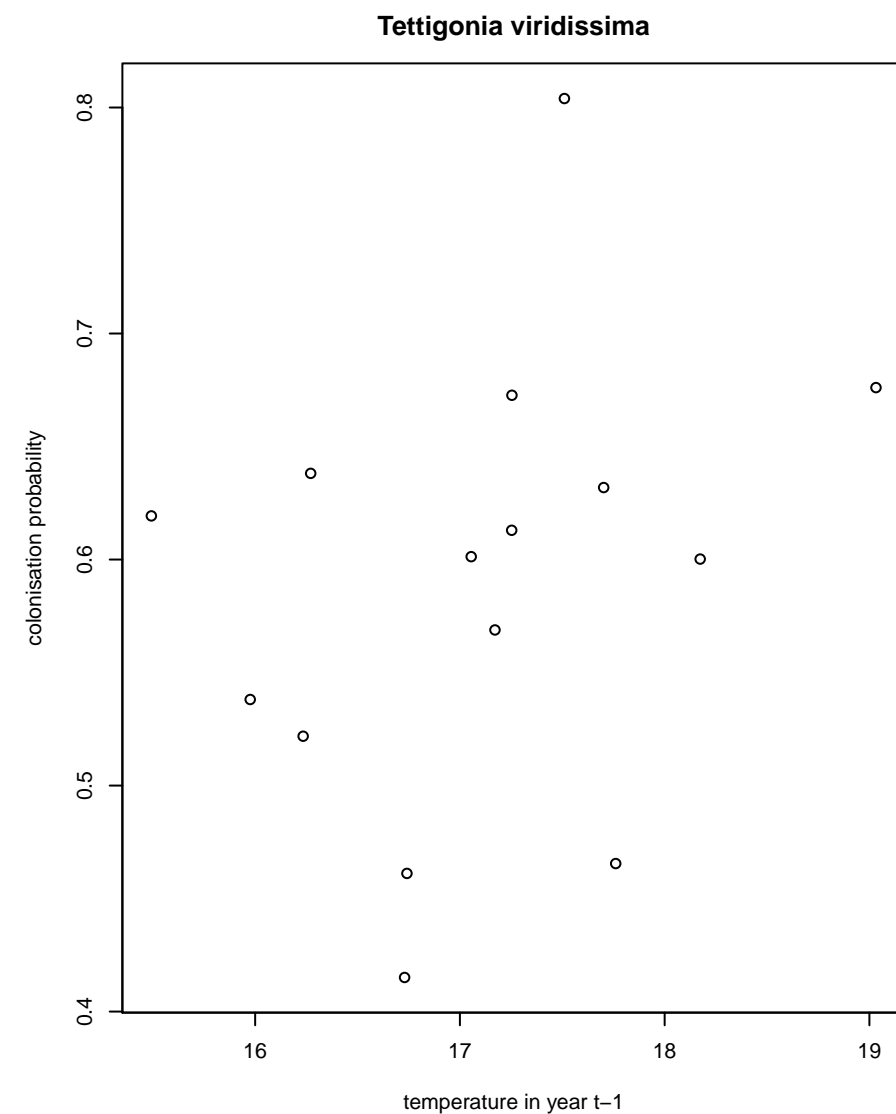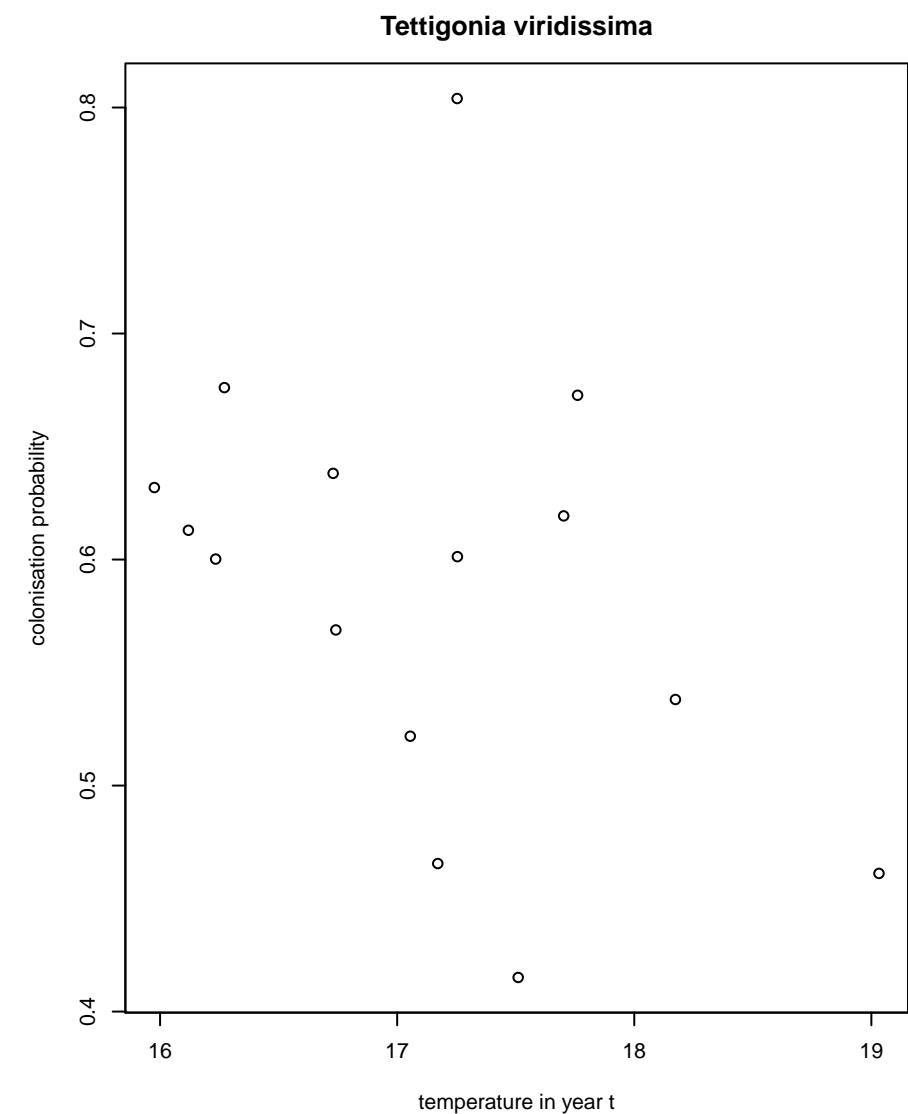

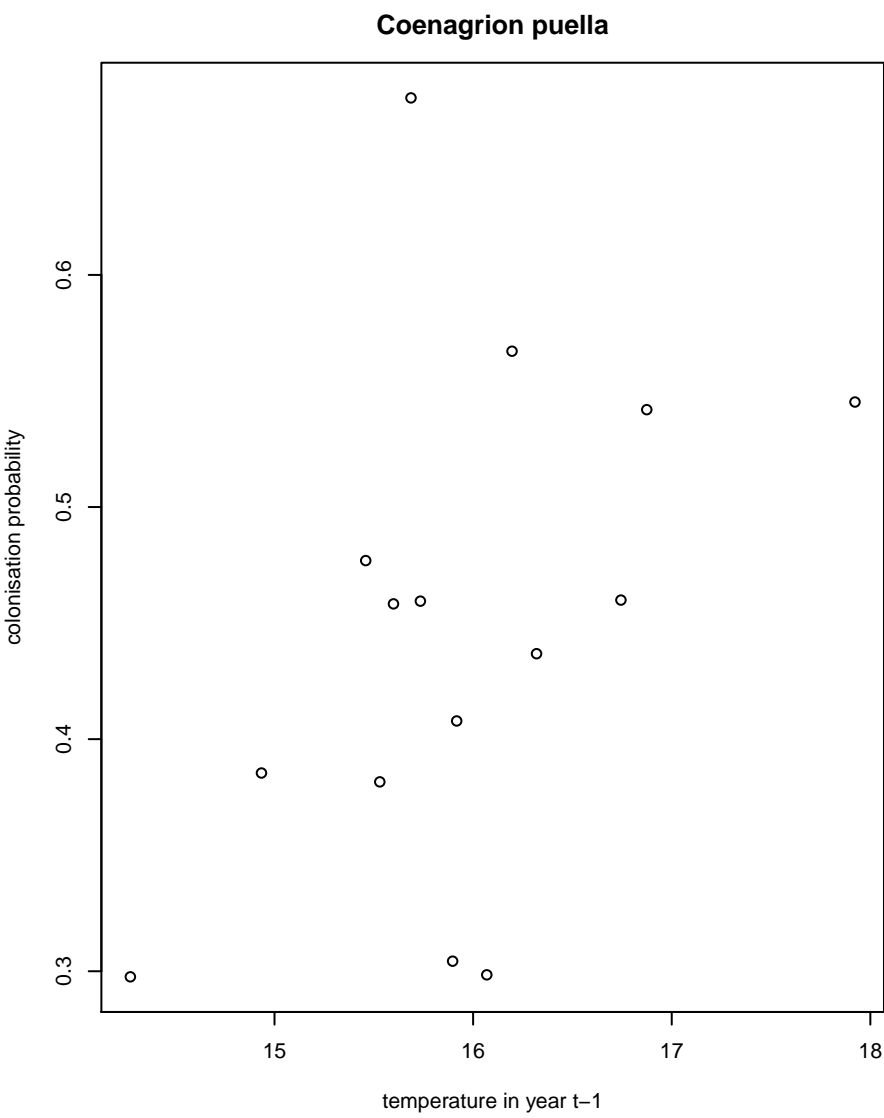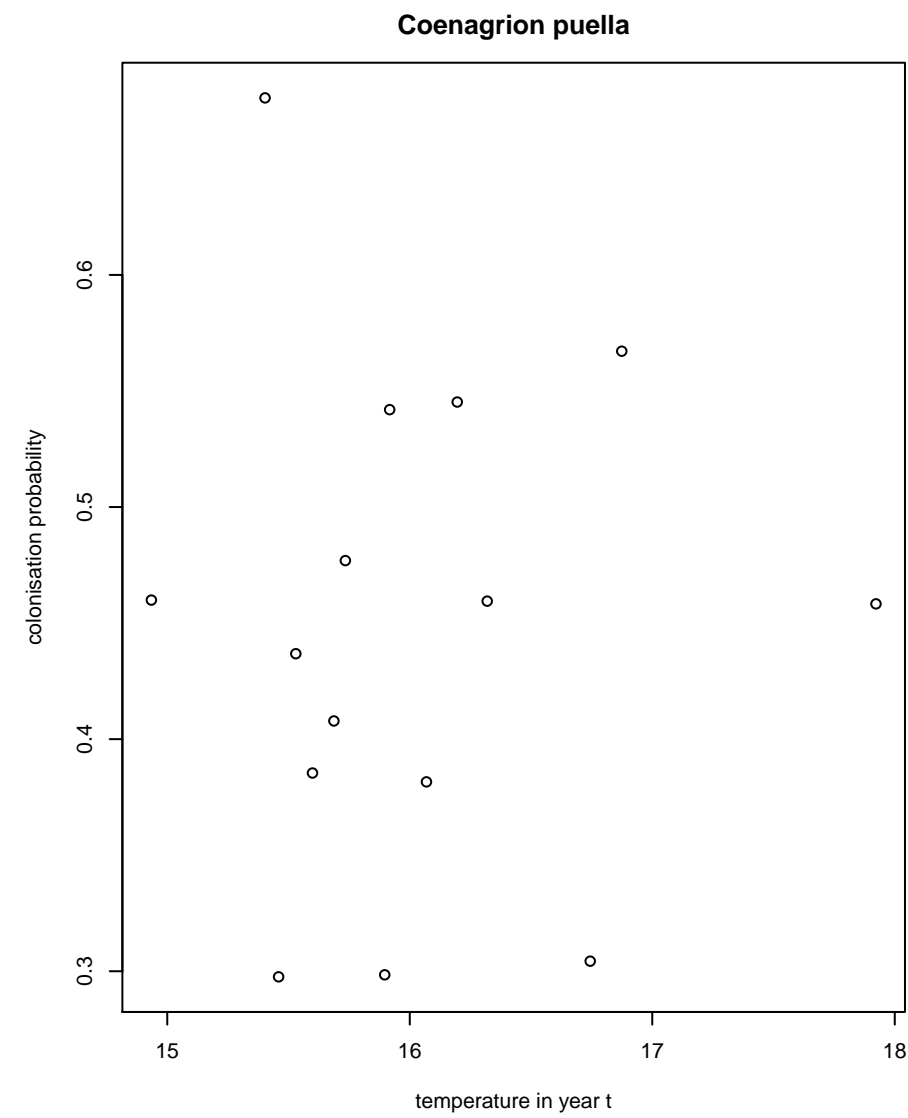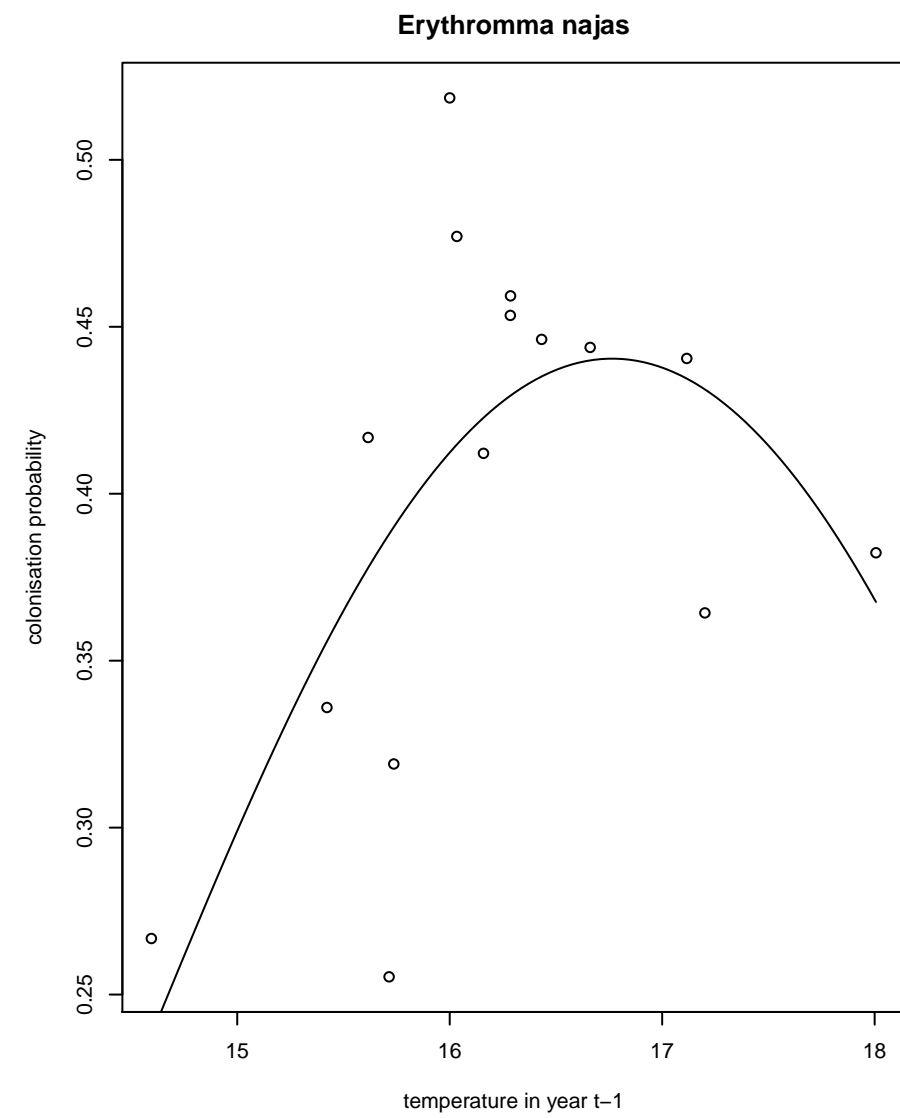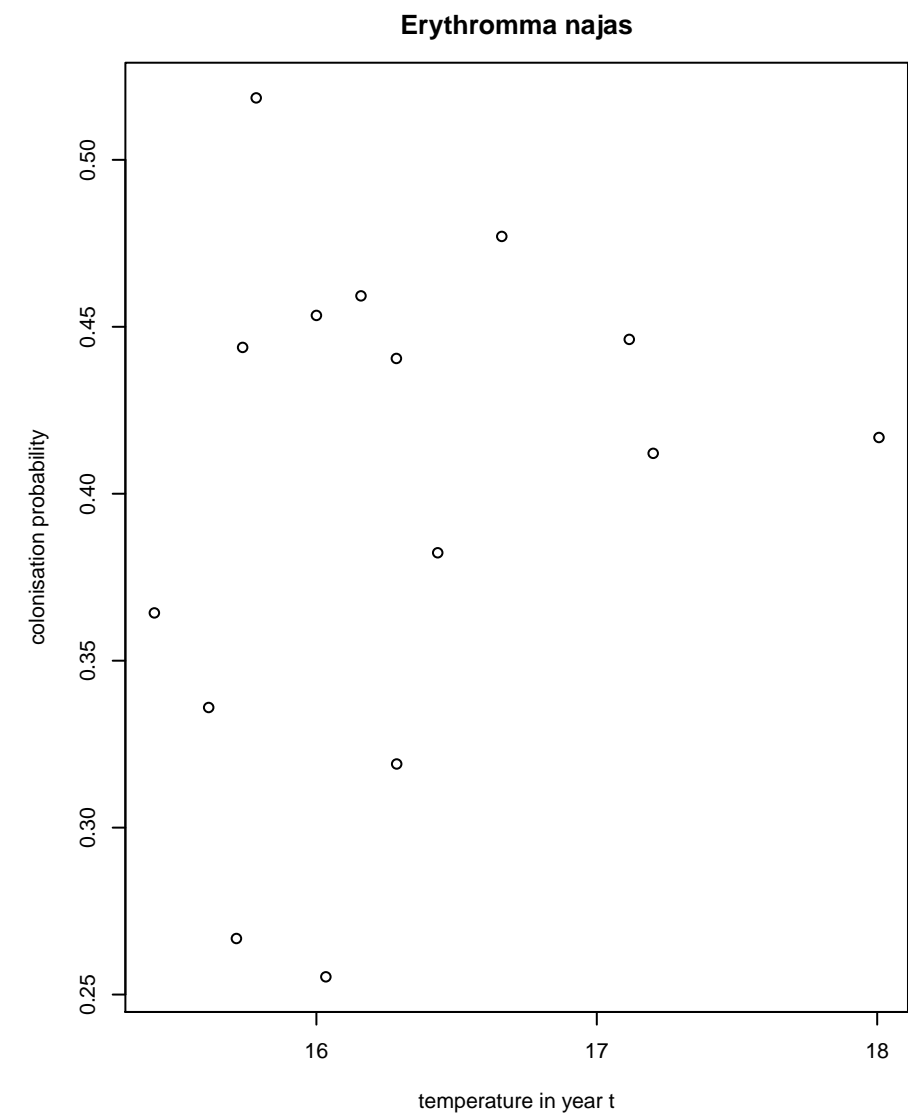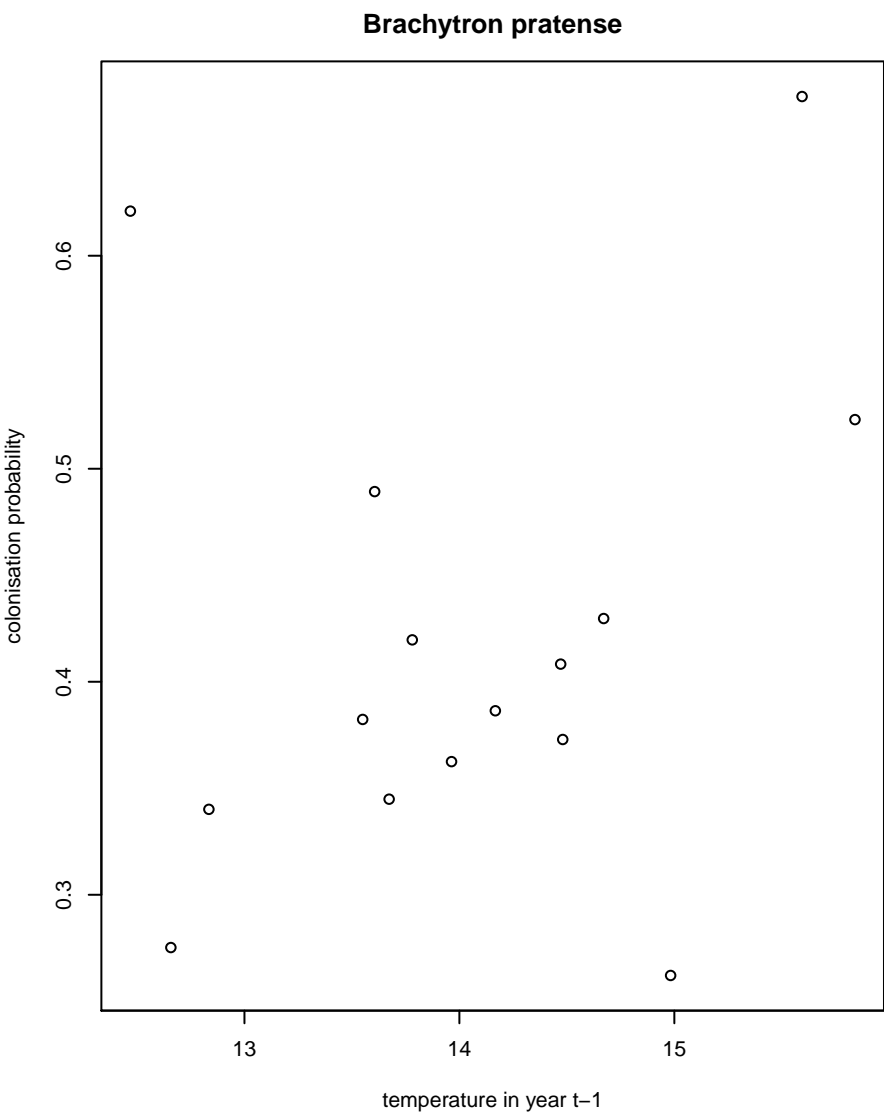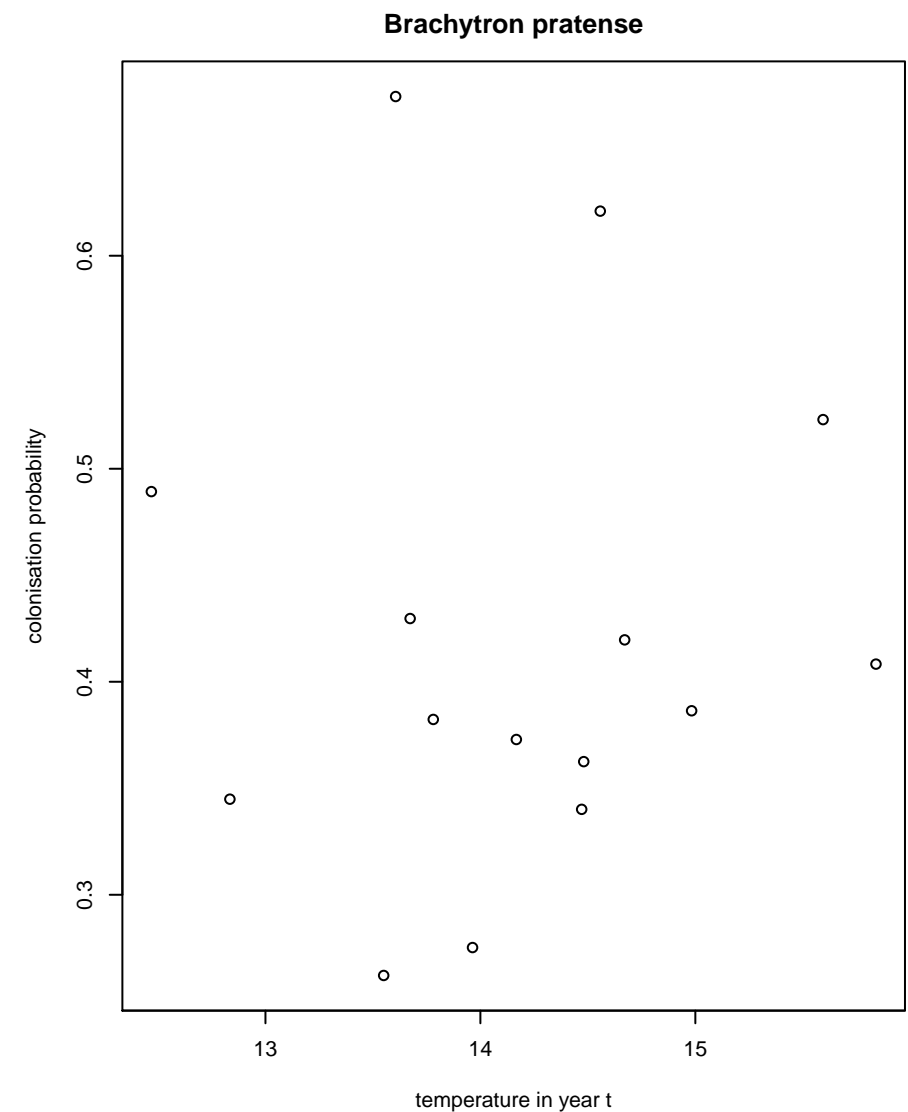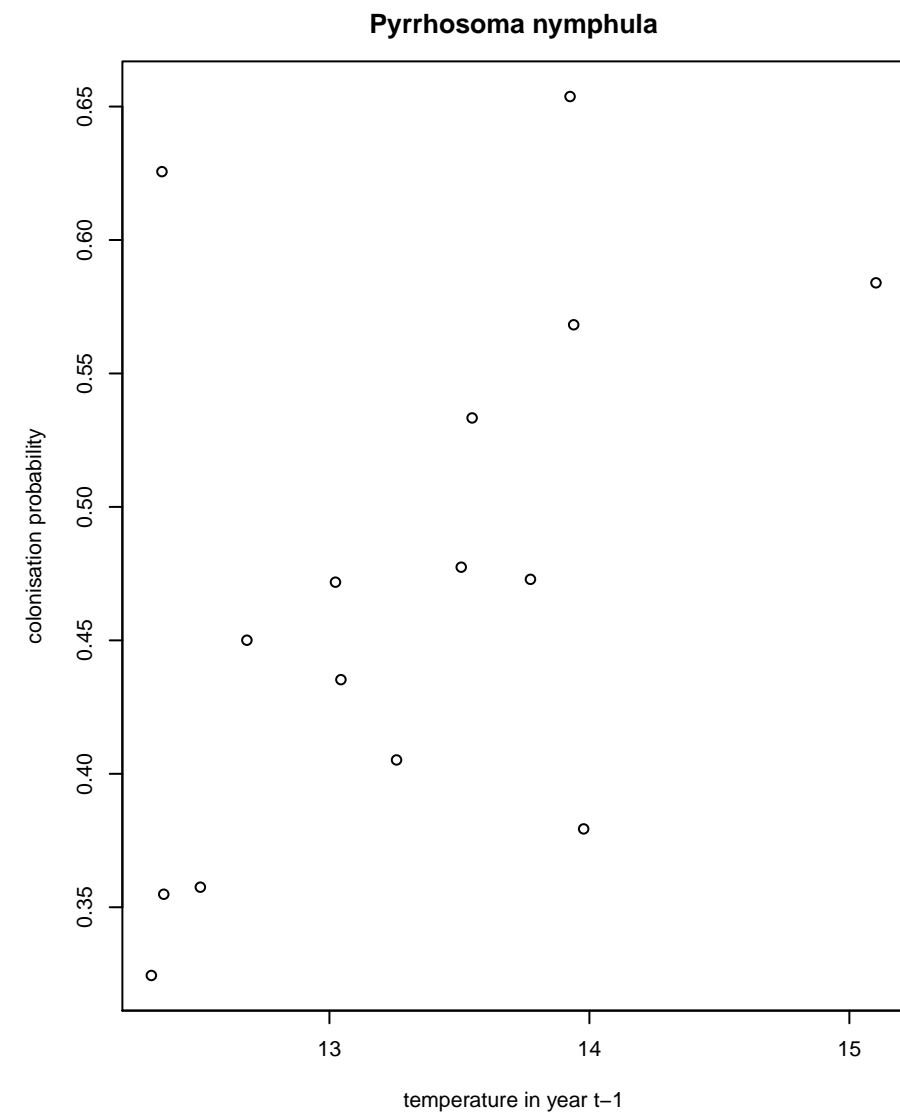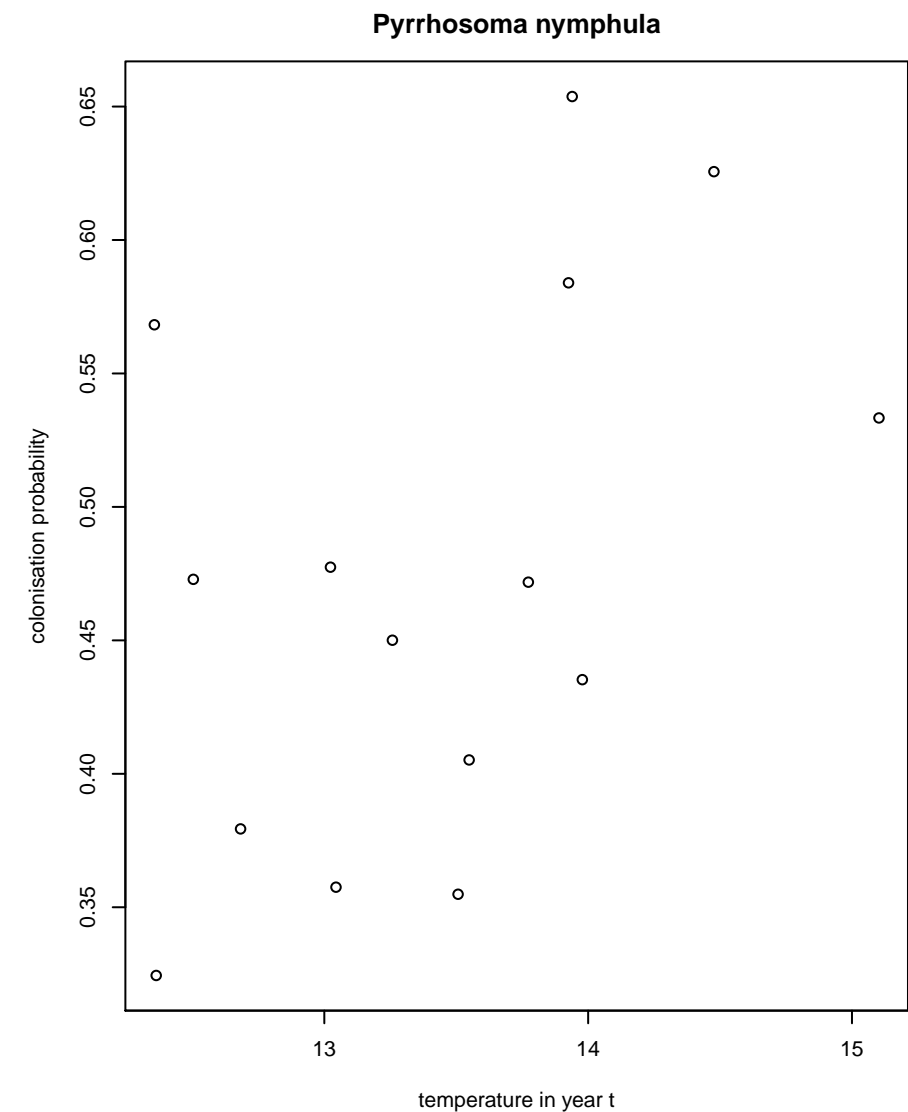

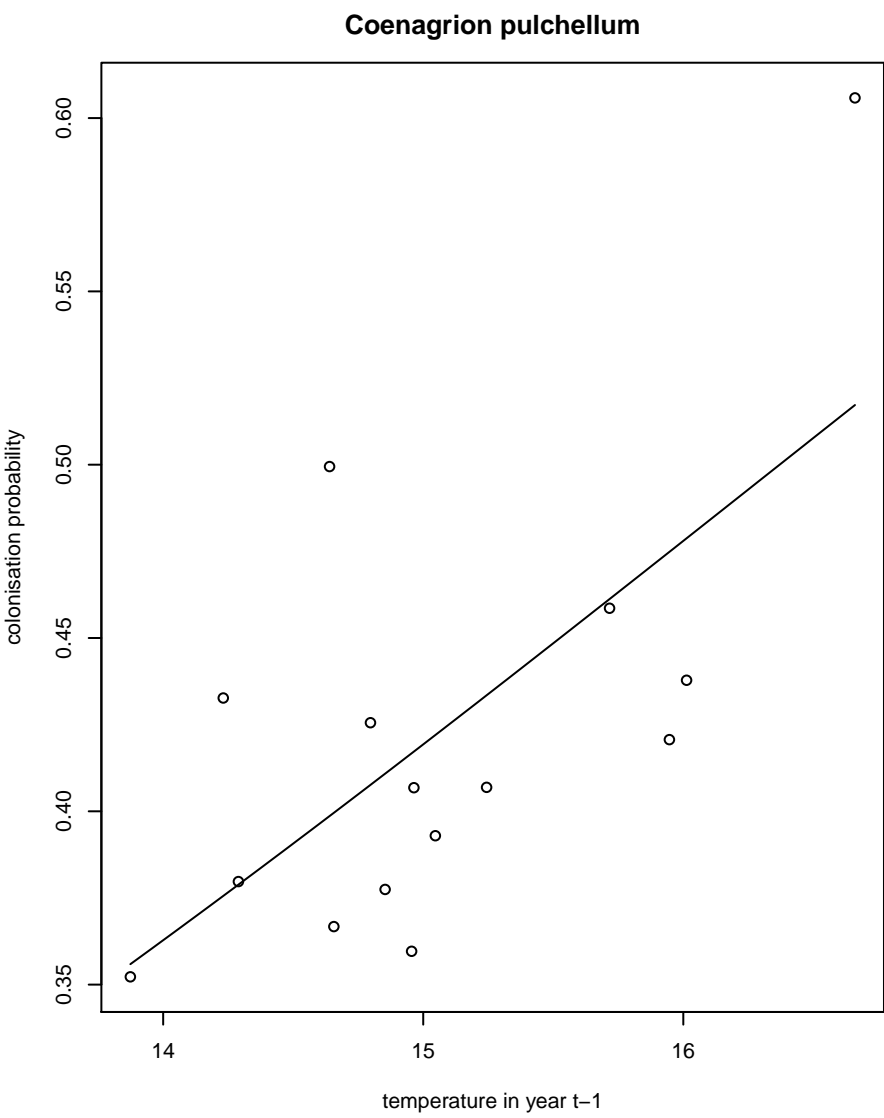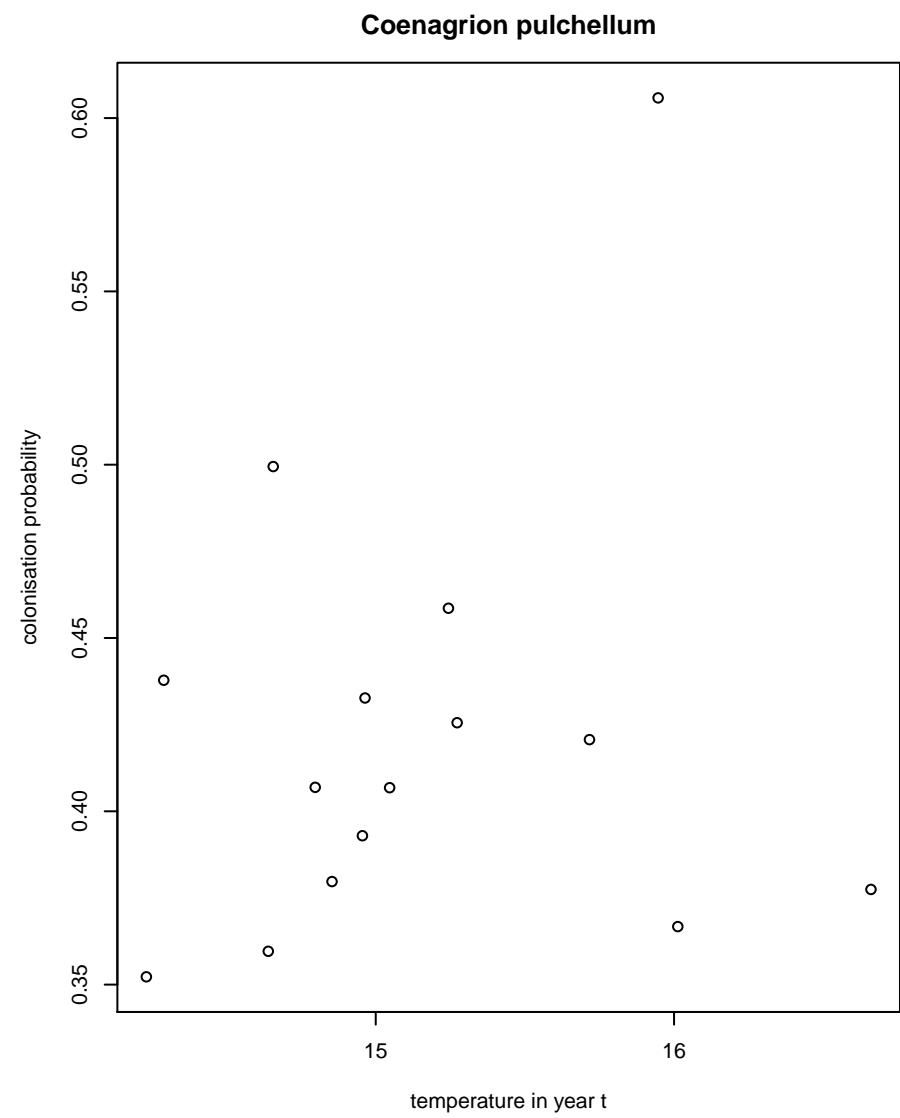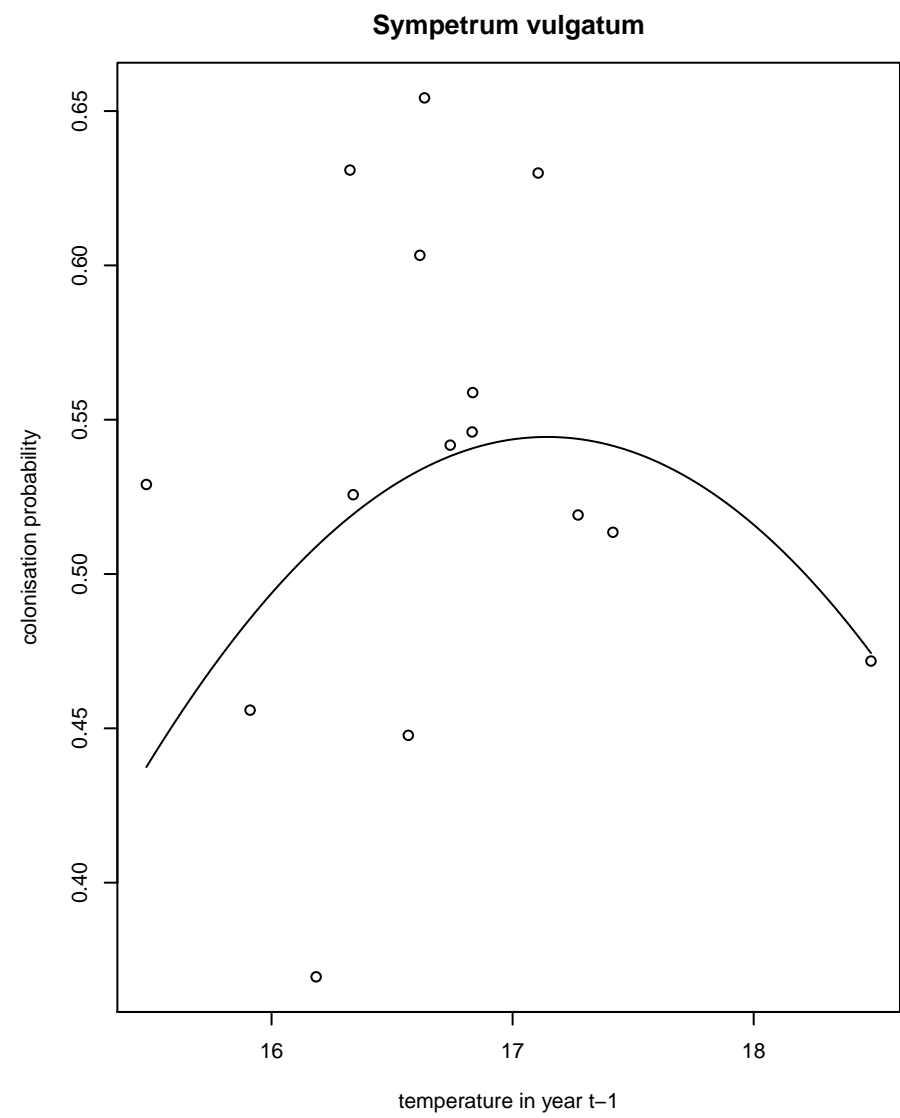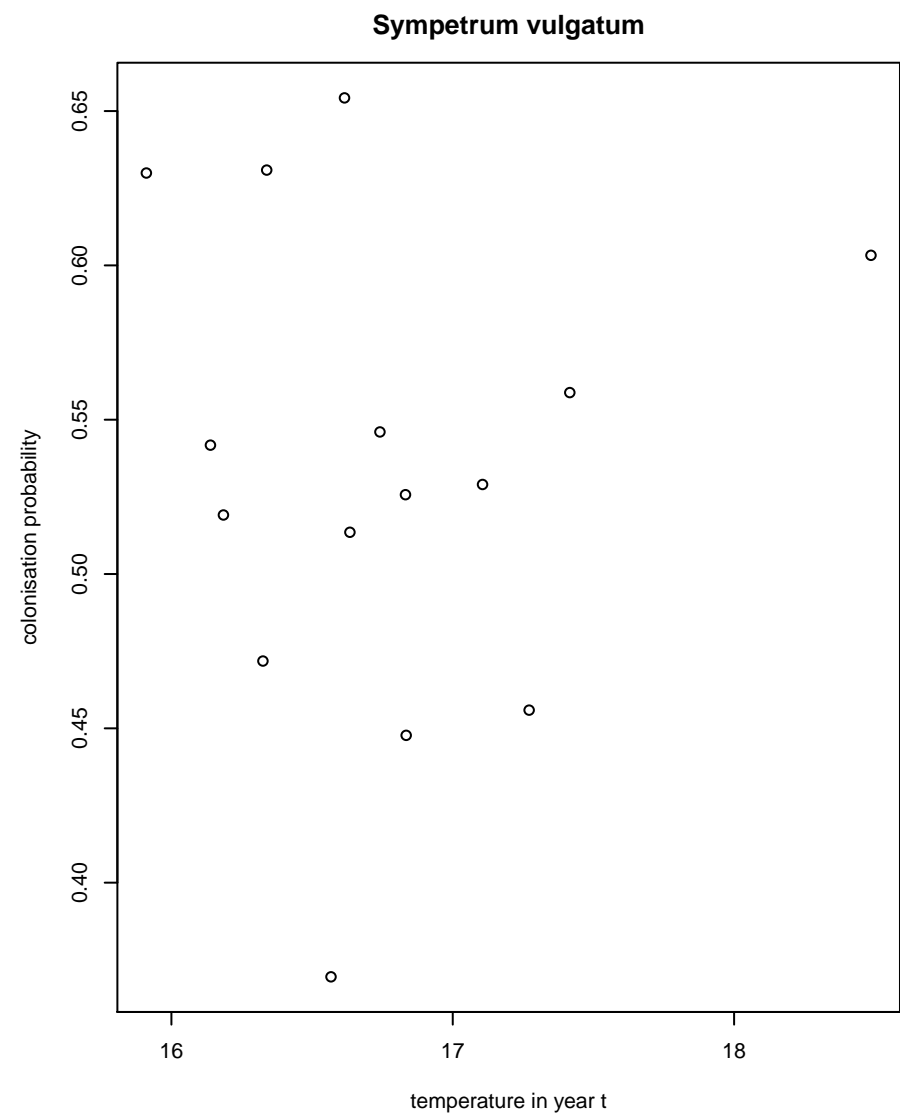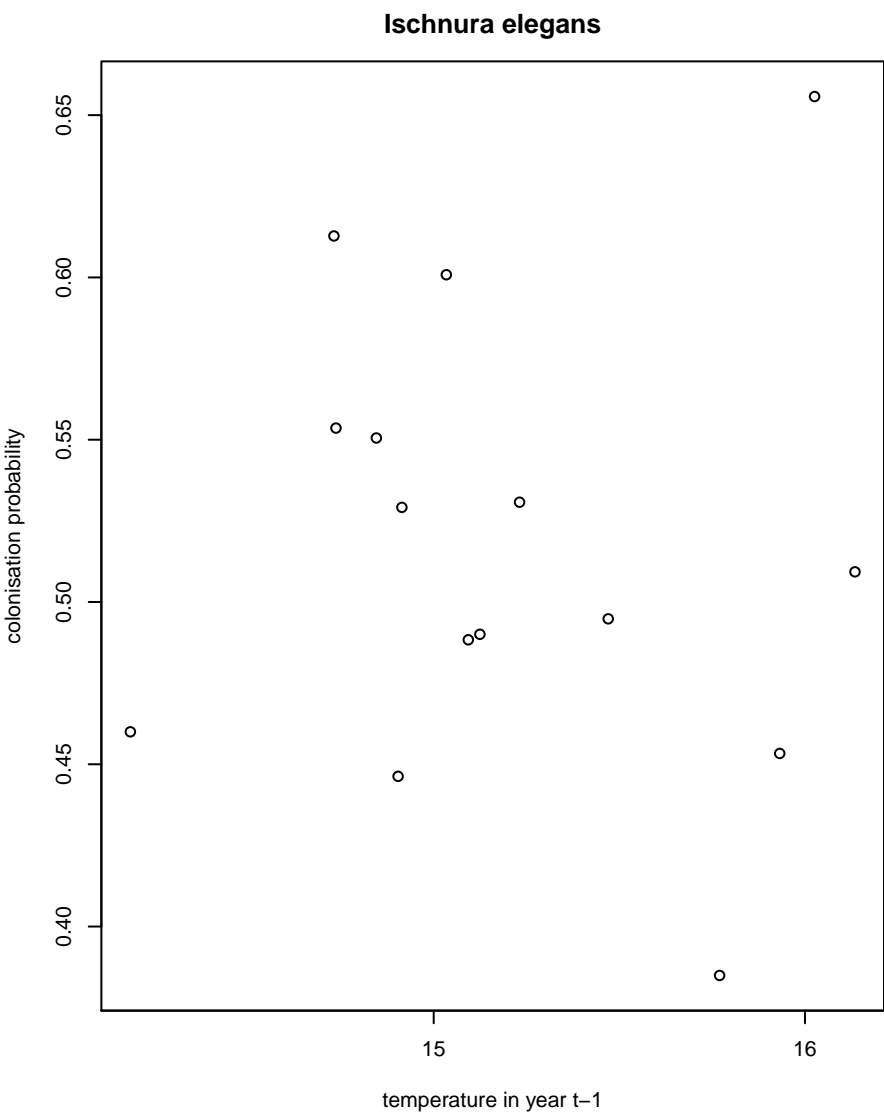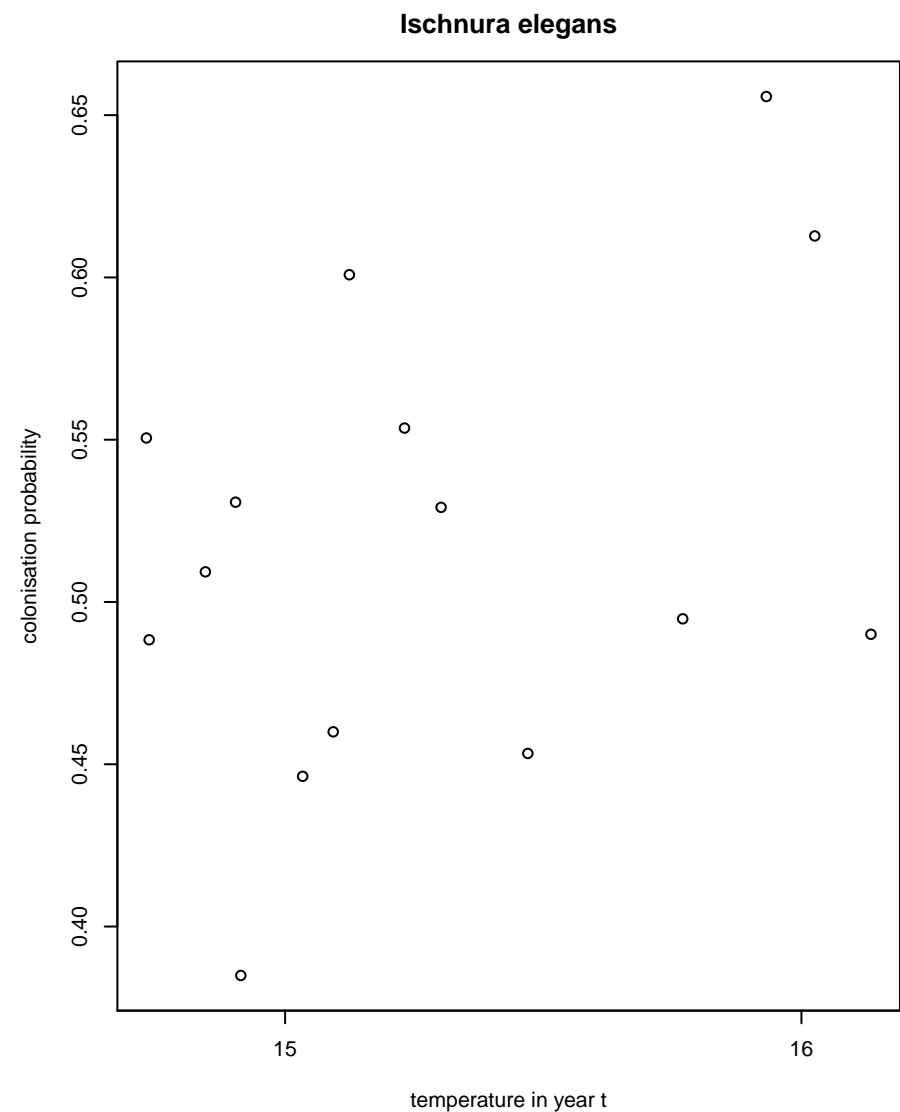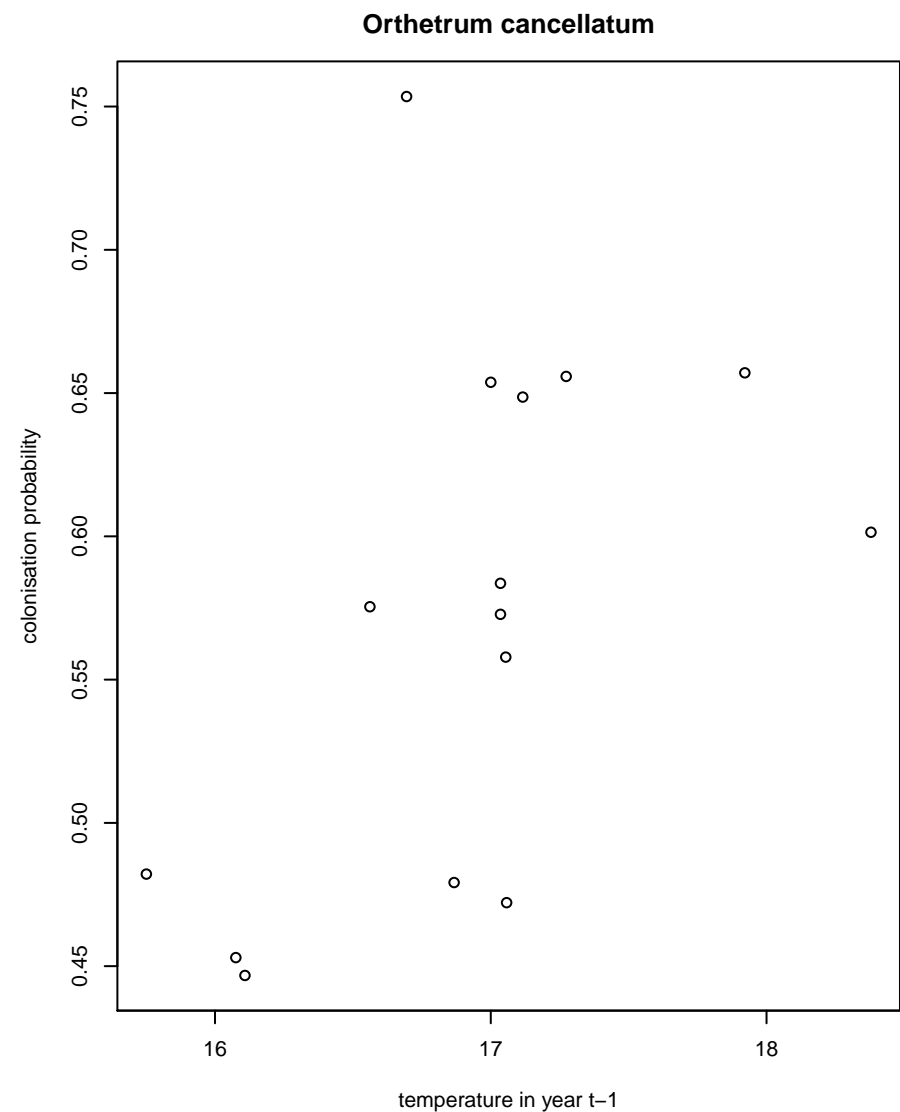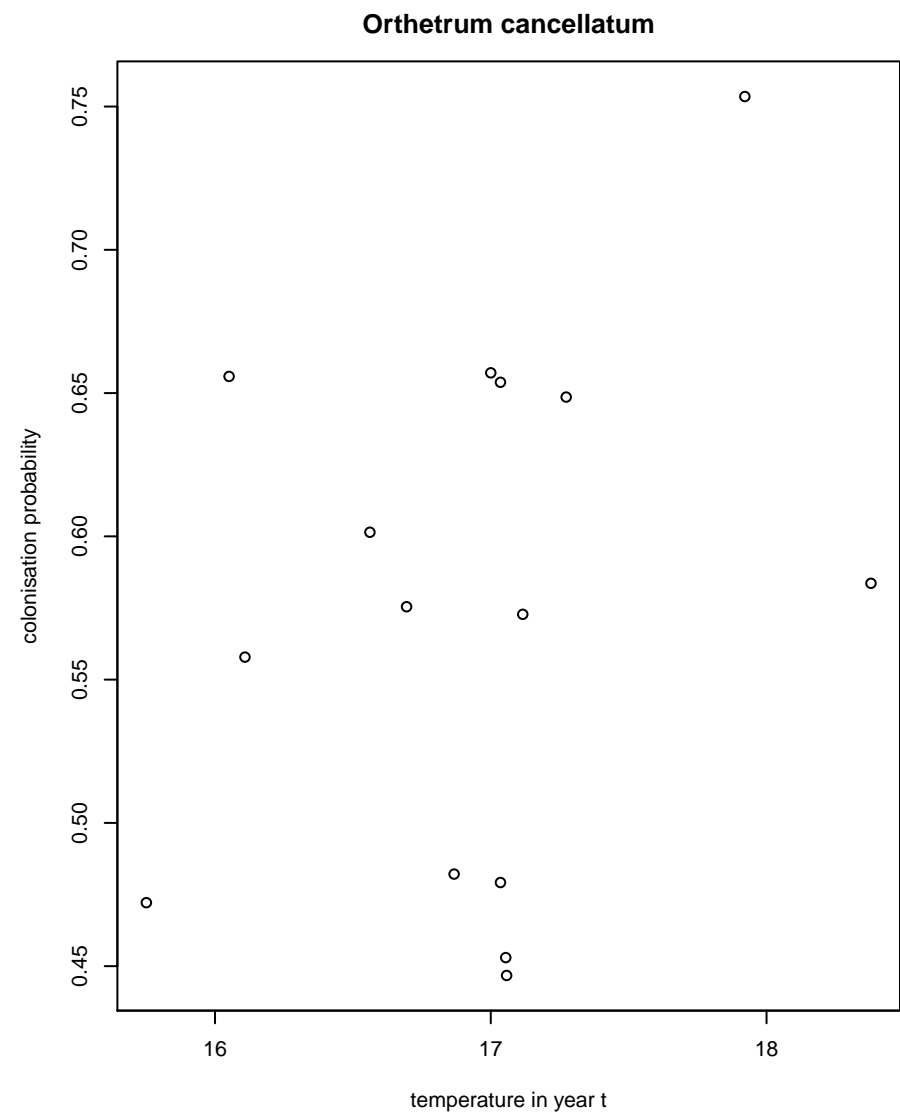

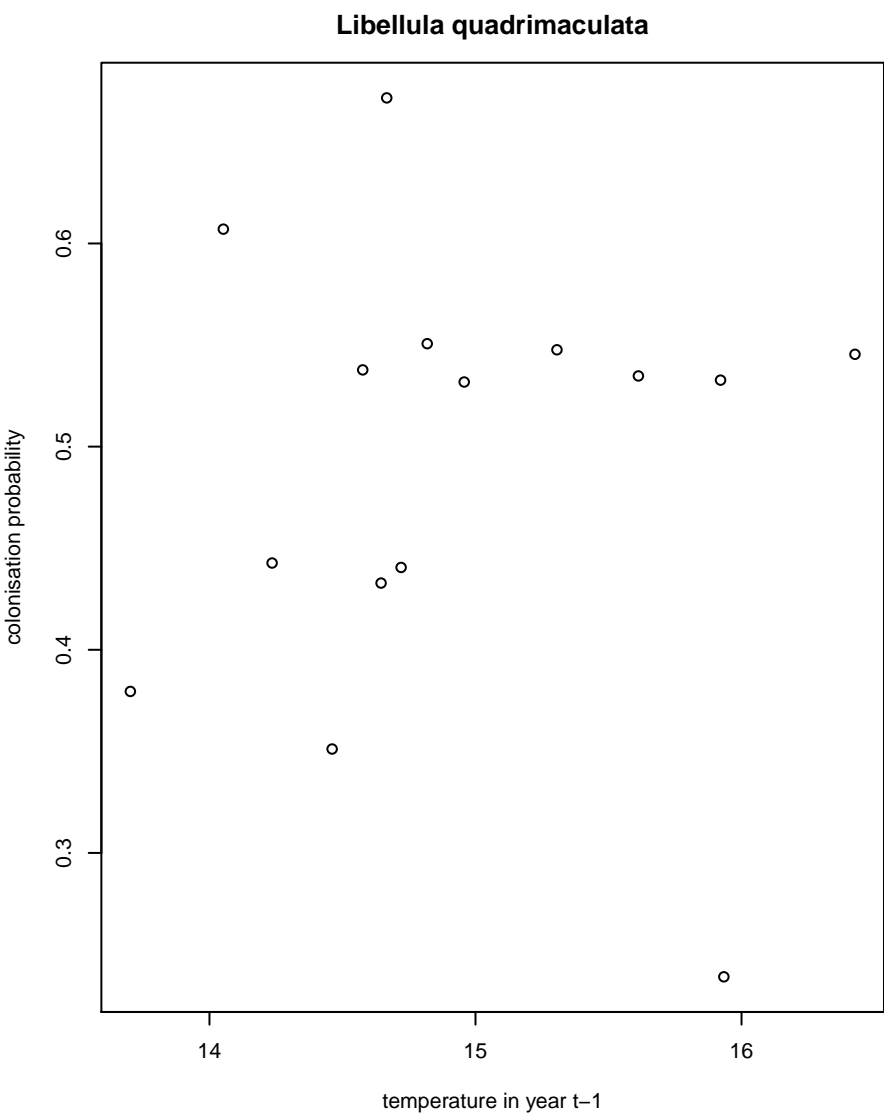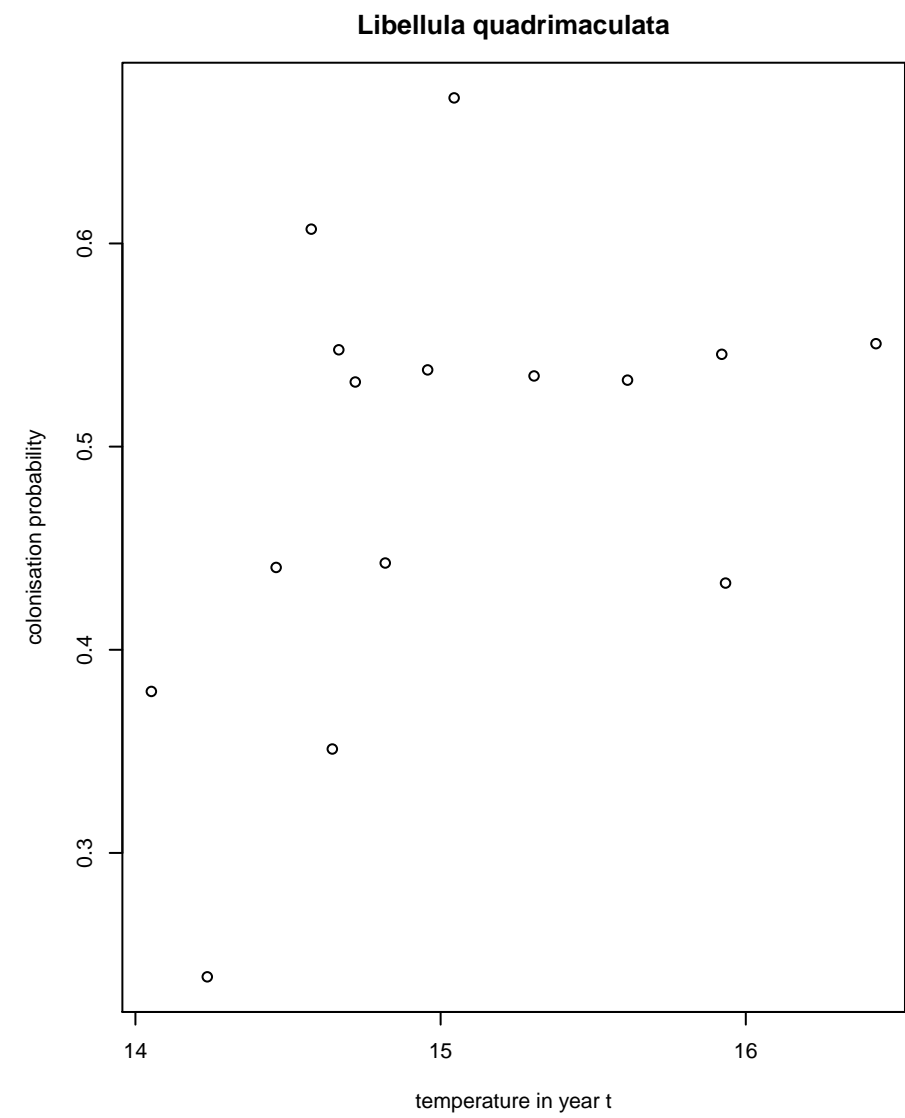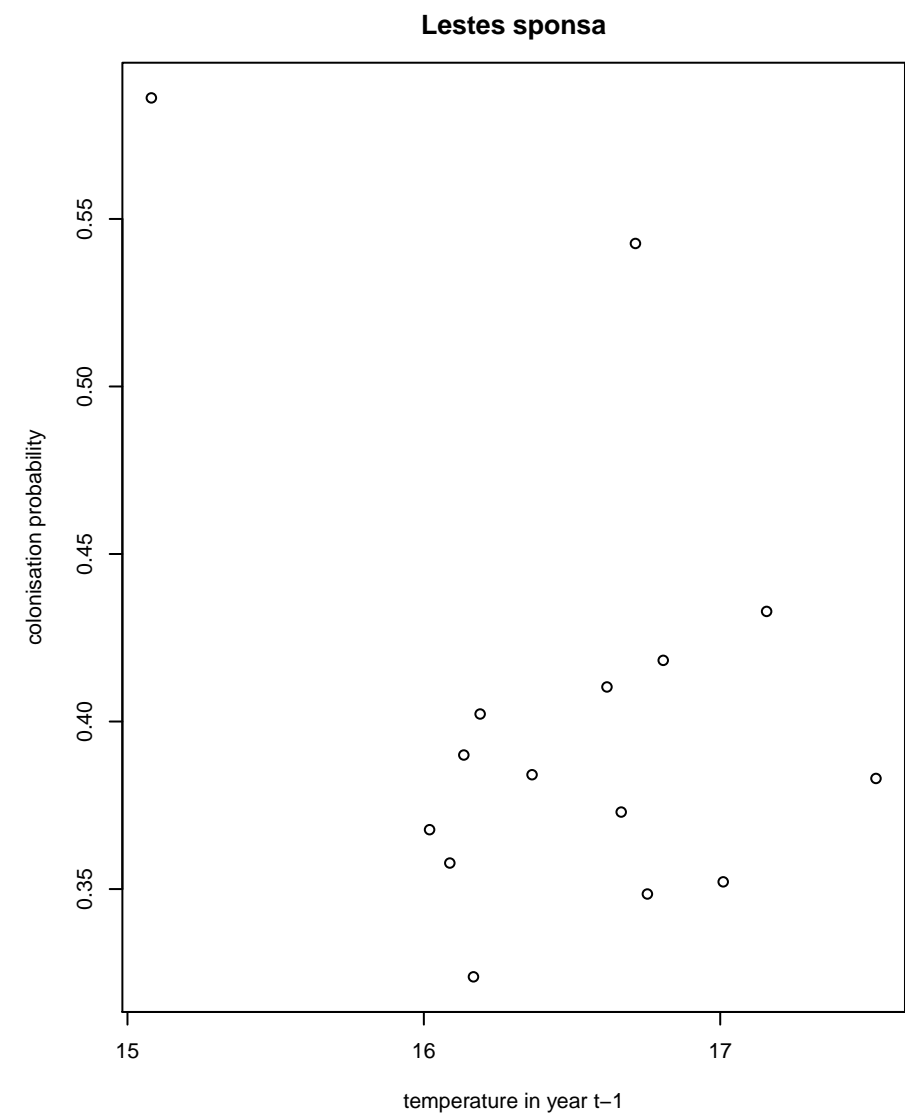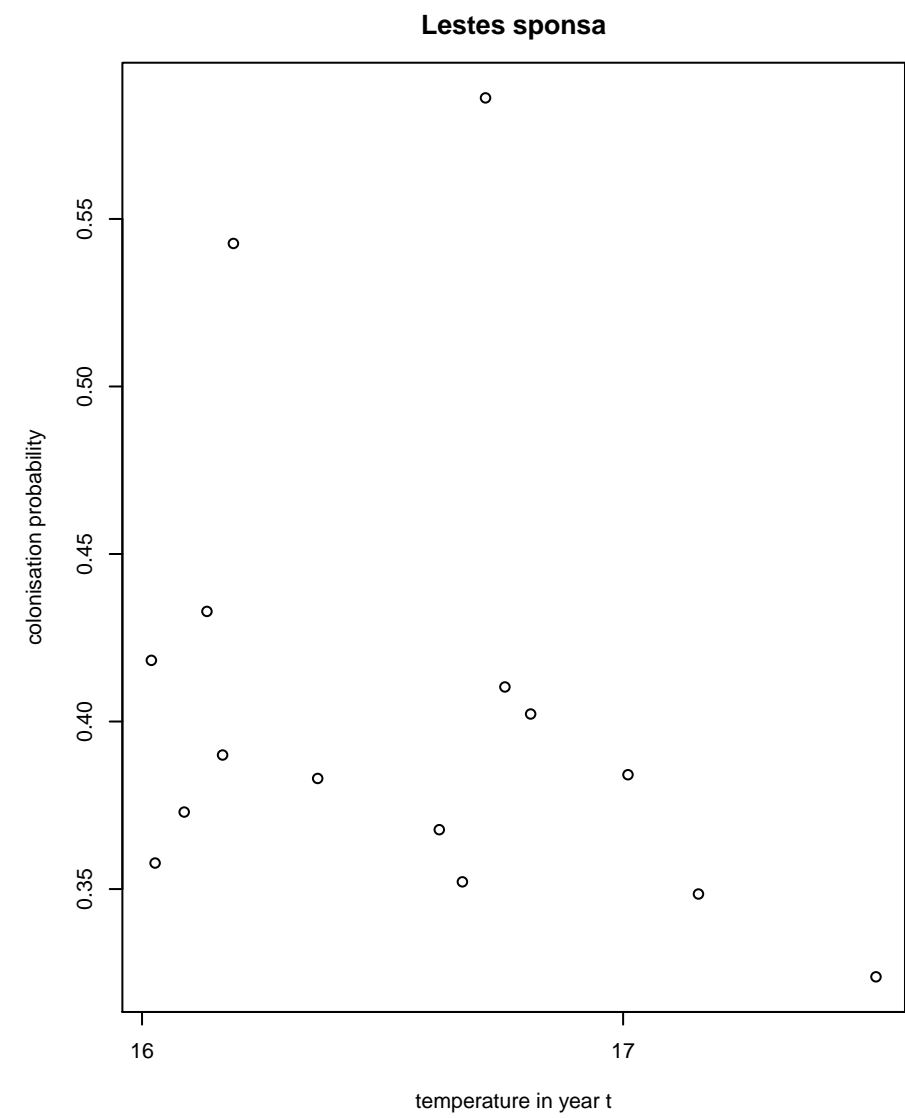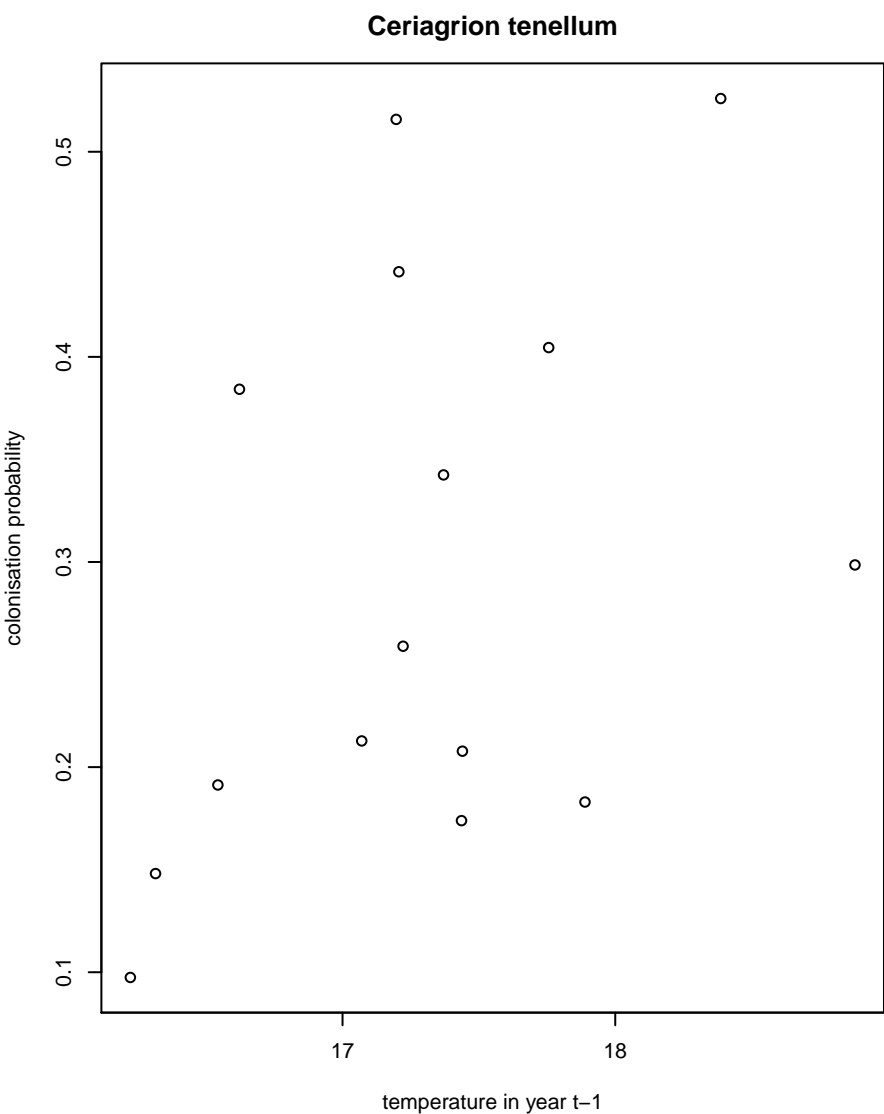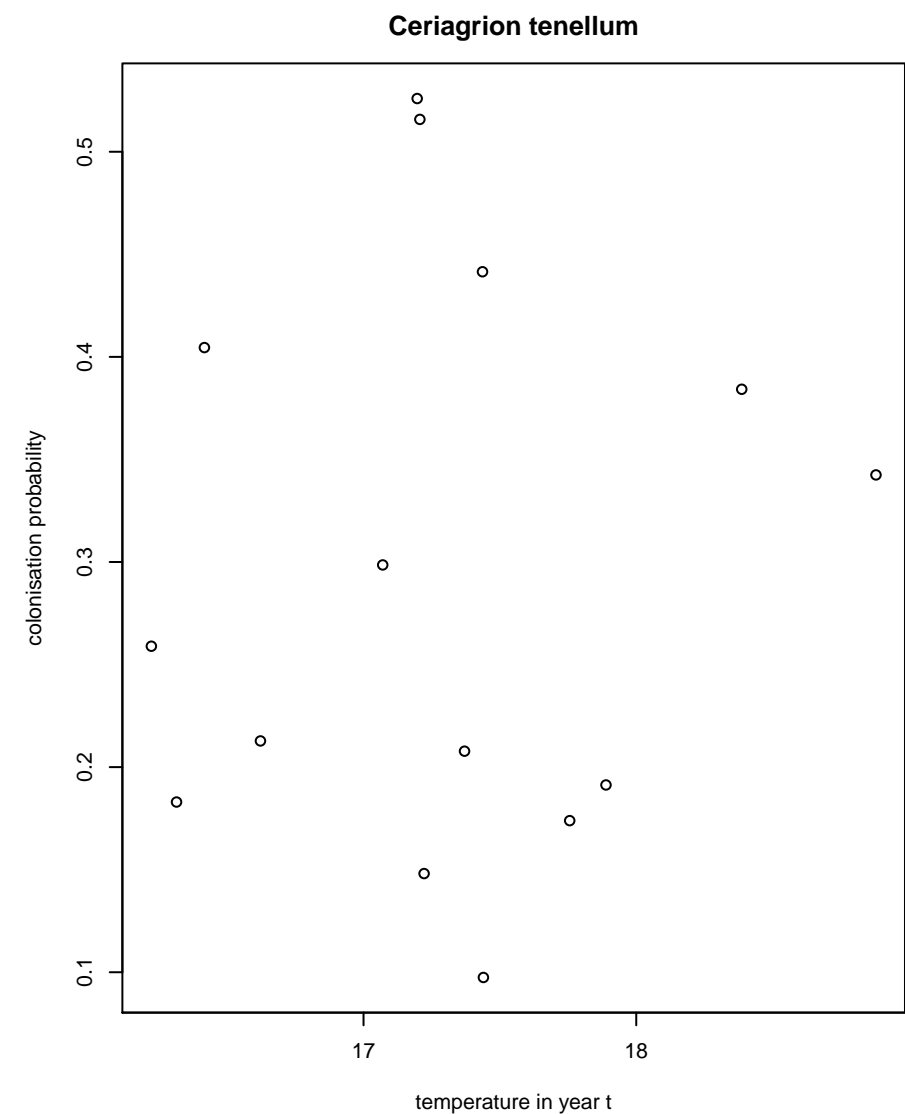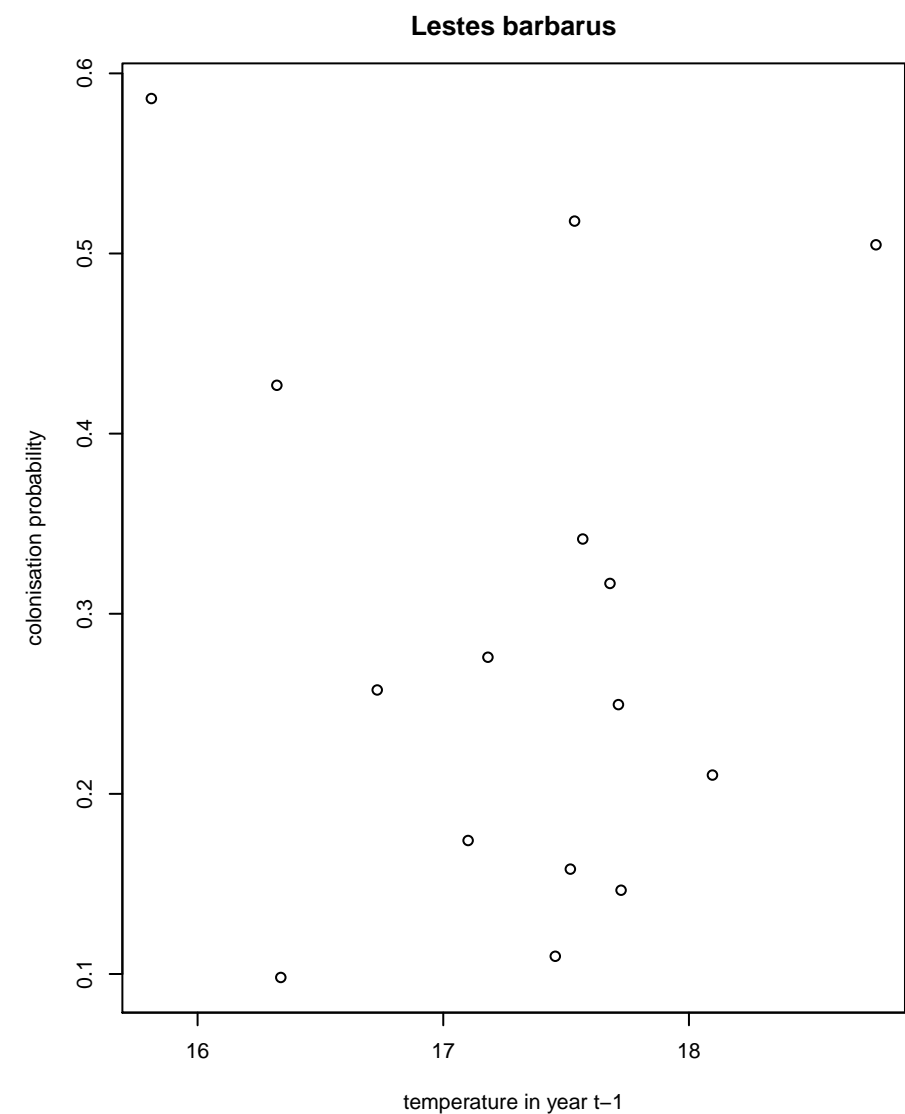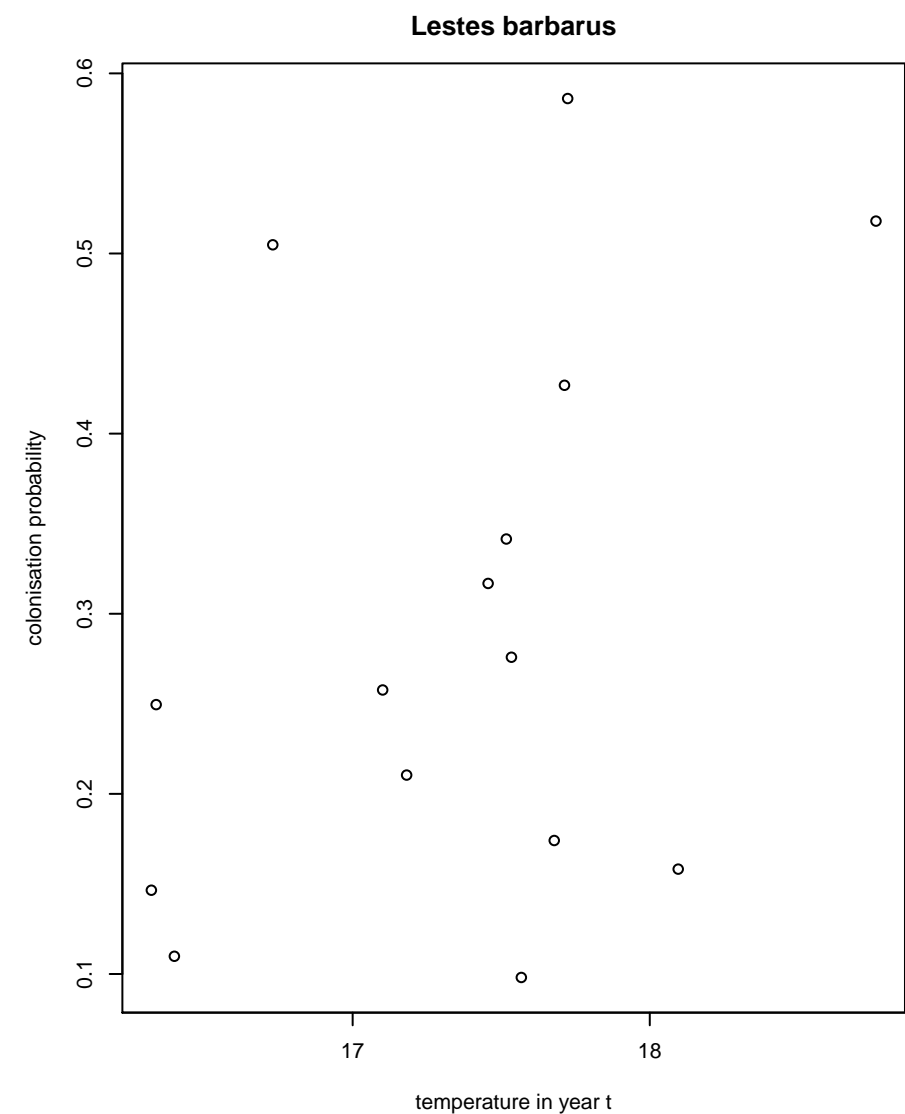

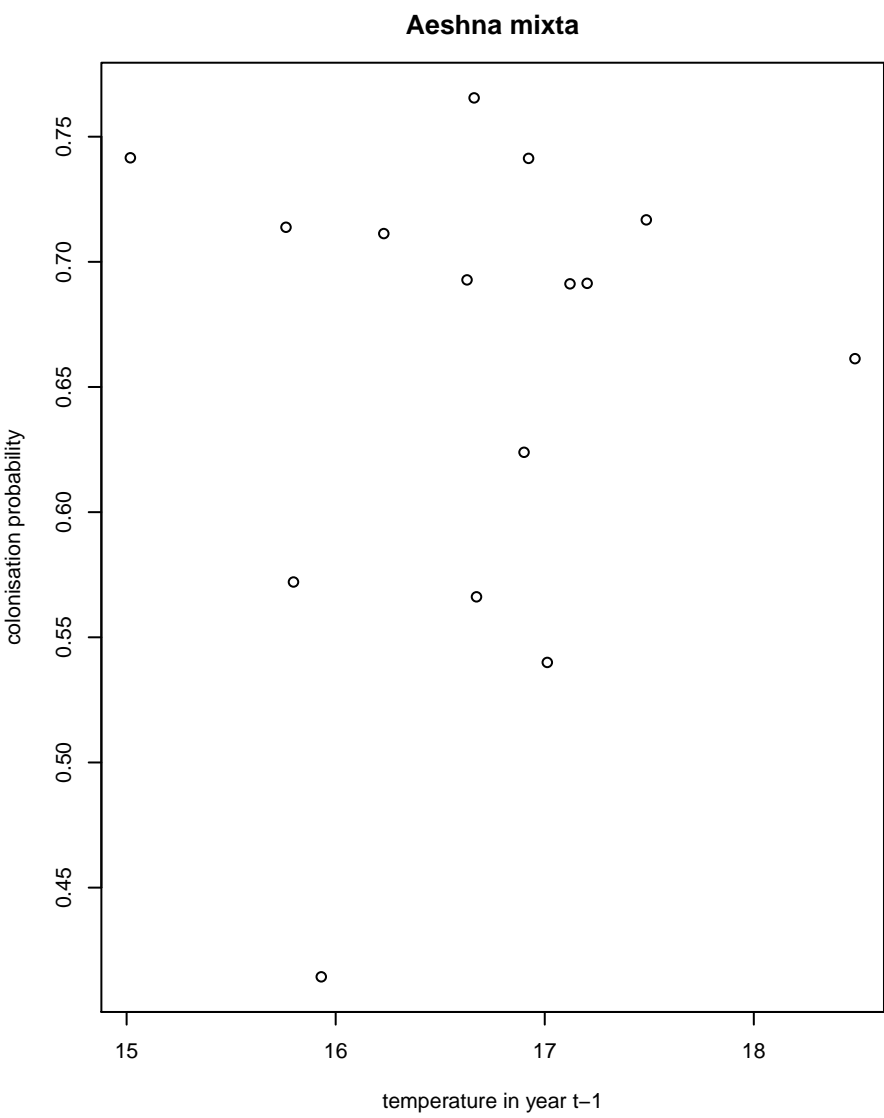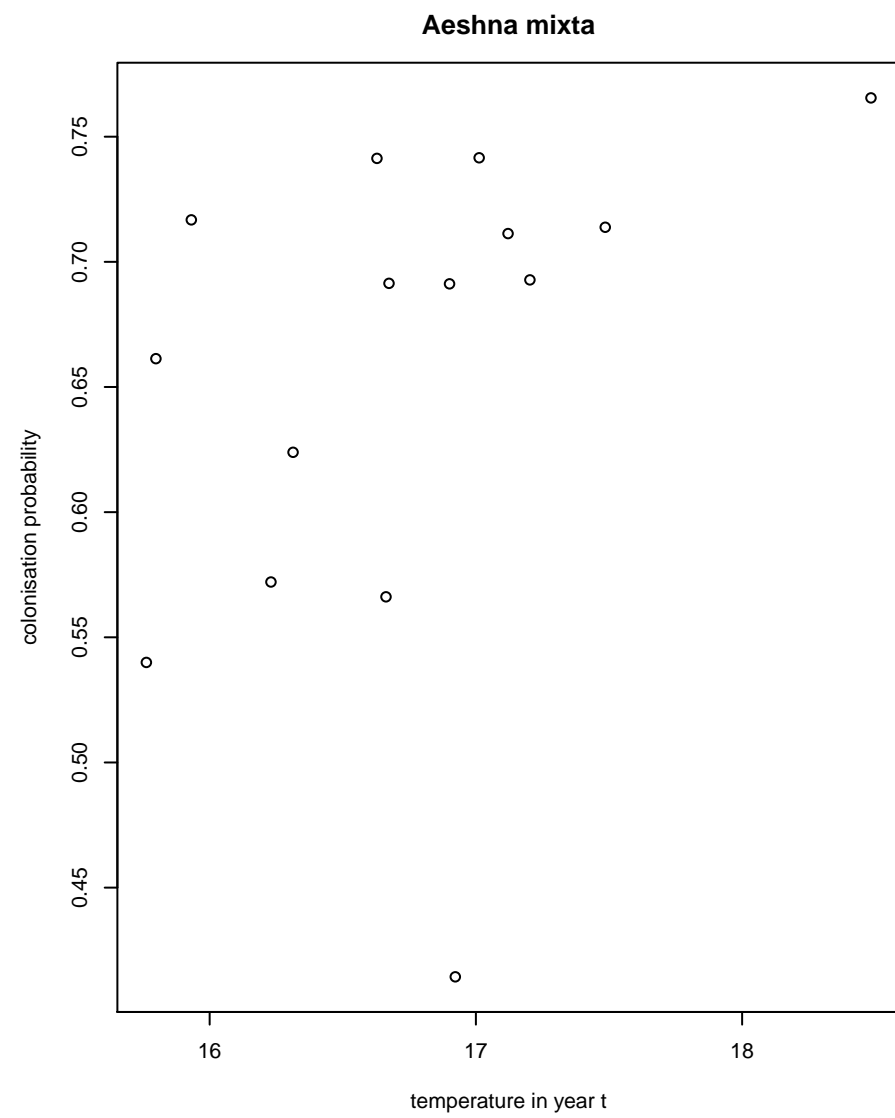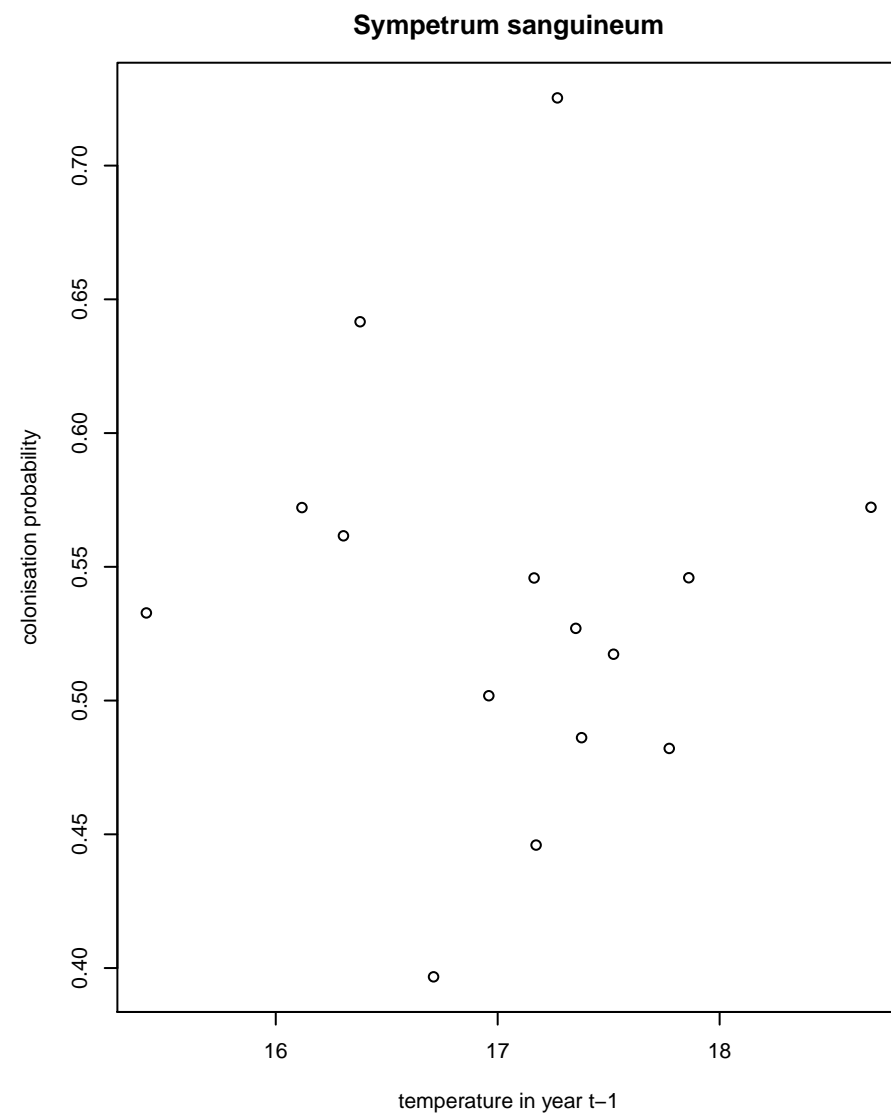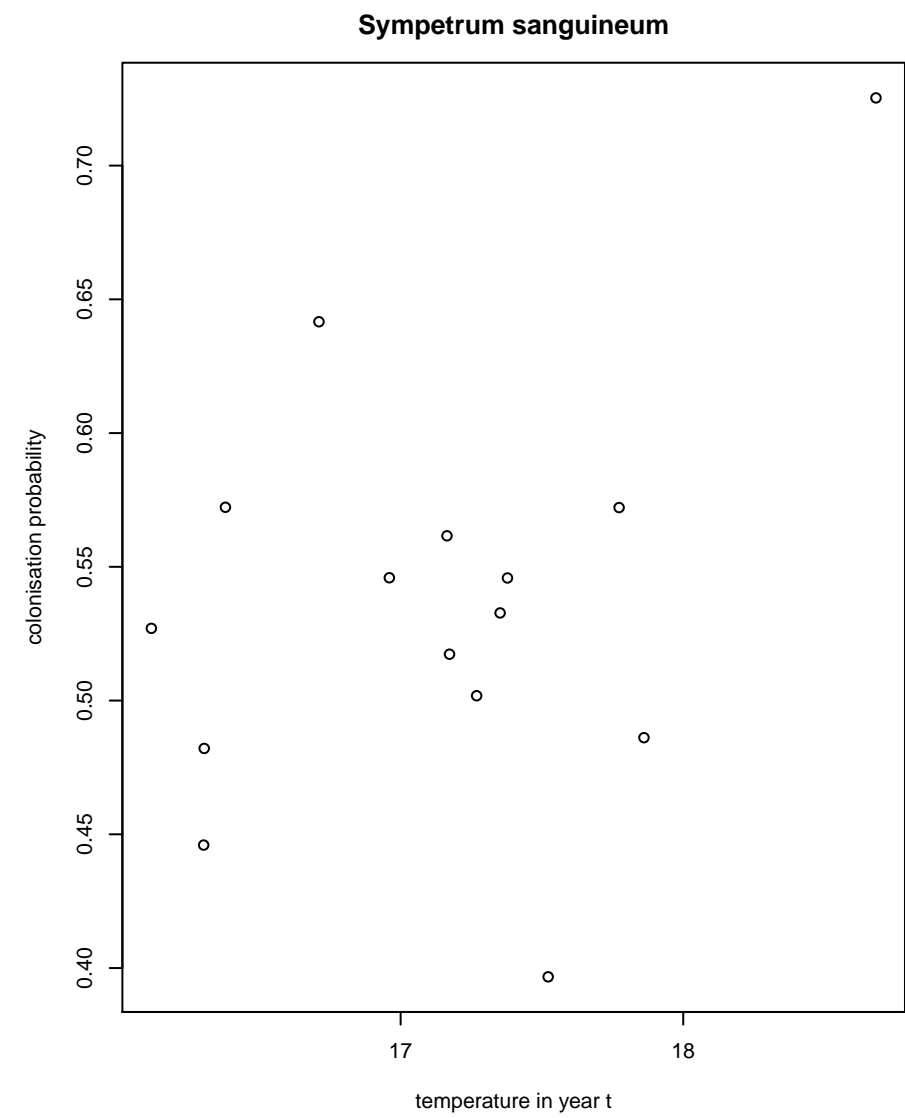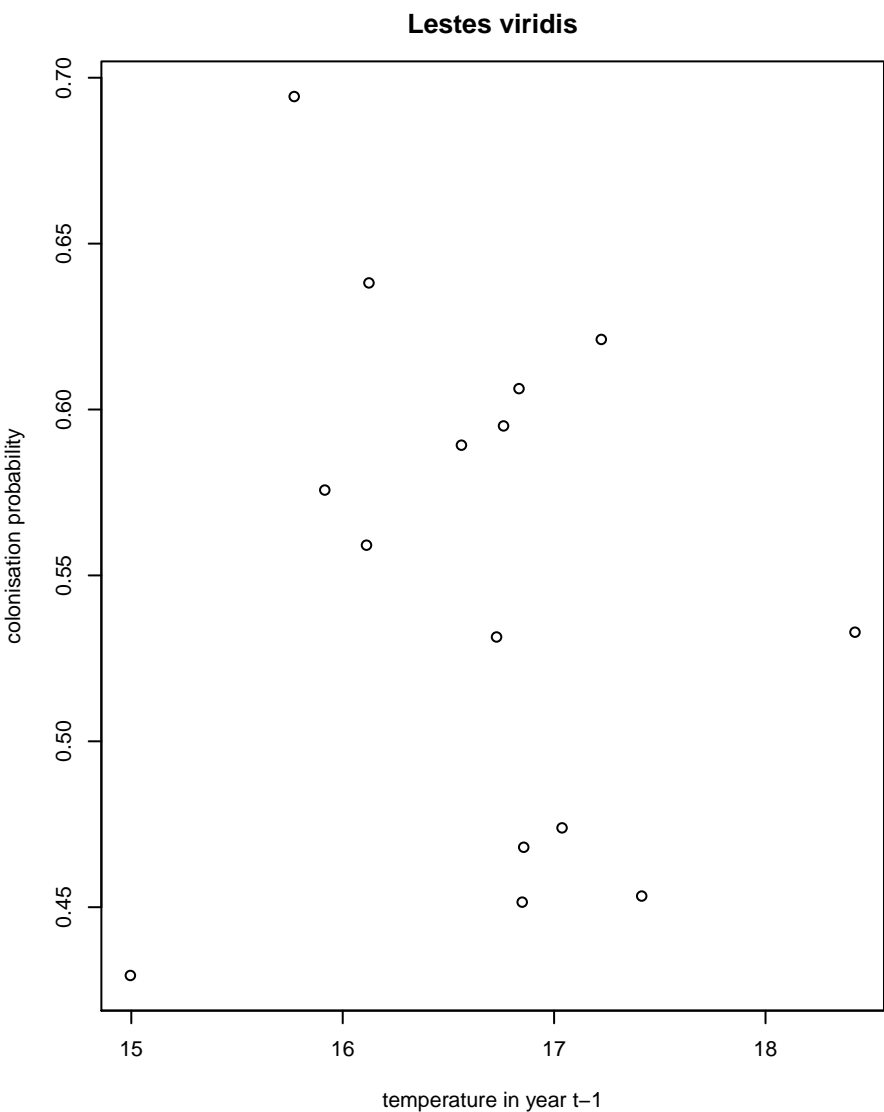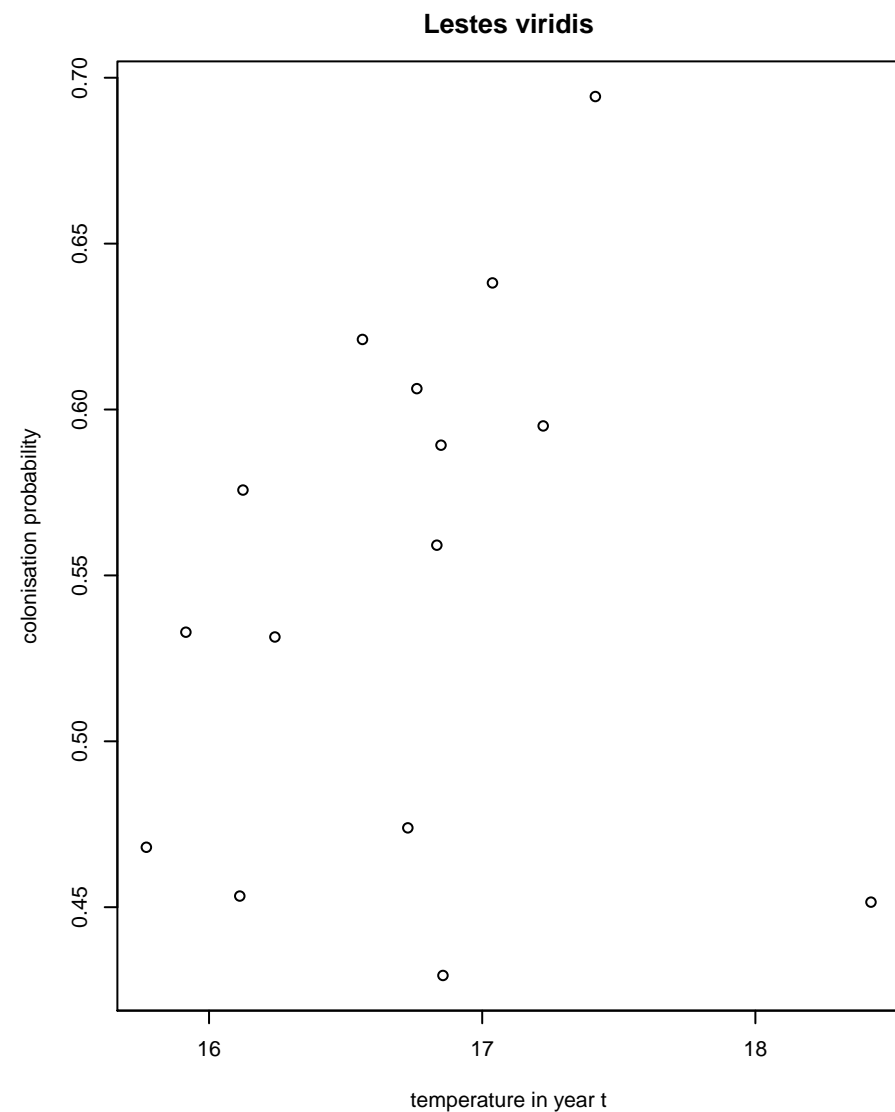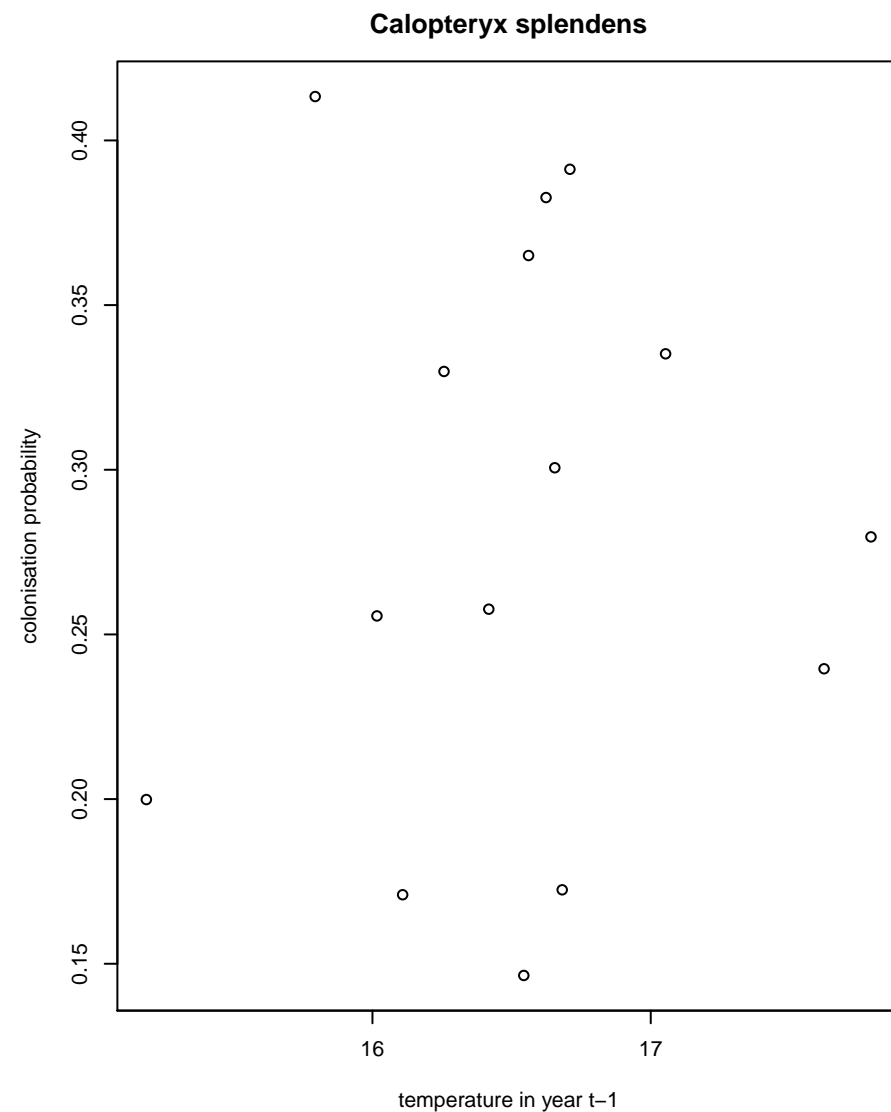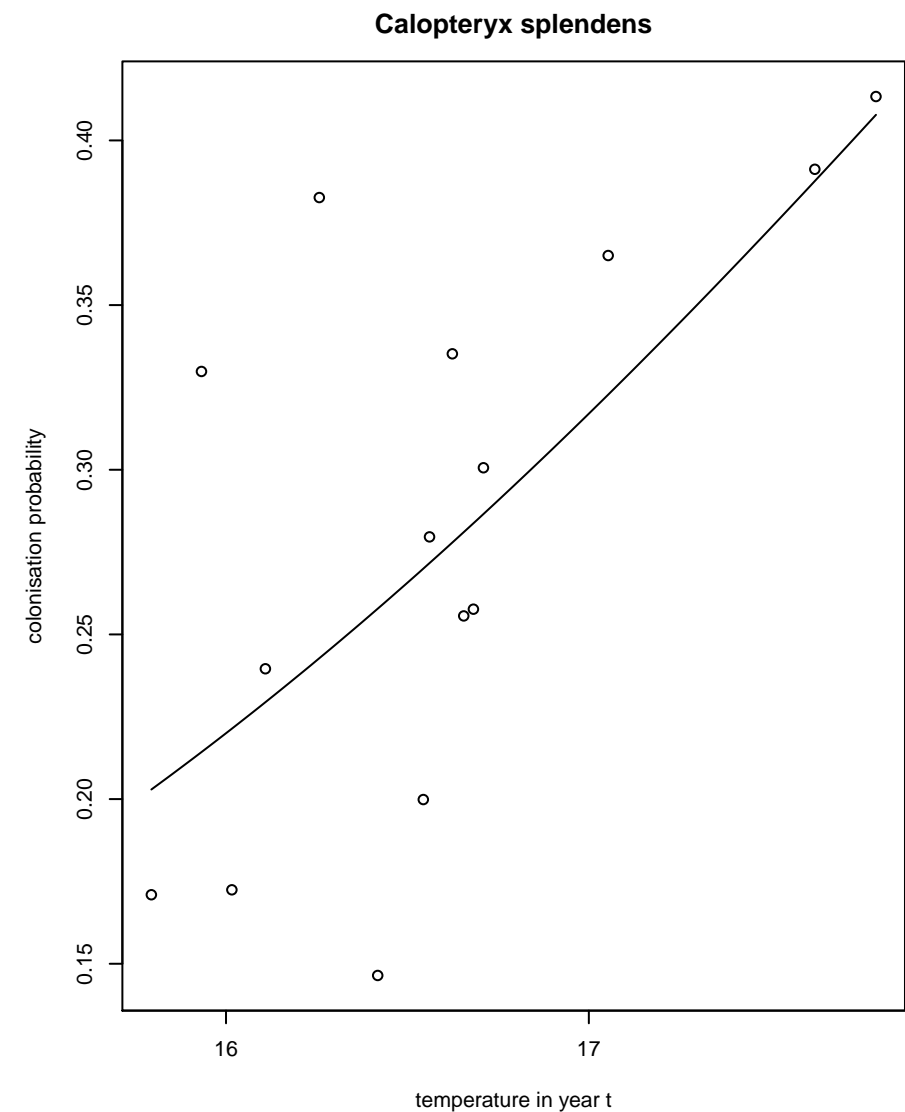

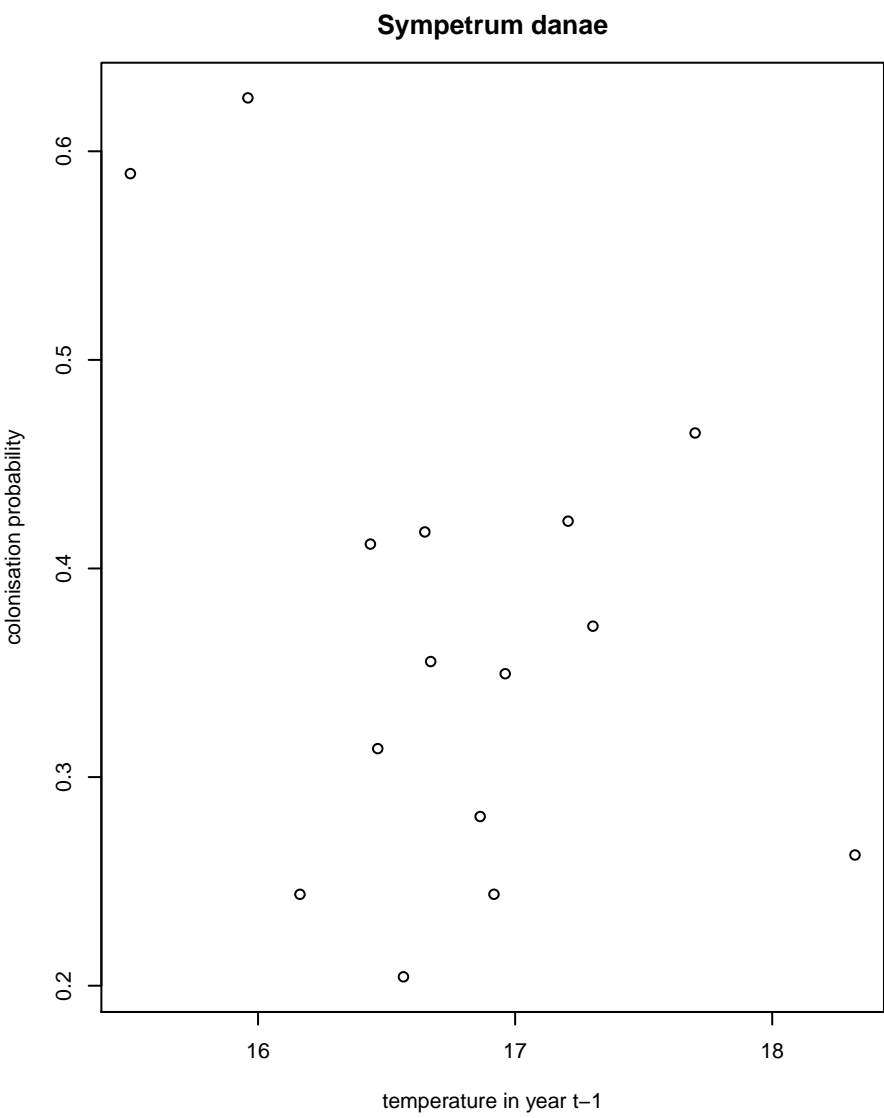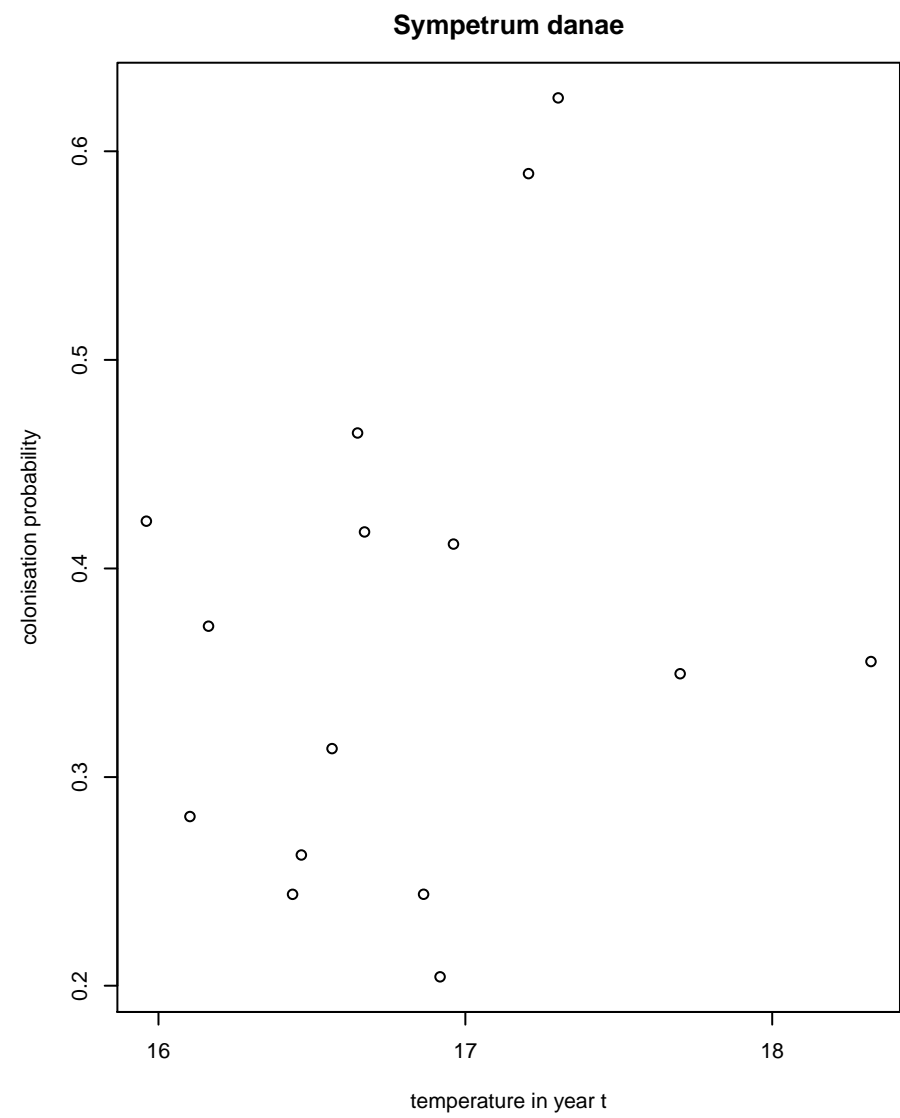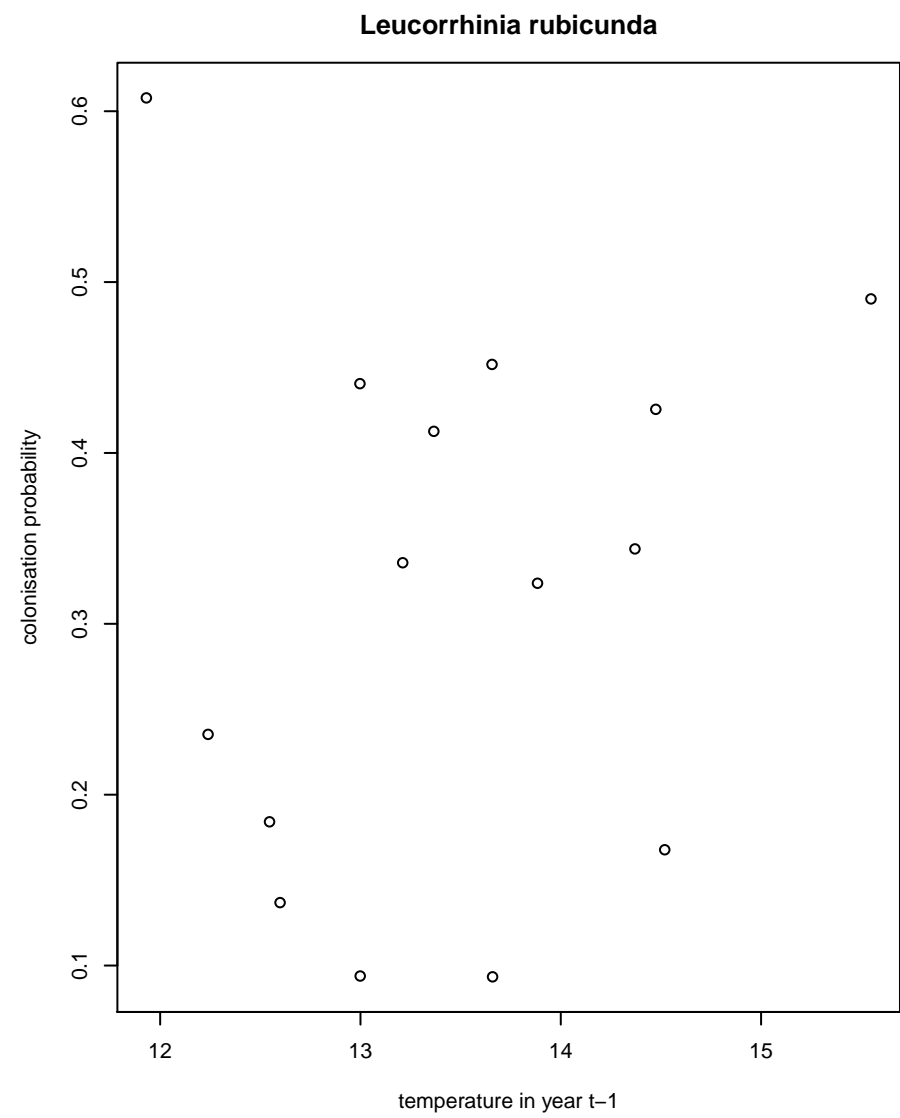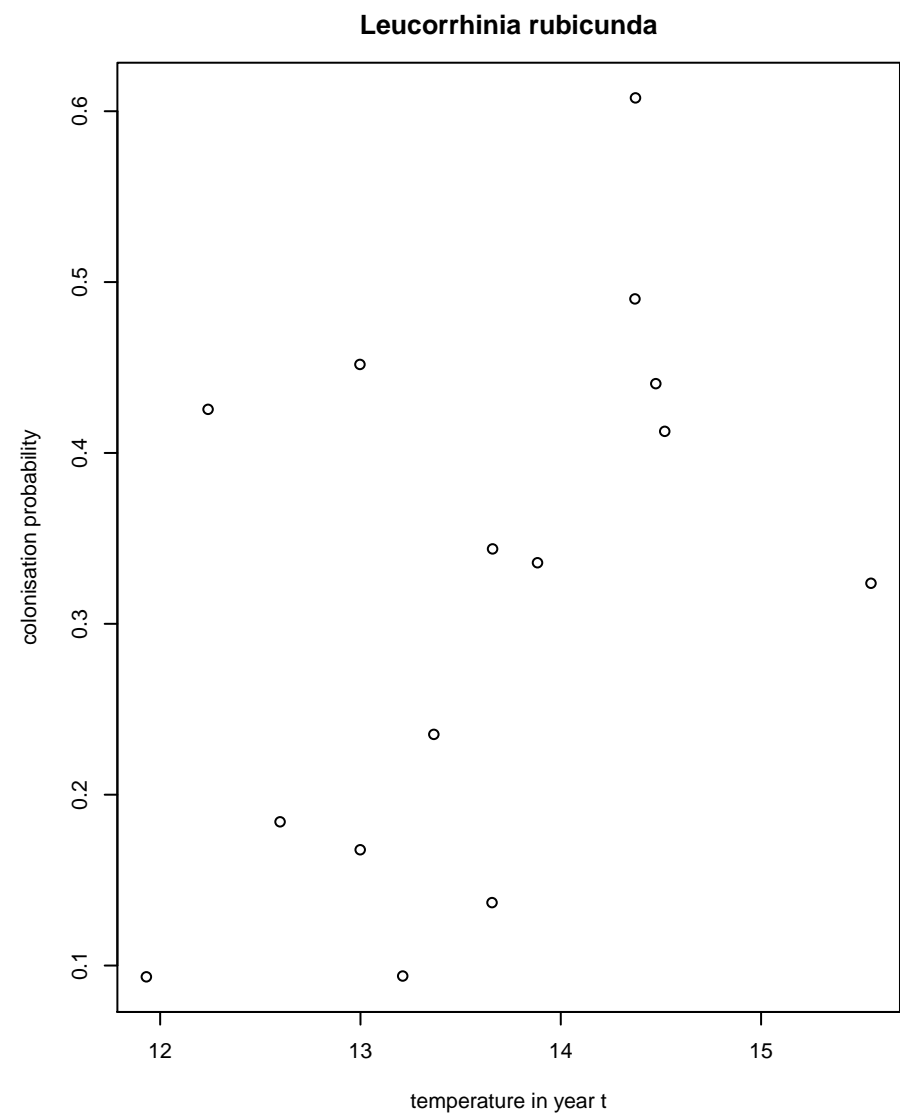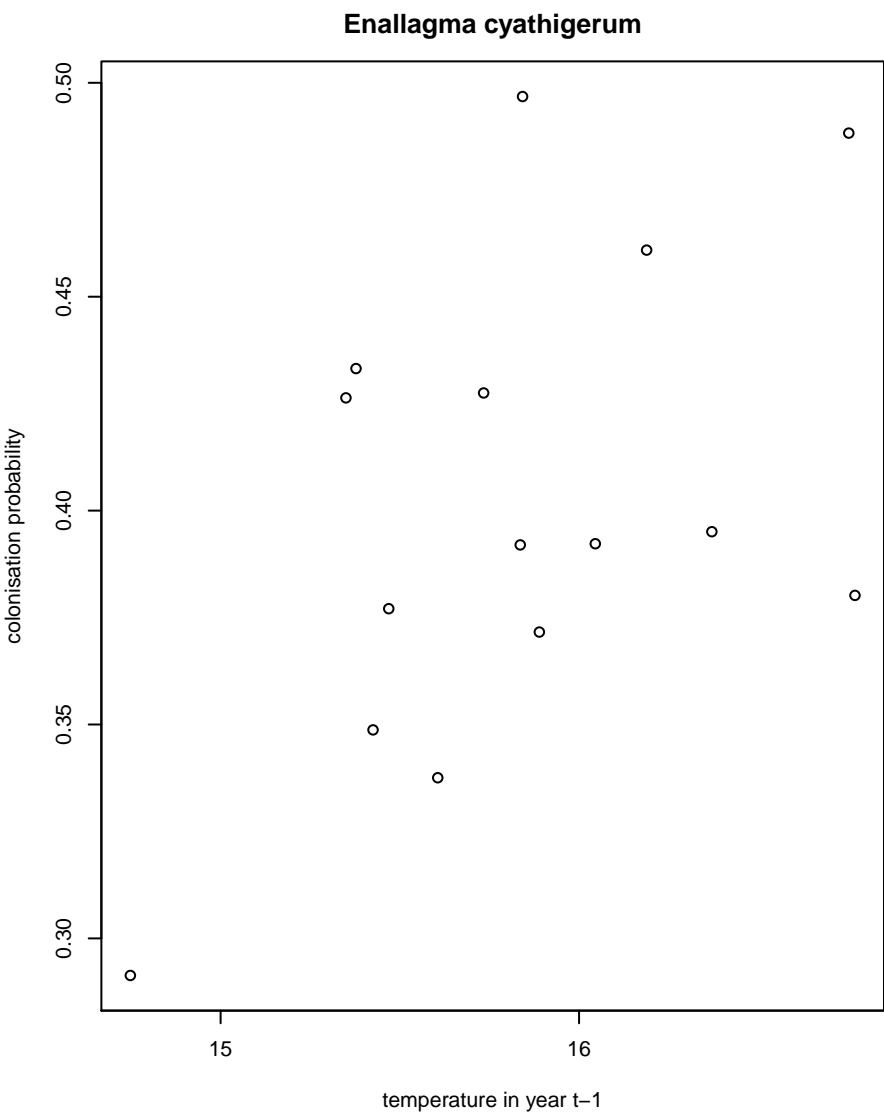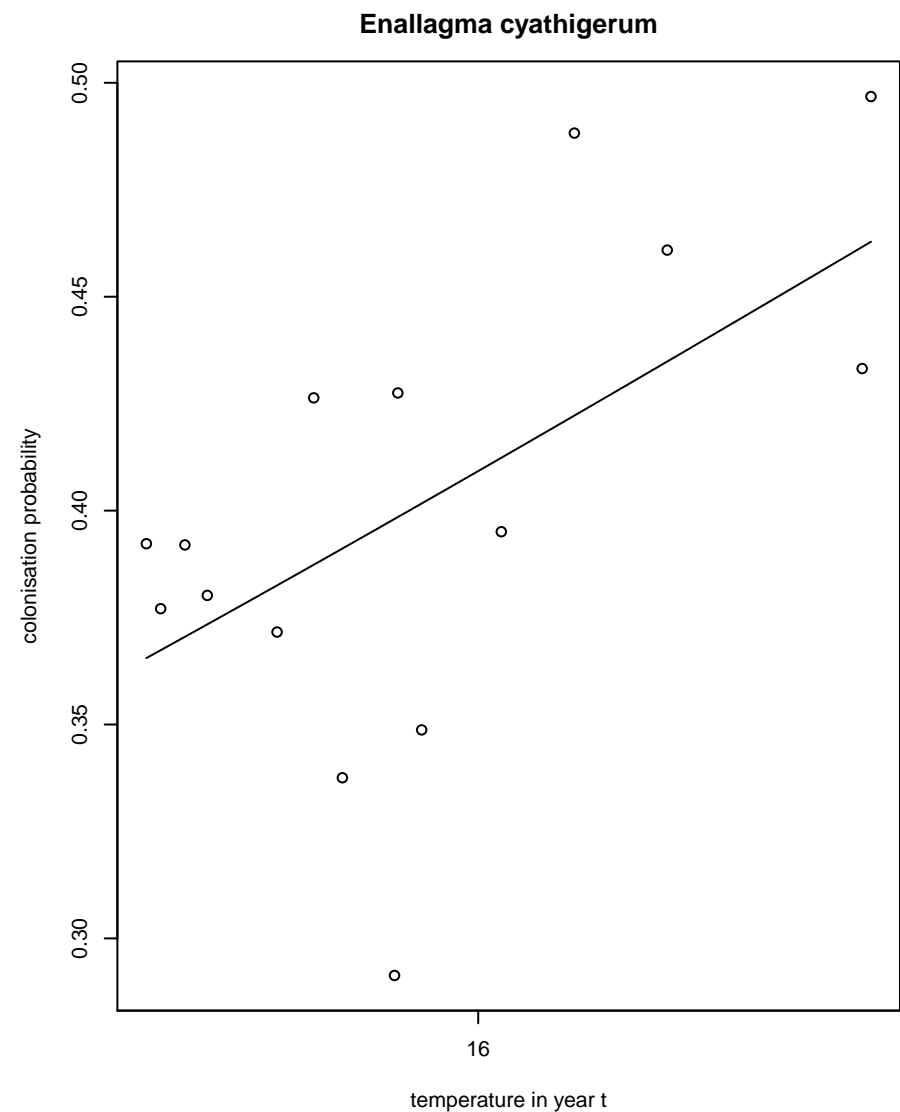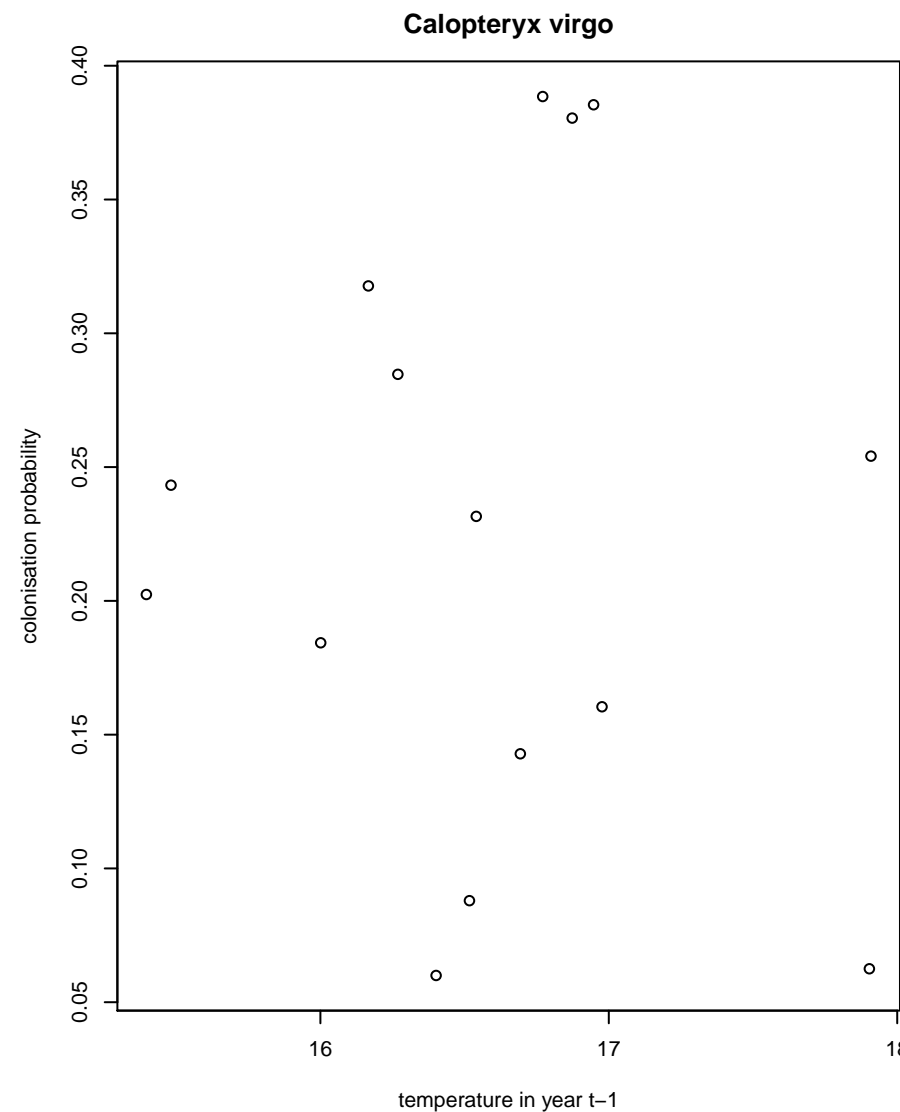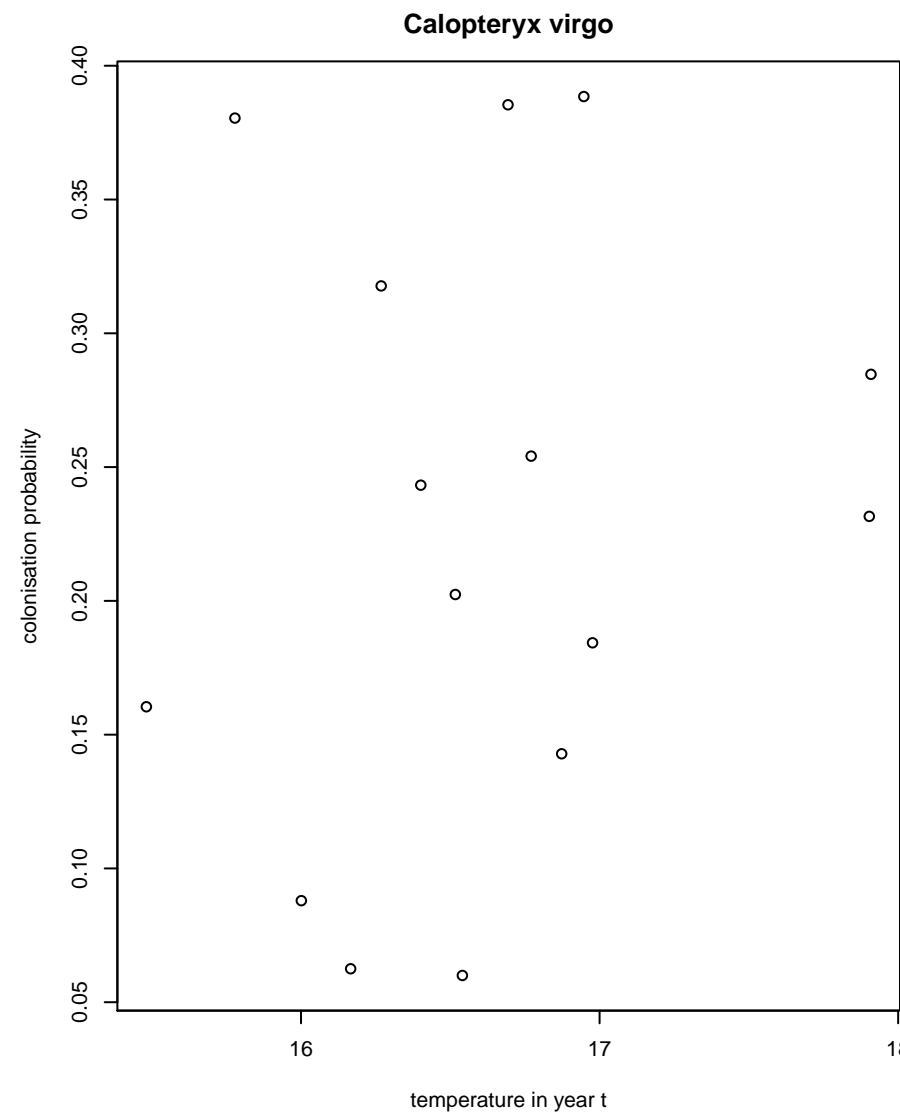

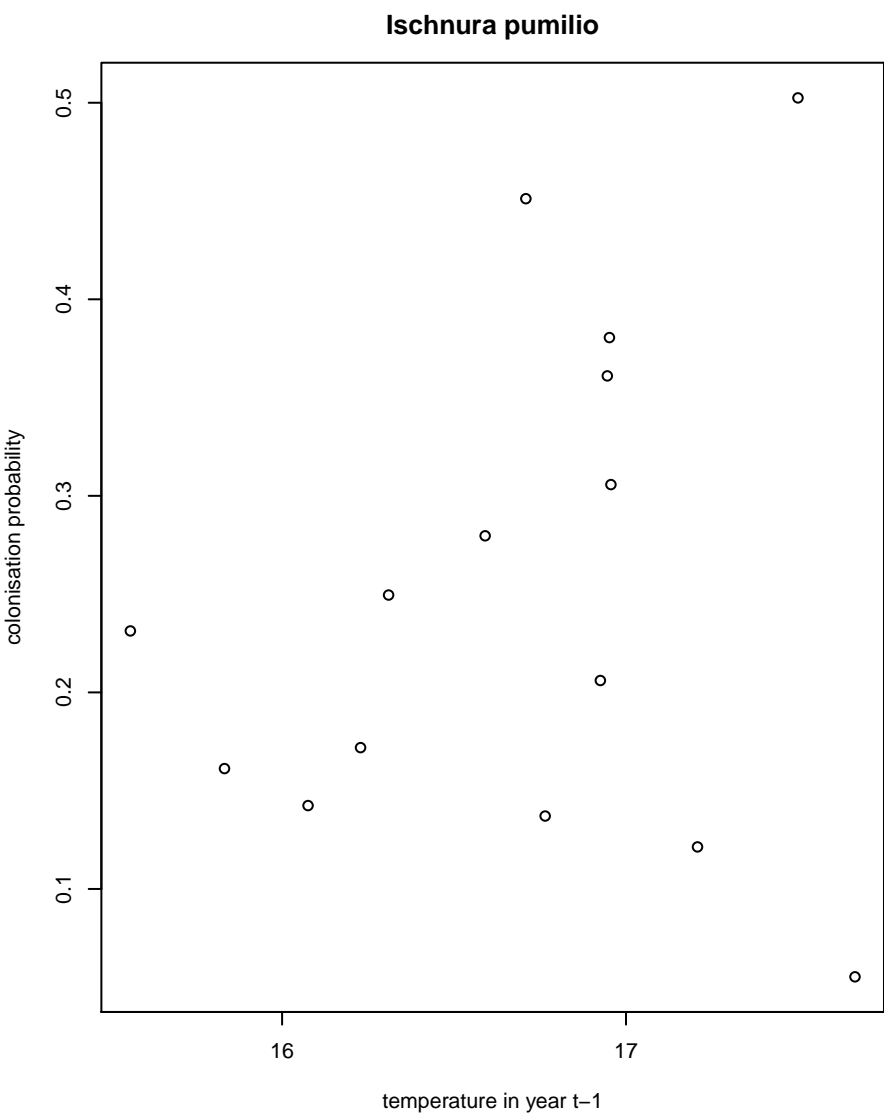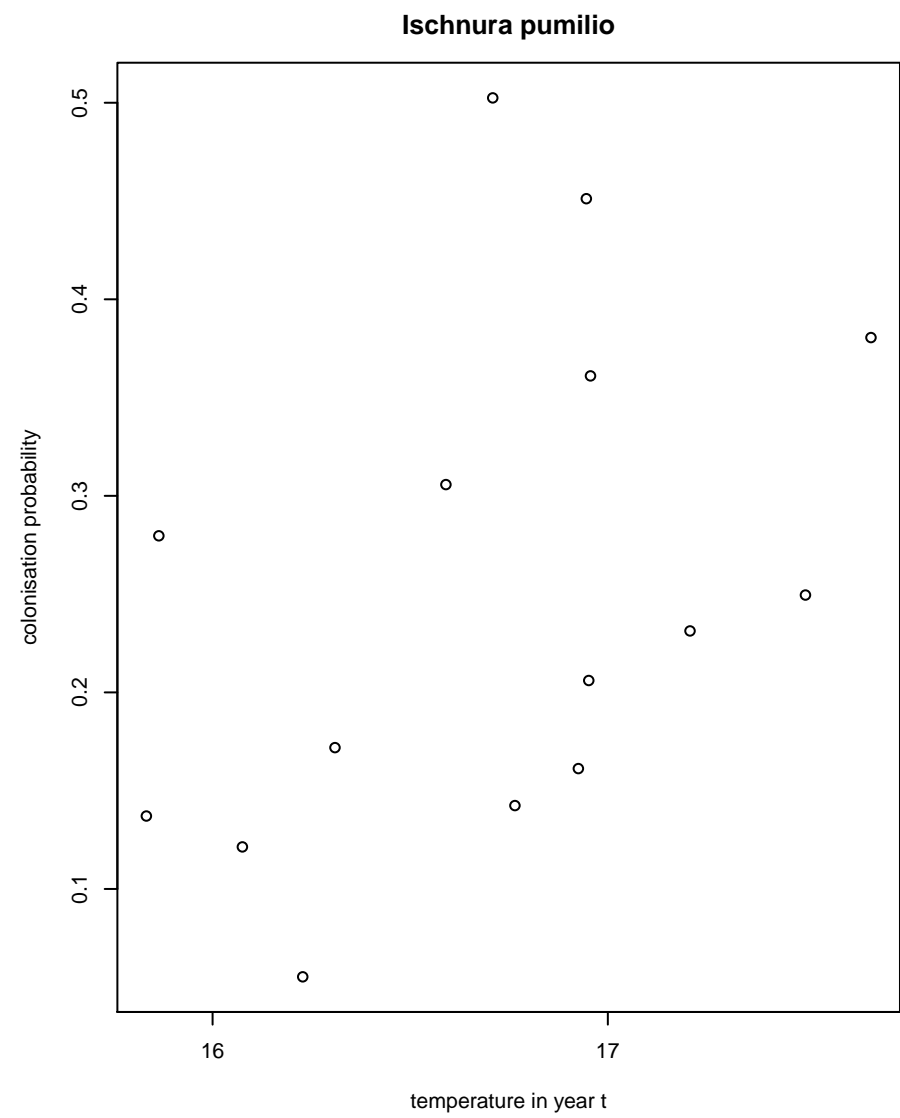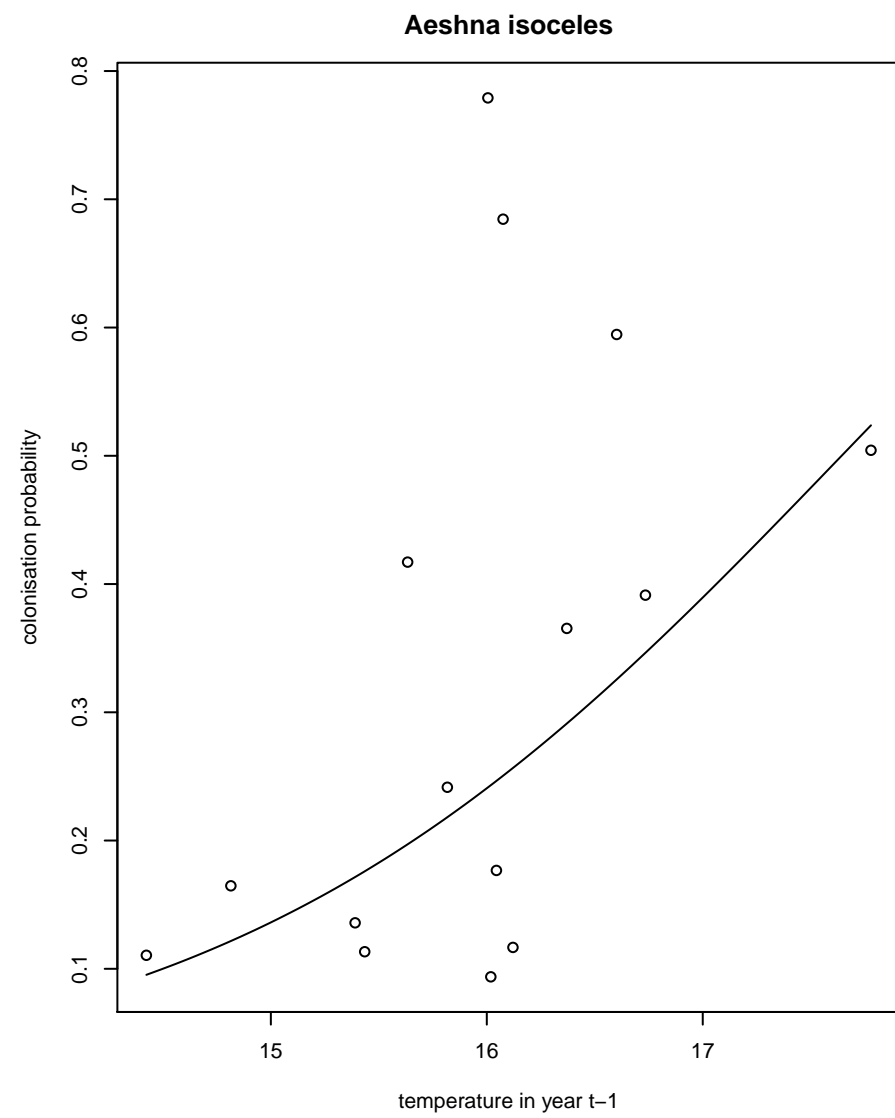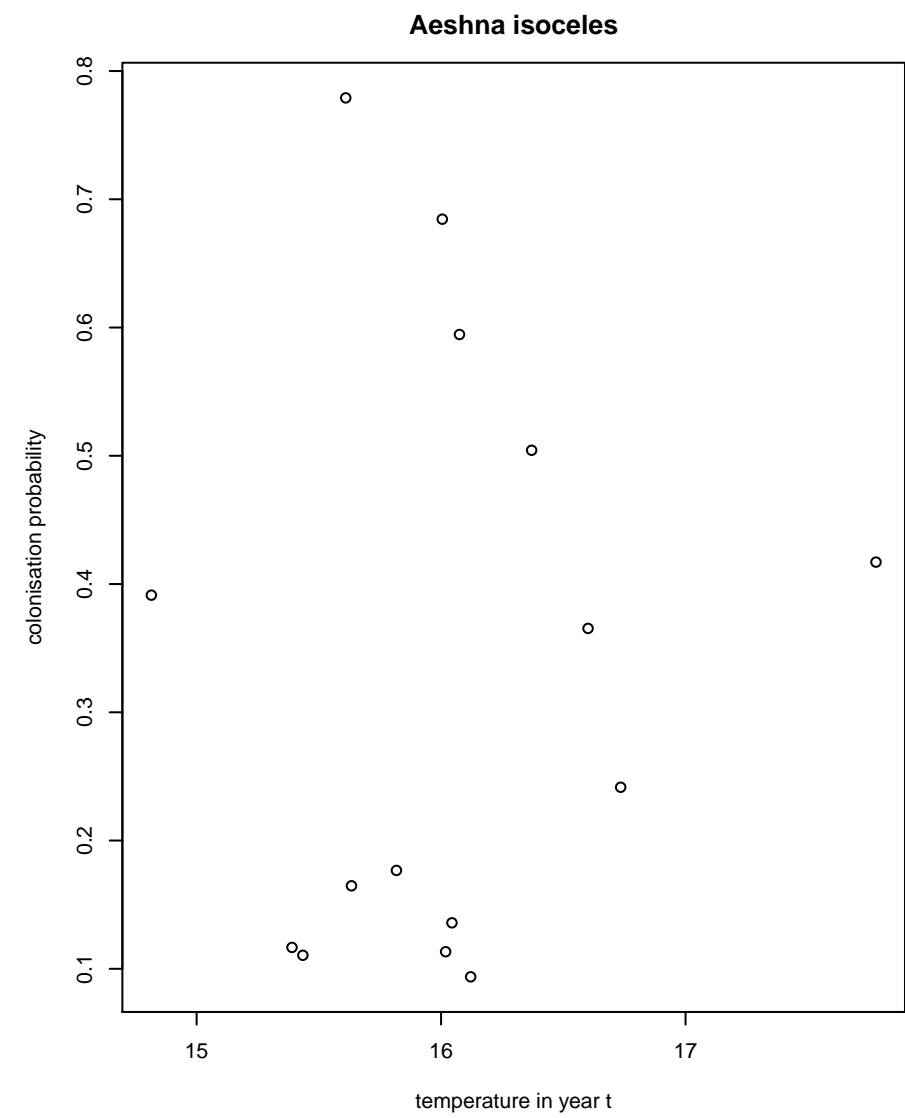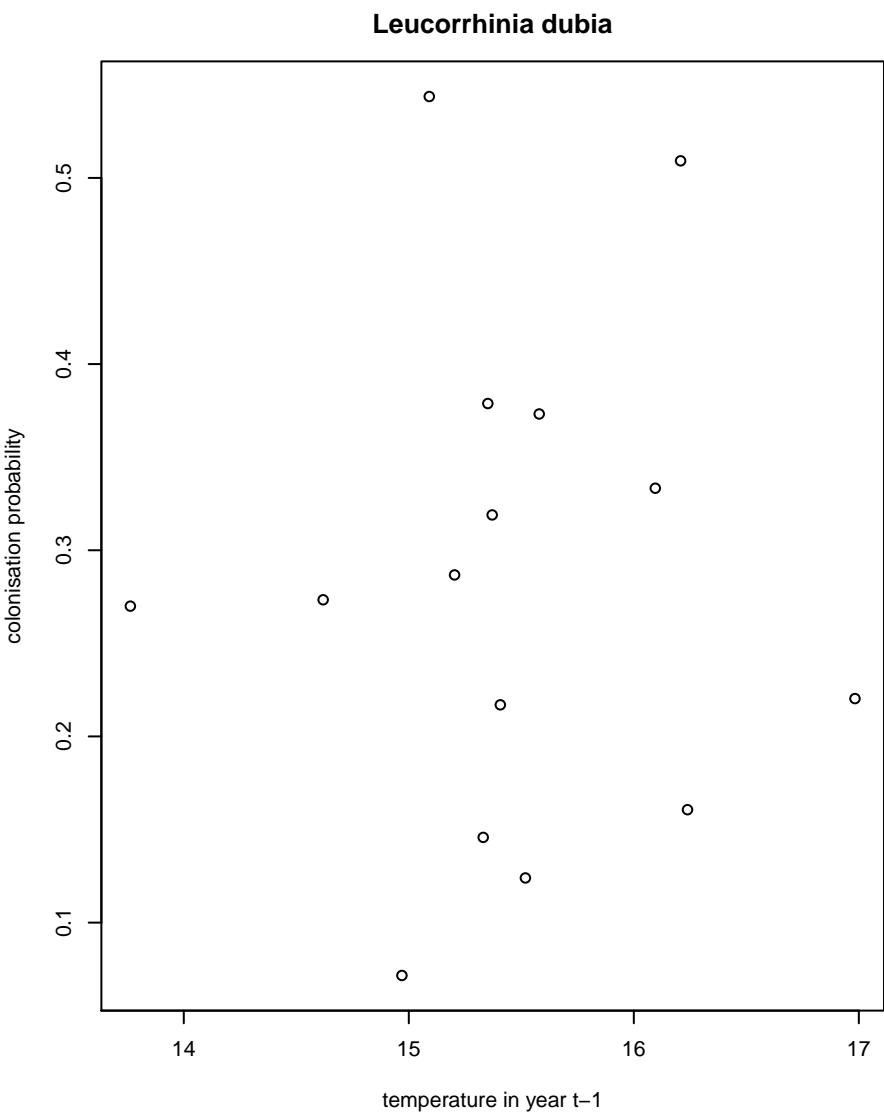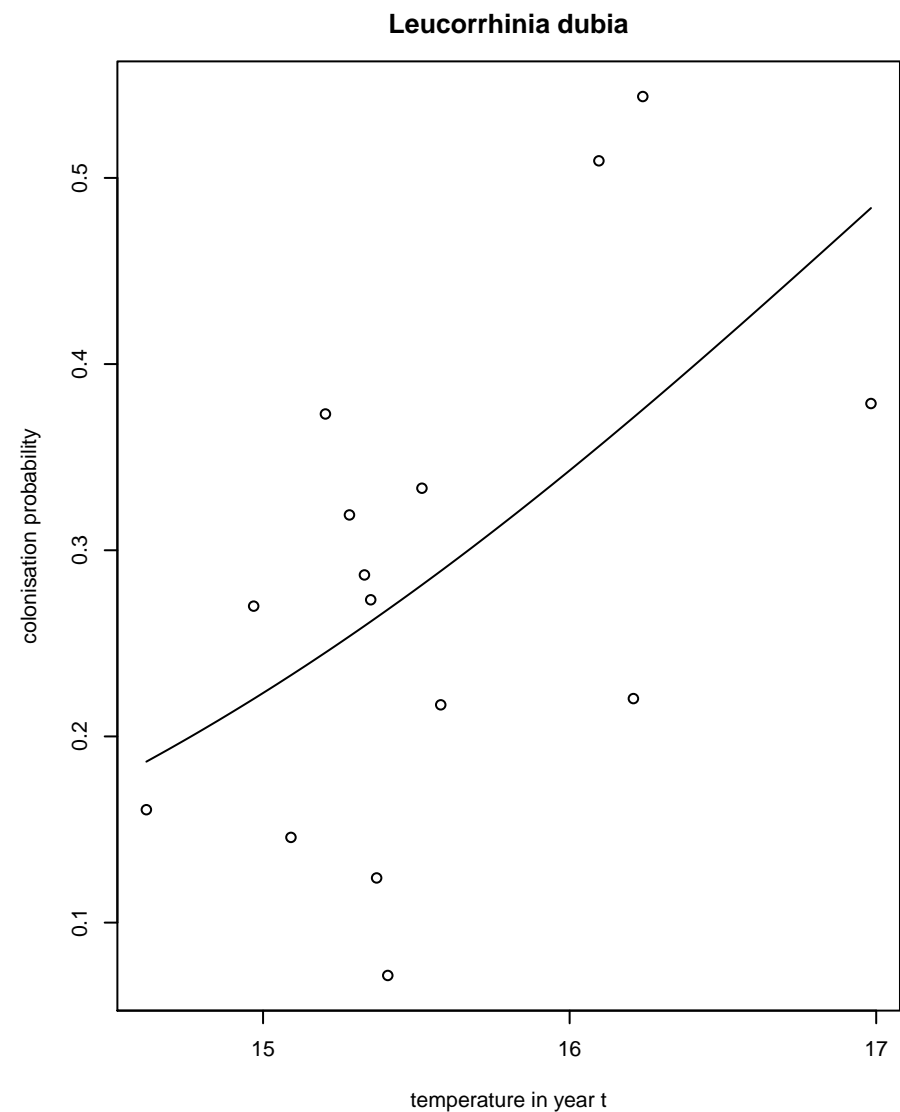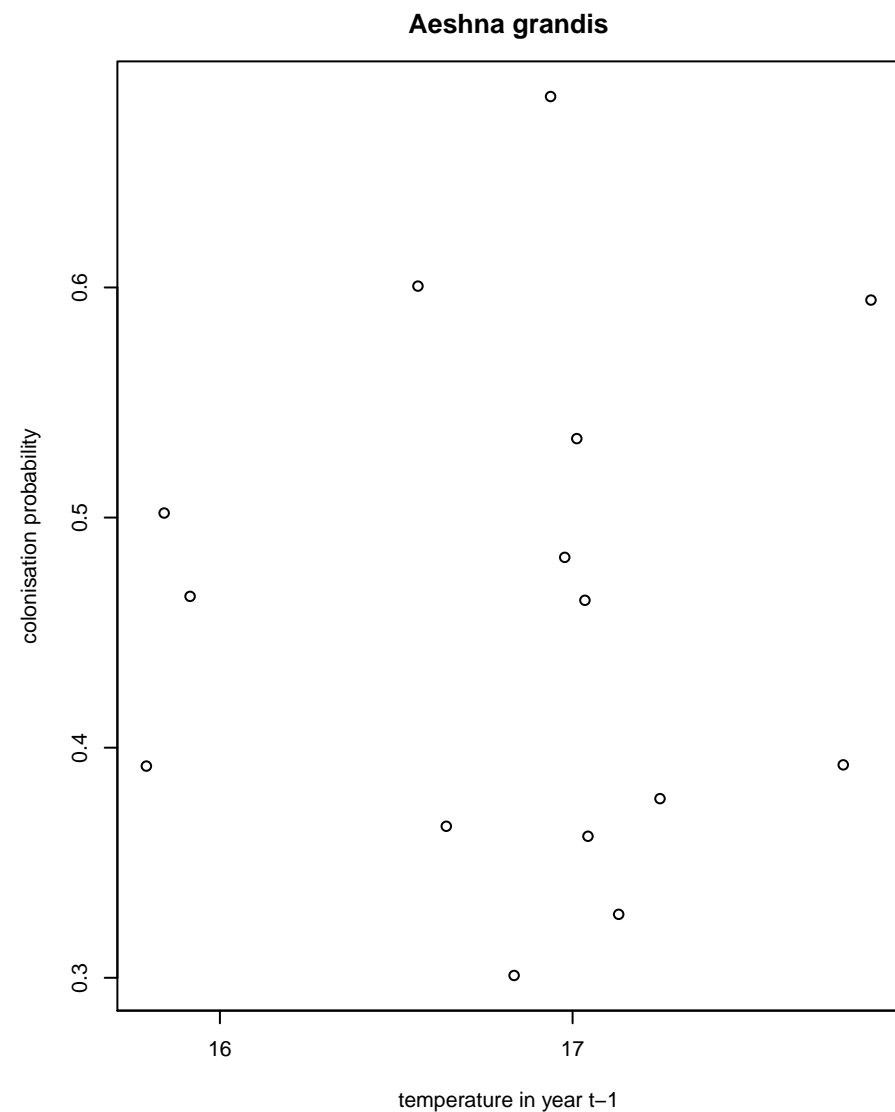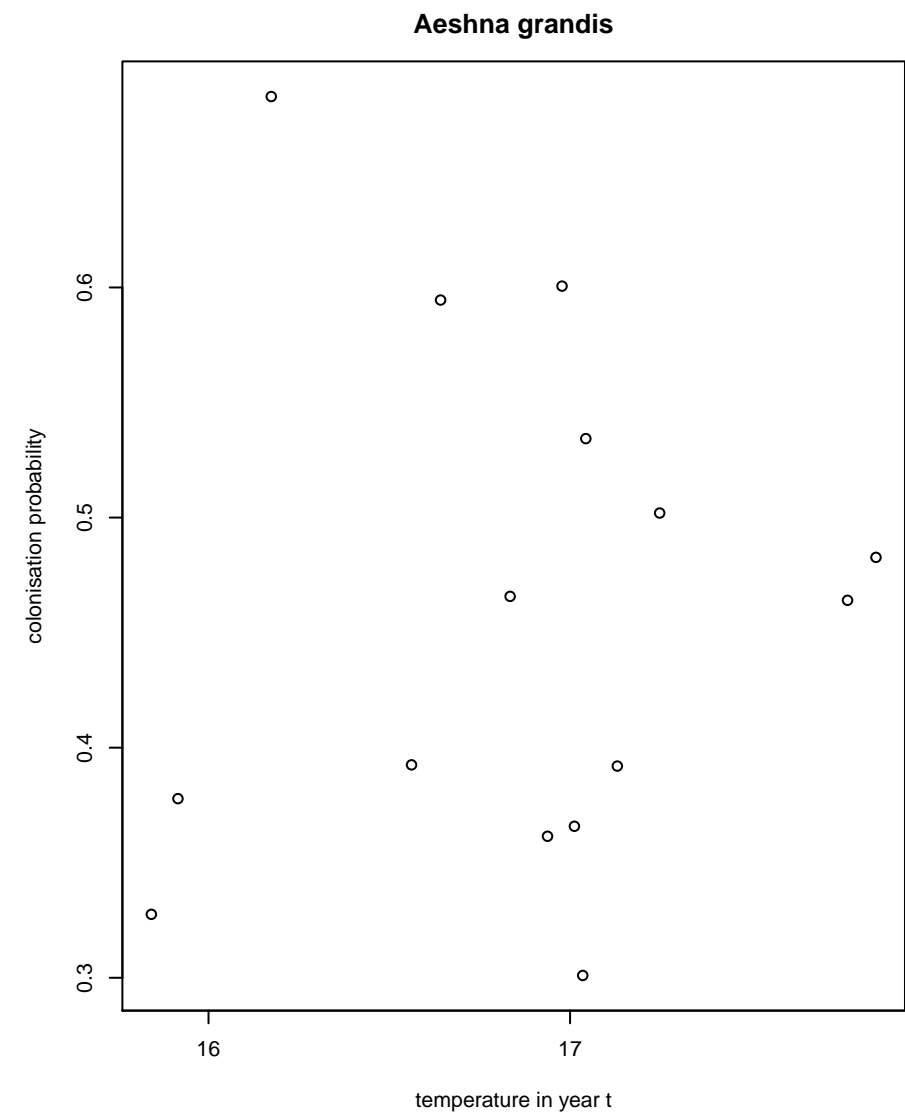

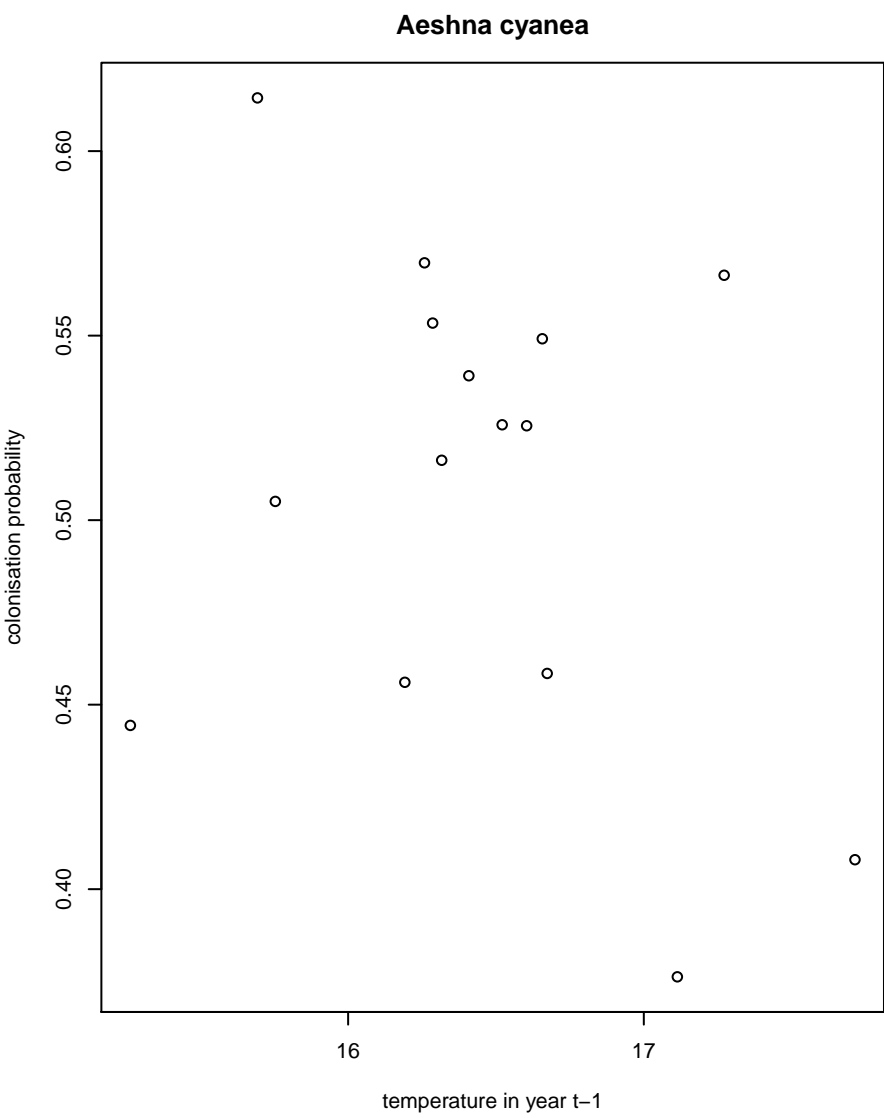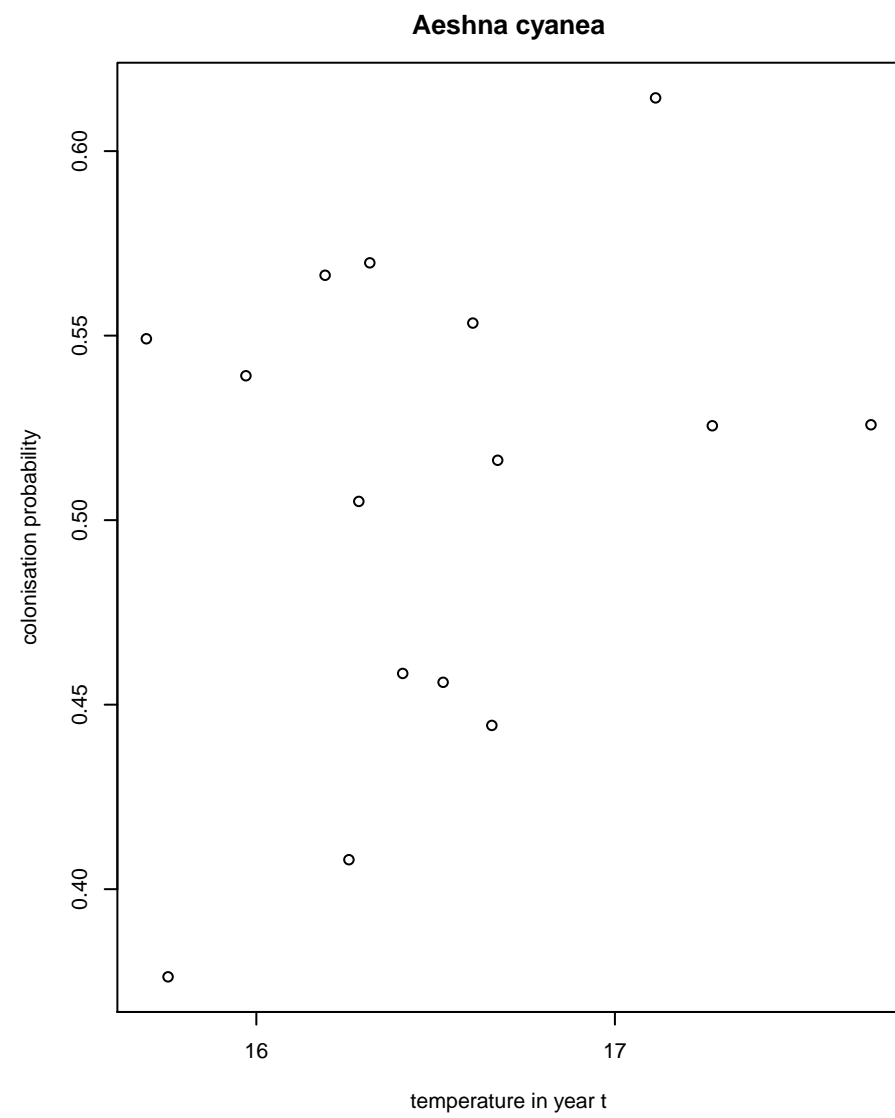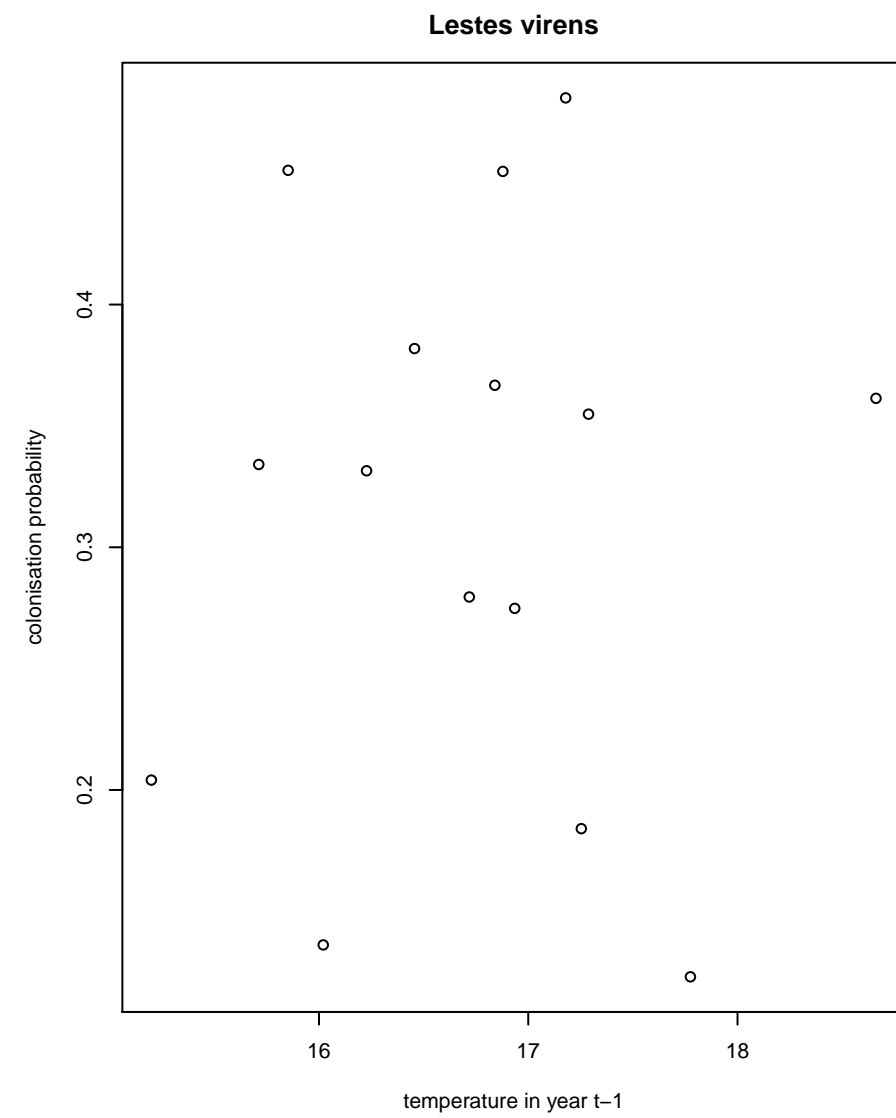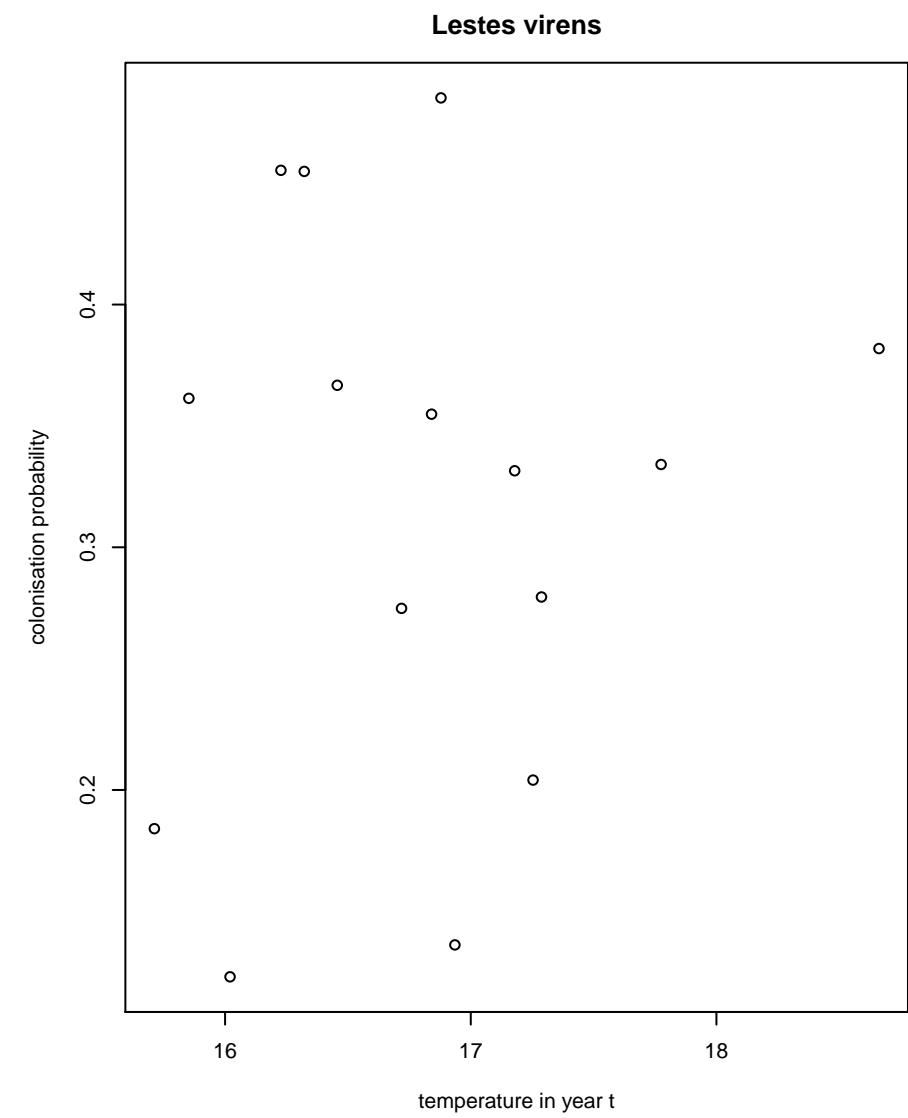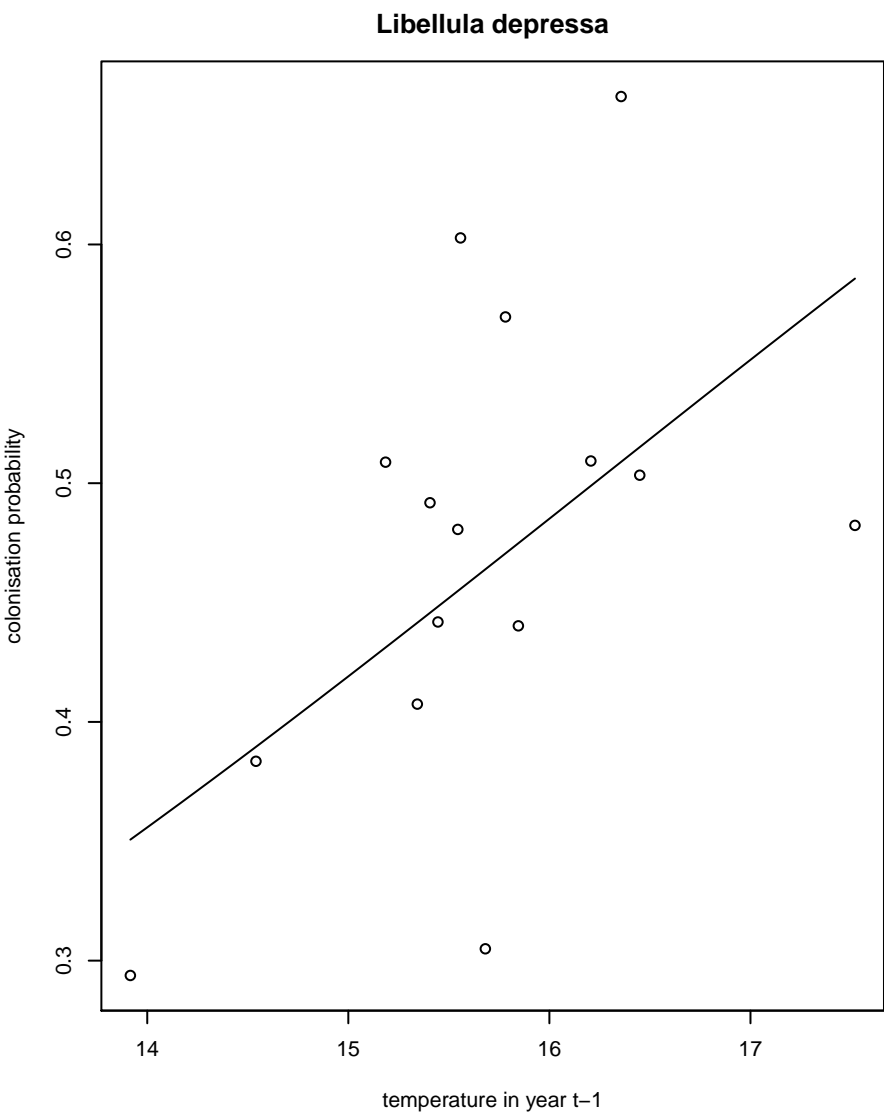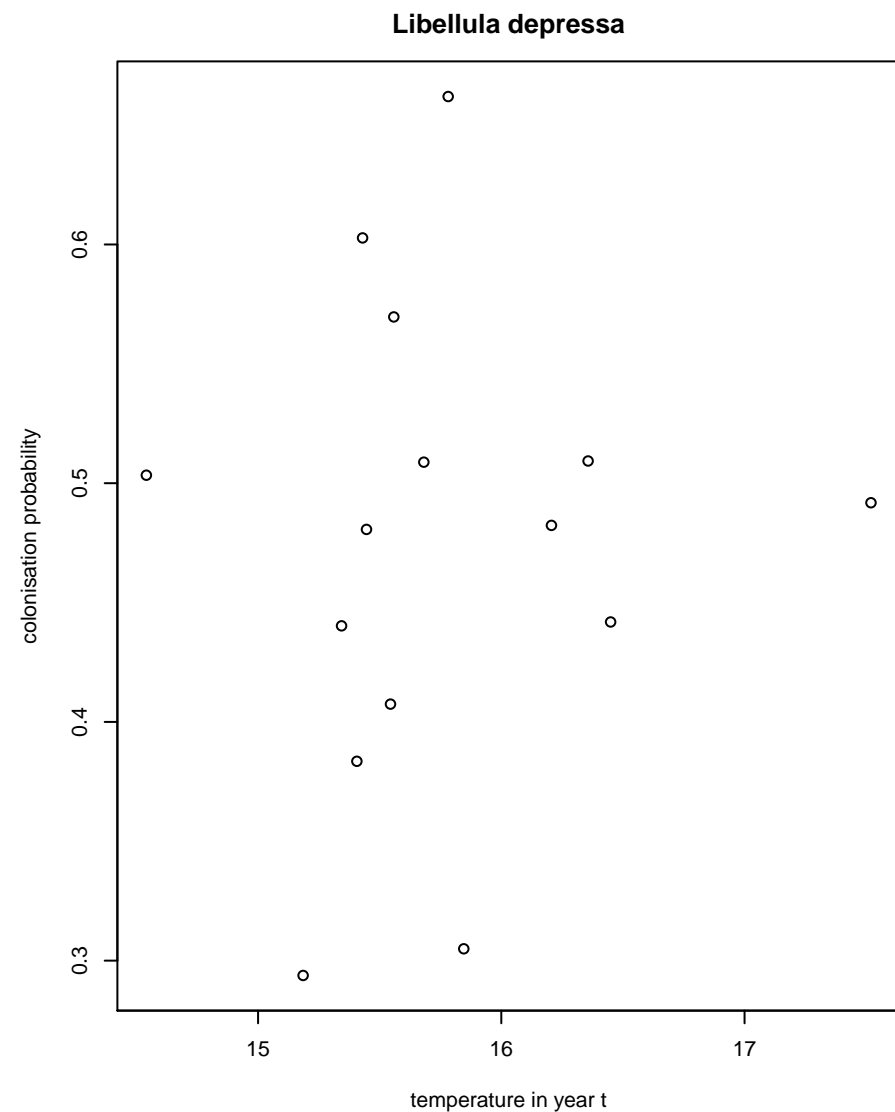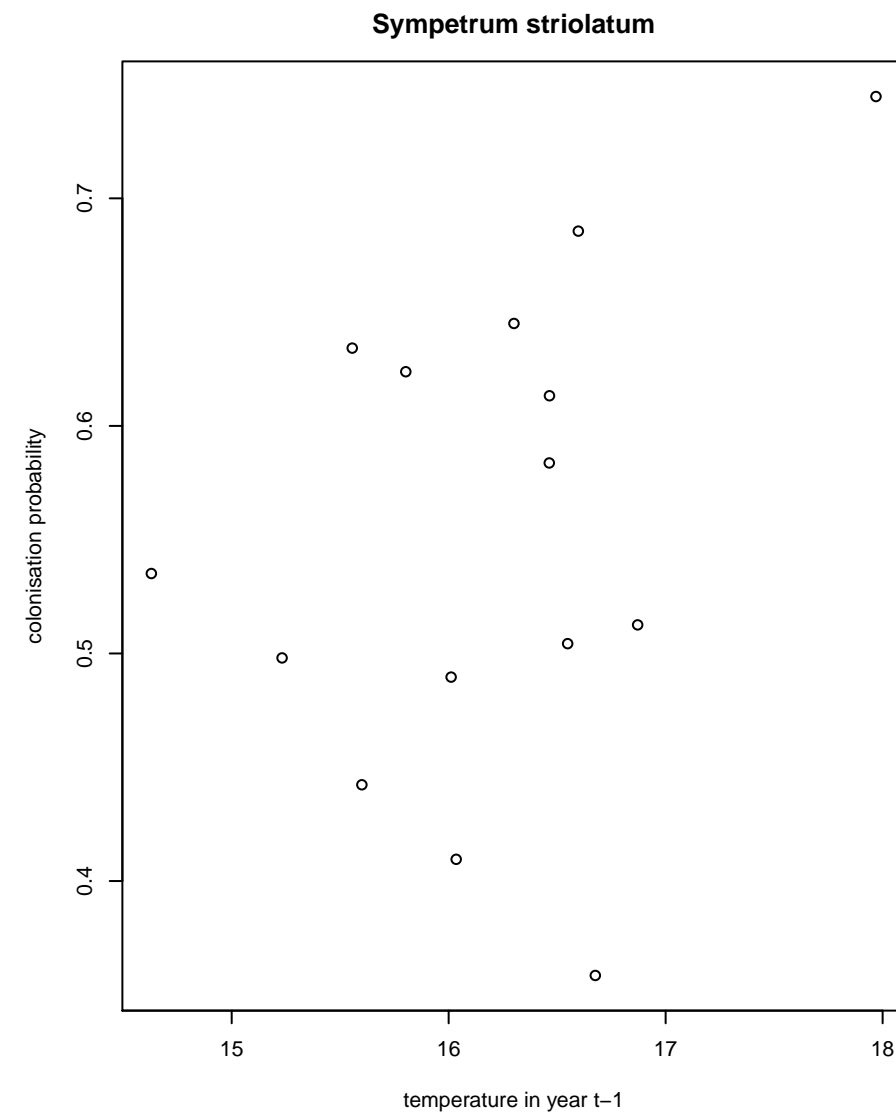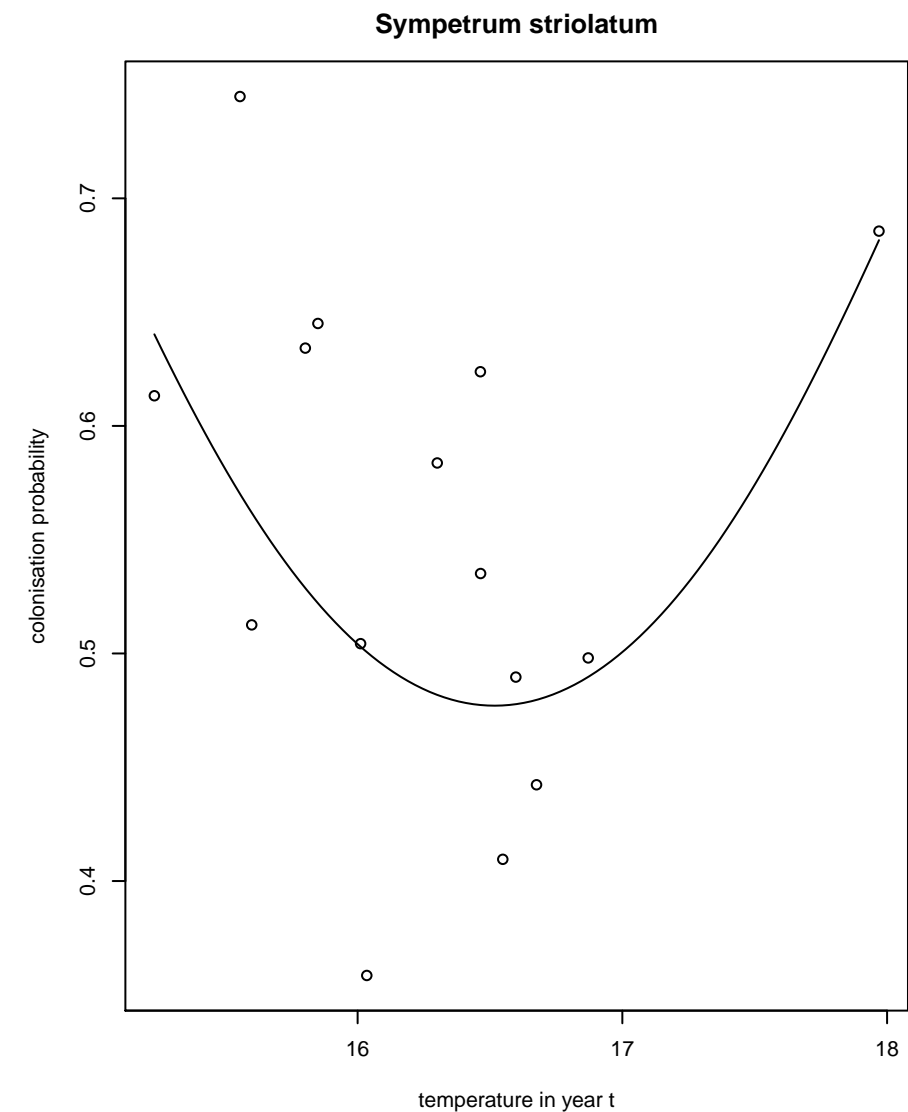

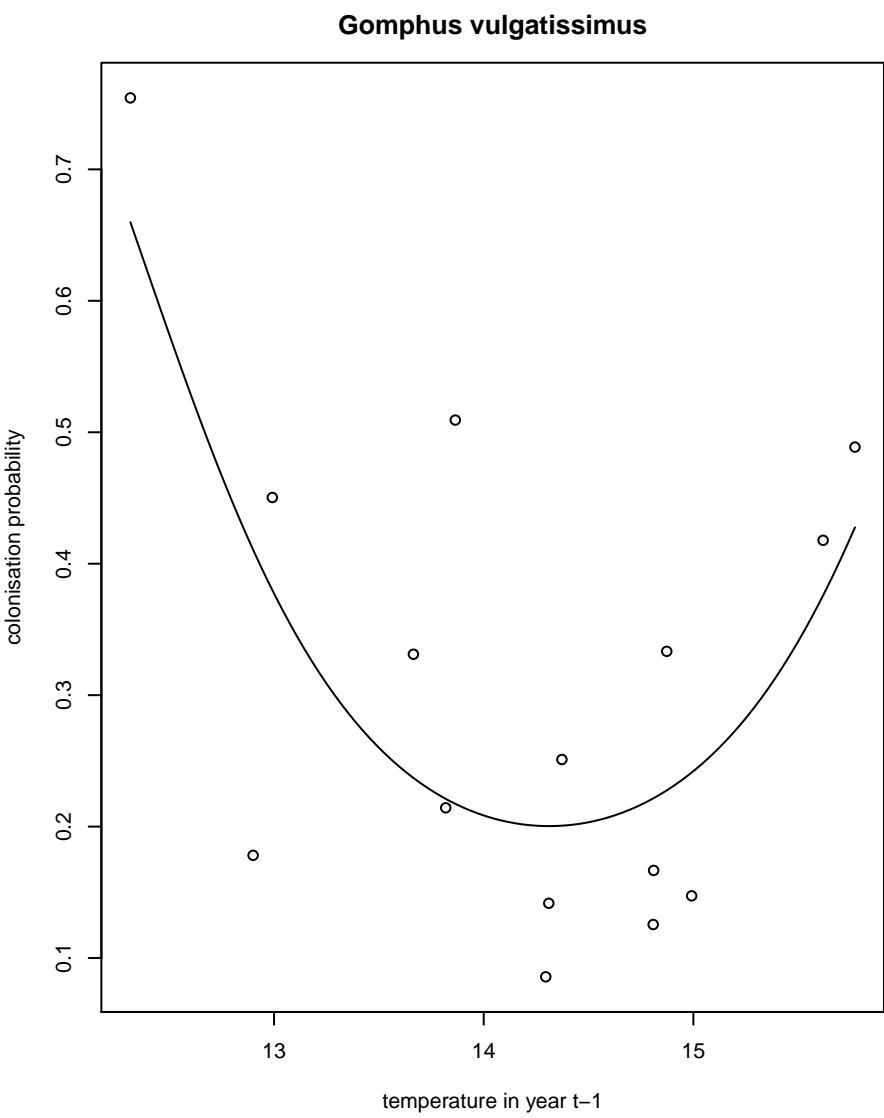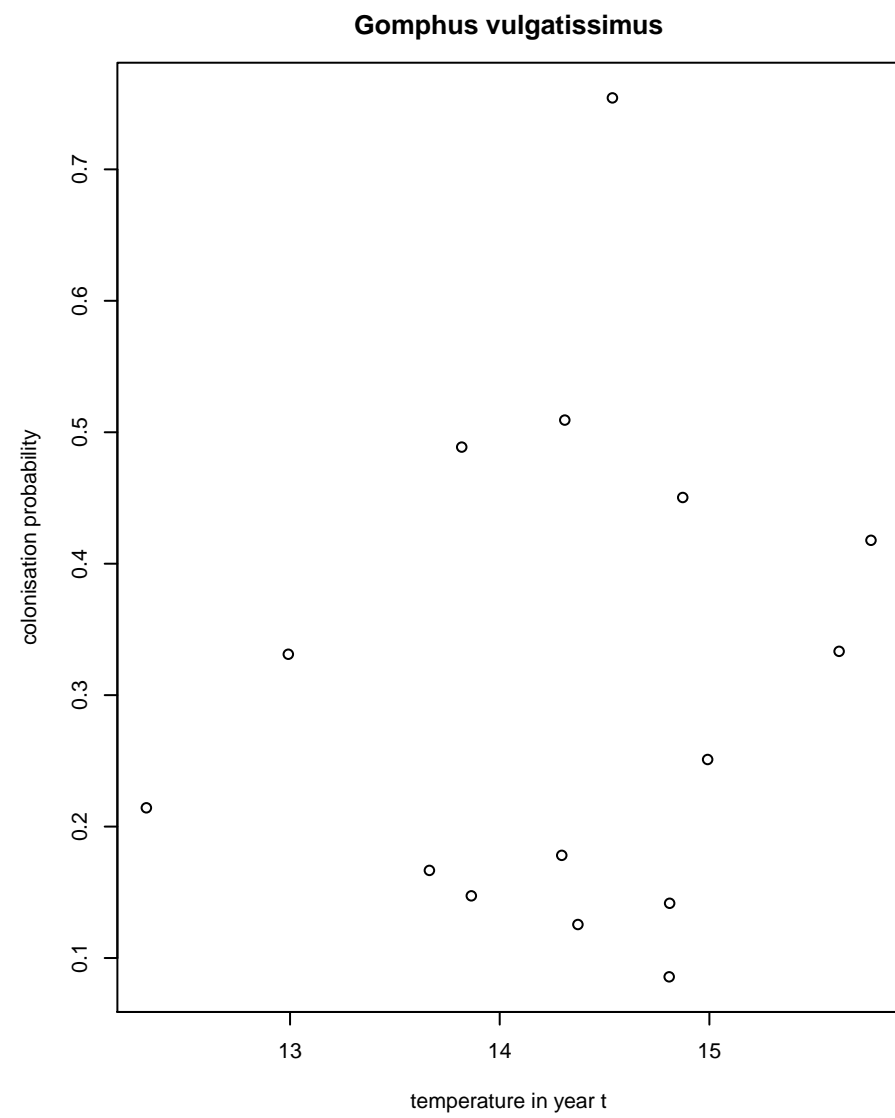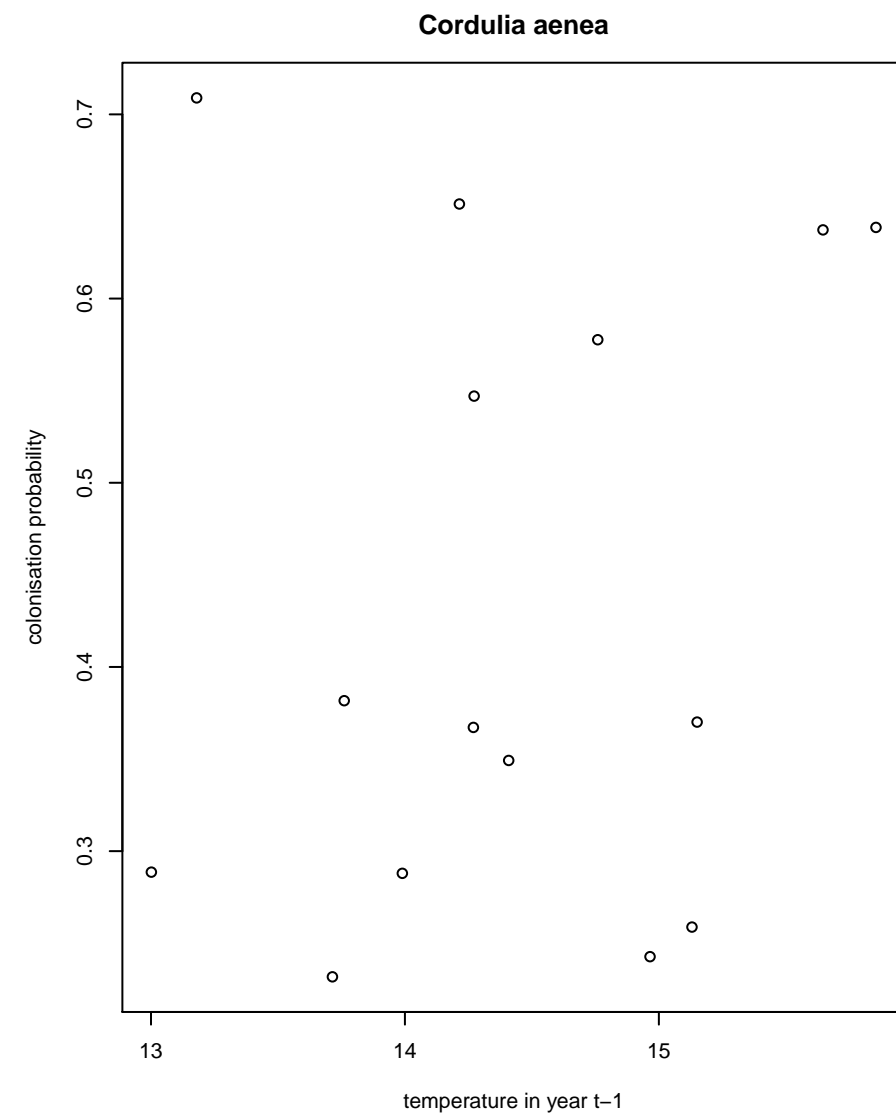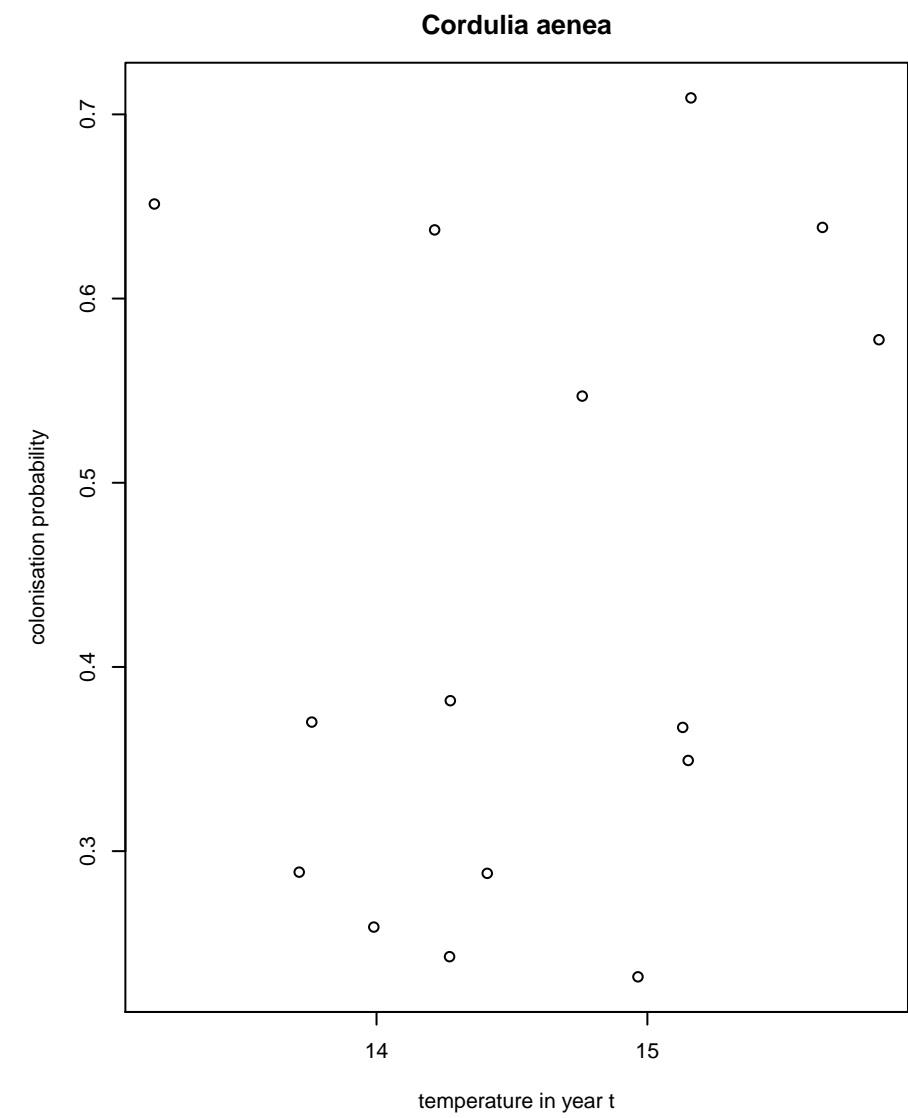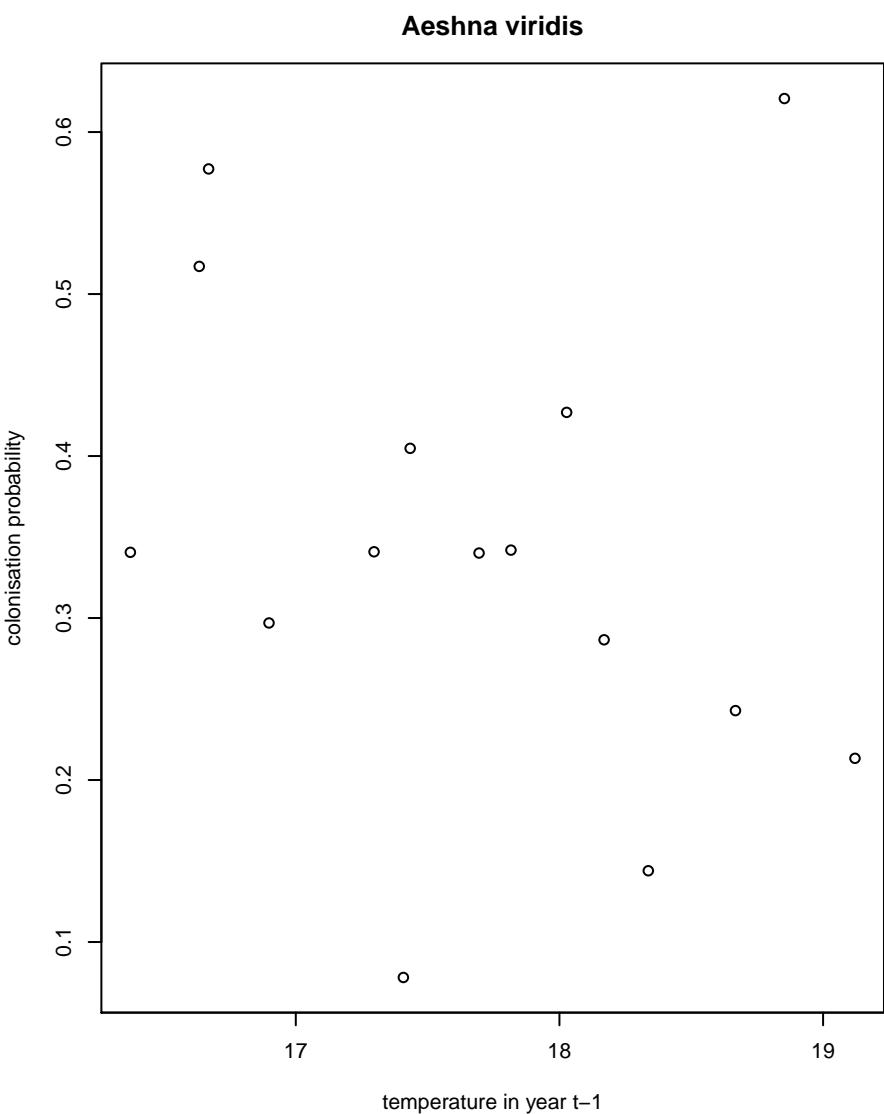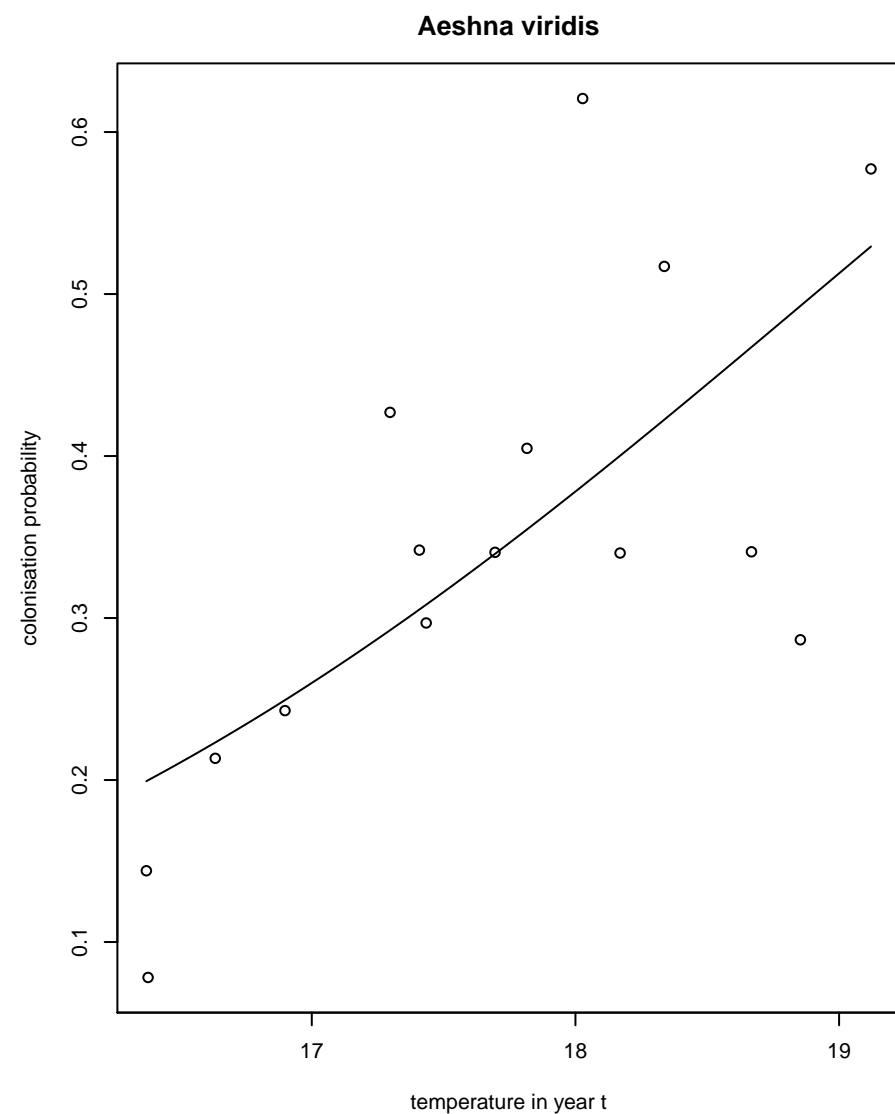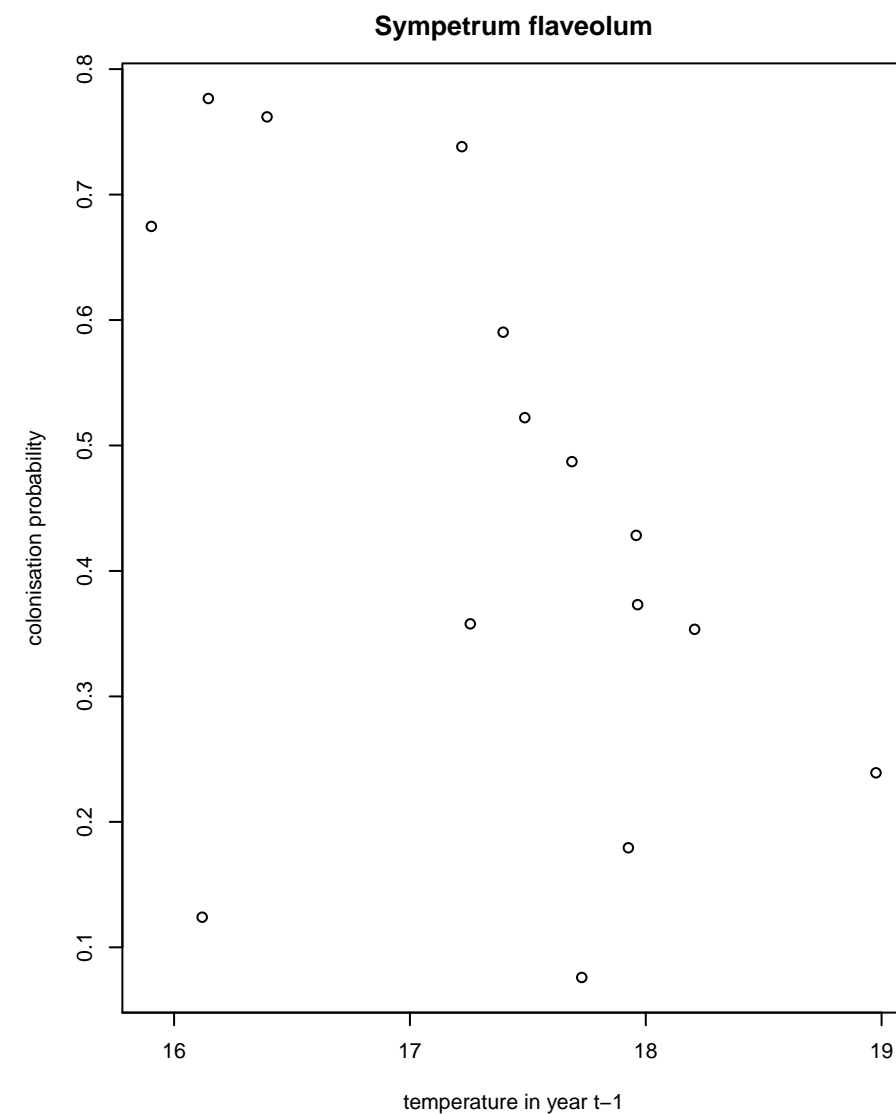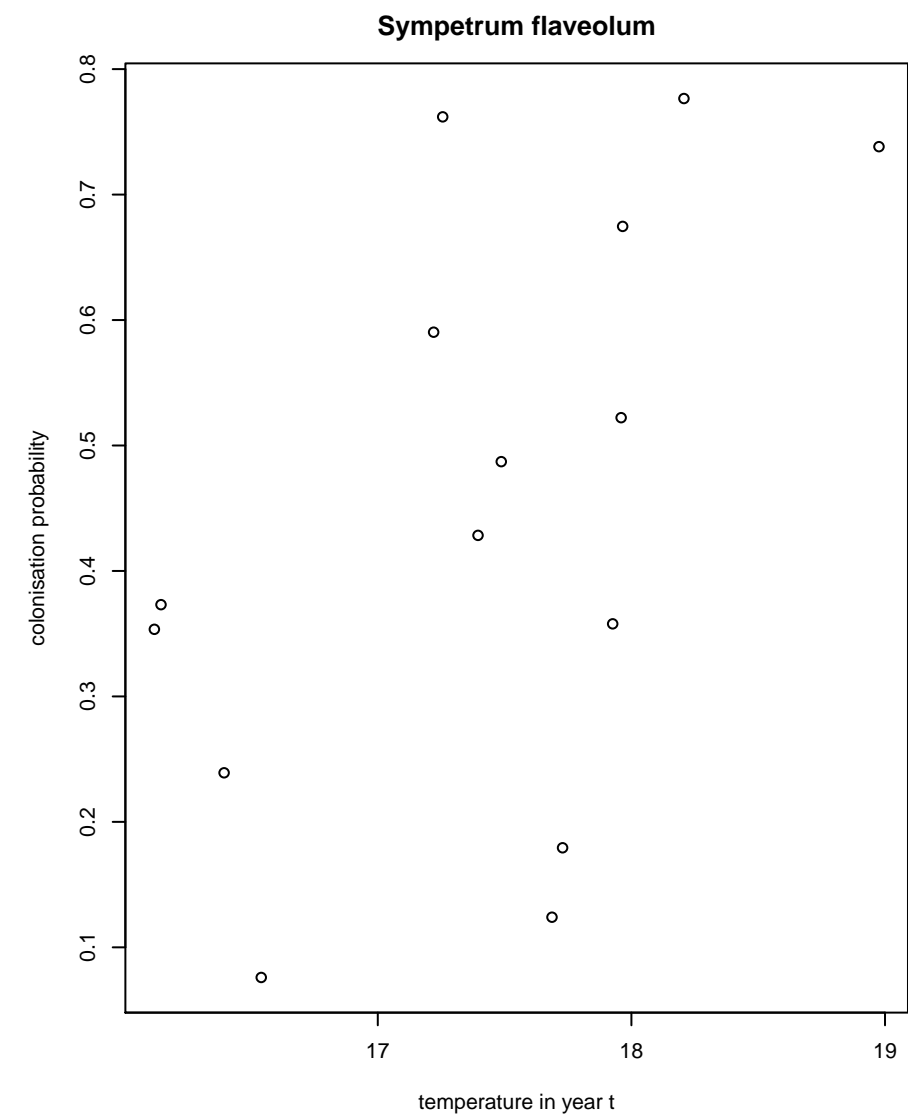

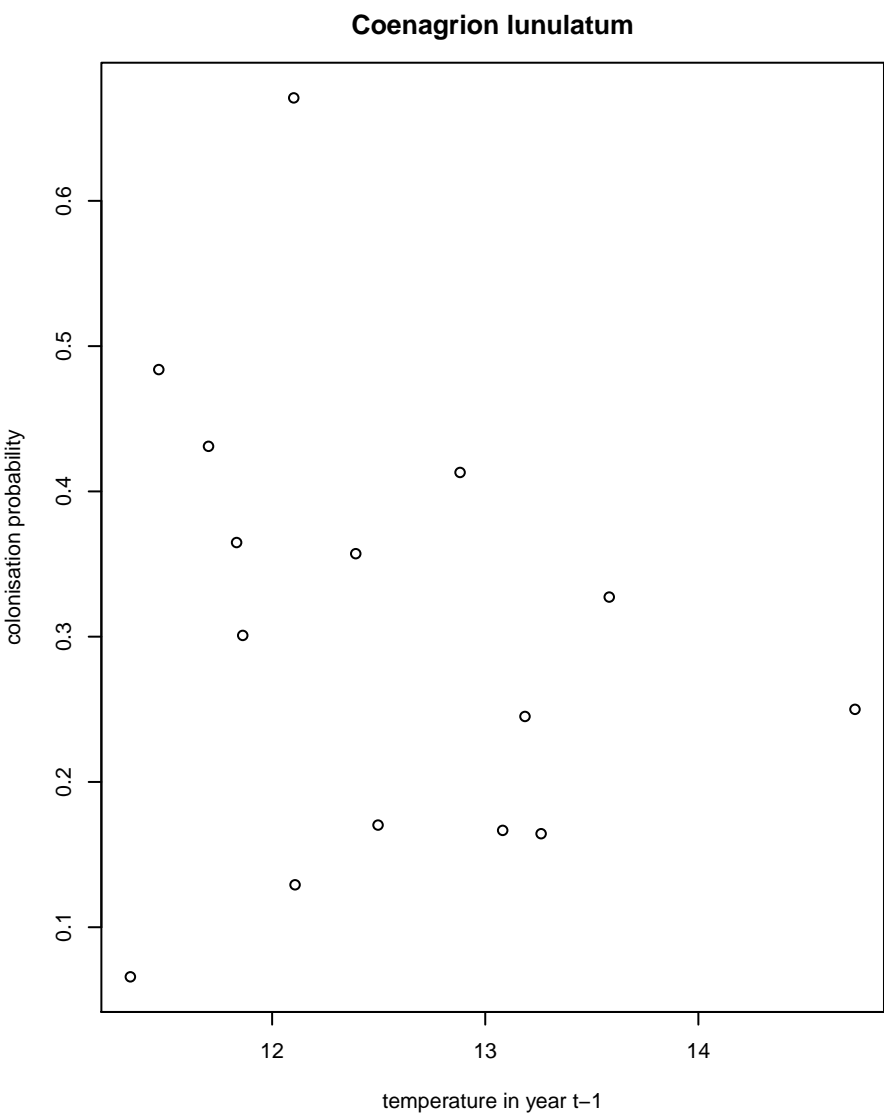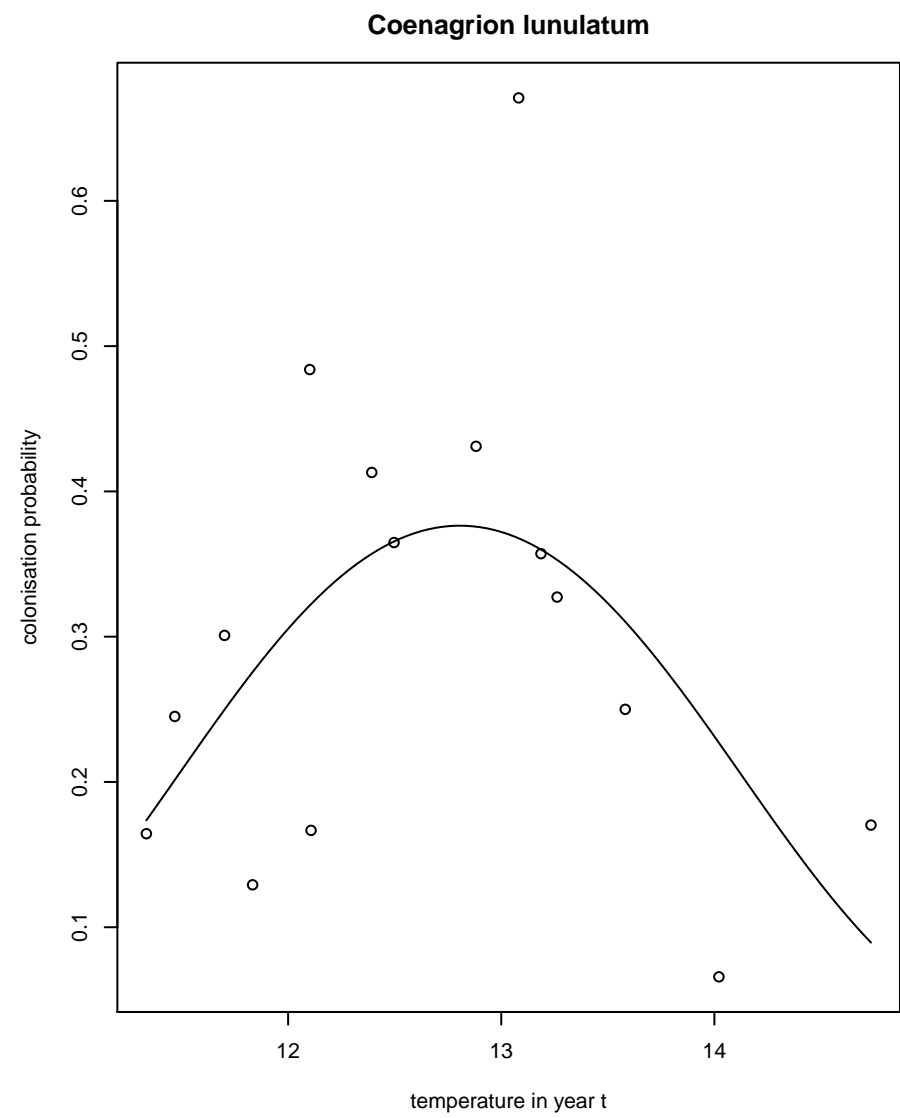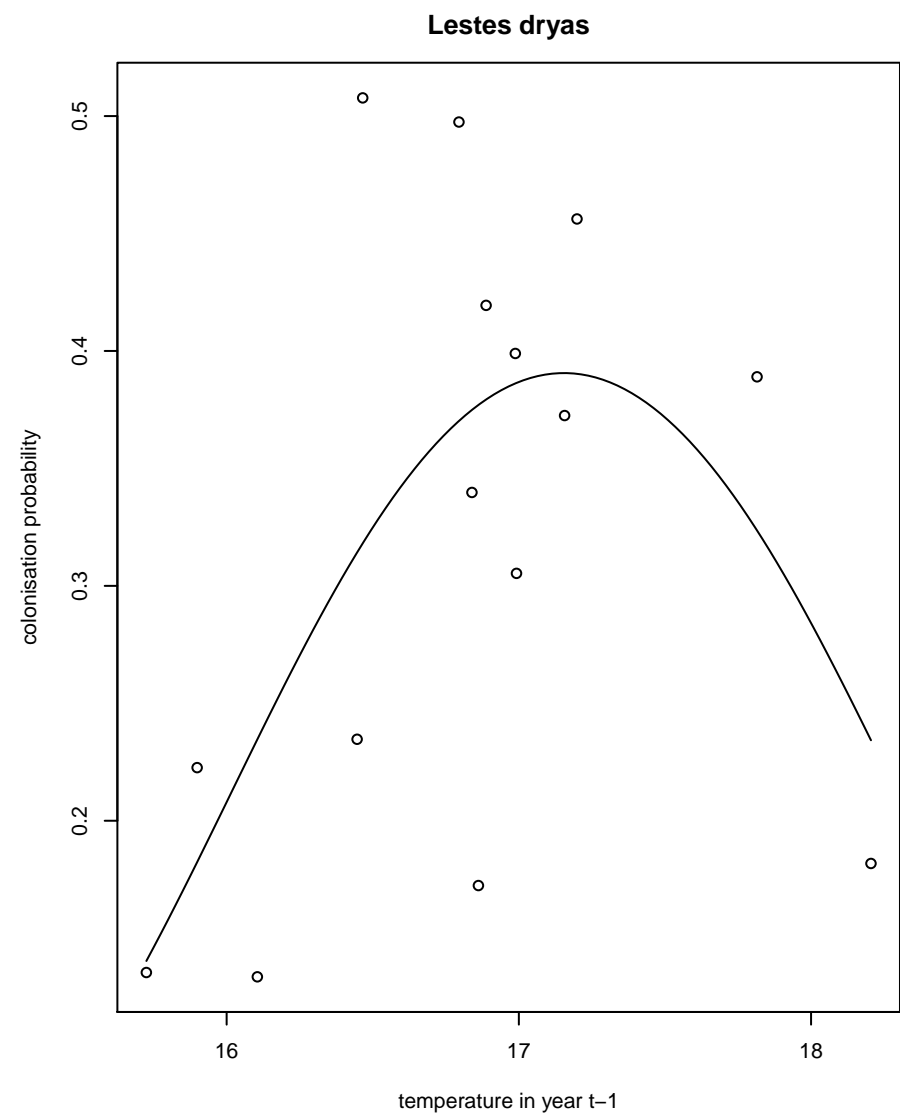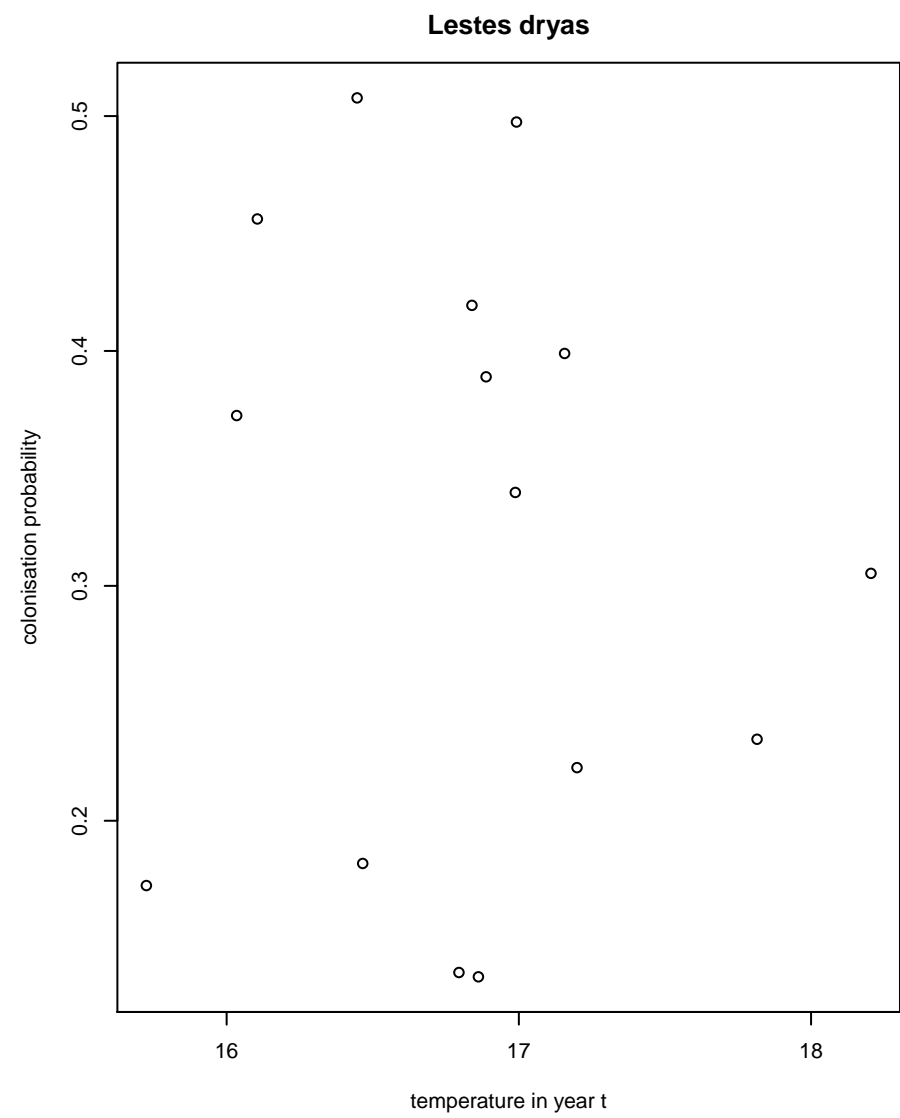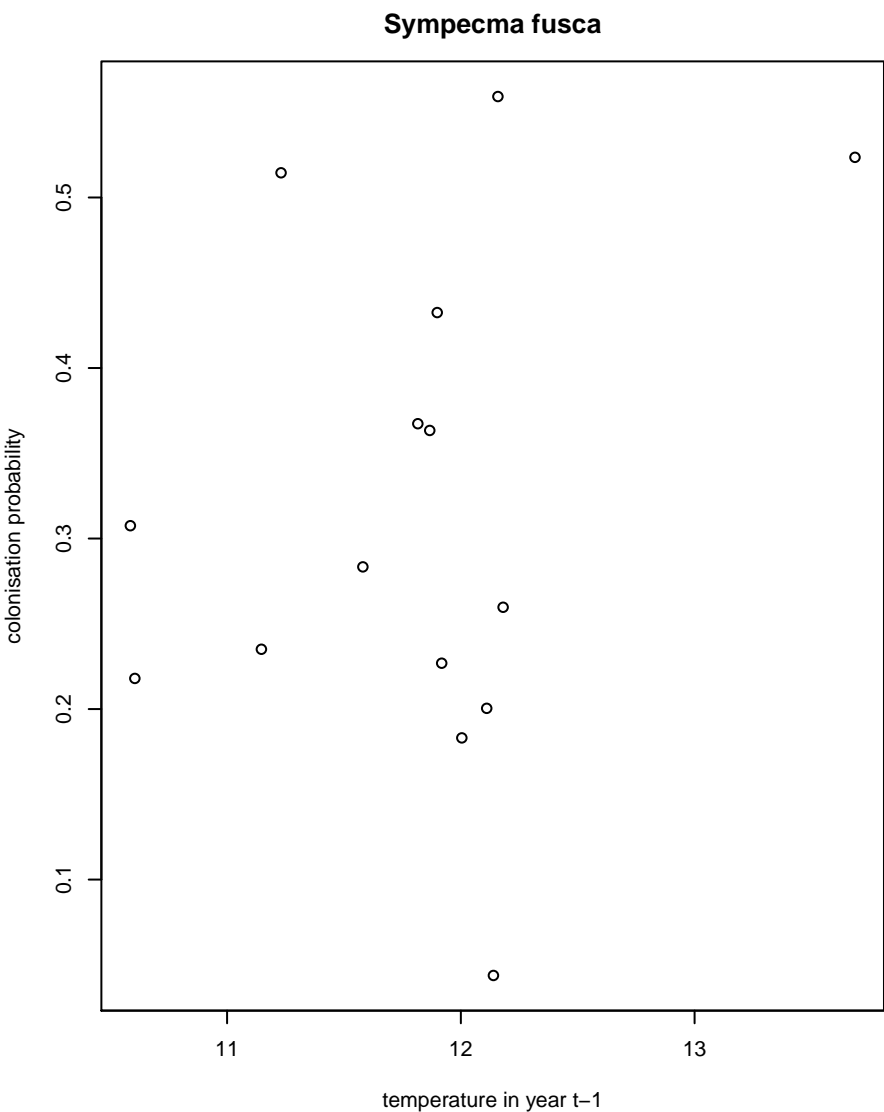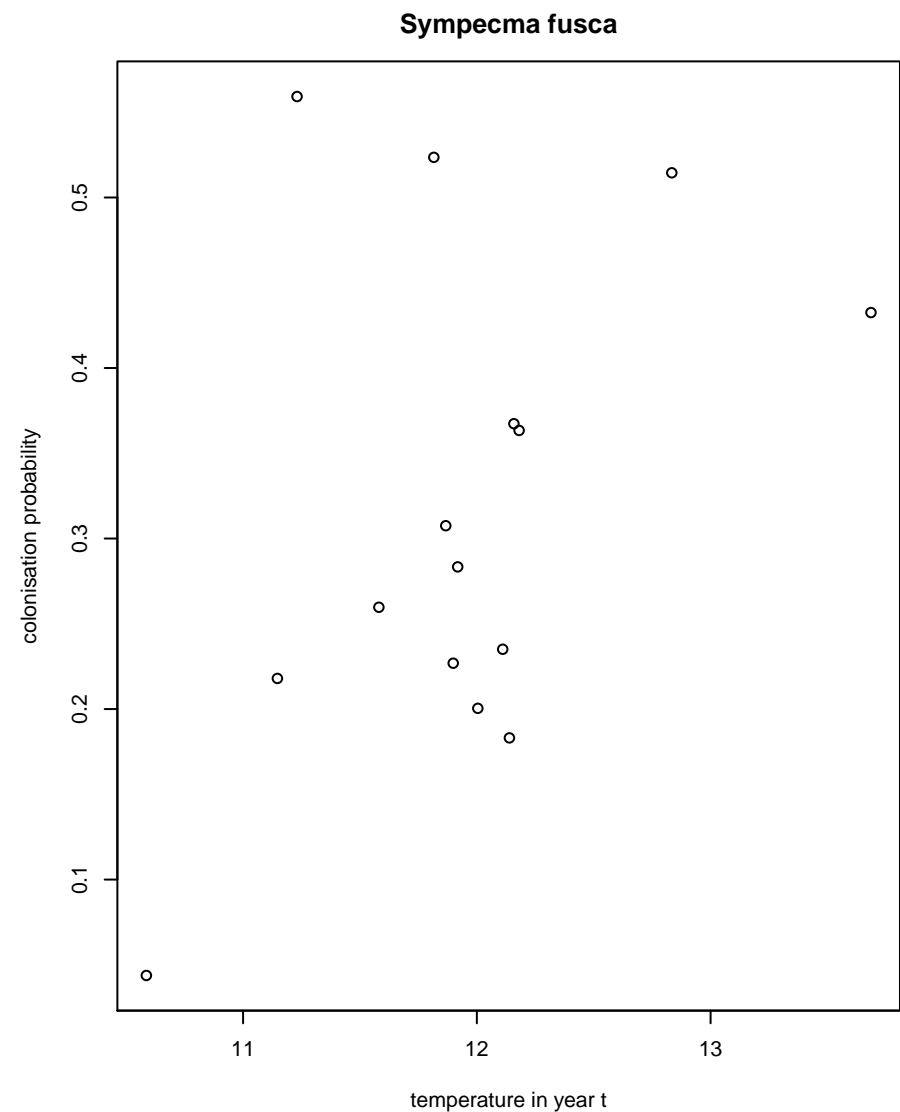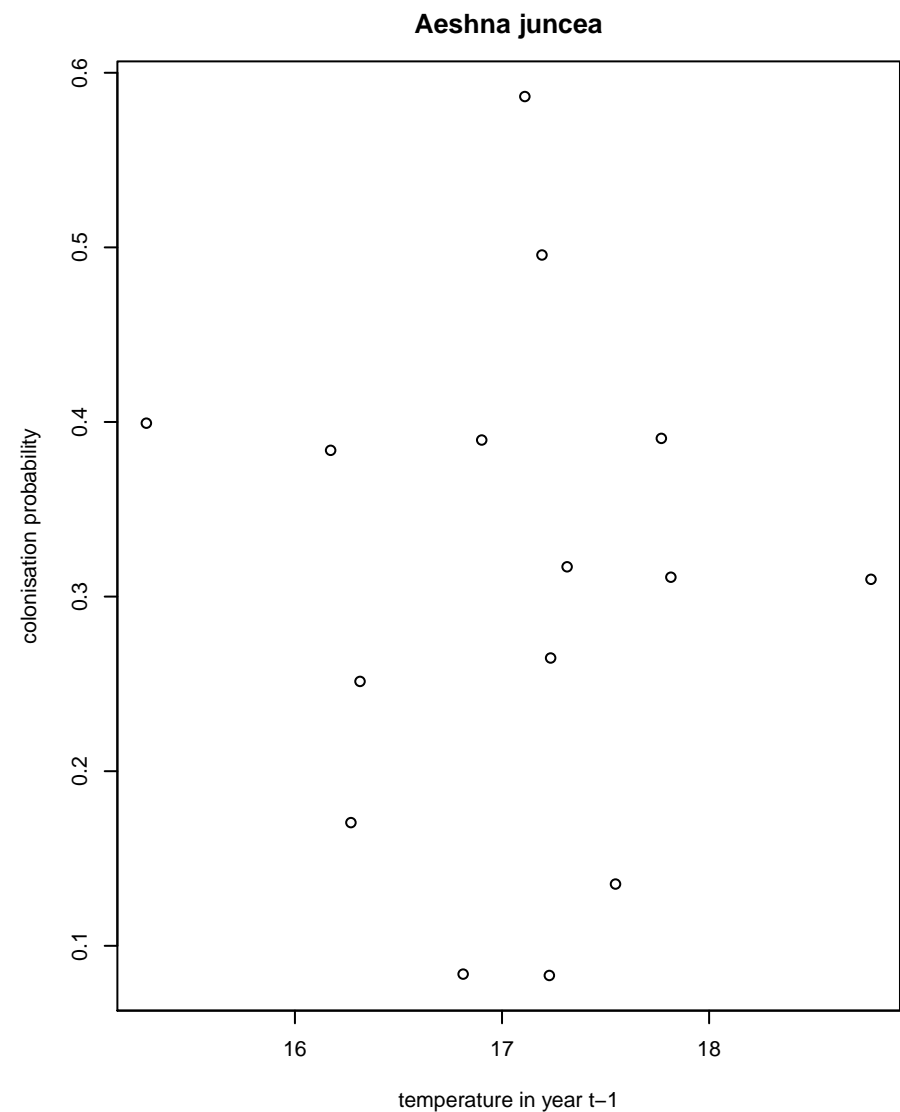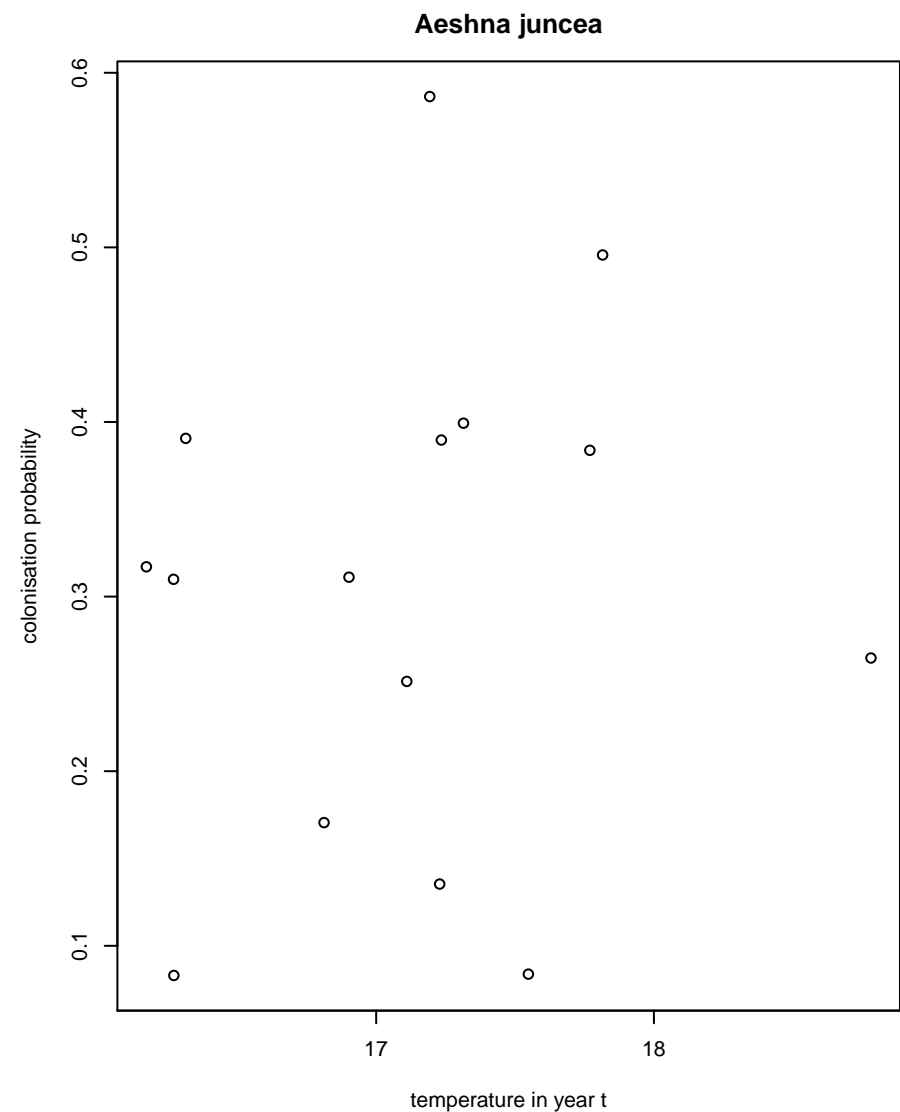

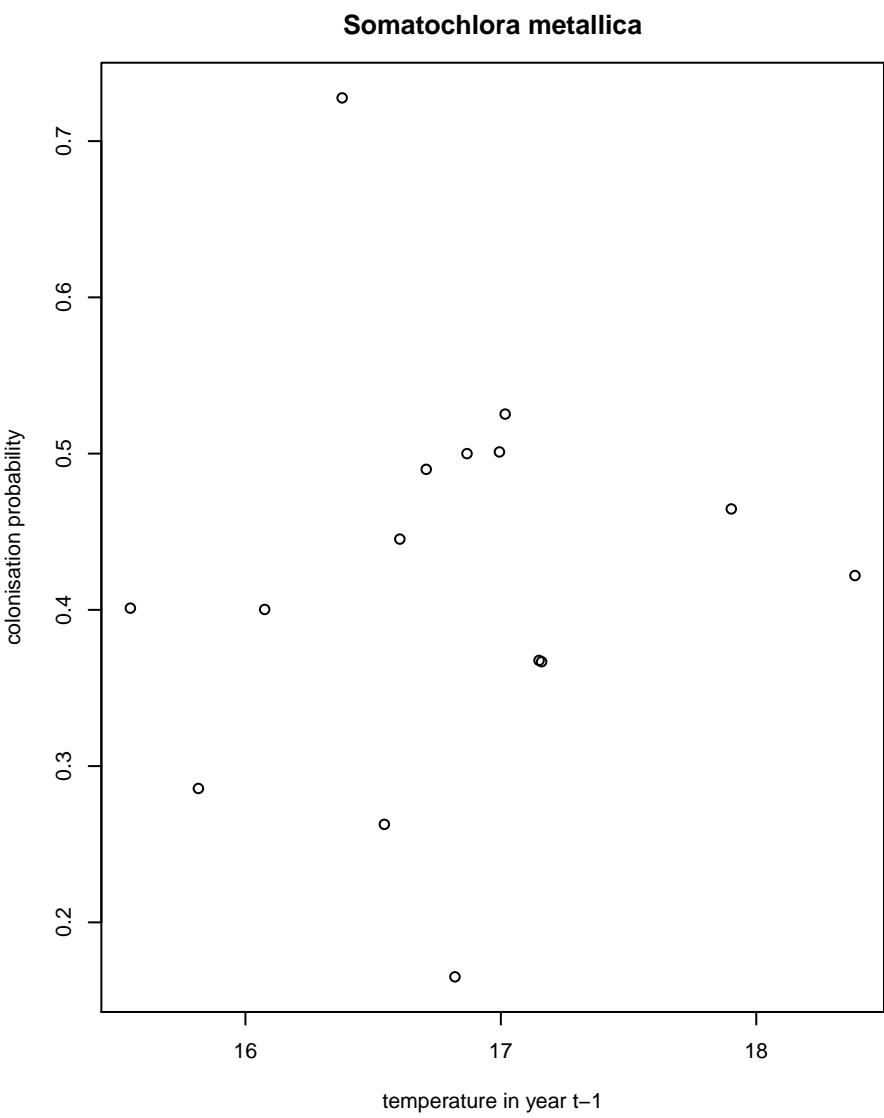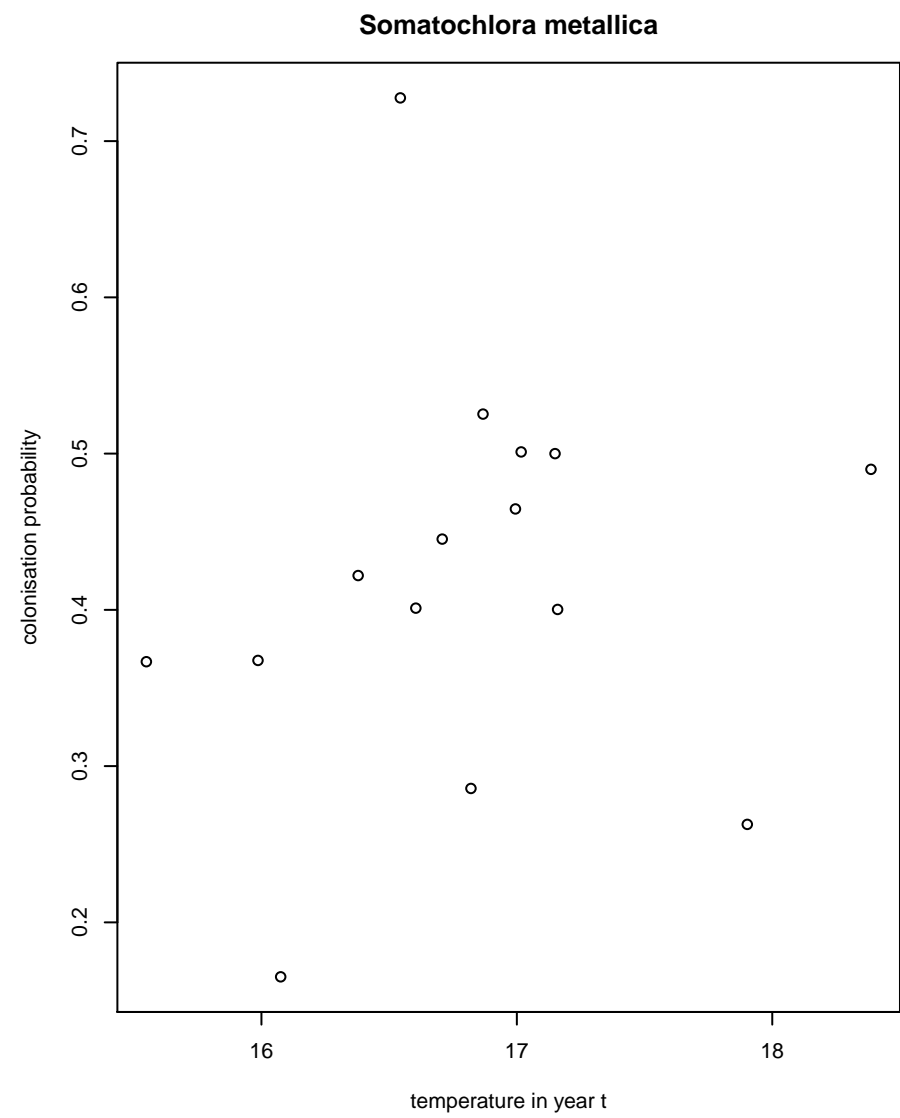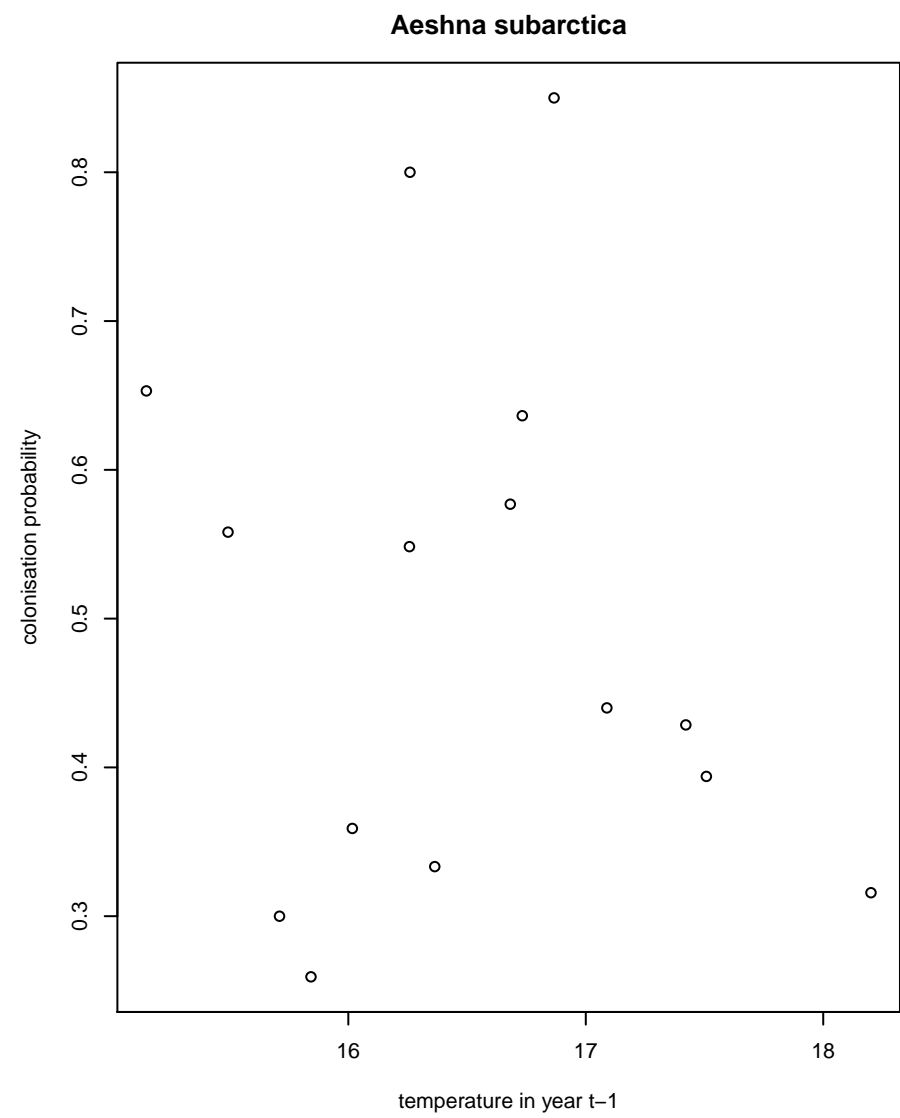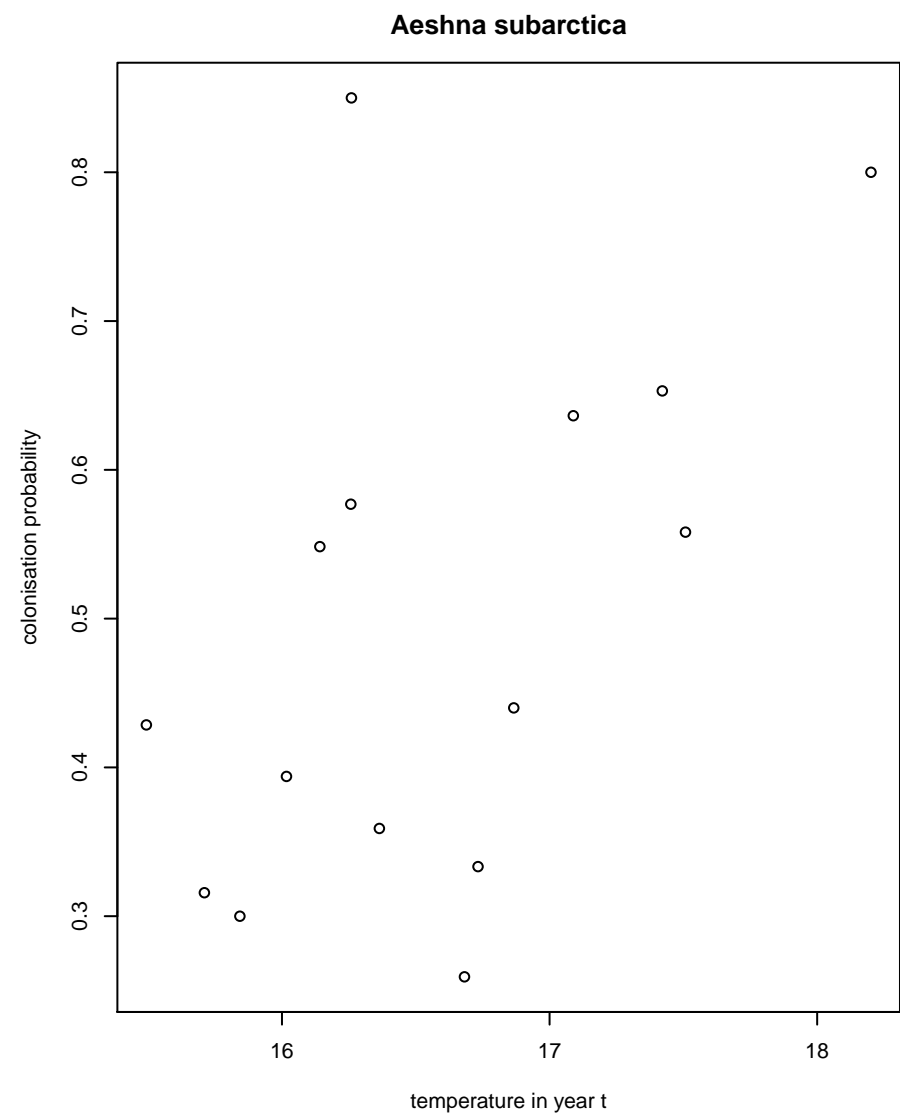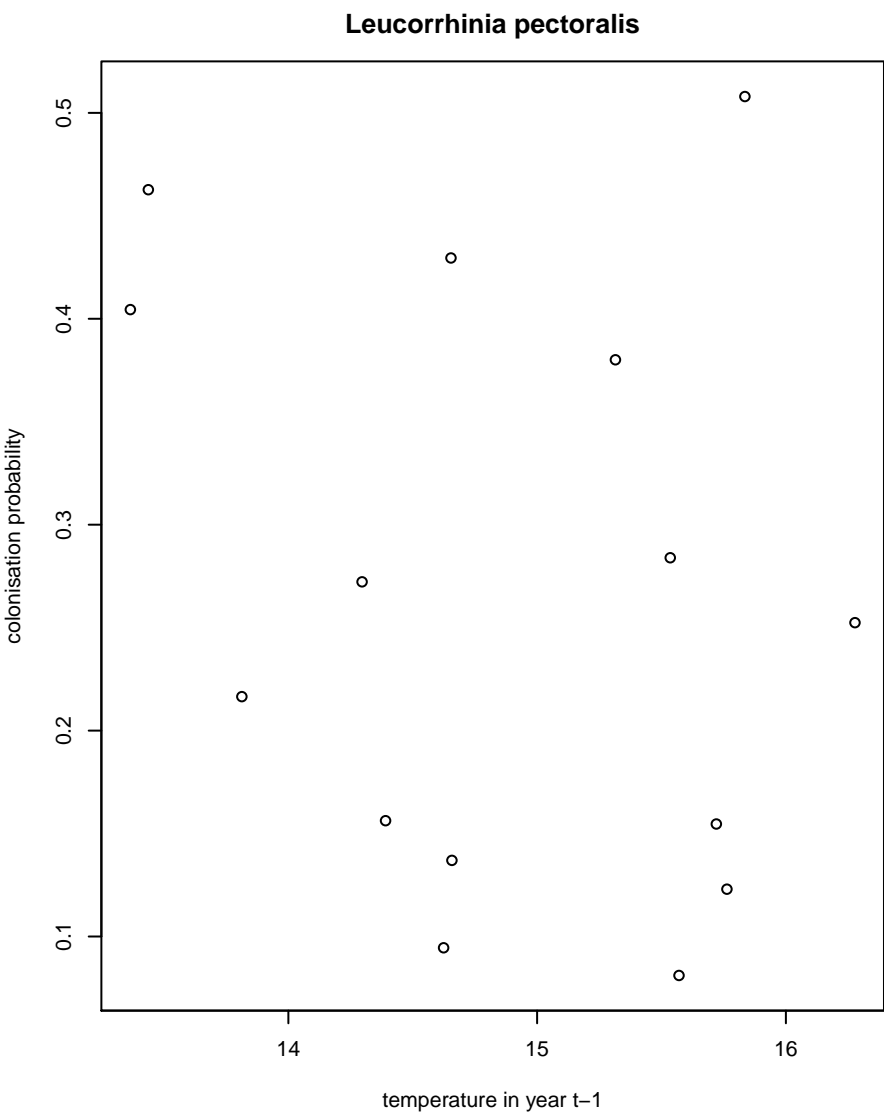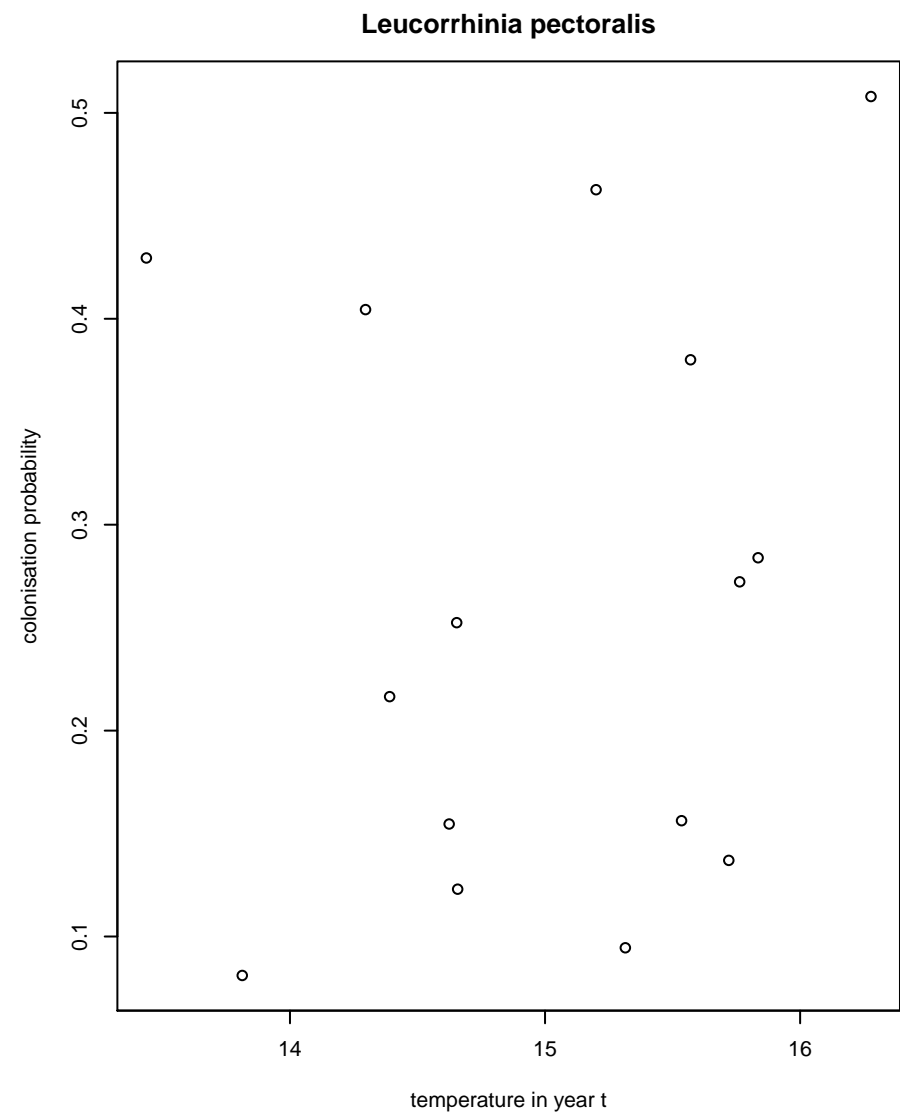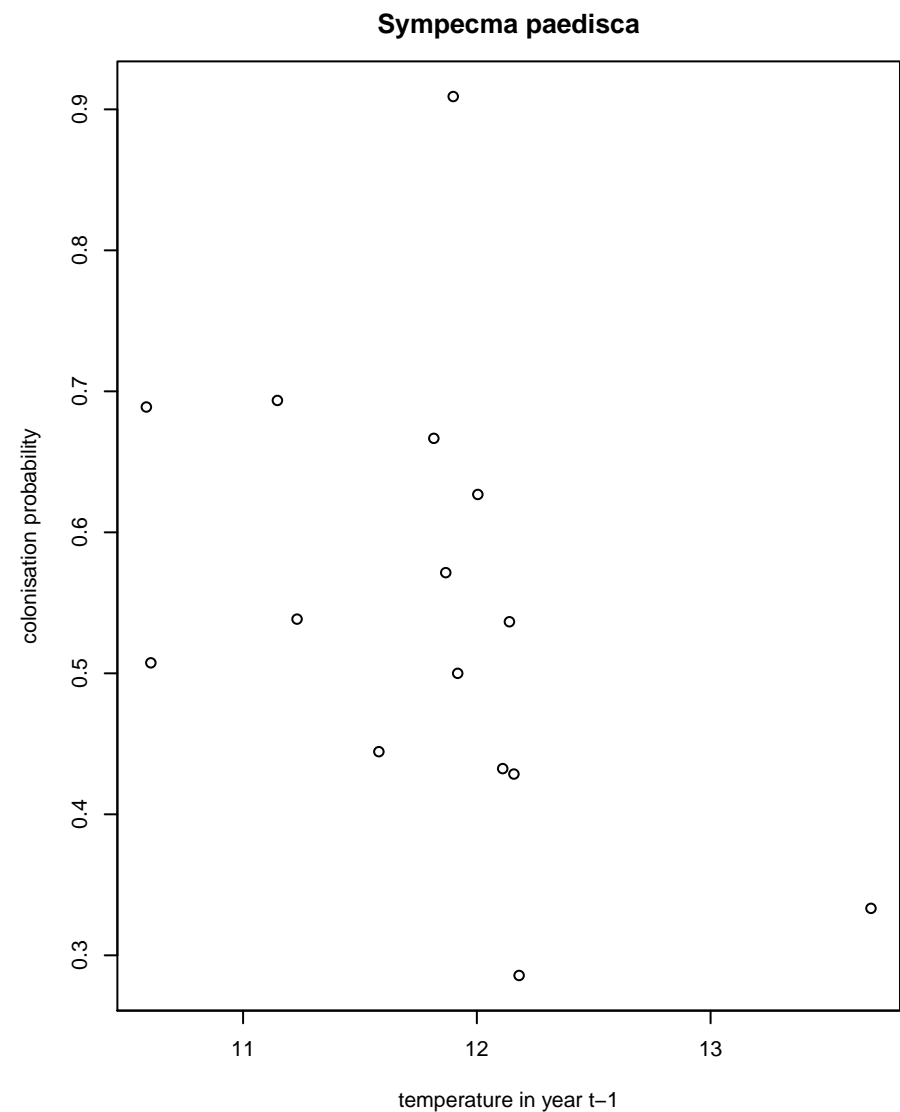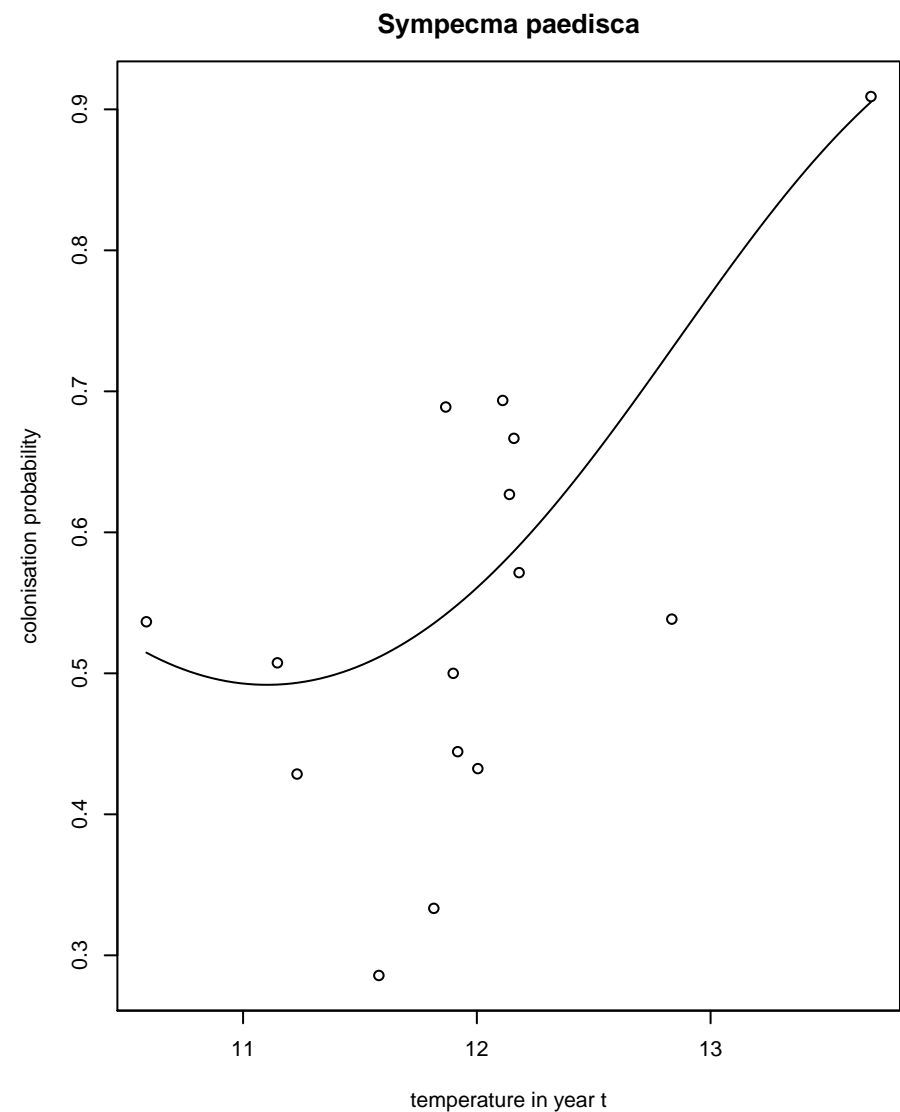

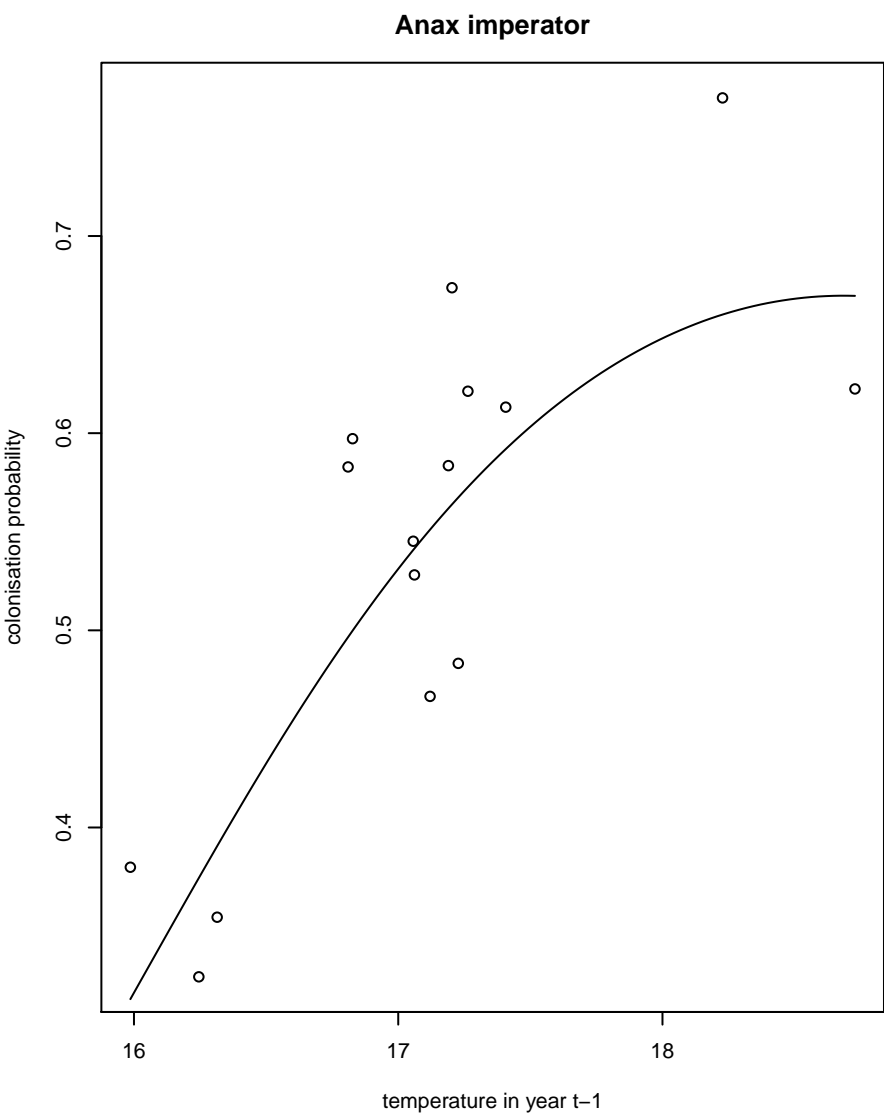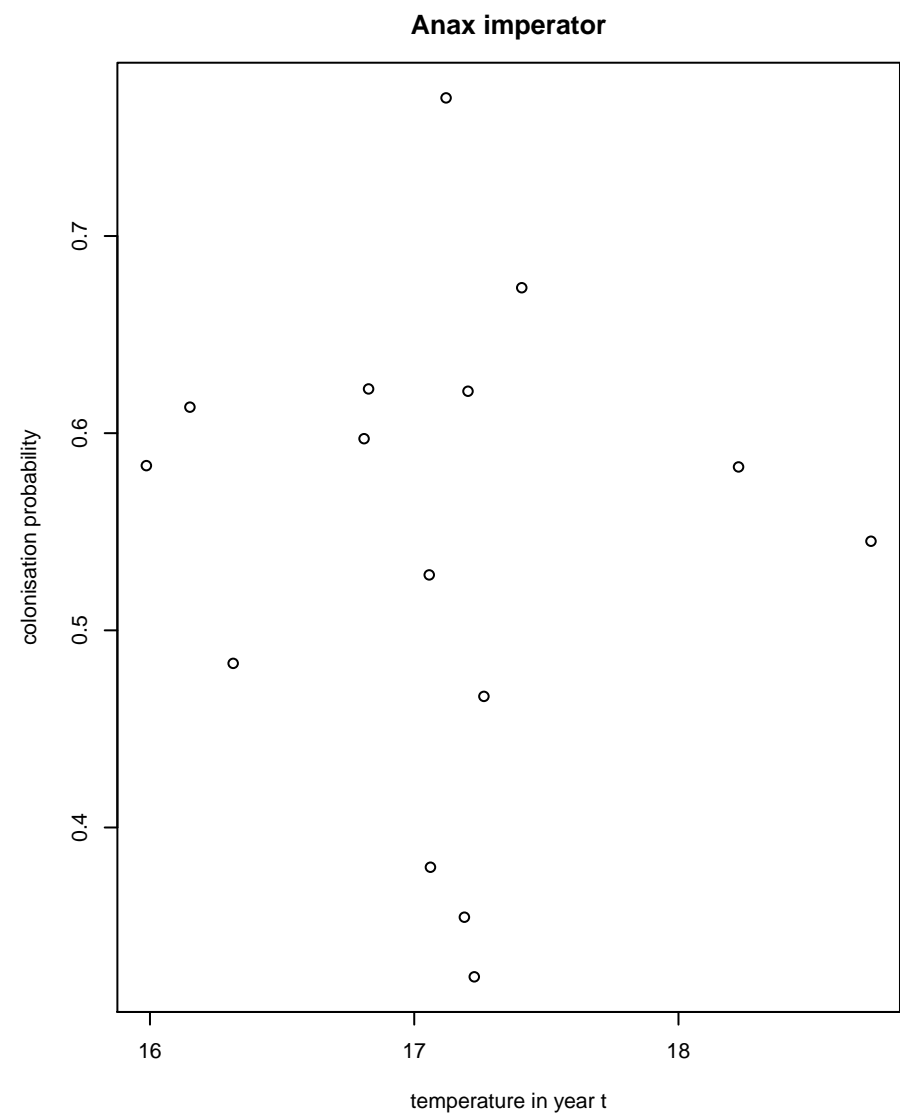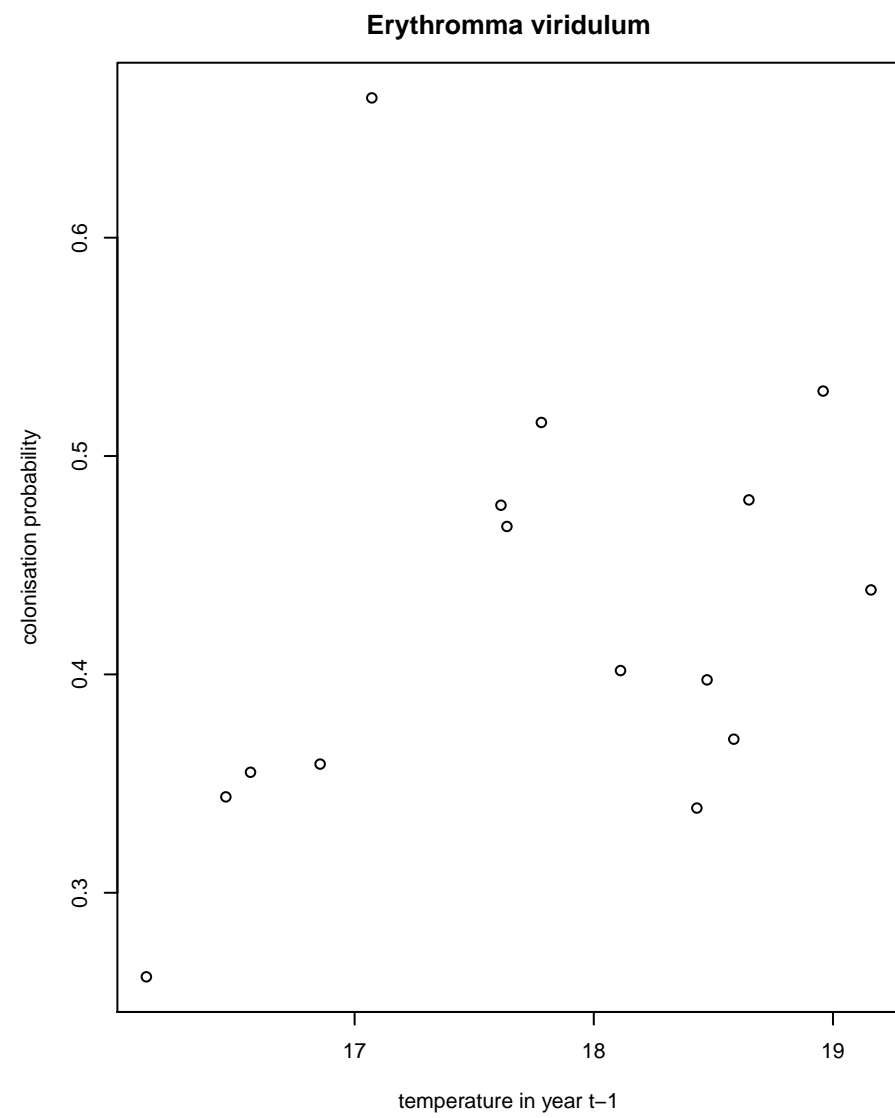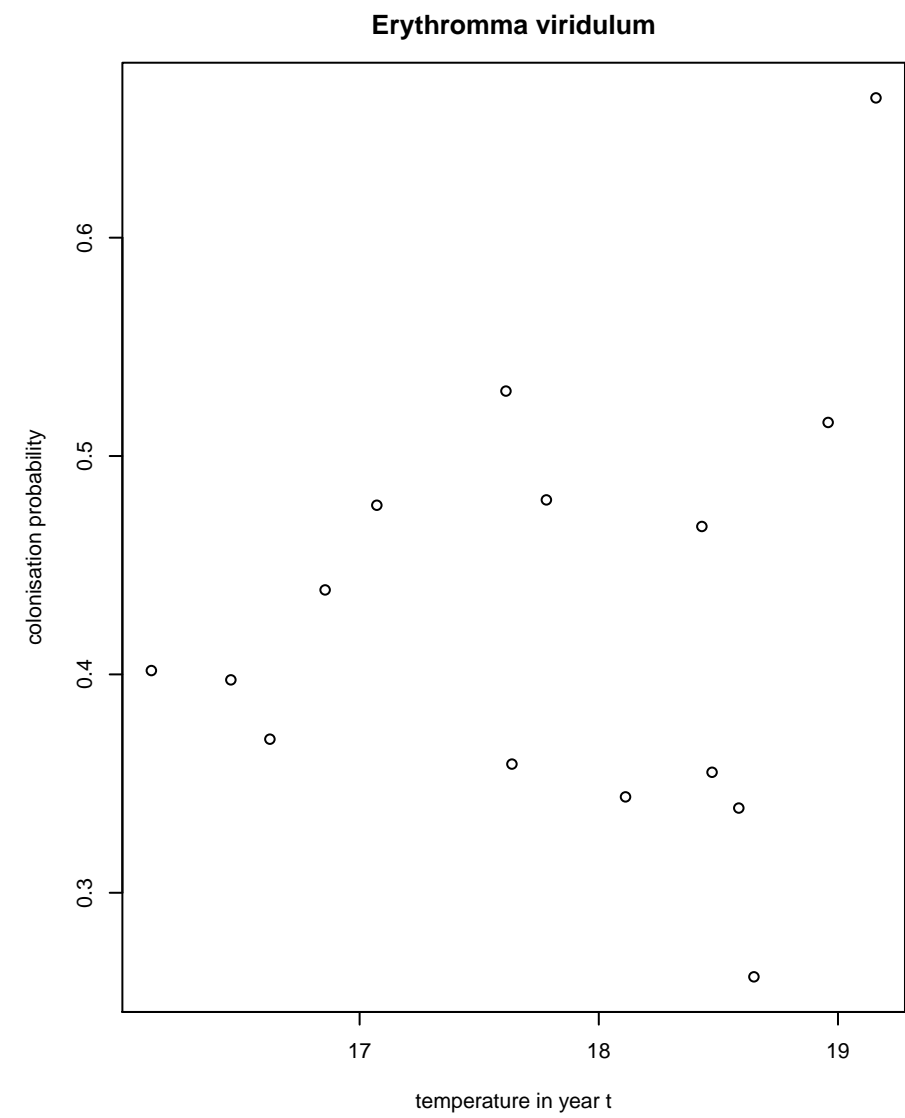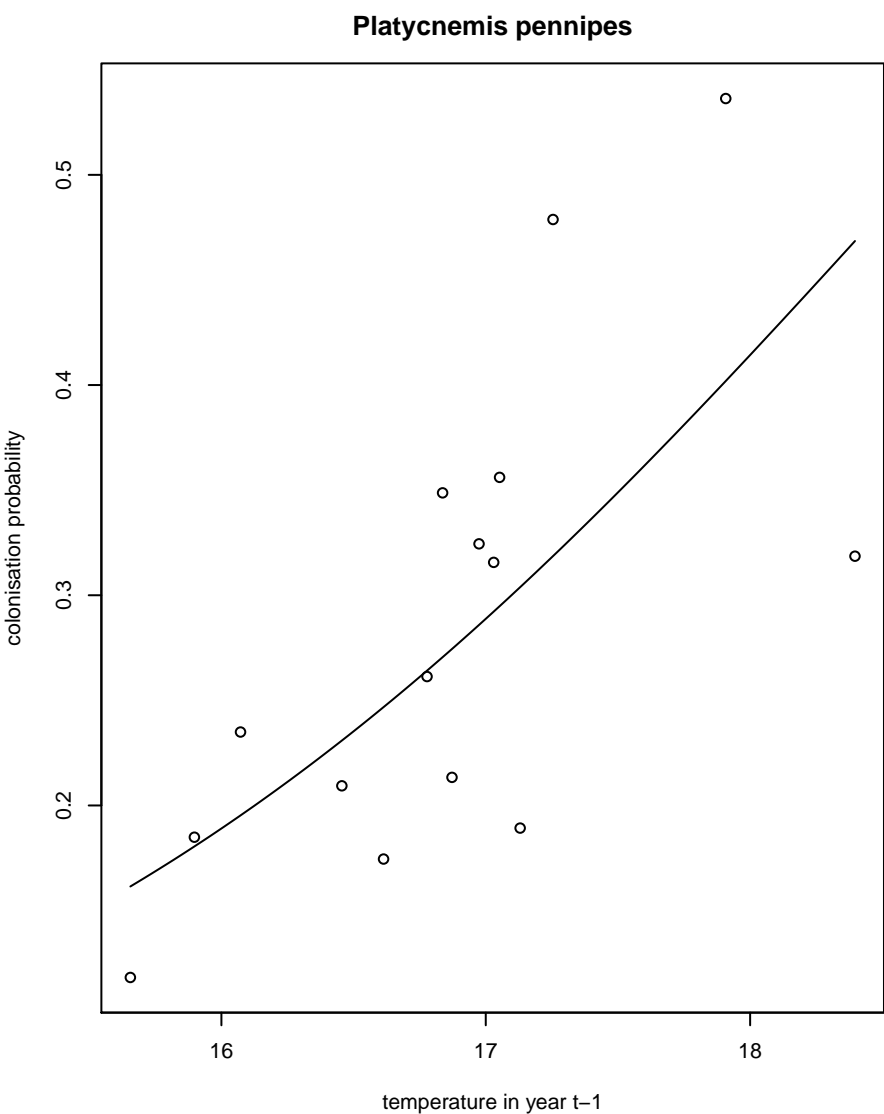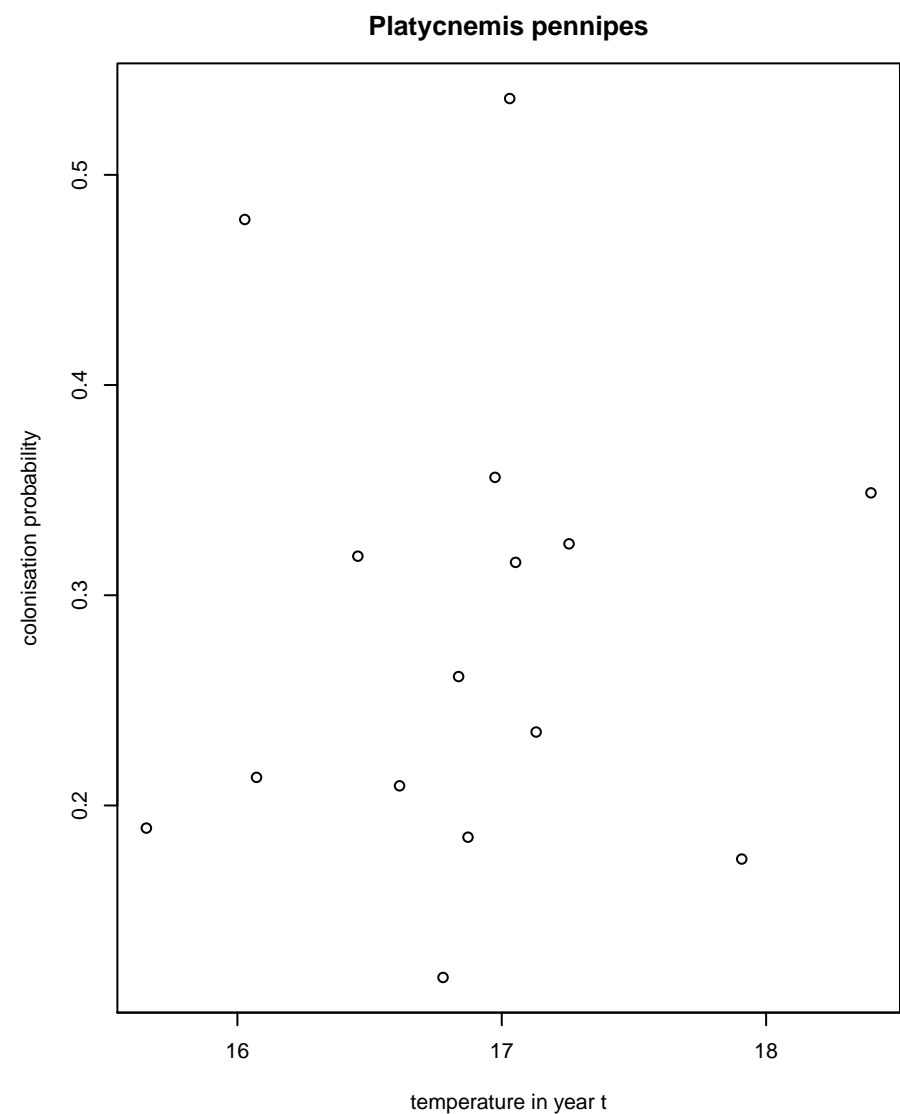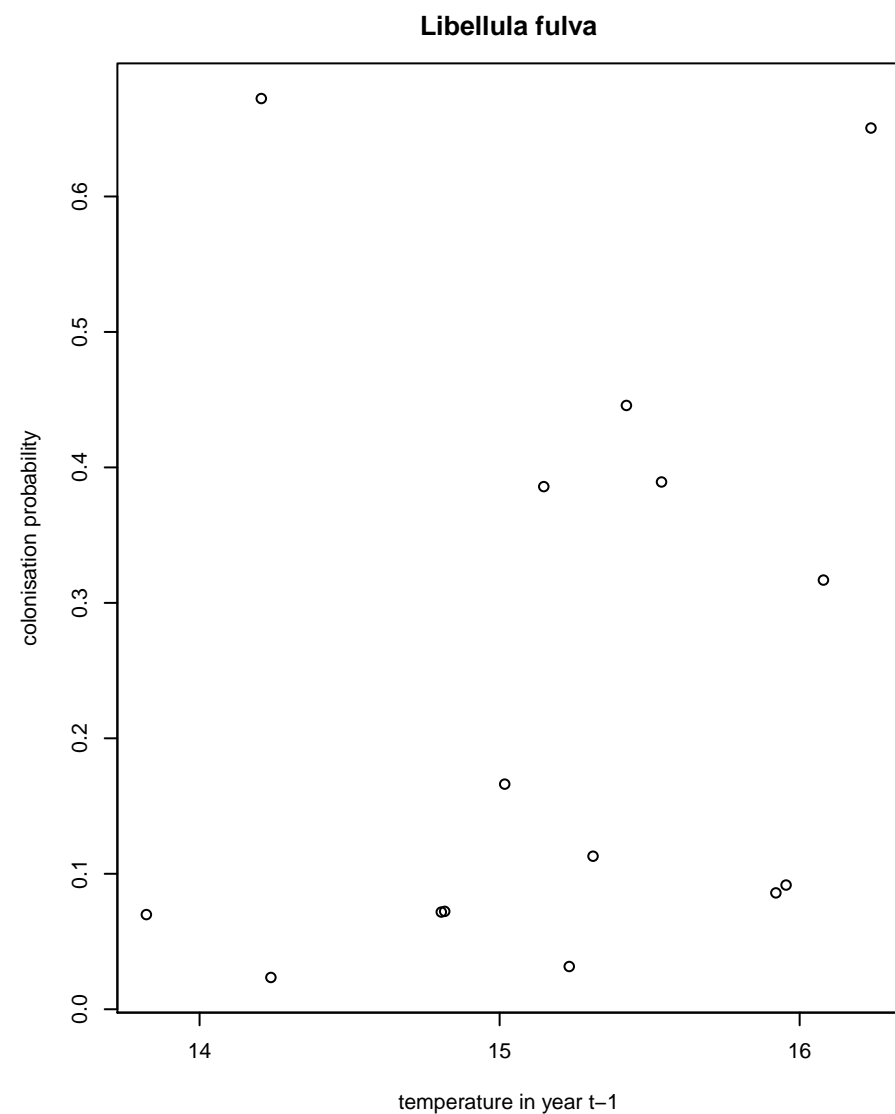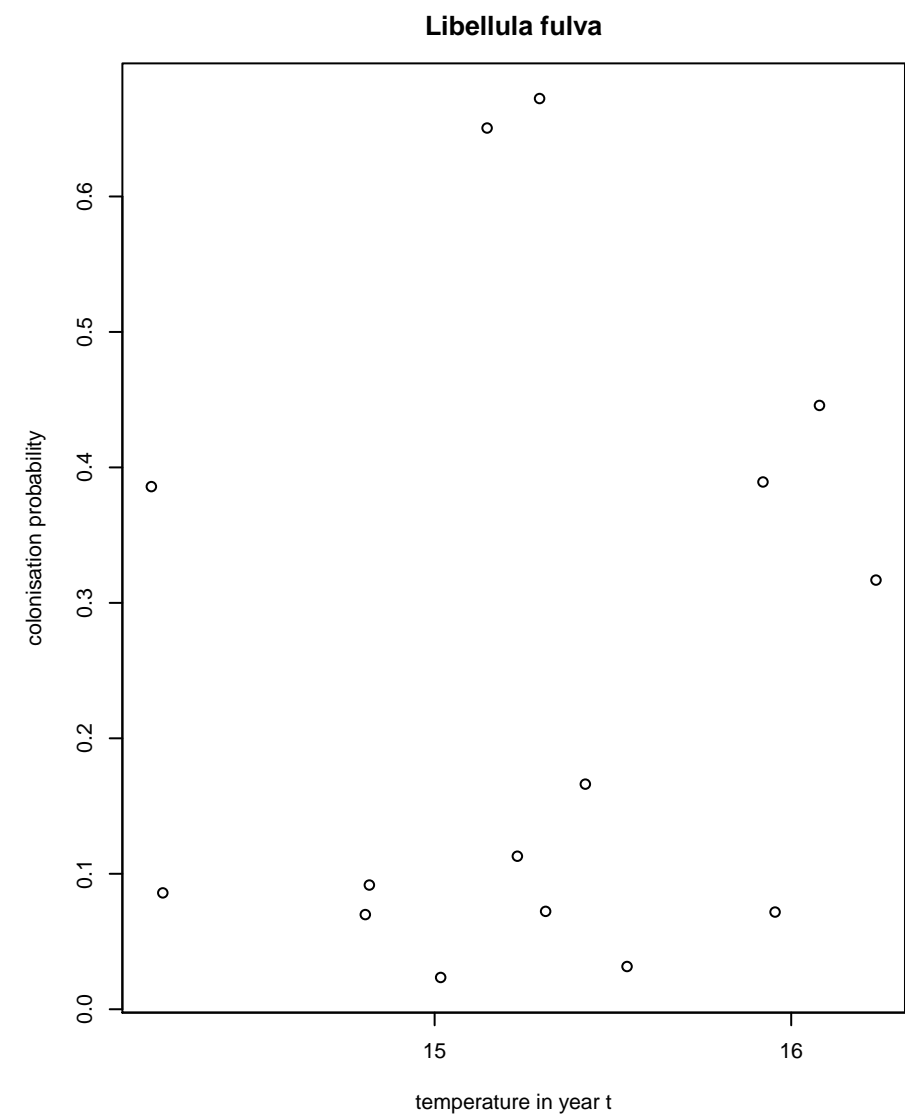

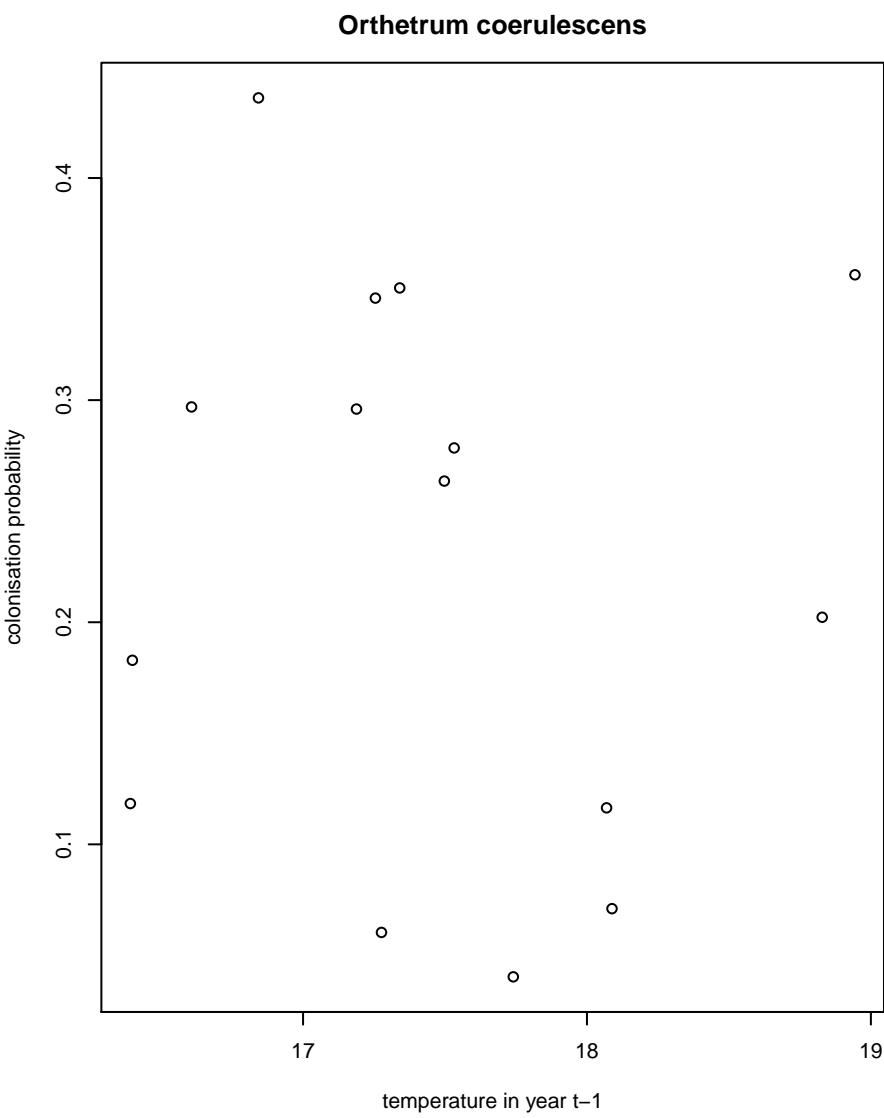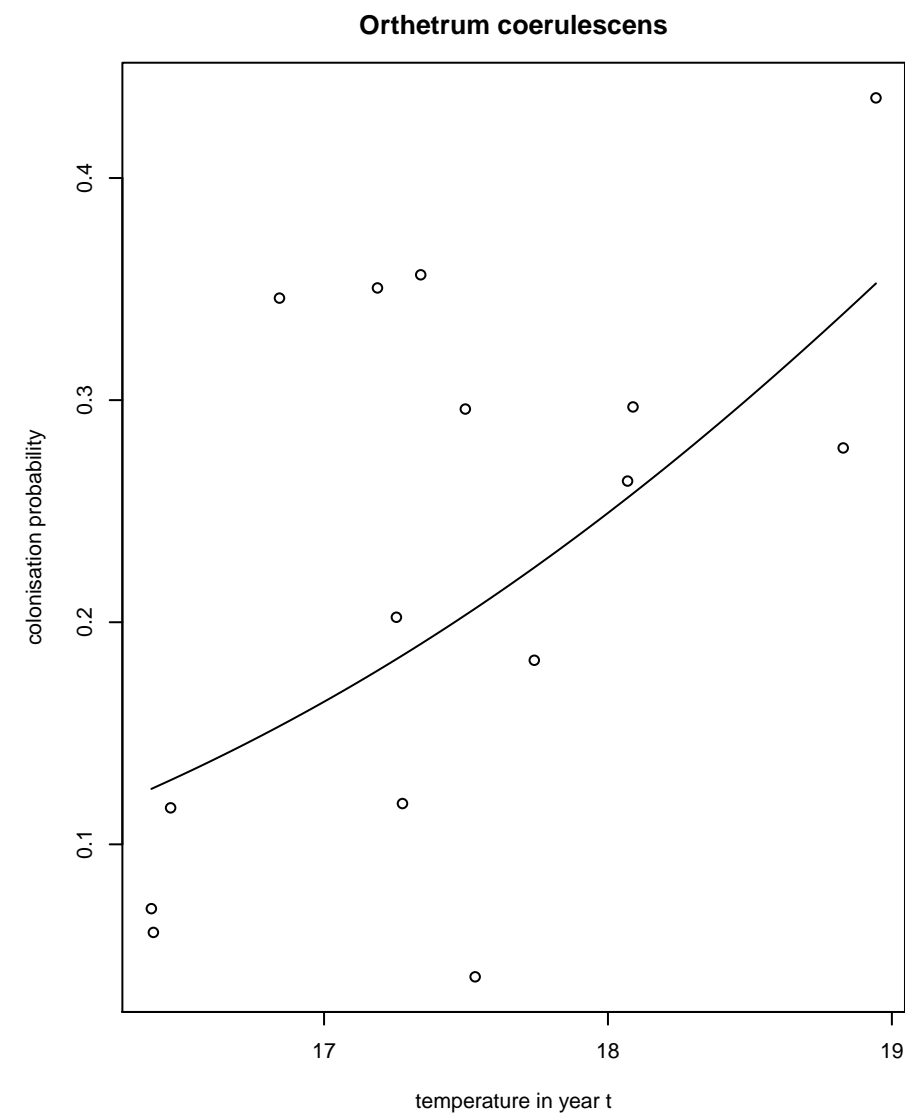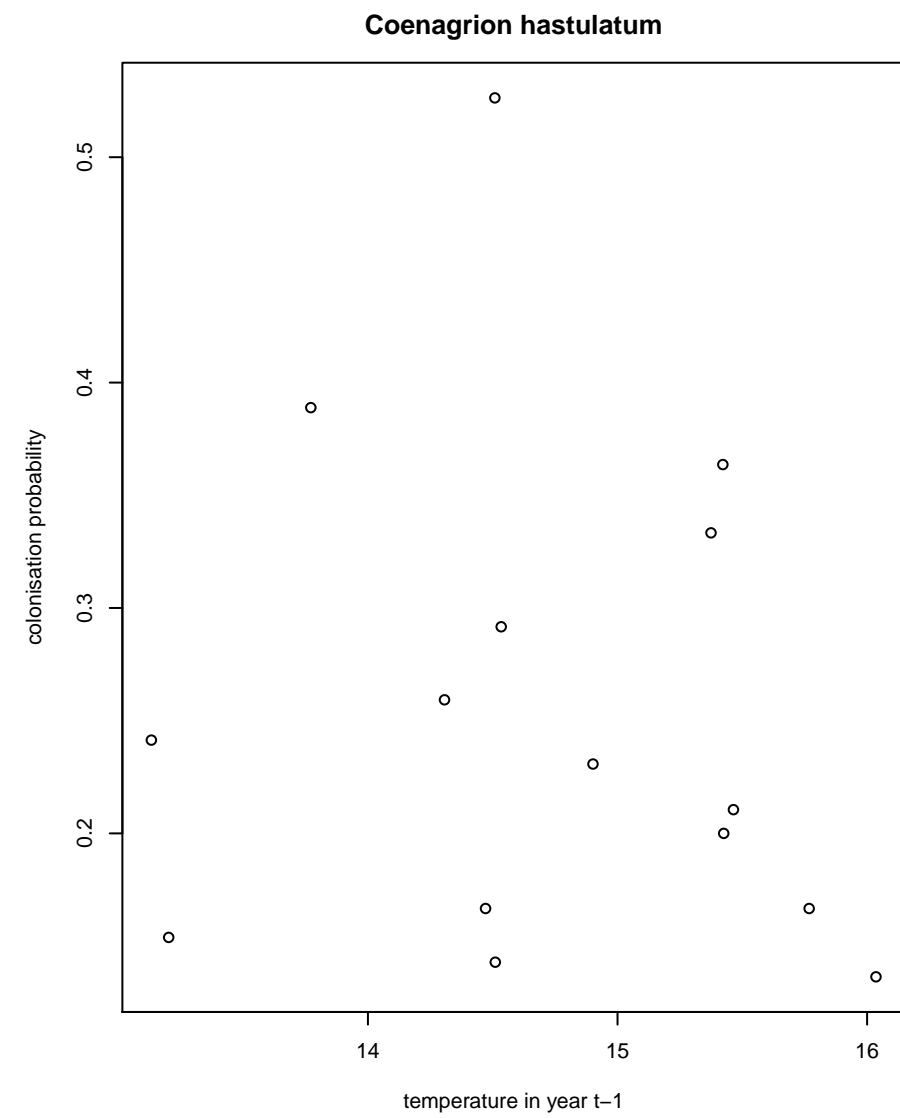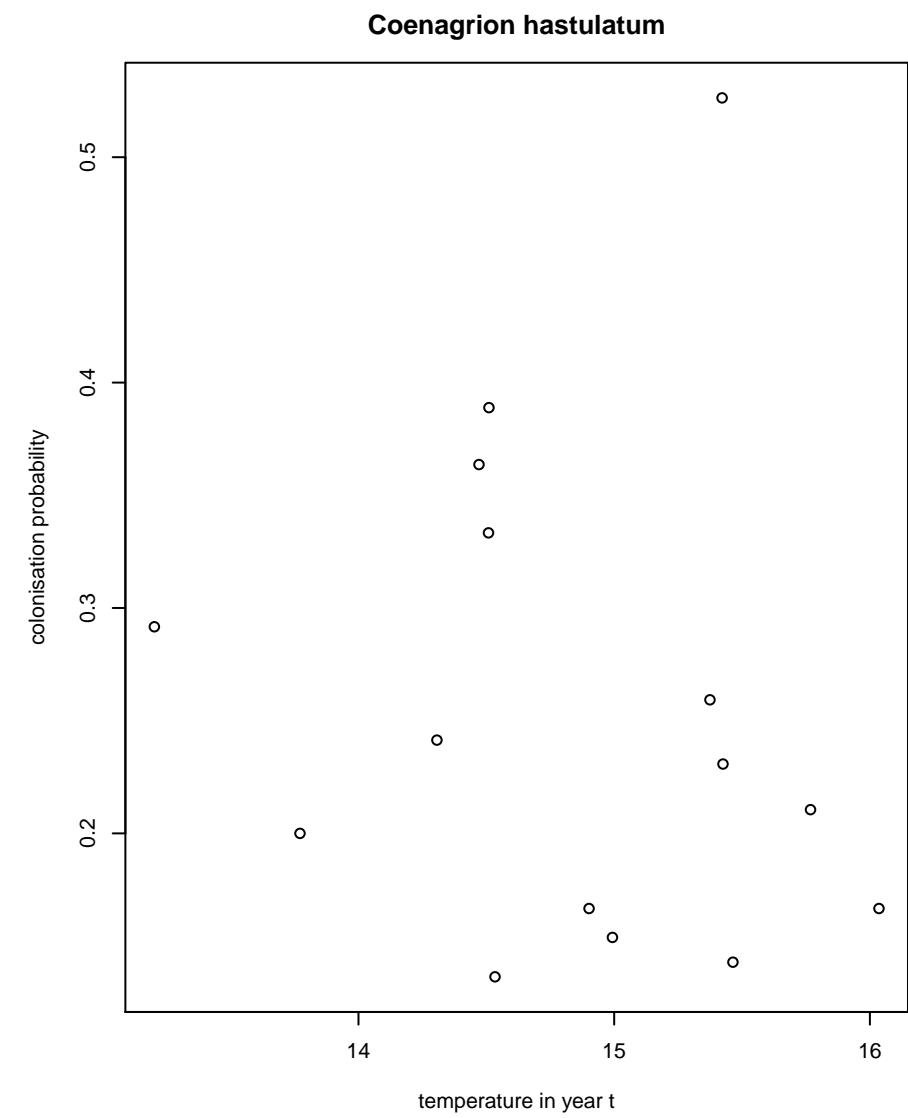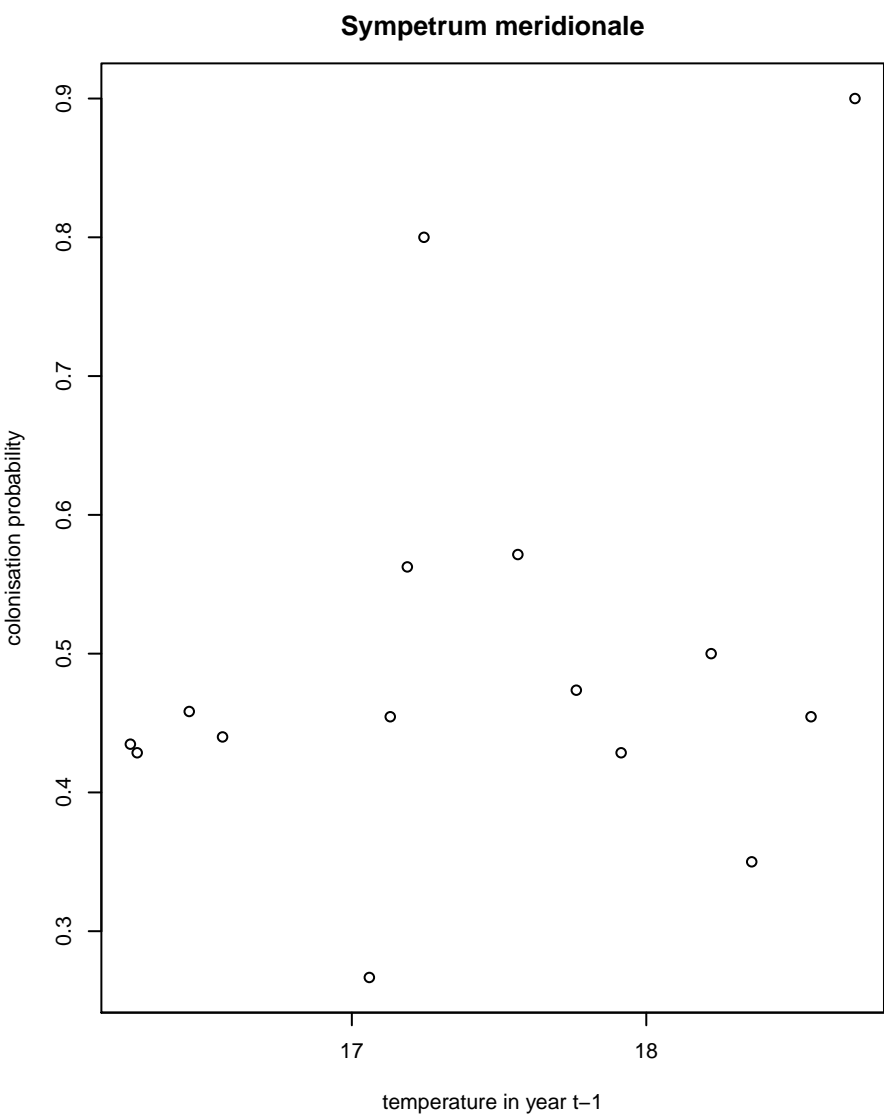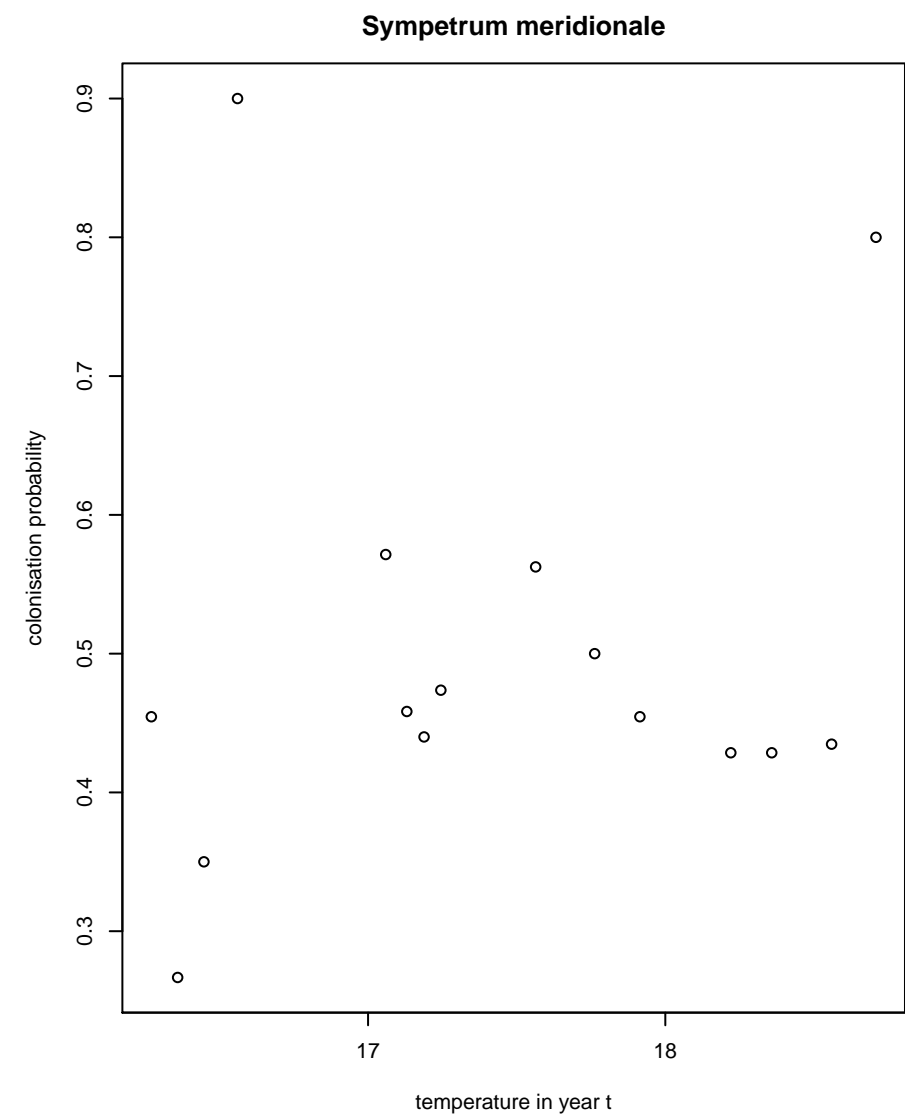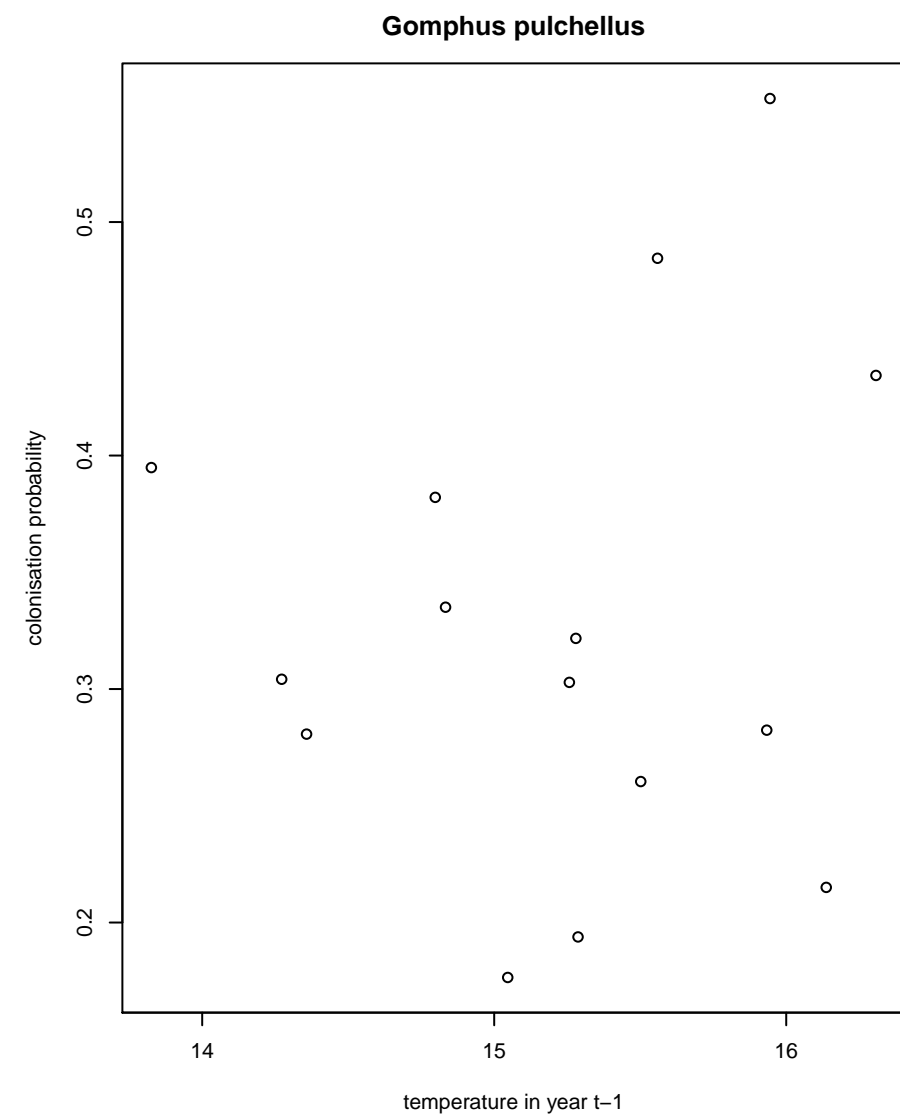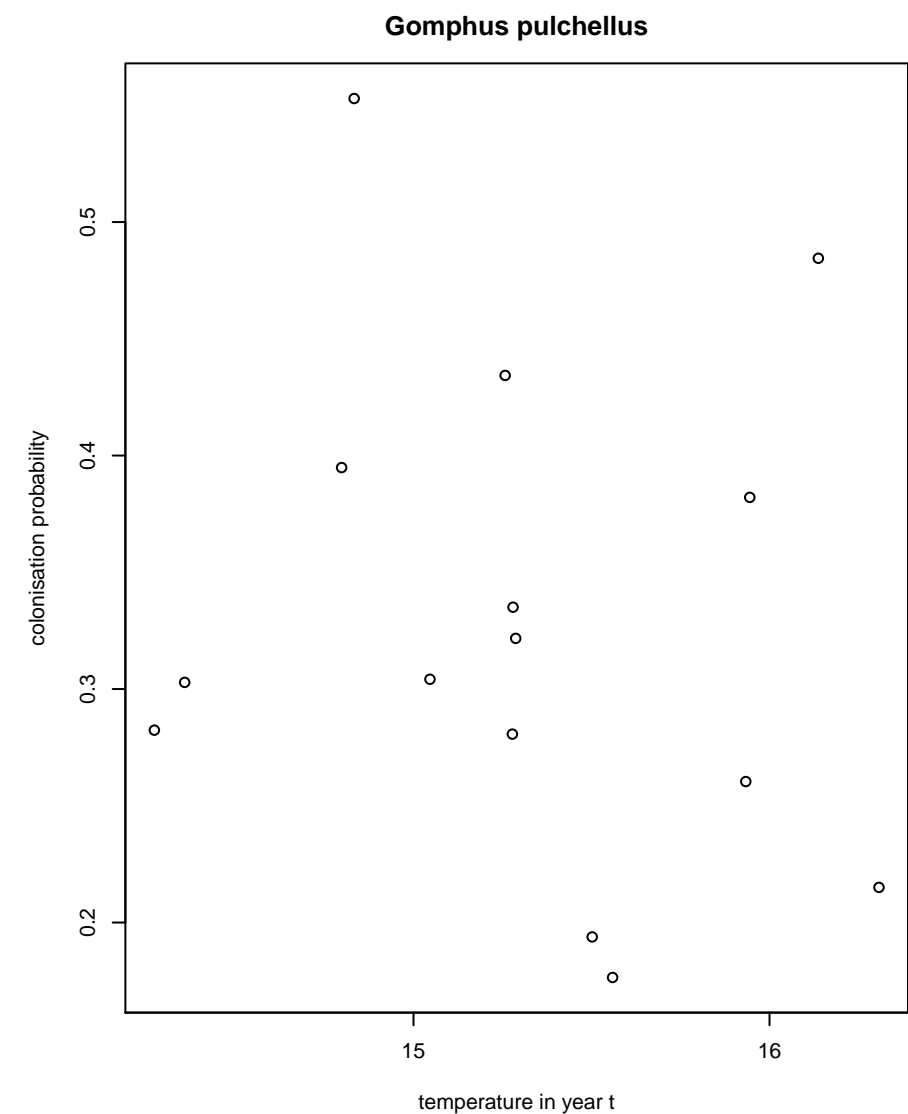

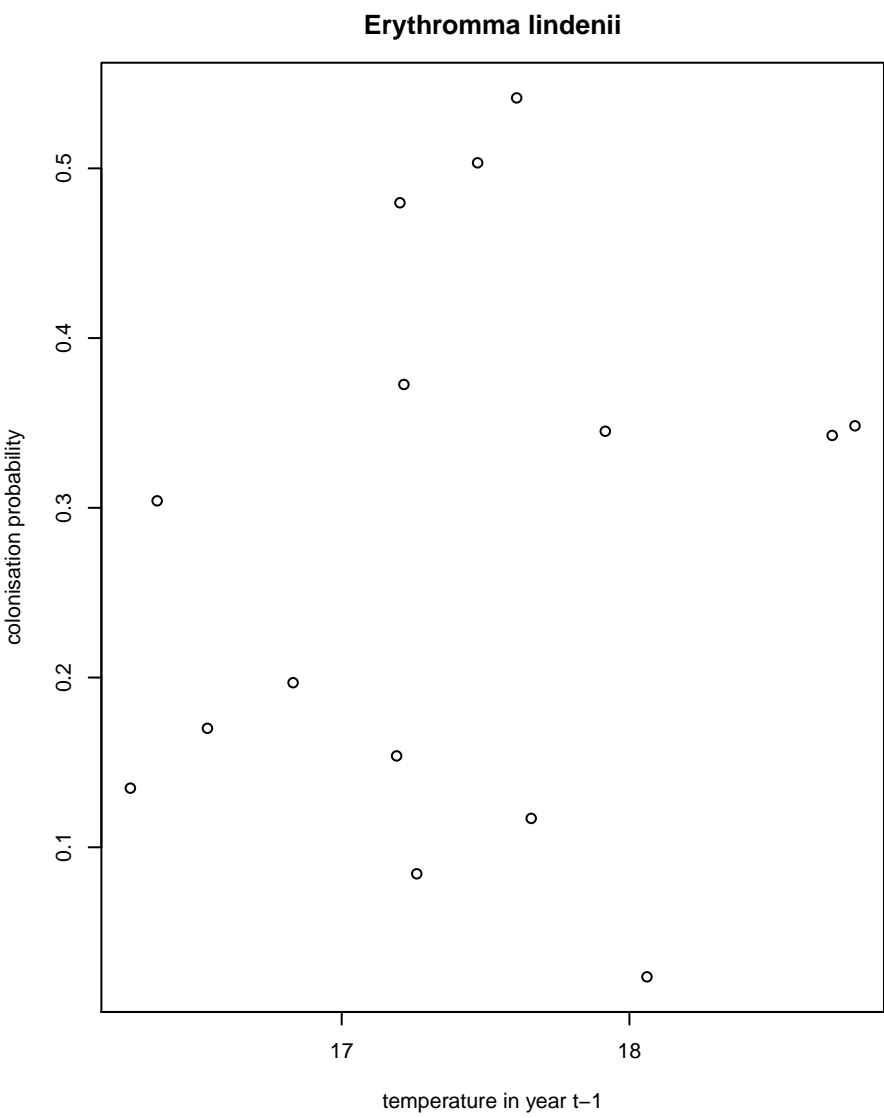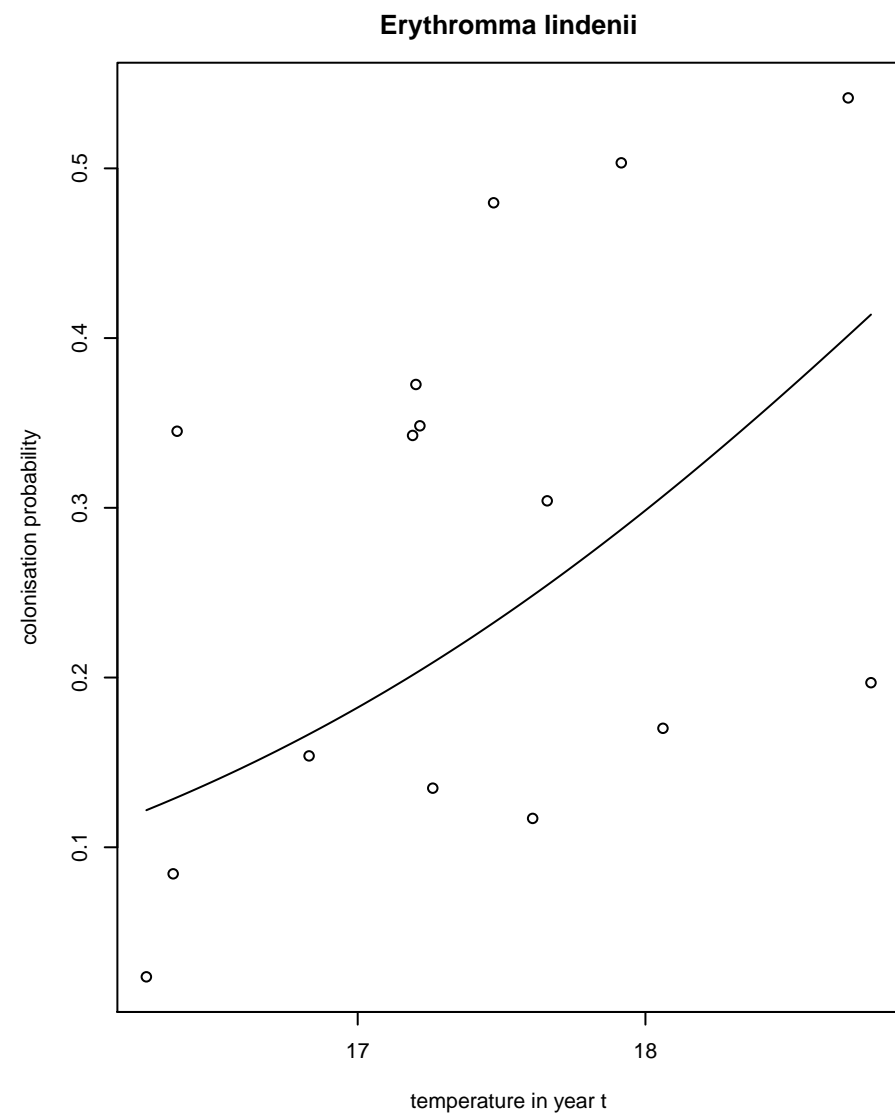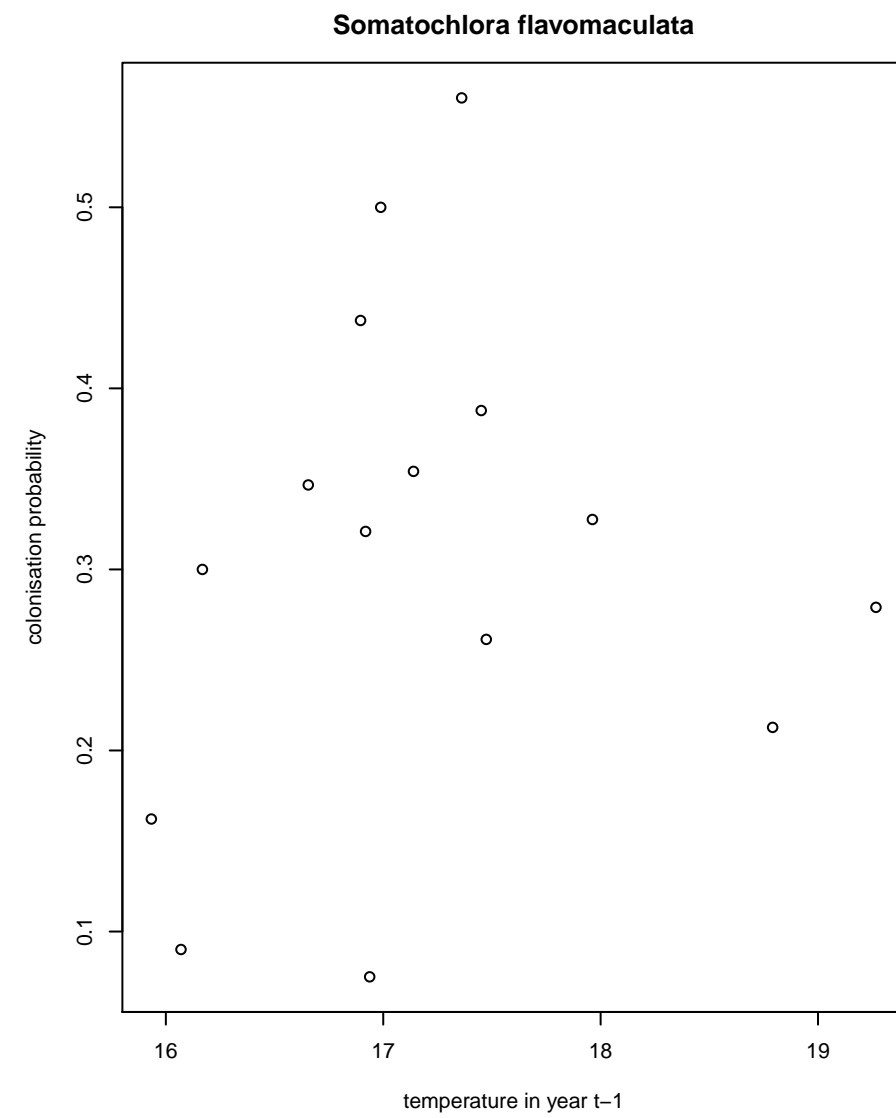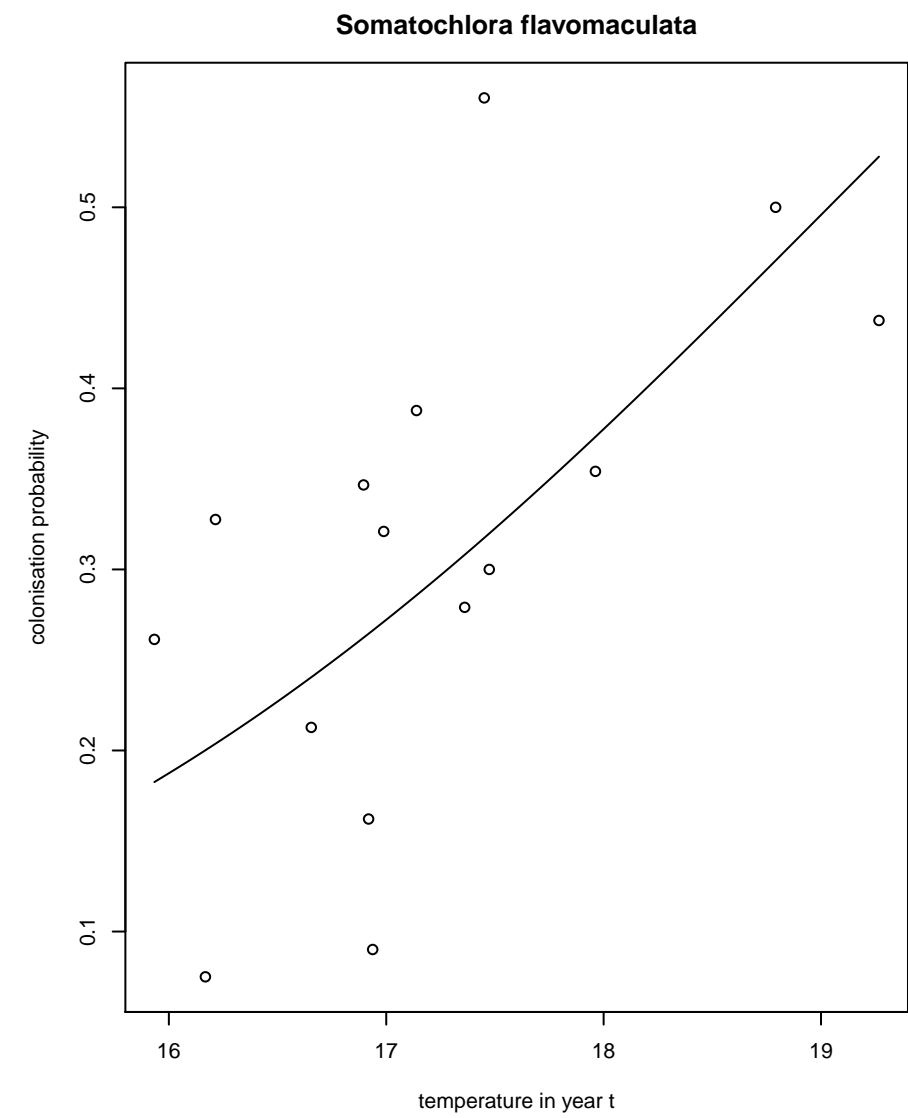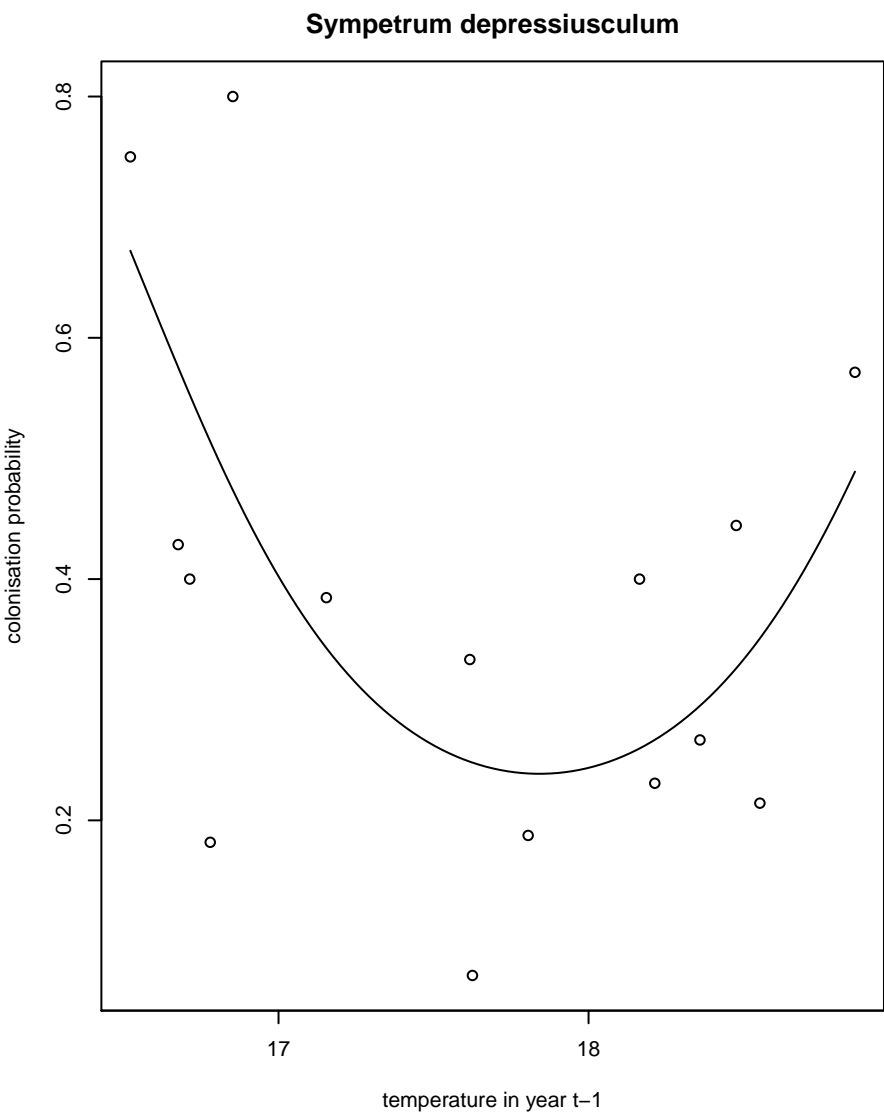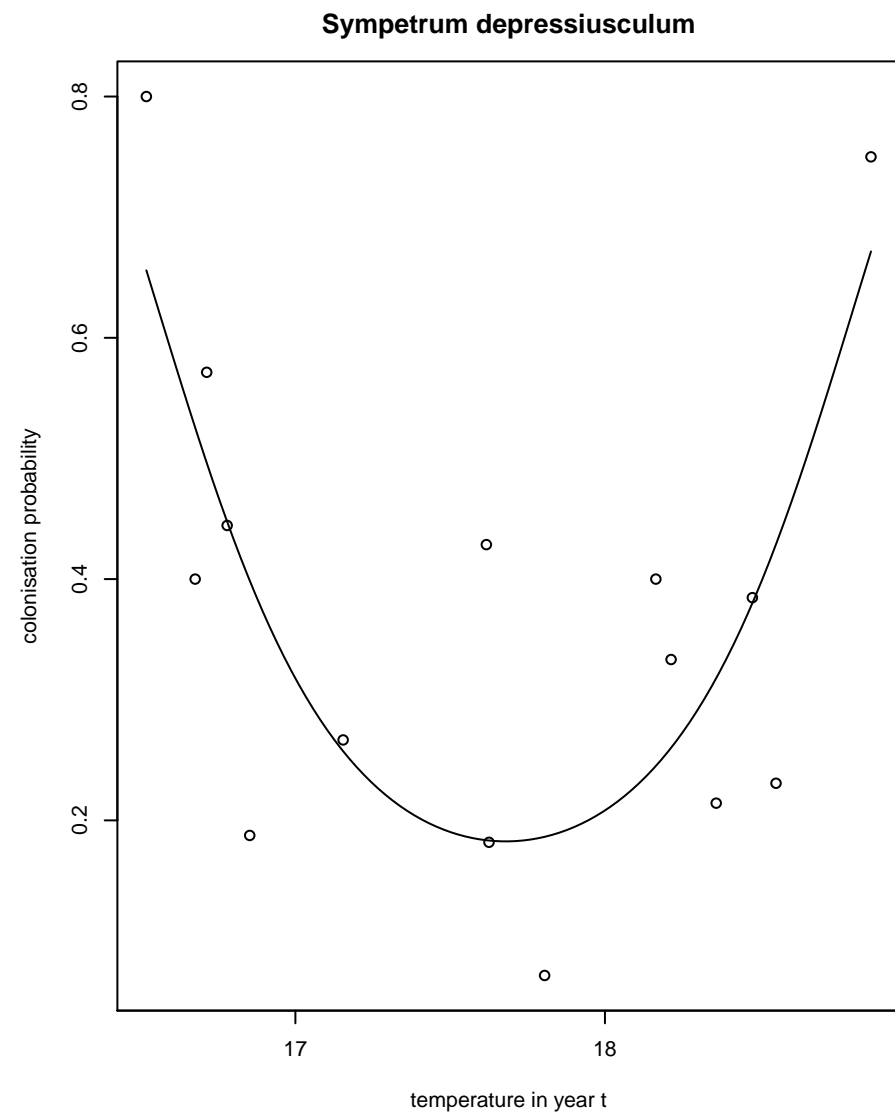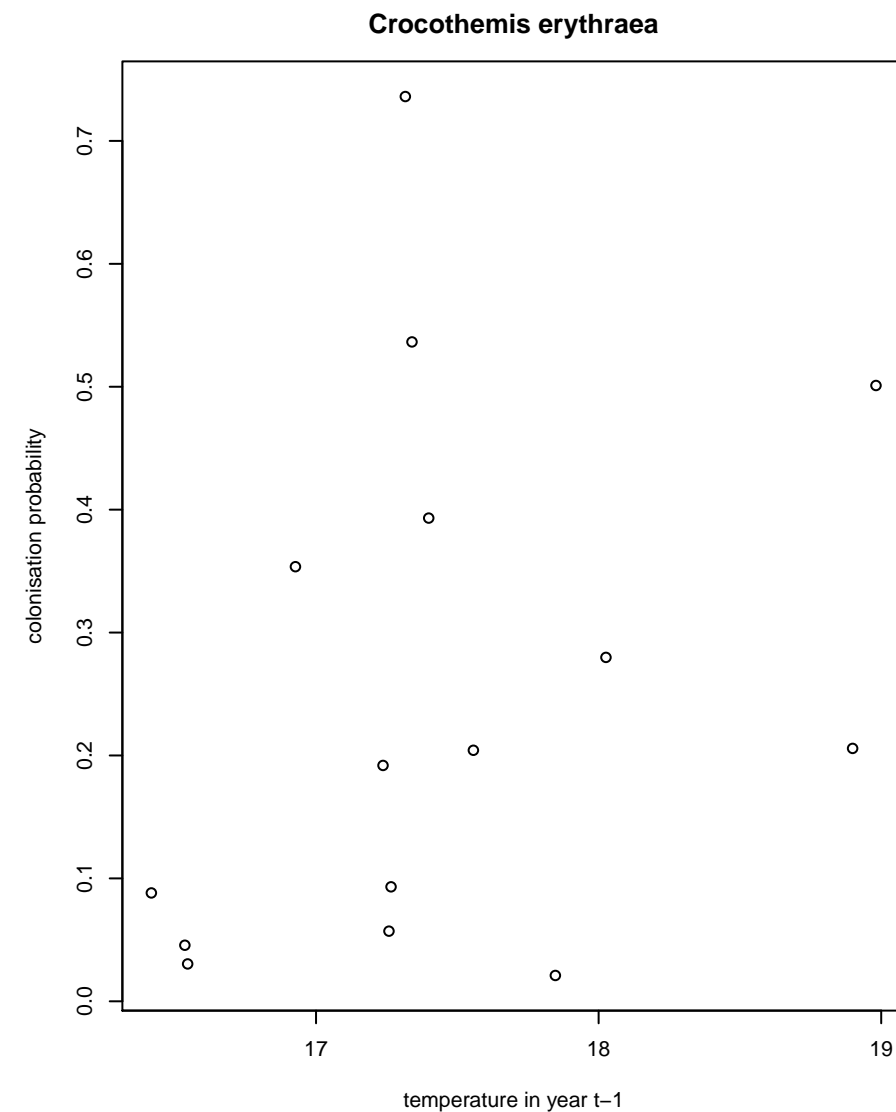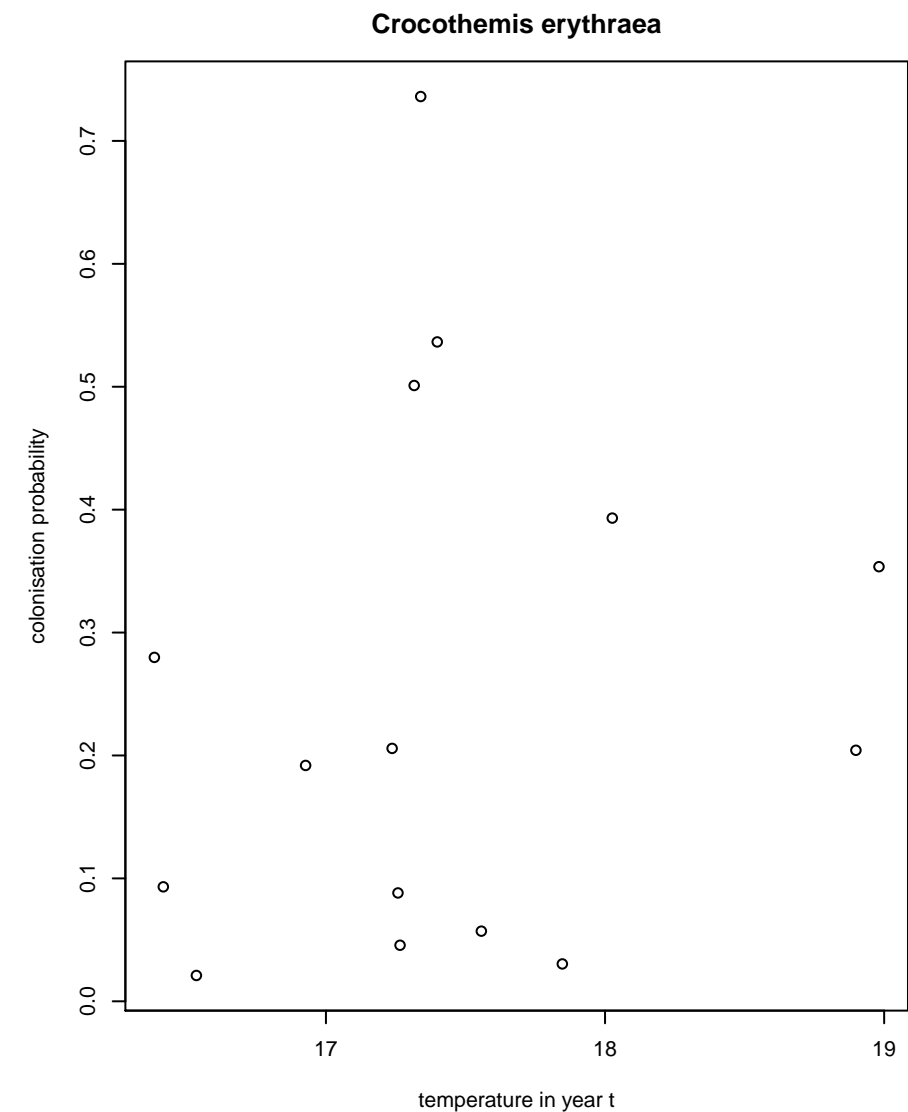

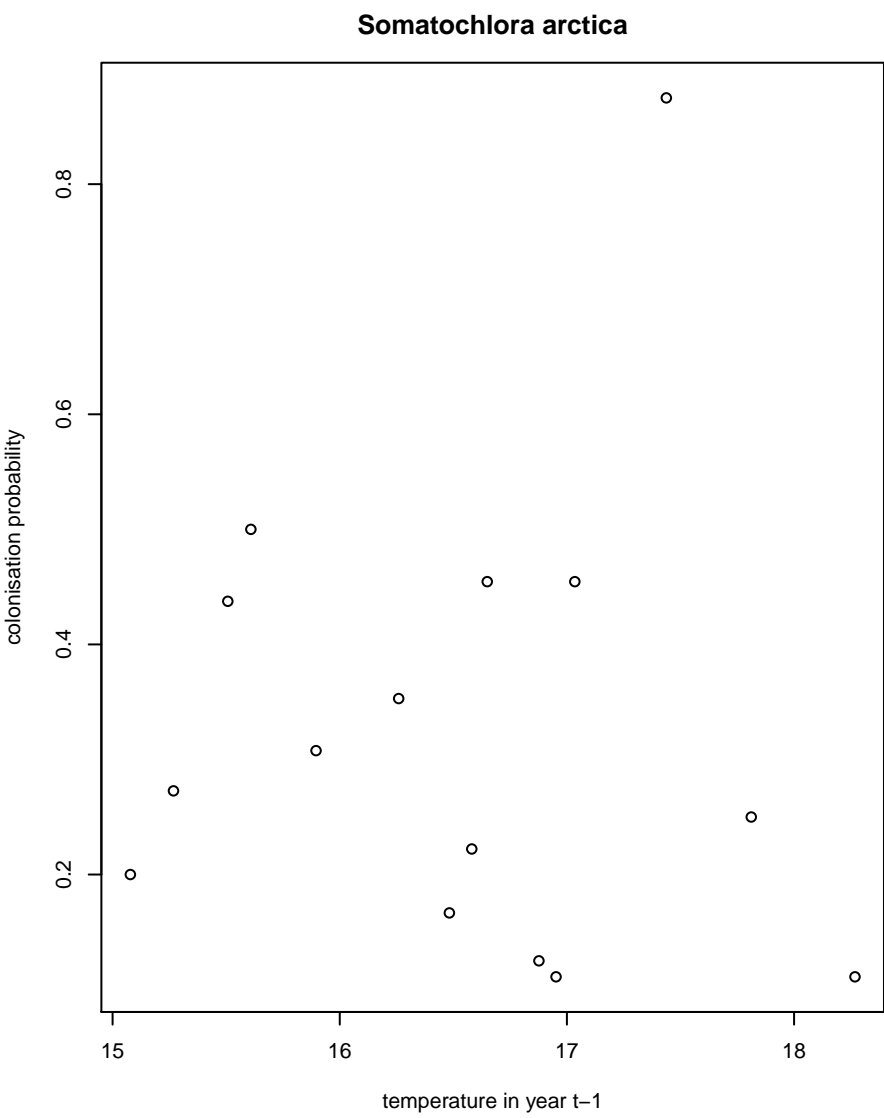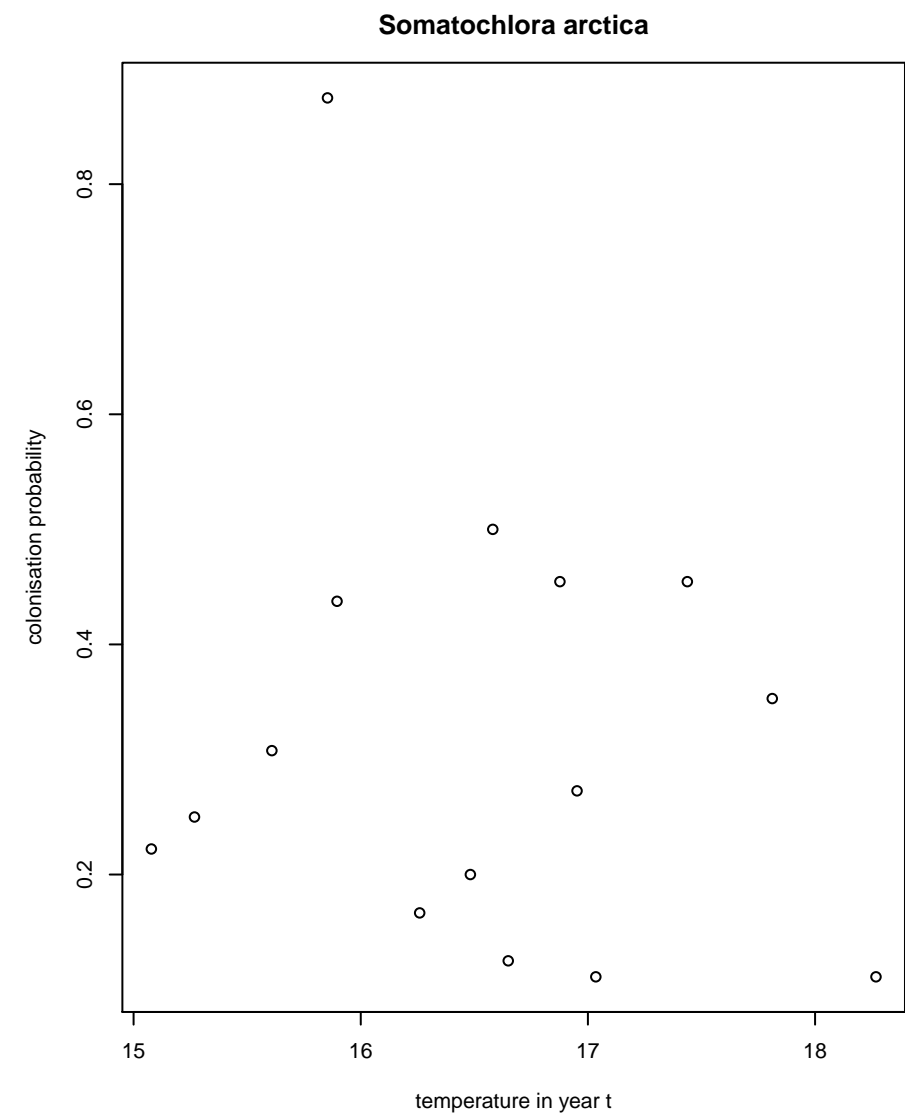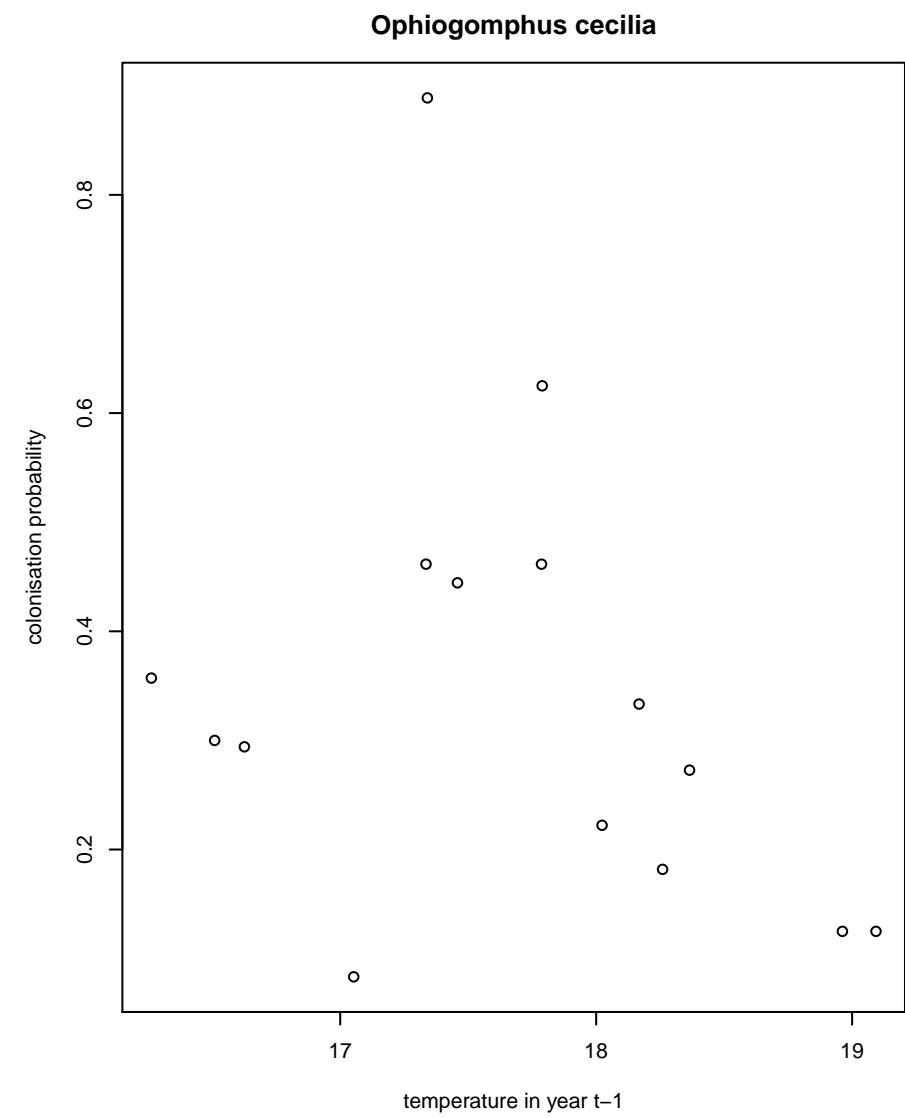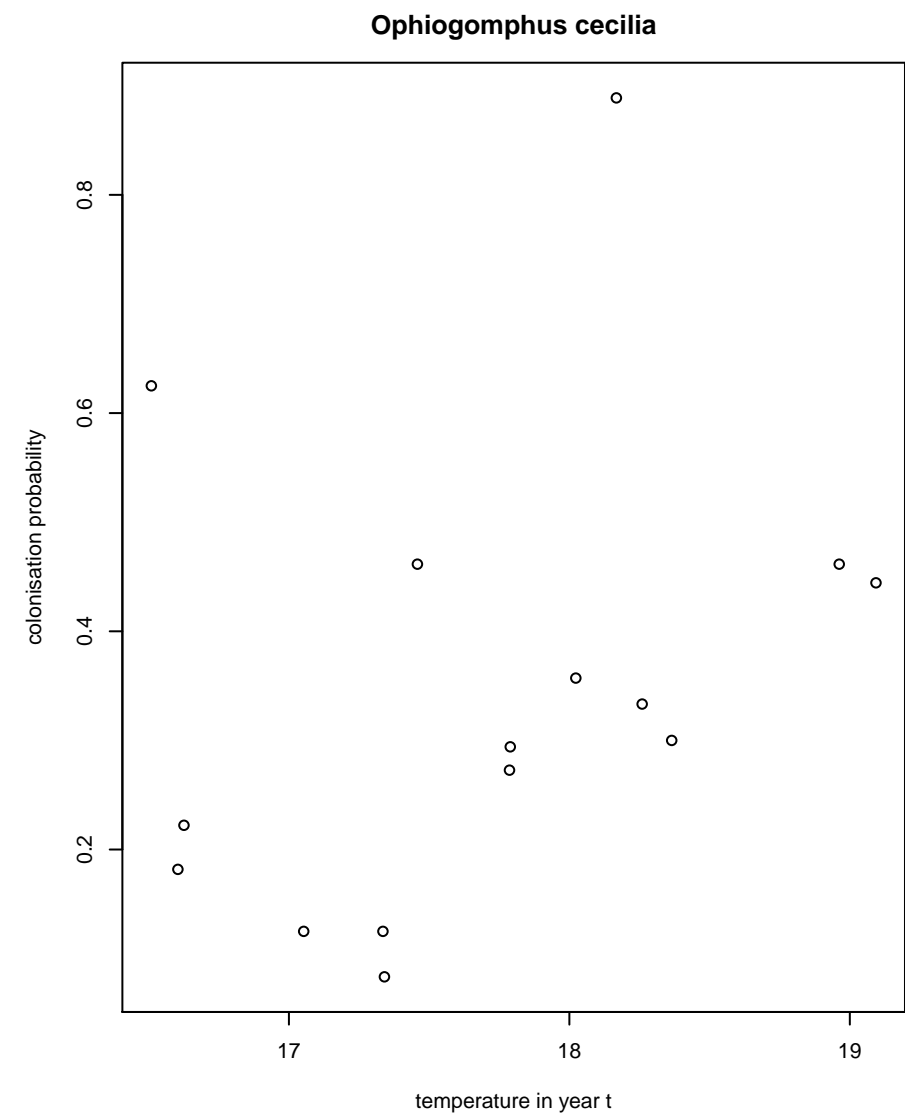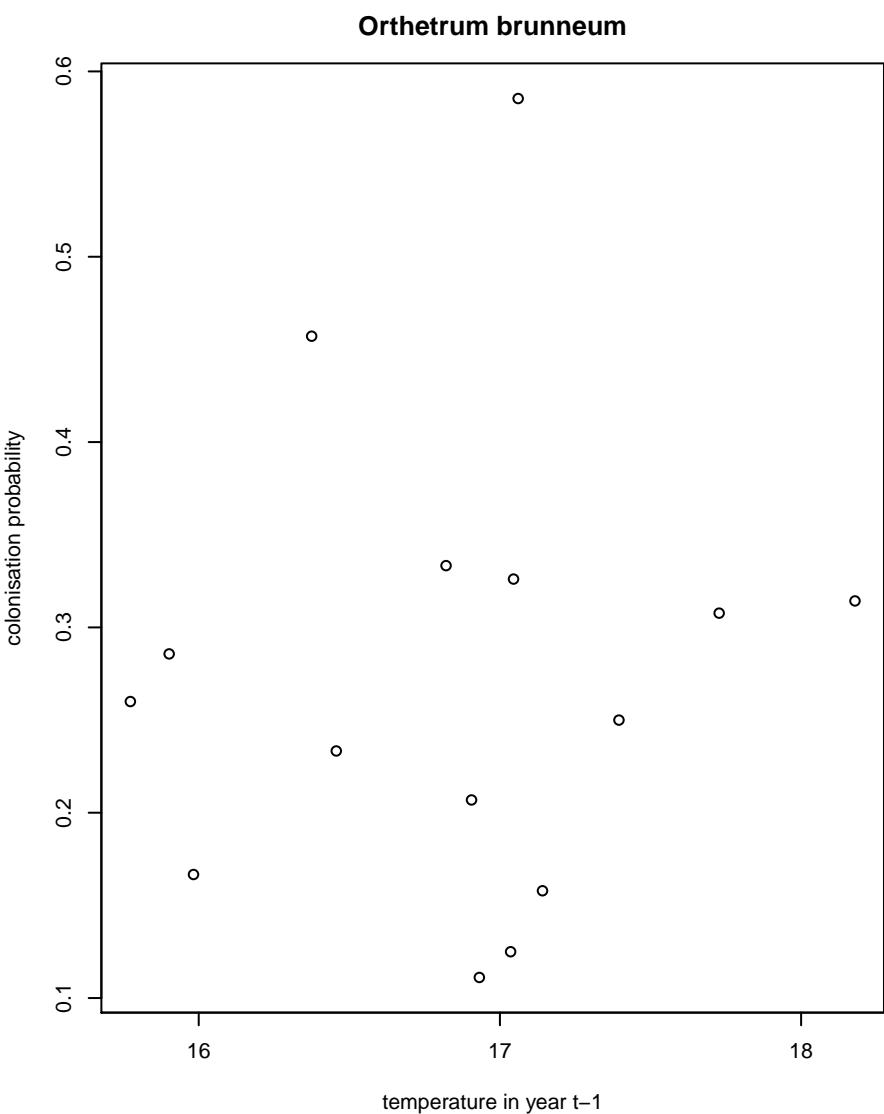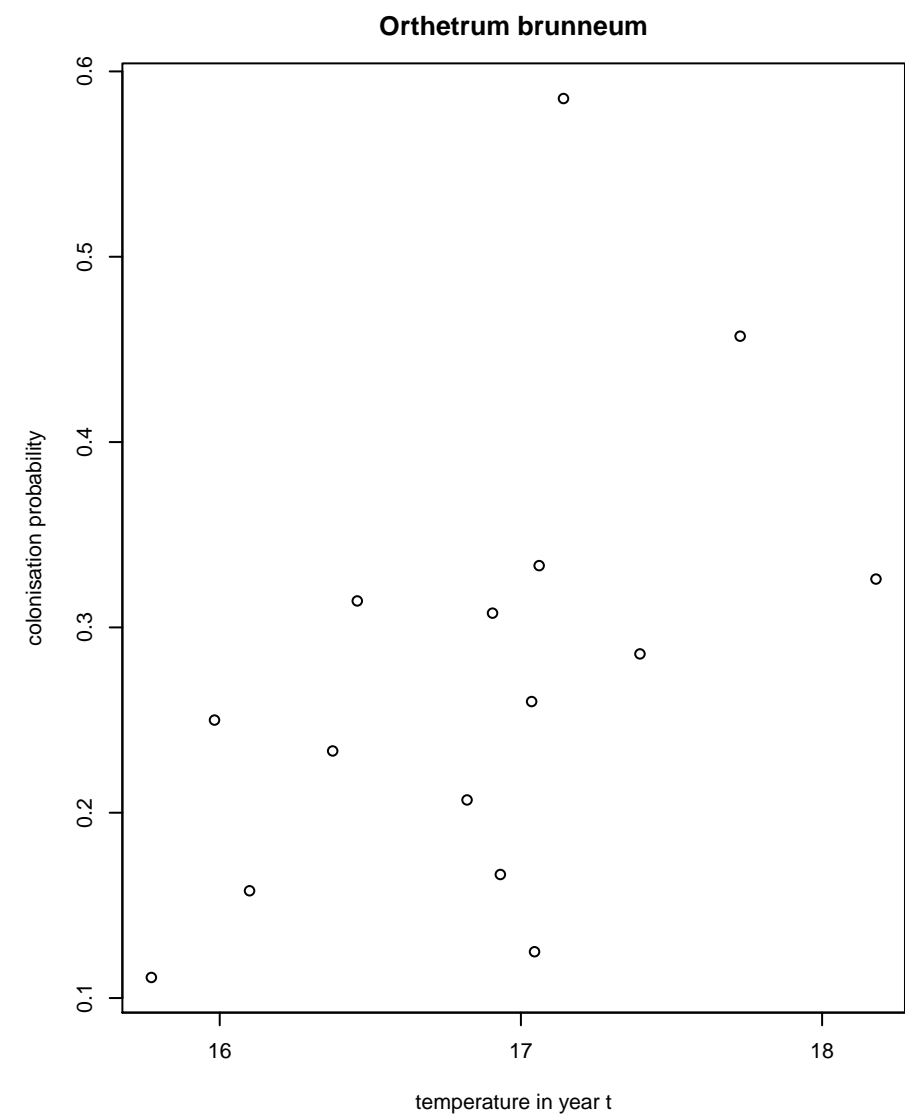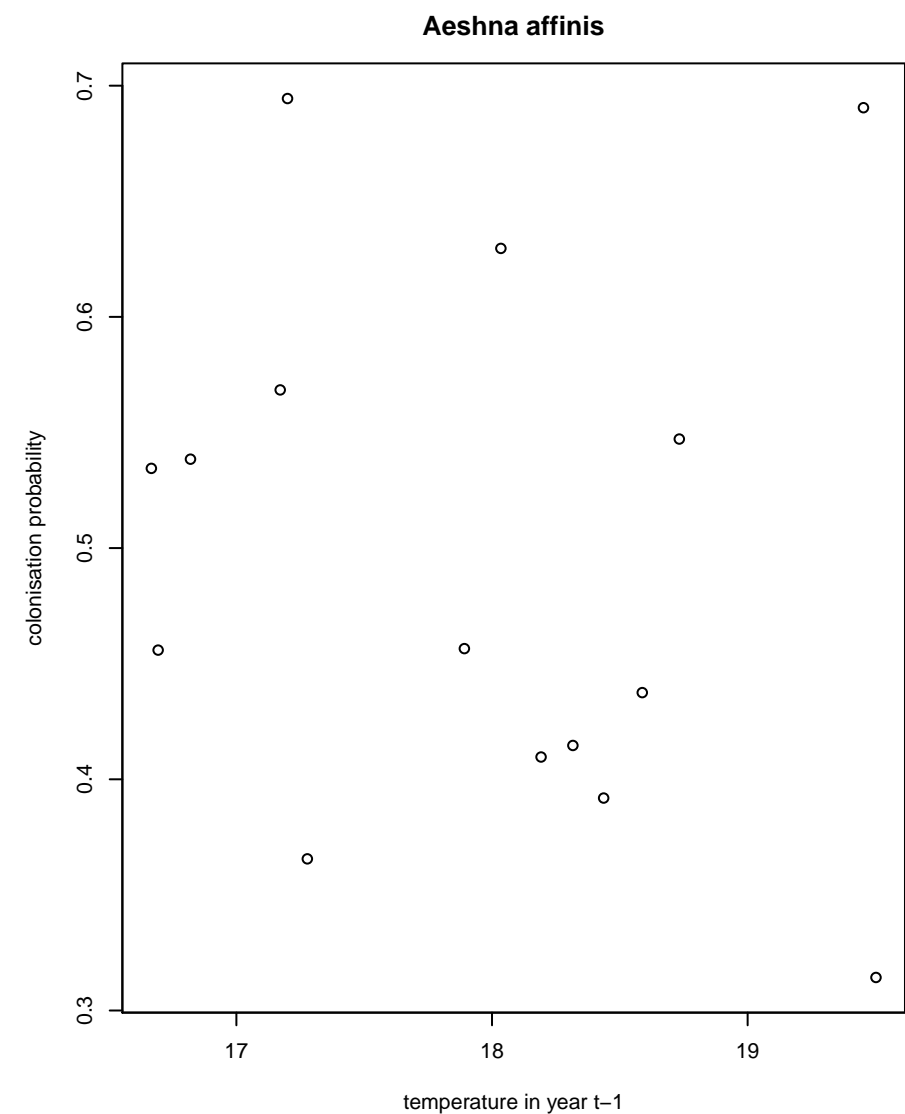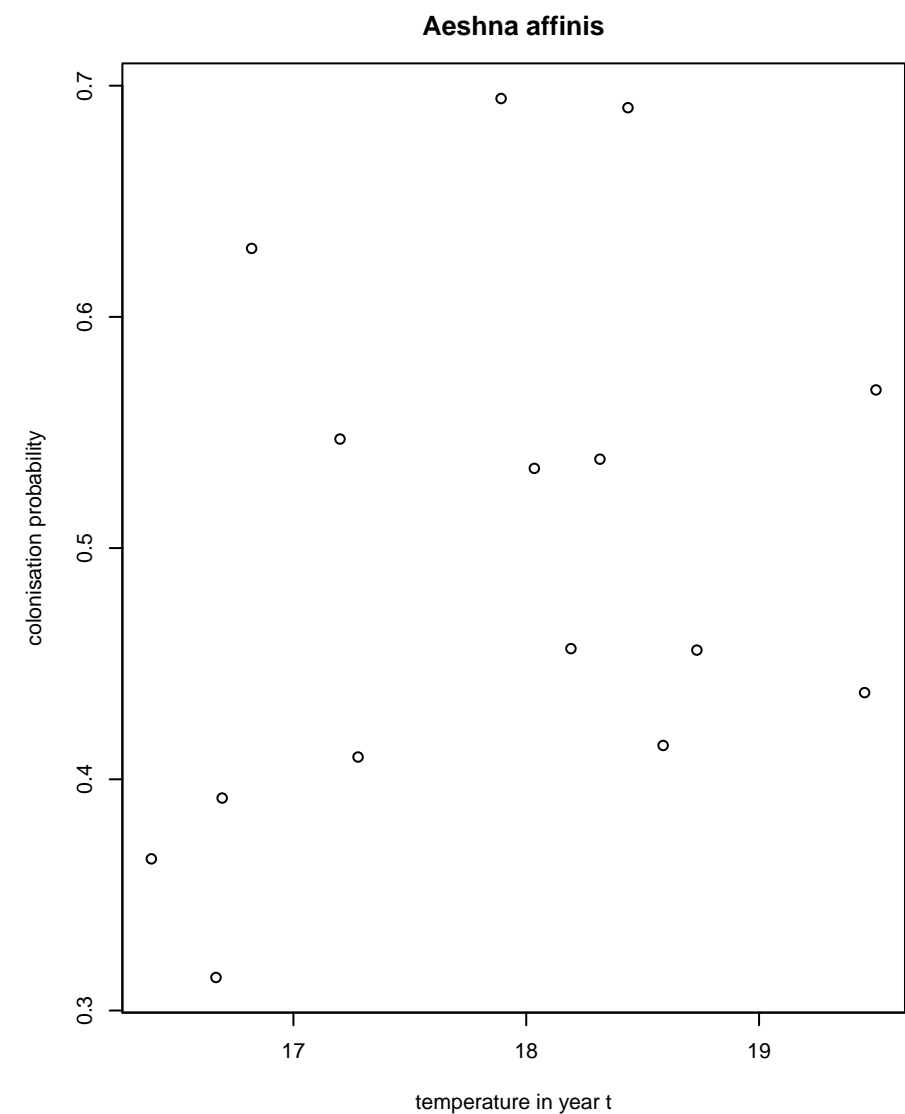

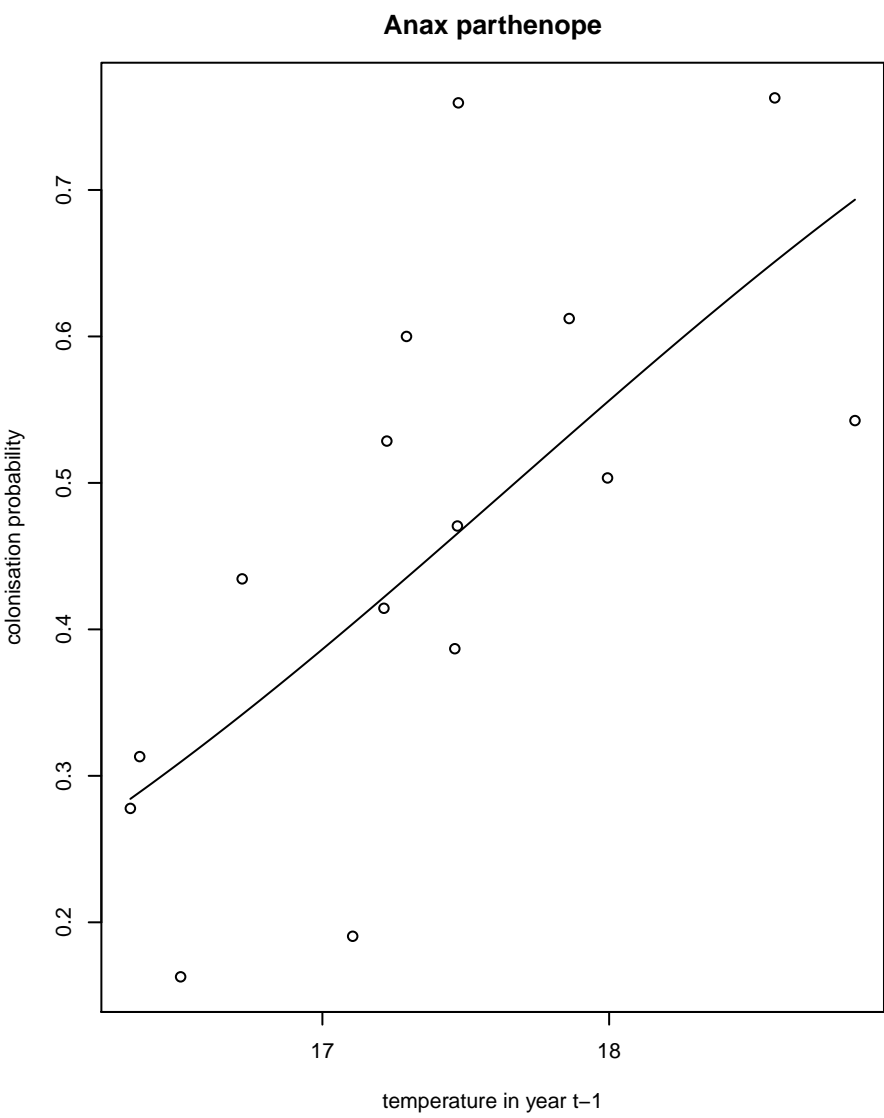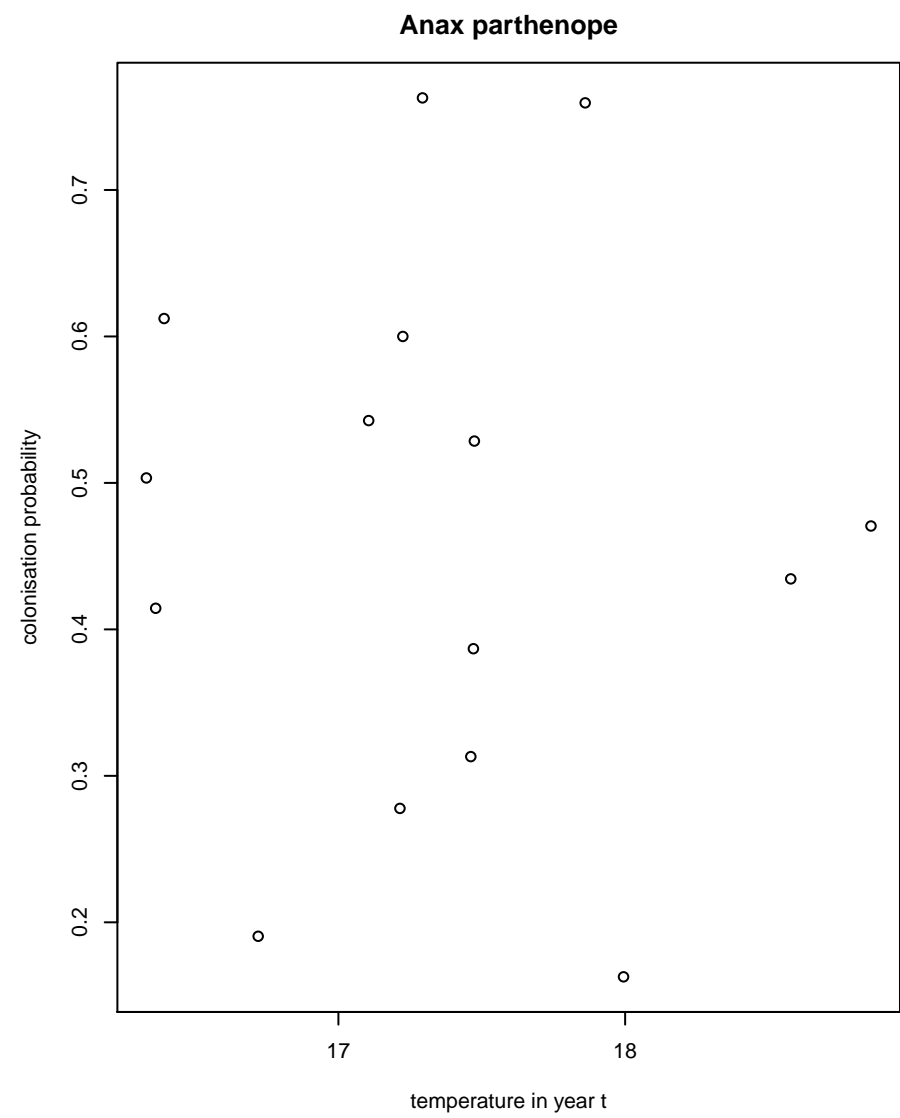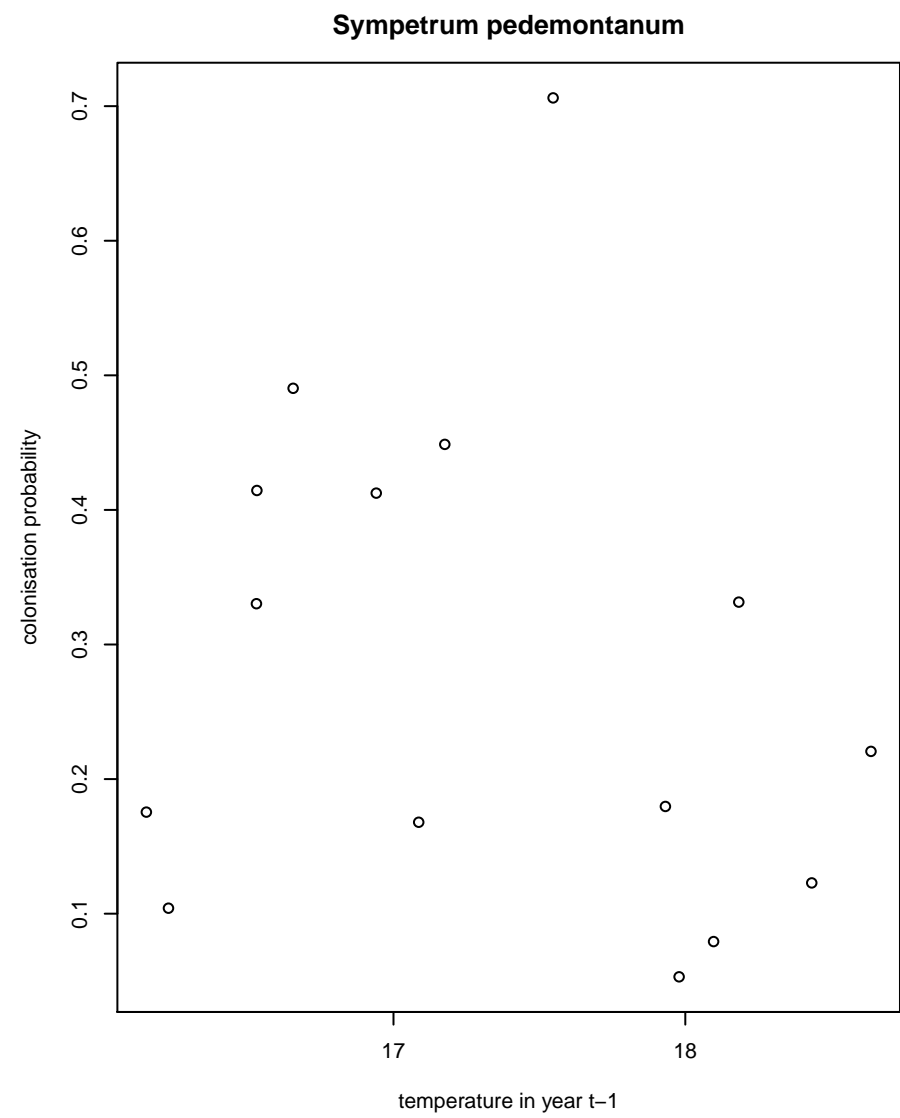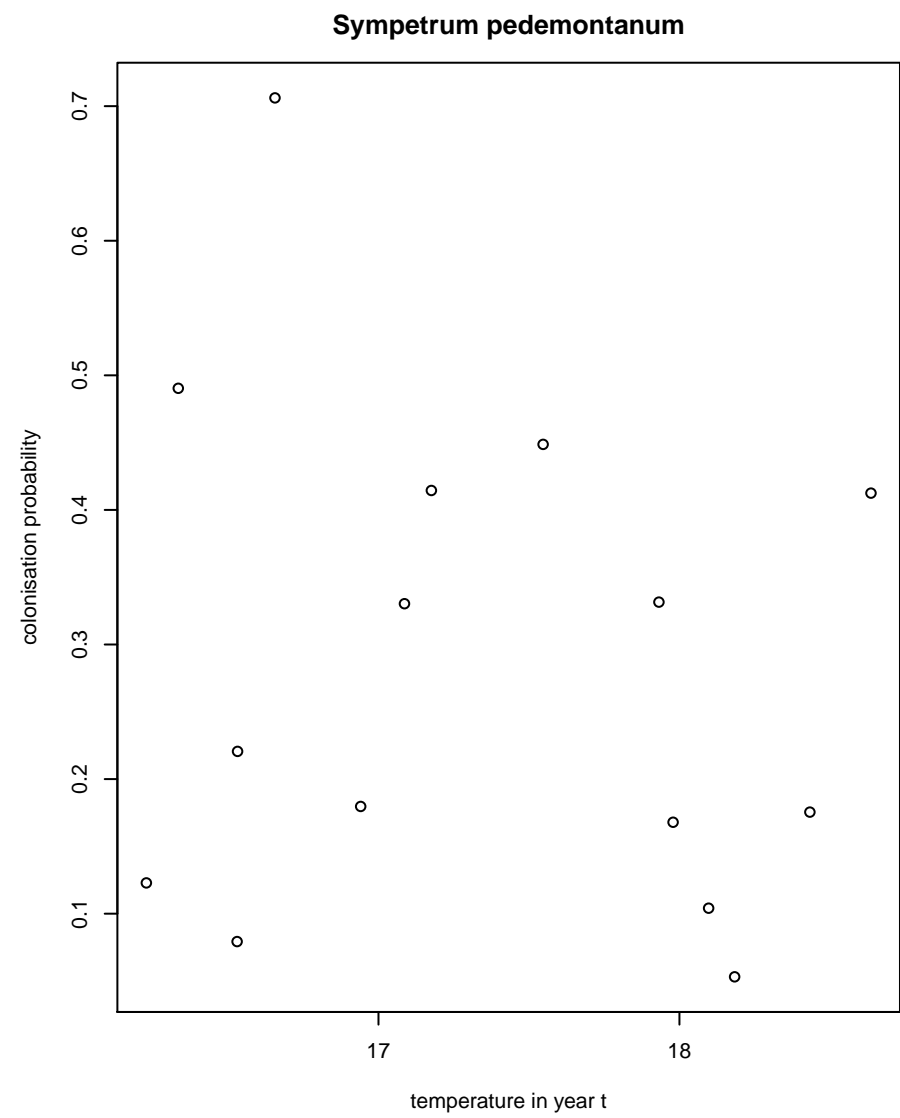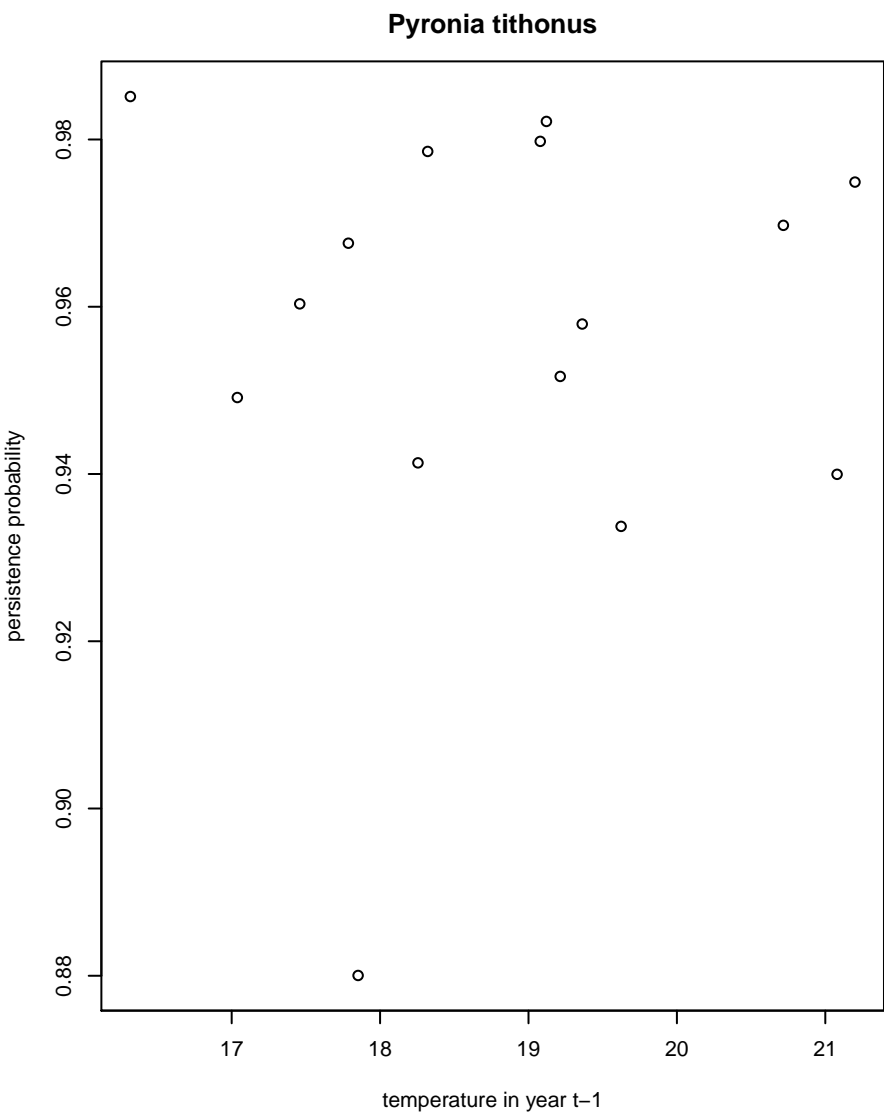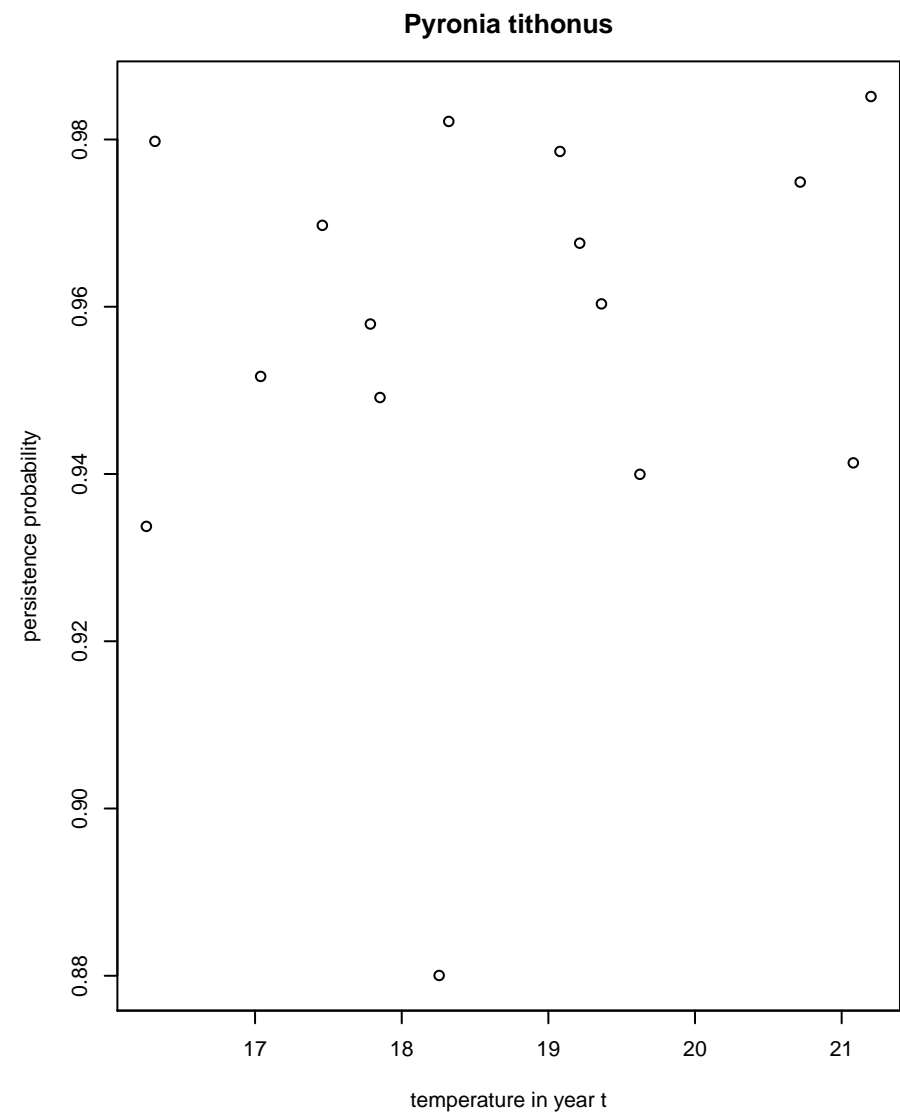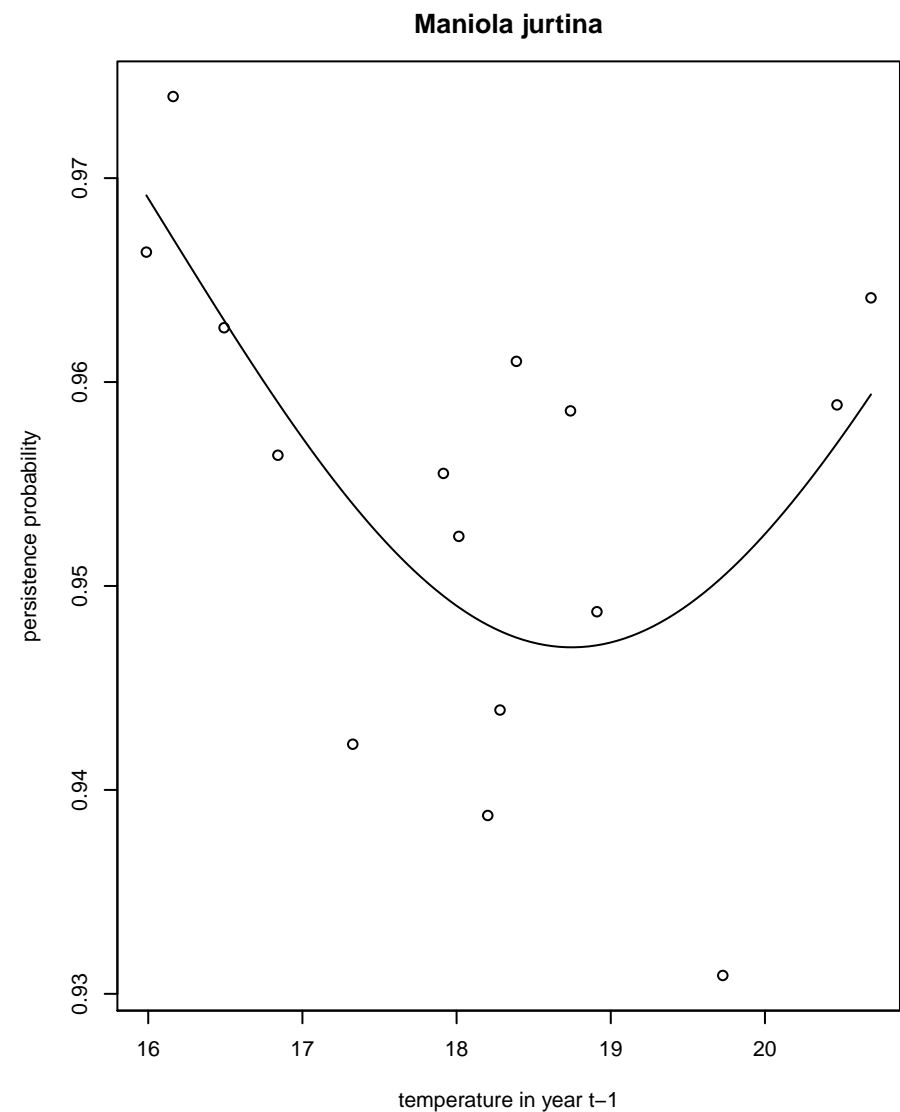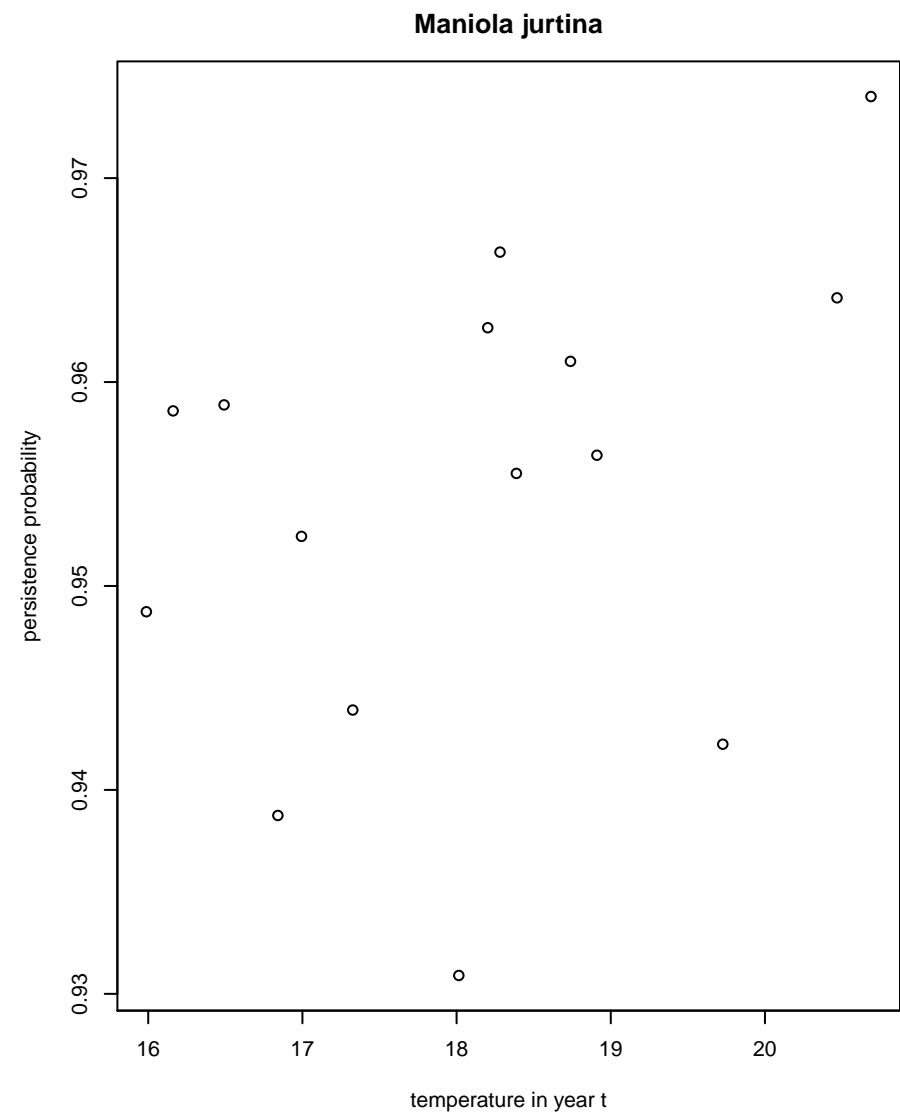

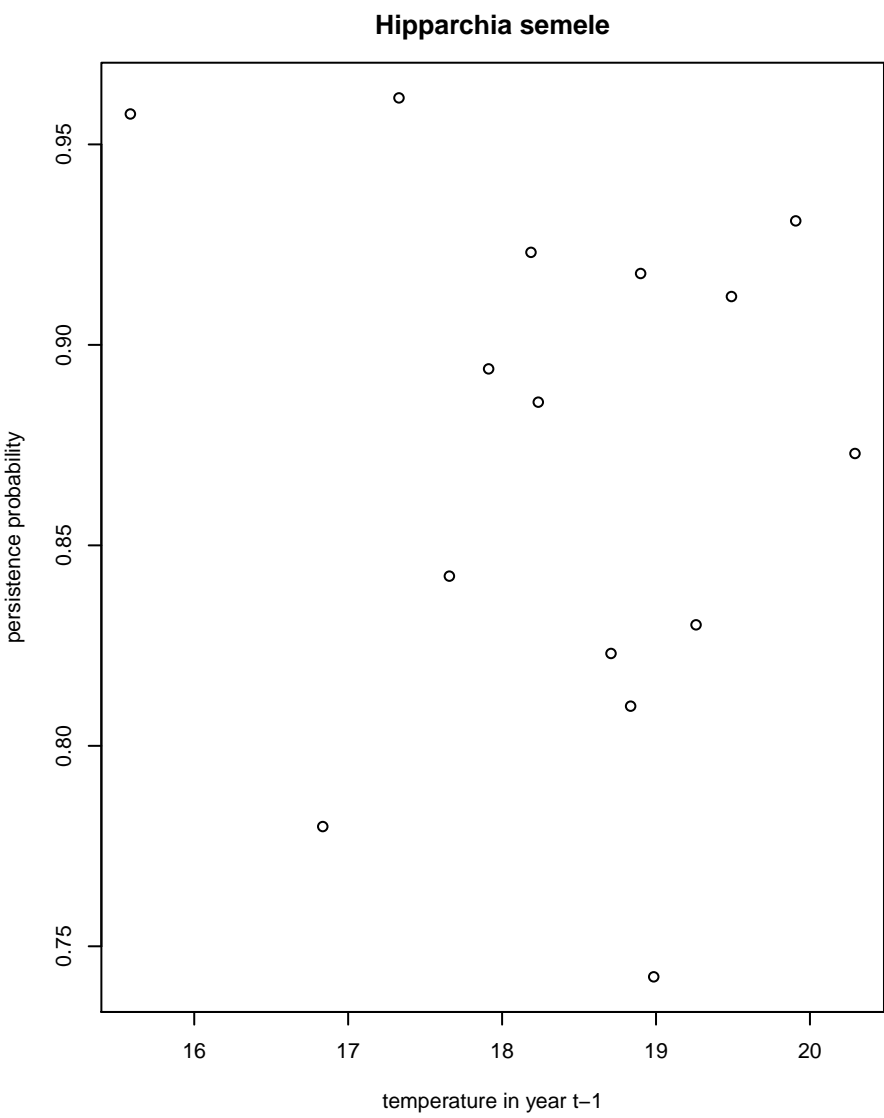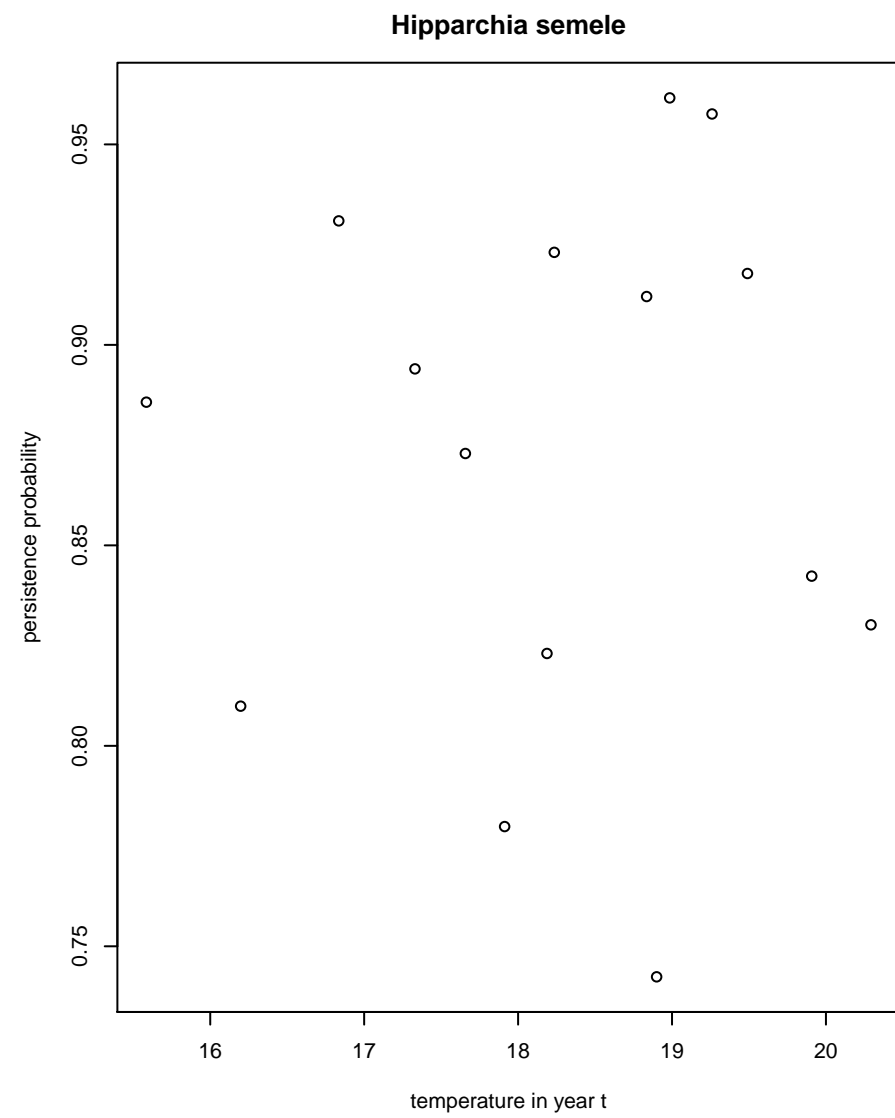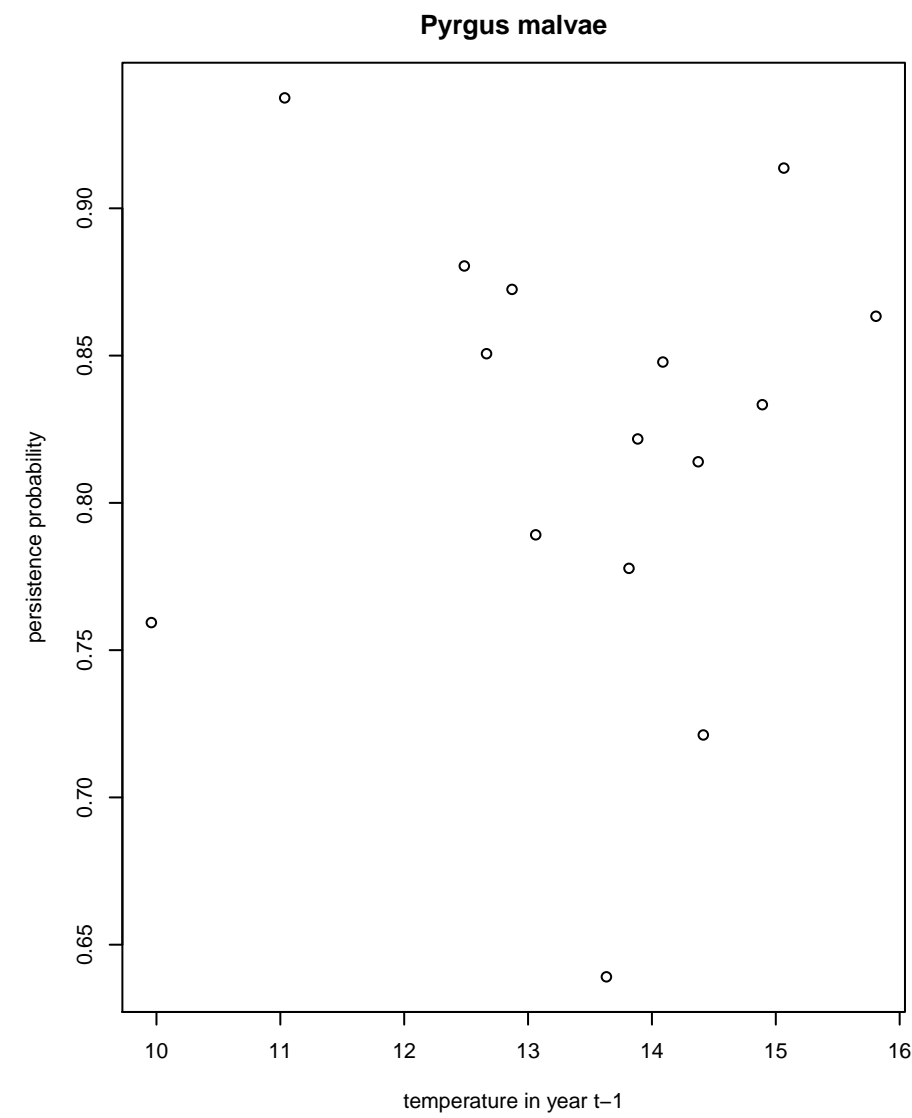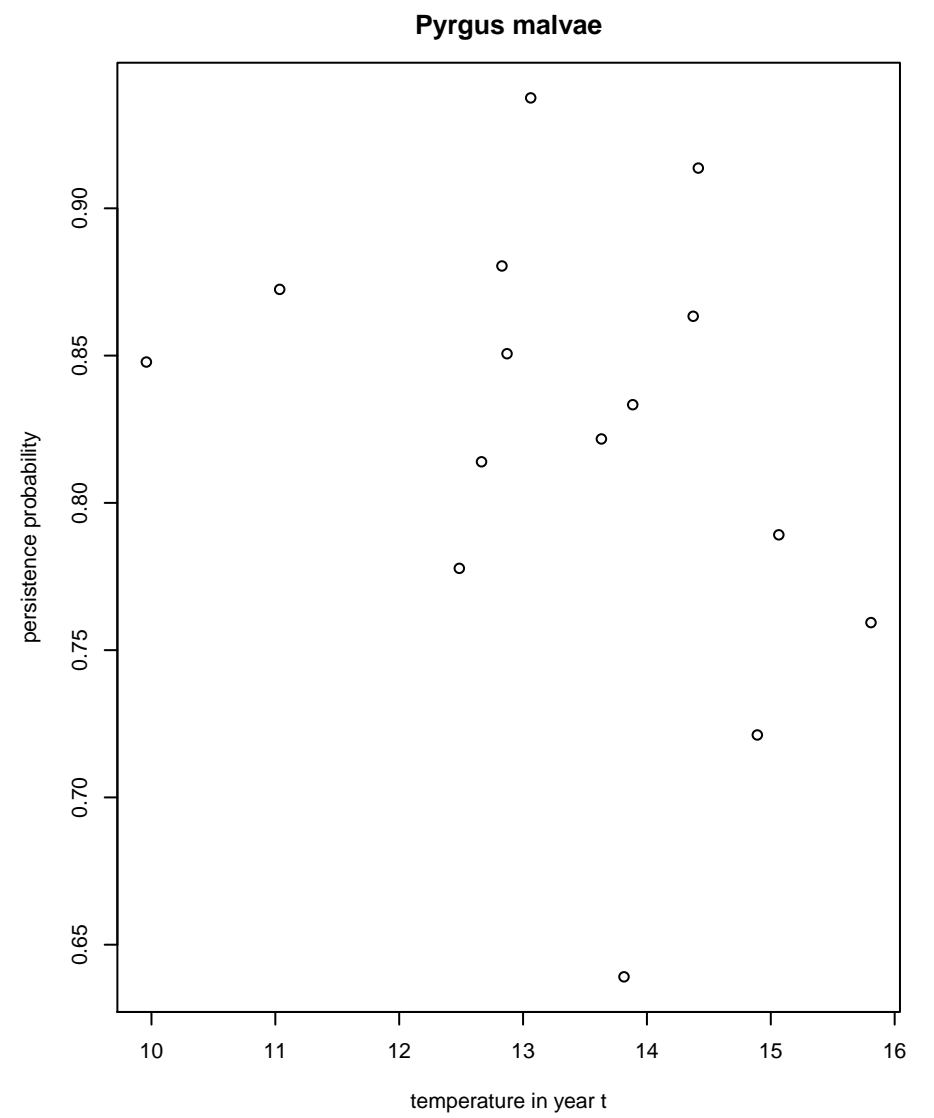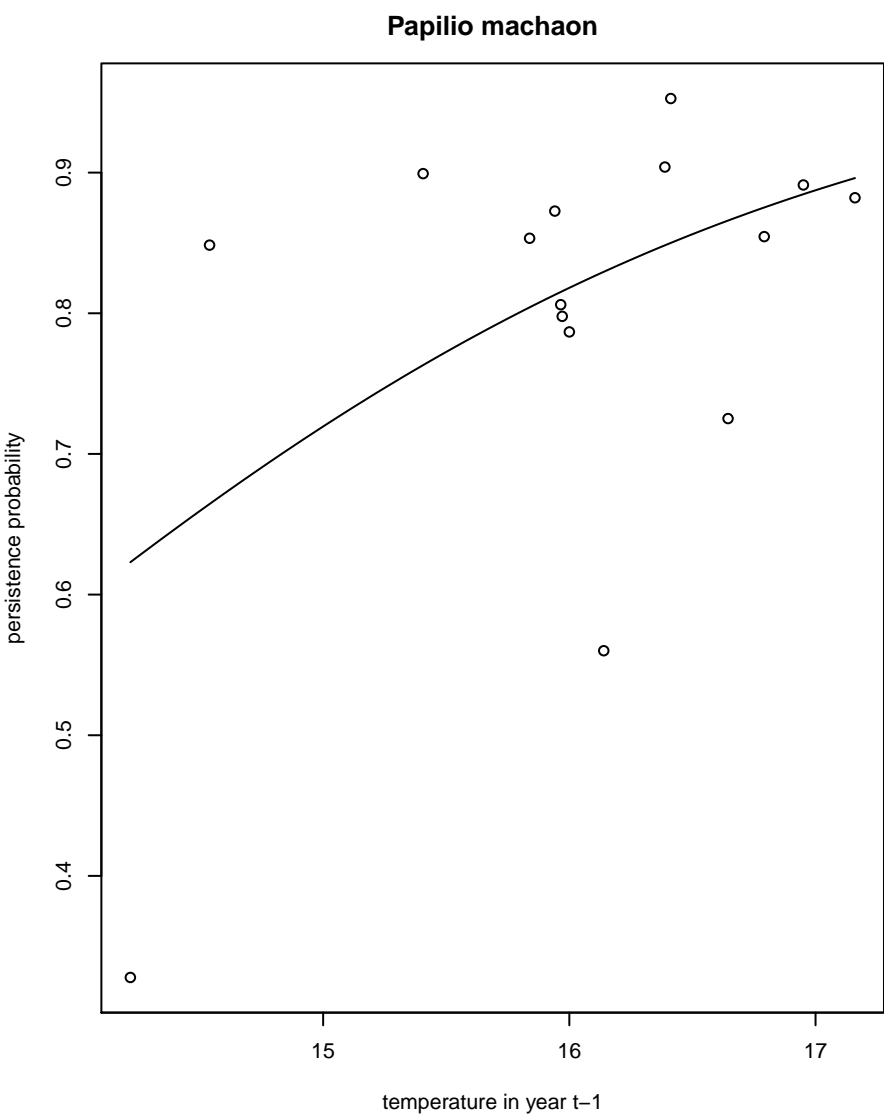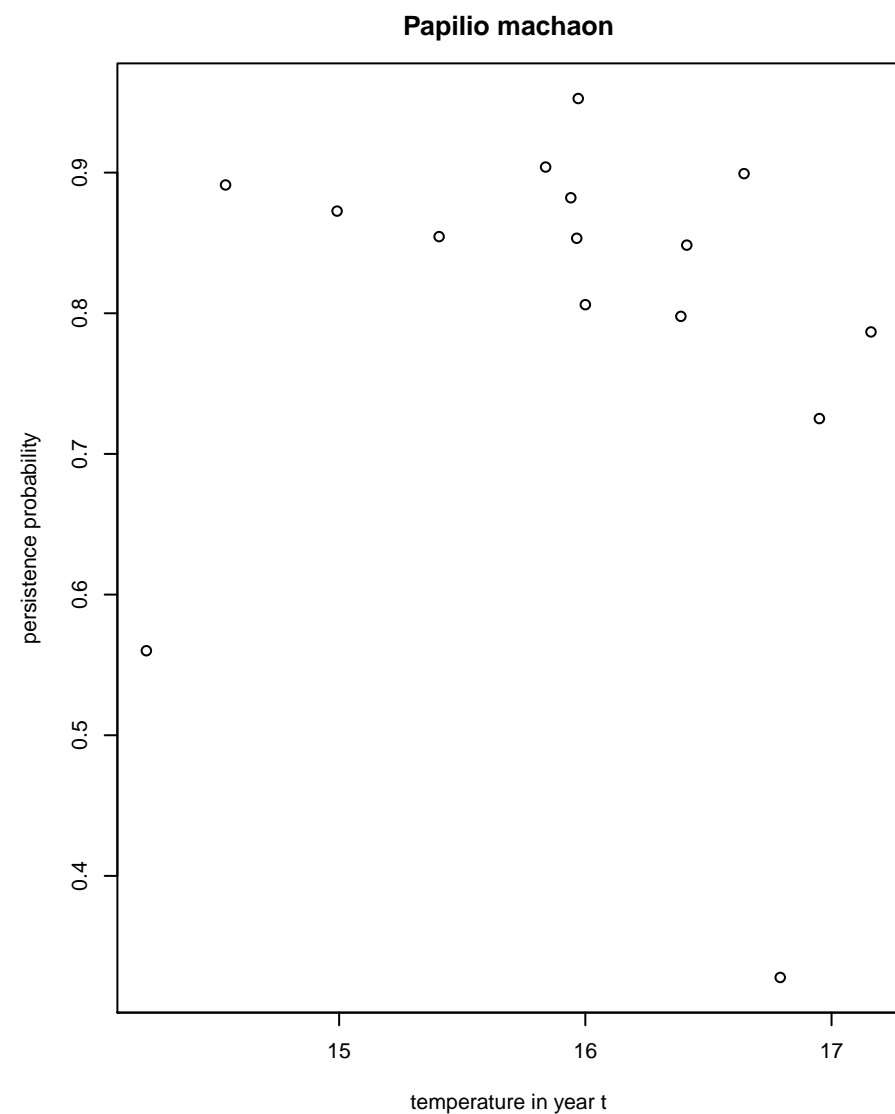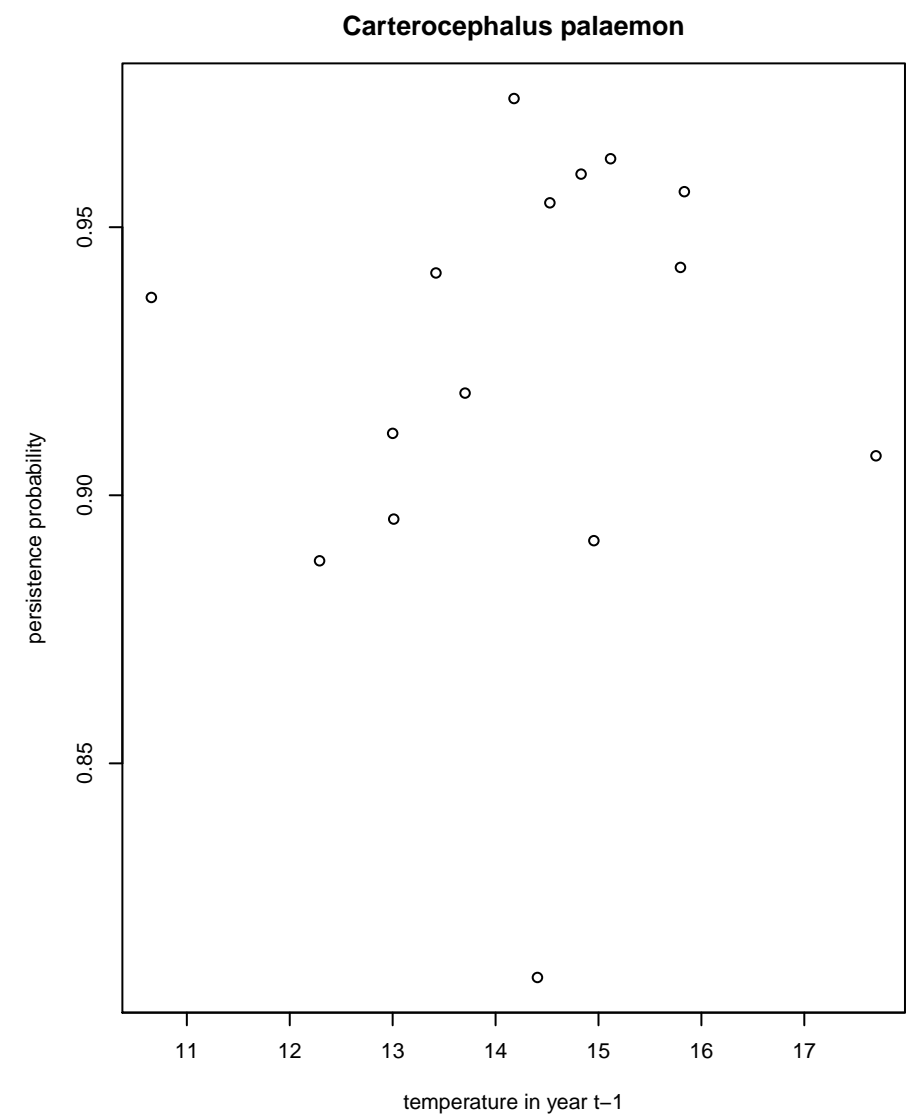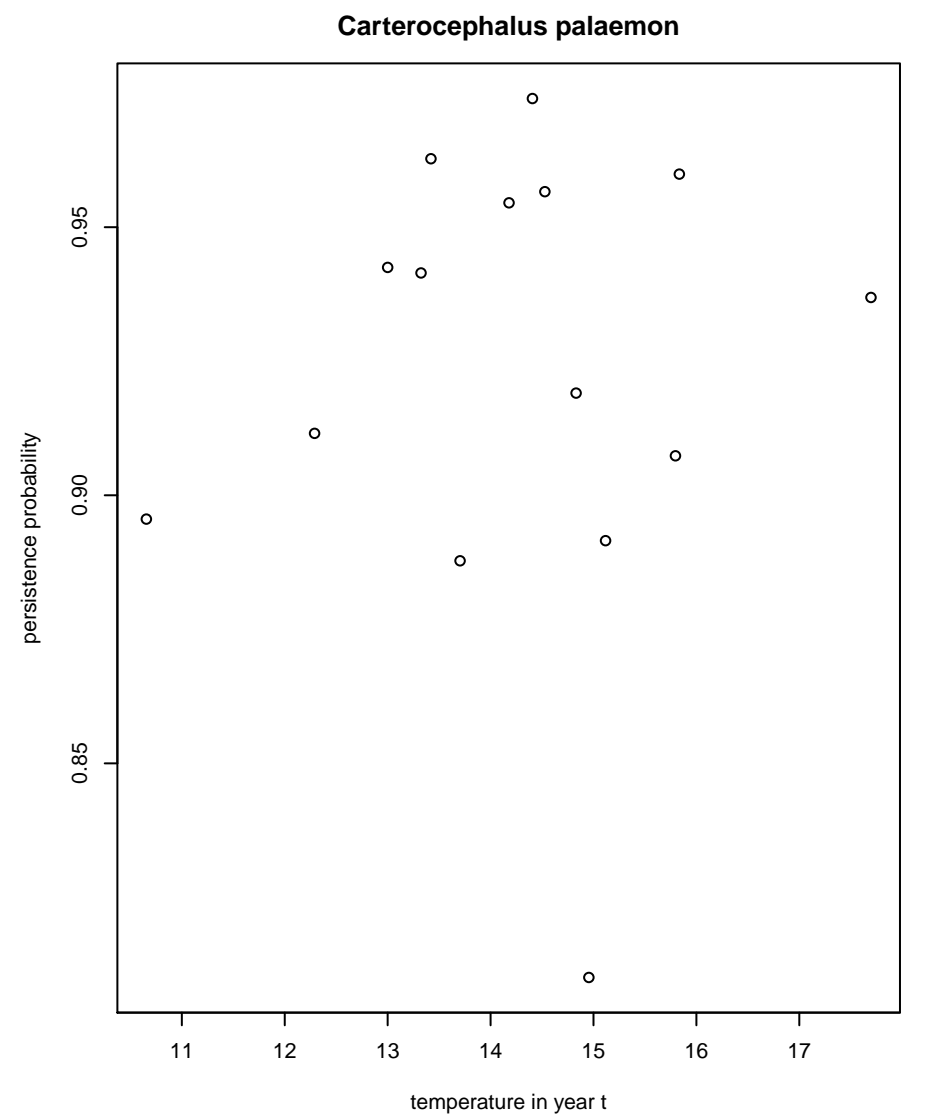

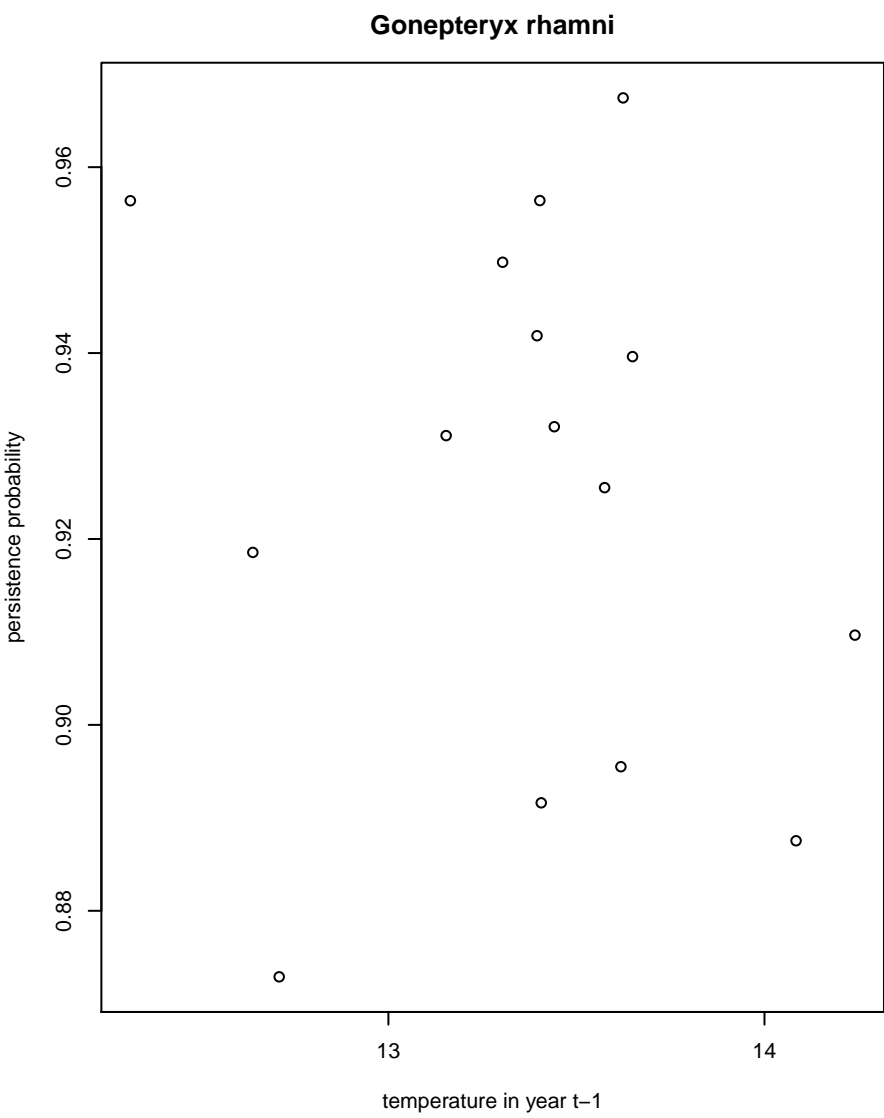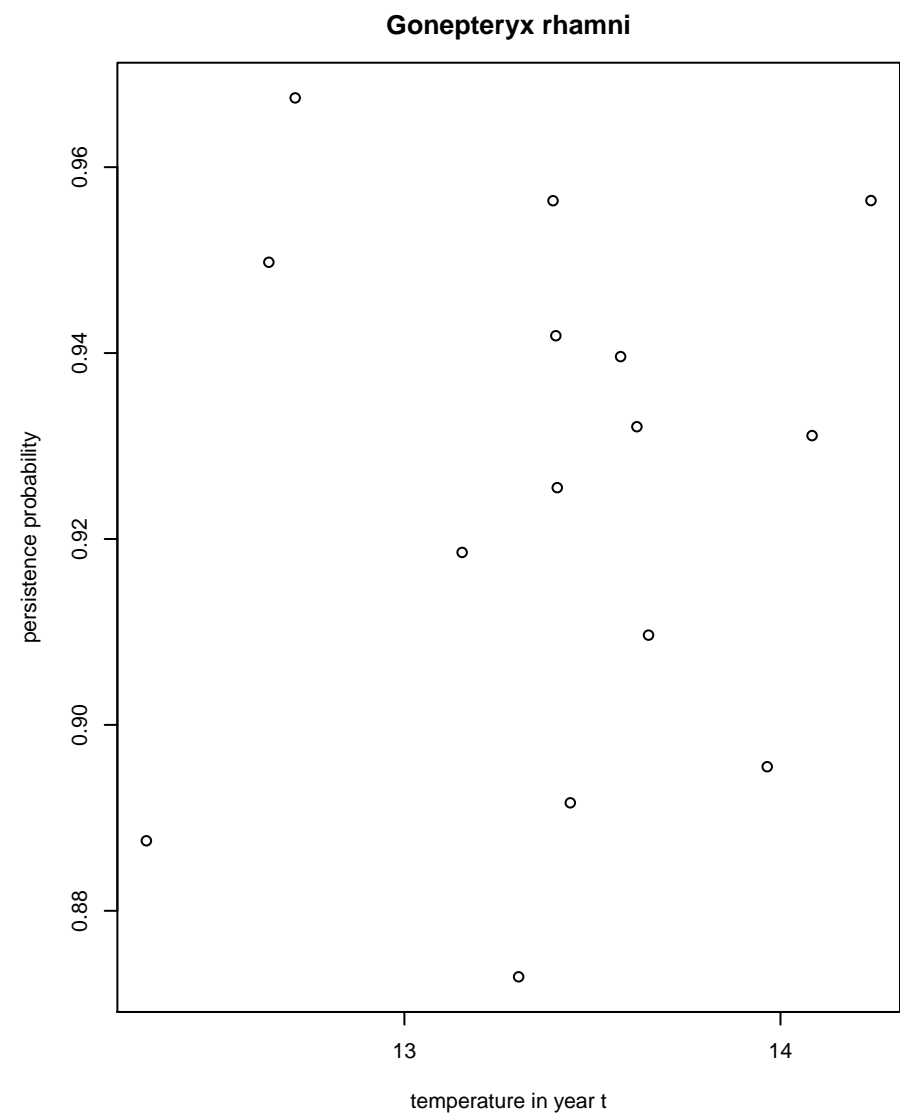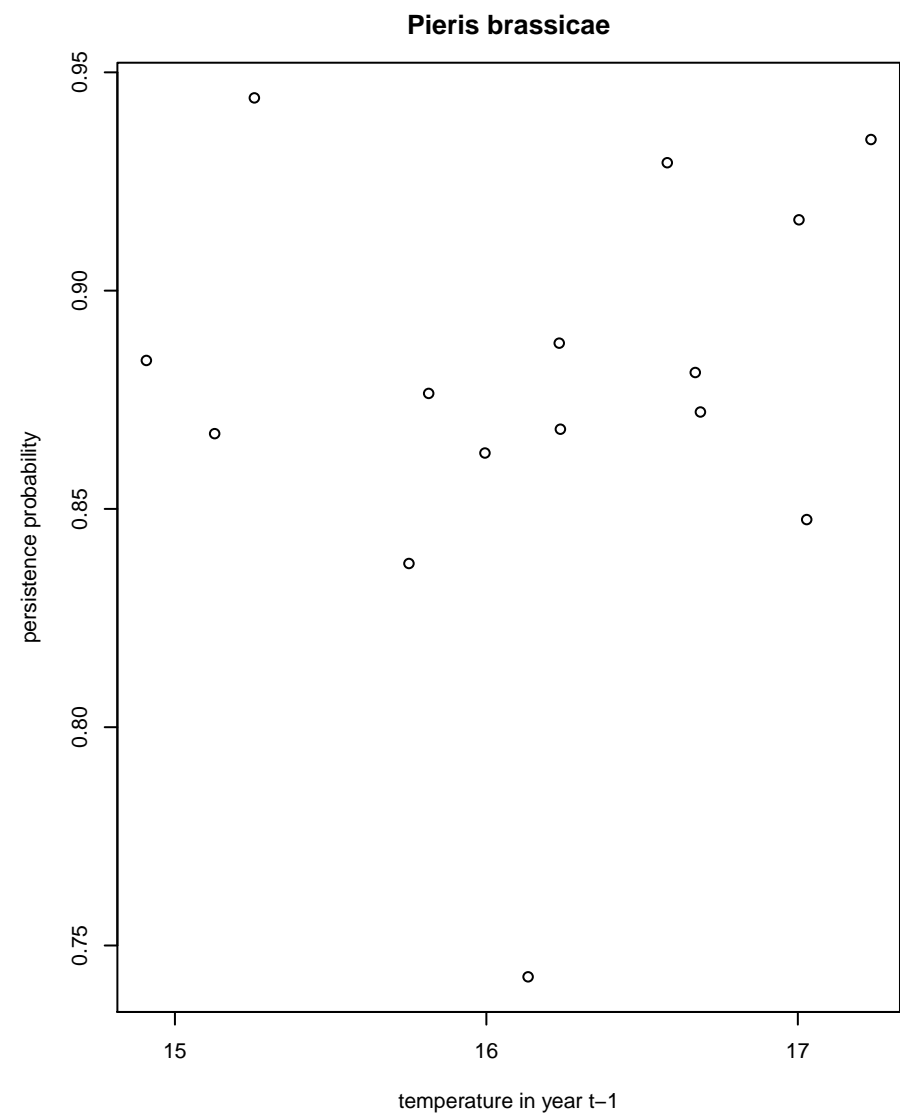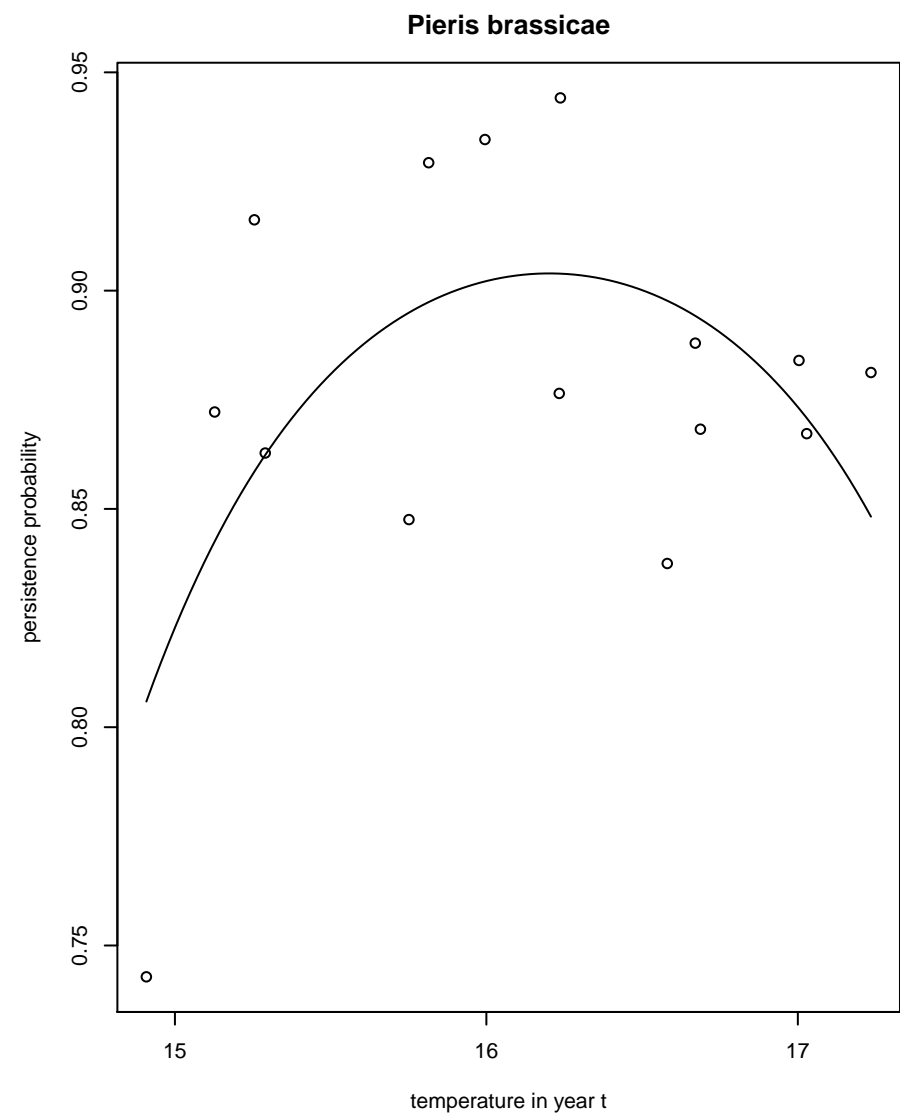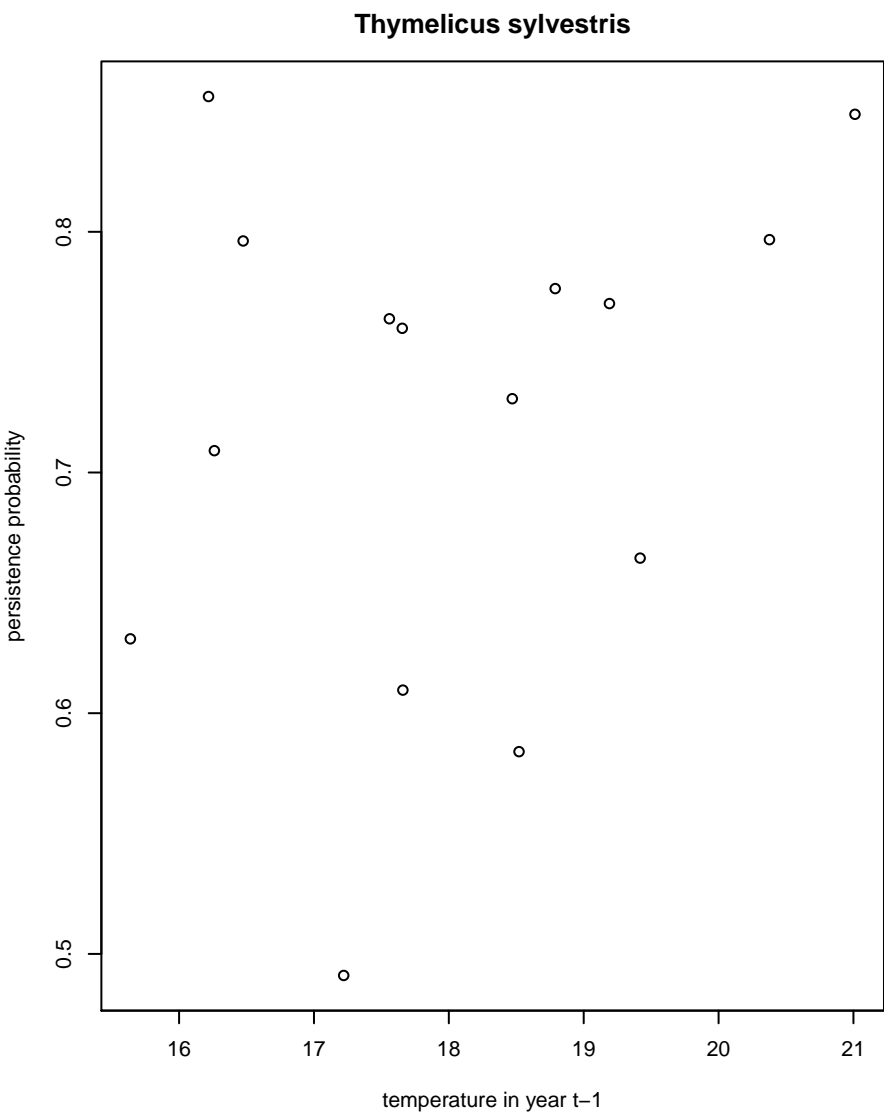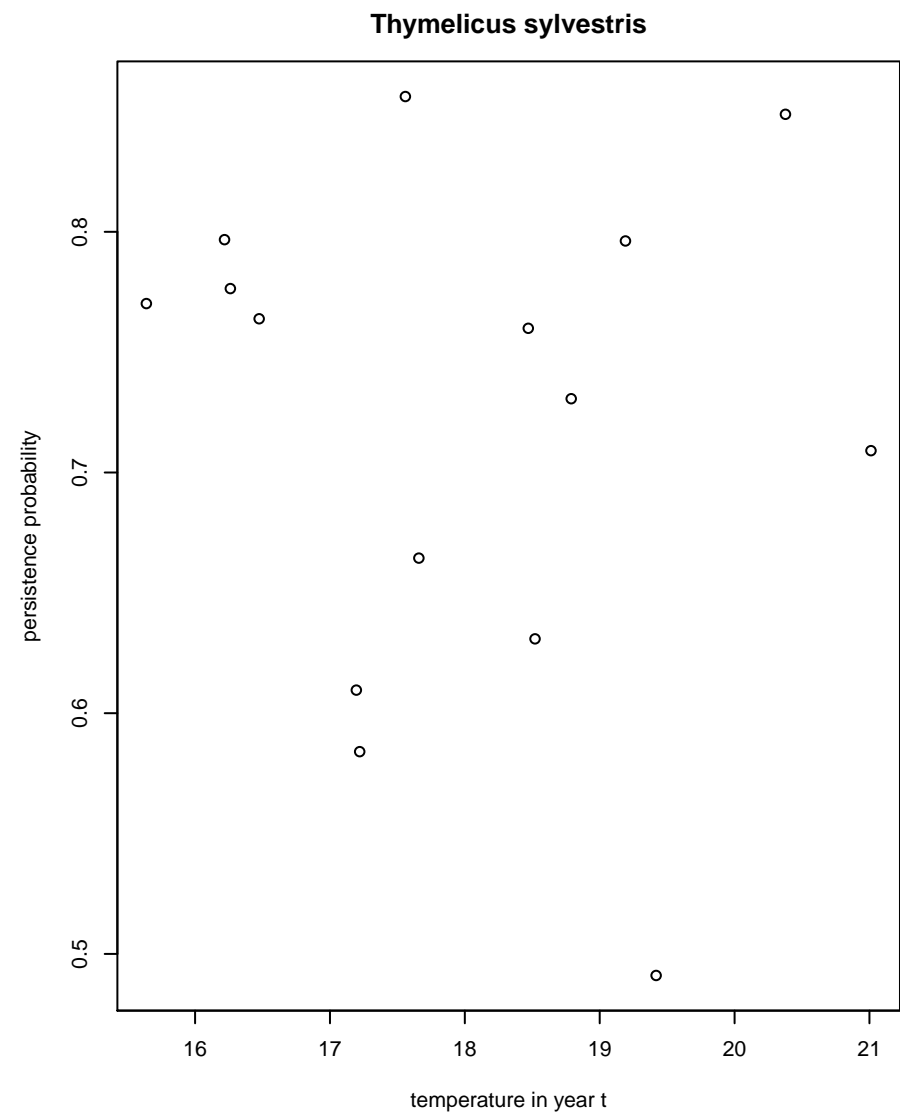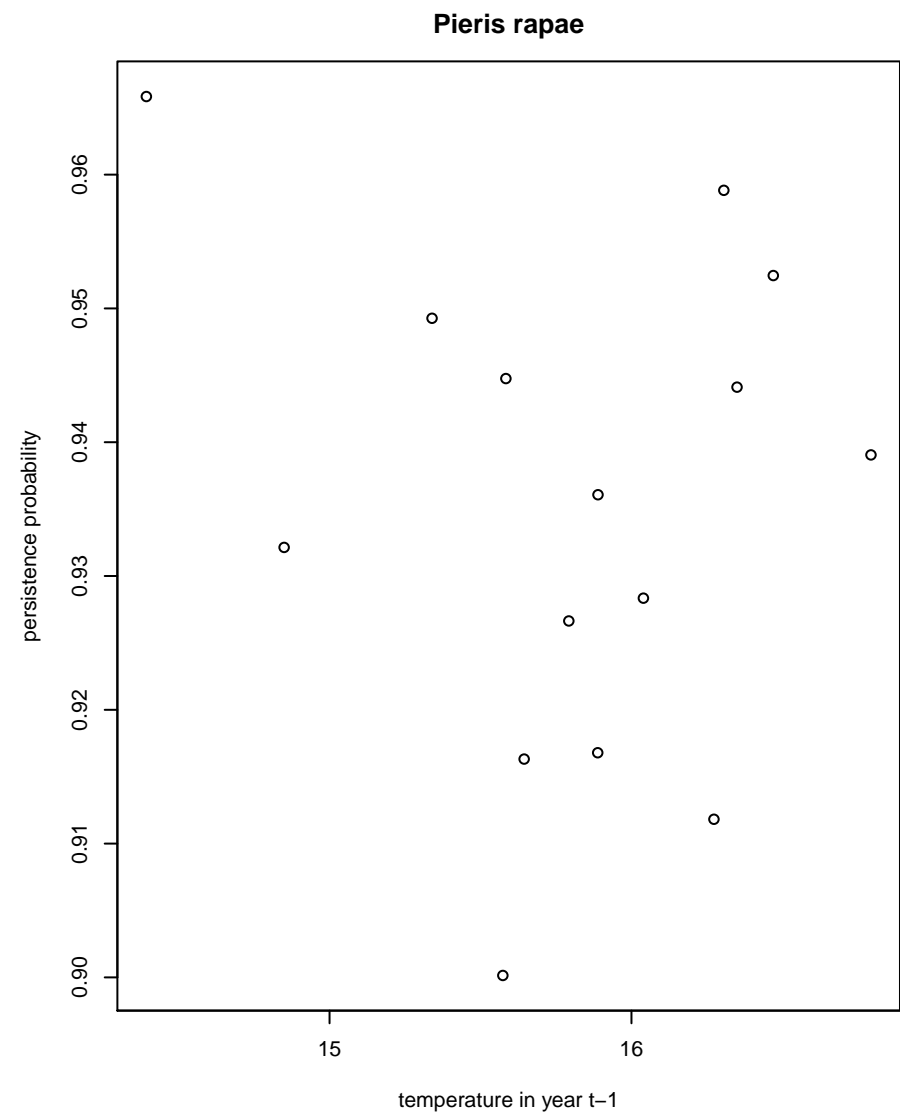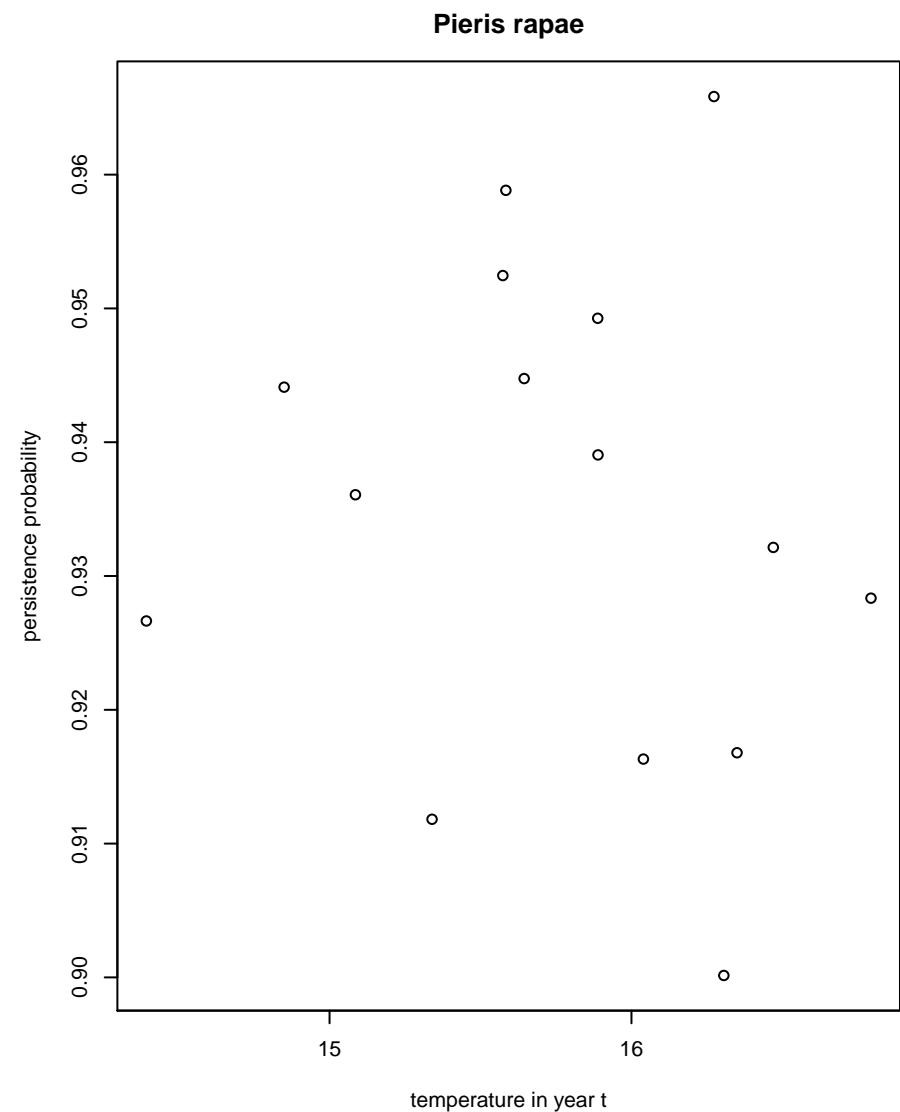

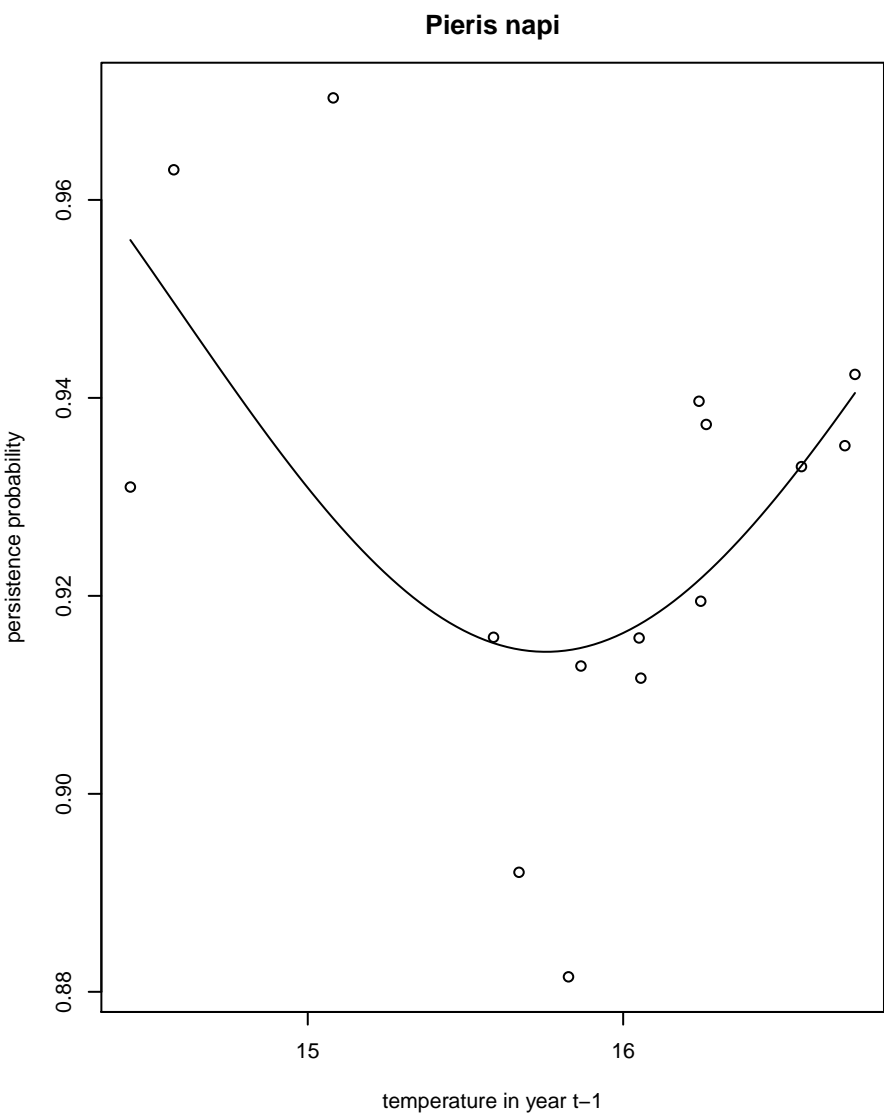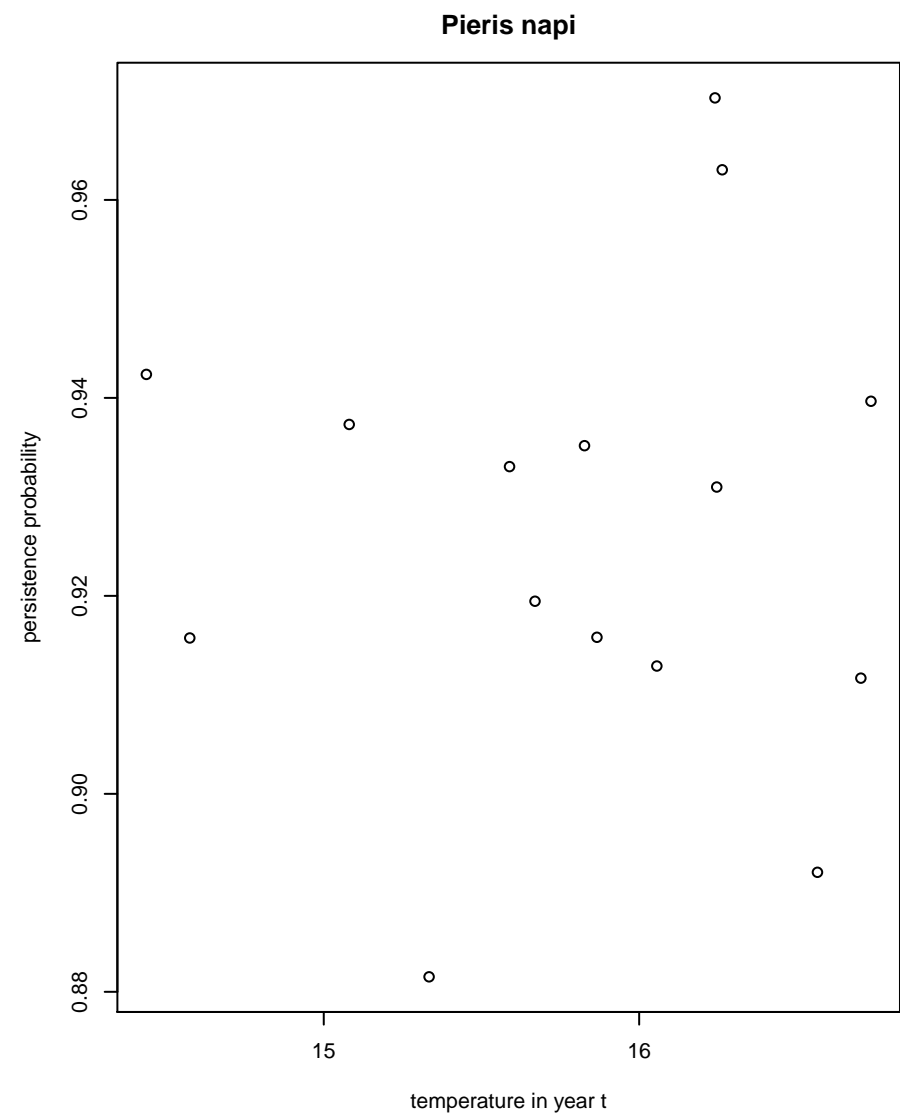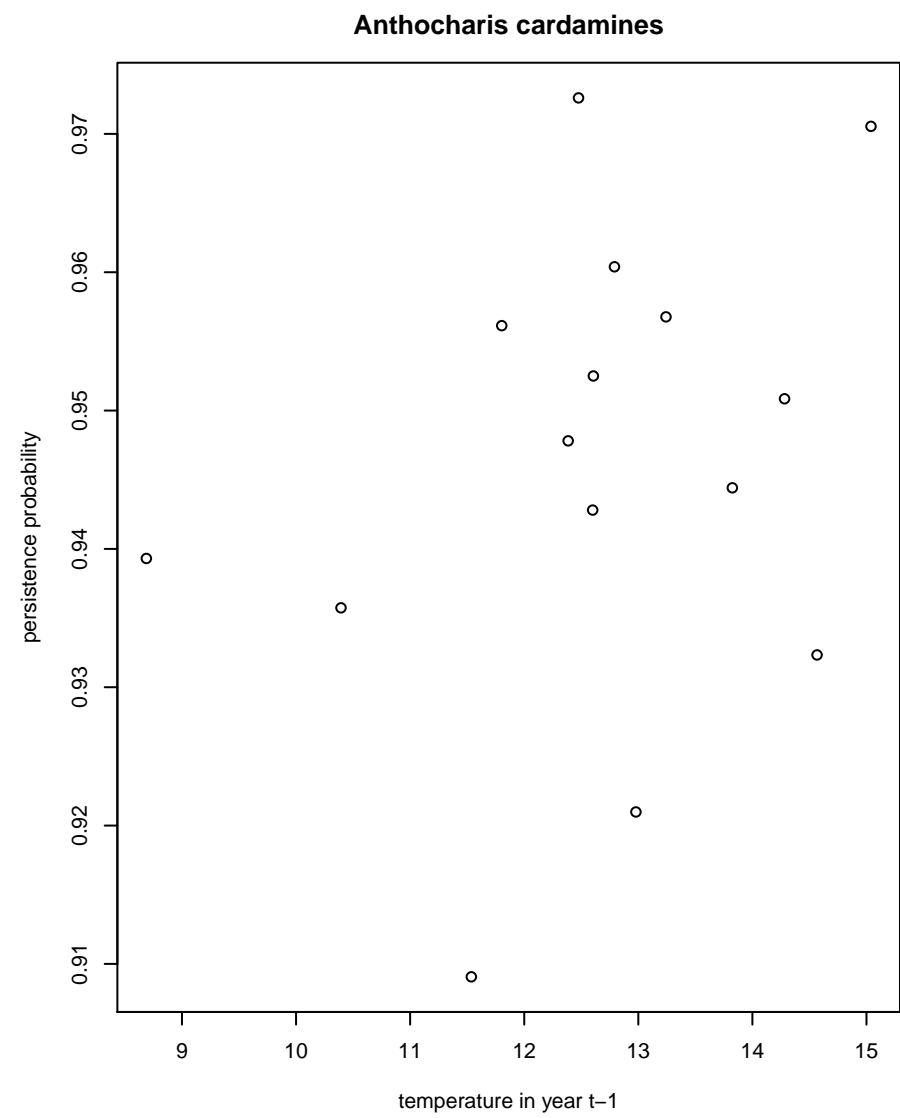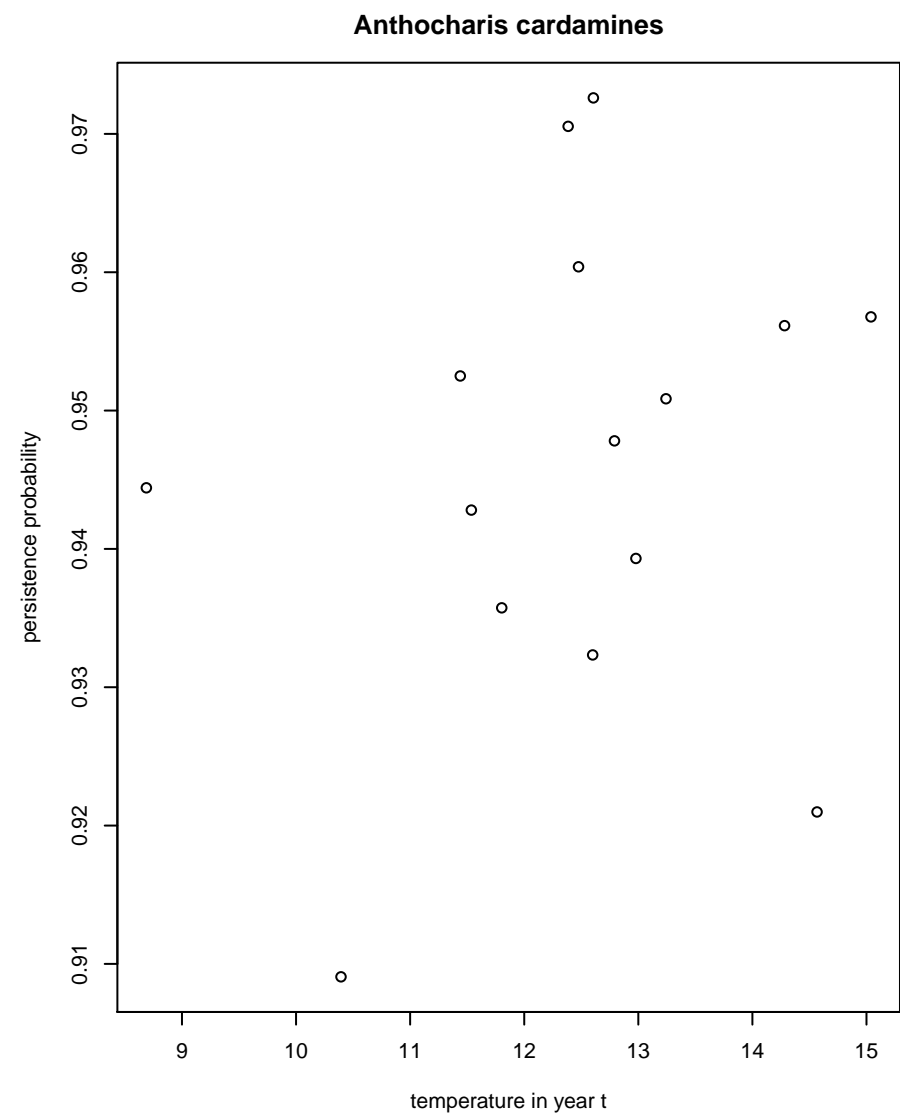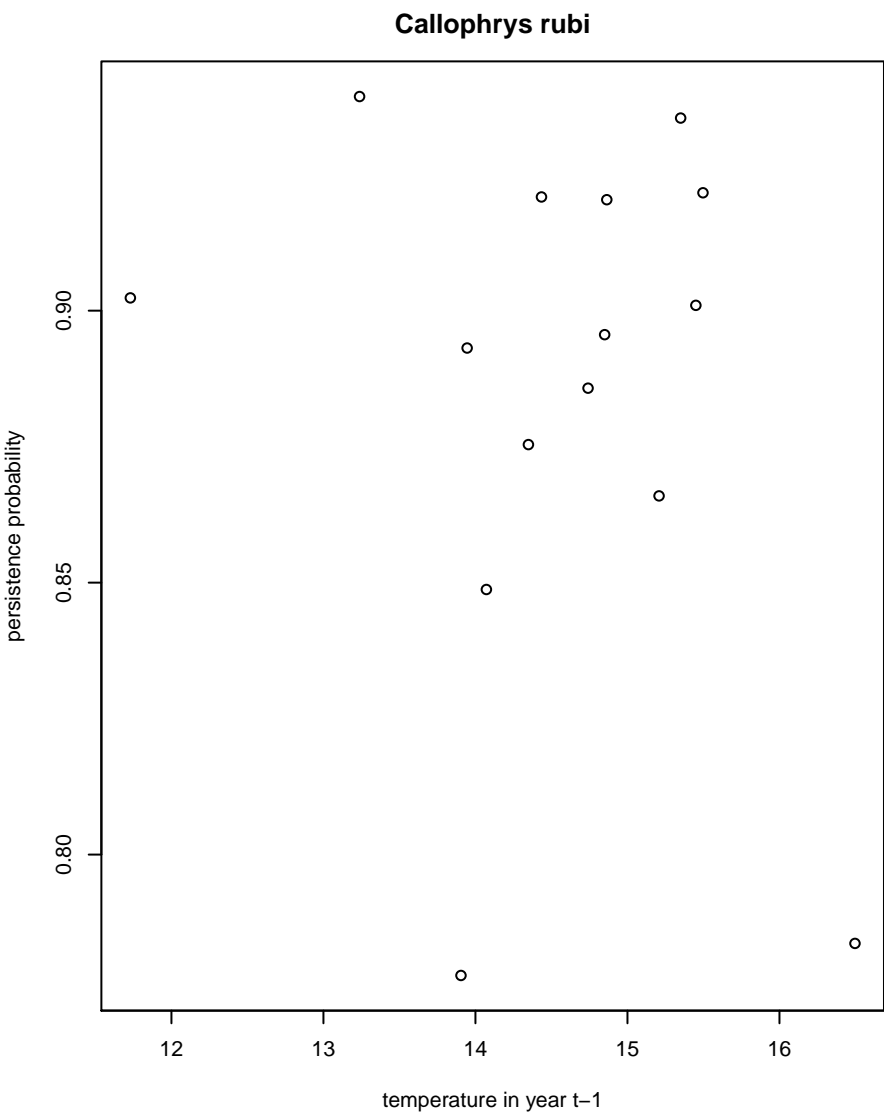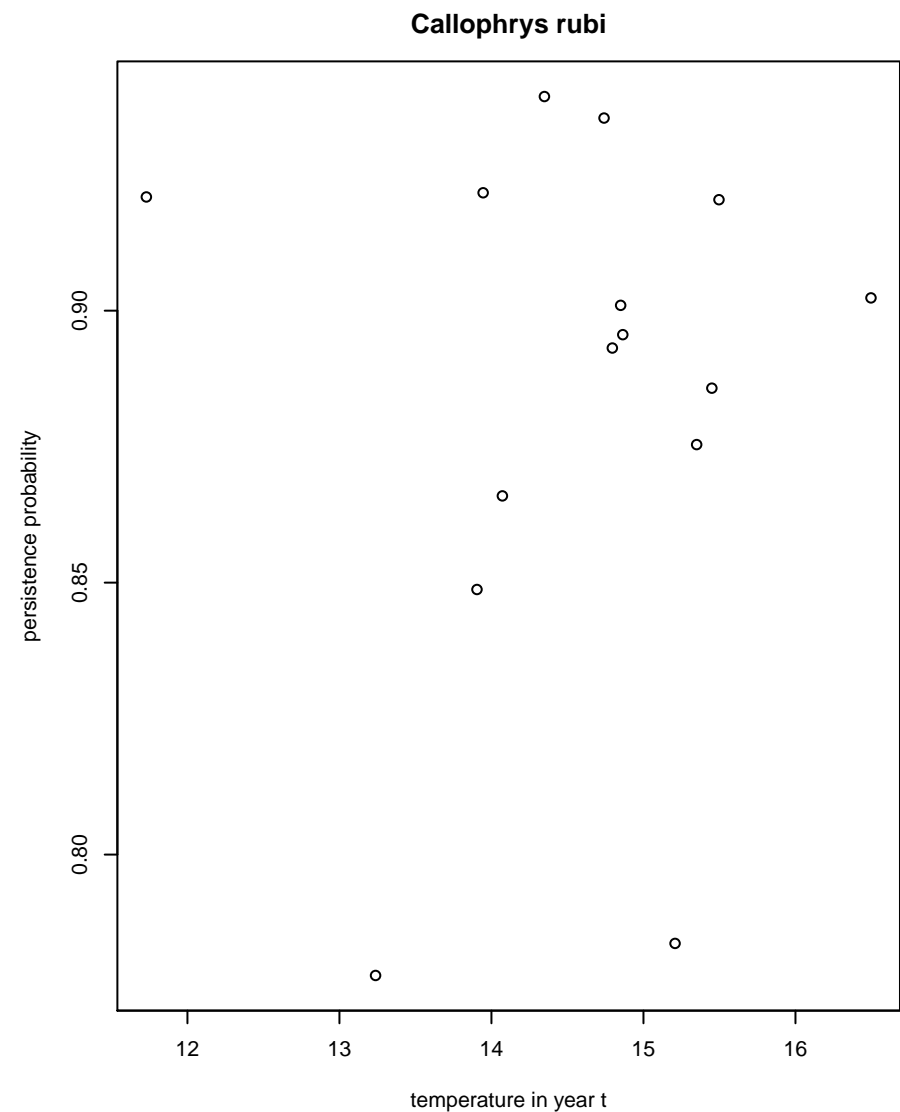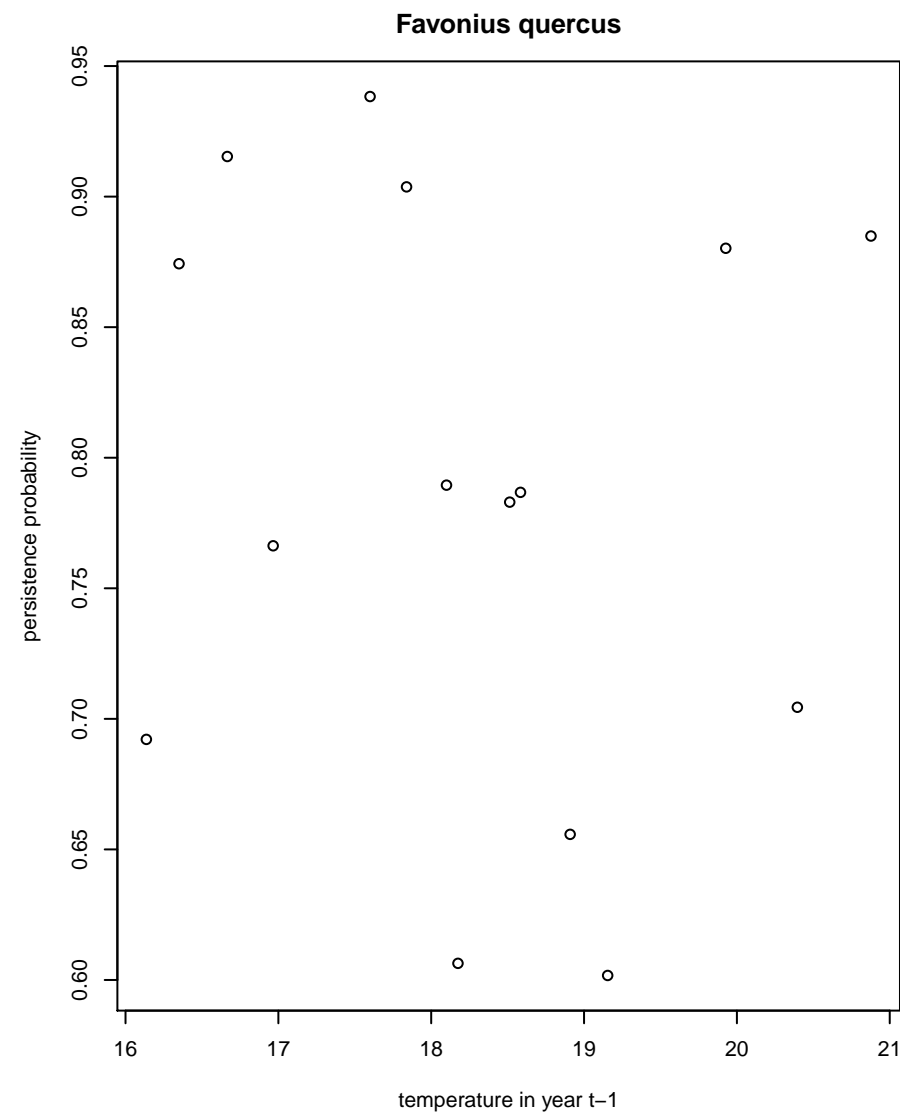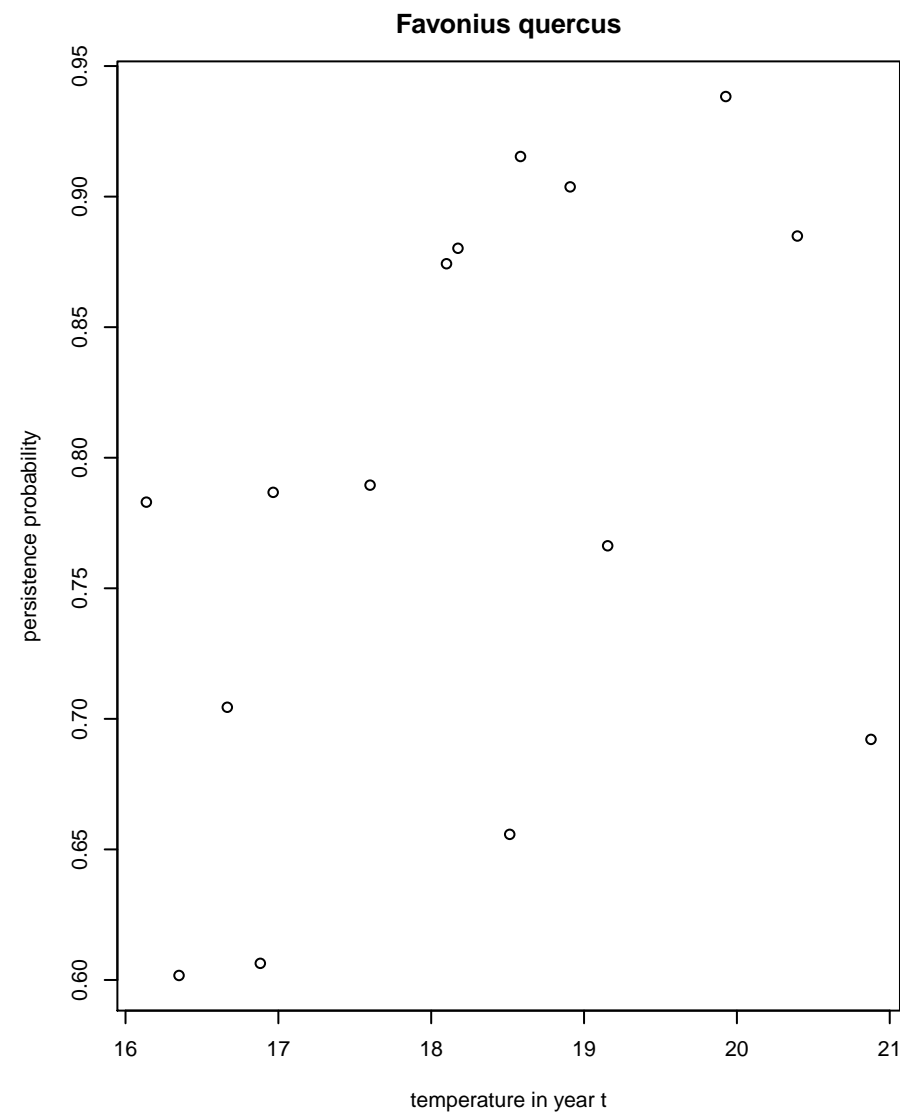

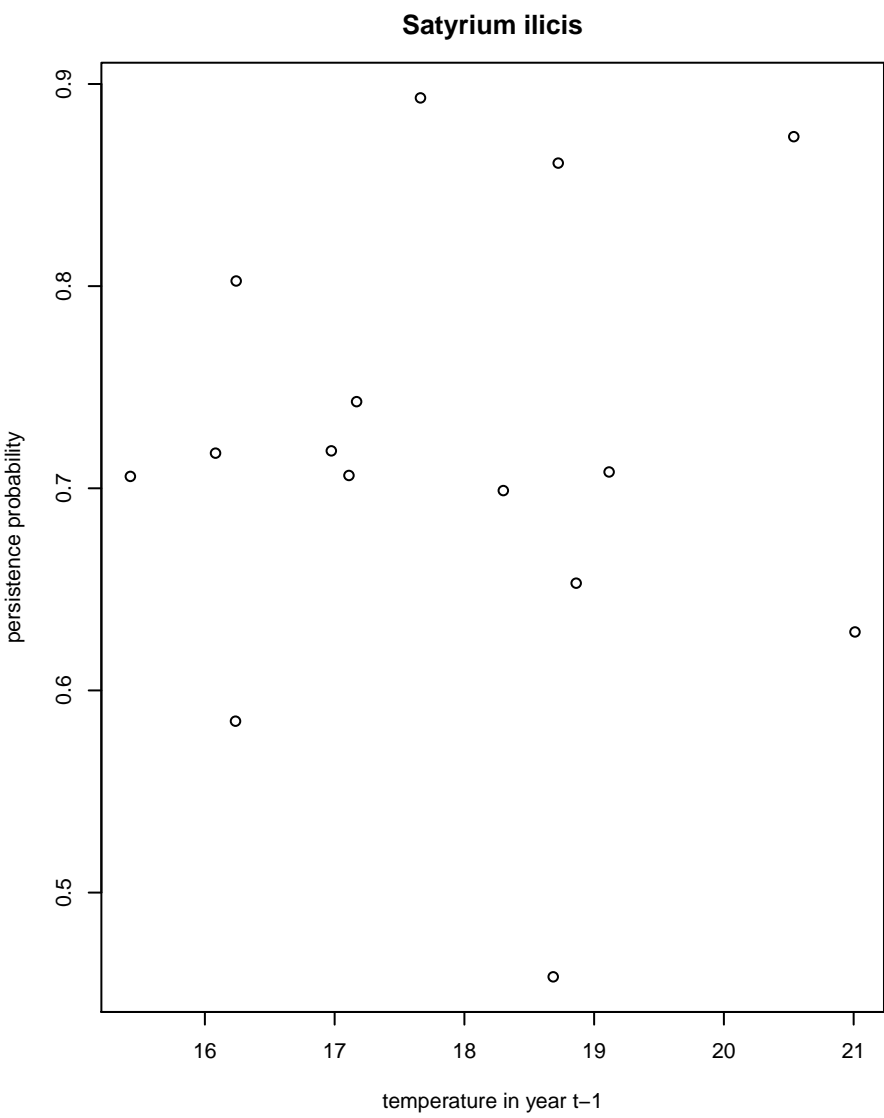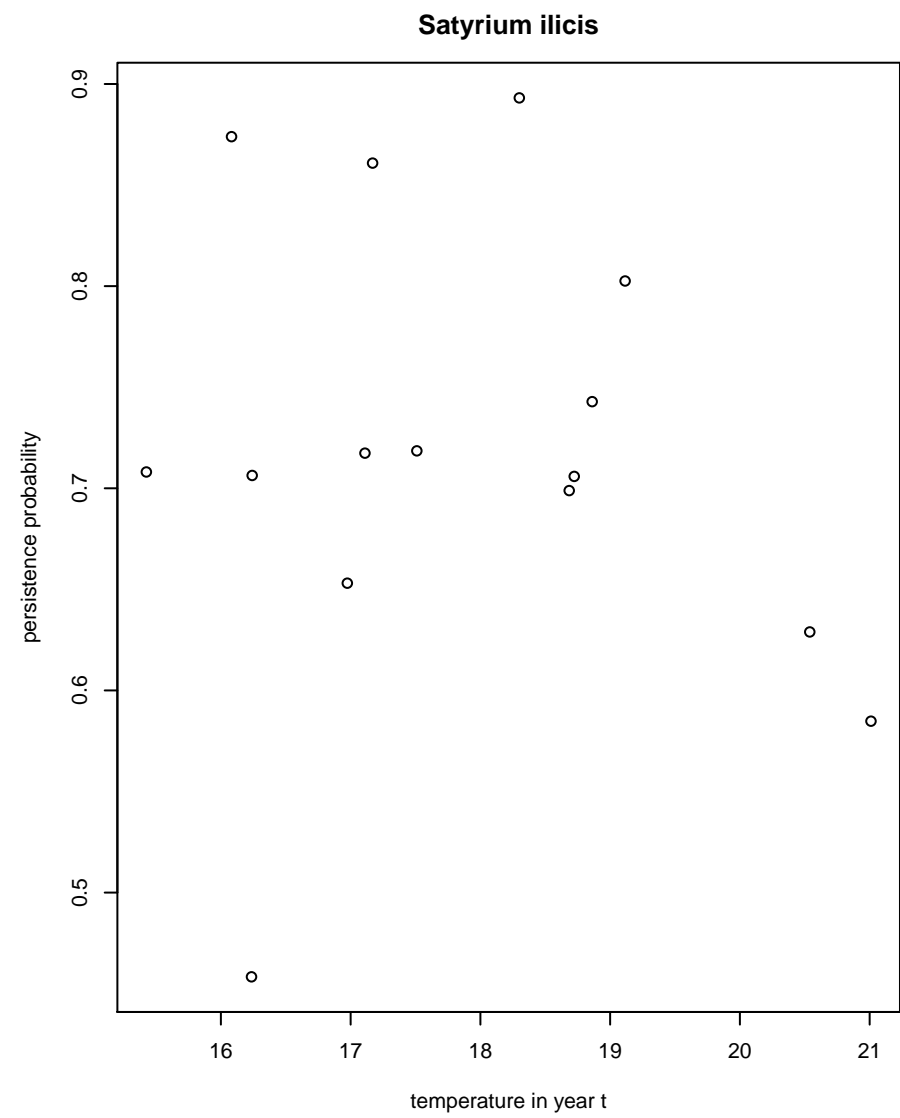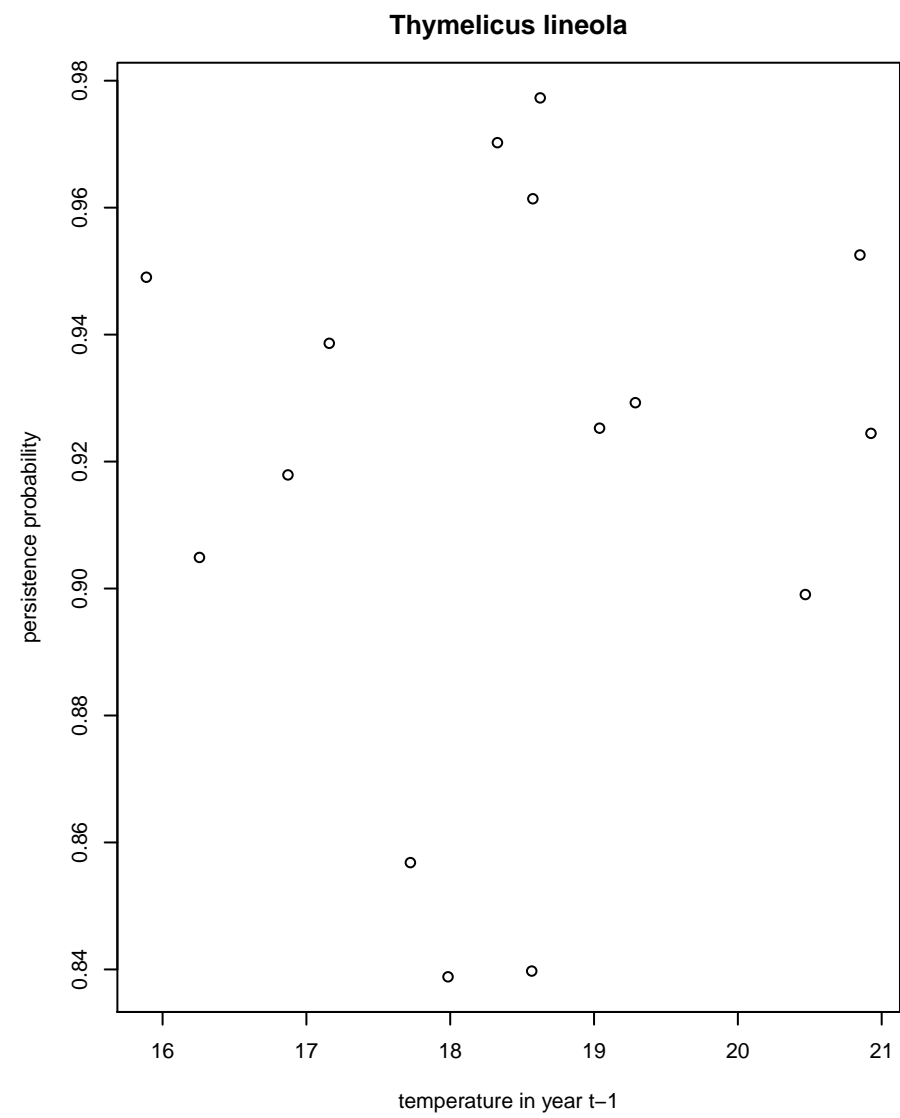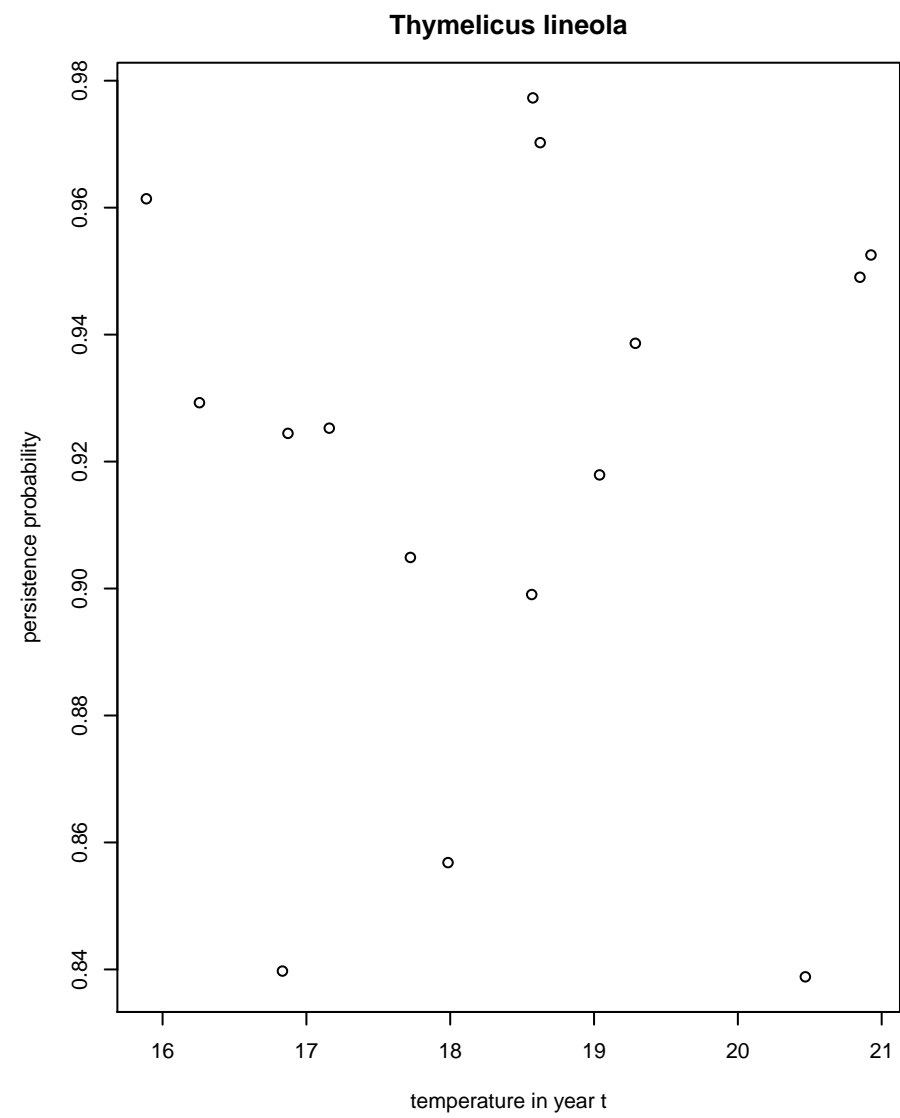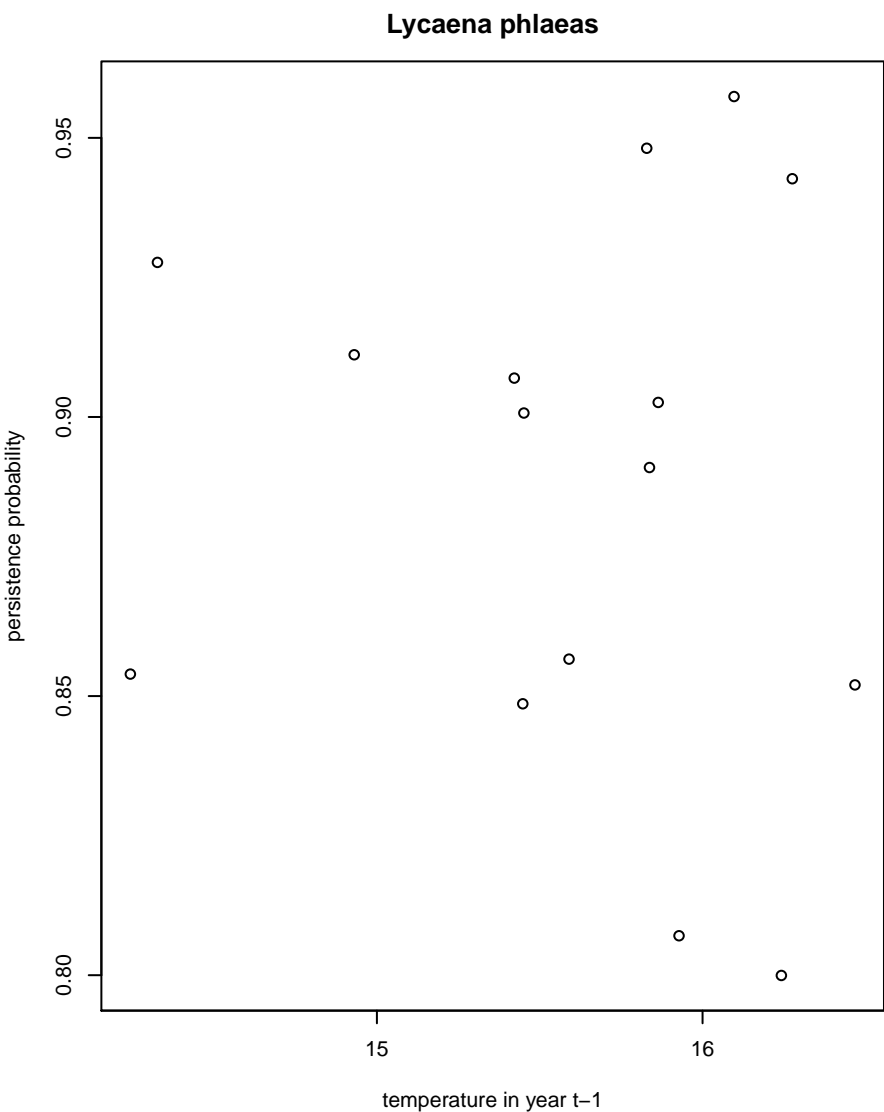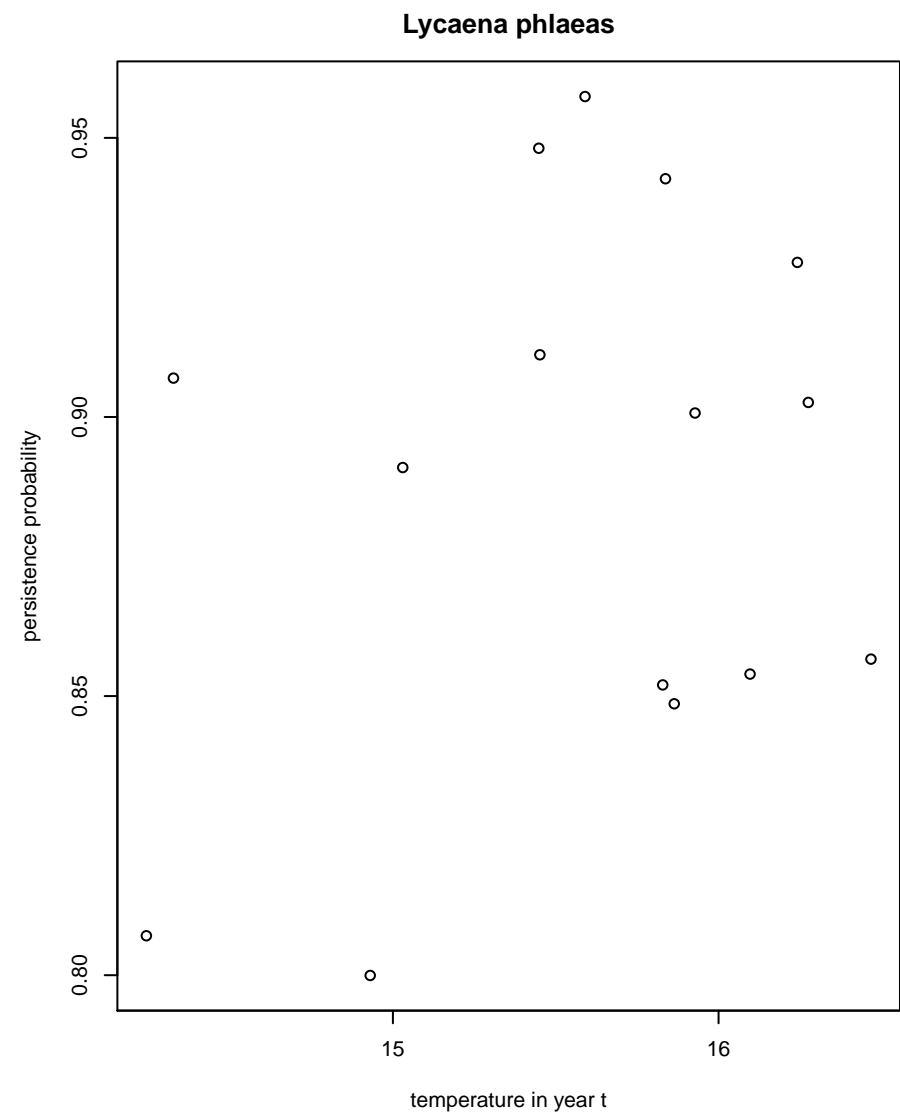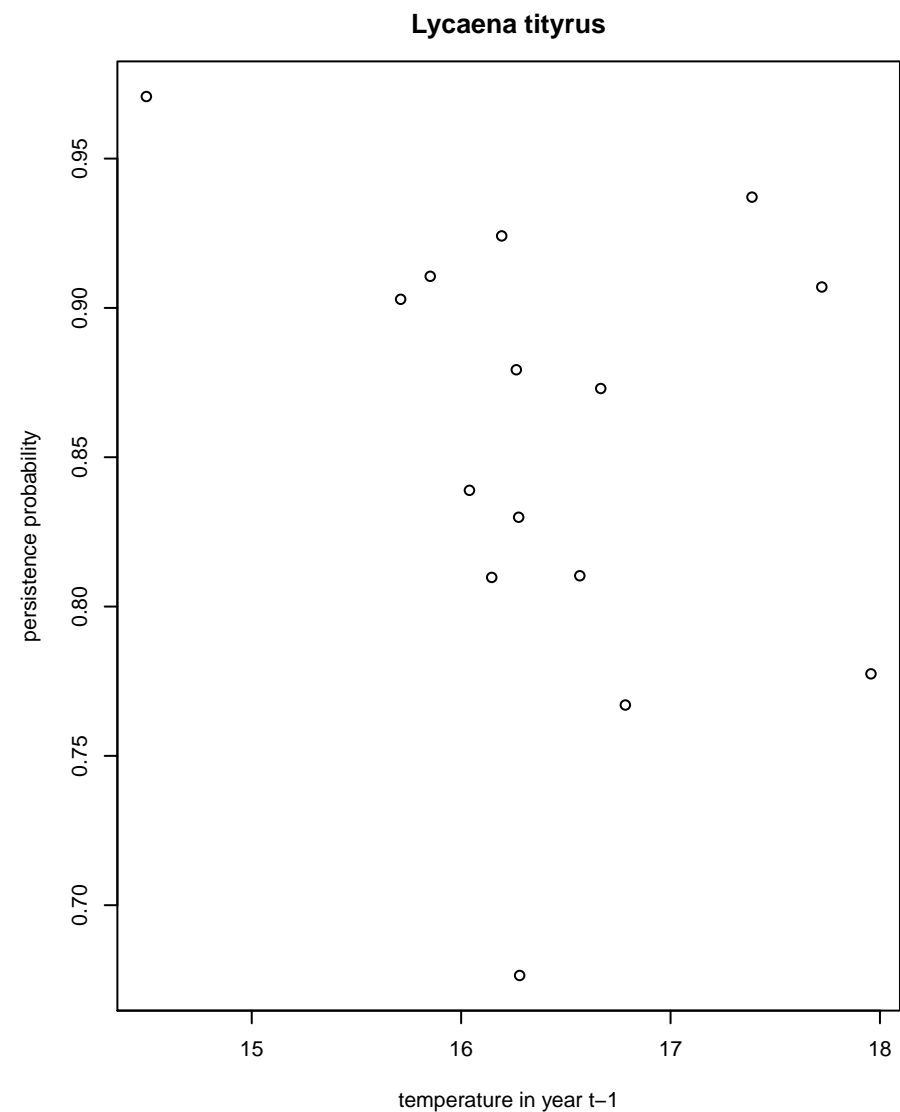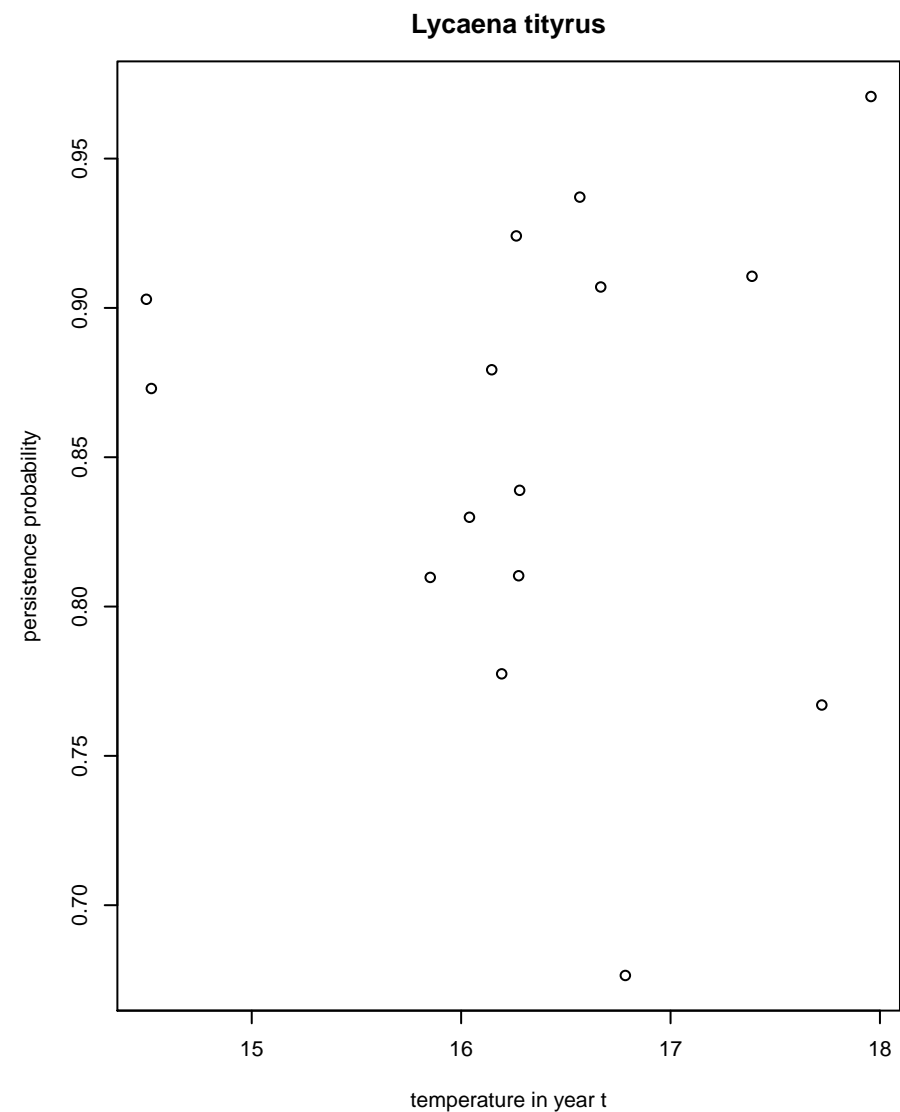

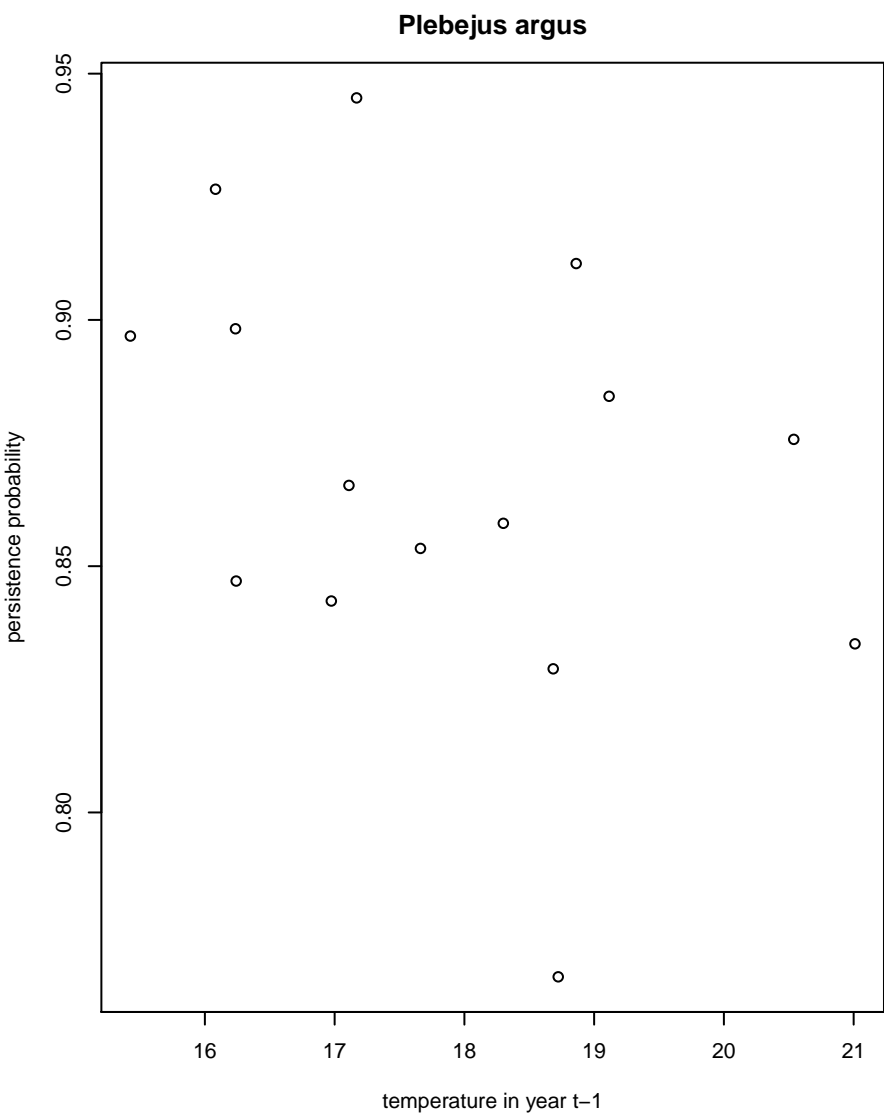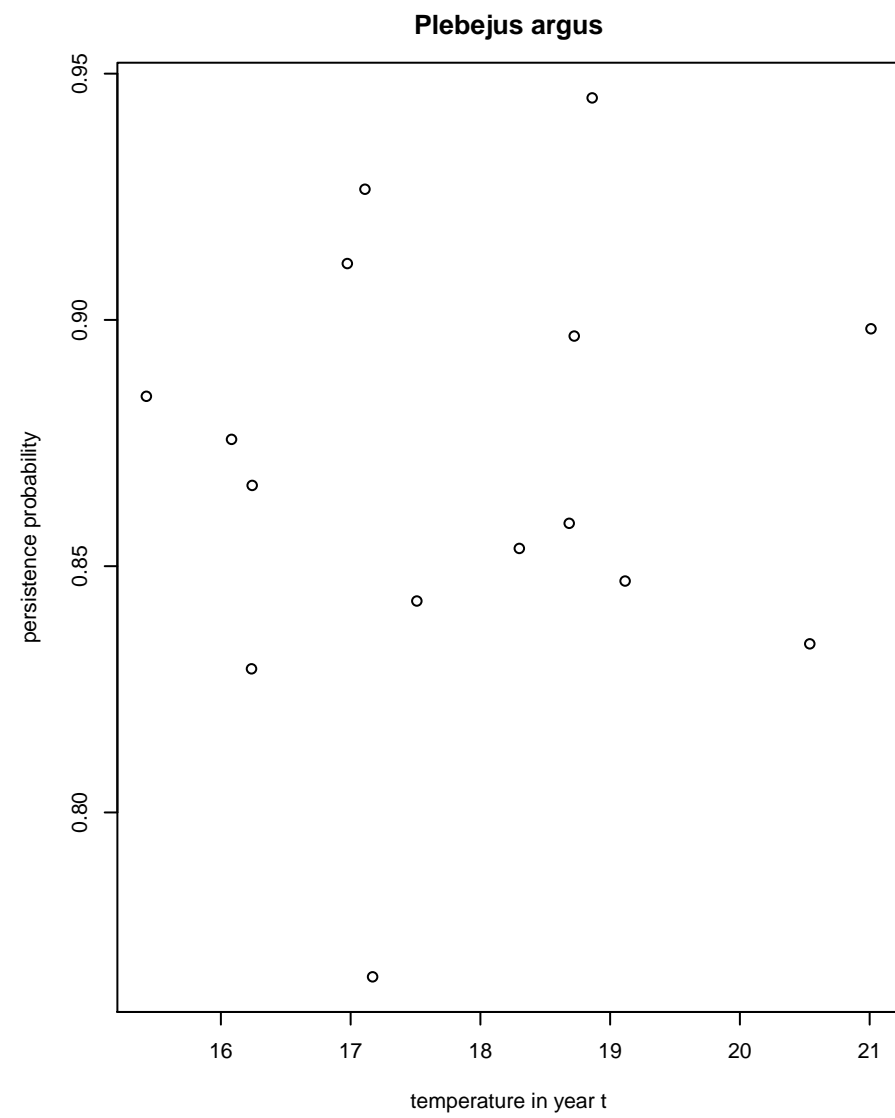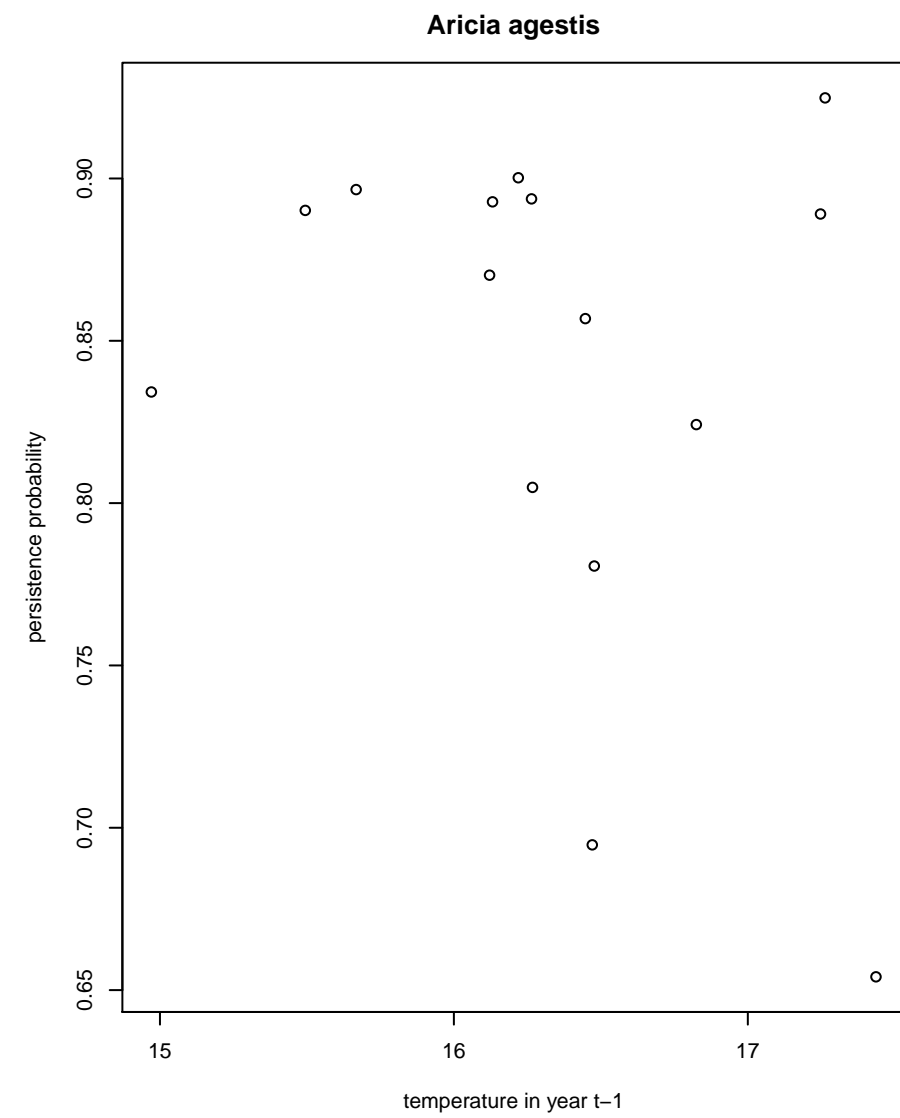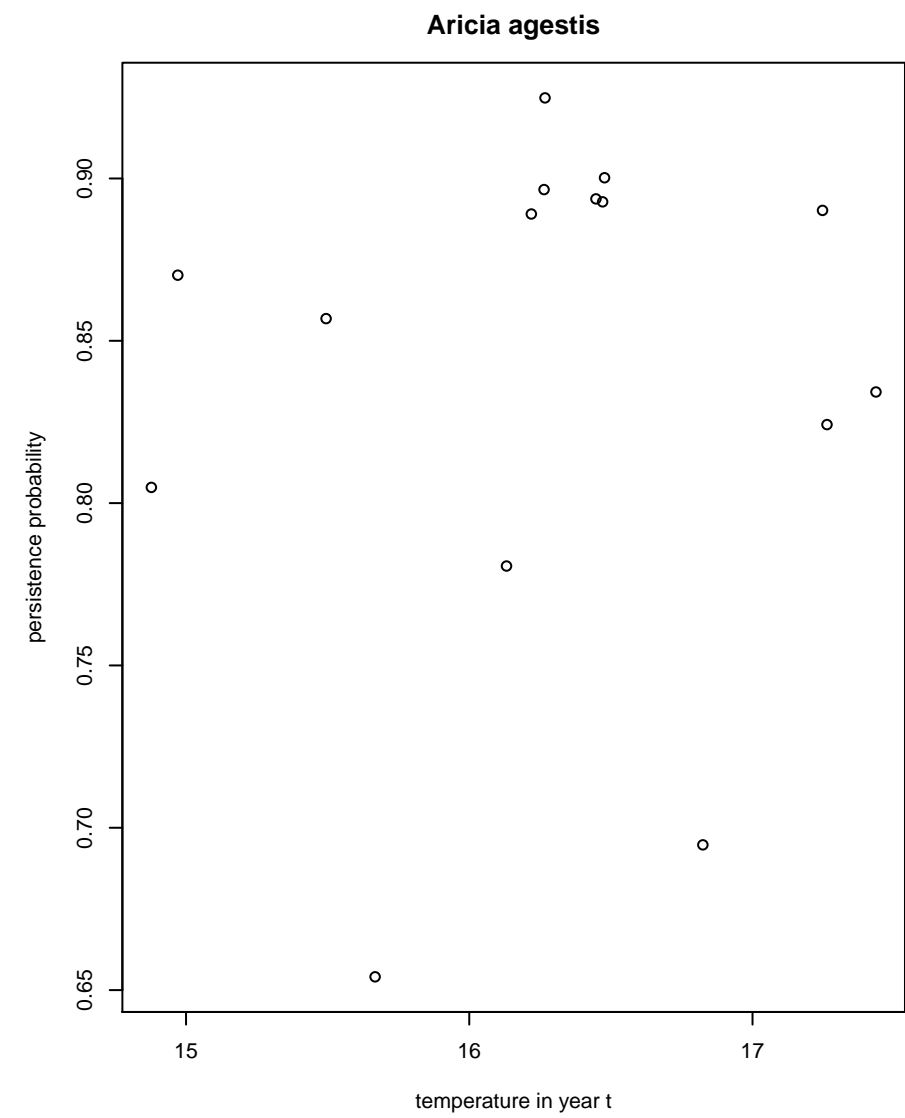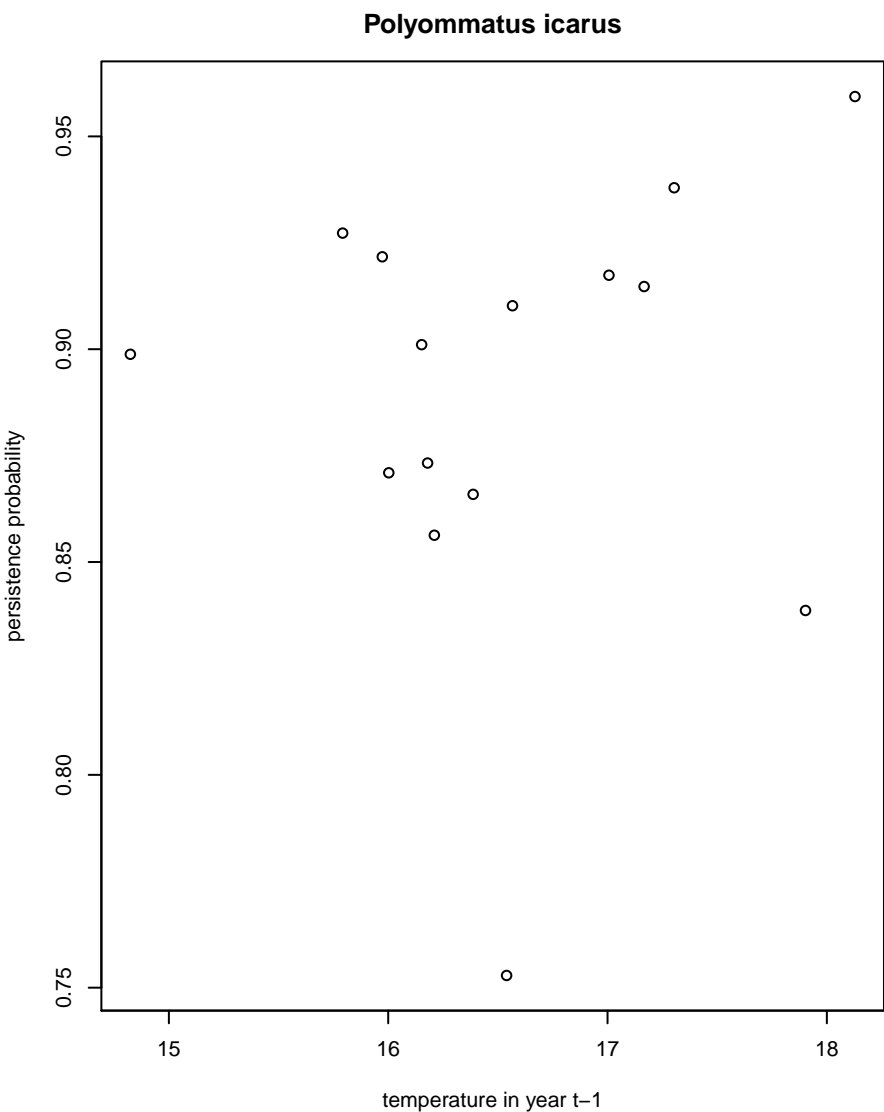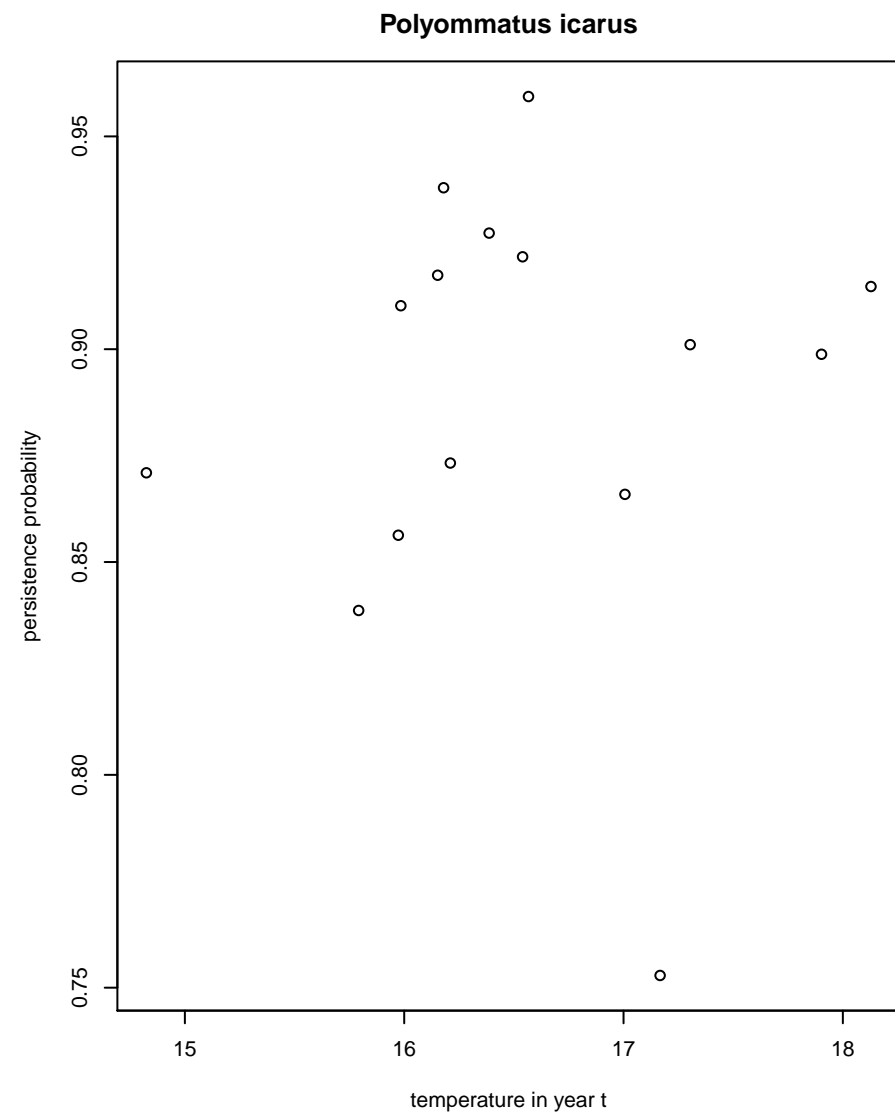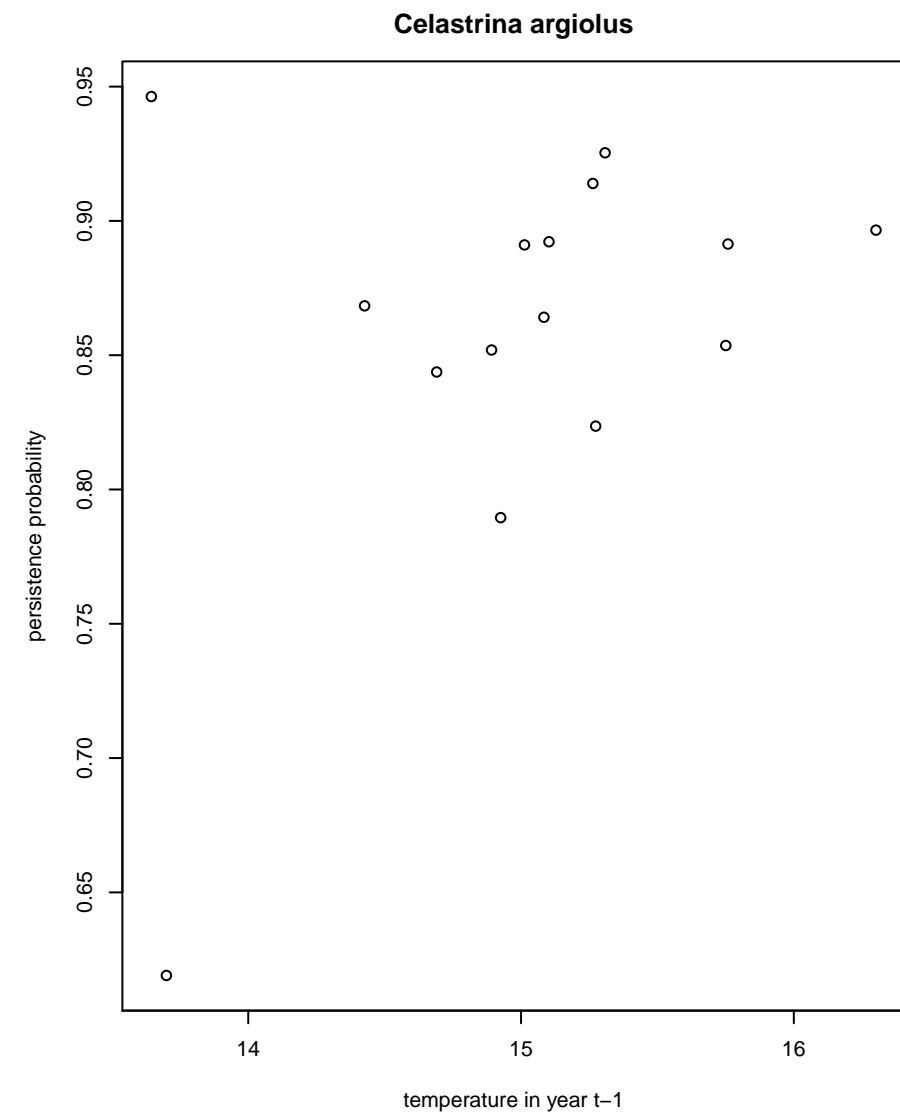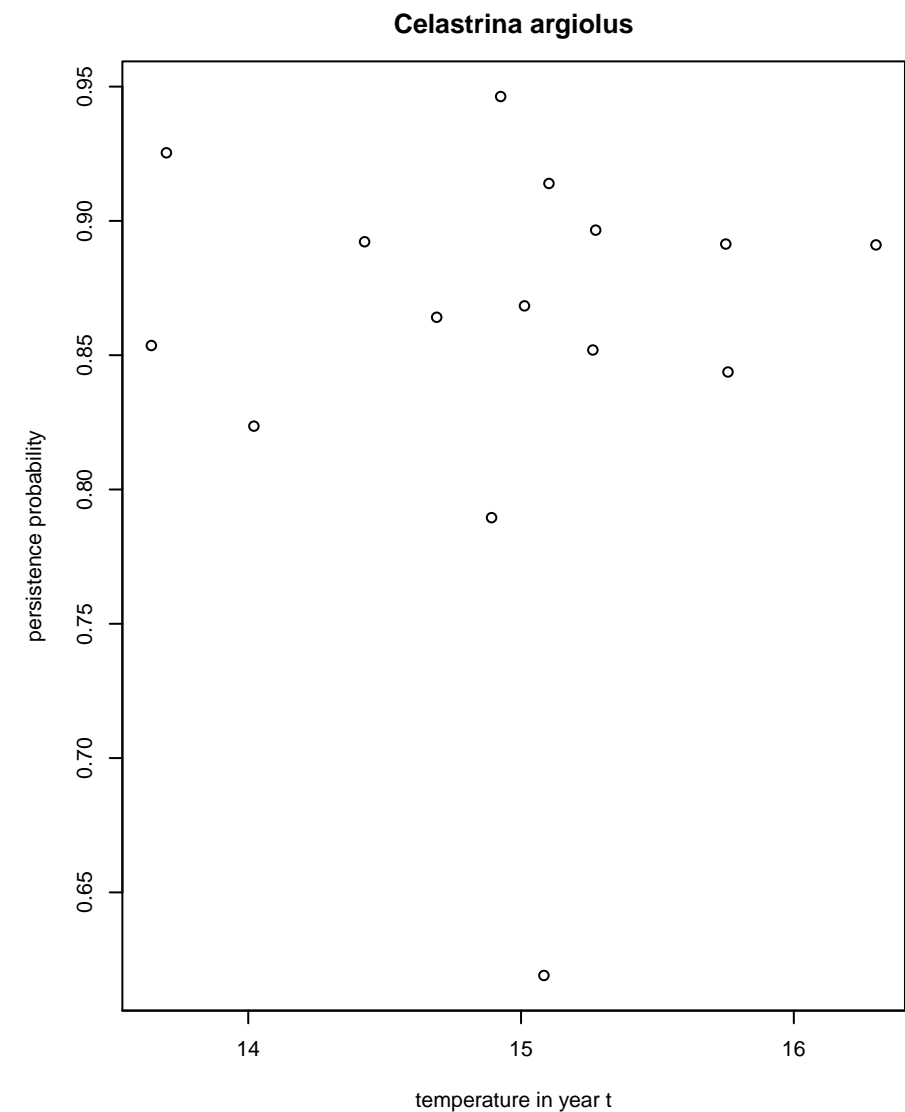

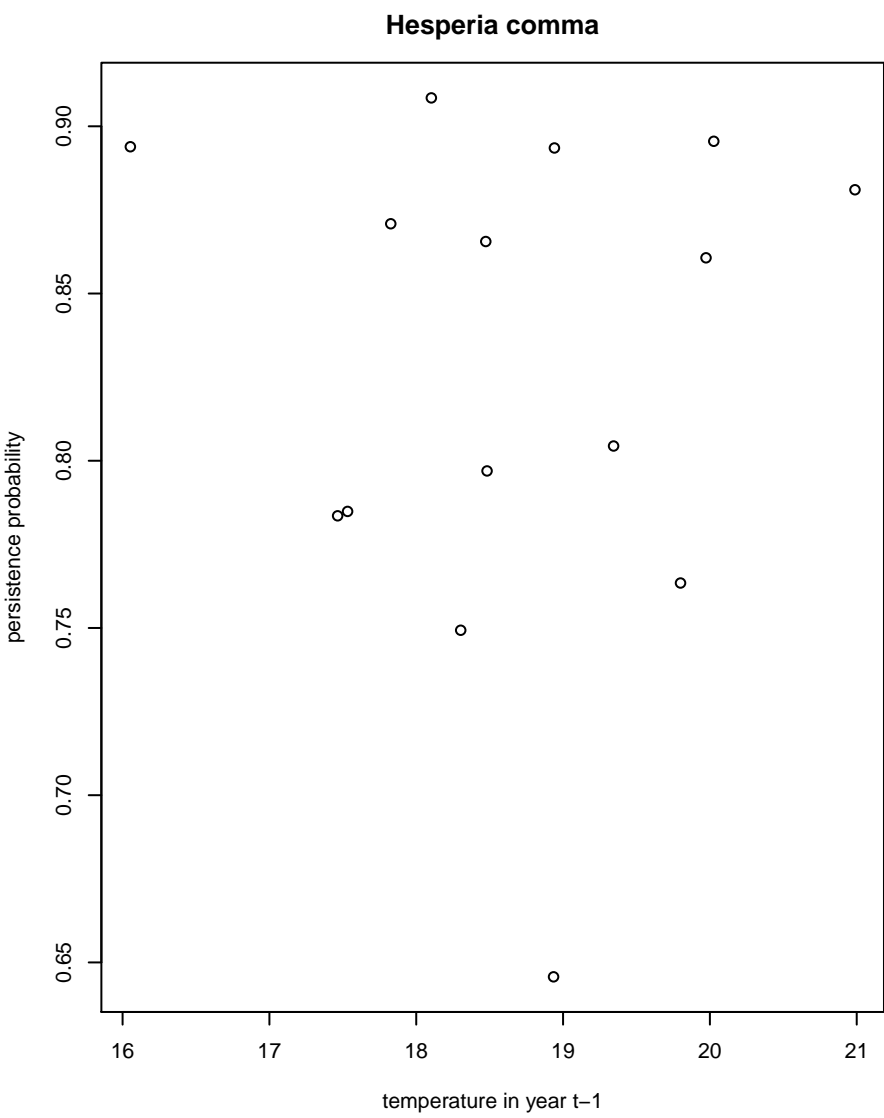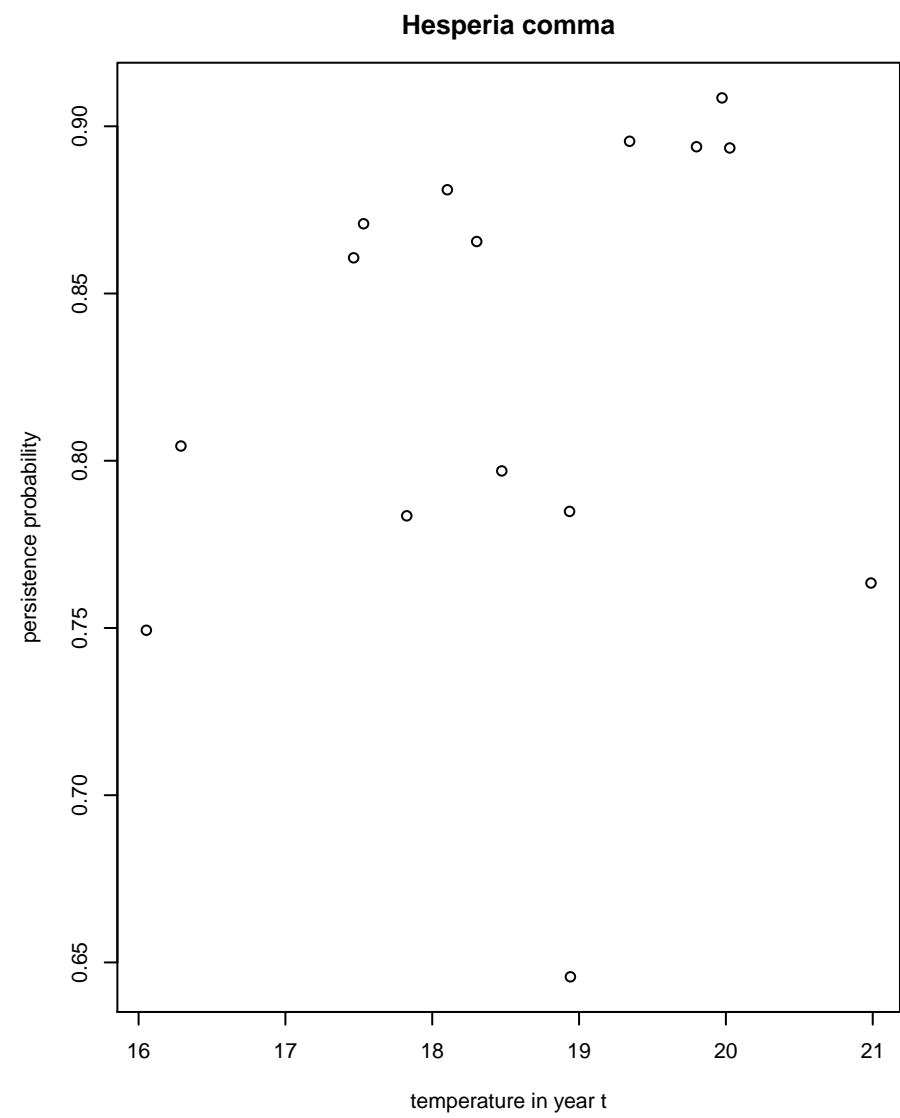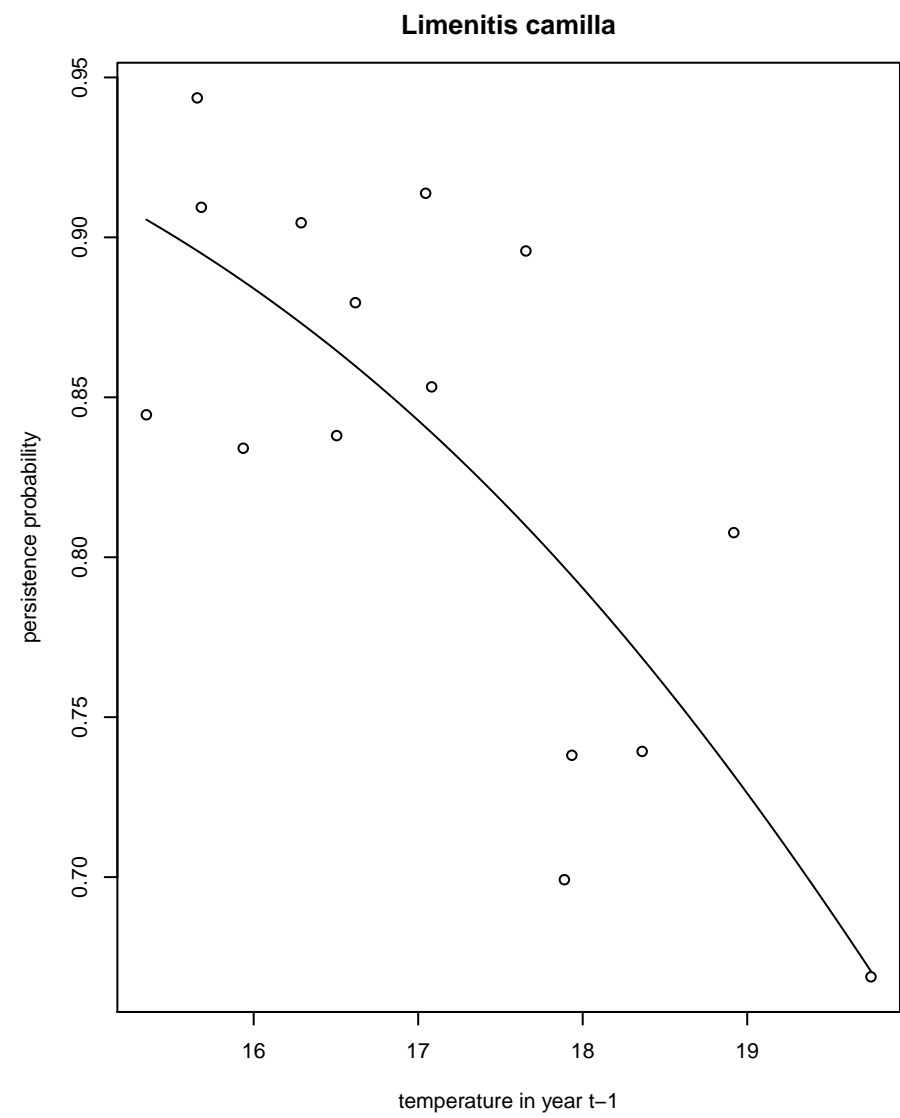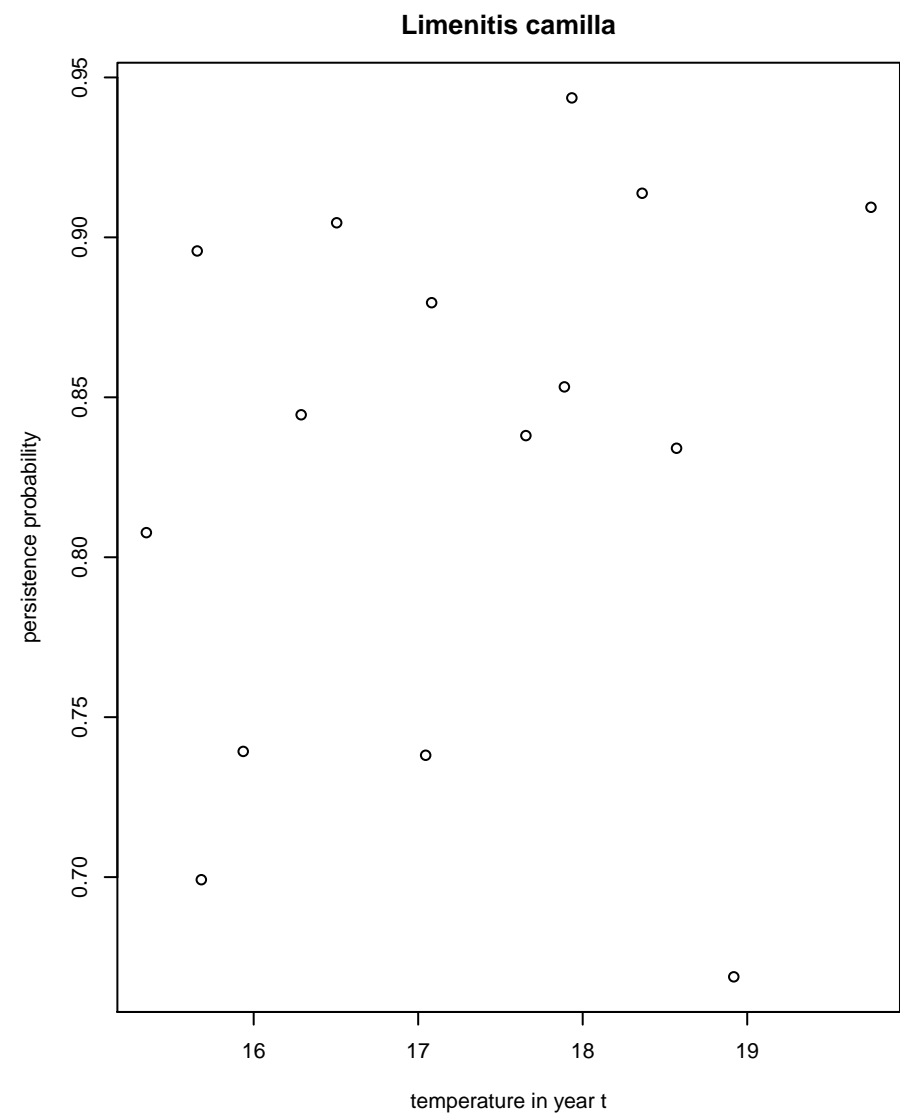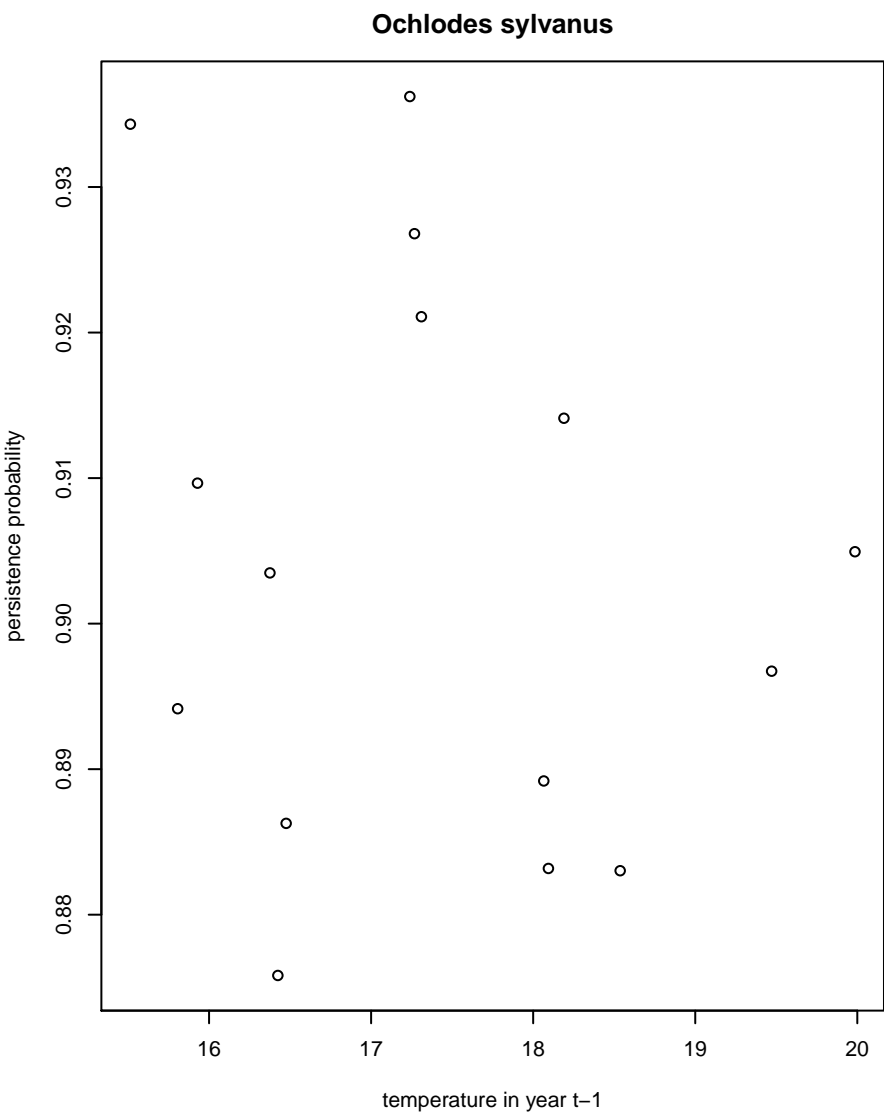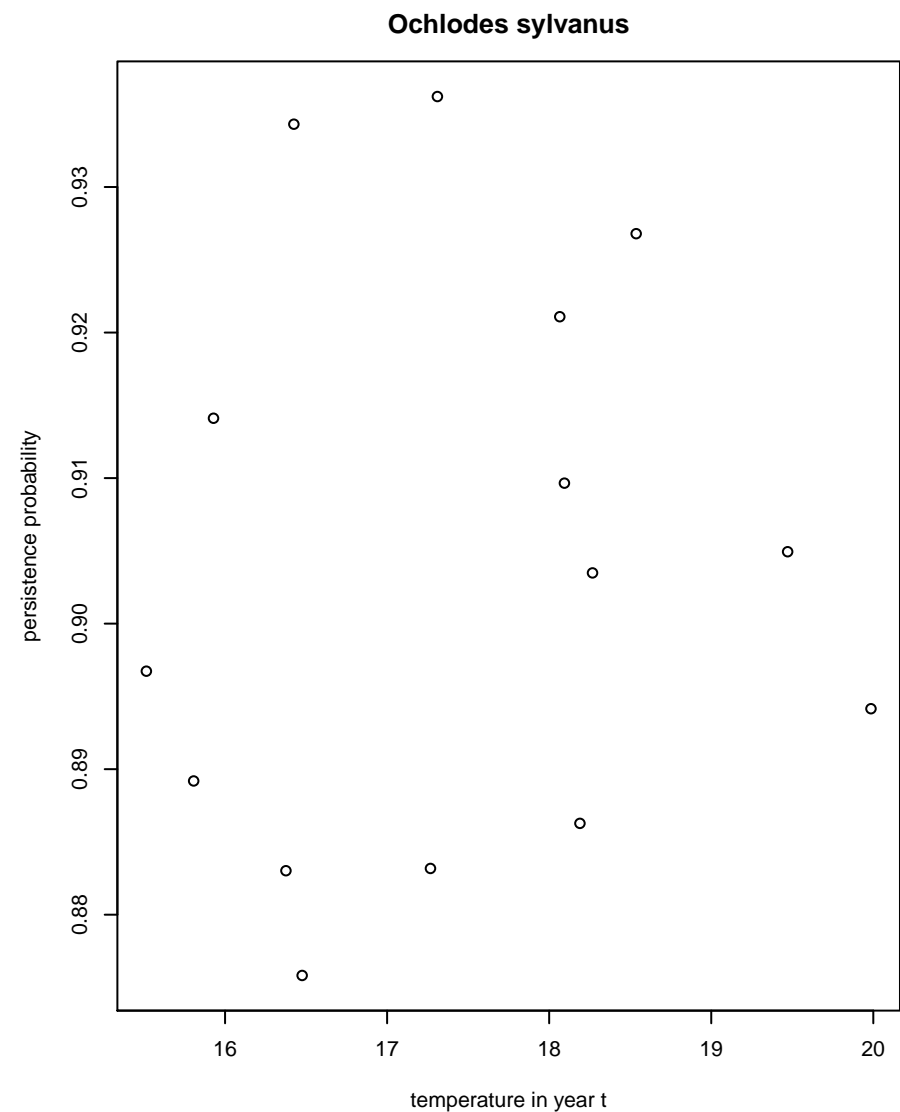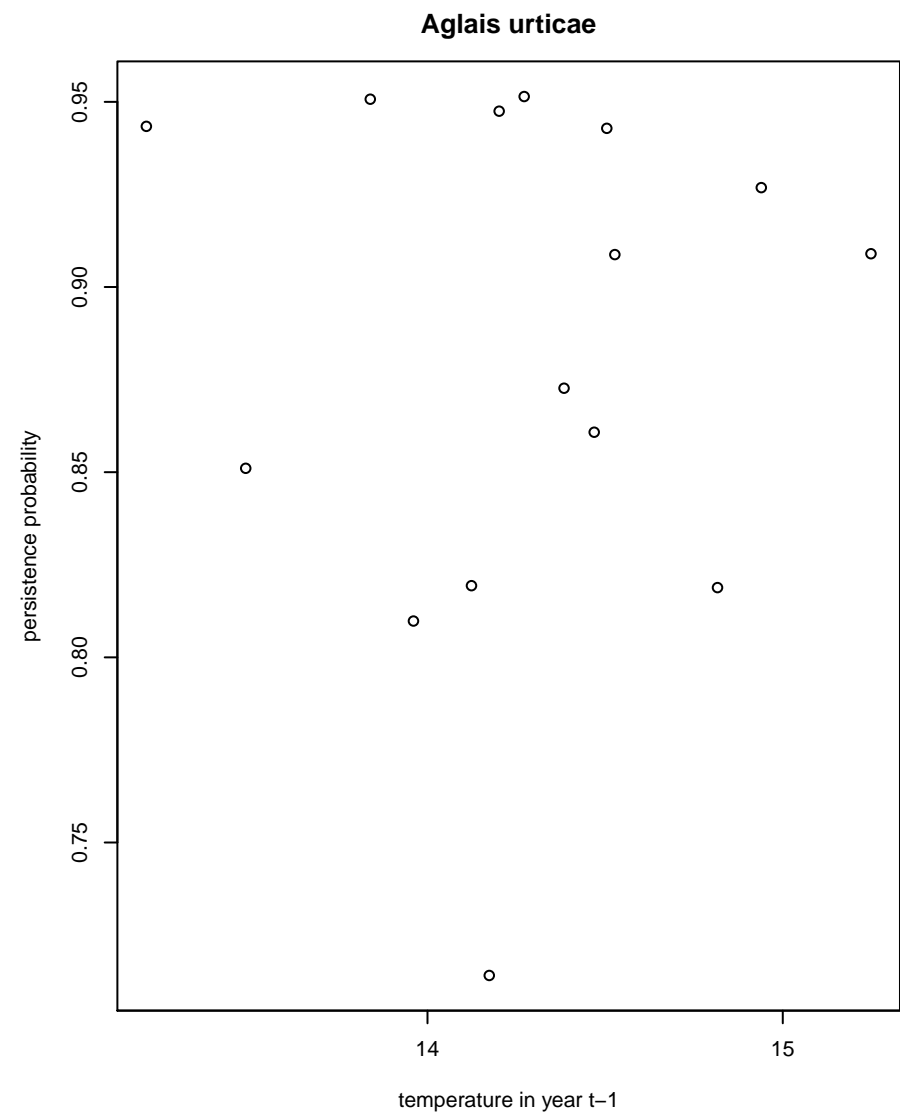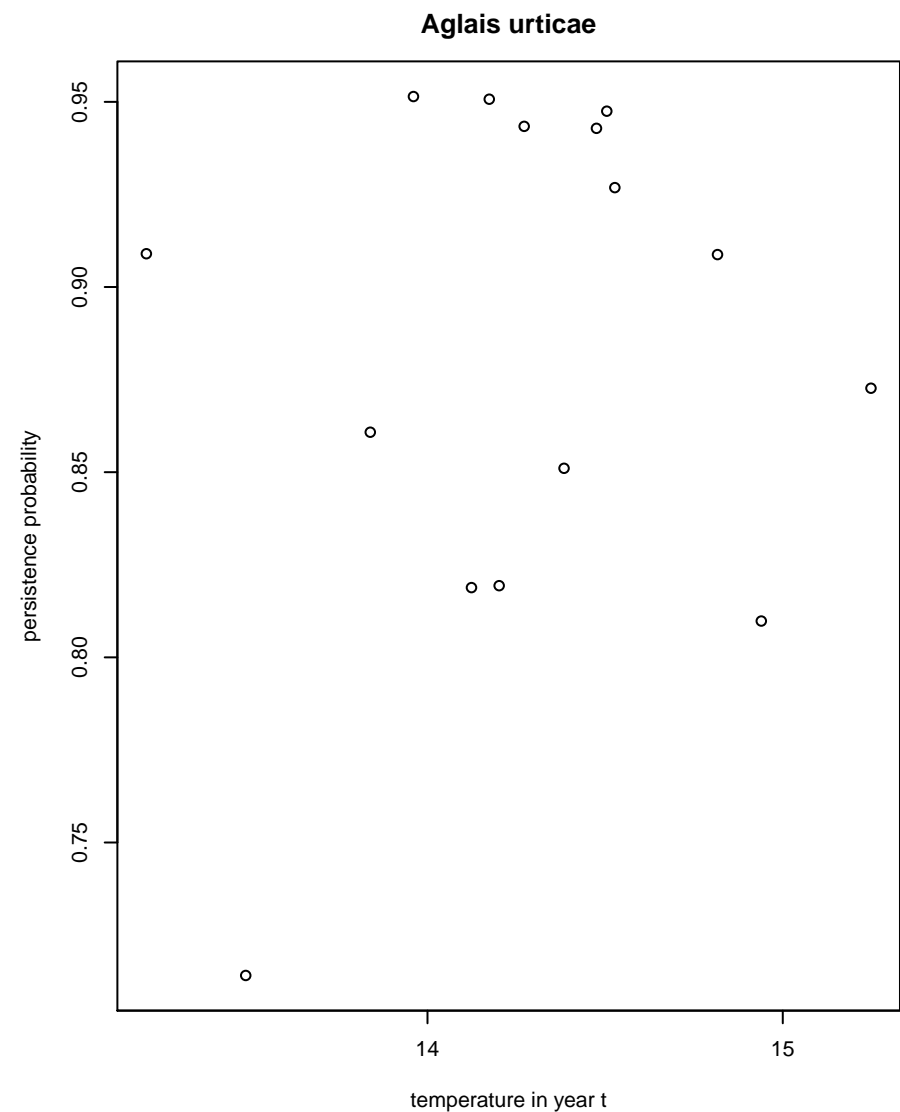

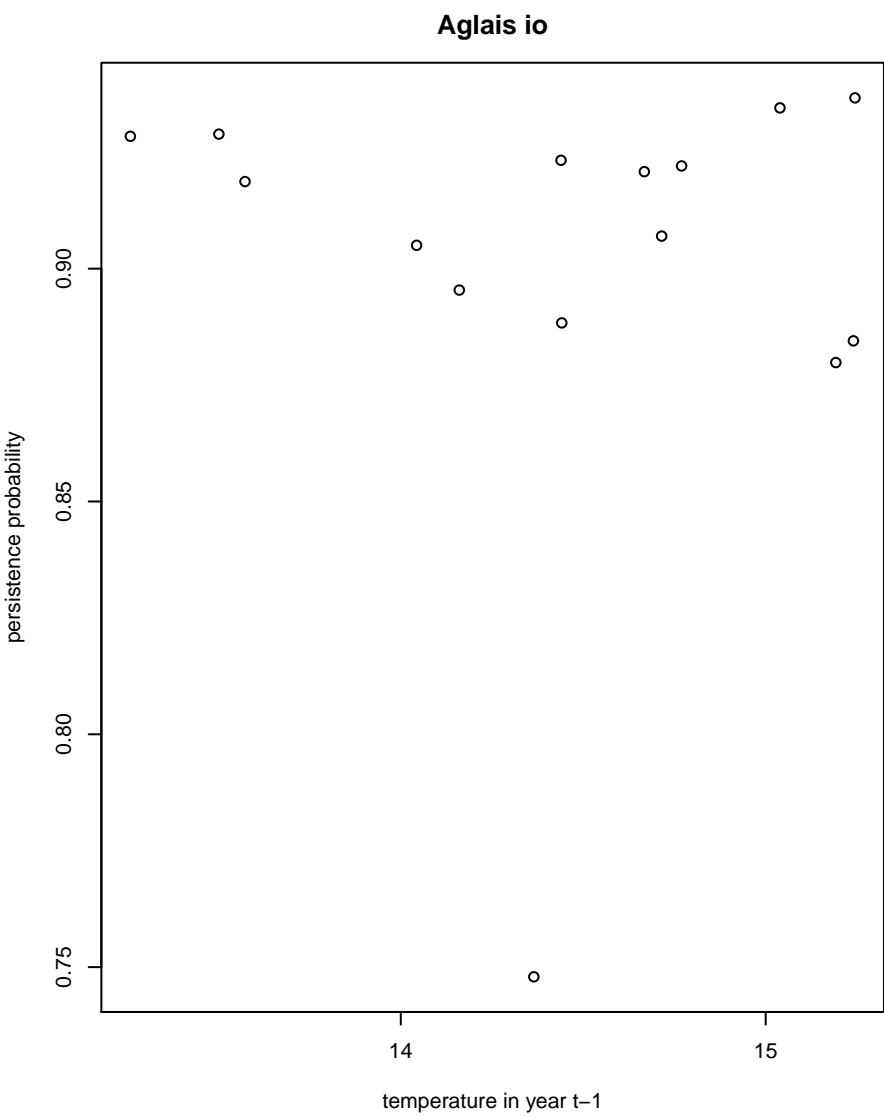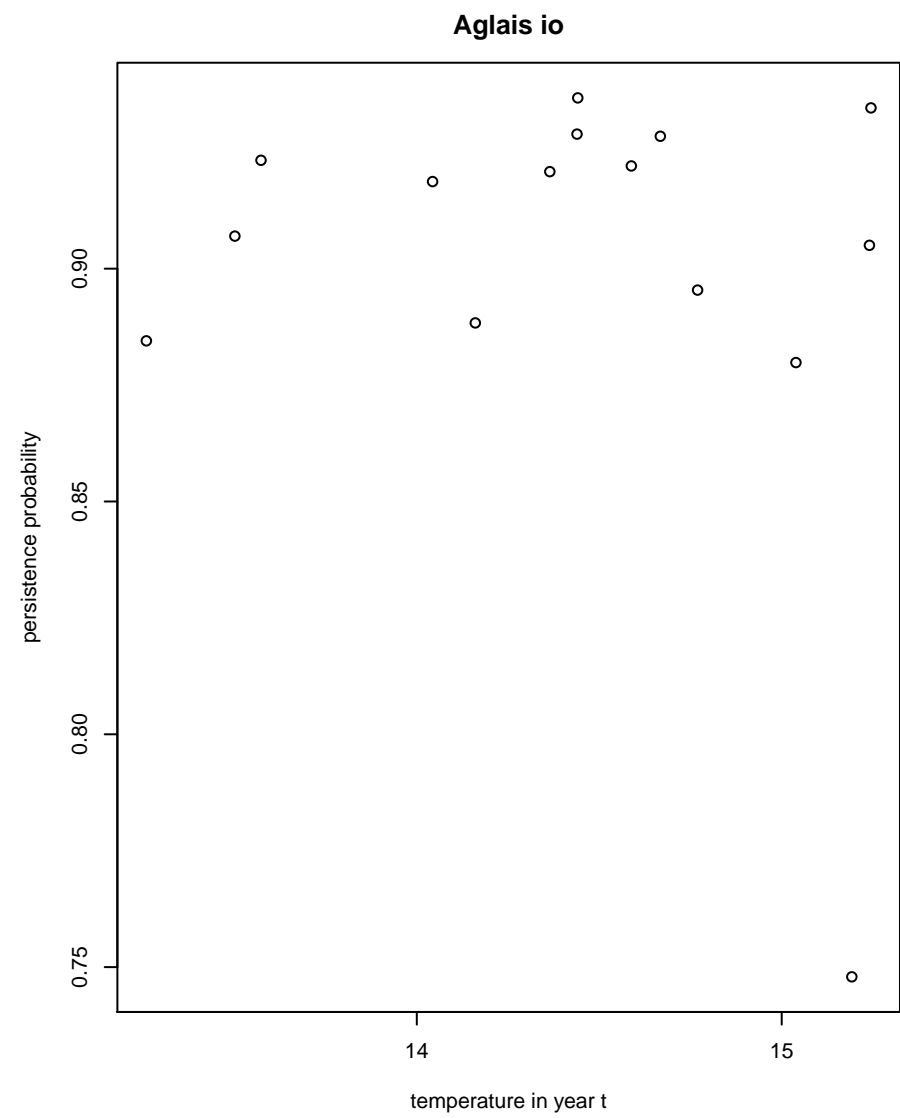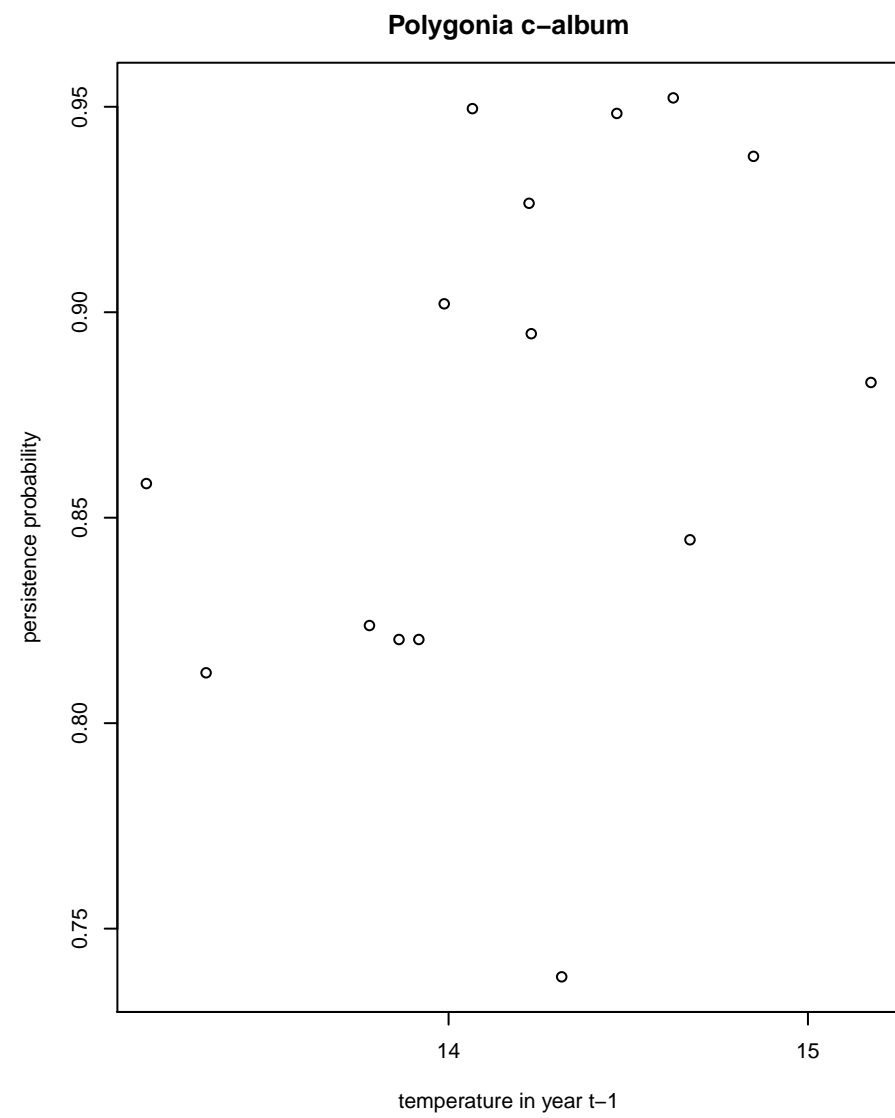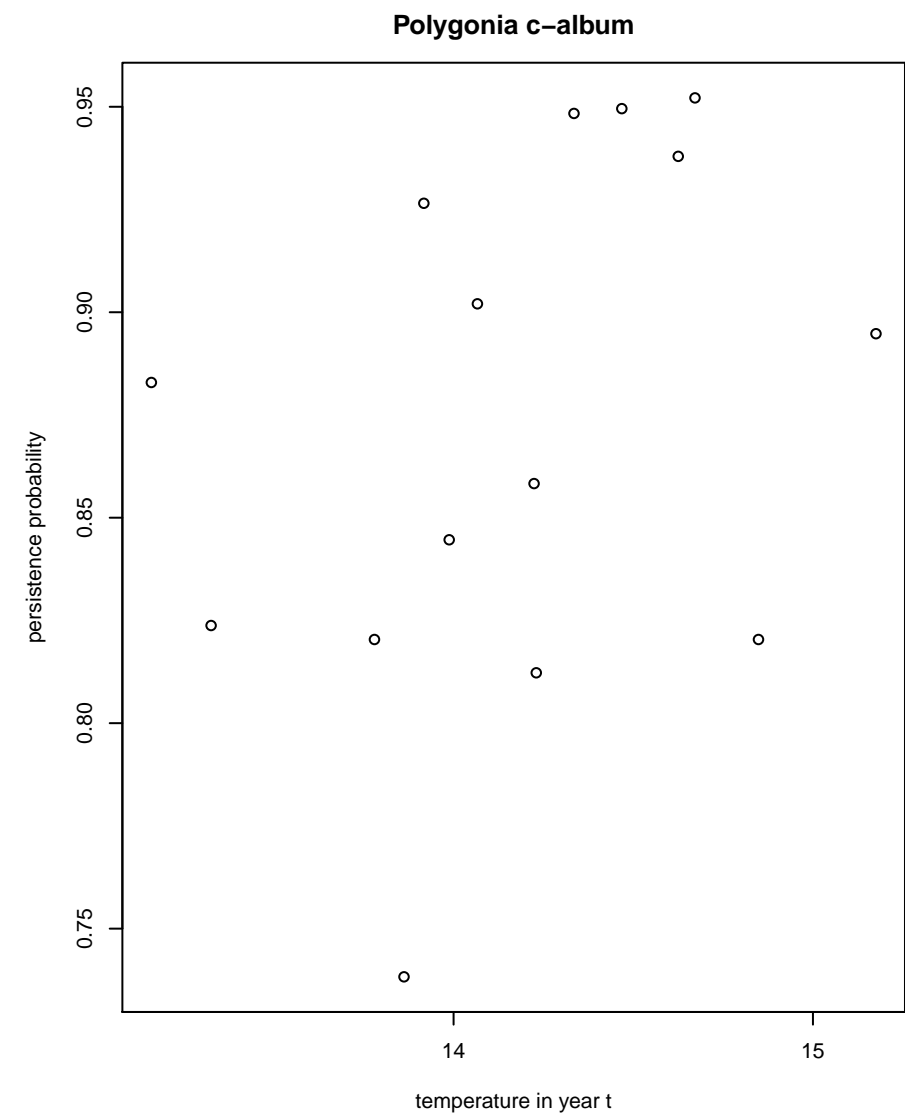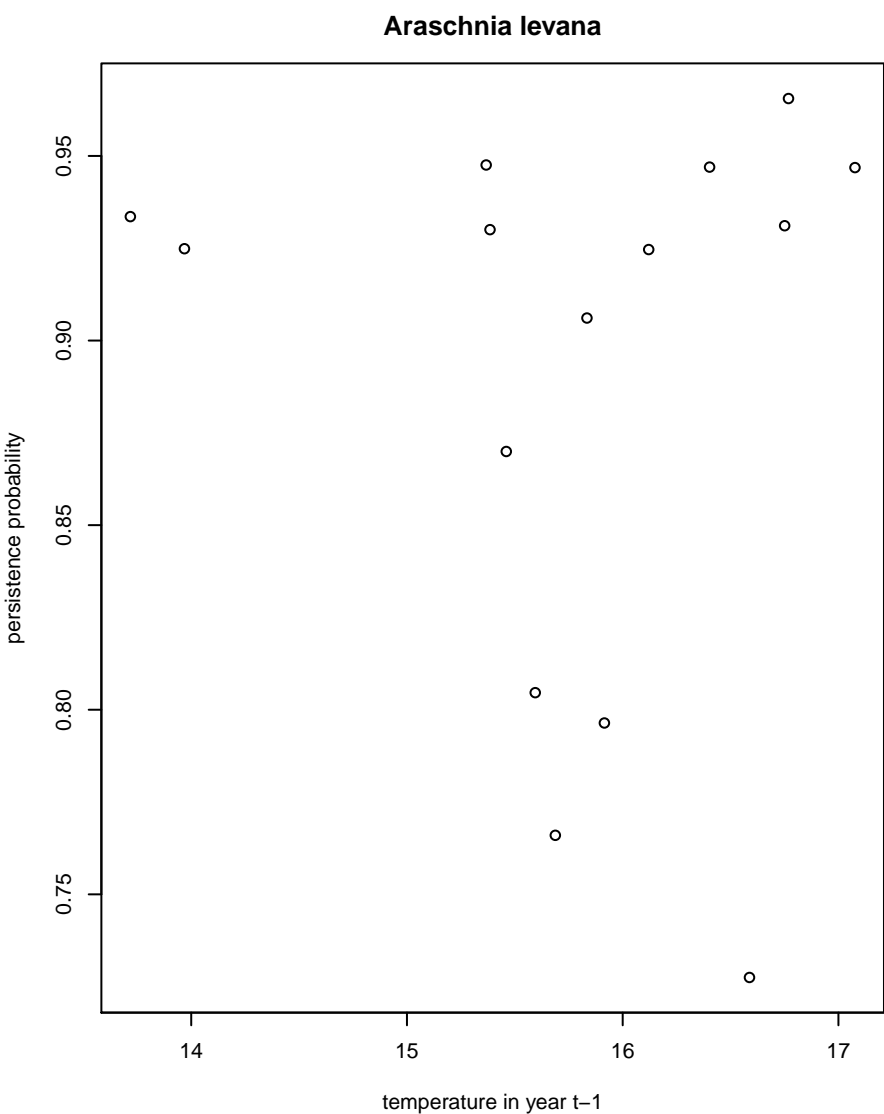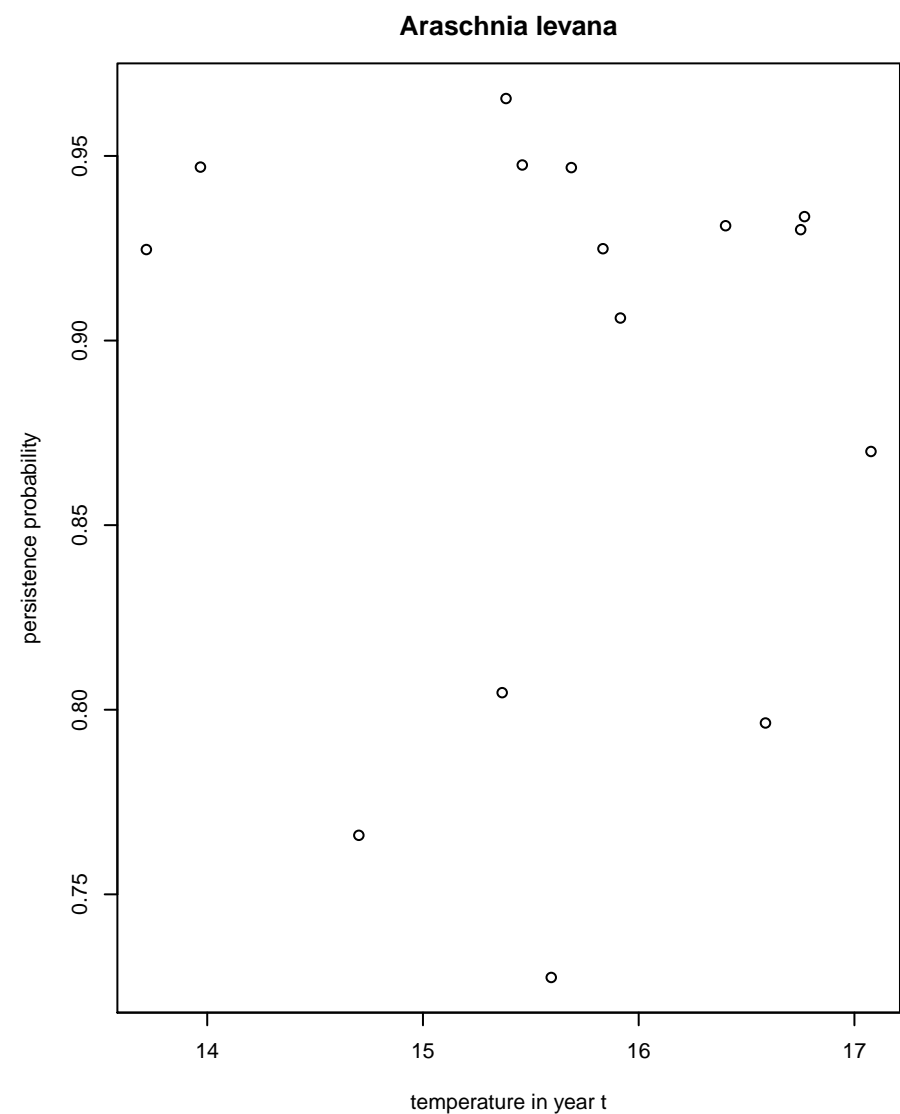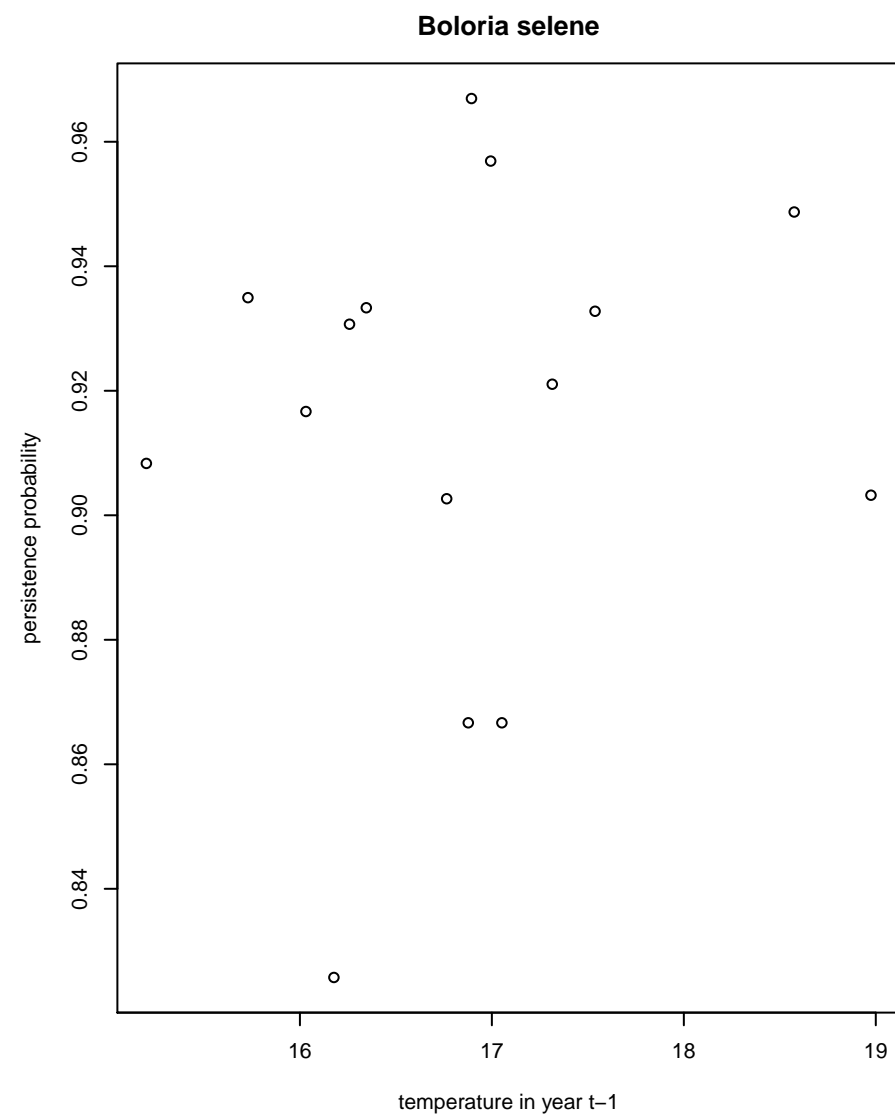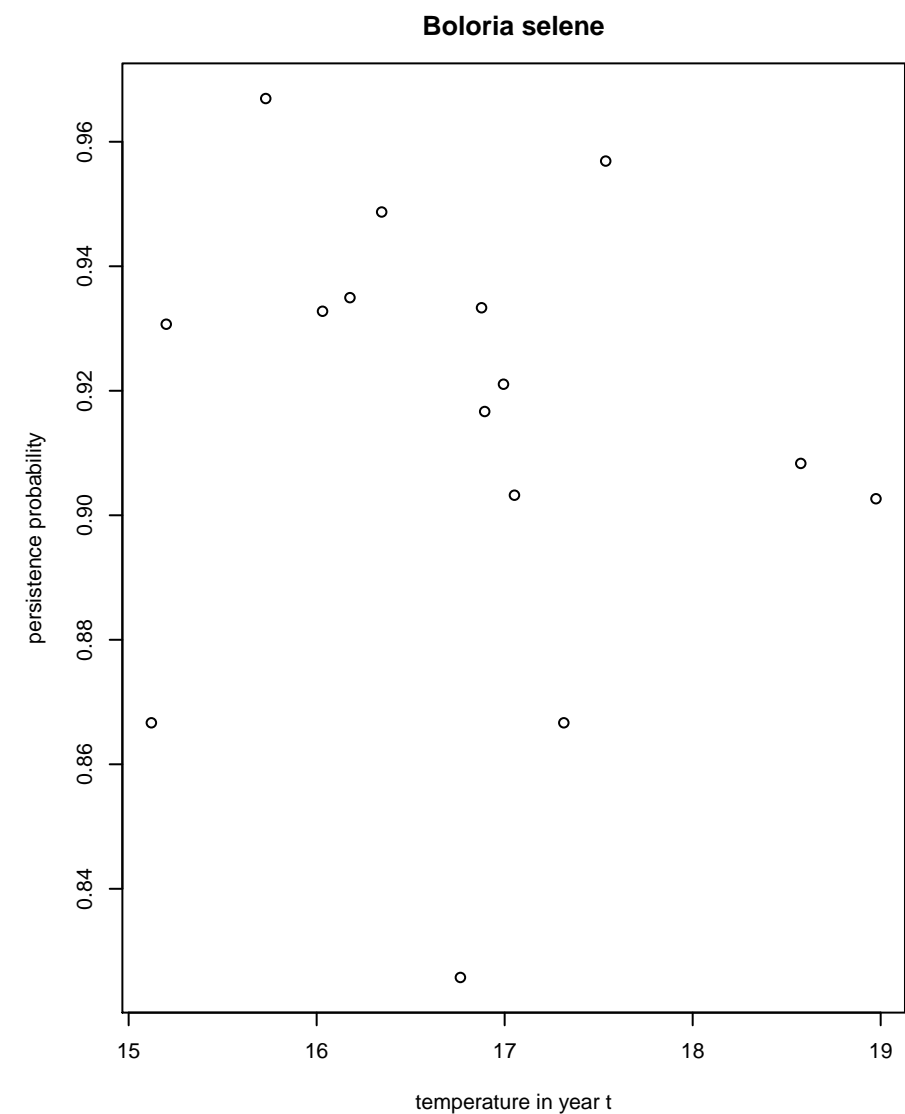

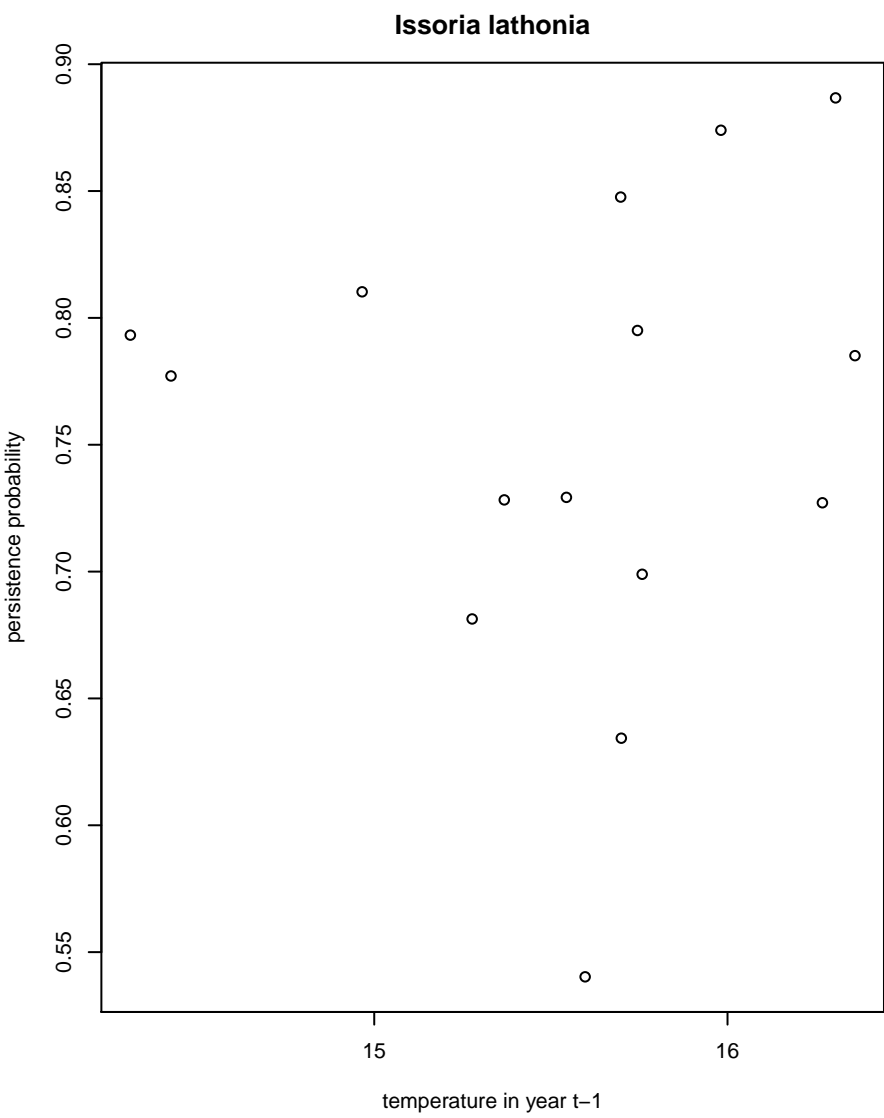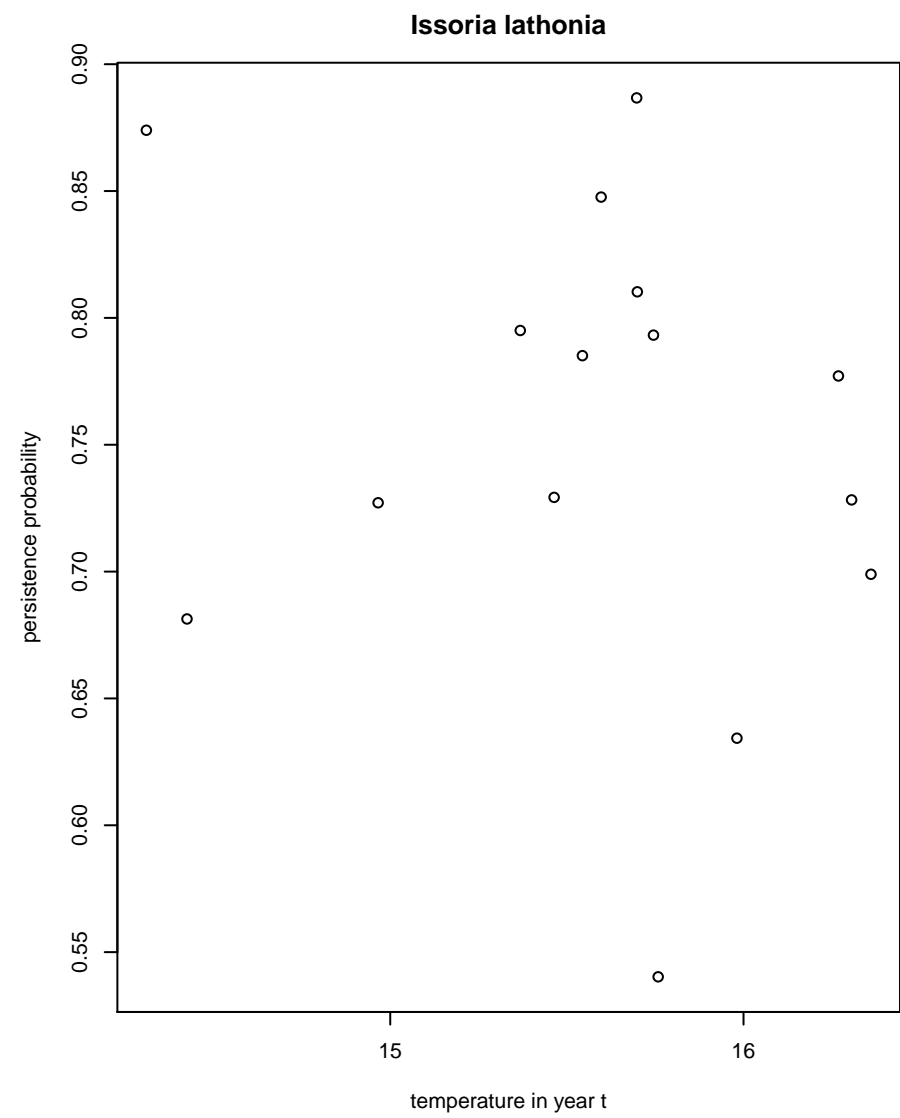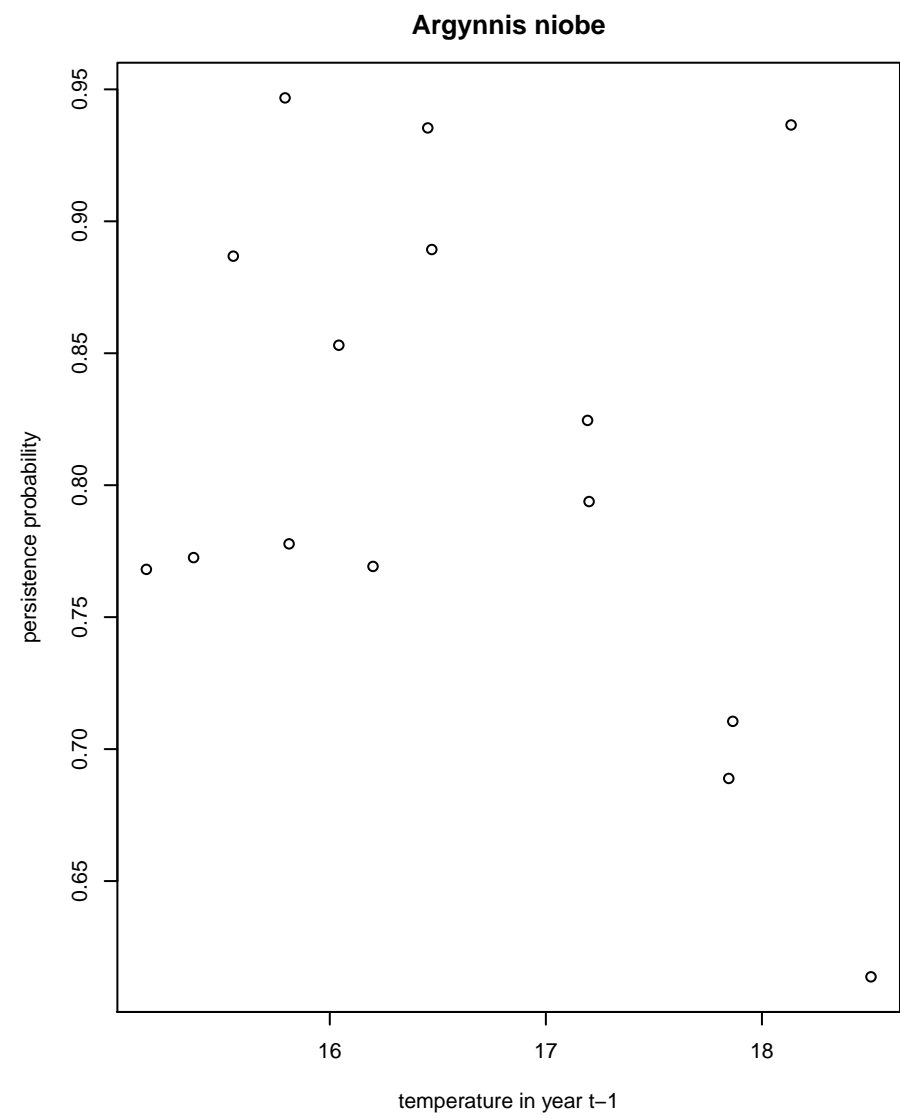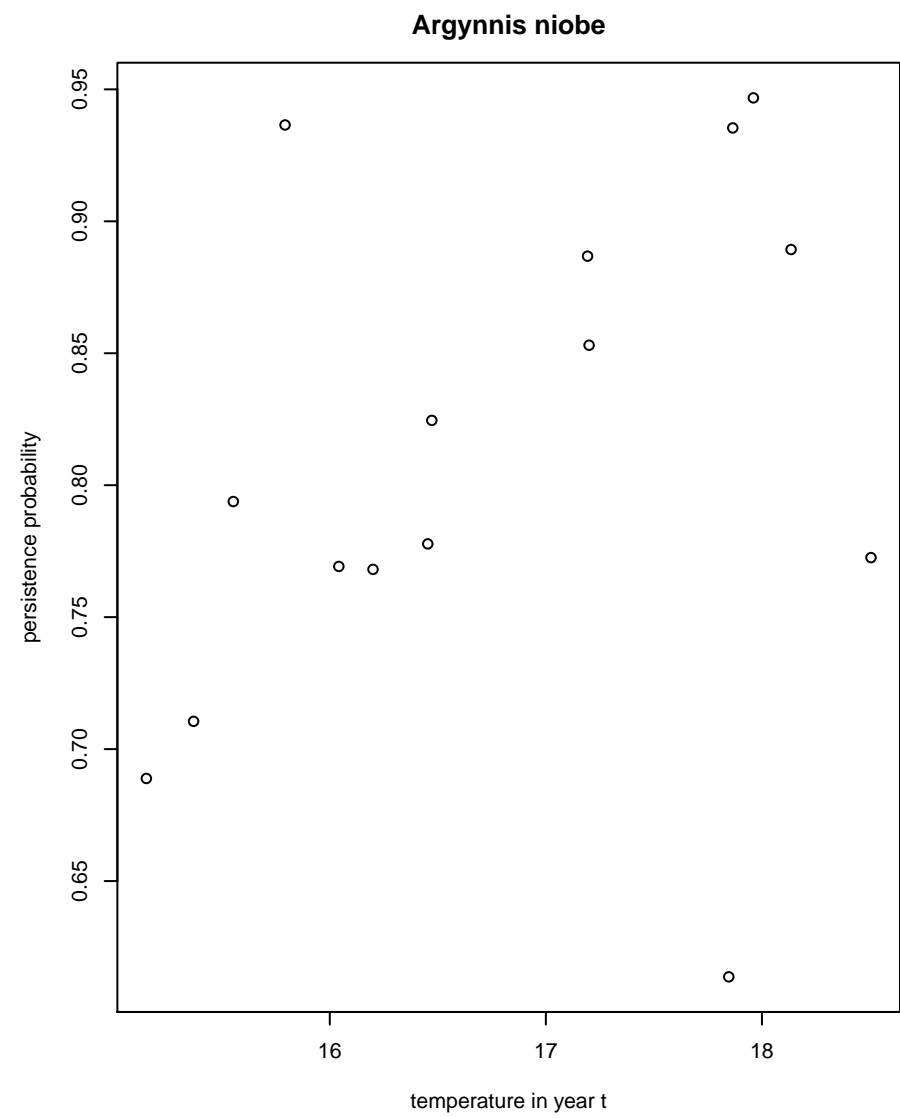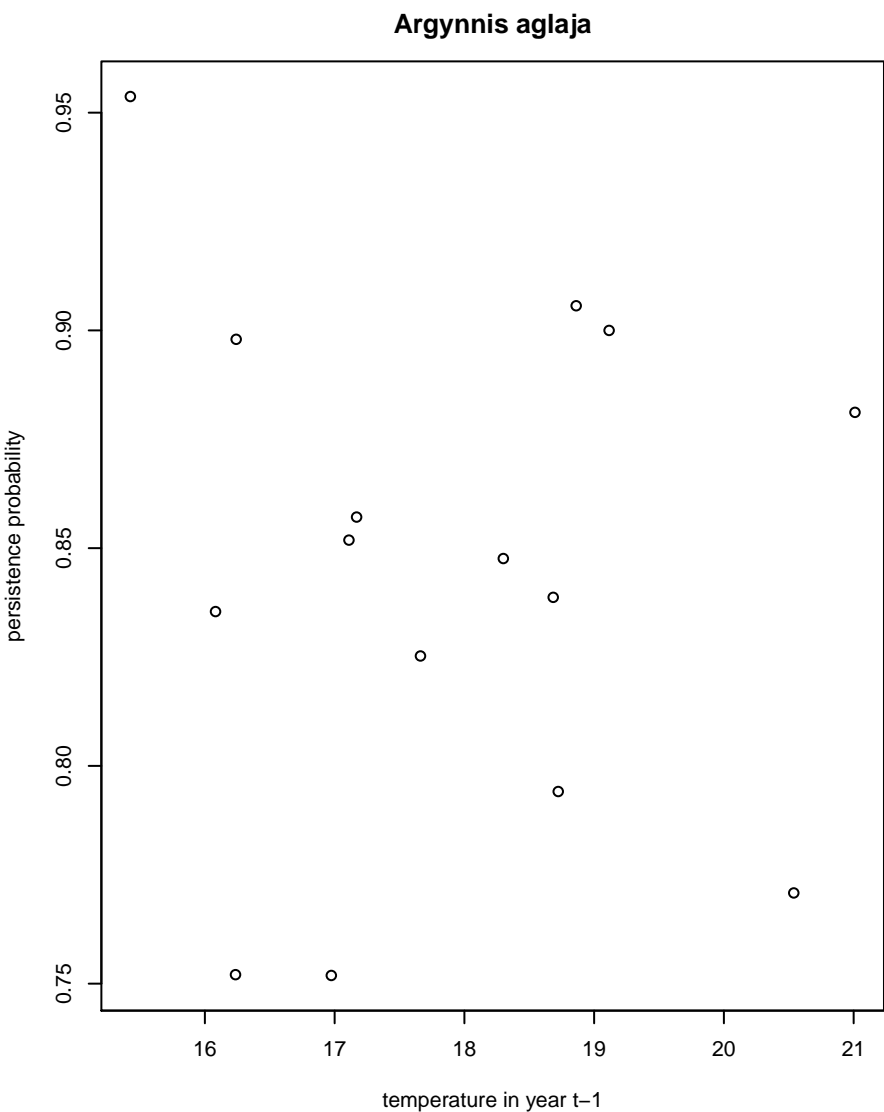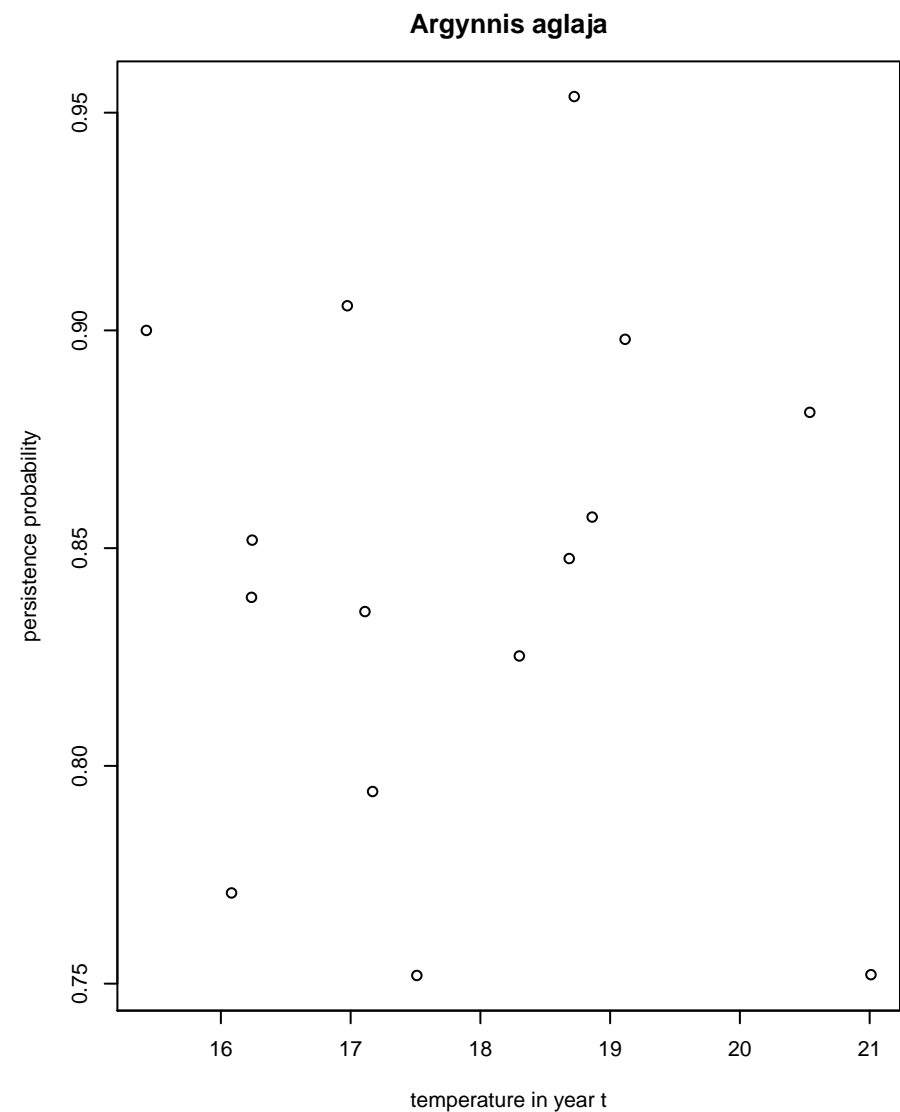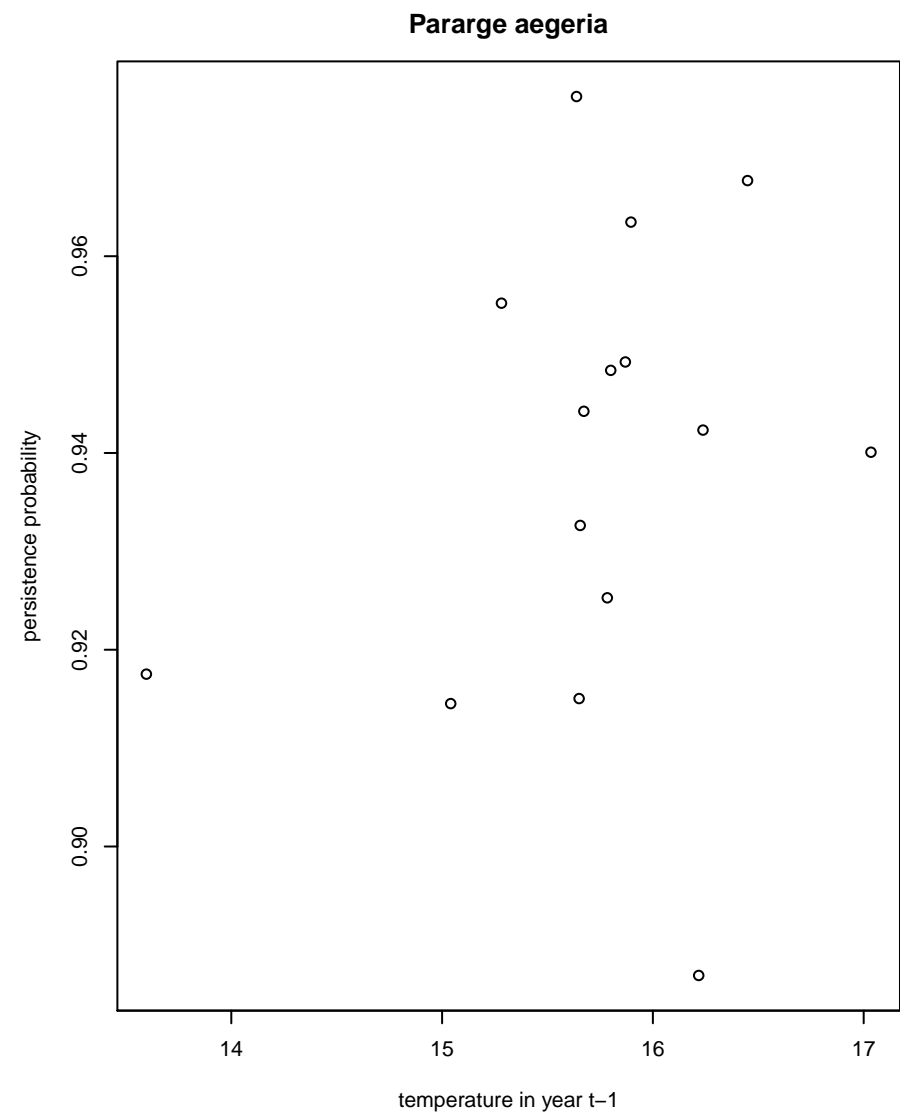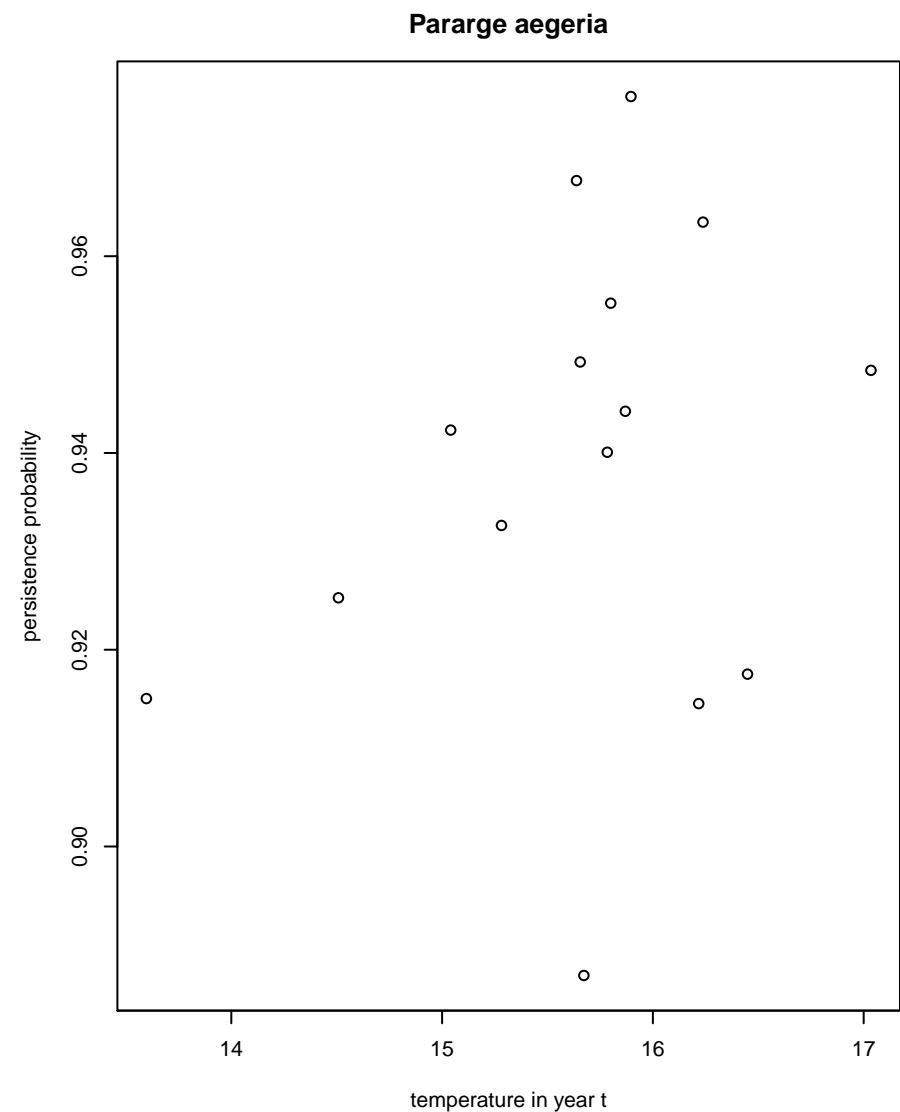

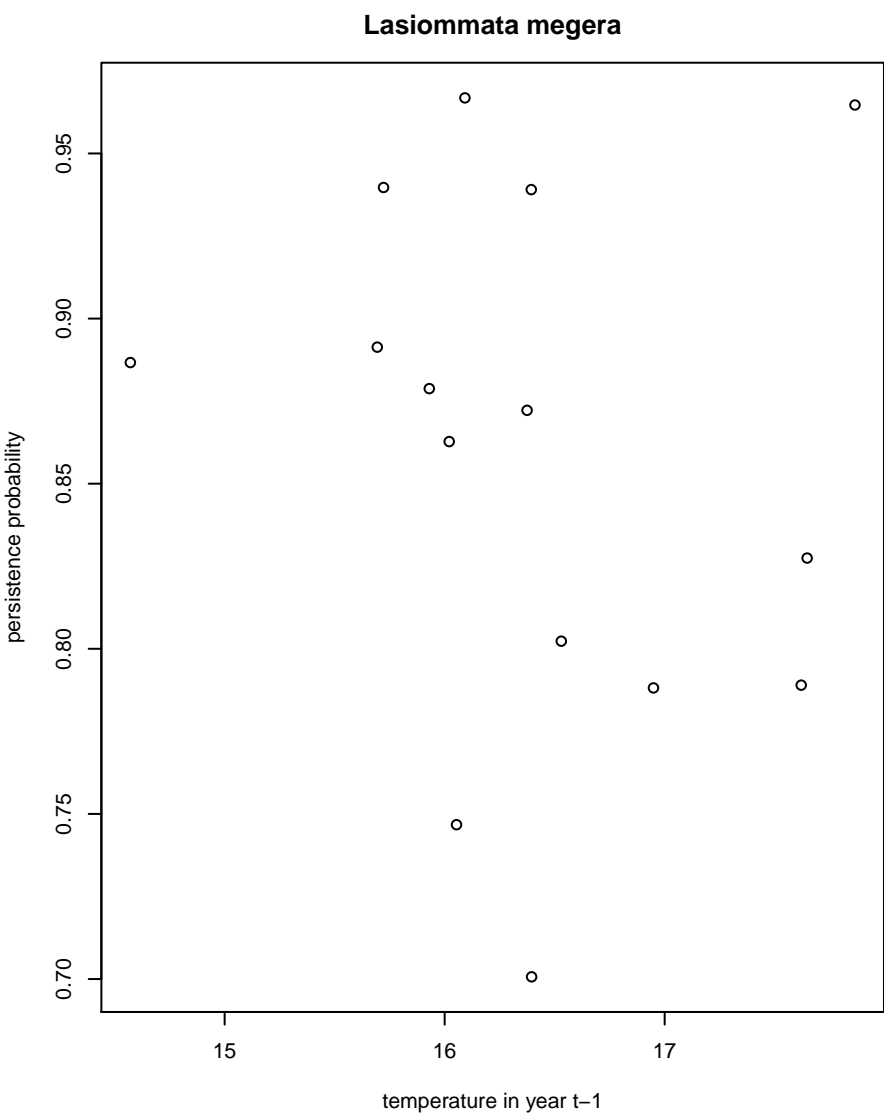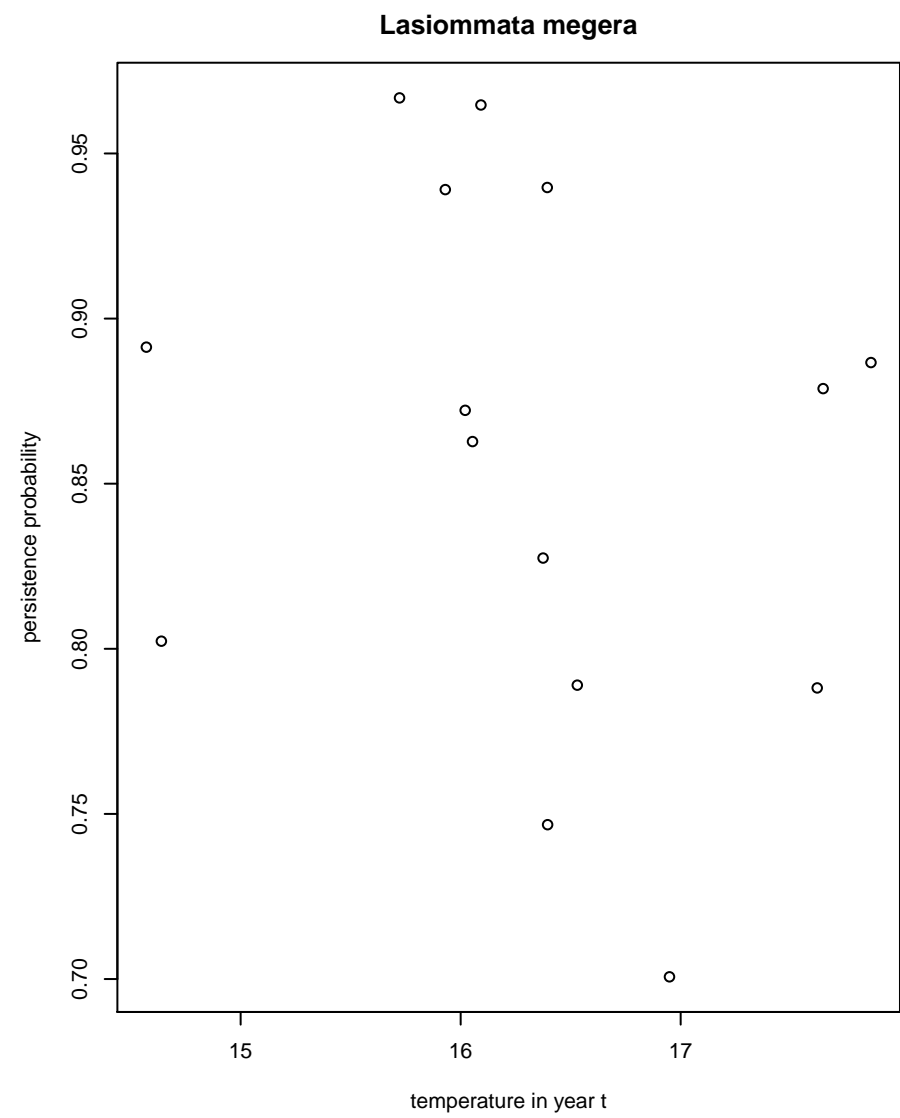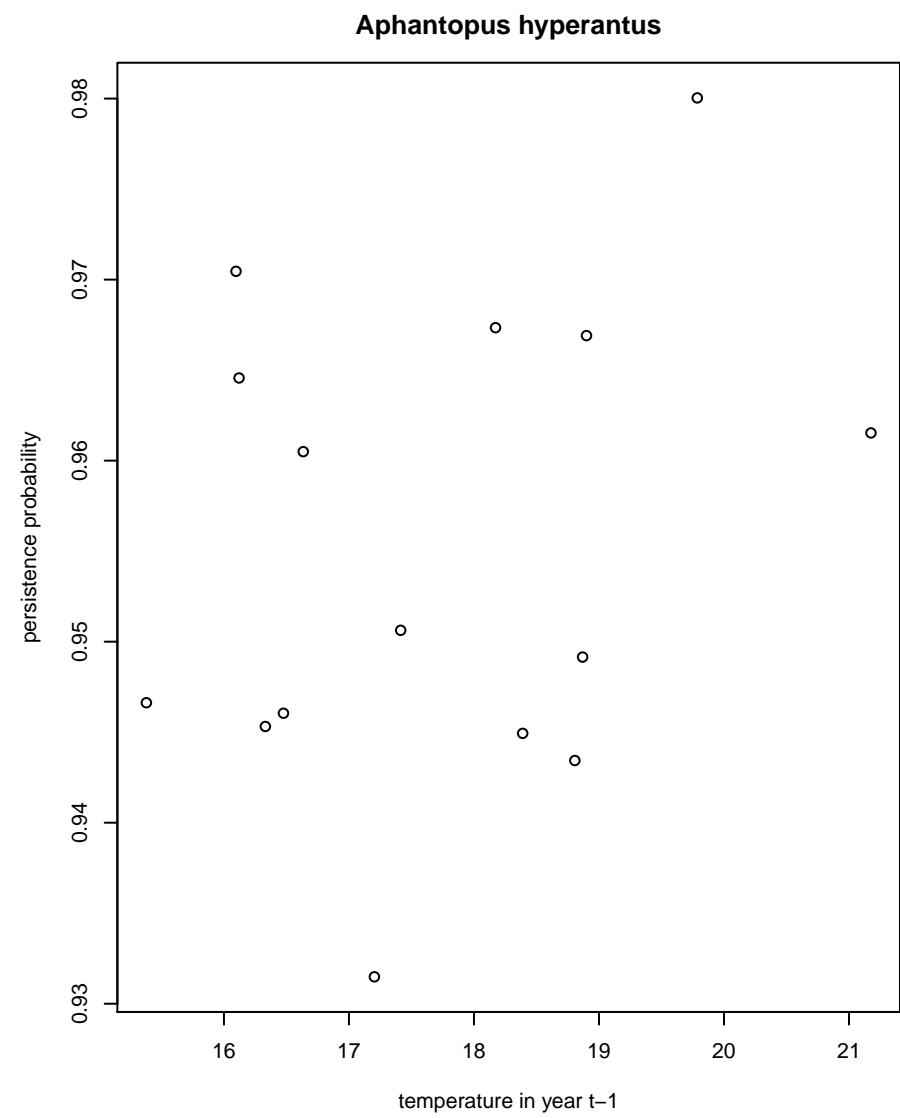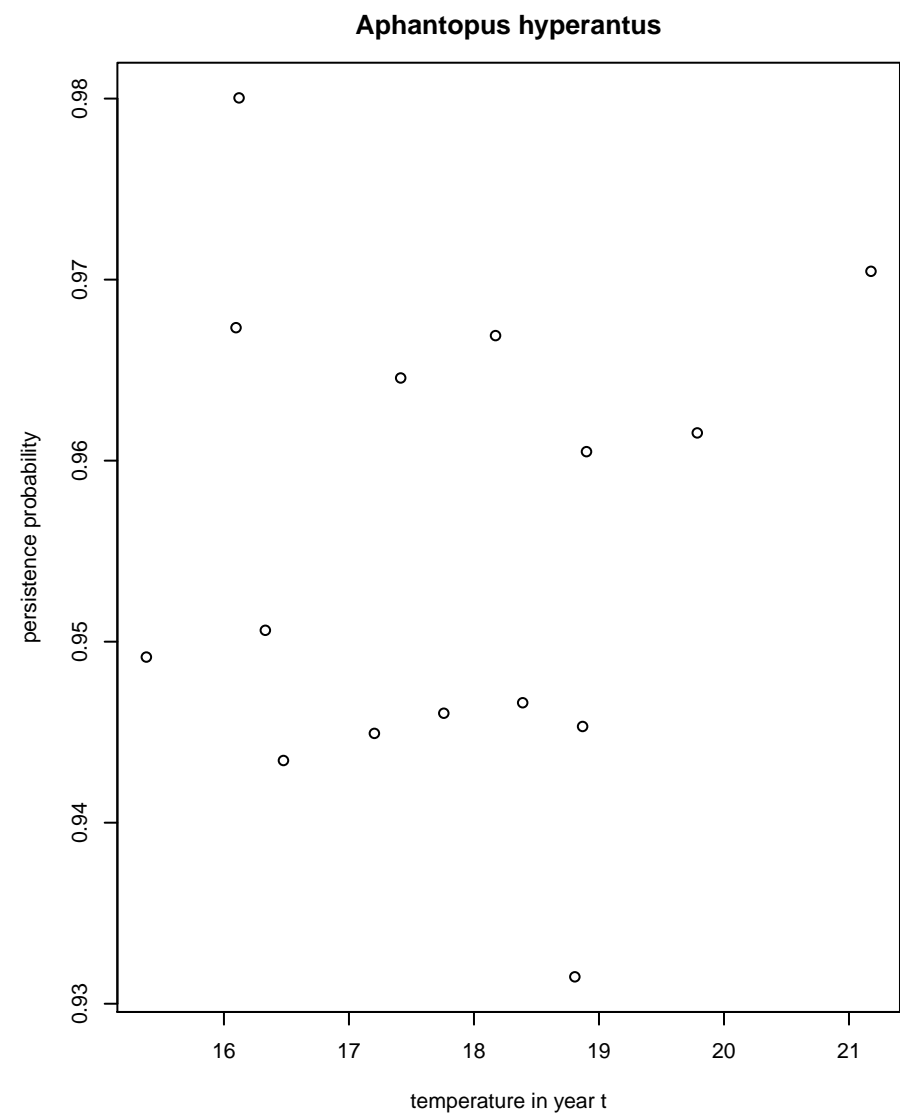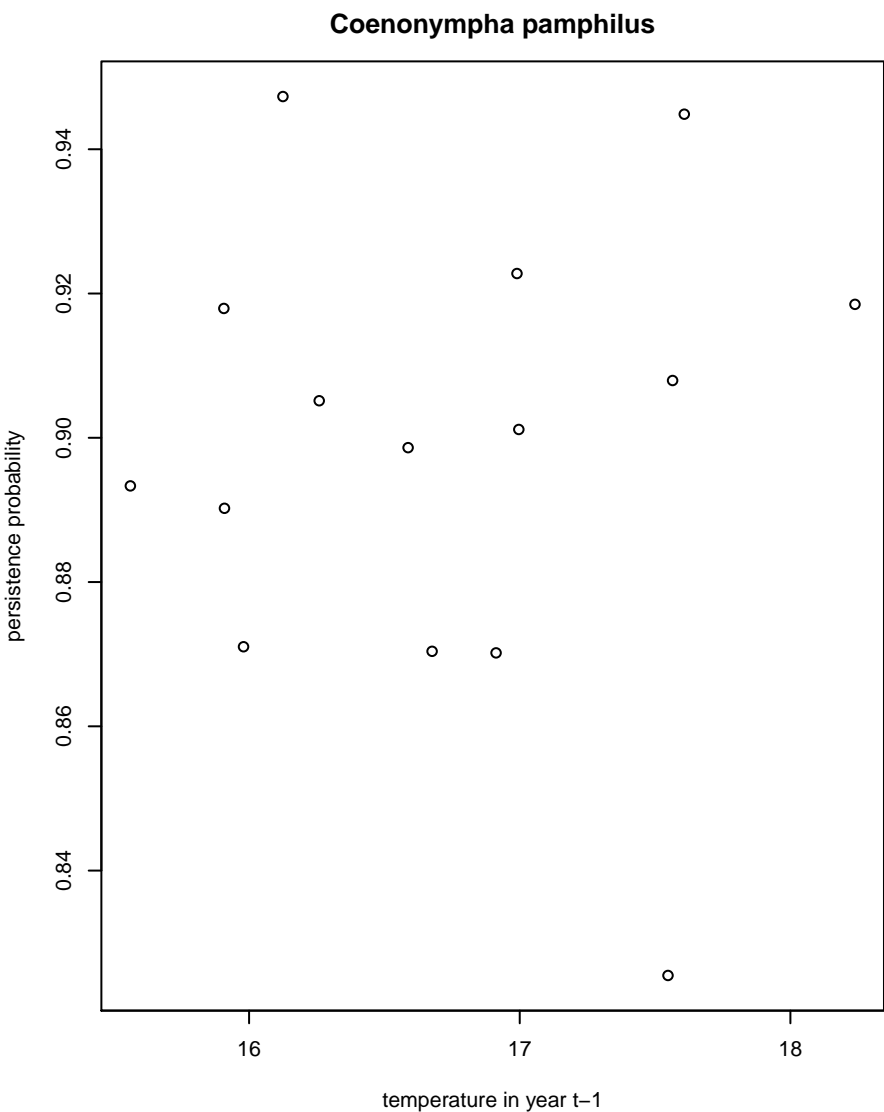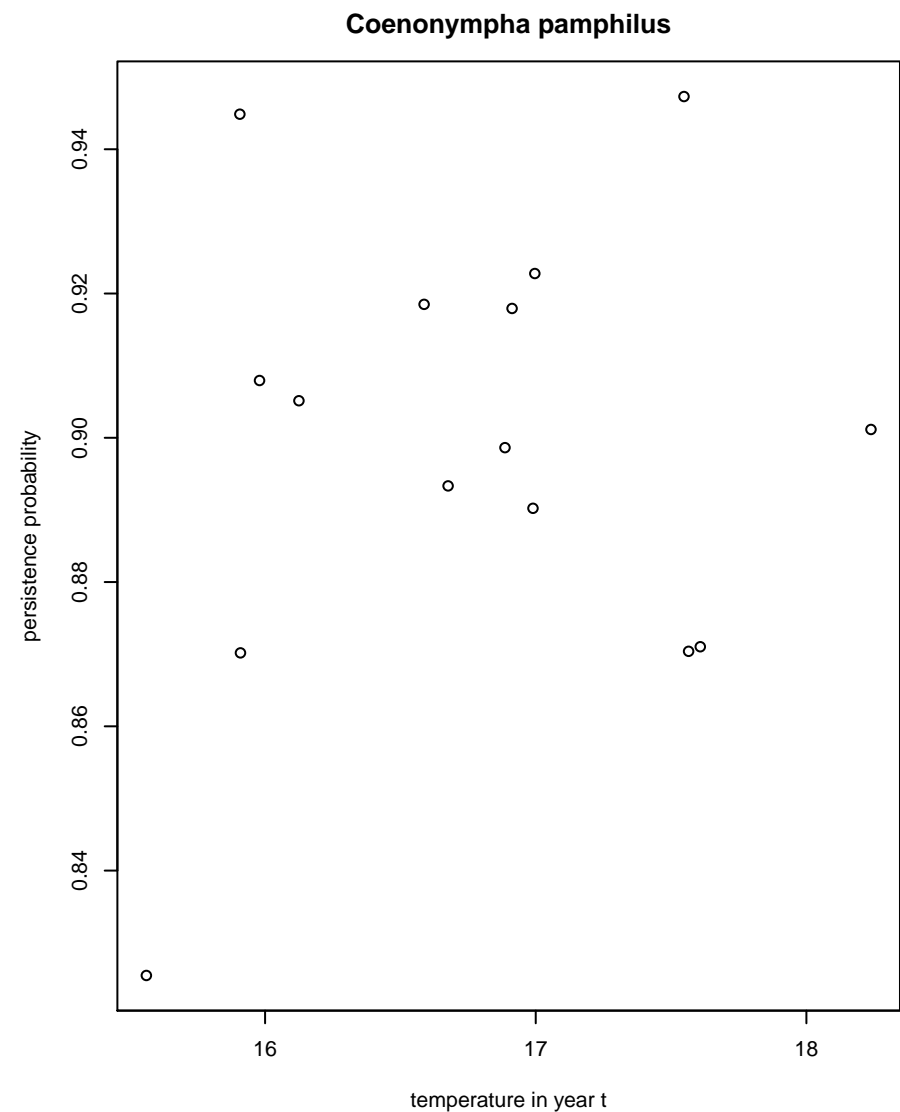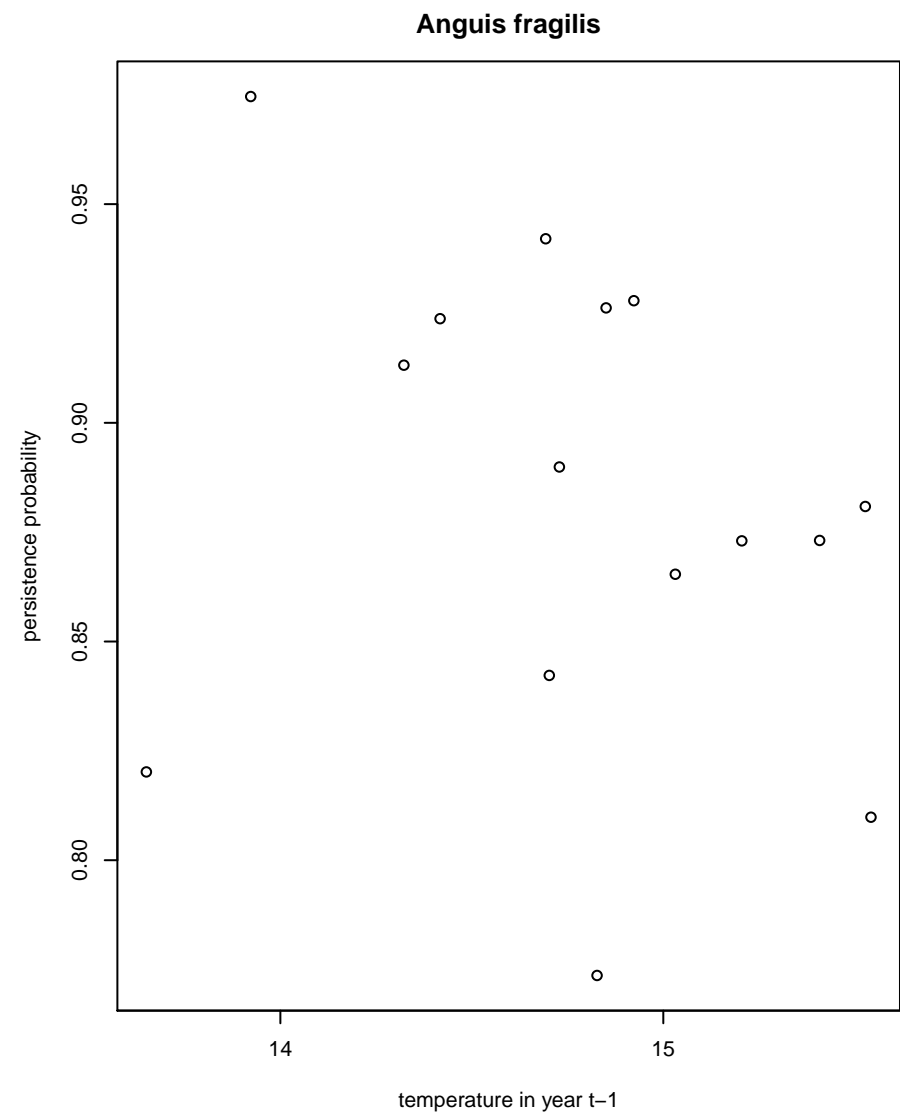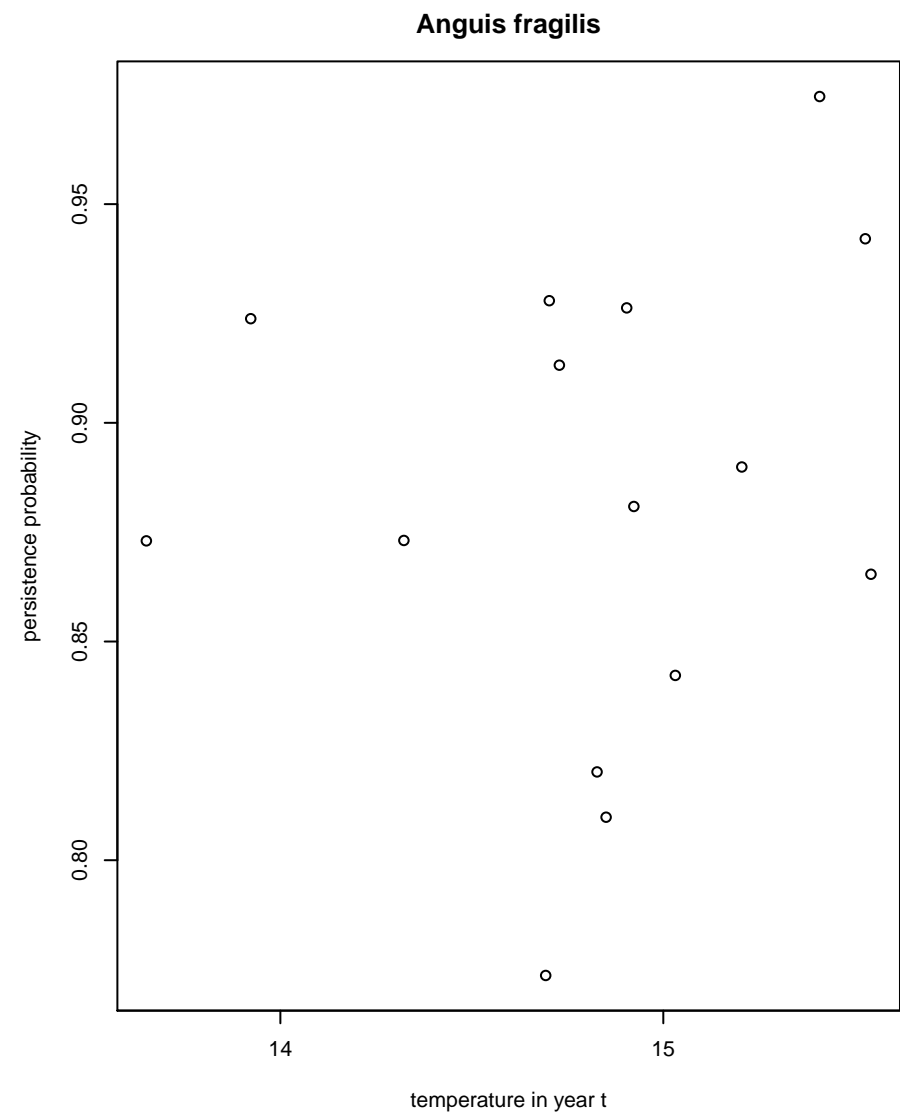

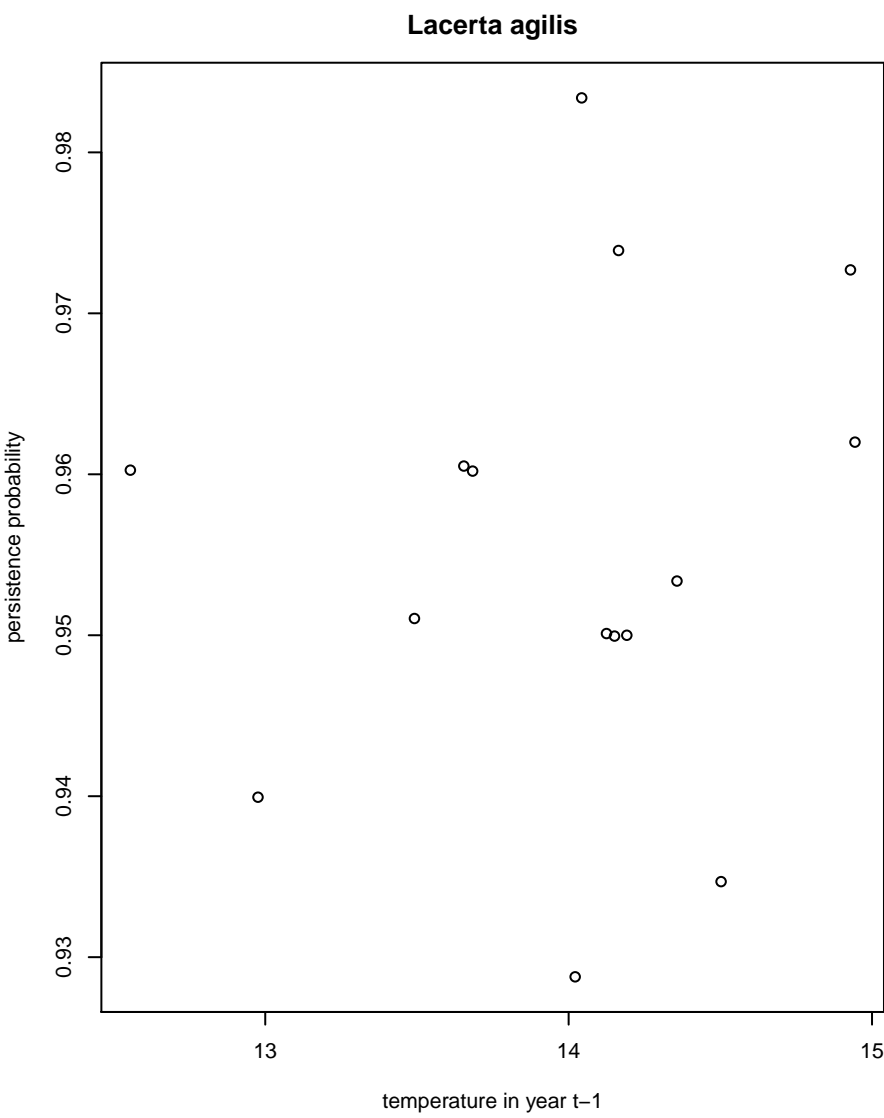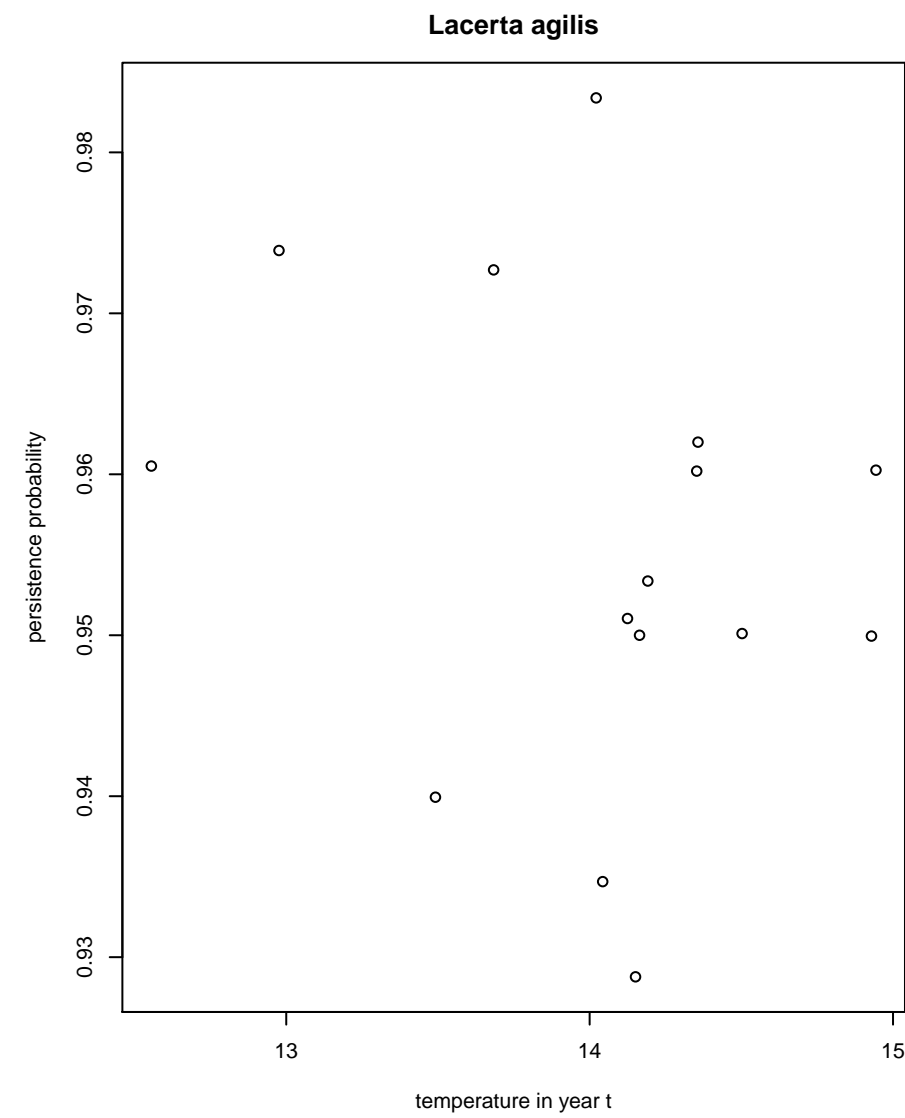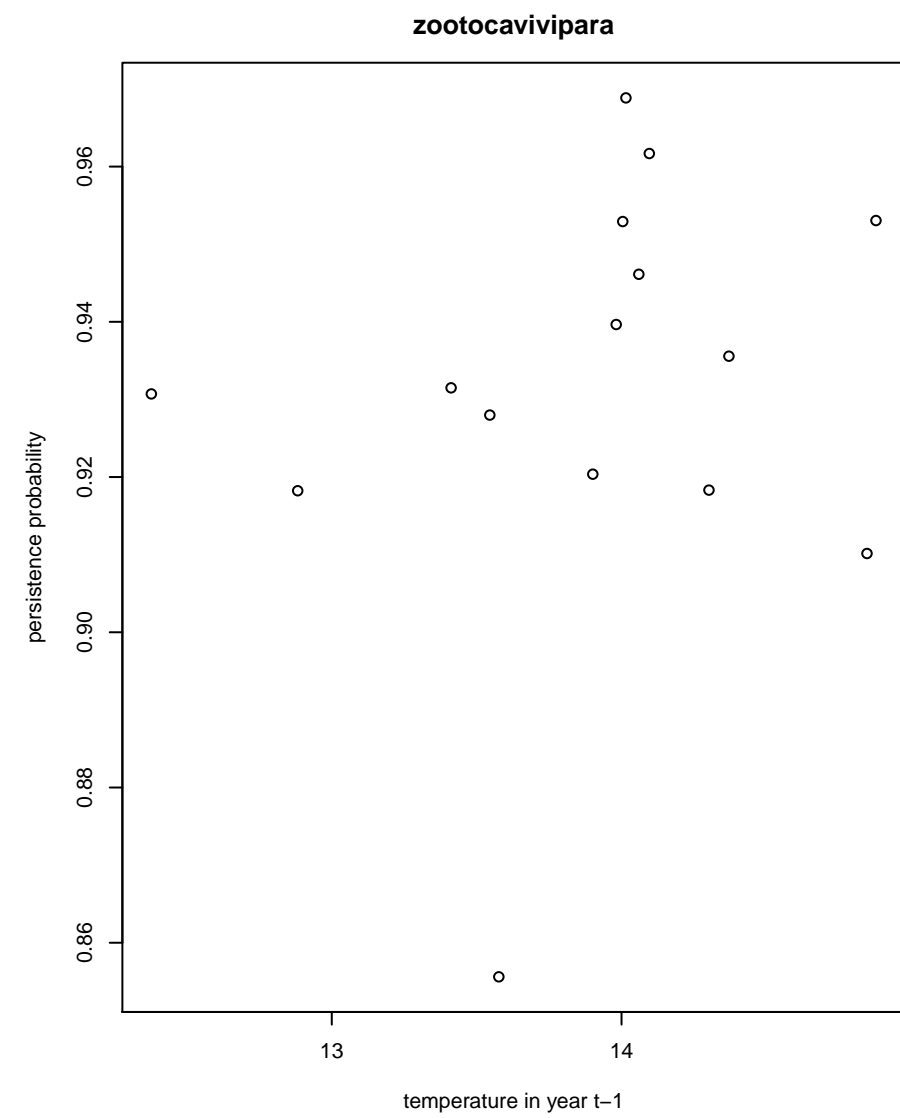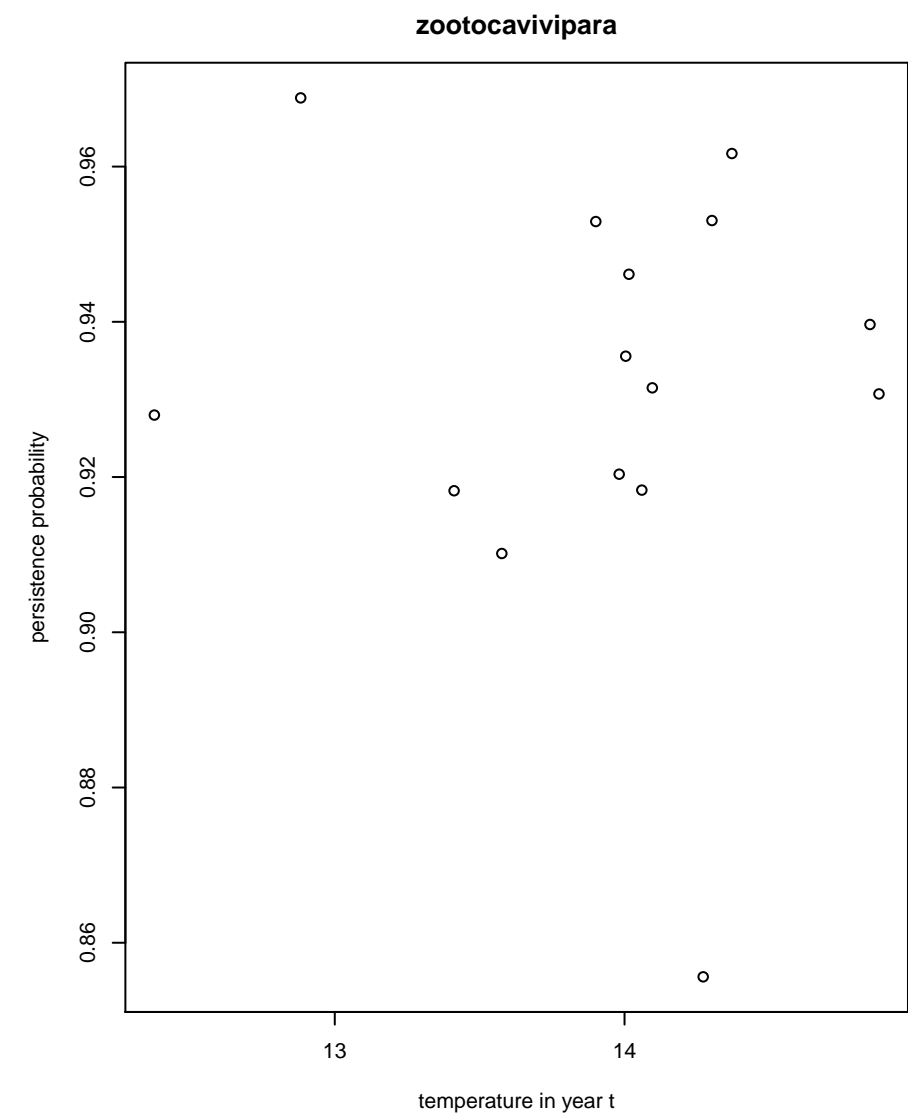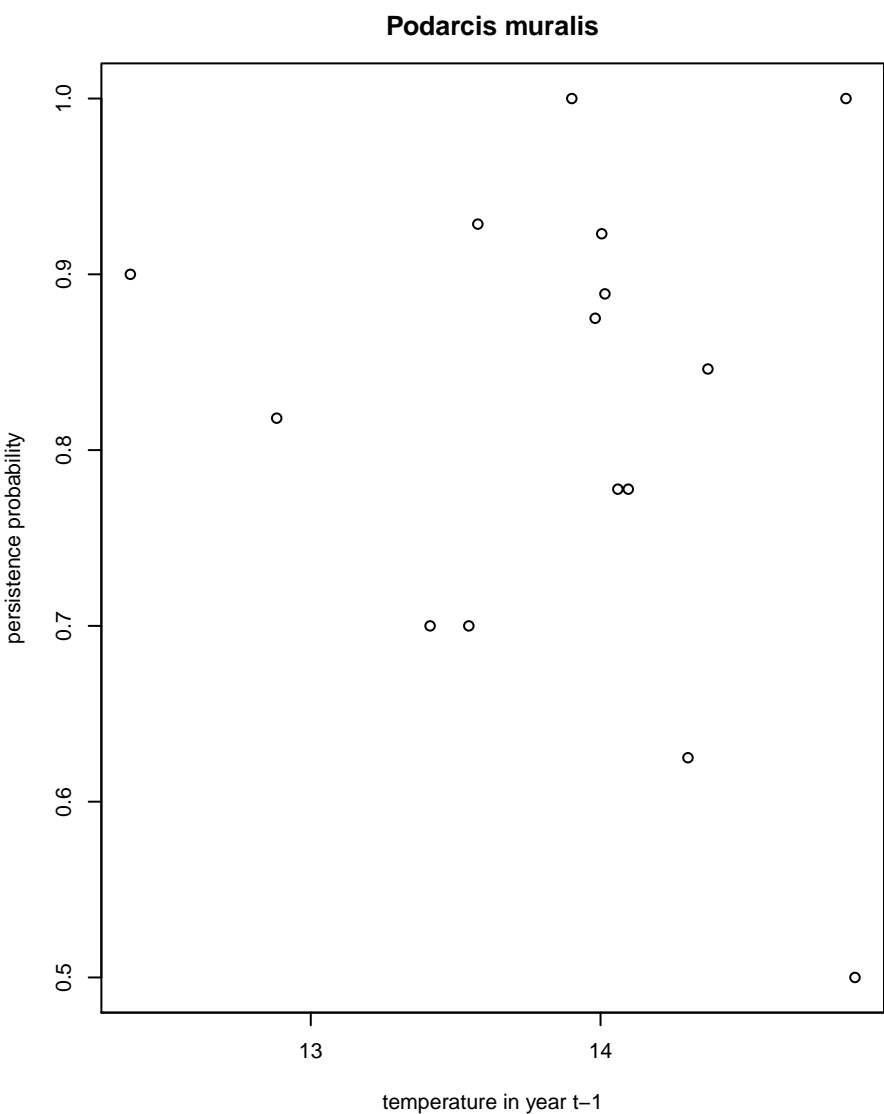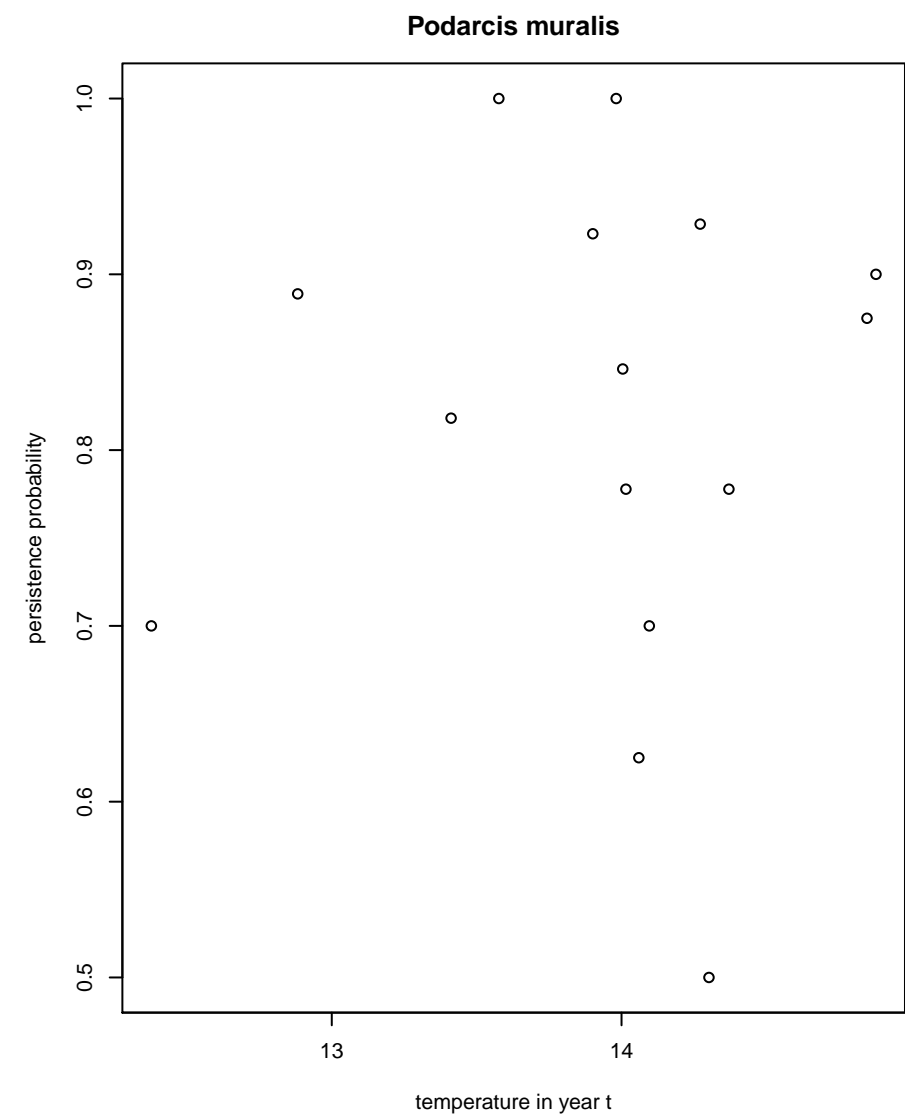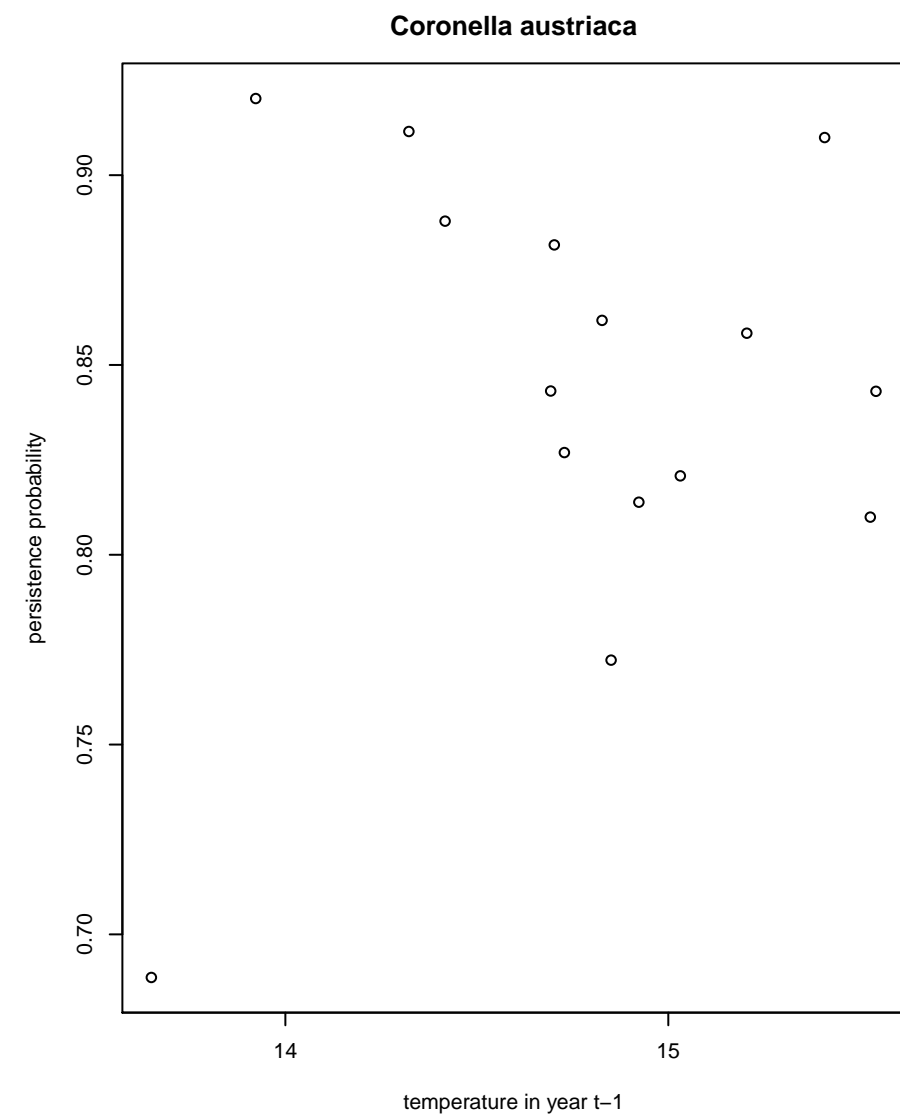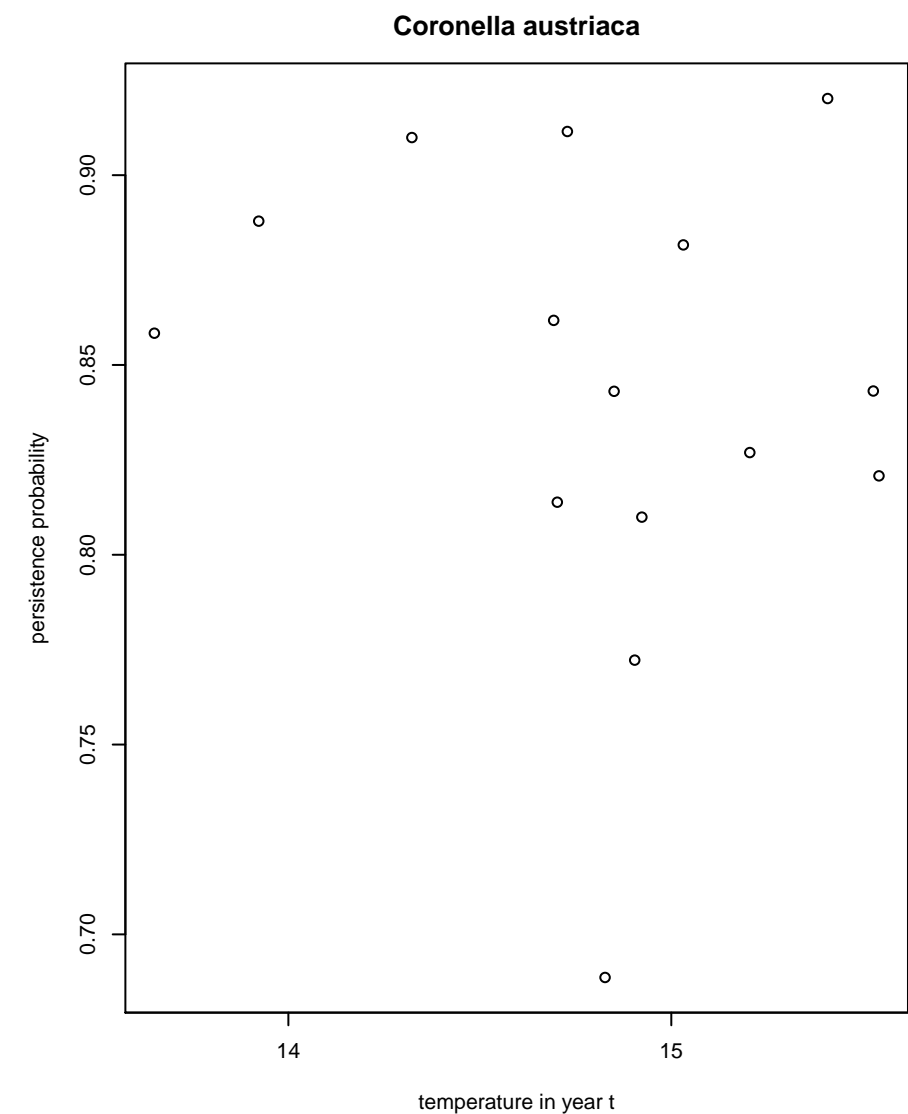

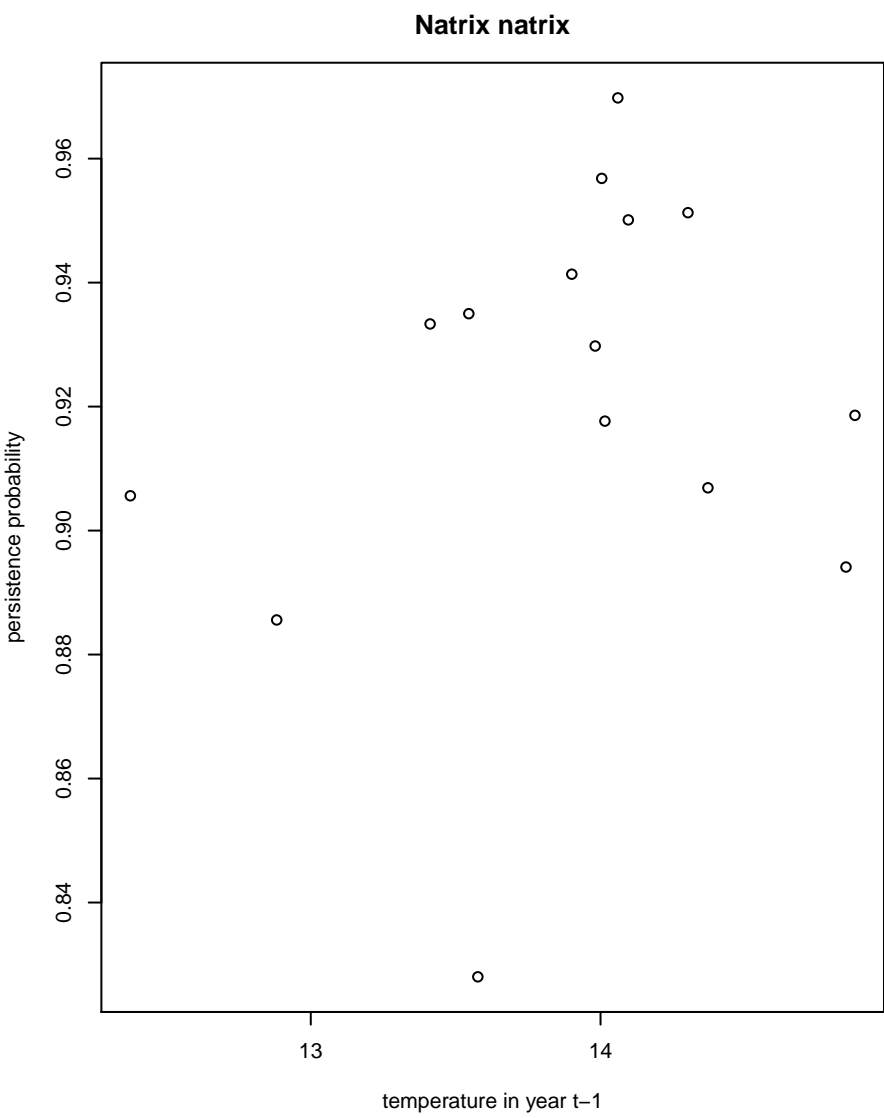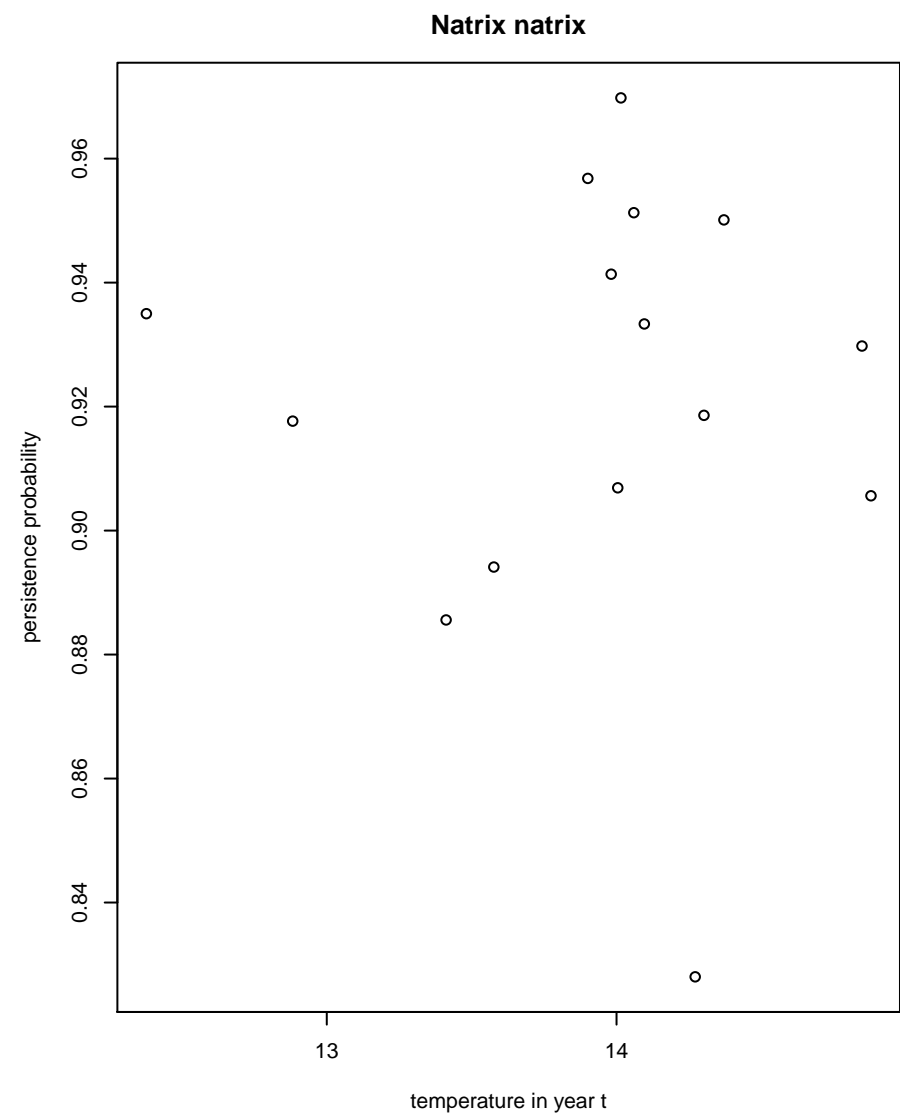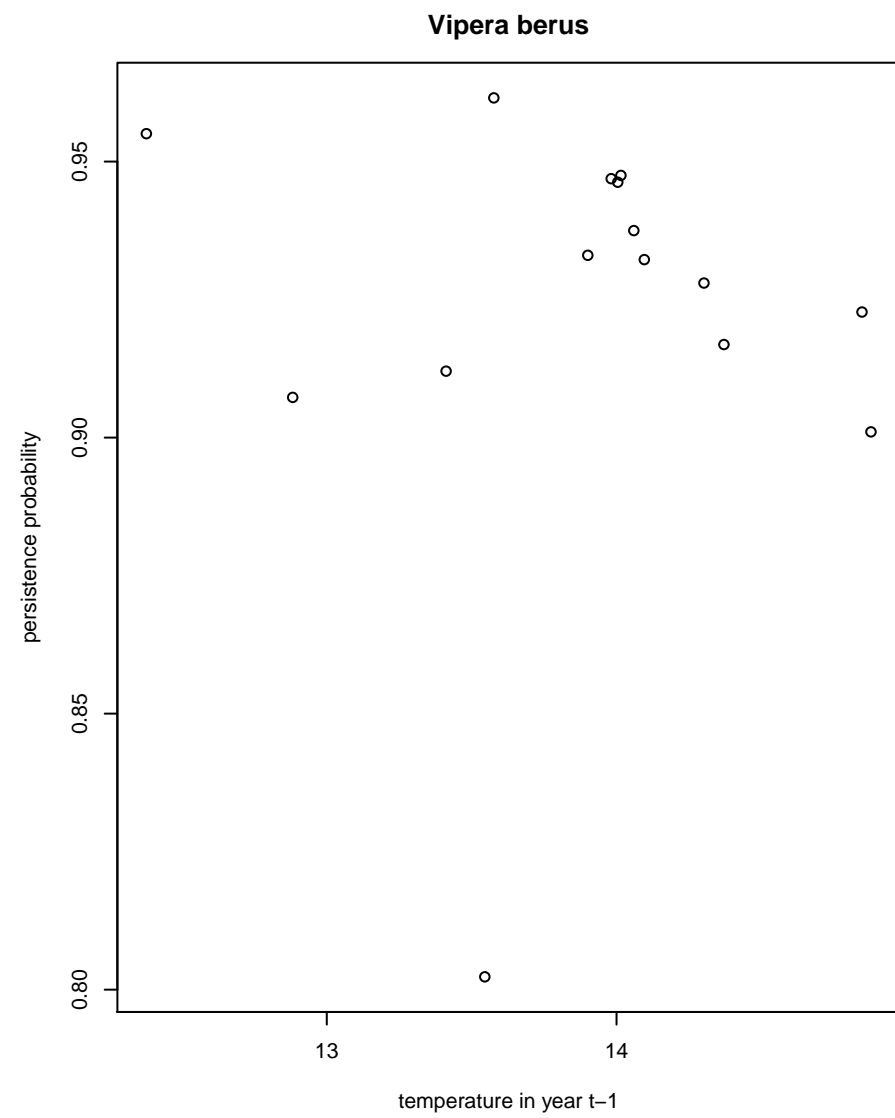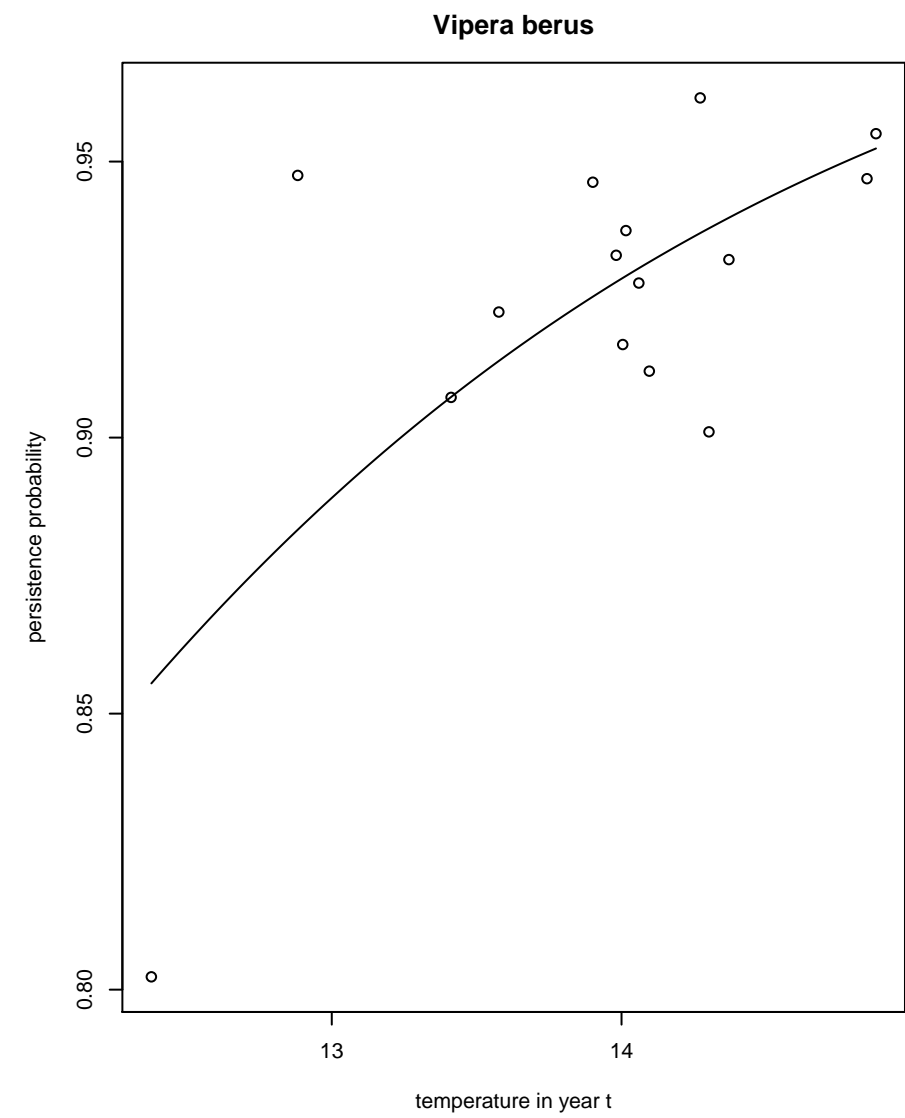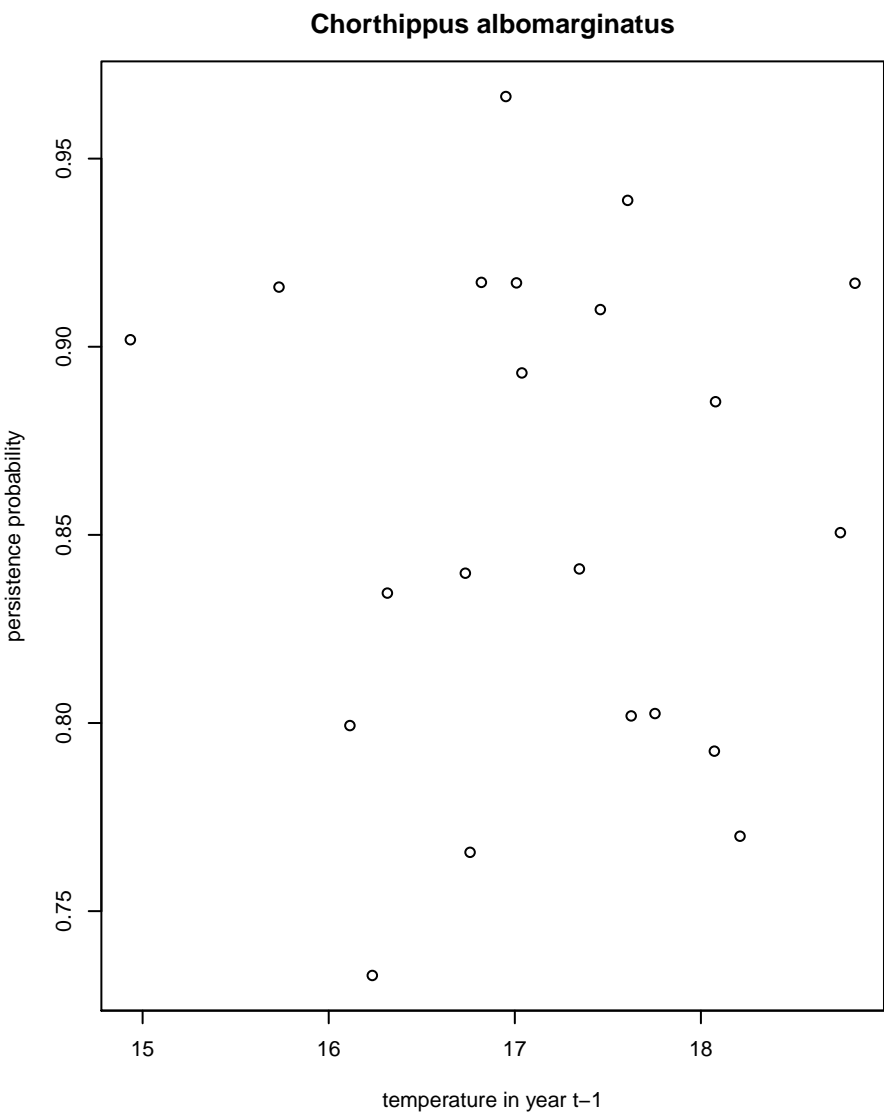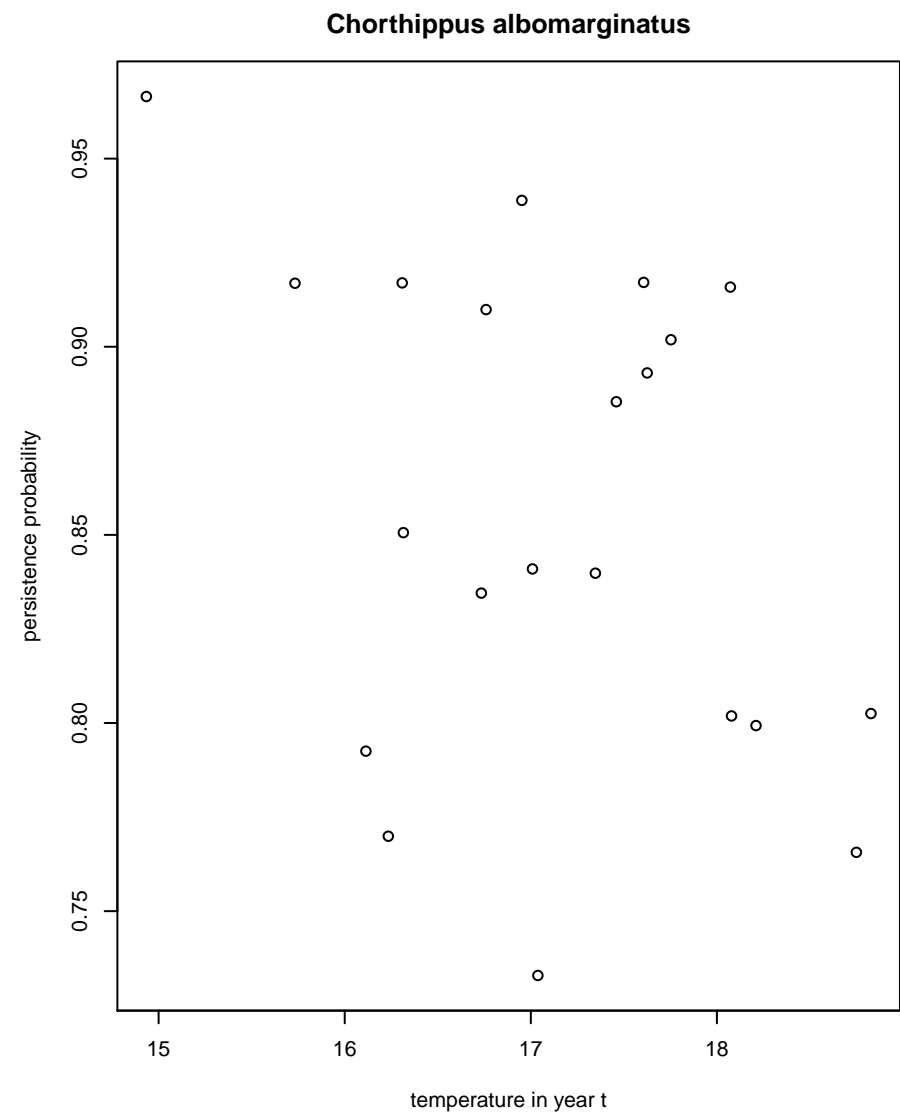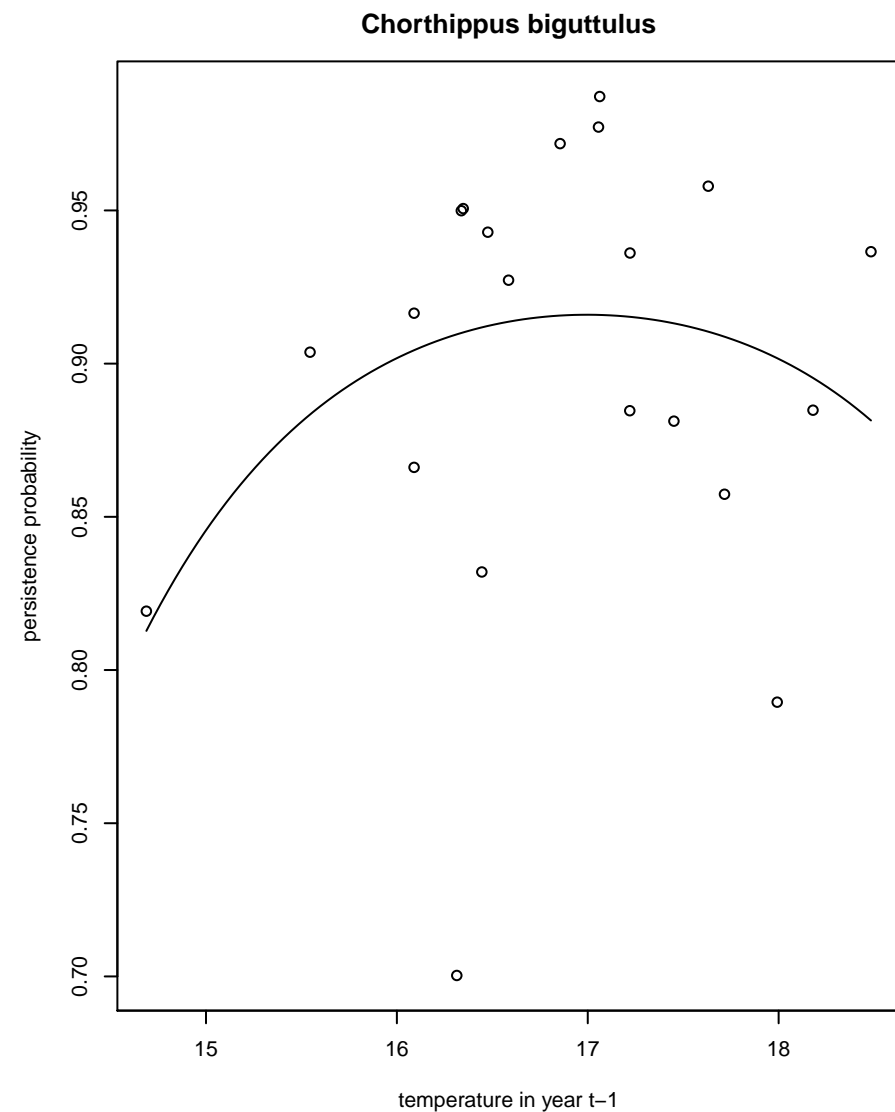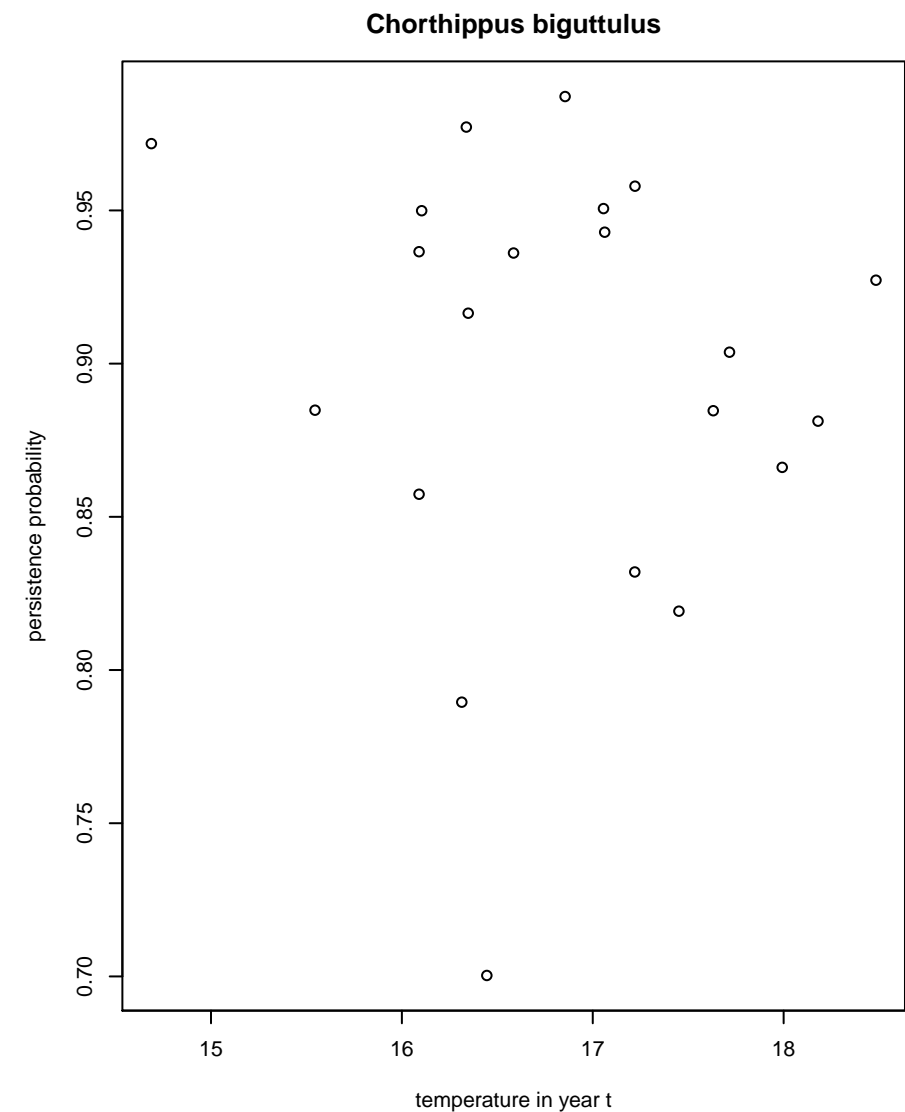

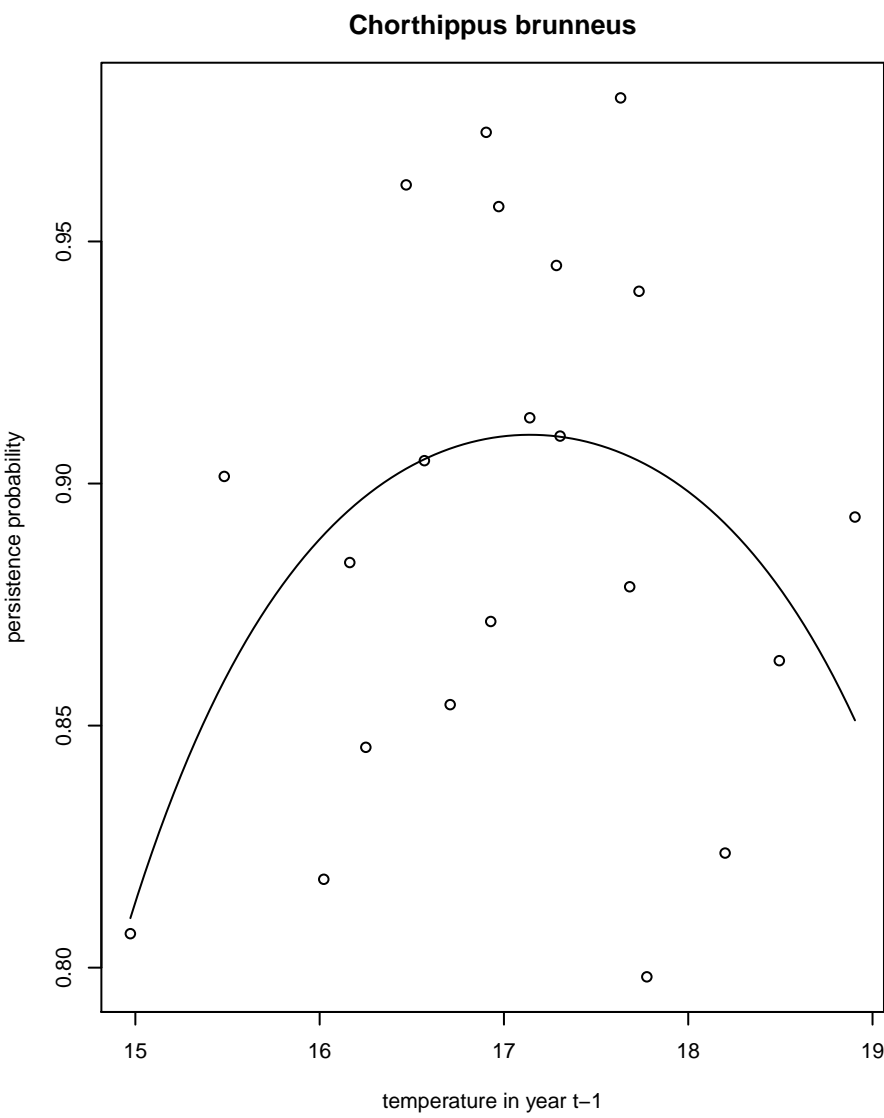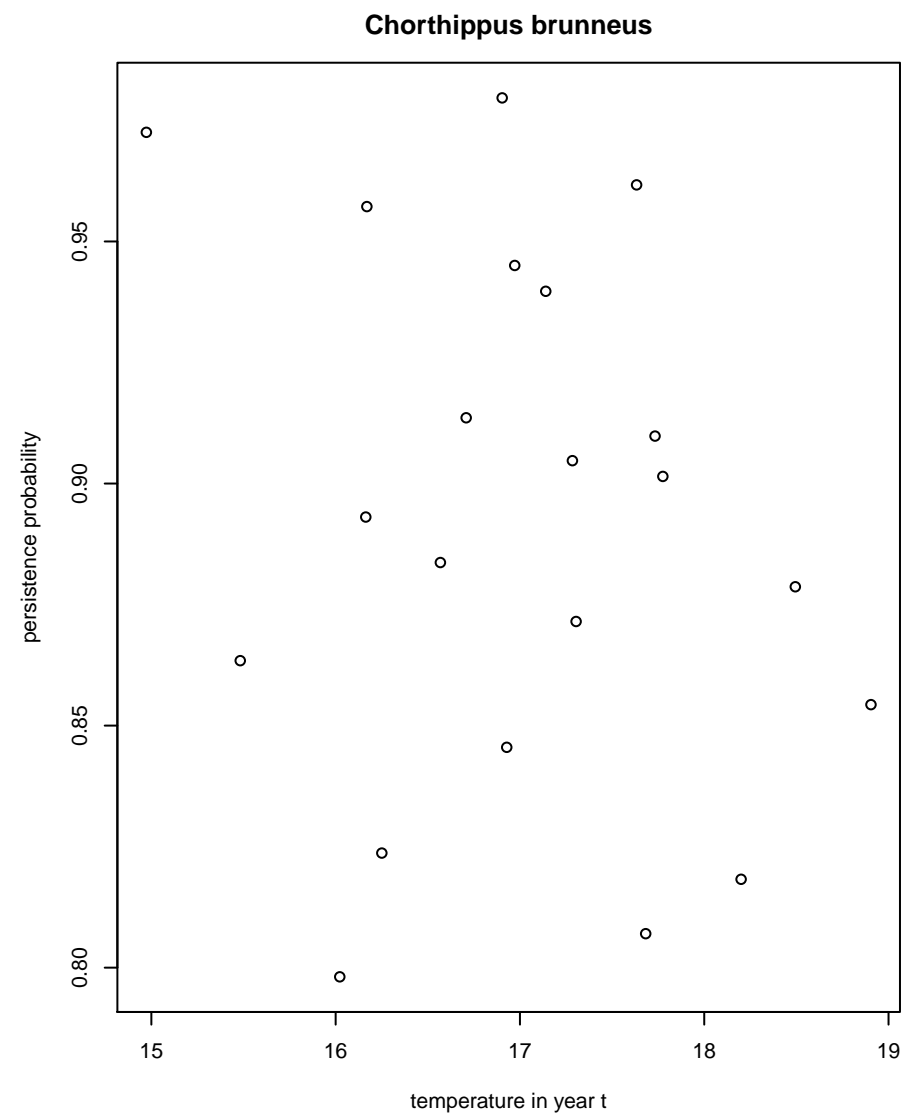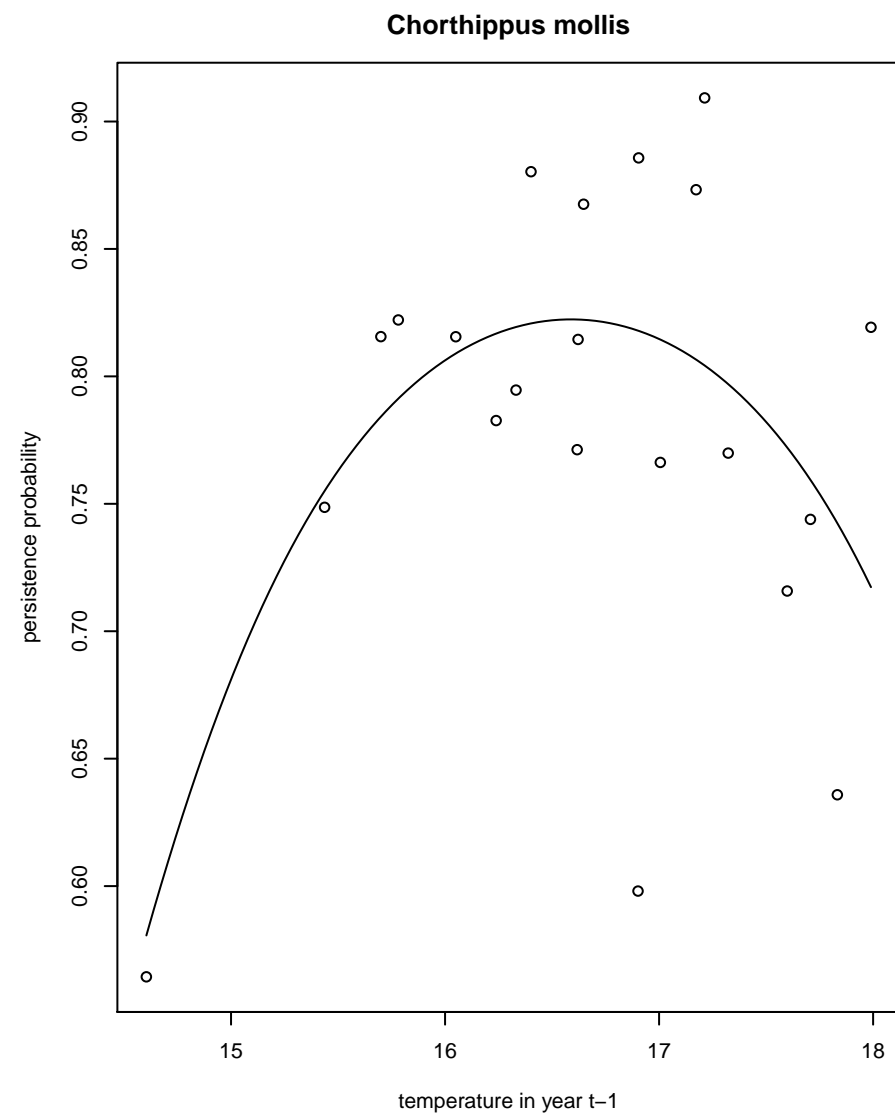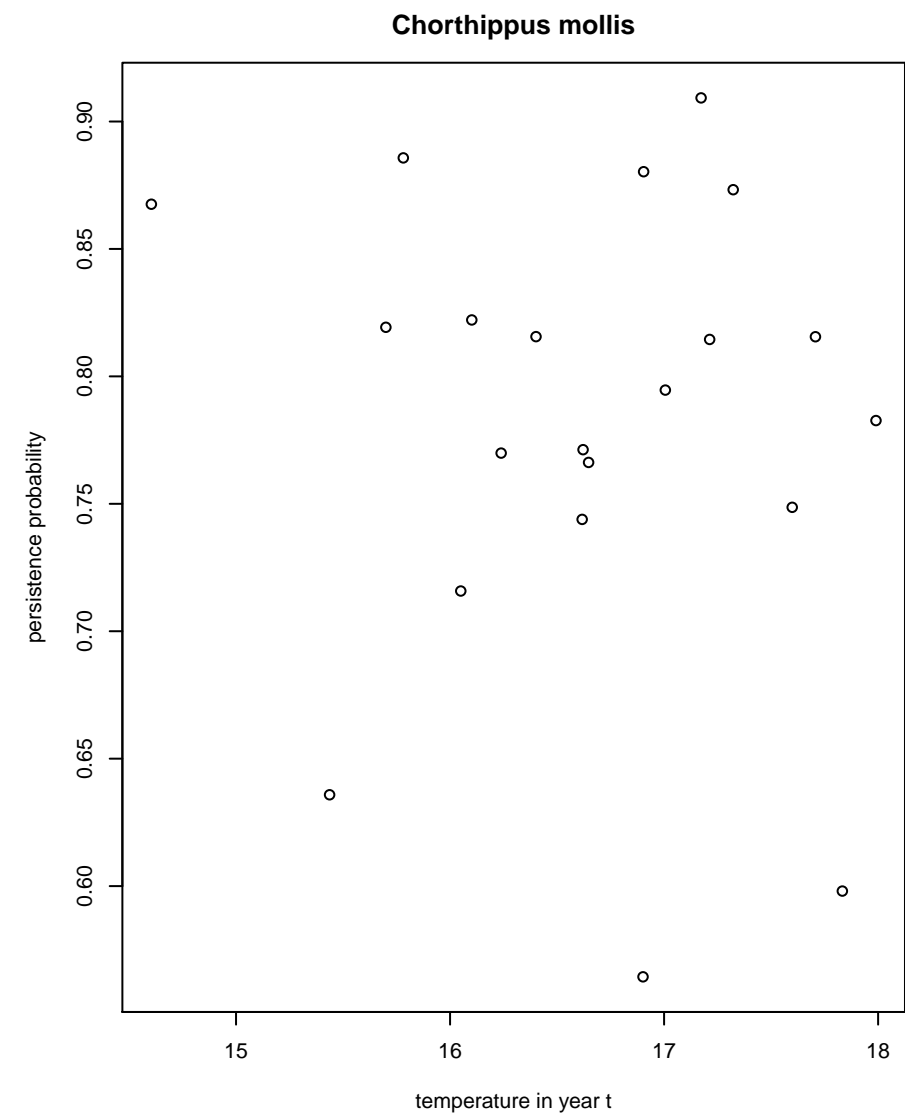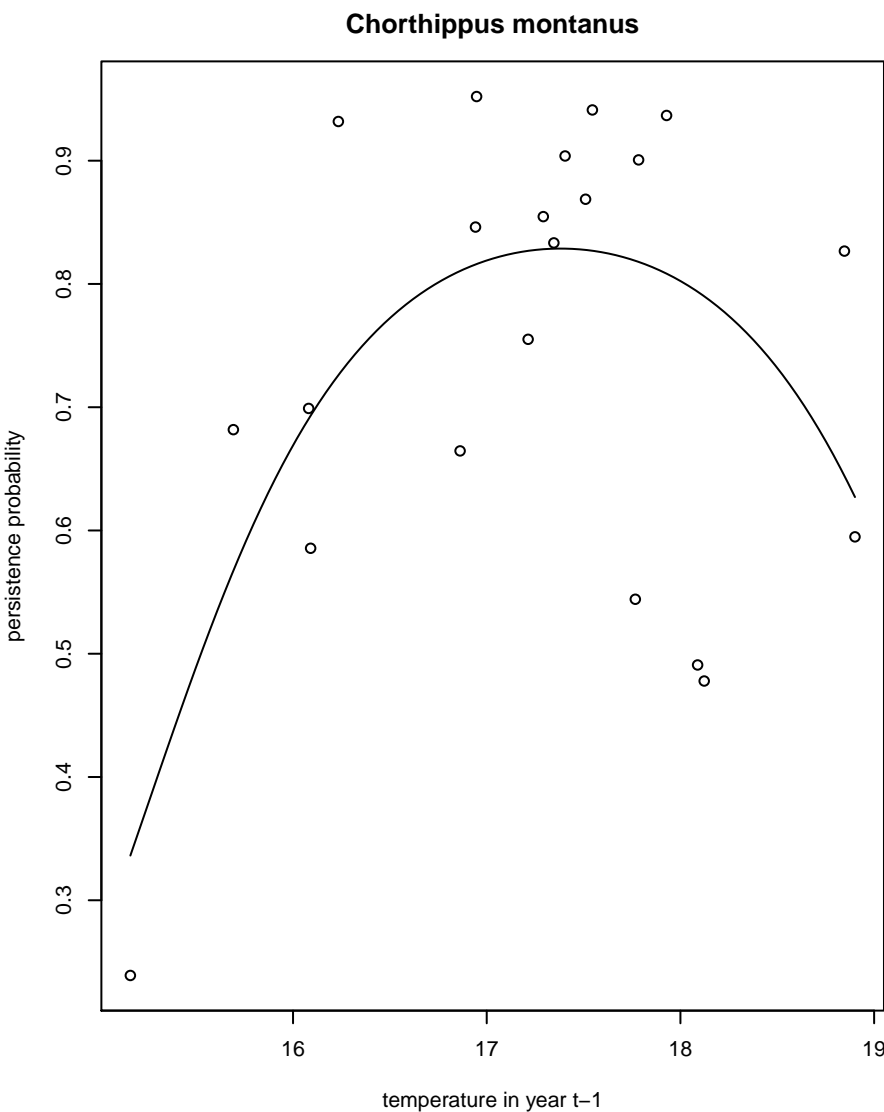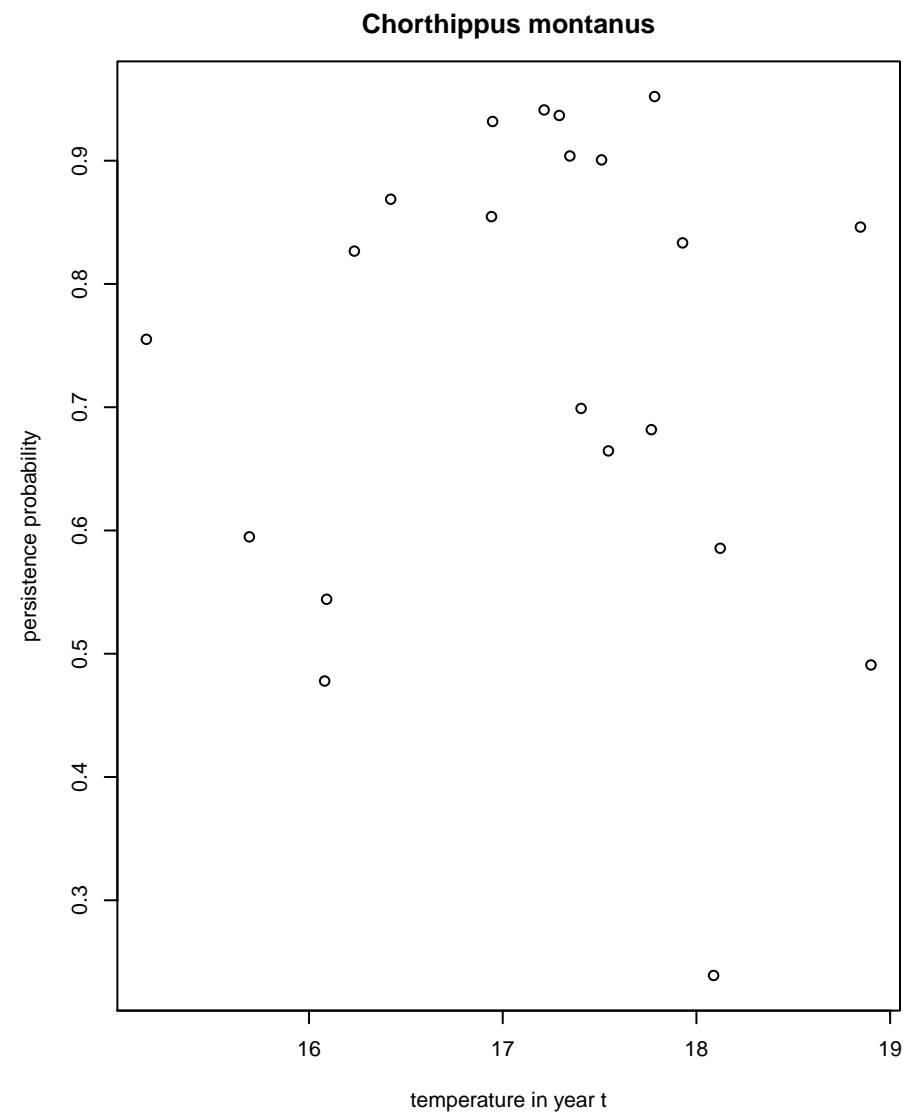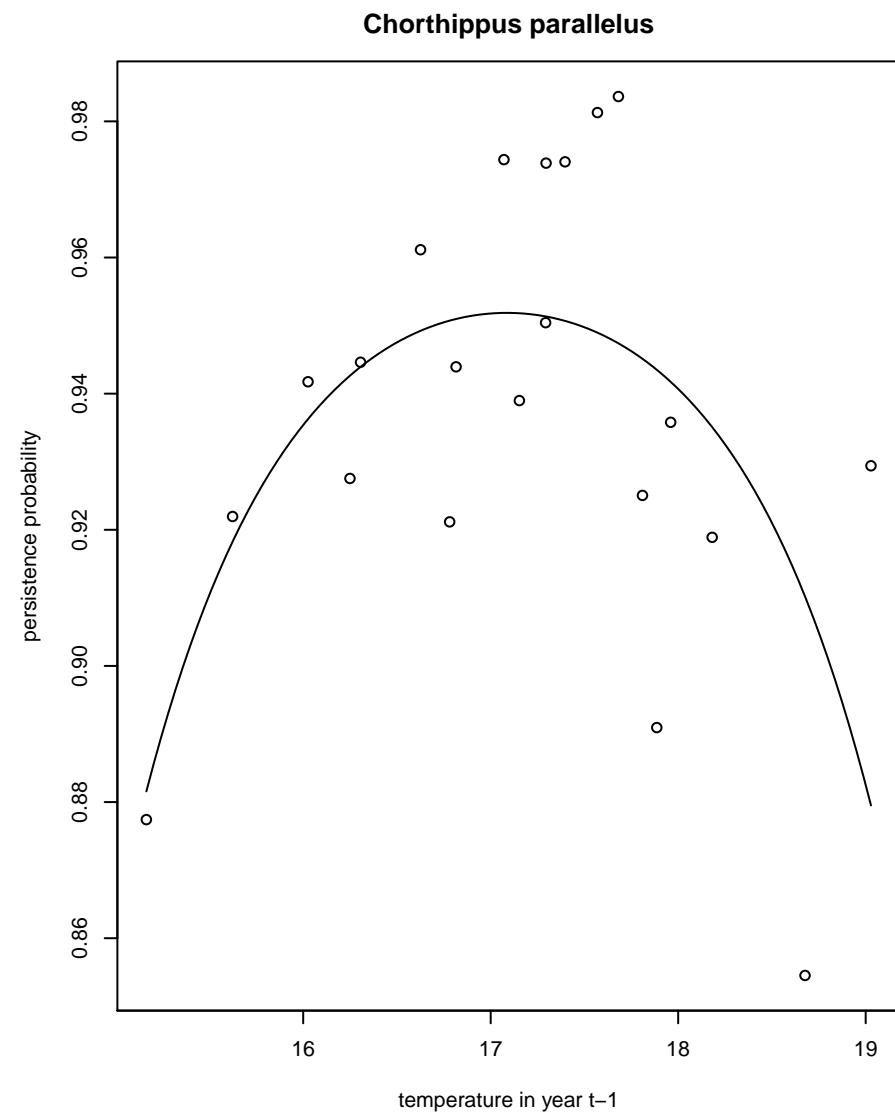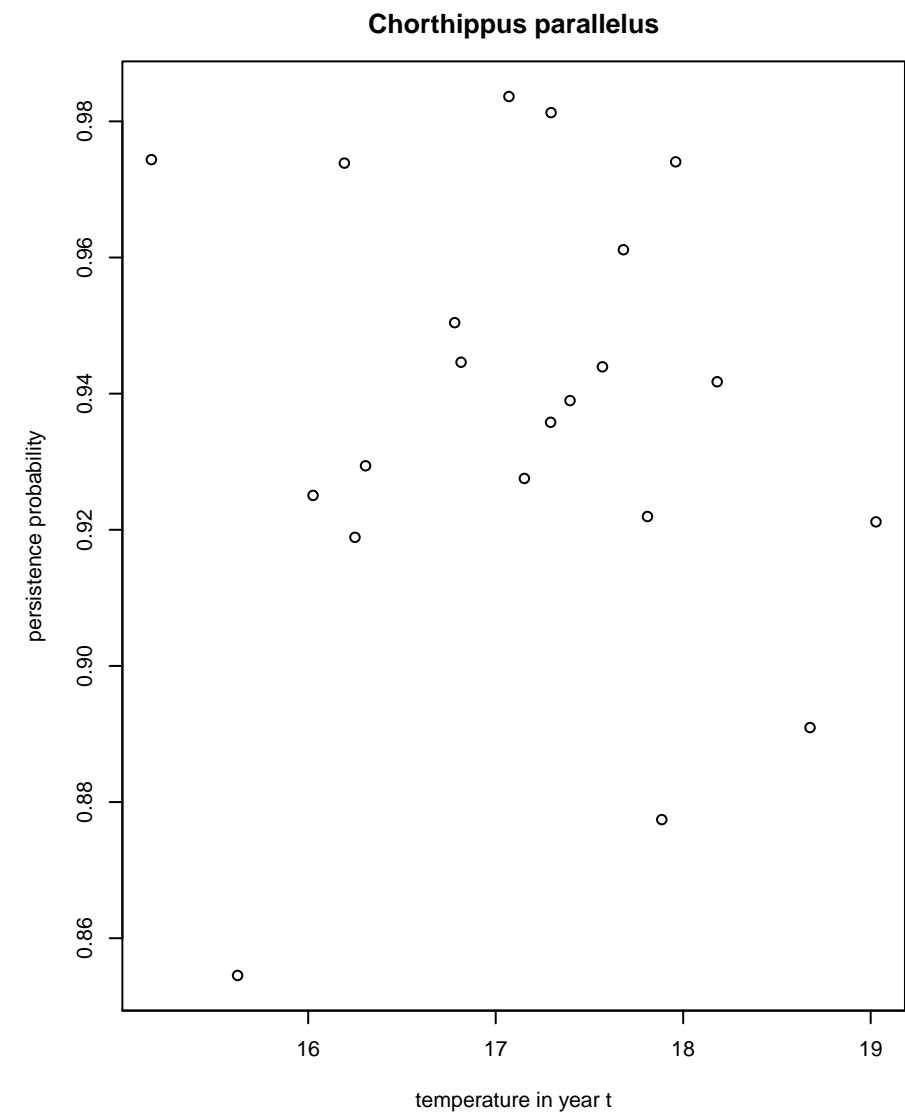

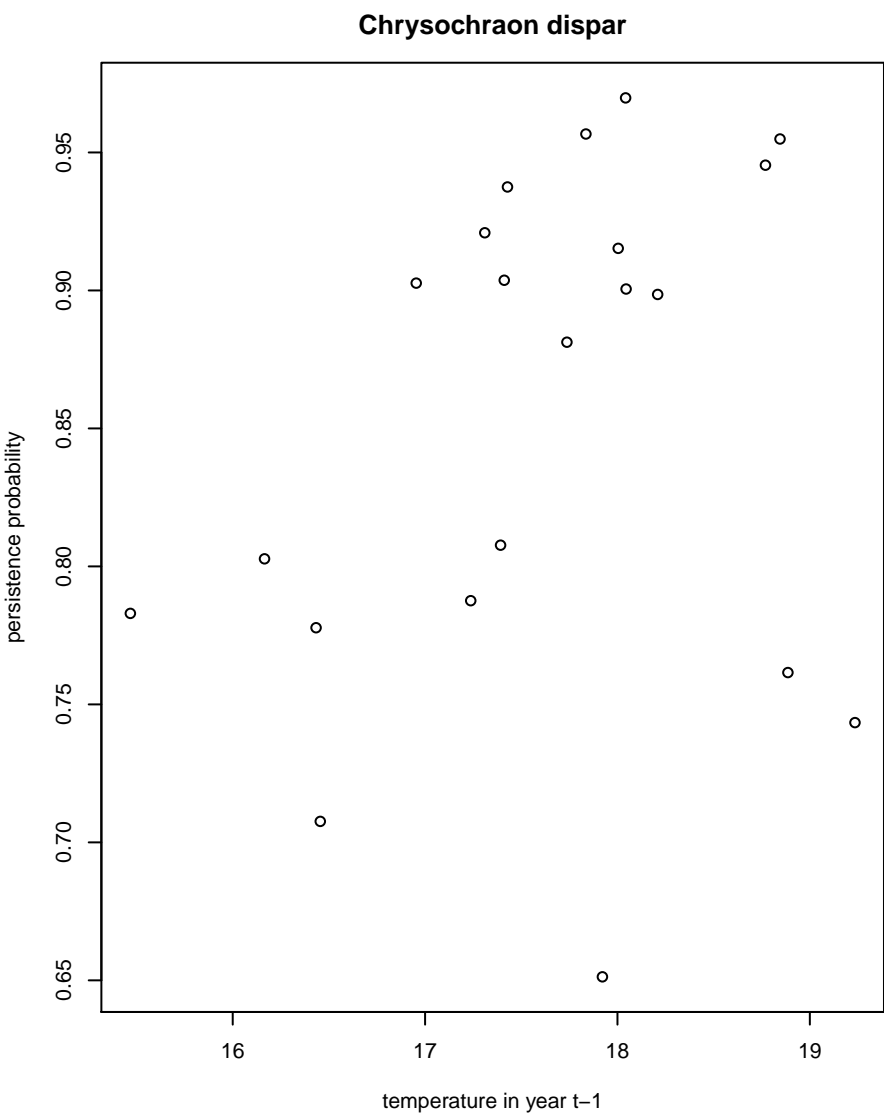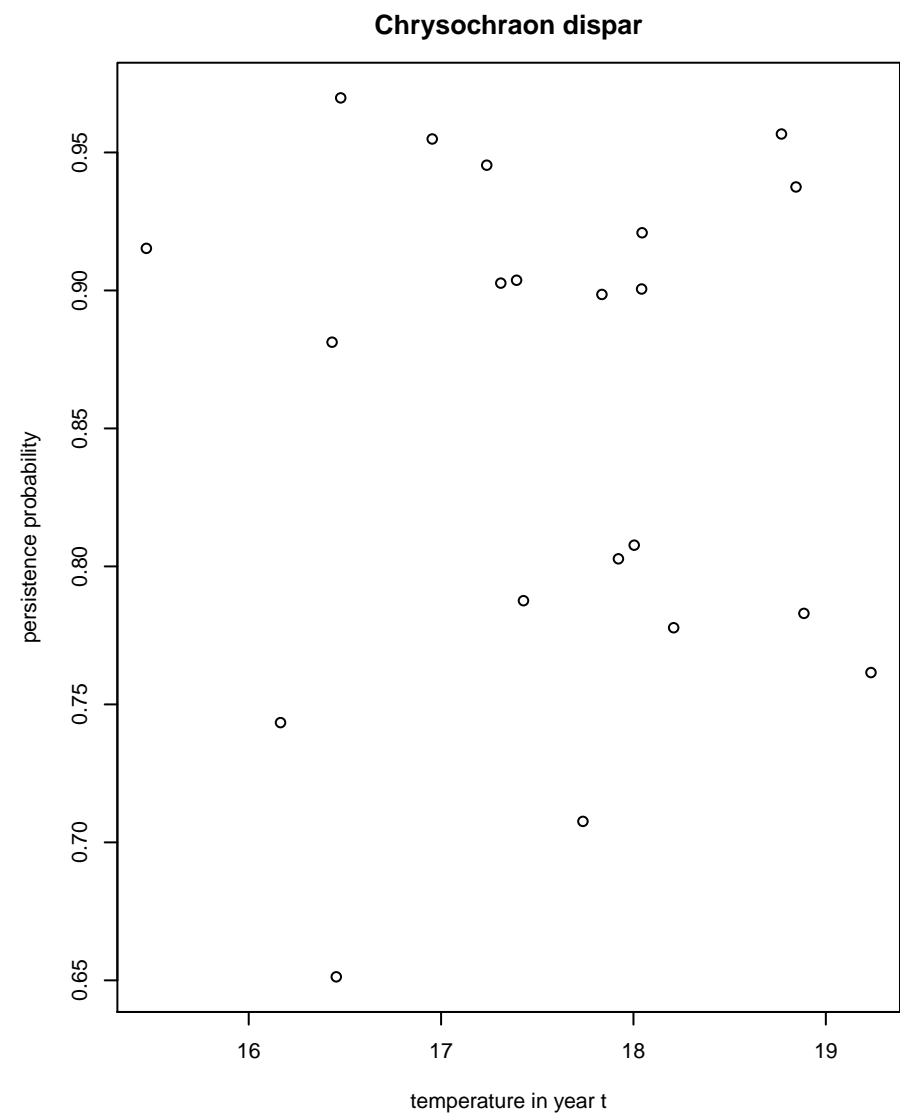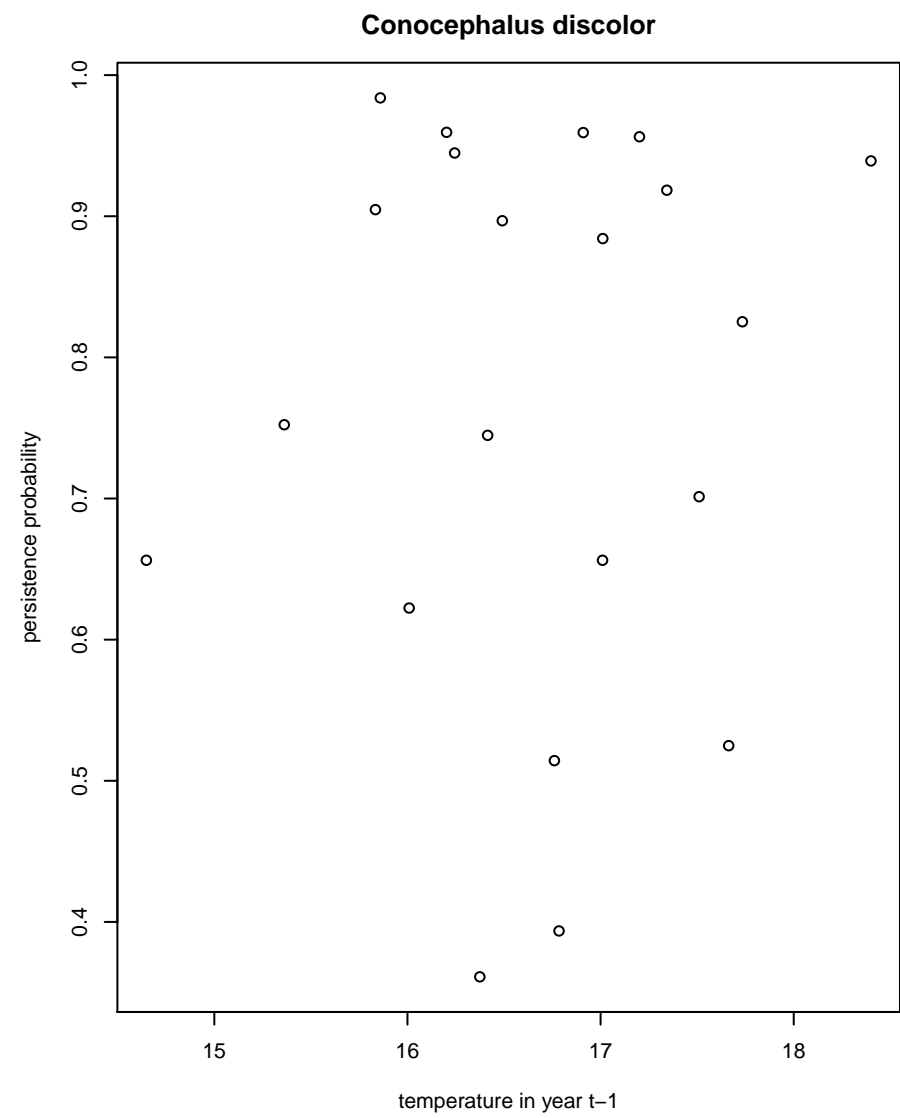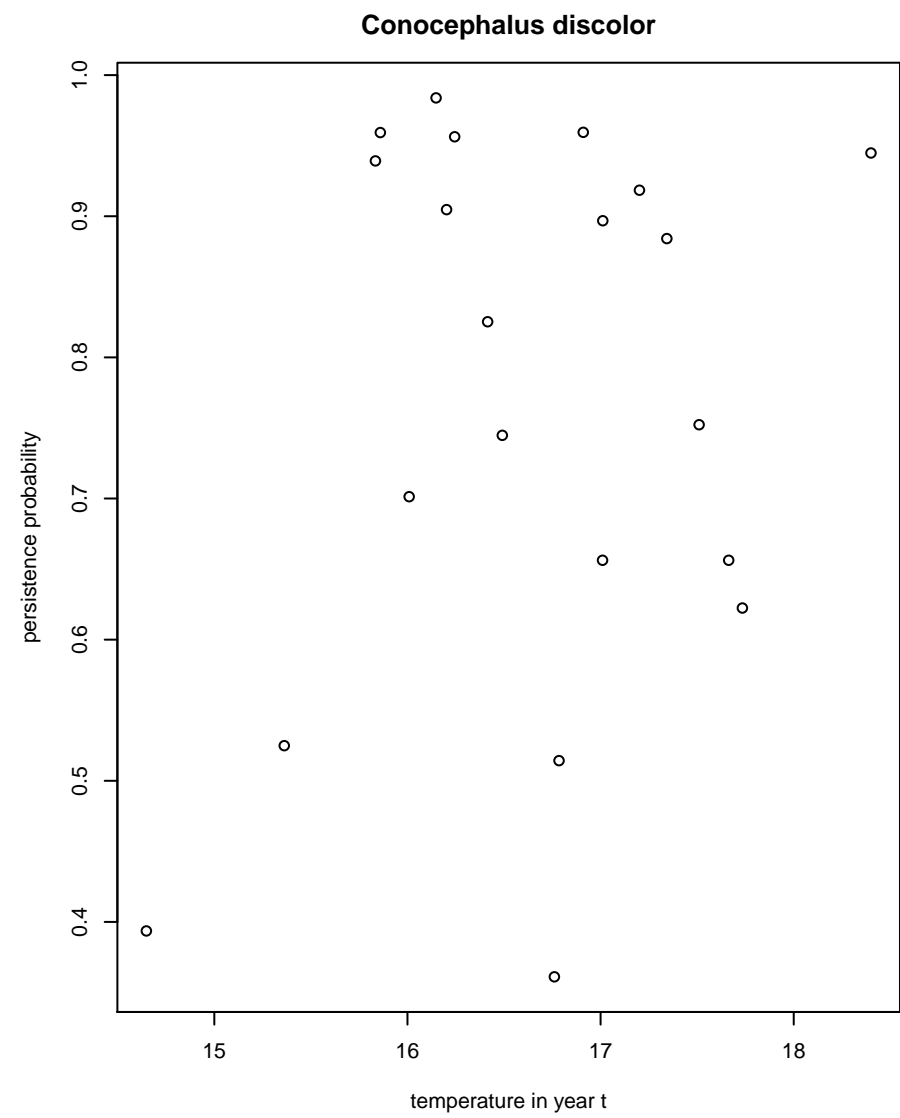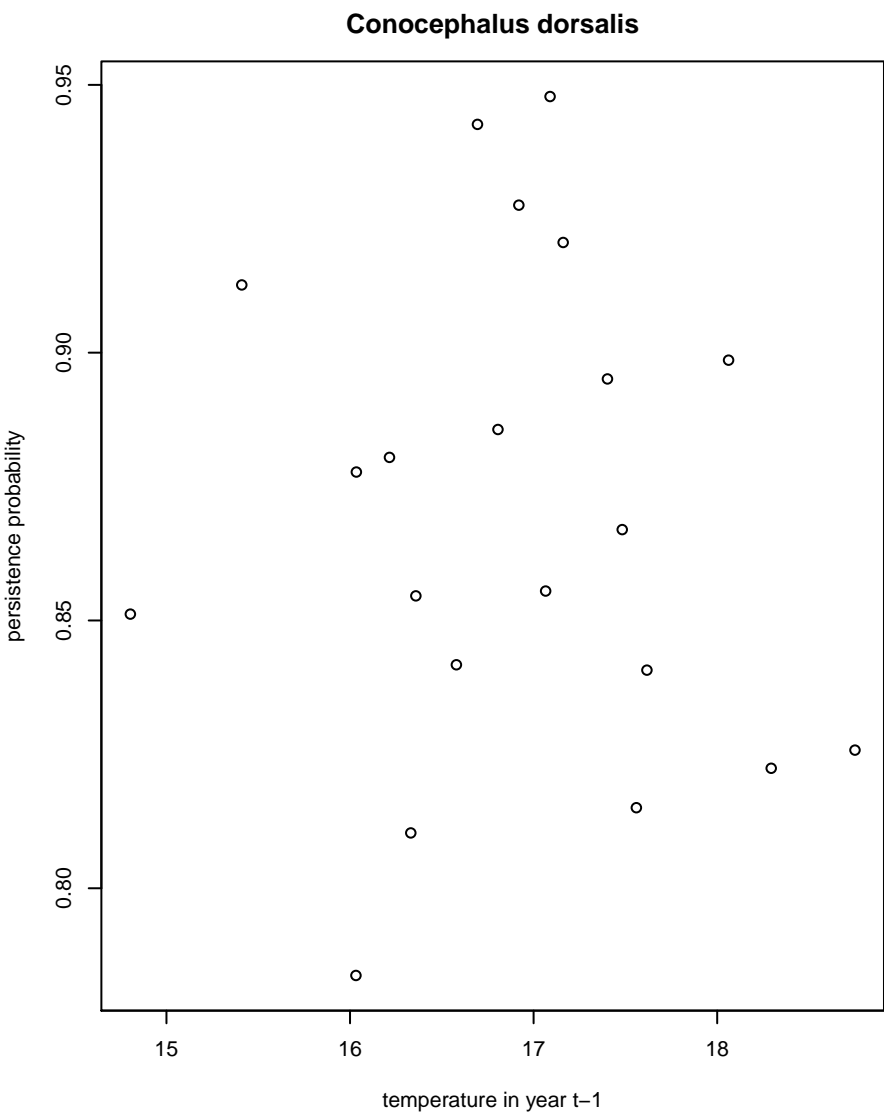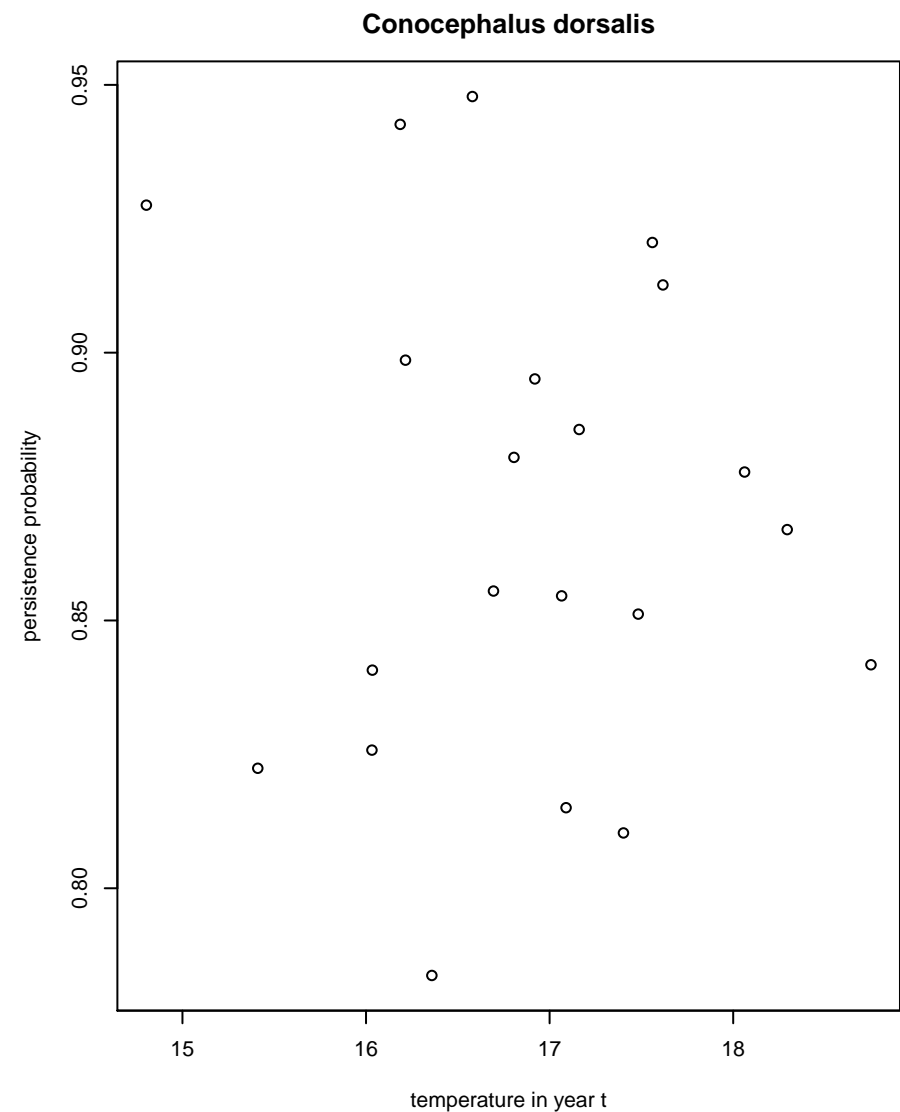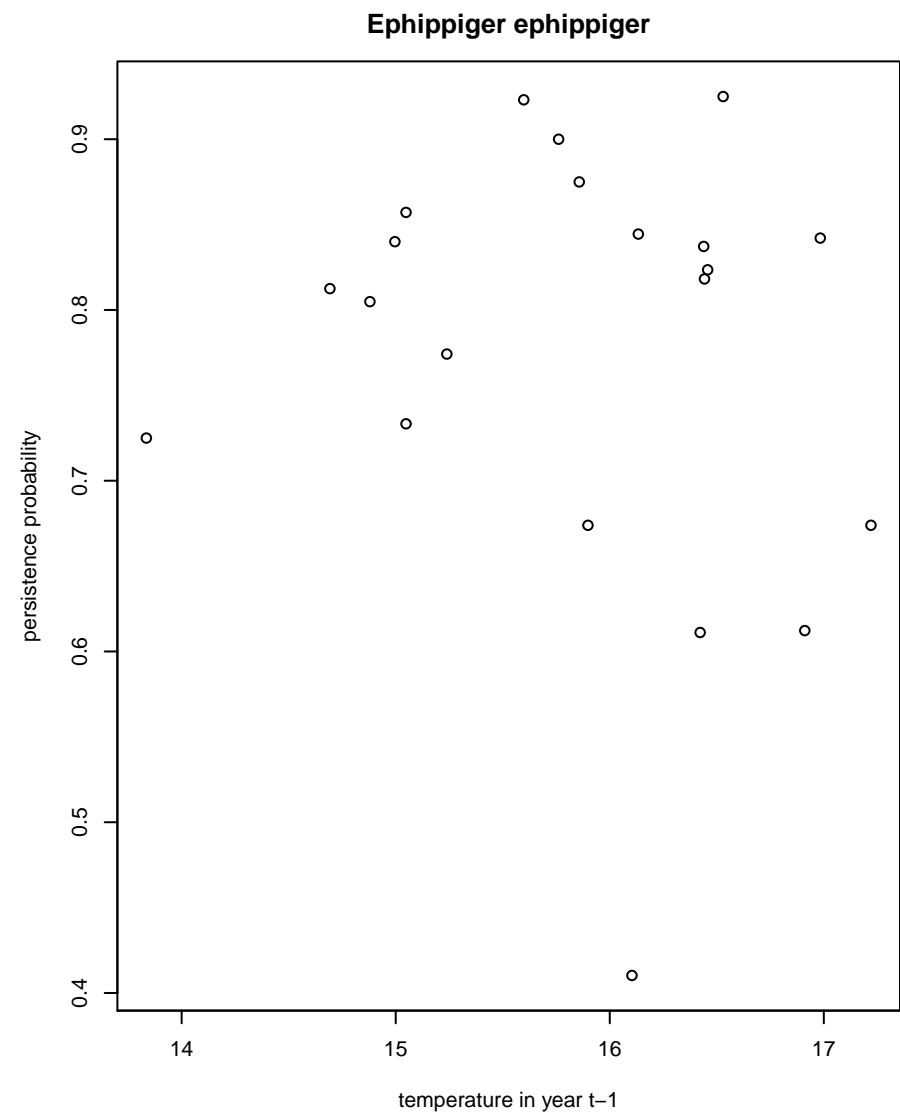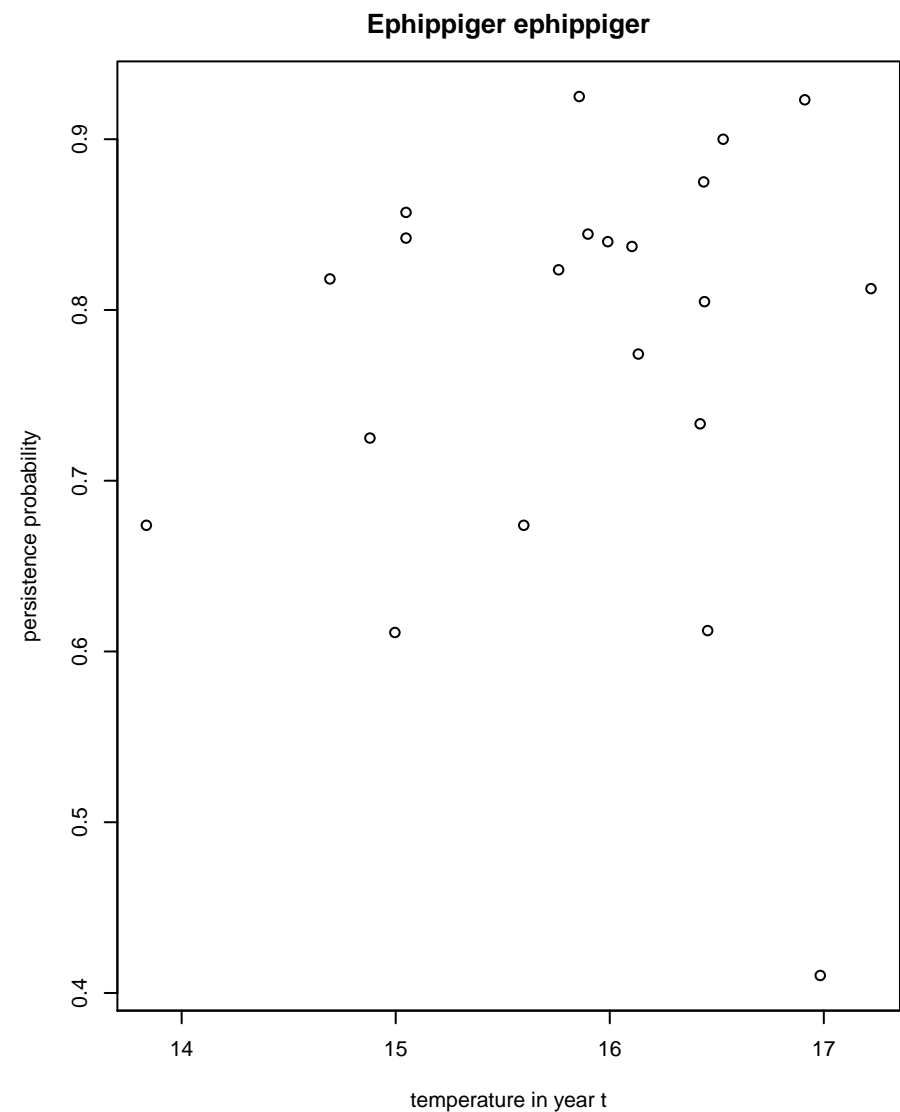

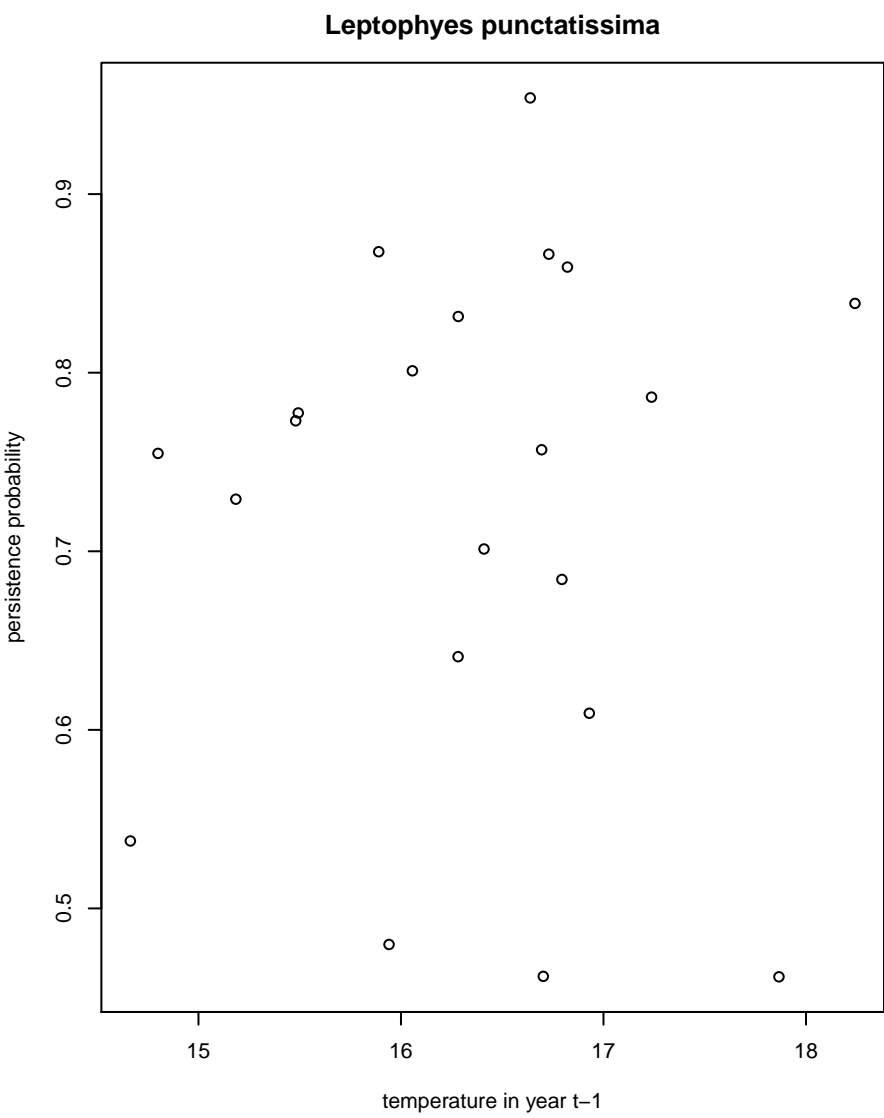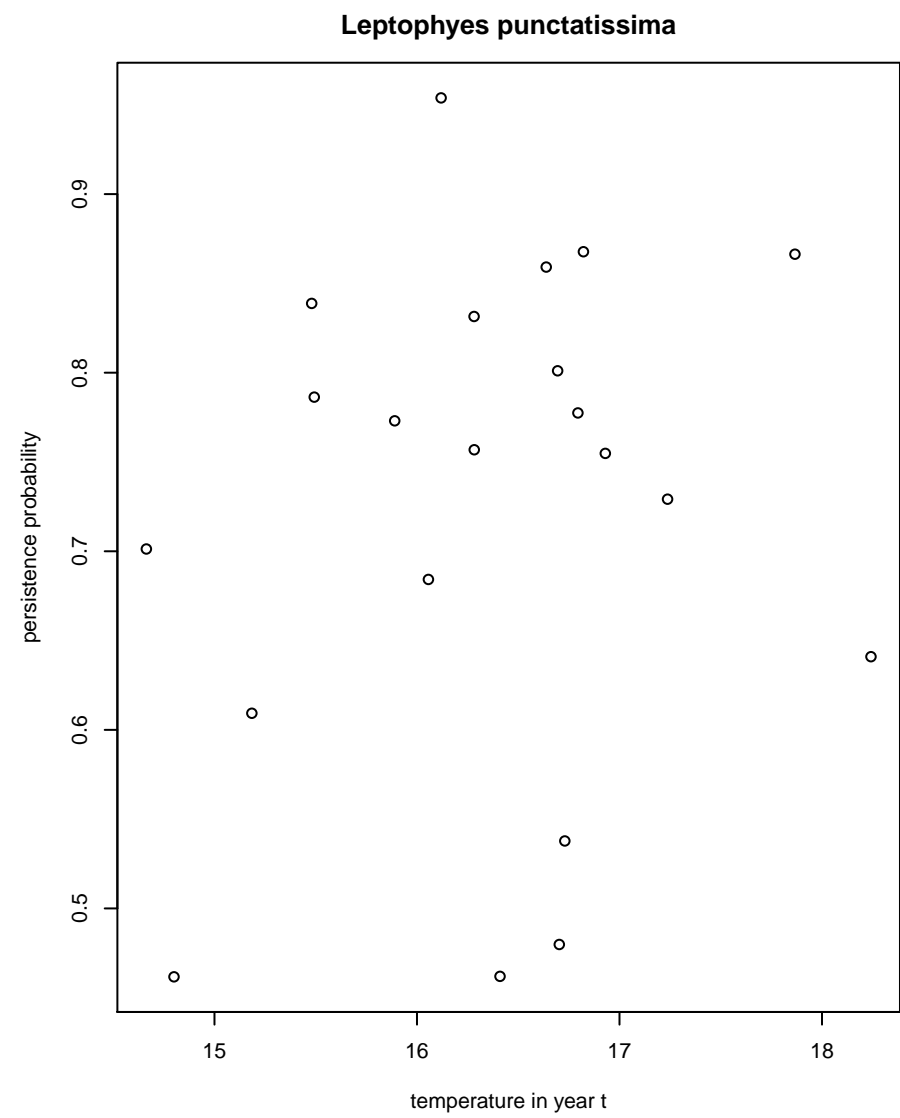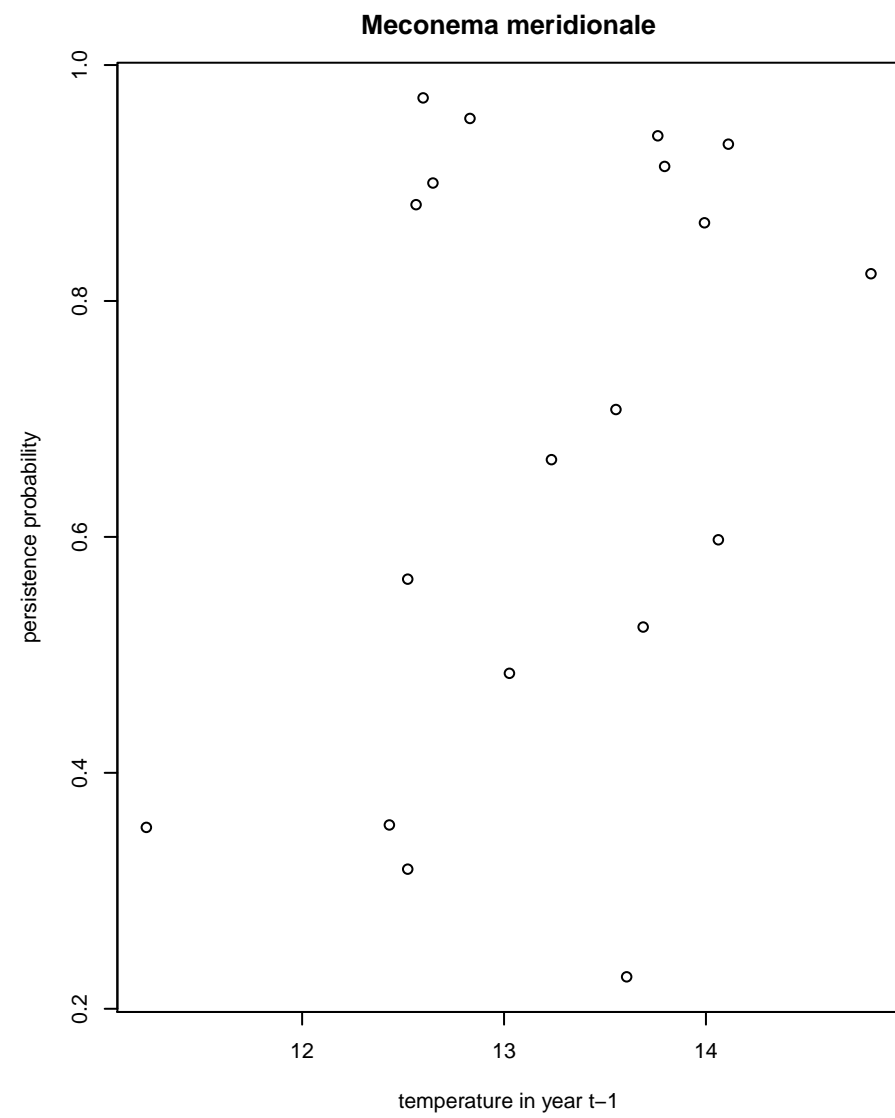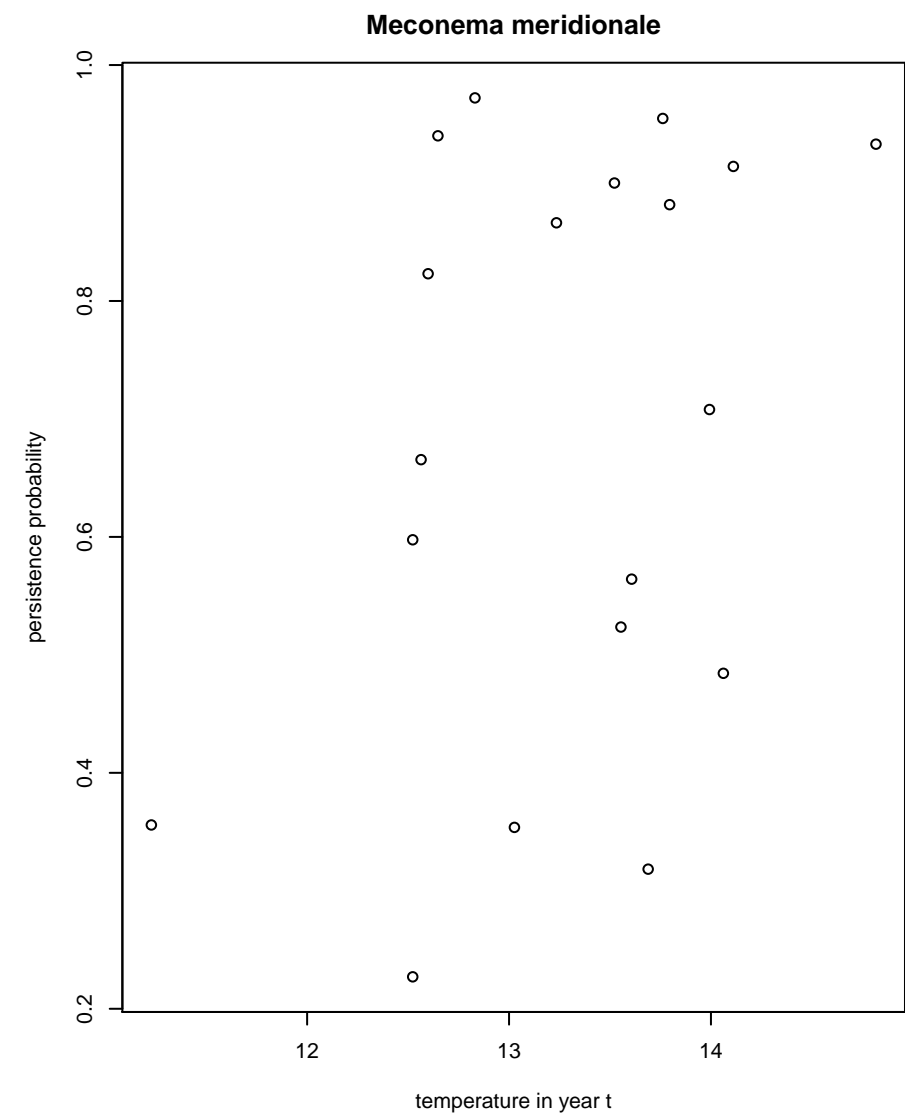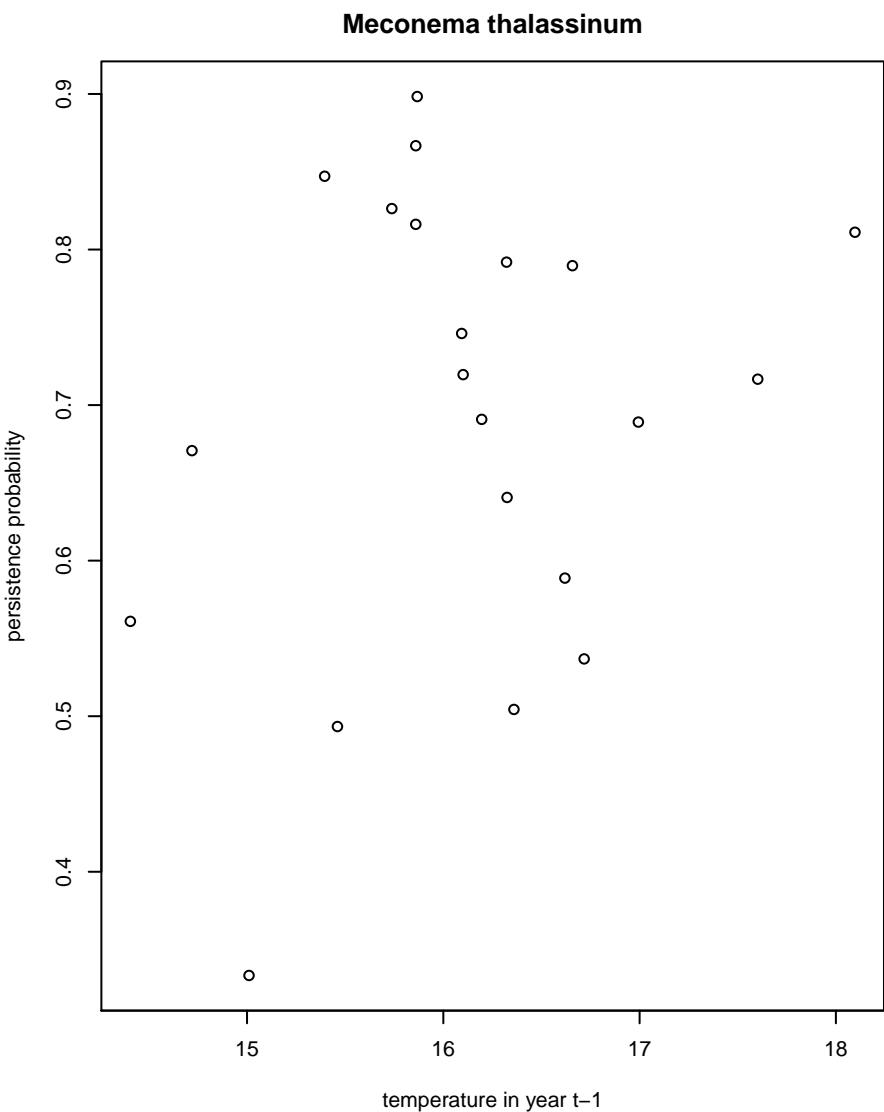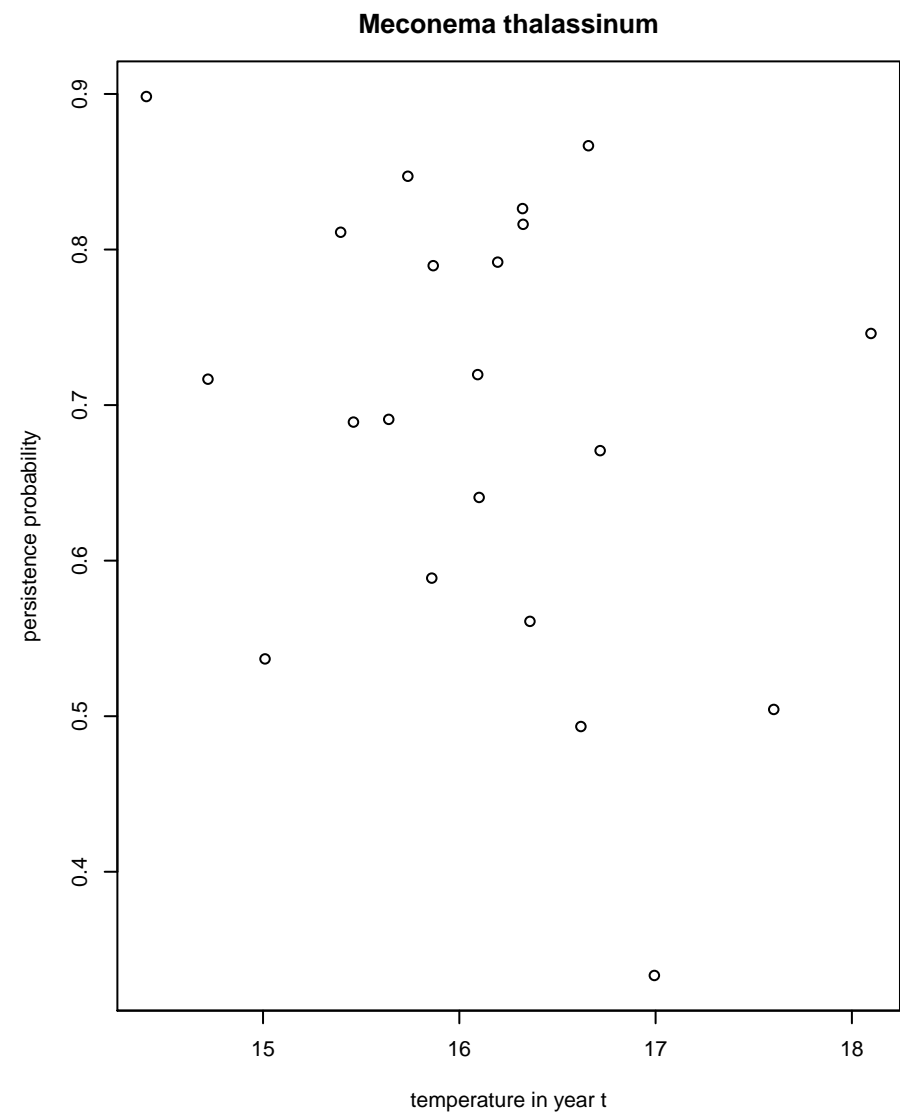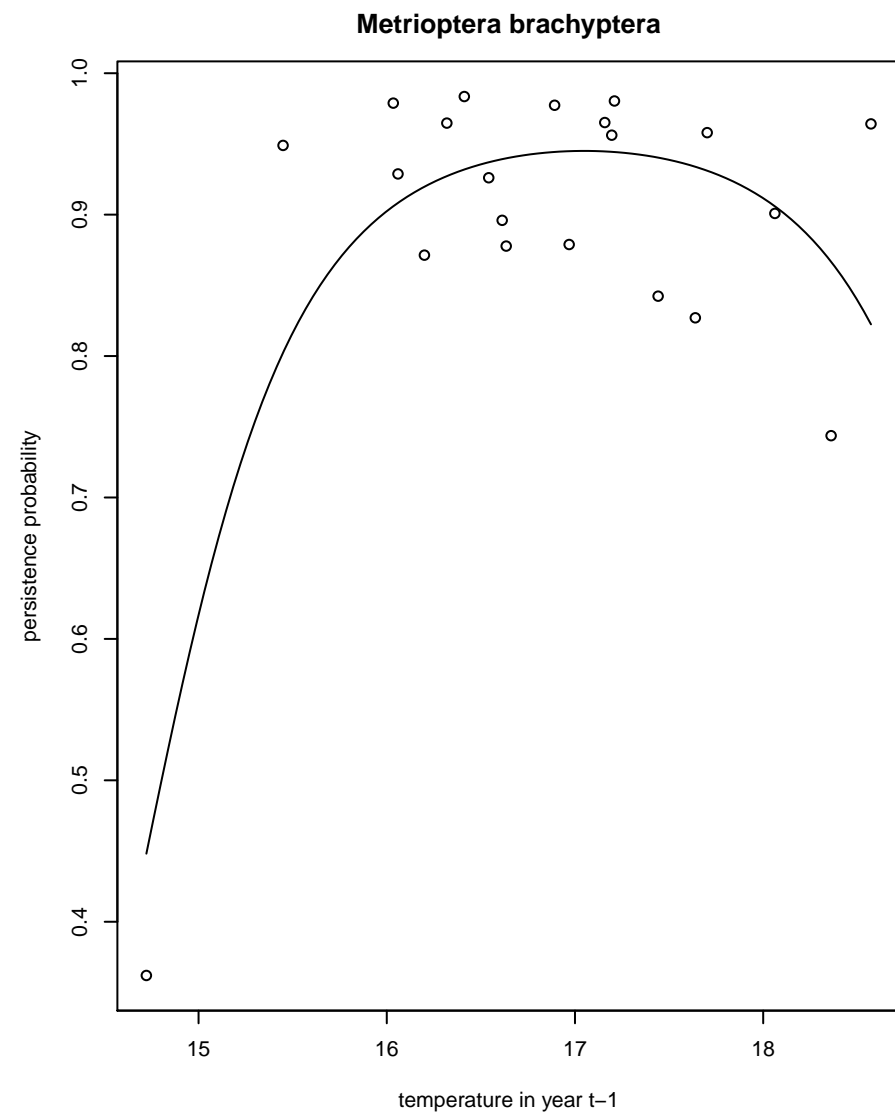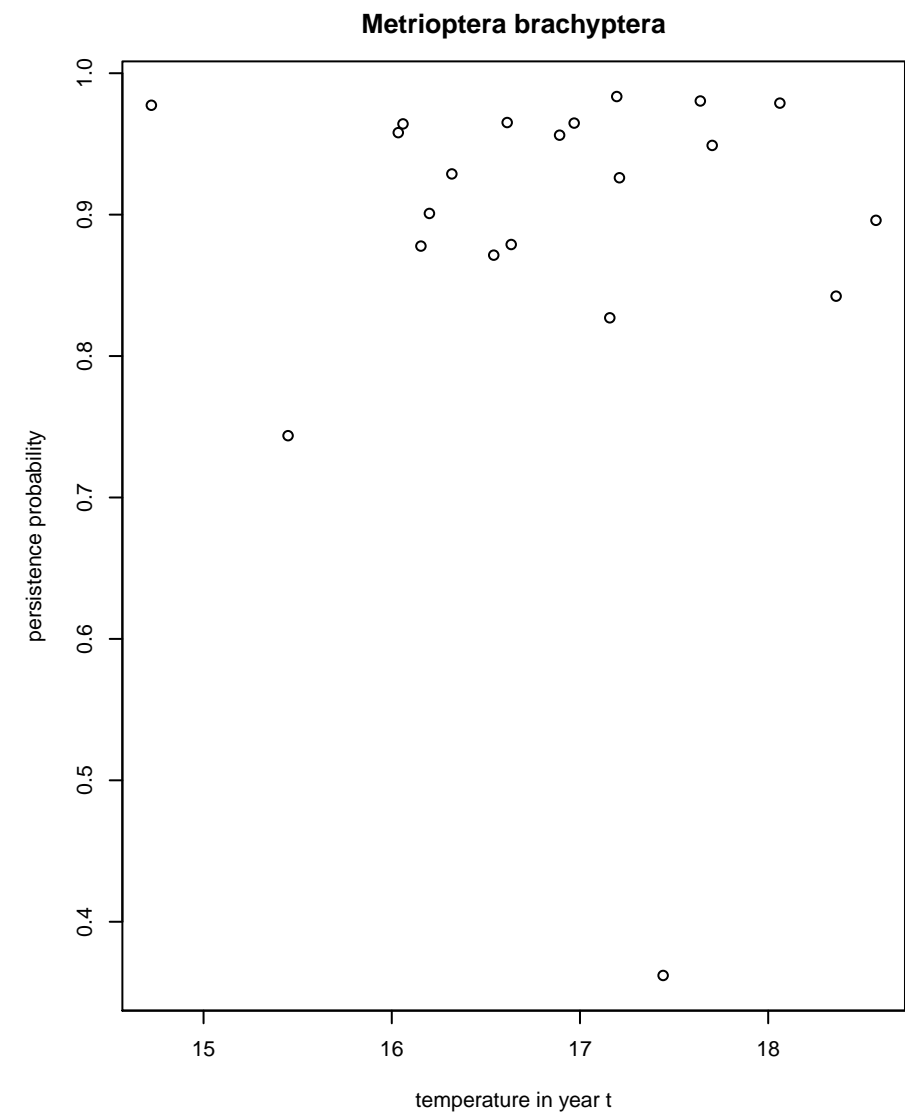

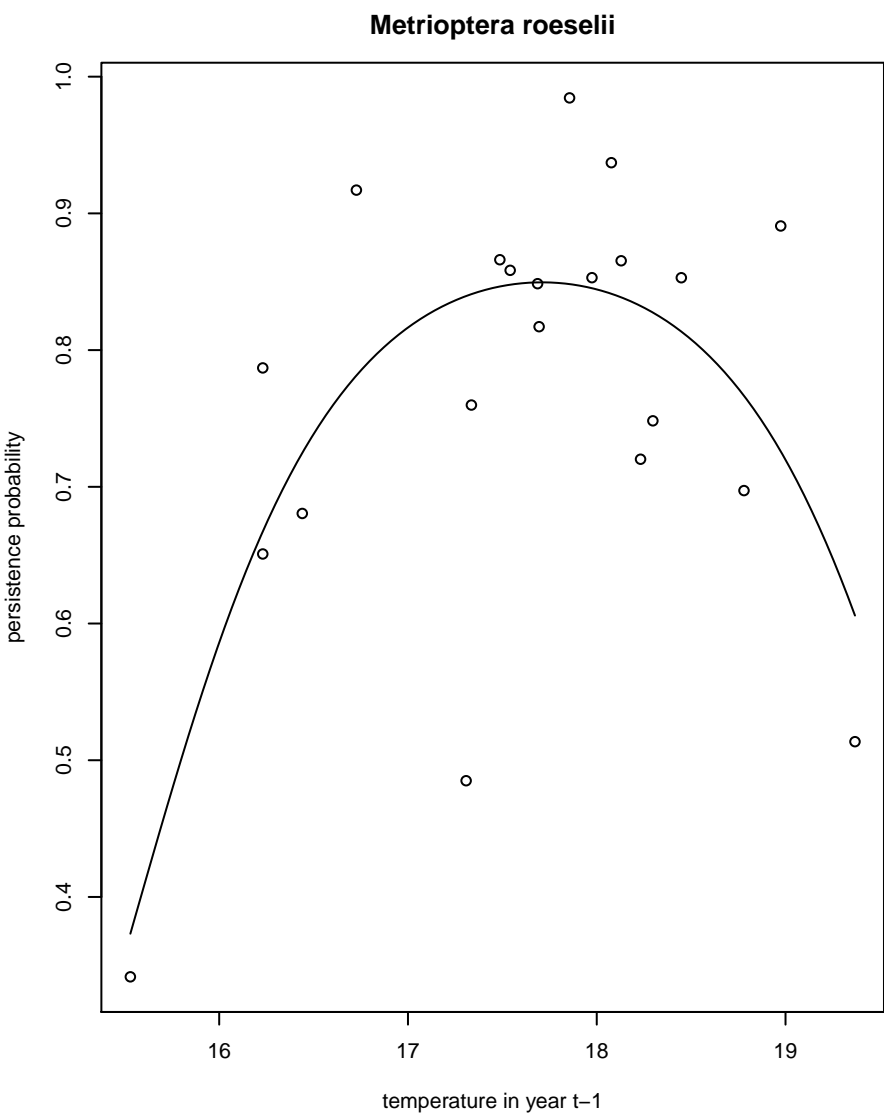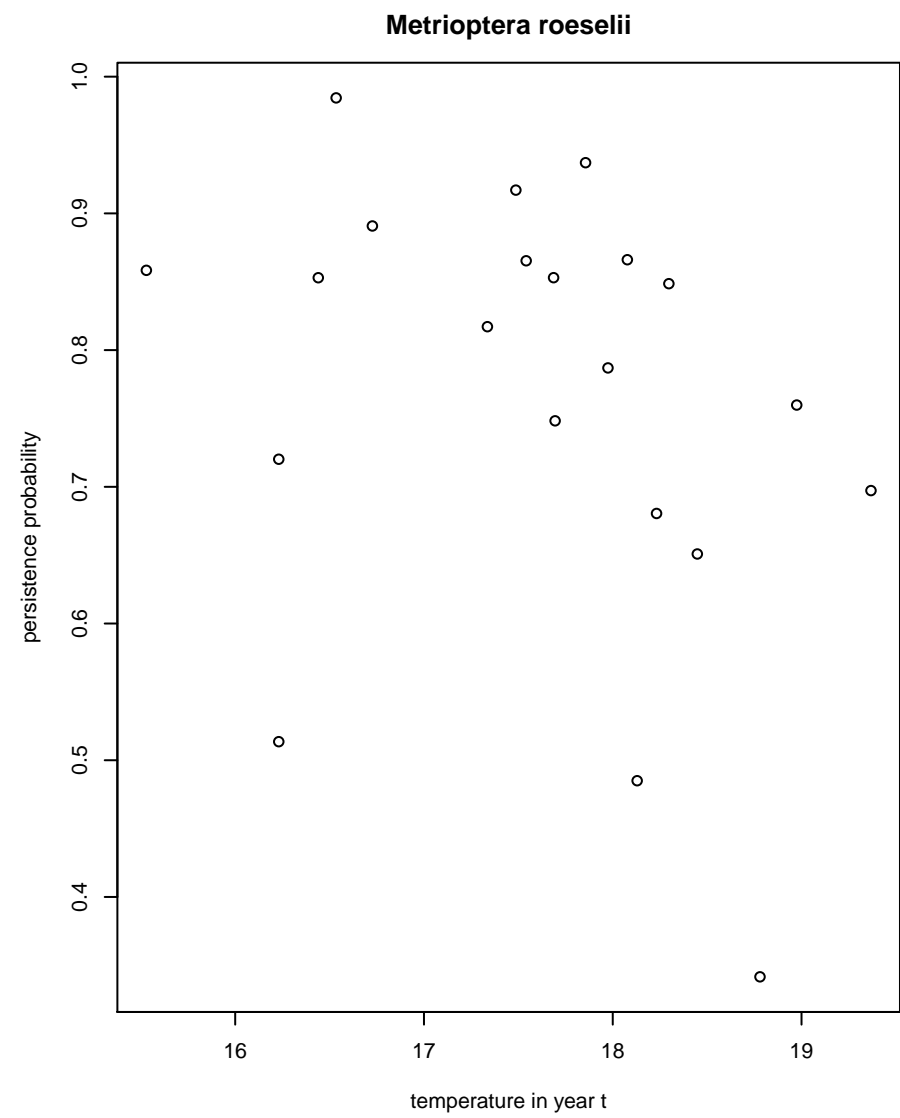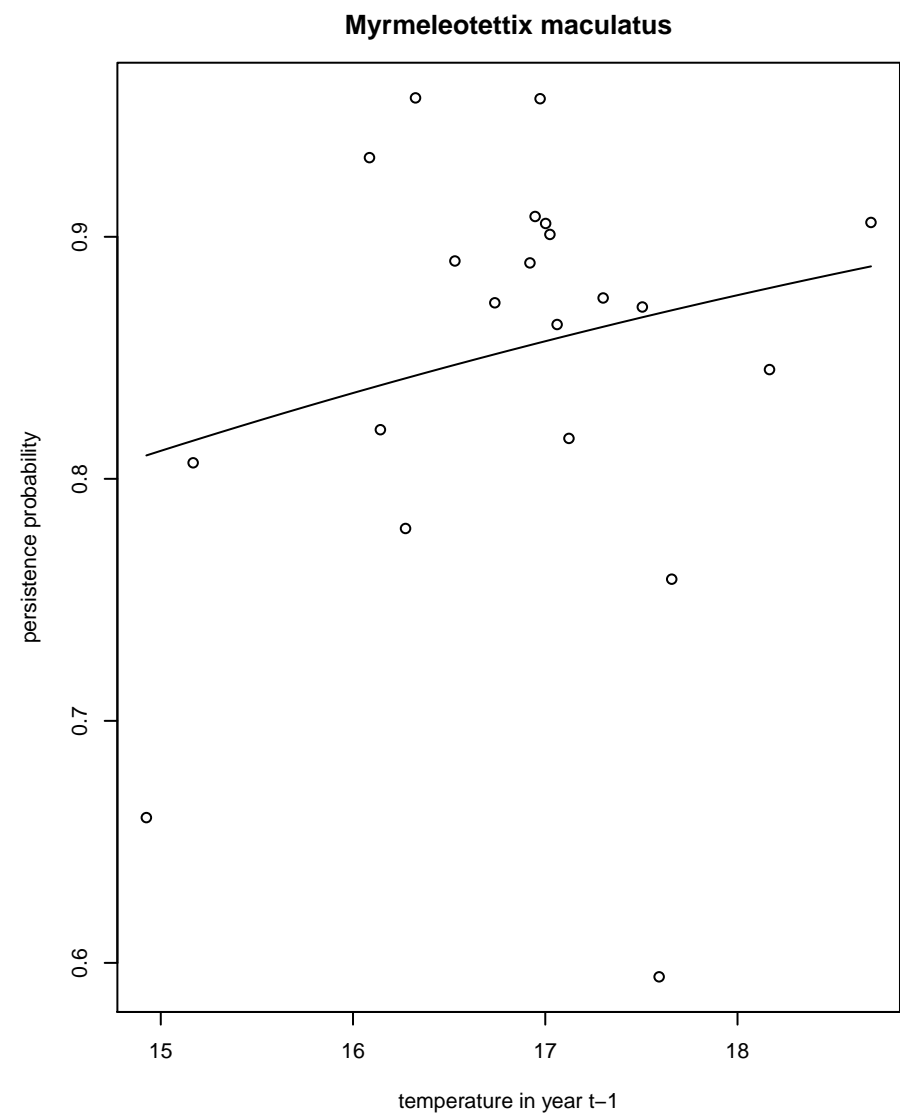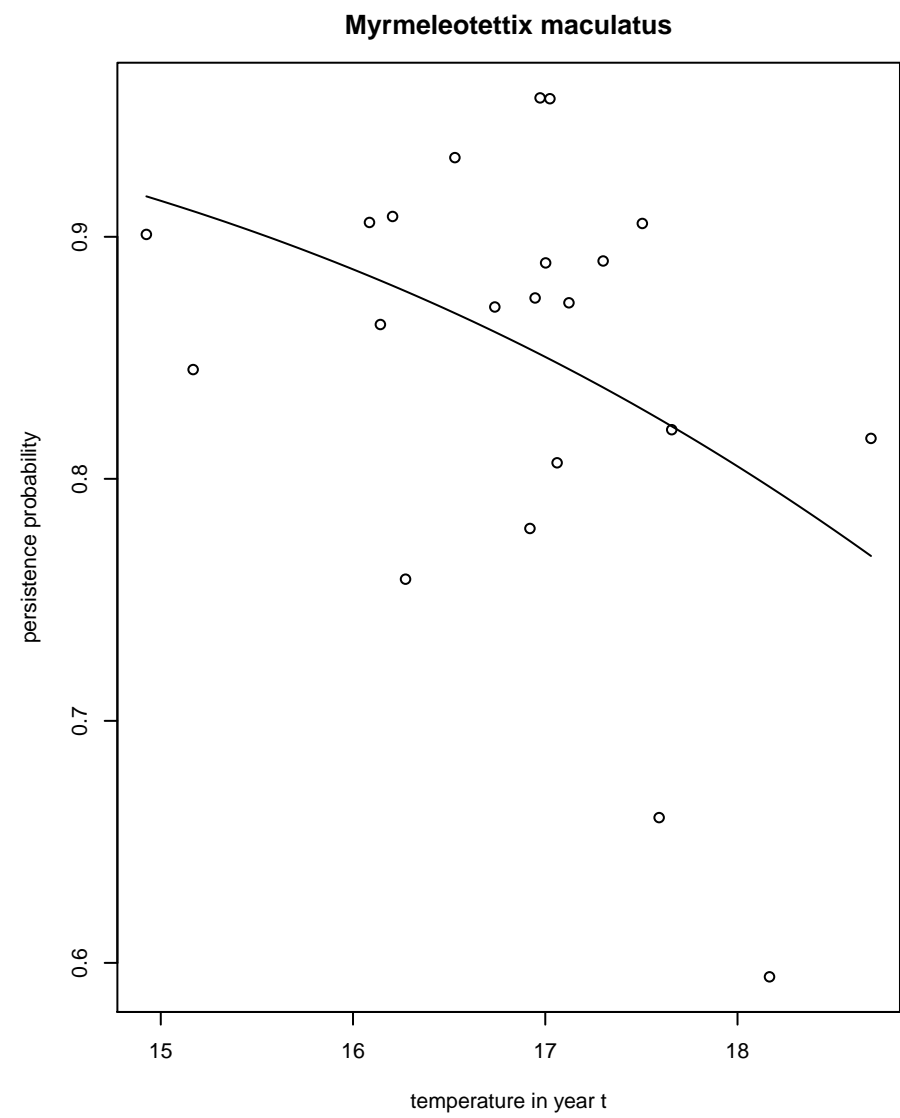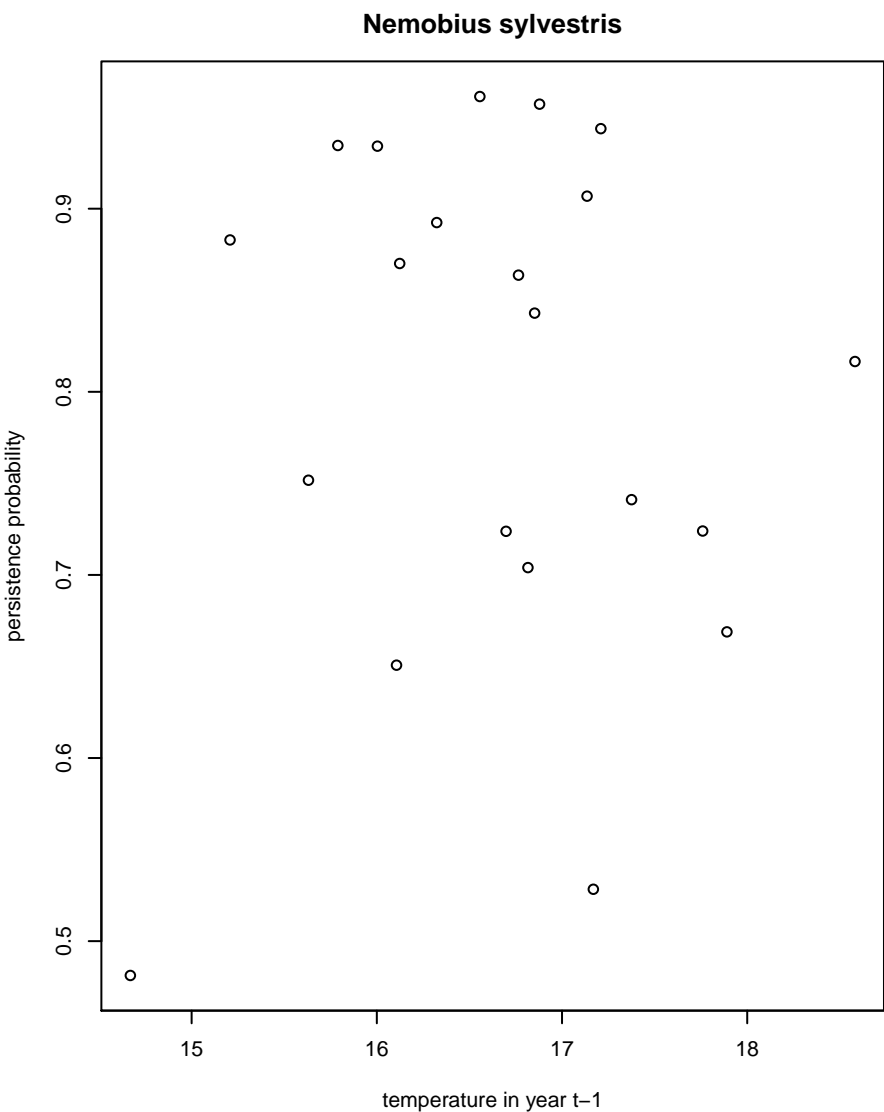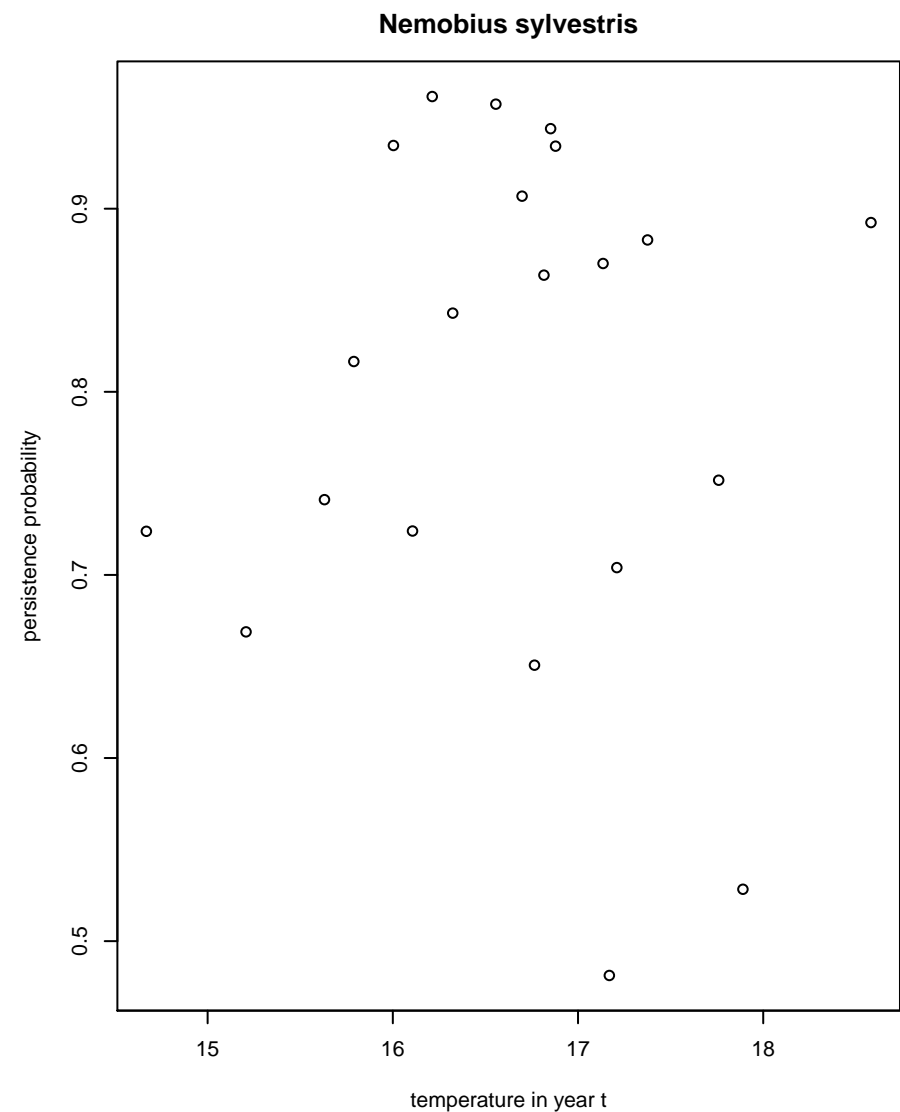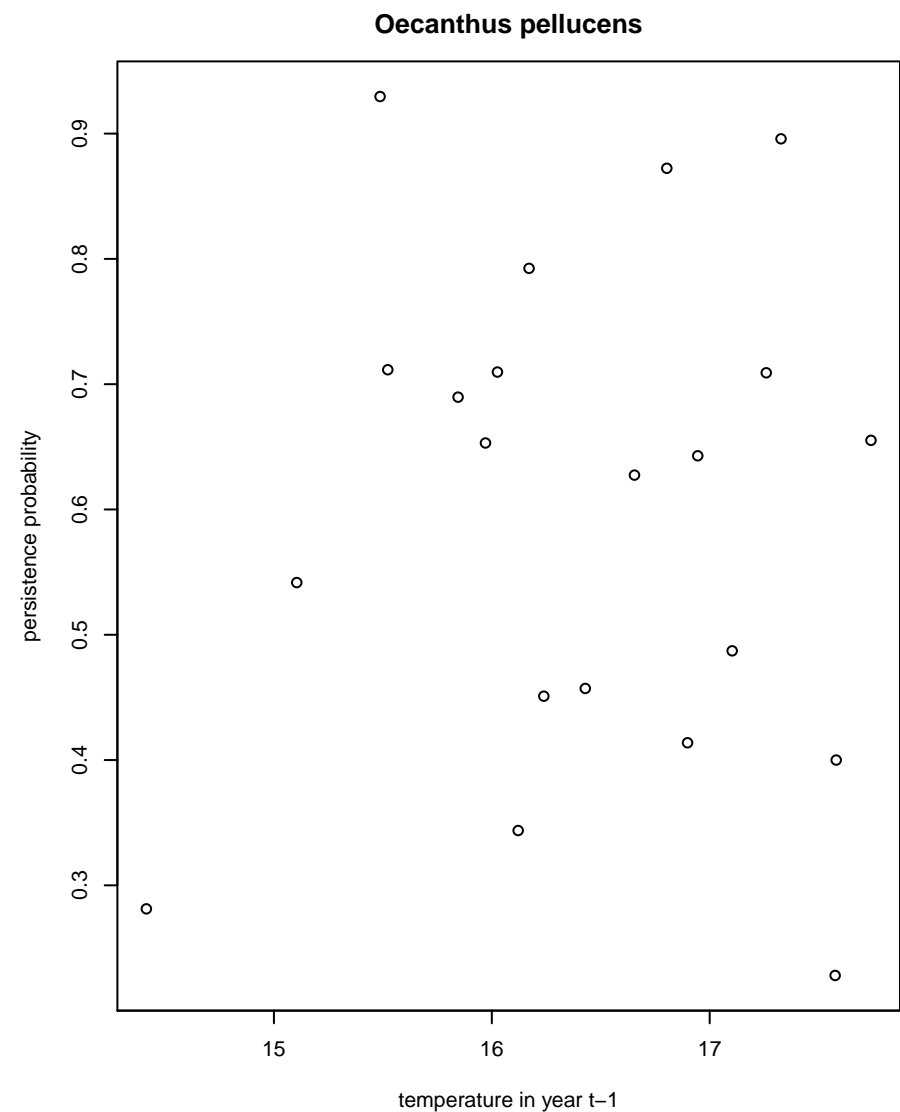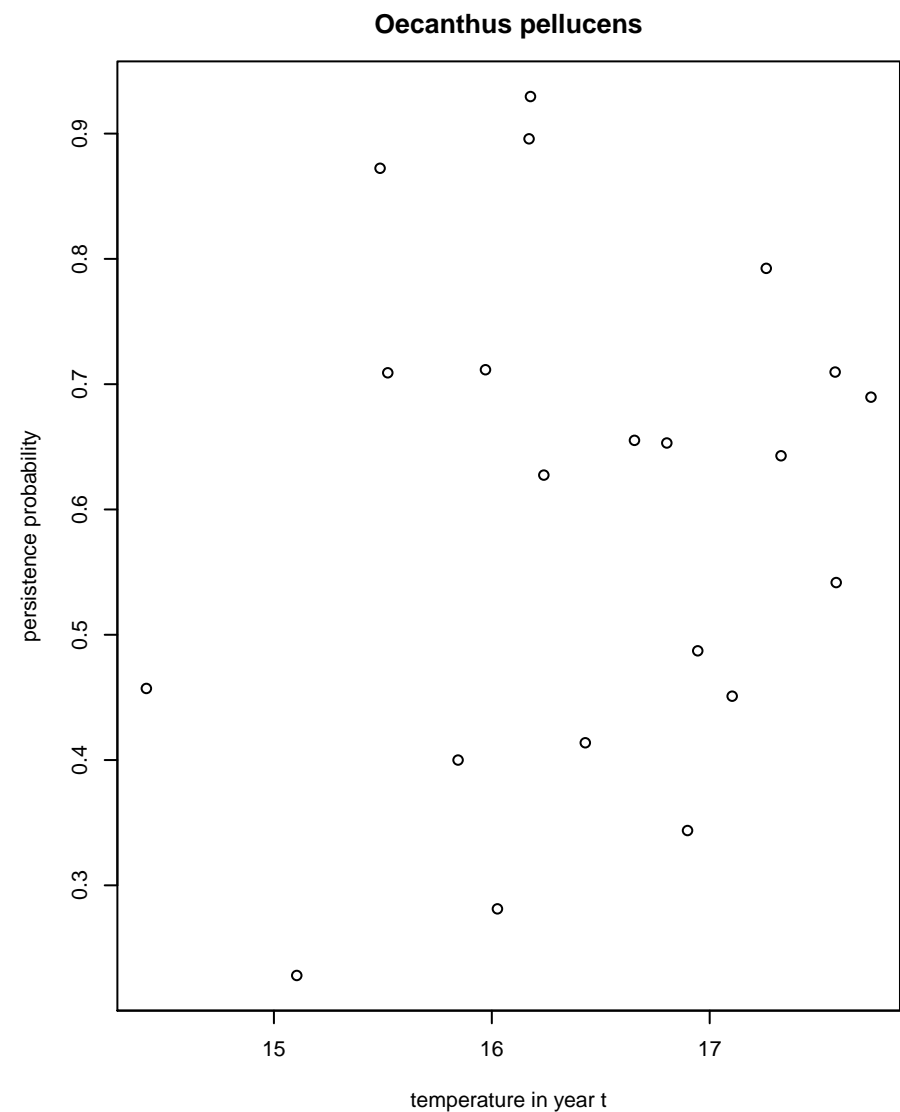

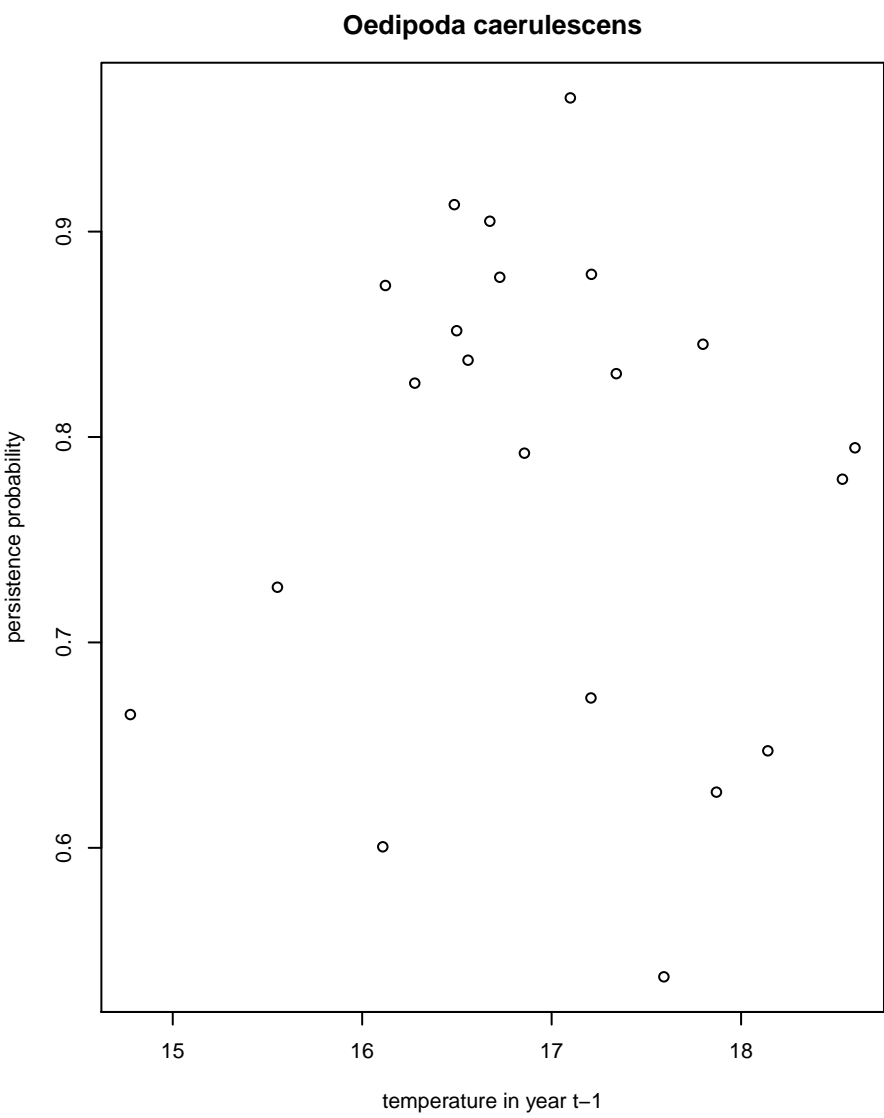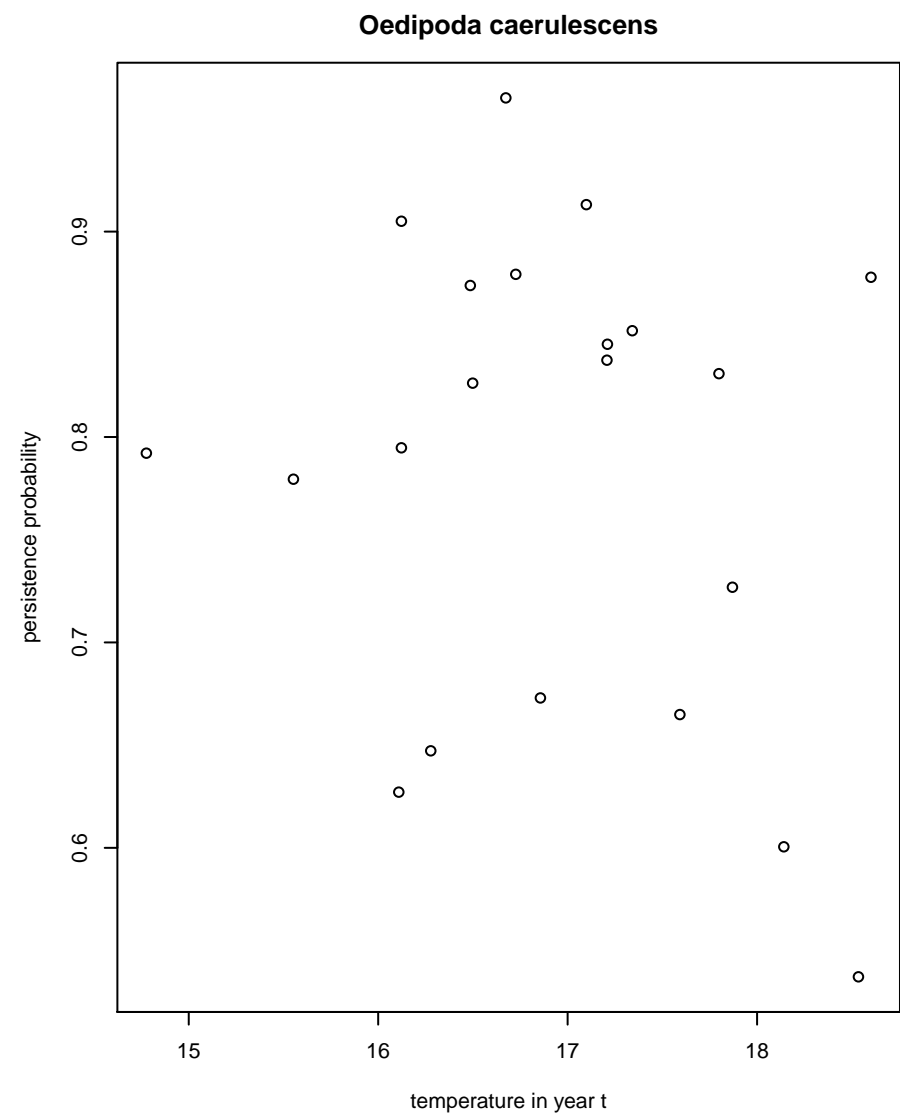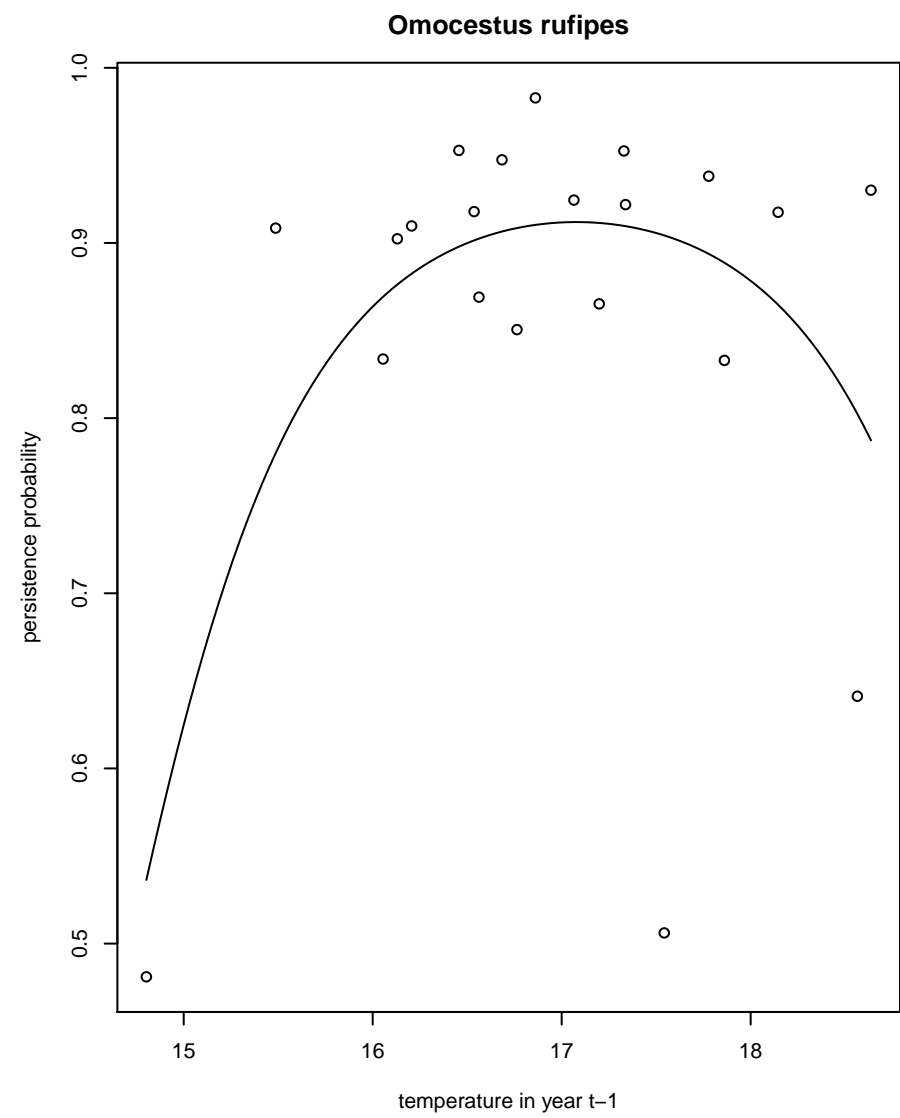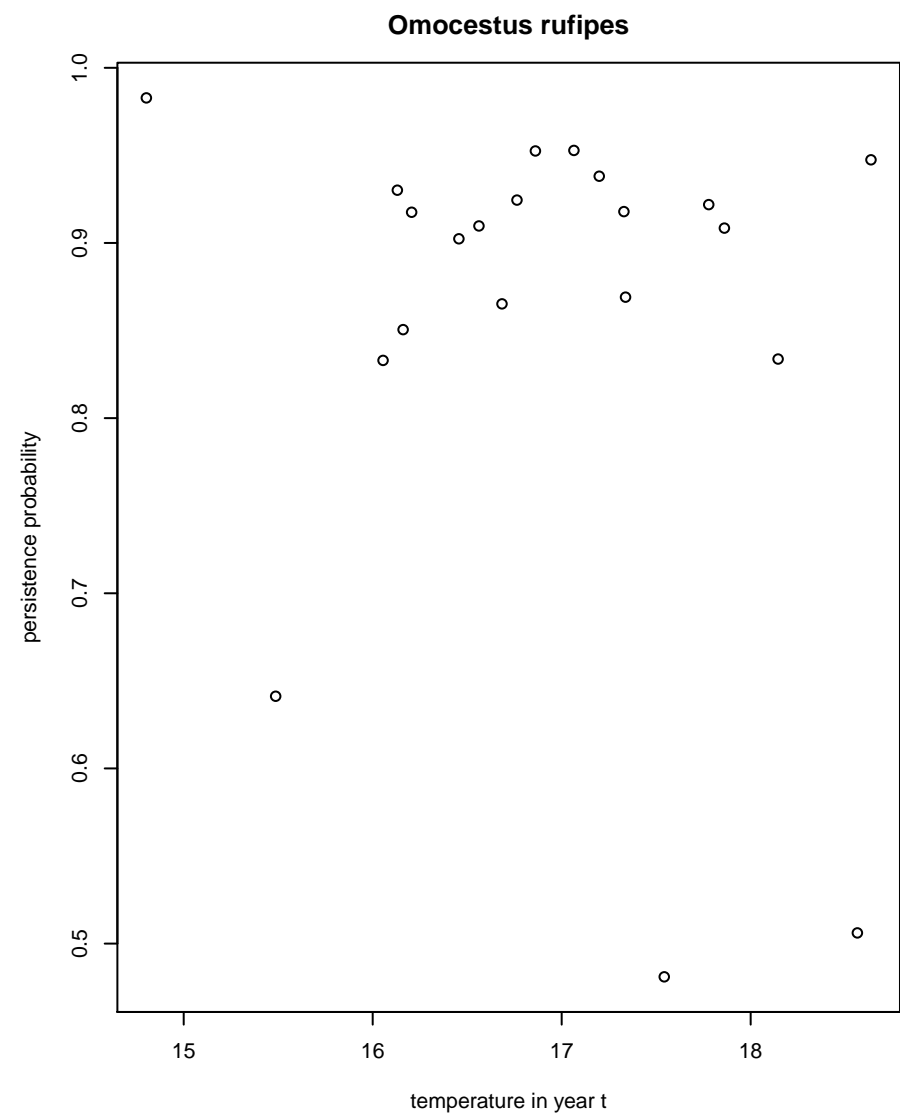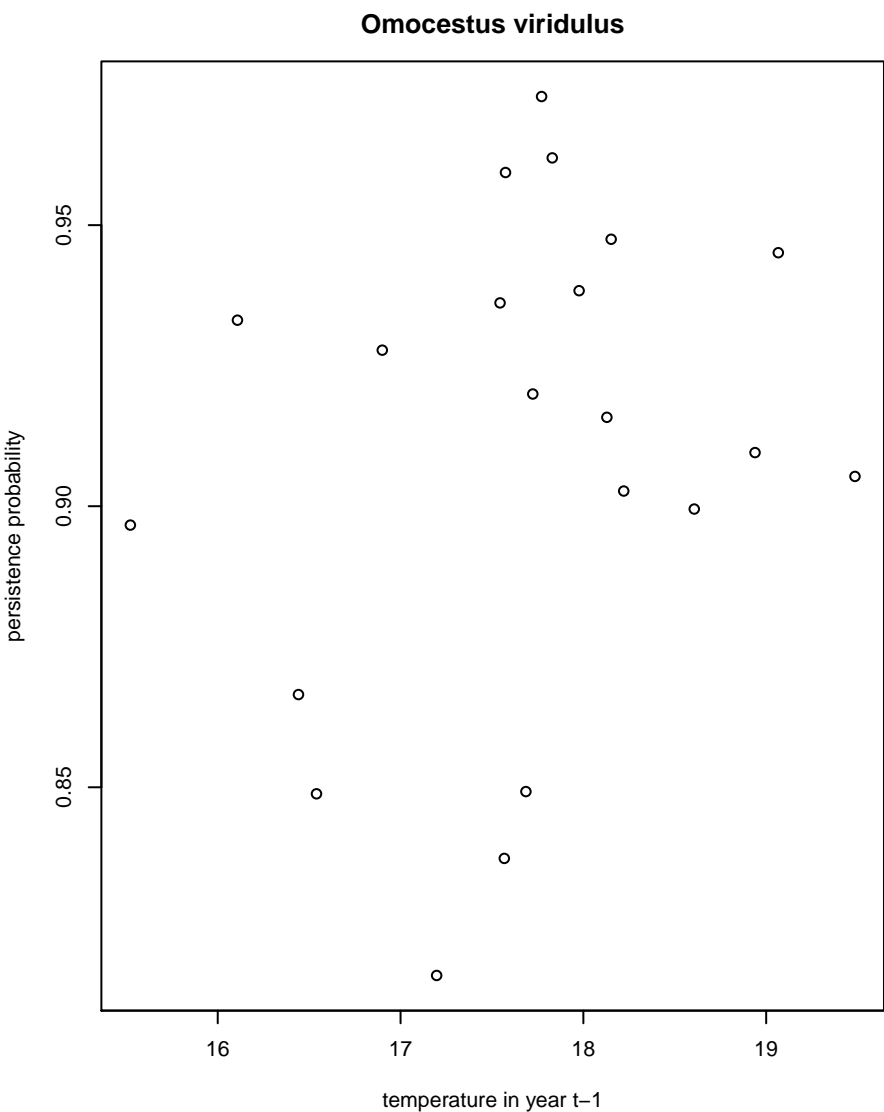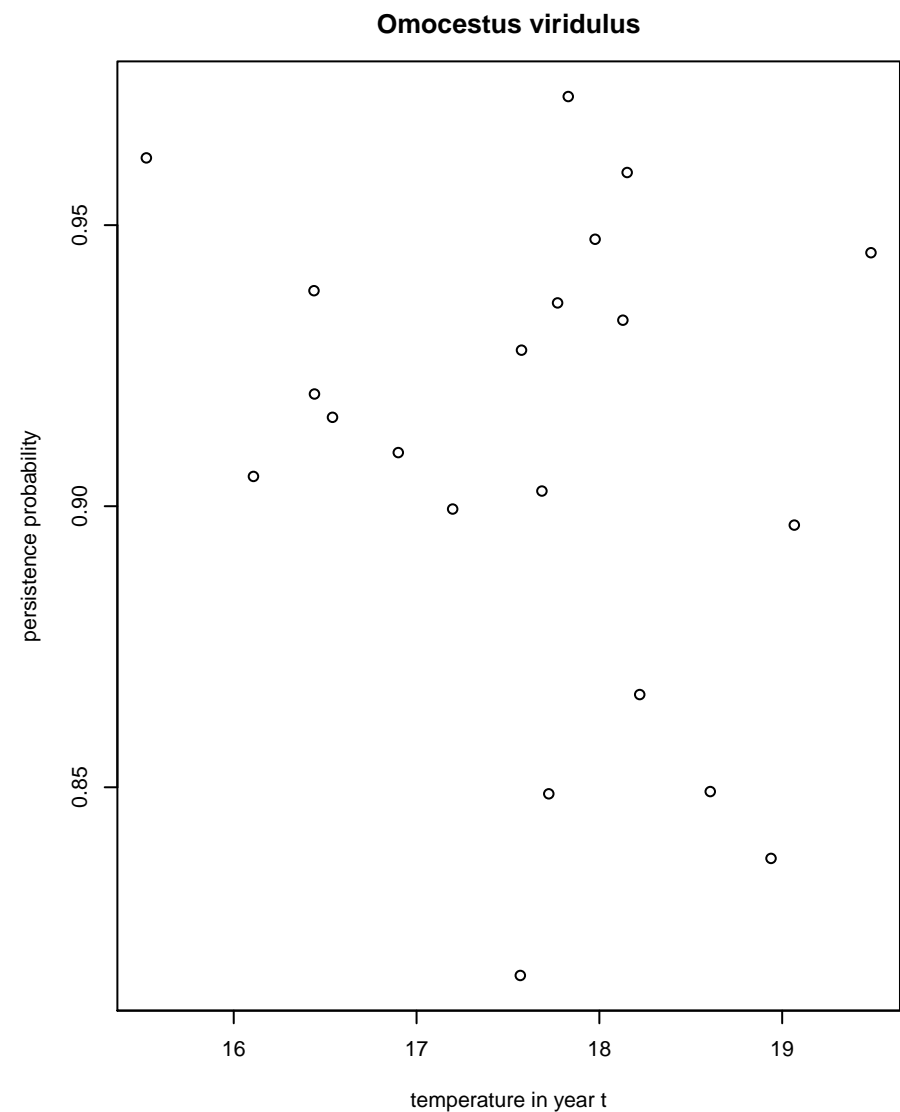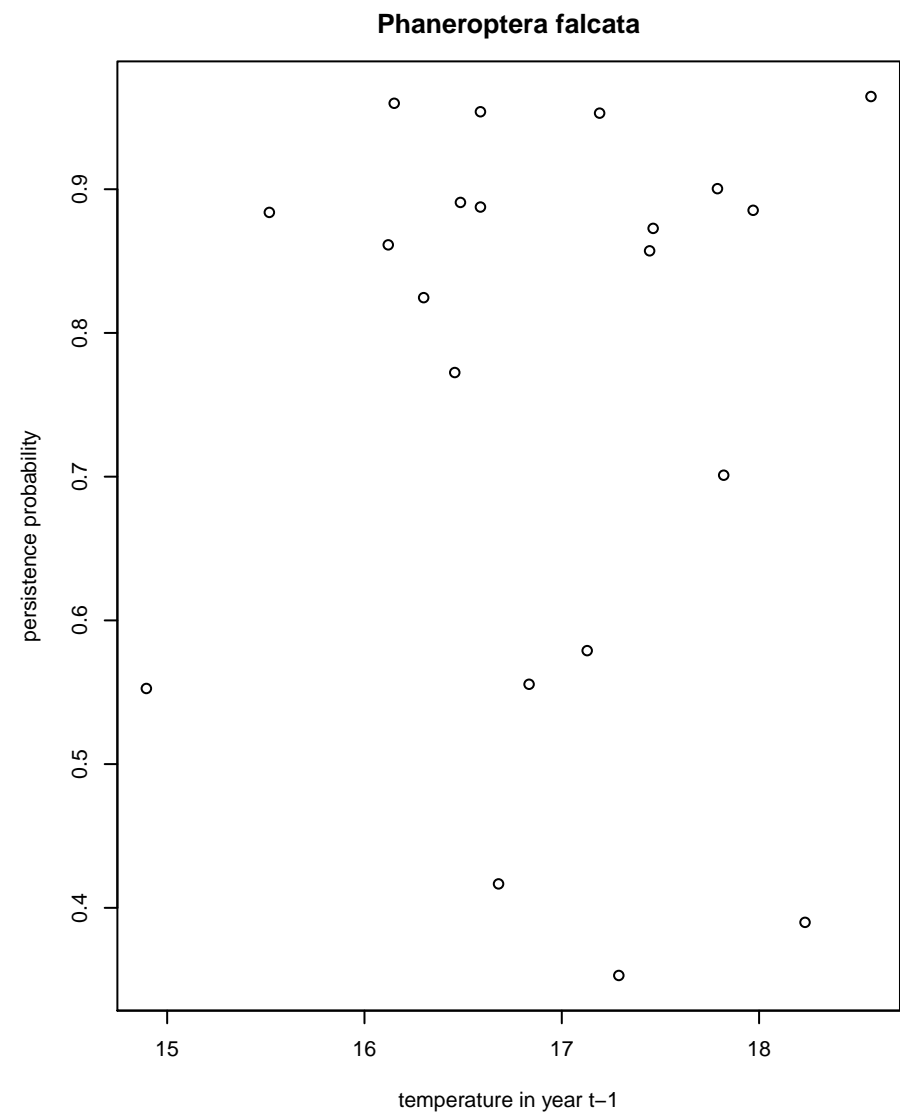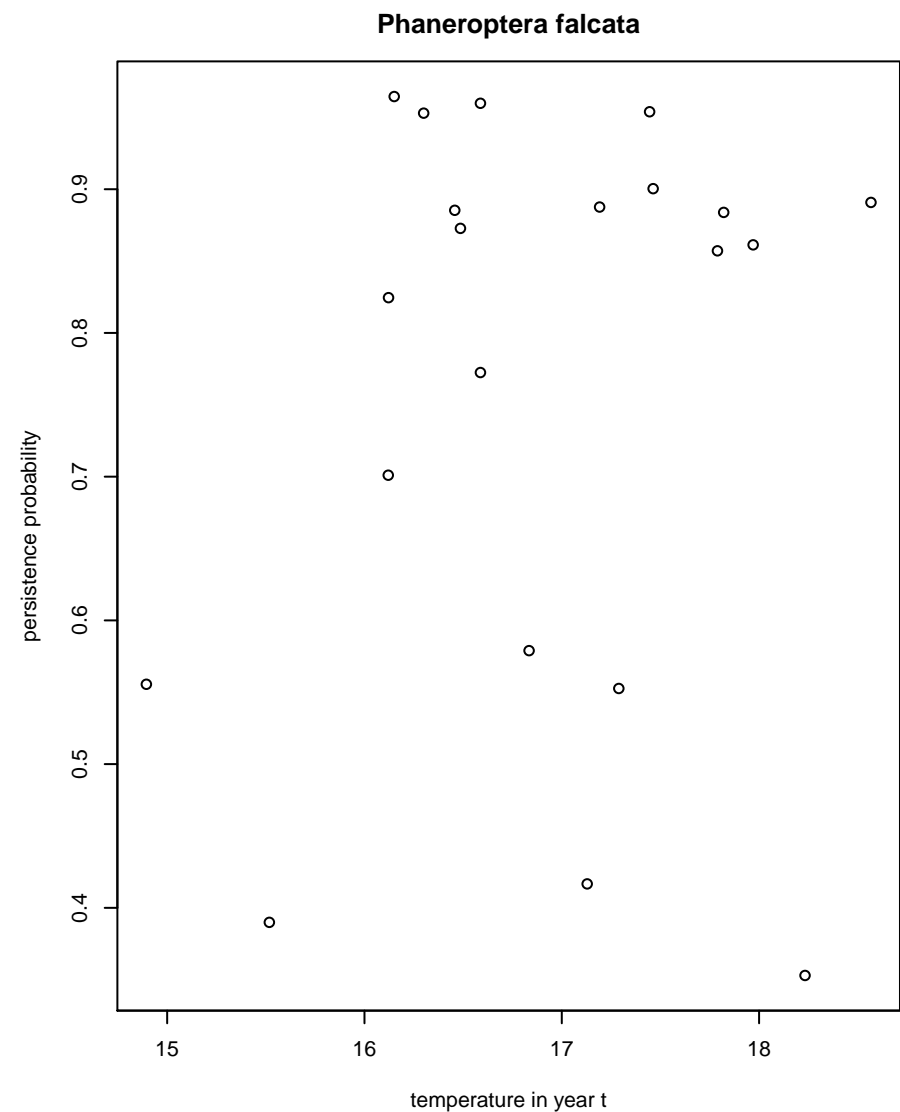

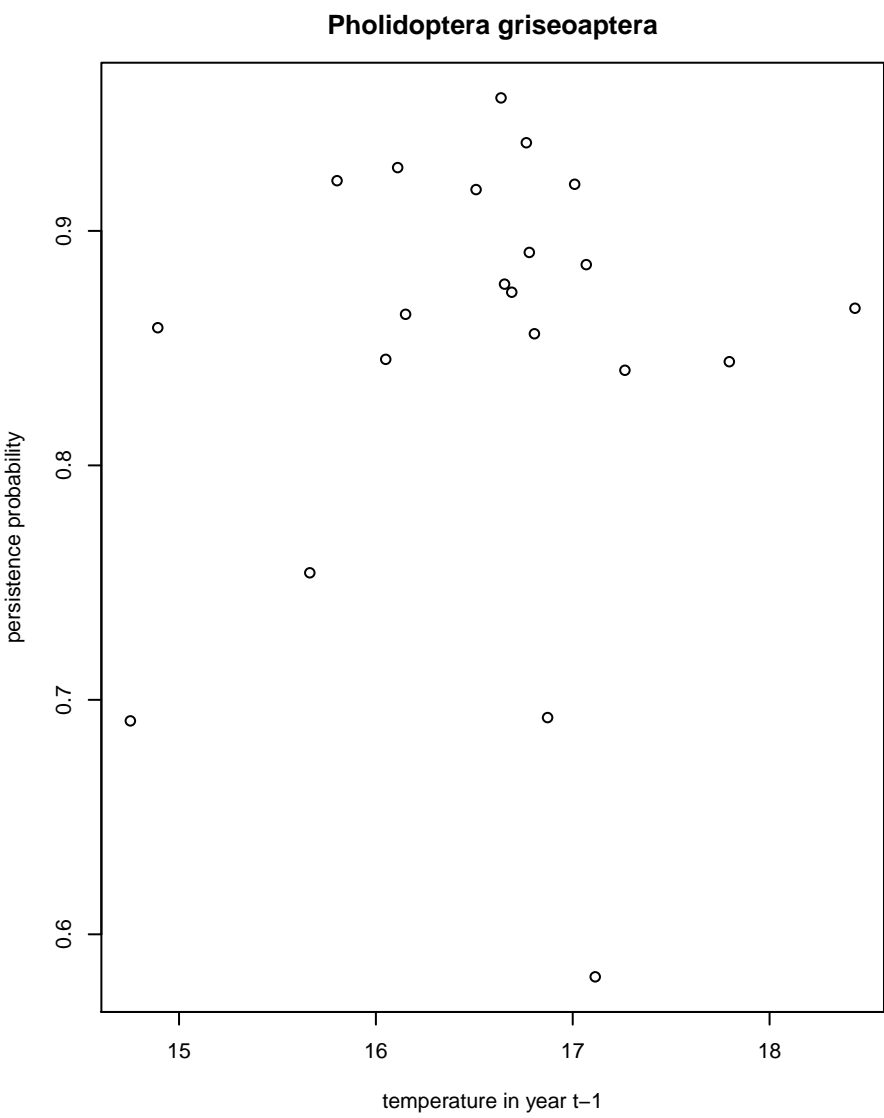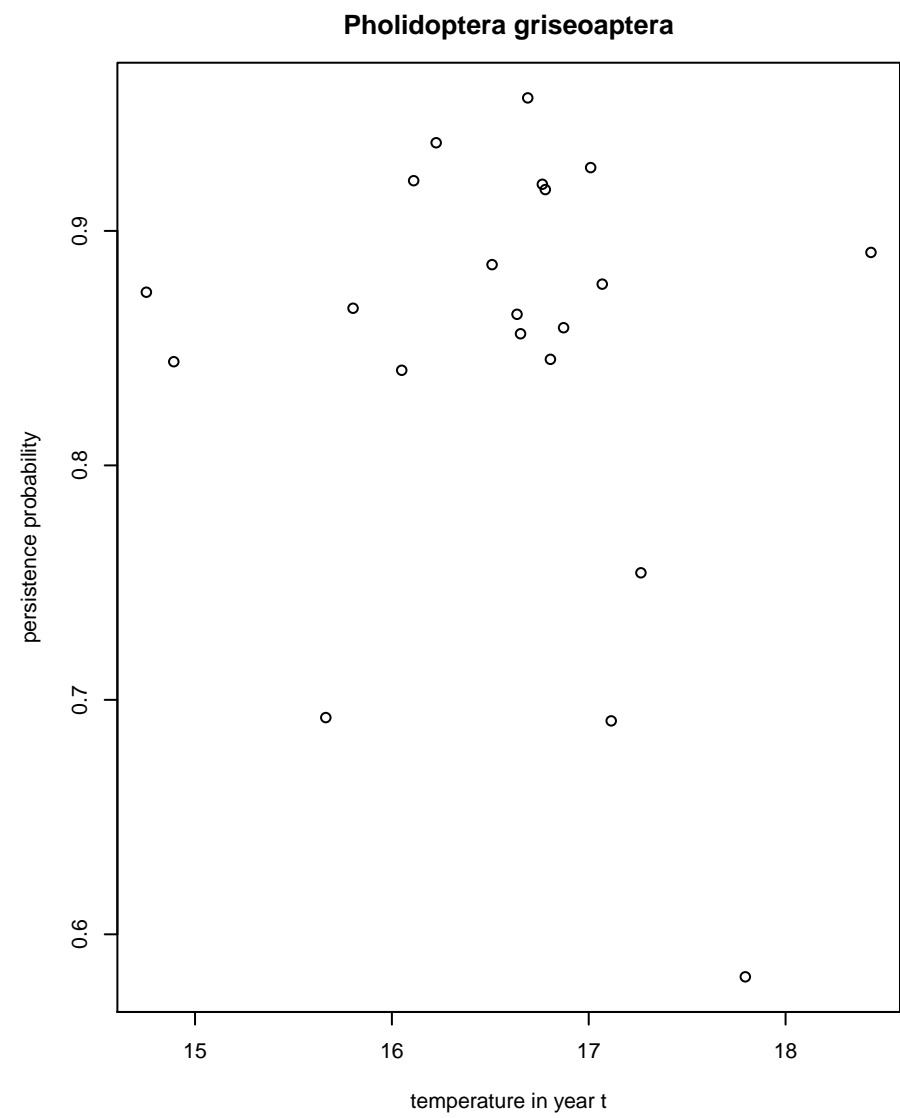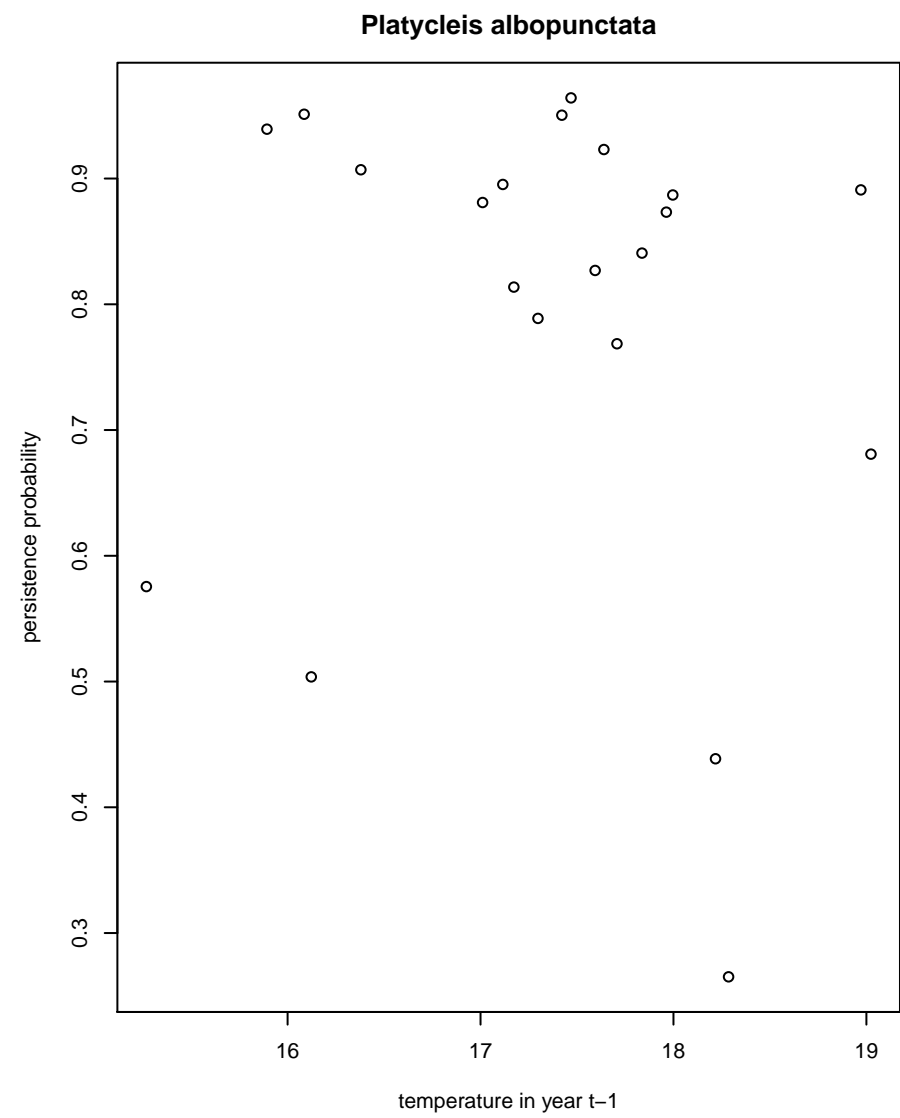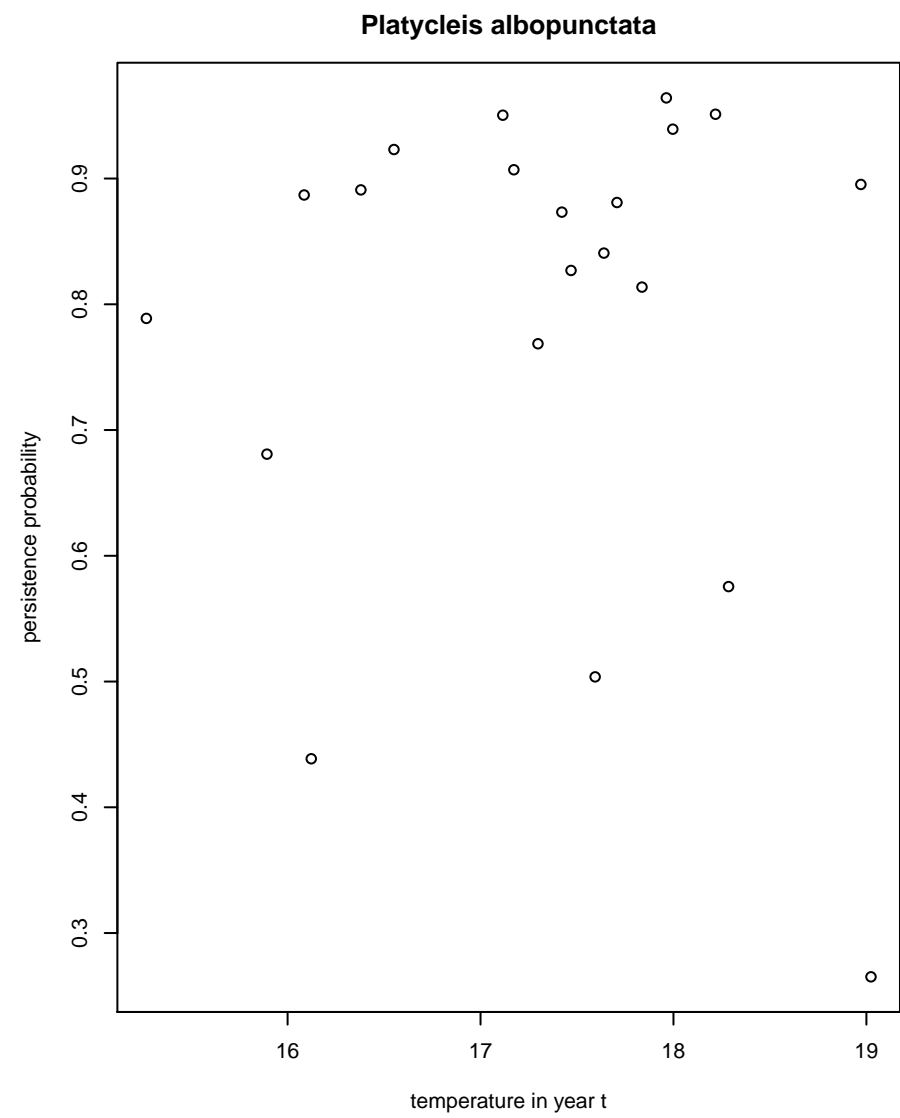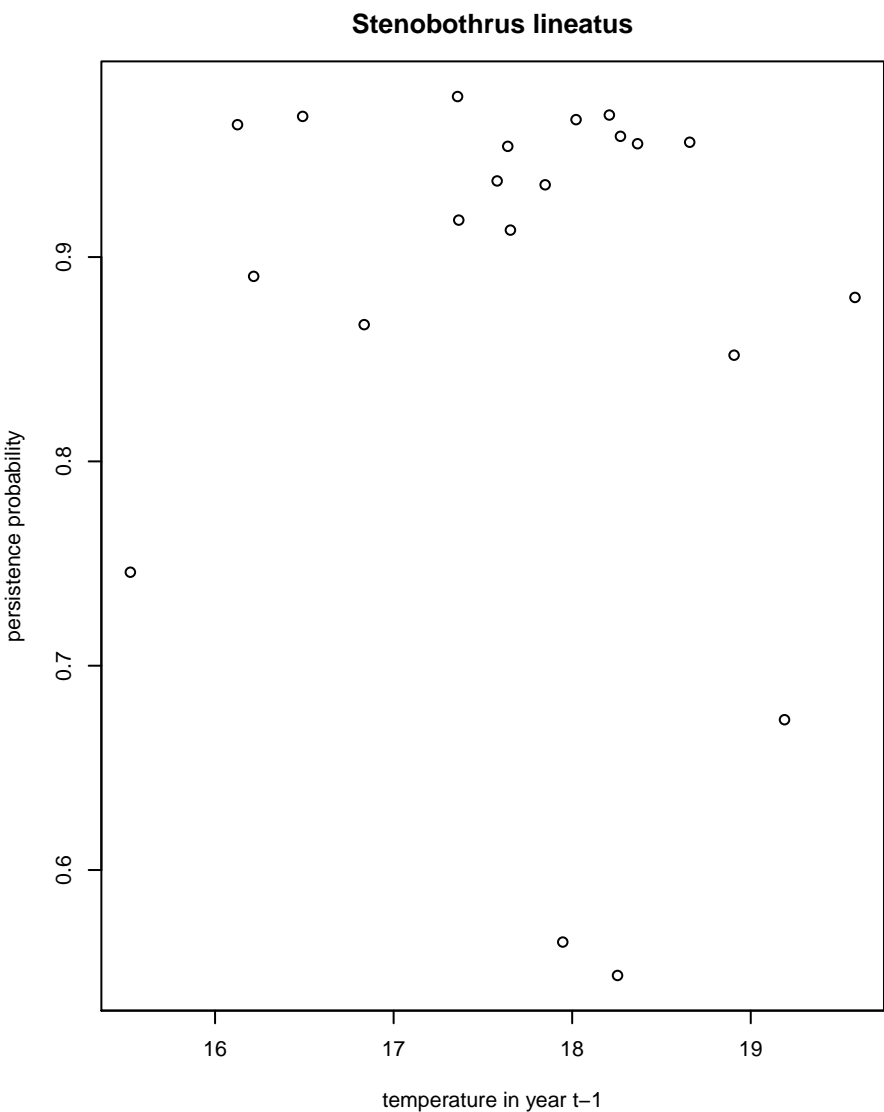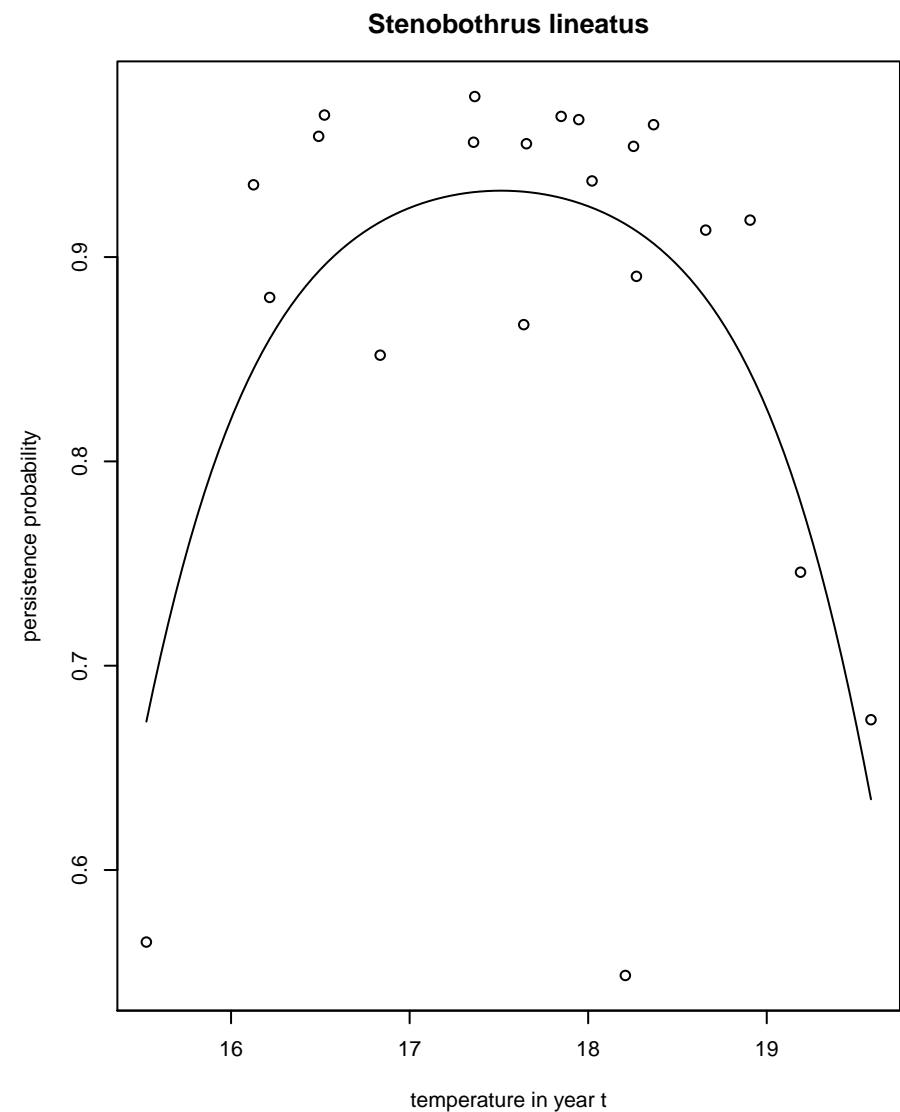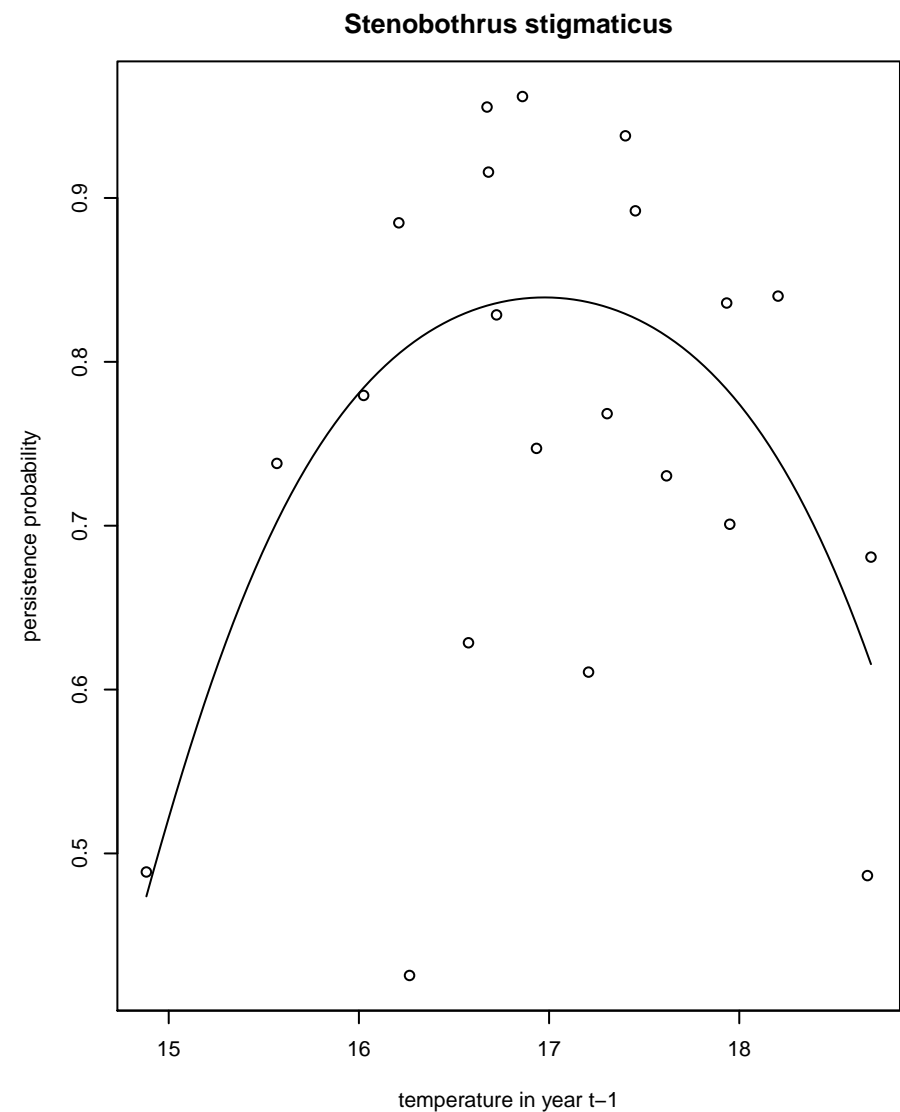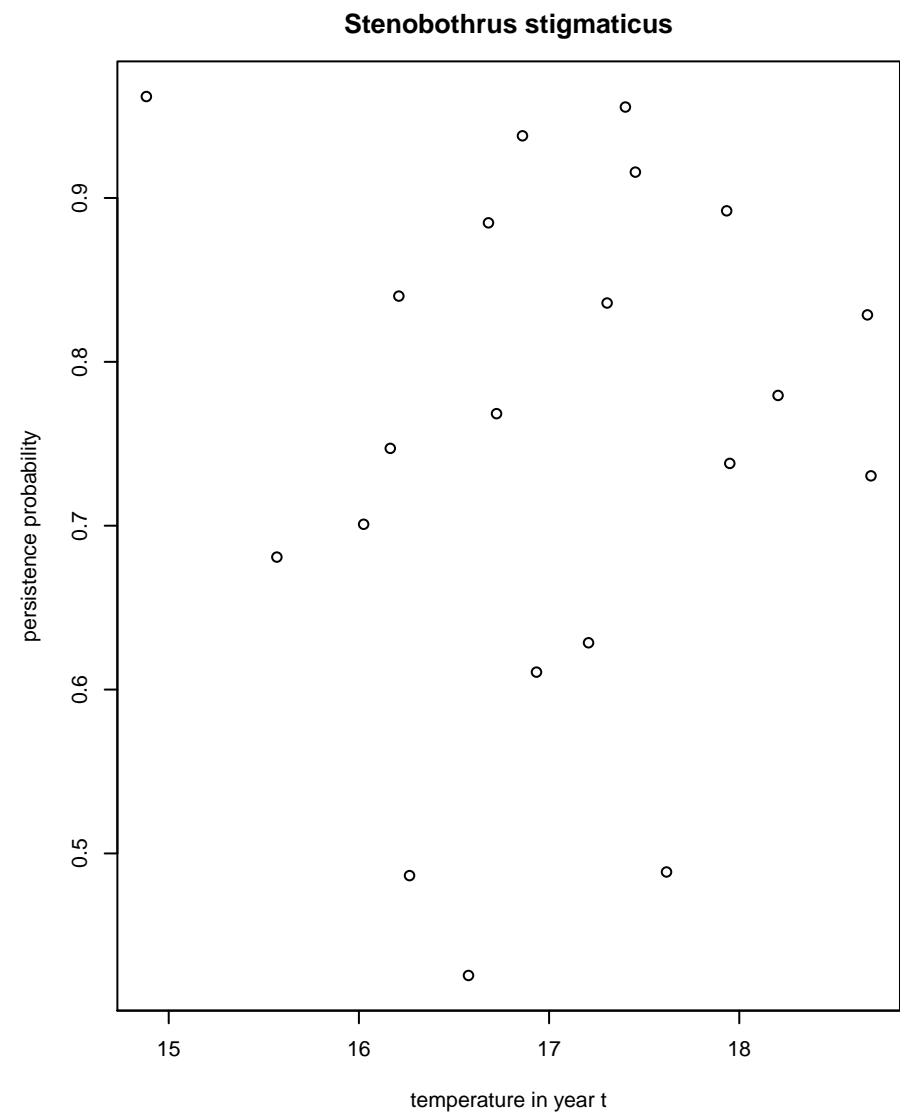

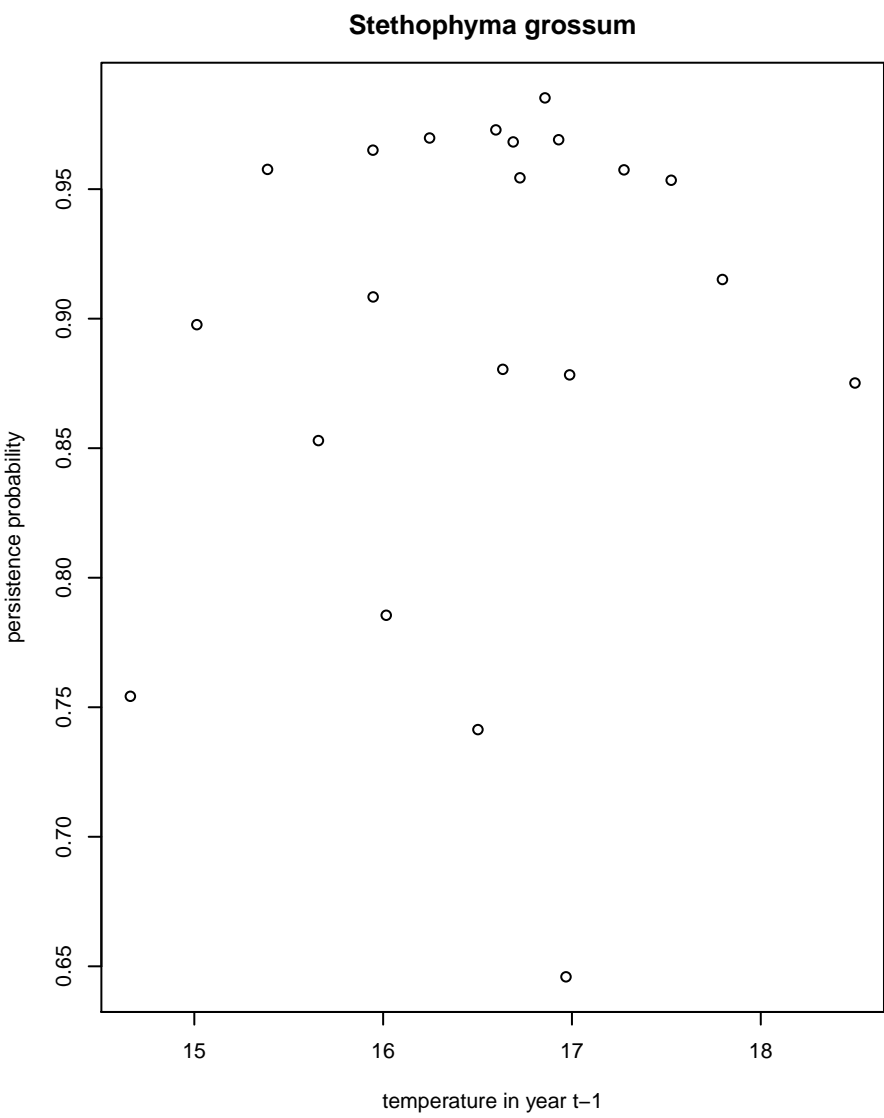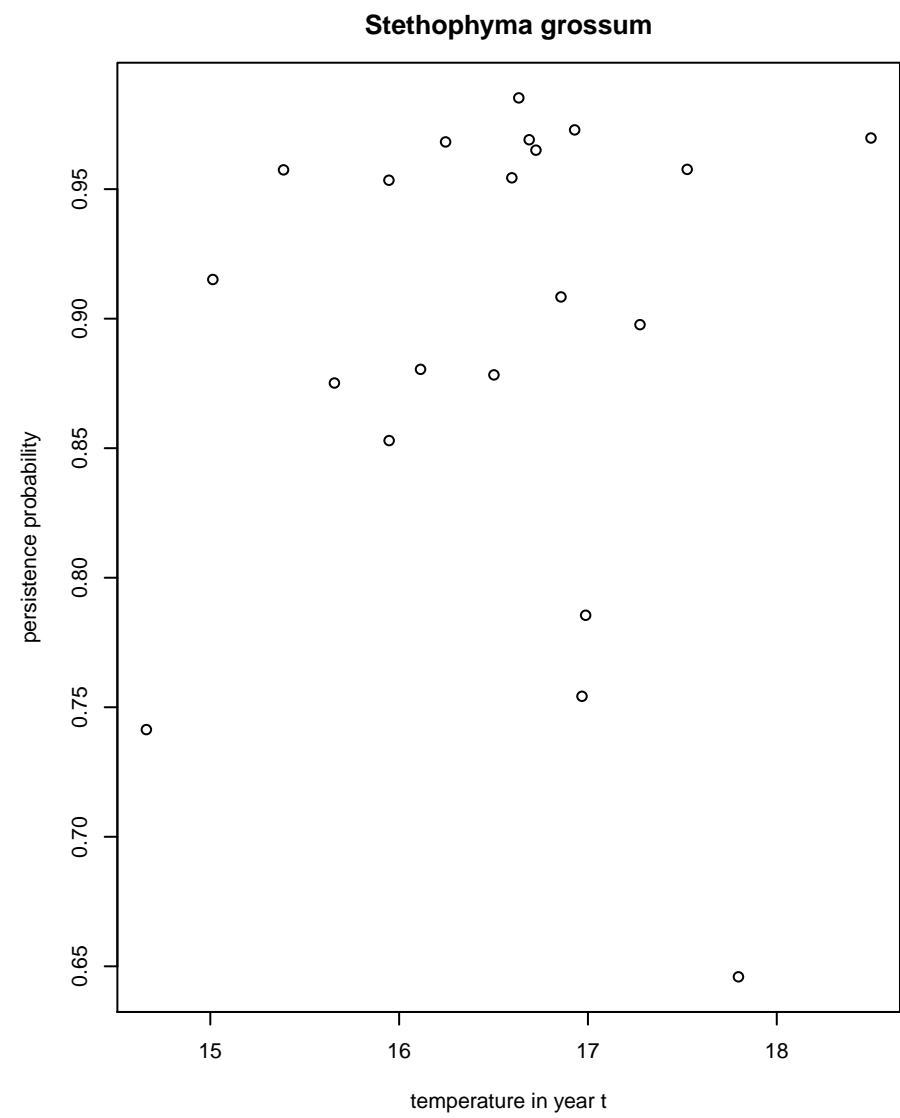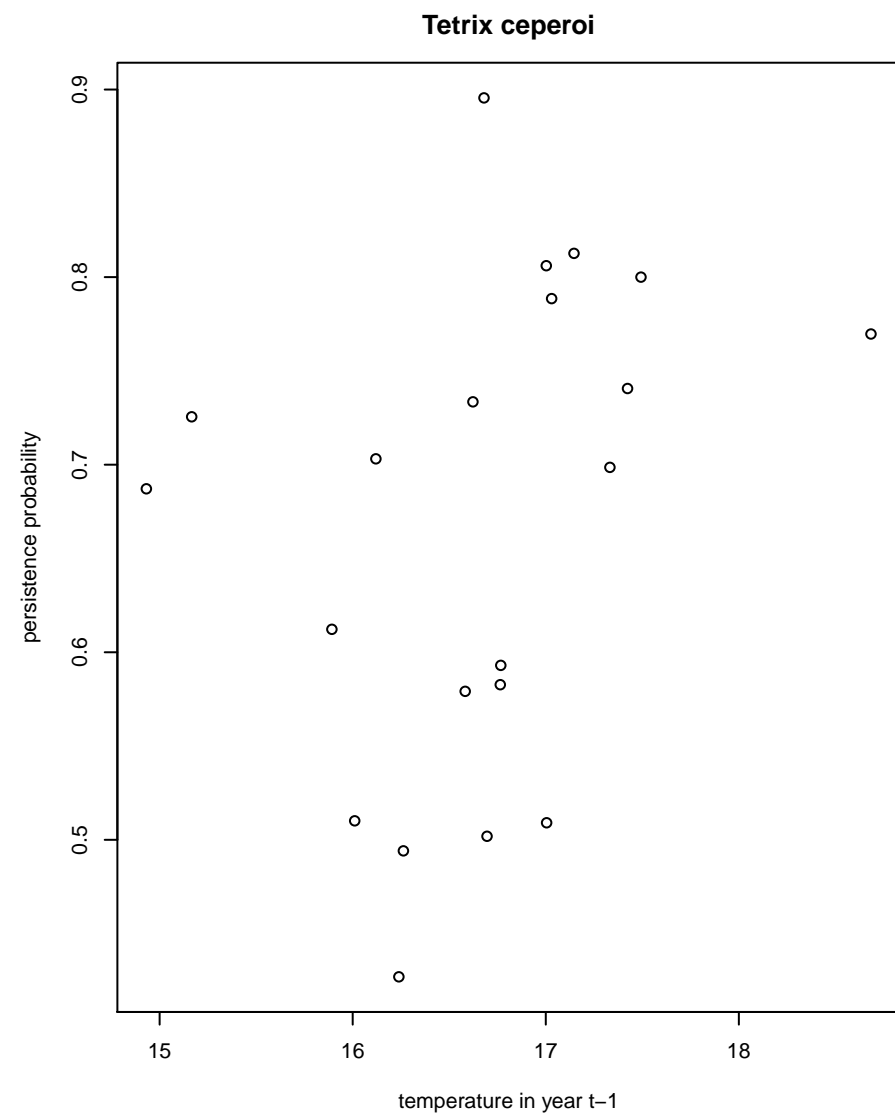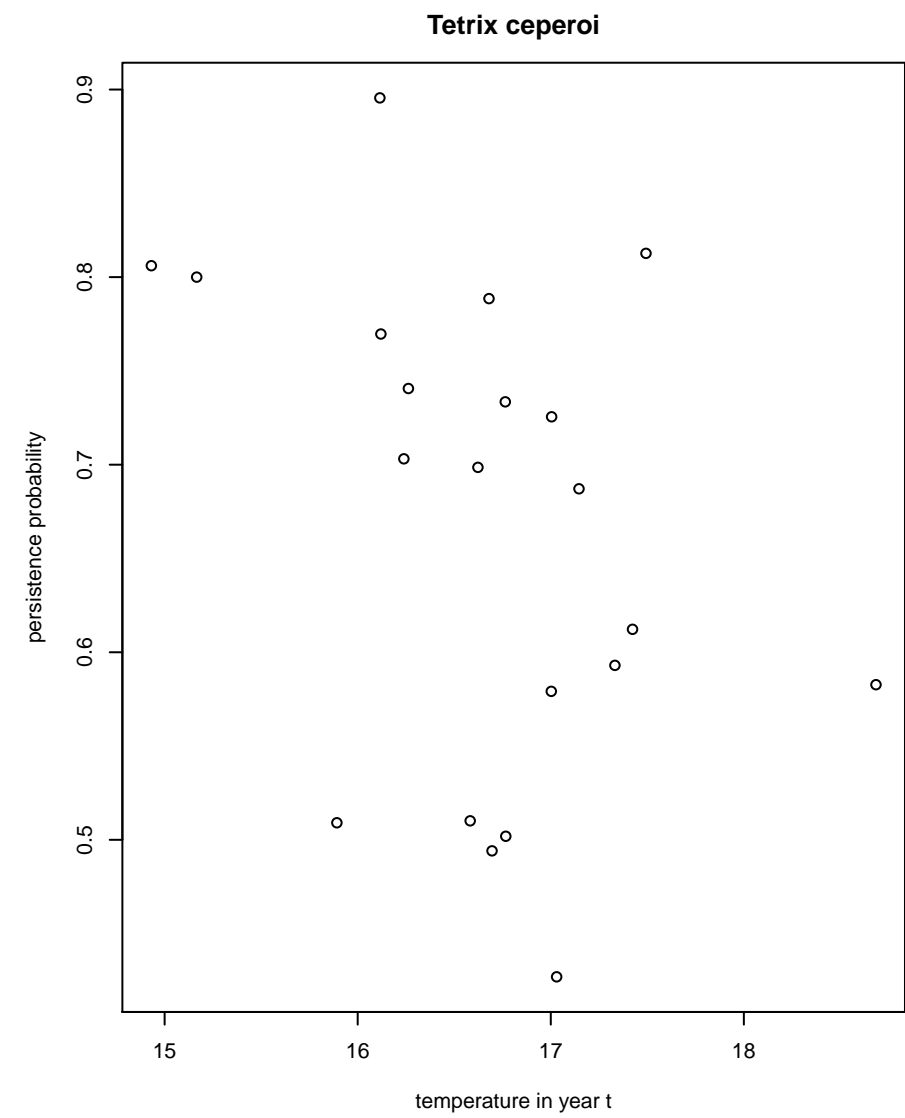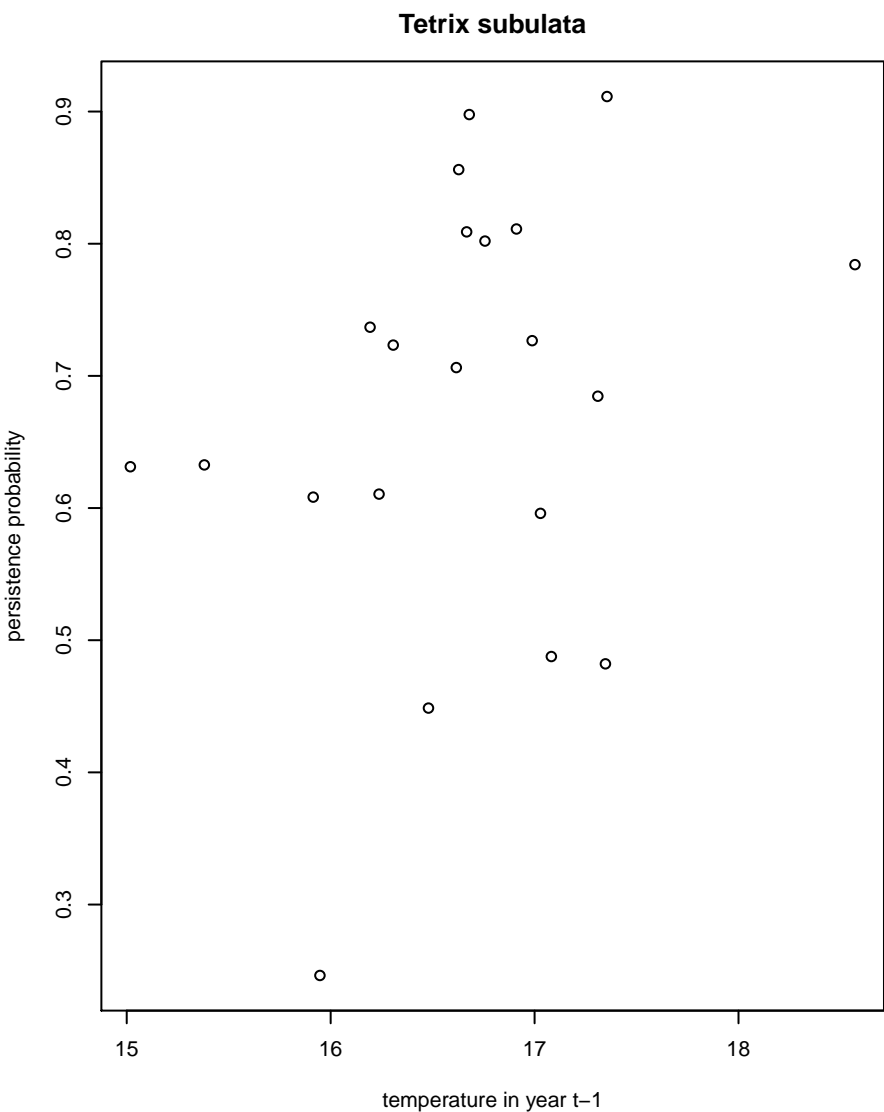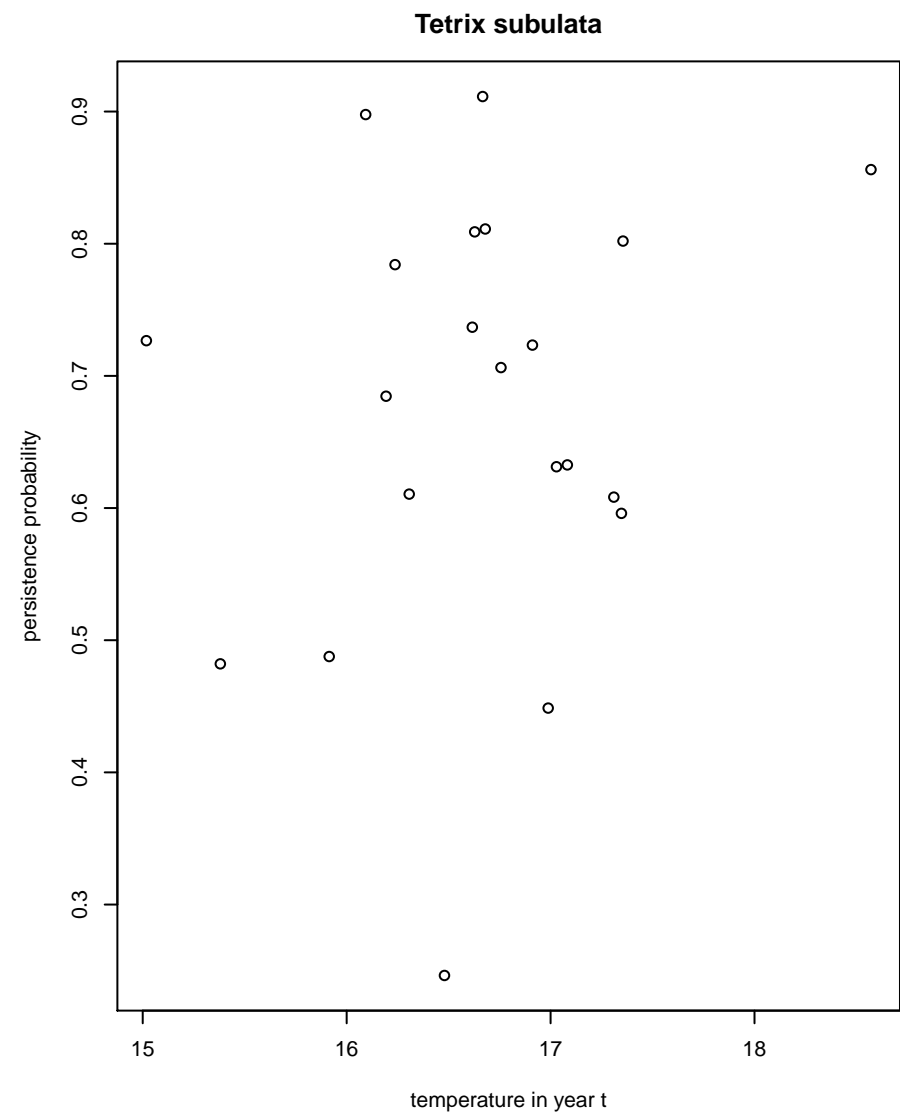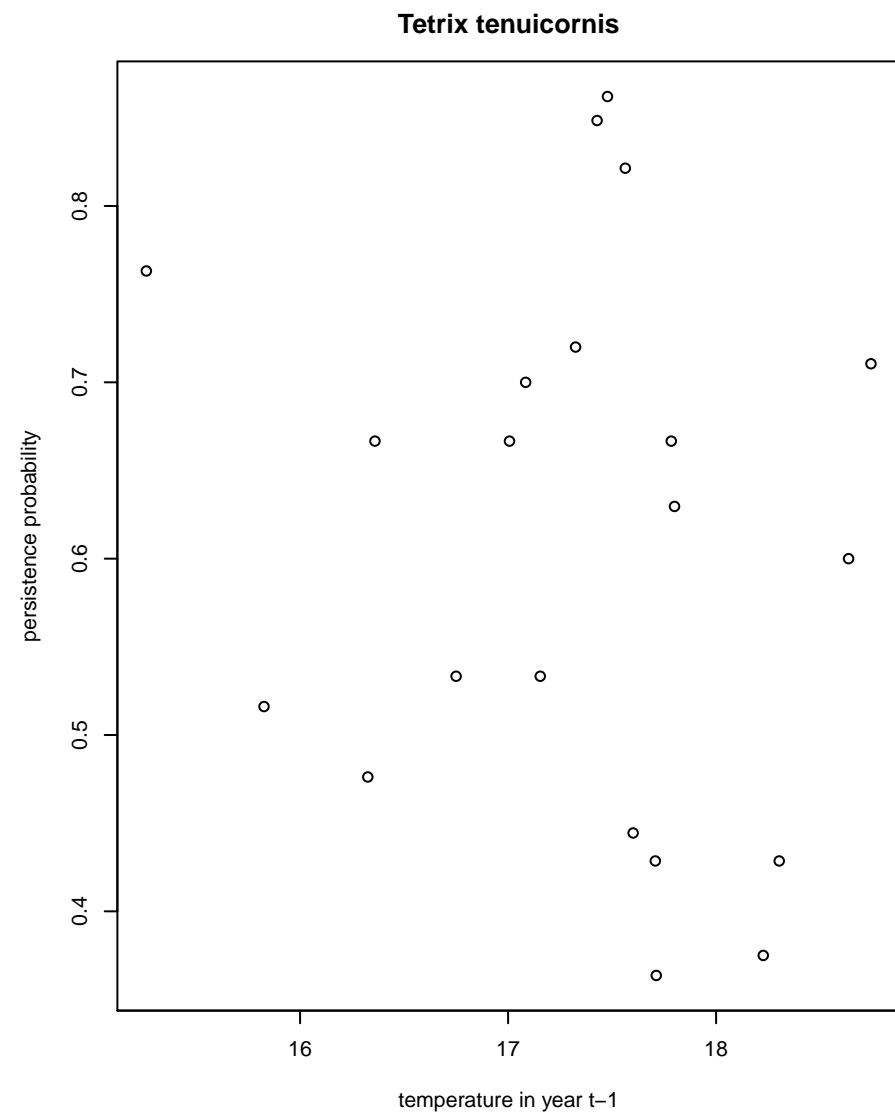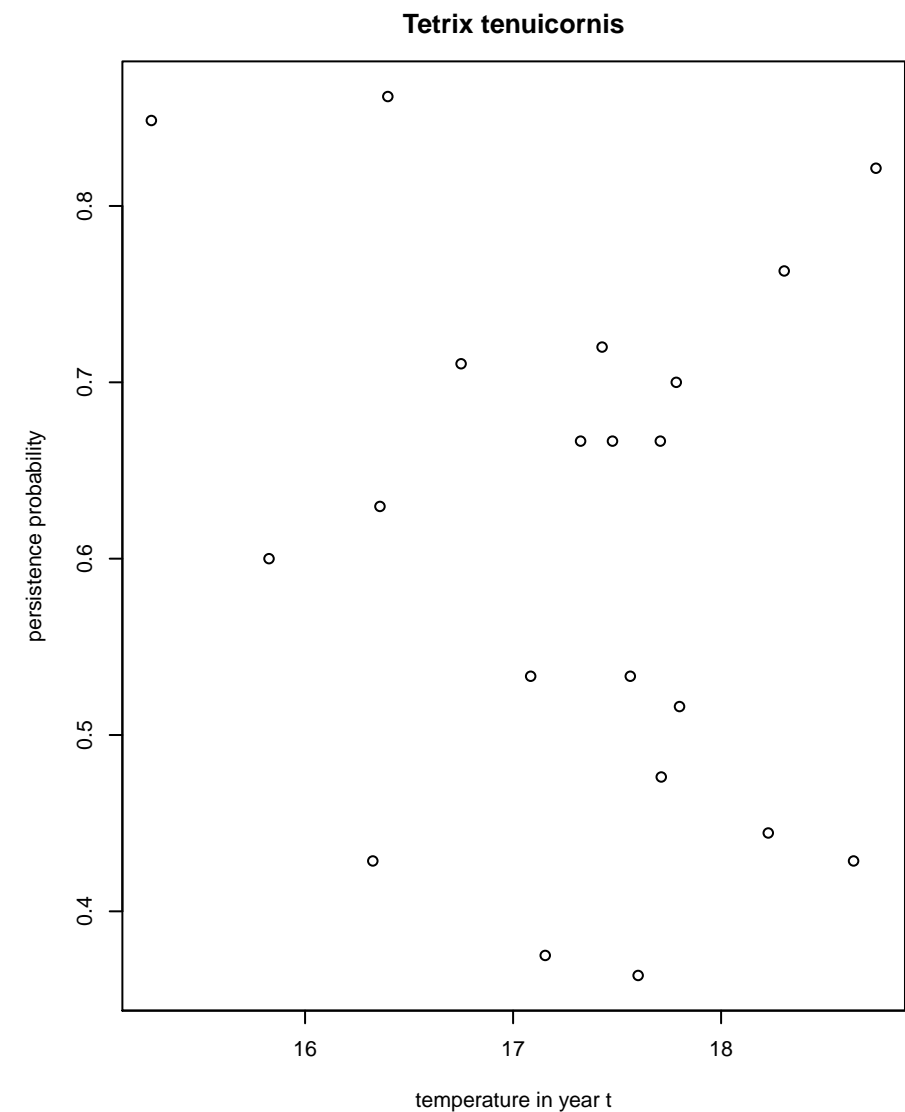

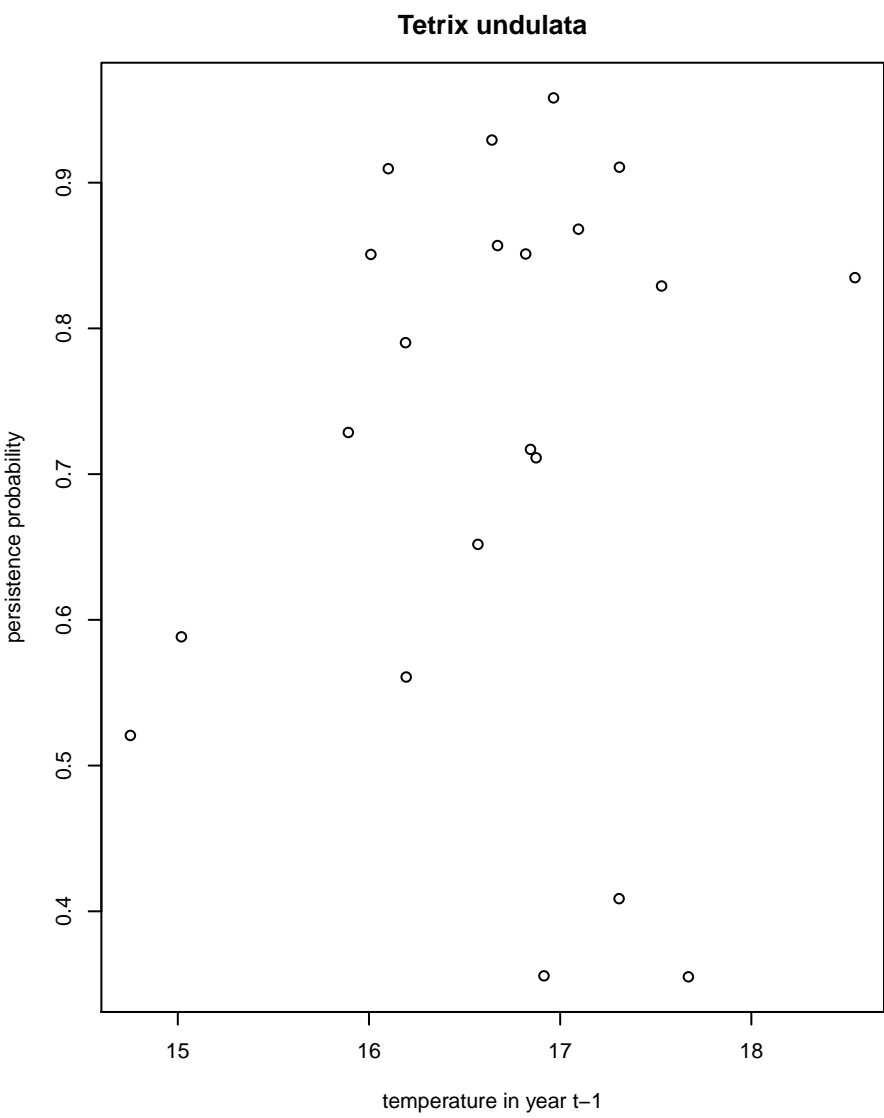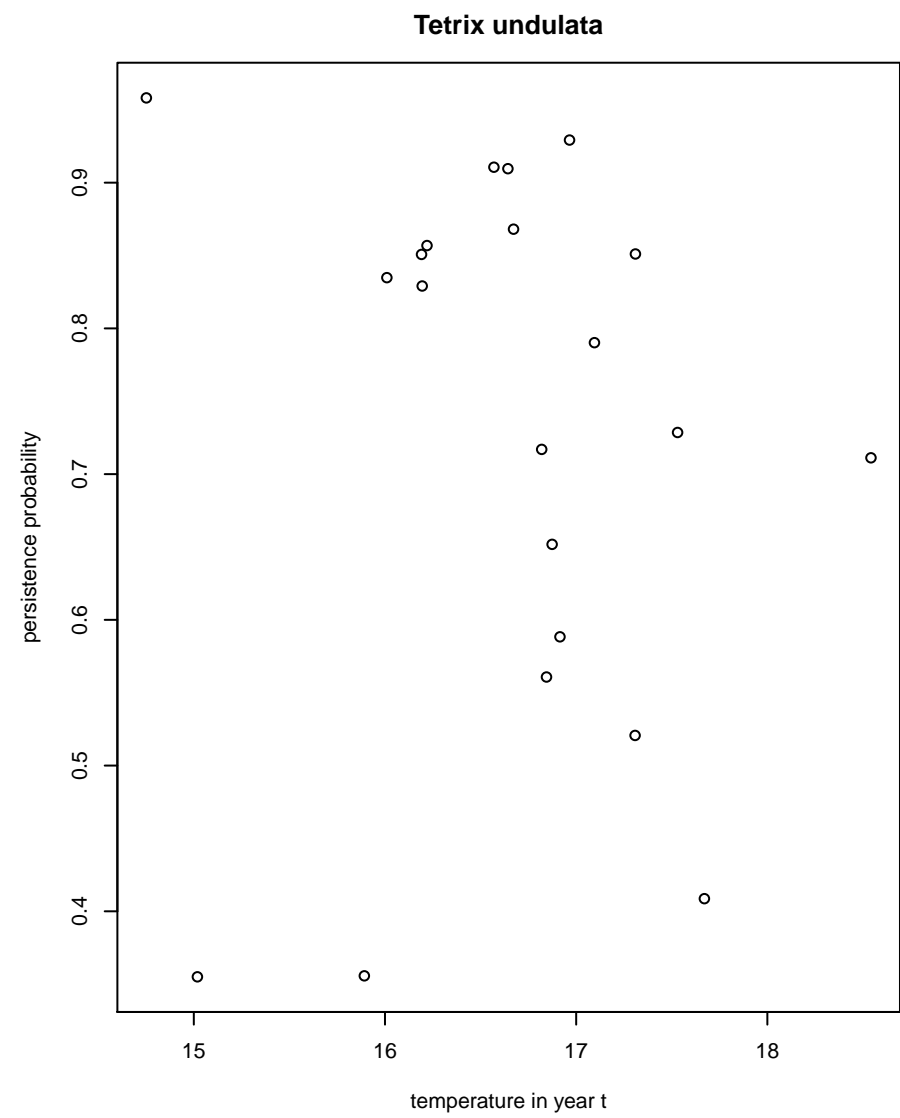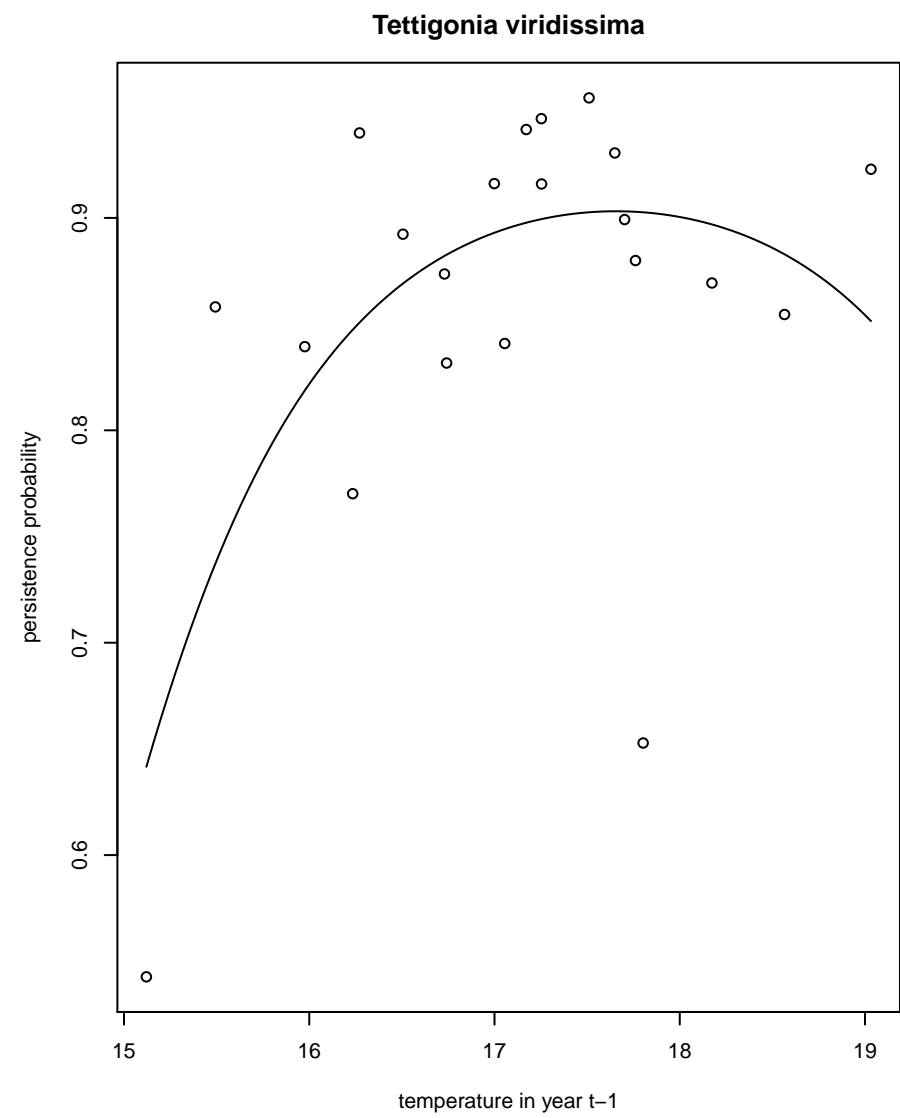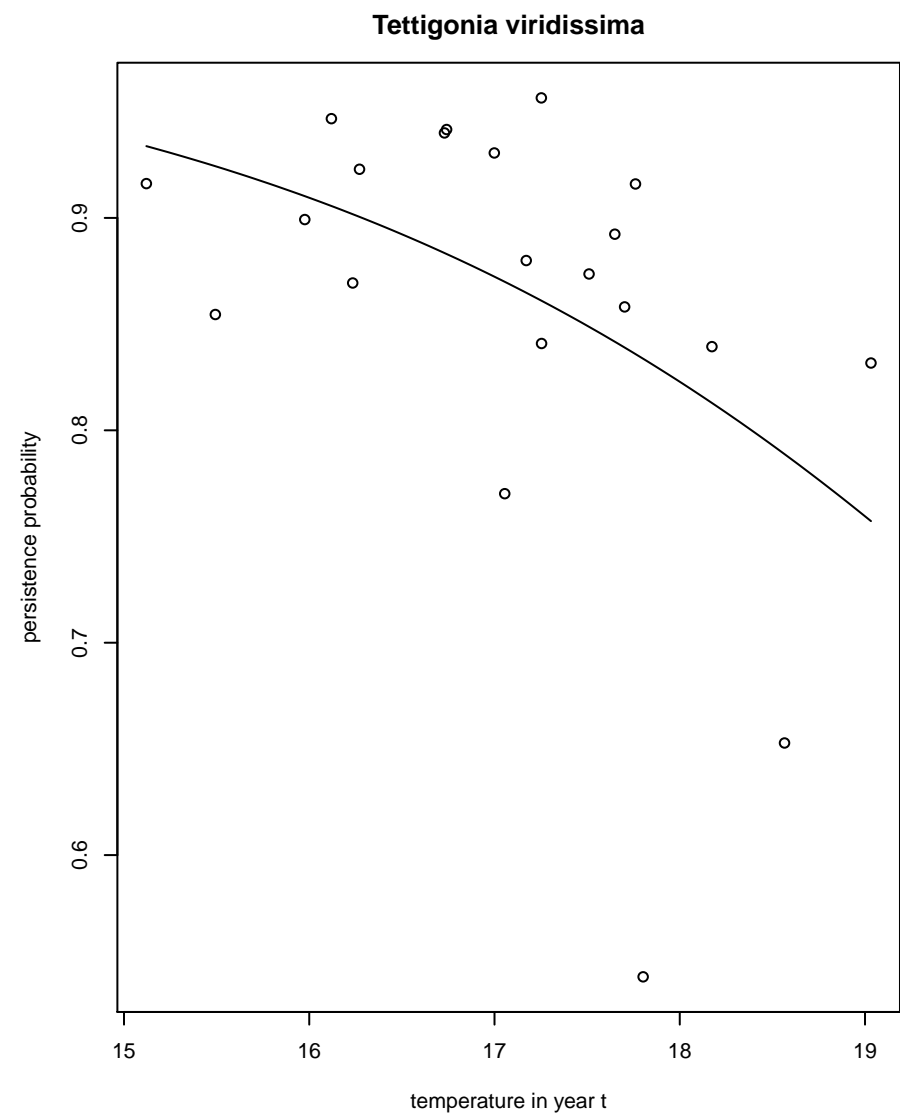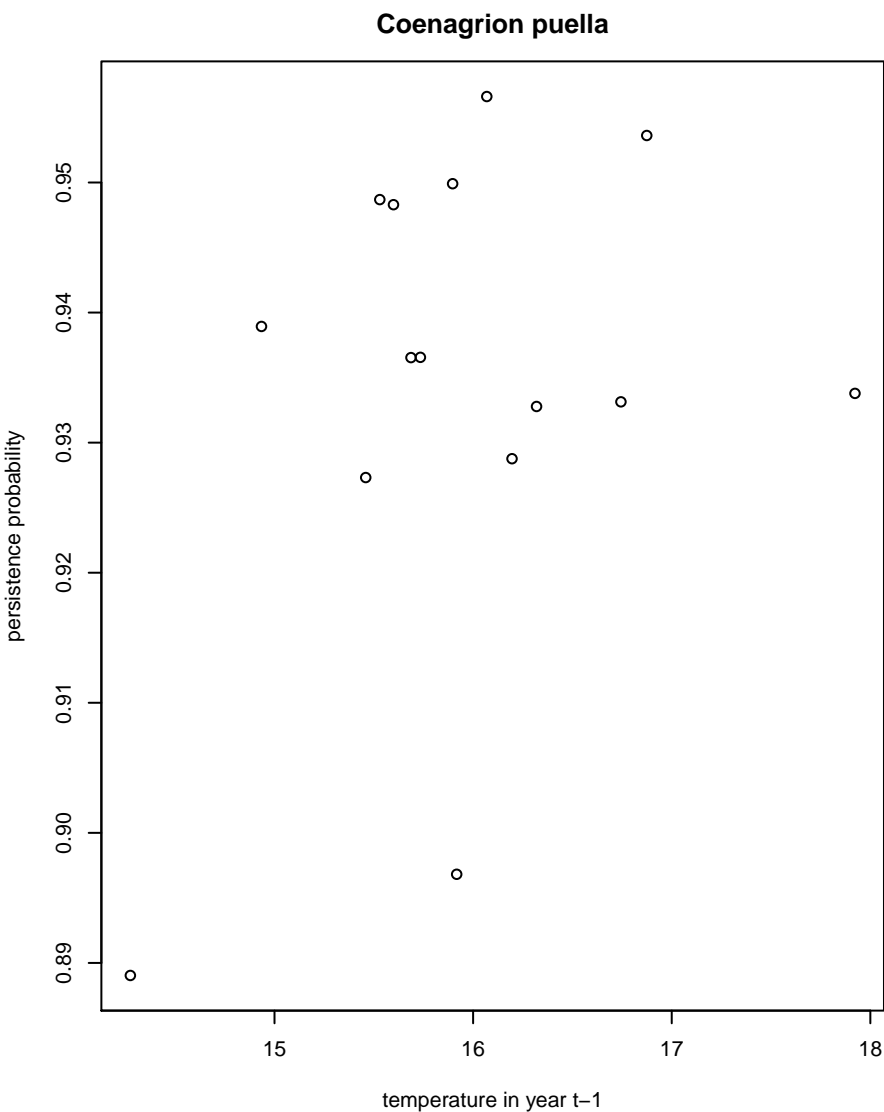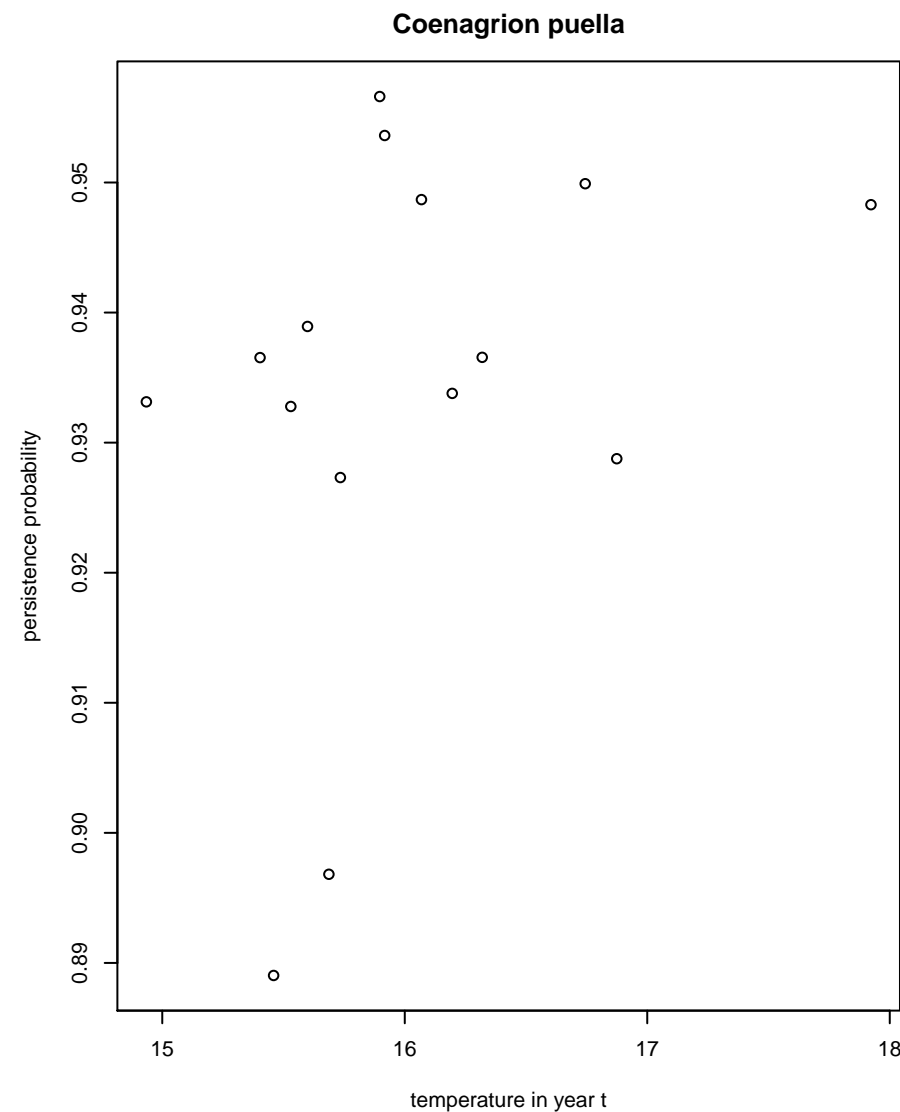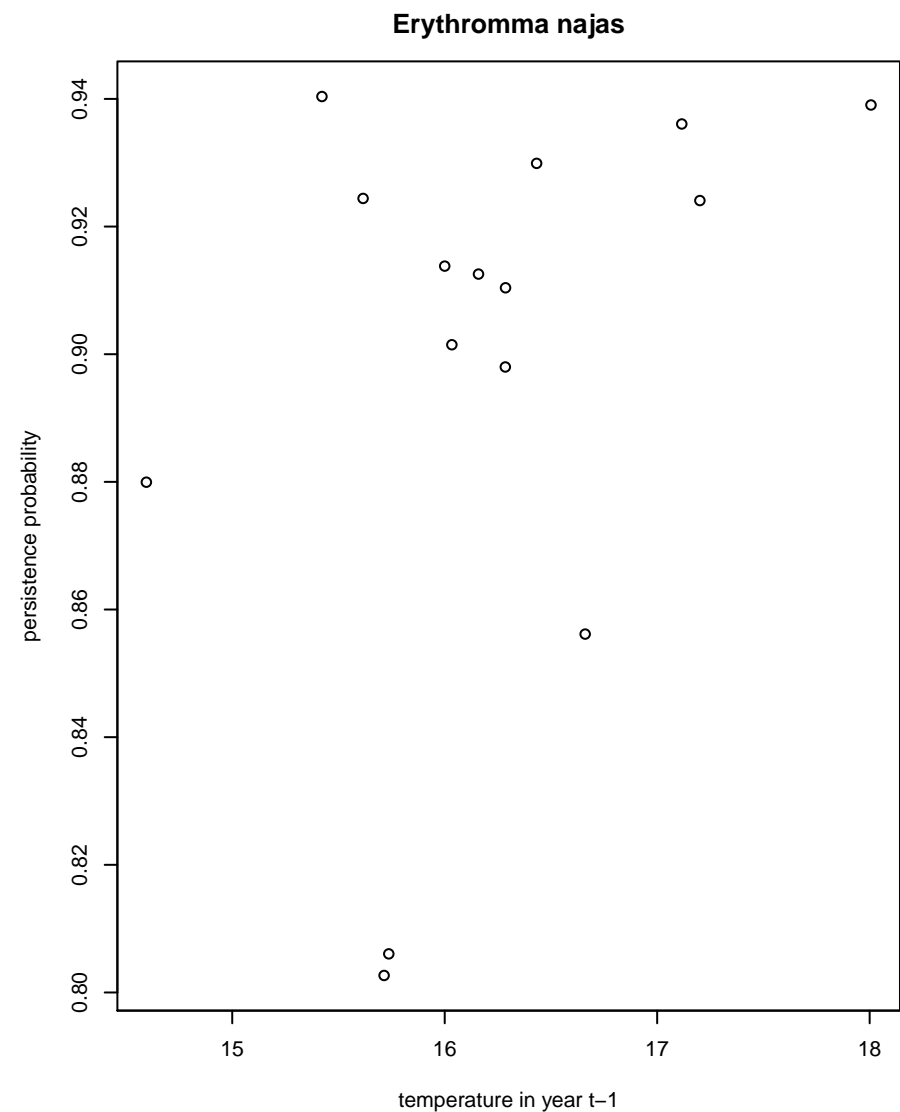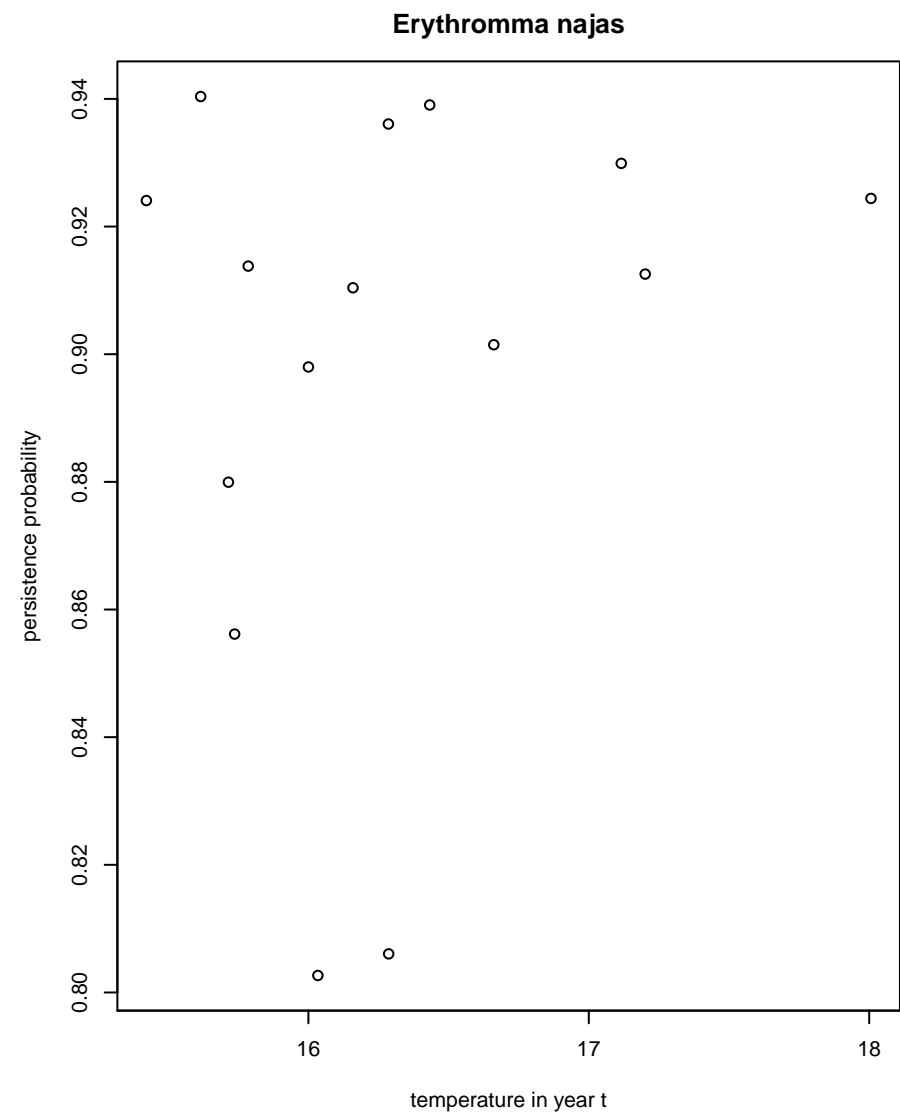

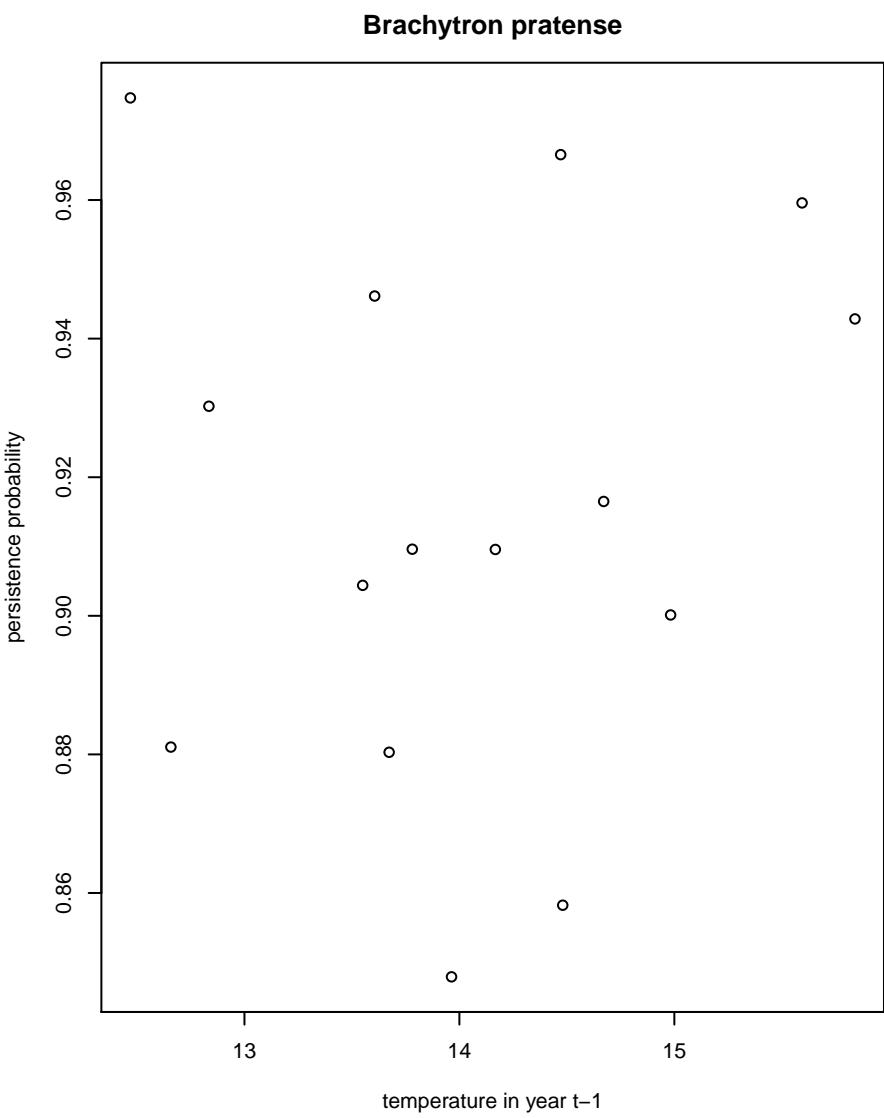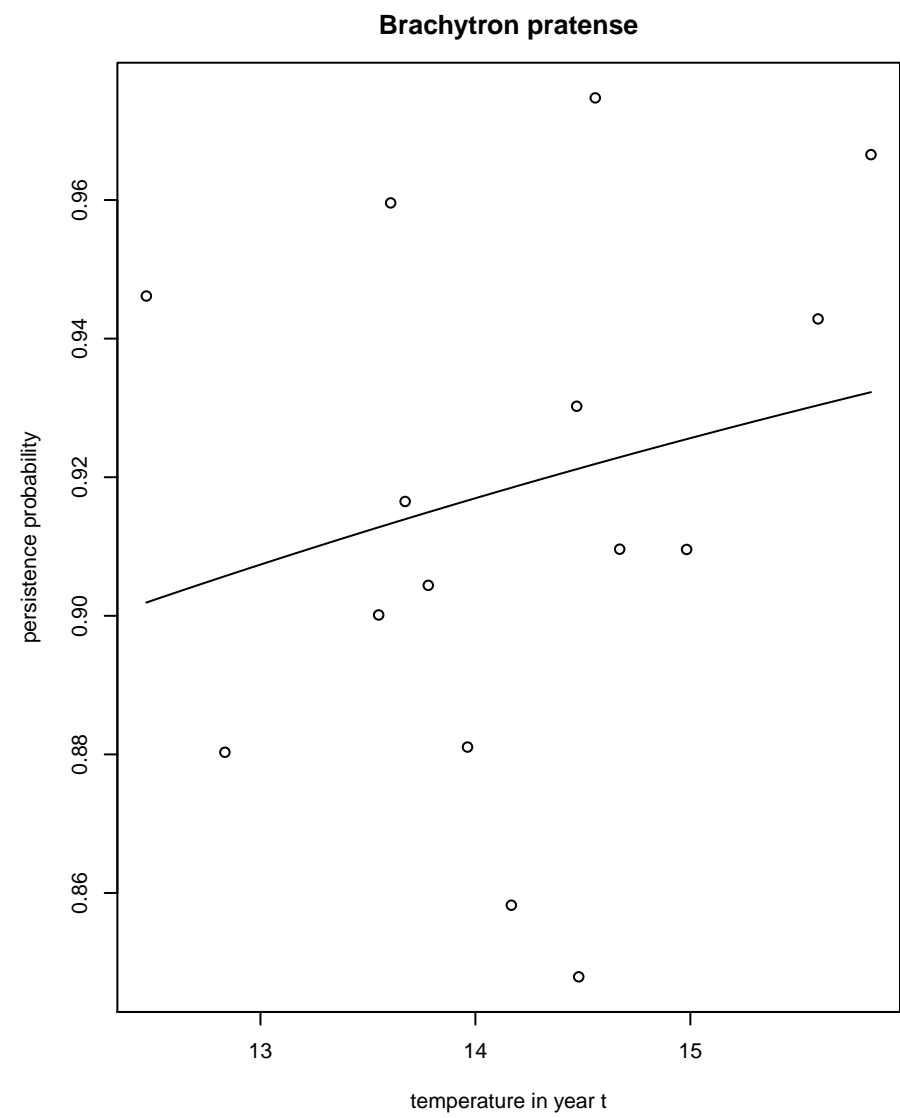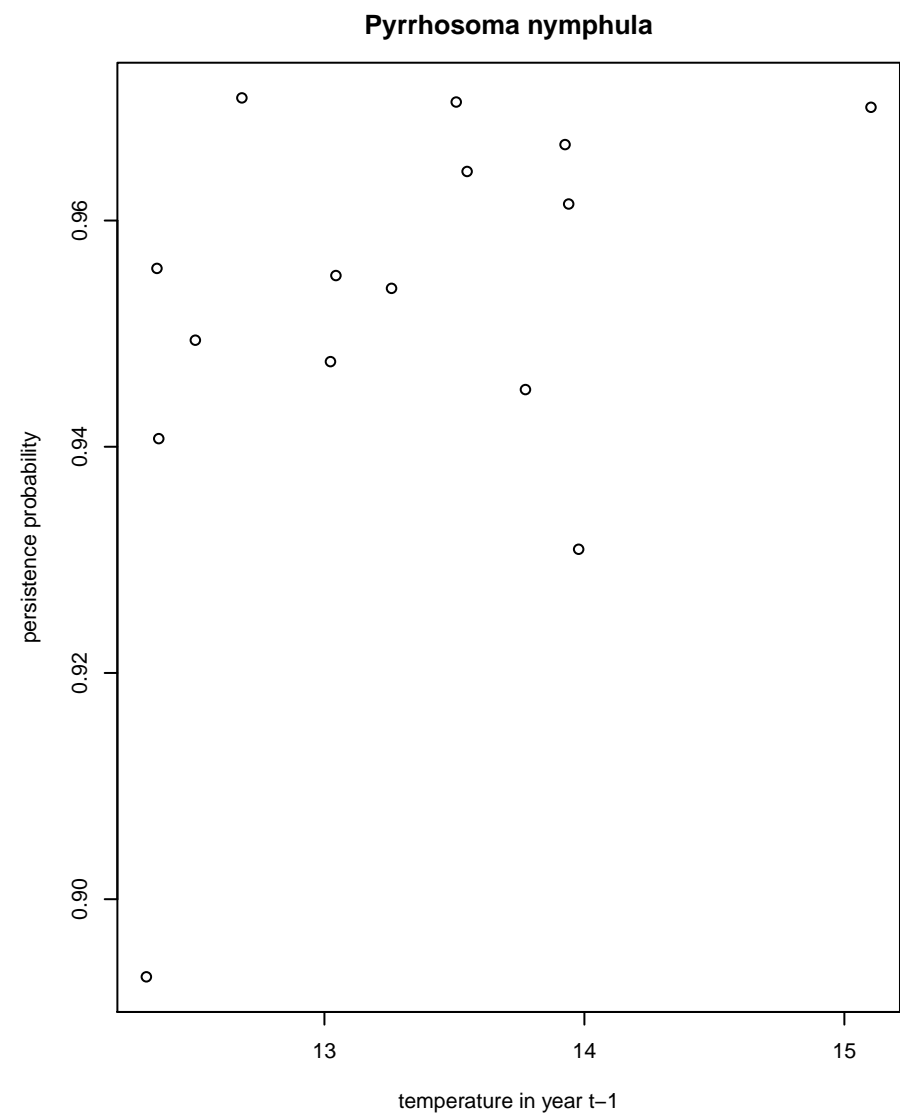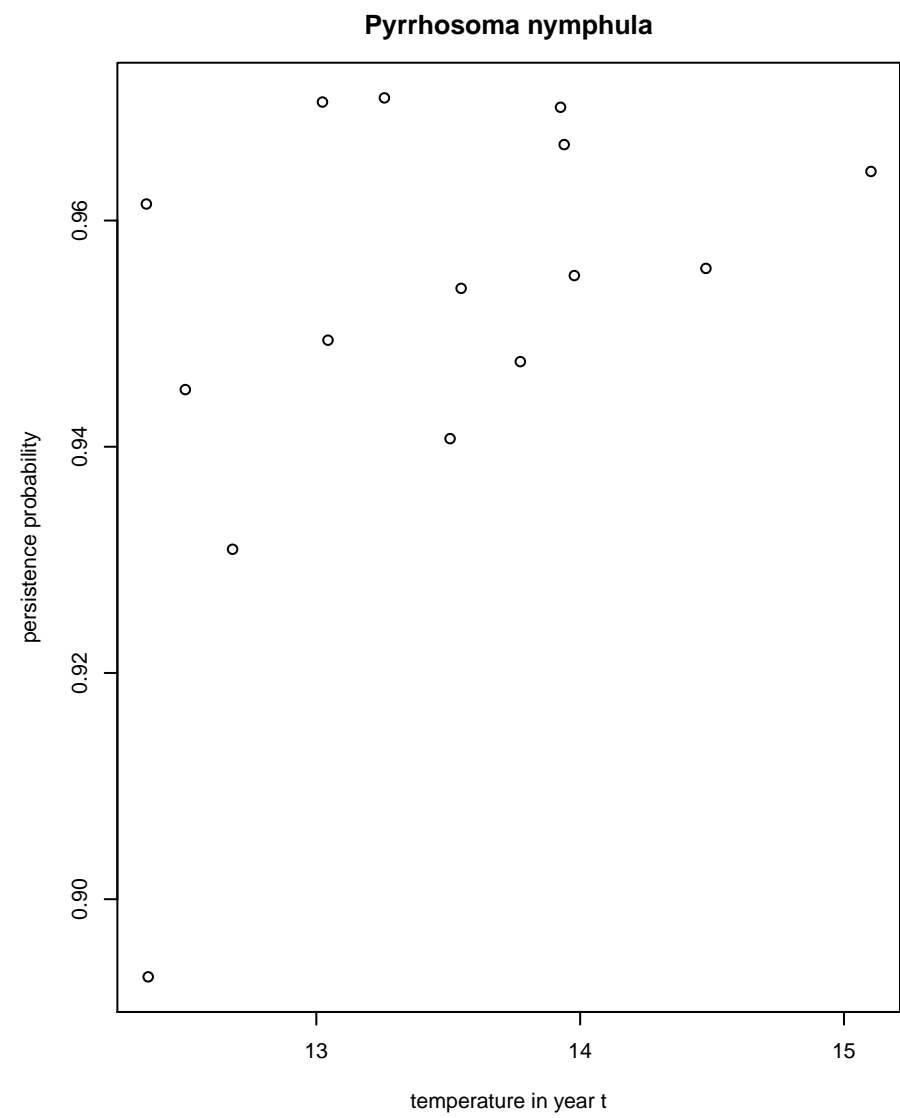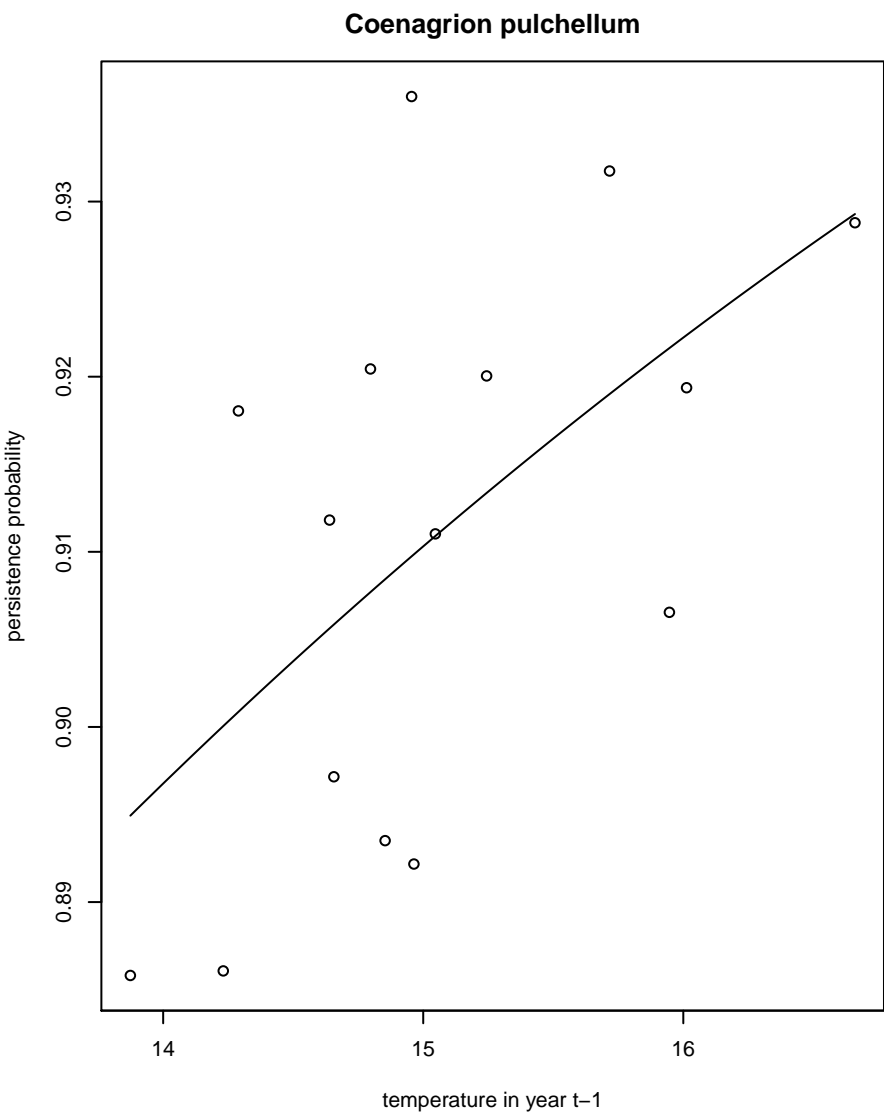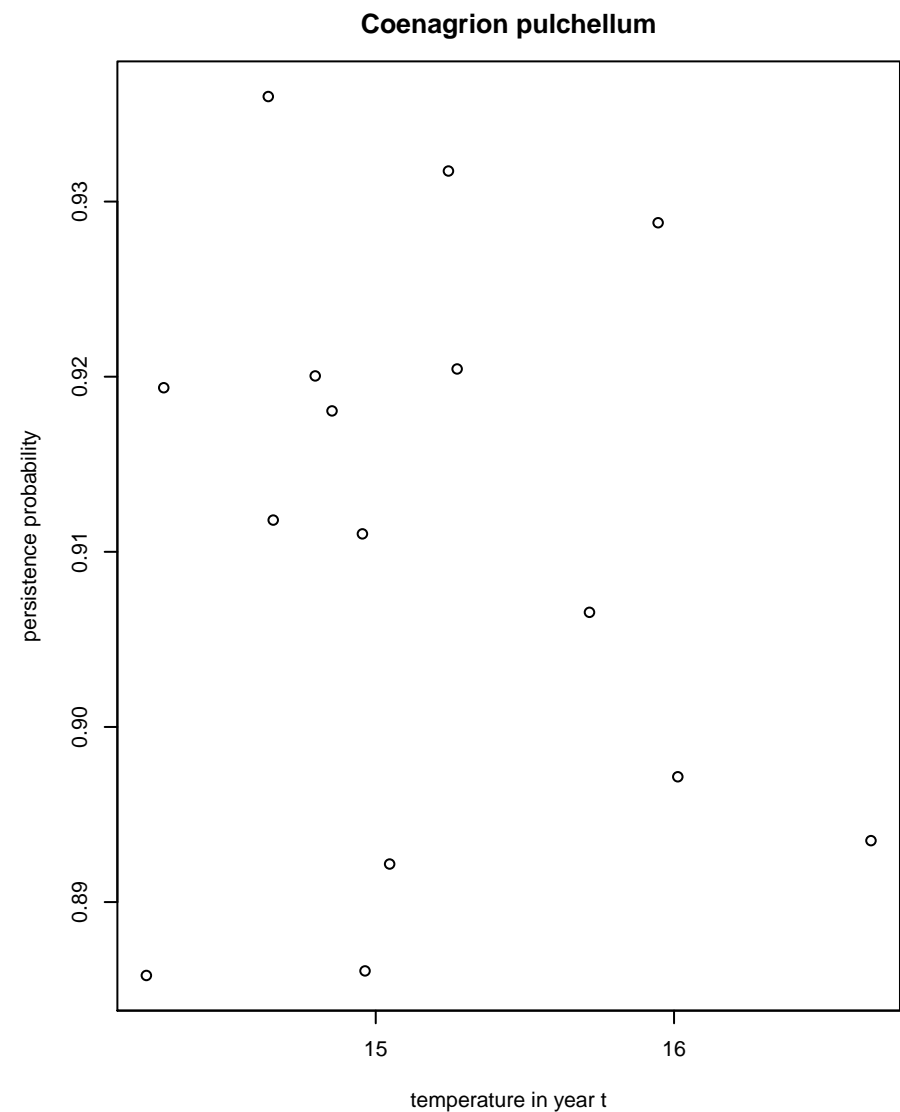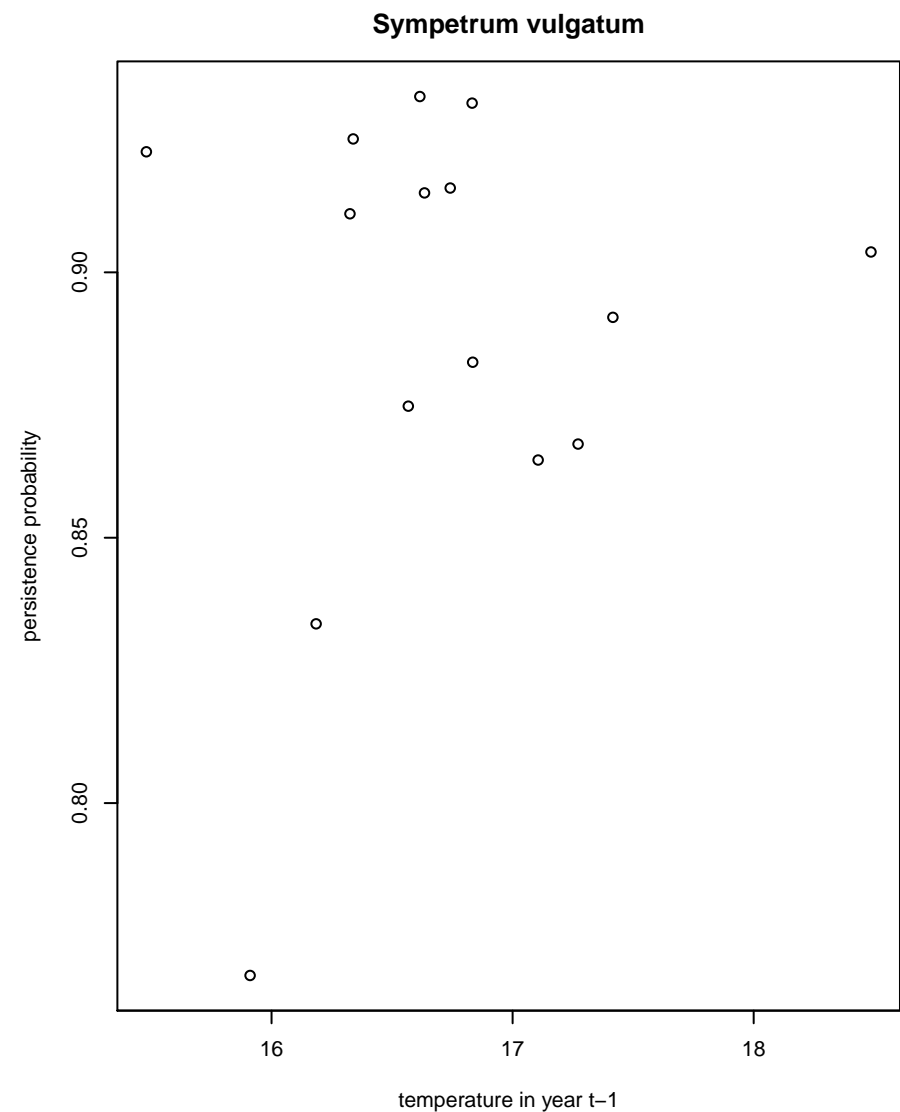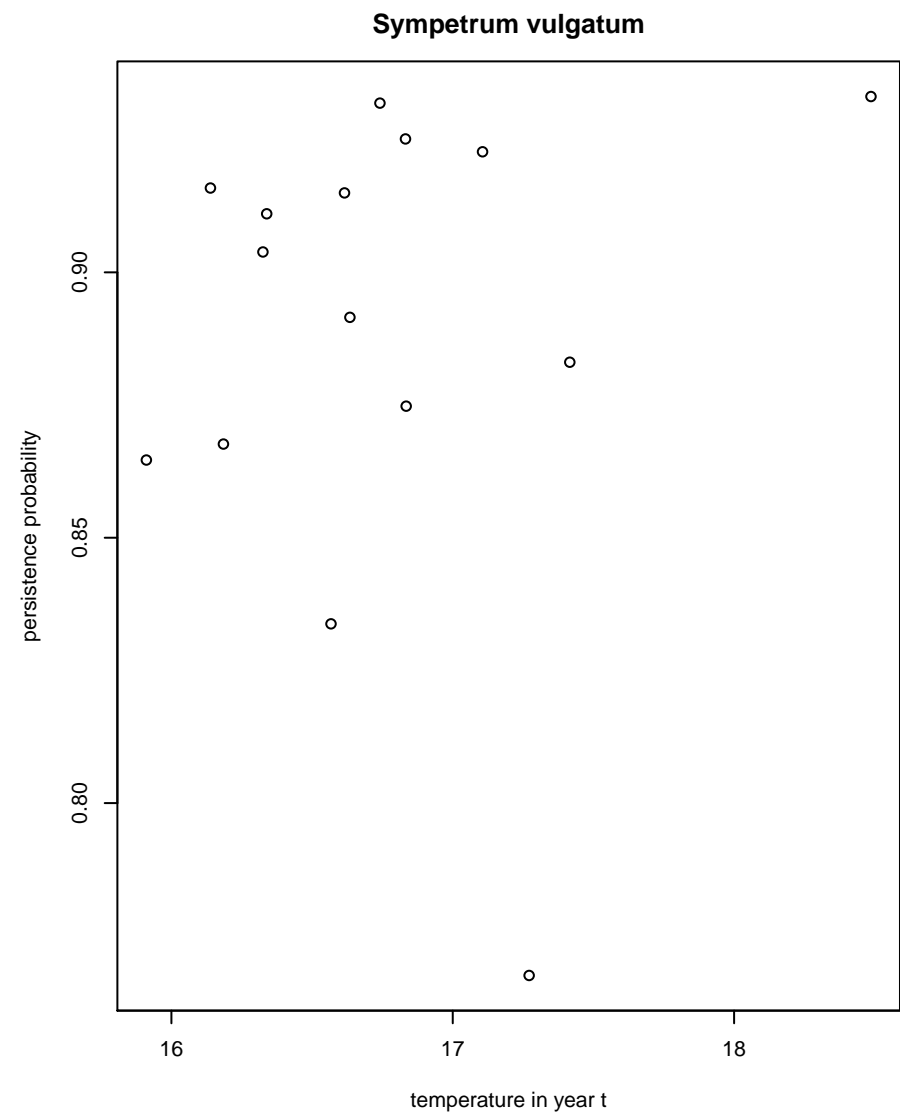

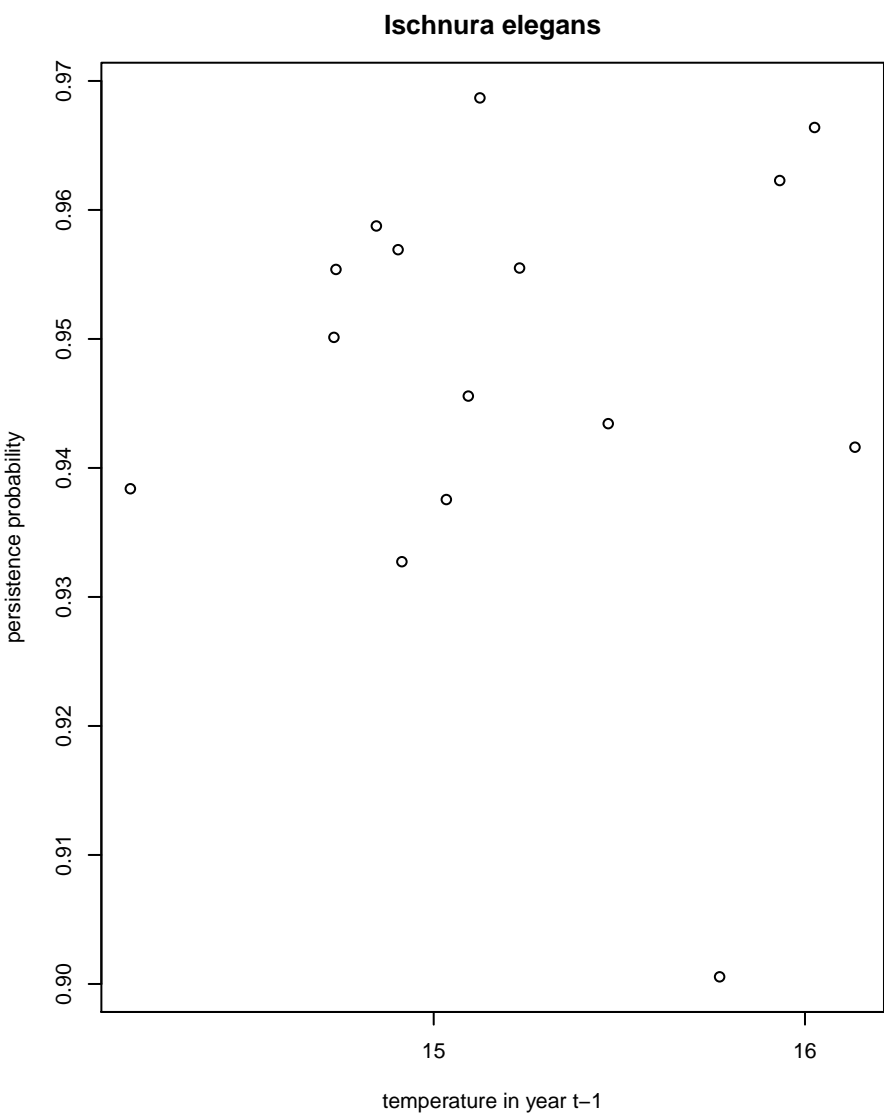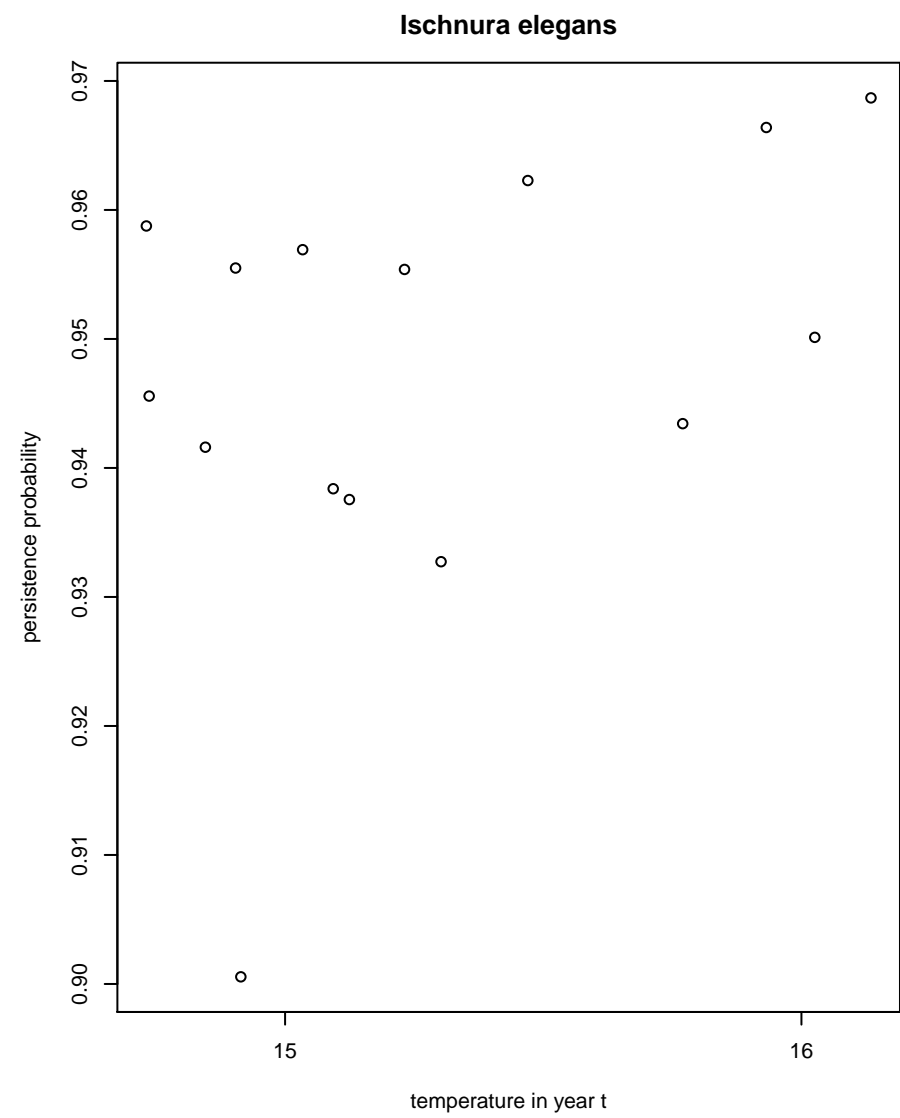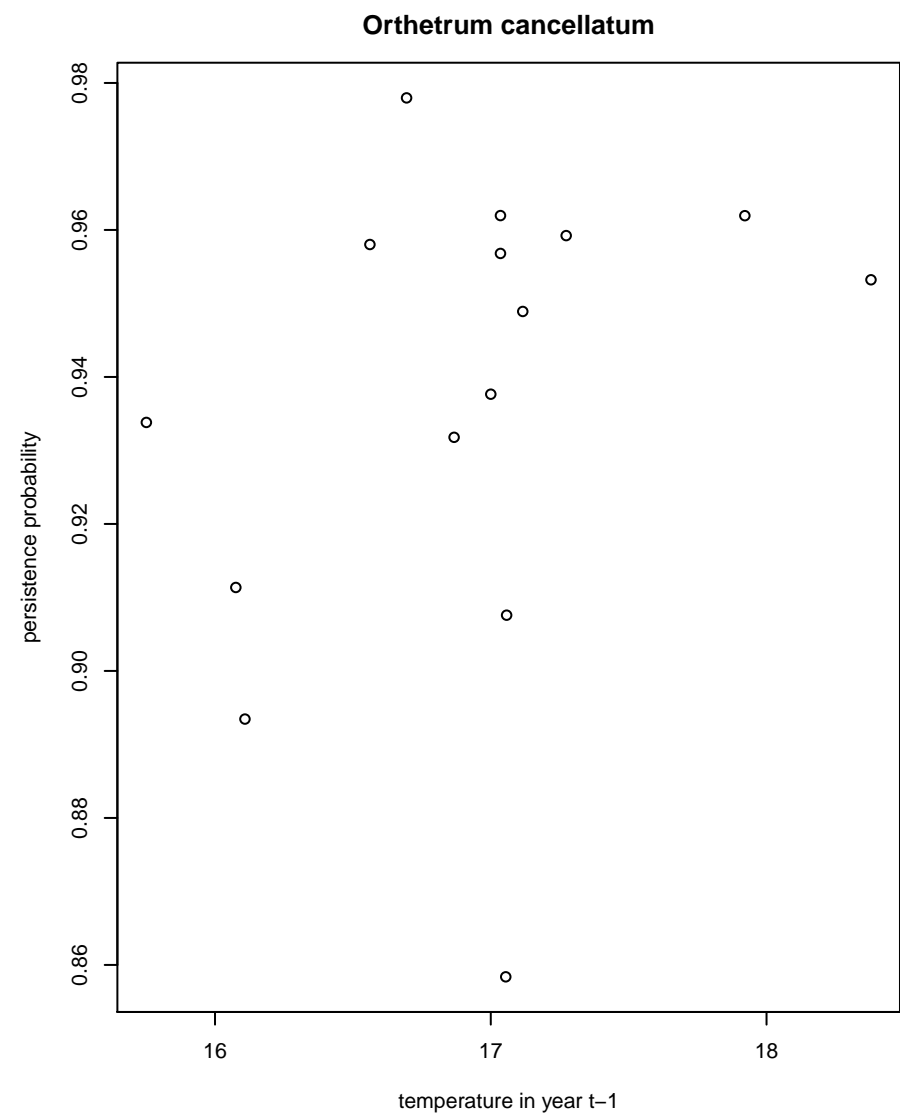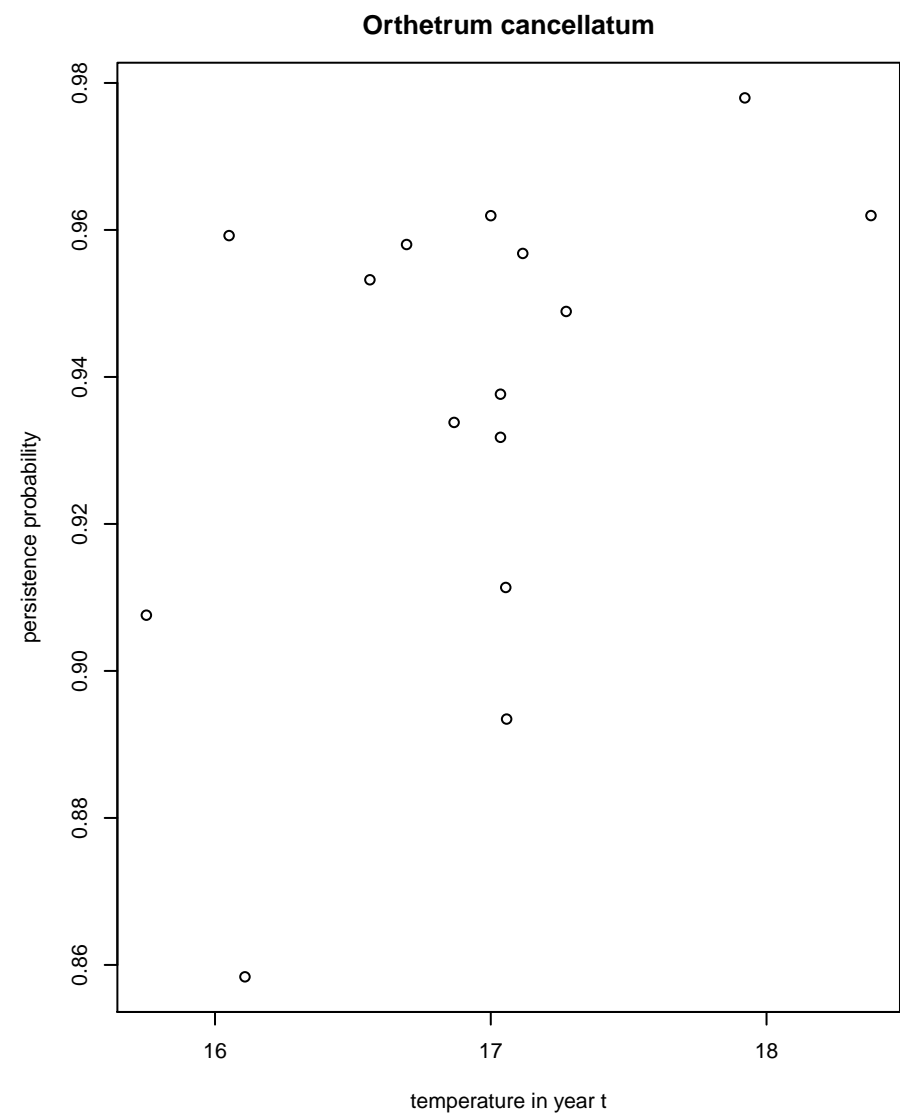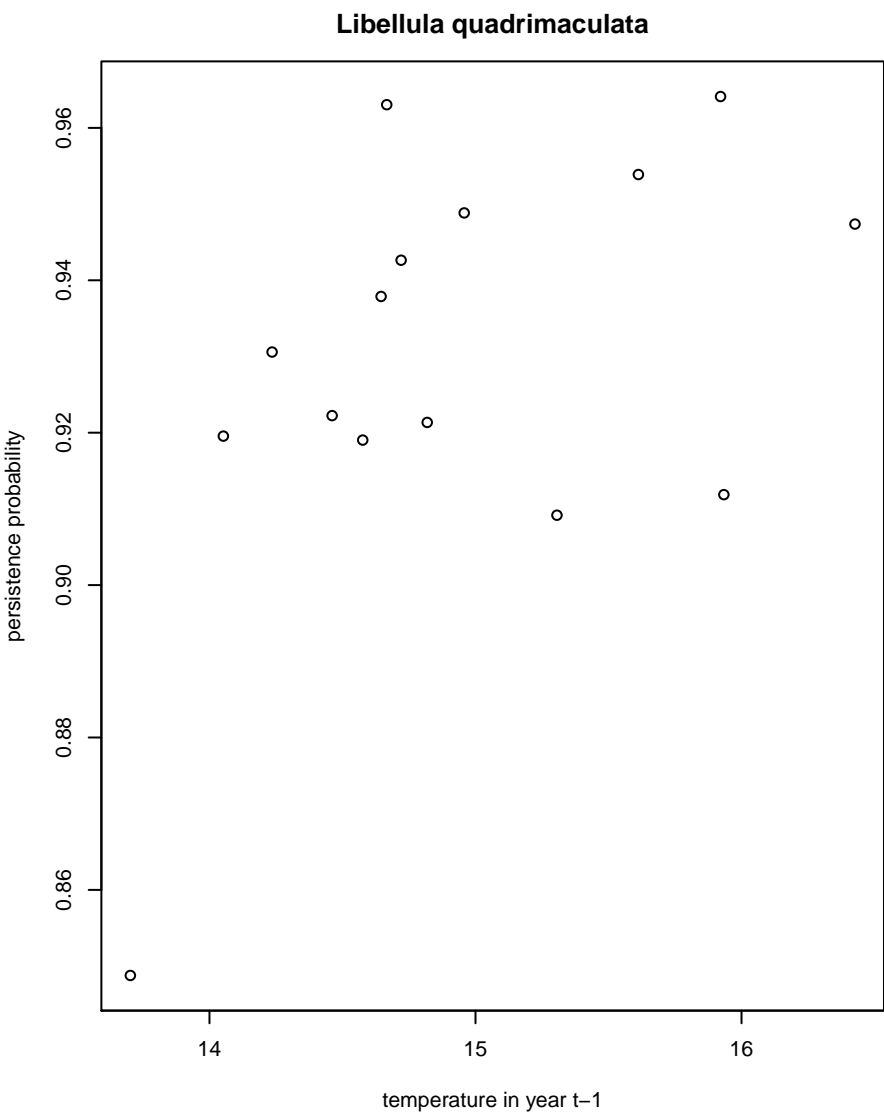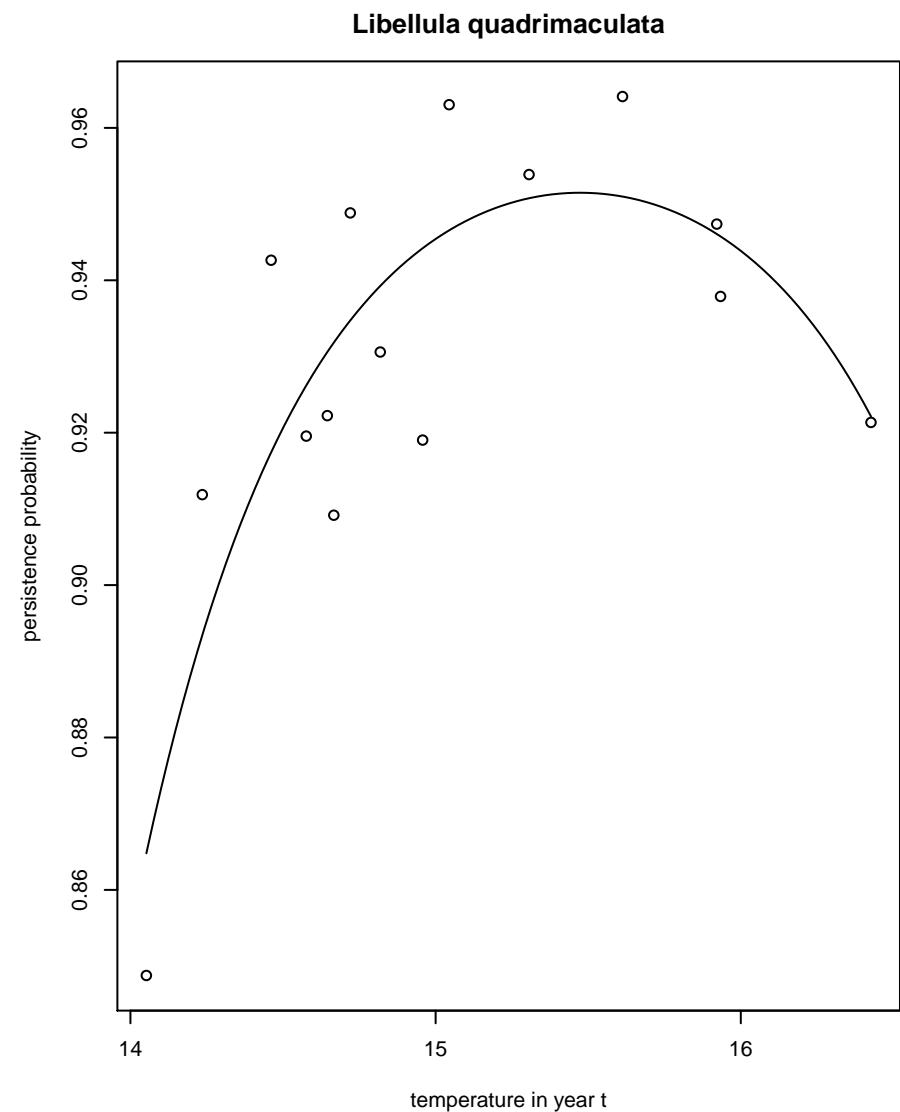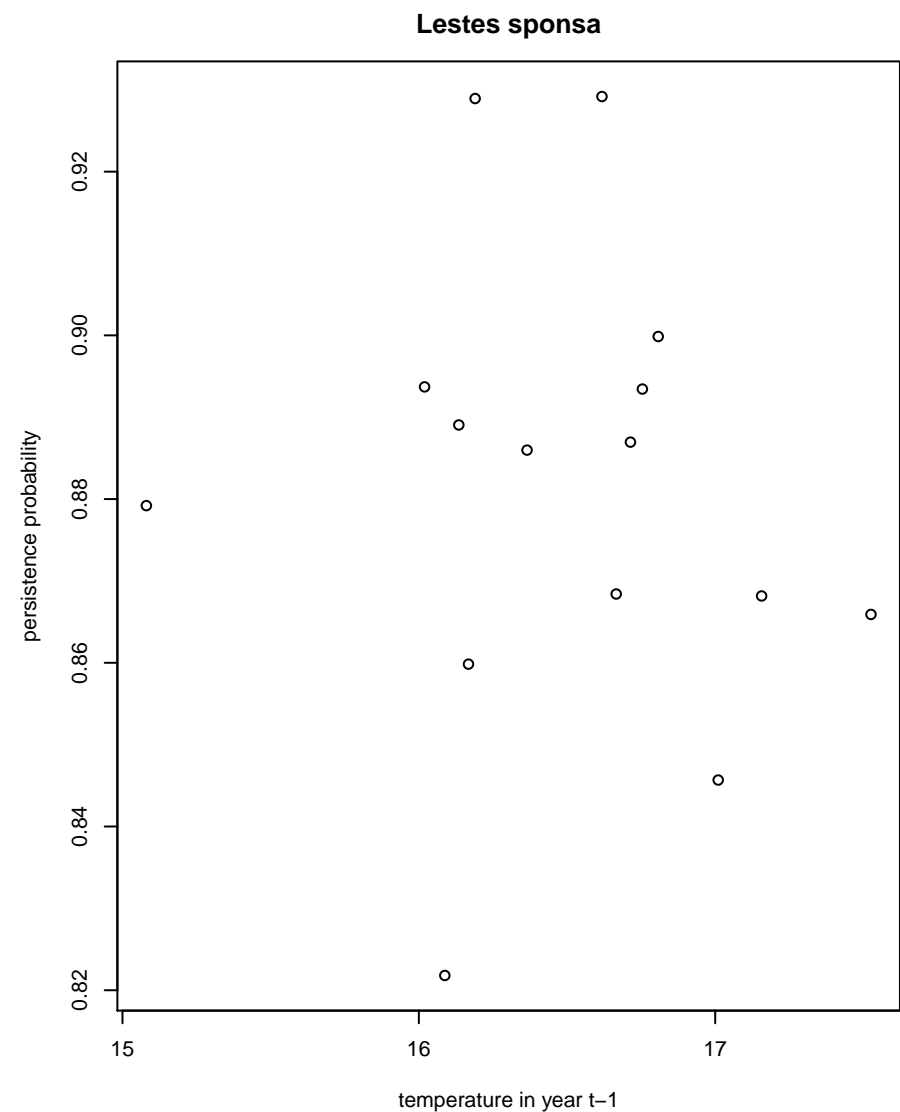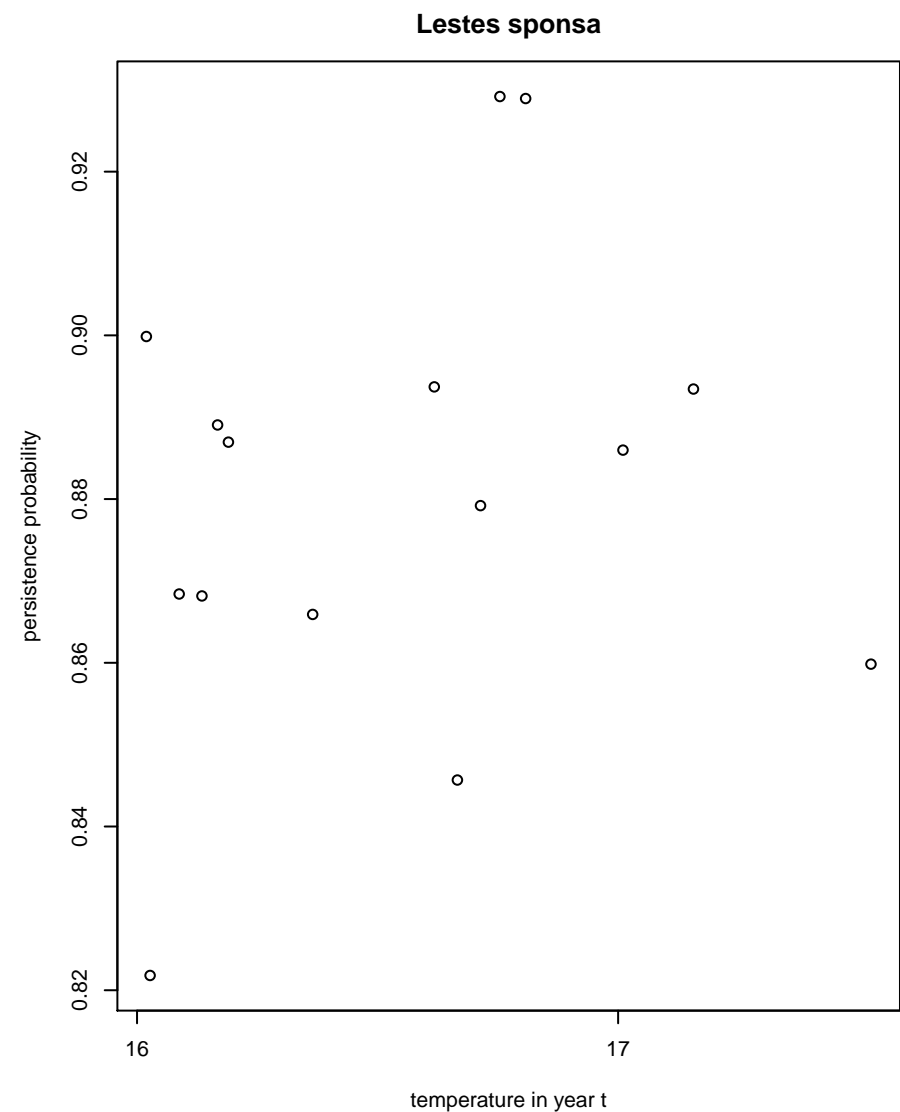

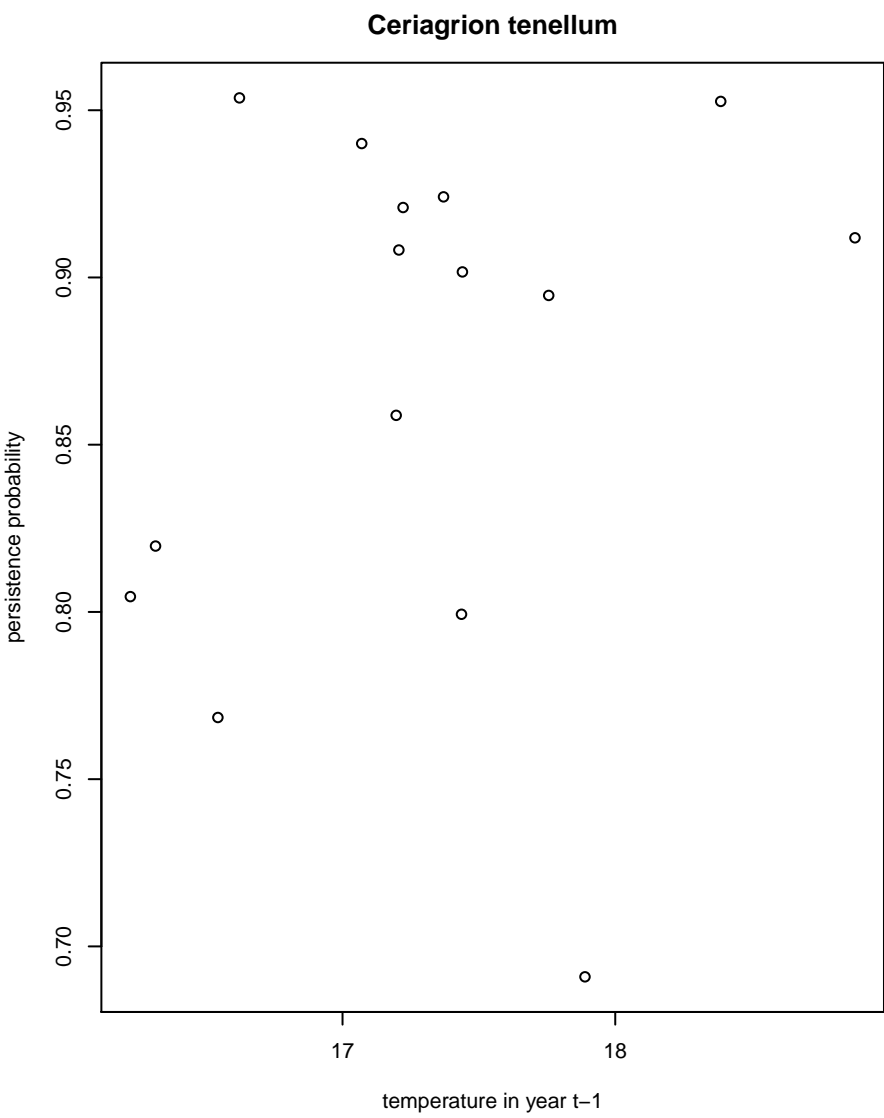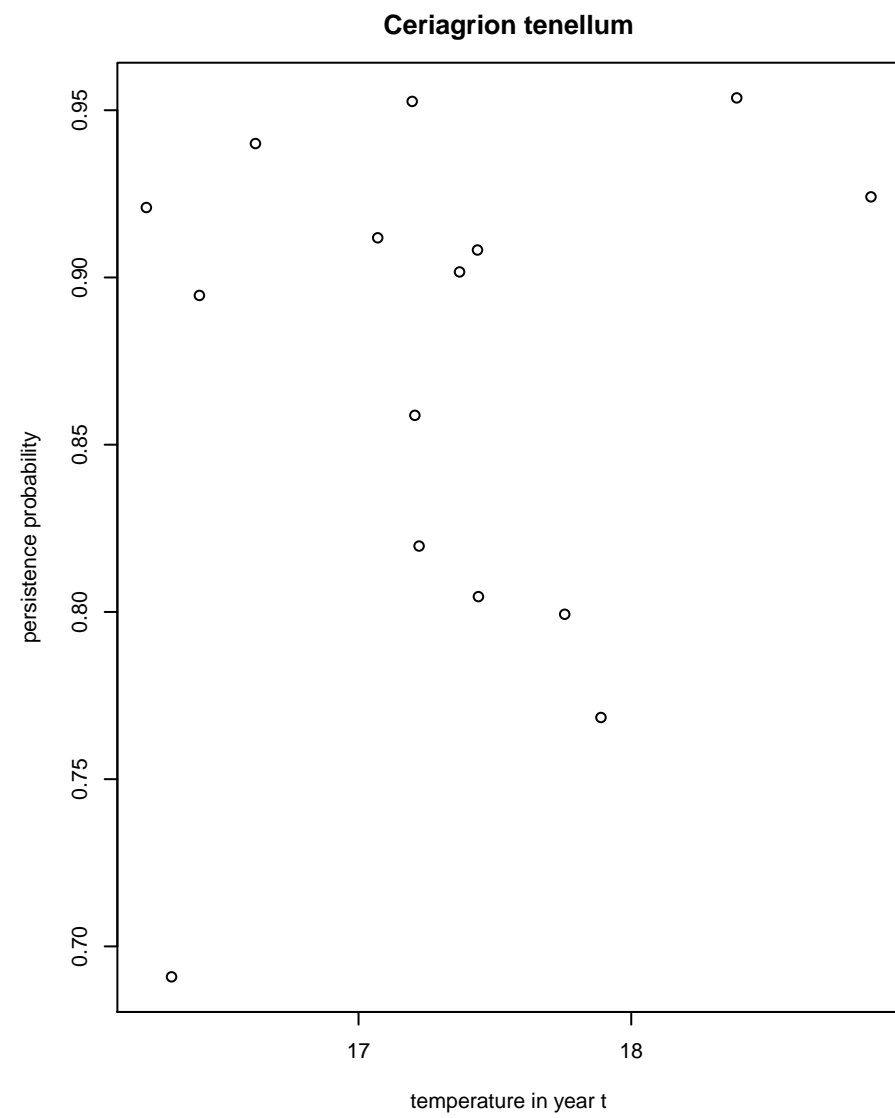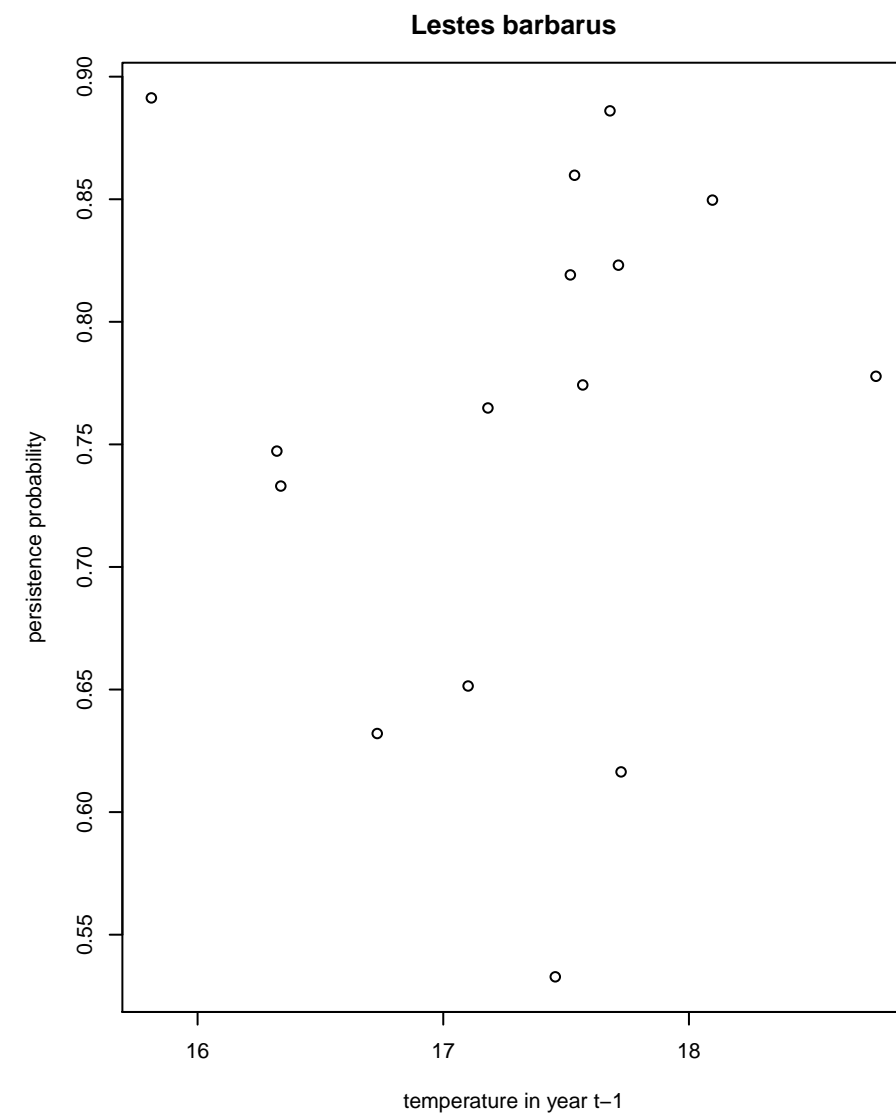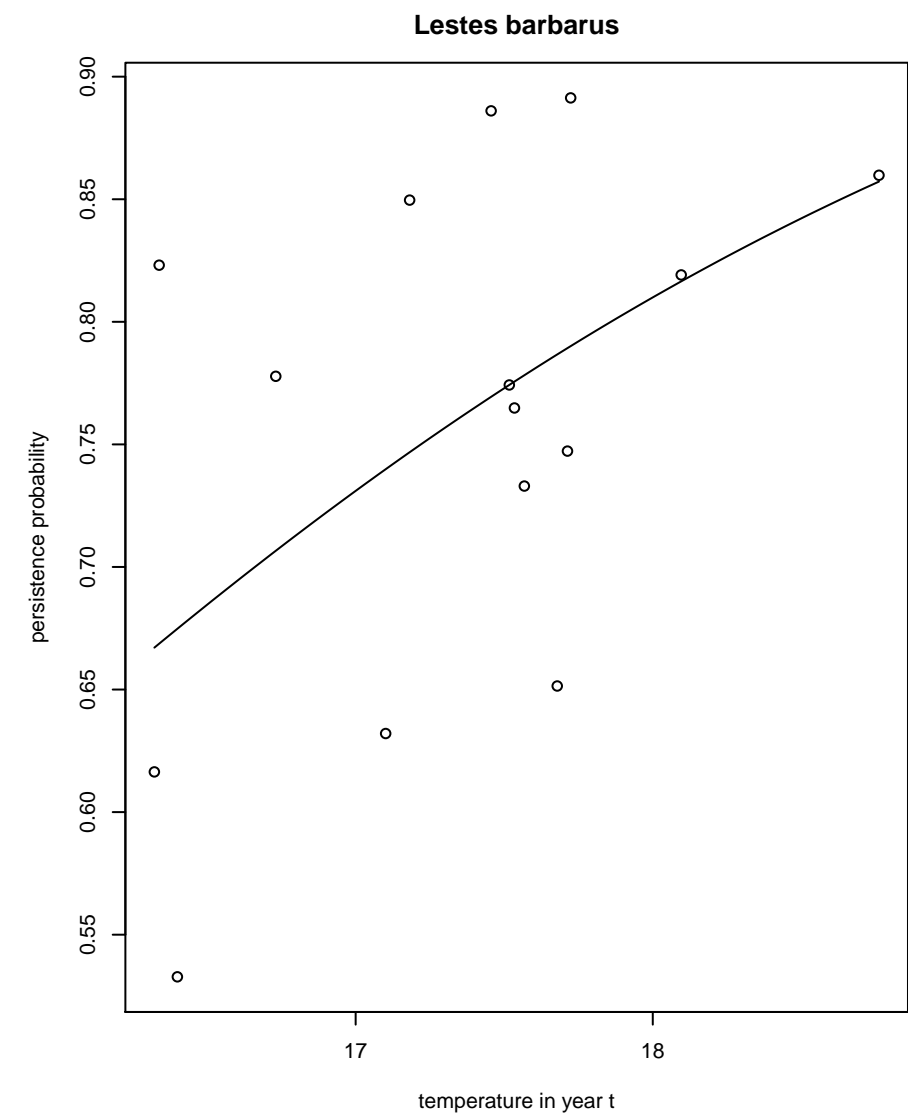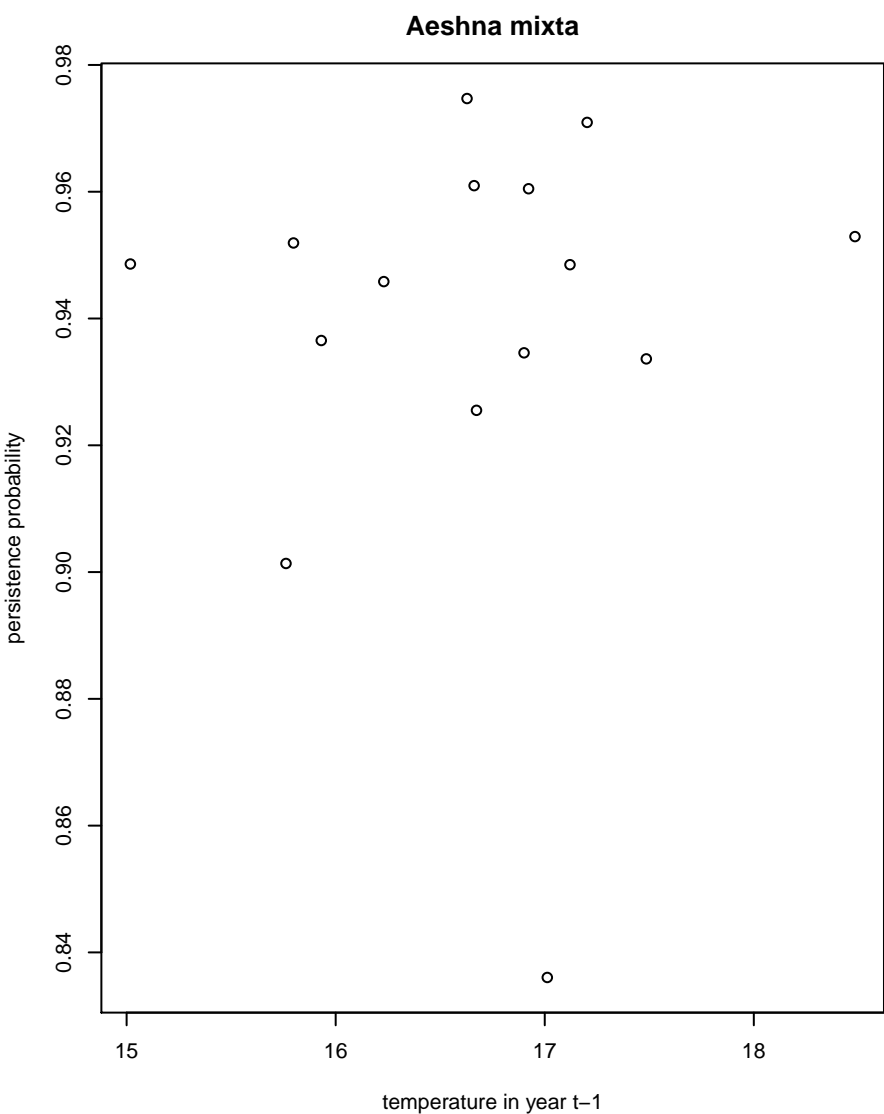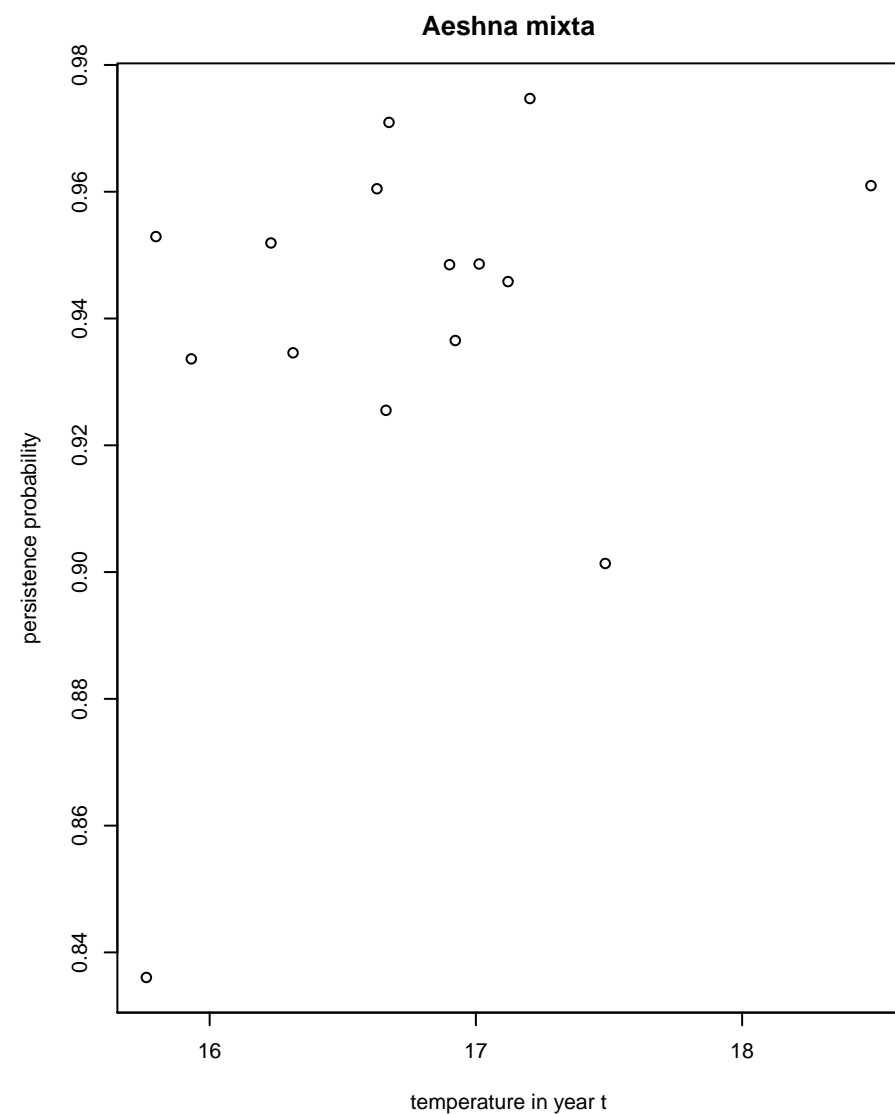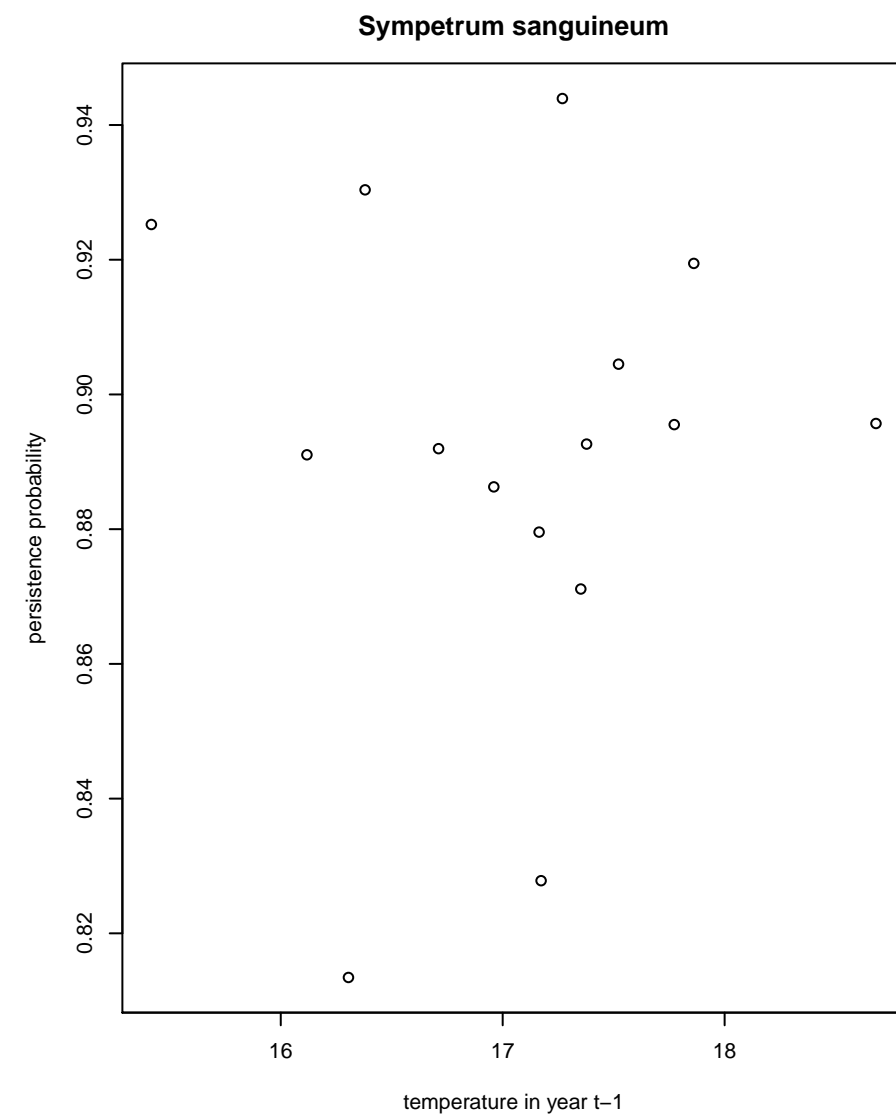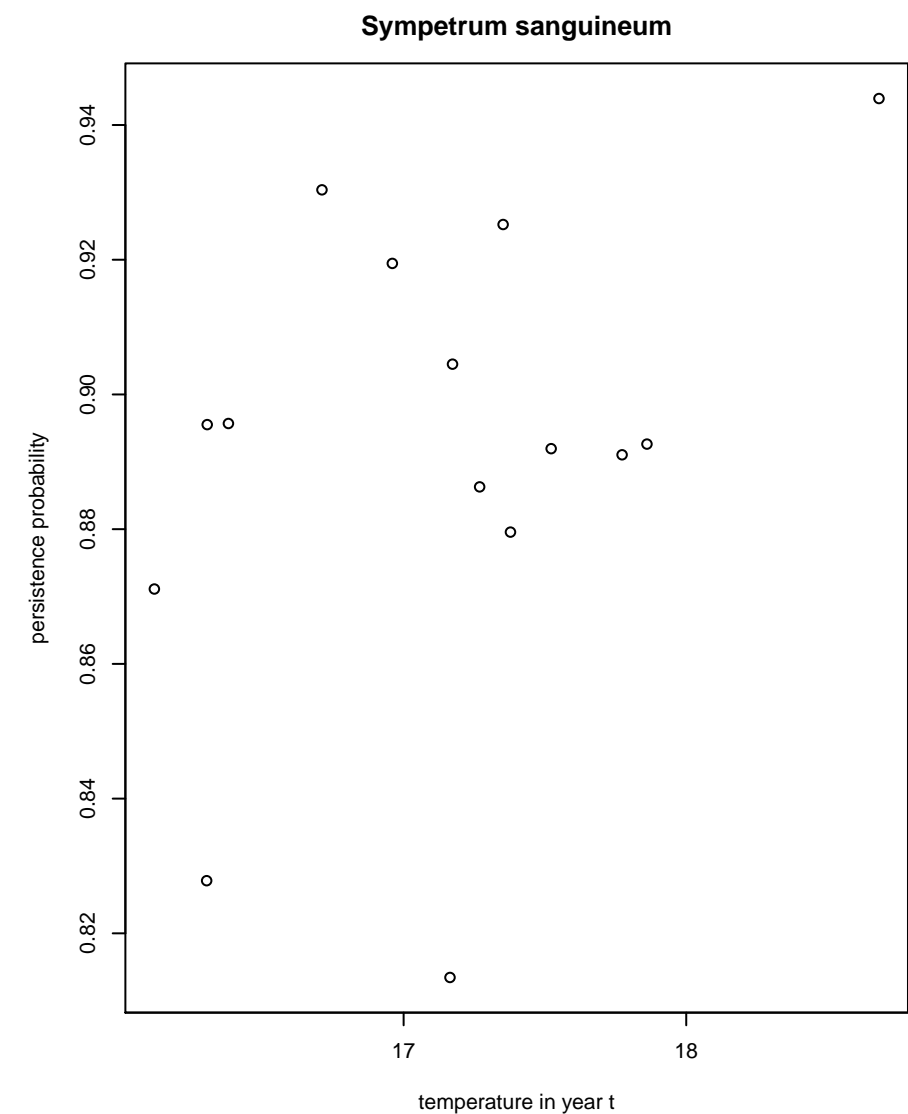

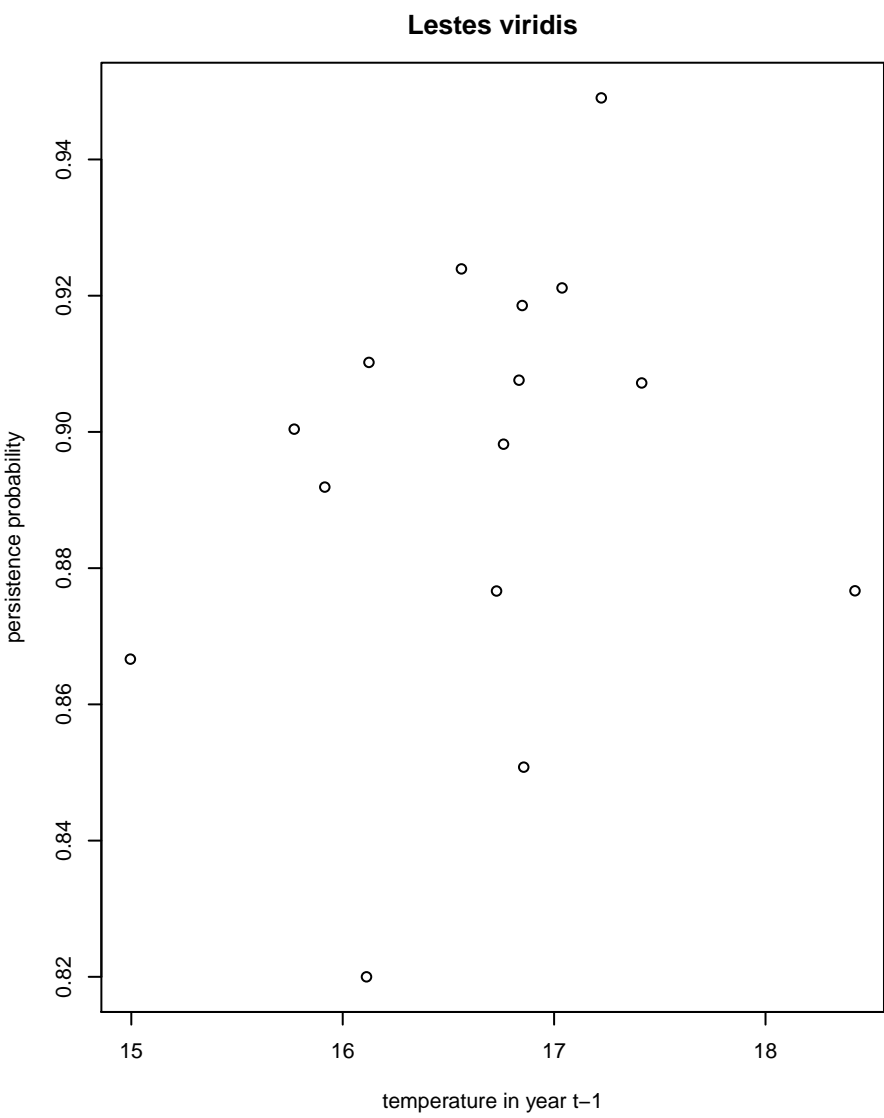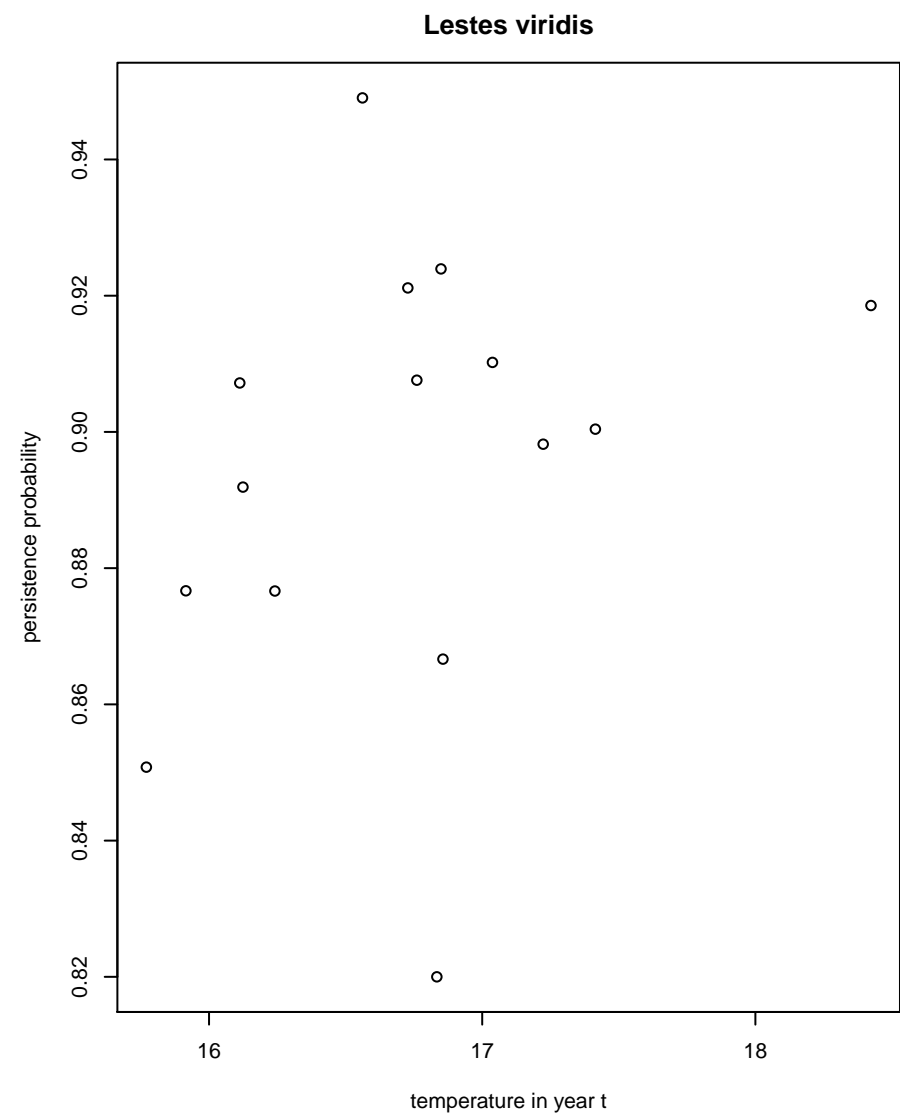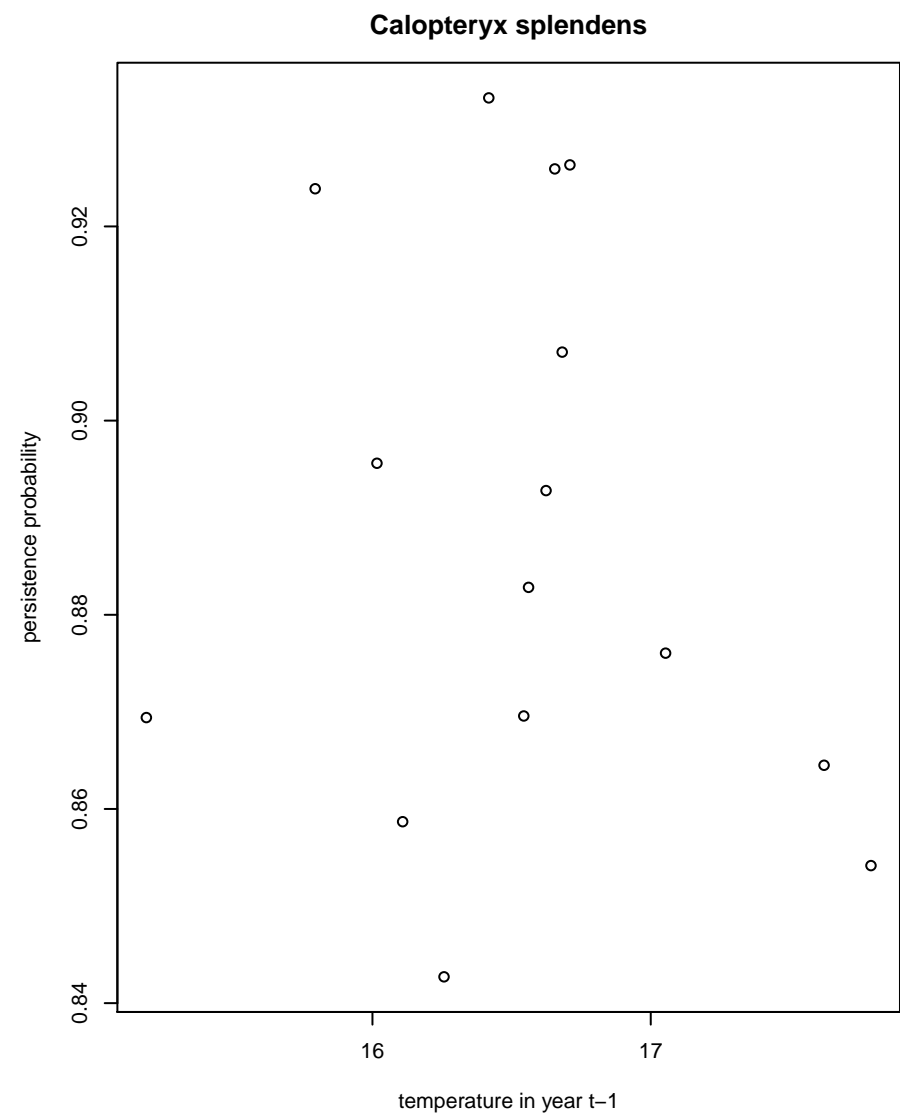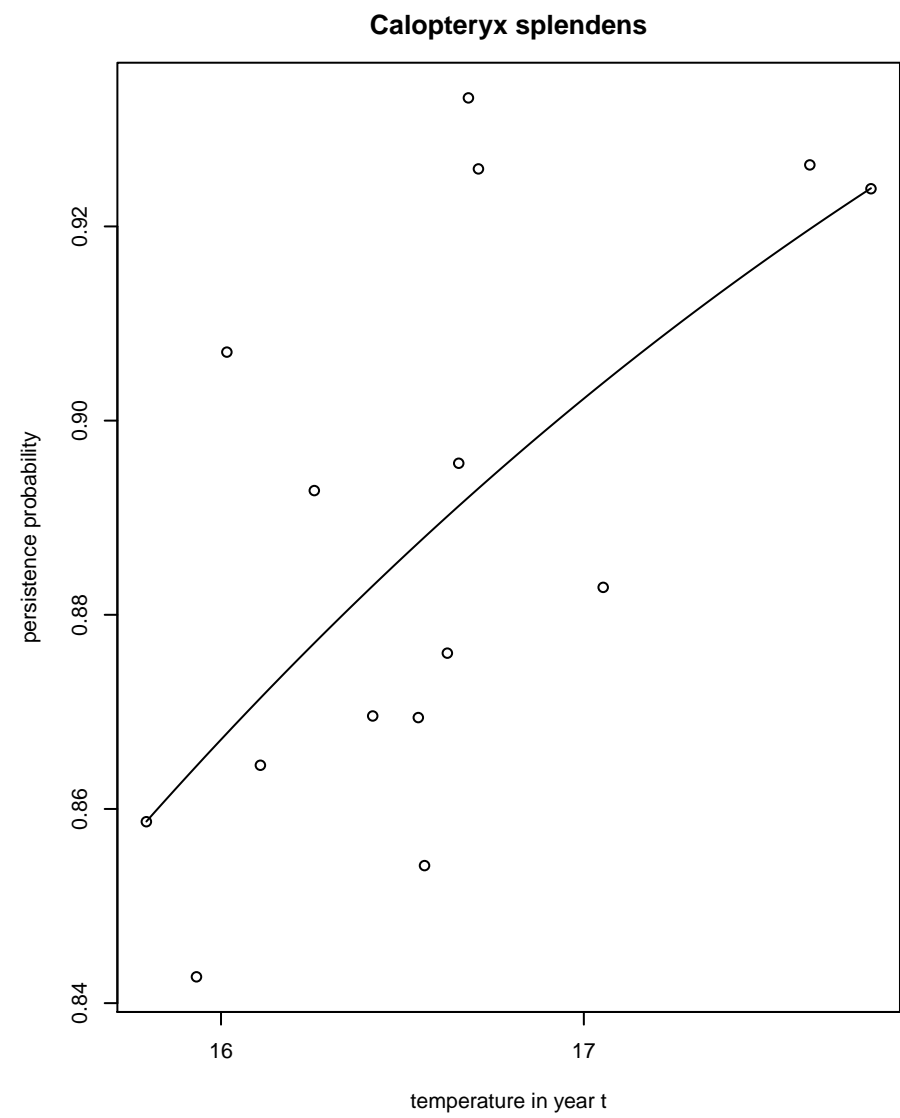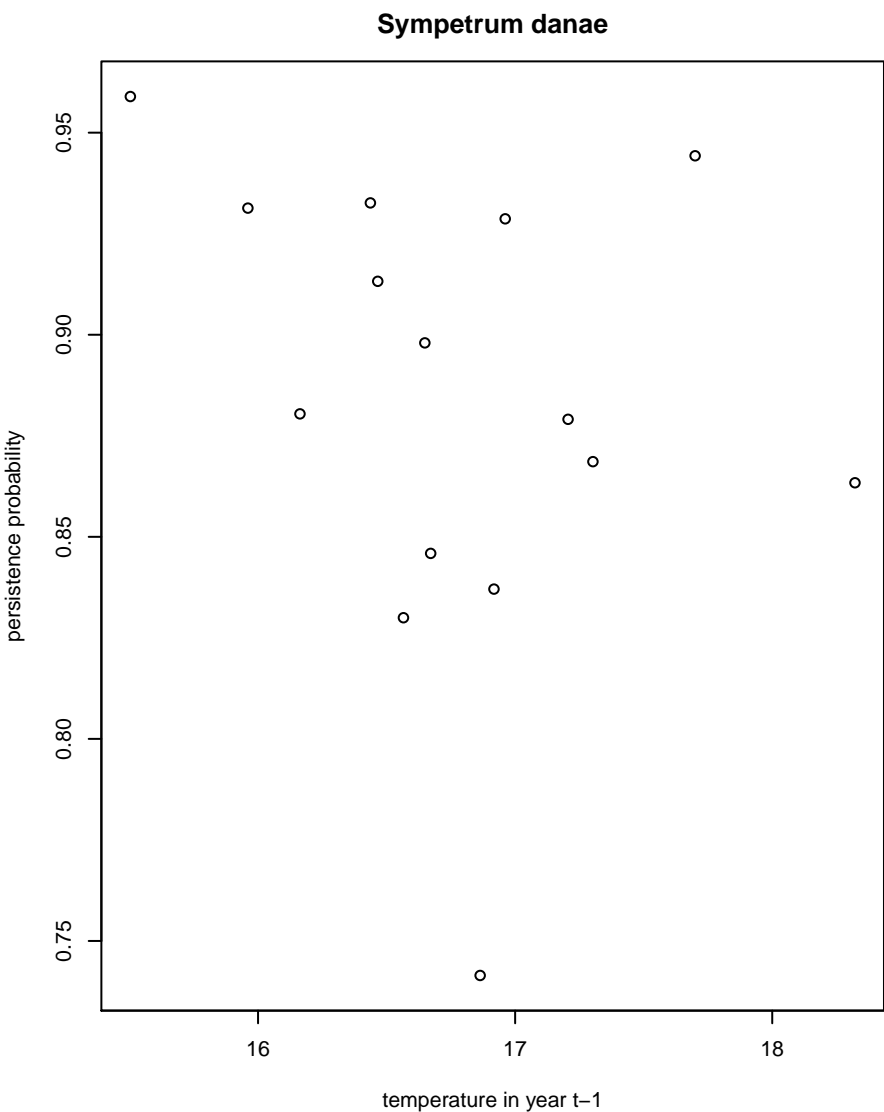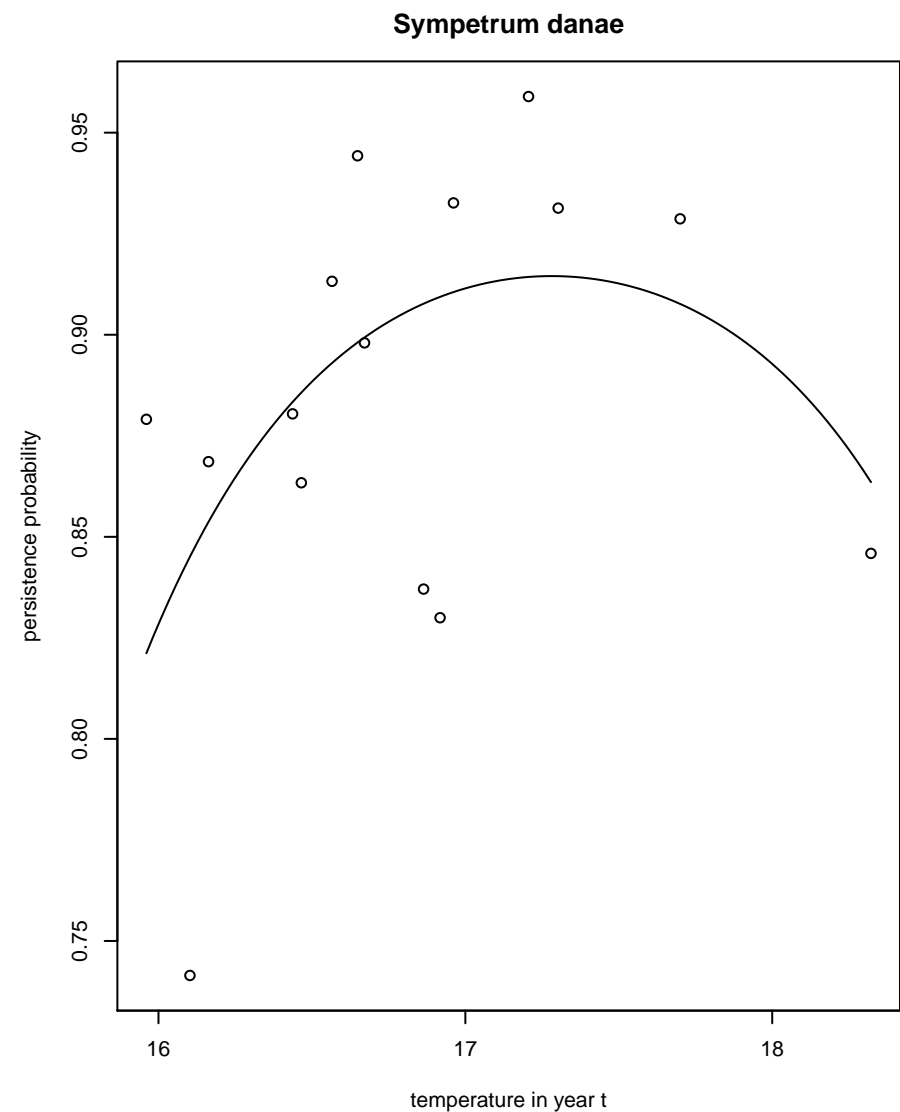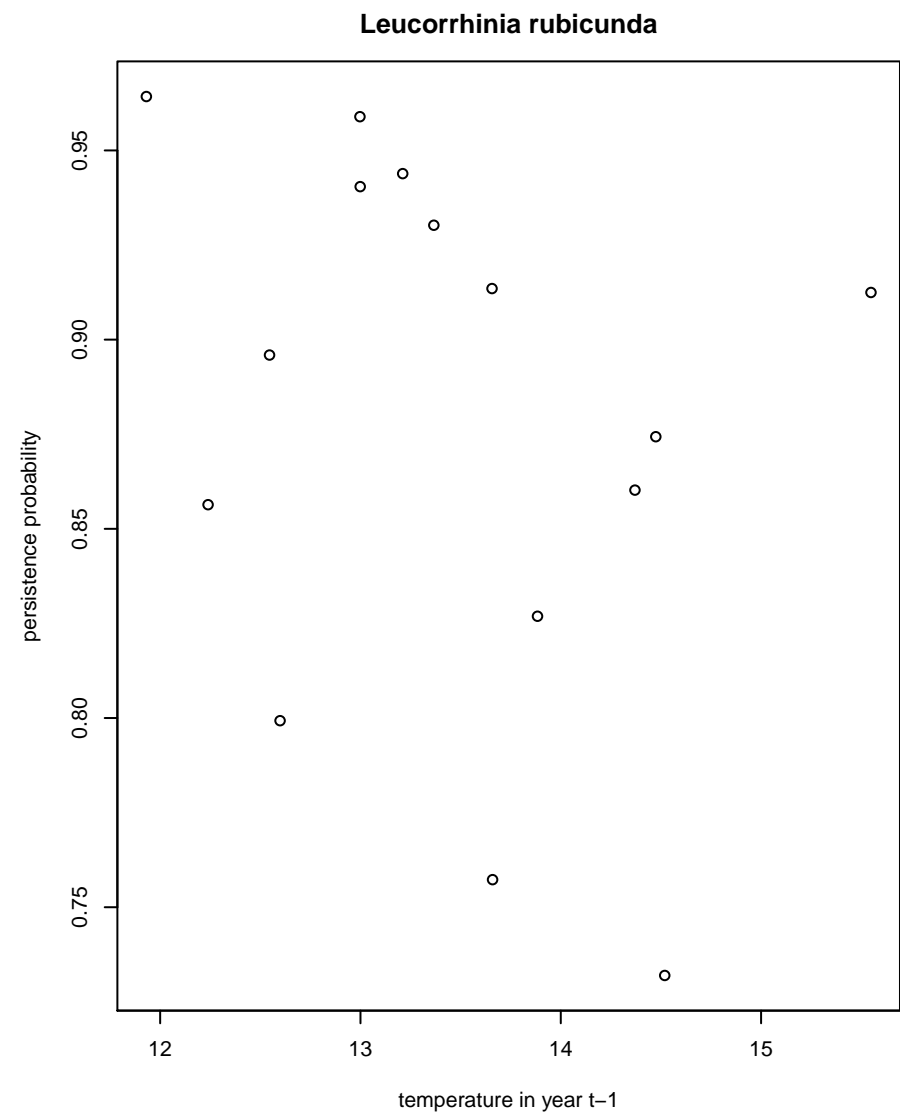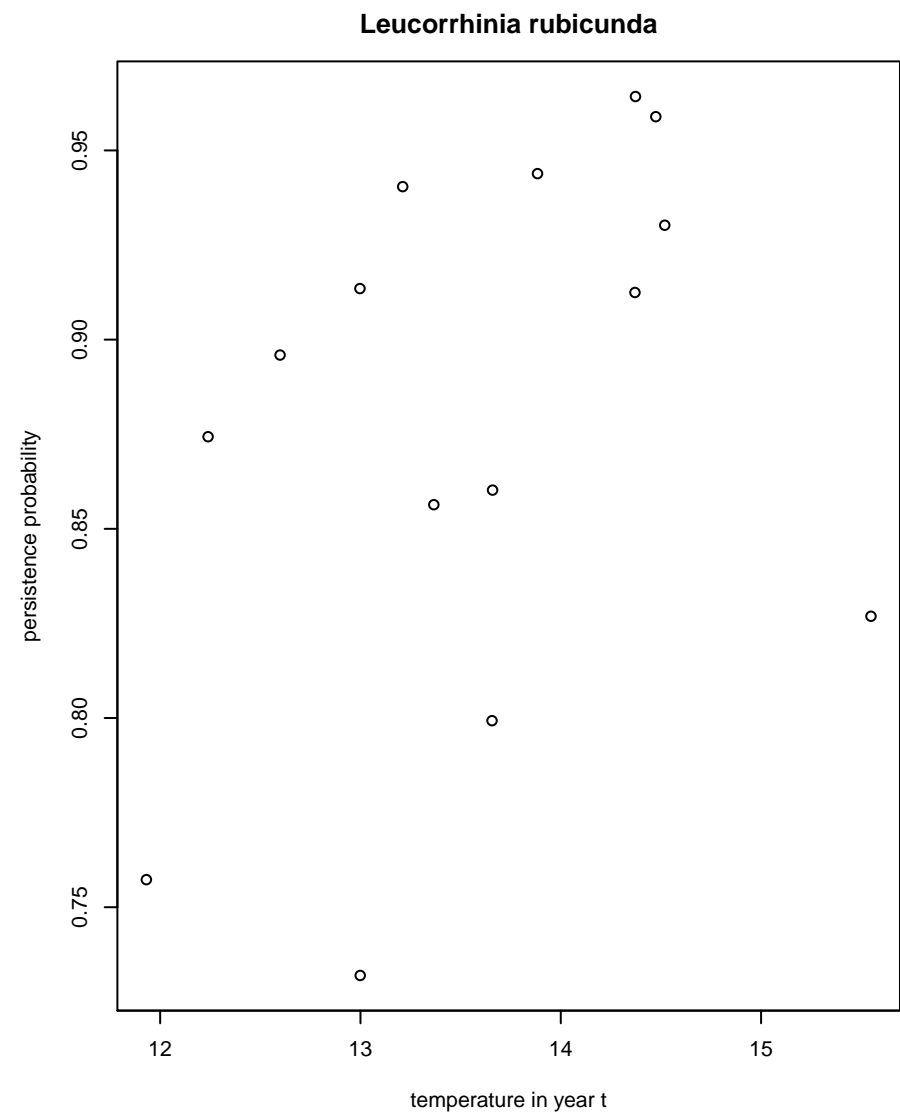

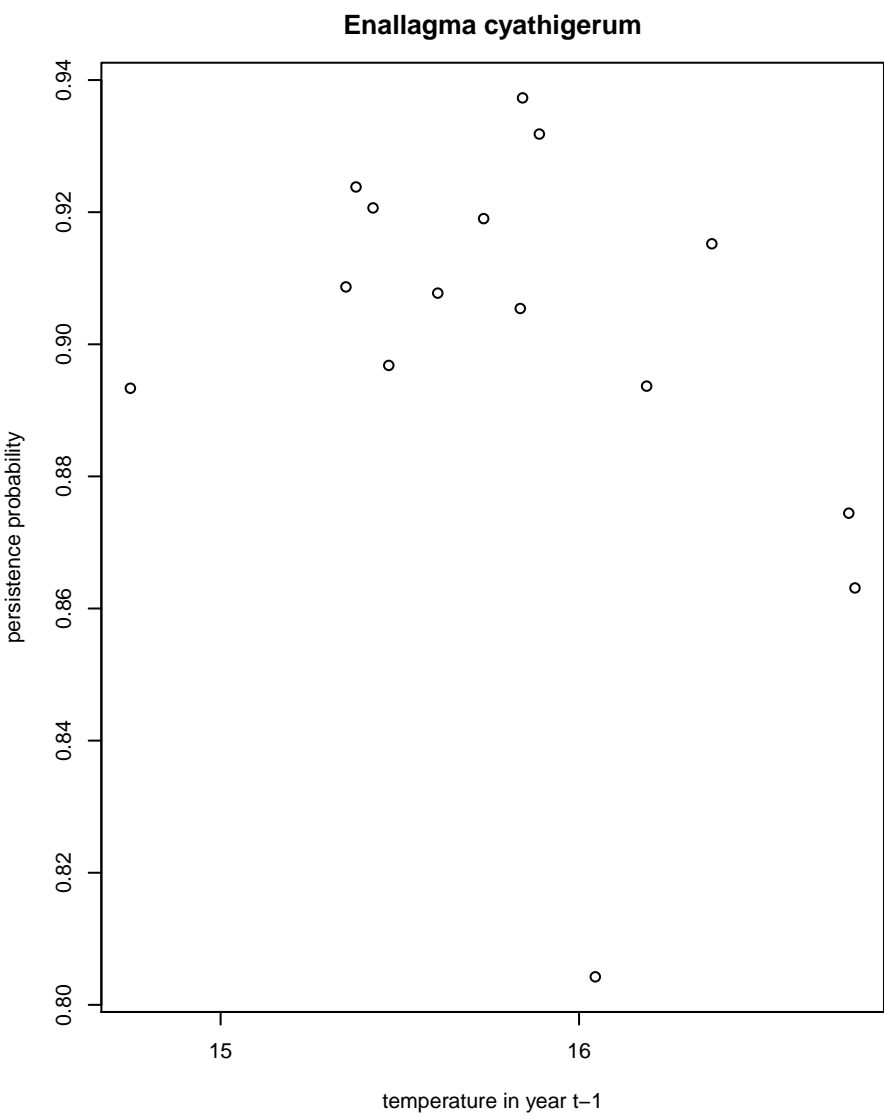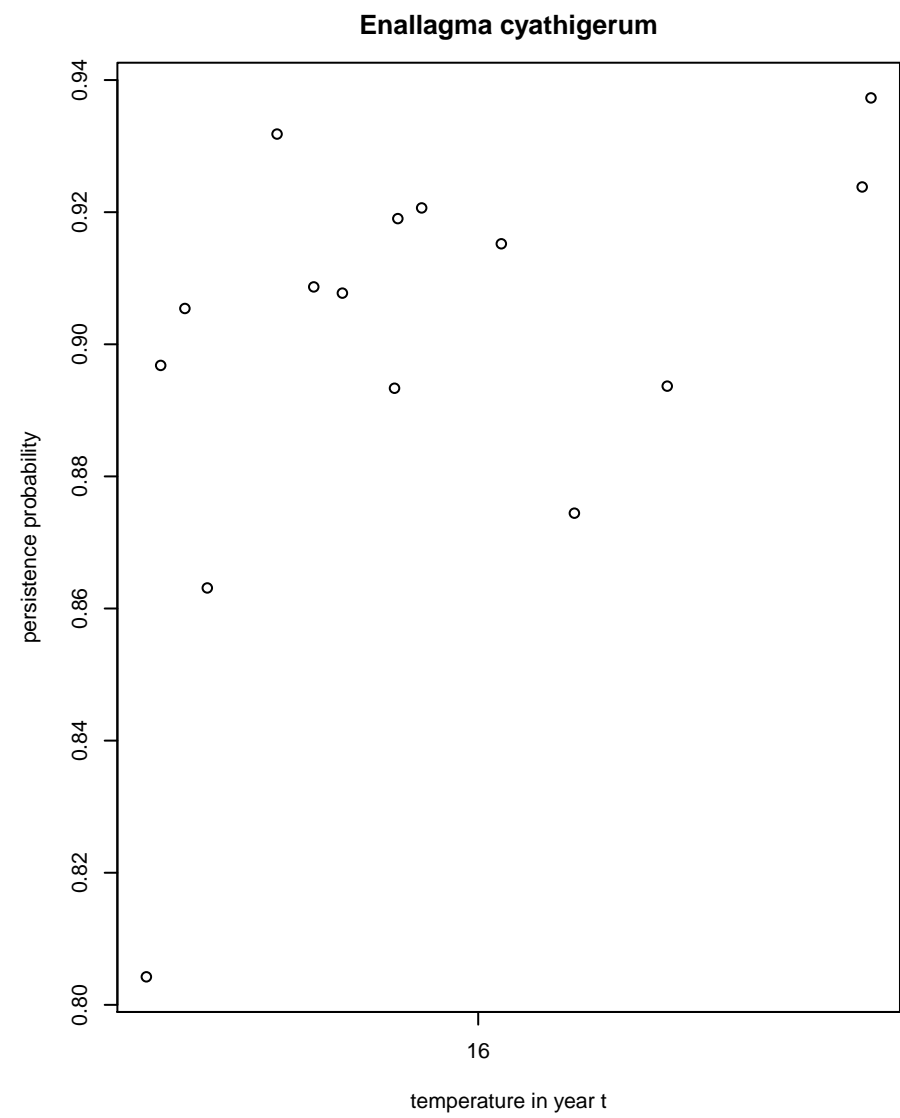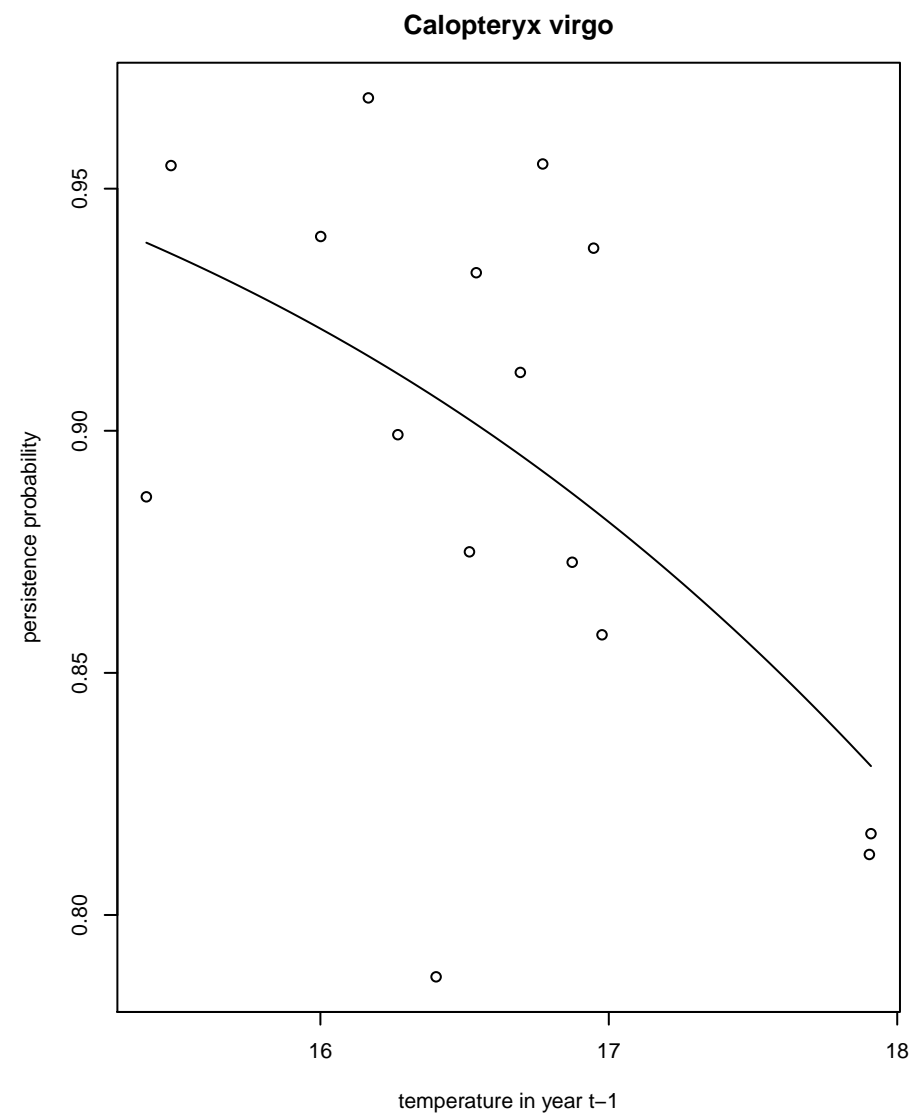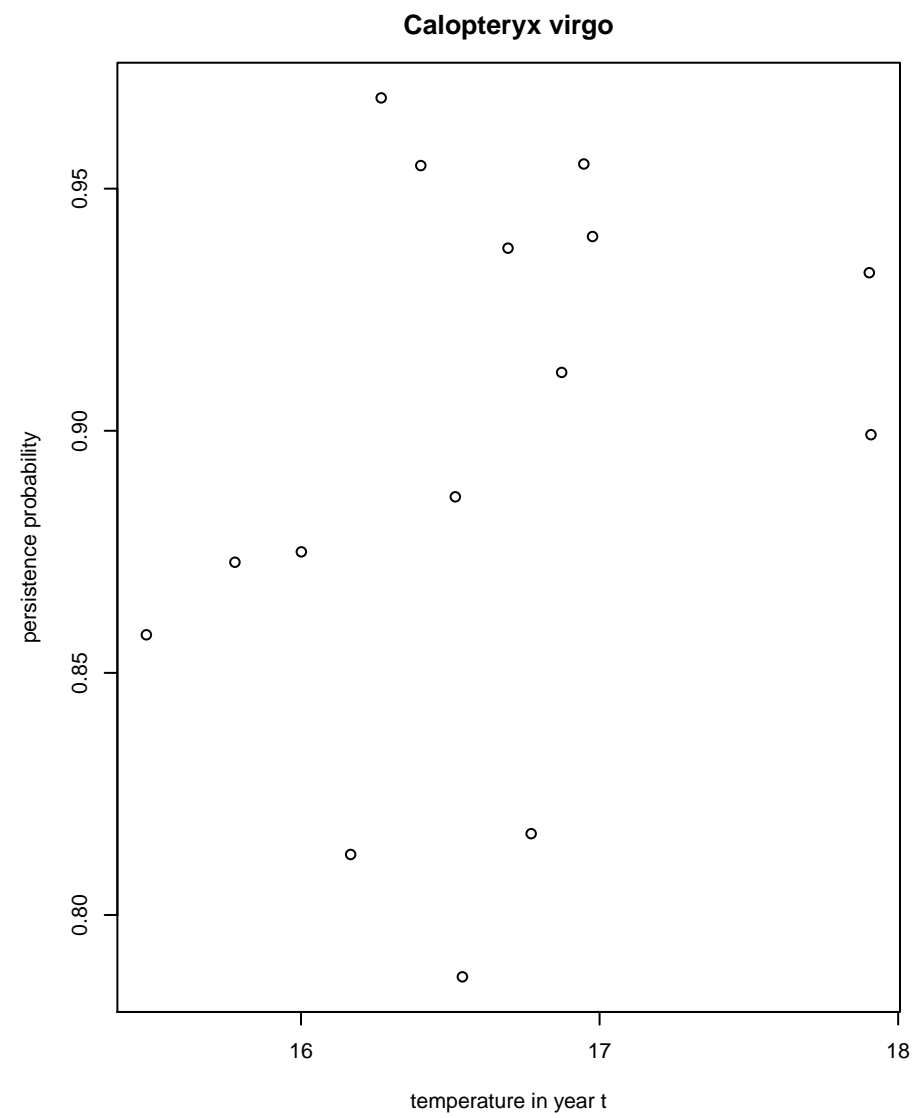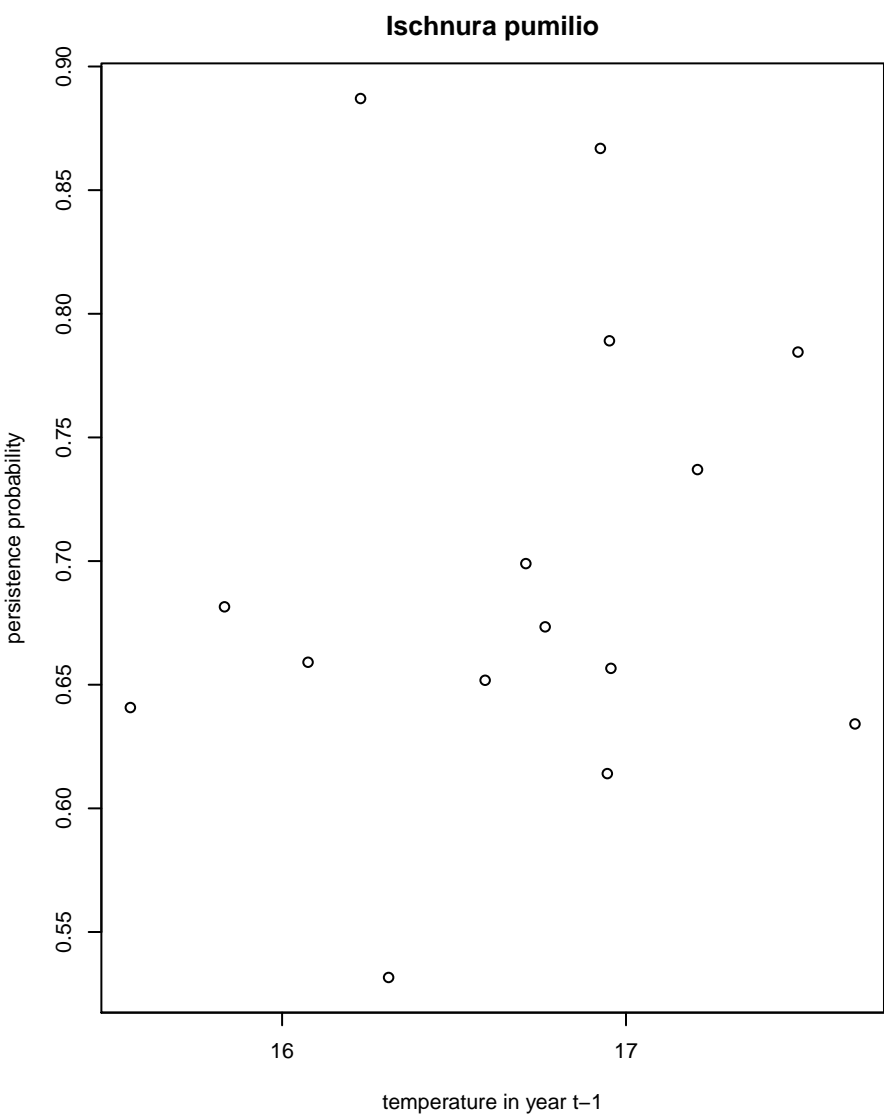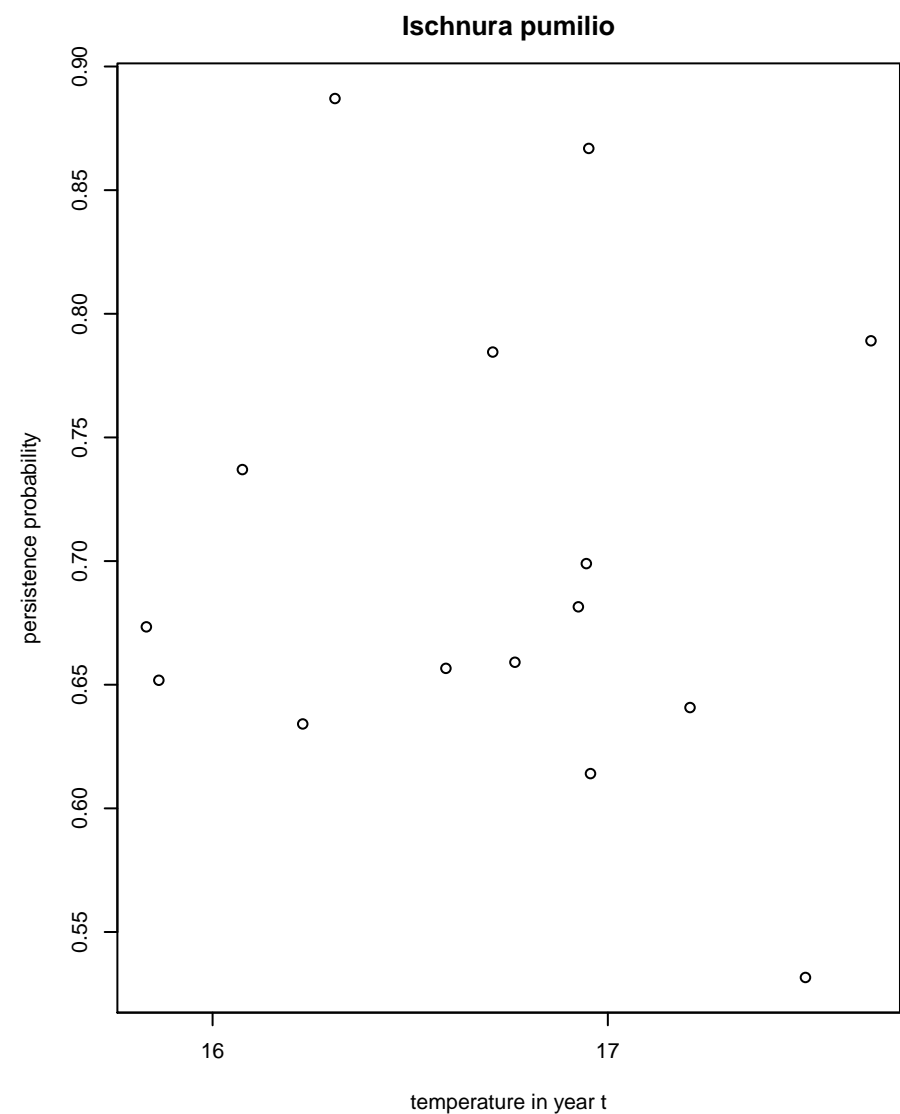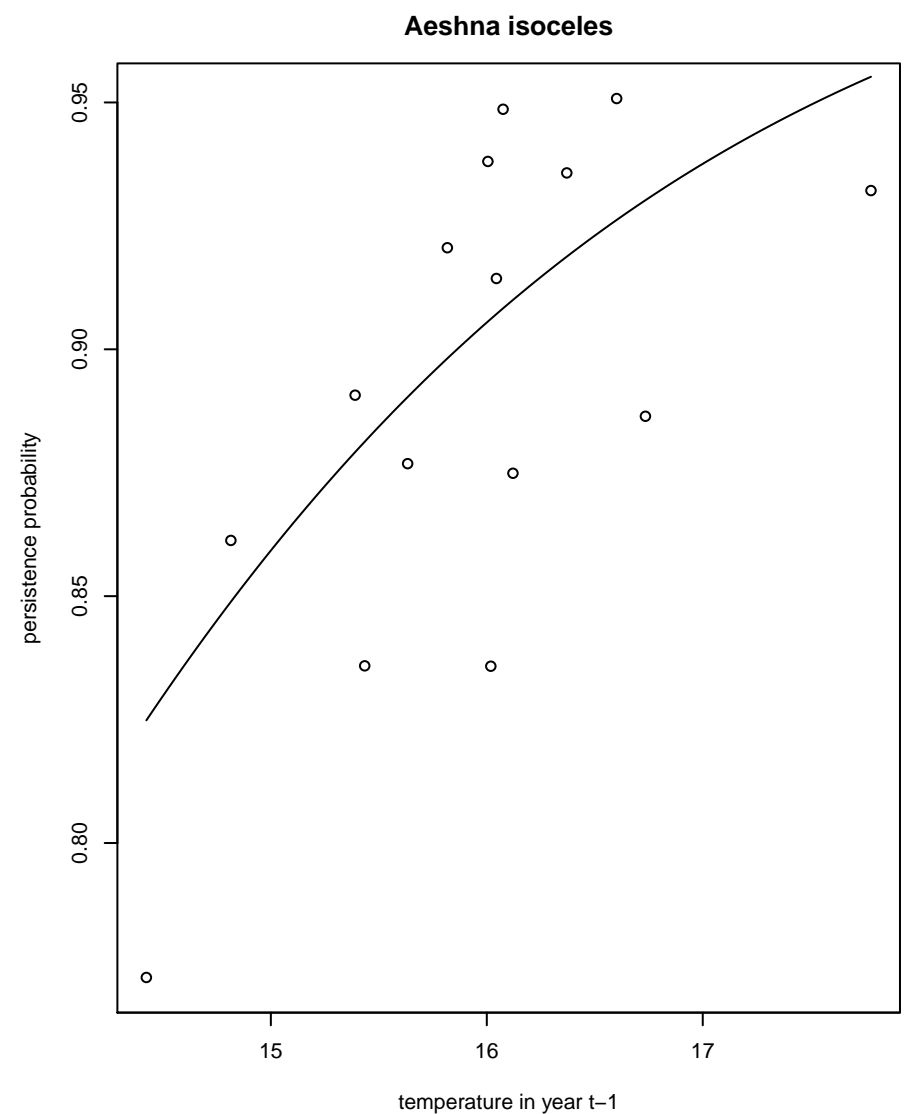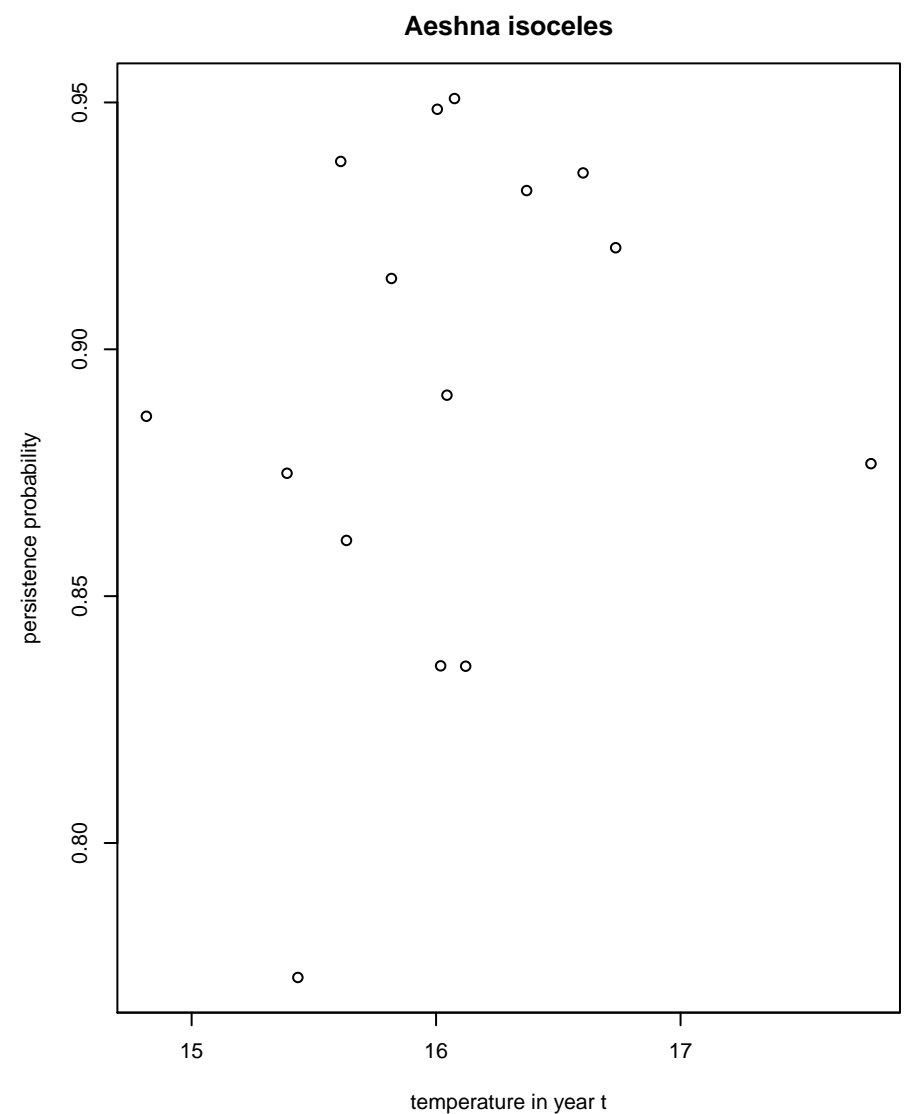

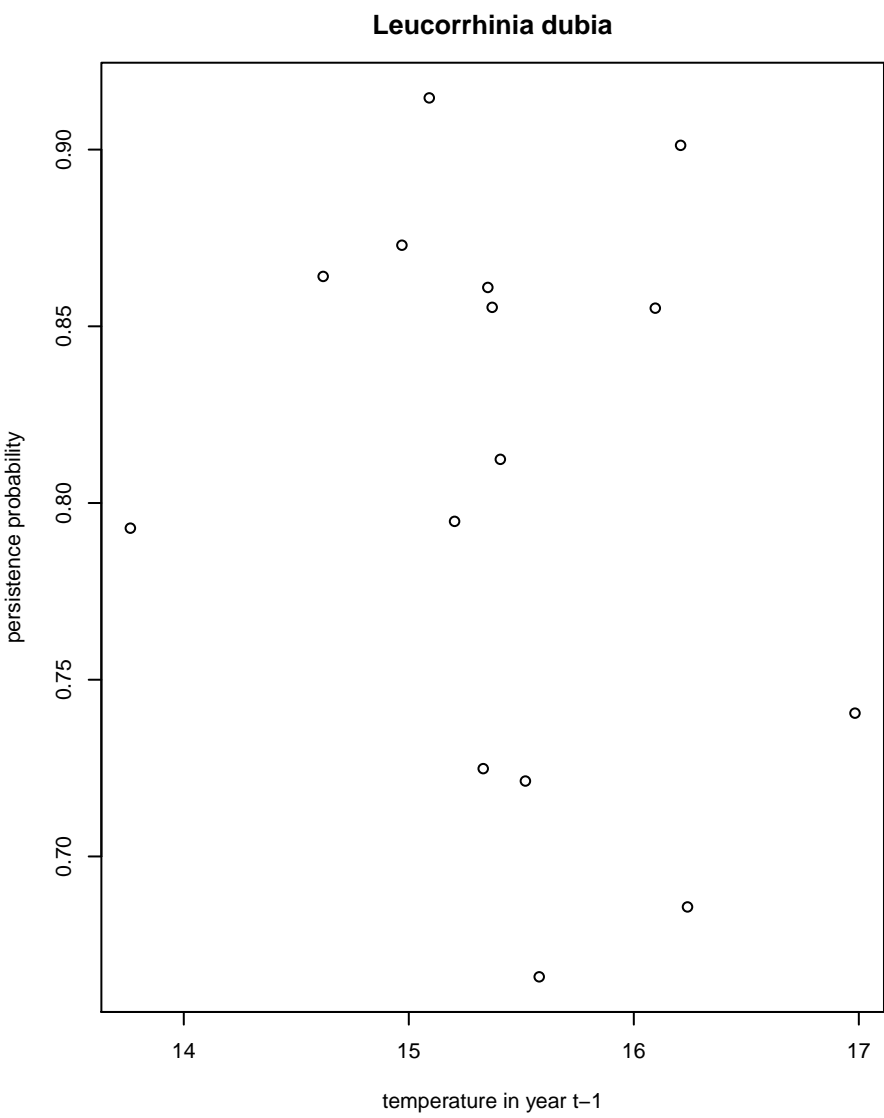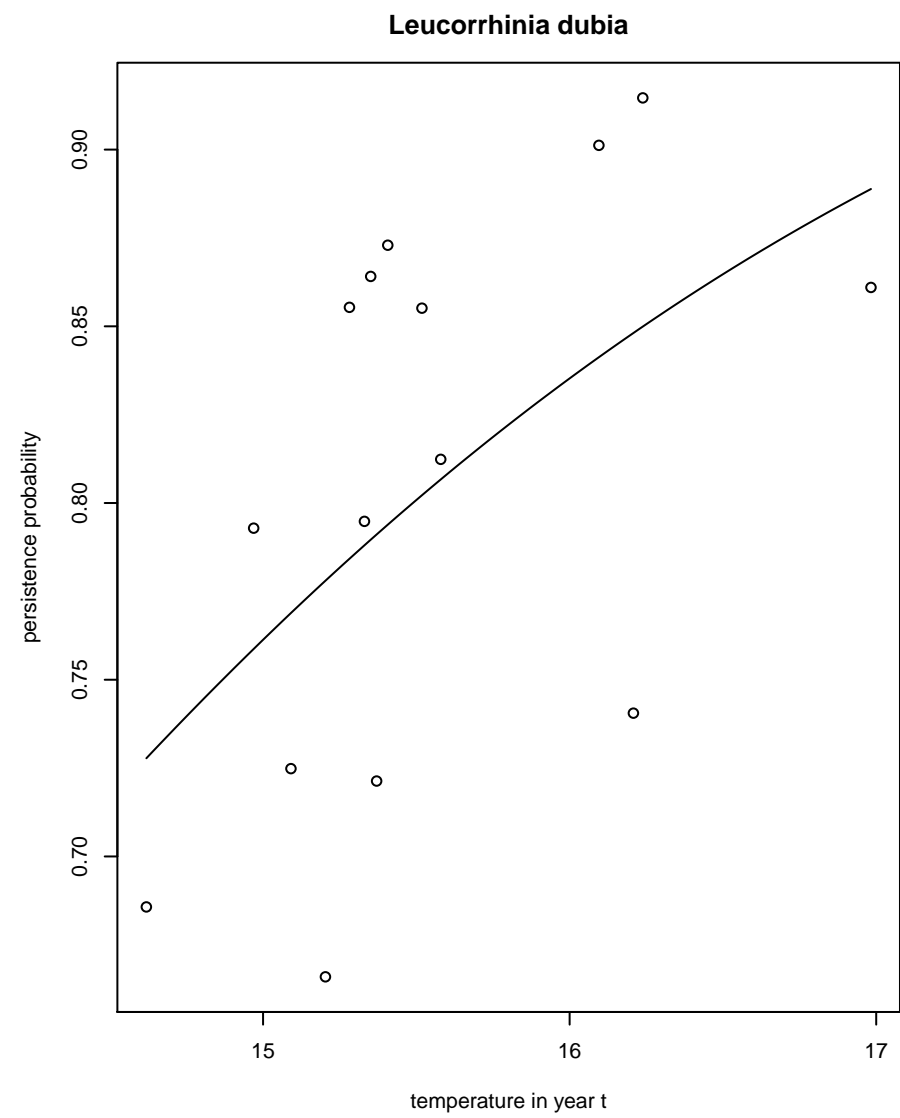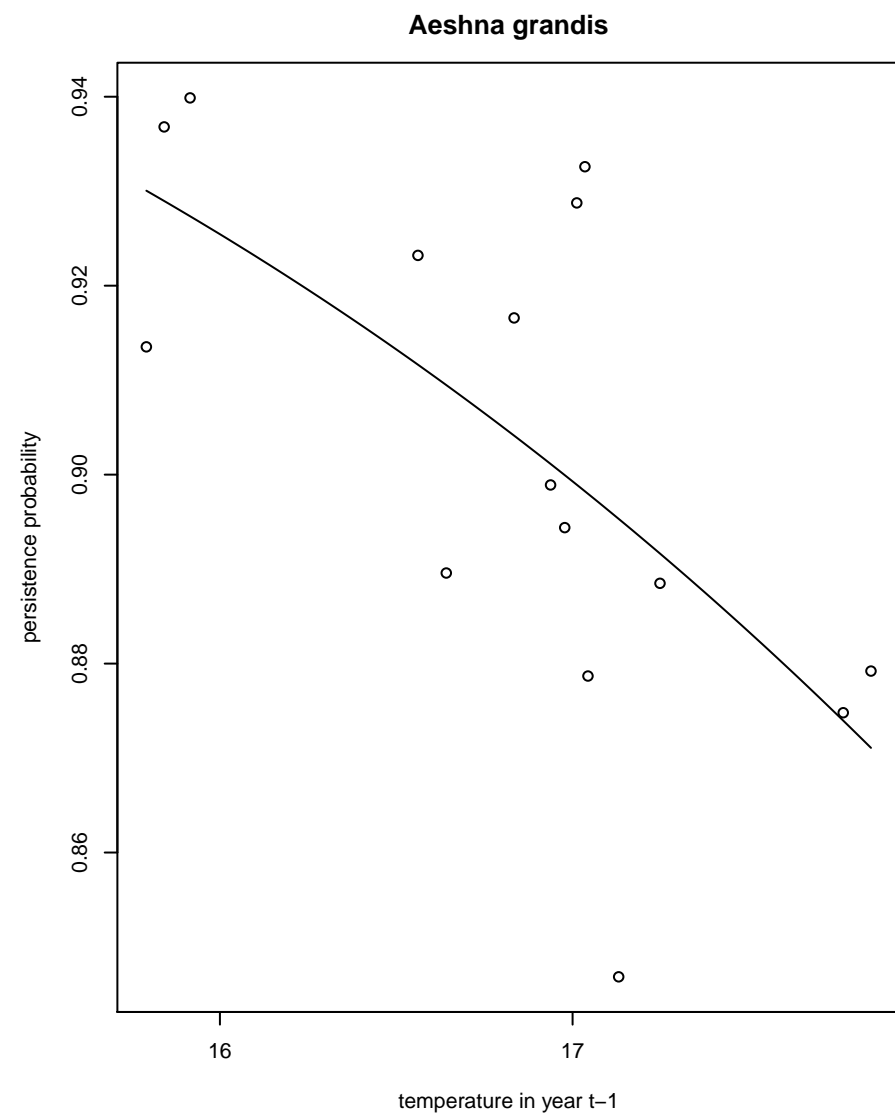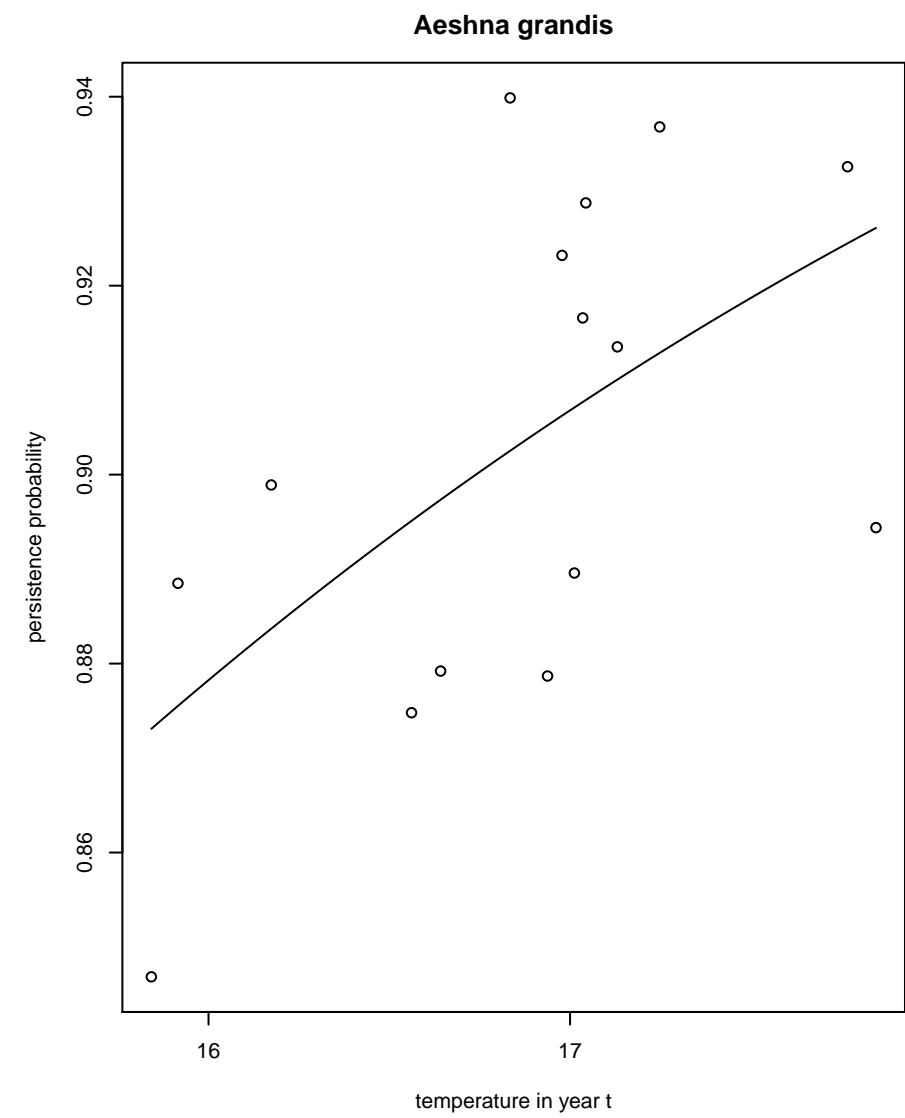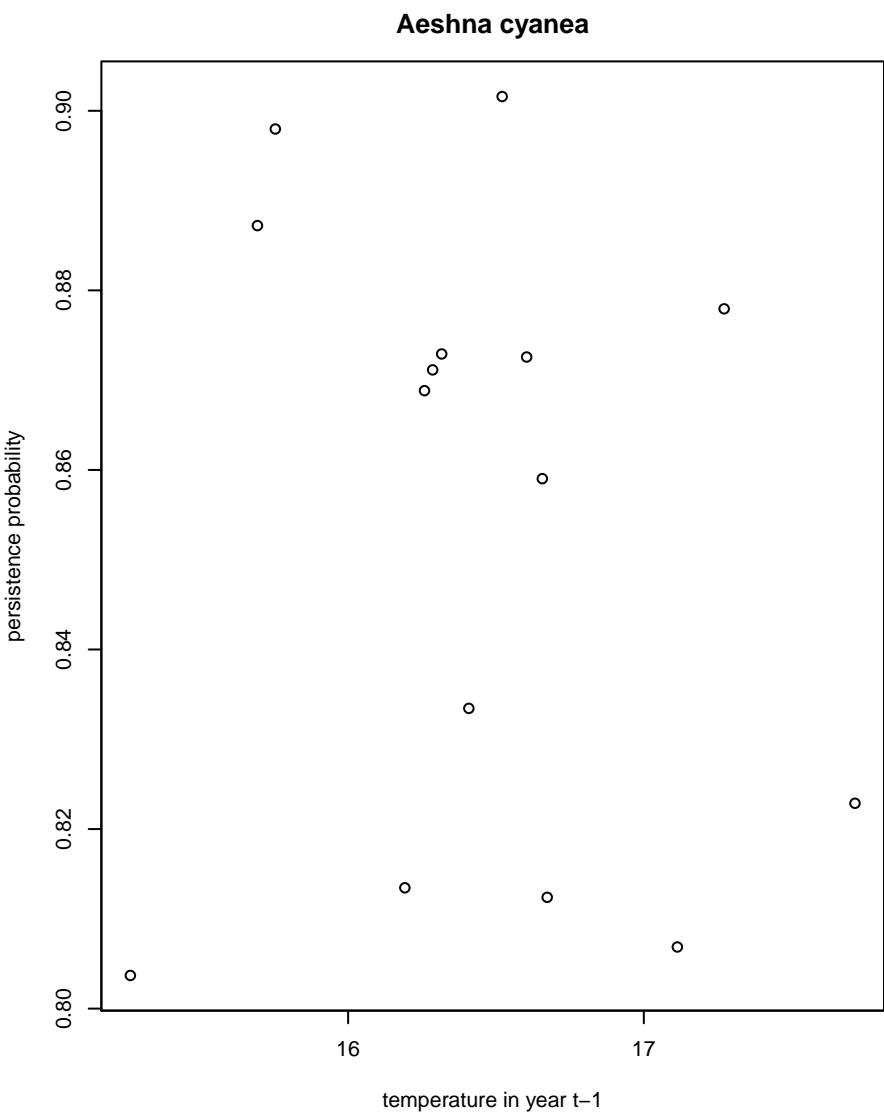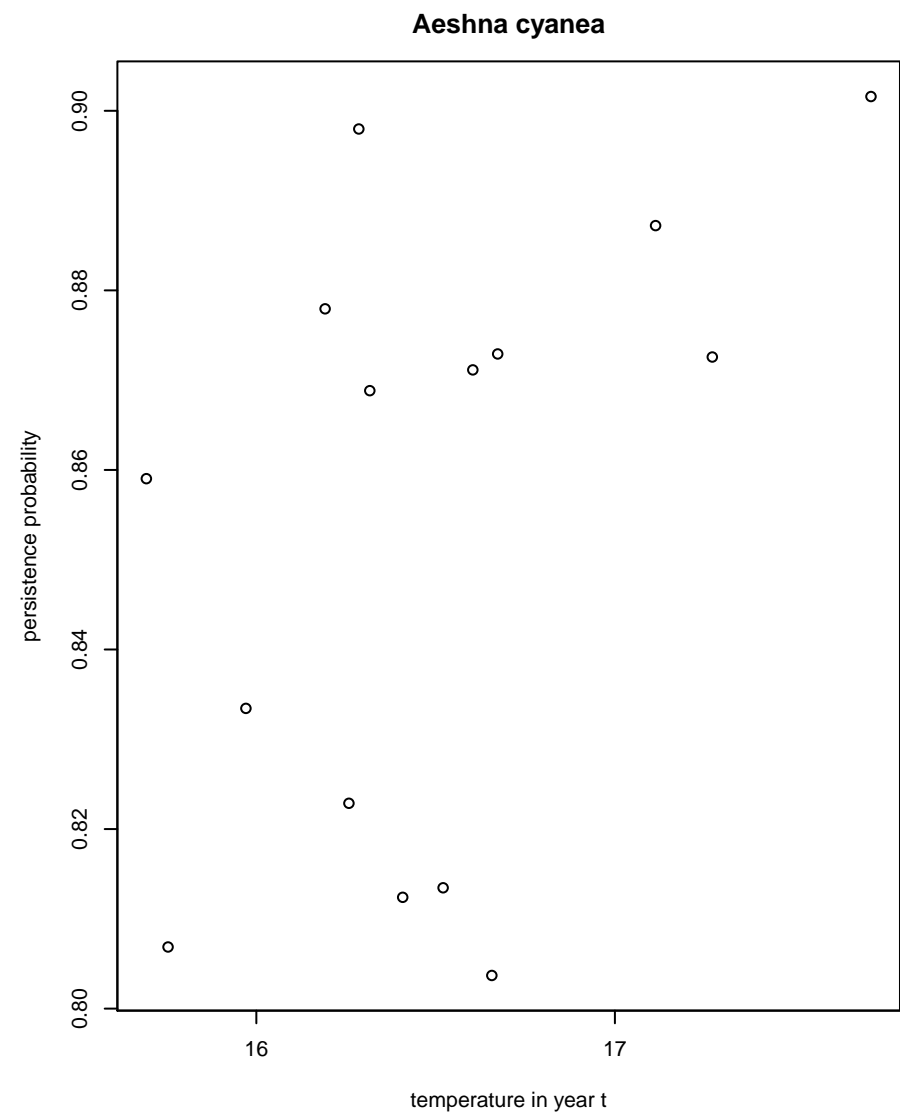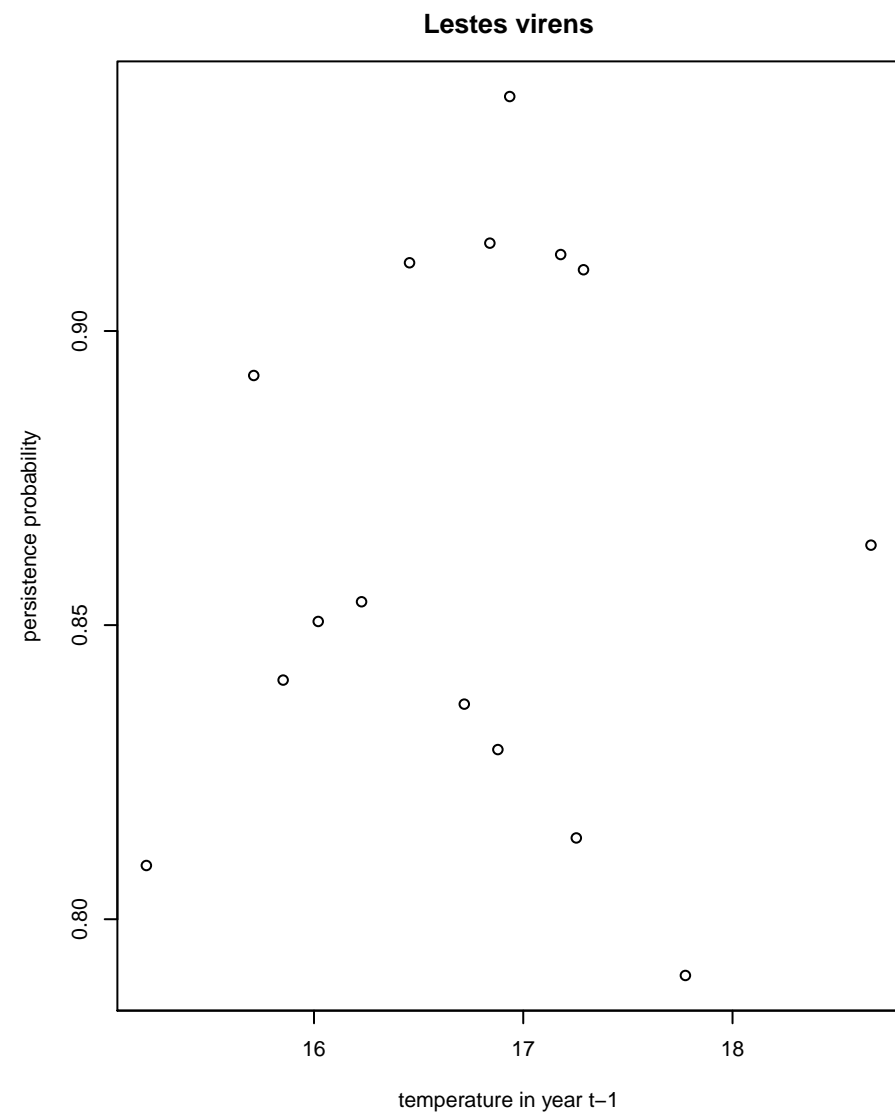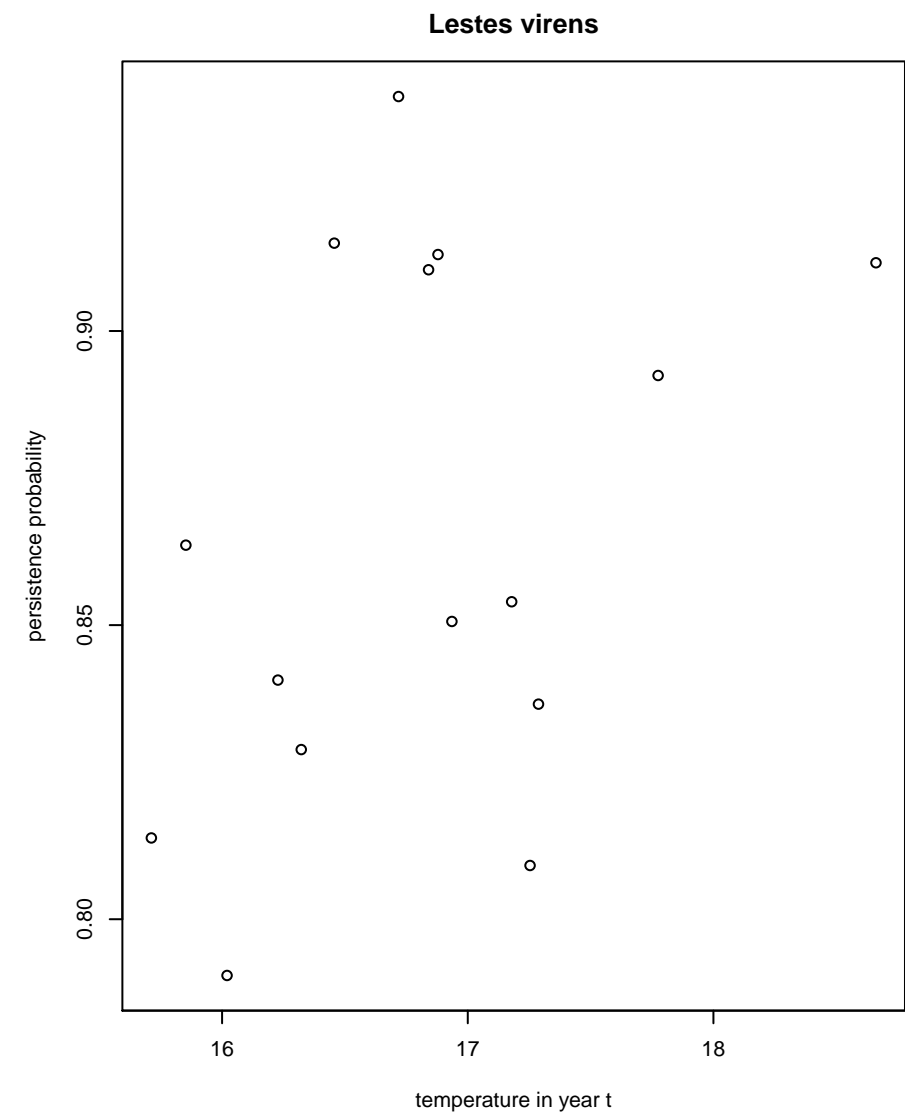

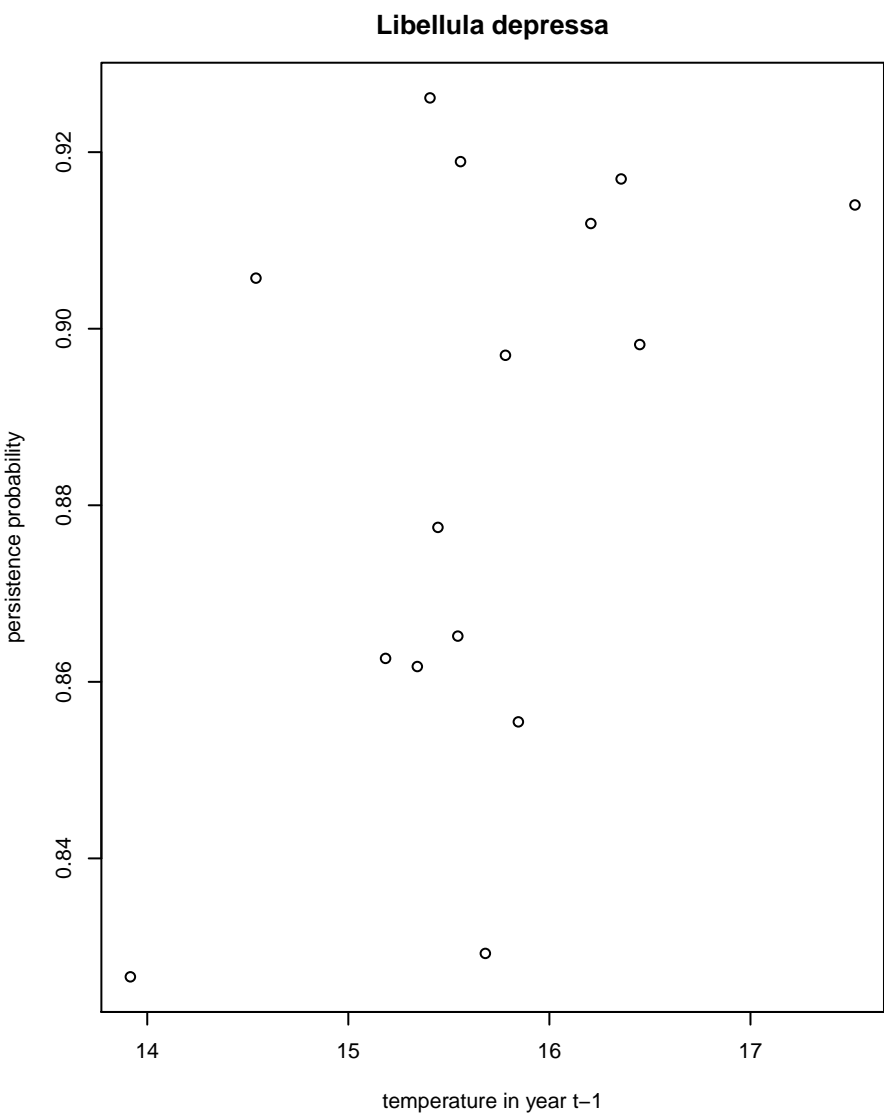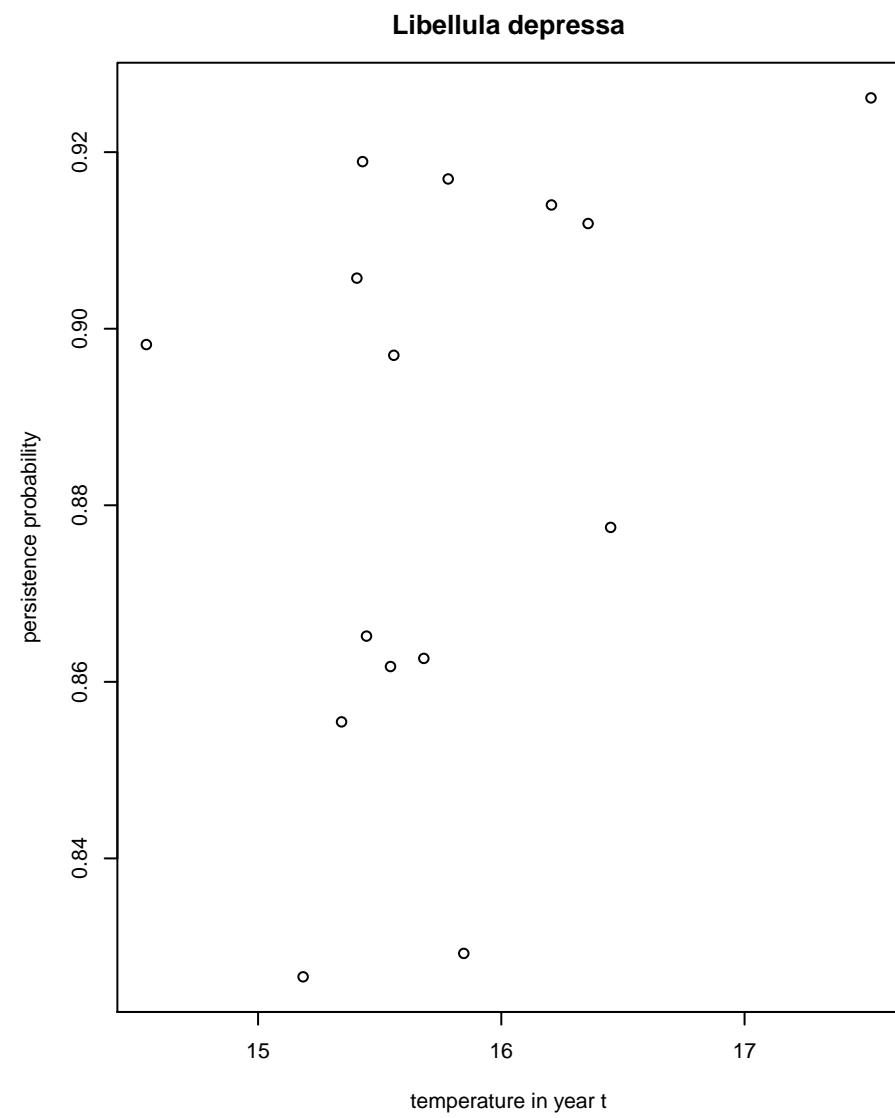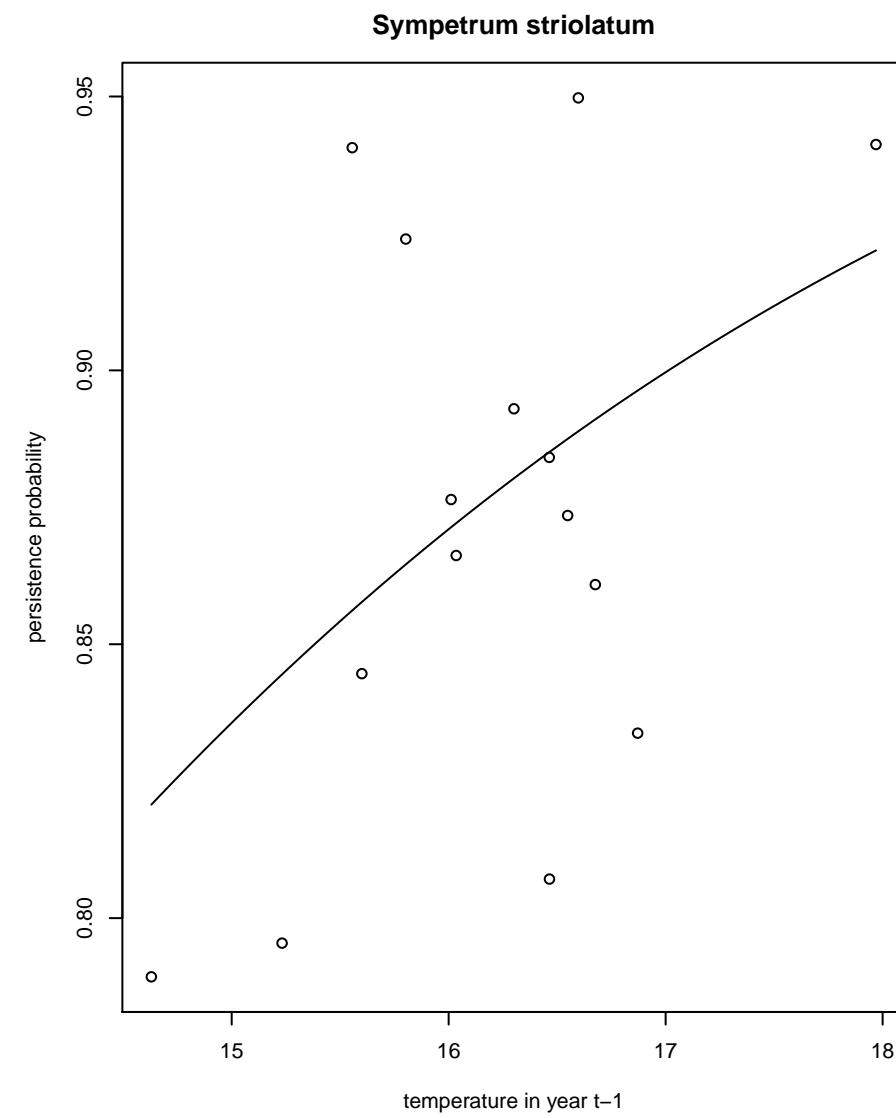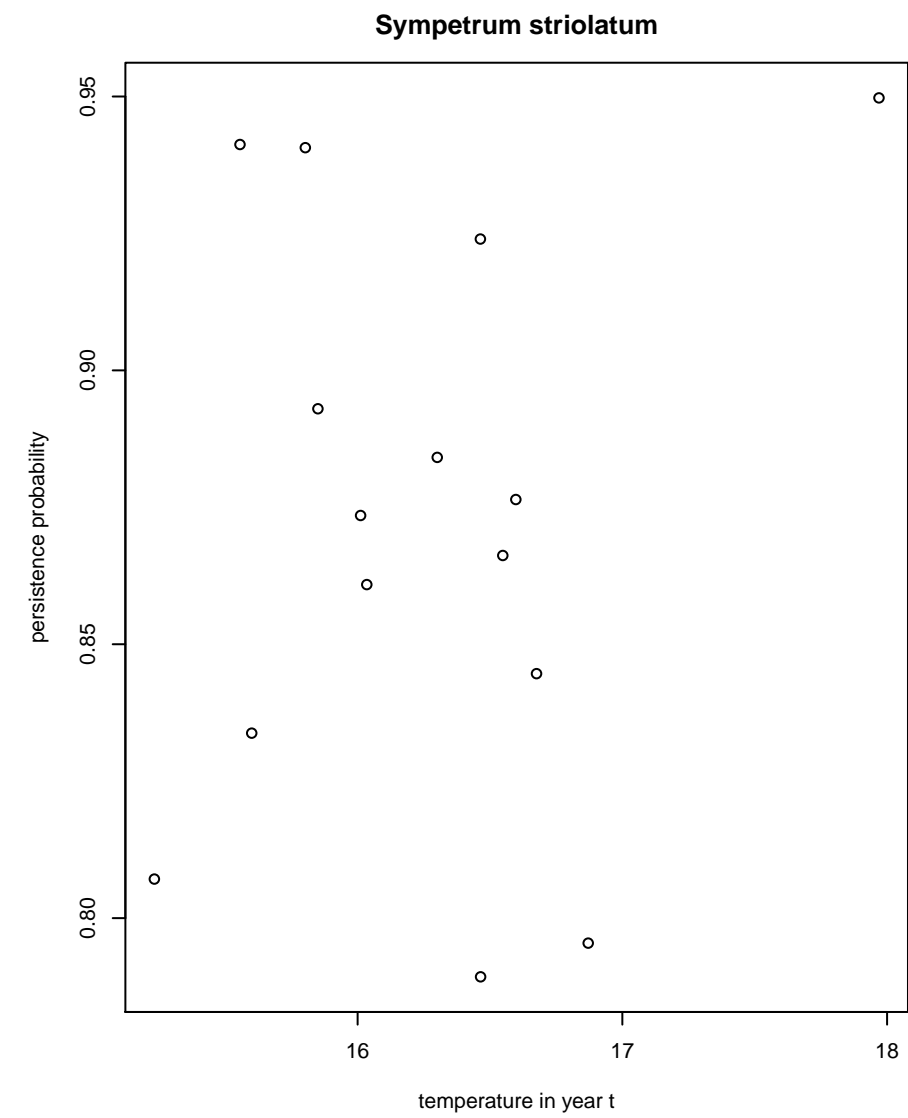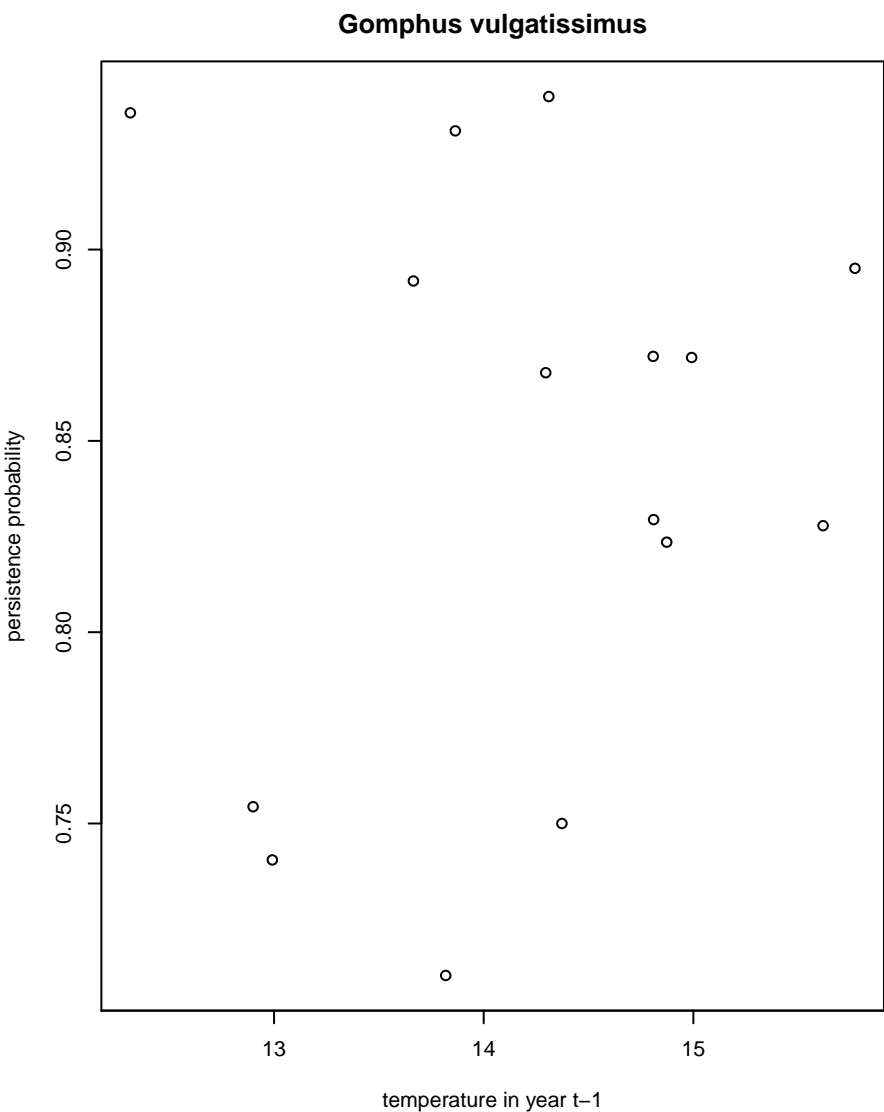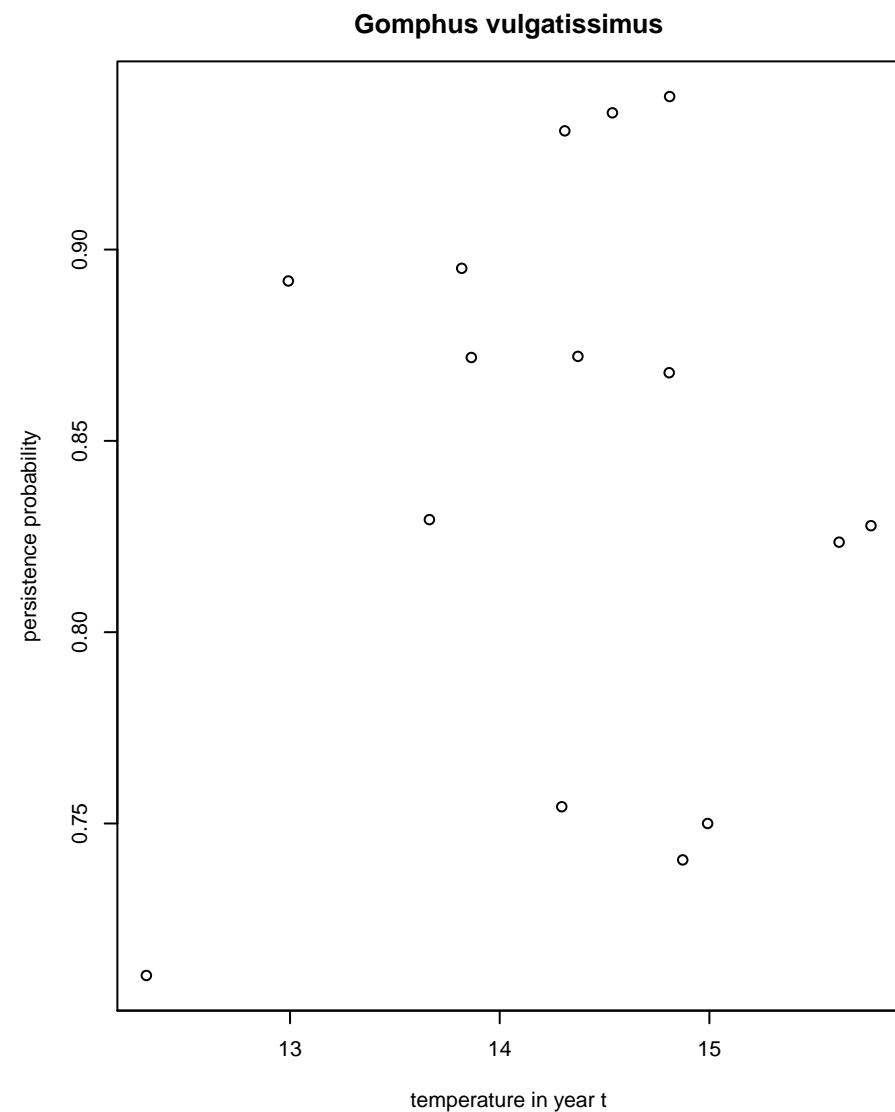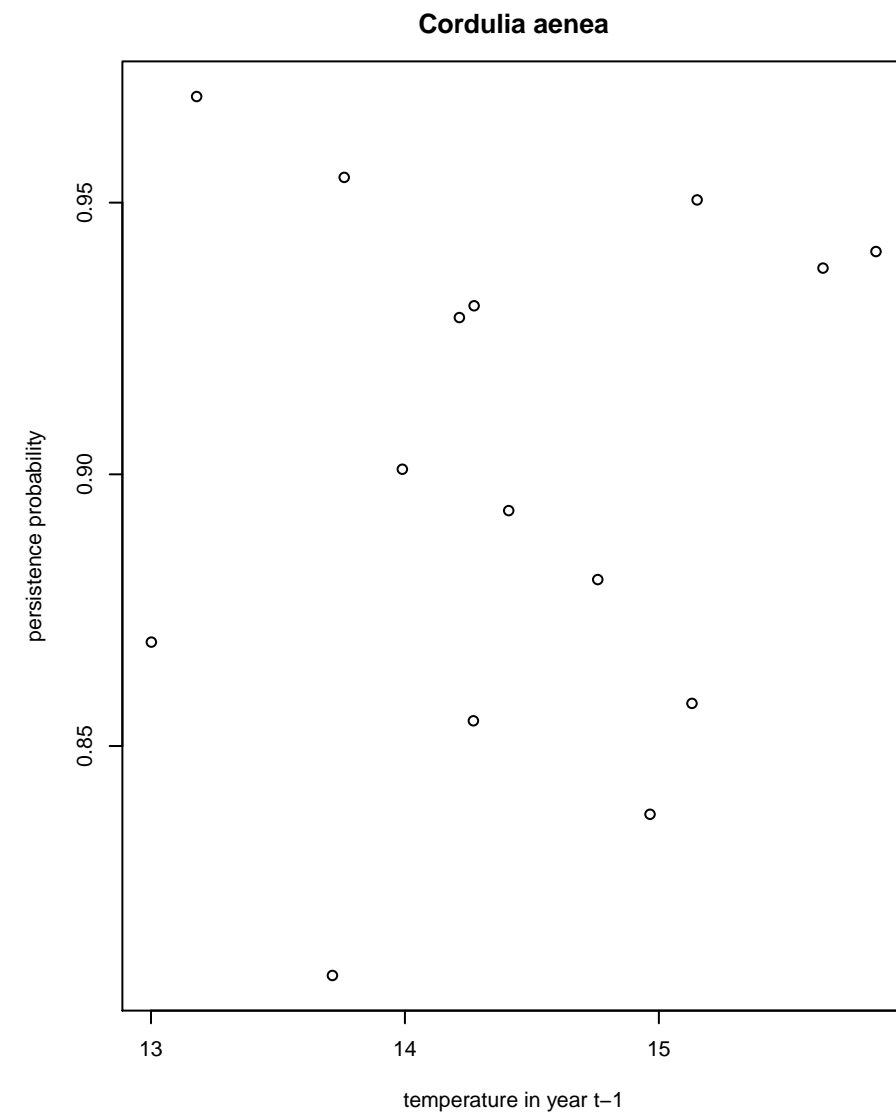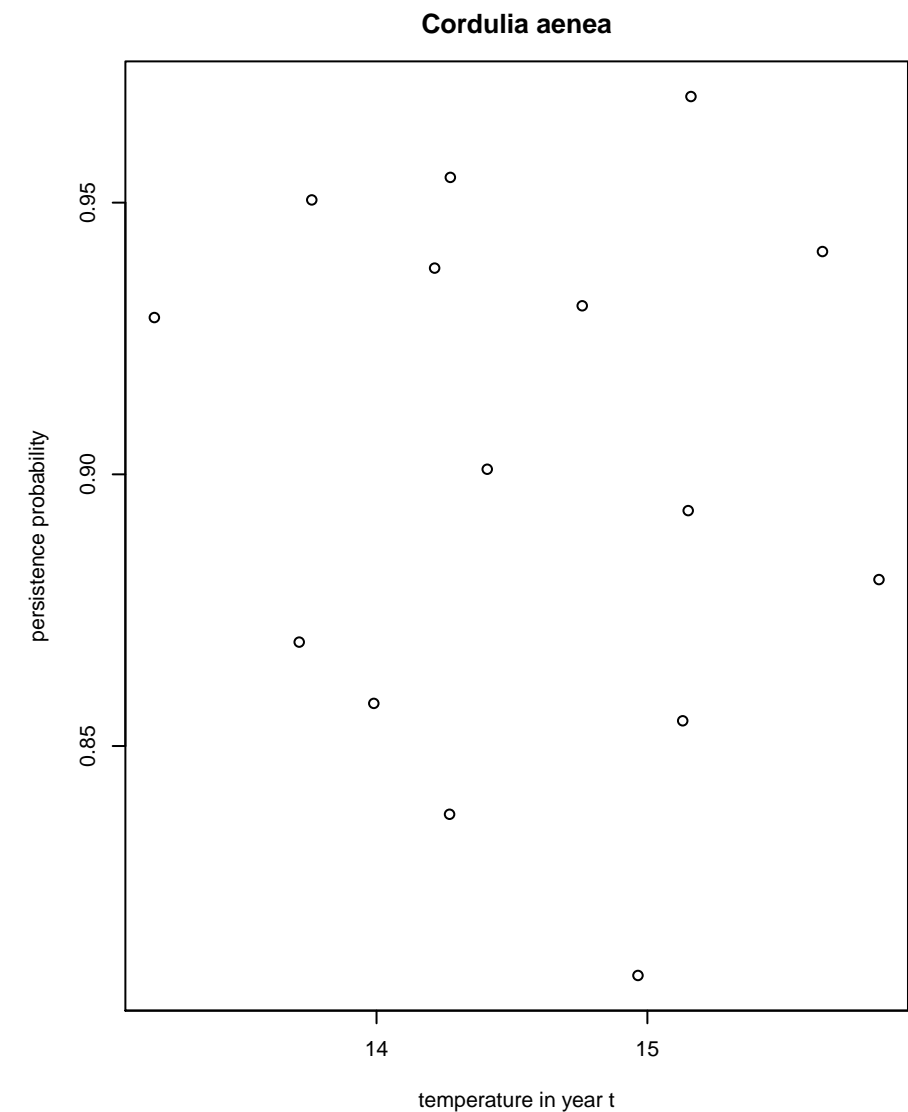

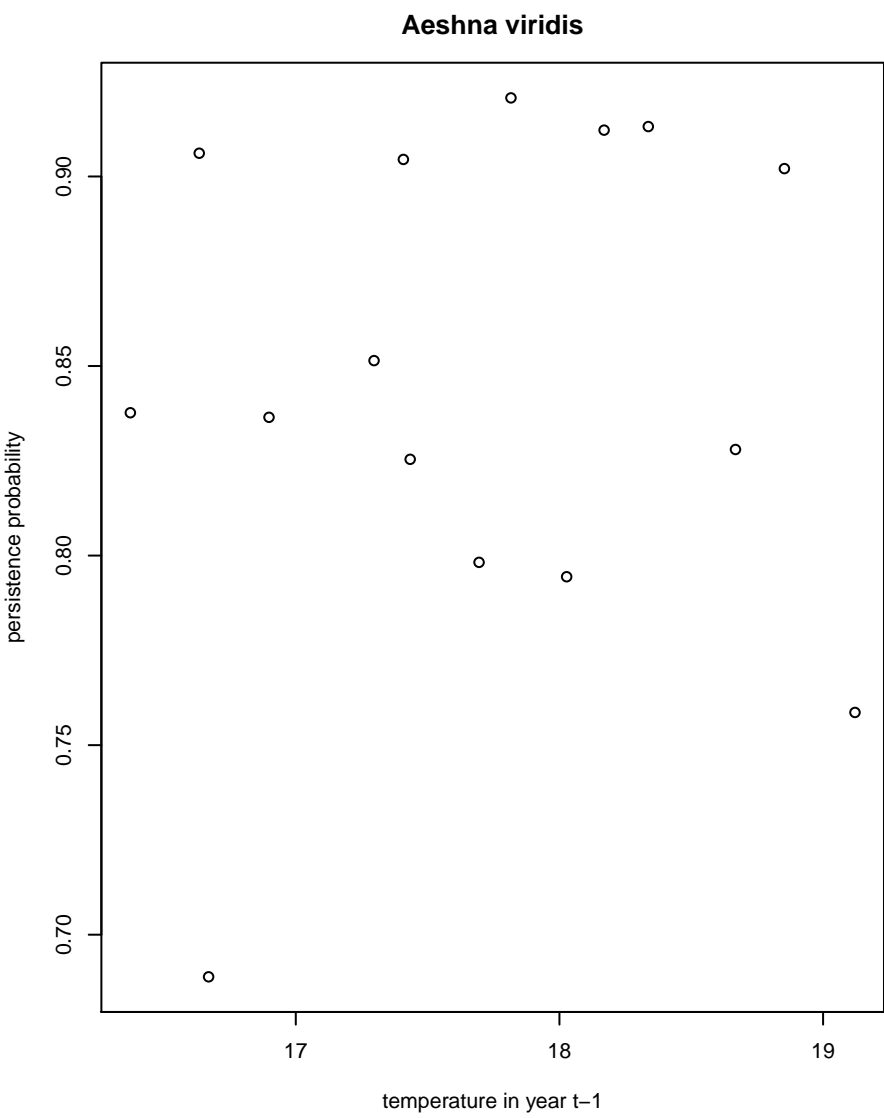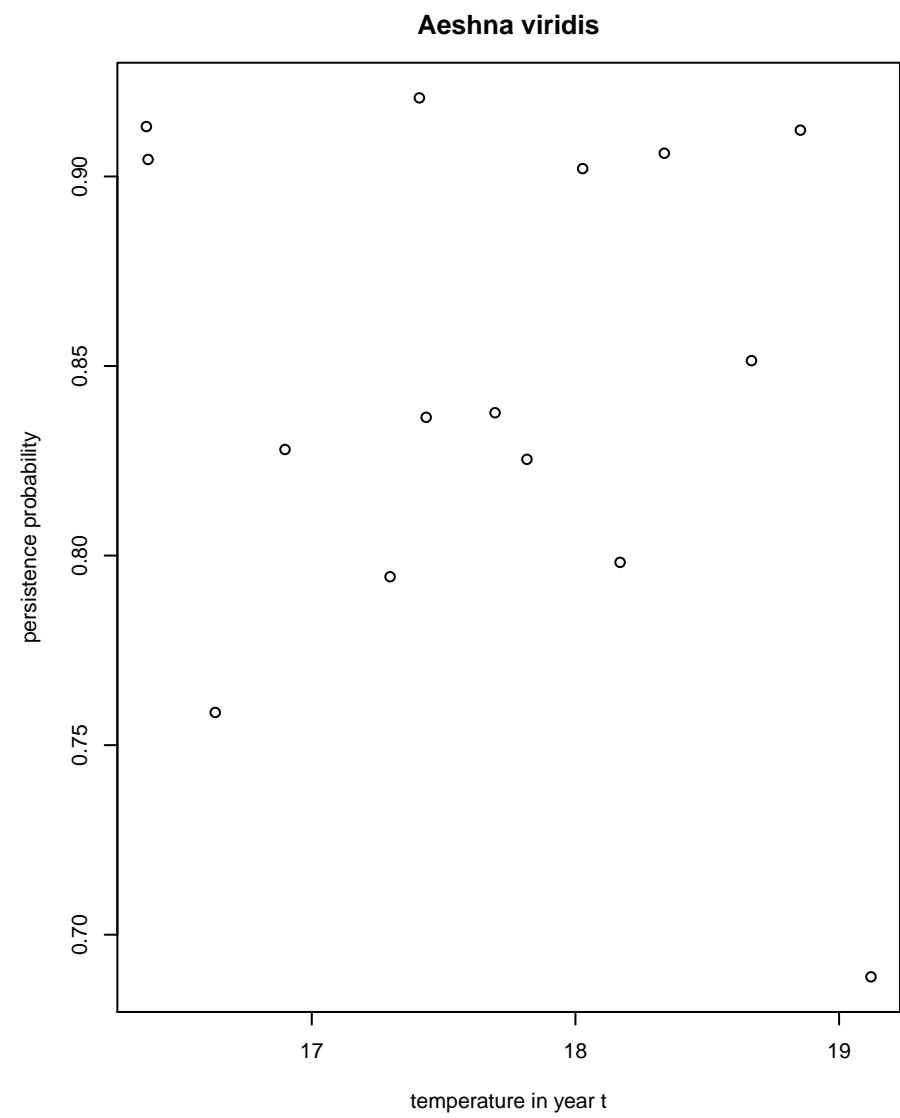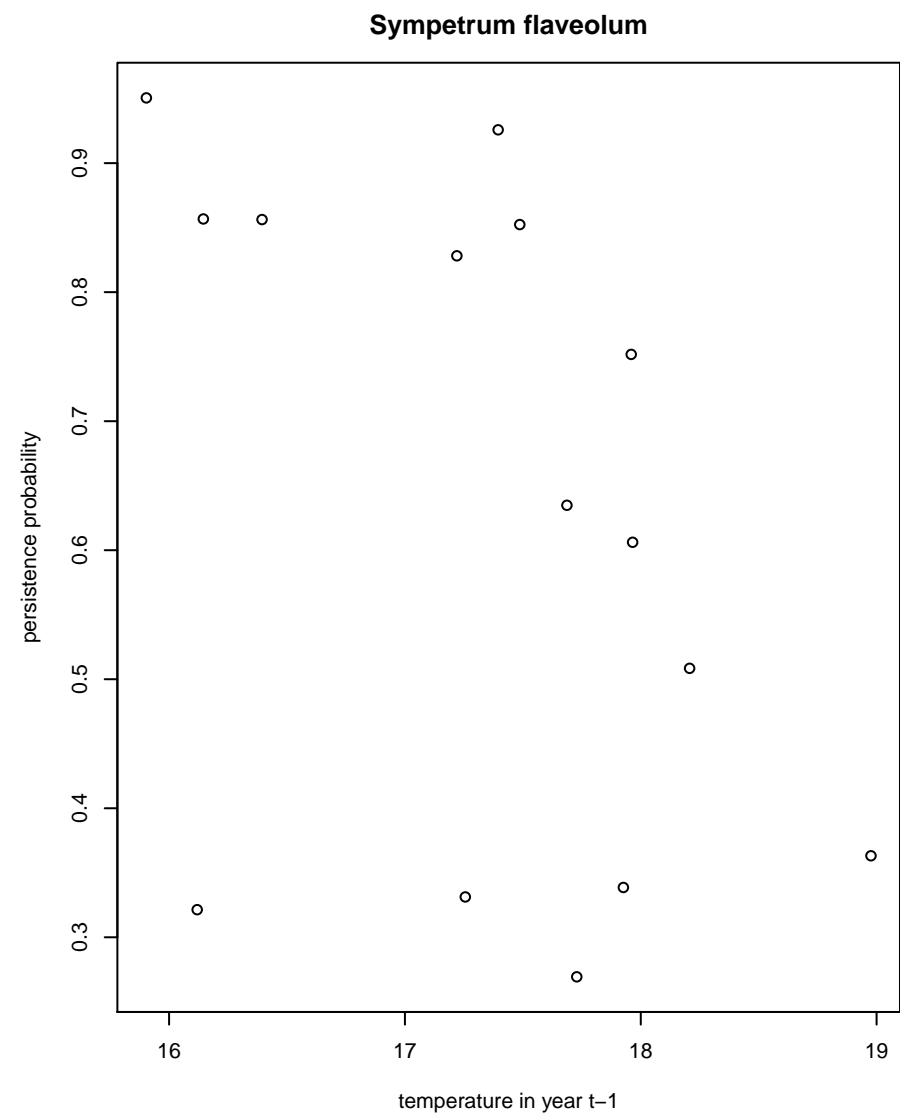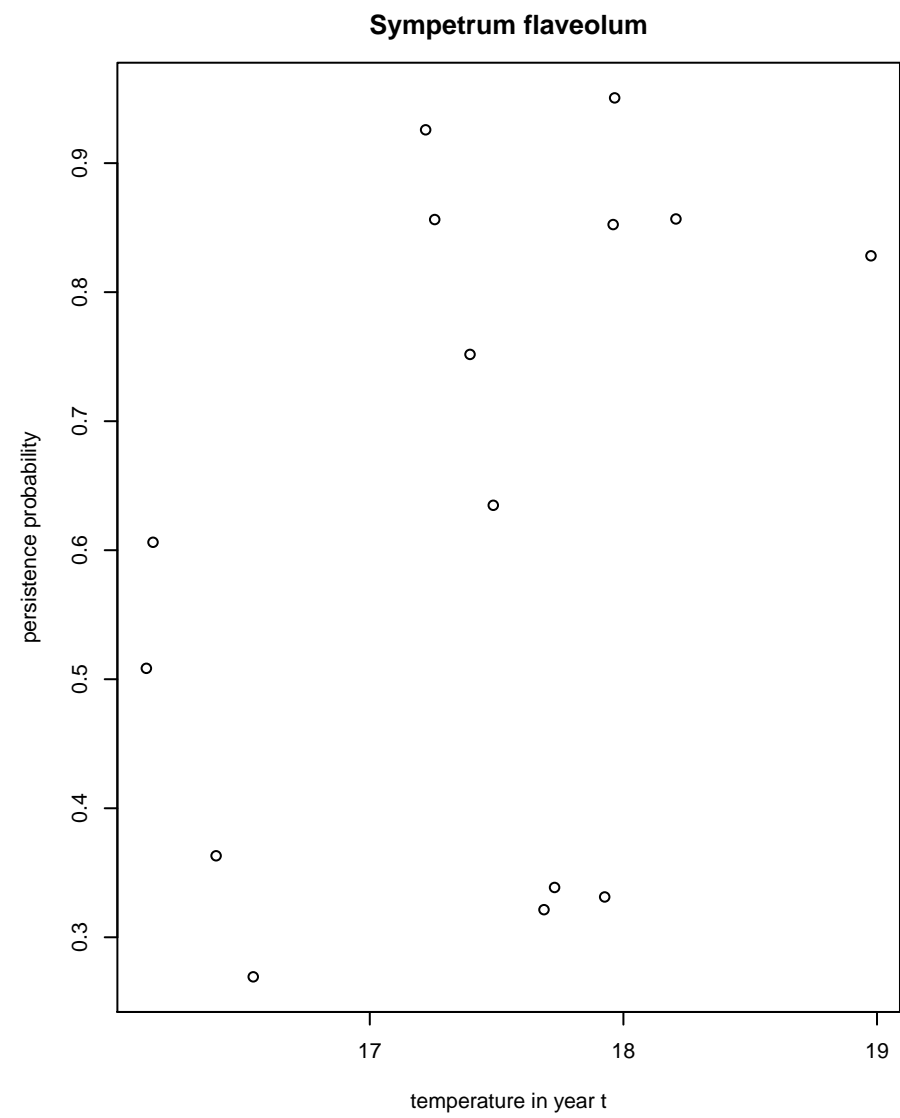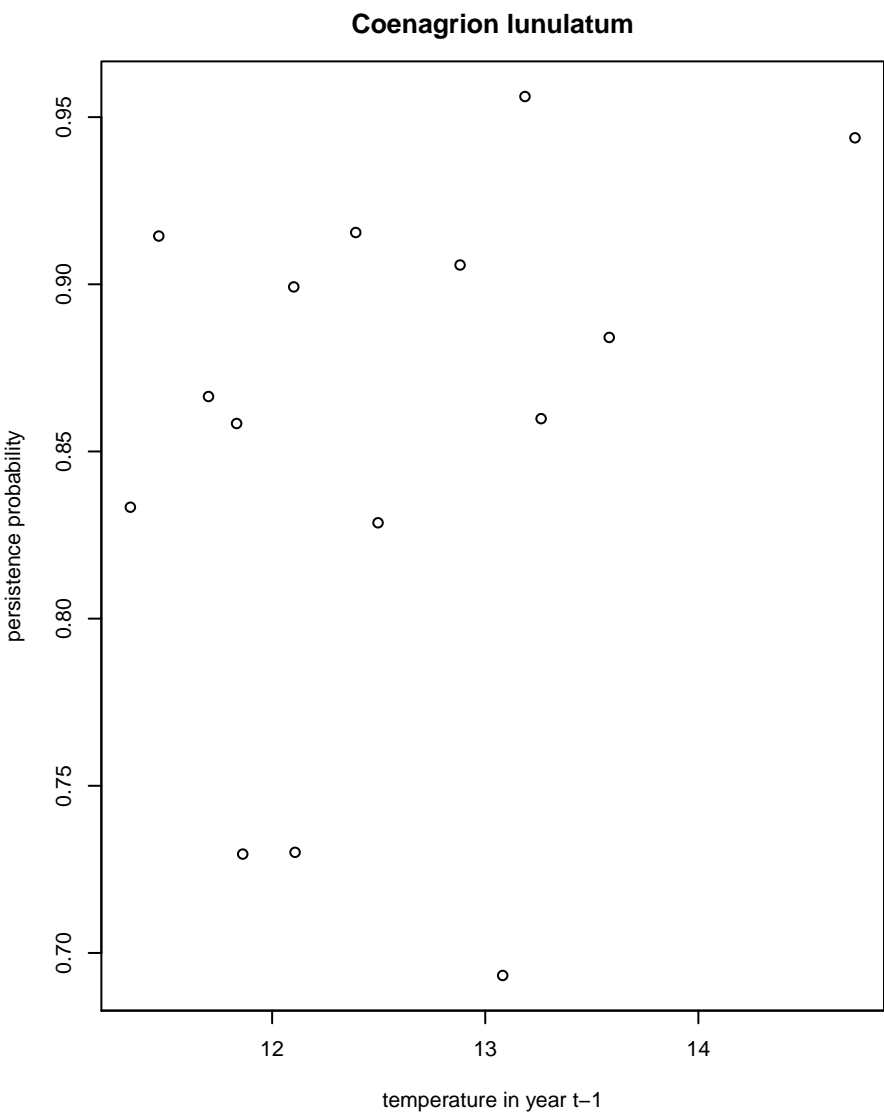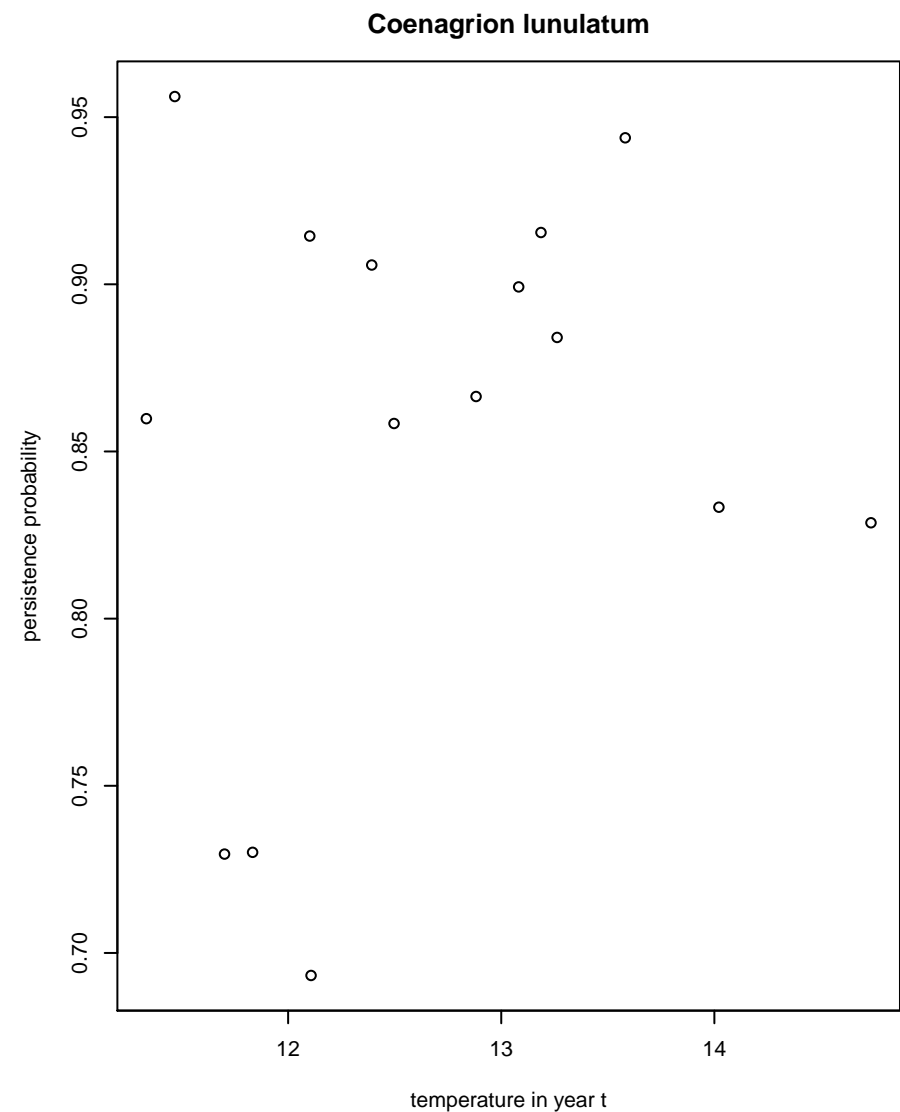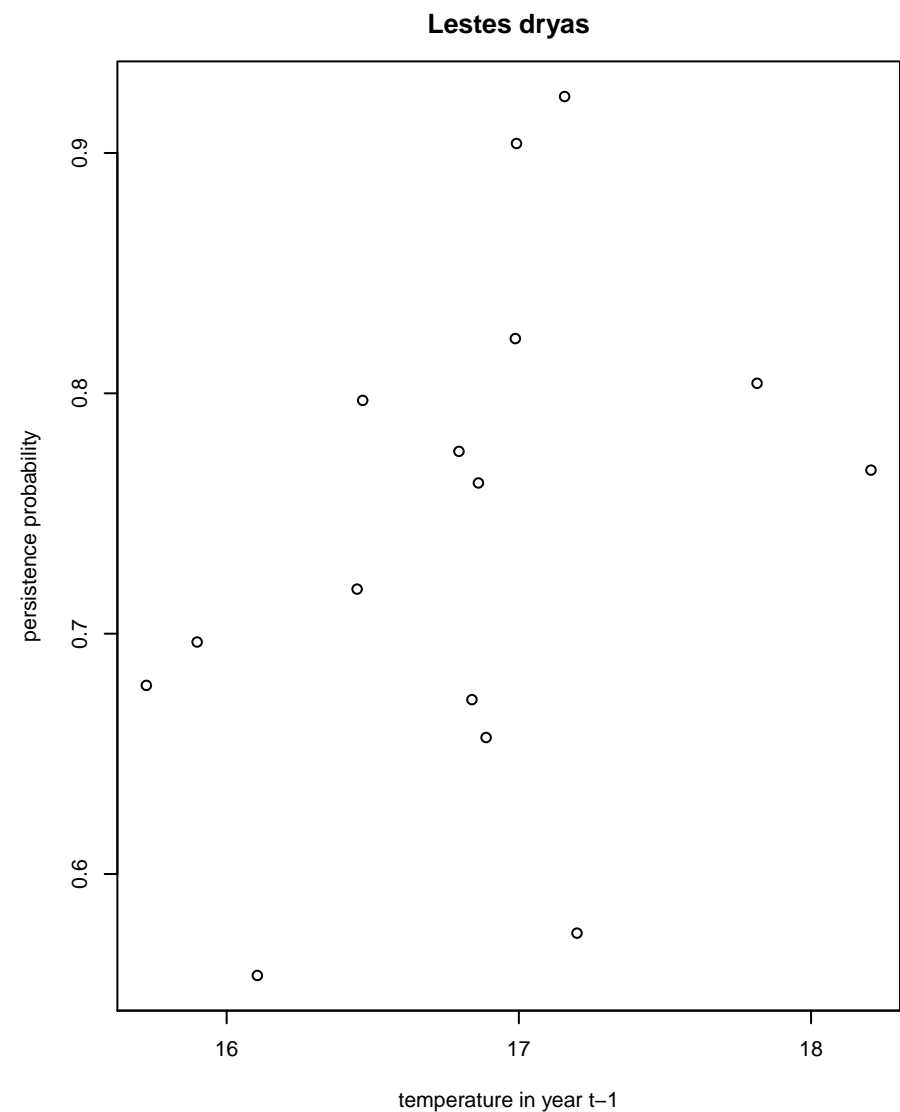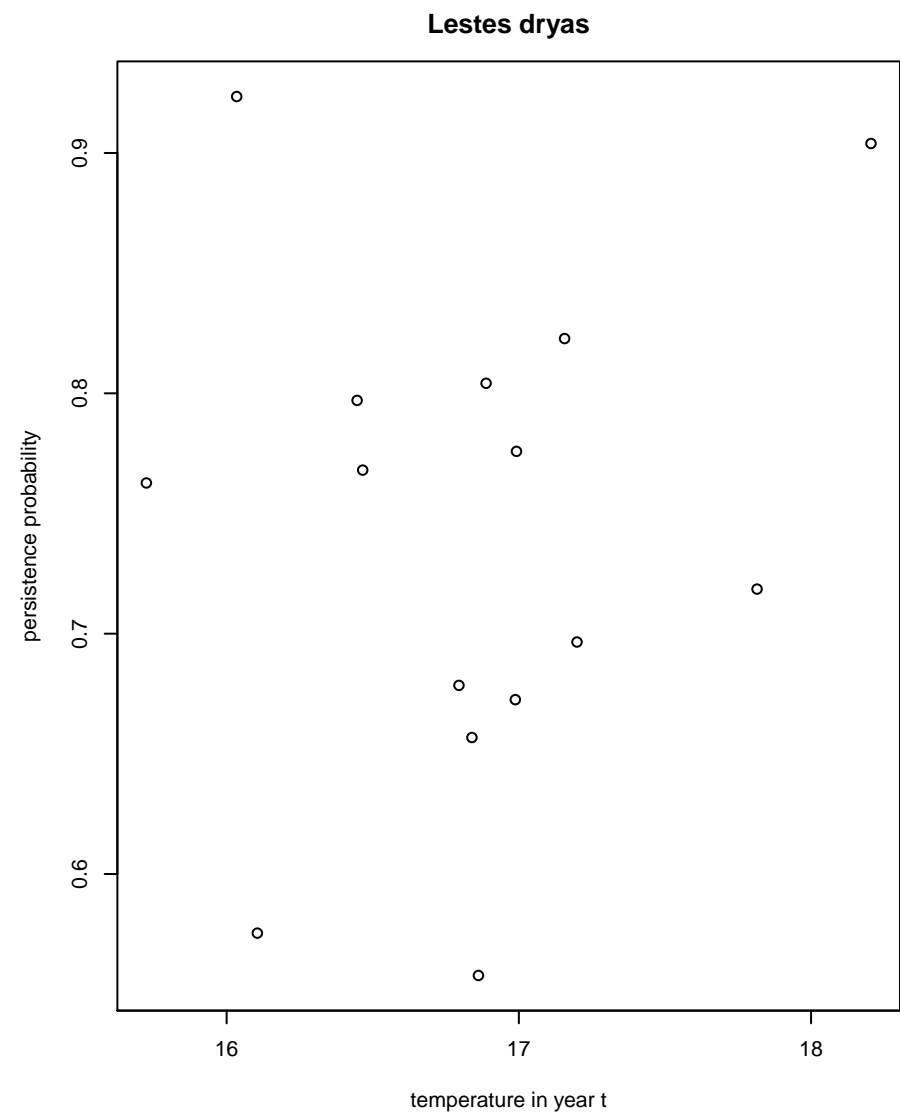

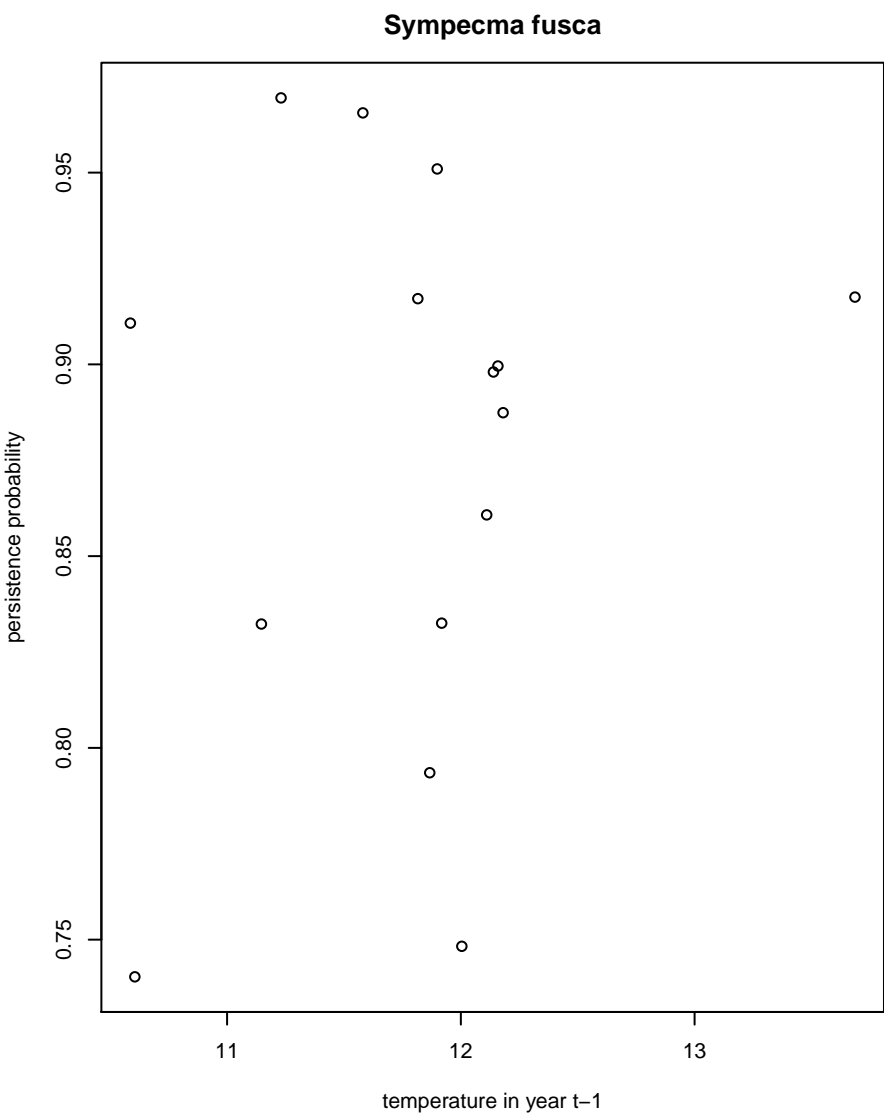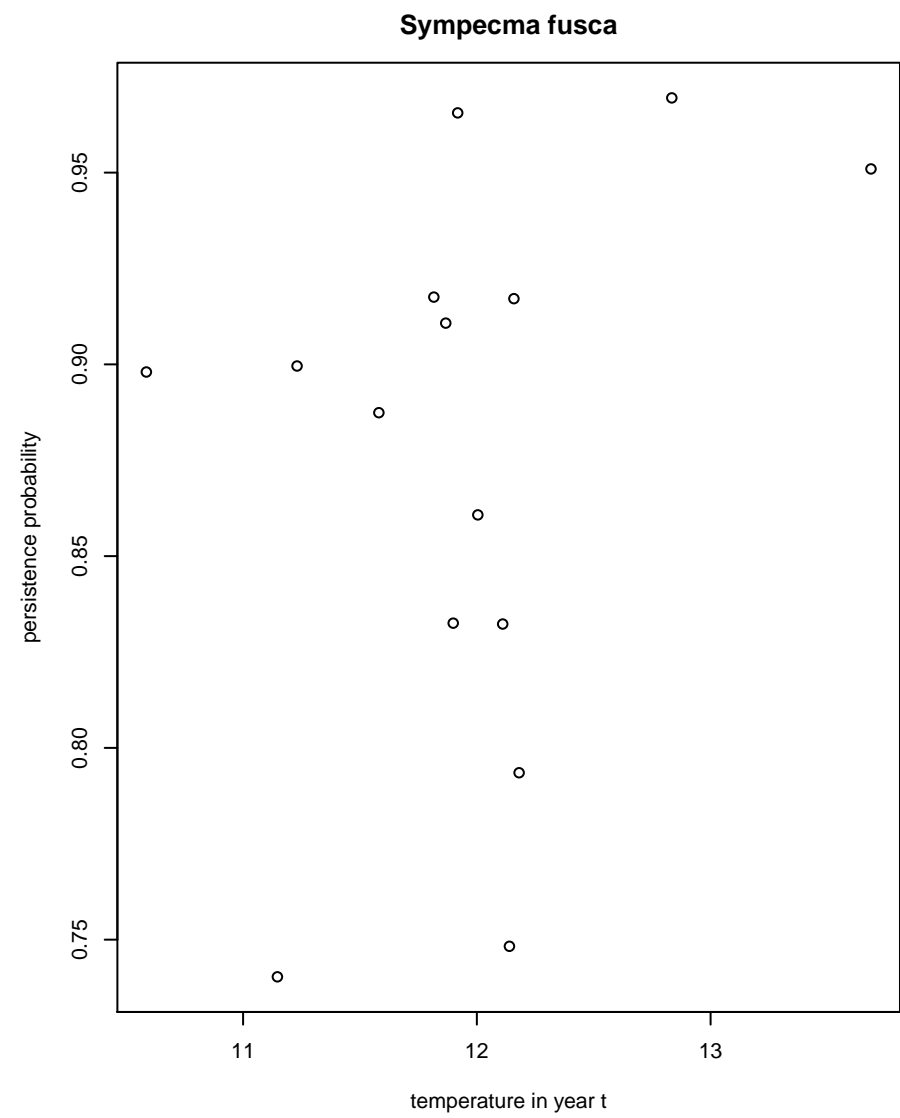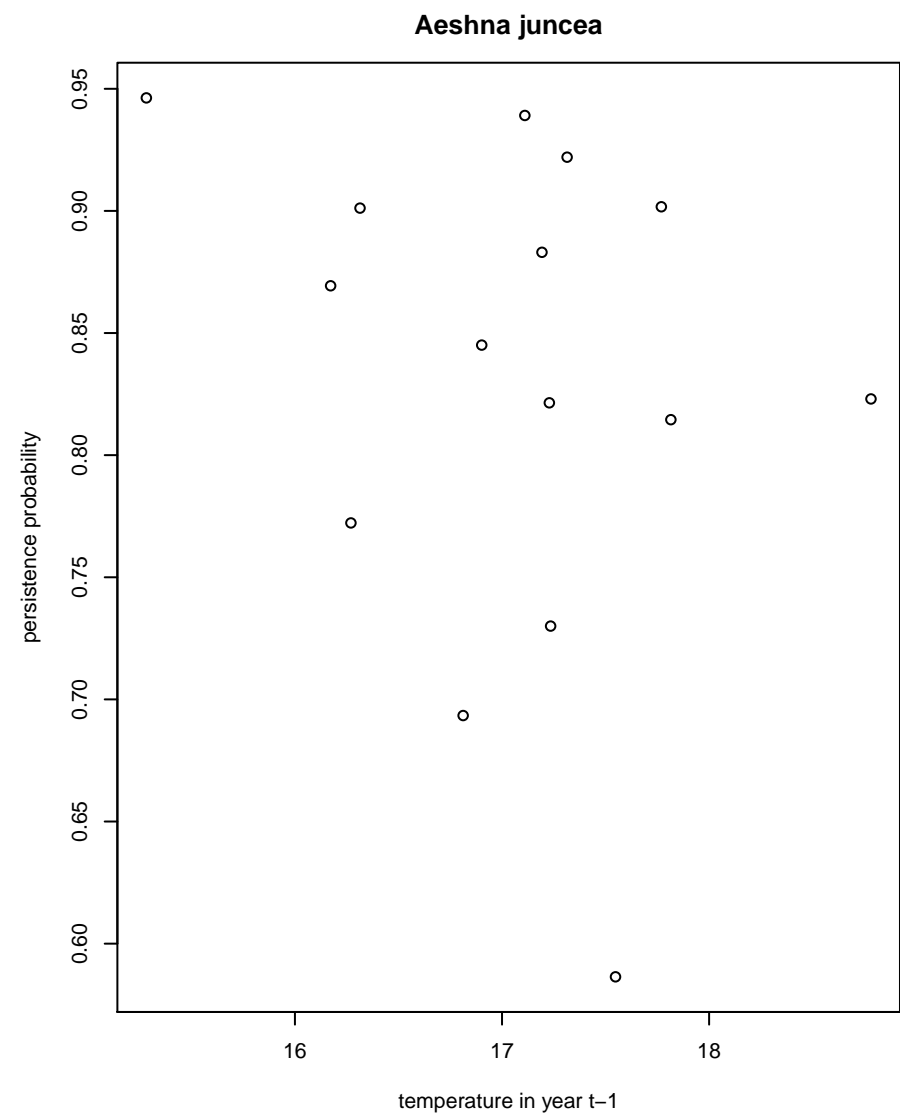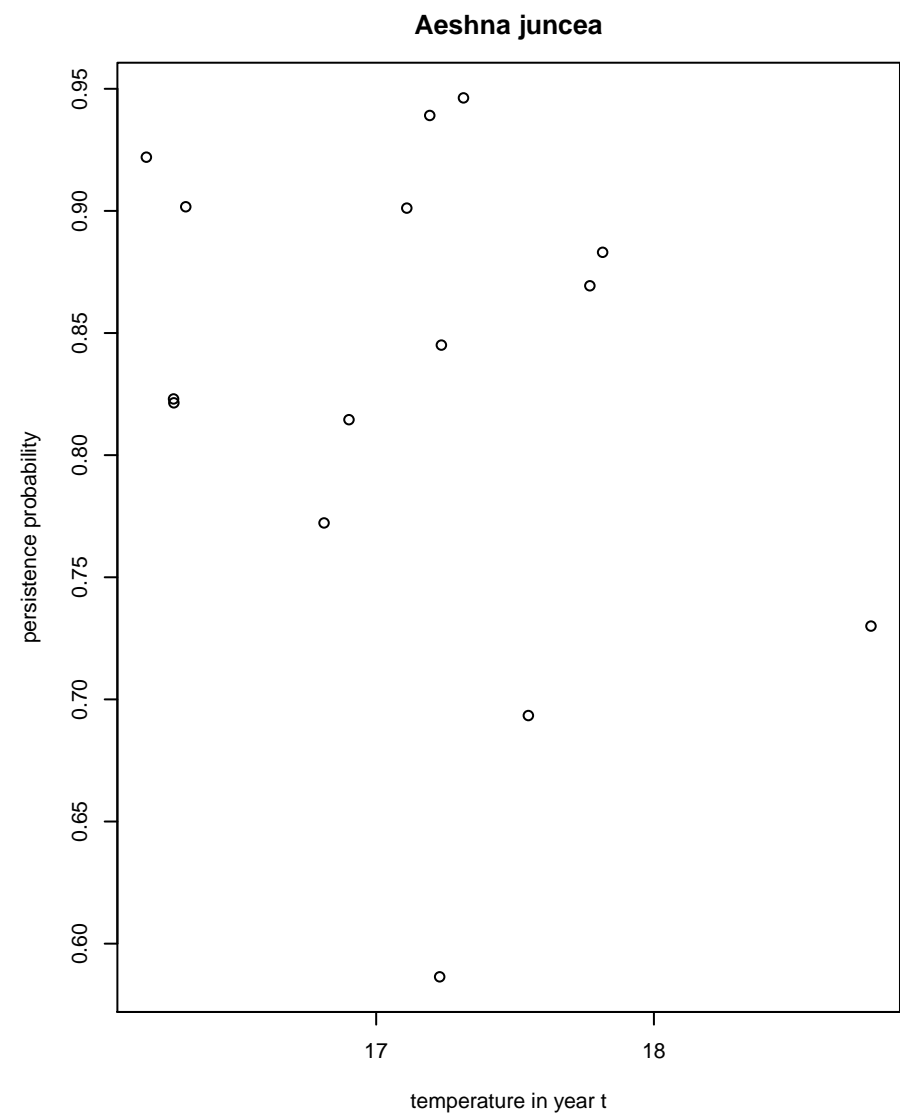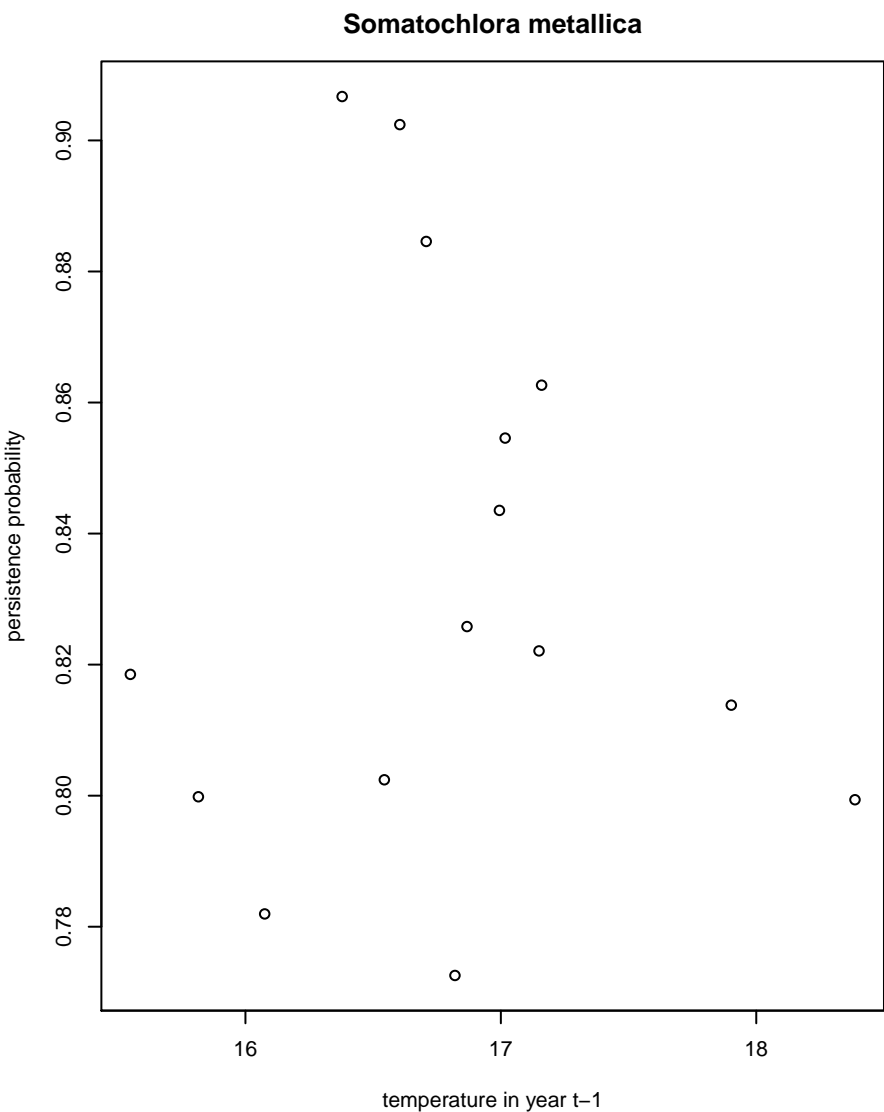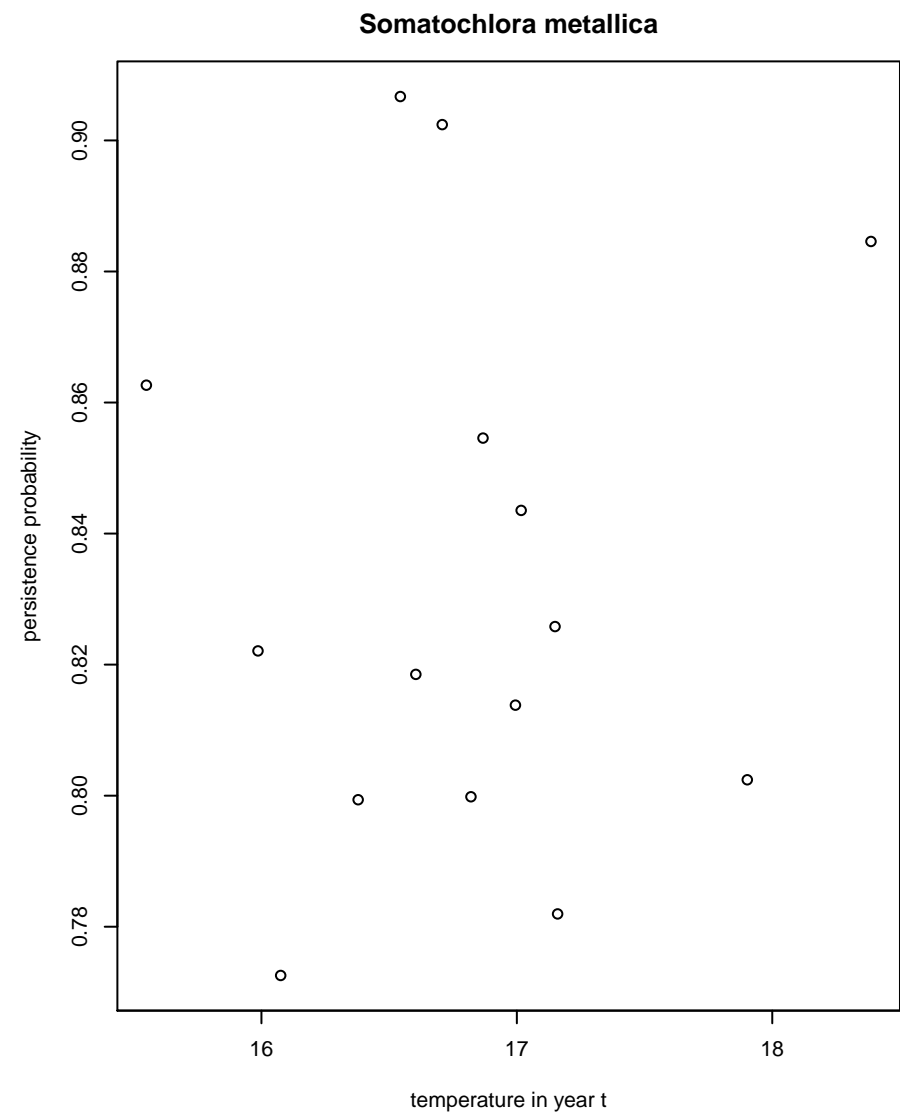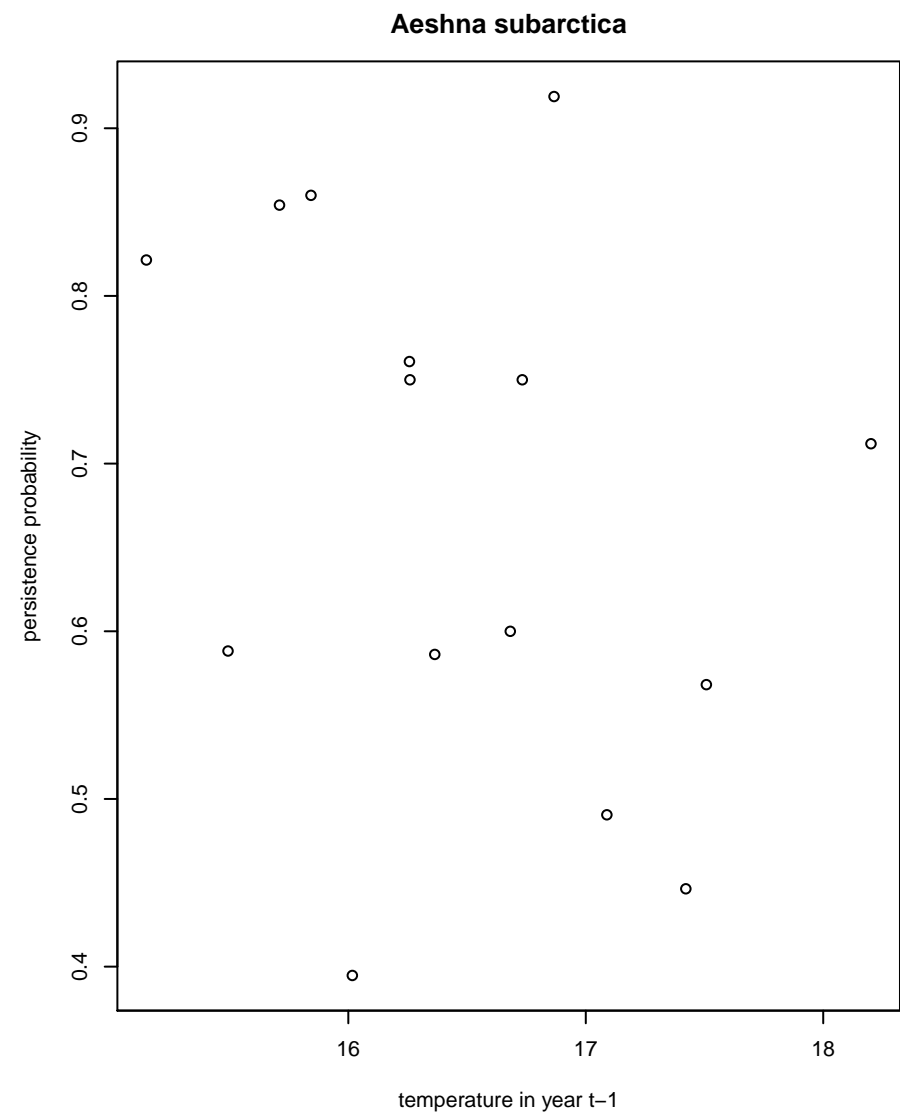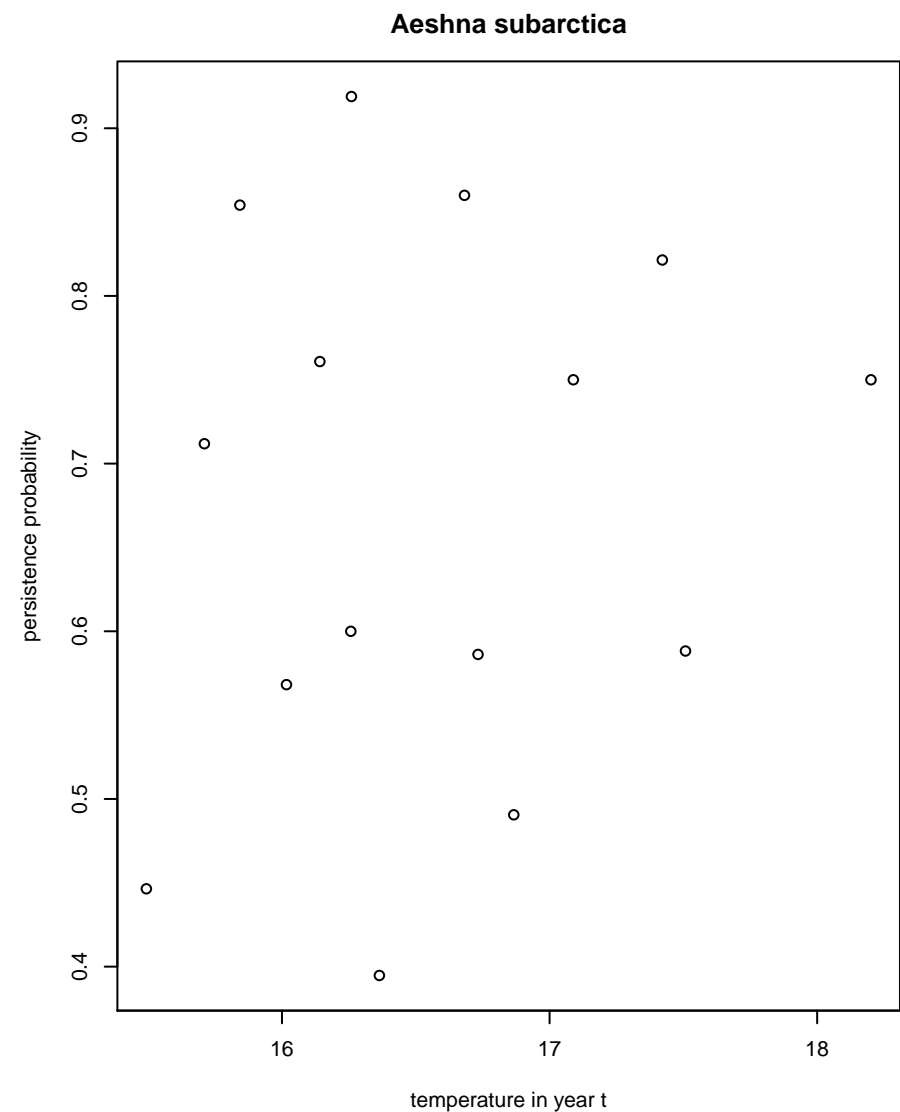

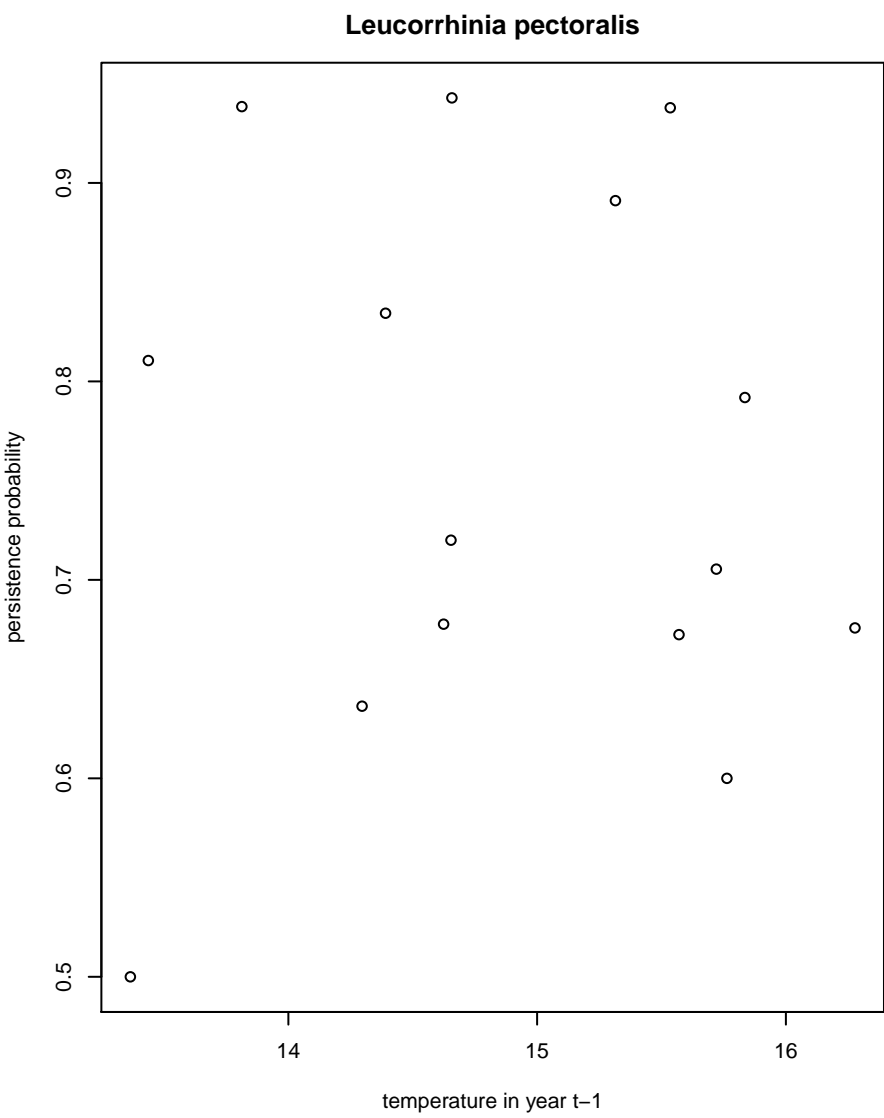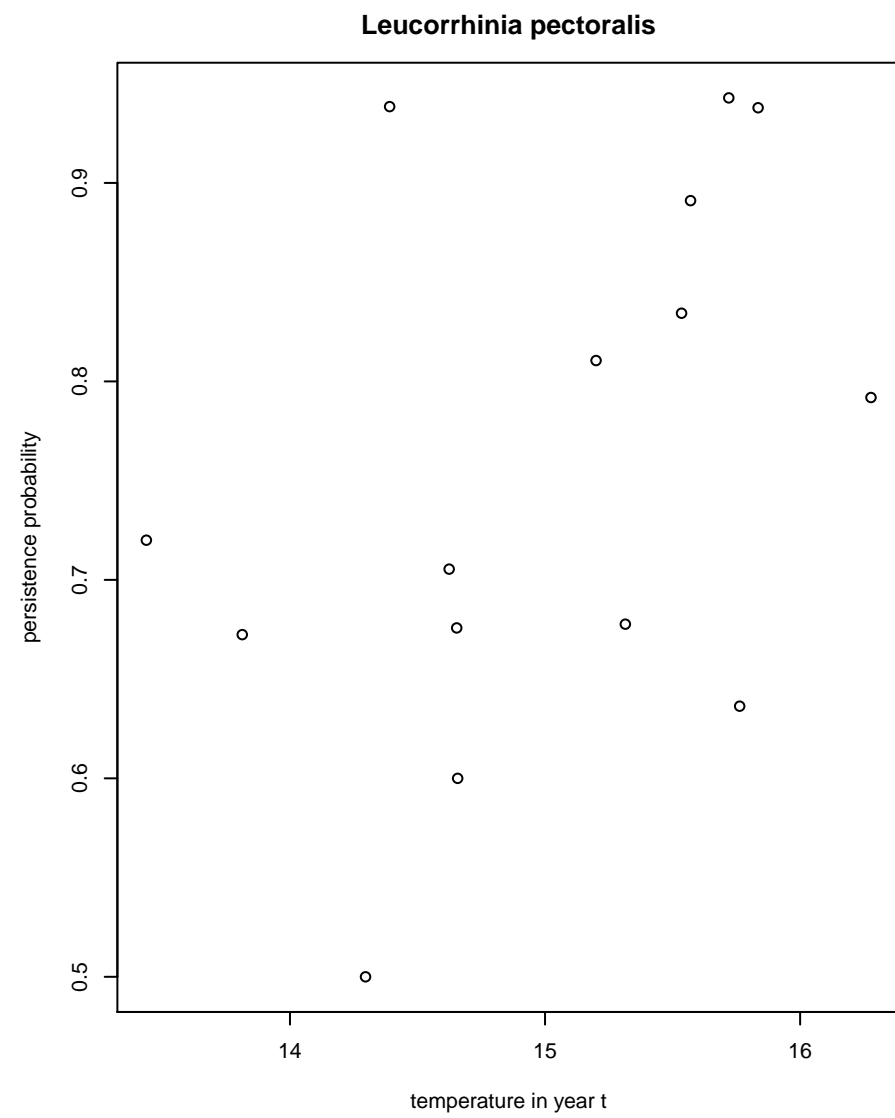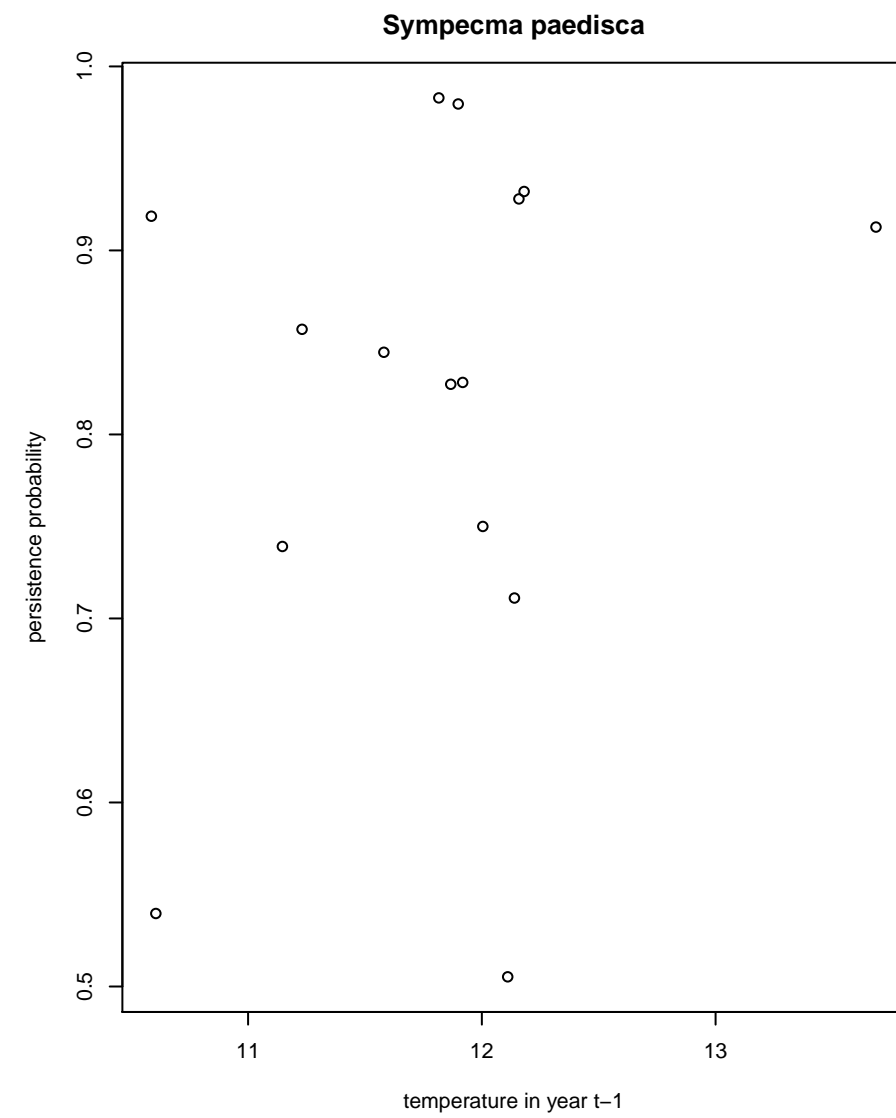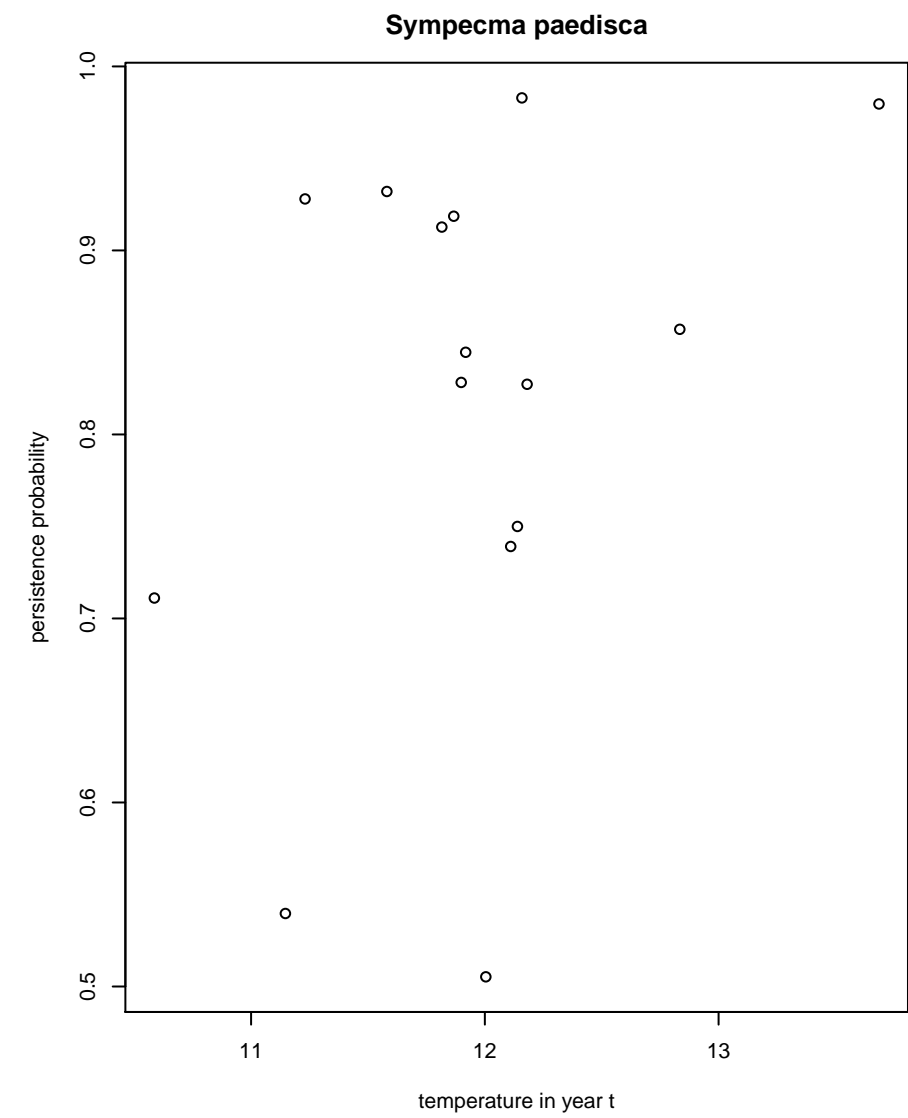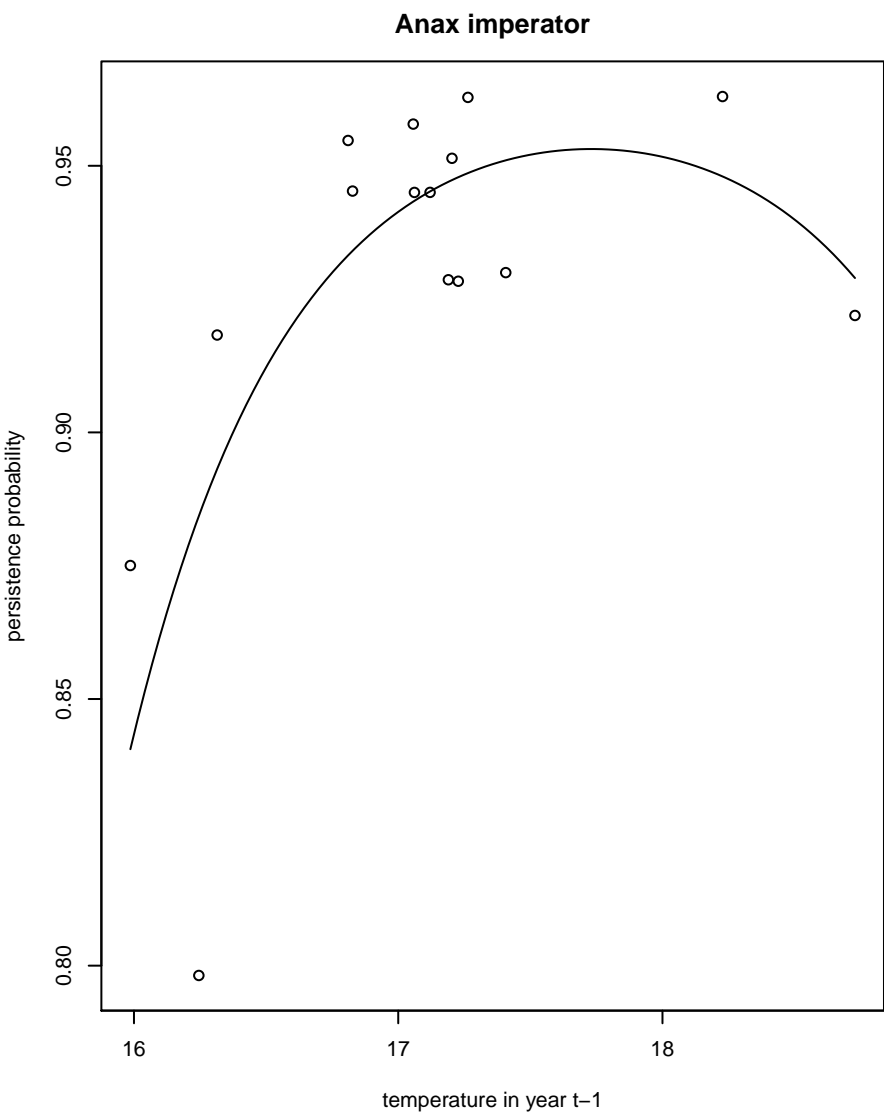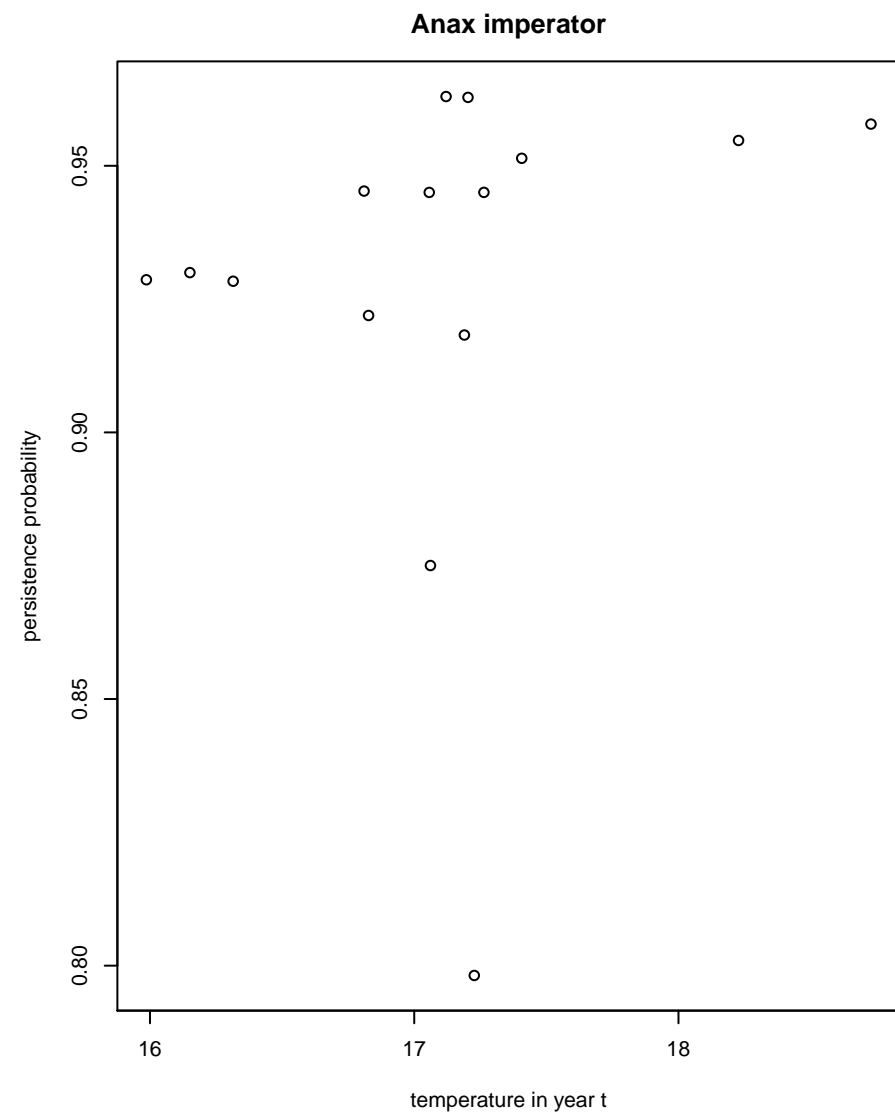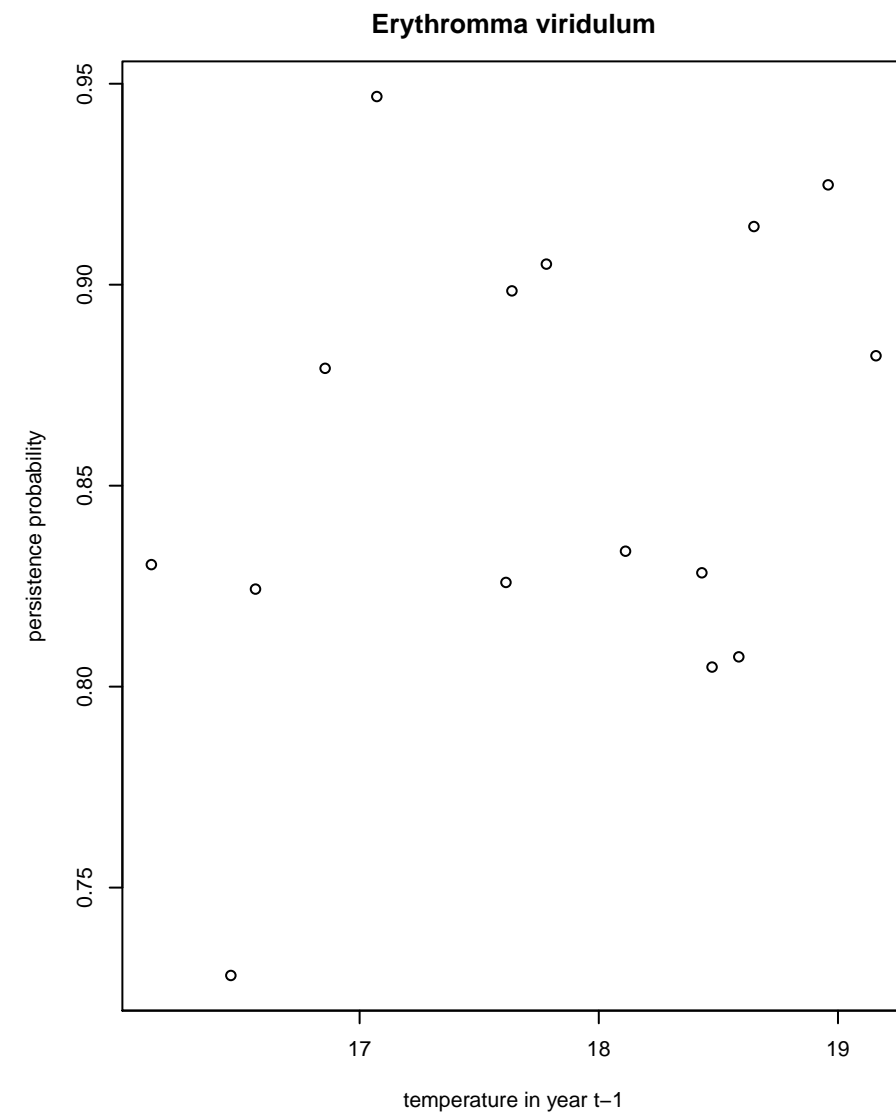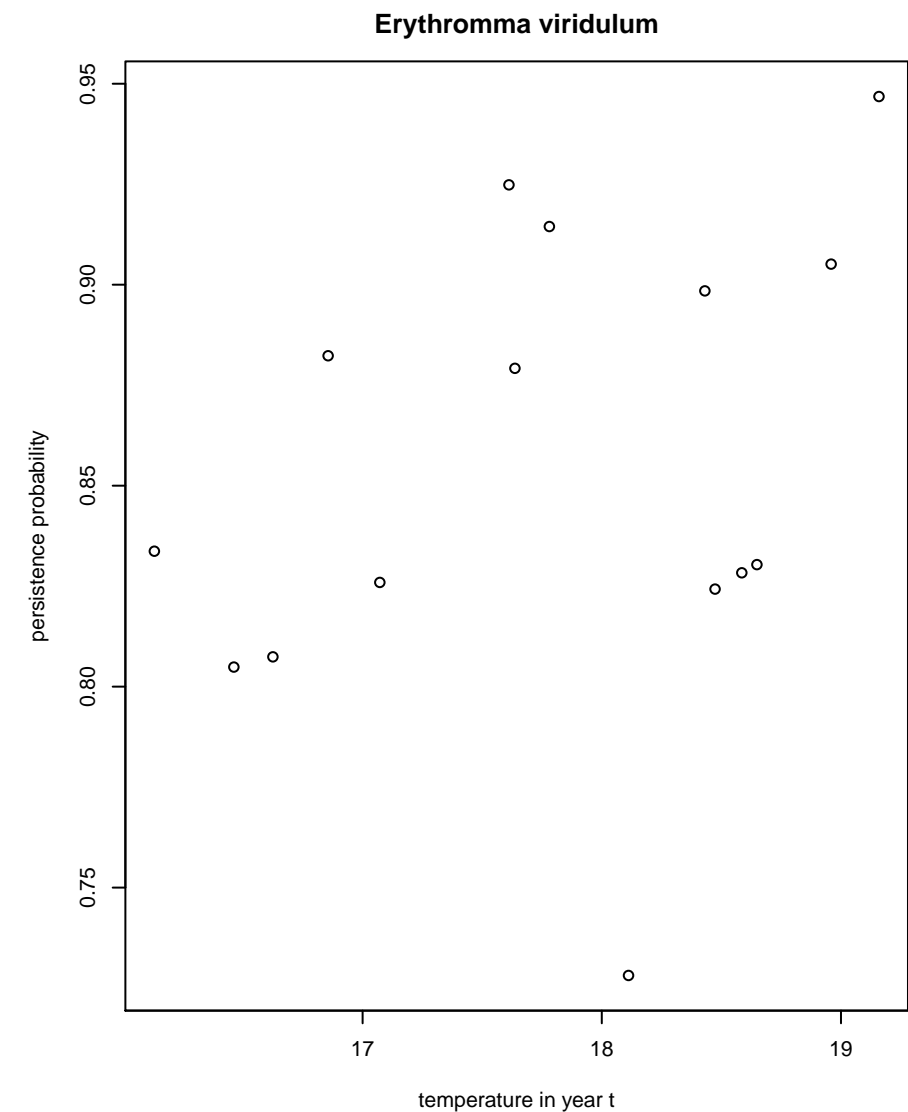

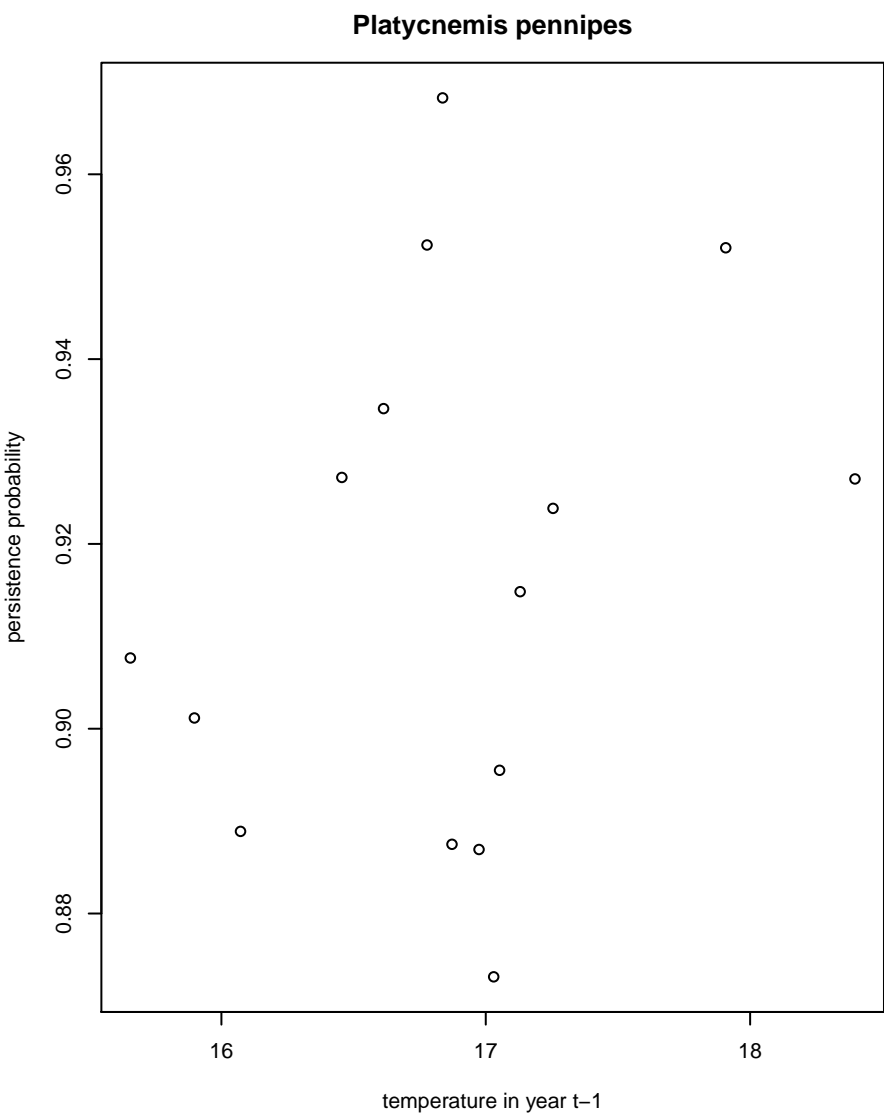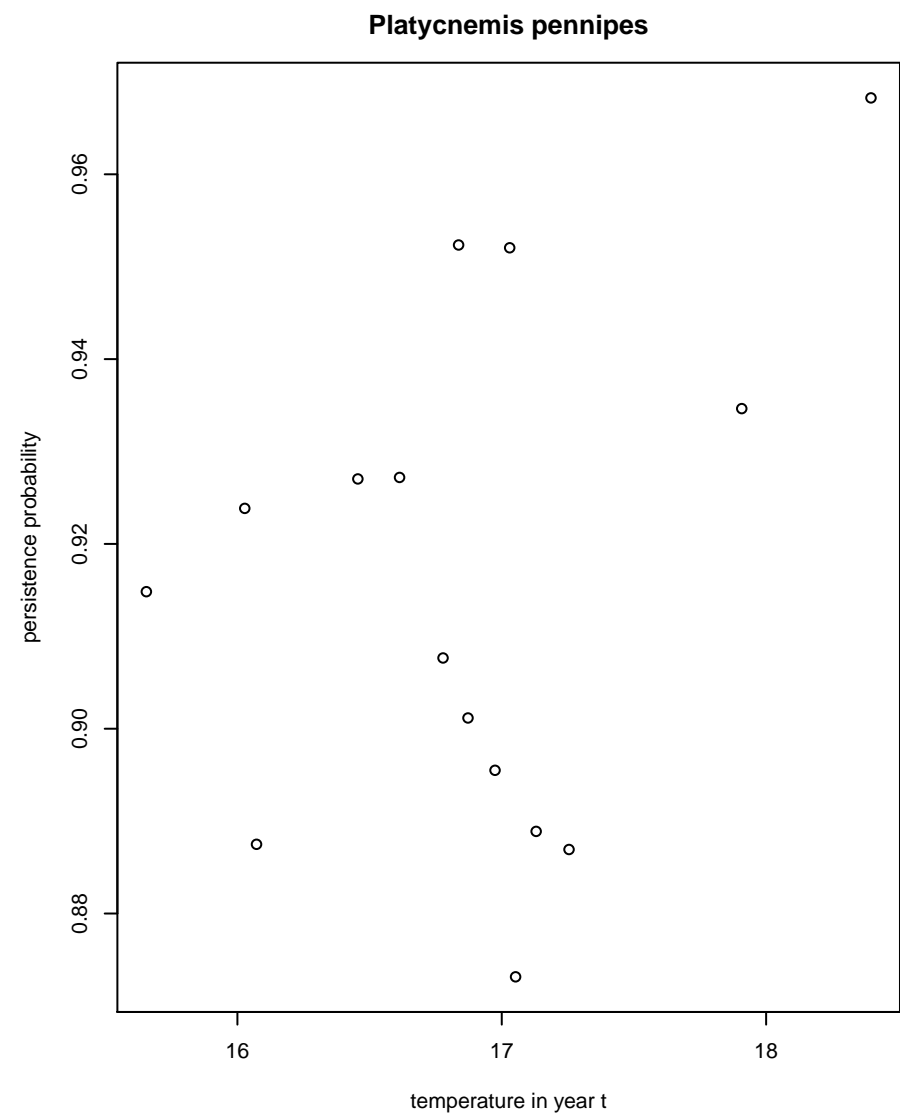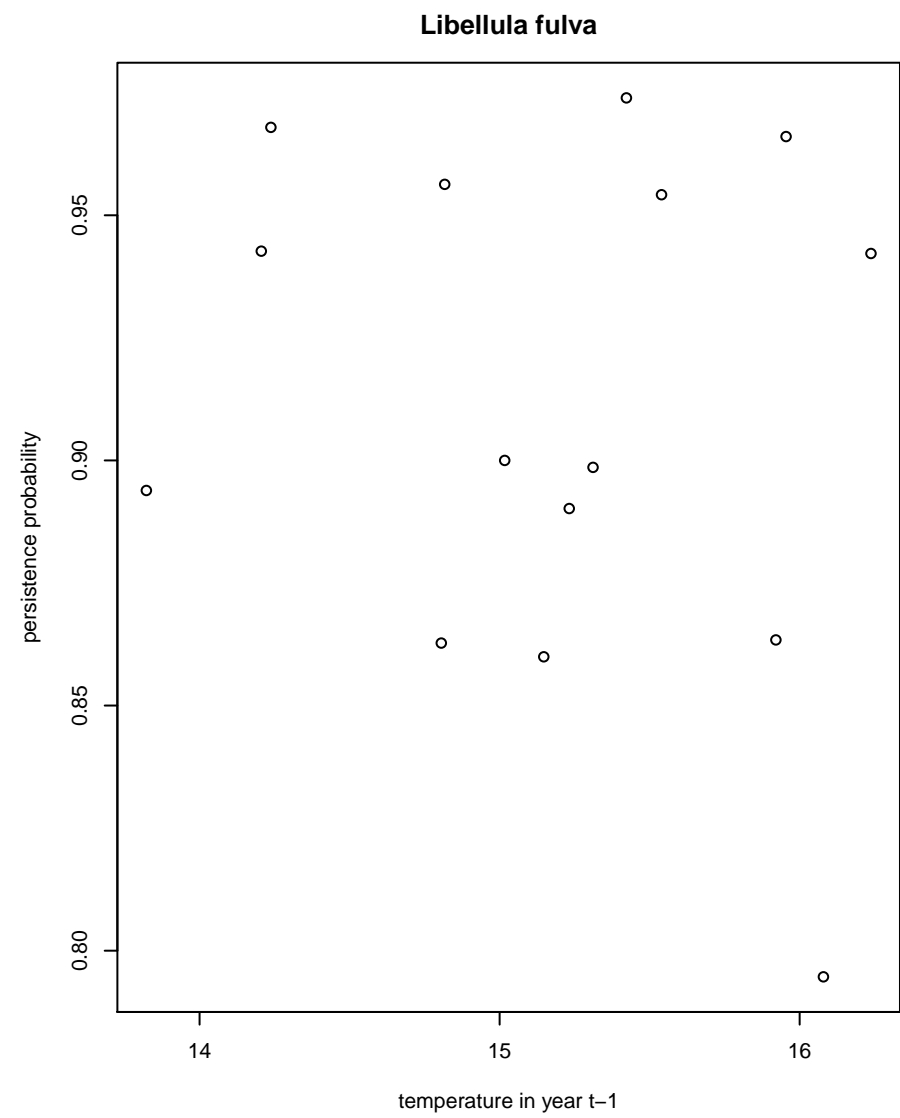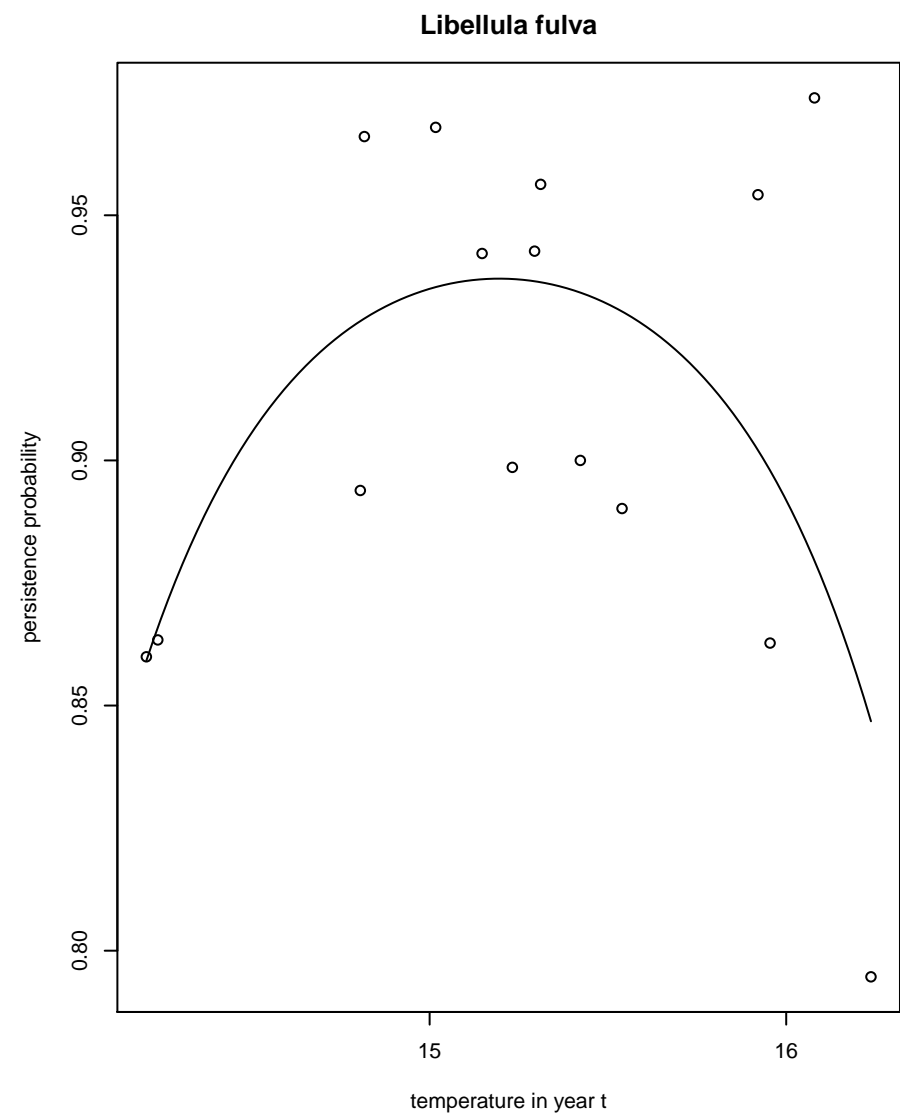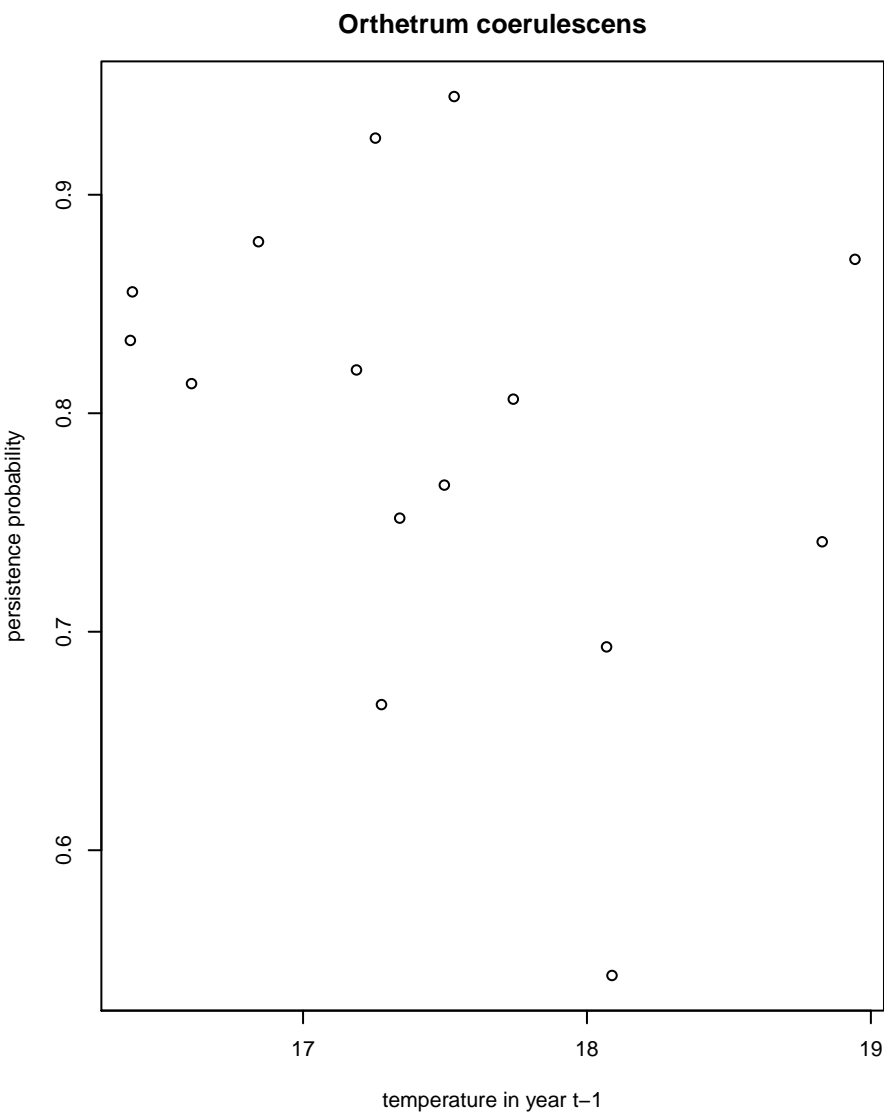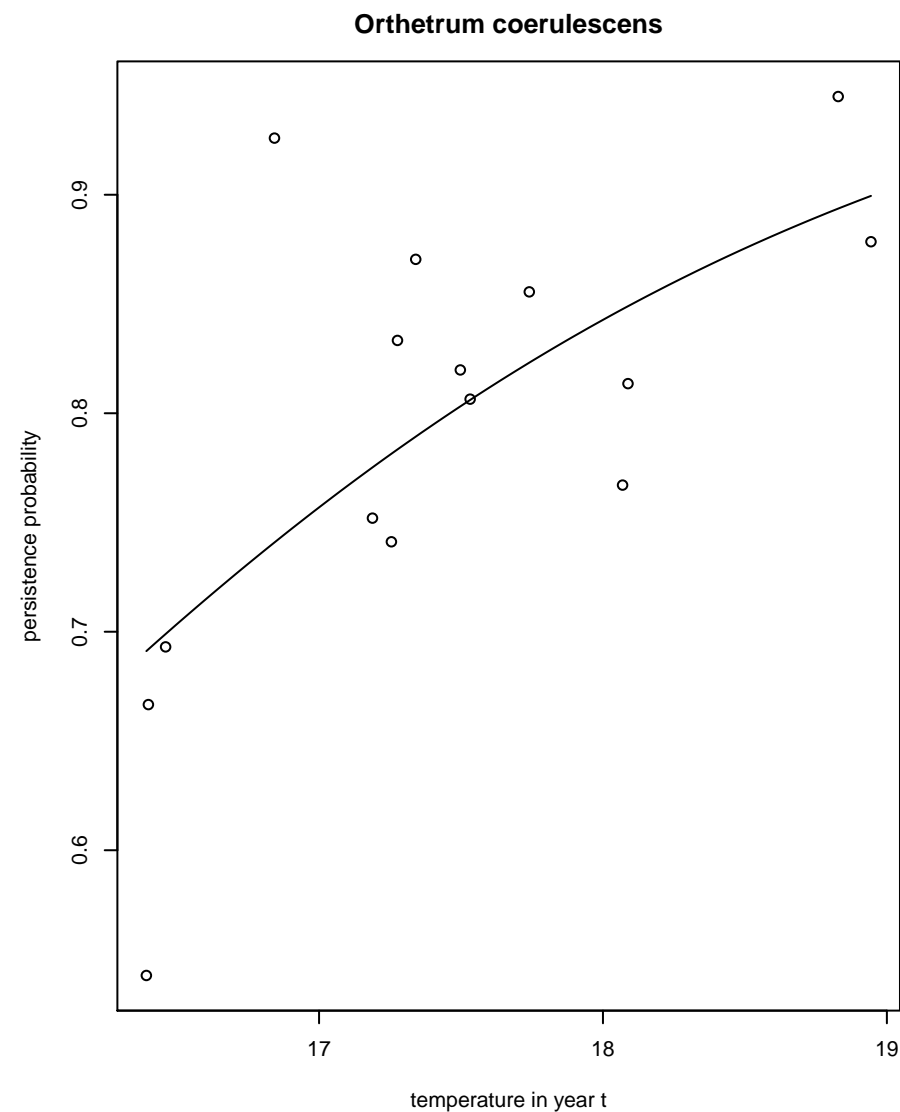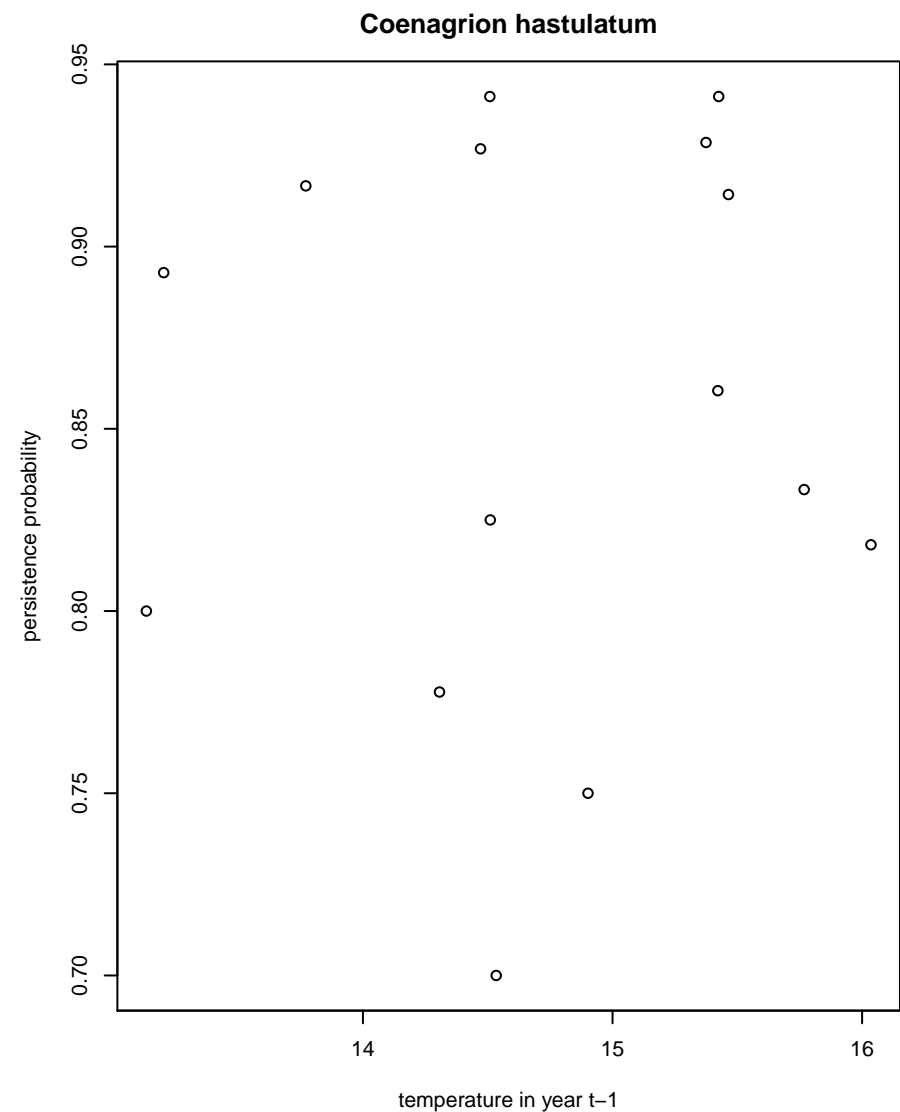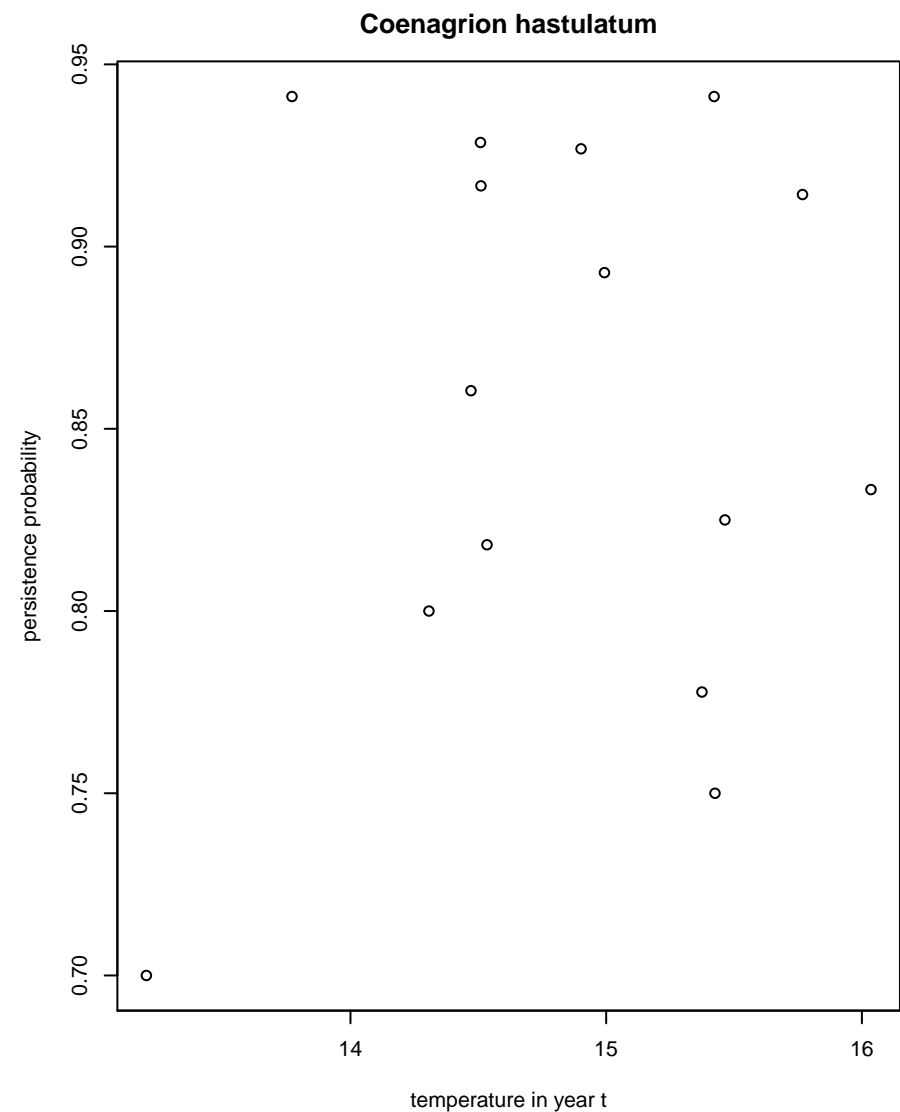

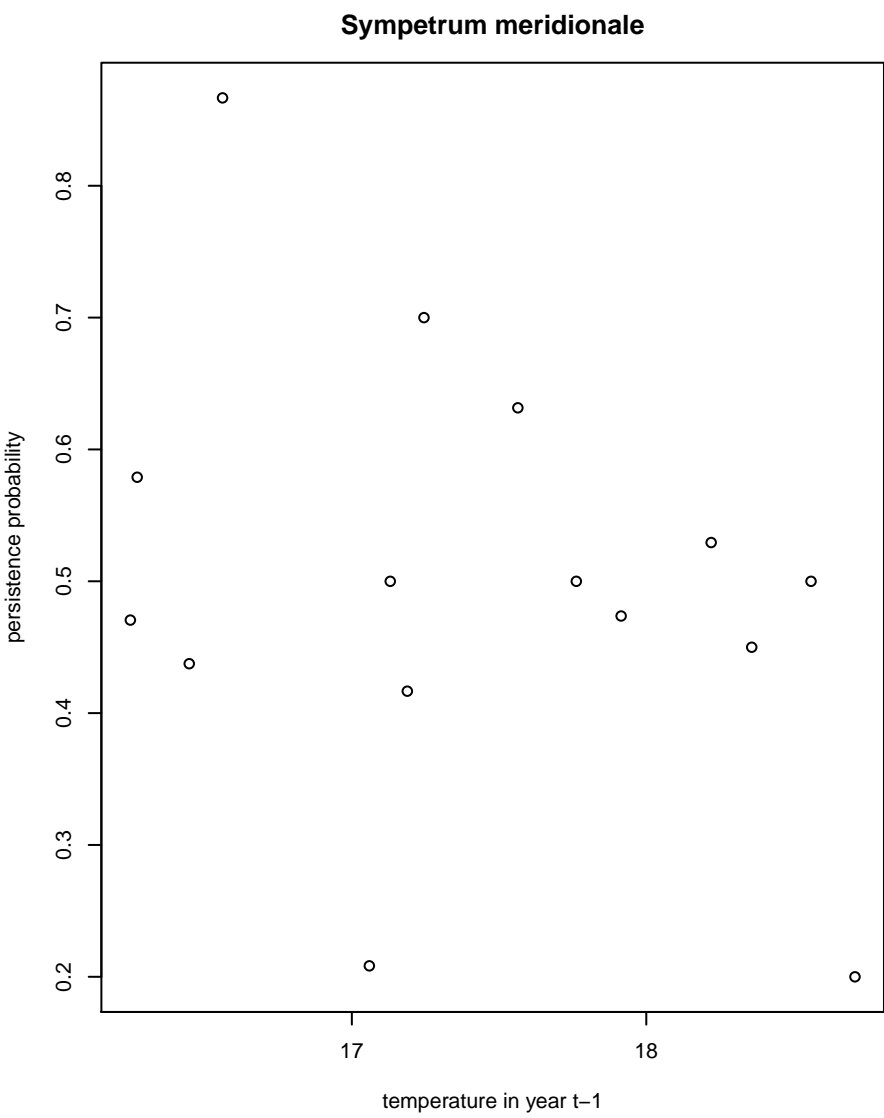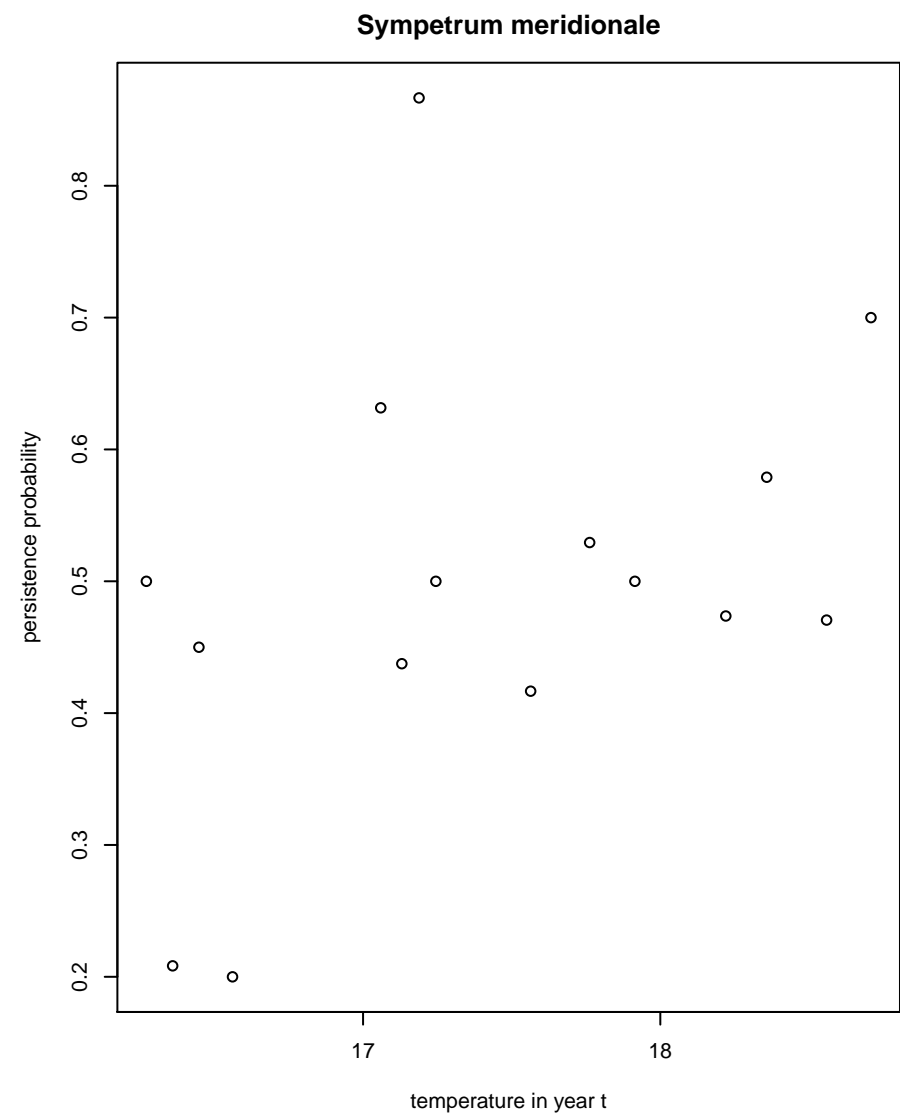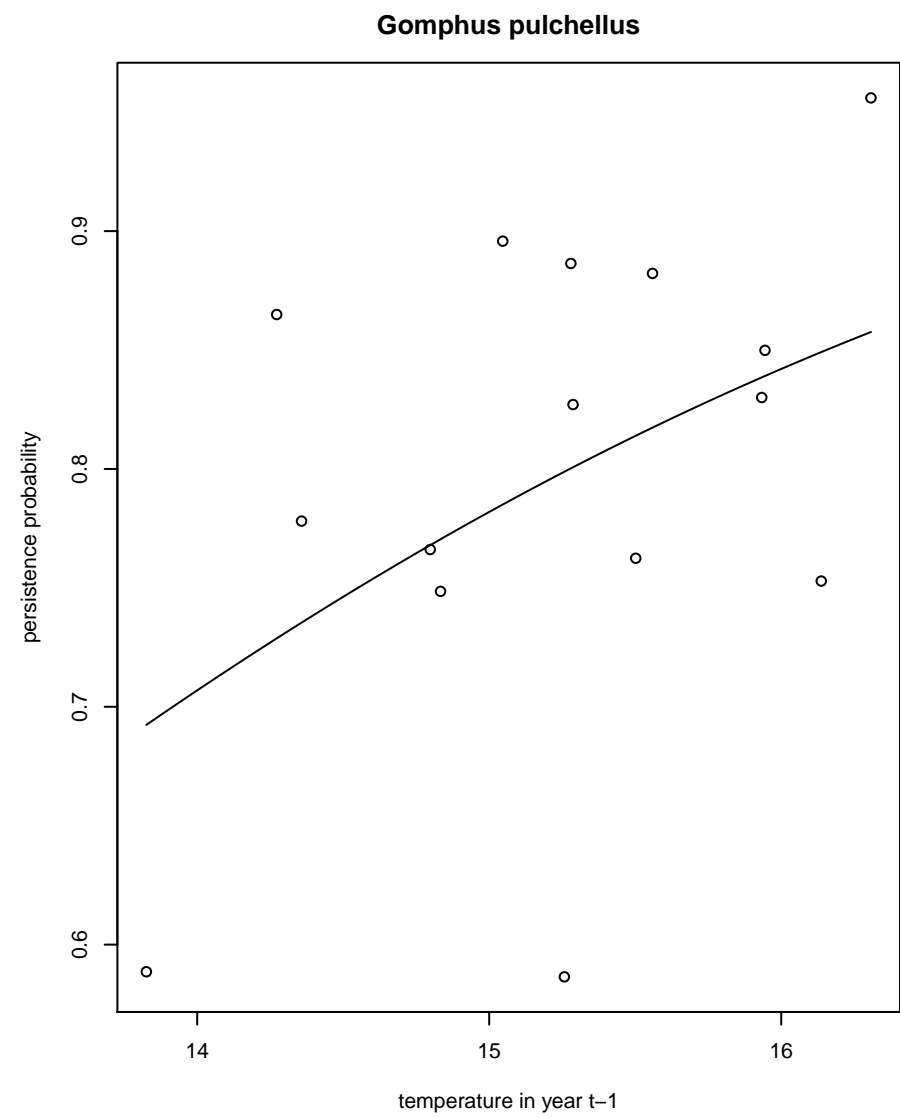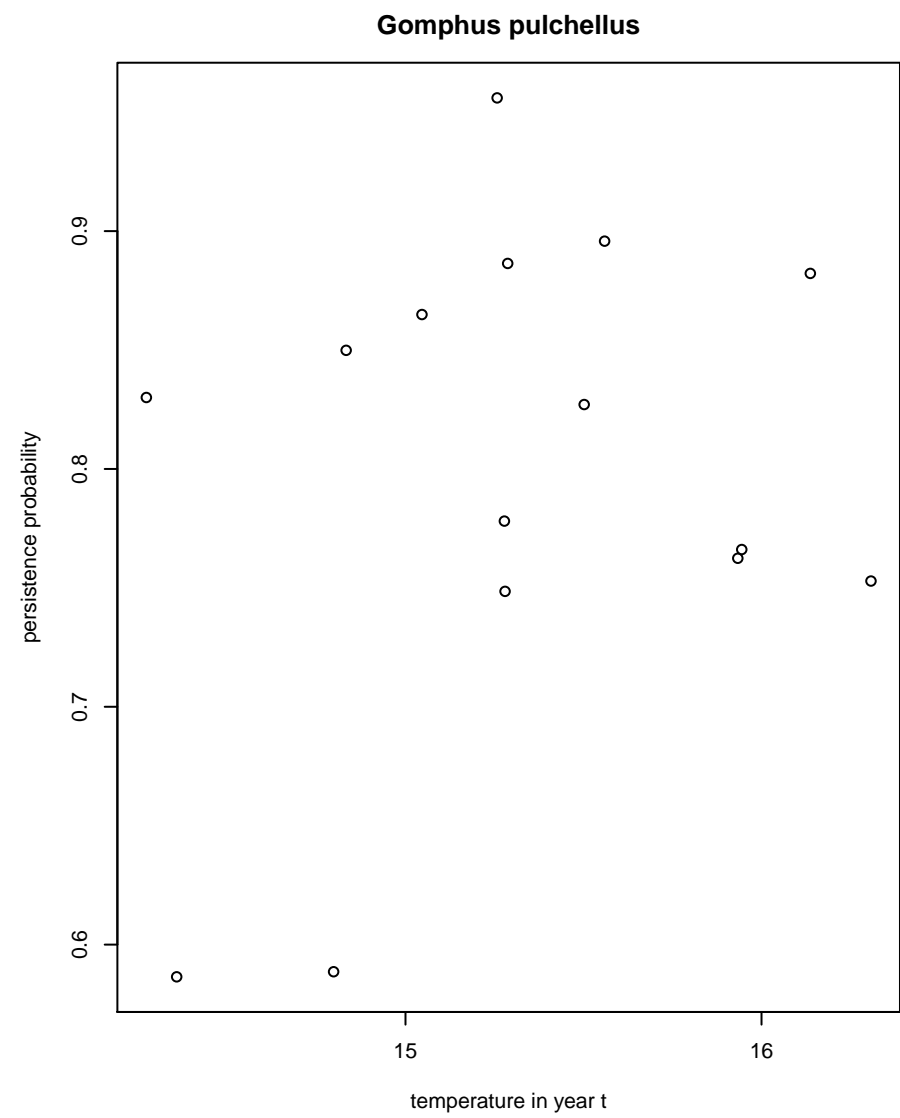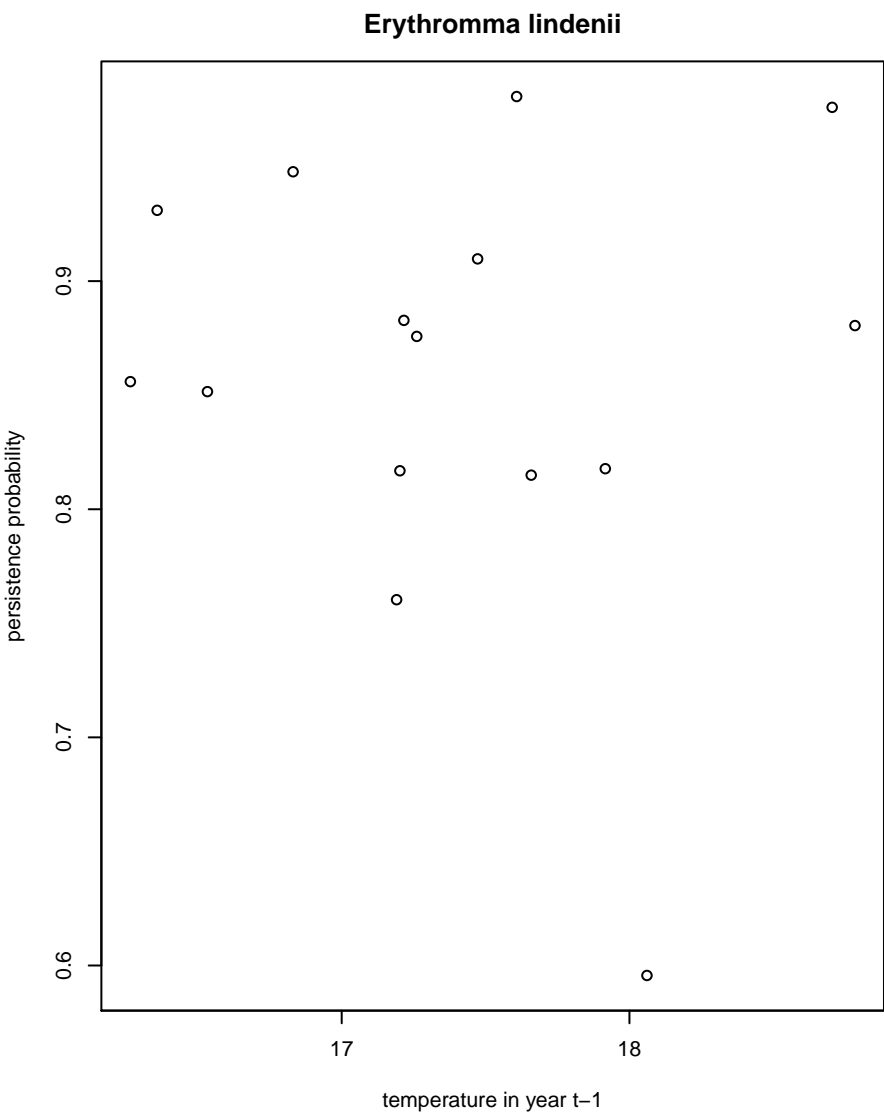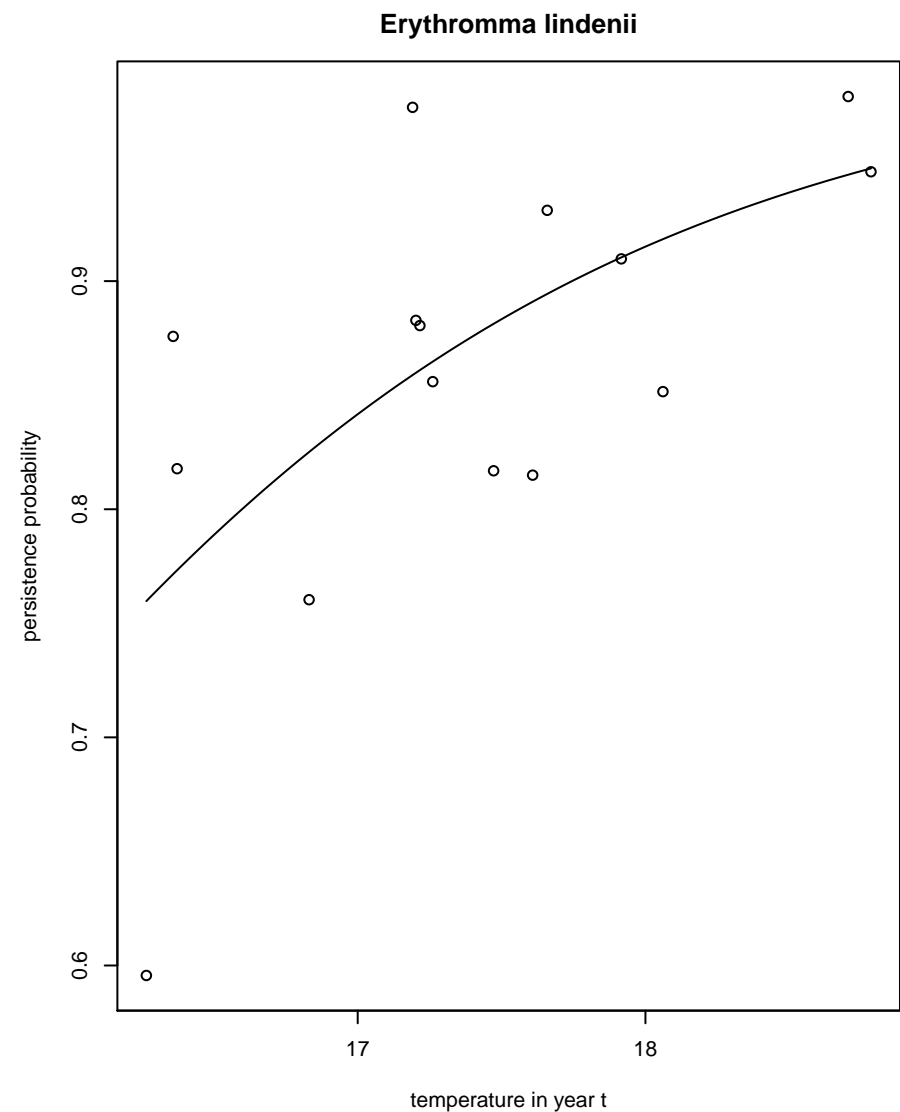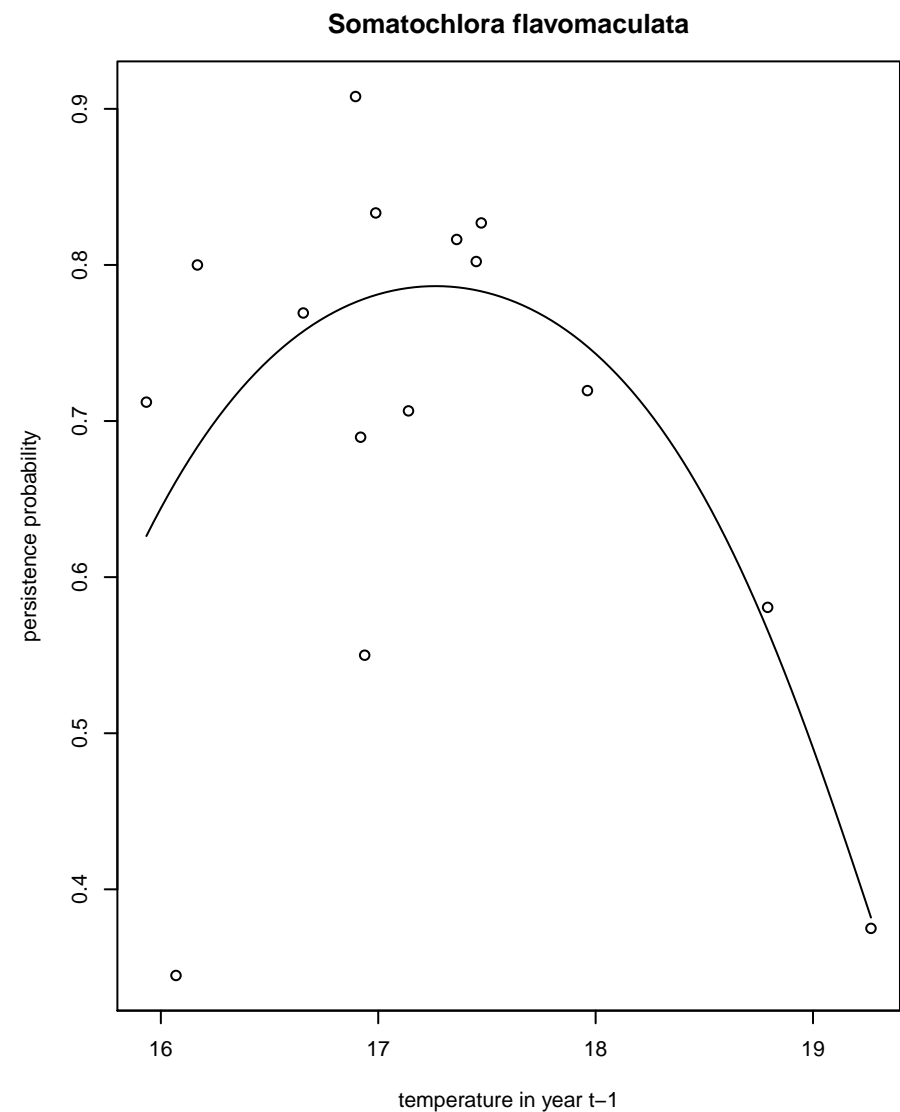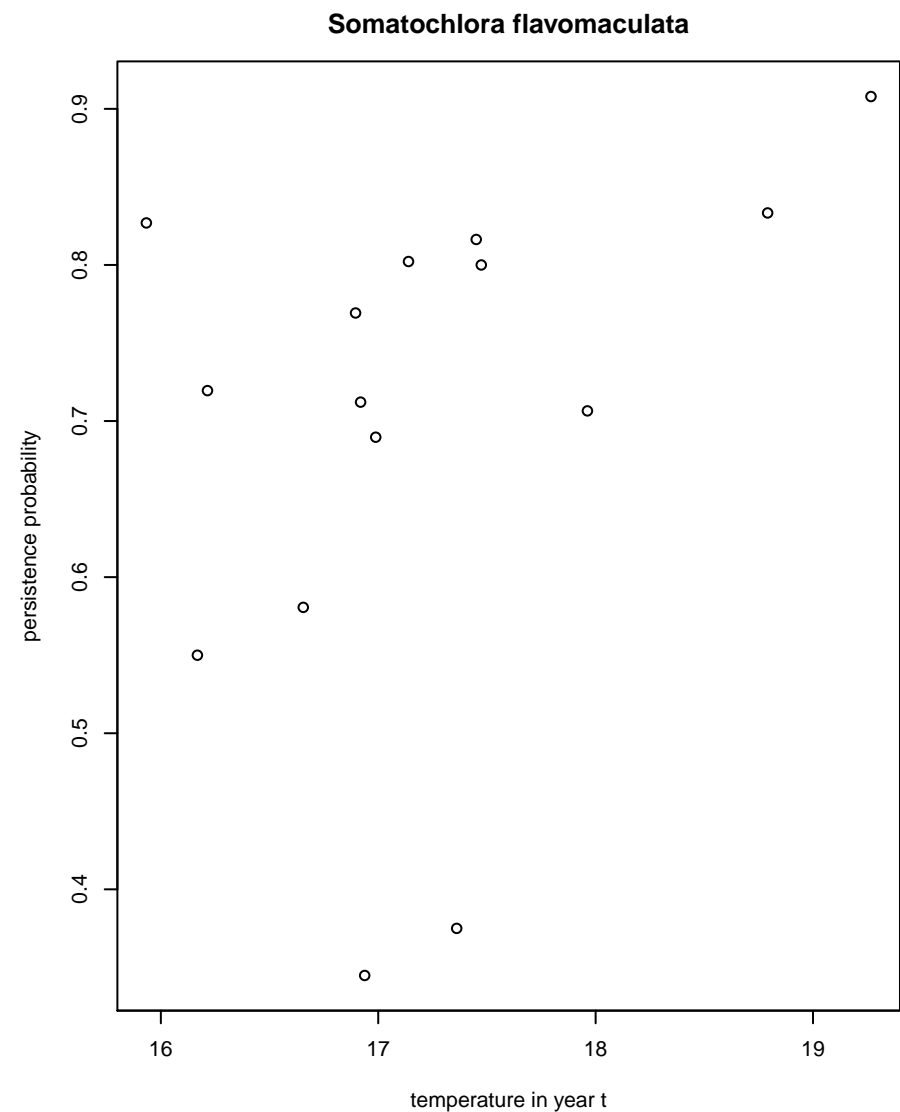

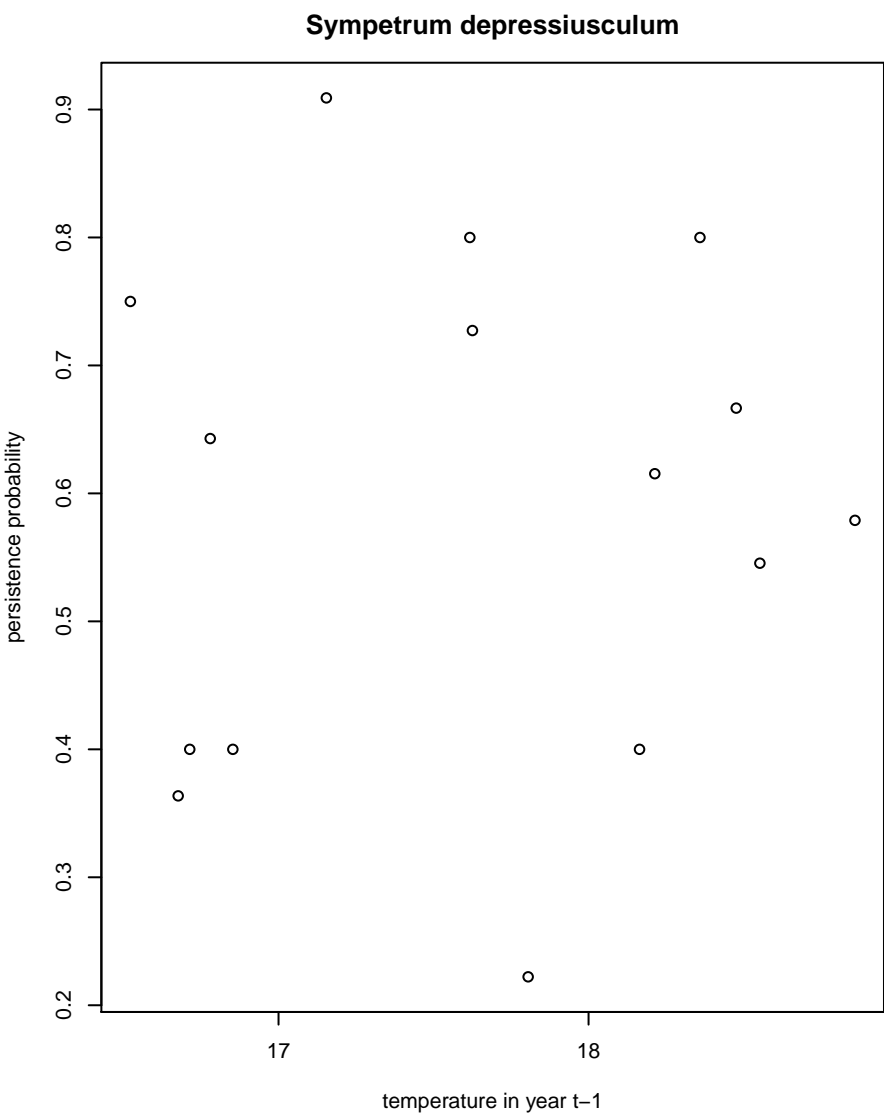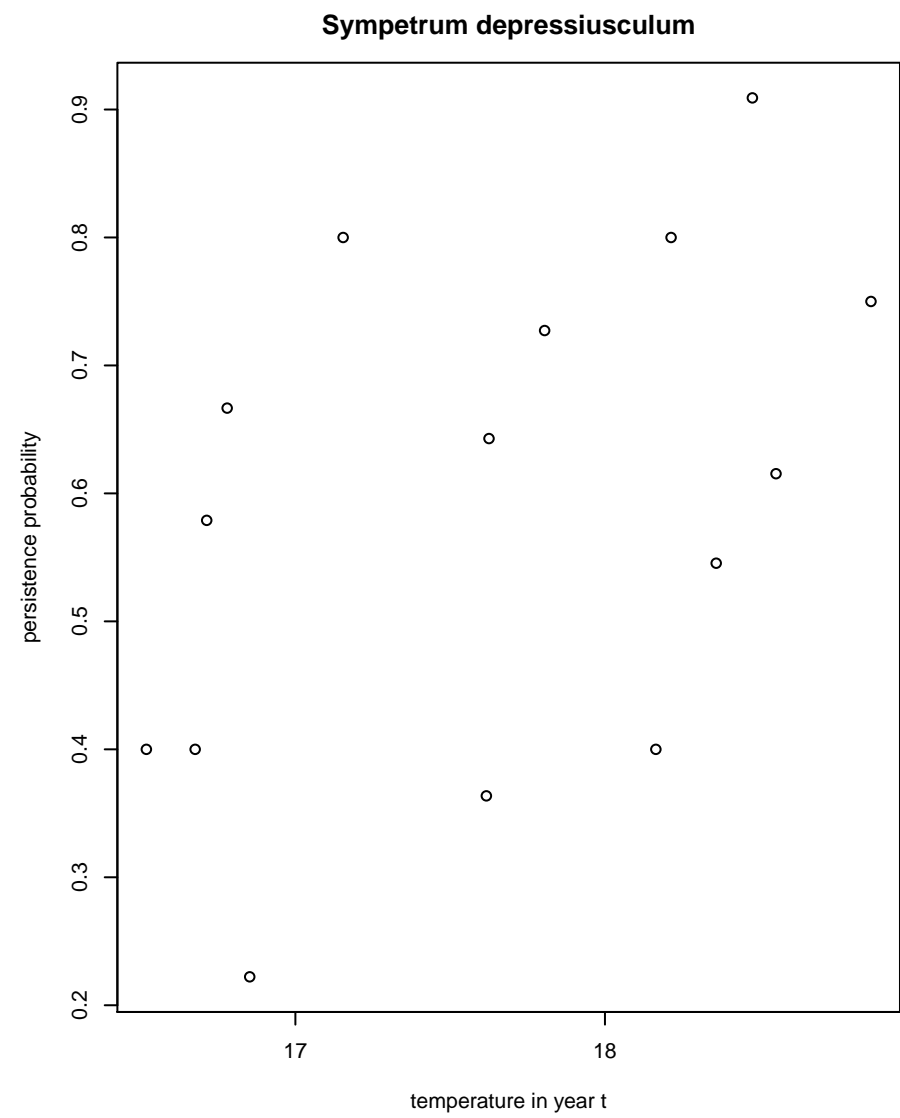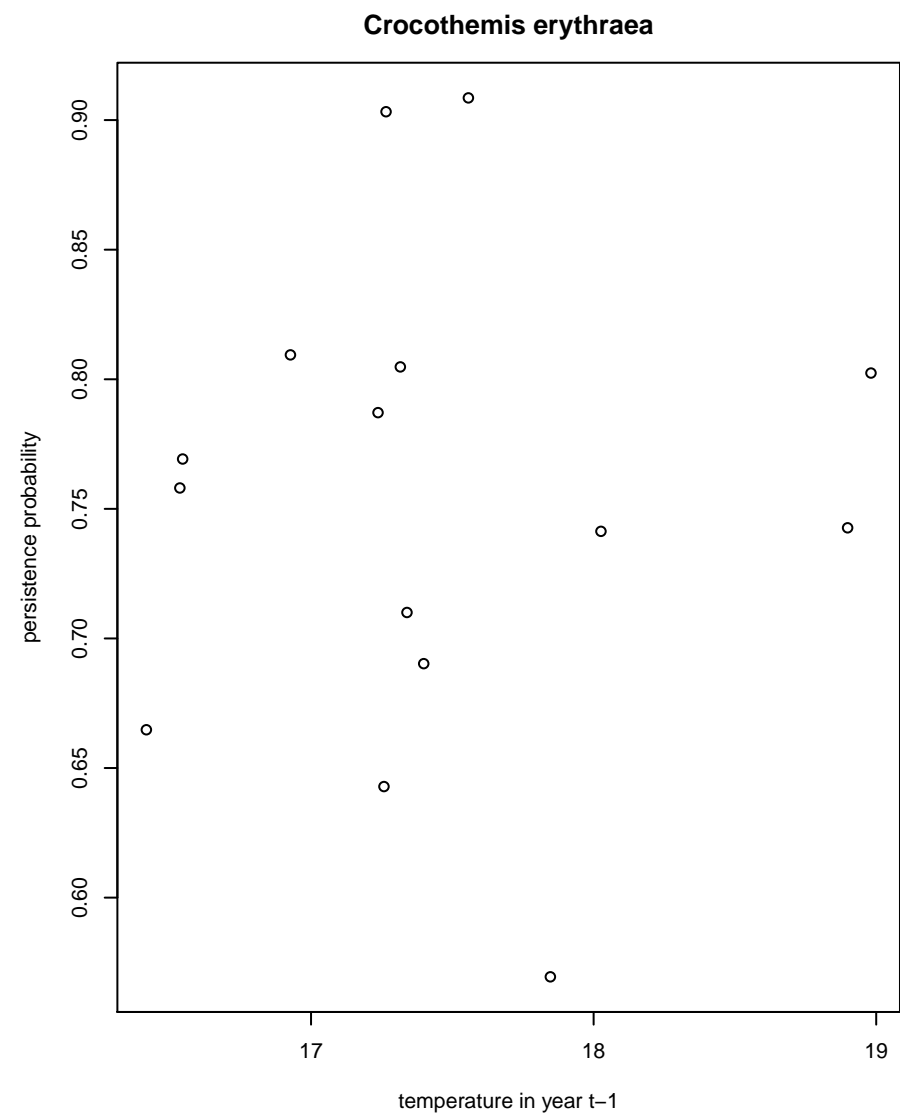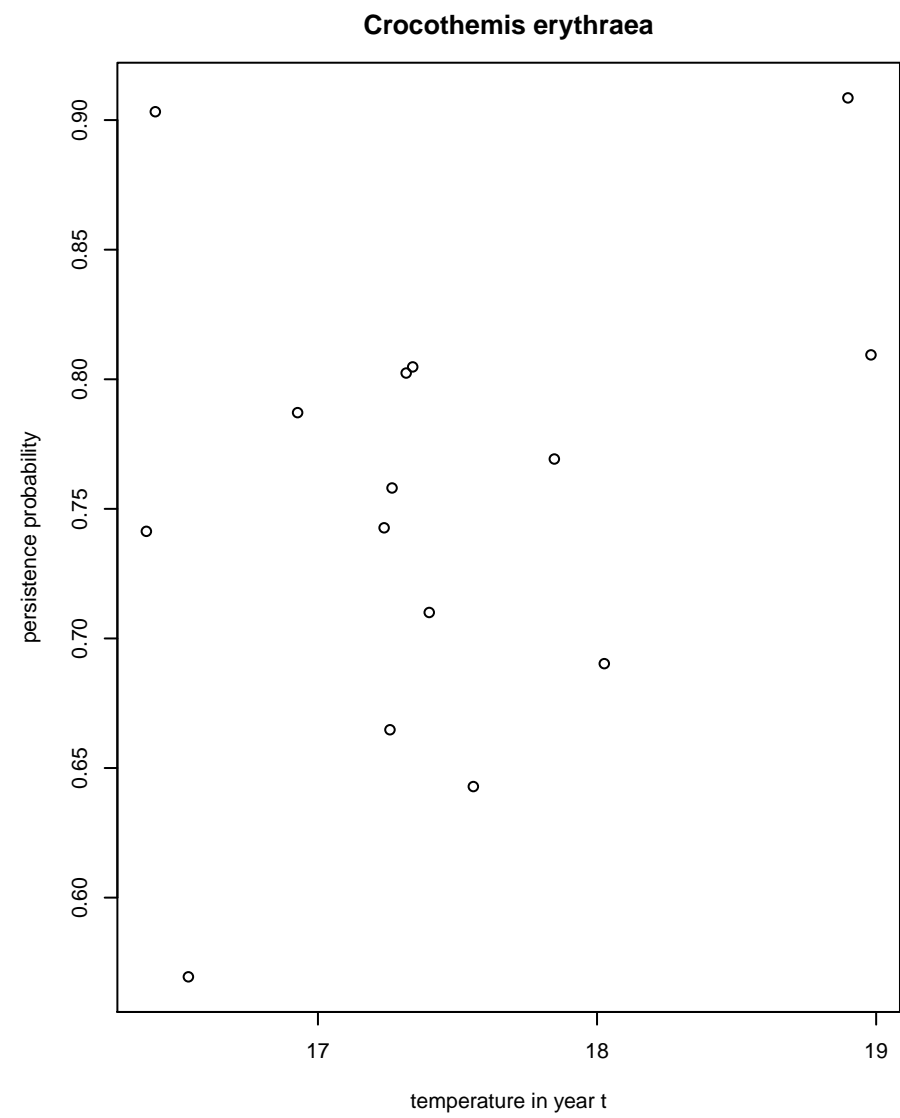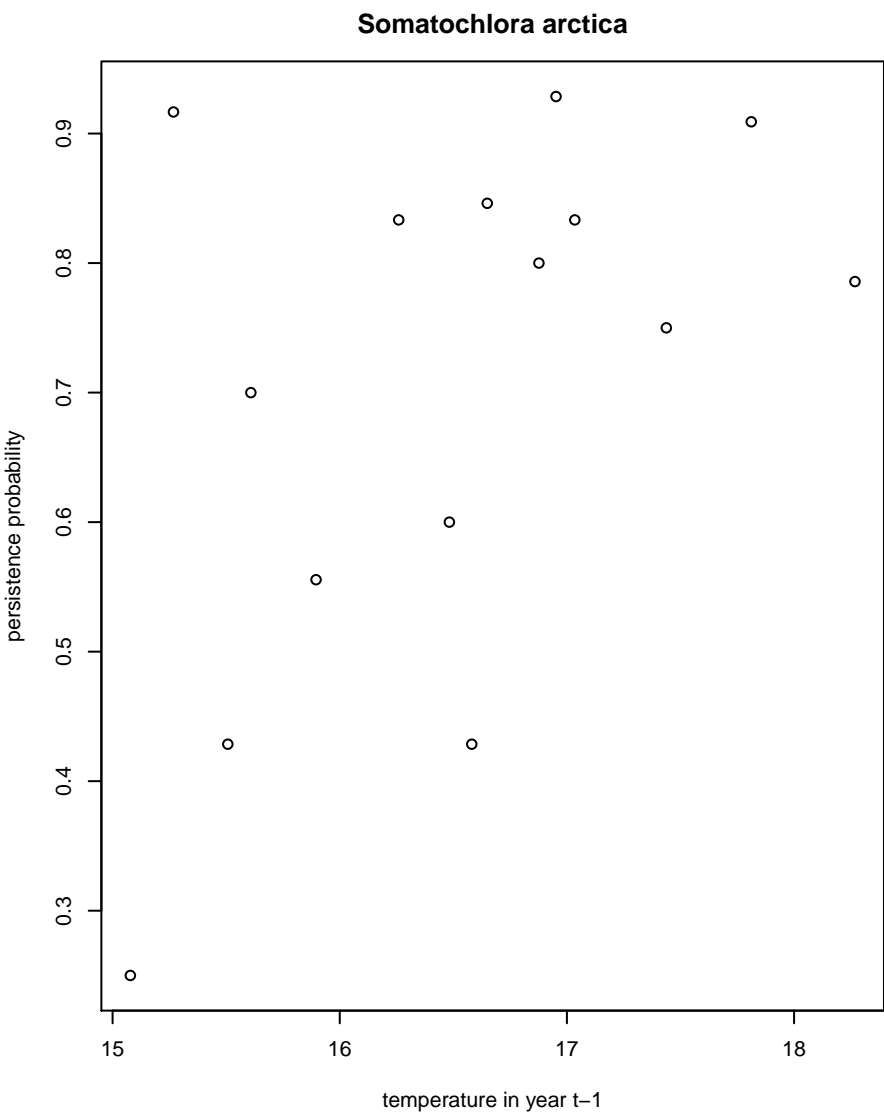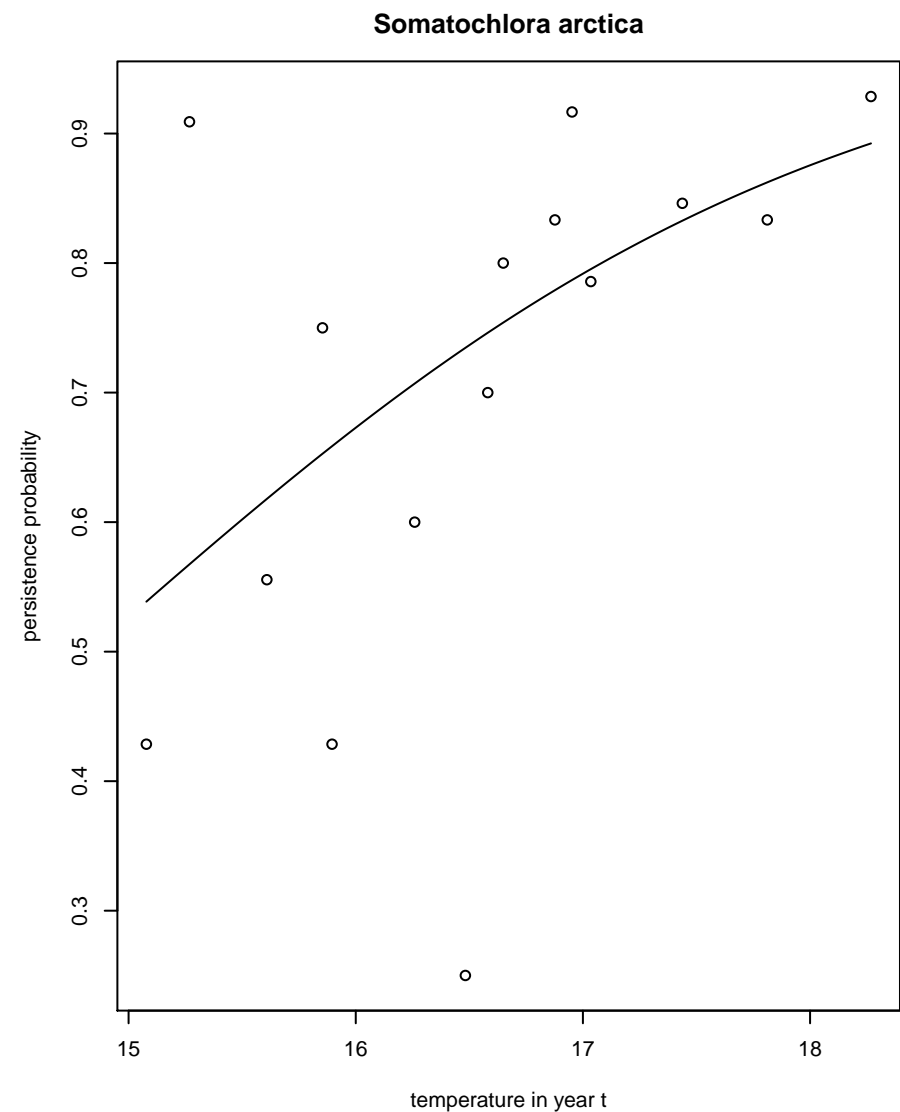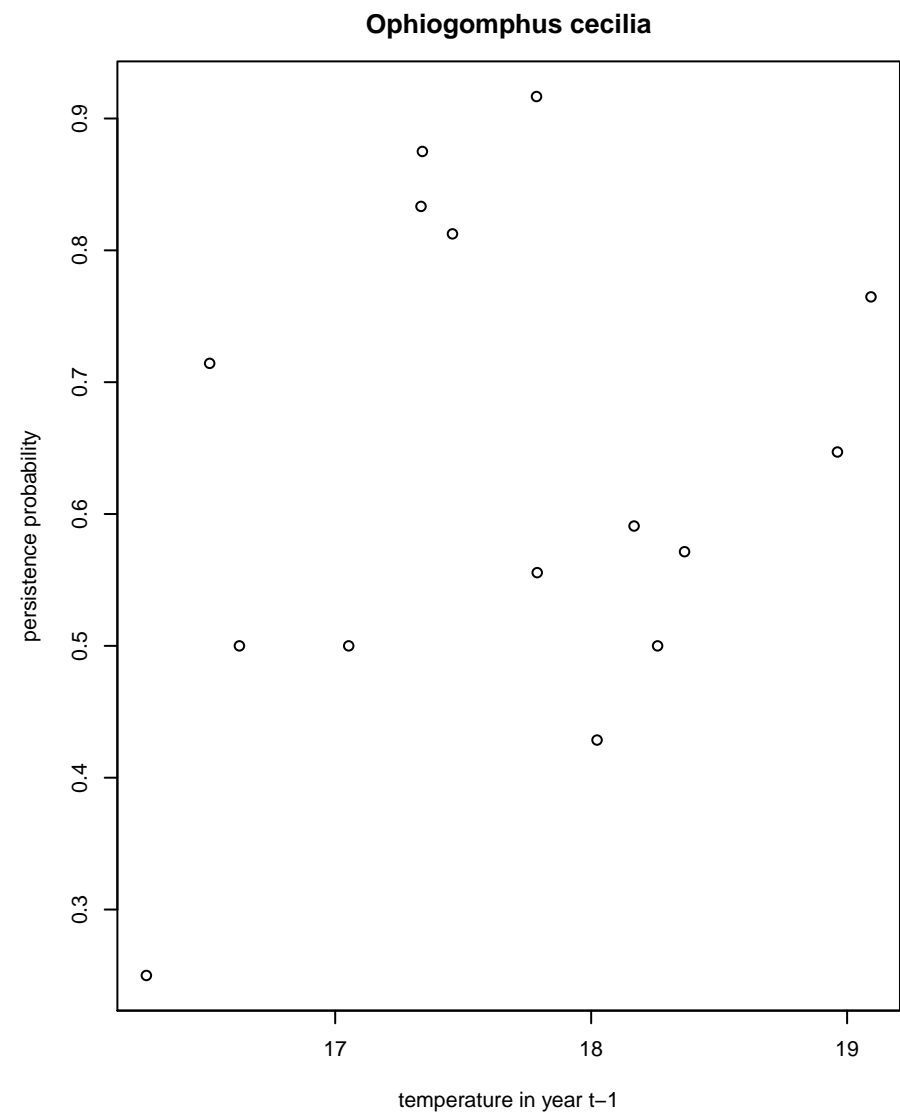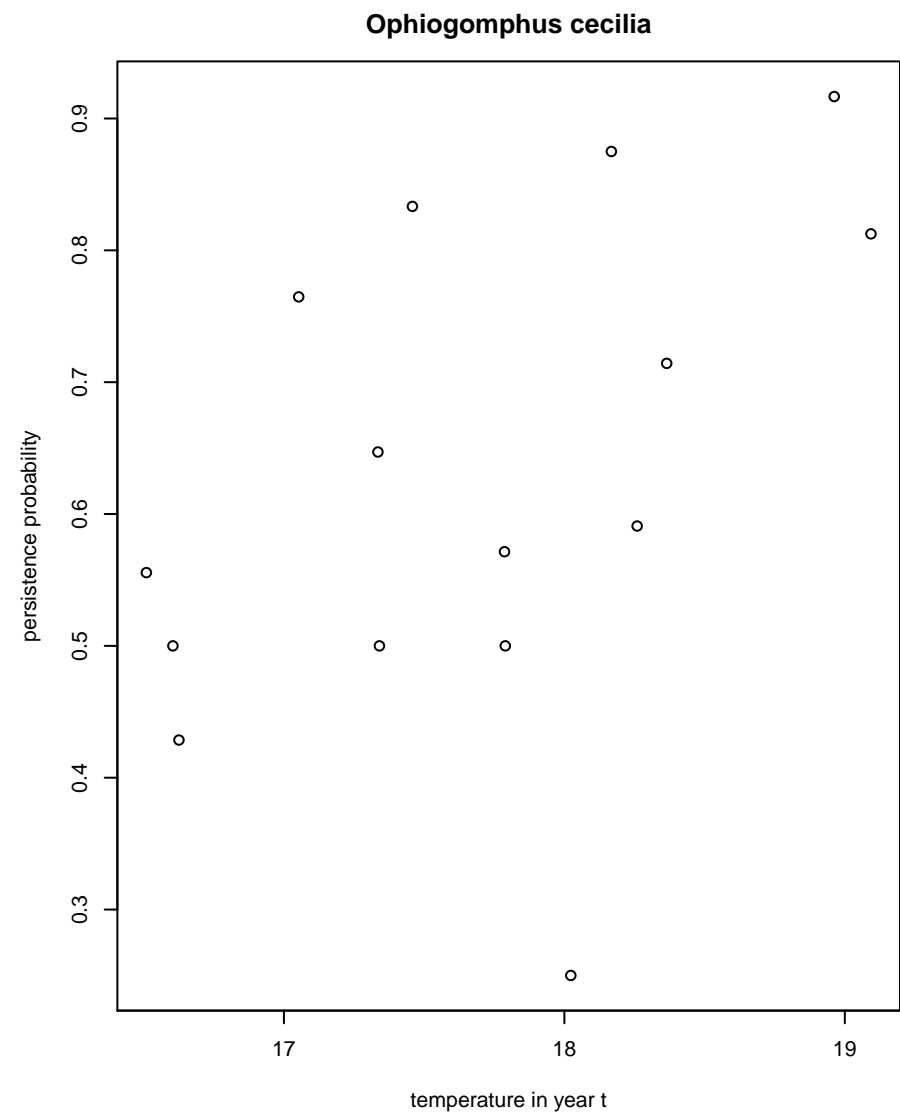

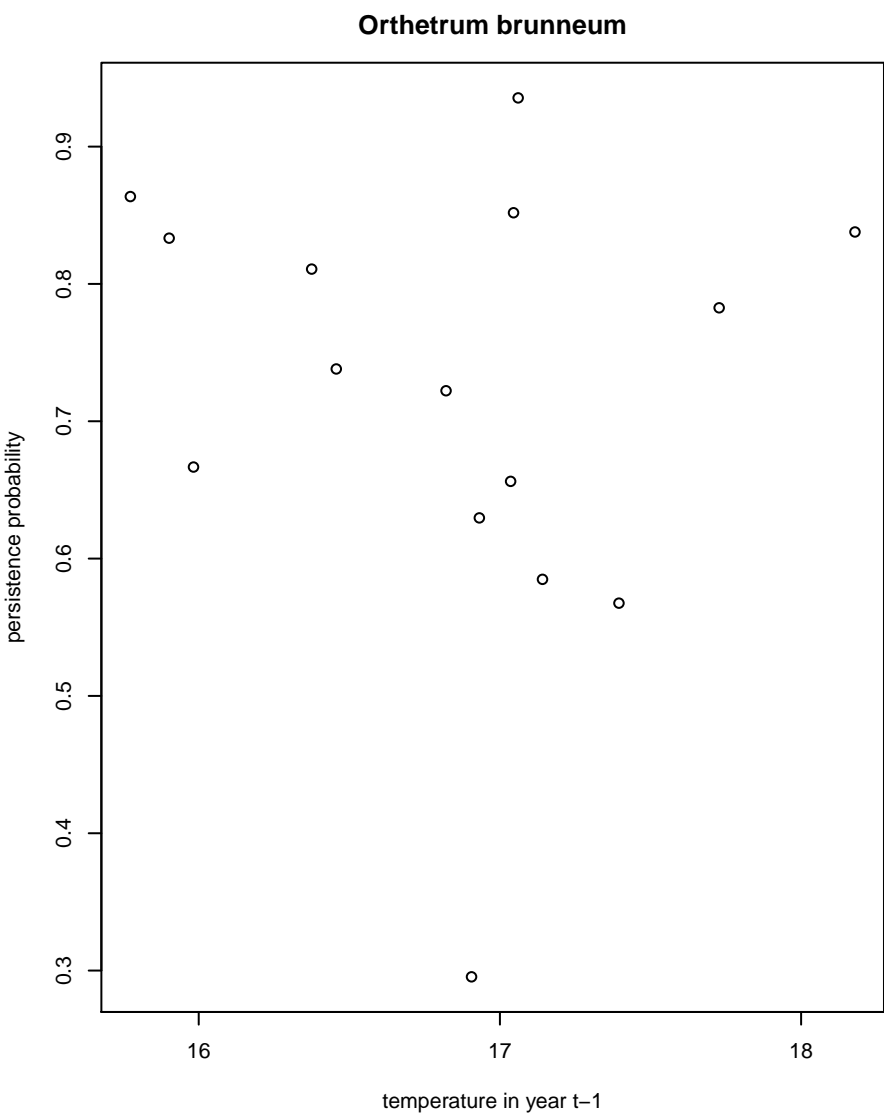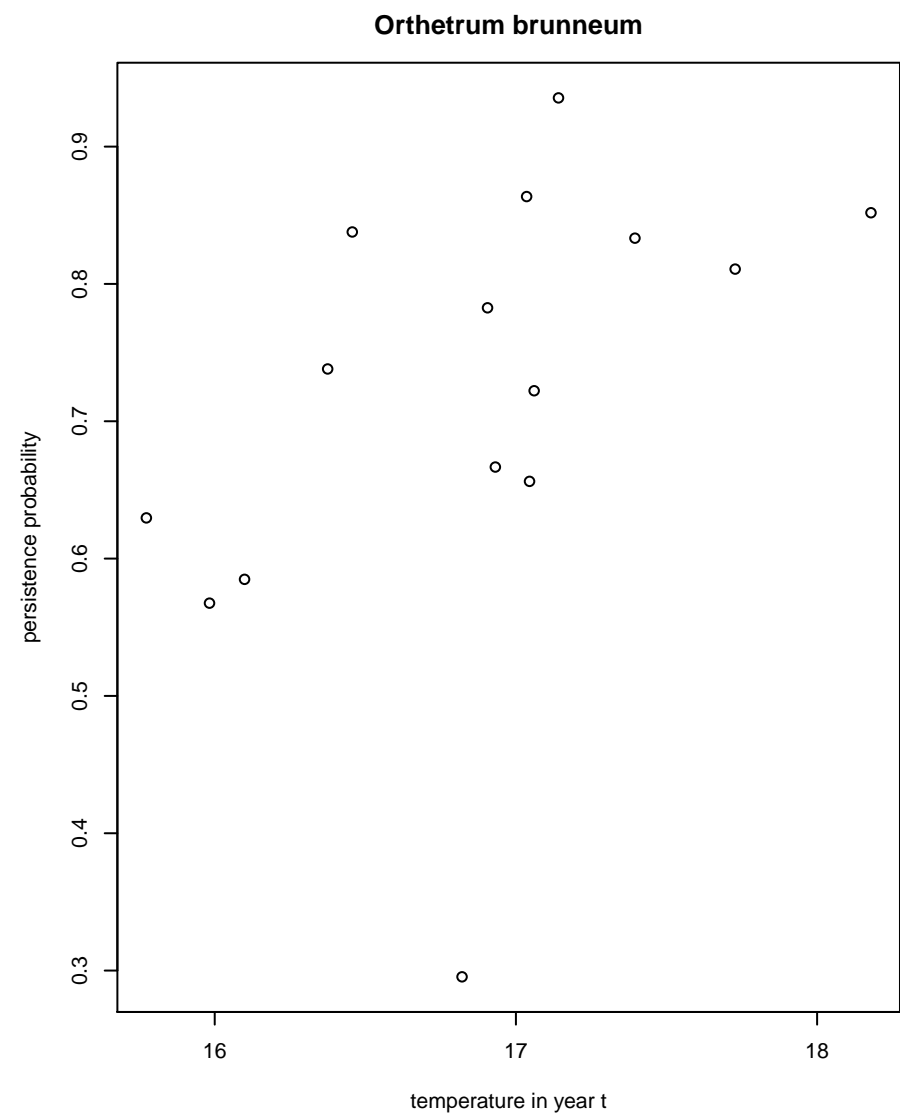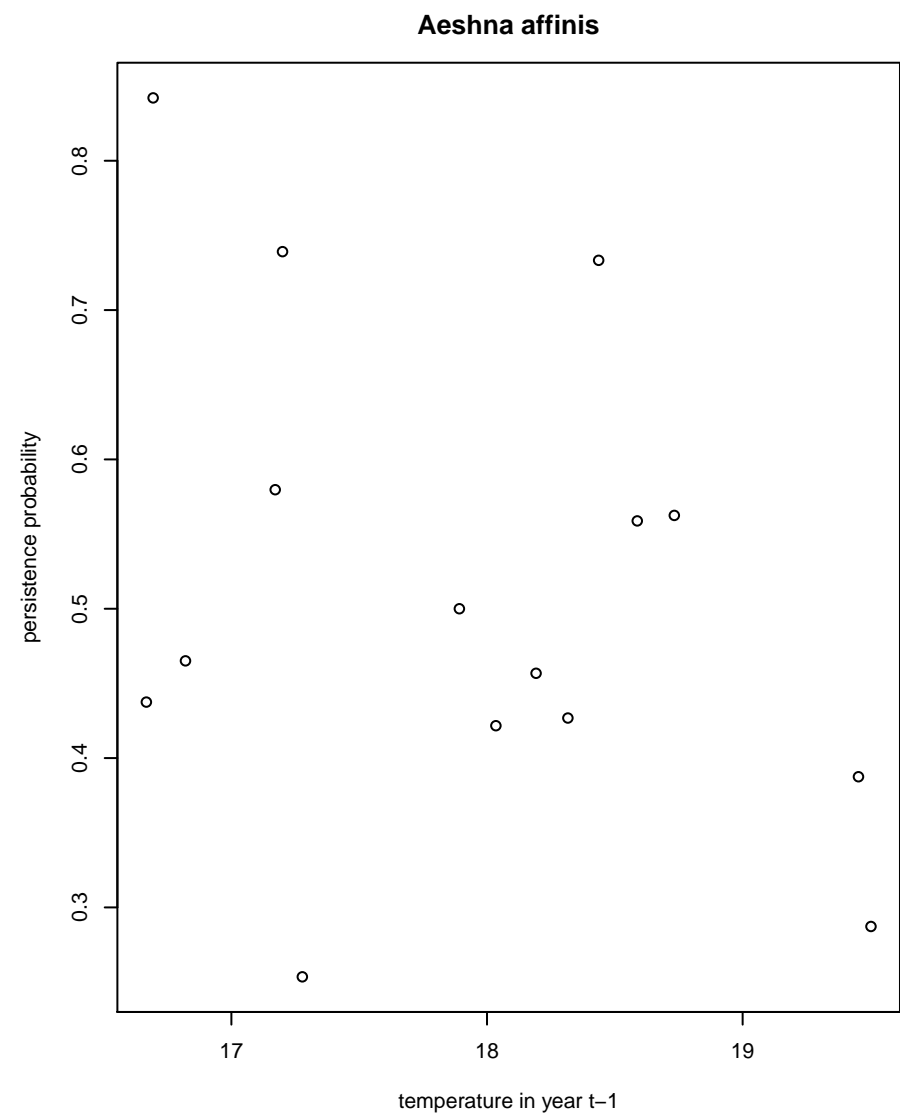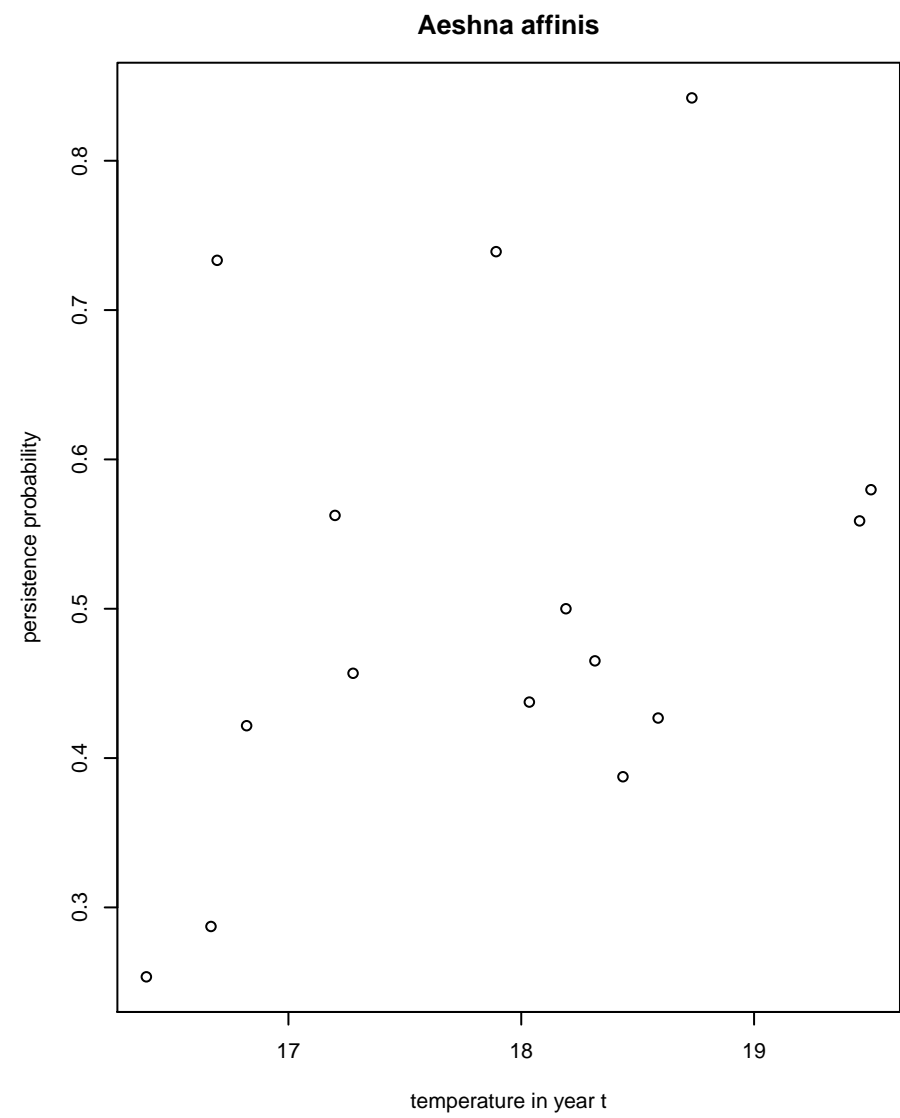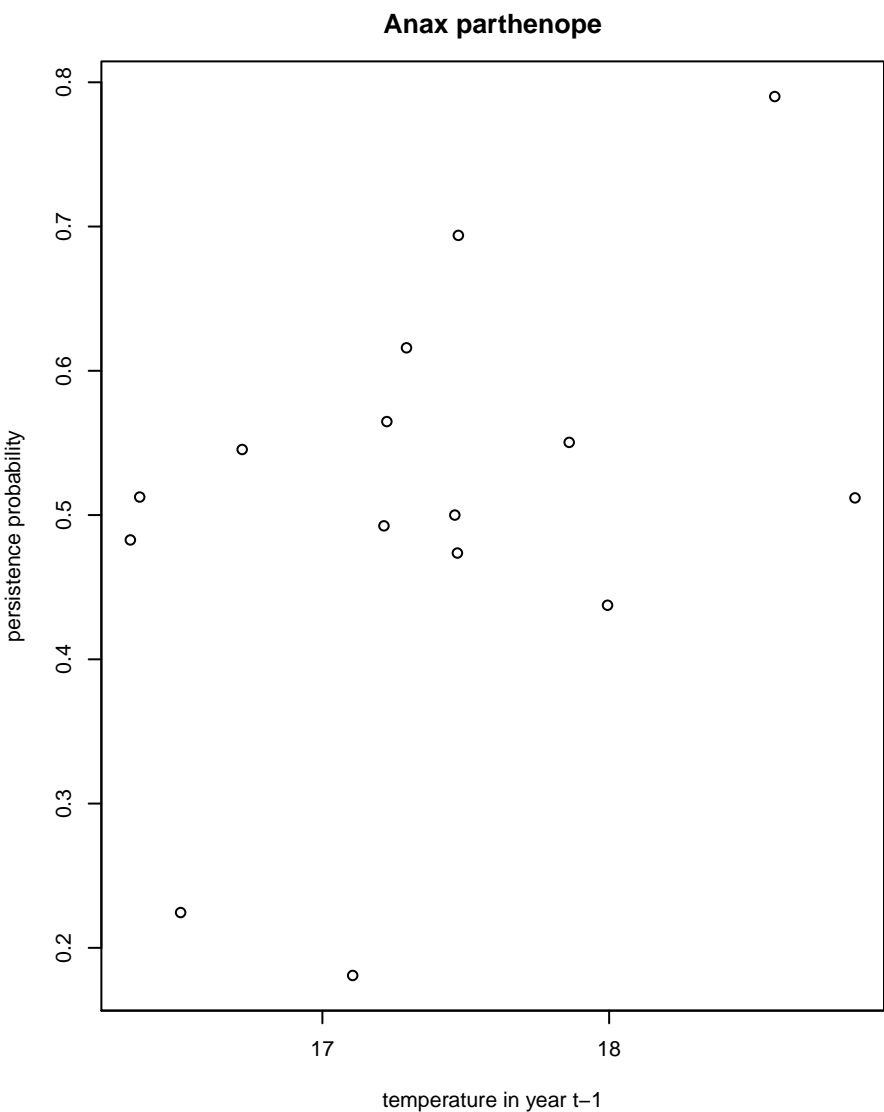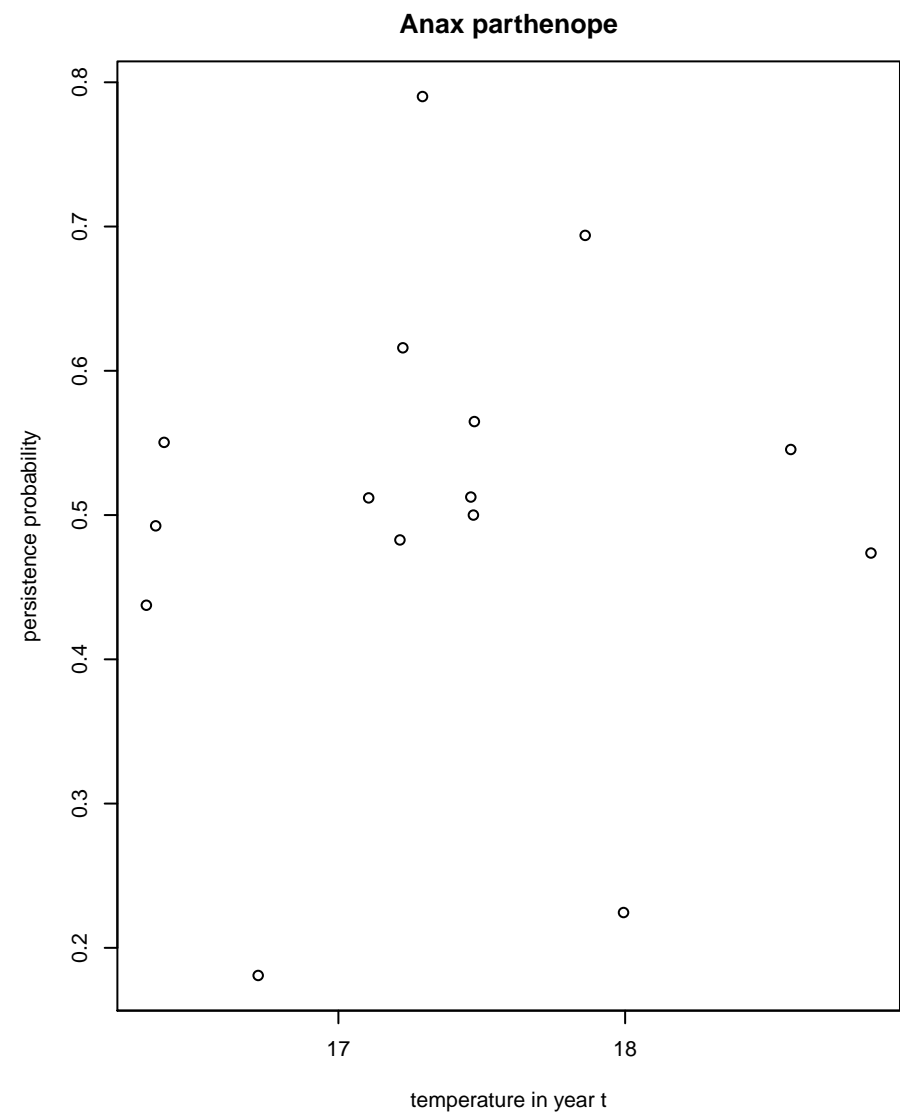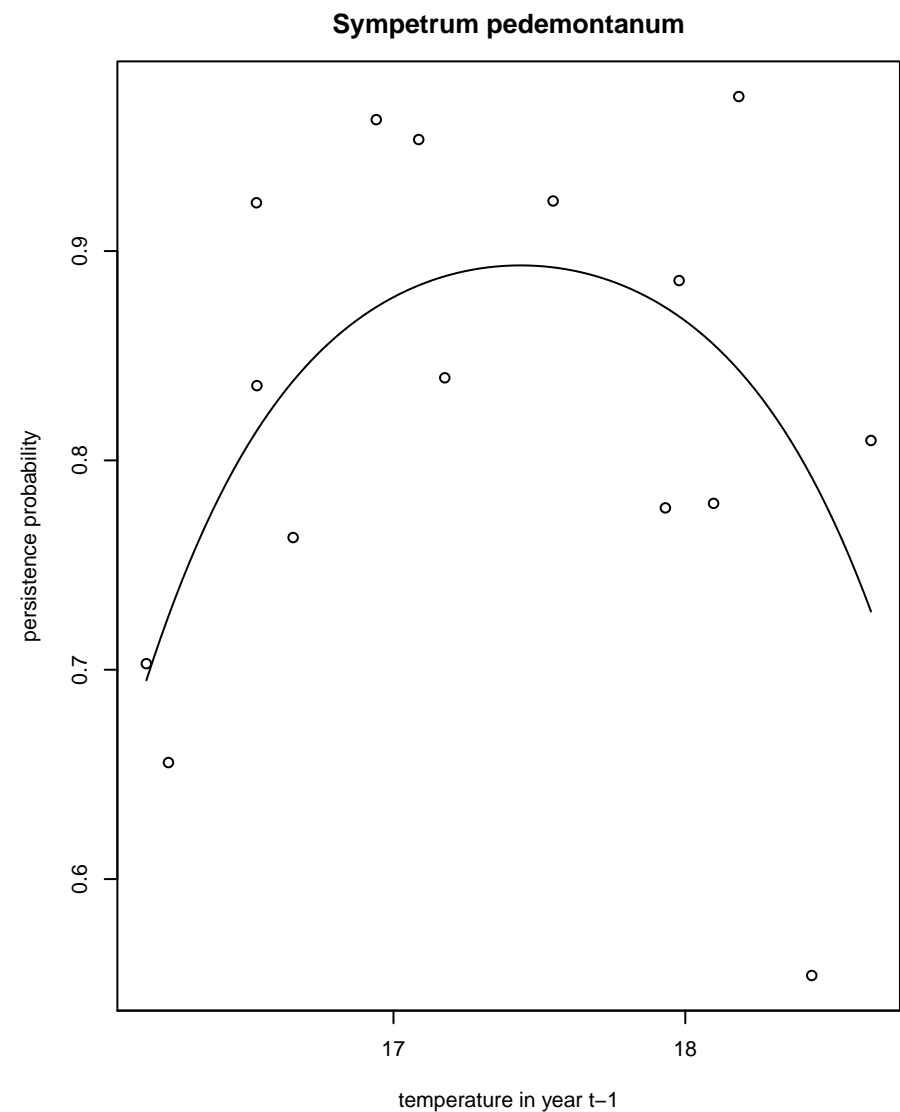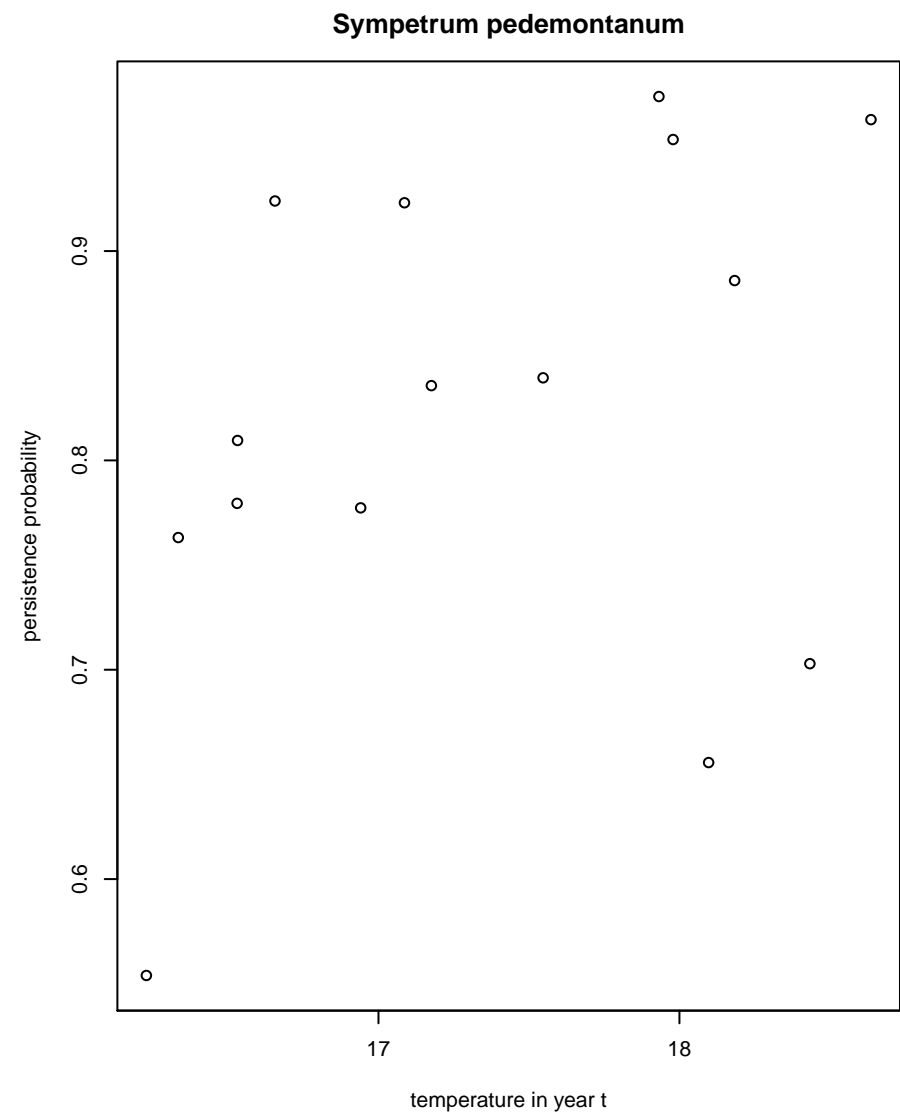

Supplement: Figure S4 — Response curves of colonisation and persistence probability in relation to temperature per species, for years 1997–2011. (PDF) [file pone.0110219.s004.pdf]
