# Supplementary material for: No Evidence of the Effect of Extreme Weather Events on Annual Occurrence of Four Groups of Ectothermic Species
Source: PLoS One. 2014 Oct 17;9(10):e110219. doi: 10.1371/journal.pone.0110219 (PMC4201516; doi:10.1371/journal.pone.0110219)
Supplement: Figure S5 — Number of species per year that show extreme values of metapopulation metrics, excluding rare species. (DOCX) [file pone.0110219.s005.docx]

Figure S2 Number of species per year that show extreme values of metapopulation metrics (de-trended) for 4 groups of animals, only for species that occupy no less than 120 sites. Positive frequencies refer to years with values greater than mean value + 1.5 times standard deviation, negative frequencies refer to years with values smaller than mean value - 1.5 times standard deviation. Dashed line marks the range if the frequencies were distributed uniformly. a) occupancy probability, b) colonisation probability, c) persistence probability, d) occurrence of extreme years. Years with extreme weather are marked as follows:

**○** hot summer, **□** dry spring, **▽** wet spring, **◊** mild winter, **❄** cold winter.
