# Supplementary material for: No Evidence of the Effect of Extreme Weather Events on Annual Occurrence of Four Groups of Ectothermic Species
Source: PLoS One. 2014 Oct 17;9(10):e110219. doi: 10.1371/journal.pone.0110219 (PMC4201516; doi:10.1371/journal.pone.0110219)
Supplement: Table S1 — Indices of extremes used in the PCA and their trends. (DOCX) [file pone.0110219.s006.docx]

Table S1 Indices of extremes used in the PCA and their trends

| index | description | retained in final analysis | number of weather stations that show a positive trend for summer months over 1979-2013 with 0.05 significance  (total number of weather stations in parentheses, dd- data deficient) | number of weather stations that show a negative trend for summer months over 1979-2013 with 00.5 significance (total number of weather stations in parentheses, dd- data deficient) |
| --- | --- | --- | --- | --- |
| CC | Mean of daily mean cloud cover |  | dd | dd |
| CC2 | Mostly sunny days |  | dd | dd |
| CC6 | Mostly cloudy days |  | dd | dd |
| GD4 | Growing degree days |  | 4(5) | 0(5) |
| CFD | No. consecutive frost days |  | 0(6) | 0(6) |
| HD17 | Heating degree days | X | 0(5) | 4(5) |
| CSDI | Cold spell duration index |  | 0(4) | 1(4) |
| TXn | Minimum of daily maximum temperature |  | 5(6) | 0(6) |
| FD | No. frost days | X | 0(6) | 0(6) |
| ID | No. ice days |  | 0(6) | 0(6) |
| TNn | Minimum of daily minimum temperature |  | 1(6) | 0(6) |
| TN10p | No. of cold nights |  | 0(4) | 2(4) |
| TX10p | No. of cold day-times |  | 0(4) | 4(4) |
| TG10p | No. of cold days |  | 0(4) | 4(4) |
| CDD | Maximum no. of consecutive dry days |  | 1(121) | 0(121) |
| PET | Potential evapotranspiration |  | dd | dd |
| SPI3 | 3-month Standardized Precipitation Index |  | 0(121) | 0(121) |
| SPI6 | 6-month Standardized Precipitation Index |  | 0(121) | 3(121) |
| SU | No. of summer days |  | 1(6) | 0(6) |
| WSDI | Warm spell duration index |  | 2(4) | 0(4) |
| TR | No. tropical nights |  | 2(6) | 0(6) |
| TN90p | No. of warm nights |  | 2(4) | 0(4) |
| TX90p | No. of warm day-times |  | 3(4) | 0(4) |
| TG90p | No. of warm days |  | 3(4) | 0(4) |
| TXx | Maximum of daily maximum temperature |  | 5(6) | 0(6) |
| TNx | Maximum of daily minimum temperature |  | 6(6) | 0(6) |
| CSU | Consecutive summer days |  | 0(6) | 0(6) |
| RH | Mean of daily mean relative humidity |  | dd | dd |
| PP | Mean of daily surface air pressure | X | 0(10) | 0(10) |
| RR | Precipitation sum | X | 30(121) | 0(121) |
| RR1 | No. of wet days |  | 0(121) | 0(121) |
| SDII | Simple daily intensity index |  | 33(121) | 0(121) |
| R10mm | Heavy precipitation days (> 10mm) |  | 31(121) | 0(121) |
| R20mm | Very heavy precipitation days (> 20mm) |  | 24(121) | 0(121) |
| CWD | Maximum no. of consecutive wet days |  | 0(121) | 1(121) |
| RX1day | Highest 1-day precipitation amount |  | 15(121) | 0(121) |
| RX5day | Highest 5-day precipitation amount |  | 19(121) | 0(121) |
| R75p | No. of moderate wet days |  | 37(121) | 0(121) |
| R95p | No. of very wet days | X | 25(121) | 0(121) |
| R99p | No. of extremely wet days |  | 17(121) | 0(121) |
| R75pTOT | Precipitation fraction due to moderate wet days |  | 39(121) | 0(121) |
| R95pTOT | Precipitation fraction due to very wet days |  | 26(121) | 0(121) |
| R99pTOT | Precipitation fraction due to extremely wet days |  | 16(121) | 0(121) |
| PRCPTOT | Precipitation amount due to wet days |  | 31(121) | 0(121) |
| SD | Mean of daily snow depth |  | dd | dd |
| SD1 | No. of snow days |  | dd | dd |
| SD5cm | No. of days with 5 cm of snow or more |  | dd | dd |
| SD50cm | No. of days with 50 cm of snow or more |  | dd | dd |
| SS | Sunshine duration | X | dd | dd |
| SSp | Sunshine duration fraction |  | dd | dd |
| TG | Mean of daily mean temperature | X | 4(5) | 0(5) |
| TN | Mean of daily minimum temperature | X | 4(6) | 0(6) |
| TX | Mean of daily maximum temperature | X | 6(6) | 0(6) |
| DTR | Mean of diurnal temperature range | X | 2(6) | 0(6) |
| ETR | Intra-period extreme temperature range |  | 2(6) | 0(6) |
| vDTR | Mean absolute day-to-day difference in DTR |  | 1(5) | 0(5) |
| FXx | Maximum value of daily maximum wind gust |  | dd | dd |
| FG6Bft | Days with FG >= 6 Bft (10.8 m/s) |  | dd | dd |
| FGcalm | Days with FG <= 2 m/s (calm days) |  | dd | dd |
| FG | Mean of daily mean wind speed |  | dd | dd |

data source for trends: <http://www.ecad.eu/utils/mapserver/trend.php#bottom>
