# Supplementary material for: No Evidence of the Effect of Extreme Weather Events on Annual Occurrence of Four Groups of Ectothermic Species
Source: PLoS One. 2014 Oct 17;9(10):e110219. doi: 10.1371/journal.pone.0110219 (PMC4201516; doi:10.1371/journal.pone.0110219)
Supplement: Table S2 — Trends excluding rare species. (DOCX) [file pone.0110219.s007.docx]

Table S2 Number of species by group showing significantly increasing trend, significantly decreasing trend or no significant trend in metapopulation metrics only for species that occupy no less than 120 sites.

|  |  | species groups | | | | |
| --- | --- | --- | --- | --- | --- | --- |
| metapopulation metric | trend | Odonata (n=40) | Orthoptera (n=25) | Lepidoptera (n=33) | Reptilia (n=6) | total (n=104) |
| occupancy | positive | 27 | 9 | 10 | 4 | 48 |
|  | negative | 4 | 3 | 13 | 1 | 21 |
|  | no trend | 9 | 13 | 10 | 1 | 33 |
|  |  |  |  |  |  |  |
| colonisation | positive | 15 | 5 | 7 | 2 | 27 |
|  | negative | 9 | 0 | 14 | 1 | 24 |
|  | no trend | 16 | 20 | 12 | 3 | 51 |
|  |  |  |  |  |  |  |
| persistence | positive | 23 | 9 | 11 | 0 | 43 |
|  | negative | 3 | 2 | 7 | 0 | 12 |
|  | no trend | 14 | 14 | 15 | 6 | 47 |
