# Supplementary material for: No Evidence of the Effect of Extreme Weather Events on Annual Occurrence of Four Groups of Ectothermic Species
Source: PLoS One. 2014 Oct 17;9(10):e110219. doi: 10.1371/journal.pone.0110219 (PMC4201516; doi:10.1371/journal.pone.0110219)
Supplement: Table S3 — Relationship between colonisation and temperature, excluding rare species. (DOCX) [file pone.0110219.s008.docx]

Table S3 Number of species by group that show given relationships between colonisation probability and temperature in current or preceding year only for species that occupy no less than 120 sites.

|  |  | relationship between colonisation probability and temperature | | |
| --- | --- | --- | --- | --- |
| species group | year | positive relation | thermal optimum | other than expected |
| Odonata (n=40) | t | 5 | 1 | 34 |
|  | t-1 | 5 | 0 | 35 |
|  |  |  |  |  |
| Orthoptera (n=25) | t | 0 | 0 | 25 |
|  | t-1 | 2 | 0 | 24 |
|  |  |  |  |  |
| Lepidoptera (n=33) | t | 5 | 0 | 28 |
|  | t-1 | 1 | 0 | 32 |
|  |  |  |  |  |
| Reptilia (n=6) | t | 1 | 0 | 5 |
|  | t-1 | 1 | 0 | 5 |
|  |  |  |  |  |
| total (n=104) | t | 11 | 1 | 92 |
|  | t-1 | 9 | 0 | 95 |
