# Supplementary material for: No Evidence of the Effect of Extreme Weather Events on Annual Occurrence of Four Groups of Ectothermic Species
Source: PLoS One. 2014 Oct 17;9(10):e110219. doi: 10.1371/journal.pone.0110219 (PMC4201516; doi:10.1371/journal.pone.0110219)
Supplement: Table S5 — Trends for years 1997–2011. (DOCX) [file pone.0110219.s010.docx]

Table S5 Number of species by group showing significantly increasing trend, significantly decreasing trend or no significant trend in metapopulation metrics for years 1997-2011

|  |  | species groups | | | | |
| --- | --- | --- | --- | --- | --- | --- |
| metapopulation metric | trend | Odonata (n=58) | Orthoptera (n=32) | Lepidoptera (n=37) | Reptilia (n=7) | total (n=134) |
| occupancy | positive | 38 | 18 | 9 | 5 | 70 |
|  | negative | 7 | 3 | 16 | 1 | 27 |
|  | no trend | 13 | 11 | 12 | 1 | 37 |
|  |  |  |  |  |  |  |
| colonisation | positive | 27 | 8 | 13 | 2 | 50 |
|  | negative | 5 | 0 | 8 | 0 | 13 |
|  | no trend | 26 | 24 | 16 | 5 | 71 |
|  |  |  |  |  |  |  |
| persistence | positive | 12 | 14 | 5 | 1 | 32 |
|  | negative | 7 | 2 | 12 | 2 | 23 |
|  | no trend | 39 | 16 | 20 | 4 | 79 |
