# Supplementary material for: No Evidence of the Effect of Extreme Weather Events on Annual Occurrence of Four Groups of Ectothermic Species
Source: PLoS One. 2014 Oct 17;9(10):e110219. doi: 10.1371/journal.pone.0110219 (PMC4201516; doi:10.1371/journal.pone.0110219)
Supplement: Table S6 — Relationship between colonisation and temperature for years 1997–2011. (DOCX) [file pone.0110219.s011.docx]

Table S6 Number of species by group that show given relationships between colonisation probability and temperature in current or preceding year for years 1997-2011

|  |  | relationship between colonisation probability and temperature | | |
| --- | --- | --- | --- | --- |
| species group | year | positive relation | thermal optimum | other than expected |
| Odonata (n=58) | t | 7 | 1 | 50 |
|  | t-1 | 6 | 3 | 49 |
|  |  |  |  |  |
| Orthoptera (n=32) | t | 2 | 1 | 29 |
|  | t-1 | 0 | 0 | 32 |
|  |  |  |  |  |
| Lepidoptera (n=37) | t | 0 | 2 | 35 |
|  | t-1 | 1 | 1 | 35 |
|  |  |  |  |  |
| Reptilia (n=7) | t | 0 | 0 | 7 |
|  | t-1 | 1 | 0 | 6 |
|  |  |  |  |  |
| total (n=134) | t | 9 | 4 | 121 |
|  | t-1 | 8 | 4 | 122 |
