# Supplementary material for: No Evidence of the Effect of Extreme Weather Events on Annual Occurrence of Four Groups of Ectothermic Species
Source: PLoS One. 2014 Oct 17;9(10):e110219. doi: 10.1371/journal.pone.0110219 (PMC4201516; doi:10.1371/journal.pone.0110219)
Supplement: Table S7 — Relationship between persistence and temperature for years 1997–2011. (DOCX) [file pone.0110219.s012.docx]

Table S7 Number of species by group that show given relationships between persistence probability and temperature in current or preceding year for years 1997-2011.

|  |  | relationship between persistence probability and temperature | | |
| --- | --- | --- | --- | --- |
| species group | year | positive relation | thermal optimum | other than expected |
| Odonata (n=58) | t | 8 | 3 | 47 |
|  | t-1 | 4 | 3 | 51 |
|  |  |  |  |  |
| Orthoptera (n=32) | t | 0 | 1 | 31 |
|  | t-1 | 1 | 9 | 22 |
|  |  |  |  |  |
| Lepidoptera (n=37) | t | 0 | 1 | 36 |
|  | t-1 | 1 | 0 | 36 |
|  |  |  |  |  |
| Reptilia (n=7) | t | 1 | 0 | 6 |
|  | t-1 | 0 | 0 | 7 |
|  |  |  |  |  |
| total (n=134) | t | 9 | 5 | 120 |
|  | t-1 | 6 | 12 | 116 |
